# Big data and computational biology strategy for personalized prognosis

**DOI:** 10.18632/oncotarget.9571

**Published:** 2016-05-24

**Authors:** Ghim Siong Ow, Zhiqun Tang, Vladimir A. Kuznetsov

**Affiliations:** ^1^ Bioinformatics Institute, Singapore 138671; ^2^ School of Computer Engineering, Nanyang Technological University, Singapore 639798

**Keywords:** personalized prognosis, risk stratification, ovarian cancer, aging, big data

## Abstract

The era of big data and precision medicine has led to accumulation of massive datasets of gene expression data and clinical information of patients. For a new patient, we propose that identification of a highly similar reference patient from an existing patient database via similarity matching of both clinical and expression data could be useful for predicting the prognostic risk or therapeutic efficacy.

Here, we propose a novel methodology to predict disease/treatment outcome via analysis of the similarity between any pair of patients who are each characterized by a certain set of pre-defined biological variables (biomarkers or clinical features) represented initially as a prognostic binary variable vector (PBVV) and subsequently transformed to a prognostic signature vector (PSV). Our analyses revealed that Euclidean distance rather correlation distance measure was effective in defining an unbiased similarity measure calculated between two PSVs.

We implemented our methods to high-grade serous ovarian cancer (HGSC) based on a 36-mRNA predictor that was previously shown to stratify patients into 3 distinct prognostic subgroups. We studied and revealed that patient's age, when converted into binary variable, was positively correlated with the overall risk of succumbing to the disease. When applied to an independent testing dataset, the inclusion of age into the molecular predictor provided more robust personalized prognosis of overall survival correlated with the therapeutic response of HGSC and provided benefit for treatment targeting of the tumors in HGSC patients.

Finally, our method can be generalized and implemented in many other diseases to accurately predict personalized patients’ outcomes.

## INTRODUCTION

Rapid technological advancement is a major driver of knowledge discovery in science today. Specifically in the field of genomics research, the development of high-throughput technologies such as microarrays, next-generation sequencing or mass spectrometry, coupled with the ability to implement computationally intensive algorithms, have led to the accumulation of varied and massive amount of biological data. The convergence of the basic sciences, along with technological and computational discipline with biomedical studies are considered as the main driver of precision medicine by refining the classification of disease, potentially with improved prognostic and therapeutic benefits [1].

While currently the state of precision and personalized medicine is still relatively immature, the promise of the field has been much discussed in detail [1–4]. Jameson and Longo defined precision medicine as “treatment targeted to the needs of individual patients on the basis of genetic, biomarker, phenotypic, or psychosocial characteristics that distinguish a given patient from other patients with similar clinical presentations. Inherent in this definition is the goal of improving clinical outcomes for individuals and minimizing unnecessary side effects for those less likely to have a response to particular treatment. [1]” Chan et al. stated that “the overarching goal of personalized medicine is to optimize medical care and outcomes for each individual, resulting in an unprecedented customization of patient care. [2]” These definitions are quite similar in the context of treated targets and they both emphasize that a given patient treatment have to be driven based on solid scientific knowledge and advanced technology, thereby providing an improvement in diagnostics, prognostics, therapy response predictions and optimal therapeutics.

Specifically with regards to disease prognosis or therapy response prediction, the use of gene expression data in discriminating between patient subgroups has been amongst the most commonly proposed [5, 6] as well as studied in many cancers [7–12]. This is typically accomplished via the classification ability of a gene signature, which in the most general term, is defined as a set of the disease-related molecular variables, whose values could be directly or indirectly detected and used for molecular characterization of certain normal and/or pathological cells, tissues, organisms, functions, processes and biomedical conditions. Research progress of the use of gene signature in prognosis and adjuvant systemic therapy recommendation is especially advanced in breast cancer, where prognostic kits such as MammaPrint®, PAM50 and Oncotype DX® have been commercially developed and subsequently used for predicting tumor recurrence risks for eligible pre-selected patients recommended by the kit's protocol [8, 13, 14]. Analyses of predictions of clinical outcomes in different cohorts suggested that each of these multi-gene expression test could assist doctors in making adjuvant systemic treatment decisions in addition to currently adopted clinical guidelines and professional judgments [15]. The kits often produce similar results for low and high-risk patients but do not agree well in their predictions for those at intermediate risk or in poorly-defined tumor subtypes. Currently, for many highly aggressive cancers, the assays/kits developed based on novel biomarkers and their computational models are not in clinical use.

Epithelial ovarian cancer (EOC), which high-grade serous ovarian carcinoma (HGSC) is the most prevalent, is one of the most lethal gynaecological diseases in the world today. The heterogeneity of HGSC tumors meant that clinical status of the patients is varied and poorly defined and that tumors are often treated sub-optimally with standard therapy. Despite progress in high-throughput biotechnology and EOC oncogenomic studies, the biomarkers for prognostic and disease prediction have not been implemented in clinical practice. Therefore, identification of high confidence molecular markers for risk of disease development and recurrence is important in prognosis and patient clinical management of HGSC.

Many published studies have investigated the tumor heterogeneity and identified biologically meaningful tumor subgroups [10, 16–18]. Based on meta-analysis of miRNA and mRNA expression profiles of more than 1100 HGSC tumors, Tang et al. identified a signature comprising 36 let-7b-correlated mRNA transcripts, that stratified patients into three high-confidence prognostic subgroups associated with cell cycle, EMT pathways and primary chemotherapy treatment outcome [10]. However, the application of this signature and that of others to personalized prognosis has not been studied yet.

Several publications have clearly demonstrated that a molecular signature, combined with patient's information related to disease risk (e.g. body mass index, ethnic group, sex or smoking status, etc.) could improve the accuracy and reproducibility of the signature. Age is often considered a risk factor for survival in cancer [19–22]. However, usually disease development risk predictors do not include this important personalized variable which plays significant roles in origin and development of cancer as well as involved in many aspects of the management of cancers in the elderly. This includes prognostic implications of advanced age on chemotherapy options, surgical considerations and geriatric assessment in predicting toxicity [20]. Despite these, older patients have often been under-represented in clinical trials, and evidence which potentially could aid clinical decisions is lacking. Studies are needed to develop strategies to determine the optimal treatment for patients of different age, including those of relative advanced age. For example, it has been reported that rapamycin could prevent cancer indirectly by slowing down the aging process [23, 24], which suggests the possibility that it could be used to slow tumor growth via the aging process.

We previously developed methods to eventually chart a path towards the desirable goal of construction of multi-gene prognostic signature that stratify cancer patients according to disease development risk and personalized prognosis scoring. These algorithms which include statistically weighted syndromes [25], data-driven grouping [26, 27] and statistically weighted voting grouping [10] have been applied to various studies such as glioblastoma [28], breast cancer [12, 29, 30] and ovarian cancer [10].

We aim to develop a method that assigns a new patient (from prospective study) based on molecular, personalized data and clinical variables profile, onto one of the reference risk groups computationally identified after training of the computational model on the reference cohort data.

With regards to the development of the personalized prognostic method, we propose that analyses of the prognostic variables in a classifier can generate a risk classification scheme, which typically for a patient can be best represented by an n-length vector where n denotes the number of variables in the classifier. In this case, a key step towards the aim of our study would rely on similarity or dissimilarity matching of a new patient to other reference patients, where each patient is suitably represented by a classifier vector.

Often, the experimental variables in the classifier may comprise of continuous values of RNA gene expression levels, copy number variation or methylation across the sample cells. Mathematical approaches to compare vectors of continuous variables have been developed and these methods of similarity or dissimilarity comparison have formed the basis of many bioinformatics analytical methods, including but not limited to applications in clustering and pattern recognition [31].

While in many cases, the vectors comprise of continuous variables that quantify biological signals, it is also likely that for practical purposes, the classifier-generated vector could contain binary-formatted values that represent one of two binary states, e.g. low or high-risk. We termed such vectors that contain binary-formatted values “prognostic binary variable vectors (PBVV)”. Methods to compare pairs of PBVV play fundamental roles in a wide range of applications, ranging from biology, bio-imaging [32] and chemistry [33] to image recognition [34, 35]. A two-by-two contingency table can be generated from the comparison of two PBFVs and the similarity measure is typically a ratio of the concordant diagonal to the discordant anti-diagonal. Despite the simplicity that analysis of a two-by-two contingency table entails, there are at least 70 methods to calculate similarity or dissimilarity (distance) measures, according to a recent review by Choi et al. [36].

From the list of similarity or distance (dissimilarity) measures reviewed by Choi et al, it appears that while most are variants of each other, there are some differences with regards to the relative importance (and hence, contribution to the value of the measure) of one binary state over the other. A simple example is illustrated in Supplementary Figure S1, where the Euclidean distance and Lance and Williams distance were calculated for two column vectors (Supplementary Figure S1A). It becomes clear that while Euclidean distance considers both binary states equally (Supplementary Figures S1B-C), Lance and Williams distance prioritizes one of the two binary states depending on how it is implemented (Supplementary Figures S1B-C). Such asymmetric distance or similarity measures that prioritize one of two binary states in the calculations are less applicable in current works as both binary states denoting low and high risk are equally important. It is therefore important to select the most appropriate similarity or dissimilarity measure that best represents the aim of their application as well as the nature and characteristic of the data.

Unlike current methods of multi-variable signature-based personalized prognosis which typically predicts patient subgroups with differential disease recurrence risks [18, 37, 38], it may be equally important to define a method where a prospective newly diagnosed patients could be compared to existing (retrospective individual) patients’ signatures with known tumor diagnostics and disease outcomes, such as to derive future clinical benefit.

Therefore in this work, we integrated age, an important prognostic and predictive factor, into our molecular prognostic signature of HGSC [10] and subsequently introduced a novel prognostic method of personalized medicine. The methods that directly measured the similarity or dissimilarity between two PBVVs were first evaluated. Subsequently, we propose a method to transform the PBVVs into a prognostic signature vector (PSV) which considers both the relative importance (via implementation of weights) as well as the order of variables in the signature. Finally, we proposed a method of personalized prognosis, where a patient's post-surgery disease recurrence risk can be reliably predicted based on the comparison of the patient's PSV with known PSVs of the training cohort.

## RESULTS

### Proposed schema and strategy for personalized diagnosis, prognosis or prediction of therapy success

With the emergence of integrated strategies and technologies, many countries are adopting the use of electronic health record systems. The potential of such integrated system has been elucidated by Chawla et al., who proposed the use of the underlying patients’ clinical histories in predicting and ranking probabilistic risk of future diseases [4]. Furthermore, the cataloging of genomic, transcriptomic, proteomic or epigenetic patients’ profile may be important in addressing the multi-factorial origins of many diseases.

**Figure 1:**
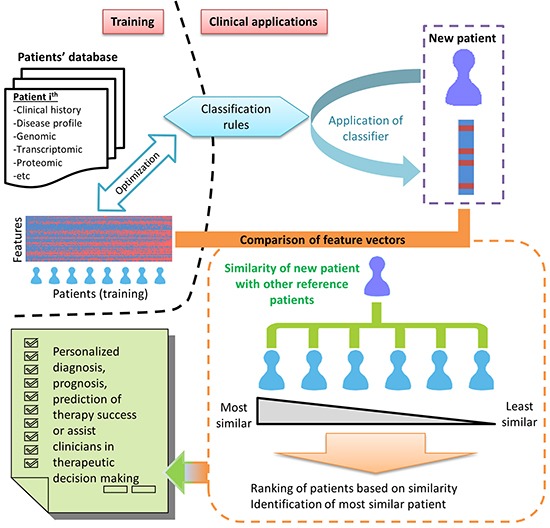
Proposed schema of big data and strategy for personalized diagnosis, prognosis or prediction of therapy success

In contrast to most personalized prognosis methods which are primarily interested in stratification of patients or diseases into prognostically relevant subgroups [18, 37, 38], we proposed that in the field of personalized medicine, it may be important to identify for each newly diagnosed patient, the most “prognostically similar” reference patients which could aid clinicians in clinical case studies when designing therapy for a newly recruited patient diagnosed with the same disease (Figure 1). Essentially, the pairwise comparison of each newly recruited patient (defined as “query” patient) with all other reference patients in the existing database form the basis of our proposed method.

### Dataset and classifier rules

We started our analysis of microarray gene expression profiles data belonging to high-grade serous ovarian carcinoma (HGSC) patients collected by The Cancer Genome Atlas (TCGA) research network [16]. Via meta-analysis of miRNA and mRNA gene expression as well as clinical data belonging to 350 HGSC patients, we had found that let-7b miRNA could be considered a master regulator of several hundreds of protein-coding genes. Subsequently, we identified a let-7b associated protein-coding gene prognostic signature, comprising 36 mRNAs that can stratify the TCGA cohort of patients into low, intermediate or high-risk subgroups with significantly distinct overall survival (OS) outcomes and sensitivity to post-surgery chemotherapy [10]. This prognostic model include the genes *ARPC1B, CALD1, CAV2, CBX3, CCL2, CCT2, CD44, CD93, CDC6, CDK4, CFD, CHEK1, COL3A1, DNMT1, EDNRA, FGFR1, FZD1, GNG12, HGF, LAMA4, MCM2, MIS12, MMP13, NCAPD2, NCAPG2, NCAPH, PDGFRA, PIK3R1, PLAUR, POLA2, POLR2D, POLR2J, TCP1, TGFBR2, TUBB* and *VCL*. Each of these genes is survival-significant and has been shown to be an independent classifier that assigned each patient a binary value of 1 or 2 representing low or high-risk subgroup respectively. Overall, the 36-gene combined prognostic classifier had been shown to provide highly significant and reproducible stratification of HGSC patients in several independent cohorts [10]. Therefore in this work, we used this classifier as the initial model of our new prognostic model of HGSC patients.

In addition, of these 350 TCGA HGSC patients, 349 have information regarding age at initial diagnosis. As age is a well-established factor of OS rates in ovarian cancer [19, 21, 22], we also consider the patient's age variable as an additional prognostic variable of our (combined) personalized patients’ prognostic model of HGSC.

**Figure 2:**
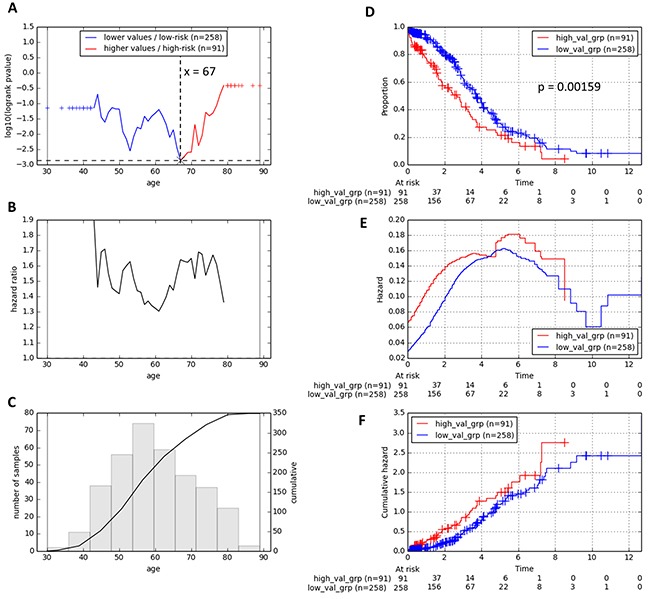
Stratification of reference patients from the TCGA training cohort into two prognostic subgroups based on their age at diagnosis One-dimensional data-driven grouping method was used as the classification method. **A.** Plot of stratification log-rank p-value (transformed y-axis) against the patients’ age cut-off value. **B.** Plot of hazard ratio against the patients’ age cut-off value. **C.** Histogram and cumulative distribution of the patients’ age at diagnosis. **D.** Survival curves, **E.** hazard curves and **F.** cumulative hazard curves of the training TCGA cohort of 349 HGSC patients.

We first assessed the prognostic significance of age in TCGA HGSC patients via the one-dimensional data-driven grouping method (1D-DDg, see Methods, see also [10, 26]). Our results revealed that age was a significant prognostic factor of OS in HGSC (Figure 2). We observed that an age cut-off value of 67 years could stratify the patients into two subgroups with most statistical significance (Figure 2A). The hazard ratios of the higher-aged patient subgroup with respect to the lower-aged patient subgroup were always greater than 1 (Figure 2B). The survival curves, as well as the hazard curves for the subgroups defined via the most optimal age cut-off were shown in Figure 2D-2F. Patients with higher age at diagnosis were faced with a poorer overall survival rate than those with lower age at diagnosis and vice versa (p = 0.00159). Therefore, we chose to incorporate the age information into our previously identified molecular signature comprising the 36 binary variables (predictors) represented by mRNAs from tumors.

In summary, our combined prognostic model comprises 37 binarized variables that include 36 mRNA expression variables and age as personalized patient clinical prognostic factor. Briefly, we used 1D-DDg for construction of the prognostic binary variable vector (PBVV), via estimating the threshold value of expression signal for each mRNA expression variable and age. Subsequently, for each variable of our combined signature, the patients were rank-ordered by their prognostic variable quantity and assigned to numerical risk value 1 or 2, depending on whether they were classified into the low or high-risk subgroups respectively (Supplementary Table S1). For each *j^th^* patient and *i^th^* variable, the risk value is denoted by r_i,j_.

### Average weighted risk value correlates with patients’ survival

We next calculated the average weighted risk (AWR) value for each reference patient *j^th^* in the training cohort using the 37 variables via:

$${\text{AWR}}_{j}=\frac{\sum_{i=1}^{n}w_{i}r_{i,j}}{\sum_{i=1}^{n}w_{i}}$$

where $w_{i}=-{\text{log}}_{10}\;{\text{p}}_{i}$ and *i* = 1, 2, 3, … n^th^ variable

**Figure 3:**
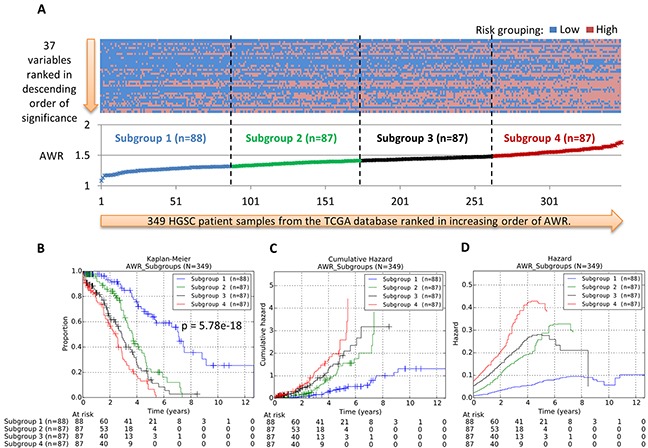
**A.** Heatmap of risk classification for 37 variables and 349 HGSC from the TCGA cohort. **B.** Kaplan-Meier survival curves of the four patient prognostic subgroups. **C–D.** Nelson-Aalen estimated cumulative hazard curves and hazard curves of the four patient prognostic subgroups. The 37 variables comprise 36 mRNA expression variables and 1 clinical variable (age). The risk classification for variable and patient was assessed using the 1D-DDg method. The average weighted risk (AWR) value for each patient across all 37 variables was calculated and used for ranking patient samples. The patient cohort was arbitrarily classified into four equal-sized sub-groups based on their AWR values.

The results of the calculations are shown in Supplementary Table S1. The heat map of the risk classification across each of the 37 variables and each of the 349 patient samples is shown in Figure 3A. To evaluate whether the AWR values provide good quantitative indication of prognostic significance, the patients were first ranked in ascending order of their AWR values and classified into four arbitrary equal-sized subgroups. Subsequently, we represented these patient subgroups on Kaplan-Meier survival curves (Figure 3B). Our results showed that patient subgroup 1, which contains the group of patients with the smallest AWR values, has the most favorable OS rates across all years. In contrast, patient subgroup 4 which contains the group of patients with the largest AWR values has the least favorable OS rates across all years. Therefore, our results suggest that AWR value could be a reliable indicator of patients’ survival patterns (Figure 3). Figure 3C and 3D show the cumulative hazard and hazard plots of the patient subgroups, additionally supporting our suggestion.

### Application of the prognostic model parameters to an independent dataset

We downloaded two independent testing datasets belonging to ovarian cancer patients, GSE9899 and GSE26712 [17, 39]. Initial analysis of expression across the TCGA reference datasets and the two independent datasets revealed the gene expression distribution within each patient cohort is different (Supplementary Figure S2A). These two independent datasets were treated as detection batches and via the pamr R package, the expressions were batch-corrected using the original TCGA data as the reference cohort. All the datasets were mean-centered and aligned to the TCGA reference cohort to allow for valid comparison across the datasets. After data preprocessing, the plots for the 0^th^, 5^th^, 25^th^, 50^th^, 75^th^, 95^th^ and 100^th^ percentiles for each sample in the three cohorts showed that their gene expression values are aligned (Supplementary Figure S2B).

For each of the 37 variables which consist of 36 mRNA expression variables and age information, we applied the quantity cut-off (threshold) value learned from the reference TCGA patient cohorts to the independent testing datasets and assign each of these patients to low or high-risk subgroups (corresponding to risk value of 1 or 2 respectively). The results after applying the variable threshold values to the independent testing datasets can be found in Supplementary File 1 and Supplementary Table S2. Consequently, each patient in either the reference or testing cohort can be represented by a PBVV based on the risk group assignment via the 37 independent variables (gene expression value or age information).

The AWR values could also be calculated for each of the patients in the independent testing datasets by using the variable weights as defined during training of the individual variable (Supplementary Table S2). However unlike the training dataset, the AWR in the testing dataset has poor association with overall survival rates (Supplementary Figure S3) which suggest that alternative methods would be required to provide prognostic prediction of the query patients based on a set of classifier variables defined *a priori*.

### Comparison of PBVVs and association with risk

**Figure 4:**
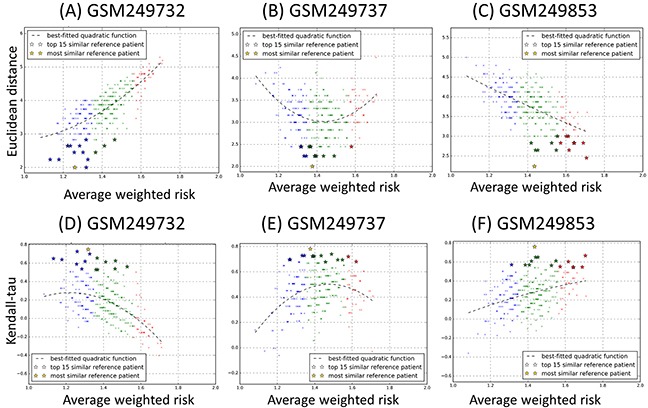
Scatter plots of **A-C.** Euclidean distance and **D-F.** Kendall's tau rank correlation coefficient against average weighted risk (AWR) values calculated for three representative testing samples GSM249732, GSM249737 and GSM249853 against each reference sample in the training cohort (n=349). Each query sample and reference sample is represented by a prognostic binary variable vector (PBVV). Each point on the plot represents each of 349 reference samples, and the y-axis represents the value of the Euclidean distance or Kendall's tau rank correlation coefficient with the testing sample. The color blue, green and red corresponds to the low, intermediate and high prognostic risk group of the reference patients. The x-axis represents the AWR values associated with each reference sample. (All results can be found in Supplementary Files 2 and 3).

Next, for each PBVV belonging to each query patient *k^th^* in the testing datasets, we calculated the measure of dissimilarity with all the other PBVVs belonging to all other reference patient *j^th^* in the training datasets (Supplementary Methods). The Euclidean distance is used as the measure of dissimilarity where a larger distance implies lower level of similarity and vice versa. The distances between the PBVVs of patients in the testing dataset against the training datasets are shown in Supplementary Table S3. The scatter plots of distances and AWR for each query-reference sample pairs can be plotted and examples for query samples GSM249732, GSM249737 and GSM249853 are shown in Figure 4A-4C (see also Supplementary File 2 for all query patients).

It should be expected that if a particular method is appropriate for comparing between a query patient and other reference sample pairs, the theoretical expected distribution of distance versus reference patients’ AWR would have quadratic characteristics (see Methods). However, our analysis here revealed that Euclidean distance between PBVVs is not strongly associated with the AWR (Figure 4A–4C). This is because the AWR values are not uniquely associated with any Euclidean distances and vice versa, especially around the intermediate AWR value range (Figure 4B). As we have earlier shown that the AWR is a strong indicator of patients’ OS prognosis (Figure 3B), our results here suggest that direct comparison of PBVVs between pairs of query and reference patients via Euclidean distances could not provide a reliable measure for identification of the most “prognostically similar” reference patients for any given query patient from the testing cohort.

Also, we compared the PBVVs between each query-reference sample pairs using Kendall's tau rank correlation coefficient measure (Supplementary Table S4). Similarly, results revealed that there is no clear relationship between the measures of similarity with the AWR of the reference sample (Figures 4D-4F, see also Supplementary File 3 for all query patients).

Thus, results suggested that an identification of the “most prognostically similar” reference patient in the training set via direct comparison of PBVVs using published measures of similarity or dissimilarity is neither effective nor informative.

**Figure 5:**
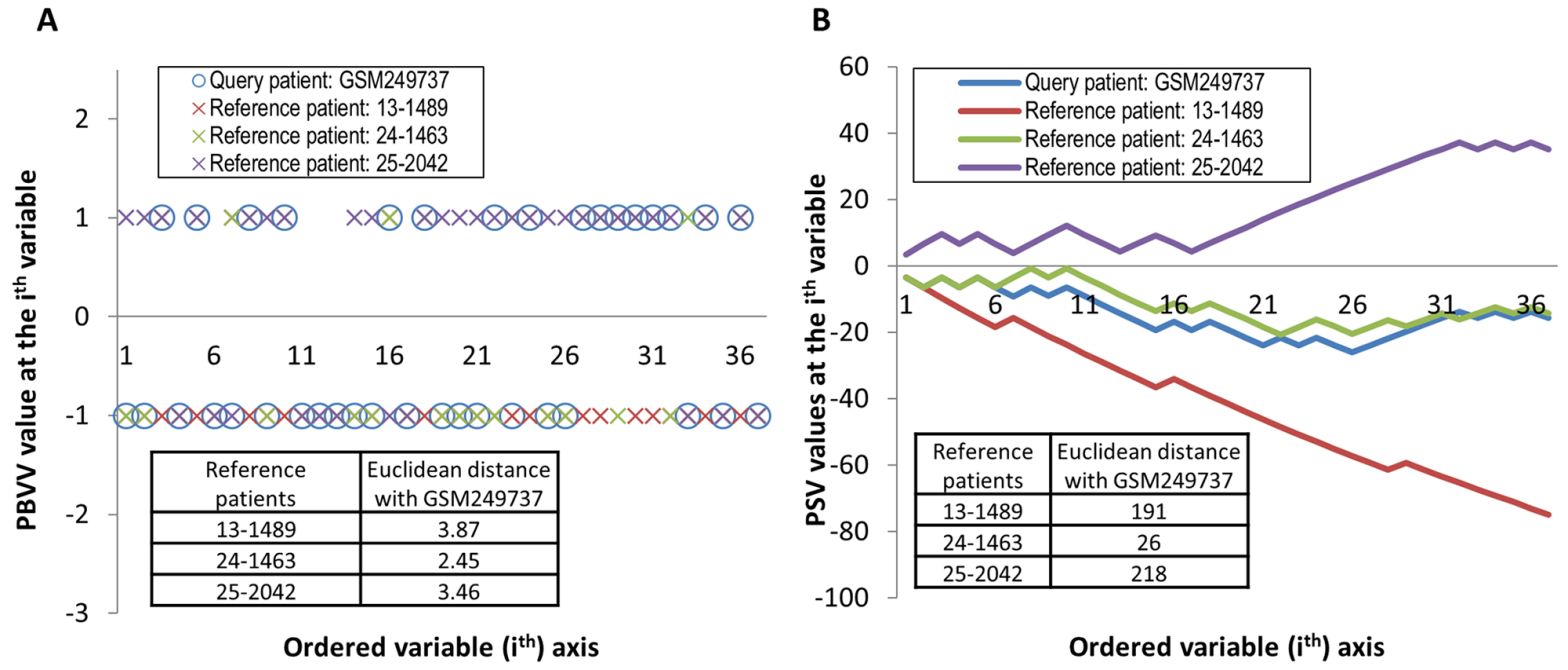
Two dimensional representation of **A.** PBVVs and **B.** PSVs across the 37 variables for one representative query patient and three representative reference patients. Euclidean distances between the query patient and the three reference patients are shown inset.

Visually, the PBVV for any patient could be represented on a two-dimensional plot where the horizontal axis represents the dimension of variables (for 37 variables) and the vertical axis represents binary values (of low-risk vs high-risk). It could be observed that assessing patients’ similarities based on PBVV could be challenging due to the lack of discriminative information of the original PBVVs (Figure 5A).

### Comparison of the PSVs and association with risk

In view of the failure of the methods which directly compare pairs of PBVVs to indicate level of similarity or dissimilarity between patients (Figure 5A), new methods should be proposed and studied. Here, we propose a novel and unbiased computational approach to assign a new patient to another patient based on firstly, the transformation of a PBVV to a more informative and higher resolution PSV and subsequently, the similarity matching of the PSVs (Supplementary Methods).

The procedures to generate the PSVs for an example reference patient *j^th^* and query patient *k^th^* are listed in Table 1. Using the procedures, each patient can be represented by a relatively unique PSV with length equal to the number of gene variables (Figure 5B). Due to the design of the origin-centered risk grouping, the positive and negative directional movement of the PSV values along the variable axis is indicative of the patient's shift towards a higher risk or lower risk prognosis respectively (Figure 5B). From Figure 5B, it can be observed that the values of the PSV of query patient (GSM249737) are most similar to that of the reference patient 24-1463 rather than patients 13-1489 and 25-2042.

**Table 1:** Conversion of prognostic binary variable vectors to prognostic signature vectors for example reference patient (j^th^) and query patient (k^th^)

Ranked variable order			Reference patient: 13-1489 = patient j^th^			Query patient: GSM249737 = patient k^th^		
Variable	Variable index (i^th^)	*W*	*D_j_*	*A_j_*	*V_j_*	*D_k_*	*A_k_*	*V_k_*
205382_s_at (*CFD*)	1	3.429	−1	−3.429	−3.429	−1	−3.429	−3.429
202246_s_at (*CDK4*)	2	3.124	−1	−3.124	−6.553	−1	−3.124	−6.553
204451_at (*FZD1*)	3	3.071	−1	−3.071	−9.625	1	3.071	−3.482
201947_s_at (*CCT2*)	4	3.024	−1	−3.024	−12.648	−1	−3.024	−6.506
205959_at (*MMP13*)	5	2.928	−1	−2.928	−15.577	1	2.928	−3.577
201954_at (*ARPC1B*)	6	2.805	−1	−2.805	−18.382	−1	−2.805	−6.382
age	7	2.800	1	2.800	−15.582	−1	−2.800	−9.182
201615_x_at (*CALD1*)	8	2.793	−1	−2.793	−18.374	1	2.793	−6.389
204464_s_at (*EDNRA*)	9	2.713	−1	−2.713	−21.088	−1	−2.713	−9.102
208944_at (*TGFBR2*)	10	2.673	−1	−2.673	−23.760	1	2.673	−6.430
203968_s_at (*CDC6*)	11	2.660	−1	−2.660	−26.420	−1	−2.660	−9.090
209026_x_at (*TUBB*)	12	2.640	−1	−2.640	−29.060	−1	−2.640	−11.730
201774_s_at (*NCAPD2*)	13	2.559	−1	−2.559	−31.619	−1	−2.559	−14.289
212239_at (*PIK3R1*)	14	2.545	−1	−2.545	−34.165	−1	−2.545	−16.834
203131_at (*PDGFRA*)	15	2.468	−1	−2.468	−36.632	−1	−2.468	−19.302
212063_at (*CD44*)	16	2.466	1	2.466	−34.166	1	2.466	−16.836
212782_x_at (*POLR2J*)	17	2.464	−1	−2.464	−36.630	−1	−2.464	−19.299
214144_at (*POLR2D*)	18	2.459	−1	−2.459	−39.089	1	2.459	−16.841
219588_s_at (*NCAPG2*)	19	2.375	−1	−2.375	−41.463	−1	−2.375	−19.215
209960_at (*HGF*)	20	2.364	−1	−2.364	−43.827	−1	−2.364	−21.579
212294_at (*GNG12*)	21	2.360	−1	−2.360	−46.187	−1	−2.360	−23.939
207822_at (*FGFR1*)	22	2.298	−1	−2.298	−48.486	1	2.298	−21.641
204441_s_at (*POLA2*)	23	2.283	−1	−2.283	−50.768	−1	−2.283	−23.924
216598_s_at (*CCL2*)	24	2.206	−1	−2.206	−52.974	1	2.206	−21.719
202107_s_at (*MCM2*)	25	2.156	−1	−2.156	−55.130	−1	−2.156	−23.875
202202_s_at (*LAMA4*)	26	2.116	−1	−2.116	−57.246	−1	−2.116	−25.991
215076_s_at (*COL3A1*)	27	2.095	−1	−2.095	−59.341	1	2.095	−23.896
210845_s_at (*PLAUR*)	28	2.081	−1	−2.081	−61.422	1	2.081	−21.815
201697_s_at (*DNMT1*)	29	2.052	1	2.052	−59.370	1	2.052	−19.763
202877_s_at (*CD93*)	30	2.044	−1	−2.044	−61.413	1	2.044	−17.719
203323_at (*CAV2*)	31	1.985	−1	−1.985	−63.398	1	1.985	−15.735
221559_s_at (*MIS12*)	32	1.961	−1	−1.961	−65.359	1	1.961	−13.774
208778_s_at (*TCP1*)	33	1.955	−1	−1.955	−67.313	−1	−1.955	−15.729
201091_s_at (*CBX3*)	34	1.921	−1	−1.921	−69.234	1	1.921	−13.808
205393_s_at (*CHEK1*)	35	1.918	−1	−1.918	−71.152	−1	−1.918	−15.727
200931_s_at (*VCL*)	36	1.902	−1	−1.902	−73.055	1	1.902	−13.824
212949_at (*NCAPH*)	37	1.889	−1	−1.889	−74.944	−1	−1.889	−15.714

Notations: W: weight vector, D: centered grouping vector (−1=“low-risk”, 1=“high-risk”), A: adjustment vector, V: signature vector.

Again, we have chosen to represent a high dimensional vector (37 dimensions of 37 variables) on two-dimensional plot where the horizontal axis represents the dimensions and the vertical axis represents value of the prognostic risk score defined by PSV (Figure 5B). This plot is possible to construct because the variables have been rank-ordered according to their weight values. Visually, the level of similarity between any pair of PSVs can be assessed by the area or vertical distance between the two curves (Figure 5B). For instance to assess the similarity between any two curves, we could define a simple metric as the sum of square differences along the horizontal axis. Quantitatively, this value is proportional to the Euclidean distance. Specifically, they differ by a square root factor, but as the quantity is only used for patient ranking, the actual value is not important. Therefore, we used the quantitative measures of Euclidean distance between the pairs of query-reference patients to rank the reference patients 24-1463, 13-1489 and 25-2042 in order of decreasing similarity with the query patient GSM249737 from the testing cohort. Subsequently, the most similar reference patient to the query patient could be identified for prognostic risk prediction.

**Figure 6:**
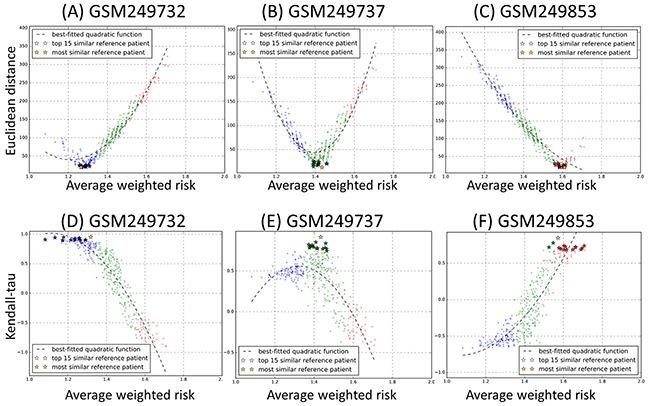
Scatter plots of **A-C.** Euclidean distance and **D-F.** Kendall's tau rank correlation coefficient against average weighted risk (AWR) values calculated for three representative testing samples GSM249732, GSM249737 and GSM249853 against each reference sample in the training cohort (n=349). Each query sample and reference sample is represented by a prognostic signature vector (PSV). Each point on the plot represents each of 349 reference samples, and the y-axis represents the value of the Euclidean distance or Kendall's tau rank correlation coefficient with the testing sample. The color blue, green and red corresponds to the low, intermediate and high prognostic risk group of the reference patients. The x-axis represents the AWR values associated with each reference sample. (All results can be found in Supplementary Files 4 and 5).

Next, using our proposed procedures as described earlier, we converted the PBVVs of all the reference patient *j^th^* from the training cohort and query patients *k^th^* from the testing cohort to their PSVs (Supplementary Tables S5–S6) and calculated the Euclidean distances between pairs of PSVs (Supplementary Table S7). We then evaluated whether Euclidean distance between pairs of PSVs can provide strong association with the AWR of the reference patients. Our results revealed that for any given query sample, there are clear associations between the Euclidean distances and the AWRs of the reference patients (Figures 6A-6C, see also Supplementary File 4 for all query patients). This suggests that our mathematical procedures of processing the PBVVs can provide a reliable way of generating relatively unique PSVs for further pair comparisons, as well as facilitating ranking and identification of the “most prognostically similar” reference patients from the training cohort.

On the other hand, the use of Kendall's tau rank correlation coefficient was observed to be less effective in identifying the “most prognostically similar” reference patients from the training cohort, specifically at the intermediate ranges of AWRs (Figures 6D-6F, see also Supplementary File 5 for all query patients).

### Distance measure of PSVs is an appropriate metric of patient similarity

We next compared summary statistics of each of the analyses performed previously, namely (i) comparison of PBVVs via Euclidean distance, (ii) comparison of PBVVs via Kendall's Tau rank correlation, (iii) comparison of PSVs via Euclidean distance, and (iv) comparison of PSVs via Kendall's Tau rank correlation.

We first analyze the relationship between the AWR of each query patient, with the AWR of its corresponding most quantitatively similar reference patient as derived via each of the discussed methods (Supplementary Figure S4A). Our analyses revealed that of the similarity measures (the Euclidean distance or the Kendall's tau rank correlation coefficient) and vectors (PBVV or PSV) used, the pairwise Euclidean distance of query patient's PSV and reference patient's PSV is most linearly correlated (Supplementary Figure S4A). On the other hand, for each query patient, other measures of similarity tend to predict reference patients that are not as similar, leading to a rather large difference between the AWR of the query patient and the AWR of the predicted most similar reference patient (Supplementary Figure S4A).

A related analysis that assessed the coefficient of variation of the top 15 quantitatively similar reference patients’ AWR also revealed that Euclidean distance measure of PSVs provide the most specific association of similarity measure with the reference patients’ AWR (Supplementary Figure S4B). In contrast, other methods tend to predict for a new query patient, AWR values that spanned across a wider range.

Next, we performed residual analysis for each of the above-mentioned methods based on an expected quadratic function that would be expected of a strong relationship between the similarity values with the AWR values (see Methods). Our results revealed Euclidean distance calculated between PSVs provided the least residuals across all query patients, which further suggests the potential of this method in the identification of the most similar reference patient (Supplementary Figure S4C).

Additionally, within the training cohort, we performed similarity calculations based on each of the above-mentioned methods on pair of patients within a particular prognostic risk group (intra-class) or between two prognostic risk groups (inter-class). The results for each of the four methods described above are presented in Supplementary Figure S5. Our results revealed that when PBVVs were used as the basis for pairwise comparison, there were no significant differences in Euclidean distance or Kendall's Tau rank correlation whether patients were from the same prognostic group or between two different prognostic groups (Supplementary Figures S5A-S5B). This suggested that PBVVs are inadequate for similarity calculation. On the other hand, when the PBVVs were converted to PSVs and subsequently compared via Euclidean distance, we observed that the distance between pairs of patients from within low, intermediate or high-risk patients were lower, and showed less variation in contrast to inter-class comparisons (Supplementary Figure S5C). Therefore, our results suggested that Euclidean distance could be a reliable measure of comparison between pairs of PSVs.

Finally, for each of the four methods, we assessed its accuracy and stability of the results via 10-fold cross validation analysis performed for the training cohort (see Methods). Our results showed that of the four methods studied, calculating the Euclidean distance between PSVs yielded the highest mean accuracy as well as the least variation across the ten cross validation analyses (Supplementary Figure S6A). This indicated the ability of our method in classifying independent patient cohort with high accuracy and robustness. Also, for the method of patient risk prediction based on Euclidean distance between PSVs, we assessed if gains in accuracy or robustness could be achieved if the prediction is based on the top few patients rather than the most quantitatively similar reference patient (Supplementary Figure S6B). Our results revealed no significant benefit of predicting the prognostic risk based on the consensus prognostic risk of the top 5, 9, 13 or 17 most similar reference patients.

### Evaluation of an entire independent testing cohort

**Figure 7:**
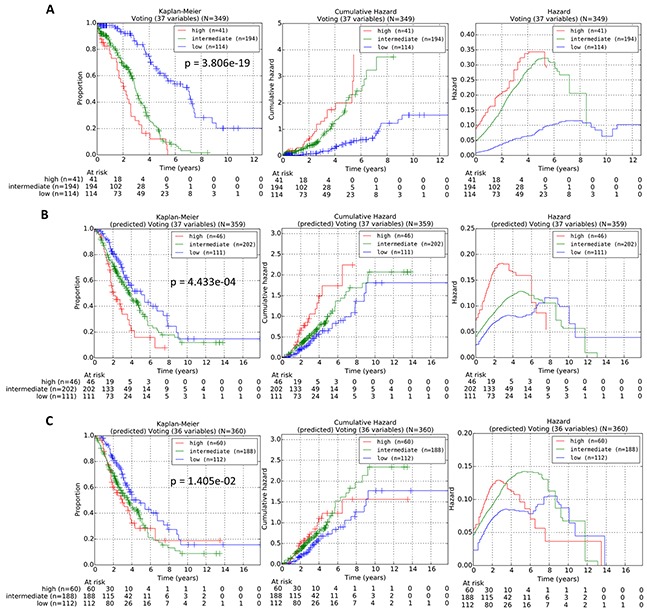
**A.** Stratification curves of the training cohort of 349 TCGA HGSC patients obtained via a 37-variable classifier comprising 36 mRNA variables and 1 age variable. **B.** Stratification curves of the testing cohort of 359 patients from GSE9899 and GSE36712 obtained via the 37-variable classifier comprising 36 mRNA variables and 1 age variable. **C.** Stratification curves of the testing cohort of 360 patients from GSE9899 and GSE36712 obtained via a 36-variable classifier comprising 36 mRNA variables. The p-values were calculated via multivariate log-rank test. Left panel: Kaplan-Meier curves; Middle panel: Cumulative hazard curves; Right panel: Hazard curves.

For each query patient from the testing cohort, the reference patients in the training cohorts can be ranked in order of decreasing similarity via our proposed procedures. The prognostic risk of each query patient can then be predicted and assigned, based on the actual risk grouping of the “prognostically most similar” reference patient which was assigned during the training of the gene classifier variables in the training cohort. The OS subgroupings of the reference patients from the training cohort into low, intermediate or high-risk subgroups was generated via the statistically-weighted grouping (SWVg) method previously developed by our group [10]. Our results following the 1D-DDg variable selection method and the SWVg method revealed that the use of 37 variables (comprising 36 mRNA variables and 1 patient clinical variable) can effectively and significantly stratify the training cohort into three distinct prognostic subgroups. The plots of Kaplan-Meier survival, cumulative hazard function and hazard function for the three subgroups for this training cohort are shown in Figure 7A. Subsequently, we implemented the above-described method of the prediction of the risk subgroups for all query patients in the testing cohort based on identification of the nearest reference patient in the training cohort. The Kaplan-Meier survival, cumulative hazard function and hazard value functions for the prediction of the risk groups for the 359 patients from the testing patient cohorts (GSE9899 and GSE26712) are shown in Figure 7B. Our results revealed that our method of matching query patients from the testing cohort to the quantitative “most similar” reference patient can be effective in assigning individual query patients into the pre-defined prognostic risk groups.

Additionally, during the classification of the training cohort of reference patients, we reduced the number of variables used to the top 35, 30, 25 and 20 variables and assessed the impact of the reduced number of variables on classification performance (Supplementary Figure S7). The reduction of the number of less significant variables (mRNAs) correlates with the number of patients in the high-risk subgroup, suggesting the loss of predictor's stability when the number of variables in the multi-variable classifier was reduced (Supplementary Figure S7). The results for the classification of the testing cohort are shown in Supplementary Figure S8. The panels of this figure showed similar patterns of reduction of the number of patients in the high-risk subgroup when the number of top predictor variables becomes smaller. Interestingly, while the use of the top 25 variables resulted in a statistically more significant stratification of the training cohort (p=7.56e-20) than the use of all 37 variables (p=3.81e-19), the use of the entire 37 variables showed a relatively stronger classification performance when applied to an independent testing dataset. This suggests that our combined 37-variable classifier is reproducible and the method of assigning query patients to the quantitatively “most similar” reference patients could be an effective computational strategy of personalized patient's prognostic risk assessment.

Next, we removed the age variable from the combined 37-variable signature, and validated the resulting 36-mRNA signature in the independent dataset. The stratification of the low, intermediate and high-risk prognostic subgroups of the testing dataset are shown in Figure 7C. Our results revealed that when age was excluded from the classifier, the predictive performance of the prognostic signature in the independent dataset worsened (Figure 7B–7C). Our results further confirmed that age, as an independent prognostic factor of overall survival of HGSC patients, when included into a combined multi-variable classifier, can improve its predictive performance in the testing cohorts.

To illustrate the improvement in stratification performance when PSV instead of PBVV was used for query-reference patient similarity matching, we assessed the stratification performance when the prediction of the testing cohort was performed via similarity matching of PBVV directly instead of converting it to PSV first (Supplementary Figure S9). Our results revealed that when PBVV is used, a large proportion of patients from the testing cohort (around 20%) could not be classified reliably. This is because there could be more than one solution (i.e. most similar reference patient from the training cohort) when PBVV is used for similarity calculations (see Supplementary File 2). Our findings clearly demonstrated the superiority of our approach when converting the PBVV to PSV before similarity matching.

In the generation of PSV from PBVV, it is intuitive to note that the variance of the signature vector value (across all patients) will increase along the variable axis *i^th^*. Therefore, we studied the use of Mahalanobis distance (rather than Euclidean distance) which is scale-invariant. Our results showed that there was no association between Mahalanobis distance with AWR (result not shown). Furthermore, patient stratification based on Mahalanobis distance was not survival significant. In summary for each query patient, the conversion of a PBVV to a PSV as stipulated by our method may have increased the variance along the variable axis (i = 1, 2, 3, … 37), but it is also a necessary feature required and designed by us for higher resolution matching to an ensemble of vectors each representing one reference patient from the training cohort.

### Using the variables’ continuous value directly as the measure of similarity

An interesting possibility would be to use the initial continuous variables (microarray gene expression signals and actual age) directly for correlation or distance calculations as measures of similarity between two patients (see Supplementary Methods). Typically, this is also analogous to the use of a clustering algorithm such as hierarchical or k-means clustering to group together the patients who have similar expression profiles. We analyzed the similarity between pairs of vectors of 37 variables from the query patients and the reference patients. However, our results indicated the lack of association between such measures and patients’ risks when either Euclidean distance or Kendall's tau rank correlation coefficient was used as the similarity metric (Supplementary Files 6–7).

### Risk groups from the training and testing cohort correlates with clinical information

To specify the clinical relevance of our signature-derived classification, we first studied the association between clinical parameters with the prognostic risk groups defined during the training of the classifier. For the training data, we studied the association of the prognostic risk groups (low, intermediate and high-risk) with tumor residual disease, and with primary therapy outcome success (Table 2A). Our results revealed a strong correlation between prognostic risks with the tumor residual size (p = 0.00801) and with primary therapy outcome success (p = 0.00104).

**Table 2:** Significant associations of our prognostic risk grouping with essential therapeutic factors in the (A) training (TCGA data) and (B) testing cohorts (GSE9899 and GSE26712) indicates the potential clinical value of our method

(A) Training (TCGA)			
Tumor Residual Disease			
Prognostic risk	No Macroscopic disease	1 to 20 mm	> 20 mm
**low**	25	51	14
**intermediate**	30	104	36
**high**	3	23	10

Kappa coefficient = 0.1501

Kappa p-value = 0.00801

**Table:** 

Primary therapy outcome success			
Prognostic risk	Complete response	Partial response	Progressive and stable disease
**low**	82	4	11
**intermediate**	88	33	32
**high**	13	10	3

Kappa coefficient = 0.183

Kappa p-value = 0.00104

**Table:** 

(B) Testing (GSE9899 and GSE26712)		
Debulking		
Prognostic risk	optimal	suboptimal
**low**	69	36
**intermediate**	92	92
**high**	21	21

Chi-sq p-value = 0. 02884

Next, we also studied the clinical relevance of the predicted prognostic risk groups in the testing cohort with the debulking information (Table 2B). Our results showed that there was a borderline association between debulking information with the prognostic risk groups (p = 0. 02884). Specifically, patients with predicted low prognostic risk have optimal debulking. It would be interesting to note that the debulking information has not been designed to be part of the classifier, and therefore the resulting association of the predicted risk group with debulking information suggests that our signature could work independently from debulking information, and could potentially be useful in pre-operative prognostic prediction of patients based on non-invasively obtained biosamples.

## DISCUSSION

Previously, we have developed computational and statistical methods to identify a robust and reliable multi-variable classifier that stratifies a retrospective reference cohort of patients into at least two subgroups with distinct prognostic risk patterns [25, 27]. The classifier information regarding the number of variables and the predictive model parameters of each variable such as stratification p-value, and expression ranges of the risk subgroups, could be applied to the expression data of a testing patient cohort, or a query patient to predict which risk groups he or she belongs to. Effectively, a new patient could be assigned to one of the reference risk curves identified from the training cohort.

However in general, sometimes for a newly recruited query patient diagnosed with the disease, it may be clinically relevant to identify a specific reference patient that is most similar based on their prognostic signature. In our current work, we propose a mathematical scheme where a newly diagnosed patient would be matched to a reference patient in the training cohort based on similarities in their prognostic signature vectors (PSVs), which in turn is derived from prognostic binary variable vector (PBVV) generated from variable selection and risk classification methods from 1D-DDg or from other similar approaches [5, 6, 11, 12, 15, 38, 40]. The ability of our algorithm to match a new query patient to a known reference patient or several known reference patients in the training cohort could be beneficial in a clinical setting where, the success or lack of success of therapeutic strategies in previous and “similar” reference patients are already known and the information might aid in the clinical intervention of this new query patient.

Our approach could also provide a non-biased priority ranking of reference patients based on similarity matching with the new query patient which could be useful for case study analysis during the therapeutic assignment. Finally, our method assumes that all training and testing data should be measured with the same detection instrument and pre-processed via identical normalization procedure and parameters.

Our survival predictive method for a query patient is based on specification of a general concept where each variable of the variables in the classifier assigns the patient to a binary risk classification based on an optimal quantity cutoff (threshold) variable quantity [25–27], leading to the generation of a PBVV. Subsequently, the method personalizes the risk of disease recurrence based on similarity matching of any pair of PBVV (each representing one patient) via the best distance measure (Euclidean distance), found in this study.

While we propose that binarization procedure could be based on our data-driven grouping (DDg) methodology, other classification rules such as mean-based or median-based gene expression value classification that similarly assign patients into binary groupings associated with either low or high risk prognostic subgroups could also be used to generate the PBVV [5, 6, 11, 12, 15, 38, 40]. Other patho-biological nature of input data, including that of copy number variation, mutation, or methylation, could also be converted to a binary risk classification schema and incorporated into the multi-gene (or multi-variable) classifier if they exhibited strong and significant stratification performance.

Additionally, clinico-pathological parameters such as estrogen receptor status, lymph node status, histologic grade or patient's age could also be integrated into the classifier since they are known to be strong predictors of clinical outcome [41]. Mathematically, our procedures could be implemented, as long as for a given experimental variable or clinical parameter, patients could be assigned values of either −1 or +1 for low-risk or high-risk subgroups respectively. We have demonstrated the possibility of combining variables of different types in our model. In our combined prognostic signature of 37 variables, it includes 36 mRNA expression-based variables and patient's clinical information (age). Our results revealed that our method can be effective in combining variables of different nature in an easy computational pipeline, thereby lending its use in effective patient risk prediction.

Mathematically, the Euclidean distance between any two points in an n-dimensional space is well-defined. However, in the context of a prognostic vector profile *(D_j_)* which is represented by a binary classification along the variable axis, the similarity or dissimilarity between the two prognostic binary variable vectors (e.g. between *D_query_* and *D_reference_*) cannot be easily determined from the Euclidean distance. In fact, there is no clear correlation between the distances with the overall risk value of the patients (Figures 4A-4C, see also Supplementary File 2), which we have shown earlier to be indicative of patients’ overall time-to-event survival curves (Figure 3).

Therefore, we propose a method to convert a vector of prognostic binary variable vector *(D)* to a prognostic signature vector *(V)* which incorporates contribution by previous variables when moving along the variable axis (Table 1). For each patient, he or she can be represented by the prognostic signature vector *(V)* which is effectively the cumulative value based on current and previous variables’ contributions (Figure 5).

Our results also suggest the importance of weights in the multi-variable classifier, where it could be used to order and weigh the variables based on their relative contribution in the overall classifier [25–27]. In this work, we have used weights at two distinct parts of the analysis. In the first part, we have incorporated variable weights as part of the definition of the term average weighted risk (AWR) which provides a summary prognostic risk score (ranging from 1 to 2 for low or high-risk respectively) for each reference patient in the training cohort. The concept of AWR has been previously applied successfully to several of our biomarker discovery studies of ovarian and breast cancers [10, 42]. In the second part, we have used variable weights to generate a prognostic signature vector (PSV) from a prognostic binary variable vector. While we have conveniently derived the variable weights using the logarithmized p-values of the stratified survival curves for each individual variable, other statistical indicators that can numerically represent the relative importance of one variable over the other could also be used to weigh each variable [6, 8, 11, 12, 15, 18, 38, 40]. In this work, we have also studied the effect of using other weights on the stratification of the test cohort. We have used inverse p-values, hazard ratios, product of logarithmized p-values and hazard ratios, summation of logarithmized p-values and hazard ratios, and even equal weights. For our studied dataset using the specified prognostic signature and classification method, our results revealed that none of the alternative weight measures could outperform the stratification performance when just negative logarithmized p-values were used (results not shown). The specific effects of incorporating multiple statistical measures into the weights of each variable should be explored for individual biomarker discovery studies.

We investigated the use of comparing continuous variables directly for comparing a query patient with the reference patients from the training cohort, but the results showed that there was a lack of association between such measures and patients’ risks whether Euclidean distance or Kendall's tau rank correlation coefficient was used as the similarity metric (Supplementary Files 6–7). This could be due to the fact that unlike expression data alone, the generation of a prognostic signature implicitly incorporates information regarding the direction of variable quantity (e.g. gene expression or age) and the directional association with lower or higher risk prognostic subgroups. Such directionality is implied, when a prognostic signature of binary values is generated for a new query patient. However, the use of continuous variable data alone would not incorporate information regarding the favorable or unfavorable outcomes and would be a cause of concern when trying to identify a patient with the most similar prognostic profile. Further studies of these findings should be analyzed using other datasets and modeled via computation simulations in separate work.

Biomedical datasets often include both continuous variables (e.g. survival time, microarray gene expression signals) and discrete/categorical variable (e.g. stage, histological type). For diagnostics, prognosis and prediction implementation, the continuous variables are often converted into categorical variables by grouping values into two or more categories. Categorizing prognostic variables is essential for their use in clinical decision-making [1, 8, 13, 18, 30, 41, 43].

Such conversion of continuous or discrete variables to dichotomized values can address the bias of dynamical ranges of the continuous variables as well as provide reduction of the dimension of the predictor space (via excluding highly noisy and uninformative variables). It can also provide a robust and synergistic multivariate descriptor of disease complexity via explicit incorporation of the interaction (synergistic) effects of the individual predictors, thereby allowing investigation of a possible classification or prediction model as well as optimizing the predictor subset in input-output interconnections and personalized dose-response relations [10, 44, 45]. Despite known loss of statistical power following dichotomization in the univariate case and in the linear multivariate regression models, it has been shown by many studies that dichotomizing continuous data can greatly improve the power of multiple testing procedures (even in false discovery rate controlling methods) [43]. The appropriate statistical-based predictive models in this case, can lead to unbiased variable selection of highly informative, robust and reproducible components of classifiers and survival predictors [10, 25, 44–46]. It was demonstrated that statistical-based optimization of dichotomous threshold of the continuous variables can be quite accurate, even with highly correlated data [10, 25, 30, 44–47].

In our strategy, dichotomization of a continuous variable, for example gene expression or age, is only a preliminary step that seeks to reduce noise and reduce the number of the variables subsequently considered during the next step of our analysis as the potential predictors in the training set. Subsequently, a follow-up complementary strategy, called statistically weighted voting grouping method SWVg [10, 26] is used to statistically synergized combinations of the predictors. Via optimization of the relatively large number of informative and robust univariate predictors, a multivariate feature predictor model could be constructed, appropriate for personalized outcome prediction.

Due to the high-dimensional and highly interconnected nature of genomic, transcriptomic and proteomic data, selections of feature lists (via different classification methods) that shares little overlap but that have similar prognostic significance or biologically relevance could be quite common. It is often necessary to evaluate both the signature (selected predictive variables, parameters of the predictive model) and the method of classification together rather than just consider the method of classification. This is because each method of classification (or prediction) would be able to identify a potentially unique small subset of features that are only useful and effective in their combination when used in the manner designed in the specific (computational or empirical-based) method. In our cases, the 1D-DDg and SWVg methods proposed have the ability to select a pathobiologically relevant sub-set of predictive variables where binarization was effective and reproducible for prognostic classification.

As such, predictors that utilize the full range of the continuous variables, as well as predictors that dichotomize the continuous variables should be considered as complementary methods which are able to identify informative and robust set of predictors unique to each predictor type, and only useful when applied to the test set in the intended designed manner.

As a result, our prognostic signature together with the classifier method (i.e. 1D-DDg) has resulted in a robust predictor of overall survival in ovarian cancer patients. In addition to matching query patients from the testing cohort directly to the most similar reference patient in the training cohort, we have also matched query patients to the reference subgroup centroids (via median or mean of the low, intermediate or high-risk prognostic subgroups) from the training cohort. It was expected that comparing with the average over a group of similar patients instead of comparing with a single patient would improve the robustness of our model. However, our findings revealed no statistical improvement when reference subgroup centroid matchings were used. In addition, averaging the prognostic risk predictions across the top 5, 9, 13 or 17 most similar reference patients also did not yield significant improvement in the mean accuracy (Supplementary Figure S6B).

Our proposal of personalized prognosis is based on retrospectively assigning patients from a reference cohort (training set) into binary states of low or high risk subgroups using clinically significant feature subset (pathobiologically relevant prognostic signature). For prospective analysis of a newly recruited patient diagnosed with the disease or a testing patient cohort, the new data under comparison is required to be adequately corrected for batch effects and aligned appropriately to the original training cohort data. In general, the specificity, sensitivity and reproducibility of the prediction of the new query patient with regards to risk subgroups is multi-factorial; it is invariably dependent on the training set, accuracy, reliability, robustness and reproducibility of the multi-feature classifier.

The notion of personalized and precise clinical therapy is a much desired goal in the field of medicine. This is especially true for complex, multi-factorial and genetic disorders such as cancers, where the heterogeneity within tumors and among patients is often a cause of worry to both clinicians and patients at all stages of clinical care such as disease diagnosis, prognosis and treatment. Here, we propose that each patient can be represented initially by a PBVV where the risk for each feature can be denoted using one of two binary states. Subsequently, our proposed algorithm can transform the PBVV to a more informative and robust PSV which allows for a better similarity or dissimilarity measure between any two patients. For any query patient from the testing cohort or a newly diagnosed patient, our algorithm facilitates the search for the most “prognostically similar” reference patient as well as provides an unbiased ranking of the reference patients from the training cohort, where analysis of historical cases could be prioritized and studied. The insights to clinical characteristics or response to any particular therapy for the most “prognostically similar” reference patients are likely to be beneficial to the prognosis of any new patients as well as represent a key step towards future personalized therapeutic intervention.

Finally, results of our analyses revealed that independently, age could be an important pro-oncogenic prognostic factor of OS in HGSC patients. Incorporating the age information into the molecular predictor provided more robust personalized prognosis of OC which correlate with the therapeutic response of HGSC. Our method could provide benefit in optimization of the treatment targeting of the tumors in HGSC patients. It may be expected that our findings would be of interest to many, including clinicians and patients.

## MATERIALS AND METHODS

### Dataset

Expression datasets belonging to high-grade serous ovarian cancer (HGSC) patients were obtained from three independent sources: 1) The Cancer Genome Atlas (TCGA) research network [16], 2) GSE9899 [17] and 3) GSE26712 [39]. Detailed clinical information was downloaded as well.

TCGA expression data comprised of 463 primary solid ovarian cancer tissue samples from 11 batches (containing between 21 to 47 samples in each batch). More than 90% of the samples were classified with Stage III tumors. Pre-processing of the mRNA (Affymetrix U133A) data were performed via quality assessment within each batch, background correction, normalization and batch effect adjustment as previously described [10]. The final expression dataset belonging to 350 patients after quality control were designated as the reference/training cohort. Of these, 349 patients in this dataset have information with respect to age at initial diagnosis.

246 and 185 patient samples from GSE9899 and GSE26712 were downloaded and assessed for their data quality as previously described [10]. The final expression dataset belonging to 230 and 130 patient samples from GSE9899 and GSE26712 were designated as the query/testing cohort. Of these, 230 and 129 patient samples from GSE9899 and GSE26712 have information with respect to age at initial diagnosis.

Systematic effects of the global expression intensities between the reference dataset (TCGA) and query datasets (GSE9899 and GSE26712) were removed via batch effect correction using the Anova method implemented from pamr R programming package [40].

### Variable selection method and training data

We briefly summarize our method of patient grouping, called one-dimensional data-driven grouping (1D-DDg) [10, 26]. In 1D-DDg, samples (e.g. patients) are ranked based on a quantifiable variable (e.g. RNA expression of gene A). Samples are categorized into two groups (i.e. low or high-risk) based on a cut-off value, identified and optimized based on maximal separation of the Kaplan-Meier survival curves which in turn is evaluated via Wald test. For each variable, samples can be arbitrarily assigned a binary value of 1 or 2 corresponding to low or high-risk respectively. The procedure is repeated for several other quantifiable variables (e.g. other probesets in a microarray platform) to identify the most prognostically significant variables. Subsequently, each sample can be represented by a prognostic binary variable vector (PBVV) containing binary states of 1 or 2 across the selected variables. In addition, statistical quantities such as p-value can provide a simple way of assessing the relative importance of one variable over the others.

Several survival-significant variables selected from 1D-DDg method could be combined into an integrative prognostic signature classifier via statistically weighted voting (SWV) as described and implemented previously [10].

### Variable conversion of PBVV

Here, we proposed a method to transform a PBVV (representing each sample) to a PSV. The details could be found in Supplementary Methods.

### Ordering the variable axis

Rank the variables in descending order of significance (correspondingly, ascending order of p-value) and select a subset of n variables to compose a signature/classifier. The variable order represented as *i* = 1, 2, 3, …, n^th^ variable is thereafter, referred to as the variable axis.

$$\text{Ranked variable order}=\left(\begin{array}{c} {\text{variable}}_{1}\\ {\text{variable}}_{2}\\ \vdots \\ {\text{variable}}_{i}\\ \vdots \\ {\text{variable}}_{n} \end{array}\right)↓\text{variable axis}$$

### Calculating the weight vector

For all patients, the weight vector is the list of negative log10 p-values as calculated during the variable selection step of the reference training patient cohort:

$$W=\left(\begin{array}{c} w_{1}\\ w_{2}\\ \vdots \\ w_{i}\\ \vdots \\ w_{n} \end{array}\right),$$

where *w_i_* ≥ *w_i+1_* for *i* = 1, 2, …, n-1^th^ variable

### Centering and rescaling the PBVV

For each reference patient j^th^, the vector of grouping information, ***G**_j_* that was calculated from the variable selection step (e.g. 1D-DDg method), is origin-centered (i.e. centered to 0) and rescaled, so that the centered grouping vector, ***D**_j_*, is now represented by −1 for low-risk and +1 for high-risk for each of the genes defined in the signature.

$$G_{j}=\left(\begin{array}{c} g_{1,j}\\ g_{2,j}\\ \vdots \\ g_{i,j}\\ \vdots \\ g_{n,j} \end{array}\right),$$

where $g_{i,j}=\{\begin{array}{c} 1,\text{ low-risk}\\ 2,\text{ high-risk} \end{array}$ and *i* = 1, 2, 3, … n^th^ variable

$$D_{j}=\left(\begin{array}{c} d_{1,j}\\ d_{2,j}\\ \vdots \\ d_{i,j}\\ \vdots \\ d_{n,j} \end{array}\right),$$

where $d_{i,j}=\{\begin{array}{c} -1,\text{ low-risk}\\ +1,\text{ high-risk} \end{array}$ and *i* = 1, 2, 3 … n^th^ variable

### Adjustment vector

For each reference patient ***j**^th^* from the training cohort, the adjustment vector ***A**_j_* is defined as:

$$A_{j}=\text{diag}(W)D_{j}=\left(\begin{array}{cccccc} w_{1} & 0 & \cdots & 0 & \cdots & 0\\ 0 & w_{2} & \ddots & 0 & \ddots & 0\\ \vdots & \ddots & \ddots & 0 & \ddots & \vdots \\ 0 & 0 & 0 & w_{i} & \ddots & 0\\ \vdots & \ddots & \ddots & \ddots & \ddots & \vdots \\ 0 & 0 & \cdots & 0 & \cdots & w_{n} \end{array}\right)\left(\begin{array}{c} d_{1,j}\\ d_{2,j}\\ \vdots \\ d_{i,j}\\ \vdots \\ d_{n,j} \end{array}\right)=\left(\begin{array}{c} a_{1,j}\\ a_{2,j}\\ \vdots \\ a_{i,j}\\ \vdots \\ a_{n,j} \end{array}\right)$$

where *i* = 1, 2, 3, … n^th^ variable.

### Prognostic signature vector (PSV)

Finally, each reference patient ***j**^th^* from the training cohort can be represented by a characteristic PSV defined as:

$$V_{j}=\left(\begin{array}{c} v_{1,j}\\ v_{2,j}\\ \vdots \\ v_{i,j}\\ \vdots \\ v_{n,j} \end{array}\right),$$

where the scalar value $v_{i,j}=\sum_{x=1}^{i}\;a_{x,\;j}$ and i = 1, 2, 3, … n^th^ variable.

### Prognostic signature vector for new patient

#### Profiling and classification for each variable

To generate a PSV for a prospective new patient, the patient is first profiled for the same variables identified in the training cohort.

Subsequently, for each variable in the signature, use the quantity cut-off (as established in the training cohort) to assign the query *k^th^* patient from the testing cohort to low or high-risk subgroups (subgroup 1 or 2 respectively). The vector of grouping information for the query patient *k^th^* is defined as *G_k_* whereas the centered design vector for the query patient *k^th^* is defined as ***D**_k_*.

#### Adjustment vector

Using the weight vector **W** previously defined in the training cohort, the adjustment vector ***A**_k_* for the query patient *k^th^* can be calculated as:

$$A_{k}=\text{diag}(W)D_{k}=\left(\begin{array}{cccccc} w_{1} & 0 & \cdots & 0 & \cdots & 0\\ 0 & w_{2} & \ddots & 0 & \ddots & 0\\ \vdots & \ddots & \ddots & 0 & \ddots & \vdots \\ 0 & 0 & 0 & w_{i} & \ddots & 0\\ \vdots & \ddots & \ddots & \ddots & \ddots & \vdots \\ 0 & 0 & \cdots & 0 & \cdots & w_{n} \end{array}\right)\left(\begin{array}{c} d_{1,k}\\ d_{2,k}\\ \vdots \\ d_{i,k}\\ \vdots \\ d_{n,k} \end{array}\right)=\left(\begin{array}{c} a_{1,k}\\ a_{2,k}\\ \vdots \\ a_{i,k}\\ \vdots \\ a_{n,k} \end{array}\right)$$

### Prognostic signature vector (PSV)

The unique PSV for the query patient *k^th^* is then defined as:

$$V_{k}=\left(\begin{array}{c} v_{1,k}\\ v_{2,k}\\ \vdots \\ v_{i,k}\\ \vdots \\ v_{n,k} \end{array}\right),$$

where the scalar value $v_{i,k}=\sum_{x=1}^{i}\;a_{x,k}$ and i = 1, 2, 3, … nth variable.

### Comparison of prognostic signature vectors

The difference in PSVs, for the query patient *k^th^* from the testing cohort with respect to a reference patient *j^th^* in the training cohort can be calculated by the Euclidean distance between the vectors ***V**_j_* and ***V**_k_* and is denoted by:

$$f\left(V_{j},V_{k}\right)=\left|V_{j}-V_{k}\right|=\left|\left(\begin{array}{c} v_{1,j}-v_{1,k}\\ v_{2,j}-v_{2,k}\\ \vdots \\ v_{i,j}-v_{i,k}\\ \vdots \\ v_{n,j}-v_{n,k} \end{array}\right)\right|=\sqrt[{2}]{\sum_{i=1}^{n}{\left(v_{i,j}-v_{i,k}\right)}^{2}}$$

### Identification of the most similar reference patient

The reference patient *j^th^* from the entire training cohort *M* that is most similar to the query patient *k^th^* can be identified via:

$$\text{j}=\text{arg }\underset{j\in M}{\text{min}}f(V_{j},\;V_{k})$$

where *M* represents all reference patients in the training cohort.

Subsequently, the risk group of the query patient *k^th^* is predicted to be similar to that of the most similar reference patient *j^th^*.

### Similarity-AWR scatter plots for PBVV-based or PSV-based approaches

Several ways of comparing patient pairs were described herein and in Supplementary Methods. We have evaluated some of the methods, e.g. (i) distance between PBVVs, (ii) correlation between PBVVs, (iii) distance between PSVs or (iv) correlation between PSVs.

For each query patient, he/she could be represented by a PBVV or PSV and compared with that from the reference patients in the training cohort. The quantitative results for each query patient could be illustrated with a scatter plot where each point is a result arising from comparison of that query patient with one reference patient. The similarity measure and the AWR of the reference sample are represented on the vertical and horizontal axes respectively. A good method (e.g. i-iv mentioned above) should yield a scatter plot with well-structured association between AWR and the similarity measure.

In each of the plots, the reference patients from the training cohort were ranked from left to right in terms of ascending AWR values, which were associated with prognostic risk. If a hypothetical query patient has intermediate prognostic risk, the expectation would be that it would be most quantitatively similar to a reference patient with intermediate AWR values (e.g. AWR=1.5). Comparison with other reference patients further away from the intermediate AWR value (e.g. AWR from 1.5 approaching 1.0, or AWR from 1.5 approaching 2.0) would yield decreasing similarity measure quantity. Therefore, the shape of such scatter plot would have quadratic characteristics with either a local minimum or maximum depending on whether Euclidean distance or Kendall's Tau rank correlation was used as the similarity measure.

To assess which of the method would be appropriate for comparing between query and reference patients, we fitted all the data to a best-fit quadratic function. The residuals of each of the plot would be used to assess the fit of the data onto the best-fitted quadratic function. A good method would be quantitatively characterized by low residual values, which in turn could be observed by well-structured association between similarity measure and AWR.

For each plot, for valid comparison across the methods, we rescaled all y-axis value representing the Euclidean distance or Kendall's Tau rank correlation coefficient to 0 and 1 before calculating the normalized residuals.

### 10-fold cross validation in the training cohort

The training cohort comprised 349 HGSC patients from TCGA. To assess the predictive accuracy and the stability of our proposed methods, we performed 10-fold cross validation analysis where the training cohort was split into 10 subgroups. For each subgroup, patients were assigned to the most quantitatively similar reference patient from the other 9 subgroups. The accuracy was calculated for each of the 10 cross validation analysis. Mean accuracy and the standard deviation of the accuracies across the 10 cross validation analyses were used as indicators of accuracy and stability.

## SUPPLEMENTARY MATERIALS FIGURES AND TABLES






























