# Supplementary material for: Big data and computational biology strategy for personalized prognosis
Source: Oncotarget. 2016 May 24;7(26):40200–20. doi: 10.18632/oncotarget.9571 (PMC5130003; doi:10.18632/oncotarget.9571)
Supplement: Supplementary file 1 [file oncotarget-07-40200-s001.pdf]

SUPPLEMENTARY MATERIALS

Introduction

Several methods of vector comparison were assessed in this study for the purpose of matching a query patient (testing cohort of patients or a new prospectively recruited diagnosed patient) to reference patients (training cohort of patients) based on quantitative measures of distance (e.g. Euclidean distance) or correlation (e.g. Kendall-tau rank correlation).

The methods are:

- Comparison by two full gene expression signal (GES) lists
- Comparison by two gene expression signal (GES) lists
- Comparison by expression of individual variables
- Comparison by prognostic binary variable vectors (PBVV)
- Comparison by prognostic signature vectors (PSV)

The first three methods are related to methods that directly compare the quantitative data that is commonly of a continuous nature. Specifically, the first two methods are commonly implemented in areas such as heatmap clustering where rows or columns (representing patients and variables, or vice versa) of expression data are grouped together based on similarity metrics.

The last two methods in the above-mentioned list are procedures consequent to our previously published methods of patient stratification into binary subgroups based on optimization of the relationships between predictive variables (input predictive data) and patients' survival data (times and events) [1-3]. In these methods, the comparisons are performed based on variables that would have been initially binarized.

It is assumed that the input predictor data or variables (e.g. gene expression level values) are appropriately pre-processed via batch correction across different patient cohorts as well as normalized across individual patients using identical mathematical method.

Methods based on direct comparison of the variable attributes

The variable attributes may include information such as experimentally detected data (e.g. mRNA expression values), age, disease stage or other clinical parameters.

Comparison by two full gene expression signal (GES) lists

Here, individual patient (j^{th}) is each represented by a gene expression signal vector $\boldsymbol{E}_j$ of length n corresponding to the full list of n microarray probesets.

For instance, the GES vector for patient j^{th} can be represented as:

$$\boldsymbol{E}_j = \begin{pmatrix} e_{1,j} \\ e_{2,j} \\ \vdots \\ e_{i,j} \\ \vdots \\ e_{n,j} \end{pmatrix}, \text{ for } i = 1, 2, \cdots, n^{th} \text{ probeset}$$

The similarity between any two such patients, for instance reference patient j^{th} from the training cohort and the query patient k^{th} from the testing cohort can be quantitatively assessed via a similarity metric calculation between $\boldsymbol{E}_j$ and $\boldsymbol{E}_k$. Commonly used metric includes distance measures including but not limited to Euclidean distance.

The mathematical representation of Euclidean distance calculation between two vectors $\boldsymbol{E}_j$ and $\boldsymbol{E}_k$ is denoted by:

$$f\left(\boldsymbol{E}_j, \boldsymbol{E}_k\right) = \left|\boldsymbol{E}_j - \boldsymbol{E}_k\right| = \left\| \begin{pmatrix} e_{1,j} - e_{1,k} \\ e_{2,j} - e_{2,k} \\ \vdots \\ e_{i,j} - e_{i,k} \\ \vdots \\ e_{n,j} - e_{n,k} \end{pmatrix} \right\| = \sqrt[2]{\sum_{i=1}^{n} \left(e_{i,j} - e_{i,k}\right)^2}$$

Commonly used metric also includes correlation measures including but not limited to Kendall-tau rank correlation.

These similarity metrics can be easily implemented in various programming languages. For instance, via open-source Python programming language and its scientific library (scipy), the similarity metric can be calculated via the following functions $f(\boldsymbol{E}_j, \boldsymbol{E}_k)$:

- Euclidean distance = scipy.spatial.distance.euclidean $(\boldsymbol{E}_j, \boldsymbol{E}_k)$
- Kendall-tau rank correlation = scipy.stats.kendalltau $(\boldsymbol{E}_j, \boldsymbol{E}_k)$

For each query patient in the testing cohort, the similarity metric with each of the reference patients in the training cohort can be assessed. Subsequently, the reference patients could be ordered based on the similarity metric to identify quantitatively the "most similar" or "most different" reference patient.

If a distance measure is used as the similarity metric, the reference j^{th} patient (from the entire training cohort M) with the smallest distance measure with the query patient is considered as quantitatively "most similar":

$$j = \arg\min_{j \in M} f\left(\boldsymbol{E}_j, \boldsymbol{E}_k\right)$$, where j = 1, 2, 3, … Mth

reference patient in the training cohort.

If a correlation measure is used as the similarity metric, the reference patient with the largest correlation measure with the query patient is considered as quantitatively "most similar":

$$k = \arg\max_{j \in M} f\left(\boldsymbol{E}_j, \boldsymbol{E}_k\right)$$, where j = 1, 2, 3, … Mth

reference patient in the training cohort.

Comparison by two gene expression signal (GES) lists

The approach is similar to comparison by two full GES lists discussed above. The only difference is that rather than the full list of n probesets, only a subset of the probesets is used to represent each patient.

The size and composition of the subset of probesets can be identified and optimized based on other variable selection methods which are beyond the scope of this current work.

Comparison by expression of individual variables

This method of comparison is contingent on the prior selection of a subset of n probesets that are the most informative with respect to prognostic significance. As mentioned in the section "comparison by two gene expression signal (GES) lists", the size and composition of the subset of probesets can be identified and optimized based on other variable selection methods which are beyond the scope of this current work.

In contrast to the method proposed in "comparison by two gene expression signal (GES) lists" which calculates the similarity metric based on direct comparison of the two GES vectors, the method proposed in this section a) calculates the similarity metric for each of the n variables independently and b) introduces a weight component for each variable that represents its relative importance within the entire signature.

The weight component for each ith variable should be defined via the variable selection step during the training of the reference cohort.

In this method, only distance measure can be used as the similarity metric.

An illustration of the procedures to calculate the overall distance between a reference j^{th} patient in the training cohort and a query k^{th} patient in the testing cohort is shown in the following table:

Variable ID (i^{th})	Weights	Reference j^{th} patient (training cohort)	Query k^{th} patient (testing cohort)	*Distance measure	Weighted distance measure
variable 1	w_1	$e_{1,j}$	$e_{1,k}$	d_1	$w_1 d_1$
variable 2	w_2	$e_{2,j}$	$e_{2,k}$	d_2	$w_2 d_2$
variable 3	w_3	$e_{3,j}$	$e_{3,k}$	d_3	$w_3 d_3$
…	…	…	…	…	…
variable i	w_i	$e_{i,j}$	$e_{i,k}$	d_i	$w_i d_i$
…	…	…	…	…	…
variable n	w_n	$e_{n,j}$	$e_{n,k}$	d_n	$w_n d_n$

sum of weights $= \sum_{i=1}^{n} w_i$

sum of weighted distance $= \sum_{i=1}^{n} w_i d_i$

$$\text{overall distance, } f(j,k) = \frac{\text{sum of weighted distance}}{\text{sum of weights}} = \frac{\sum_{i=1}^{n} w_i d_i}{\sum_{i=1}^{N} w_i}$$

*distance measure of individual variable i^{th}, d_i = distance($e_{i,j}$, $e_{i,k}$)

For a given query k^{th} patient, after evaluation of the overall distances, f(j,k) with several reference j^{th} patients (from the entire reference cohort M), the reference j^{th} patient with the smallest distance measure with the query k^{th} patient is considered as quantitatively "most similar":

$$j = \arg\min_{j \in M} f(j, k), \text{ where j} = 1, 2, 3, \ldots \text{ M}^{\text{th}}$$

reference patient in the training cohort.

Methods based on comparison of processed variable attributes

The following methods introduced in this section is contingent on the prior use of any relevant quantitative-based variable selection methods to select a subset of the most informative and important variables, as well as to provide binarization of each continuous variable (e.g. gene expression, age information, or other clinical information) to represent low or high prognostic risk subgroups.

Details of the choice, as well as the individual merits of each variable selection methods are beyond the scope of the current work. However, we here provide a brief summary of our choice of variable selection method, called one-dimensional data-driven grouping method (1D-DDg) which was previously developed by our group and applied in various studies including glioblastoma and ovarian cancer [1-3].

Briefly, in 1D-DDg, samples (e.g. from patients) are ranked based on a quantifiable variable (e.g. RNA expression of a particular gene). Samples are categorized into two groups (i.e. low or high-risk) based on a cut-off value, identified and optimized based on maximal separation of the Kaplan-Meier survival curves which in turn is evaluated via the log-rank test. For each variable, samples can be arbitrarily assigned a binary value of 1 or 2 corresponding to low or high-risk respectively. The procedures may be repeated for several other quantifiable variables (e.g. other probesets in a microarray platform) to identify the most prognostically significant variables. Subsequently, each sample can be represented by a prognostic binary variable vector (PBVV) containing binary states of 1 or 2 across the selected variables.

In addition, log-rank p-values can provide a simple way of assessing the relative importance of one variable over the others.

$$\text{Ranked variable order} = \begin{pmatrix} variable_1 \\ variable_2 \\ \vdots \\ variable_i \\ \vdots \\ variable_n \end{pmatrix} \downarrow \text{variable axis}$$

Comparison by prognostic binary variable vectors (PBVV)

Using a variable selection method such as 1D-DDg, each variable (e.g. probeset expression) is independently assessed for its prognostic value across all reference patients in the training cohort. Subsequently, for each jth reference patient from the training cohort, it can be represented by an n-length vector (corresponding to i = 1, 2, 3, …, nth variable) of grouping information $\boldsymbol{G_j}$.

$$\text{group vector for } j^{th} \text{reference patient, } \boldsymbol{G_j} = \begin{pmatrix} g_{1,j} \\ g_{2,j} \\ \vdots \\ g_{i,j} \\ \vdots \\ g_{n,j} \end{pmatrix},$$

$$\text{where } g_{i,j} = \begin{cases} 1, \text{ low-risk} \\ 2, \text{ high-risk} \end{cases} \text{ and } i = 1, 2, 3, \ldots \text{ n}^{\text{th}} \text{ variable}$$

For each kth query patient from a testing cohort, or a prospective newly diagnosed patient, he/she is first profiled for similar variables established in the training cohort. Subsequently, for each variable, the parameters of the variable selection method (in our case, the parameters include the cut-off value) as established from the training cohort were used to assign the kth query patient to low or high-risk (group 1 or 2 respectively). Thereafter, the query k^{th} patient can be represented by an n-length vector (corresponding to i = 1, 2, 3, …, nth variable) of grouping information $\boldsymbol{G_k}$.

$$\text{group vector for } k^{th} \text{query patient, } \boldsymbol{G_k} = \begin{pmatrix} g_{1,k} \\ g_{2,k} \\ \vdots \\ g_{i,k} \\ \vdots \\ g_{n,k} \end{pmatrix},$$

$$\text{where } g_{i,k} = \begin{cases} 1, \text{ low-risk} \\ 2, \text{ high-risk} \end{cases} \text{ and } i = 1, 2, 3, \ldots \text{ n}^{\text{th}} \text{ variable}$$

As described in the section "Comparison by two full gene expression signal (GES) lists", the similarity metric between the jth reference patient and k^{th} query patient can be easily calculated via the following functions $f(\boldsymbol{G_j}, \boldsymbol{G_k})$:

- Euclidean distance = scipy.spatial.distance.euclidean ($\boldsymbol{G_j}$, $\boldsymbol{G_k}$)

- Kendall-tau rank correlation = scipy.stats.kendalltau ($\boldsymbol{G_j}$, $\boldsymbol{G_k}$)

Comparison by prognostic signature vectors (PSV)

This analysis follows the section on "comparison of prognostic binary variable vector (PBVV)" where the group vector for j^{th} patient, $\boldsymbol{G_j}$ was defined.

Centering and rescaling the prognostic binary variable vector for j^{th} reference patient

Here for each jth patient, the group vector, $\boldsymbol{G_j}$ that was calculated from the variable selection step (e.g. 1D-DDg method), is origin-centered (i.e. centered to 0) and rescaled, so that the centered grouping vector, $\boldsymbol{D_j}$, is now represented by -1 for low-risk and +1 for high-risk for each of the variables defined in the signature.

$$\text{centered grouping vector for } j^{th} \text{reference patient, } \boldsymbol{D_j} = \begin{pmatrix} d_{1,j} \\ d_{2,j} \\ \vdots \\ d_{i,j} \\ \vdots \\ d_{n,j} \end{pmatrix},$$

where $d_{i,j} = \begin{cases} -1, \text{ low-risk} \\ +1, \text{ high-risk} \end{cases}$ and i = 1, 2, 3, … nth variable

Weight vector for all reference patients

Several parameters could be options for the choice of weights to rank the contribution of the individual variables in the signature. Such parameters could include p-value, fold change, hazard ratio or any other relevant quantifiable variable.

In our current study, for all patients, the weight vector is the list of negative log10 log-rank p-values as calculated during the variable selection step.

$$\text{weight vector, } \boldsymbol{W} = \begin{pmatrix} w_1 \\ w_2 \\ \vdots \\ w_i \\ \vdots \\ w_n \end{pmatrix},$$

where $w_i \geq w_{i+1}$ for i = 1, 2, …, n-1th variable

Adjustment vector for j^{th} reference patient

For each jth reference patient in the training cohort, the adjustment vector $\boldsymbol{A_j}$ is defined as:

$$\text{adjustment vector for } j^{th} \text{ reference patient, } \boldsymbol{A_j} = \text{diag}(\boldsymbol{W})\boldsymbol{D_j}$$

$$= \begin{pmatrix} w_1 & 0 & \cdots & 0 & \cdots & 0 \\ 0 & w_2 & \ddots & 0 & \ddots & 0 \\ \vdots & \ddots & \ddots & 0 & \ddots & \vdots \\ 0 & 0 & 0 & w_i & \ddots & 0 \\ \vdots & \ddots & \ddots & \ddots & \ddots & \vdots \\ 0 & 0 & \cdots & 0 & \cdots & w_n \end{pmatrix} \begin{pmatrix} d_{1,j} \\ d_{2,j} \\ \vdots \\ d_{i,j} \\ \vdots \\ d_{n,j} \end{pmatrix} = \begin{pmatrix} a_{1,j} \\ a_{2,j} \\ \vdots \\ a_{i,j} \\ \vdots \\ a_{n,j} \end{pmatrix}$$

where i = 1, 2, 3, … nth variable.

Prognostic signature vector (PSV) for j^{th} reference patient

Finally, each jth reference patient in the training cohort can be represented by a characteristic PSV defined as:

$$\text{prognostic signature vector for } j^{th} \text{ reference patient, } \boldsymbol{V_j} = \begin{pmatrix} v_{1,j} \\ v_{2,j} \\ \vdots \\ v_{i,j} \\ \vdots \\ v_{n,j} \end{pmatrix},$$

where the scalar value $v_{i,j} = \sum_{x=1}^{i} a_{x,\,j}$ and i = 1, 2, 3, ... nth variable.

Prognostic signature vector (PSV) for k^{th} query patient

The centered grouping vector for the kth query patient, $\boldsymbol{D_k}$ could be calculated from the group vector for the same query patient, $\boldsymbol{G_k}$.

Using the same weight vector $\boldsymbol{W}$ previously defined from the training cohort, the adjustment vector $\boldsymbol{A_k}$ for the kth query patient can be calculated as:

$$\boldsymbol{A_k} = diag(\boldsymbol{W})\boldsymbol{D_k} =$$

$$\begin{pmatrix} w_1 & 0 & \cdots & 0 & \cdots & 0 \\ 0 & w_2 & \ddots & 0 & \ddots & 0 \\ \vdots & \ddots & \ddots & 0 & \ddots & \vdots \\ 0 & 0 & 0 & w_i & \ddots & 0 \\ \vdots & \ddots & \ddots & \ddots & \ddots & \vdots \\ 0 & 0 & \cdots & 0 & \cdots & w_n \end{pmatrix} \begin{pmatrix} d_{1,k} \\ d_{2,k} \\ \vdots \\ d_{i,k} \\ \vdots \\ d_{n,k} \end{pmatrix} = \begin{pmatrix} a_{1,k} \\ a_{2,k} \\ \vdots \\ a_{i,k} \\ \vdots \\ a_{n,k} \end{pmatrix}$$

The PSV for the kth query patient is then defined as:

$$\boldsymbol{V_k} = \begin{pmatrix} v_{1,k} \\ v_{2,k} \\ \vdots \\ v_{i,k} \\ \vdots \\ v_{n,k} \end{pmatrix},$$

where the scalar value $v_{i,k} = \sum_{x=1}^{i} a_{x,k}$ and $i = 1, 2, 3, \ldots$ n^{th} variable.

Similarity metric between prognostic signature vectors

As described in the section "Comparison by two full gene expression signal (GES) lists", the similarity metric between the jth reference patient and k^{th} query patient can be easily calculated via the following functions $f(\boldsymbol{V_j}, \boldsymbol{V_k})$:

- euclidean distance = scipy.spatial.distance.euclidean (V_j, V_k)
- kendall-tau correlation = scipy.stats.kendalltau (V_j, V_k)

Identification of most similar reference jth patient for a query kth patient

For a given query k^{th} patient, after evaluation of the similarity metric $f(V_j, V_k)$ with several reference j^{th} patients (j = 1, 2, 3, … M^{th} reference patient in the training cohort), the reference j^{th} patient that is quantitatively "most similar" to the query k^{th} patient can be defined as:

$j = \arg\min_{j \in M} f\left(\boldsymbol{V_j}, \boldsymbol{V_k}\right)$, where the similarity metric is a distance measure

or

$j = \arg\max_{j \in M} f\left(\boldsymbol{V_j}, \boldsymbol{V_k}\right)$, where the similarity metric is a correlation measure

REFERENCES

1. Motakis E, Ivshina AV and Kuznetsov VA. Data-driven approach to predict survival of cancer patients: estimation of microarray genes' prediction significance by Cox proportional hazard regression model. IEEE Eng Med Biol Mag. 2009; 28:58-66.
2. Tang Z, Ow GS, Thiery JP, Ivshina AV and Kuznetsov VA. Meta-analysis of transcriptome reveals let-7b as an unfavorable prognostic biomarker and predicts molecular and clinical sub-classes in high-grade serous ovarian carcinoma. Int J Cancer. 2013; 134:306-318.
3. Chan XH, Nama S, Gopal F, Rizk P, Ramasamy S, Sundaram G, Ow GS, Ivshina AV, Tanavde V, Haybaeck J, Kuznetsov V and Sampath P. Targeting glioma stem cells by functional inhibition of a prosurvival oncomiR-138 in malignant gliomas. Cell Rep. 2012; 2:591-602.

SUPPLEMENTARY FIGURES, TABLES AND FILES

A

Variable index	Binary variable vector 1	Binary variable vector 2
1:	1	1
2:	1	1
3:	1	2
4:	2	2
5:	1	2
6:	2	1
7:	2	2
8:	1	1
9:	1	1
10:	1	1
11:	1	2
12:	2	1
13:	1	2
14:	1	1
15:	1	2

B

Binary variable vector 2 \ Binary variable vector 1	Binary state 1	Binary state 2
Binary state 1	a = 6	b = 5
Binary state 2	c = 2	d = 2

$*D_E = 2.646$
$**D_{LW} = 0.368$

C

Binary variable vector 2 \ Binary variable vector 1	Binary state 2	Binary state 1
Binary state 2	a' = 2	b' = 2
Binary state 1	c' = 5	d' = 6

$*D_E' = 2.646$
$**D_{LW}' = 0.636$

* Euclidean distance, $D_E = \sqrt{b + c}$

**Lance and Williams distance, $D_{LW} = \frac{b+c}{(2a+b+c)}$

Supplementary Figure S1: Example of distance measure calculations between two binary variable vectors. A. Two binary variable column vectors containing binary states 1 or 2. **B-C.** Contingency tables corresponding to the number of matches and mismatches for the two vectors along the variable axis. The dissimilarity between the two vectors were measured via Euclidean (D_E) as well as Lance and Williams distance (D_{LW}). Euclidean distance considers both binary states equally. On the other hand, Lance and Williams distance prioritizes one of the two binary states, depending on the arrangement of the columns and rows of the contingency table.

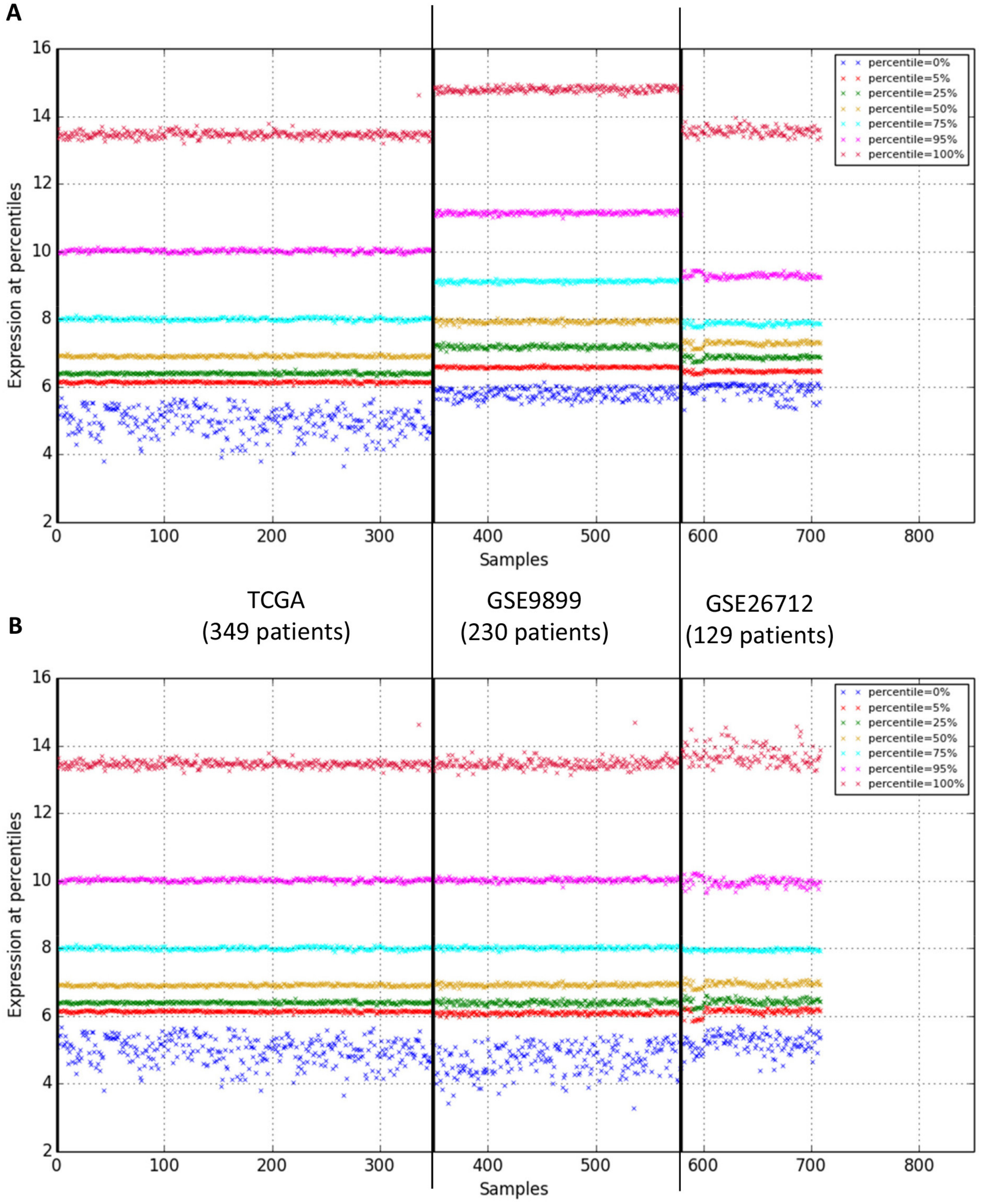

Supplementary Figure S2: Percentile plots across HGSC patients from the TCGA, GSE9899 and GSE26712 cohorts **A.** before batch correction and **B.** after Anova batch-correction and mean centering to align to the TCGA reference patients.

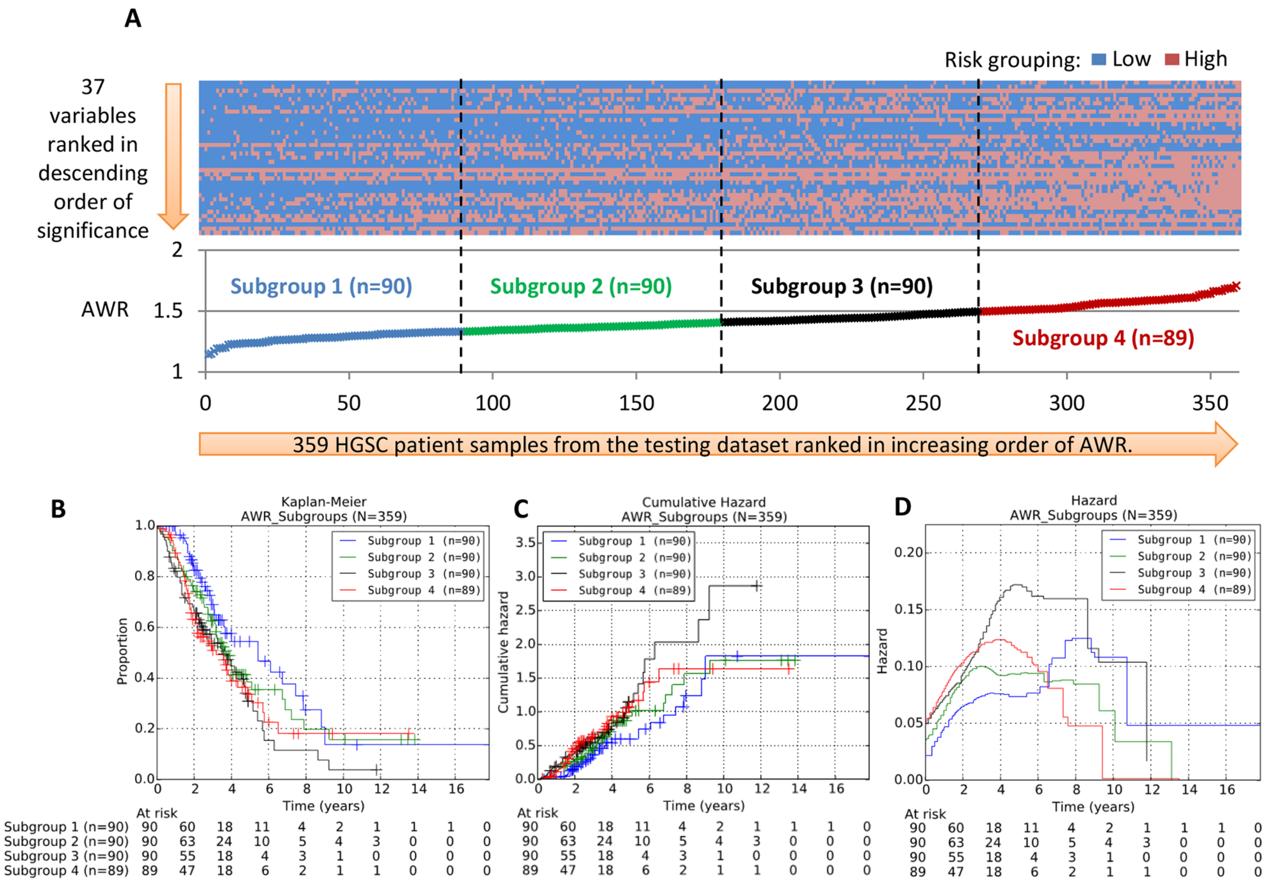

Supplementary Figure S3: A. Heatmap of predicted risk classification for 37 variables and 359 HGSC from the testing cohort (GSE9899 and GSE26712). **B.** Kaplan-Meier survival curves of the four patient prognostic subgroups. **C-D.** Nelson-Aalen estimated cumulative hazard curves and hazard curves of the four patient prognostic subgroups. The 37 variables comprise 36 mRNA expression variables and 1 clinical variable (age). The risk classification for variable and patient was obtained via the parameters learned from the training cohort. The average weighted risk (AWR) value for each patient across all 37 variables was calculated and used for ranking patient samples. The patient cohort was arbitrarily classified into four equal-sized subgroups based on their AWR values.

A

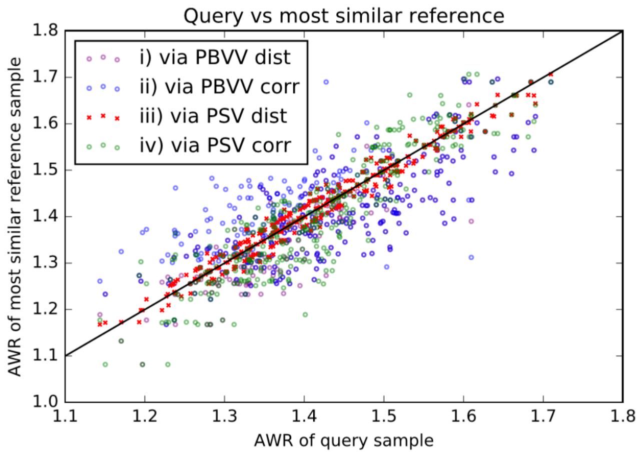

B

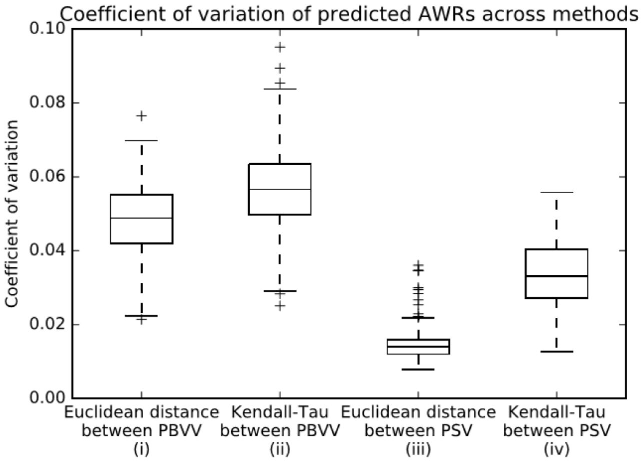

C

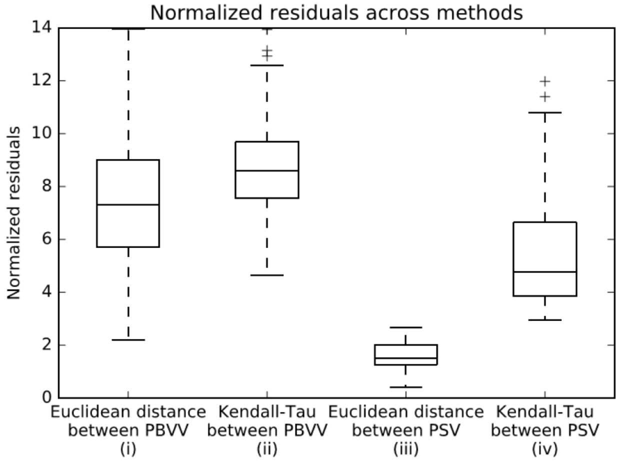

Supplementary Figure S4: A. Relationships between the average weighted risk of each query sample with its corresponding most similar reference sample obtained via various methods. Each point represents each query sample from the testing cohort. **B.** Coefficient of variation of predicted AWRs for query patients across different methods. **C.** Residuals of curve fitting for query patients across different methods. Methods: (i) comparison of PBVVs via euclidean distance, (ii) comparison of PBVVs via Kendall's Tau rank correlation, (iii) comparison of PSVs via euclidean distance, (iv) comparison of PSVs via Kendall's Tau rank correlation. Abbreviations: PBVV: Prognostic binary variable vector; PSV: Prognostic signature vector; AWR: average-weighted risk; dist: distance; corr: correlation.

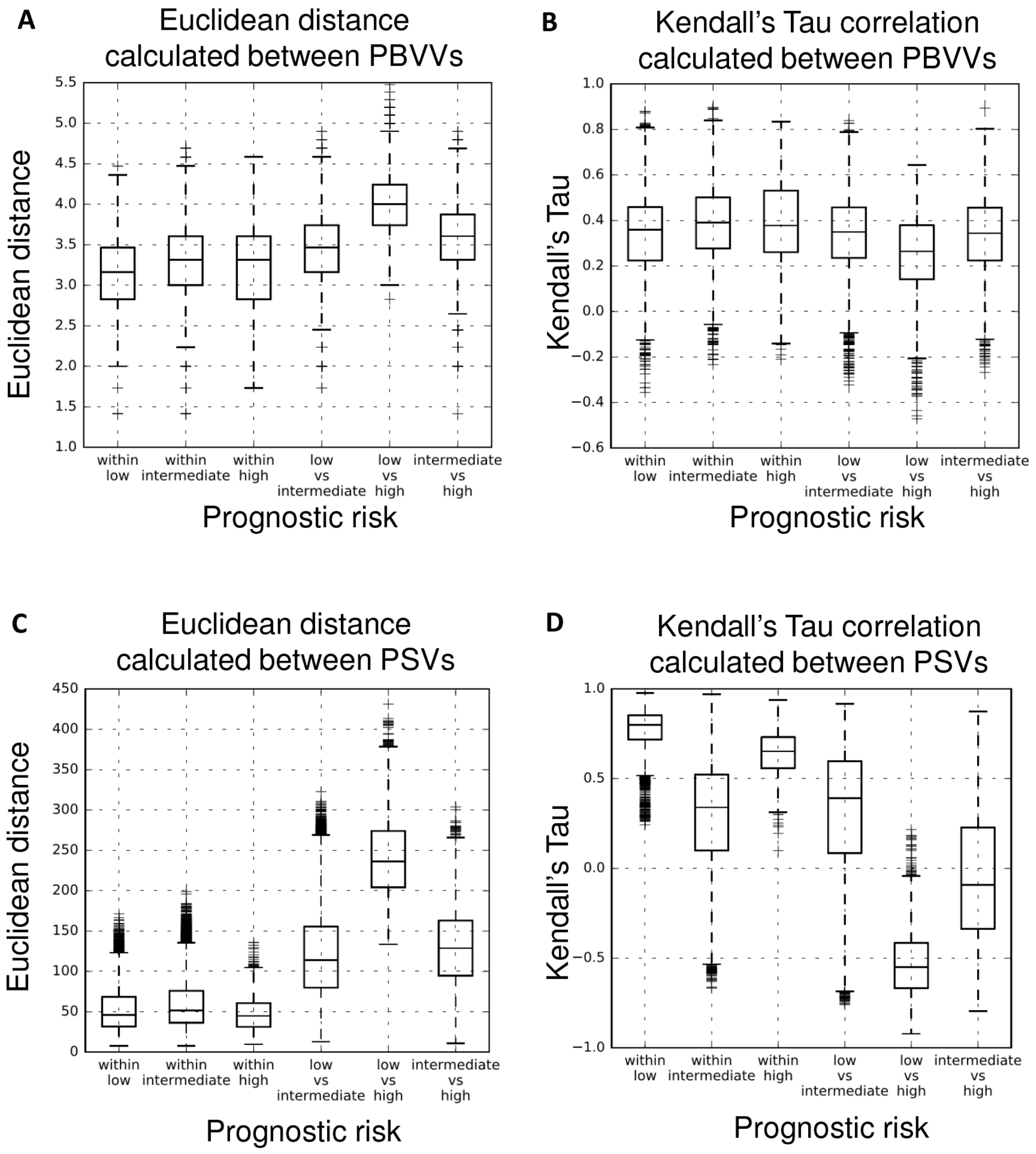

Supplementary Figure S5: Boxplots of similarity measures calculated for reference patient pairs within a specific prognostic class (low, intermediate or high-risk) or for patient pairs between two prognostic class. A. Euclidean distance was calculated between two prognostic binary variable vector (PBVVs). **B.** Kendall's Tau rank correlation was calculated between two PBVVs. **C.** Euclidean distance was calculated between two prognostic signature vector (PSVs). **D.** Kendall's Tau rank correlation was calculated between two PSVs. The prognostic risk groups (low, intermediate, or high-risk) were classified during training of the classifier.

A

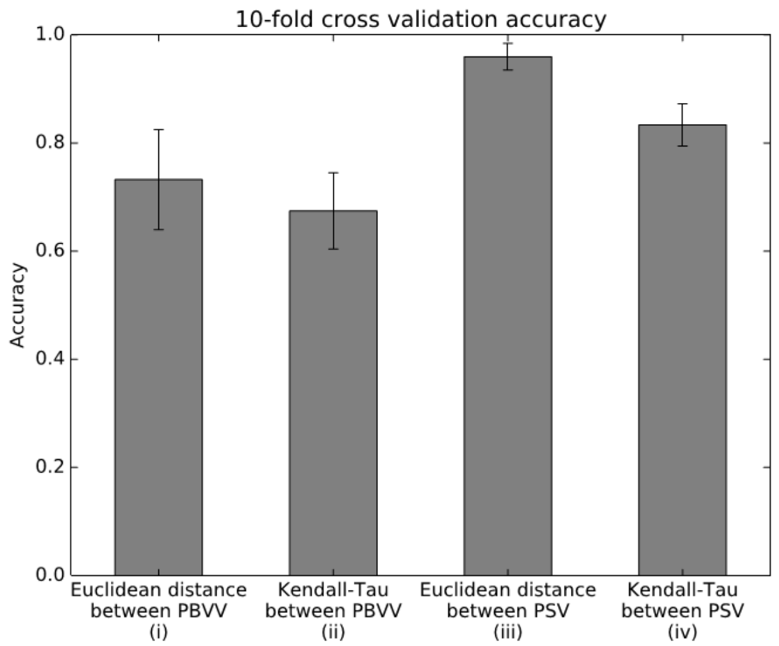

B

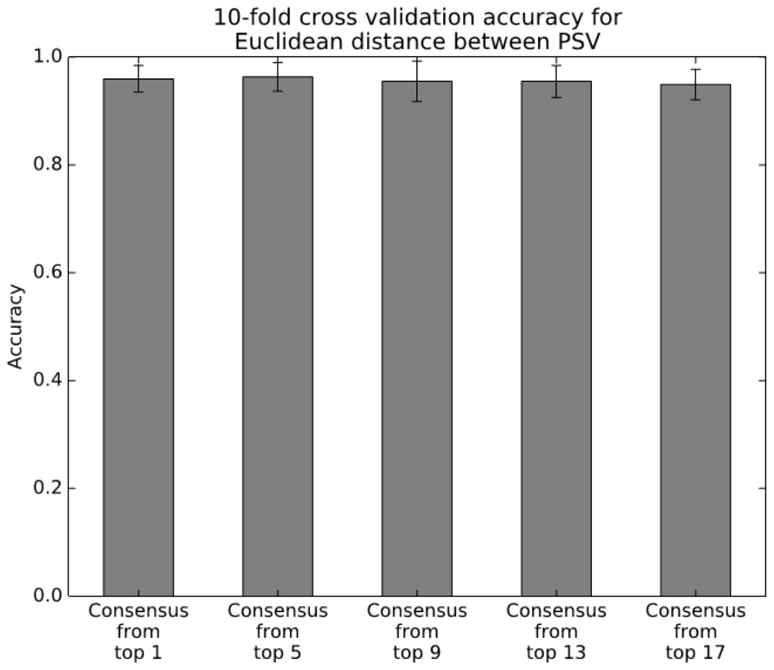

Supplementary Figure S6: A. Accuracies of 10-fold cross validation calculated across each of four methods. The mean accuracy across the 10 cross validation analyses is shown. The standard deviation across the 10 cross validation analysis is shown as error bars. **B.** Accuracies of 10-fold cross validation analyses calculated for the method which compares patient pairs via euclidean distance of PSVs. The patient in the validation cohort is assigned a prognostic risk group based on the most similar reference patient (top 1), or based on the consensus assignment from the top N similar reference patient (top N where N = 5, 9, 13 or 17).

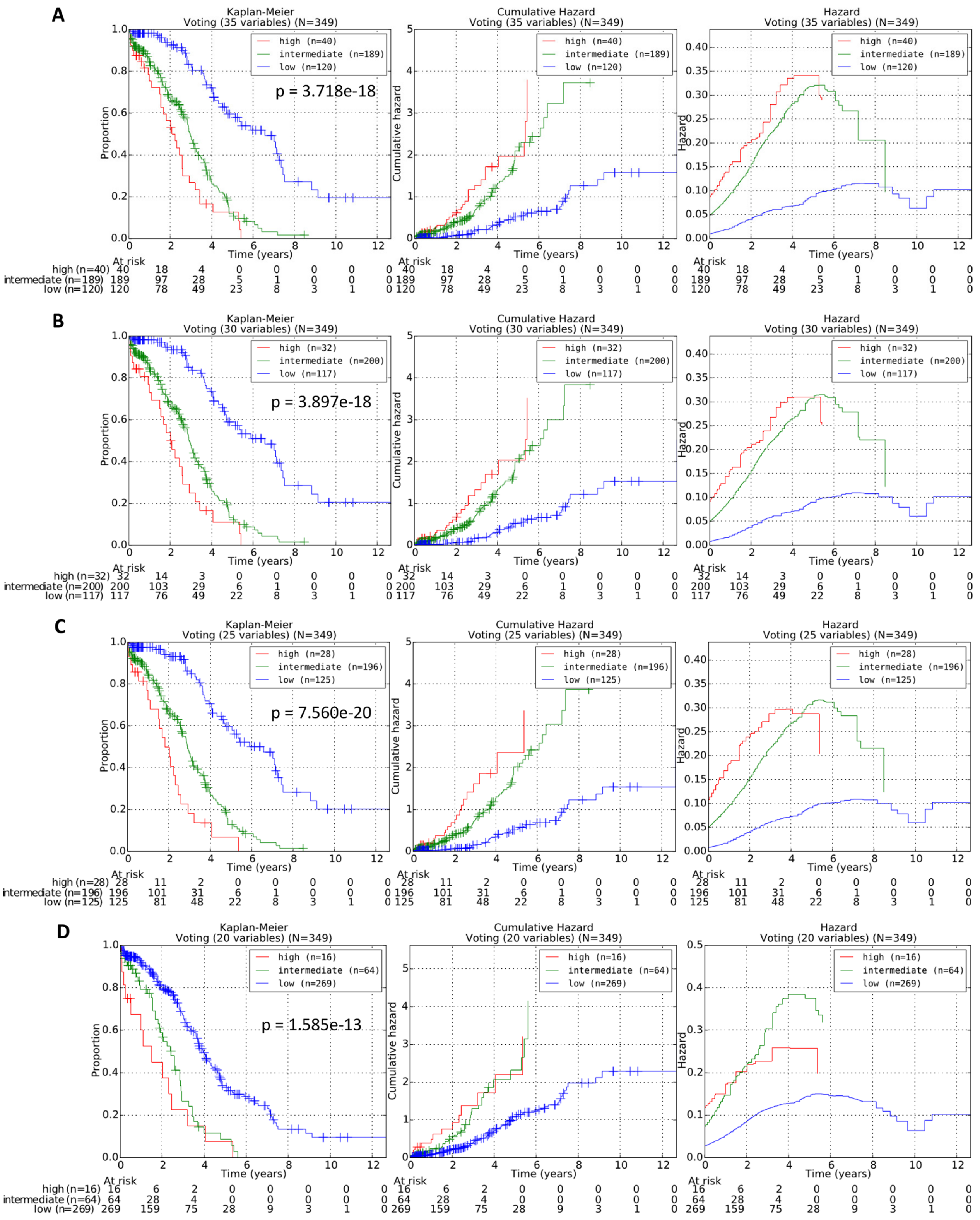

Supplementary Figure S7: Kaplan-Meier survival curves, Nelson-Aalen estimated cumulative hazard curves and hazard curves of risk groups of 349 reference patients from TCGA using **A.** 35 variables, **B.** 30 variables, **C.** 25 variables and **D.** 20 variables. The p-values were calculated via the multivariate log-rank test.

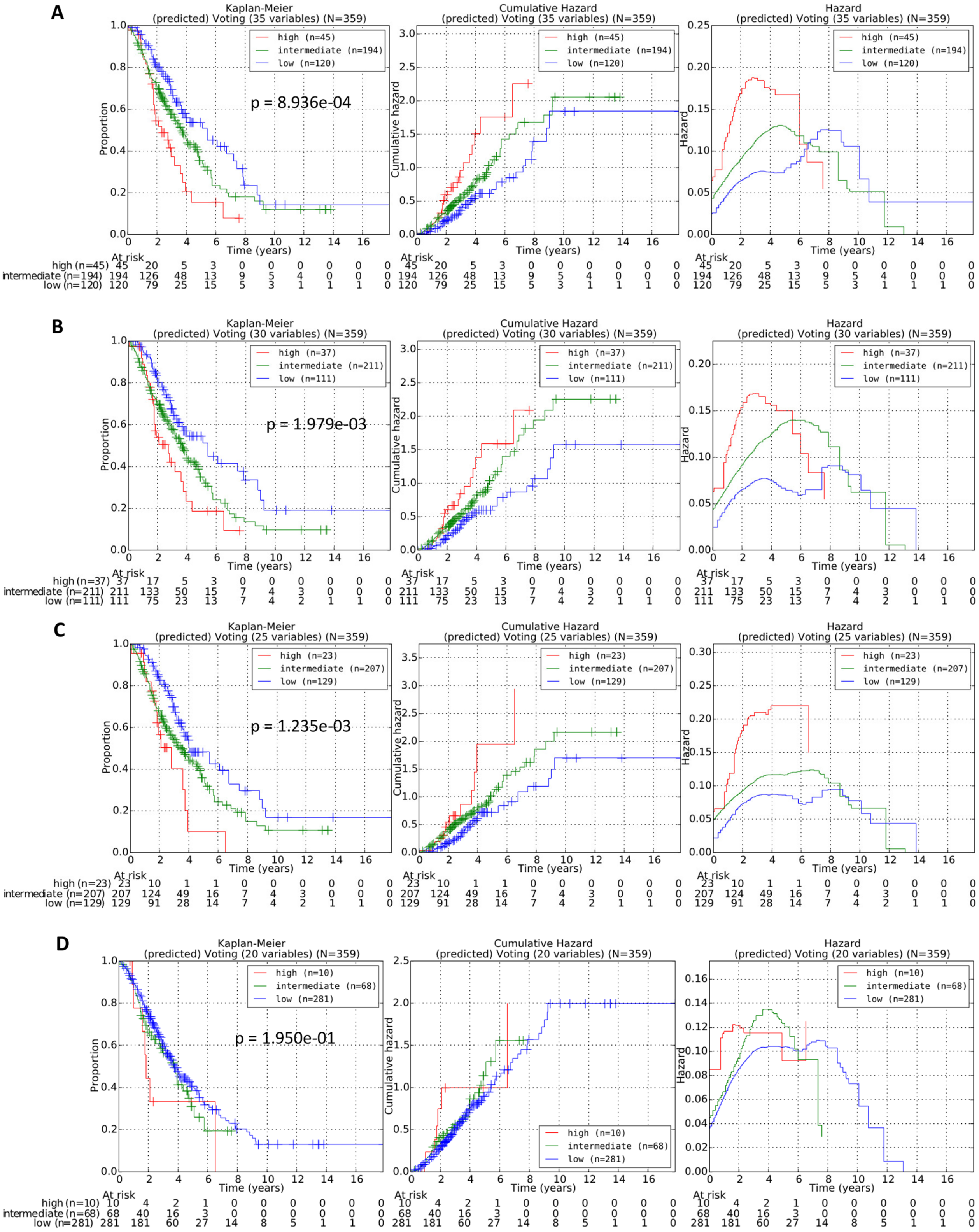

Supplementary Figure S8: Kaplan-Meier survival curves, Nelson-Aalen estimated cumulative hazard curves and hazard curves of risk groups of 359 query patients from GSE9899 and GSE26712 using **A.** 35 variables, **B.** 30 variables, **C.** 25 variables and **D.** 20 variables. The p-values were calculated via the multivariate logrank test. Classification prediction was performed via euclidean distance measures of PSVs.

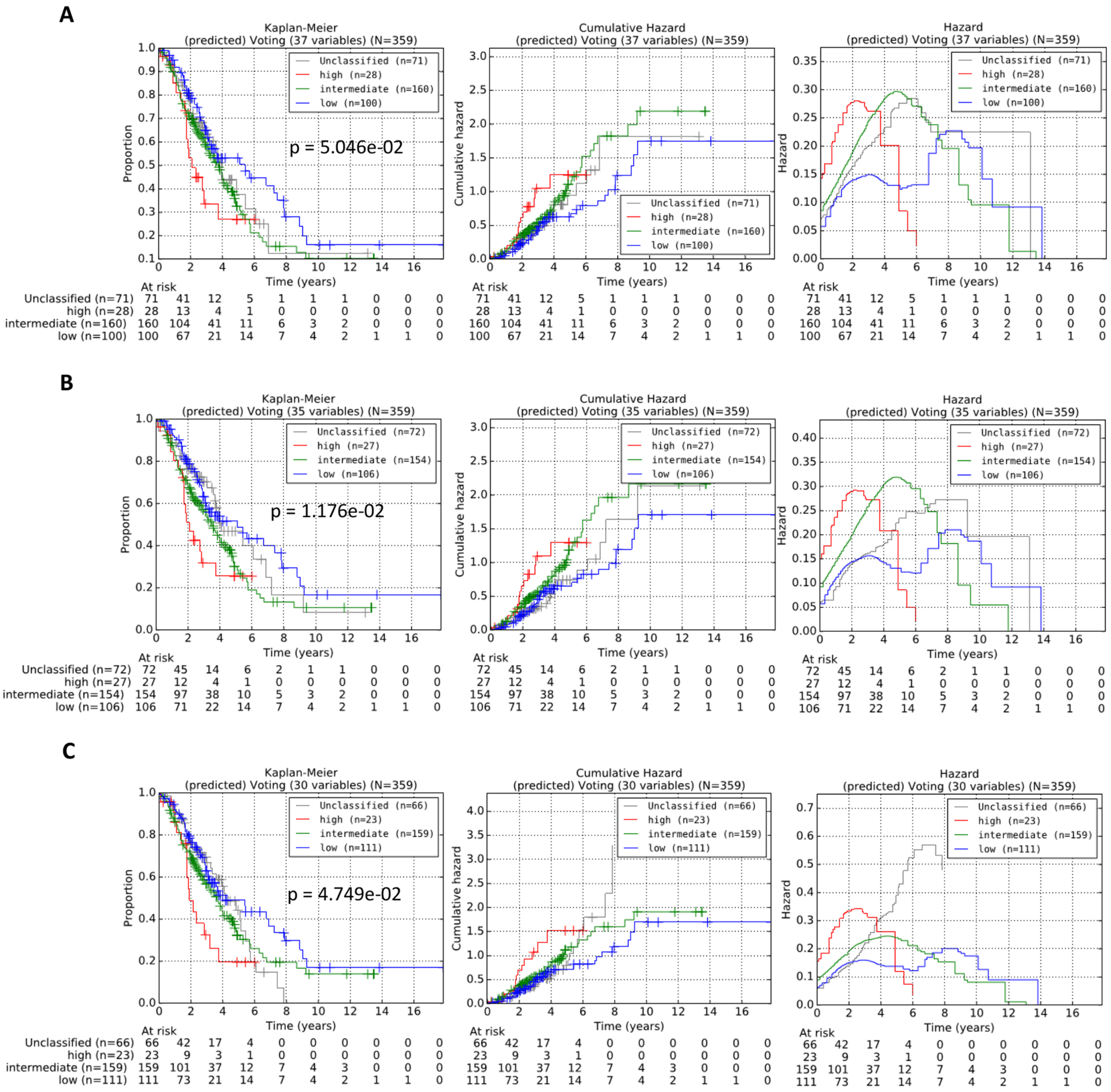

Supplementary Figure S9: Kaplan-Meier survival curves, Nelson-Aalen estimated cumulative hazard curves and hazard curves of risk groups of 359 query patients from GSE9899 and GSE26712 using **A.** 37 variables, **B.** 35 variables, and **C.** 30 variables. The p-values were calculated via the multivariate log-rank test. Classification prediction was performed via euclidean distance measures of PBVV.

Supplementary Table S1: Prognostic binary variable vectors (PBVV) for 349 reference high-grade serous ovarian carcinoma patients from the TCGA training cohort. Risk classifications for 37 variables (comprising 36 mRNA variables and 1 patient clinical information) across the 349 patients were generated by the 1D-DDg algorithm. The numerical values 1 and 2 denotes low and high-risk respectively. The p-values for each variable, represents the statistical significance of stratification between the low and high-risk subgroups. The average weighted risk was calculated by using the negative log10 of the p-values as weights for each variable.

See Supplementary Table S1 (.xlsx)

Supplementary Table S2: Prognostic binary variable vectors (PBVV) for 359 query patients from the testing cohorts (GSE9899 and GSE26712). Risk classification for each variable and patient was determined via the use of the variable parameters trained from the training dataset. The p-values for each variable, represents the statistical significance of stratification between the low and high-risk subgroups as obtained from the reference training patient cohort. The numerical values 1 and 2 denotes low and high-risk respectively. The average weighted risk was calculated by using the negative log10 of the p-values as weights for each variable.

See Supplementary Table S2 (.xlsx)

Supplementary Table S3: Measure of distance between query patient k^{th} and reference patient j^{th} by direct comparison of their prognostic binary variable vectors (PBVV).

See Supplementary Table S3 (.xlsx)

Supplementary Table S4: Measure of correlation between query patient k^{th} and reference patient j^{th} by direct comparison of their prognostic binary variable vectors (PBVV).

See Supplementary Table S4 (.xlsx)

Supplementary Table S5: Prognostic signature vectors (PSV) of the reference patients in the training cohort.

See Supplementary Table S5 (.xlsx)

Supplementary Table S6: Prognostic signature vectors (PSV) of the query patients in the testing cohort.

See Supplementary Table S6 (.xlsx)

Supplementary Table S7: Measure of distance between query patient k^{th} and reference patient j^{th} by comparison of their prognostic signature vectors (PSV).

See Supplementary Table S7 (.xlsx)

Supplementary File 1 (37 pages): Identification of 1D-DDg cutoff for each variable in the training cohort (comprising 349 reference patients) and subsequent application of the 1D-DDg cutoff to the testing cohort (comprising 359 query patients).

See Supplementary File 1 (.pdf)

Supplementary File 2: Scatter plot of Euclidean distance against average weighted risk (AWR) calculated via comparison of prognostic binary variable vectors (PBVVs).

See Supplementary File 2 (.pdf)

Supplementary File 3: Scatter plot of Kendall's tau correlation against average weighted risk (AWR) calculated via comparison of prognostic binary variable vector (PBVVs).

See Supplementary File 3 (.pdf)

Supplementary File 4: Scatter plot of Euclidean distance against average weighted risk (AWR) calculated via comparison of prognostic signature vectors (PSVs).

See Supplementary File 4 (.pdf)

Supplementary File 5: Scatter plot of Kendall's tau correlation against average weighted risk (AWR) calculated via comparison of prognostic signature vectors (PSVs).

See Supplementary File 5 (.pdf)

Supplementary File 6: Scatter plot of Euclidean distance against average weighted risk (AWR) calculated via direct comparison of expression vectors.

See Supplementary File 6 (.pdf)

Supplementary File 7: Scatter plot of Kendall's tau correlation against average weighted risk (AWR) calculated via direct comparison of expression vectors.

See Supplementary File 7 (.pdf)

Supplementary Files 2 to 7 contain 359 pages each - one for each query patient from the testing cohort. Each point on the scatter plot represents each of 349 reference samples, and the y-axis represents the value of the similarity measure (either Euclidean distance or Kendall's Tau correlation) with the testing sample. The color blue, green and red corresponds to the low, intermediate and high prognostic risk group of the reference patients. The x-axis represents the AWR values associated with each reference sample.
