# Supplementary material for: Big data and computational biology strategy for personalized prognosis
Source: Oncotarget. 2016 May 24;7(26):40200–20. doi: 10.18632/oncotarget.9571 (PMC5130003; doi:10.18632/oncotarget.9571)
Supplement: Supplementary file 9 [file oncotarget-07-40200-s009.pdf]

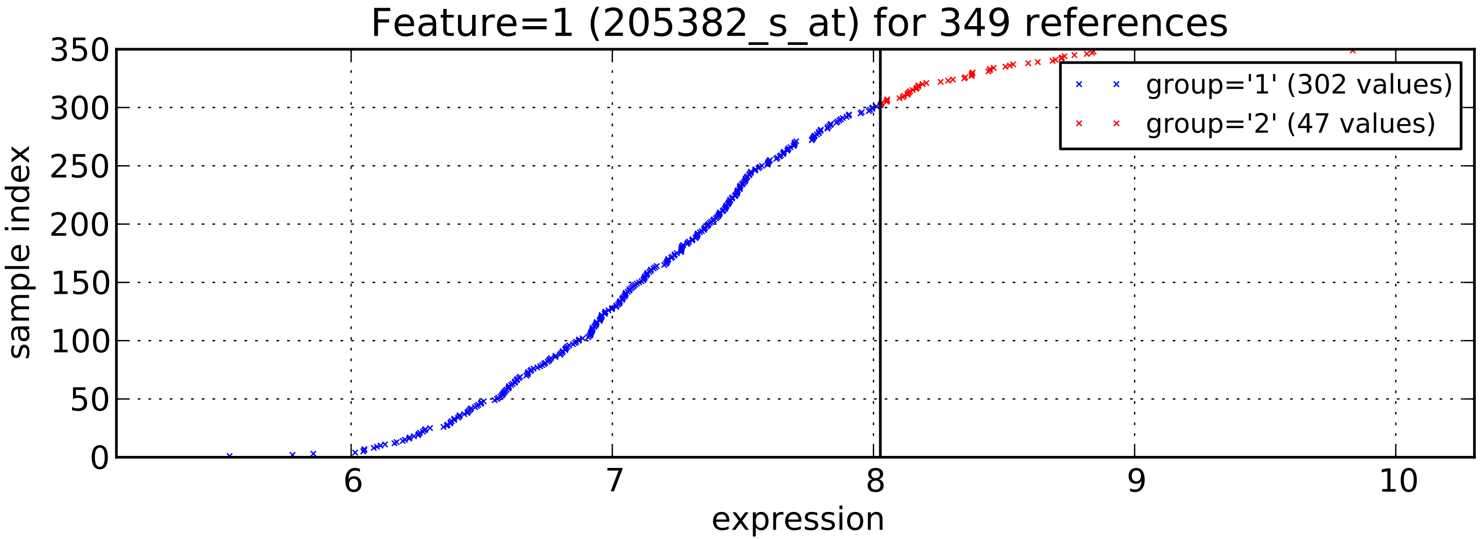

Feature=1 (205382_s_at) for 349 references
sample index
expression
group='1' (302 values)
group='2' (47 values)
0
50
100
150
200
250
300
350
6
7
8
9
10

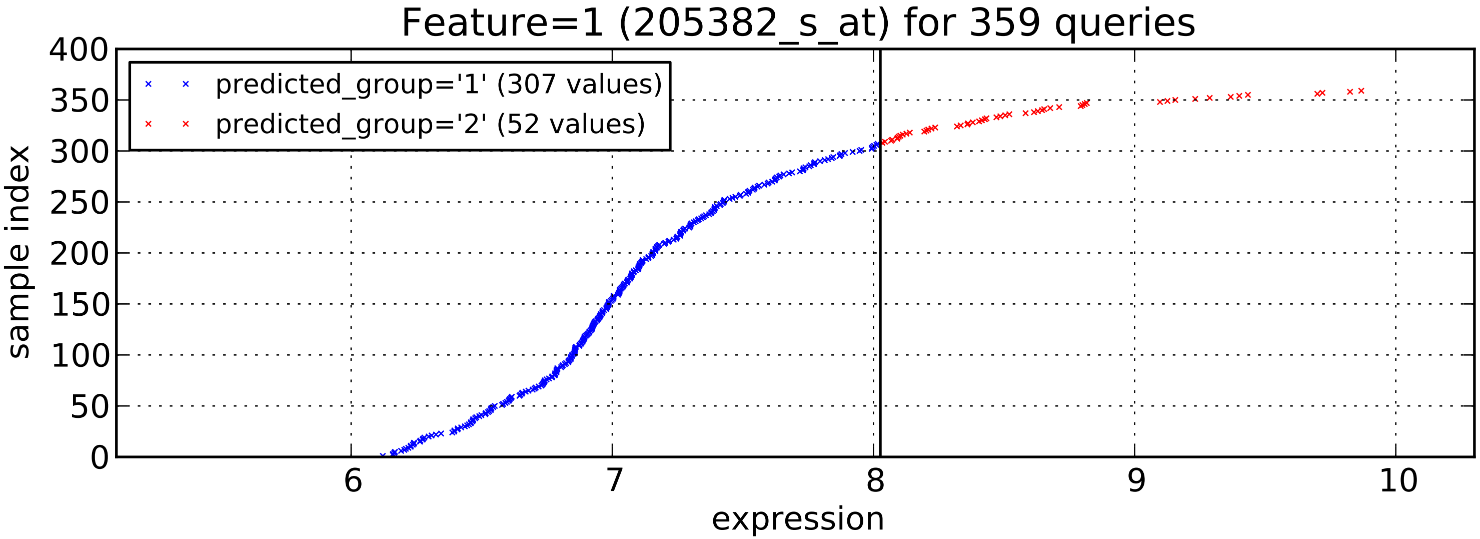

Feature=1 (205382_s_at) for 359 queries
sample index
expression
predicted_group='1' (307 values)
predicted_group='2' (52 values)
0
50
100
150
200
250
300
350
400
6
7
8
9
10

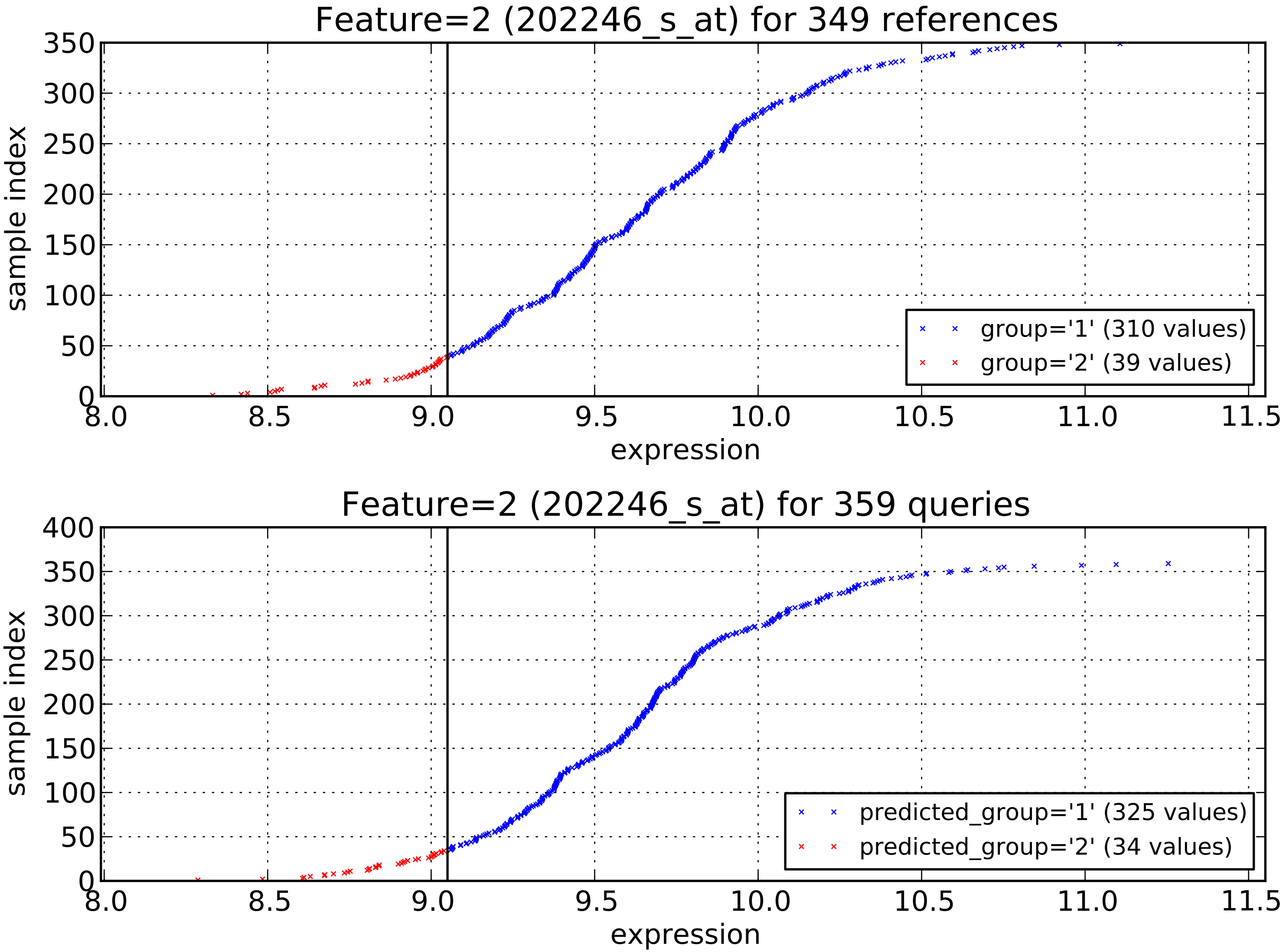

Feature=2 (202246_s_at) for 349 references
sample index
expression
group='1' (310 values)
group='2' (39 values)
Feature=2 (202246_s_at) for 359 queries
sample index
expression
predicted_group='1' (325 values)
predicted_group='2' (34 values)

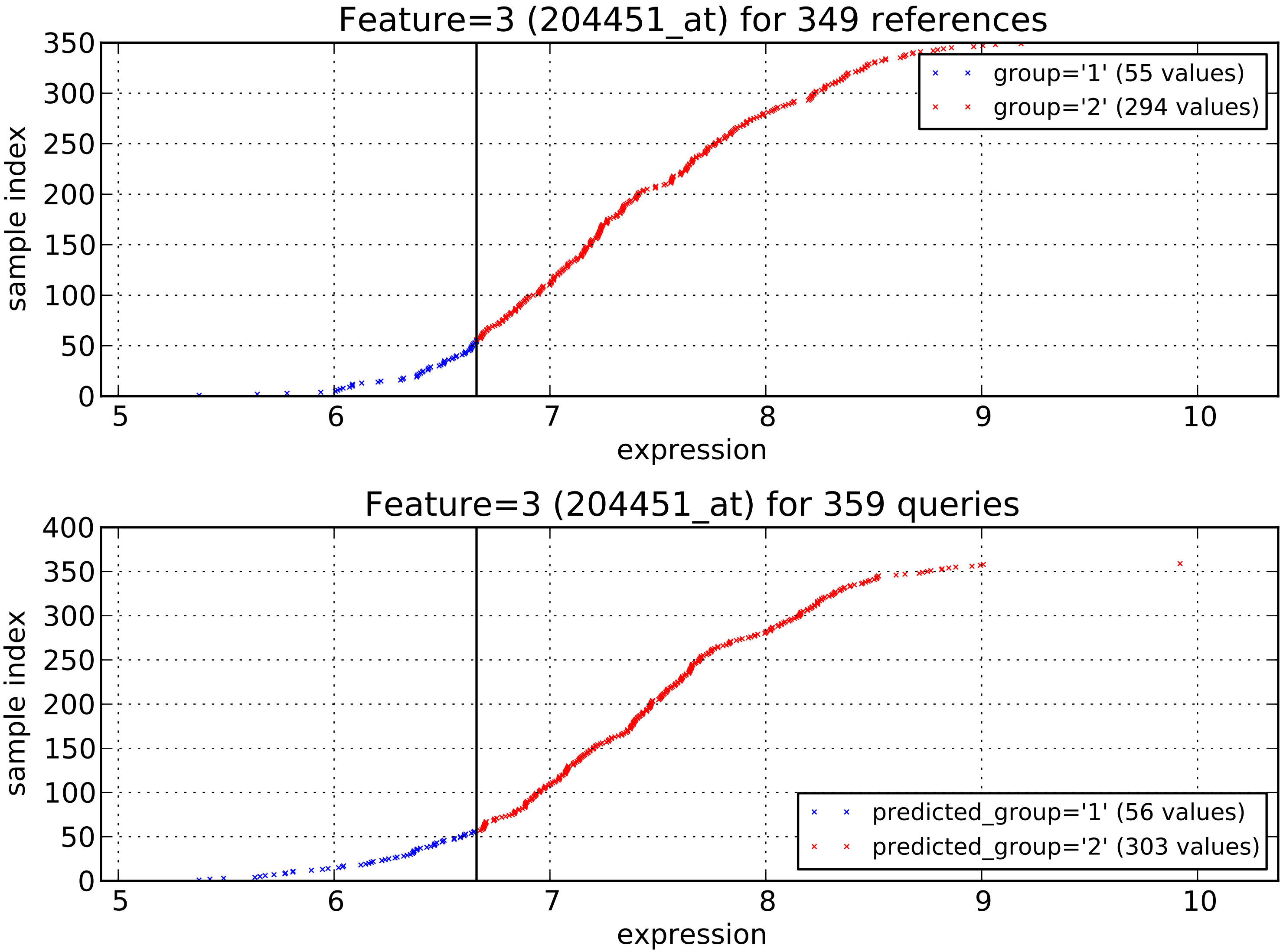

Feature=3 (204451_at) for 349 references
sample index
expression
group='1' (55 values)
group='2' (294 values)
Feature=3 (204451_at) for 359 queries
sample index
expression
predicted_group='1' (56 values)
predicted_group='2' (303 values)

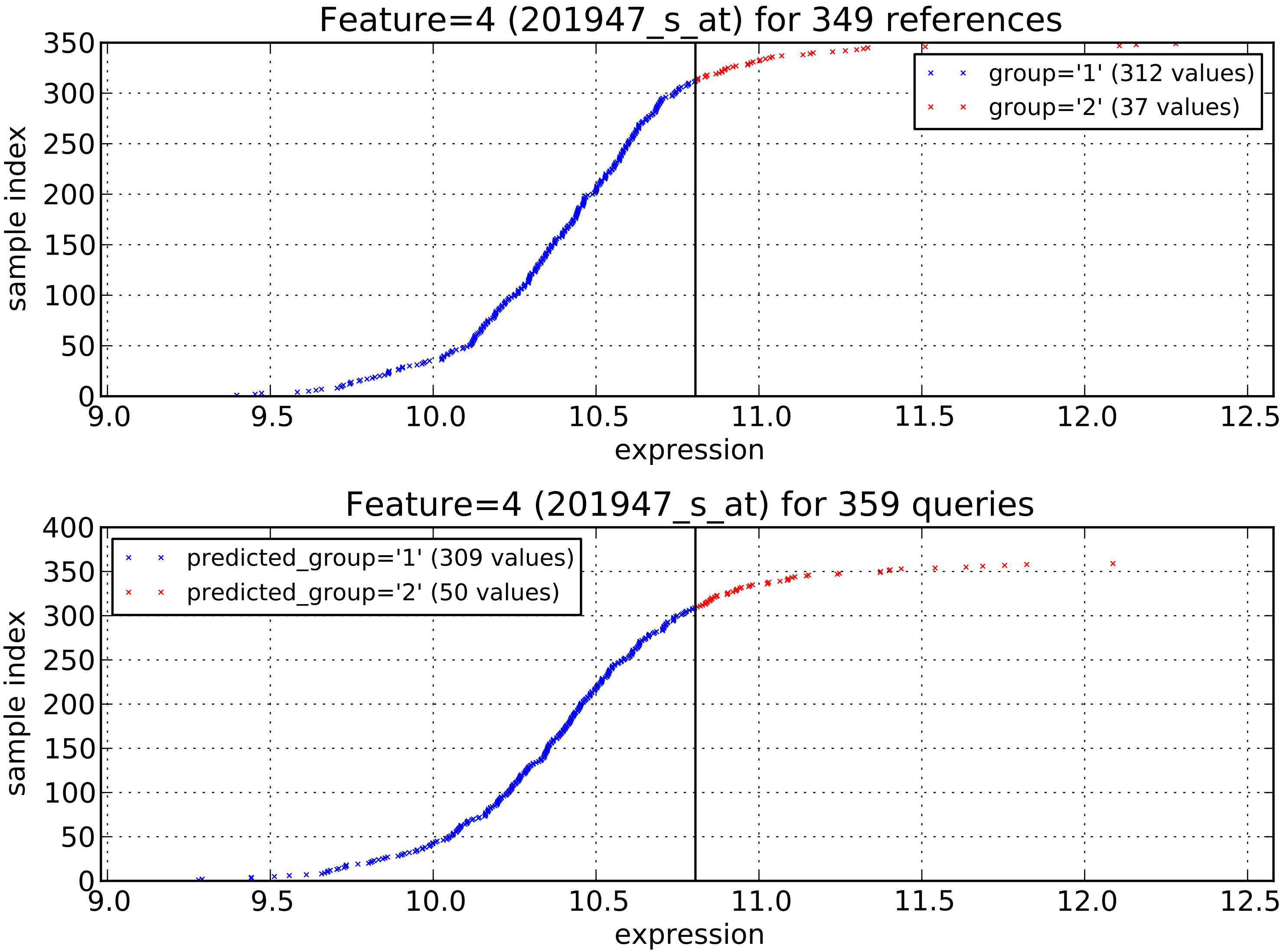
Feature=4 (201947_s_at) for 349 references
sample index
expression
group='1' (312 values)
group='2' (37 values)
Feature=4 (201947_s_at) for 359 queries
sample index
expression
predicted_group='1' (309 values)
predicted_group='2' (50 values)

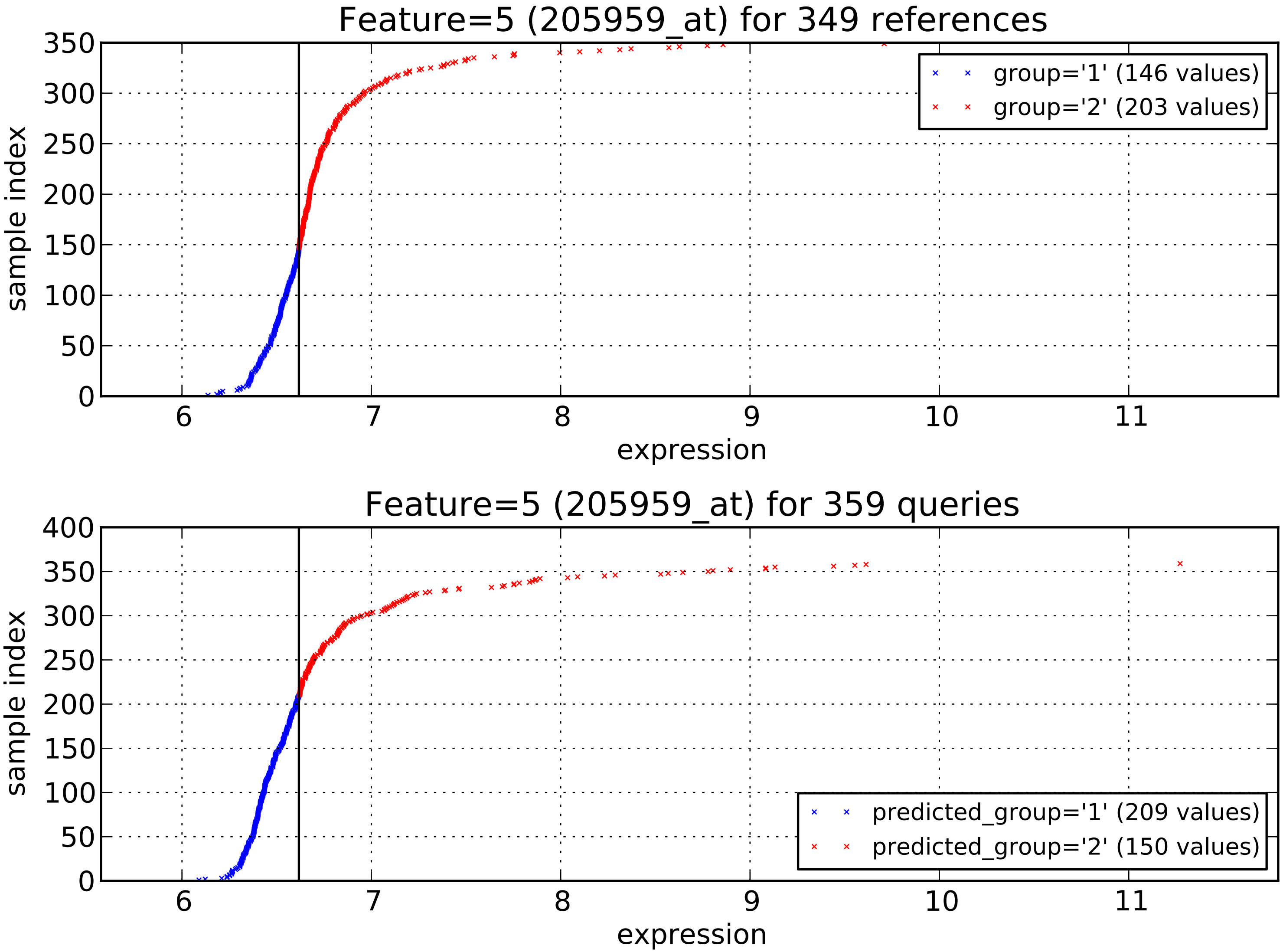
Feature=5 (205959_at) for 349 references
sample index
expression
group='1' (146 values)
group='2' (203 values)
0
50
100
150
200
250
300
350
6
7
8
9
10
11
Feature=5 (205959_at) for 359 queries
sample index
expression
predicted_group='1' (209 values)
predicted_group='2' (150 values)
0
50
100
150
200
250
300
350
400
6
7
8
9
10
11

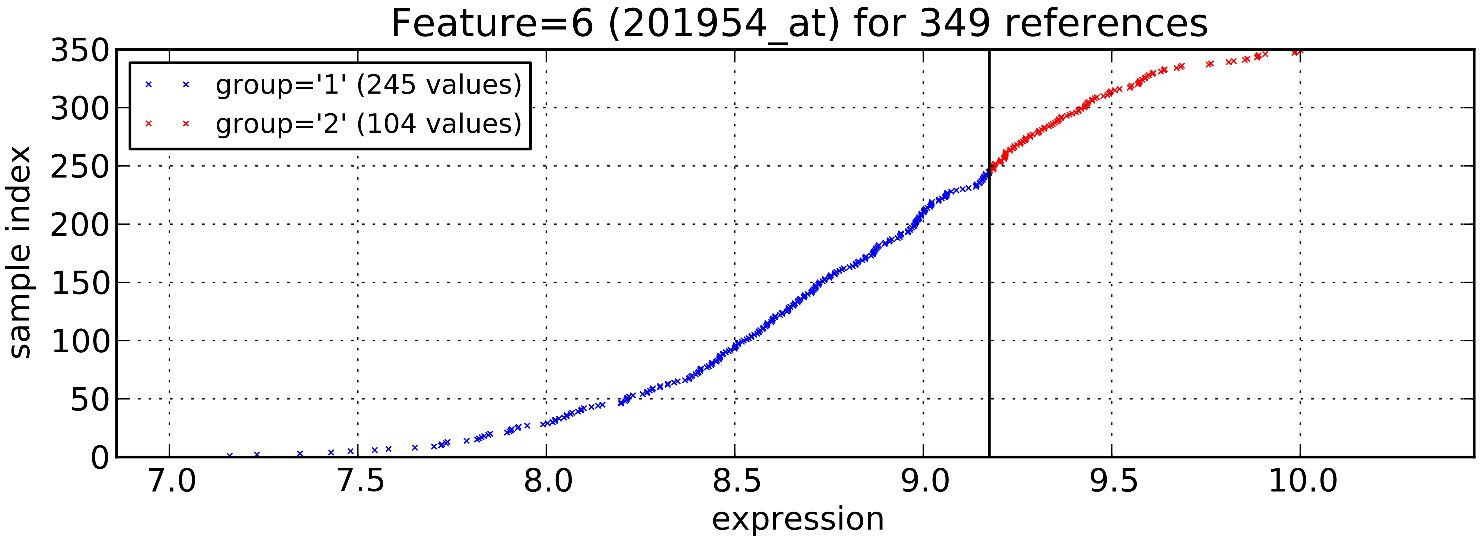
Feature=6 (201954_at) for 349 references
group='1' (245 values)
group='2' (104 values)
sample index
expression
0
50
100
150
200
250
300
350
7.0
7.5
8.0
8.5
9.0
9.5
10.0

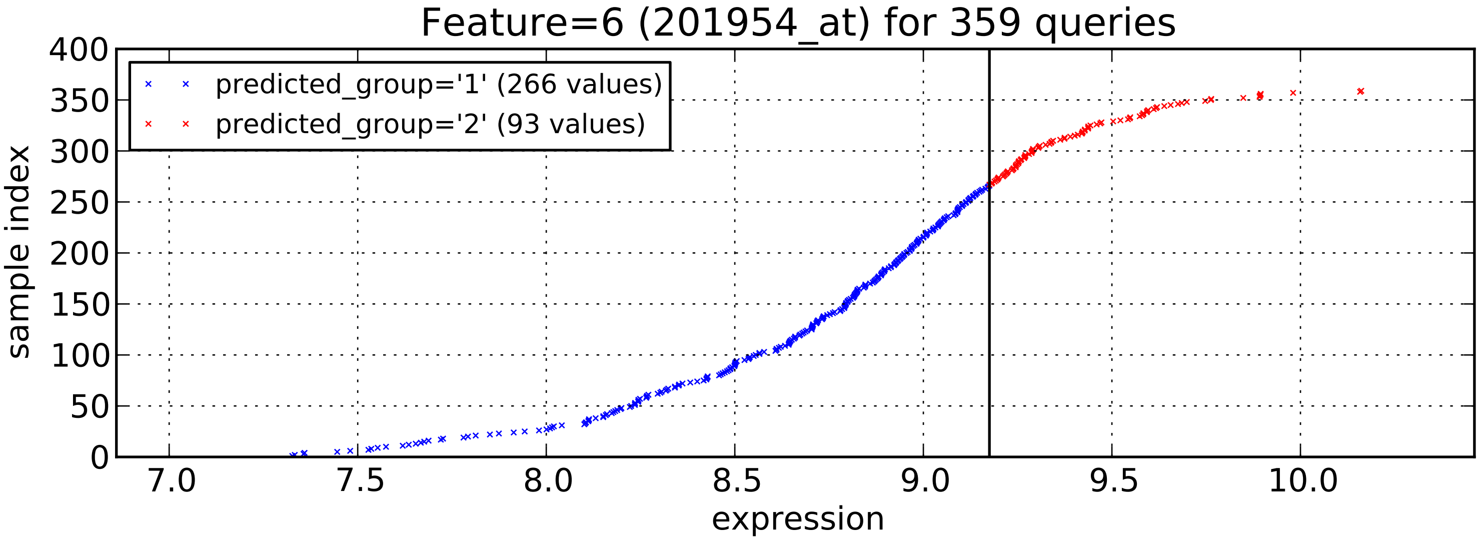
Feature=6 (201954_at) for 359 queries
predicted_group='1' (266 values)
predicted_group='2' (93 values)
sample index
expression
0
50
100
150
200
250
300
350
400
7.0
7.5
8.0
8.5
9.0
9.5
10.0

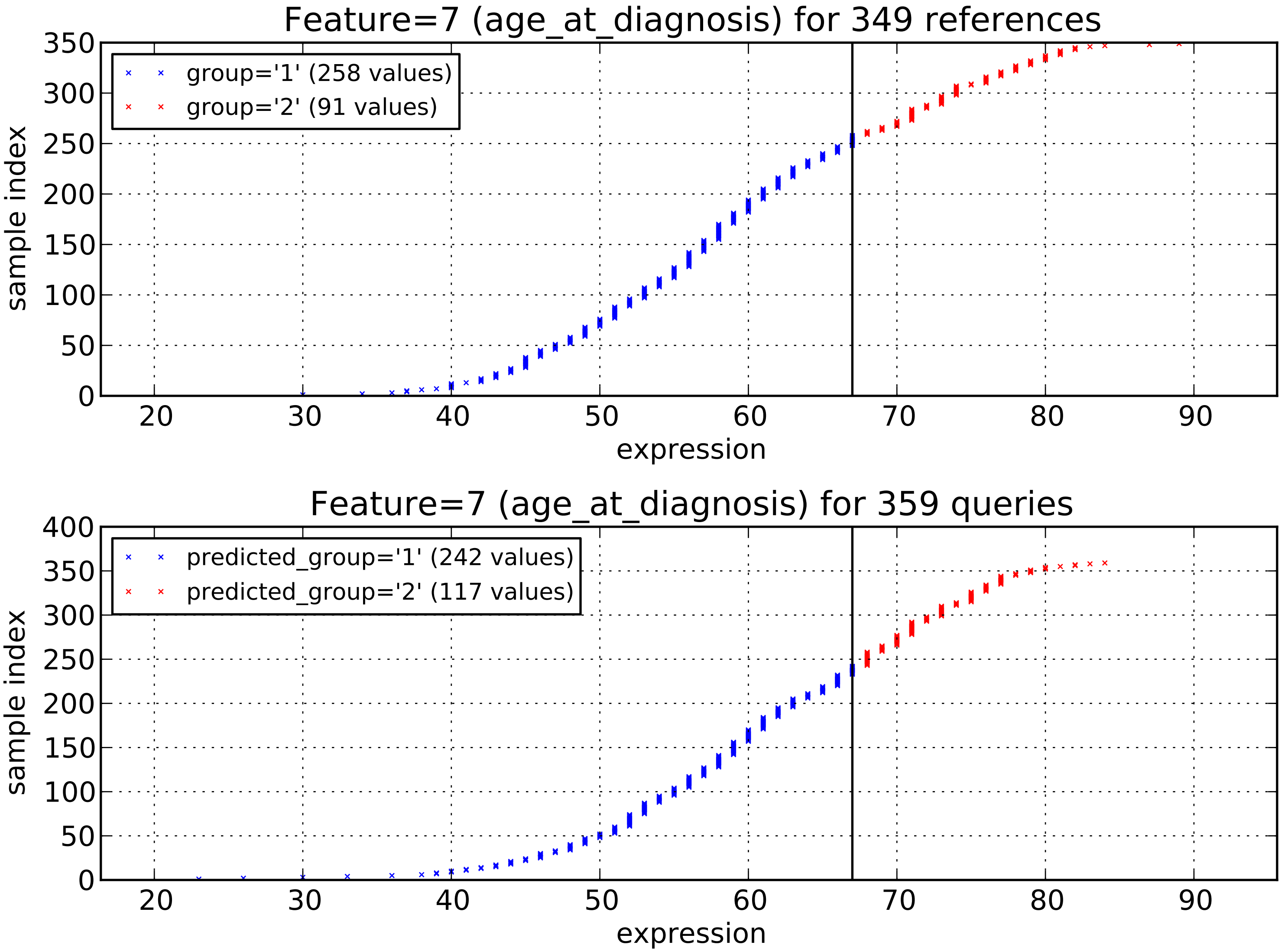

Feature=7 (age_at_diagnosis) for 349 references
group='1' (258 values)
group='2' (91 values)
sample index
expression
0
50
100
150
200
250
300
350
20
30
40
50
60
70
80
90
Feature=7 (age_at_diagnosis) for 359 queries
predicted_group='1' (242 values)
predicted_group='2' (117 values)
sample index
expression
0
50
100
150
200
250
300
350
400
20
30
40
50
60
70
80
90

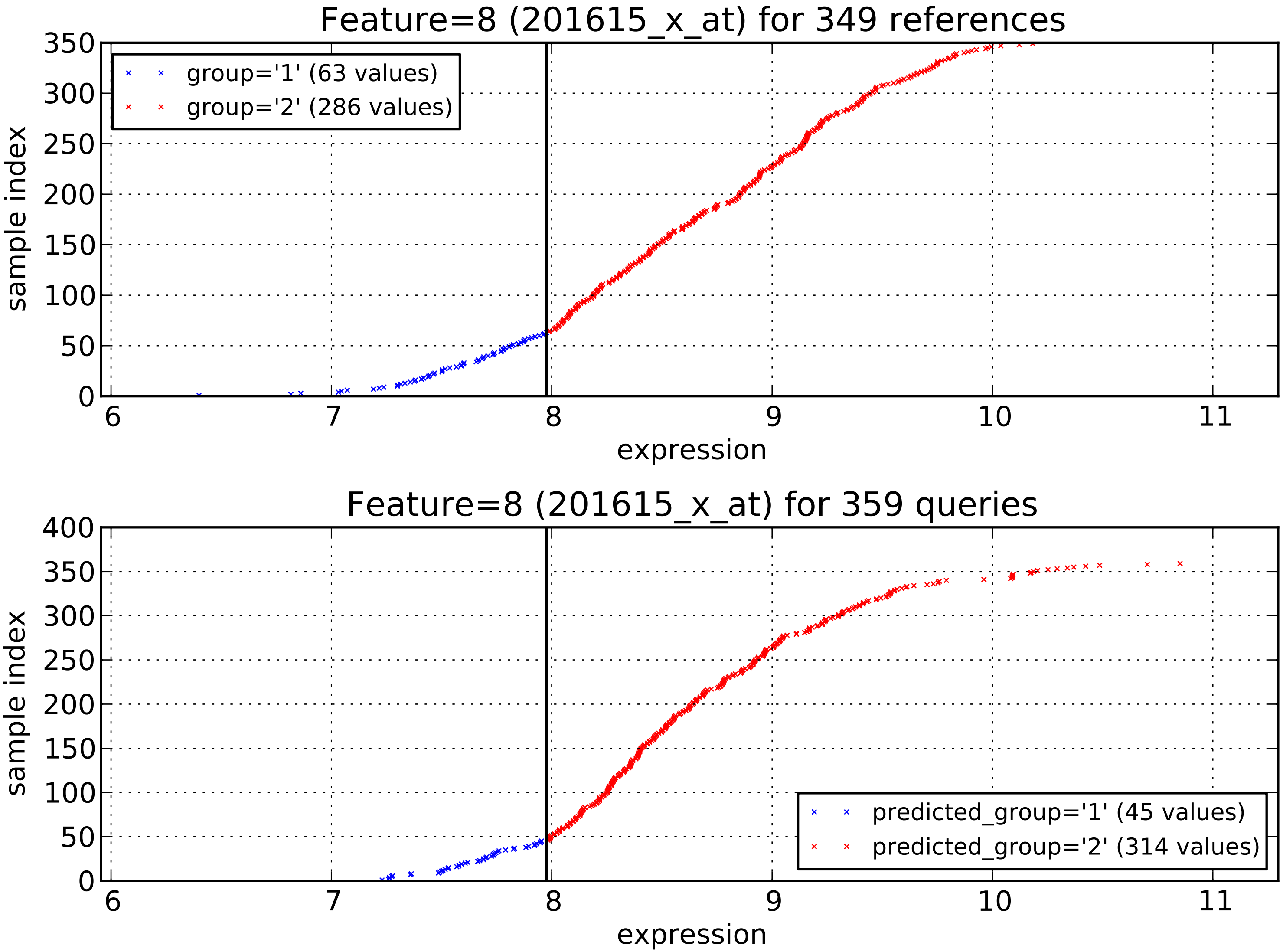

Feature=8 (201615_x_at) for 349 references
group='1' (63 values)
group='2' (286 values)
sample index
expression
0
50
100
150
200
250
300
350
6
7
8
9
10
11
Feature=8 (201615_x_at) for 359 queries
predicted_group='1' (45 values)
predicted_group='2' (314 values)
sample index
expression
0
50
100
150
200
250
300
350
400
6
7
8
9
10
11

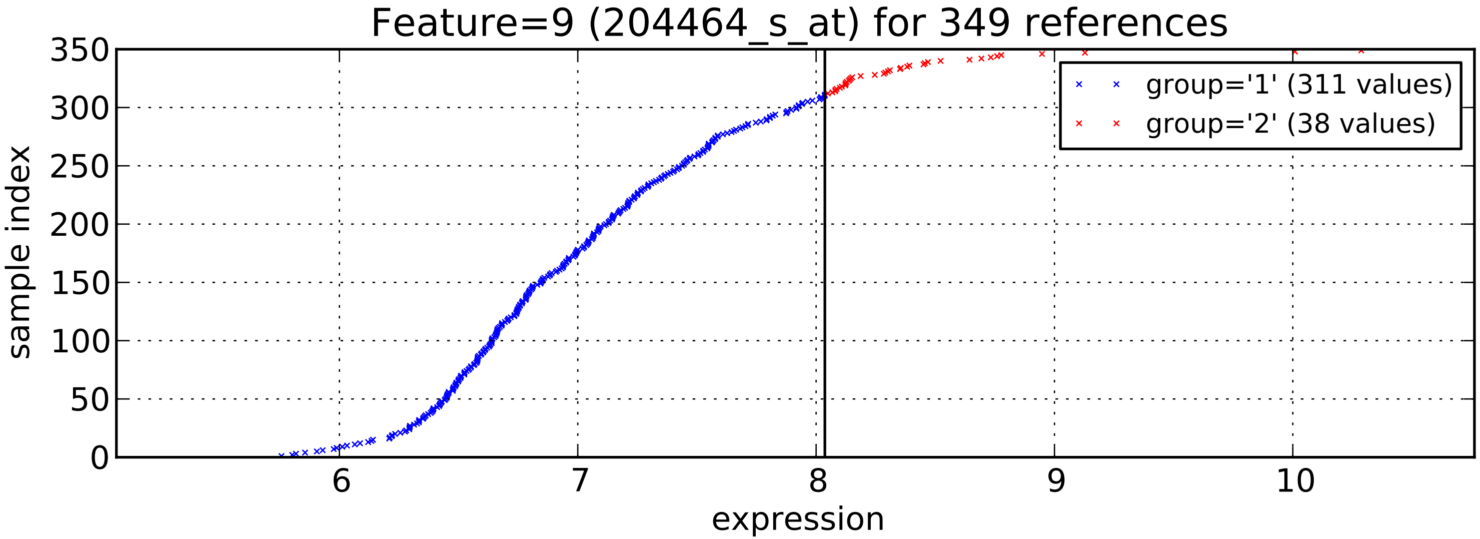
Feature=9 (204464_s_at) for 349 references
sample index
expression
group='1' (311 values)
group='2' (38 values)
0
50
100
150
200
250
300
350
6
7
8
9
10

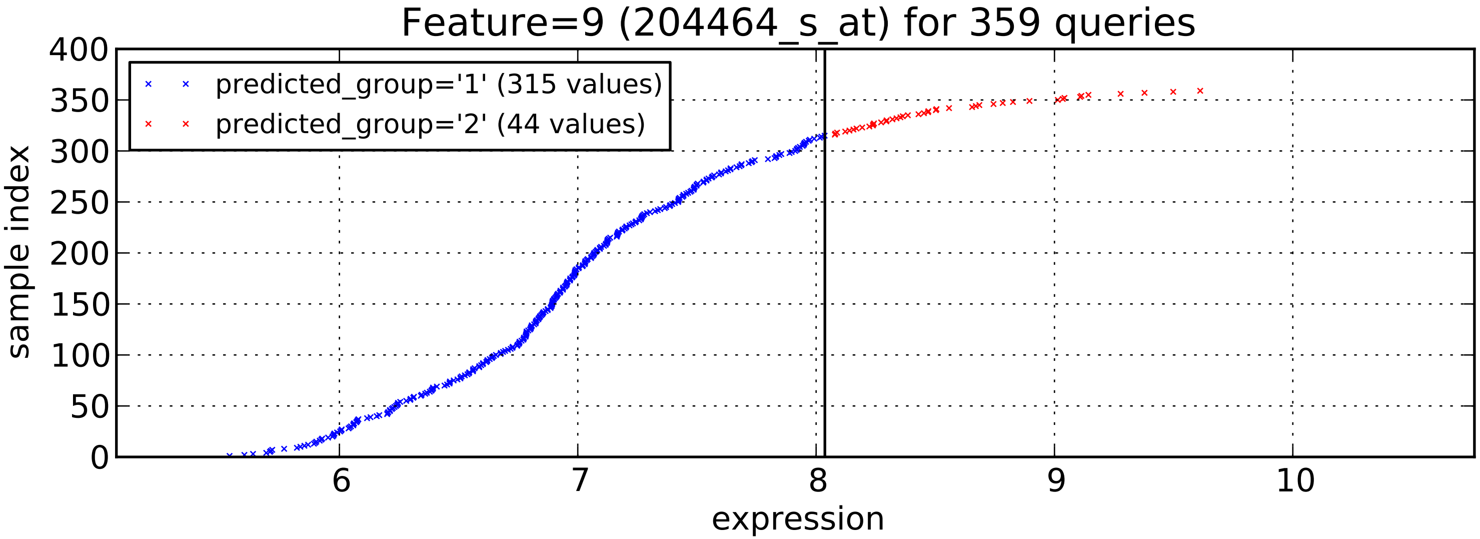
Feature=9 (204464_s_at) for 359 queries
sample index
expression
predicted_group='1' (315 values)
predicted_group='2' (44 values)
0
50
100
150
200
250
300
350
400
6
7
8
9
10

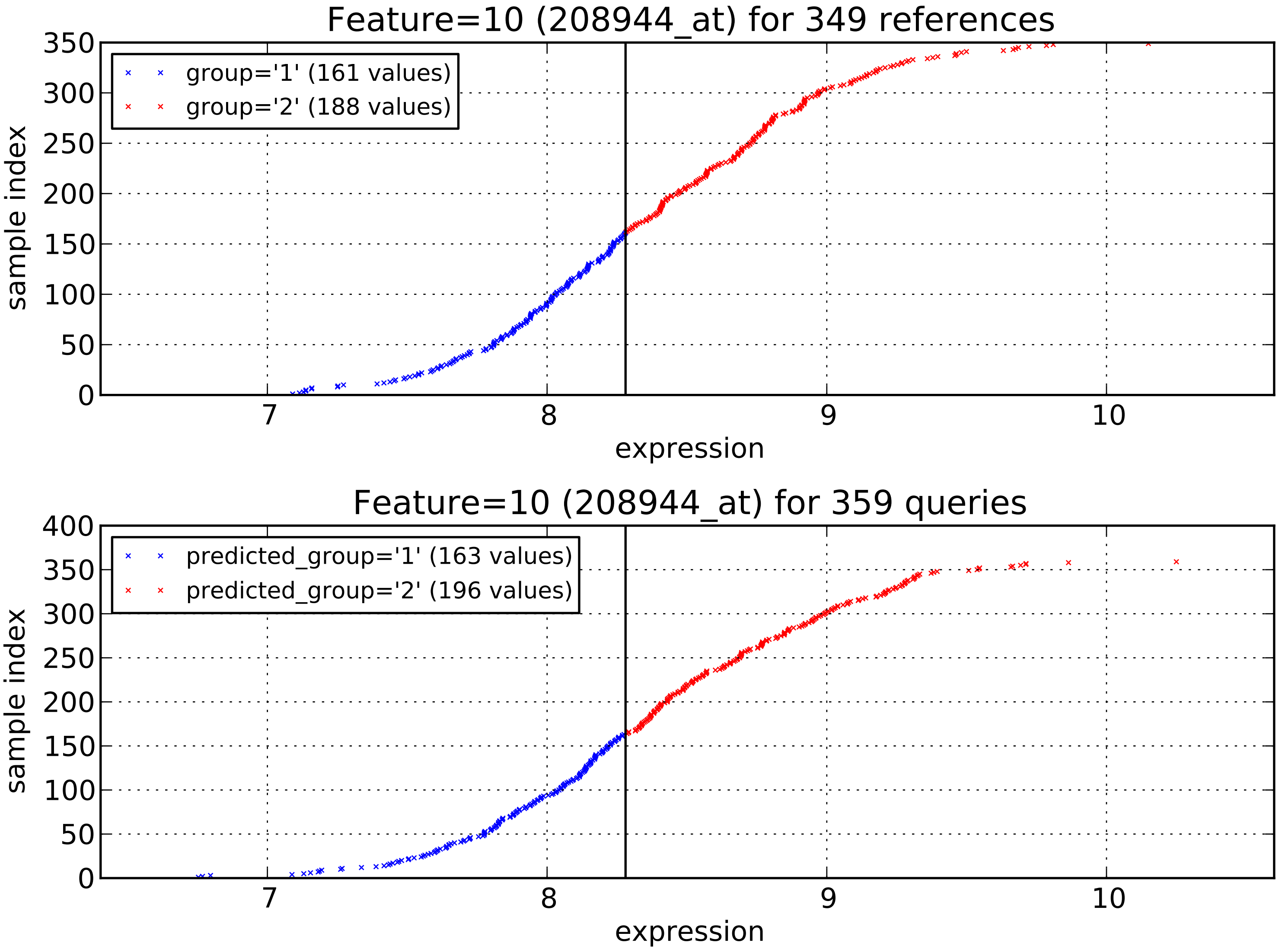

Feature=10 (208944_at) for 349 references
group='1' (161 values)
group='2' (188 values)
sample index
expression
0
50
100
150
200
250
300
350
7
8
9
10
Feature=10 (208944_at) for 359 queries
predicted_group='1' (163 values)
predicted_group='2' (196 values)
sample index
expression
0
50
100
150
200
250
300
350
400
7
8
9
10

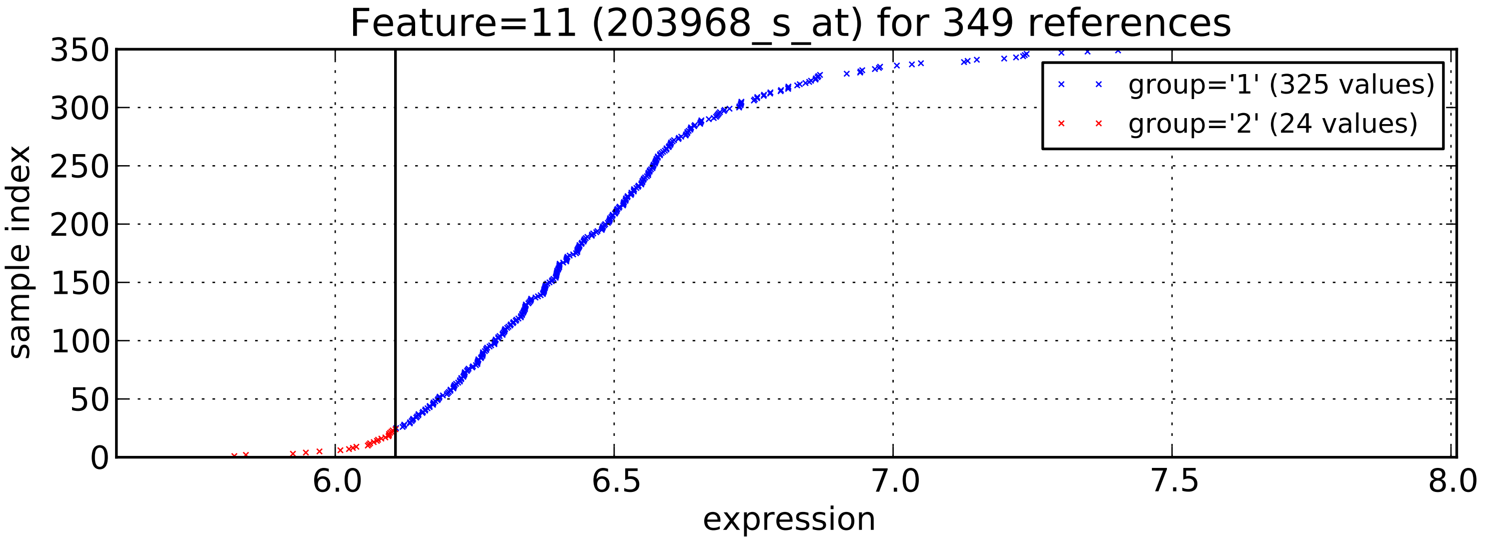

Feature=11 (203968_s_at) for 349 references
sample index
expression
group='1' (325 values)
group='2' (24 values)
0
50
100
150
200
250
300
350
6.0
6.5
7.0
7.5
8.0

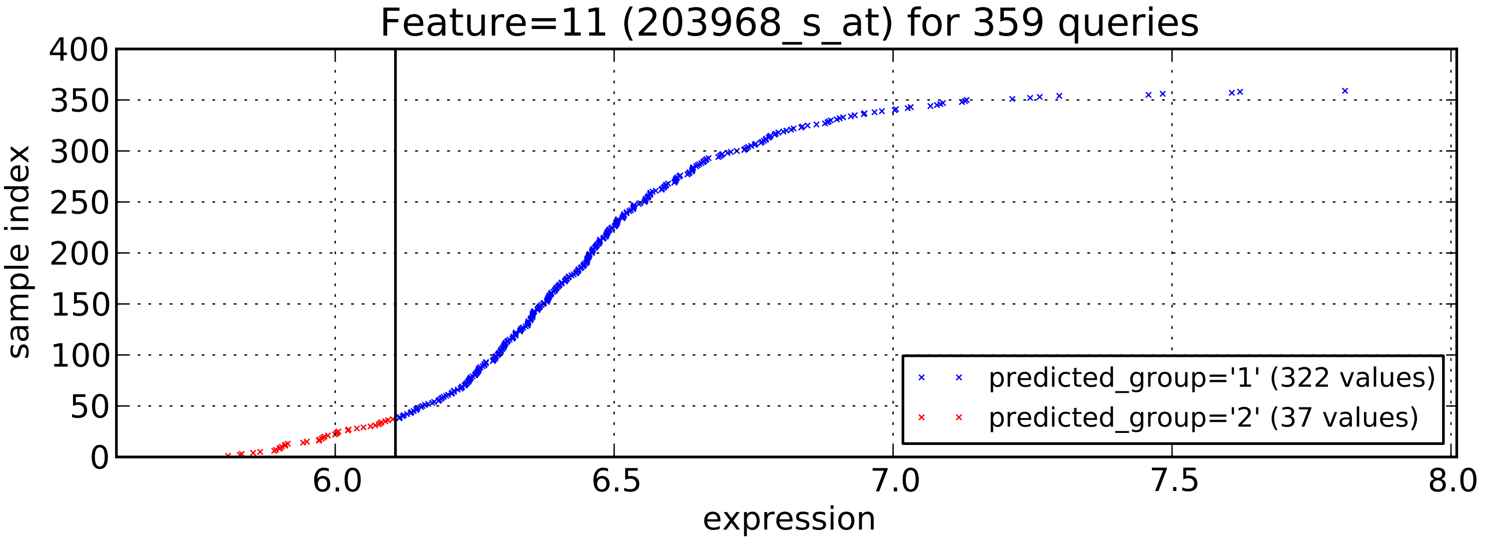

Feature=11 (203968_s_at) for 359 queries
sample index
expression
predicted_group='1' (322 values)
predicted_group='2' (37 values)
0
50
100
150
200
250
300
350
400
6.0
6.5
7.0
7.5
8.0

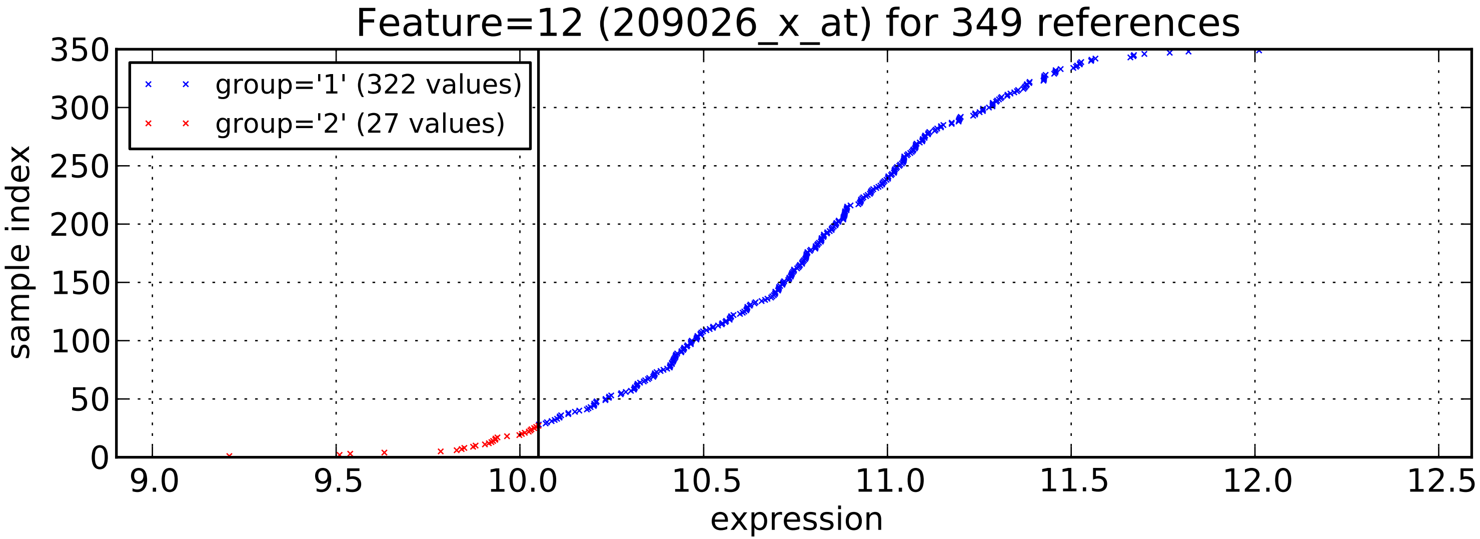

Feature=12 (209026_x_at) for 349 references
sample index
expression
group='1' (322 values)
group='2' (27 values)
0
50
100
150
200
250
300
350
9.0
9.5
10.0
10.5
11.0
11.5
12.0
12.5

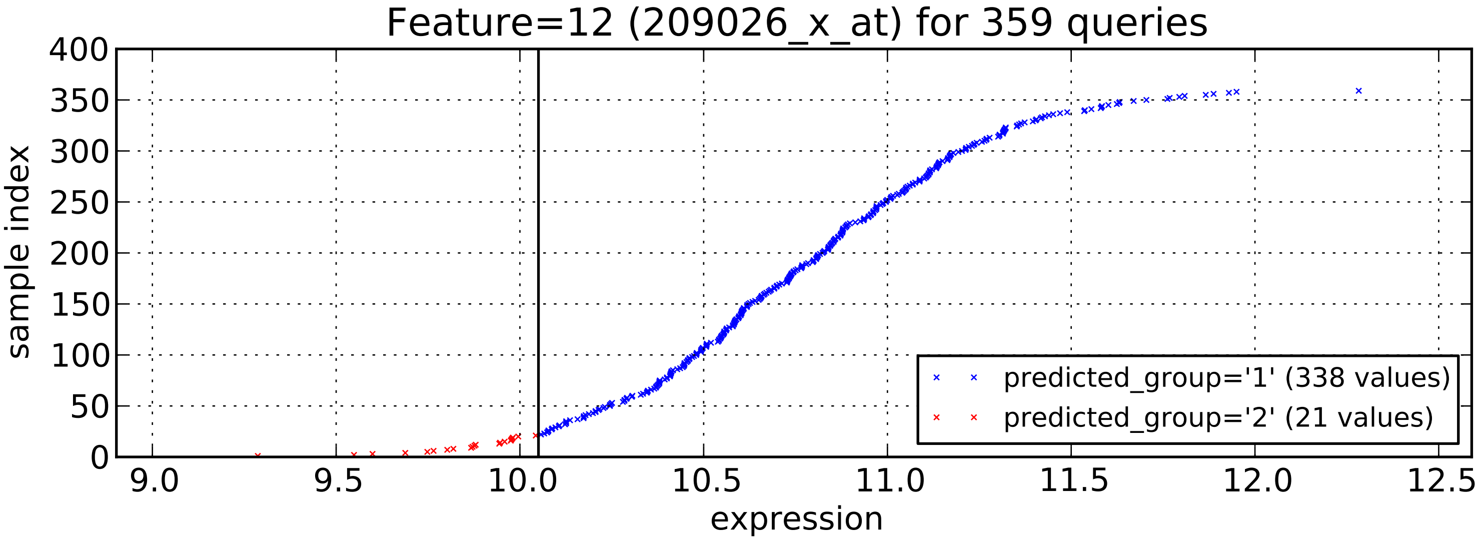

Feature=12 (209026_x_at) for 359 queries
sample index
expression
predicted_group='1' (338 values)
predicted_group='2' (21 values)
0
50
100
150
200
250
300
350
400
9.0
9.5
10.0
10.5
11.0
11.5
12.0
12.5

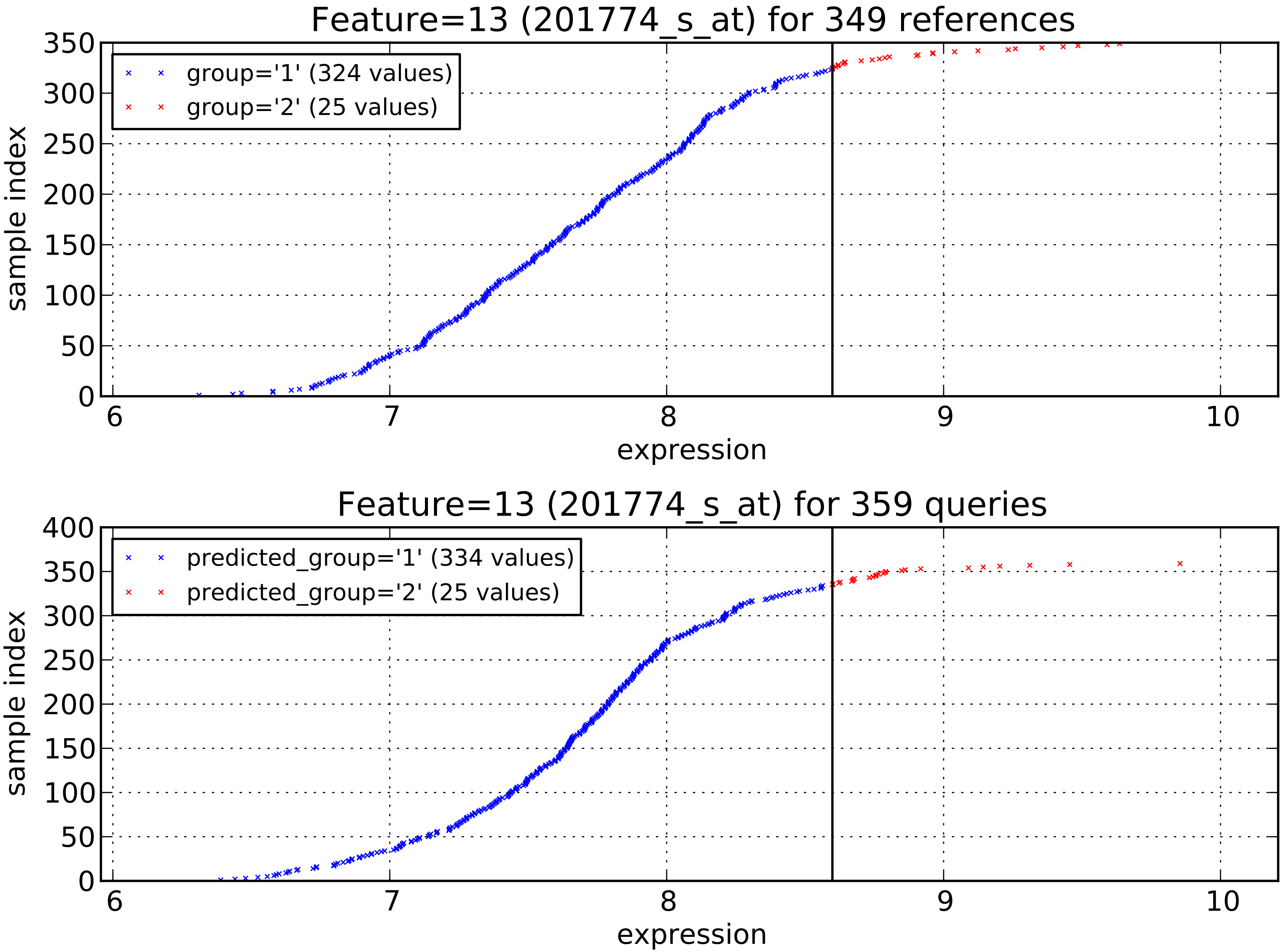
Feature=13 (201774_s_at) for 349 references
group='1' (324 values)
group='2' (25 values)
sample index
expression
0
50
100
150
200
250
300
350
6
7
8
9
10
Feature=13 (201774_s_at) for 359 queries
predicted_group='1' (334 values)
predicted_group='2' (25 values)
sample index
expression
0
50
100
150
200
250
300
350
400
6
7
8
9
10

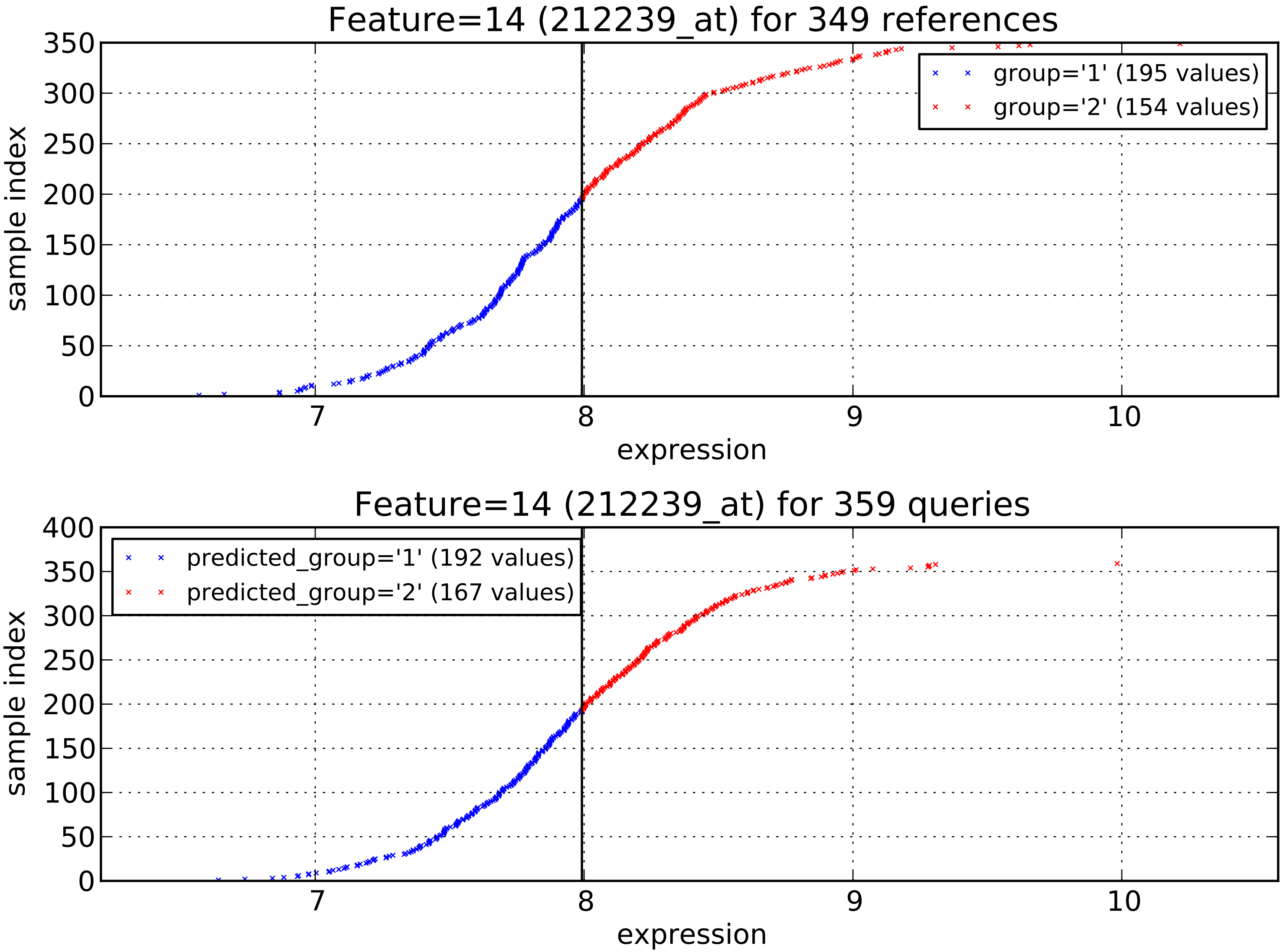

Feature=14 (212239_at) for 349 references
group='1' (195 values)
group='2' (154 values)
sample index
expression
0
50
100
150
200
250
300
350
7
8
9
10
Feature=14 (212239_at) for 359 queries
predicted_group='1' (192 values)
predicted_group='2' (167 values)
sample index
expression
0
50
100
150
200
250
300
350
400
7
8
9
10

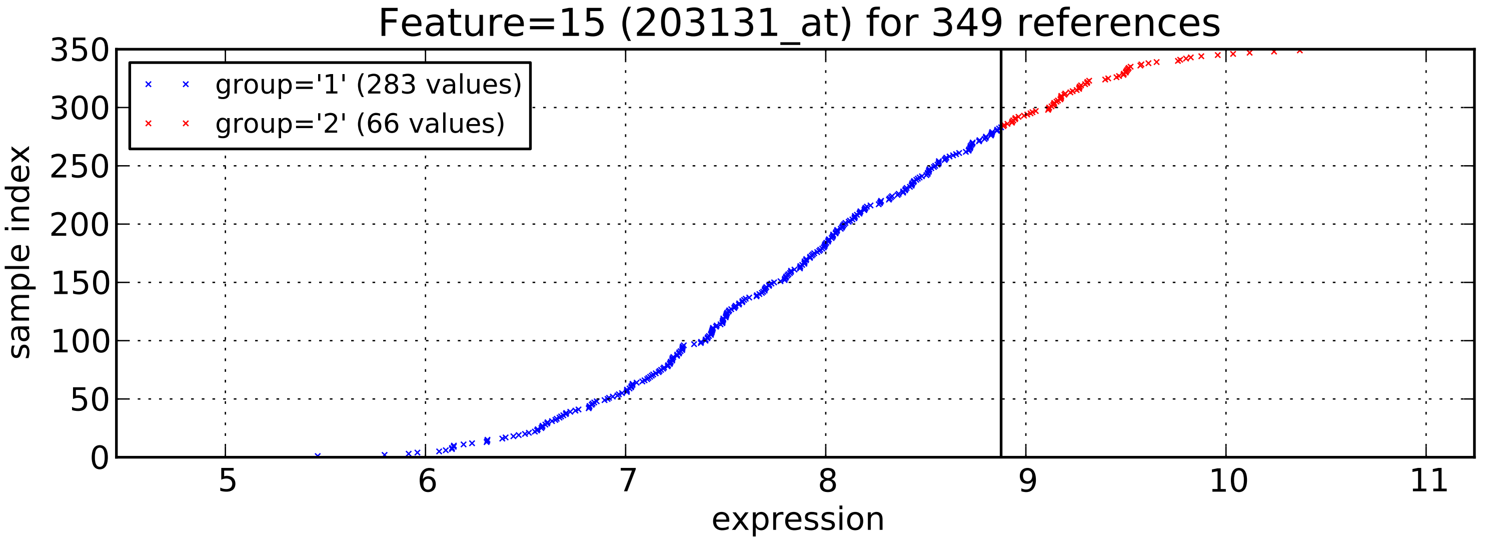
Feature=15 (203131_at) for 349 references
group='1' (283 values)
group='2' (66 values)
sample index
expression
0
50
100
150
200
250
300
350
5
6
7
8
9
10
11

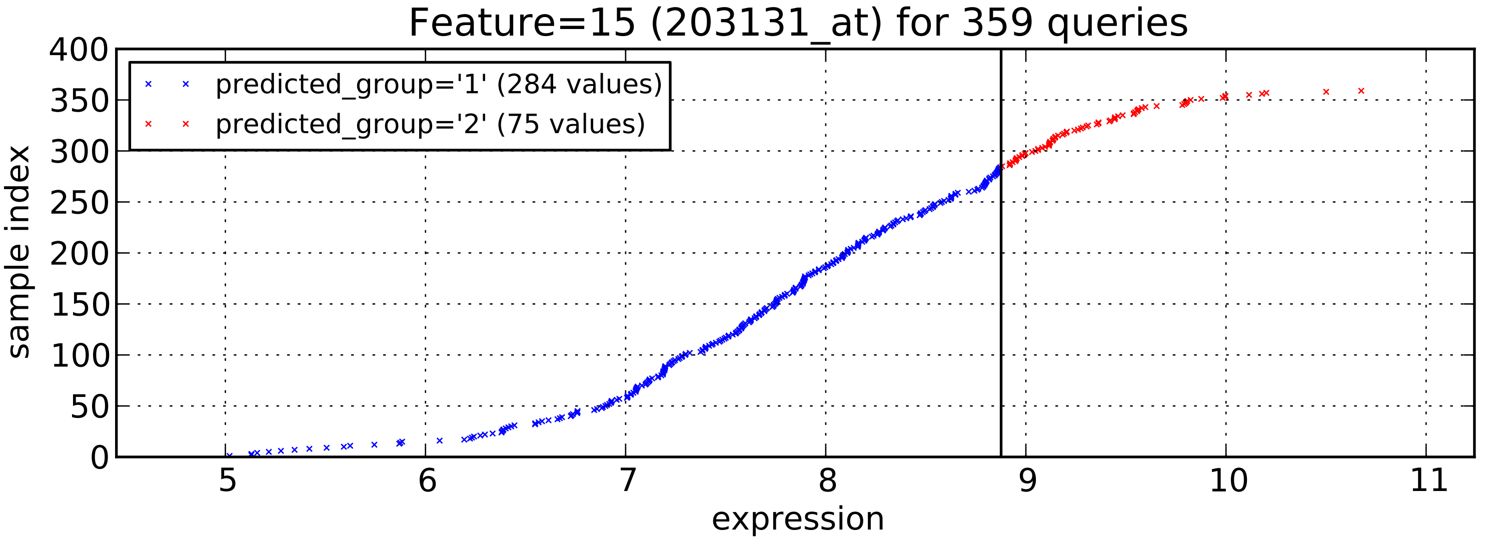
Feature=15 (203131_at) for 359 queries
predicted_group='1' (284 values)
predicted_group='2' (75 values)
sample index
expression
0
50
100
150
200
250
300
350
400
5
6
7
8
9
10
11

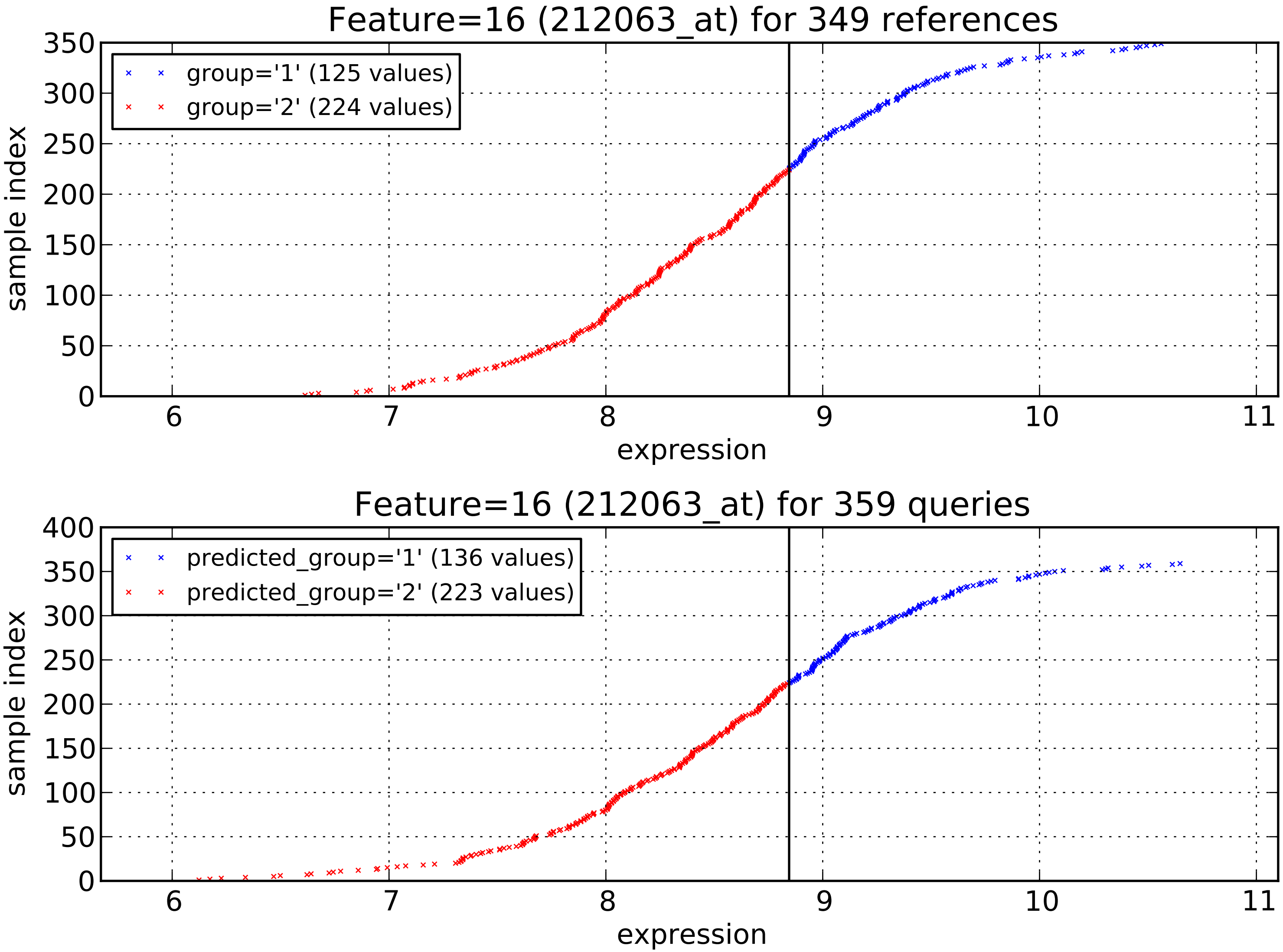

Feature=16 (212063_at) for 349 references
group='1' (125 values)
group='2' (224 values)
sample index
expression
Feature=16 (212063_at) for 359 queries
predicted_group='1' (136 values)
predicted_group='2' (223 values)
sample index
expression

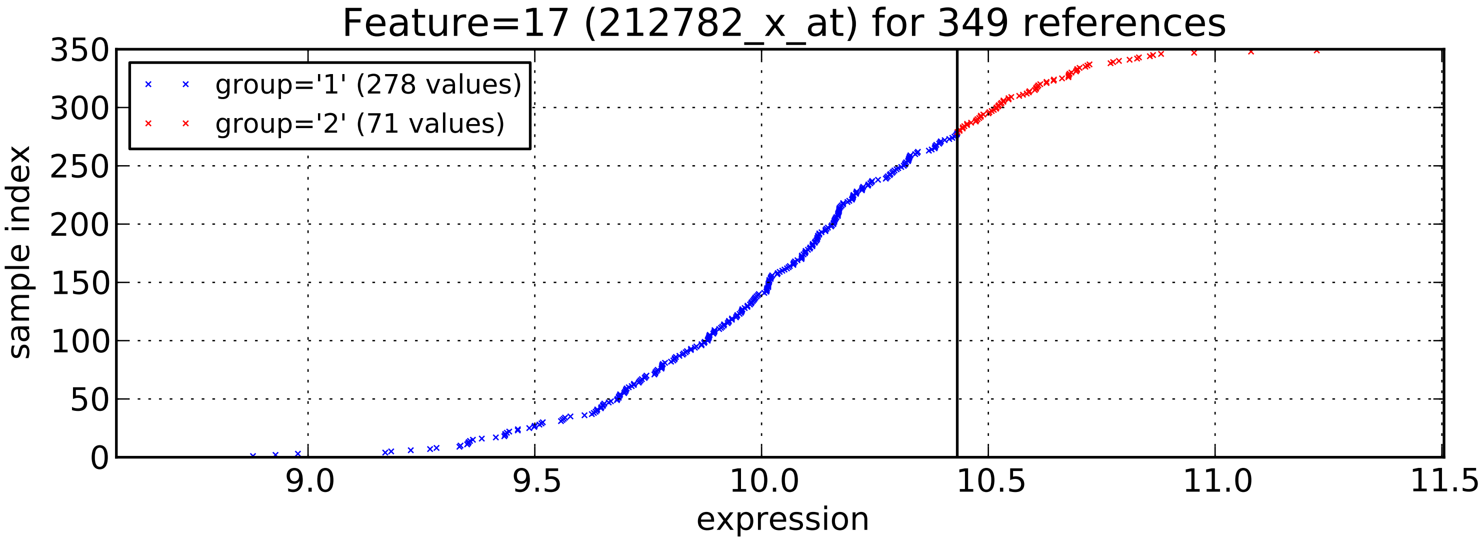
Feature=17 (212782_x_at) for 349 references
group='1' (278 values)
group='2' (71 values)
sample index
0
50
100
150
200
250
300
350
9.0
9.5
10.0
10.5
11.0
11.5
expression

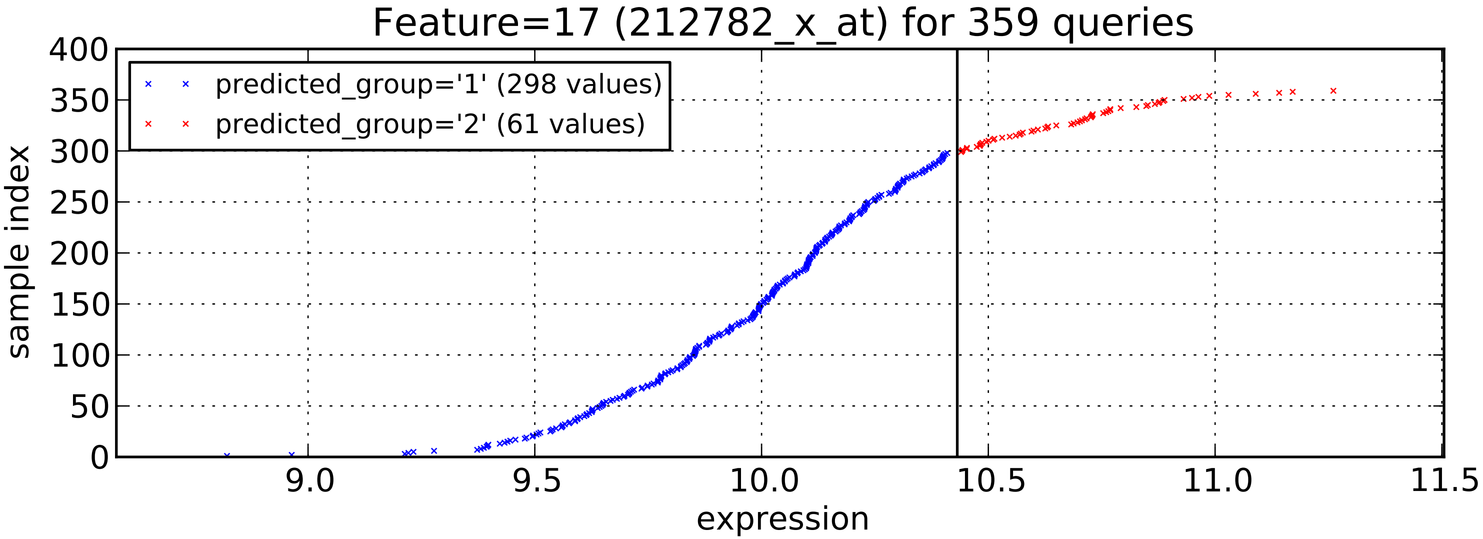
Feature=17 (212782_x_at) for 359 queries
predicted_group='1' (298 values)
predicted_group='2' (61 values)
sample index
0
50
100
150
200
250
300
350
400
9.0
9.5
10.0
10.5
11.0
11.5
expression

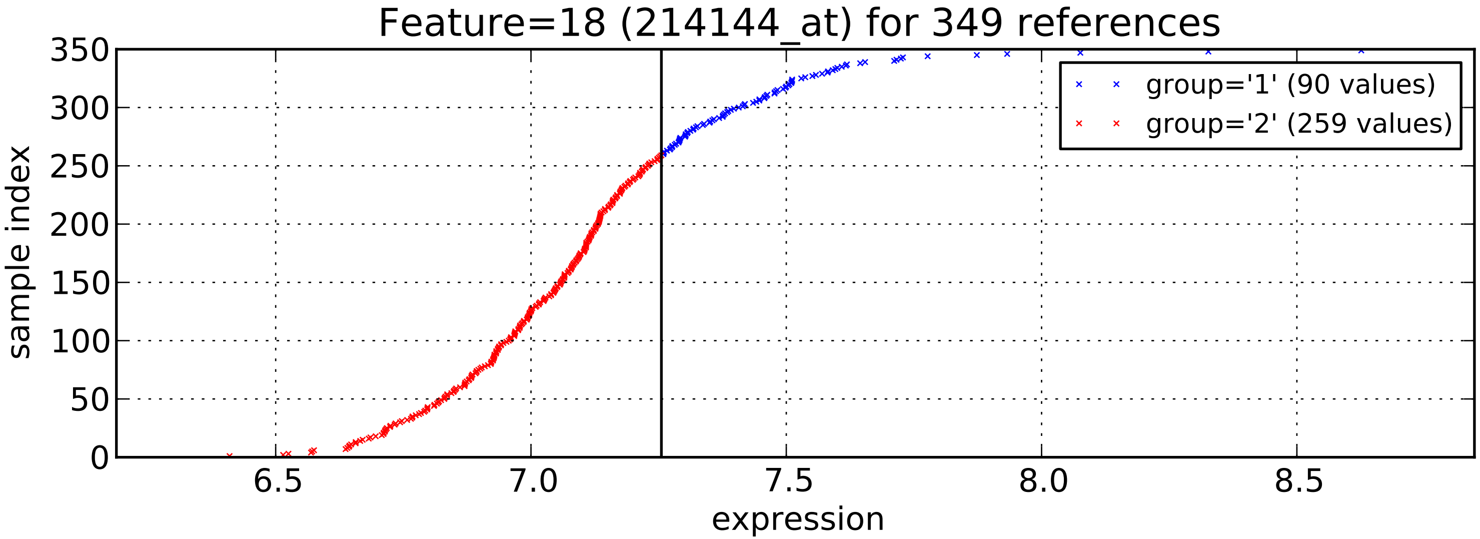
Feature=18 (214144_at) for 349 references
sample index
expression
group='1' (90 values)
group='2' (259 values)
0
50
100
150
200
250
300
350
6.5
7.0
7.5
8.0
8.5
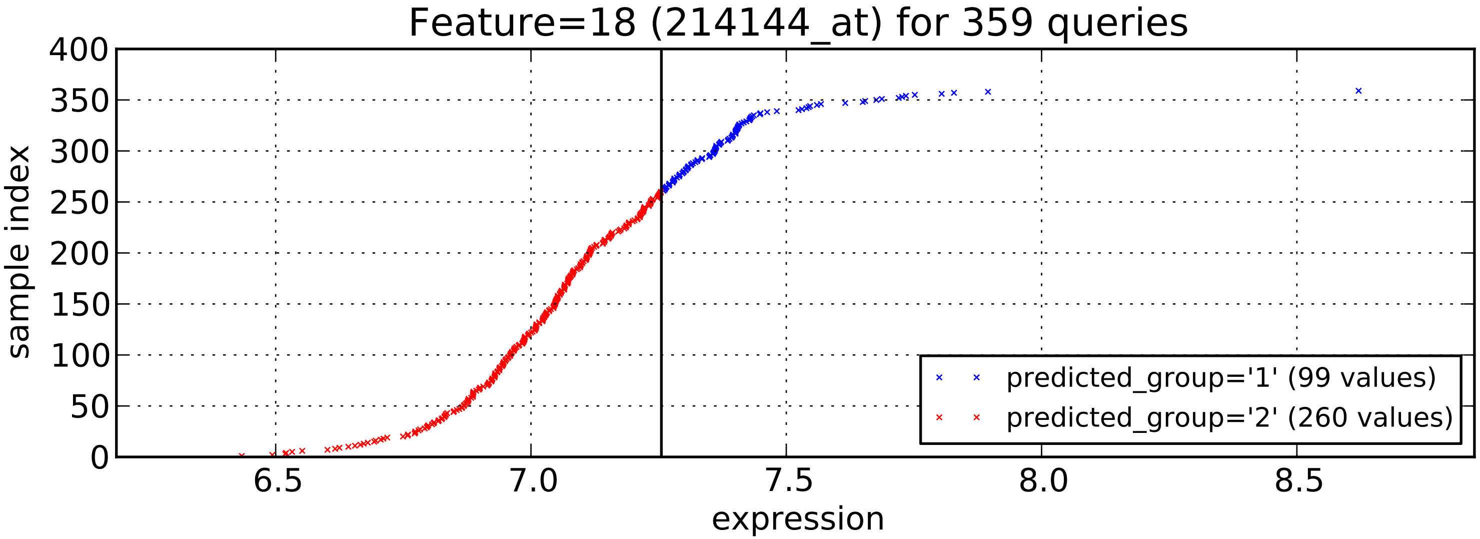
Feature=18 (214144_at) for 359 queries
sample index
expression
predicted_group='1' (99 values)
predicted_group='2' (260 values)
0
50
100
150
200
250
300
350
400
6.5
7.0
7.5
8.0
8.5

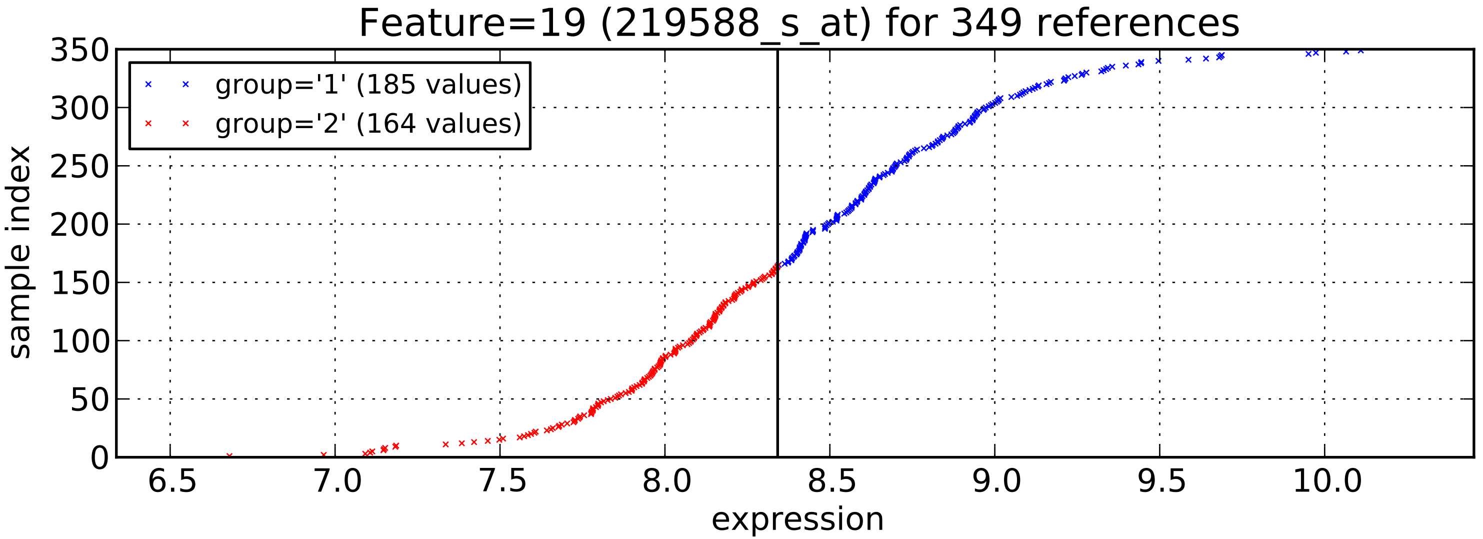
Feature=19 (219588_s_at) for 349 references
group='1' (185 values)
group='2' (164 values)
sample index
expression
0
50
100
150
200
250
300
350
6.5
7.0
7.5
8.0
8.5
9.0
9.5
10.0

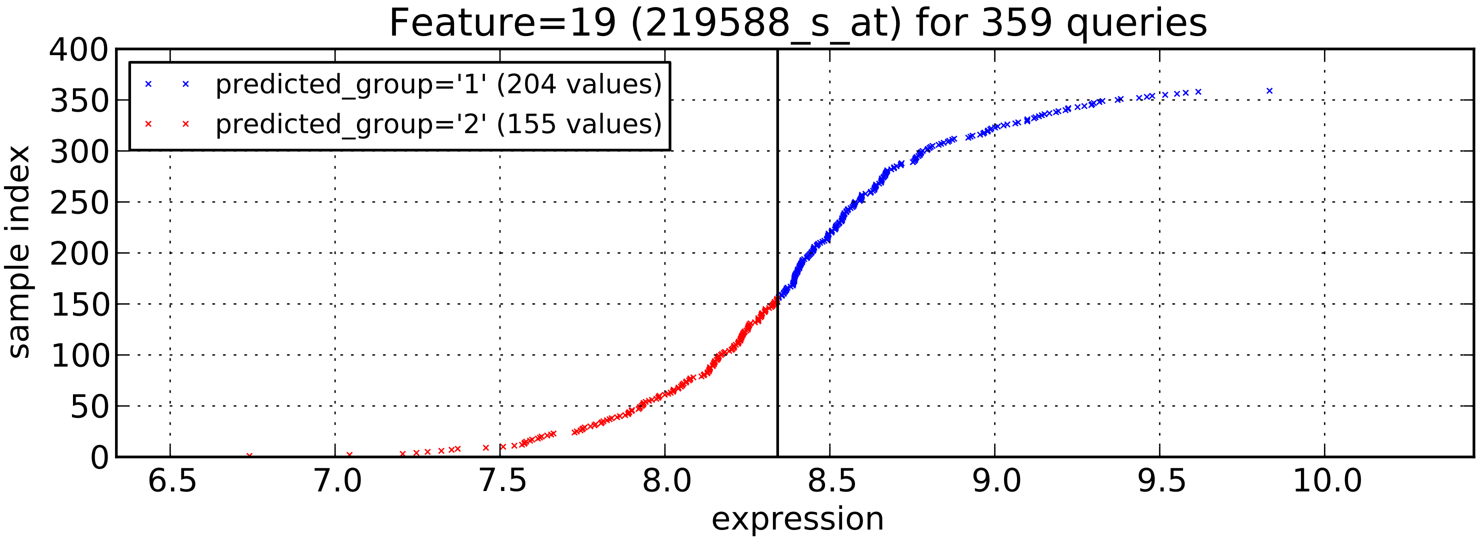
Feature=19 (219588_s_at) for 359 queries
predicted_group='1' (204 values)
predicted_group='2' (155 values)
sample index
expression
0
50
100
150
200
250
300
350
400
6.5
7.0
7.5
8.0
8.5
9.0
9.5
10.0

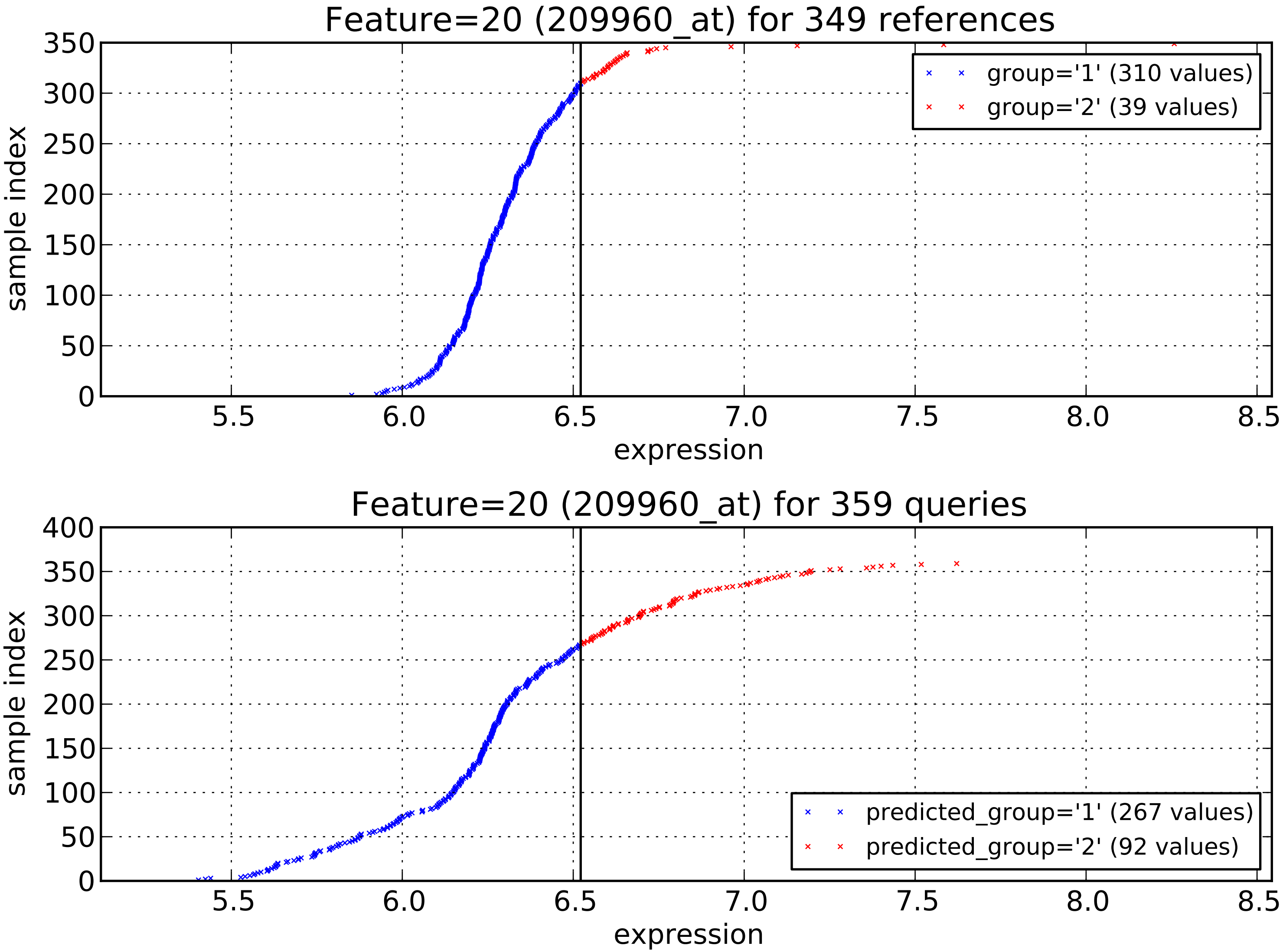

Feature=20 (209960_at) for 349 references
sample index
expression
group='1' (310 values)
group='2' (39 values)
Feature=20 (209960_at) for 359 queries
sample index
expression
predicted_group='1' (267 values)
predicted_group='2' (92 values)

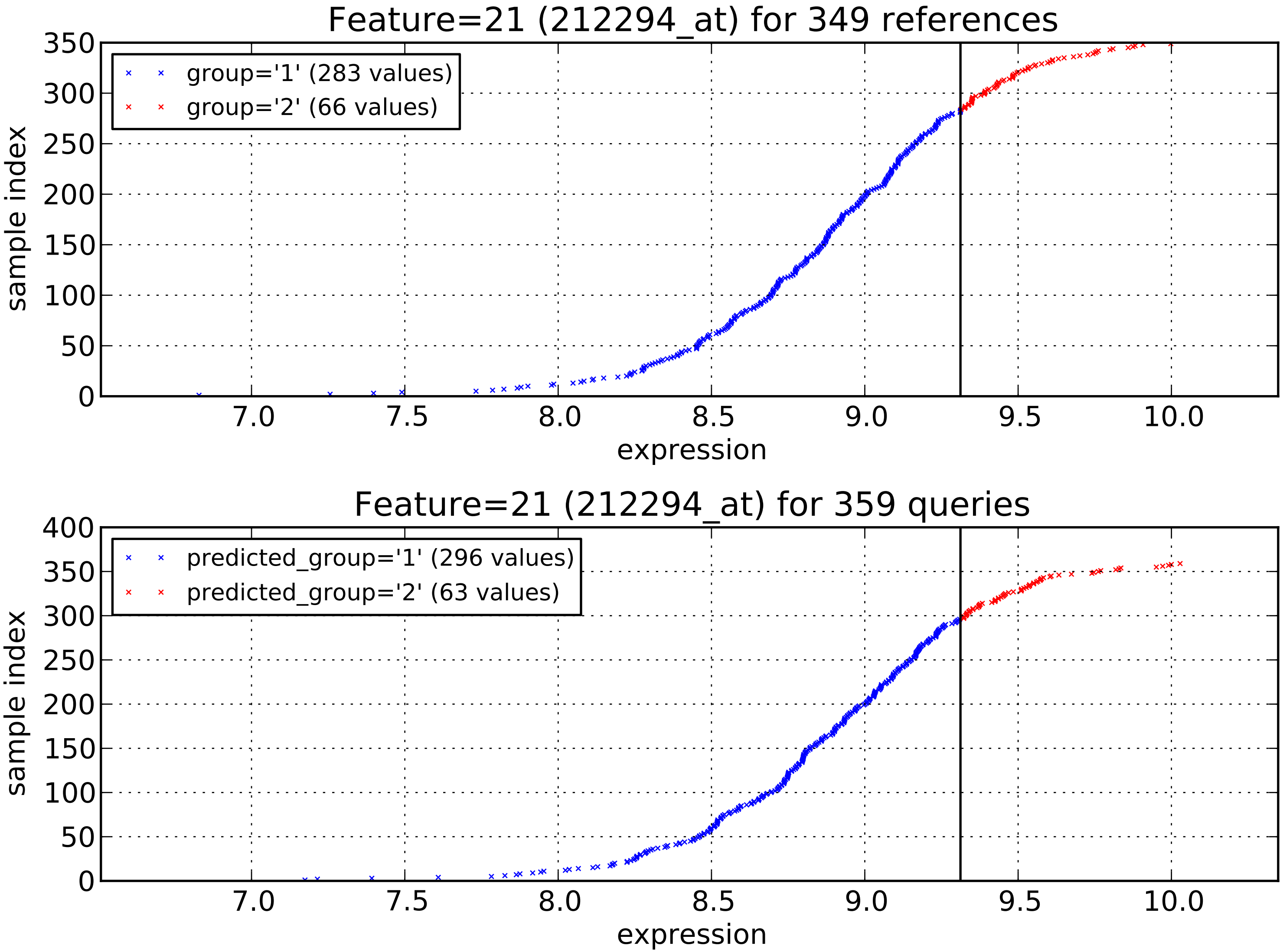
Feature=21 (212294_at) for 349 references
group='1' (283 values)
group='2' (66 values)
sample index
expression
Feature=21 (212294_at) for 359 queries
predicted_group='1' (296 values)
predicted_group='2' (63 values)
sample index
expression

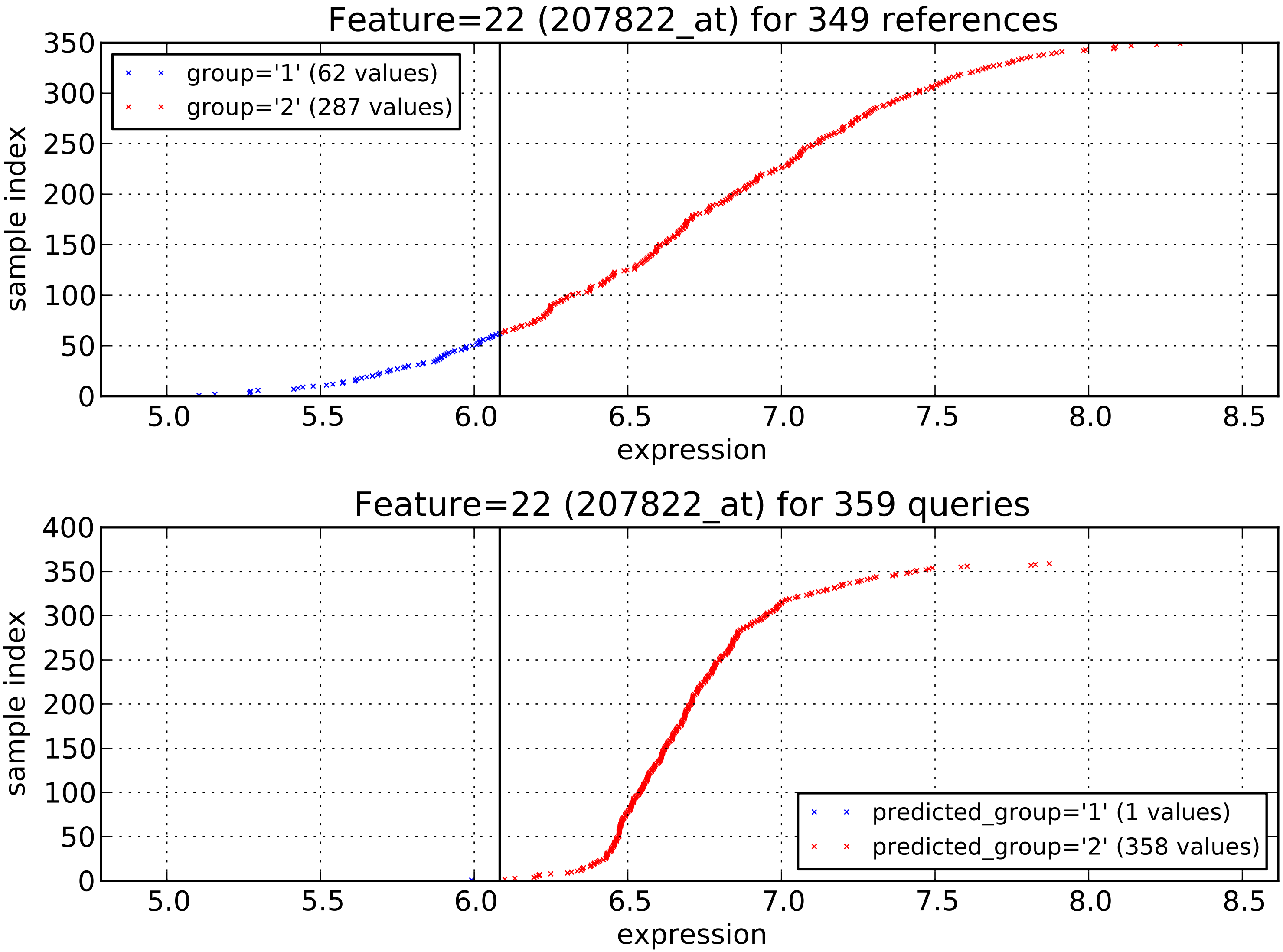
Feature=22 (207822_at) for 349 references
group='1' (62 values)
group='2' (287 values)
sample index
expression
0
50
100
150
200
250
300
350
5.0
5.5
6.0
6.5
7.0
7.5
8.0
8.5
Feature=22 (207822_at) for 359 queries
predicted_group='1' (1 values)
predicted_group='2' (358 values)
sample index
expression
0
50
100
150
200
250
300
350
400
5.0
5.5
6.0
6.5
7.0
7.5
8.0
8.5

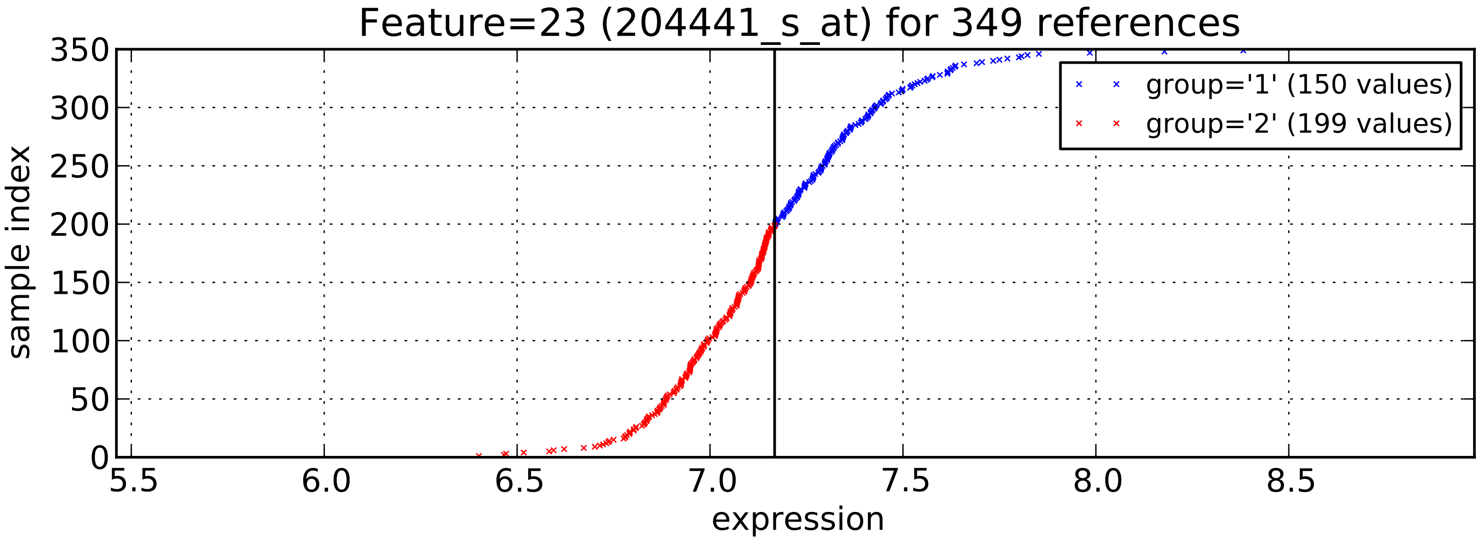
Feature=23 (204441_s_at) for 349 references
sample index
expression
group='1' (150 values)
group='2' (199 values)
0
50
100
150
200
250
300
350
5.5
6.0
6.5
7.0
7.5
8.0
8.5

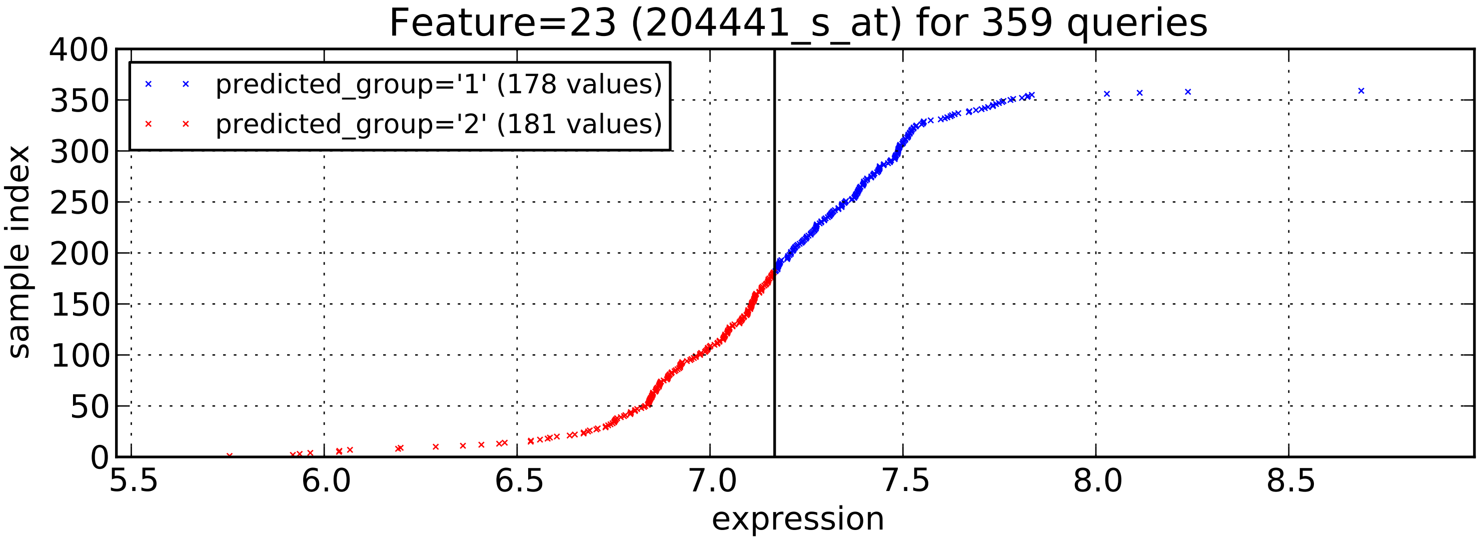
Feature=23 (204441_s_at) for 359 queries
sample index
expression
predicted_group='1' (178 values)
predicted_group='2' (181 values)
0
50
100
150
200
250
300
350
400
5.5
6.0
6.5
7.0
7.5
8.0
8.5

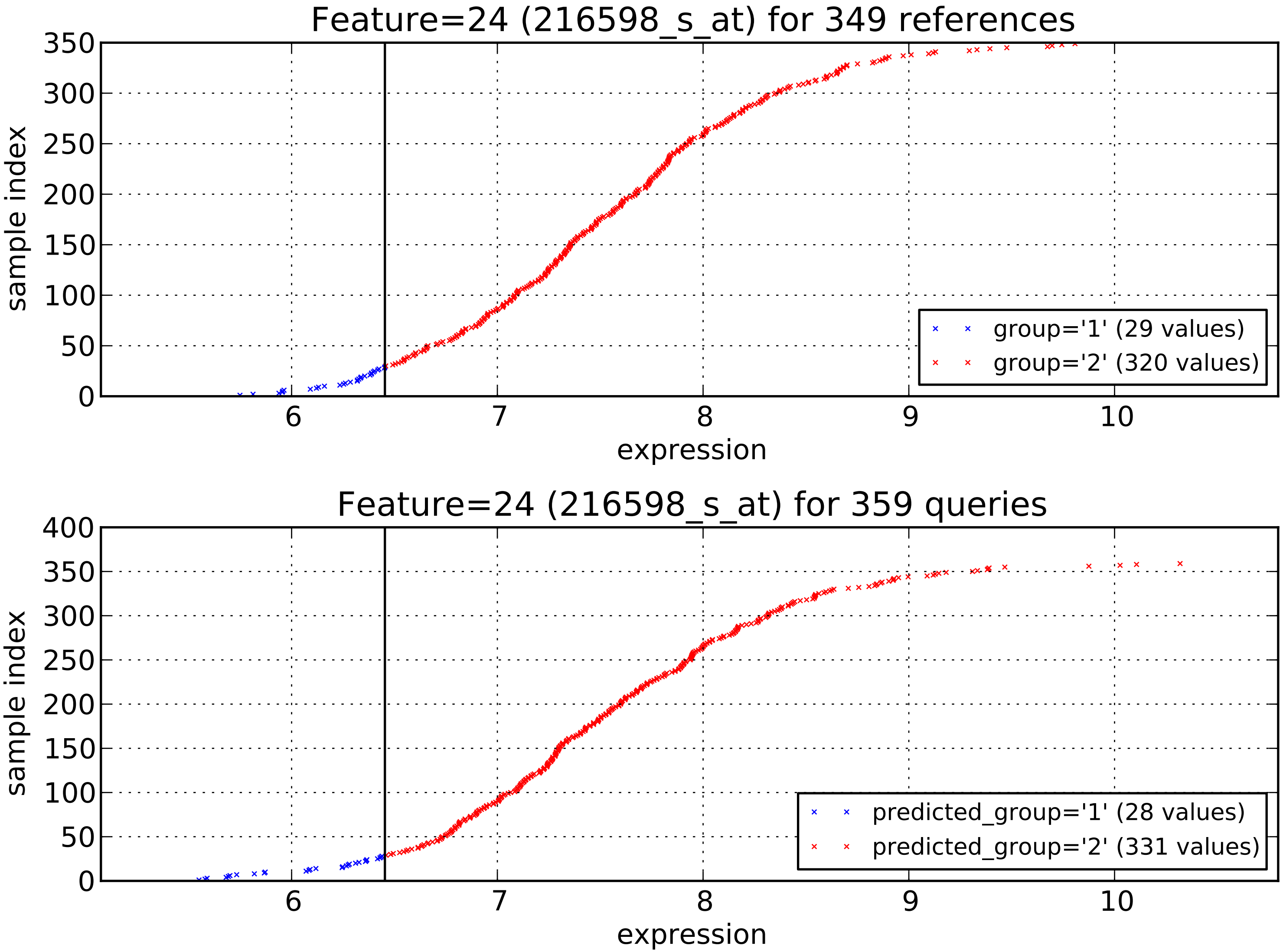
Feature=24 (216598_s_at) for 349 references
sample index
expression
group='1' (29 values)
group='2' (320 values)
Feature=24 (216598_s_at) for 359 queries
sample index
expression
predicted_group='1' (28 values)
predicted_group='2' (331 values)

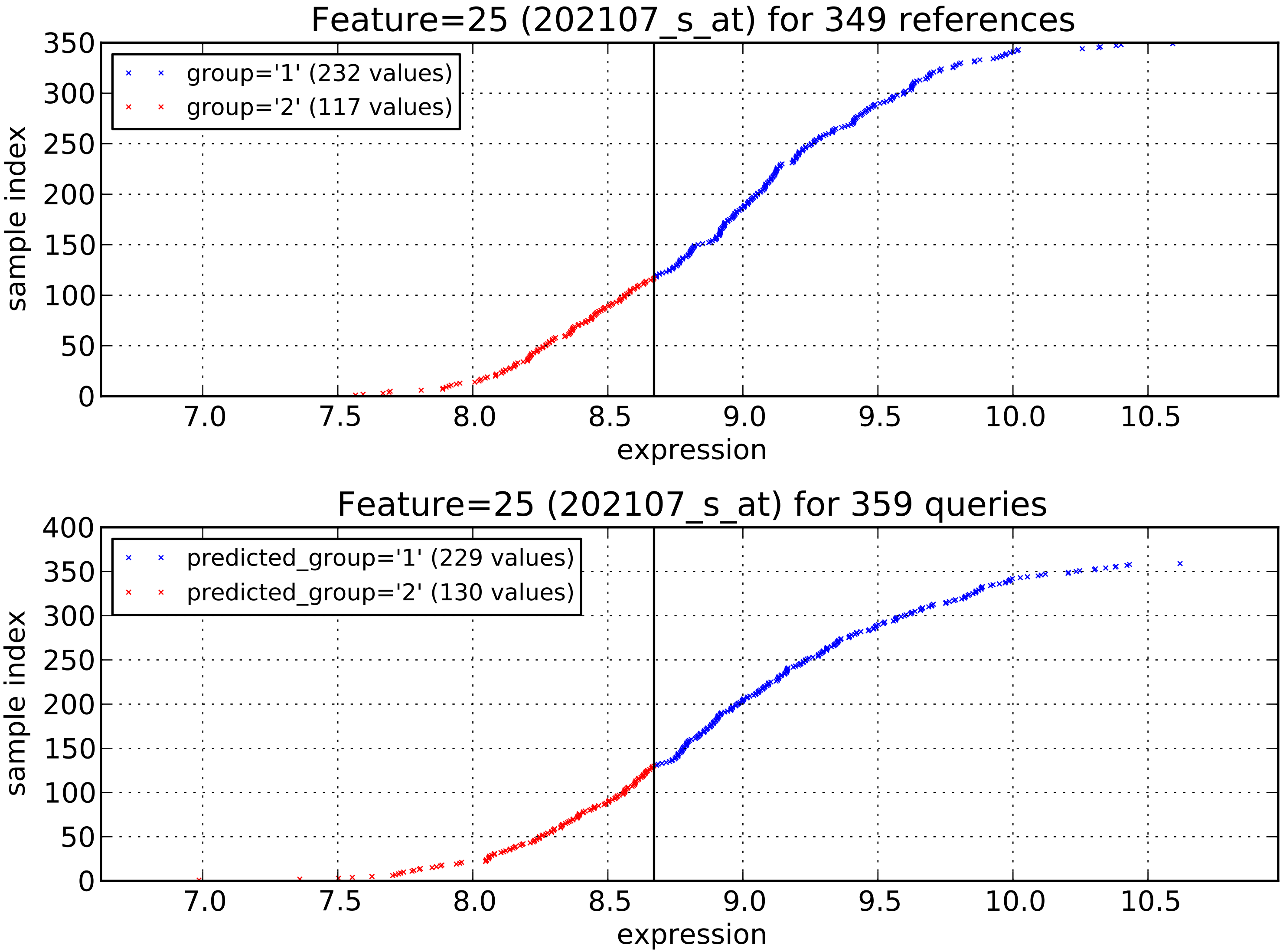

Feature=25 (202107_s_at) for 349 references
group='1' (232 values)
group='2' (117 values)
sample index
expression
0
50
100
150
200
250
300
350
7.0
7.5
8.0
8.5
9.0
9.5
10.0
10.5
Feature=25 (202107_s_at) for 359 queries
predicted_group='1' (229 values)
predicted_group='2' (130 values)
sample index
expression
400

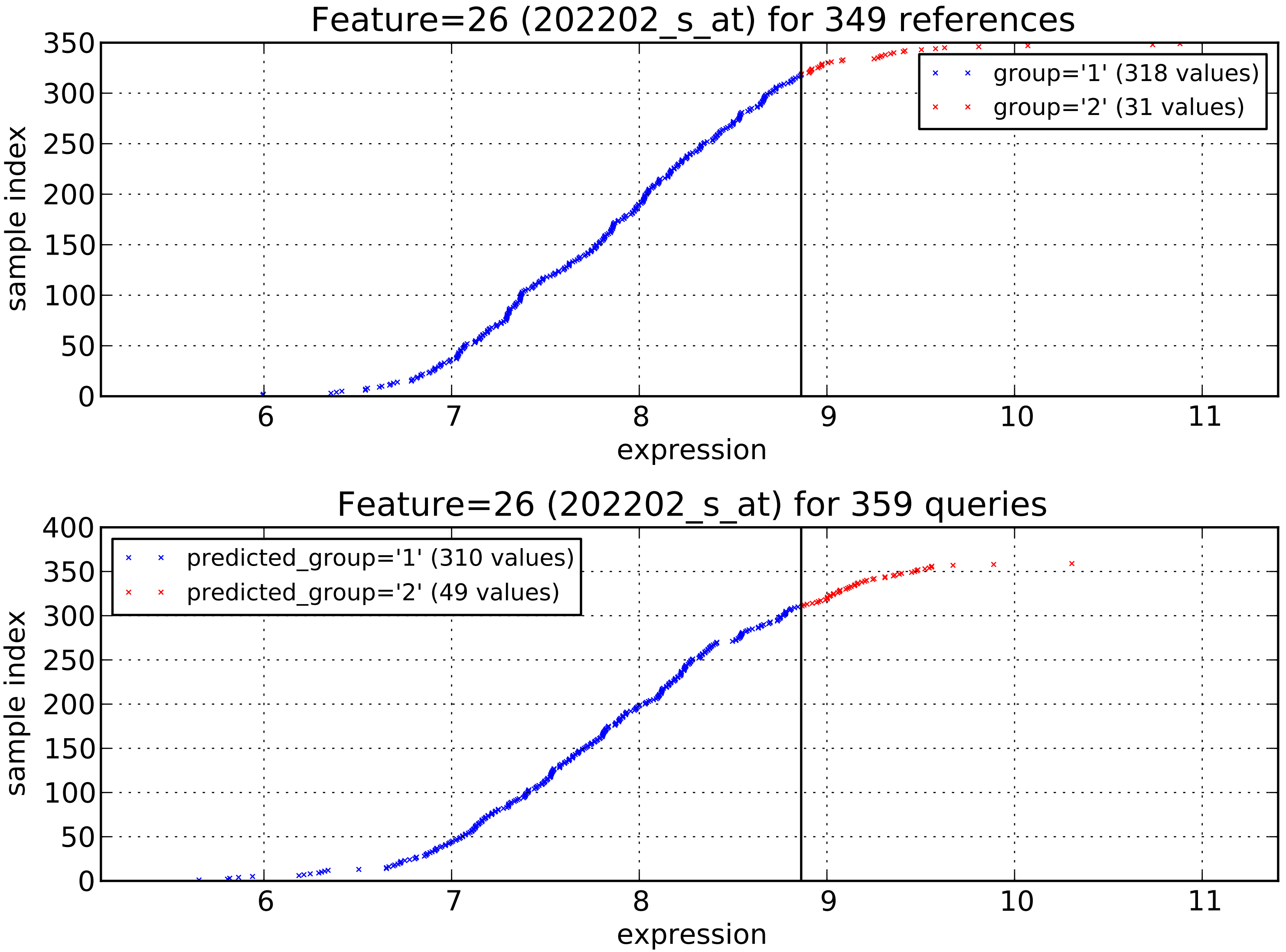

Feature=26 (202202_s_at) for 349 references
sample index
expression
group='1' (318 values)
group='2' (31 values)
Feature=26 (202202_s_at) for 359 queries
sample index
expression
predicted_group='1' (310 values)
predicted_group='2' (49 values)

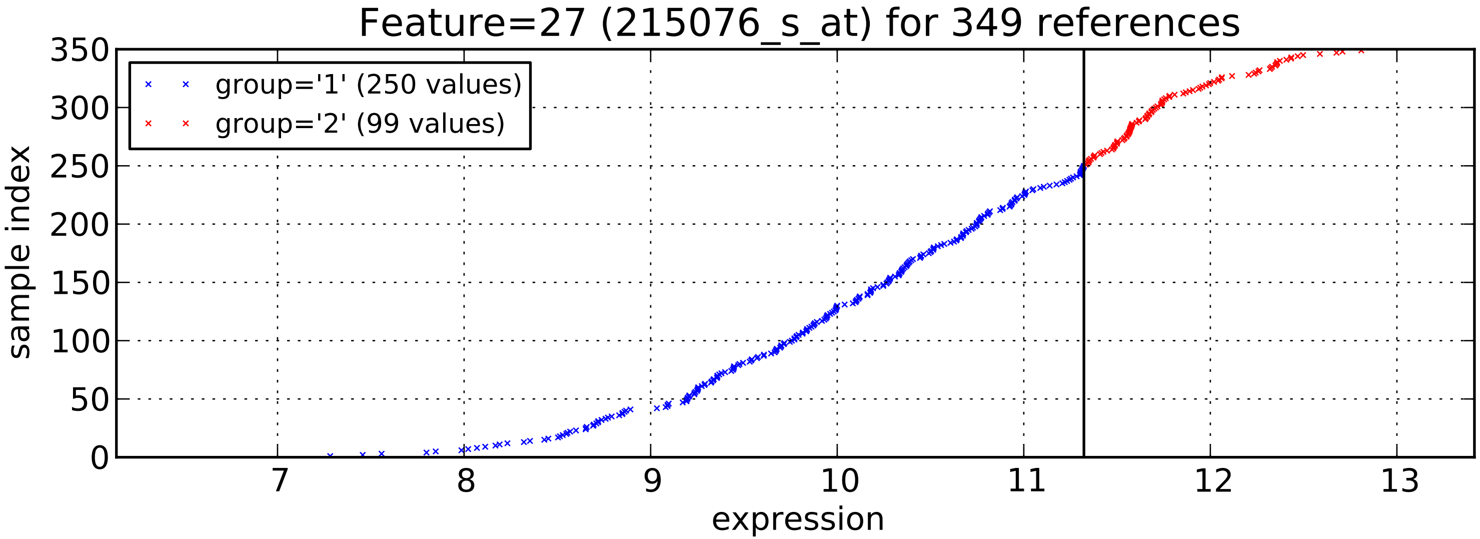
Feature=27 (215076_s_at) for 349 references
sample index
expression
group='1' (250 values)
group='2' (99 values)
0
50
100
150
200
250
300
350
7
8
9
10
11
12
13

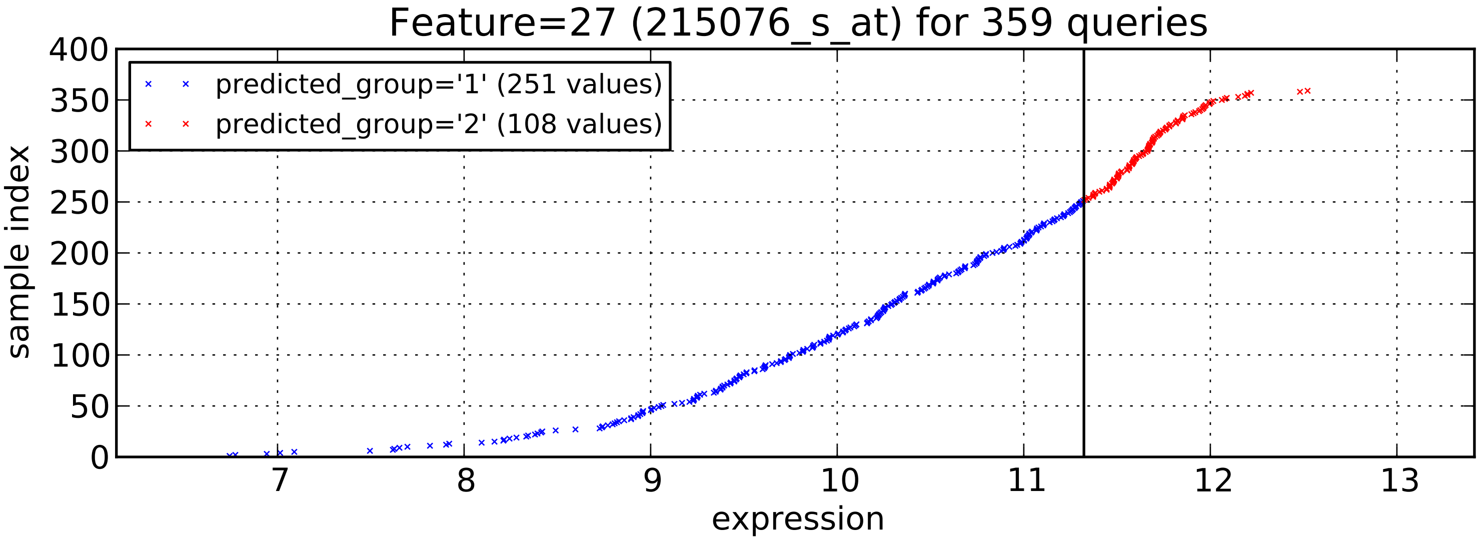
Feature=27 (215076_s_at) for 359 queries
sample index
expression
predicted_group='1' (251 values)
predicted_group='2' (108 values)
0
50
100
150
200
250
300
350
400
7
8
9
10
11
12
13

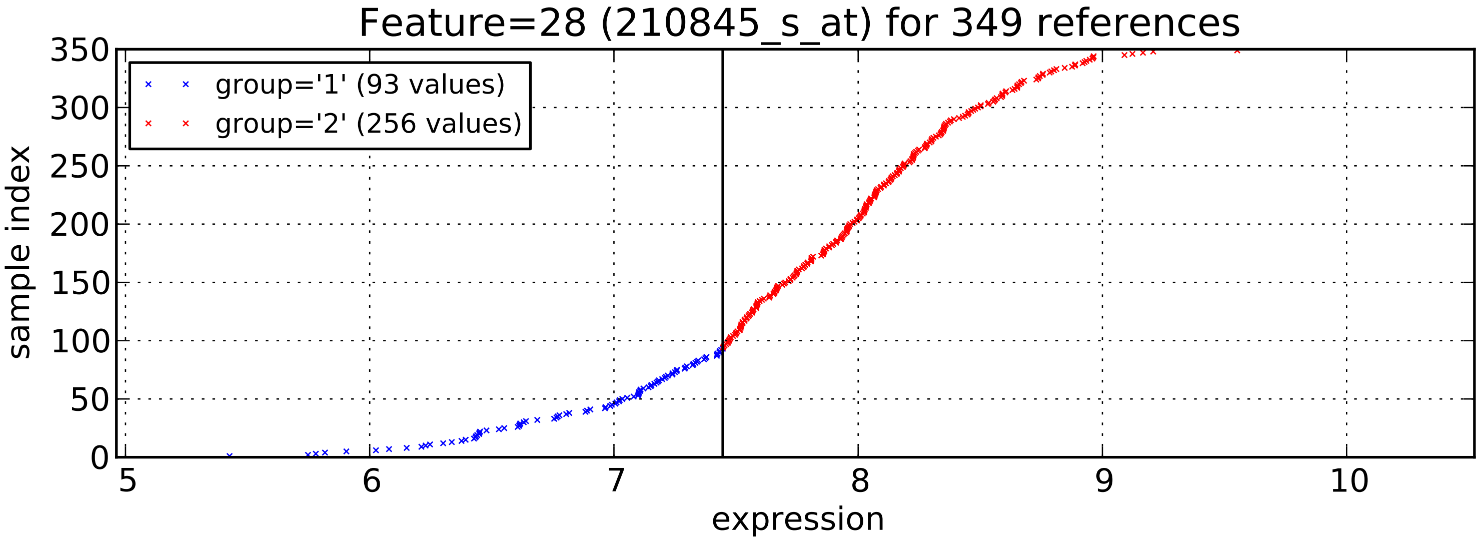
Feature=28 (210845_s_at) for 349 references
group='1' (93 values)
group='2' (256 values)
sample index
expression
0
50
100
150
200
250
300
350
5
6
7
8
9
10

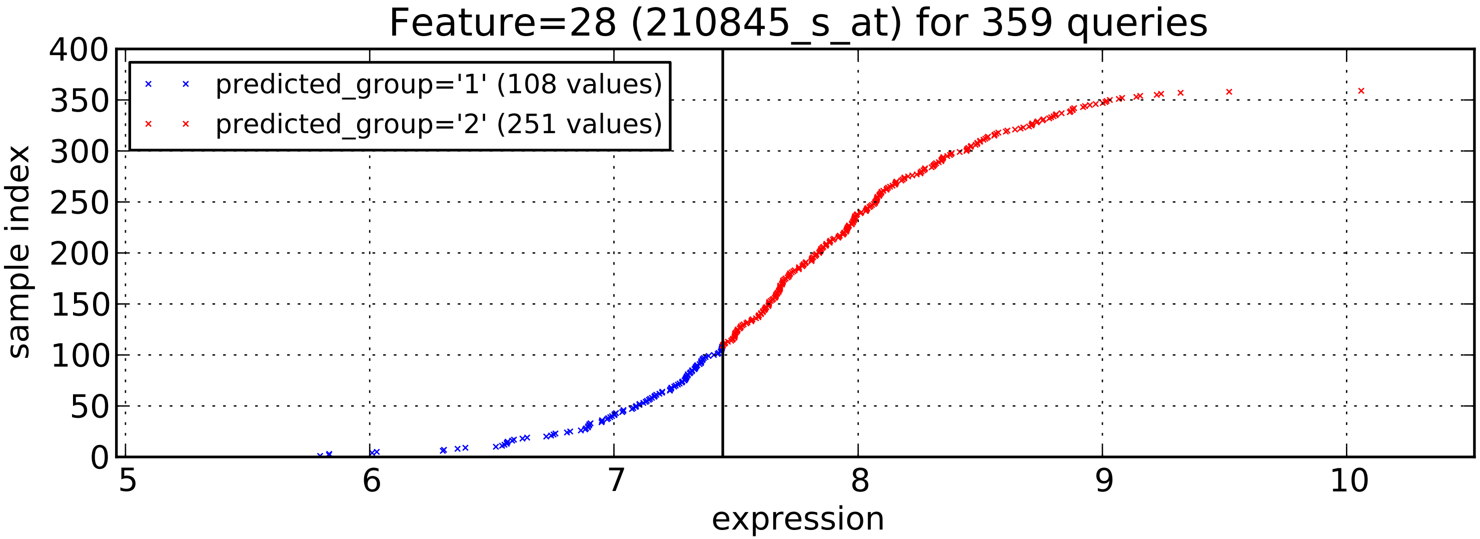
Feature=28 (210845_s_at) for 359 queries
predicted_group='1' (108 values)
predicted_group='2' (251 values)
sample index
expression
0
50
100
150
200
250
300
350
400
5
6
7
8
9
10

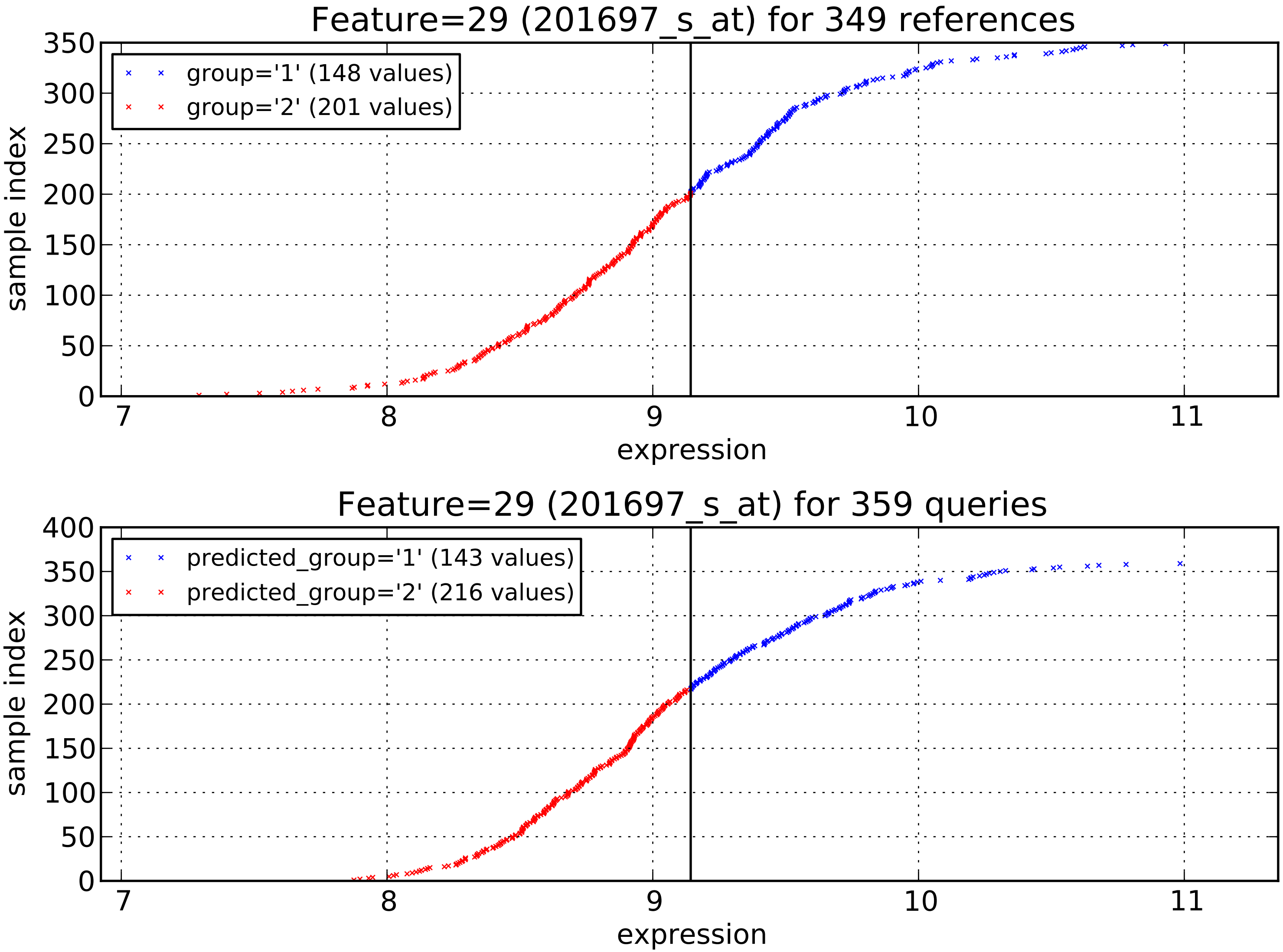

Feature=29 (201697_s_at) for 349 references
group='1' (148 values)
group='2' (201 values)
sample index
expression
0
50
100
150
200
250
300
350
7
8
9
10
11
Feature=29 (201697_s_at) for 359 queries
predicted_group='1' (143 values)
predicted_group='2' (216 values)
sample index
expression
0
50
100
150
200
250
300
350
400
7
8
9
10
11

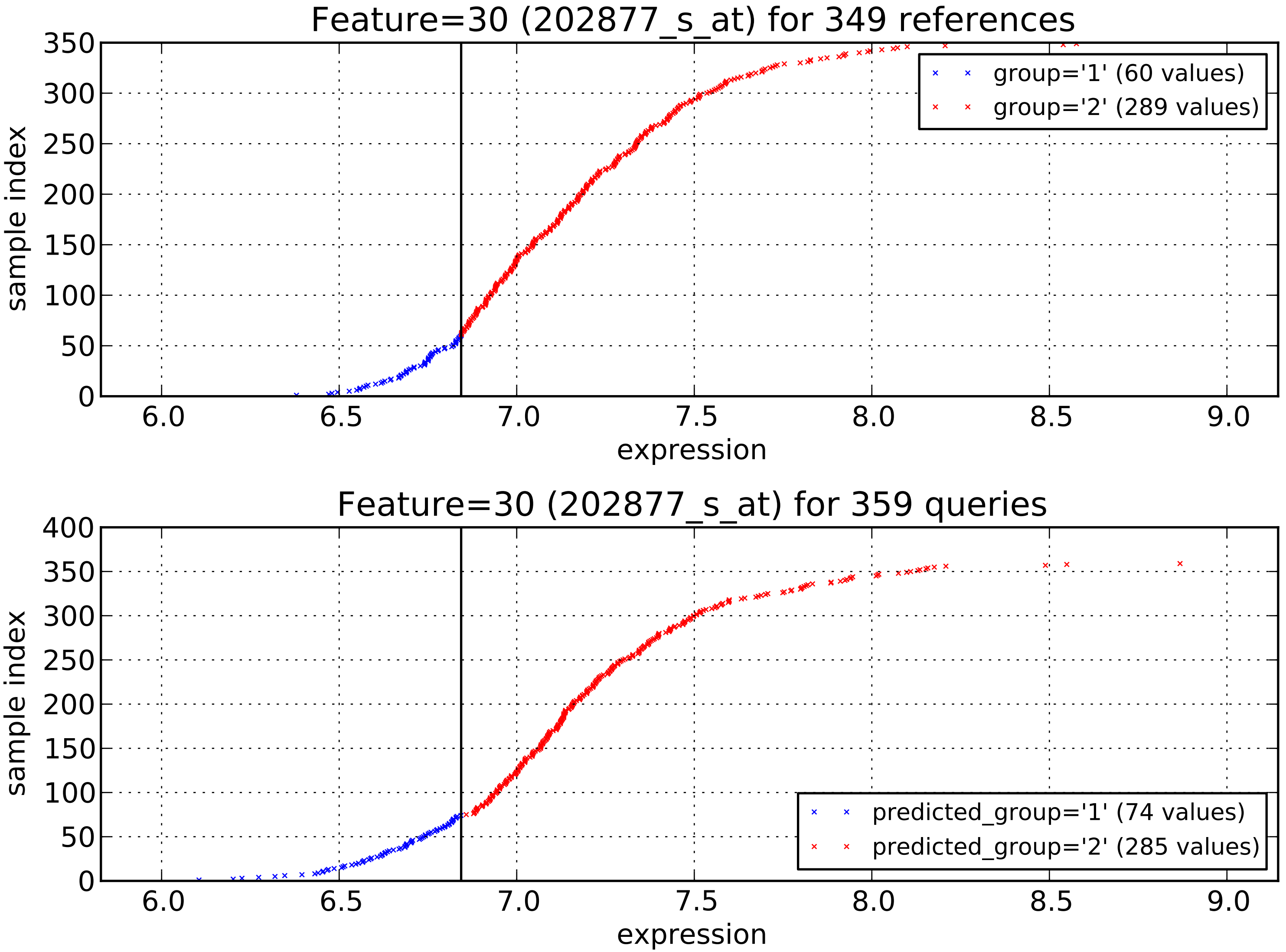

Feature=30 (202877_s_at) for 349 references
sample index
expression
group='1' (60 values)
group='2' (289 values)
0
50
100
150
200
250
300
350
6.0
6.5
7.0
7.5
8.0
8.5
9.0
Feature=30 (202877_s_at) for 359 queries
sample index
expression
predicted_group='1' (74 values)
predicted_group='2' (285 values)
0
50
100
150
200
250
300
350
400
6.0
6.5
7.0
7.5
8.0
8.5
9.0

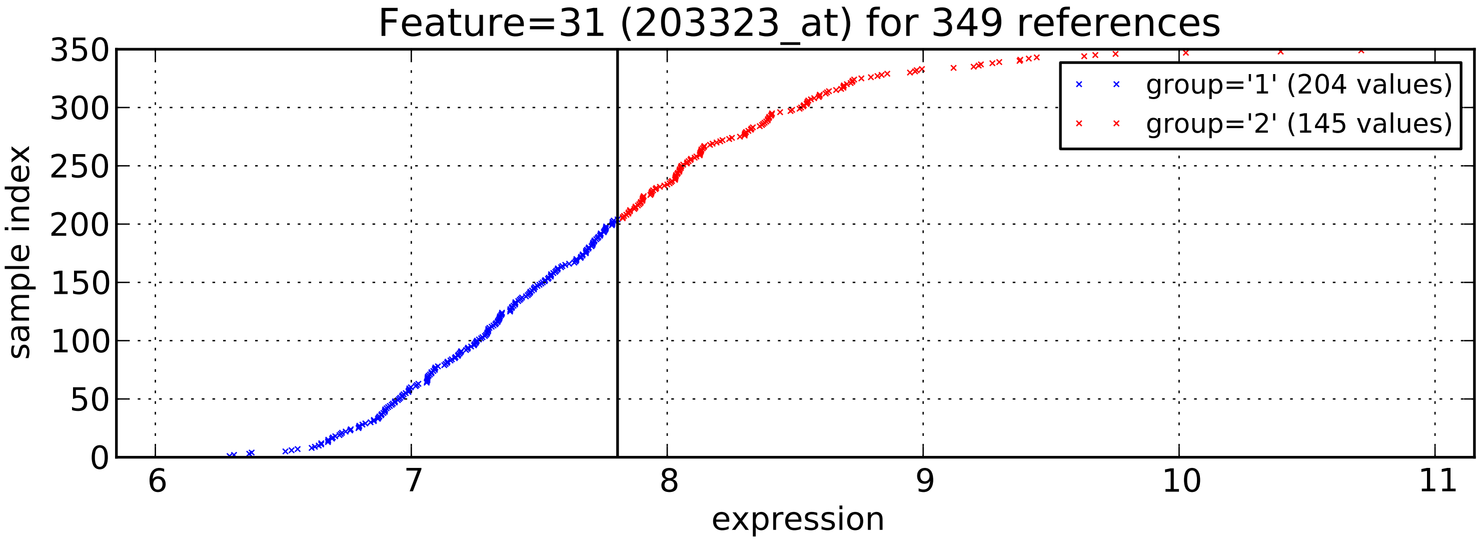

Feature=31 (203323_at) for 349 references
sample index
expression
group='1' (204 values)
group='2' (145 values)
0
50
100
150
200
250
300
350
6
7
8
9
10
11

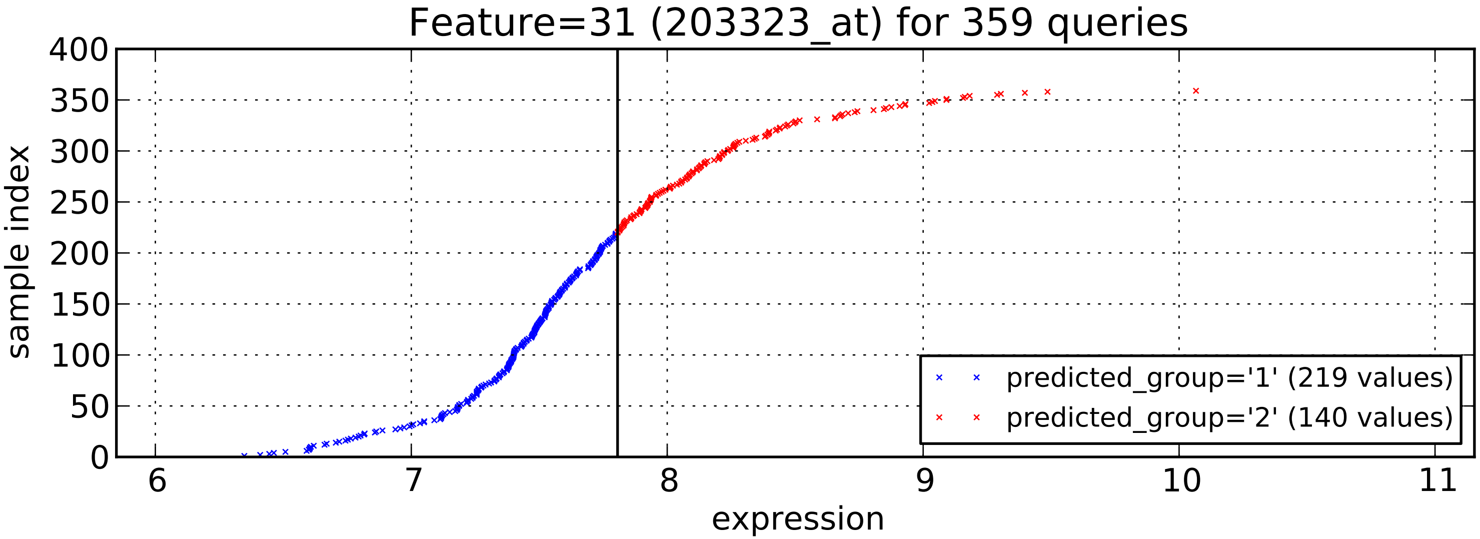

Feature=31 (203323_at) for 359 queries
sample index
expression
predicted_group='1' (219 values)
predicted_group='2' (140 values)
0
50
100
150
200
250
300
350
400
6
7
8
9
10
11

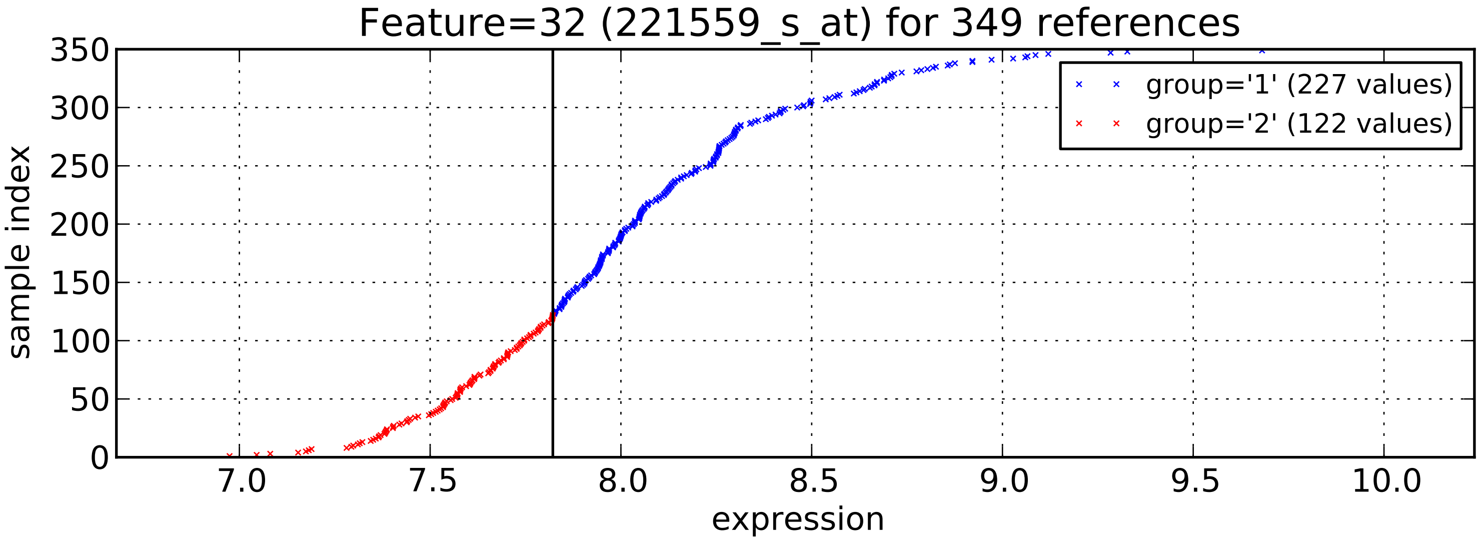
Feature=32 (221559_s_at) for 349 references
sample index
expression
group='1' (227 values)
group='2' (122 values)
0
50
100
150
200
250
300
350
7.0
7.5
8.0
8.5
9.0
9.5
10.0

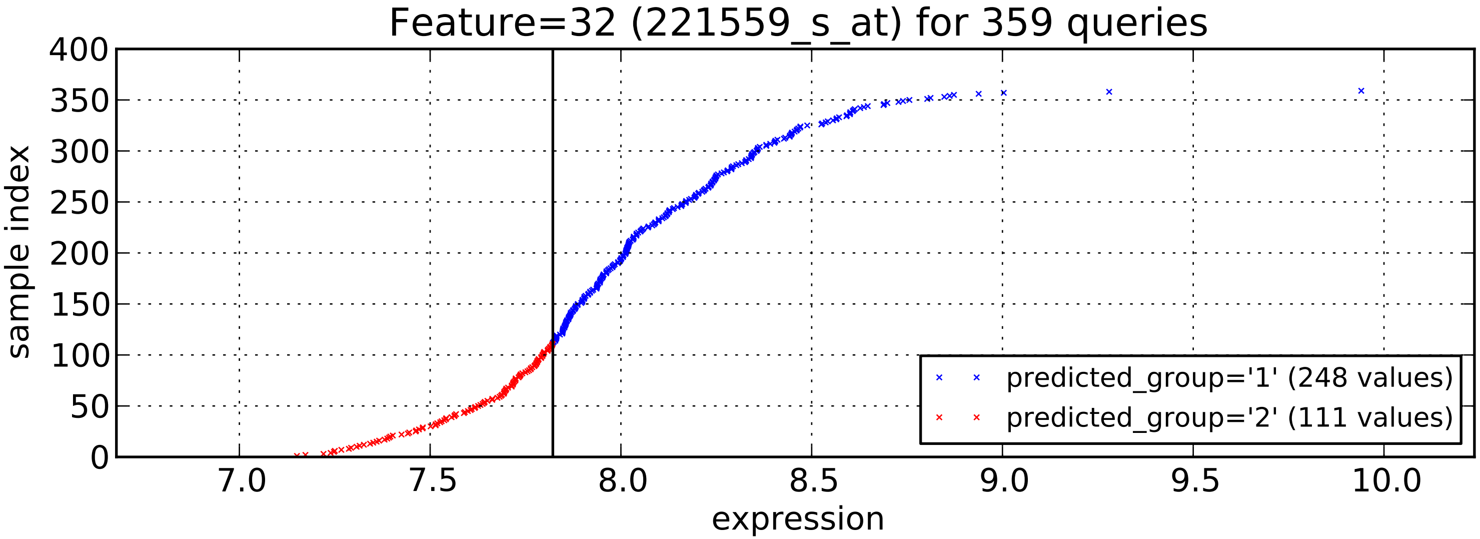
Feature=32 (221559_s_at) for 359 queries
sample index
expression
predicted_group='1' (248 values)
predicted_group='2' (111 values)
0
50
100
150
200
250
300
350
400
7.0
7.5
8.0
8.5
9.0
9.5
10.0

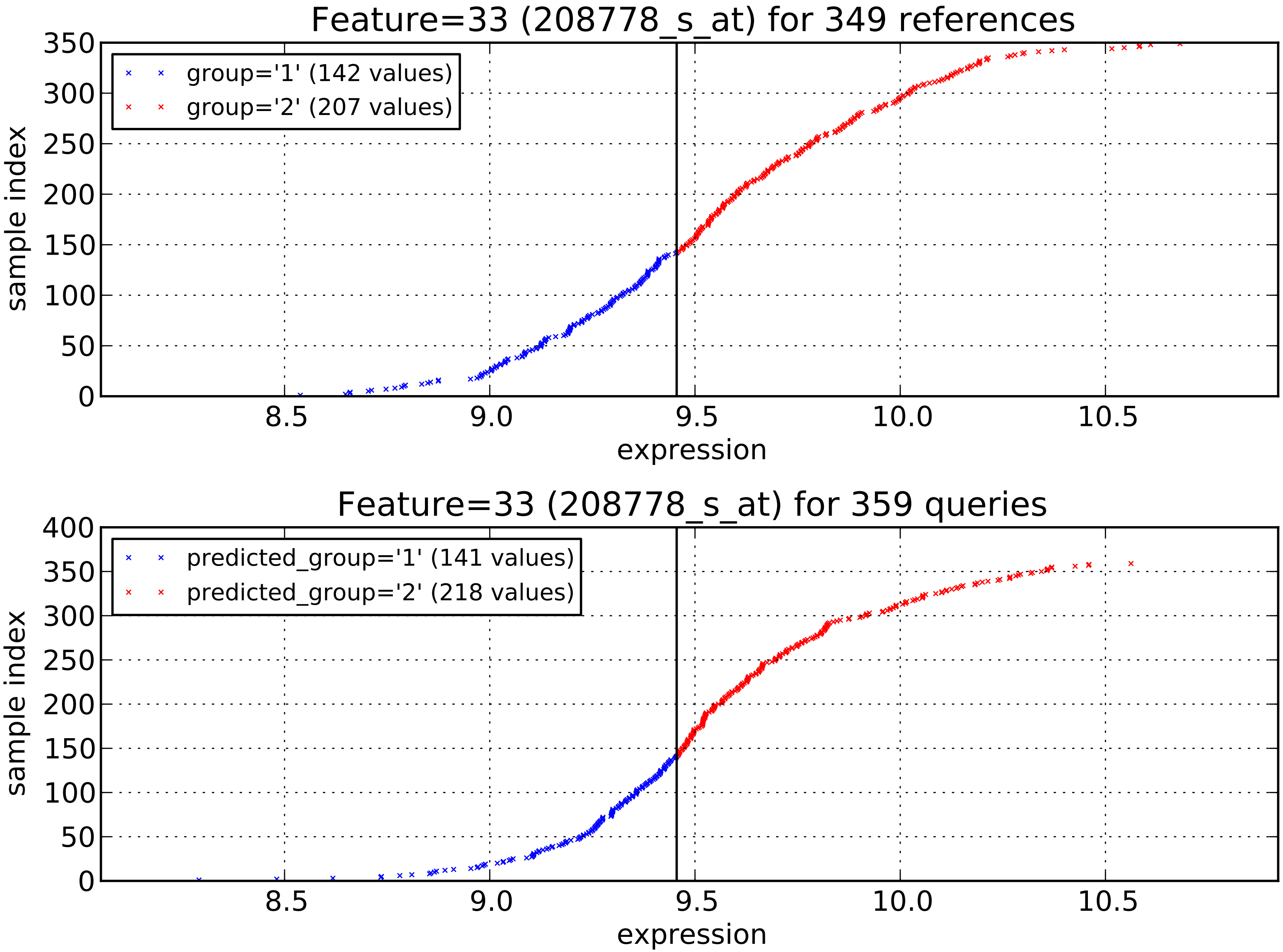
Feature=33 (208778_s_at) for 349 references
group='1' (142 values)
group='2' (207 values)
sample index
expression
0
50
100
150
200
250
300
350
8.5
9.0
9.5
10.0
10.5
Feature=33 (208778_s_at) for 359 queries
predicted_group='1' (141 values)
predicted_group='2' (218 values)
sample index
expression
0
50
100
150
200
250
300
350
400
8.5
9.0
9.5
10.0
10.5

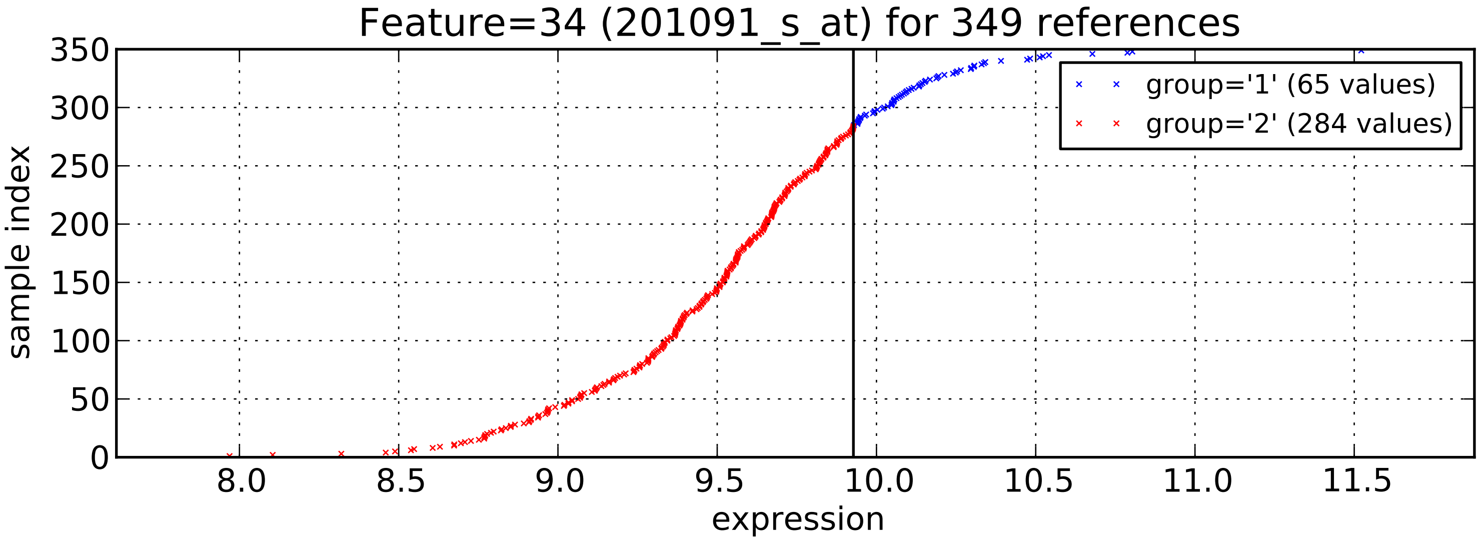
Feature=34 (201091_s_at) for 349 references
sample index
expression
group='1' (65 values)
group='2' (284 values)
0
50
100
150
200
250
300
350
8.0
8.5
9.0
9.5
10.0
10.5
11.0
11.5

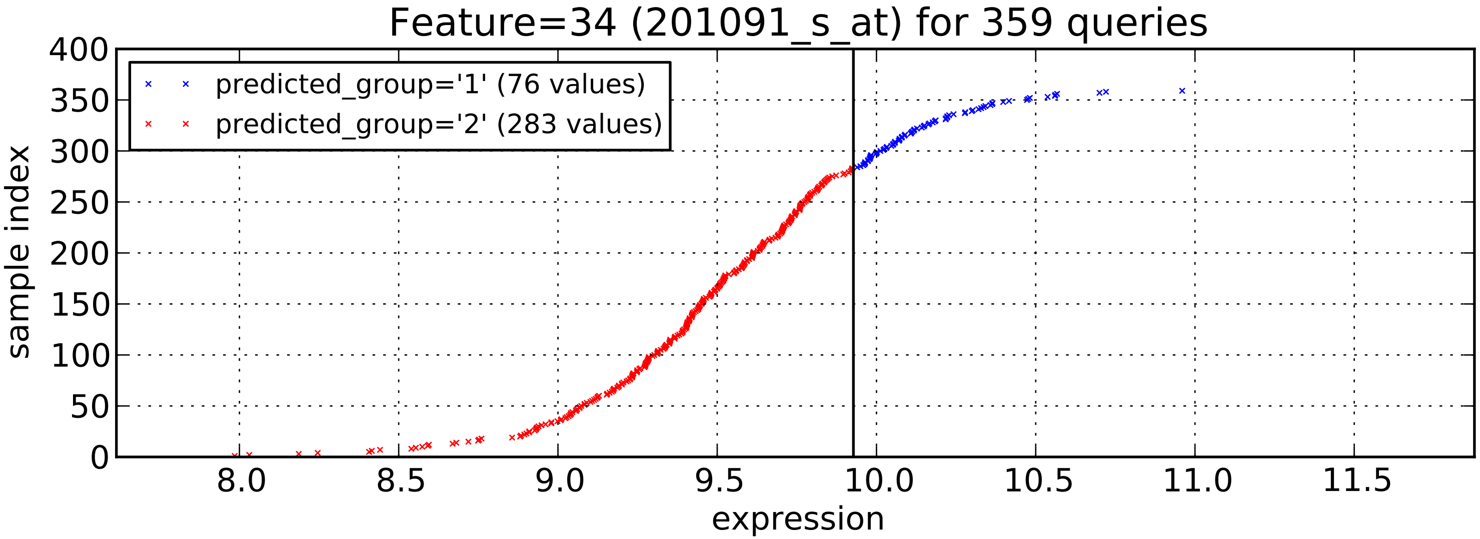
Feature=34 (201091_s_at) for 359 queries
sample index
expression
predicted_group='1' (76 values)
predicted_group='2' (283 values)
0
50
100
150
200
250
300
350
400
8.0
8.5
9.0
9.5
10.0
10.5
11.0
11.5

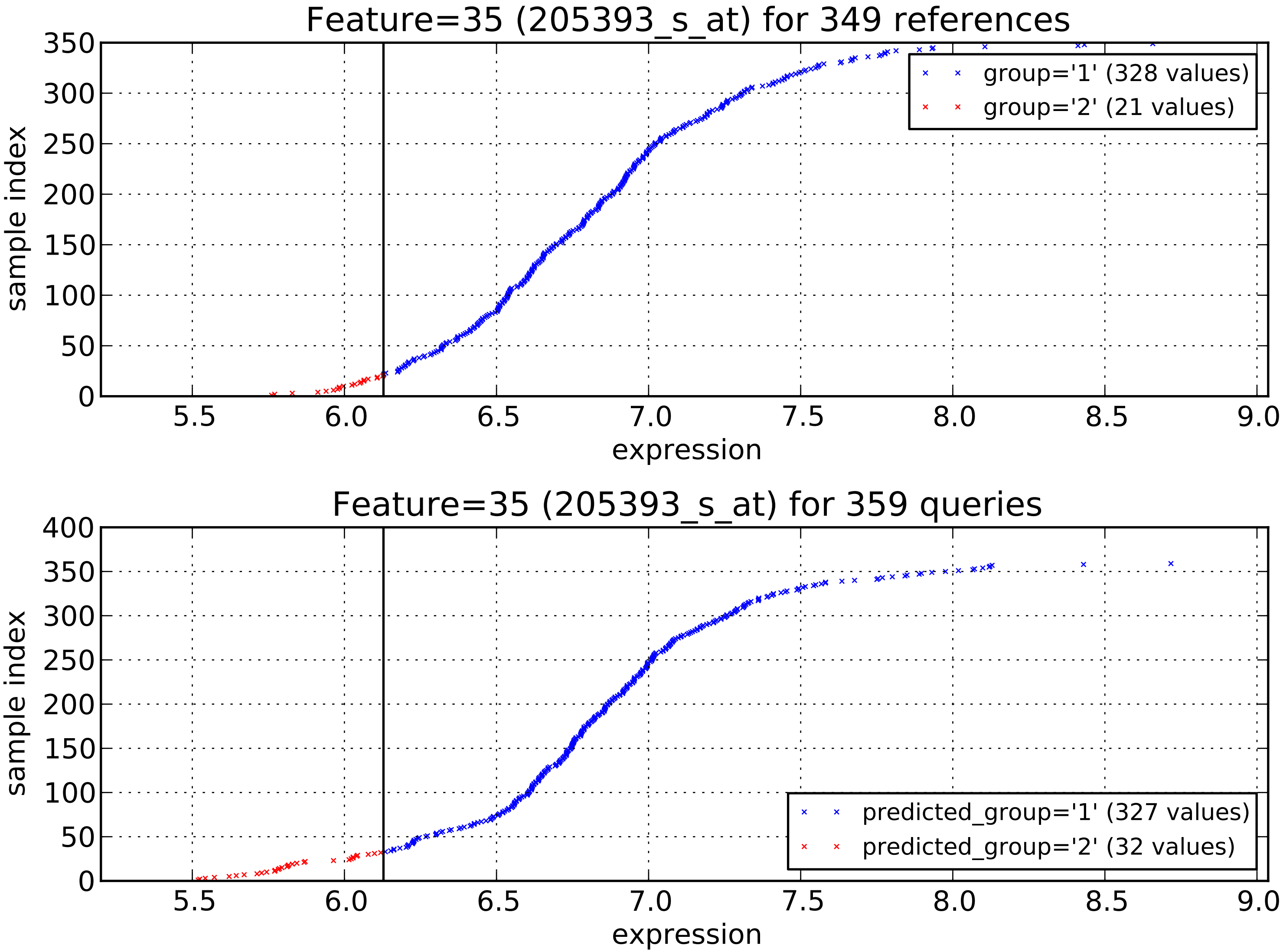

Feature=35 (205393_s_at) for 349 references
sample index
expression
group='1' (328 values)
group='2' (21 values)
Feature=35 (205393_s_at) for 359 queries
sample index
expression
predicted_group='1' (327 values)
predicted_group='2' (32 values)

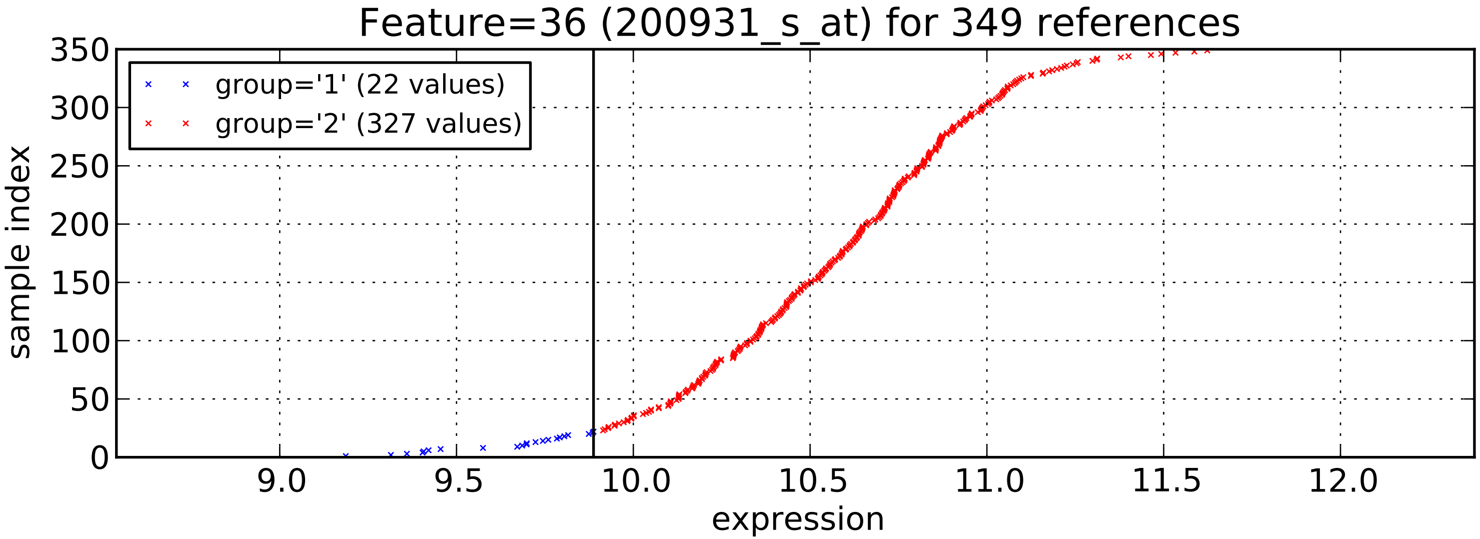

Feature=36 (200931_s_at) for 349 references
sample index
expression
group='1' (22 values)
group='2' (327 values)
0
50
100
150
200
250
300
350
9.0
9.5
10.0
10.5
11.0
11.5
12.0

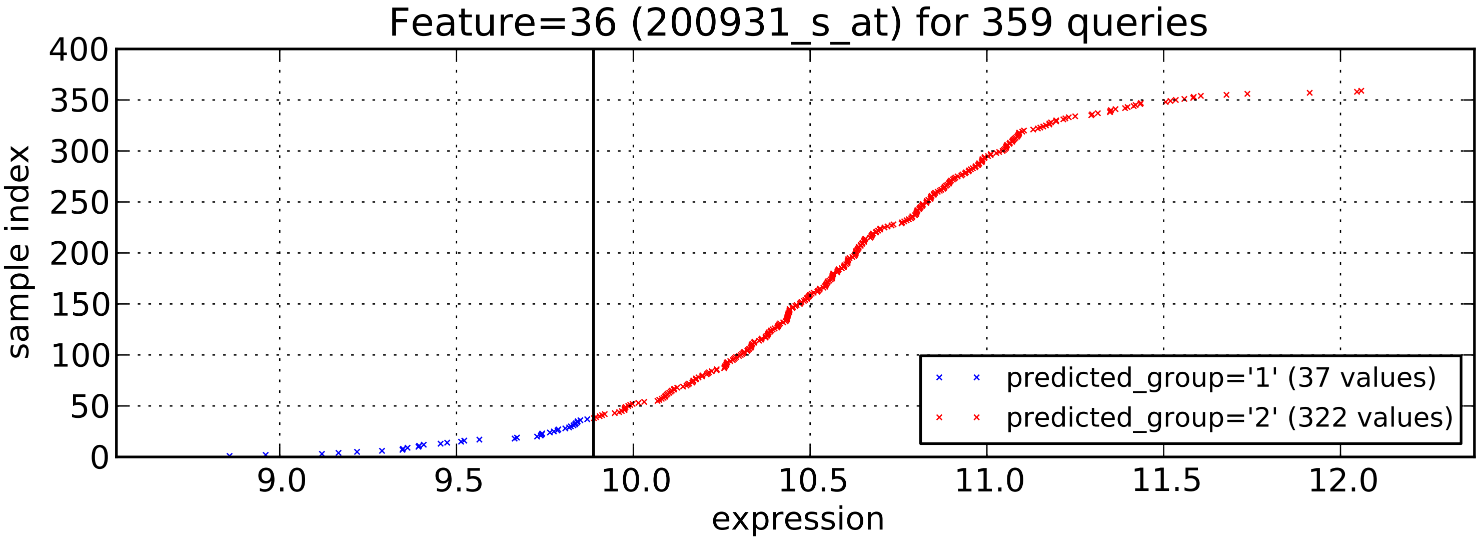

Feature=36 (200931_s_at) for 359 queries
sample index
expression
predicted_group='1' (37 values)
predicted_group='2' (322 values)
0
50
100
150
200
250
300
350
400
9.0
9.5
10.0
10.5
11.0
11.5
12.0

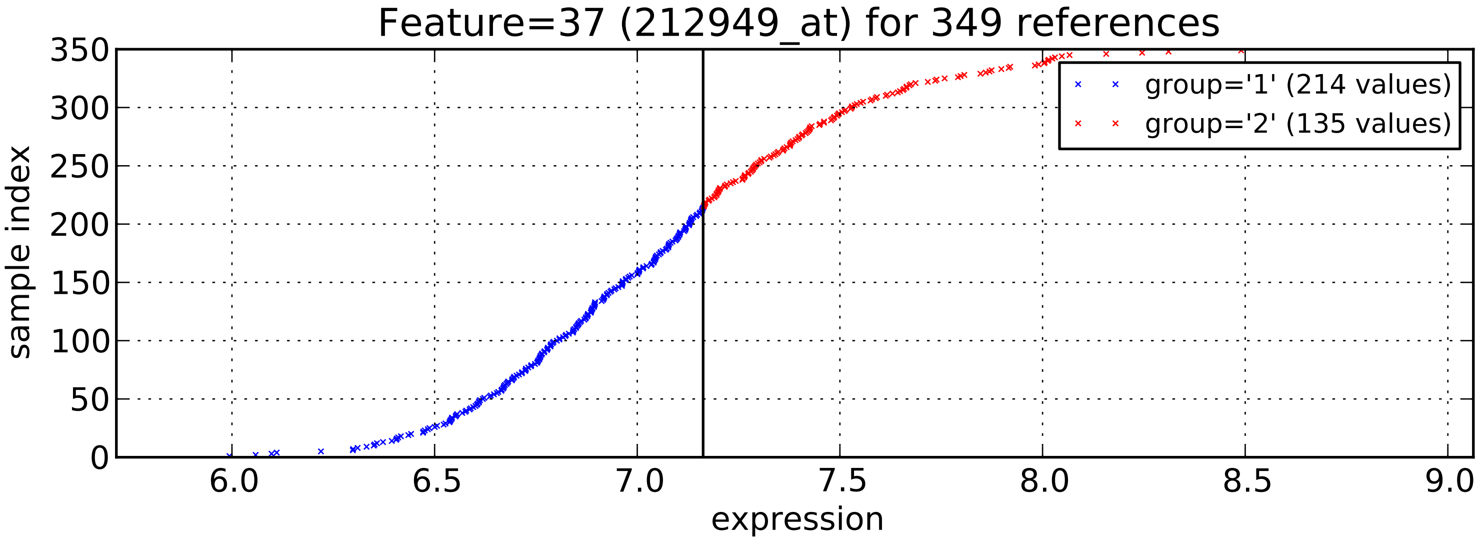
Feature=37 (212949_at) for 349 references
sample index
expression
group='1' (214 values)
group='2' (135 values)
0
50
100
150
200
250
300
350
6.0
6.5
7.0
7.5
8.0
8.5
9.0

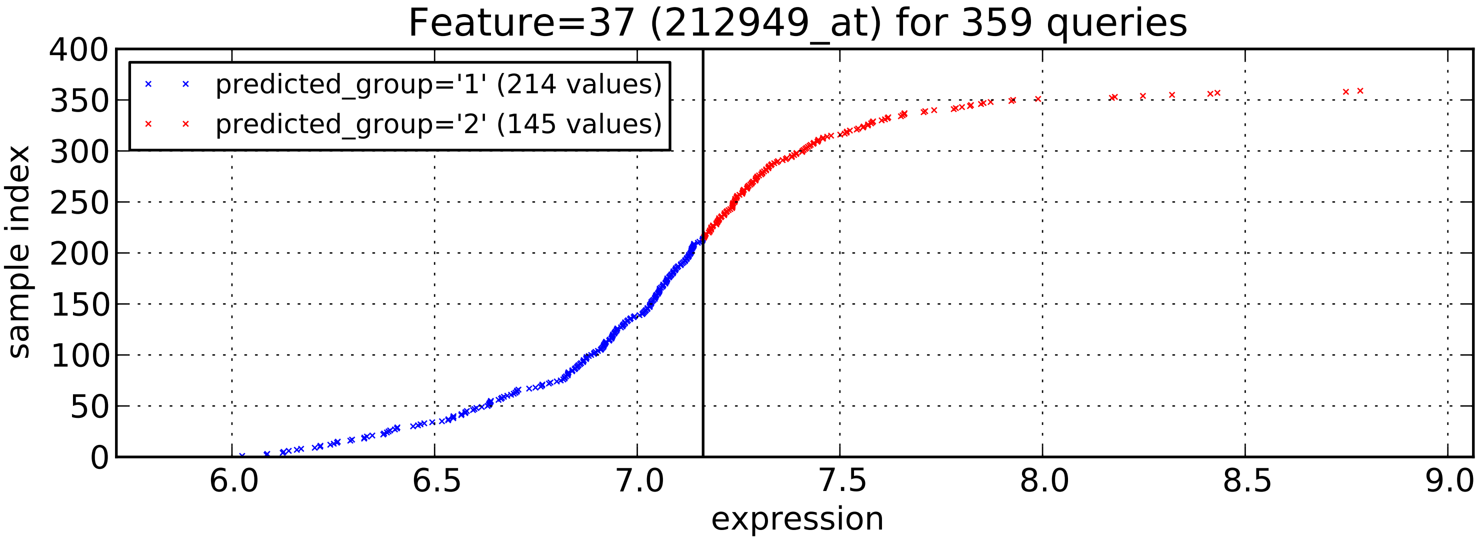
Feature=37 (212949_at) for 359 queries
sample index
expression
predicted_group='1' (214 values)
predicted_group='2' (145 values)
0
50
100
150
200
250
300
350
400
6.0
6.5
7.0
7.5
8.0
8.5
9.0
