# Supplementary material for: Big data and computational biology strategy for personalized prognosis
Source: Oncotarget. 2016 May 24;7(26):40200–20. doi: 10.18632/oncotarget.9571 (PMC5130003; doi:10.18632/oncotarget.9571)
Supplement: Supplementary file 12 [file oncotarget-07-40200-s012.pdf]

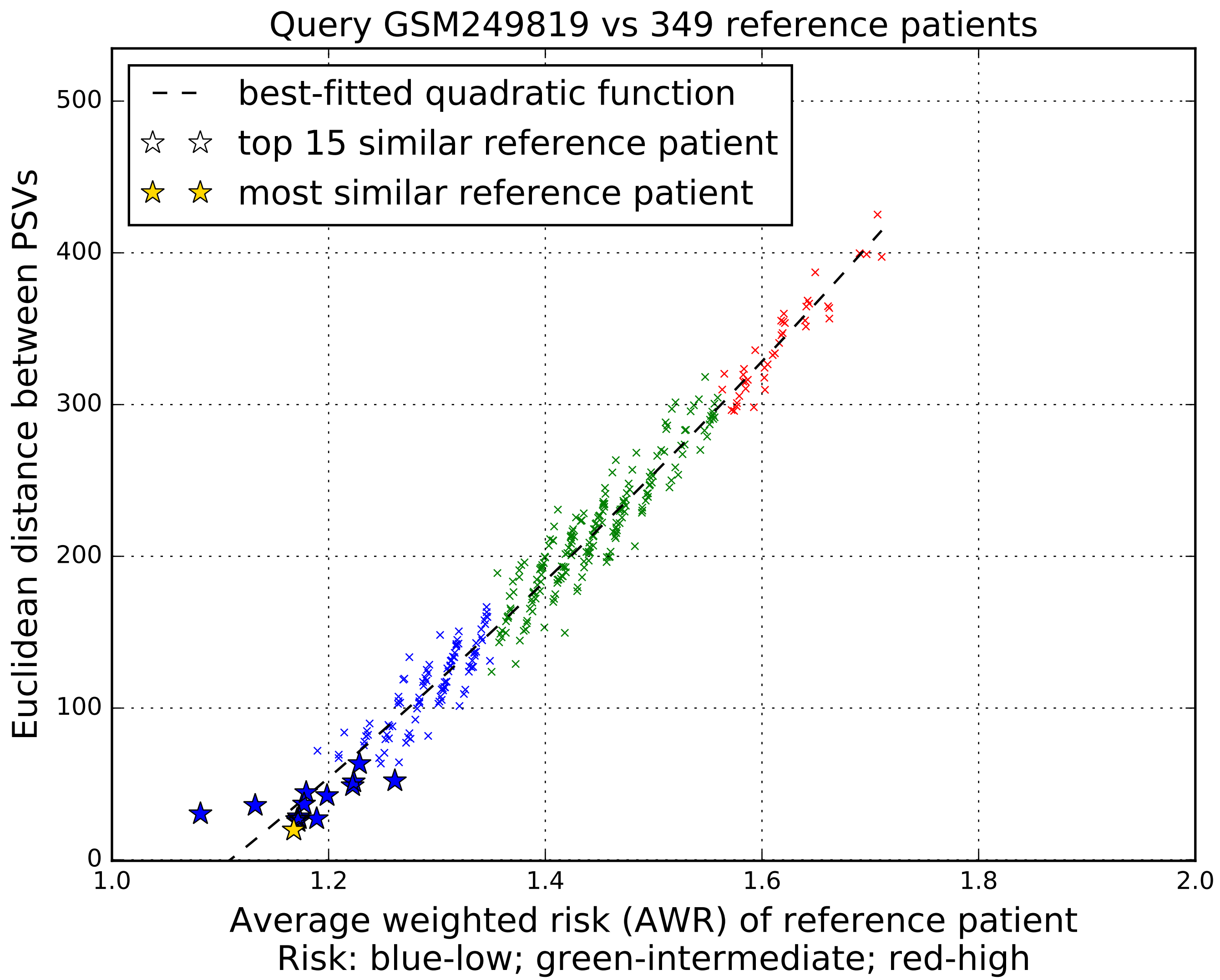

Query GSM249819 vs 349 reference patients
best-fitted quadratic function
top 15 similar reference patient
most similar reference patient
Euclidean distance between PSVs
0
100
200
300
400
500
1.0
1.2
1.4
1.6
1.8
2.0
Average weighted risk (AWR) of reference patient
Risk: blue-low; green-intermediate; red-high

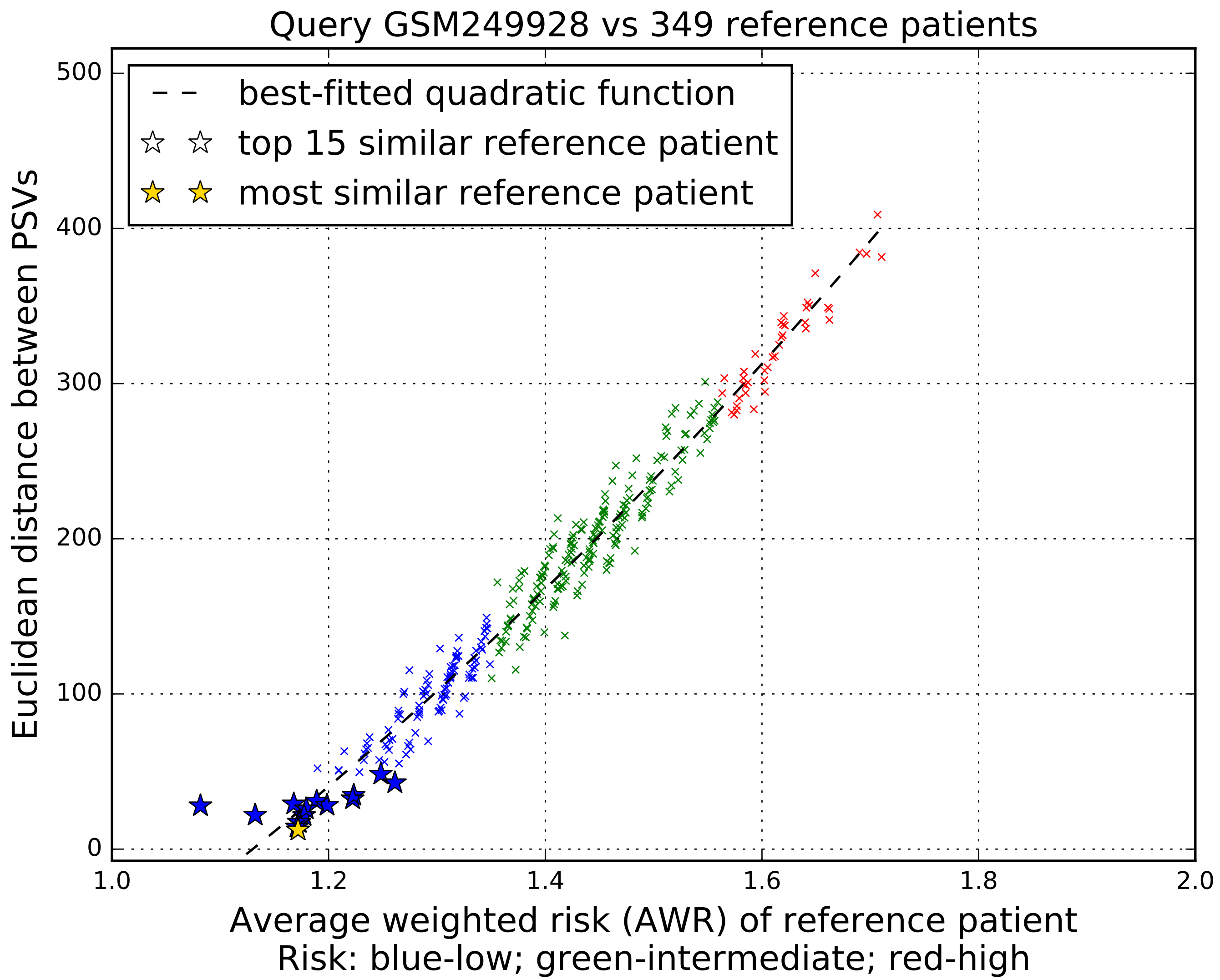
Query GSM249928 vs 349 reference patients
best-fitted quadratic function
top 15 similar reference patient
most similar reference patient
Euclidean distance between PSVs
0
100
200
300
400
500
1.0
1.2
1.4
1.6
1.8
2.0
Average weighted risk (AWR) of reference patient
Risk: blue-low; green-intermediate; red-high

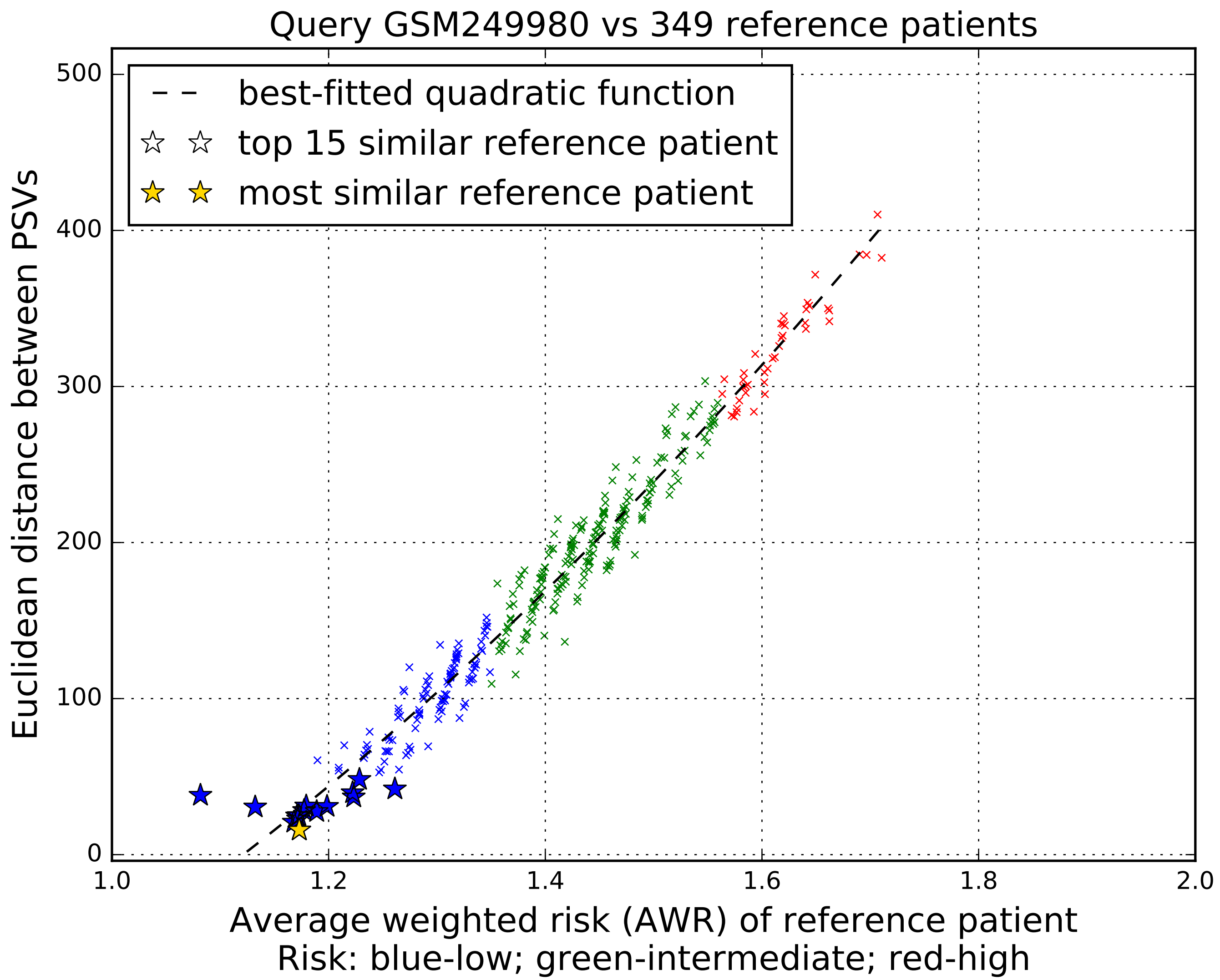
Query GSM249980 vs 349 reference patients
best-fitted quadratic function
top 15 similar reference patient
most similar reference patient
Euclidean distance between PSVs
0
100
200
300
400
500
1.0
1.2
1.4
1.6
1.8
2.0
Average weighted risk (AWR) of reference patient
Risk: blue-low; green-intermediate; red-high

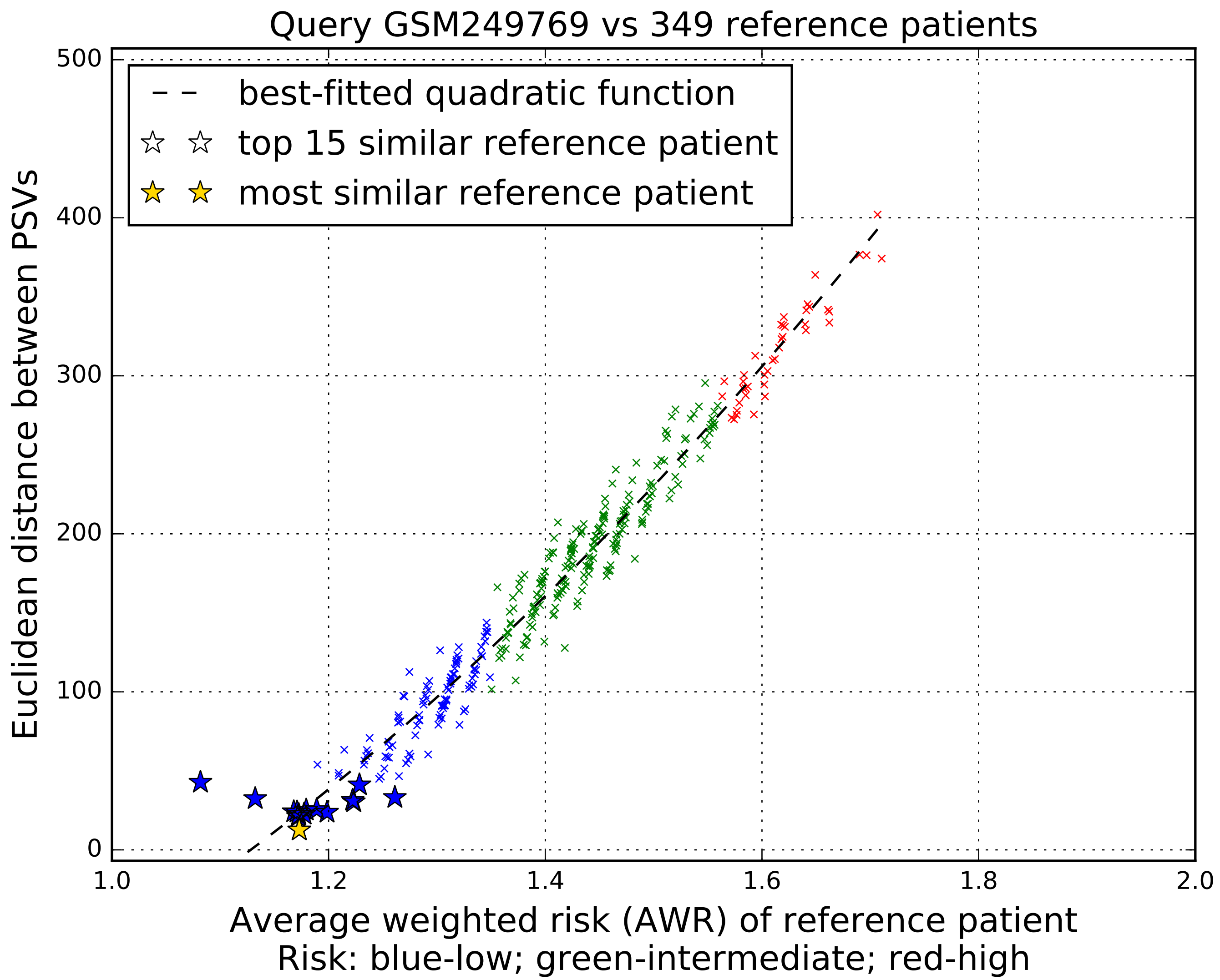

Query GSM249769 vs 349 reference patients
best-fitted quadratic function
top 15 similar reference patient
most similar reference patient
Euclidean distance between PSVs
0
100
200
300
400
500
1.0
1.2
1.4
1.6
1.8
2.0
Average weighted risk (AWR) of reference patient
Risk: blue-low; green-intermediate; red-high

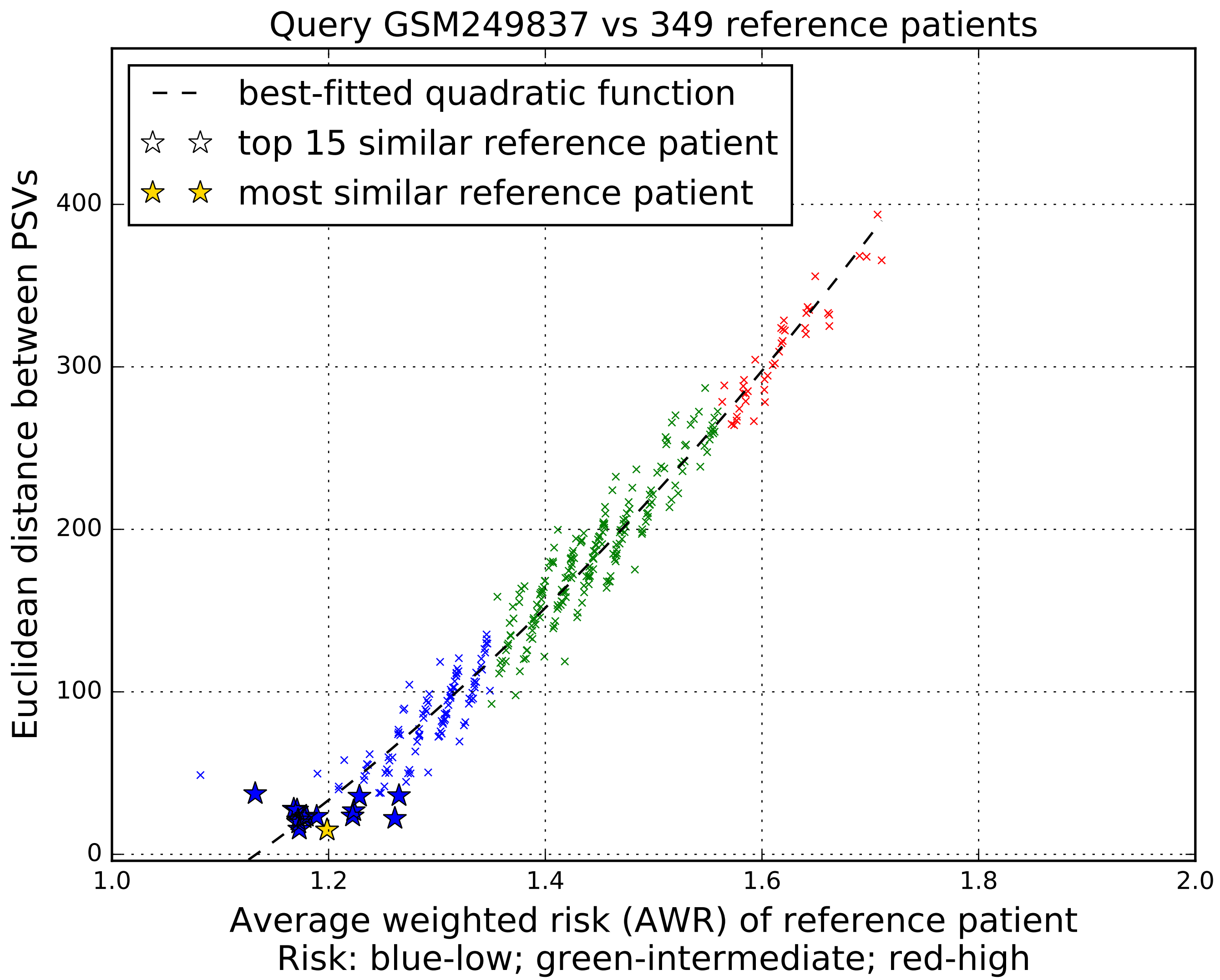

Query GSM249837 vs 349 reference patients
best-fitted quadratic function
top 15 similar reference patient
most similar reference patient
Euclidean distance between PSVs
0
100
200
300
400
1.0
1.2
1.4
1.6
1.8
2.0
Average weighted risk (AWR) of reference patient
Risk: blue-low; green-intermediate; red-high

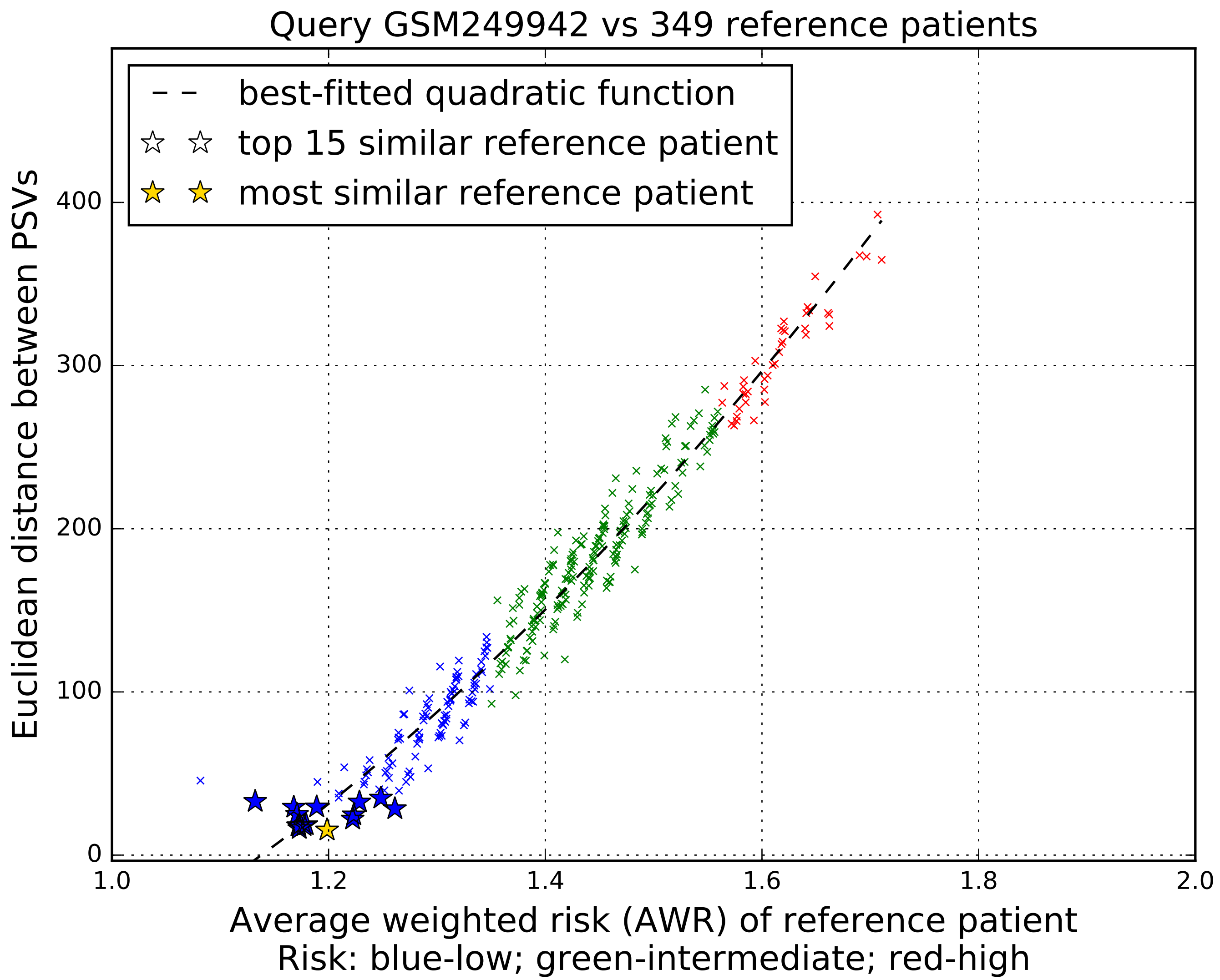
Query GSM249942 vs 349 reference patients
best-fitted quadratic function
top 15 similar reference patient
most similar reference patient
Euclidean distance between PSVs
Average weighted risk (AWR) of reference patient
Risk: blue-low; green-intermediate; red-high
0
100
200
300
400
1.0
1.2
1.4
1.6
1.8
2.0

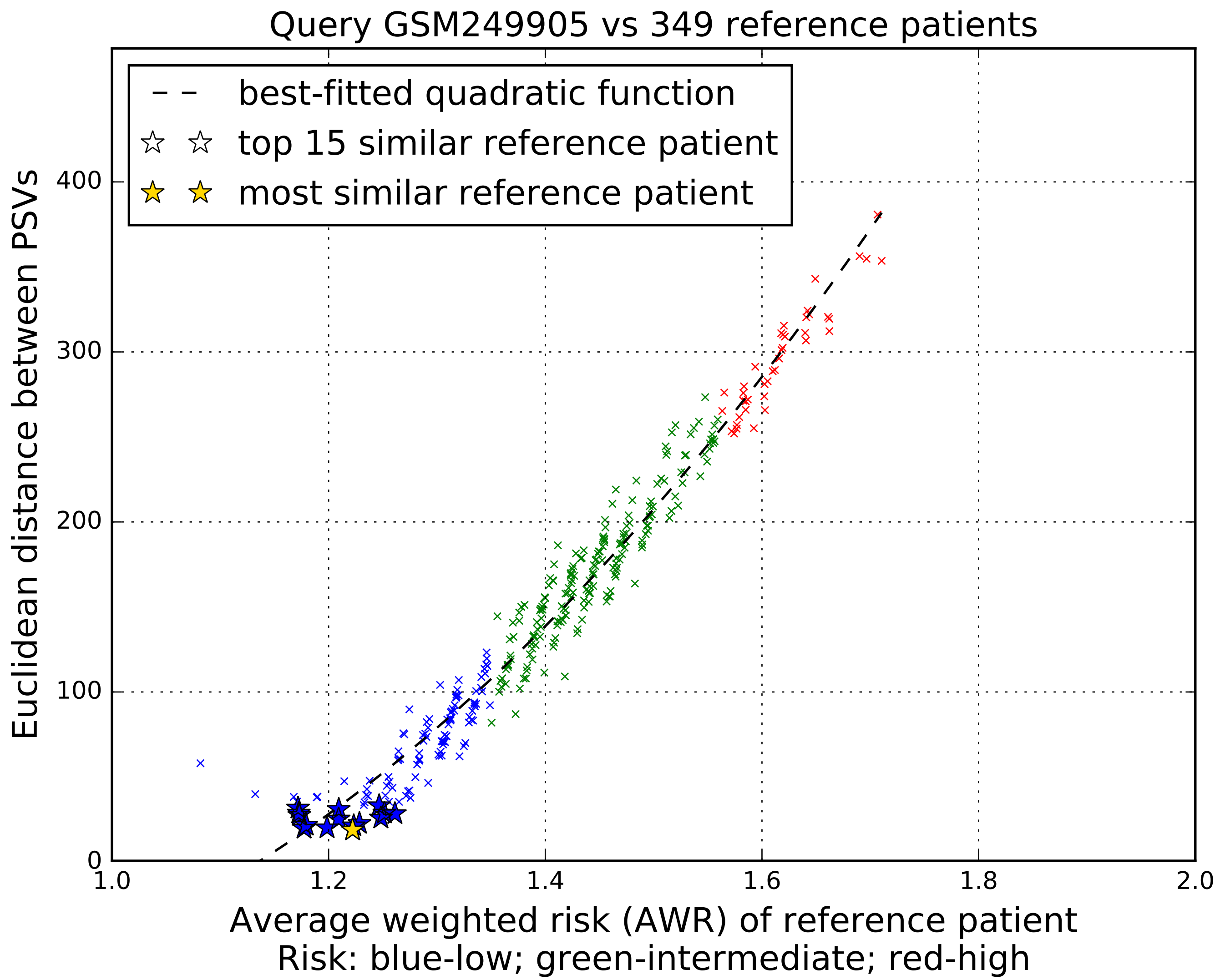

Query GSM249905 vs 349 reference patients
best-fitted quadratic function
top 15 similar reference patient
most similar reference patient
Euclidean distance between PSVs
0
100
200
300
400
1.0
1.2
1.4
1.6
1.8
2.0
Average weighted risk (AWR) of reference patient
Risk: blue-low; green-intermediate; red-high

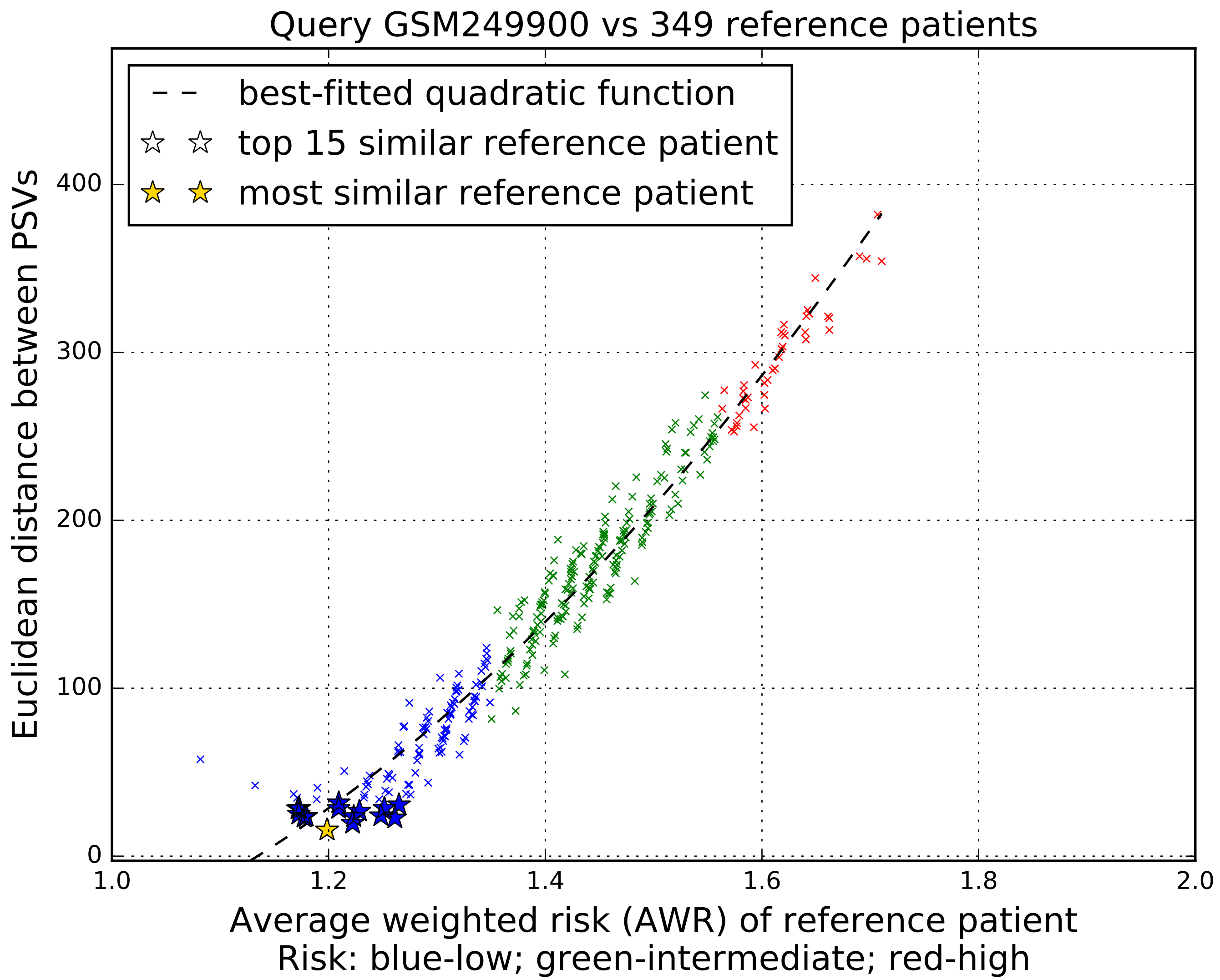
Query GSM249900 vs 349 reference patients
best-fitted quadratic function
top 15 similar reference patient
most similar reference patient
Euclidean distance between PSVs
Average weighted risk (AWR) of reference patient
Risk: blue-low; green-intermediate; red-high
0
100
200
300
400
1.0
1.2
1.4
1.6
1.8
2.0

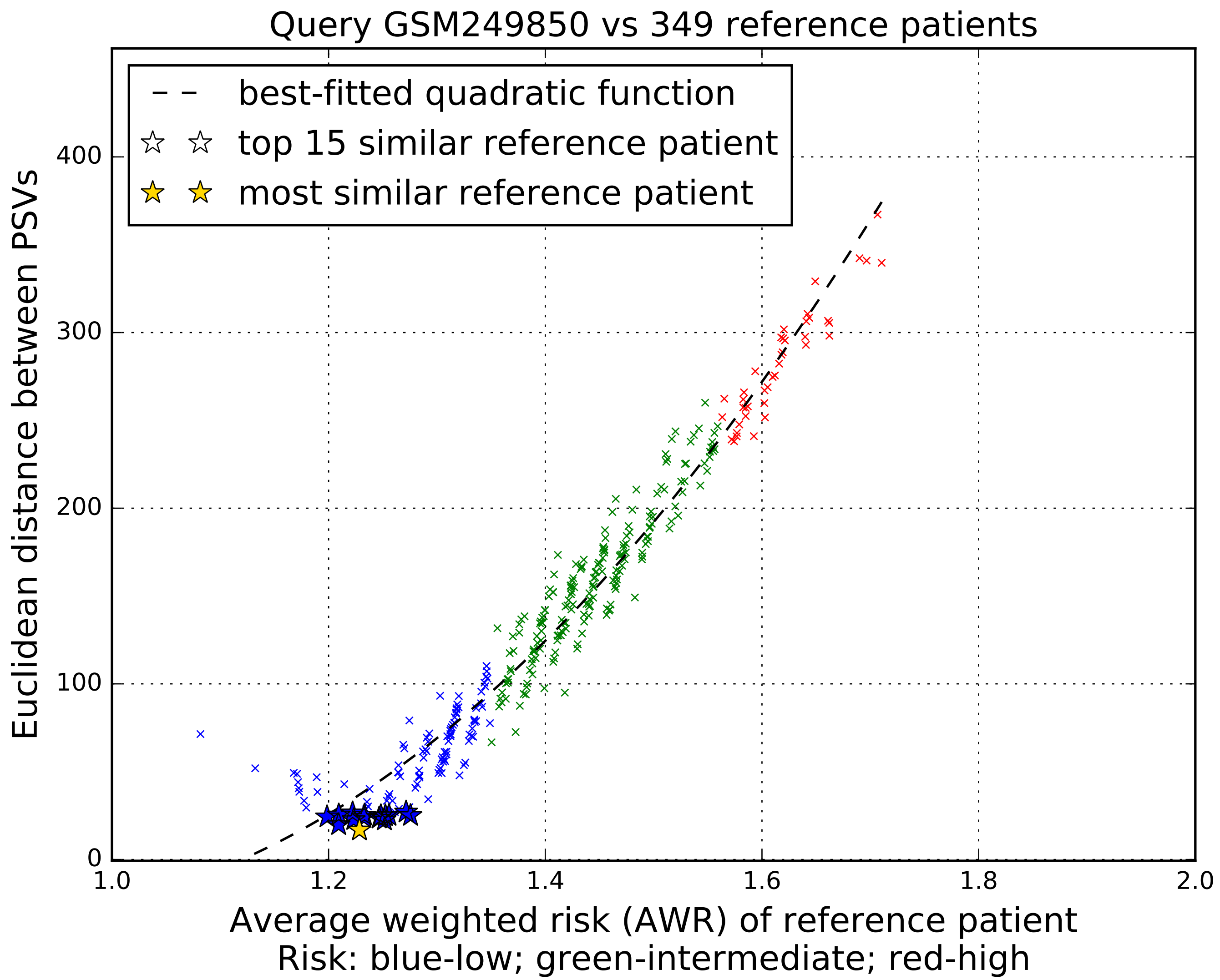

Query GSM249850 vs 349 reference patients
best-fitted quadratic function
top 15 similar reference patient
most similar reference patient
Euclidean distance between PSVs
0
100
200
300
400
1.0
1.2
1.4
1.6
1.8
2.0
Average weighted risk (AWR) of reference patient
Risk: blue-low; green-intermediate; red-high

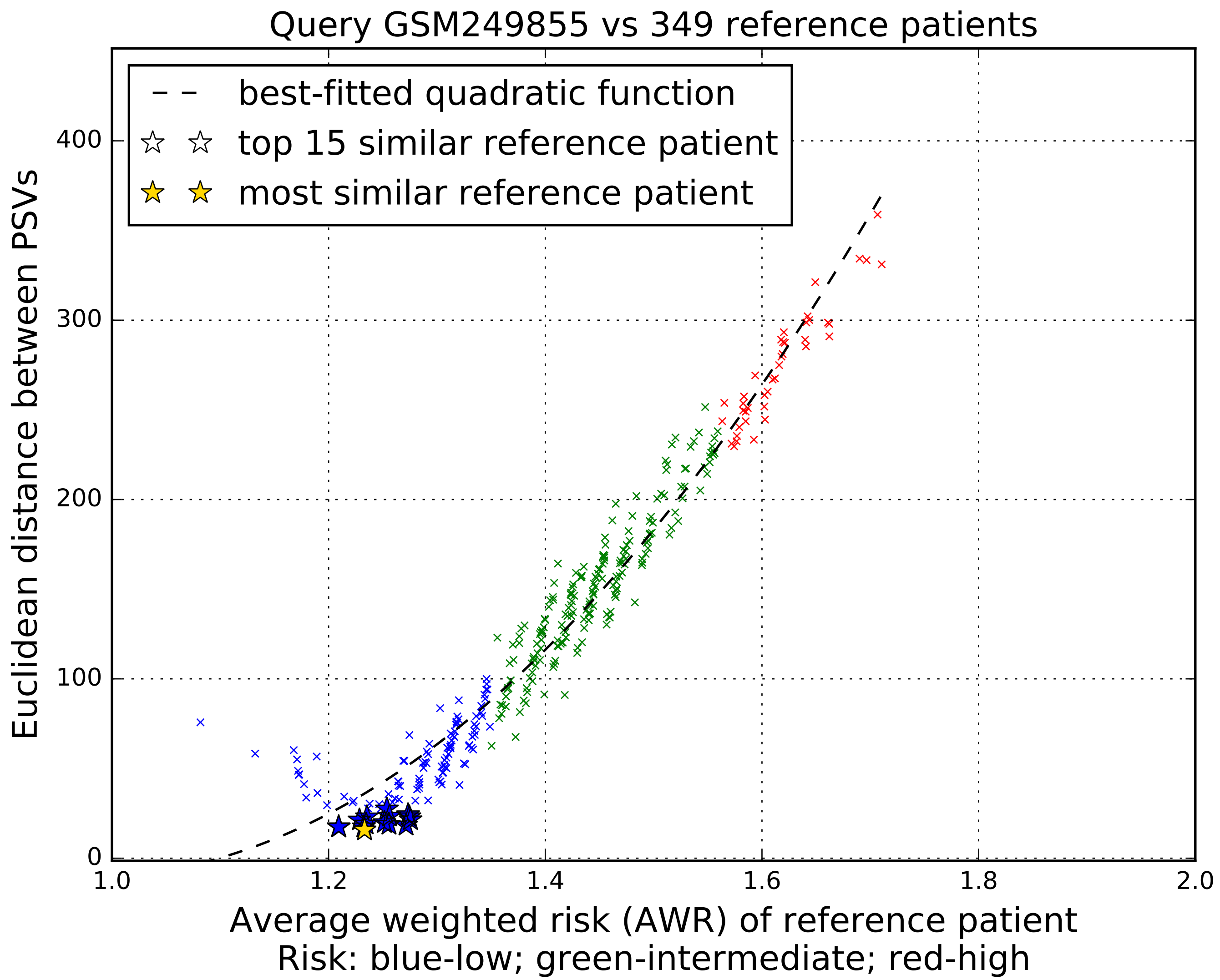
Query GSM249855 vs 349 reference patients
best-fitted quadratic function
top 15 similar reference patient
most similar reference patient
Euclidean distance between PSVs
0
100
200
300
400
1.0
1.2
1.4
1.6
1.8
2.0
Average weighted risk (AWR) of reference patient
Risk: blue-low; green-intermediate; red-high

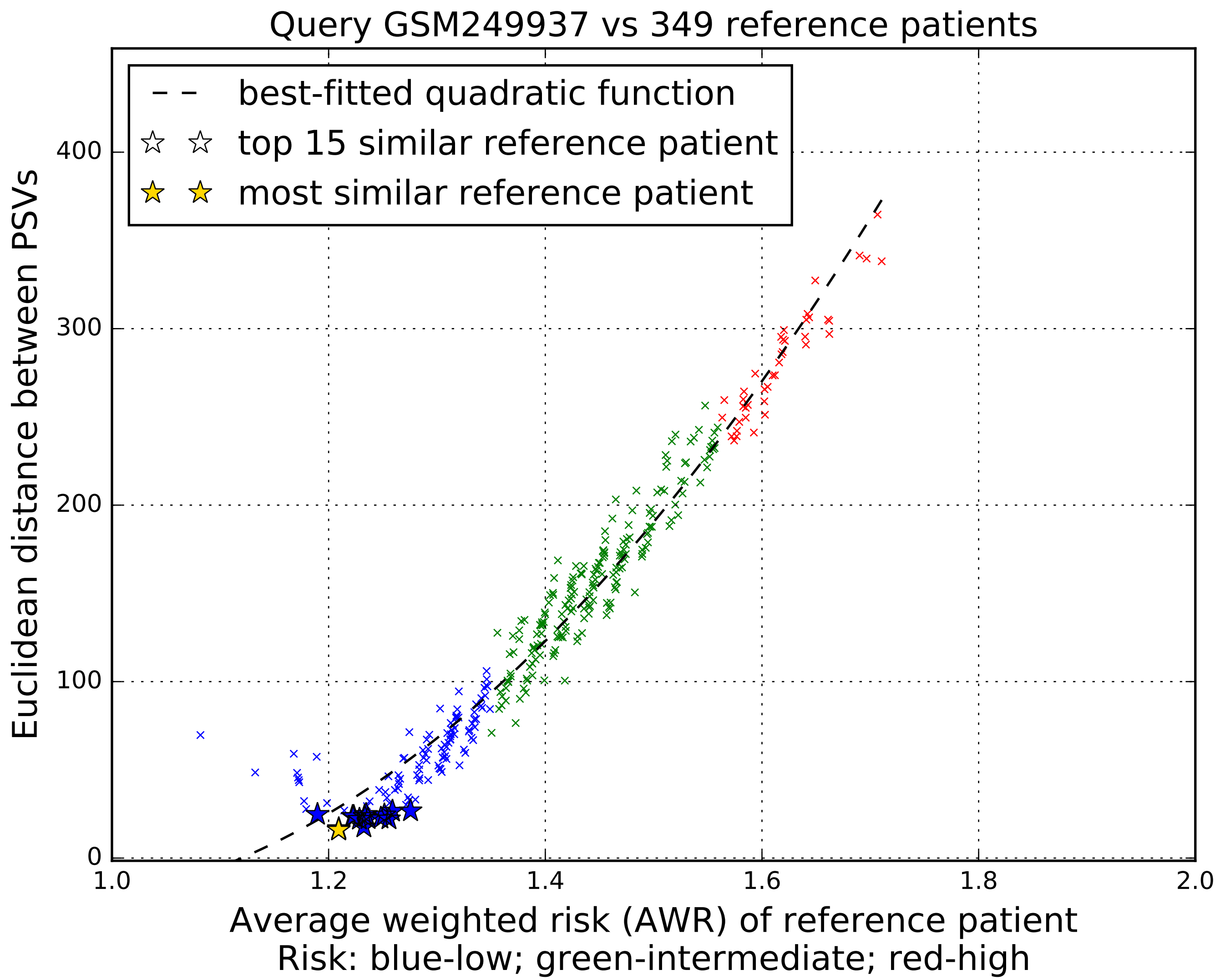
Query GSM249937 vs 349 reference patients
best-fitted quadratic function
top 15 similar reference patient
most similar reference patient
Euclidean distance betweeen PSVs
Average weighted risk (AWR) of reference patient
Risk: blue-low; green-intermediate; red-high
0
100
200
300
400
1.0
1.2
1.4
1.6
1.8
2.0

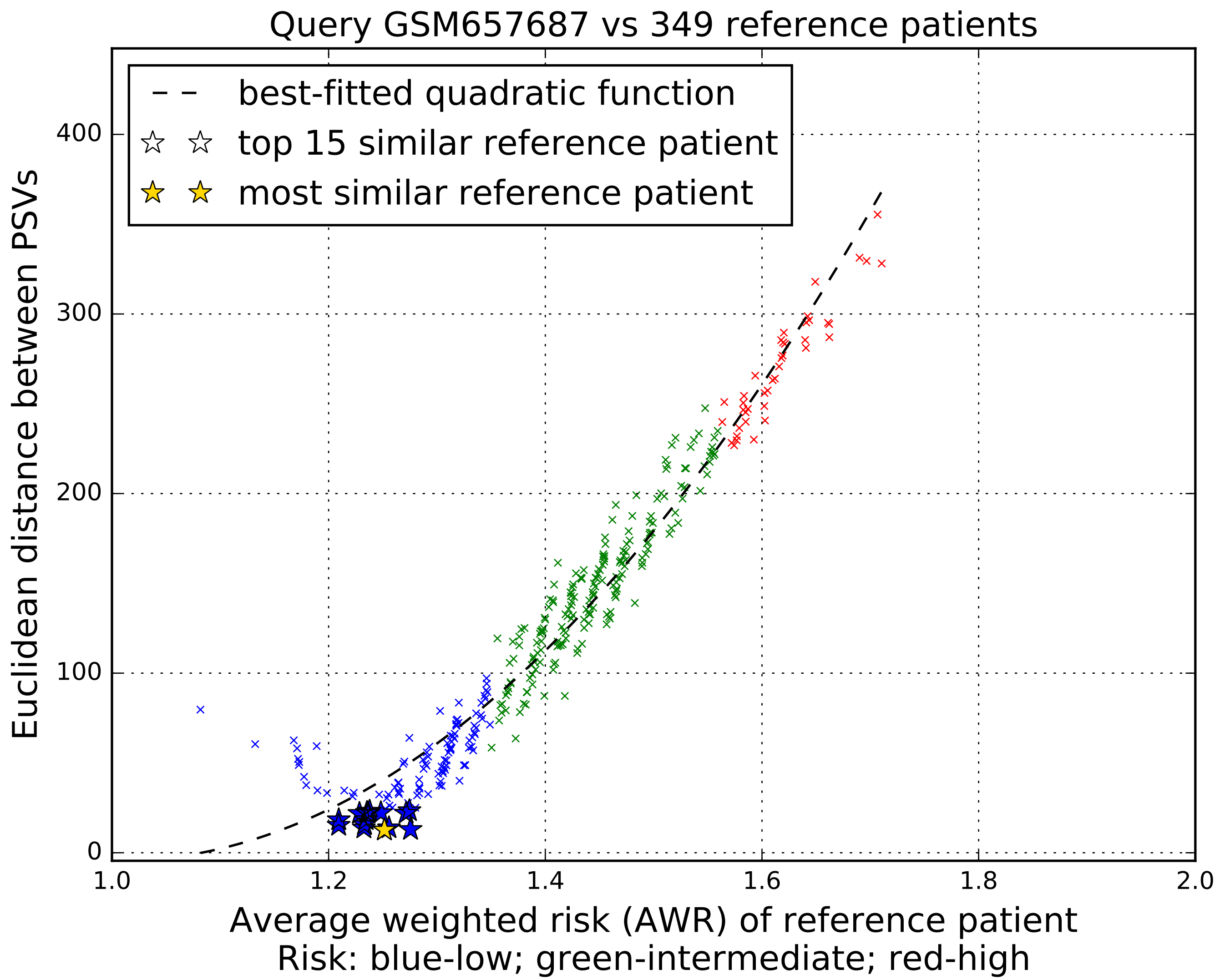

Query GSM657687 vs 349 reference patients
best-fitted quadratic function
top 15 similar reference patient
most similar reference patient
Euclidean distance between PSVs
0
100
200
300
400
1.0
1.2
1.4
1.6
1.8
2.0
Average weighted risk (AWR) of reference patient
Risk: blue-low; green-intermediate; red-high

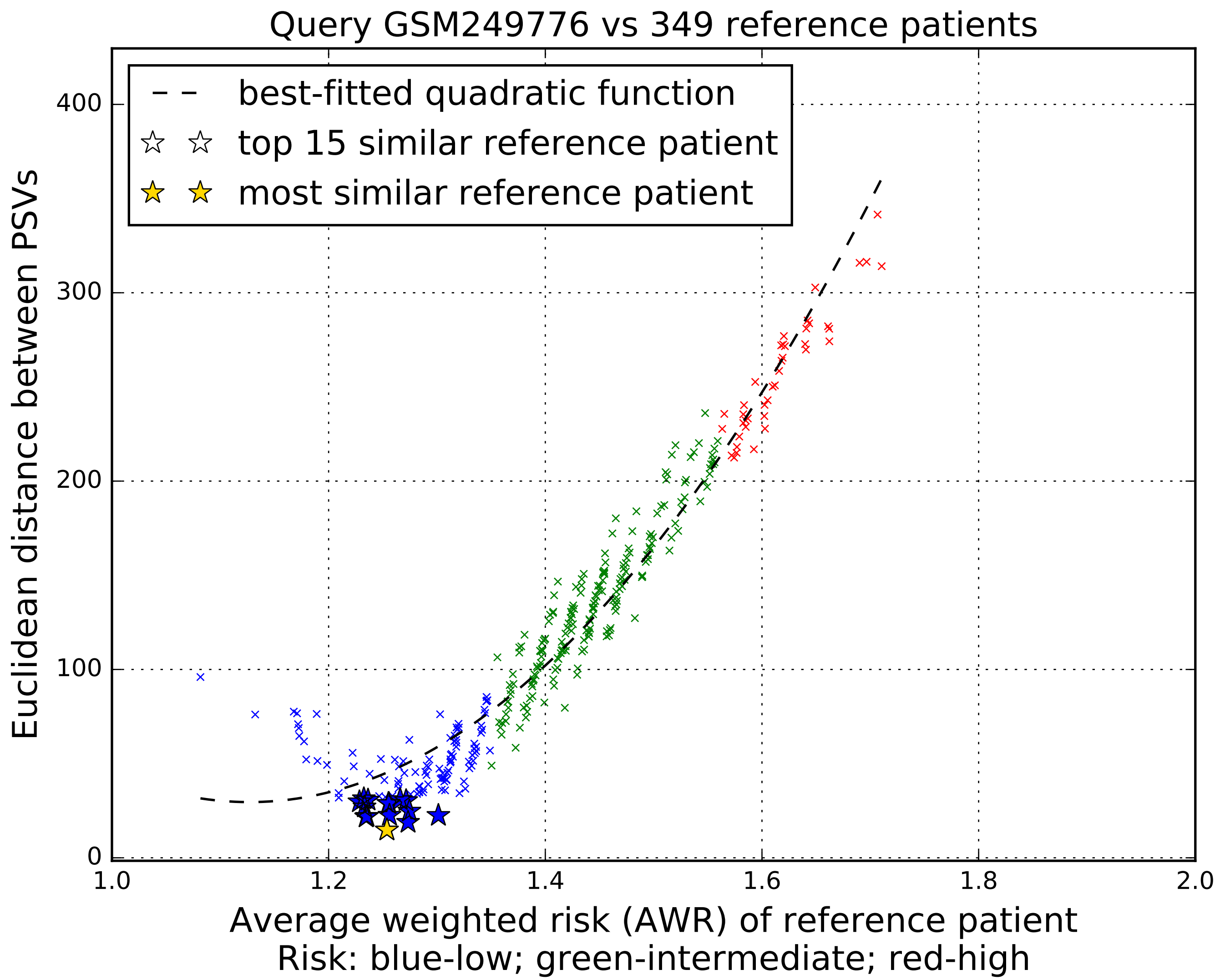
Query GSM249776 vs 349 reference patients
best-fitted quadratic function
top 15 similar reference patient
most similar reference patient
Euclidean distance between PSVs
Average weighted risk (AWR) of reference patient
Risk: blue-low; green-intermediate; red-high
0
100
200
300
400
1.0
1.2
1.4
1.6
1.8
2.0

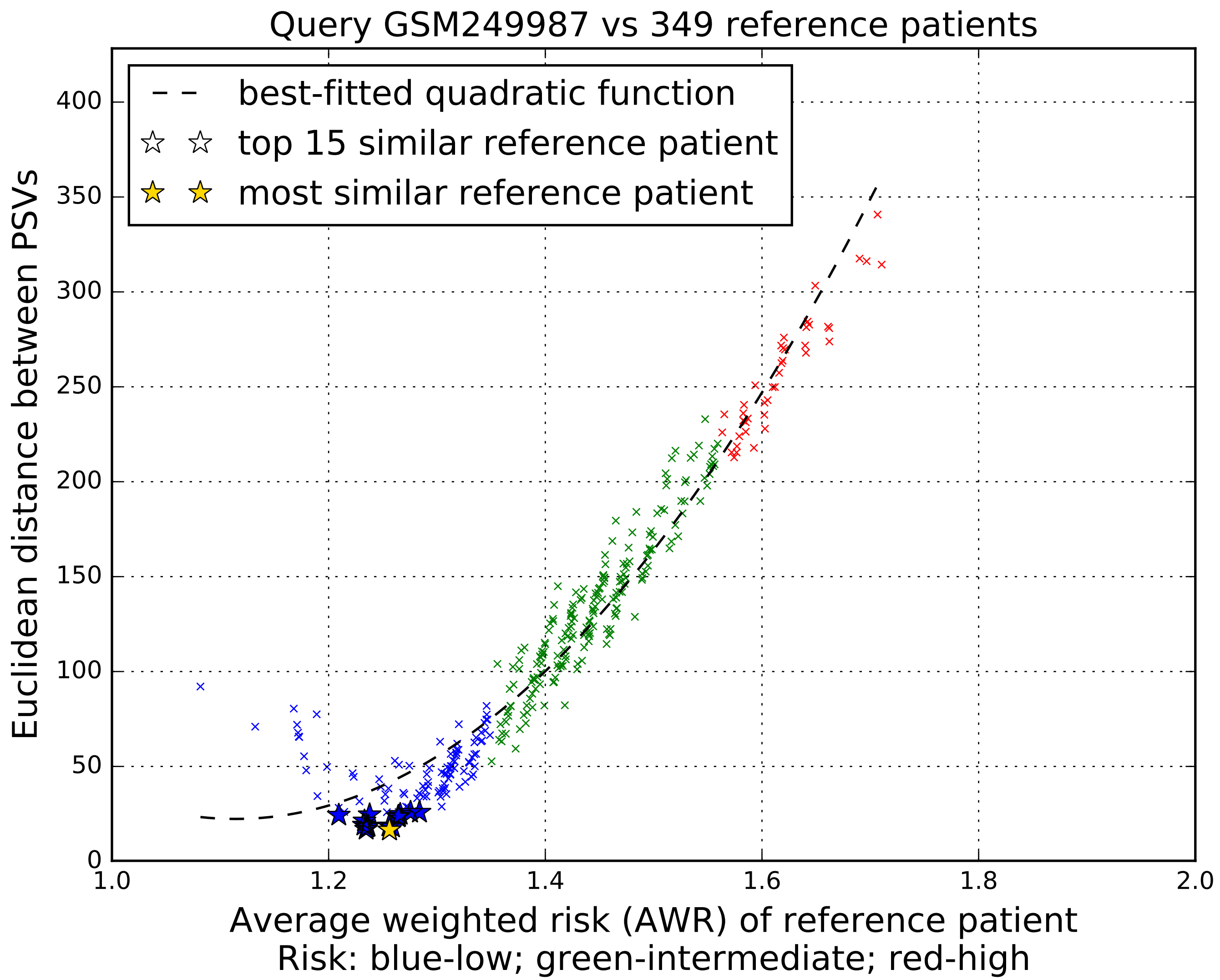

Query GSM249987 vs 349 reference patients
best-fitted quadratic function
top 15 similar reference patient
most similar reference patient
Euclidean distance between PSVs
0
50
100
150
200
250
300
350
400
1.0
1.2
1.4
1.6
1.8
2.0
Average weighted risk (AWR) of reference patient
Risk: blue-low; green-intermediate; red-high

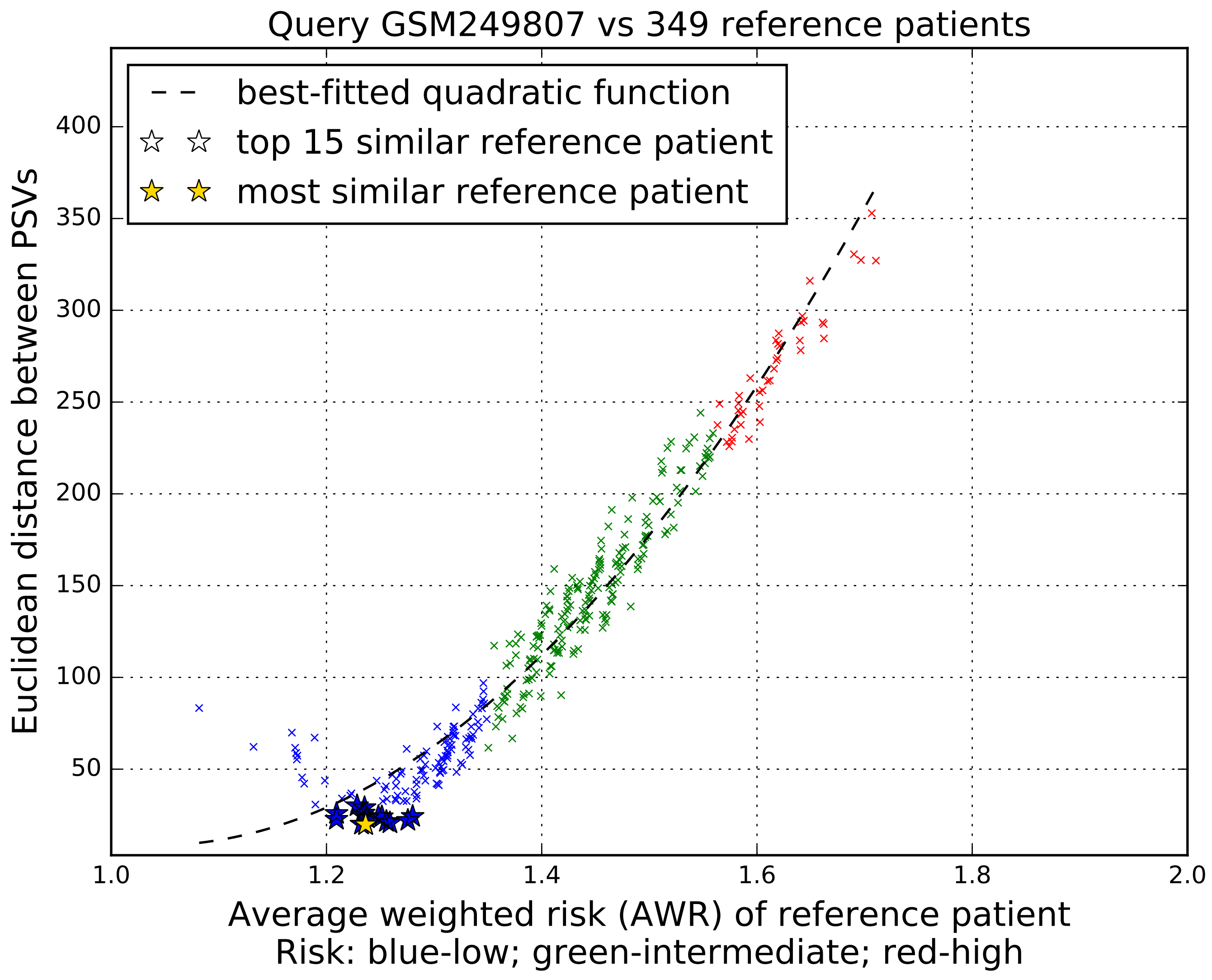

Query GSM249807 vs 349 reference patients
best-fitted quadratic function
top 15 similar reference patient
most similar reference patient
Euclidean distance between PSVs
400
350
300
250
200
150
100
50
1.0
1.2
1.4
1.6
1.8
2.0
Average weighted risk (AWR) of reference patient
Risk: blue-low; green-intermediate; red-high

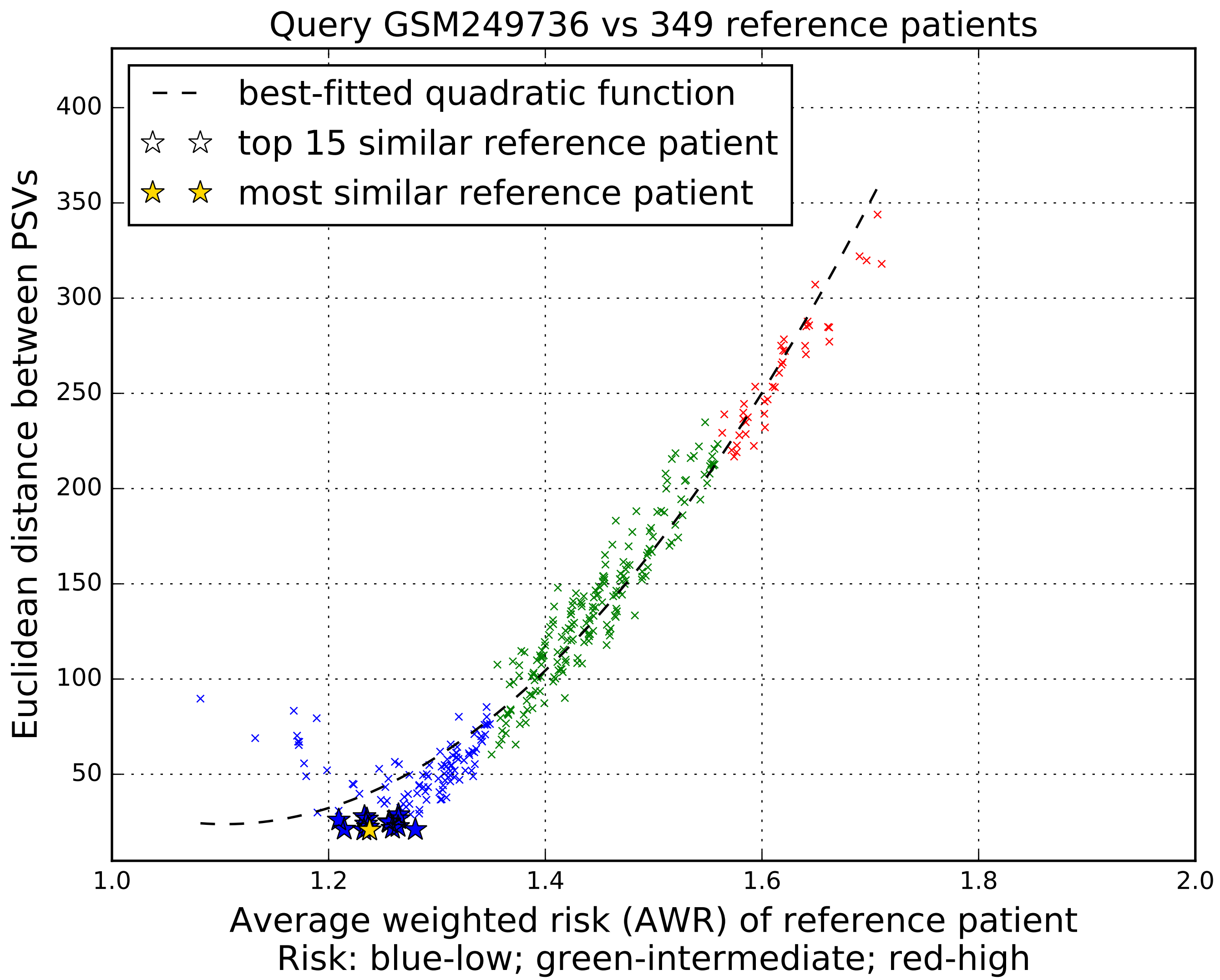
Query GSM249736 vs 349 reference patients
best-fitted quadratic function
top 15 similar reference patient
most similar reference patient
Euclidean distance between PSVs
400
350
300
250
200
150
100
50
1.0
1.2
1.4
1.6
1.8
2.0
Average weighted risk (AWR) of reference patient
Risk: blue-low; green-intermediate; red-high

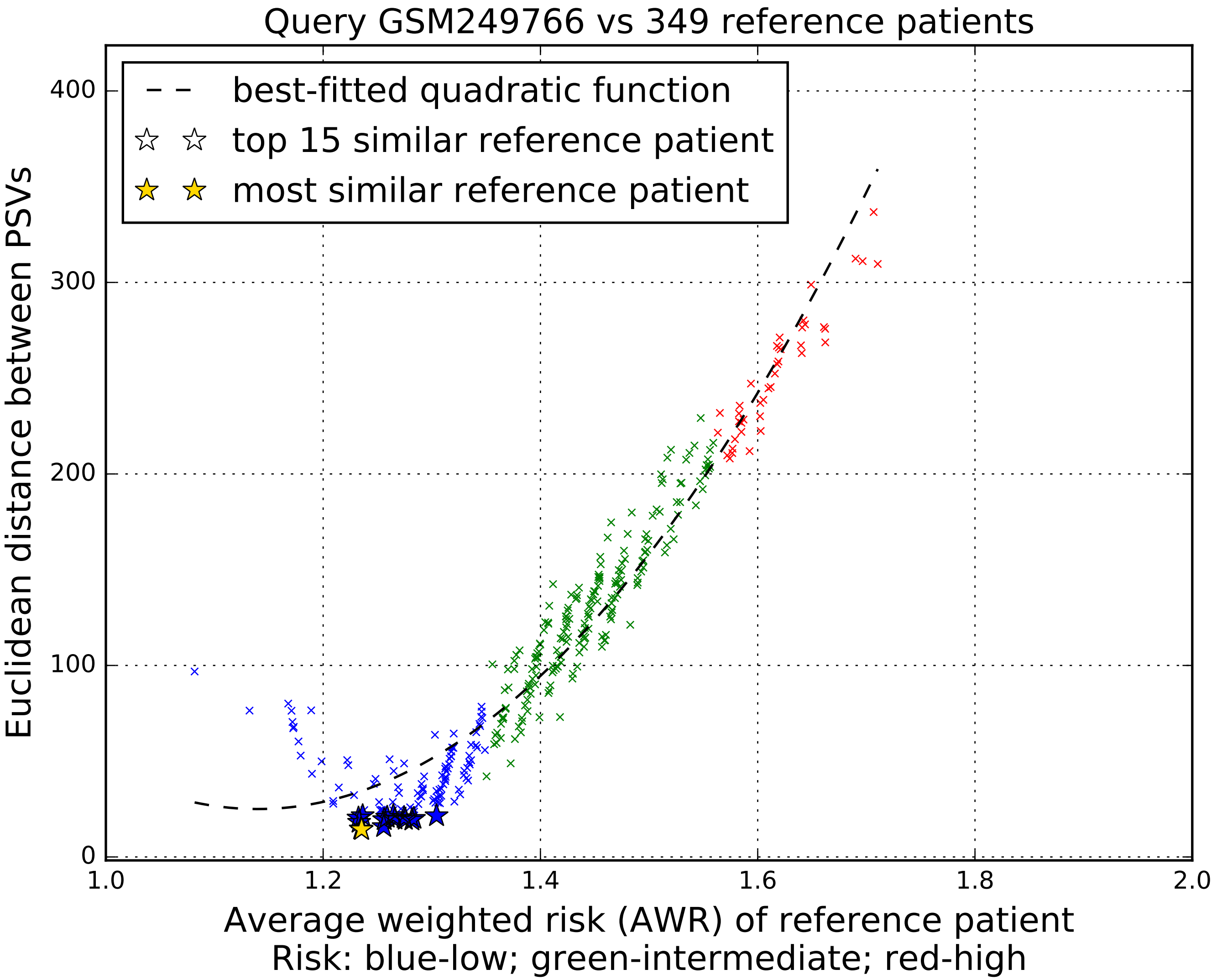
Query GSM249766 vs 349 reference patients
best-fitted quadratic function
top 15 similar reference patient
most similar reference patient
400
300
200
100
0
1.0
1.2
1.4
1.6
1.8
2.0
Euclidean distance between PSVs
Average weighted risk (AWR) of reference patient
Risk: blue-low; green-intermediate; red-high

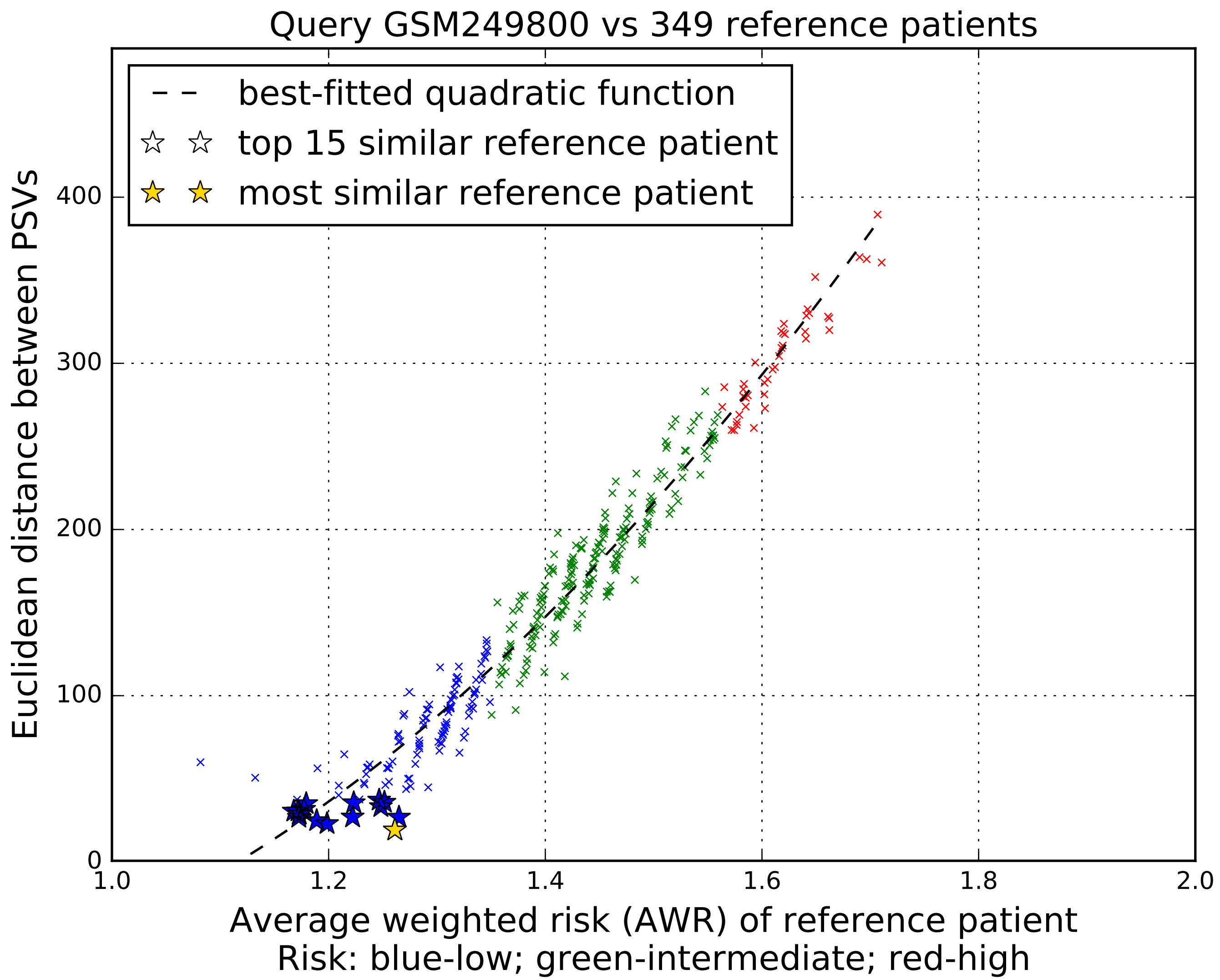

Query GSM249800 vs 349 reference patients
best-fitted quadratic function
top 15 similar reference patient
most similar reference patient
Euclidean distance between PSVs
0
100
200
300
400
1.0
1.2
1.4
1.6
1.8
2.0
Average weighted risk (AWR) of reference patient
Risk: blue-low; green-intermediate; red-high

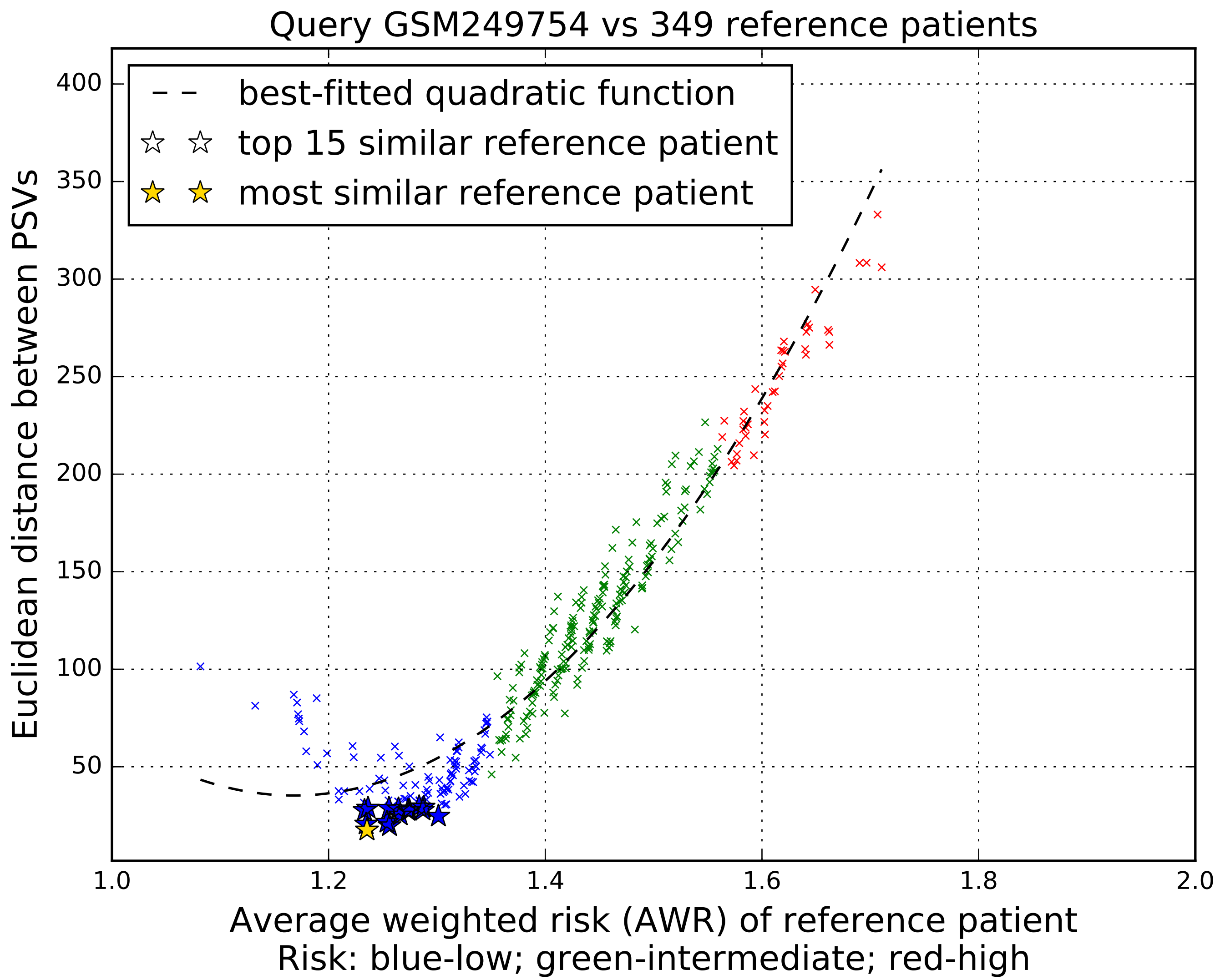
Query GSM249754 vs 349 reference patients
best-fitted quadratic function
top 15 similar reference patient
most similar reference patient
Euclidean distance between PSVs
400
350
300
250
200
150
100
50
1.0
1.2
1.4
1.6
1.8
2.0
Average weighted risk (AWR) of reference patient
Risk: blue-low; green-intermediate; red-high

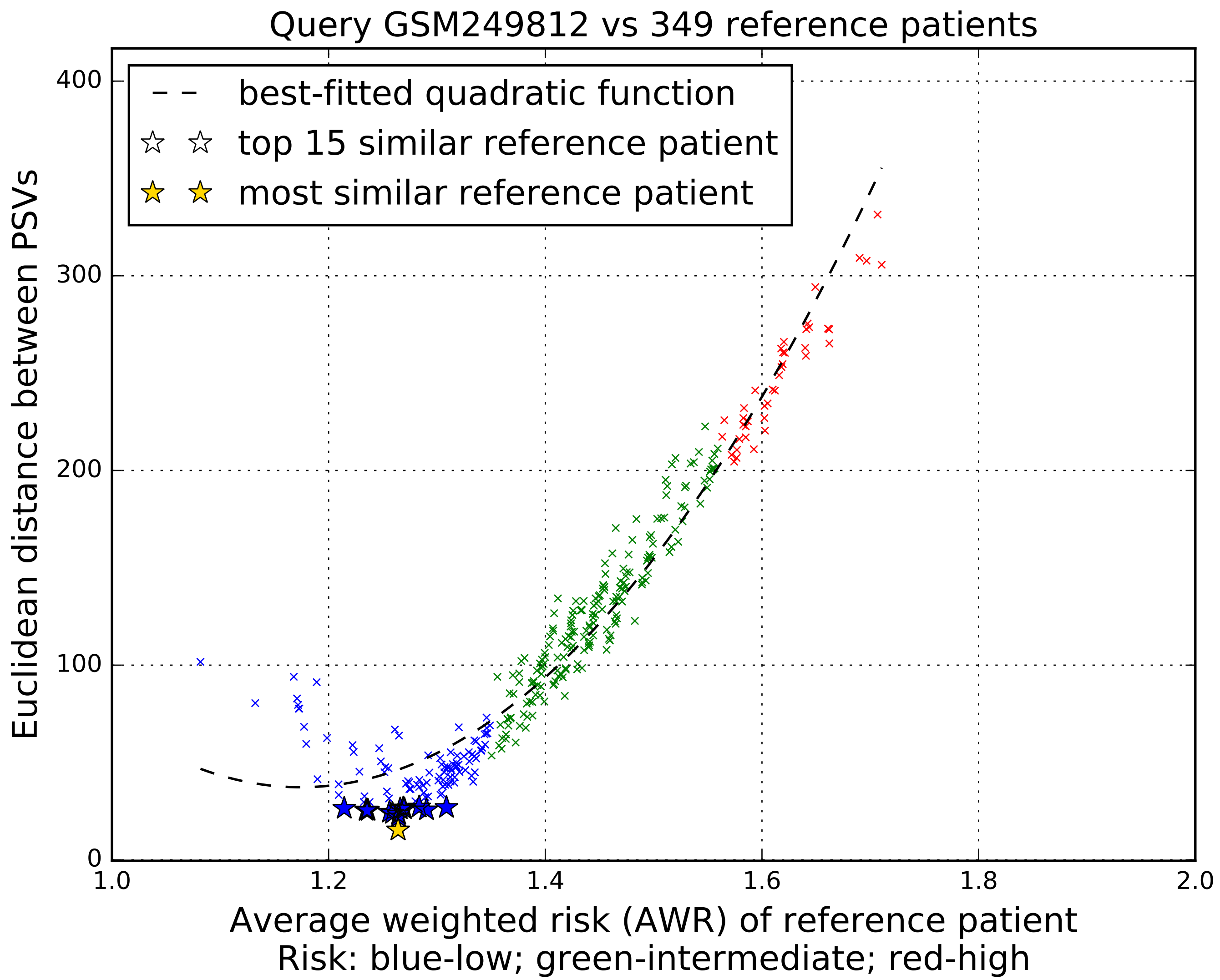
Query GSM249812 vs 349 reference patients
best-fitted quadratic function
top 15 similar reference patient
most similar reference patient
Euclidean distance between PSVs
Average weighted risk (AWR) of reference patient
Risk: blue-low; green-intermediate; red-high
0
100
200
300
400
1.0
1.2
1.4
1.6
1.8
2.0

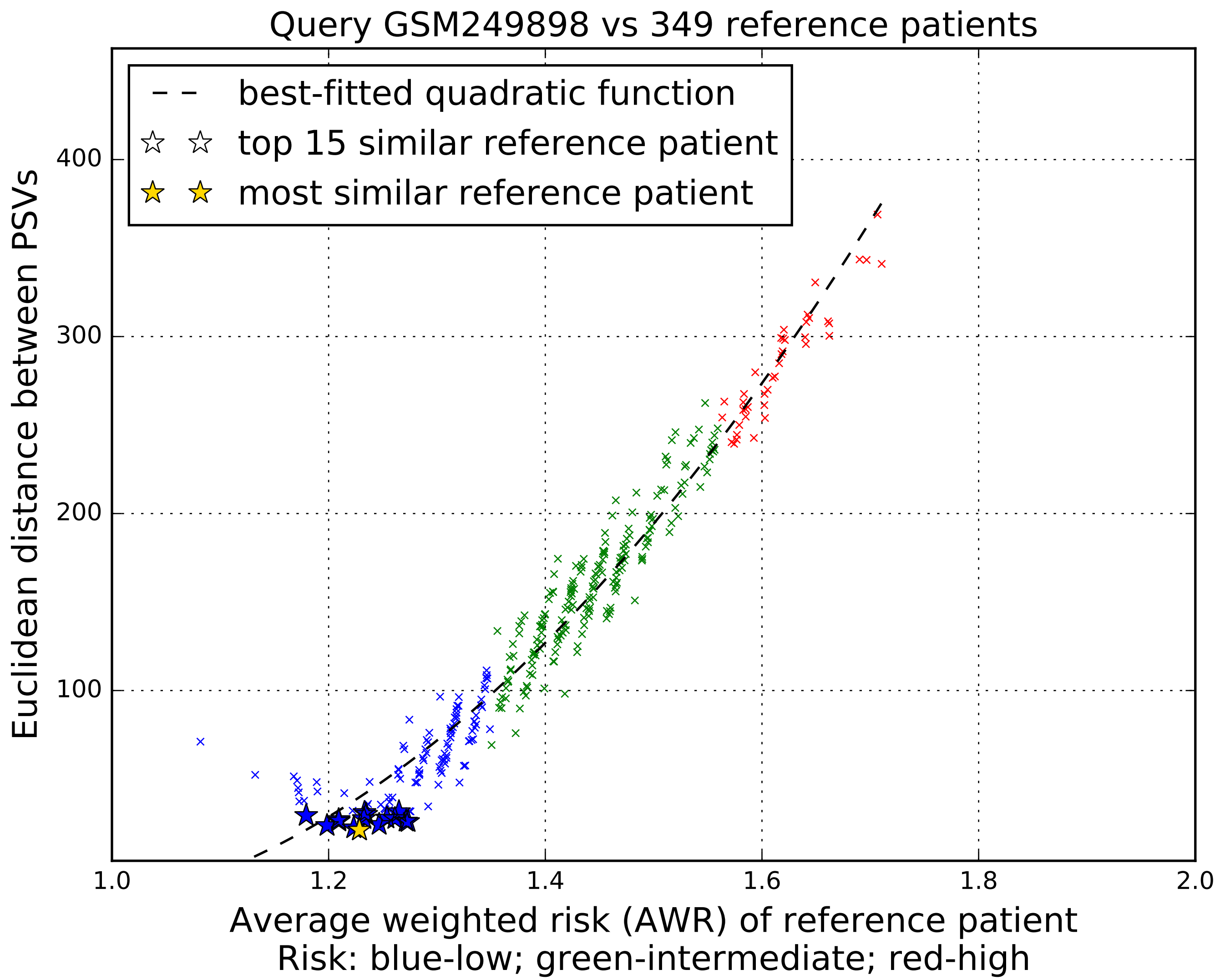

Query GSM249898 vs 349 reference patients
best-fitted quadratic function
top 15 similar reference patient
most similar reference patient
Euclidean distance between PSVs
400
300
200
100
1.0
1.2
1.4
1.6
1.8
2.0
Average weighted risk (AWR) of reference patient
Risk: blue-low; green-intermediate; red-high

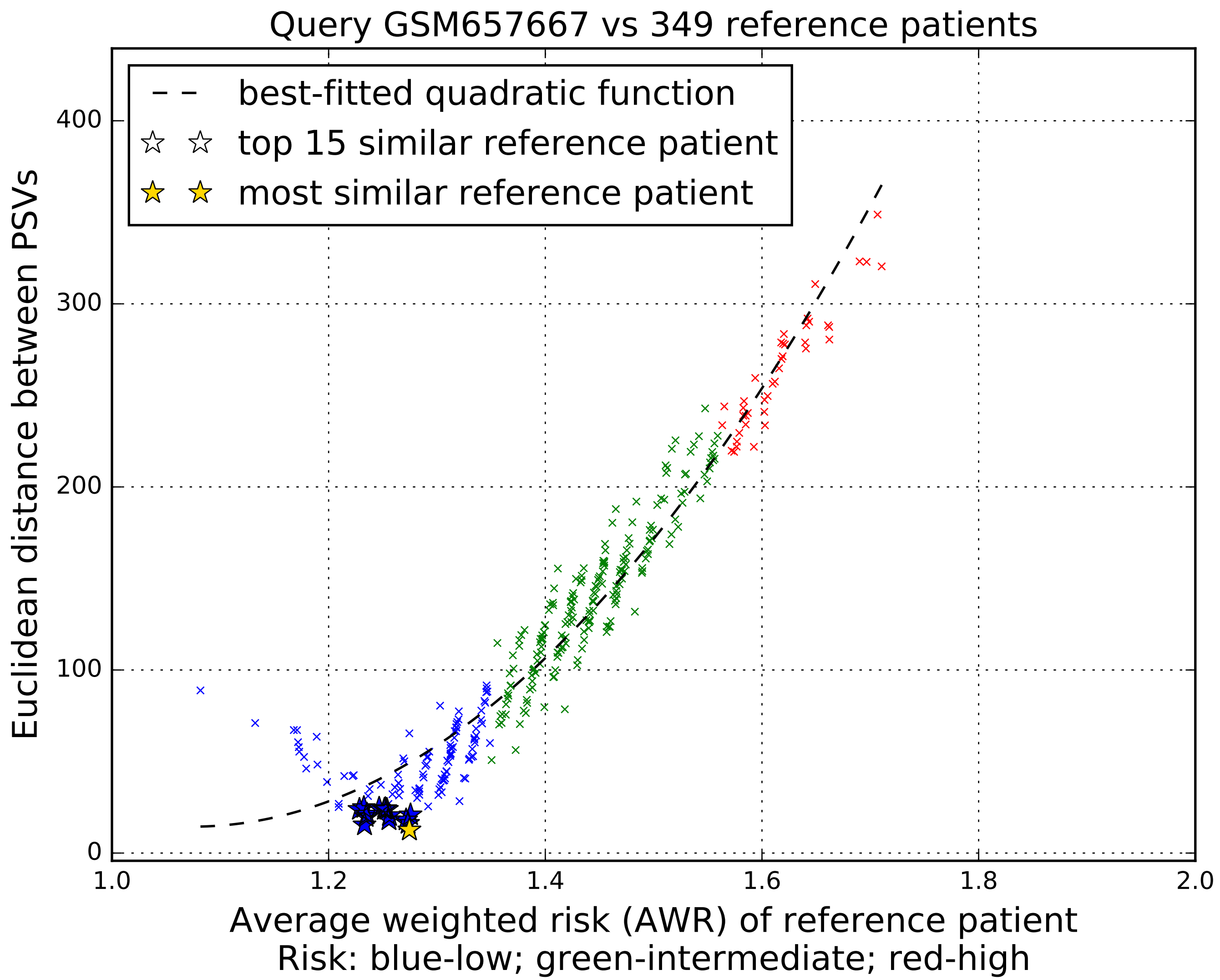

Query GSM657667 vs 349 reference patients
best-fitted quadratic function
top 15 similar reference patient
most similar reference patient
Euclidean distance between PSVs
0
100
200
300
400
1.0
1.2
1.4
1.6
1.8
2.0
Average weighted risk (AWR) of reference patient
Risk: blue-low; green-intermediate; red-high

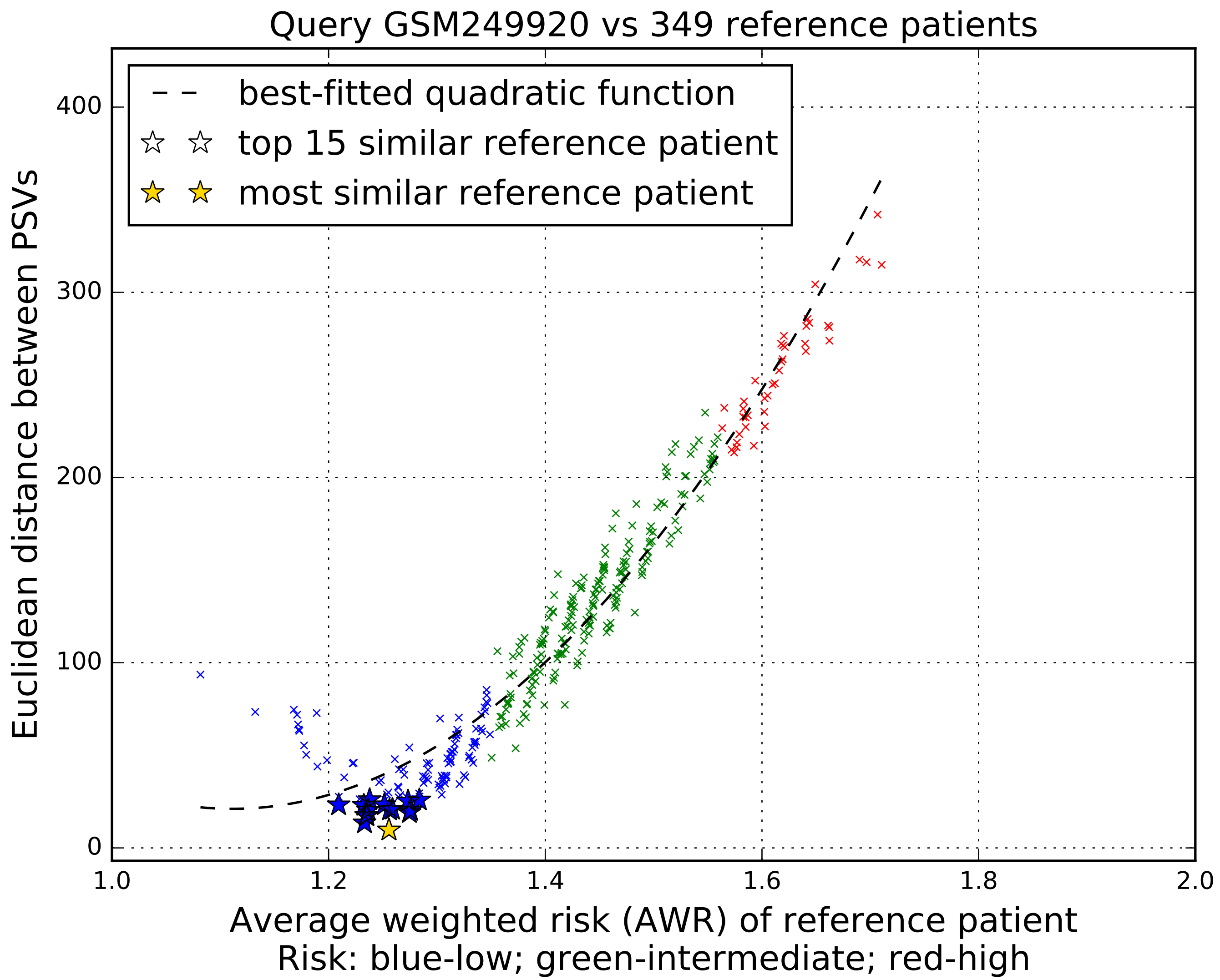

Query GSM249920 vs 349 reference patients
best-fitted quadratic function
top 15 similar reference patient
most similar reference patient
Euclidean distance between PSVs
0
100
200
300
400
1.0
1.2
1.4
1.6
1.8
2.0
Average weighted risk (AWR) of reference patient
Risk: blue-low; green-intermediate; red-high

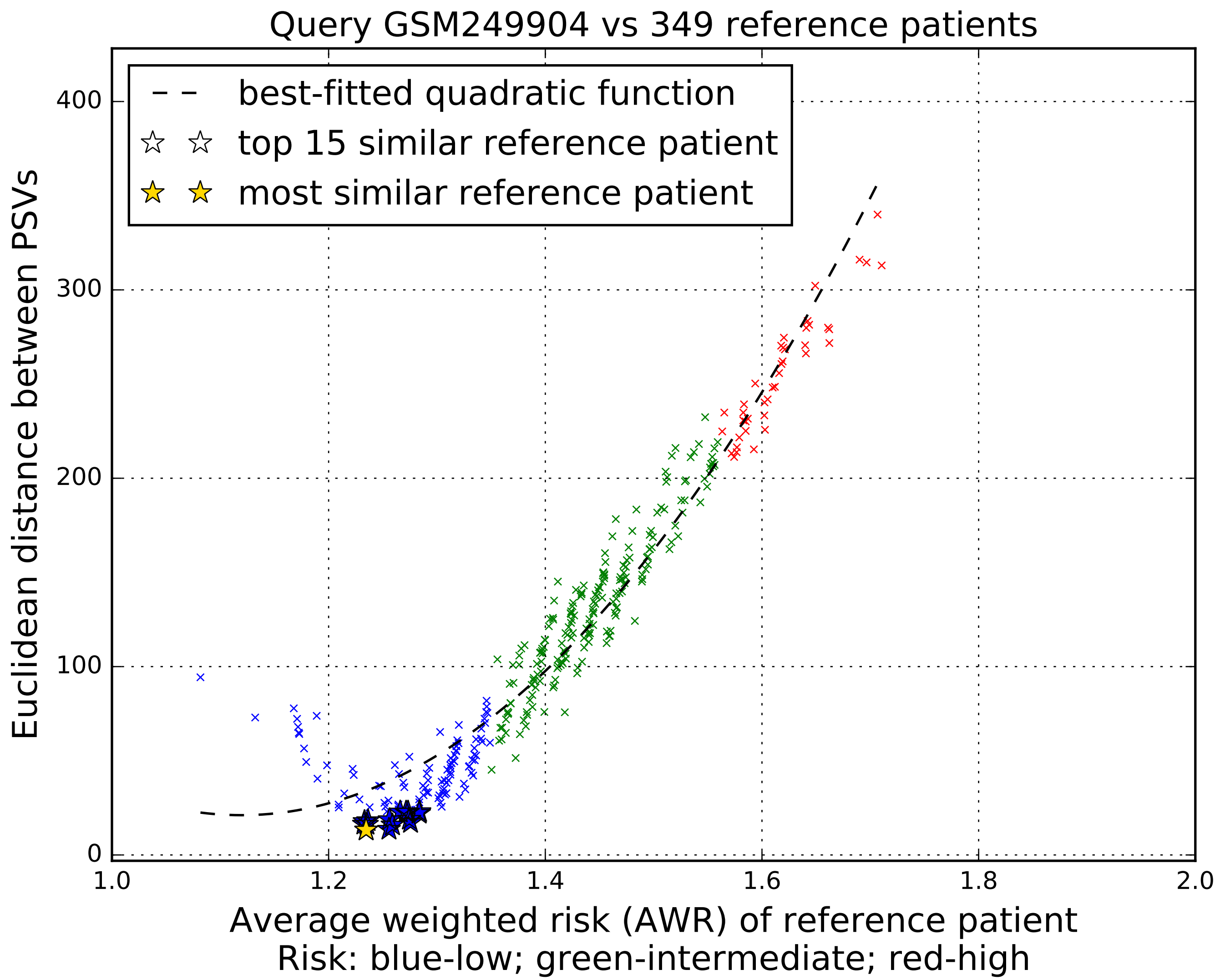

Query GSM249904 vs 349 reference patients
best-fitted quadratic function
top 15 similar reference patient
most similar reference patient
Euclidean distance between PSVs
0
100
200
300
400
1.0
1.2
1.4
1.6
1.8
2.0
Average weighted risk (AWR) of reference patient
Risk: blue-low; green-intermediate; red-high

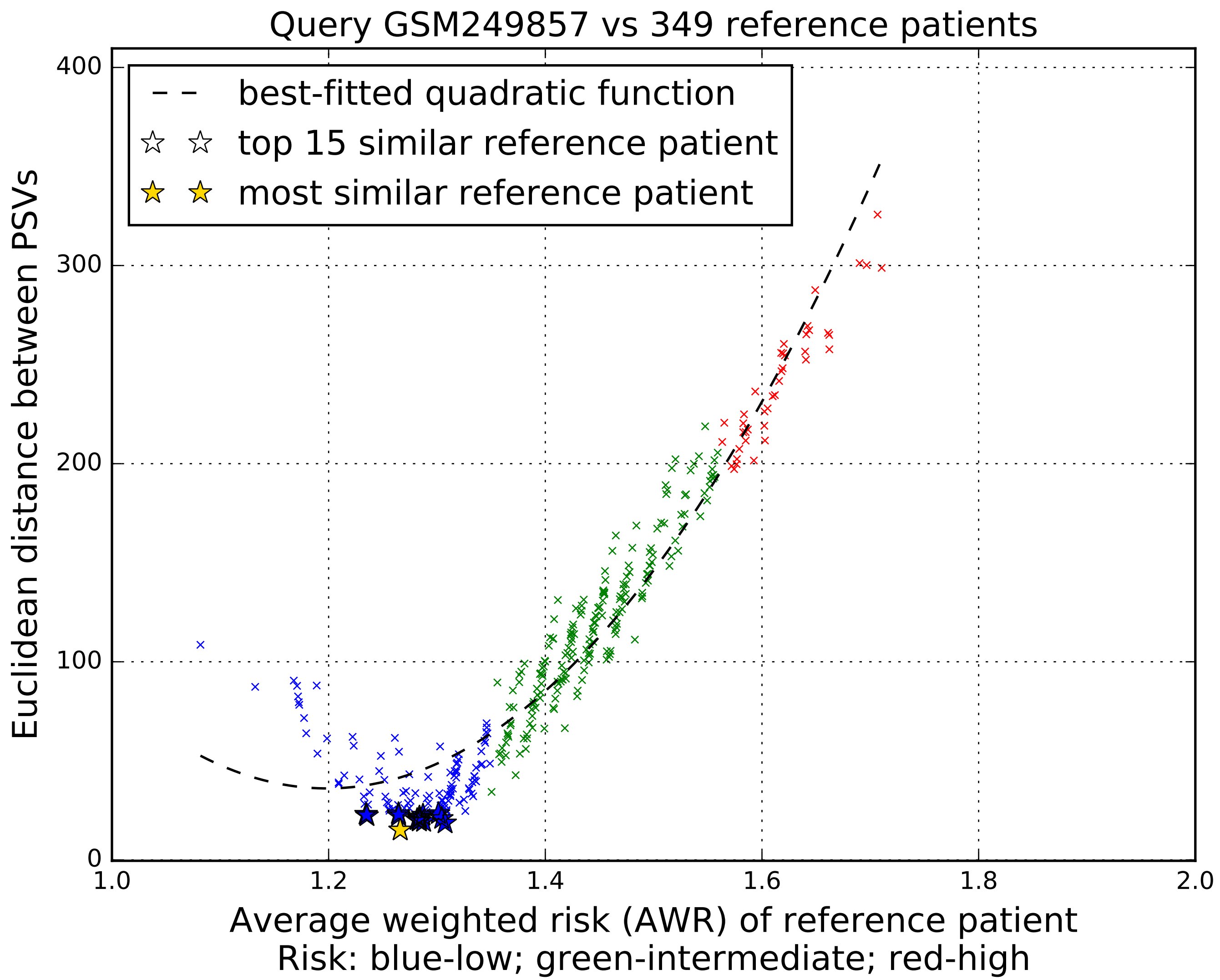

Query GSM249857 vs 349 reference patients
best-fitted quadratic function
top 15 similar reference patient
most similar reference patient
Euclidean distance between PSVs
0
100
200
300
400
1.0
1.2
1.4
1.6
1.8
2.0
Average weighted risk (AWR) of reference patient
Risk: blue-low; green-intermediate; red-high

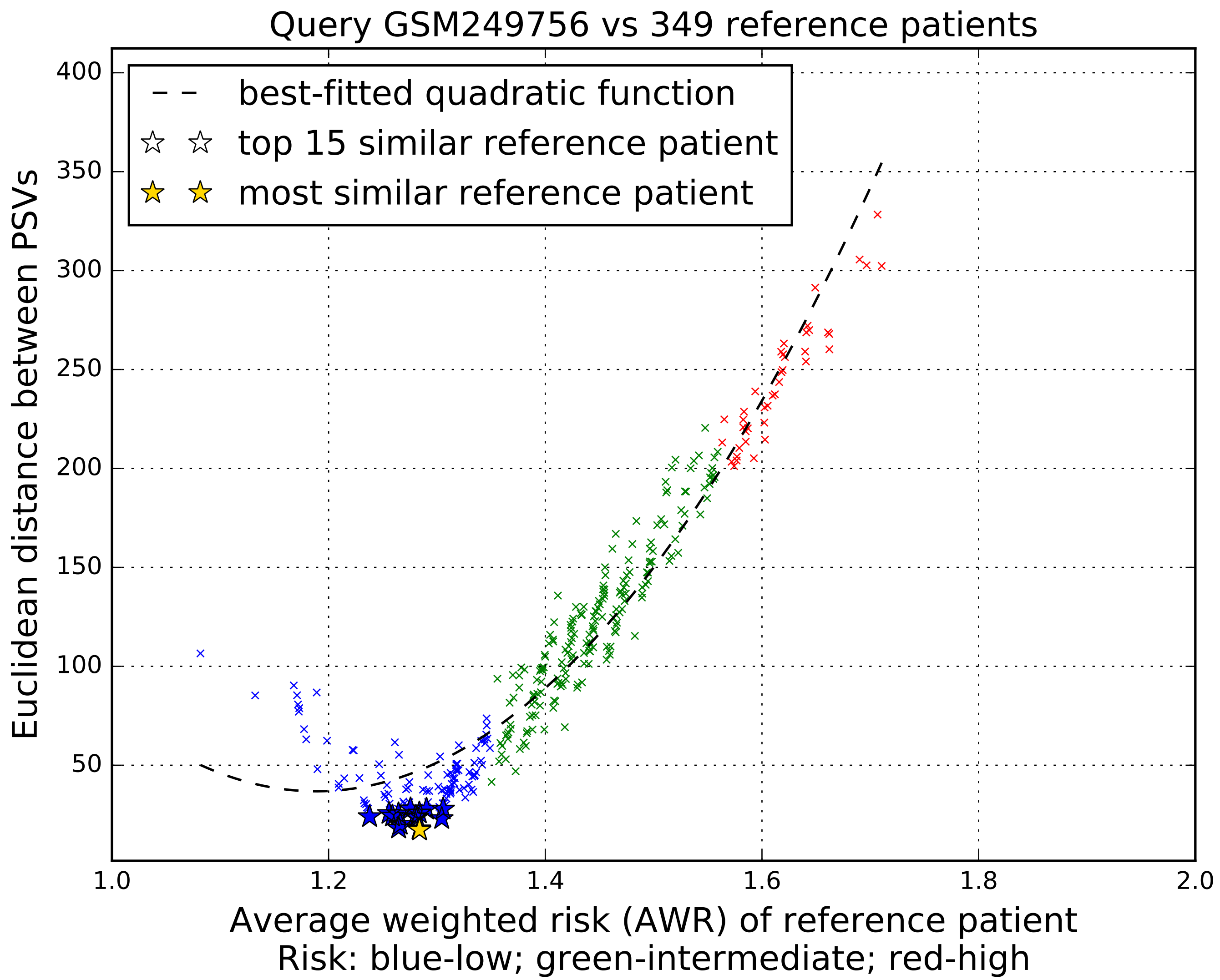

Query GSM249756 vs 349 reference patients
best-fitted quadratic function
top 15 similar reference patient
most similar reference patient
Euclidean distance between PSVs
400
350
300
250
200
150
100
50
1.0
1.2
1.4
1.6
1.8
2.0
Average weighted risk (AWR) of reference patient
Risk: blue-low; green-intermediate; red-high

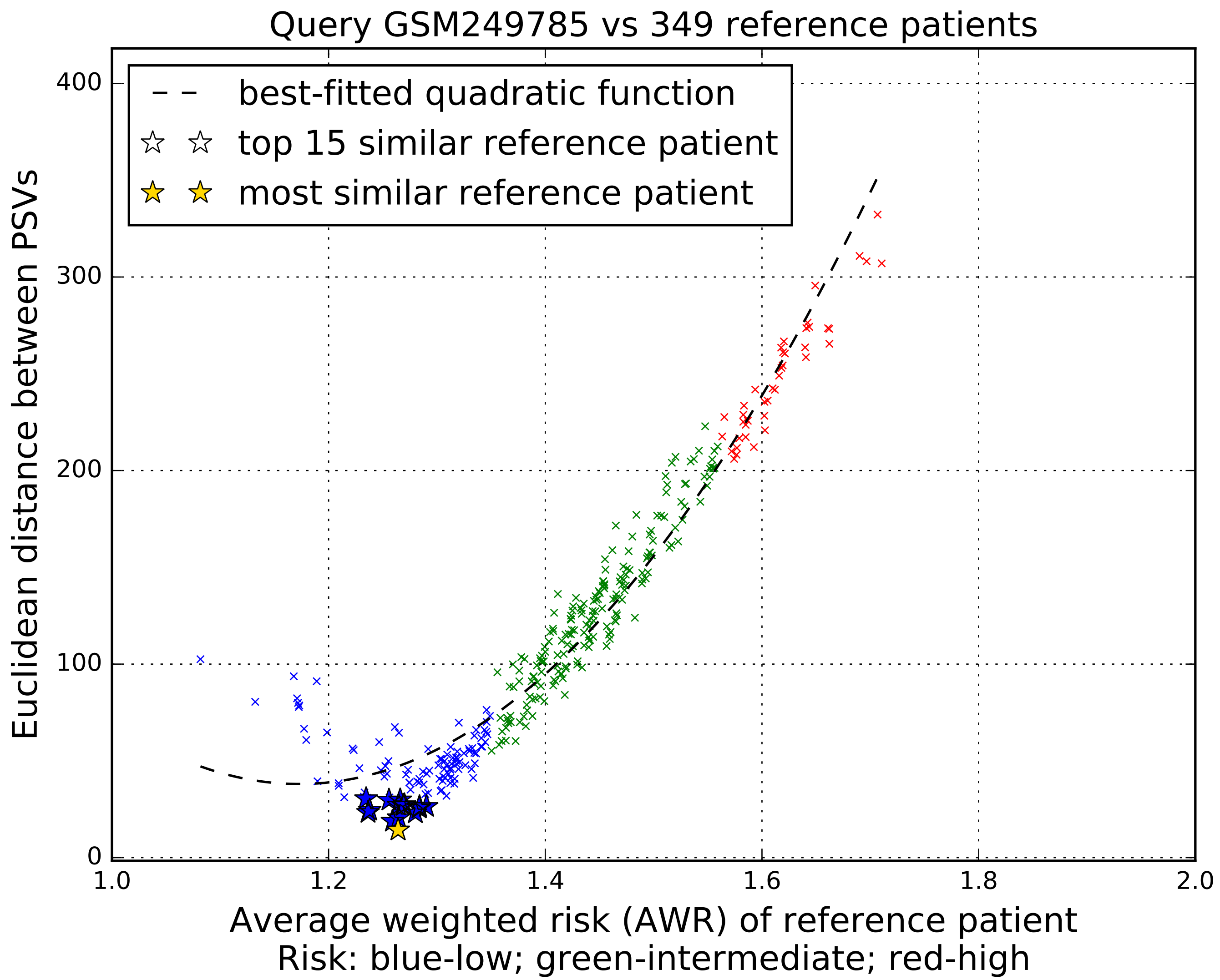
Query GSM249785 vs 349 reference patients
best-fitted quadratic function
top 15 similar reference patient
most similar reference patient
Euclidean distance between PSVs
0
100
200
300
400
1.0
1.2
1.4
1.6
1.8
2.0
Average weighted risk (AWR) of reference patient
Risk: blue-low; green-intermediate; red-high

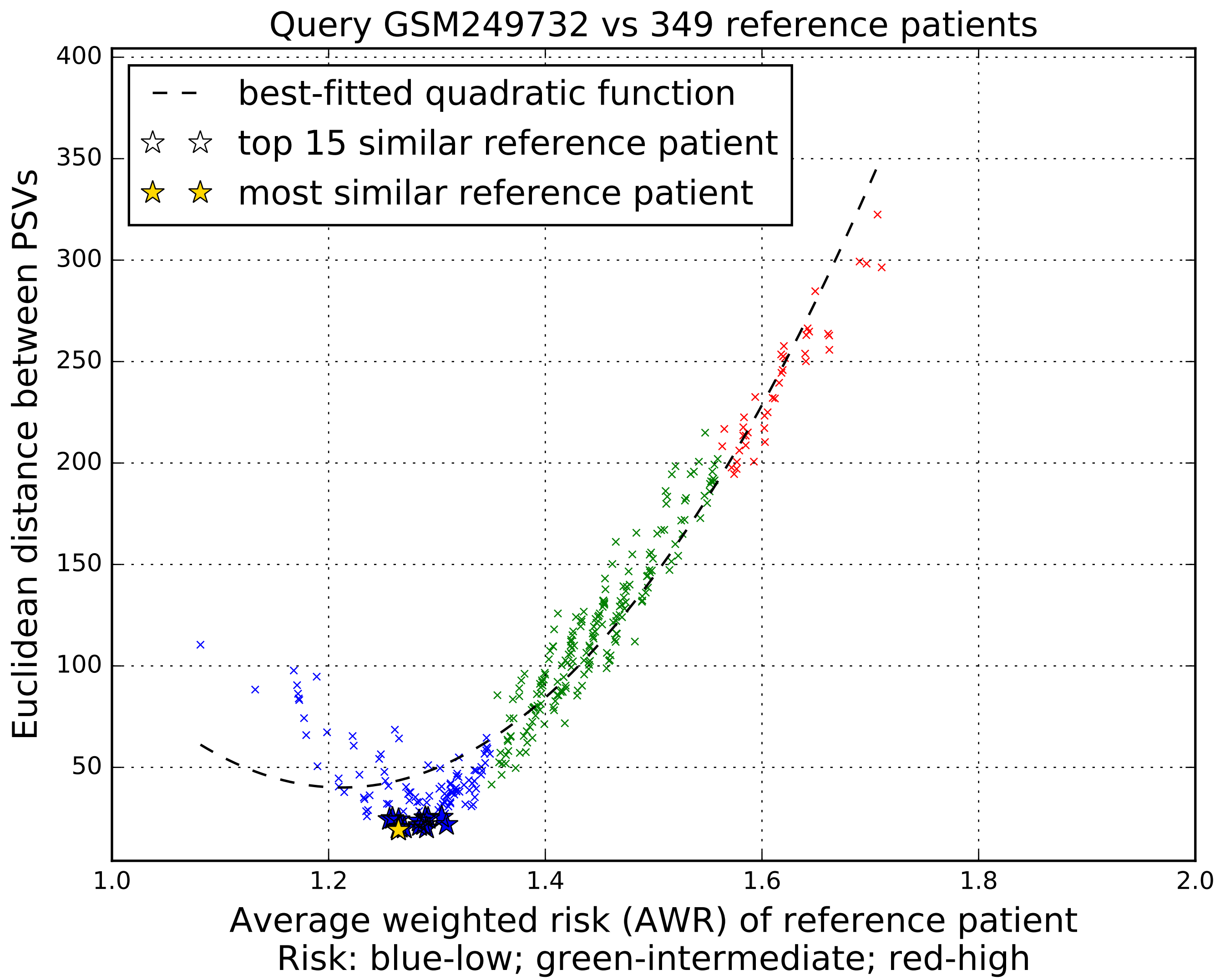

Query GSM249732 vs 349 reference patients
best-fitted quadratic function
top 15 similar reference patient
most similar reference patient
Euclidean distance between PSVs
400
350
300
250
200
150
100
50
1.0
1.2
1.4
1.6
1.8
2.0
Average weighted risk (AWR) of reference patient
Risk: blue-low; green-intermediate; red-high

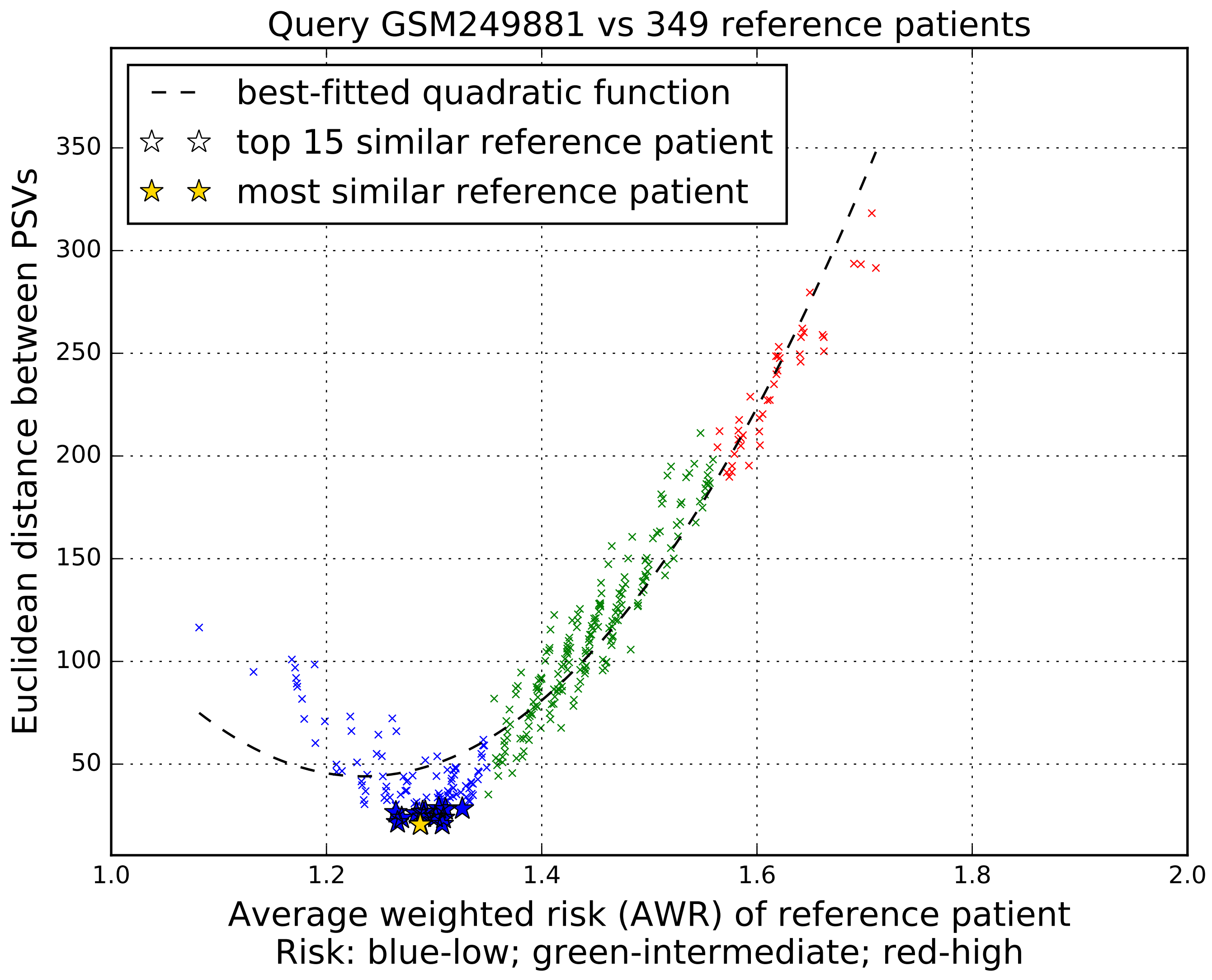

Query GSM249881 vs 349 reference patients
best-fitted quadratic function
top 15 similar reference patient
most similar reference patient
Euclidean distance between PSVs
350
300
250
200
150
100
50
1.0
1.2
1.4
1.6
1.8
2.0
Average weighted risk (AWR) of reference patient
Risk: blue-low; green-intermediate; red-high

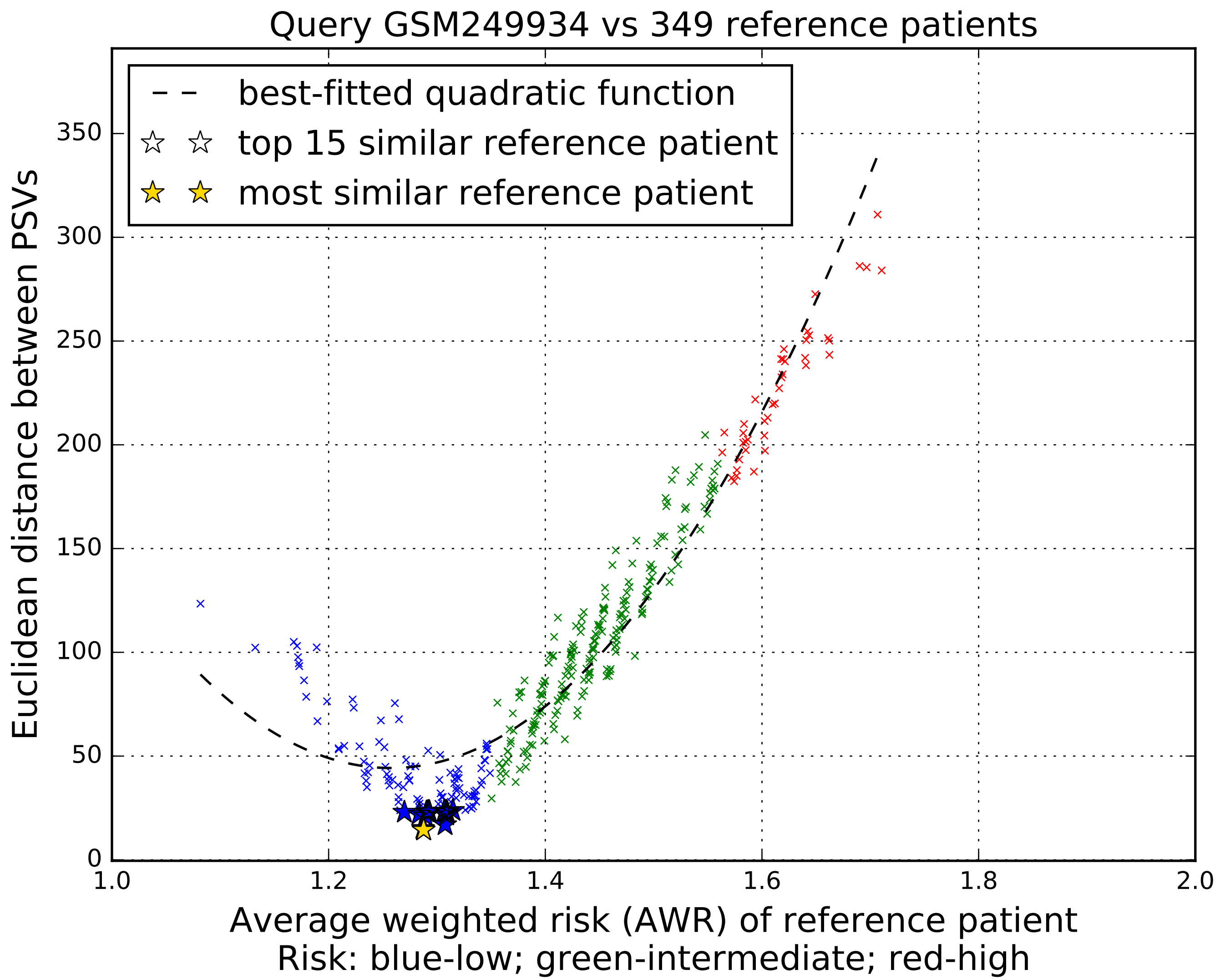
Query GSM249934 vs 349 reference patients
best-fitted quadratic function
top 15 similar reference patient
most similar reference patient
Euclidean distance between PSVs
0
50
100
150
200
250
300
350
1.0
1.2
1.4
1.6
1.8
2.0
Average weighted risk (AWR) of reference patient
Risk: blue-low; green-intermediate; red-high

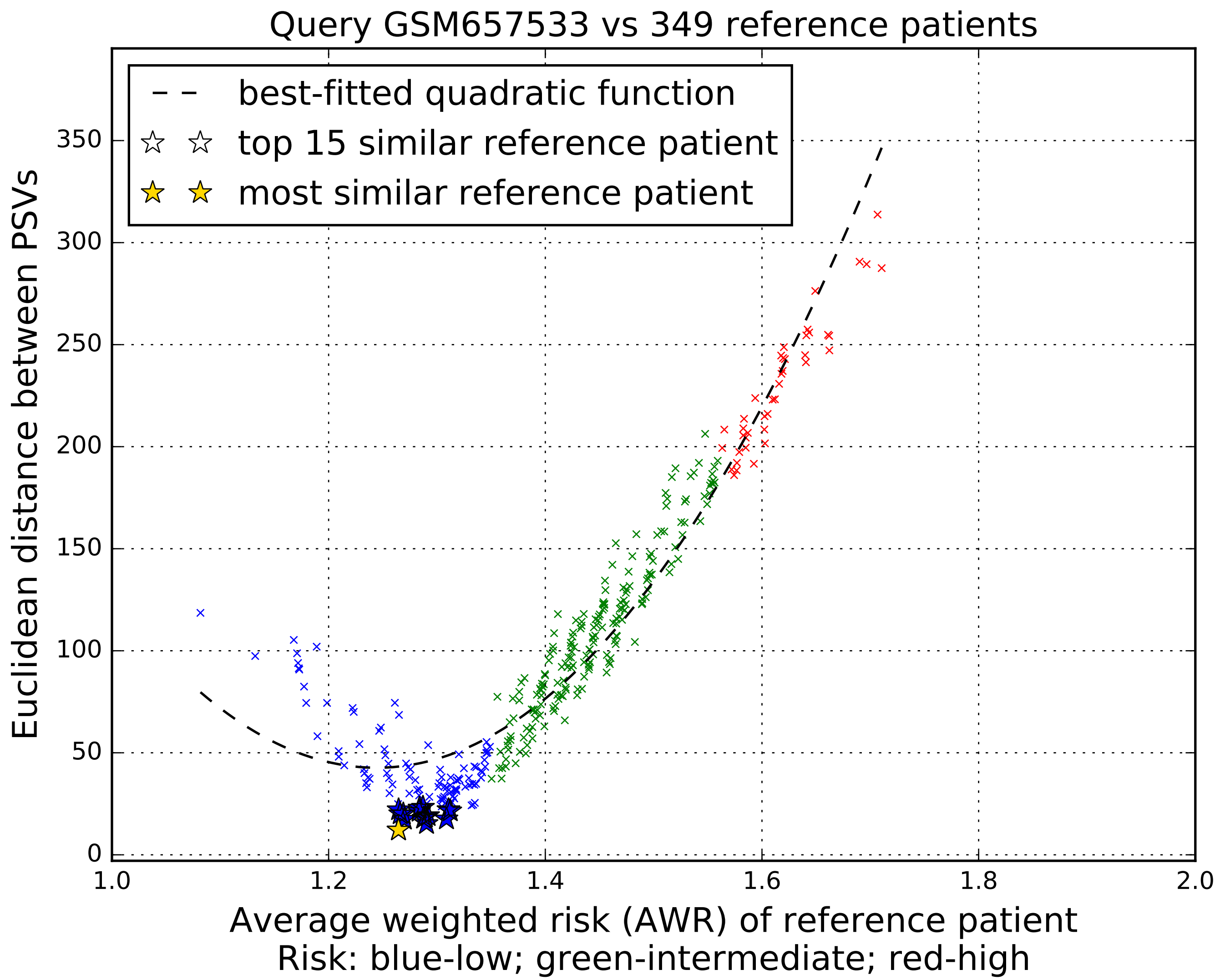
Query GSM657533 vs 349 reference patients
best-fitted quadratic function
top 15 similar reference patient
most similar reference patient
Euclidean distance between PSVs
0
50
100
150
200
250
300
350
1.0
1.2
1.4
1.6
1.8
2.0
Average weighted risk (AWR) of reference patient
Risk: blue-low; green-intermediate; red-high

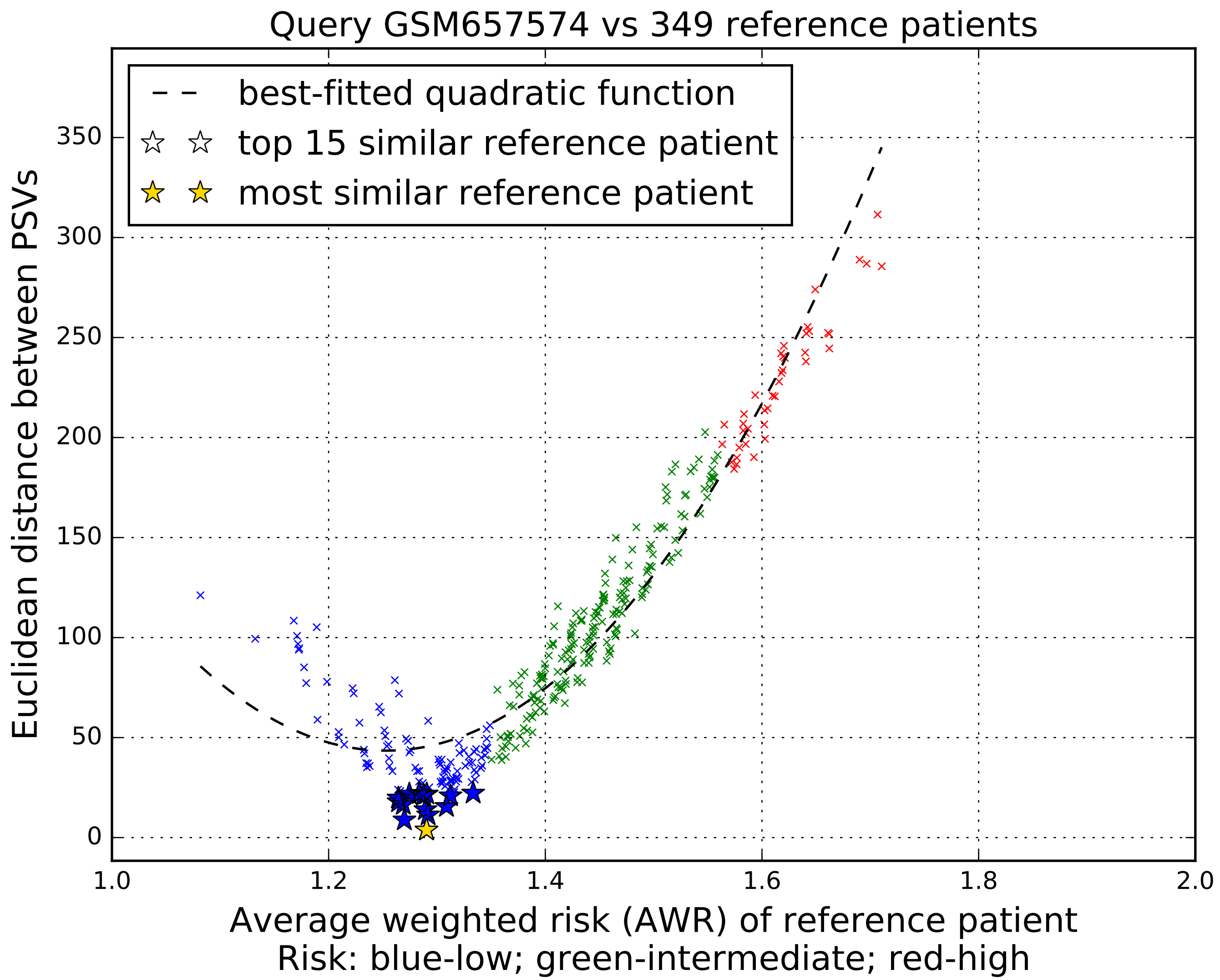

Query GSM657574 vs 349 reference patients
best-fitted quadratic function
top 15 similar reference patient
most similar reference patient
Euclidean distance between PSVs
0
50
100
150
200
250
300
350
1.0
1.2
1.4
1.6
1.8
2.0
Average weighted risk (AWR) of reference patient
Risk: blue-low; green-intermediate; red-high

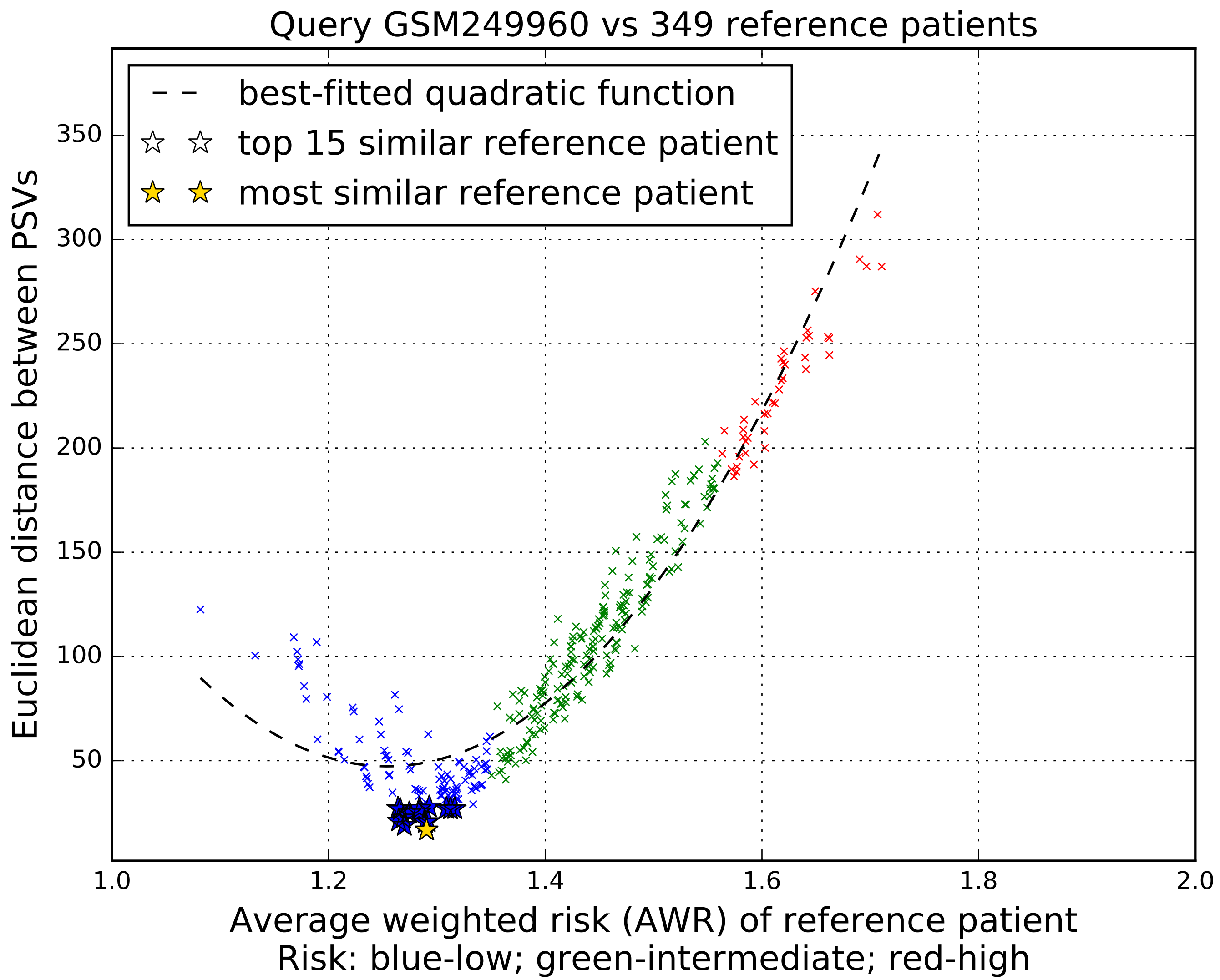
Query GSM249960 vs 349 reference patients
best-fitted quadratic function
top 15 similar reference patient
most similar reference patient
Euclidean distance between PSVs
Average weighted risk (AWR) of reference patient
Risk: blue-low; green-intermediate; red-high
350
300
250
200
150
100
50
1.0
1.2
1.4
1.6
1.8
2.0

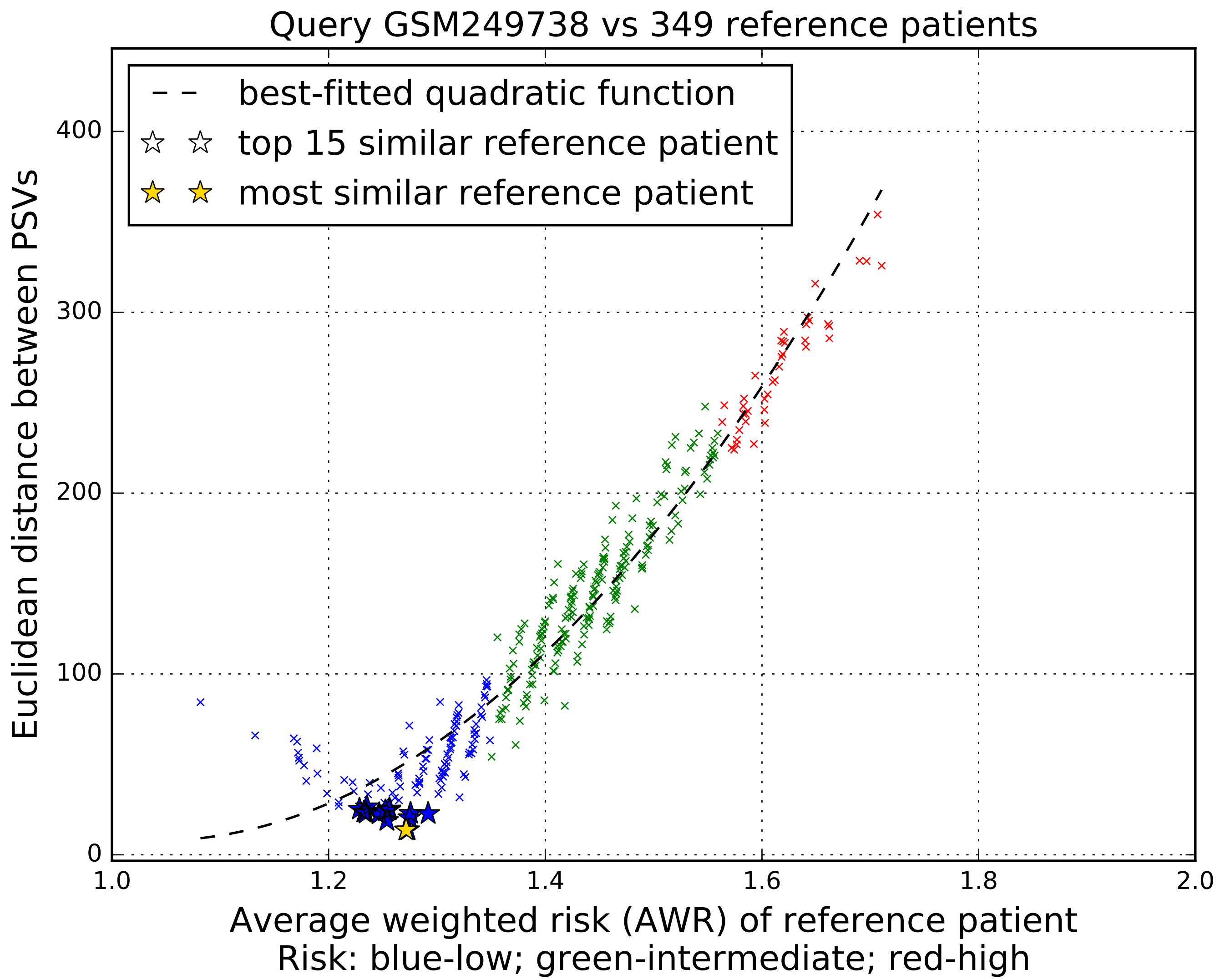
Query GSM249738 vs 349 reference patients
best-fitted quadratic function
top 15 similar reference patient
most similar reference patient
Euclidean distance between PSVs
0
100
200
300
400
1.0
1.2
1.4
1.6
1.8
2.0
Average weighted risk (AWR) of reference patient
Risk: blue-low; green-intermediate; red-high

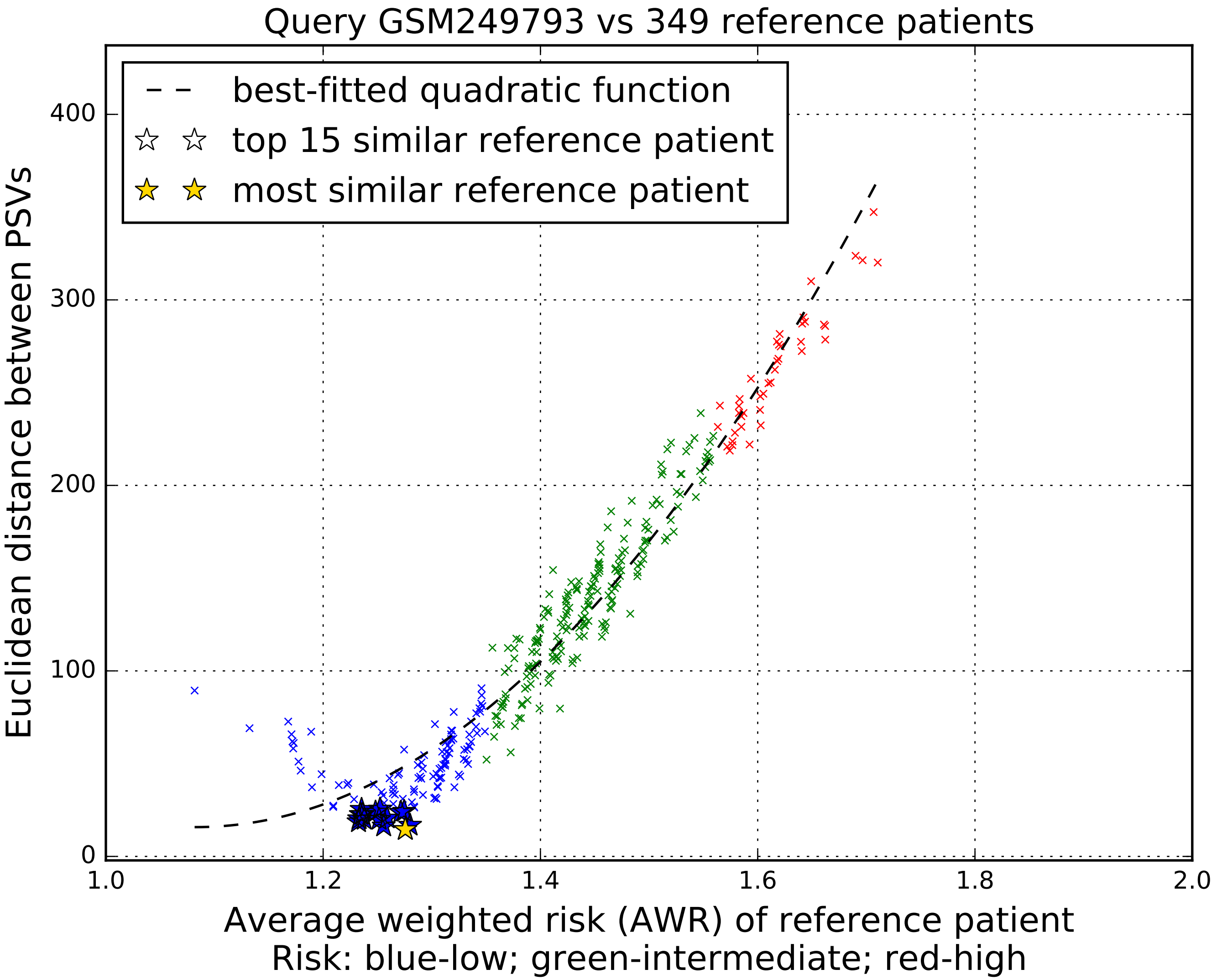
Query GSM249793 vs 349 reference patients
best-fitted quadratic function
top 15 similar reference patient
most similar reference patient
Euclidean distance between PSVs
Average weighted risk (AWR) of reference patient
Risk: blue-low; green-intermediate; red-high
0
100
200
300
400
1.0
1.2
1.4
1.6
1.8
2.0

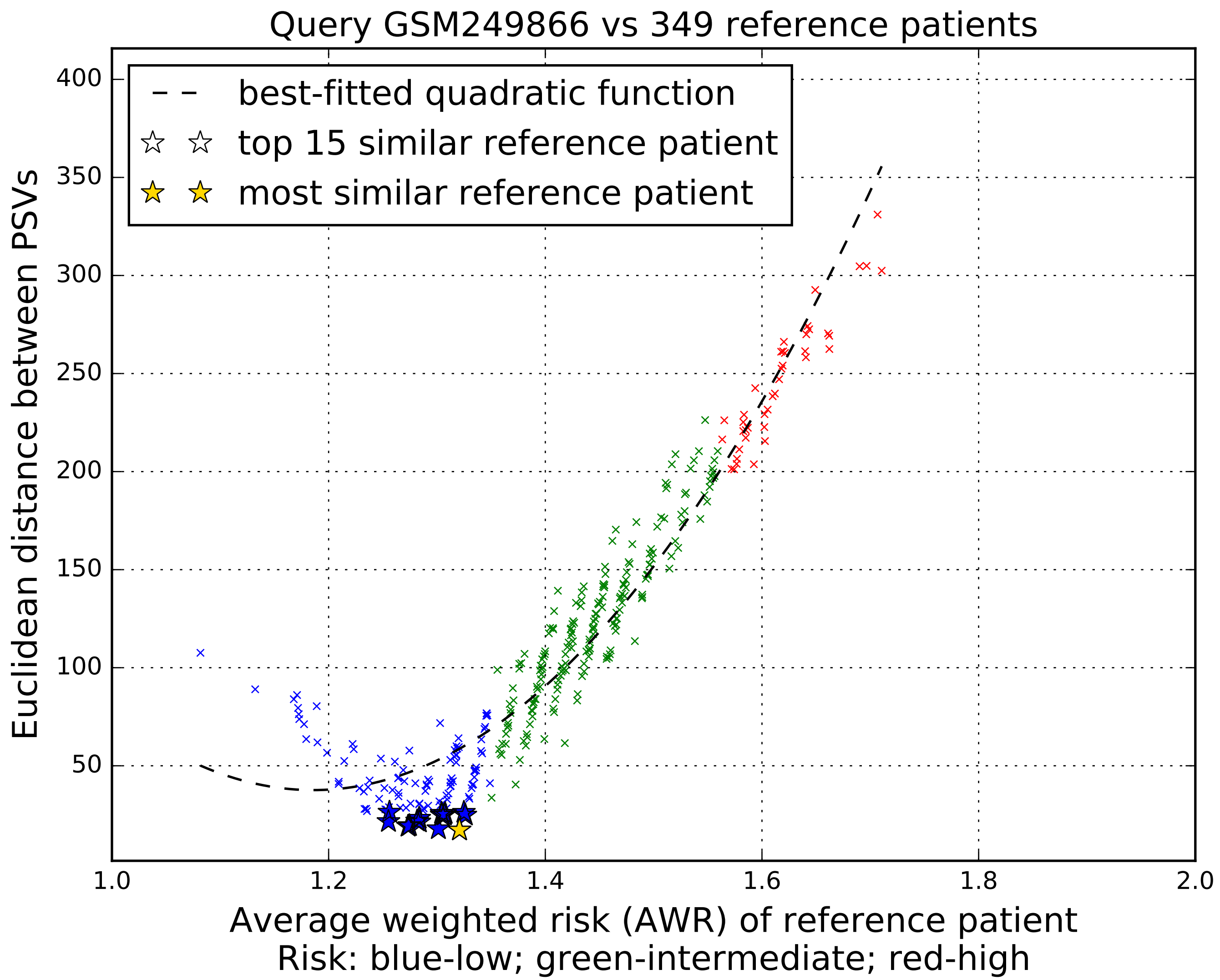

Query GSM249866 vs 349 reference patients
best-fitted quadratic function
top 15 similar reference patient
most similar reference patient
Euclidean distance between PSVs
400
350
300
250
200
150
100
50
1.0
1.2
1.4
1.6
1.8
2.0
Average weighted risk (AWR) of reference patient
Risk: blue-low; green-intermediate; red-high

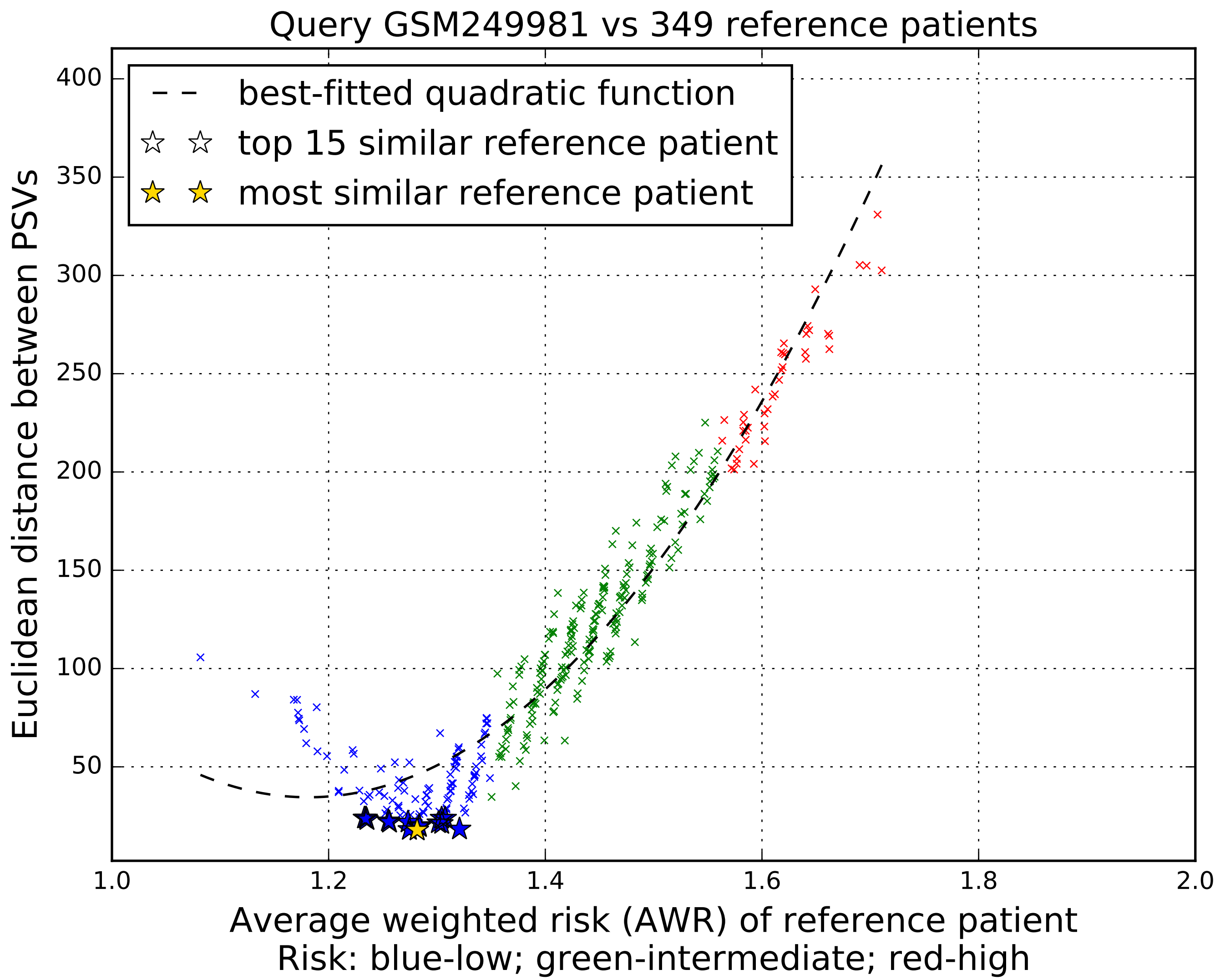
Query GSM249981 vs 349 reference patients
best-fitted quadratic function
top 15 similar reference patient
most similar reference patient
Euclidean distance between PSVs
400
350
300
250
200
150
100
50
1.0
1.2
1.4
1.6
1.8
2.0
Average weighted risk (AWR) of reference patient
Risk: blue-low; green-intermediate; red-high

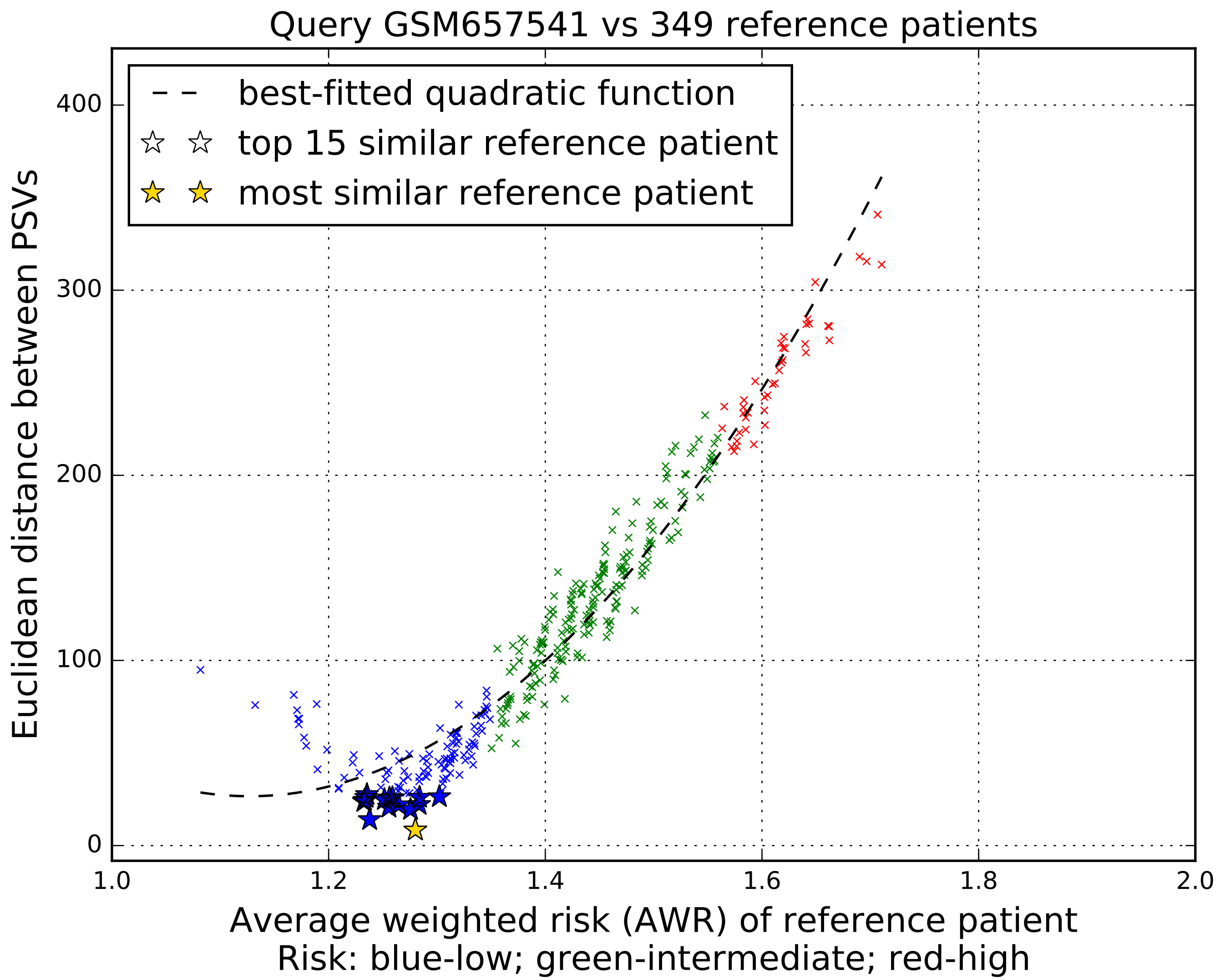

Query GSM657541 vs 349 reference patients
best-fitted quadratic function
top 15 similar reference patient
most similar reference patient
Euclidean distance between PSVs
0
100
200
300
400
1.0
1.2
1.4
1.6
1.8
2.0
Average weighted risk (AWR) of reference patient
Risk: blue-low; green-intermediate; red-high

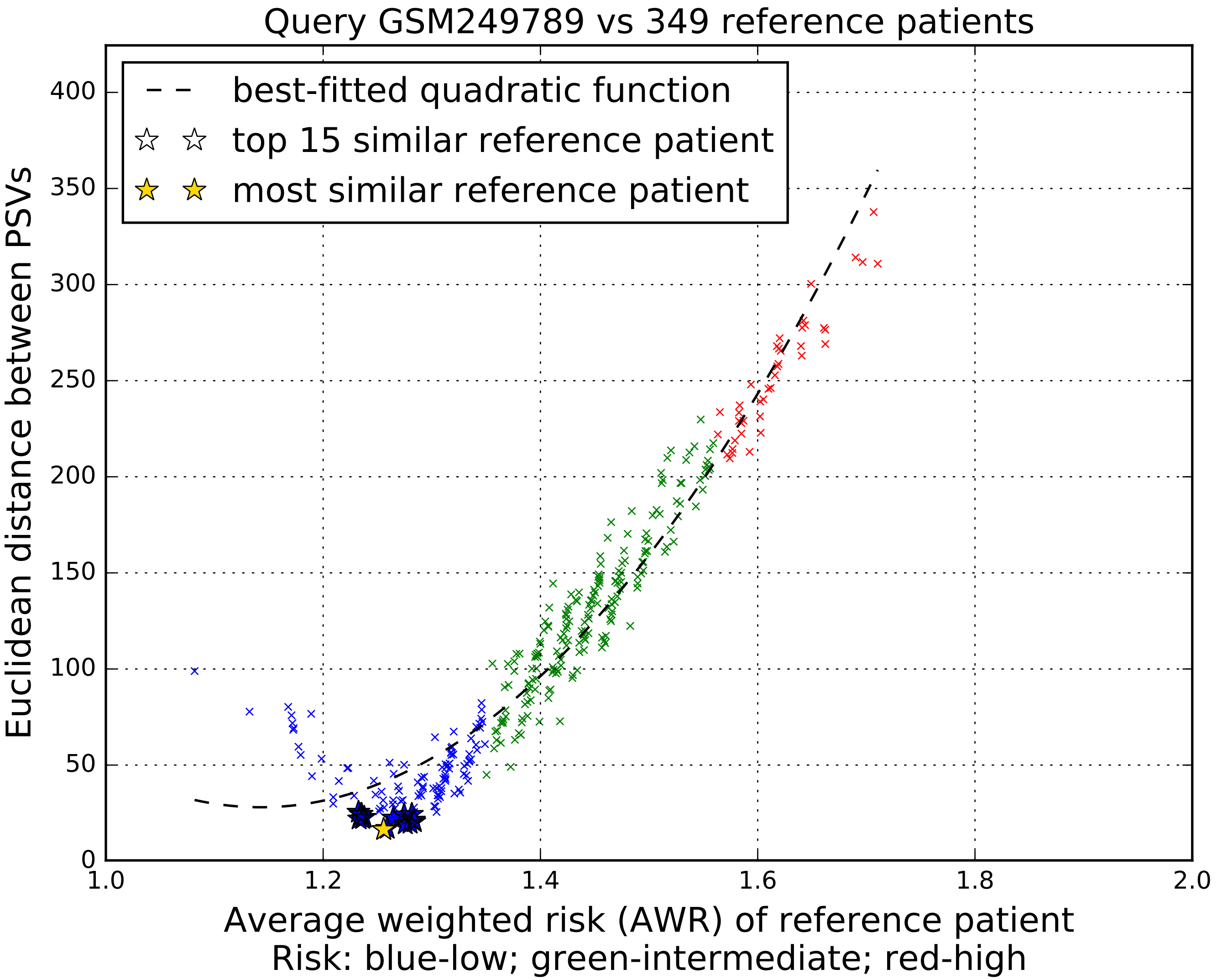

Query GSM249789 vs 349 reference patients
best-fitted quadratic function
top 15 similar reference patient
most similar reference patient
Euclidean distance between PSVs
0
50
100
150
200
250
300
350
400
1.0
1.2
1.4
1.6
1.8
2.0
Average weighted risk (AWR) of reference patient
Risk: blue-low; green-intermediate; red-high

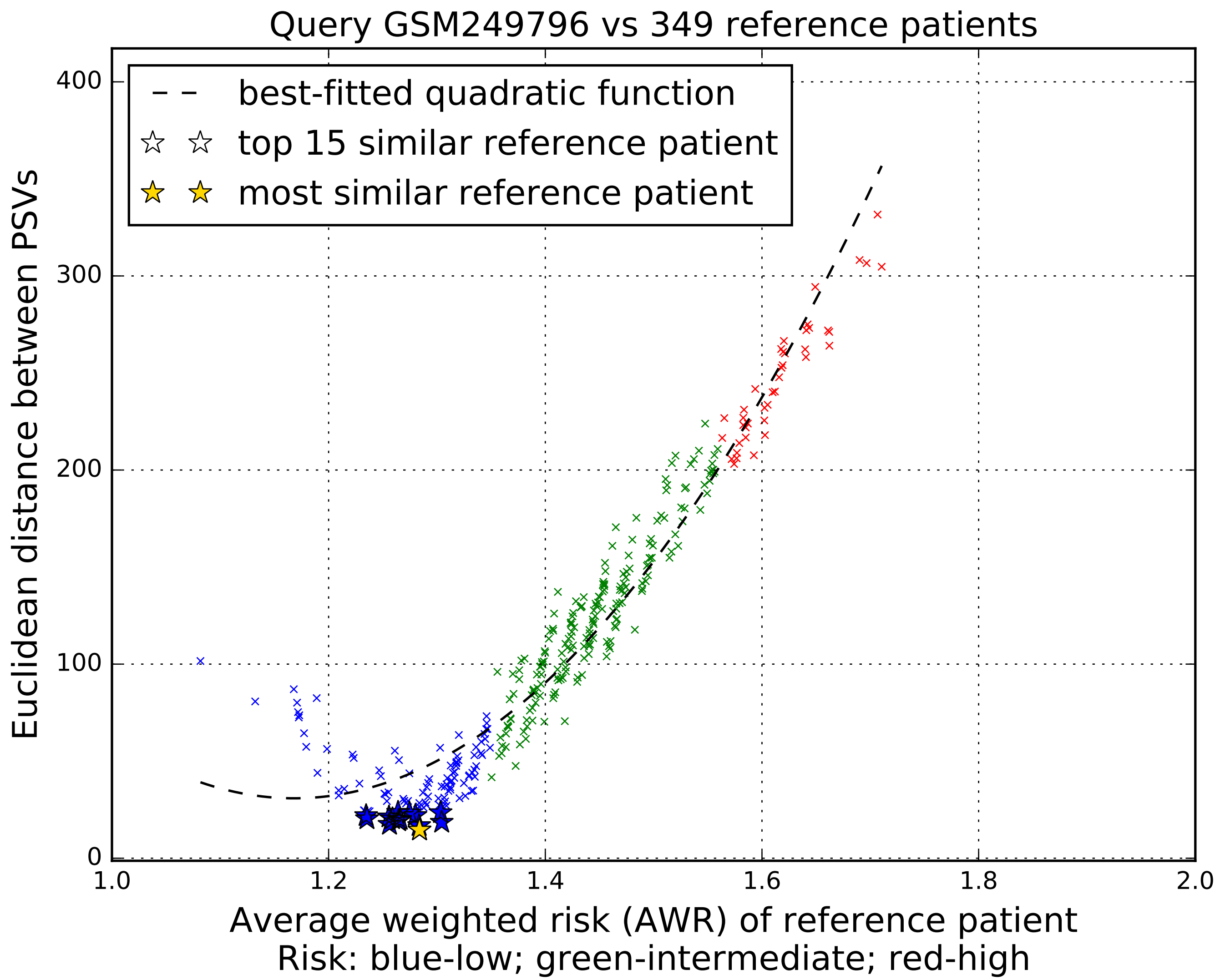
Query GSM249796 vs 349 reference patients
best-fitted quadratic function
top 15 similar reference patient
most similar reference patient
Euclidean distance between PSVs
0
100
200
300
400
1.0
1.2
1.4
1.6
1.8
2.0
Average weighted risk (AWR) of reference patient
Risk: blue-low; green-intermediate; red-high

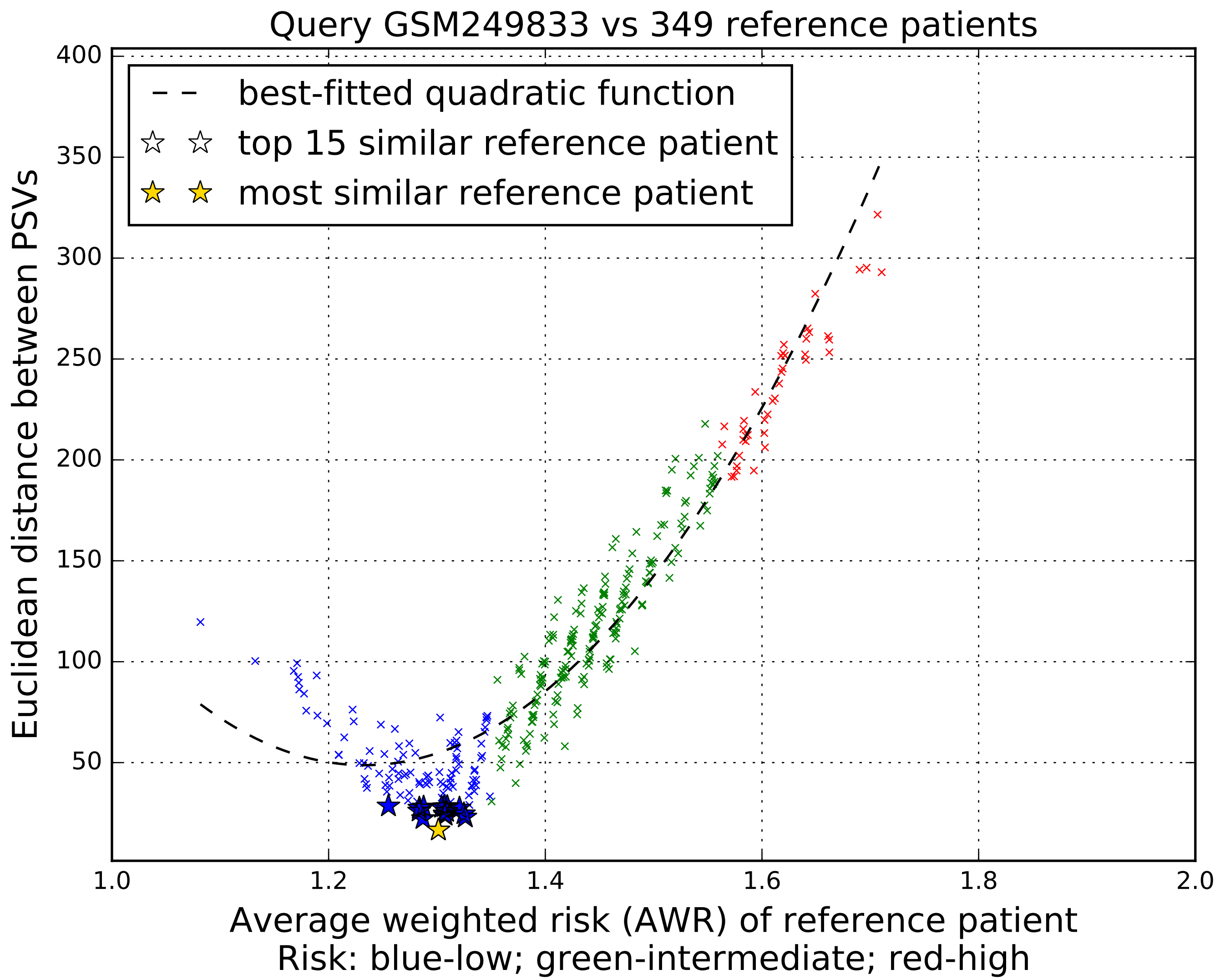
Query GSM249833 vs 349 reference patients
best-fitted quadratic function
top 15 similar reference patient
most similar reference patient
Euclidean distance between PSVs
400
350
300
250
200
150
100
50
1.0
1.2
1.4
1.6
1.8
2.0
Average weighted risk (AWR) of reference patient
Risk: blue-low; green-intermediate; red-high

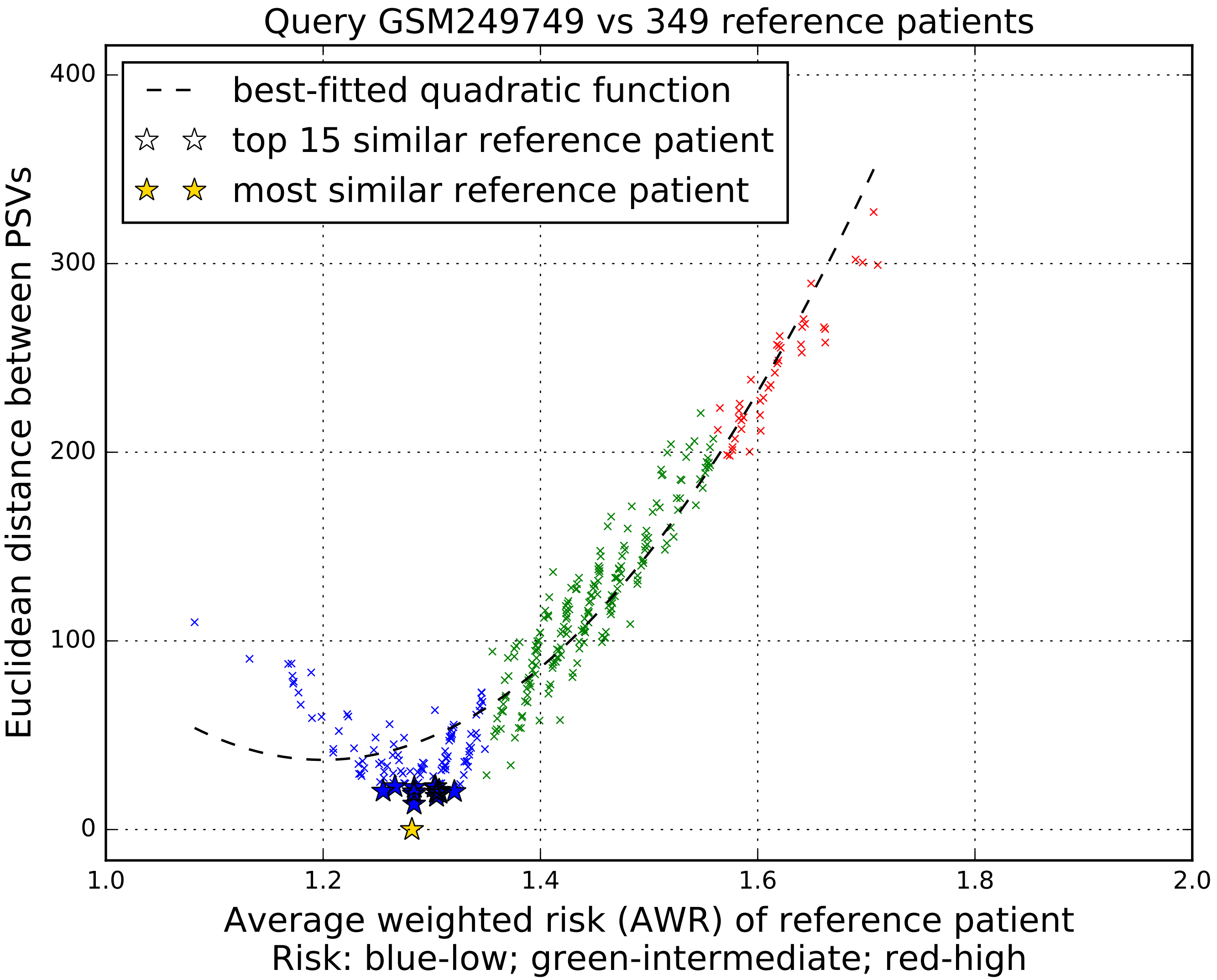
Query GSM249749 vs 349 reference patients
best-fitted quadratic function
top 15 similar reference patient
most similar reference patient
Euclidean distance between PSVs
0
100
200
300
400
1.0
1.2
1.4
1.6
1.8
2.0
Average weighted risk (AWR) of reference patient
Risk: blue-low; green-intermediate; red-high

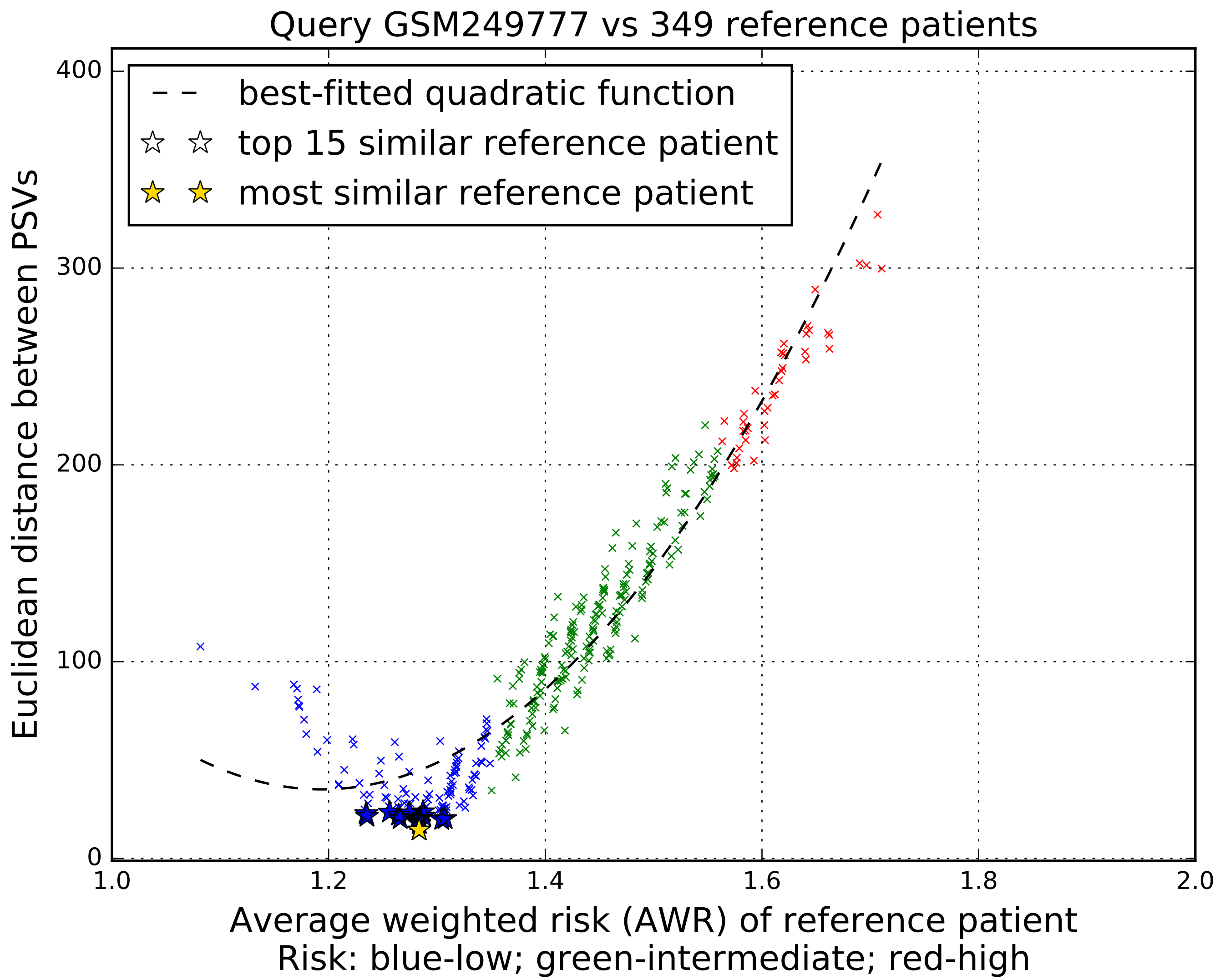

Query GSM249777 vs 349 reference patients
best-fitted quadratic function
top 15 similar reference patient
most similar reference patient
Euclidean distance between PSVs
Average weighted risk (AWR) of reference patient
Risk: blue-low; green-intermediate; red-high
400
300
200
100
0
1.0
1.2
1.4
1.6
1.8
2.0

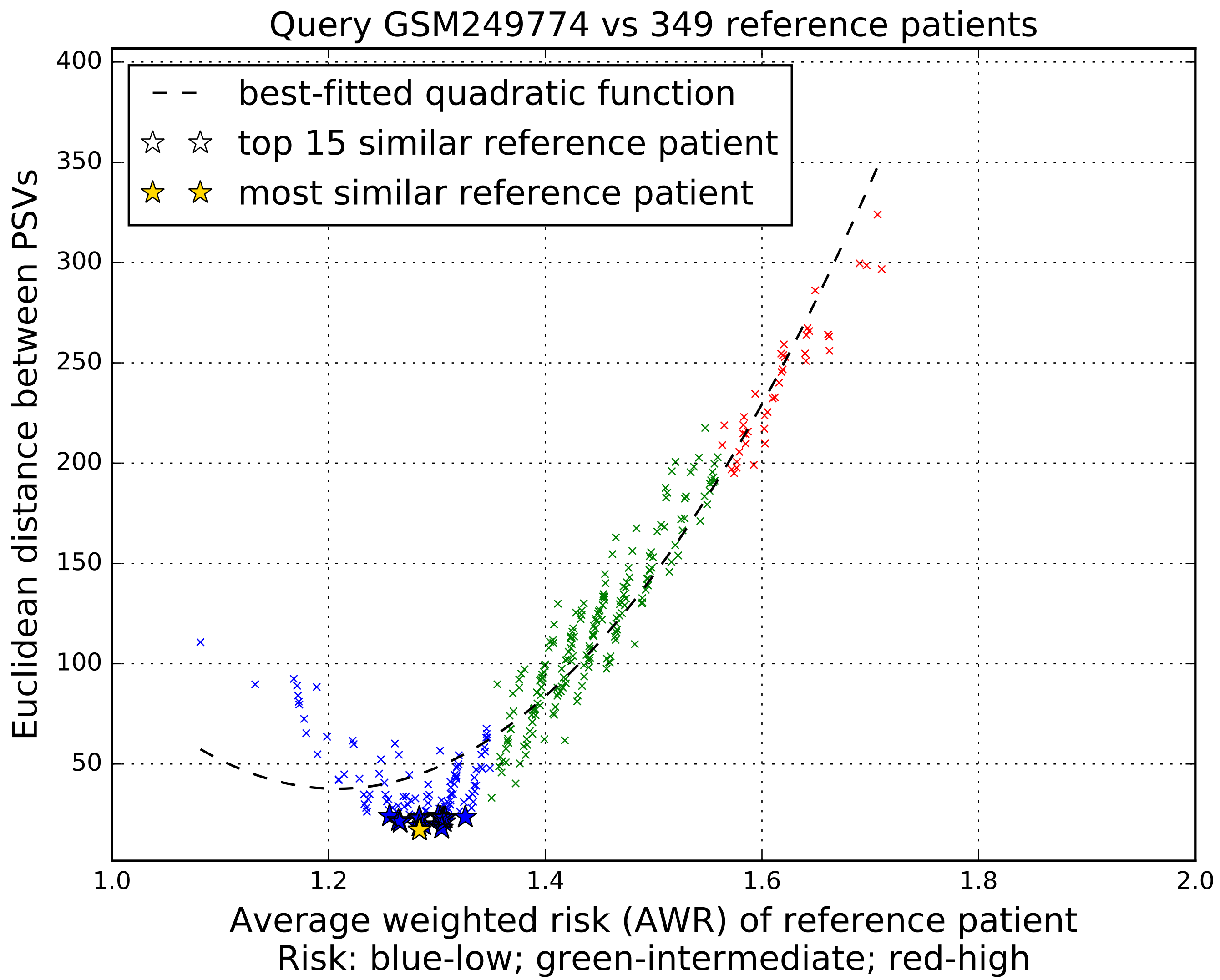

Query GSM249774 vs 349 reference patients
best-fitted quadratic function
top 15 similar reference patient
most similar reference patient
Euclidean distance between PSVs
400
350
300
250
200
150
100
50
1.0
1.2
1.4
1.6
1.8
2.0
Average weighted risk (AWR) of reference patient
Risk: blue-low; green-intermediate; red-high

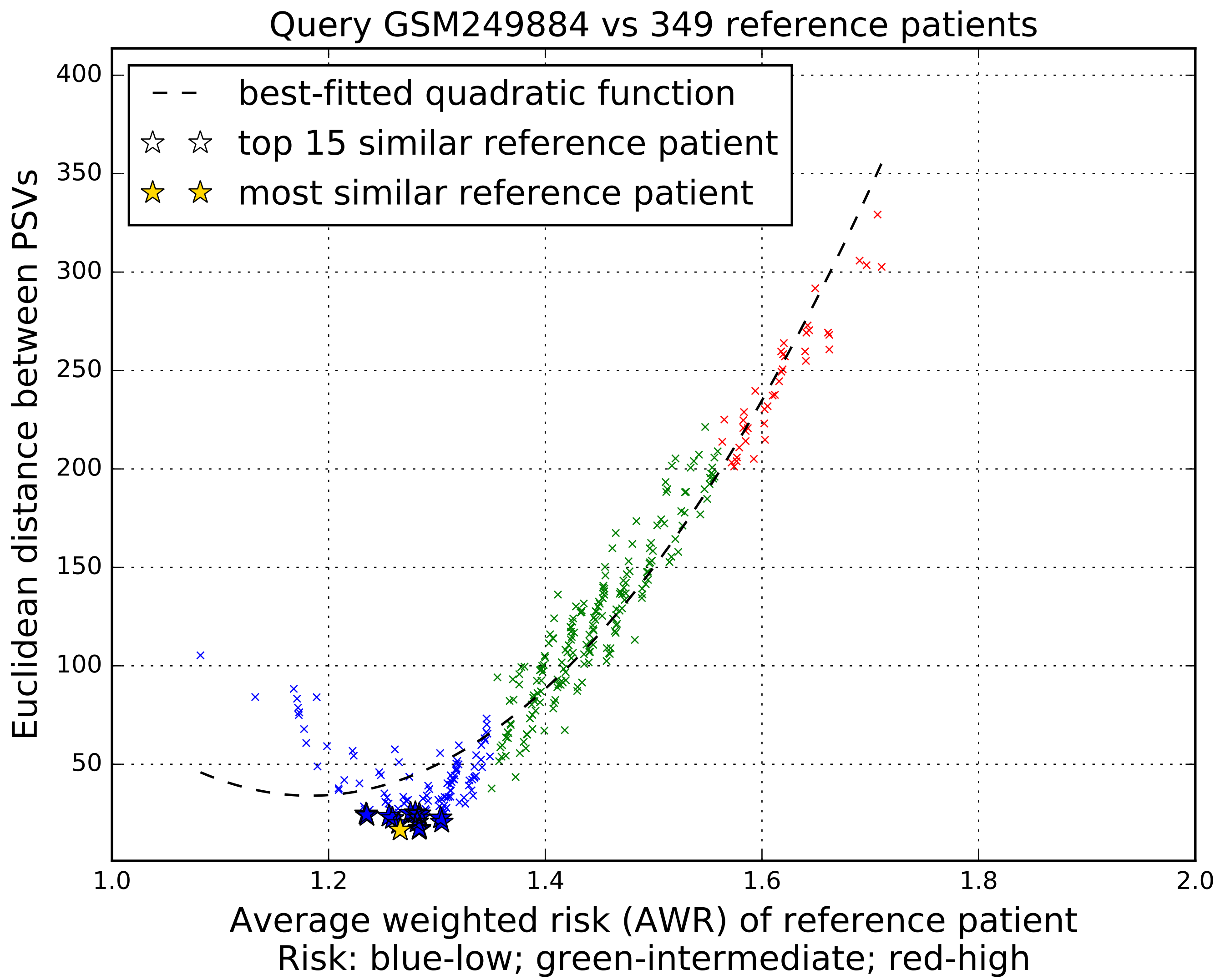
Query GSM249884 vs 349 reference patients
best-fitted quadratic function
top 15 similar reference patient
most similar reference patient
Euclidean distance between PSVs
400
350
300
250
200
150
100
50
1.0
1.2
1.4
1.6
1.8
2.0
Average weighted risk (AWR) of reference patient
Risk: blue-low; green-intermediate; red-high

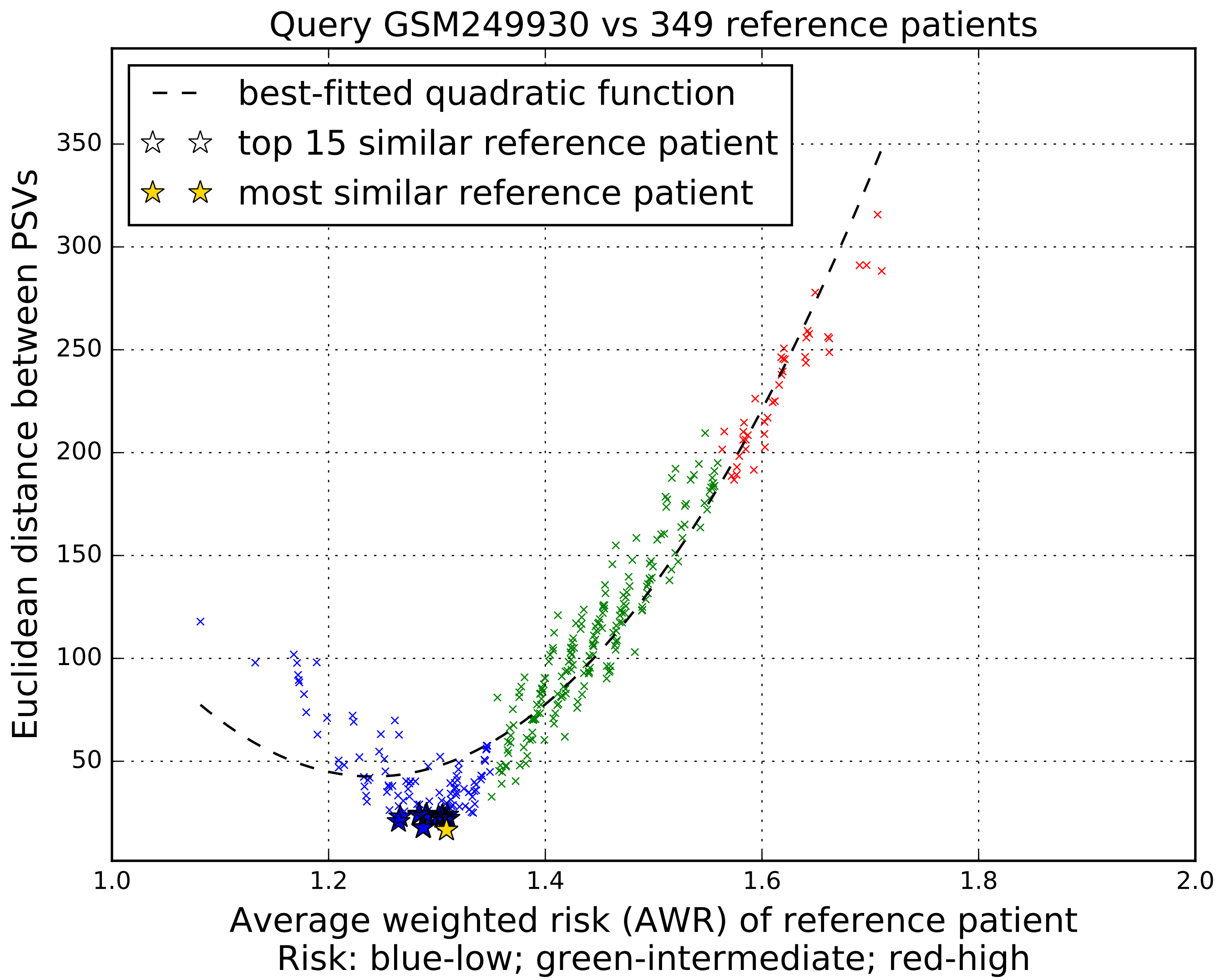

Query GSM249930 vs 349 reference patients
best-fitted quadratic function
top 15 similar reference patient
most similar reference patient
Euclidean distance between PSVs
350
300
250
200
150
100
50
1.0
1.2
1.4
1.6
1.8
2.0
Average weighted risk (AWR) of reference patient
Risk: blue-low; green-intermediate; red-high

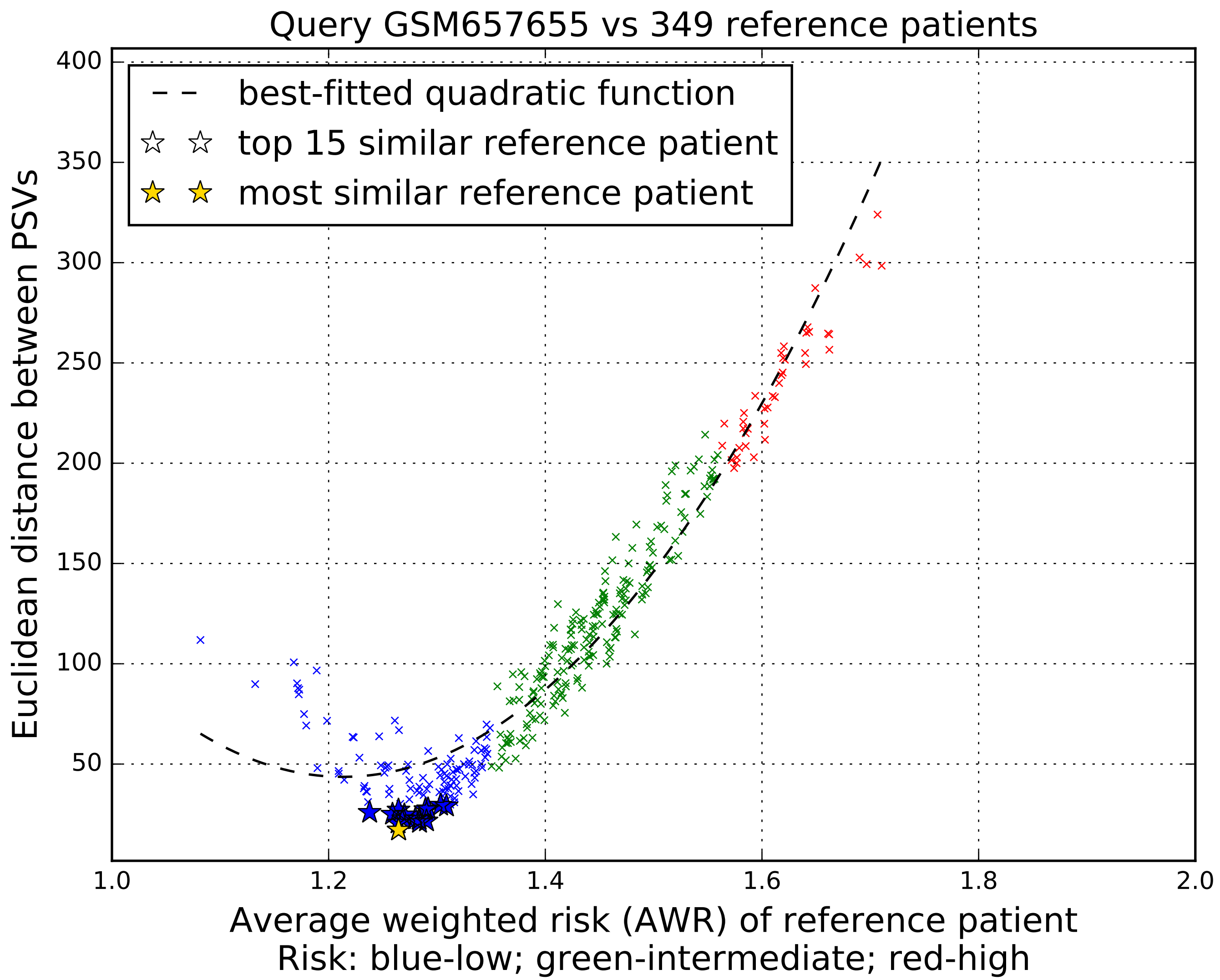

Query GSM657655 vs 349 reference patients
best-fitted quadratic function
top 15 similar reference patient
most similar reference patient
Euclidean distance between PSVs
Average weighted risk (AWR) of reference patient
Risk: blue-low; green-intermediate; red-high
400
350
300
250
200
150
100
50
1.0
1.2
1.4
1.6
1.8
2.0

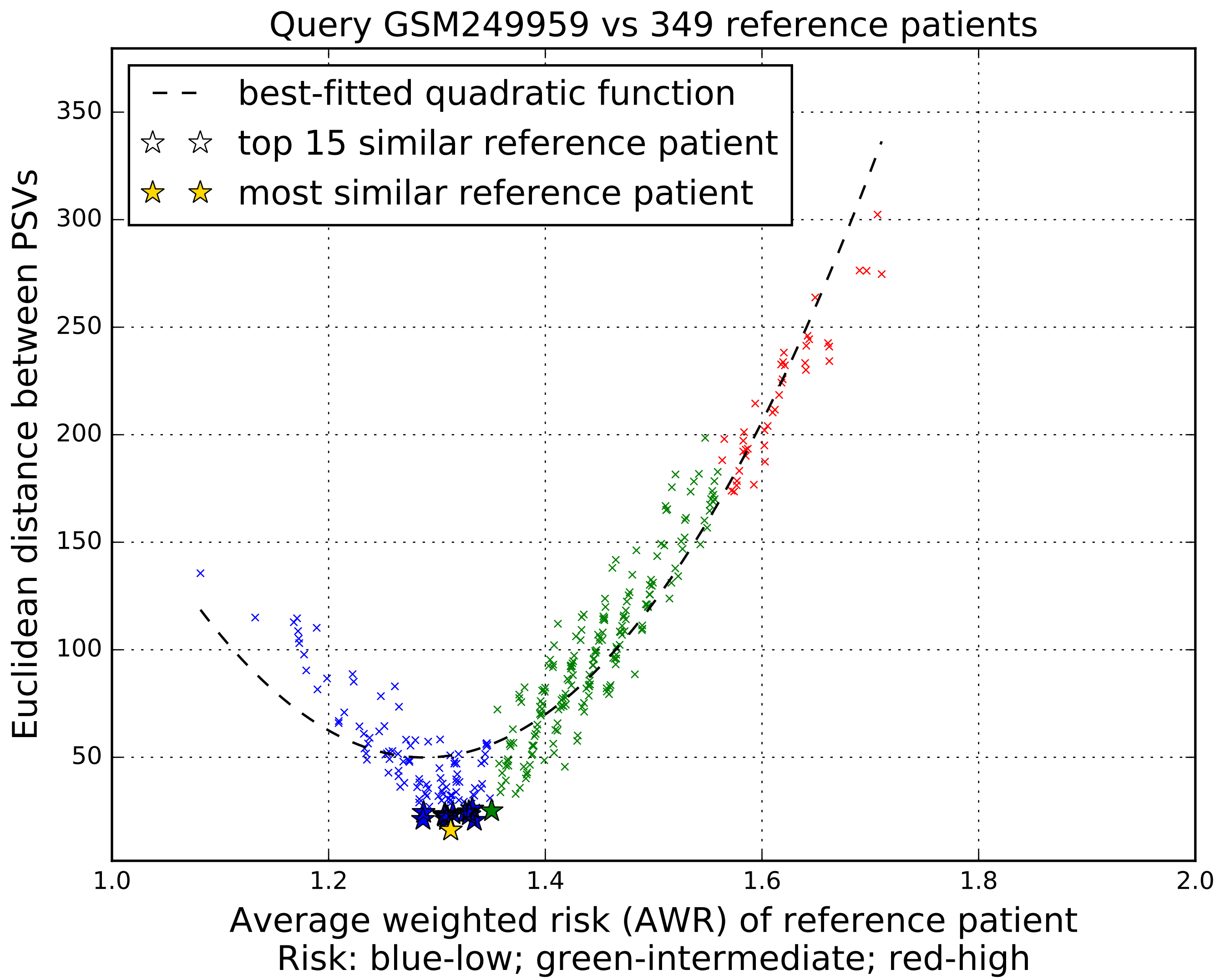

Query GSM249959 vs 349 reference patients
best-fitted quadratic function
top 15 similar reference patient
most similar reference patient
Euclidean distance between PSVs
350
300
250
200
150
100
50
1.0
1.2
1.4
1.6
1.8
2.0
Average weighted risk (AWR) of reference patient
Risk: blue-low; green-intermediate; red-high

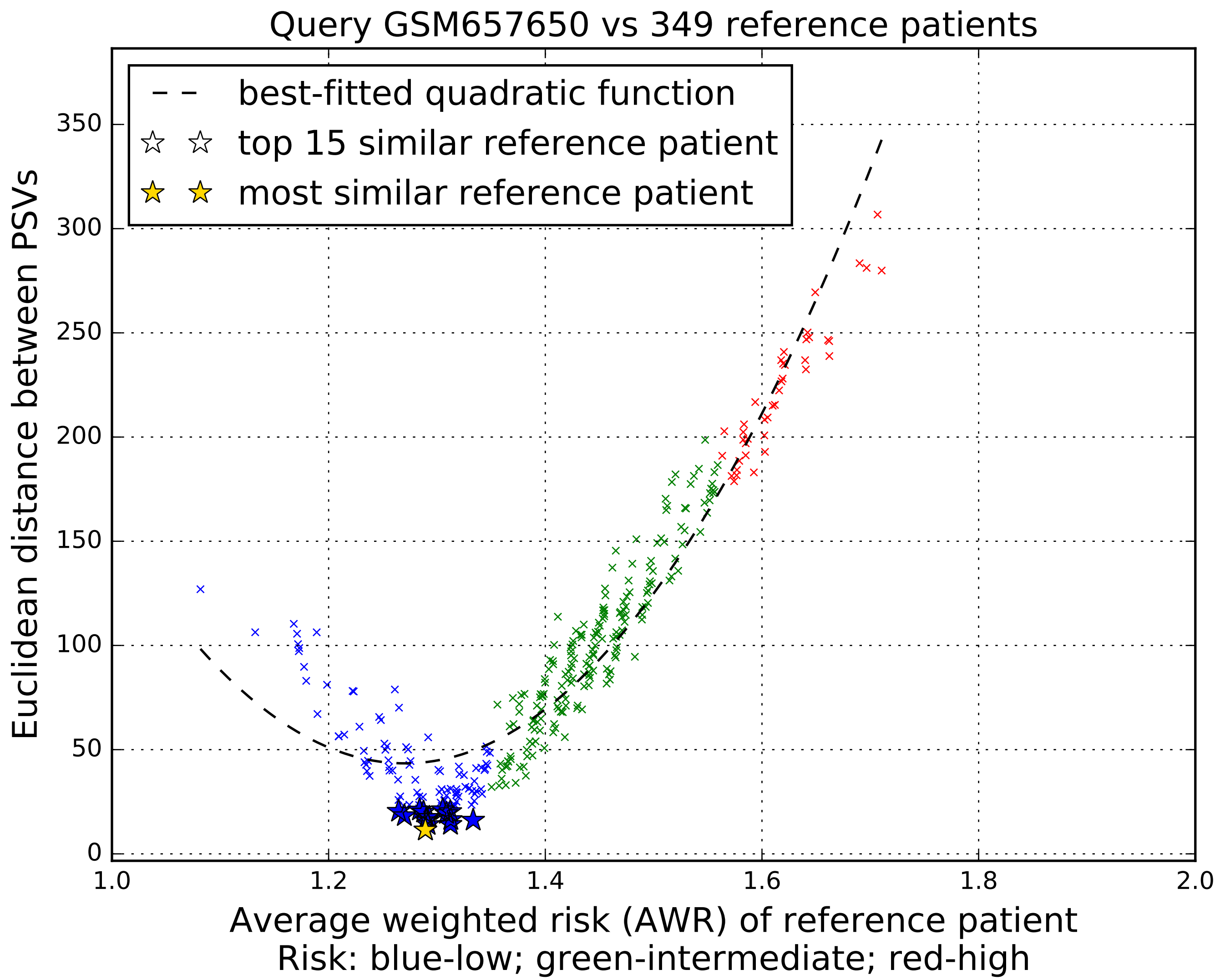
Query GSM657650 vs 349 reference patients
best-fitted quadratic function
top 15 similar reference patient
most similar reference patient
Euclidean distance between PSVs
0
50
100
150
200
250
300
350
1.0
1.2
1.4
1.6
1.8
2.0
Average weighted risk (AWR) of reference patient
Risk: blue-low; green-intermediate; red-high

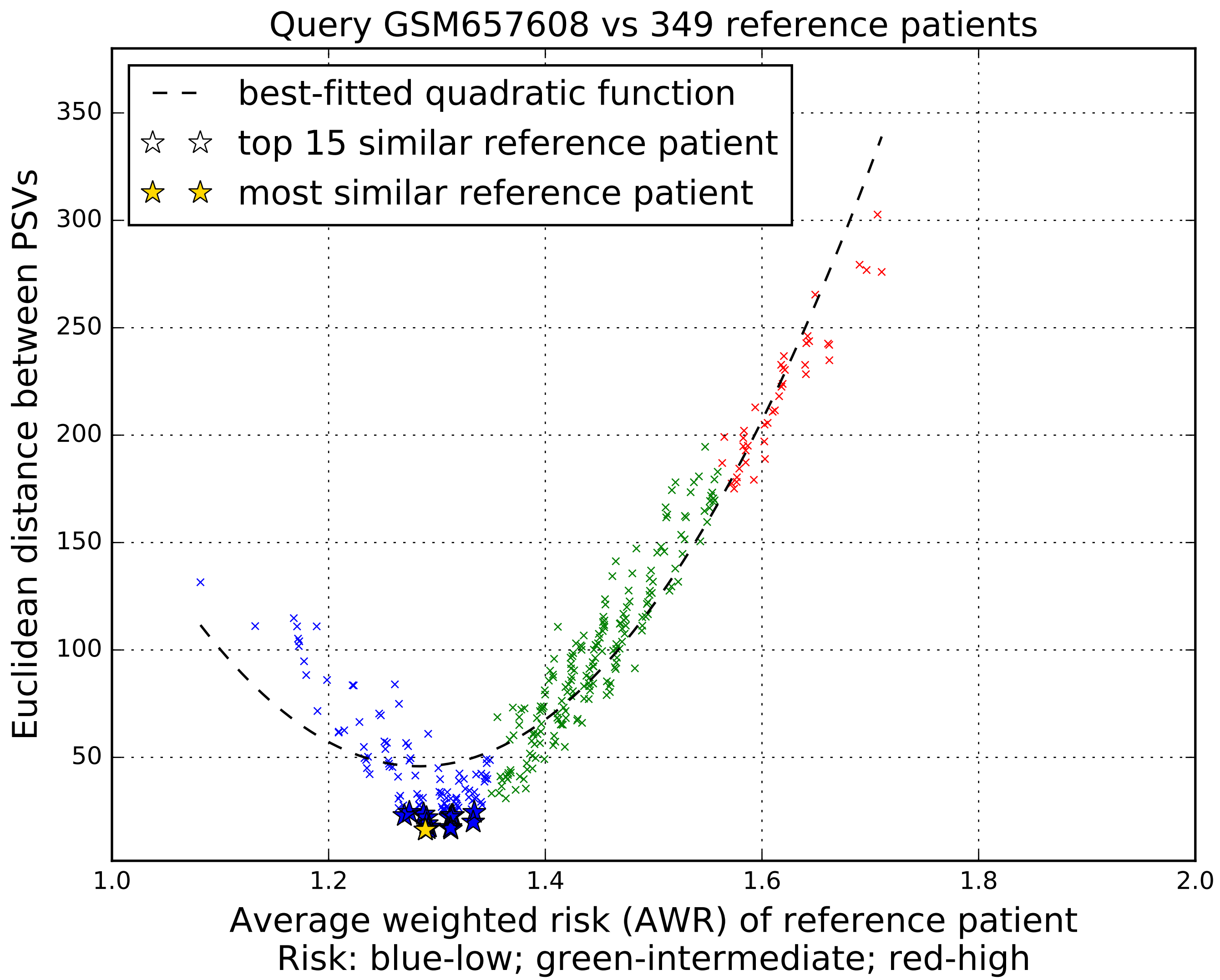
Query GSM657608 vs 349 reference patients
best-fitted quadratic function
top 15 similar reference patient
most similar reference patient
Euclidean distance between PSVs
350
300
250
200
150
100
50
1.0
1.2
1.4
1.6
1.8
2.0
Average weighted risk (AWR) of reference patient
Risk: blue-low; green-intermediate; red-high

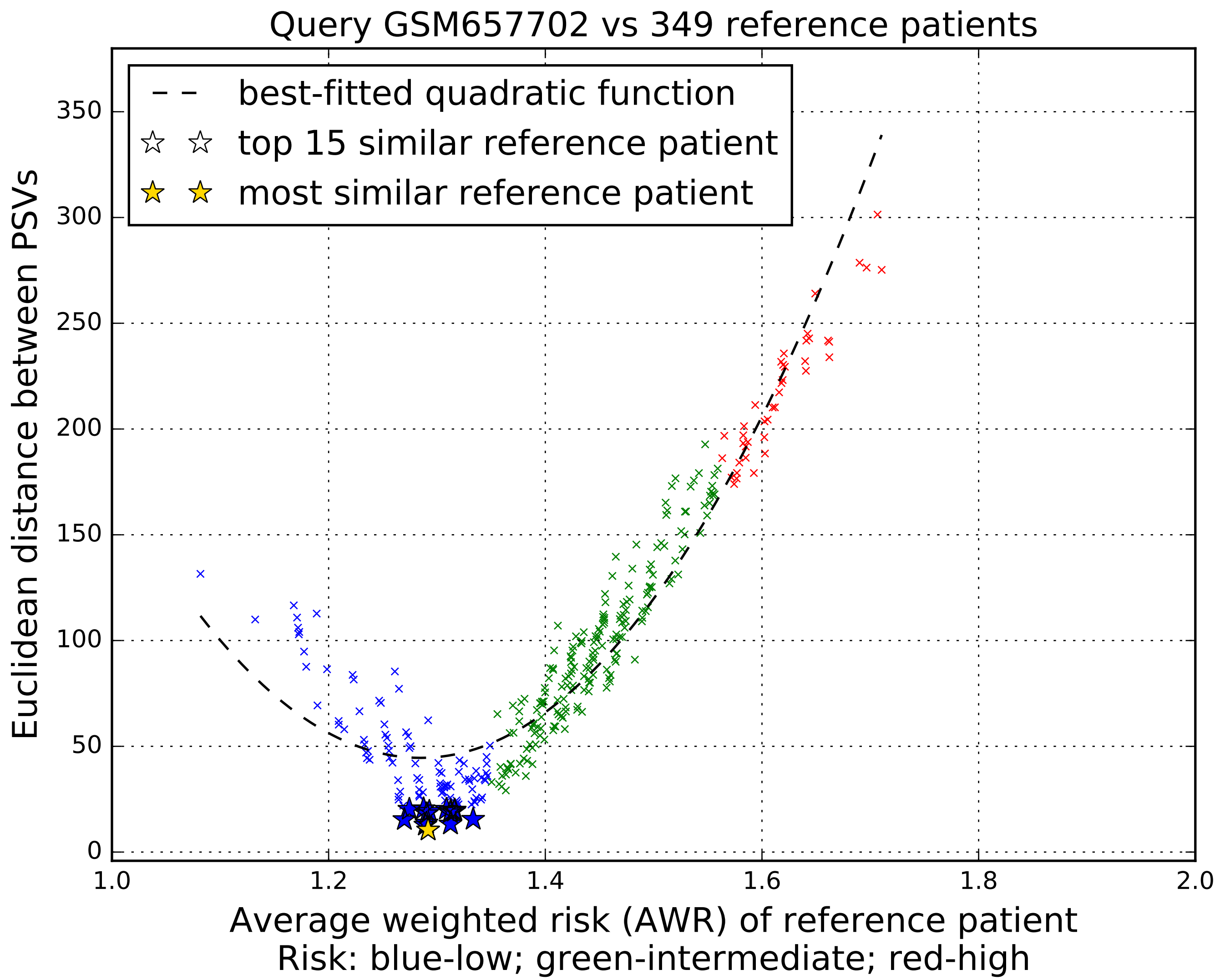
Query GSM657702 vs 349 reference patients
best-fitted quadratic function
top 15 similar reference patient
most similar reference patient
Euclidean distance between PSVs
0
50
100
150
200
250
300
350
1.0
1.2
1.4
1.6
1.8
2.0
Average weighted risk (AWR) of reference patient
Risk: blue-low; green-intermediate; red-high

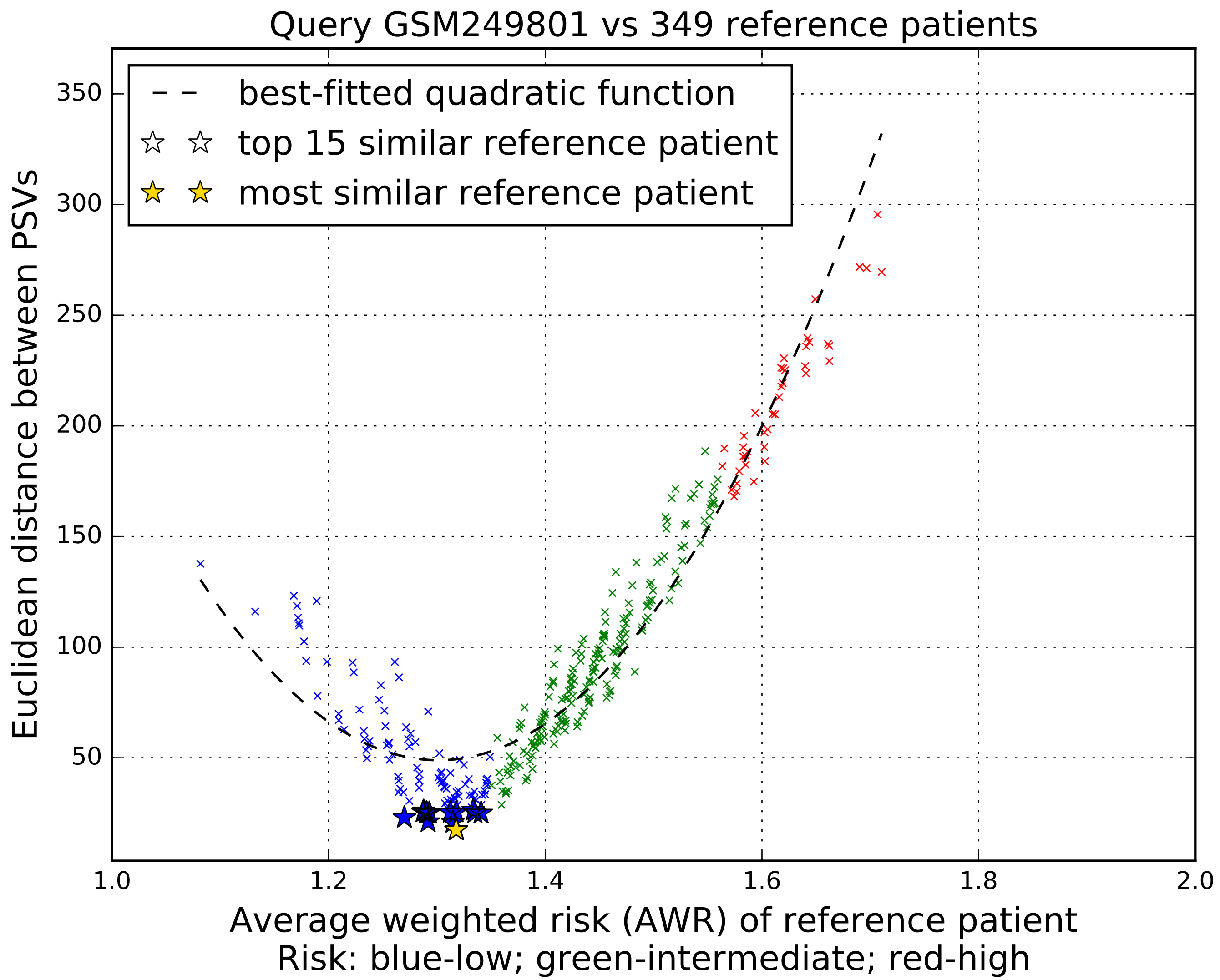
Query GSM249801 vs 349 reference patients
best-fitted quadratic function
top 15 similar reference patient
most similar reference patient
Euclidean distance between PSVs
Average weighted risk (AWR) of reference patient
Risk: blue-low; green-intermediate; red-high
350
300
250
200
150
100
50
1.0
1.2
1.4
1.6
1.8
2.0

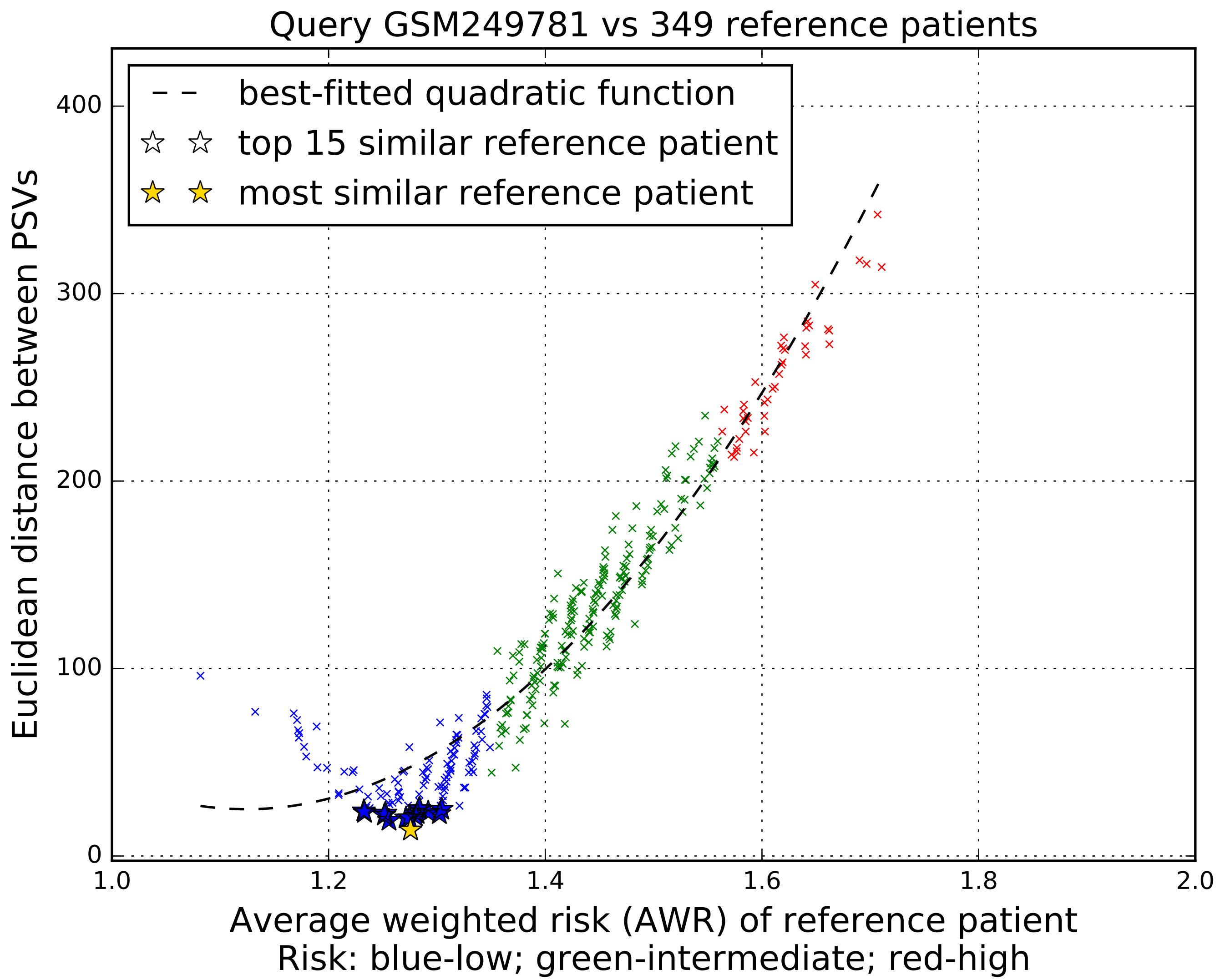

Query GSM249781 vs 349 reference patients
best-fitted quadratic function
top 15 similar reference patient
most similar reference patient
Euclidean distance between PSVs
0
100
200
300
400
1.0
1.2
1.4
1.6
1.8
2.0
Average weighted risk (AWR) of reference patient
Risk: blue-low; green-intermediate; red-high

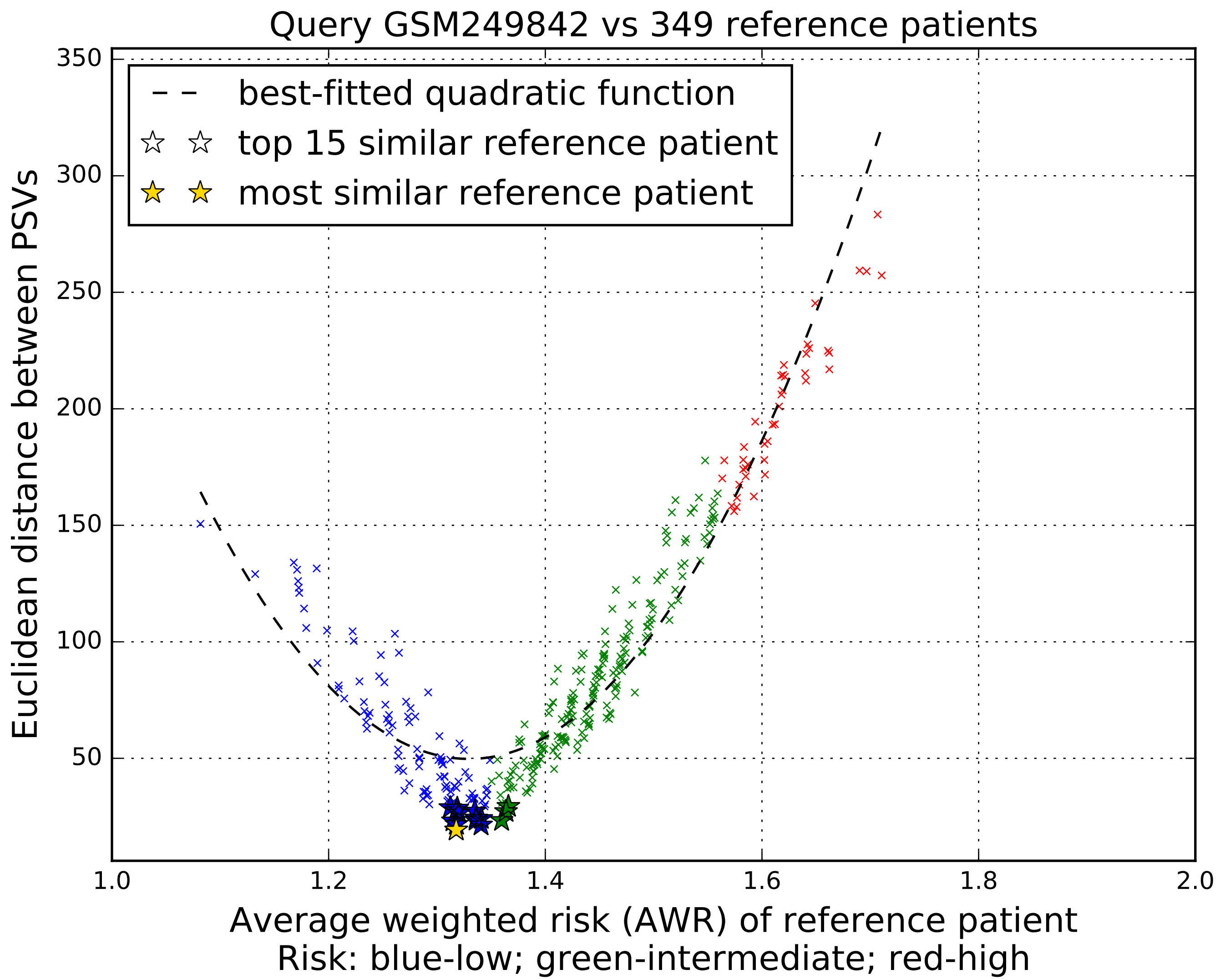
Query GSM249842 vs 349 reference patients
best-fitted quadratic function
top 15 similar reference patient
most similar reference patient
Euclidean distance between PSVs
350
300
250
200
150
100
50
1.0
1.2
1.4
1.6
1.8
2.0
Average weighted risk (AWR) of reference patient
Risk: blue-low; green-intermediate; red-high

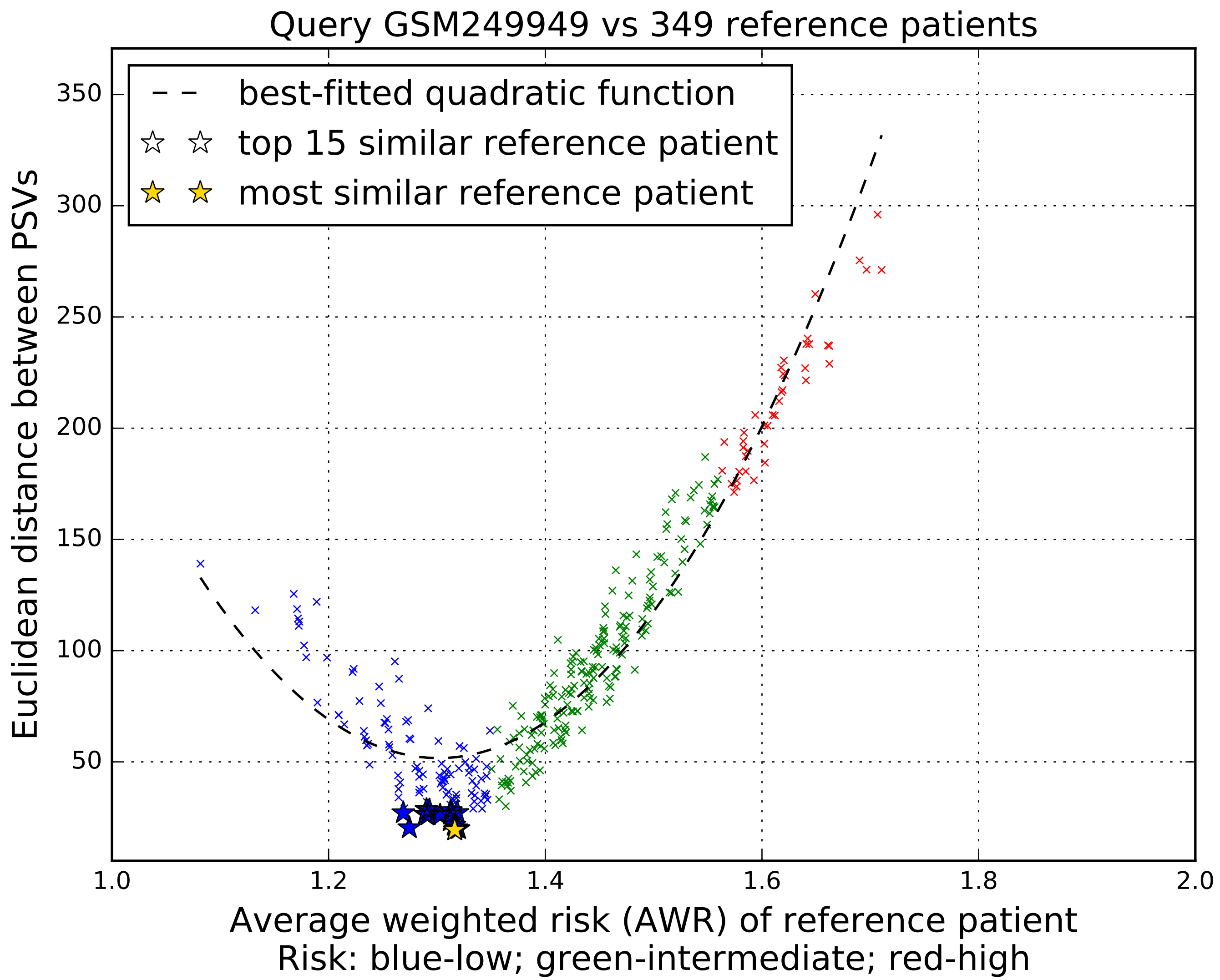
Query GSM249949 vs 349 reference patients
best-fitted quadratic function
top 15 similar reference patient
most similar reference patient
Euclidean distance between PSVs
350
300
250
200
150
100
50
1.0
1.2
1.4
1.6
1.8
2.0
Average weighted risk (AWR) of reference patient
Risk: blue-low; green-intermediate; red-high

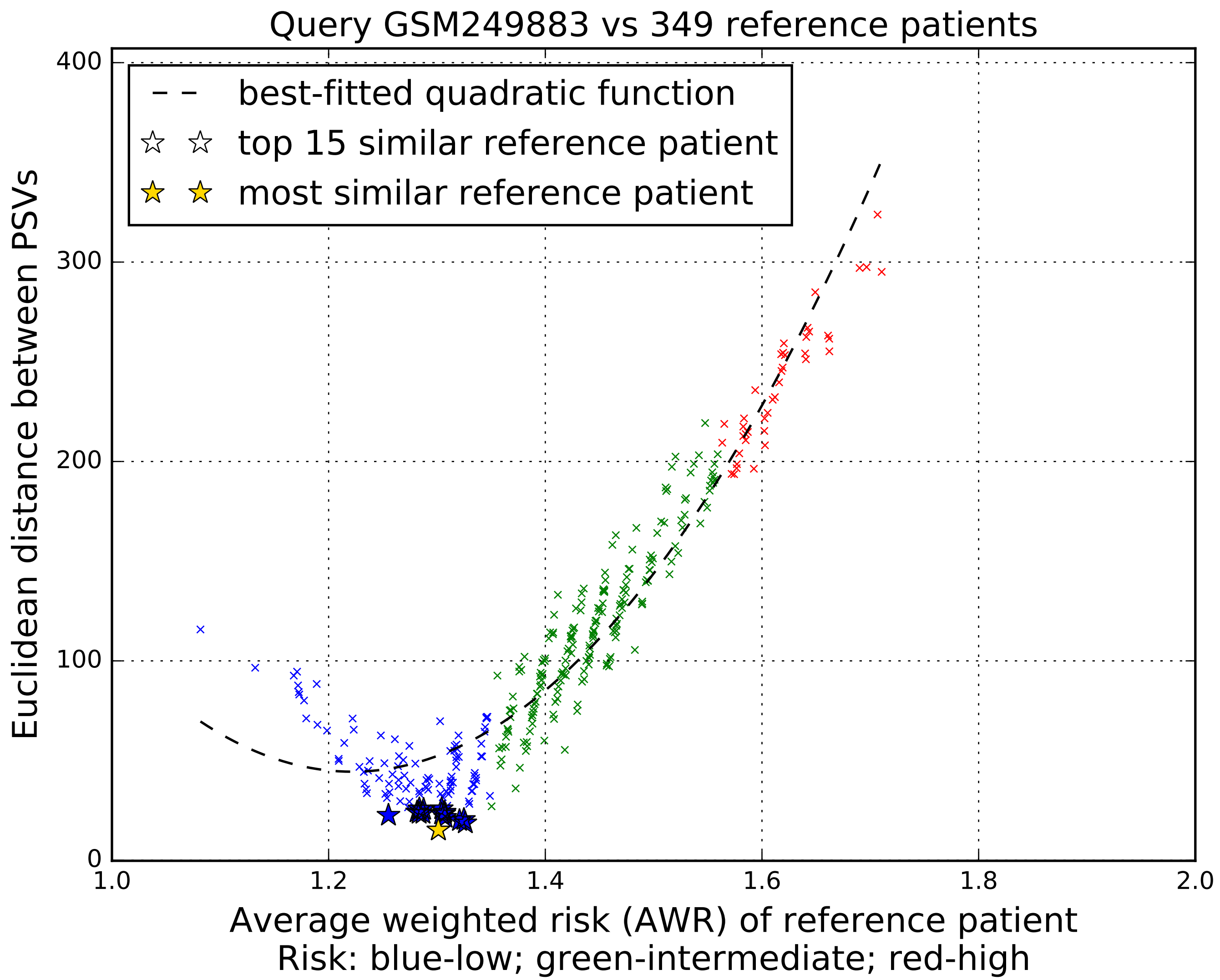

Query GSM249883 vs 349 reference patients
best-fitted quadratic function
top 15 similar reference patient
most similar reference patient
Euclidean distance between PSVs
400
300
200
100
0
1.0
1.2
1.4
1.6
1.8
2.0
Average weighted risk (AWR) of reference patient
Risk: blue-low; green-intermediate; red-high

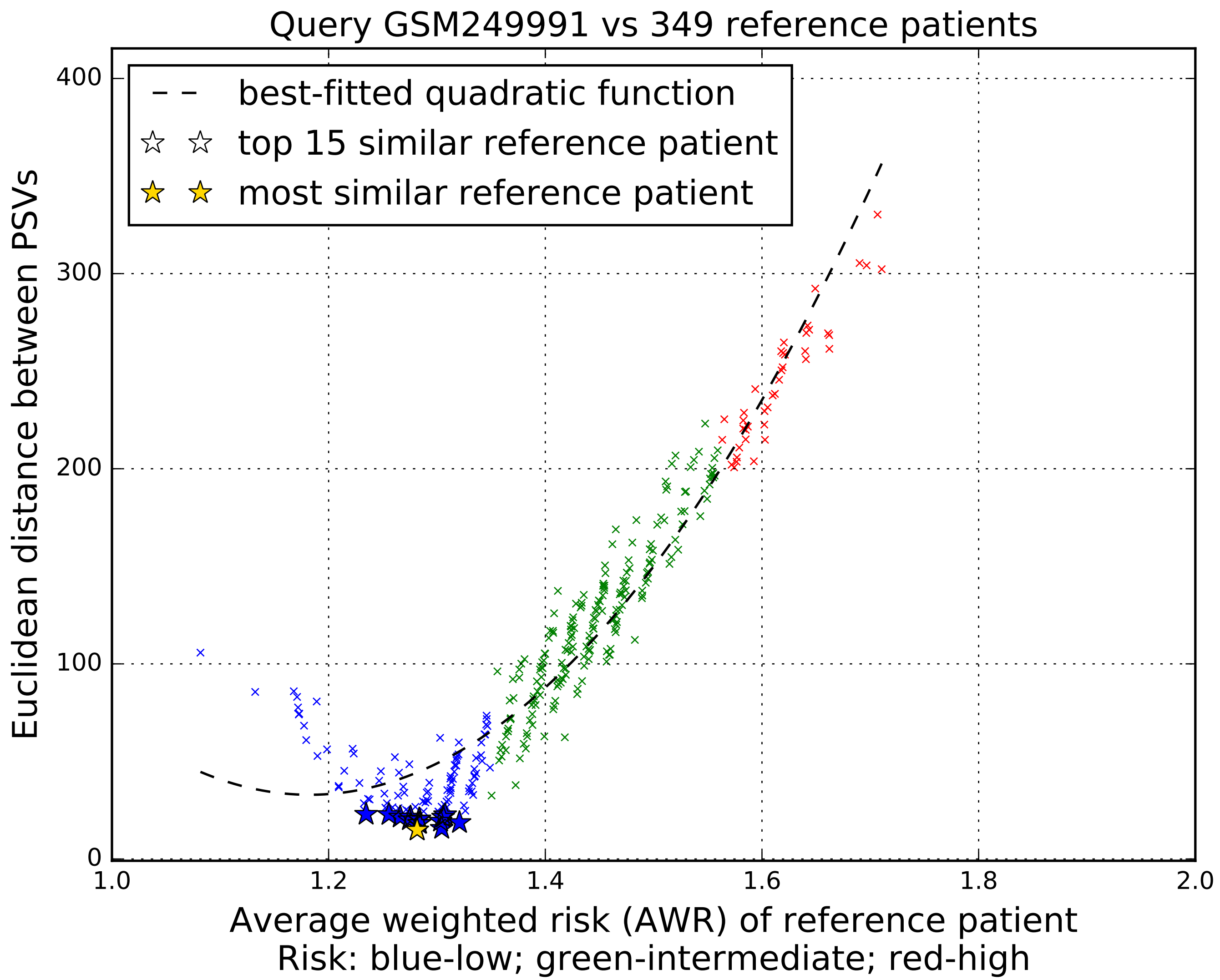
Query GSM249991 vs 349 reference patients
best-fitted quadratic function
top 15 similar reference patient
most similar reference patient
Euclidean distance between PSVs
Average weighted risk (AWR) of reference patient
Risk: blue-low; green-intermediate; red-high
0
100
200
300
400
1.0
1.2
1.4
1.6
1.8
2.0

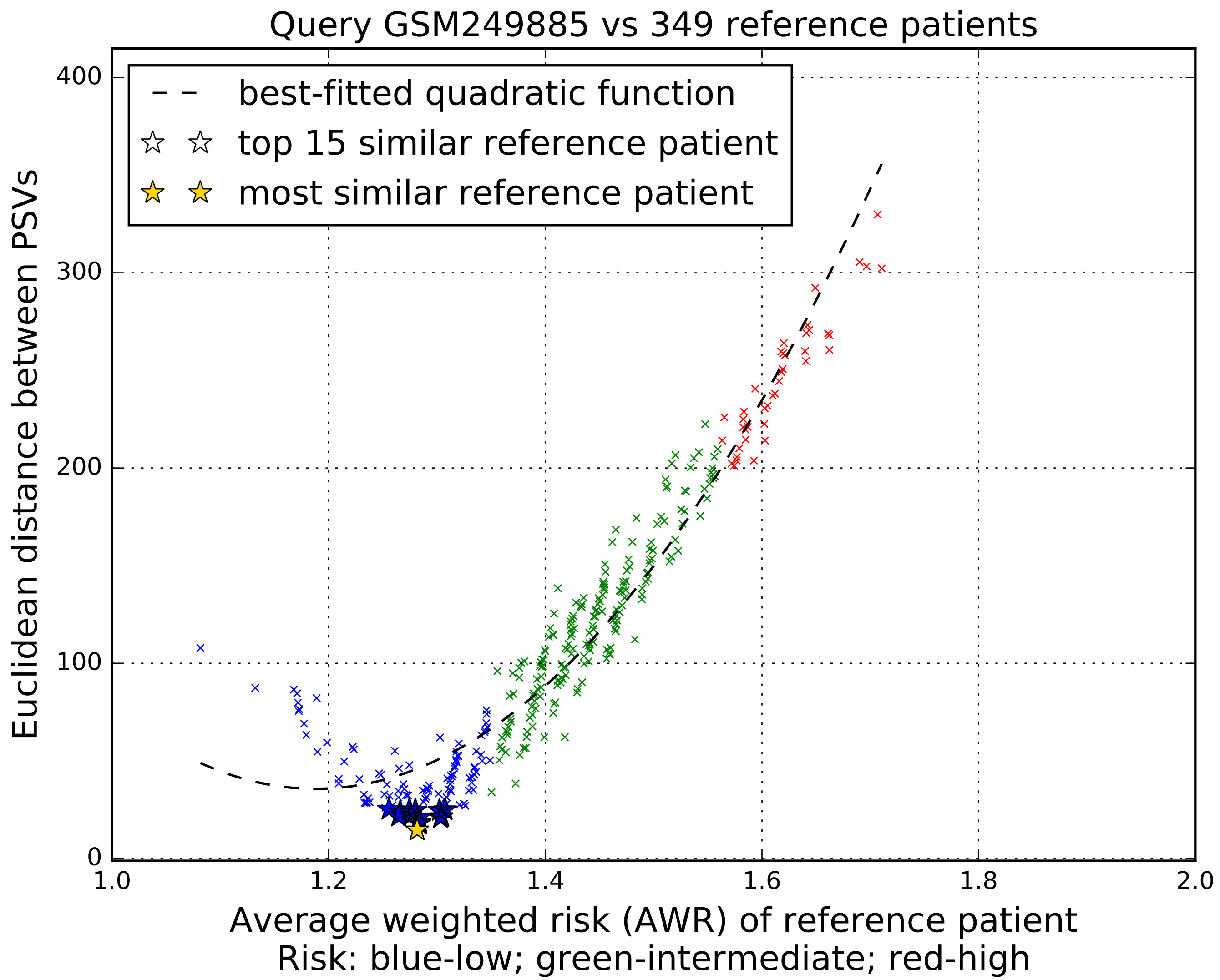
Query GSM249885 vs 349 reference patients
best-fitted quadratic function
top 15 similar reference patient
most similar reference patient
Euclidean distance between PSVs
Average weighted risk (AWR) of reference patient
Risk: blue-low; green-intermediate; red-high
0
100
200
300
400
1.0
1.2
1.4
1.6
1.8
2.0

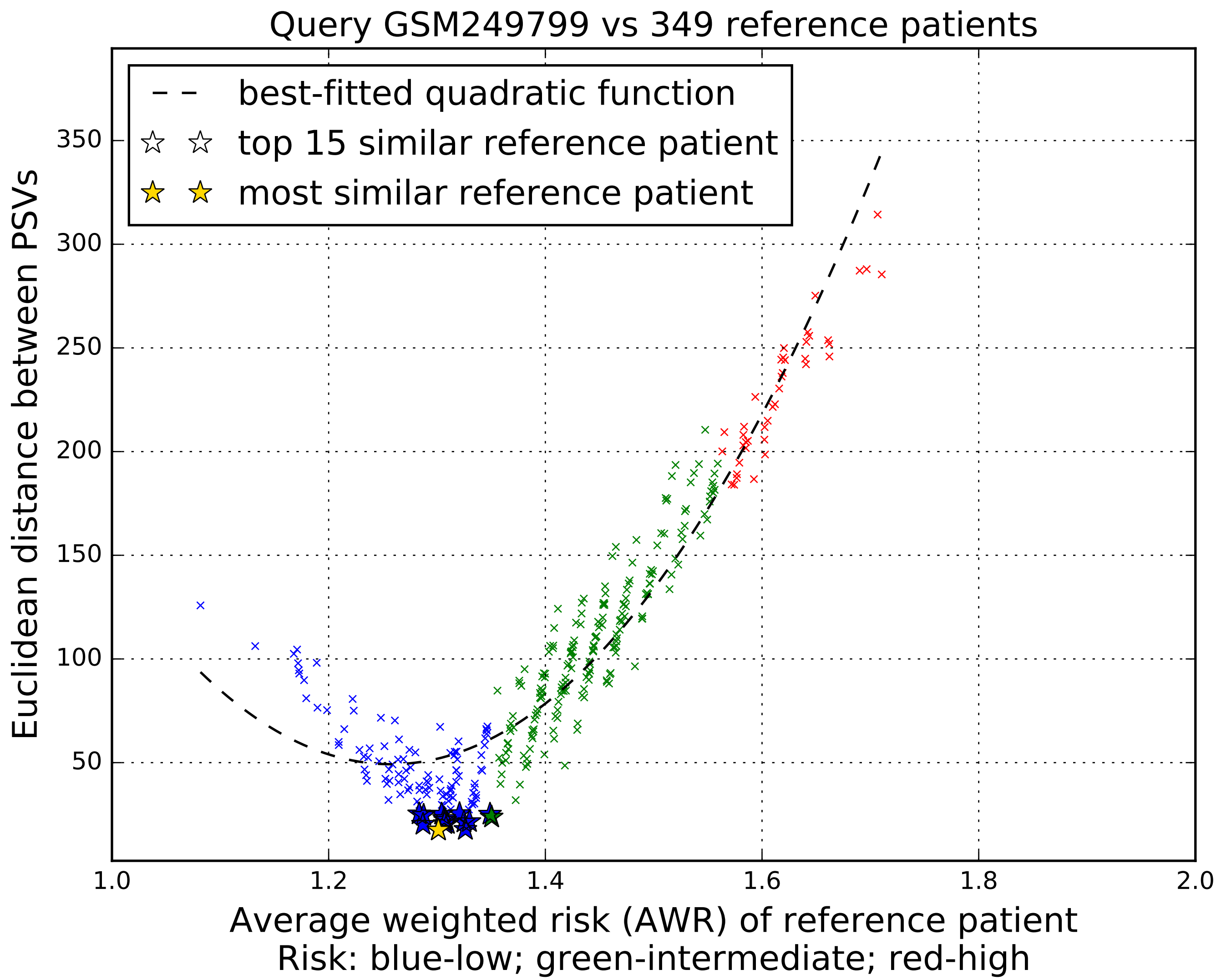
Query GSM249799 vs 349 reference patients
best-fitted quadratic function
top 15 similar reference patient
most similar reference patient
Euclidean distance between PSVs
50
100
150
200
250
300
350
1.0
1.2
1.4
1.6
1.8
2.0
Average weighted risk (AWR) of reference patient
Risk: blue-low; green-intermediate; red-high

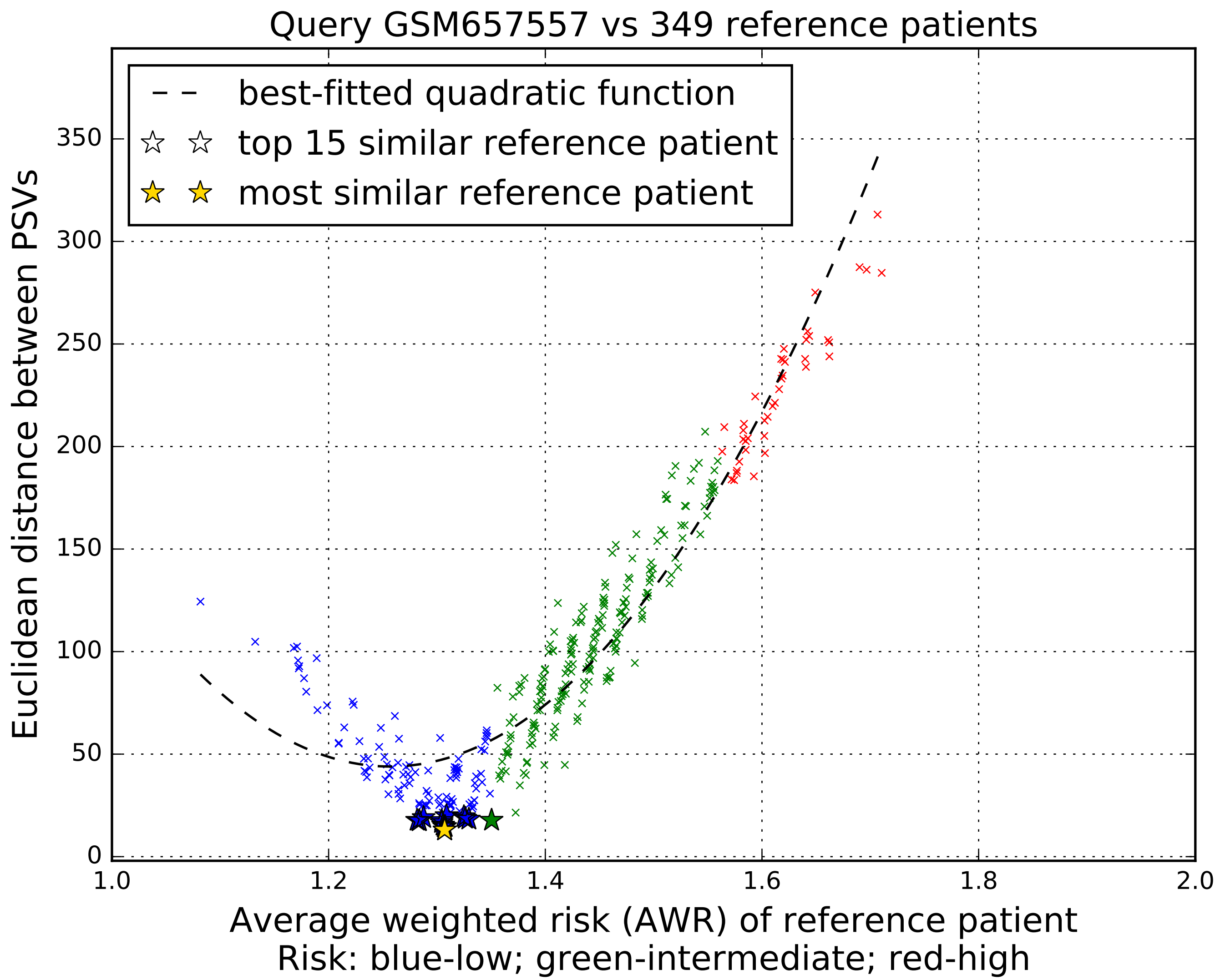
Query GSM657557 vs 349 reference patients
best-fitted quadratic function
top 15 similar reference patient
most similar reference patient
Euclidean distance between PSVs
0
50
100
150
200
250
300
350
1.0
1.2
1.4
1.6
1.8
2.0
Average weighted risk (AWR) of reference patient
Risk: blue-low; green-intermediate; red-high

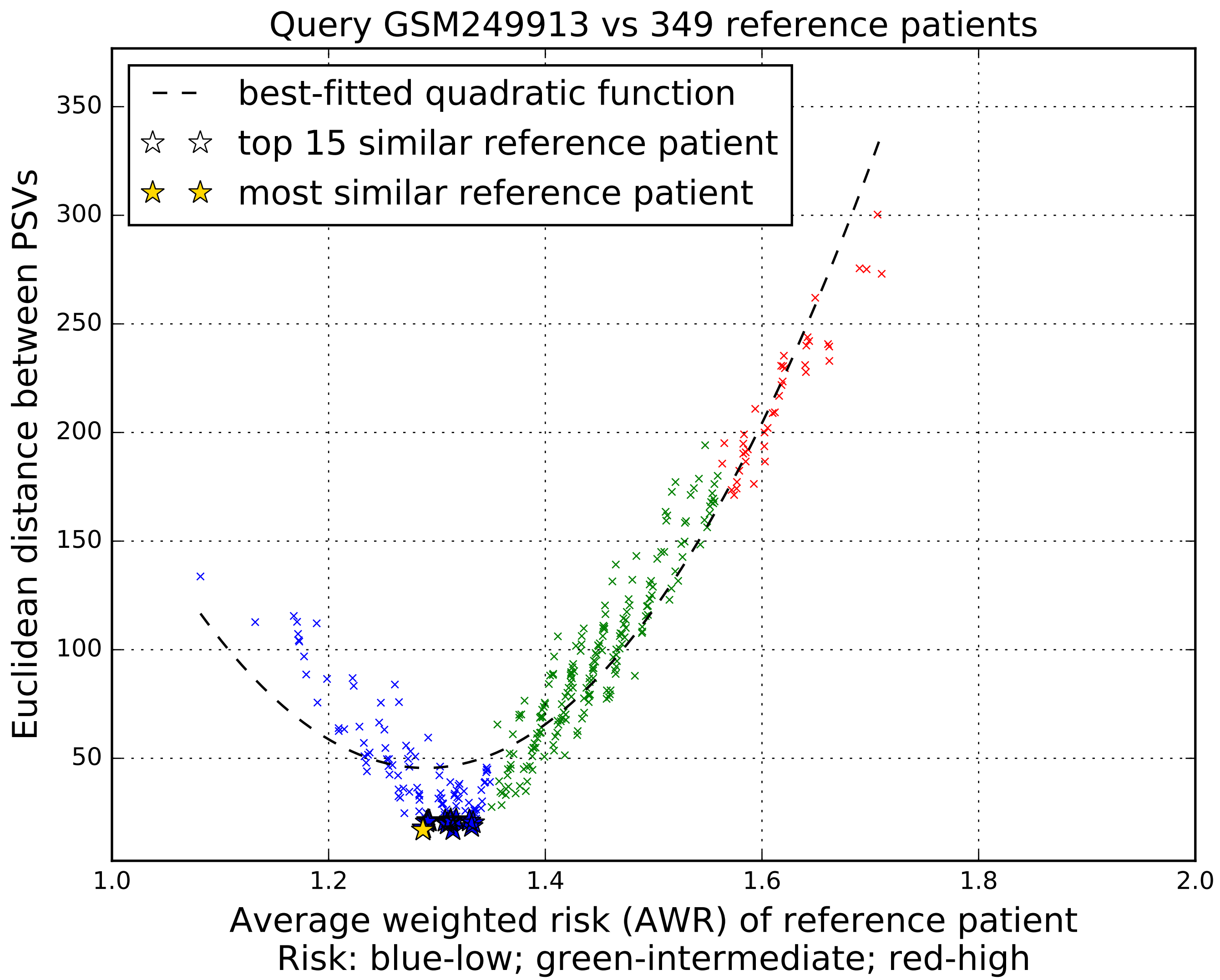

Query GSM249913 vs 349 reference patients
best-fitted quadratic function
top 15 similar reference patient
most similar reference patient
Euclidean distance between PSVs
350
300
250
200
150
100
50
1.0
1.2
1.4
1.6
1.8
2.0
Average weighted risk (AWR) of reference patient
Risk: blue-low; green-intermediate; red-high

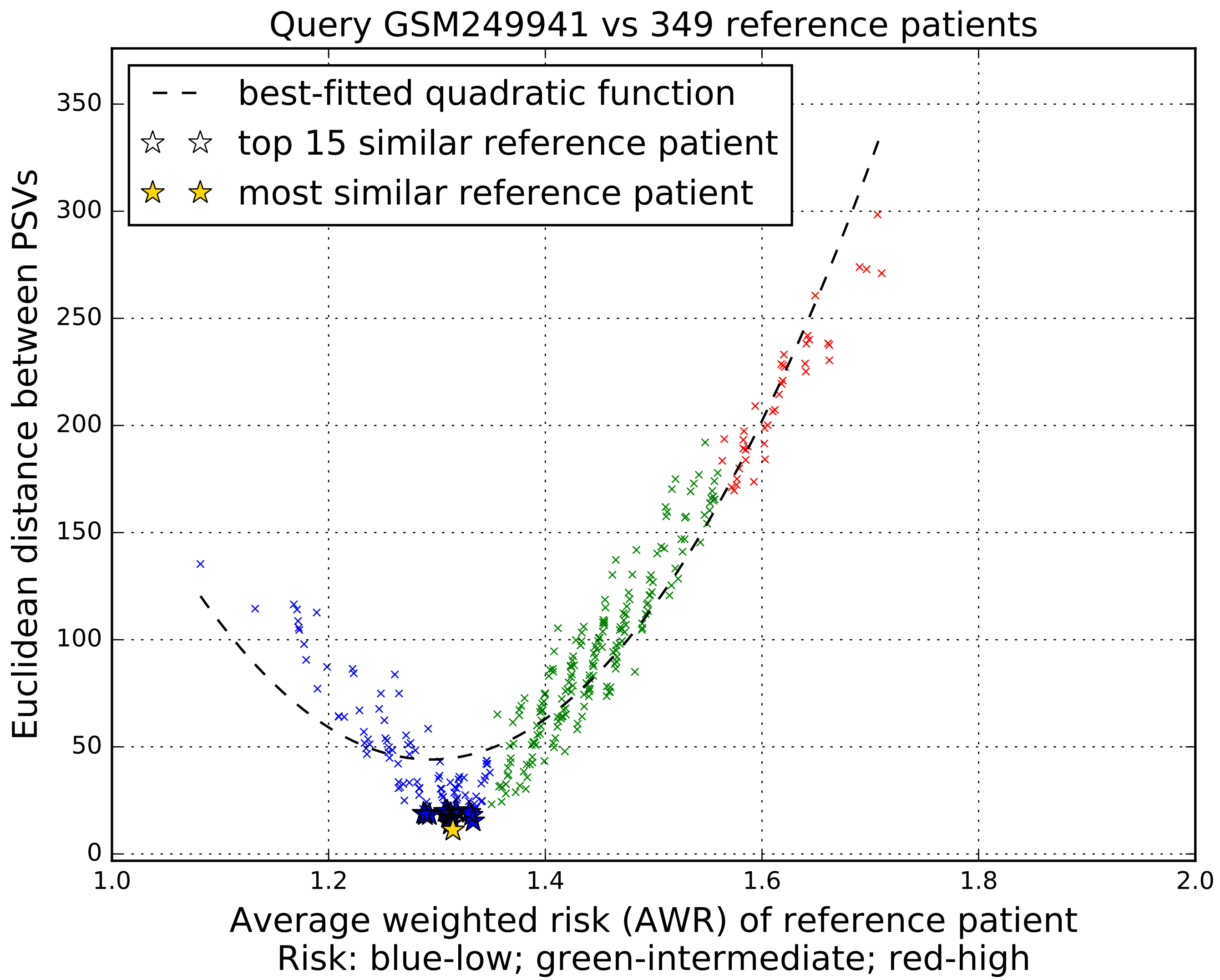

Query GSM249941 vs 349 reference patients
best-fitted quadratic function
top 15 similar reference patient
most similar reference patient
Euclidean distance between PSVs
Average weighted risk (AWR) of reference patient
Risk: blue-low; green-intermediate; red-high
0
50
100
150
200
250
300
350
1.0
1.2
1.4
1.6
1.8
2.0

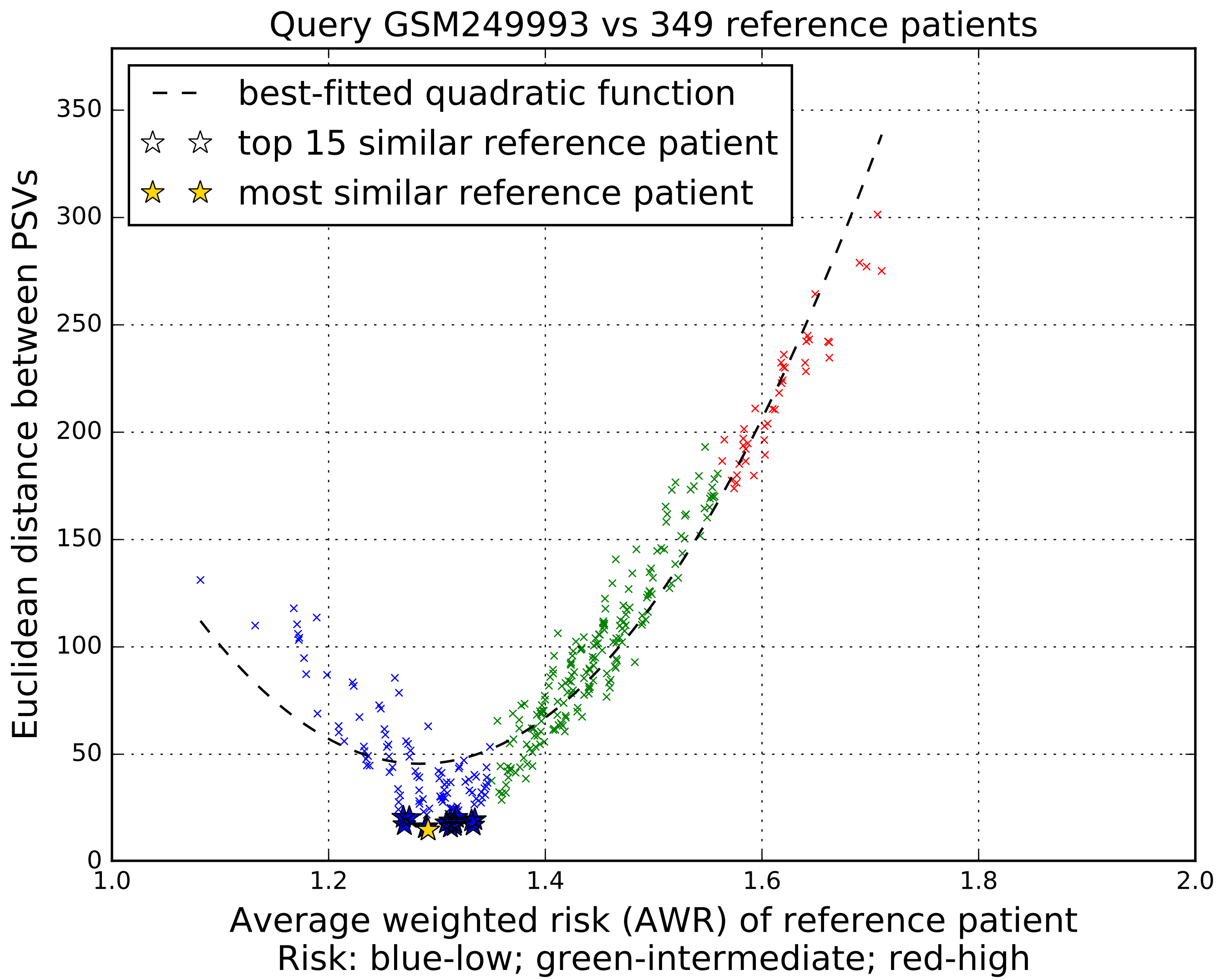
Query GSM249993 vs 349 reference patients
best-fitted quadratic function
top 15 similar reference patient
most similar reference patient
Euclidean distance between PSVs
0
50
100
150
200
250
300
350
1.0
1.2
1.4
1.6
1.8
2.0
Average weighted risk (AWR) of reference patient
Risk: blue-low; green-intermediate; red-high

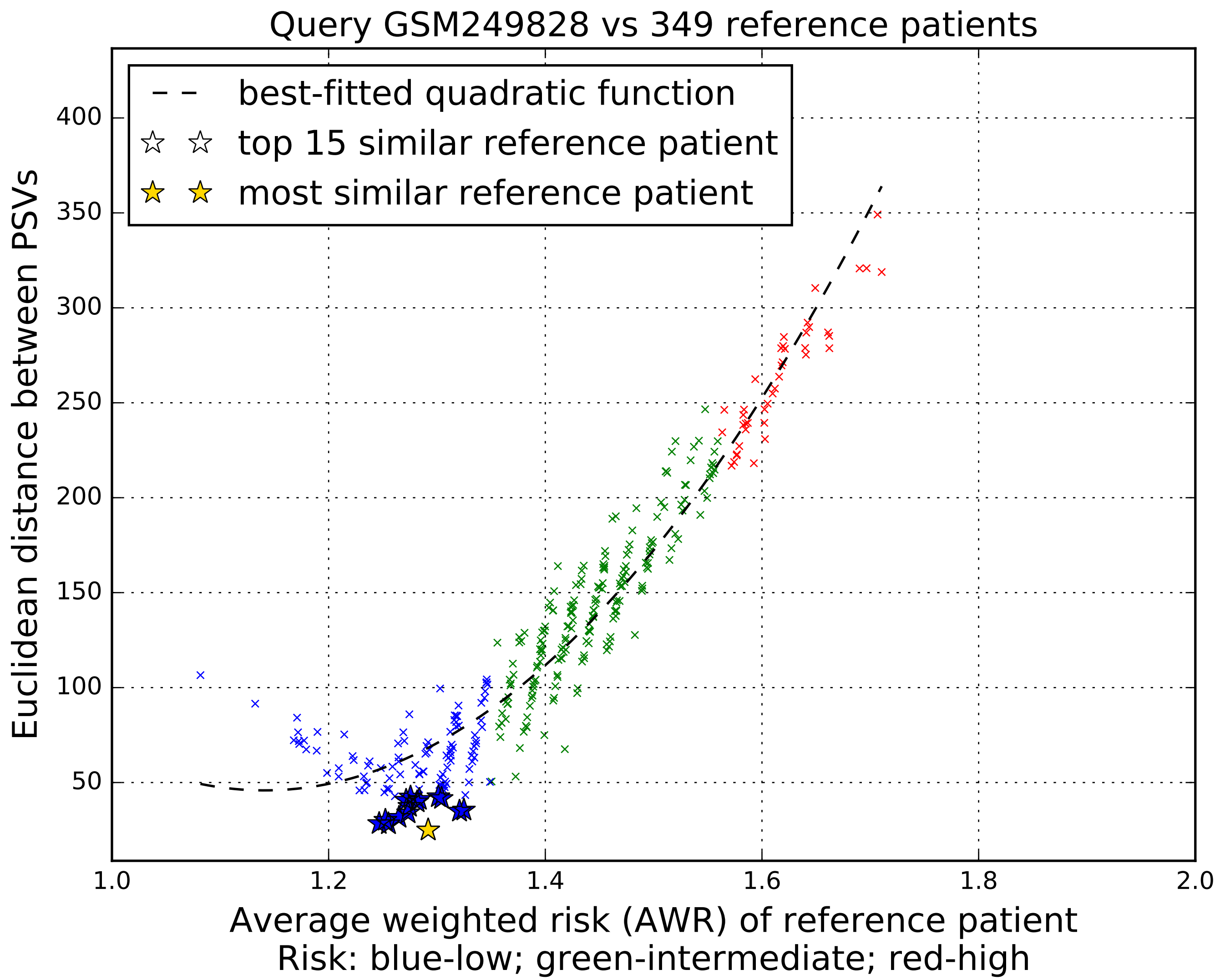

Query GSM249828 vs 349 reference patients
best-fitted quadratic function
top 15 similar reference patient
most similar reference patient
Euclidean distance between PSVs
400
350
300
250
200
150
100
50
1.0
1.2
1.4
1.6
1.8
2.0
Average weighted risk (AWR) of reference patient
Risk: blue-low; green-intermediate; red-high

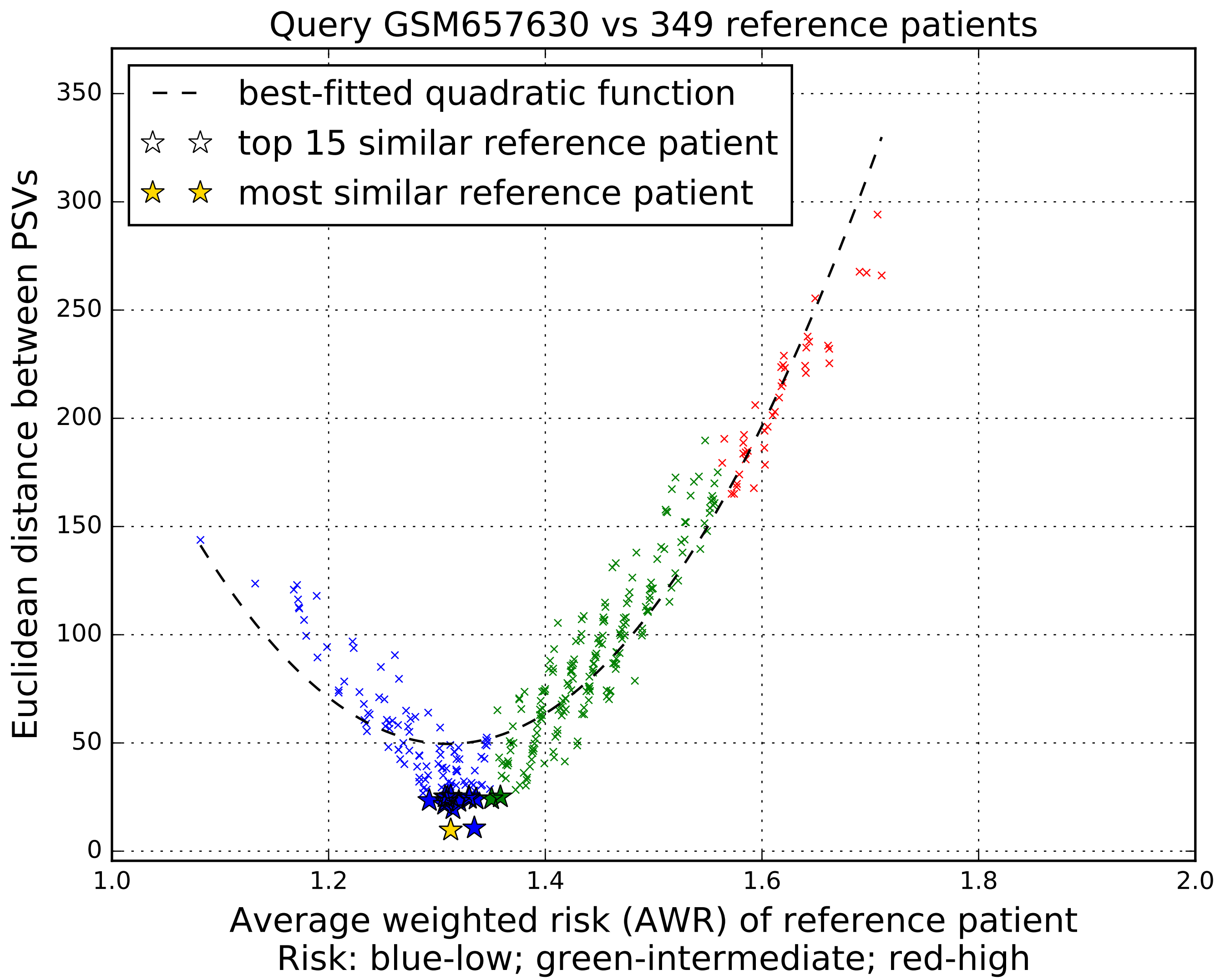
Query GSM657630 vs 349 reference patients
best-fitted quadratic function
top 15 similar reference patient
most similar reference patient
Euclidean distance between PSVs
Average weighted risk (AWR) of reference patient
Risk: blue-low; green-intermediate; red-high
0
50
100
150
200
250
300
350
1.0
1.2
1.4
1.6
1.8
2.0

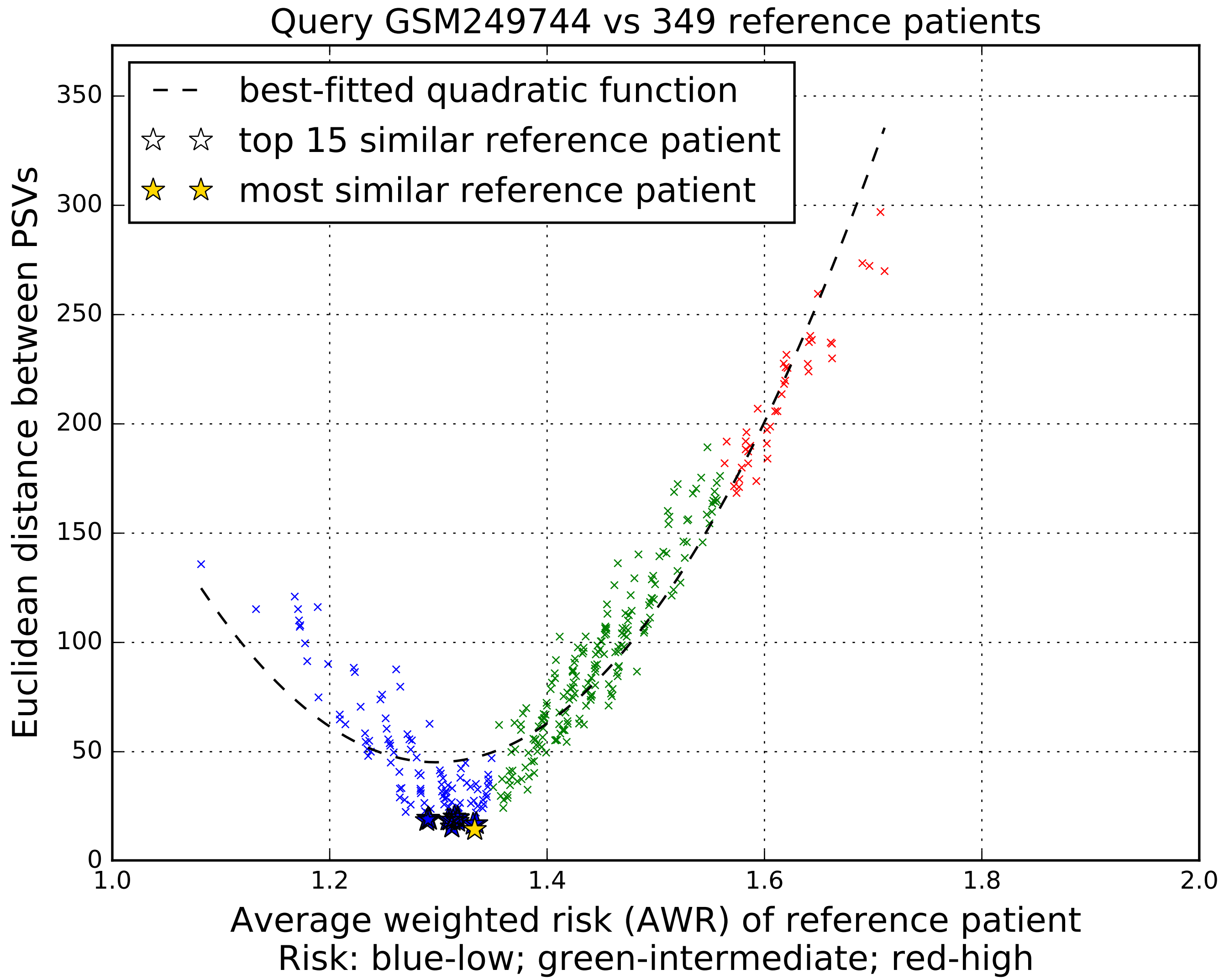

Query GSM249744 vs 349 reference patients
best-fitted quadratic function
top 15 similar reference patient
most similar reference patient
Euclidean distance between PSVs
0
50
100
150
200
250
300
350
1.0
1.2
1.4
1.6
1.8
2.0
Average weighted risk (AWR) of reference patient
Risk: blue-low; green-intermediate; red-high

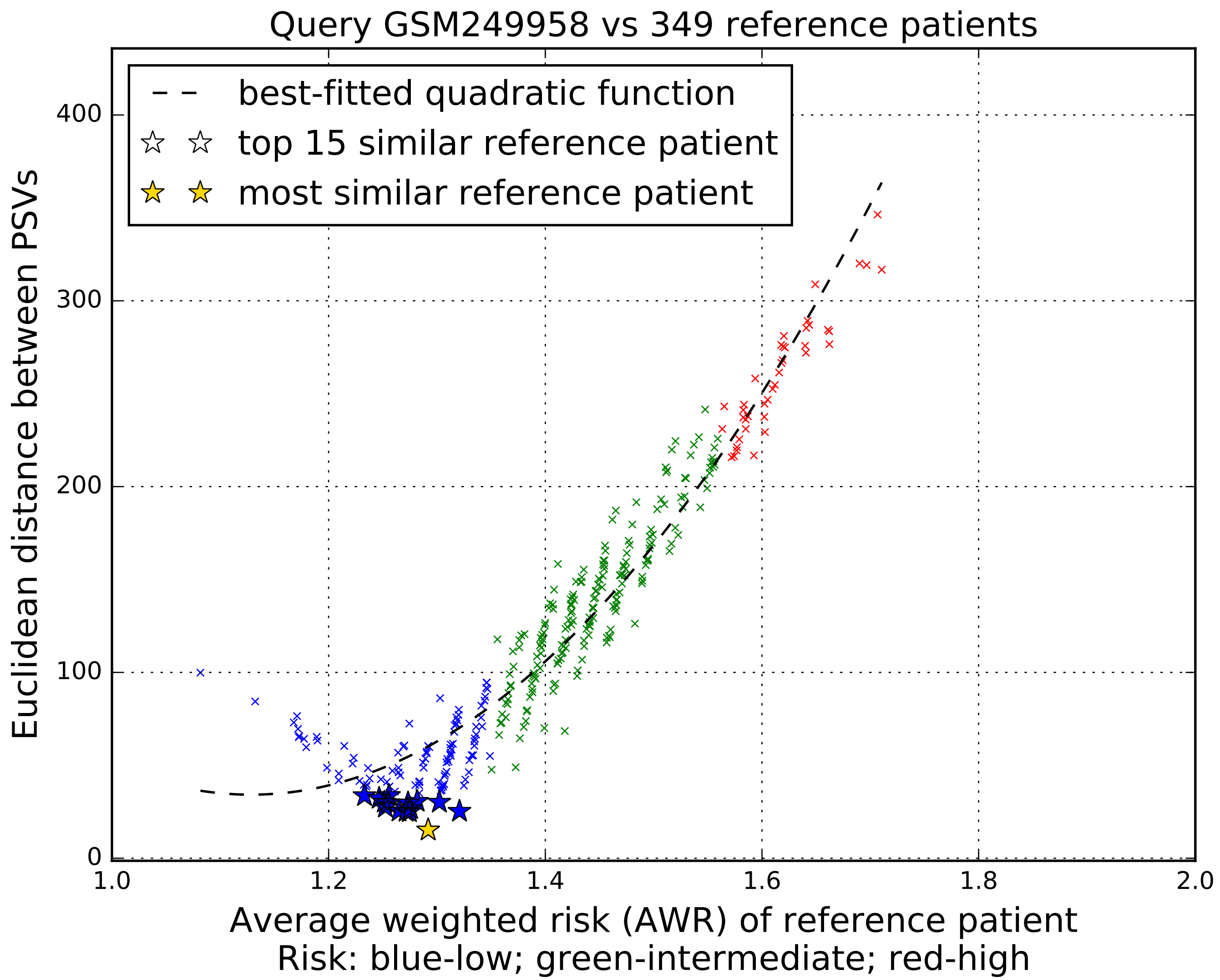

Query GSM249958 vs 349 reference patients
best-fitted quadratic function
top 15 similar reference patient
most similar reference patient
Euclidean distance between PSVs
0
100
200
300
400
1.0
1.2
1.4
1.6
1.8
2.0
Average weighted risk (AWR) of reference patient
Risk: blue-low; green-intermediate; red-high

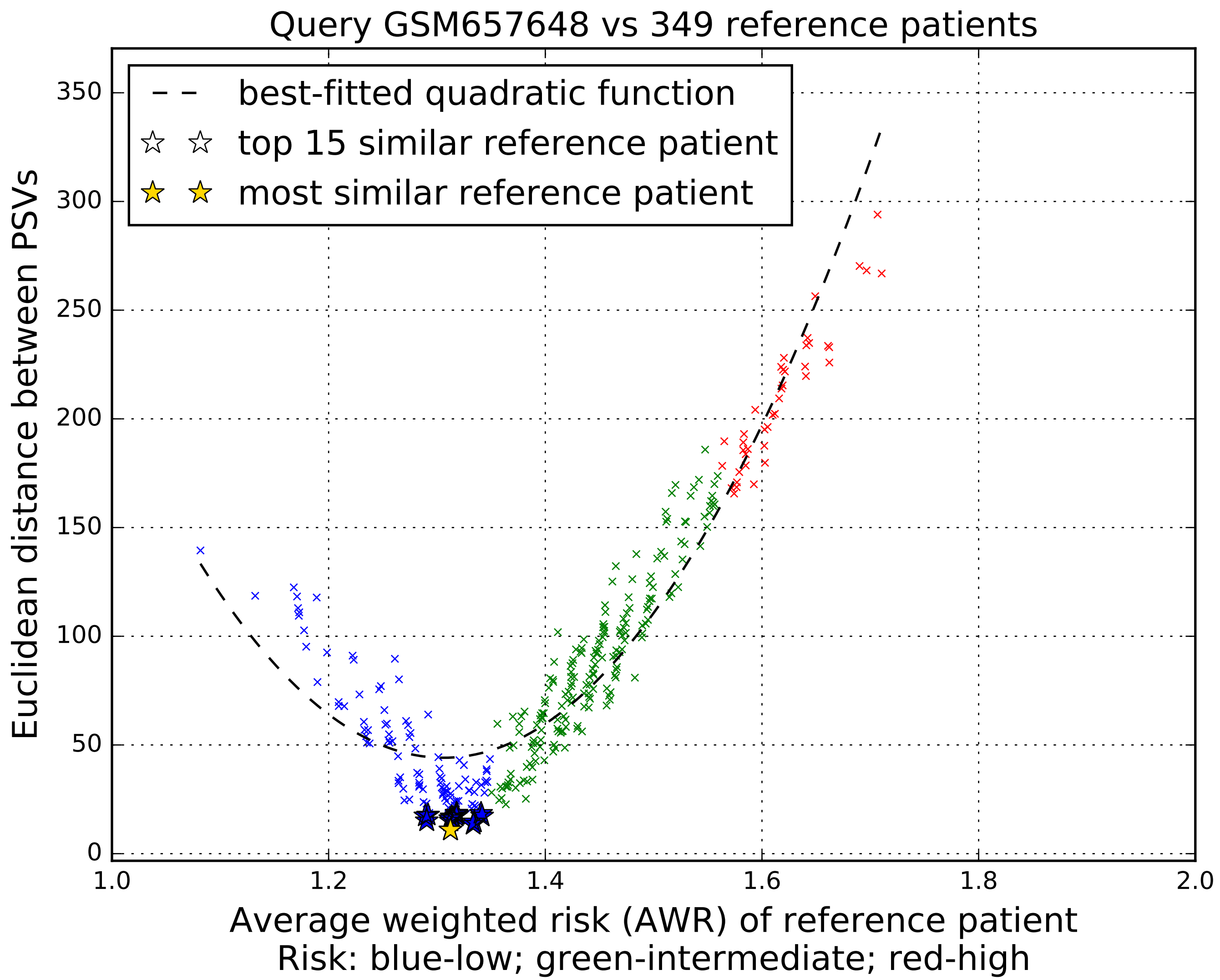
Query GSM657648 vs 349 reference patients
best-fitted quadratic function
top 15 similar reference patient
most similar reference patient
Euclidean distance between PSVs
Average weighted risk (AWR) of reference patient
Risk: blue-low; green-intermediate; red-high
0
50
100
150
200
250
300
350
1.0
1.2
1.4
1.6
1.8
2.0

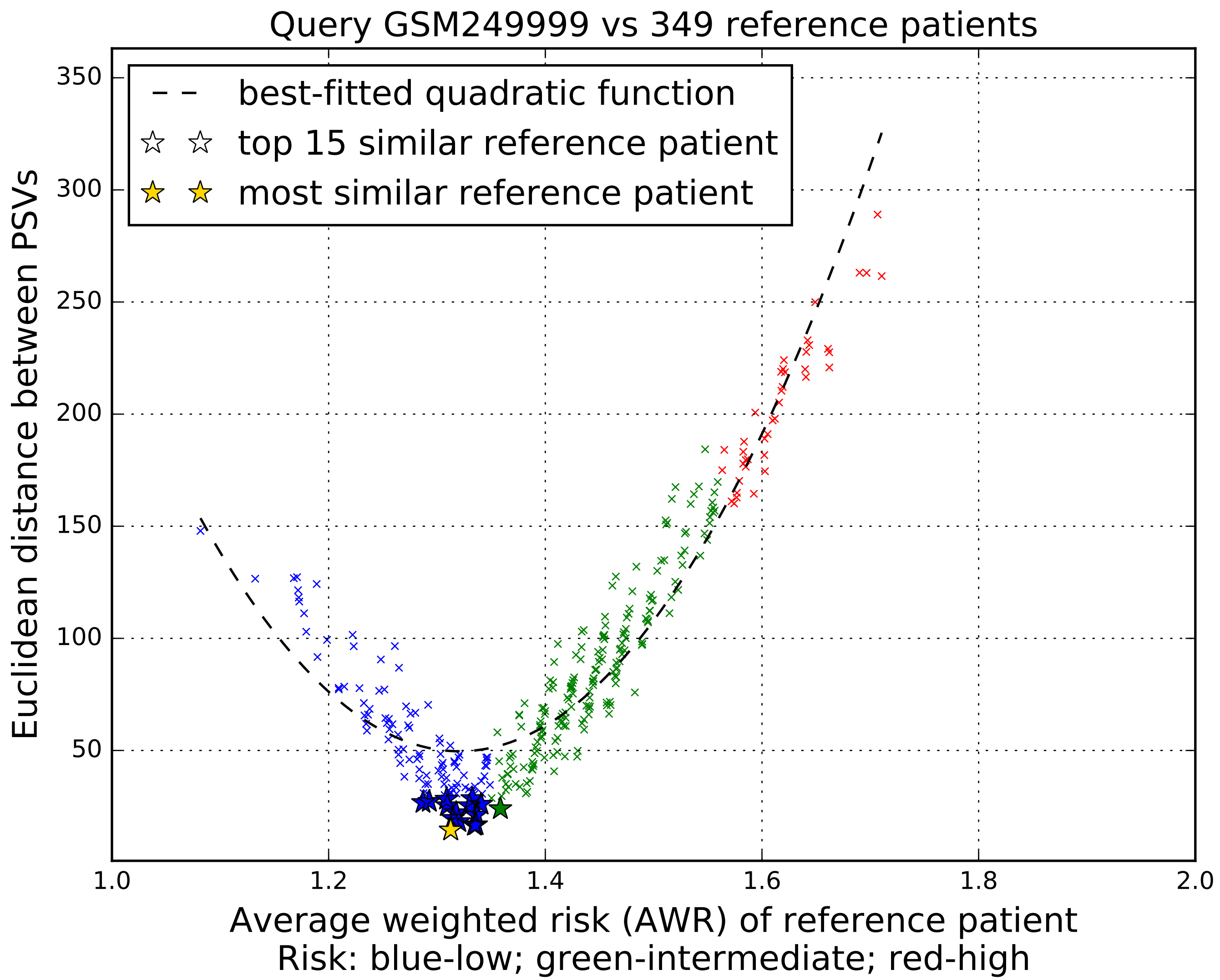
Query GSM249999 vs 349 reference patients
best-fitted quadratic function
top 15 similar reference patient
most similar reference patient
Euclidean distance between PSVs
350
300
250
200
150
100
50
1.0
1.2
1.4
1.6
1.8
2.0
Average weighted risk (AWR) of reference patient
Risk: blue-low; green-intermediate; red-high

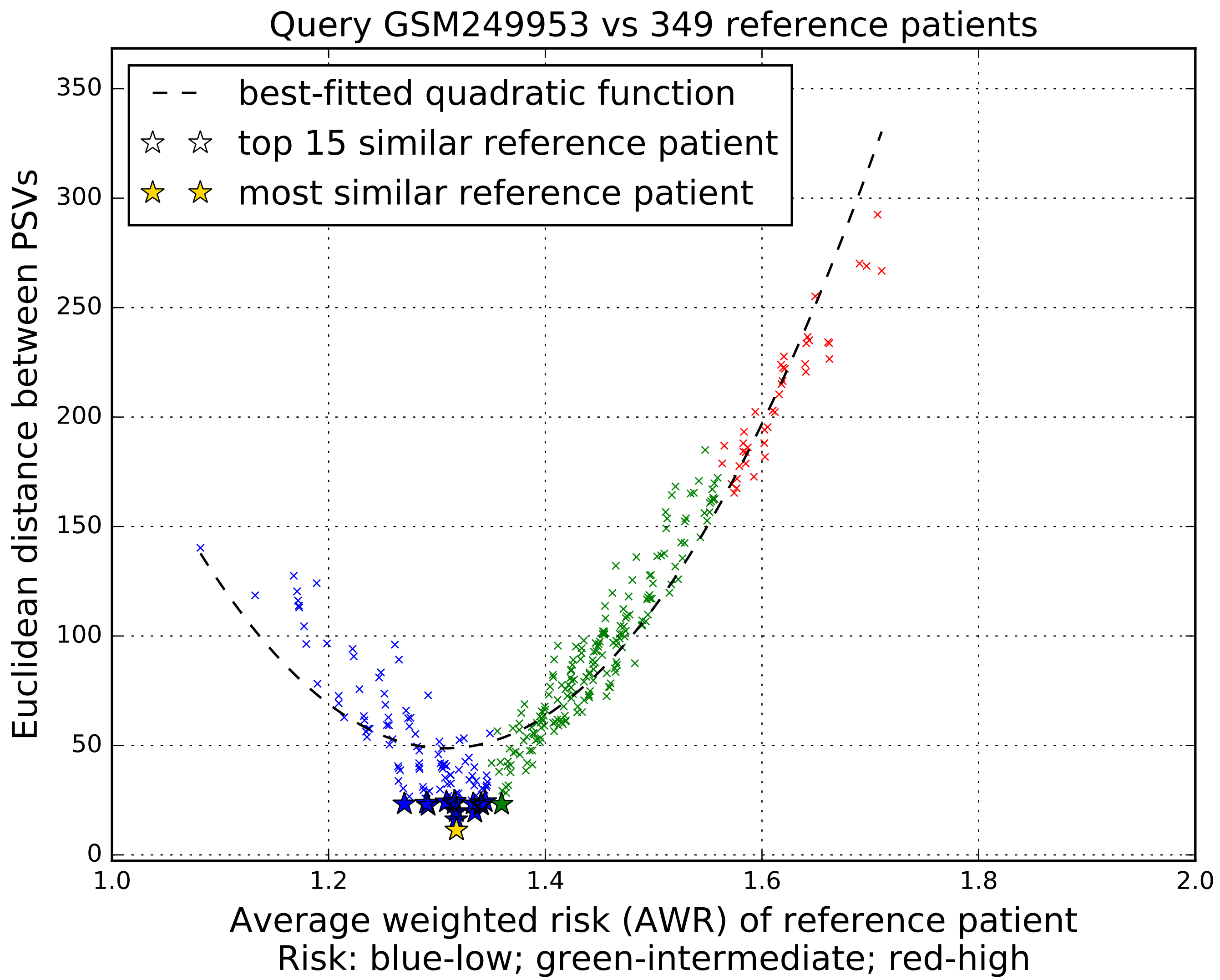

Query GSM249953 vs 349 reference patients
best-fitted quadratic function
top 15 similar reference patient
most similar reference patient
Euclidean distance between PSVs
Average weighted risk (AWR) of reference patient
Risk: blue-low; green-intermediate; red-high
0
50
100
150
200
250
300
350
1.0
1.2
1.4
1.6
1.8
2.0

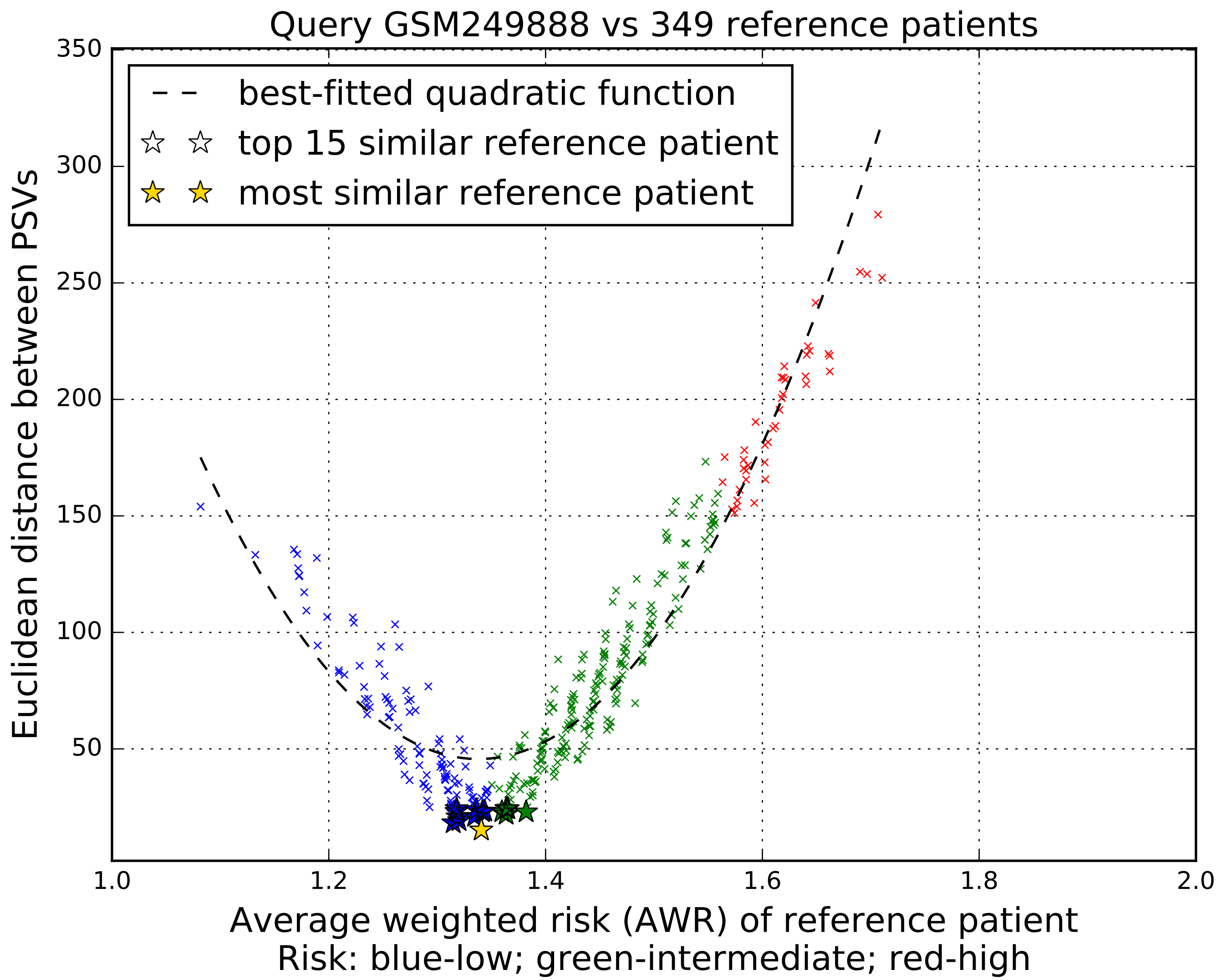

Query GSM249888 vs 349 reference patients
best-fitted quadratic function
top 15 similar reference patient
most similar reference patient
Euclidean distance between PSVs
350
300
250
200
150
100
50
1.0
1.2
1.4
1.6
1.8
2.0
Average weighted risk (AWR) of reference patient
Risk: blue-low; green-intermediate; red-high

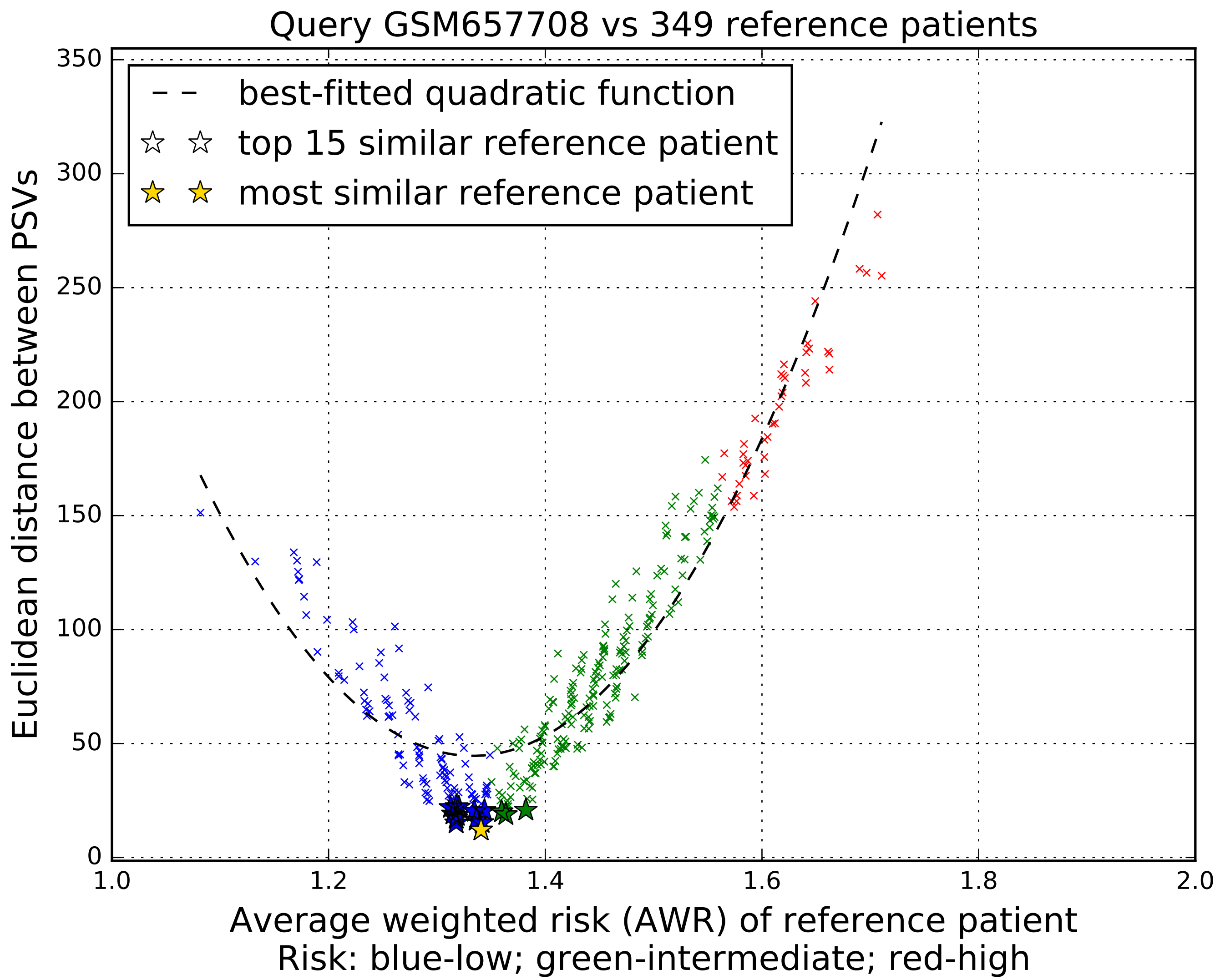
Query GSM657708 vs 349 reference patients
best-fitted quadratic function
top 15 similar reference patient
most similar reference patient
Euclidean distance between PSVs
0
50
100
150
200
250
300
350
1.0
1.2
1.4
1.6
1.8
2.0
Average weighted risk (AWR) of reference patient
Risk: blue-low; green-intermediate; red-high

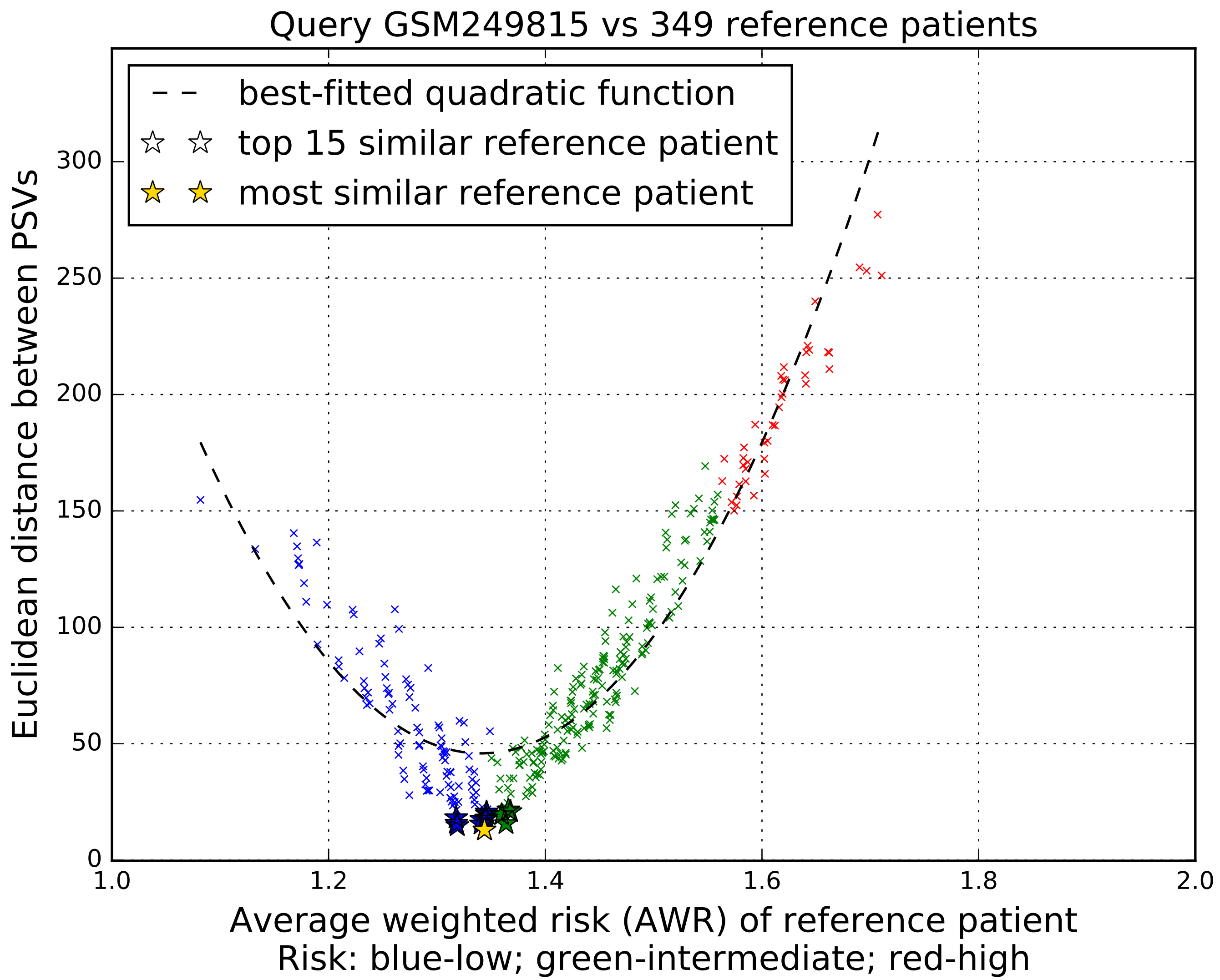
Query GSM249815 vs 349 reference patients
best-fitted quadratic function
top 15 similar reference patient
most similar reference patient
Euclidean distance between PSVs
0
50
100
150
200
250
300
1.0
1.2
1.4
1.6
1.8
2.0
Average weighted risk (AWR) of reference patient
Risk: blue-low; green-intermediate; red-high

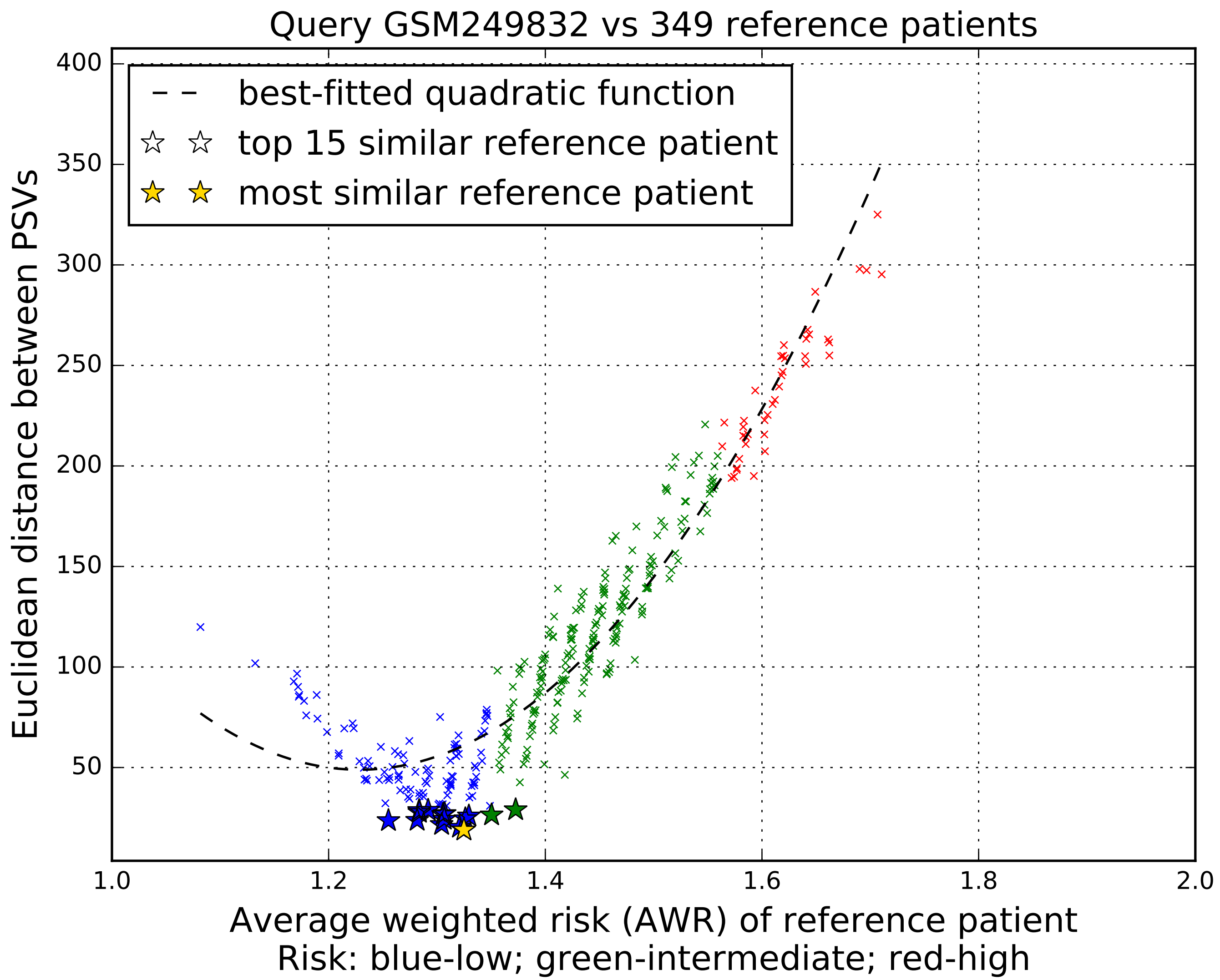

Query GSM249832 vs 349 reference patients
best-fitted quadratic function
top 15 similar reference patient
most similar reference patient
Euclidean distance between PSVs
400
350
300
250
200
150
100
50
1.0
1.2
1.4
1.6
1.8
2.0
Average weighted risk (AWR) of reference patient
Risk: blue-low; green-intermediate; red-high

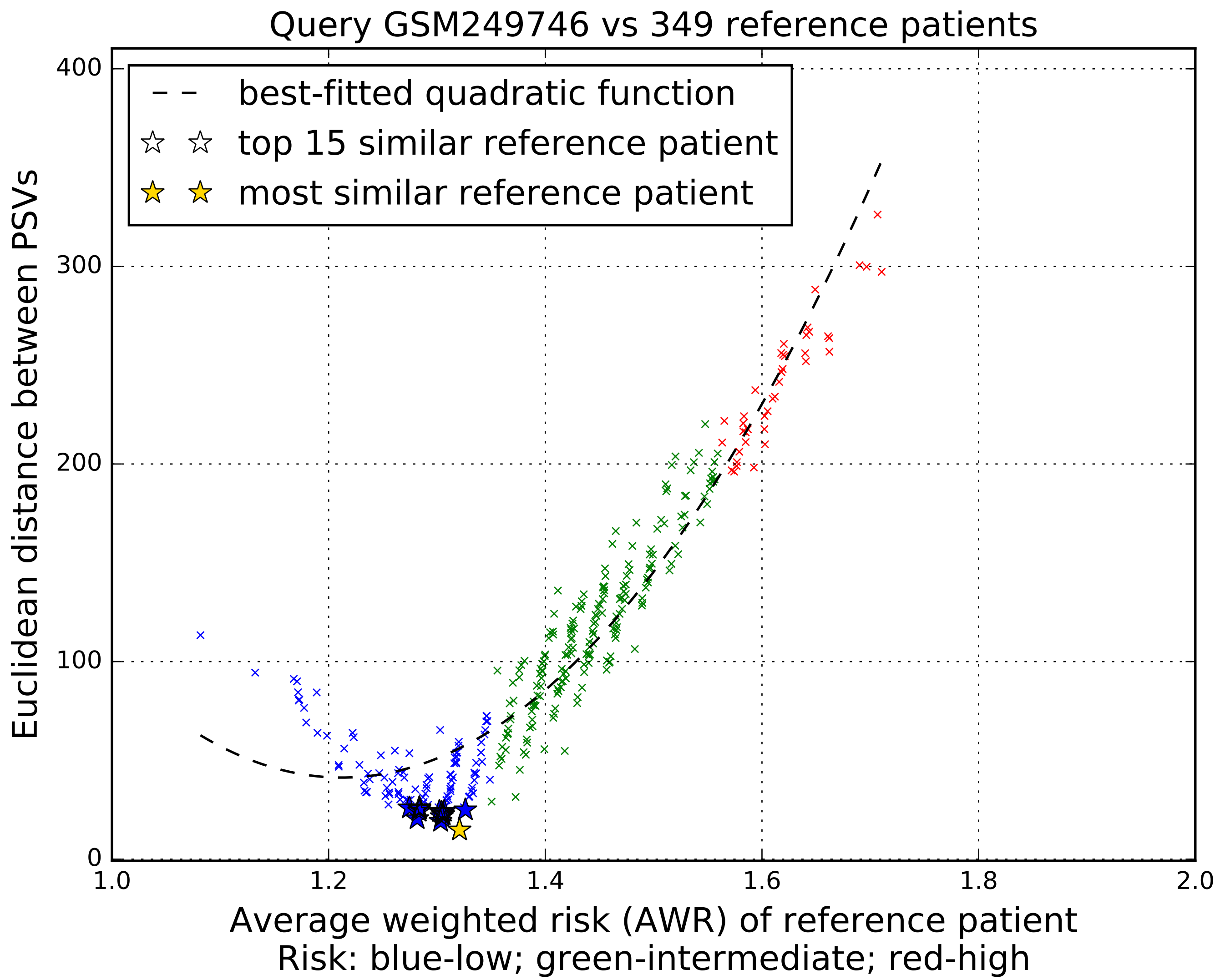

Query GSM249746 vs 349 reference patients
best-fitted quadratic function
top 15 similar reference patient
most similar reference patient
Euclidean distance between PSVs
Average weighted risk (AWR) of reference patient
Risk: blue-low; green-intermediate; red-high
0
100
200
300
400
1.0
1.2
1.4
1.6
1.8
2.0

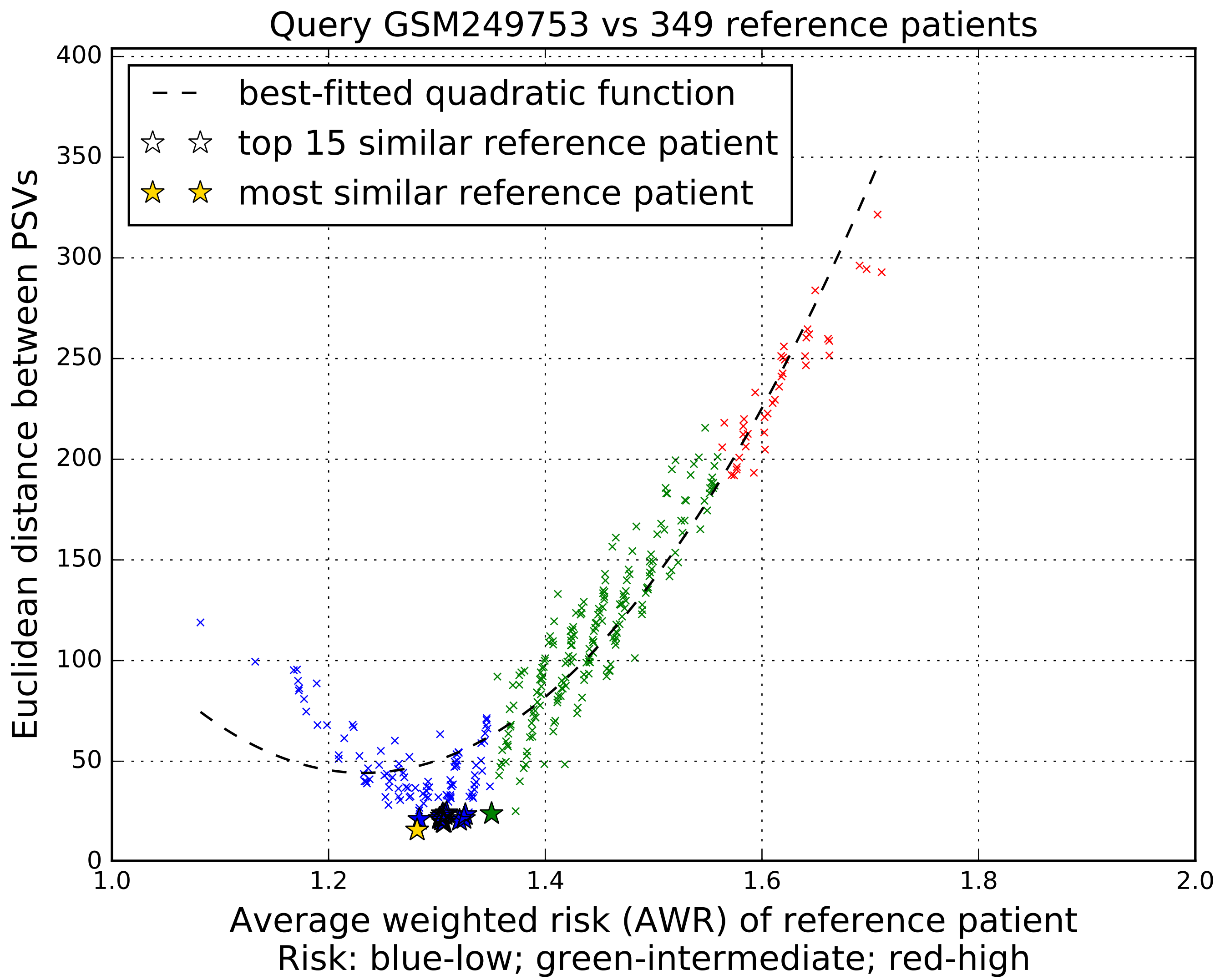

Query GSM249753 vs 349 reference patients
best-fitted quadratic function
top 15 similar reference patient
most similar reference patient
Euclidean distance between PSVs
0
50
100
150
200
250
300
350
400
1.0
1.2
1.4
1.6
1.8
2.0
Average weighted risk (AWR) of reference patient
Risk: blue-low; green-intermediate; red-high

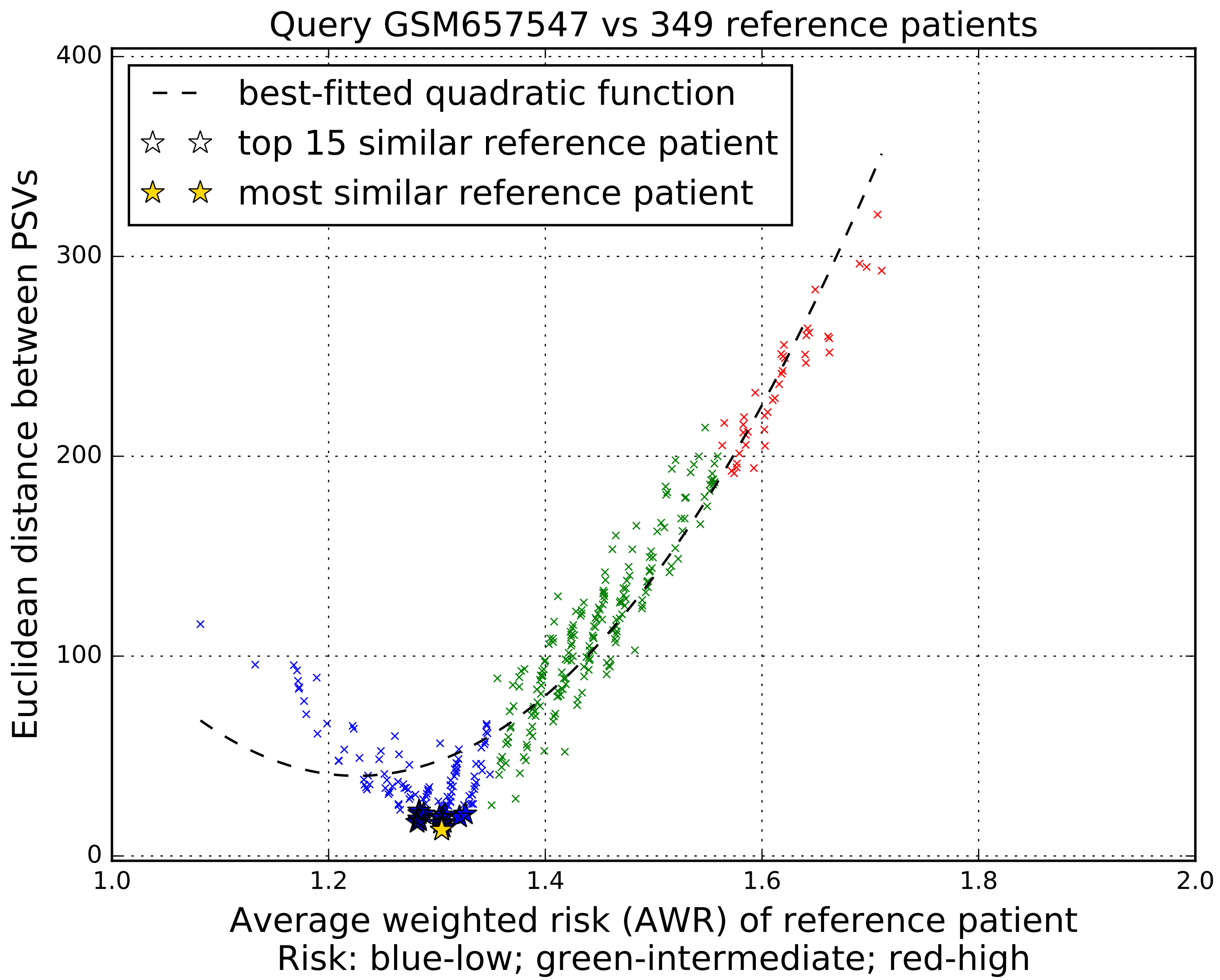

Query GSM657547 vs 349 reference patients
best-fitted quadratic function
top 15 similar reference patient
most similar reference patient
Euclidean distance between PSVs
0
100
200
300
400
1.0
1.2
1.4
1.6
1.8
2.0
Average weighted risk (AWR) of reference patient
Risk: blue-low; green-intermediate; red-high

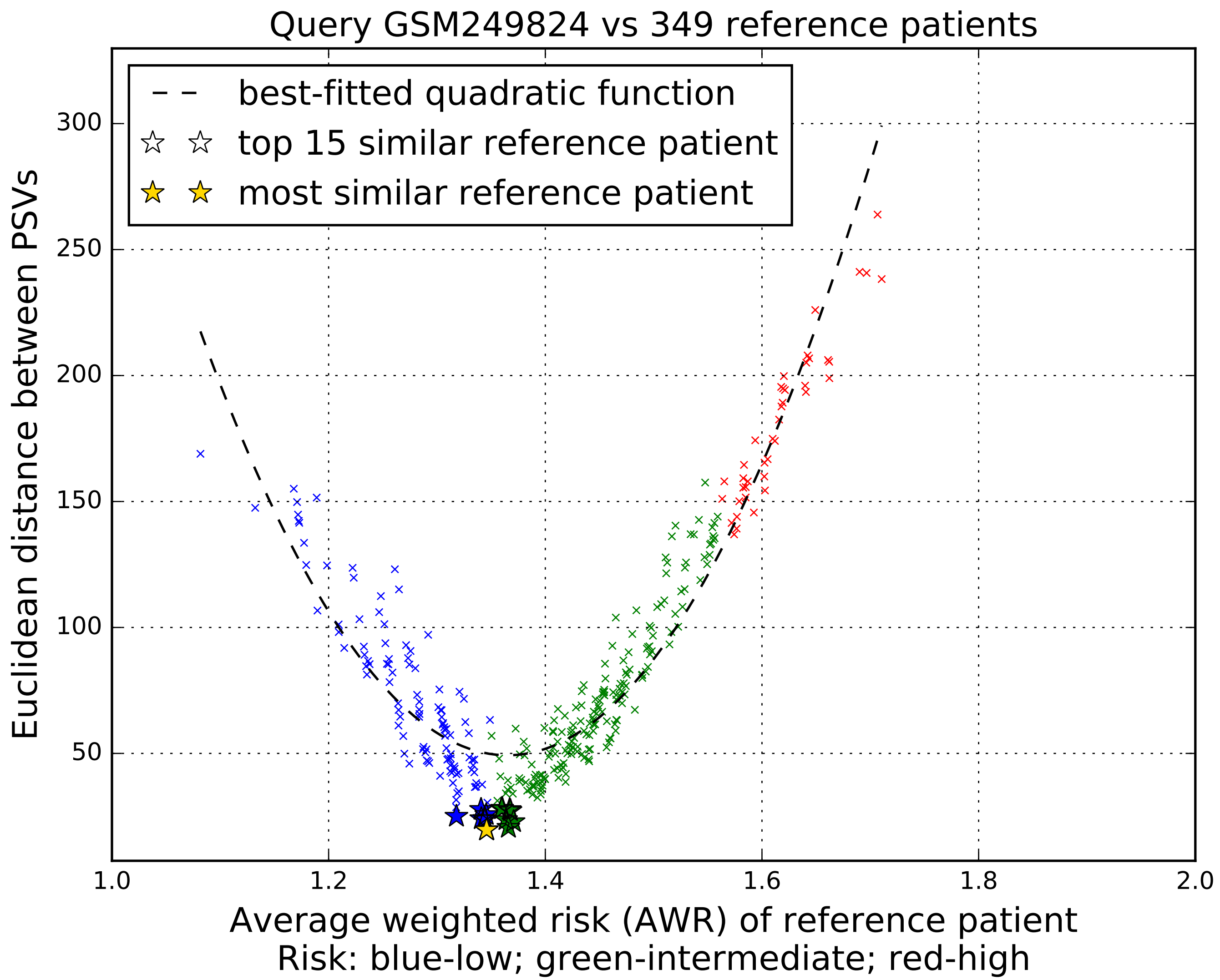

Query GSM249824 vs 349 reference patients
best-fitted quadratic function
top 15 similar reference patient
most similar reference patient
300
250
200
150
100
50
1.0
1.2
1.4
1.6
1.8
2.0
Euclidean distance between PSVs
Average weighted risk (AWR) of reference patient
Risk: blue-low; green-intermediate; red-high

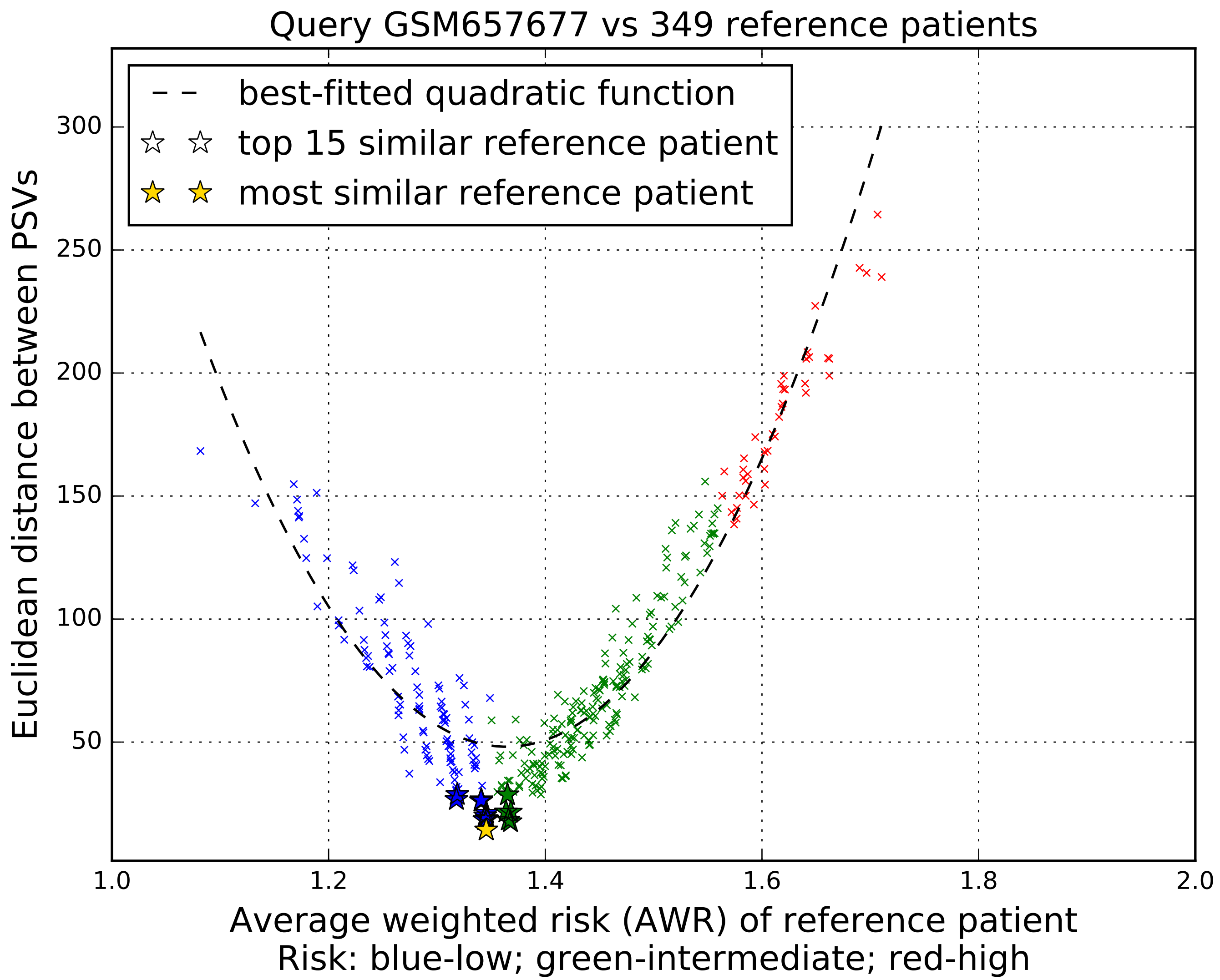
Query GSM657677 vs 349 reference patients
best-fitted quadratic function
top 15 similar reference patient
most similar reference patient
Euclidean distance between PSVs
300
250
200
150
100
50
1.0
1.2
1.4
1.6
1.8
2.0
Average weighted risk (AWR) of reference patient
Risk: blue-low; green-intermediate; red-high

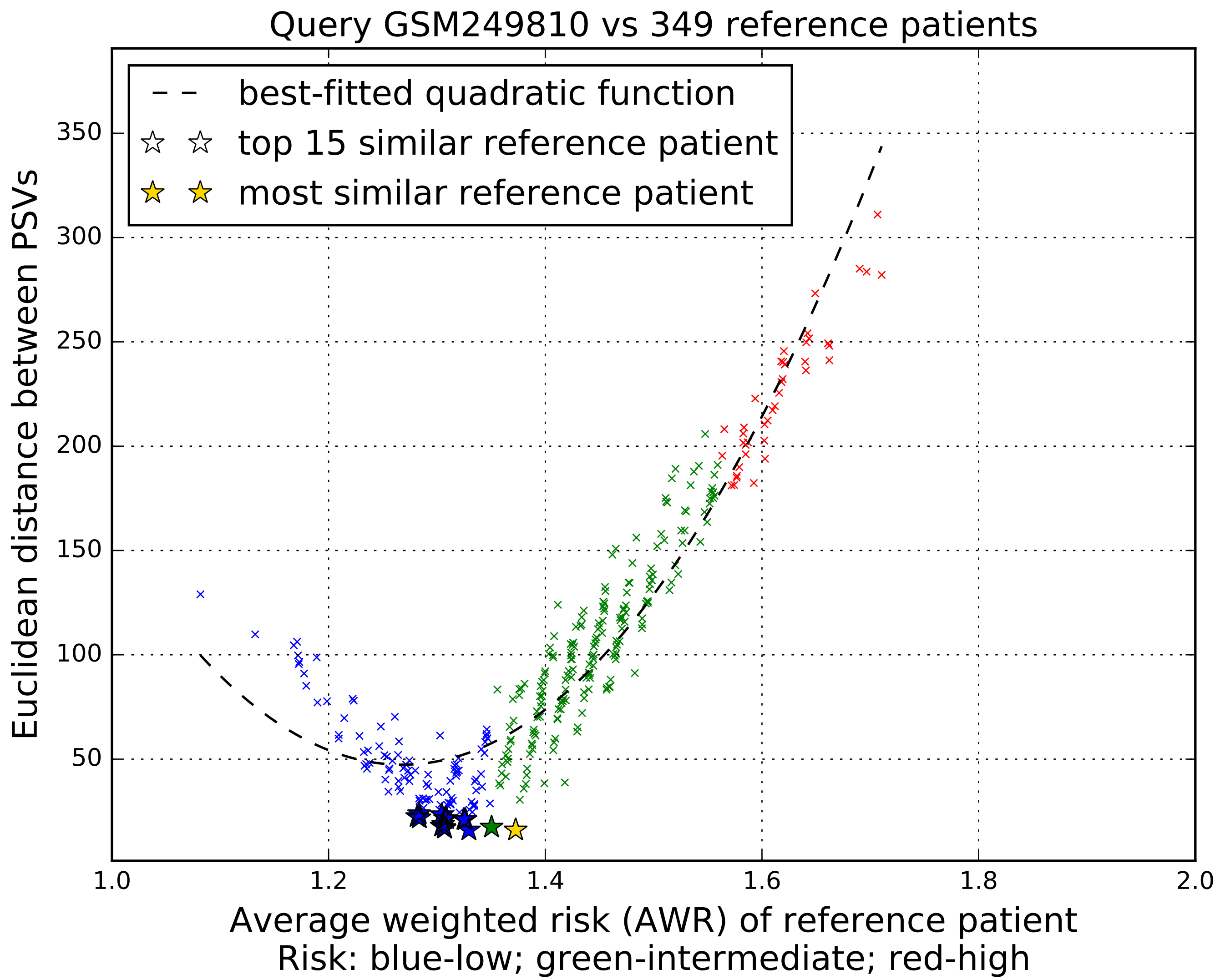

Query GSM249810 vs 349 reference patients
best-fitted quadratic function
top 15 similar reference patient
most similar reference patient
350
300
250
200
150
100
50
1.0
1.2
1.4
1.6
1.8
2.0
Euclidean distance between PSVs
Average weighted risk (AWR) of reference patient
Risk: blue-low; green-intermediate; red-high

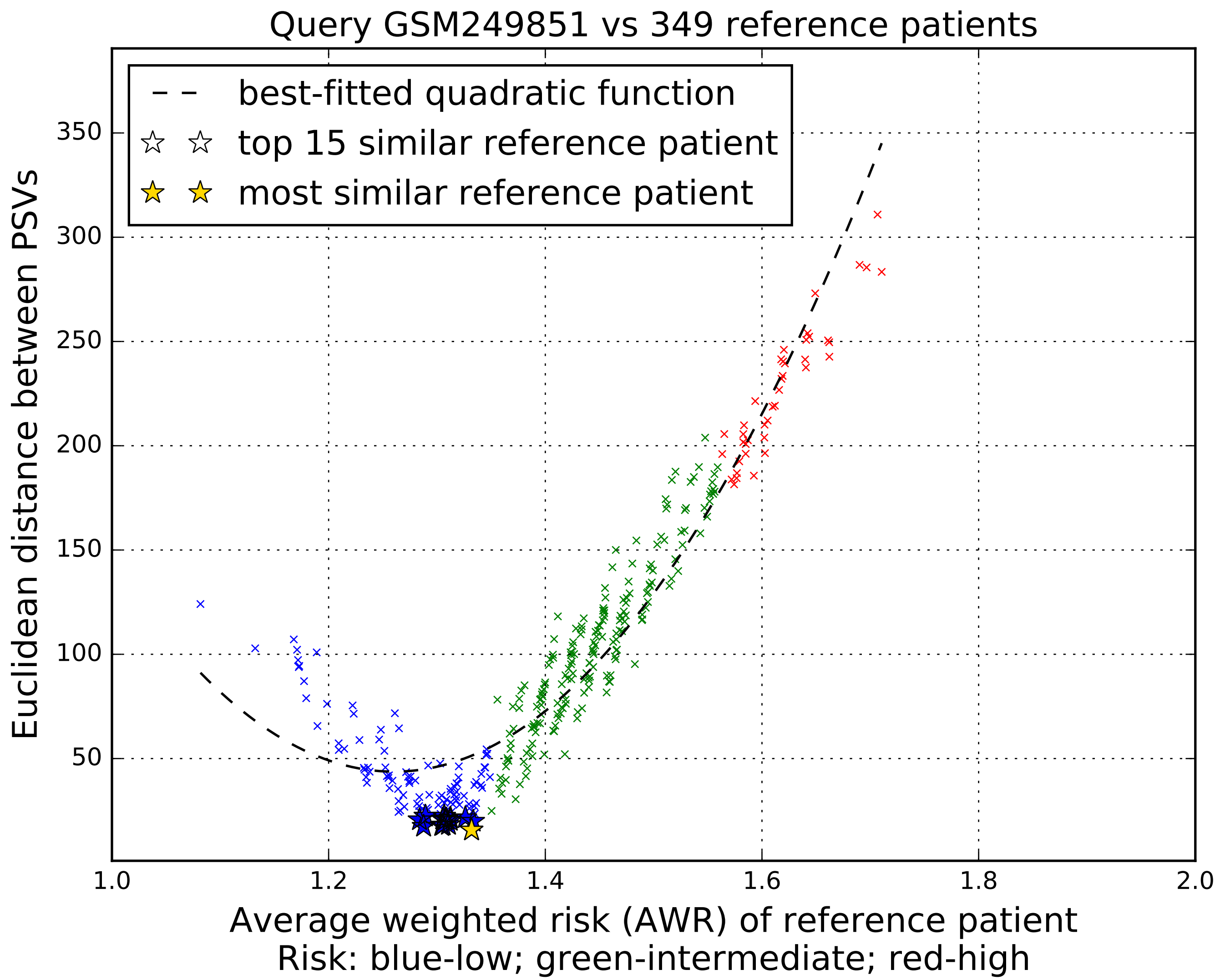
Query GSM249851 vs 349 reference patients
best-fitted quadratic function
top 15 similar reference patient
most similar reference patient
Euclidean distance between PSVs
350
300
250
200
150
100
50
1.0
1.2
1.4
1.6
1.8
2.0
Average weighted risk (AWR) of reference patient
Risk: blue-low; green-intermediate; red-high

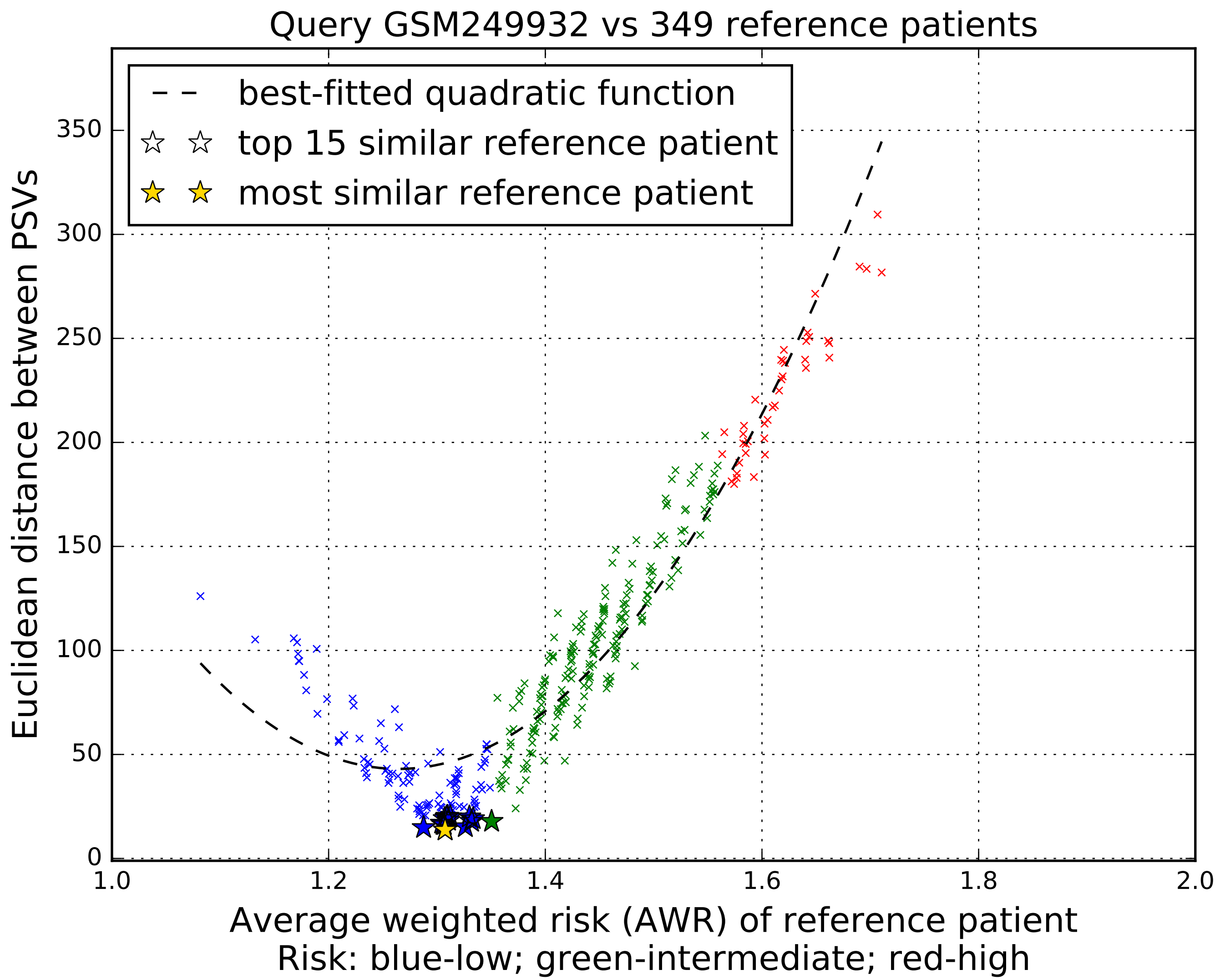
Query GSM249932 vs 349 reference patients
best-fitted quadratic function
top 15 similar reference patient
most similar reference patient
Euclidean distance between PSVs
0
50
100
150
200
250
300
350
1.0
1.2
1.4
1.6
1.8
2.0
Average weighted risk (AWR) of reference patient
Risk: blue-low; green-intermediate; red-high

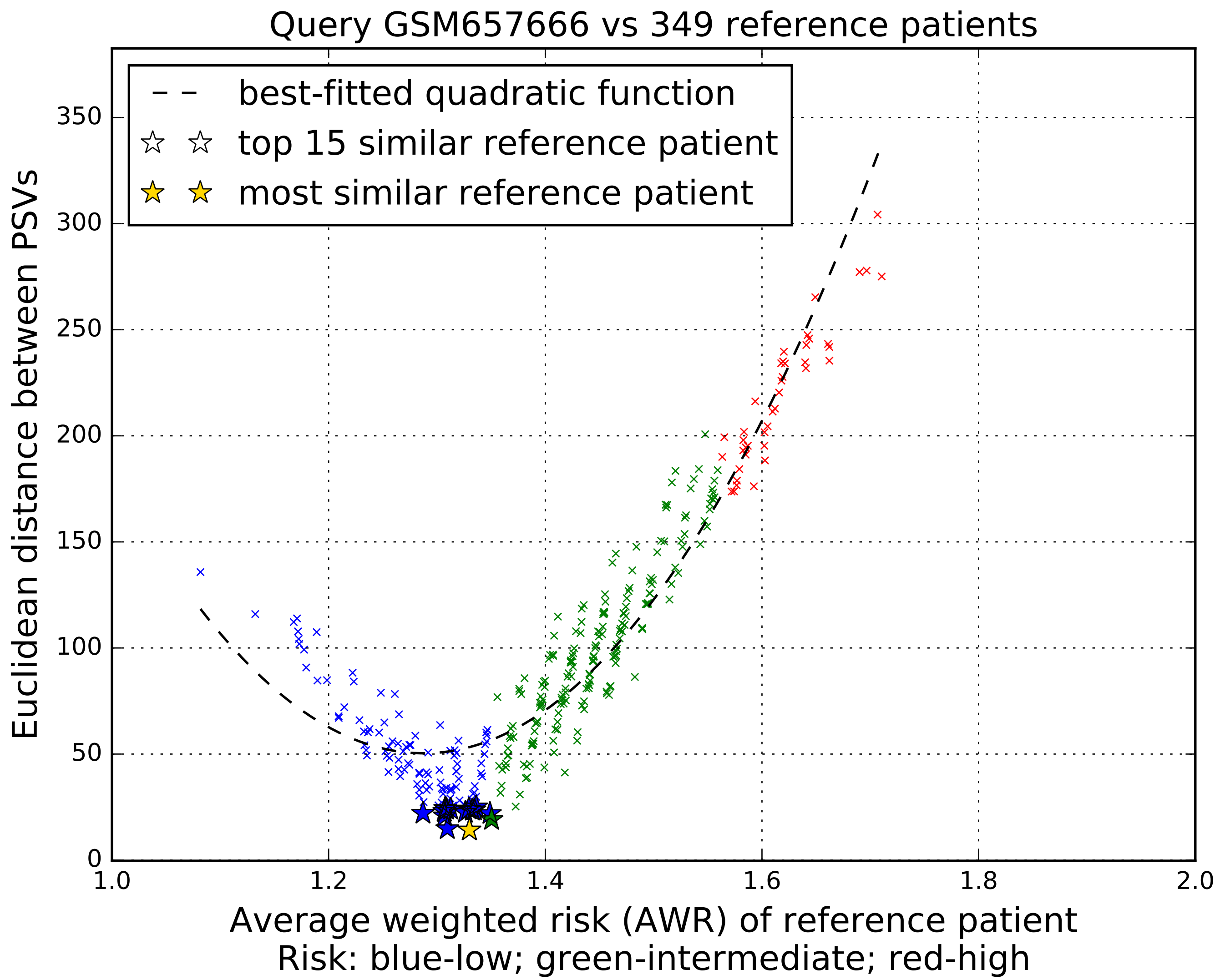
Query GSM657666 vs 349 reference patients
best-fitted quadratic function
top 15 similar reference patient
most similar reference patient
Euclidean distance between PSVs
0
50
100
150
200
250
300
350
1.0
1.2
1.4
1.6
1.8
2.0
Average weighted risk (AWR) of reference patient
Risk: blue-low; green-intermediate; red-high

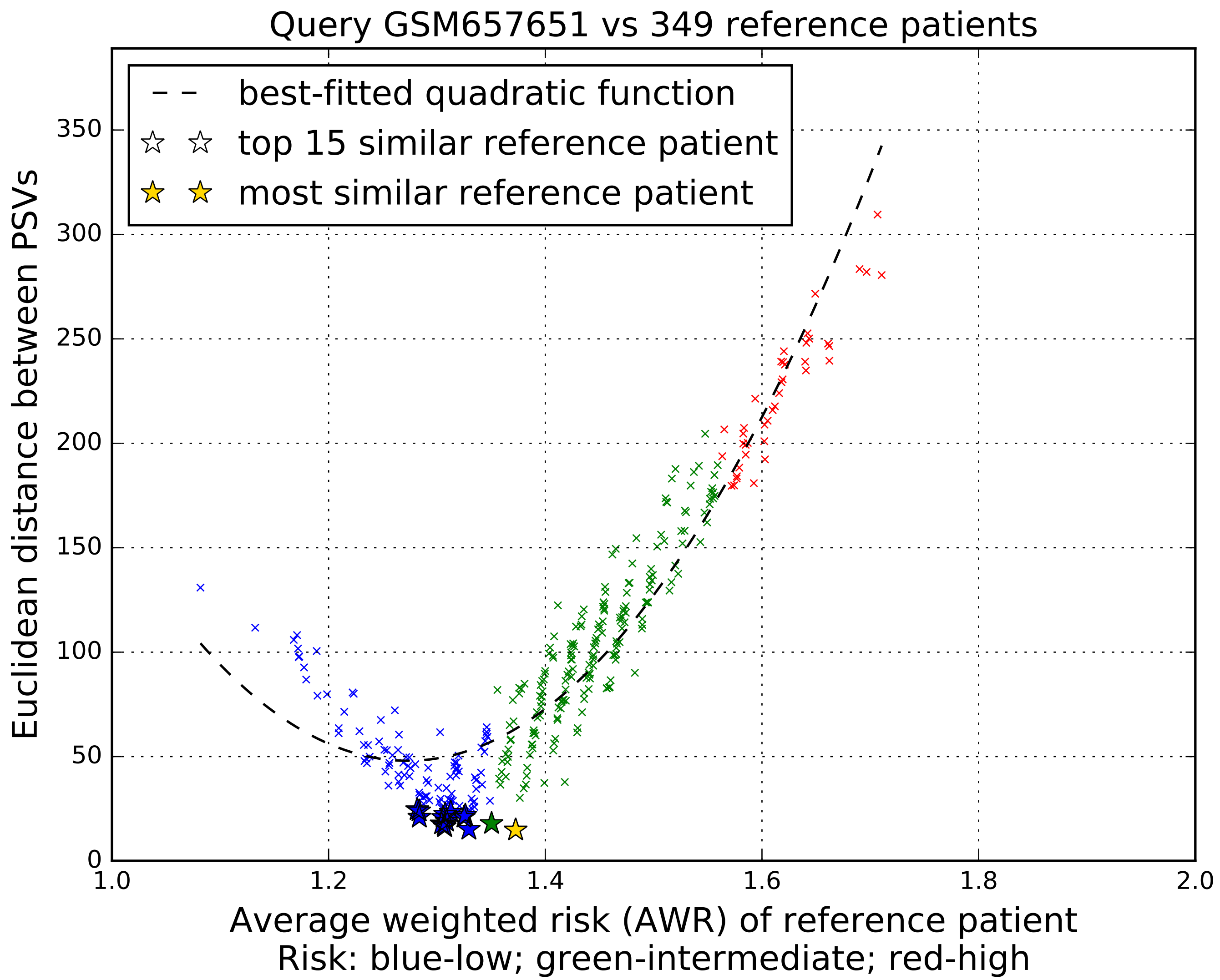

Query GSM657651 vs 349 reference patients
best-fitted quadratic function
top 15 similar reference patient
most similar reference patient
Euclidean distance between PSVs
0
50
100
150
200
250
300
350
1.0
1.2
1.4
1.6
1.8
2.0
Average weighted risk (AWR) of reference patient
Risk: blue-low; green-intermediate; red-high

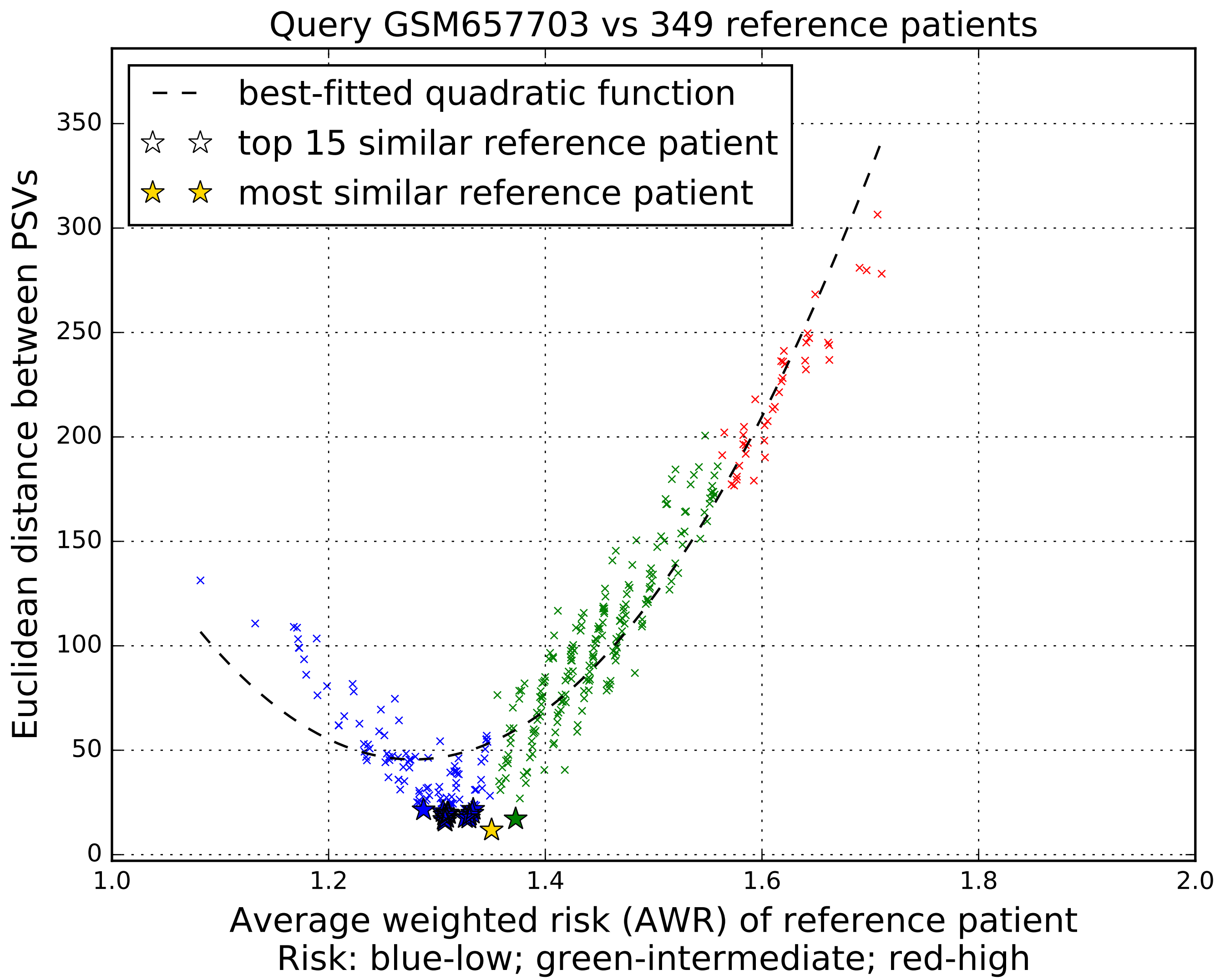

Query GSM657703 vs 349 reference patients
best-fitted quadratic function
top 15 similar reference patient
most similar reference patient
Euclidean distance between PSVs
Average weighted risk (AWR) of reference patient
Risk: blue-low; green-intermediate; red-high
0
50
100
150
200
250
300
350
1.0
1.2
1.4
1.6
1.8
2.0

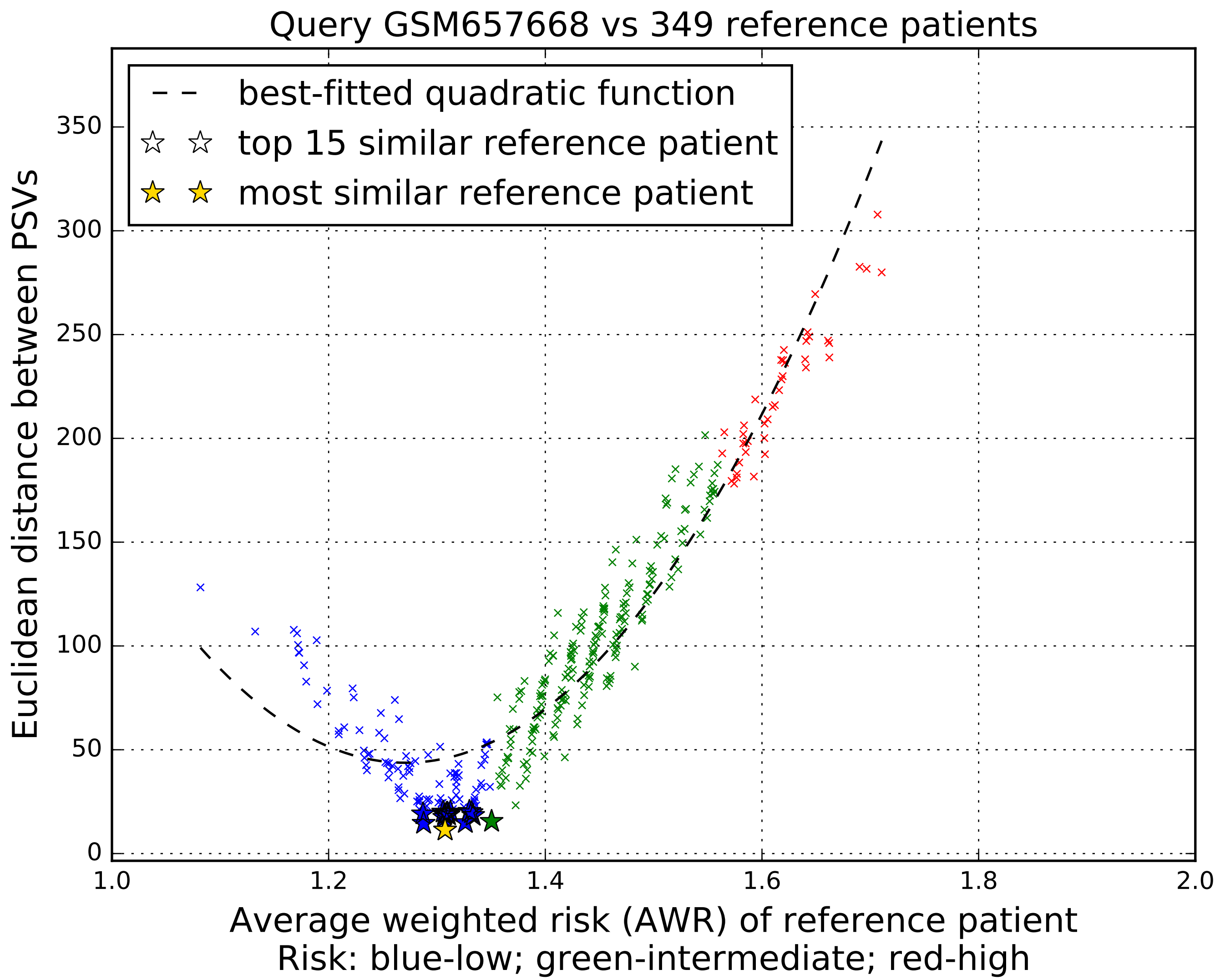
Query GSM657668 vs 349 reference patients
best-fitted quadratic function
top 15 similar reference patient
most similar reference patient
Euclidean distance between PSVs
0
50
100
150
200
250
300
350
1.0
1.2
1.4
1.6
1.8
2.0
Average weighted risk (AWR) of reference patient
Risk: blue-low; green-intermediate; red-high

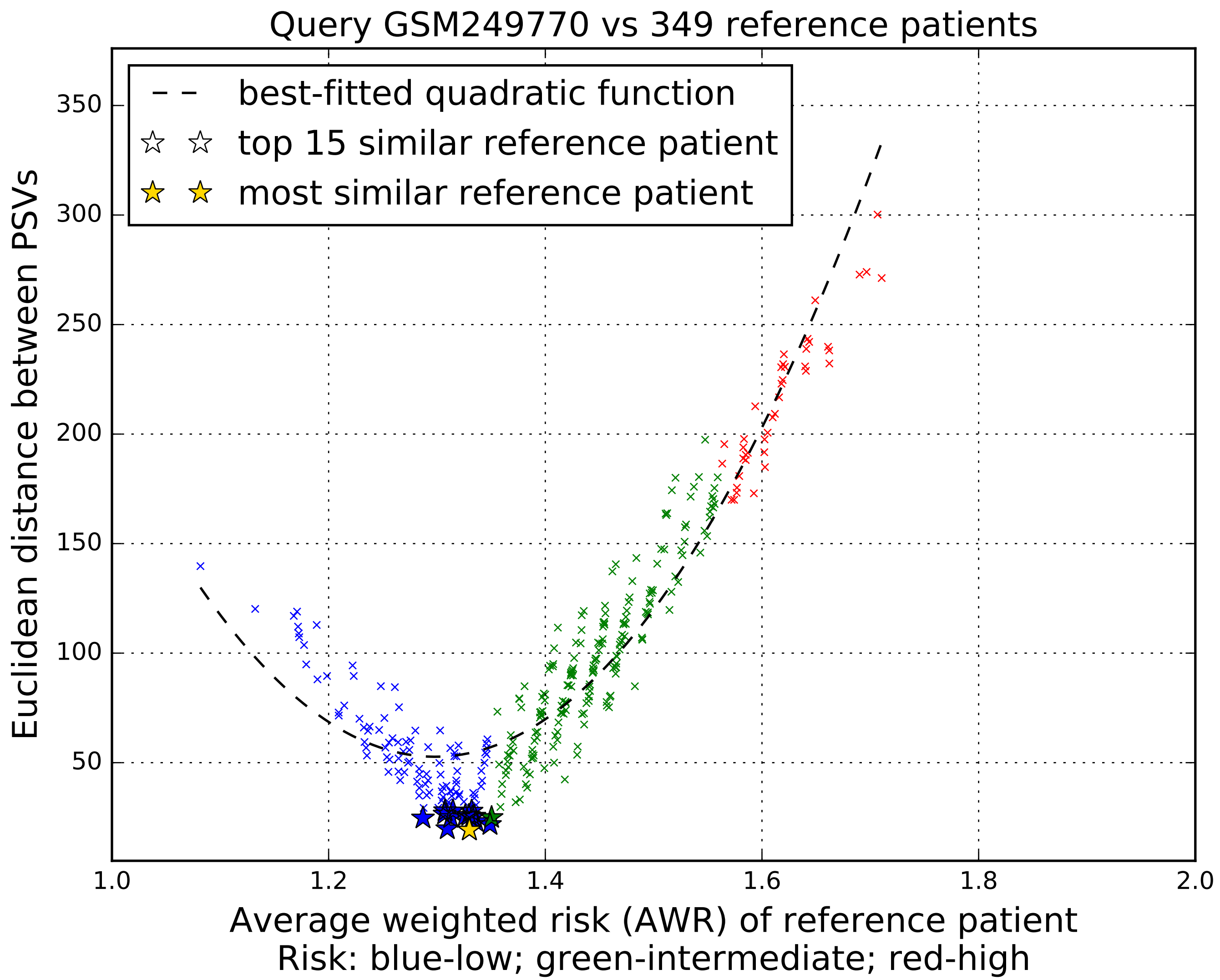

Query GSM249770 vs 349 reference patients
best-fitted quadratic function
top 15 similar reference patient
most similar reference patient
Euclidean distance between PSVs
50
100
150
200
250
300
350
1.0
1.2
1.4
1.6
1.8
2.0
Average weighted risk (AWR) of reference patient
Risk: blue-low; green-intermediate; red-high

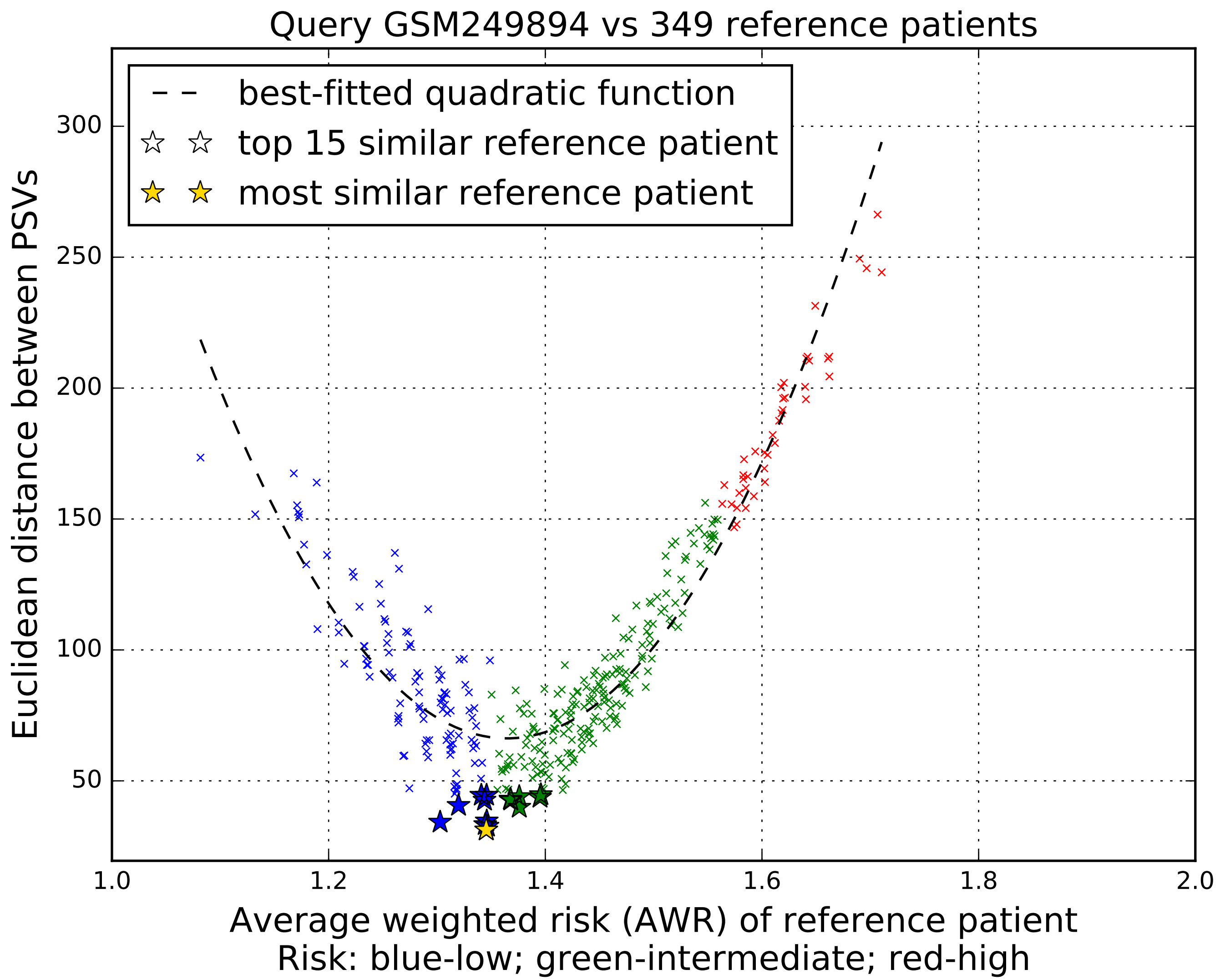
Query GSM249894 vs 349 reference patients
best-fitted quadratic function
top 15 similar reference patient
most similar reference patient
Euclidean distance between PSVs
300
250
200
150
100
50
1.0
1.2
1.4
1.6
1.8
2.0
Average weighted risk (AWR) of reference patient
Risk: blue-low; green-intermediate; red-high

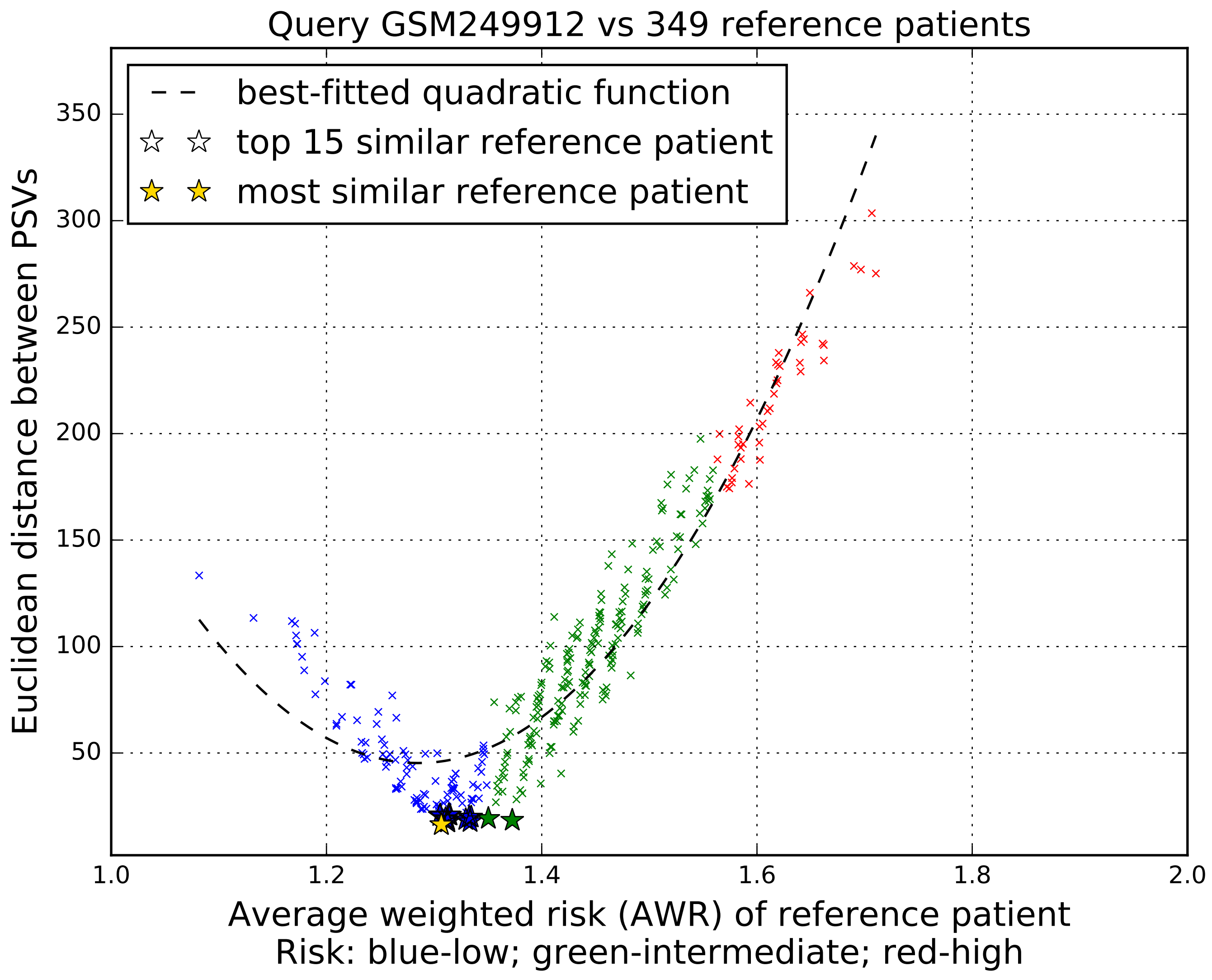

Query GSM249912 vs 349 reference patients
best-fitted quadratic function
top 15 similar reference patient
most similar reference patient
Euclidean distance between PSVs
350
300
250
200
150
100
50
1.0
1.2
1.4
1.6
1.8
2.0
Average weighted risk (AWR) of reference patient
Risk: blue-low; green-intermediate; red-high

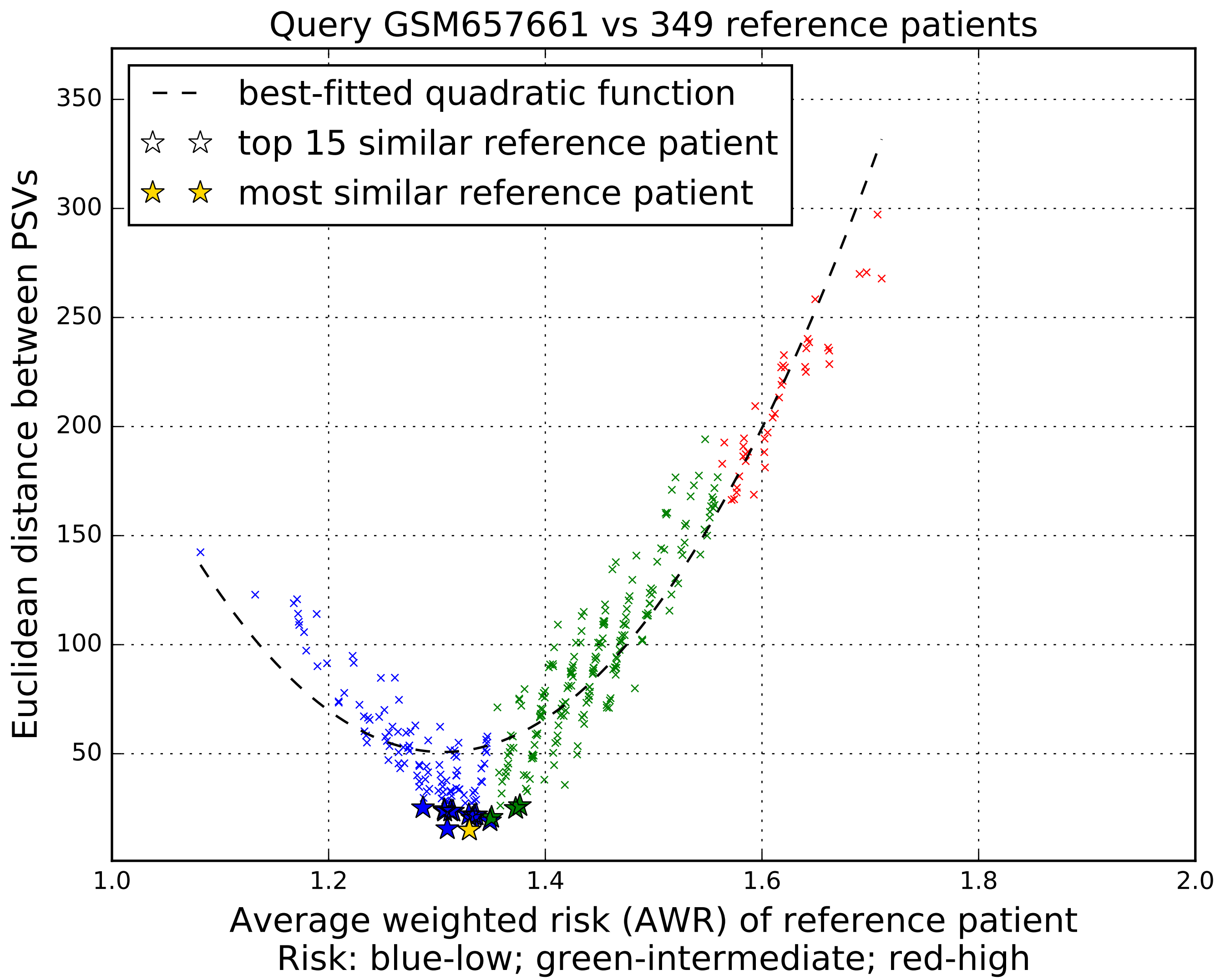
Query GSM657661 vs 349 reference patients
best-fitted quadratic function
top 15 similar reference patient
most similar reference patient
Euclidean distance between PSVs
350
300
250
200
150
100
50
1.0
1.2
1.4
1.6
1.8
2.0
Average weighted risk (AWR) of reference patient
Risk: blue-low; green-intermediate; red-high

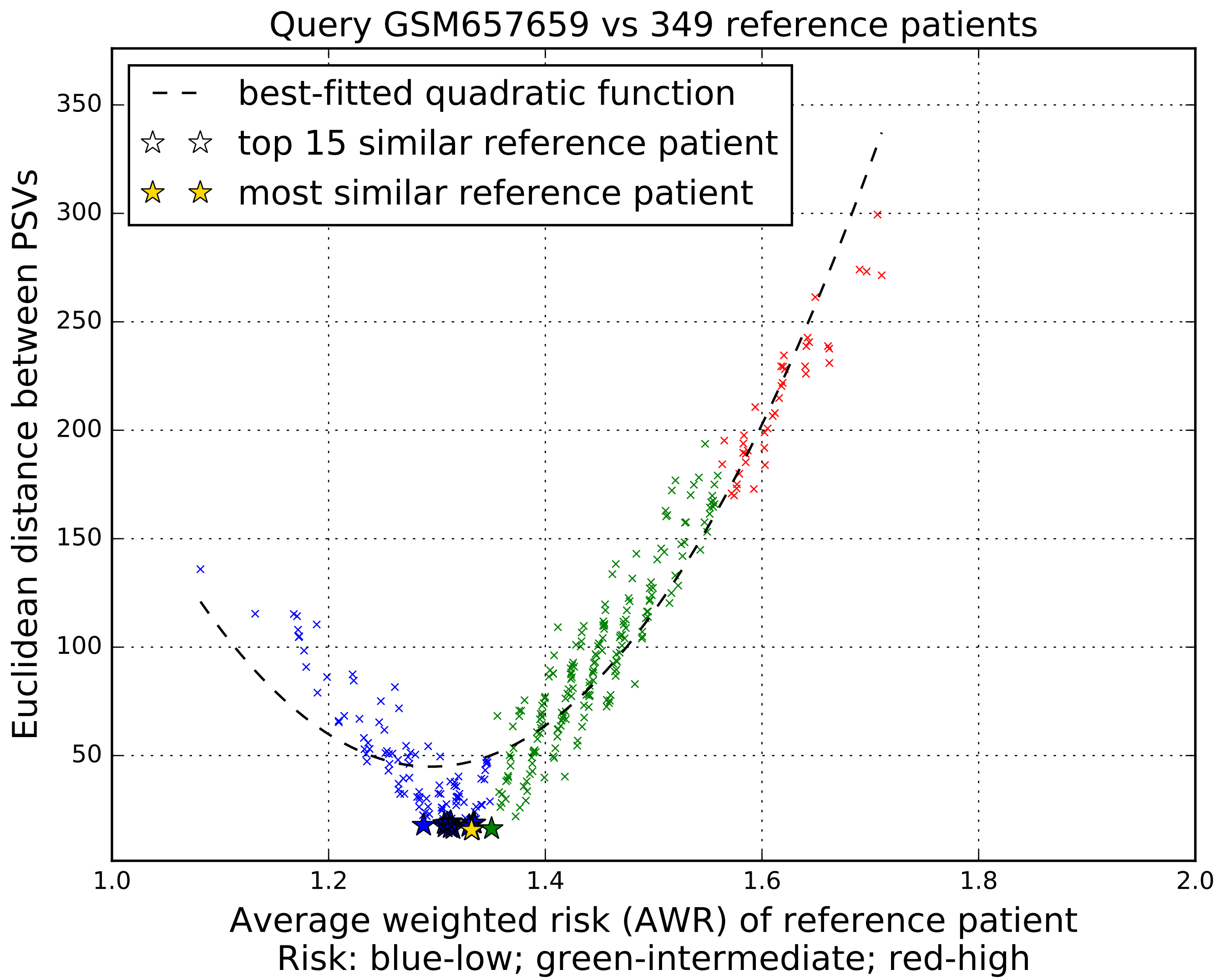

Query GSM657659 vs 349 reference patients
best-fitted quadratic function
top 15 similar reference patient
most similar reference patient
Euclidean distance between PSVs
350
300
250
200
150
100
50
1.0
1.2
1.4
1.6
1.8
2.0
Average weighted risk (AWR) of reference patient
Risk: blue-low; green-intermediate; red-high

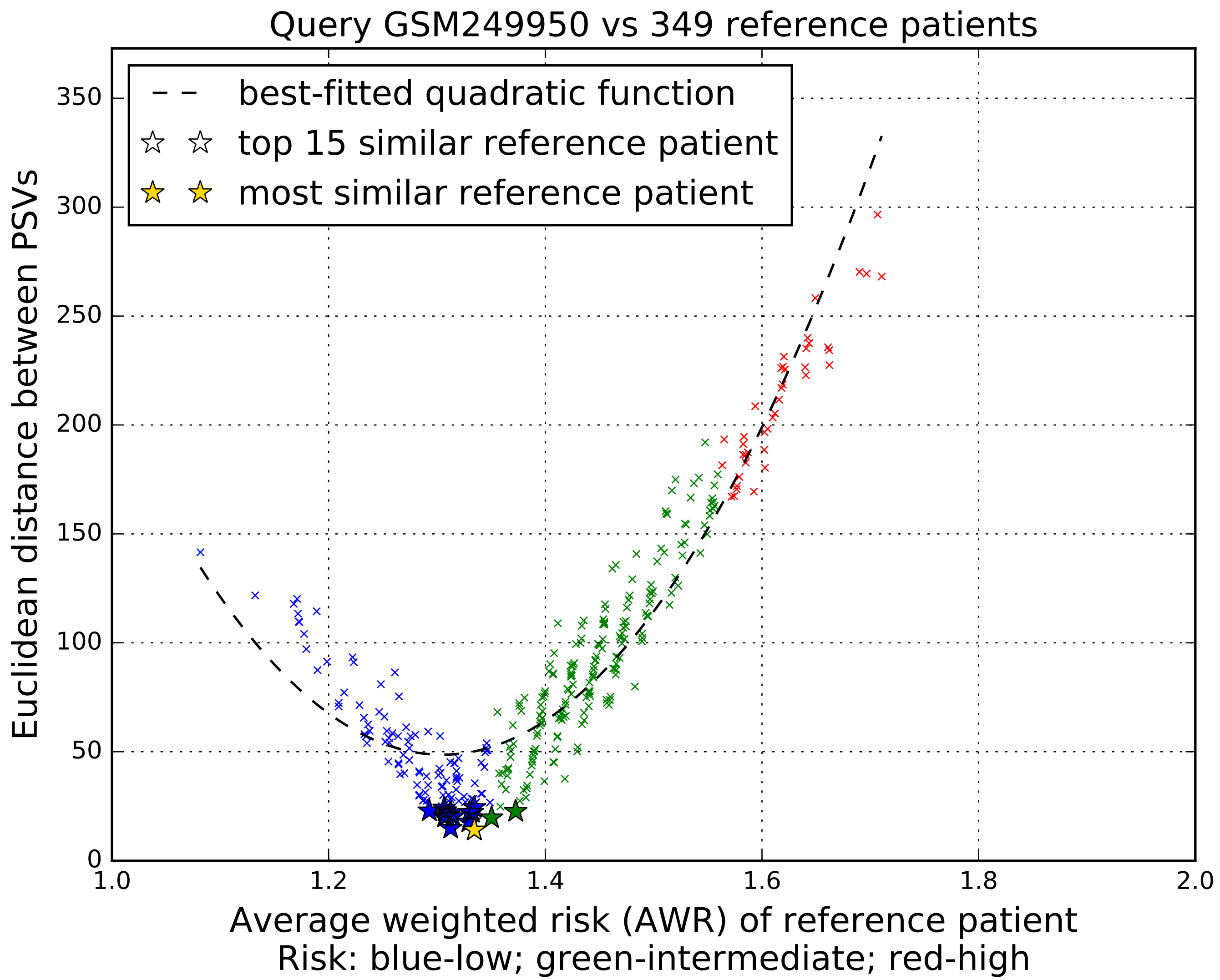

Query GSM249950 vs 349 reference patients
best-fitted quadratic function
top 15 similar reference patient
most similar reference patient
Euclidean distance between PSVs
Average weighted risk (AWR) of reference patient
Risk: blue-low; green-intermediate; red-high
0
50
100
150
200
250
300
350
1.0
1.2
1.4
1.6
1.8
2.0

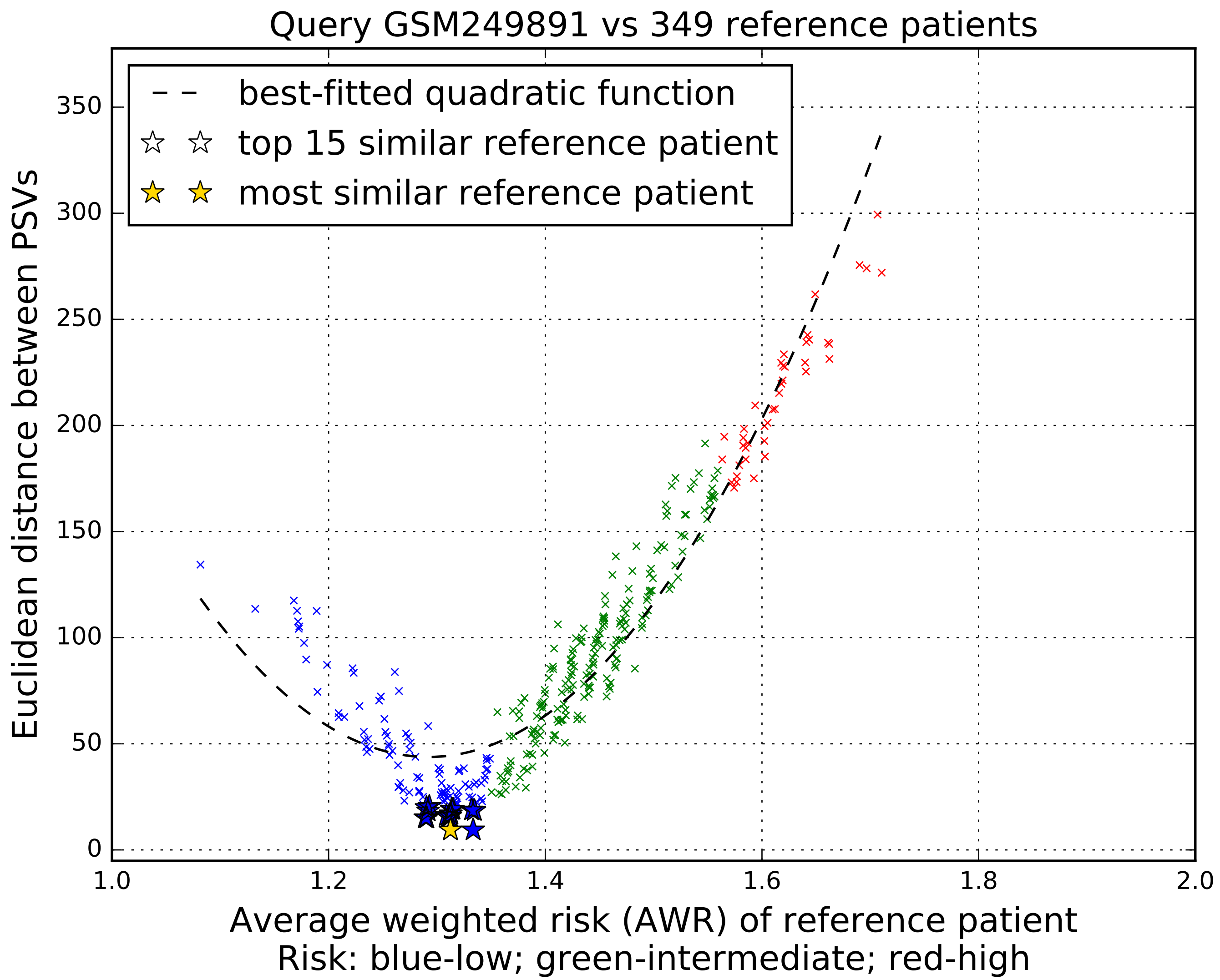

Query GSM249891 vs 349 reference patients
best-fitted quadratic function
top 15 similar reference patient
most similar reference patient
Euclidean distance between PSVs
0
50
100
150
200
250
300
350
1.0
1.2
1.4
1.6
1.8
2.0
Average weighted risk (AWR) of reference patient
Risk: blue-low; green-intermediate; red-high

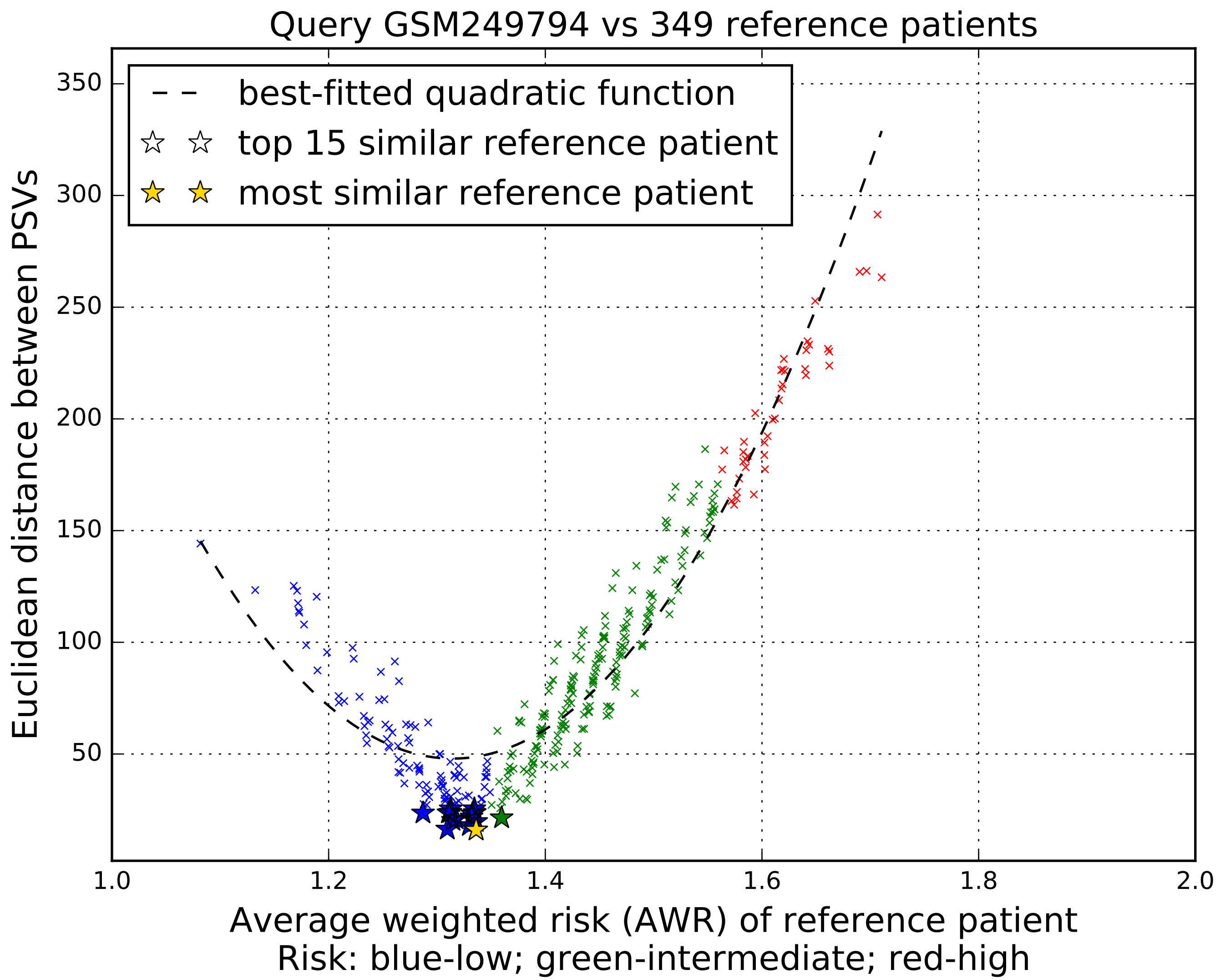
Query GSM249794 vs 349 reference patients
best-fitted quadratic function
top 15 similar reference patient
most similar reference patient
Euclidean distance between PSVs
350
300
250
200
150
100
50
1.0
1.2
1.4
1.6
1.8
2.0
Average weighted risk (AWR) of reference patient
Risk: blue-low; green-intermediate; red-high

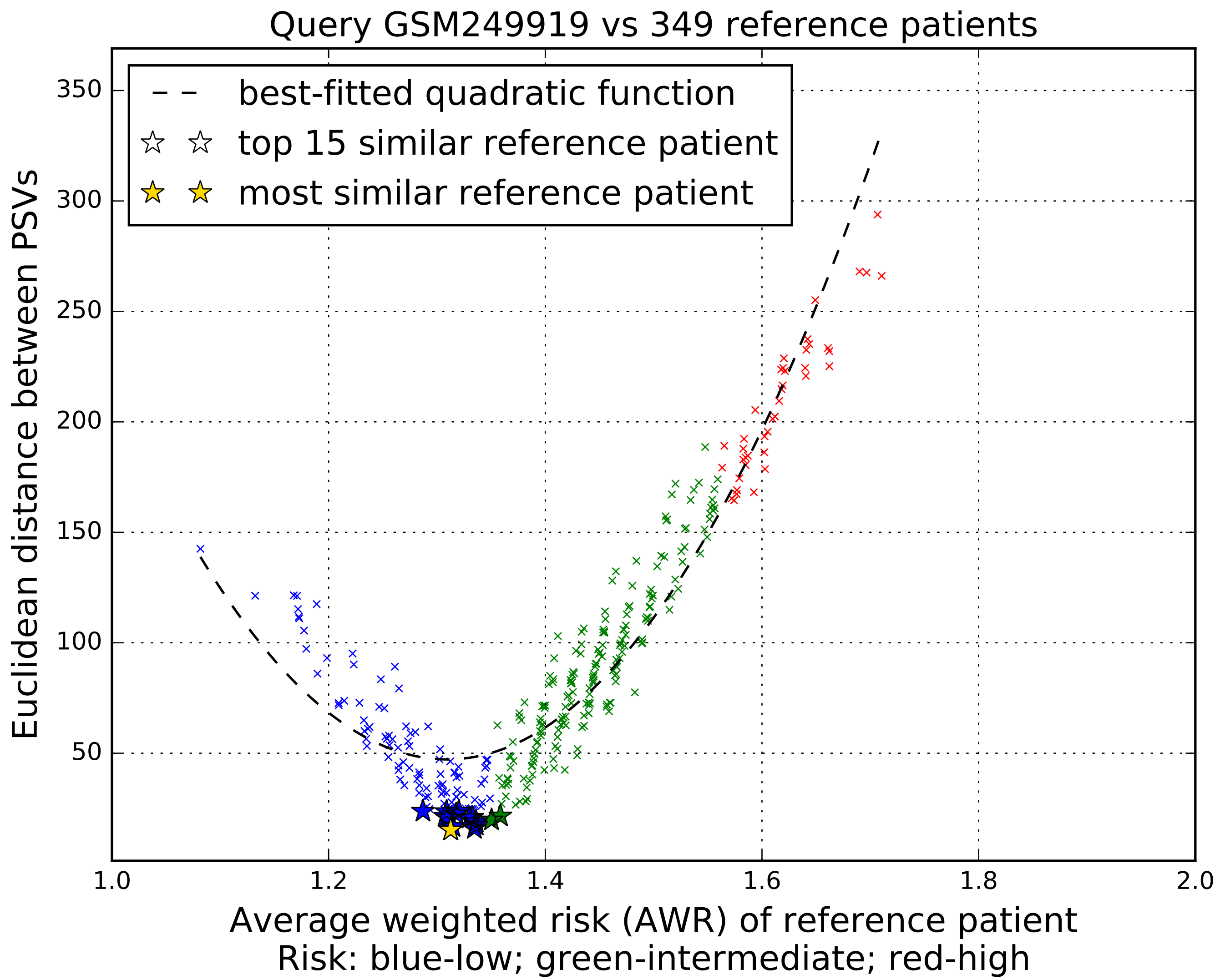

Query GSM249919 vs 349 reference patients
best-fitted quadratic function
top 15 similar reference patient
most similar reference patient
Euclidean distance between PSVs
350
300
250
200
150
100
50
1.0
1.2
1.4
1.6
1.8
2.0
Average weighted risk (AWR) of reference patient
Risk: blue-low; green-intermediate; red-high

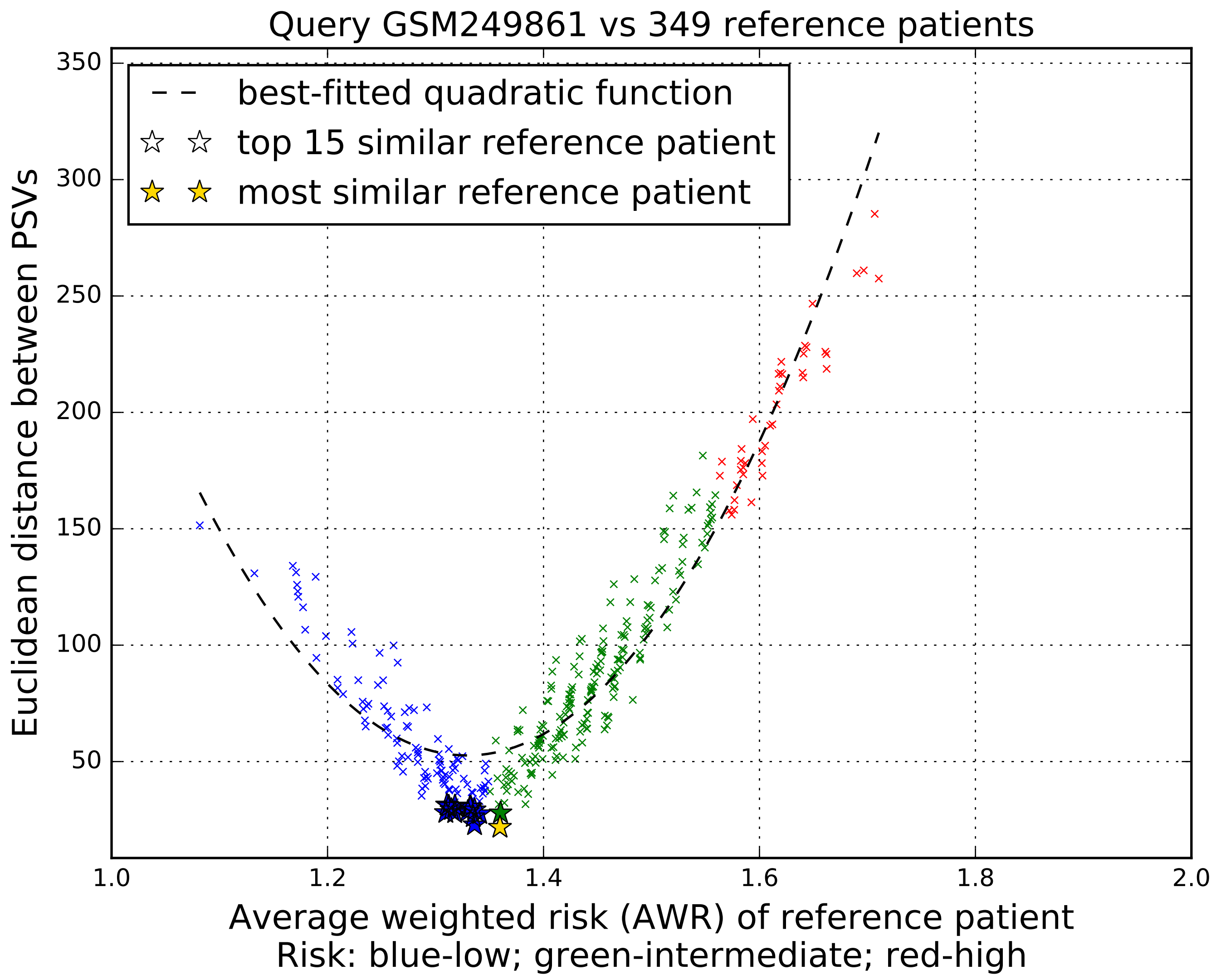

Query GSM249861 vs 349 reference patients
best-fitted quadratic function
top 15 similar reference patient
most similar reference patient
Euclidean distance between PSVs
350
300
250
200
150
100
50
1.0
1.2
1.4
1.6
1.8
2.0
Average weighted risk (AWR) of reference patient
Risk: blue-low; green-intermediate; red-high

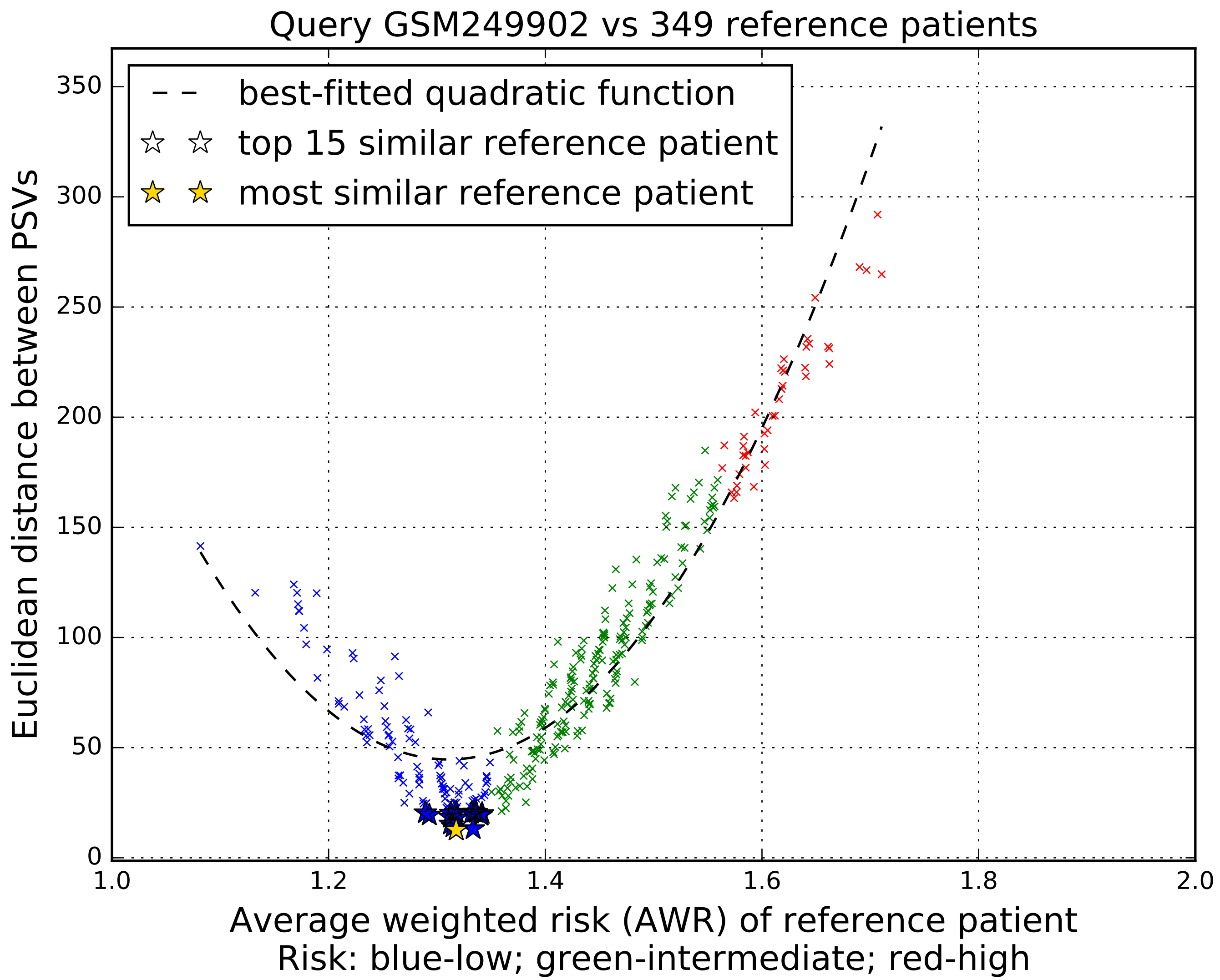

Query GSM249902 vs 349 reference patients
best-fitted quadratic function
top 15 similar reference patient
most similar reference patient
Euclidean distance between PSVs
Average weighted risk (AWR) of reference patient
Risk: blue-low; green-intermediate; red-high
0
50
100
150
200
250
300
350
1.0
1.2
1.4
1.6
1.8
2.0

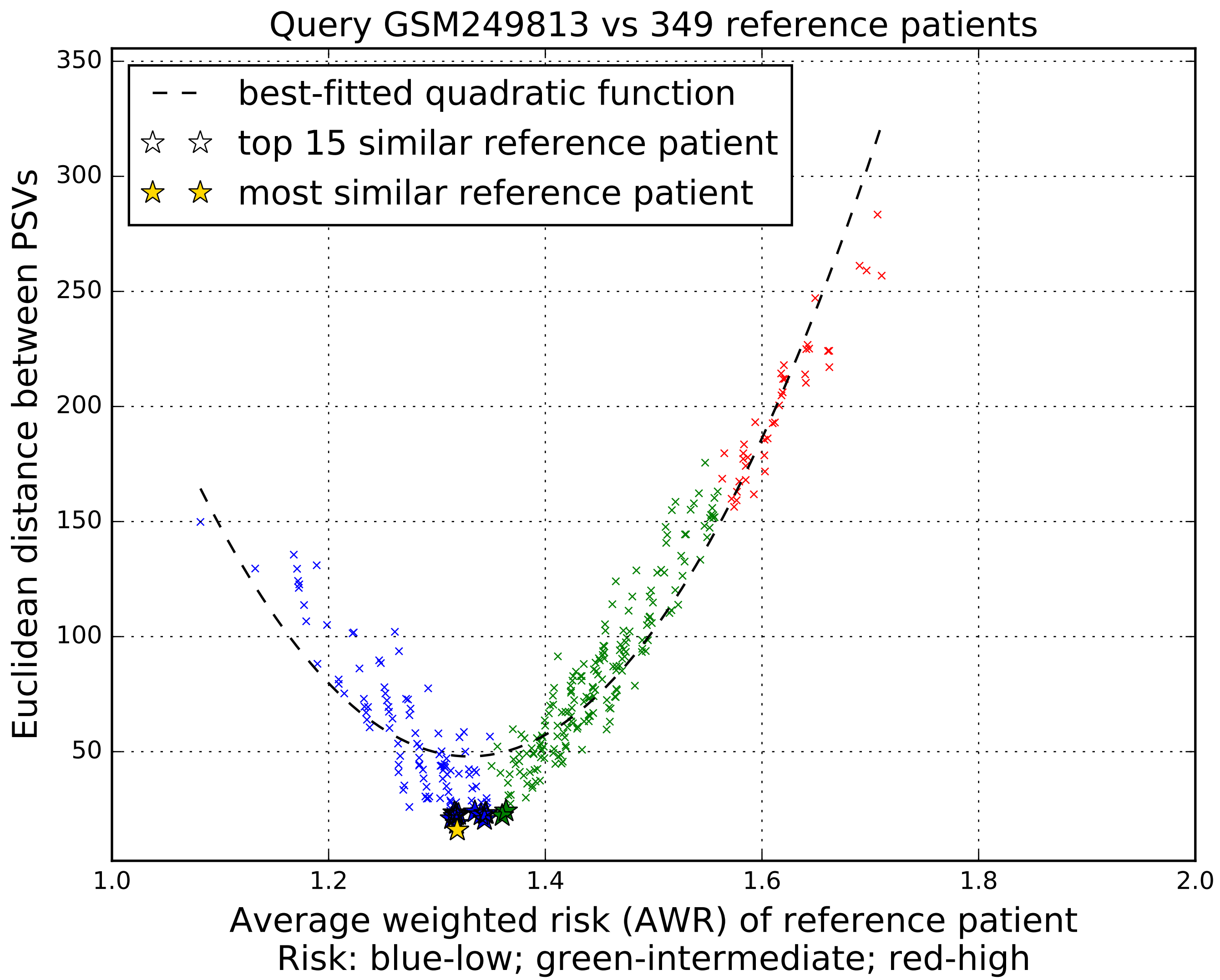

Query GSM249813 vs 349 reference patients
best-fitted quadratic function
top 15 similar reference patient
most similar reference patient
Euclidean distance between PSVs
Average weighted risk (AWR) of reference patient
Risk: blue-low; green-intermediate; red-high
350
300
250
200
150
100
50
1.0
1.2
1.4
1.6
1.8
2.0

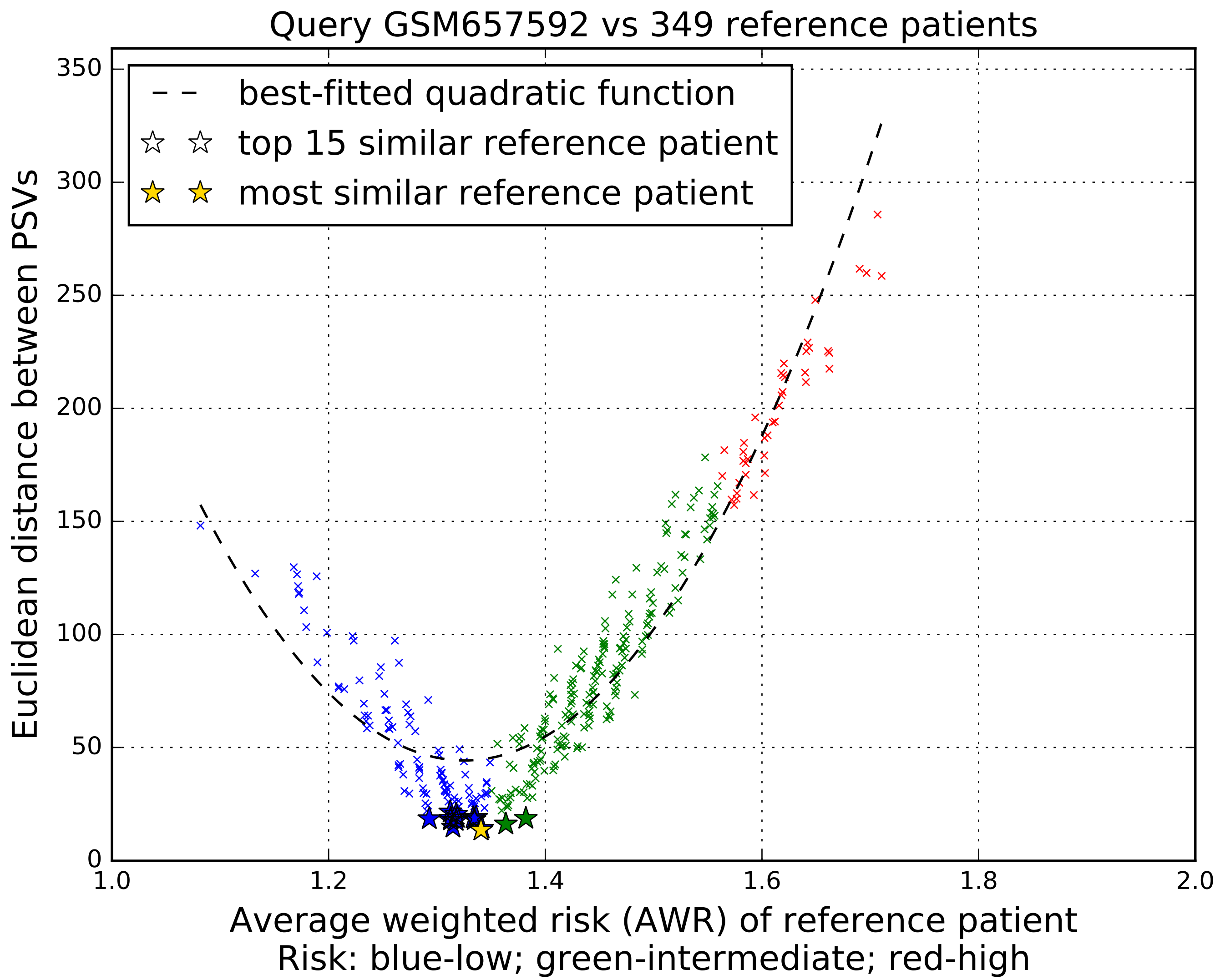

Query GSM657592 vs 349 reference patients
best-fitted quadratic function
top 15 similar reference patient
most similar reference patient
Euclidean distance between PSVs
Average weighted risk (AWR) of reference patient
Risk: blue-low; green-intermediate; red-high
0
50
100
150
200
250
300
350
1.0
1.2
1.4
1.6
1.8
2.0

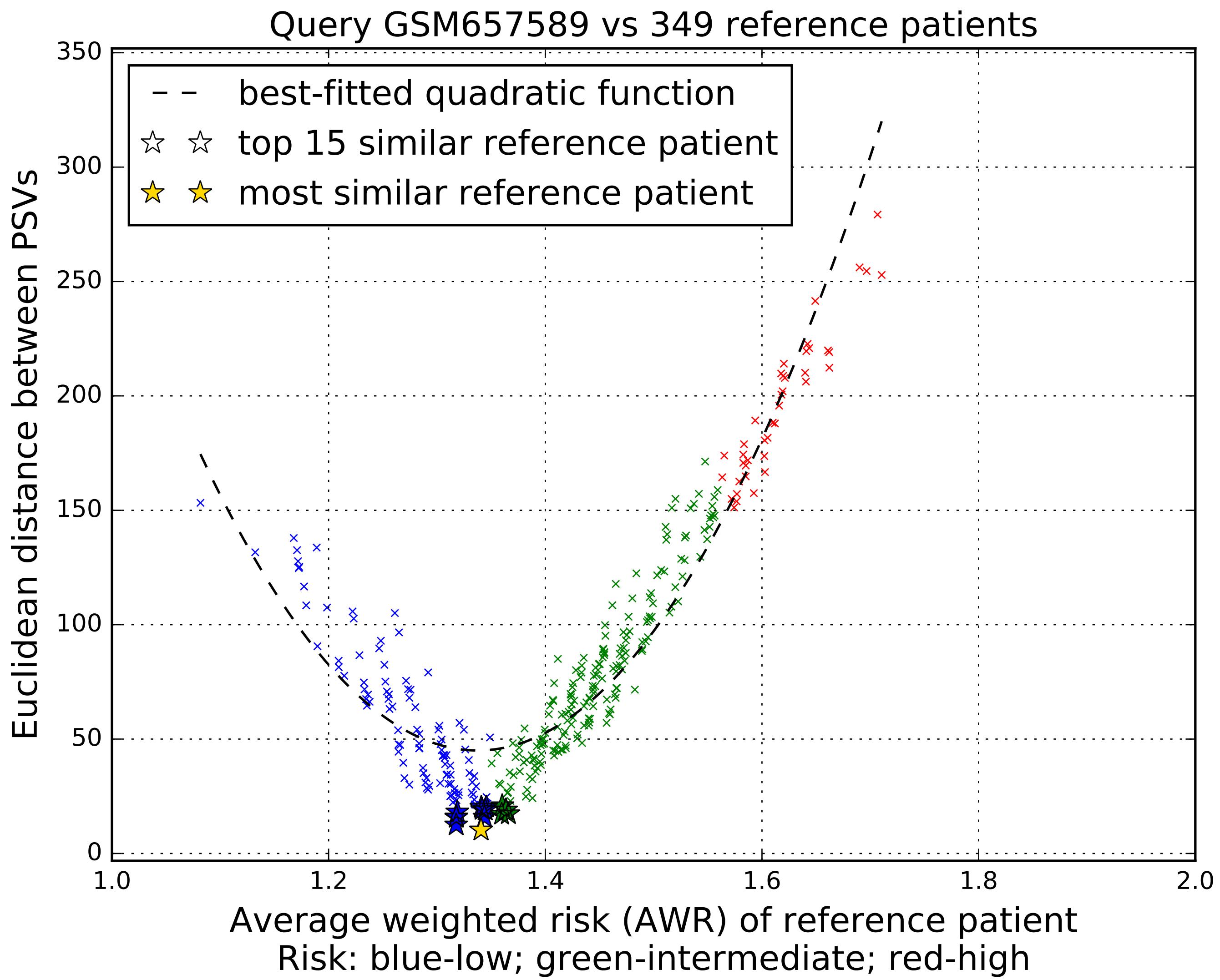

Query GSM657589 vs 349 reference patients
best-fitted quadratic function
top 15 similar reference patient
most similar reference patient
Euclidean distance between PSVs
Average weighted risk (AWR) of reference patient
Risk: blue-low; green-intermediate; red-high
0
50
100
150
200
250
300
350
1.0
1.2
1.4
1.6
1.8
2.0

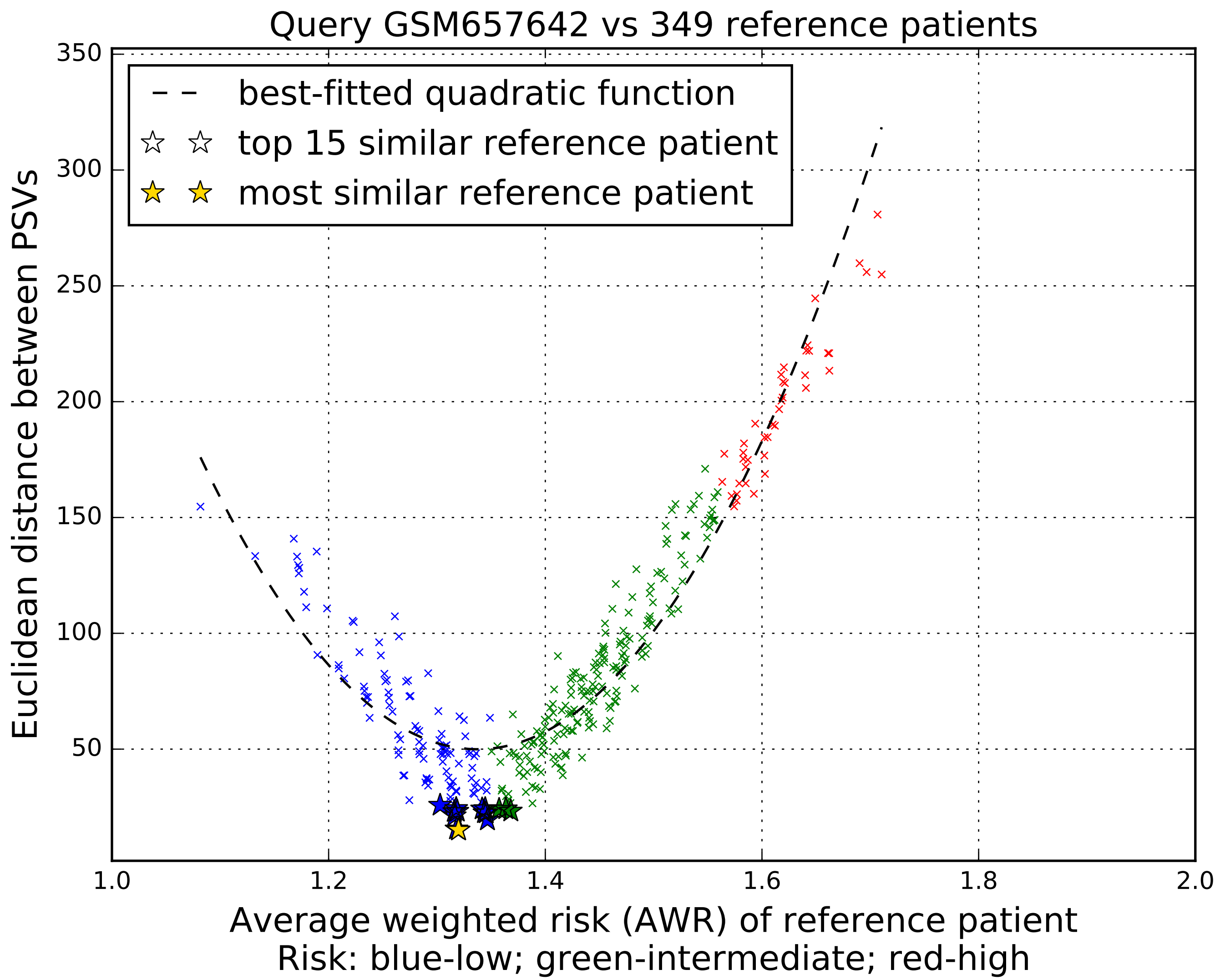

Query GSM657642 vs 349 reference patients
best-fitted quadratic function
top 15 similar reference patient
most similar reference patient
Euclidean distance between PSVs
Average weighted risk (AWR) of reference patient
Risk: blue-low; green-intermediate; red-high
350
300
250
200
150
100
50
1.0
1.2
1.4
1.6
1.8
2.0

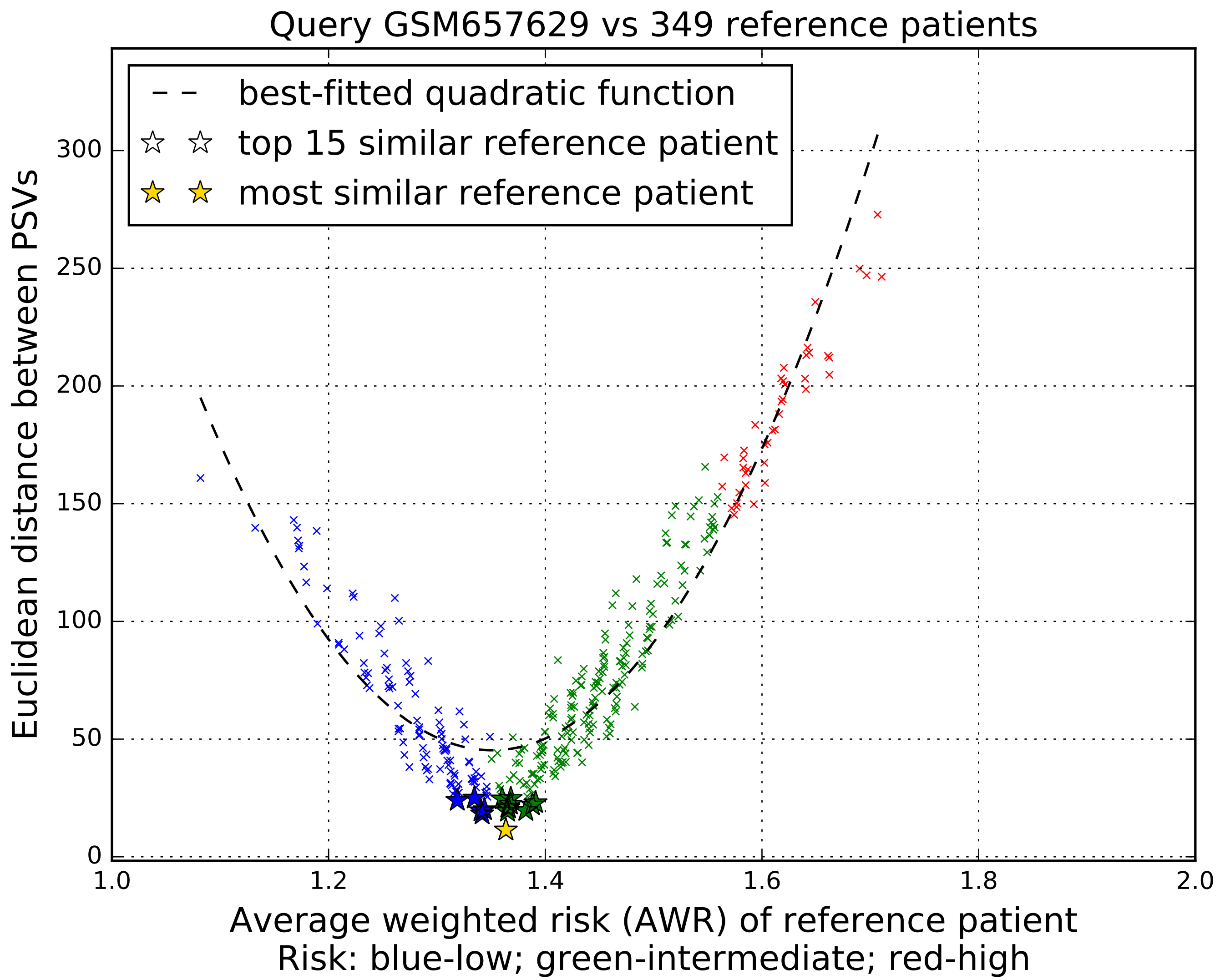
Query GSM657629 vs 349 reference patients
best-fitted quadratic function
top 15 similar reference patient
most similar reference patient
Euclidean distance between PSVs
0
50
100
150
200
250
300
1.0
1.2
1.4
1.6
1.8
2.0
Average weighted risk (AWR) of reference patient
Risk: blue-low; green-intermediate; red-high

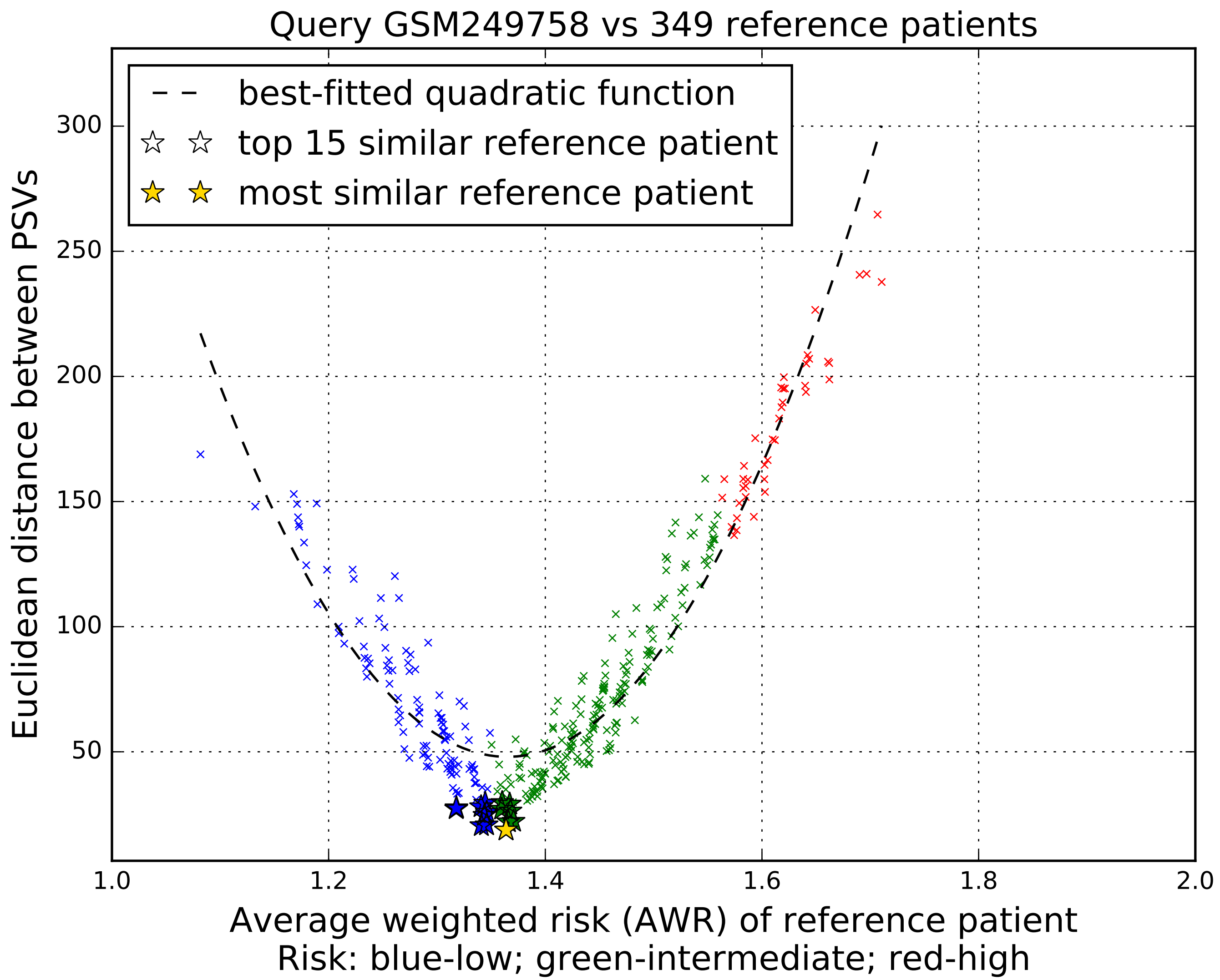

Query GSM249758 vs 349 reference patients
best-fitted quadratic function
top 15 similar reference patient
most similar reference patient
Euclidean distance between PSVs
300
250
200
150
100
50
1.0
1.2
1.4
1.6
1.8
2.0
Average weighted risk (AWR) of reference patient
Risk: blue-low; green-intermediate; red-high

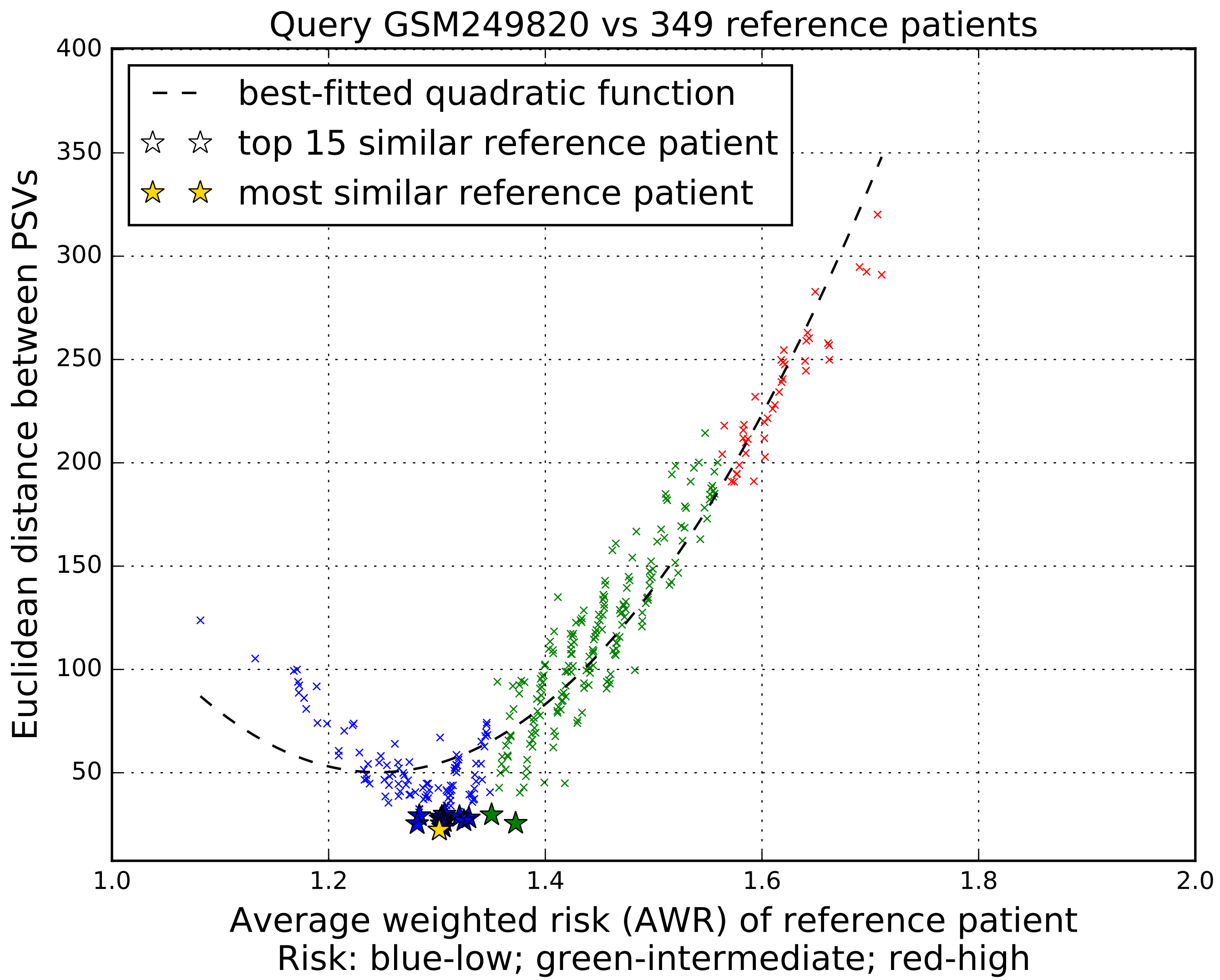
Query GSM249820 vs 349 reference patients
best-fitted quadratic function
top 15 similar reference patient
most similar reference patient
Euclidean distance between PSVs
400
350
300
250
200
150
100
50
1.0
1.2
1.4
1.6
1.8
2.0
Average weighted risk (AWR) of reference patient
Risk: blue-low; green-intermediate; red-high

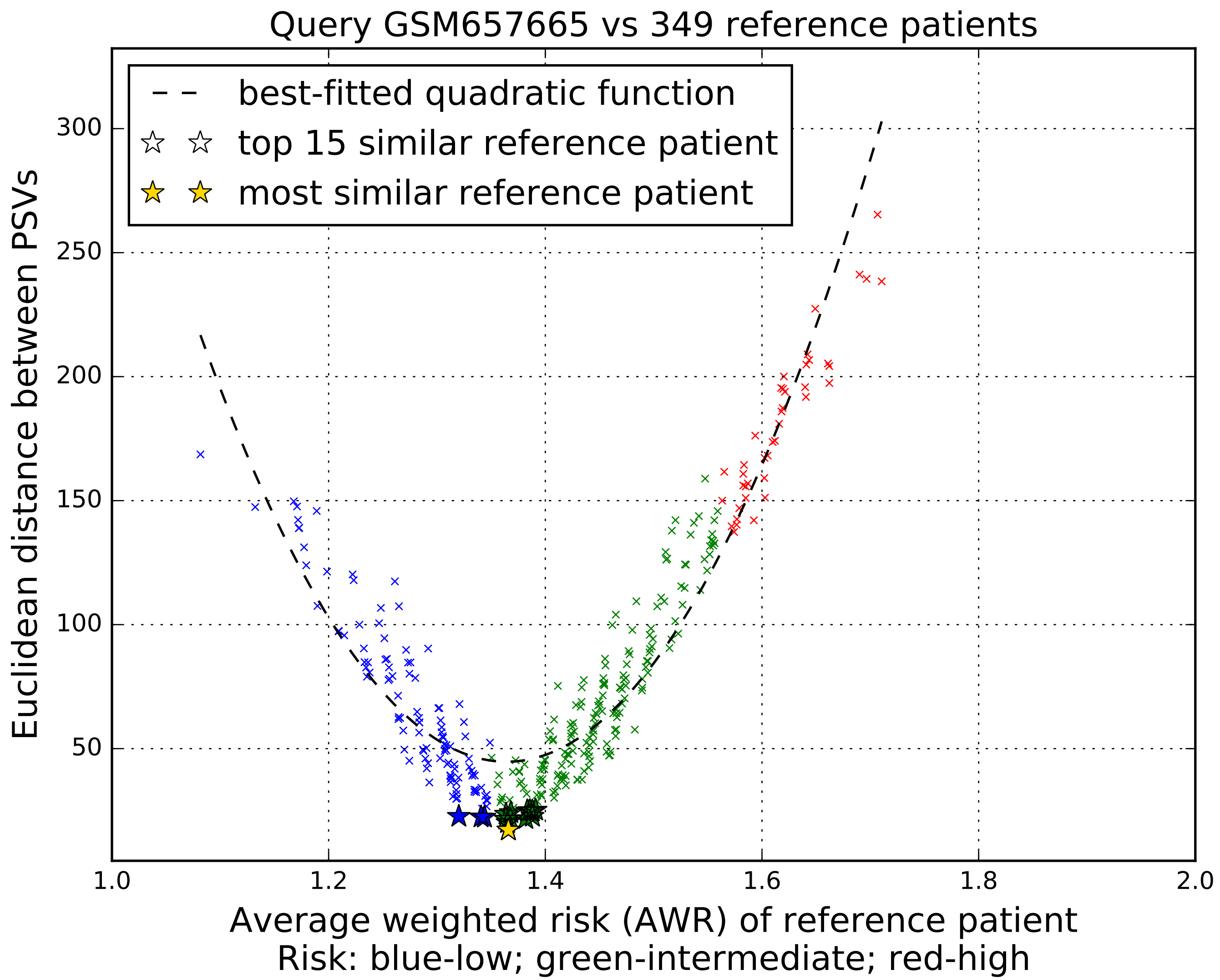
Query GSM657665 vs 349 reference patients
best-fitted quadratic function
top 15 similar reference patient
most similar reference patient
Euclidean distance between PSVs
300
250
200
150
100
50
1.0
1.2
1.4
1.6
1.8
2.0
Average weighted risk (AWR) of reference patient
Risk: blue-low; green-intermediate; red-high

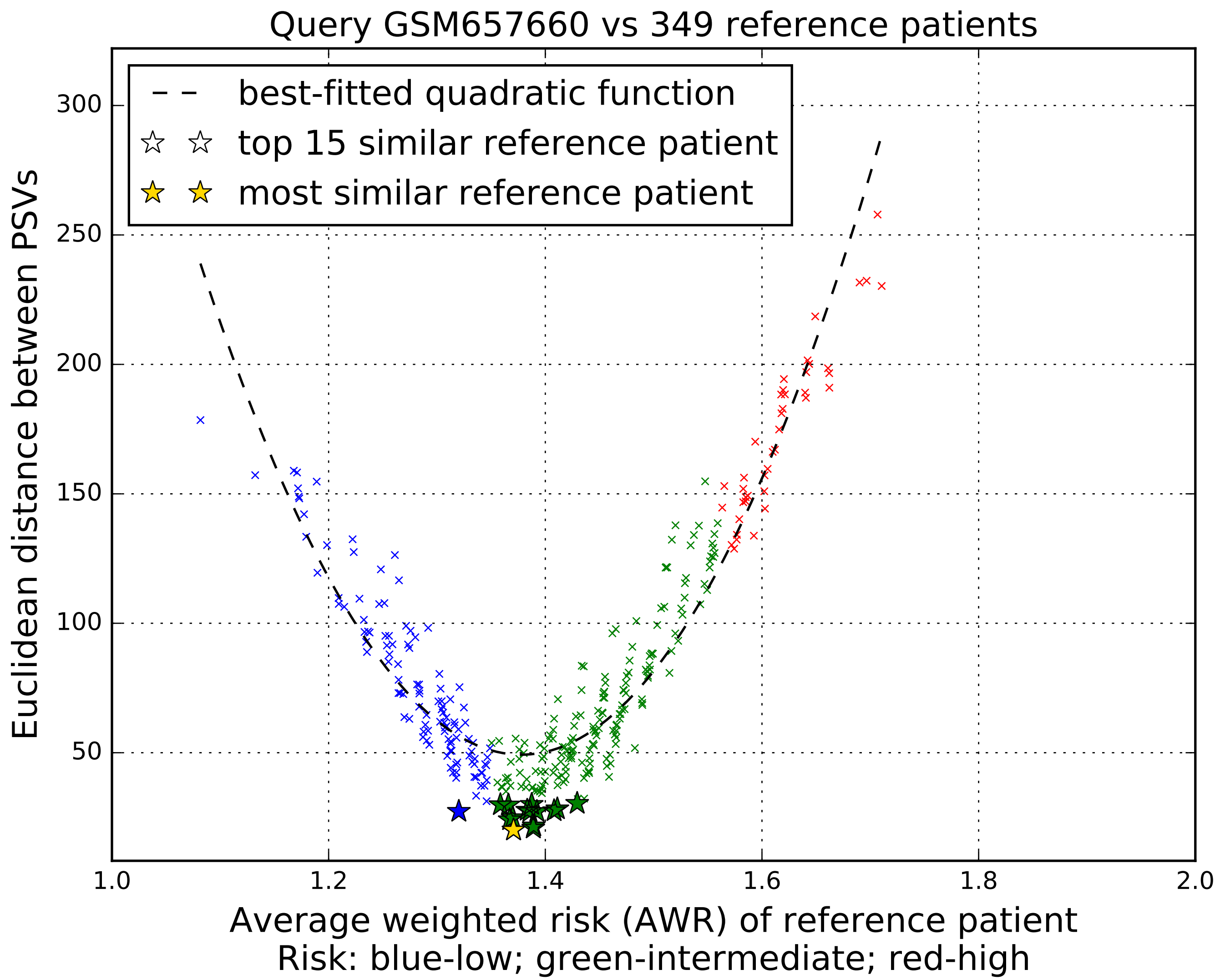
Query GSM657660 vs 349 reference patients
best-fitted quadratic function
top 15 similar reference patient
most similar reference patient
Euclidean distance between PSVs
300
250
200
150
100
50
1.0
1.2
1.4
1.6
1.8
2.0
Average weighted risk (AWR) of reference patient
Risk: blue-low; green-intermediate; red-high

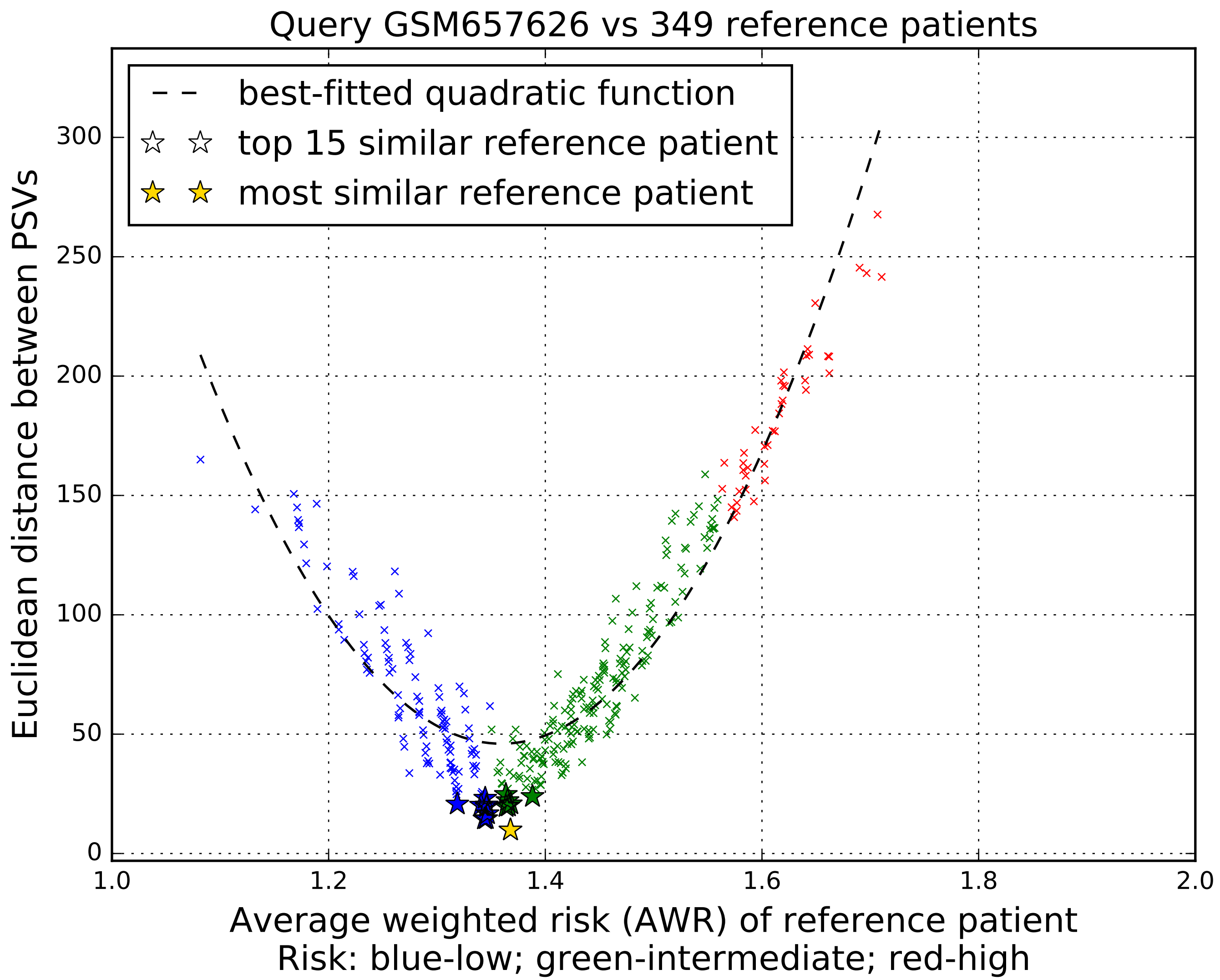

Query GSM657626 vs 349 reference patients
best-fitted quadratic function
top 15 similar reference patient
most similar reference patient
Euclidean distance between PSVs
0
50
100
150
200
250
300
1.0
1.2
1.4
1.6
1.8
2.0
Average weighted risk (AWR) of reference patient
Risk: blue-low; green-intermediate; red-high

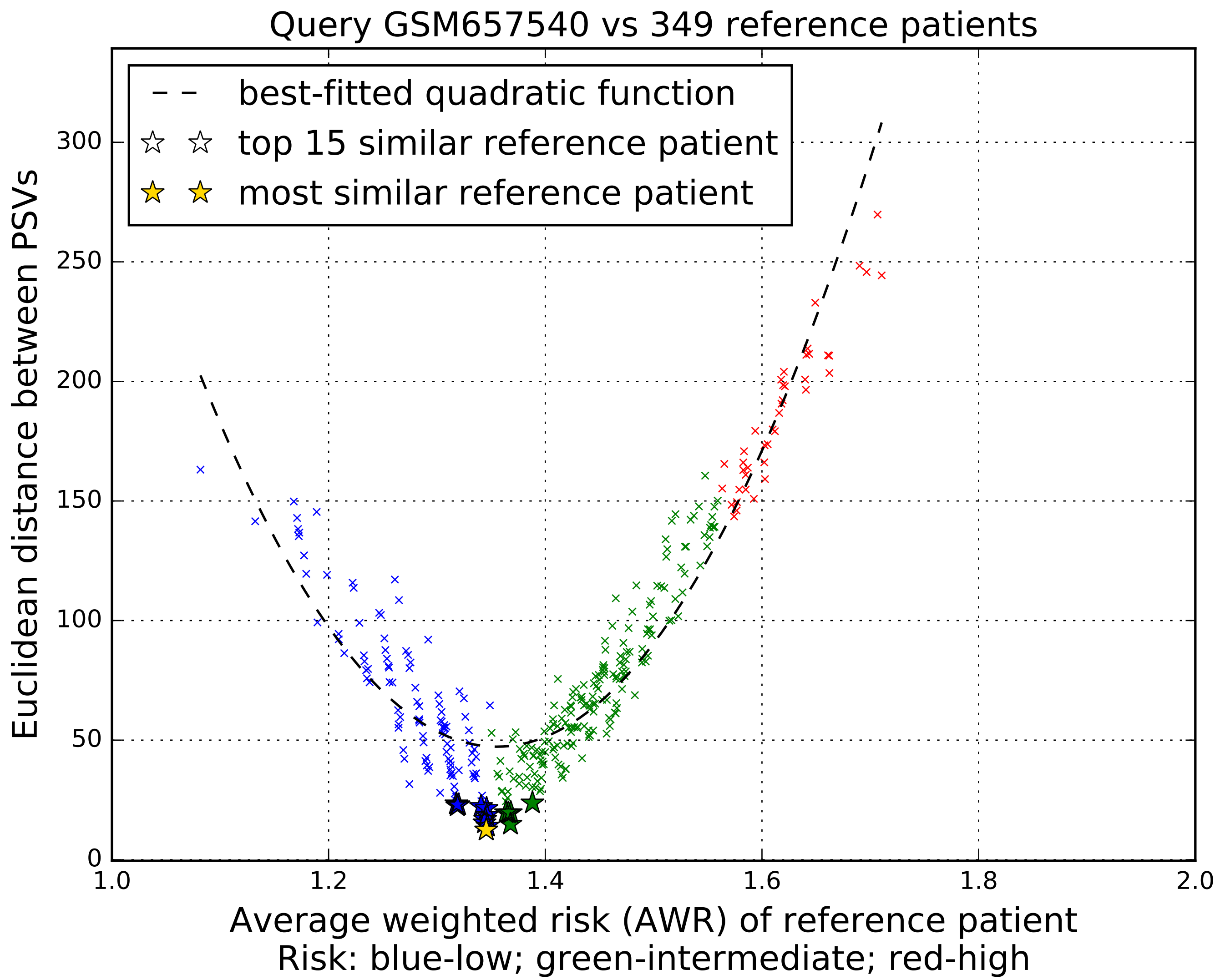
Query GSM657540 vs 349 reference patients
best-fitted quadratic function
top 15 similar reference patient
most similar reference patient
Euclidean distance between PSVs
0
50
100
150
200
250
300
1.0
1.2
1.4
1.6
1.8
2.0
Average weighted risk (AWR) of reference patient
Risk: blue-low; green-intermediate; red-high

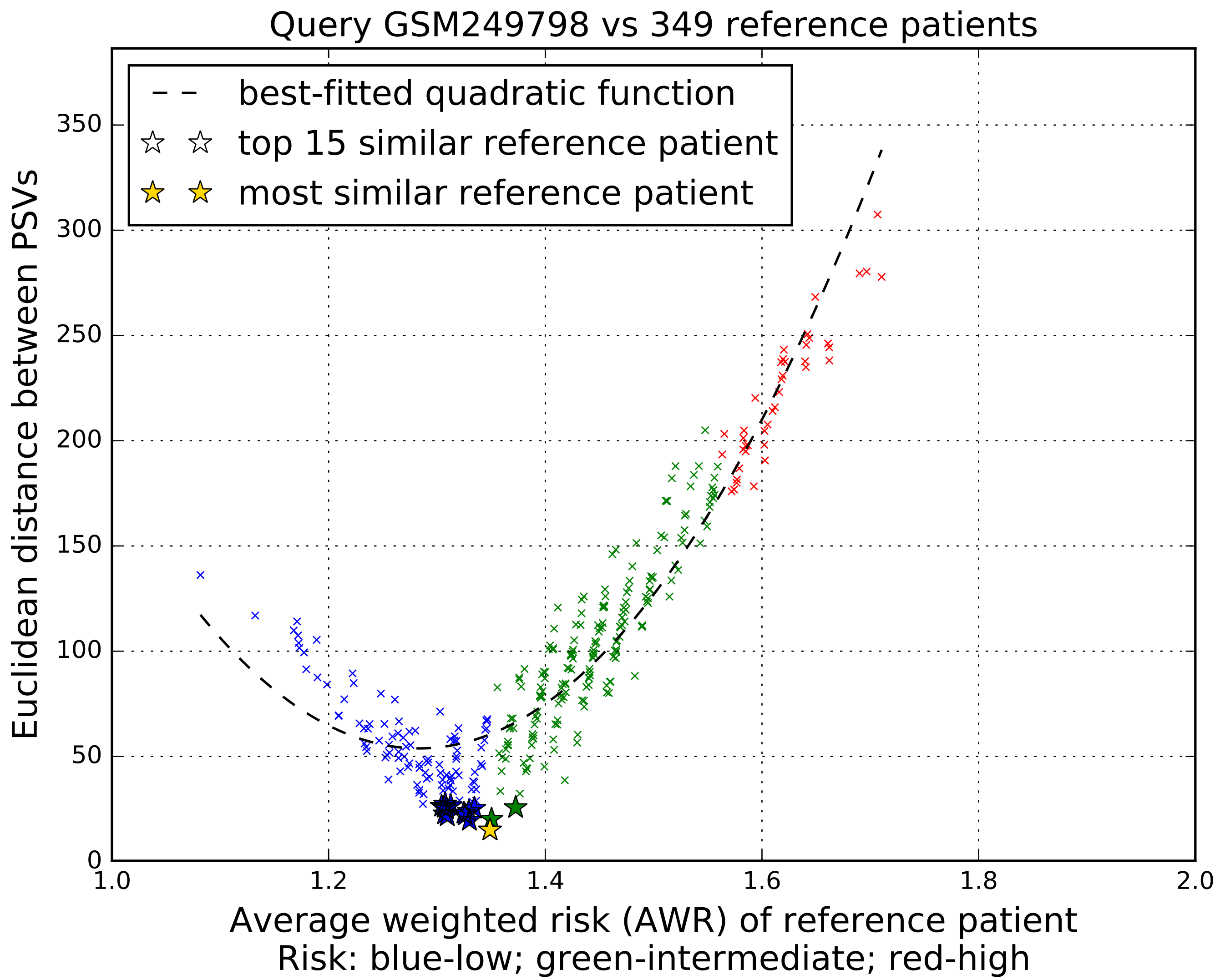
Query GSM249798 vs 349 reference patients
best-fitted quadratic function
top 15 similar reference patient
most similar reference patient
Euclidean distance between PSVs
0
50
100
150
200
250
300
350
1.0
1.2
1.4
1.6
1.8
2.0
Average weighted risk (AWR) of reference patient
Risk: blue-low; green-intermediate; red-high

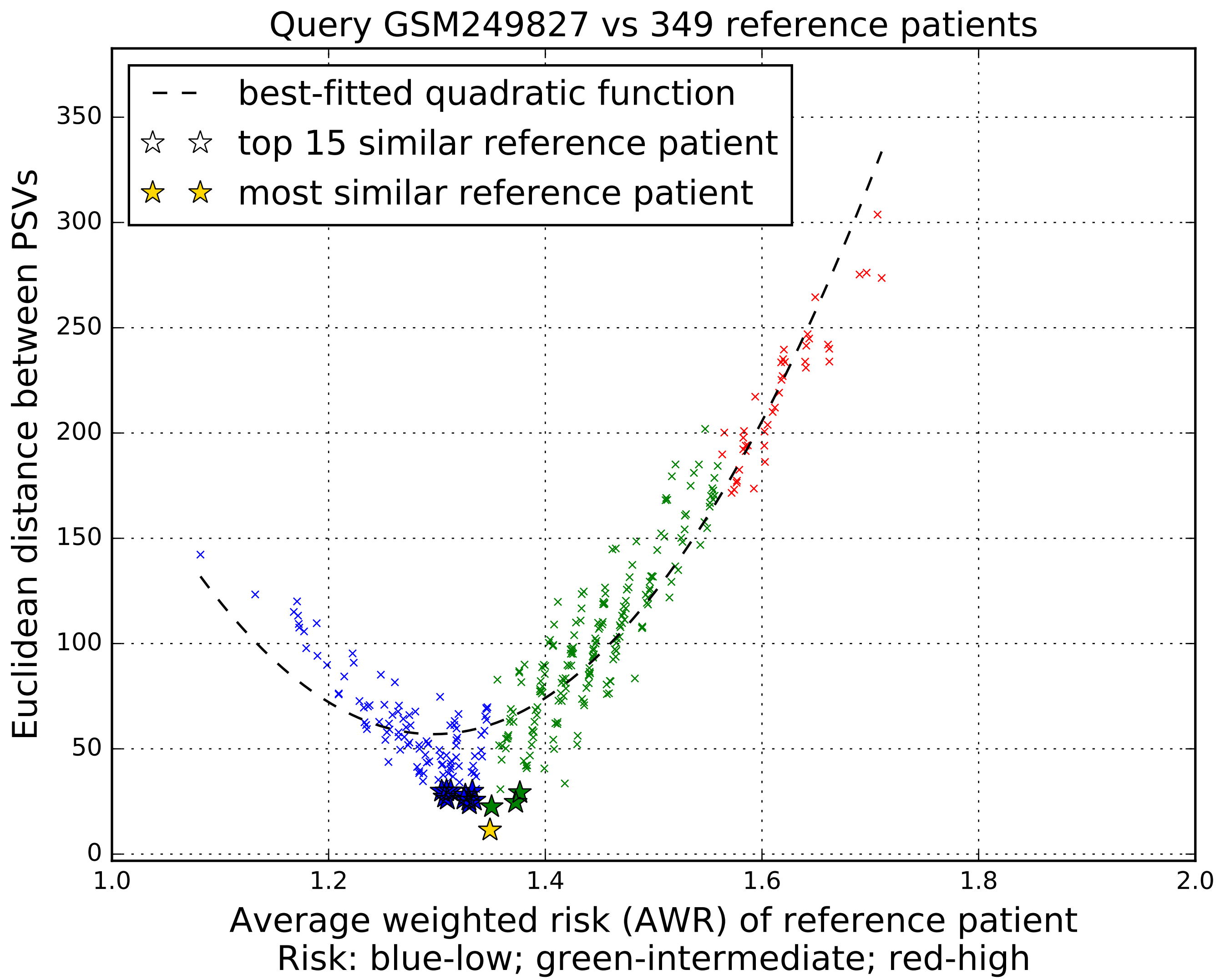

Query GSM249827 vs 349 reference patients
best-fitted quadratic function
top 15 similar reference patient
most similar reference patient
Euclidean distance between PSVs
Average weighted risk (AWR) of reference patient
Risk: blue-low; green-intermediate; red-high
0
50
100
150
200
250
300
350
1.0
1.2
1.4
1.6
1.8
2.0

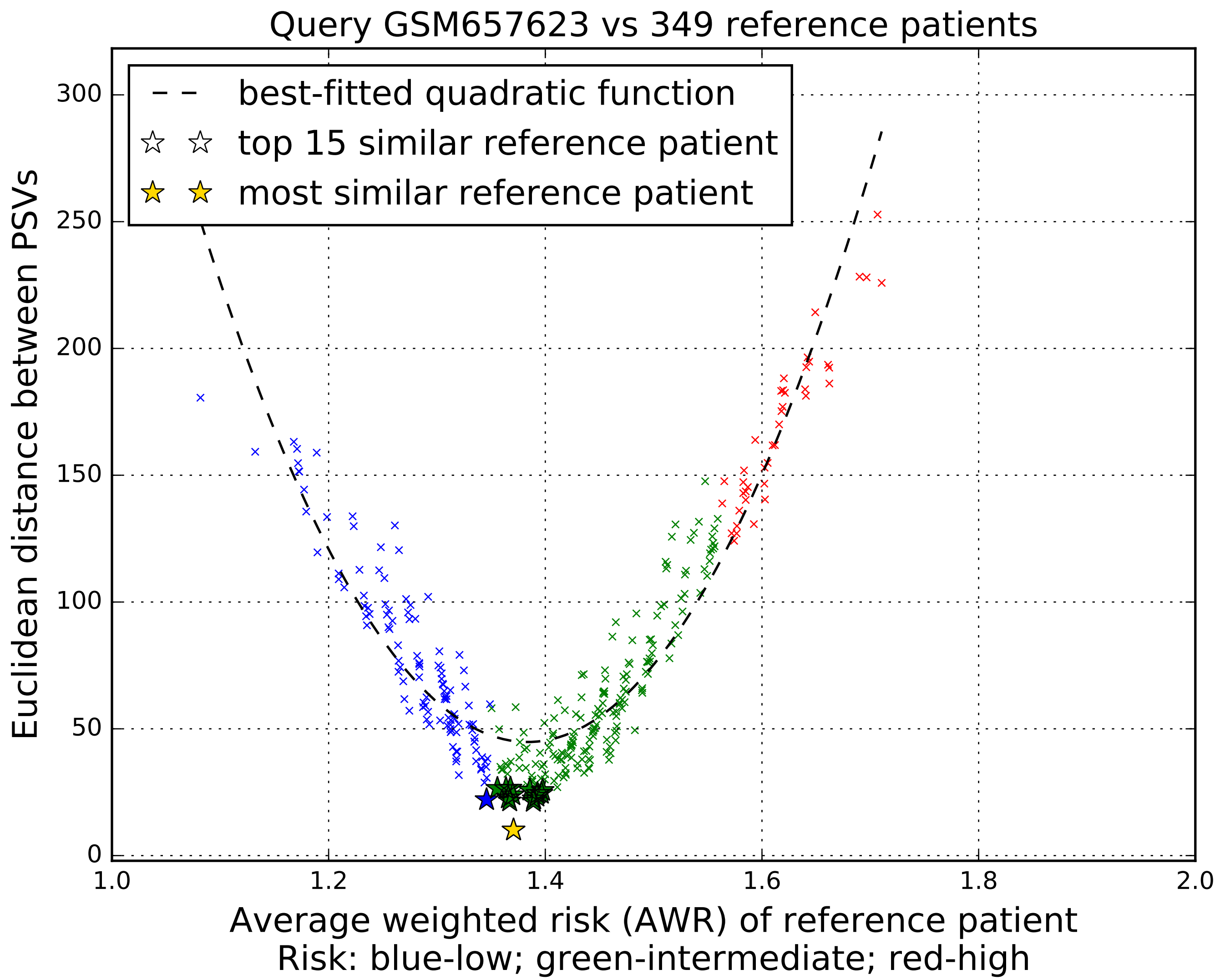
Query GSM657623 vs 349 reference patients
best-fitted quadratic function
top 15 similar reference patient
most similar reference patient
Euclidean distance between PSVs
0
50
100
150
200
250
300
1.0
1.2
1.4
1.6
1.8
2.0
Average weighted risk (AWR) of reference patient
Risk: blue-low; green-intermediate; red-high

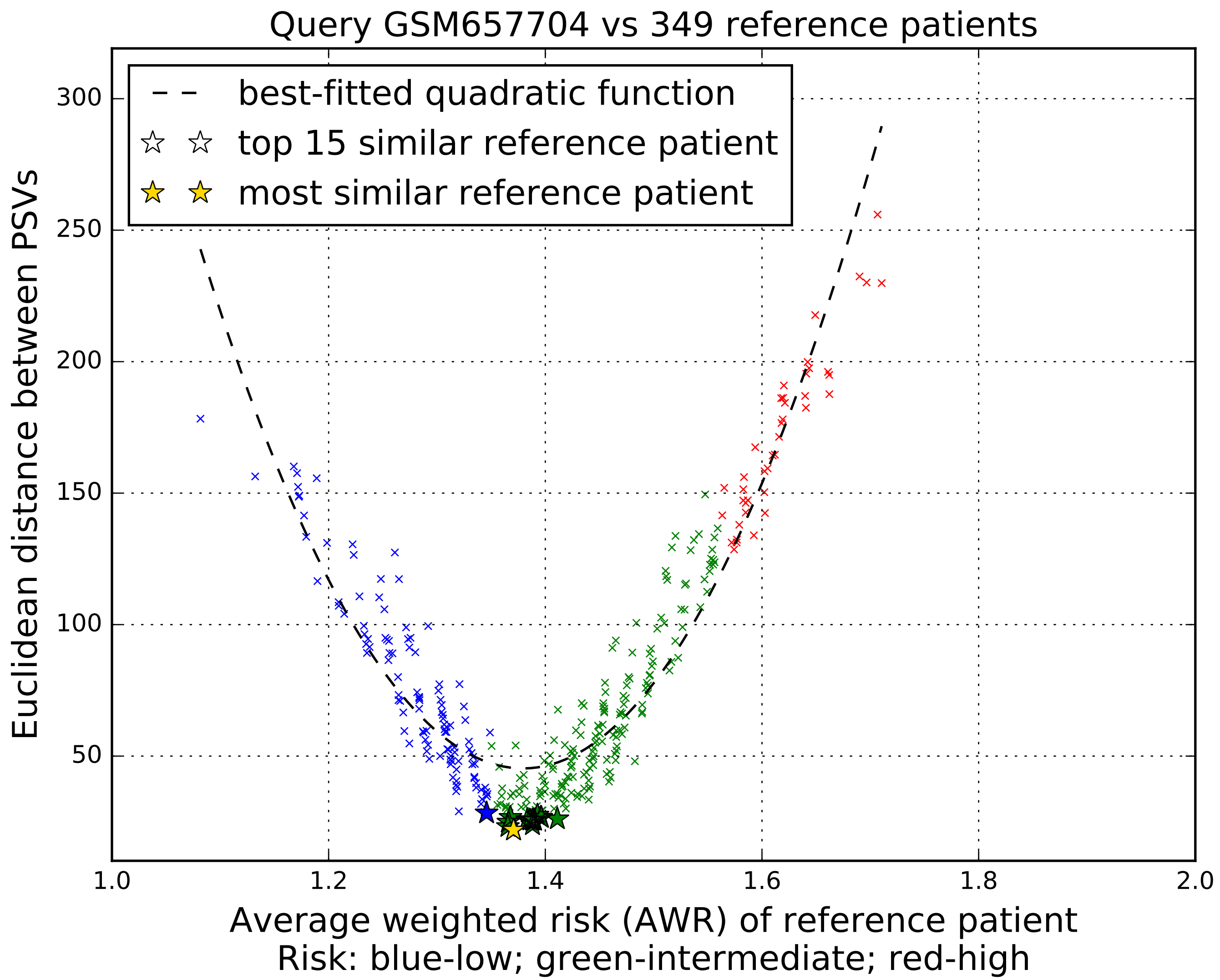

Query GSM657704 vs 349 reference patients
best-fitted quadratic function
top 15 similar reference patient
most similar reference patient
Euclidean distance between PSVs
300
250
200
150
100
50
1.0
1.2
1.4
1.6
1.8
2.0
Average weighted risk (AWR) of reference patient
Risk: blue-low; green-intermediate; red-high

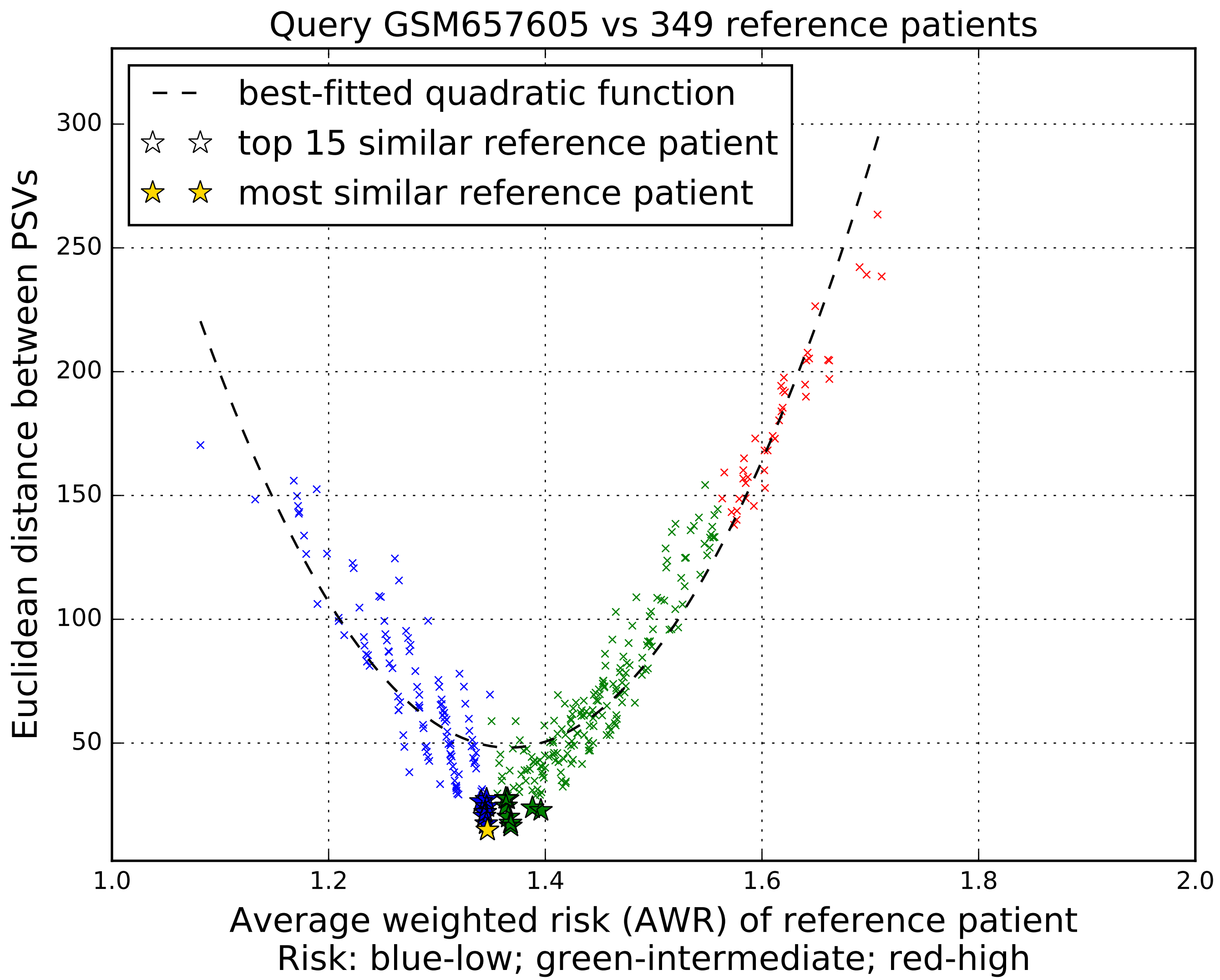

Query GSM657605 vs 349 reference patients
best-fitted quadratic function
top 15 similar reference patient
most similar reference patient
Euclidean distance between PSVs
300
250
200
150
100
50
1.0
1.2
1.4
1.6
1.8
2.0
Average weighted risk (AWR) of reference patient
Risk: blue-low; green-intermediate; red-high

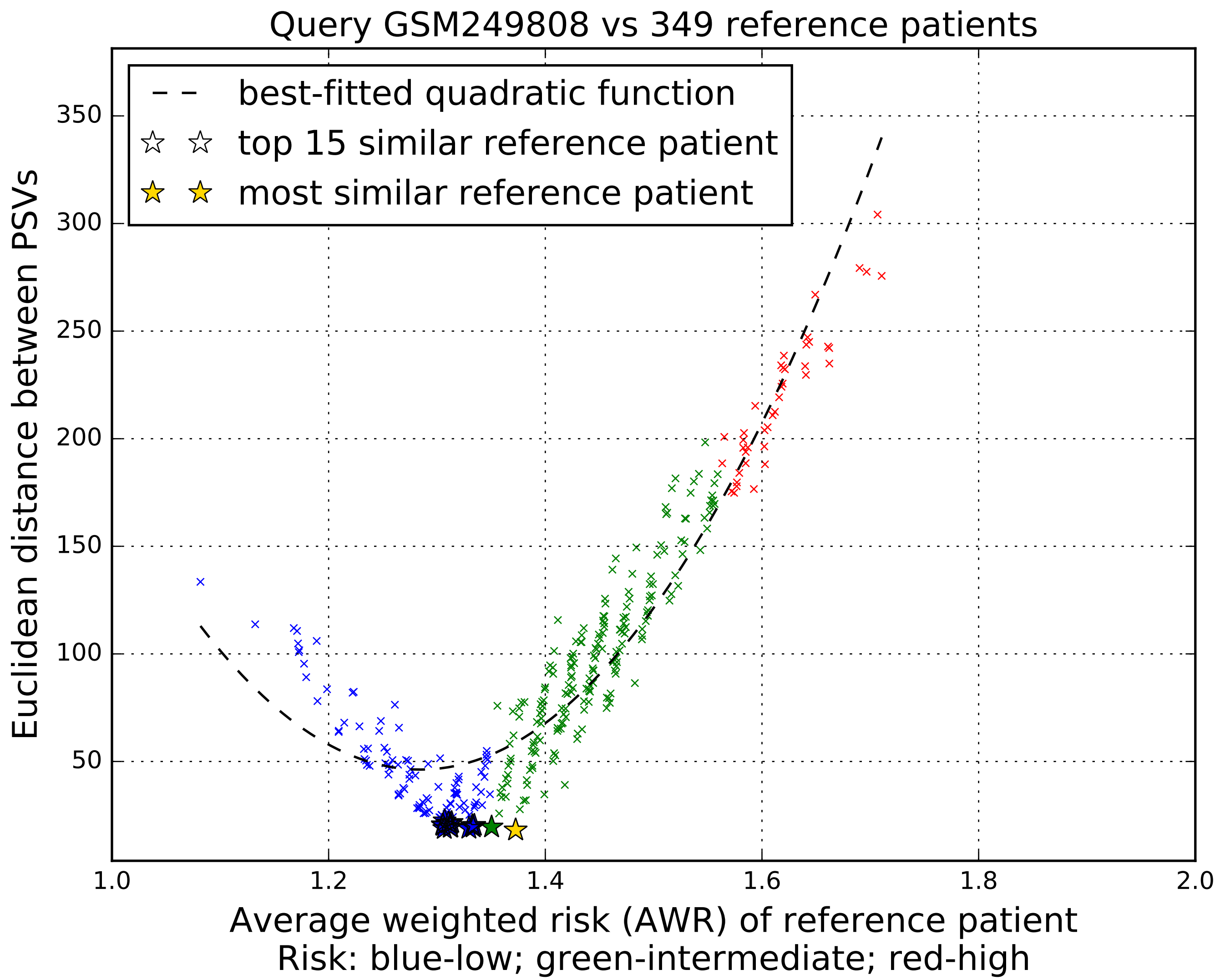
Query GSM249808 vs 349 reference patients
best-fitted quadratic function
top 15 similar reference patient
most similar reference patient
Euclidean distance between PSVs
350
300
250
200
150
100
50
1.0
1.2
1.4
1.6
1.8
2.0
Average weighted risk (AWR) of reference patient
Risk: blue-low; green-intermediate; red-high

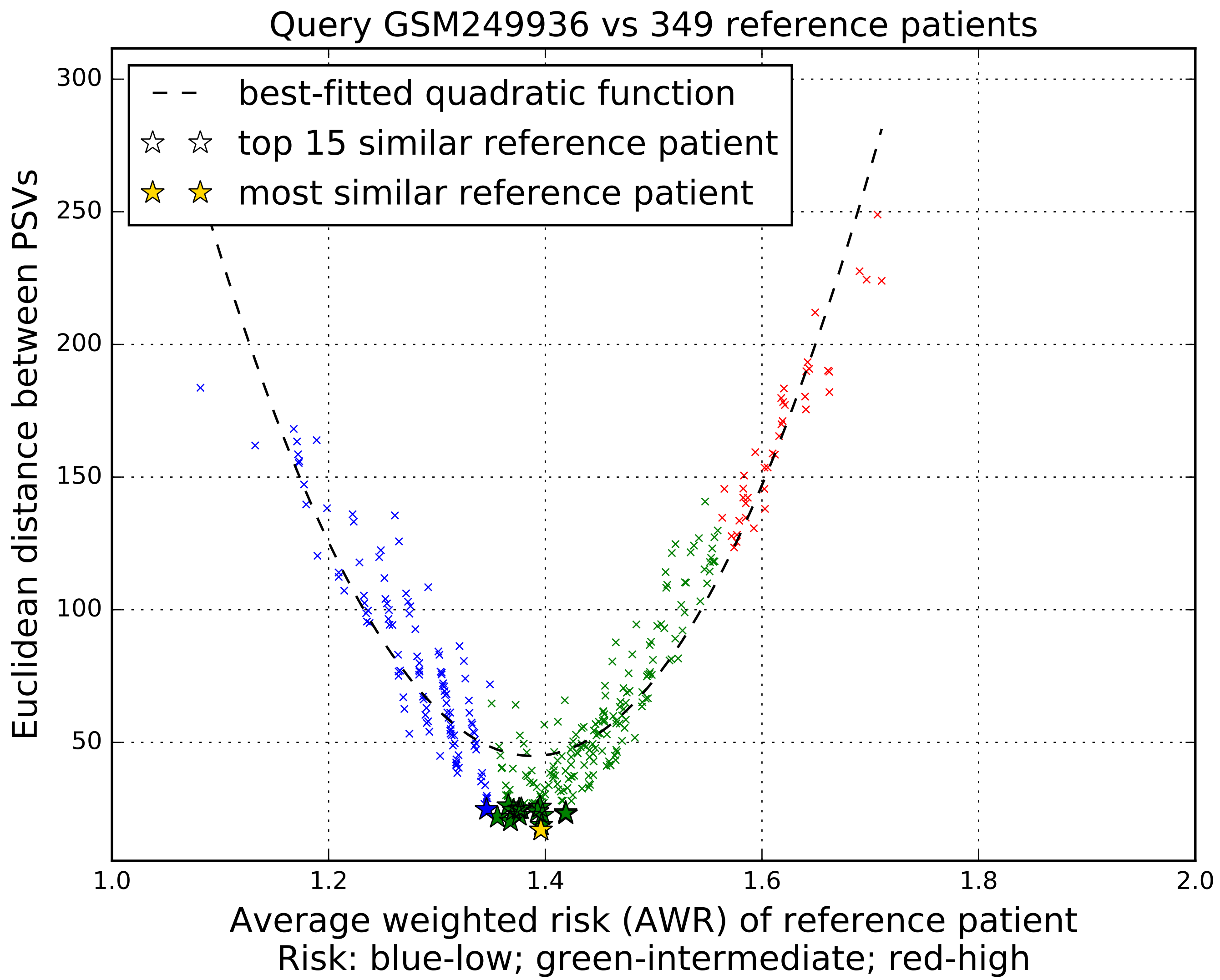

Query GSM249936 vs 349 reference patients
best-fitted quadratic function
top 15 similar reference patient
most similar reference patient
Euclidean distance between PSVs
300
250
200
150
100
50
1.0
1.2
1.4
1.6
1.8
2.0
Average weighted risk (AWR) of reference patient
Risk: blue-low; green-intermediate; red-high

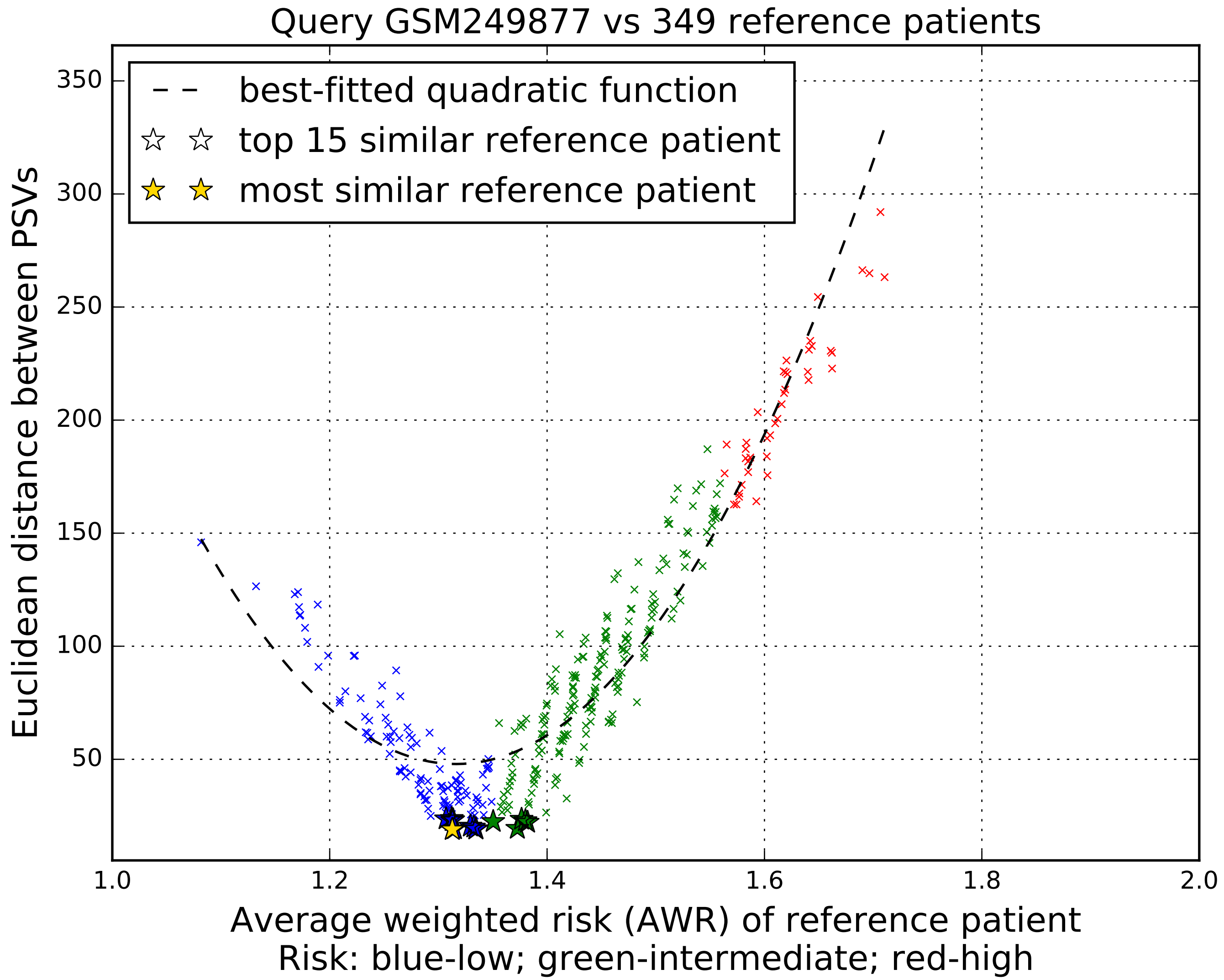
Query GSM249877 vs 349 reference patients
best-fitted quadratic function
top 15 similar reference patient
most similar reference patient
Euclidean distance between PSVs
Average weighted risk (AWR) of reference patient
Risk: blue-low; green-intermediate; red-high
350
300
250
200
150
100
50
1.0
1.2
1.4
1.6
1.8
2.0

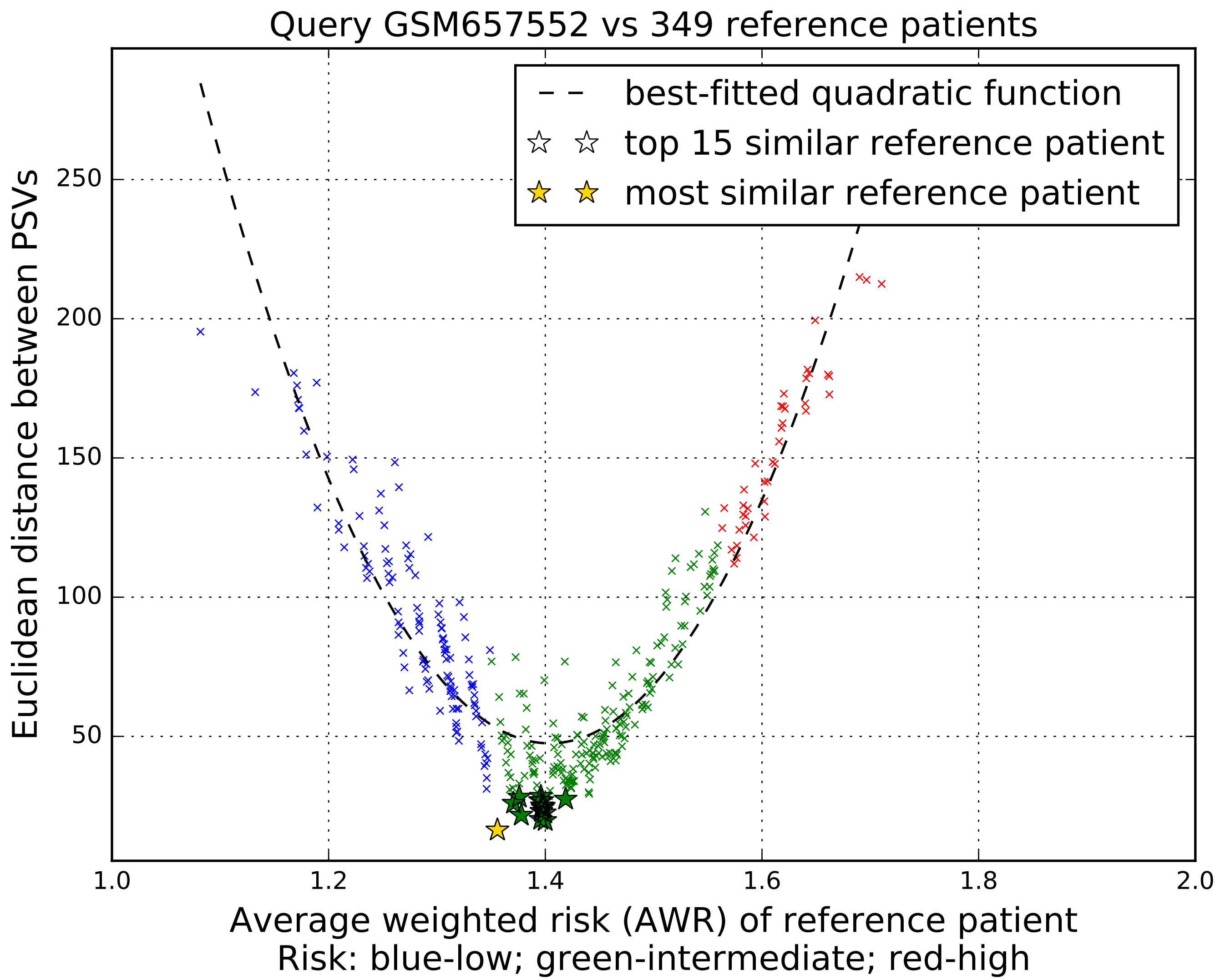

Query GSM657552 vs 349 reference patients
best-fitted quadratic function
top 15 similar reference patient
most similar reference patient
Euclidean distance between PSVs
50
100
150
200
250
1.0
1.2
1.4
1.6
1.8
2.0
Average weighted risk (AWR) of reference patient
Risk: blue-low; green-intermediate; red-high

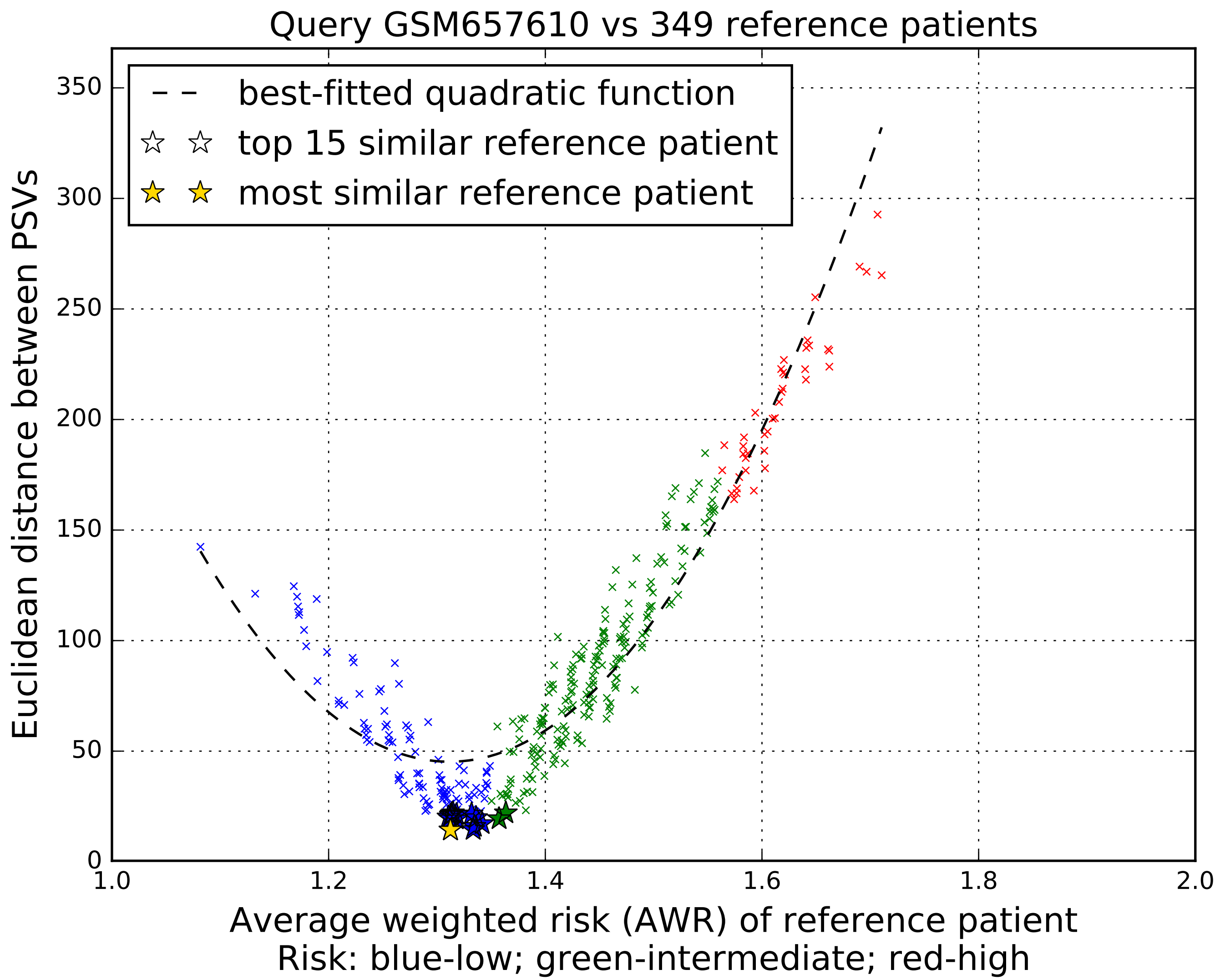

Query GSM657610 vs 349 reference patients
best-fitted quadratic function
top 15 similar reference patient
most similar reference patient
Euclidean distance between PSVs
0
50
100
150
200
250
300
350
1.0
1.2
1.4
1.6
1.8
2.0
Average weighted risk (AWR) of reference patient
Risk: blue-low; green-intermediate; red-high

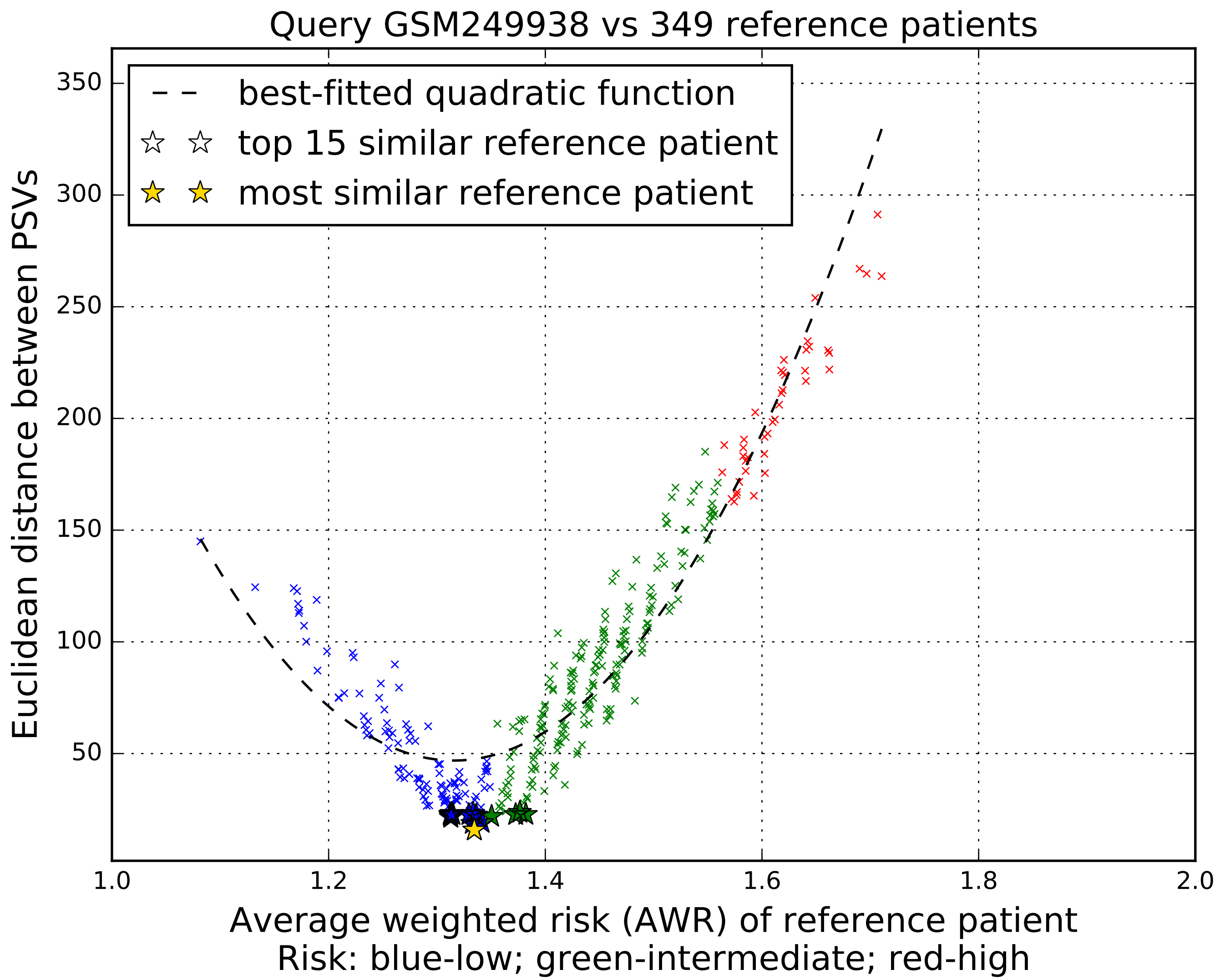
Query GSM249938 vs 349 reference patients
best-fitted quadratic function
top 15 similar reference patient
most similar reference patient
Euclidean distance between PSVs
Average weighted risk (AWR) of reference patient
Risk: blue-low; green-intermediate; red-high
350
300
250
200
150
100
50
1.0
1.2
1.4
1.6
1.8
2.0

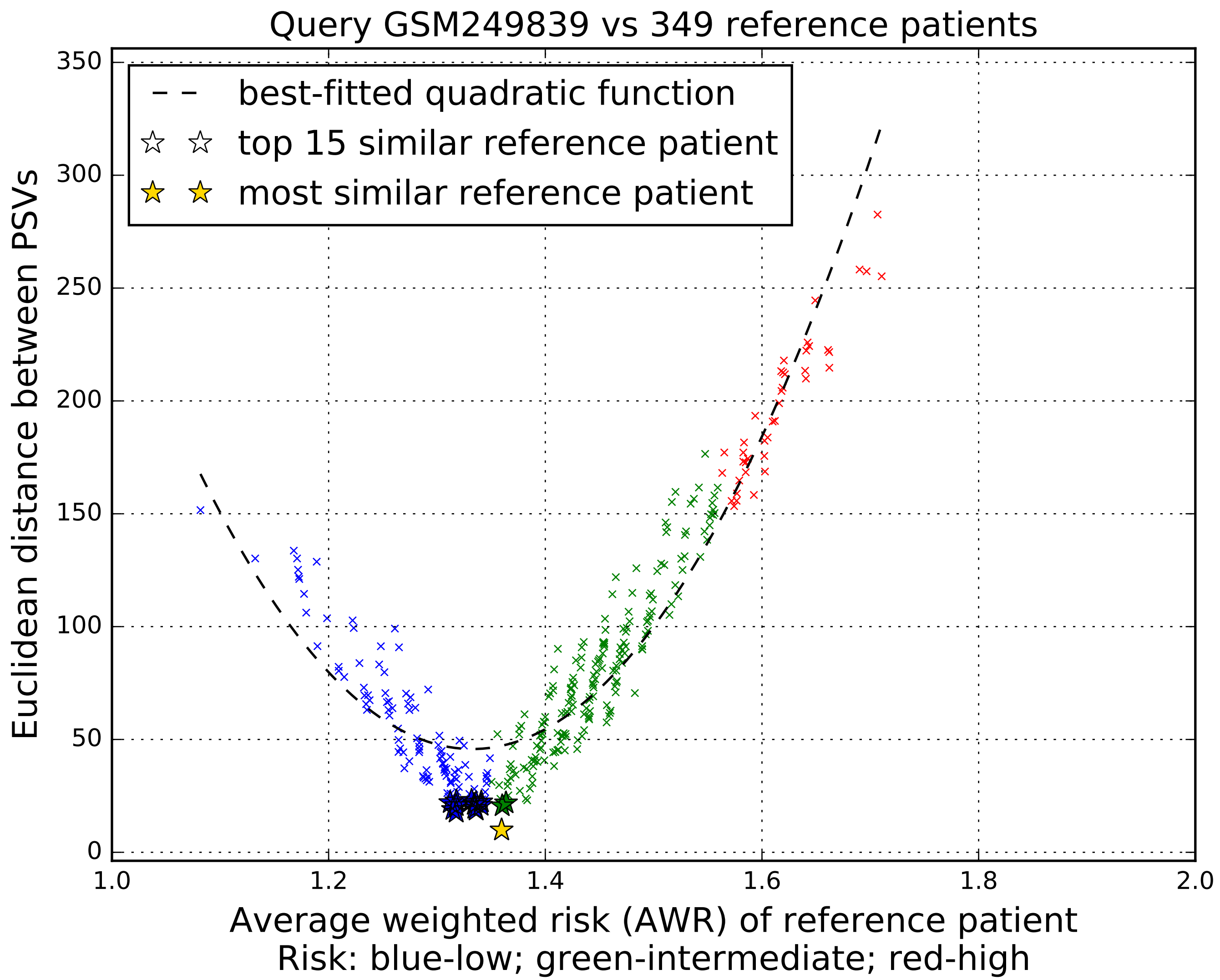

Query GSM249839 vs 349 reference patients
best-fitted quadratic function
top 15 similar reference patient
most similar reference patient
Euclidean distance between PSVs
350
300
250
200
150
100
50
0
1.0
1.2
1.4
1.6
1.8
2.0
Average weighted risk (AWR) of reference patient
Risk: blue-low; green-intermediate; red-high

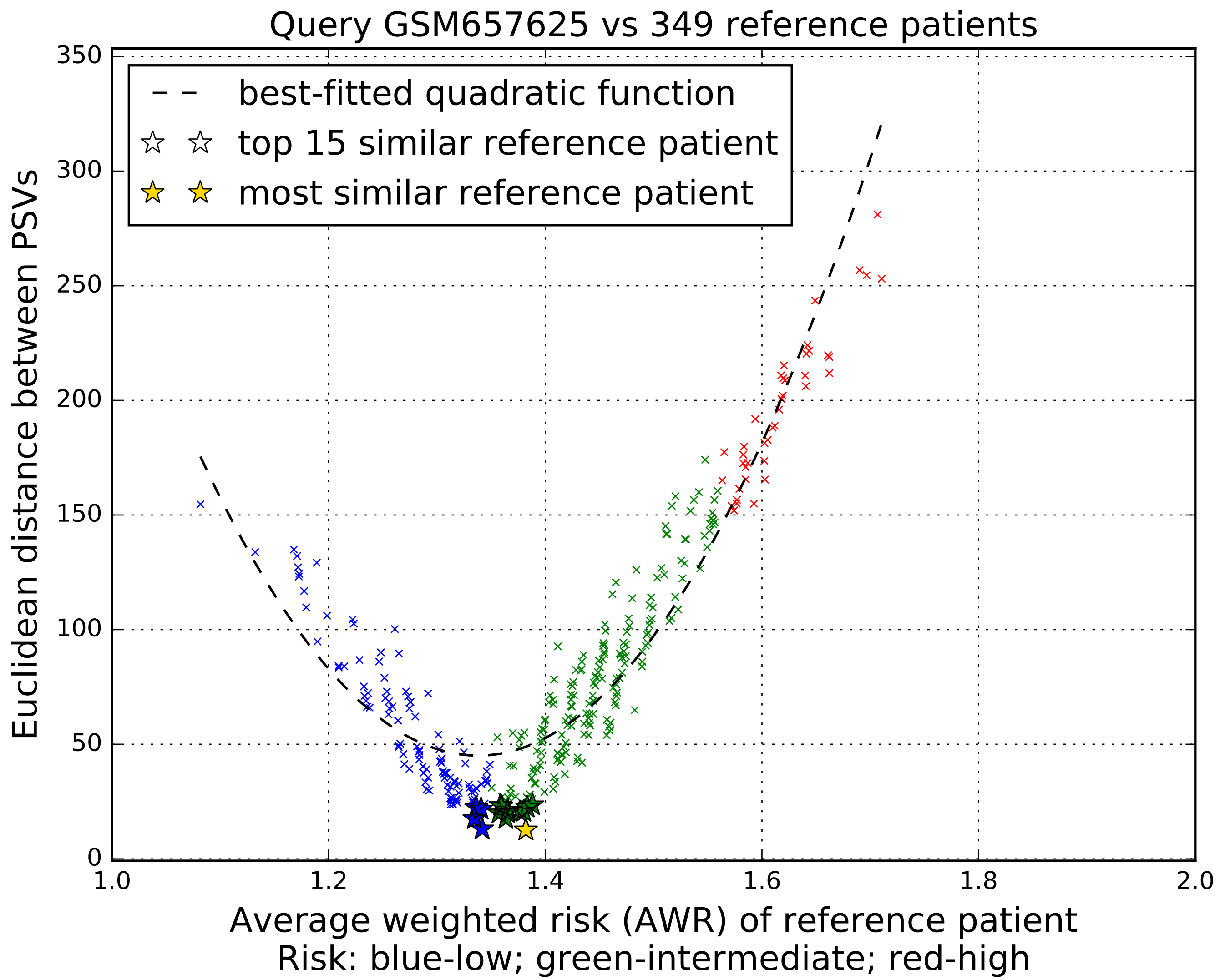

Query GSM657625 vs 349 reference patients
best-fitted quadratic function
top 15 similar reference patient
most similar reference patient
Euclidean distance between PSVs
Average weighted risk (AWR) of reference patient
Risk: blue-low; green-intermediate; red-high
0
50
100
150
200
250
300
350
1.0
1.2
1.4
1.6
1.8
2.0

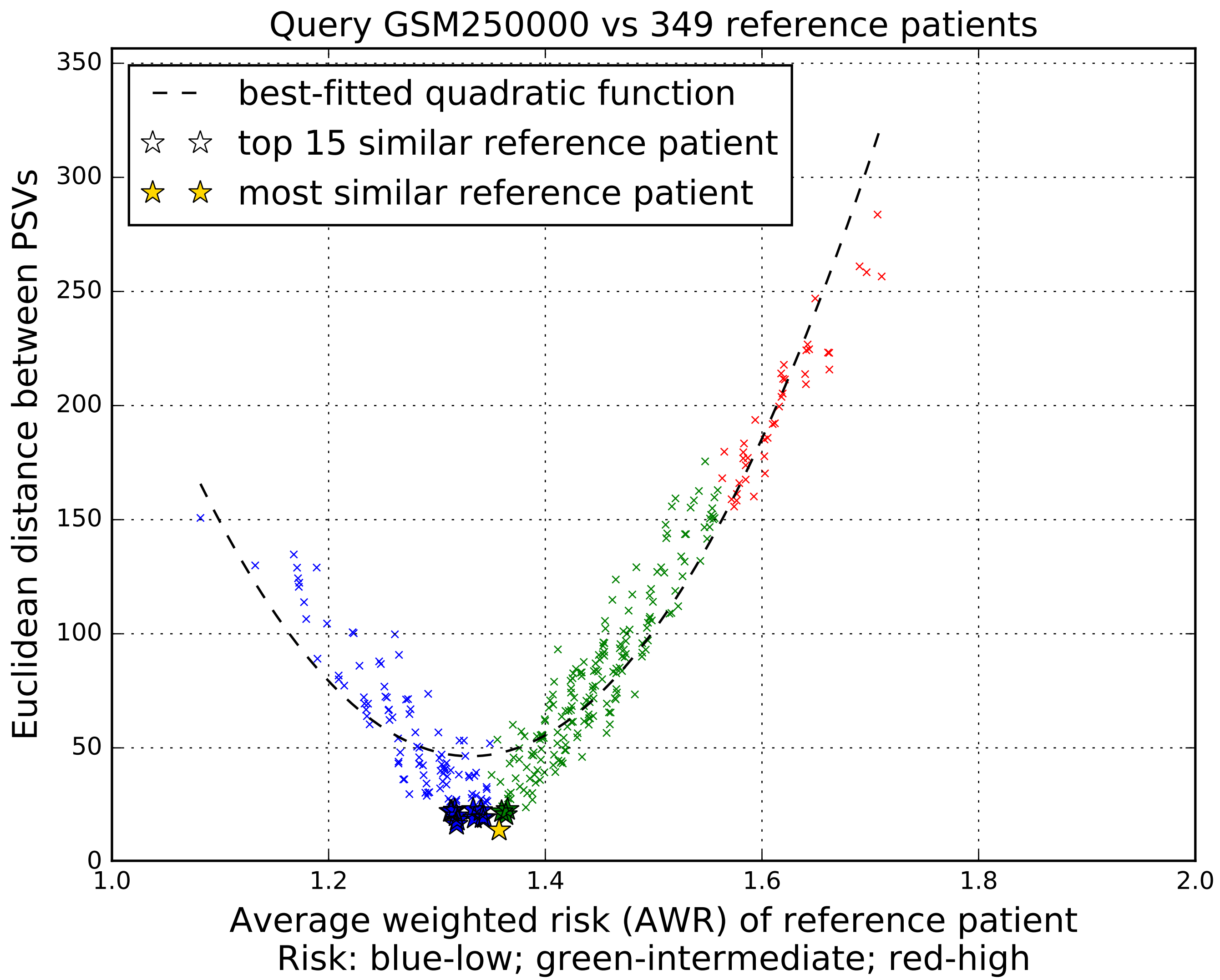
Query GSM250000 vs 349 reference patients
best-fitted quadratic function
top 15 similar reference patient
most similar reference patient
Euclidean distance between PSVs
0
50
100
150
200
250
300
350
1.0
1.2
1.4
1.6
1.8
2.0
Average weighted risk (AWR) of reference patient
Risk: blue-low; green-intermediate; red-high

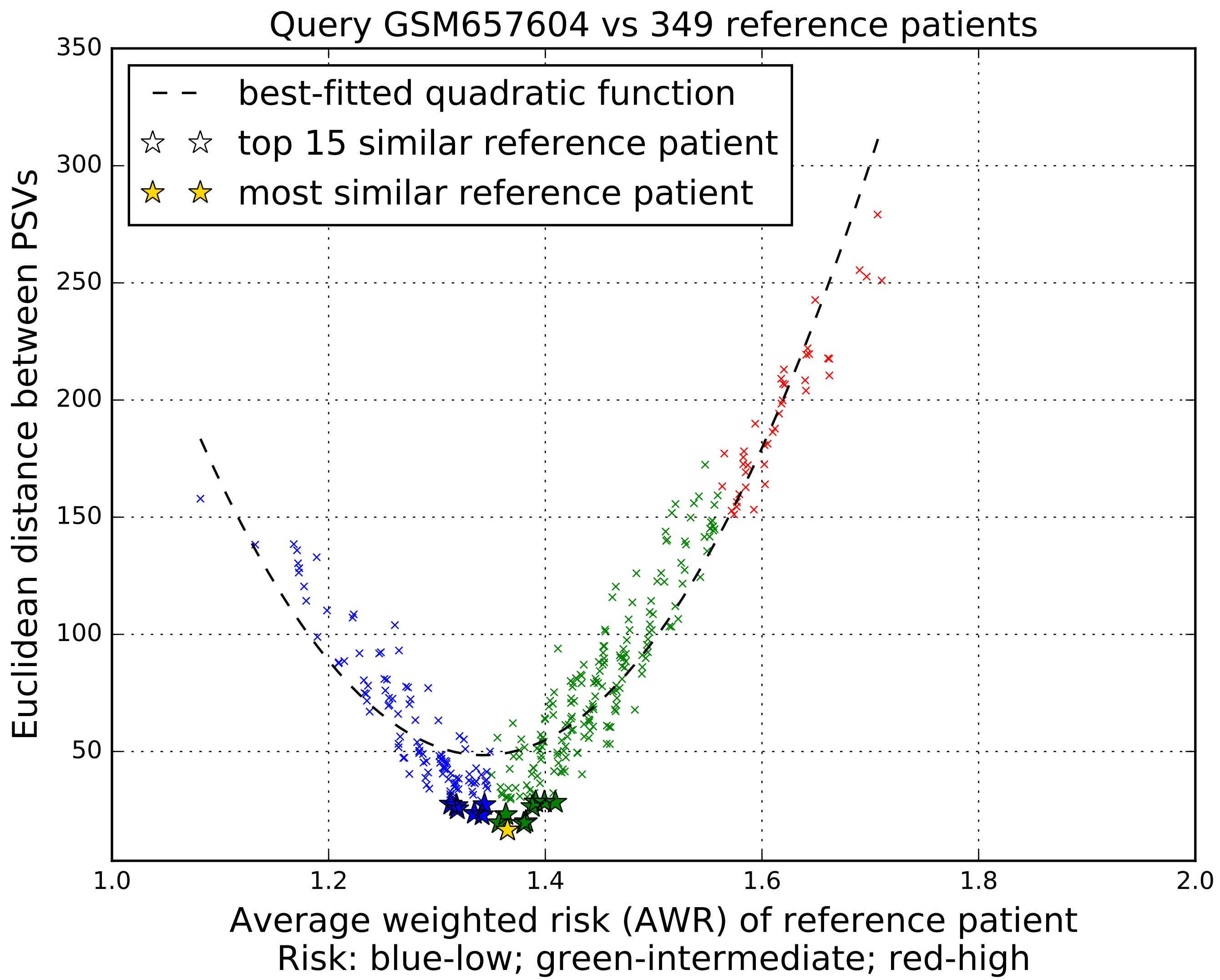
Query GSM657604 vs 349 reference patients
best-fitted quadratic function
top 15 similar reference patient
most similar reference patient
Euclidean distance between PSVs
Average weighted risk (AWR) of reference patient
Risk: blue-low; green-intermediate; red-high
350
300
250
200
150
100
50
1.0
1.2
1.4
1.6
1.8
2.0

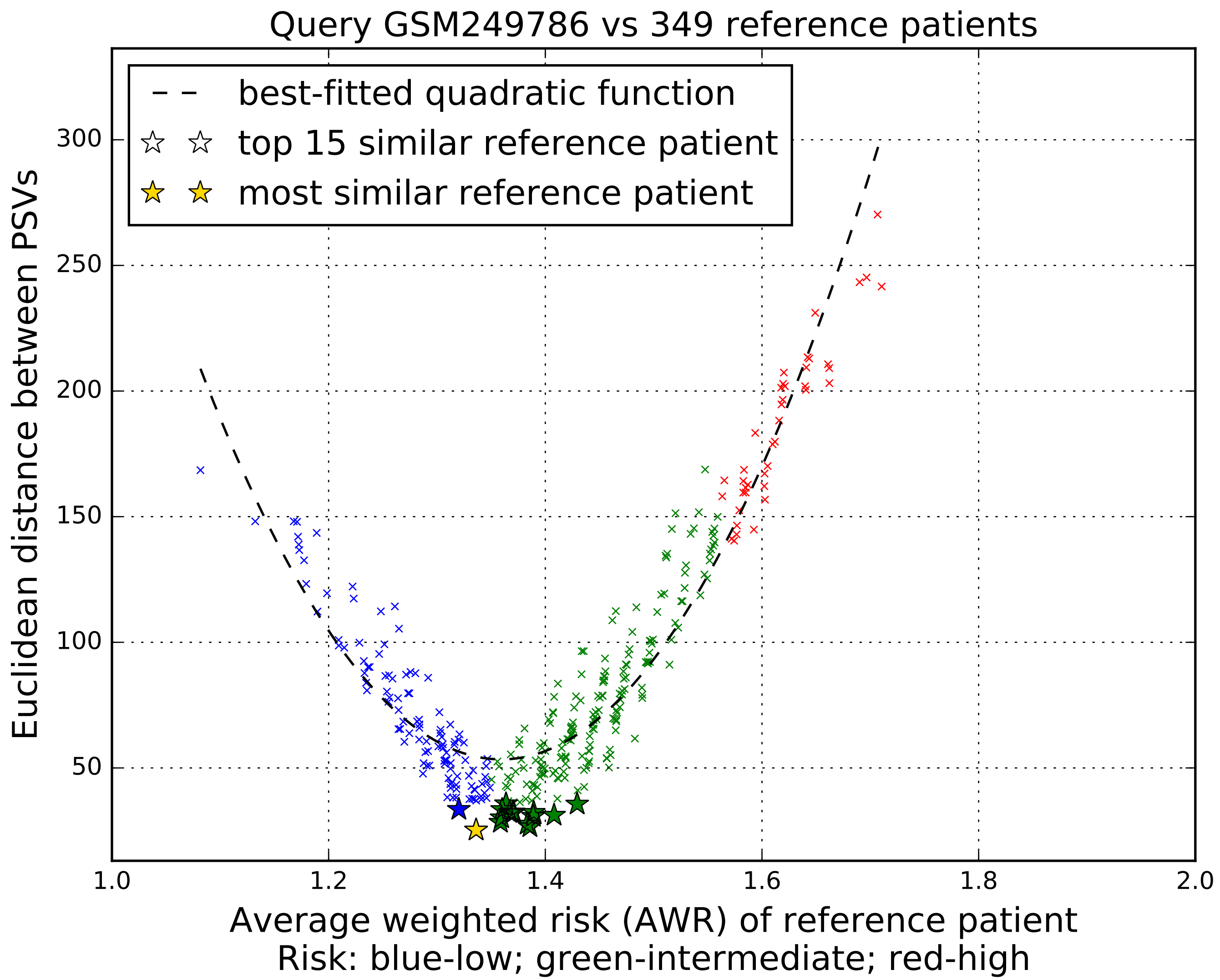
Query GSM249786 vs 349 reference patients
best-fitted quadratic function
top 15 similar reference patient
most similar reference patient
Euclidean distance between PSVs
300
250
200
150
100
50
1.0
1.2
1.4
1.6
1.8
2.0
Average weighted risk (AWR) of reference patient
Risk: blue-low; green-intermediate; red-high

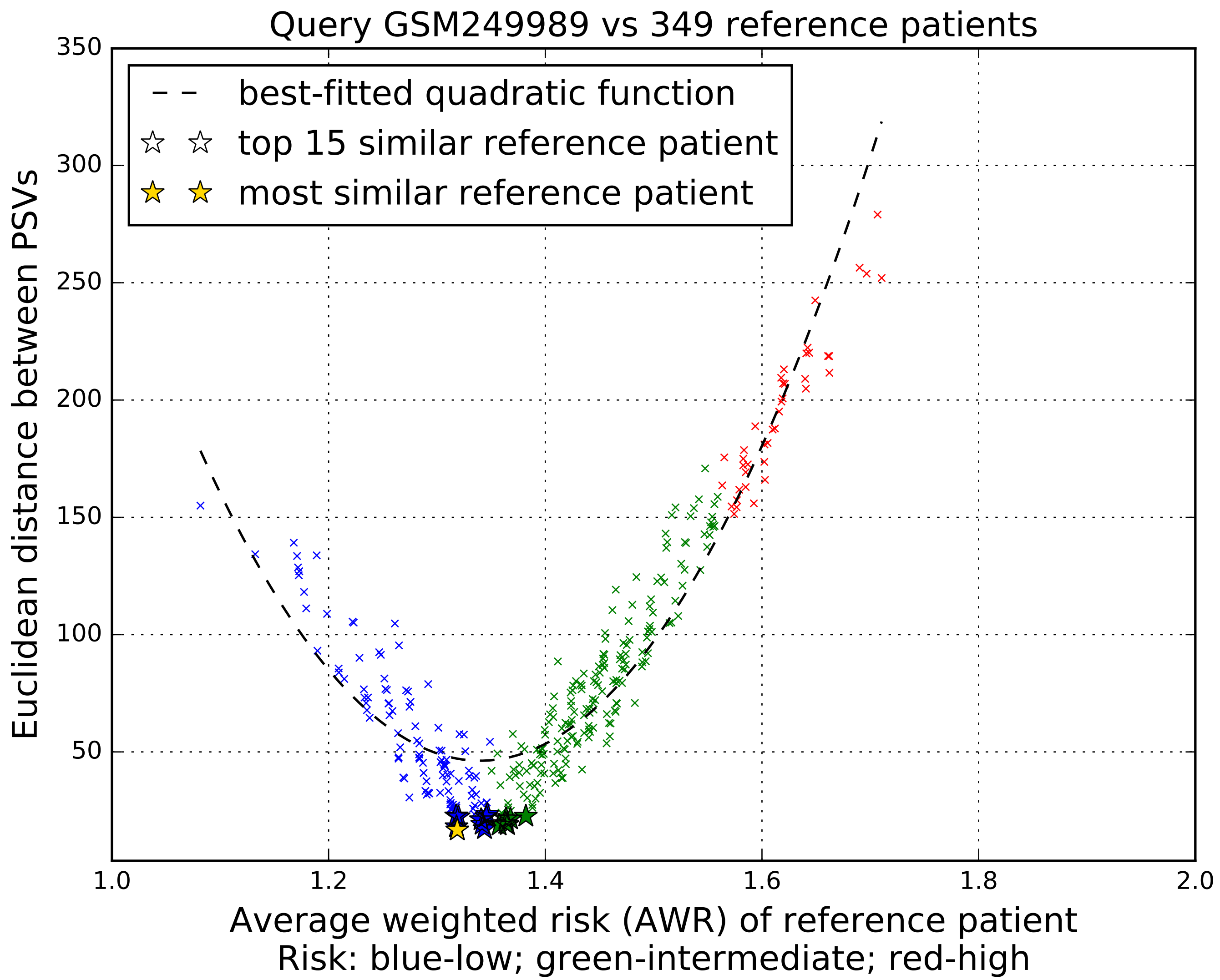

Query GSM249989 vs 349 reference patients
best-fitted quadratic function
top 15 similar reference patient
most similar reference patient
Euclidean distance between PSVs
350
300
250
200
150
100
50
1.0
1.2
1.4
1.6
1.8
2.0
Average weighted risk (AWR) of reference patient
Risk: blue-low; green-intermediate; red-high

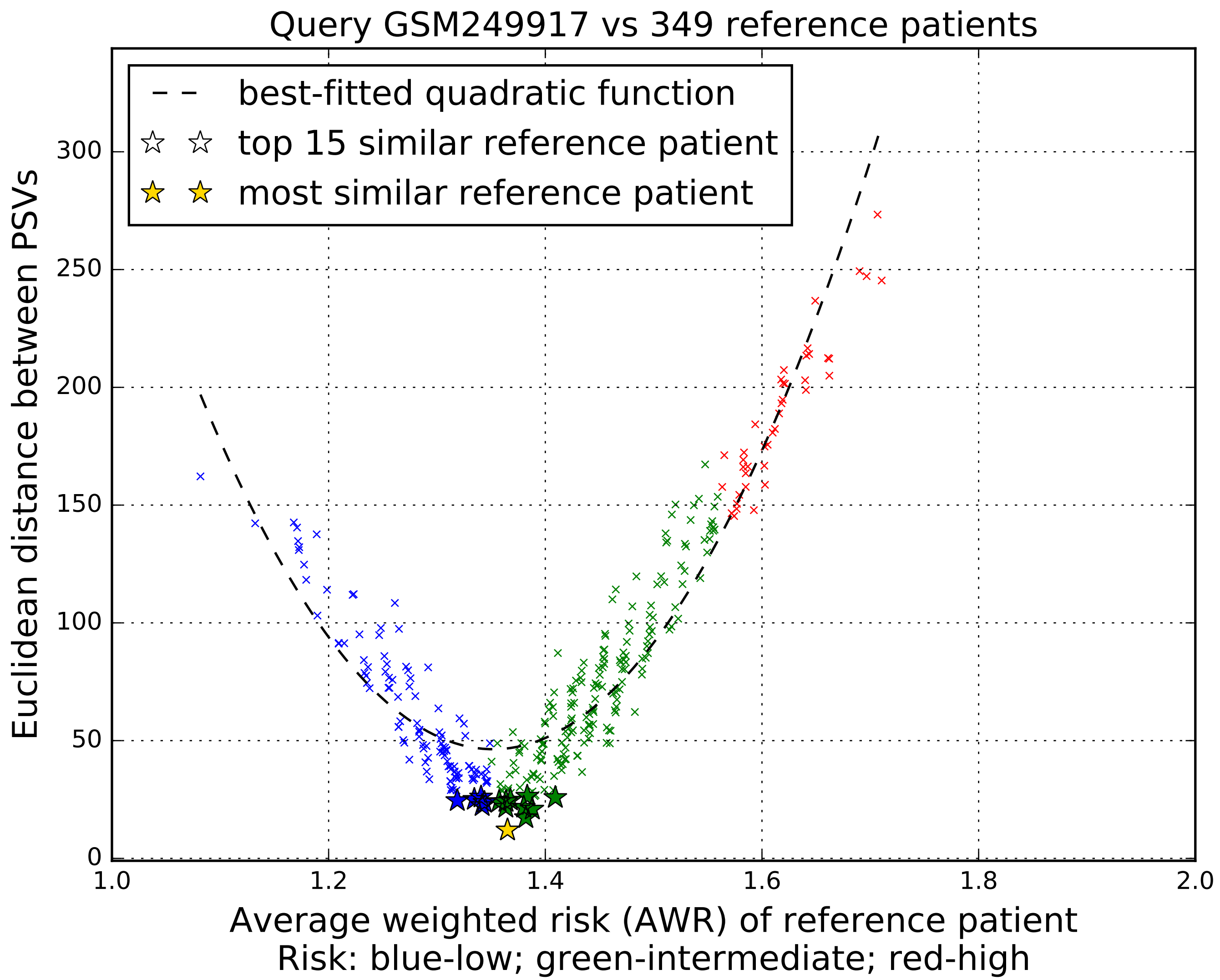

Query GSM249917 vs 349 reference patients
best-fitted quadratic function
top 15 similar reference patient
most similar reference patient
Euclidean distance between PSVs
0
50
100
150
200
250
300
1.0
1.2
1.4
1.6
1.8
2.0
Average weighted risk (AWR) of reference patient
Risk: blue-low; green-intermediate; red-high

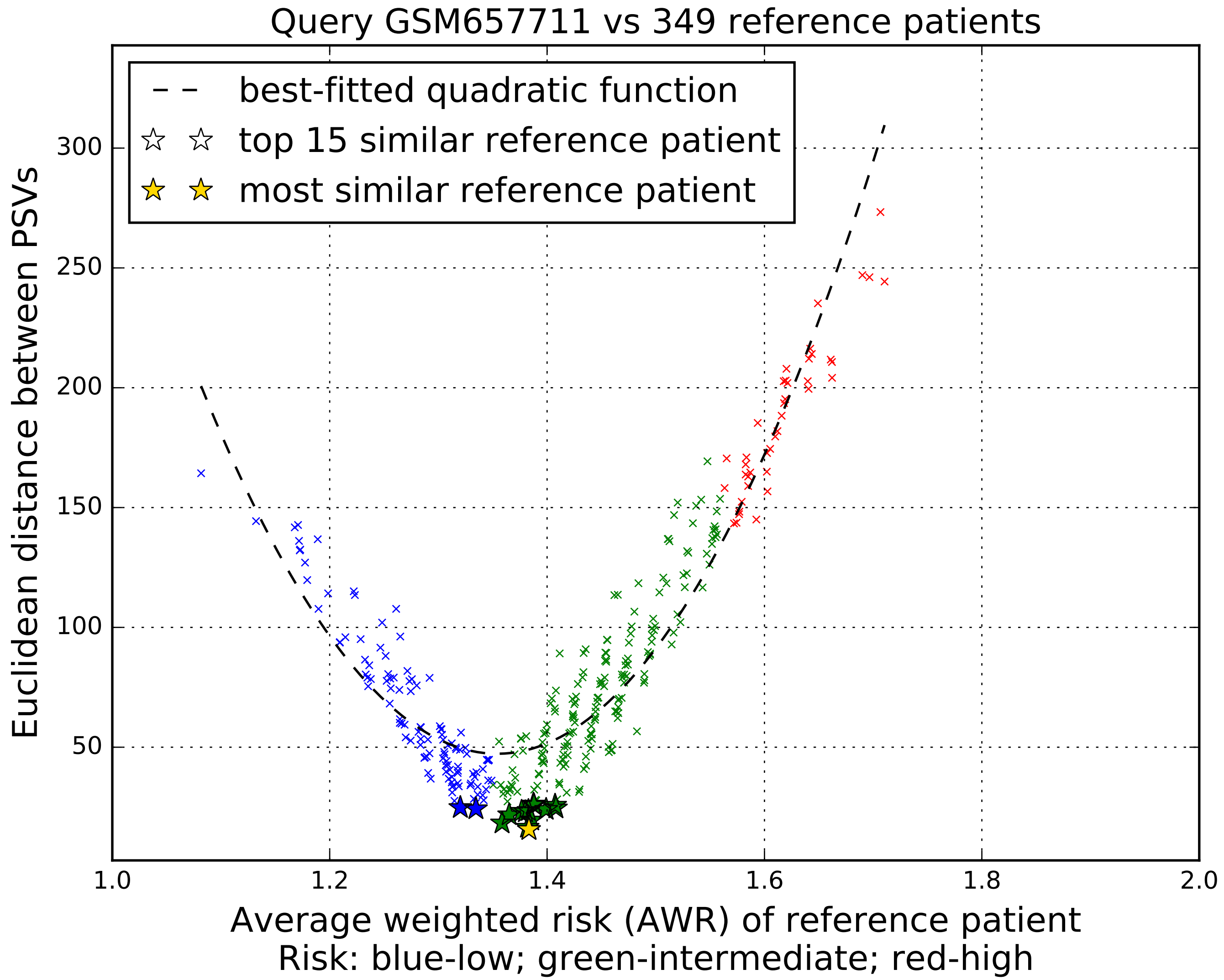
Query GSM657711 vs 349 reference patients
best-fitted quadratic function
top 15 similar reference patient
most similar reference patient
Euclidean distance between PSVs
300
250
200
150
100
50
1.0
1.2
1.4
1.6
1.8
2.0
Average weighted risk (AWR) of reference patient
Risk: blue-low; green-intermediate; red-high

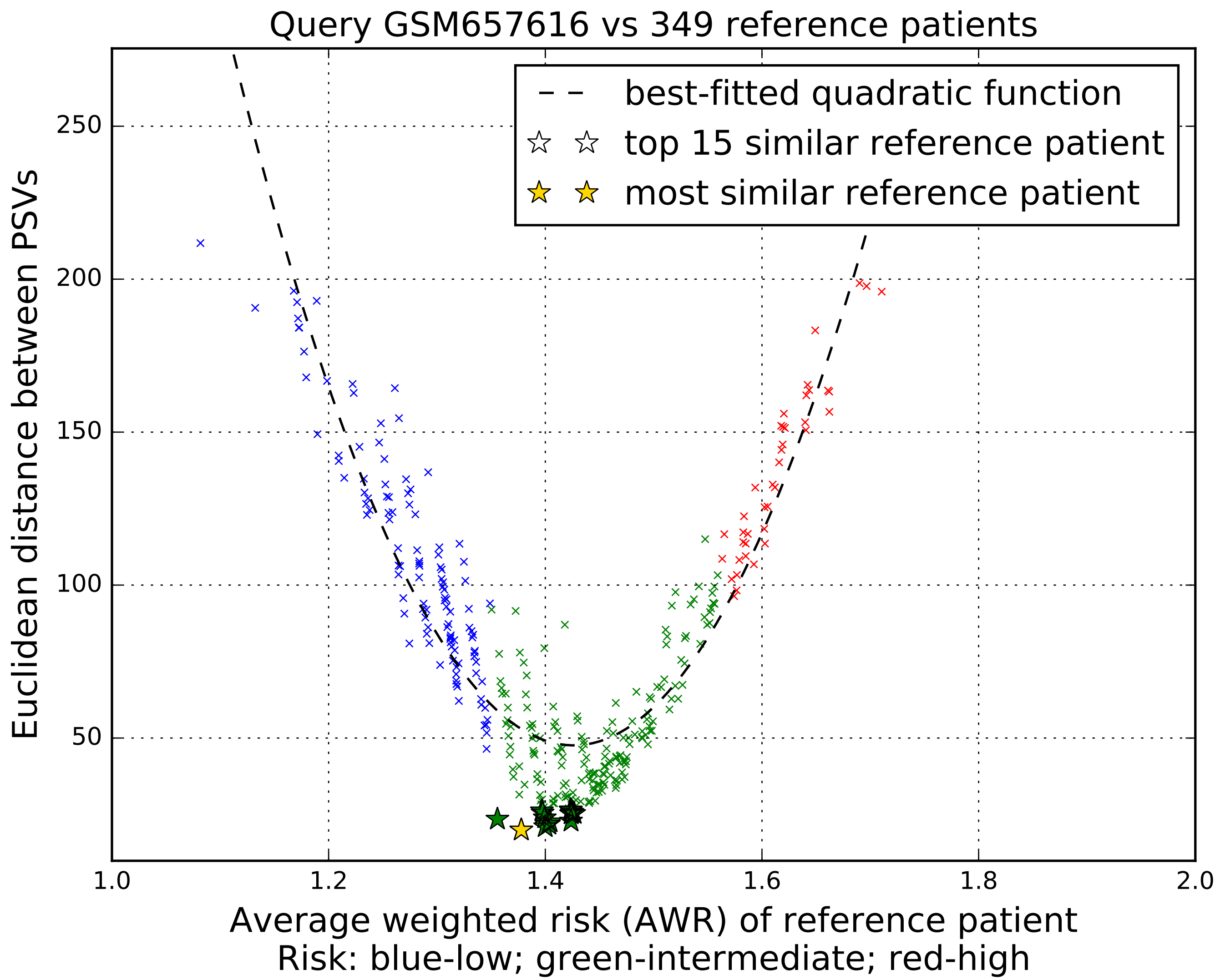

Query GSM657616 vs 349 reference patients
best-fitted quadratic function
top 15 similar reference patient
most similar reference patient
Euclidean distance between PSVs
250
200
150
100
50
1.0
1.2
1.4
1.6
1.8
2.0
Average weighted risk (AWR) of reference patient
Risk: blue-low; green-intermediate; red-high

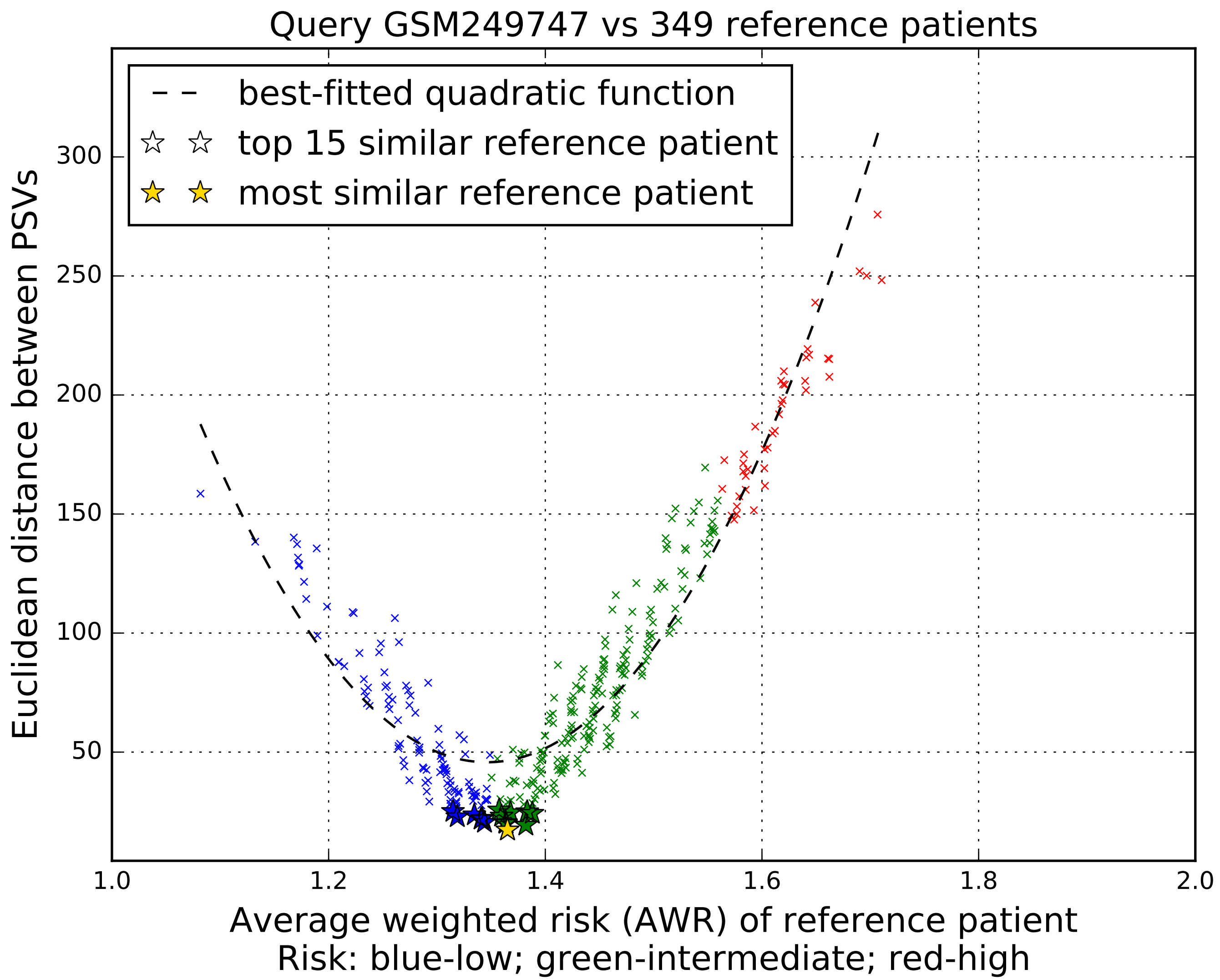

Query GSM249747 vs 349 reference patients
best-fitted quadratic function
top 15 similar reference patient
most similar reference patient
Euclidean distance between PSVs
300
250
200
150
100
50
1.0
1.2
1.4
1.6
1.8
2.0
Average weighted risk (AWR) of reference patient
Risk: blue-low; green-intermediate; red-high

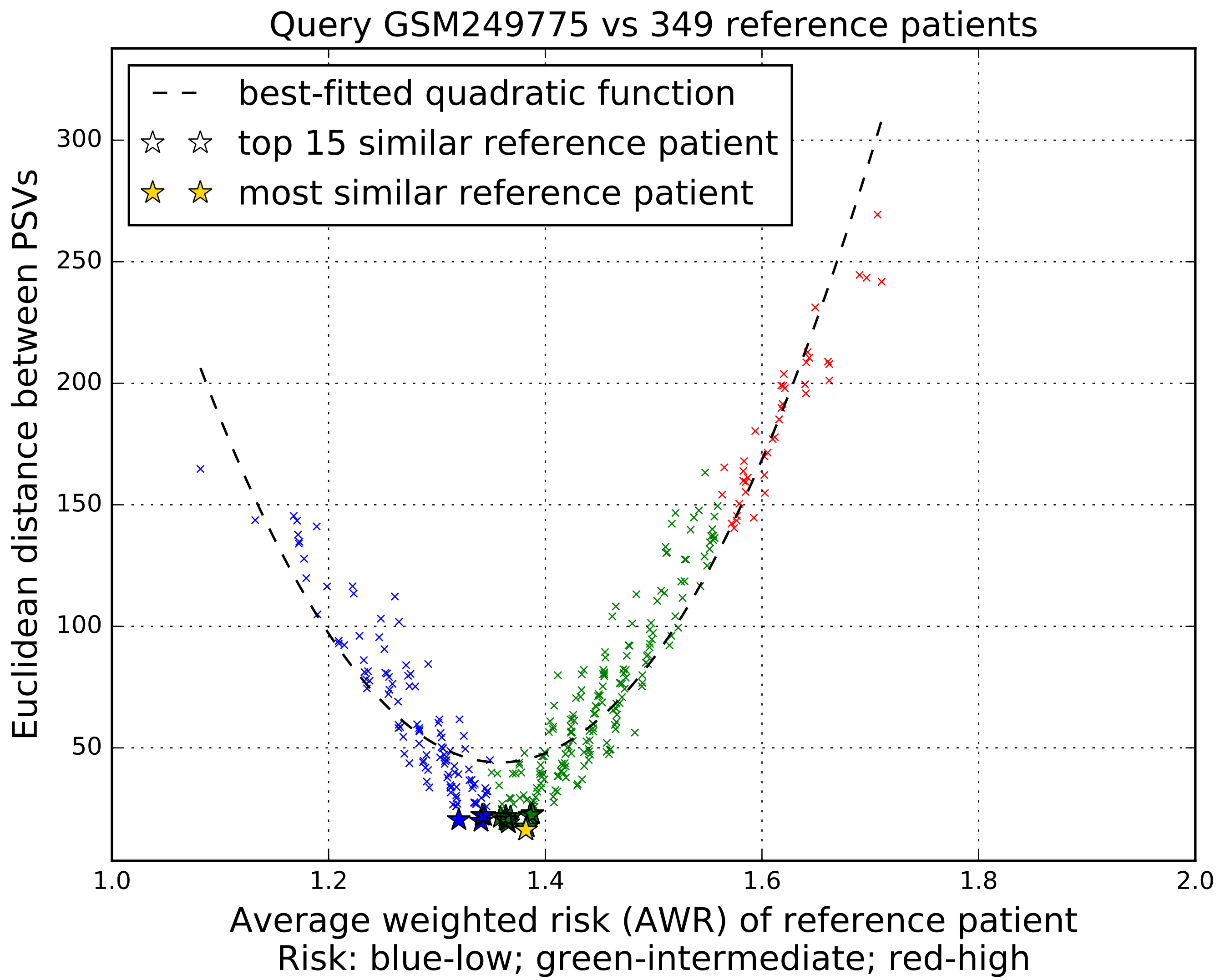

Query GSM249775 vs 349 reference patients
best-fitted quadratic function
top 15 similar reference patient
most similar reference patient
300
250
200
150
100
50
1.0
1.2
1.4
1.6
1.8
2.0
Euclidean distance between PSVs
Average weighted risk (AWR) of reference patient
Risk: blue-low; green-intermediate; red-high

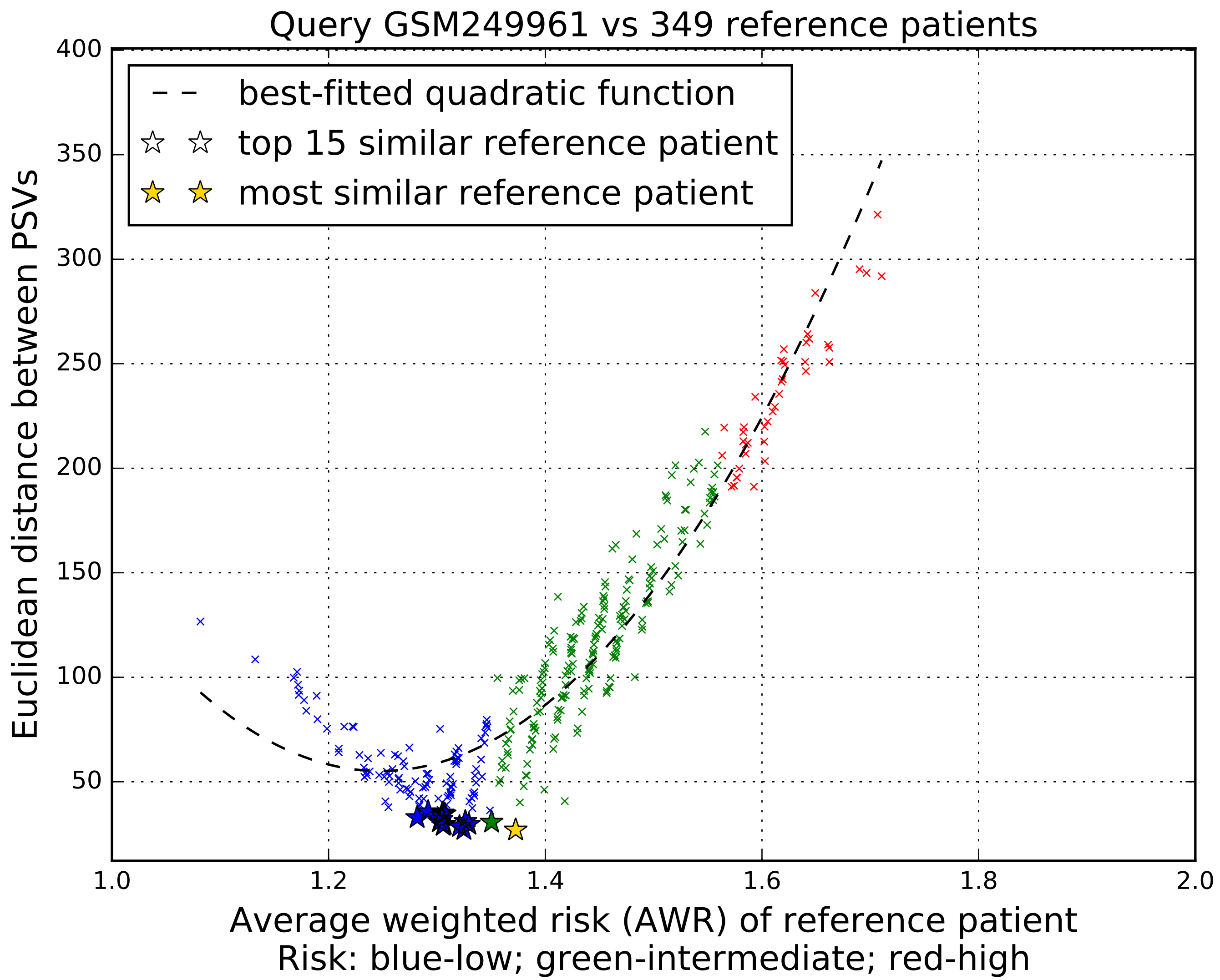

Query GSM249961 vs 349 reference patients
best-fitted quadratic function
top 15 similar reference patient
most similar reference patient
Euclidean distance between PSVs
Average weighted risk (AWR) of reference patient
Risk: blue-low; green-intermediate; red-high
400
350
300
250
200
150
100
50
1.0
1.2
1.4
1.6
1.8
2.0

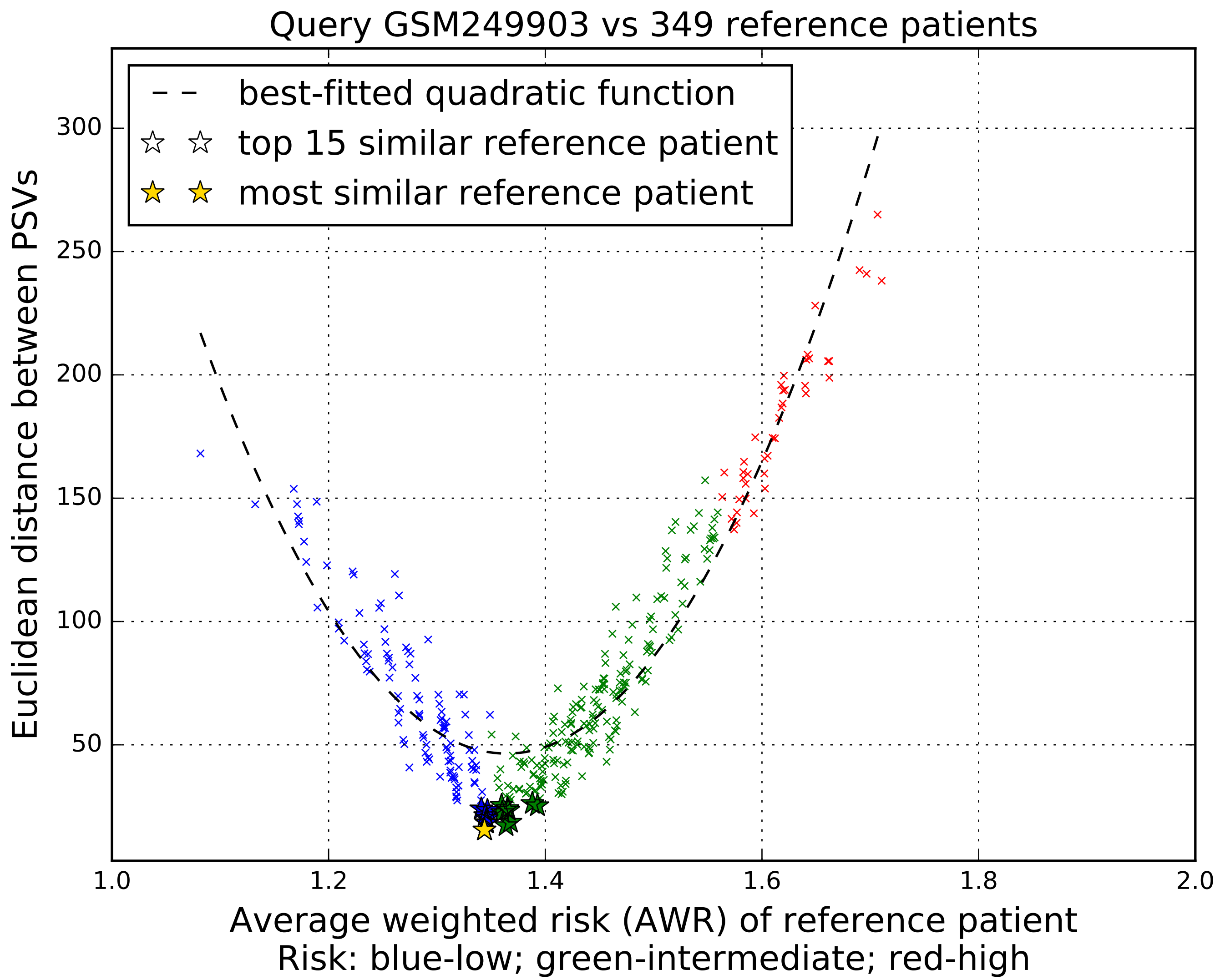

Query GSM249903 vs 349 reference patients
best-fitted quadratic function
top 15 similar reference patient
most similar reference patient
Euclidean distance between PSVs
300
250
200
150
100
50
1.0
1.2
1.4
1.6
1.8
2.0
Average weighted risk (AWR) of reference patient
Risk: blue-low; green-intermediate; red-high

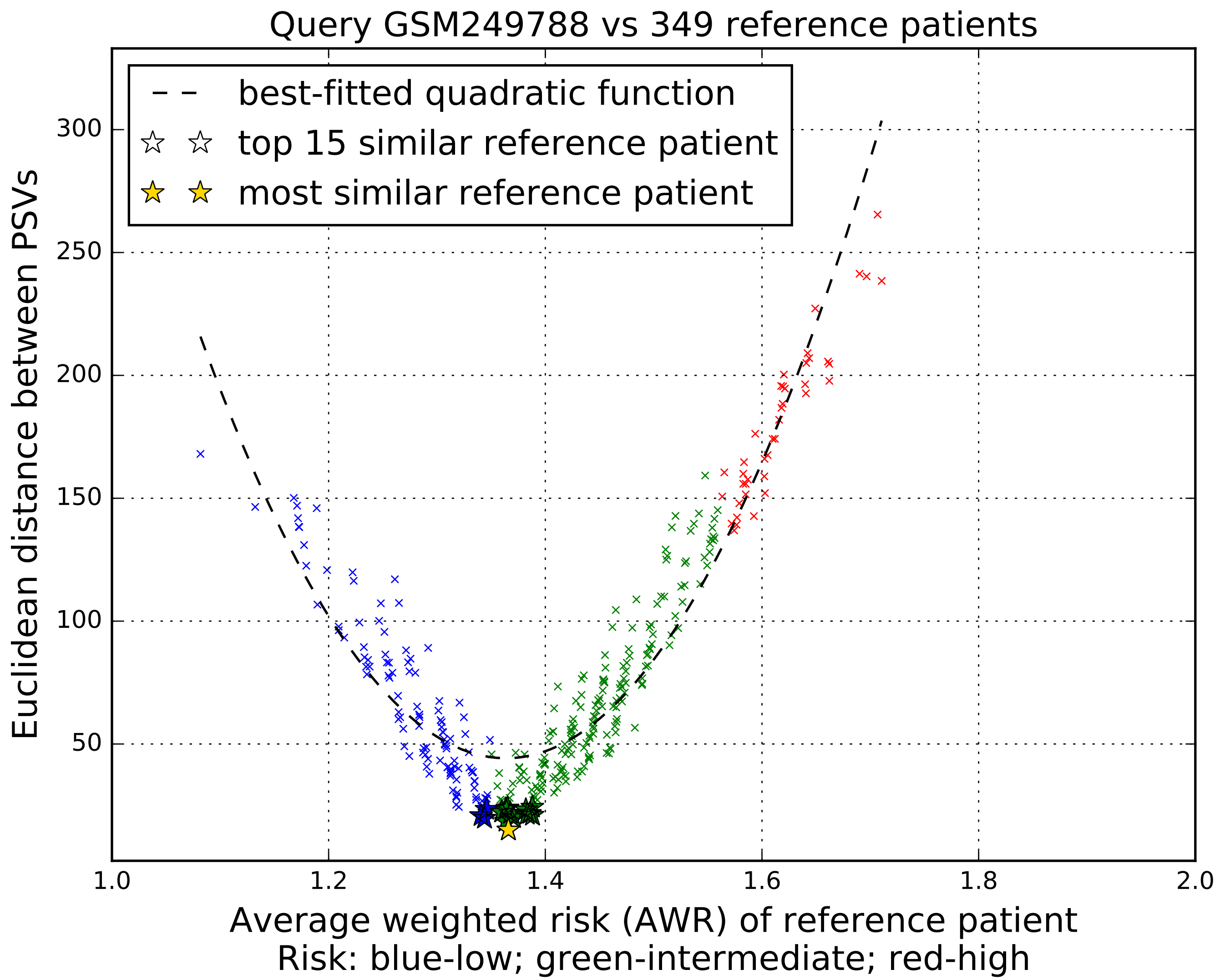
Query GSM249788 vs 349 reference patients
best-fitted quadratic function
top 15 similar reference patient
most similar reference patient
Euclidean distance between PSVs
300
250
200
150
100
50
1.0
1.2
1.4
1.6
1.8
2.0
Average weighted risk (AWR) of reference patient
Risk: blue-low; green-intermediate; red-high

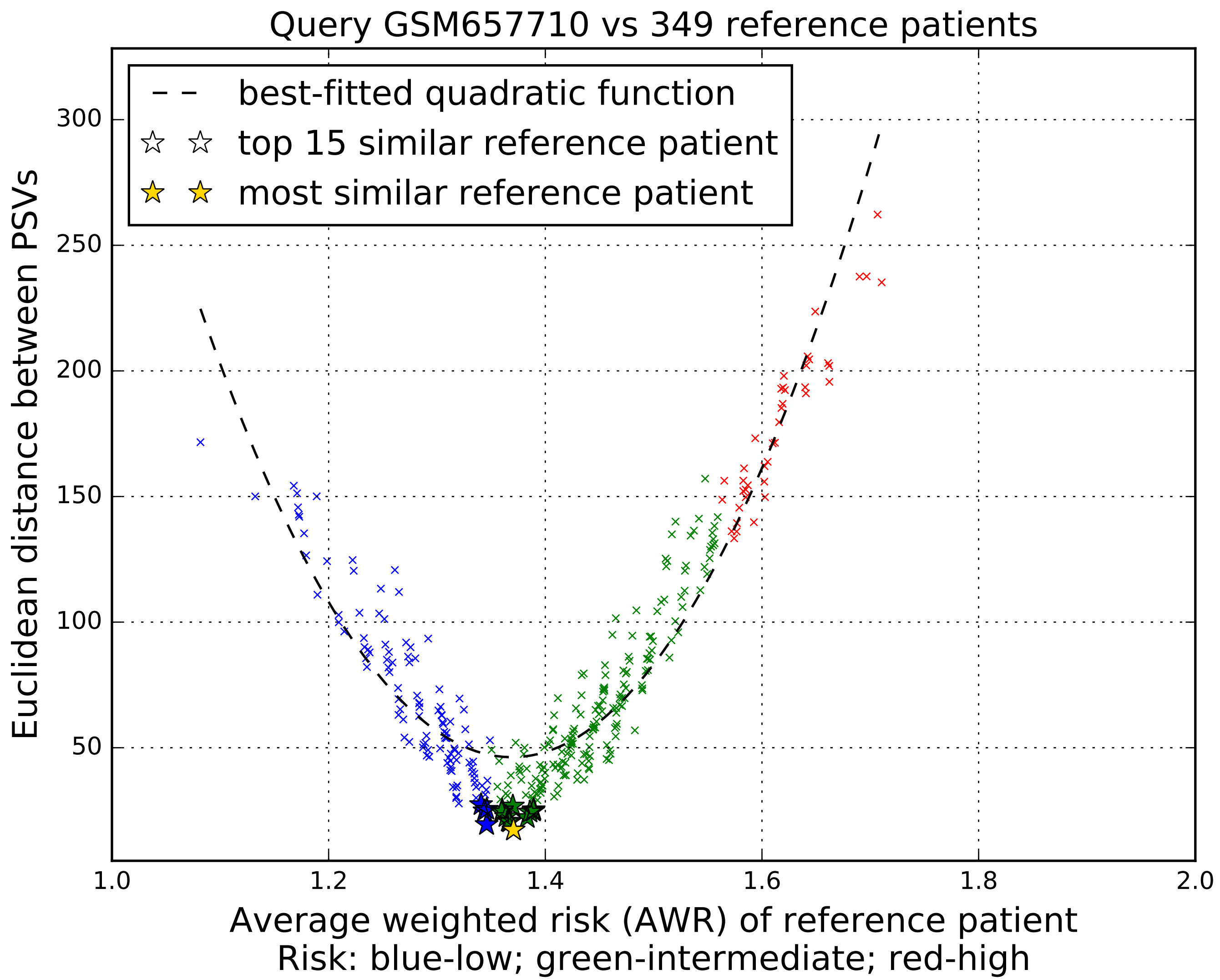

Query GSM657710 vs 349 reference patients
best-fitted quadratic function
top 15 similar reference patient
most similar reference patient
Euclidean distance between PSVs
300
250
200
150
100
50
1.0
1.2
1.4
1.6
1.8
2.0
Average weighted risk (AWR) of reference patient
Risk: blue-low; green-intermediate; red-high

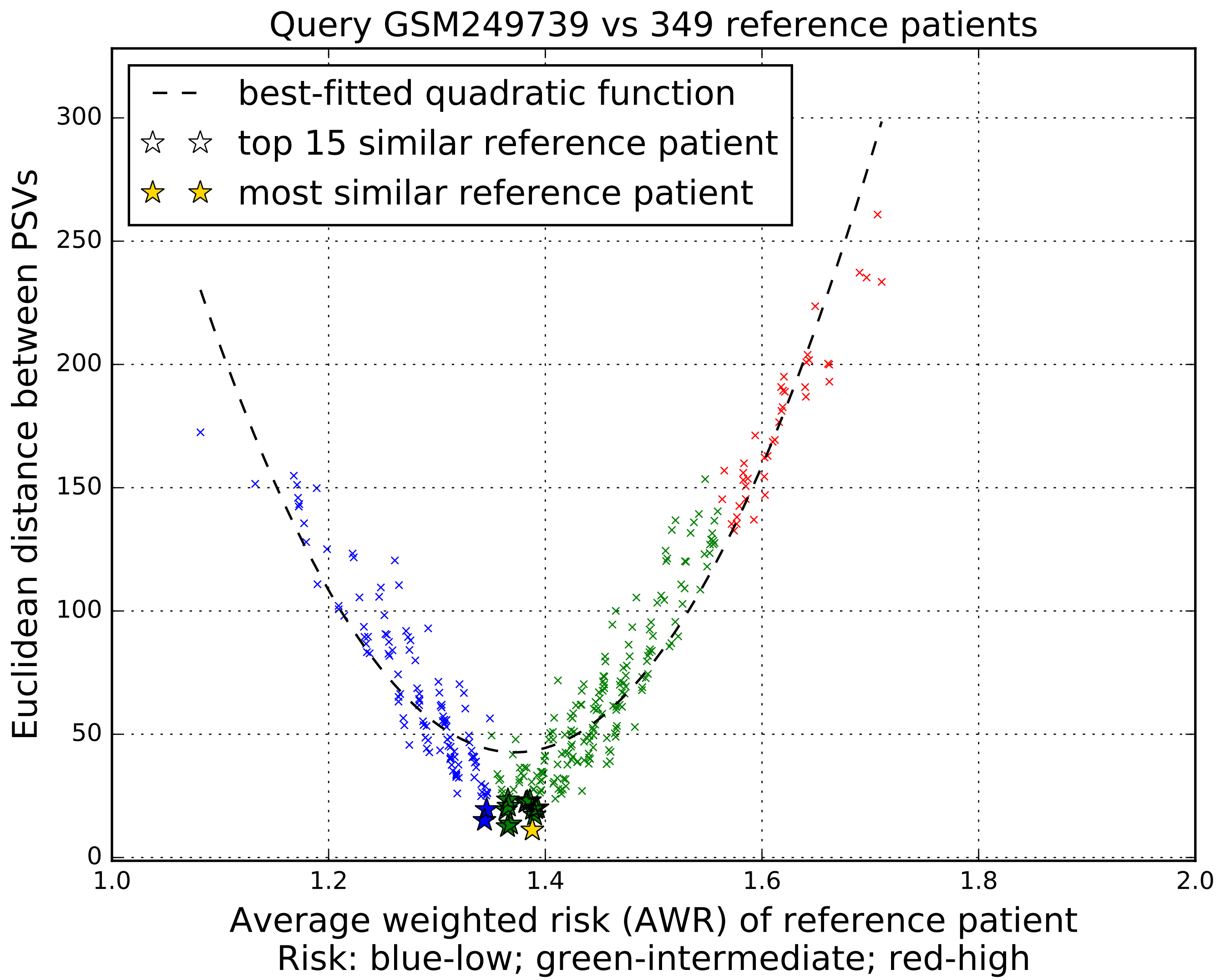

Query GSM249739 vs 349 reference patients
best-fitted quadratic function
top 15 similar reference patient
most similar reference patient
Euclidean distance between PSVs
0
50
100
150
200
250
300
1.0
1.2
1.4
1.6
1.8
2.0
Average weighted risk (AWR) of reference patient
Risk: blue-low; green-intermediate; red-high

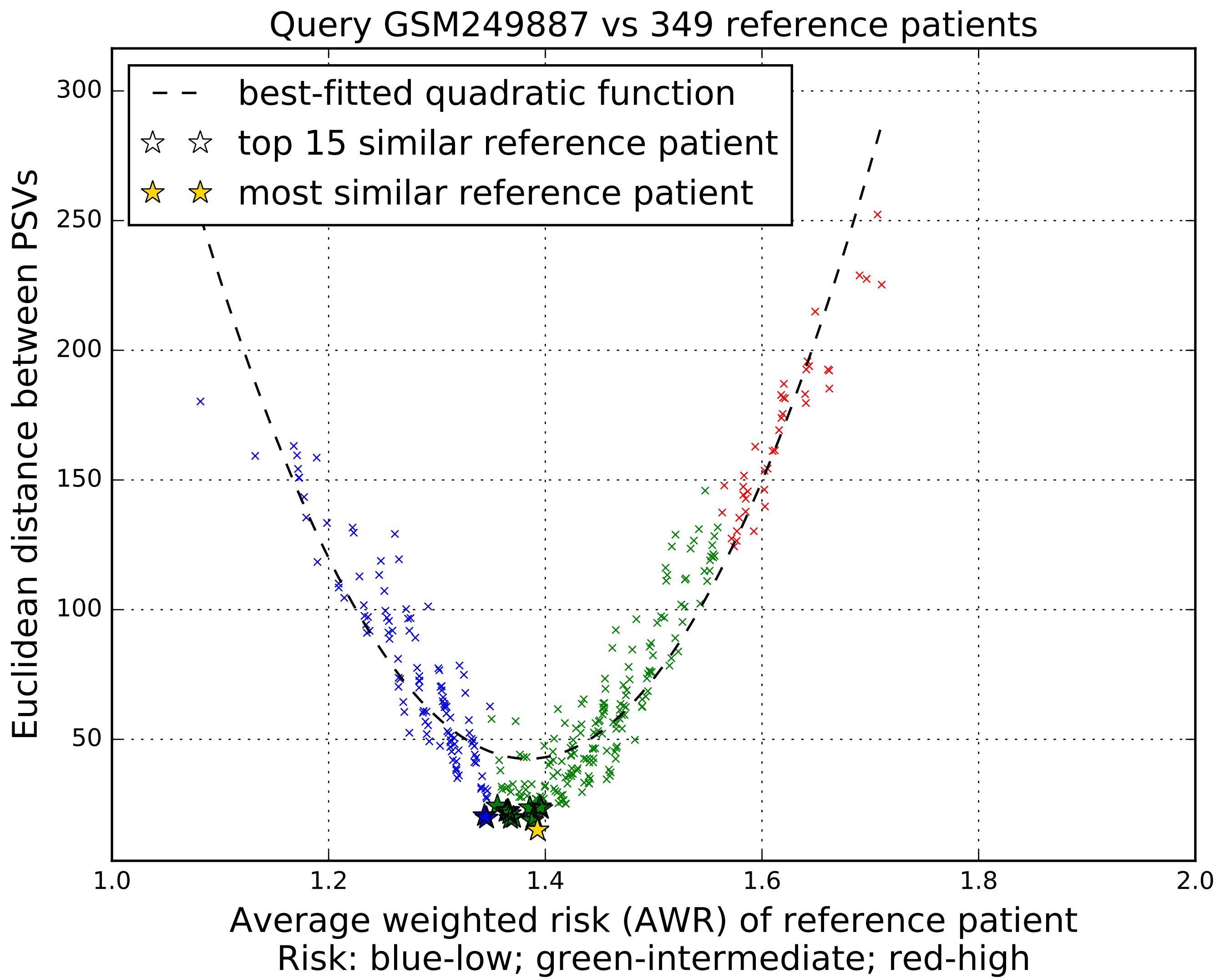
Query GSM249887 vs 349 reference patients
best-fitted quadratic function
top 15 similar reference patient
most similar reference patient
Euclidean distance between PSVs
300
250
200
150
100
50
1.0
1.2
1.4
1.6
1.8
2.0
Average weighted risk (AWR) of reference patient
Risk: blue-low; green-intermediate; red-high

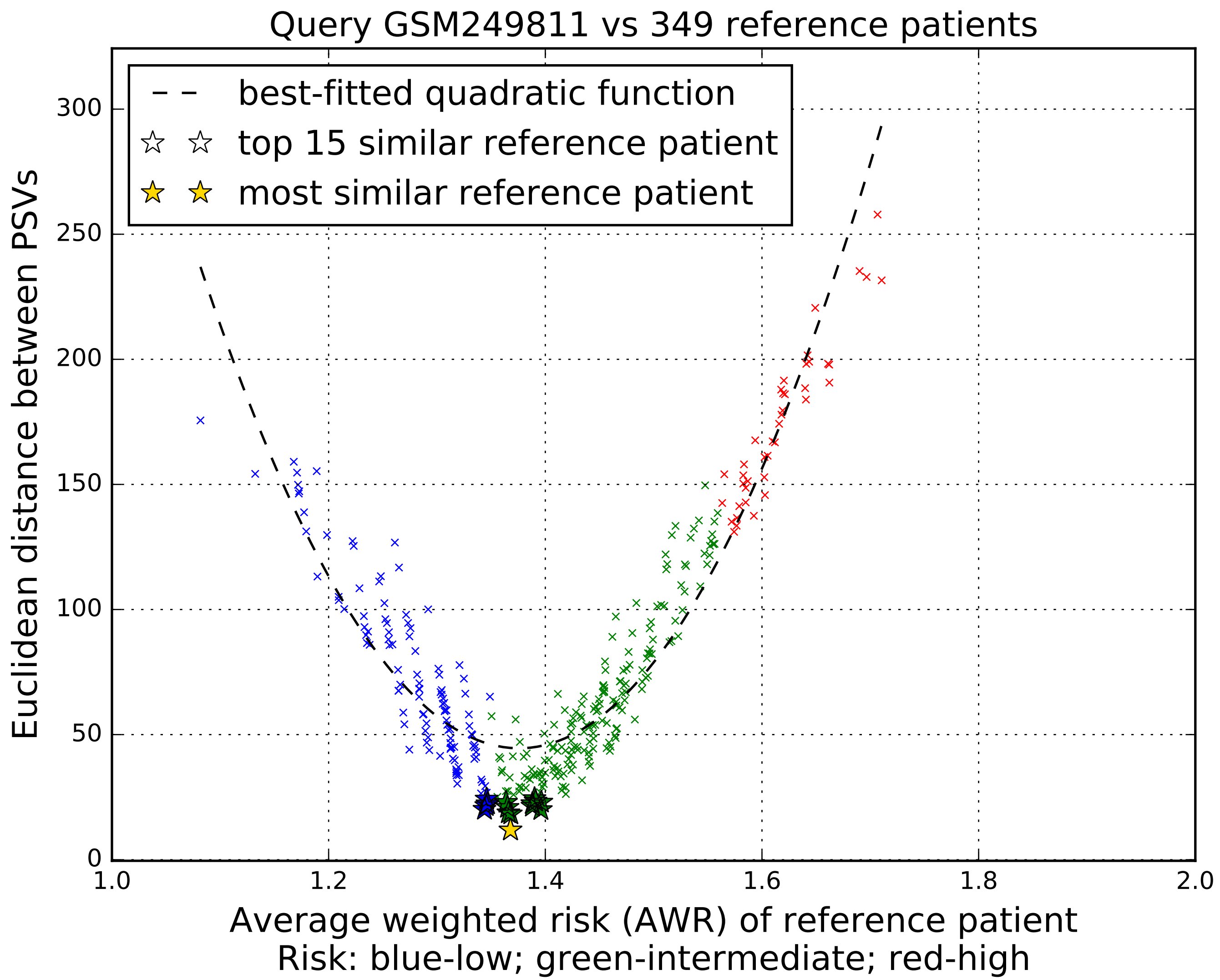
Query GSM249811 vs 349 reference patients
best-fitted quadratic function
top 15 similar reference patient
most similar reference patient
Euclidean distance between PSVs
0
50
100
150
200
250
300
1.0
1.2
1.4
1.6
1.8
2.0
Average weighted risk (AWR) of reference patient
Risk: blue-low; green-intermediate; red-high

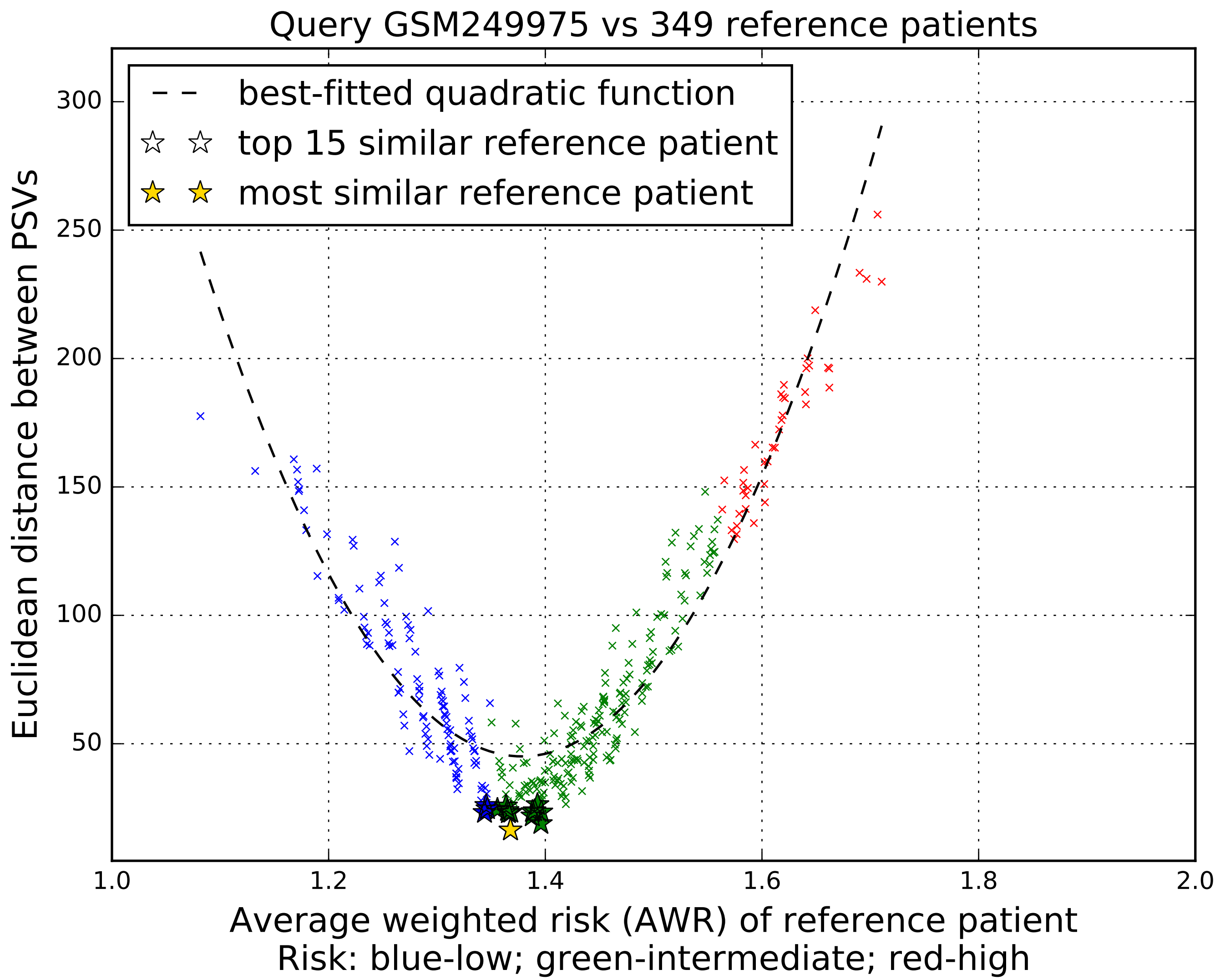

Query GSM249975 vs 349 reference patients
best-fitted quadratic function
top 15 similar reference patient
most similar reference patient
Euclidean distance between PSVs
300
250
200
150
100
50
1.0
1.2
1.4
1.6
1.8
2.0
Average weighted risk (AWR) of reference patient
Risk: blue-low; green-intermediate; red-high

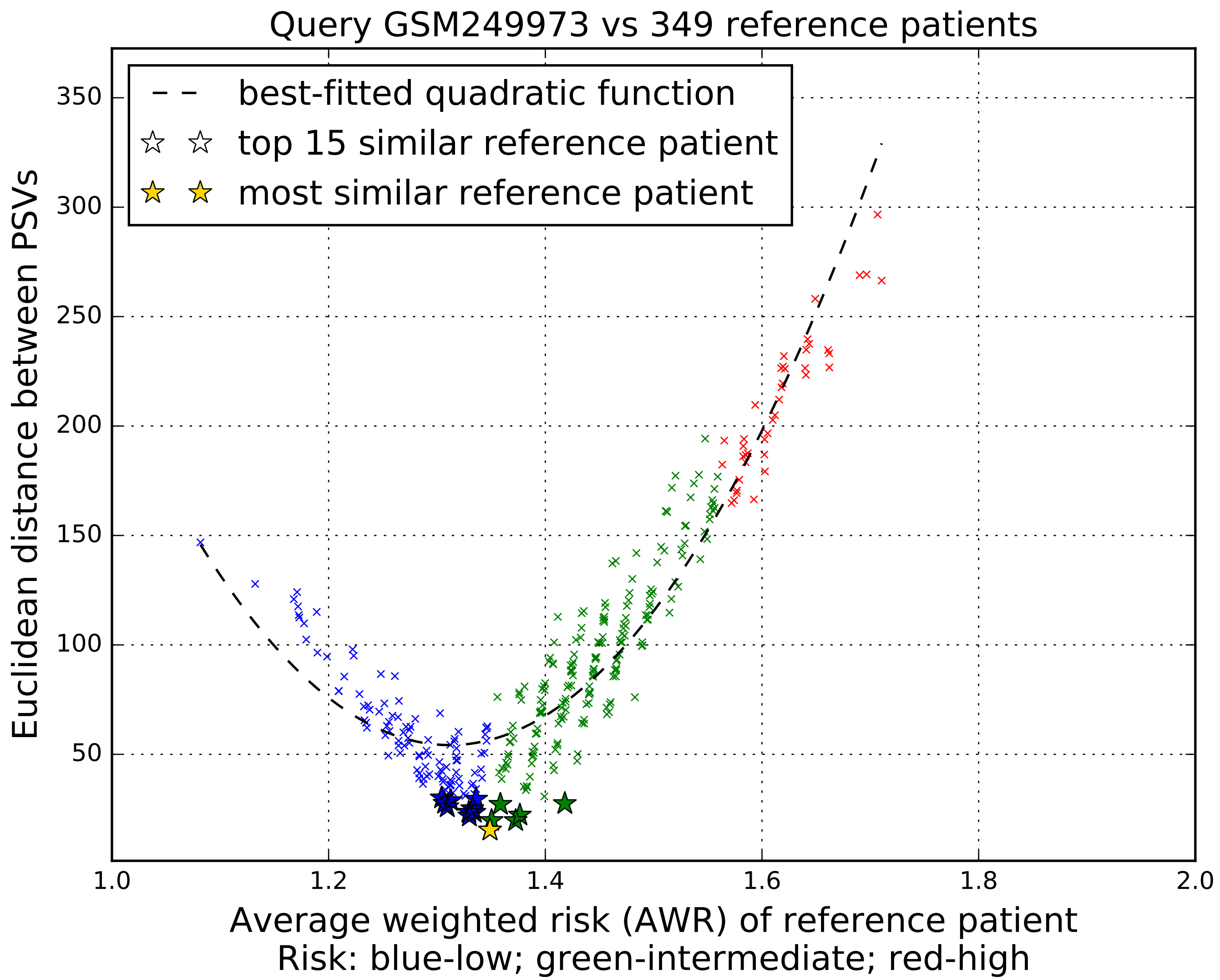
Query GSM249973 vs 349 reference patients
best-fitted quadratic function
top 15 similar reference patient
most similar reference patient
Euclidean distance between PSVs
350
300
250
200
150
100
50
1.0
1.2
1.4
1.6
1.8
2.0
Average weighted risk (AWR) of reference patient
Risk: blue-low; green-intermediate; red-high

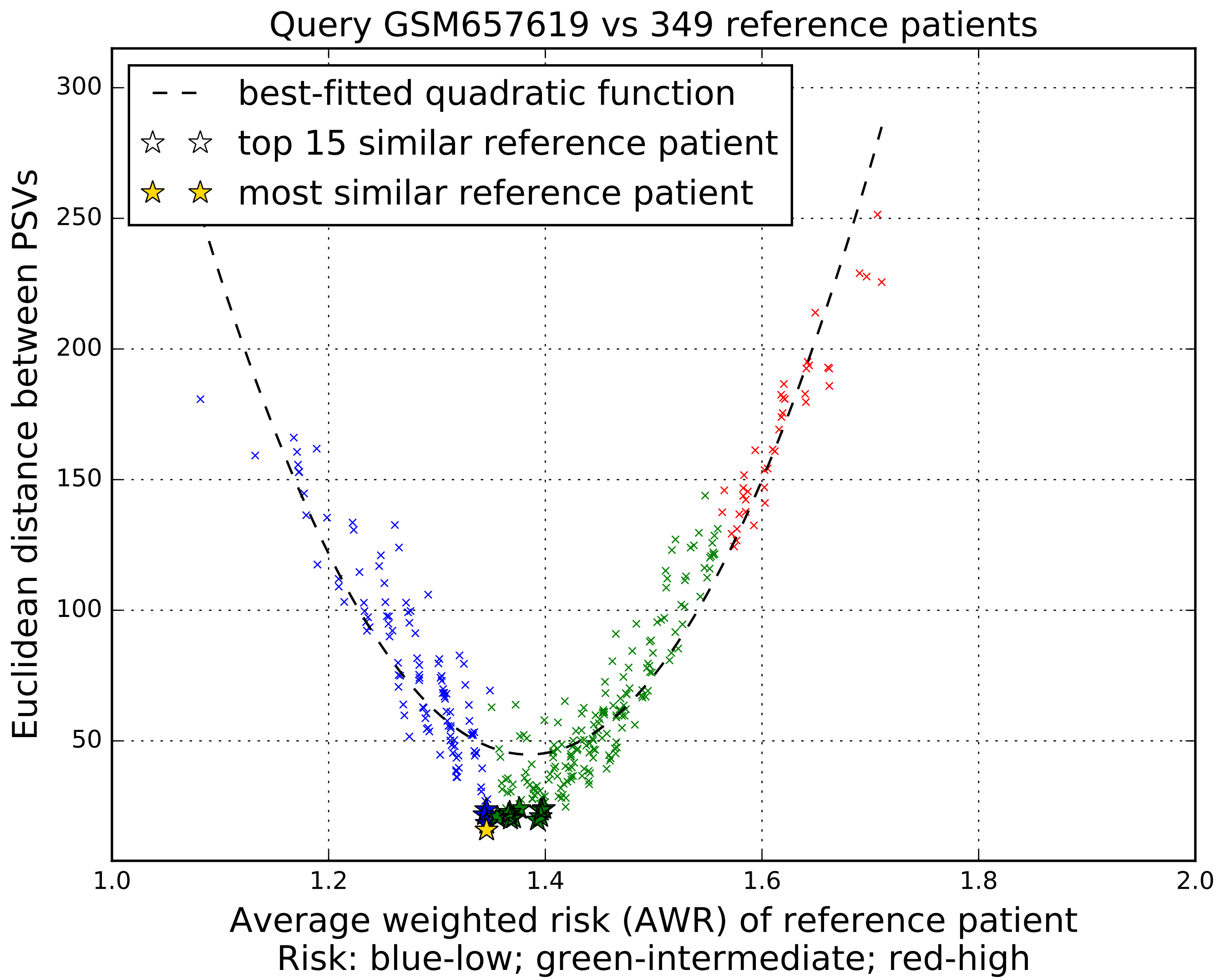

Query GSM657619 vs 349 reference patients
best-fitted quadratic function
top 15 similar reference patient
most similar reference patient
Euclidean distance between PSVs
300
250
200
150
100
50
1.0
1.2
1.4
1.6
1.8
2.0
Average weighted risk (AWR) of reference patient
Risk: blue-low; green-intermediate; red-high

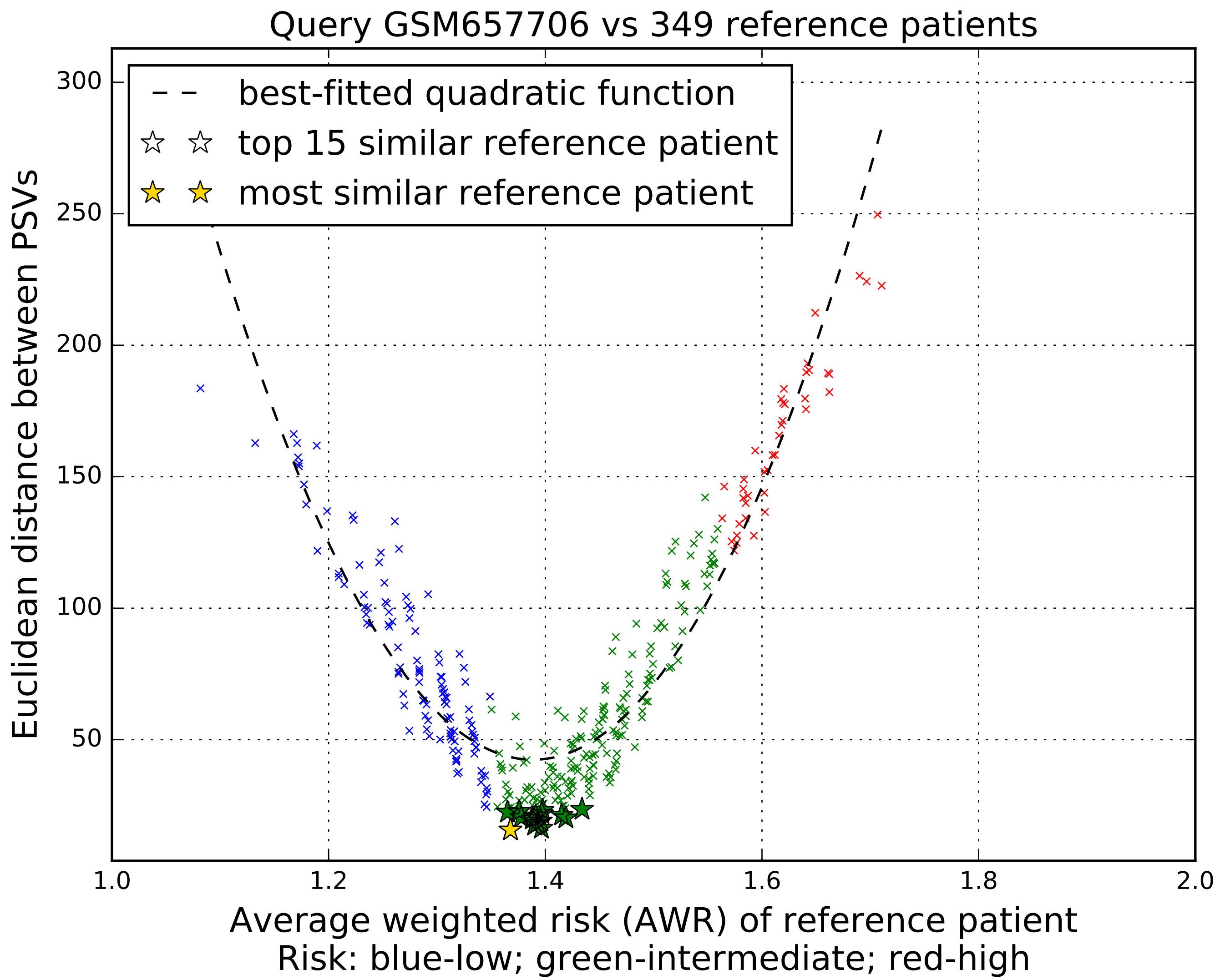

Query GSM657706 vs 349 reference patients
best-fitted quadratic function
top 15 similar reference patient
most similar reference patient
Euclidean distance between PSVs
300
250
200
150
100
50
1.0
1.2
1.4
1.6
1.8
2.0
Average weighted risk (AWR) of reference patient
Risk: blue-low; green-intermediate; red-high

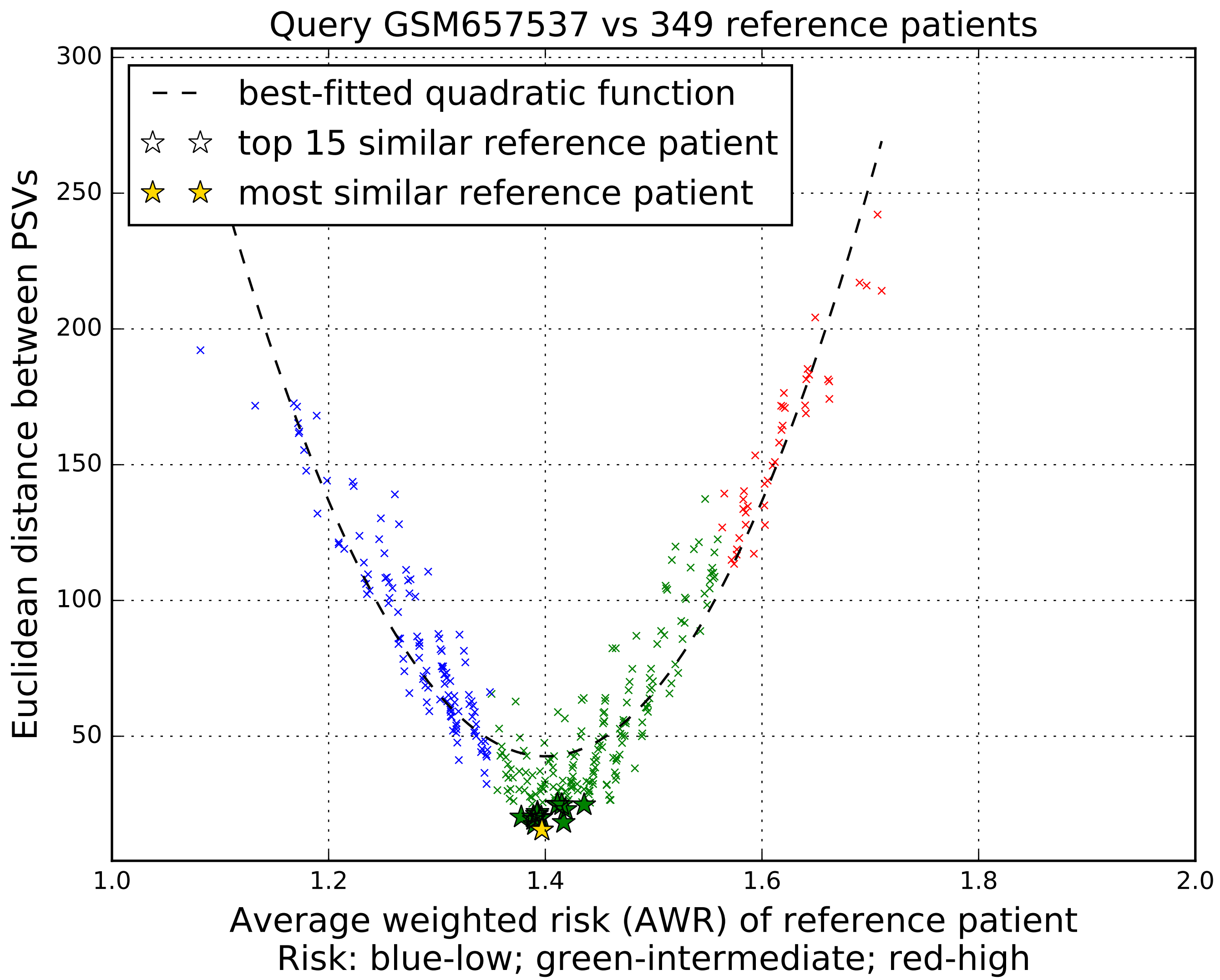

Query GSM657537 vs 349 reference patients
best-fitted quadratic function
top 15 similar reference patient
most similar reference patient
Euclidean distance between PSVs
300
250
200
150
100
50
1.0
1.2
1.4
1.6
1.8
2.0
Average weighted risk (AWR) of reference patient
Risk: blue-low; green-intermediate; red-high

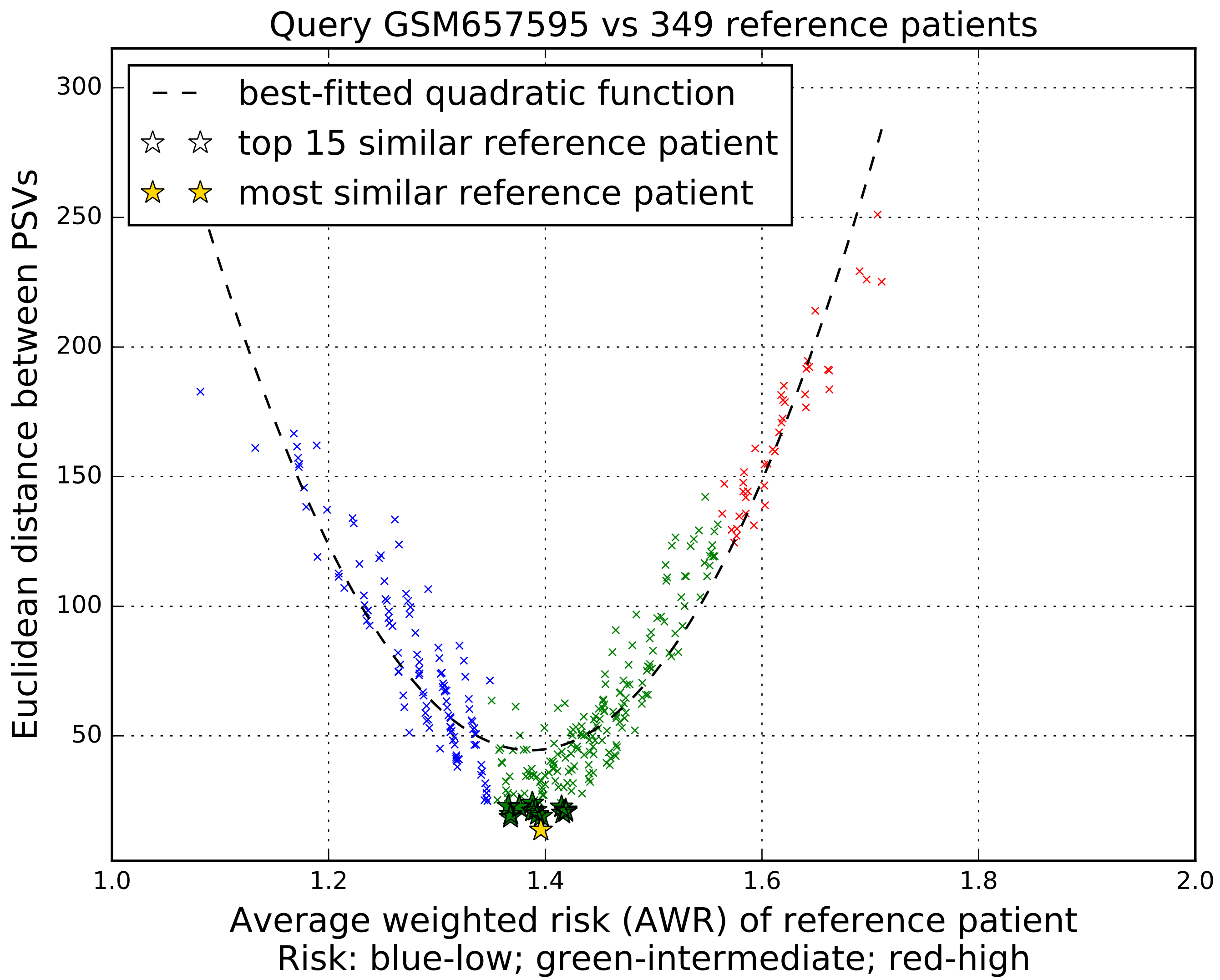

Query GSM657595 vs 349 reference patients
best-fitted quadratic function
top 15 similar reference patient
most similar reference patient
Euclidean distance between PSVs
300
250
200
150
100
50
1.0
1.2
1.4
1.6
1.8
2.0
Average weighted risk (AWR) of reference patient
Risk: blue-low; green-intermediate; red-high

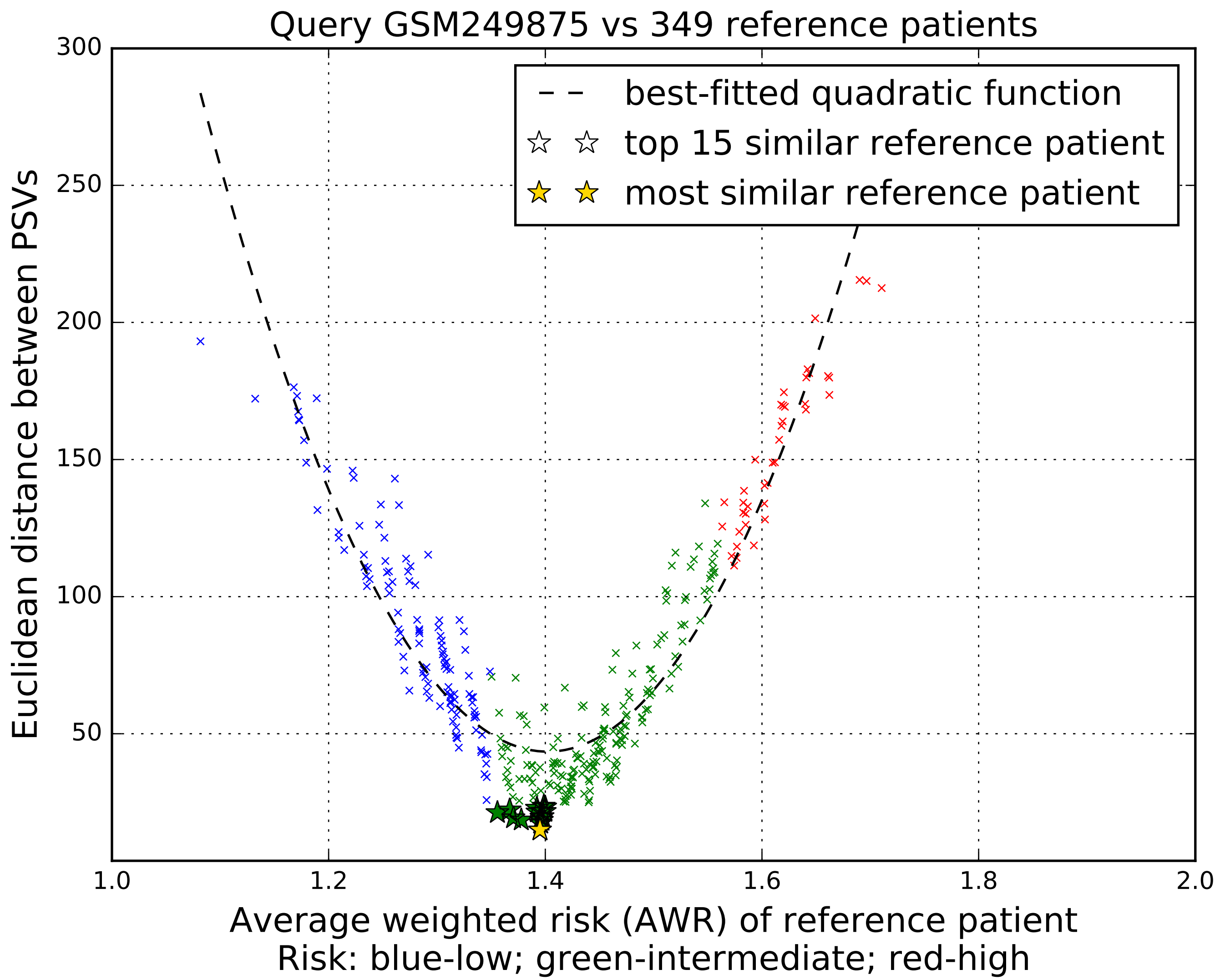
Query GSM249875 vs 349 reference patients
best-fitted quadratic function
top 15 similar reference patient
most similar reference patient
Euclidean distance between PSVs
300
250
200
150
100
50
1.0
1.2
1.4
1.6
1.8
2.0
Average weighted risk (AWR) of reference patient
Risk: blue-low; green-intermediate; red-high

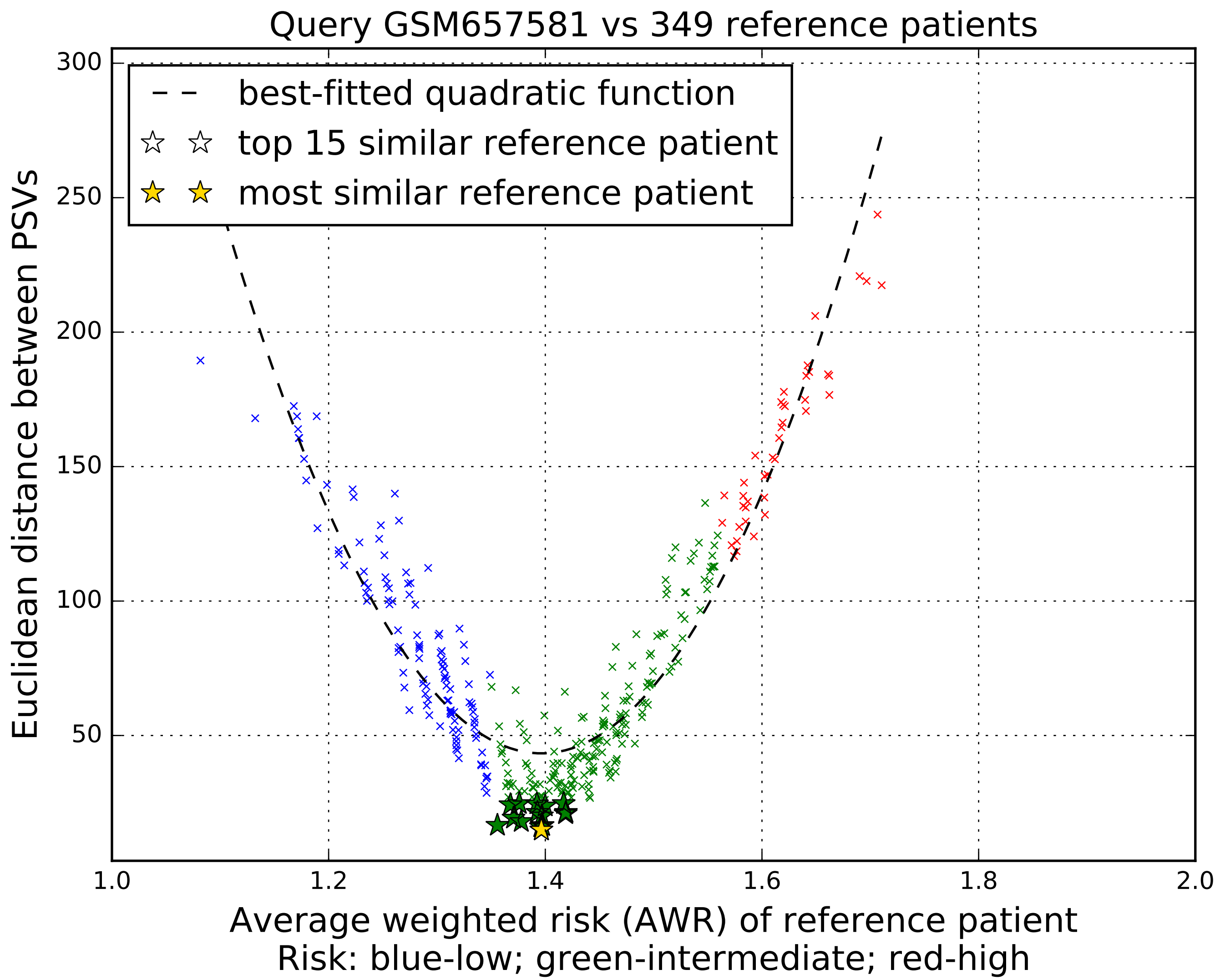

Query GSM657581 vs 349 reference patients
best-fitted quadratic function
top 15 similar reference patient
most similar reference patient
Euclidean distance between PSVs
Average weighted risk (AWR) of reference patient
Risk: blue-low; green-intermediate; red-high
300
250
200
150
100
50
1.0
1.2
1.4
1.6
1.8
2.0

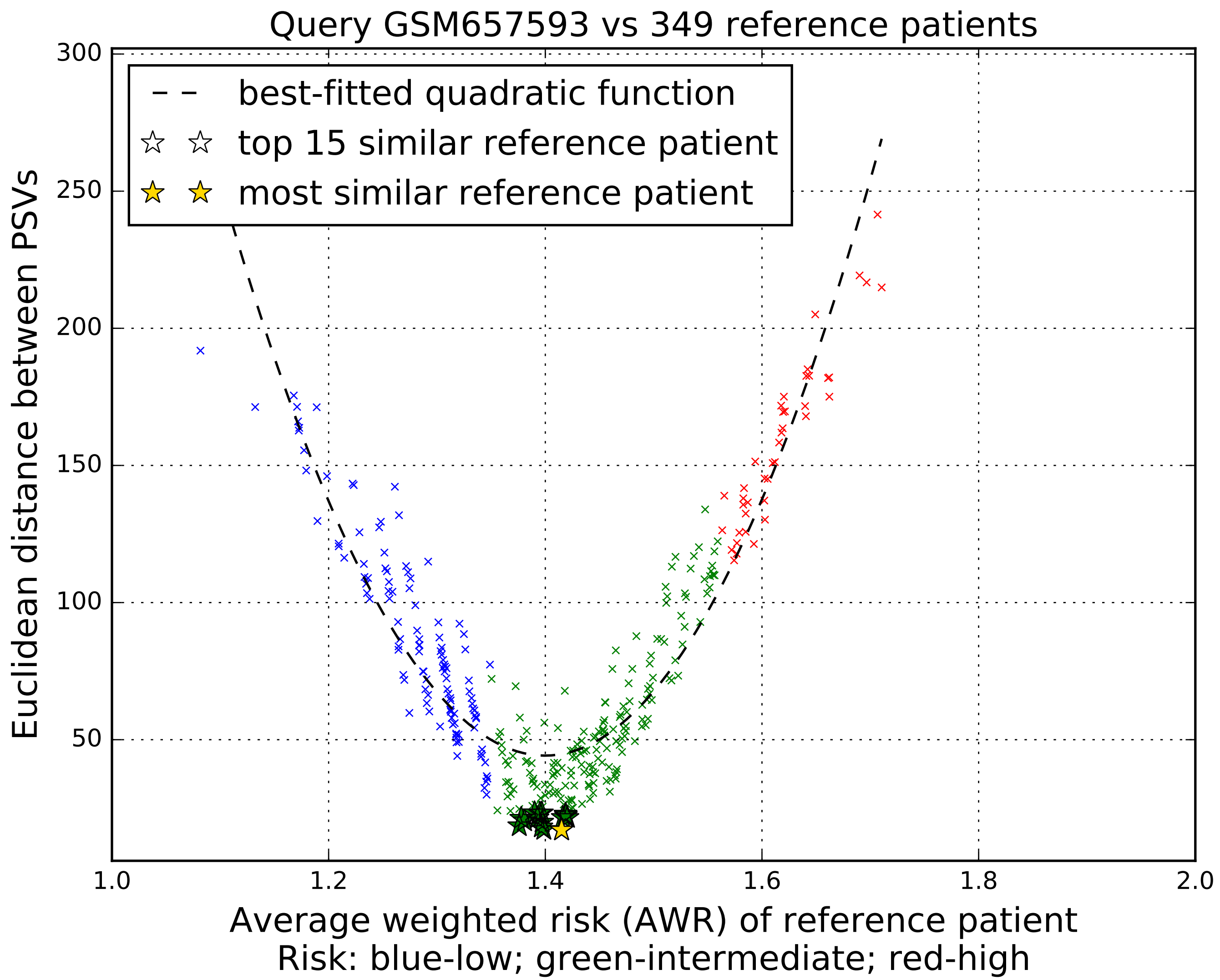
Query GSM657593 vs 349 reference patients
best-fitted quadratic function
top 15 similar reference patient
most similar reference patient
Euclidean distance between PSVs
300
250
200
150
100
50
1.0
1.2
1.4
1.6
1.8
2.0
Average weighted risk (AWR) of reference patient
Risk: blue-low; green-intermediate; red-high

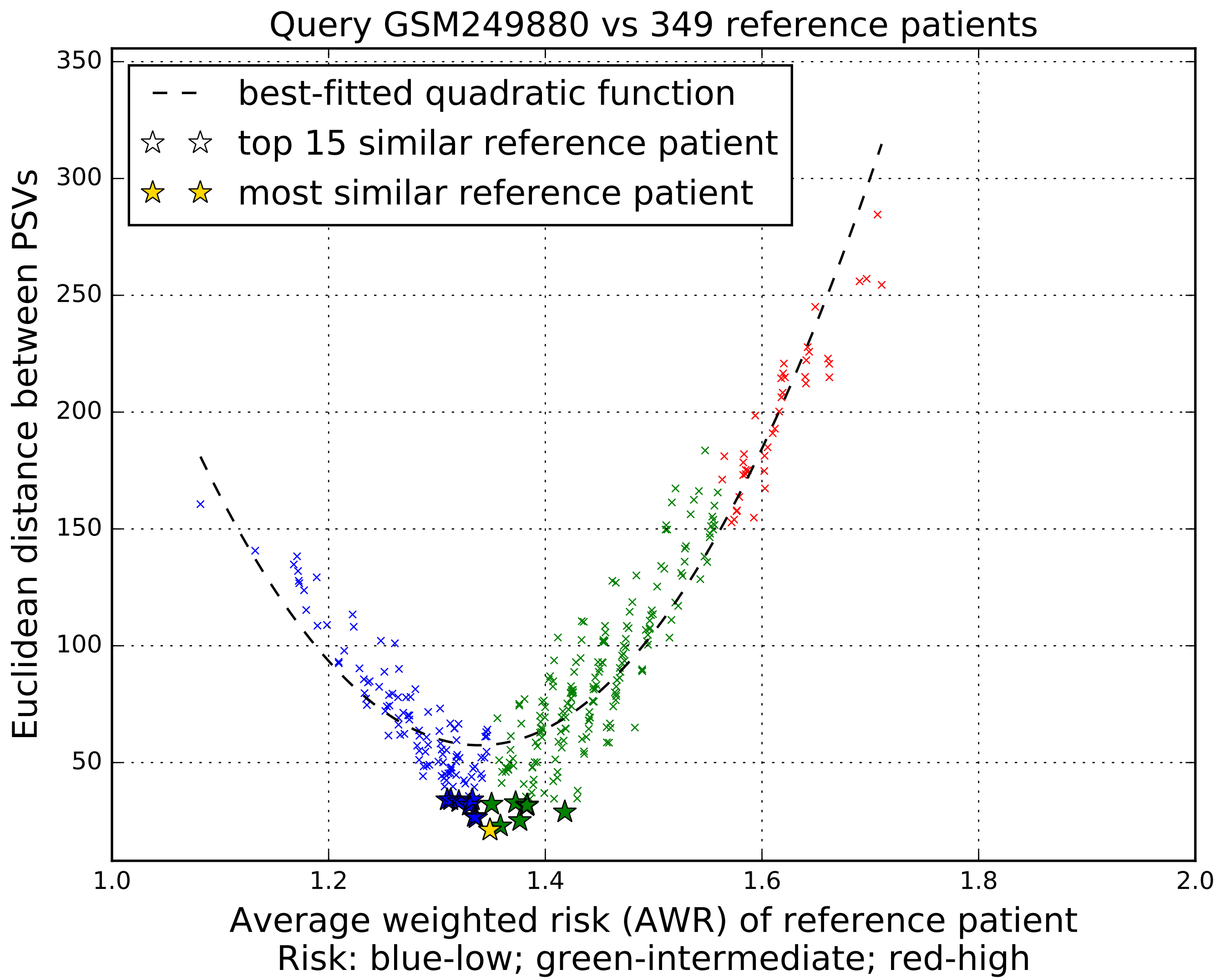
Query GSM249880 vs 349 reference patients
best-fitted quadratic function
top 15 similar reference patient
most similar reference patient
Euclidean distance between PSVs
350
300
250
200
150
100
50
1.0
1.2
1.4
1.6
1.8
2.0
Average weighted risk (AWR) of reference patient
Risk: blue-low; green-intermediate; red-high

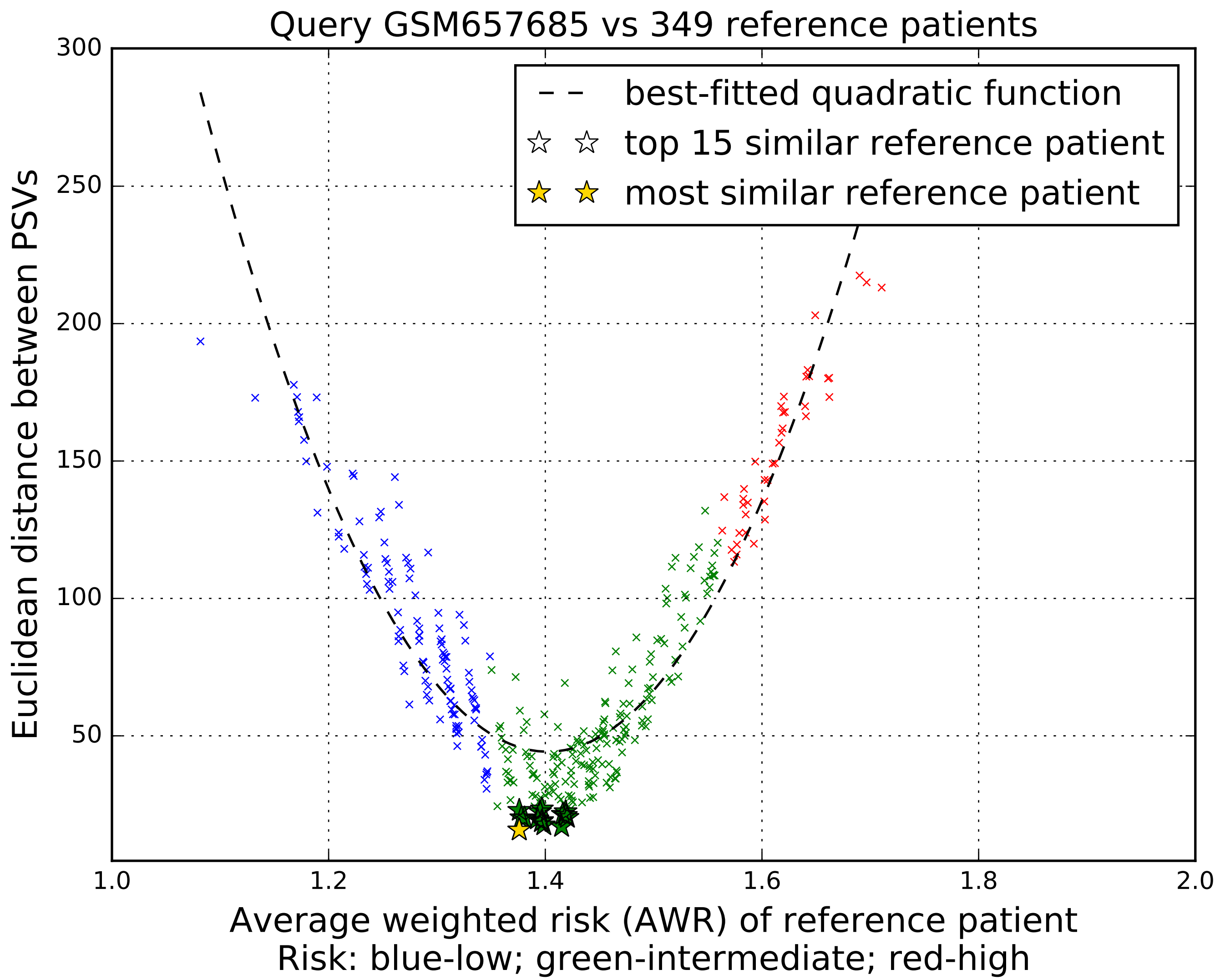

Query GSM657685 vs 349 reference patients
best-fitted quadratic function
top 15 similar reference patient
most similar reference patient
Euclidean distance between PSVs
300
250
200
150
100
50
1.0
1.2
1.4
1.6
1.8
2.0
Average weighted risk (AWR) of reference patient
Risk: blue-low; green-intermediate; red-high

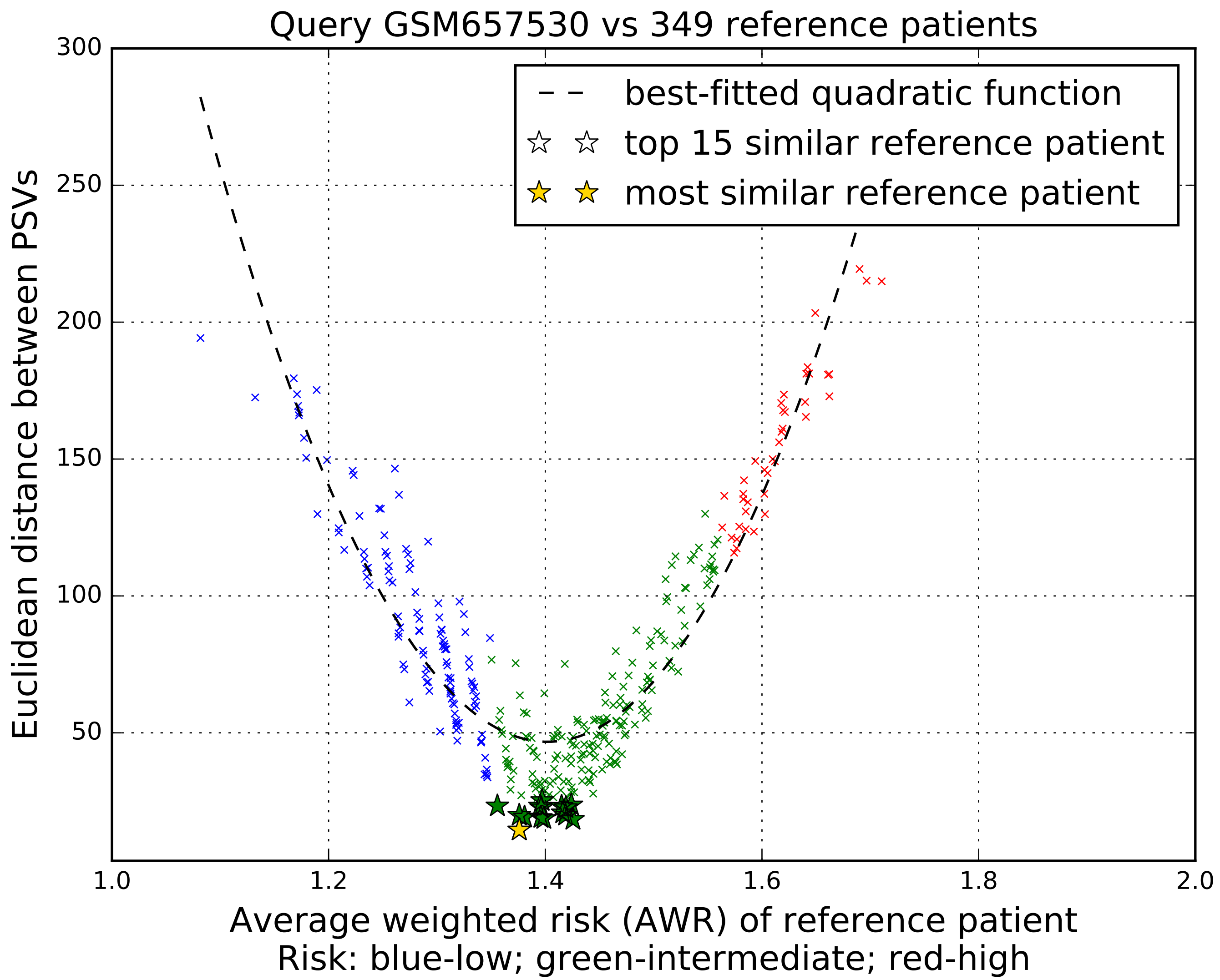
Query GSM657530 vs 349 reference patients
best-fitted quadratic function
top 15 similar reference patient
most similar reference patient
Euclidean distance between PSVs
300
250
200
150
100
50
1.0
1.2
1.4
1.6
1.8
2.0
Average weighted risk (AWR) of reference patient
Risk: blue-low; green-intermediate; red-high

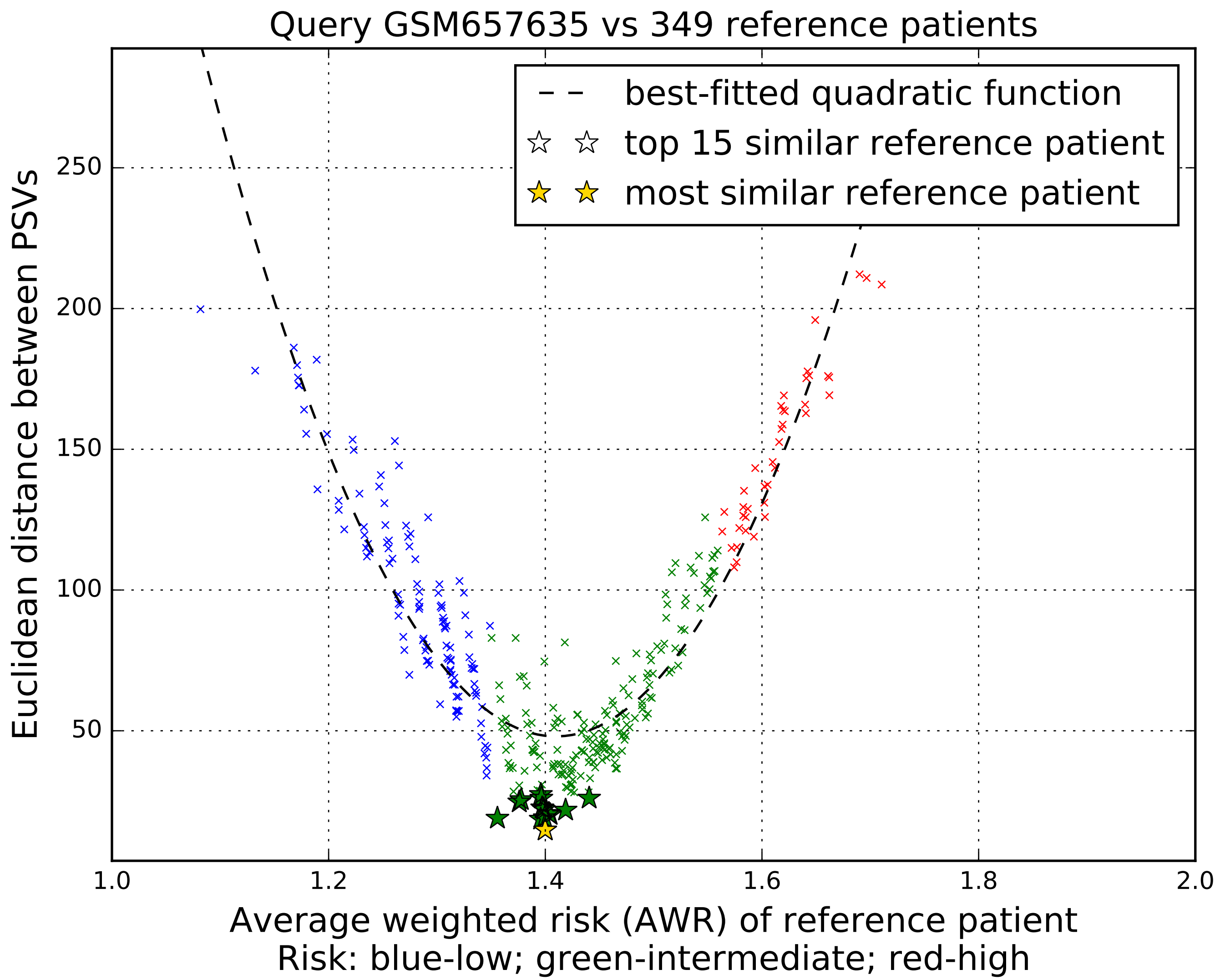

Query GSM657635 vs 349 reference patients
best-fitted quadratic function
top 15 similar reference patient
most similar reference patient
Euclidean distance between PSVs
250
200
150
100
50
1.0
1.2
1.4
1.6
1.8
2.0
Average weighted risk (AWR) of reference patient
Risk: blue-low; green-intermediate; red-high

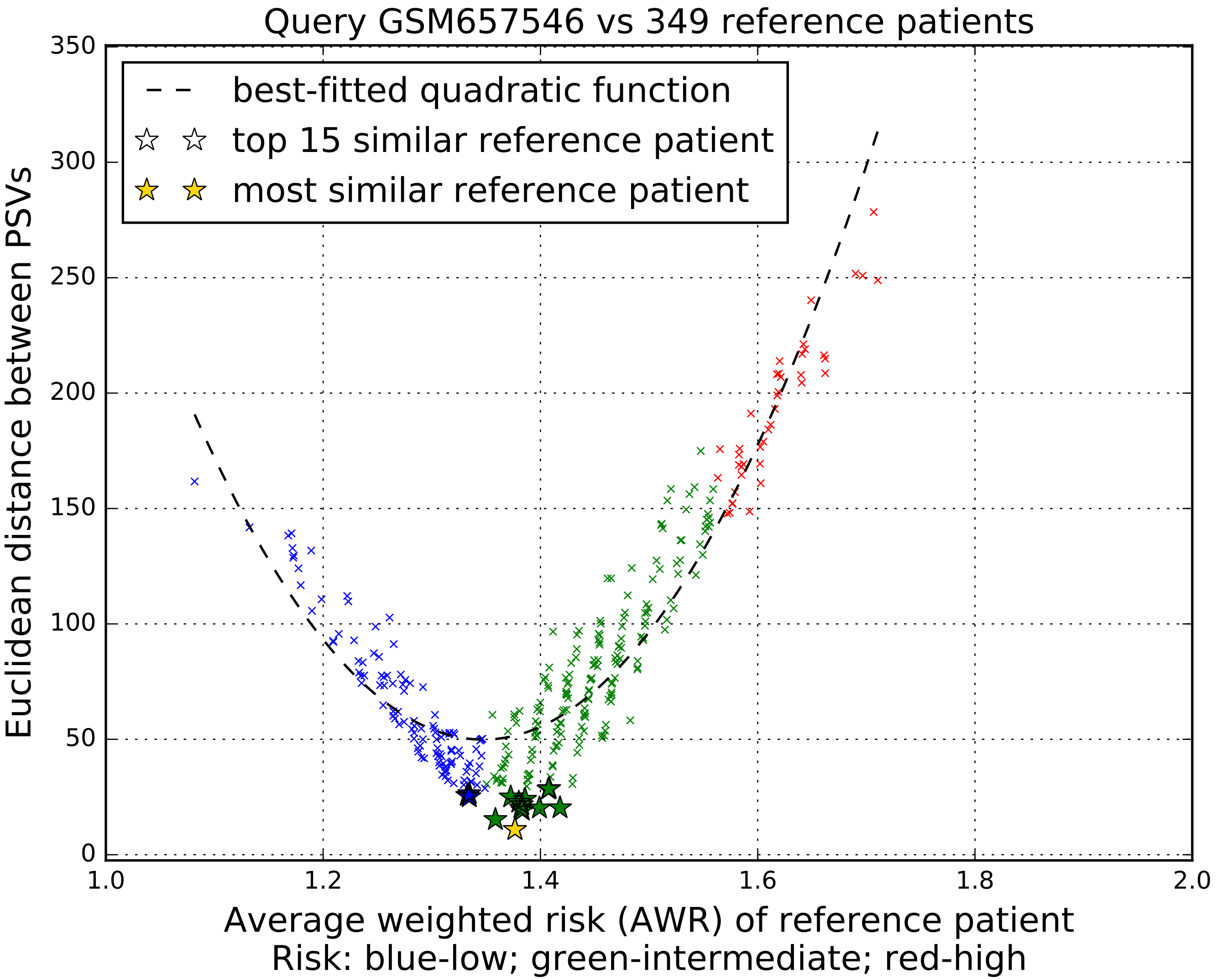

Query GSM657546 vs 349 reference patients
best-fitted quadratic function
top 15 similar reference patient
most similar reference patient
Euclidean distance between PSVs
0
50
100
150
200
250
300
350
1.0
1.2
1.4
1.6
1.8
2.0
Average weighted risk (AWR) of reference patient
Risk: blue-low; green-intermediate; red-high

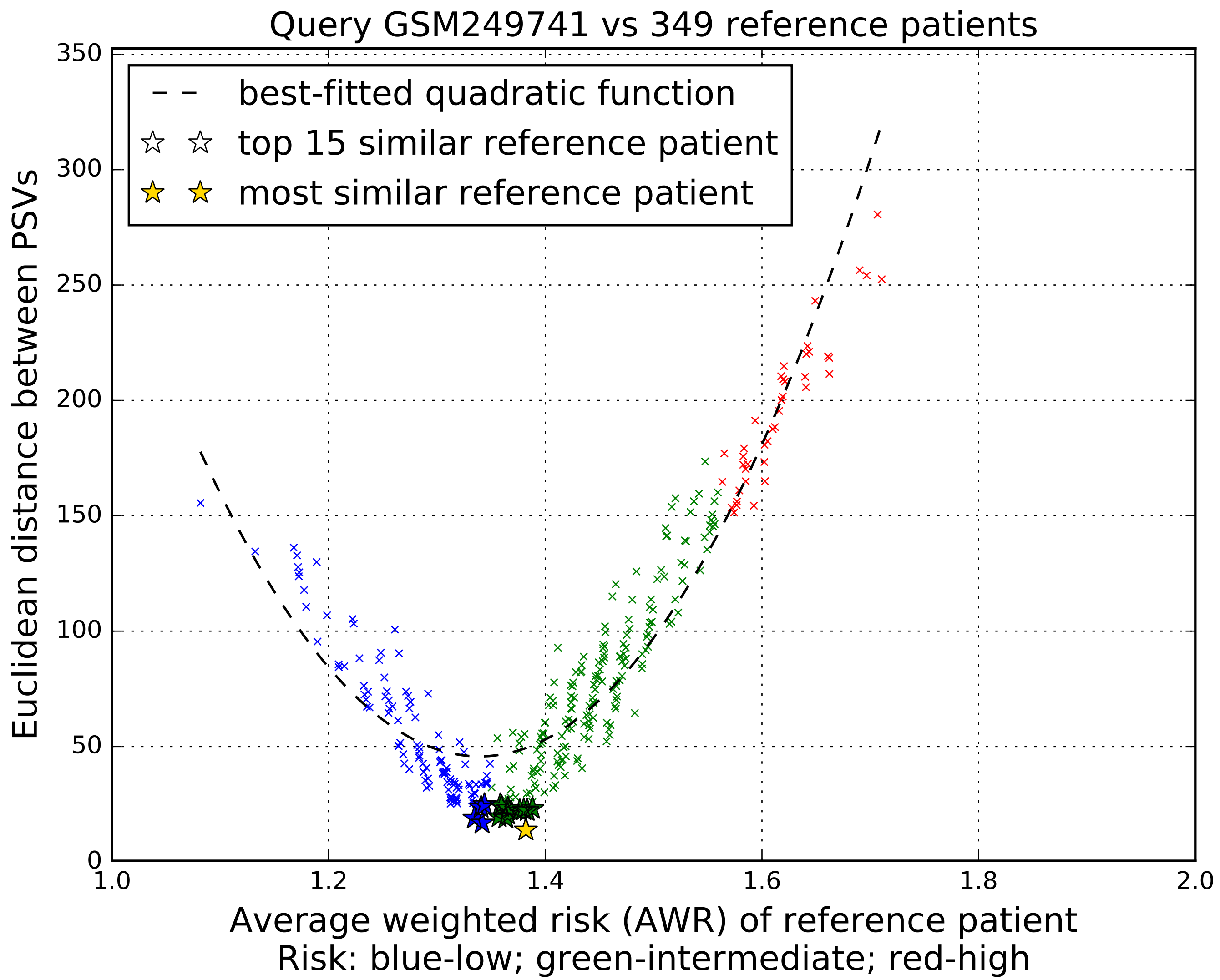

Query GSM249741 vs 349 reference patients
best-fitted quadratic function
top 15 similar reference patient
most similar reference patient
Euclidean distance between PSVs
0
50
100
150
200
250
300
350
1.0
1.2
1.4
1.6
1.8
2.0
Average weighted risk (AWR) of reference patient
Risk: blue-low; green-intermediate; red-high

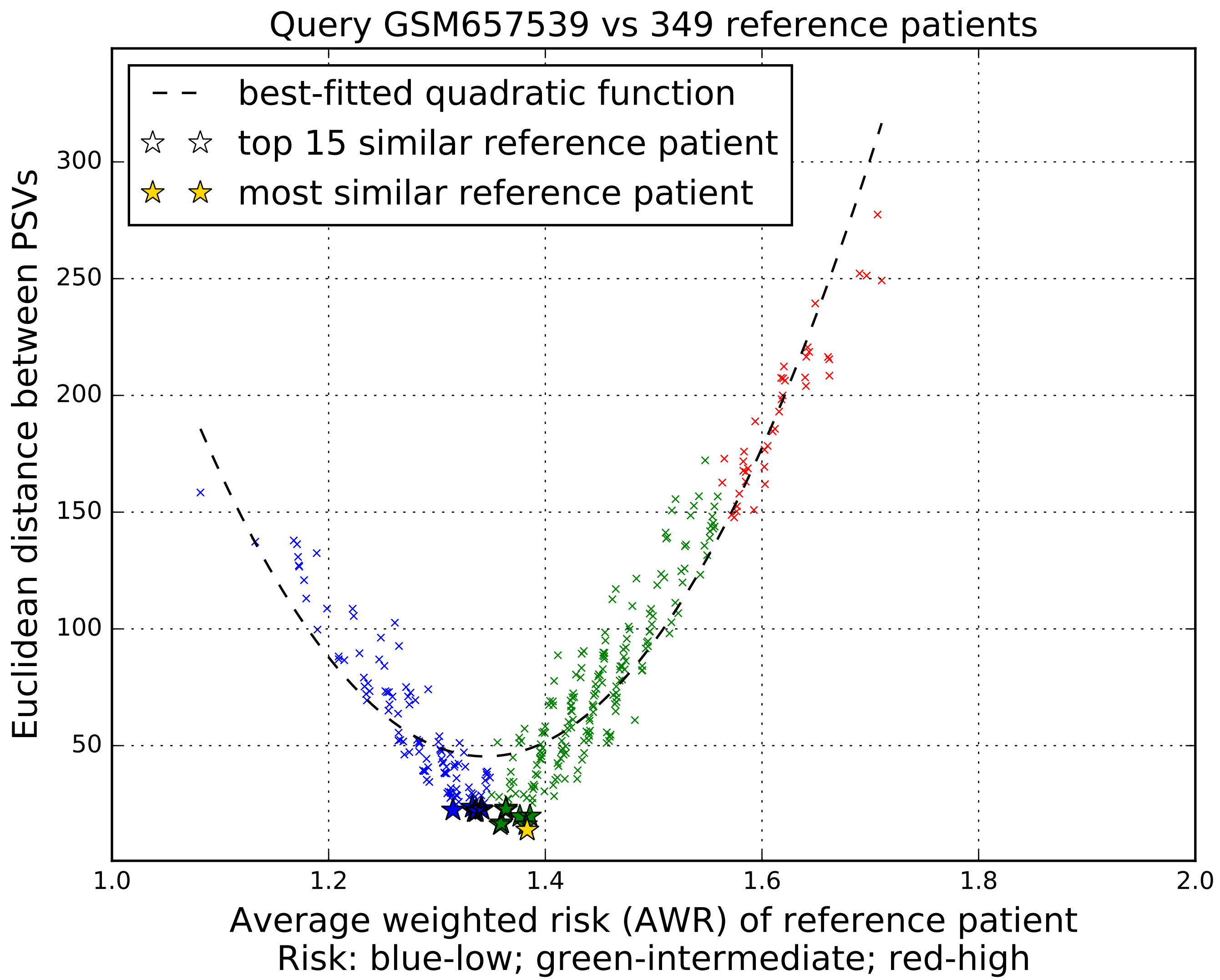
Query GSM657539 vs 349 reference patients
best-fitted quadratic function
top 15 similar reference patient
most similar reference patient
Euclidean distance between PSVs
300
250
200
150
100
50
1.0
1.2
1.4
1.6
1.8
2.0
Average weighted risk (AWR) of reference patient
Risk: blue-low; green-intermediate; red-high

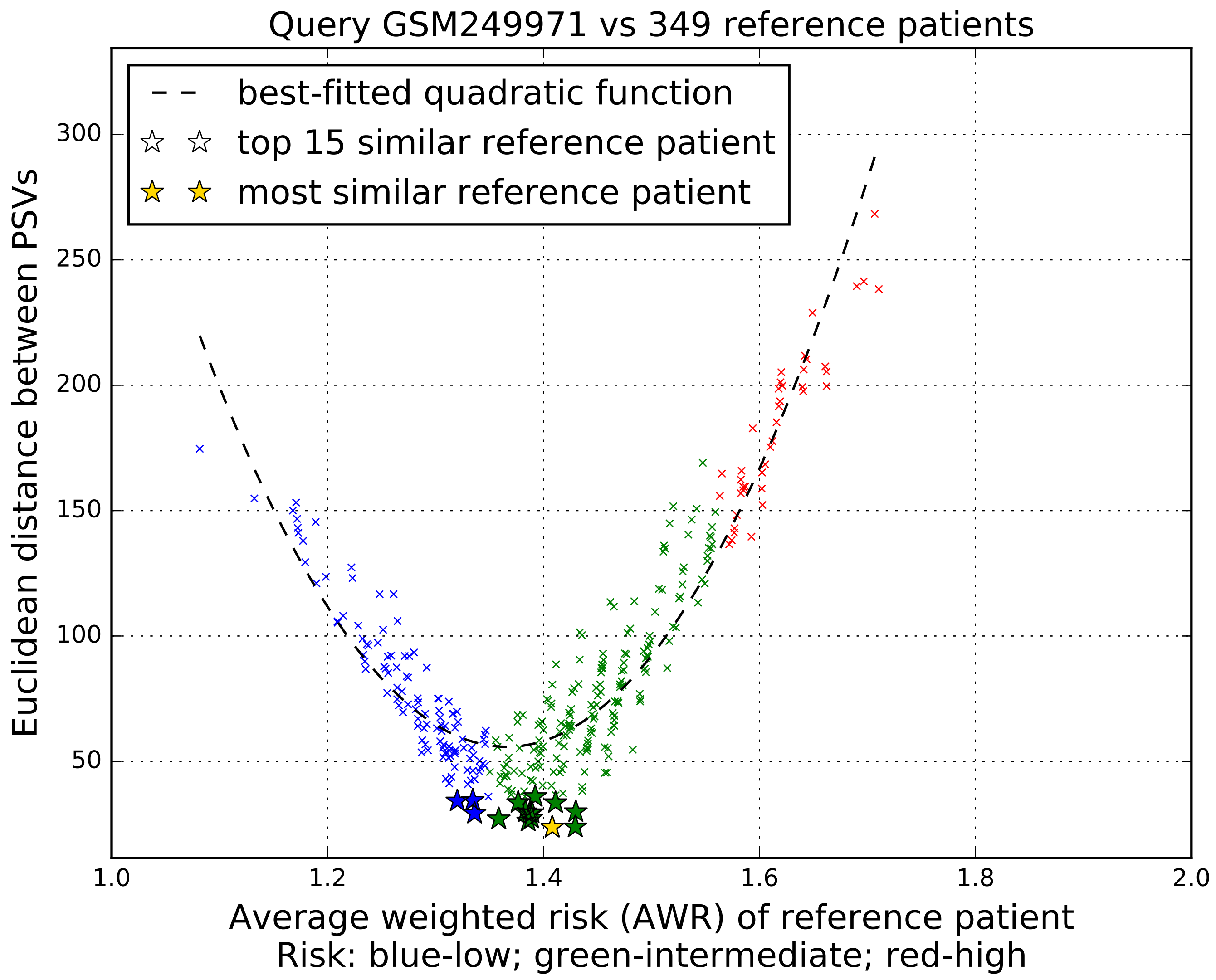

Query GSM249971 vs 349 reference patients
best-fitted quadratic function
top 15 similar reference patient
most similar reference patient
Euclidean distance between PSVs
300
250
200
150
100
50
1.0
1.2
1.4
1.6
1.8
2.0
Average weighted risk (AWR) of reference patient
Risk: blue-low; green-intermediate; red-high

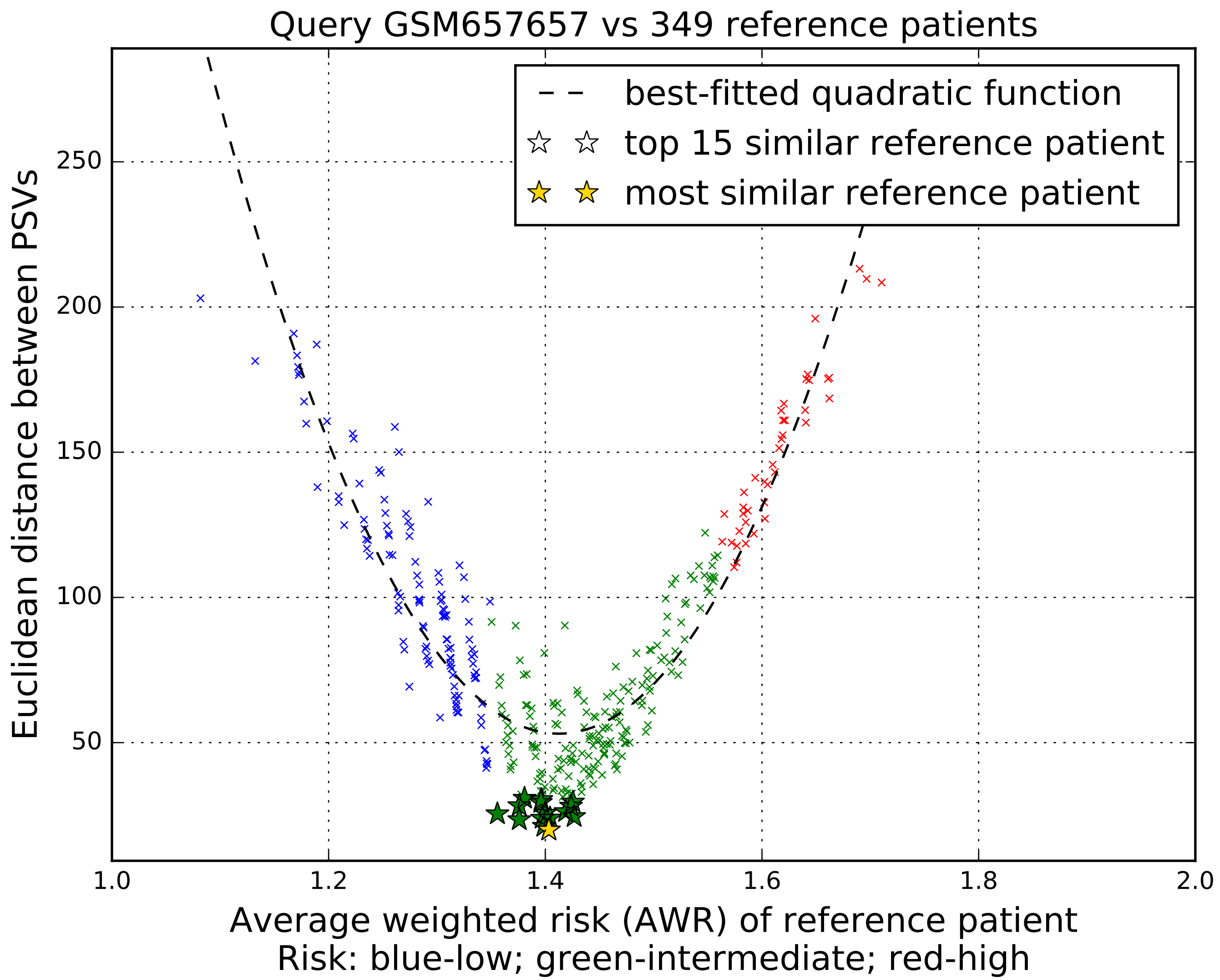

Query GSM657657 vs 349 reference patients
best-fitted quadratic function
top 15 similar reference patient
most similar reference patient
Euclidean distance between PSVs
250
200
150
100
50
1.0
1.2
1.4
1.6
1.8
2.0
Average weighted risk (AWR) of reference patient
Risk: blue-low; green-intermediate; red-high

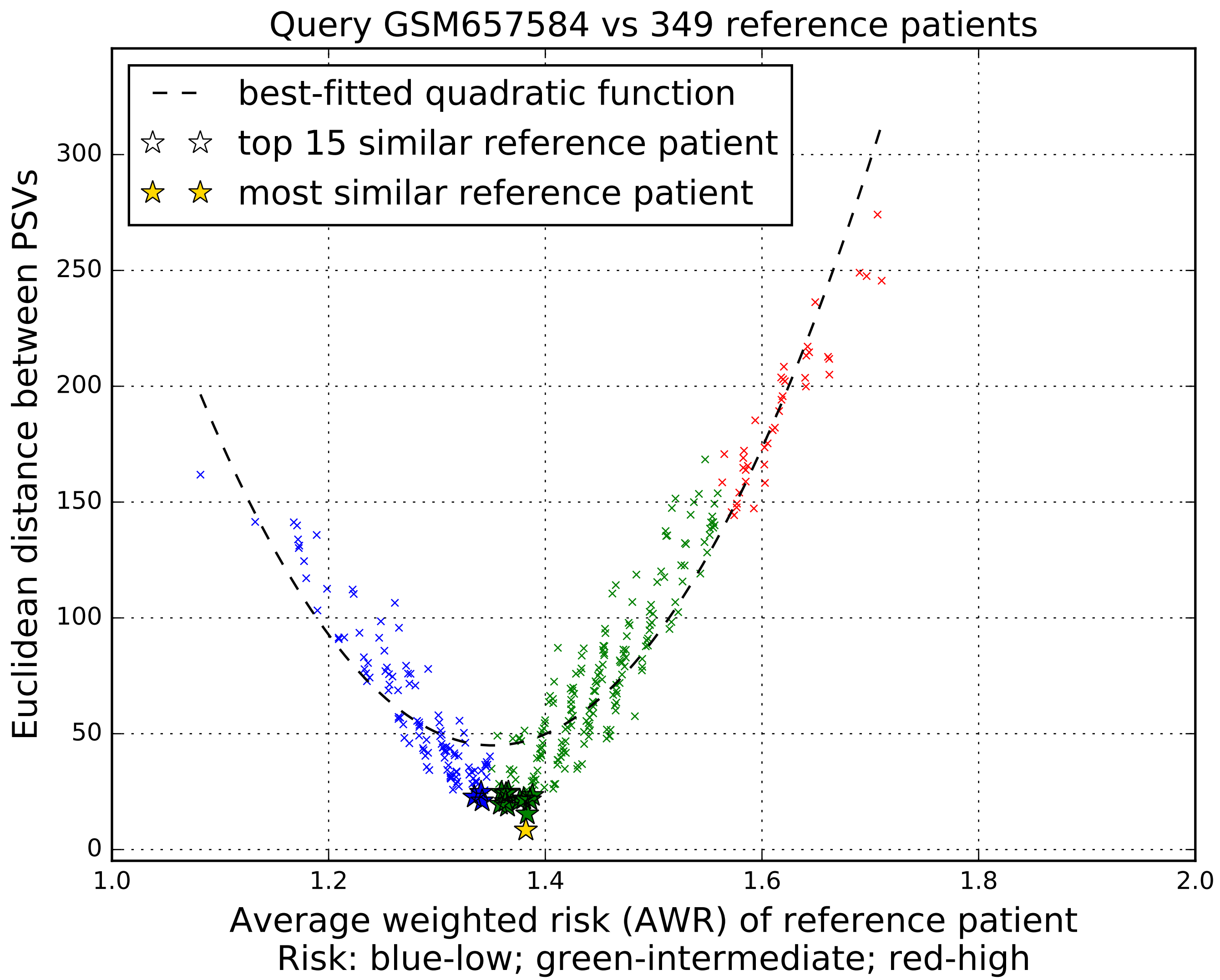
Query GSM657584 vs 349 reference patients
best-fitted quadratic function
top 15 similar reference patient
most similar reference patient
Euclidean distance between PSVs
0
50
100
150
200
250
300
1.0
1.2
1.4
1.6
1.8
2.0
Average weighted risk (AWR) of reference patient
Risk: blue-low; green-intermediate; red-high

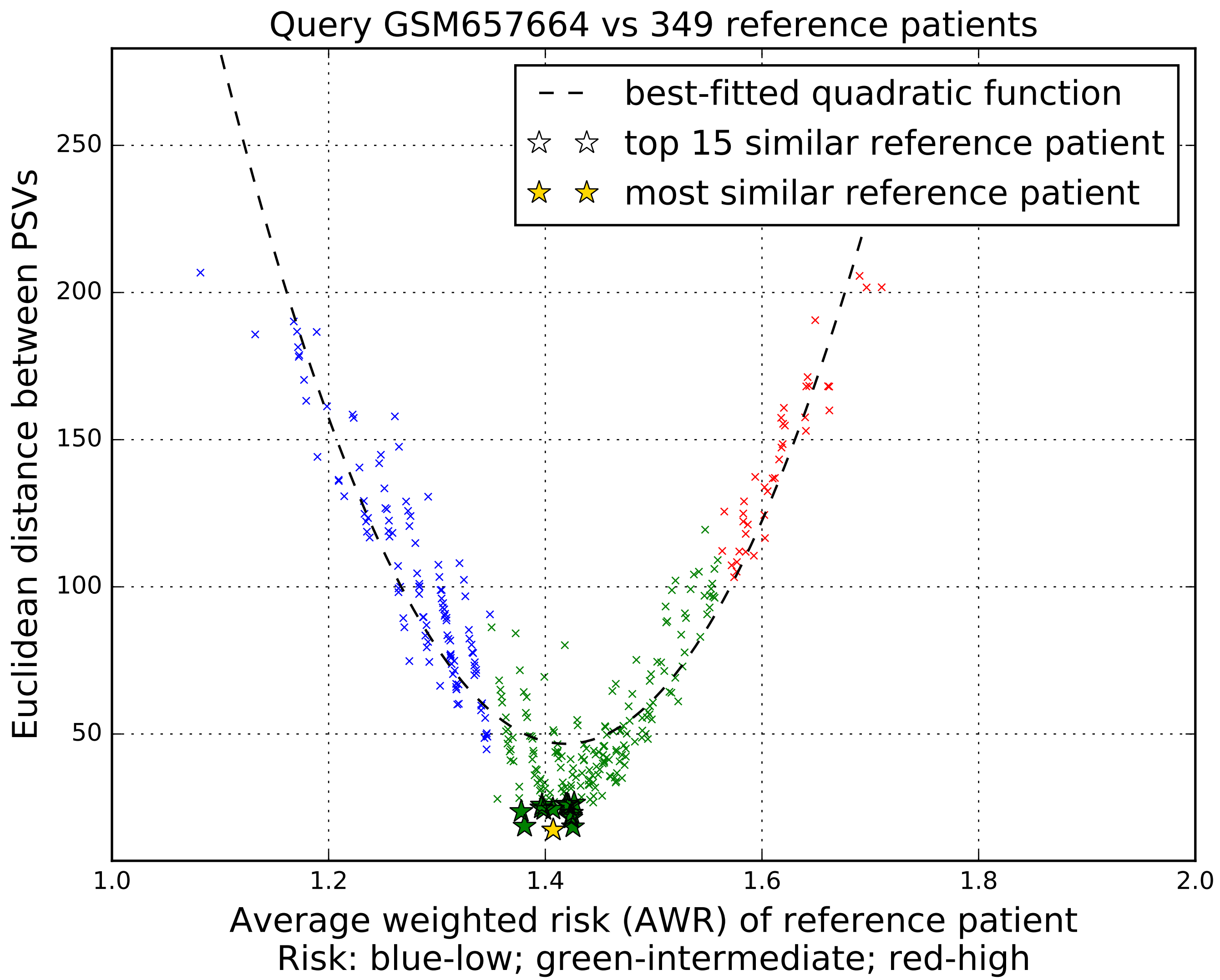
Query GSM657664 vs 349 reference patients
best-fitted quadratic function
top 15 similar reference patient
most similar reference patient
Euclidean distance between PSVs
Average weighted risk (AWR) of reference patient
Risk: blue-low; green-intermediate; red-high
250
200
150
100
50
1.0
1.2
1.4
1.6
1.8
2.0

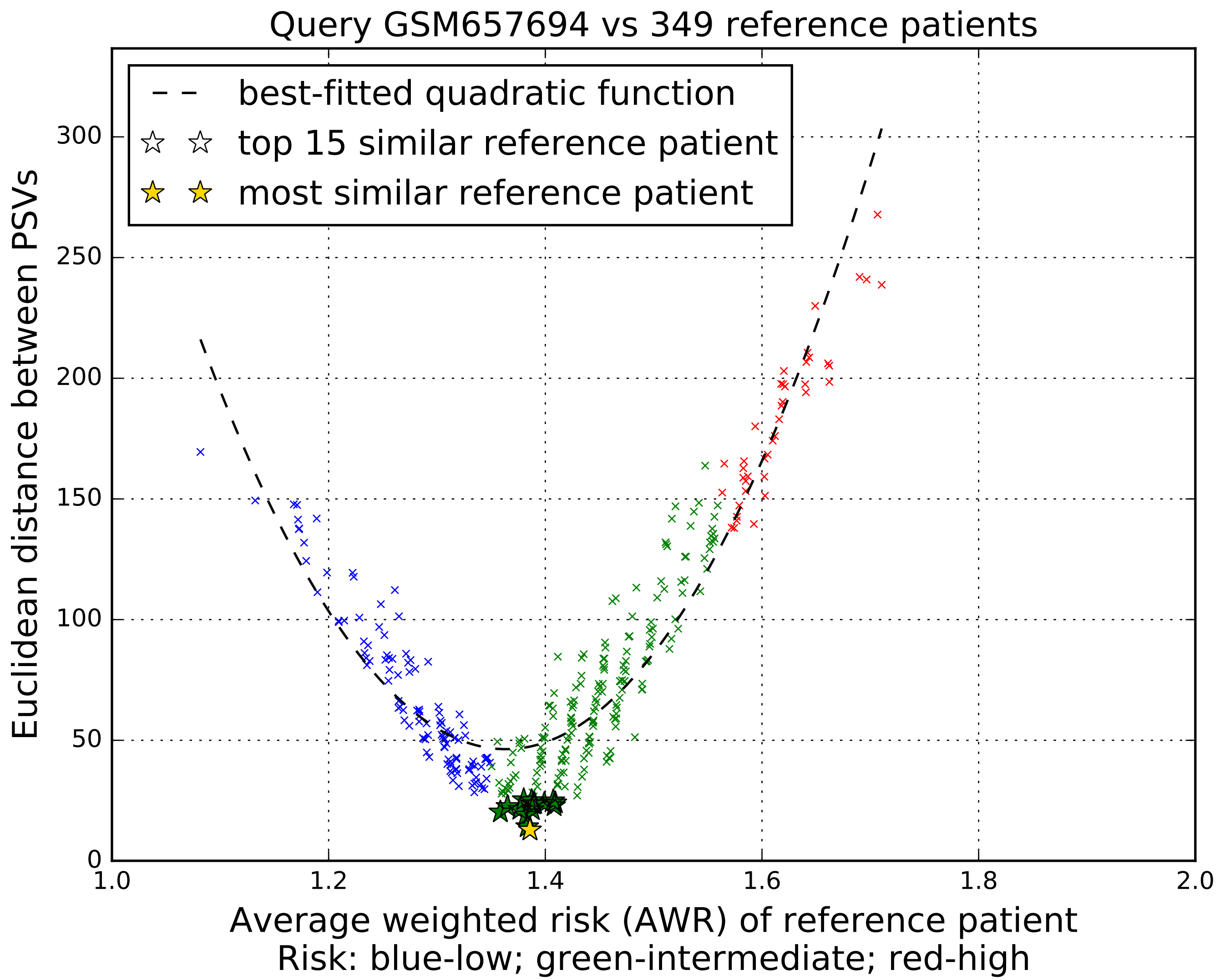
Query GSM657694 vs 349 reference patients
best-fitted quadratic function
top 15 similar reference patient
most similar reference patient
Euclidean distance between PSVs
0
50
100
150
200
250
300
1.0
1.2
1.4
1.6
1.8
2.0
Average weighted risk (AWR) of reference patient
Risk: blue-low; green-intermediate; red-high

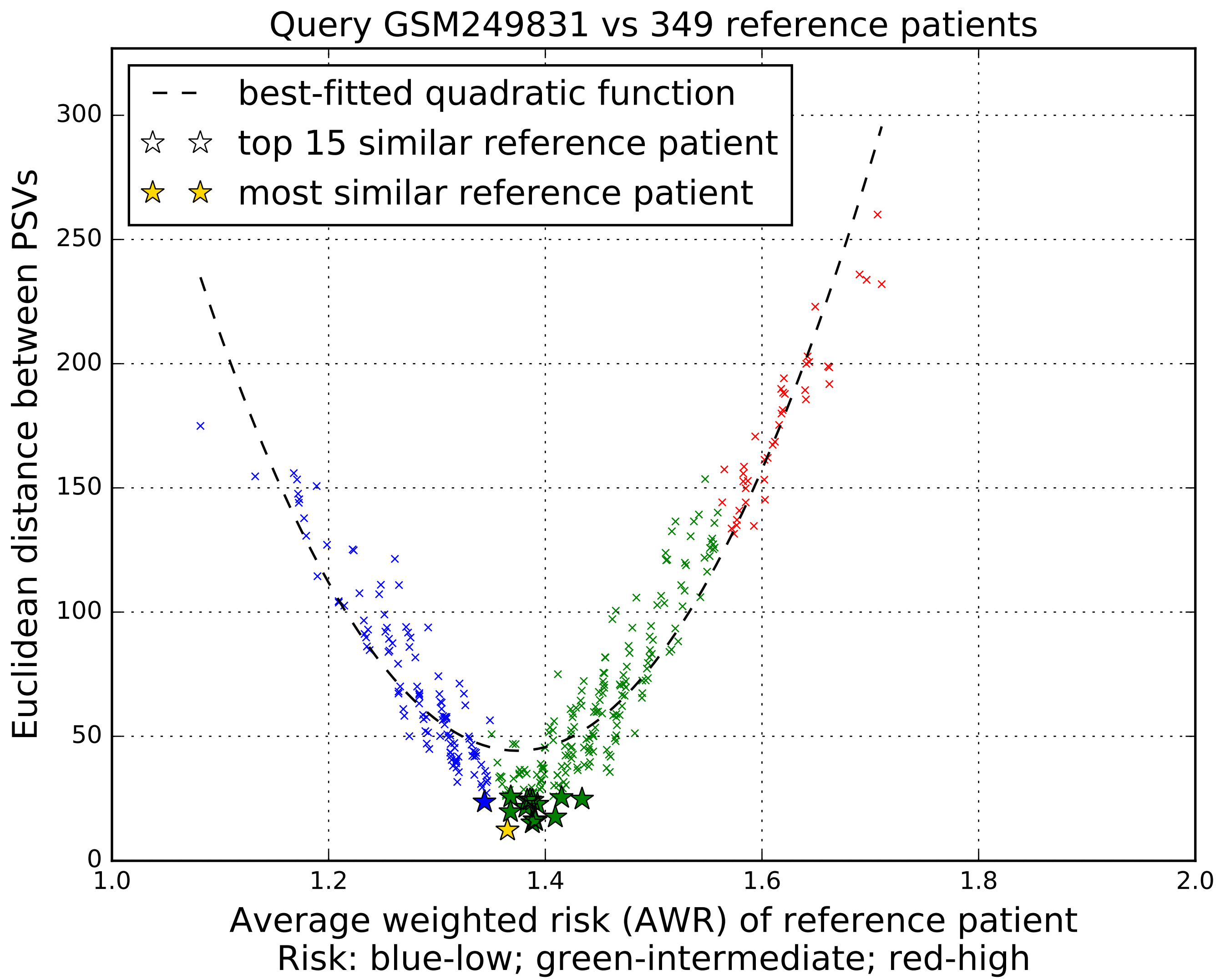

Query GSM249831 vs 349 reference patients
best-fitted quadratic function
top 15 similar reference patient
most similar reference patient
Euclidean distance between PSVs
Average weighted risk (AWR) of reference patient
Risk: blue-low; green-intermediate; red-high
0
50
100
150
200
250
300
1.0
1.2
1.4
1.6
1.8
2.0

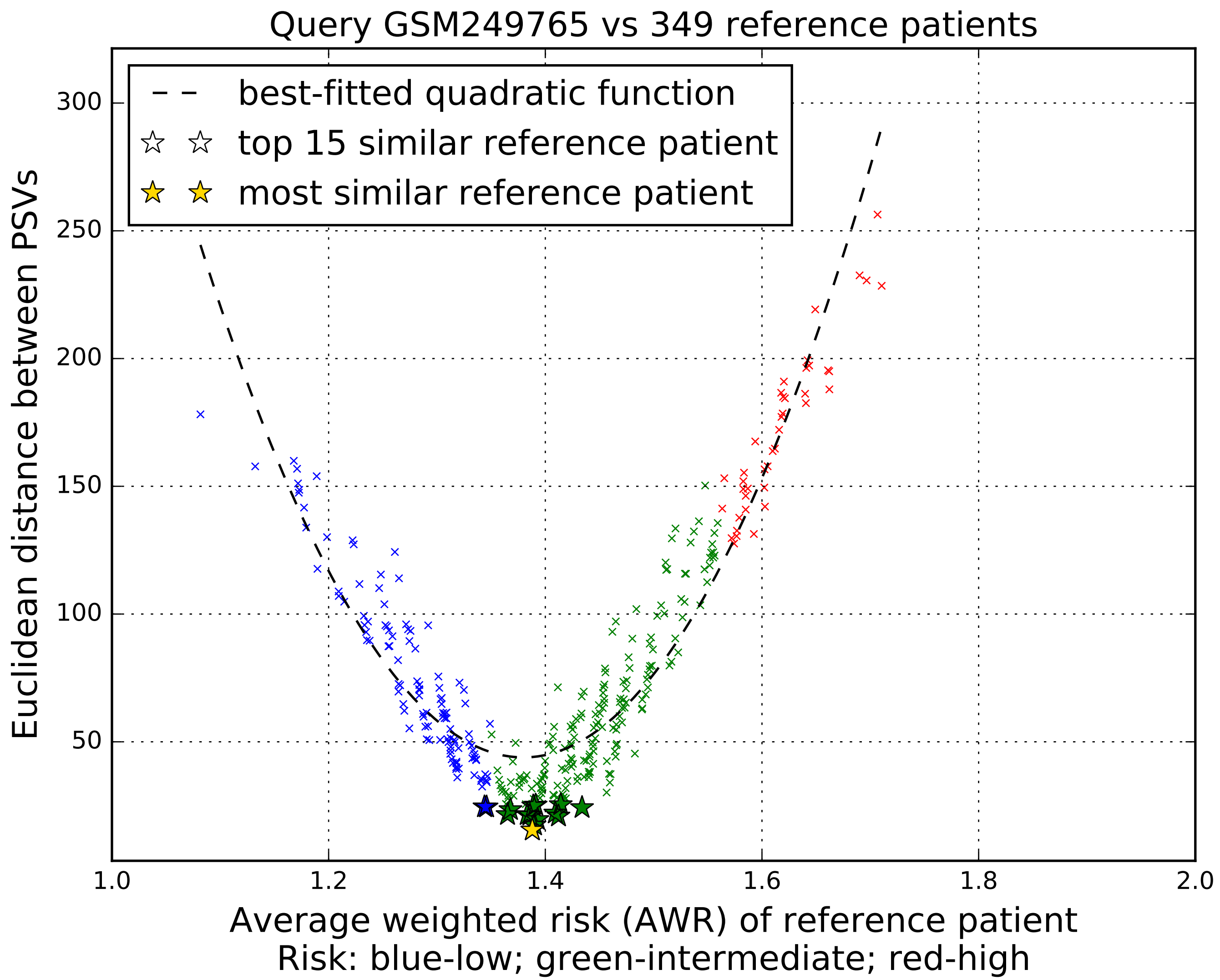

Query GSM249765 vs 349 reference patients
best-fitted quadratic function
top 15 similar reference patient
most similar reference patient
Euclidean distance between PSVs
300
250
200
150
100
50
1.0
1.2
1.4
1.6
1.8
2.0
Average weighted risk (AWR) of reference patient
Risk: blue-low; green-intermediate; red-high

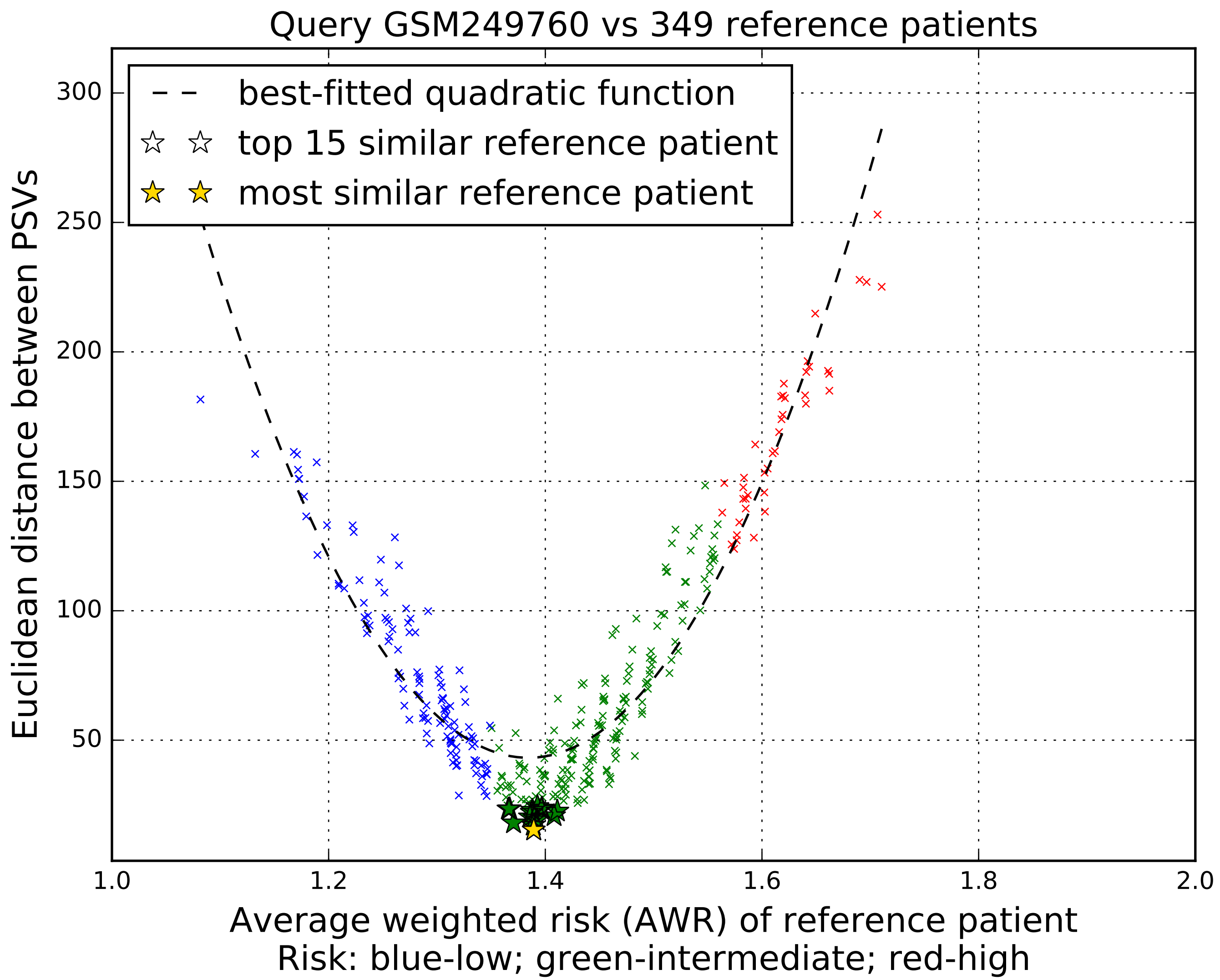
Query GSM249760 vs 349 reference patients
best-fitted quadratic function
top 15 similar reference patient
most similar reference patient
Euclidean distance between PSVs
300
250
200
150
100
50
1.0
1.2
1.4
1.6
1.8
2.0
Average weighted risk (AWR) of reference patient
Risk: blue-low; green-intermediate; red-high

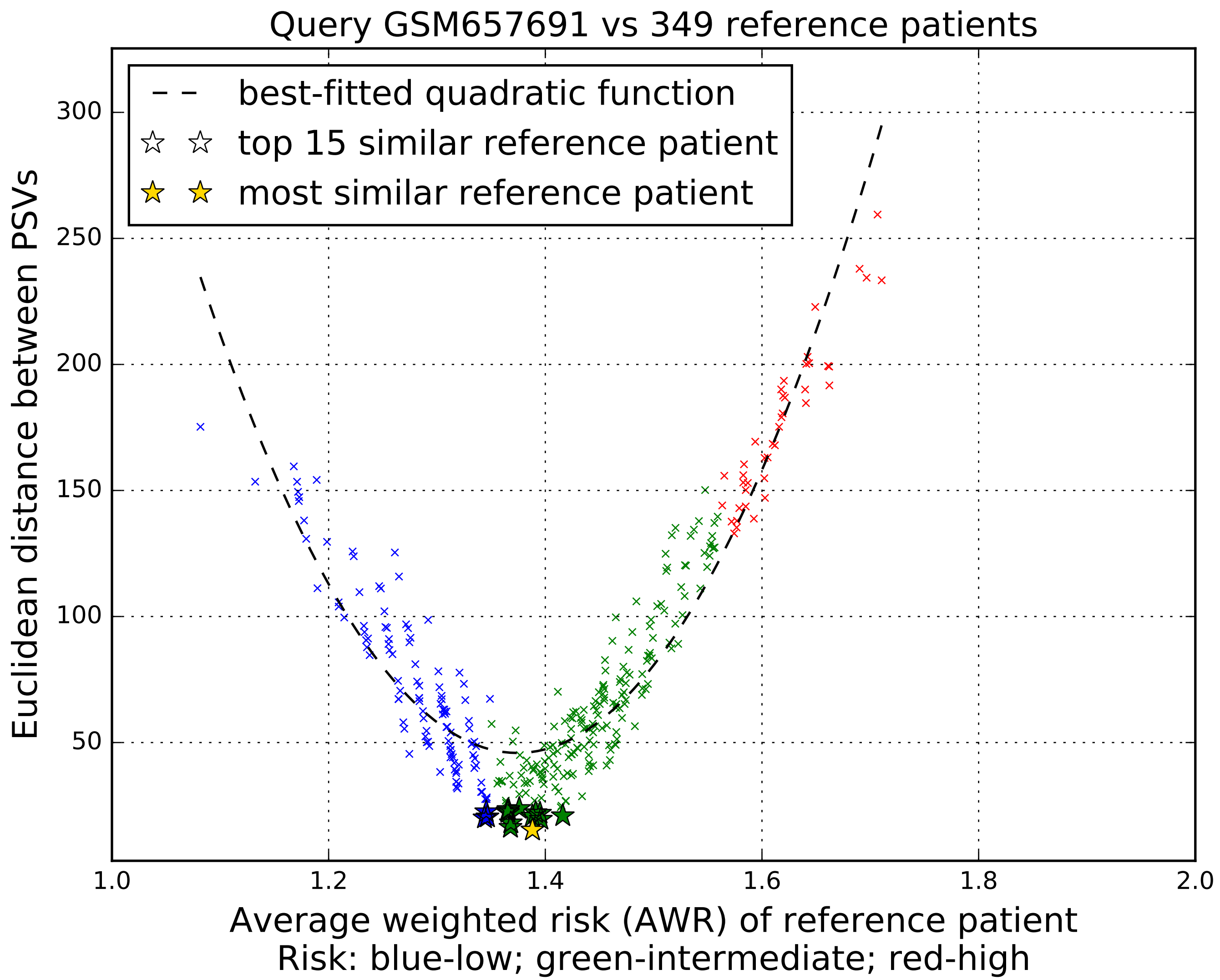
Query GSM657691 vs 349 reference patients
best-fitted quadratic function
top 15 similar reference patient
most similar reference patient
Euclidean distance between PSVs
300
250
200
150
100
50
1.0
1.2
1.4
1.6
1.8
2.0
Average weighted risk (AWR) of reference patient
Risk: blue-low; green-intermediate; red-high

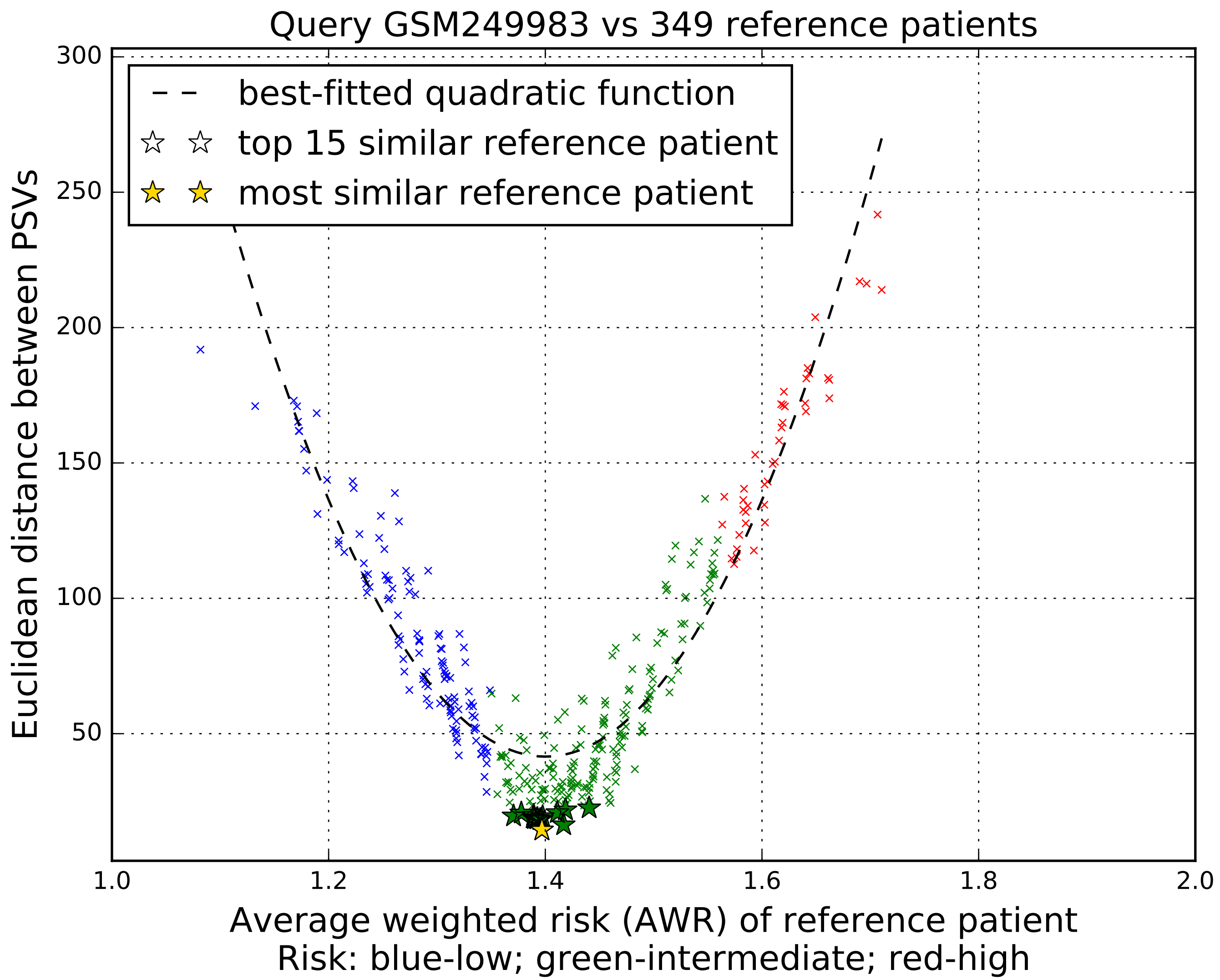

Query GSM249983 vs 349 reference patients
best-fitted quadratic function
top 15 similar reference patient
most similar reference patient
Euclidean distance between PSVs
300
250
200
150
100
50
1.0
1.2
1.4
1.6
1.8
2.0
Average weighted risk (AWR) of reference patient
Risk: blue-low; green-intermediate; red-high

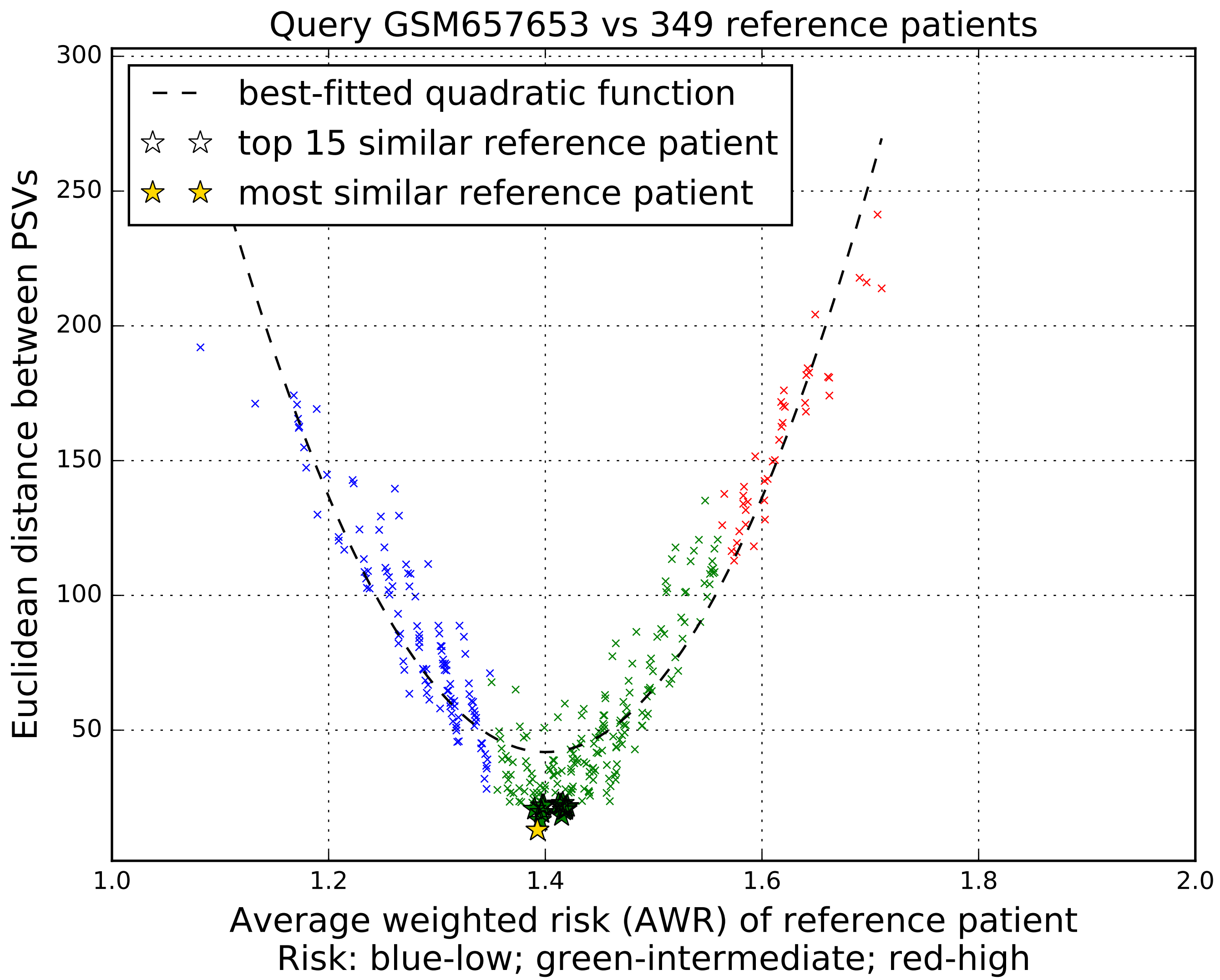

Query GSM657653 vs 349 reference patients
best-fitted quadratic function
top 15 similar reference patient
most similar reference patient
Euclidean distance between PSVs
300
250
200
150
100
50
1.0
1.2
1.4
1.6
1.8
2.0
Average weighted risk (AWR) of reference patient
Risk: blue-low; green-intermediate; red-high

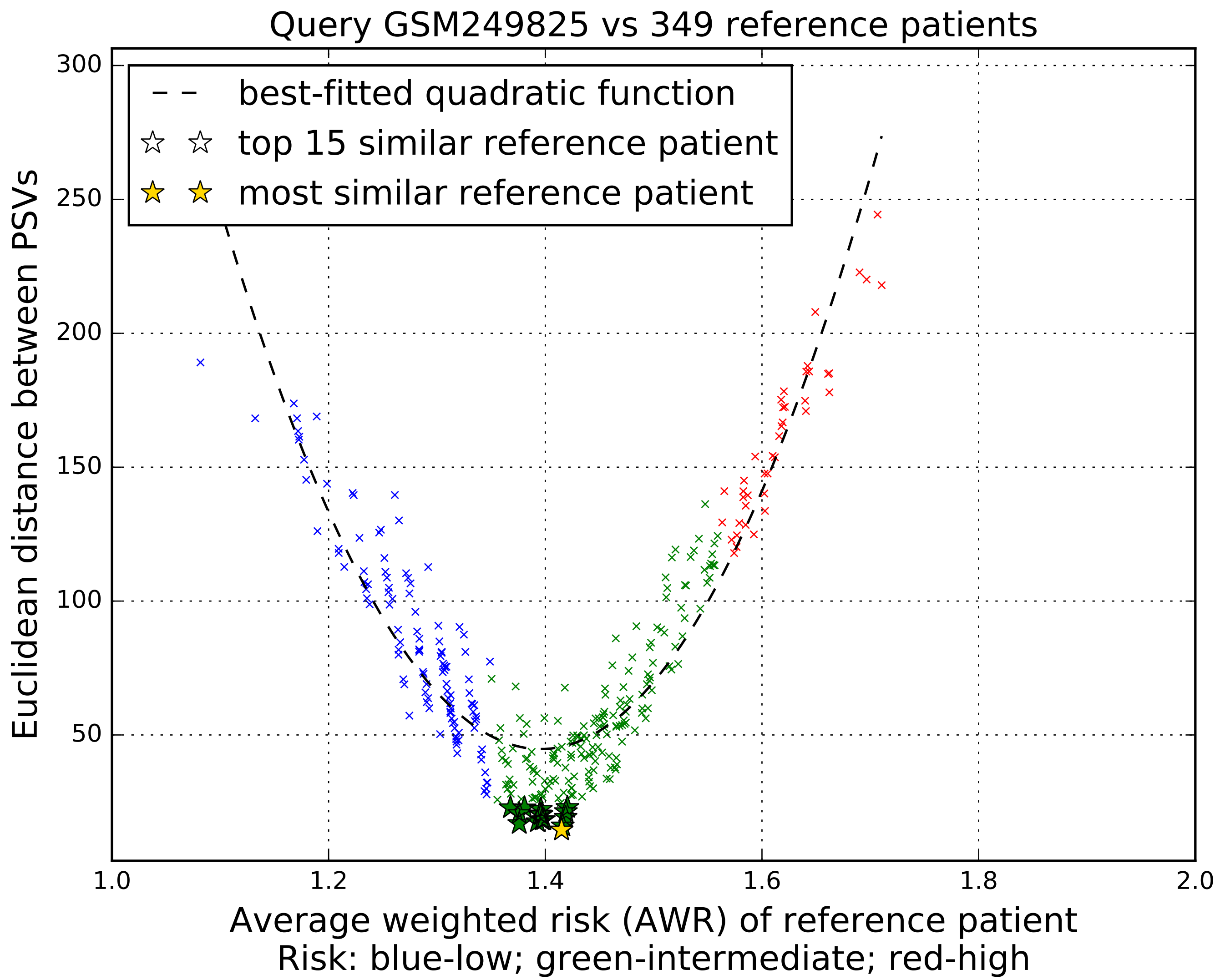

Query GSM249825 vs 349 reference patients
best-fitted quadratic function
top 15 similar reference patient
most similar reference patient
Euclidean distance between PSVs
Average weighted risk (AWR) of reference patient
Risk: blue-low; green-intermediate; red-high
300
250
200
150
100
50
1.0
1.2
1.4
1.6
1.8
2.0

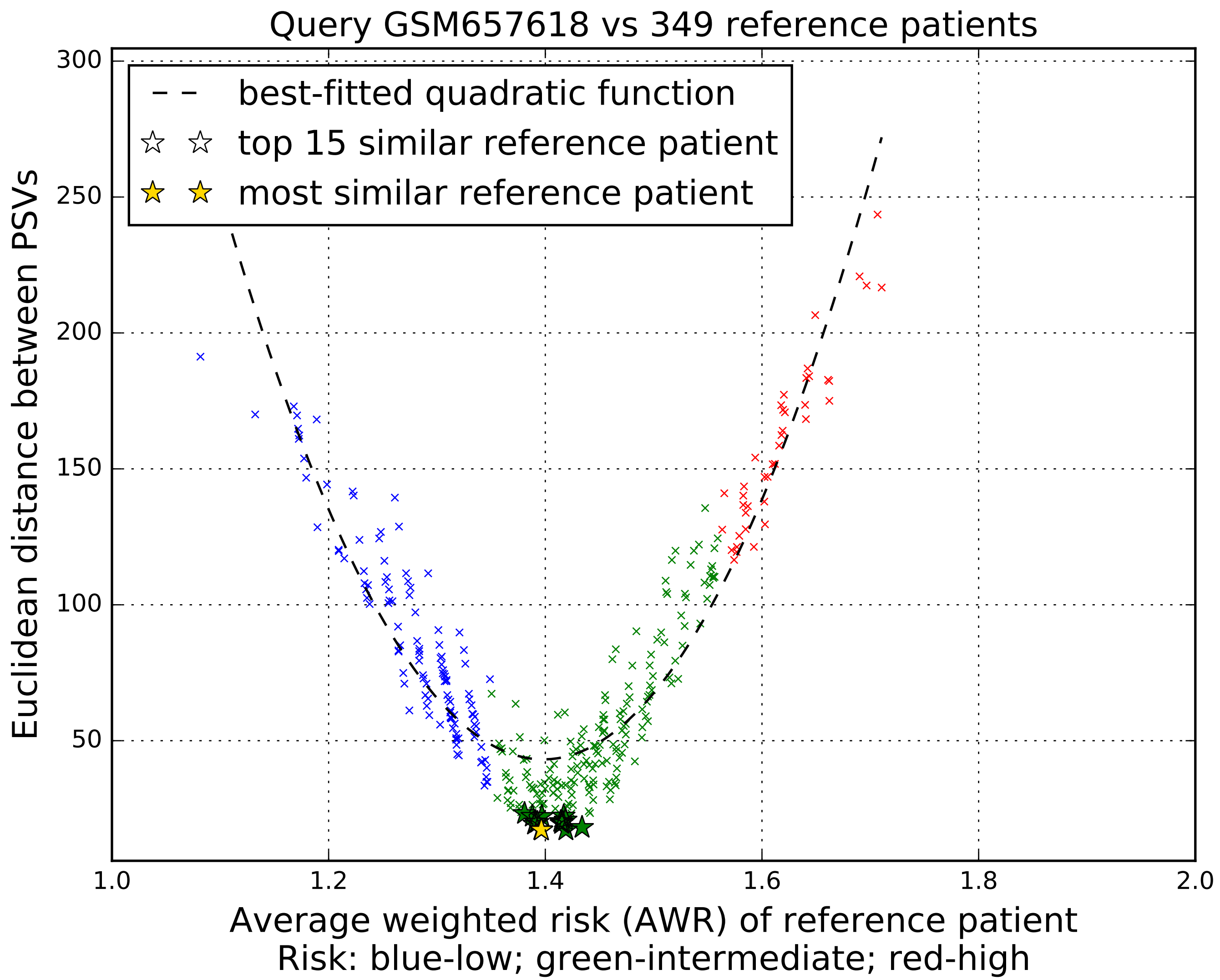

Query GSM657618 vs 349 reference patients
best-fitted quadratic function
top 15 similar reference patient
most similar reference patient
Euclidean distance between PSVs
300
250
200
150
100
50
1.0
1.2
1.4
1.6
1.8
2.0
Average weighted risk (AWR) of reference patient
Risk: blue-low; green-intermediate; red-high

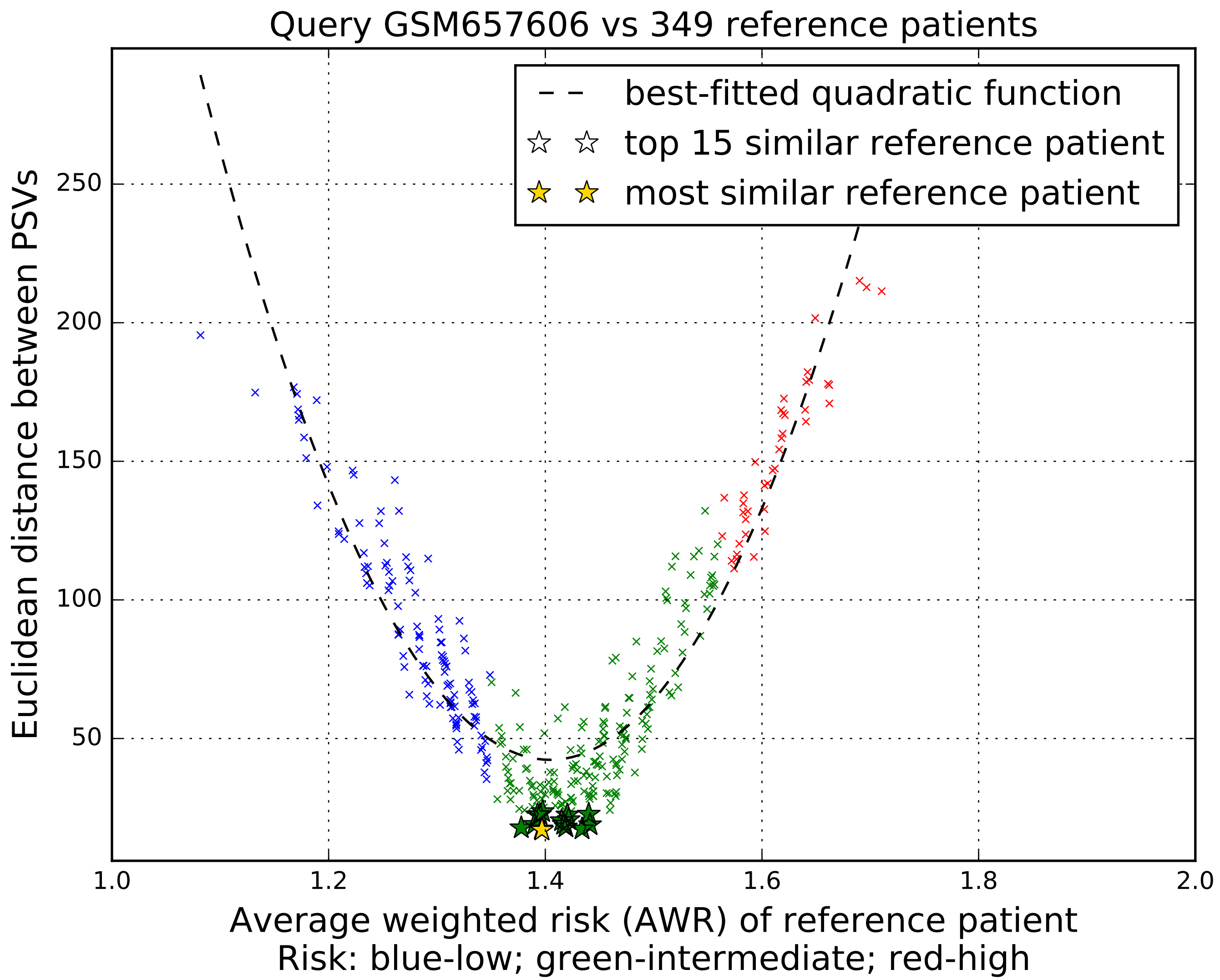

Query GSM657606 vs 349 reference patients
best-fitted quadratic function
top 15 similar reference patient
most similar reference patient
Euclidean distance between PSVs
50
100
150
200
250
1.0
1.2
1.4
1.6
1.8
2.0
Average weighted risk (AWR) of reference patient
Risk: blue-low; green-intermediate; red-high

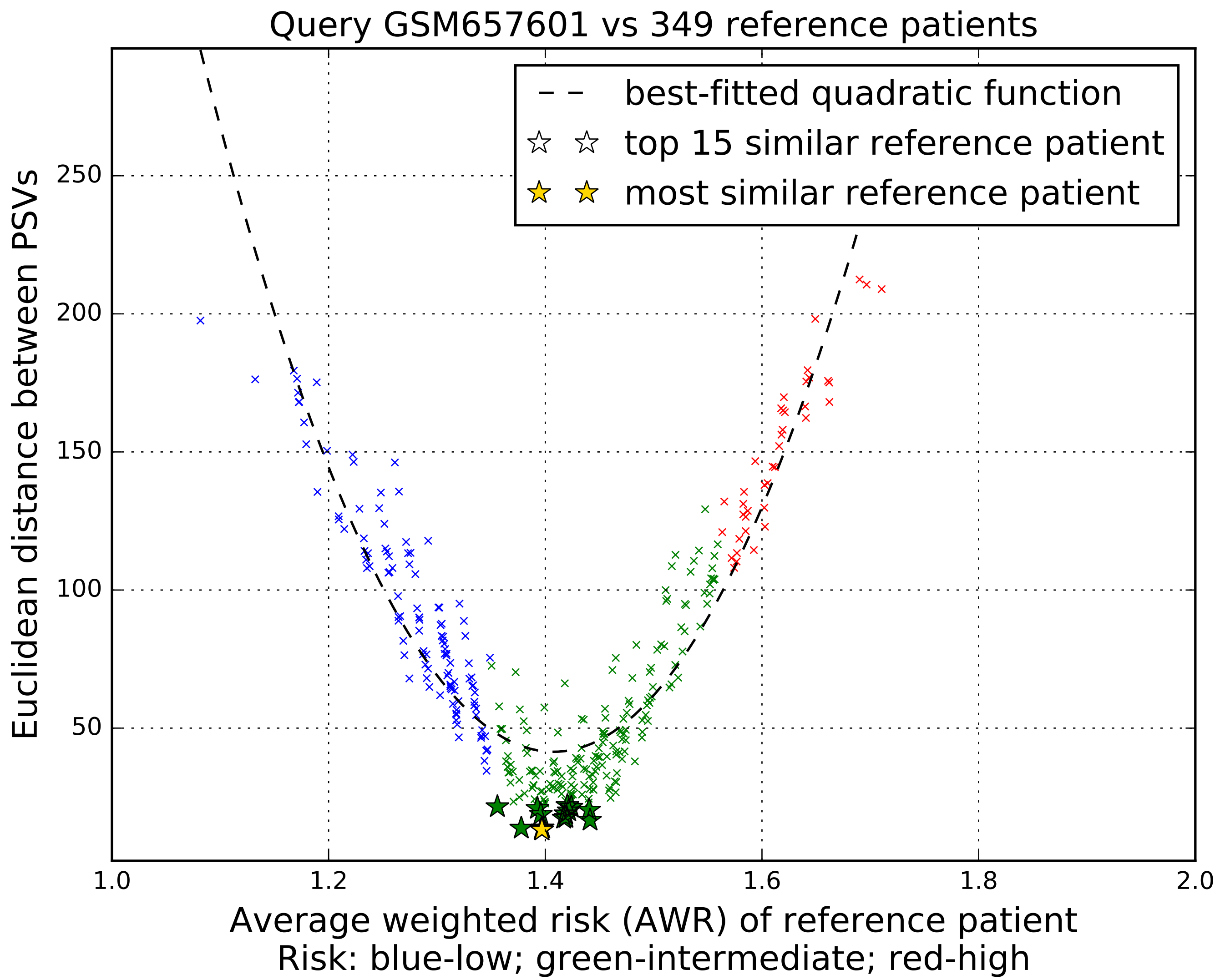
Query GSM657601 vs 349 reference patients
best-fitted quadratic function
top 15 similar reference patient
most similar reference patient
Euclidean distance between PSVs
250
200
150
100
50
1.0
1.2
1.4
1.6
1.8
2.0
Average weighted risk (AWR) of reference patient
Risk: blue-low; green-intermediate; red-high

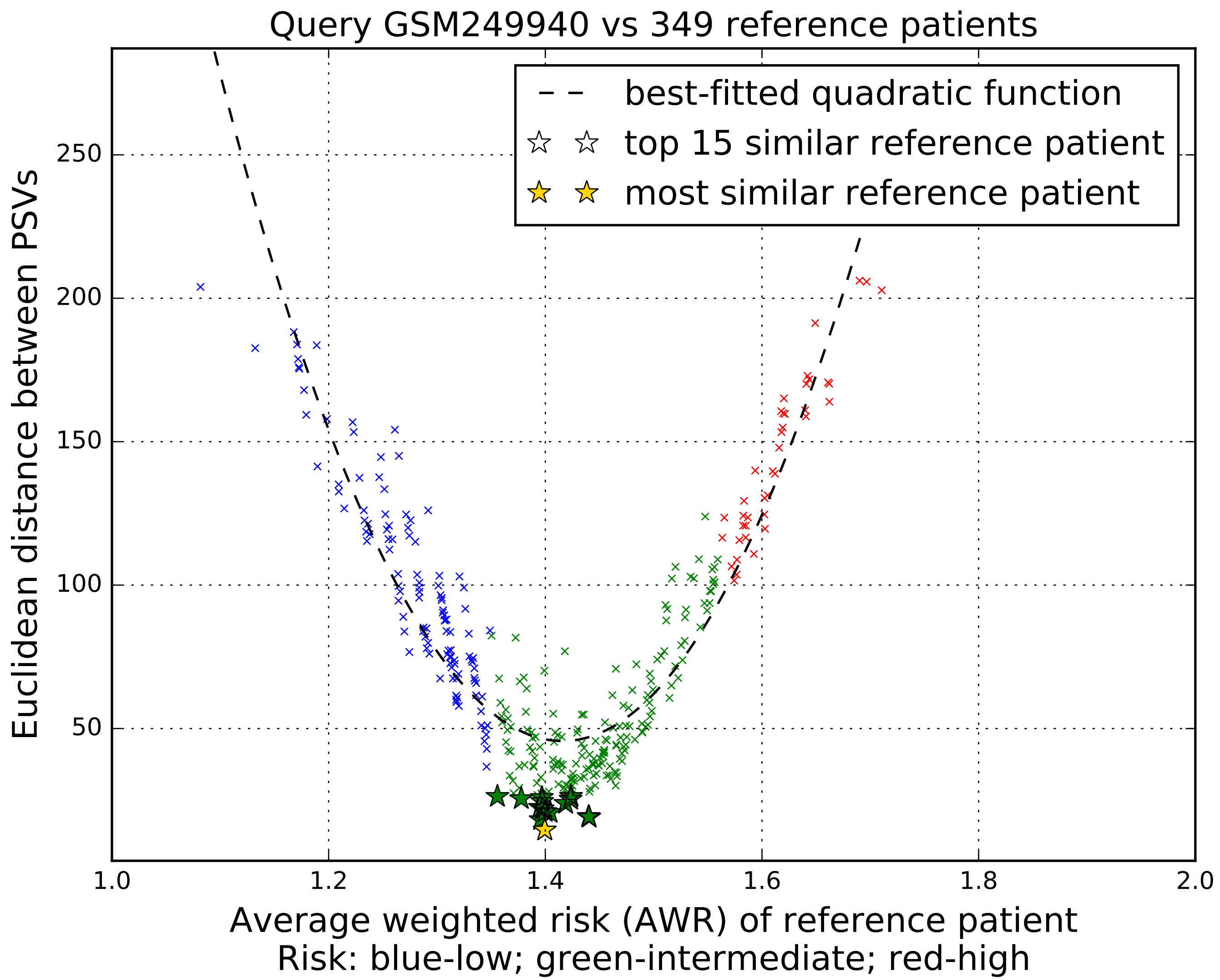
Query GSM249940 vs 349 reference patients
best-fitted quadratic function
top 15 similar reference patient
most similar reference patient
Euclidean distance between PSVs
250
200
150
100
50
1.0
1.2
1.4
1.6
1.8
2.0
Average weighted risk (AWR) of reference patient
Risk: blue-low; green-intermediate; red-high

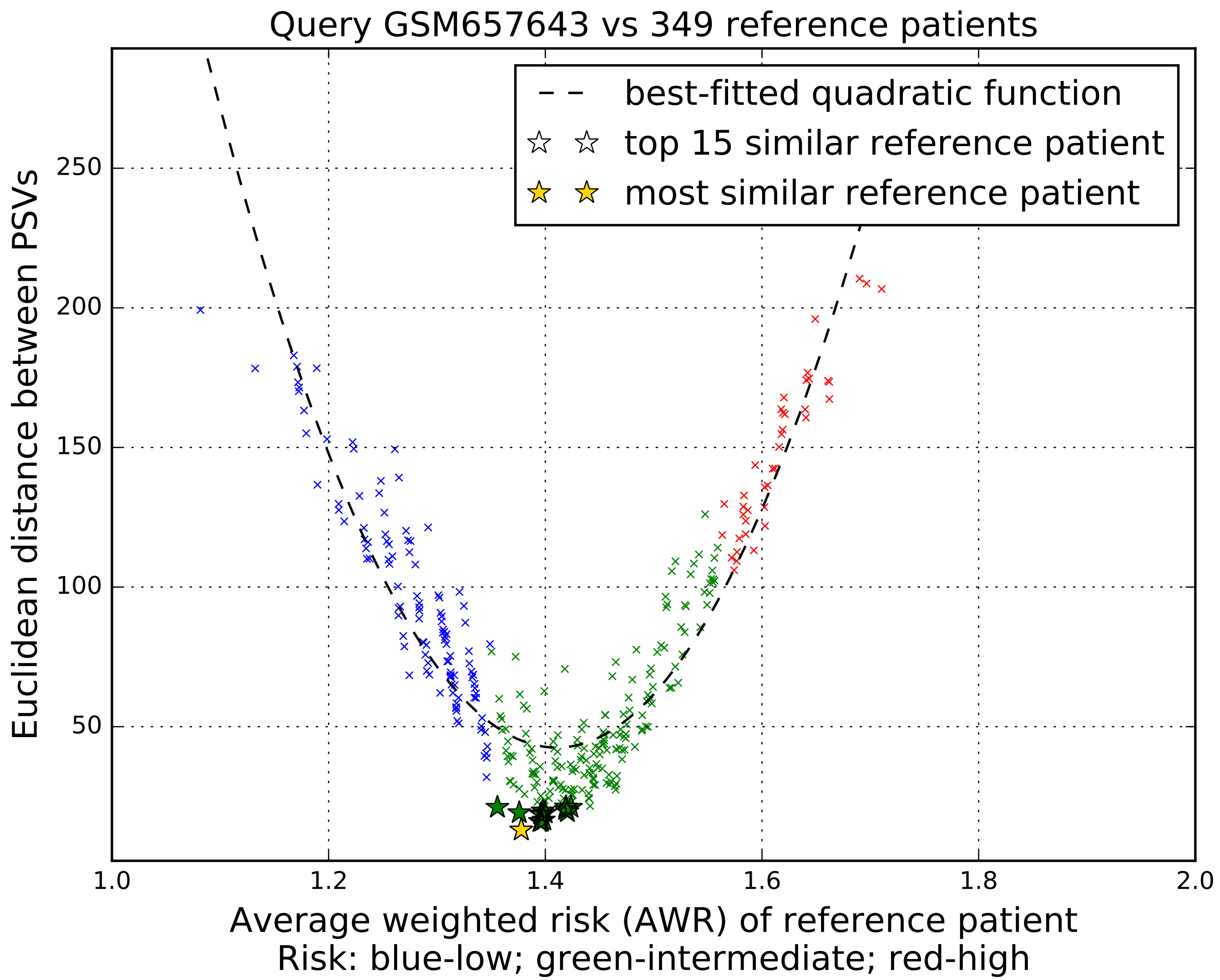

Query GSM657643 vs 349 reference patients
best-fitted quadratic function
top 15 similar reference patient
most similar reference patient
Euclidean distance between PSVs
250
200
150
100
50
1.0
1.2
1.4
1.6
1.8
2.0
Average weighted risk (AWR) of reference patient
Risk: blue-low; green-intermediate; red-high

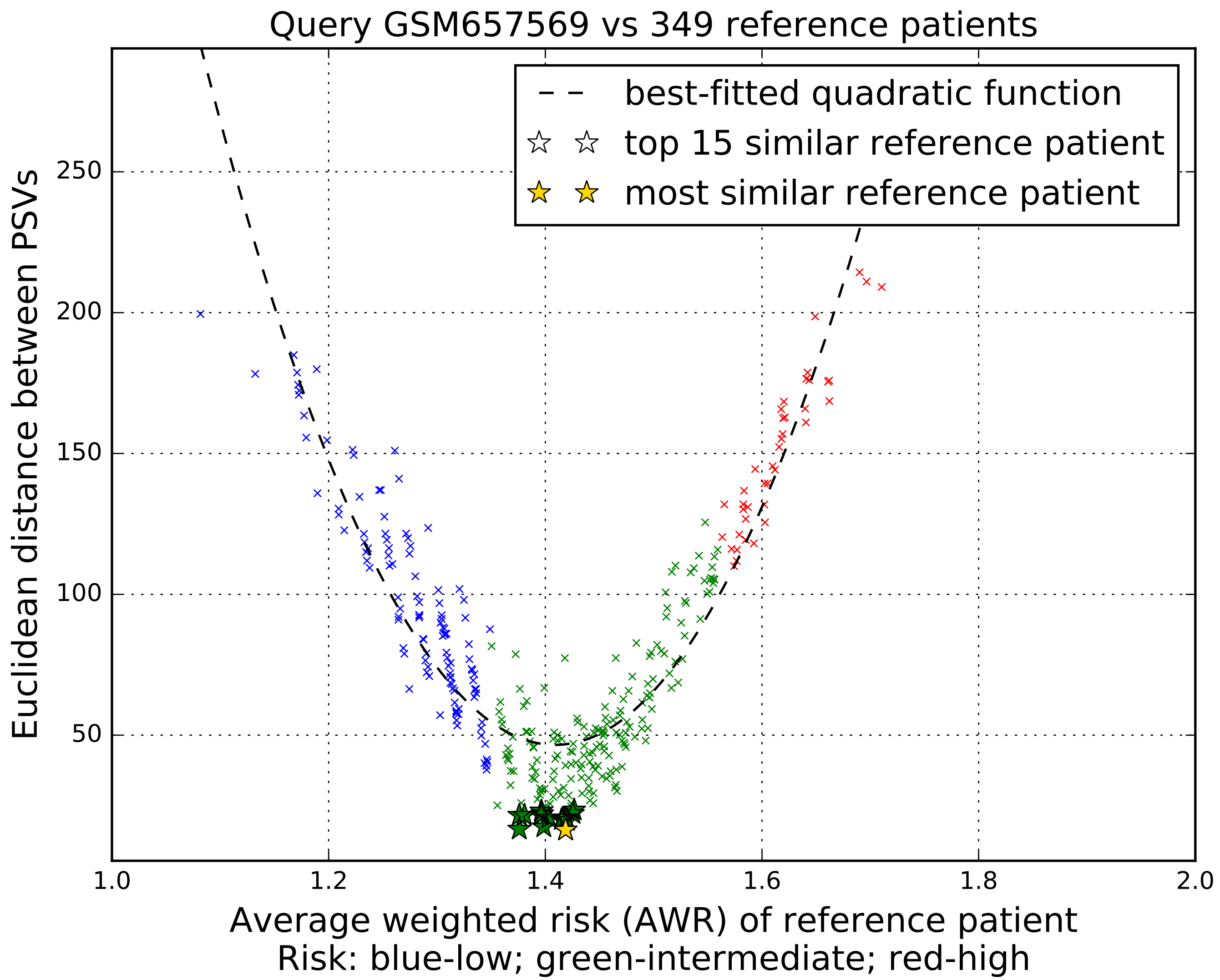

Query GSM657569 vs 349 reference patients
best-fitted quadratic function
top 15 similar reference patient
most similar reference patient
Euclidean distance between PSVs
250
200
150
100
50
1.0
1.2
1.4
1.6
1.8
2.0
Average weighted risk (AWR) of reference patient
Risk: blue-low; green-intermediate; red-high

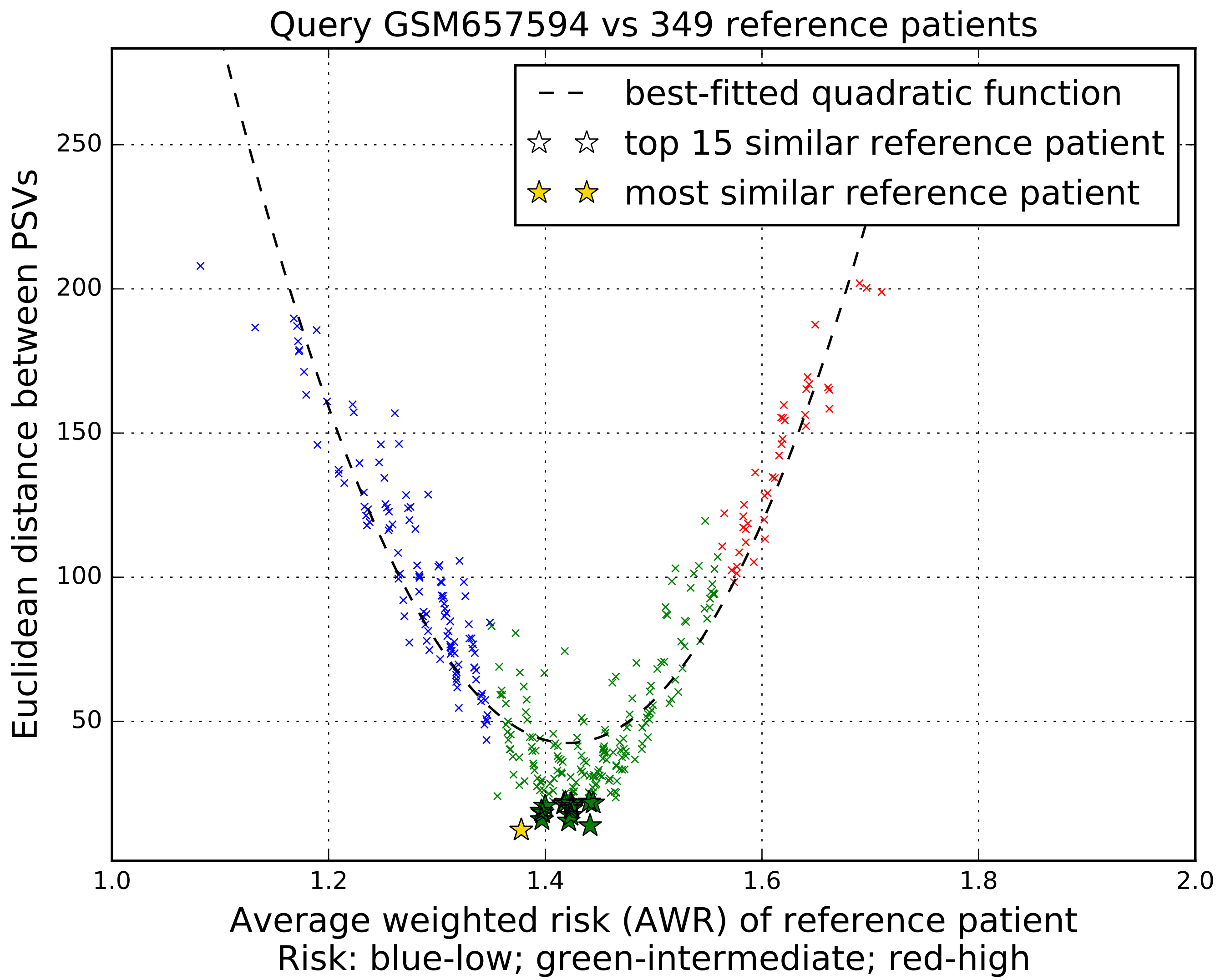
Query GSM657594 vs 349 reference patients
best-fitted quadratic function
top 15 similar reference patient
most similar reference patient
Euclidean distance between PSVs
250
200
150
100
50
1.0
1.2
1.4
1.6
1.8
2.0
Average weighted risk (AWR) of reference patient
Risk: blue-low; green-intermediate; red-high

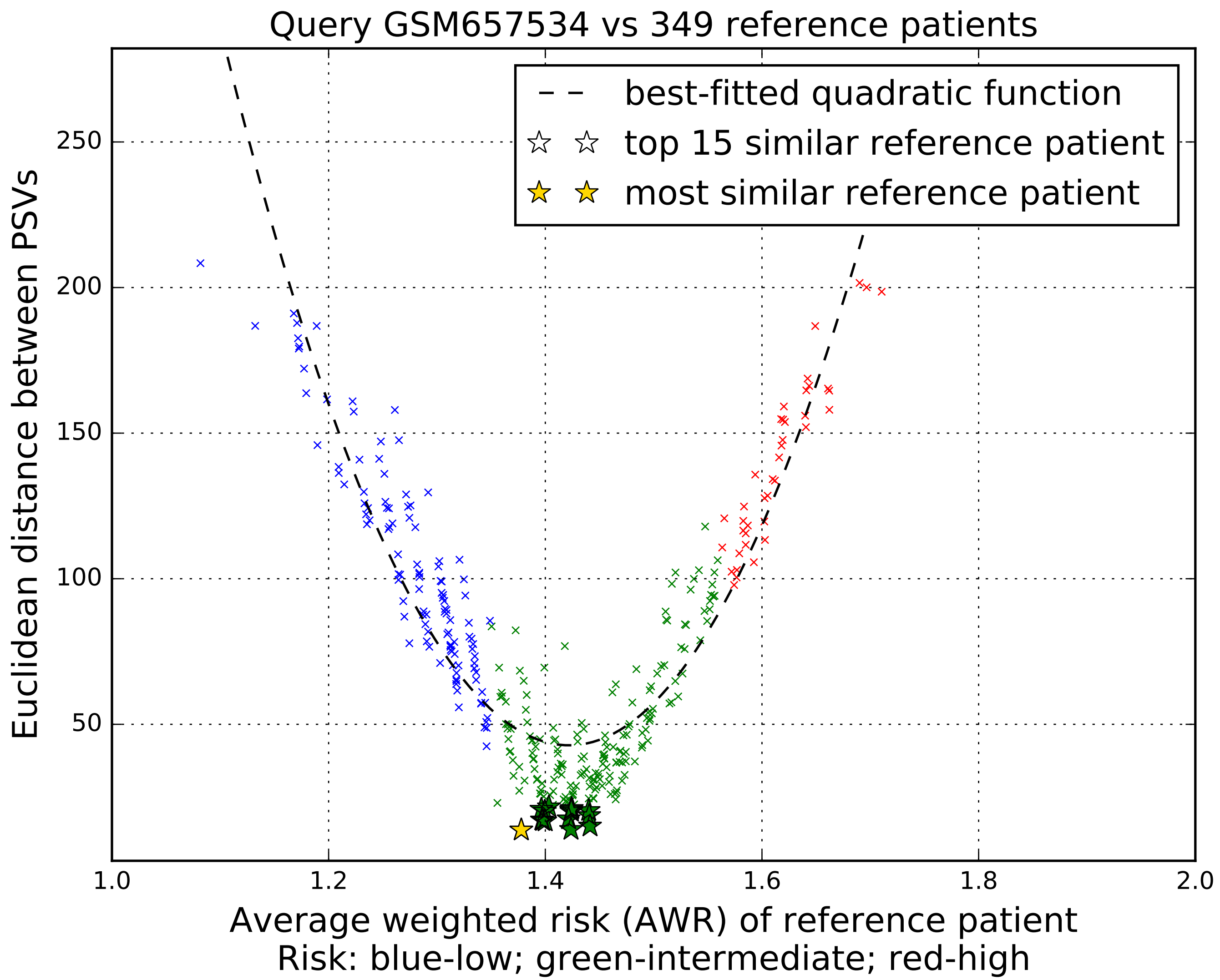
Query GSM657534 vs 349 reference patients
best-fitted quadratic function
top 15 similar reference patient
most similar reference patient
Euclidean distance between PSVs
250
200
150
100
50
1.0
1.2
1.4
1.6
1.8
2.0
Average weighted risk (AWR) of reference patient
Risk: blue-low; green-intermediate; red-high

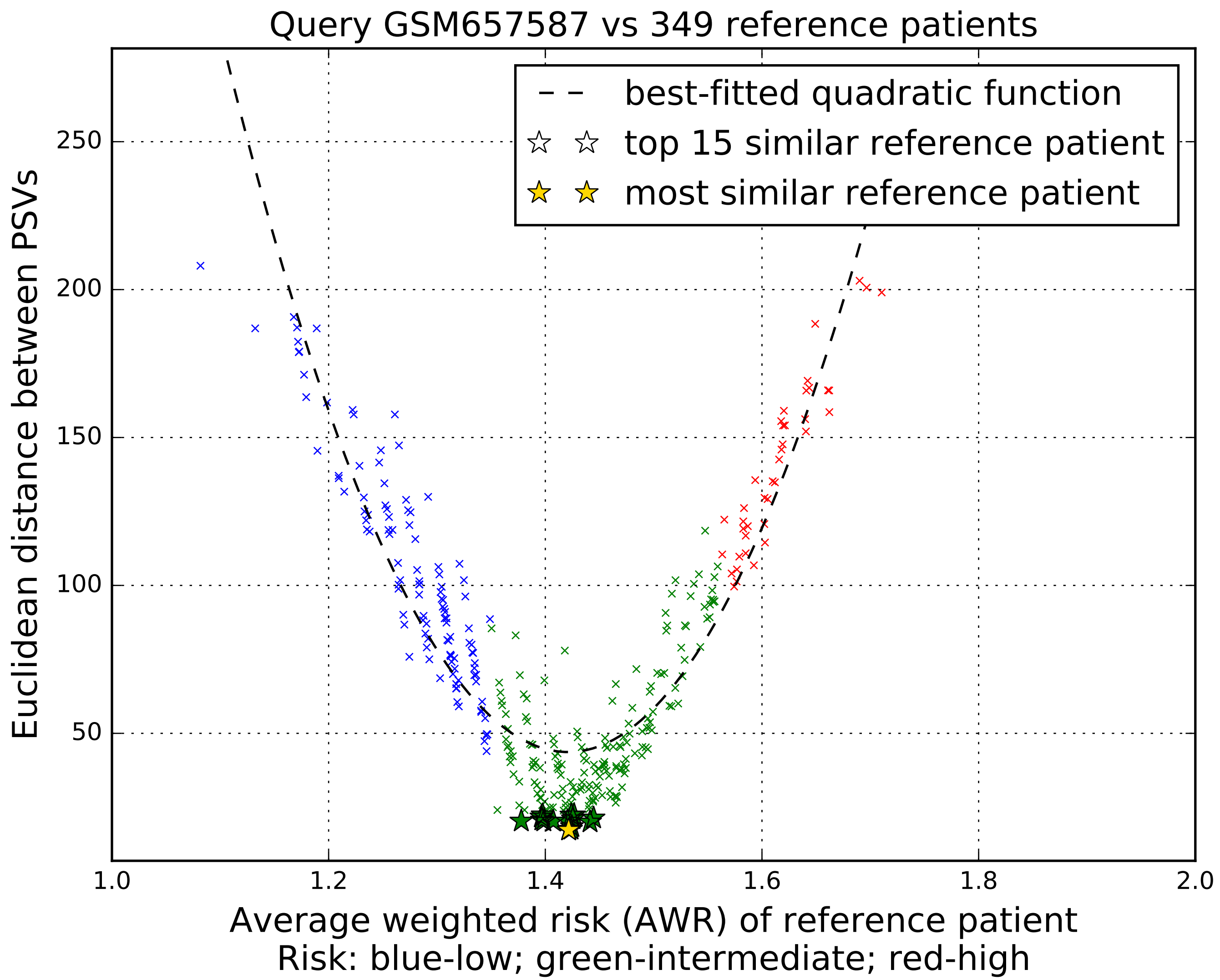

Query GSM657587 vs 349 reference patients
best-fitted quadratic function
top 15 similar reference patient
most similar reference patient
Euclidean distance between PSVs
250
200
150
100
50
1.0
1.2
1.4
1.6
1.8
2.0
Average weighted risk (AWR) of reference patient
Risk: blue-low; green-intermediate; red-high

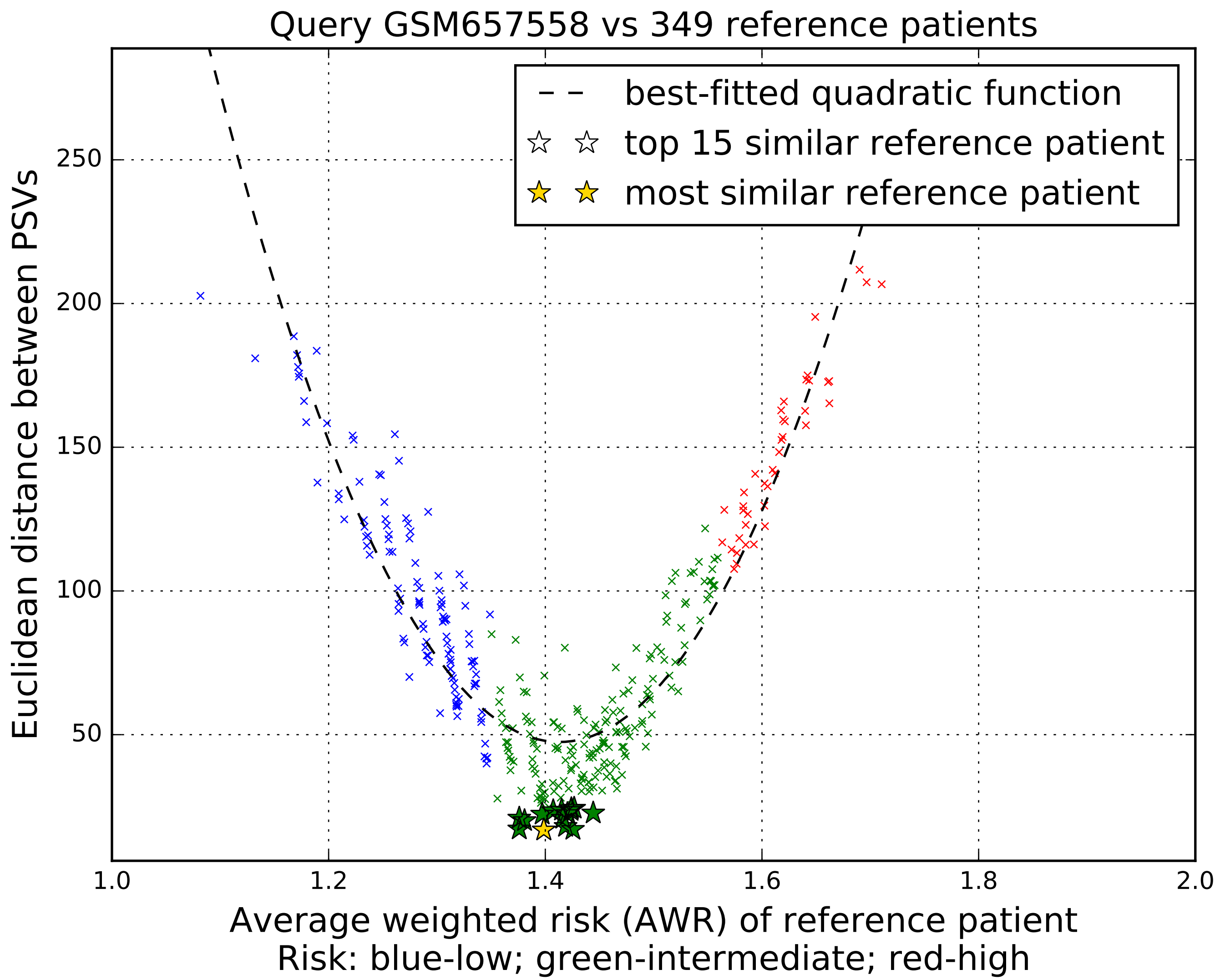

Query GSM657558 vs 349 reference patients
best-fitted quadratic function
top 15 similar reference patient
most similar reference patient
Euclidean distance between PSVs
50
100
150
200
250
1.0
1.2
1.4
1.6
1.8
2.0
Average weighted risk (AWR) of reference patient
Risk: blue-low; green-intermediate; red-high

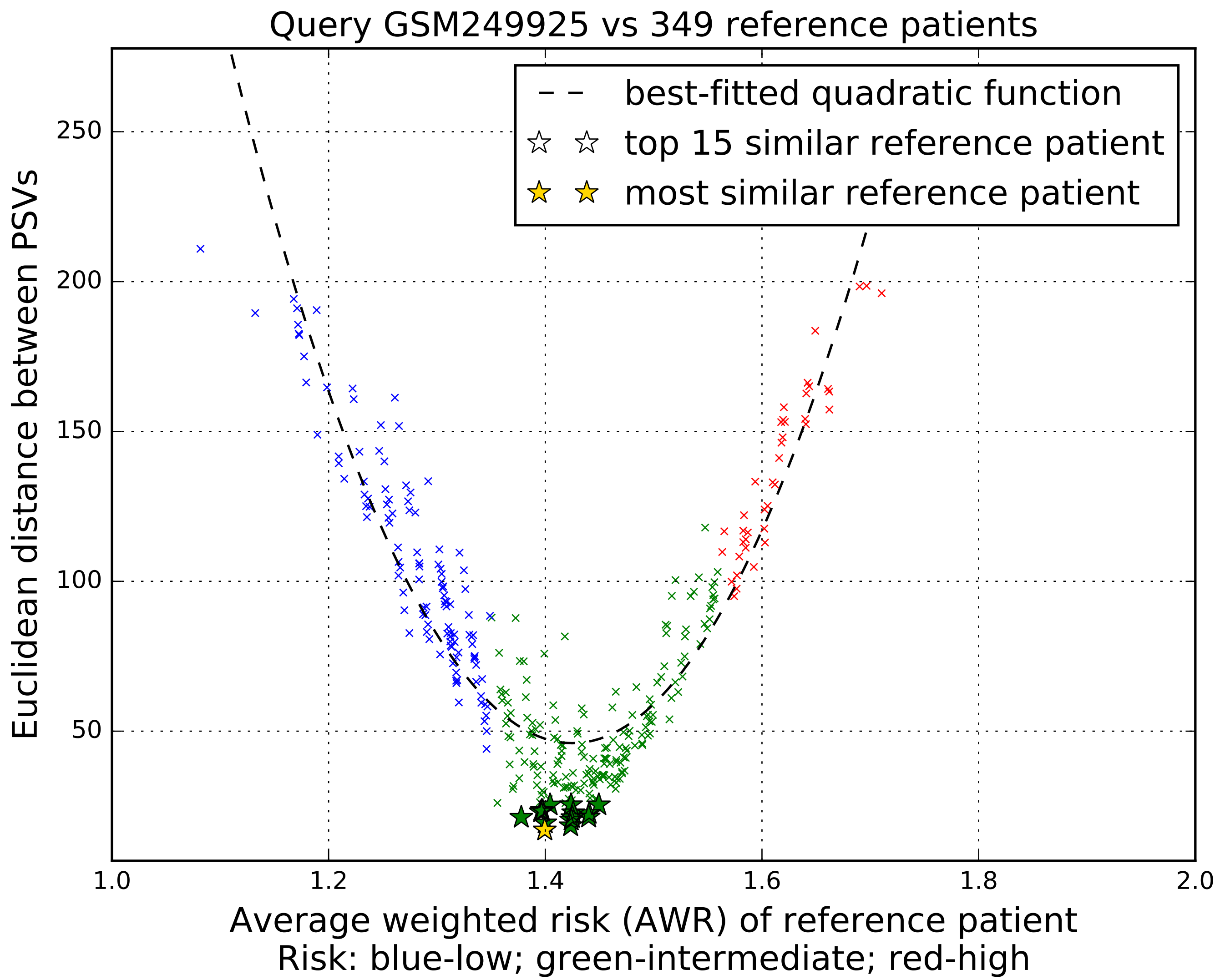

Query GSM249925 vs 349 reference patients
best-fitted quadratic function
top 15 similar reference patient
most similar reference patient
Euclidean distance between PSVs
250
200
150
100
50
1.0
1.2
1.4
1.6
1.8
2.0
Average weighted risk (AWR) of reference patient
Risk: blue-low; green-intermediate; red-high

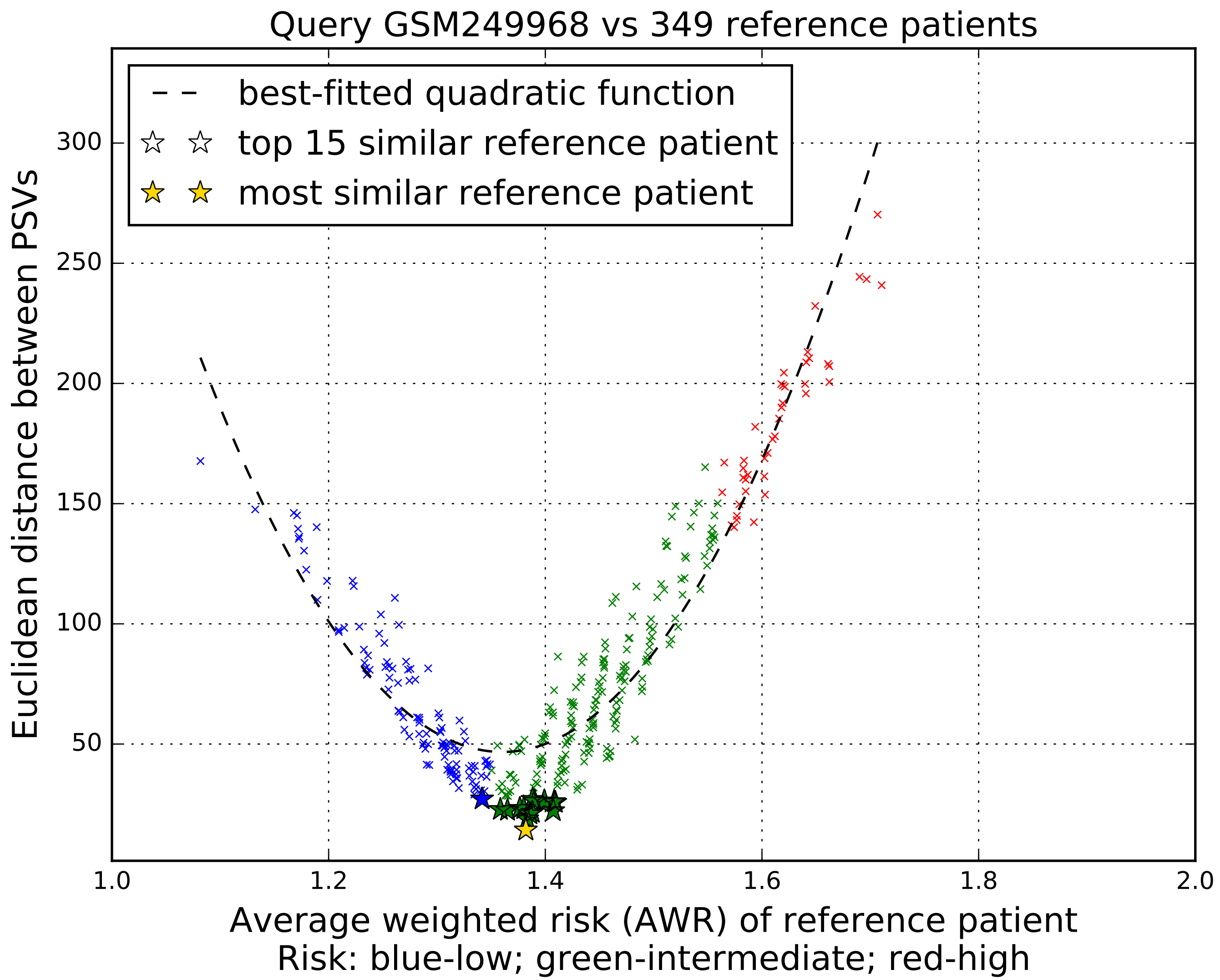

Query GSM249968 vs 349 reference patients
best-fitted quadratic function
top 15 similar reference patient
most similar reference patient
Euclidean distance between PSVs
300
250
200
150
100
50
1.0
1.2
1.4
1.6
1.8
2.0
Average weighted risk (AWR) of reference patient
Risk: blue-low; green-intermediate; red-high

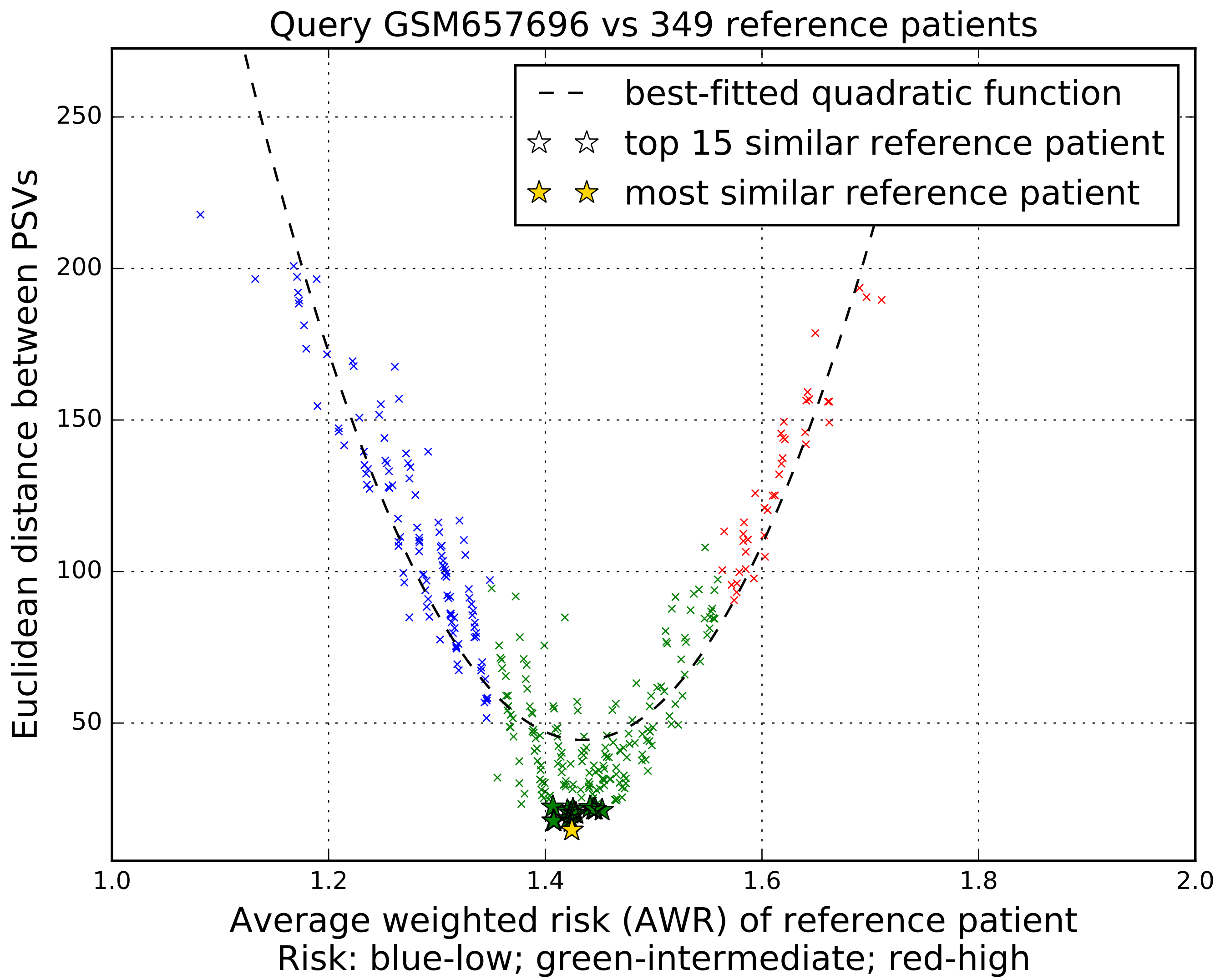

Query GSM657696 vs 349 reference patients
best-fitted quadratic function
top 15 similar reference patient
most similar reference patient
Euclidean distance between PSVs
250
200
150
100
50
1.0
1.2
1.4
1.6
1.8
2.0
Average weighted risk (AWR) of reference patient
Risk: blue-low; green-intermediate; red-high

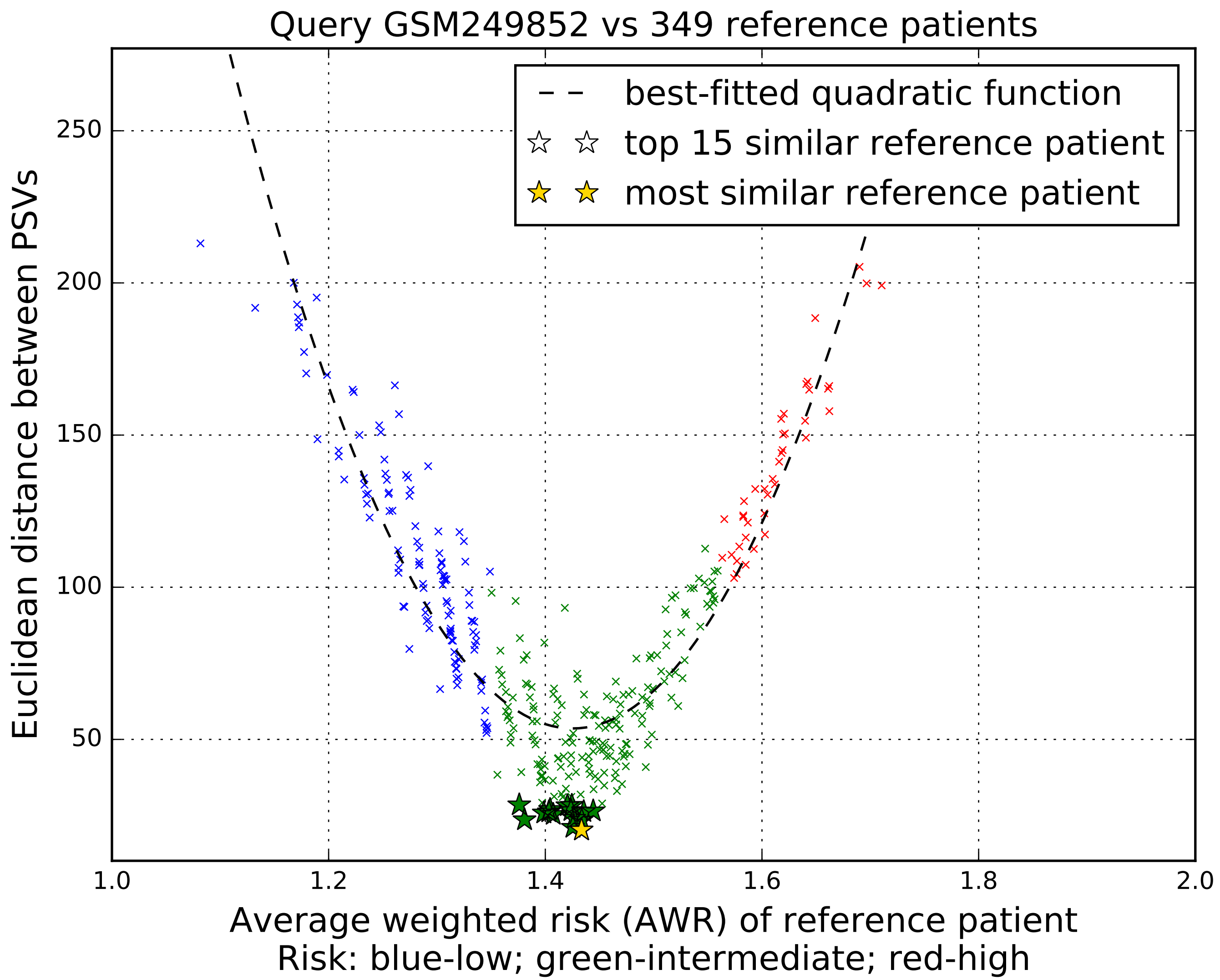

Query GSM249852 vs 349 reference patients
best-fitted quadratic function
top 15 similar reference patient
most similar reference patient
Euclidean distance between PSVs
250
200
150
100
50
1.0
1.2
1.4
1.6
1.8
2.0
Average weighted risk (AWR) of reference patient
Risk: blue-low; green-intermediate; red-high

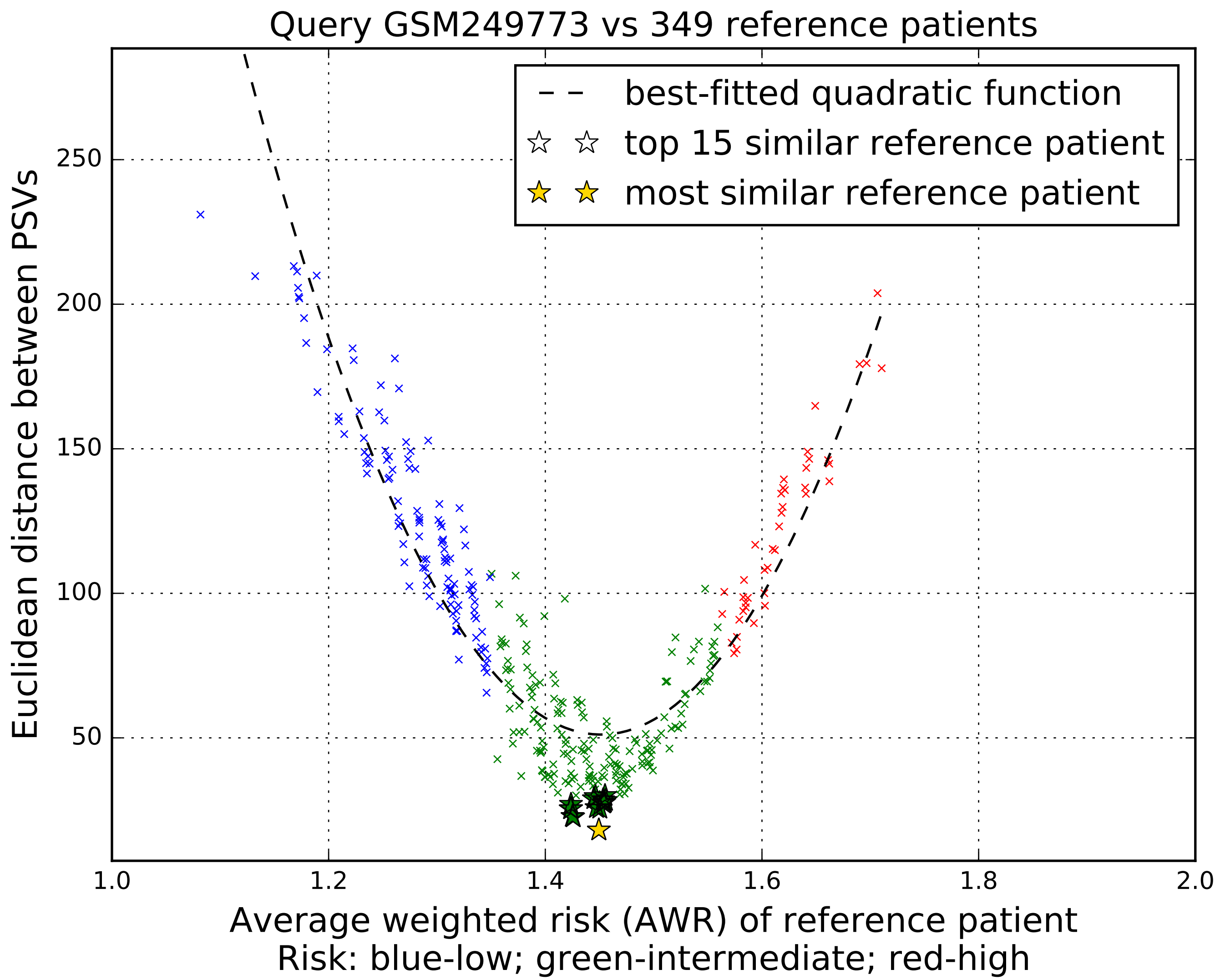
Query GSM249773 vs 349 reference patients
best-fitted quadratic function
top 15 similar reference patient
most similar reference patient
Euclidean distance between PSVs
250
200
150
100
50
1.0
1.2
1.4
1.6
1.8
2.0
Average weighted risk (AWR) of reference patient
Risk: blue-low; green-intermediate; red-high

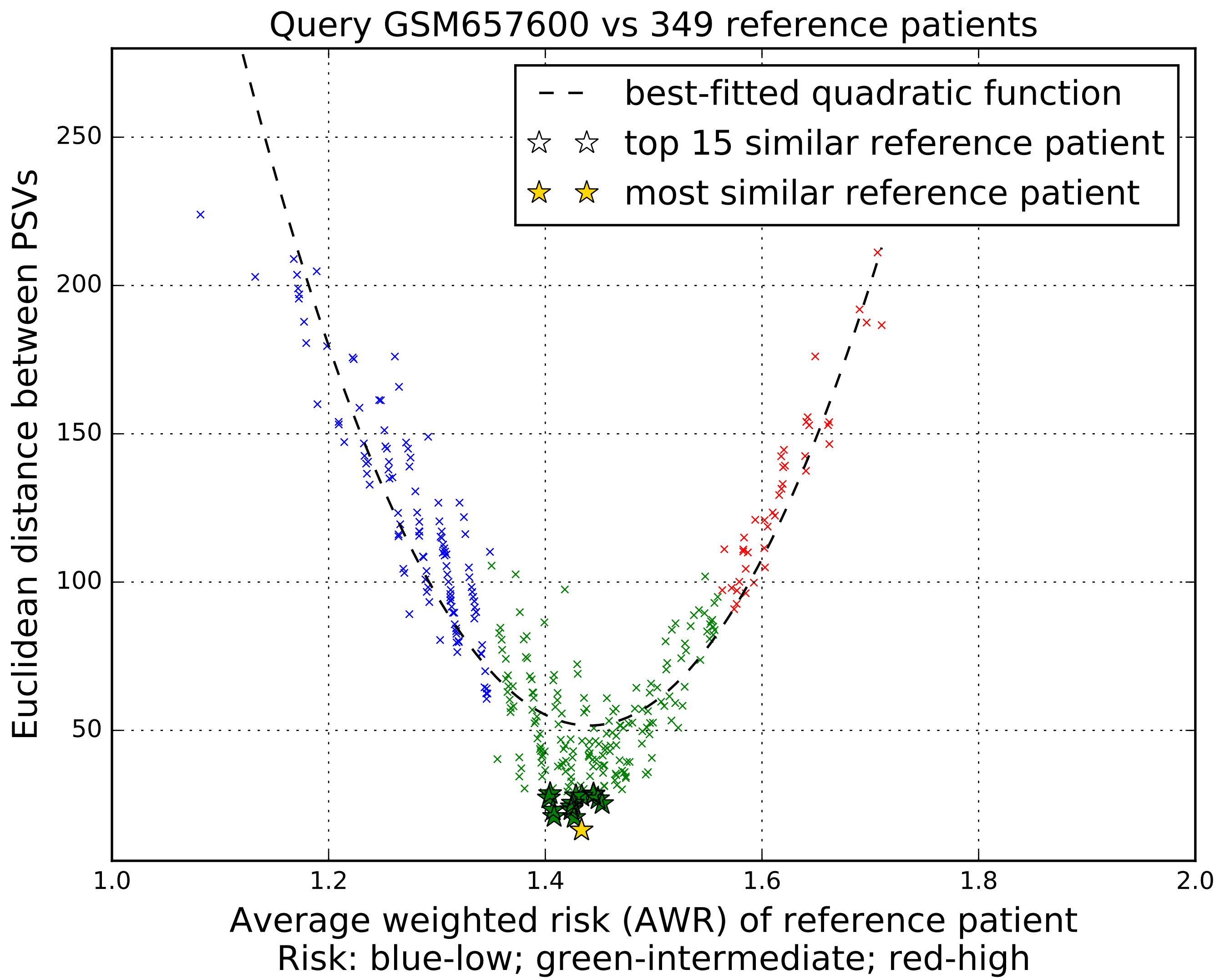

Query GSM657600 vs 349 reference patients
best-fitted quadratic function
top 15 similar reference patient
most similar reference patient
Euclidean distance between PSVs
250
200
150
100
50
1.0
1.2
1.4
1.6
1.8
2.0
Average weighted risk (AWR) of reference patient
Risk: blue-low; green-intermediate; red-high

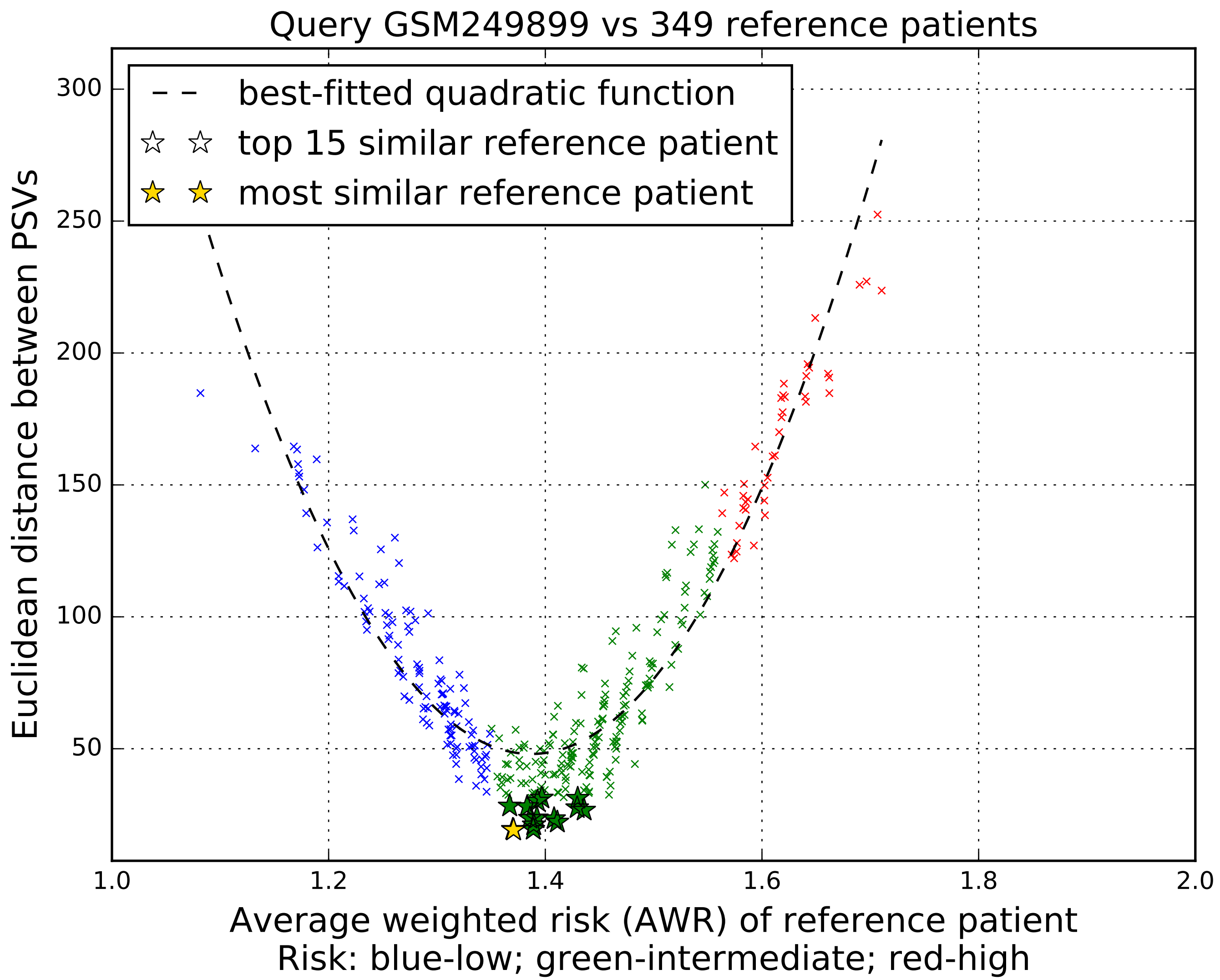
Query GSM249899 vs 349 reference patients
best-fitted quadratic function
top 15 similar reference patient
most similar reference patient
Euclidean distance between PSVs
300
250
200
150
100
50
1.0
1.2
1.4
1.6
1.8
2.0
Average weighted risk (AWR) of reference patient
Risk: blue-low; green-intermediate; red-high

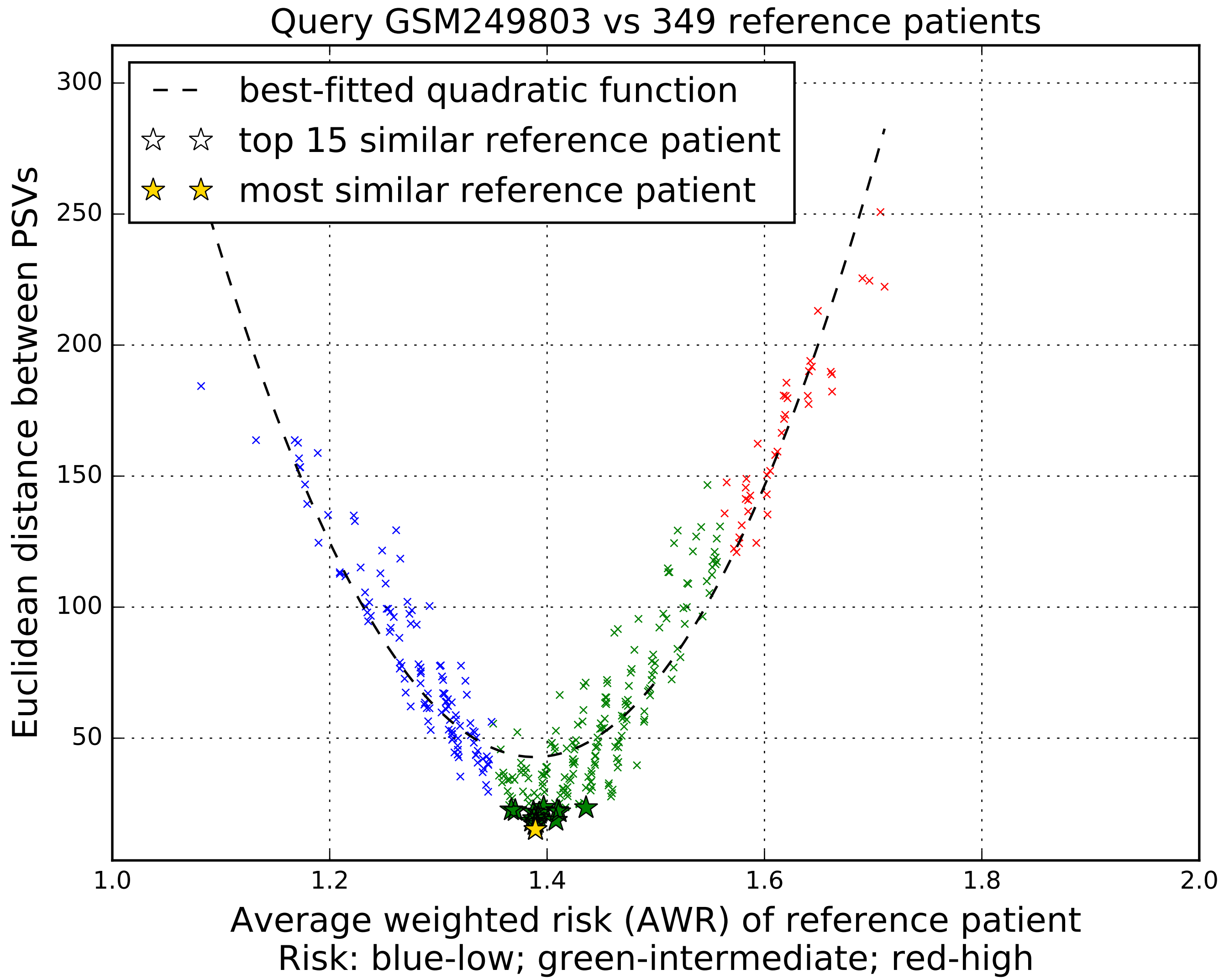
Query GSM249803 vs 349 reference patients
best-fitted quadratic function
top 15 similar reference patient
most similar reference patient
Euclidean distance between PSVs
300
250
200
150
100
50
1.0
1.2
1.4
1.6
1.8
2.0
Average weighted risk (AWR) of reference patient
Risk: blue-low; green-intermediate; red-high

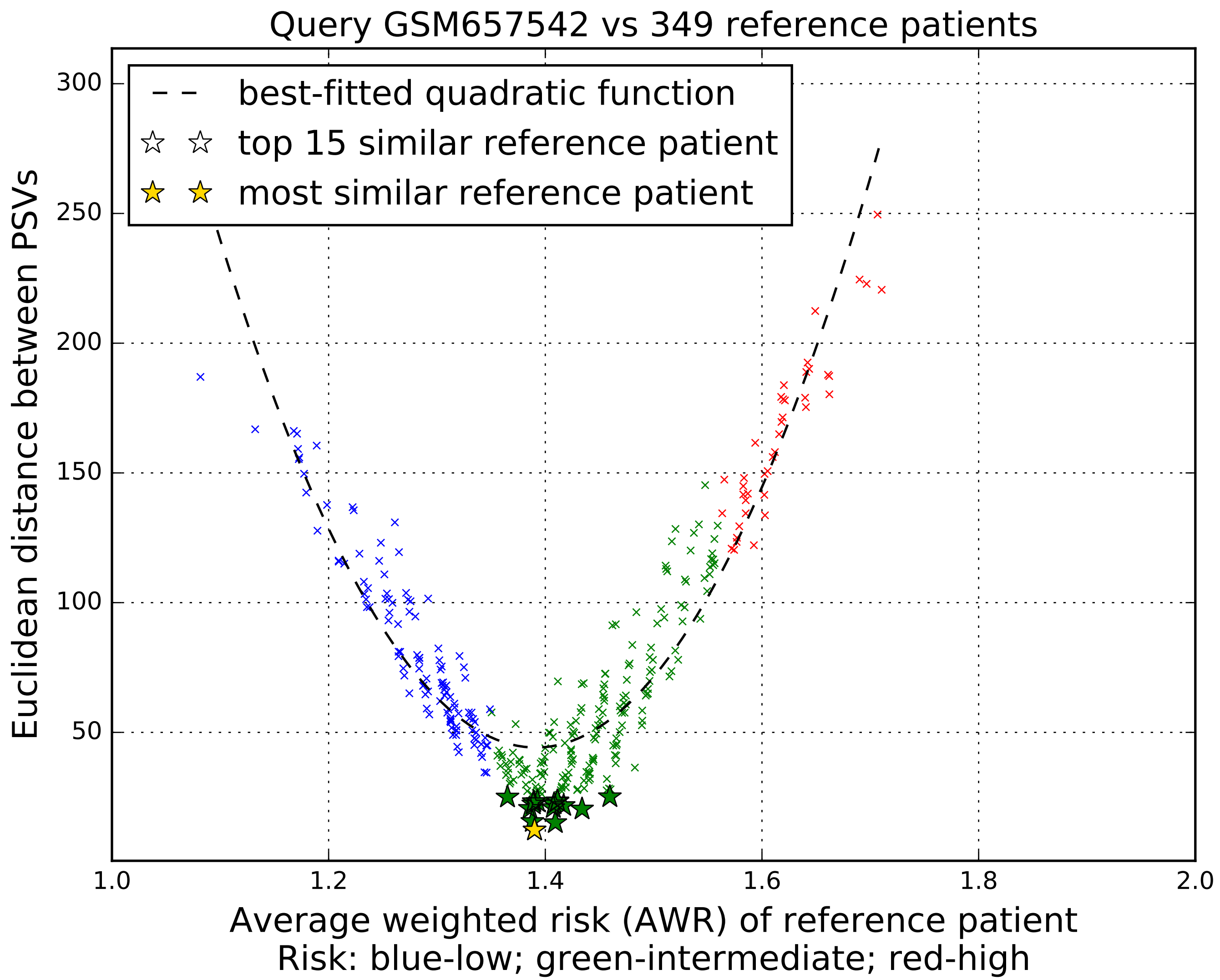

Query GSM657542 vs 349 reference patients
best-fitted quadratic function
top 15 similar reference patient
most similar reference patient
Euclidean distance between PSVs
300
250
200
150
100
50
1.0
1.2
1.4
1.6
1.8
2.0
Average weighted risk (AWR) of reference patient
Risk: blue-low; green-intermediate; red-high

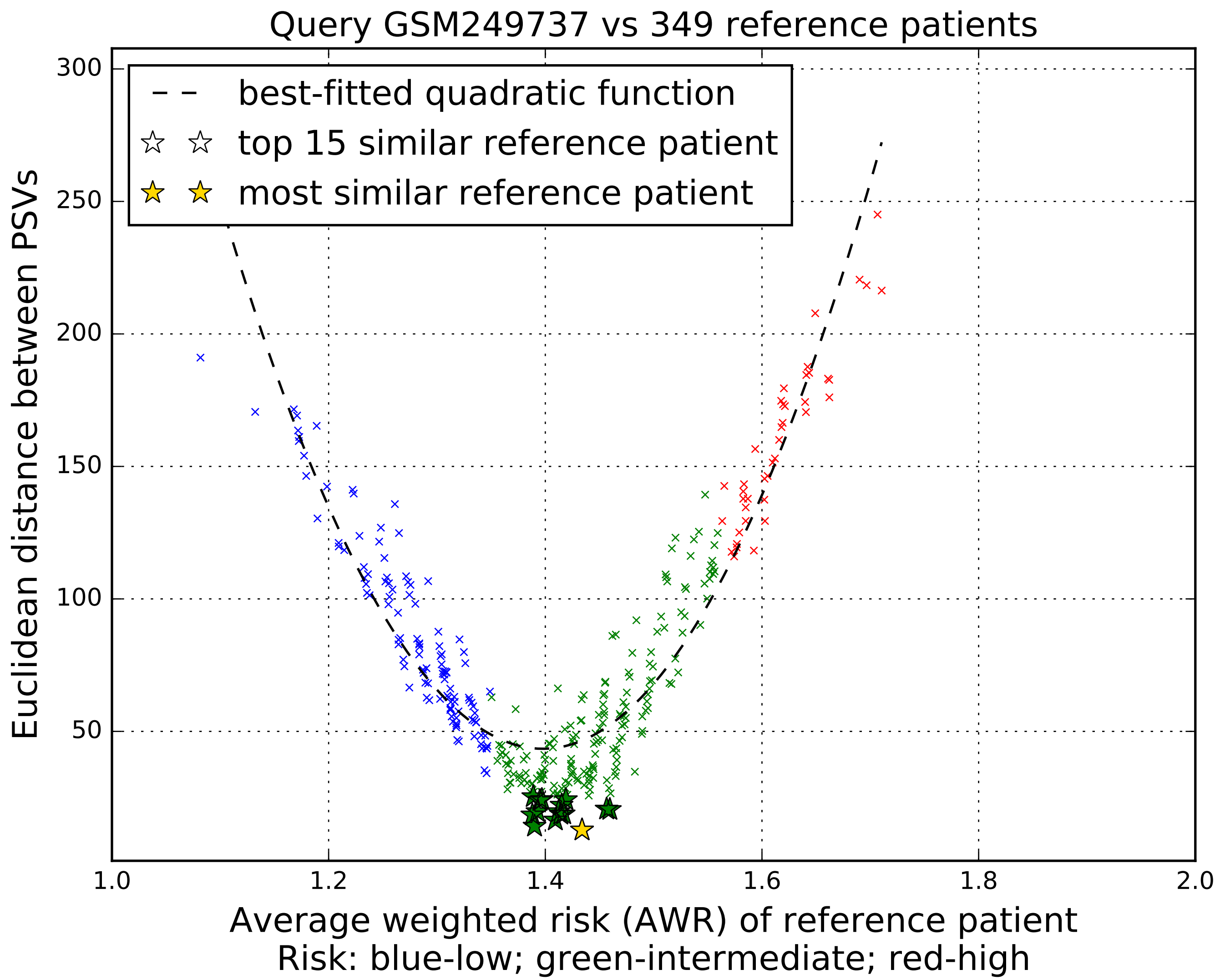
Query GSM249737 vs 349 reference patients
best-fitted quadratic function
top 15 similar reference patient
most similar reference patient
Euclidean distance between PSVs
300
250
200
150
100
50
1.0
1.2
1.4
1.6
1.8
2.0
Average weighted risk (AWR) of reference patient
Risk: blue-low; green-intermediate; red-high

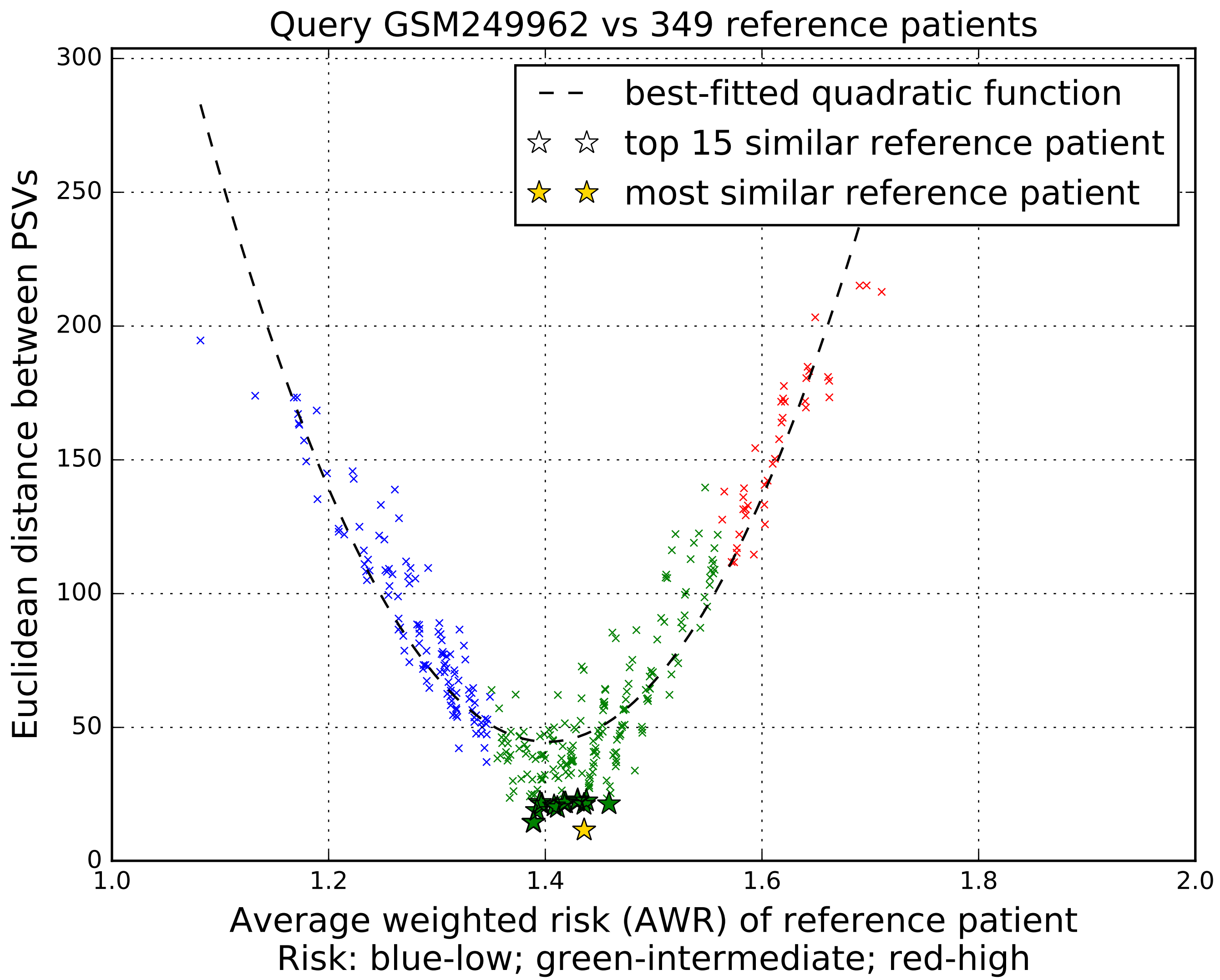

Query GSM249962 vs 349 reference patients
best-fitted quadratic function
top 15 similar reference patient
most similar reference patient
Euclidean distance between PSVs
300
250
200
150
100
50
0
1.0
1.2
1.4
1.6
1.8
2.0
Average weighted risk (AWR) of reference patient
Risk: blue-low; green-intermediate; red-high

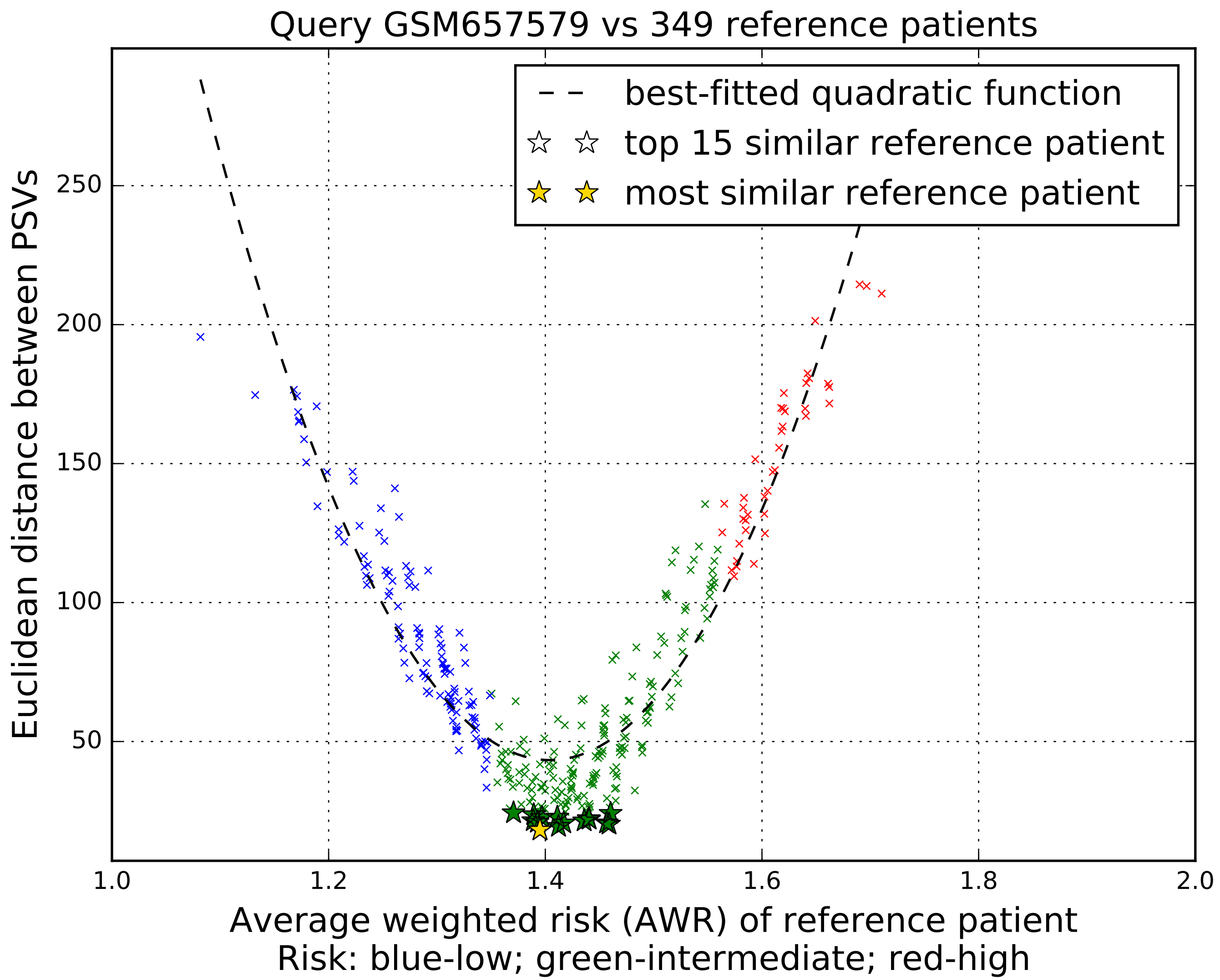
Query GSM657579 vs 349 reference patients
best-fitted quadratic function
top 15 similar reference patient
most similar reference patient
Euclidean distance between PSVs
250
200
150
100
50
1.0
1.2
1.4
1.6
1.8
2.0
Average weighted risk (AWR) of reference patient
Risk: blue-low; green-intermediate; red-high

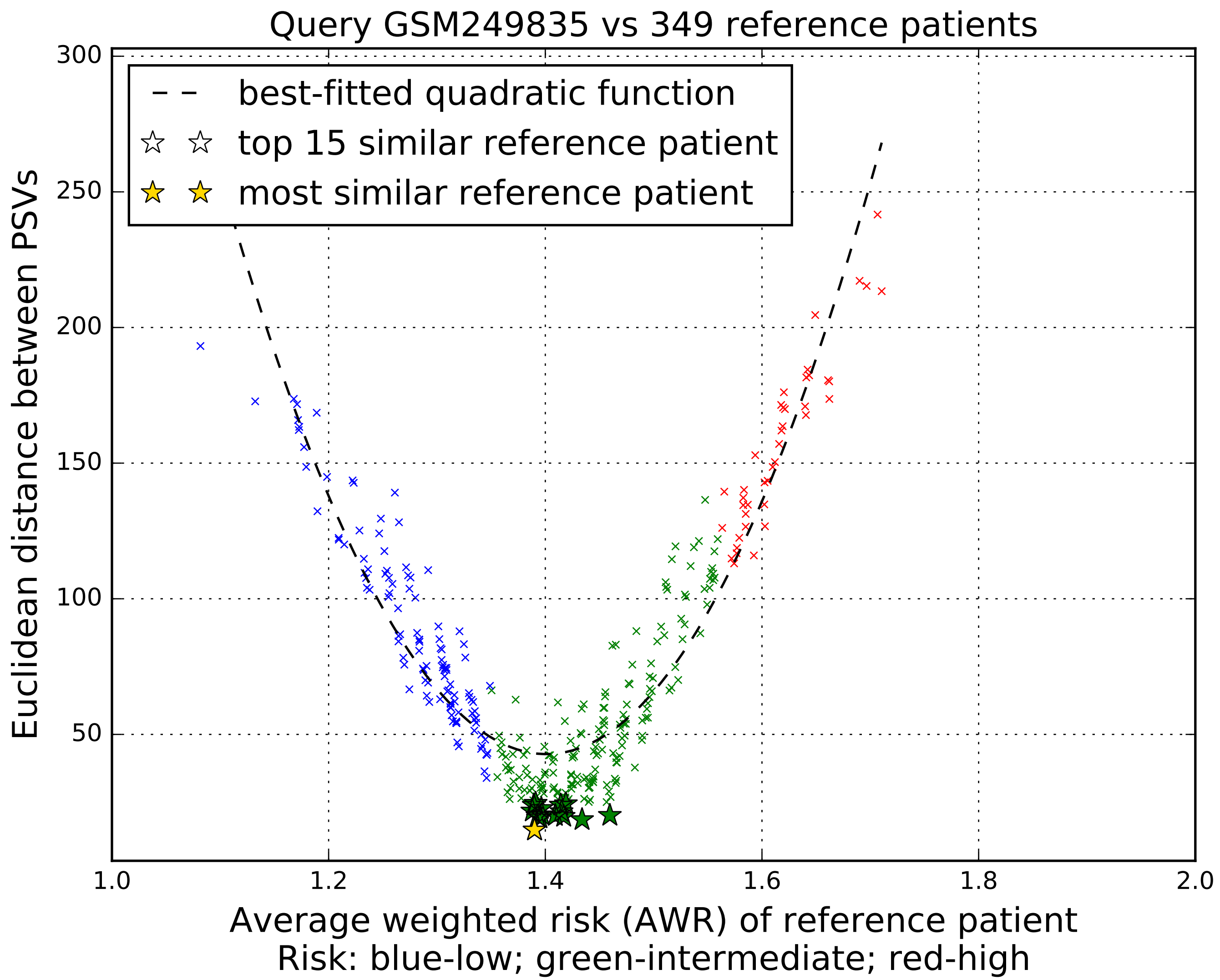

Query GSM249835 vs 349 reference patients
best-fitted quadratic function
top 15 similar reference patient
most similar reference patient
Euclidean distance between PSVs
300
250
200
150
100
50
1.0
1.2
1.4
1.6
1.8
2.0
Average weighted risk (AWR) of reference patient
Risk: blue-low; green-intermediate; red-high

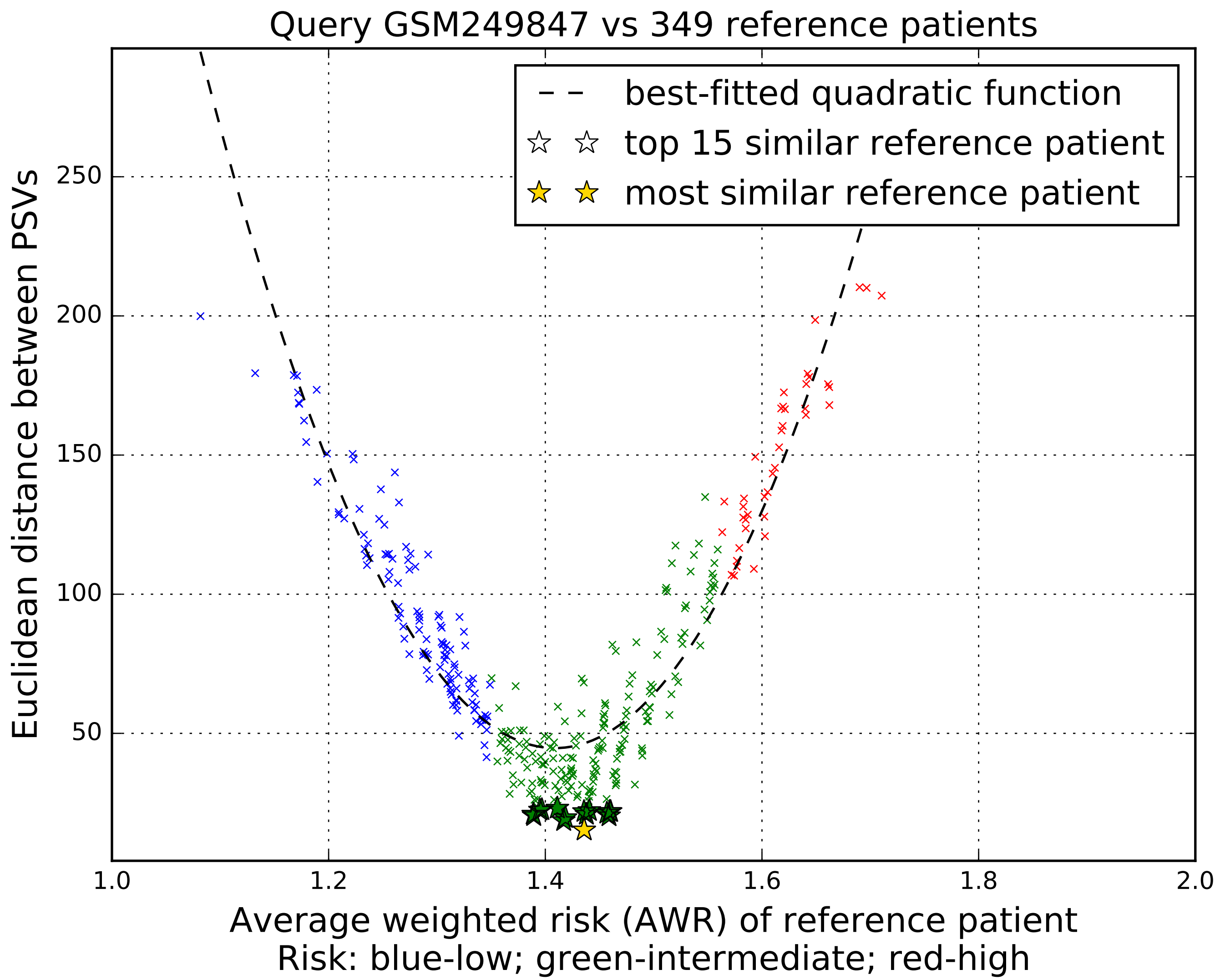

Query GSM249847 vs 349 reference patients
best-fitted quadratic function
top 15 similar reference patient
most similar reference patient
Euclidean distance between PSVs
250
200
150
100
50
1.0
1.2
1.4
1.6
1.8
2.0
Average weighted risk (AWR) of reference patient
Risk: blue-low; green-intermediate; red-high

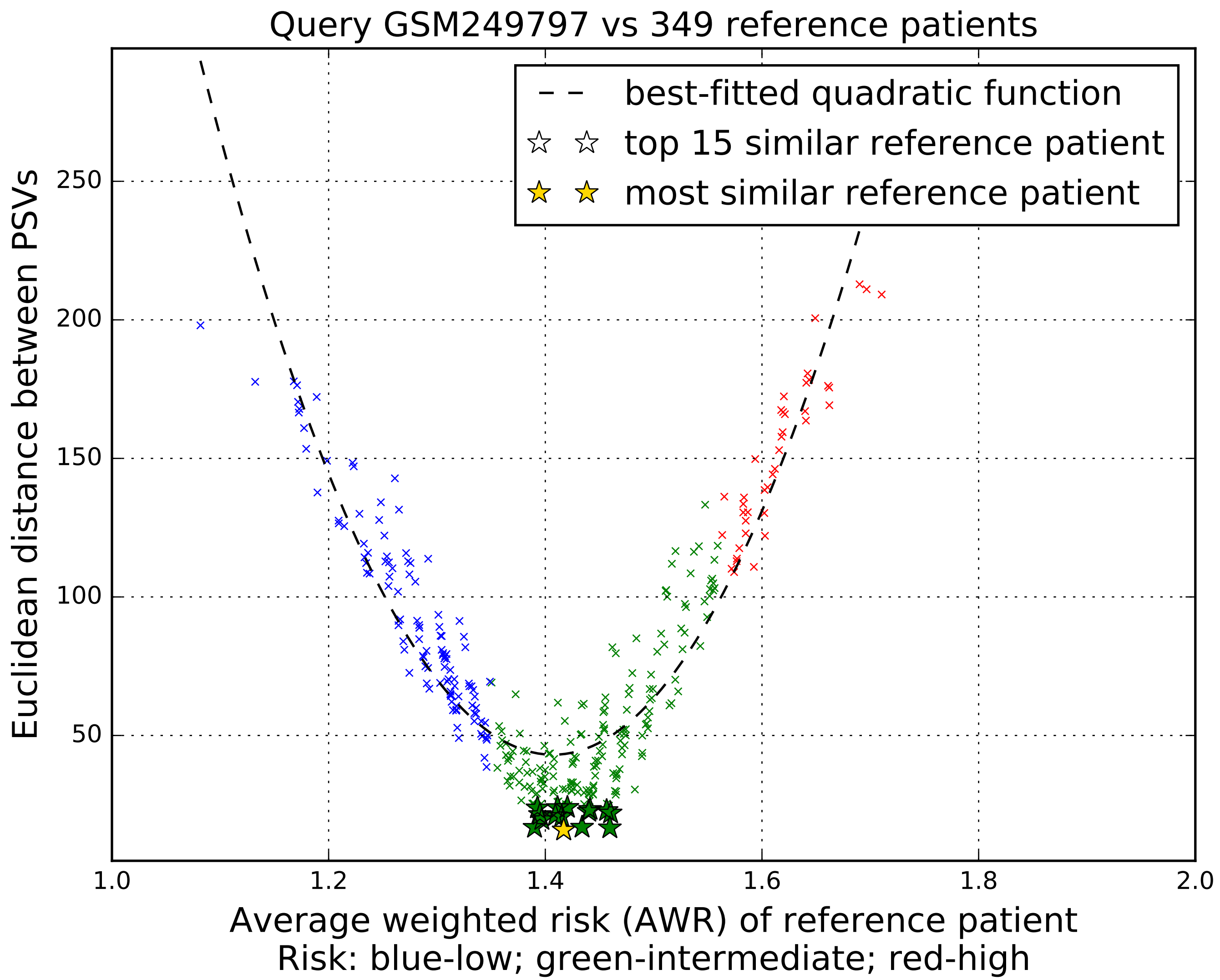
Query GSM249797 vs 349 reference patients
best-fitted quadratic function
top 15 similar reference patient
most similar reference patient
Euclidean distance between PSVs
50
100
150
200
250
1.0
1.2
1.4
1.6
1.8
2.0
Average weighted risk (AWR) of reference patient
Risk: blue-low; green-intermediate; red-high

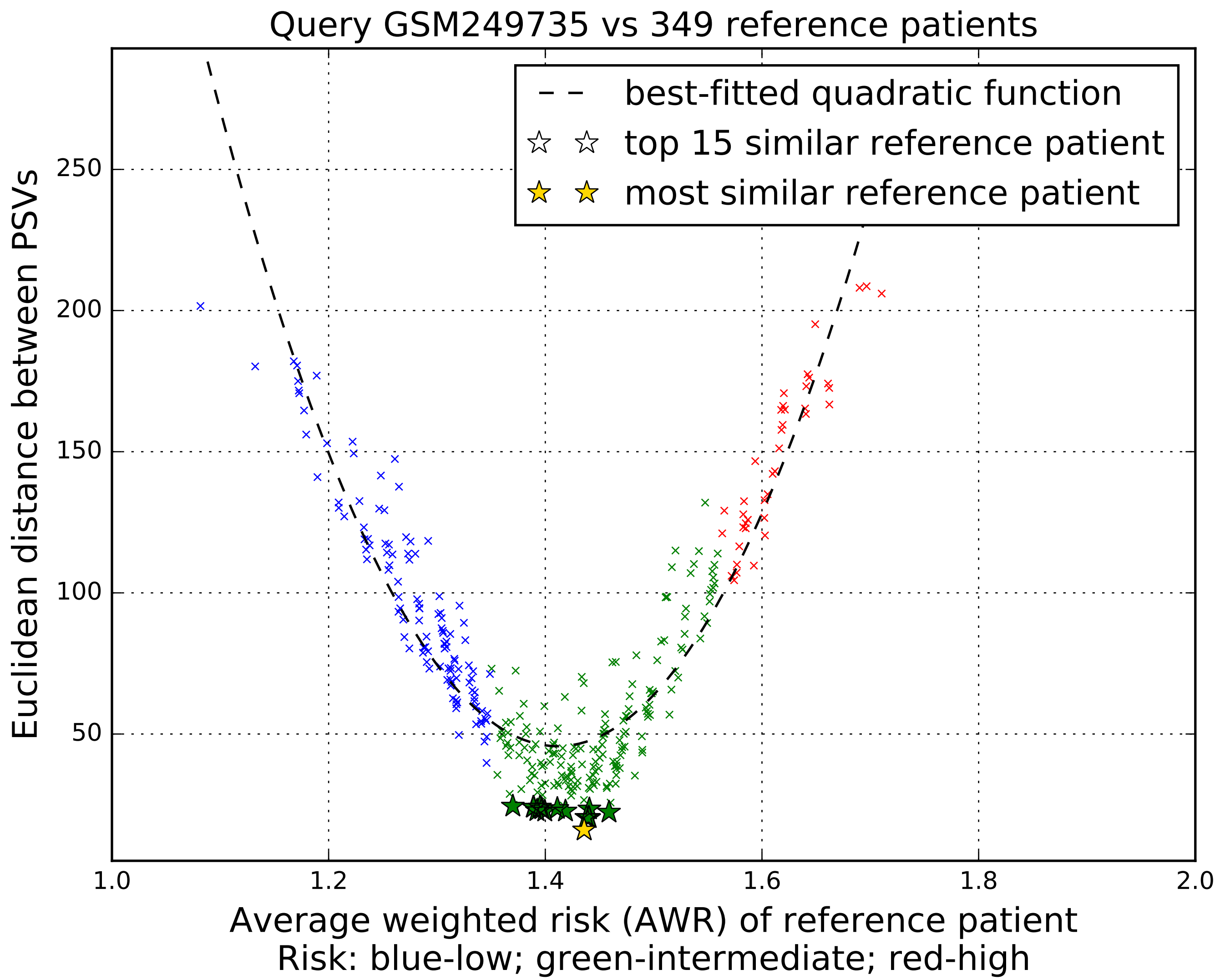

Query GSM249735 vs 349 reference patients
best-fitted quadratic function
top 15 similar reference patient
most similar reference patient
Euclidean distance between PSVs
Average weighted risk (AWR) of reference patient
Risk: blue-low; green-intermediate; red-high
250
200
150
100
50
1.0
1.2
1.4
1.6
1.8
2.0

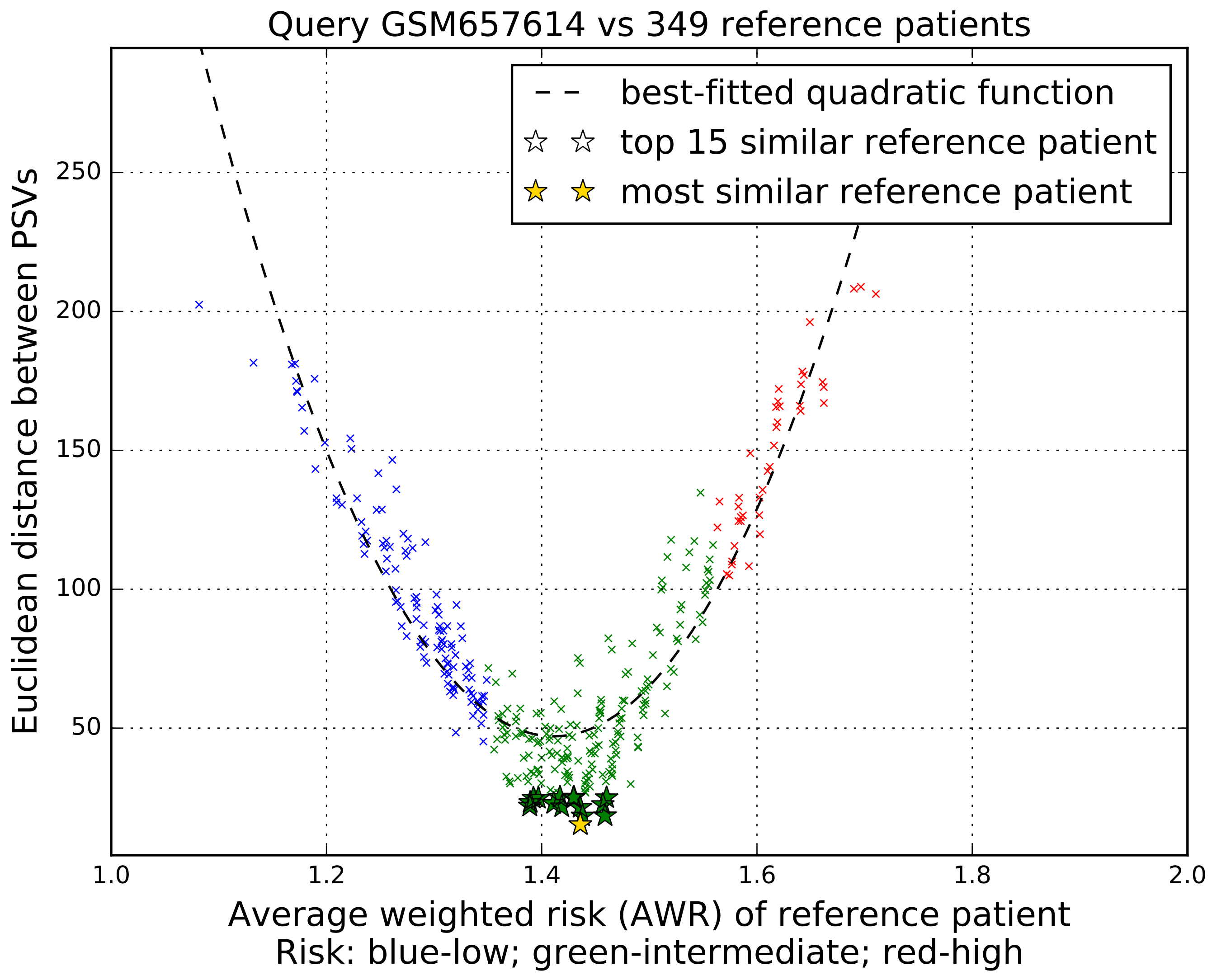
Query GSM657614 vs 349 reference patients
best-fitted quadratic function
top 15 similar reference patient
most similar reference patient
Euclidean distance between PSVs
250
200
150
100
50
1.0
1.2
1.4
1.6
1.8
2.0
Average weighted risk (AWR) of reference patient
Risk: blue-low; green-intermediate; red-high

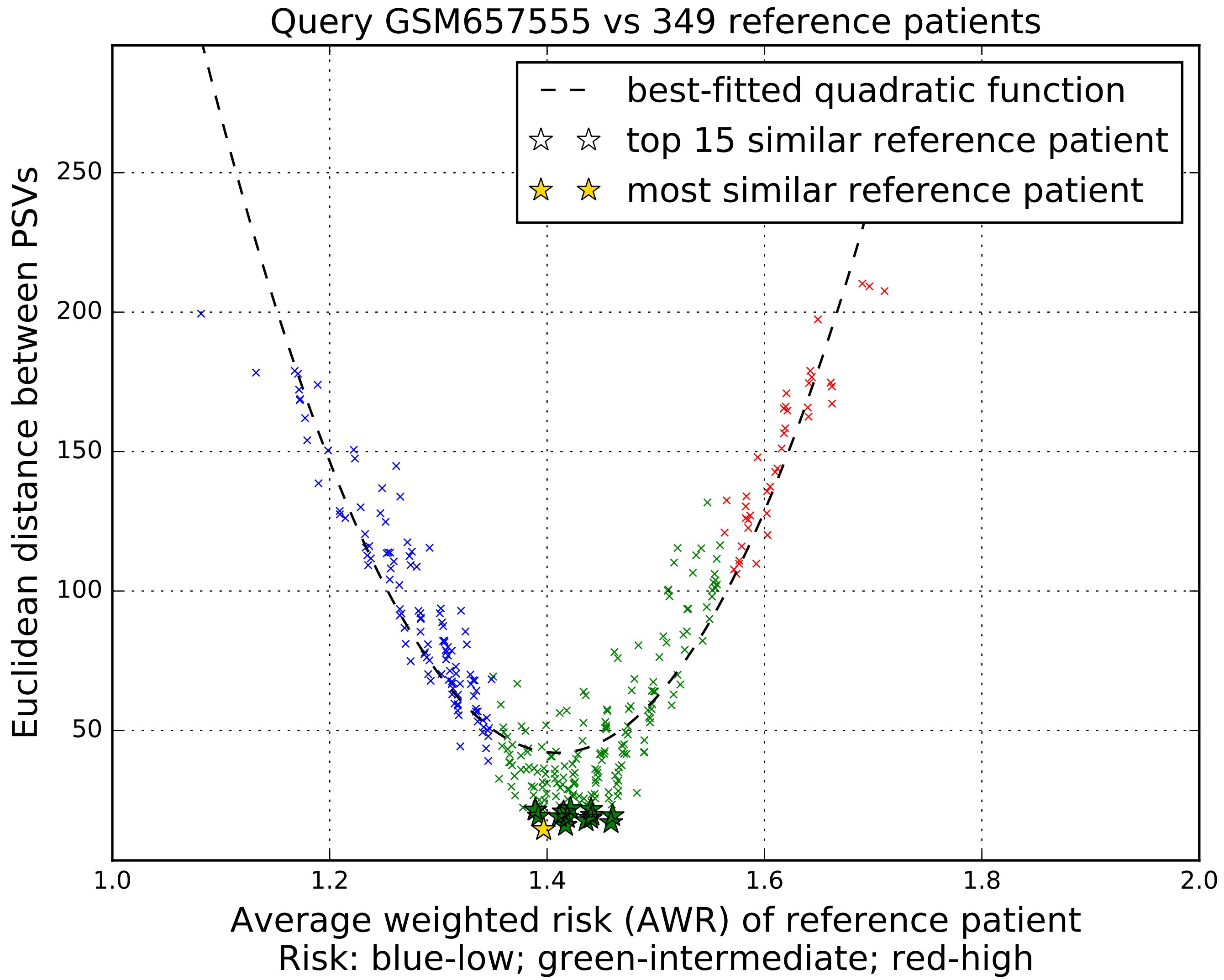
Query GSM657555 vs 349 reference patients
best-fitted quadratic function
top 15 similar reference patient
most similar reference patient
Euclidean distance between PSVs
50
100
150
200
250
1.0
1.2
1.4
1.6
1.8
2.0
Average weighted risk (AWR) of reference patient
Risk: blue-low; green-intermediate; red-high

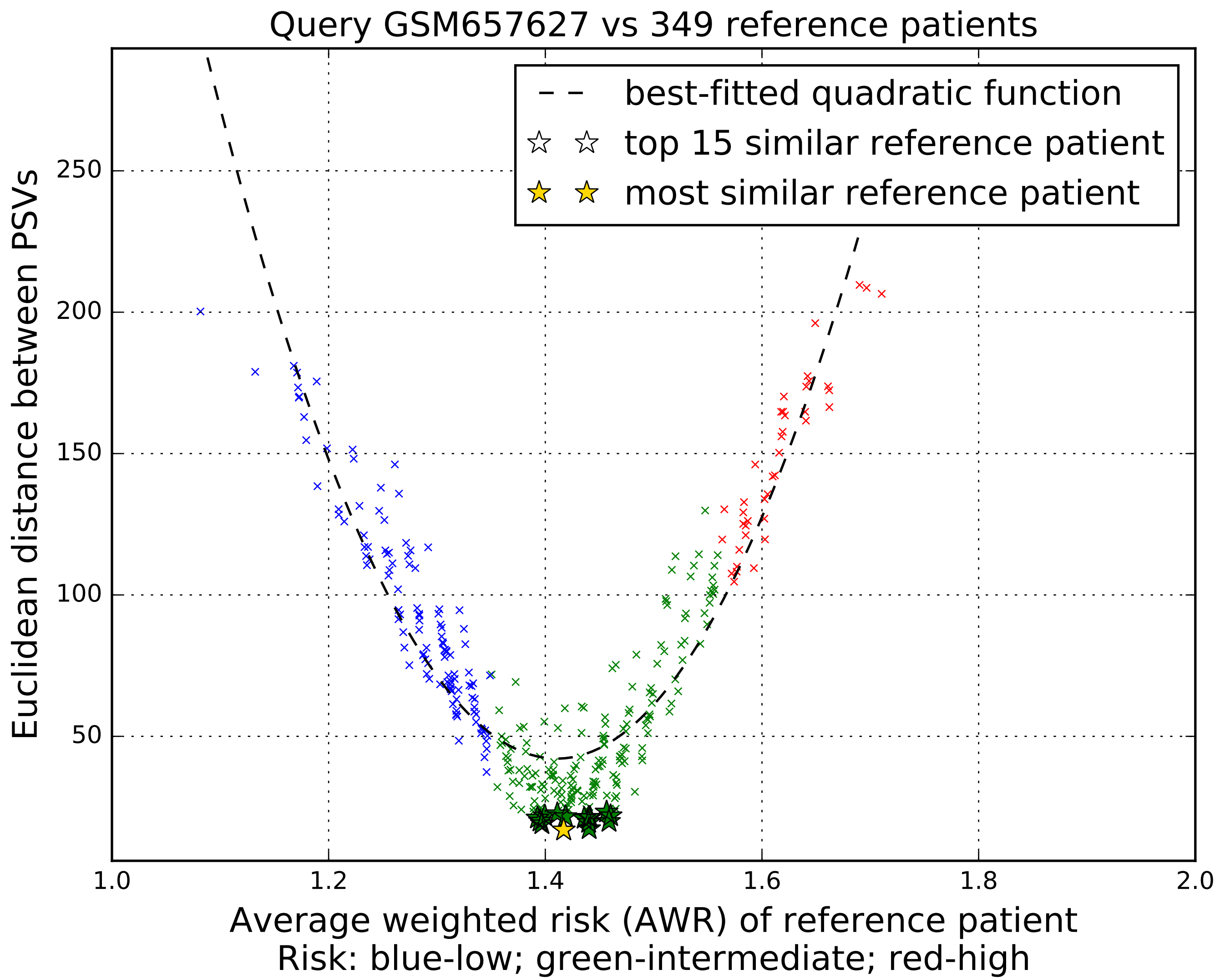

Query GSM657627 vs 349 reference patients
best-fitted quadratic function
top 15 similar reference patient
most similar reference patient
Euclidean distance between PSVs
250
200
150
100
50
1.0
1.2
1.4
1.6
1.8
2.0
Average weighted risk (AWR) of reference patient
Risk: blue-low; green-intermediate; red-high

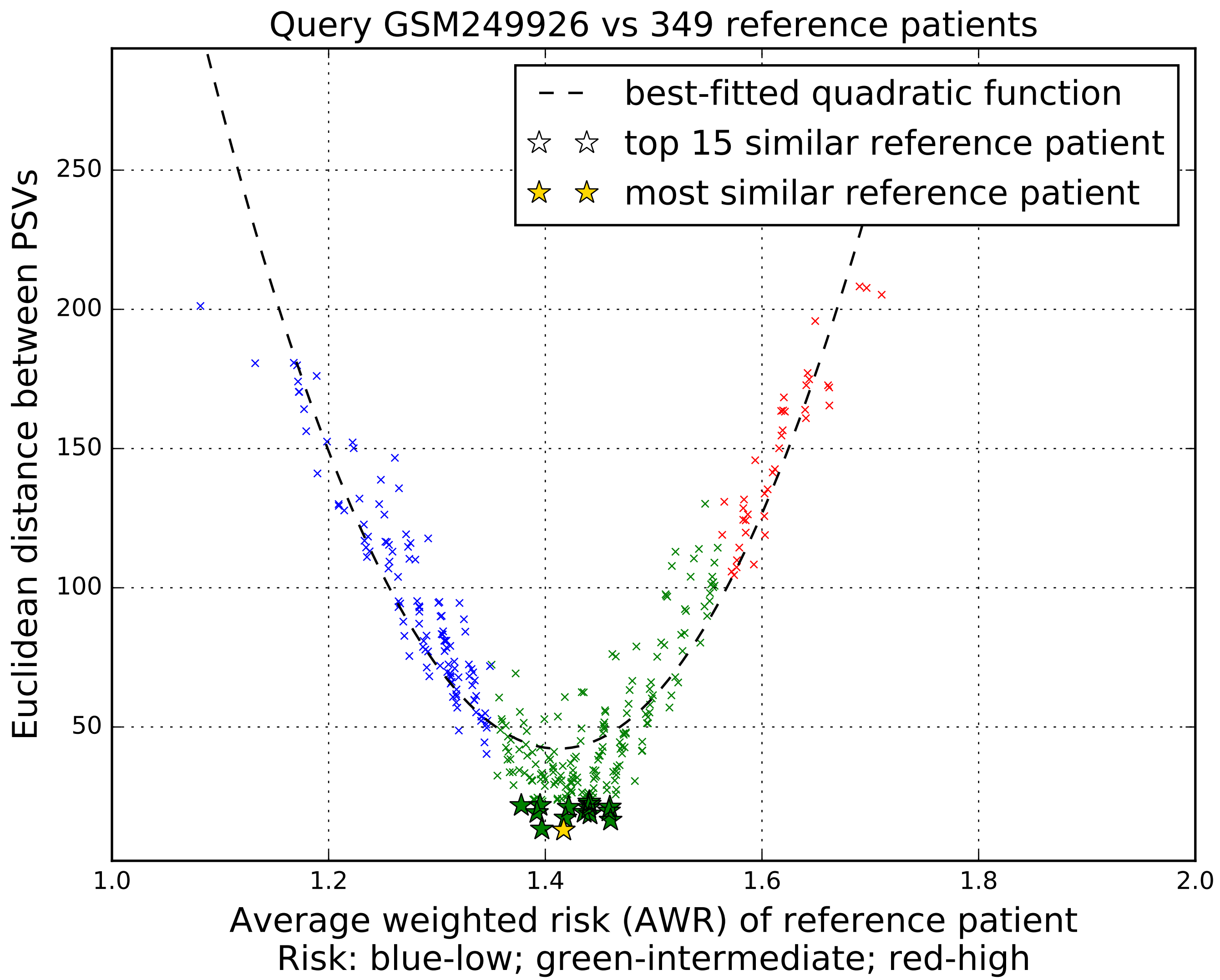
Query GSM249926 vs 349 reference patients
best-fitted quadratic function
top 15 similar reference patient
most similar reference patient
Euclidean distance between PSVs
250
200
150
100
50
1.0
1.2
1.4
1.6
1.8
2.0
Average weighted risk (AWR) of reference patient
Risk: blue-low; green-intermediate; red-high

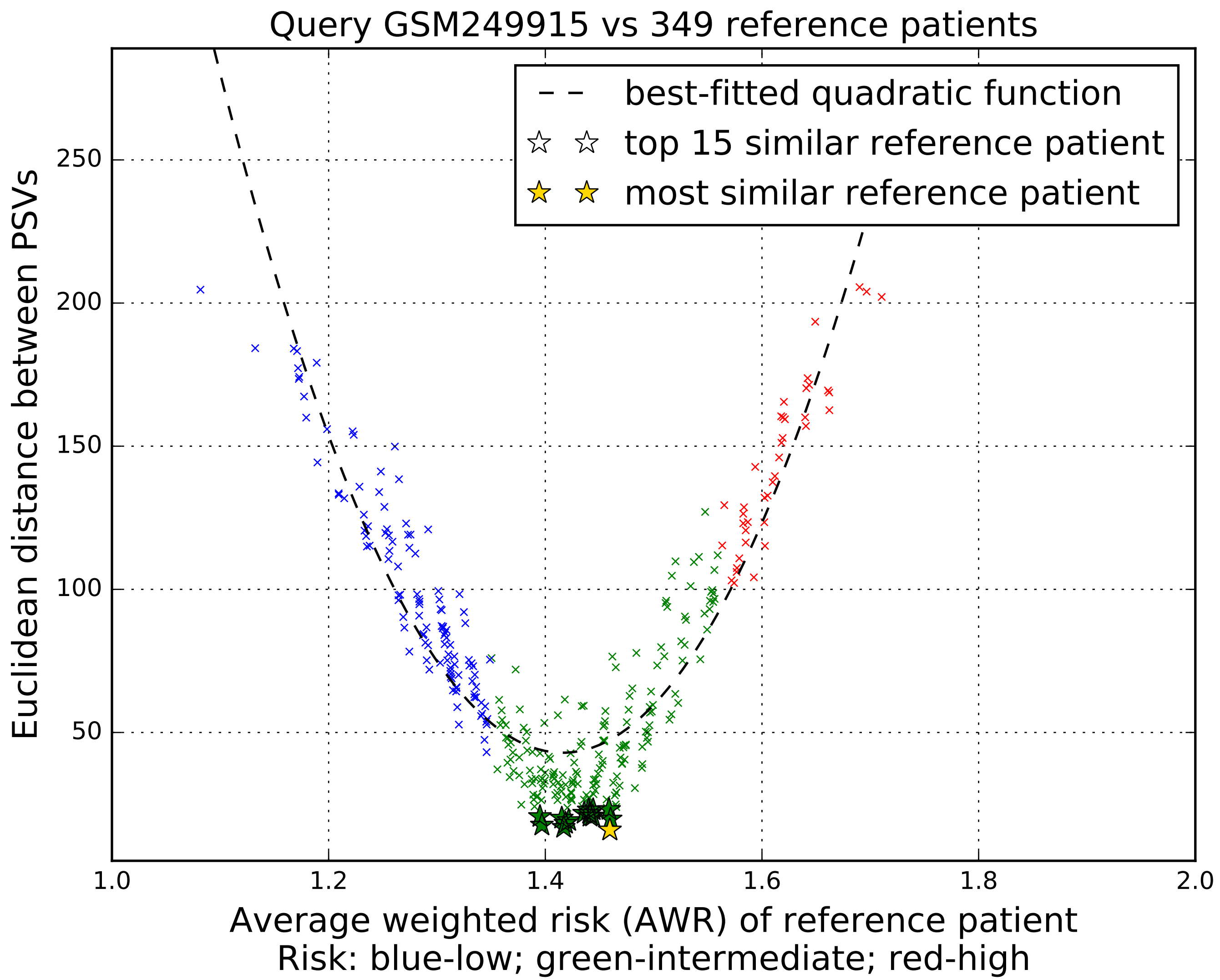
Query GSM249915 vs 349 reference patients
best-fitted quadratic function
top 15 similar reference patient
most similar reference patient
Euclidean distance between PSVs
250
200
150
100
50
1.0
1.2
1.4
1.6
1.8
2.0
Average weighted risk (AWR) of reference patient
Risk: blue-low; green-intermediate; red-high

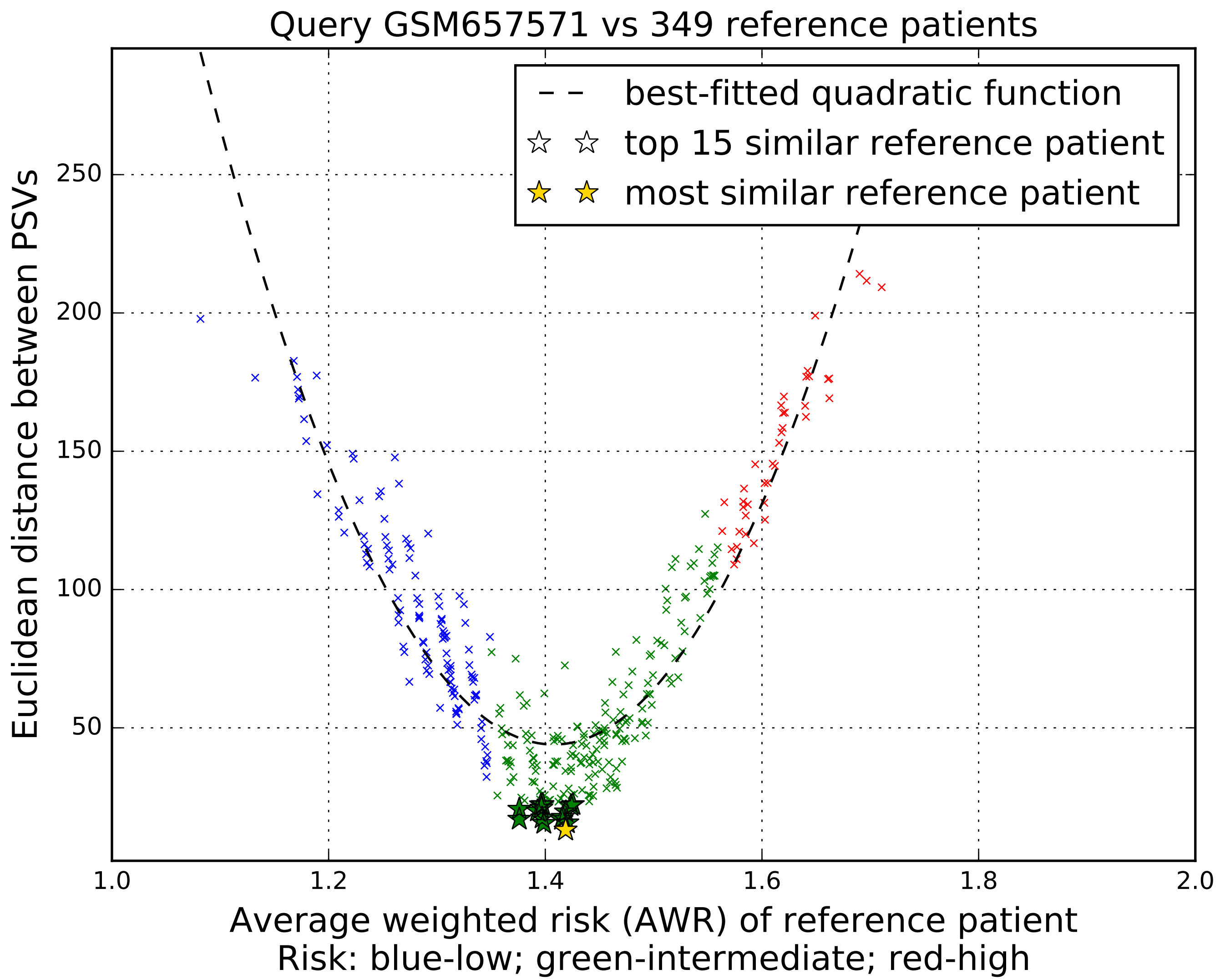
Query GSM657571 vs 349 reference patients
best-fitted quadratic function
top 15 similar reference patient
most similar reference patient
Euclidean distance between PSVs
50
100
150
200
250
1.0
1.2
1.4
1.6
1.8
2.0
Average weighted risk (AWR) of reference patient
Risk: blue-low; green-intermediate; red-high

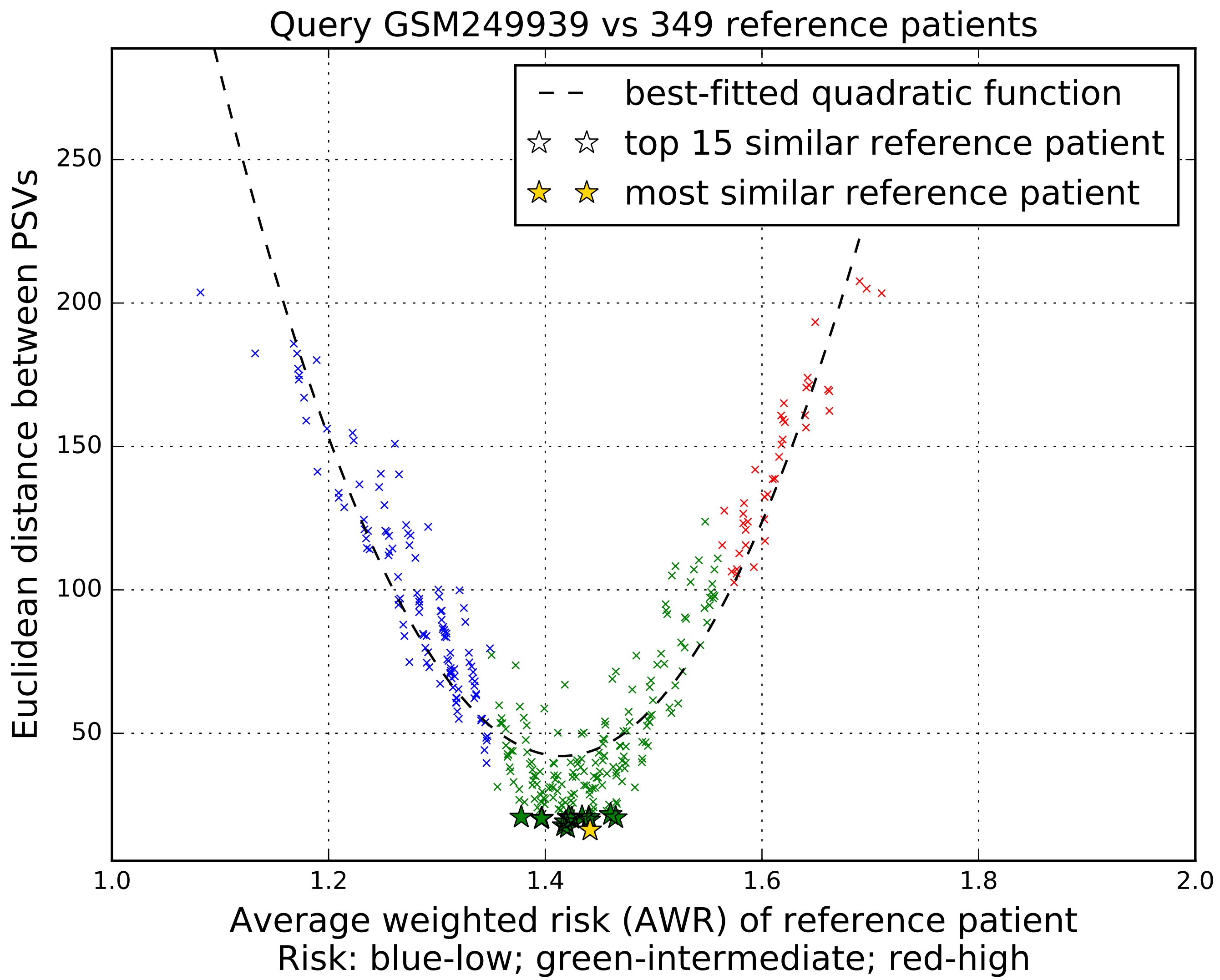

Query GSM249939 vs 349 reference patients
best-fitted quadratic function
top 15 similar reference patient
most similar reference patient
Euclidean distance between PSVs
250
200
150
100
50
1.0
1.2
1.4
1.6
1.8
2.0
Average weighted risk (AWR) of reference patient
Risk: blue-low; green-intermediate; red-high

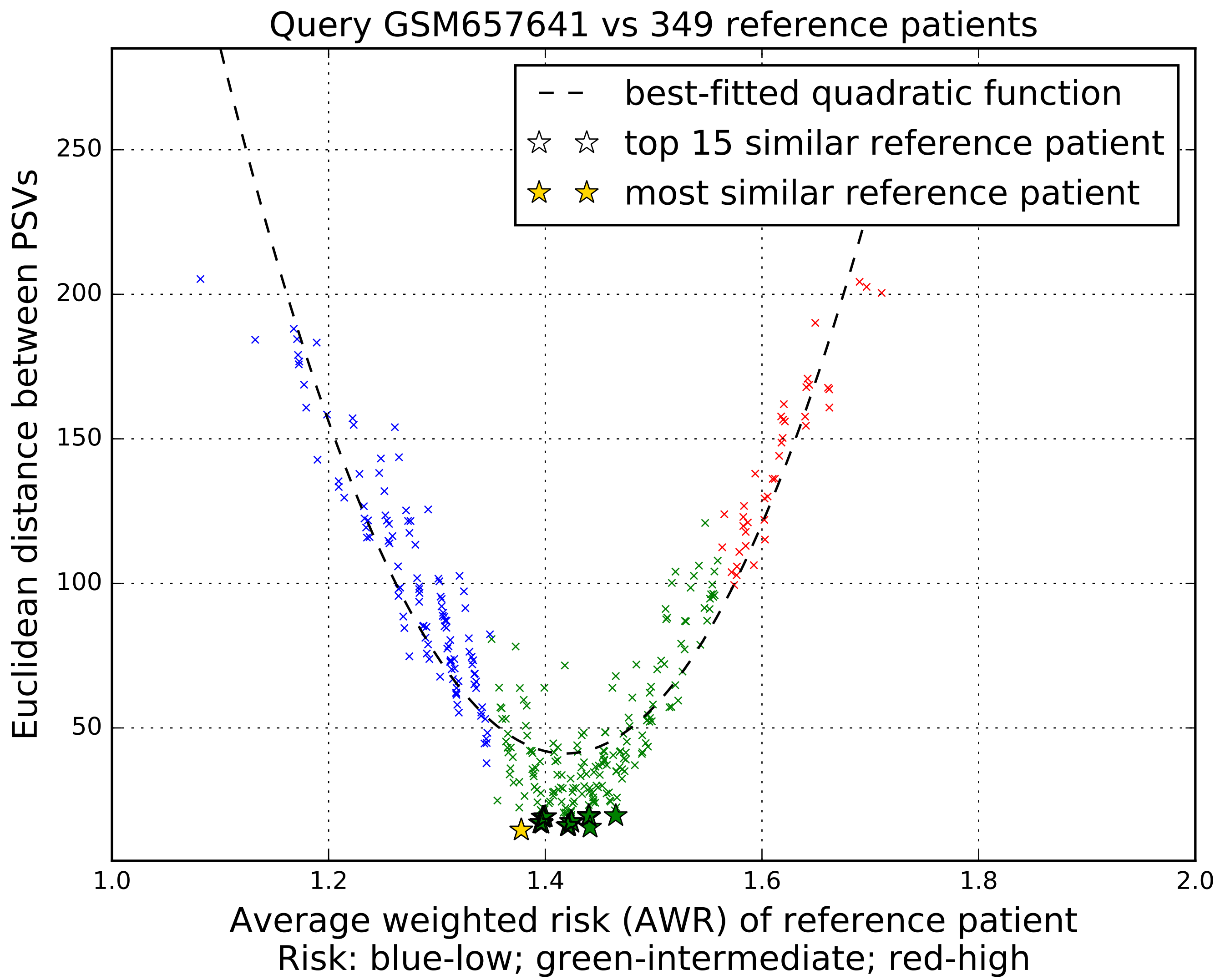

Query GSM657641 vs 349 reference patients
best-fitted quadratic function
top 15 similar reference patient
most similar reference patient
Euclidean distance between PSVs
50
100
150
200
250
1.0
1.2
1.4
1.6
1.8
2.0
Average weighted risk (AWR) of reference patient
Risk: blue-low; green-intermediate; red-high

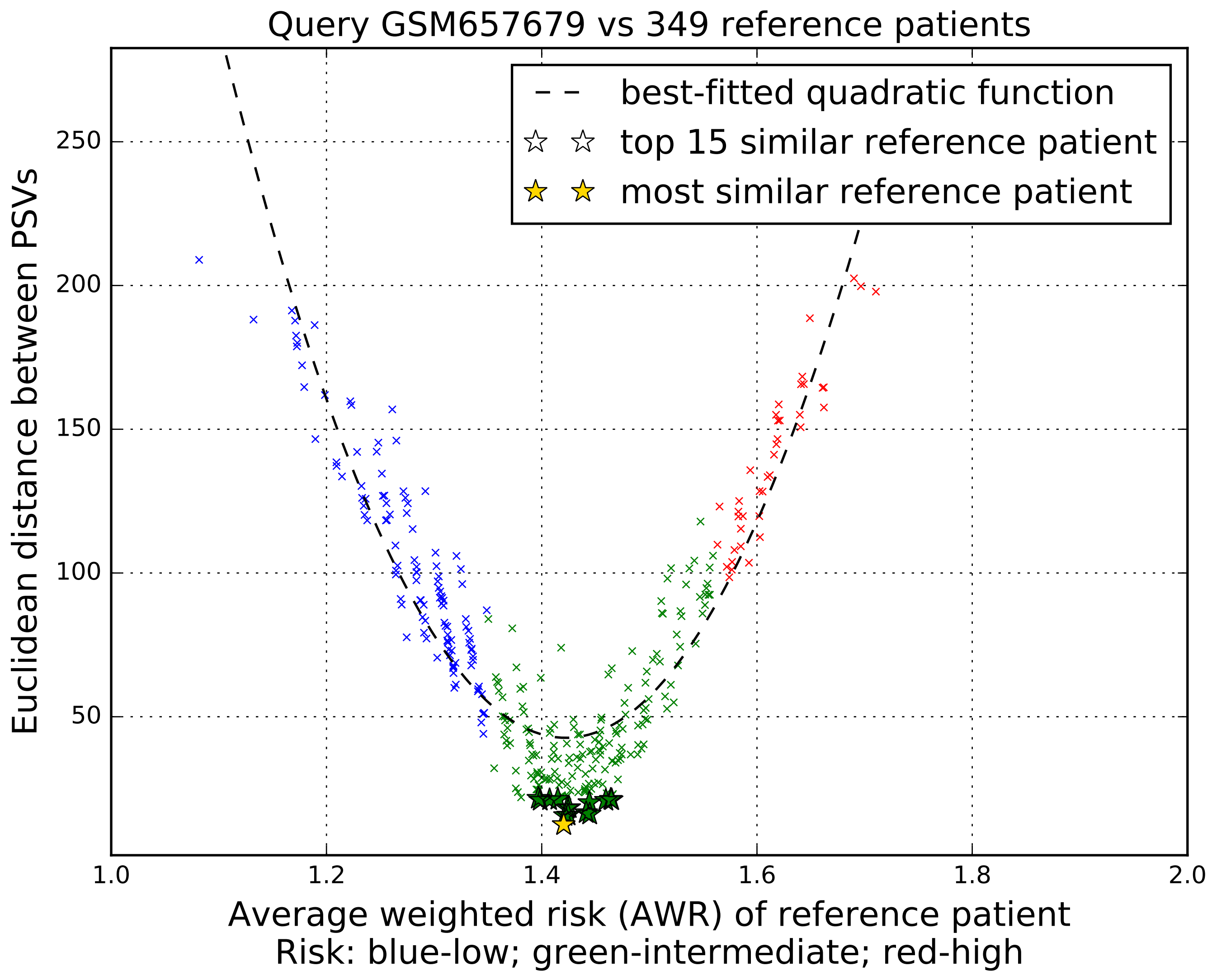

Query GSM657679 vs 349 reference patients
best-fitted quadratic function
top 15 similar reference patient
most similar reference patient
Euclidean distance between PSVs
50
100
150
200
250
1.0
1.2
1.4
1.6
1.8
2.0
Average weighted risk (AWR) of reference patient
Risk: blue-low; green-intermediate; red-high

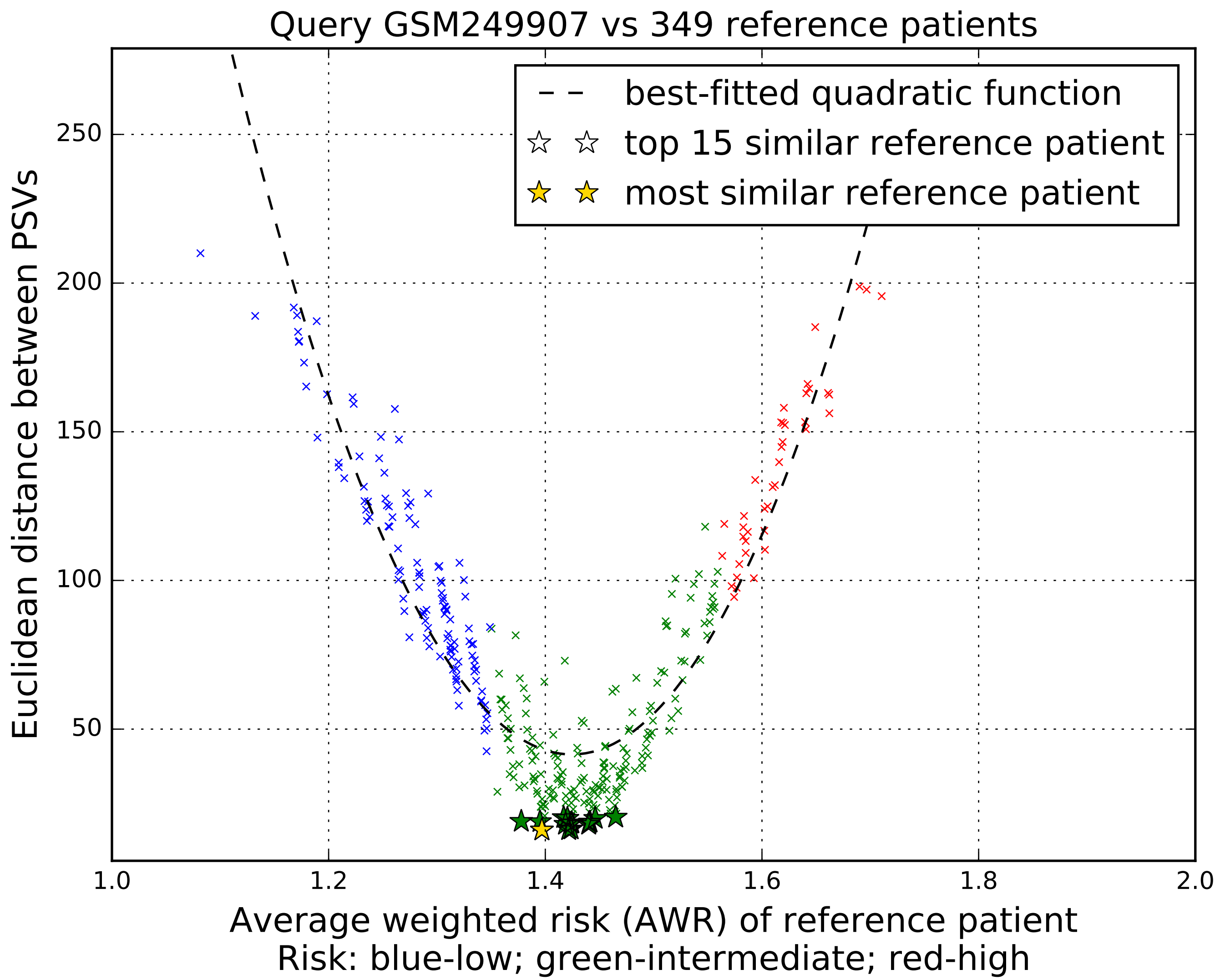

Query GSM249907 vs 349 reference patients
best-fitted quadratic function
top 15 similar reference patient
most similar reference patient
Euclidean distance between PSVs
250
200
150
100
50
1.0
1.2
1.4
1.6
1.8
2.0
Average weighted risk (AWR) of reference patient
Risk: blue-low; green-intermediate; red-high

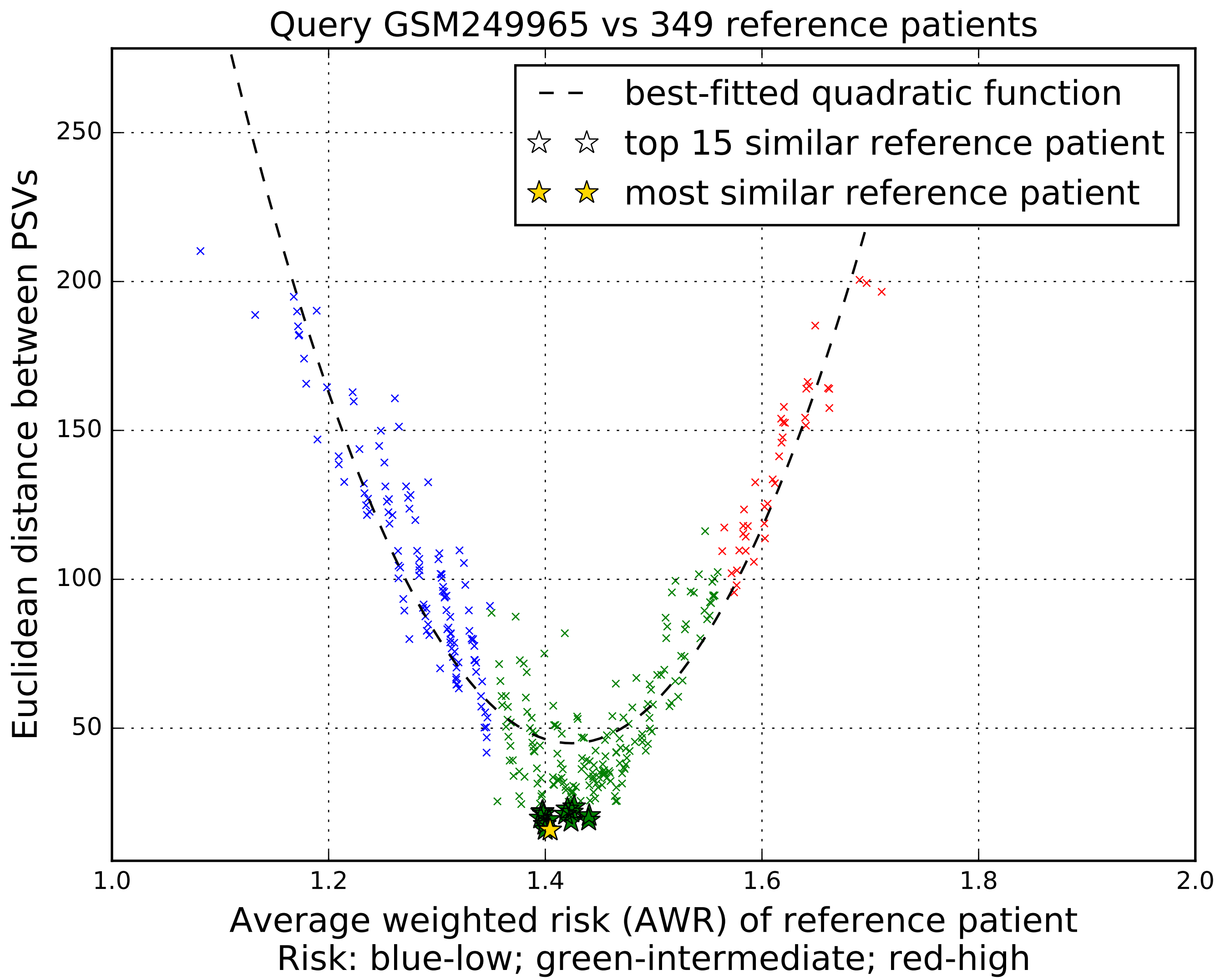

Query GSM249965 vs 349 reference patients
best-fitted quadratic function
top 15 similar reference patient
most similar reference patient
Euclidean distance between PSVs
250
200
150
100
50
1.0
1.2
1.4
1.6
1.8
2.0
Average weighted risk (AWR) of reference patient
Risk: blue-low; green-intermediate; red-high

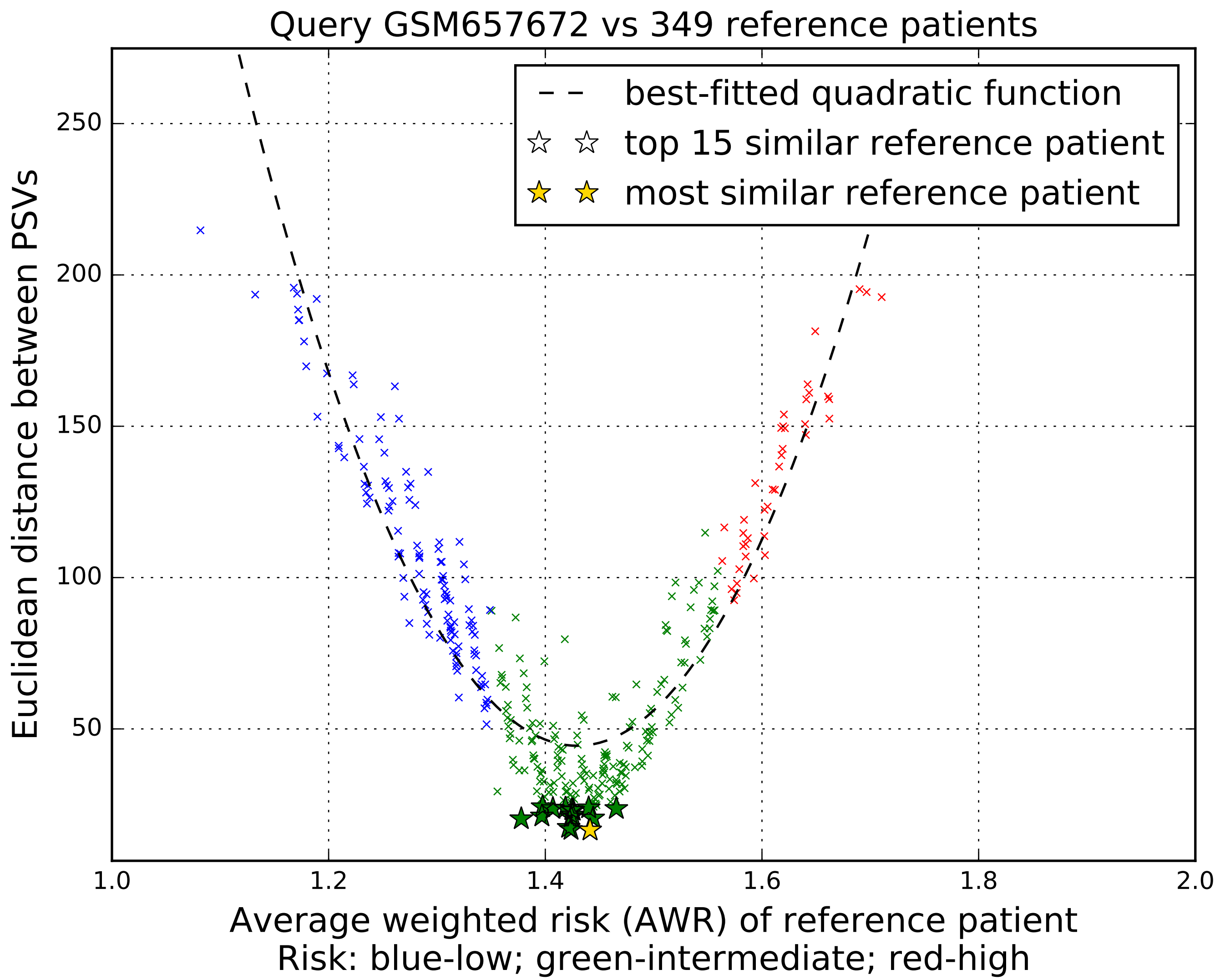
Query GSM657672 vs 349 reference patients
best-fitted quadratic function
top 15 similar reference patient
most similar reference patient
Euclidean distance between PSVs
250
200
150
100
50
1.0
1.2
1.4
1.6
1.8
2.0
Average weighted risk (AWR) of reference patient
Risk: blue-low; green-intermediate; red-high

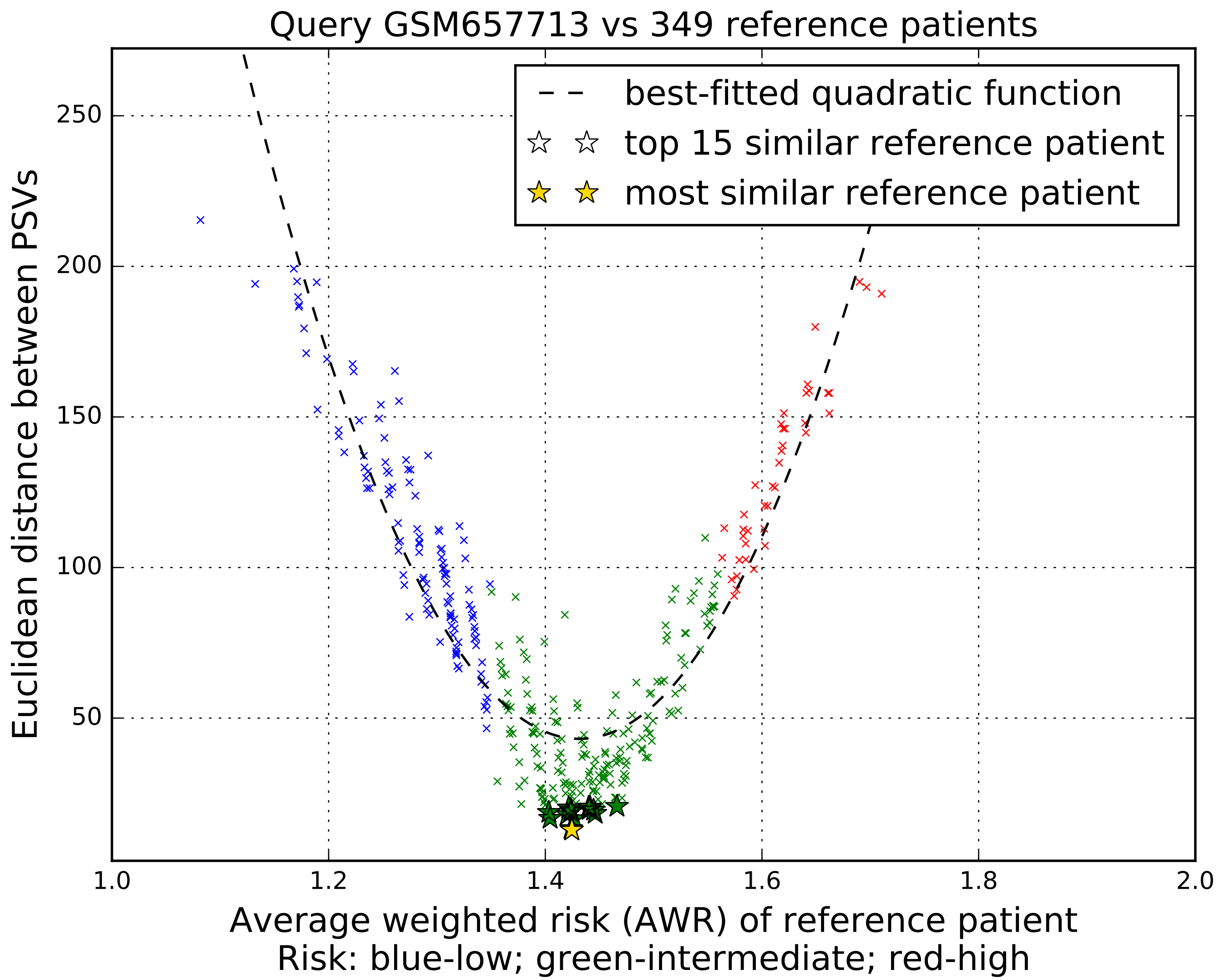

Query GSM657713 vs 349 reference patients
best-fitted quadratic function
top 15 similar reference patient
most similar reference patient
Euclidean distance between PSVs
250
200
150
100
50
1.0
1.2
1.4
1.6
1.8
2.0
Average weighted risk (AWR) of reference patient
Risk: blue-low; green-intermediate; red-high

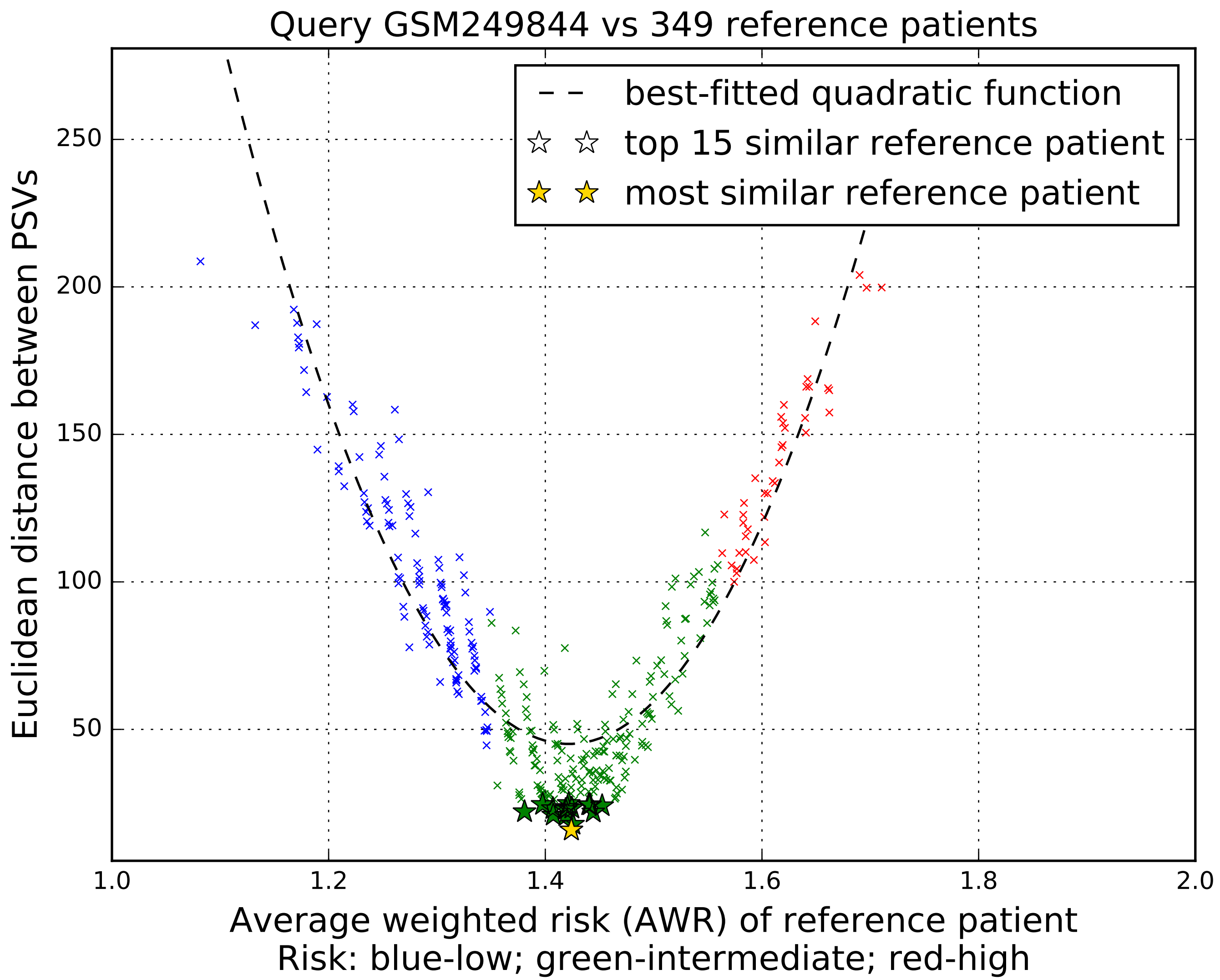
Query GSM249844 vs 349 reference patients
best-fitted quadratic function
top 15 similar reference patient
most similar reference patient
Euclidean distance between PSVs
250
200
150
100
50
1.0
1.2
1.4
1.6
1.8
2.0
Average weighted risk (AWR) of reference patient
Risk: blue-low; green-intermediate; red-high

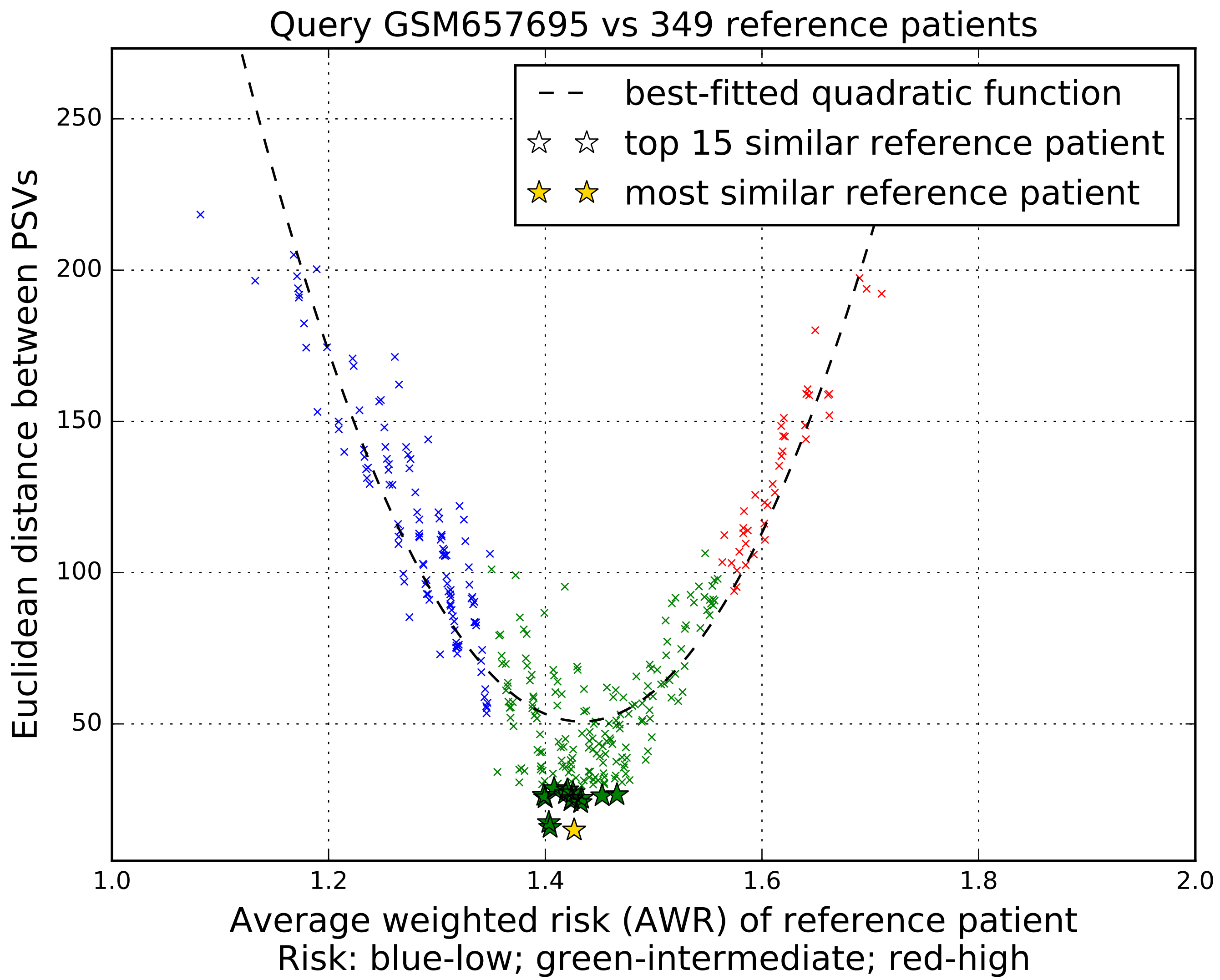
Query GSM657695 vs 349 reference patients
best-fitted quadratic function
top 15 similar reference patient
most similar reference patient
Euclidean distance between PSVs
250
200
150
100
50
1.0
1.2
1.4
1.6
1.8
2.0
Average weighted risk (AWR) of reference patient
Risk: blue-low; green-intermediate; red-high

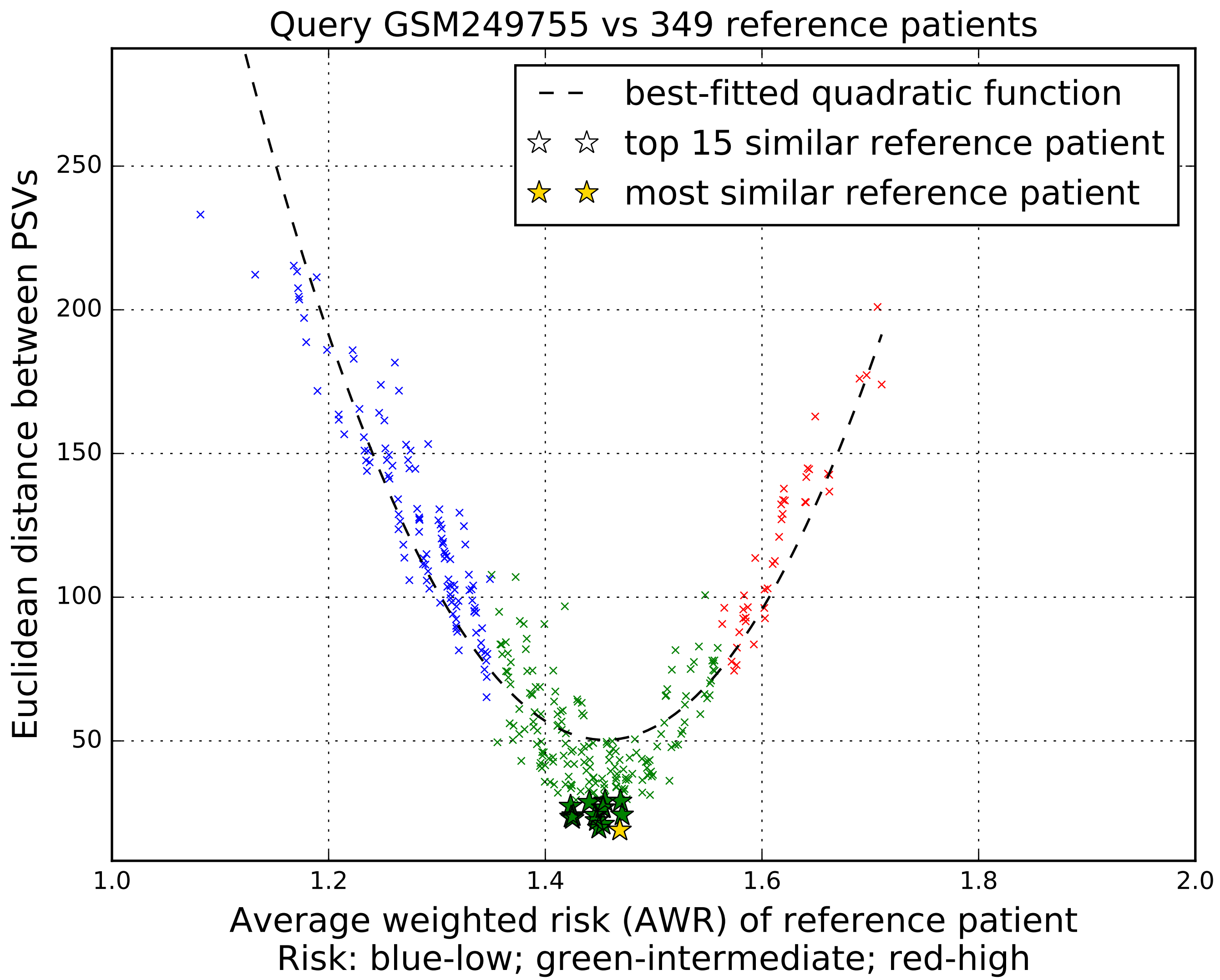
Query GSM249755 vs 349 reference patients
best-fitted quadratic function
top 15 similar reference patient
most similar reference patient
Euclidean distance between PSVs
250
200
150
100
50
1.0
1.2
1.4
1.6
1.8
2.0
Average weighted risk (AWR) of reference patient
Risk: blue-low; green-intermediate; red-high

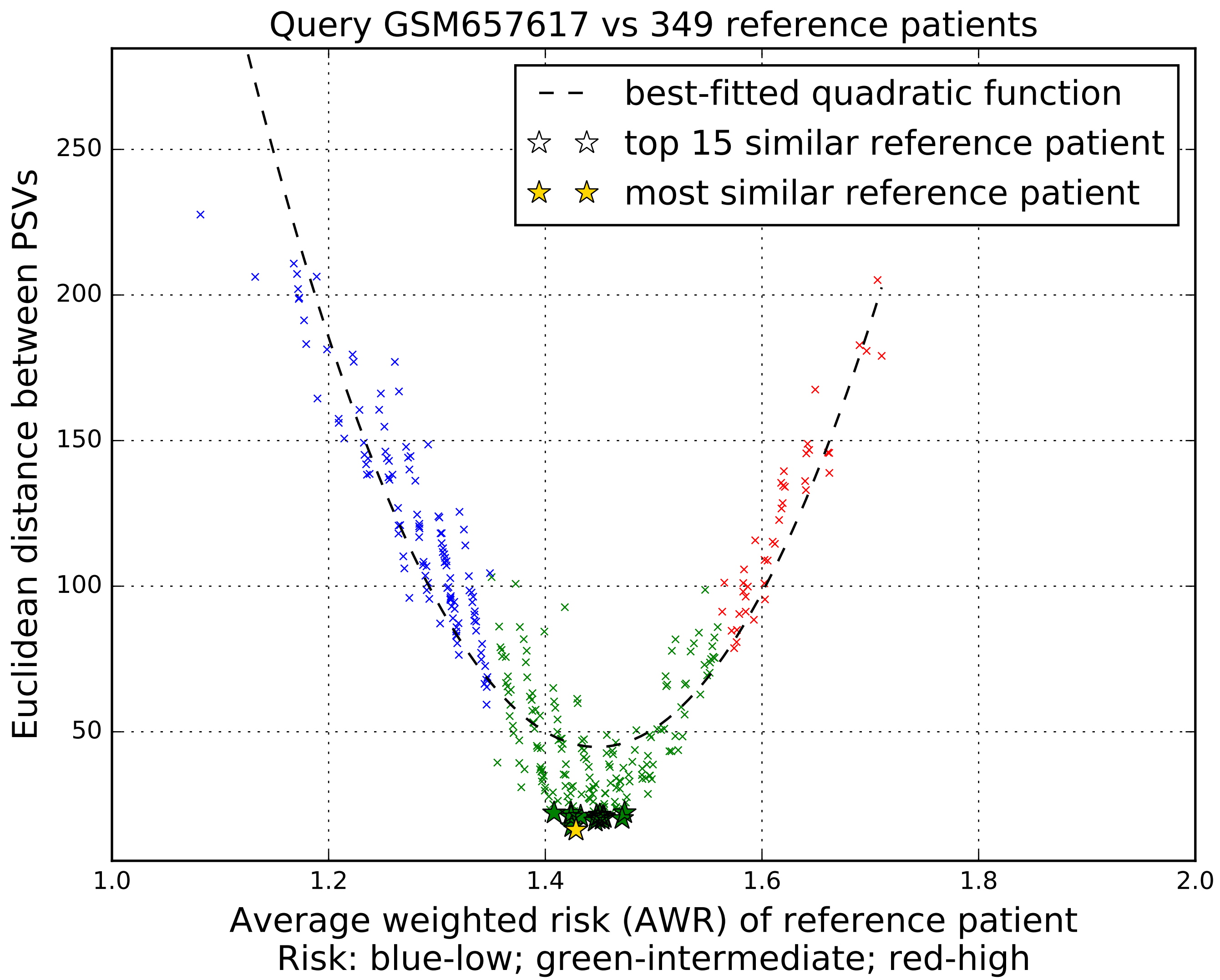
Query GSM657617 vs 349 reference patients
best-fitted quadratic function
top 15 similar reference patient
most similar reference patient
Euclidean distance between PSVs
250
200
150
100
50
1.0
1.2
1.4
1.6
1.8
2.0
Average weighted risk (AWR) of reference patient
Risk: blue-low; green-intermediate; red-high

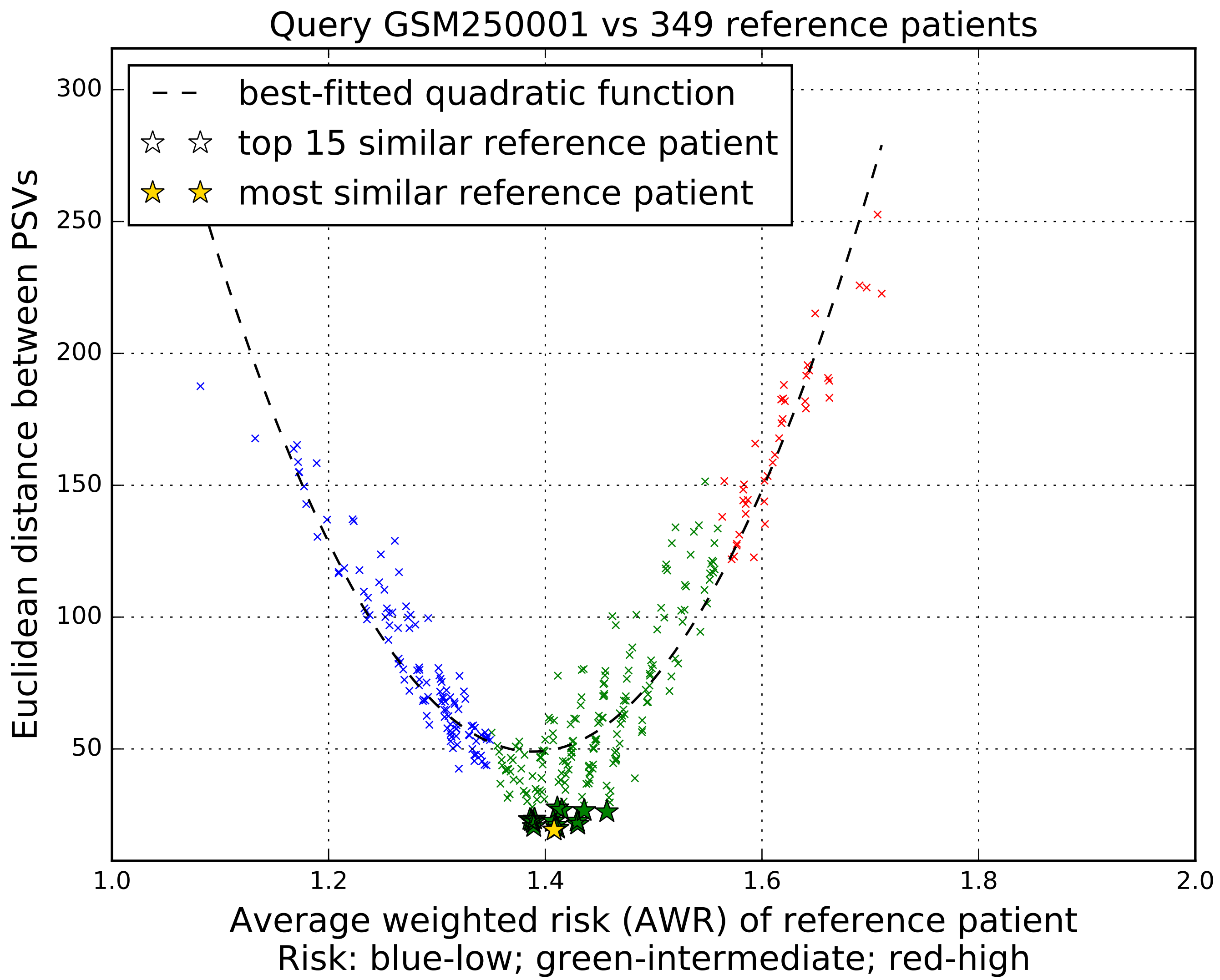

Query GSM250001 vs 349 reference patients
best-fitted quadratic function
top 15 similar reference patient
most similar reference patient
300
250
200
150
100
50
1.0
1.2
1.4
1.6
1.8
2.0
Euclidean distance between PSVs
Average weighted risk (AWR) of reference patient
Risk: blue-low; green-intermediate; red-high

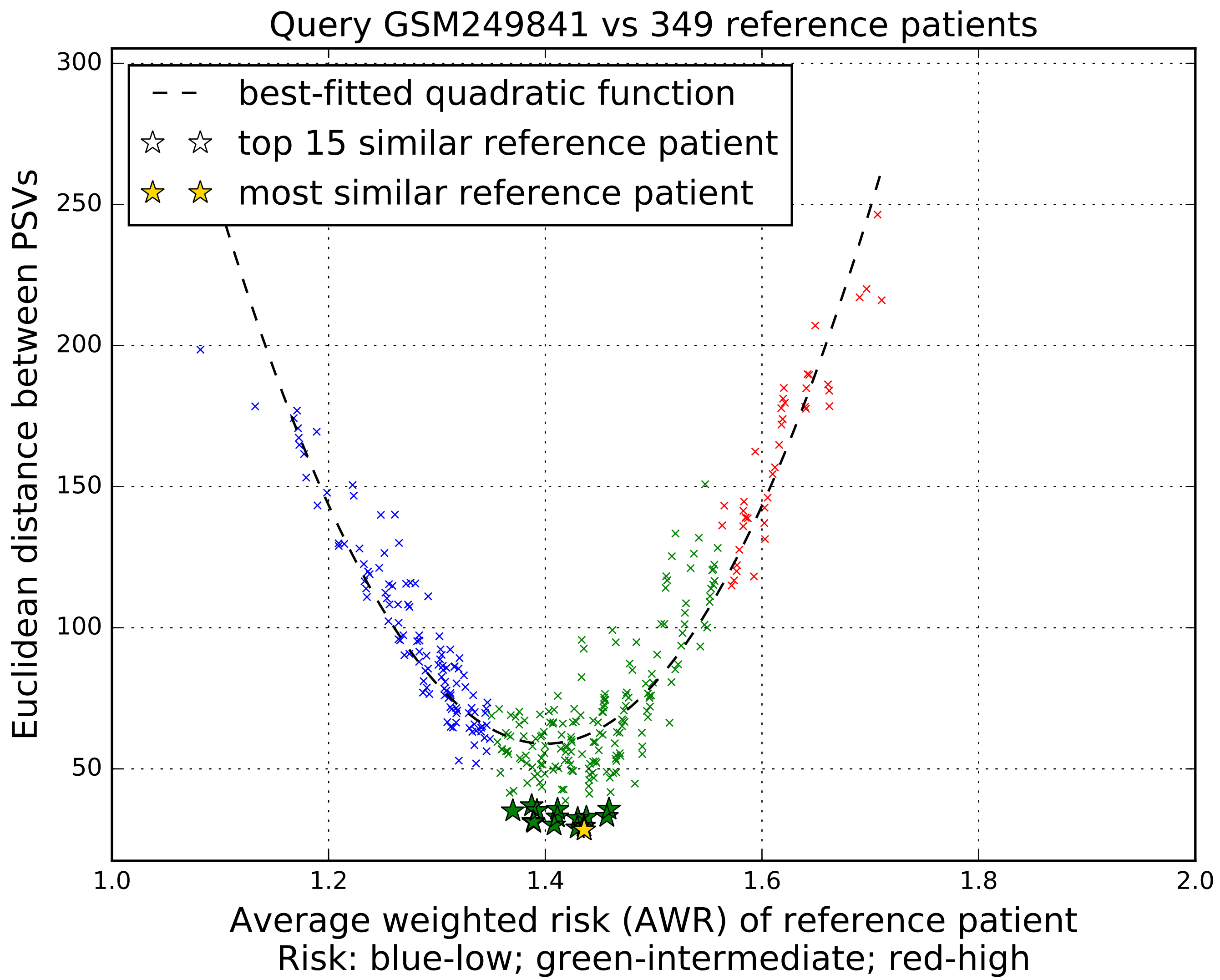
Query GSM249841 vs 349 reference patients
best-fitted quadratic function
top 15 similar reference patient
most similar reference patient
Euclidean distance between PSVs
Average weighted risk (AWR) of reference patient
Risk: blue-low; green-intermediate; red-high
300
250
200
150
100
50
1.0
1.2
1.4
1.6
1.8
2.0

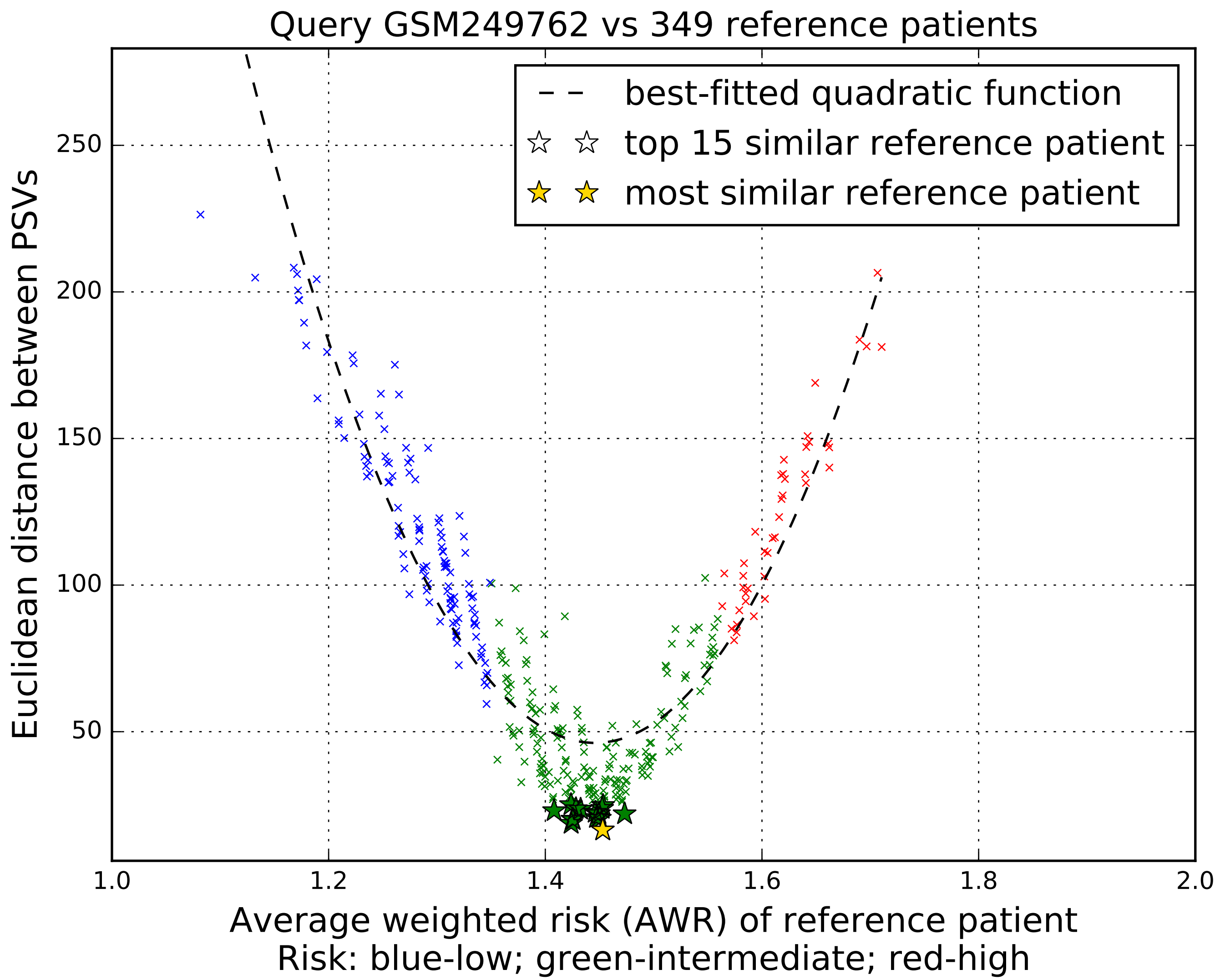

Query GSM249762 vs 349 reference patients
best-fitted quadratic function
top 15 similar reference patient
most similar reference patient
Euclidean distance between PSVs
250
200
150
100
50
1.0
1.2
1.4
1.6
1.8
2.0
Average weighted risk (AWR) of reference patient
Risk: blue-low; green-intermediate; red-high

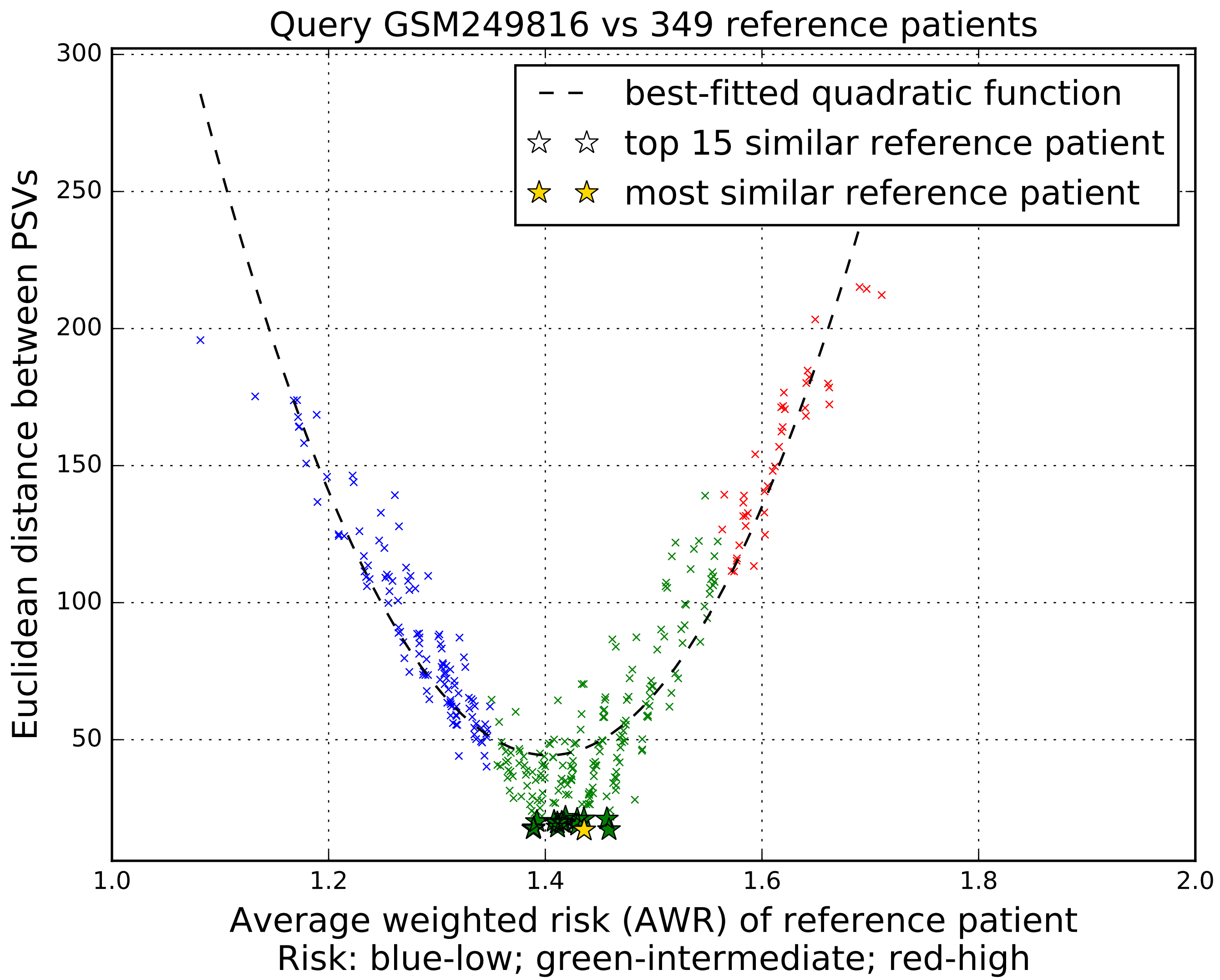
Query GSM249816 vs 349 reference patients
best-fitted quadratic function
top 15 similar reference patient
most similar reference patient
Euclidean distance between PSVs
Average weighted risk (AWR) of reference patient
Risk: blue-low; green-intermediate; red-high
300
250
200
150
100
50
1.0
1.2
1.4
1.6
1.8
2.0

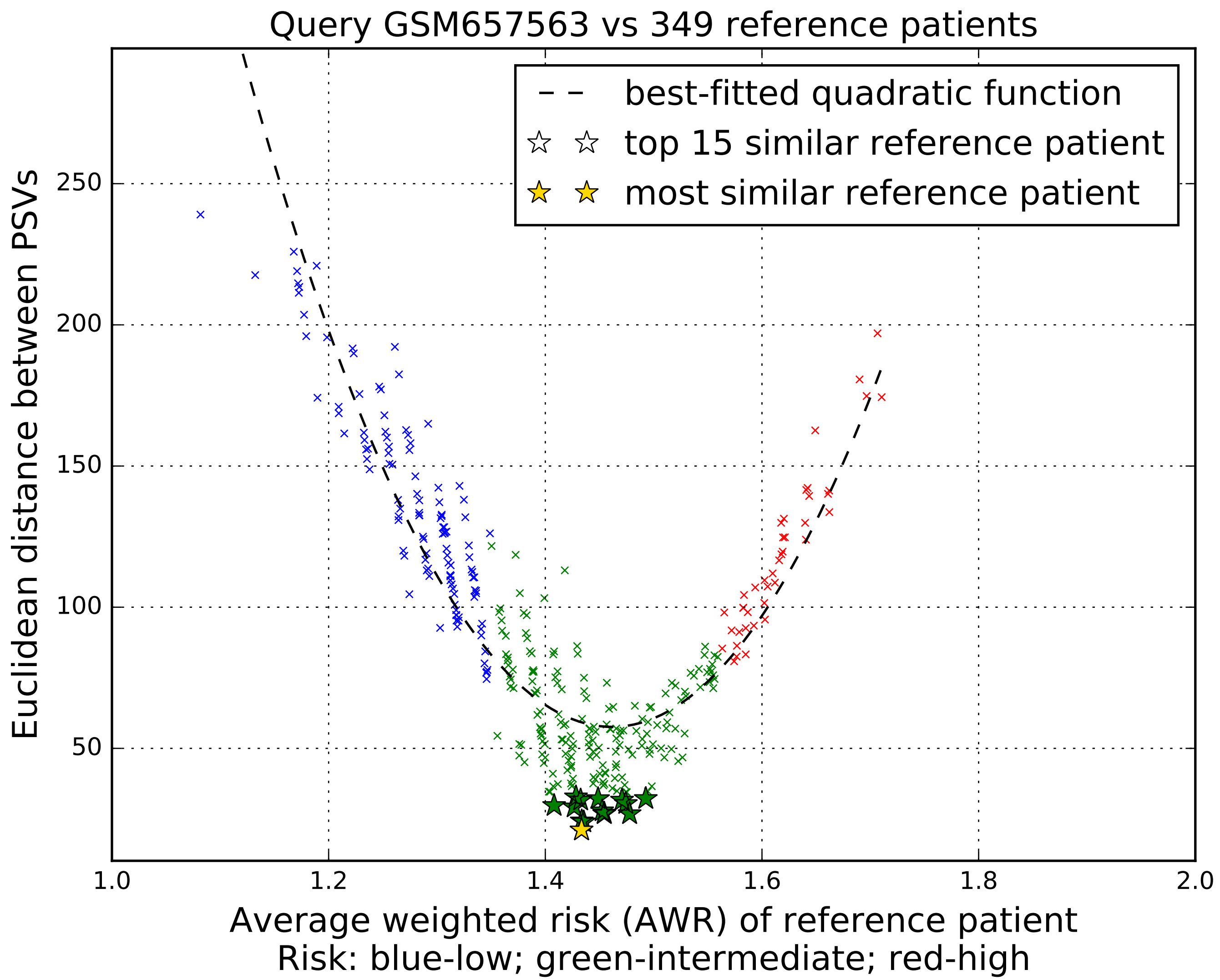

Query GSM657563 vs 349 reference patients
best-fitted quadratic function
top 15 similar reference patient
most similar reference patient
Euclidean distance between PSVs
250
200
150
100
50
1.0
1.2
1.4
1.6
1.8
2.0
Average weighted risk (AWR) of reference patient
Risk: blue-low; green-intermediate; red-high

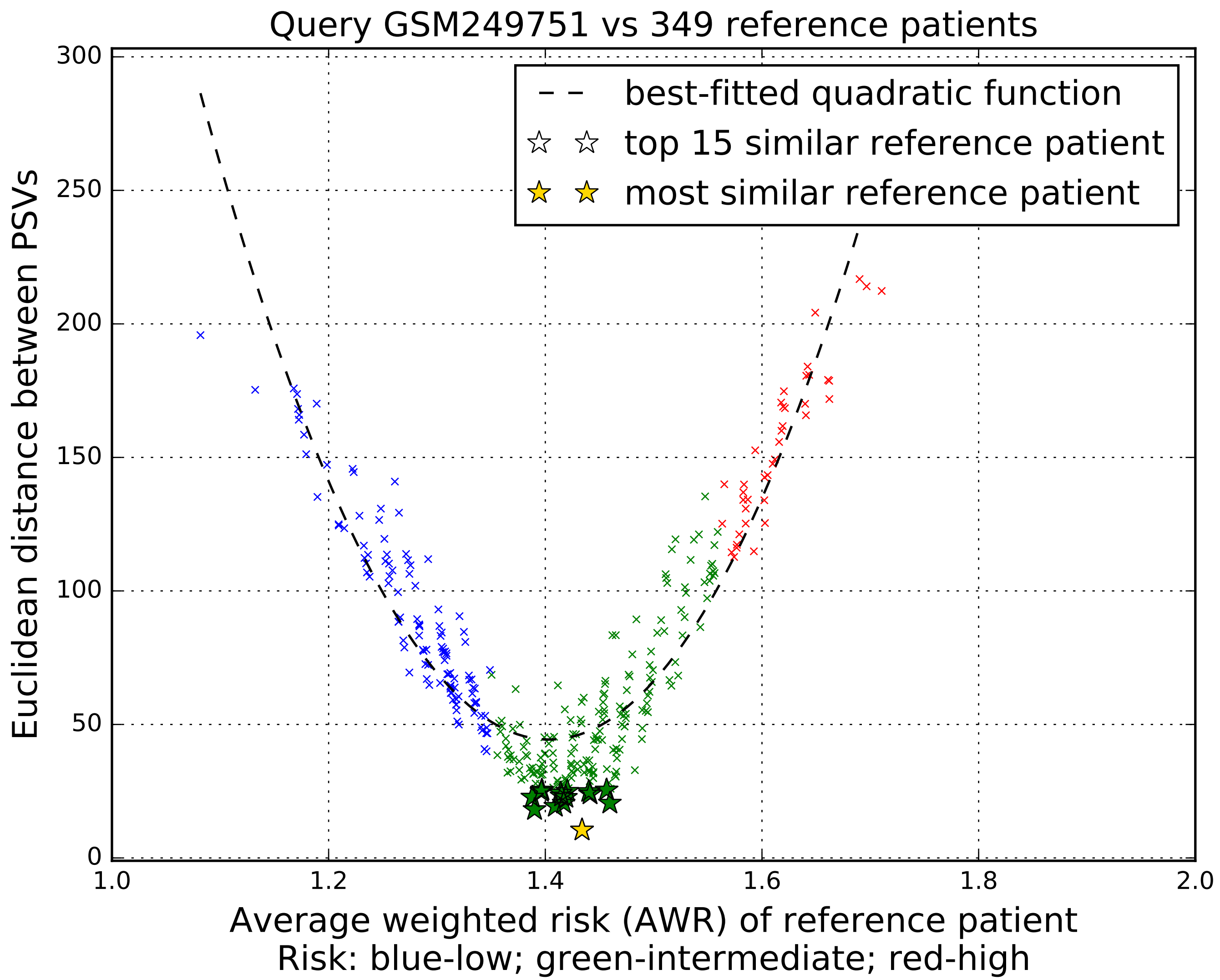
Query GSM249751 vs 349 reference patients
best-fitted quadratic function
top 15 similar reference patient
most similar reference patient
Euclidean distance between PSVs
Average weighted risk (AWR) of reference patient
Risk: blue-low; green-intermediate; red-high
0
50
100
150
200
250
300
1.0
1.2
1.4
1.6
1.8
2.0

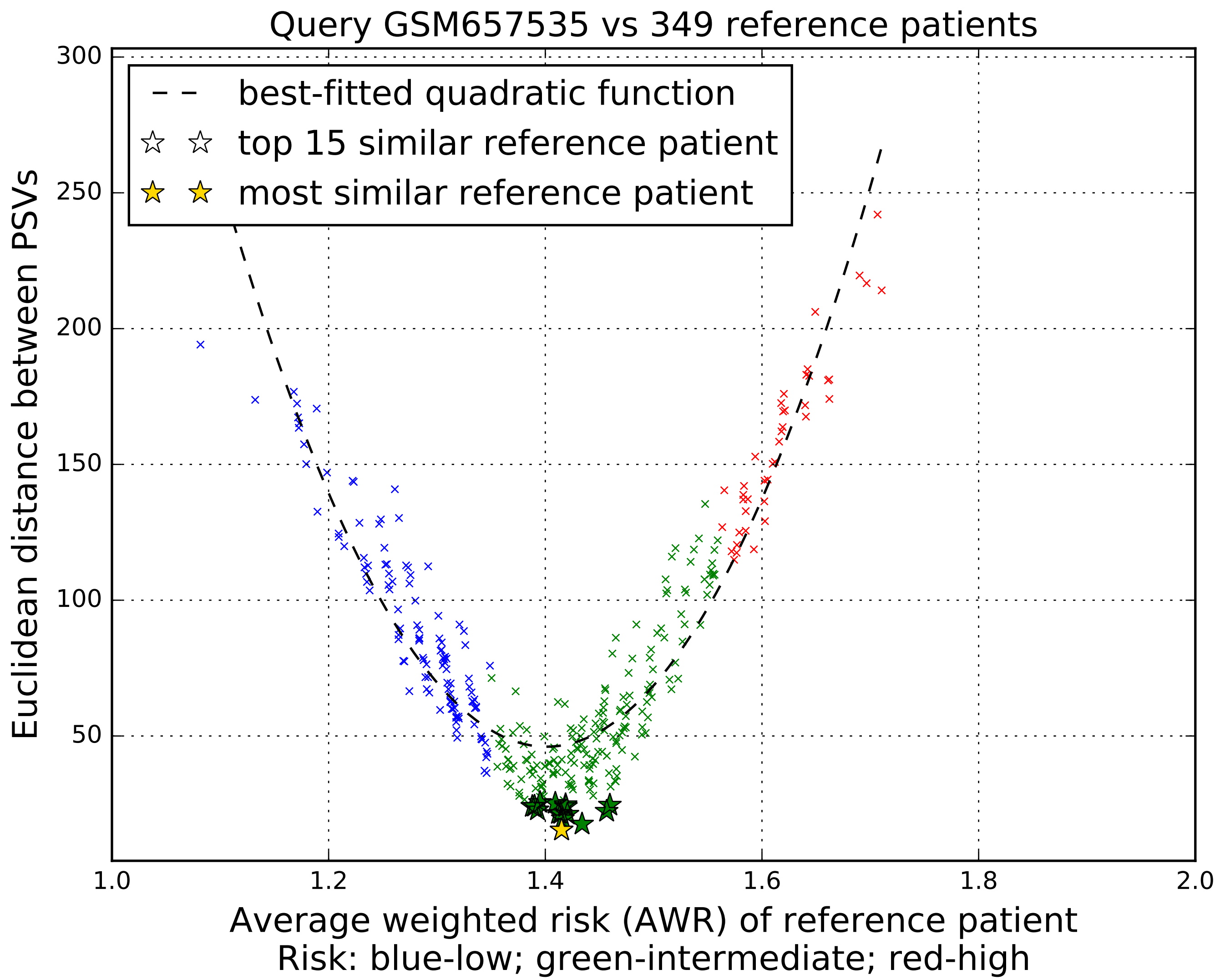
Query GSM657535 vs 349 reference patients
best-fitted quadratic function
top 15 similar reference patient
most similar reference patient
Euclidean distance between PSVs
300
250
200
150
100
50
1.0
1.2
1.4
1.6
1.8
2.0
Average weighted risk (AWR) of reference patient
Risk: blue-low; green-intermediate; red-high

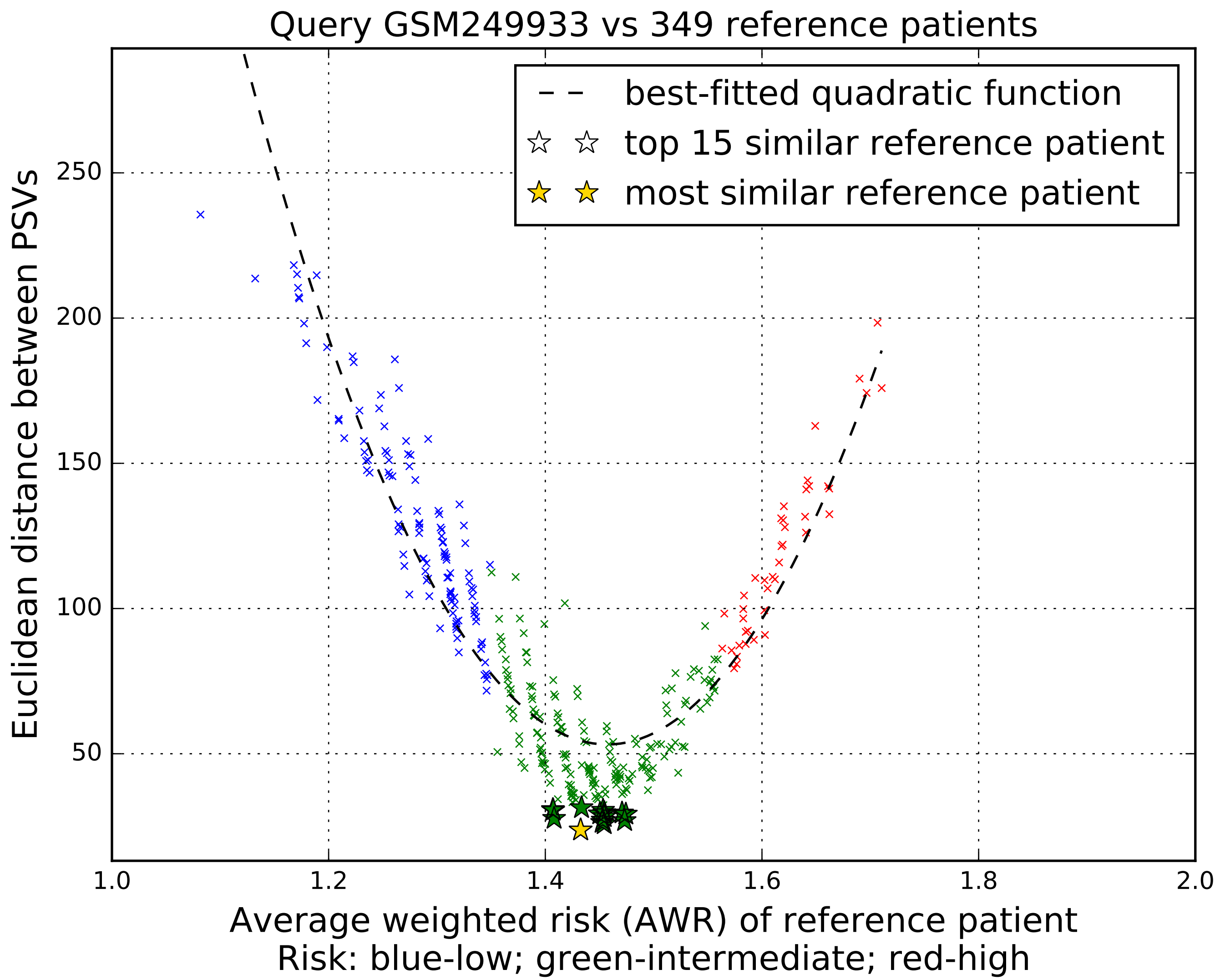

Query GSM249933 vs 349 reference patients
best-fitted quadratic function
top 15 similar reference patient
most similar reference patient
Euclidean distance between PSVs
250
200
150
100
50
1.0
1.2
1.4
1.6
1.8
2.0
Average weighted risk (AWR) of reference patient
Risk: blue-low; green-intermediate; red-high

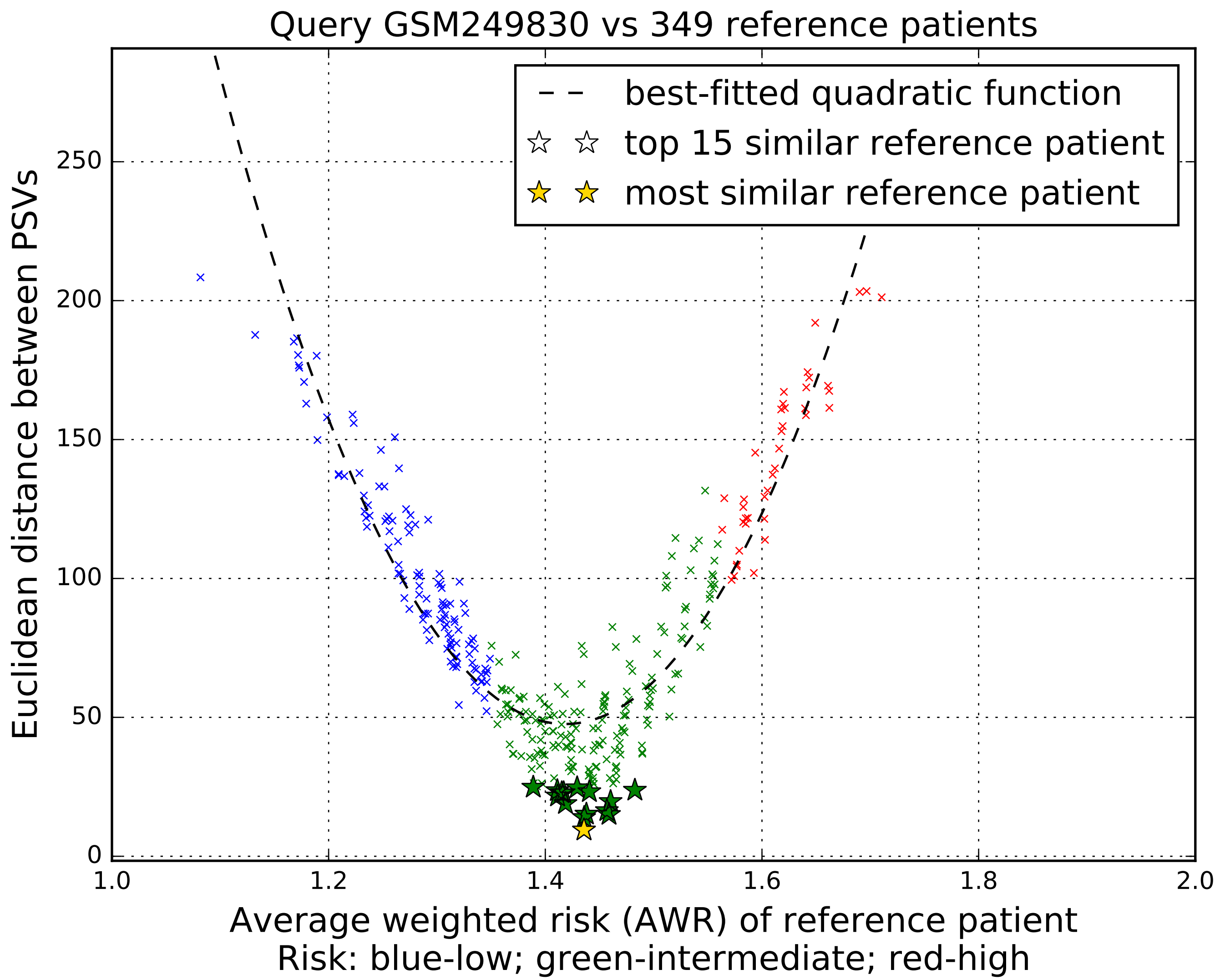
Query GSM249830 vs 349 reference patients
best-fitted quadratic function
top 15 similar reference patient
most similar reference patient
Euclidean distance between PSVs
0
50
100
150
200
250
1.0
1.2
1.4
1.6
1.8
2.0
Average weighted risk (AWR) of reference patient
Risk: blue-low; green-intermediate; red-high

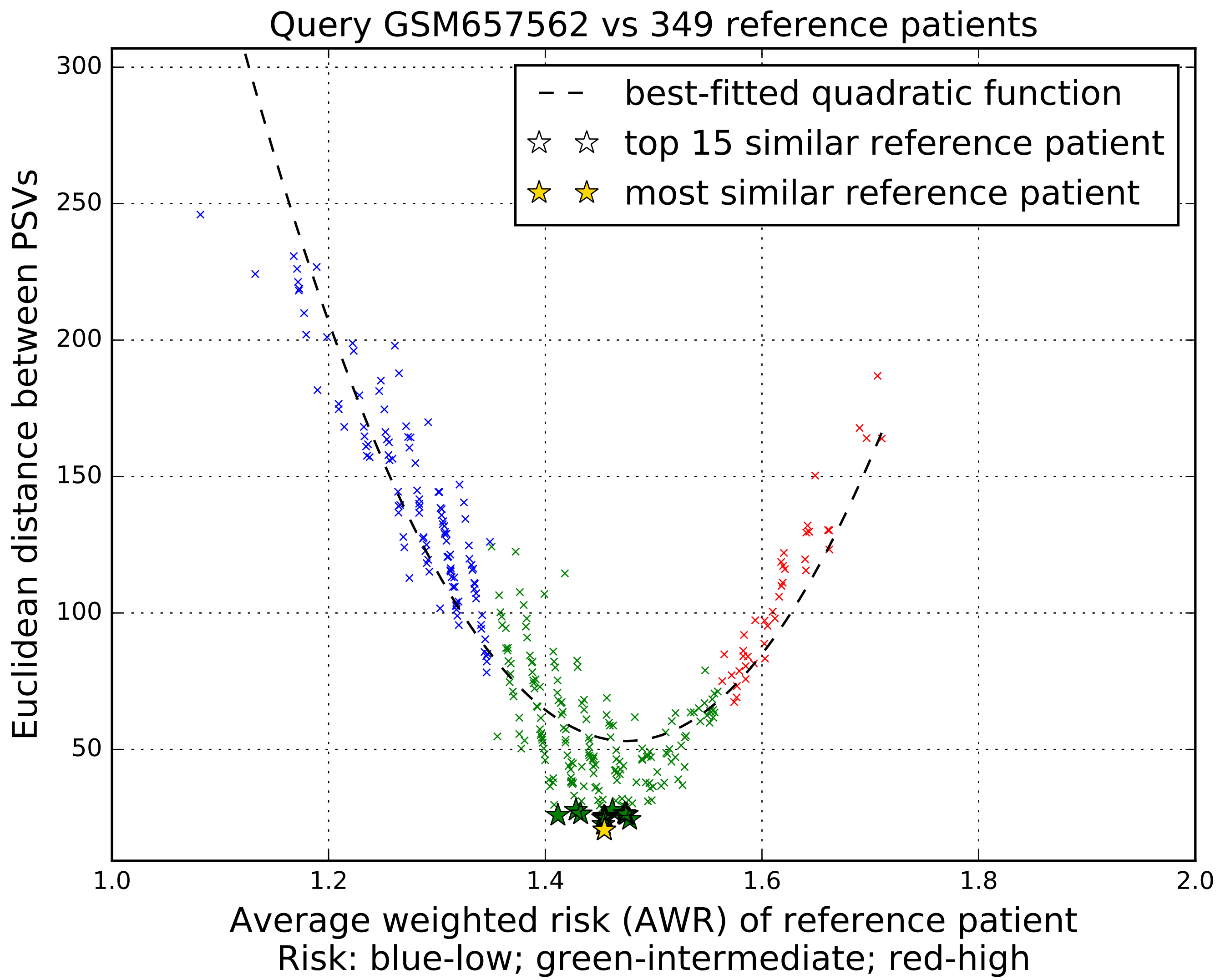
Query GSM657562 vs 349 reference patients
best-fitted quadratic function
top 15 similar reference patient
most similar reference patient
Euclidean distance between PSVs
300
250
200
150
100
50
1.0
1.2
1.4
1.6
1.8
2.0
Average weighted risk (AWR) of reference patient
Risk: blue-low; green-intermediate; red-high

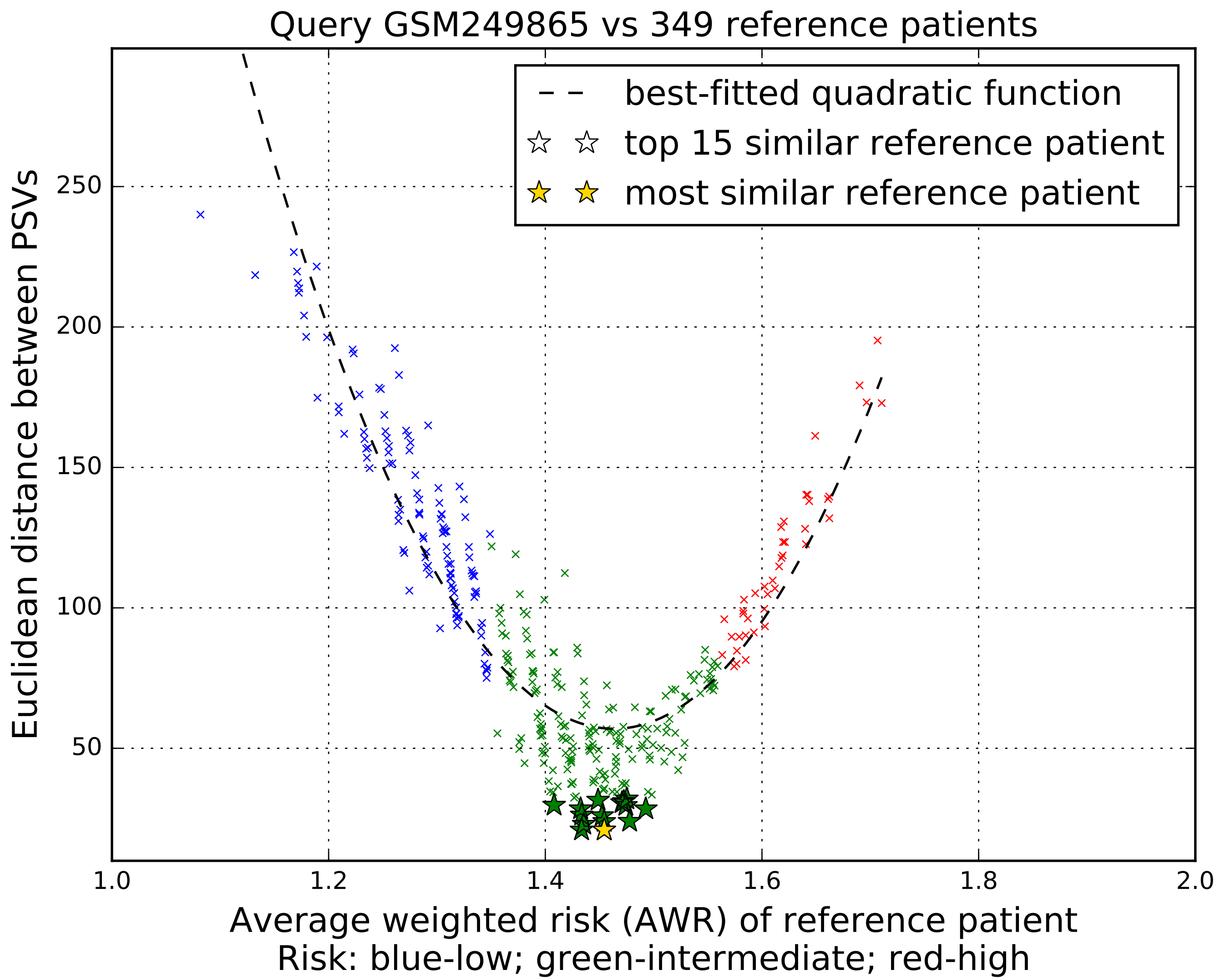
Query GSM249865 vs 349 reference patients
best-fitted quadratic function
top 15 similar reference patient
most similar reference patient
Euclidean distance between PSVs
250
200
150
100
50
1.0
1.2
1.4
1.6
1.8
2.0
Average weighted risk (AWR) of reference patient
Risk: blue-low; green-intermediate; red-high

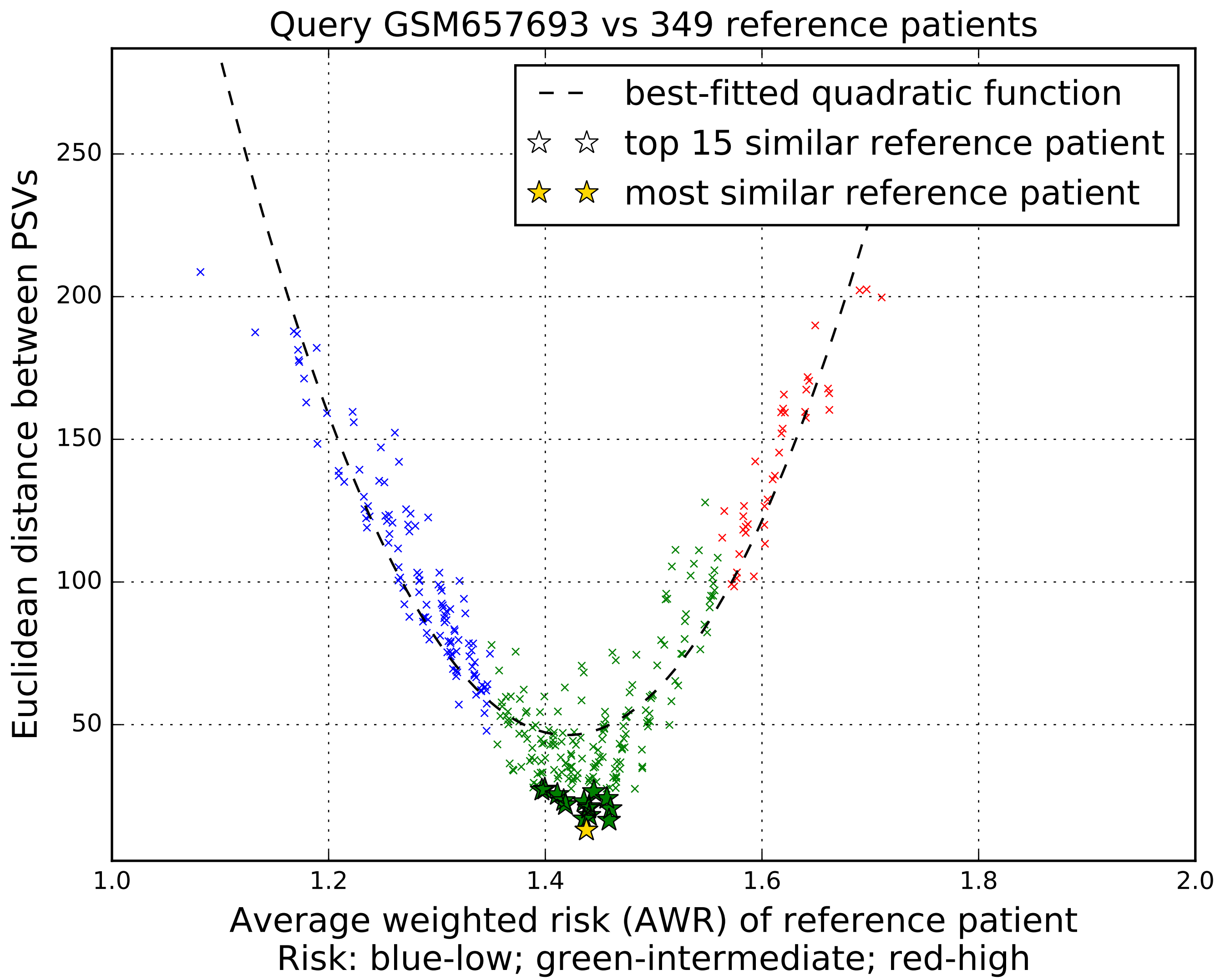
Query GSM657693 vs 349 reference patients
best-fitted quadratic function
top 15 similar reference patient
most similar reference patient
Euclidean distance between PSVs
250
200
150
100
50
1.0
1.2
1.4
1.6
1.8
2.0
Average weighted risk (AWR) of reference patient
Risk: blue-low; green-intermediate; red-high

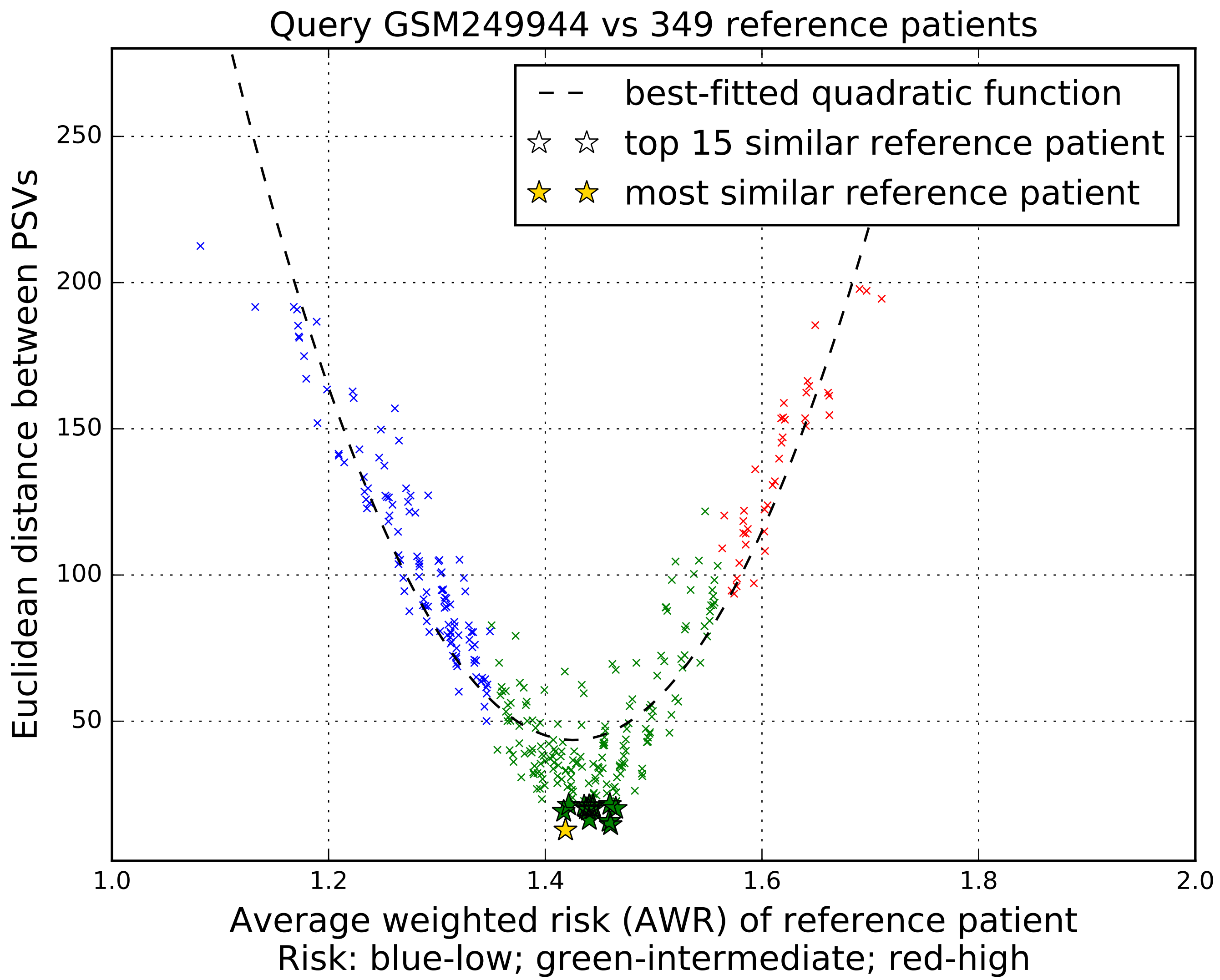
Query GSM249944 vs 349 reference patients
best-fitted quadratic function
top 15 similar reference patient
most similar reference patient
Euclidean distance between PSVs
Average weighted risk (AWR) of reference patient
Risk: blue-low; green-intermediate; red-high
250
200
150
100
50
1.0
1.2
1.4
1.6
1.8
2.0

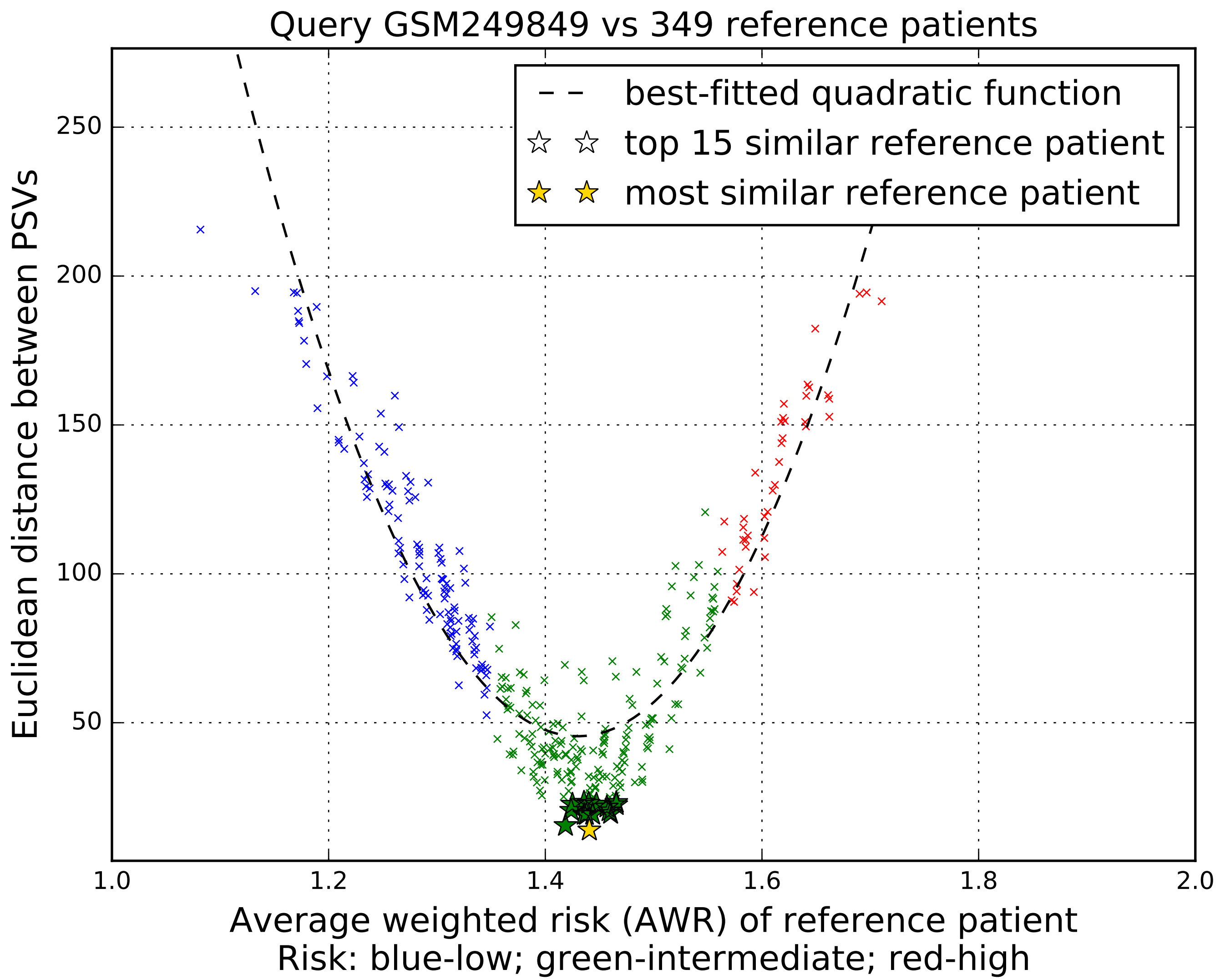

Query GSM249849 vs 349 reference patients
best-fitted quadratic function
top 15 similar reference patient
most similar reference patient
250
200
150
100
50
1.0
1.2
1.4
1.6
1.8
2.0
Euclidean distance between PSVs
Average weighted risk (AWR) of reference patient
Risk: blue-low; green-intermediate; red-high

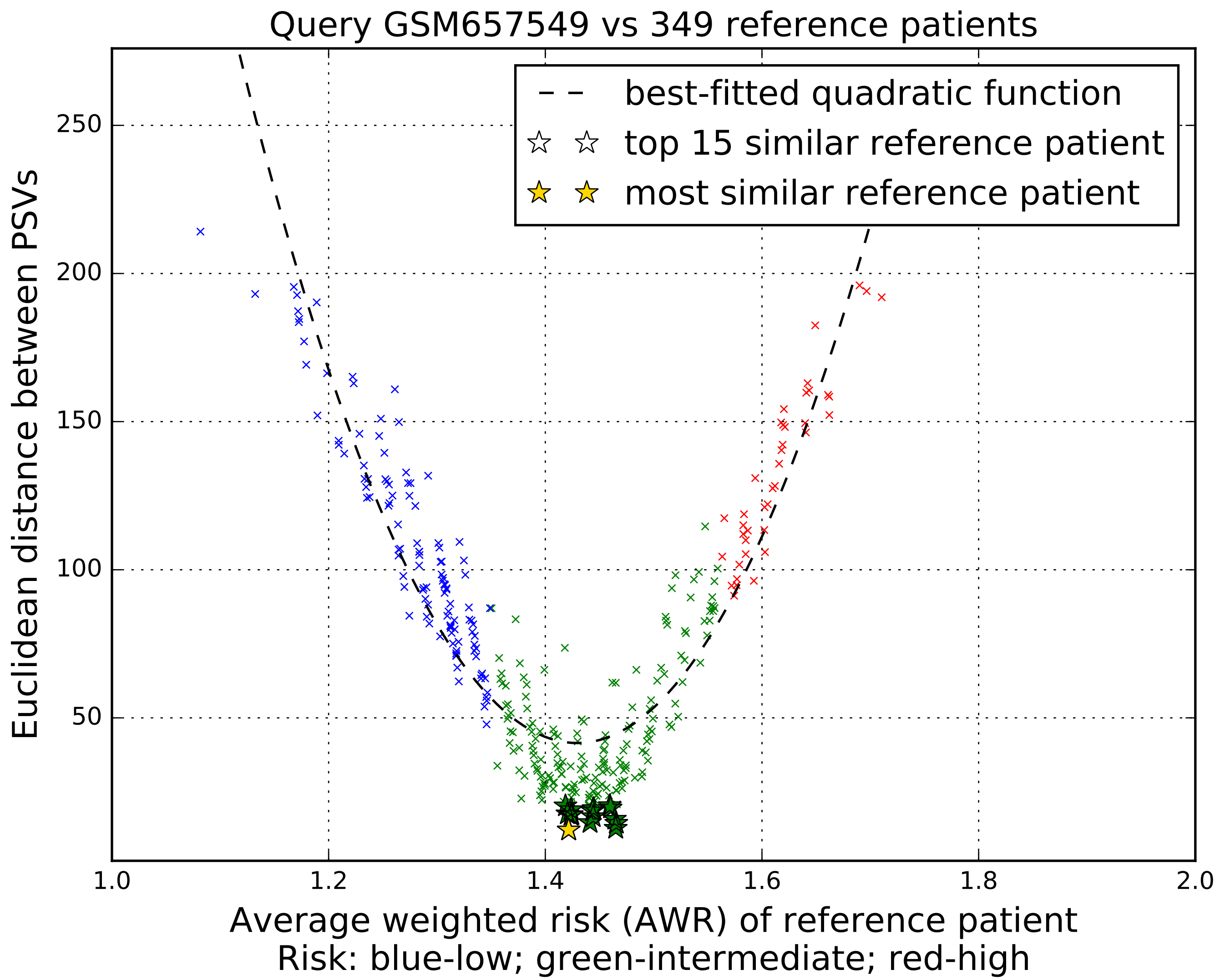
Query GSM657549 vs 349 reference patients
best-fitted quadratic function
top 15 similar reference patient
most similar reference patient
Euclidean distance between PSVs
250
200
150
100
50
1.0
1.2
1.4
1.6
1.8
2.0
Average weighted risk (AWR) of reference patient
Risk: blue-low; green-intermediate; red-high

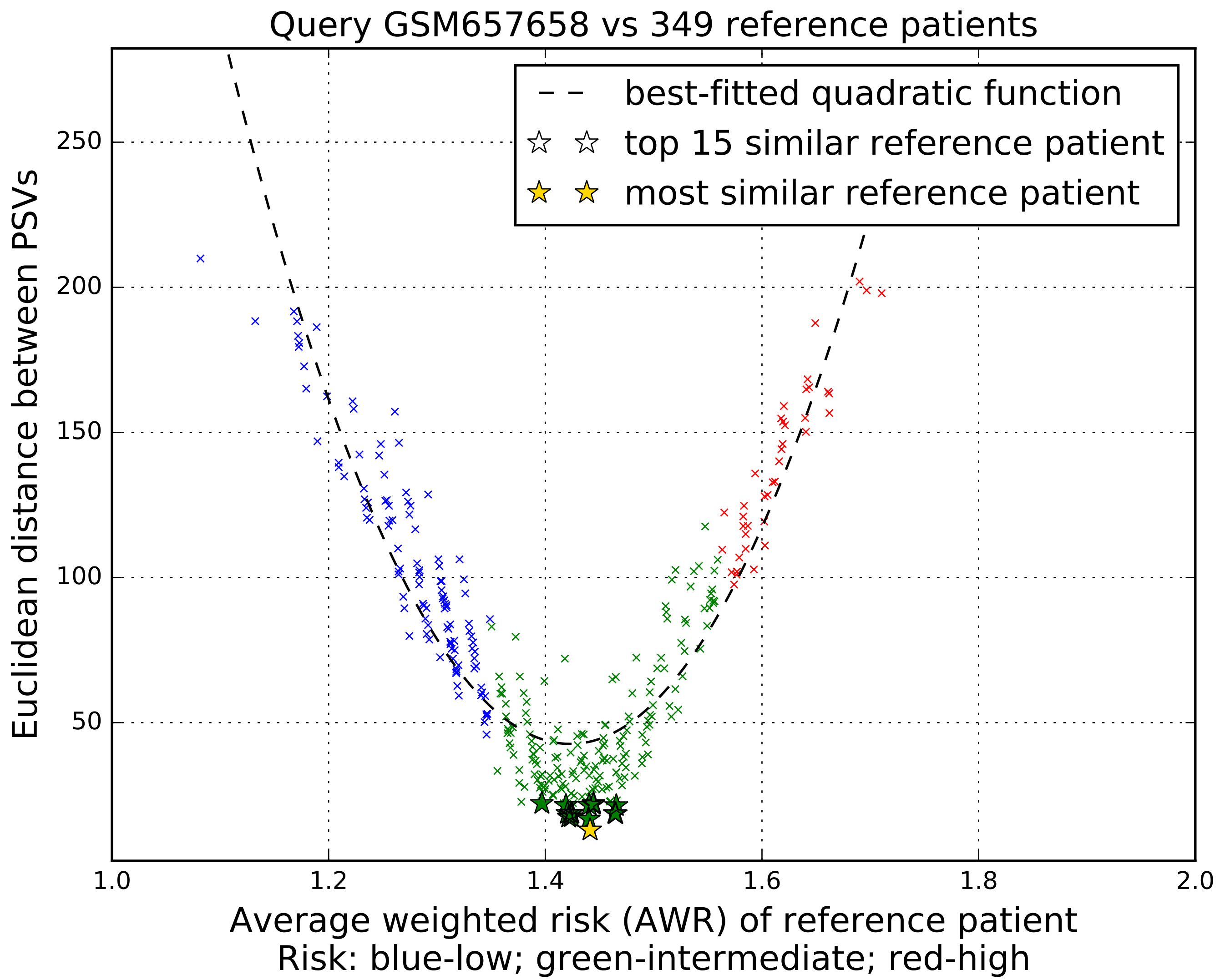

Query GSM657658 vs 349 reference patients
best-fitted quadratic function
top 15 similar reference patient
most similar reference patient
Euclidean distance between PSVs
250
200
150
100
50
1.0
1.2
1.4
1.6
1.8
2.0
Average weighted risk (AWR) of reference patient
Risk: blue-low; green-intermediate; red-high

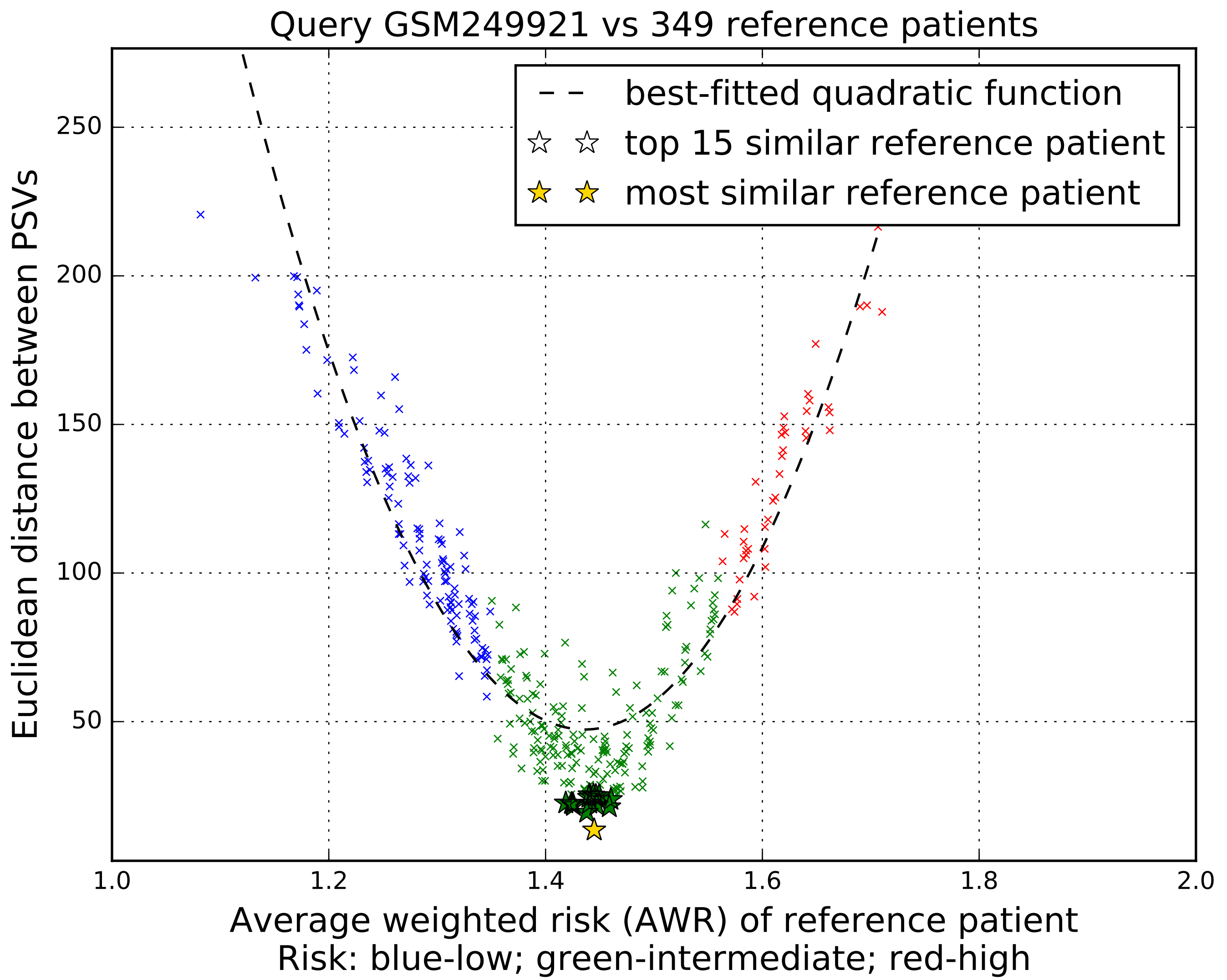

Query GSM249921 vs 349 reference patients
best-fitted quadratic function
top 15 similar reference patient
most similar reference patient
Euclidean distance between PSVs
250
200
150
100
50
1.0
1.2
1.4
1.6
1.8
2.0
Average weighted risk (AWR) of reference patient
Risk: blue-low; green-intermediate; red-high

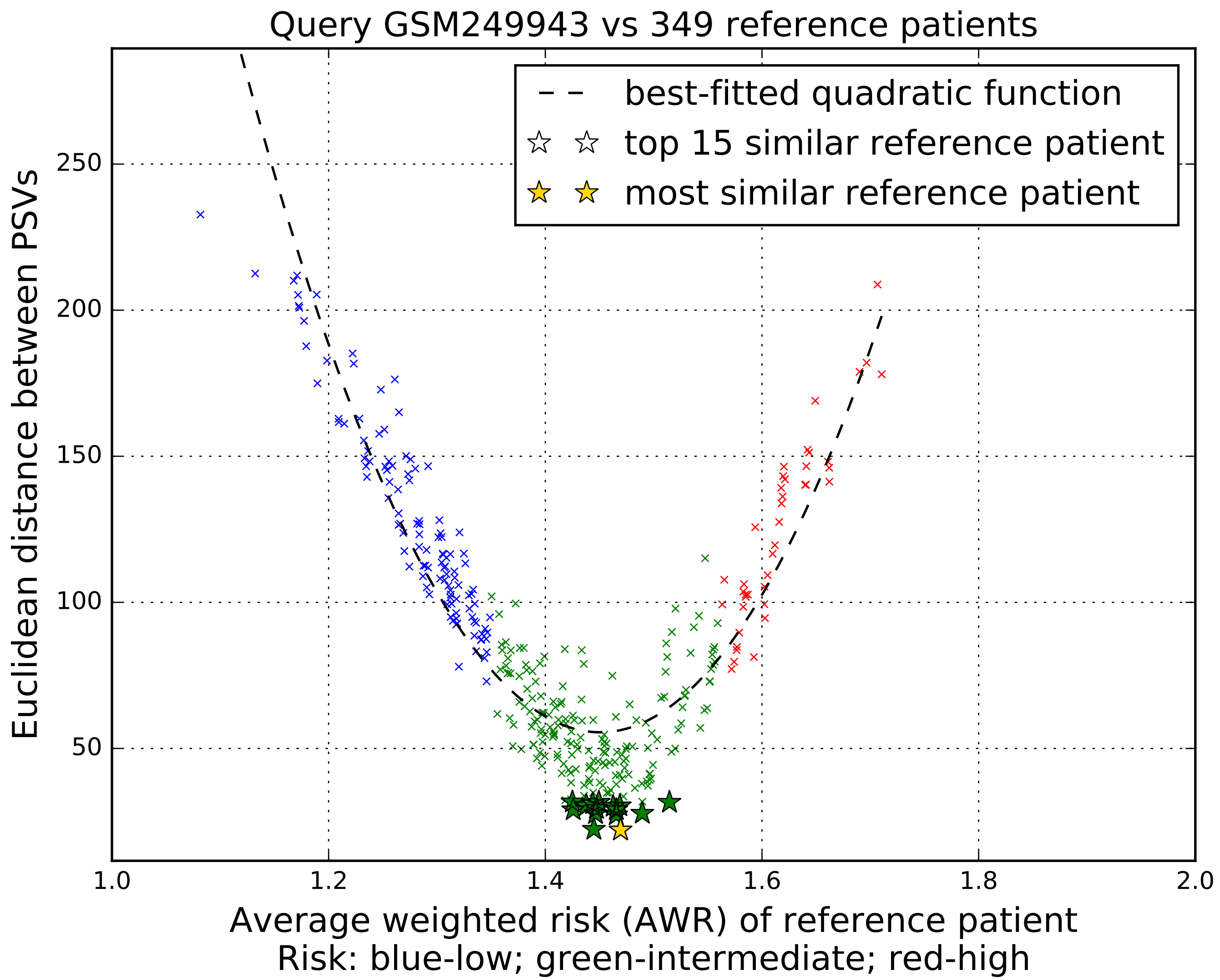

Query GSM249943 vs 349 reference patients
best-fitted quadratic function
top 15 similar reference patient
most similar reference patient
Euclidean distance between PSVs
250
200
150
100
50
1.0
1.2
1.4
1.6
1.8
2.0
Average weighted risk (AWR) of reference patient
Risk: blue-low; green-intermediate; red-high

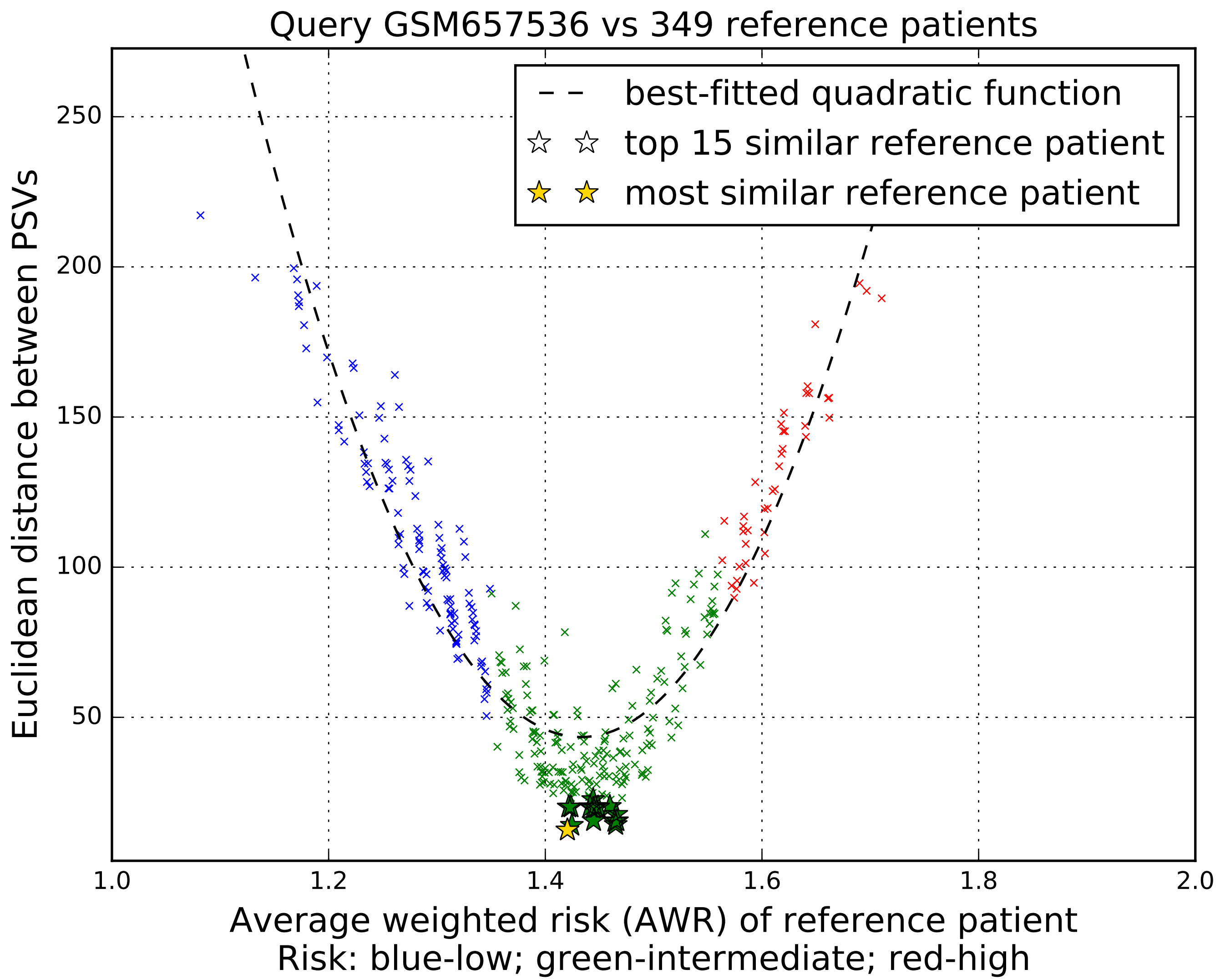

Query GSM657536 vs 349 reference patients
best-fitted quadratic function
top 15 similar reference patient
most similar reference patient
Euclidean distance between PSVs
250
200
150
100
50
1.0
1.2
1.4
1.6
1.8
2.0
Average weighted risk (AWR) of reference patient
Risk: blue-low; green-intermediate; red-high

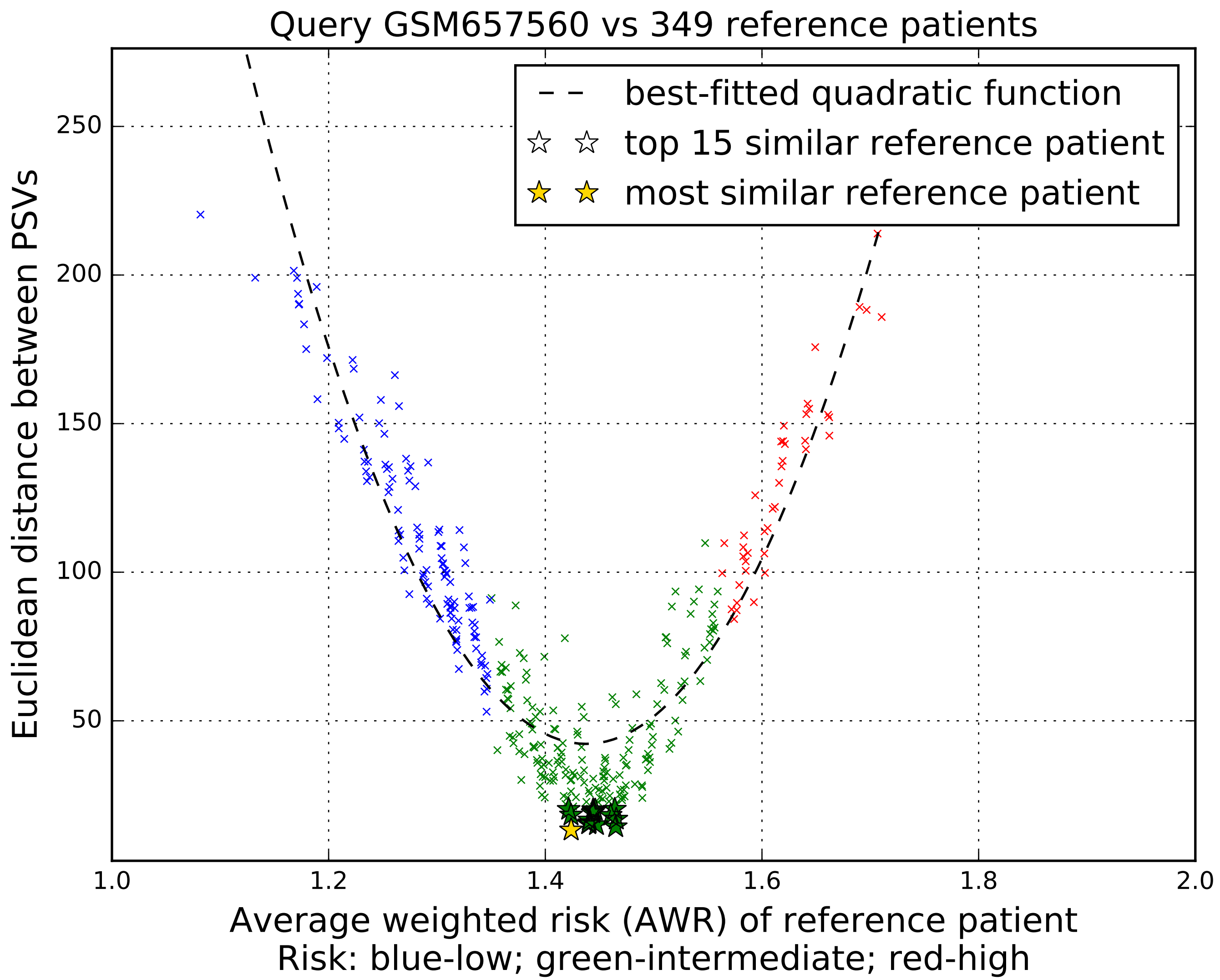

Query GSM657560 vs 349 reference patients
best-fitted quadratic function
top 15 similar reference patient
most similar reference patient
Euclidean distance between PSVs
250
200
150
100
50
1.0
1.2
1.4
1.6
1.8
2.0
Average weighted risk (AWR) of reference patient
Risk: blue-low; green-intermediate; red-high

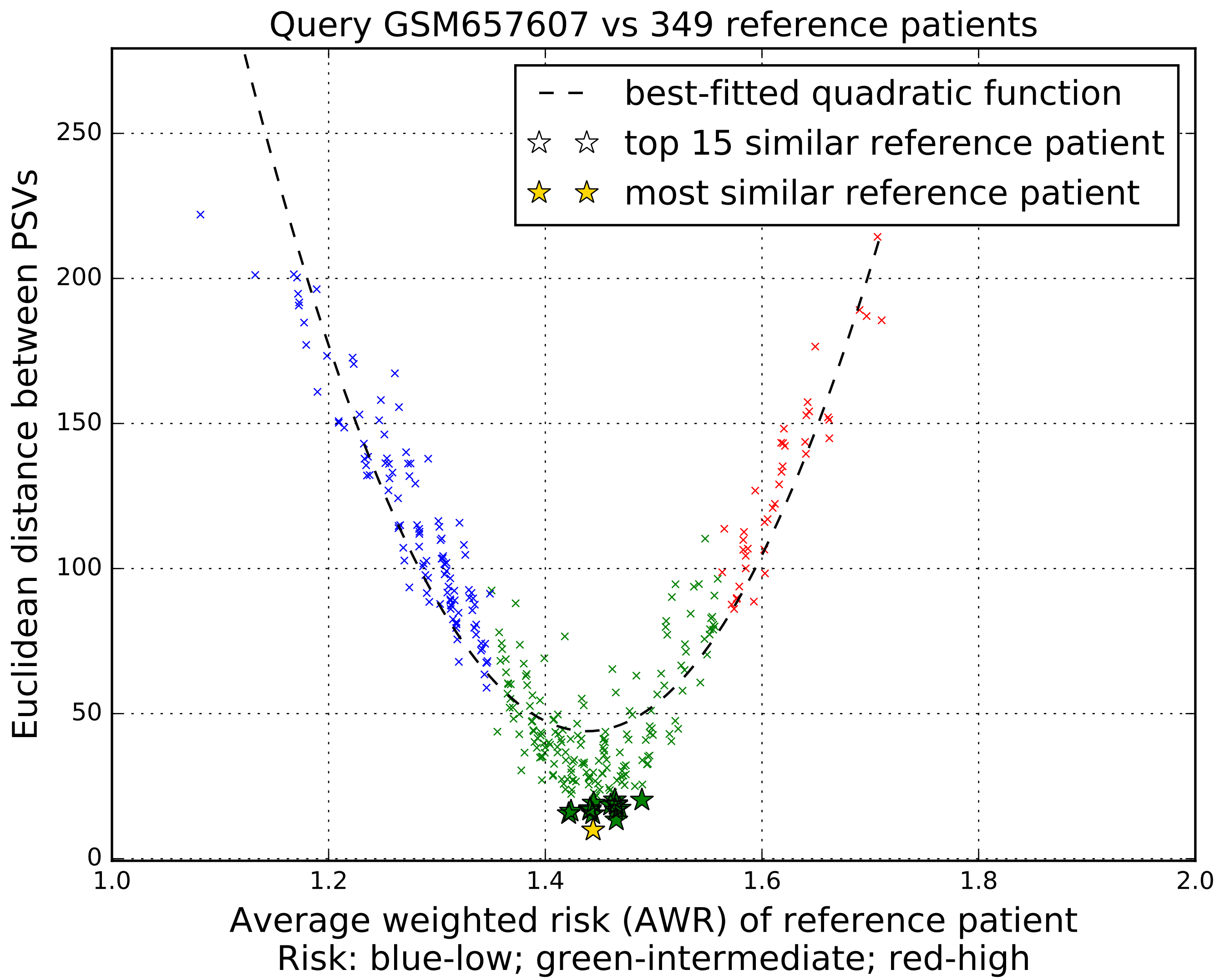
Query GSM657607 vs 349 reference patients
best-fitted quadratic function
top 15 similar reference patient
most similar reference patient
Euclidean distance between PSVs
0
50
100
150
200
250
1.0
1.2
1.4
1.6
1.8
2.0
Average weighted risk (AWR) of reference patient
Risk: blue-low; green-intermediate; red-high

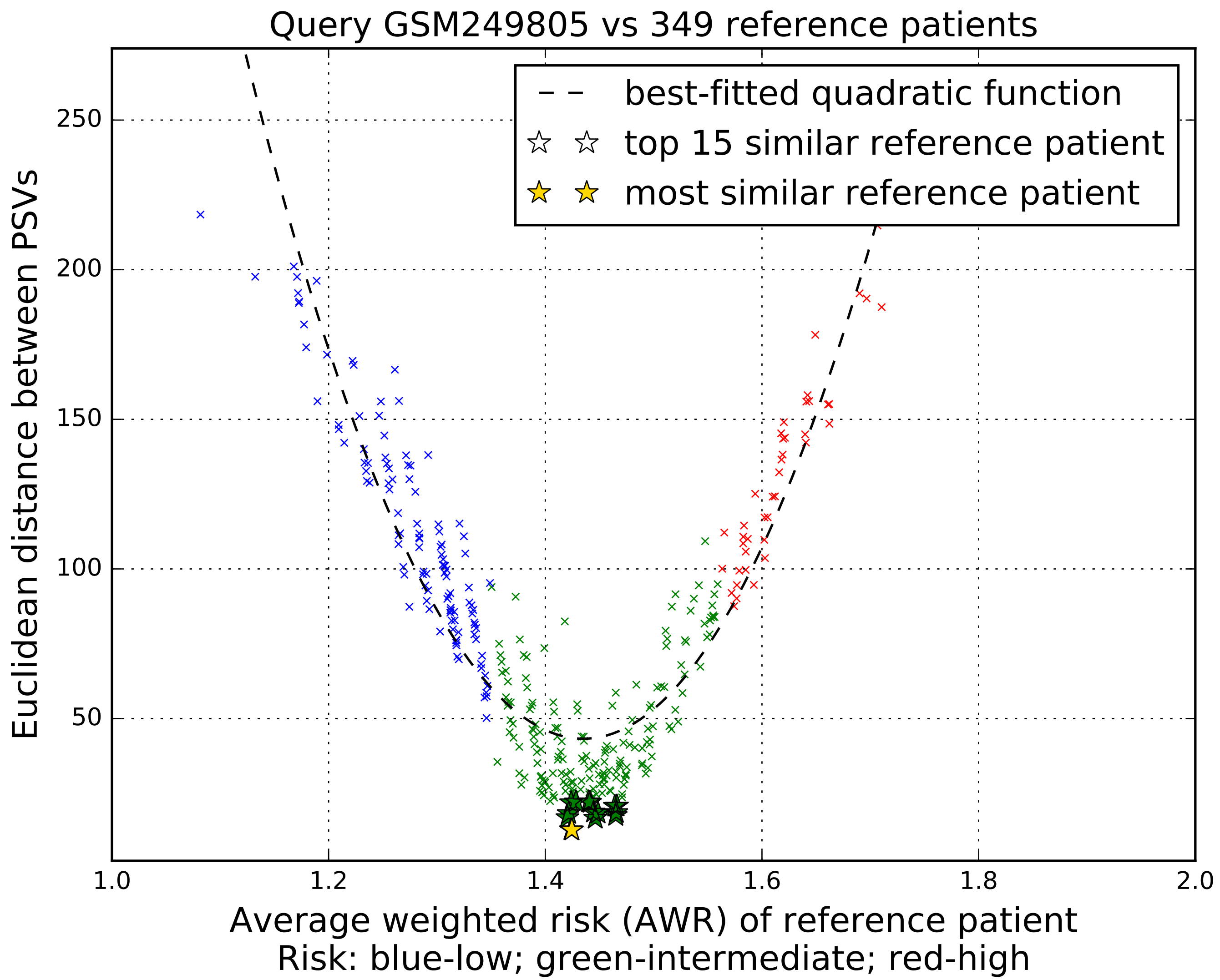
Query GSM249805 vs 349 reference patients
best-fitted quadratic function
top 15 similar reference patient
most similar reference patient
Euclidean distance between PSVs
250
200
150
100
50
1.0
1.2
1.4
1.6
1.8
2.0
Average weighted risk (AWR) of reference patient
Risk: blue-low; green-intermediate; red-high

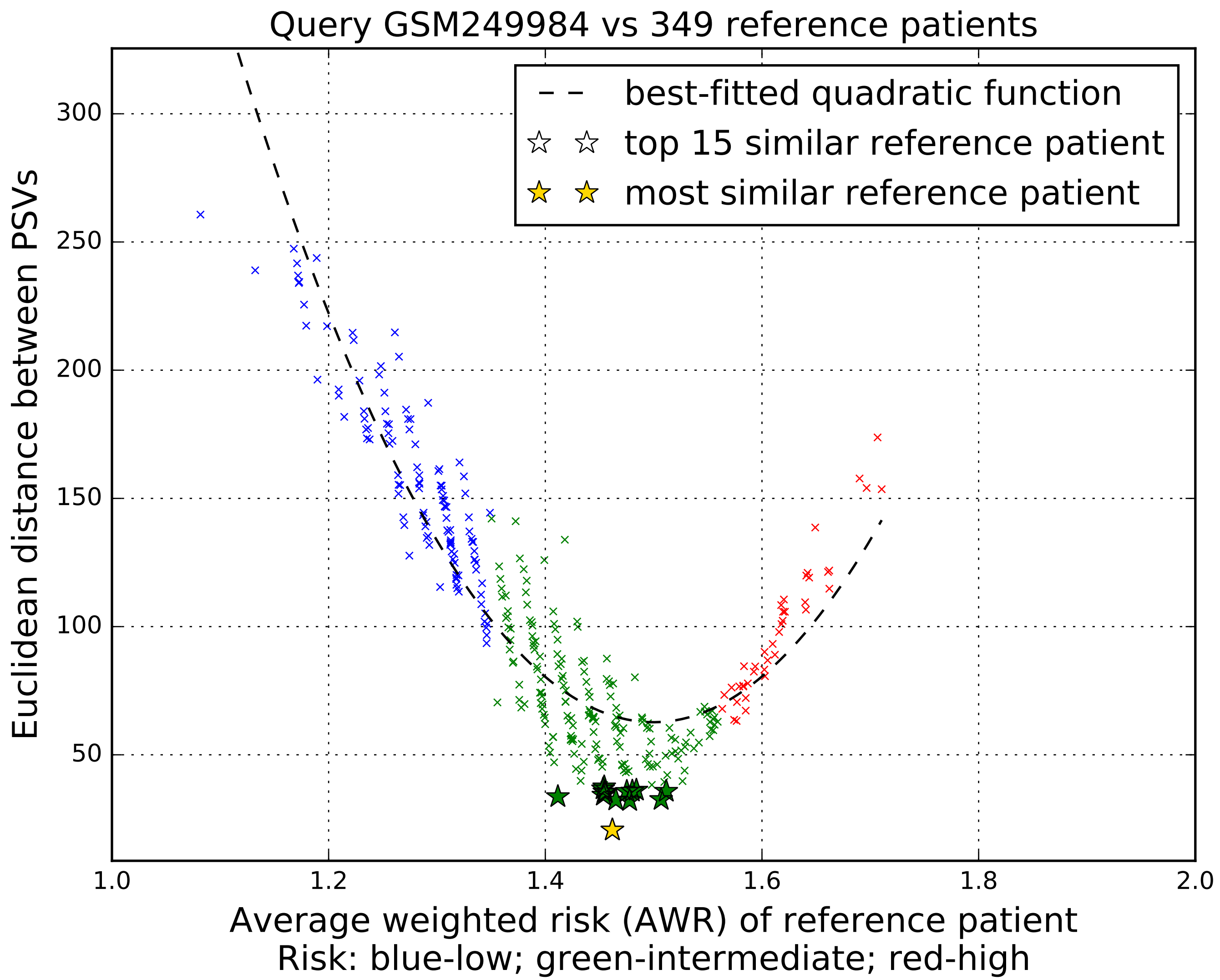

Query GSM249984 vs 349 reference patients
best-fitted quadratic function
top 15 similar reference patient
most similar reference patient
Euclidean distance between PSVs
300
250
200
150
100
50
1.0
1.2
1.4
1.6
1.8
2.0
Average weighted risk (AWR) of reference patient
Risk: blue-low; green-intermediate; red-high

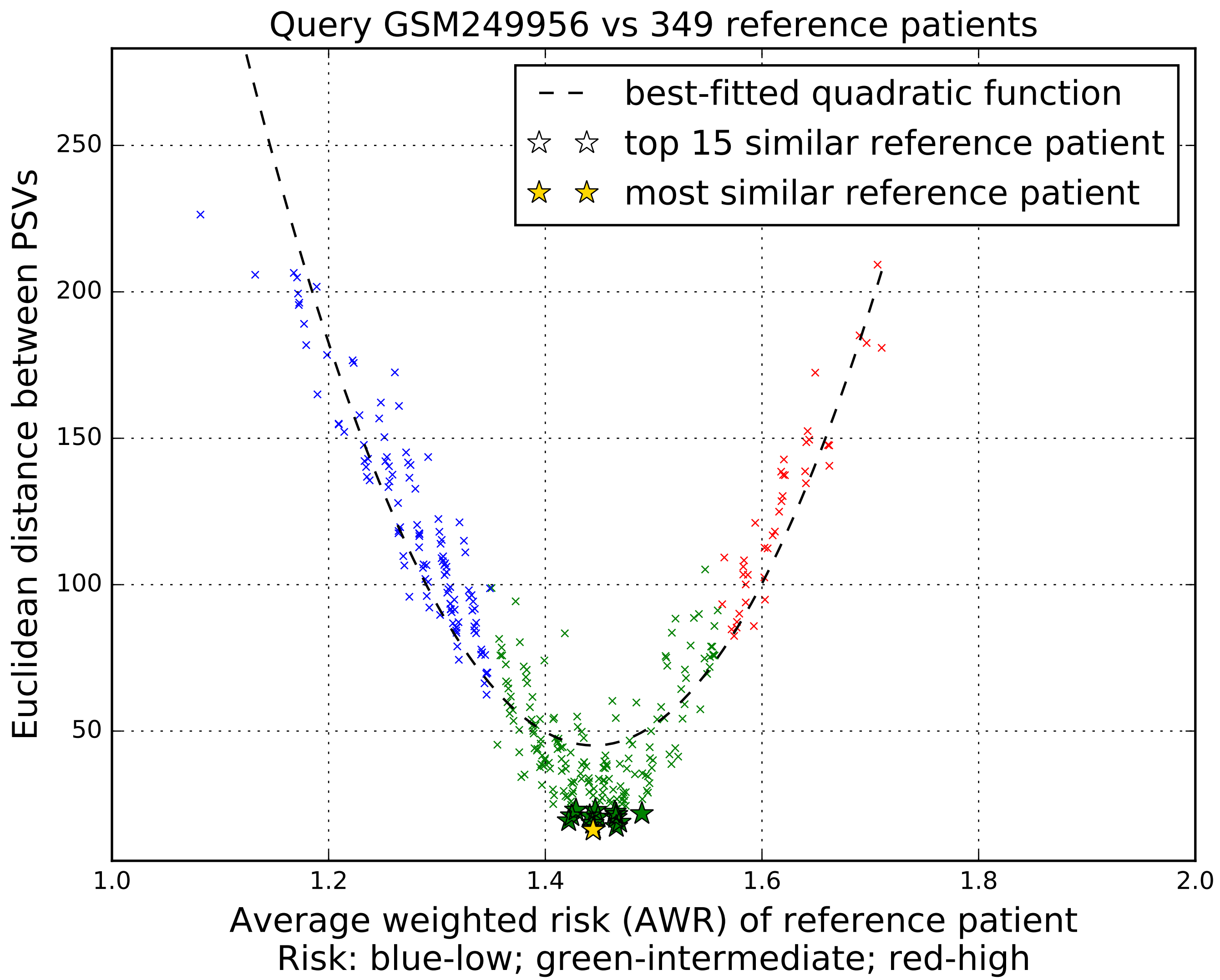
Query GSM249956 vs 349 reference patients
best-fitted quadratic function
top 15 similar reference patient
most similar reference patient
Euclidean distance between PSVs
250
200
150
100
50
1.0
1.2
1.4
1.6
1.8
2.0
Average weighted risk (AWR) of reference patient
Risk: blue-low; green-intermediate; red-high

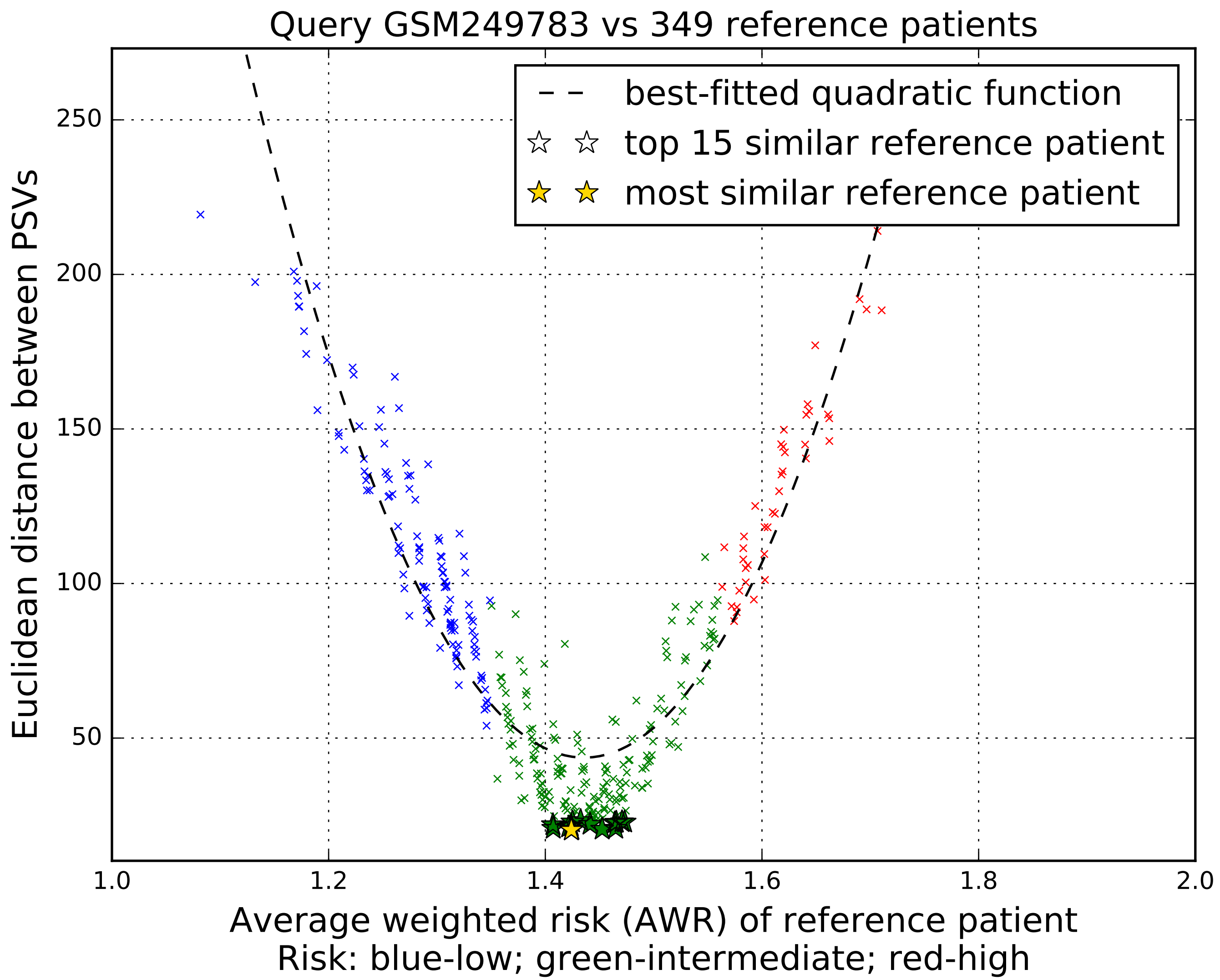

Query GSM249783 vs 349 reference patients
best-fitted quadratic function
top 15 similar reference patient
most similar reference patient
Euclidean distance between PSVs
250
200
150
100
50
1.0
1.2
1.4
1.6
1.8
2.0
Average weighted risk (AWR) of reference patient
Risk: blue-low; green-intermediate; red-high

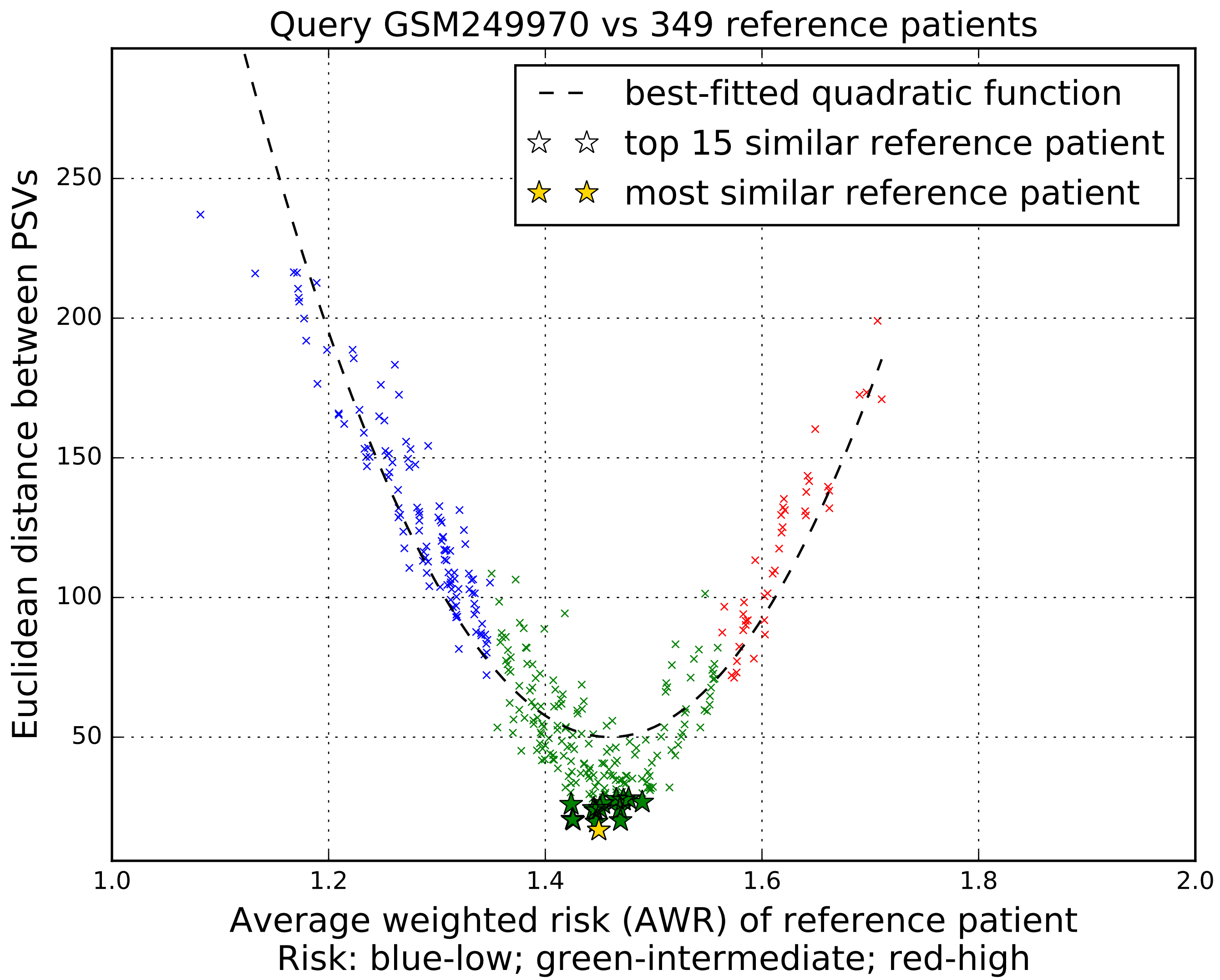

Query GSM249970 vs 349 reference patients
best-fitted quadratic function
top 15 similar reference patient
most similar reference patient
Euclidean distance between PSVs
250
200
150
100
50
1.0
1.2
1.4
1.6
1.8
2.0
Average weighted risk (AWR) of reference patient
Risk: blue-low; green-intermediate; red-high

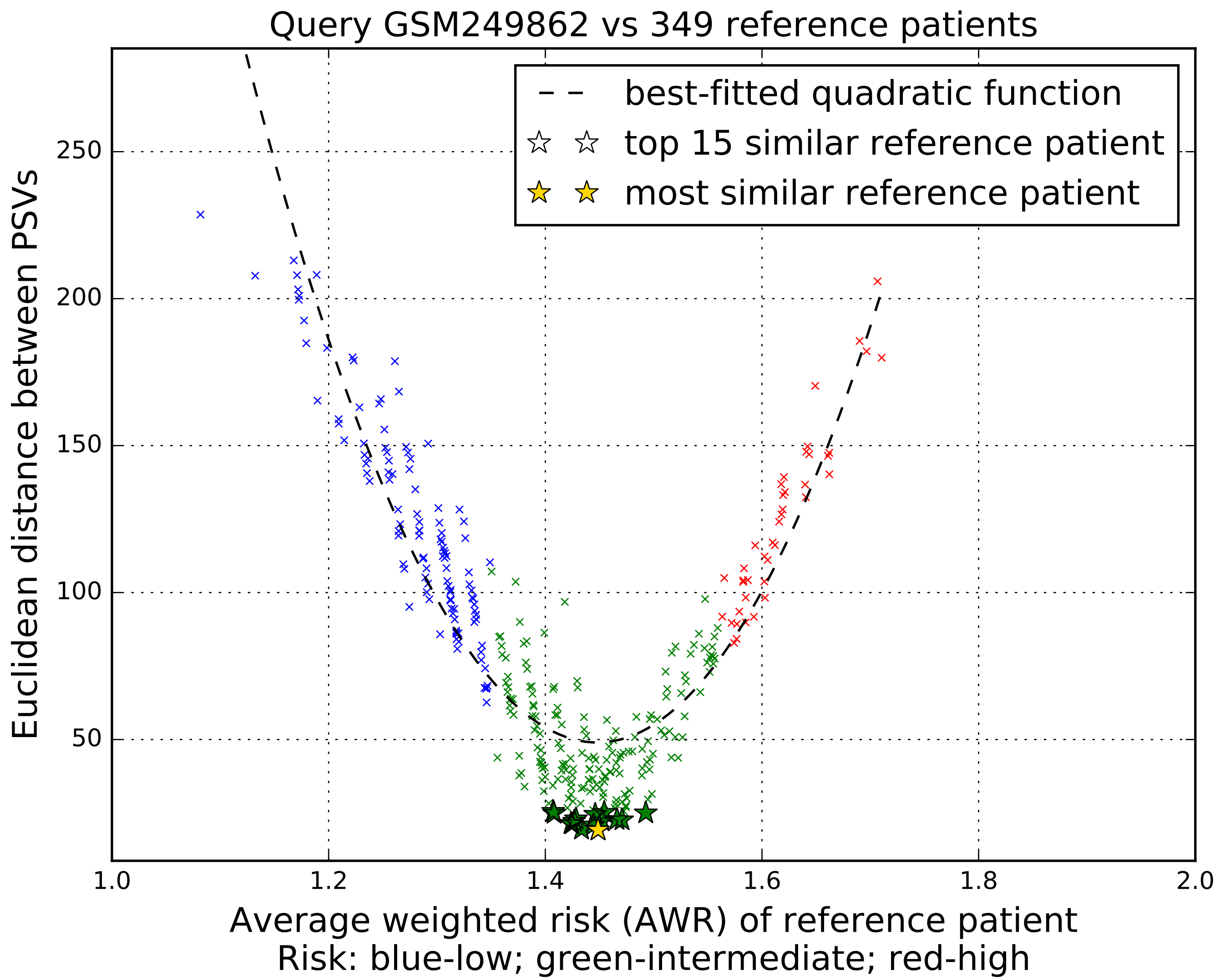
Query GSM249862 vs 349 reference patients
best-fitted quadratic function
top 15 similar reference patient
most similar reference patient
Euclidean distance between PSVs
250
200
150
100
50
1.0
1.2
1.4
1.6
1.8
2.0
Average weighted risk (AWR) of reference patient
Risk: blue-low; green-intermediate; red-high

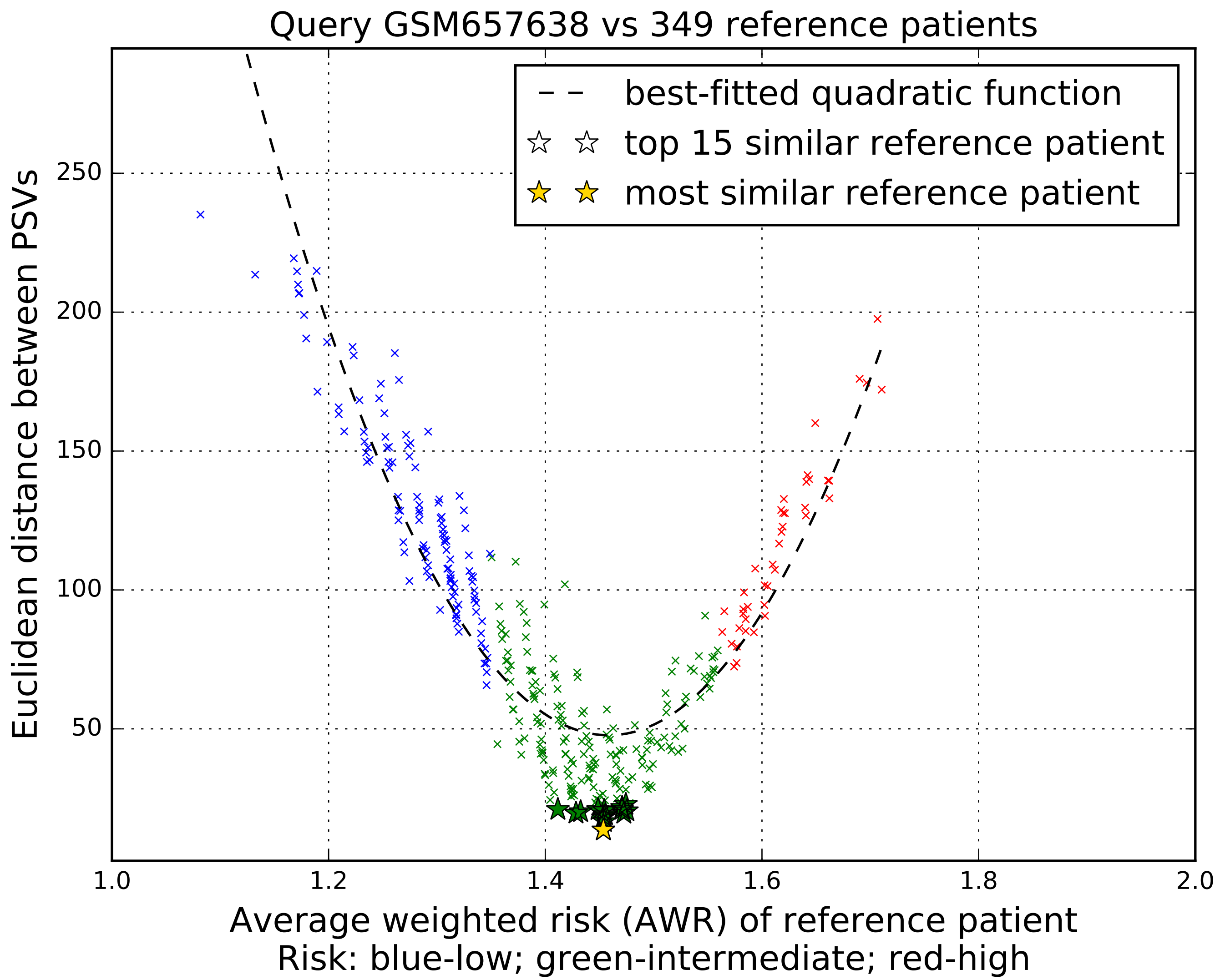

Query GSM657638 vs 349 reference patients
best-fitted quadratic function
top 15 similar reference patient
most similar reference patient
Euclidean distance between PSVs
250
200
150
100
50
1.0
1.2
1.4
1.6
1.8
2.0
Average weighted risk (AWR) of reference patient
Risk: blue-low; green-intermediate; red-high

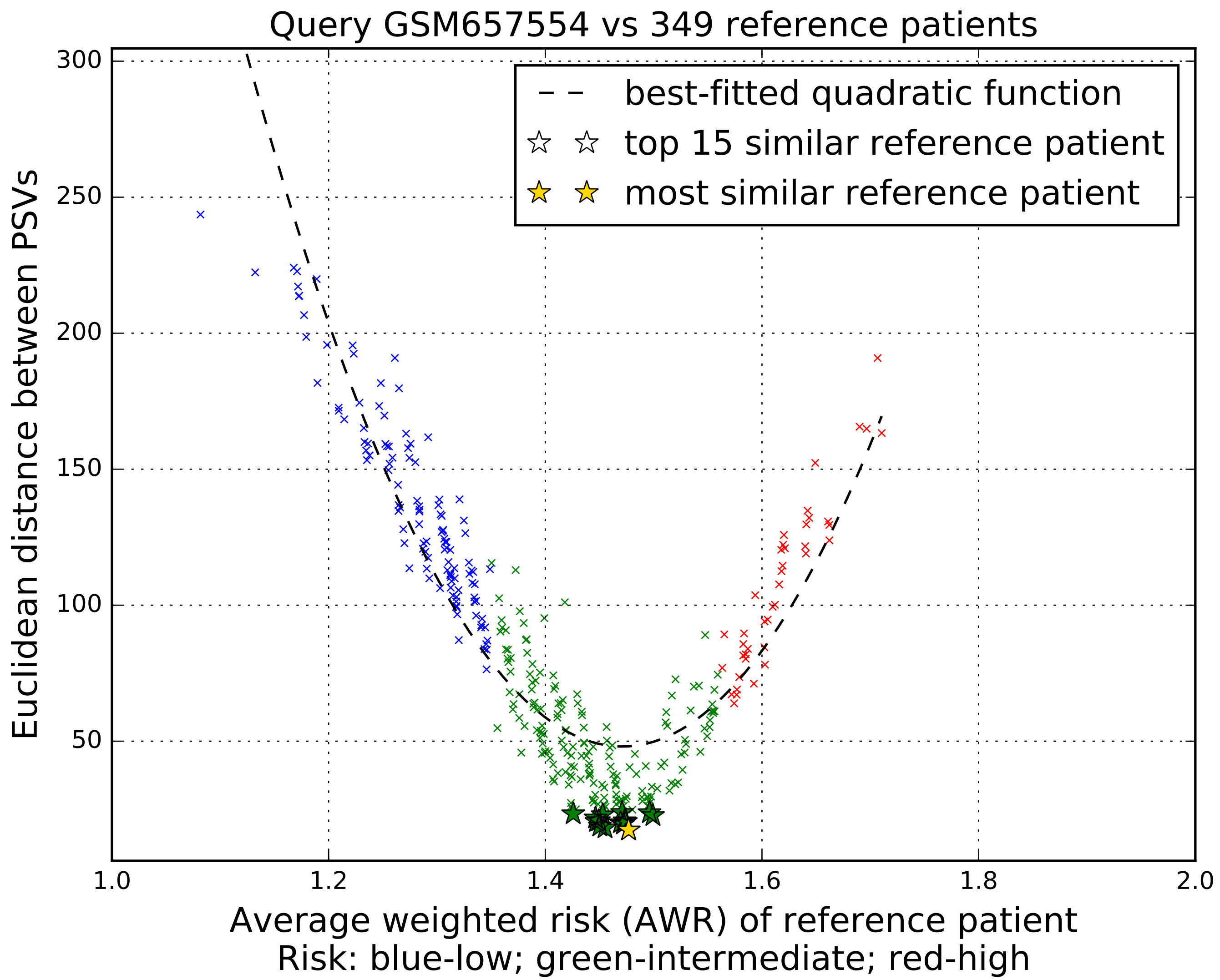

Query GSM657554 vs 349 reference patients
best-fitted quadratic function
top 15 similar reference patient
most similar reference patient
Euclidean distance between PSVs
300
250
200
150
100
50
1.0
1.2
1.4
1.6
1.8
2.0
Average weighted risk (AWR) of reference patient
Risk: blue-low; green-intermediate; red-high

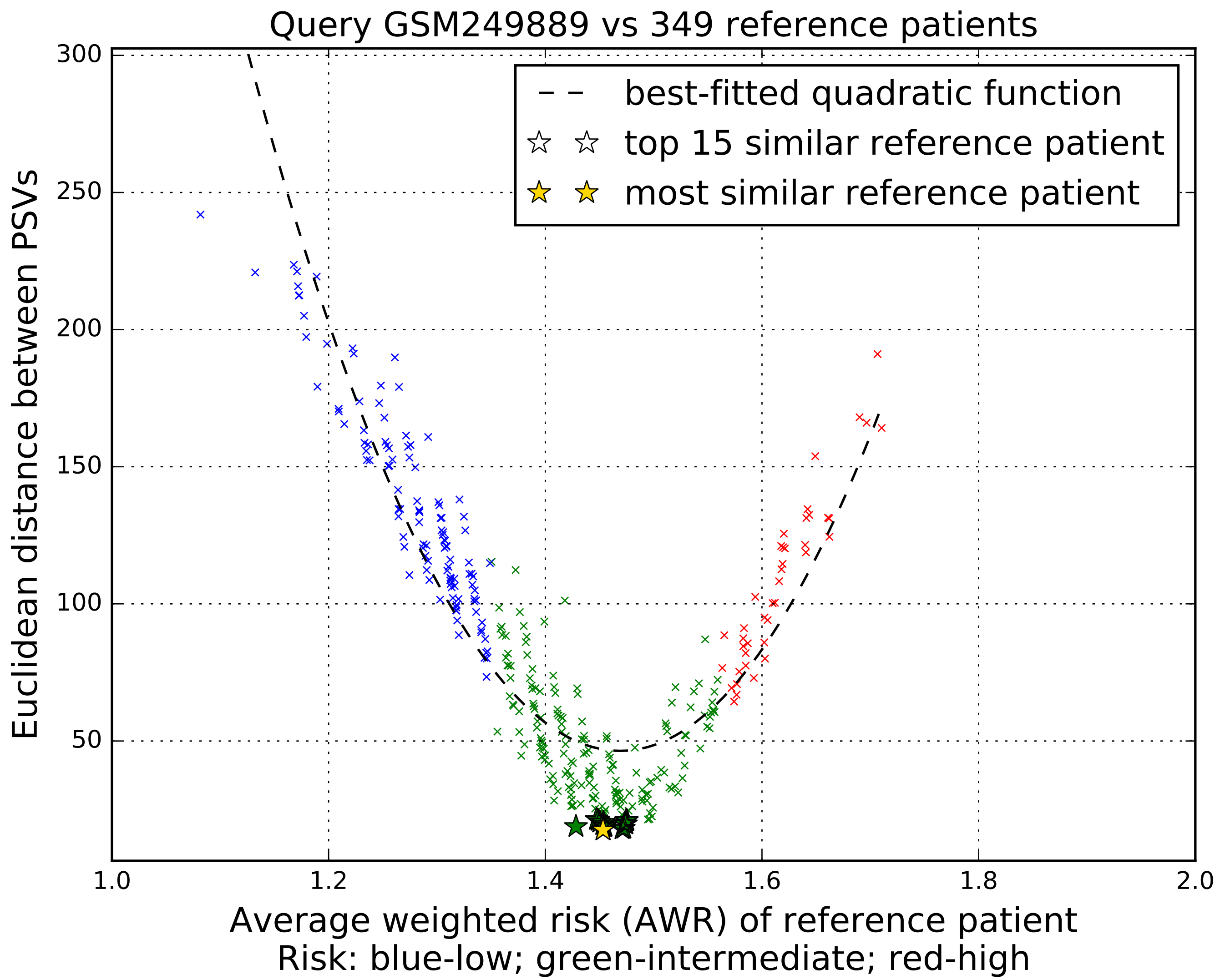
Query GSM249889 vs 349 reference patients
best-fitted quadratic function
top 15 similar reference patient
most similar reference patient
300
250
200
150
100
50
1.0
1.2
1.4
1.6
1.8
2.0
Euclidean distance between PSVs
Average weighted risk (AWR) of reference patient
Risk: blue-low; green-intermediate; red-high

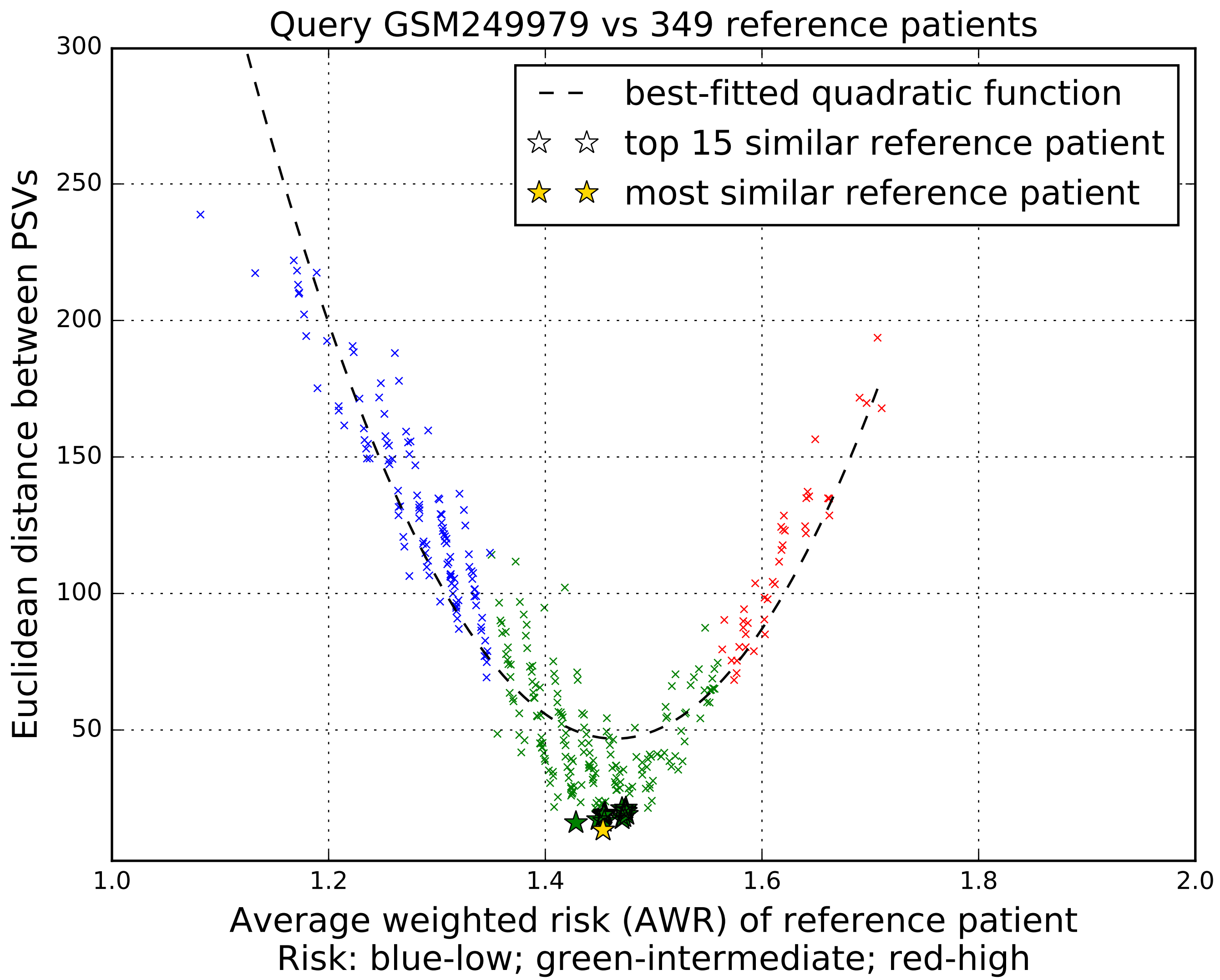

Query GSM249979 vs 349 reference patients
best-fitted quadratic function
top 15 similar reference patient
most similar reference patient
Euclidean distance between PSVs
300
250
200
150
100
50
1.0
1.2
1.4
1.6
1.8
2.0
Average weighted risk (AWR) of reference patient
Risk: blue-low; green-intermediate; red-high

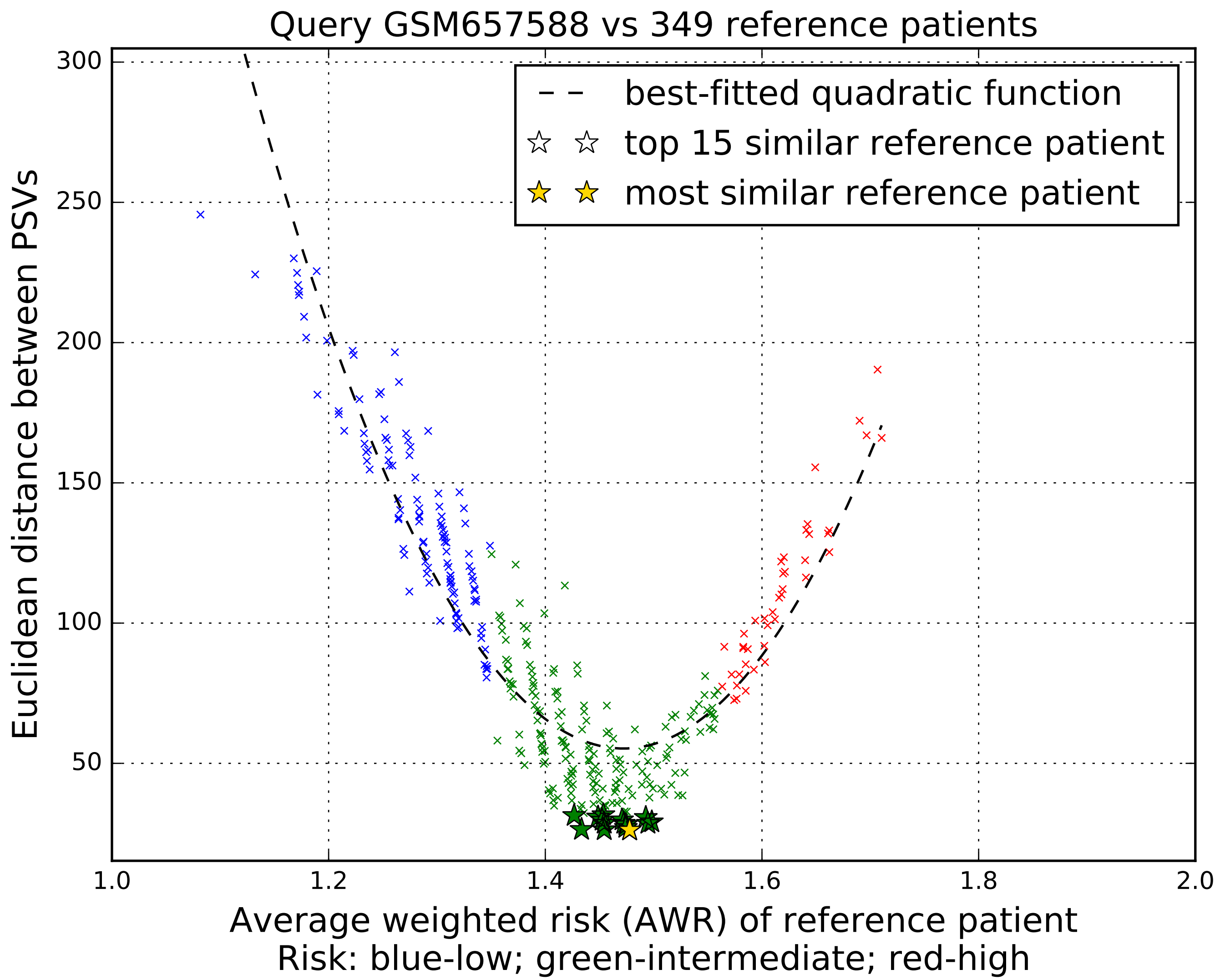

Query GSM657588 vs 349 reference patients
best-fitted quadratic function
top 15 similar reference patient
most similar reference patient
Euclidean distance between PSVs
300
250
200
150
100
50
1.0
1.2
1.4
1.6
1.8
2.0
Average weighted risk (AWR) of reference patient
Risk: blue-low; green-intermediate; red-high

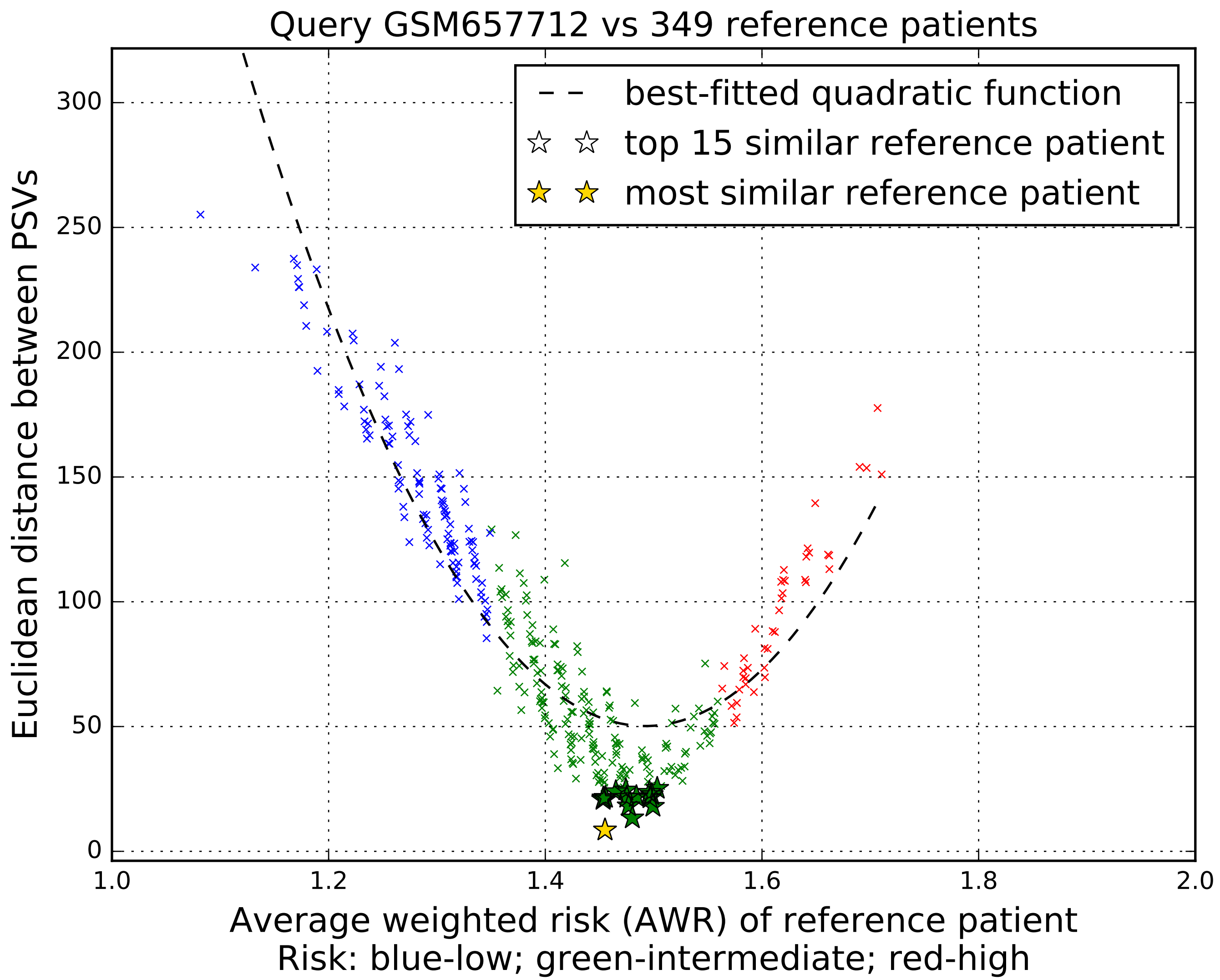

Query GSM657712 vs 349 reference patients
best-fitted quadratic function
top 15 similar reference patient
most similar reference patient
Euclidean distance between PSVs
0
50
100
150
200
250
300
1.0
1.2
1.4
1.6
1.8
2.0
Average weighted risk (AWR) of reference patient
Risk: blue-low; green-intermediate; red-high

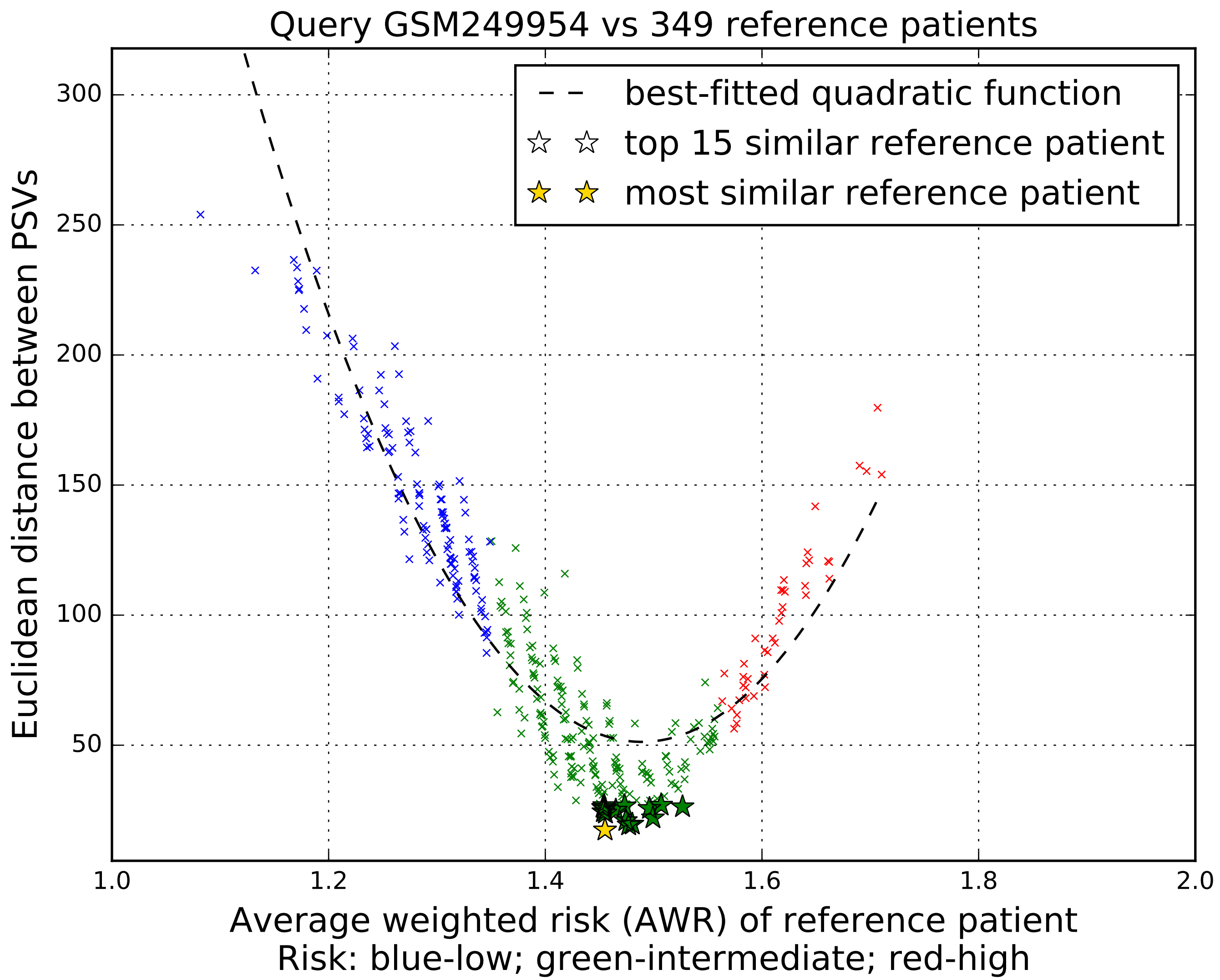

Query GSM249954 vs 349 reference patients
best-fitted quadratic function
top 15 similar reference patient
most similar reference patient
Euclidean distance between PSVs
300
250
200
150
100
50
1.0
1.2
1.4
1.6
1.8
2.0
Average weighted risk (AWR) of reference patient
Risk: blue-low; green-intermediate; red-high

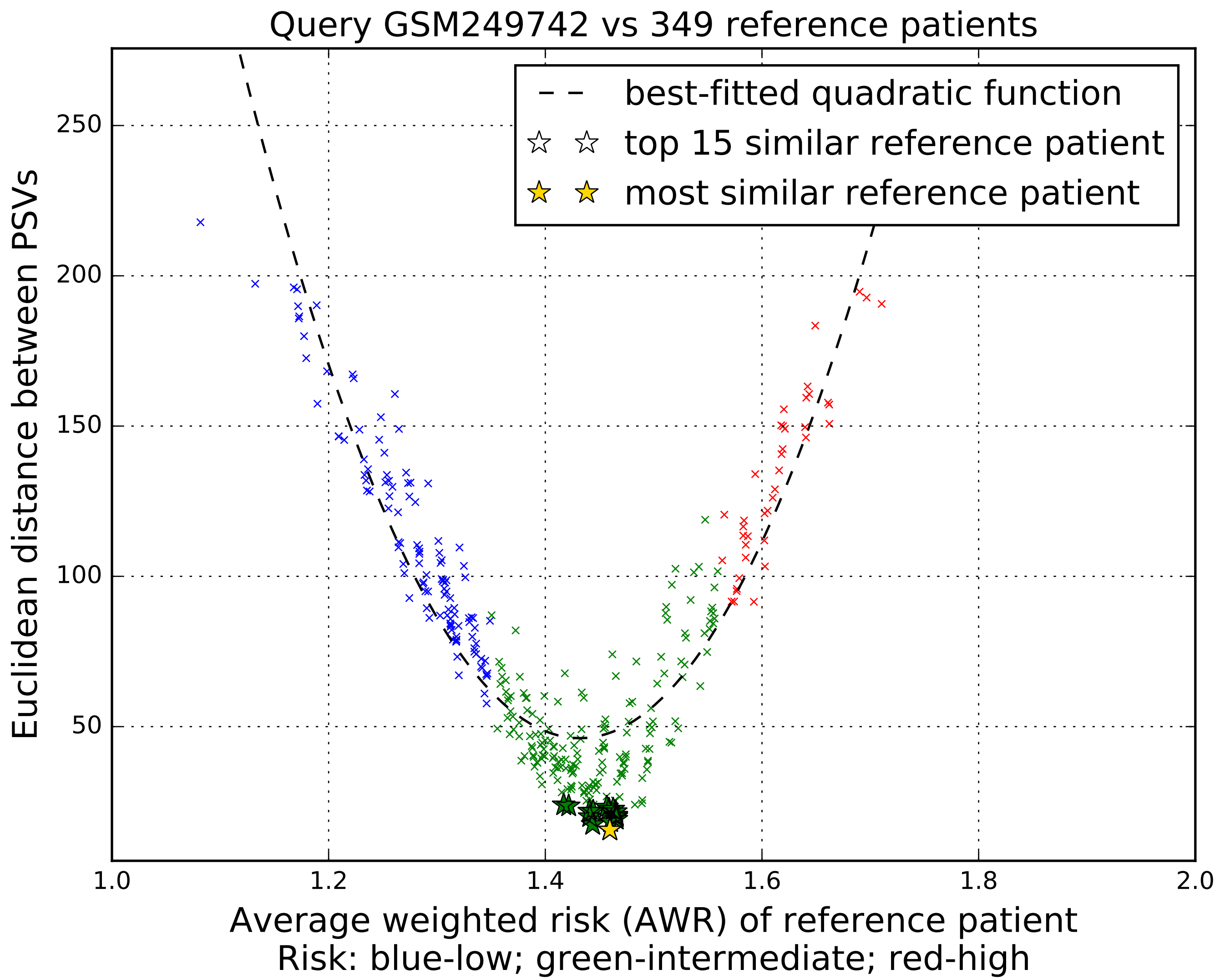

Query GSM249742 vs 349 reference patients
best-fitted quadratic function
top 15 similar reference patient
most similar reference patient
Euclidean distance between PSVs
250
200
150
100
50
1.0
1.2
1.4
1.6
1.8
2.0
Average weighted risk (AWR) of reference patient
Risk: blue-low; green-intermediate; red-high

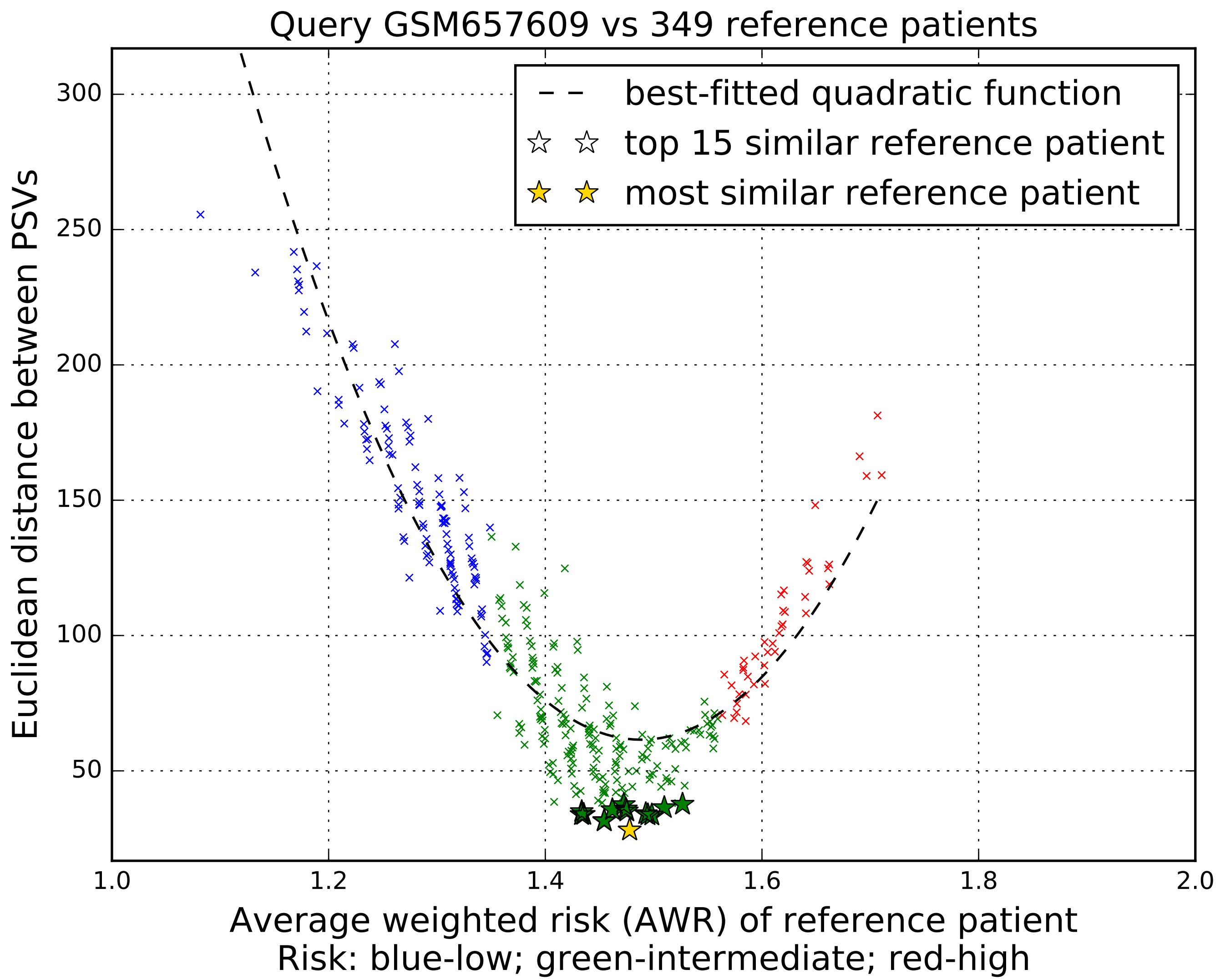
Query GSM657609 vs 349 reference patients
best-fitted quadratic function
top 15 similar reference patient
most similar reference patient
Euclidean distance between PSVs
300
250
200
150
100
50
1.0
1.2
1.4
1.6
1.8
2.0
Average weighted risk (AWR) of reference patient
Risk: blue-low; green-intermediate; red-high

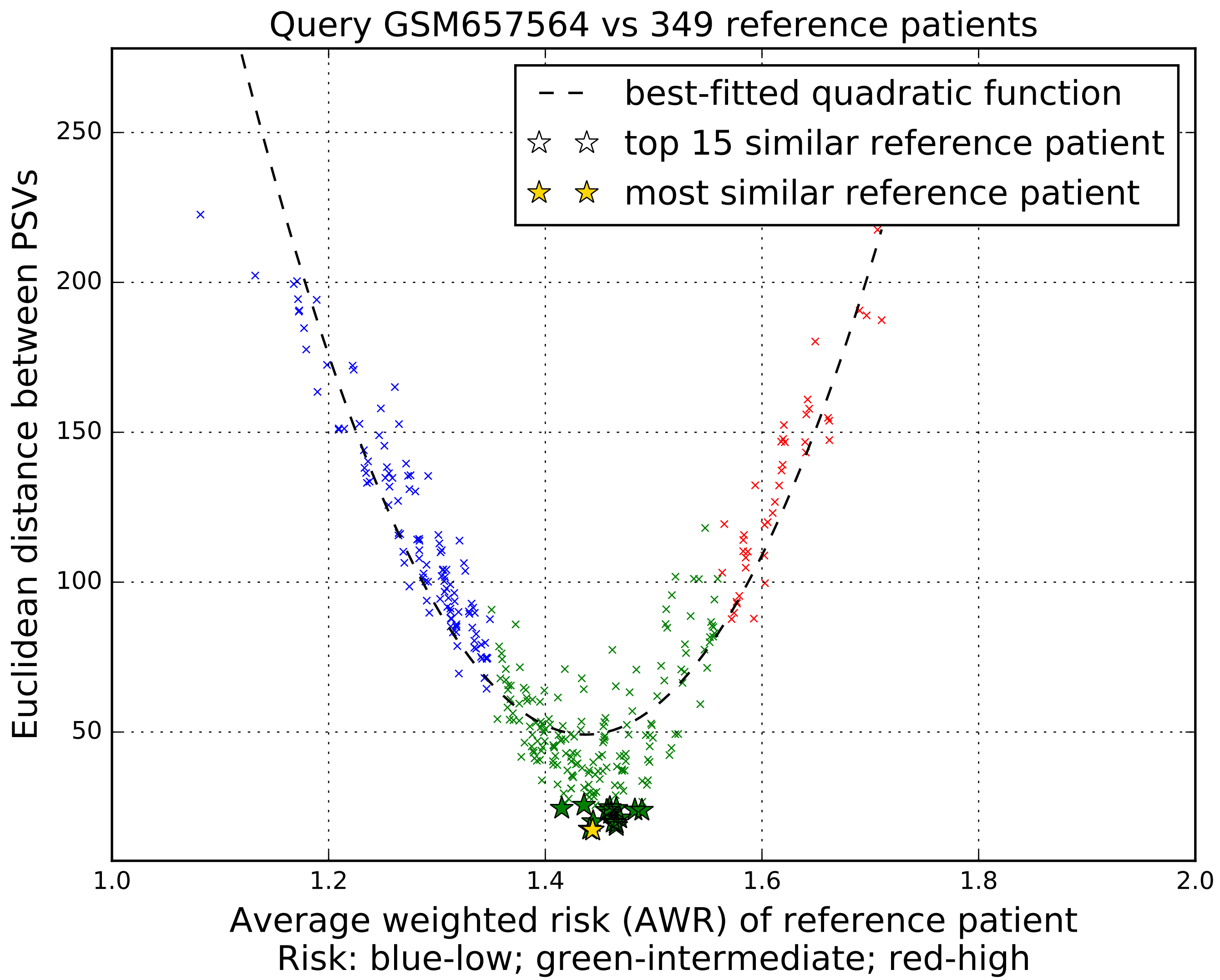

Query GSM657564 vs 349 reference patients
best-fitted quadratic function
top 15 similar reference patient
most similar reference patient
Euclidean distance between PSVs
250
200
150
100
50
1.0
1.2
1.4
1.6
1.8
2.0
Average weighted risk (AWR) of reference patient
Risk: blue-low; green-intermediate; red-high

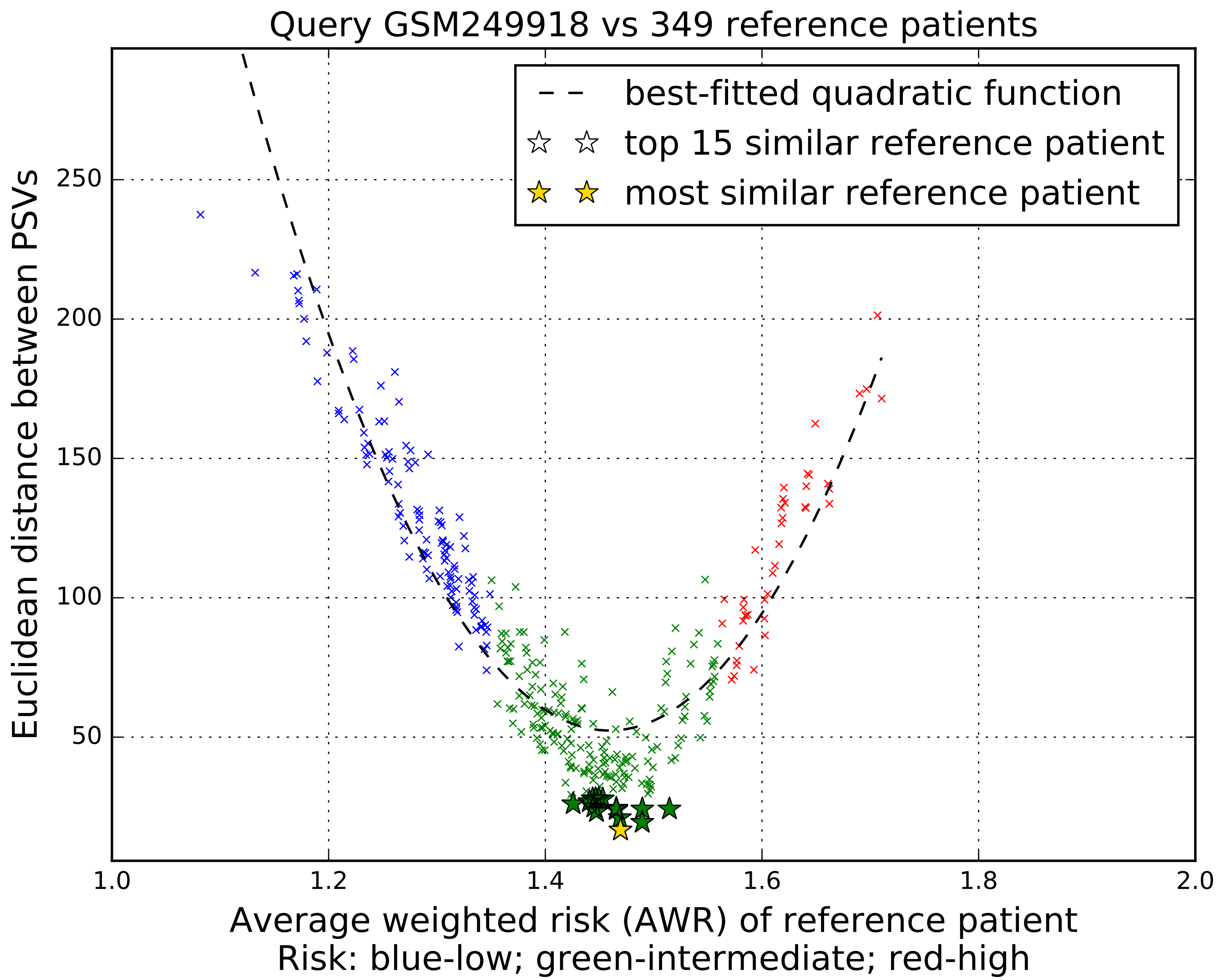
Query GSM249918 vs 349 reference patients
best-fitted quadratic function
top 15 similar reference patient
most similar reference patient
Euclidean distance between PSVs
250
200
150
100
50
1.0
1.2
1.4
1.6
1.8
2.0
Average weighted risk (AWR) of reference patient
Risk: blue-low; green-intermediate; red-high

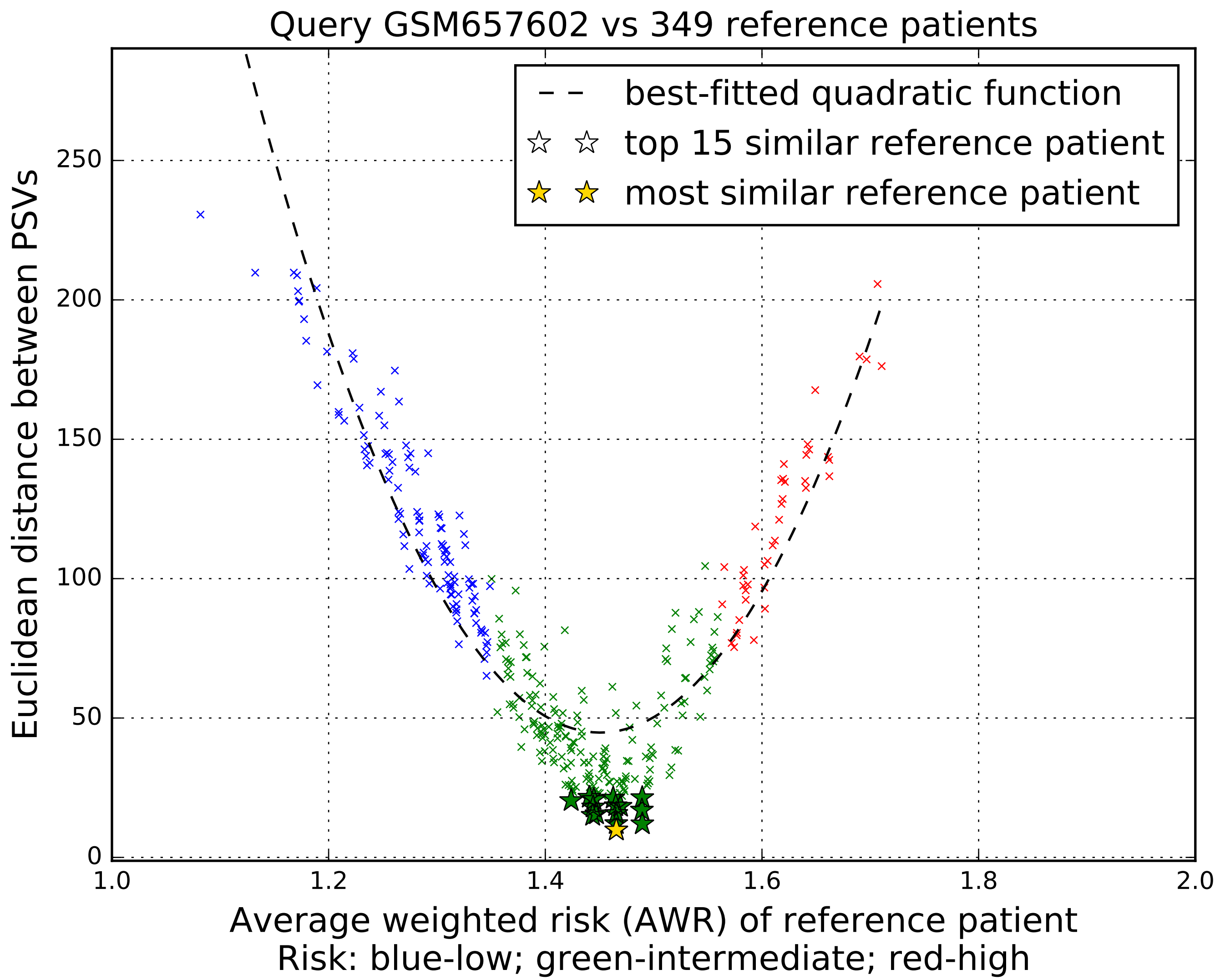

Query GSM657602 vs 349 reference patients
best-fitted quadratic function
top 15 similar reference patient
most similar reference patient
Euclidean distance between PSVs
0
50
100
150
200
250
1.0
1.2
1.4
1.6
1.8
2.0
Average weighted risk (AWR) of reference patient
Risk: blue-low; green-intermediate; red-high

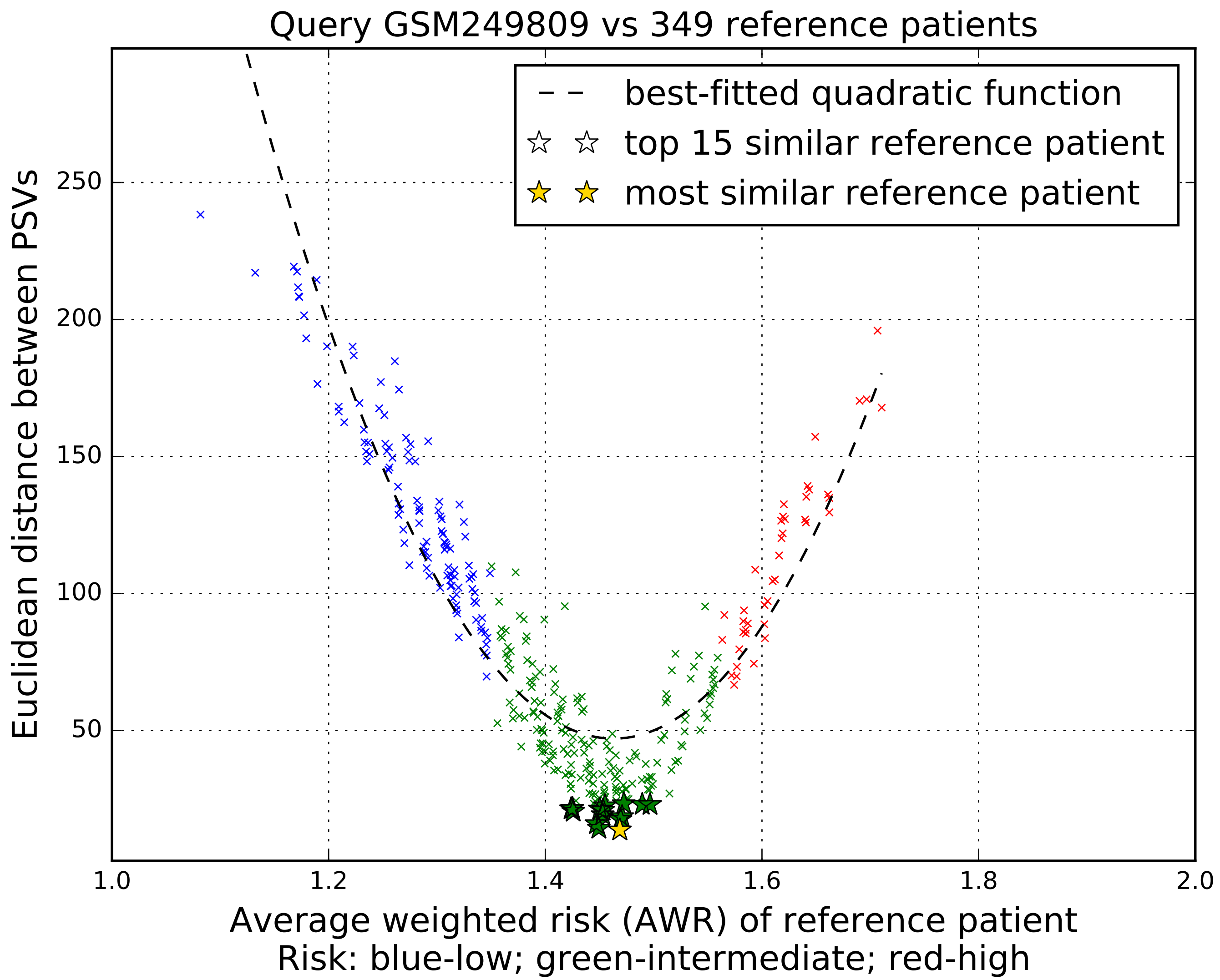

Query GSM249809 vs 349 reference patients
best-fitted quadratic function
top 15 similar reference patient
most similar reference patient
Euclidean distance between PSVs
250
200
150
100
50
1.0
1.2
1.4
1.6
1.8
2.0
Average weighted risk (AWR) of reference patient
Risk: blue-low; green-intermediate; red-high

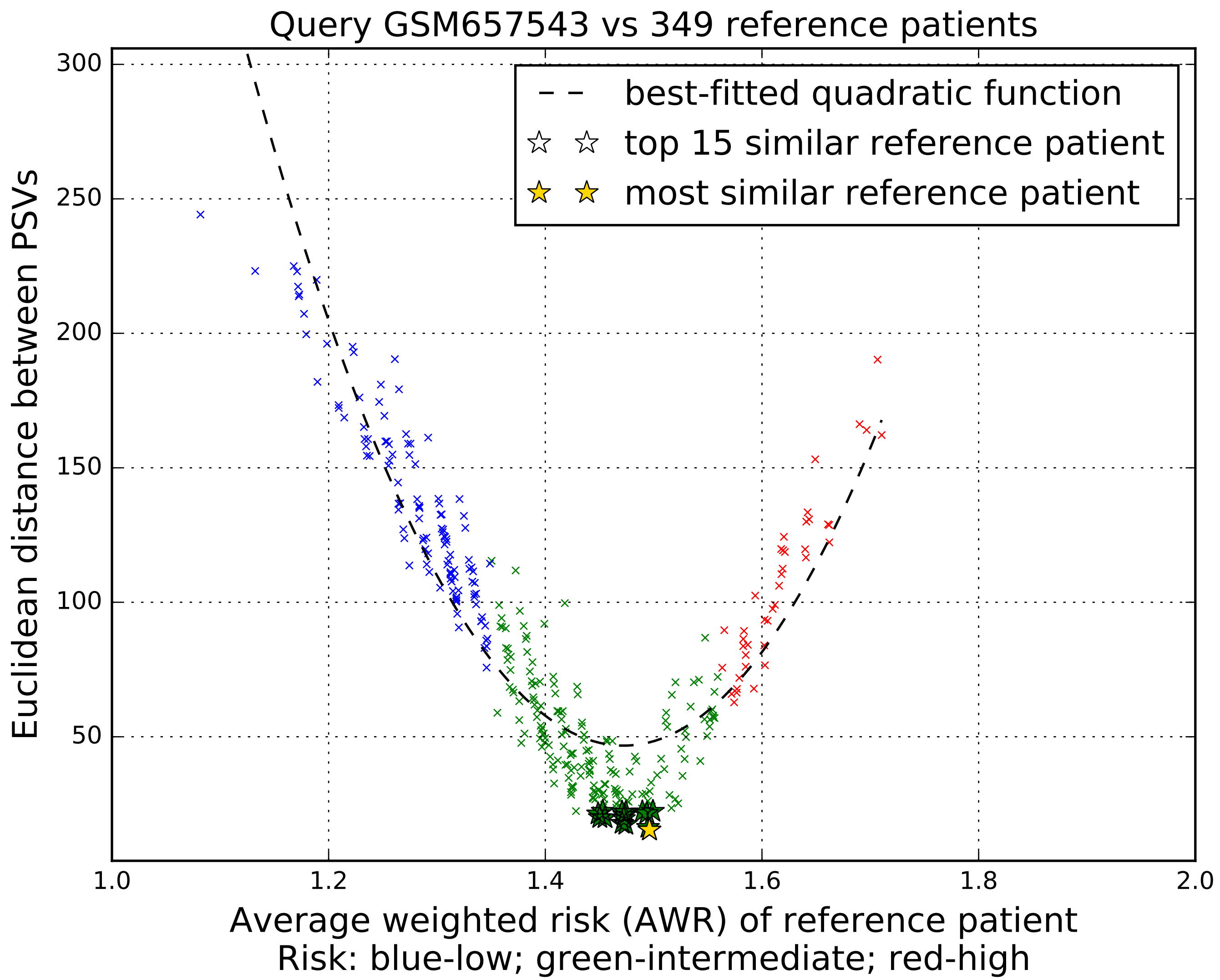
Query GSM657543 vs 349 reference patients
best-fitted quadratic function
top 15 similar reference patient
most similar reference patient
Euclidean distance between PSVs
300
250
200
150
100
50
1.0
1.2
1.4
1.6
1.8
2.0
Average weighted risk (AWR) of reference patient
Risk: blue-low; green-intermediate; red-high

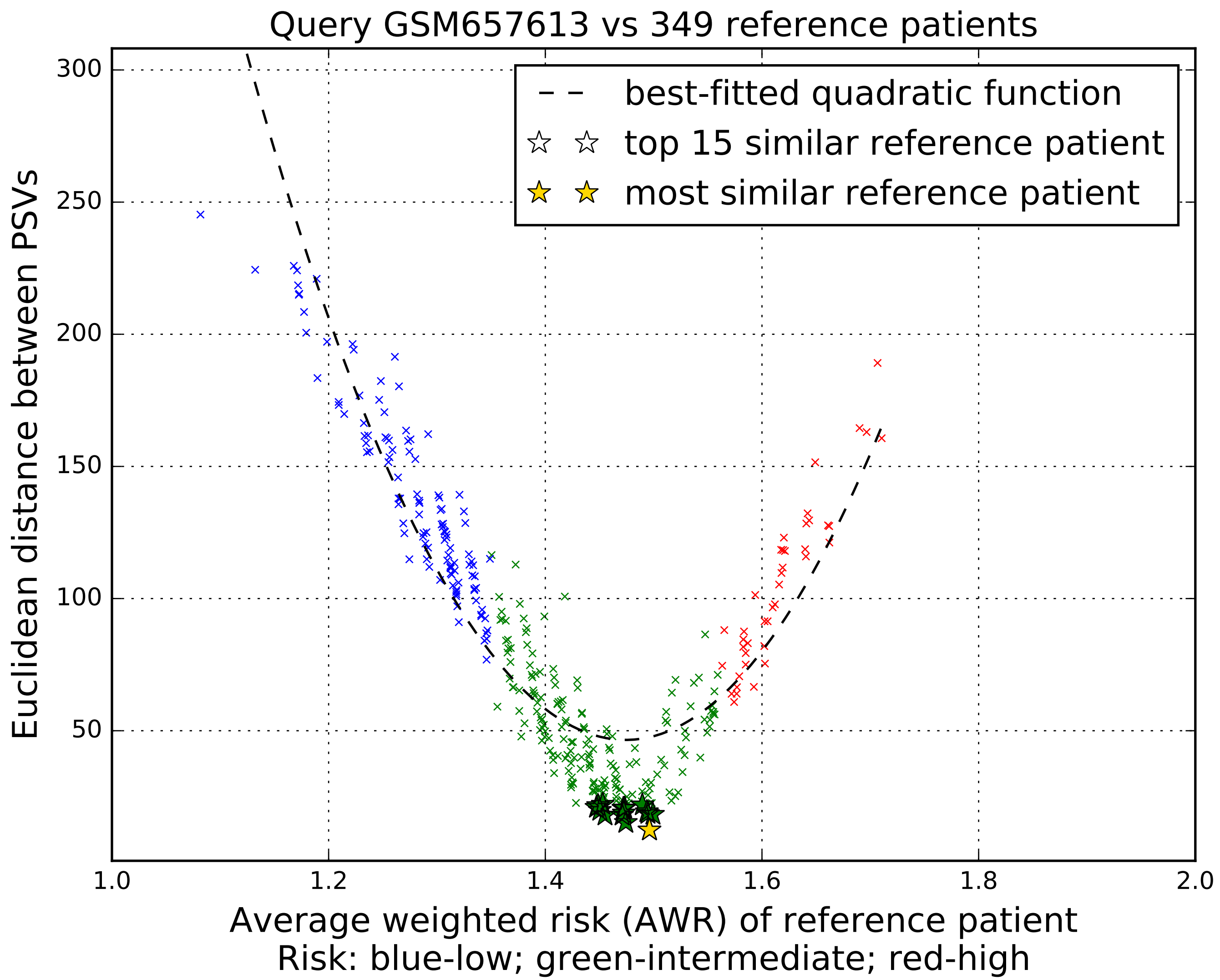
Query GSM657613 vs 349 reference patients
best-fitted quadratic function
top 15 similar reference patient
most similar reference patient
Euclidean distance between PSVs
300
250
200
150
100
50
1.0
1.2
1.4
1.6
1.8
2.0
Average weighted risk (AWR) of reference patient
Risk: blue-low; green-intermediate; red-high

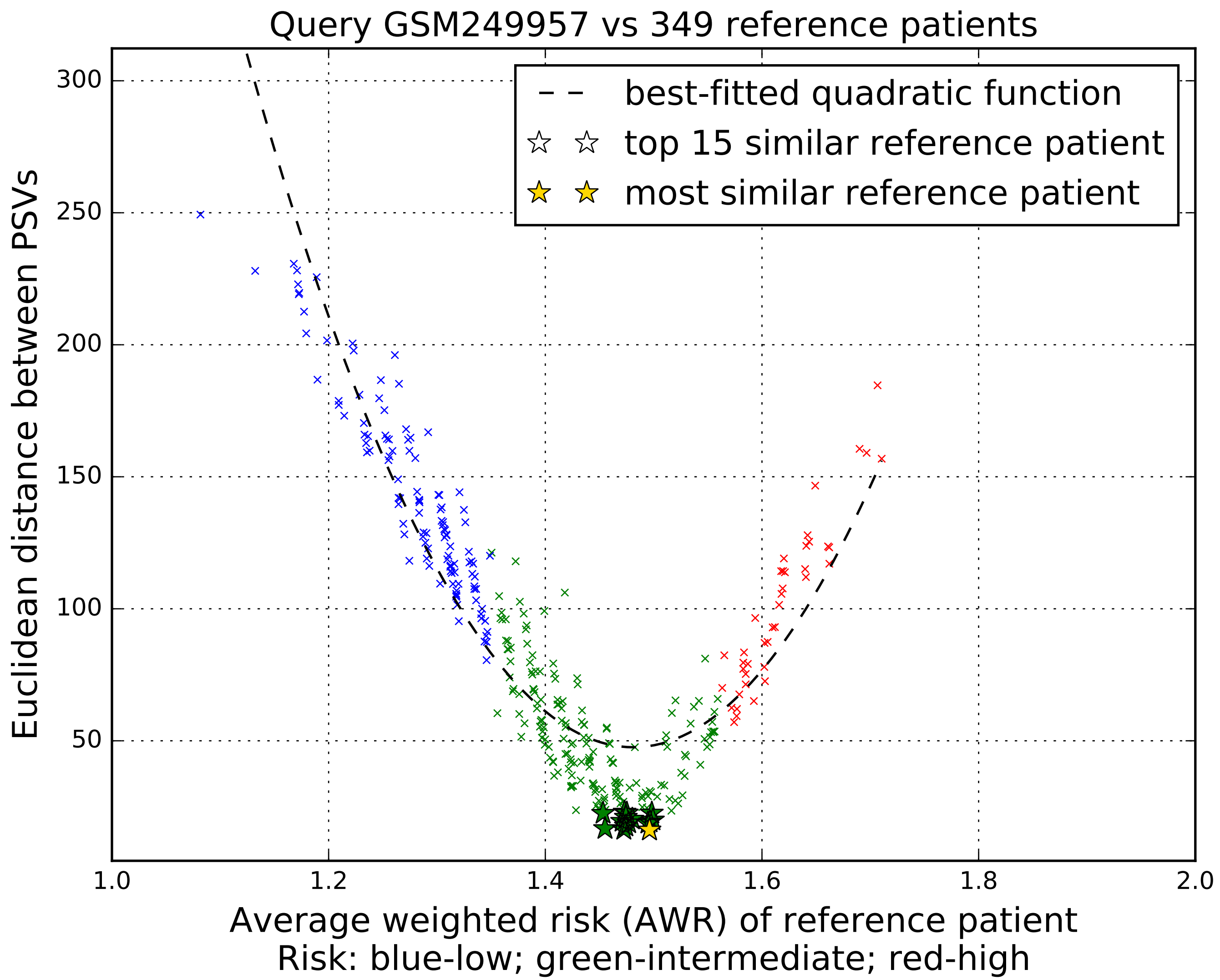

Query GSM249957 vs 349 reference patients
best-fitted quadratic function
top 15 similar reference patient
most similar reference patient
Euclidean distance between PSVs
300
250
200
150
100
50
1.0
1.2
1.4
1.6
1.8
2.0
Average weighted risk (AWR) of reference patient
Risk: blue-low; green-intermediate; red-high

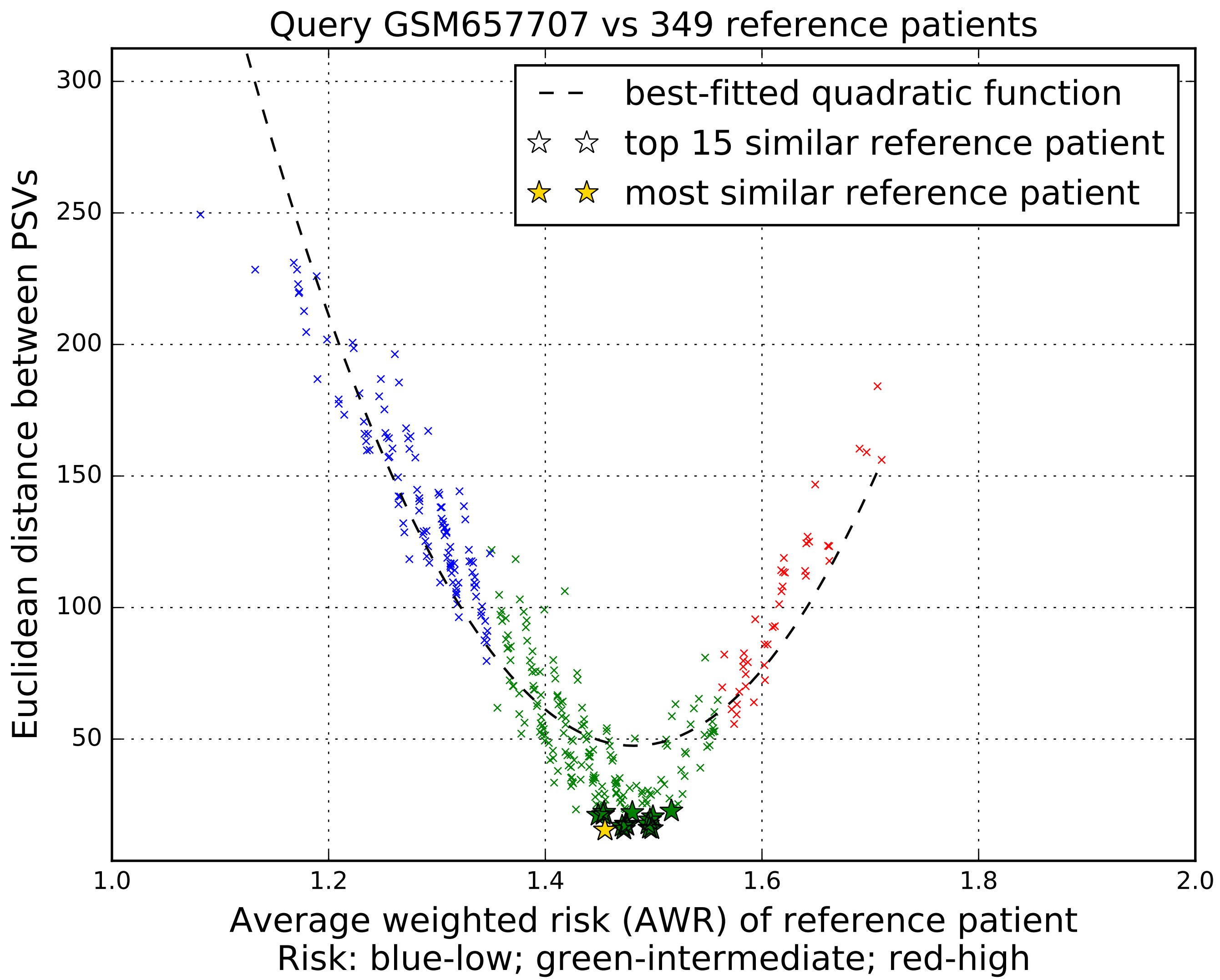

Query GSM657707 vs 349 reference patients
best-fitted quadratic function
top 15 similar reference patient
most similar reference patient
Euclidean distance between PSVs
300
250
200
150
100
50
1.0
1.2
1.4
1.6
1.8
2.0
Average weighted risk (AWR) of reference patient
Risk: blue-low; green-intermediate; red-high

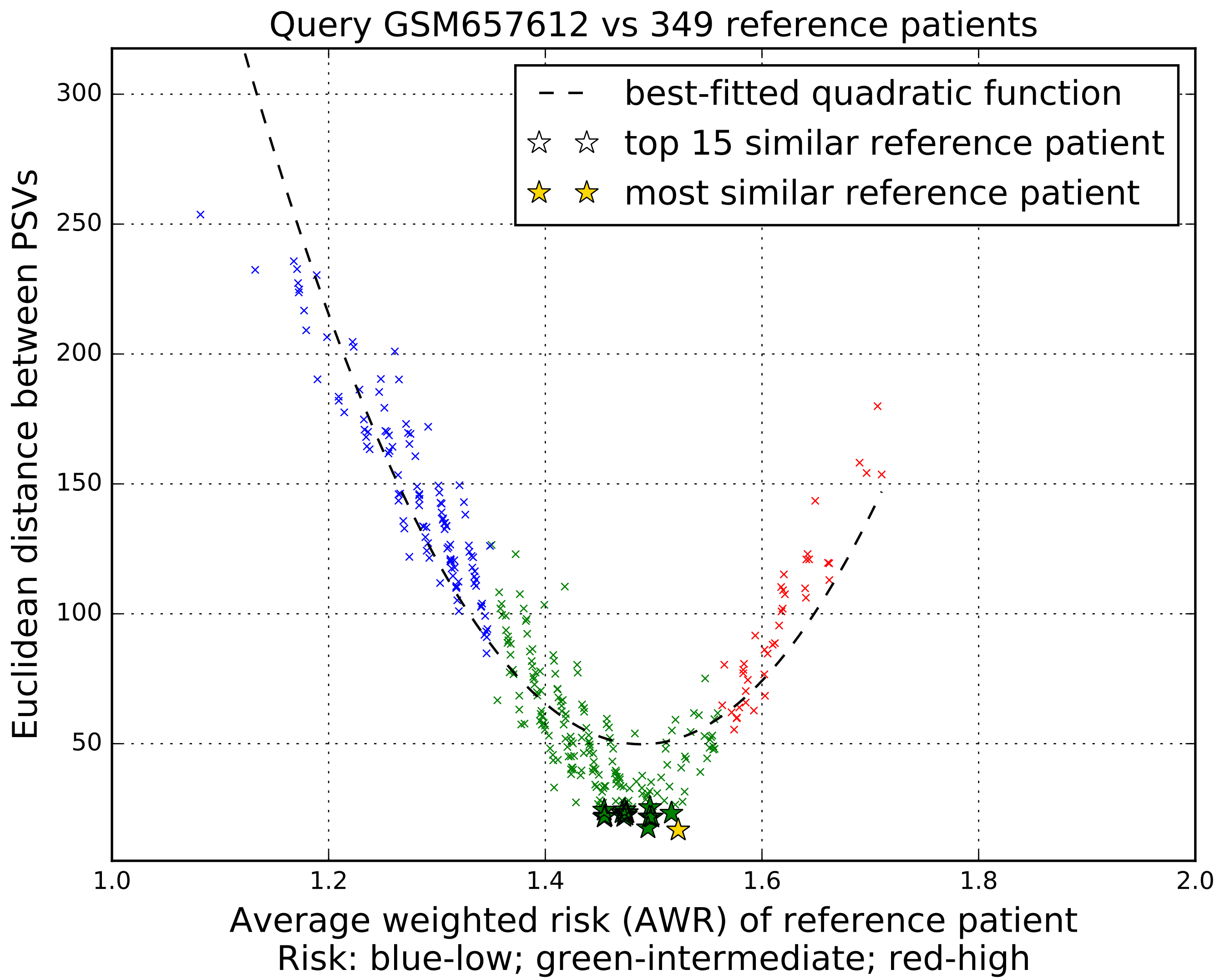

Query GSM657612 vs 349 reference patients
best-fitted quadratic function
top 15 similar reference patient
most similar reference patient
Euclidean distance between PSVs
300
250
200
150
100
50
1.0
1.2
1.4
1.6
1.8
2.0
Average weighted risk (AWR) of reference patient
Risk: blue-low; green-intermediate; red-high

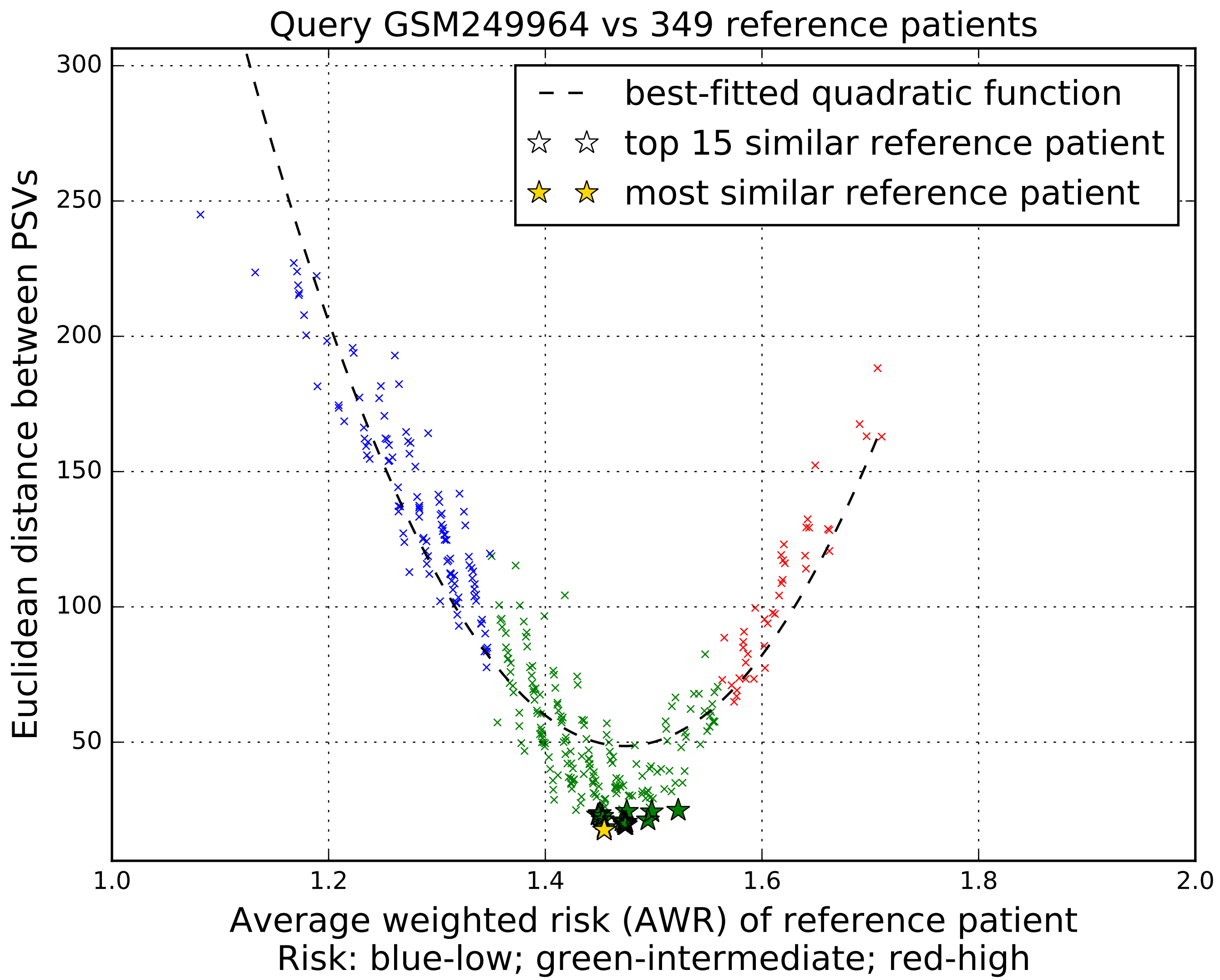

Query GSM249964 vs 349 reference patients
best-fitted quadratic function
top 15 similar reference patient
most similar reference patient
Euclidean distance between PSVs
300
250
200
150
100
50
1.0
1.2
1.4
1.6
1.8
2.0
Average weighted risk (AWR) of reference patient
Risk: blue-low; green-intermediate; red-high

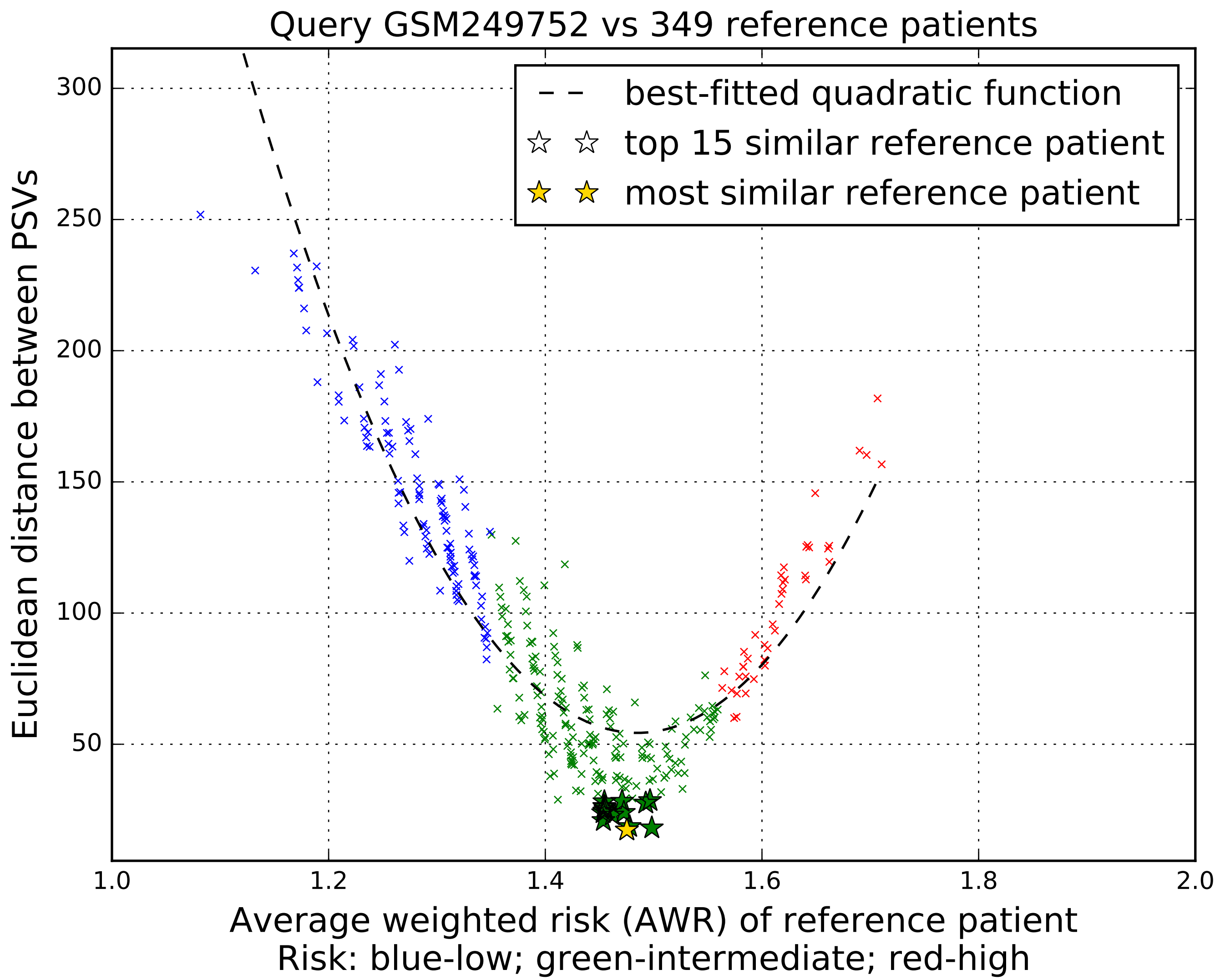

Query GSM249752 vs 349 reference patients
best-fitted quadratic function
top 15 similar reference patient
most similar reference patient
Euclidean distance between PSVs
300
250
200
150
100
50
1.0
1.2
1.4
1.6
1.8
2.0
Average weighted risk (AWR) of reference patient
Risk: blue-low; green-intermediate; red-high

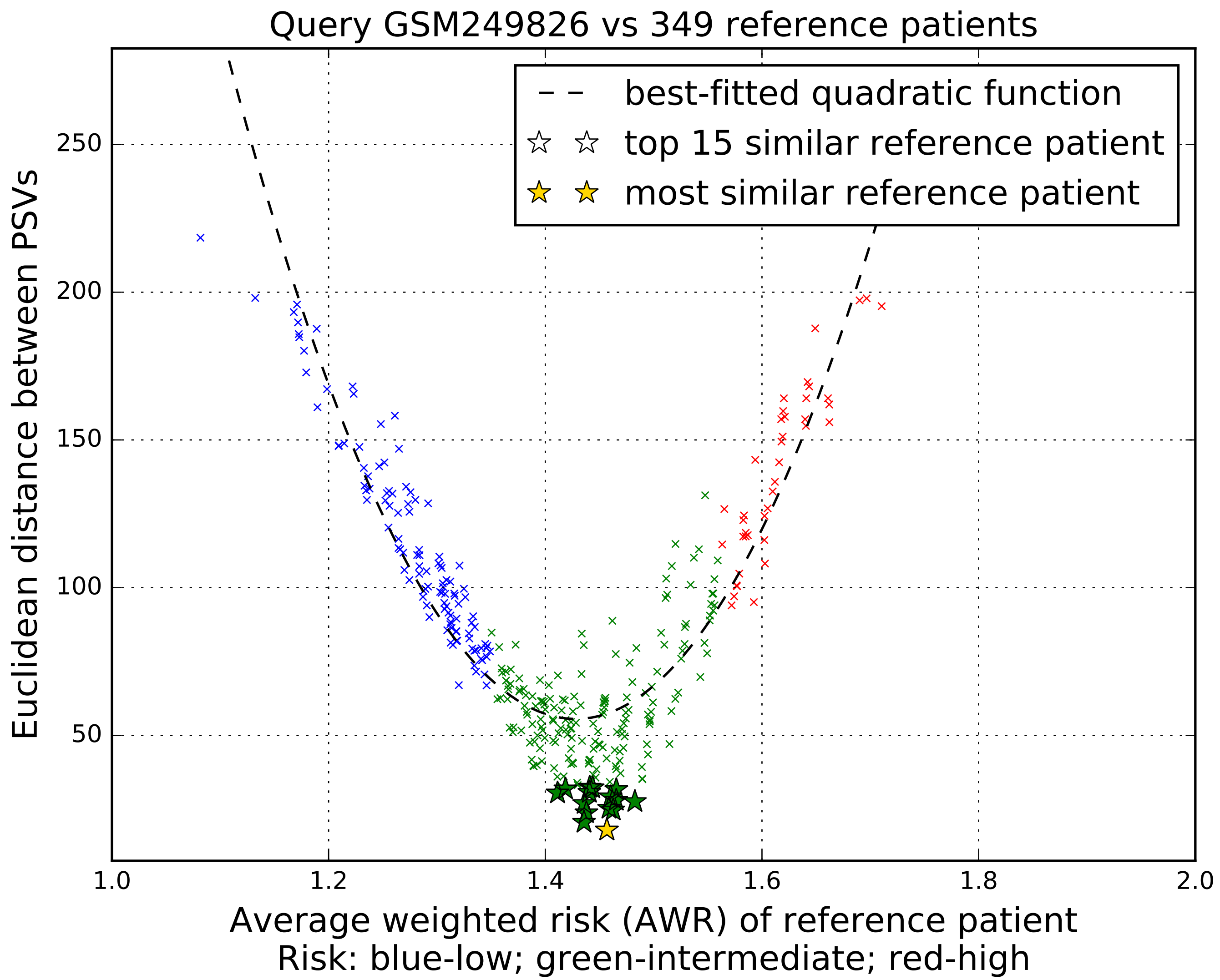
Query GSM249826 vs 349 reference patients
best-fitted quadratic function
top 15 similar reference patient
most similar reference patient
Euclidean distance between PSVs
250
200
150
100
50
1.0
1.2
1.4
1.6
1.8
2.0
Average weighted risk (AWR) of reference patient
Risk: blue-low; green-intermediate; red-high

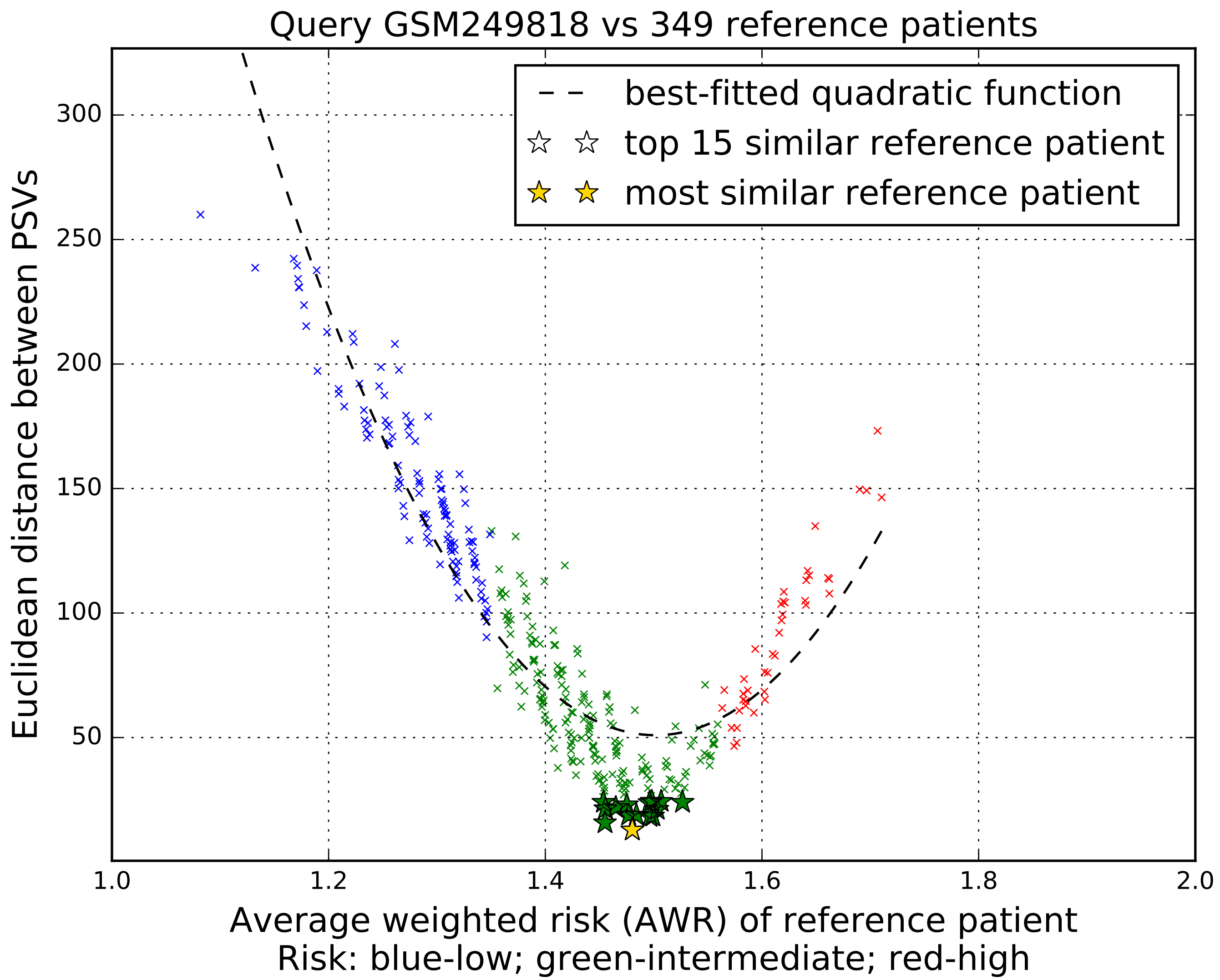

Query GSM249818 vs 349 reference patients
best-fitted quadratic function
top 15 similar reference patient
most similar reference patient
Euclidean distance between PSVs
300
250
200
150
100
50
1.0
1.2
1.4
1.6
1.8
2.0
Average weighted risk (AWR) of reference patient
Risk: blue-low; green-intermediate; red-high

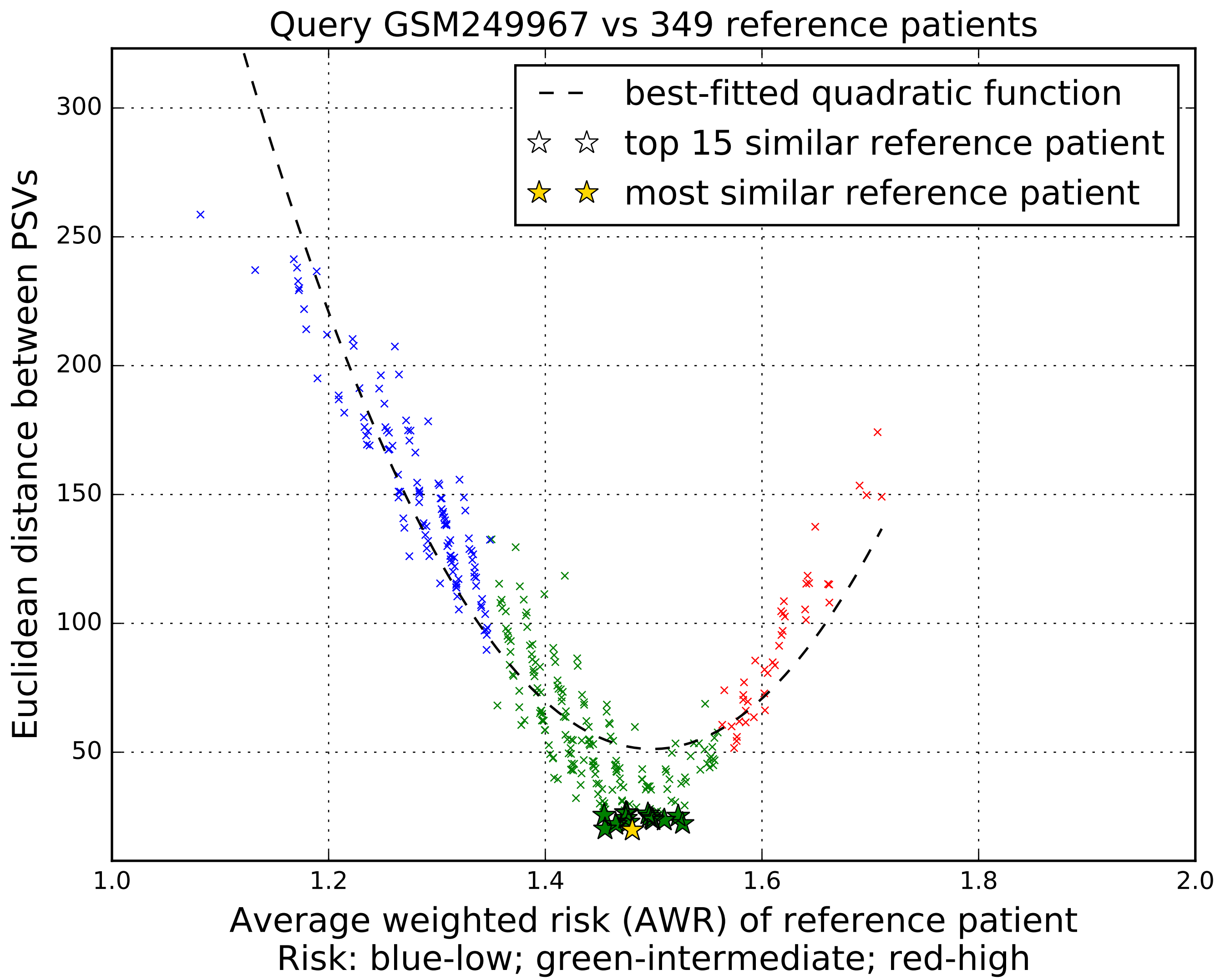
Query GSM249967 vs 349 reference patients
best-fitted quadratic function
top 15 similar reference patient
most similar reference patient
Euclidean distance between PSVs
300
250
200
150
100
50
1.0
1.2
1.4
1.6
1.8
2.0
Average weighted risk (AWR) of reference patient
Risk: blue-low; green-intermediate; red-high

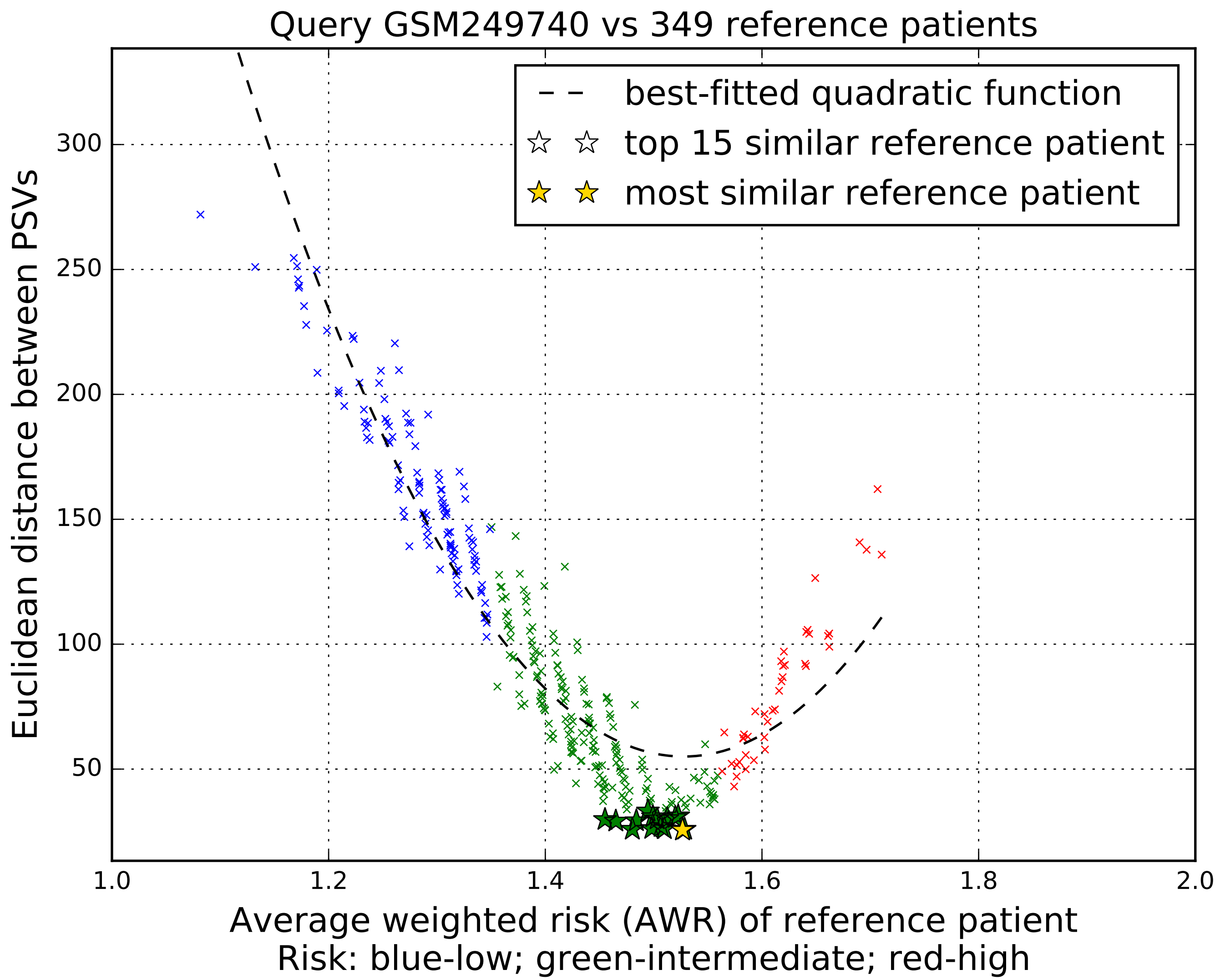

Query GSM249740 vs 349 reference patients
best-fitted quadratic function
top 15 similar reference patient
most similar reference patient
Euclidean distance between PSVs
300
250
200
150
100
50
1.0
1.2
1.4
1.6
1.8
2.0
Average weighted risk (AWR) of reference patient
Risk: blue-low; green-intermediate; red-high

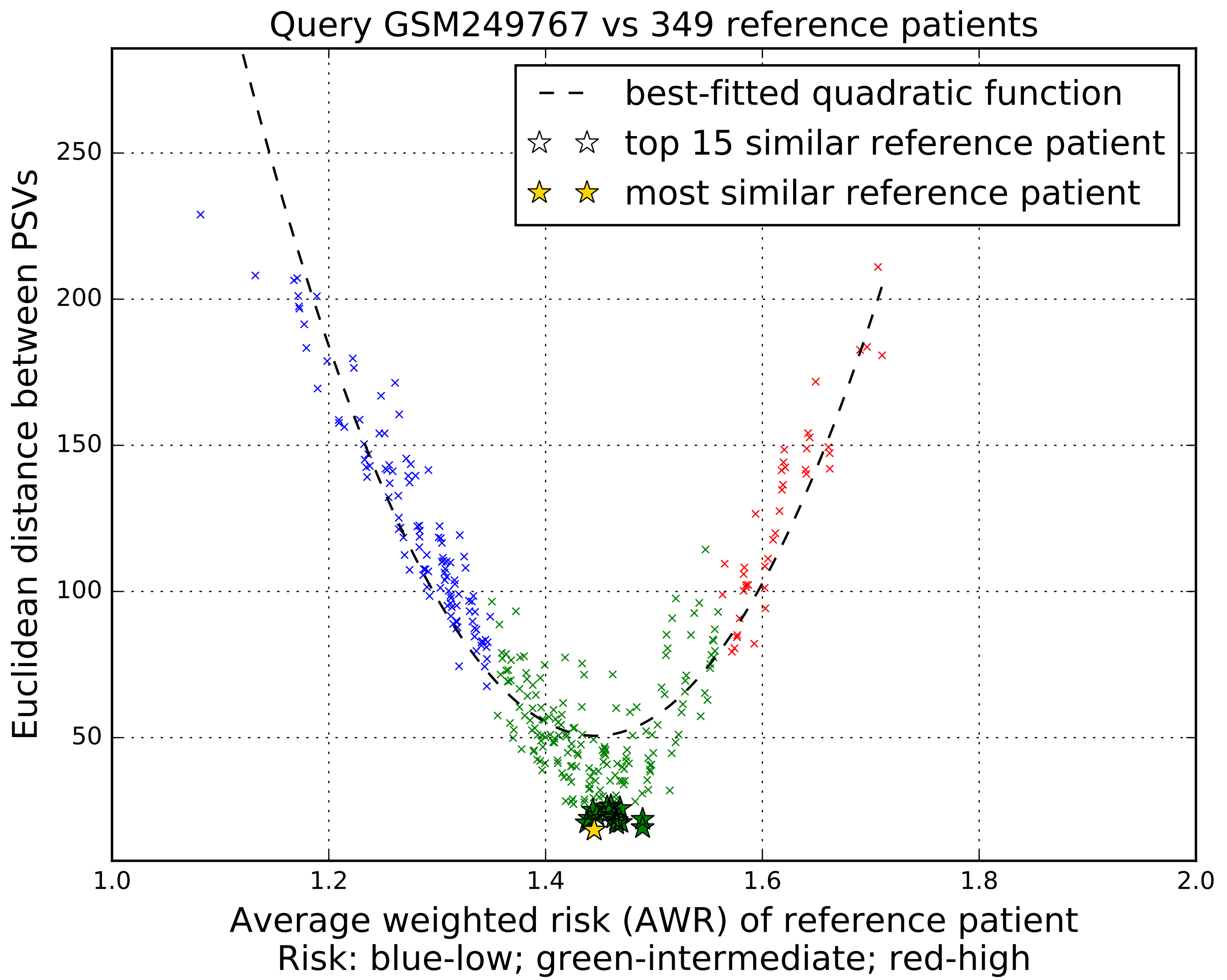

Query GSM249767 vs 349 reference patients
best-fitted quadratic function
top 15 similar reference patient
most similar reference patient
Euclidean distance between PSVs
250
200
150
100
50
1.0
1.2
1.4
1.6
1.8
2.0
Average weighted risk (AWR) of reference patient
Risk: blue-low; green-intermediate; red-high

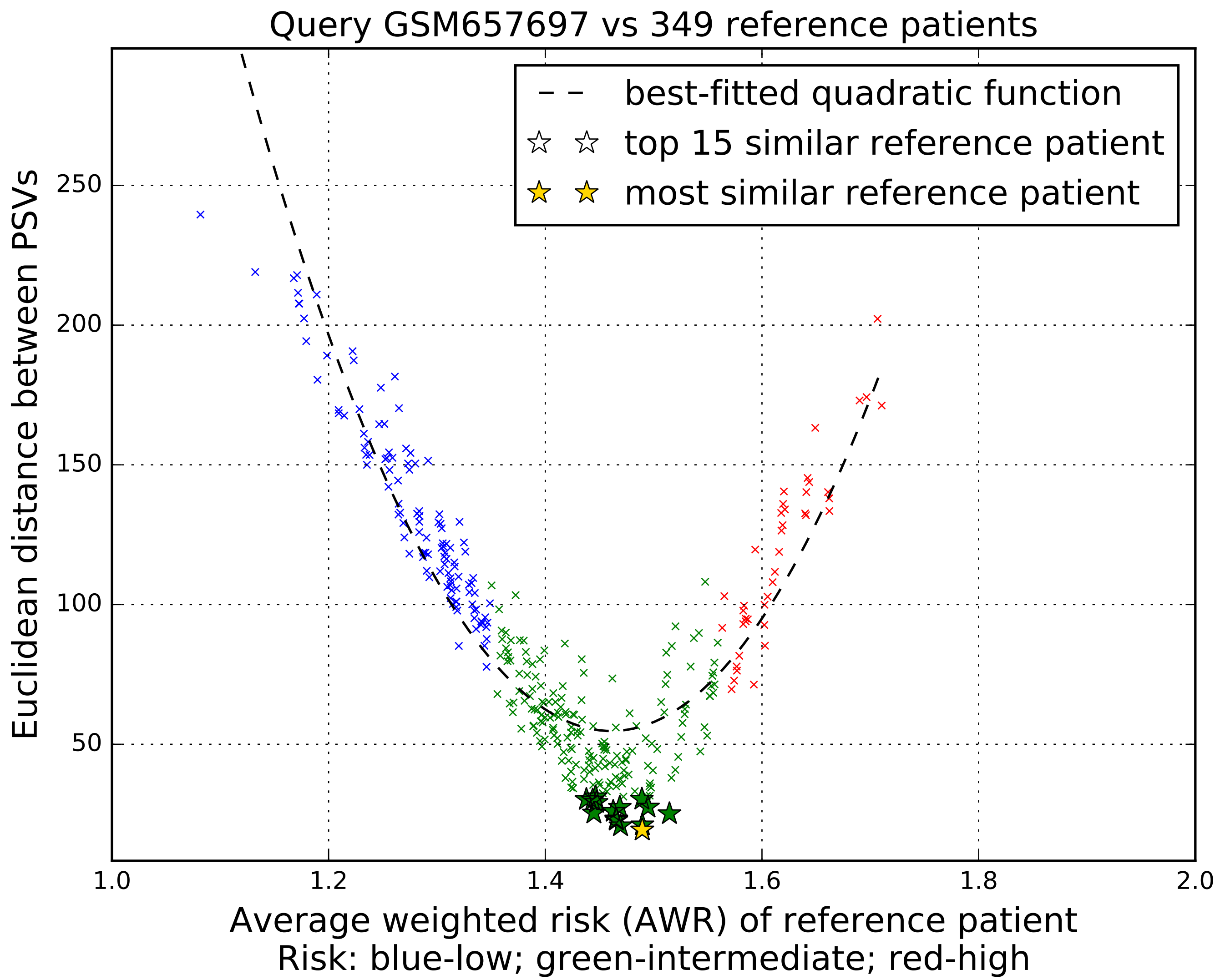

Query GSM657697 vs 349 reference patients
best-fitted quadratic function
top 15 similar reference patient
most similar reference patient
Euclidean distance between PSVs
50
100
150
200
250
1.0
1.2
1.4
1.6
1.8
2.0
Average weighted risk (AWR) of reference patient
Risk: blue-low; green-intermediate; red-high

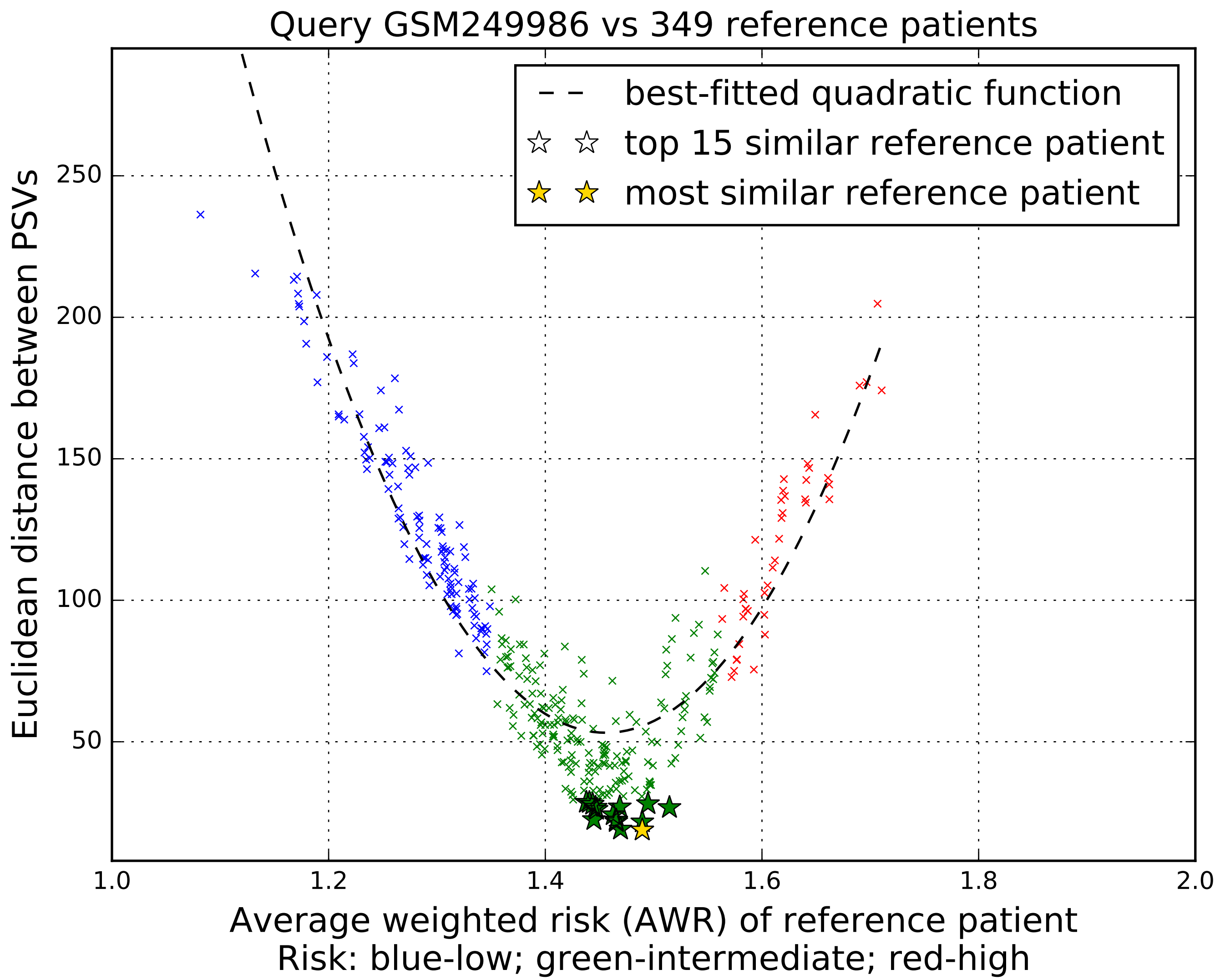
Query GSM249986 vs 349 reference patients
best-fitted quadratic function
top 15 similar reference patient
most similar reference patient
Euclidean distance between PSVs
250
200
150
100
50
1.0
1.2
1.4
1.6
1.8
2.0
Average weighted risk (AWR) of reference patient
Risk: blue-low; green-intermediate; red-high

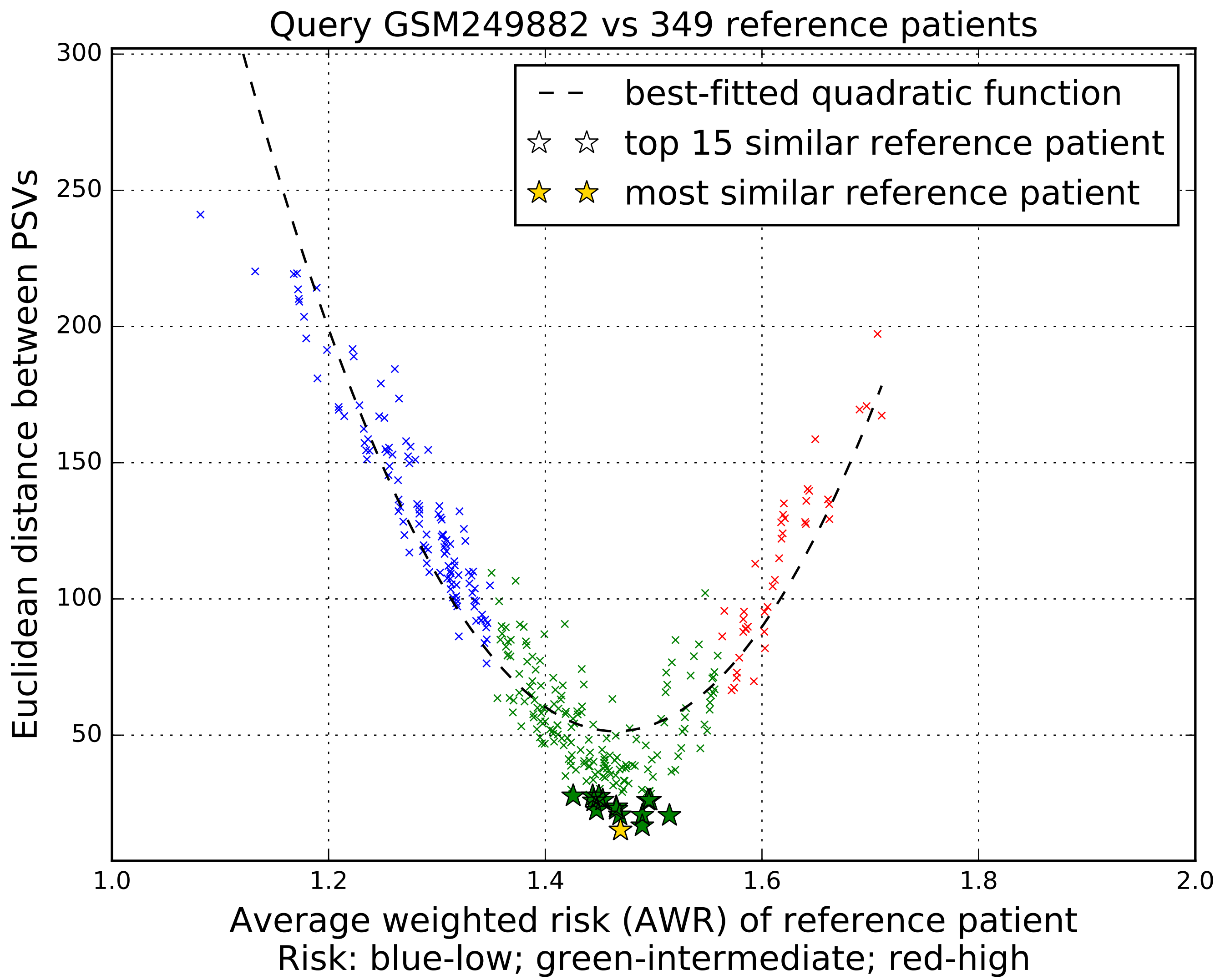

Query GSM249882 vs 349 reference patients
best-fitted quadratic function
top 15 similar reference patient
most similar reference patient
Euclidean distance between PSVs
300
250
200
150
100
50
1.0
1.2
1.4
1.6
1.8
2.0
Average weighted risk (AWR) of reference patient
Risk: blue-low; green-intermediate; red-high

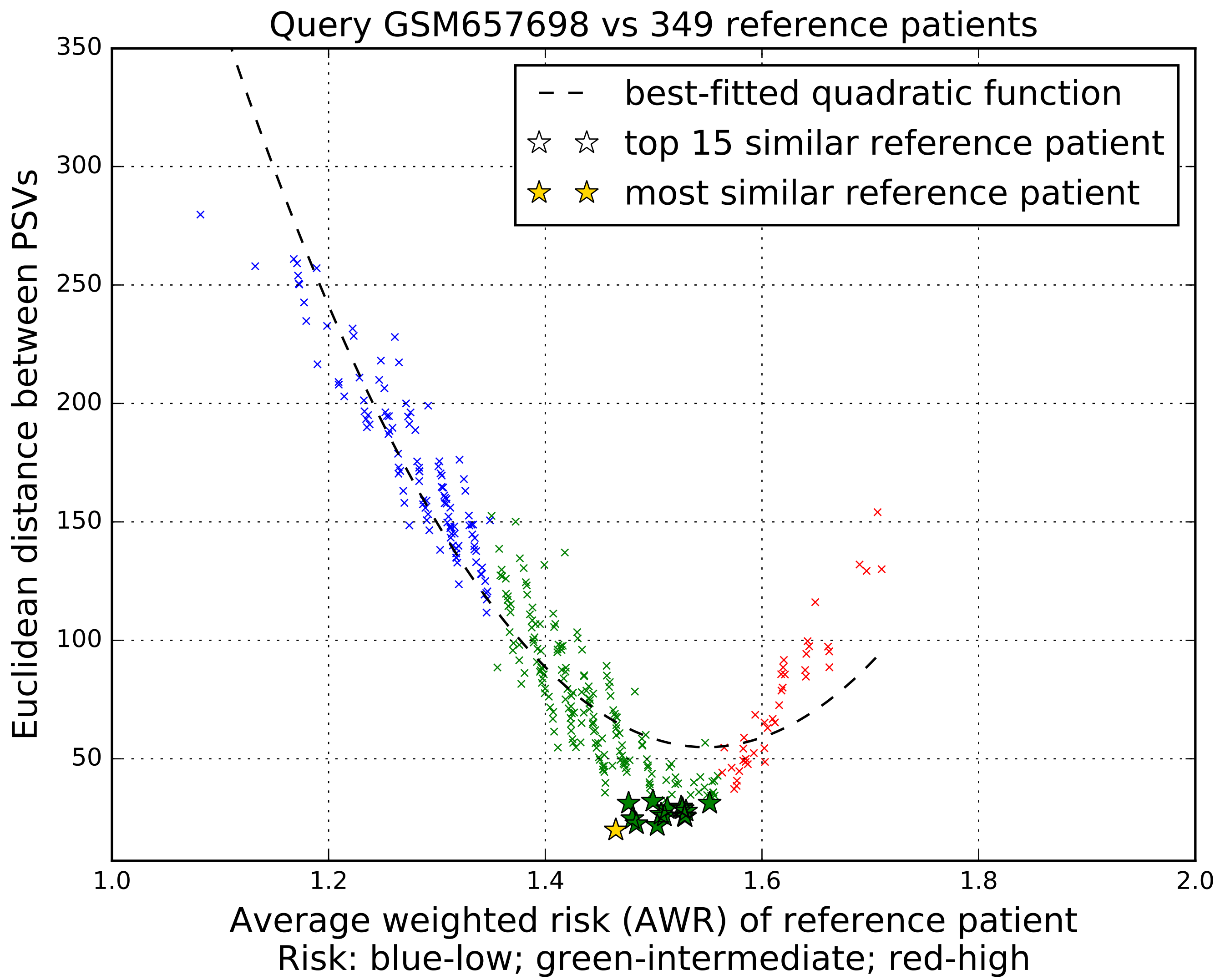
Query GSM657698 vs 349 reference patients
best-fitted quadratic function
top 15 similar reference patient
most similar reference patient
Euclidean distance between PSVs
350
300
250
200
150
100
50
1.0
1.2
1.4
1.6
1.8
2.0
Average weighted risk (AWR) of reference patient
Risk: blue-low; green-intermediate; red-high

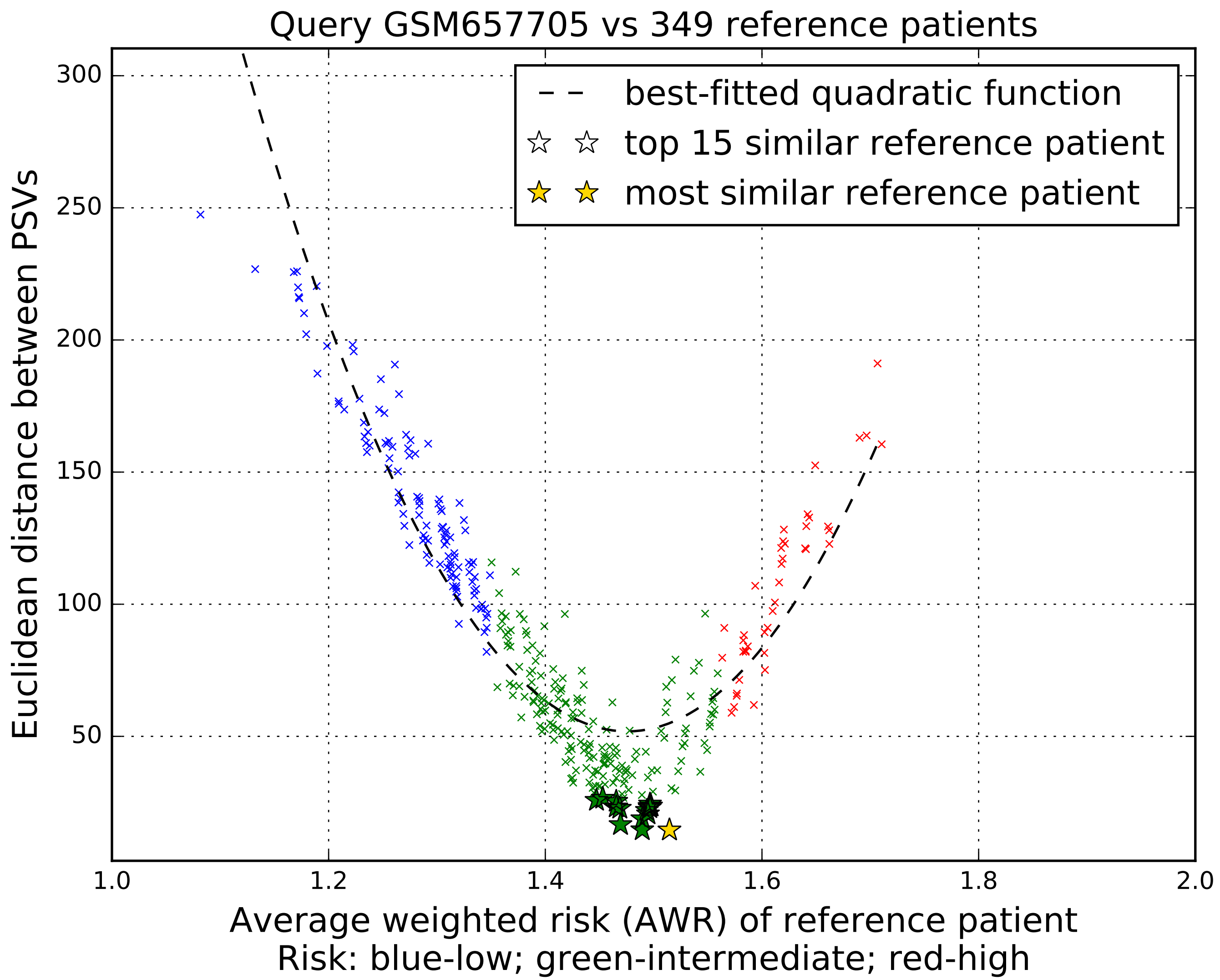

Query GSM657705 vs 349 reference patients
best-fitted quadratic function
top 15 similar reference patient
most similar reference patient
Euclidean distance between PSVs
300
250
200
150
100
50
1.0
1.2
1.4
1.6
1.8
2.0
Average weighted risk (AWR) of reference patient
Risk: blue-low; green-intermediate; red-high

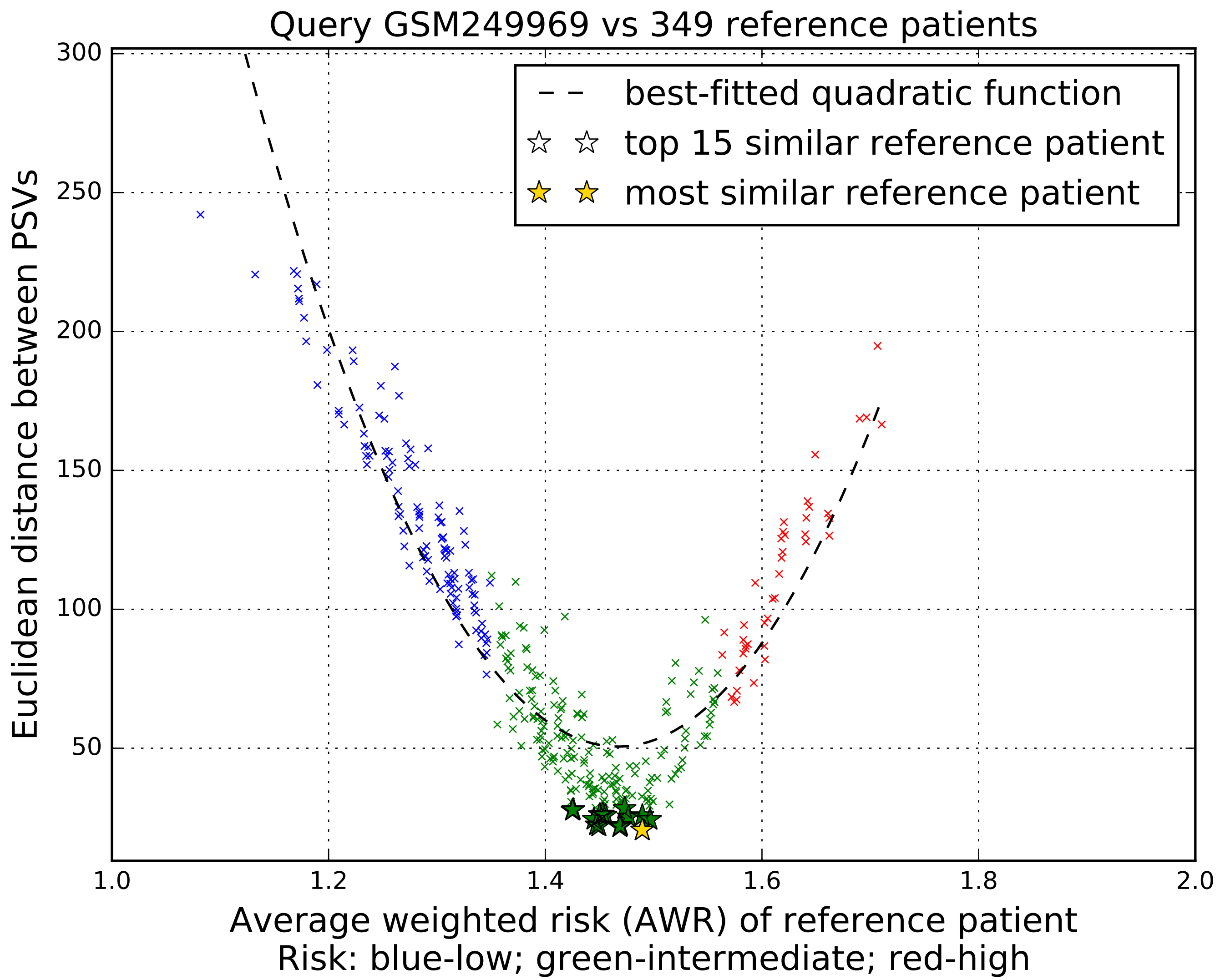

Query GSM249969 vs 349 reference patients
best-fitted quadratic function
top 15 similar reference patient
most similar reference patient
Euclidean distance between PSVs
300
250
200
150
100
50
1.0
1.2
1.4
1.6
1.8
2.0
Average weighted risk (AWR) of reference patient
Risk: blue-low; green-intermediate; red-high

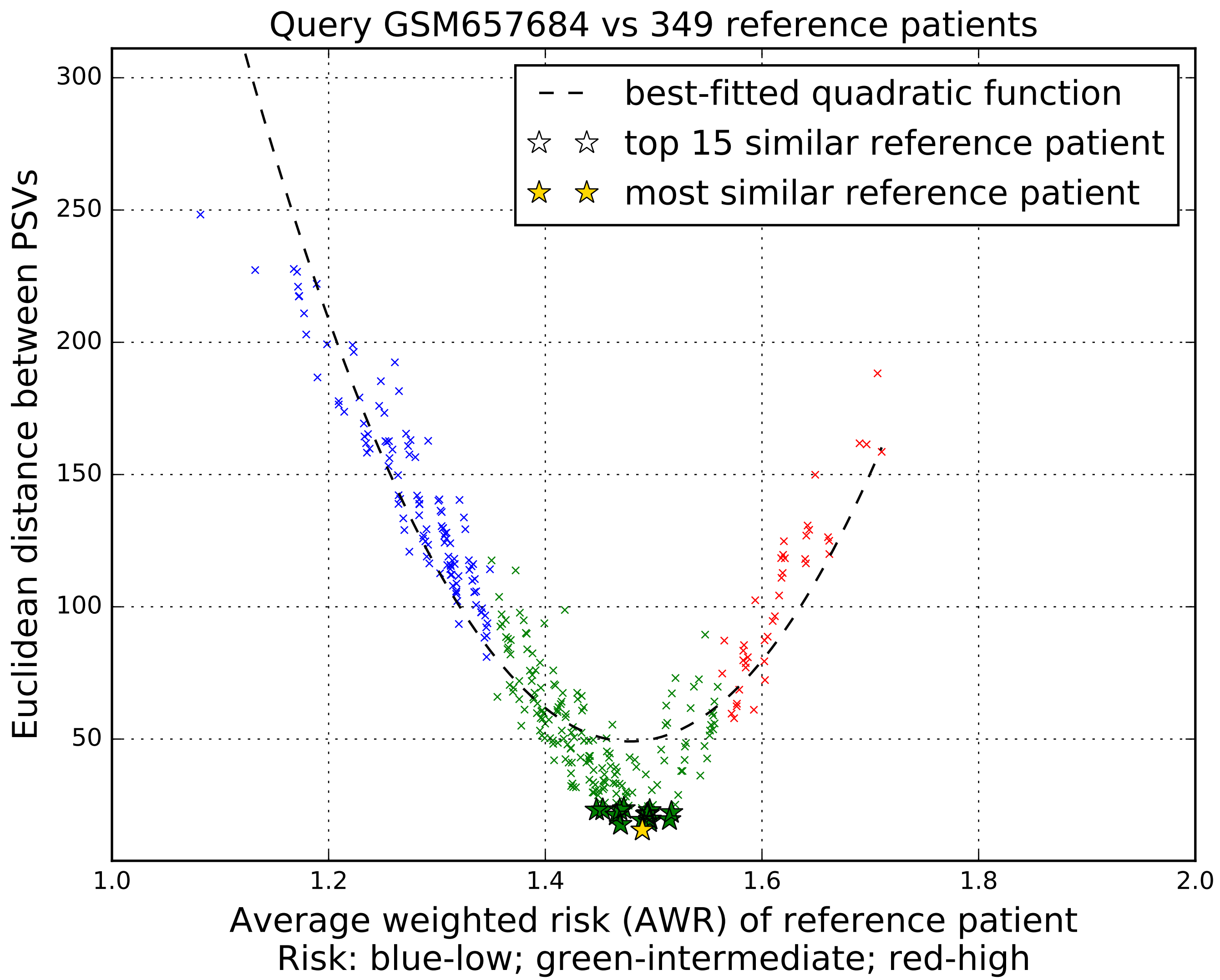

Query GSM657684 vs 349 reference patients
best-fitted quadratic function
top 15 similar reference patient
most similar reference patient
Euclidean distance between PSVs
300
250
200
150
100
50
1.0
1.2
1.4
1.6
1.8
2.0
Average weighted risk (AWR) of reference patient
Risk: blue-low; green-intermediate; red-high

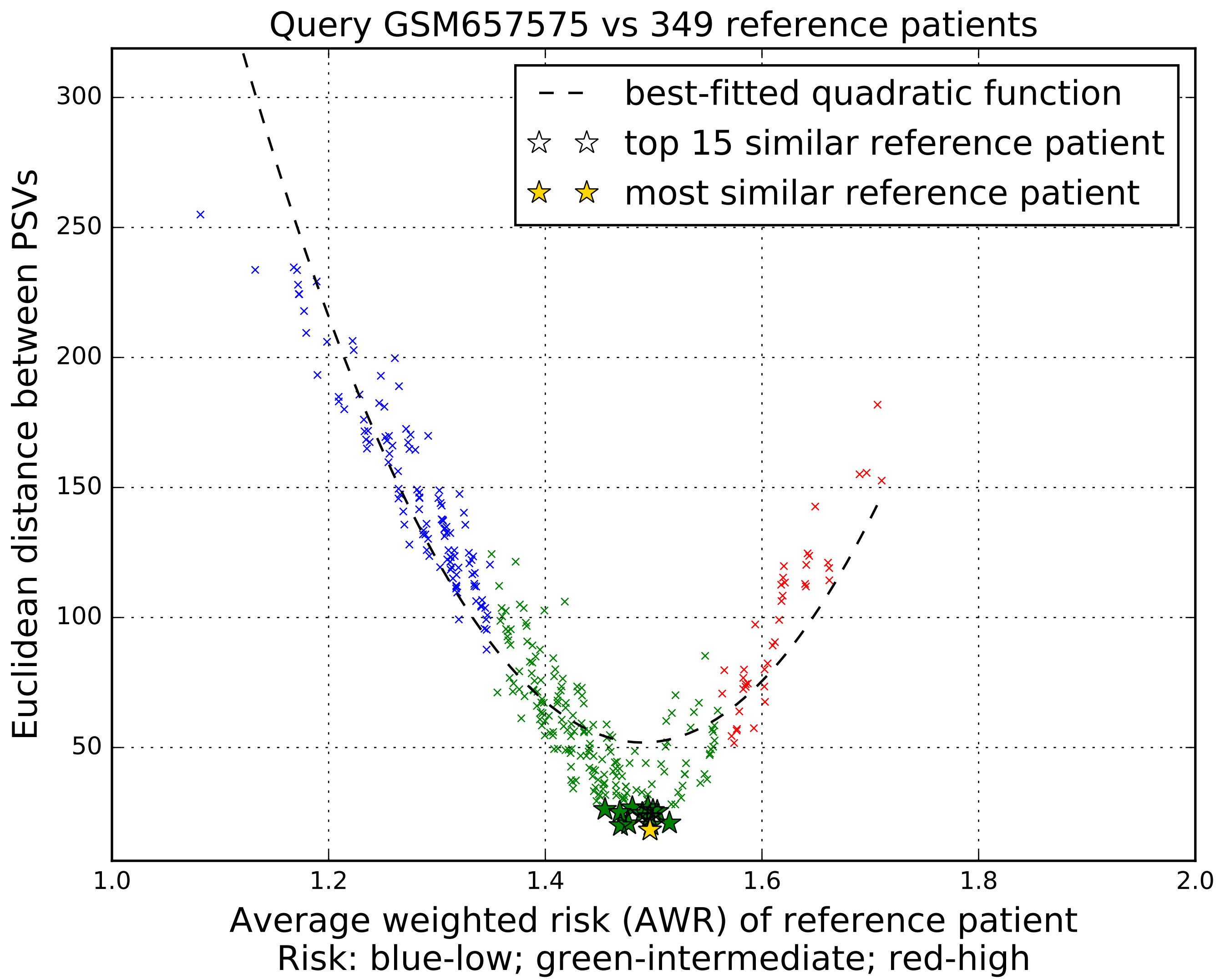

Query GSM657575 vs 349 reference patients
best-fitted quadratic function
top 15 similar reference patient
most similar reference patient
Euclidean distance between PSVs
300
250
200
150
100
50
1.0
1.2
1.4
1.6
1.8
2.0
Average weighted risk (AWR) of reference patient
Risk: blue-low; green-intermediate; red-high

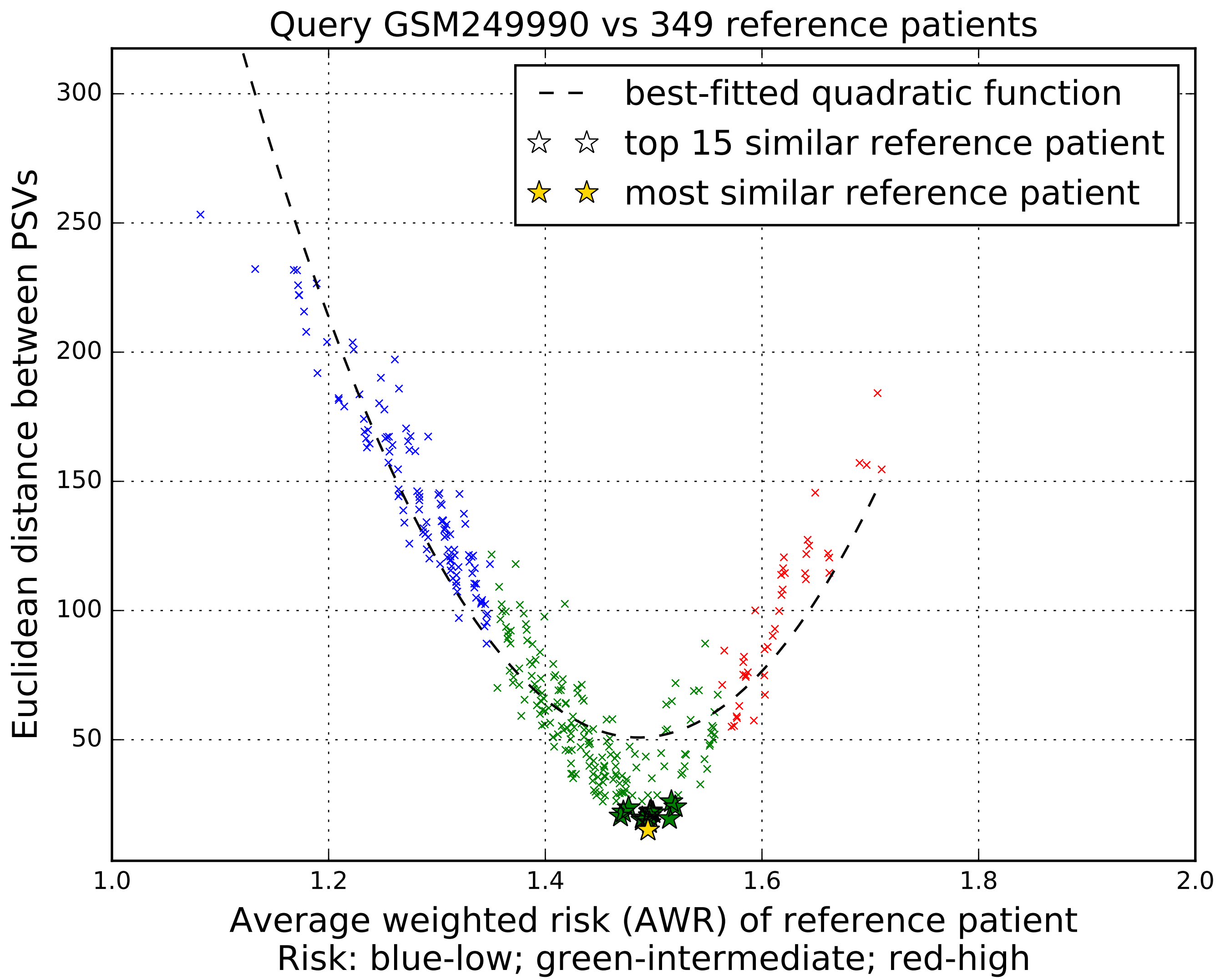

Query GSM249990 vs 349 reference patients
best-fitted quadratic function
top 15 similar reference patient
most similar reference patient
Euclidean distance between PSVs
300
250
200
150
100
50
1.0
1.2
1.4
1.6
1.8
2.0
Average weighted risk (AWR) of reference patient
Risk: blue-low; green-intermediate; red-high

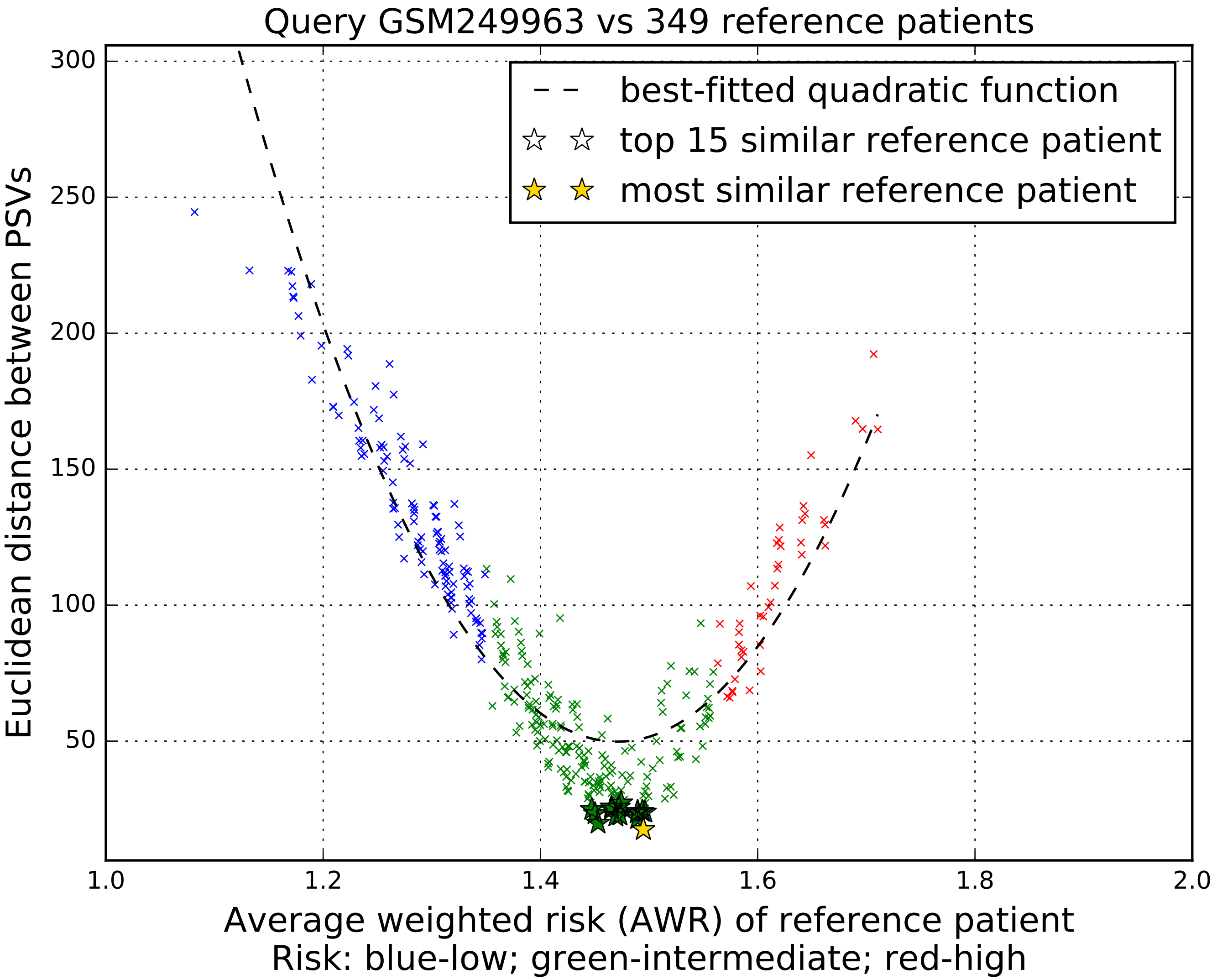
Query GSM249963 vs 349 reference patients
best-fitted quadratic function
top 15 similar reference patient
most similar reference patient
Euclidean distance between PSVs
300
250
200
150
100
50
1.0
1.2
1.4
1.6
1.8
2.0
Average weighted risk (AWR) of reference patient
Risk: blue-low; green-intermediate; red-high

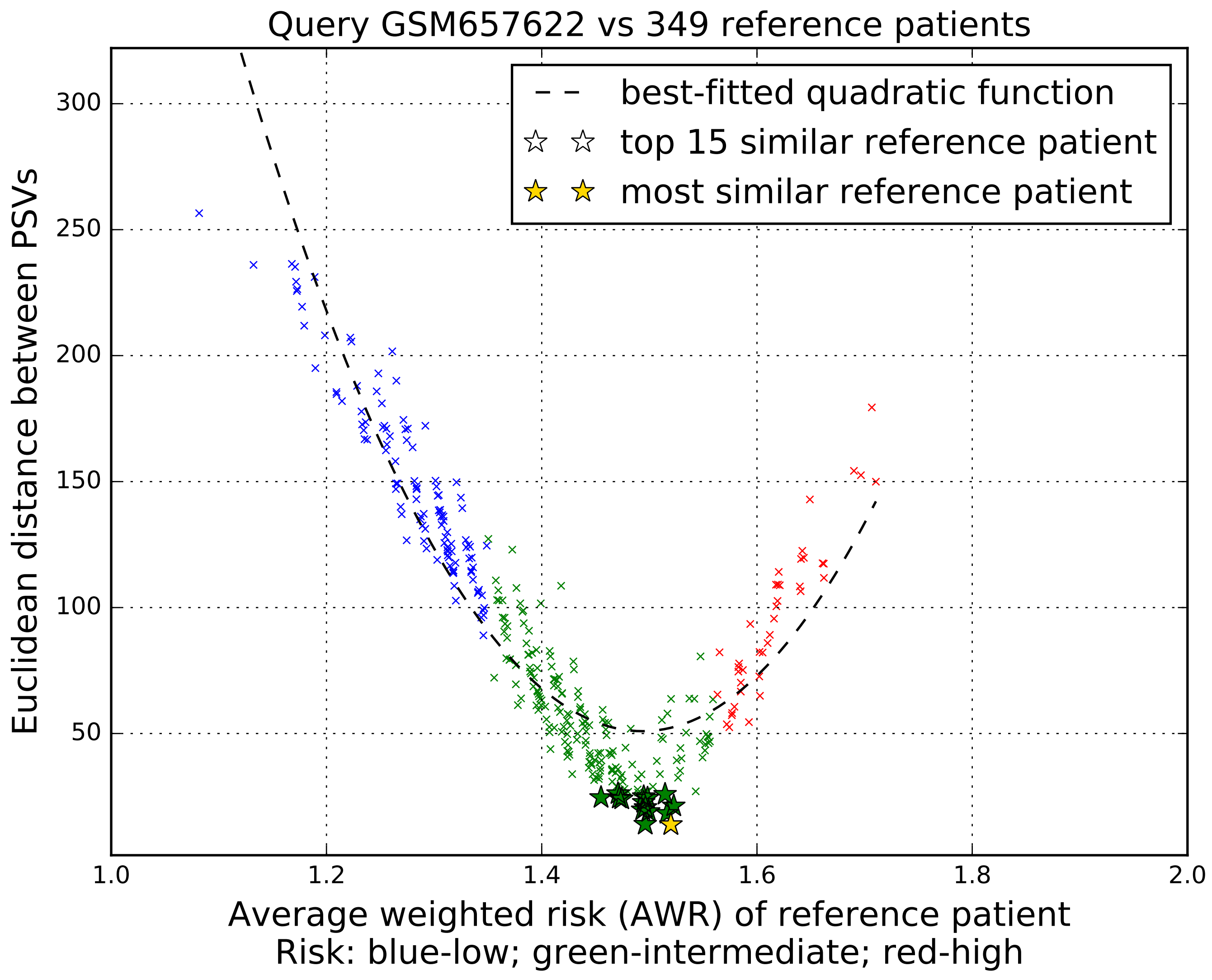

Query GSM657622 vs 349 reference patients
best-fitted quadratic function
top 15 similar reference patient
most similar reference patient
Euclidean distance between PSVs
300
250
200
150
100
50
1.0
1.2
1.4
1.6
1.8
2.0
Average weighted risk (AWR) of reference patient
Risk: blue-low; green-intermediate; red-high

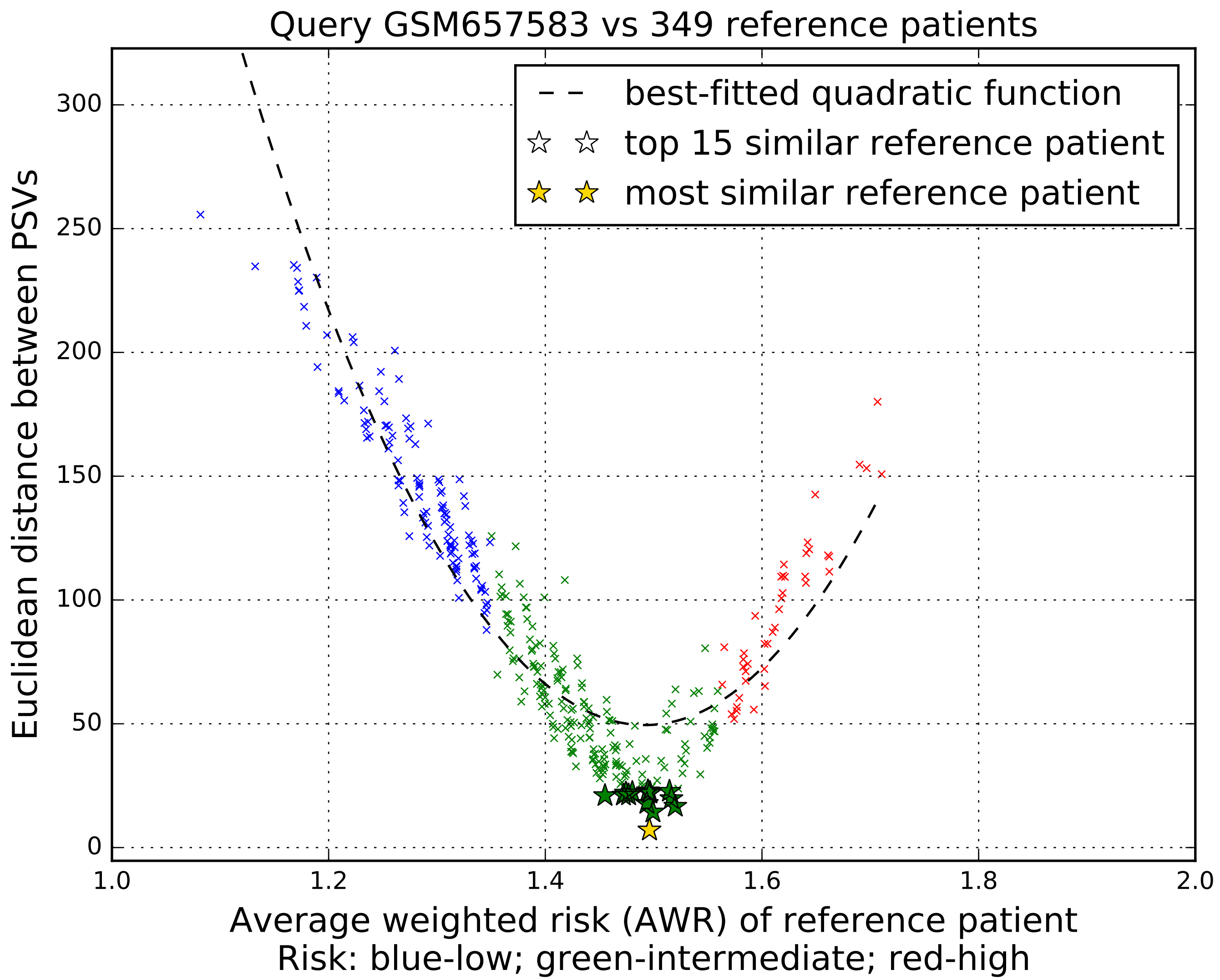
Query GSM657583 vs 349 reference patients
best-fitted quadratic function
top 15 similar reference patient
most similar reference patient
Euclidean distance between PSVs
0
50
100
150
200
250
300
1.0
1.2
1.4
1.6
1.8
2.0
Average weighted risk (AWR) of reference patient
Risk: blue-low; green-intermediate; red-high

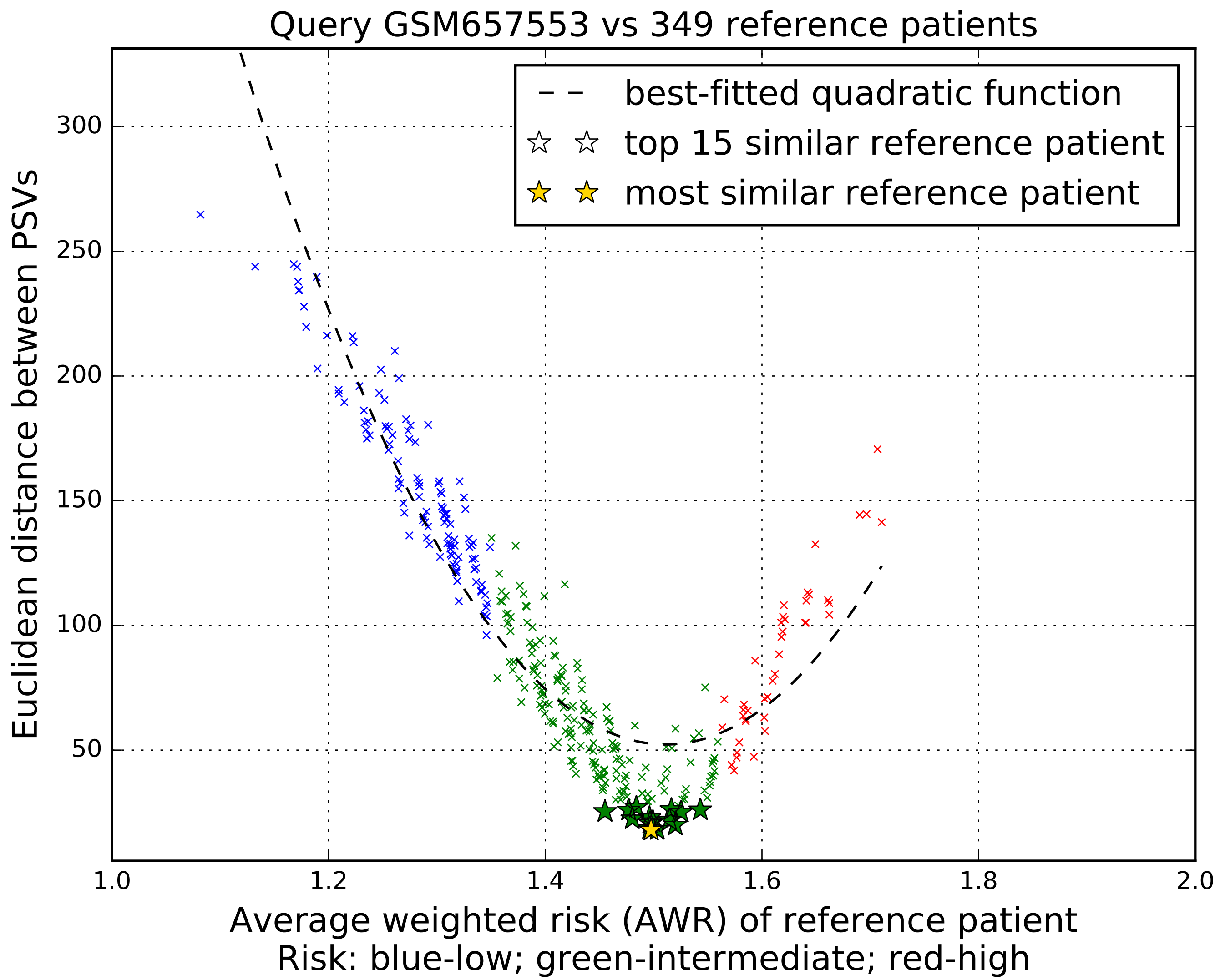

Query GSM657553 vs 349 reference patients
best-fitted quadratic function
top 15 similar reference patient
most similar reference patient
Euclidean distance between PSVs
300
250
200
150
100
50
1.0
1.2
1.4
1.6
1.8
2.0
Average weighted risk (AWR) of reference patient
Risk: blue-low; green-intermediate; red-high

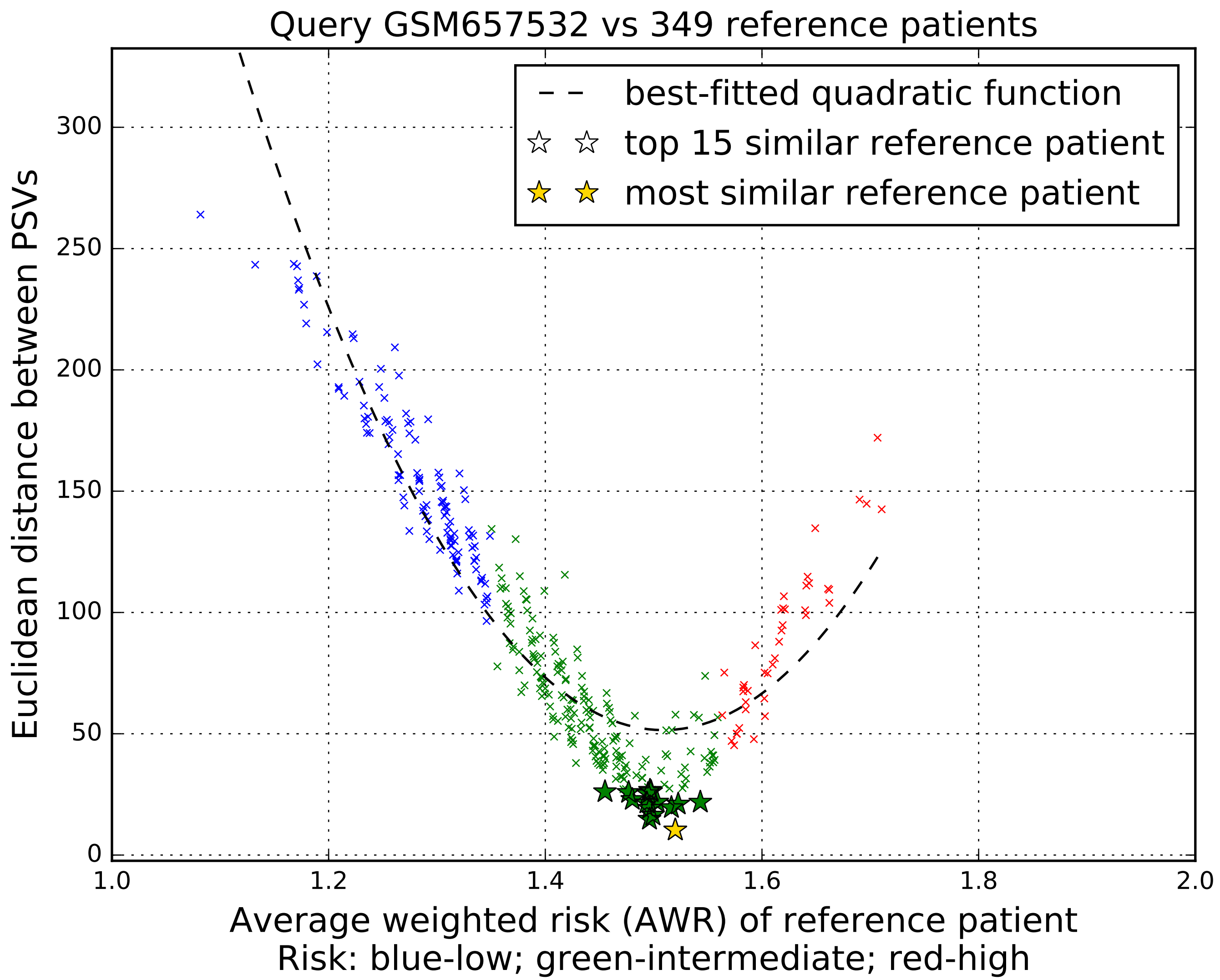
Query GSM657532 vs 349 reference patients
best-fitted quadratic function
top 15 similar reference patient
most similar reference patient
Euclidean distance between PSVs
0
50
100
150
200
250
300
1.0
1.2
1.4
1.6
1.8
2.0
Average weighted risk (AWR) of reference patient
Risk: blue-low; green-intermediate; red-high

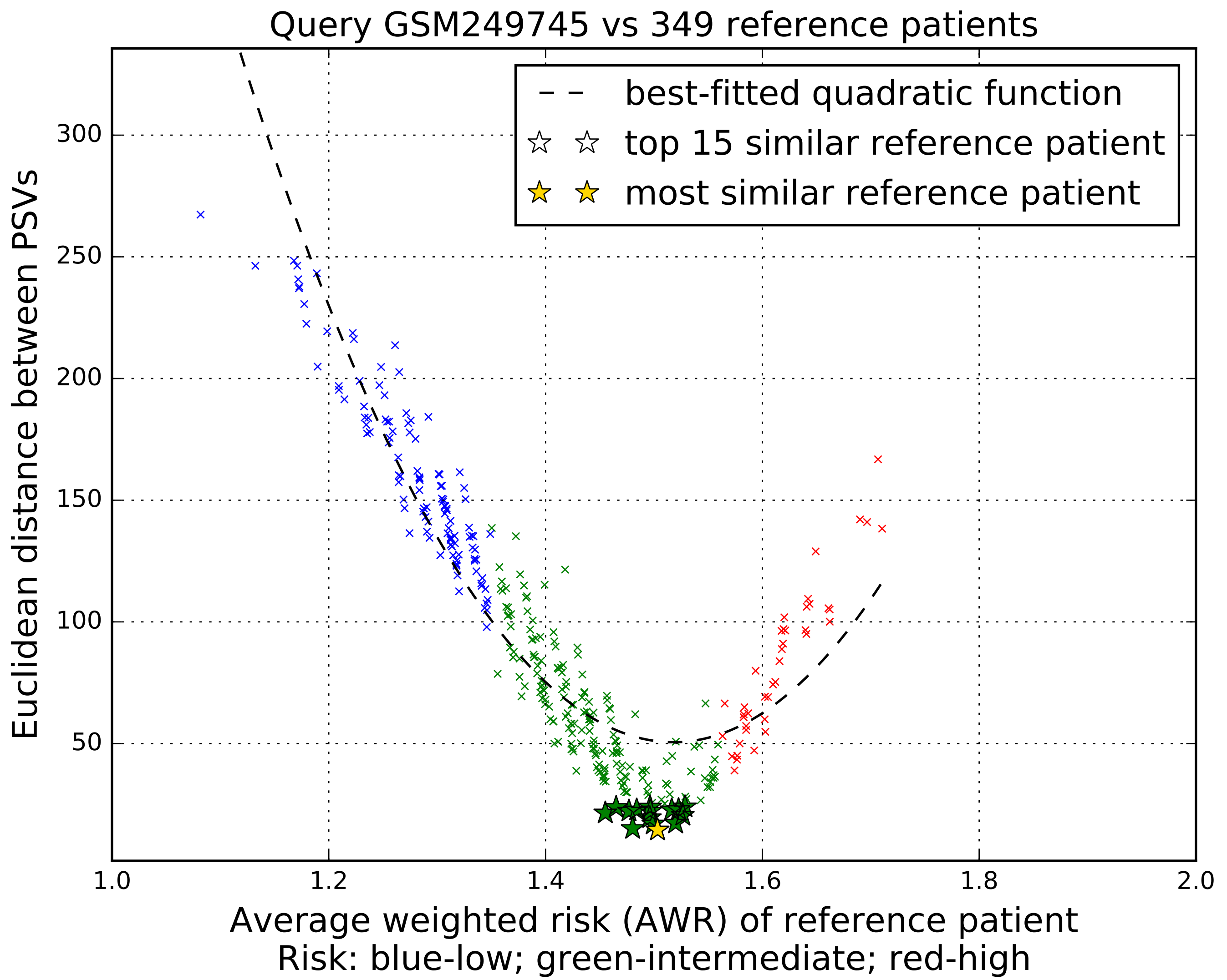
Query GSM249745 vs 349 reference patients
best-fitted quadratic function
top 15 similar reference patient
most similar reference patient
Euclidean distance between PSVs
300
250
200
150
100
50
1.0
1.2
1.4
1.6
1.8
2.0
Average weighted risk (AWR) of reference patient
Risk: blue-low; green-intermediate; red-high

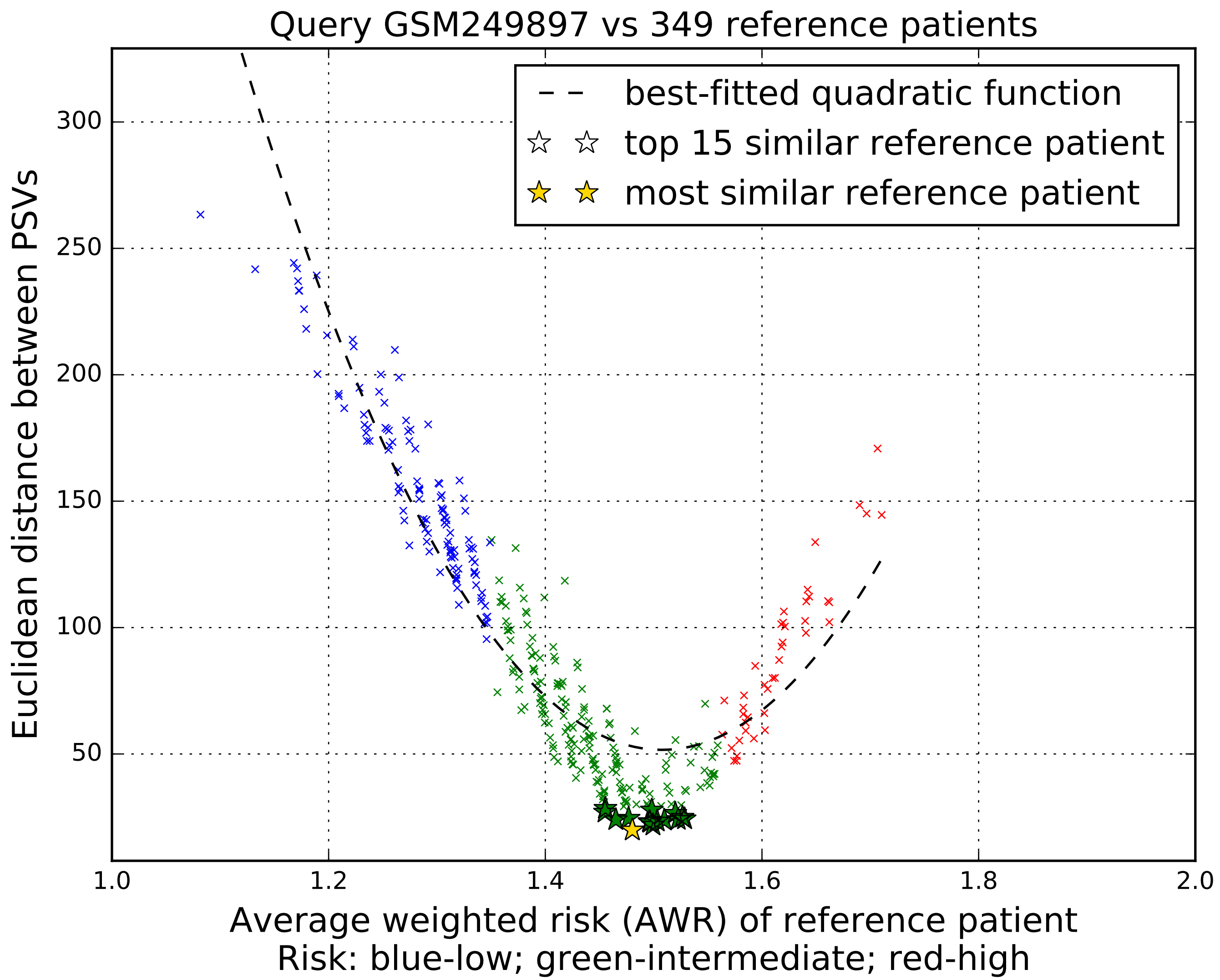
Query GSM249897 vs 349 reference patients
best-fitted quadratic function
top 15 similar reference patient
most similar reference patient
Euclidean distance between PSVs
300
250
200
150
100
50
1.0
1.2
1.4
1.6
1.8
2.0
Average weighted risk (AWR) of reference patient
Risk: blue-low; green-intermediate; red-high

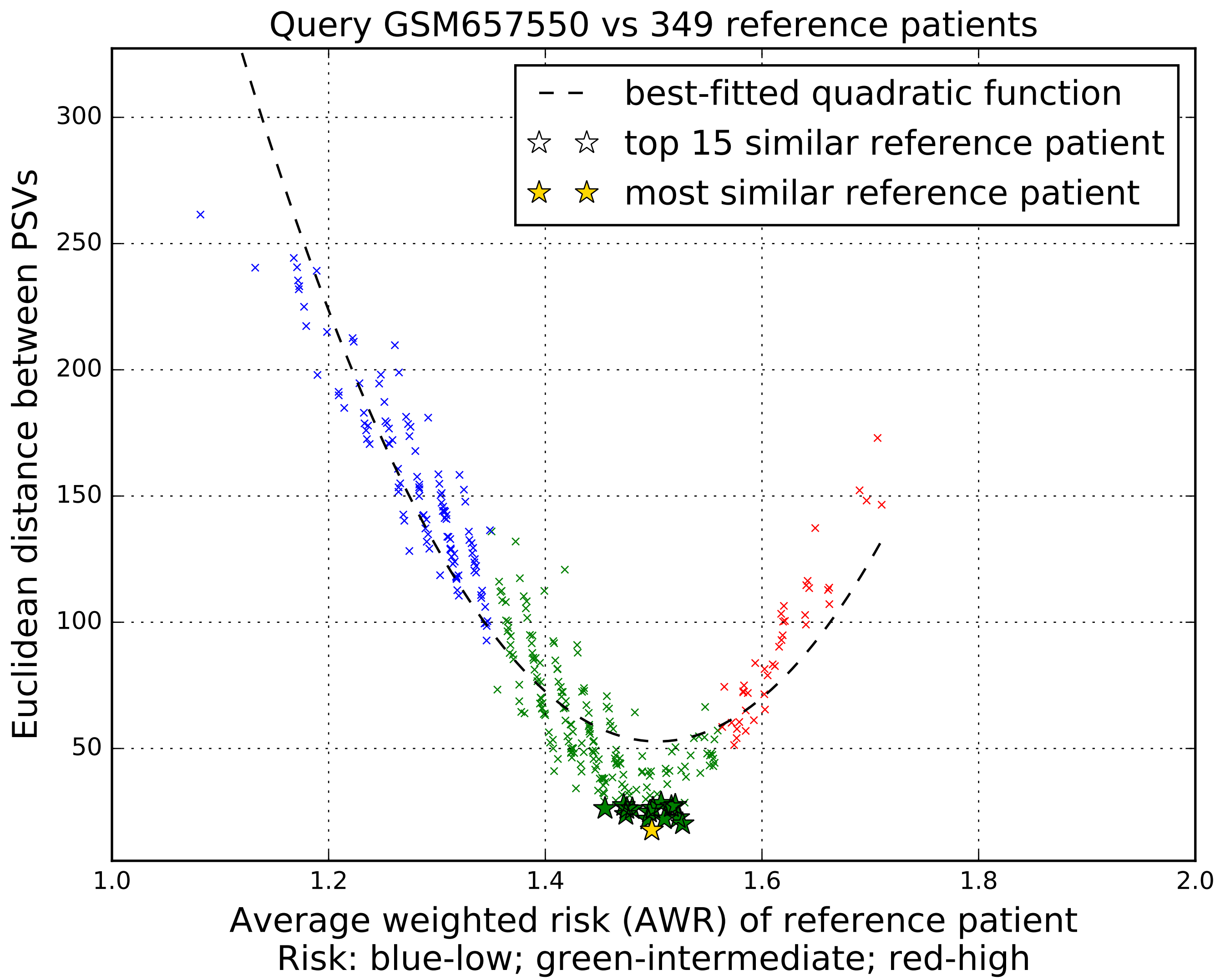
Query GSM657550 vs 349 reference patients
best-fitted quadratic function
top 15 similar reference patient
most similar reference patient
Euclidean distance between PSVs
Average weighted risk (AWR) of reference patient
Risk: blue-low; green-intermediate; red-high
300
250
200
150
100
50
1.0
1.2
1.4
1.6
1.8
2.0

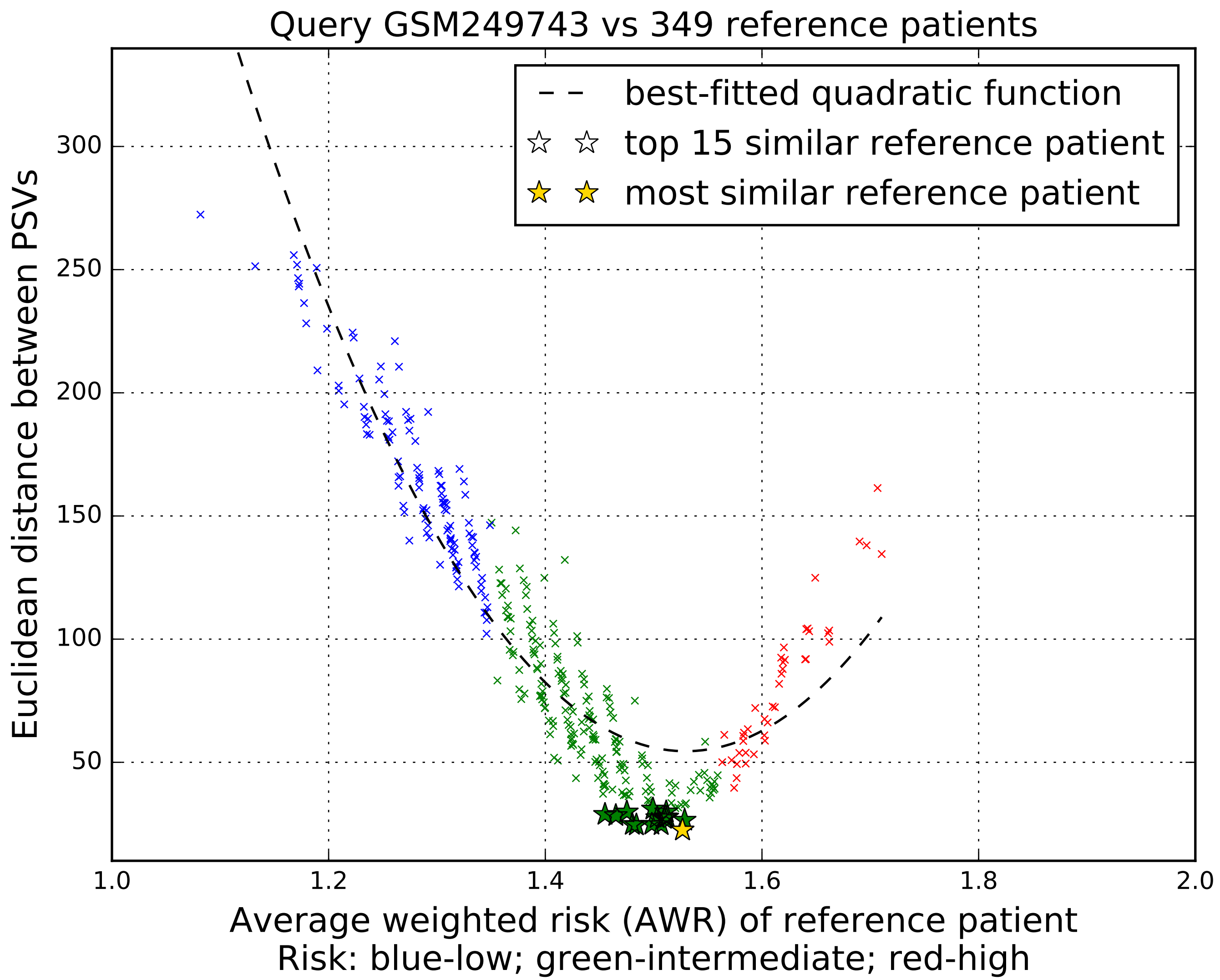
Query GSM249743 vs 349 reference patients
best-fitted quadratic function
top 15 similar reference patient
most similar reference patient
Euclidean distance between PSVs
300
250
200
150
100
50
1.0
1.2
1.4
1.6
1.8
2.0
Average weighted risk (AWR) of reference patient
Risk: blue-low; green-intermediate; red-high

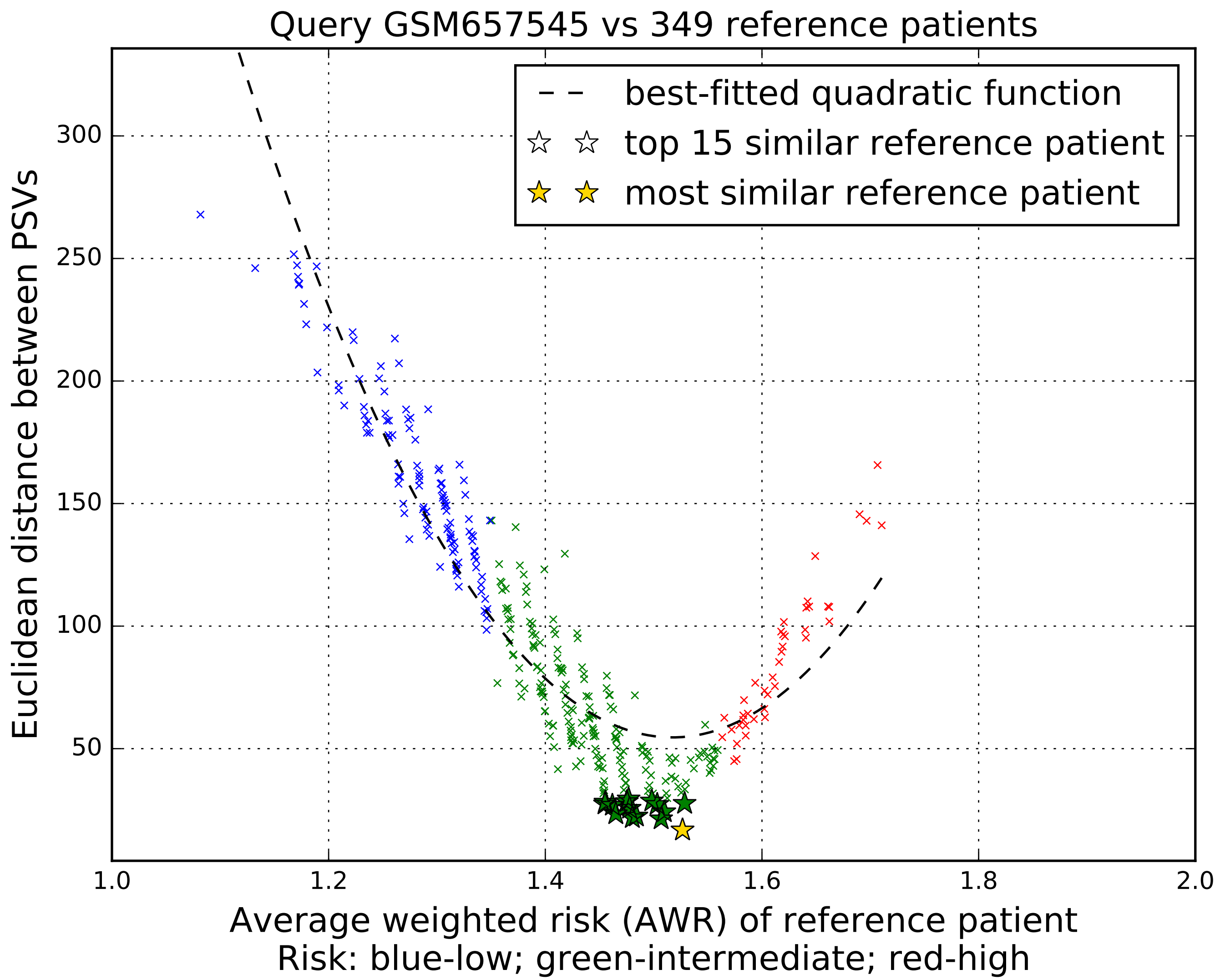
Query GSM657545 vs 349 reference patients
best-fitted quadratic function
top 15 similar reference patient
most similar reference patient
Euclidean distance between PSVs
300
250
200
150
100
50
1.0
1.2
1.4
1.6
1.8
2.0
Average weighted risk (AWR) of reference patient
Risk: blue-low; green-intermediate; red-high

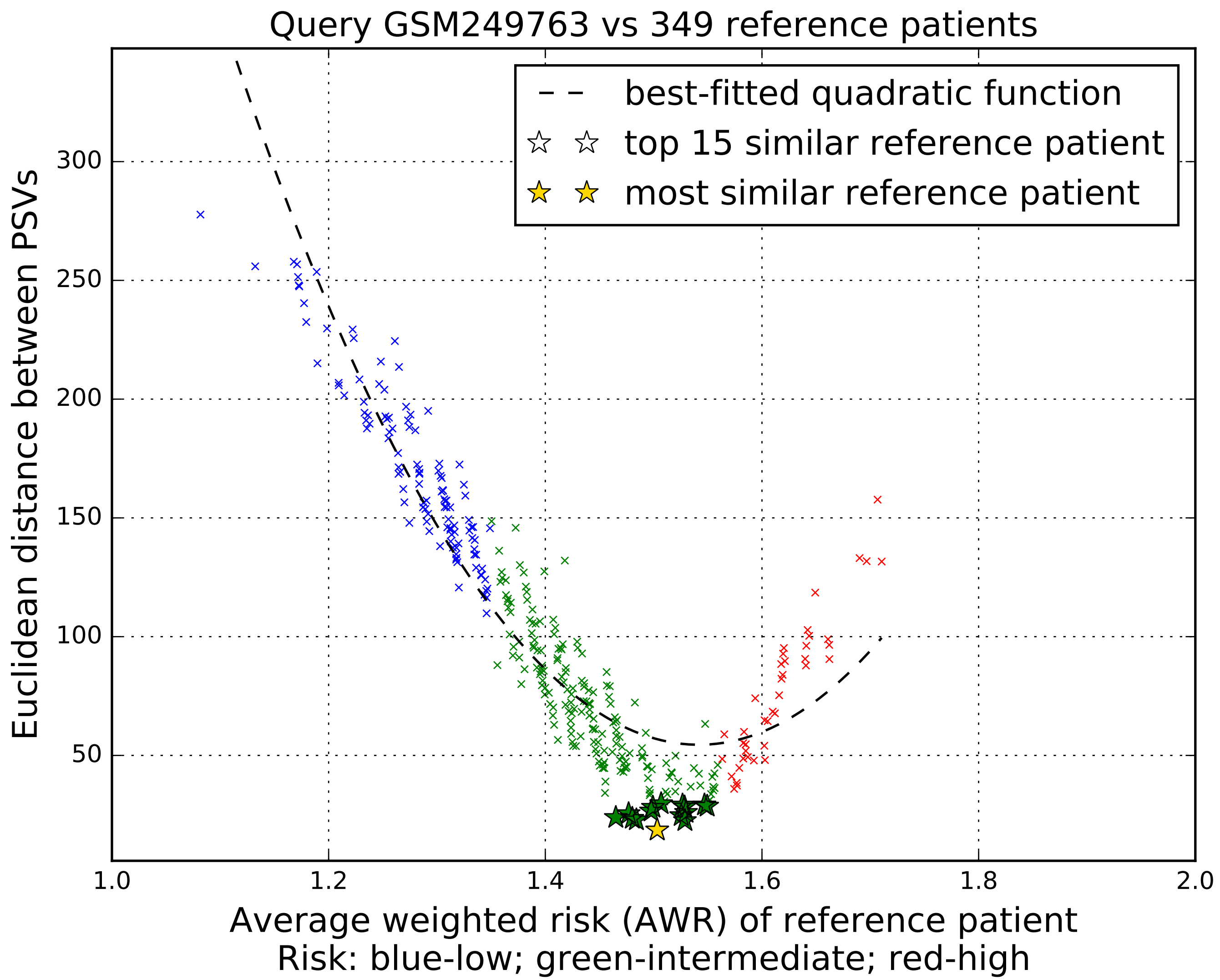
Query GSM249763 vs 349 reference patients
best-fitted quadratic function
top 15 similar reference patient
most similar reference patient
Euclidean distance between PSVs
300
250
200
150
100
50
1.0
1.2
1.4
1.6
1.8
2.0
Average weighted risk (AWR) of reference patient
Risk: blue-low; green-intermediate; red-high

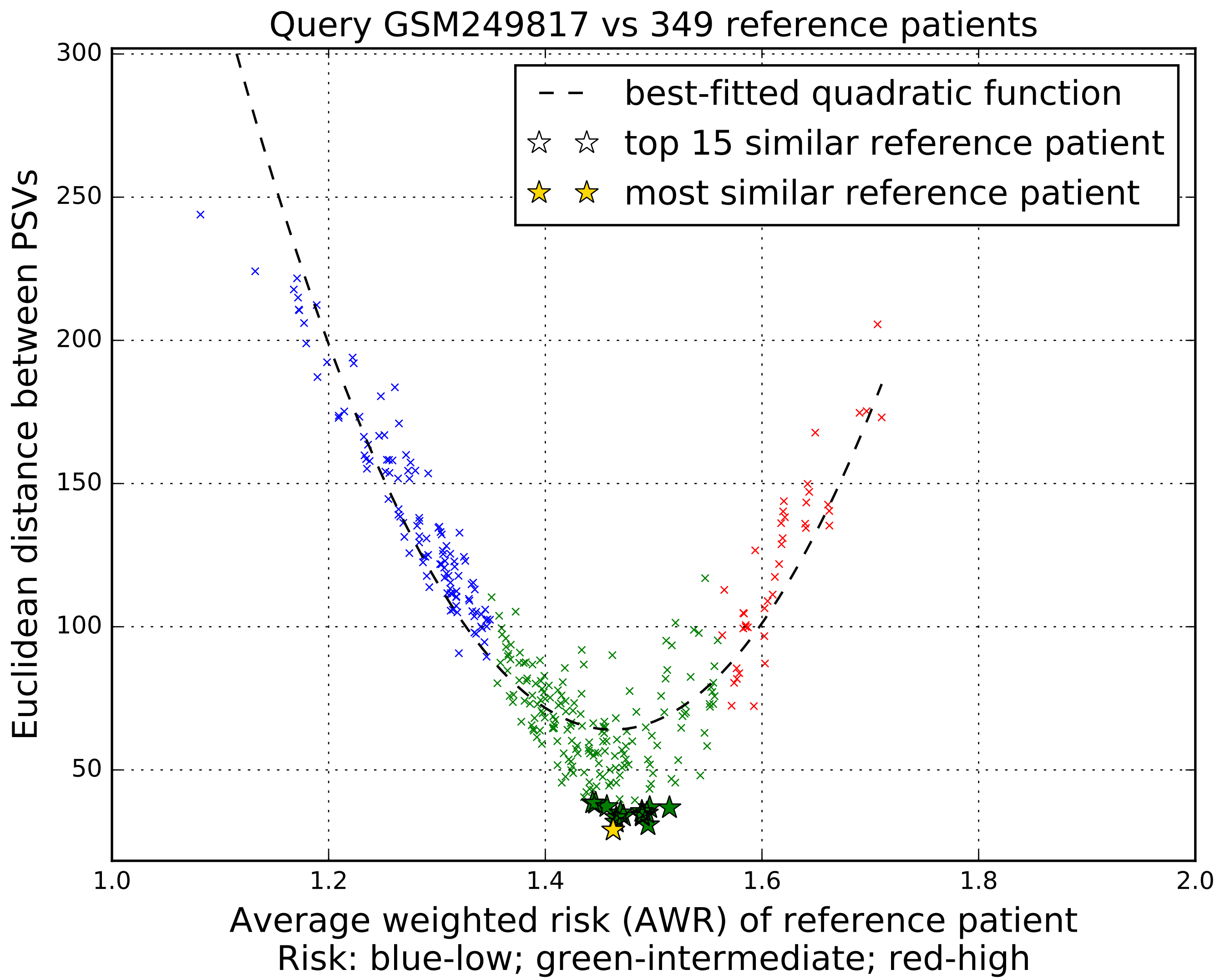
Query GSM249817 vs 349 reference patients
best-fitted quadratic function
top 15 similar reference patient
most similar reference patient
Euclidean distance between PSVs
300
250
200
150
100
50
1.0
1.2
1.4
1.6
1.8
2.0
Average weighted risk (AWR) of reference patient
Risk: blue-low; green-intermediate; red-high

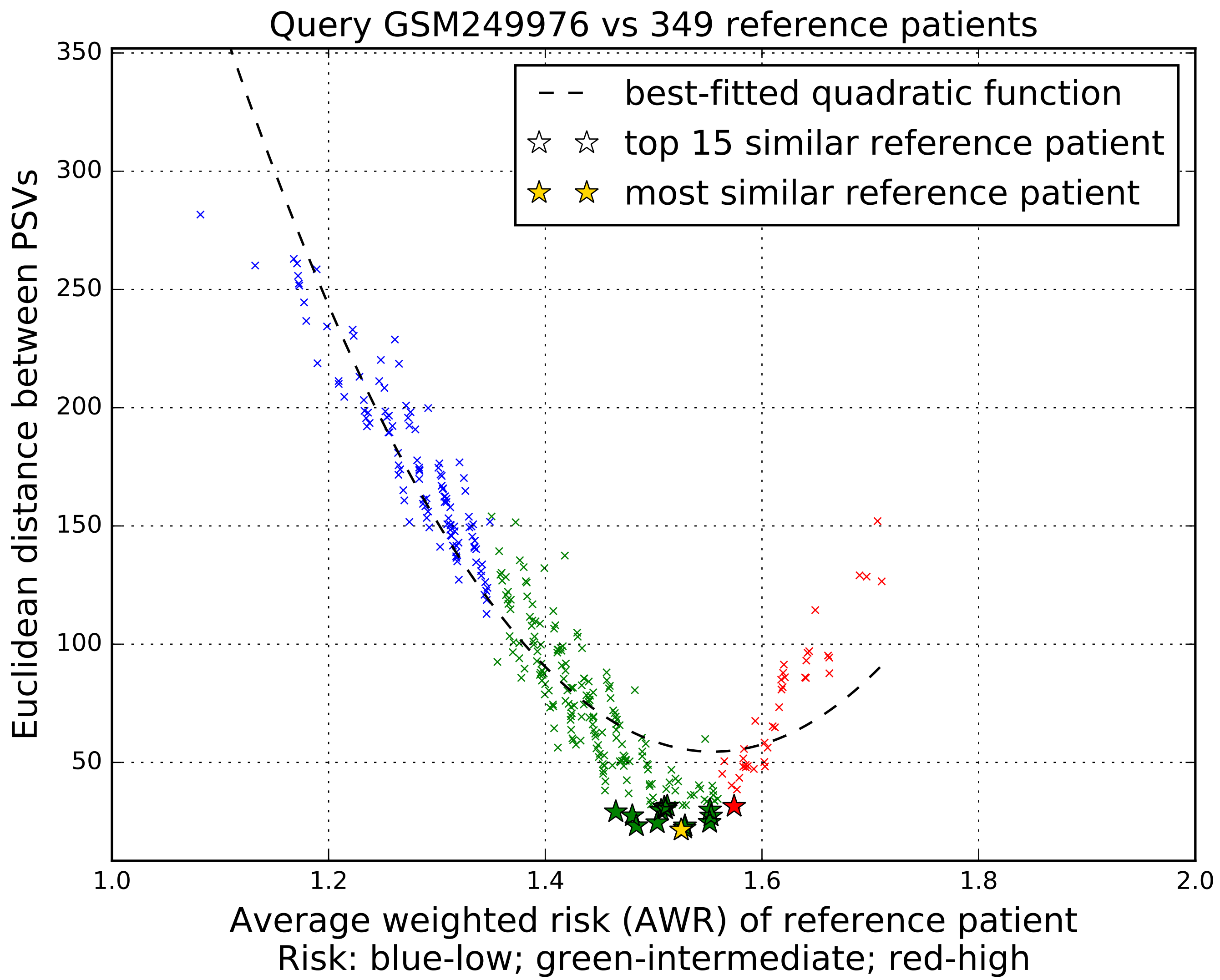

Query GSM249976 vs 349 reference patients
best-fitted quadratic function
top 15 similar reference patient
most similar reference patient
Euclidean distance between PSVs
350
300
250
200
150
100
50
1.0
1.2
1.4
1.6
1.8
2.0
Average weighted risk (AWR) of reference patient
Risk: blue-low; green-intermediate; red-high

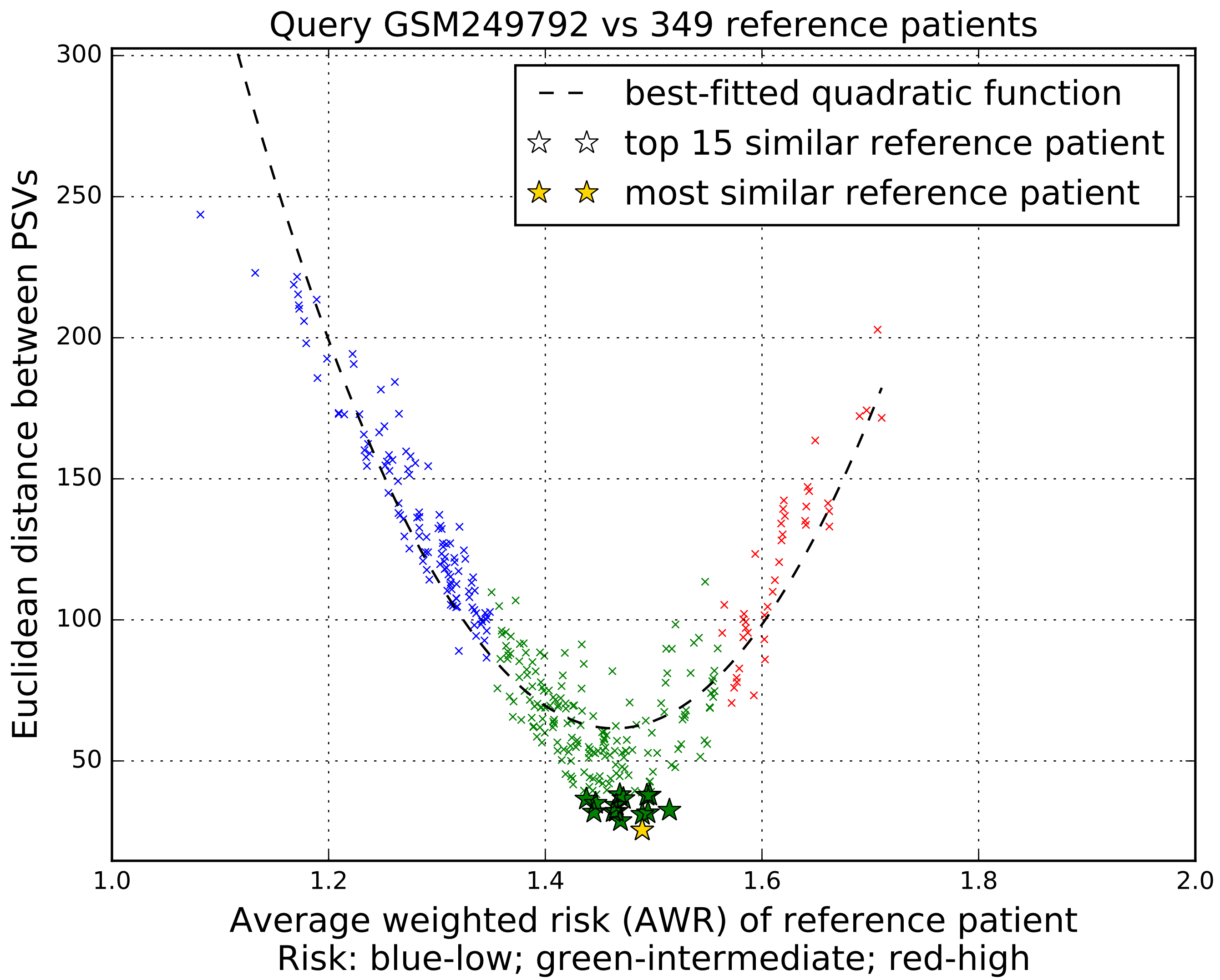
Query GSM249792 vs 349 reference patients
best-fitted quadratic function
top 15 similar reference patient
most similar reference patient
Euclidean distance between PSVs
300
250
200
150
100
50
1.0
1.2
1.4
1.6
1.8
2.0
Average weighted risk (AWR) of reference patient
Risk: blue-low; green-intermediate; red-high

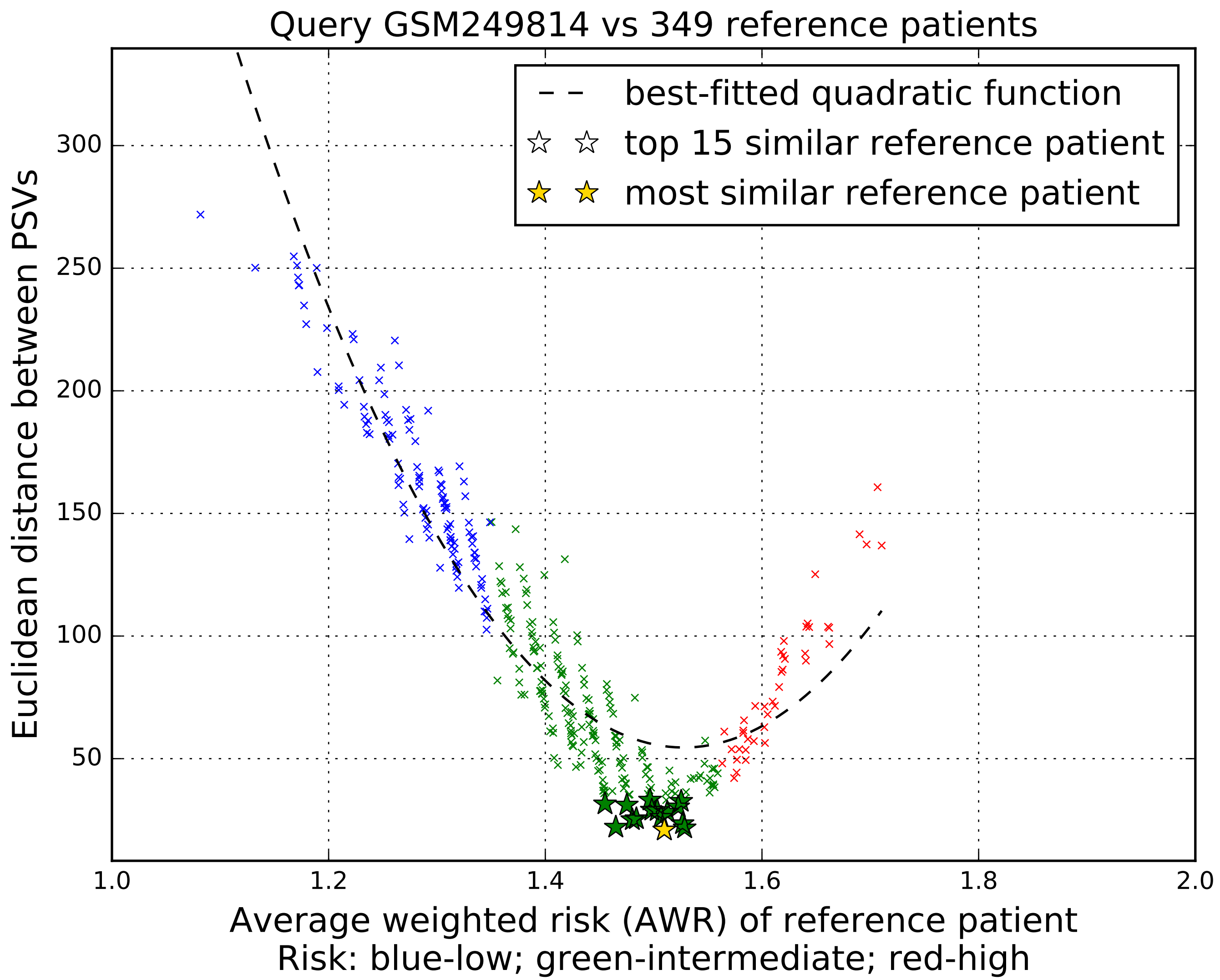
Query GSM249814 vs 349 reference patients
best-fitted quadratic function
top 15 similar reference patient
most similar reference patient
Euclidean distance between PSVs
300
250
200
150
100
50
1.0
1.2
1.4
1.6
1.8
2.0
Average weighted risk (AWR) of reference patient
Risk: blue-low; green-intermediate; red-high

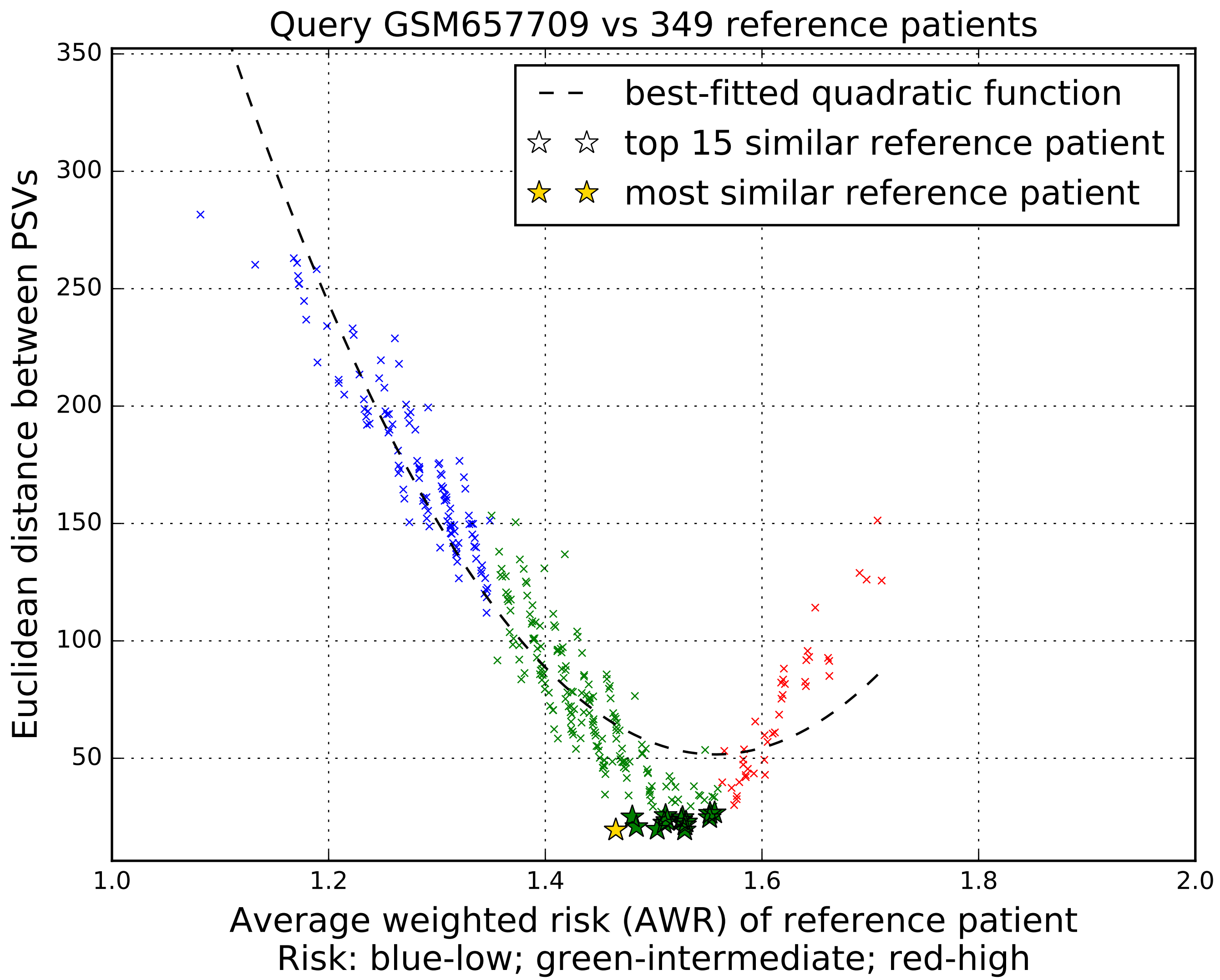
Query GSM657709 vs 349 reference patients
350
300
250
200
150
100
50
1.0
1.2
1.4
1.6
1.8
2.0
Euclidean distance between PSVs
Average weighted risk (AWR) of reference patient
Risk: blue-low; green-intermediate; red-high
best-fitted quadratic function
top 15 similar reference patient
most similar reference patient

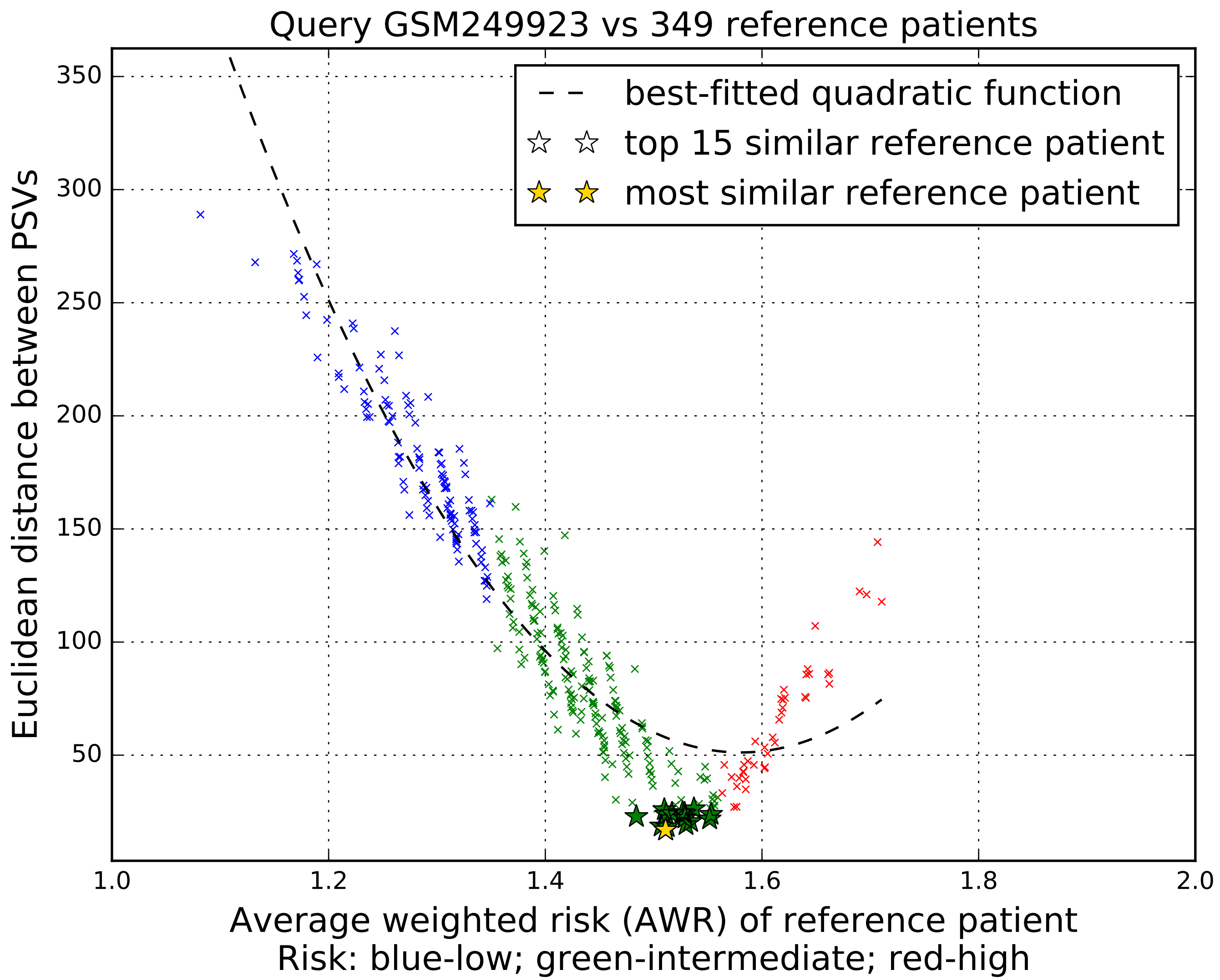
Query GSM249923 vs 349 reference patients
best-fitted quadratic function
top 15 similar reference patient
most similar reference patient
Euclidean distance between PSVs
350
300
250
200
150
100
50
1.0
1.2
1.4
1.6
1.8
2.0
Average weighted risk (AWR) of reference patient
Risk: blue-low; green-intermediate; red-high

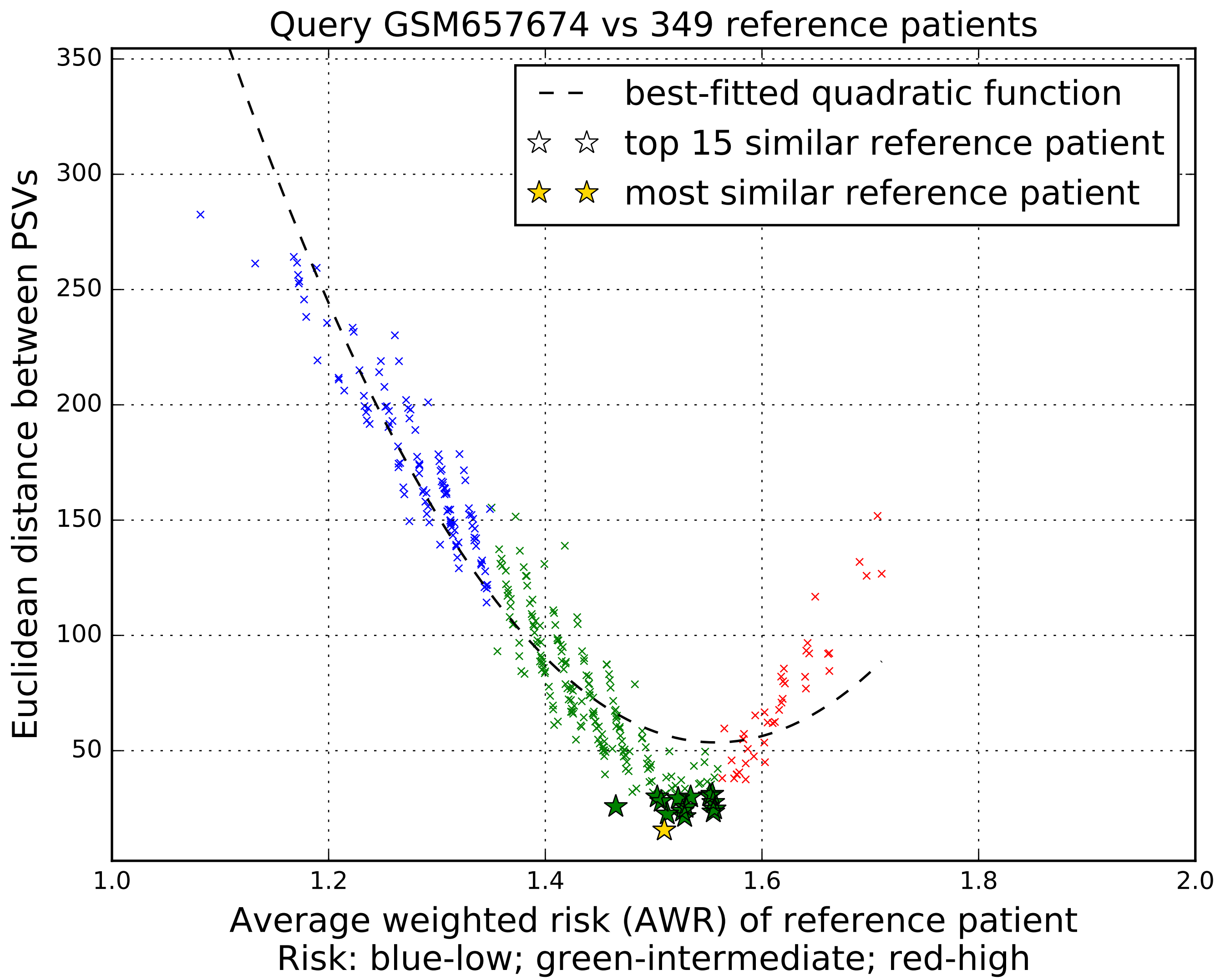

Query GSM657674 vs 349 reference patients
best-fitted quadratic function
top 15 similar reference patient
most similar reference patient
Euclidean distance between PSVs
Average weighted risk (AWR) of reference patient
Risk: blue-low; green-intermediate; red-high
350
300
250
200
150
100
50
1.0
1.2
1.4
1.6
1.8
2.0

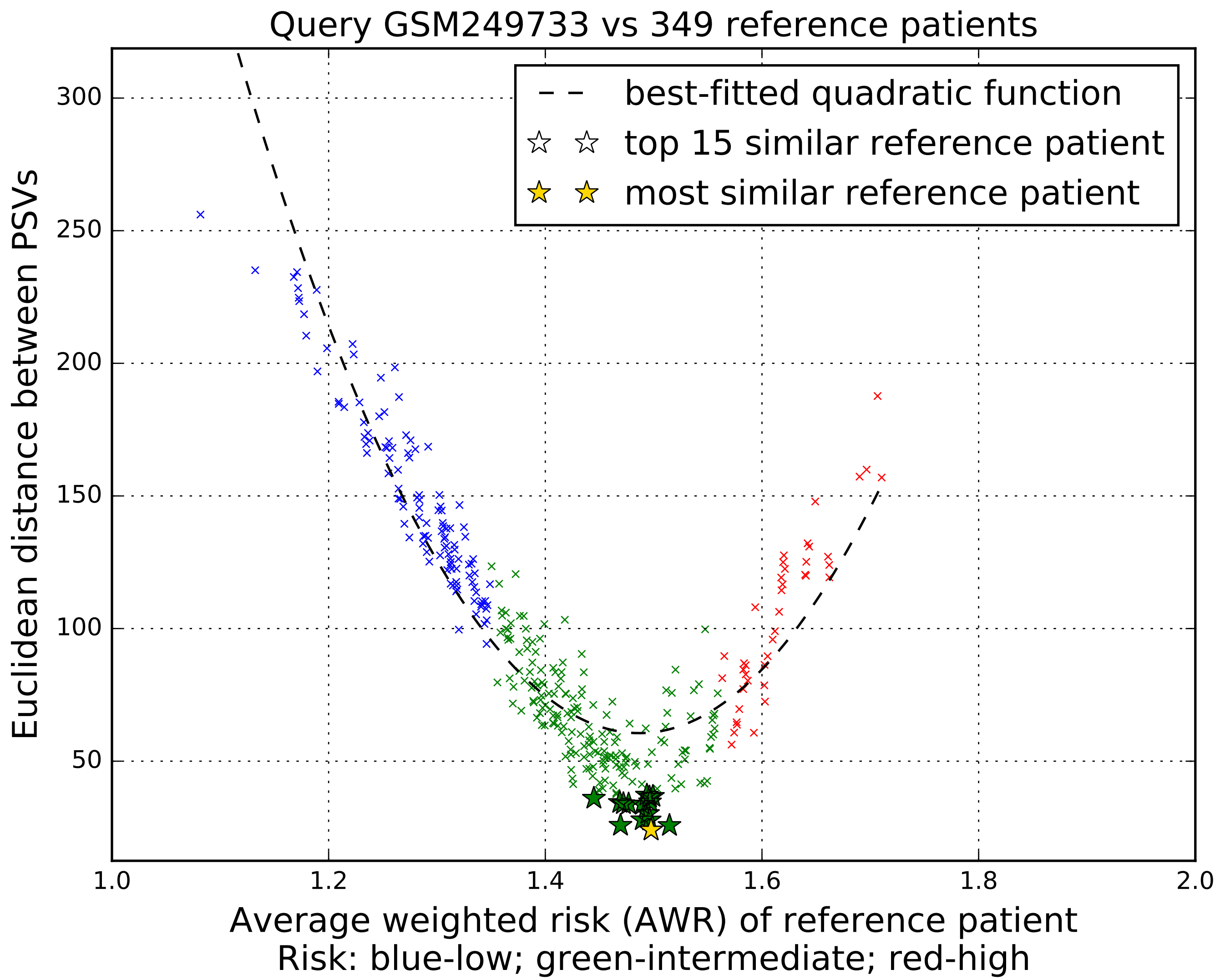

Query GSM249733 vs 349 reference patients
best-fitted quadratic function
top 15 similar reference patient
most similar reference patient
Euclidean distance between PSVs
300
250
200
150
100
50
1.0
1.2
1.4
1.6
1.8
2.0
Average weighted risk (AWR) of reference patient
Risk: blue-low; green-intermediate; red-high

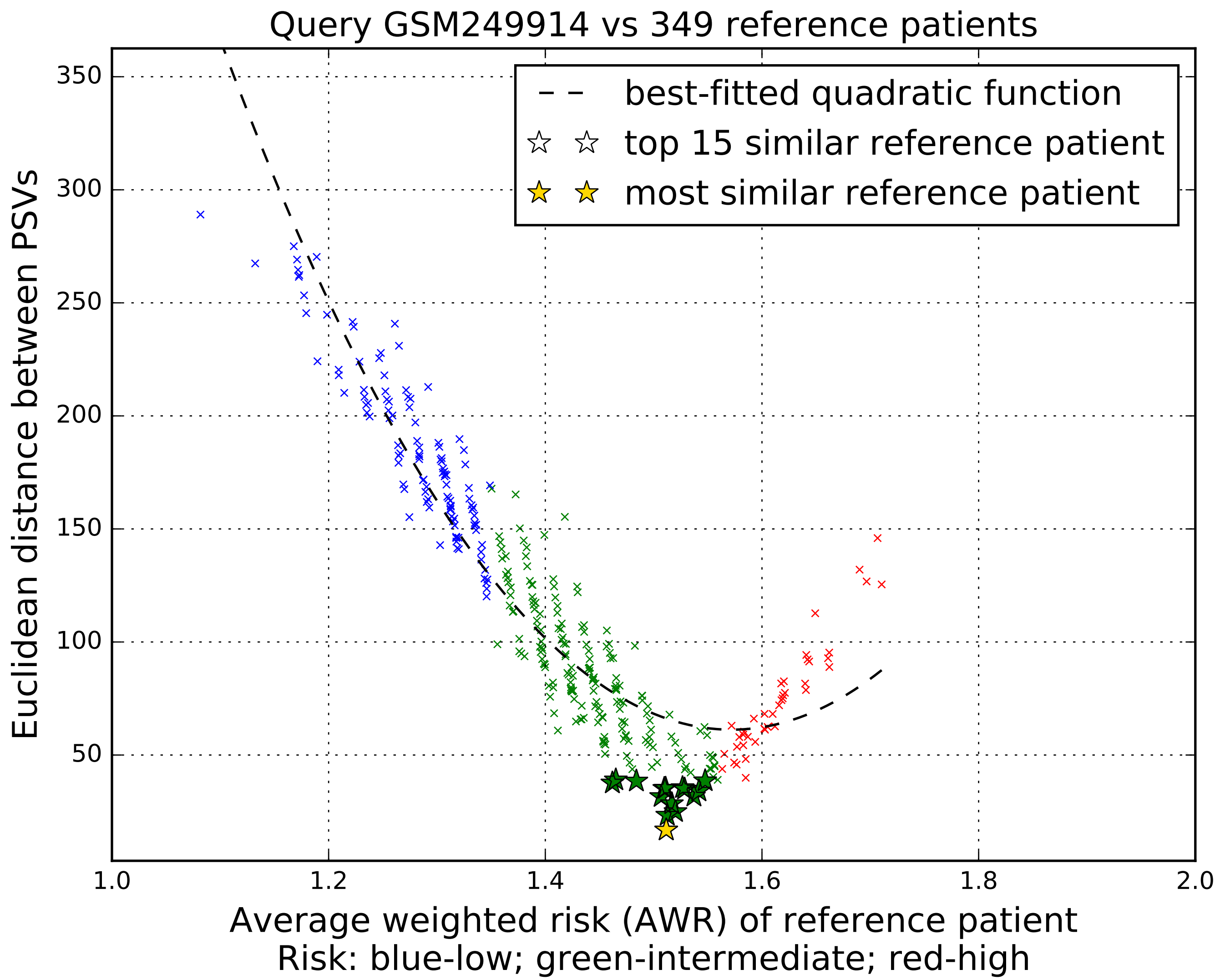
Query GSM249914 vs 349 reference patients
best-fitted quadratic function
top 15 similar reference patient
most similar reference patient
Euclidean distance between PSVs
350
300
250
200
150
100
50
1.0
1.2
1.4
1.6
1.8
2.0
Average weighted risk (AWR) of reference patient
Risk: blue-low; green-intermediate; red-high

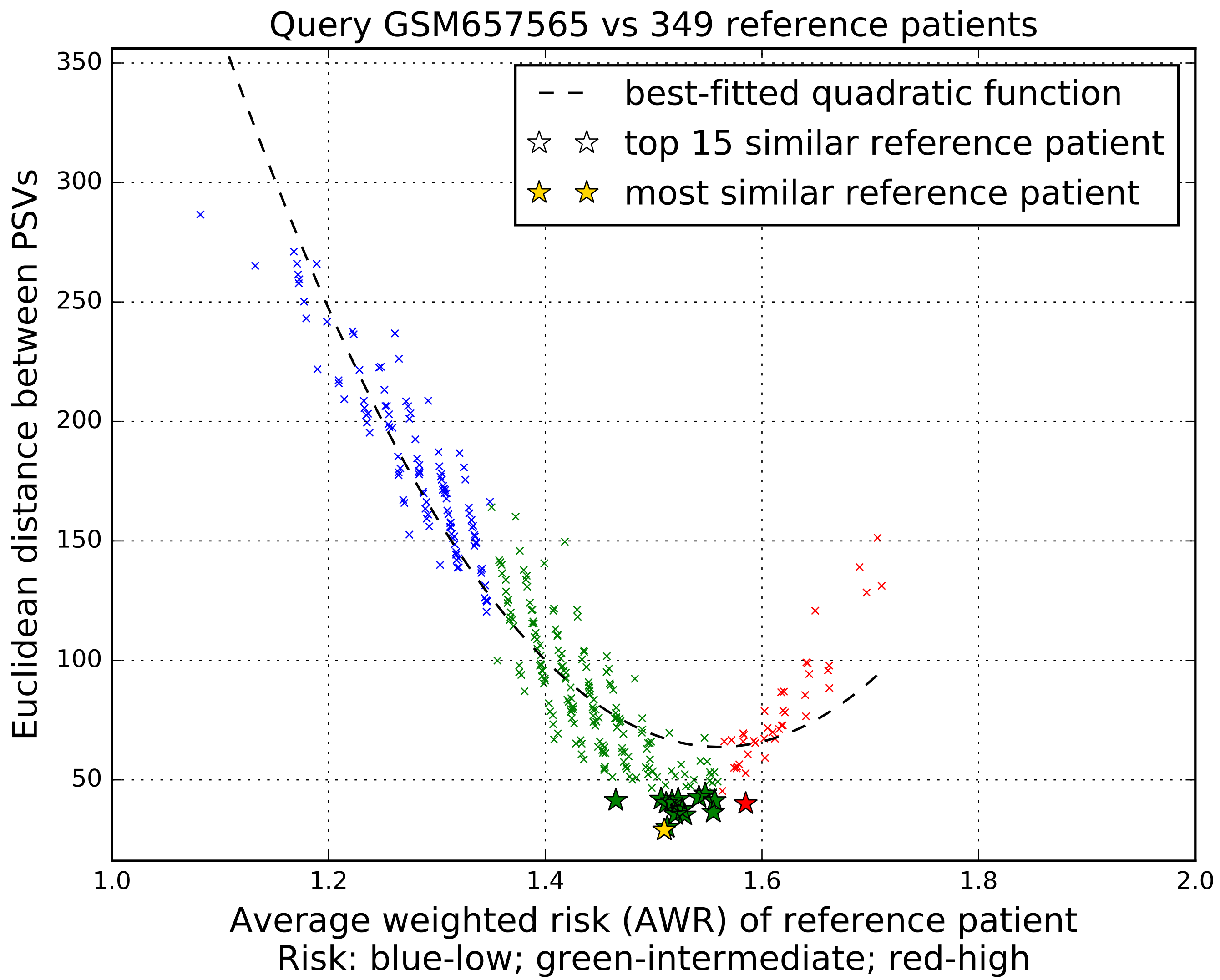

Query GSM657565 vs 349 reference patients
best-fitted quadratic function
top 15 similar reference patient
most similar reference patient
Euclidean distance between PSVs
350
300
250
200
150
100
50
1.0
1.2
1.4
1.6
1.8
2.0
Average weighted risk (AWR) of reference patient
Risk: blue-low; green-intermediate; red-high

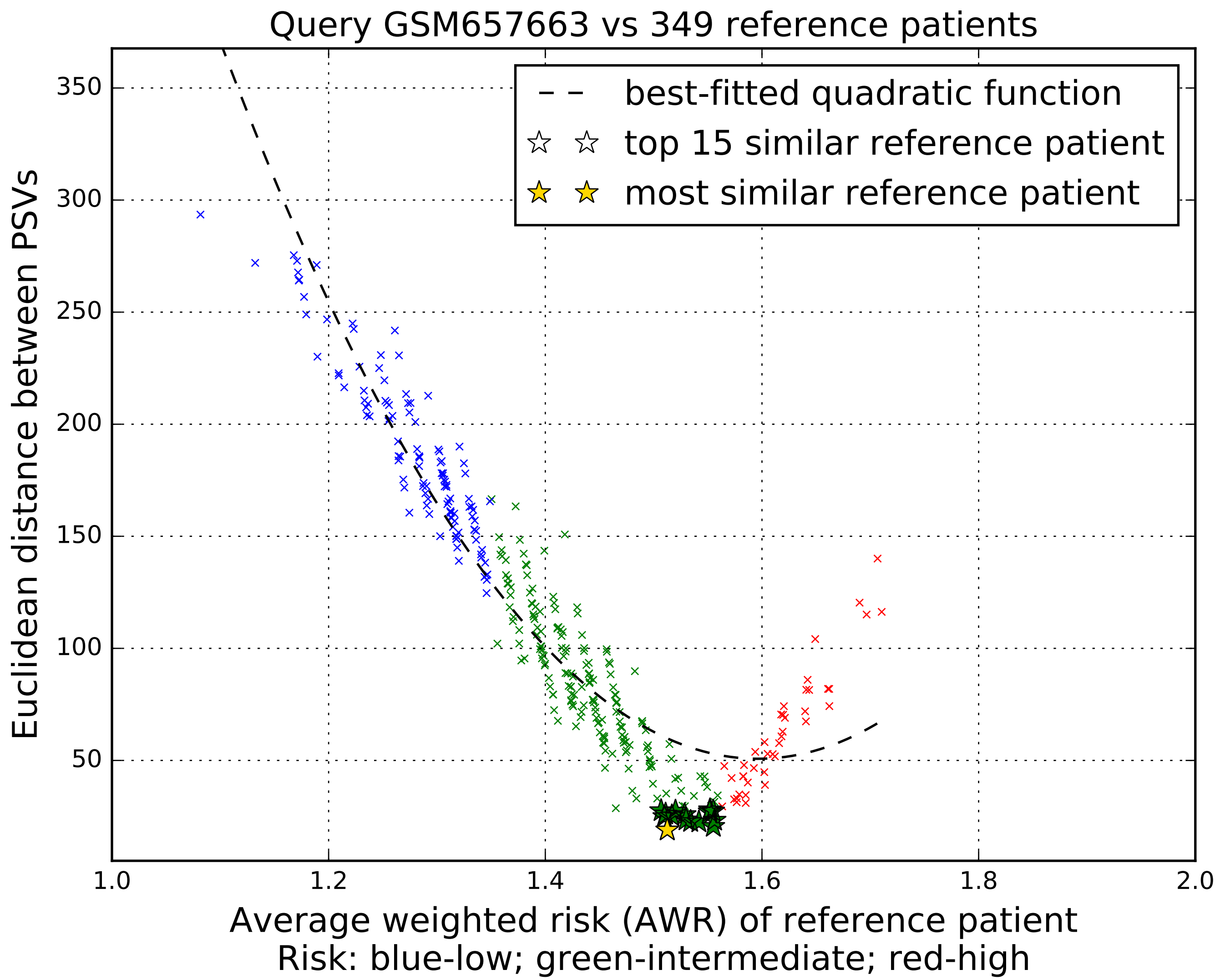
Query GSM657663 vs 349 reference patients
best-fitted quadratic function
top 15 similar reference patient
most similar reference patient
Euclidean distance between PSVs
Average weighted risk (AWR) of reference patient
Risk: blue-low; green-intermediate; red-high
350
300
250
200
150
100
50
1.0
1.2
1.4
1.6
1.8
2.0

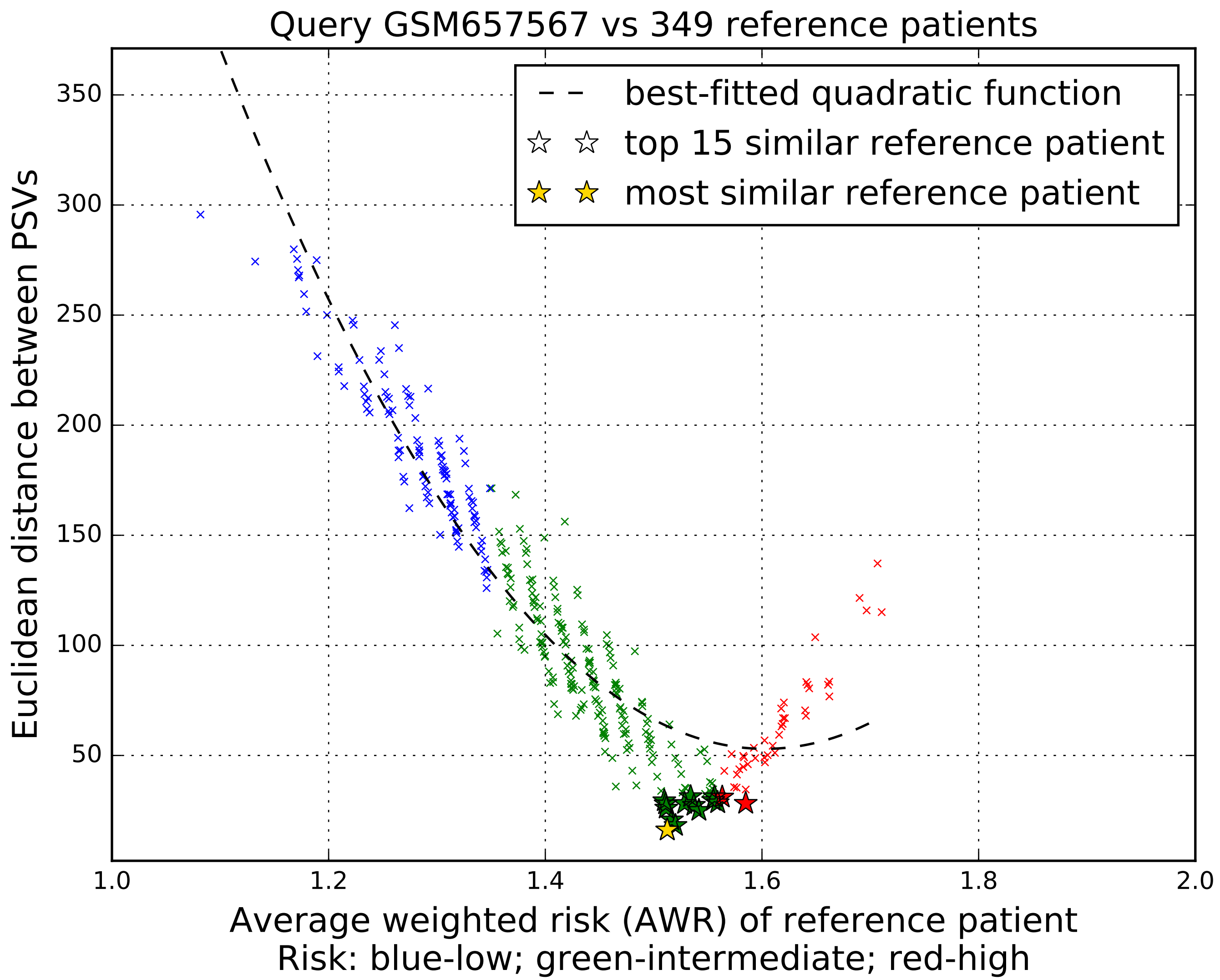

Query GSM657567 vs 349 reference patients
best-fitted quadratic function
top 15 similar reference patient
most similar reference patient
Euclidean distance between PSVs
350
300
250
200
150
100
50
1.0
1.2
1.4
1.6
1.8
2.0
Average weighted risk (AWR) of reference patient
Risk: blue-low; green-intermediate; red-high

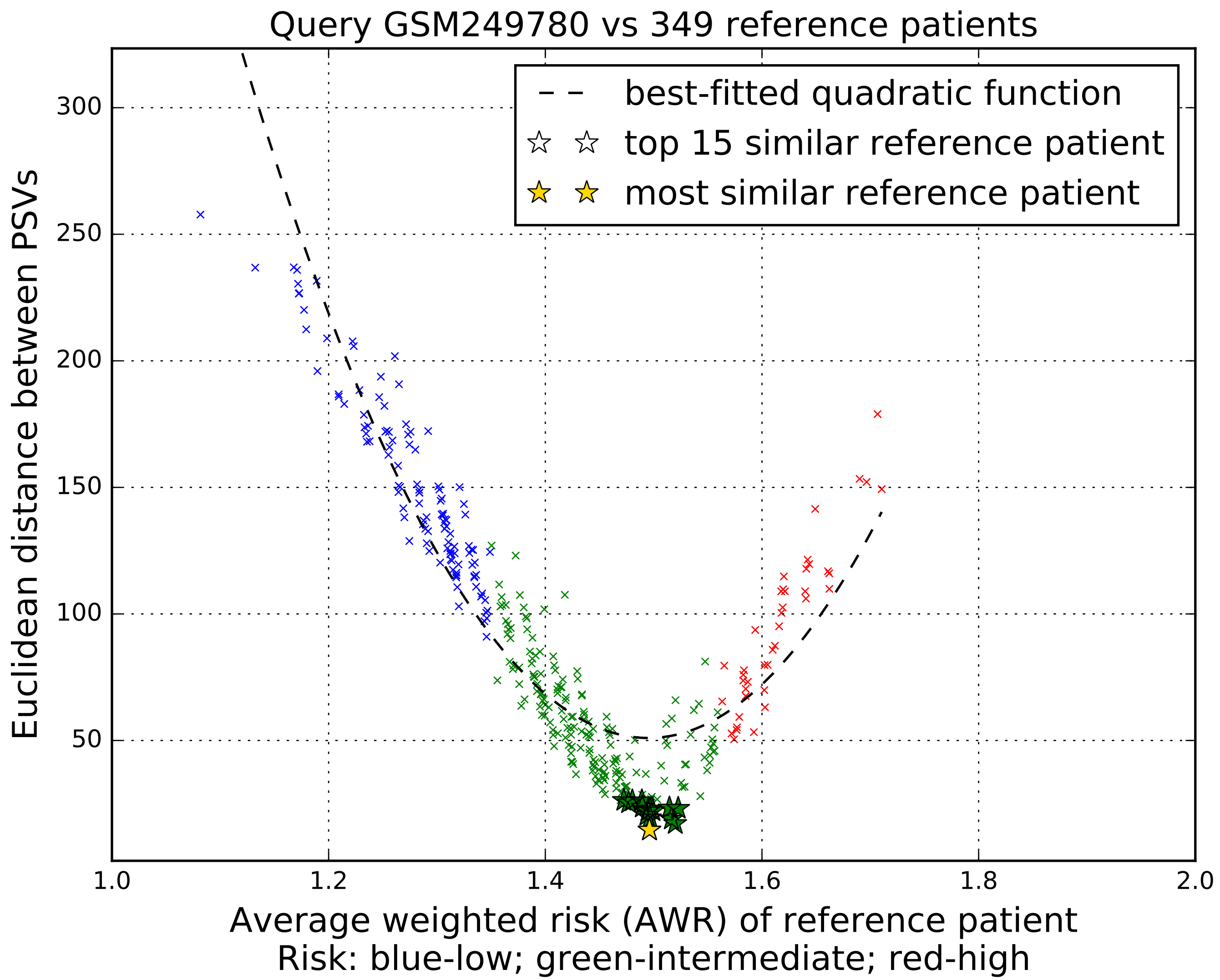

Query GSM249780 vs 349 reference patients
best-fitted quadratic function
top 15 similar reference patient
most similar reference patient
Euclidean distance between PSVs
300
250
200
150
100
50
1.0
1.2
1.4
1.6
1.8
2.0
Average weighted risk (AWR) of reference patient
Risk: blue-low; green-intermediate; red-high

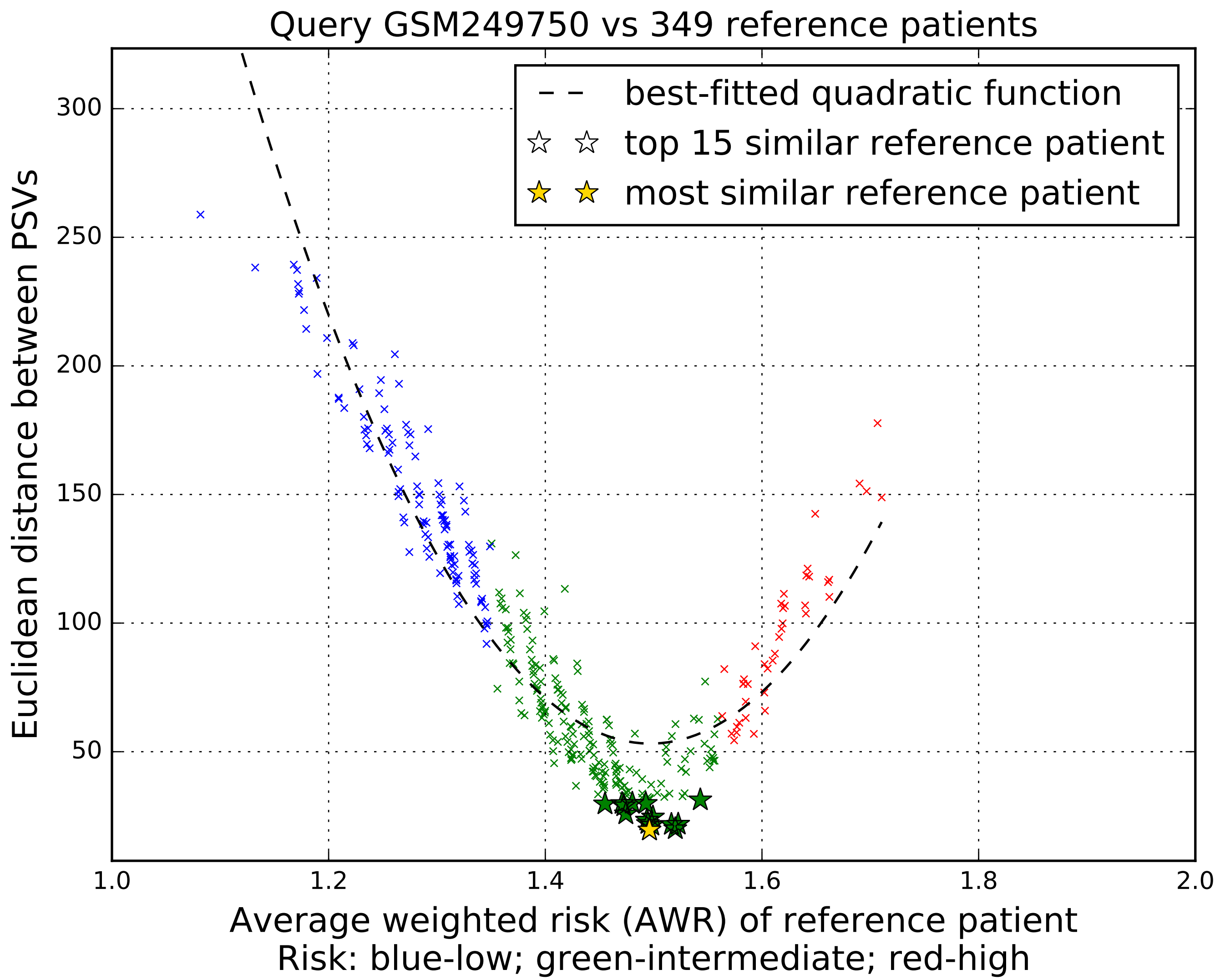

Query GSM249750 vs 349 reference patients
best-fitted quadratic function
top 15 similar reference patient
most similar reference patient
Euclidean distance between PSVs
300
250
200
150
100
50
1.0
1.2
1.4
1.6
1.8
2.0
Average weighted risk (AWR) of reference patient
Risk: blue-low; green-intermediate; red-high

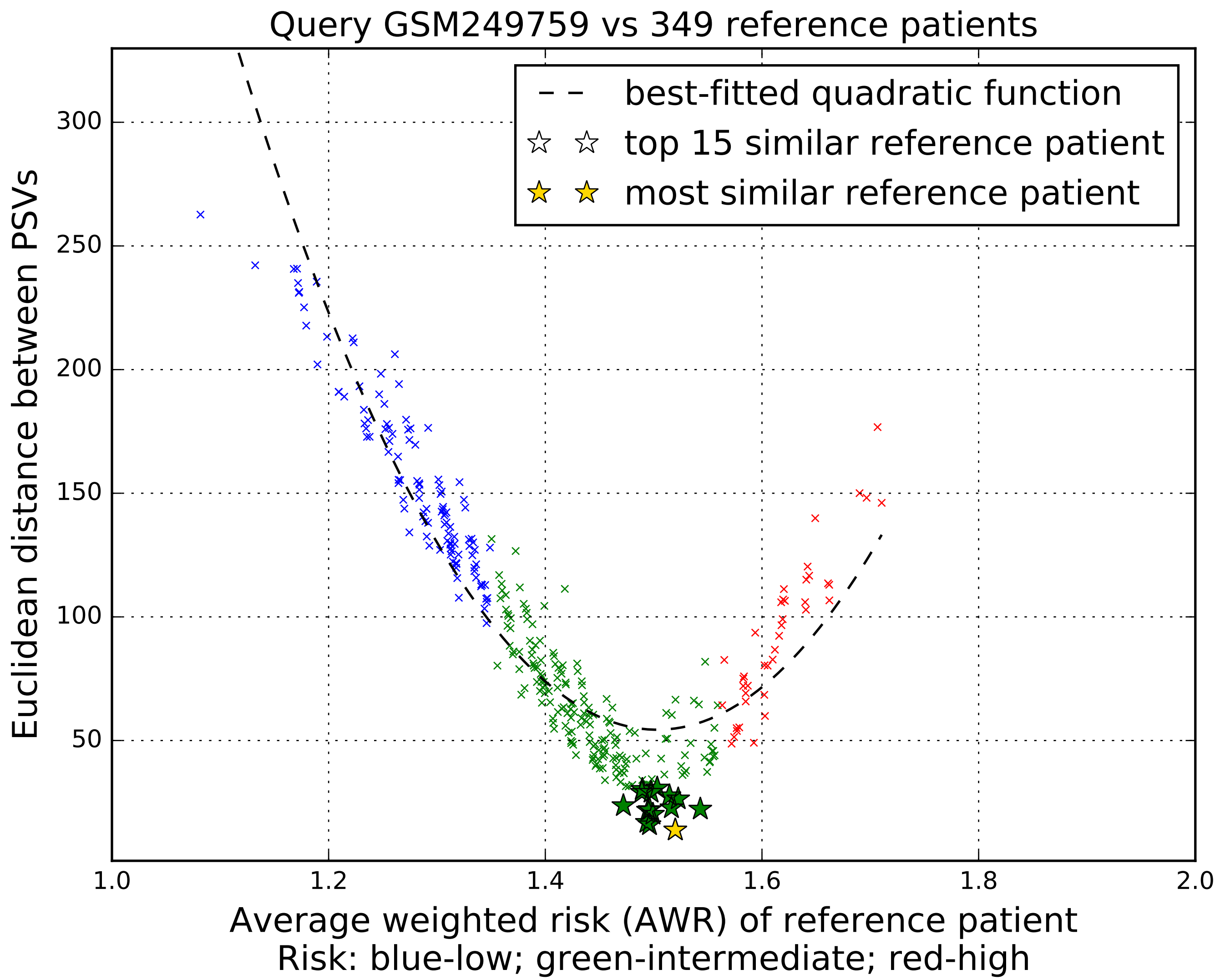

Query GSM249759 vs 349 reference patients
best-fitted quadratic function
top 15 similar reference patient
most similar reference patient
Euclidean distance between PSVs
300
250
200
150
100
50
1.0
1.2
1.4
1.6
1.8
2.0
Average weighted risk (AWR) of reference patient
Risk: blue-low; green-intermediate; red-high

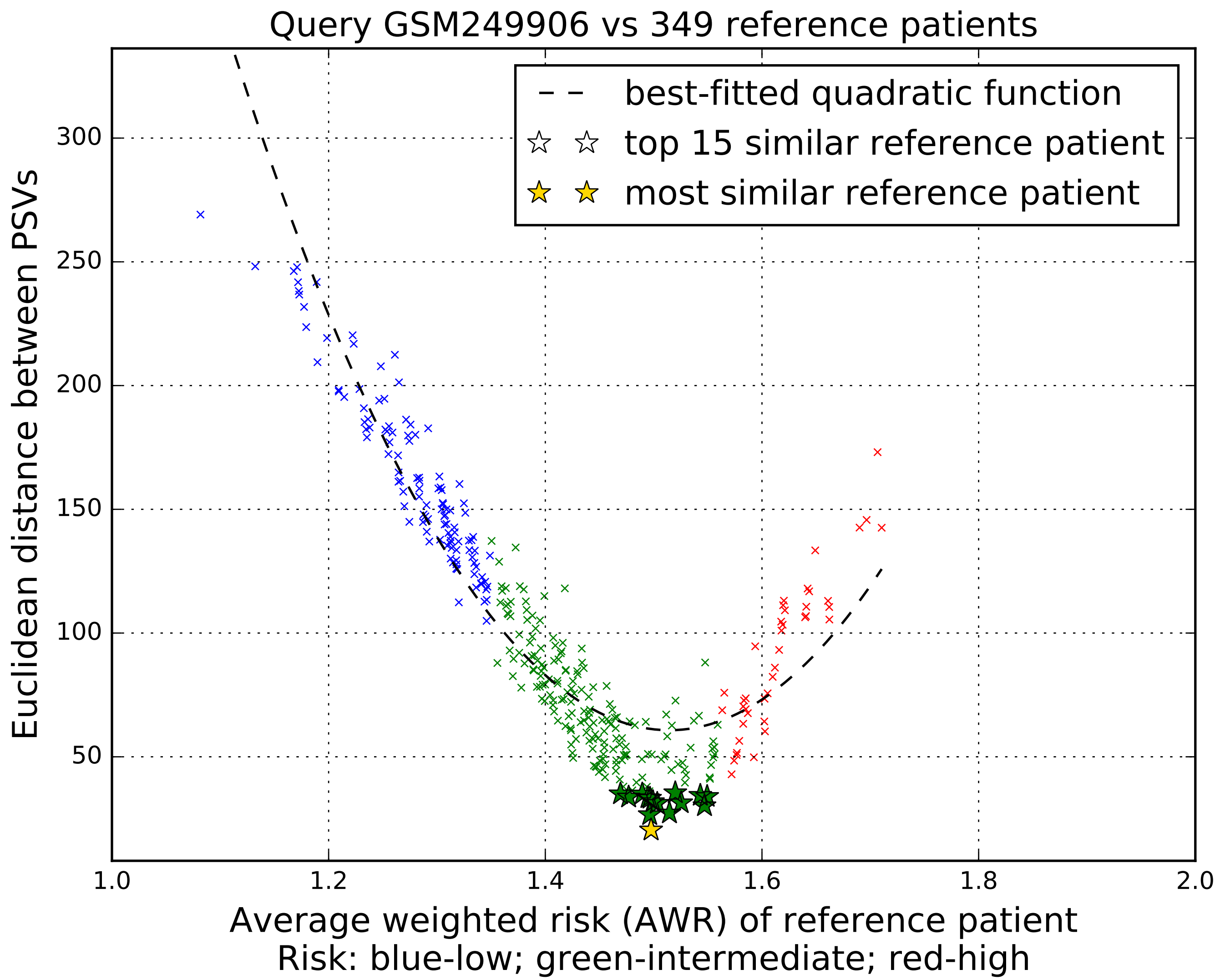
Query GSM249906 vs 349 reference patients
best-fitted quadratic function
top 15 similar reference patient
most similar reference patient
Euclidean distance between PSVs
300
250
200
150
100
50
1.0
1.2
1.4
1.6
1.8
2.0
Average weighted risk (AWR) of reference patient
Risk: blue-low; green-intermediate; red-high

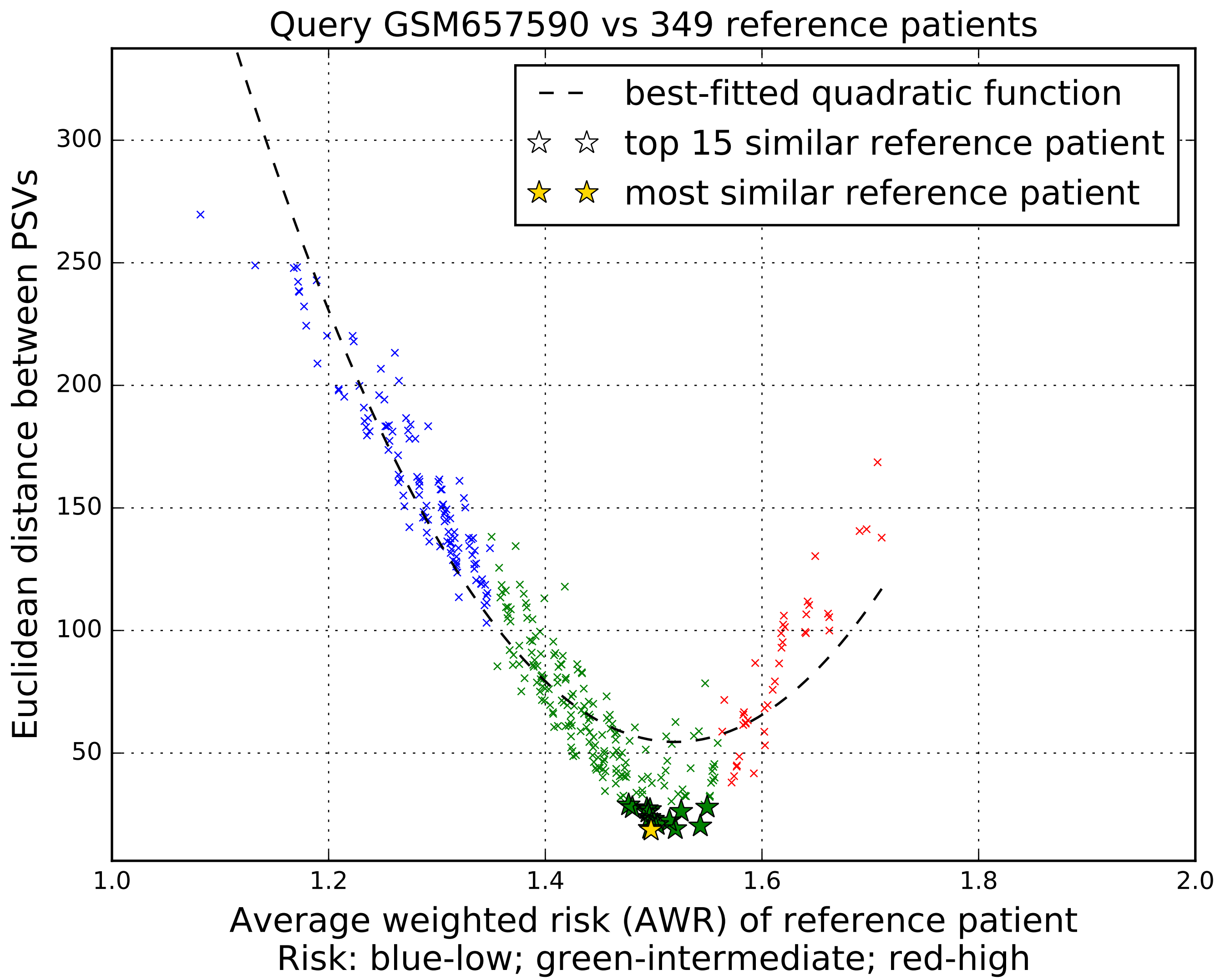
Query GSM657590 vs 349 reference patients
best-fitted quadratic function
top 15 similar reference patient
most similar reference patient
Euclidean distance between PSVs
300
250
200
150
100
50
1.0
1.2
1.4
1.6
1.8
2.0
Average weighted risk (AWR) of reference patient
Risk: blue-low; green-intermediate; red-high

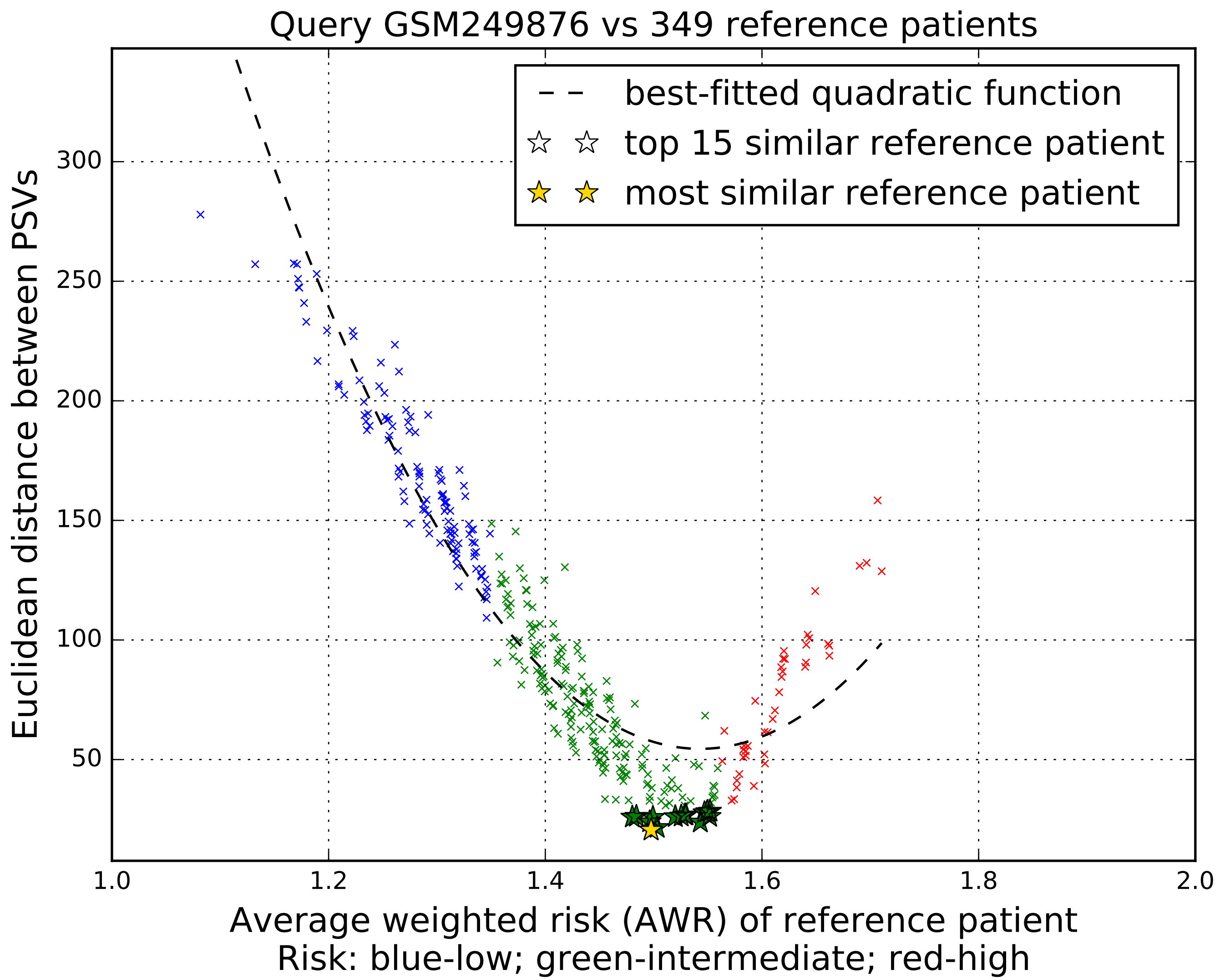

Query GSM249876 vs 349 reference patients
best-fitted quadratic function
top 15 similar reference patient
most similar reference patient
Euclidean distance between PSVs
300
250
200
150
100
50
1.0
1.2
1.4
1.6
1.8
2.0
Average weighted risk (AWR) of reference patient
Risk: blue-low; green-intermediate; red-high

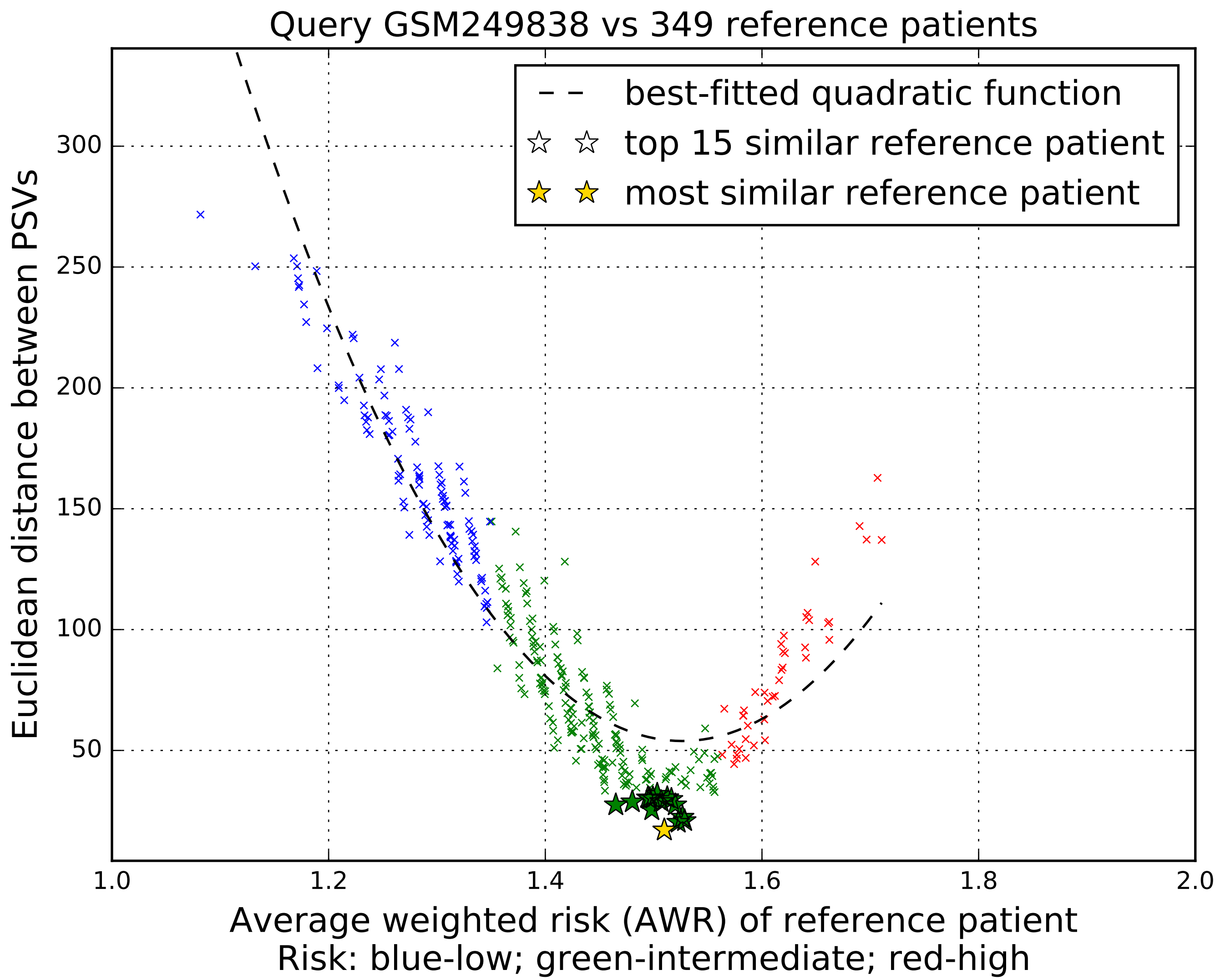
Query GSM249838 vs 349 reference patients
best-fitted quadratic function
top 15 similar reference patient
most similar reference patient
Euclidean distance between PSVs
300
250
200
150
100
50
1.0
1.2
1.4
1.6
1.8
2.0
Average weighted risk (AWR) of reference patient
Risk: blue-low; green-intermediate; red-high

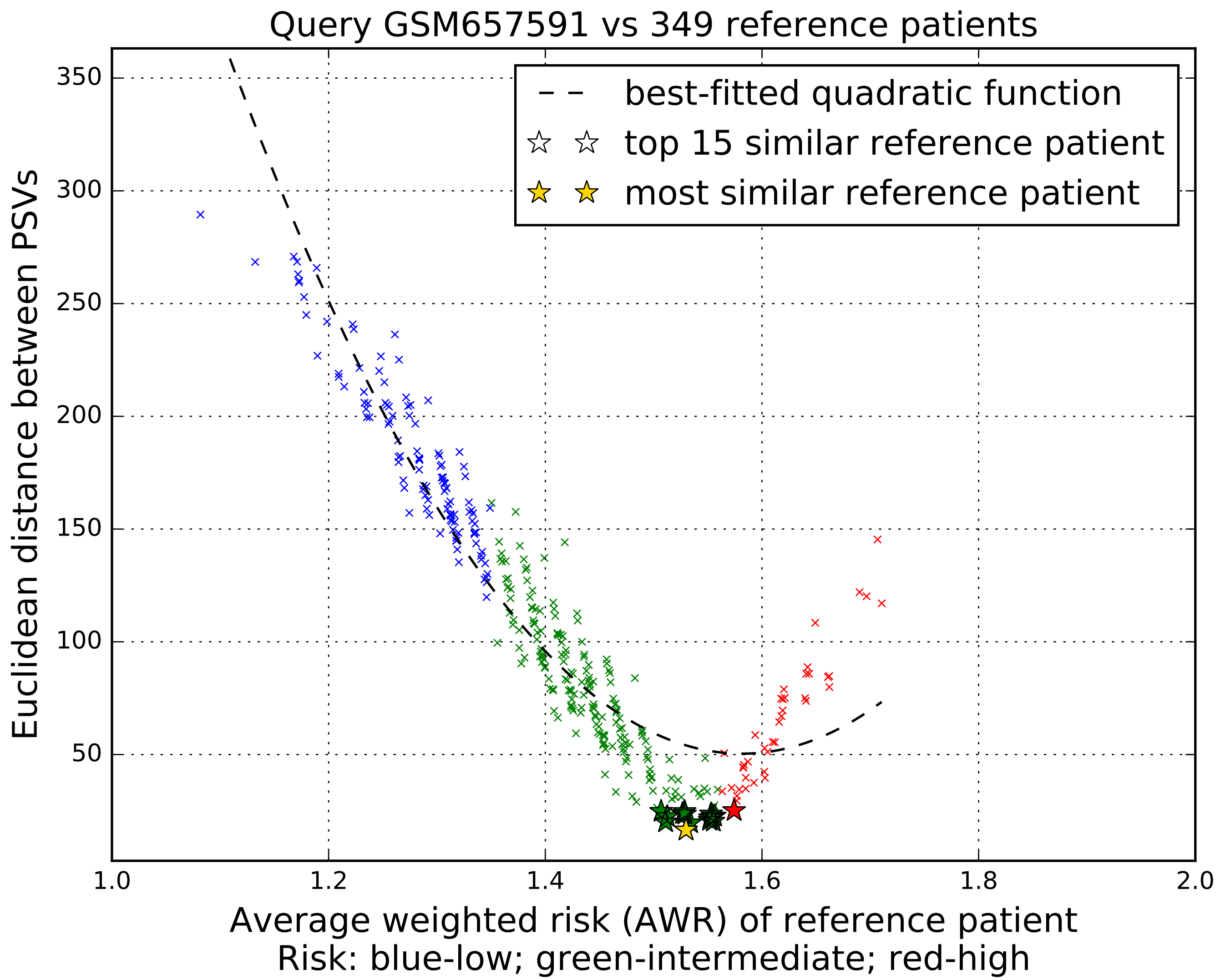

Query GSM657591 vs 349 reference patients
best-fitted quadratic function
top 15 similar reference patient
most similar reference patient
Euclidean distance between PSVs
350
300
250
200
150
100
50
1.0
1.2
1.4
1.6
1.8
2.0
Average weighted risk (AWR) of reference patient
Risk: blue-low; green-intermediate; red-high

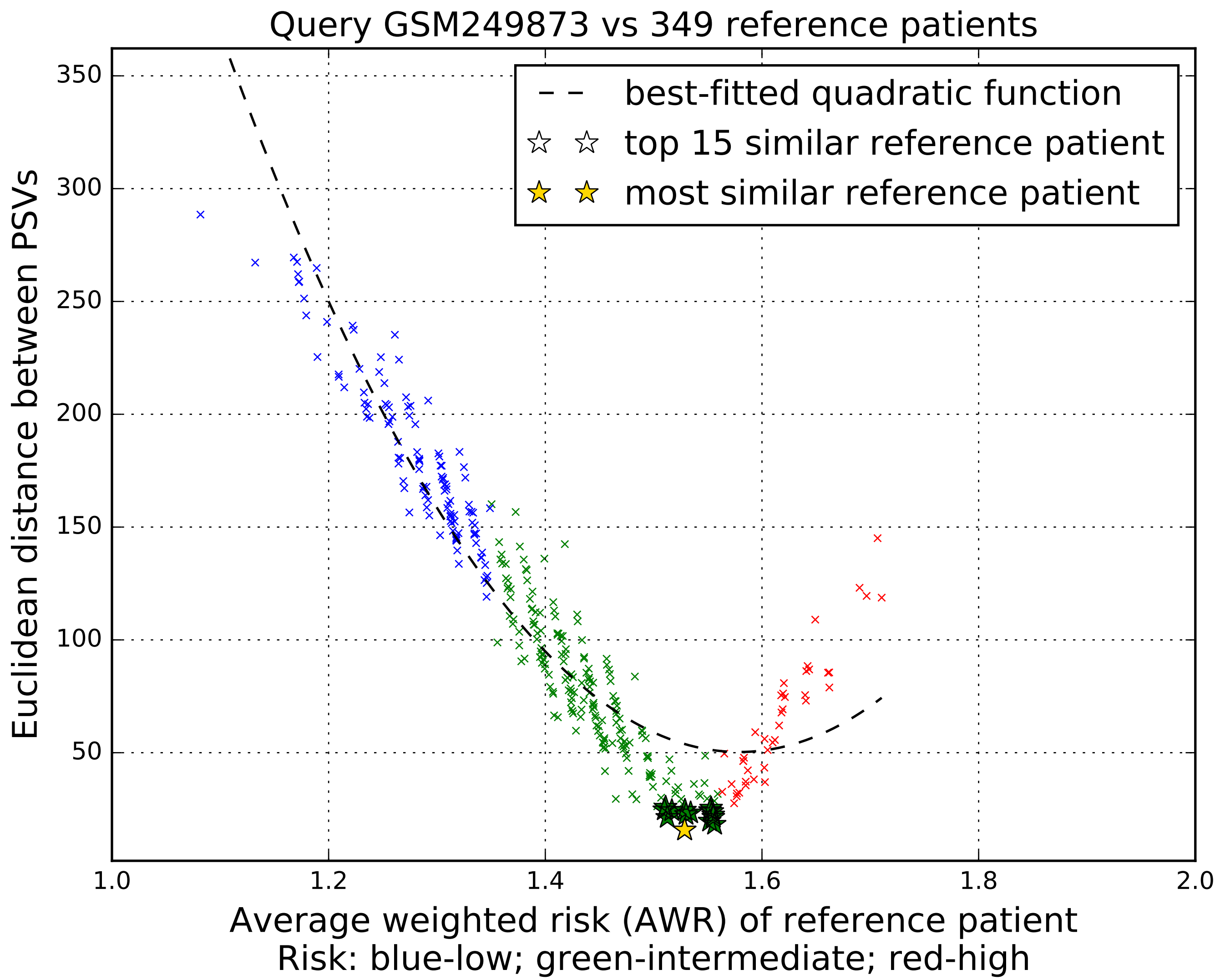
Query GSM249873 vs 349 reference patients
best-fitted quadratic function
top 15 similar reference patient
most similar reference patient
Euclidean distance between PSVs
350
300
250
200
150
100
50
1.0
1.2
1.4
1.6
1.8
2.0
Average weighted risk (AWR) of reference patient
Risk: blue-low; green-intermediate; red-high

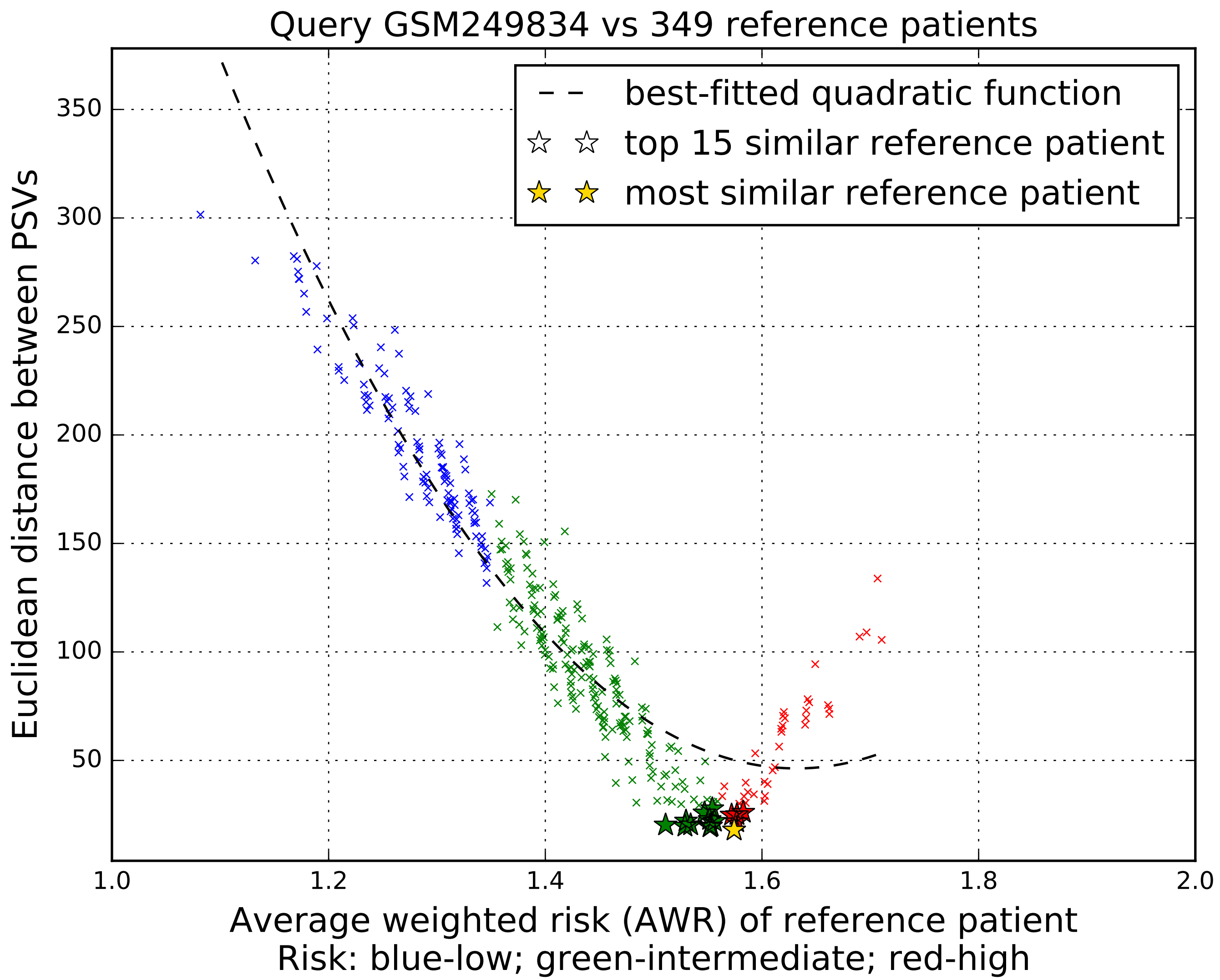
Query GSM249834 vs 349 reference patients
best-fitted quadratic function
top 15 similar reference patient
most similar reference patient
Euclidean distance between PSVs
350
300
250
200
150
100
50
1.0
1.2
1.4
1.6
1.8
2.0
Average weighted risk (AWR) of reference patient
Risk: blue-low; green-intermediate; red-high

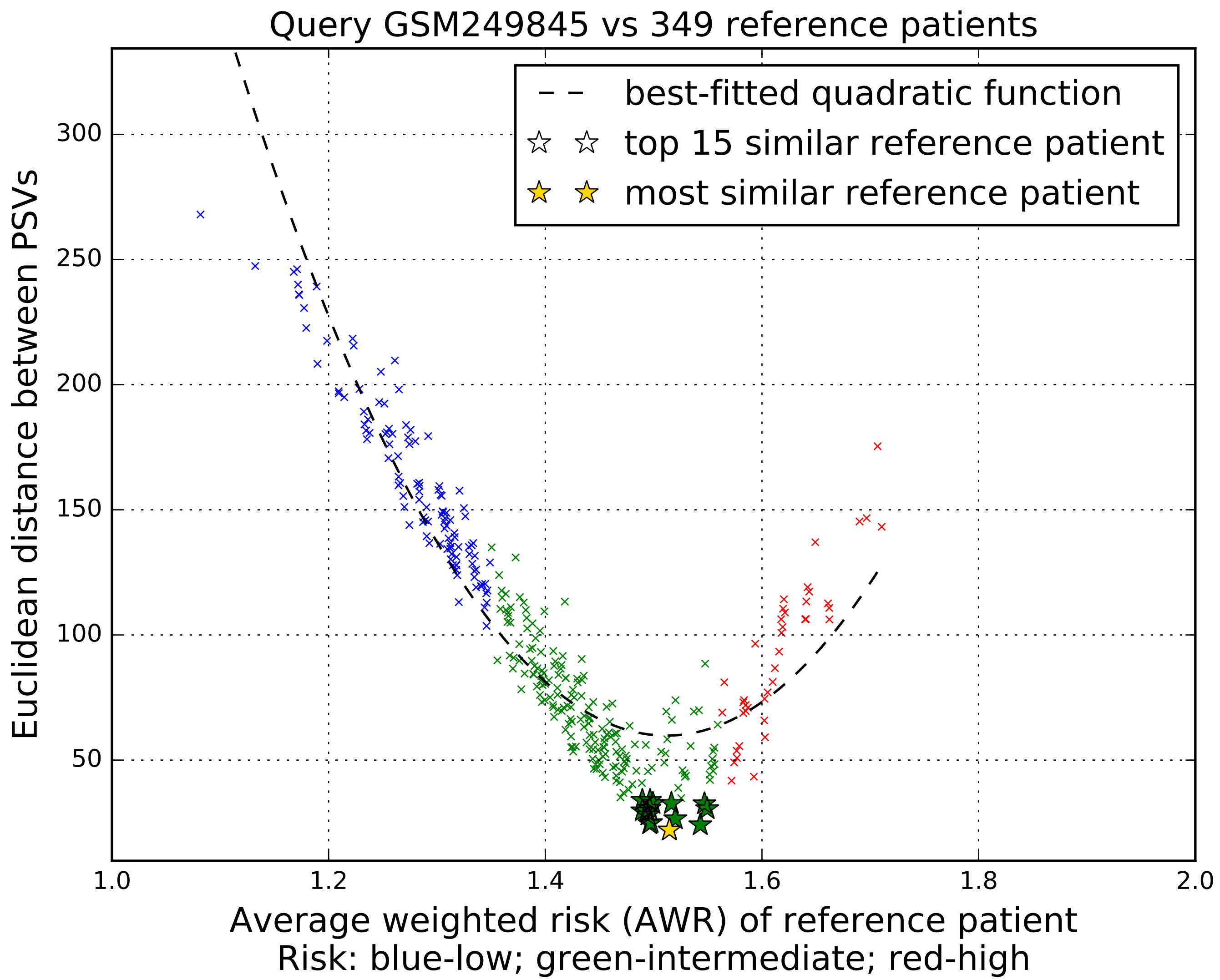

Query GSM249845 vs 349 reference patients
best-fitted quadratic function
top 15 similar reference patient
most similar reference patient
Euclidean distance between PSVs
300
250
200
150
100
50
1.0
1.2
1.4
1.6
1.8
2.0
Average weighted risk (AWR) of reference patient
Risk: blue-low; green-intermediate; red-high

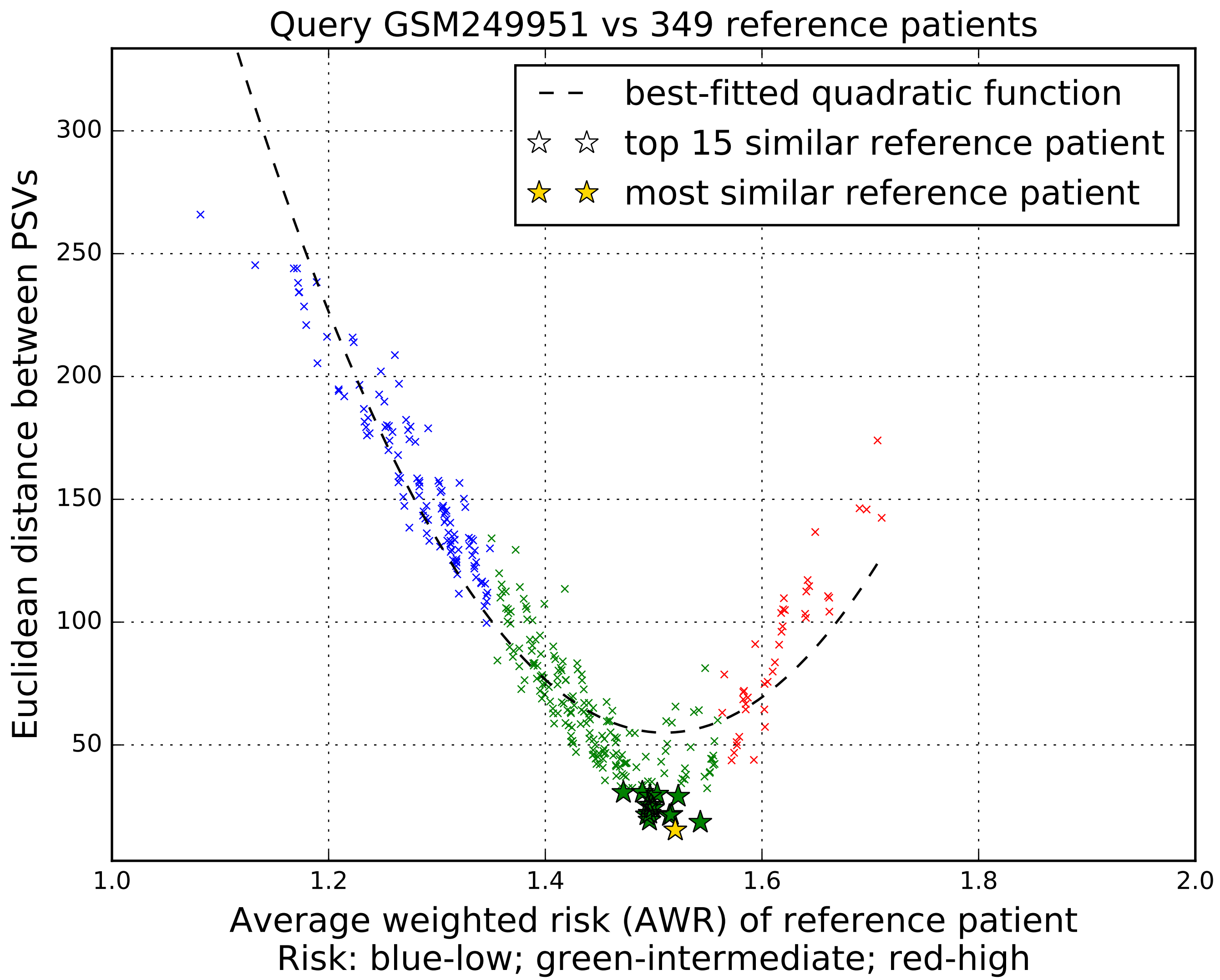

Query GSM249951 vs 349 reference patients
best-fitted quadratic function
top 15 similar reference patient
most similar reference patient
Euclidean distance between PSVs
300
250
200
150
100
50
1.0
1.2
1.4
1.6
1.8
2.0
Average weighted risk (AWR) of reference patient
Risk: blue-low; green-intermediate; red-high

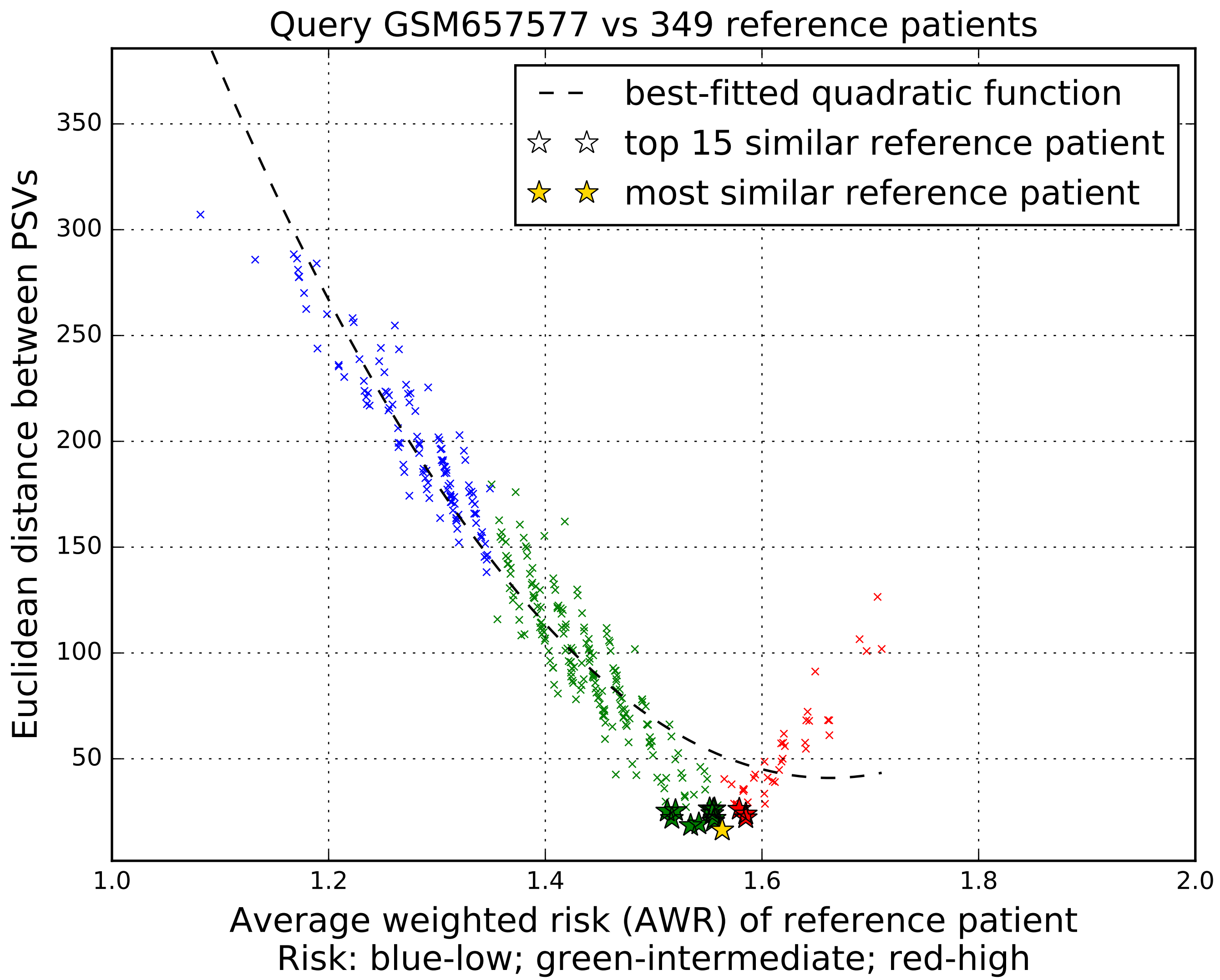

Query GSM657577 vs 349 reference patients
best-fitted quadratic function
top 15 similar reference patient
most similar reference patient
Euclidean distance between PSVs
50
100
150
200
250
300
350
1.0
1.2
1.4
1.6
1.8
2.0
Average weighted risk (AWR) of reference patient
Risk: blue-low; green-intermediate; red-high

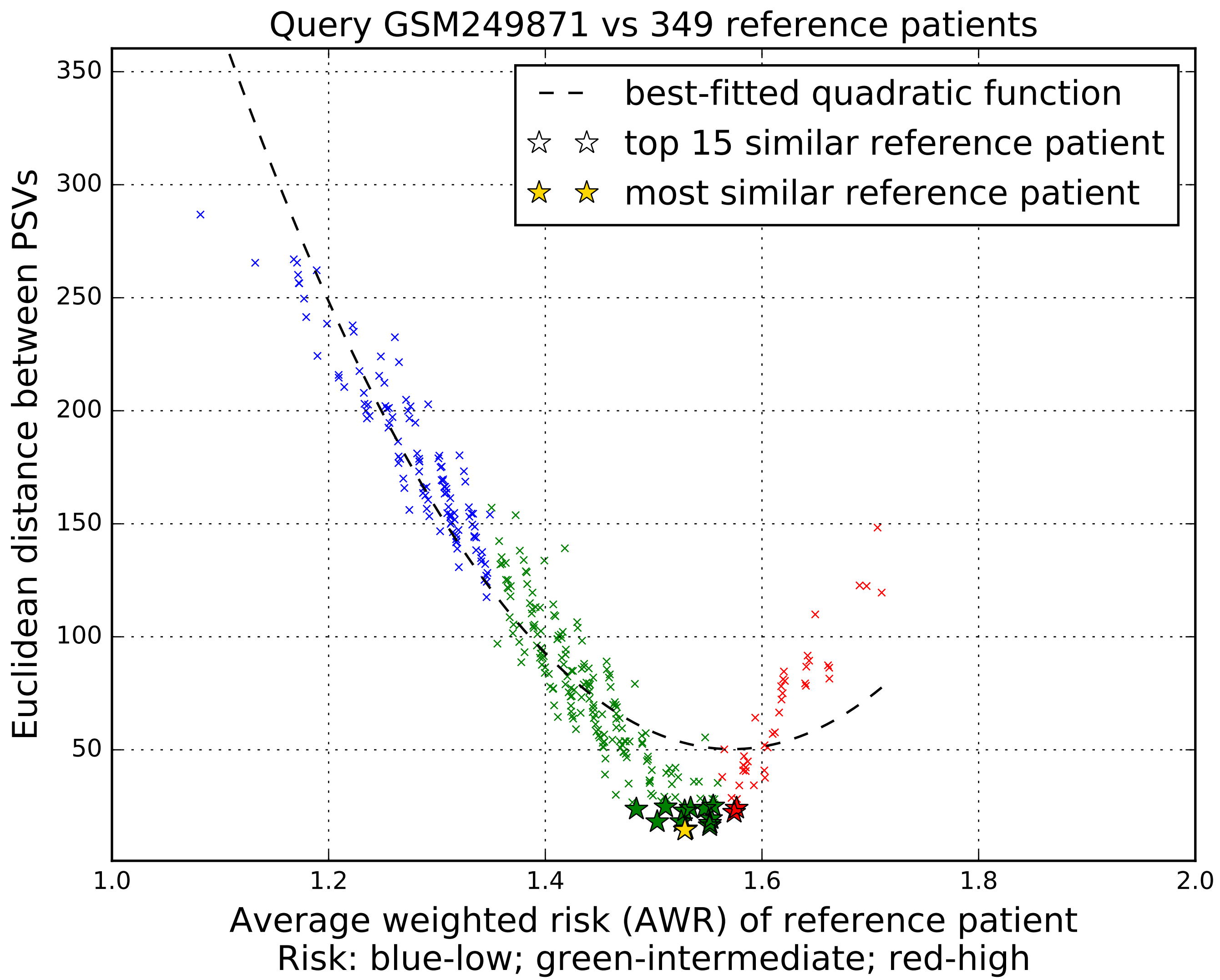
Query GSM249871 vs 349 reference patients
best-fitted quadratic function
top 15 similar reference patient
most similar reference patient
Euclidean distance between PSVs
350
300
250
200
150
100
50
1.0
1.2
1.4
1.6
1.8
2.0
Average weighted risk (AWR) of reference patient
Risk: blue-low; green-intermediate; red-high

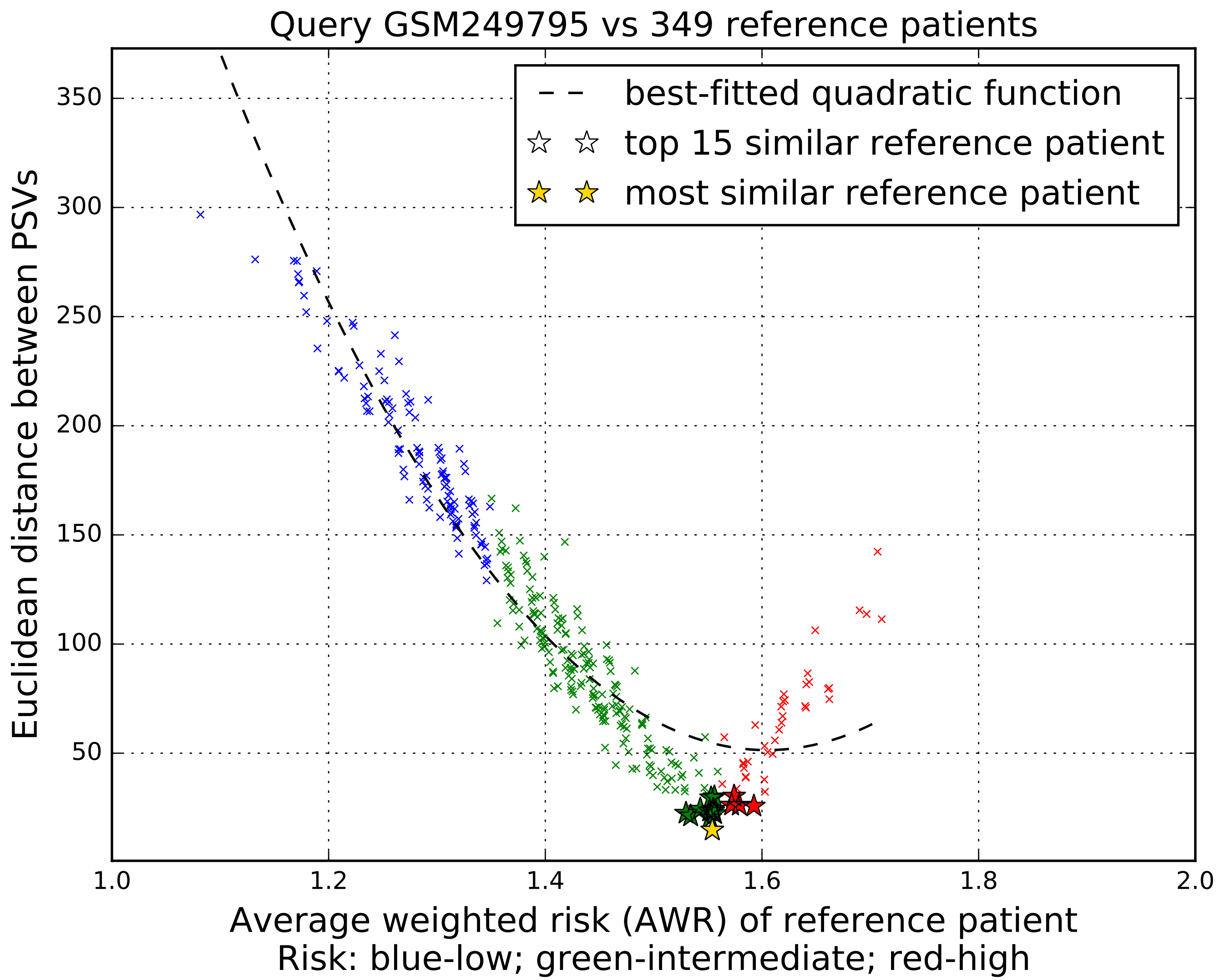

Query GSM249795 vs 349 reference patients
best-fitted quadratic function
top 15 similar reference patient
most similar reference patient
Euclidean distance between PSVs
350
300
250
200
150
100
50
1.0
1.2
1.4
1.6
1.8
2.0
Average weighted risk (AWR) of reference patient
Risk: blue-low; green-intermediate; red-high

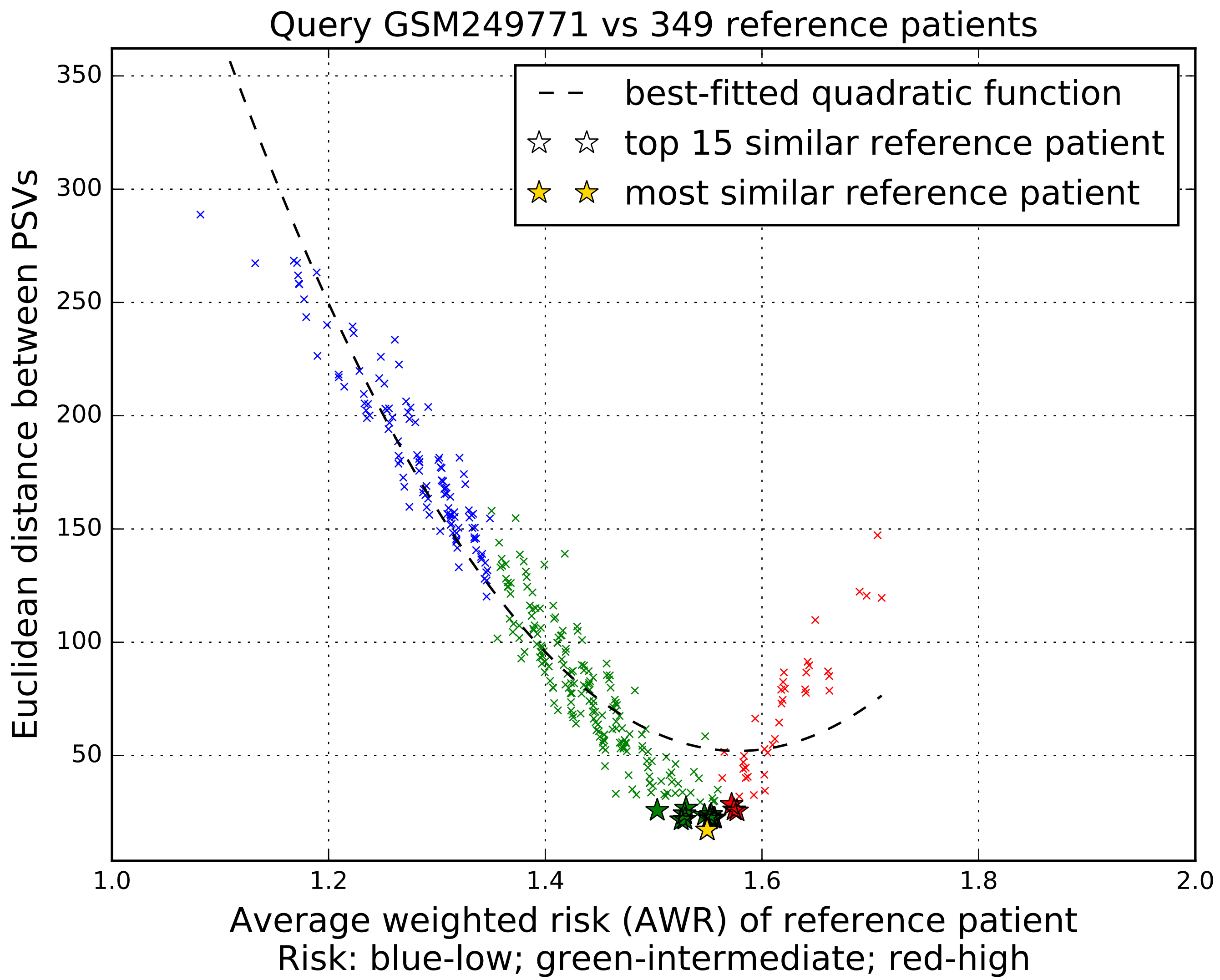

Query GSM249771 vs 349 reference patients
best-fitted quadratic function
top 15 similar reference patient
most similar reference patient
Euclidean distance between PSVs
350
300
250
200
150
100
50
1.0
1.2
1.4
1.6
1.8
2.0
Average weighted risk (AWR) of reference patient
Risk: blue-low; green-intermediate; red-high

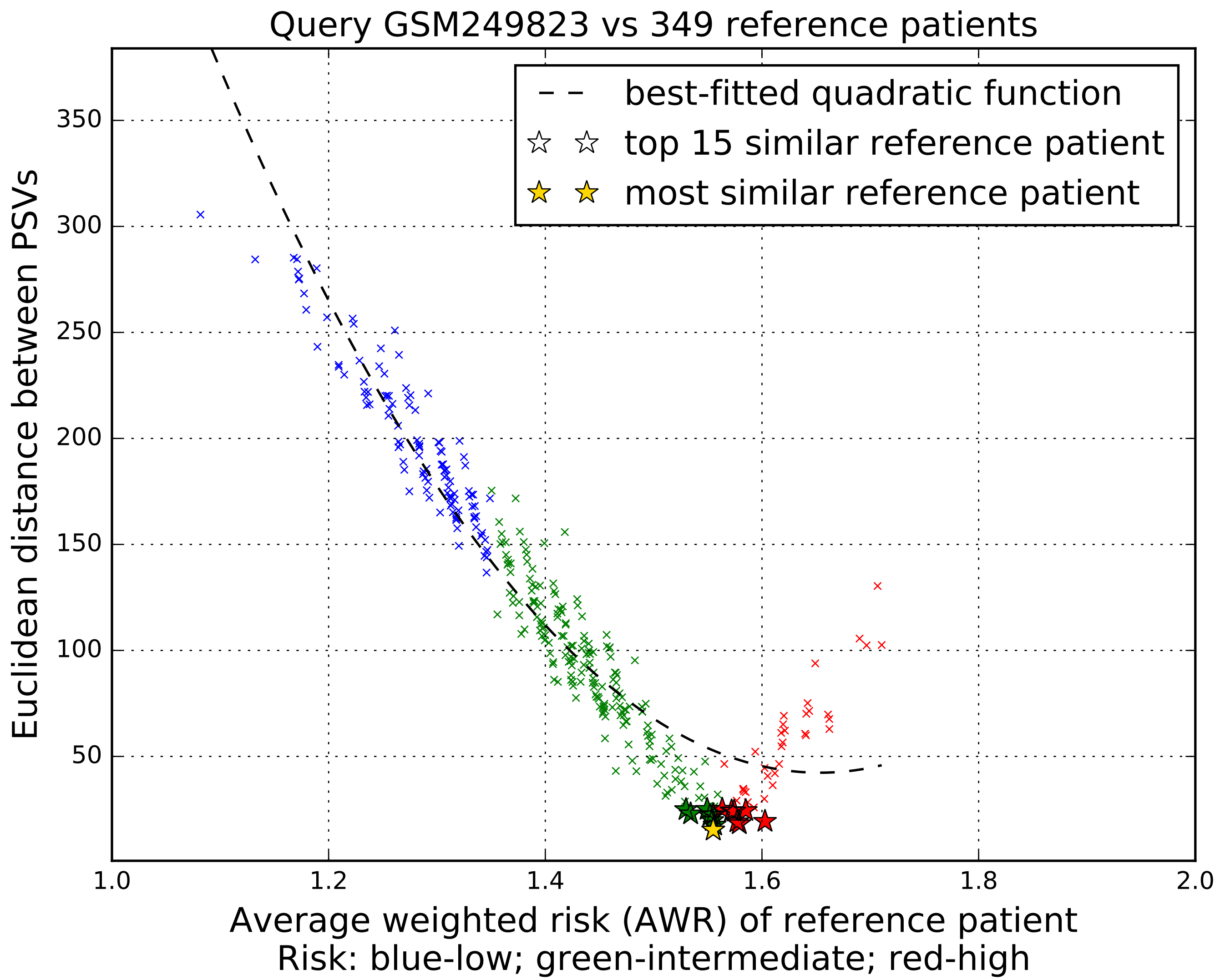

Query GSM249823 vs 349 reference patients
best-fitted quadratic function
top 15 similar reference patient
most similar reference patient
Euclidean distance between PSVs
Average weighted risk (AWR) of reference patient
Risk: blue-low; green-intermediate; red-high
350
300
250
200
150
100
50
1.0
1.2
1.4
1.6
1.8
2.0

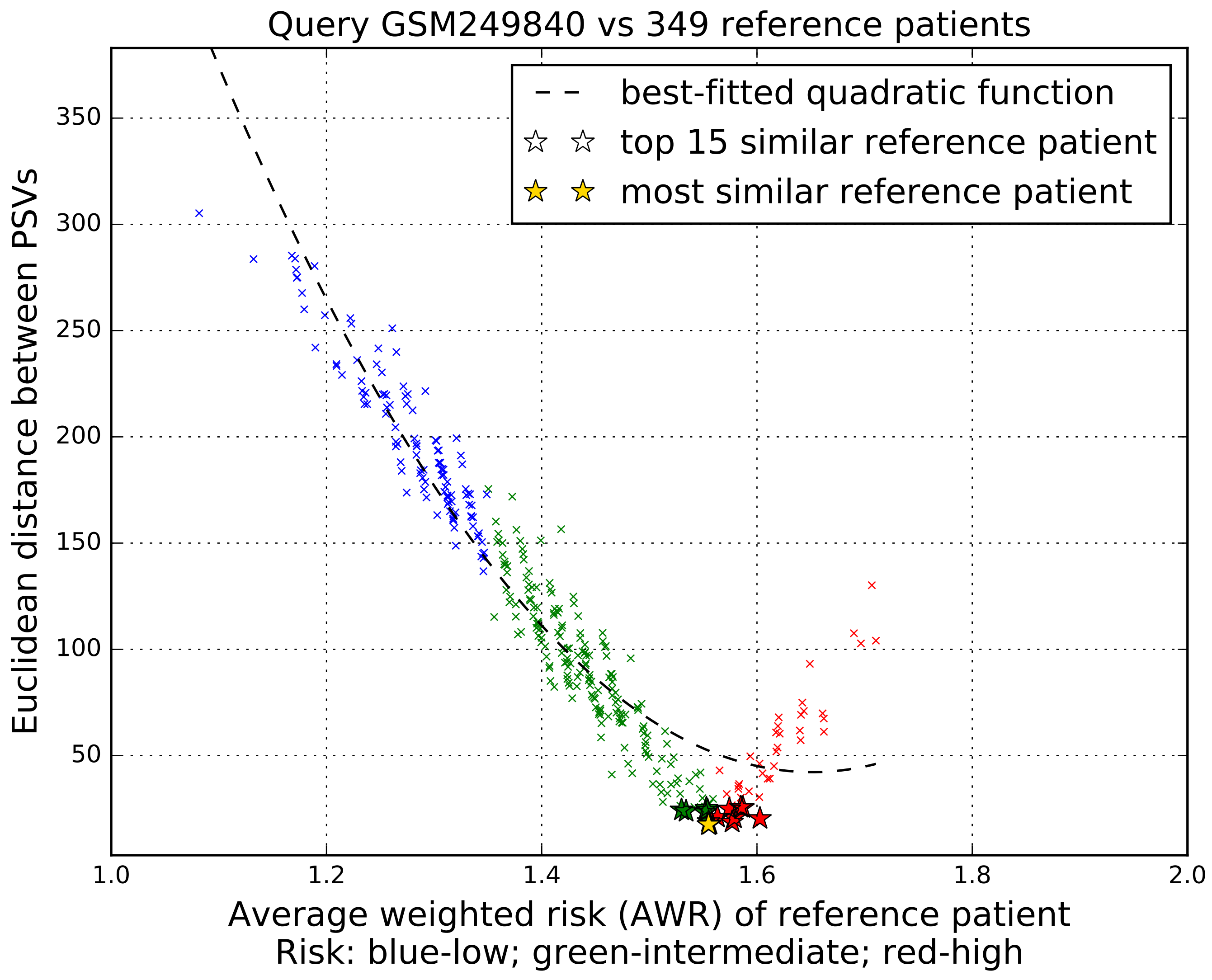

Query GSM249840 vs 349 reference patients
best-fitted quadratic function
top 15 similar reference patient
most similar reference patient
Euclidean distance between PSVs
50
100
150
200
250
300
350
1.0
1.2
1.4
1.6
1.8
2.0
Average weighted risk (AWR) of reference patient
Risk: blue-low; green-intermediate; red-high

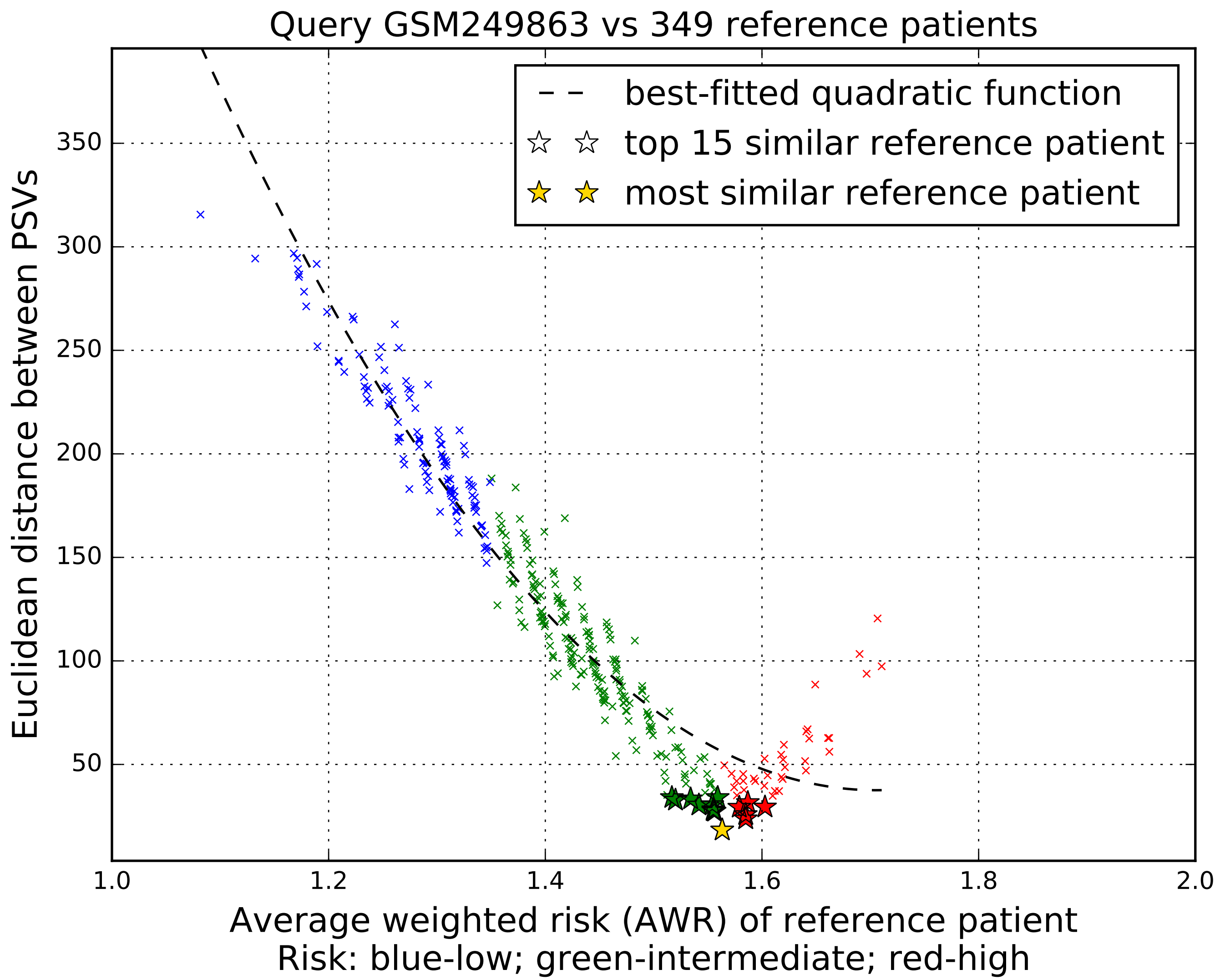

Query GSM249863 vs 349 reference patients
best-fitted quadratic function
top 15 similar reference patient
most similar reference patient
Euclidean distance between PSVs
350
300
250
200
150
100
50
1.0
1.2
1.4
1.6
1.8
2.0
Average weighted risk (AWR) of reference patient
Risk: blue-low; green-intermediate; red-high

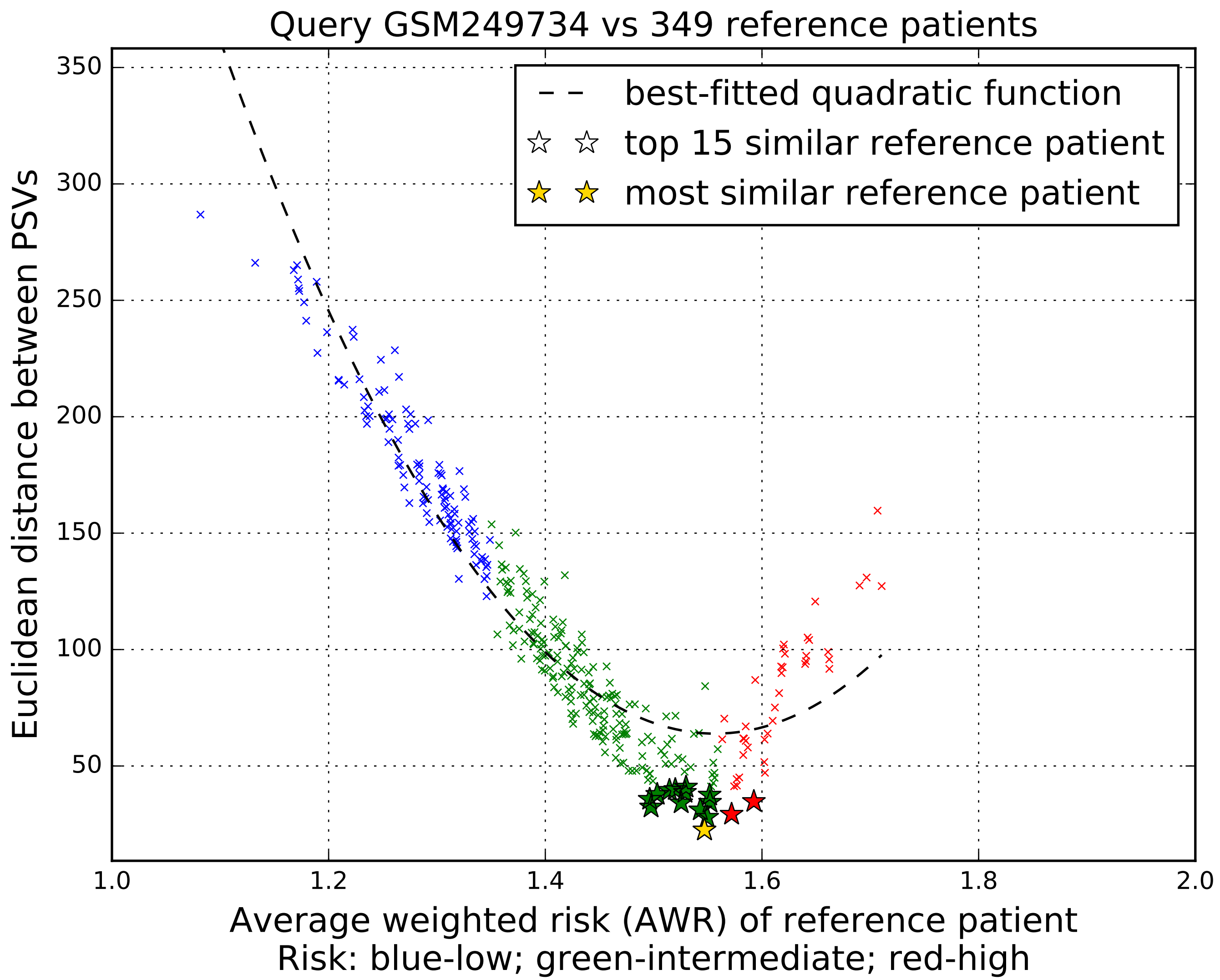

Query GSM249734 vs 349 reference patients
best-fitted quadratic function
top 15 similar reference patient
most similar reference patient
Euclidean distance between PSVs
350
300
250
200
150
100
50
1.0
1.2
1.4
1.6
1.8
2.0
Average weighted risk (AWR) of reference patient
Risk: blue-low; green-intermediate; red-high

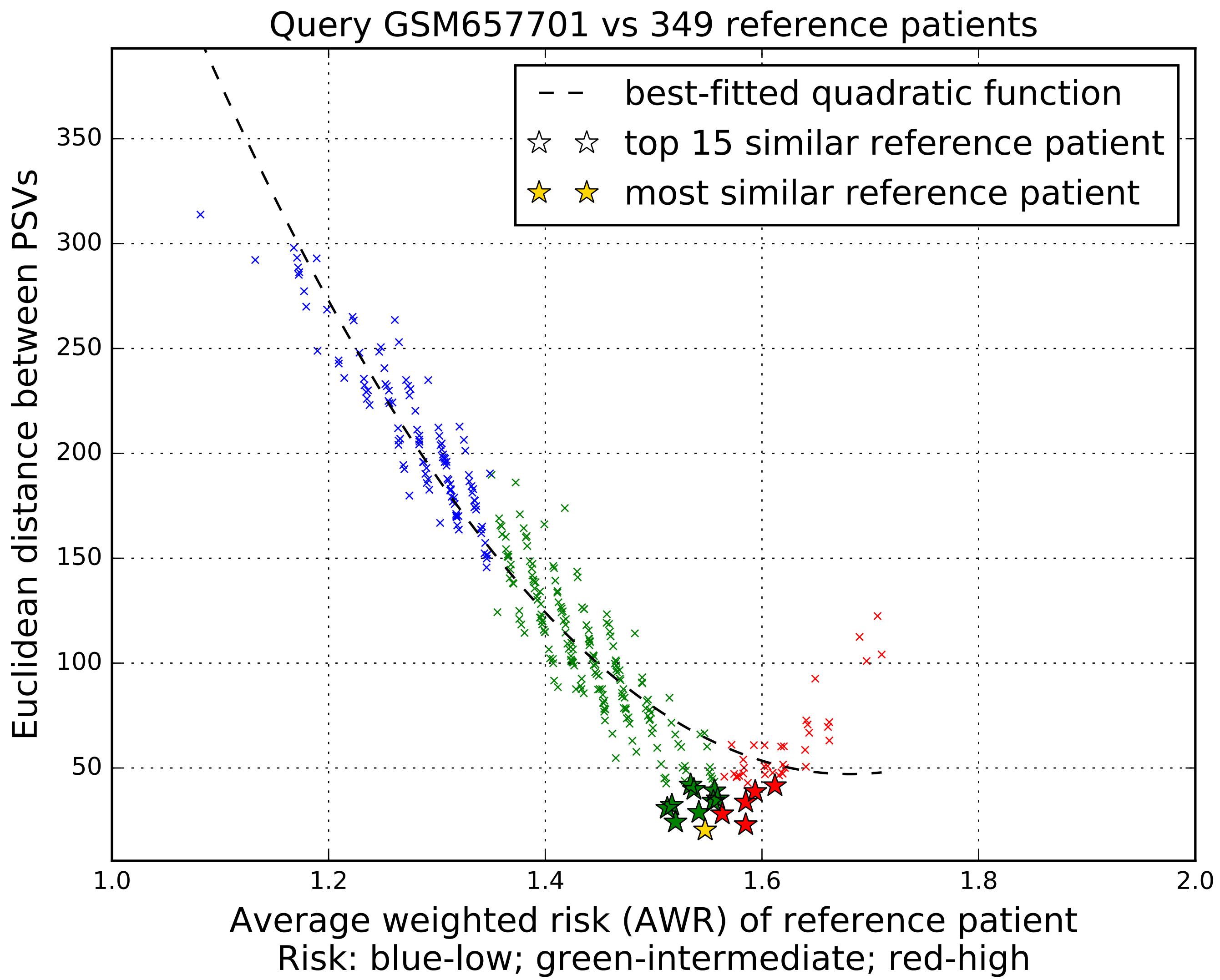
Query GSM657701 vs 349 reference patients
best-fitted quadratic function
top 15 similar reference patient
most similar reference patient
Euclidean distance between PSVs
350
300
250
200
150
100
50
1.0
1.2
1.4
1.6
1.8
2.0
Average weighted risk (AWR) of reference patient
Risk: blue-low; green-intermediate; red-high

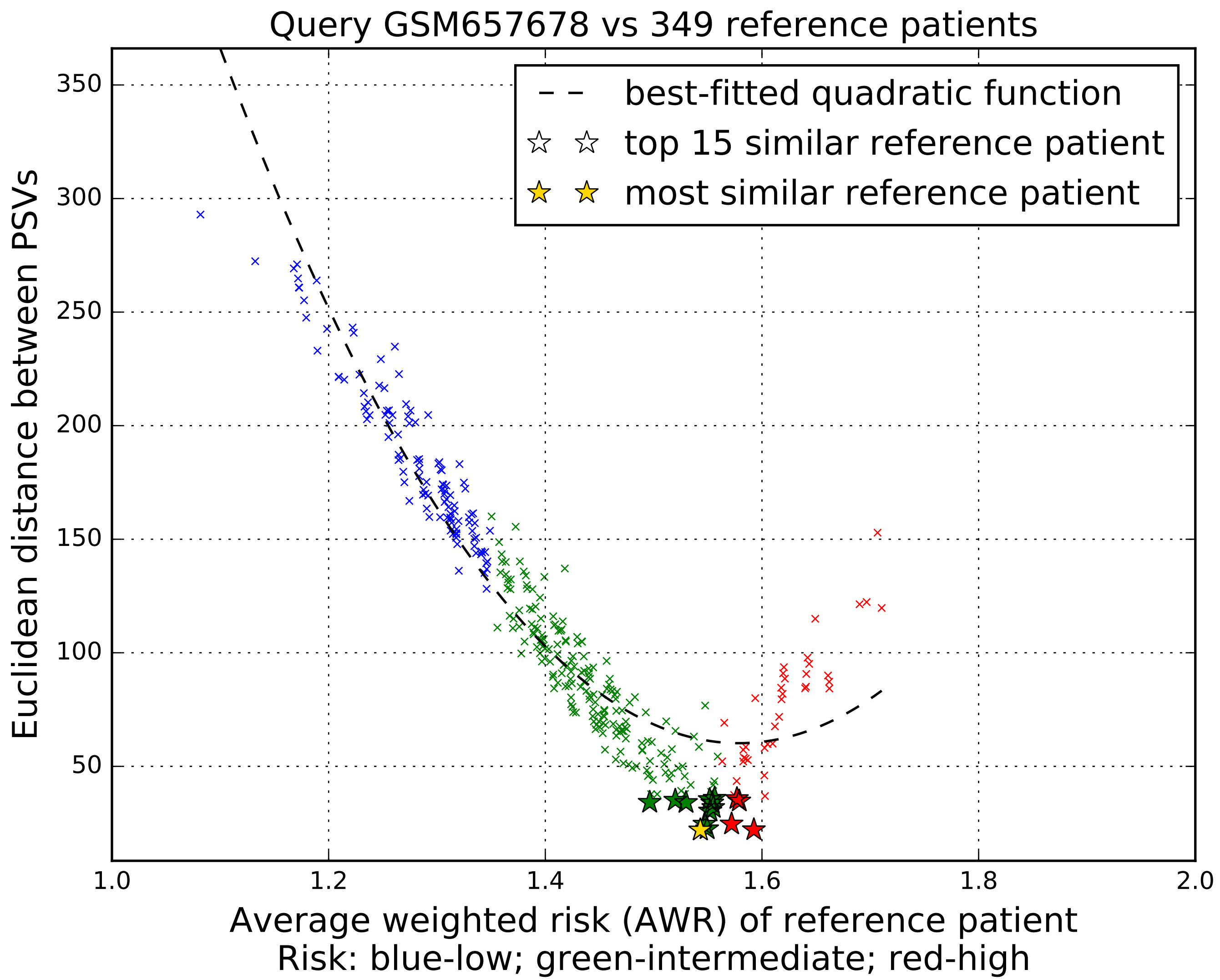

Query GSM657678 vs 349 reference patients
best-fitted quadratic function
top 15 similar reference patient
most similar reference patient
Euclidean distance between PSVs
Average weighted risk (AWR) of reference patient
Risk: blue-low; green-intermediate; red-high
350
300
250
200
150
100
50
1.0
1.2
1.4
1.6
1.8
2.0

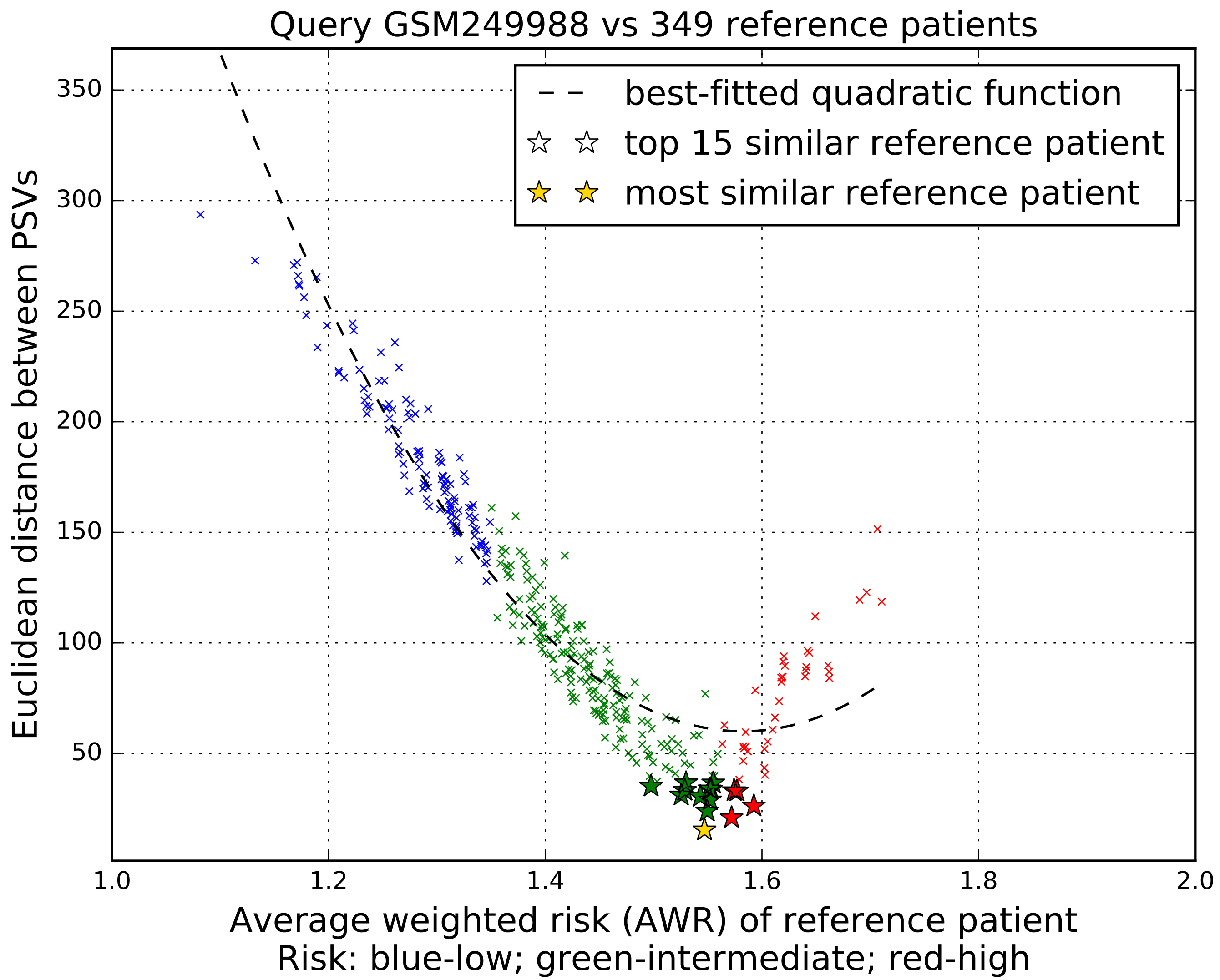

Query GSM249988 vs 349 reference patients
best-fitted quadratic function
top 15 similar reference patient
most similar reference patient
Euclidean distance between PSVs
350
300
250
200
150
100
50
1.0
1.2
1.4
1.6
1.8
2.0
Average weighted risk (AWR) of reference patient
Risk: blue-low; green-intermediate; red-high

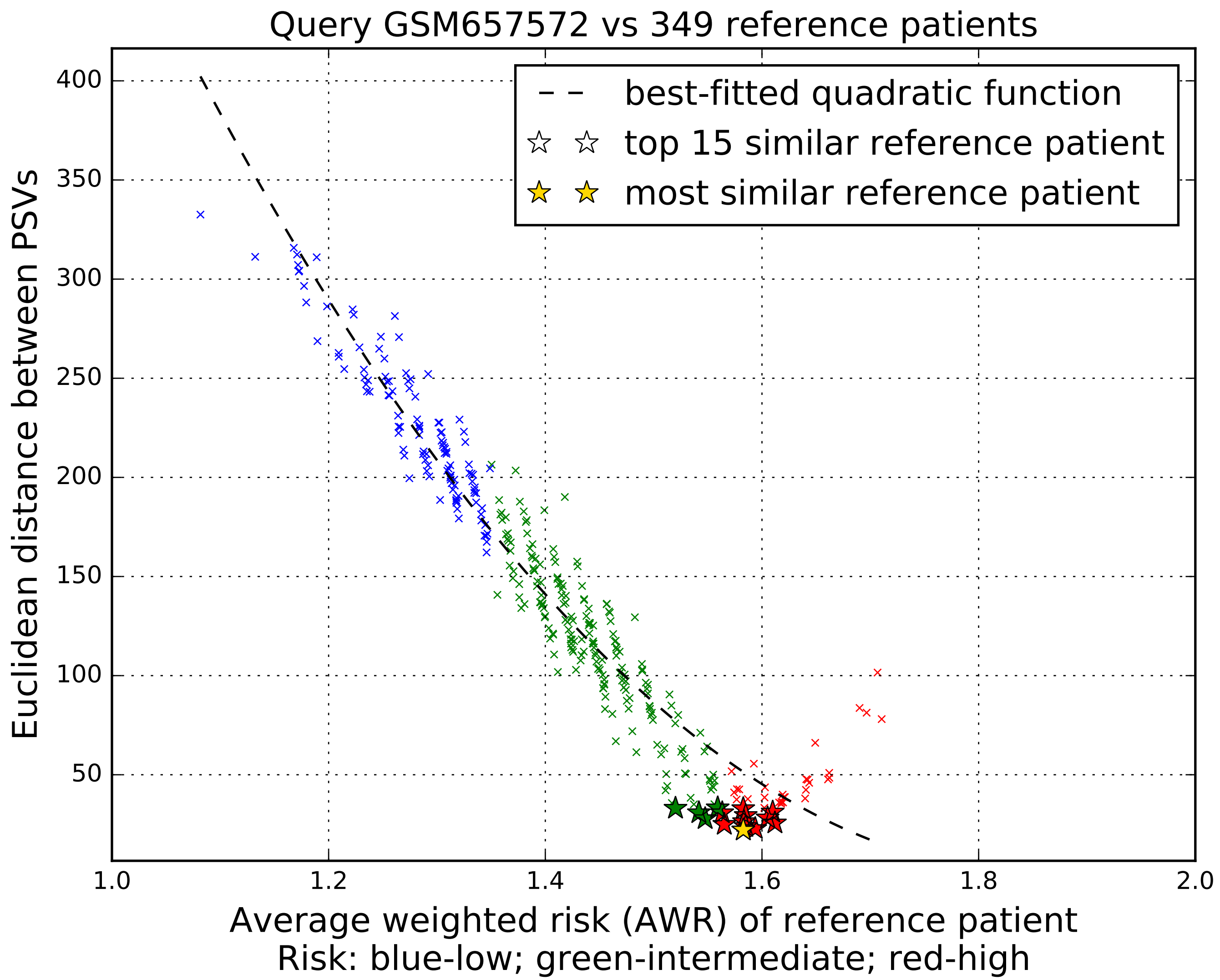
Query GSM657572 vs 349 reference patients
best-fitted quadratic function
top 15 similar reference patient
most similar reference patient
Euclidean distance between PSVs
400
350
300
250
200
150
100
50
1.0
1.2
1.4
1.6
1.8
2.0
Average weighted risk (AWR) of reference patient
Risk: blue-low; green-intermediate; red-high

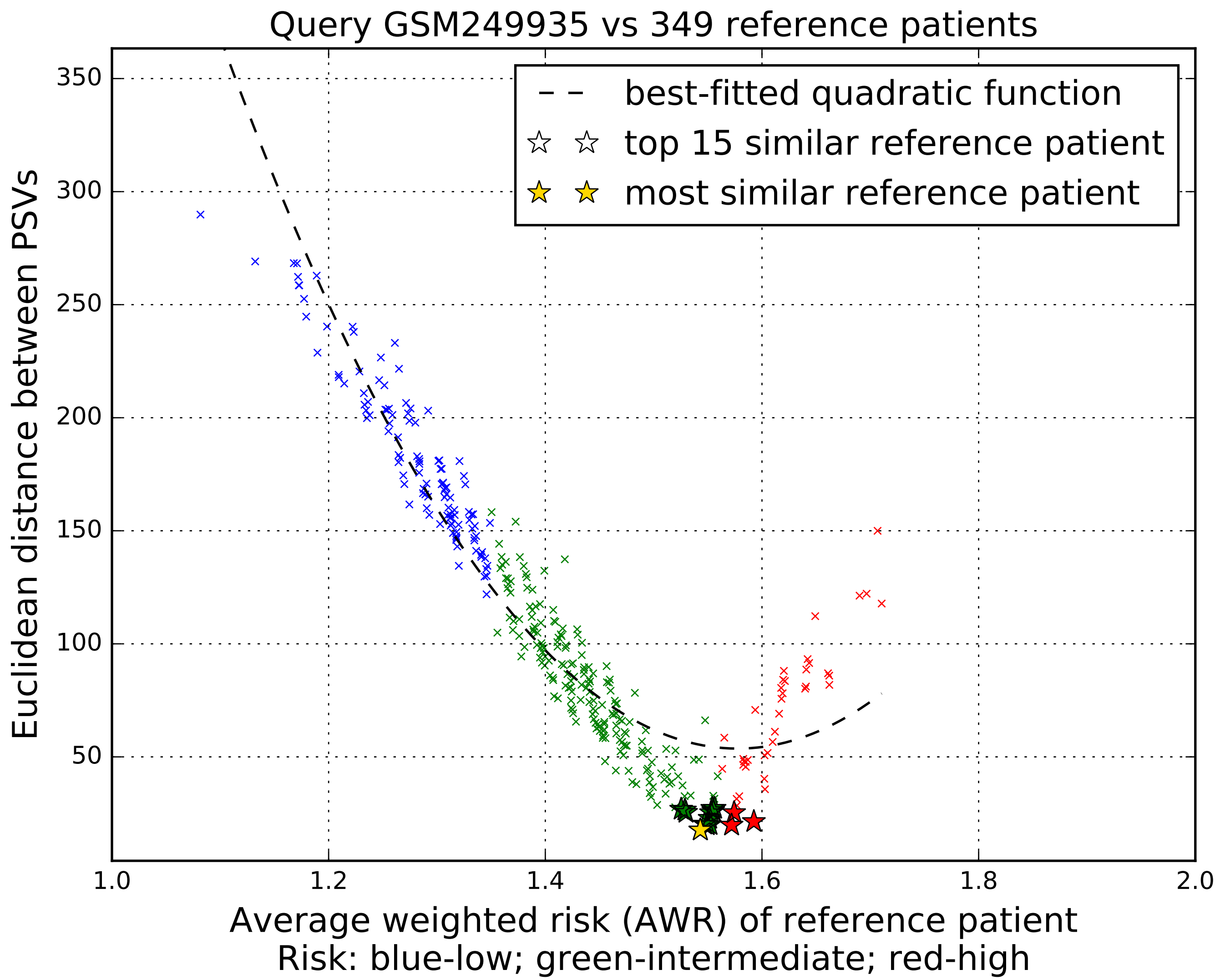
Query GSM249935 vs 349 reference patients
best-fitted quadratic function
top 15 similar reference patient
most similar reference patient
Euclidean distance between PSVs
350
300
250
200
150
100
50
1.0
1.2
1.4
1.6
1.8
2.0
Average weighted risk (AWR) of reference patient
Risk: blue-low; green-intermediate; red-high

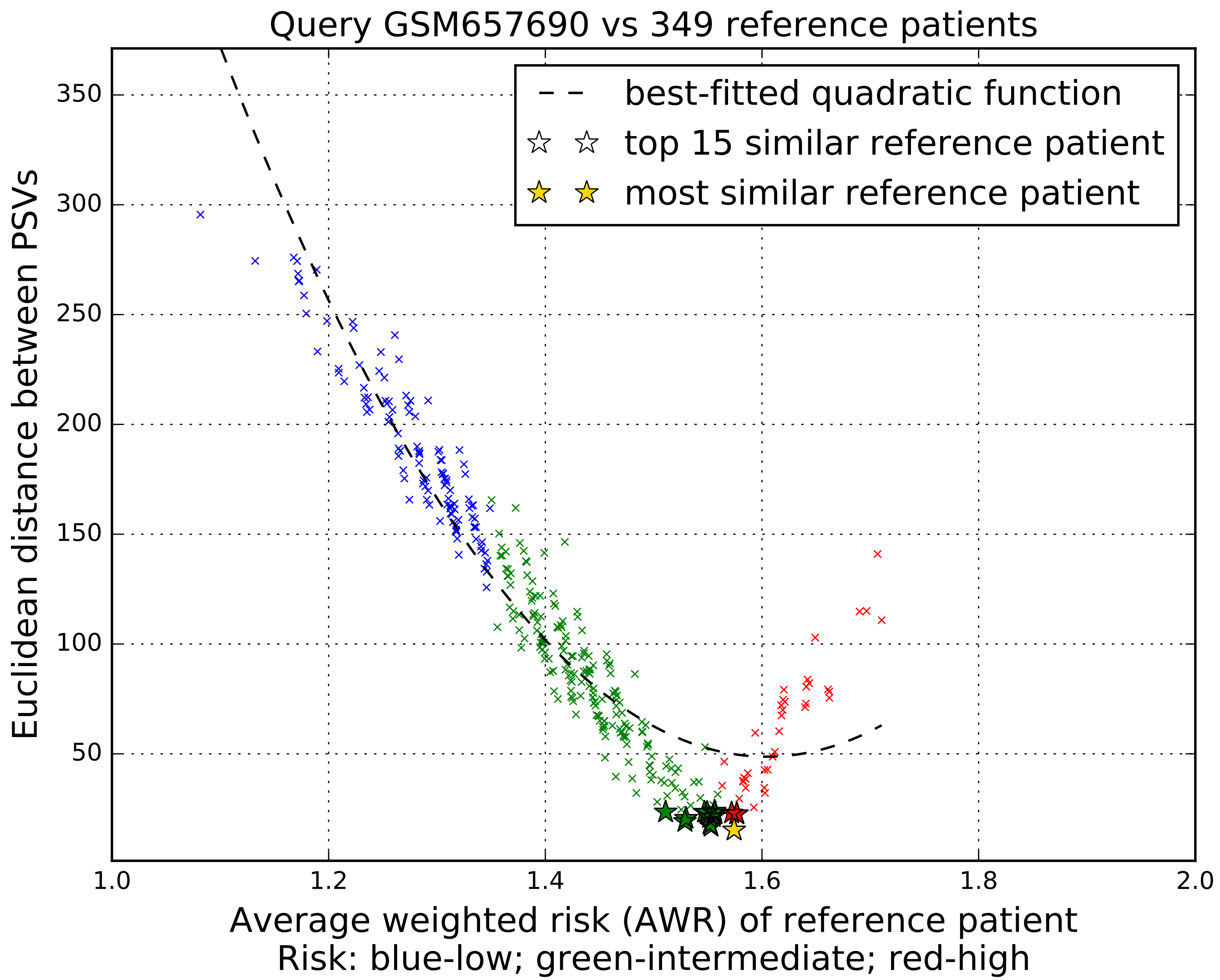

Query GSM657690 vs 349 reference patients
best-fitted quadratic function
top 15 similar reference patient
most similar reference patient
Euclidean distance between PSVs
Average weighted risk (AWR) of reference patient
Risk: blue-low; green-intermediate; red-high
350
300
250
200
150
100
50
1.0
1.2
1.4
1.6
1.8
2.0

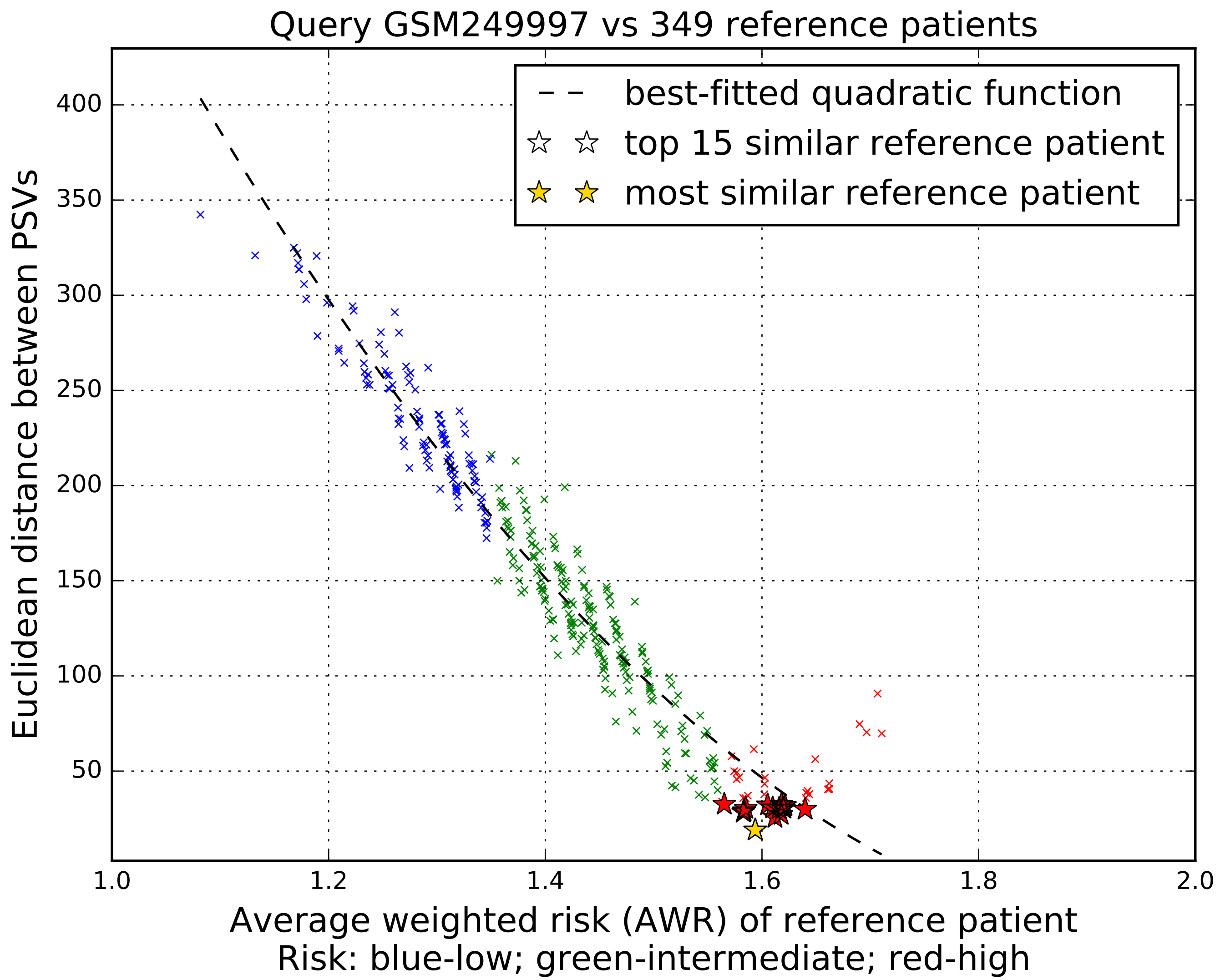

Query GSM249997 vs 349 reference patients
best-fitted quadratic function
top 15 similar reference patient
most similar reference patient
Euclidean distance between PSVs
400
350
300
250
200
150
100
50
1.0
1.2
1.4
1.6
1.8
2.0
Average weighted risk (AWR) of reference patient
Risk: blue-low; green-intermediate; red-high

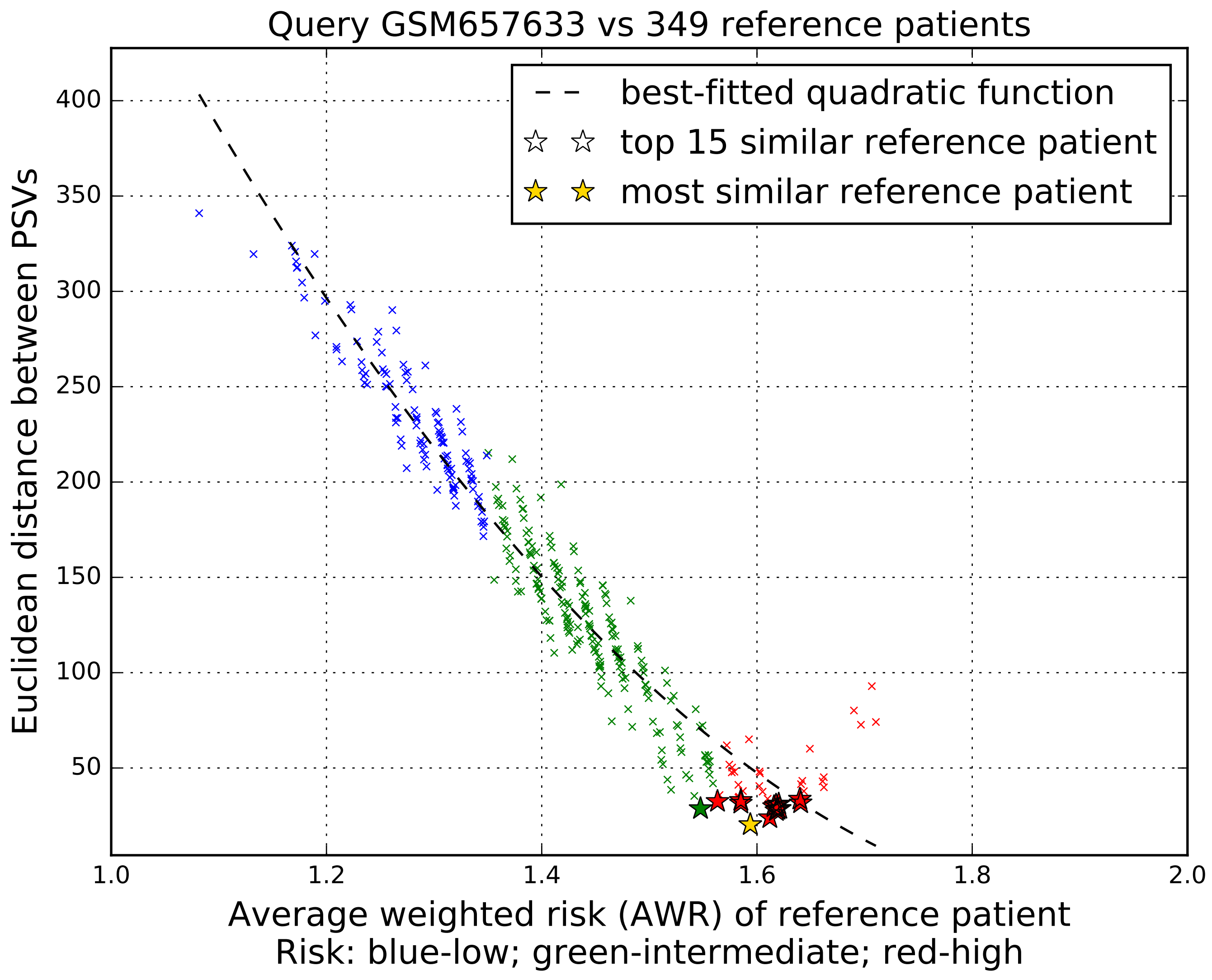
Query GSM657633 vs 349 reference patients
best-fitted quadratic function
top 15 similar reference patient
most similar reference patient
Euclidean distance between PSVs
400
350
300
250
200
150
100
50
1.0
1.2
1.4
1.6
1.8
2.0
Average weighted risk (AWR) of reference patient
Risk: blue-low; green-intermediate; red-high

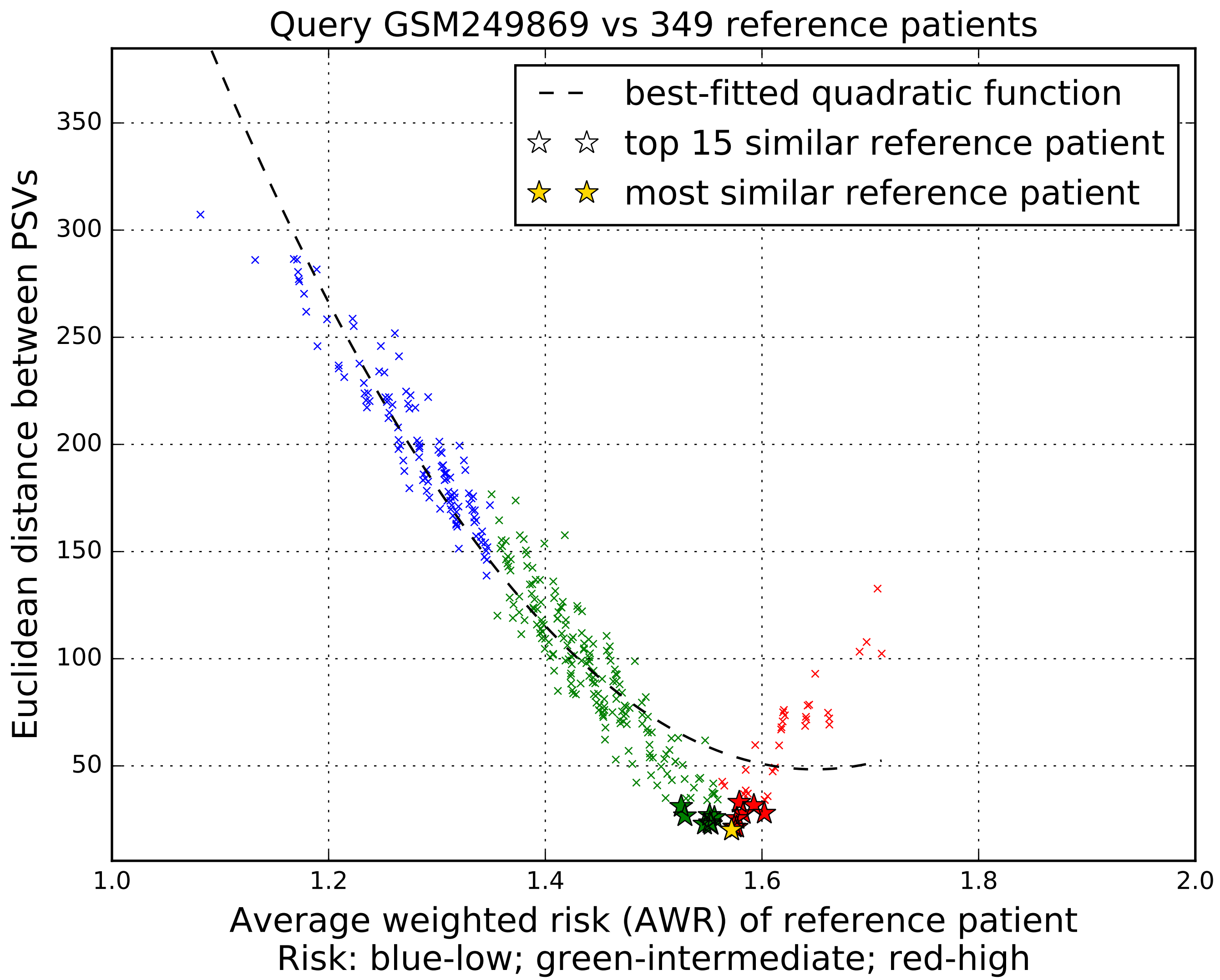
Query GSM249869 vs 349 reference patients
best-fitted quadratic function
top 15 similar reference patient
most similar reference patient
Euclidean distance between PSVs
350
300
250
200
150
100
50
1.0
1.2
1.4
1.6
1.8
2.0
Average weighted risk (AWR) of reference patient
Risk: blue-low; green-intermediate; red-high

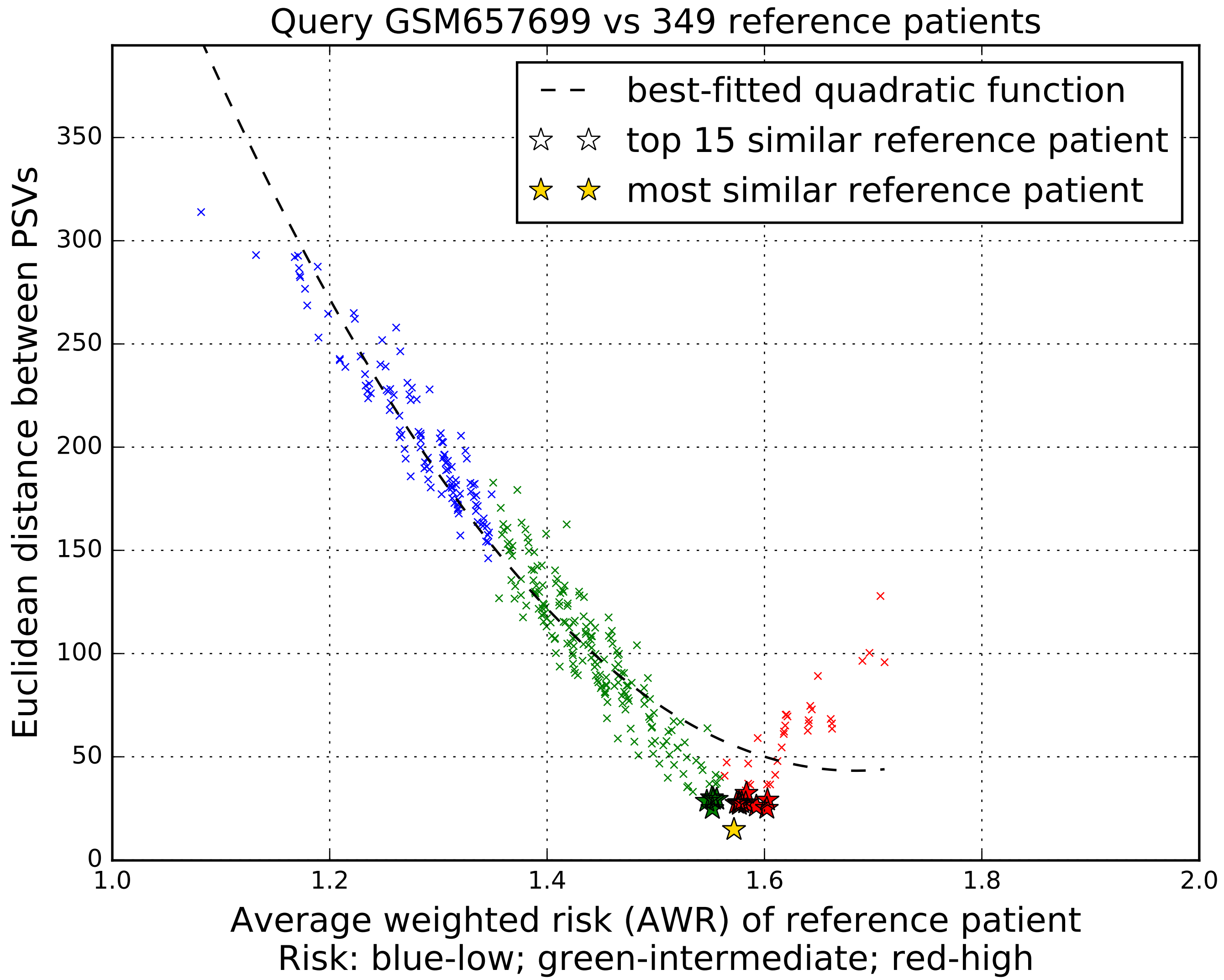
Query GSM657699 vs 349 reference patients
best-fitted quadratic function
top 15 similar reference patient
most similar reference patient
Euclidean distance between PSVs
0
50
100
150
200
250
300
350
1.0
1.2
1.4
1.6
1.8
2.0
Average weighted risk (AWR) of reference patient
Risk: blue-low; green-intermediate; red-high

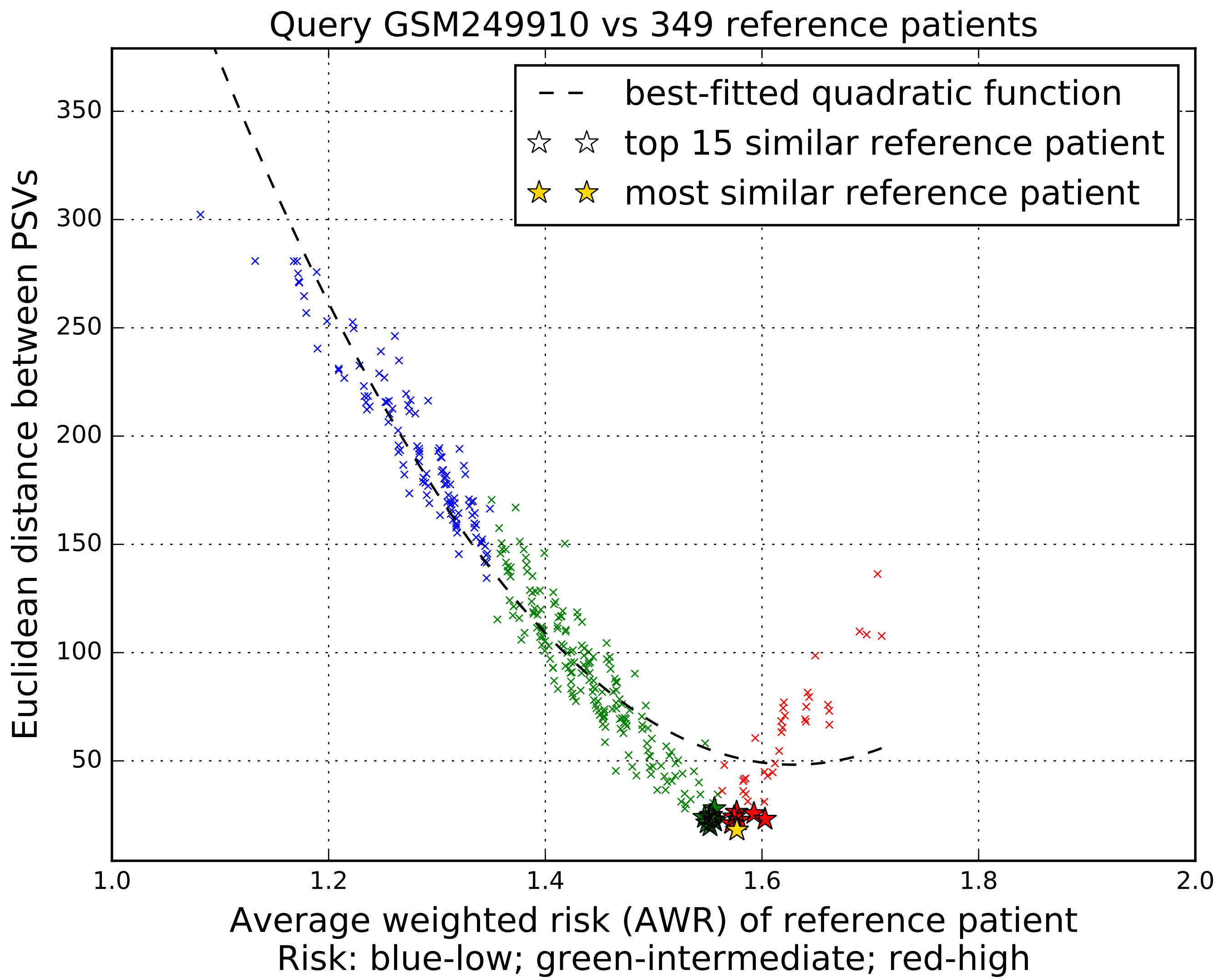

Query GSM249910 vs 349 reference patients
best-fitted quadratic function
top 15 similar reference patient
most similar reference patient
Euclidean distance between PSVs
350
300
250
200
150
100
50
1.0
1.2
1.4
1.6
1.8
2.0
Average weighted risk (AWR) of reference patient
Risk: blue-low; green-intermediate; red-high

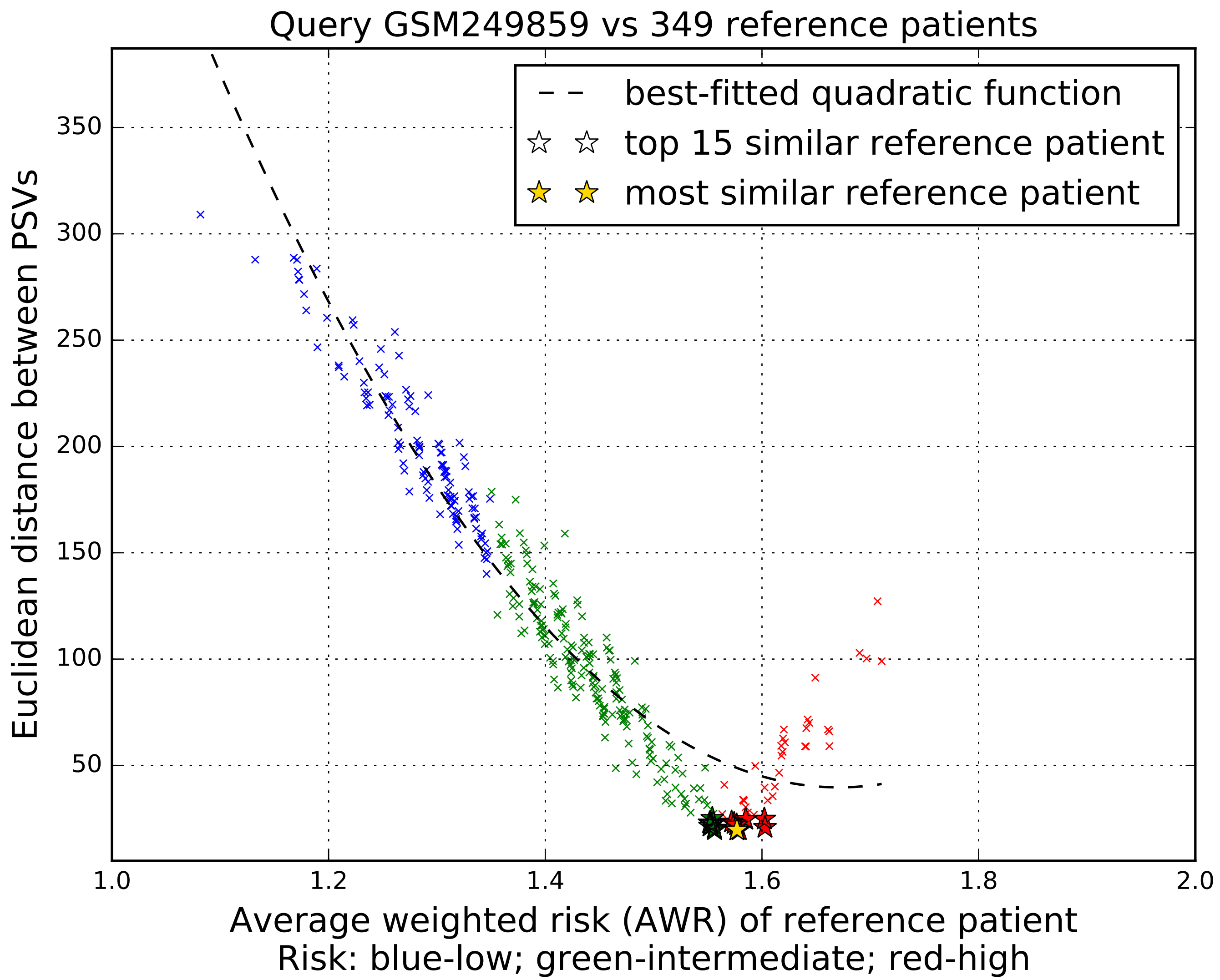

Query GSM249859 vs 349 reference patients
best-fitted quadratic function
top 15 similar reference patient
most similar reference patient
Euclidean distance between PSVs
350
300
250
200
150
100
50
1.0
1.2
1.4
1.6
1.8
2.0
Average weighted risk (AWR) of reference patient
Risk: blue-low; green-intermediate; red-high

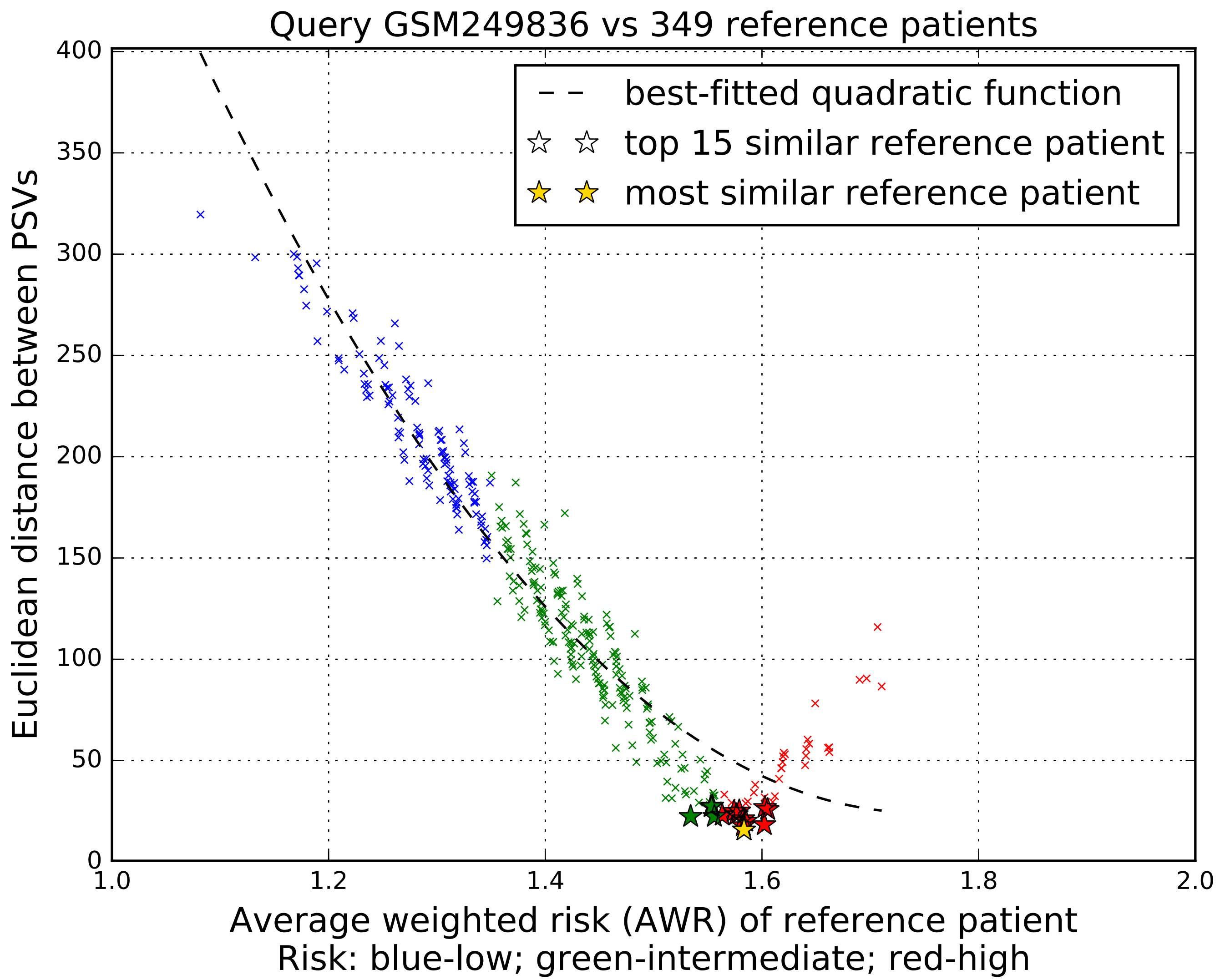
Query GSM249836 vs 349 reference patients
best-fitted quadratic function
top 15 similar reference patient
most similar reference patient
Euclidean distance between PSVs
Average weighted risk (AWR) of reference patient
Risk: blue-low; green-intermediate; red-high
0
50
100
150
200
250
300
350
400
1.0
1.2
1.4
1.6
1.8
2.0

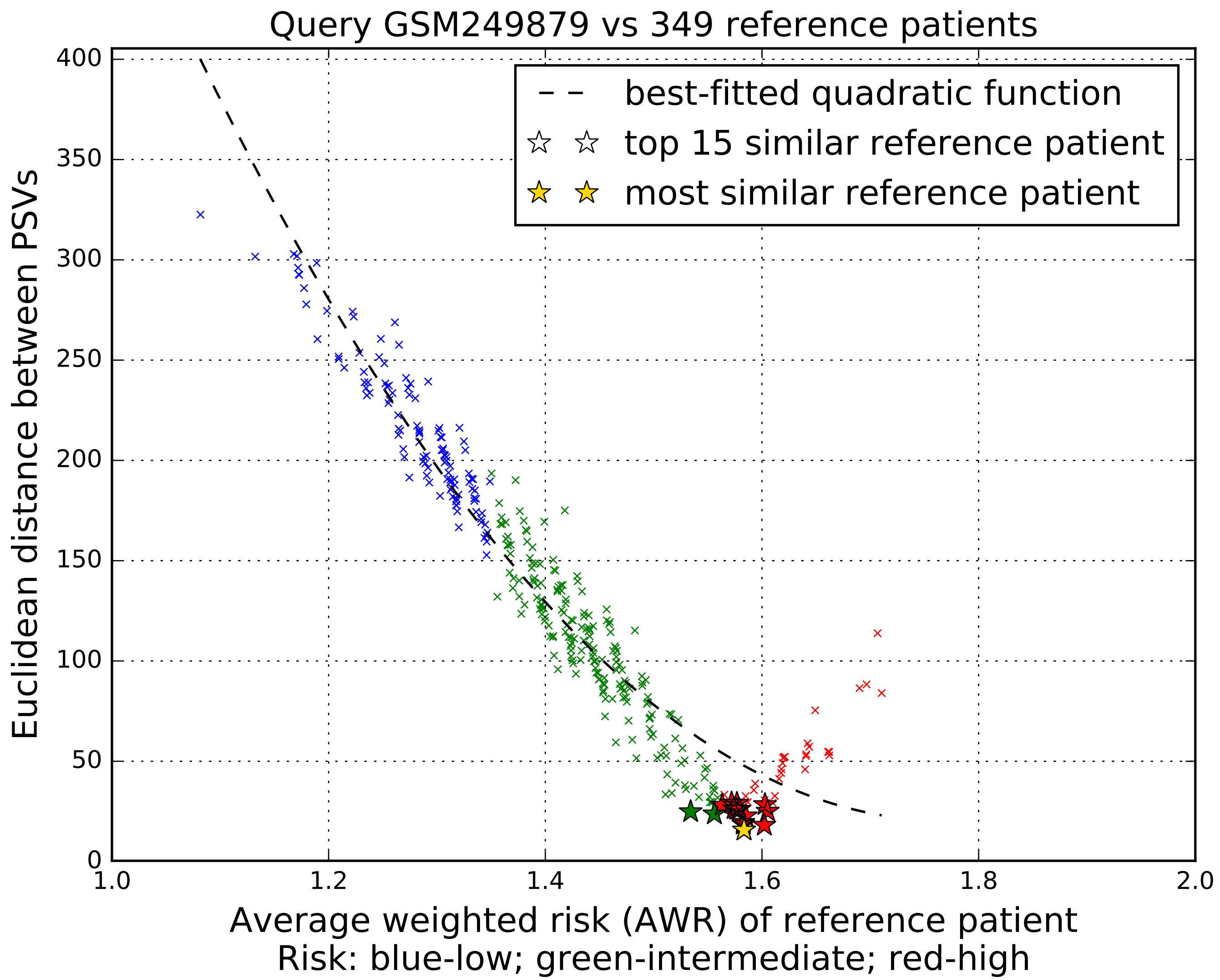
Query GSM249879 vs 349 reference patients
best-fitted quadratic function
top 15 similar reference patient
most similar reference patient
Euclidean distance between PSVs
Average weighted risk (AWR) of reference patient
Risk: blue-low; green-intermediate; red-high
400
350
300
250
200
150
100
50
0
1.0
1.2
1.4
1.6
1.8
2.0

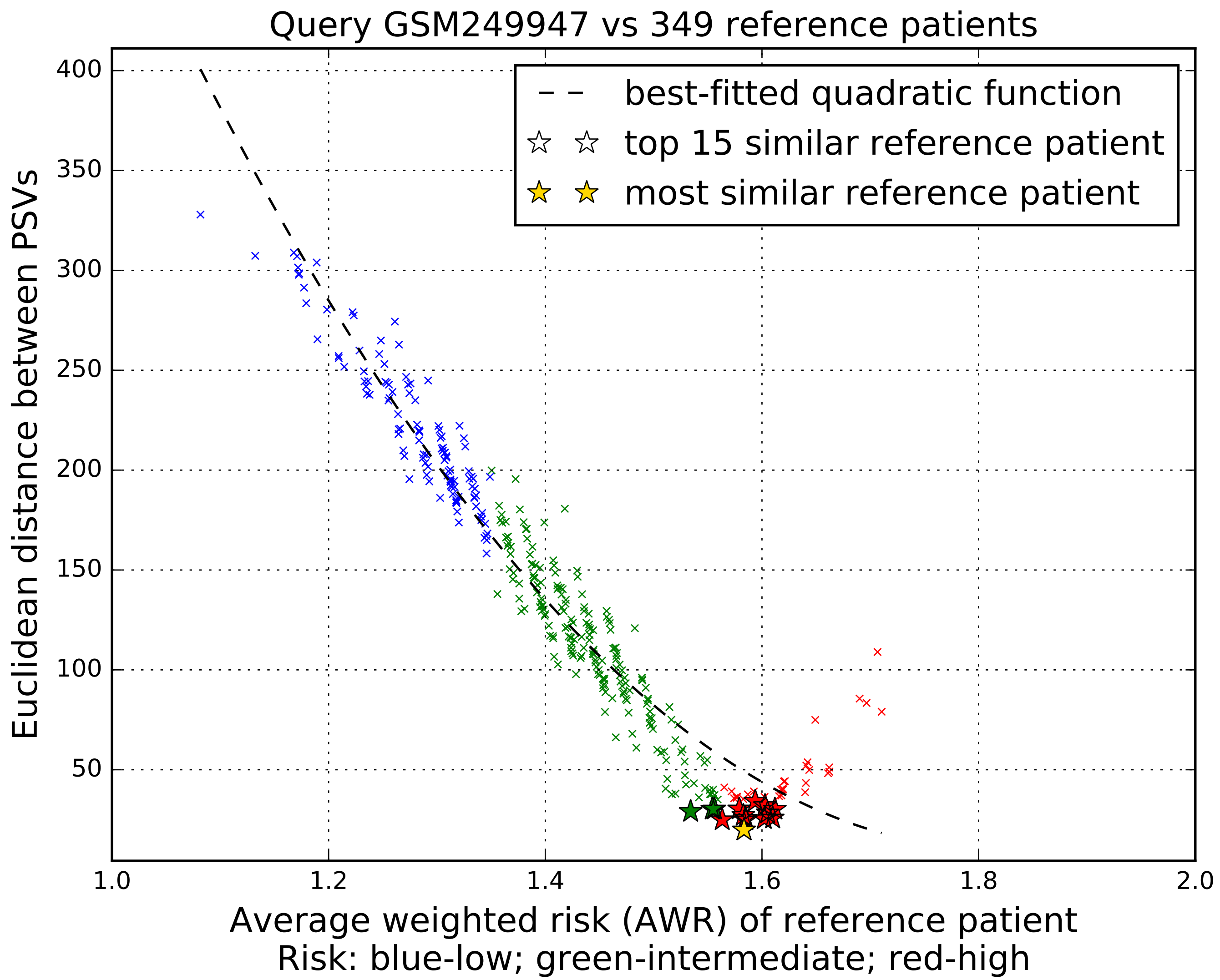

Query GSM249947 vs 349 reference patients
best-fitted quadratic function
top 15 similar reference patient
most similar reference patient
Euclidean distance between PSVs
400
350
300
250
200
150
100
50
1.0
1.2
1.4
1.6
1.8
2.0
Average weighted risk (AWR) of reference patient
Risk: blue-low; green-intermediate; red-high

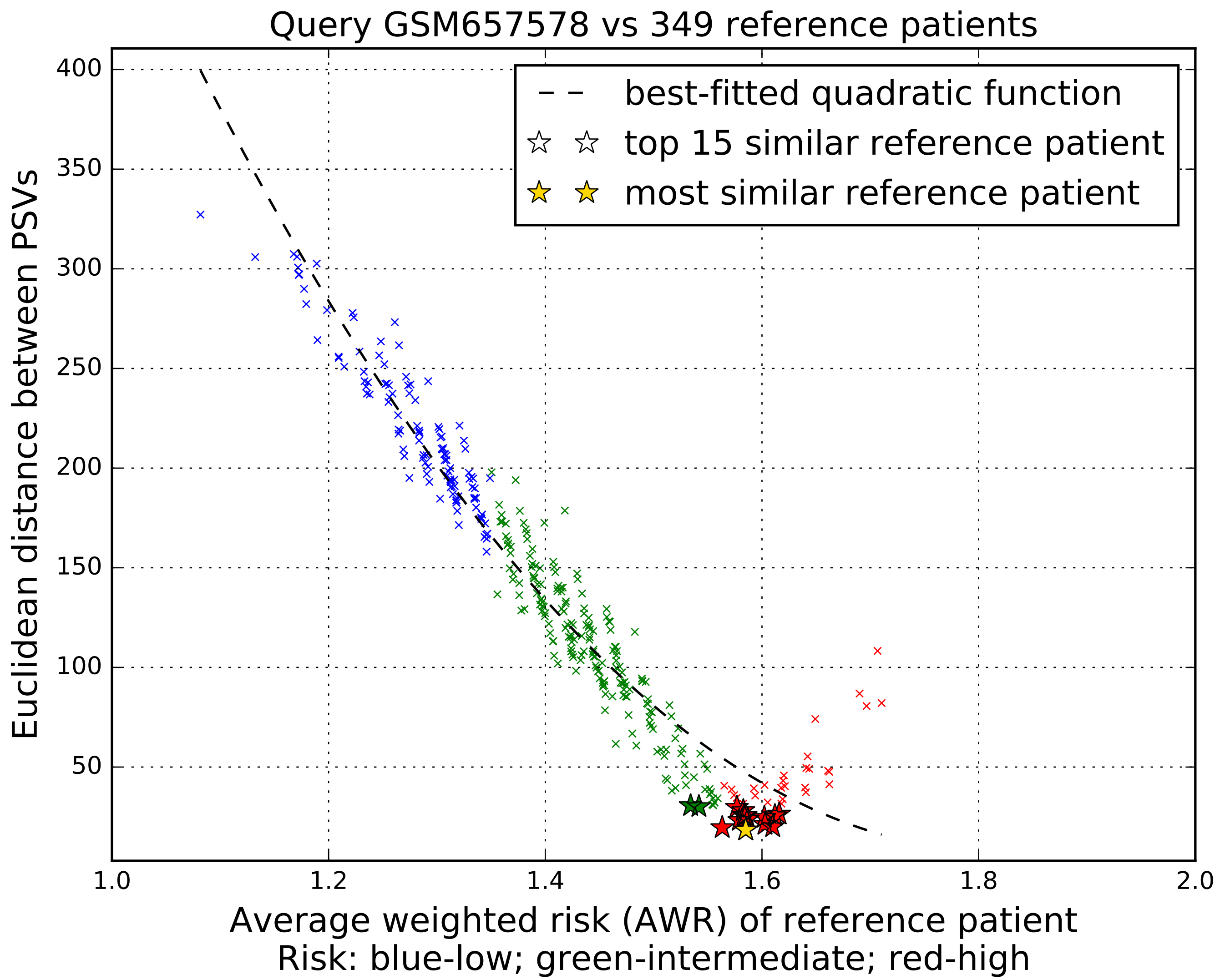
Query GSM657578 vs 349 reference patients
best-fitted quadratic function
top 15 similar reference patient
most similar reference patient
Euclidean distance between PSVs
400
350
300
250
200
150
100
50
1.0
1.2
1.4
1.6
1.8
2.0
Average weighted risk (AWR) of reference patient
Risk: blue-low; green-intermediate; red-high

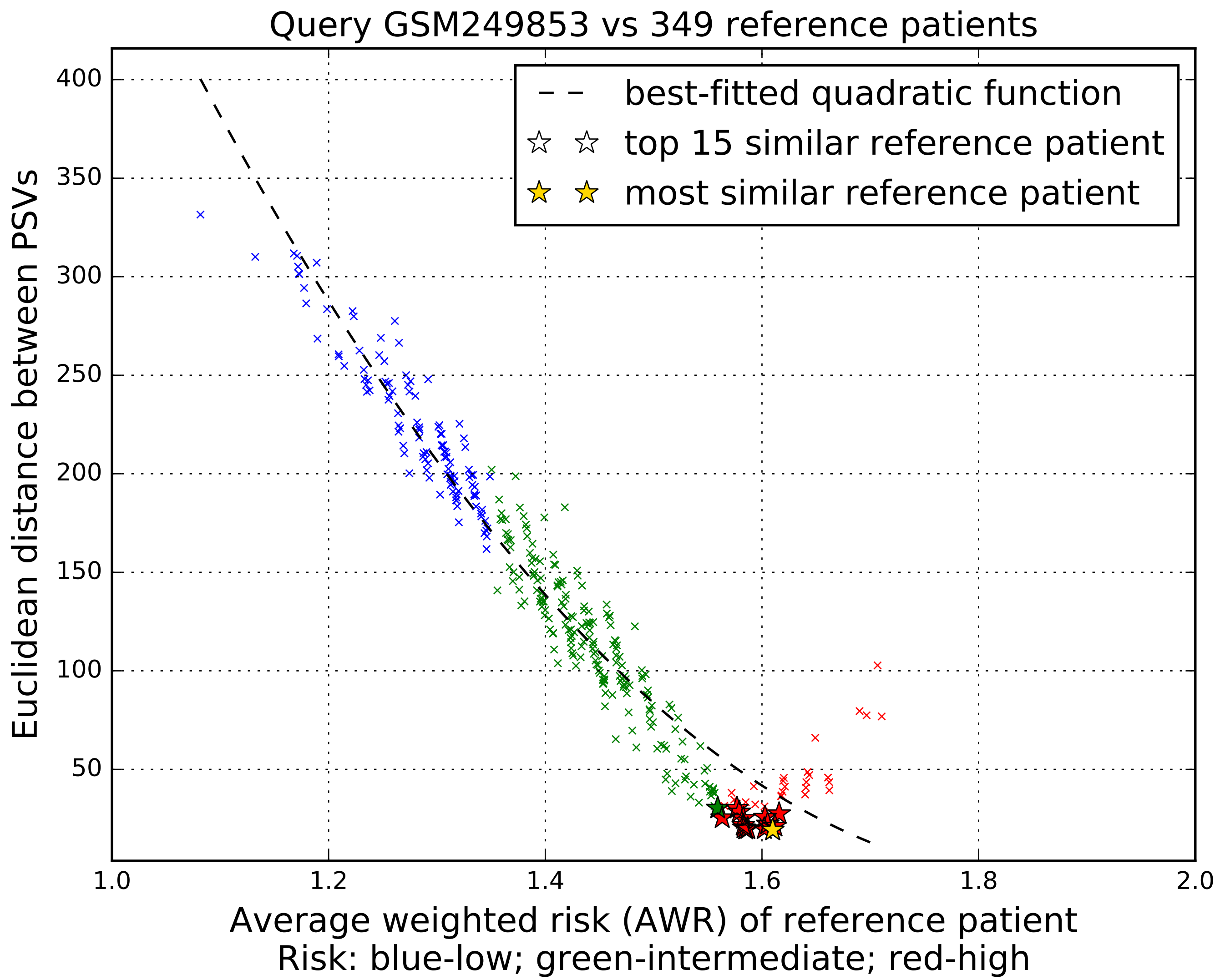

Query GSM249853 vs 349 reference patients
best-fitted quadratic function
top 15 similar reference patient
most similar reference patient
Euclidean distance between PSVs
400
350
300
250
200
150
100
50
1.0
1.2
1.4
1.6
1.8
2.0
Average weighted risk (AWR) of reference patient
Risk: blue-low; green-intermediate; red-high

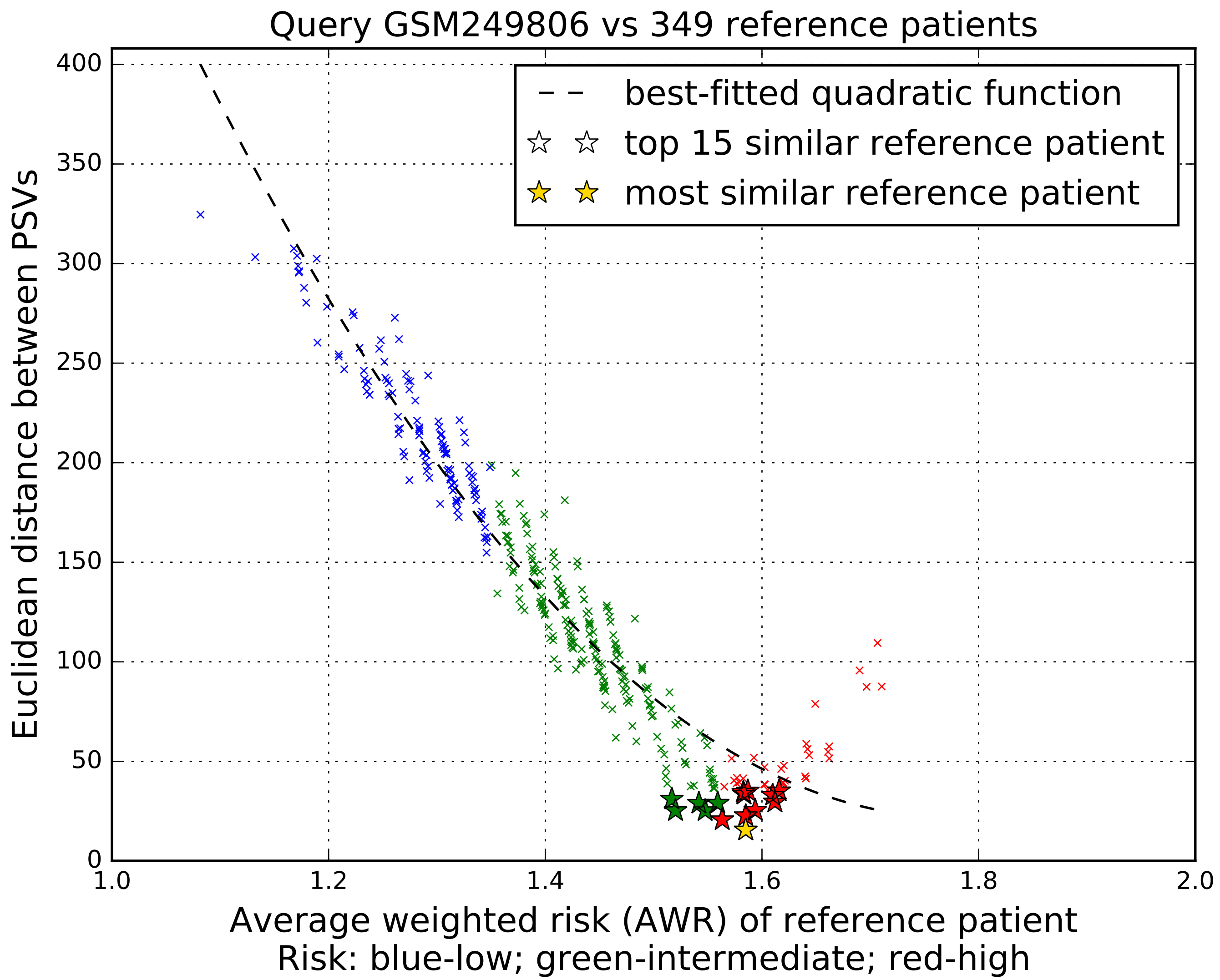
Query GSM249806 vs 349 reference patients
best-fitted quadratic function
top 15 similar reference patient
most similar reference patient
Euclidean distance between PSVs
Average weighted risk (AWR) of reference patient
Risk: blue-low; green-intermediate; red-high
0
50
100
150
200
250
300
350
400
1.0
1.2
1.4
1.6
1.8
2.0

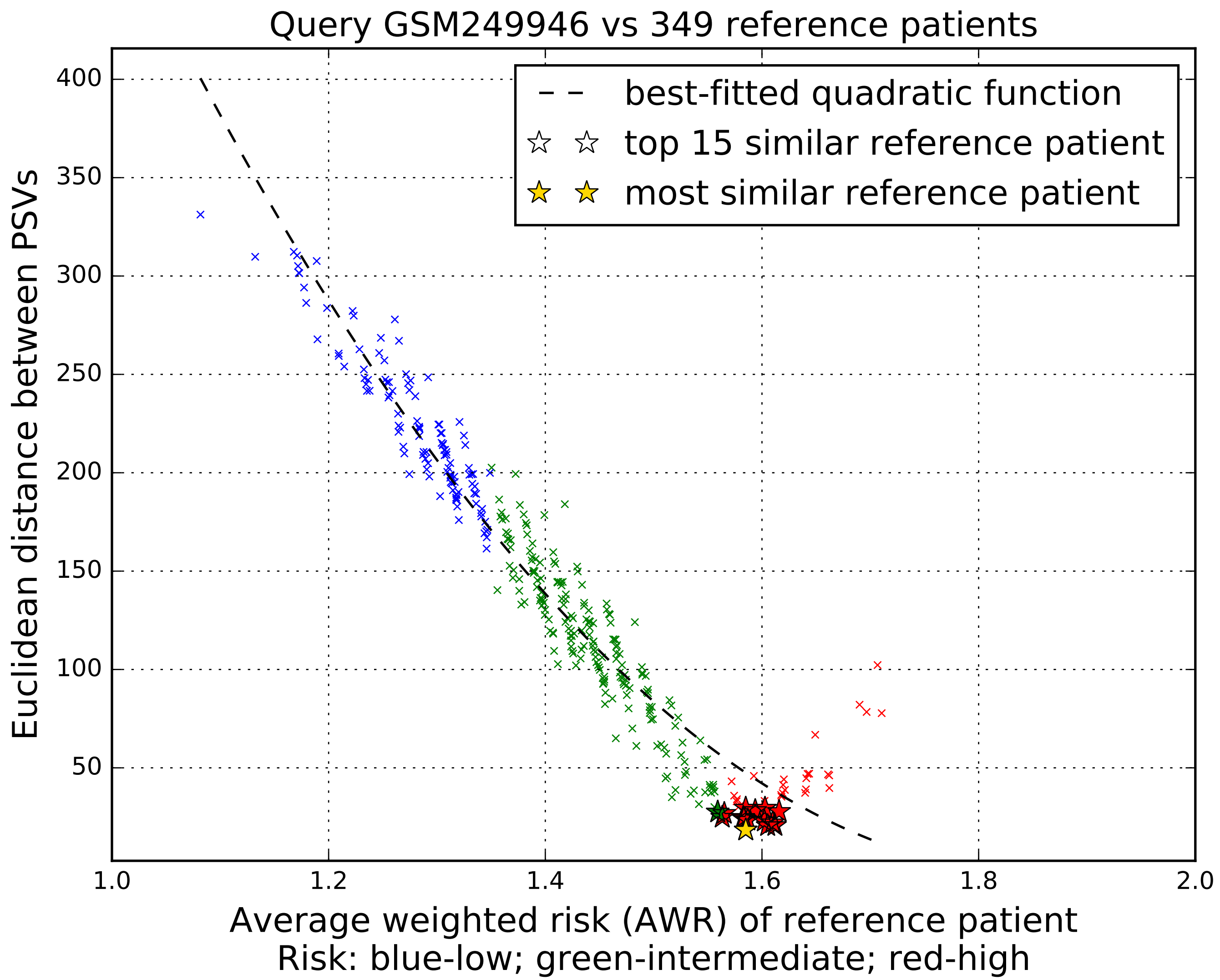

Query GSM249946 vs 349 reference patients
best-fitted quadratic function
top 15 similar reference patient
most similar reference patient
Euclidean distance between PSVs
400
350
300
250
200
150
100
50
1.0
1.2
1.4
1.6
1.8
2.0
Average weighted risk (AWR) of reference patient
Risk: blue-low; green-intermediate; red-high

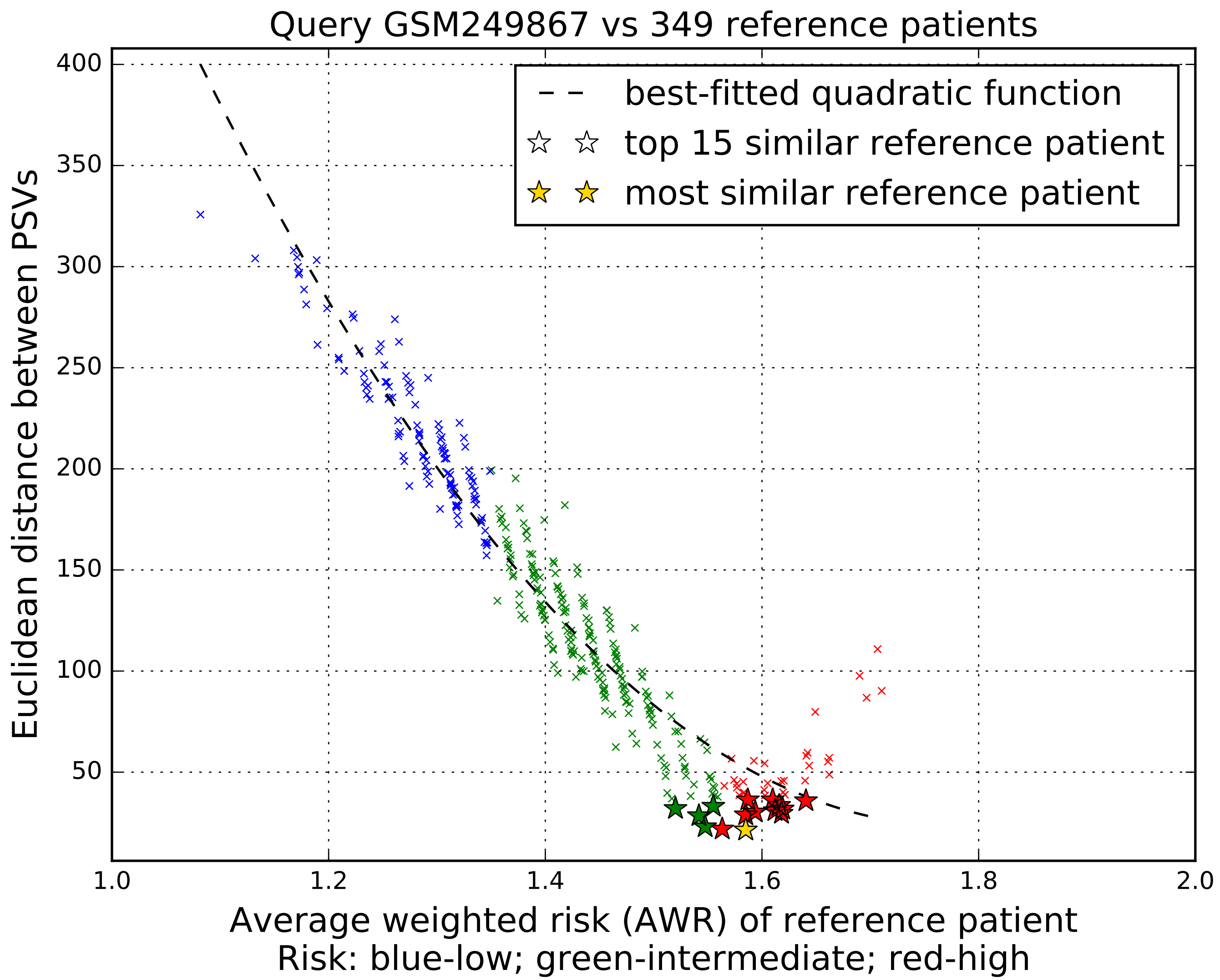

Query GSM249867 vs 349 reference patients
best-fitted quadratic function
top 15 similar reference patient
most similar reference patient
Euclidean distance between PSVs
Average weighted risk (AWR) of reference patient
Risk: blue-low; green-intermediate; red-high
400
350
300
250
200
150
100
50
1.0
1.2
1.4
1.6
1.8
2.0

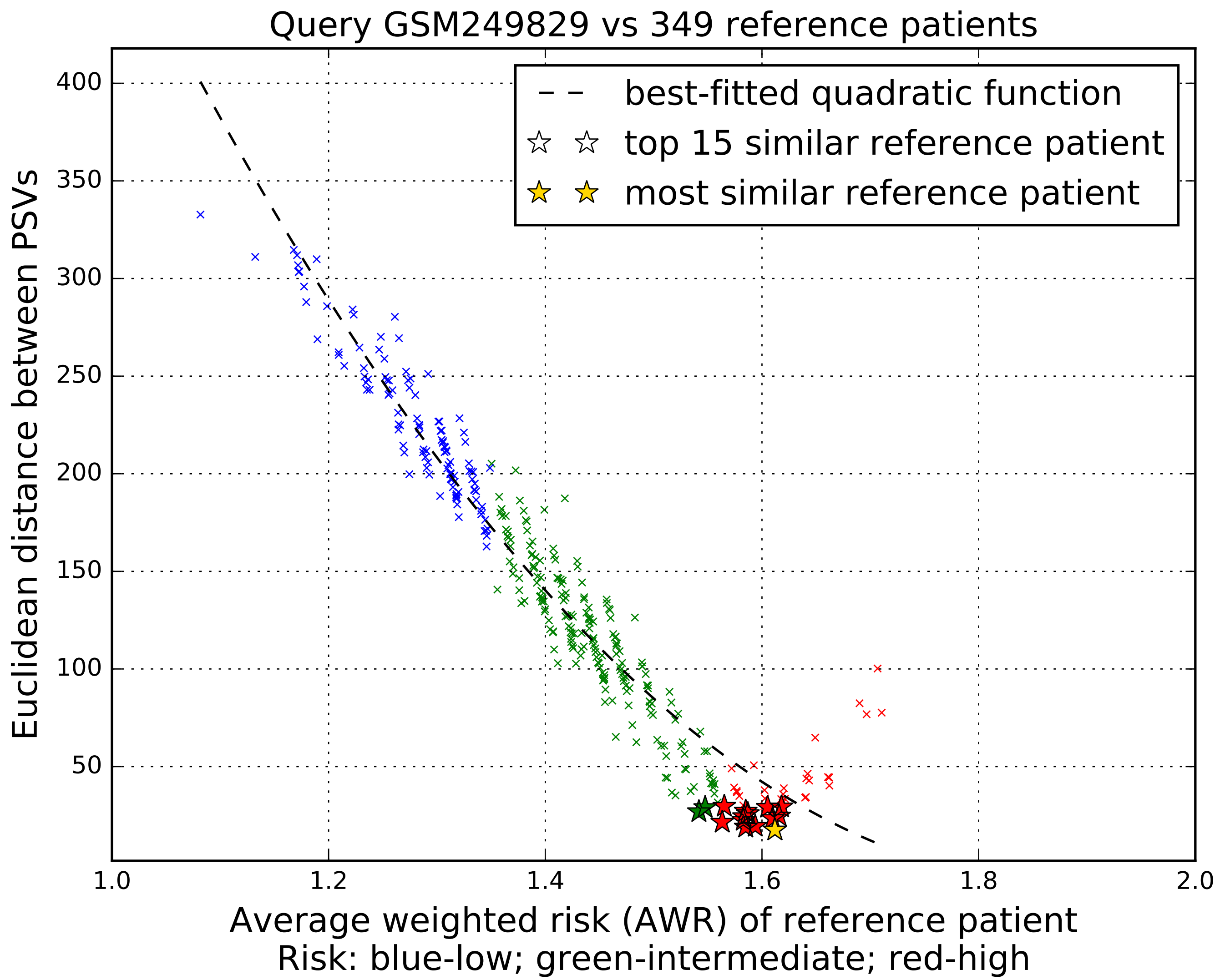

Query GSM249829 vs 349 reference patients
best-fitted quadratic function
top 15 similar reference patient
most similar reference patient
Euclidean distance between PSVs
400
350
300
250
200
150
100
50
1.0
1.2
1.4
1.6
1.8
2.0
Average weighted risk (AWR) of reference patient
Risk: blue-low; green-intermediate; red-high

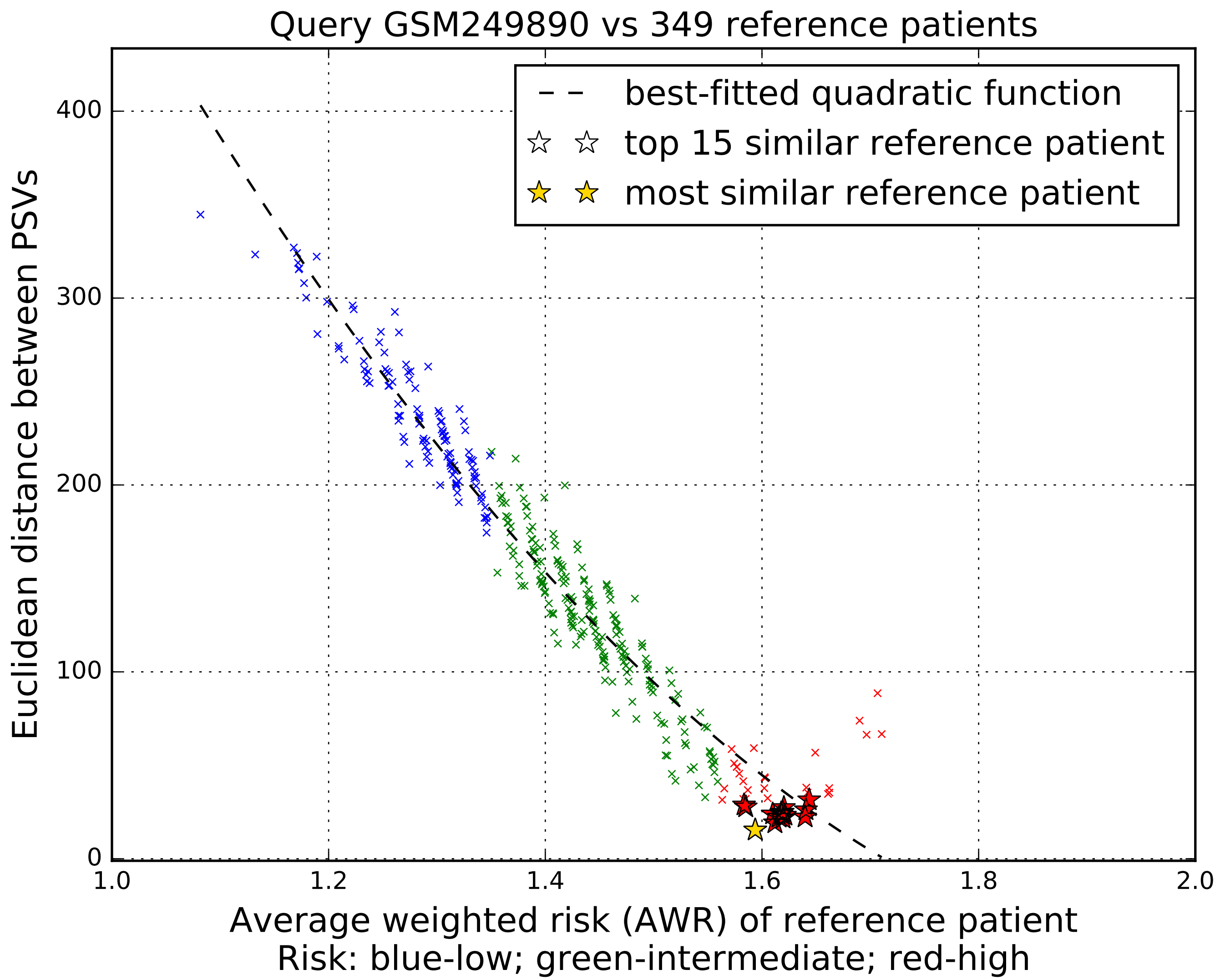
Query GSM249890 vs 349 reference patients
best-fitted quadratic function
top 15 similar reference patient
most similar reference patient
Euclidean distance between PSVs
0
100
200
300
400
1.0
1.2
1.4
1.6
1.8
2.0
Average weighted risk (AWR) of reference patient
Risk: blue-low; green-intermediate; red-high

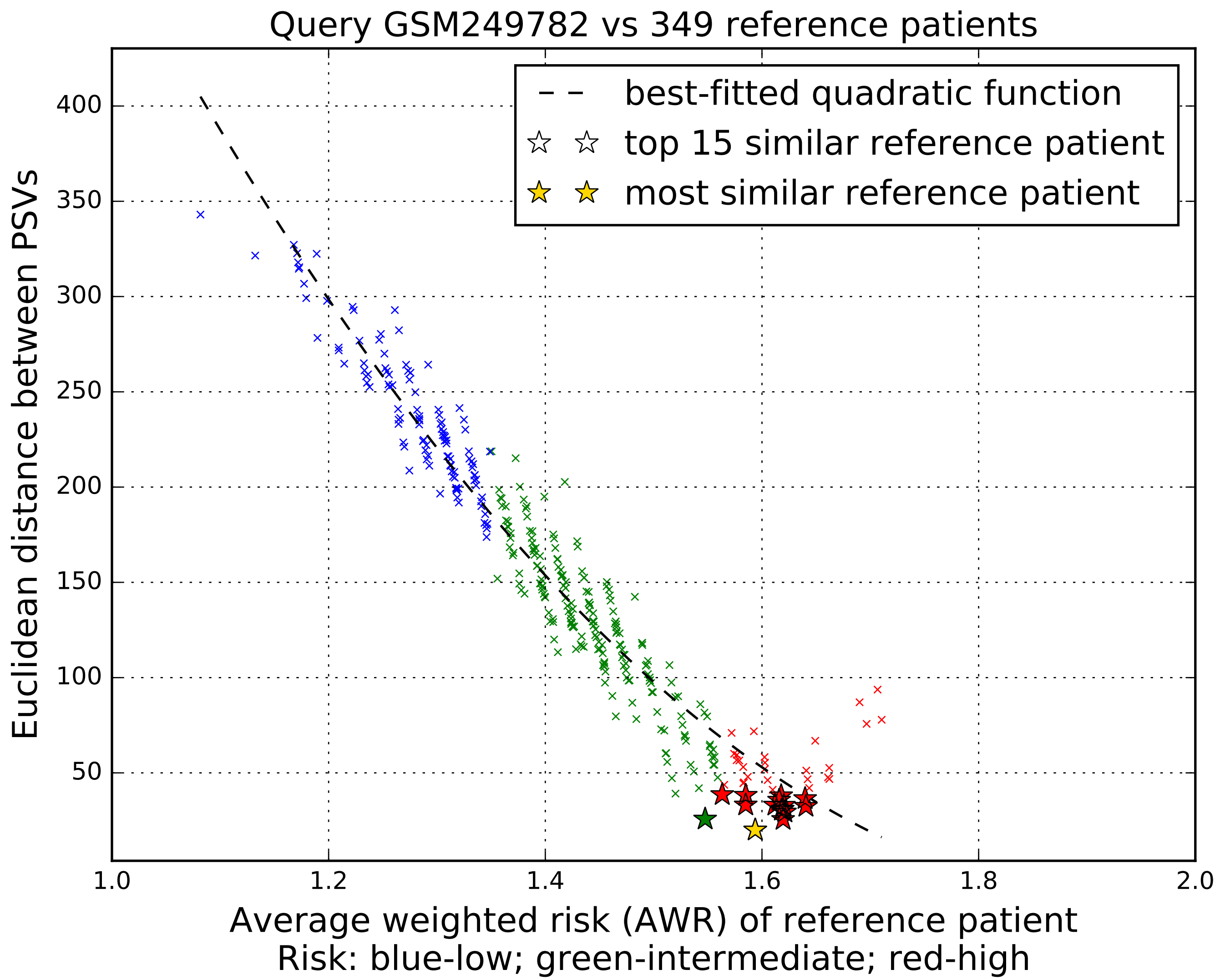
Query GSM249782 vs 349 reference patients
best-fitted quadratic function
top 15 similar reference patient
most similar reference patient
Euclidean distance between PSVs
400
350
300
250
200
150
100
50
1.0
1.2
1.4
1.6
1.8
2.0
Average weighted risk (AWR) of reference patient
Risk: blue-low; green-intermediate; red-high

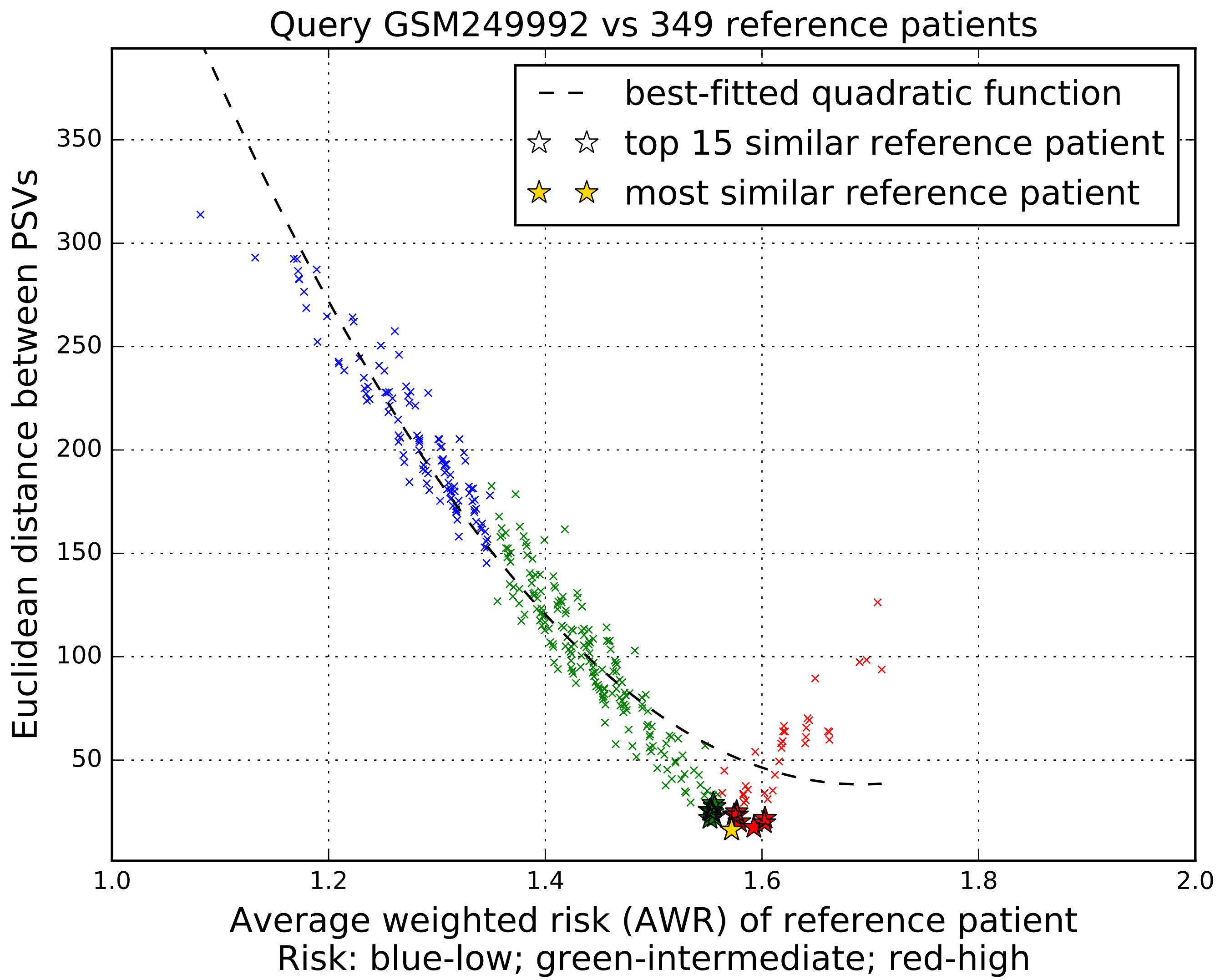

Query GSM249992 vs 349 reference patients
best-fitted quadratic function
top 15 similar reference patient
most similar reference patient
Euclidean distance between PSVs
350
300
250
200
150
100
50
1.0
1.2
1.4
1.6
1.8
2.0
Average weighted risk (AWR) of reference patient
Risk: blue-low; green-intermediate; red-high

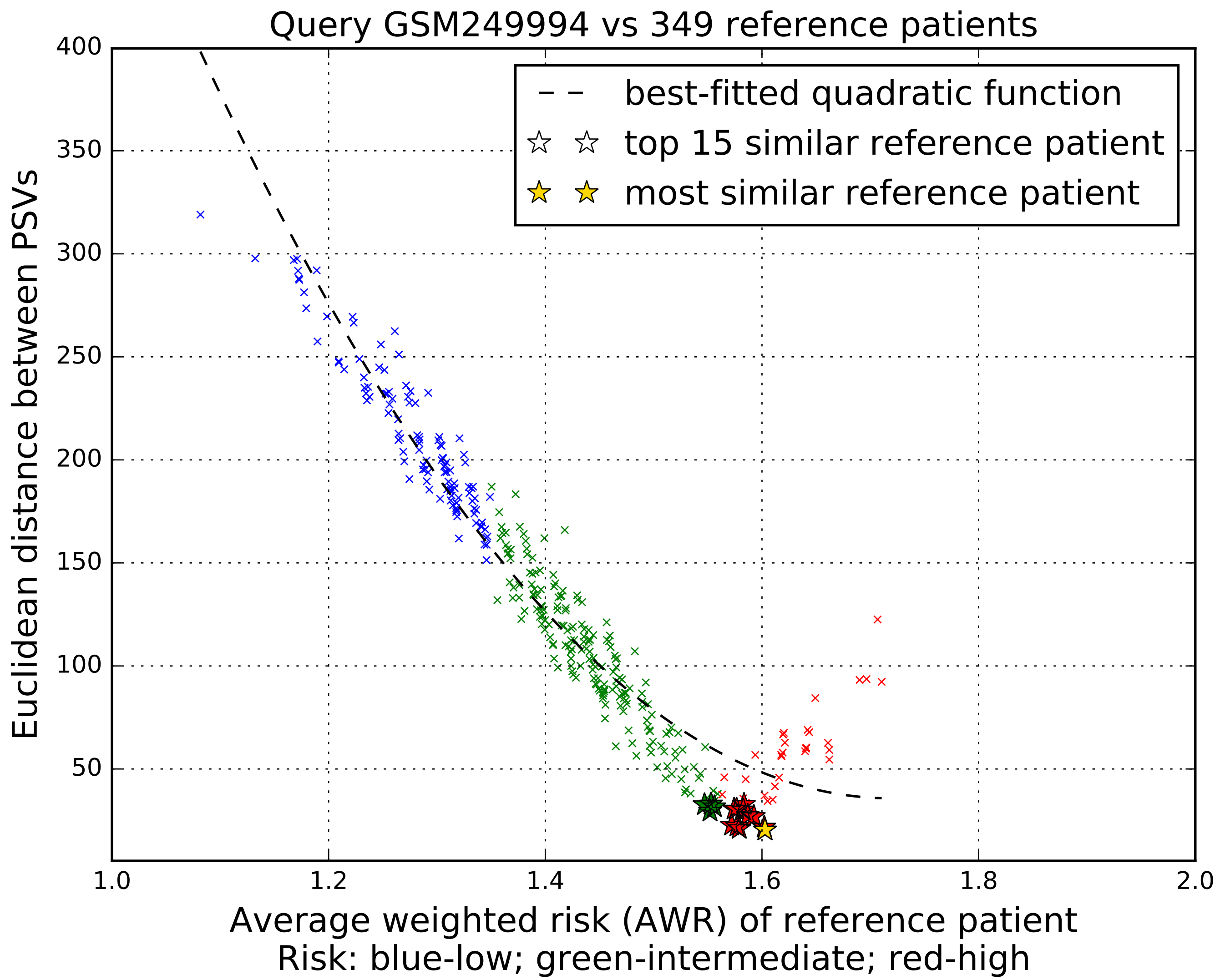
Query GSM249994 vs 349 reference patients
best-fitted quadratic function
top 15 similar reference patient
most similar reference patient
Euclidean distance between PSVs
400
350
300
250
200
150
100
50
1.0
1.2
1.4
1.6
1.8
2.0
Average weighted risk (AWR) of reference patient
Risk: blue-low; green-intermediate; red-high

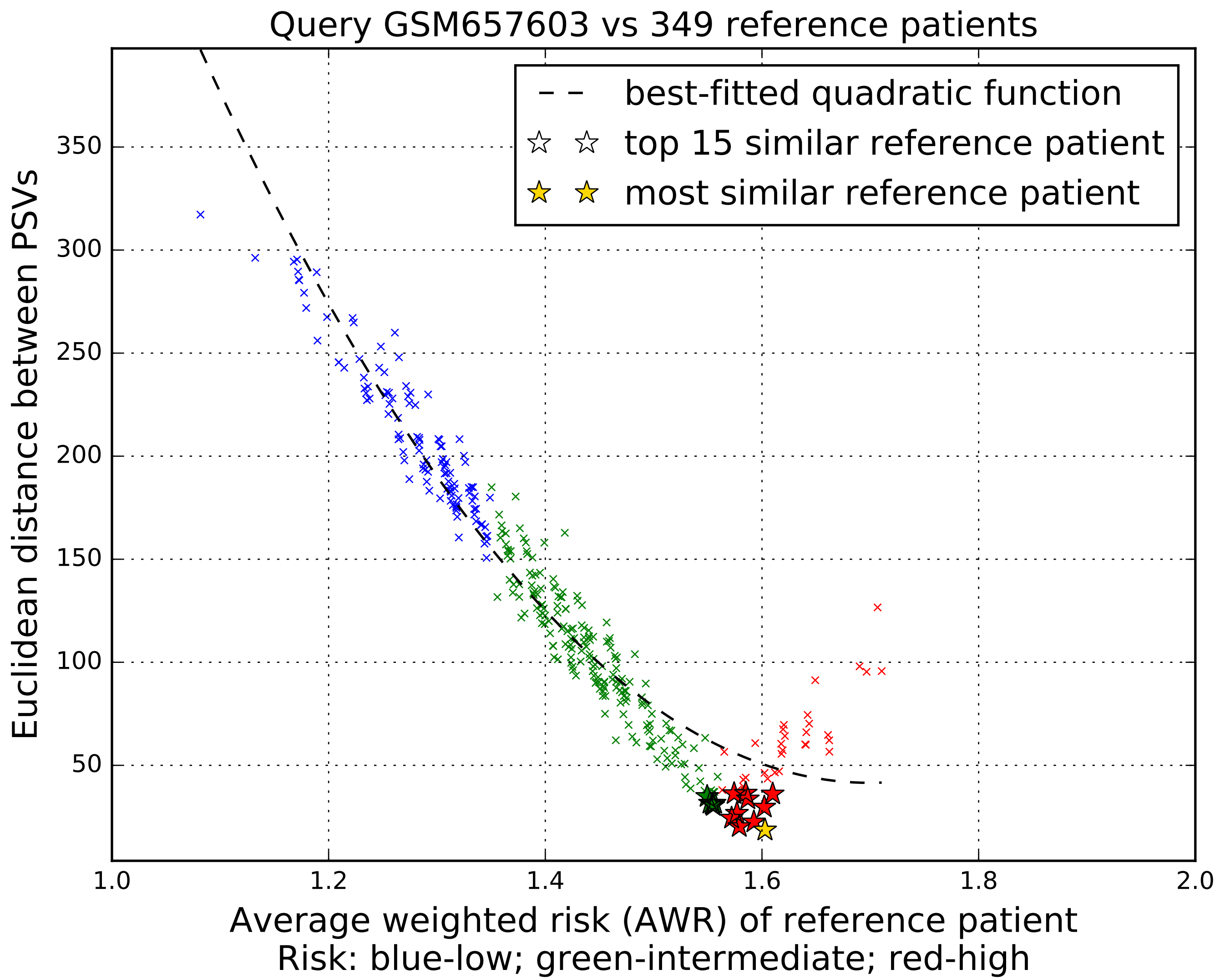

Query GSM657603 vs 349 reference patients
best-fitted quadratic function
top 15 similar reference patient
most similar reference patient
Euclidean distance between PSVs
50
100
150
200
250
300
350
1.0
1.2
1.4
1.6
1.8
2.0
Average weighted risk (AWR) of reference patient
Risk: blue-low; green-intermediate; red-high

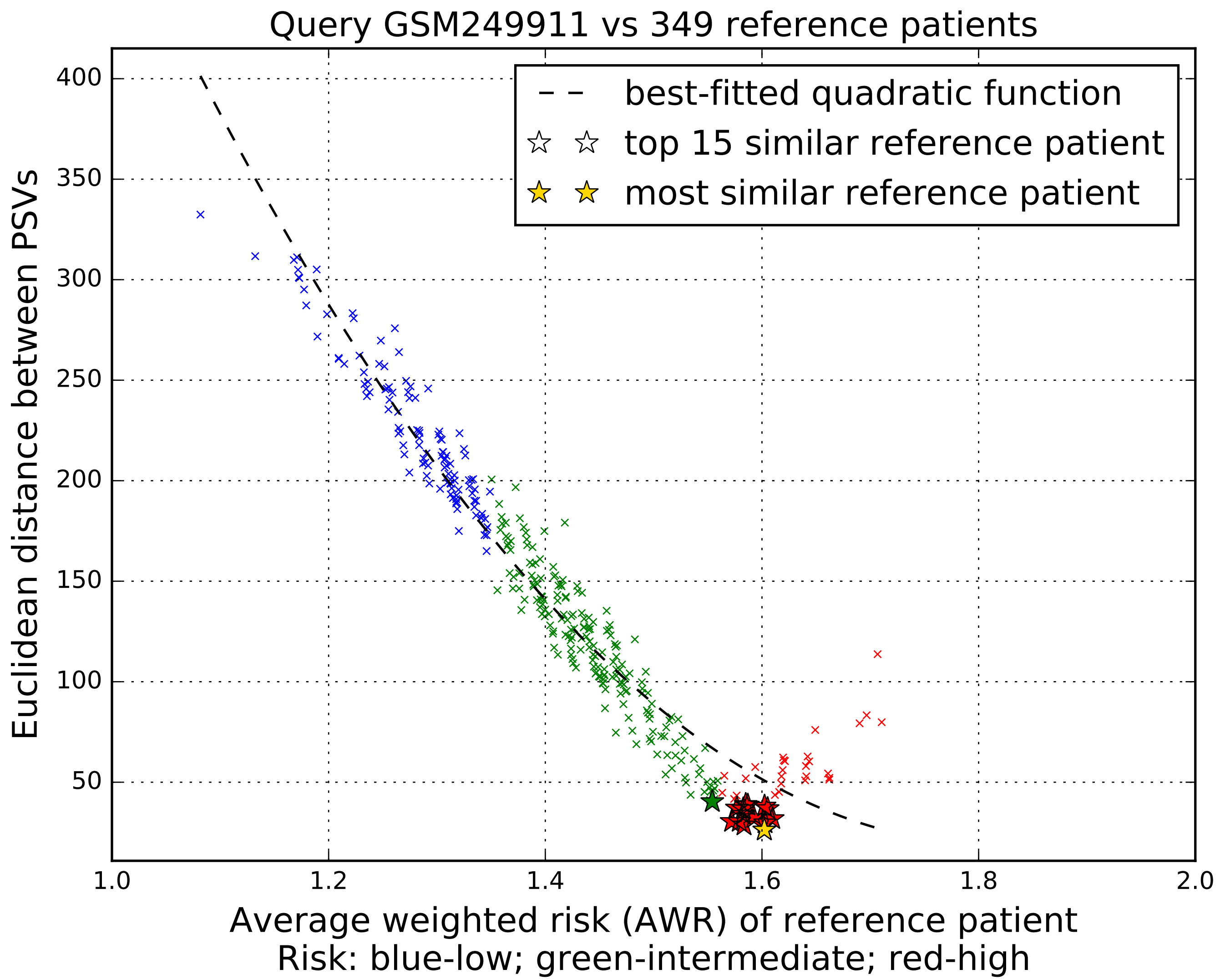

Query GSM249911 vs 349 reference patients
best-fitted quadratic function
top 15 similar reference patient
most similar reference patient
Euclidean distance between PSVs
400
350
300
250
200
150
100
50
1.0
1.2
1.4
1.6
1.8
2.0
Average weighted risk (AWR) of reference patient
Risk: blue-low; green-intermediate; red-high

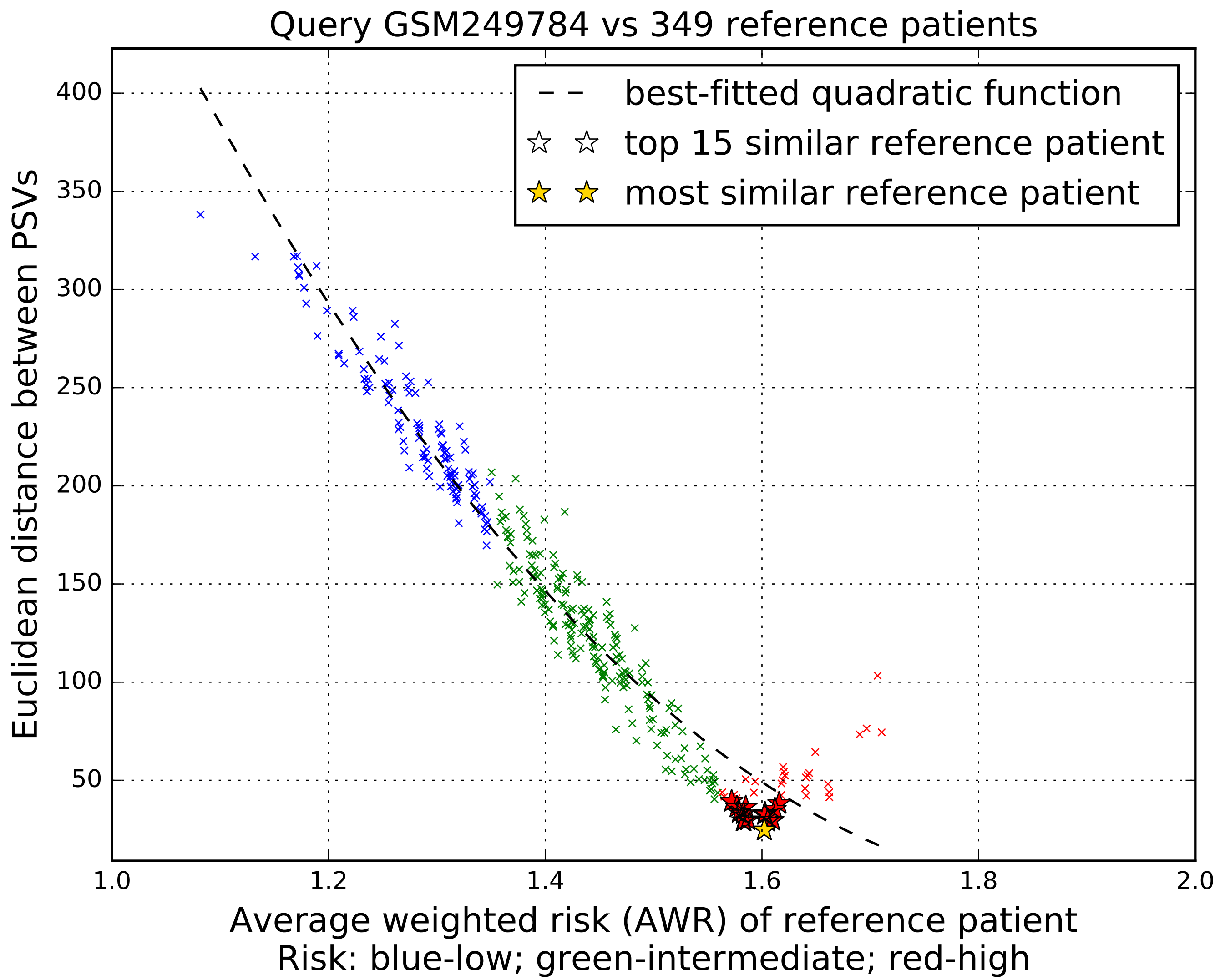
Query GSM249784 vs 349 reference patients
best-fitted quadratic function
top 15 similar reference patient
most similar reference patient
Euclidean distance between PSVs
400
350
300
250
200
150
100
50
1.0
1.2
1.4
1.6
1.8
2.0
Average weighted risk (AWR) of reference patient
Risk: blue-low; green-intermediate; red-high

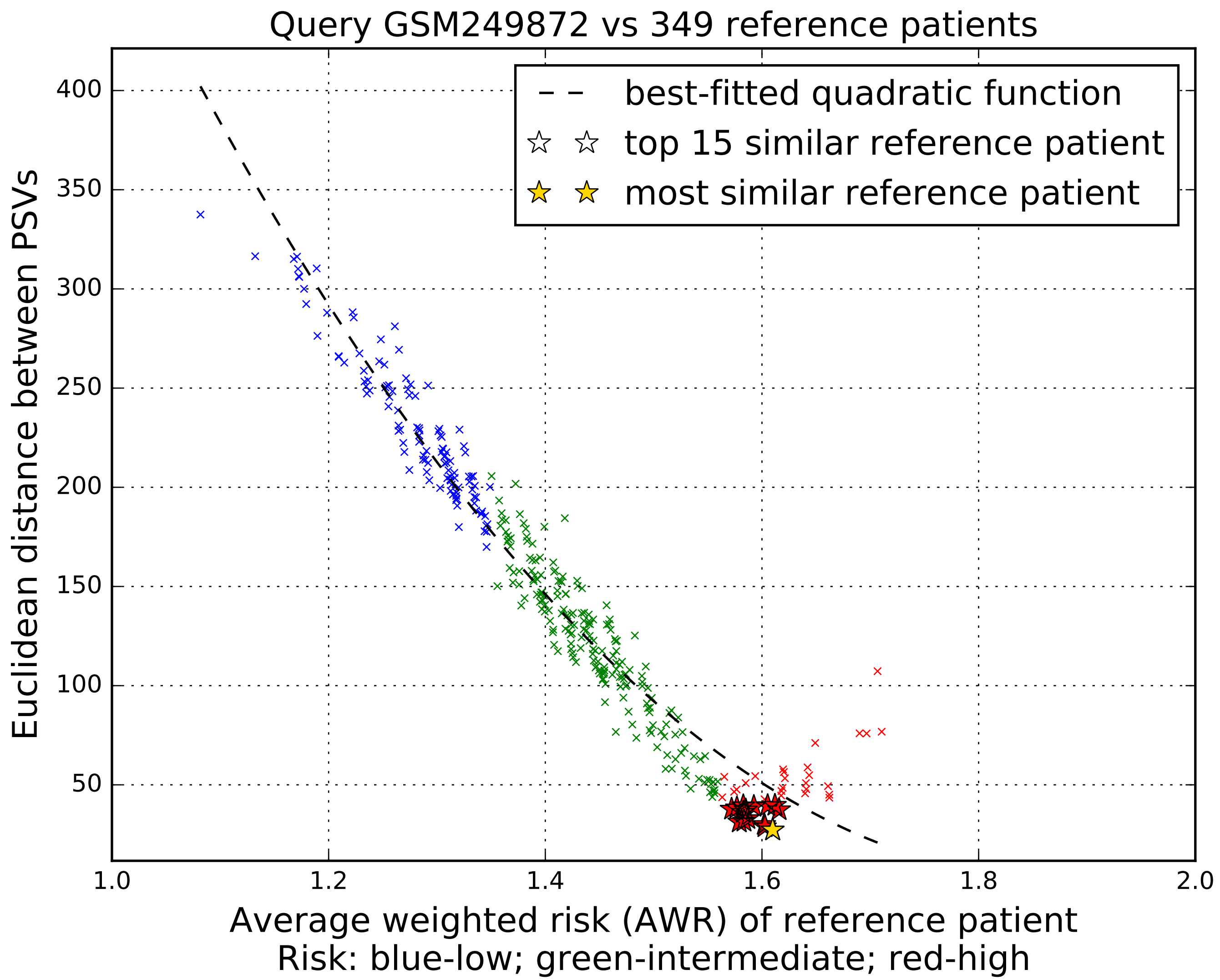

Query GSM249872 vs 349 reference patients
best-fitted quadratic function
top 15 similar reference patient
most similar reference patient
Euclidean distance between PSVs
400
350
300
250
200
150
100
50
1.0
1.2
1.4
1.6
1.8
2.0
Average weighted risk (AWR) of reference patient
Risk: blue-low; green-intermediate; red-high

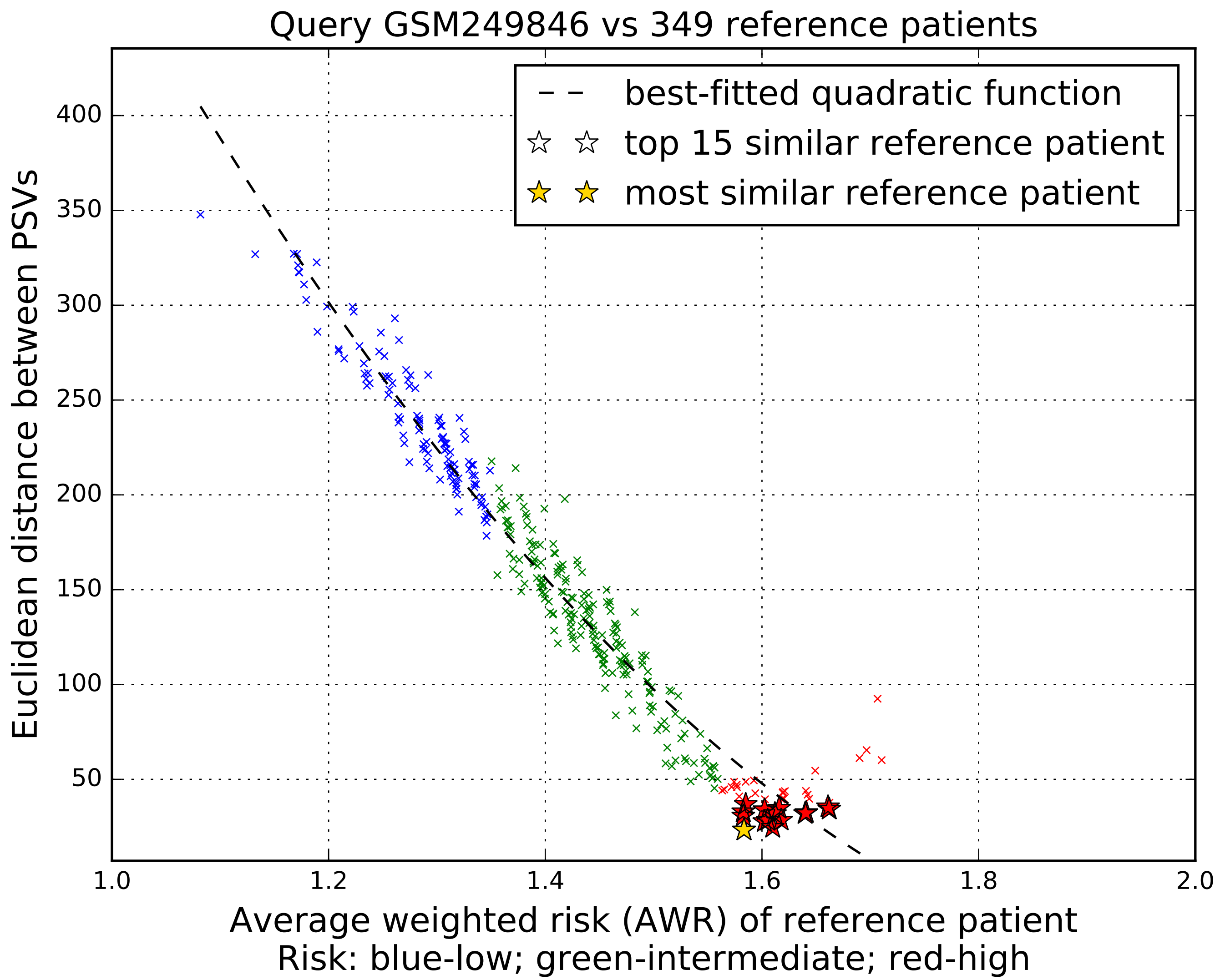
Query GSM249846 vs 349 reference patients
best-fitted quadratic function
top 15 similar reference patient
most similar reference patient
Euclidean distance between PSVs
400
350
300
250
200
150
100
50
1.0
1.2
1.4
1.6
1.8
2.0
Average weighted risk (AWR) of reference patient
Risk: blue-low; green-intermediate; red-high

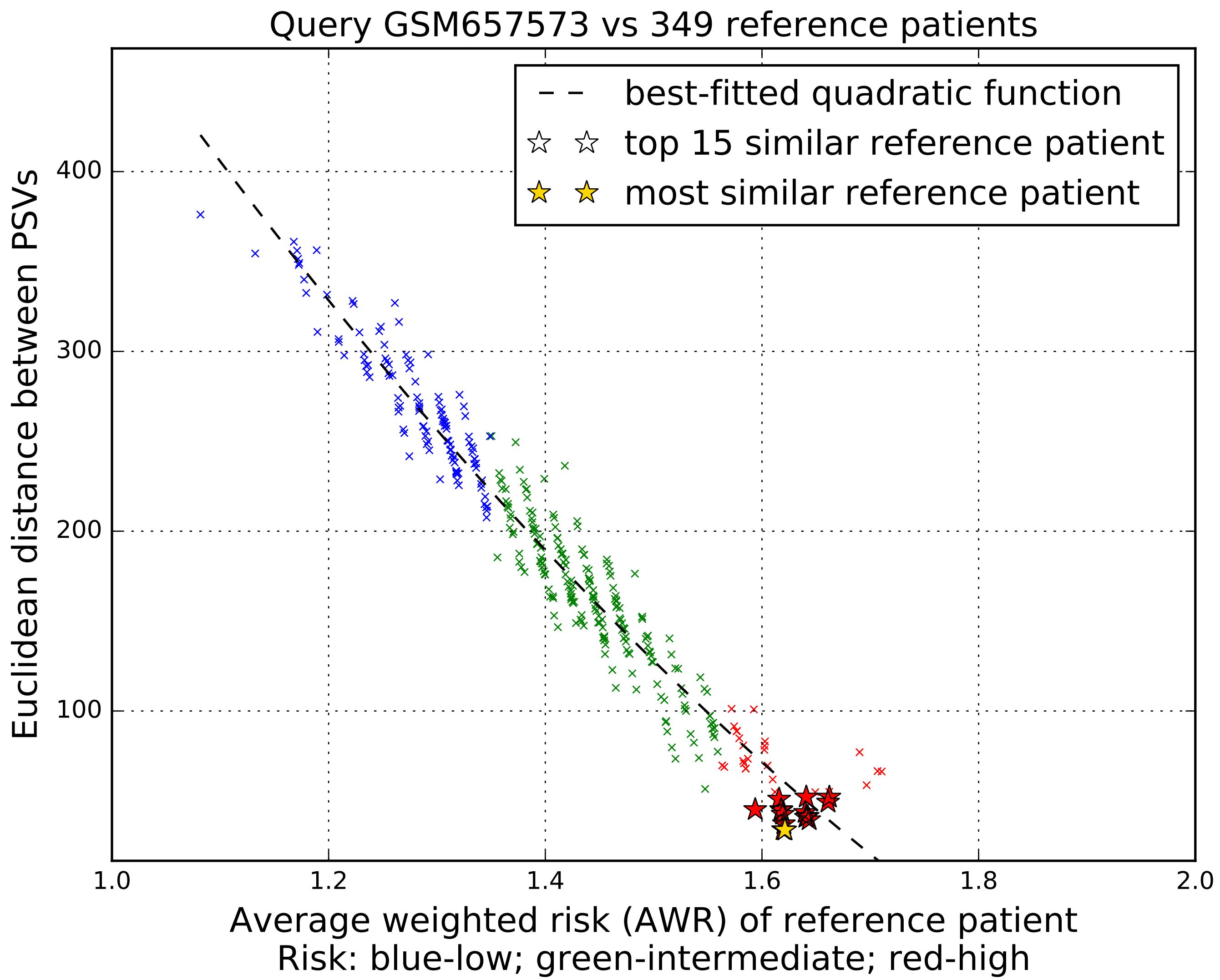
Query GSM657573 vs 349 reference patients
best-fitted quadratic function
top 15 similar reference patient
most similar reference patient
Euclidean distance between PSVs
400
300
200
100
1.0
1.2
1.4
1.6
1.8
2.0
Average weighted risk (AWR) of reference patient
Risk: blue-low; green-intermediate; red-high

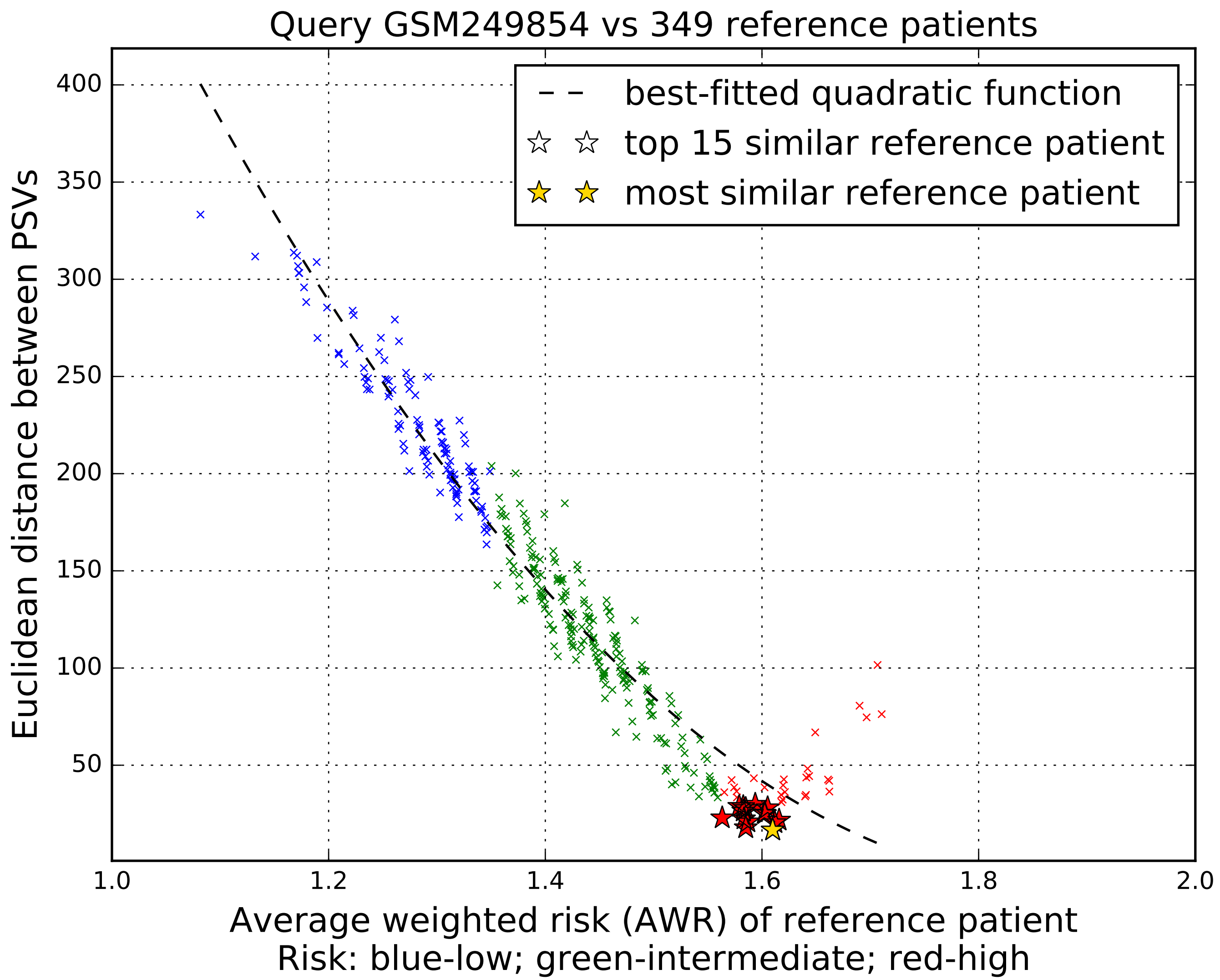
Query GSM249854 vs 349 reference patients
best-fitted quadratic function
top 15 similar reference patient
most similar reference patient
Euclidean distance between PSVs
400
350
300
250
200
150
100
50
1.0
1.2
1.4
1.6
1.8
2.0
Average weighted risk (AWR) of reference patient
Risk: blue-low; green-intermediate; red-high

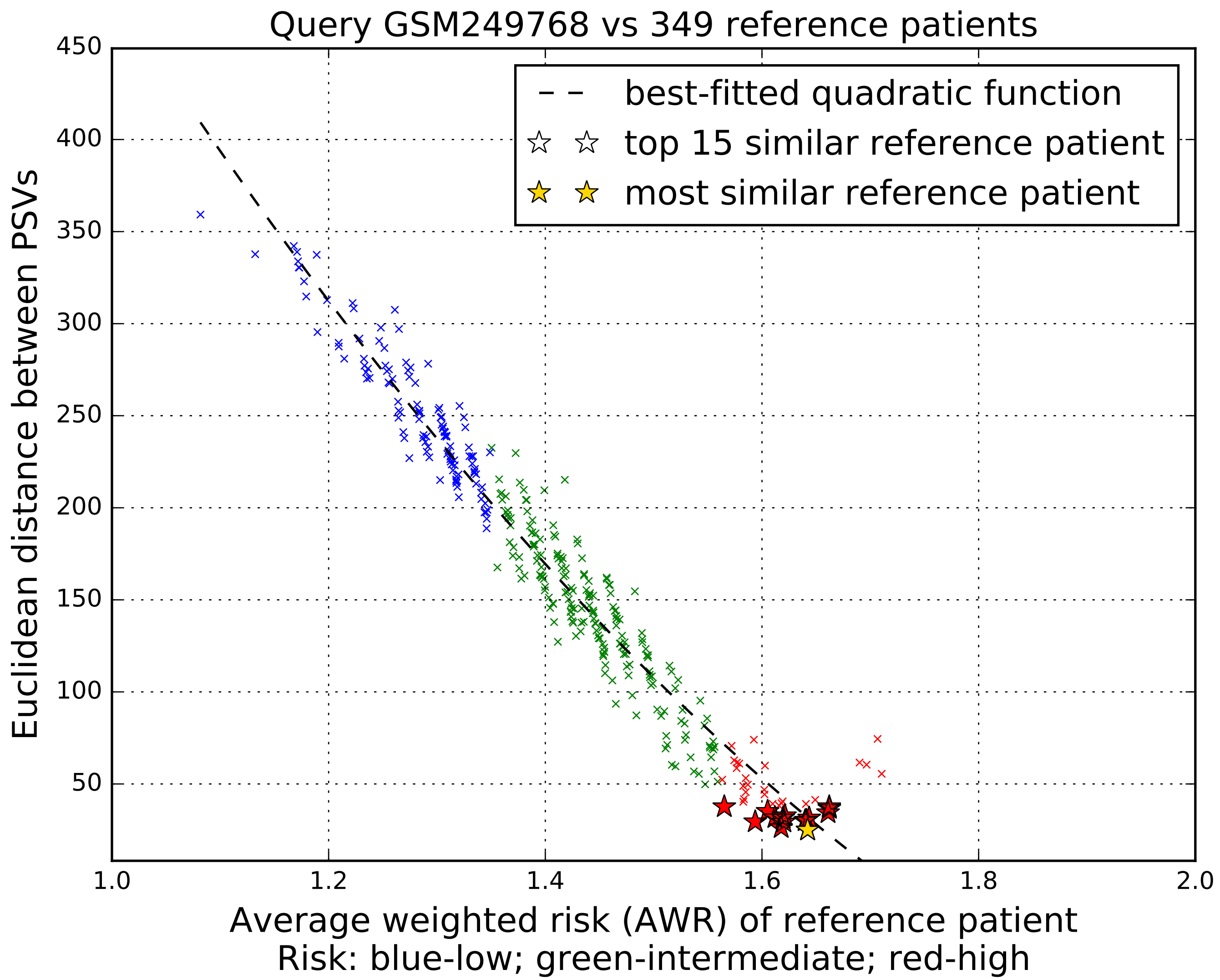
Query GSM249768 vs 349 reference patients
best-fitted quadratic function
top 15 similar reference patient
most similar reference patient
Euclidean distance between PSVs
450
400
350
300
250
200
150
100
50
1.0
1.2
1.4
1.6
1.8
2.0
Average weighted risk (AWR) of reference patient
Risk: blue-low; green-intermediate; red-high

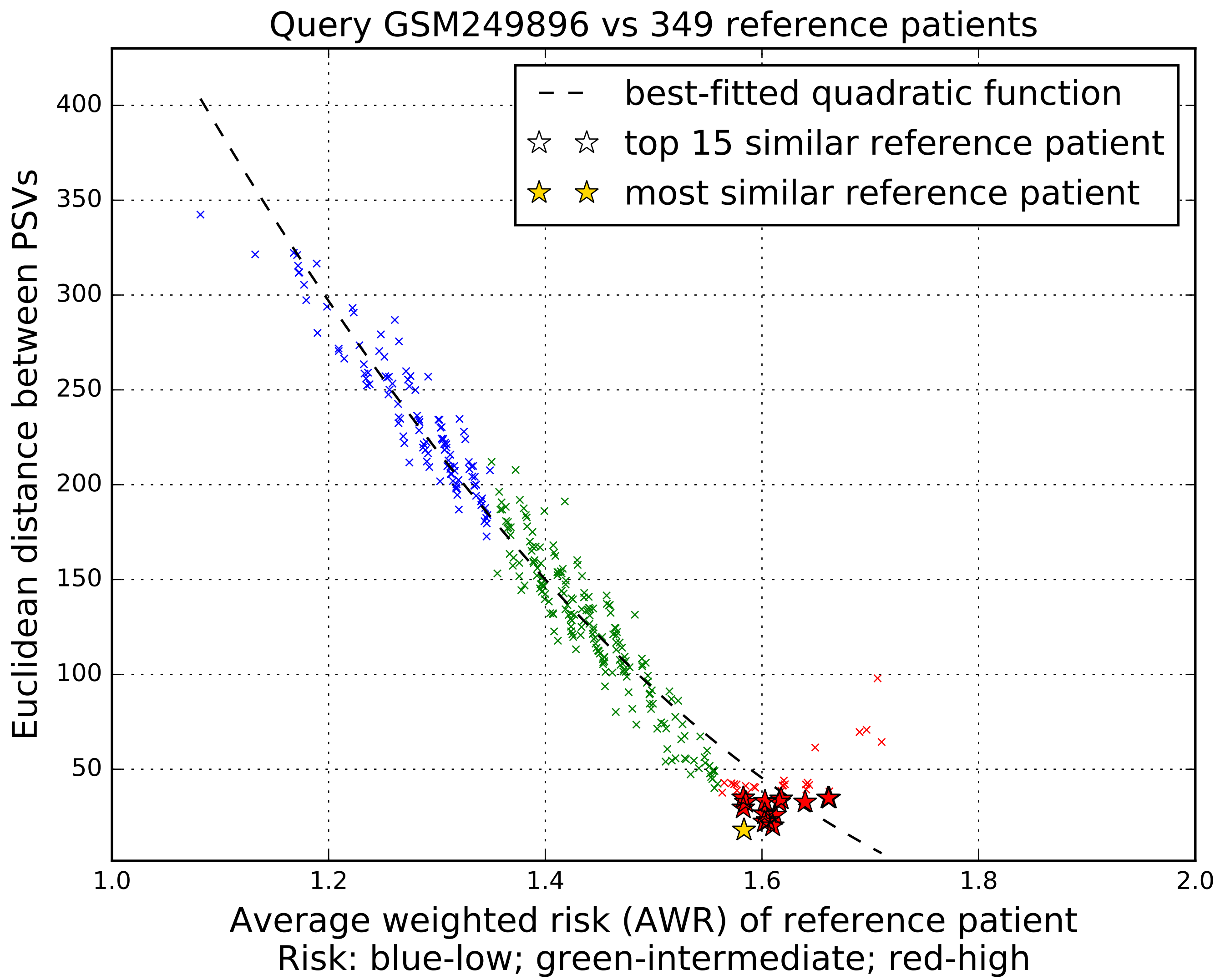

Query GSM249896 vs 349 reference patients
best-fitted quadratic function
top 15 similar reference patient
most similar reference patient
Euclidean distance between PSVs
400
350
300
250
200
150
100
50
1.0
1.2
1.4
1.6
1.8
2.0
Average weighted risk (AWR) of reference patient
Risk: blue-low; green-intermediate; red-high

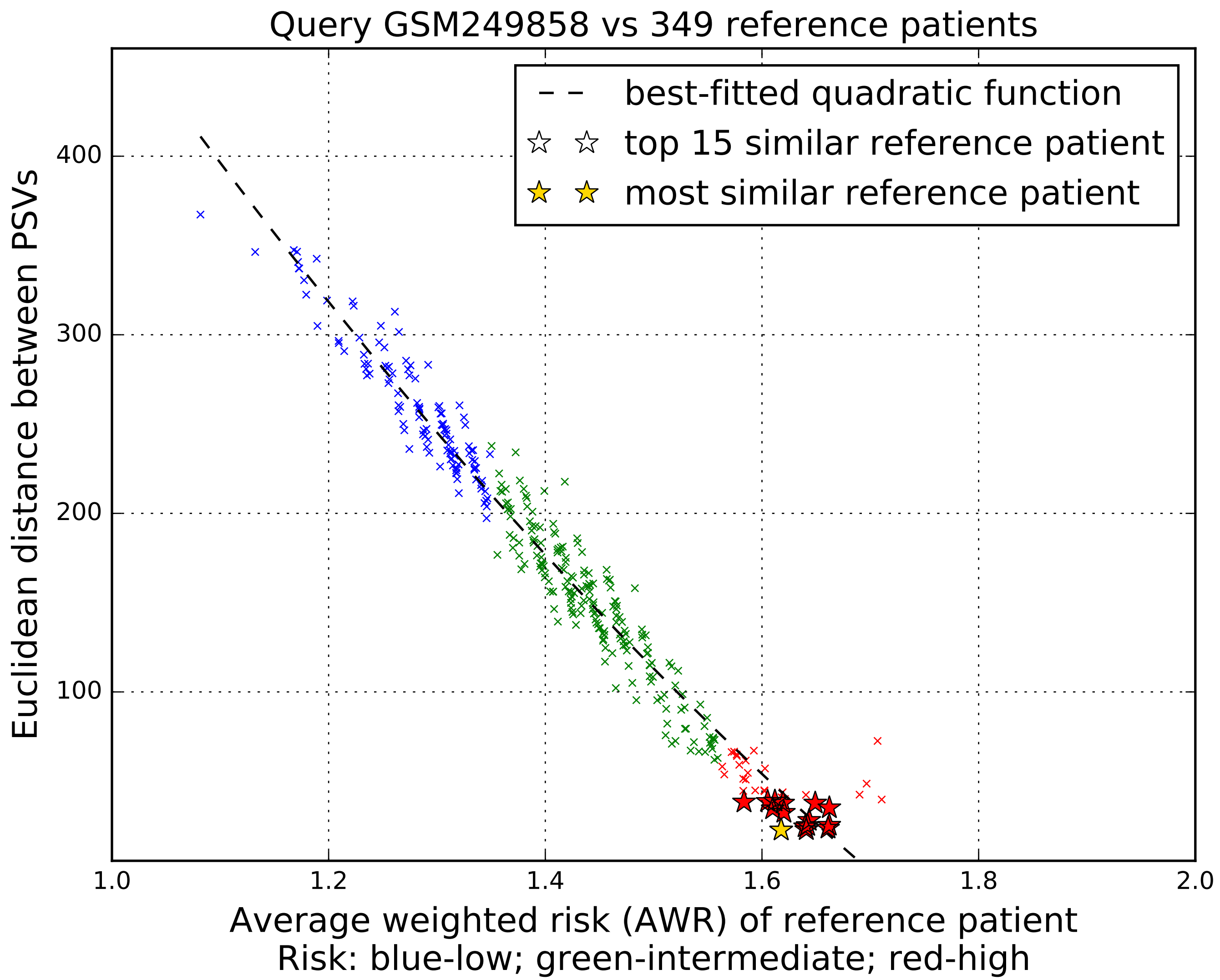

Query GSM249858 vs 349 reference patients
best-fitted quadratic function
top 15 similar reference patient
most similar reference patient
Euclidean distance between PSVs
400
300
200
100
1.0
1.2
1.4
1.6
1.8
2.0
Average weighted risk (AWR) of reference patient
Risk: blue-low; green-intermediate; red-high

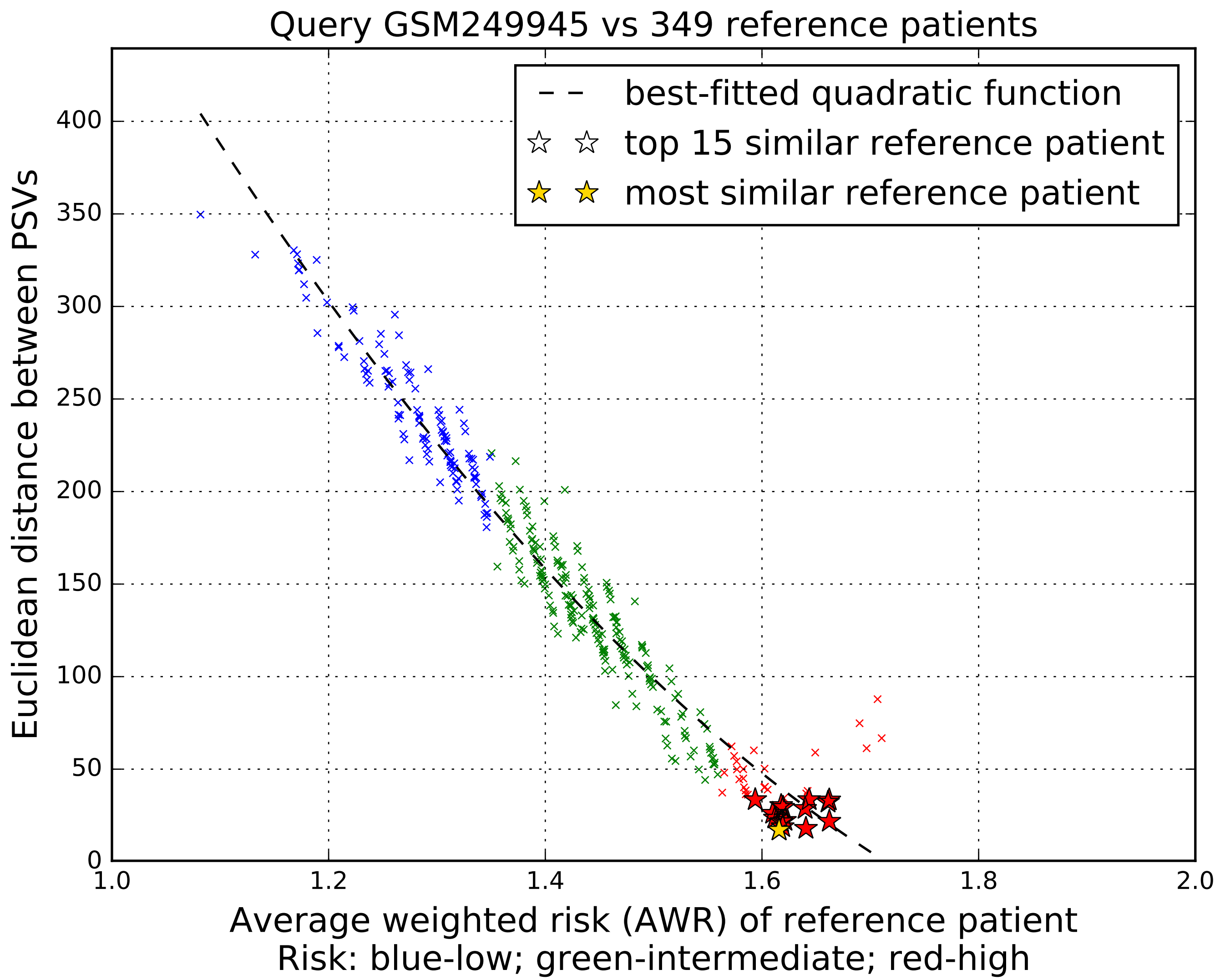

Query GSM249945 vs 349 reference patients
best-fitted quadratic function
top 15 similar reference patient
most similar reference patient
Euclidean distance between PSVs
0
50
100
150
200
250
300
350
400
1.0
1.2
1.4
1.6
1.8
2.0
Average weighted risk (AWR) of reference patient
Risk: blue-low; green-intermediate; red-high

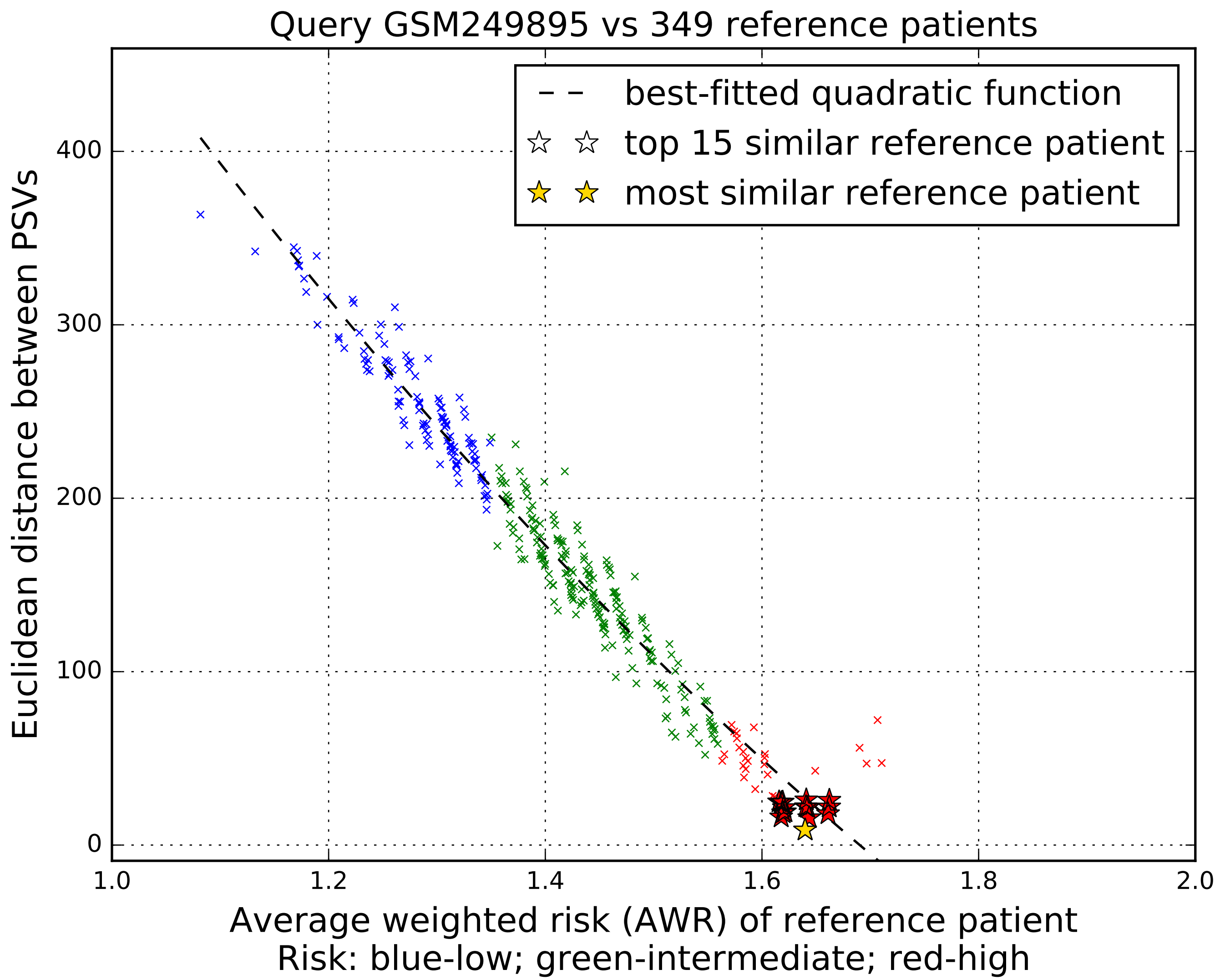

Query GSM249895 vs 349 reference patients
best-fitted quadratic function
top 15 similar reference patient
most similar reference patient
Euclidean distance between PSVs
0
100
200
300
400
1.0
1.2
1.4
1.6
1.8
2.0
Average weighted risk (AWR) of reference patient
Risk: blue-low; green-intermediate; red-high

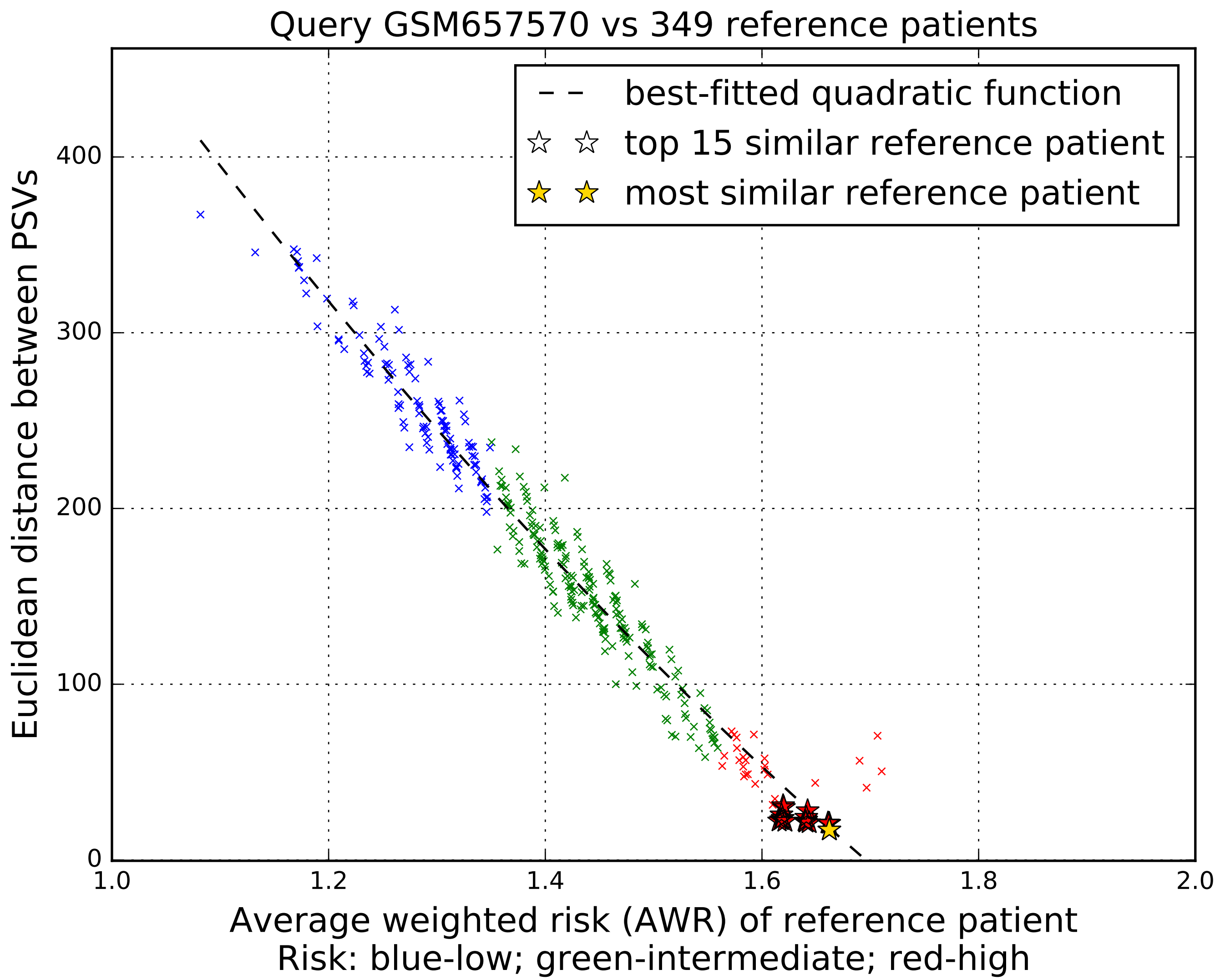

Query GSM657570 vs 349 reference patients
best-fitted quadratic function
top 15 similar reference patient
most similar reference patient
Euclidean distance between PSVs
0
100
200
300
400
1.0
1.2
1.4
1.6
1.8
2.0
Average weighted risk (AWR) of reference patient
Risk: blue-low; green-intermediate; red-high

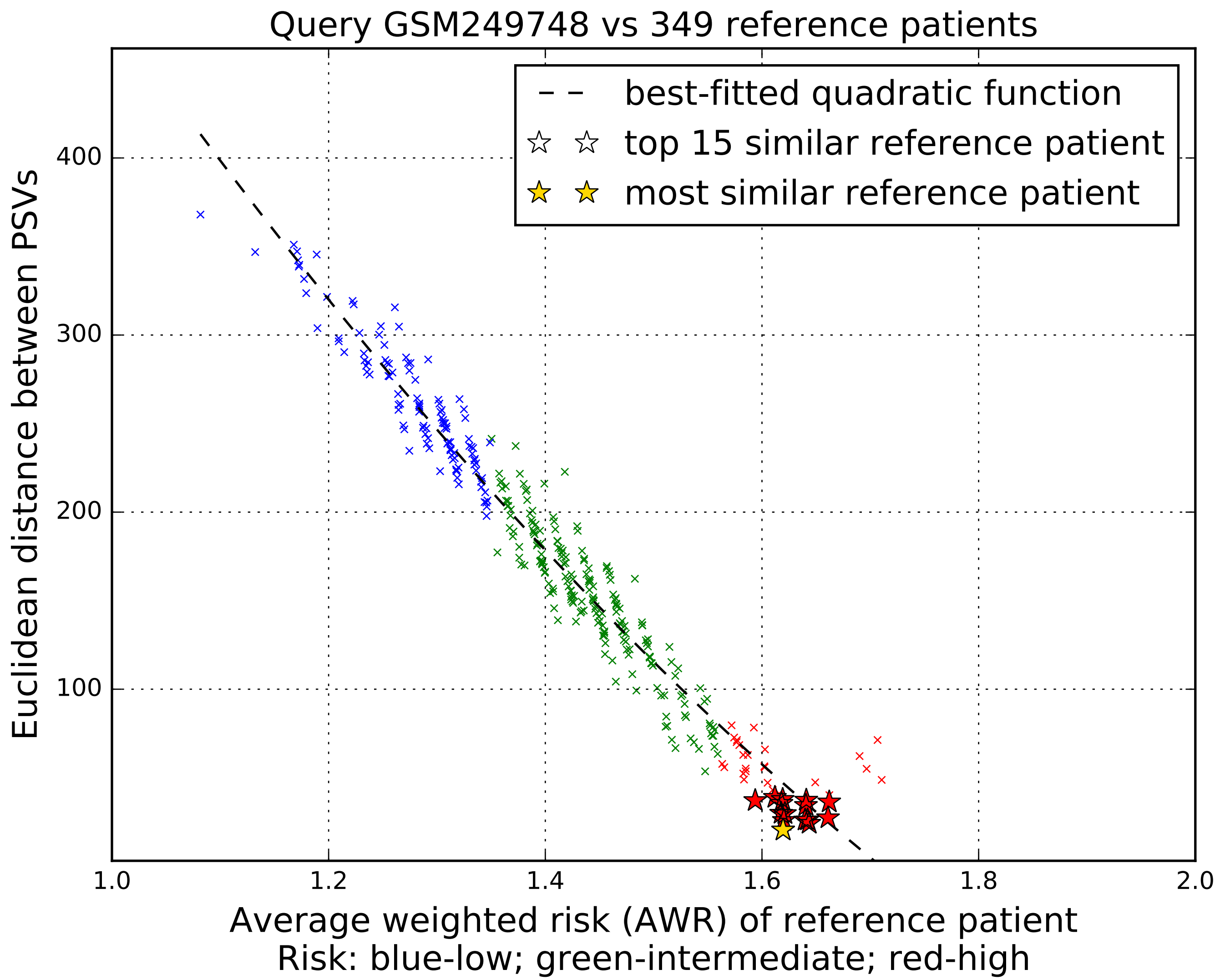
Query GSM249748 vs 349 reference patients
best-fitted quadratic function
top 15 similar reference patient
most similar reference patient
Euclidean distance between PSVs
400
300
200
100
1.0
1.2
1.4
1.6
1.8
2.0
Average weighted risk (AWR) of reference patient
Risk: blue-low; green-intermediate; red-high

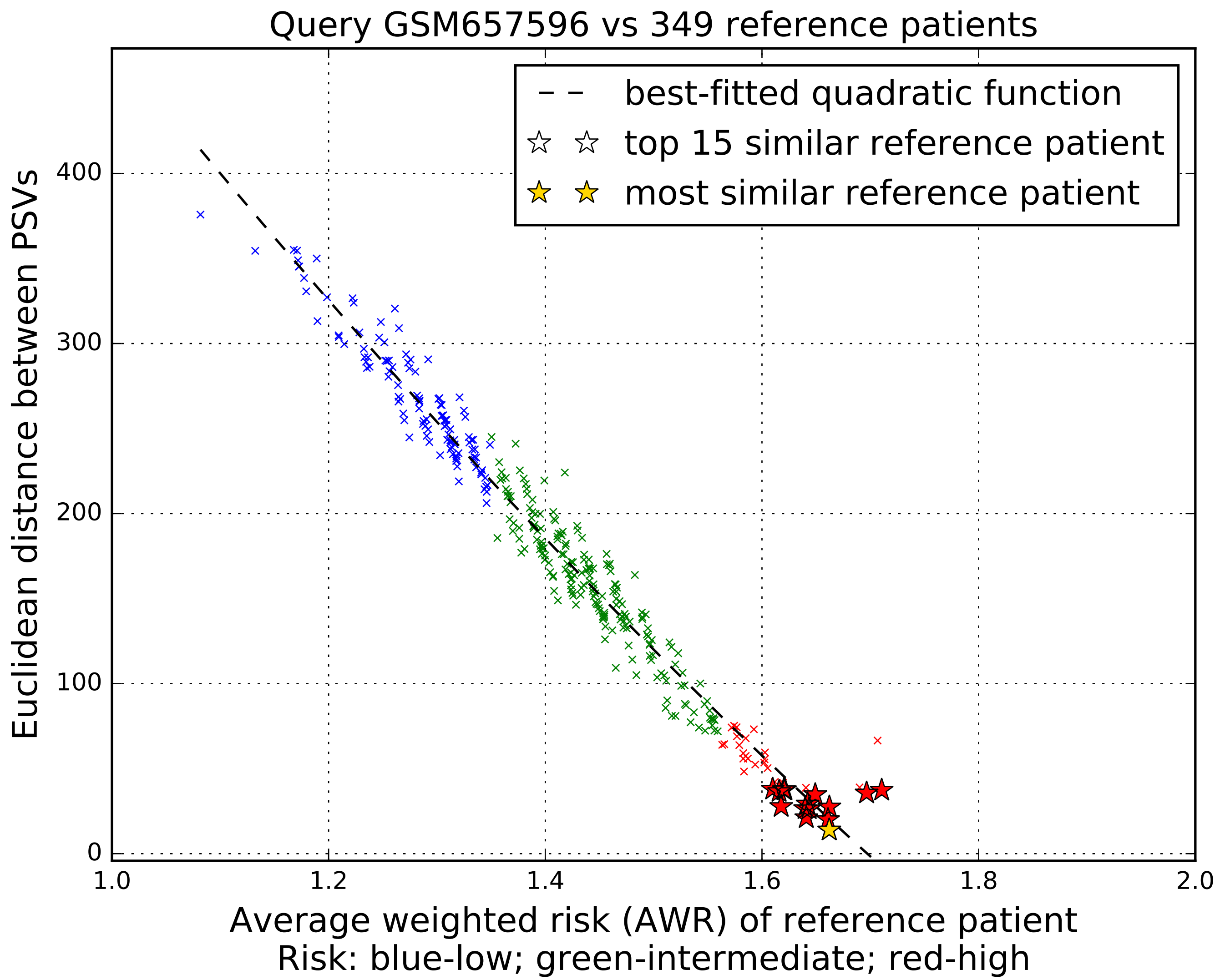

Query GSM657596 vs 349 reference patients
best-fitted quadratic function
top 15 similar reference patient
most similar reference patient
Euclidean distance between PSVs
0
100
200
300
400
1.0
1.2
1.4
1.6
1.8
2.0
Average weighted risk (AWR) of reference patient
Risk: blue-low; green-intermediate; red-high

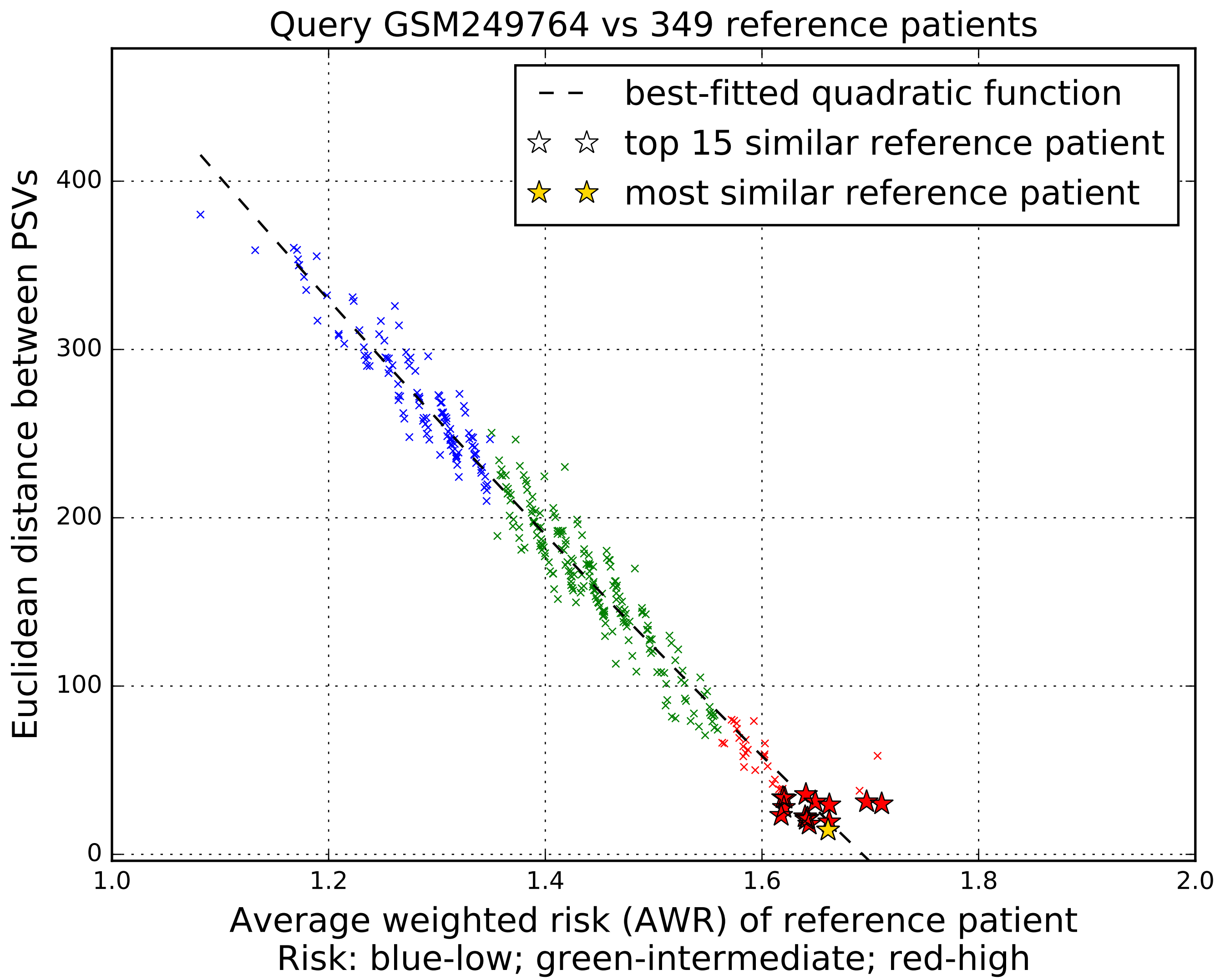
Query GSM249764 vs 349 reference patients
best-fitted quadratic function
top 15 similar reference patient
most similar reference patient
Euclidean distance between PSVs
0
100
200
300
400
1.0
1.2
1.4
1.6
1.8
2.0
Average weighted risk (AWR) of reference patient
Risk: blue-low; green-intermediate; red-high

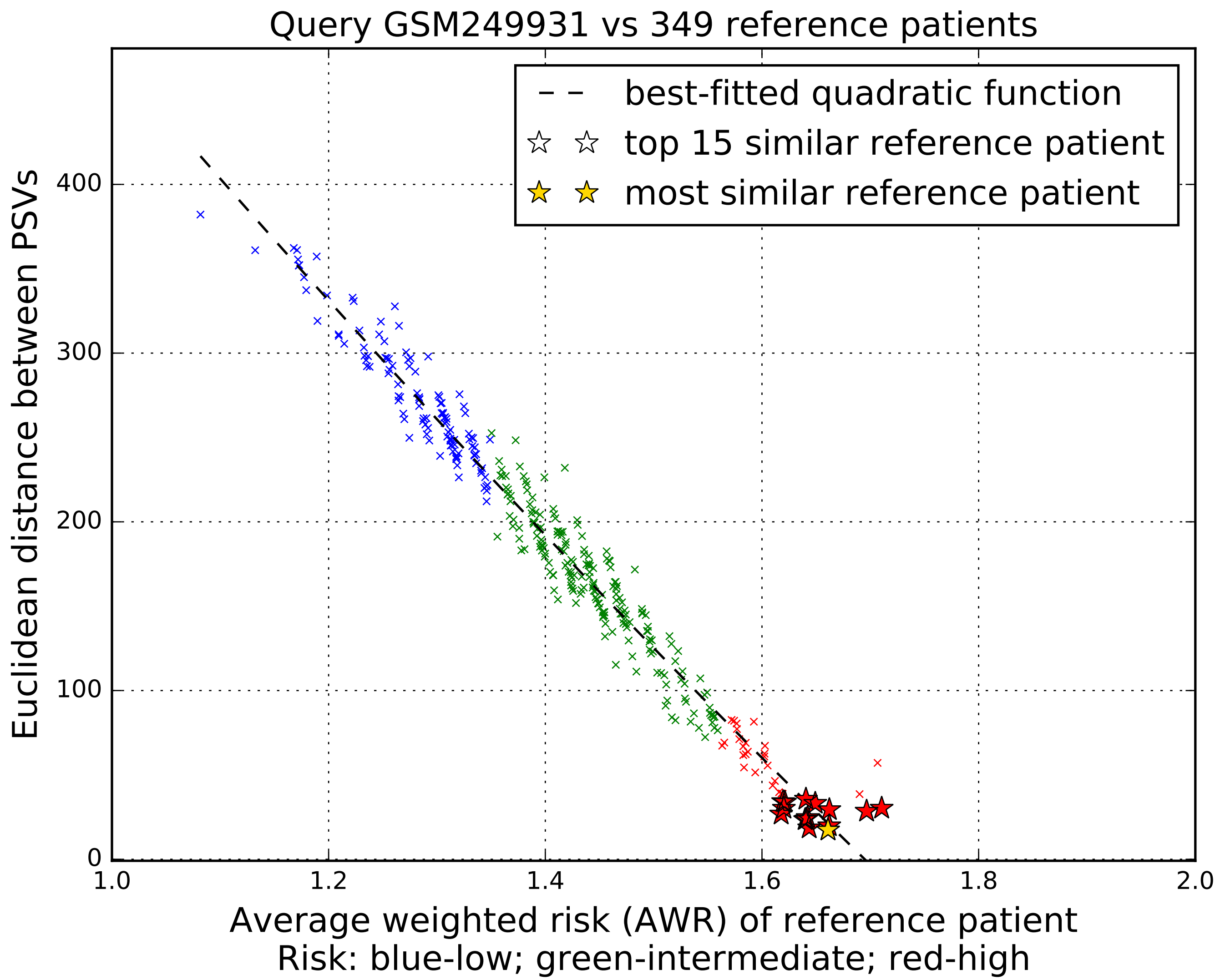

Query GSM249931 vs 349 reference patients
best-fitted quadratic function
top 15 similar reference patient
most similar reference patient
Euclidean distance between PSVs
0
100
200
300
400
1.0
1.2
1.4
1.6
1.8
2.0
Average weighted risk (AWR) of reference patient
Risk: blue-low; green-intermediate; red-high

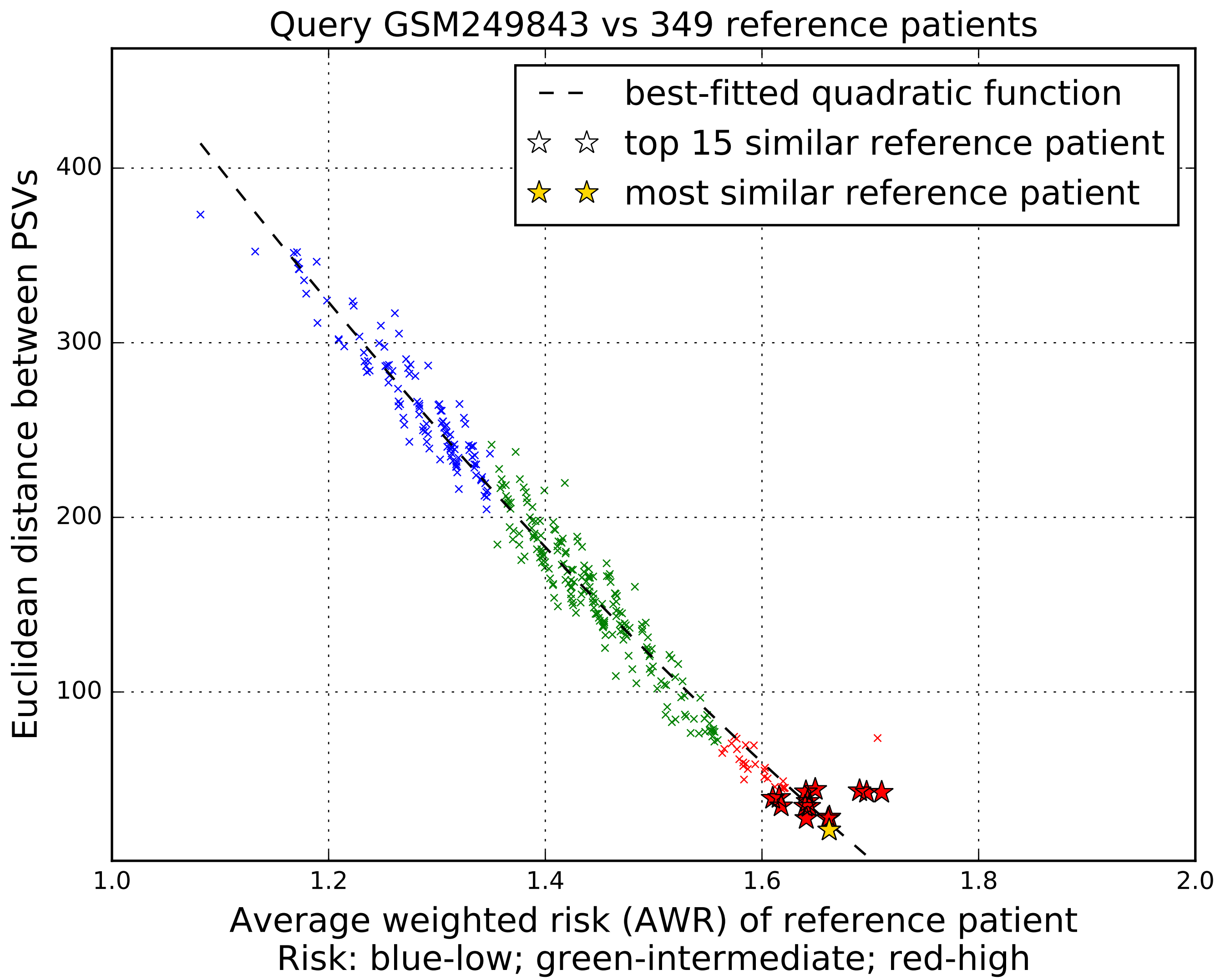
Query GSM249843 vs 349 reference patients
best-fitted quadratic function
top 15 similar reference patient
most similar reference patient
Euclidean distance between PSVs
400
300
200
100
1.0
1.2
1.4
1.6
1.8
2.0
Average weighted risk (AWR) of reference patient
Risk: blue-low; green-intermediate; red-high

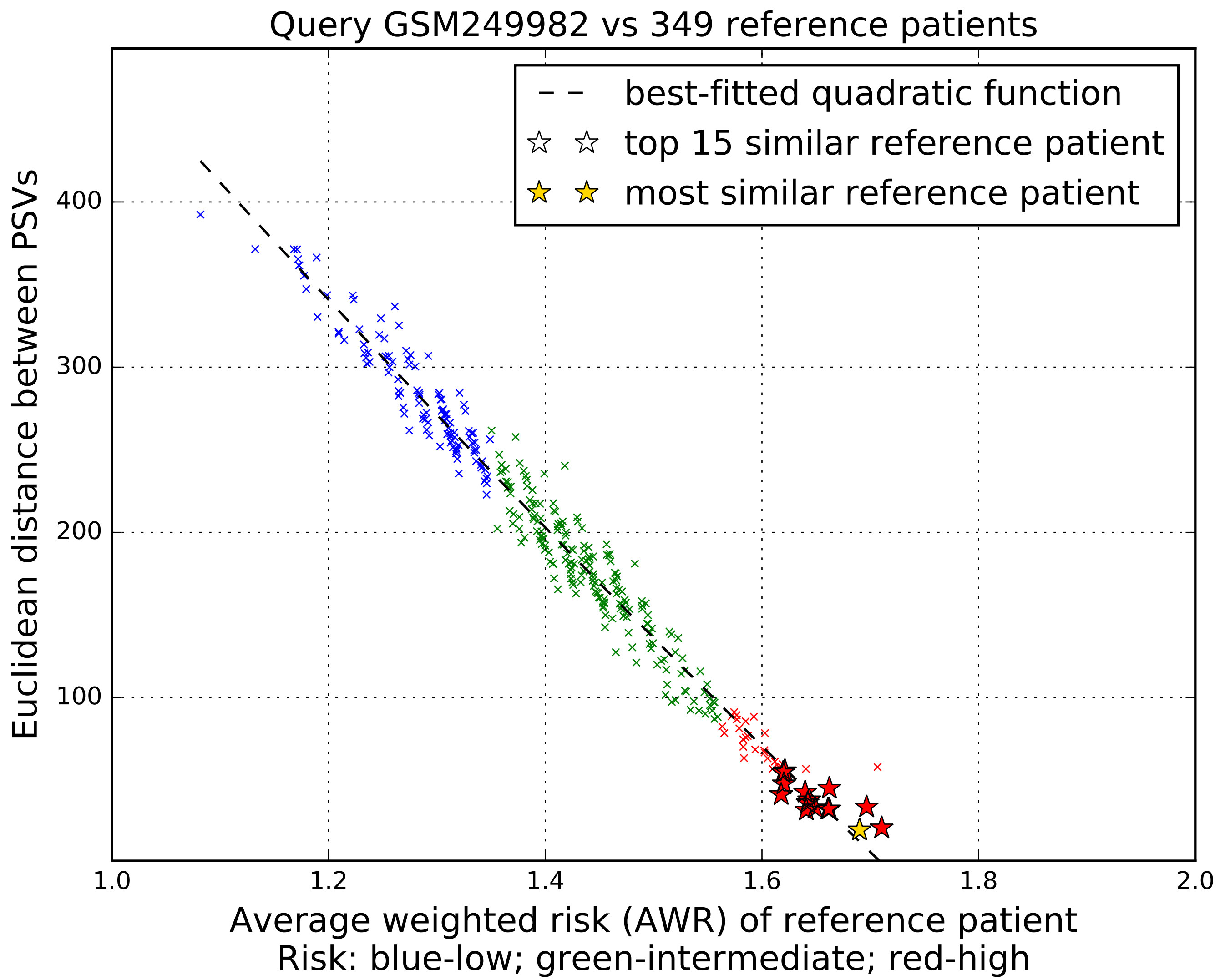
Query GSM249982 vs 349 reference patients
best-fitted quadratic function
top 15 similar reference patient
most similar reference patient
Euclidean distance between PSVs
400
300
200
100
1.0
1.2
1.4
1.6
1.8
2.0
Average weighted risk (AWR) of reference patient
Risk: blue-low; green-intermediate; red-high

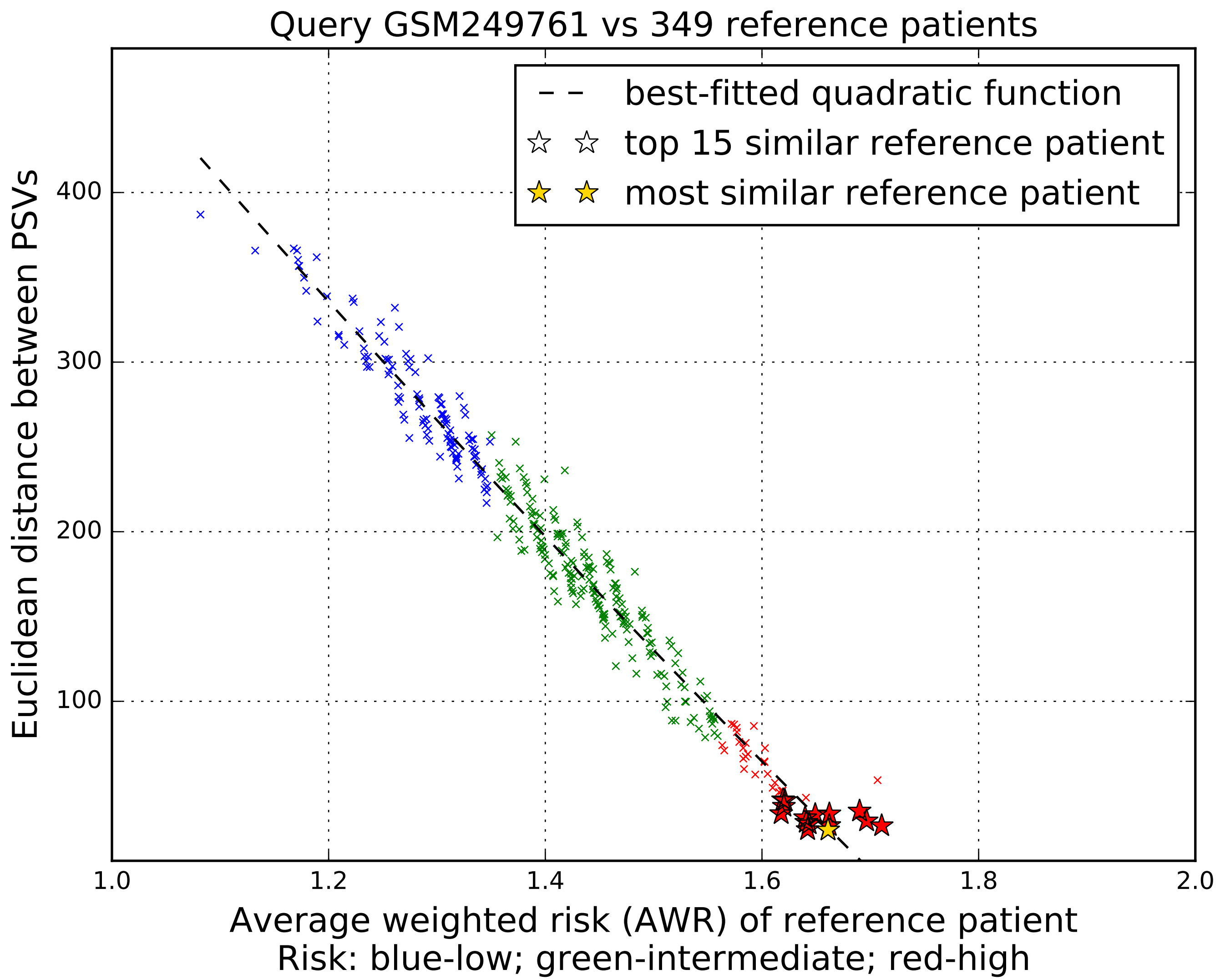
Query GSM249761 vs 349 reference patients
best-fitted quadratic function
top 15 similar reference patient
most similar reference patient
400
300
200
100
Euclidean distance between PSVs
1.0
1.2
1.4
1.6
1.8
2.0
Average weighted risk (AWR) of reference patient
Risk: blue-low; green-intermediate; red-high

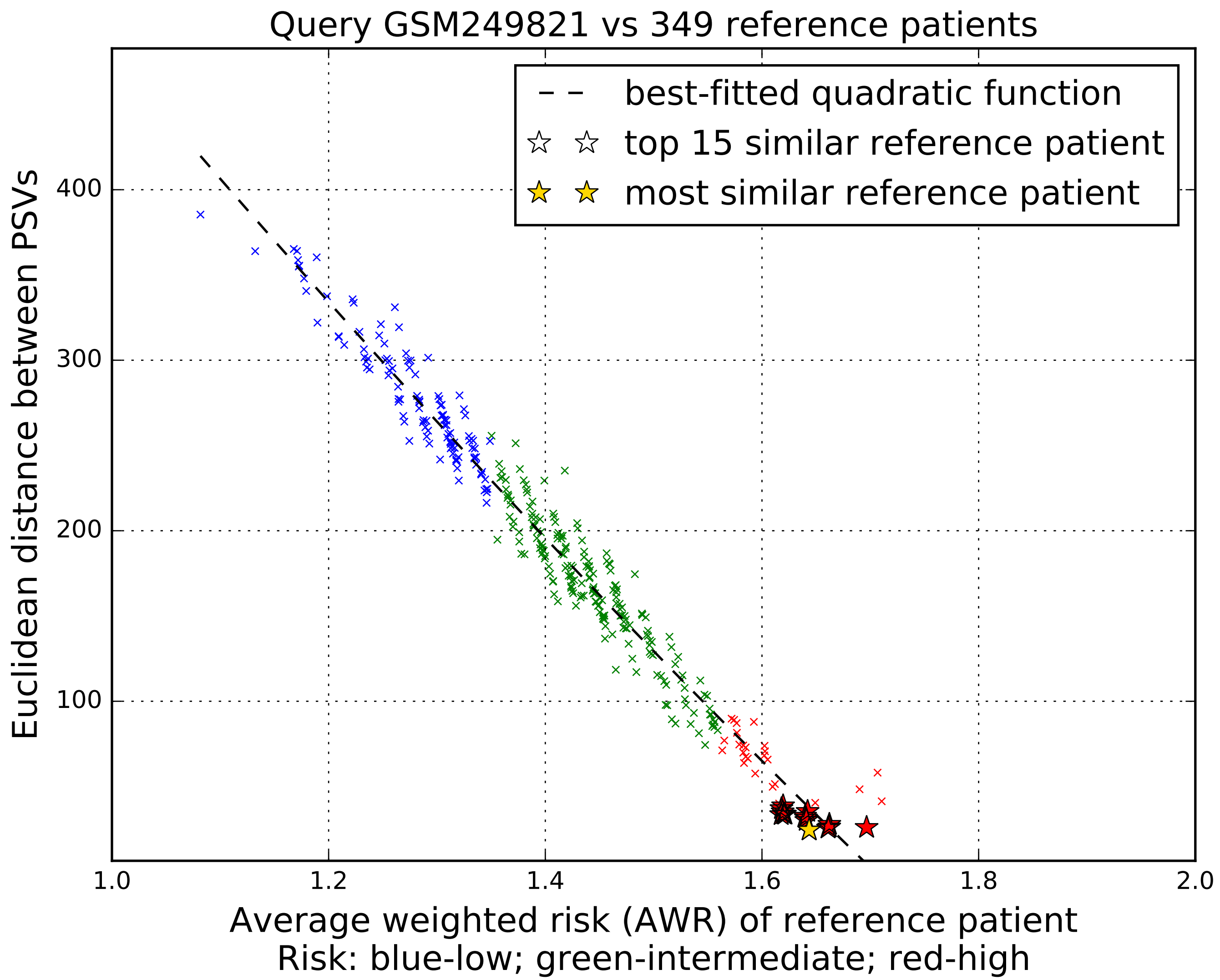
Query GSM249821 vs 349 reference patients
best-fitted quadratic function
top 15 similar reference patient
most similar reference patient
Euclidean distance between PSVs
400
300
200
100
1.0
1.2
1.4
1.6
1.8
2.0
Average weighted risk (AWR) of reference patient
Risk: blue-low; green-intermediate; red-high

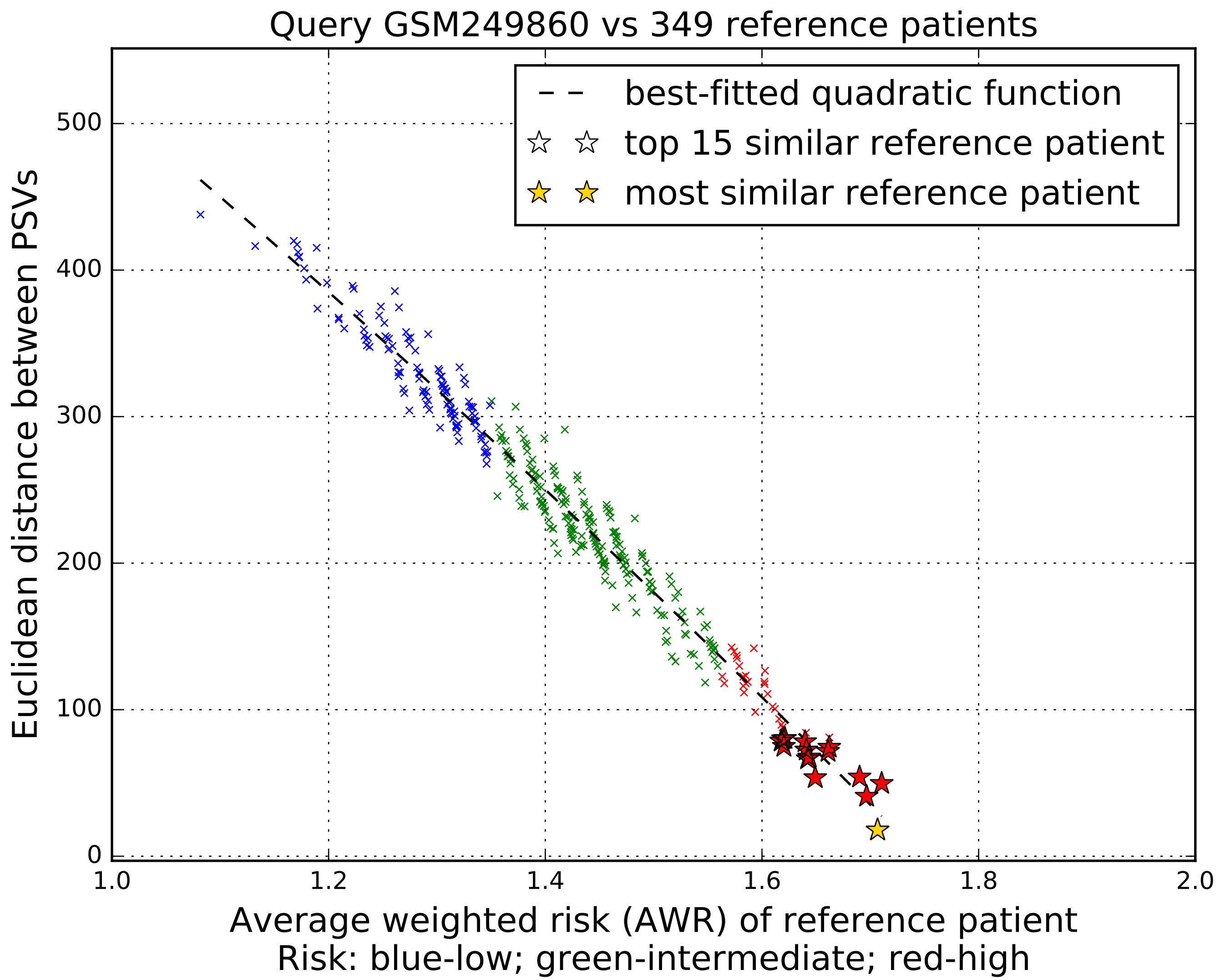

Query GSM249860 vs 349 reference patients
best-fitted quadratic function
top 15 similar reference patient
most similar reference patient
Euclidean distance between PSVs
0
100
200
300
400
500
1.0
1.2
1.4
1.6
1.8
2.0
Average weighted risk (AWR) of reference patient
Risk: blue-low; green-intermediate; red-high
