# Supplementary material for: Big data and computational biology strategy for personalized prognosis
Source: Oncotarget. 2016 May 24;7(26):40200–20. doi: 10.18632/oncotarget.9571 (PMC5130003; doi:10.18632/oncotarget.9571)
Supplement: Supplementary file 13 [file oncotarget-07-40200-s013.pdf]

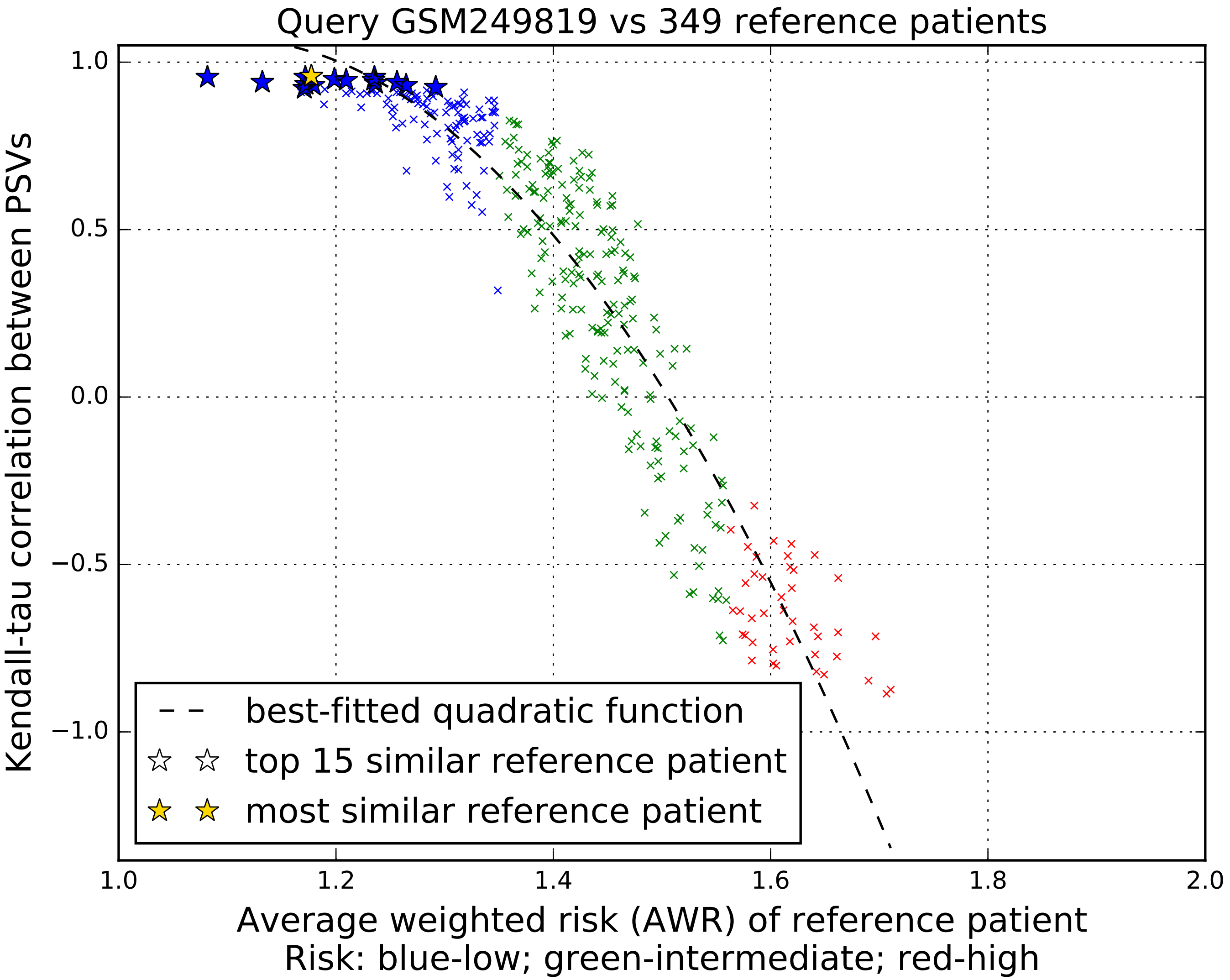
Query GSM249819 vs 349 reference patients
Kendall-tau correlation between PSVs
1.0
0.5
0.0
−0.5
−1.0
1.0
1.2
1.4
1.6
1.8
2.0
Average weighted risk (AWR) of reference patient
Risk: blue-low; green-intermediate; red-high
best-fitted quadratic function
top 15 similar reference patient
most similar reference patient

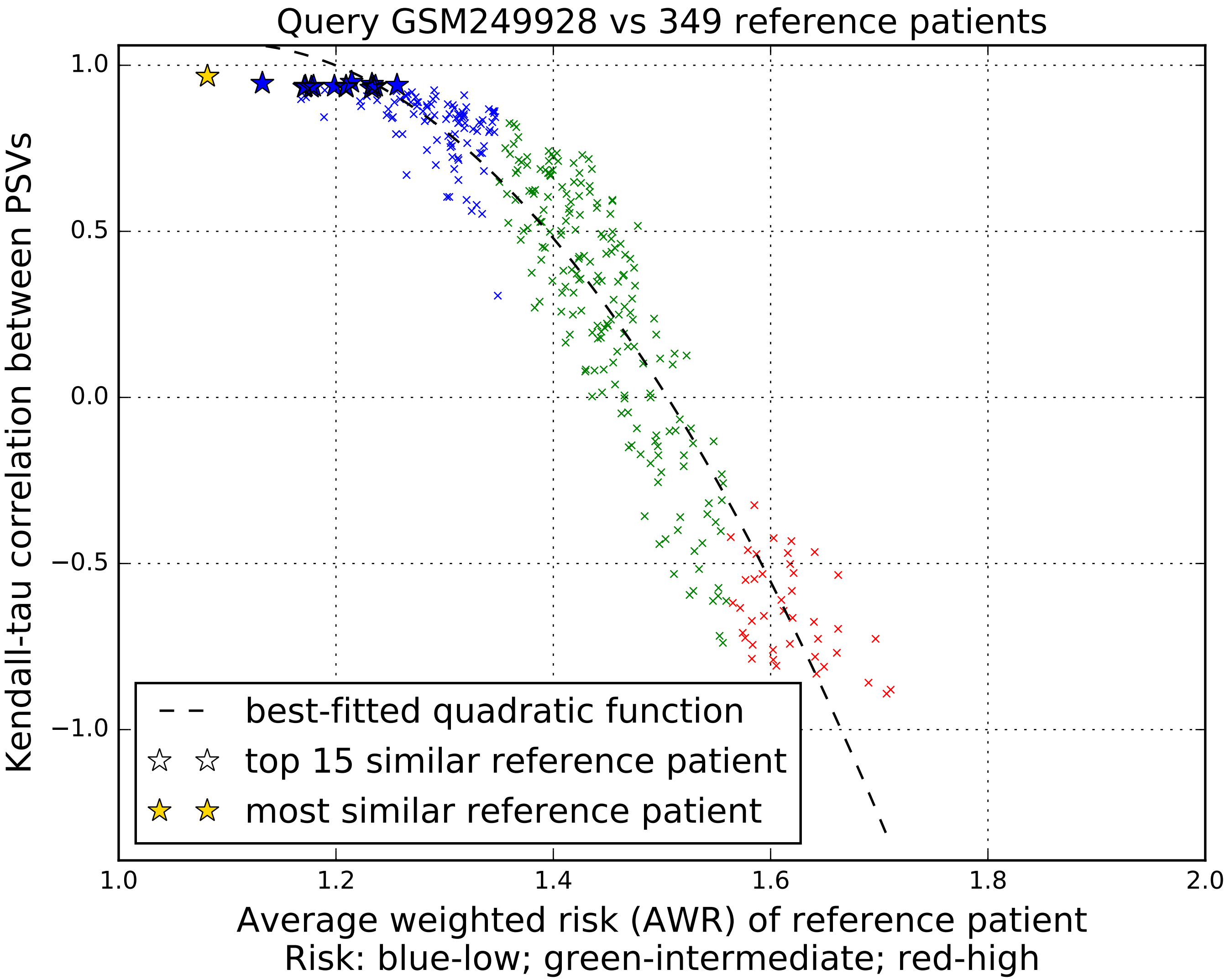

Query GSM249928 vs 349 reference patients
Kendall-tau correlation between PSVs
Average weighted risk (AWR) of reference patient
Risk: blue-low; green-intermediate; red-high
best-fitted quadratic function
top 15 similar reference patient
most similar reference patient
1.0
0.5
0.0
−0.5
−1.0
1.0
1.2
1.4
1.6
1.8
2.0

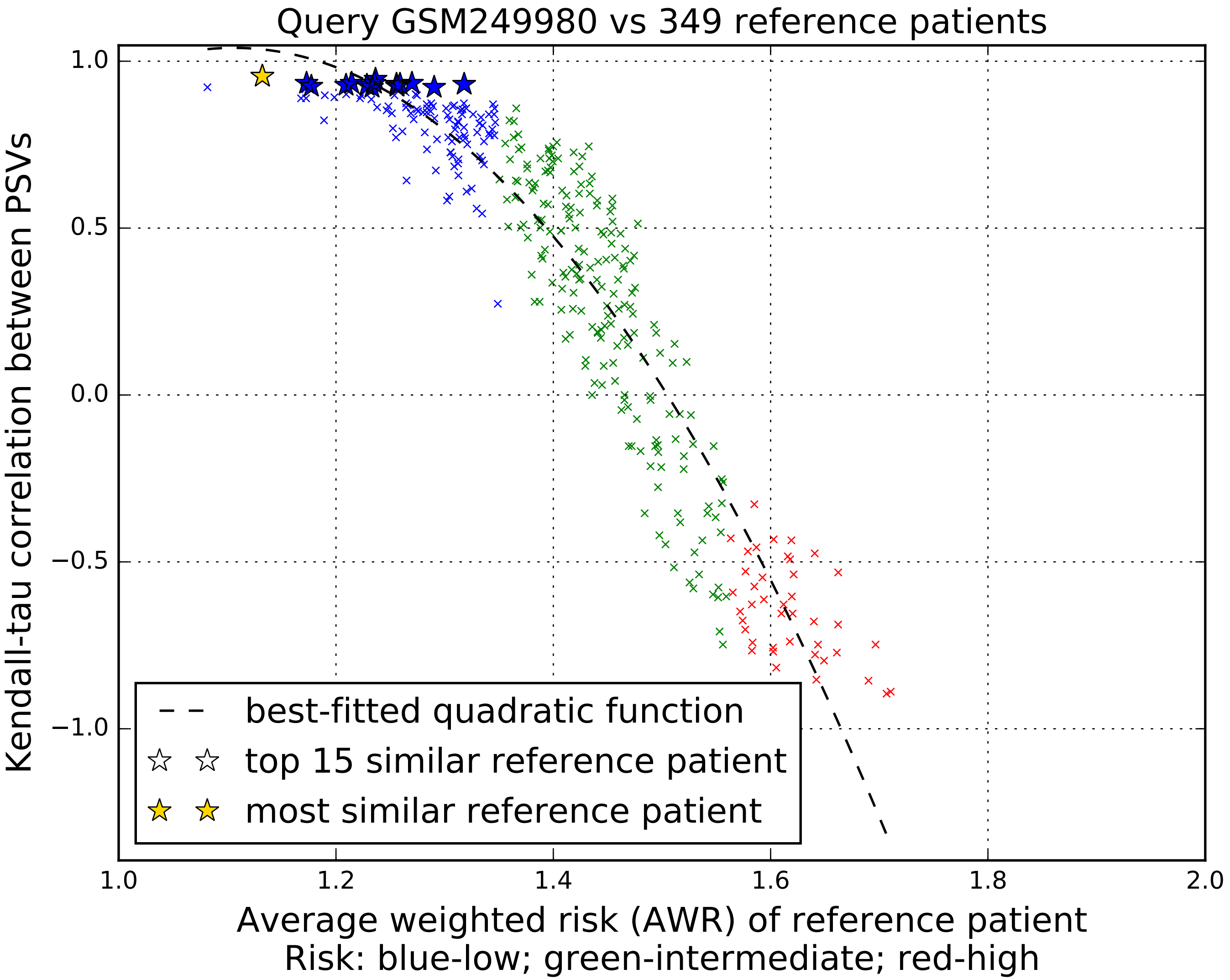

Query GSM249980 vs 349 reference patients
Kendall-tau correlation between PSVs
Average weighted risk (AWR) of reference patient
Risk: blue-low; green-intermediate; red-high
best-fitted quadratic function
top 15 similar reference patient
most similar reference patient
1.0
0.5
0.0
−0.5
−1.0
1.0
1.2
1.4
1.6
1.8
2.0

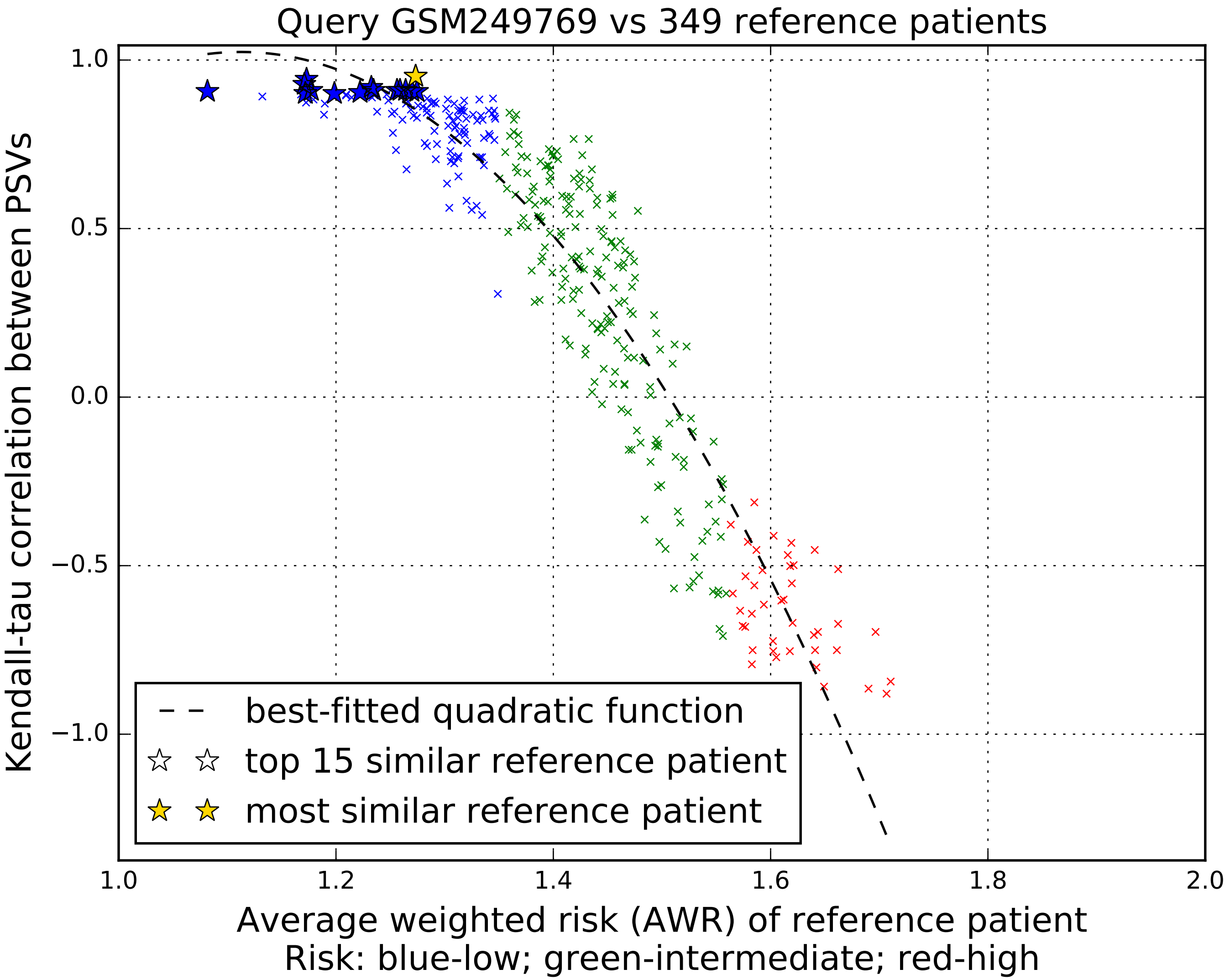

Query GSM249769 vs 349 reference patients
Kendall-tau correlation between PSVs
1.0
0.5
0.0
−0.5
−1.0
1.0
1.2
1.4
1.6
1.8
2.0
best-fitted quadratic function
top 15 similar reference patient
most similar reference patient
Average weighted risk (AWR) of reference patient
Risk: blue-low; green-intermediate; red-high

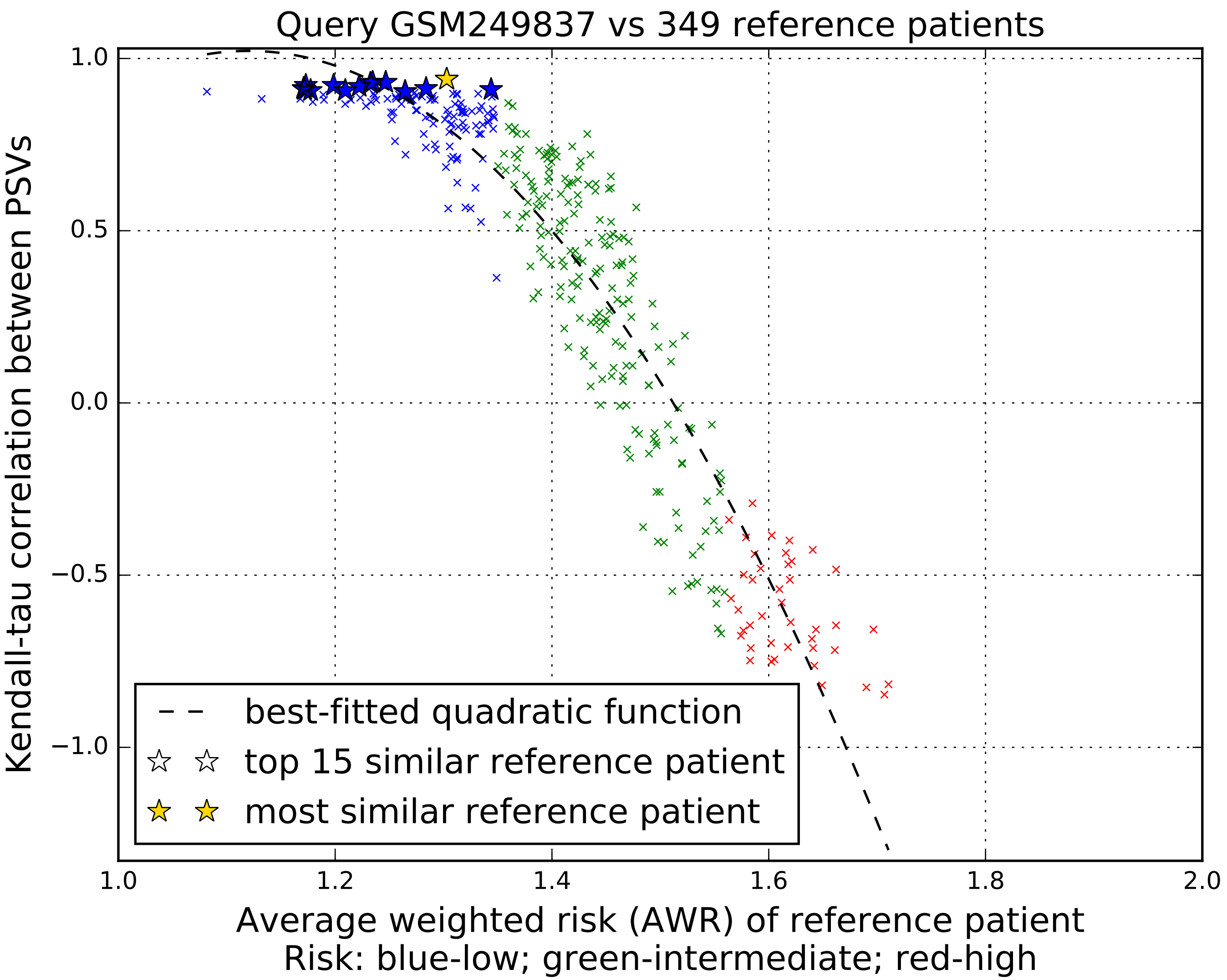
Query GSM249837 vs 349 reference patients
Kendall-tau correlation between PSVs
1.0
0.5
0.0
−0.5
−1.0
1.0
1.2
1.4
1.6
1.8
2.0
best-fitted quadratic function
top 15 similar reference patient
most similar reference patient
Average weighted risk (AWR) of reference patient
Risk: blue-low; green-intermediate; red-high

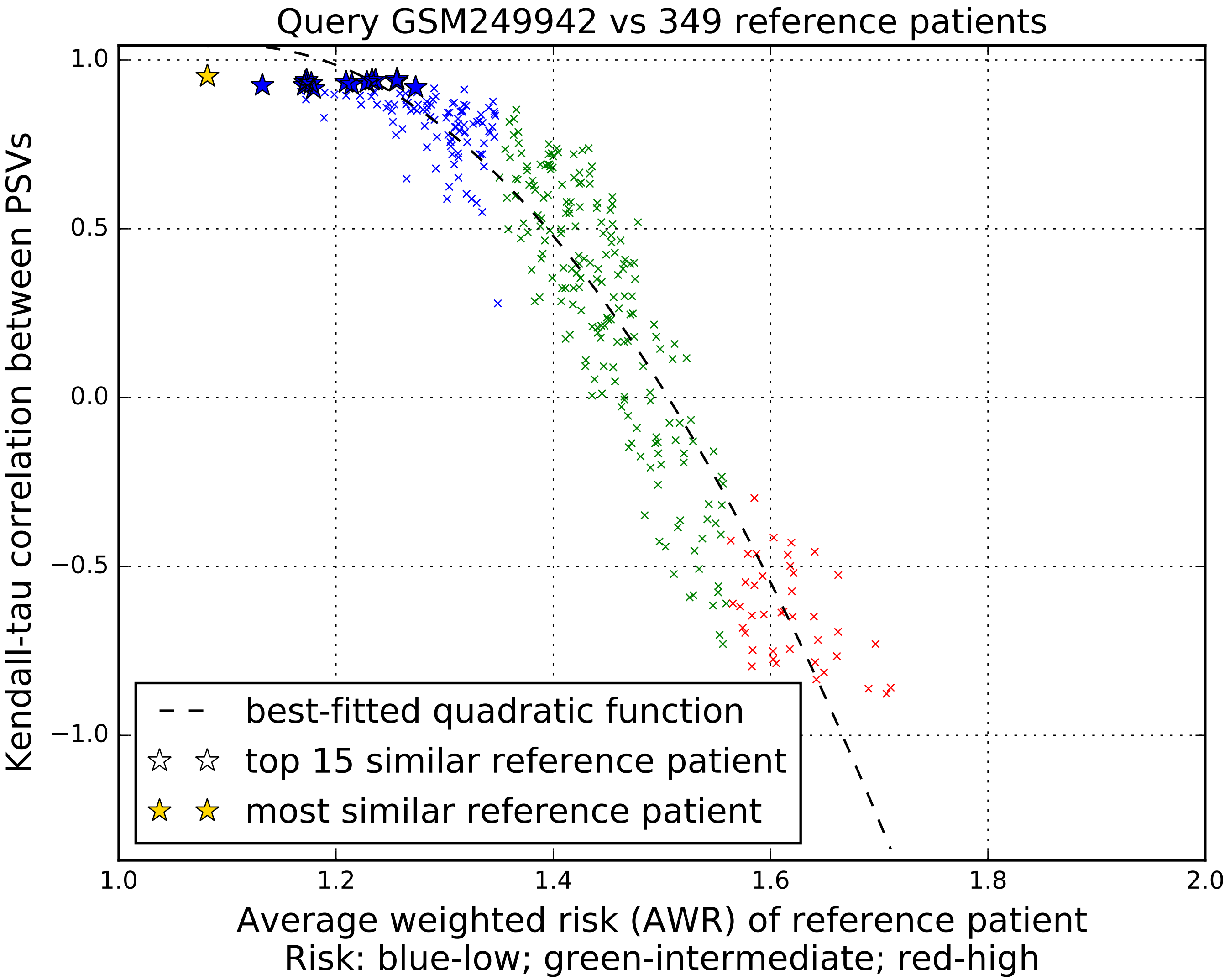

Query GSM249942 vs 349 reference patients
Kendall-tau correlation between PSVs
1.0
0.5
0.0
−0.5
−1.0
1.0
1.2
1.4
1.6
1.8
2.0
best-fitted quadratic function
top 15 similar reference patient
most similar reference patient
Average weighted risk (AWR) of reference patient
Risk: blue-low; green-intermediate; red-high

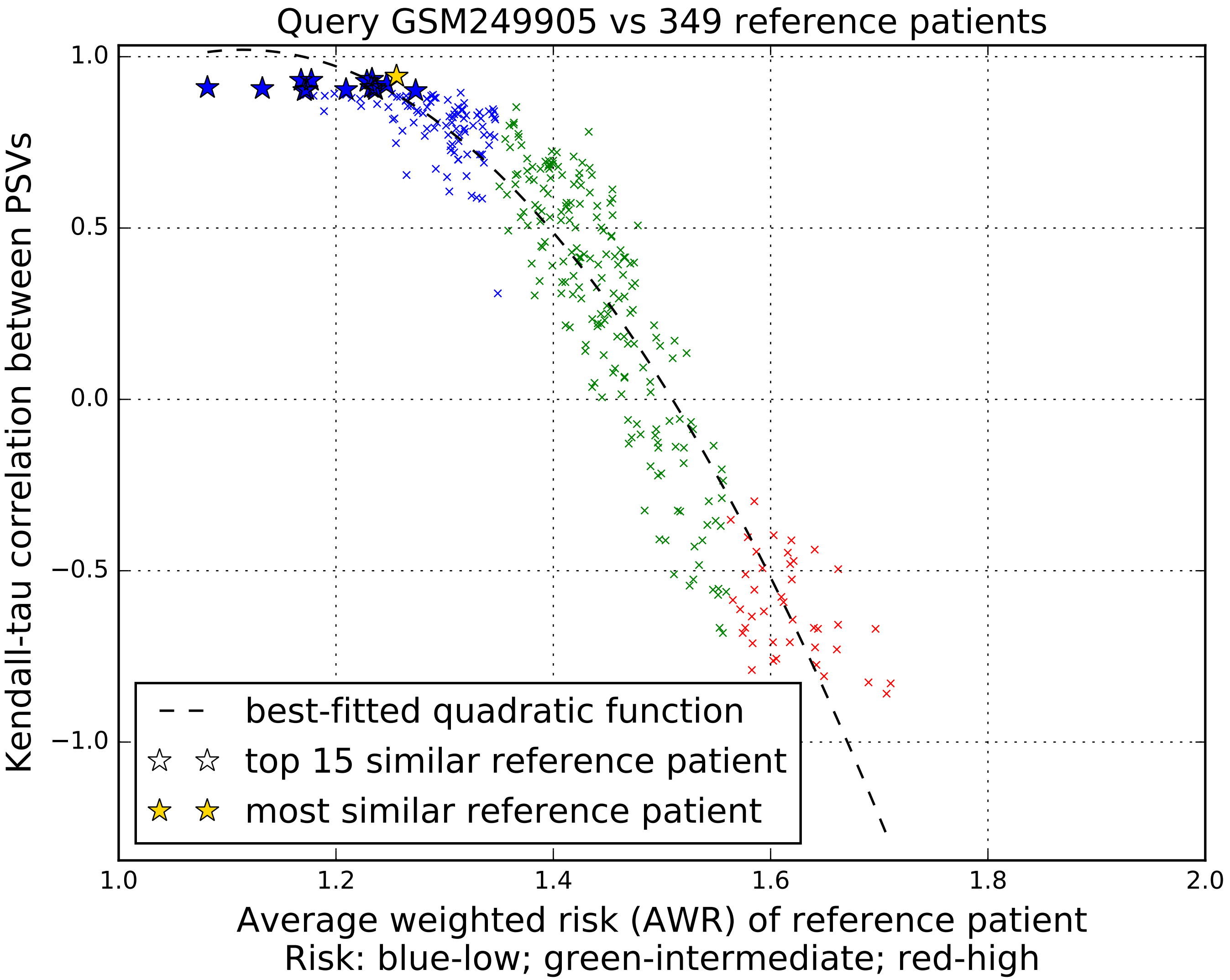

Query GSM249905 vs 349 reference patients
Kendall-tau correlation between PSVs
1.0
0.5
0.0
−0.5
−1.0
1.0
1.2
1.4
1.6
1.8
2.0
best-fitted quadratic function
top 15 similar reference patient
most similar reference patient
Average weighted risk (AWR) of reference patient
Risk: blue-low; green-intermediate; red-high

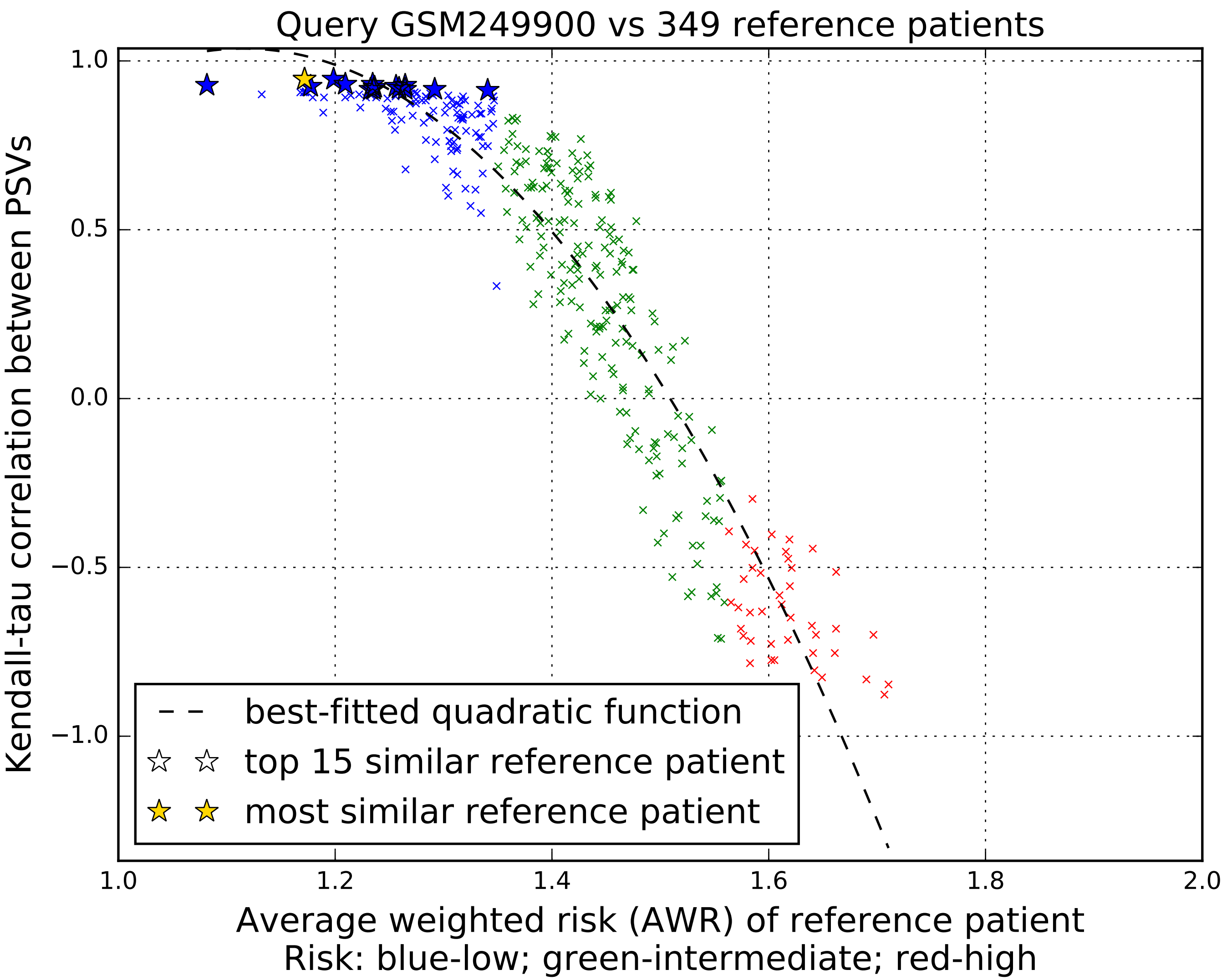

Query GSM249900 vs 349 reference patients
Kendall-tau correlation between PSVs
Average weighted risk (AWR) of reference patient
Risk: blue-low; green-intermediate; red-high
best-fitted quadratic function
top 15 similar reference patient
most similar reference patient
1.0
0.5
0.0
−0.5
−1.0
1.0
1.2
1.4
1.6
1.8
2.0

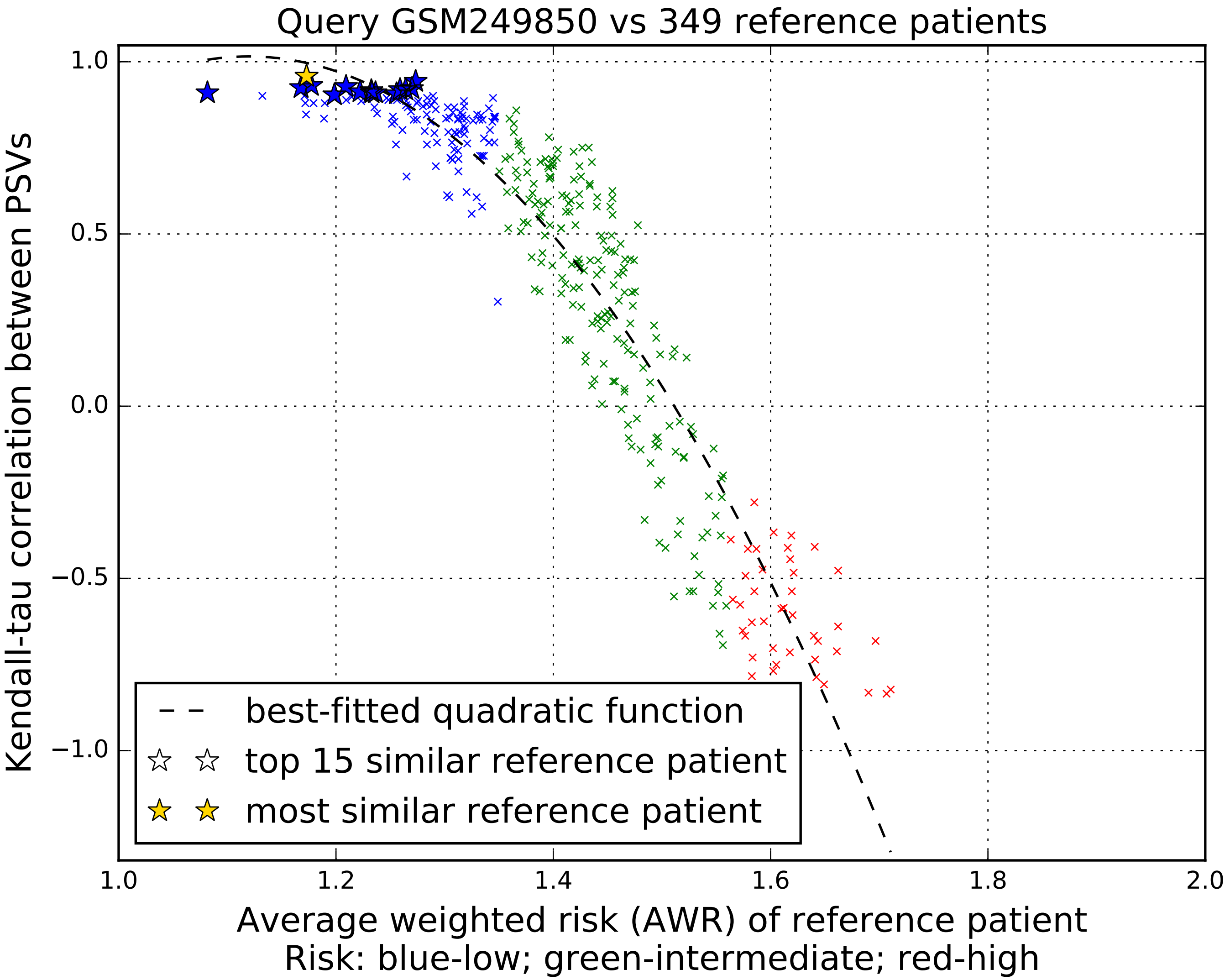

Query GSM249850 vs 349 reference patients
Kendall-tau correlation between PSVs
1.0
0.5
0.0
−0.5
−1.0
1.0
1.2
1.4
1.6
1.8
2.0
best-fitted quadratic function
top 15 similar reference patient
most similar reference patient
Average weighted risk (AWR) of reference patient
Risk: blue-low; green-intermediate; red-high

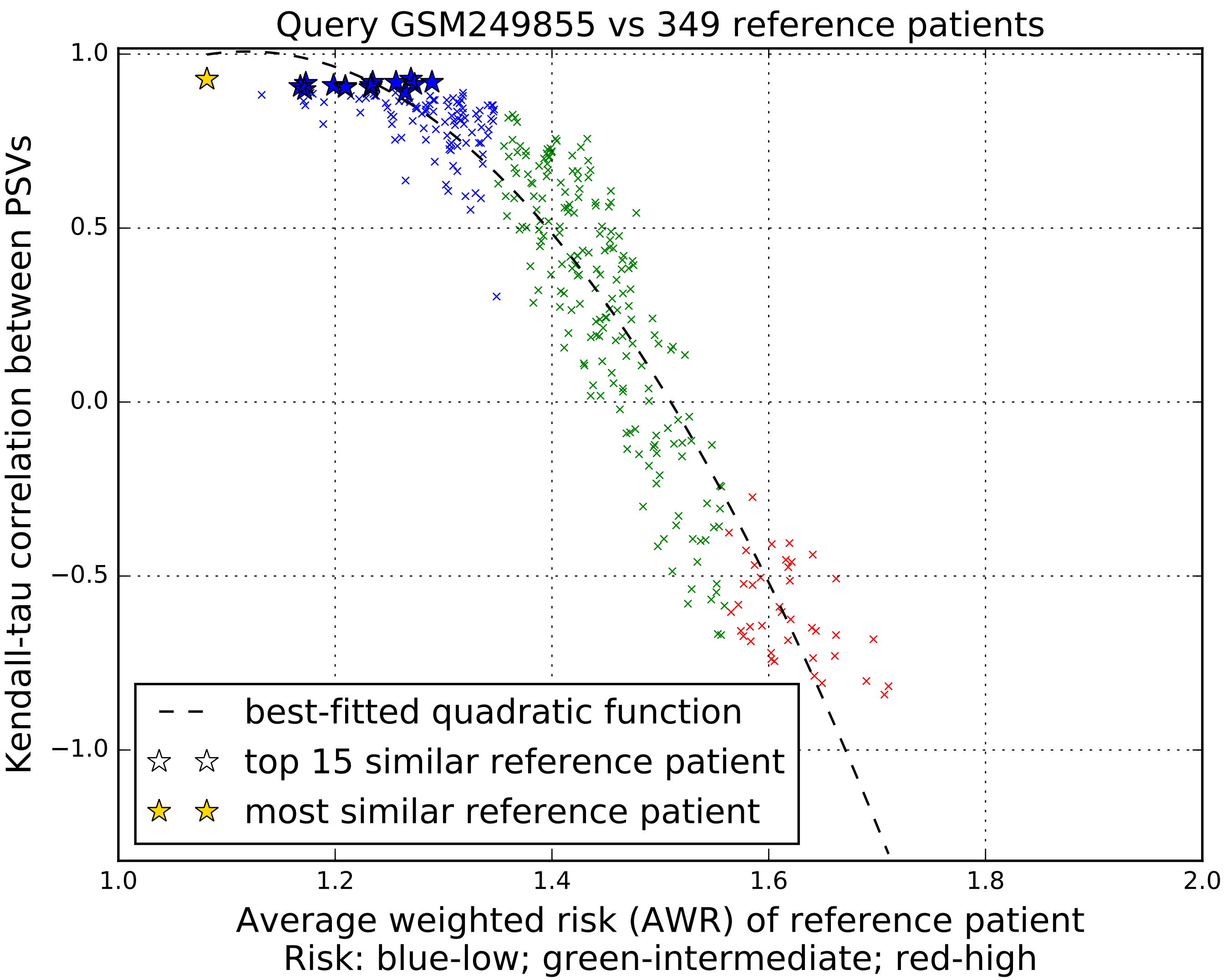

Query GSM249855 vs 349 reference patients
Kendall-tau correlation between PSVs
Average weighted risk (AWR) of reference patient
Risk: blue-low; green-intermediate; red-high
best-fitted quadratic function
top 15 similar reference patient
most similar reference patient
1.0
0.5
0.0
−0.5
−1.0
1.0
1.2
1.4
1.6
1.8
2.0

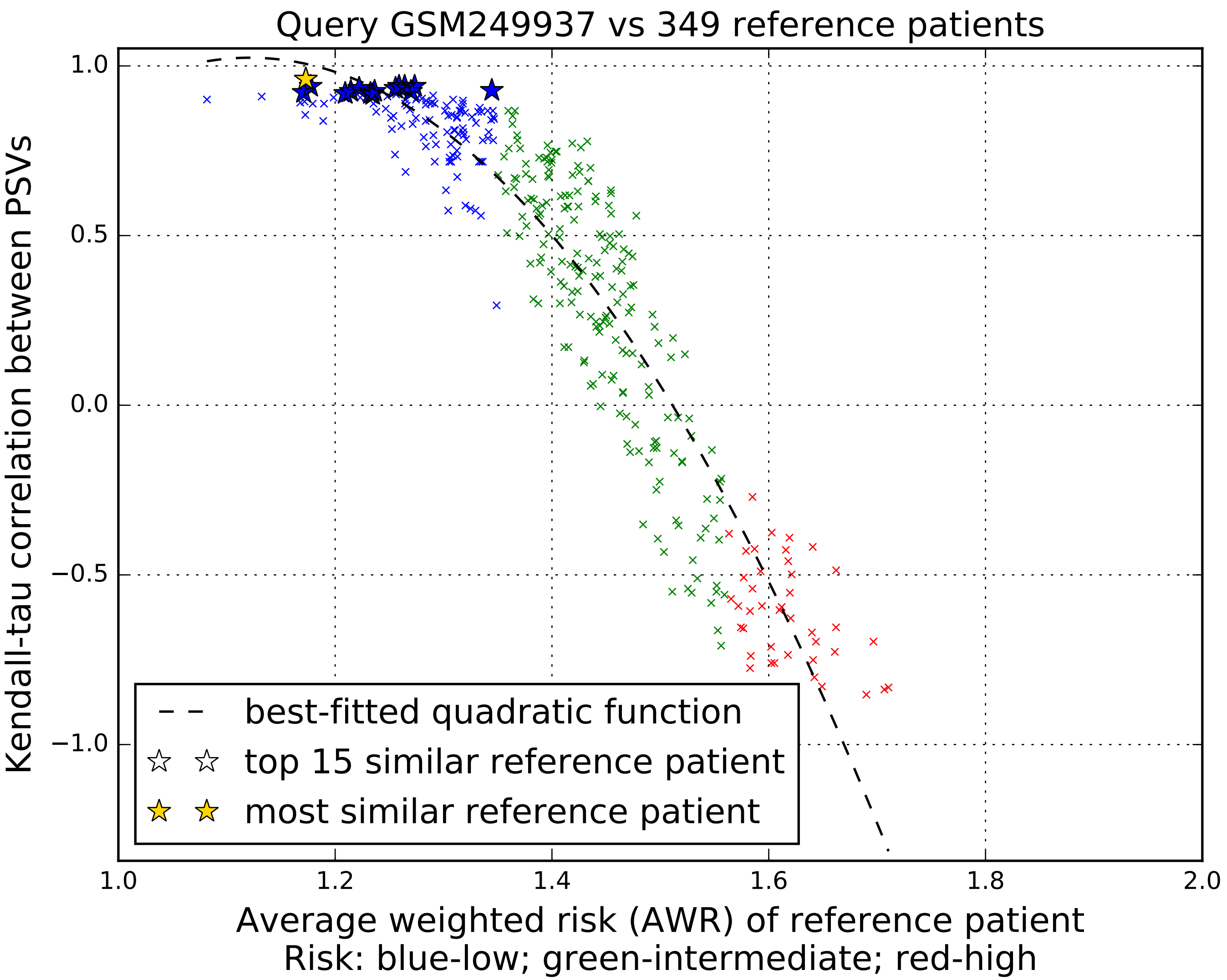

Query GSM249937 vs 349 reference patients
1.0
0.5
0.0
−0.5
−1.0
Kendall-tau correlation between PSVs
1.0
1.2
1.4
1.6
1.8
2.0
Average weighted risk (AWR) of reference patient
Risk: blue-low; green-intermediate; red-high
best-fitted quadratic function
top 15 similar reference patient
most similar reference patient

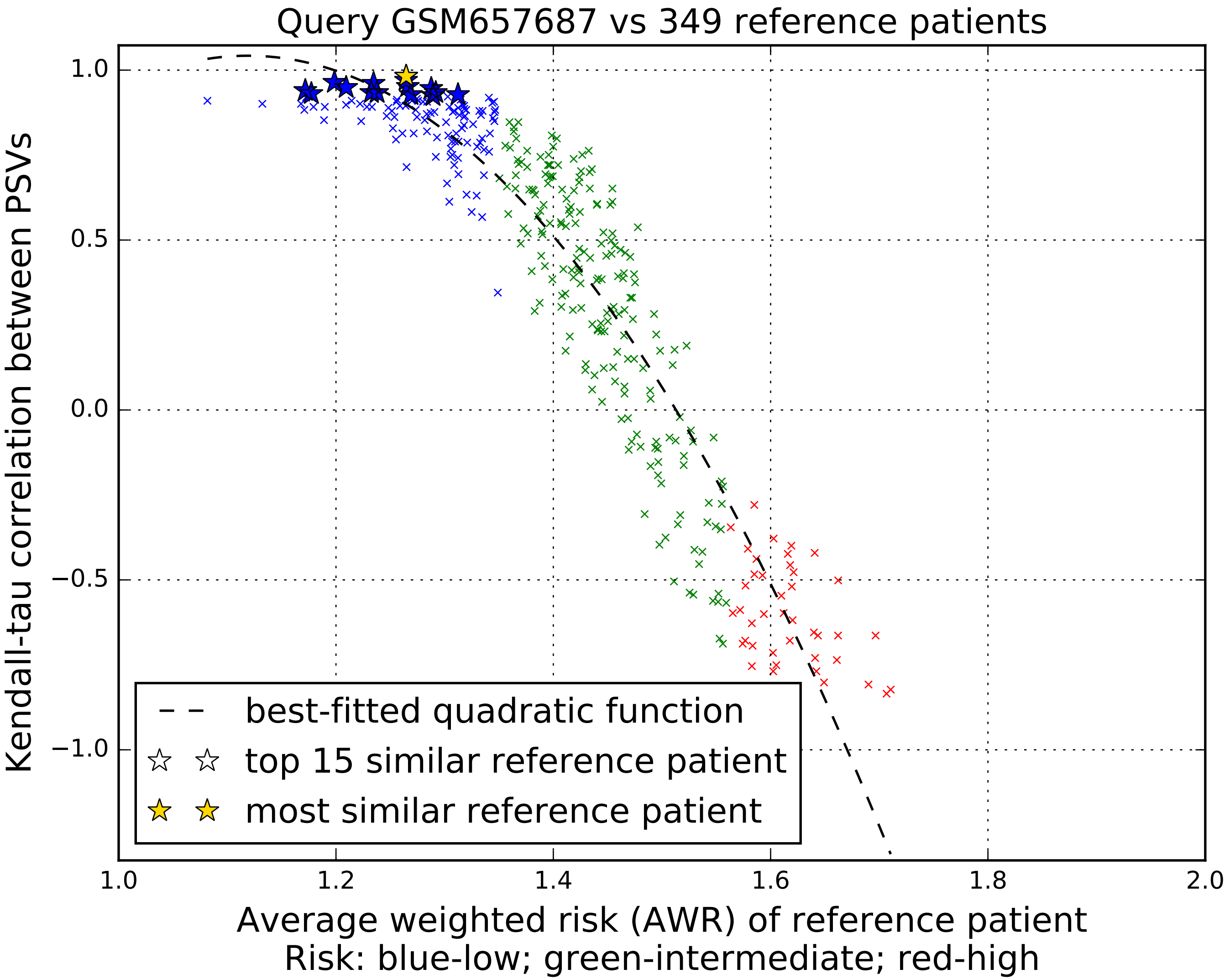

Query GSM657687 vs 349 reference patients
Kendall-tau correlation between PSVs
1.0
0.5
0.0
−0.5
−1.0
1.0
1.2
1.4
1.6
1.8
2.0
best-fitted quadratic function
top 15 similar reference patient
most similar reference patient
Average weighted risk (AWR) of reference patient
Risk: blue-low; green-intermediate; red-high

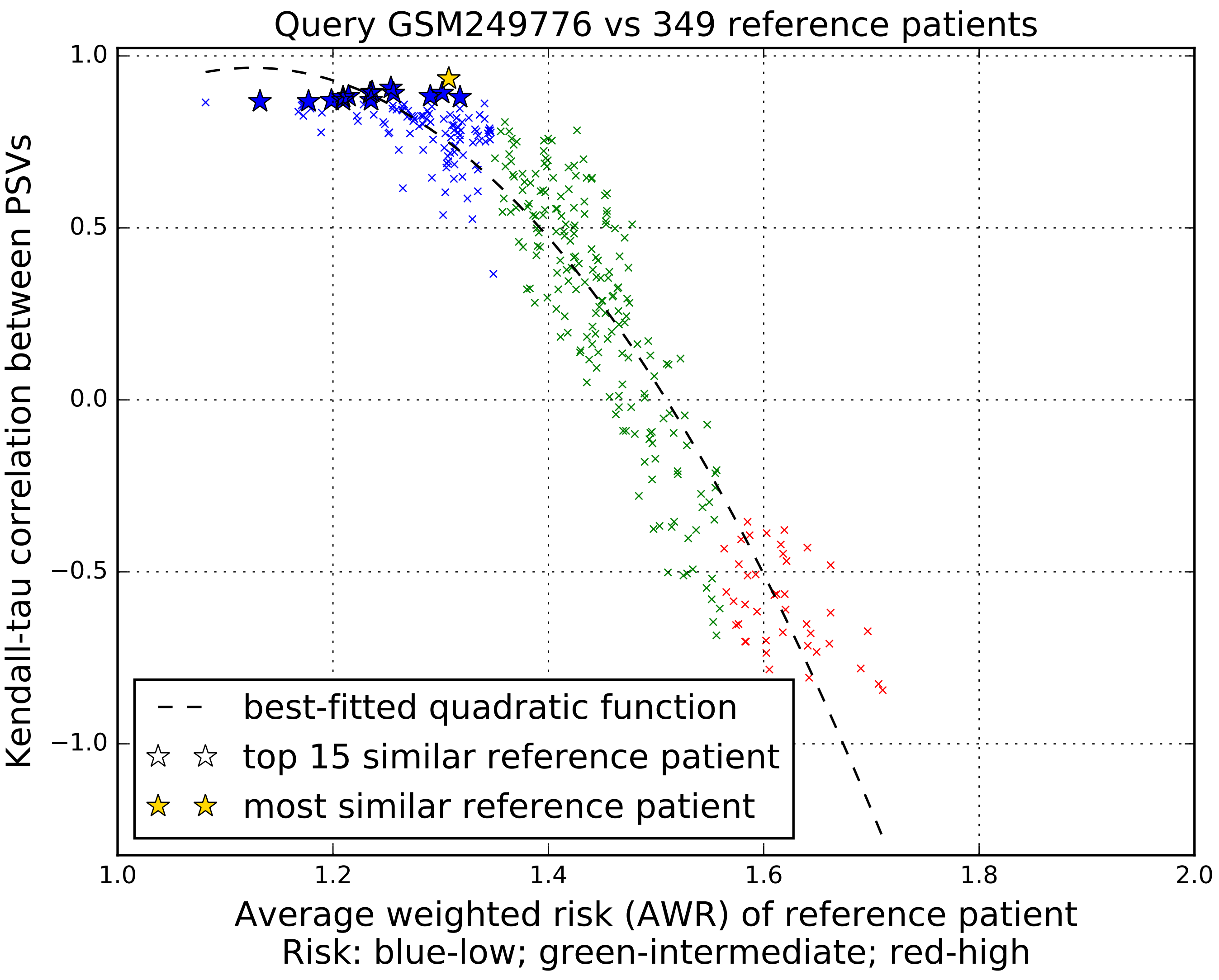
Query GSM249776 vs 349 reference patients
Kendall-tau correlation between PSVs
1.0
0.5
0.0
−0.5
−1.0
1.0
1.2
1.4
1.6
1.8
2.0
best-fitted quadratic function
top 15 similar reference patient
most similar reference patient
Average weighted risk (AWR) of reference patient
Risk: blue-low; green-intermediate; red-high

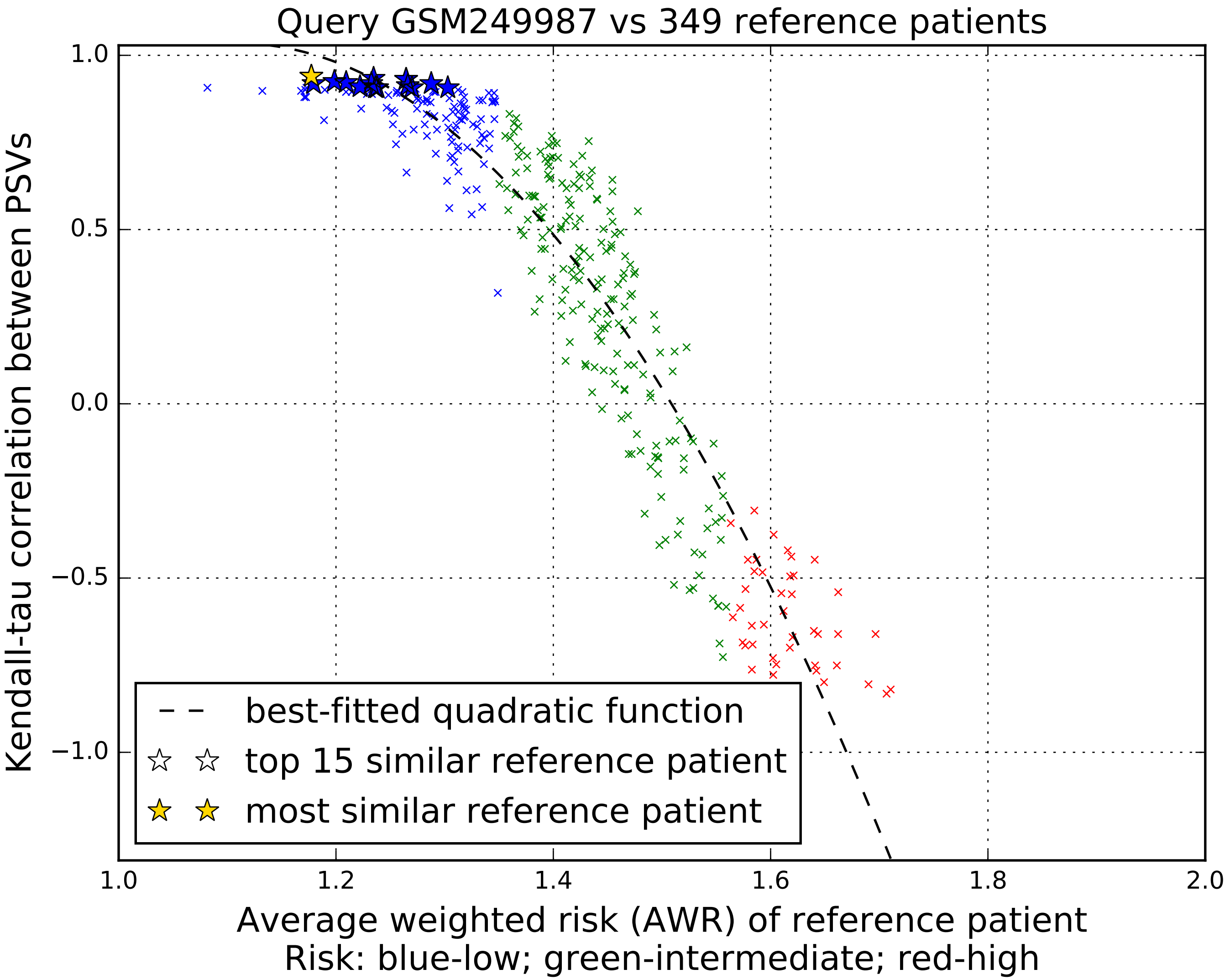
Query GSM249987 vs 349 reference patients
1.0
0.5
0.0
−0.5
−1.0
Kendall-tau correlation between PSVs
1.0
1.2
1.4
1.6
1.8
2.0
best-fitted quadratic function
top 15 similar reference patient
most similar reference patient
Average weighted risk (AWR) of reference patient
Risk: blue-low; green-intermediate; red-high

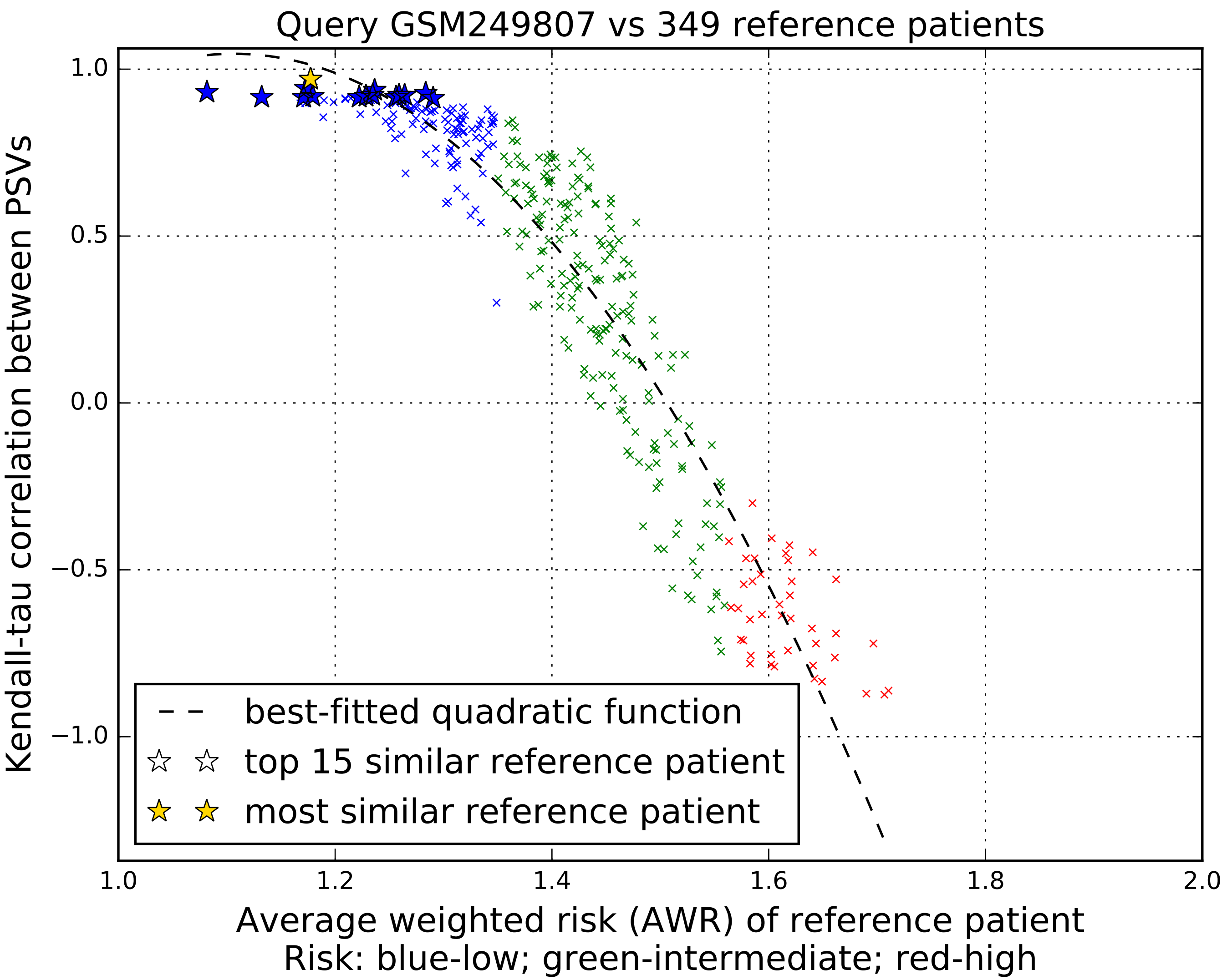
Query GSM249807 vs 349 reference patients
Kendall-tau correlation between PSVs
1.0
0.5
0.0
−0.5
−1.0
1.0
1.2
1.4
1.6
1.8
2.0
best-fitted quadratic function
top 15 similar reference patient
most similar reference patient
Average weighted risk (AWR) of reference patient
Risk: blue-low; green-intermediate; red-high

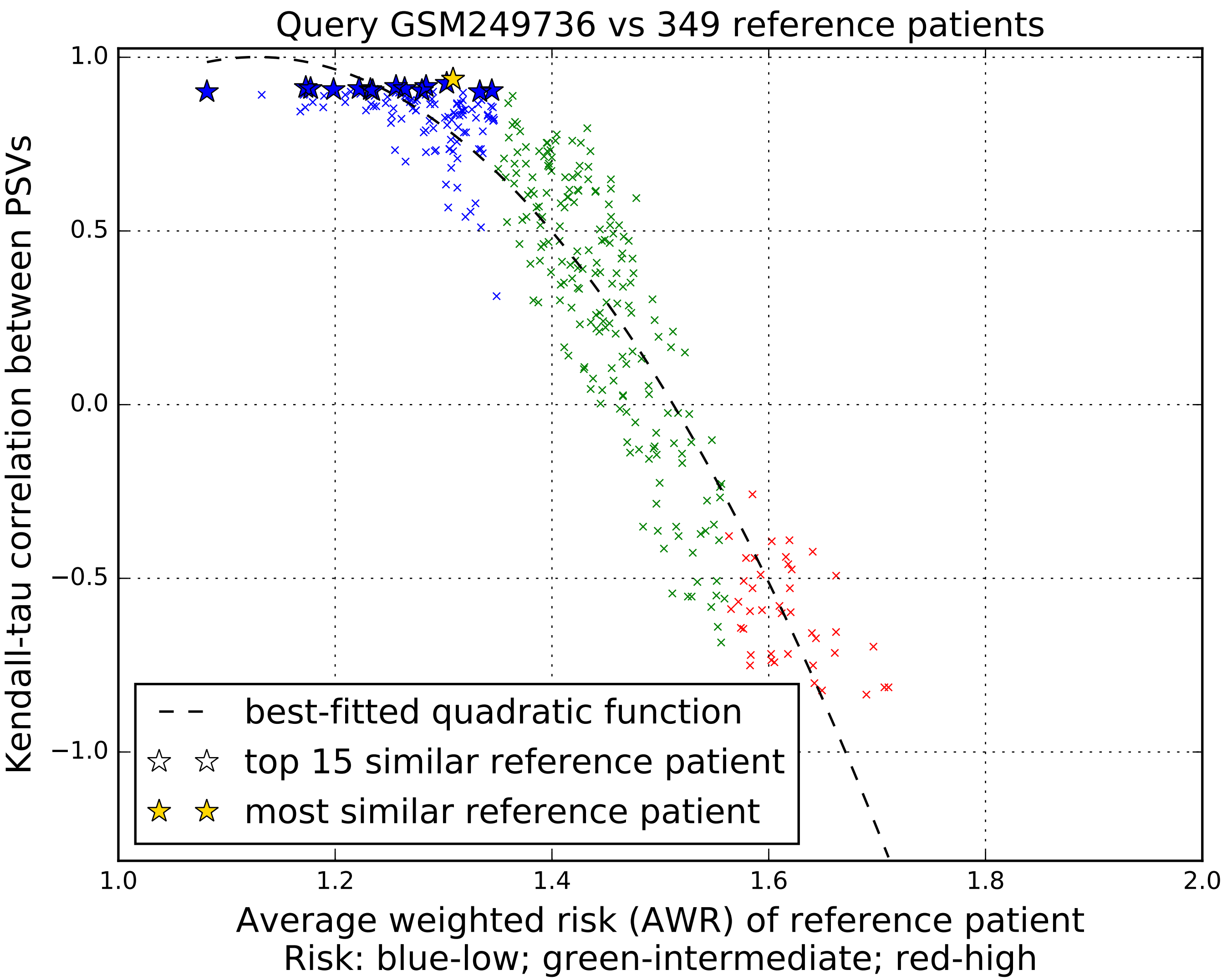
Query GSM249736 vs 349 reference patients
Kendall-tau correlation between PSVs
Average weighted risk (AWR) of reference patient
Risk: blue-low; green-intermediate; red-high
1.0
0.5
0.0
−0.5
−1.0
1.0
1.2
1.4
1.6
1.8
2.0
best-fitted quadratic function
top 15 similar reference patient
most similar reference patient

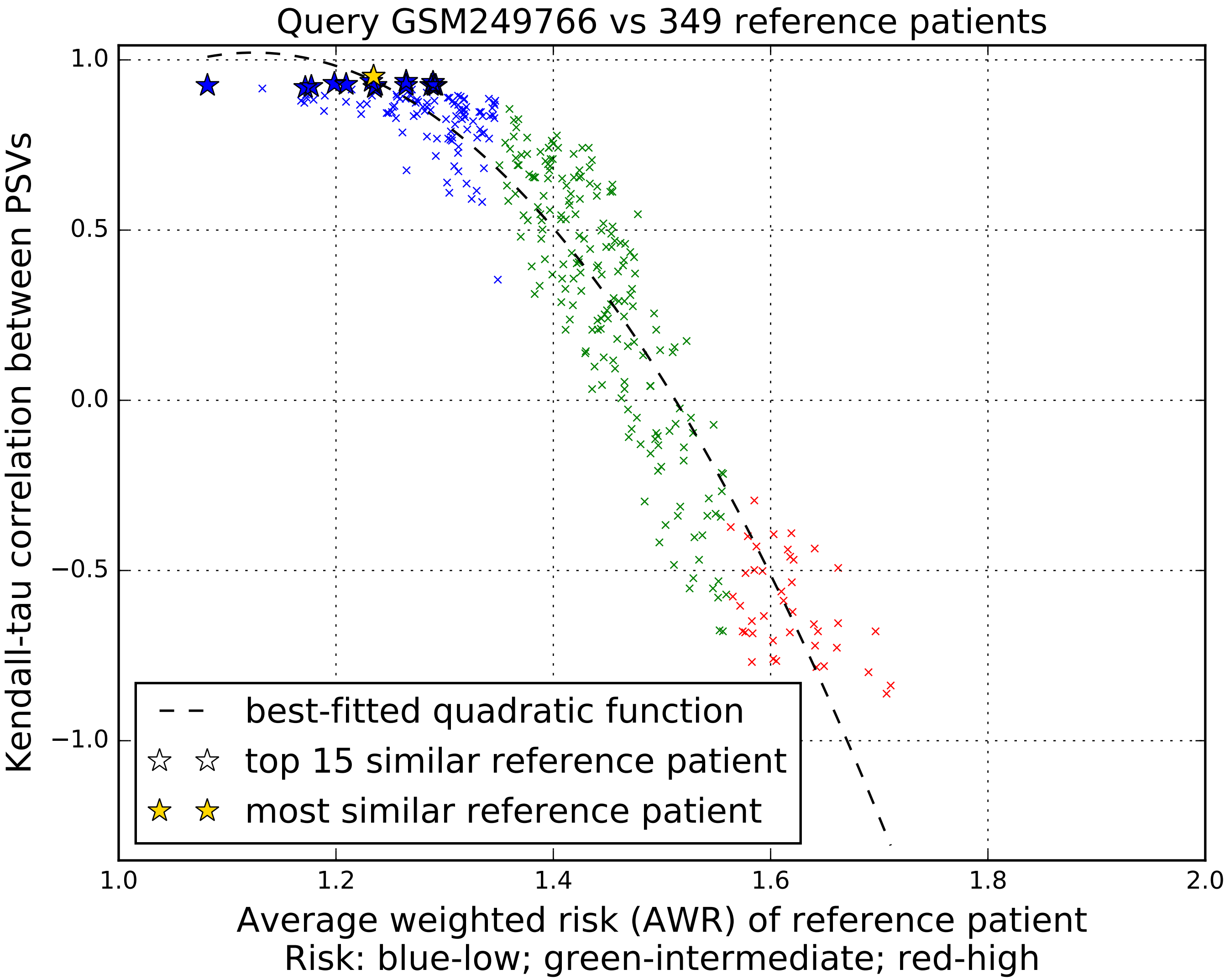

Query GSM249766 vs 349 reference patients
Kendall-tau correlation between PSVs
1.0
0.5
0.0
−0.5
−1.0
1.0
1.2
1.4
1.6
1.8
2.0
best-fitted quadratic function
top 15 similar reference patient
most similar reference patient
Average weighted risk (AWR) of reference patient
Risk: blue-low; green-intermediate; red-high

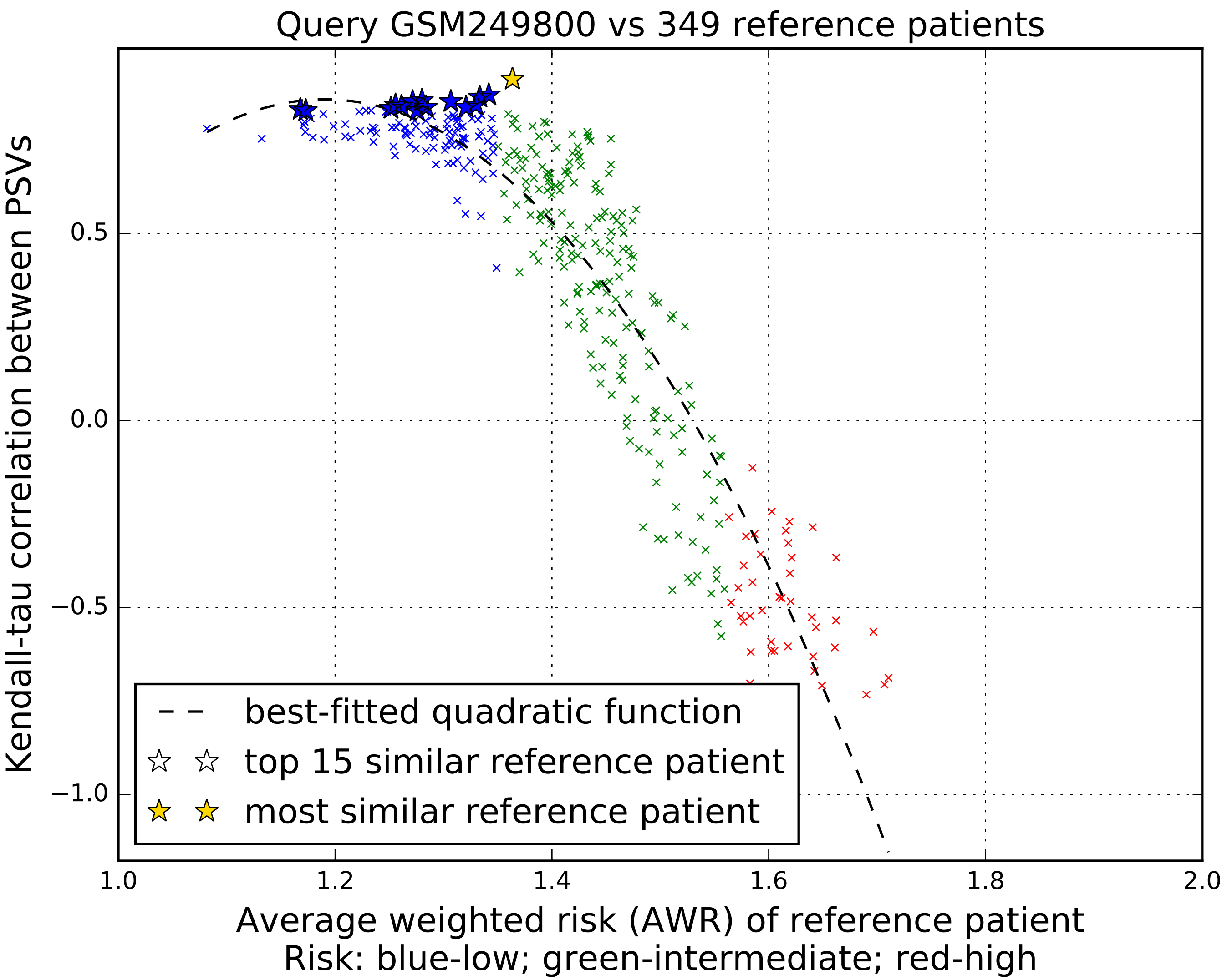
Query GSM249800 vs 349 reference patients
Kendall-tau correlation between PSVs
0.5
0.0
−0.5
−1.0
1.0
1.2
1.4
1.6
1.8
2.0
Average weighted risk (AWR) of reference patient
Risk: blue-low; green-intermediate; red-high
best-fitted quadratic function
top 15 similar reference patient
most similar reference patient

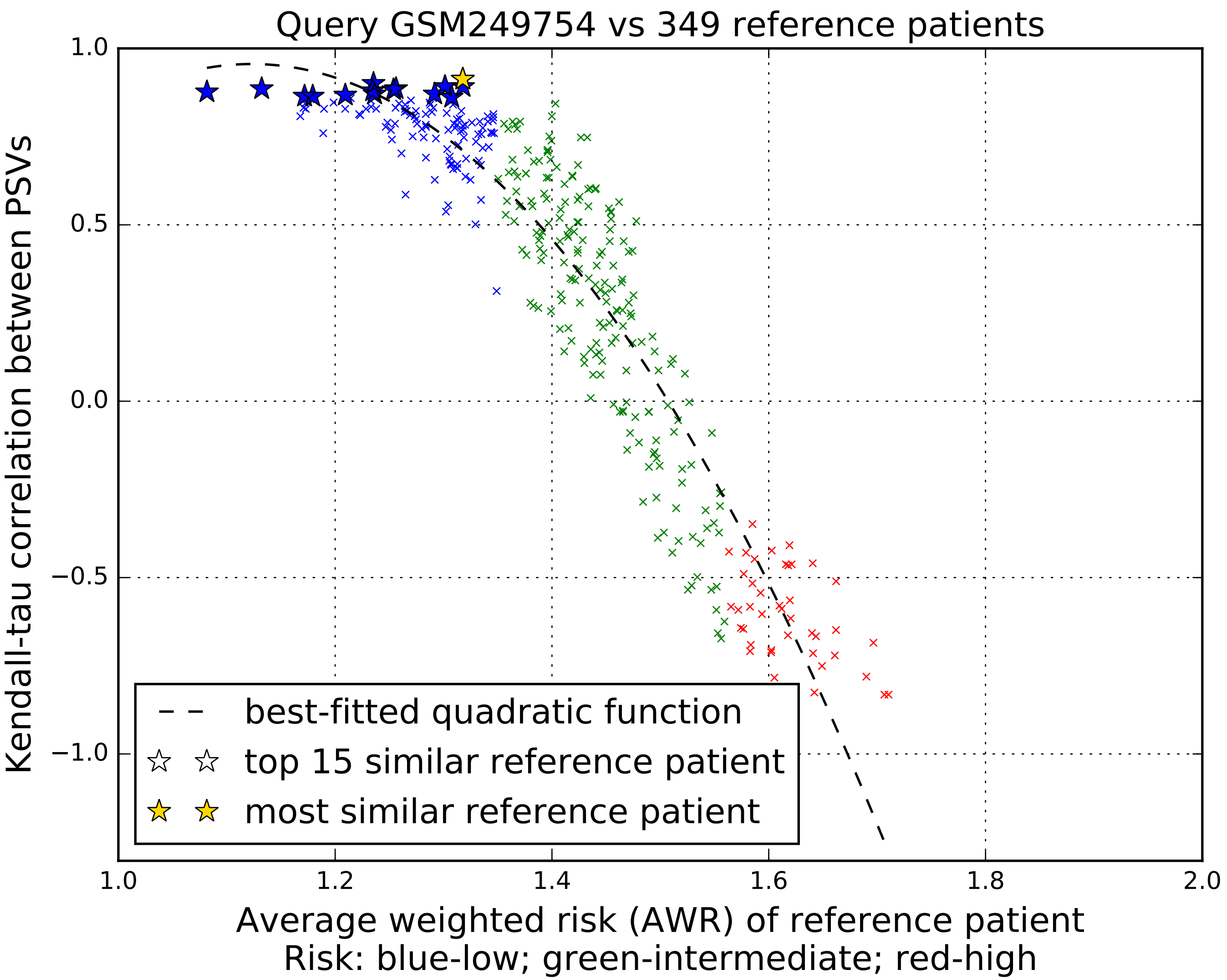
Query GSM249754 vs 349 reference patients
Kendall-tau correlation between PSVs
Average weighted risk (AWR) of reference patient
Risk: blue-low; green-intermediate; red-high
best-fitted quadratic function
top 15 similar reference patient
most similar reference patient
1.0
0.5
0.0
−0.5
−1.0
1.2
1.4
1.6
1.8
2.0

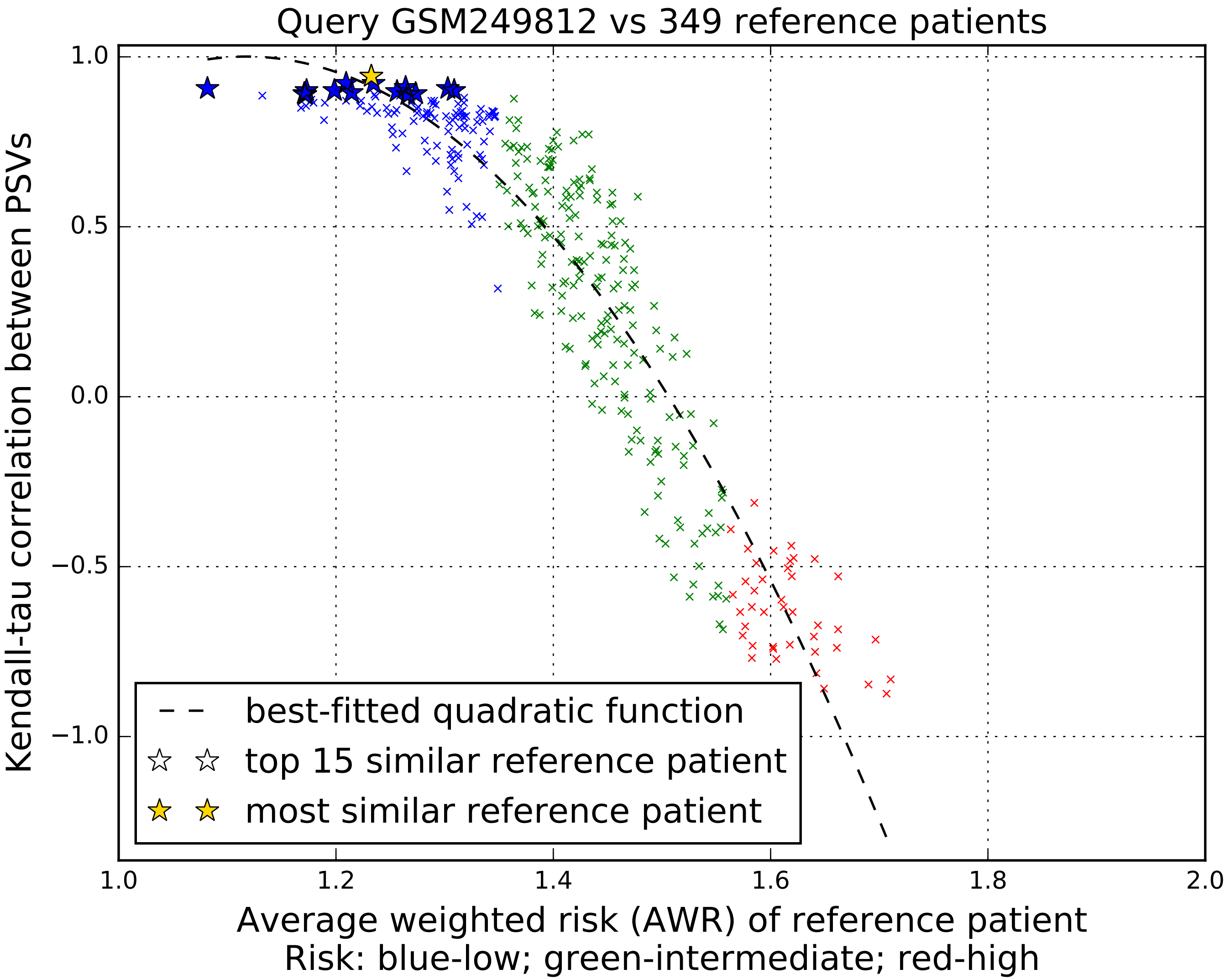
Query GSM249812 vs 349 reference patients
Kendall-tau correlation between PSVs
1.0
0.5
0.0
−0.5
−1.0
1.0
1.2
1.4
1.6
1.8
2.0
best-fitted quadratic function
top 15 similar reference patient
most similar reference patient
Average weighted risk (AWR) of reference patient
Risk: blue-low; green-intermediate; red-high

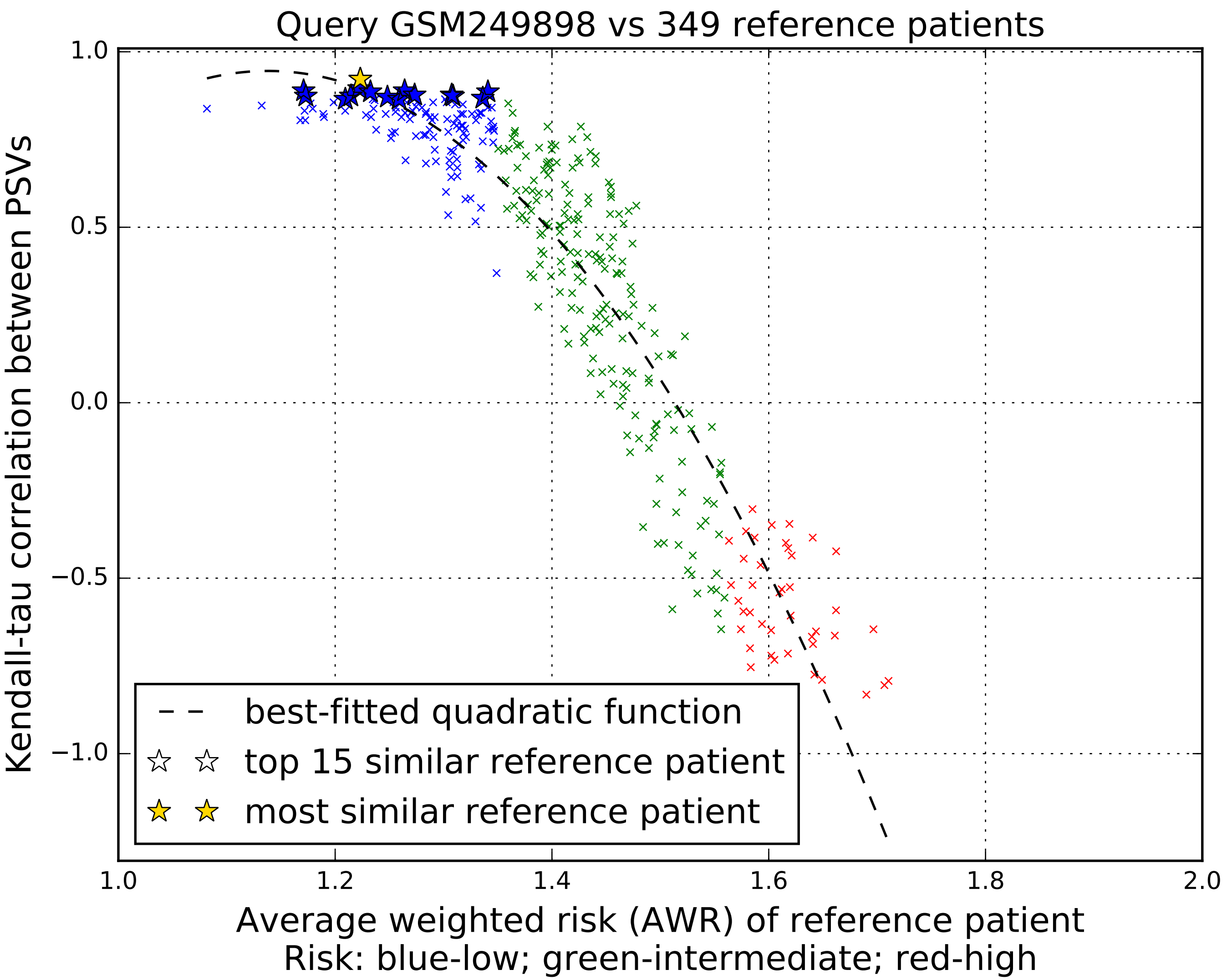

Query GSM249898 vs 349 reference patients
Kendall-tau correlation between PSVs
1.0
0.5
0.0
−0.5
−1.0
1.0
1.2
1.4
1.6
1.8
2.0
best-fitted quadratic function
top 15 similar reference patient
most similar reference patient
Average weighted risk (AWR) of reference patient
Risk: blue-low; green-intermediate; red-high

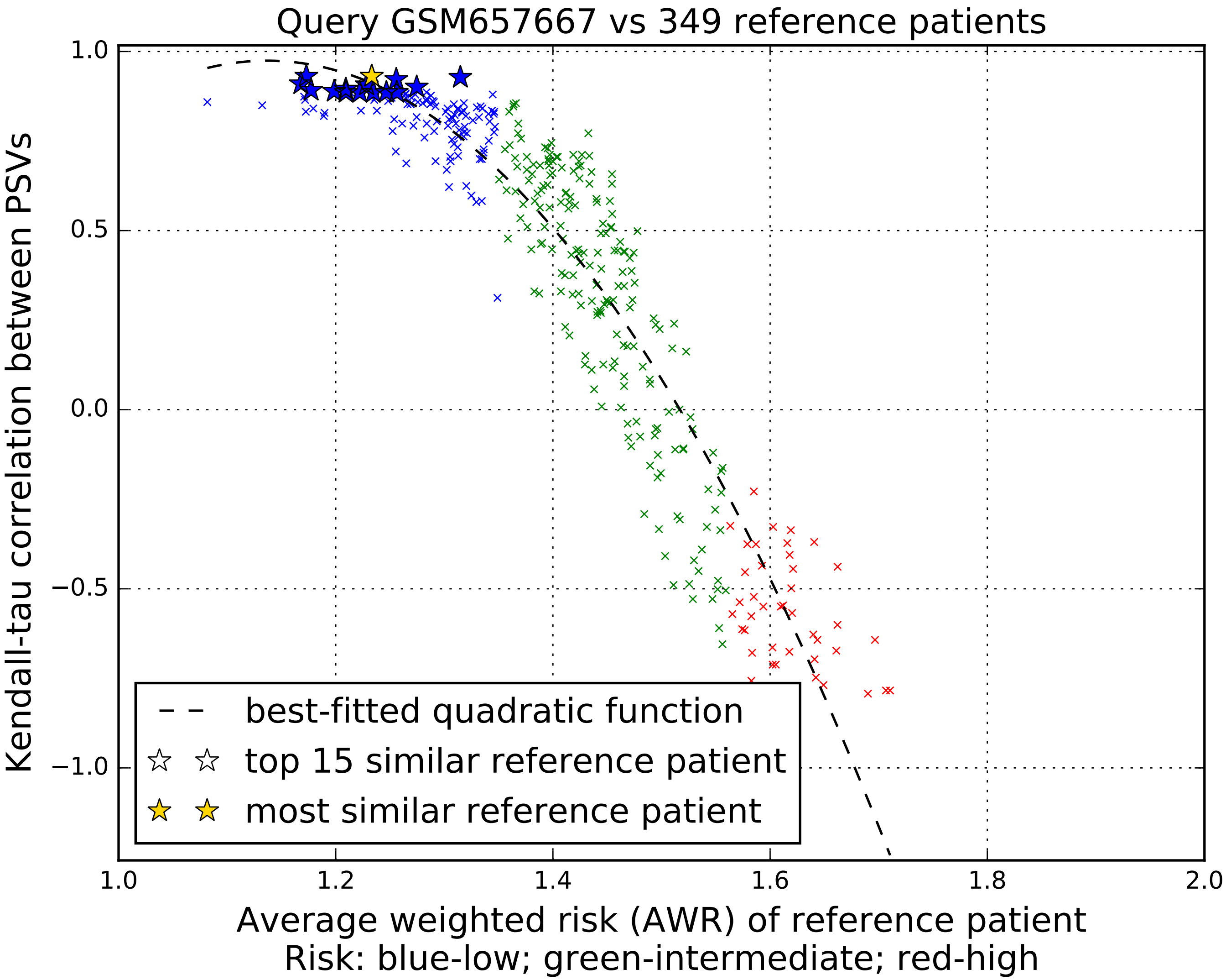

Query GSM657667 vs 349 reference patients
Kendall-tau correlation between PSVs
1.0
0.5
0.0
−0.5
−1.0
1.0
1.2
1.4
1.6
1.8
2.0
best-fitted quadratic function
top 15 similar reference patient
most similar reference patient
Average weighted risk (AWR) of reference patient
Risk: blue-low; green-intermediate; red-high

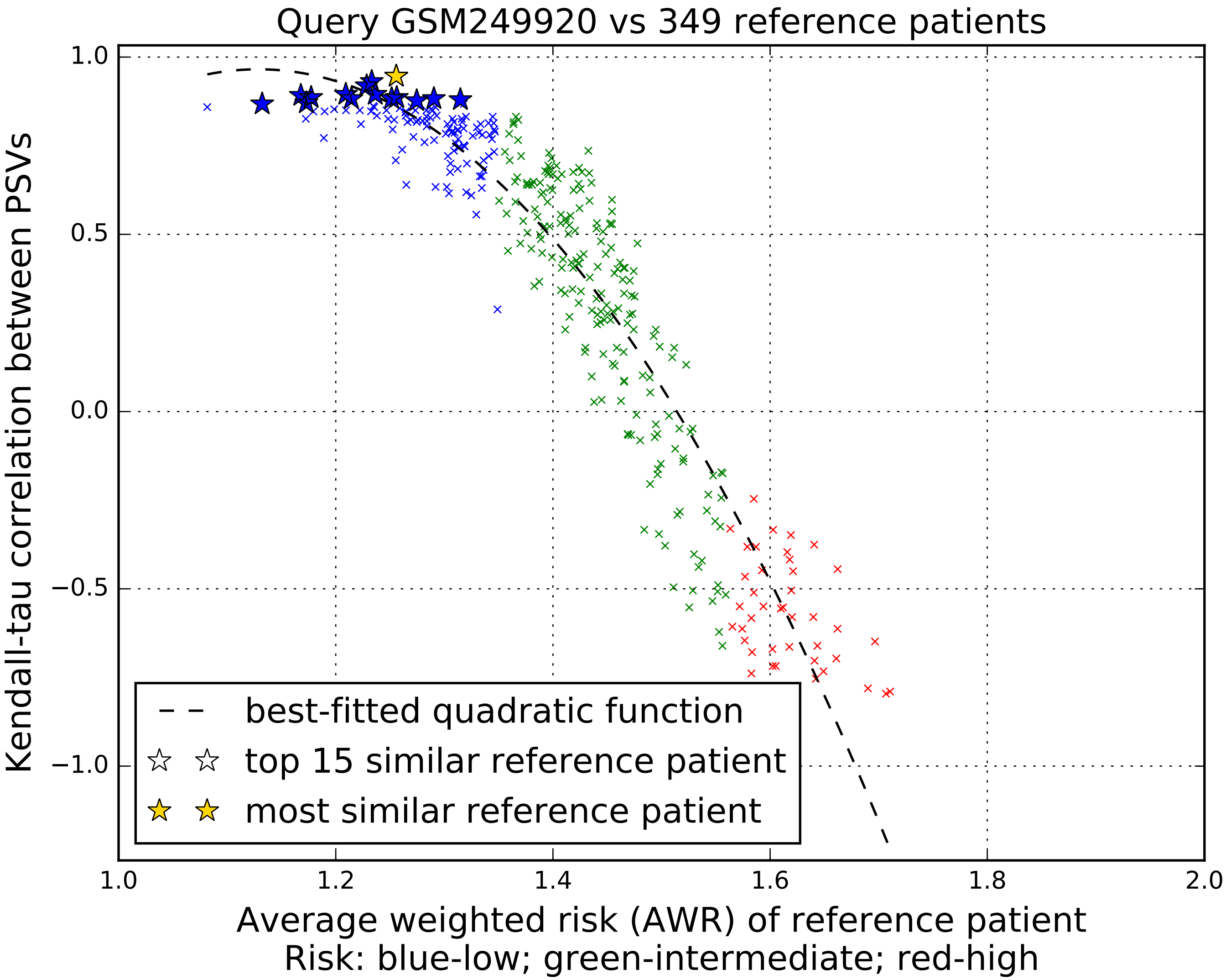

Query GSM249920 vs 349 reference patients
Kendall-tau correlation between PSVs
1.0
0.5
0.0
−0.5
−1.0
1.0
1.2
1.4
1.6
1.8
2.0
best-fitted quadratic function
top 15 similar reference patient
most similar reference patient
Average weighted risk (AWR) of reference patient
Risk: blue-low; green-intermediate; red-high

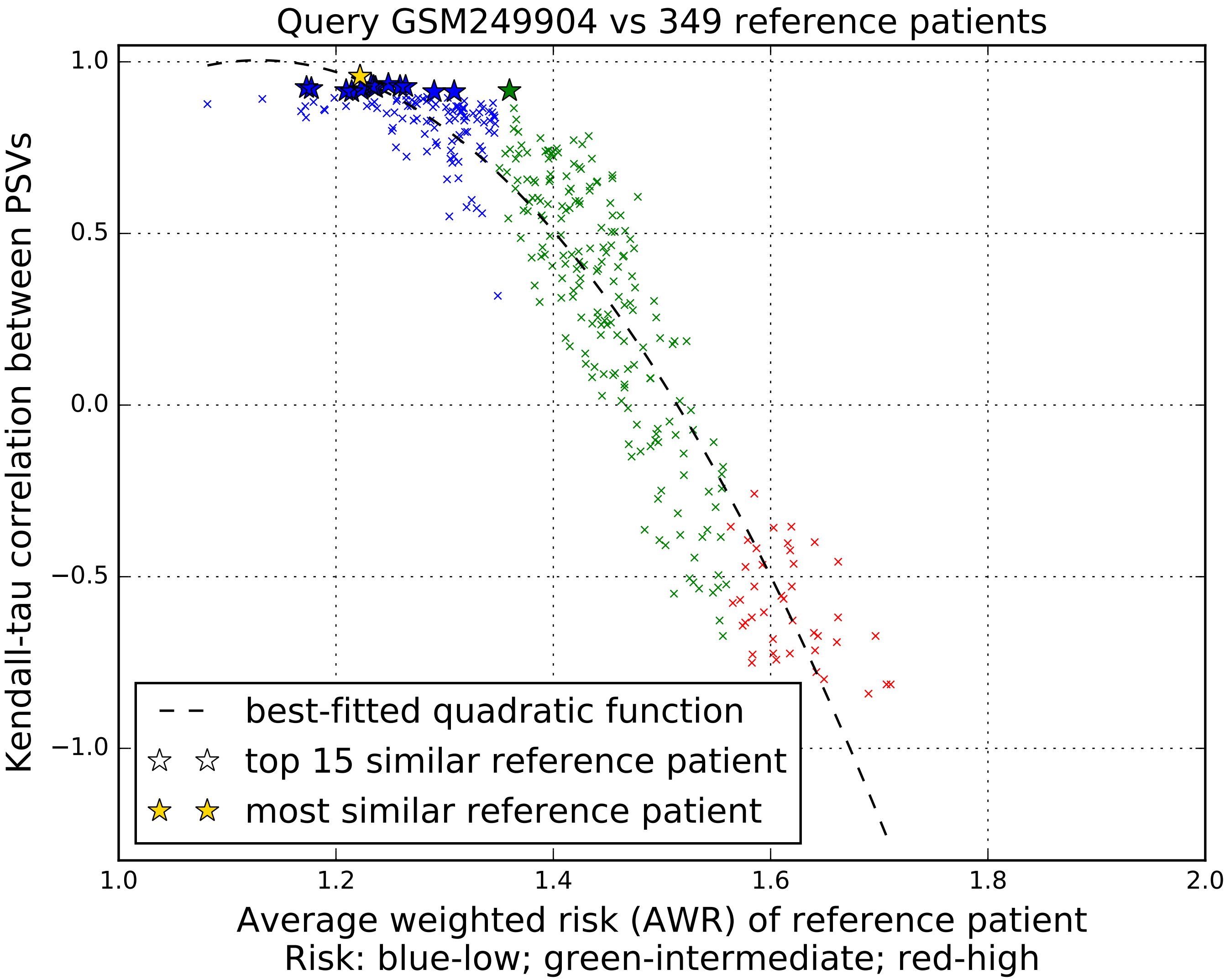

Query GSM249904 vs 349 reference patients
Kendall-tau correlation between PSVs
1.0
0.5
0.0
−0.5
−1.0
1.0
1.2
1.4
1.6
1.8
2.0
best-fitted quadratic function
top 15 similar reference patient
most similar reference patient
Average weighted risk (AWR) of reference patient
Risk: blue-low; green-intermediate; red-high

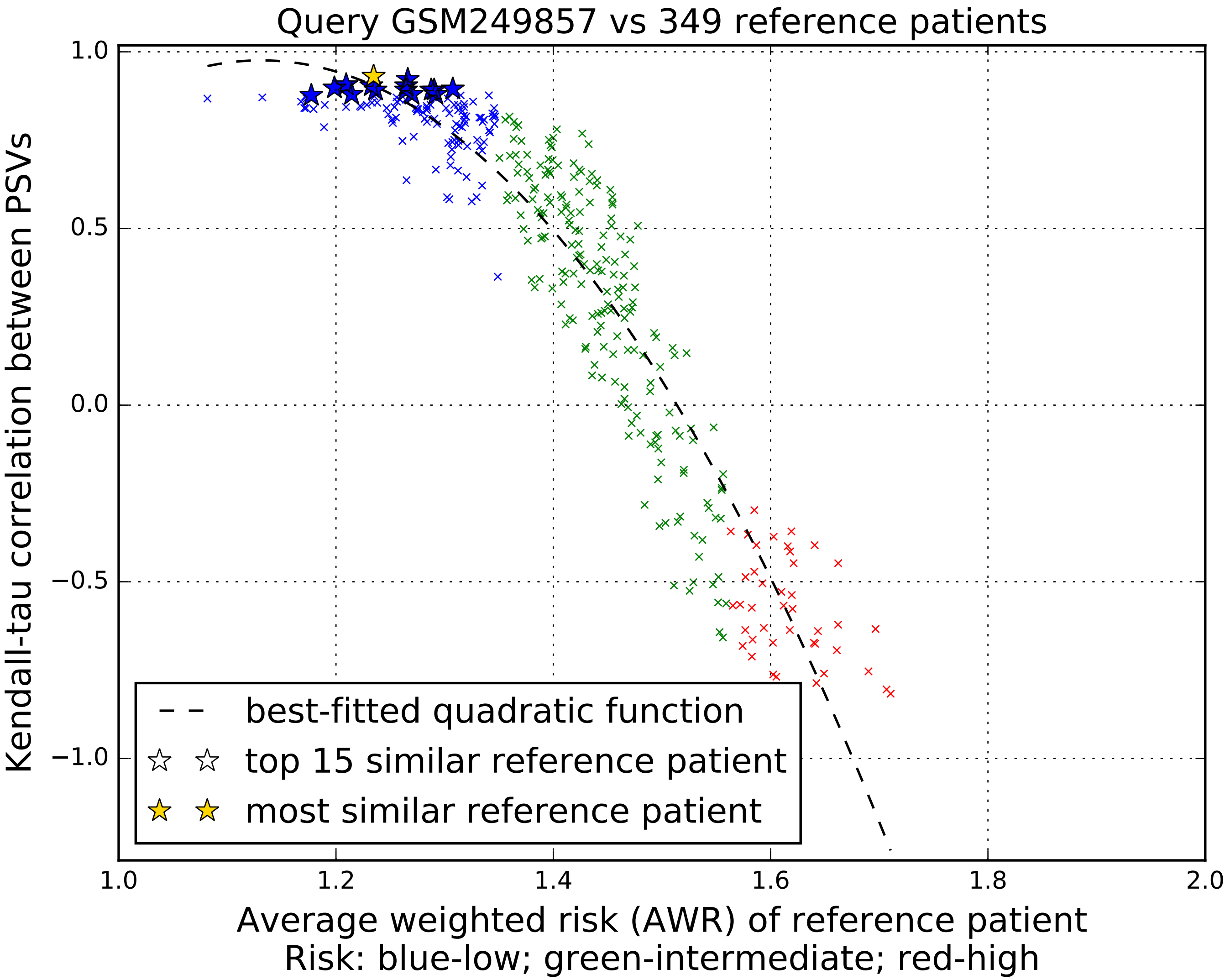

Query GSM249857 vs 349 reference patients
1.0
0.5
0.0
−0.5
−1.0
1.0
1.2
1.4
1.6
1.8
2.0
Kendall-tau correlation between PSVs
Average weighted risk (AWR) of reference patient
Risk: blue-low; green-intermediate; red-high
best-fitted quadratic function
top 15 similar reference patient
most similar reference patient

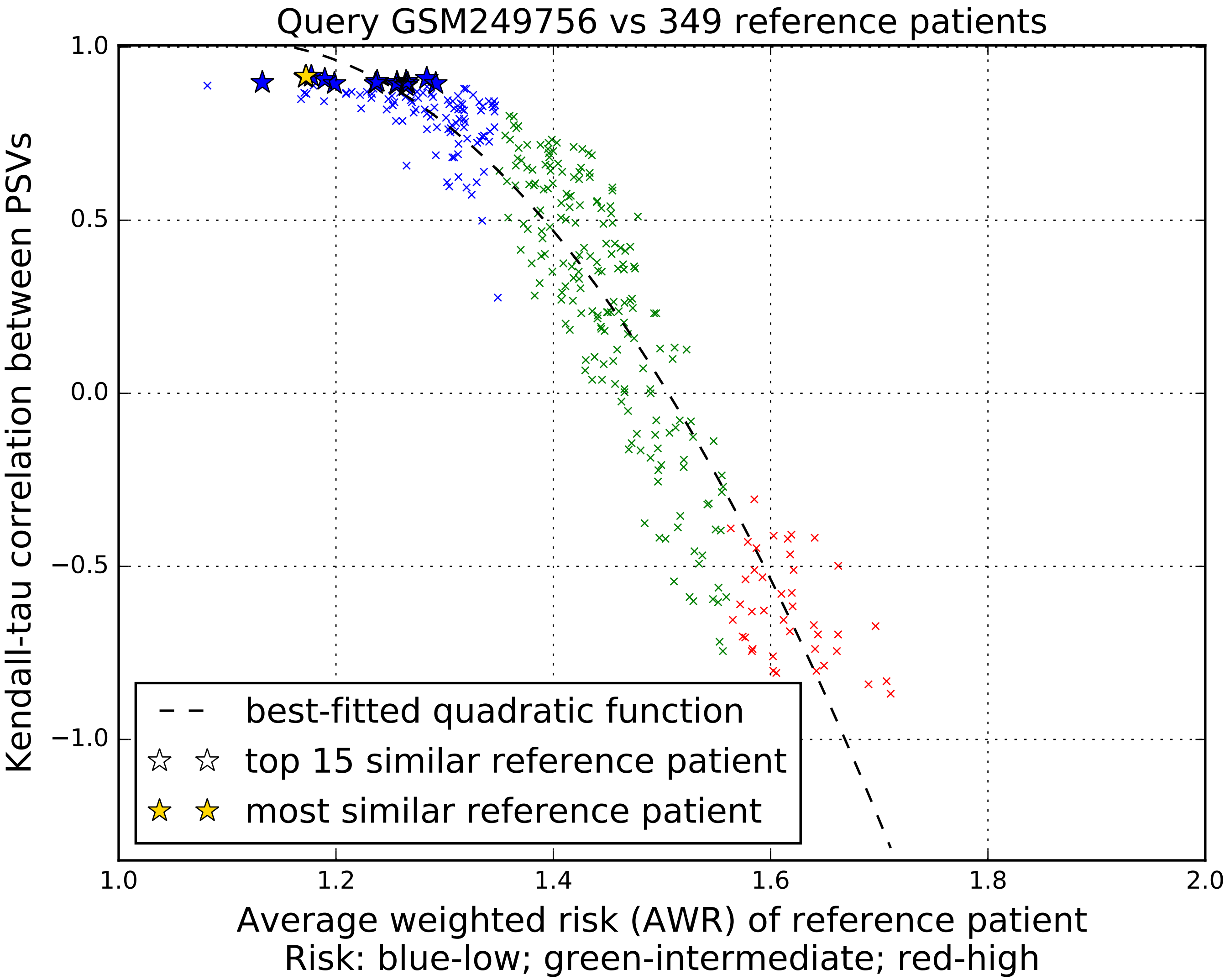

Query GSM249756 vs 349 reference patients
Kendall-tau correlation between PSVs
1.0
0.5
0.0
−0.5
−1.0
1.0
1.2
1.4
1.6
1.8
2.0
best-fitted quadratic function
top 15 similar reference patient
most similar reference patient
Average weighted risk (AWR) of reference patient
Risk: blue-low; green-intermediate; red-high

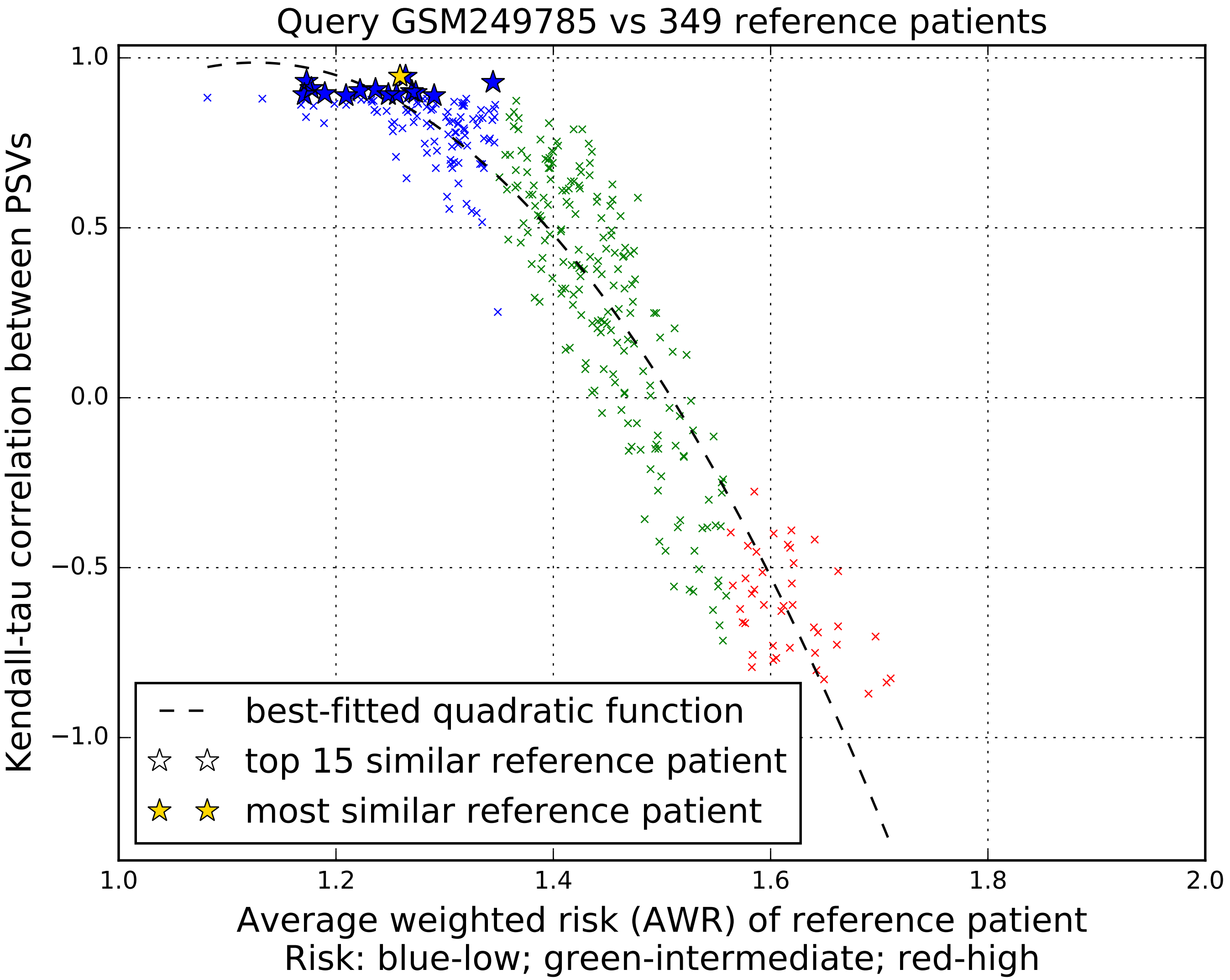
Query GSM249785 vs 349 reference patients
Kendall-tau correlation between PSVs
Average weighted risk (AWR) of reference patient
Risk: blue-low; green-intermediate; red-high
best-fitted quadratic function
top 15 similar reference patient
most similar reference patient
1.0
0.5
0.0
−0.5
−1.0
1.0
1.2
1.4
1.6
1.8
2.0

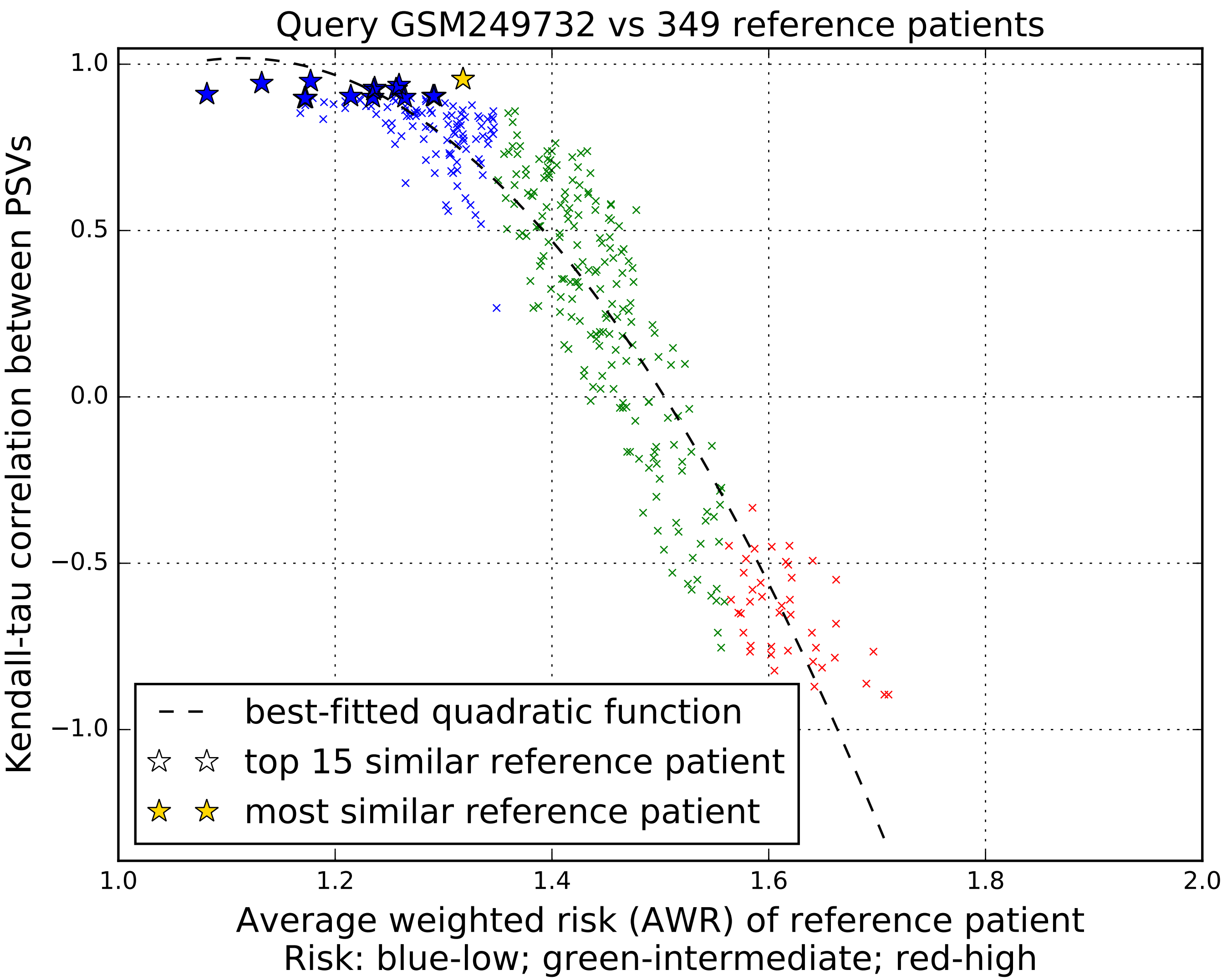
Query GSM249732 vs 349 reference patients
Kendall-tau correlation between PSVs
Average weighted risk (AWR) of reference patient
Risk: blue-low; green-intermediate; red-high
best-fitted quadratic function
top 15 similar reference patient
most similar reference patient
1.0
0.5
0.0
−0.5
−1.0
1.0
1.2
1.4
1.6
1.8
2.0

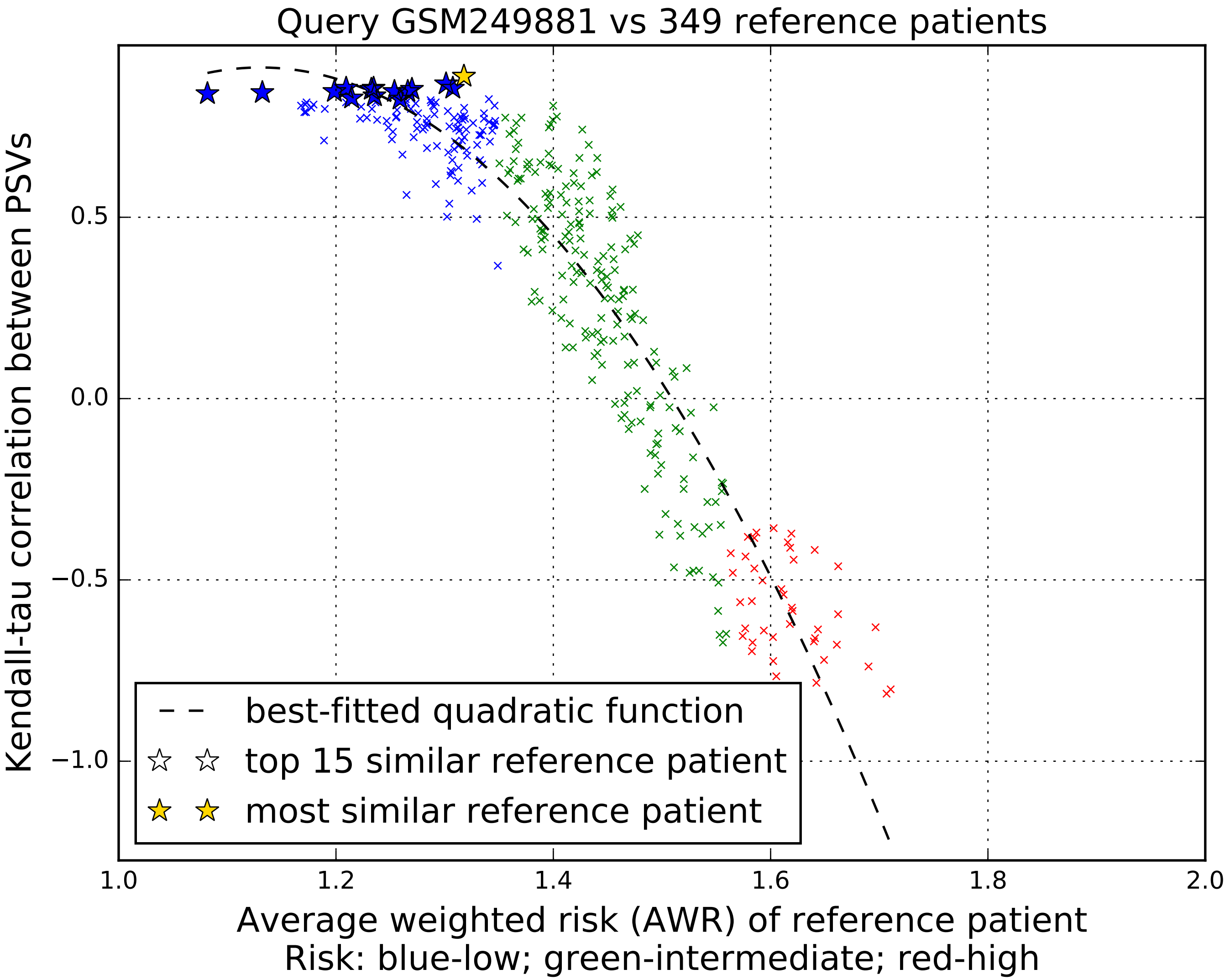
Query GSM249881 vs 349 reference patients
Kendall-tau correlation between PSVs
0.5
0.0
−0.5
−1.0
1.0
1.2
1.4
1.6
1.8
2.0
best-fitted quadratic function
top 15 similar reference patient
most similar reference patient
Average weighted risk (AWR) of reference patient
Risk: blue-low; green-intermediate; red-high

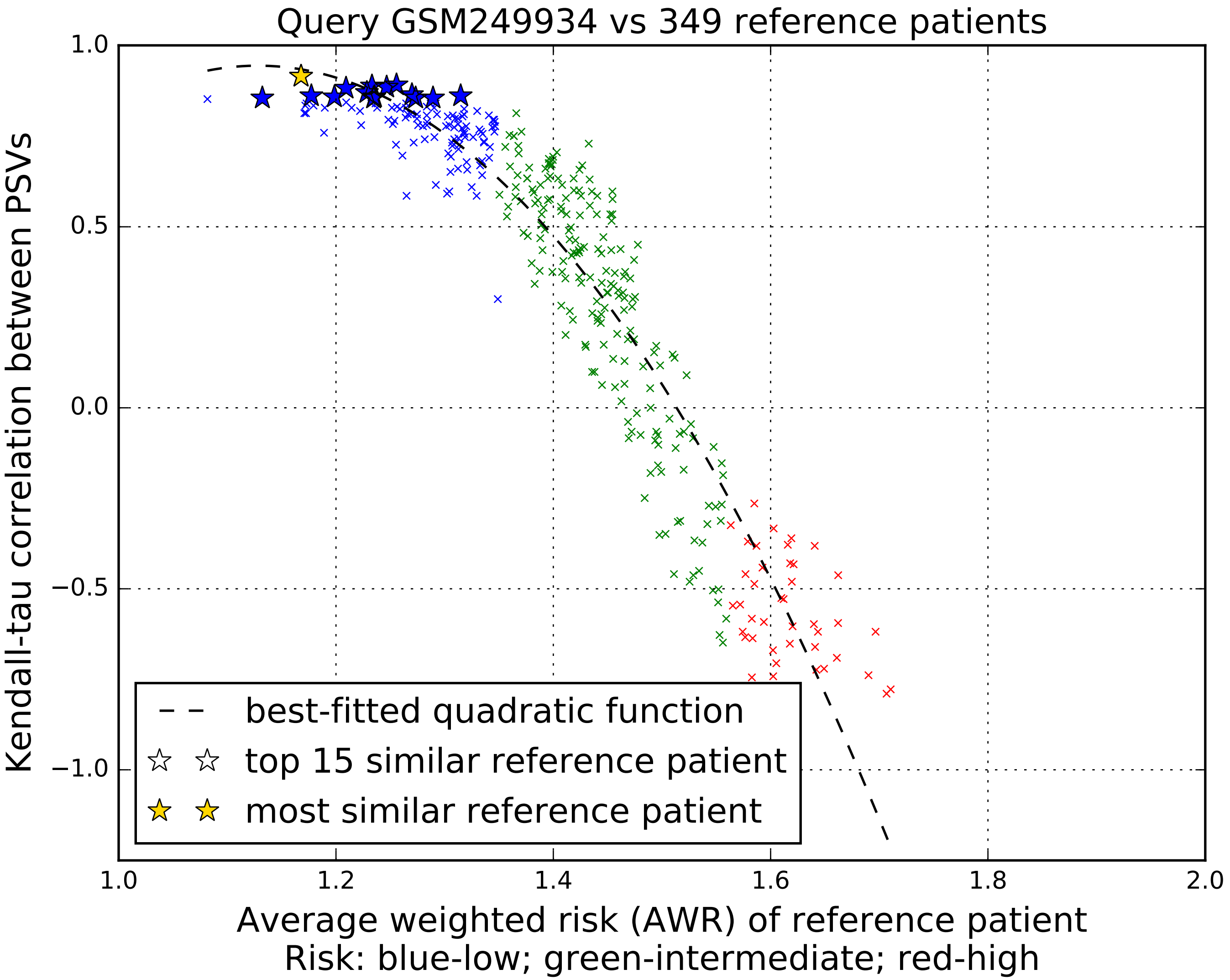

Query GSM249934 vs 349 reference patients
Kendall-tau correlation between PSVs
Average weighted risk (AWR) of reference patient
Risk: blue-low; green-intermediate; red-high
best-fitted quadratic function
top 15 similar reference patient
most similar reference patient
1.0
0.5
0.0
−0.5
−1.0
1.0
1.2
1.4
1.6
1.8
2.0

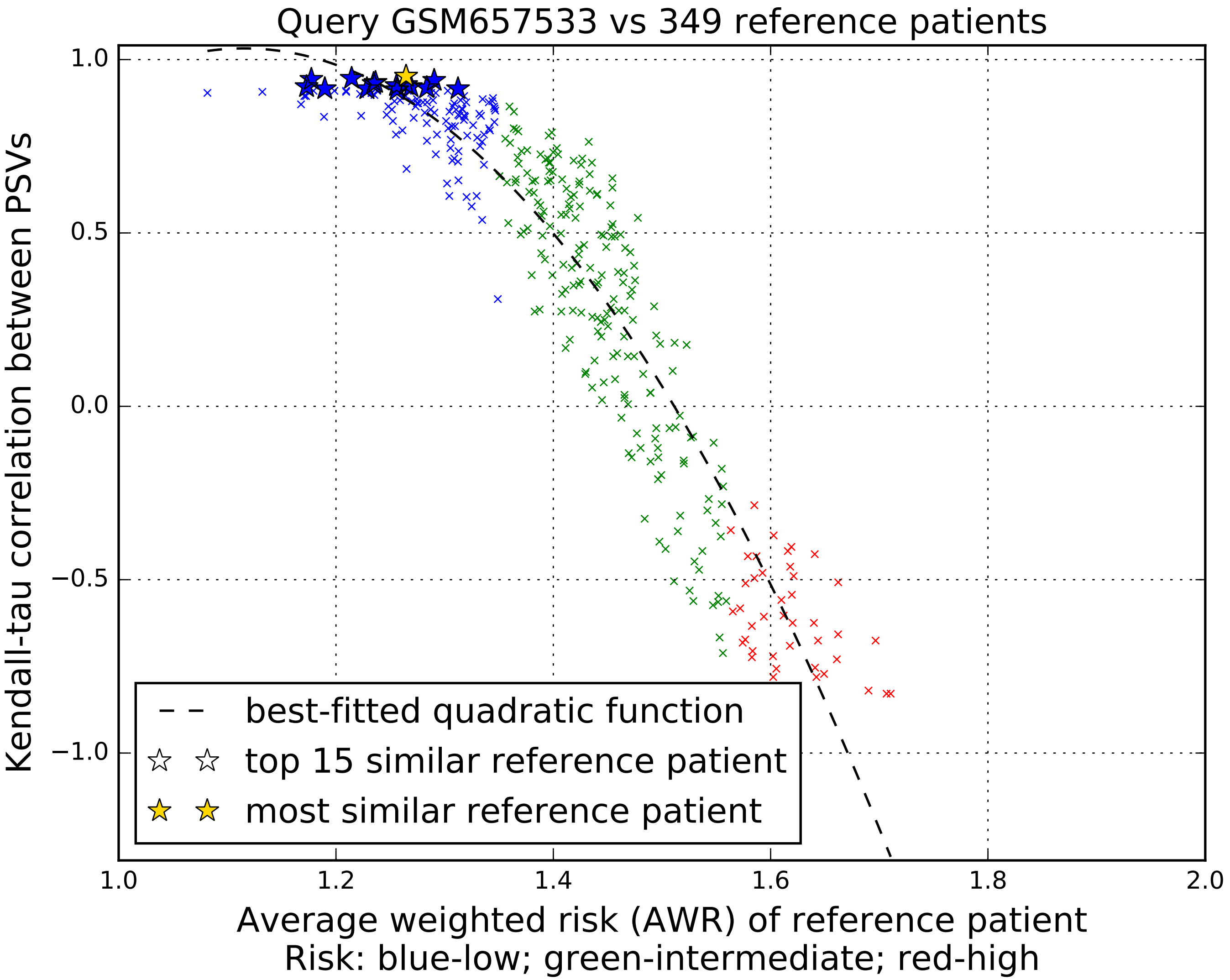

Query GSM657533 vs 349 reference patients
1.0
0.5
0.0
−0.5
−1.0
Kendall-tau correlation between PSVs
best-fitted quadratic function
top 15 similar reference patient
most similar reference patient
1.0
1.2
1.4
1.6
1.8
2.0
Average weighted risk (AWR) of reference patient
Risk: blue-low; green-intermediate; red-high

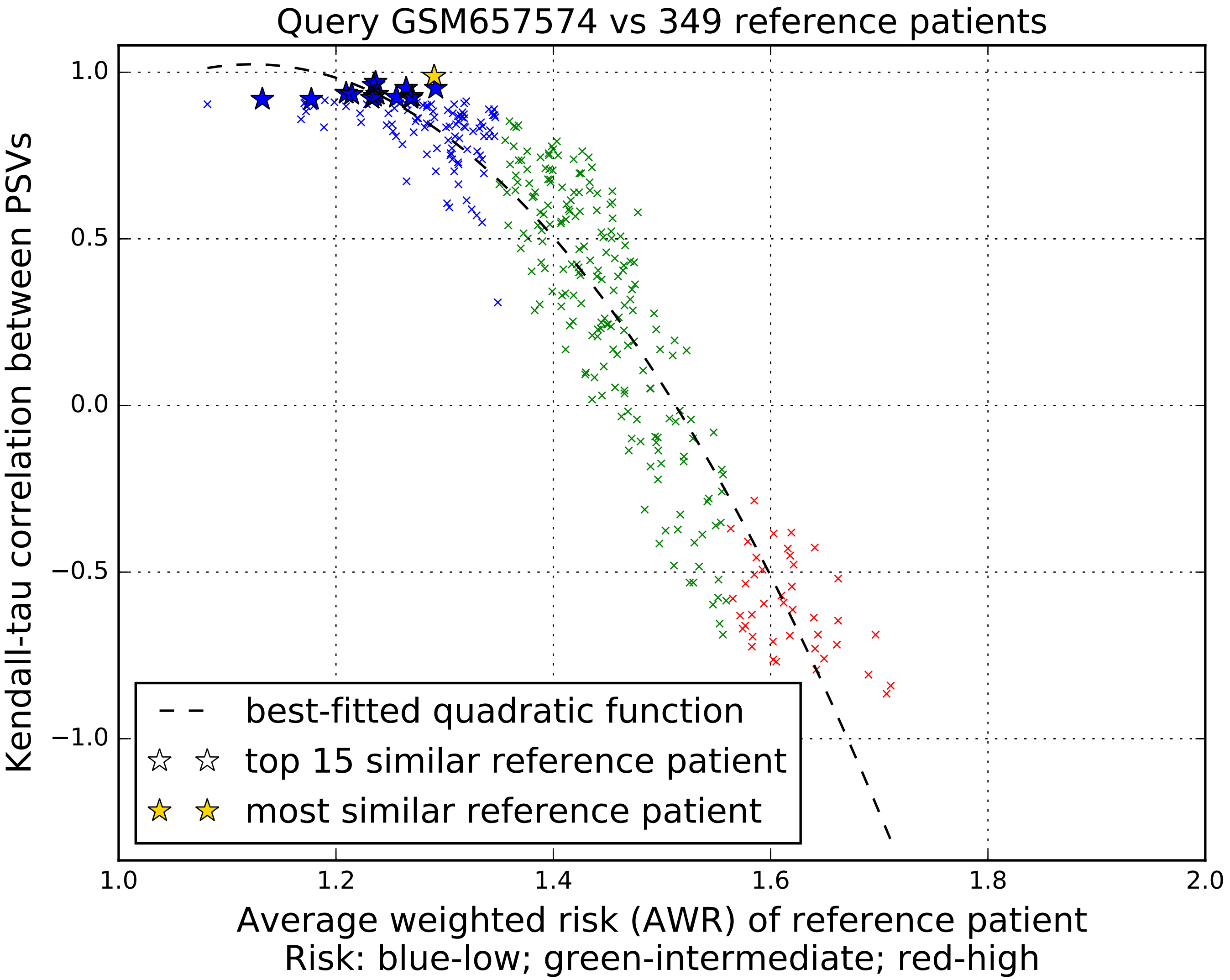
Query GSM657574 vs 349 reference patients
Kendall-tau correlation between PSVs
1.0
0.5
0.0
−0.5
−1.0
1.0
1.2
1.4
1.6
1.8
2.0
best-fitted quadratic function
top 15 similar reference patient
most similar reference patient
Average weighted risk (AWR) of reference patient
Risk: blue-low; green-intermediate; red-high

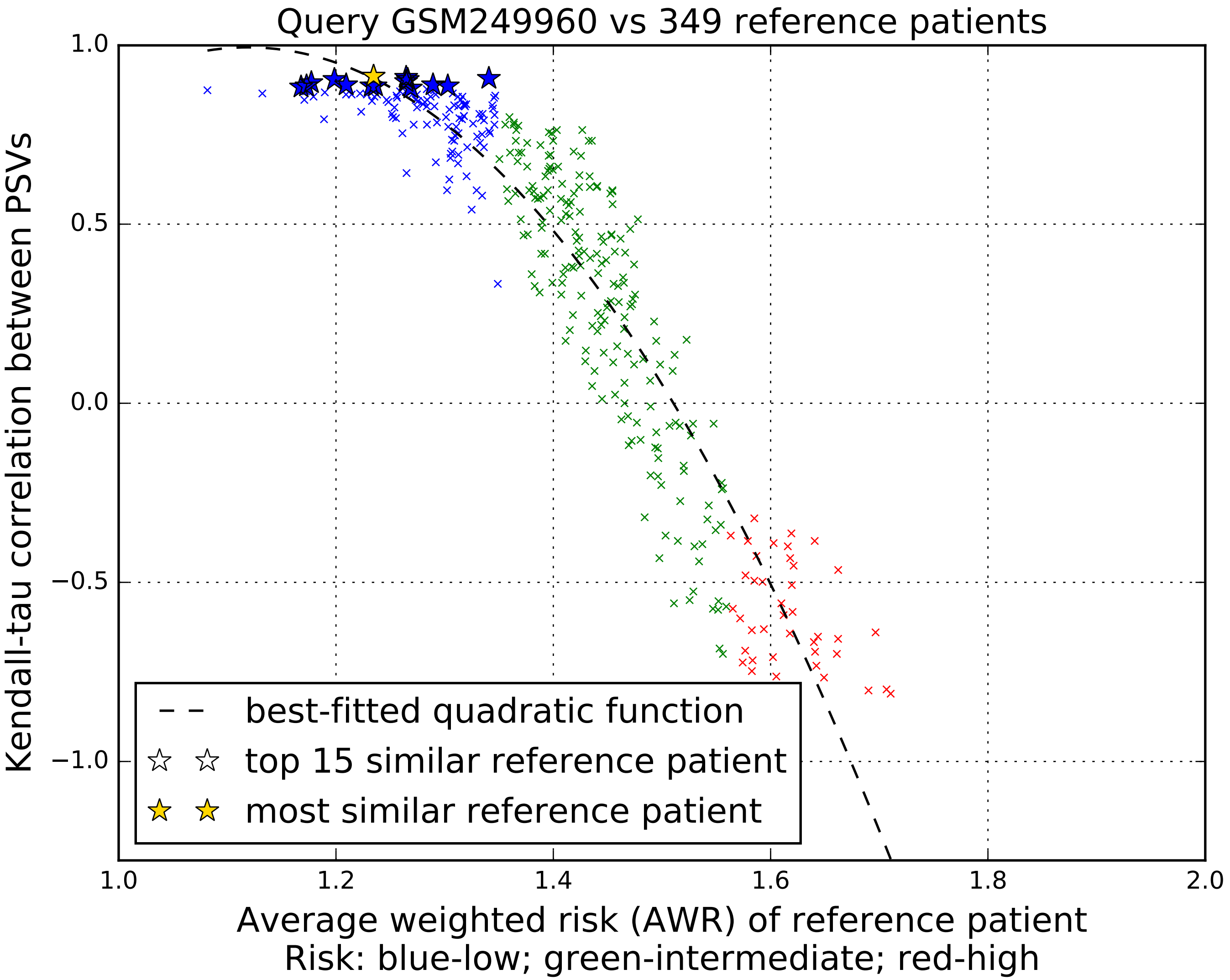

Query GSM249960 vs 349 reference patients
Kendall-tau correlation between PSVs
1.0
0.5
0.0
−0.5
−1.0
1.0
1.2
1.4
1.6
1.8
2.0
best-fitted quadratic function
top 15 similar reference patient
most similar reference patient
Average weighted risk (AWR) of reference patient
Risk: blue-low; green-intermediate; red-high

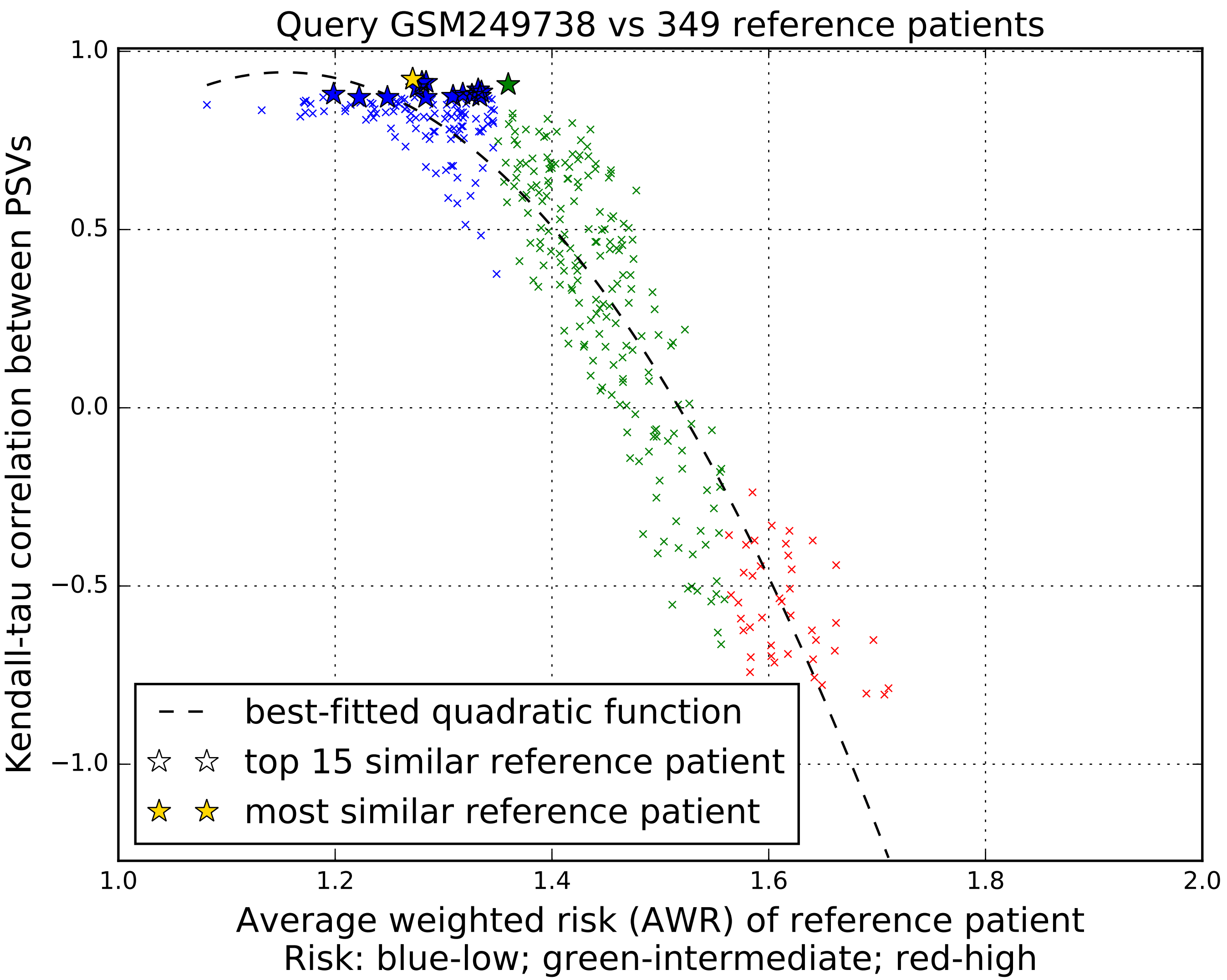
Query GSM249738 vs 349 reference patients
1.0
0.5
0.0
−0.5
−1.0
Kendall-tau correlation between PSVs
1.0
1.2
1.4
1.6
1.8
2.0
best-fitted quadratic function
top 15 similar reference patient
most similar reference patient
Average weighted risk (AWR) of reference patient
Risk: blue-low; green-intermediate; red-high

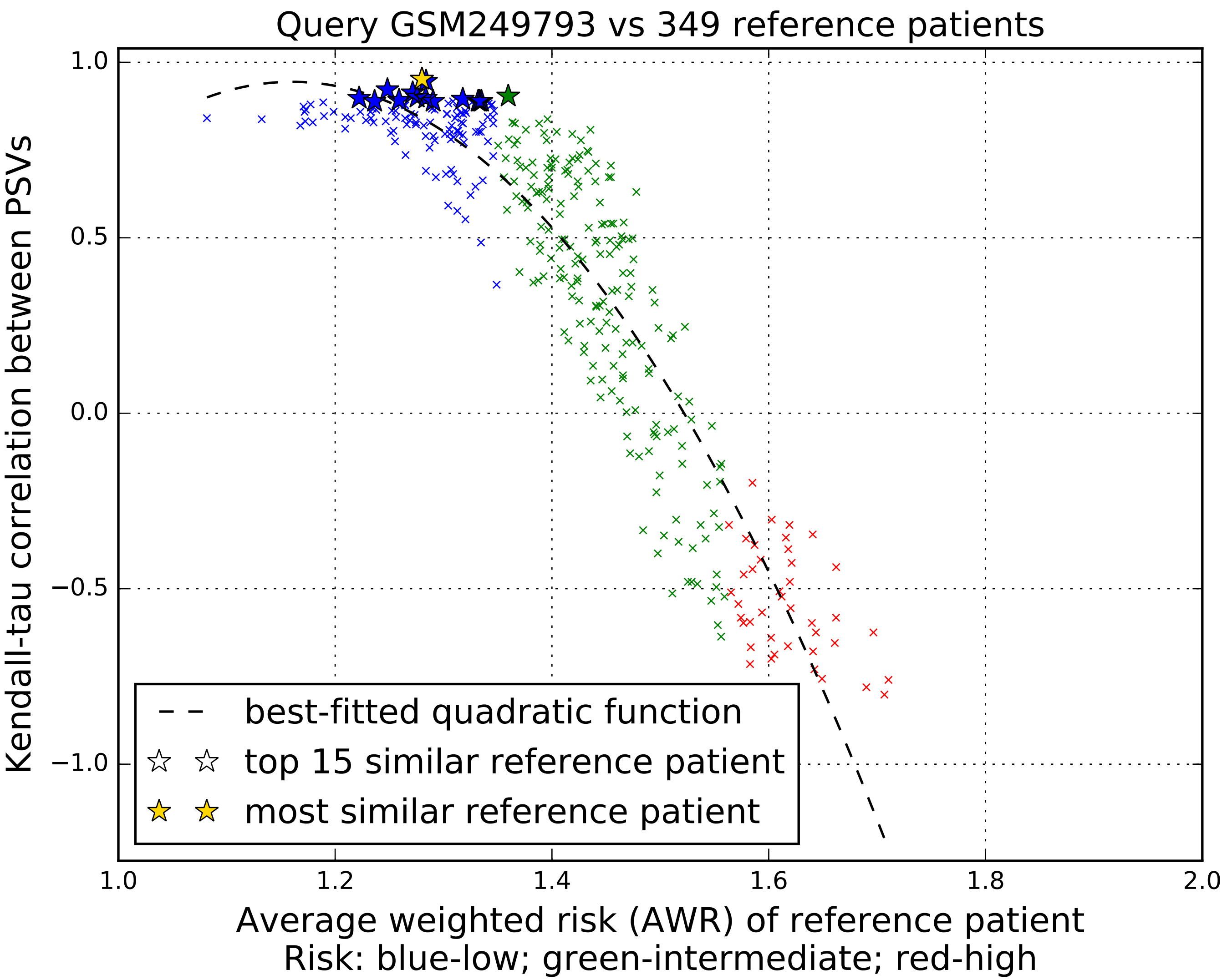

Query GSM249793 vs 349 reference patients
1.0
0.5
0.0
−0.5
−1.0
Kendall-tau correlation between PSVs
1.0
1.2
1.4
1.6
1.8
2.0
Average weighted risk (AWR) of reference patient
Risk: blue-low; green-intermediate; red-high
best-fitted quadratic function
top 15 similar reference patient
most similar reference patient

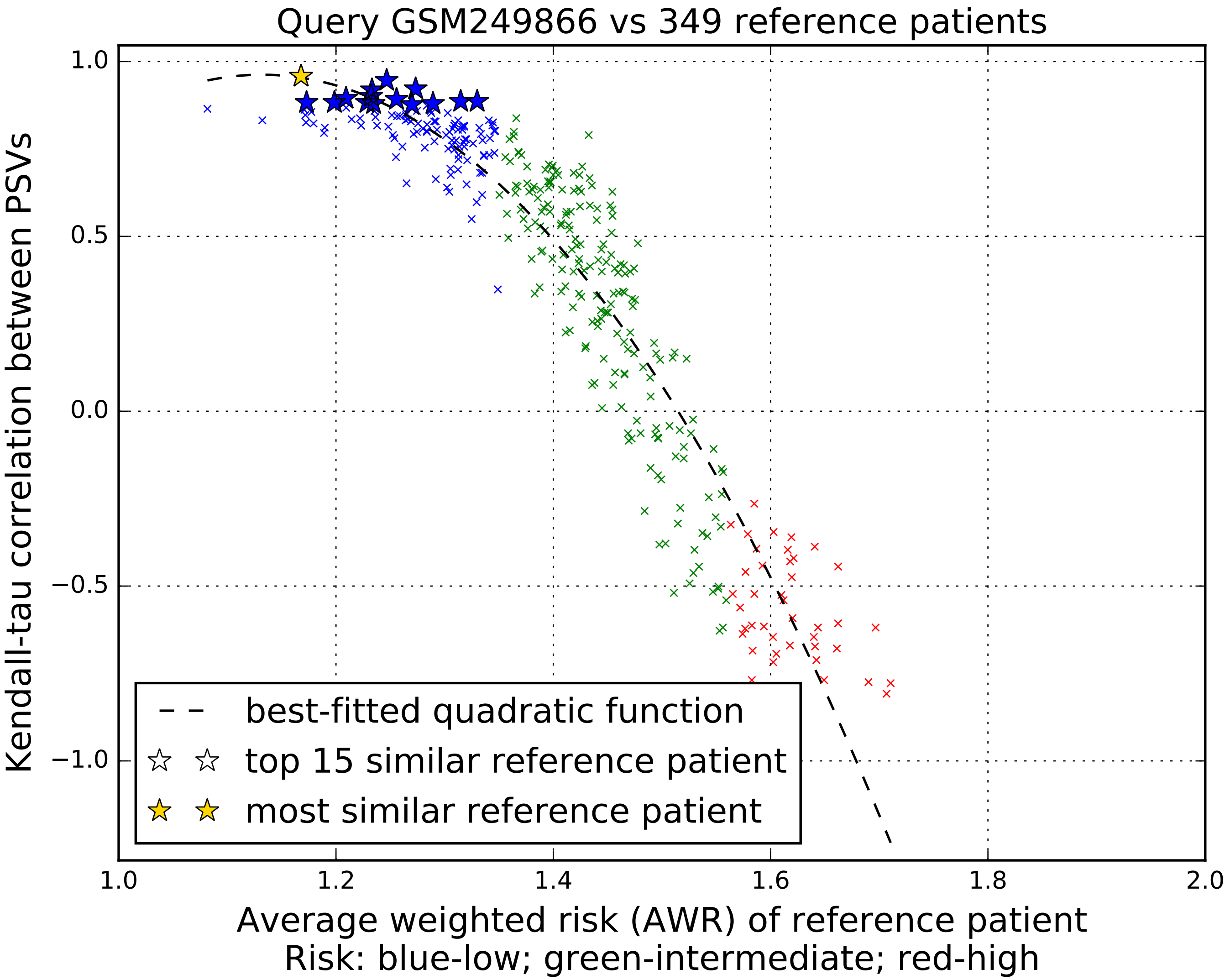

Query GSM249866 vs 349 reference patients
Kendall-tau correlation between PSVs
1.0
0.5
0.0
−0.5
−1.0
1.0
1.2
1.4
1.6
1.8
2.0
best-fitted quadratic function
top 15 similar reference patient
most similar reference patient
Average weighted risk (AWR) of reference patient
Risk: blue-low; green-intermediate; red-high

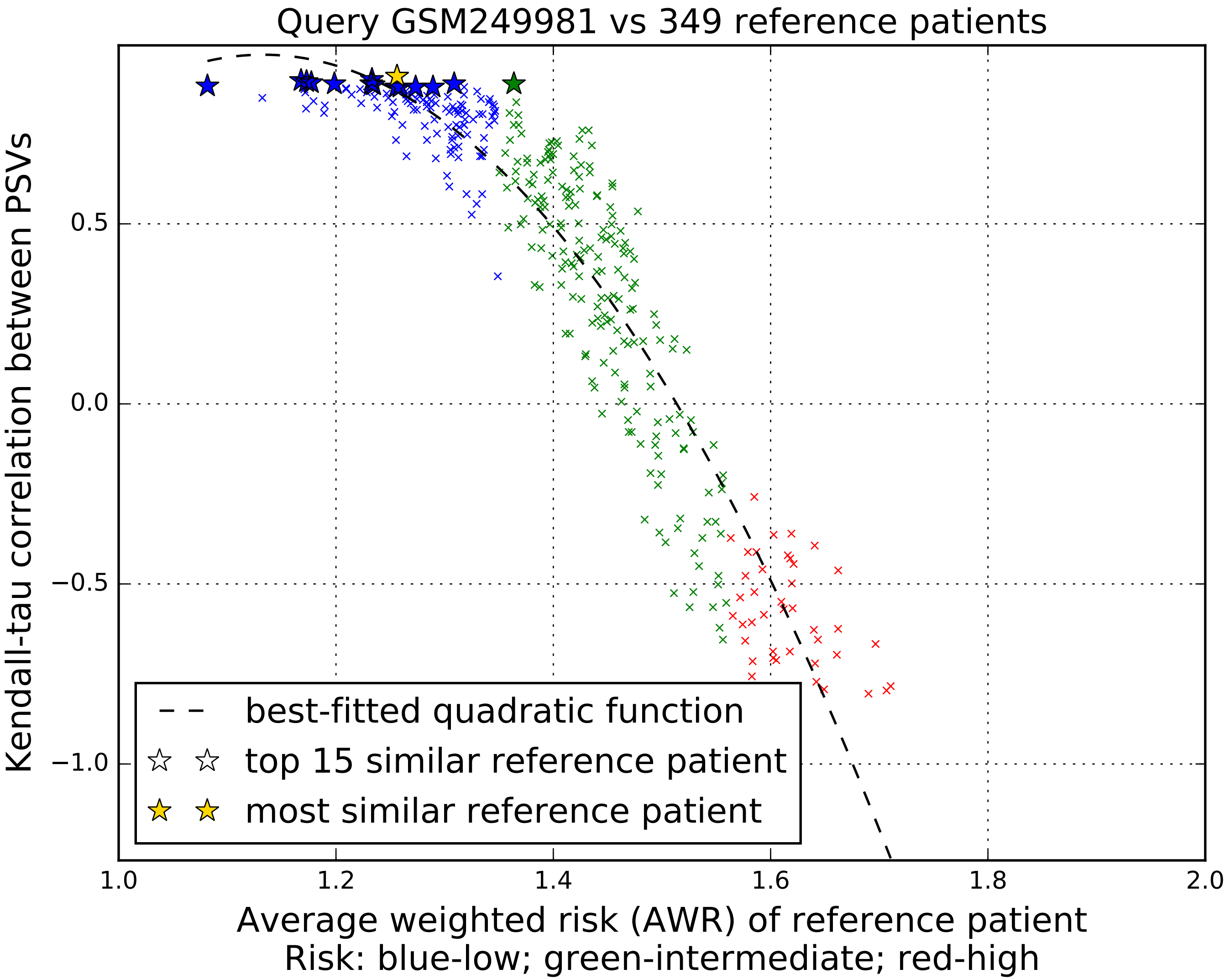
Query GSM249981 vs 349 reference patients
Kendall-tau correlation between PSVs
0.5
0.0
−0.5
−1.0
1.0
1.2
1.4
1.6
1.8
2.0
best-fitted quadratic function
top 15 similar reference patient
most similar reference patient
Average weighted risk (AWR) of reference patient
Risk: blue-low; green-intermediate; red-high

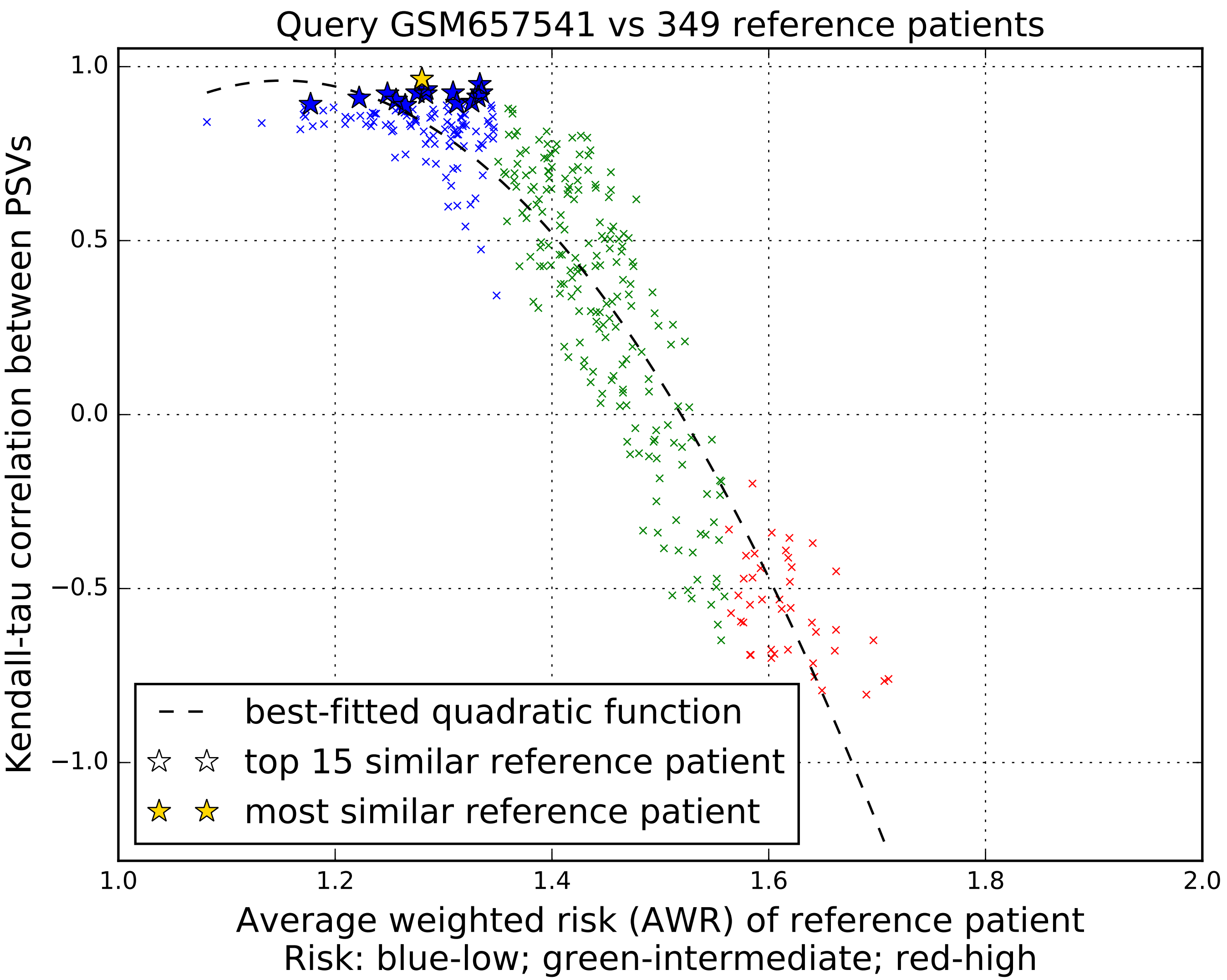

Query GSM657541 vs 349 reference patients
Kendall-tau correlation between PSVs
1.0
0.5
0.0
−0.5
−1.0
1.0
1.2
1.4
1.6
1.8
2.0
best-fitted quadratic function
top 15 similar reference patient
most similar reference patient
Average weighted risk (AWR) of reference patient
Risk: blue-low; green-intermediate; red-high

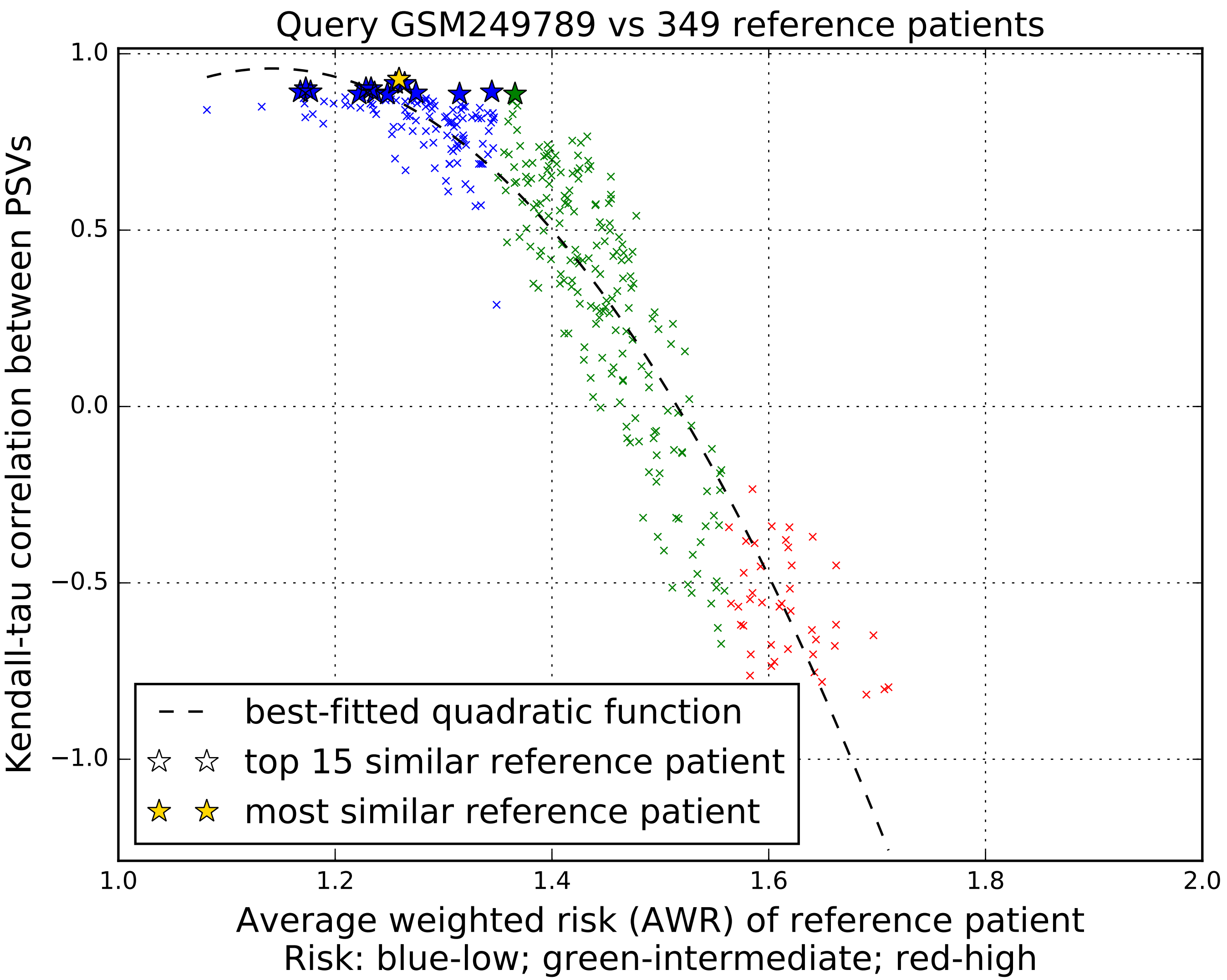
Query GSM249789 vs 349 reference patients
Kendall-tau correlation between PSVs
1.0
0.5
0.0
−0.5
−1.0
1.0
1.2
1.4
1.6
1.8
2.0
best-fitted quadratic function
top 15 similar reference patient
most similar reference patient
Average weighted risk (AWR) of reference patient
Risk: blue-low; green-intermediate; red-high

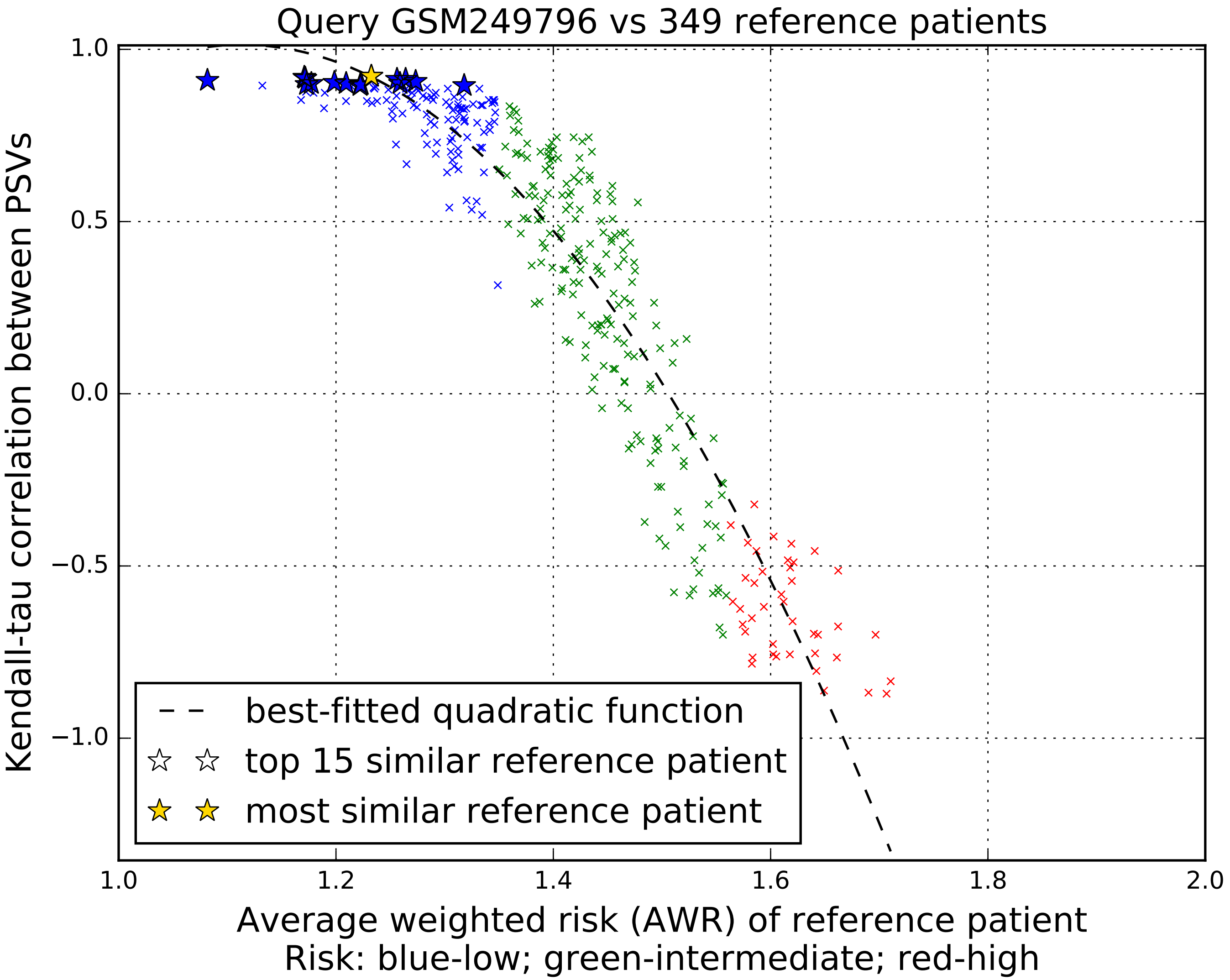

Query GSM249796 vs 349 reference patients
Kendall-tau correlation between PSVs
Average weighted risk (AWR) of reference patient
Risk: blue-low; green-intermediate; red-high
best-fitted quadratic function
top 15 similar reference patient
most similar reference patient
1.0
0.5
0.0
−0.5
−1.0
1.0
1.2
1.4
1.6
1.8
2.0

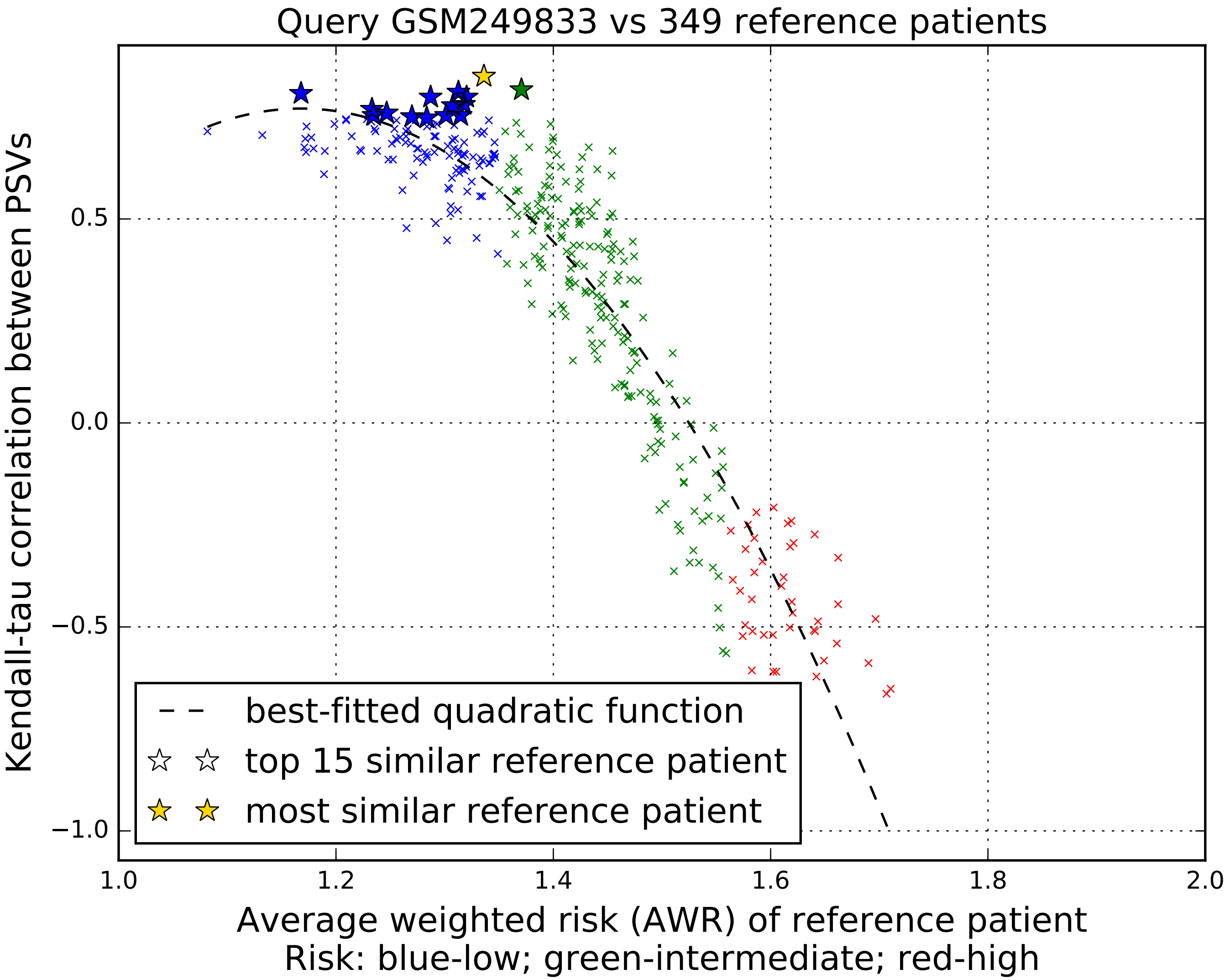

Query GSM249833 vs 349 reference patients
Kendall-tau correlation between PSVs
0.5
0.0
−0.5
−1.0
1.0
1.2
1.4
1.6
1.8
2.0
best-fitted quadratic function
top 15 similar reference patient
most similar reference patient
Average weighted risk (AWR) of reference patient
Risk: blue-low; green-intermediate; red-high

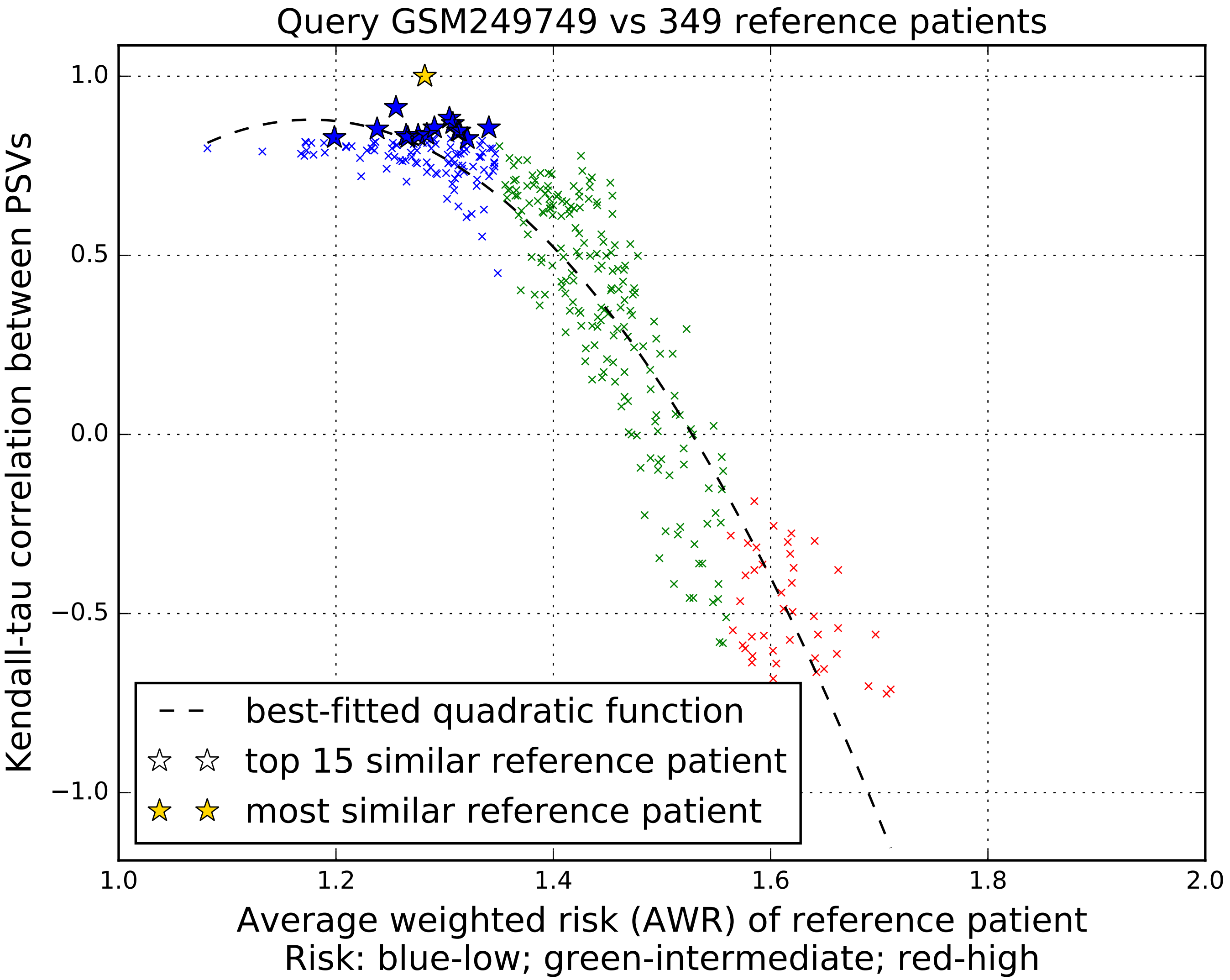
Query GSM249749 vs 349 reference patients
Kendall-tau correlation between PSVs
1.0
0.5
0.0
−0.5
−1.0
1.0
1.2
1.4
1.6
1.8
2.0
Average weighted risk (AWR) of reference patient
Risk: blue-low; green-intermediate; red-high
best-fitted quadratic function
top 15 similar reference patient
most similar reference patient

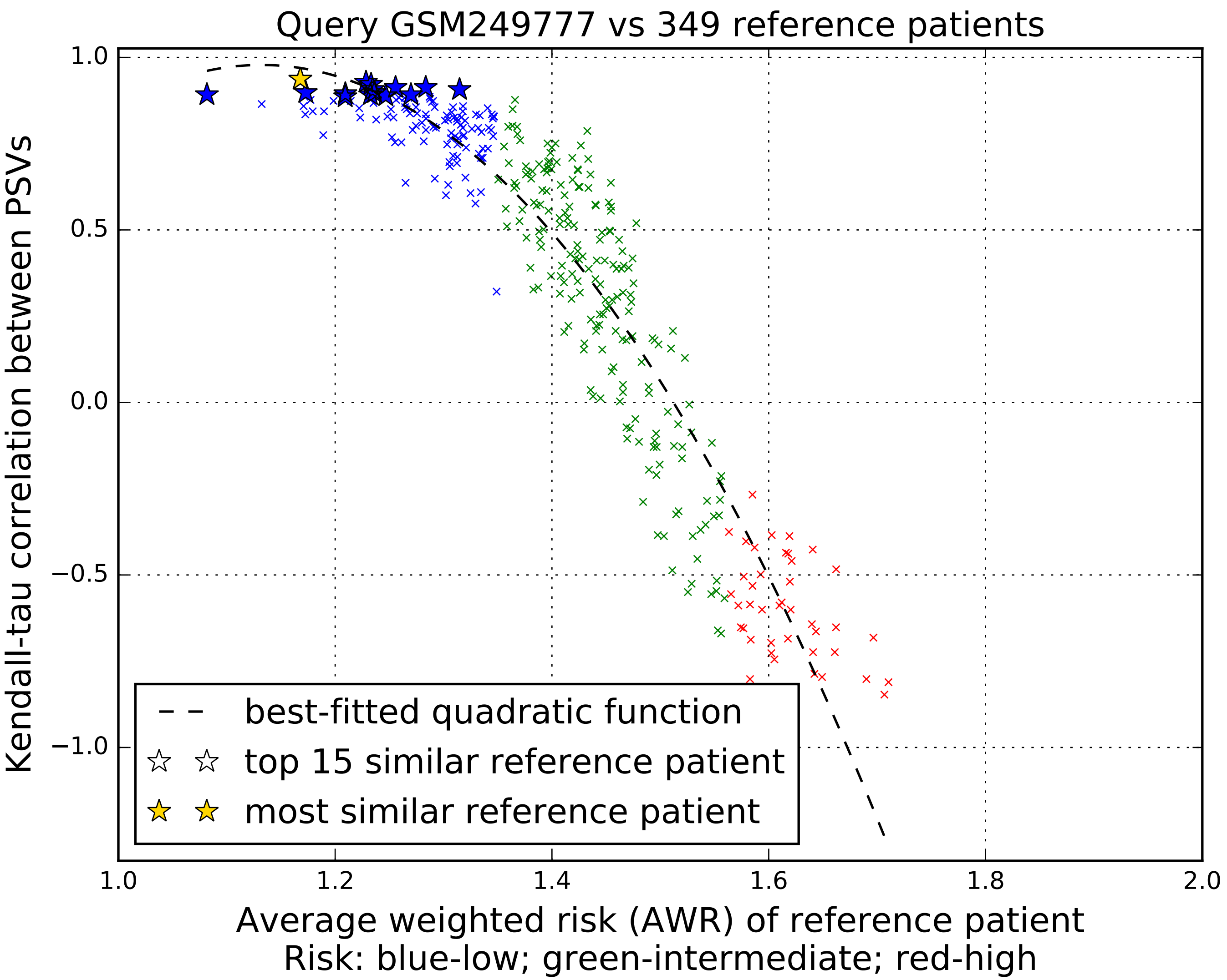
Query GSM249777 vs 349 reference patients
Kendall-tau correlation between PSVs
1.0
0.5
0.0
−0.5
−1.0
1.0
1.2
1.4
1.6
1.8
2.0
best-fitted quadratic function
top 15 similar reference patient
most similar reference patient
Average weighted risk (AWR) of reference patient
Risk: blue-low; green-intermediate; red-high

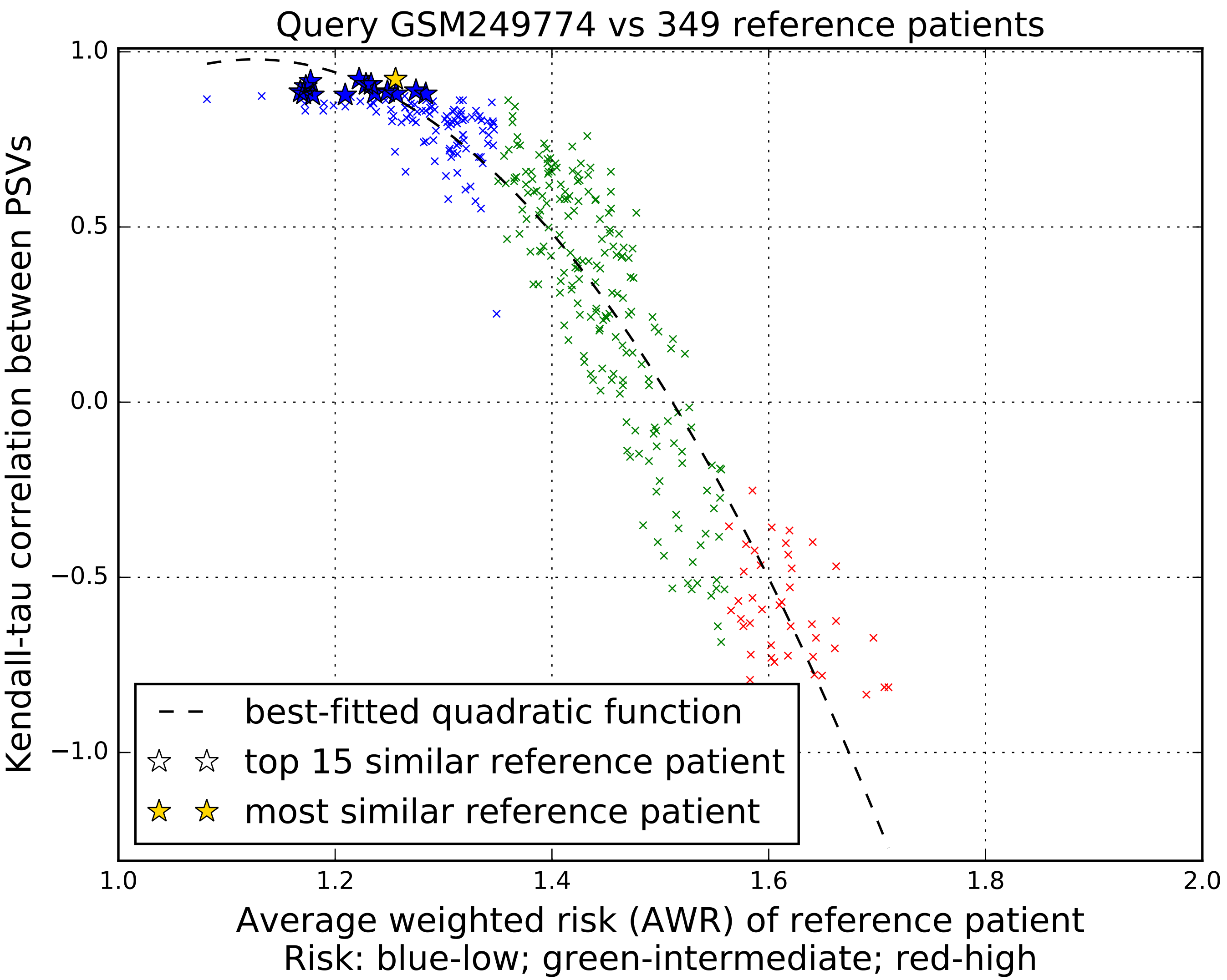

Query GSM249774 vs 349 reference patients
1.0
0.5
0.0
−0.5
−1.0
Kendall-tau correlation between PSVs
1.0
1.2
1.4
1.6
1.8
2.0
Average weighted risk (AWR) of reference patient
Risk: blue-low; green-intermediate; red-high
best-fitted quadratic function
top 15 similar reference patient
most similar reference patient

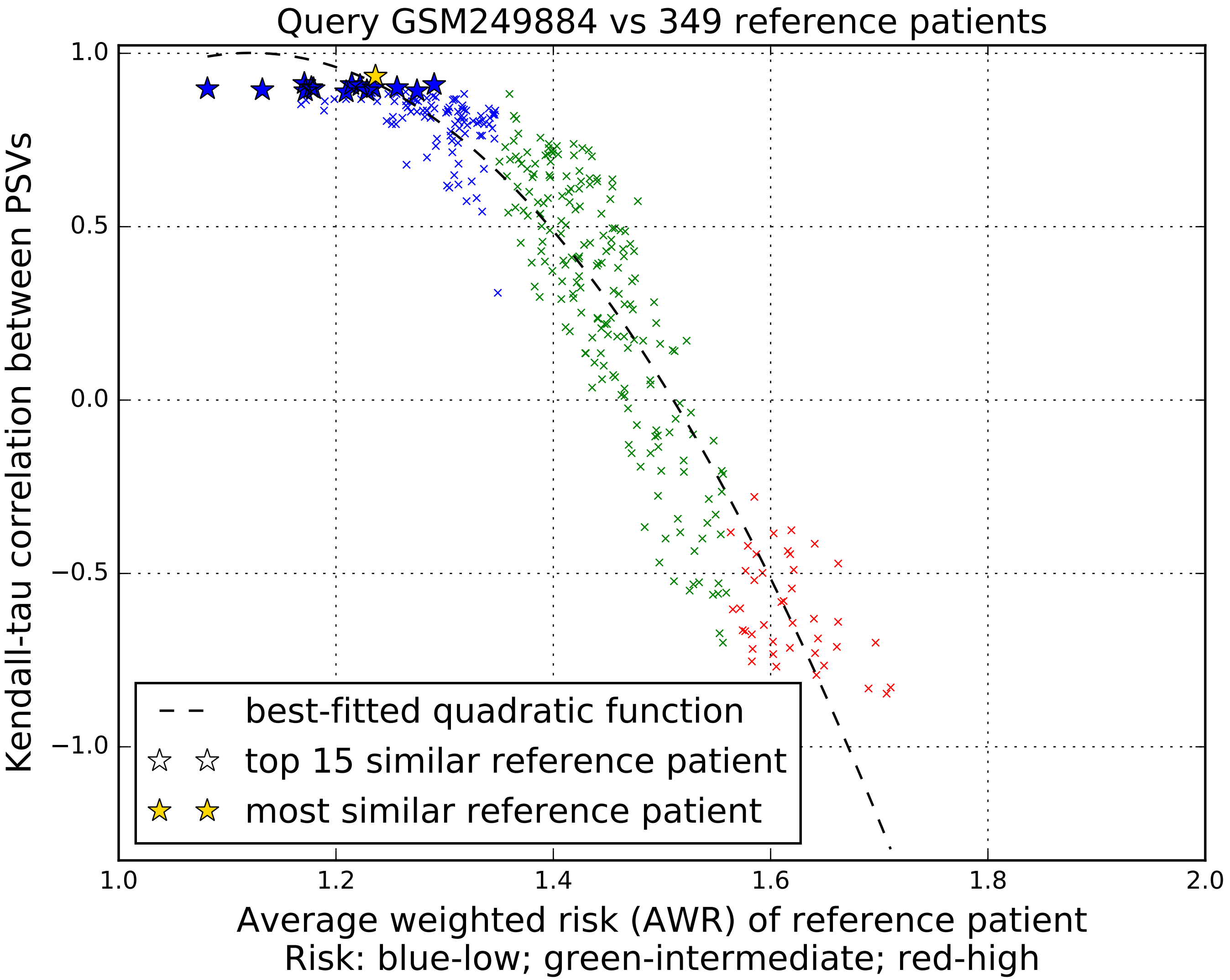
Query GSM249884 vs 349 reference patients
Kendall-tau correlation between PSVs
Average weighted risk (AWR) of reference patient
Risk: blue-low; green-intermediate; red-high
best-fitted quadratic function
top 15 similar reference patient
most similar reference patient
1.0
0.5
0.0
−0.5
−1.0
1.0
1.2
1.4
1.6
1.8
2.0

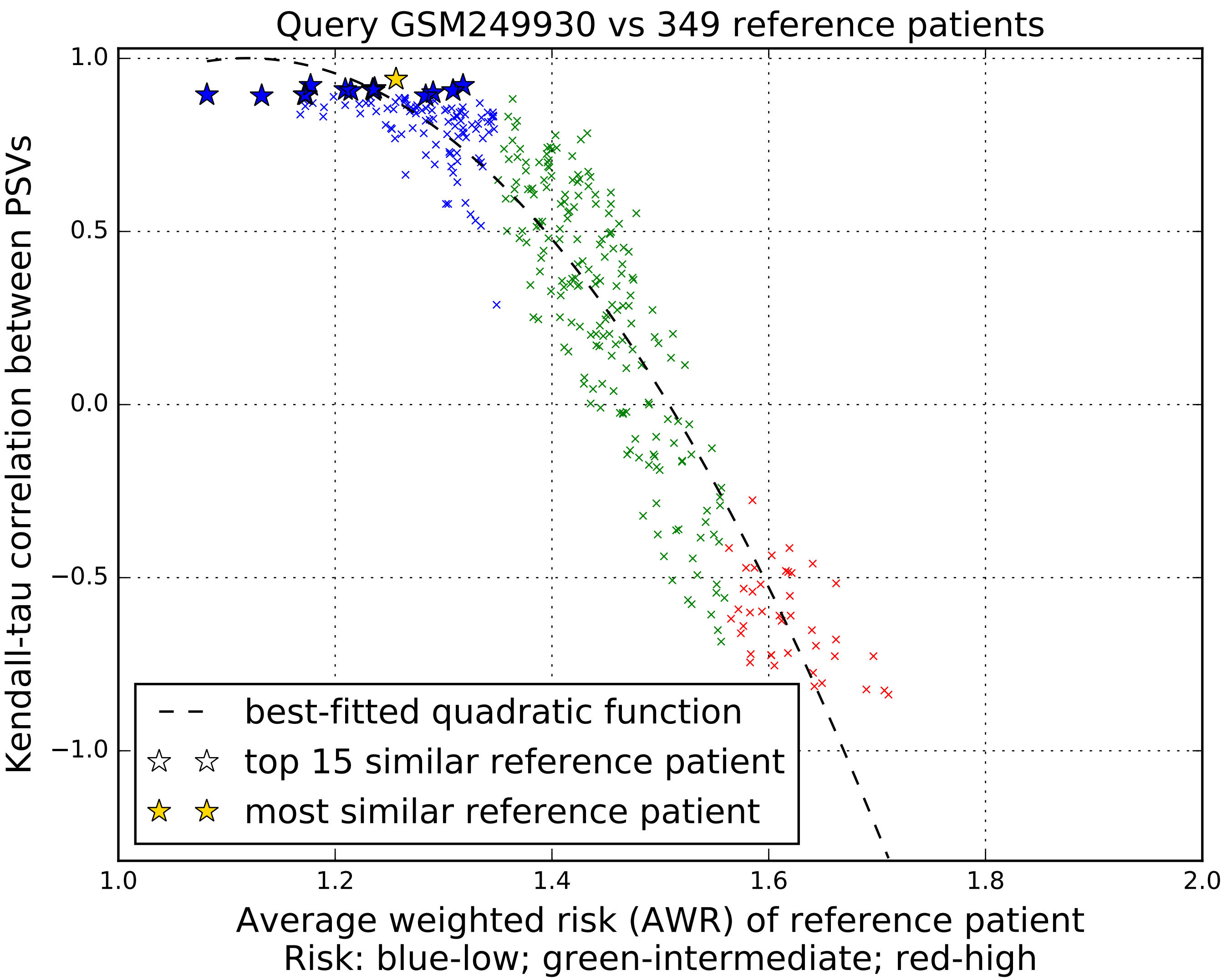

Query GSM249930 vs 349 reference patients
Kendall-tau correlation between PSVs
Average weighted risk (AWR) of reference patient
Risk: blue-low; green-intermediate; red-high
best-fitted quadratic function
top 15 similar reference patient
most similar reference patient
1.0
0.5
0.0
−0.5
−1.0
1.0
1.2
1.4
1.6
1.8
2.0

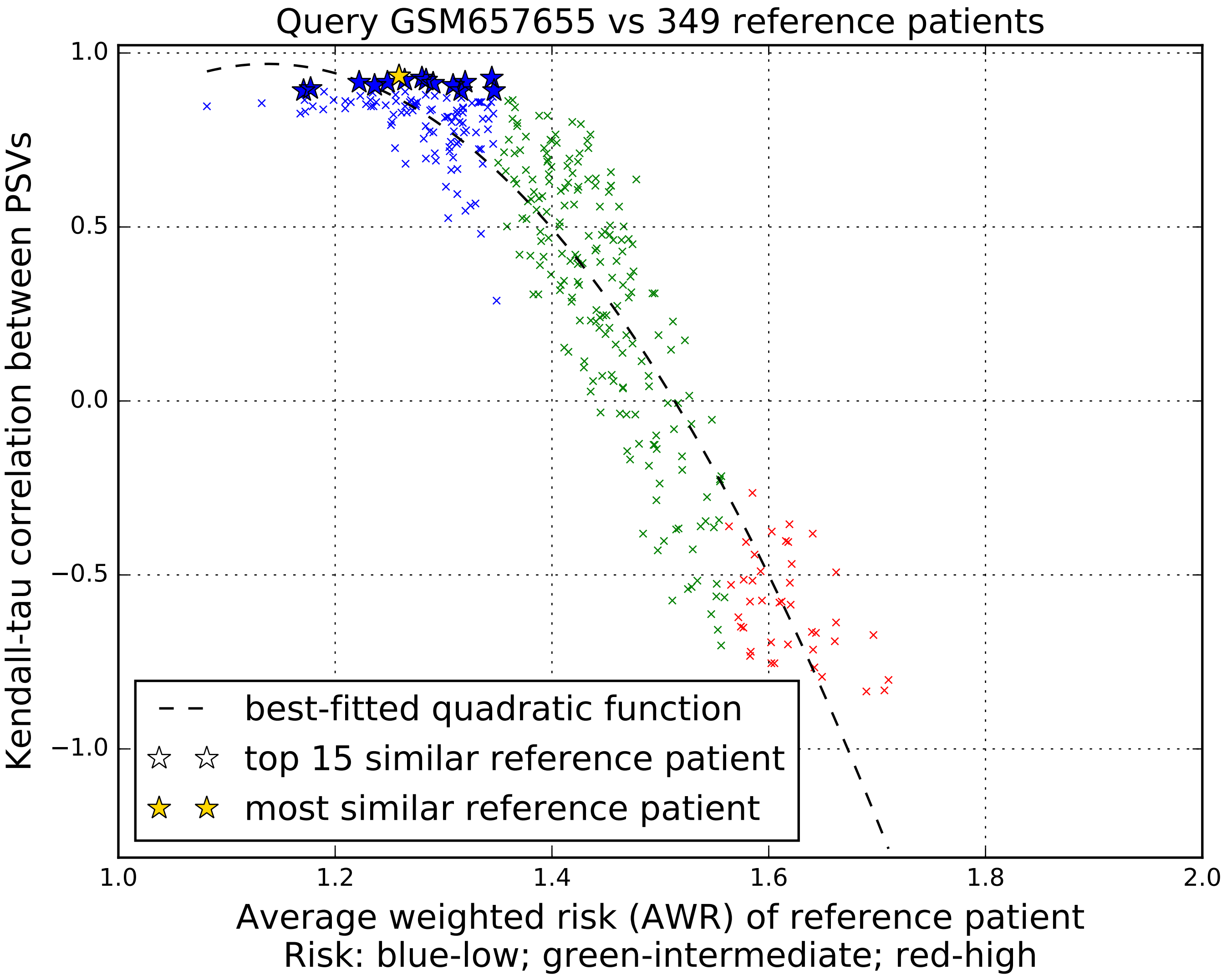

Query GSM657655 vs 349 reference patients
Kendall-tau correlation between PSVs
1.0
0.5
0.0
−0.5
−1.0
1.0
1.2
1.4
1.6
1.8
2.0
best-fitted quadratic function
top 15 similar reference patient
most similar reference patient
Average weighted risk (AWR) of reference patient
Risk: blue-low; green-intermediate; red-high

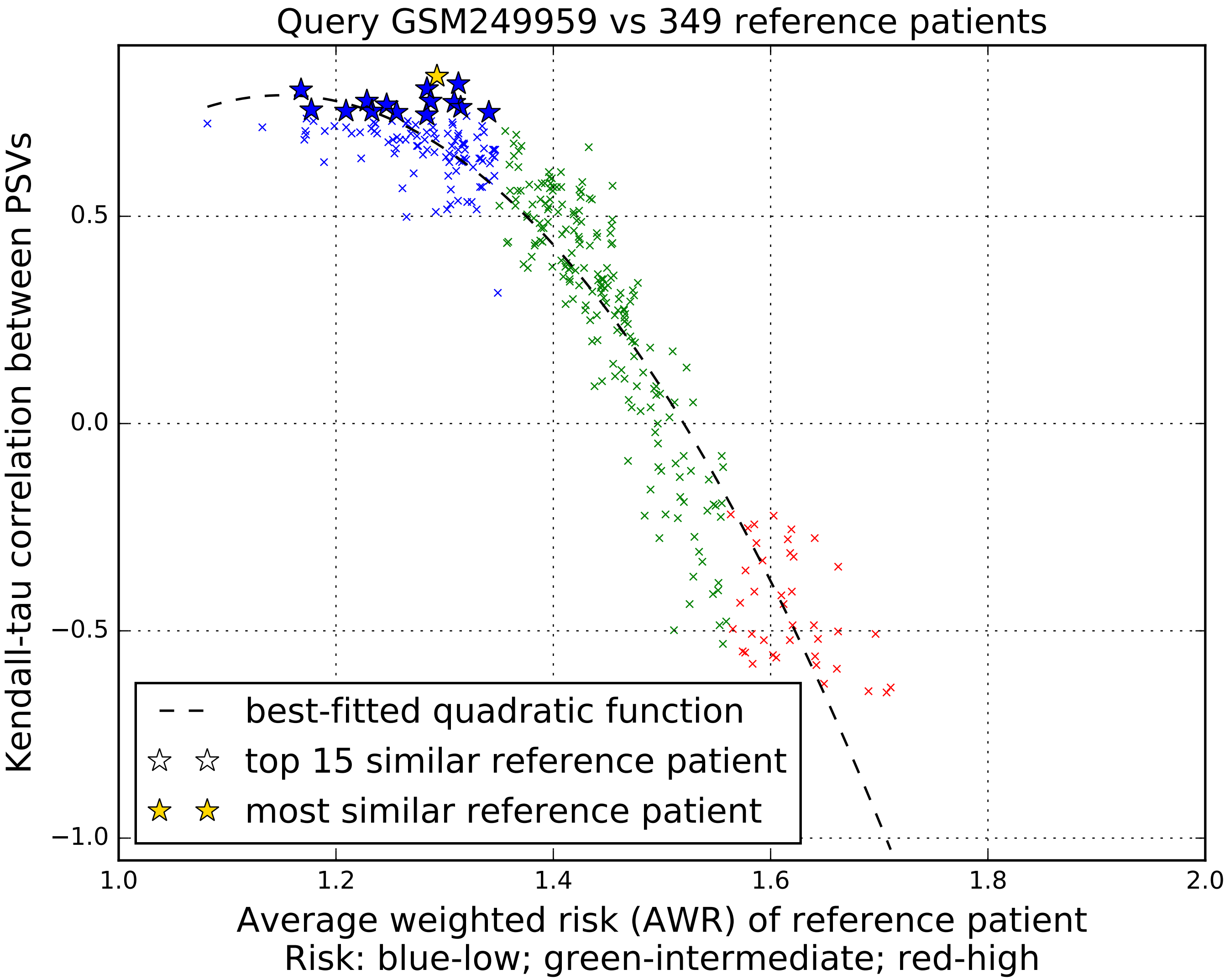

Query GSM249959 vs 349 reference patients
Kendall-tau correlation between PSVs
0.5
0.0
−0.5
−1.0
1.0
1.2
1.4
1.6
1.8
2.0
best-fitted quadratic function
top 15 similar reference patient
most similar reference patient
Average weighted risk (AWR) of reference patient
Risk: blue-low; green-intermediate; red-high

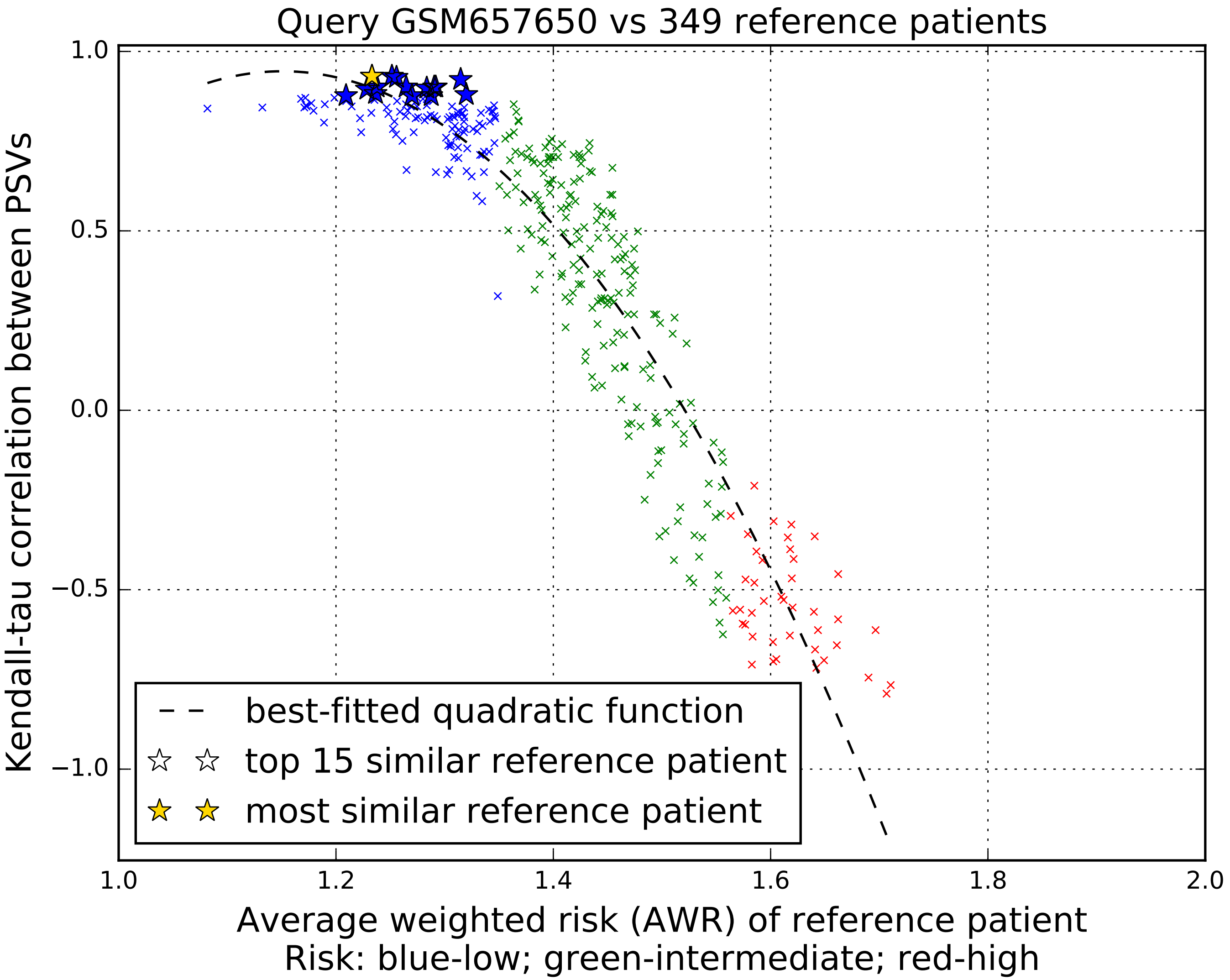

Query GSM657650 vs 349 reference patients
Kendall-tau correlation between PSVs
1.0
0.5
0.0
−0.5
−1.0
1.0
1.2
1.4
1.6
1.8
2.0
best-fitted quadratic function
top 15 similar reference patient
most similar reference patient
Average weighted risk (AWR) of reference patient
Risk: blue-low; green-intermediate; red-high

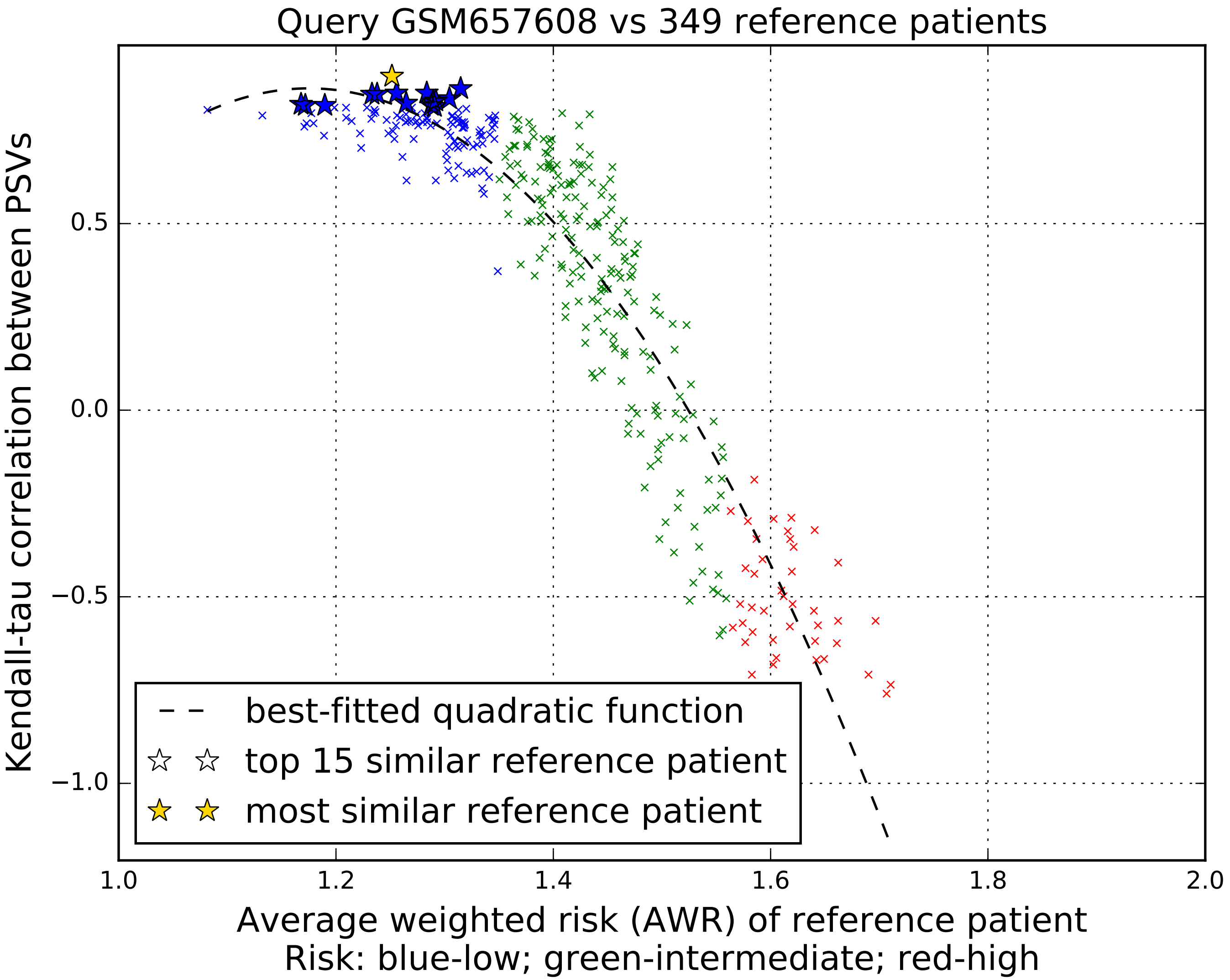
Query GSM657608 vs 349 reference patients
Kendall-tau correlation between PSVs
0.5
0.0
−0.5
−1.0
1.0
1.2
1.4
1.6
1.8
2.0
best-fitted quadratic function
top 15 similar reference patient
most similar reference patient
Average weighted risk (AWR) of reference patient
Risk: blue-low; green-intermediate; red-high

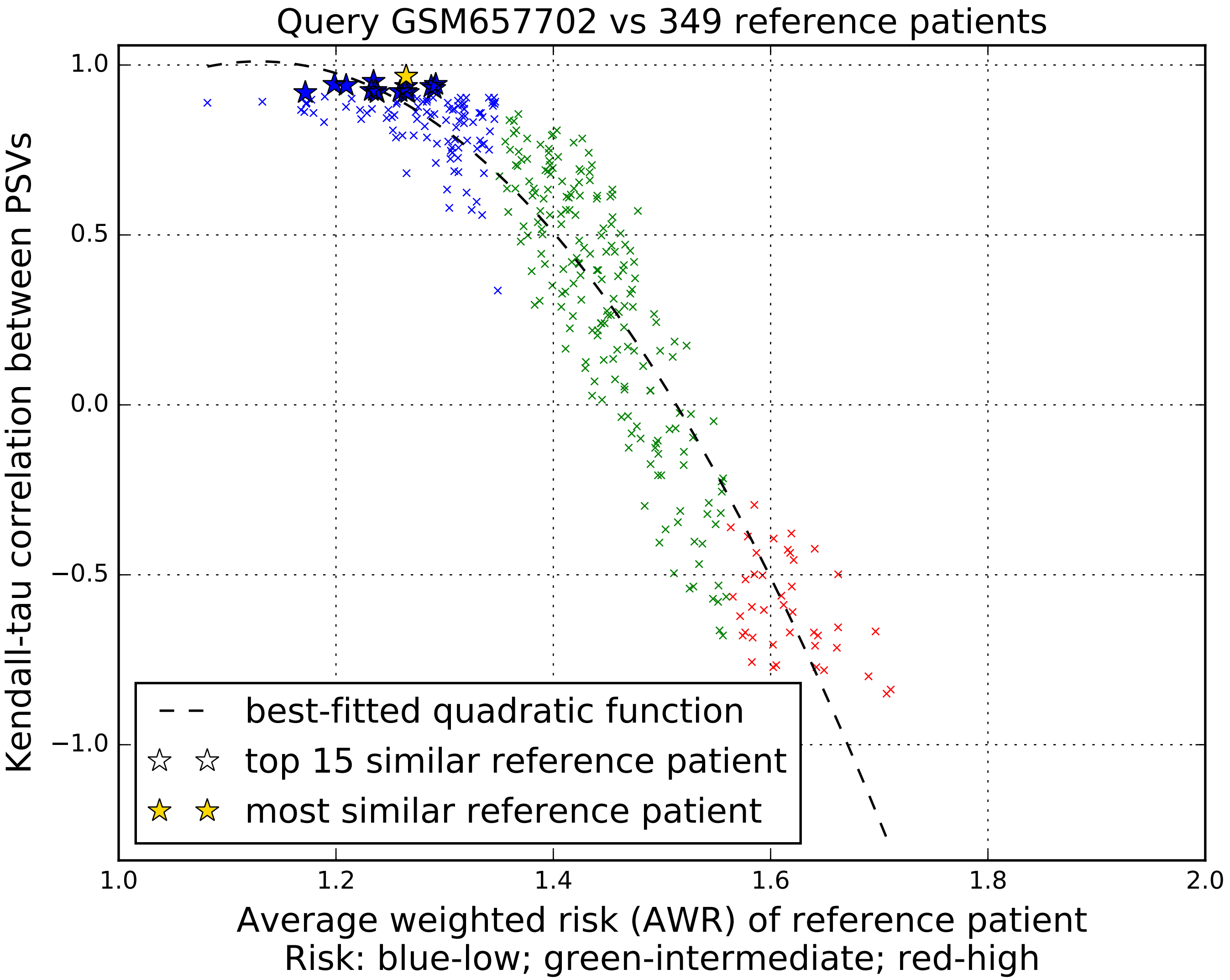
Query GSM657702 vs 349 reference patients
Kendall-tau correlation between PSVs
1.0
0.5
0.0
−0.5
−1.0
1.0
1.2
1.4
1.6
1.8
2.0
best-fitted quadratic function
top 15 similar reference patient
most similar reference patient
Average weighted risk (AWR) of reference patient
Risk: blue-low; green-intermediate; red-high

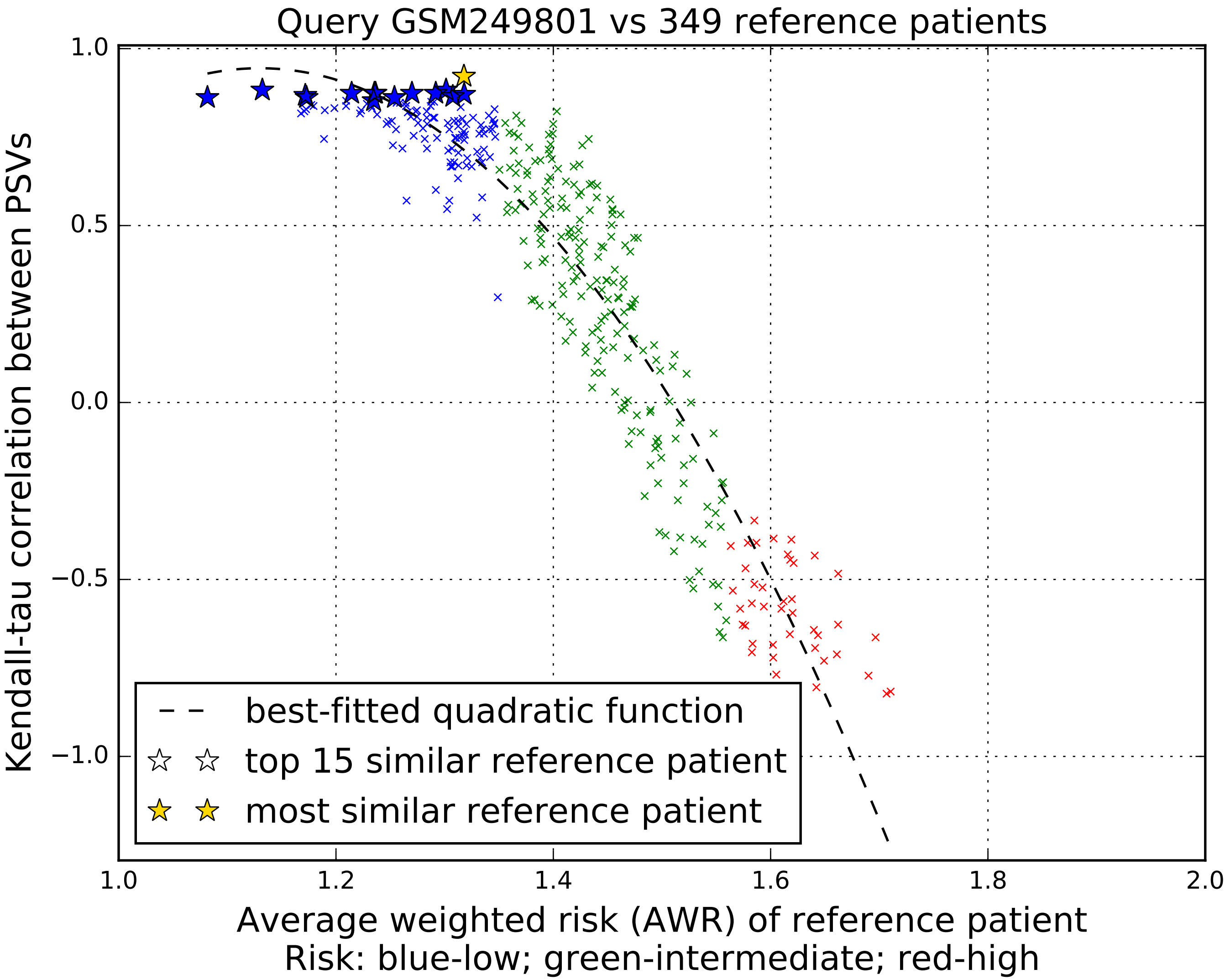

Query GSM249801 vs 349 reference patients
Kendall-tau correlation between PSVs
1.0
0.5
0.0
−0.5
−1.0
1.0
1.2
1.4
1.6
1.8
2.0
best-fitted quadratic function
top 15 similar reference patient
most similar reference patient
Average weighted risk (AWR) of reference patient
Risk: blue-low; green-intermediate; red-high

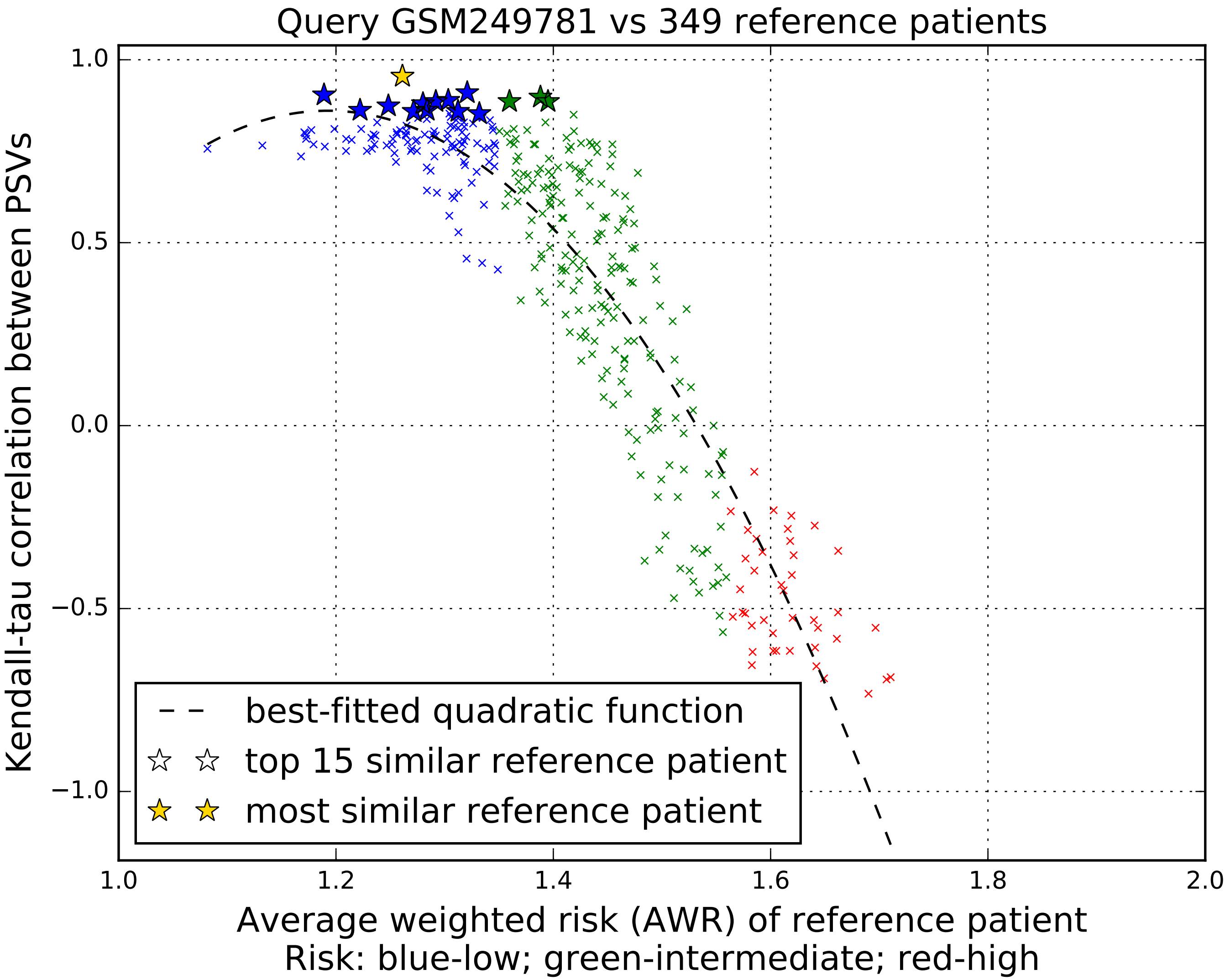

Query GSM249781 vs 349 reference patients
Kendall-tau correlation between PSVs
1.0
0.5
0.0
−0.5
−1.0
1.0
1.2
1.4
1.6
1.8
2.0
best-fitted quadratic function
top 15 similar reference patient
most similar reference patient
Average weighted risk (AWR) of reference patient
Risk: blue-low; green-intermediate; red-high

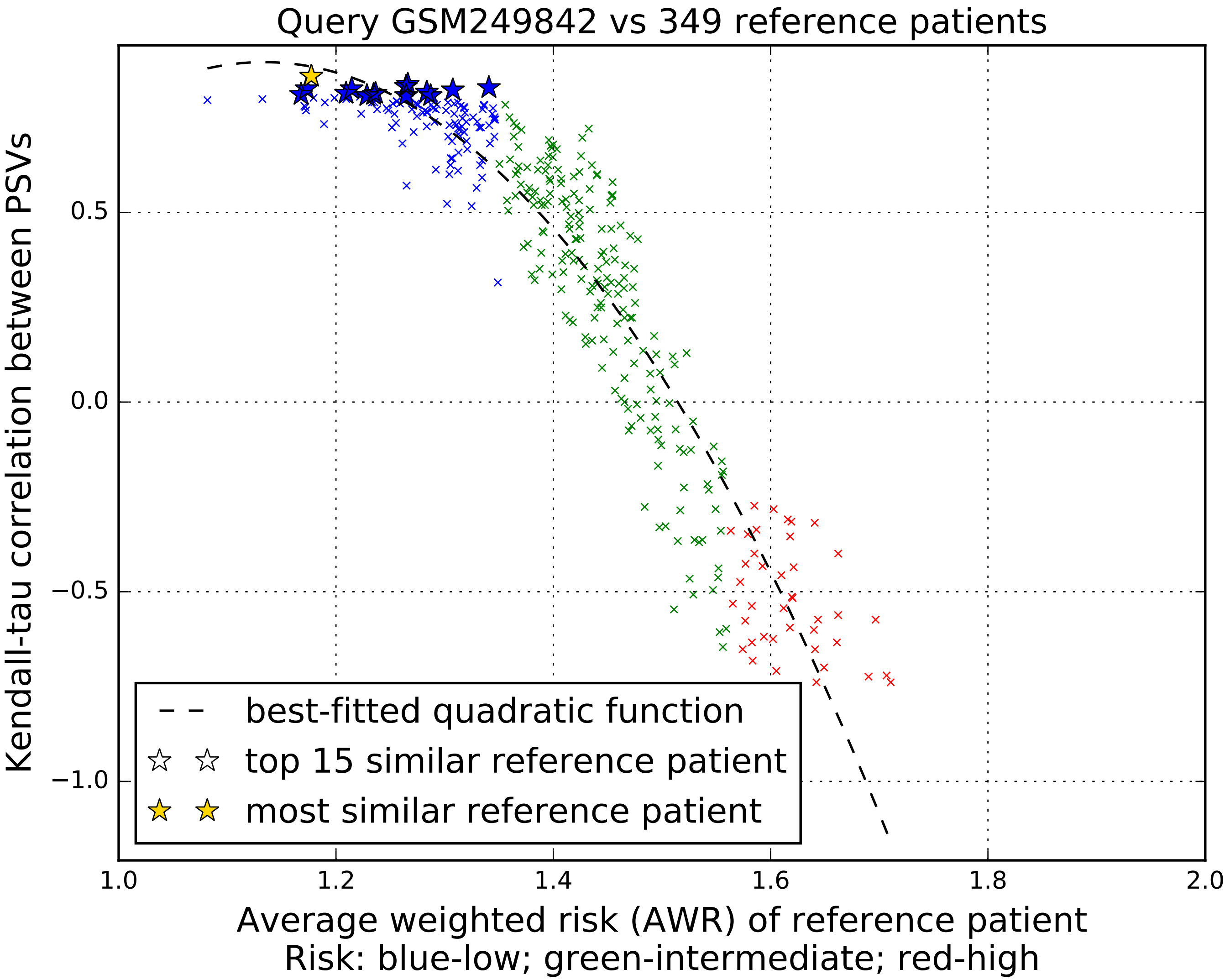

Query GSM249842 vs 349 reference patients
Kendall-tau correlation between PSVs
0.5
0.0
−0.5
−1.0
1.0
1.2
1.4
1.6
1.8
2.0
best-fitted quadratic function
top 15 similar reference patient
most similar reference patient
Average weighted risk (AWR) of reference patient
Risk: blue-low; green-intermediate; red-high

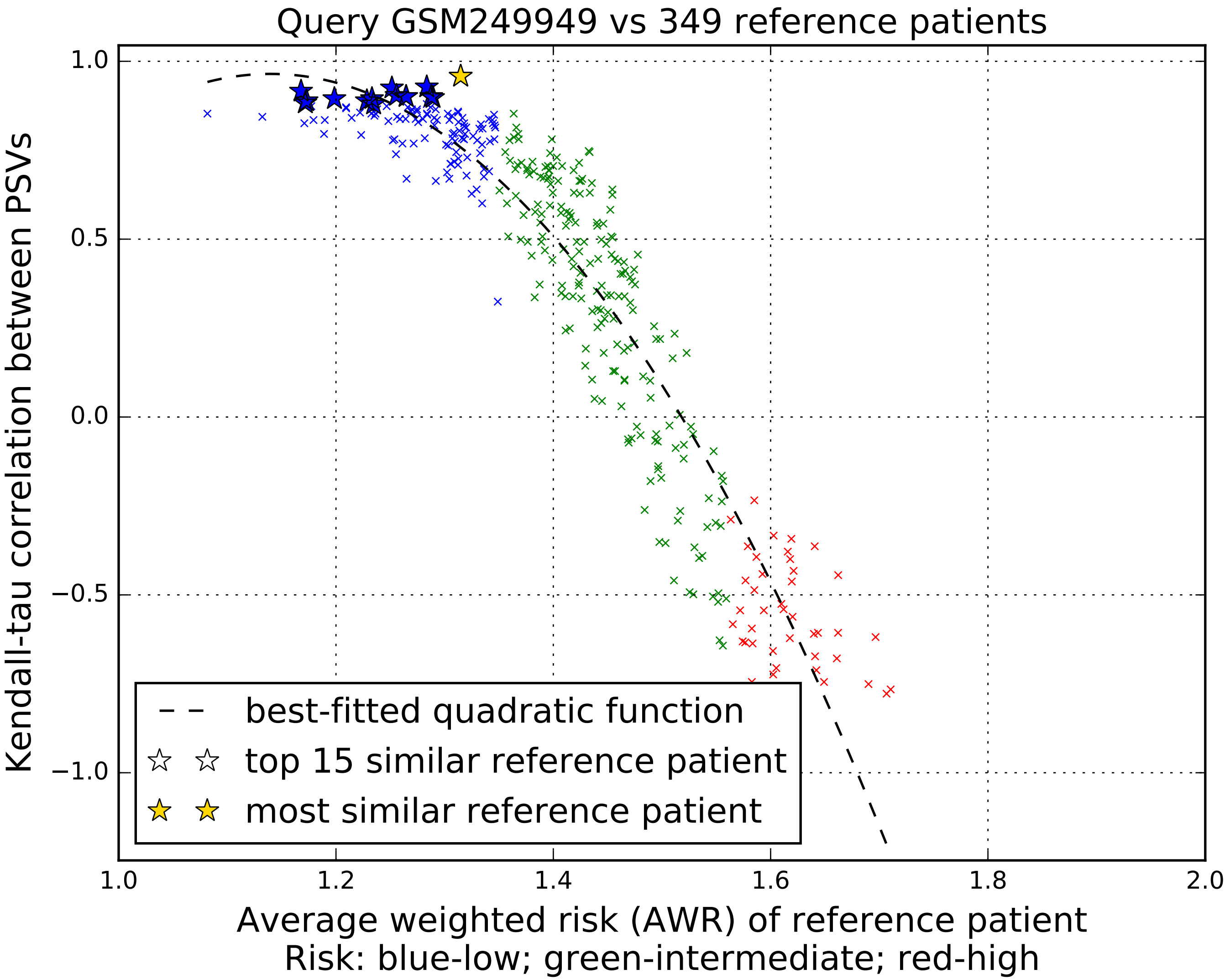
Query GSM249949 vs 349 reference patients
Kendall-tau correlation between PSVs
1.0
0.5
0.0
−0.5
−1.0
1.0
1.2
1.4
1.6
1.8
2.0
best-fitted quadratic function
top 15 similar reference patient
most similar reference patient
Average weighted risk (AWR) of reference patient
Risk: blue-low; green-intermediate; red-high

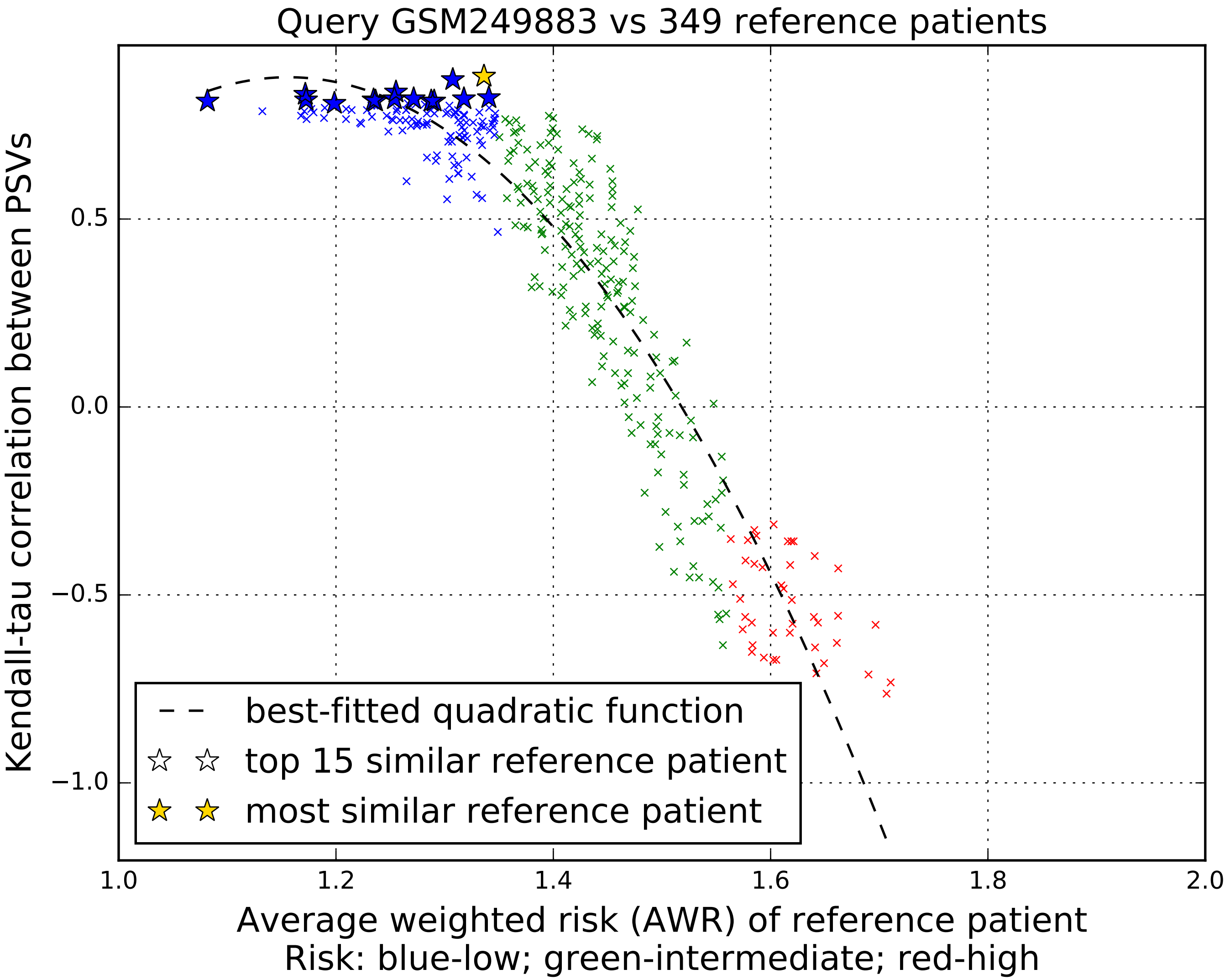

Query GSM249883 vs 349 reference patients
Kendall-tau correlation between PSVs
0.5
0.0
−0.5
−1.0
1.0
1.2
1.4
1.6
1.8
2.0
best-fitted quadratic function
top 15 similar reference patient
most similar reference patient
Average weighted risk (AWR) of reference patient
Risk: blue-low; green-intermediate; red-high

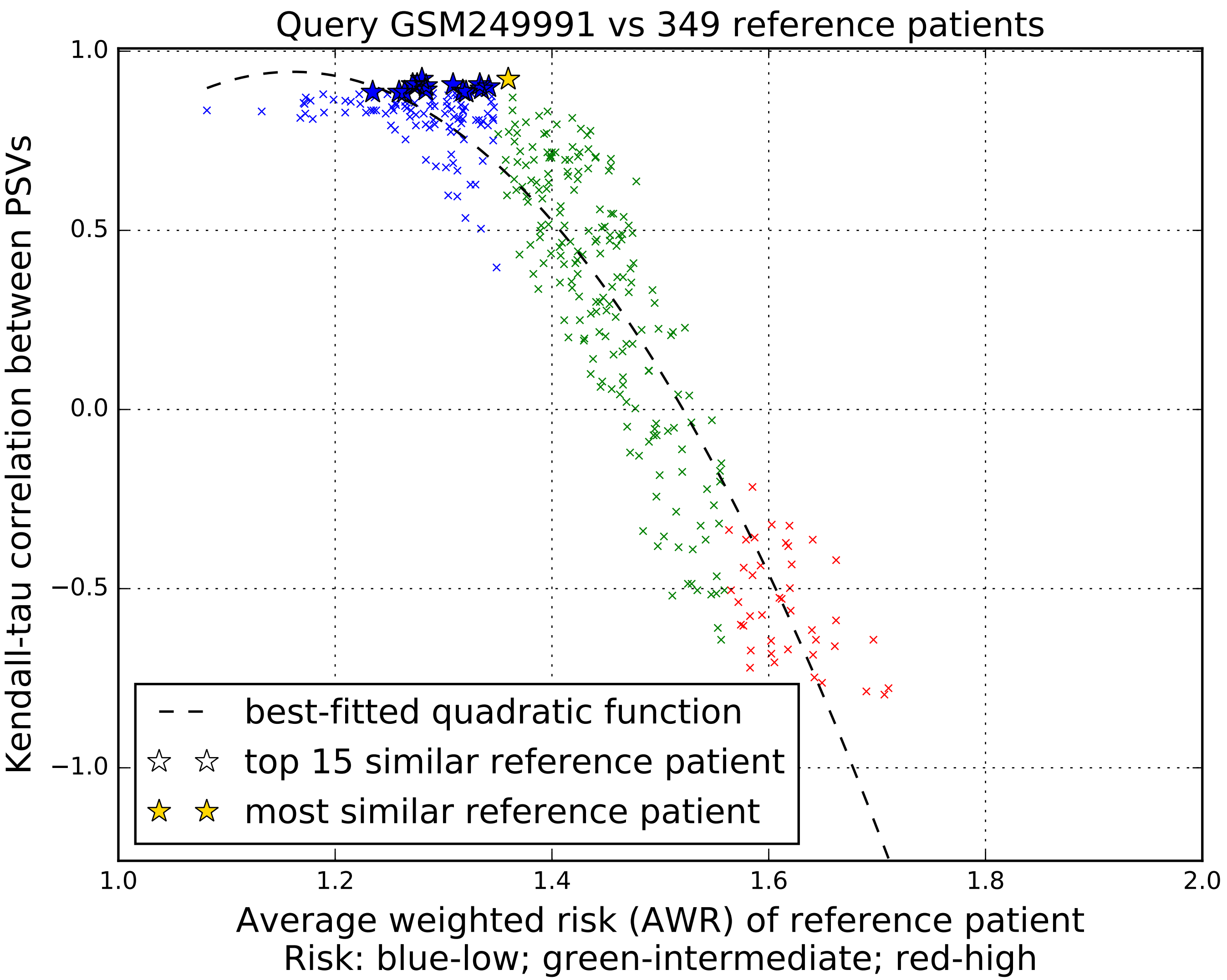

Query GSM249991 vs 349 reference patients
Kendall-tau correlation between PSVs
Average weighted risk (AWR) of reference patient
Risk: blue-low; green-intermediate; red-high
1.0
0.5
0.0
−0.5
−1.0
1.0
1.2
1.4
1.6
1.8
2.0
best-fitted quadratic function
top 15 similar reference patient
most similar reference patient

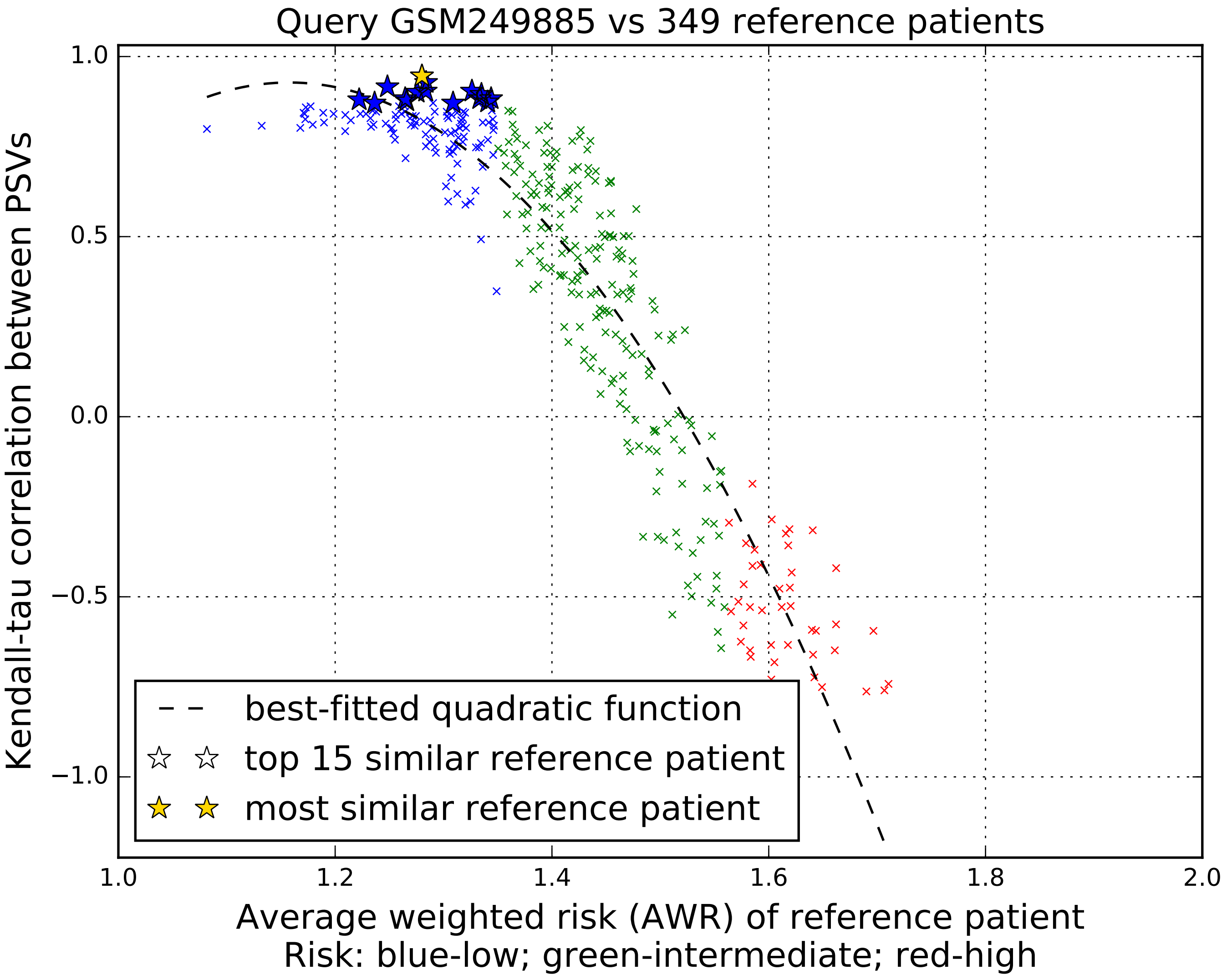
Query GSM249885 vs 349 reference patients
1.0
0.5
0.0
−0.5
−1.0
Kendall-tau correlation between PSVs
best-fitted quadratic function
top 15 similar reference patient
most similar reference patient
1.0
1.2
1.4
1.6
1.8
2.0
Average weighted risk (AWR) of reference patient
Risk: blue-low; green-intermediate; red-high

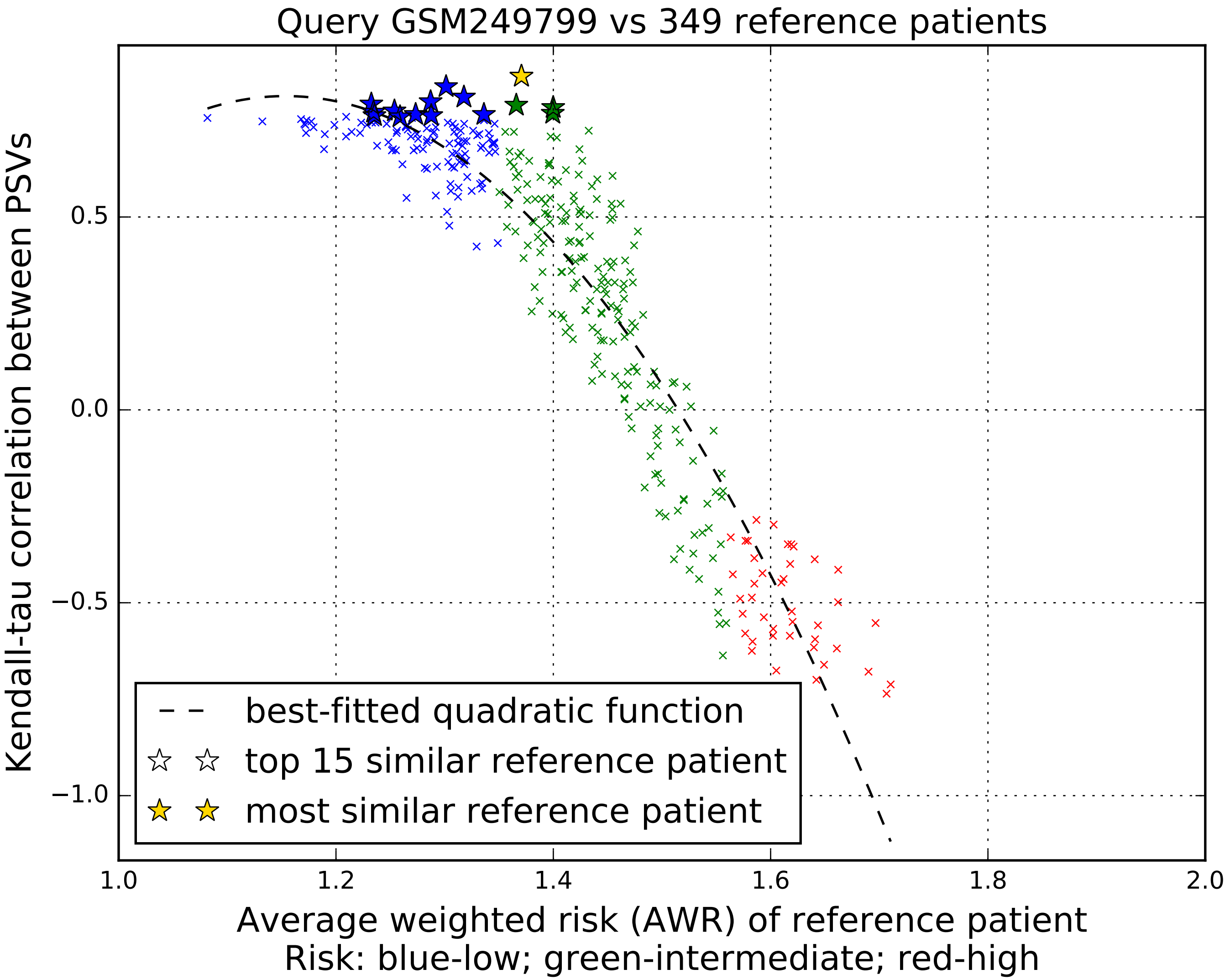
Query GSM249799 vs 349 reference patients
Kendall-tau correlation between PSVs
0.5
0.0
−0.5
−1.0
1.0
1.2
1.4
1.6
1.8
2.0
best-fitted quadratic function
top 15 similar reference patient
most similar reference patient
Average weighted risk (AWR) of reference patient
Risk: blue-low; green-intermediate; red-high

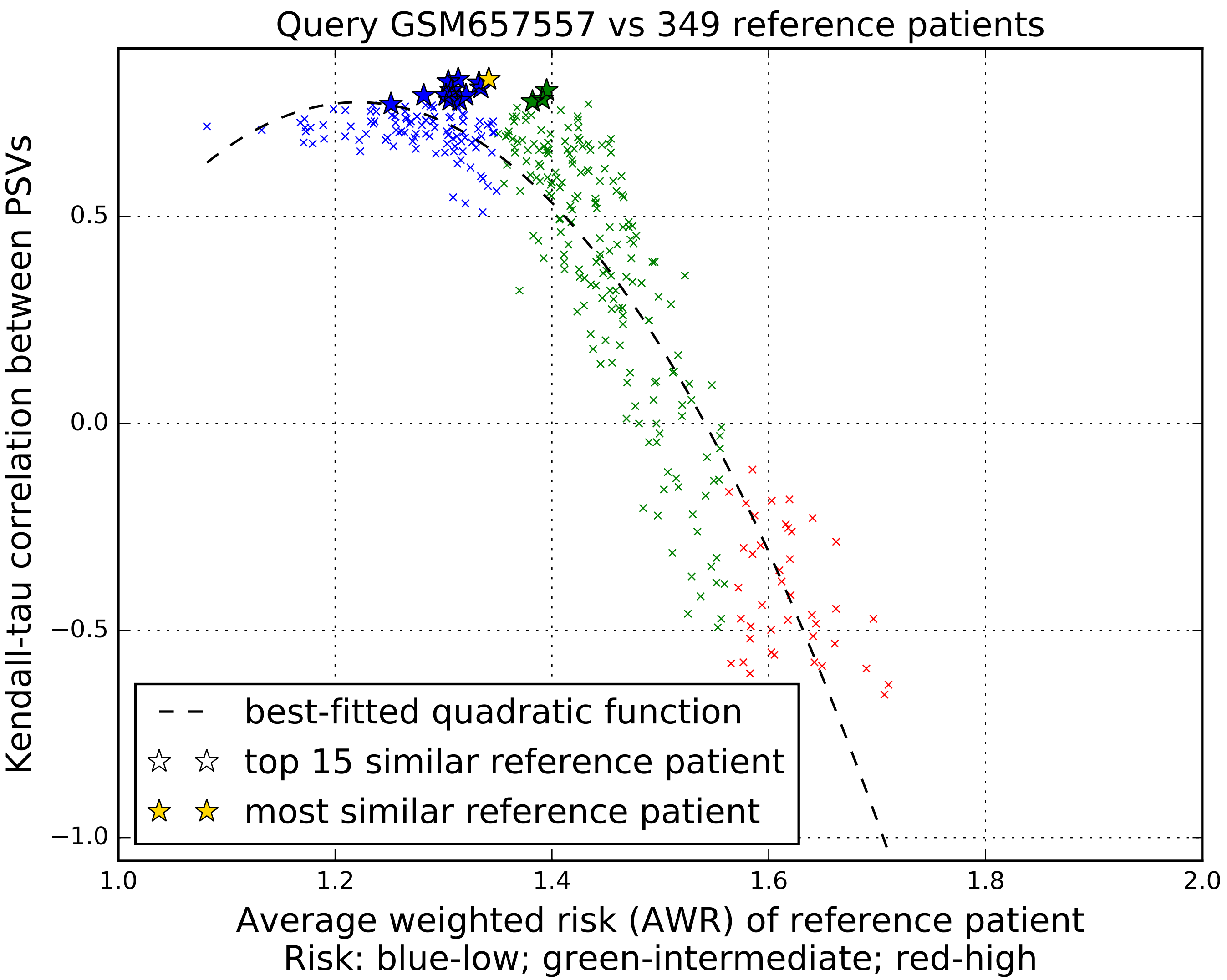

Query GSM657557 vs 349 reference patients
Kendall-tau correlation between PSVs
0.5
0.0
−0.5
−1.0
1.0
1.2
1.4
1.6
1.8
2.0
Average weighted risk (AWR) of reference patient
Risk: blue-low; green-intermediate; red-high
best-fitted quadratic function
top 15 similar reference patient
most similar reference patient

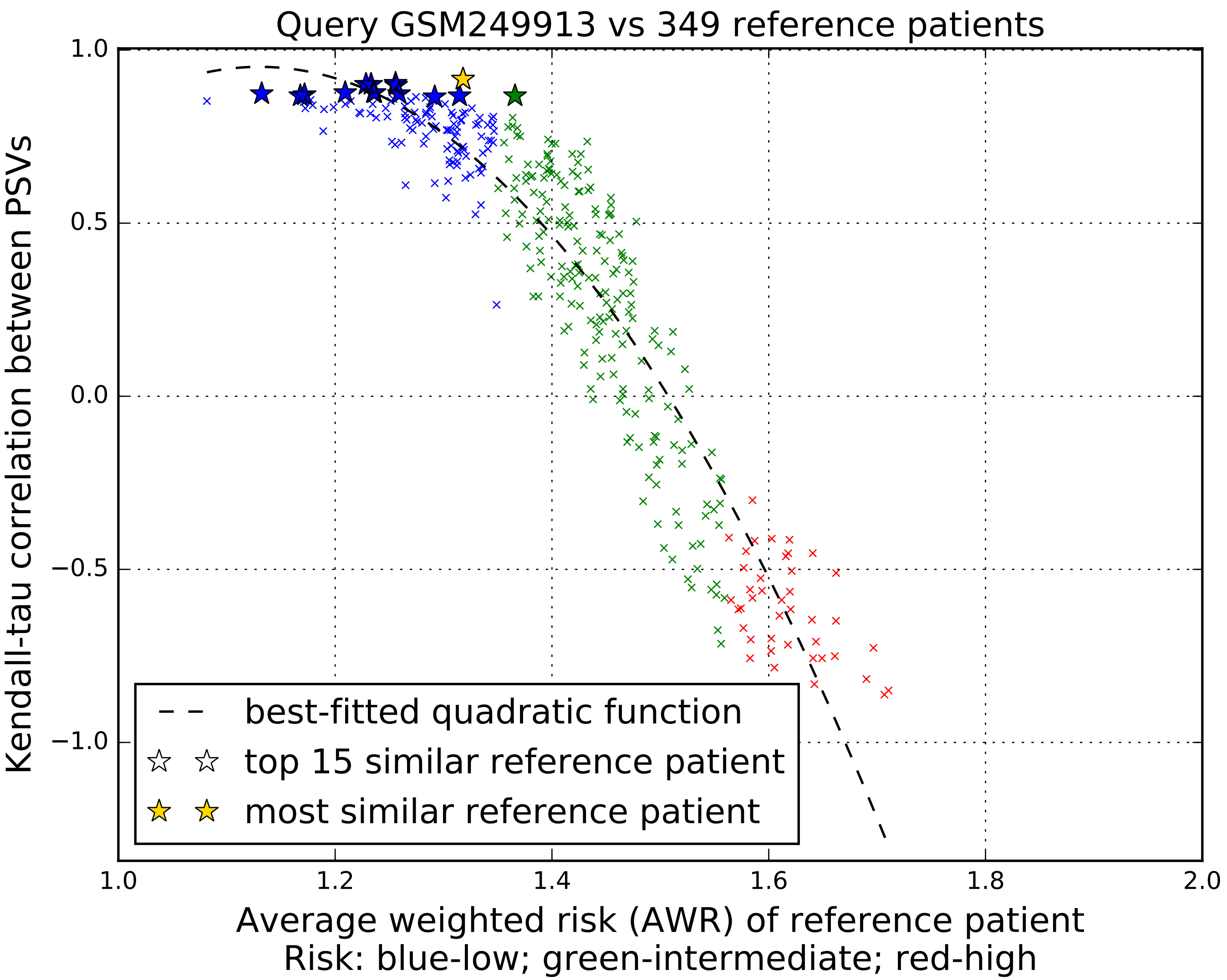
Query GSM249913 vs 349 reference patients
Kendall-tau correlation between PSVs
1.0
0.5
0.0
−0.5
−1.0
1.0
1.2
1.4
1.6
1.8
2.0
best-fitted quadratic function
top 15 similar reference patient
most similar reference patient
Average weighted risk (AWR) of reference patient
Risk: blue-low; green-intermediate; red-high

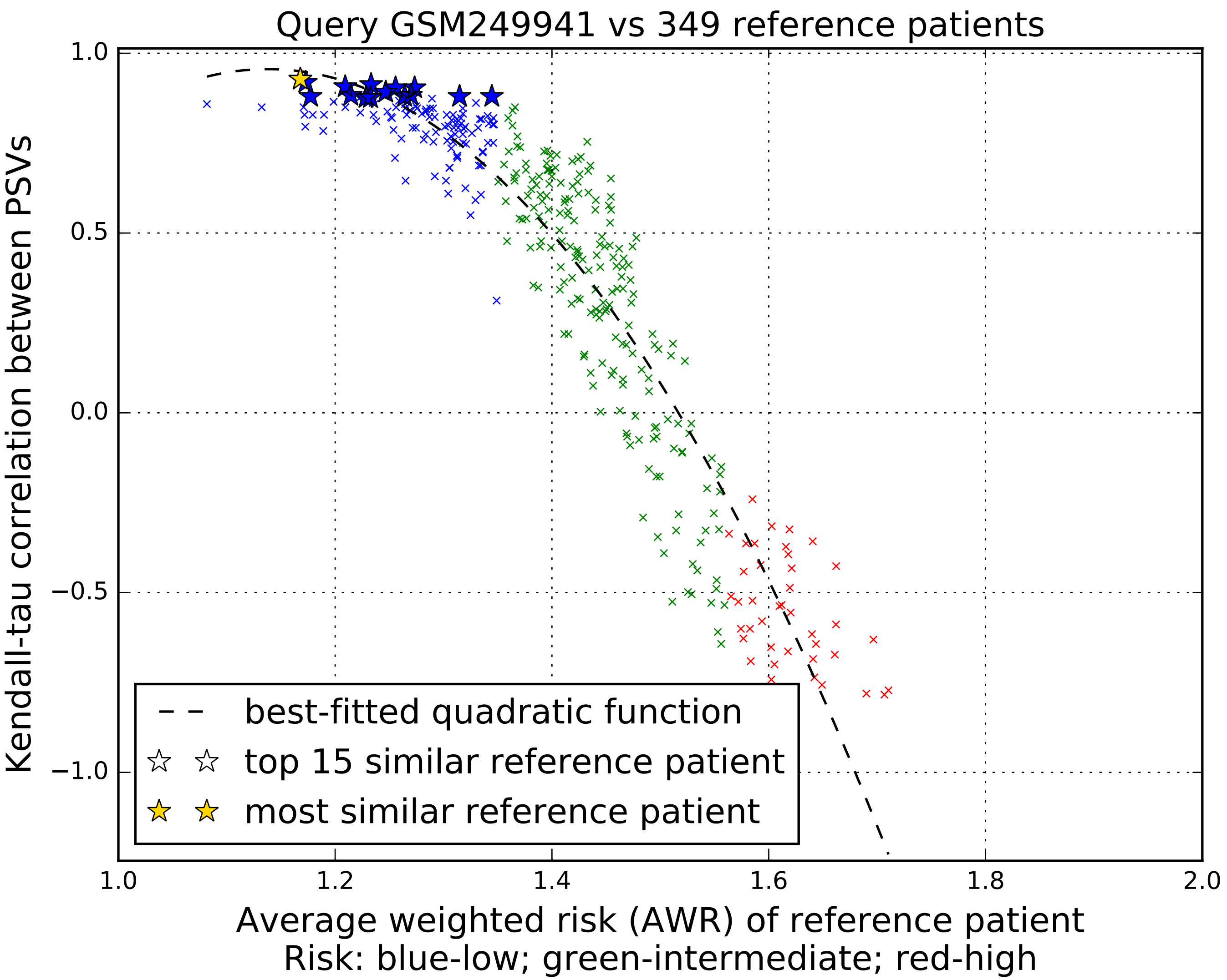

Query GSM249941 vs 349 reference patients
Kendall-tau correlation between PSVs
Average weighted risk (AWR) of reference patient
Risk: blue-low; green-intermediate; red-high
best-fitted quadratic function
top 15 similar reference patient
most similar reference patient
1.0
0.5
0.0
−0.5
−1.0
1.0
1.2
1.4
1.6
1.8
2.0

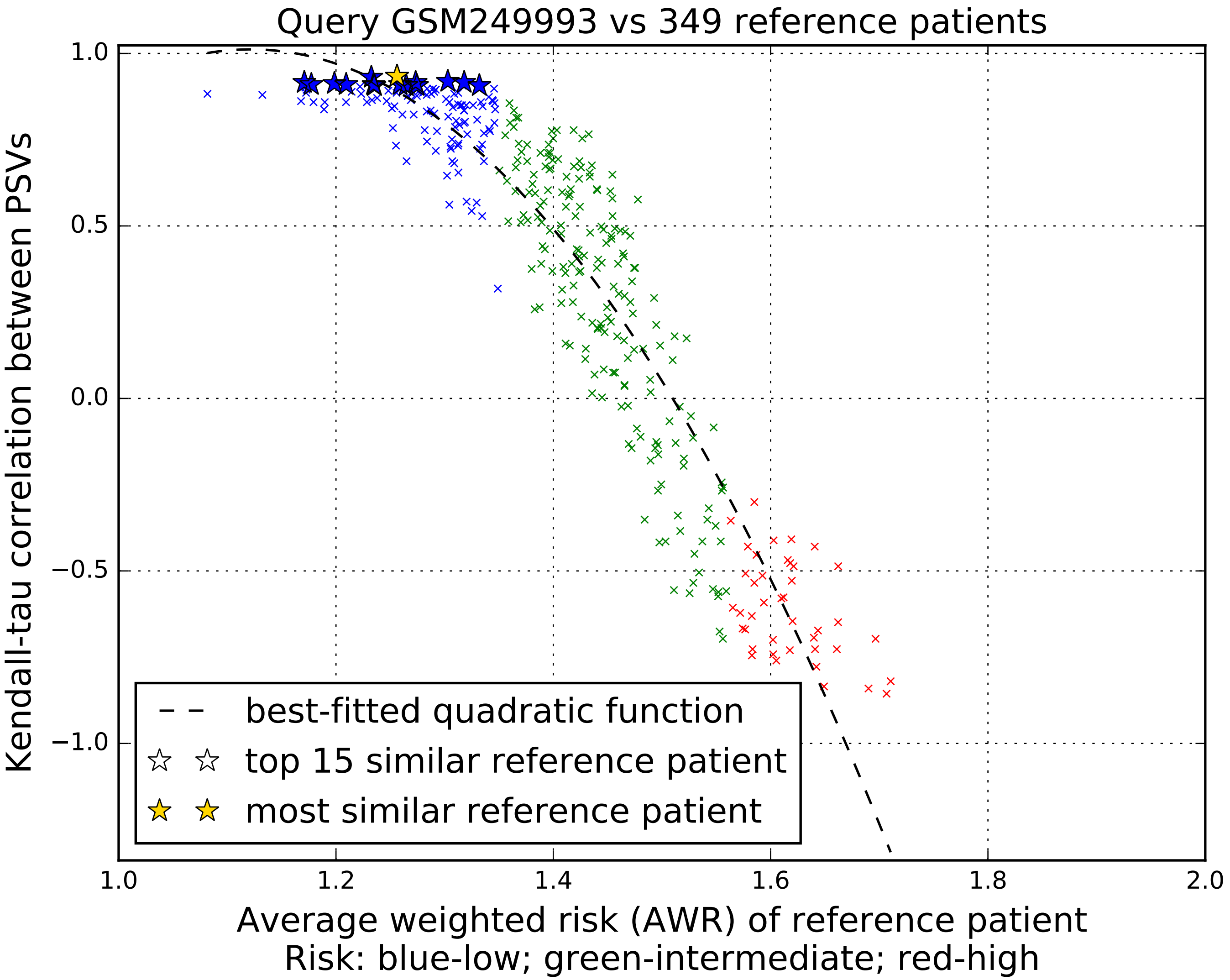

Query GSM249993 vs 349 reference patients
1.0
0.5
0.0
−0.5
−1.0
Kendall-tau correlation between PSVs
1.0
1.2
1.4
1.6
1.8
2.0
Average weighted risk (AWR) of reference patient
Risk: blue-low; green-intermediate; red-high
best-fitted quadratic function
top 15 similar reference patient
most similar reference patient

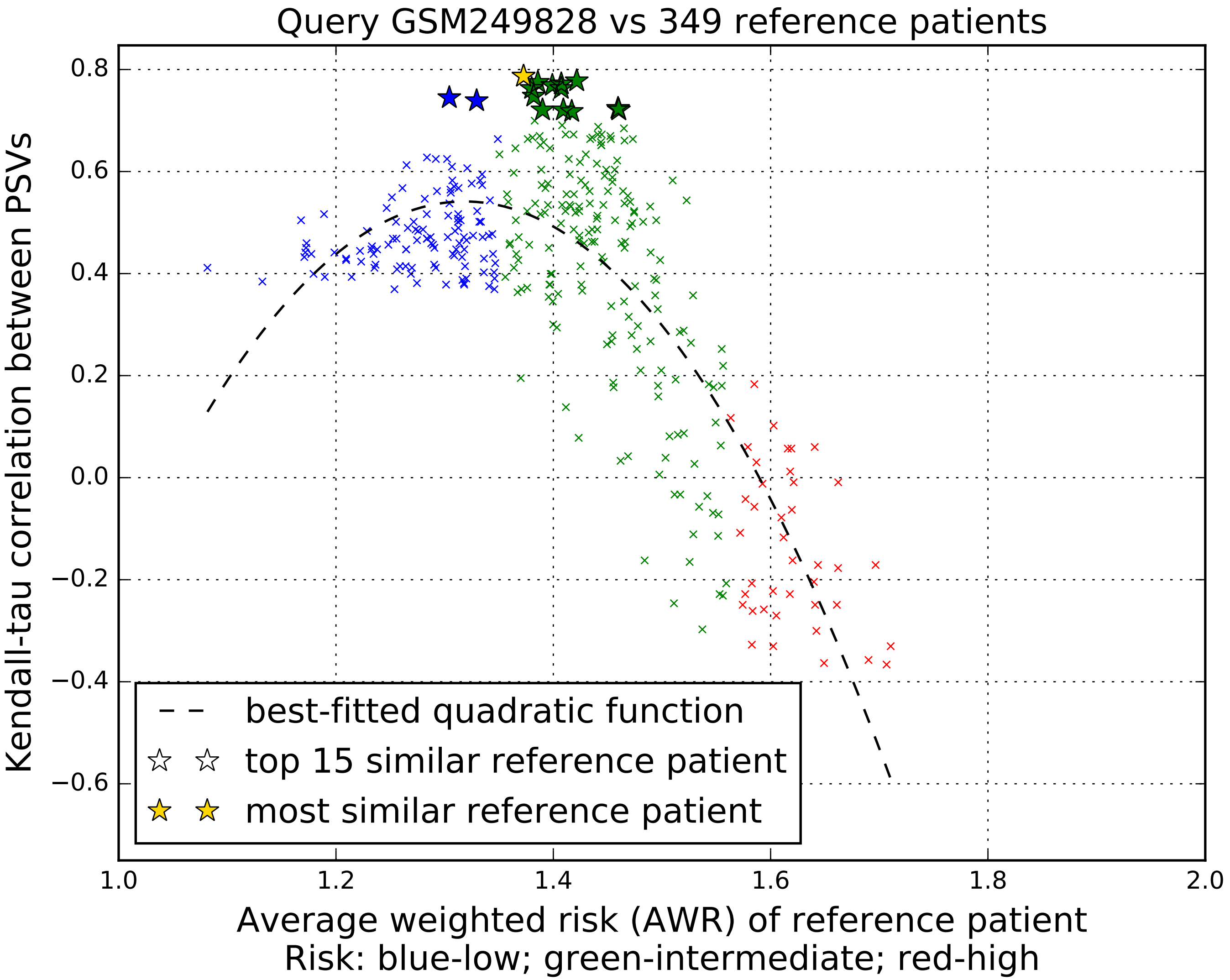
Query GSM249828 vs 349 reference patients
Kendall-tau correlation between PSVs
Average weighted risk (AWR) of reference patient
Risk: blue-low; green-intermediate; red-high
best-fitted quadratic function
top 15 similar reference patient
most similar reference patient
0.8
0.6
0.4
0.2
0.0
−0.2
−0.4
−0.6
1.0
1.2
1.4
1.6
1.8
2.0

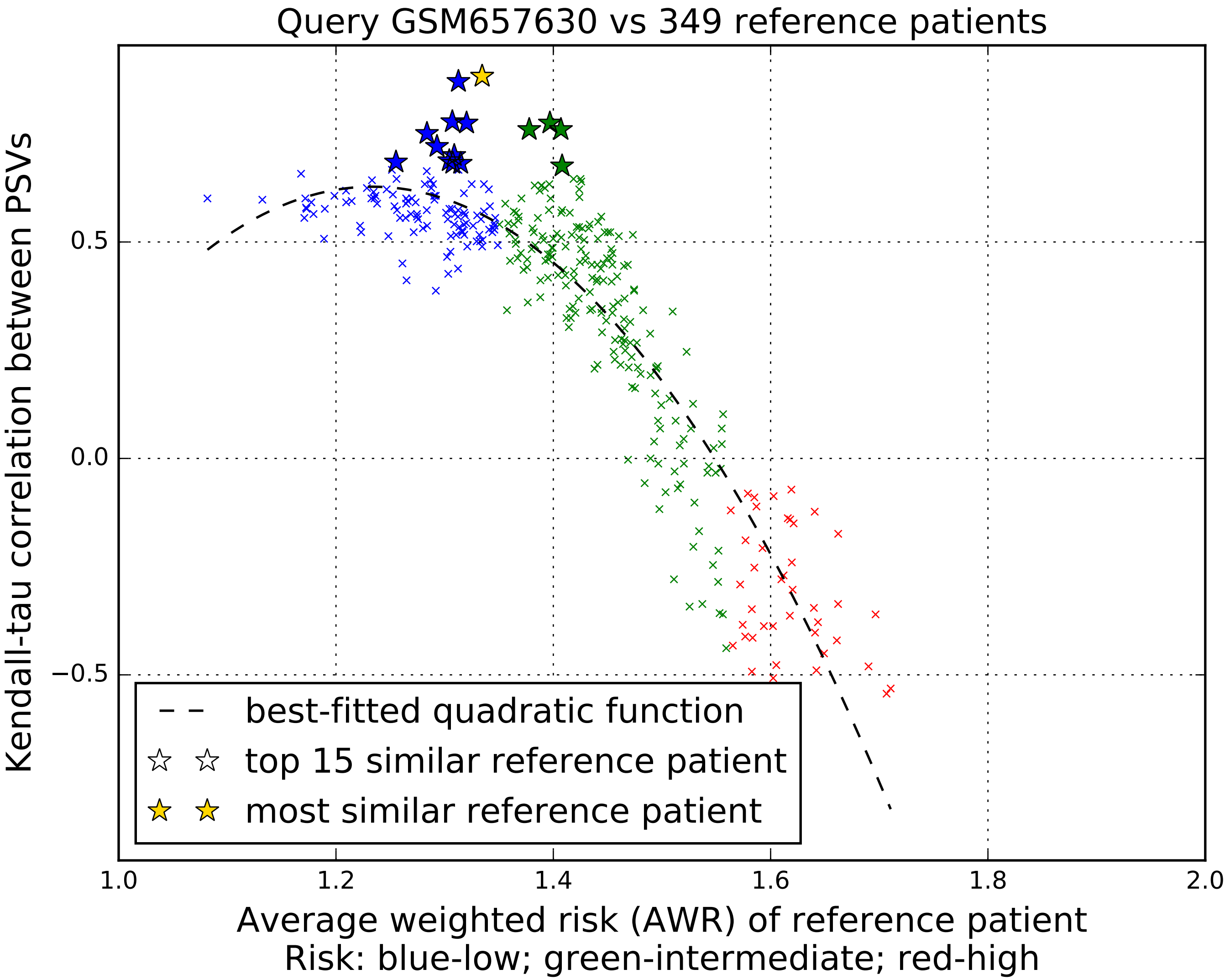

Query GSM657630 vs 349 reference patients
Kendall-tau correlation between PSVs
0.5
0.0
−0.5
1.0
1.2
1.4
1.6
1.8
2.0
best-fitted quadratic function
top 15 similar reference patient
most similar reference patient
Average weighted risk (AWR) of reference patient
Risk: blue-low; green-intermediate; red-high

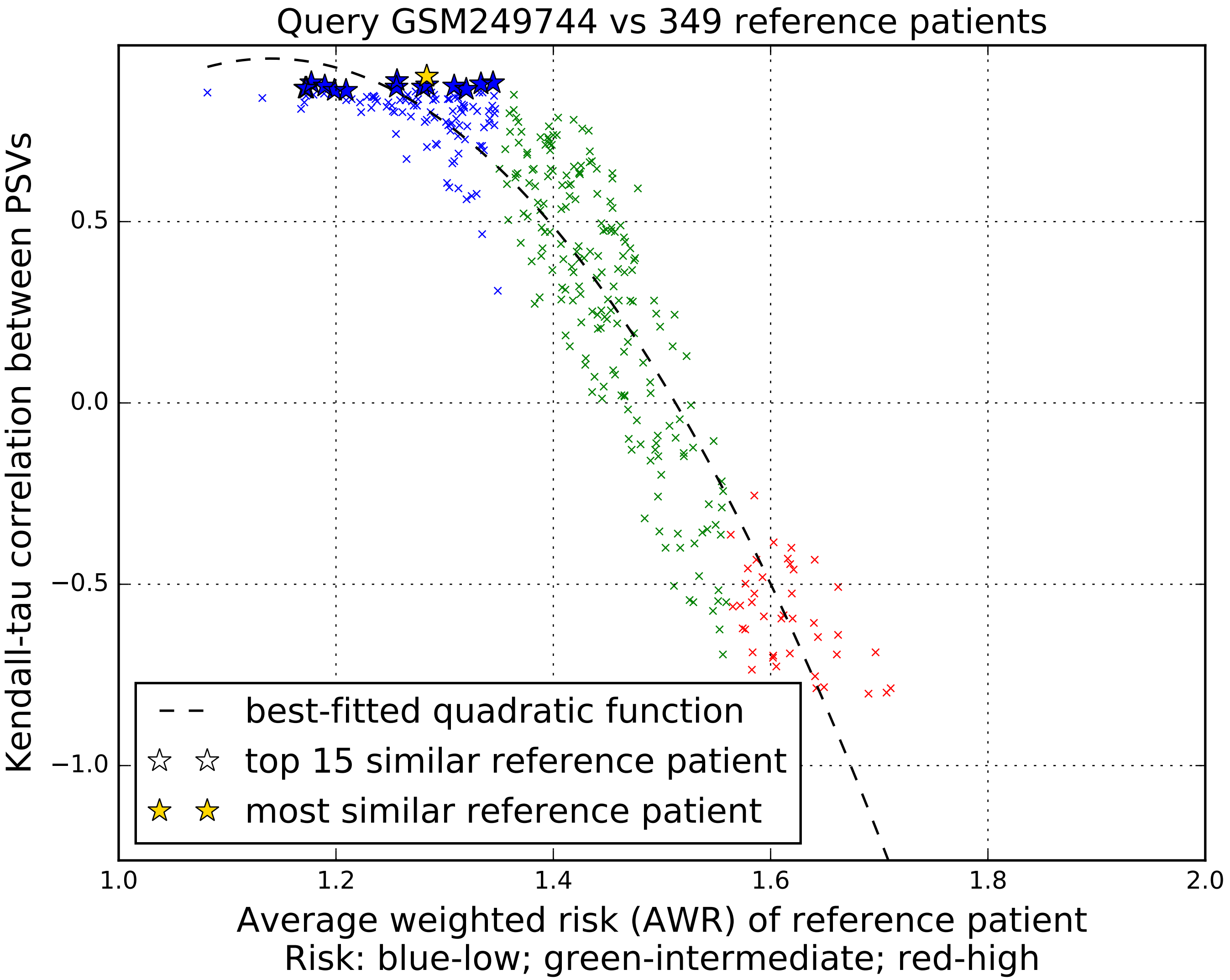
Query GSM249744 vs 349 reference patients
Kendall-tau correlation between PSVs
0.5
0.0
−0.5
−1.0
1.0
1.2
1.4
1.6
1.8
2.0
best-fitted quadratic function
top 15 similar reference patient
most similar reference patient
Average weighted risk (AWR) of reference patient
Risk: blue-low; green-intermediate; red-high

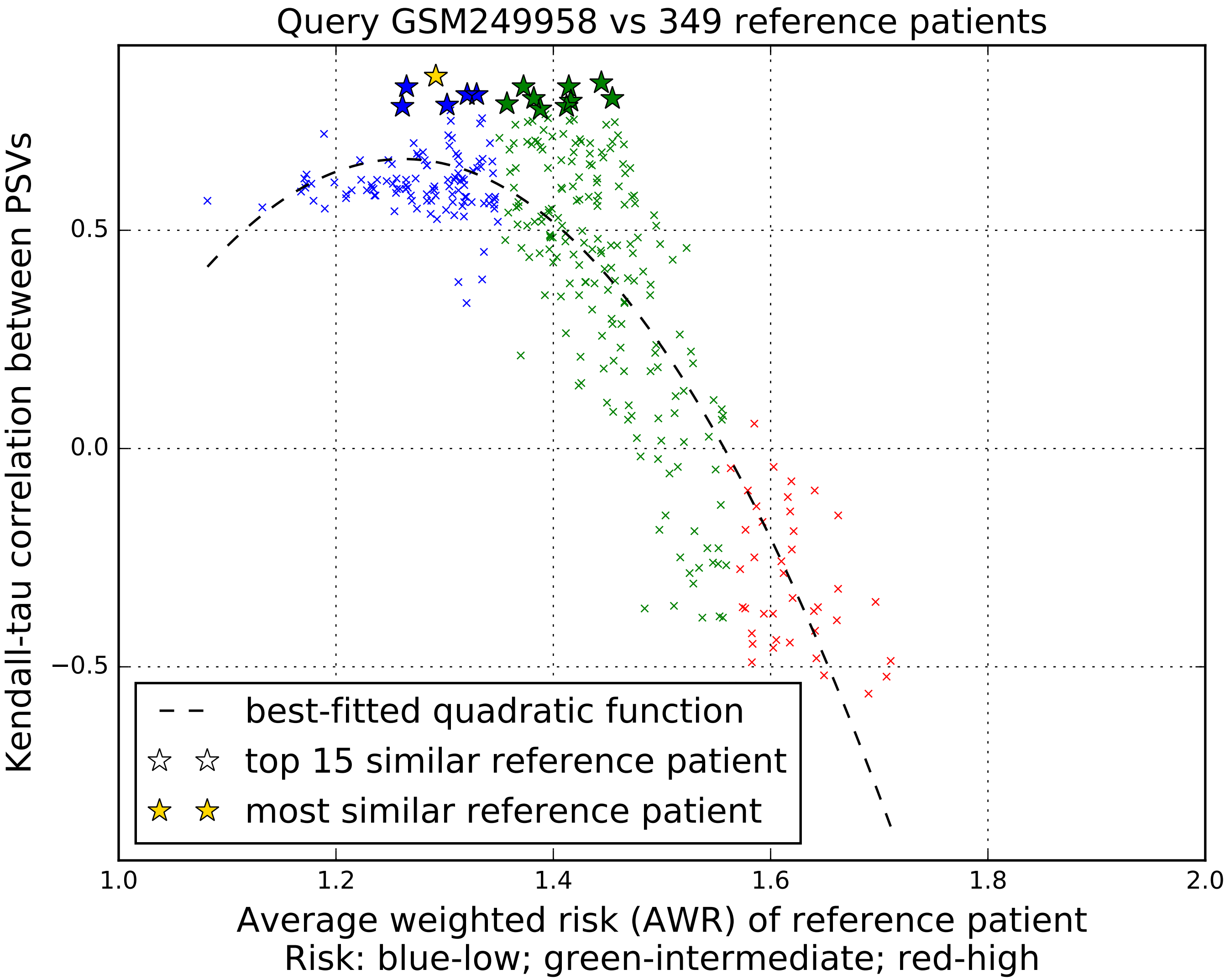

Query GSM249958 vs 349 reference patients
Kendall-tau correlation between PSVs
0.5
0.0
−0.5
1.0
1.2
1.4
1.6
1.8
2.0
best-fitted quadratic function
top 15 similar reference patient
most similar reference patient
Average weighted risk (AWR) of reference patient
Risk: blue-low; green-intermediate; red-high

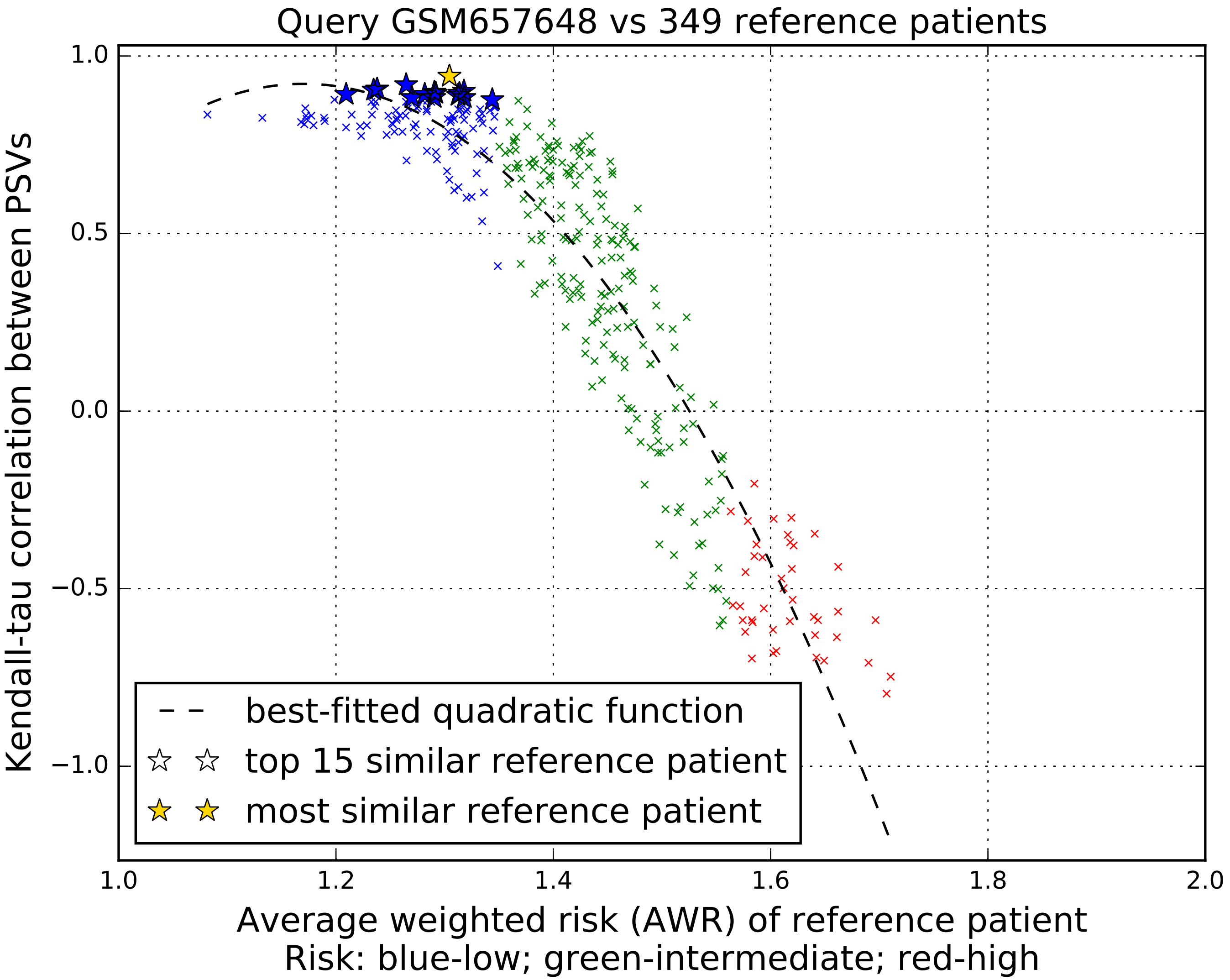
Query GSM657648 vs 349 reference patients
Kendall-tau correlation between PSVs
1.0
0.5
0.0
−0.5
−1.0
1.0
1.2
1.4
1.6
1.8
2.0
best-fitted quadratic function
top 15 similar reference patient
most similar reference patient
Average weighted risk (AWR) of reference patient
Risk: blue-low; green-intermediate; red-high

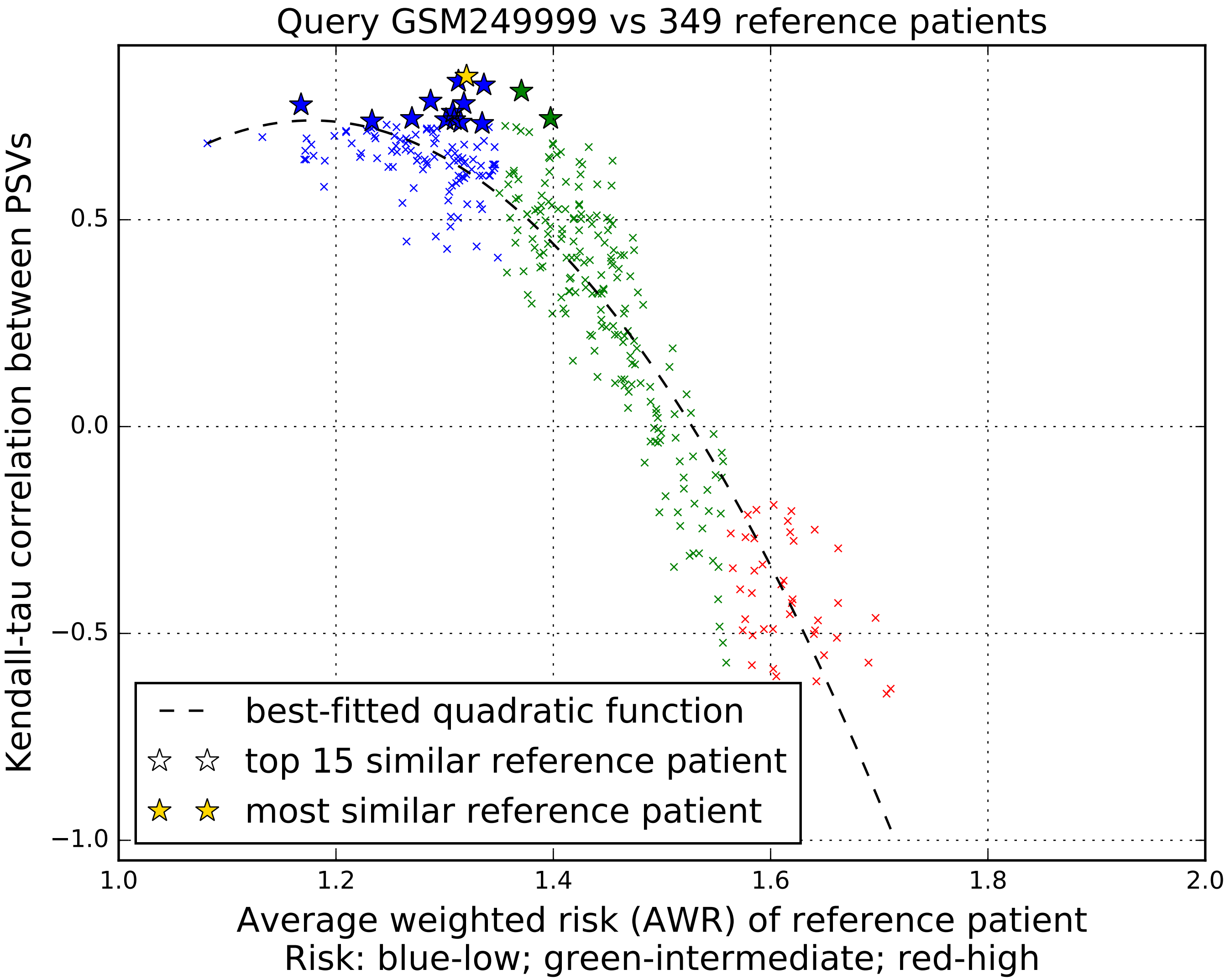

Query GSM249999 vs 349 reference patients
Kendall-tau correlation between PSVs
0.5
0.0
−0.5
−1.0
1.0
1.2
1.4
1.6
1.8
2.0
best-fitted quadratic function
top 15 similar reference patient
most similar reference patient
Average weighted risk (AWR) of reference patient
Risk: blue-low; green-intermediate; red-high

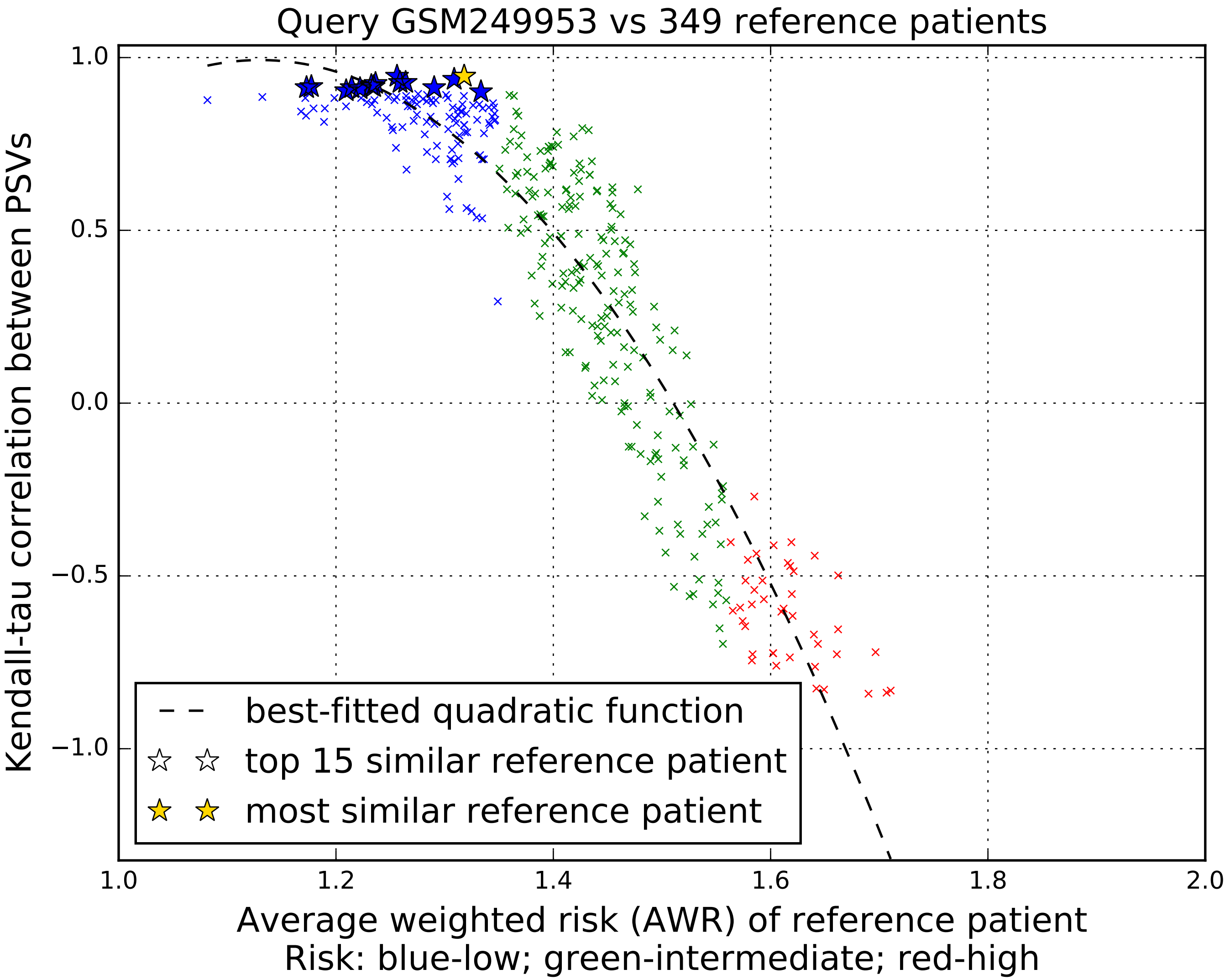
Query GSM249953 vs 349 reference patients
Kendall-tau correlation between PSVs
1.0
0.5
0.0
−0.5
−1.0
1.0
1.2
1.4
1.6
1.8
2.0
best-fitted quadratic function
top 15 similar reference patient
most similar reference patient
Average weighted risk (AWR) of reference patient
Risk: blue-low; green-intermediate; red-high

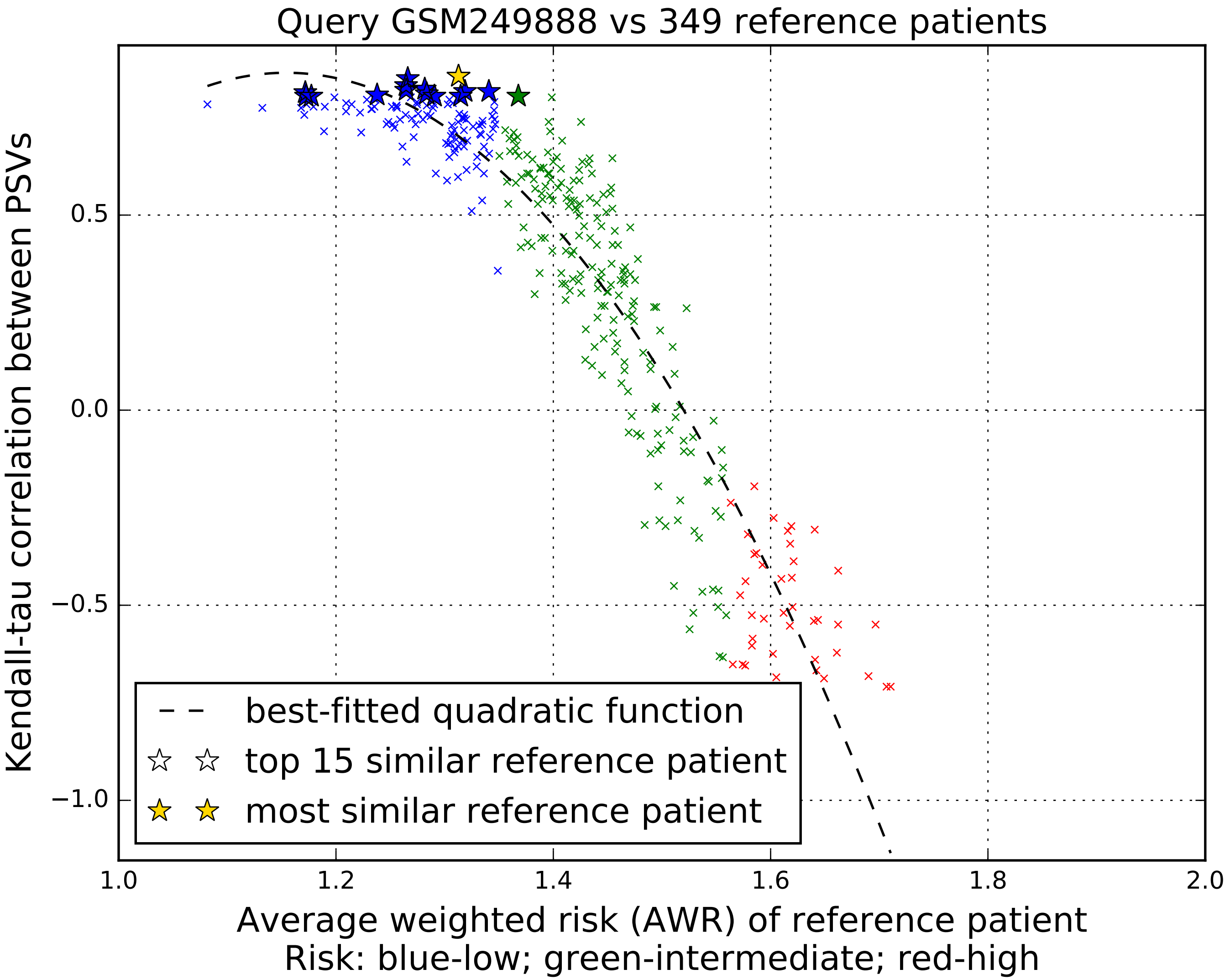
Query GSM249888 vs 349 reference patients
Kendall-tau correlation between PSVs
0.5
0.0
−0.5
−1.0
1.0
1.2
1.4
1.6
1.8
2.0
best-fitted quadratic function
top 15 similar reference patient
most similar reference patient
Average weighted risk (AWR) of reference patient
Risk: blue-low; green-intermediate; red-high

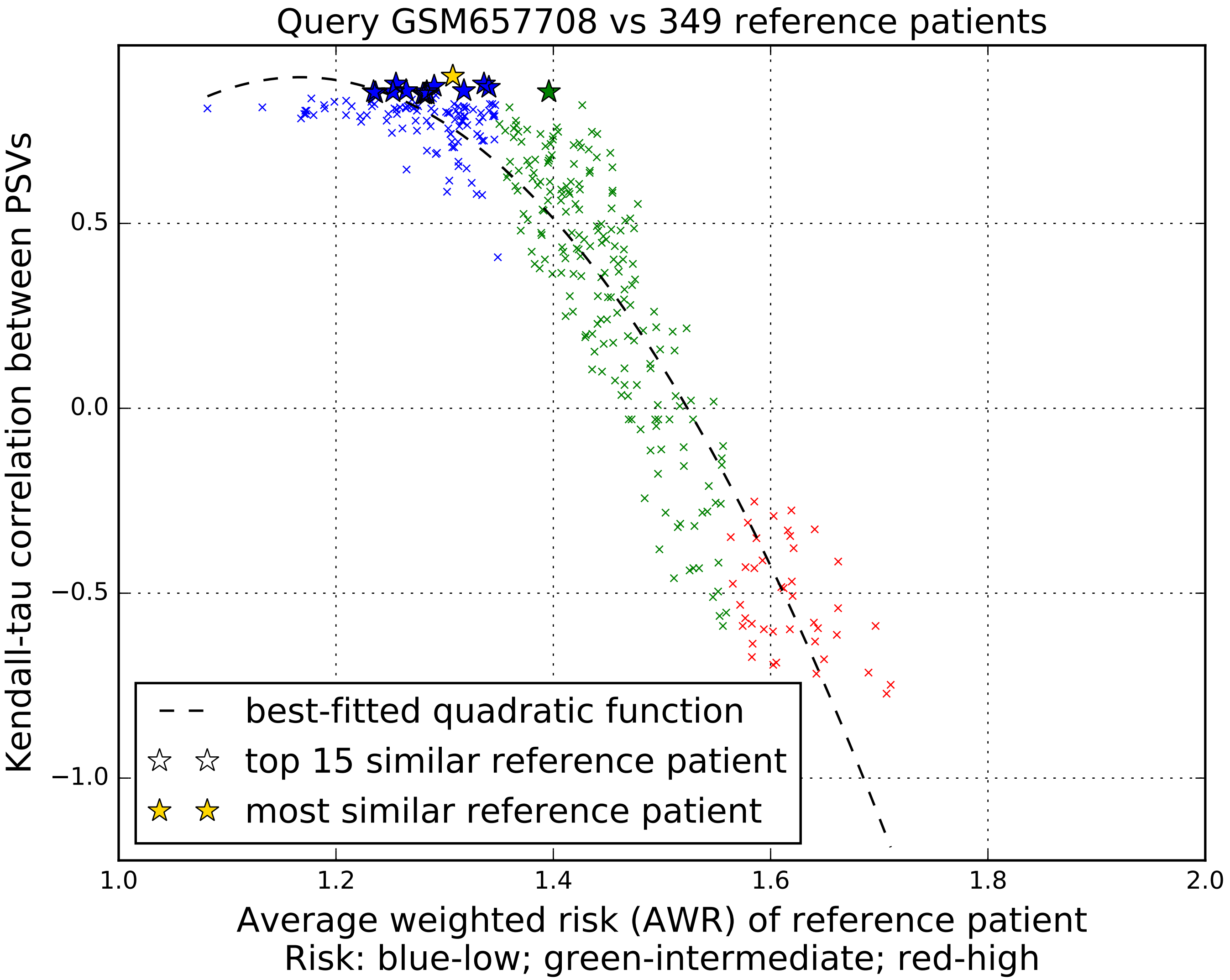

Query GSM657708 vs 349 reference patients
Kendall-tau correlation between PSVs
0.5
0.0
−0.5
−1.0
1.0
1.2
1.4
1.6
1.8
2.0
Average weighted risk (AWR) of reference patient
Risk: blue-low; green-intermediate; red-high
best-fitted quadratic function
top 15 similar reference patient
most similar reference patient

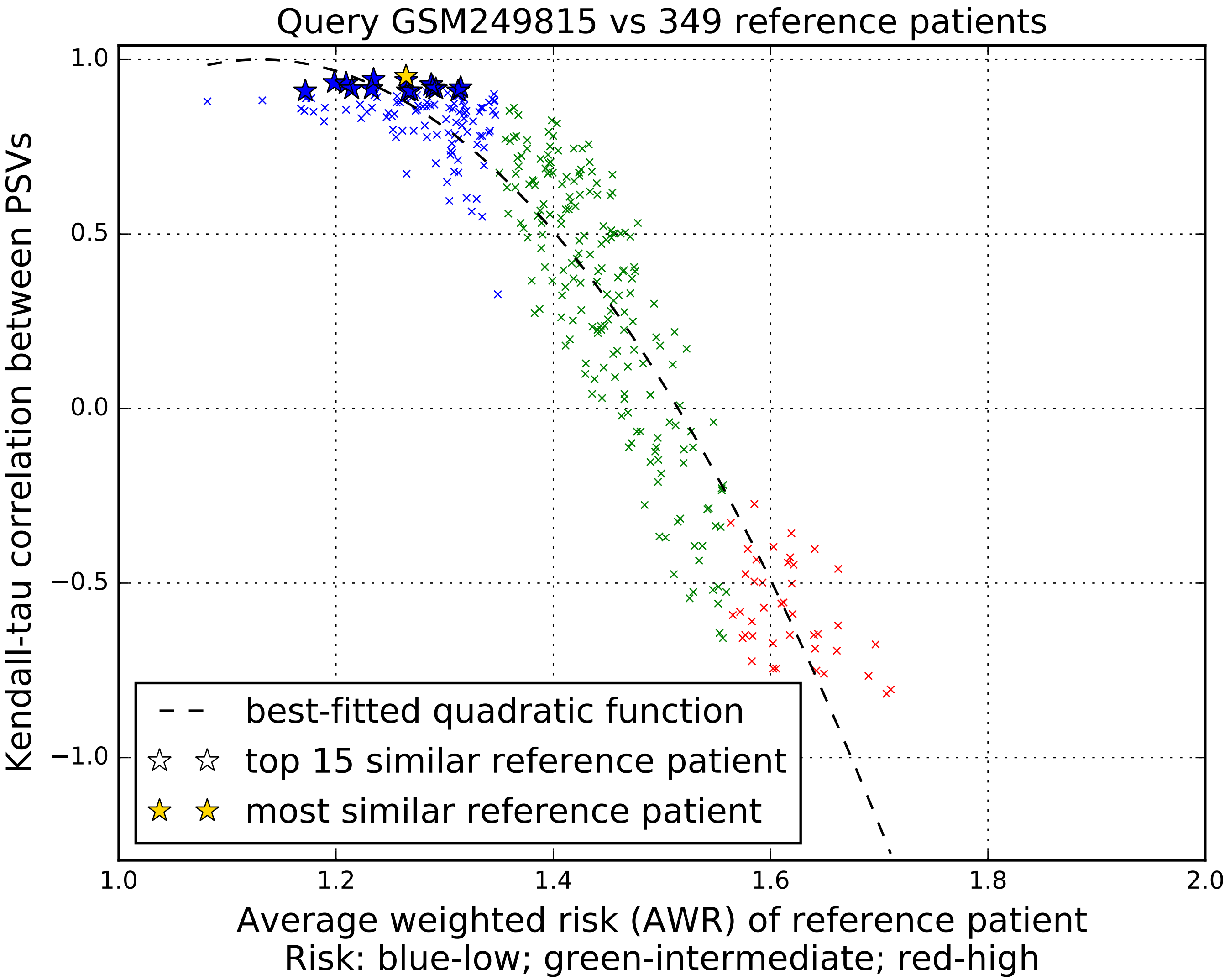

Query GSM249815 vs 349 reference patients
Kendall-tau correlation between PSVs
1.0
0.5
0.0
−0.5
−1.0
1.0
1.2
1.4
1.6
1.8
2.0
best-fitted quadratic function
top 15 similar reference patient
most similar reference patient
Average weighted risk (AWR) of reference patient
Risk: blue-low; green-intermediate; red-high

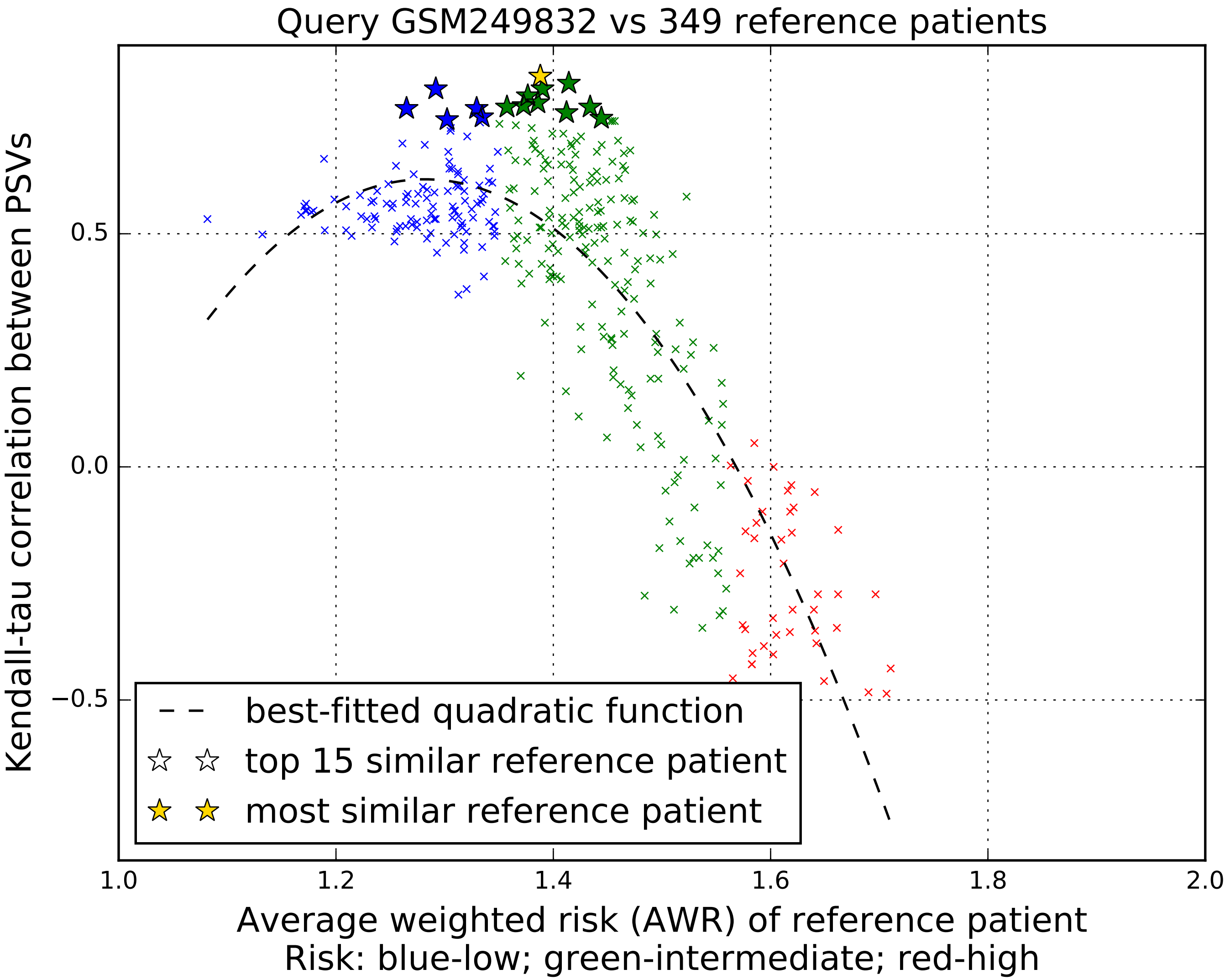

Query GSM249832 vs 349 reference patients
Kendall-tau correlation between PSVs
0.5
0.0
−0.5
1.0
1.2
1.4
1.6
1.8
2.0
best-fitted quadratic function
top 15 similar reference patient
most similar reference patient
Average weighted risk (AWR) of reference patient
Risk: blue-low; green-intermediate; red-high

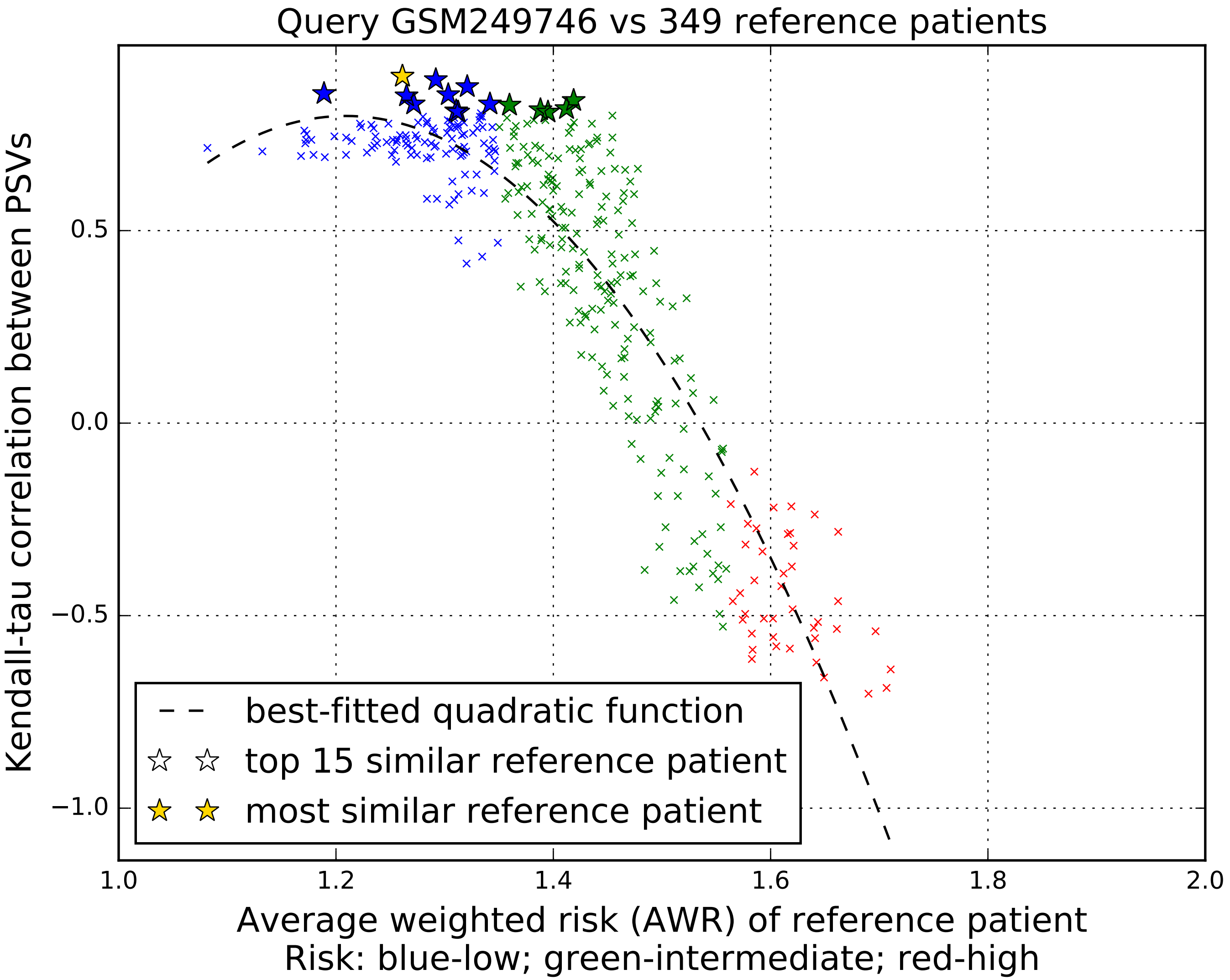

Query GSM249746 vs 349 reference patients
Kendall-tau correlation between PSVs
0.5
0.0
−0.5
−1.0
1.0
1.2
1.4
1.6
1.8
2.0
best-fitted quadratic function
top 15 similar reference patient
most similar reference patient
Average weighted risk (AWR) of reference patient
Risk: blue-low; green-intermediate; red-high

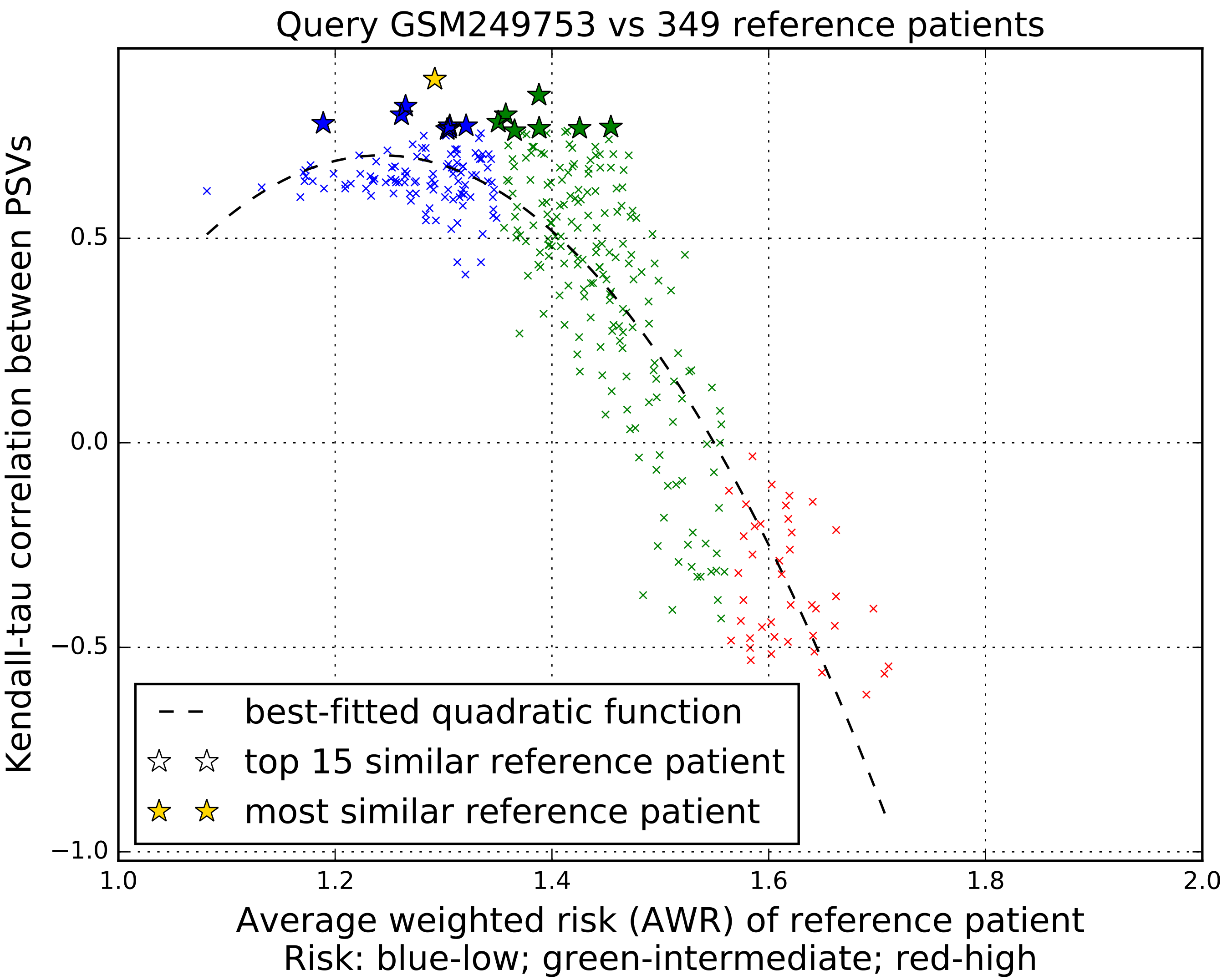

Query GSM249753 vs 349 reference patients
Kendall-tau correlation between PSVs
0.5
0.0
−0.5
−1.0
1.0
1.2
1.4
1.6
1.8
2.0
best-fitted quadratic function
top 15 similar reference patient
most similar reference patient
Average weighted risk (AWR) of reference patient
Risk: blue-low; green-intermediate; red-high

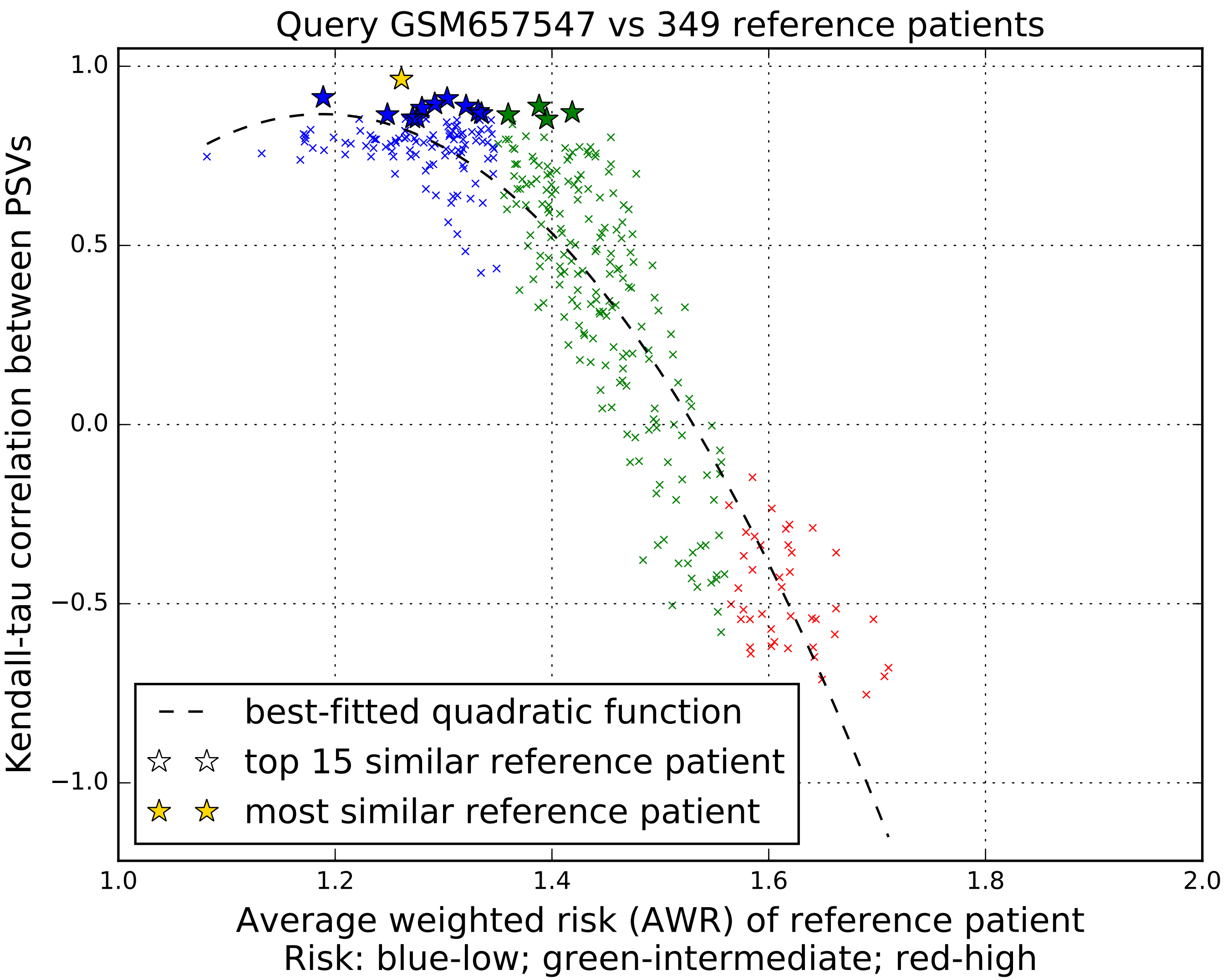
Query GSM657547 vs 349 reference patients
1.0
0.5
0.0
−0.5
−1.0
Kendall-tau correlation between PSVs
1.0
1.2
1.4
1.6
1.8
2.0
Average weighted risk (AWR) of reference patient
Risk: blue-low; green-intermediate; red-high
best-fitted quadratic function
top 15 similar reference patient
most similar reference patient

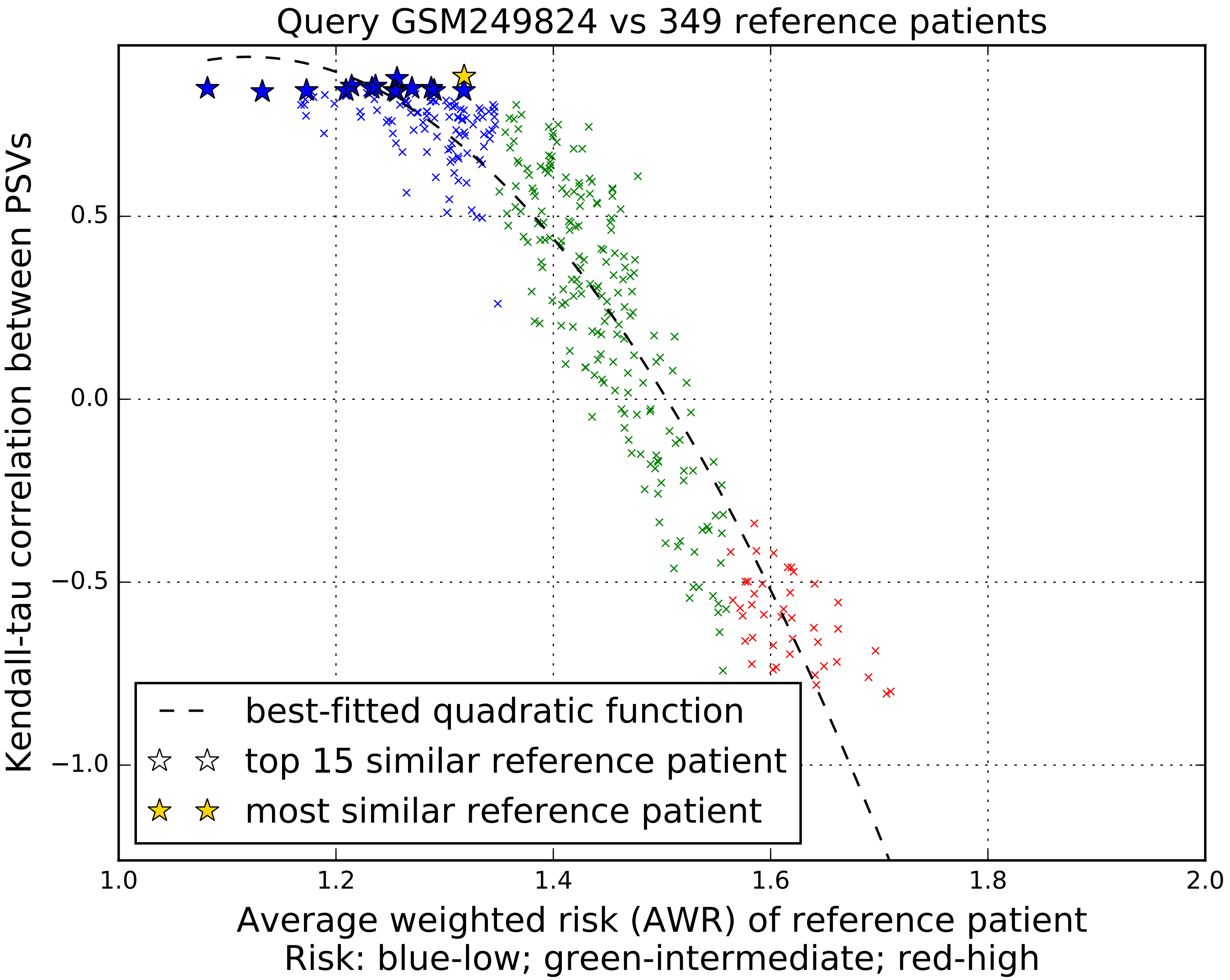
Query GSM249824 vs 349 reference patients
Kendall-tau correlation between PSVs
Average weighted risk (AWR) of reference patient
Risk: blue-low; green-intermediate; red-high
best-fitted quadratic function
top 15 similar reference patient
most similar reference patient
0.5
0.0
−0.5
−1.0
1.0
1.2
1.4
1.6
1.8
2.0

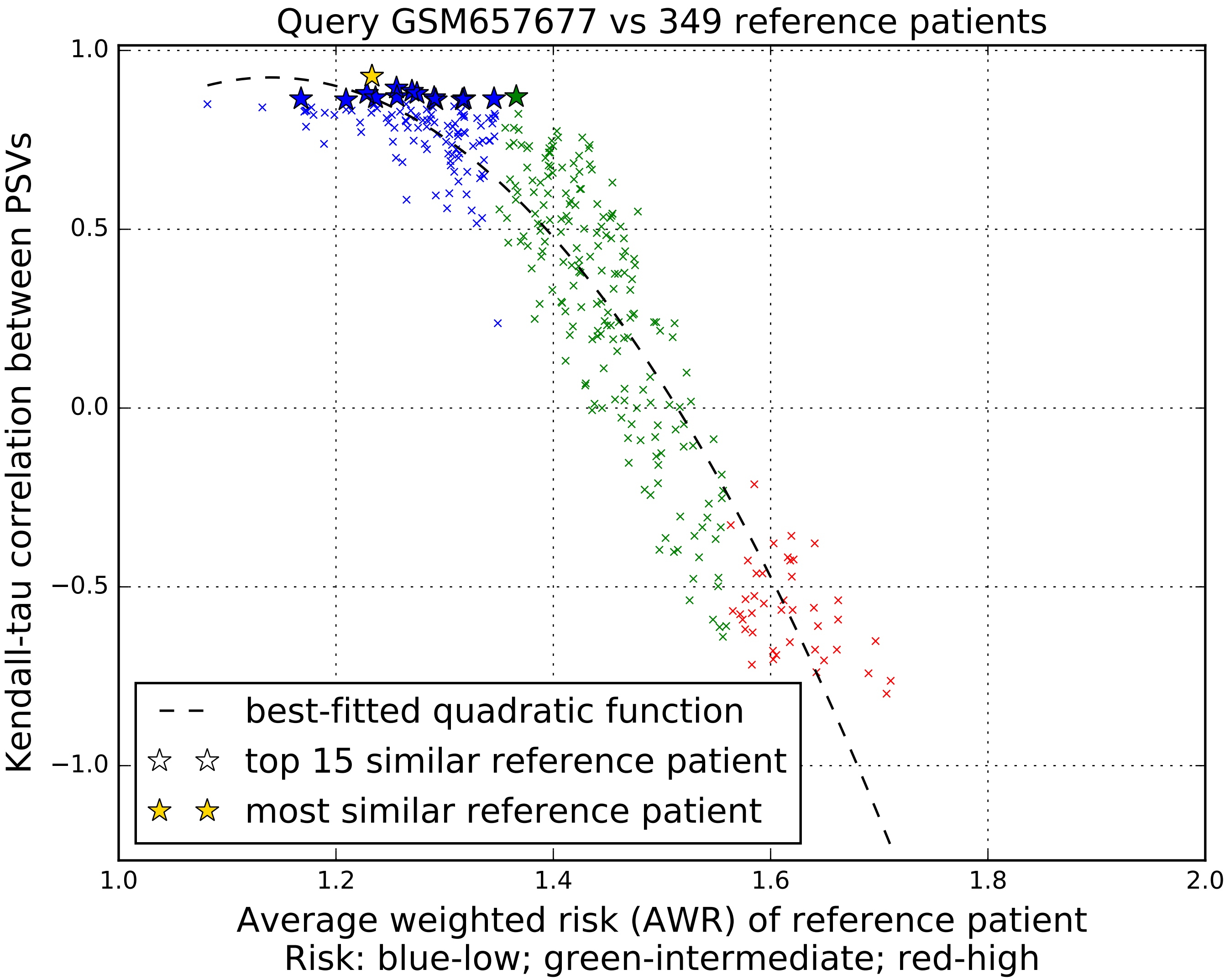

Query GSM657677 vs 349 reference patients
Kendall-tau correlation between PSVs
1.0
0.5
0.0
−0.5
−1.0
1.0
1.2
1.4
1.6
1.8
2.0
best-fitted quadratic function
top 15 similar reference patient
most similar reference patient
Average weighted risk (AWR) of reference patient
Risk: blue-low; green-intermediate; red-high

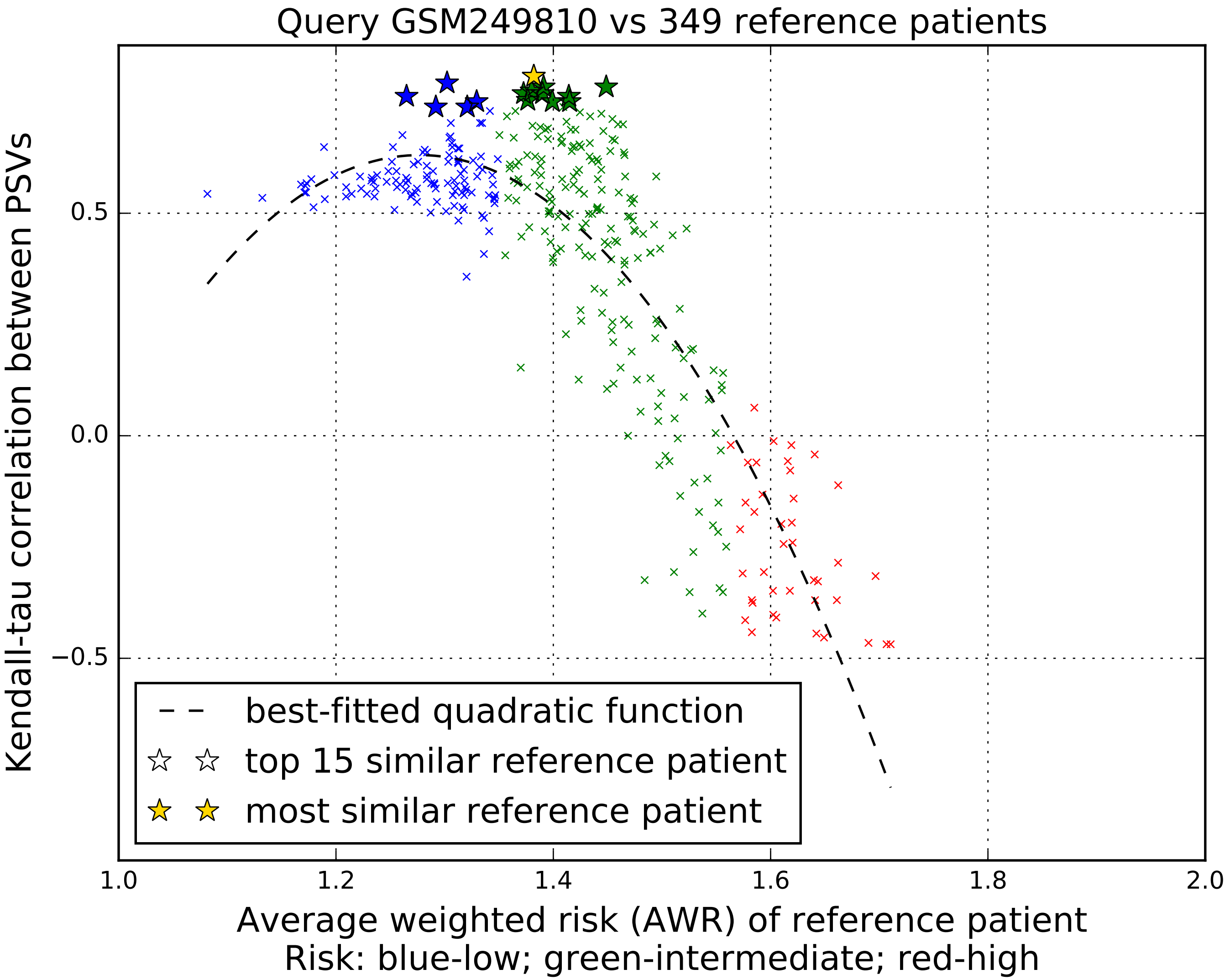

Query GSM249810 vs 349 reference patients
Kendall-tau correlation between PSVs
0.5
0.0
−0.5
1.0
1.2
1.4
1.6
1.8
2.0
best-fitted quadratic function
top 15 similar reference patient
most similar reference patient
Average weighted risk (AWR) of reference patient
Risk: blue-low; green-intermediate; red-high

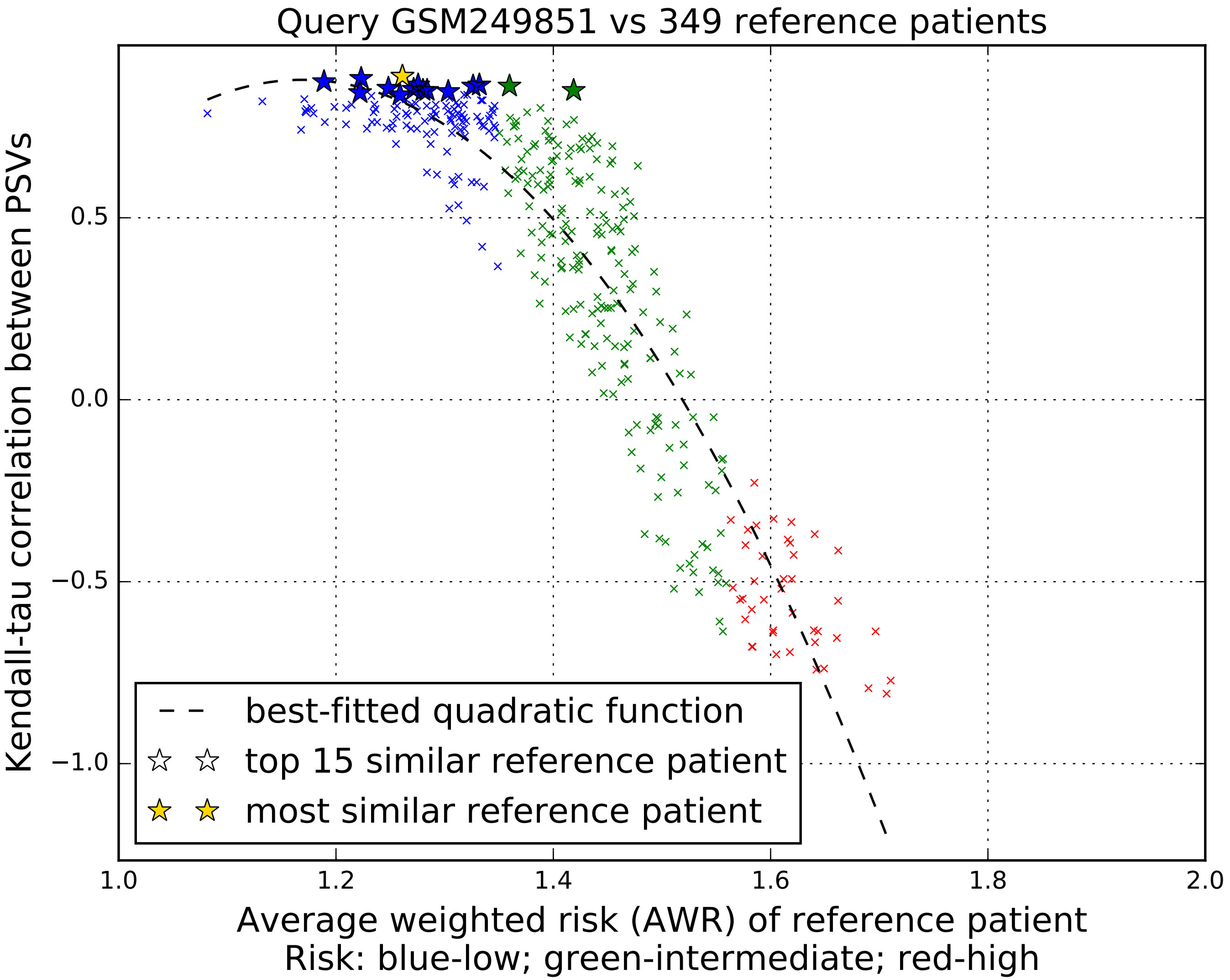

Query GSM249851 vs 349 reference patients
Kendall-tau correlation between PSVs
0.5
0.0
−0.5
−1.0
1.0
1.2
1.4
1.6
1.8
2.0
best-fitted quadratic function
top 15 similar reference patient
most similar reference patient
Average weighted risk (AWR) of reference patient
Risk: blue-low; green-intermediate; red-high

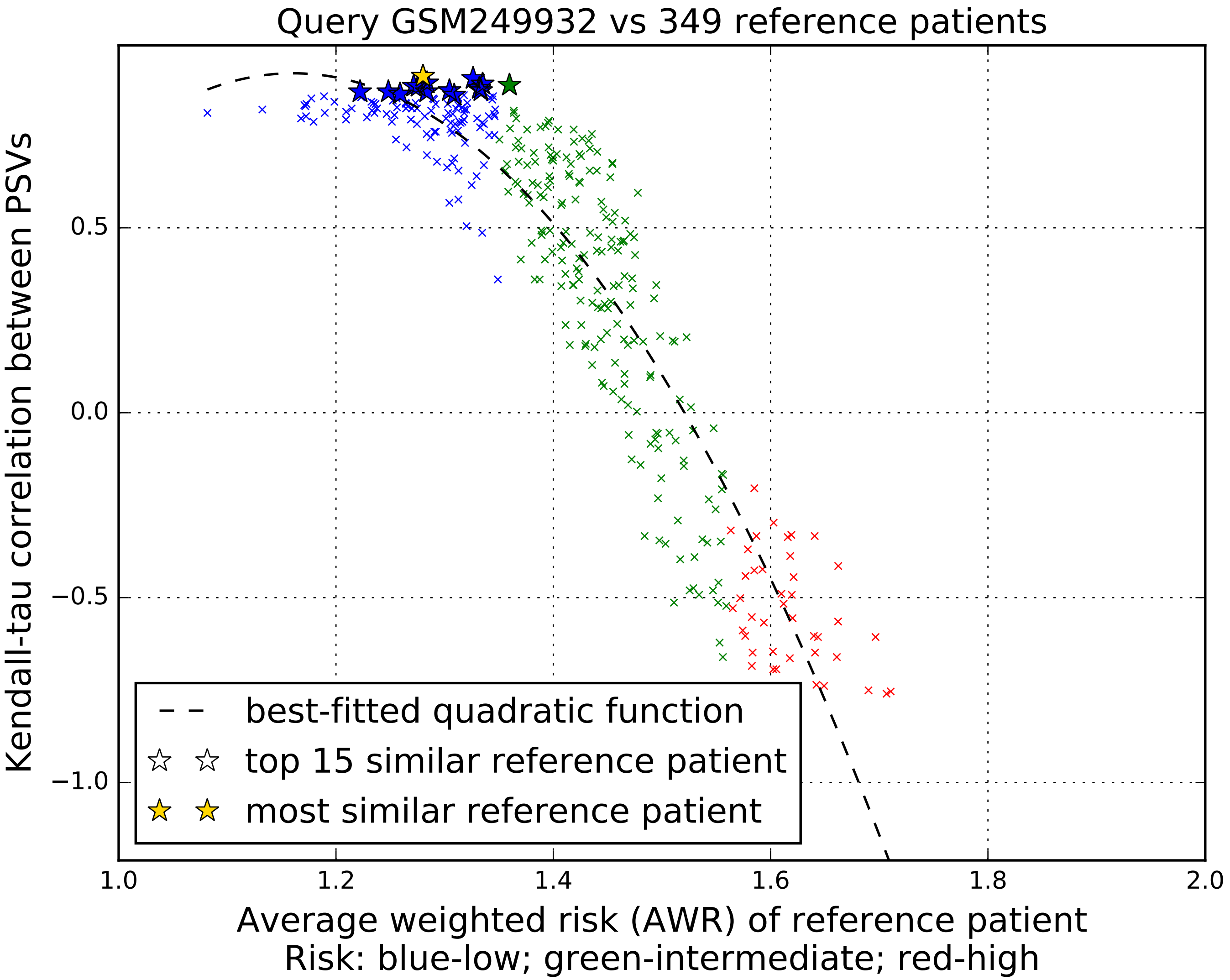

Query GSM249932 vs 349 reference patients
Kendall-tau correlation between PSVs
0.5
0.0
−0.5
−1.0
1.0
1.2
1.4
1.6
1.8
2.0
best-fitted quadratic function
top 15 similar reference patient
most similar reference patient
Average weighted risk (AWR) of reference patient
Risk: blue-low; green-intermediate; red-high

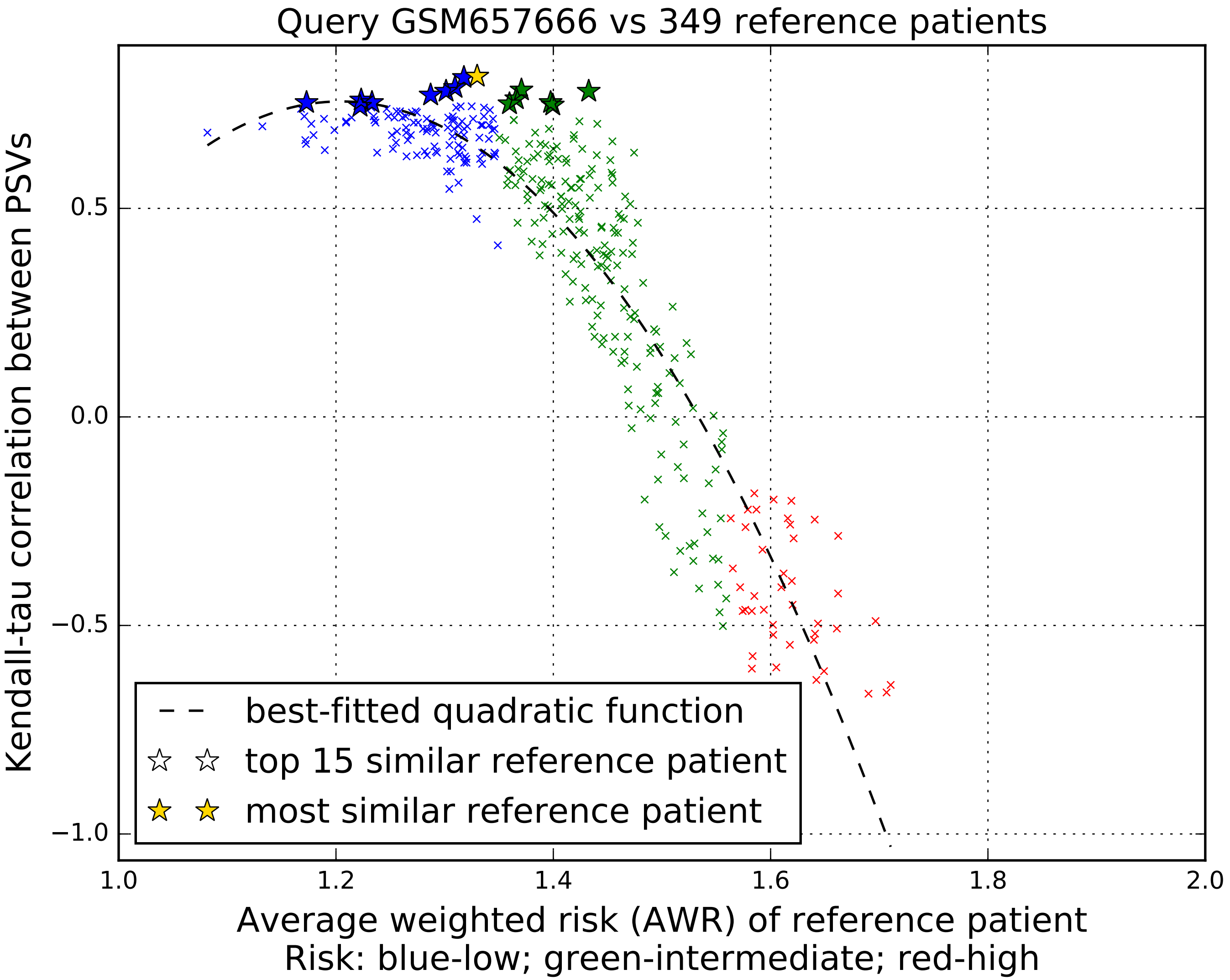

Query GSM657666 vs 349 reference patients
Kendall-tau correlation between PSVs
0.5
0.0
−0.5
−1.0
1.0
1.2
1.4
1.6
1.8
2.0
best-fitted quadratic function
top 15 similar reference patient
most similar reference patient
Average weighted risk (AWR) of reference patient
Risk: blue-low; green-intermediate; red-high

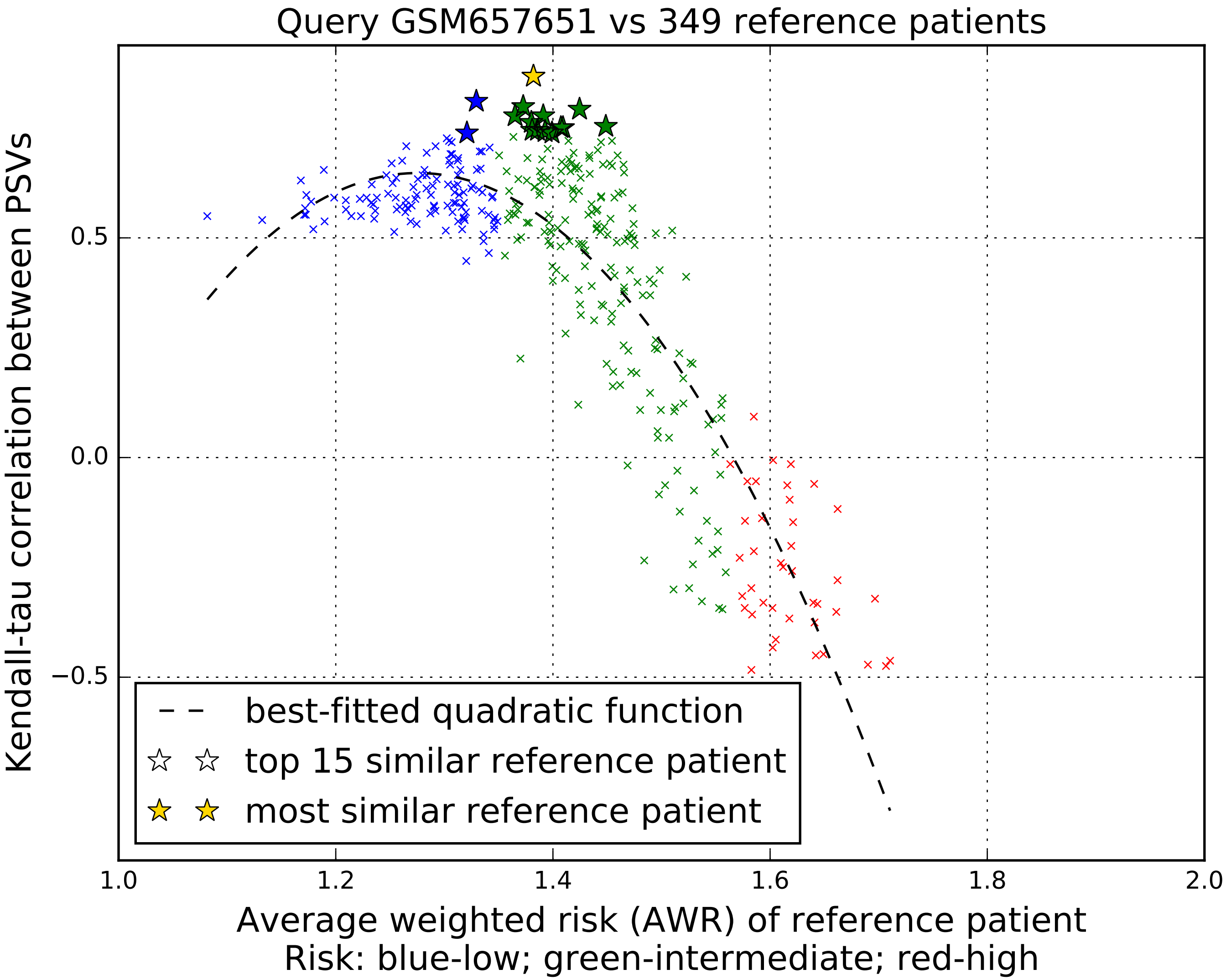

Query GSM657651 vs 349 reference patients
Kendall-tau correlation between PSVs
0.5
0.0
−0.5
1.0
1.2
1.4
1.6
1.8
2.0
best-fitted quadratic function
top 15 similar reference patient
most similar reference patient
Average weighted risk (AWR) of reference patient
Risk: blue-low; green-intermediate; red-high

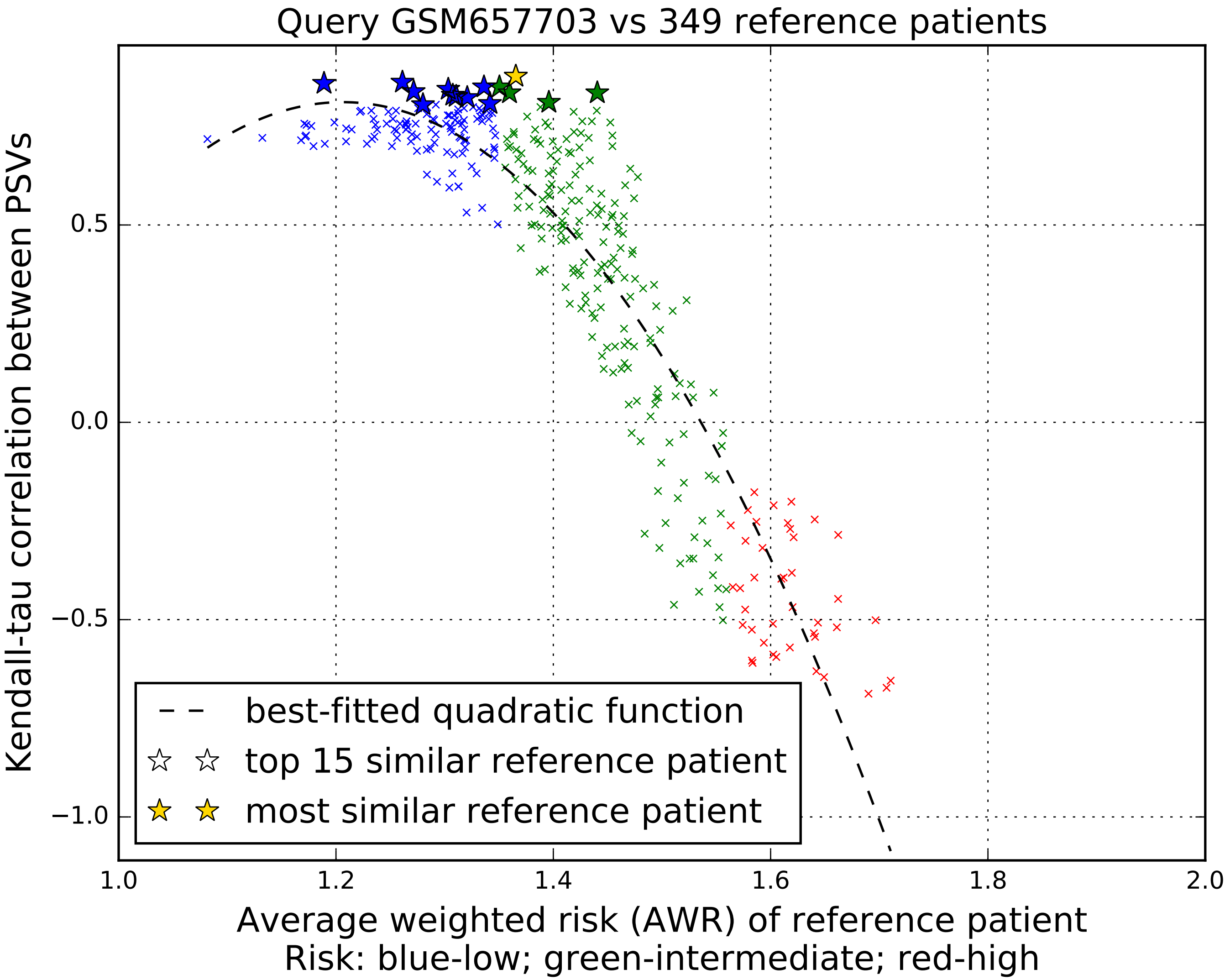
Query GSM657703 vs 349 reference patients
Kendall-tau correlation between PSVs
0.5
0.0
−0.5
−1.0
1.0
1.2
1.4
1.6
1.8
2.0
best-fitted quadratic function
top 15 similar reference patient
most similar reference patient
Average weighted risk (AWR) of reference patient
Risk: blue-low; green-intermediate; red-high

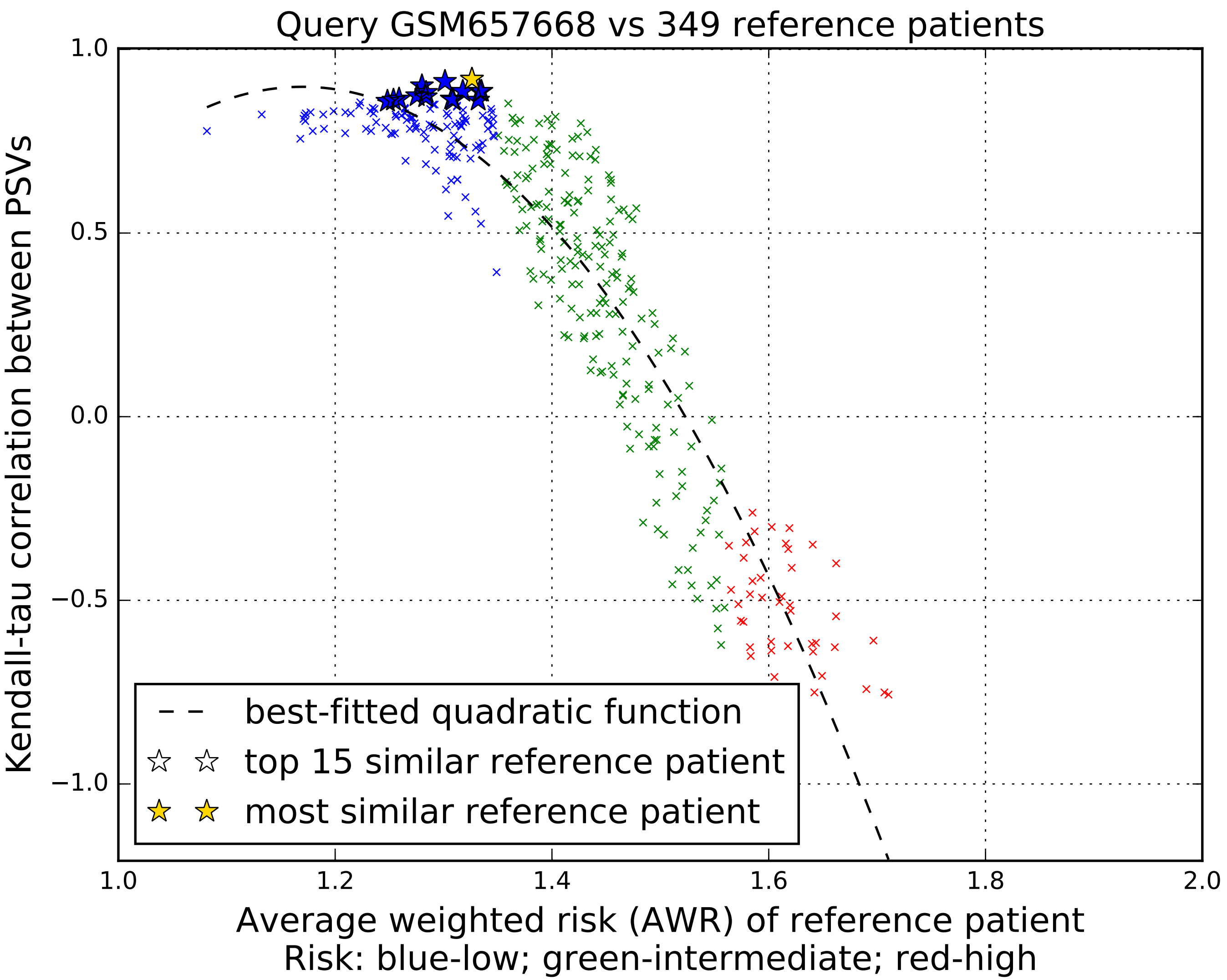

Query GSM657668 vs 349 reference patients
Kendall-tau correlation between PSVs
1.0
0.5
0.0
−0.5
−1.0
1.0
1.2
1.4
1.6
1.8
2.0
best-fitted quadratic function
top 15 similar reference patient
most similar reference patient
Average weighted risk (AWR) of reference patient
Risk: blue-low; green-intermediate; red-high

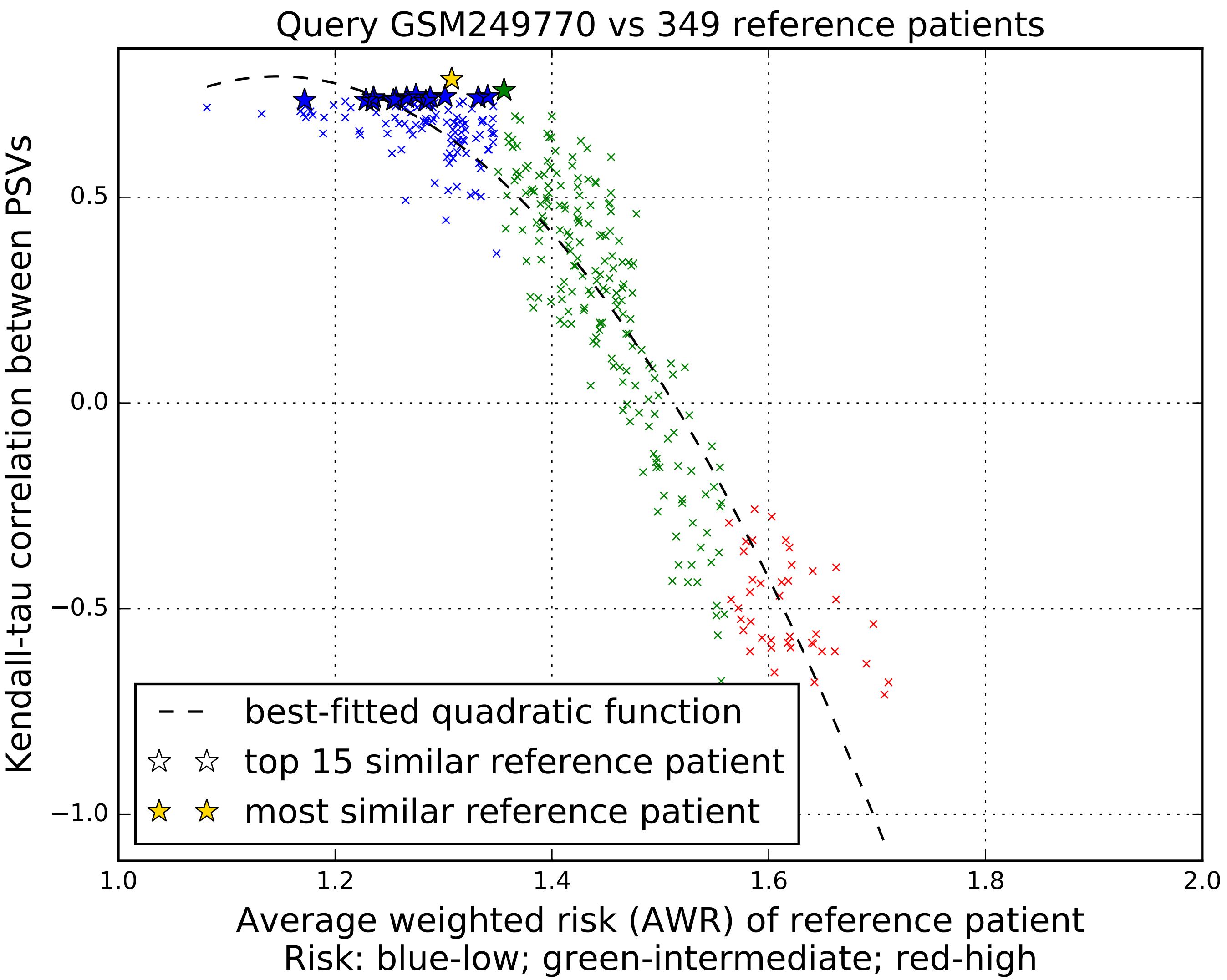
Query GSM249770 vs 349 reference patients
Kendall-tau correlation between PSVs
0.5
0.0
−0.5
−1.0
1.0
1.2
1.4
1.6
1.8
2.0
best-fitted quadratic function
top 15 similar reference patient
most similar reference patient
Average weighted risk (AWR) of reference patient
Risk: blue-low; green-intermediate; red-high

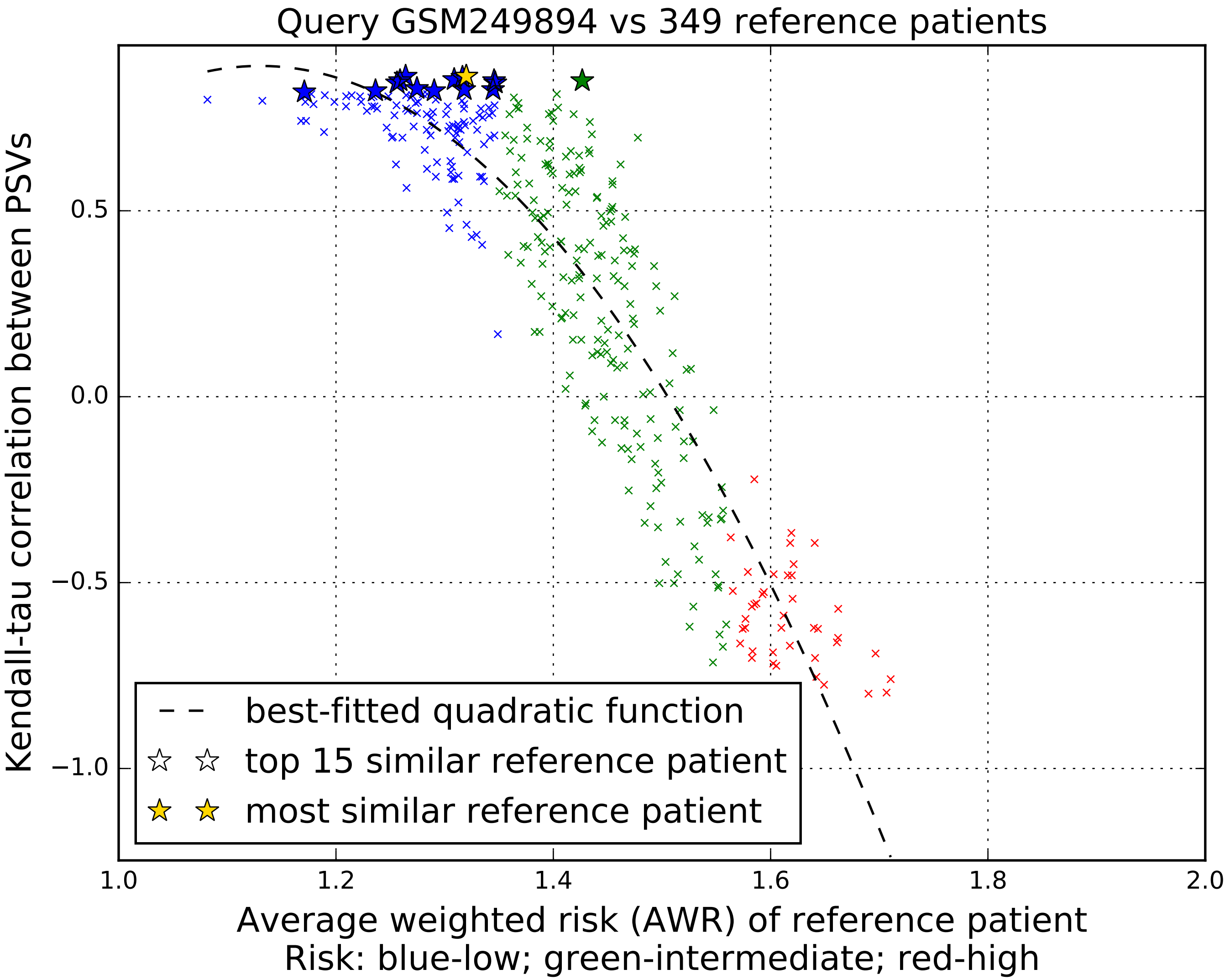
Query GSM249894 vs 349 reference patients
Kendall-tau correlation between PSVs
0.5
0.0
−0.5
−1.0
1.0
1.2
1.4
1.6
1.8
2.0
Average weighted risk (AWR) of reference patient
Risk: blue-low; green-intermediate; red-high
best-fitted quadratic function
top 15 similar reference patient
most similar reference patient

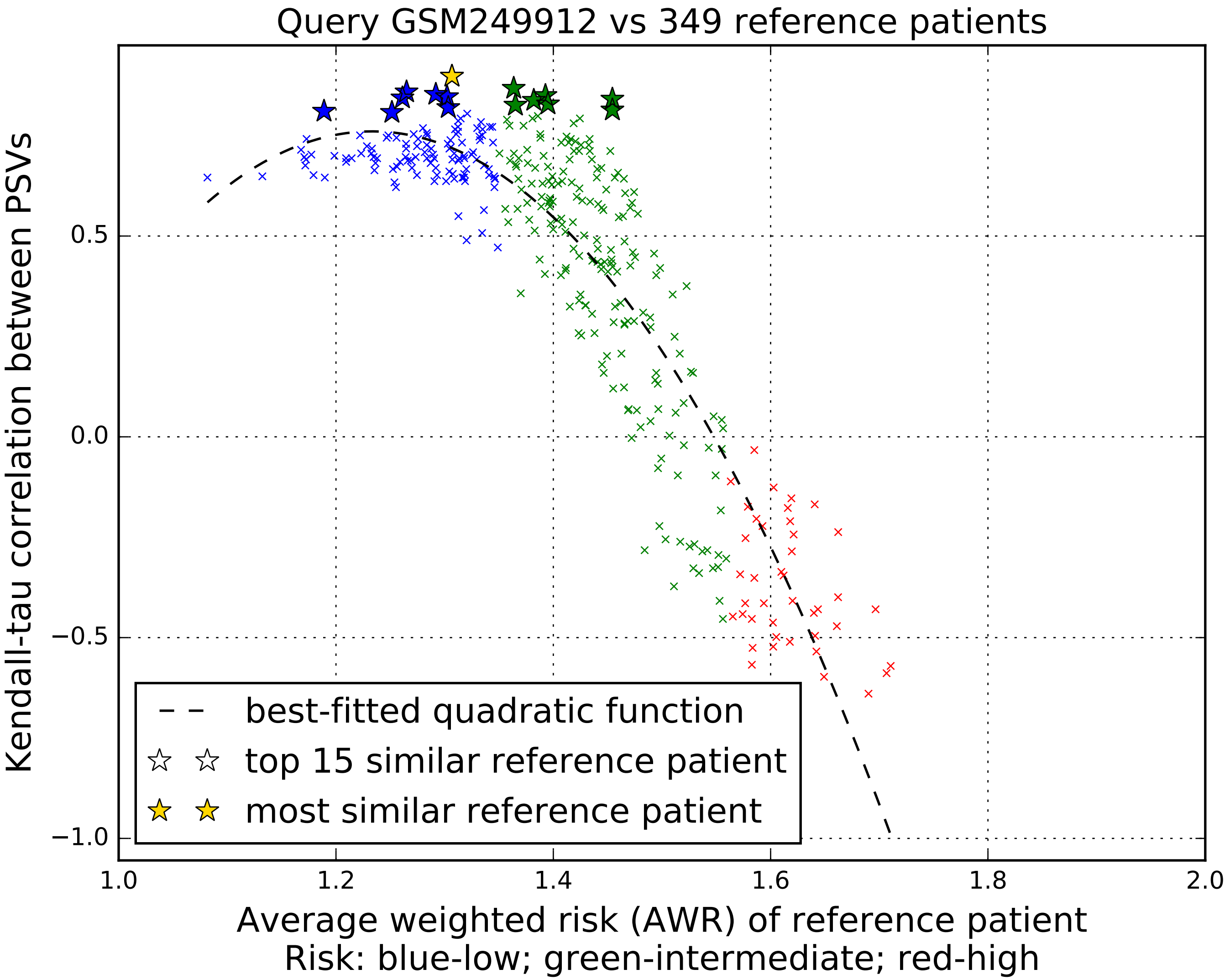
Query GSM249912 vs 349 reference patients
Kendall-tau correlation between PSVs
0.5
0.0
−0.5
−1.0
1.0
1.2
1.4
1.6
1.8
2.0
best-fitted quadratic function
top 15 similar reference patient
most similar reference patient
Average weighted risk (AWR) of reference patient
Risk: blue-low; green-intermediate; red-high

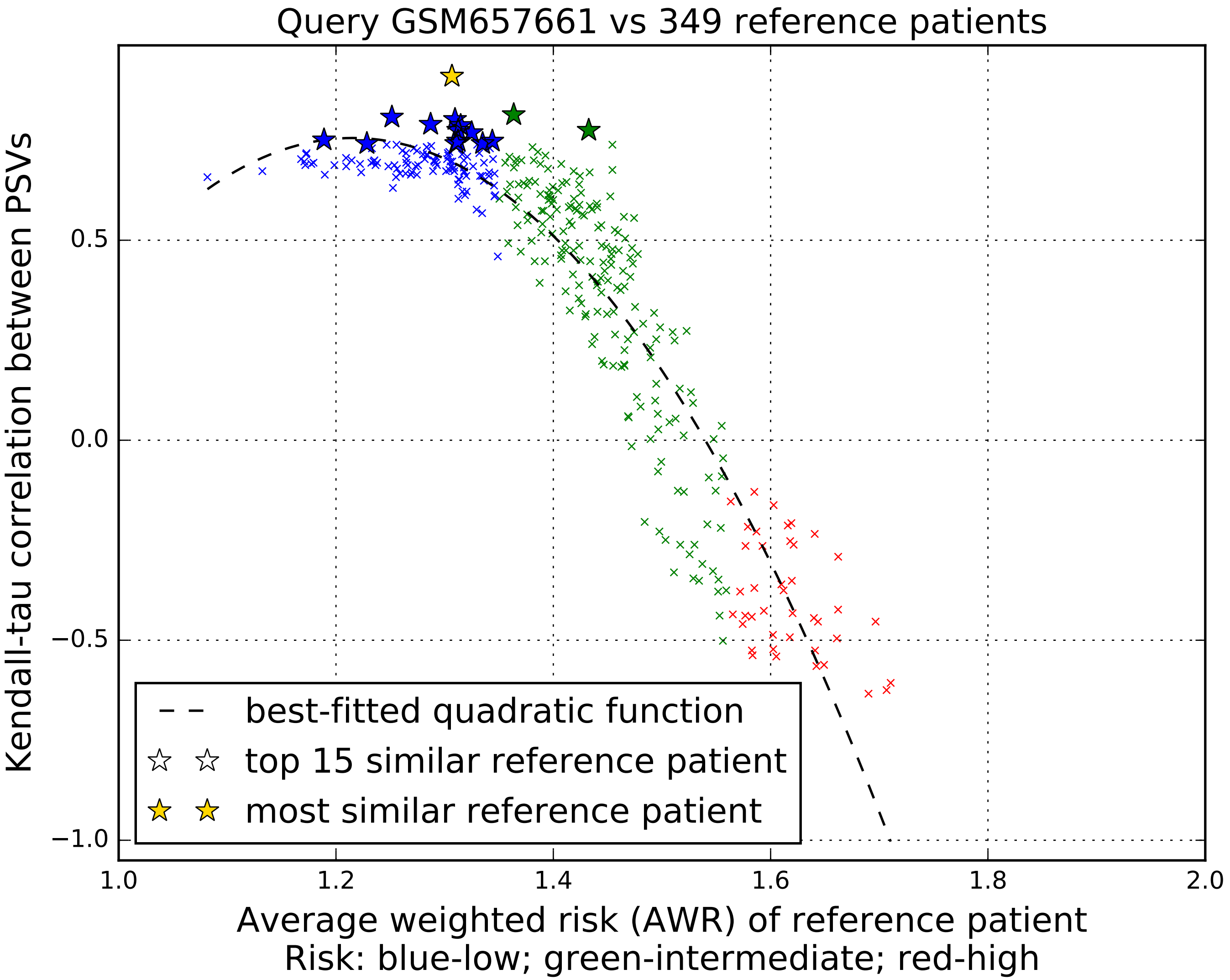

Query GSM657661 vs 349 reference patients
Kendall-tau correlation between PSVs
Average weighted risk (AWR) of reference patient
Risk: blue-low; green-intermediate; red-high
best-fitted quadratic function
top 15 similar reference patient
most similar reference patient
0.5
0.0
−0.5
−1.0
1.0
1.2
1.4
1.6
1.8
2.0

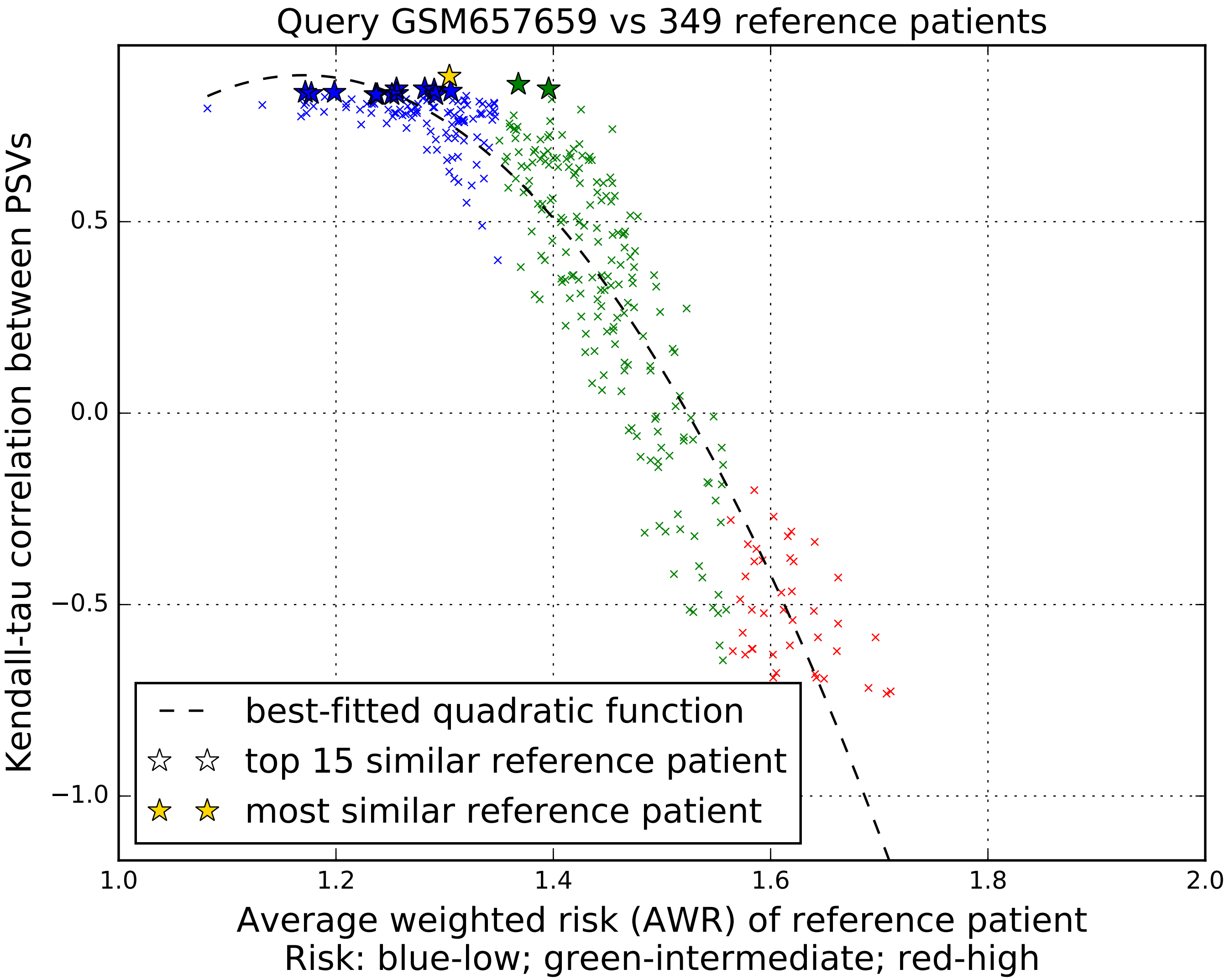
Query GSM657659 vs 349 reference patients
Kendall-tau correlation between PSVs
0.5
0.0
−0.5
−1.0
1.0
1.2
1.4
1.6
1.8
2.0
best-fitted quadratic function
top 15 similar reference patient
most similar reference patient
Average weighted risk (AWR) of reference patient
Risk: blue-low; green-intermediate; red-high

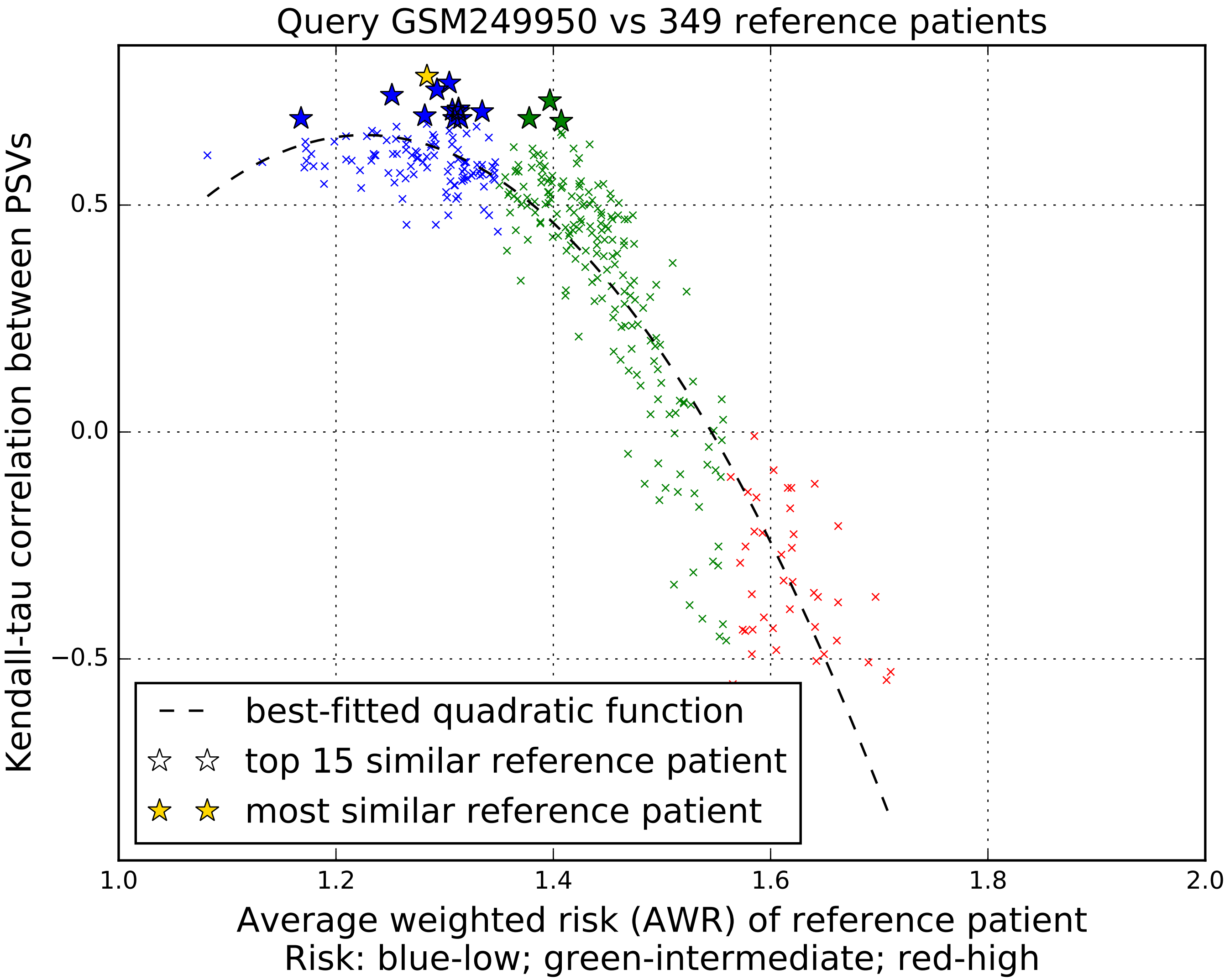
Query GSM249950 vs 349 reference patients
Kendall-tau correlation between PSVs
0.5
0.0
−0.5
1.0
1.2
1.4
1.6
1.8
2.0
best-fitted quadratic function
top 15 similar reference patient
most similar reference patient
Average weighted risk (AWR) of reference patient
Risk: blue-low; green-intermediate; red-high

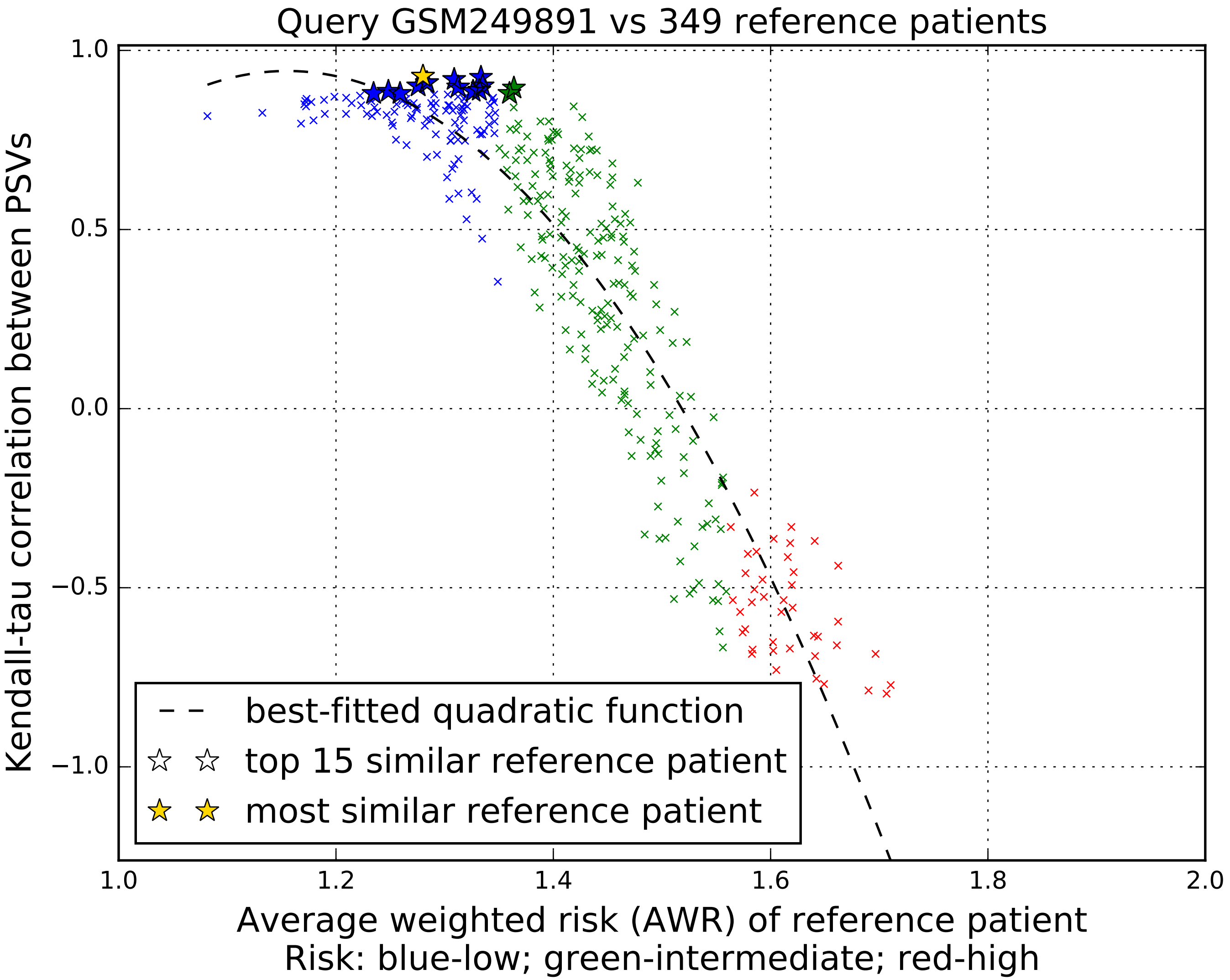

Query GSM249891 vs 349 reference patients
Kendall-tau correlation between PSVs
1.0
0.5
0.0
−0.5
−1.0
1.0
1.2
1.4
1.6
1.8
2.0
best-fitted quadratic function
top 15 similar reference patient
most similar reference patient
Average weighted risk (AWR) of reference patient
Risk: blue-low; green-intermediate; red-high

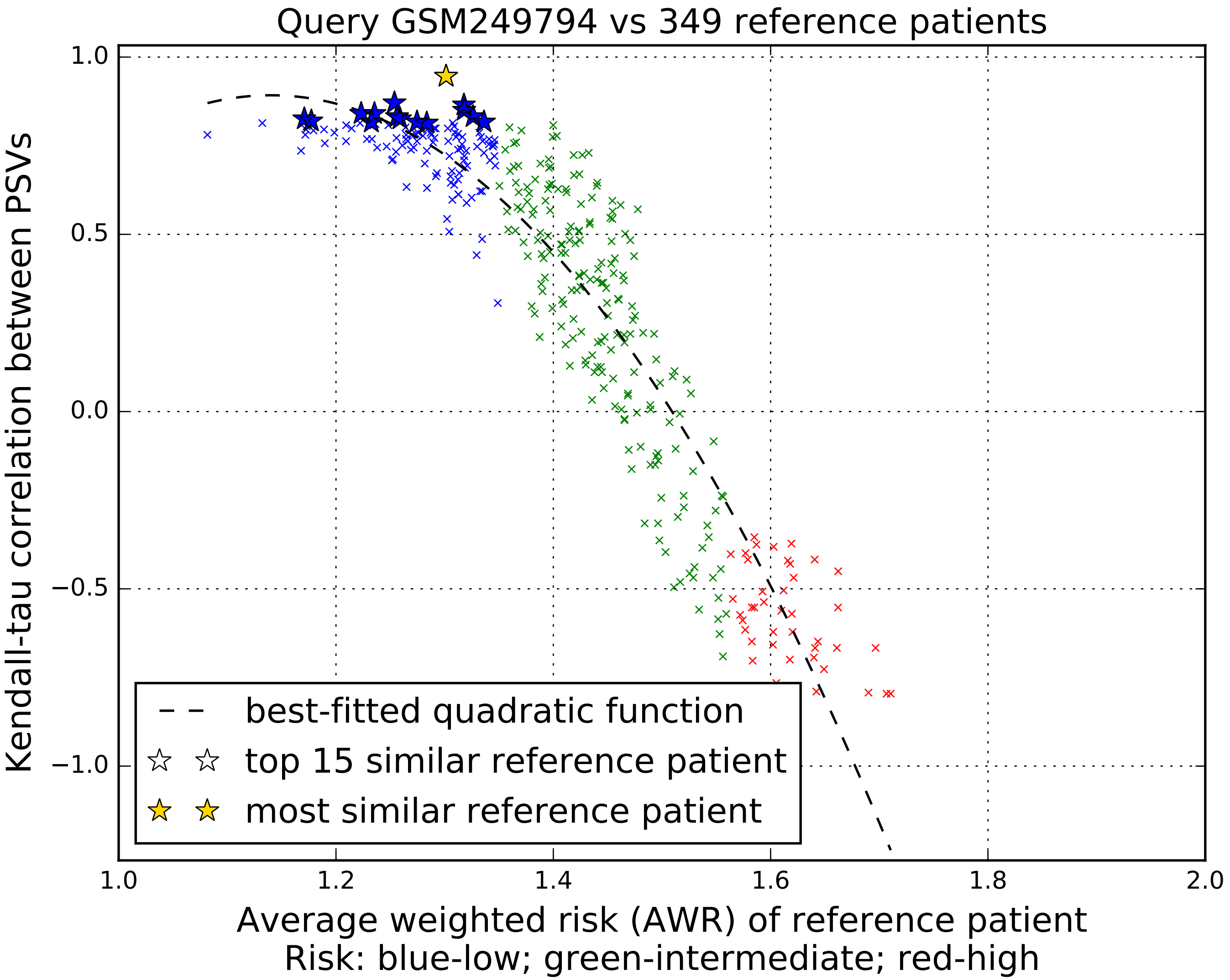

Query GSM249794 vs 349 reference patients
Kendall-tau correlation between PSVs
1.0
0.5
0.0
−0.5
−1.0
1.0
1.2
1.4
1.6
1.8
2.0
best-fitted quadratic function
top 15 similar reference patient
most similar reference patient
Average weighted risk (AWR) of reference patient
Risk: blue-low; green-intermediate; red-high

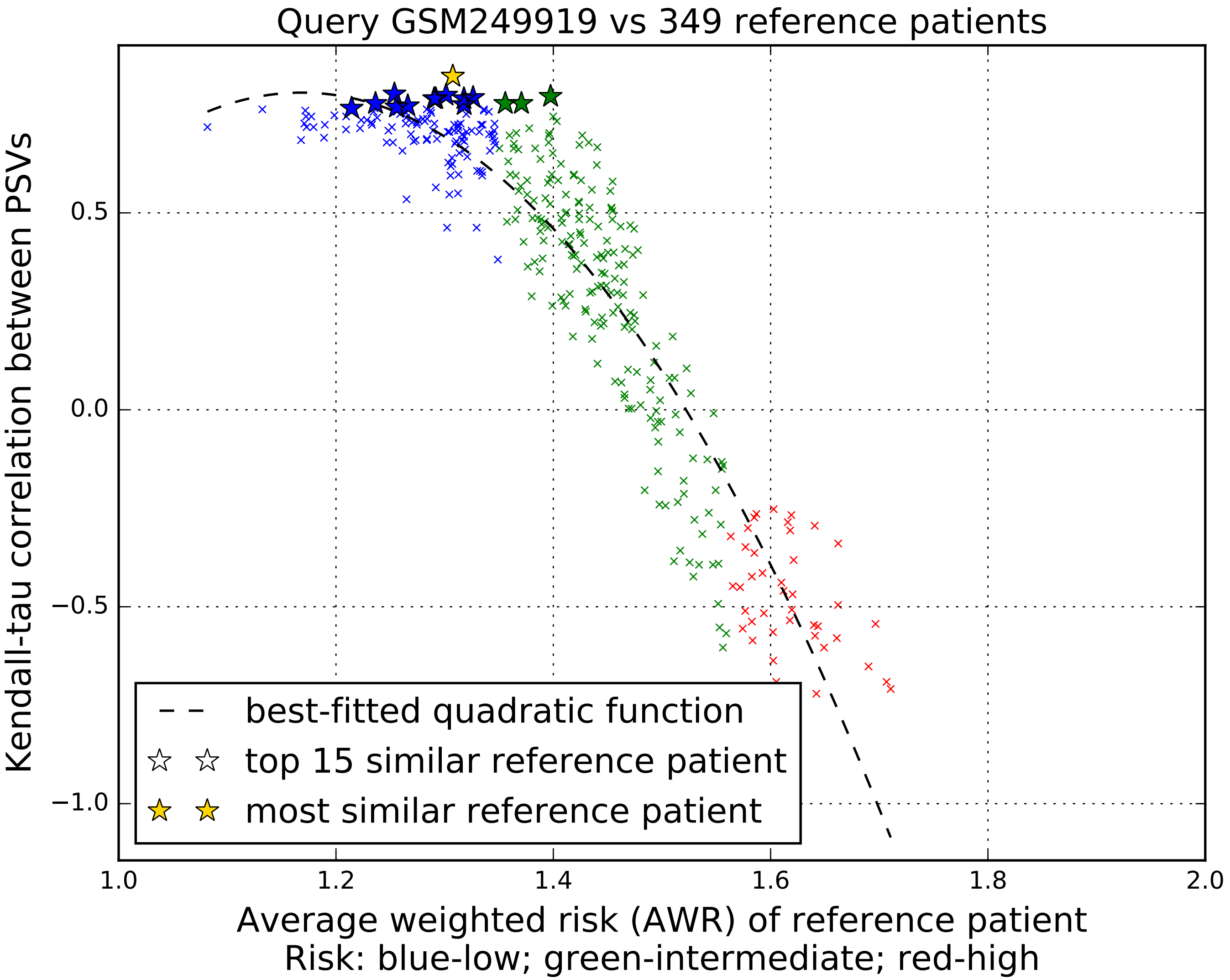
Query GSM249919 vs 349 reference patients
Kendall-tau correlation between PSVs
0.5
0.0
−0.5
−1.0
1.0
1.2
1.4
1.6
1.8
2.0
Average weighted risk (AWR) of reference patient
Risk: blue-low; green-intermediate; red-high
best-fitted quadratic function
top 15 similar reference patient
most similar reference patient

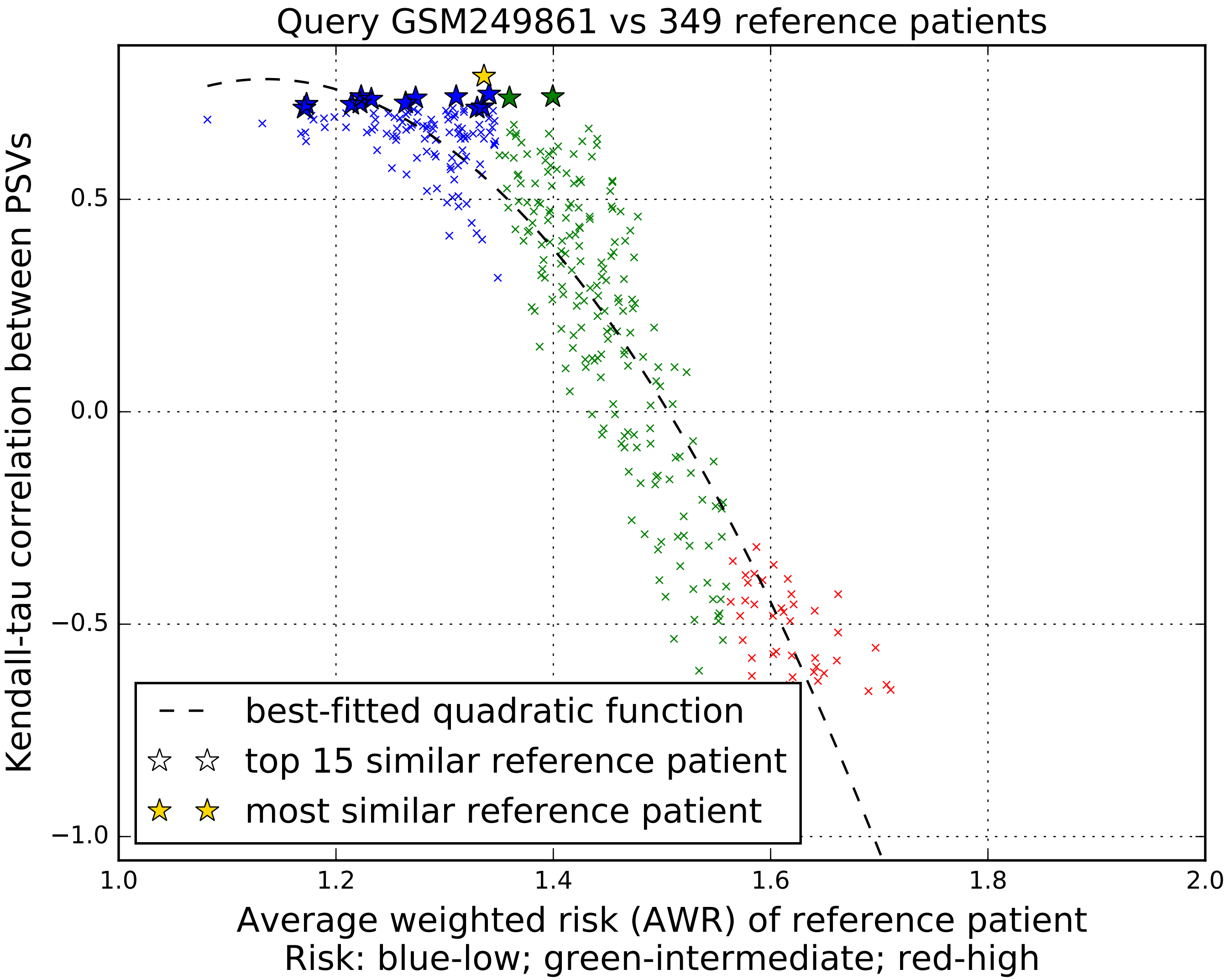

Query GSM249861 vs 349 reference patients
Kendall-tau correlation between PSVs
0.5
0.0
−0.5
−1.0
1.0
1.2
1.4
1.6
1.8
2.0
best-fitted quadratic function
top 15 similar reference patient
most similar reference patient
Average weighted risk (AWR) of reference patient
Risk: blue-low; green-intermediate; red-high

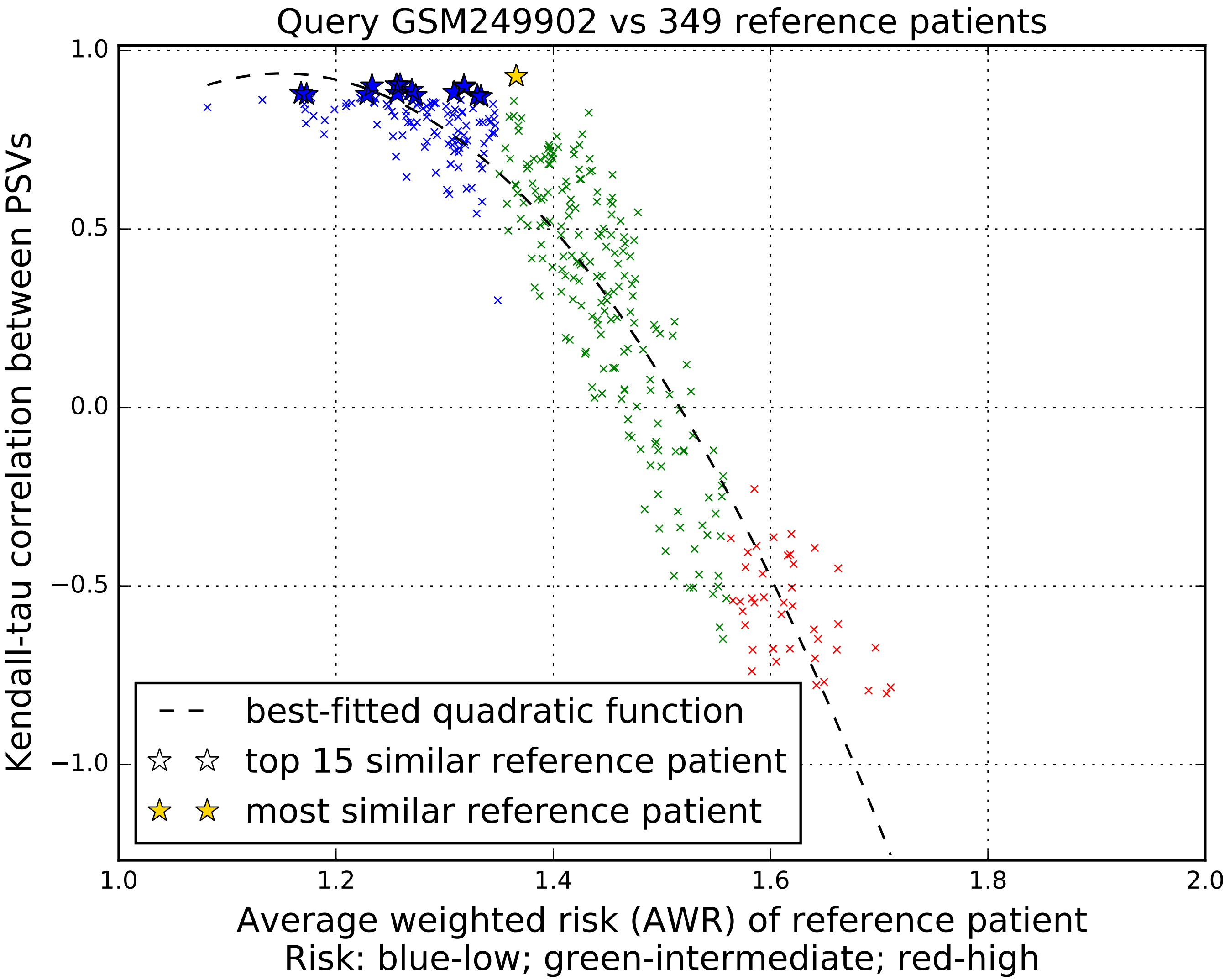

Query GSM249902 vs 349 reference patients
Kendall-tau correlation between PSVs
1.0
0.5
0.0
−0.5
−1.0
1.0
1.2
1.4
1.6
1.8
2.0
best-fitted quadratic function
top 15 similar reference patient
most similar reference patient
Average weighted risk (AWR) of reference patient
Risk: blue-low; green-intermediate; red-high

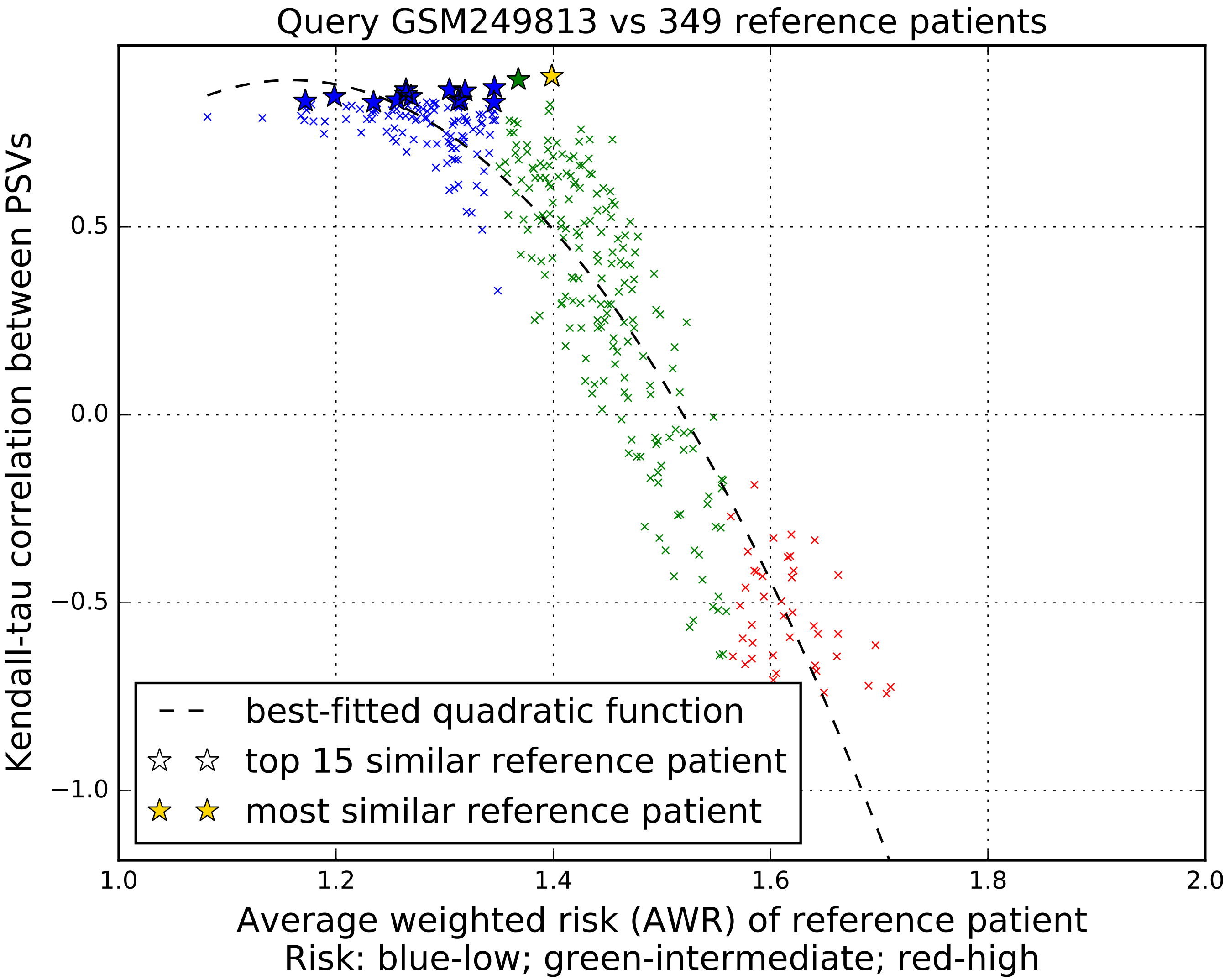
Query GSM249813 vs 349 reference patients
Kendall-tau correlation between PSVs
Average weighted risk (AWR) of reference patient
Risk: blue-low; green-intermediate; red-high
best-fitted quadratic function
top 15 similar reference patient
most similar reference patient
0.5
0.0
−0.5
−1.0
1.0
1.2
1.4
1.6
1.8
2.0

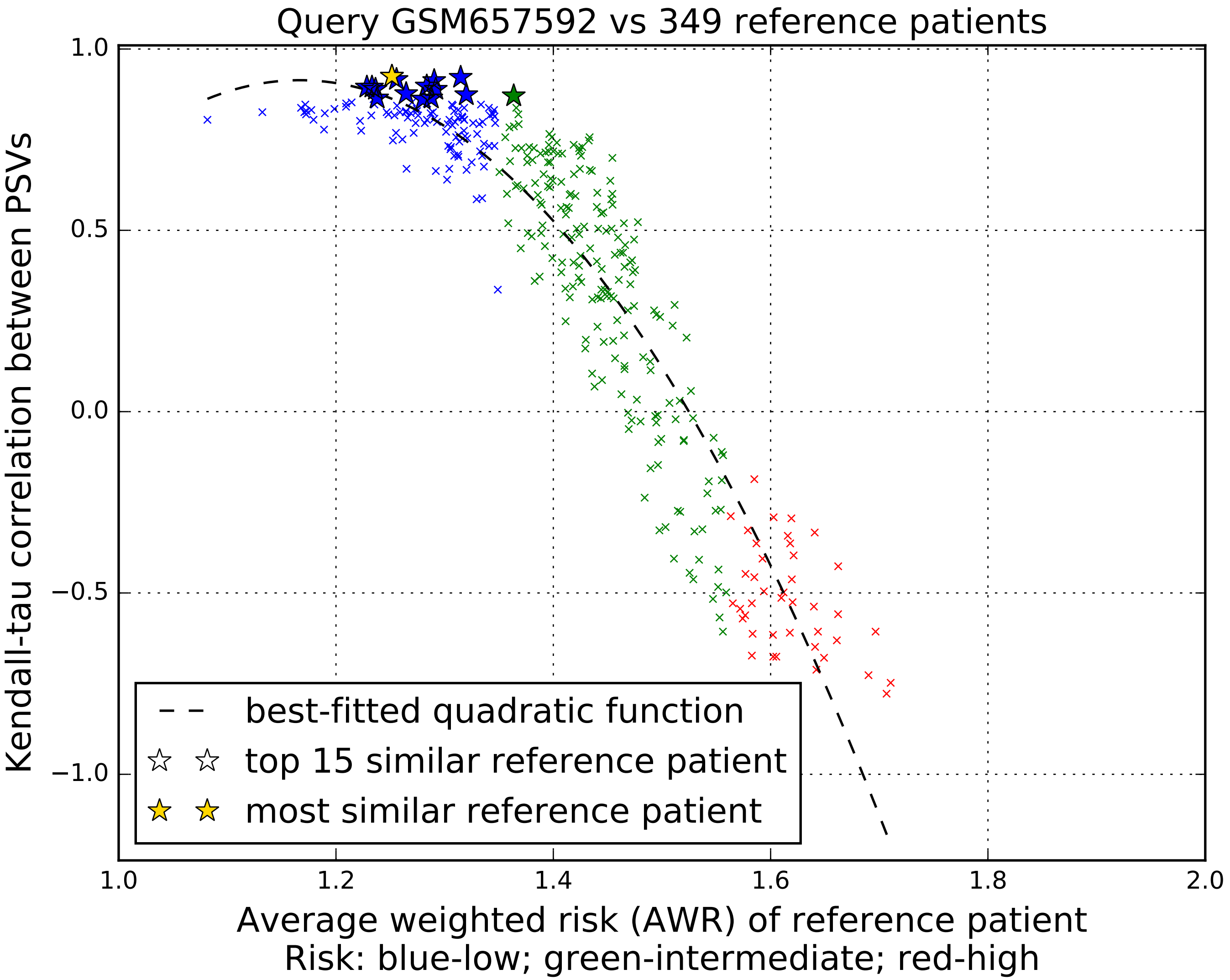

Query GSM657592 vs 349 reference patients
Kendall-tau correlation between PSVs
1.0
0.5
0.0
−0.5
−1.0
1.0
1.2
1.4
1.6
1.8
2.0
best-fitted quadratic function
top 15 similar reference patient
most similar reference patient
Average weighted risk (AWR) of reference patient
Risk: blue-low; green-intermediate; red-high

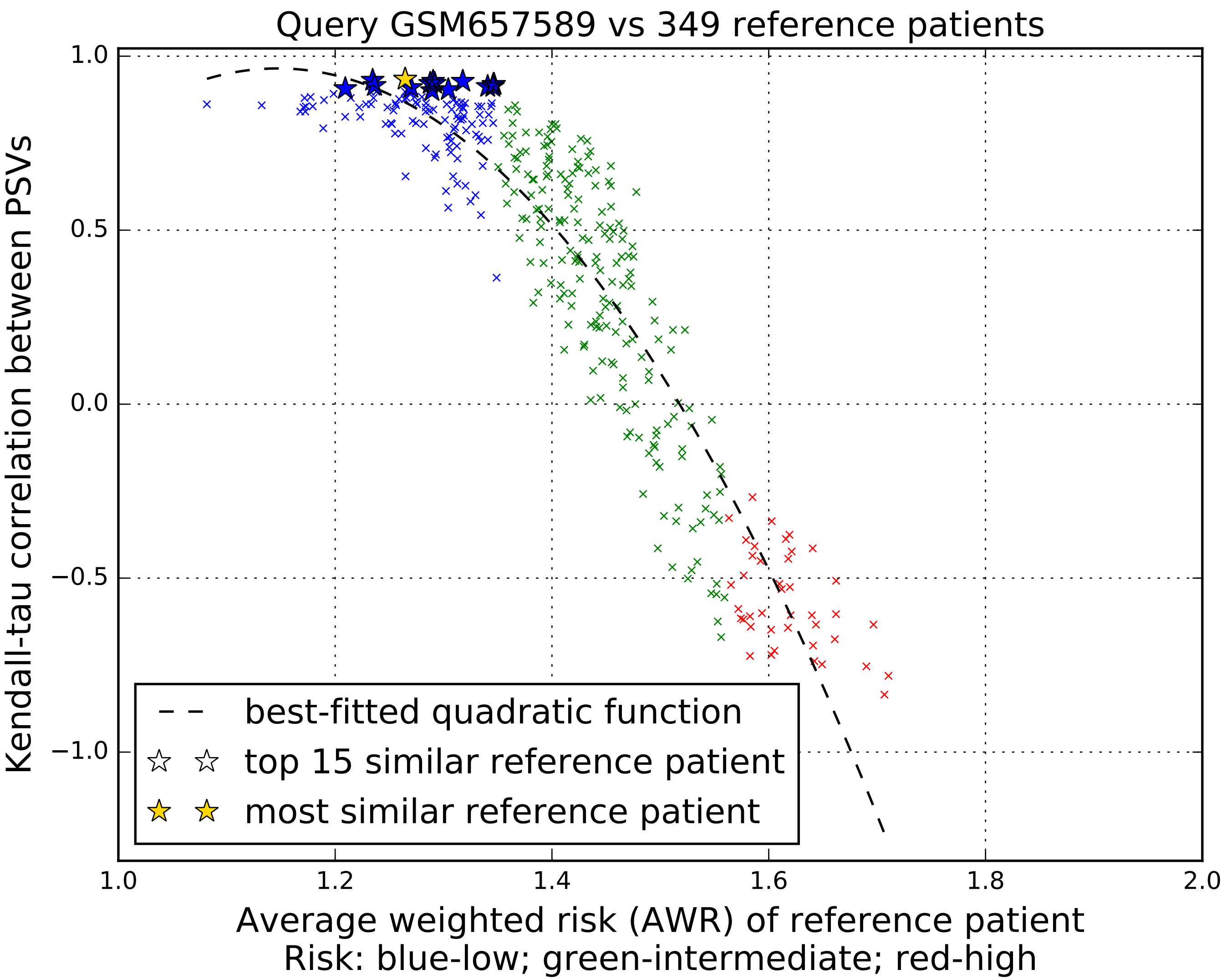

Query GSM657589 vs 349 reference patients
Kendall-tau correlation between PSVs
Average weighted risk (AWR) of reference patient
Risk: blue-low; green-intermediate; red-high
best-fitted quadratic function
top 15 similar reference patient
most similar reference patient
1.0
0.5
0.0
−0.5
−1.0
1.0
1.2
1.4
1.6
1.8
2.0

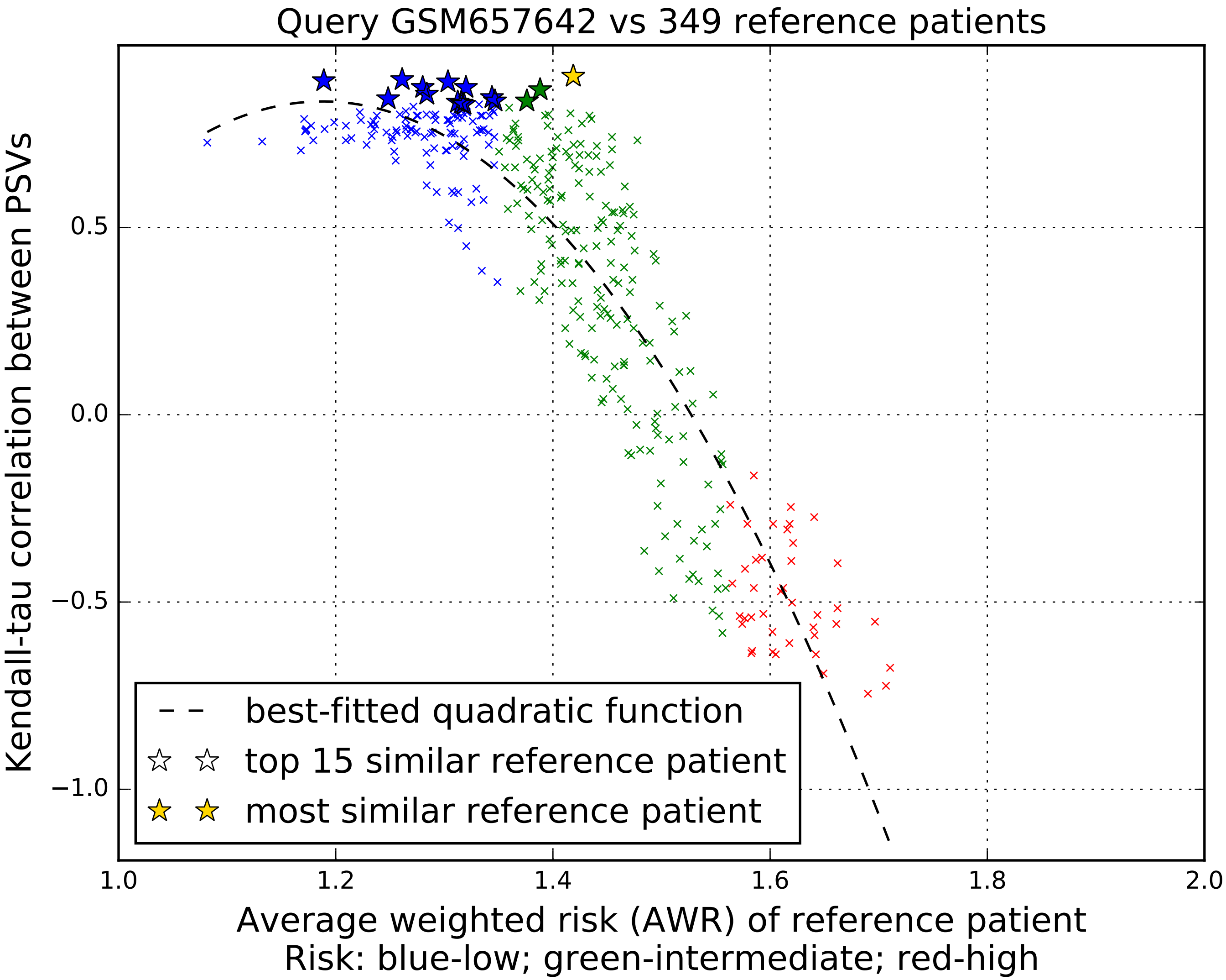

Query GSM657642 vs 349 reference patients
Kendall-tau correlation between PSVs
0.5
0.0
−0.5
−1.0
1.0
1.2
1.4
1.6
1.8
2.0
best-fitted quadratic function
top 15 similar reference patient
most similar reference patient
Average weighted risk (AWR) of reference patient
Risk: blue-low; green-intermediate; red-high

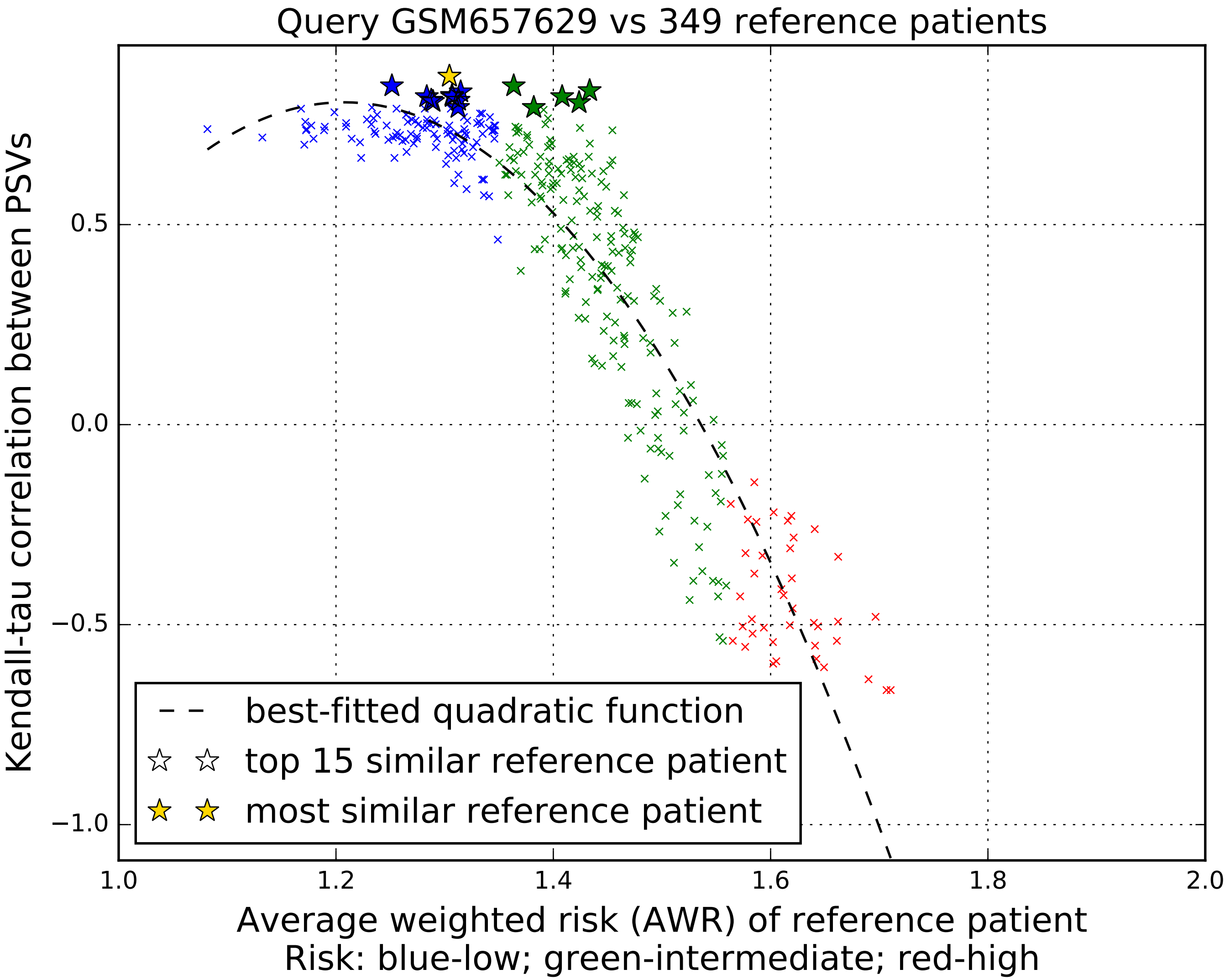
Query GSM657629 vs 349 reference patients
Kendall-tau correlation between PSVs
0.5
0.0
−0.5
−1.0
1.0
1.2
1.4
1.6
1.8
2.0
best-fitted quadratic function
top 15 similar reference patient
most similar reference patient
Average weighted risk (AWR) of reference patient
Risk: blue-low; green-intermediate; red-high

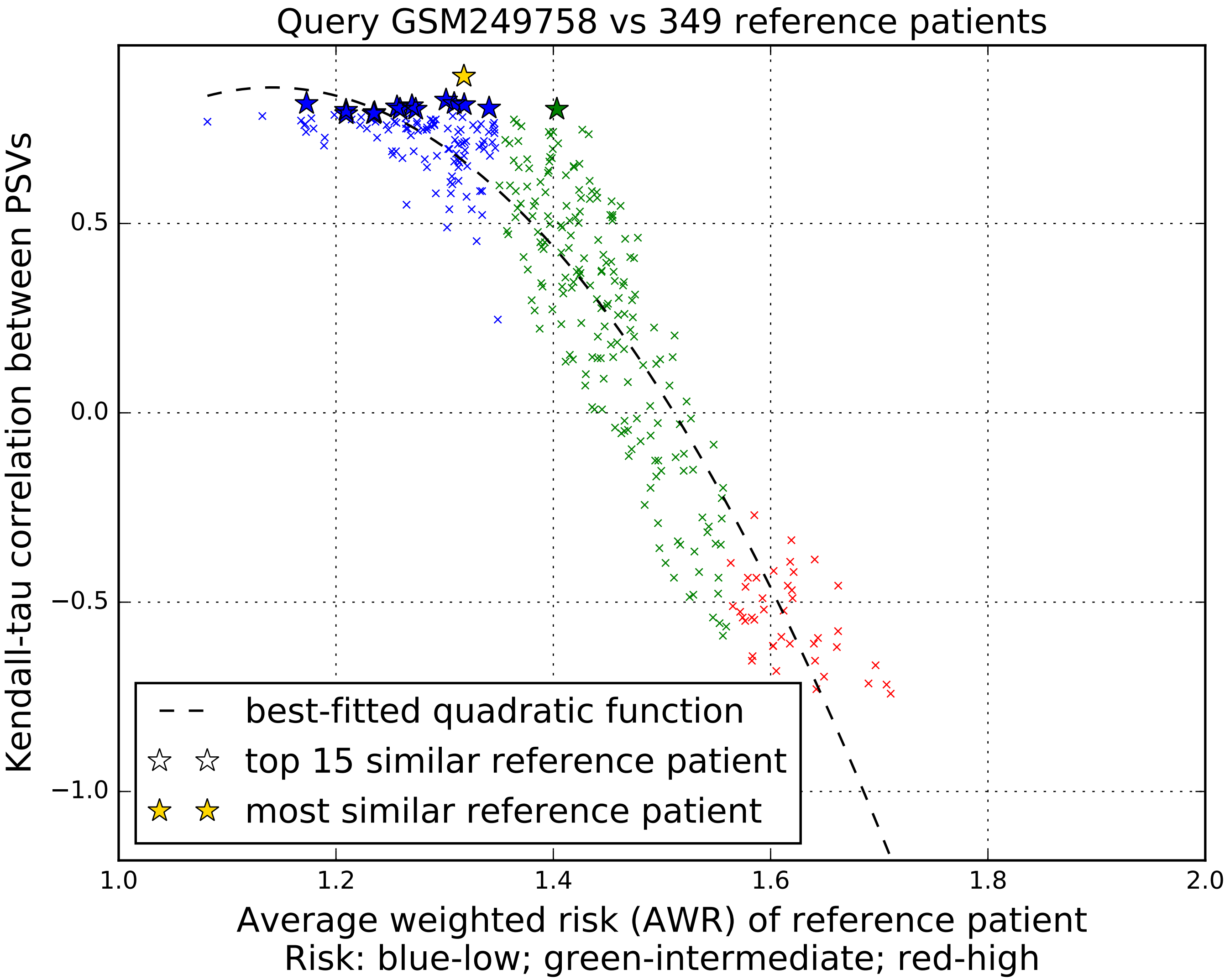
Query GSM249758 vs 349 reference patients
Kendall-tau correlation between PSVs
Average weighted risk (AWR) of reference patient
Risk: blue-low; green-intermediate; red-high
best-fitted quadratic function
top 15 similar reference patient
most similar reference patient
0.5
0.0
−0.5
−1.0
1.0
1.2
1.4
1.6
1.8
2.0

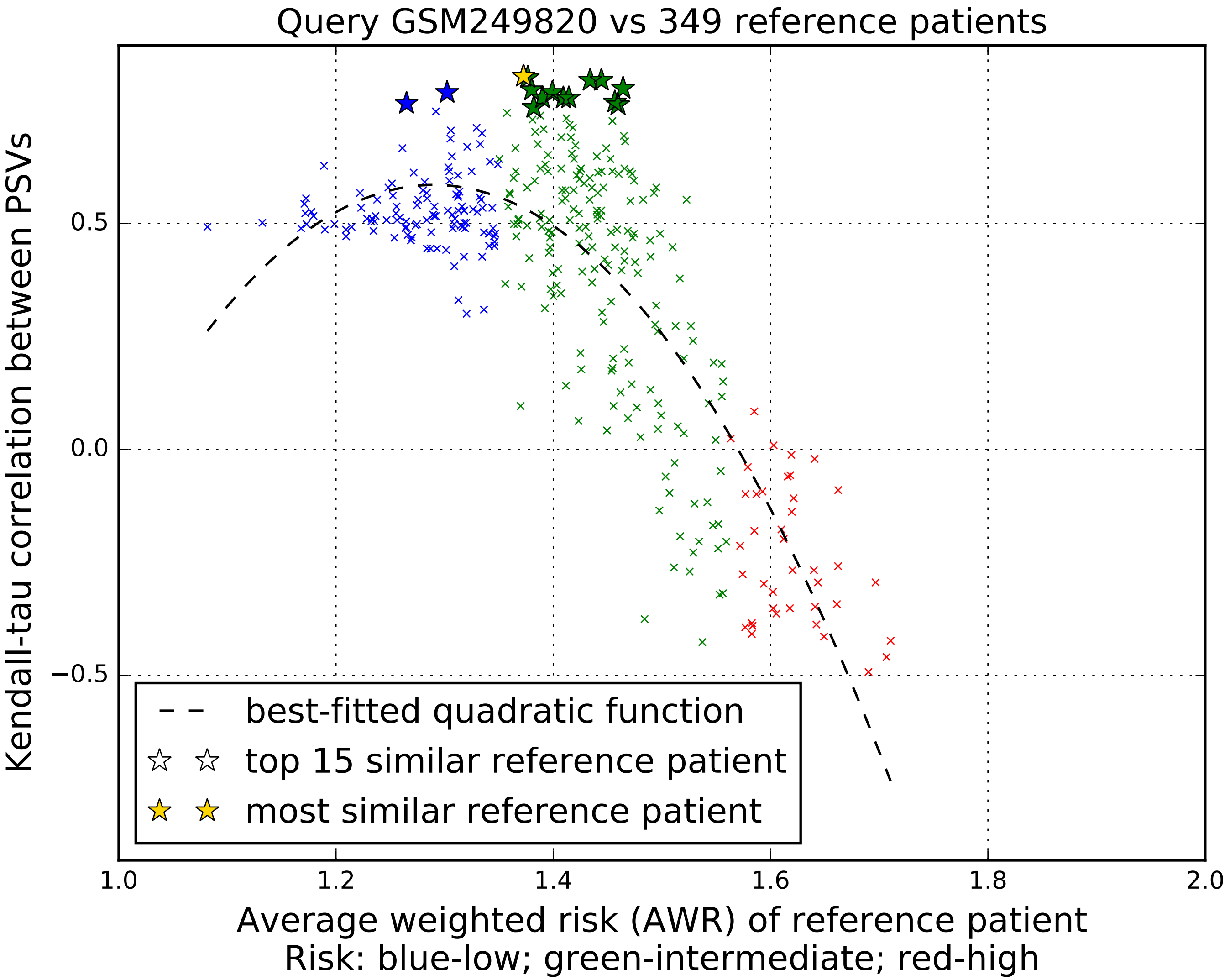

Query GSM249820 vs 349 reference patients
Kendall-tau correlation between PSVs
0.5
0.0
−0.5
best-fitted quadratic function
top 15 similar reference patient
most similar reference patient
1.0
1.2
1.4
1.6
1.8
2.0
Average weighted risk (AWR) of reference patient
Risk: blue-low; green-intermediate; red-high

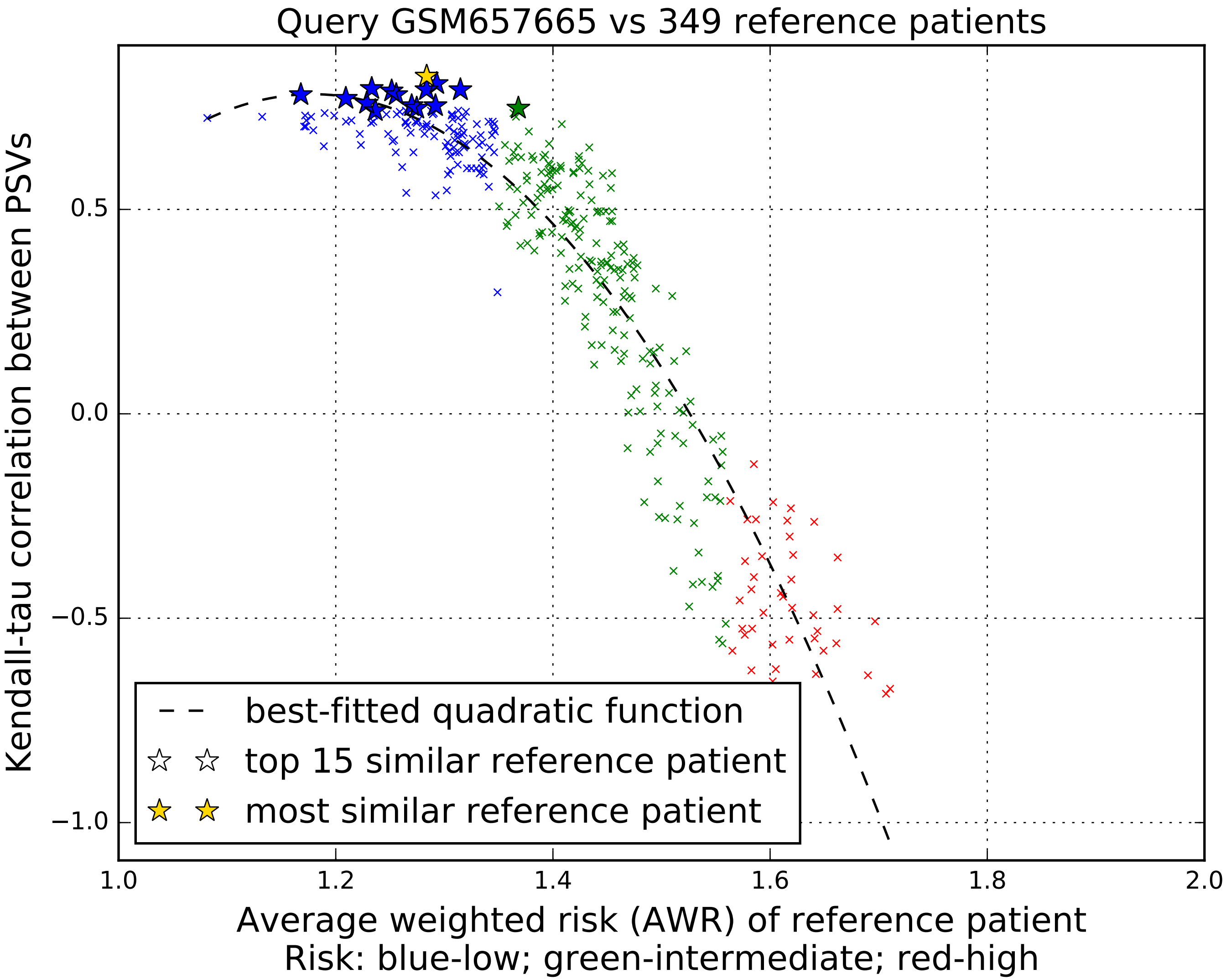

Query GSM657665 vs 349 reference patients
Kendall-tau correlation between PSVs
0.5
0.0
−0.5
−1.0
1.0
1.2
1.4
1.6
1.8
2.0
best-fitted quadratic function
top 15 similar reference patient
most similar reference patient
Average weighted risk (AWR) of reference patient
Risk: blue-low; green-intermediate; red-high

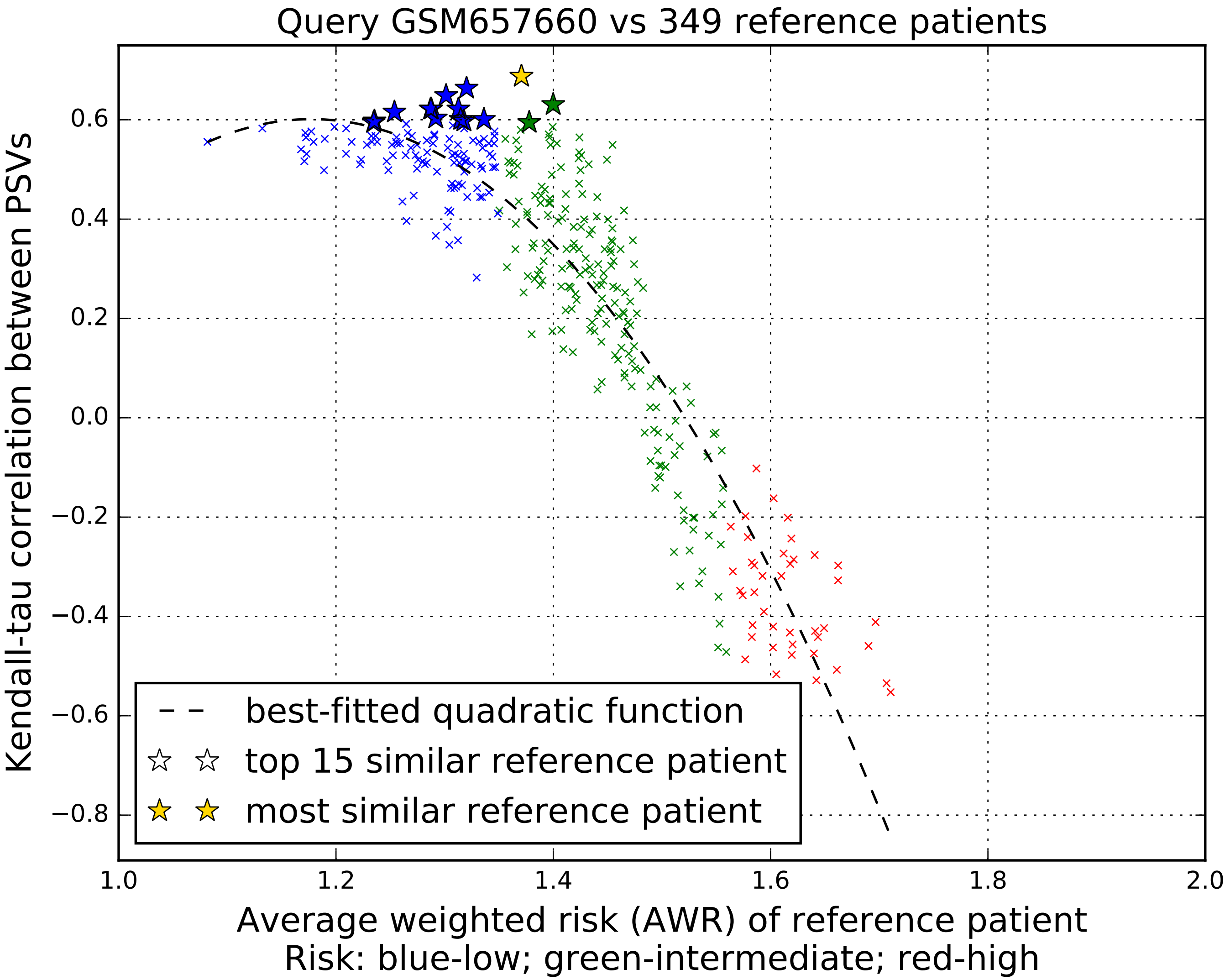
Query GSM657660 vs 349 reference patients
Kendall-tau correlation between PSVs
0.6
0.4
0.2
0.0
−0.2
−0.4
−0.6
−0.8
1.0
1.2
1.4
1.6
1.8
2.0
best-fitted quadratic function
top 15 similar reference patient
most similar reference patient
Average weighted risk (AWR) of reference patient
Risk: blue-low; green-intermediate; red-high

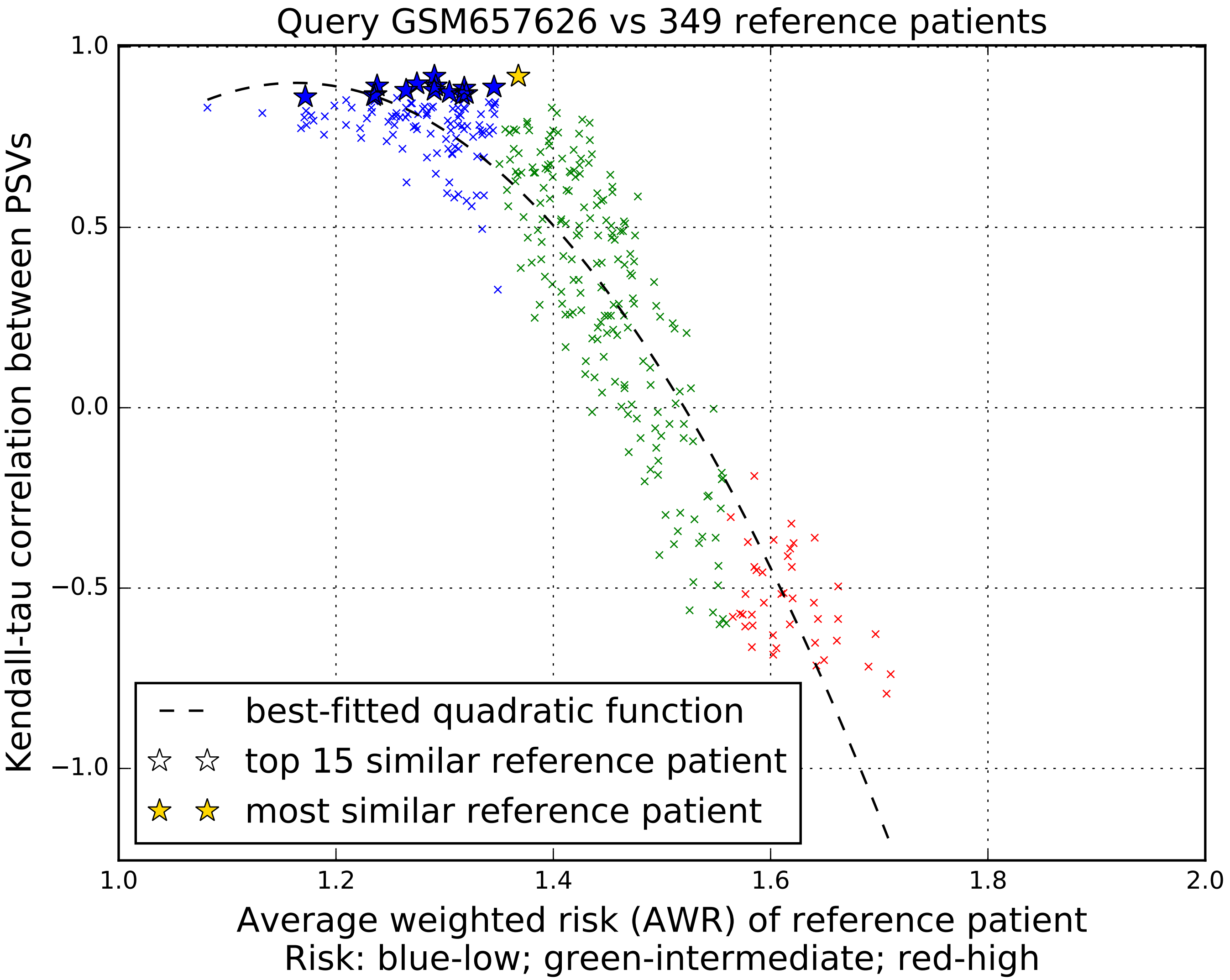

Query GSM657626 vs 349 reference patients
Kendall-tau correlation between PSVs
1.0
0.5
0.0
−0.5
−1.0
1.0
1.2
1.4
1.6
1.8
2.0
best-fitted quadratic function
top 15 similar reference patient
most similar reference patient
Average weighted risk (AWR) of reference patient
Risk: blue-low; green-intermediate; red-high

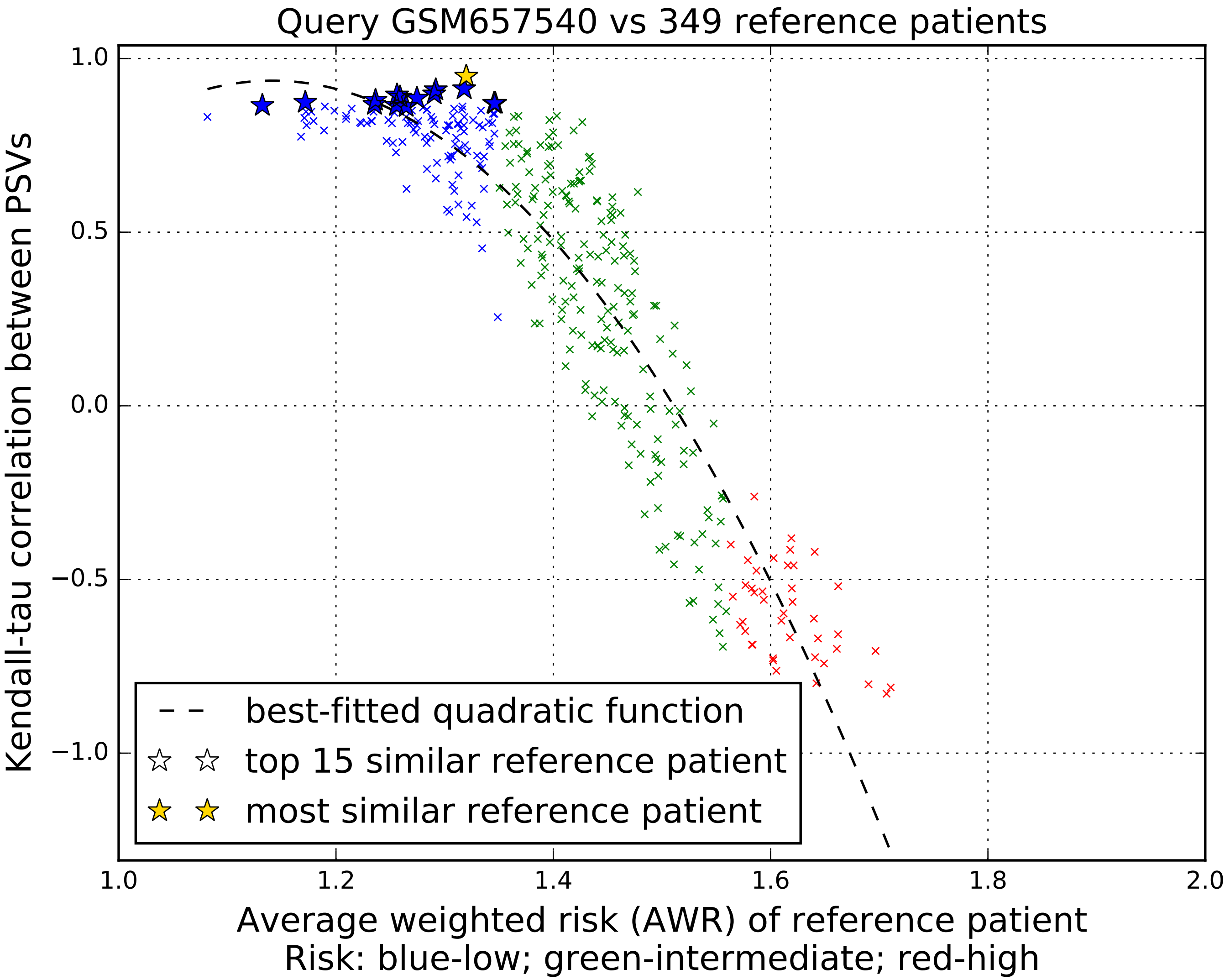

Query GSM657540 vs 349 reference patients
Kendall-tau correlation between PSVs
1.0
0.5
0.0
−0.5
−1.0
1.0
1.2
1.4
1.6
1.8
2.0
best-fitted quadratic function
top 15 similar reference patient
most similar reference patient
Average weighted risk (AWR) of reference patient
Risk: blue-low; green-intermediate; red-high

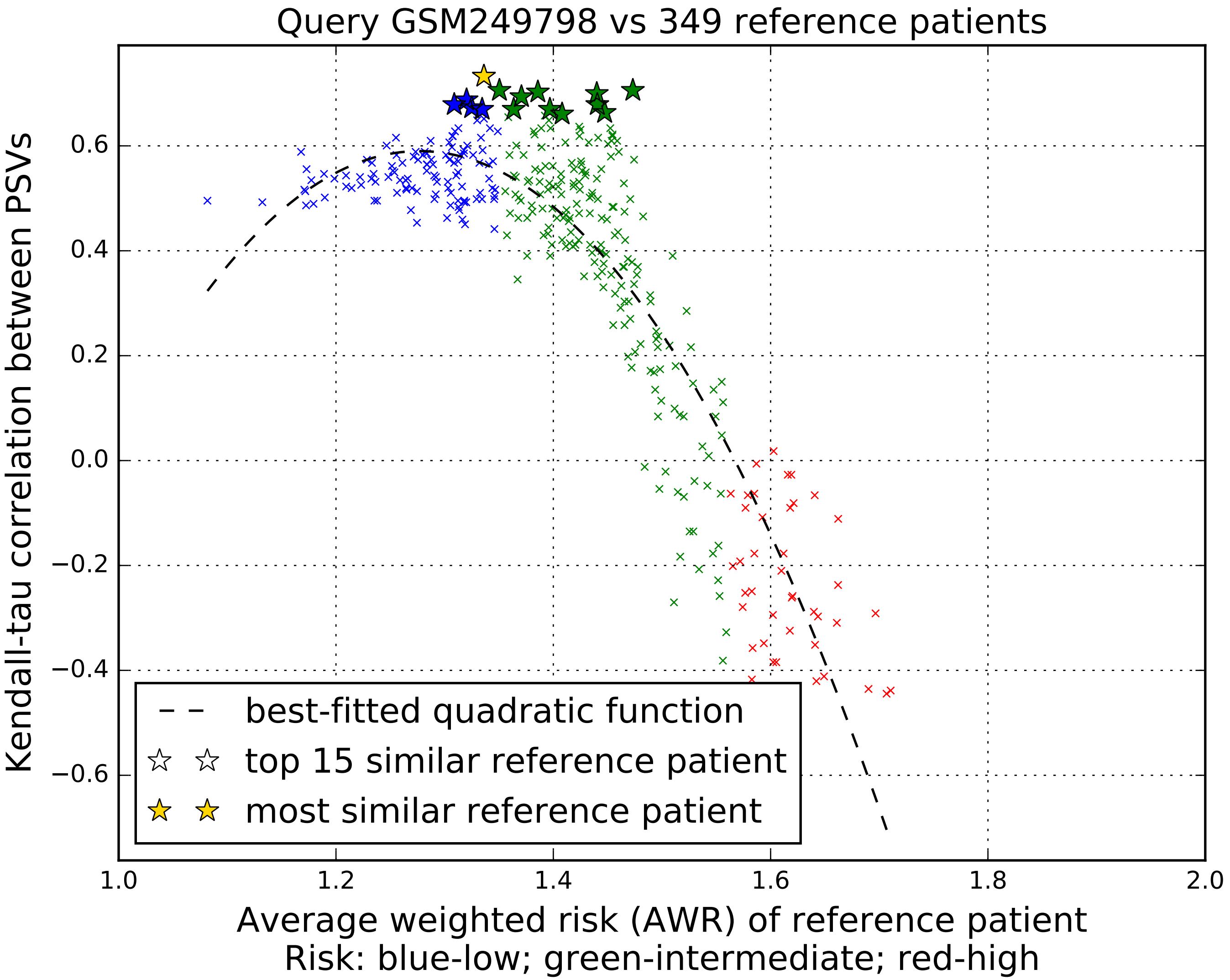

Query GSM249798 vs 349 reference patients
Kendall-tau correlation between PSVs
0.6
0.4
0.2
0.0
−0.2
−0.4
−0.6
1.0
1.2
1.4
1.6
1.8
2.0
best-fitted quadratic function
top 15 similar reference patient
most similar reference patient
Average weighted risk (AWR) of reference patient
Risk: blue-low; green-intermediate; red-high

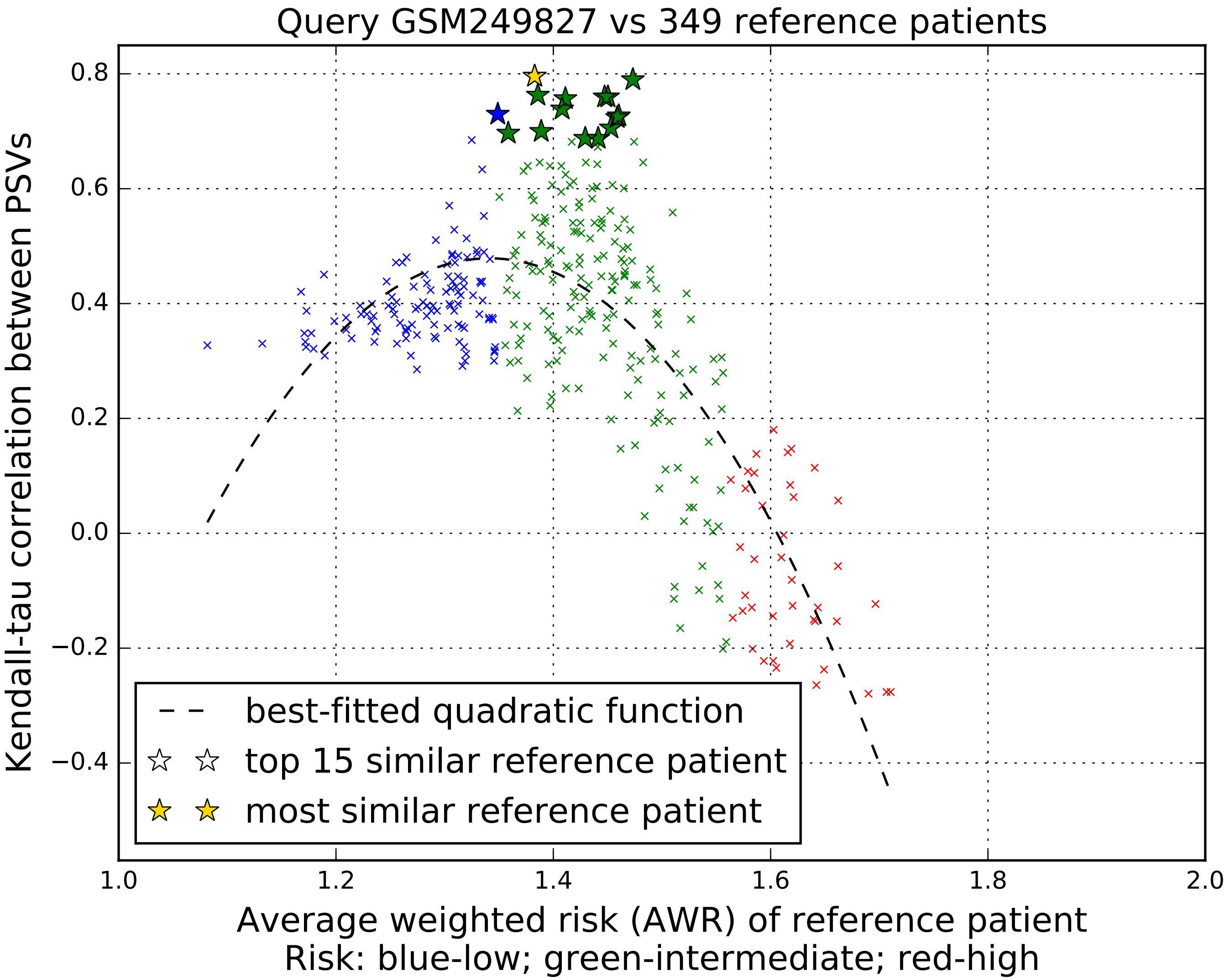

Query GSM249827 vs 349 reference patients
Kendall-tau correlation between PSVs
0.8
0.6
0.4
0.2
0.0
−0.2
−0.4
1.0
1.2
1.4
1.6
1.8
2.0
best-fitted quadratic function
top 15 similar reference patient
most similar reference patient
Average weighted risk (AWR) of reference patient
Risk: blue-low; green-intermediate; red-high

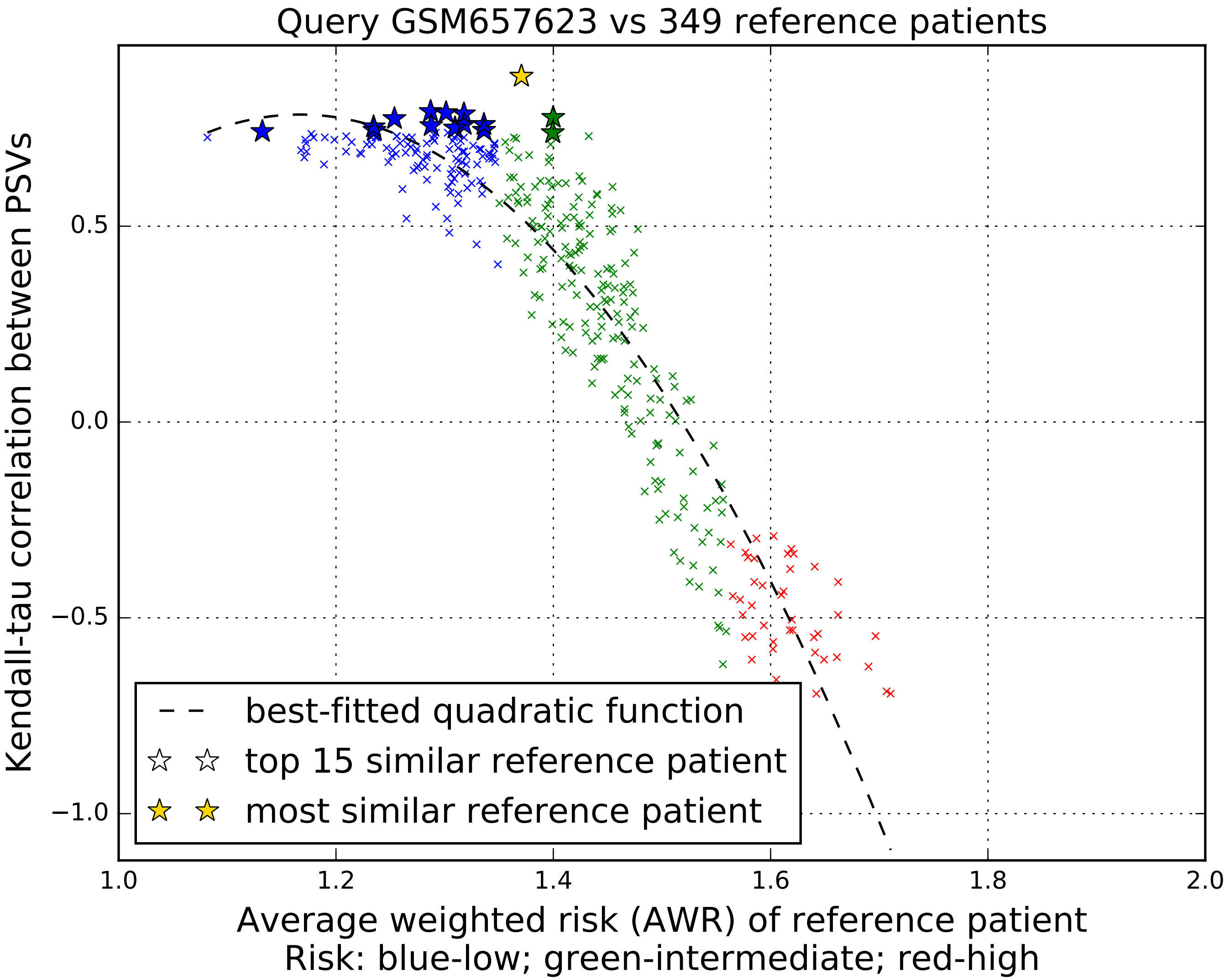

Query GSM657623 vs 349 reference patients
Kendall-tau correlation between PSVs
0.5
0.0
−0.5
−1.0
1.0
1.2
1.4
1.6
1.8
2.0
best-fitted quadratic function
top 15 similar reference patient
most similar reference patient
Average weighted risk (AWR) of reference patient
Risk: blue-low; green-intermediate; red-high

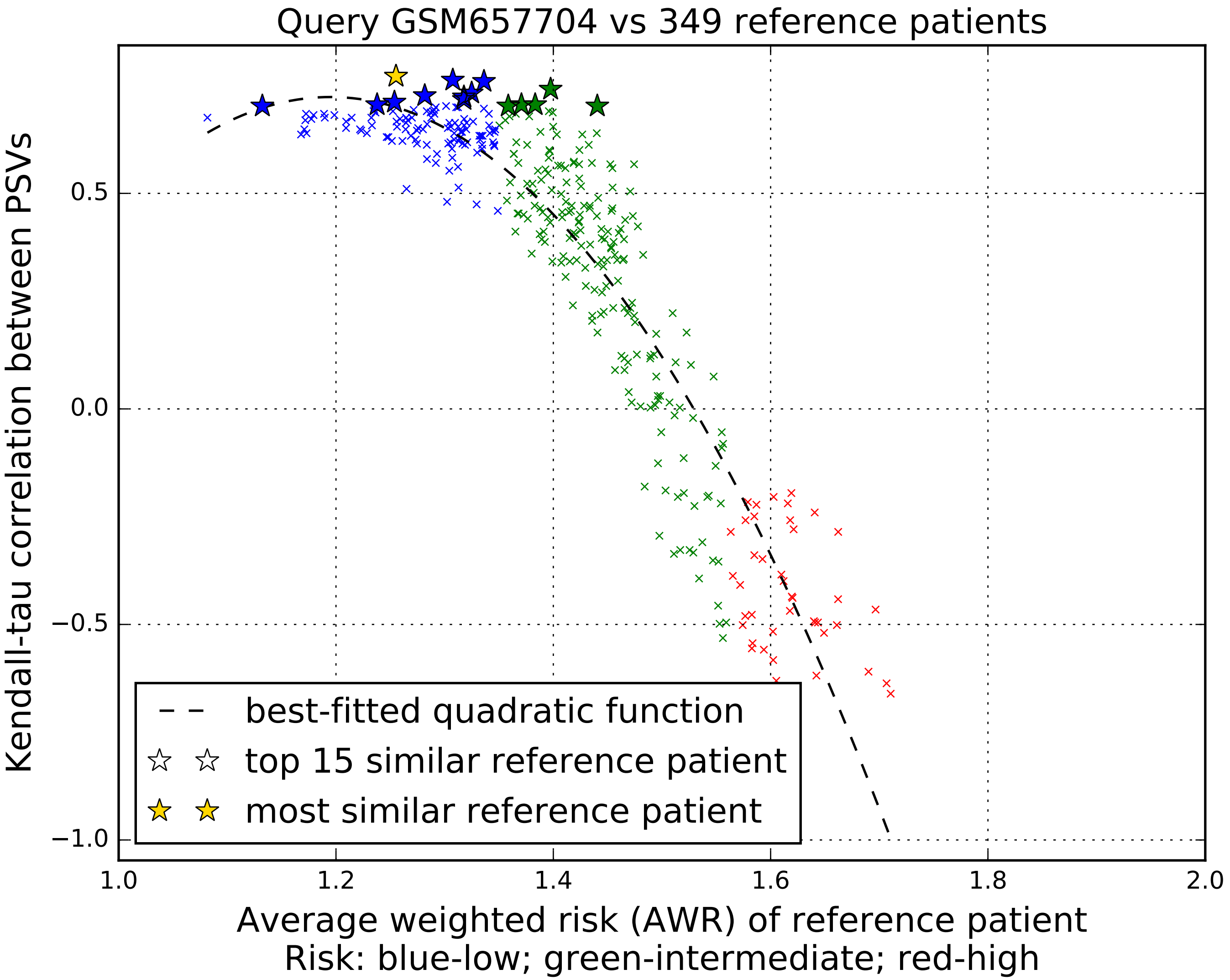
Query GSM657704 vs 349 reference patients
Kendall-tau correlation between PSVs
0.5
0.0
−0.5
−1.0
1.0
1.2
1.4
1.6
1.8
2.0
best-fitted quadratic function
top 15 similar reference patient
most similar reference patient
Average weighted risk (AWR) of reference patient
Risk: blue-low; green-intermediate; red-high

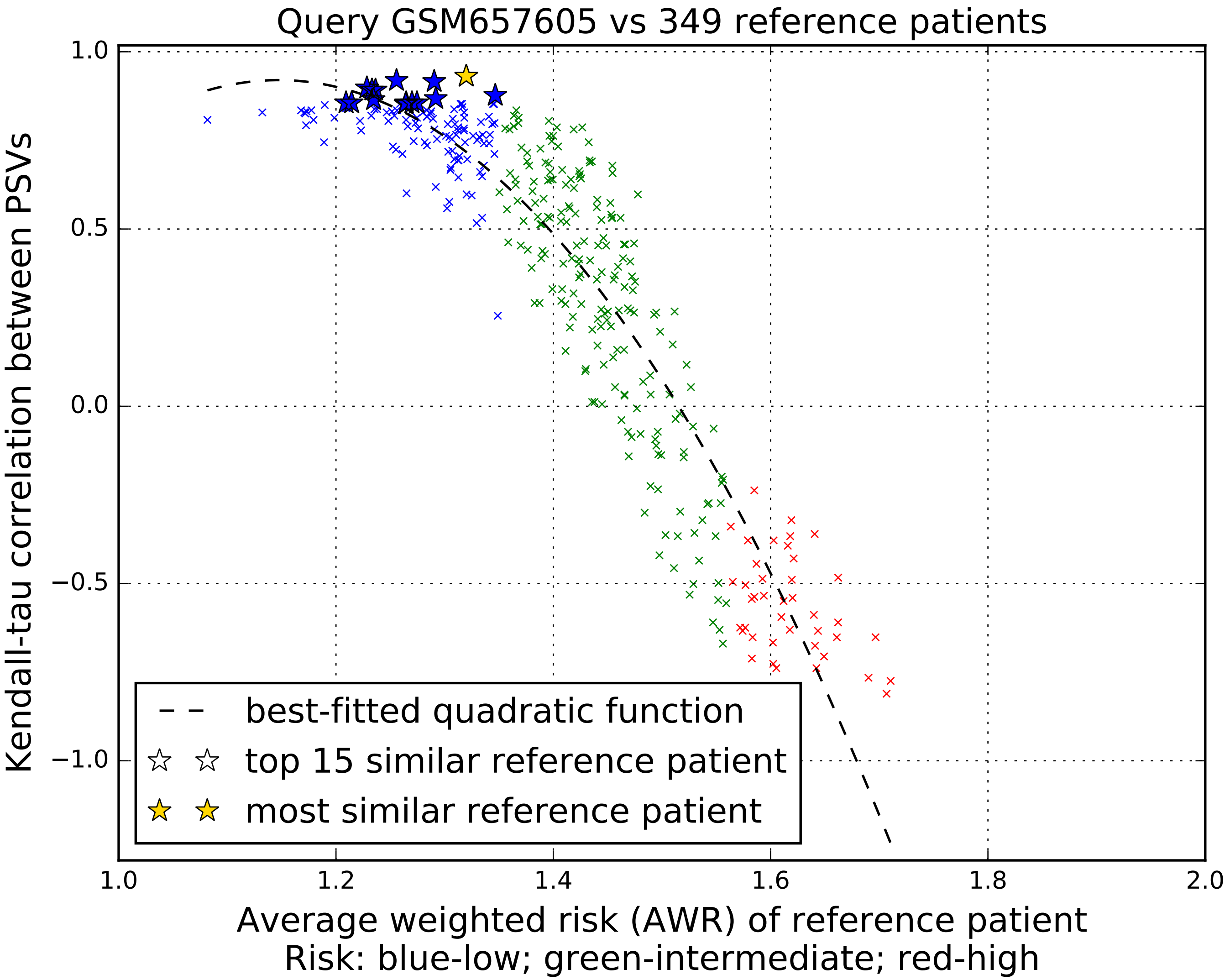

Query GSM657605 vs 349 reference patients
Kendall-tau correlation between PSVs
1.0
0.5
0.0
−0.5
−1.0
1.0
1.2
1.4
1.6
1.8
2.0
best-fitted quadratic function
top 15 similar reference patient
most similar reference patient
Average weighted risk (AWR) of reference patient
Risk: blue-low; green-intermediate; red-high

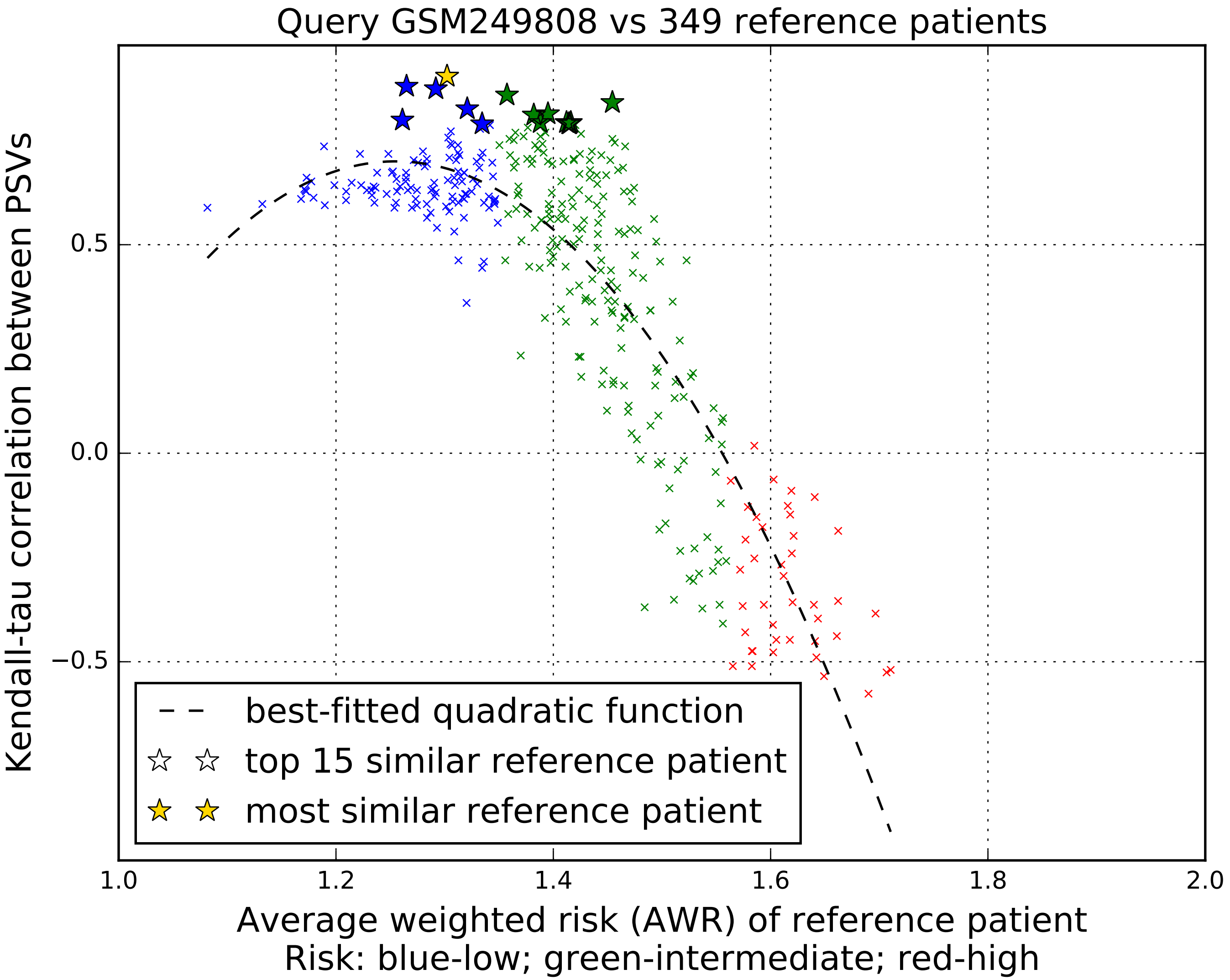
Query GSM249808 vs 349 reference patients
Kendall-tau correlation between PSVs
0.5
0.0
−0.5
1.0
1.2
1.4
1.6
1.8
2.0
best-fitted quadratic function
top 15 similar reference patient
most similar reference patient
Average weighted risk (AWR) of reference patient
Risk: blue-low; green-intermediate; red-high

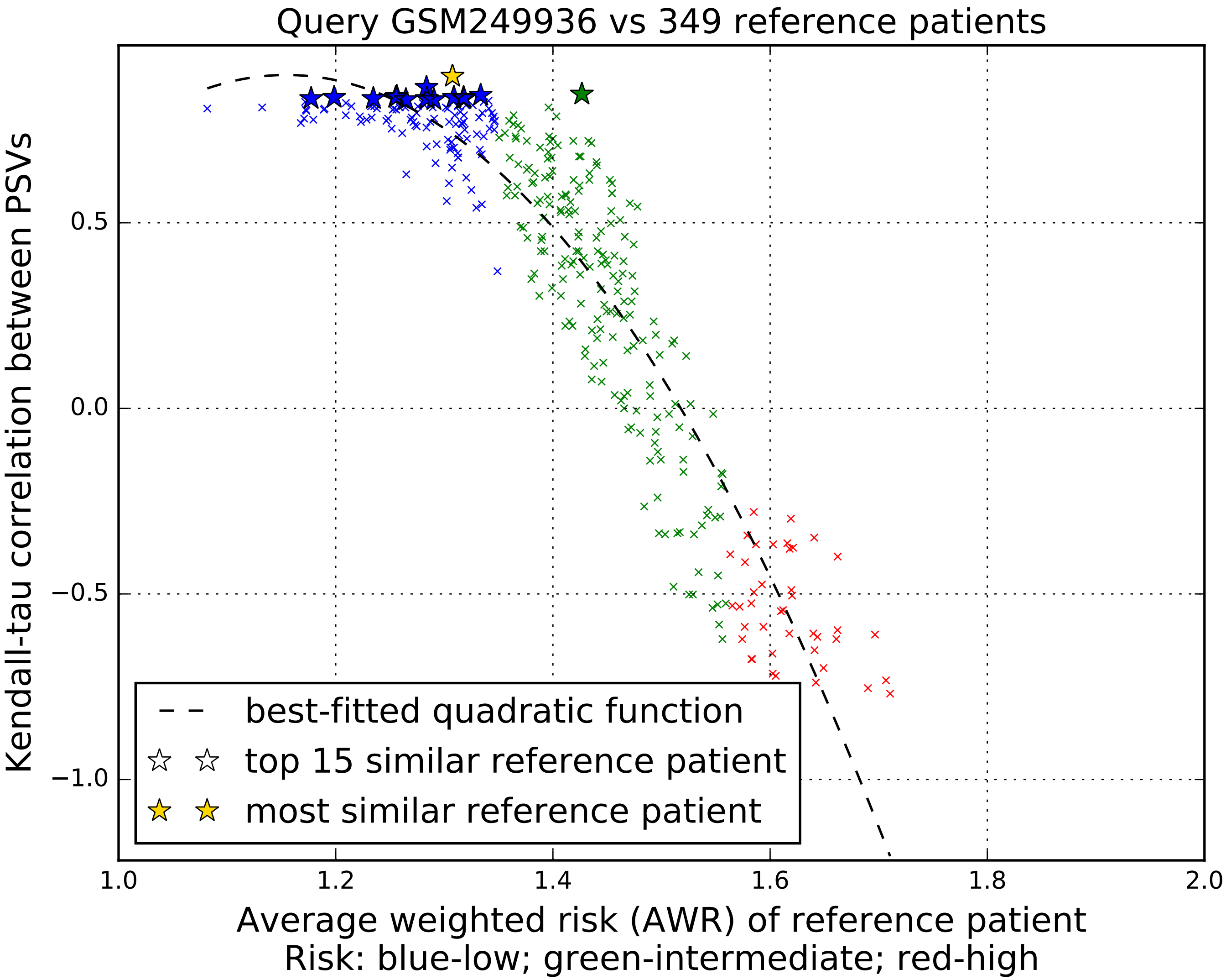
Query GSM249936 vs 349 reference patients
Kendall-tau correlation between PSVs
0.5
0.0
−0.5
−1.0
1.0
1.2
1.4
1.6
1.8
2.0
Average weighted risk (AWR) of reference patient
Risk: blue-low; green-intermediate; red-high
best-fitted quadratic function
top 15 similar reference patient
most similar reference patient

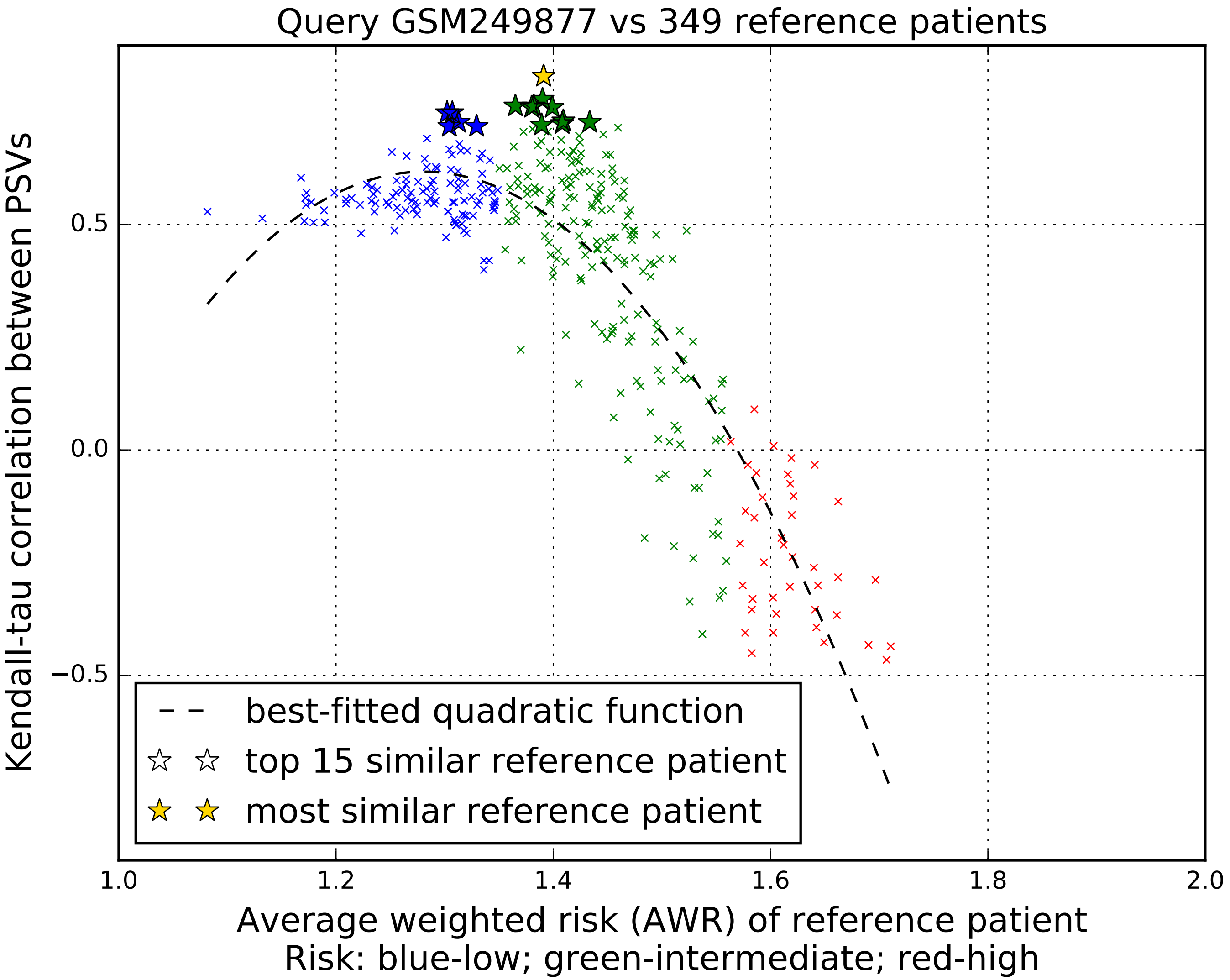
Query GSM249877 vs 349 reference patients
Kendall-tau correlation between PSVs
0.5
0.0
−0.5
1.0
1.2
1.4
1.6
1.8
2.0
best-fitted quadratic function
top 15 similar reference patient
most similar reference patient
Average weighted risk (AWR) of reference patient
Risk: blue-low; green-intermediate; red-high

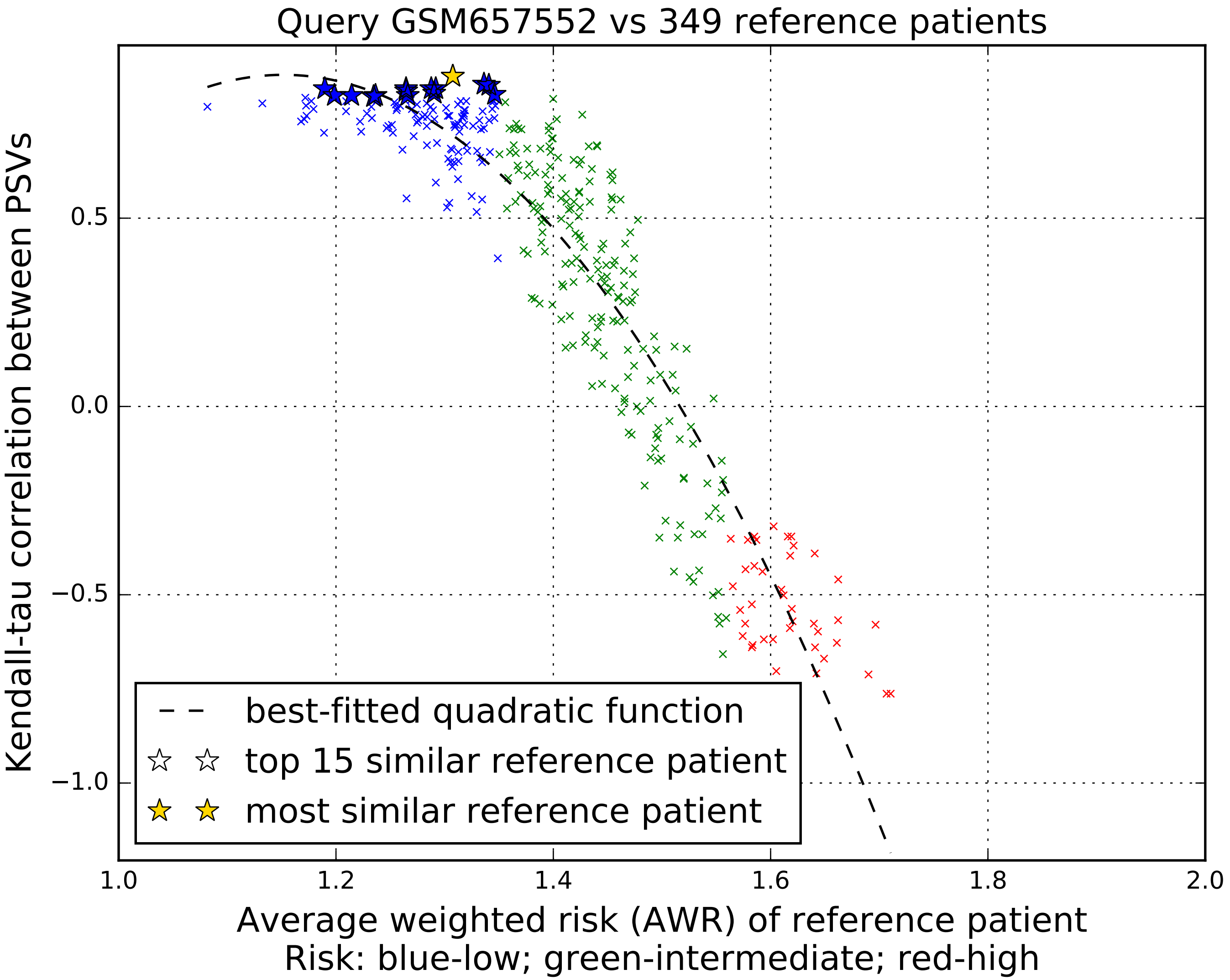

Query GSM657552 vs 349 reference patients
Kendall-tau correlation between PSVs
0.5
0.0
−0.5
−1.0
1.0
1.2
1.4
1.6
1.8
2.0
best-fitted quadratic function
top 15 similar reference patient
most similar reference patient
Average weighted risk (AWR) of reference patient
Risk: blue-low; green-intermediate; red-high

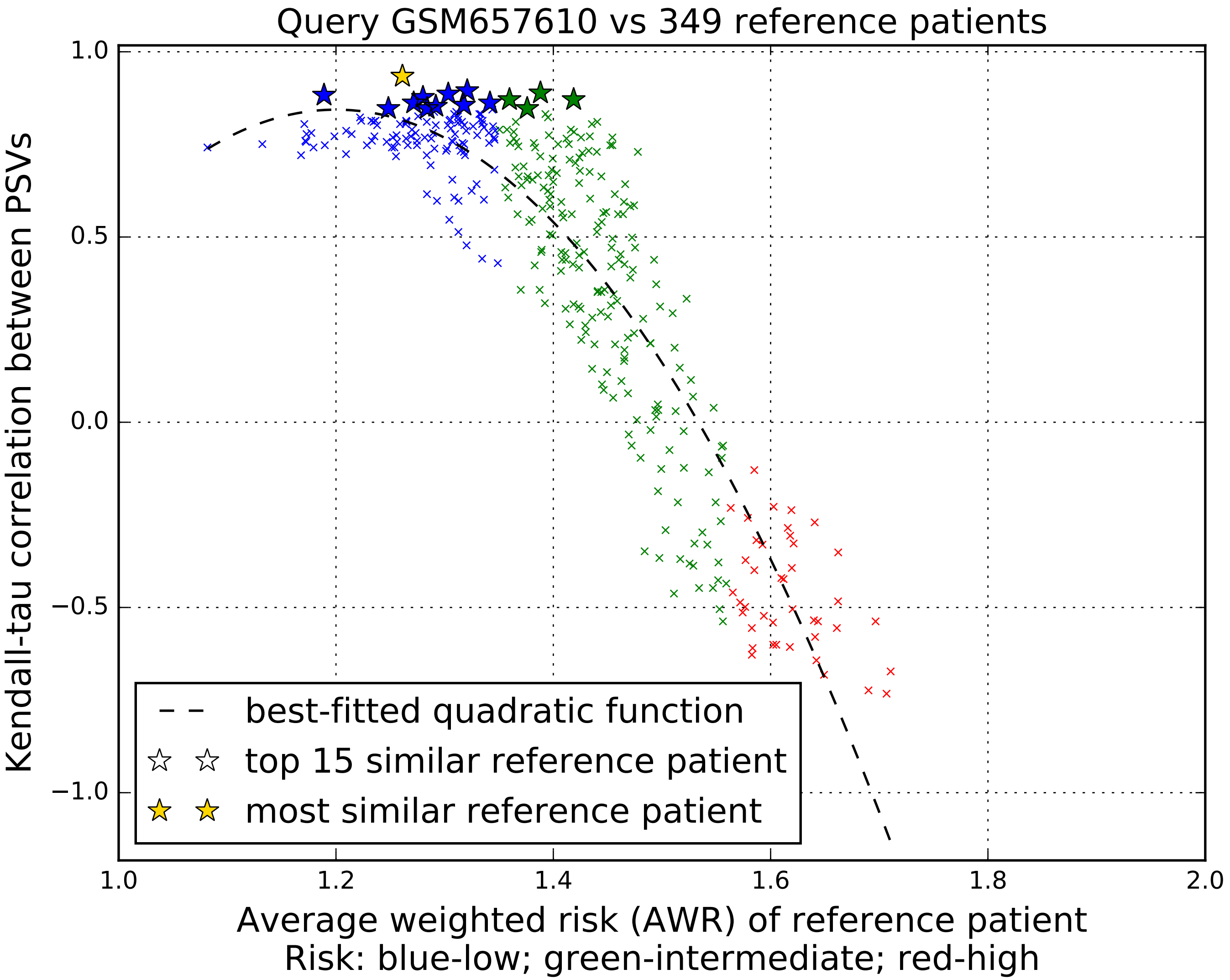

Query GSM657610 vs 349 reference patients
Kendall-tau correlation between PSVs
1.0
0.5
0.0
−0.5
−1.0
1.0
1.2
1.4
1.6
1.8
2.0
Average weighted risk (AWR) of reference patient
Risk: blue-low; green-intermediate; red-high
best-fitted quadratic function
top 15 similar reference patient
most similar reference patient

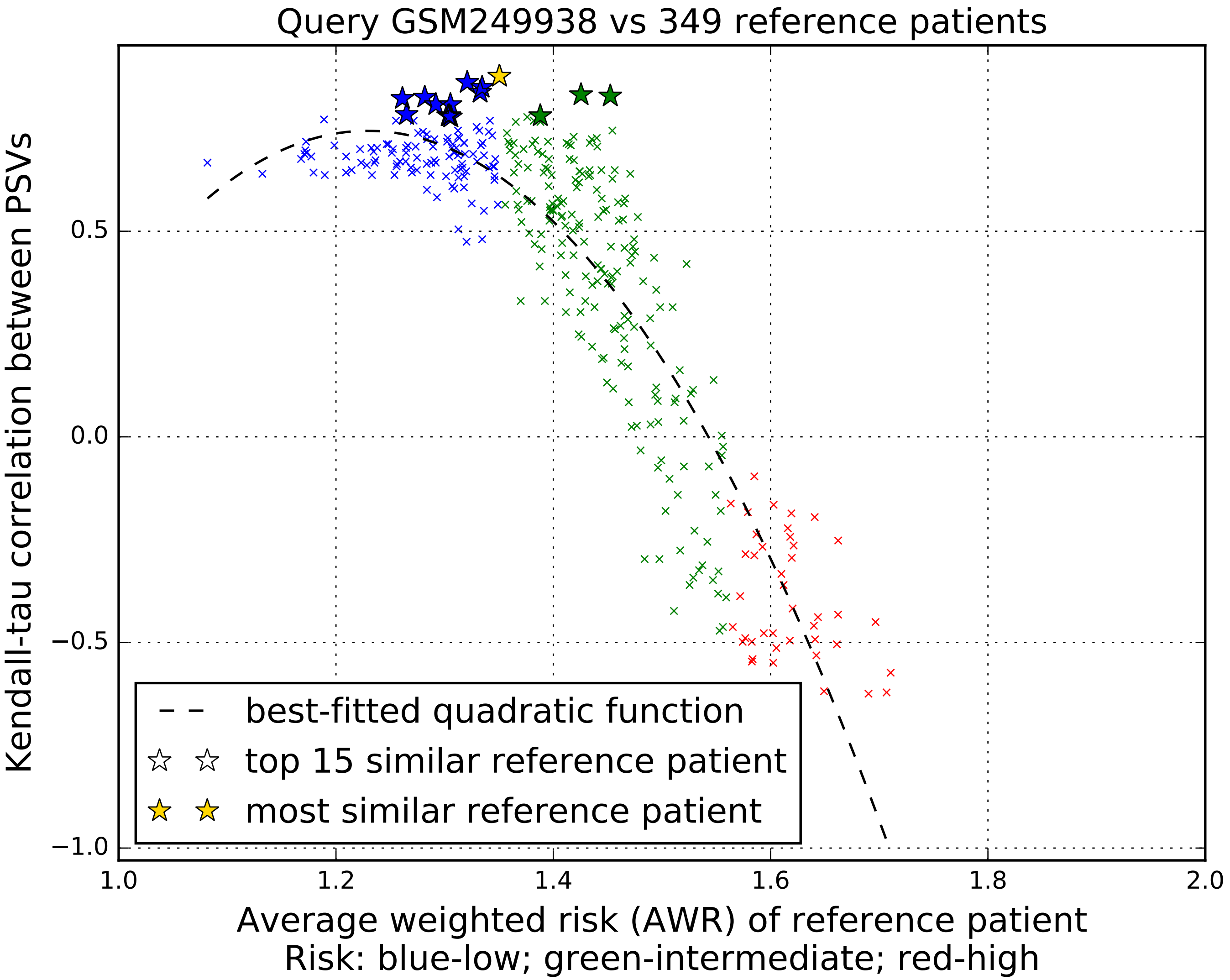

Query GSM249938 vs 349 reference patients
Kendall-tau correlation between PSVs
0.5
0.0
−0.5
−1.0
1.0
1.2
1.4
1.6
1.8
2.0
best-fitted quadratic function
top 15 similar reference patient
most similar reference patient
Average weighted risk (AWR) of reference patient
Risk: blue-low; green-intermediate; red-high

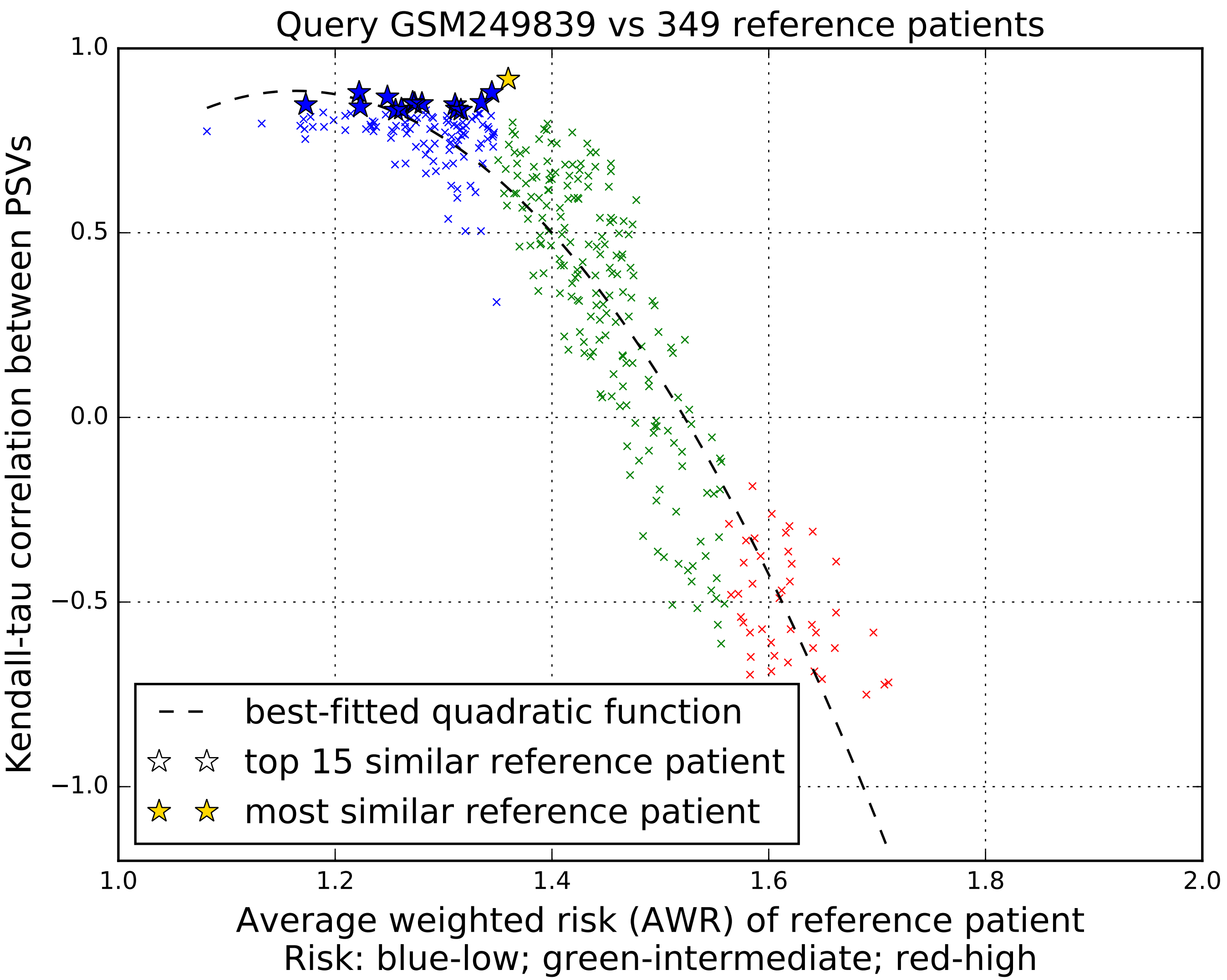
Query GSM249839 vs 349 reference patients
Kendall-tau correlation between PSVs
Average weighted risk (AWR) of reference patient
Risk: blue-low; green-intermediate; red-high
best-fitted quadratic function
top 15 similar reference patient
most similar reference patient
1.0
0.5
0.0
−0.5
−1.0
1.0
1.2
1.4
1.6
1.8
2.0

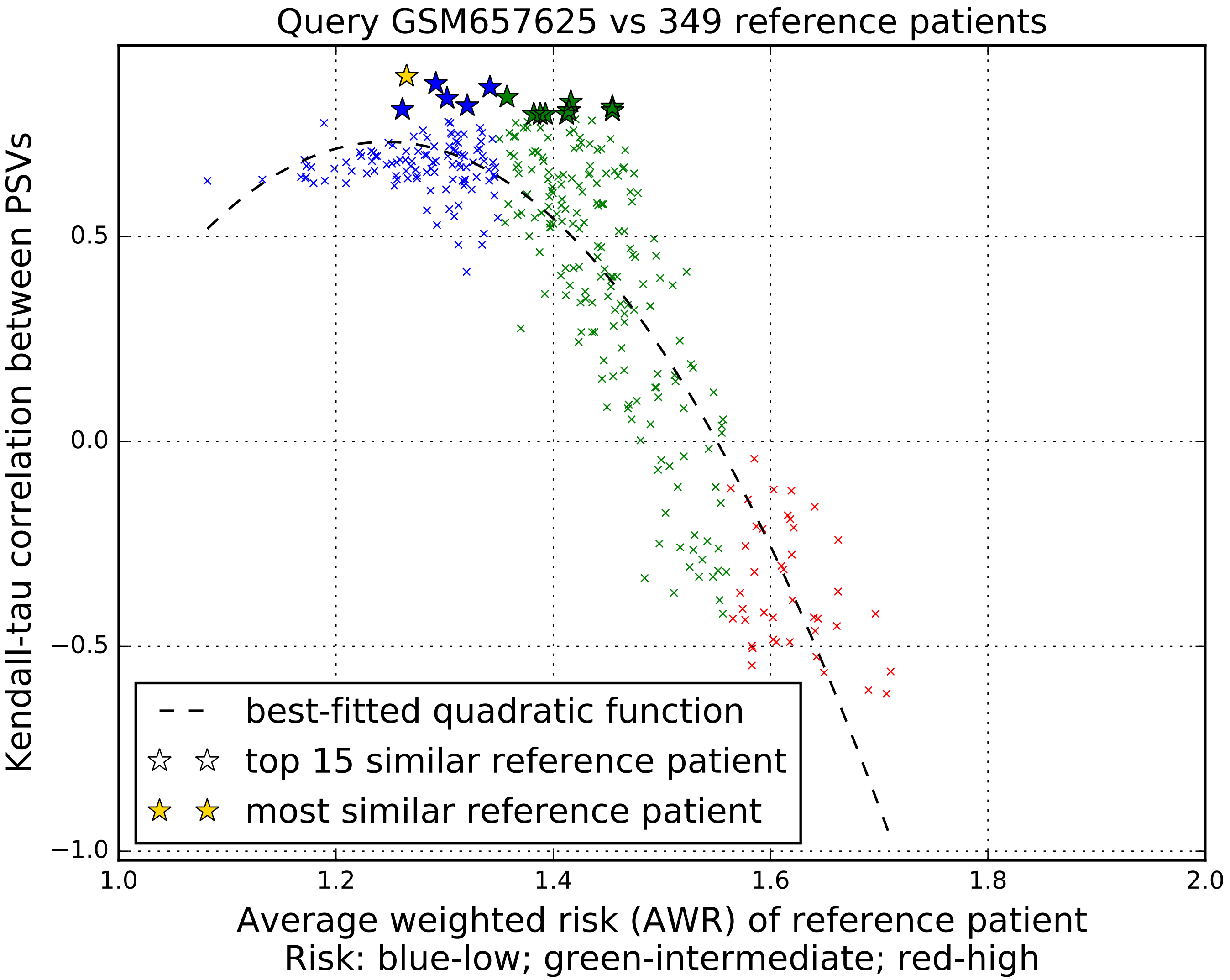

Query GSM657625 vs 349 reference patients
Kendall-tau correlation between PSVs
0.5
0.0
−0.5
−1.0
1.0
1.2
1.4
1.6
1.8
2.0
best-fitted quadratic function
top 15 similar reference patient
most similar reference patient
Average weighted risk (AWR) of reference patient
Risk: blue-low; green-intermediate; red-high

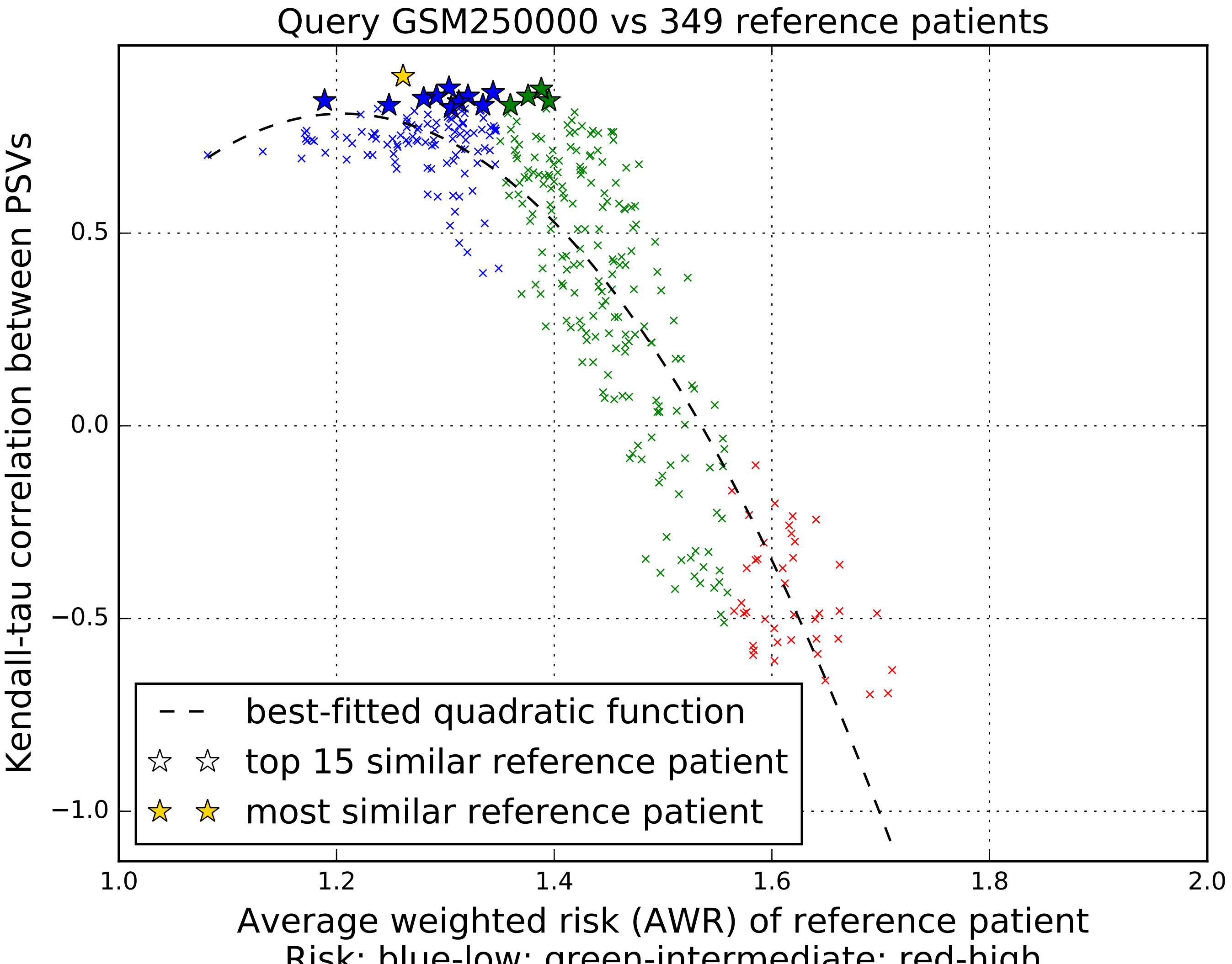
Query GSM250000 vs 349 reference patients
Kendall-tau correlation between PSVs
0.5
0.0
−0.5
−1.0
1.0
1.2
1.4
1.6
1.8
2.0
best-fitted quadratic function
top 15 similar reference patient
most similar reference patient
Average weighted risk (AWR) of reference patient
Risk: blue-low; green-intermediate; red-high

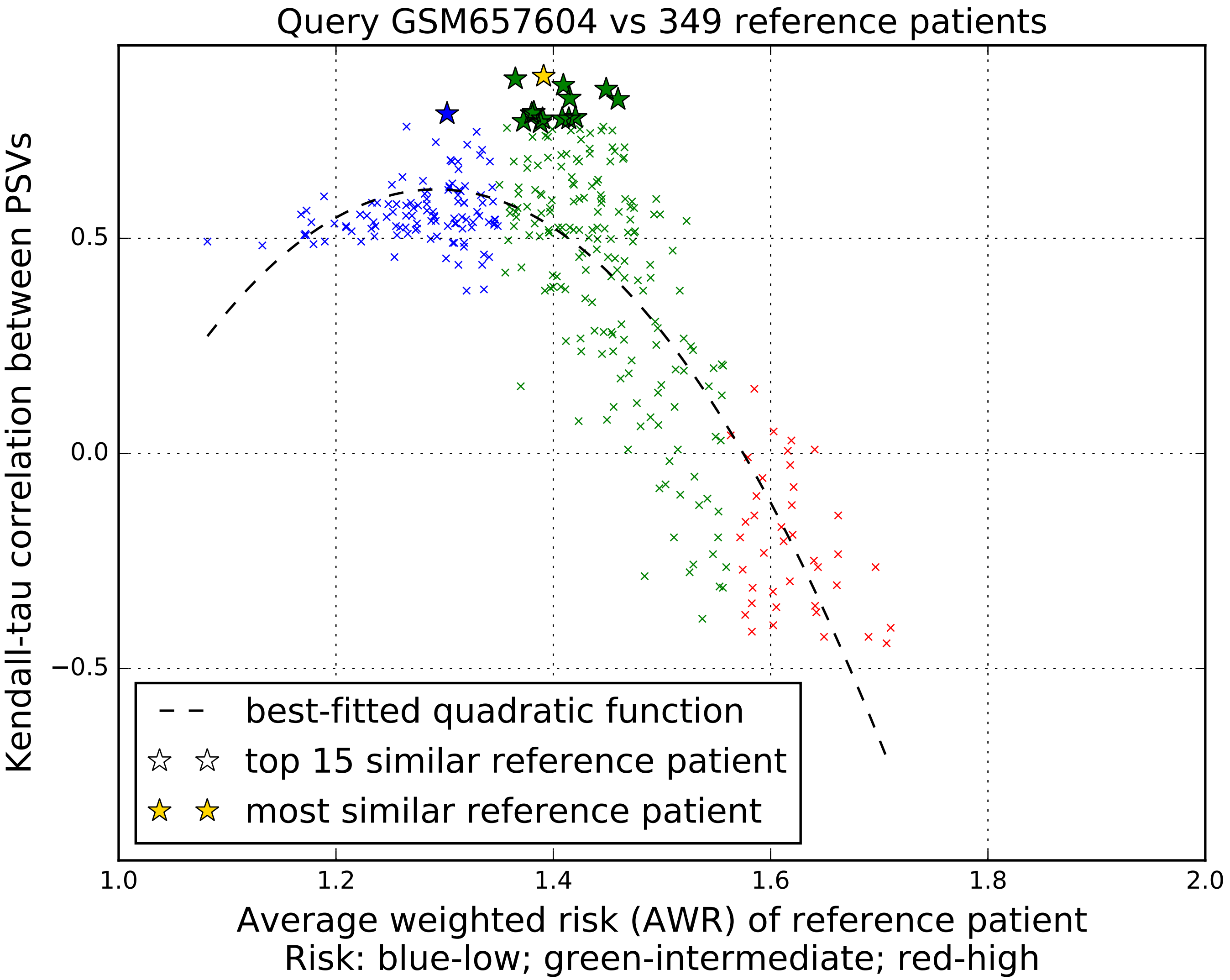

Query GSM657604 vs 349 reference patients
Kendall-tau correlation between PSVs
0.5
0.0
−0.5
1.0
1.2
1.4
1.6
1.8
2.0
best-fitted quadratic function
top 15 similar reference patient
most similar reference patient
Average weighted risk (AWR) of reference patient
Risk: blue-low; green-intermediate; red-high

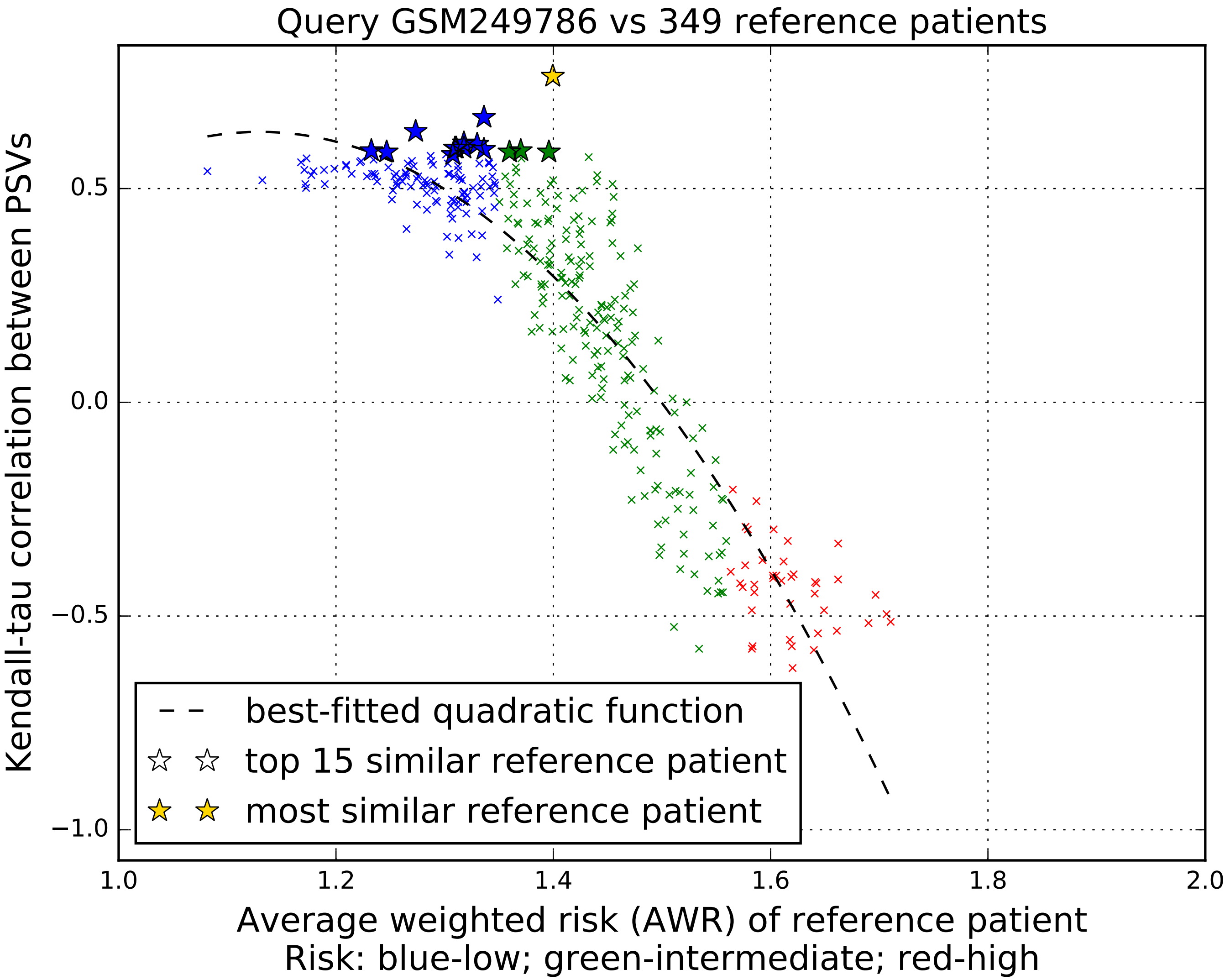
Query GSM249786 vs 349 reference patients
Kendall-tau correlation between PSVs
0.5
0.0
−0.5
−1.0
1.0
1.2
1.4
1.6
1.8
2.0
best-fitted quadratic function
top 15 similar reference patient
most similar reference patient
Average weighted risk (AWR) of reference patient
Risk: blue-low; green-intermediate; red-high

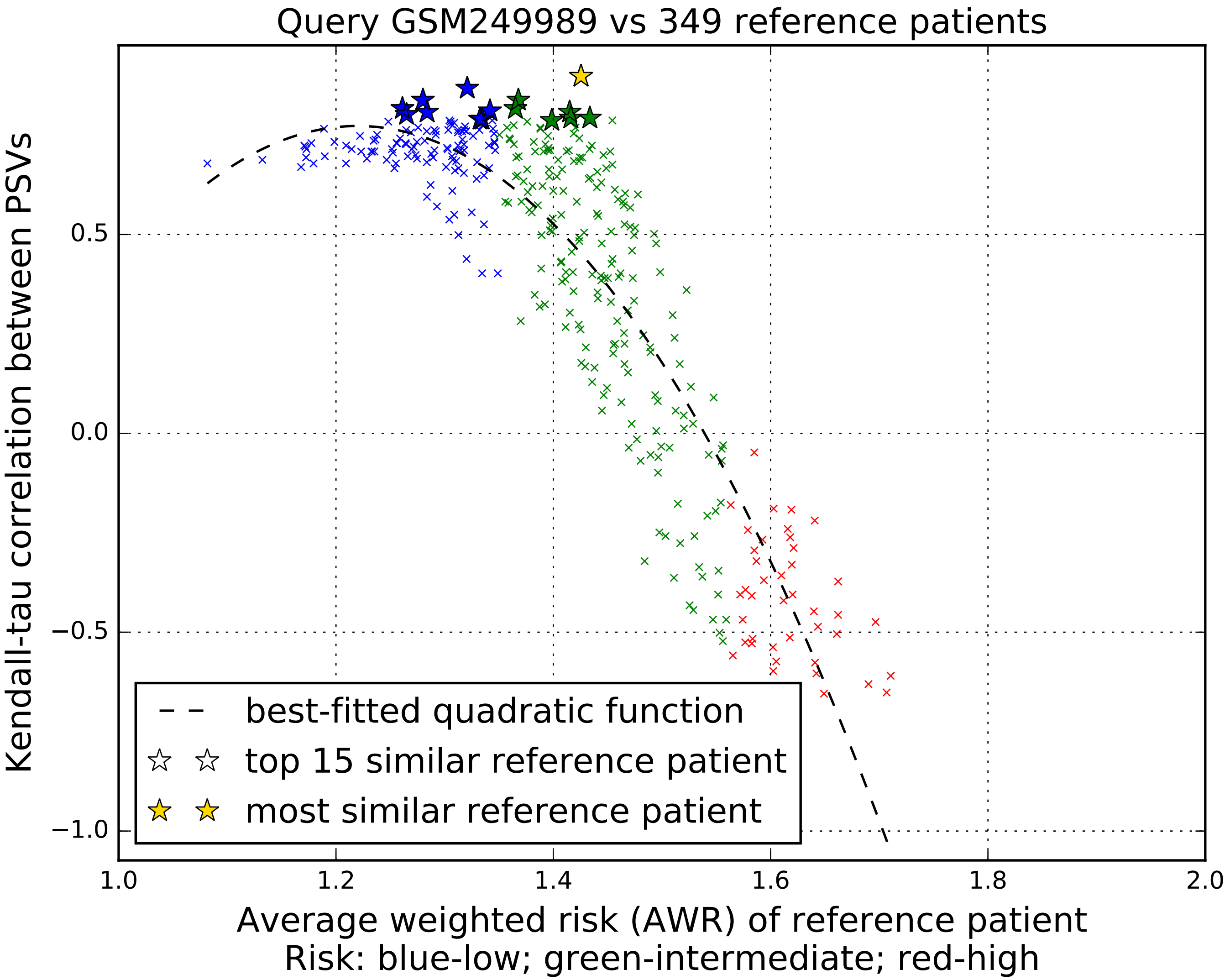

Query GSM249989 vs 349 reference patients
Kendall-tau correlation between PSVs
0.5
0.0
−0.5
−1.0
1.0
1.2
1.4
1.6
1.8
2.0
best-fitted quadratic function
top 15 similar reference patient
most similar reference patient
Average weighted risk (AWR) of reference patient
Risk: blue-low; green-intermediate; red-high

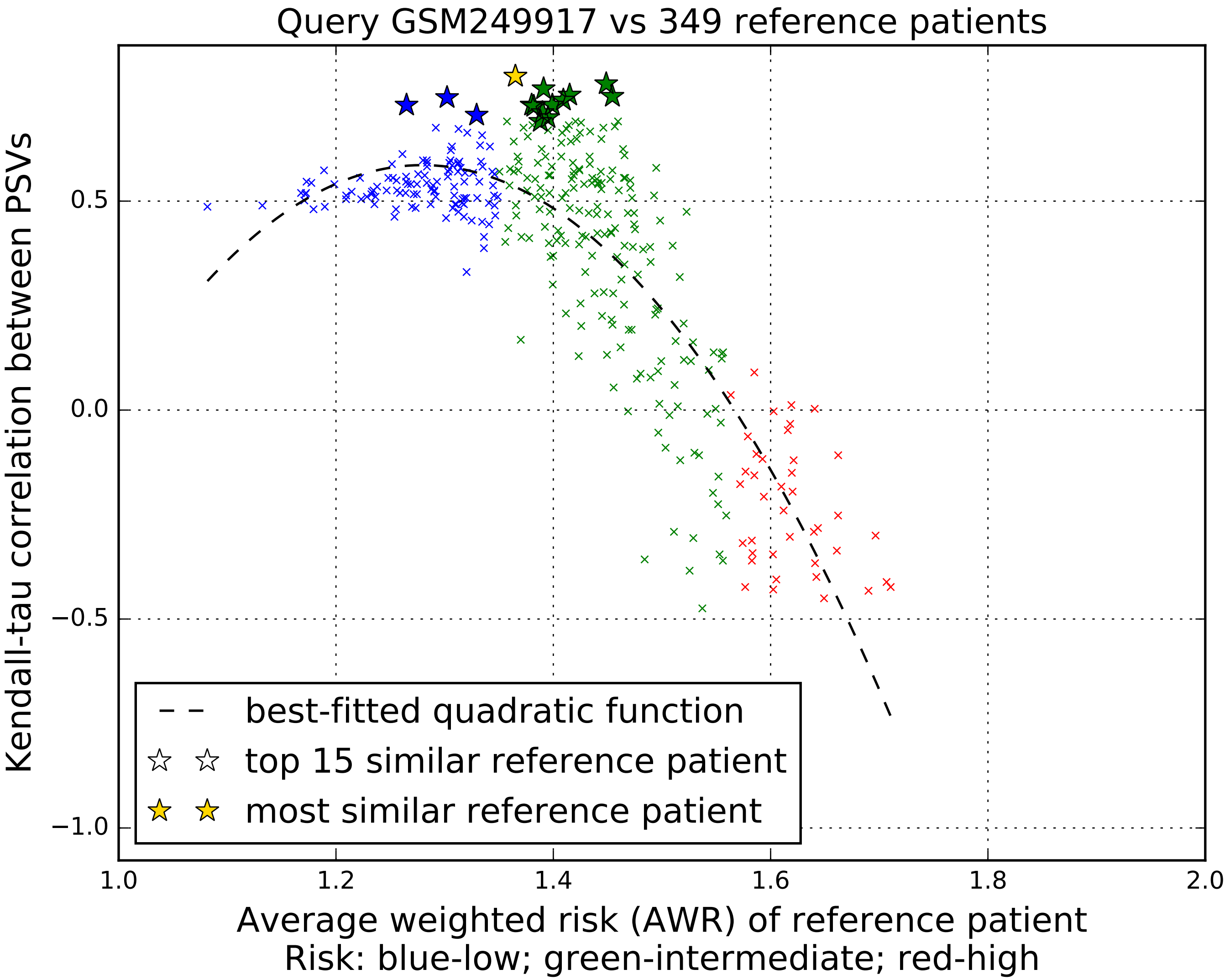
Query GSM249917 vs 349 reference patients
Kendall-tau correlation between PSVs
0.5
0.0
−0.5
−1.0
1.0
1.2
1.4
1.6
1.8
2.0
best-fitted quadratic function
top 15 similar reference patient
most similar reference patient
Average weighted risk (AWR) of reference patient
Risk: blue-low; green-intermediate; red-high

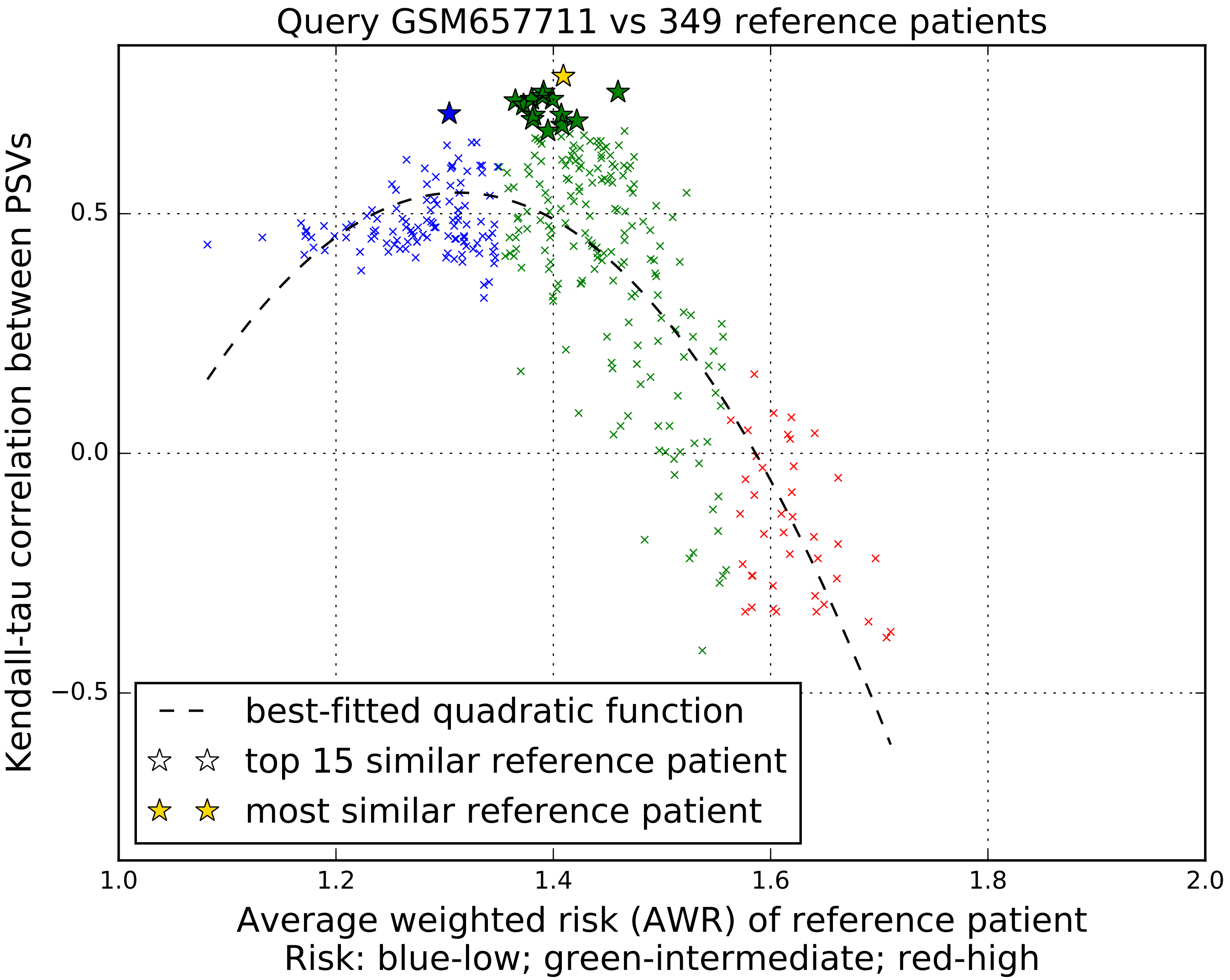
Query GSM657711 vs 349 reference patients
Kendall-tau correlation between PSVs
0.5
0.0
−0.5
1.0
1.2
1.4
1.6
1.8
2.0
best-fitted quadratic function
top 15 similar reference patient
most similar reference patient
Average weighted risk (AWR) of reference patient
Risk: blue-low; green-intermediate; red-high

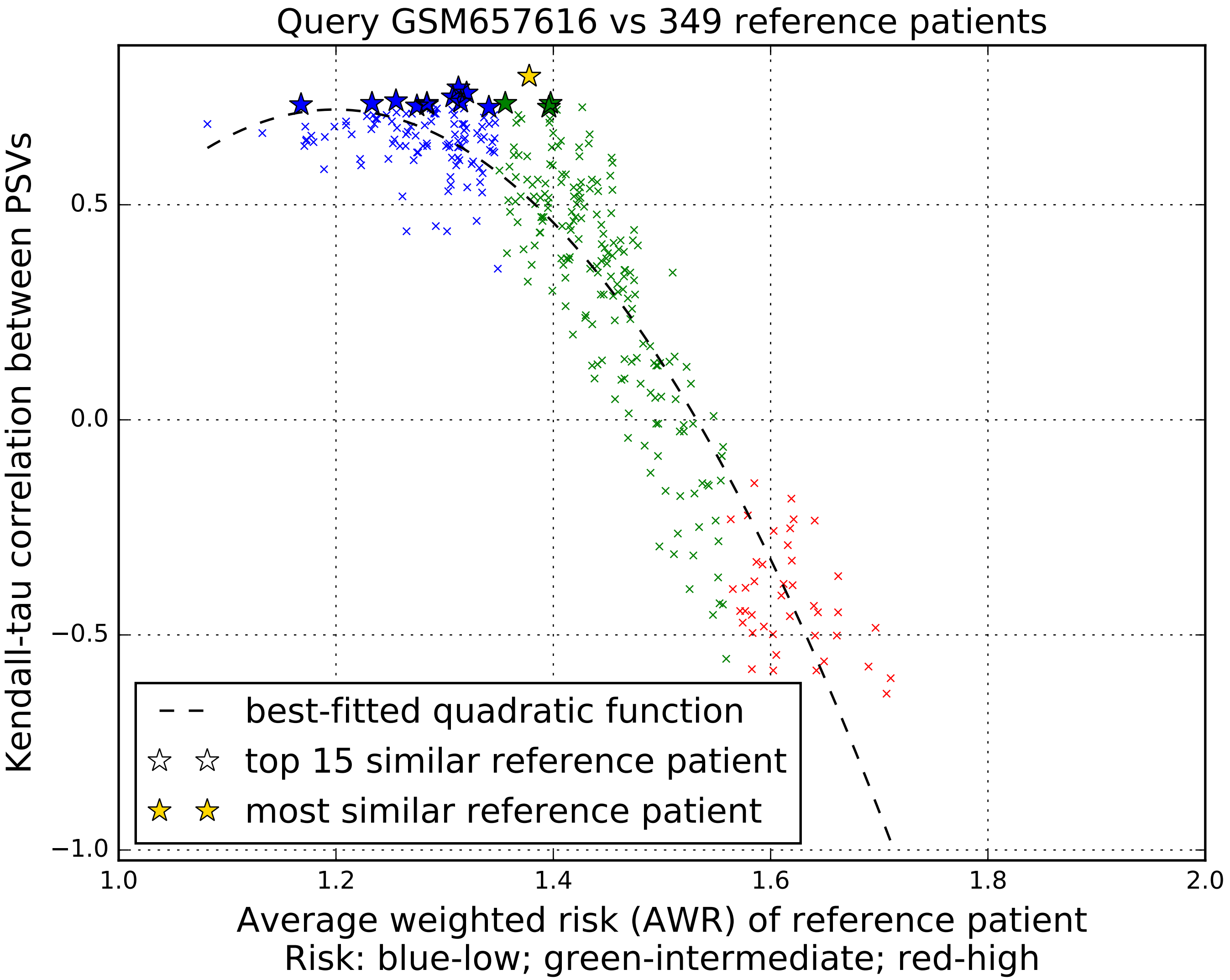

Query GSM657616 vs 349 reference patients
Kendall-tau correlation between PSVs
Average weighted risk (AWR) of reference patient
Risk: blue-low; green-intermediate; red-high
best-fitted quadratic function
top 15 similar reference patient
most similar reference patient
0.5
0.0
−0.5
−1.0
1.0
1.2
1.4
1.6
1.8
2.0

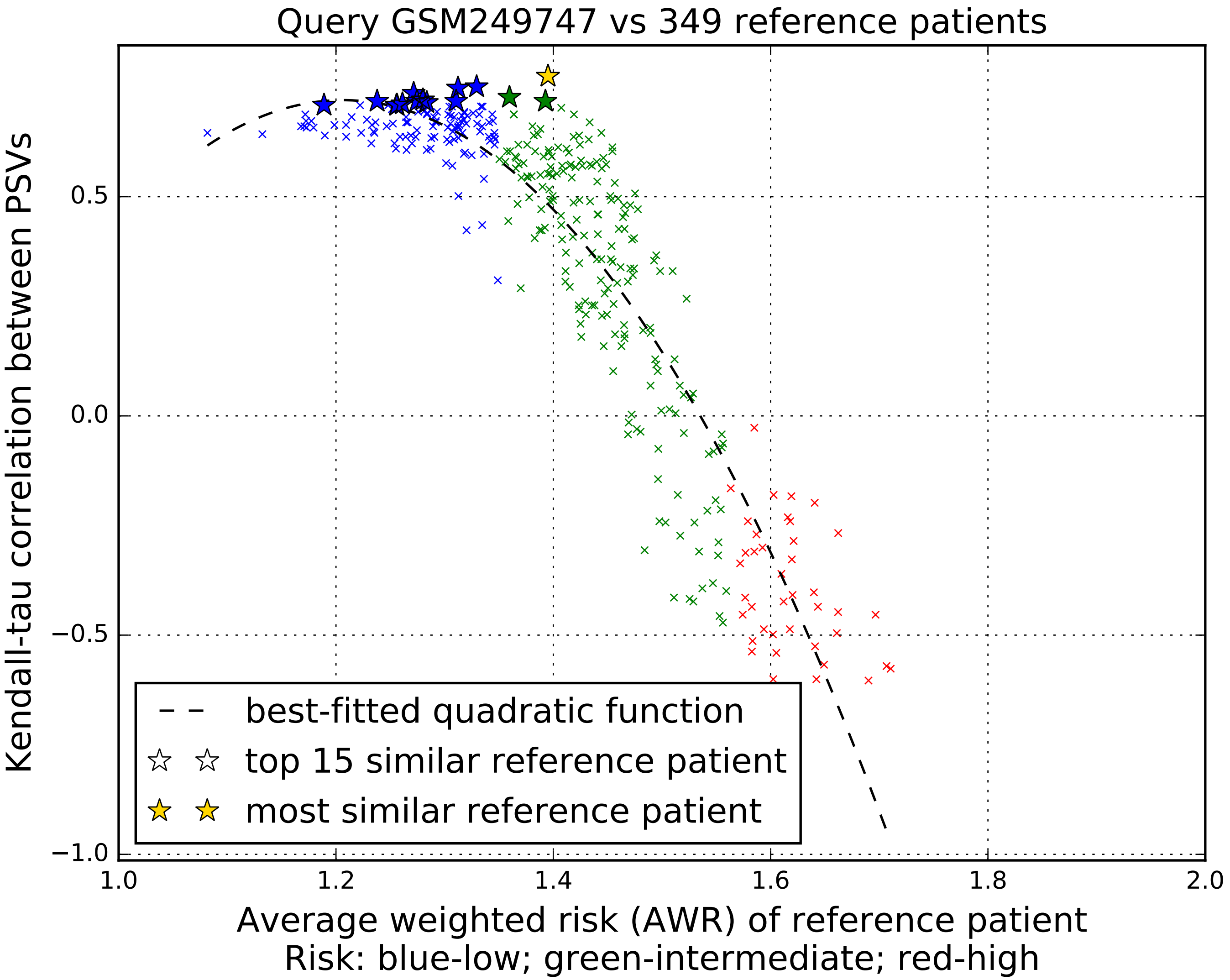
Query GSM249747 vs 349 reference patients
Kendall-tau correlation between PSVs
0.5
0.0
−0.5
−1.0
1.0
1.2
1.4
1.6
1.8
2.0
best-fitted quadratic function
top 15 similar reference patient
most similar reference patient
Average weighted risk (AWR) of reference patient
Risk: blue-low; green-intermediate; red-high

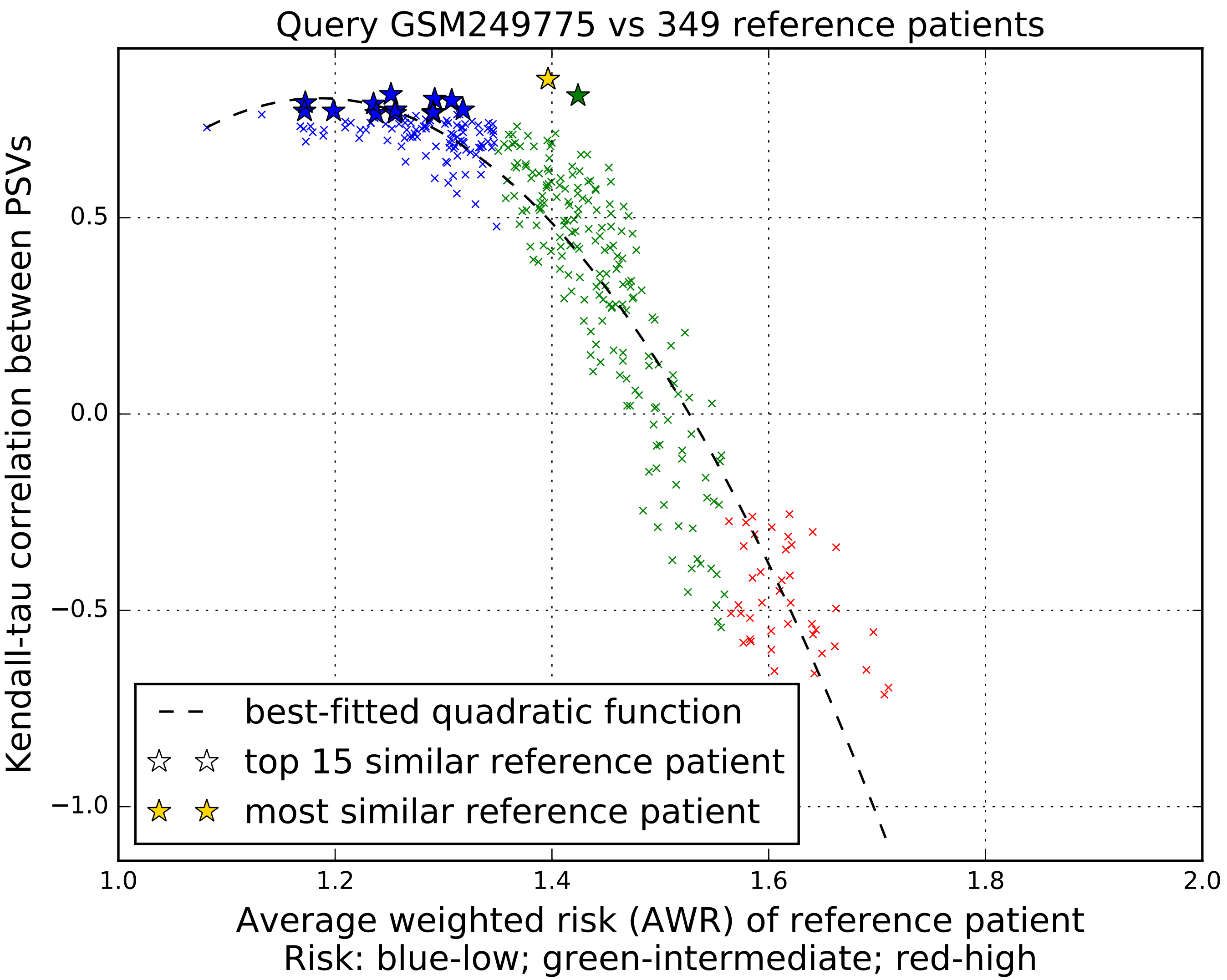

Query GSM249775 vs 349 reference patients
Kendall-tau correlation between PSVs
0.5
0.0
−0.5
−1.0
1.0
1.2
1.4
1.6
1.8
2.0
best-fitted quadratic function
top 15 similar reference patient
most similar reference patient
Average weighted risk (AWR) of reference patient
Risk: blue-low; green-intermediate; red-high

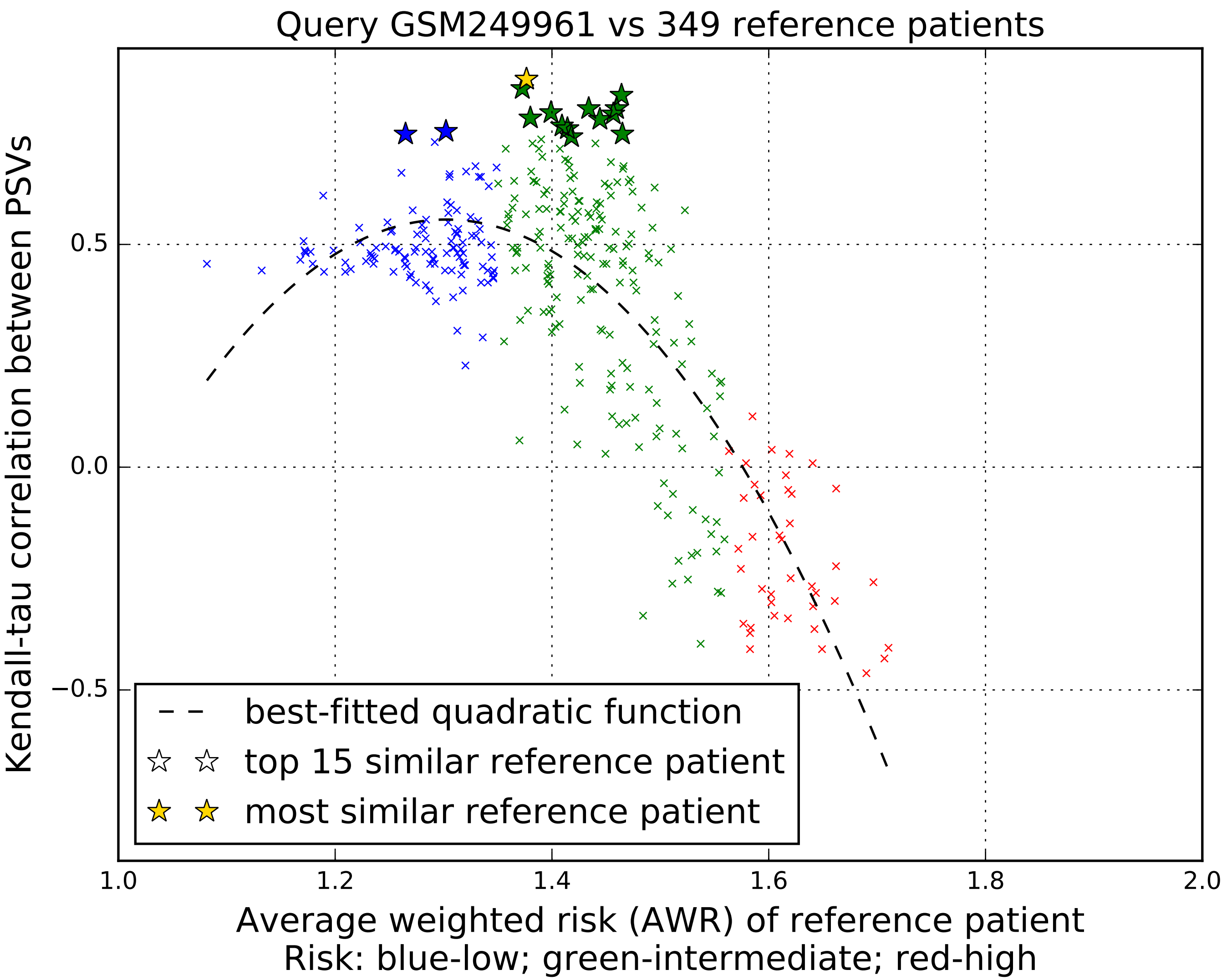

Query GSM249961 vs 349 reference patients
Kendall-tau correlation between PSVs
0.5
0.0
−0.5
1.0
1.2
1.4
1.6
1.8
2.0
Average weighted risk (AWR) of reference patient
Risk: blue-low; green-intermediate; red-high
best-fitted quadratic function
top 15 similar reference patient
most similar reference patient

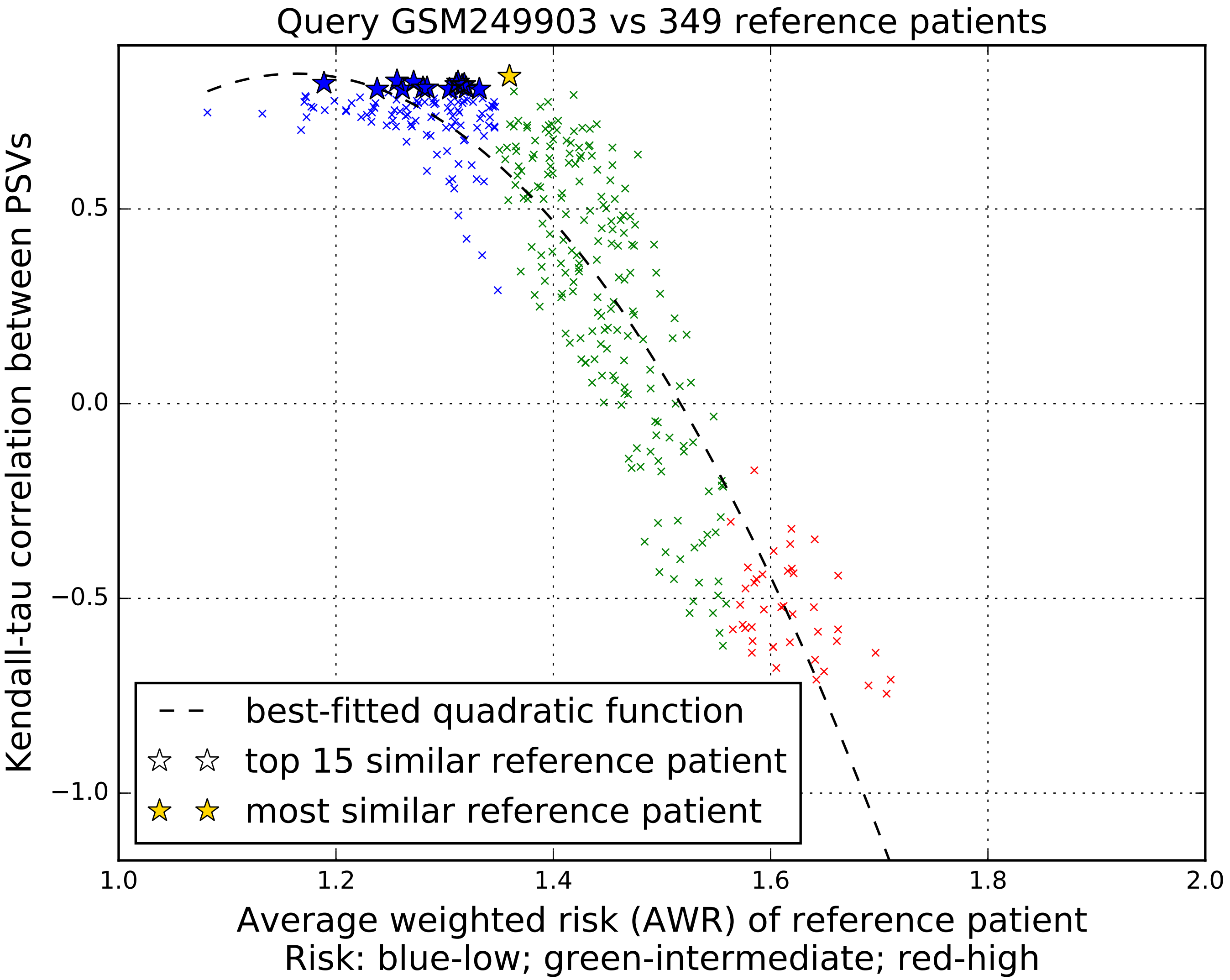

Query GSM249903 vs 349 reference patients
Kendall-tau correlation between PSVs
0.5
0.0
−0.5
−1.0
1.0
1.2
1.4
1.6
1.8
2.0
best-fitted quadratic function
top 15 similar reference patient
most similar reference patient
Average weighted risk (AWR) of reference patient
Risk: blue-low; green-intermediate; red-high

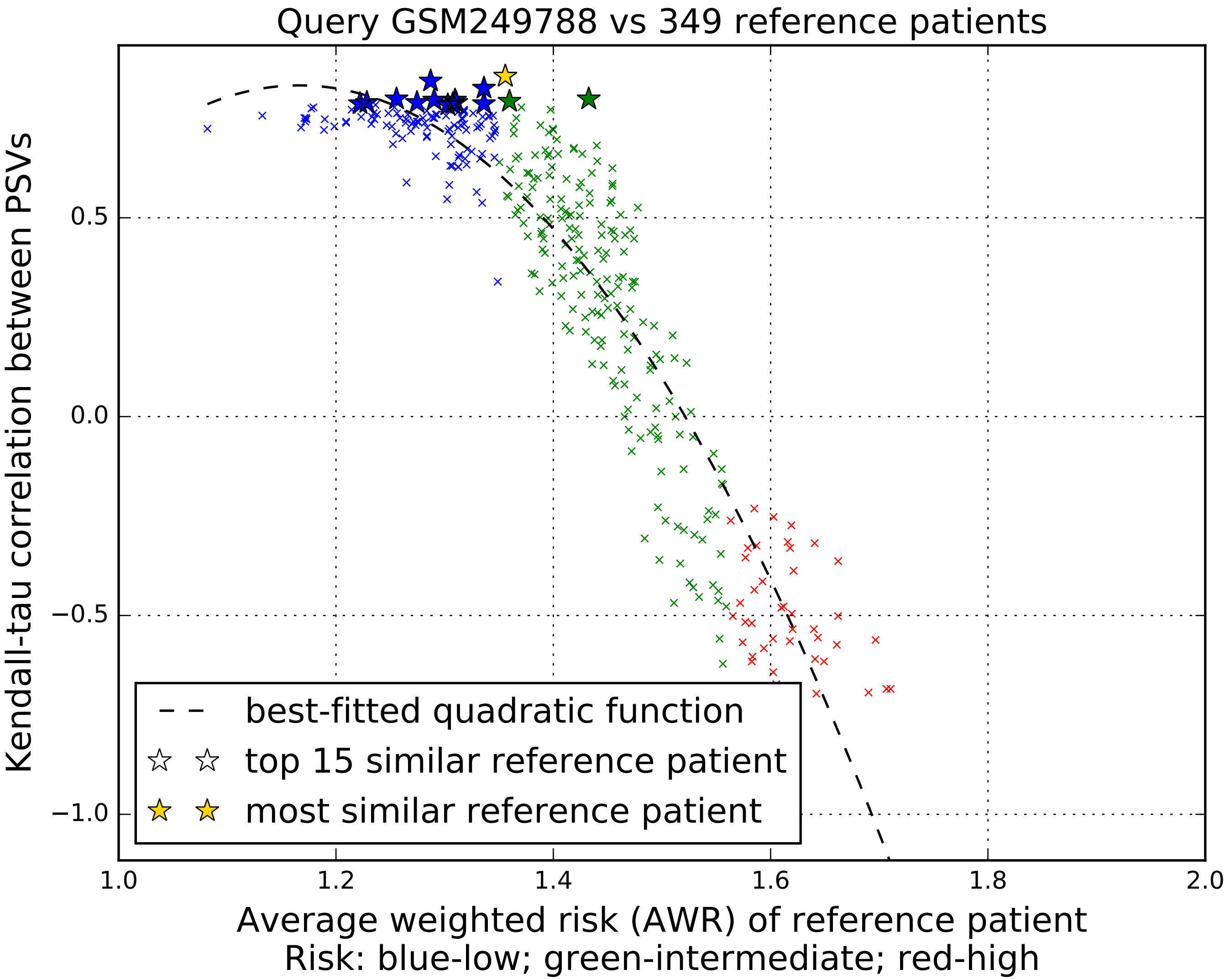

Query GSM249788 vs 349 reference patients
Kendall-tau correlation between PSVs
0.5
0.0
−0.5
−1.0
1.0
1.2
1.4
1.6
1.8
2.0
best-fitted quadratic function
top 15 similar reference patient
most similar reference patient
Average weighted risk (AWR) of reference patient
Risk: blue-low; green-intermediate; red-high

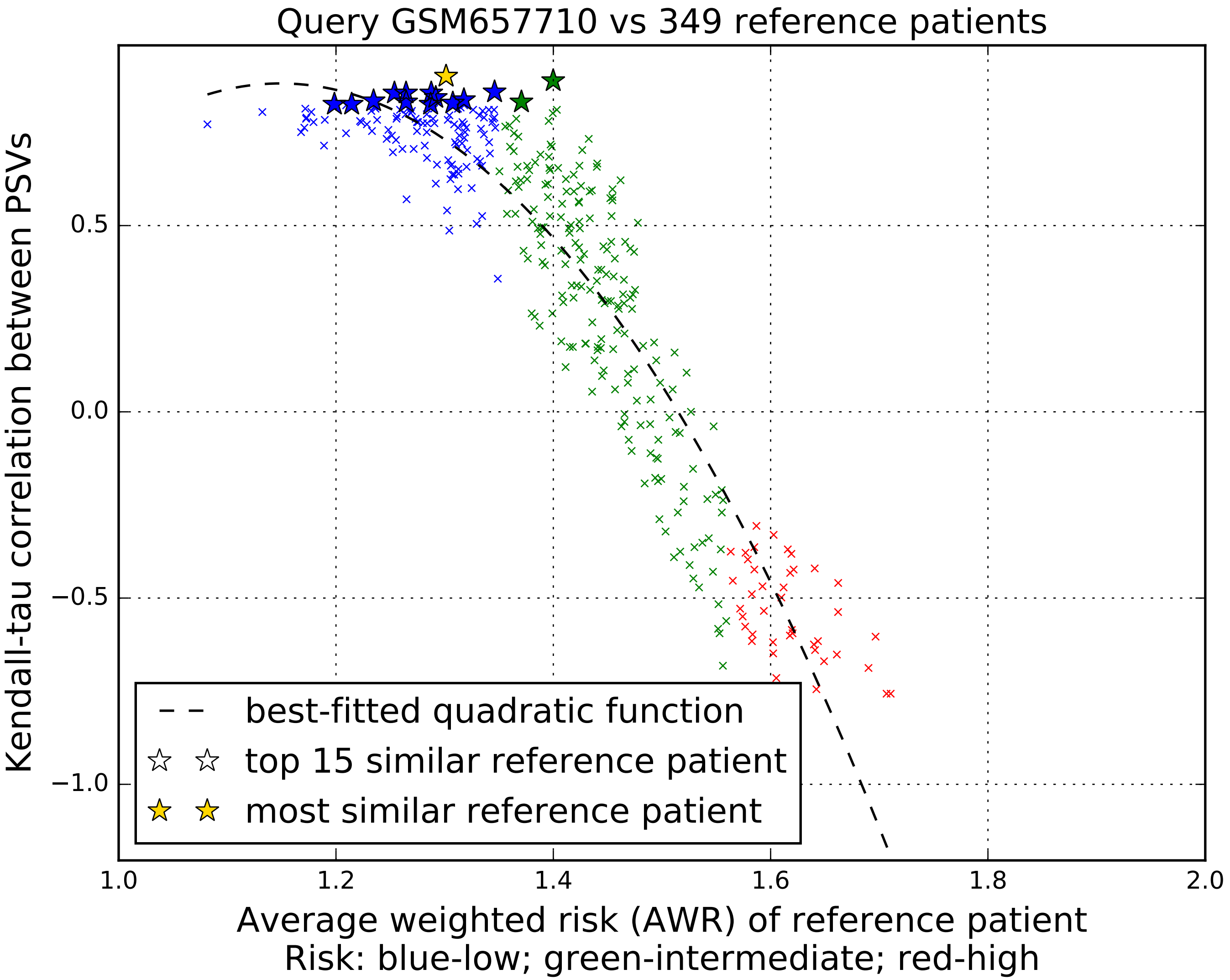

Query GSM657710 vs 349 reference patients
Kendall-tau correlation between PSVs
0.5
0.0
−0.5
−1.0
1.0
1.2
1.4
1.6
1.8
2.0
best-fitted quadratic function
top 15 similar reference patient
most similar reference patient
Average weighted risk (AWR) of reference patient
Risk: blue-low; green-intermediate; red-high

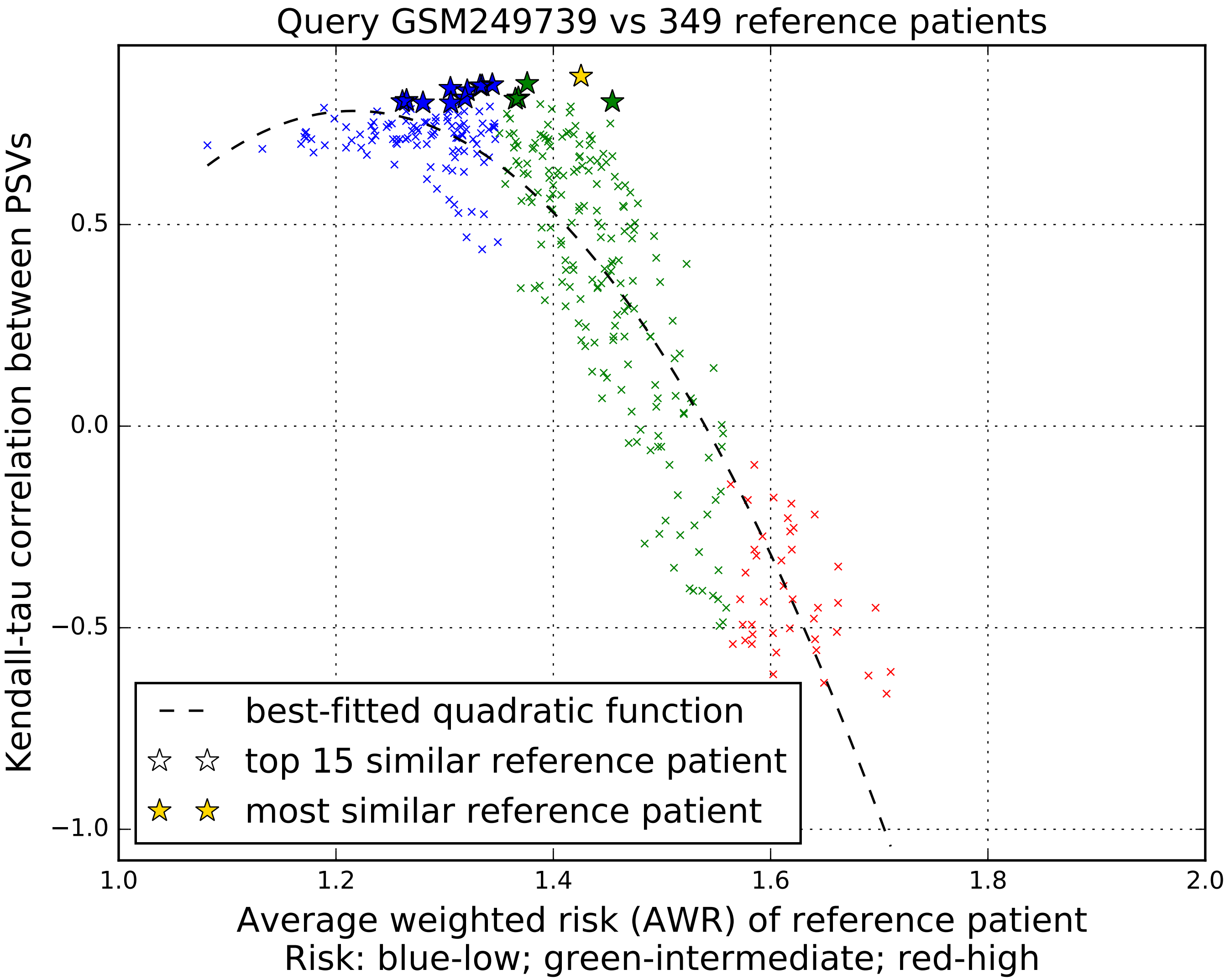

Query GSM249739 vs 349 reference patients
Kendall-tau correlation between PSVs
0.5
0.0
−0.5
−1.0
1.0
1.2
1.4
1.6
1.8
2.0
best-fitted quadratic function
top 15 similar reference patient
most similar reference patient
Average weighted risk (AWR) of reference patient
Risk: blue-low; green-intermediate; red-high

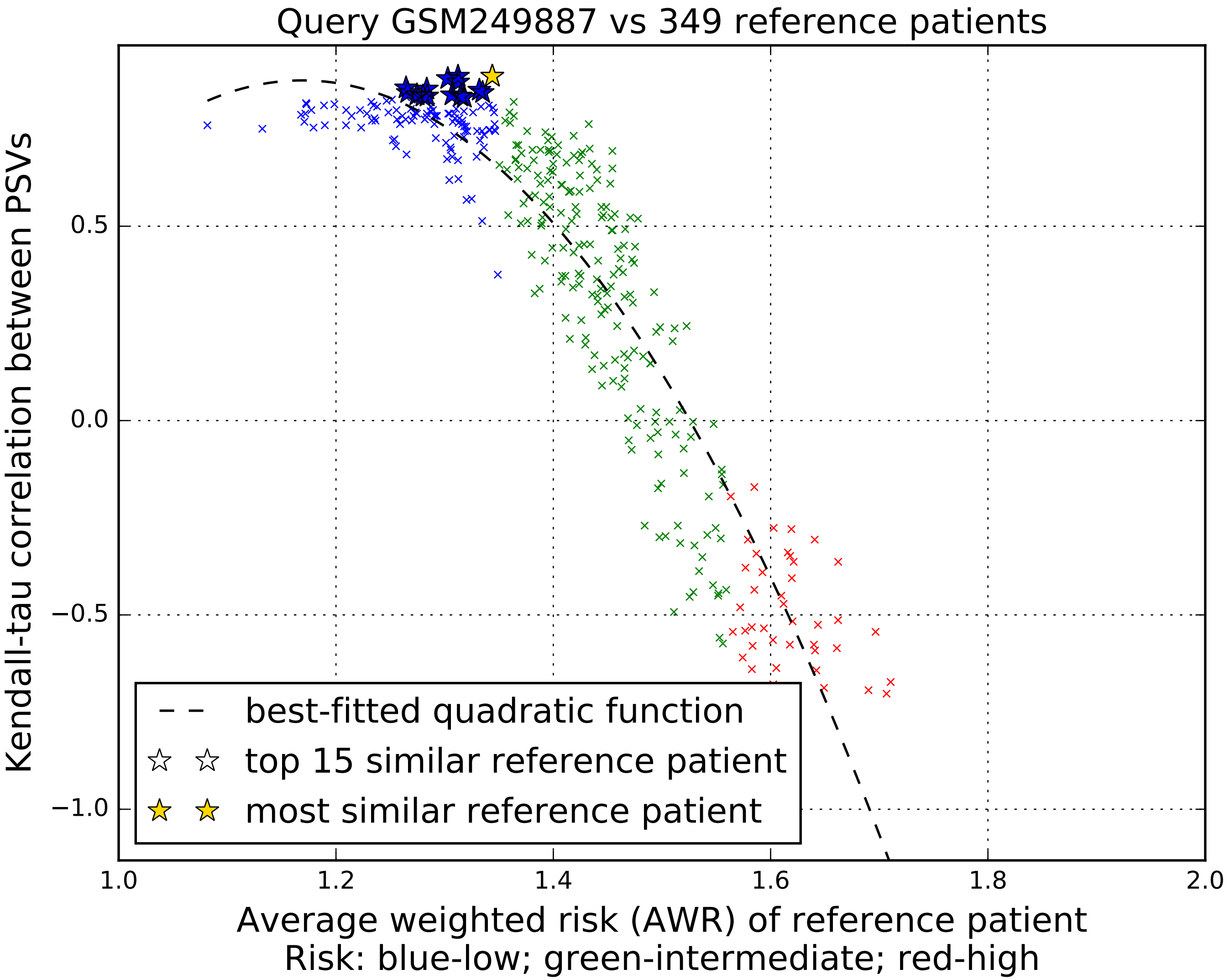

Query GSM249887 vs 349 reference patients
Kendall-tau correlation between PSVs
0.5
0.0
−0.5
−1.0
1.0
1.2
1.4
1.6
1.8
2.0
best-fitted quadratic function
top 15 similar reference patient
most similar reference patient
Average weighted risk (AWR) of reference patient
Risk: blue-low; green-intermediate; red-high

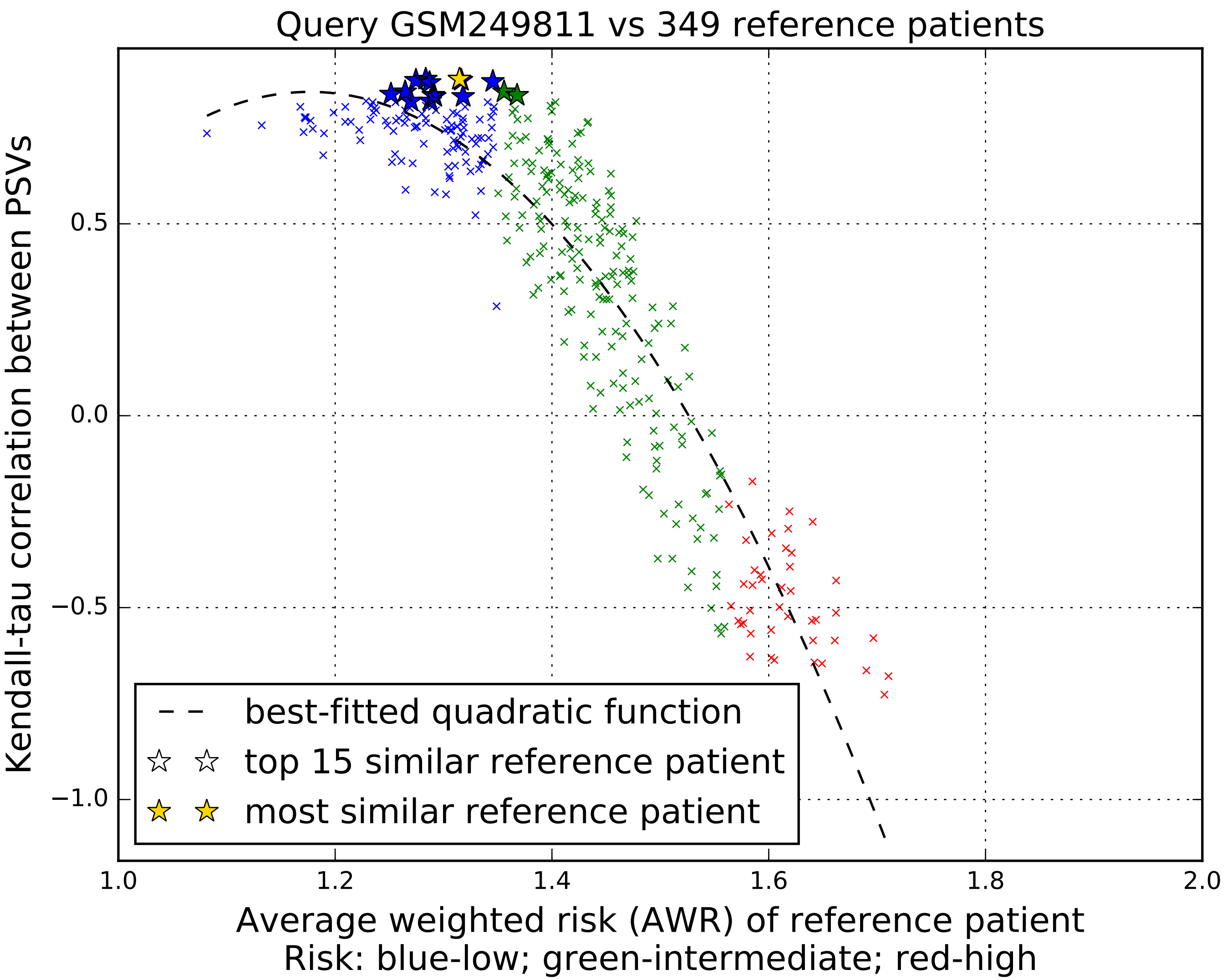

Query GSM249811 vs 349 reference patients
Kendall-tau correlation between PSVs
0.5
0.0
−0.5
−1.0
1.0
1.2
1.4
1.6
1.8
2.0
best-fitted quadratic function
top 15 similar reference patient
most similar reference patient
Average weighted risk (AWR) of reference patient
Risk: blue-low; green-intermediate; red-high

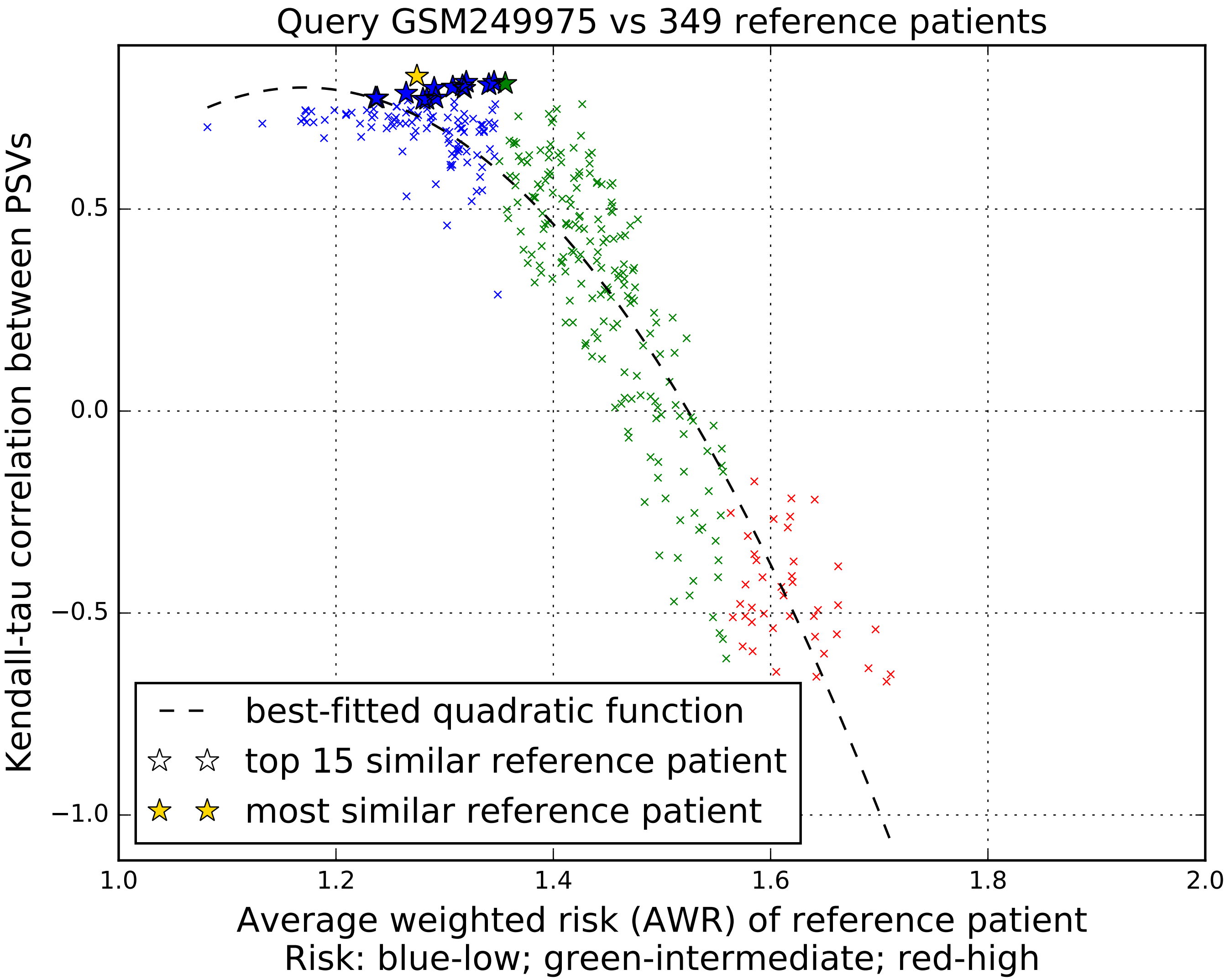
Query GSM249975 vs 349 reference patients
Kendall-tau correlation between PSVs
0.5
0.0
−0.5
−1.0
1.0
1.2
1.4
1.6
1.8
2.0
best-fitted quadratic function
top 15 similar reference patient
most similar reference patient
Average weighted risk (AWR) of reference patient
Risk: blue-low; green-intermediate; red-high

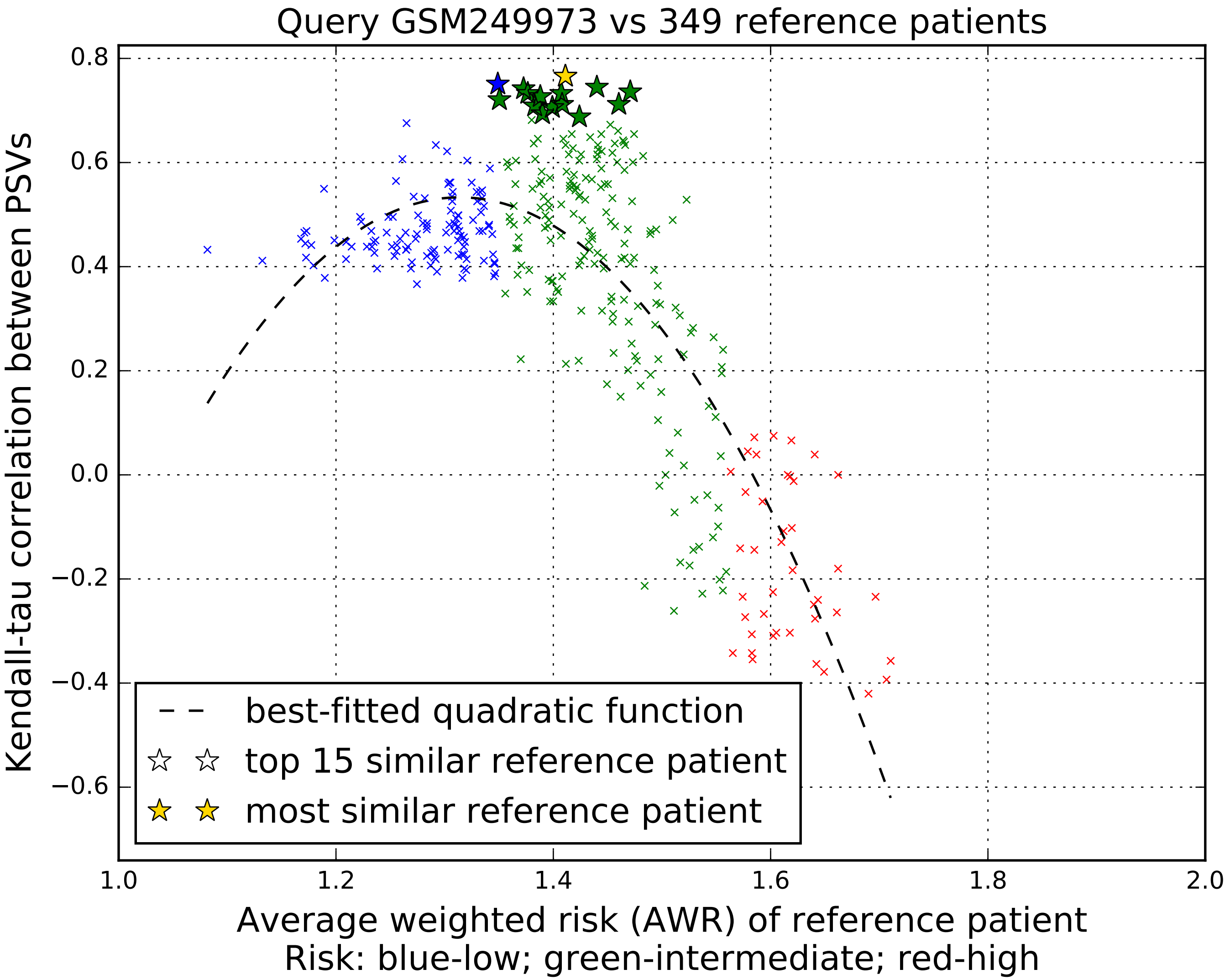
Query GSM249973 vs 349 reference patients
Kendall-tau correlation between PSVs
0.8
0.6
0.4
0.2
0.0
−0.2
−0.4
−0.6
1.0
1.2
1.4
1.6
1.8
2.0
best-fitted quadratic function
top 15 similar reference patient
most similar reference patient
Average weighted risk (AWR) of reference patient
Risk: blue-low; green-intermediate; red-high

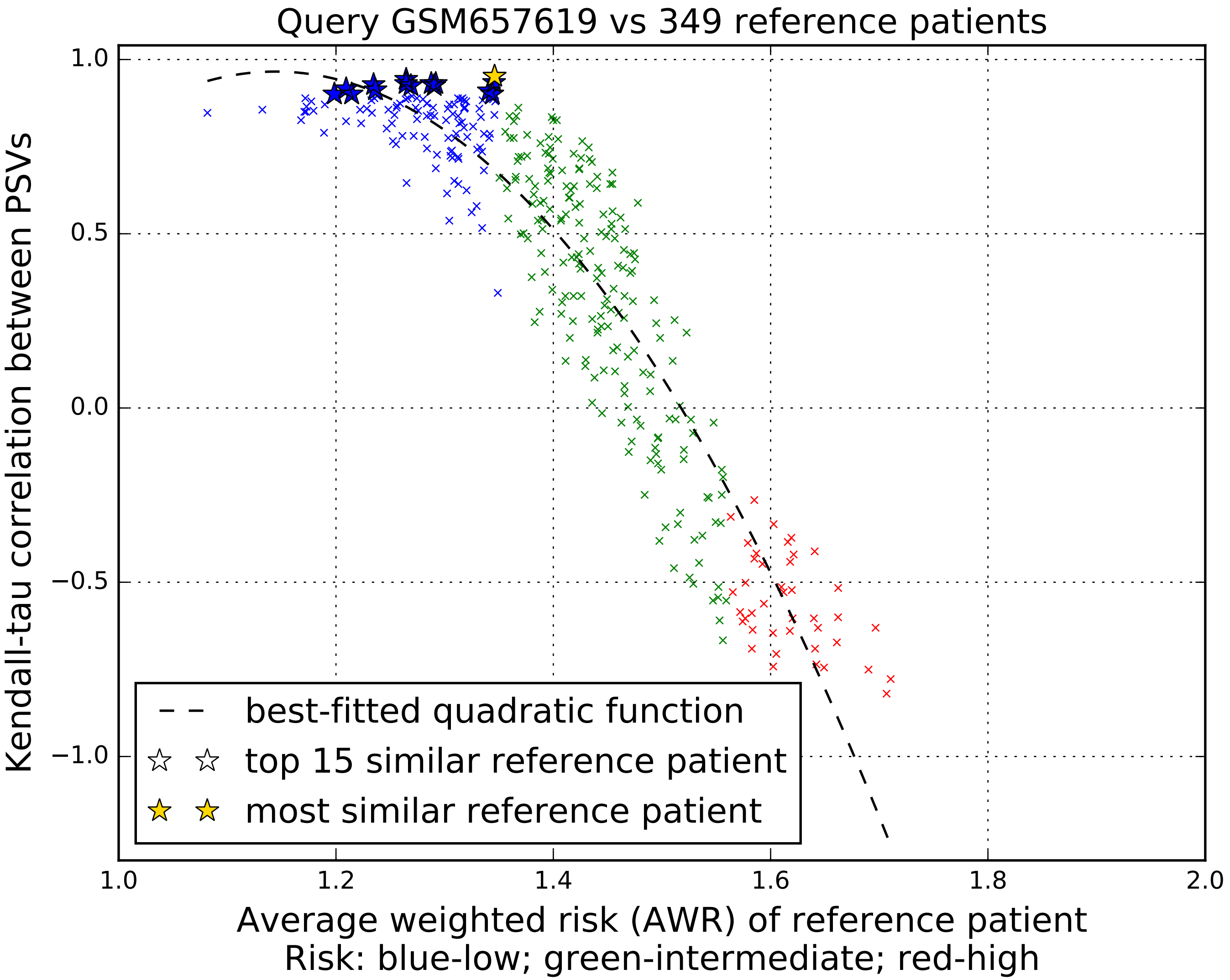

Query GSM657619 vs 349 reference patients
Kendall-tau correlation between PSVs
Average weighted risk (AWR) of reference patient
Risk: blue-low; green-intermediate; red-high
best-fitted quadratic function
top 15 similar reference patient
most similar reference patient
1.0
0.5
0.0
−0.5
−1.0
1.0
1.2
1.4
1.6
1.8
2.0

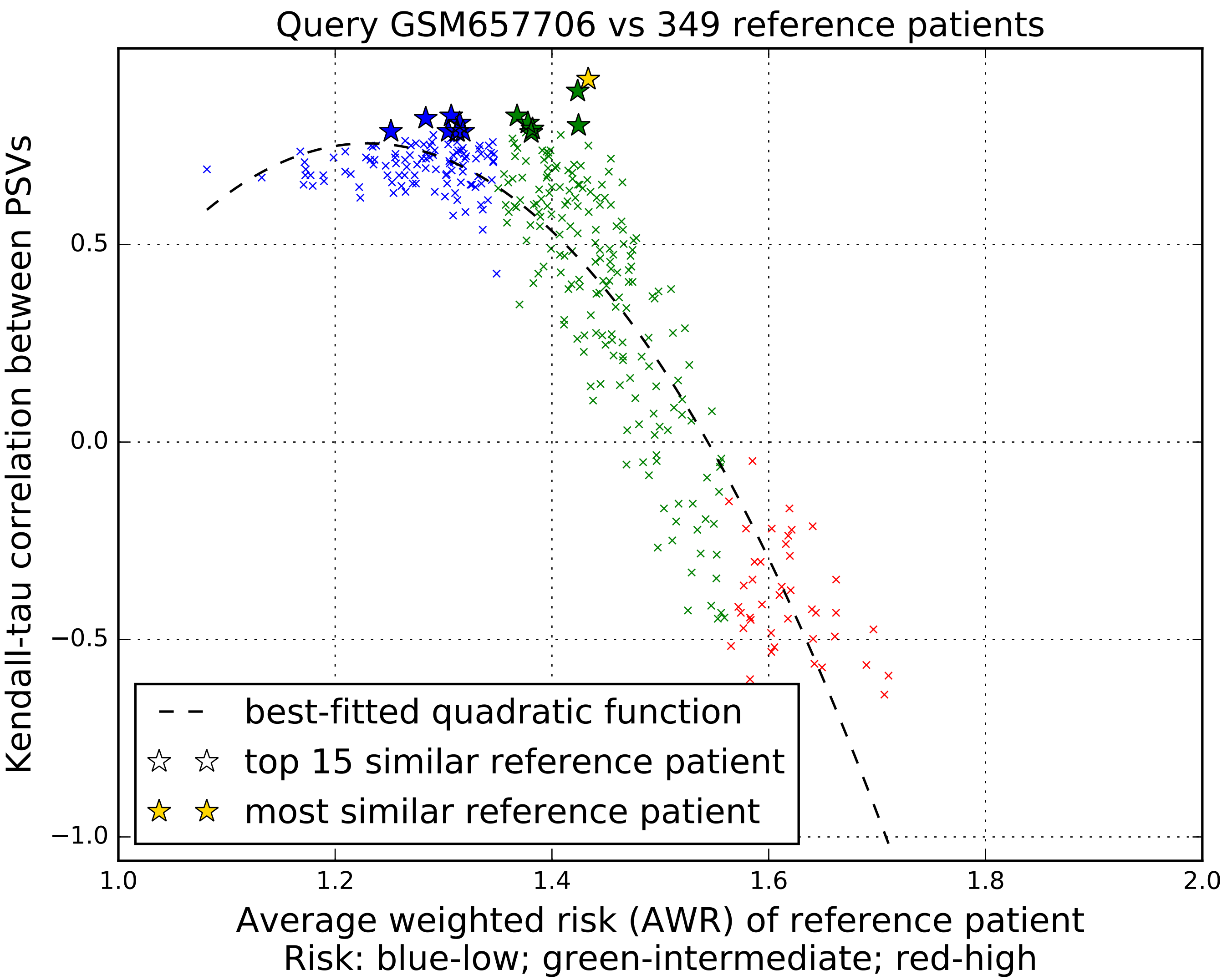
Query GSM657706 vs 349 reference patients
Kendall-tau correlation between PSVs
0.5
0.0
−0.5
−1.0
1.0
1.2
1.4
1.6
1.8
2.0
Average weighted risk (AWR) of reference patient
Risk: blue-low; green-intermediate; red-high
best-fitted quadratic function
top 15 similar reference patient
most similar reference patient

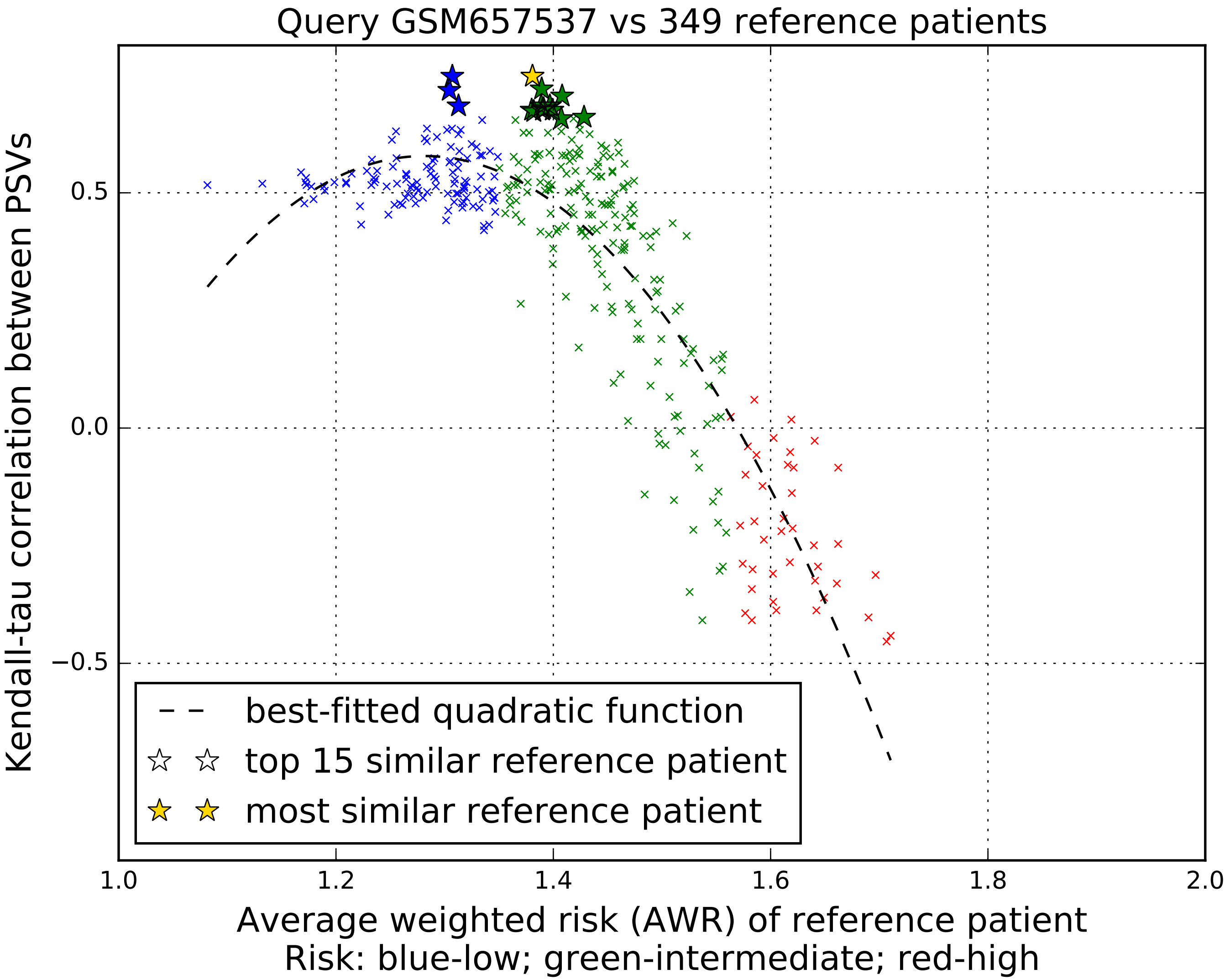

Query GSM657537 vs 349 reference patients
Kendall-tau correlation between PSVs
0.5
0.0
−0.5
1.0
1.2
1.4
1.6
1.8
2.0
best-fitted quadratic function
top 15 similar reference patient
most similar reference patient
Average weighted risk (AWR) of reference patient
Risk: blue-low; green-intermediate; red-high

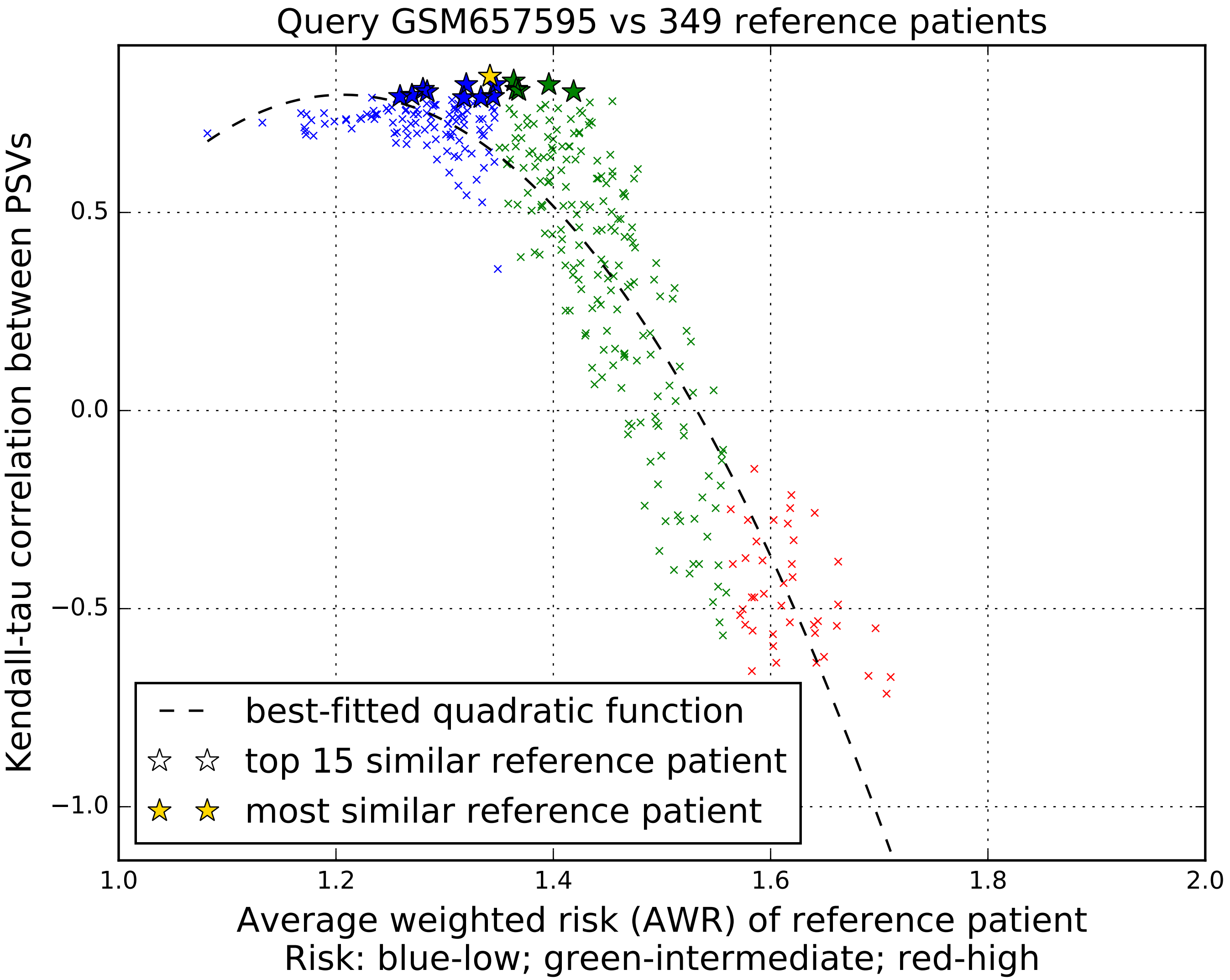

Query GSM657595 vs 349 reference patients
Kendall-tau correlation between PSVs
0.5
0.0
−0.5
−1.0
1.0
1.2
1.4
1.6
1.8
2.0
best-fitted quadratic function
top 15 similar reference patient
most similar reference patient
Average weighted risk (AWR) of reference patient
Risk: blue-low; green-intermediate; red-high

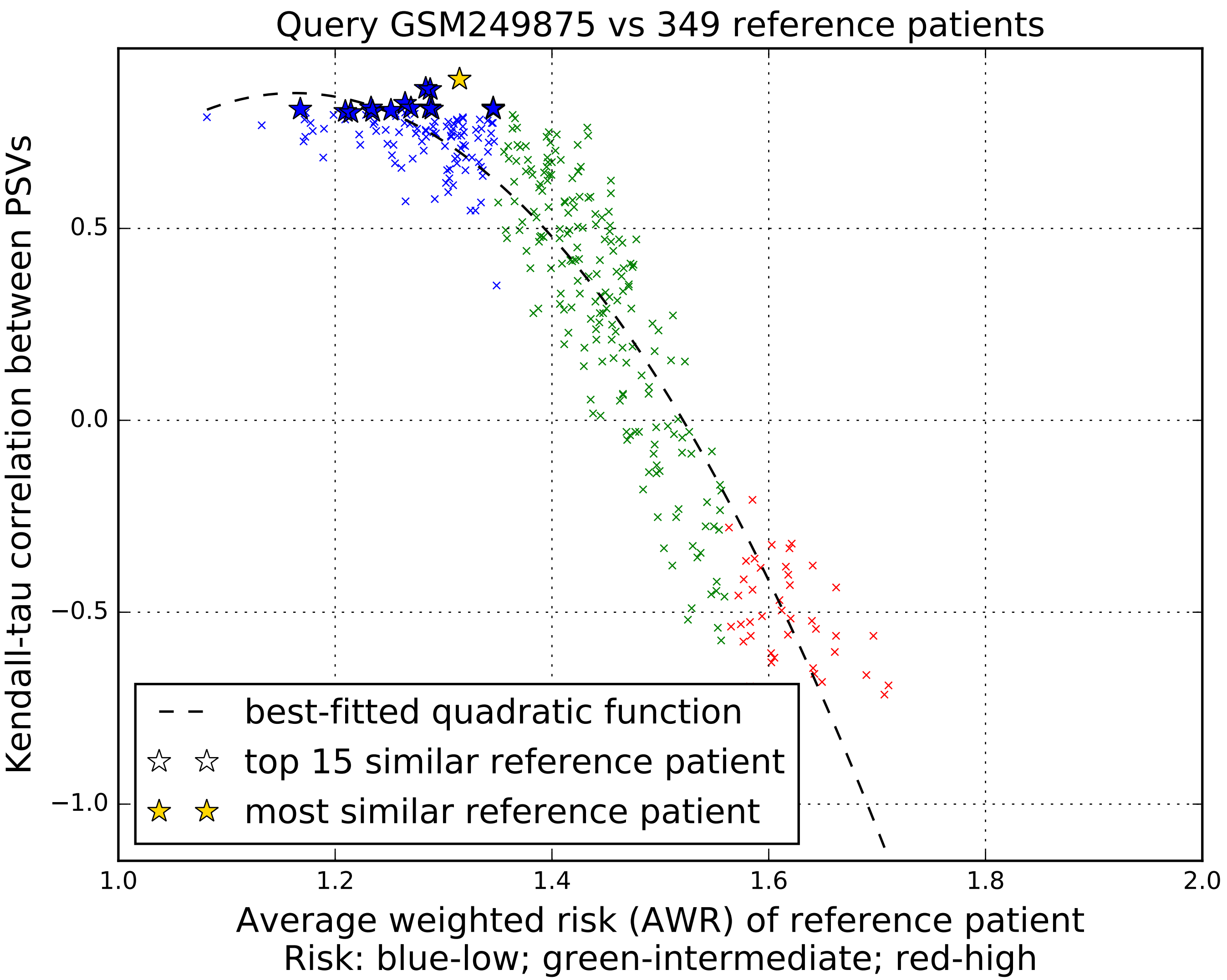

Query GSM249875 vs 349 reference patients
Kendall-tau correlation between PSVs
0.5
0.0
−0.5
−1.0
1.0
1.2
1.4
1.6
1.8
2.0
Average weighted risk (AWR) of reference patient
Risk: blue-low; green-intermediate; red-high
best-fitted quadratic function
top 15 similar reference patient
most similar reference patient

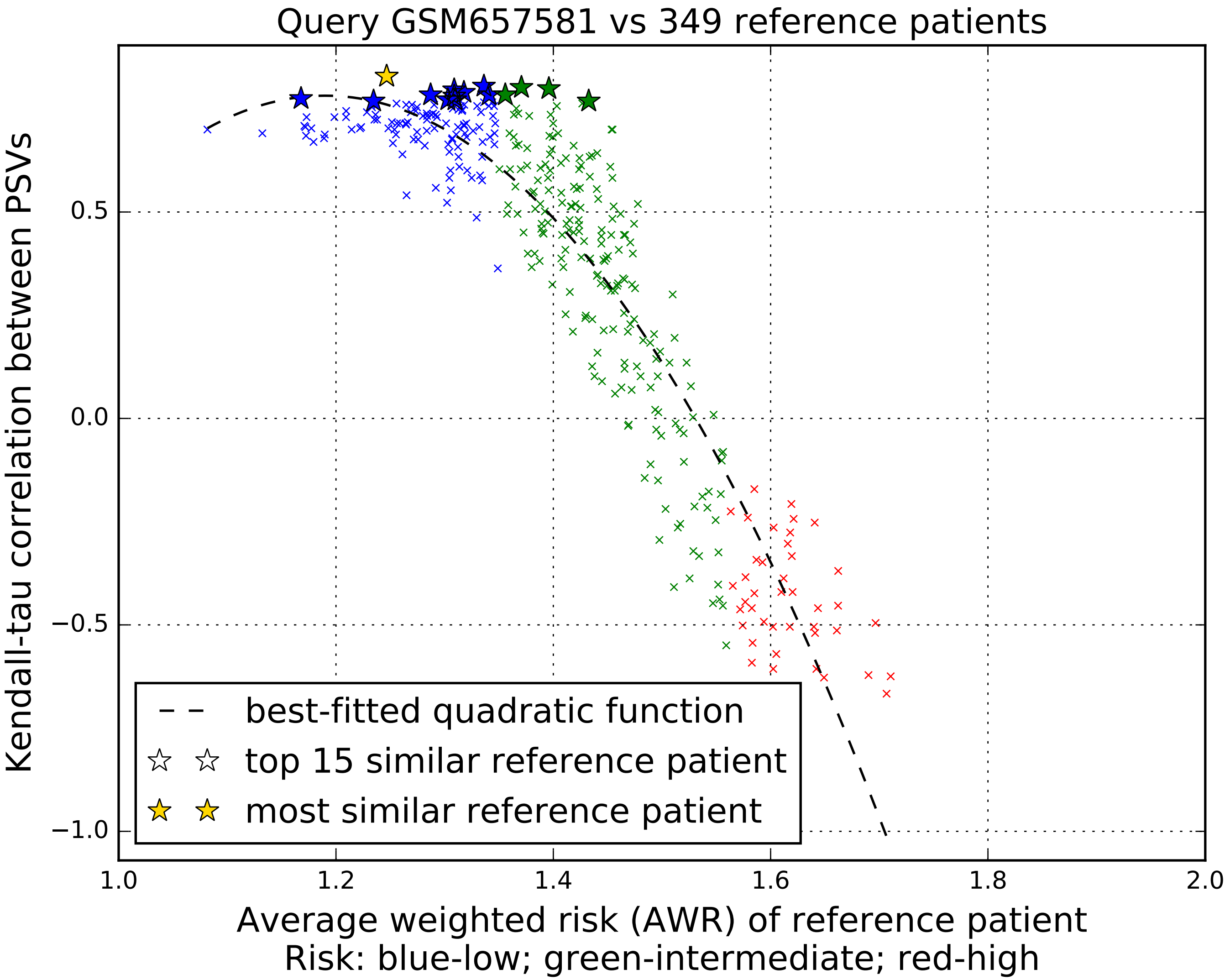
Query GSM657581 vs 349 reference patients
Kendall-tau correlation between PSVs
0.5
0.0
−0.5
−1.0
1.0
1.2
1.4
1.6
1.8
2.0
best-fitted quadratic function
top 15 similar reference patient
most similar reference patient
Average weighted risk (AWR) of reference patient
Risk: blue-low; green-intermediate; red-high

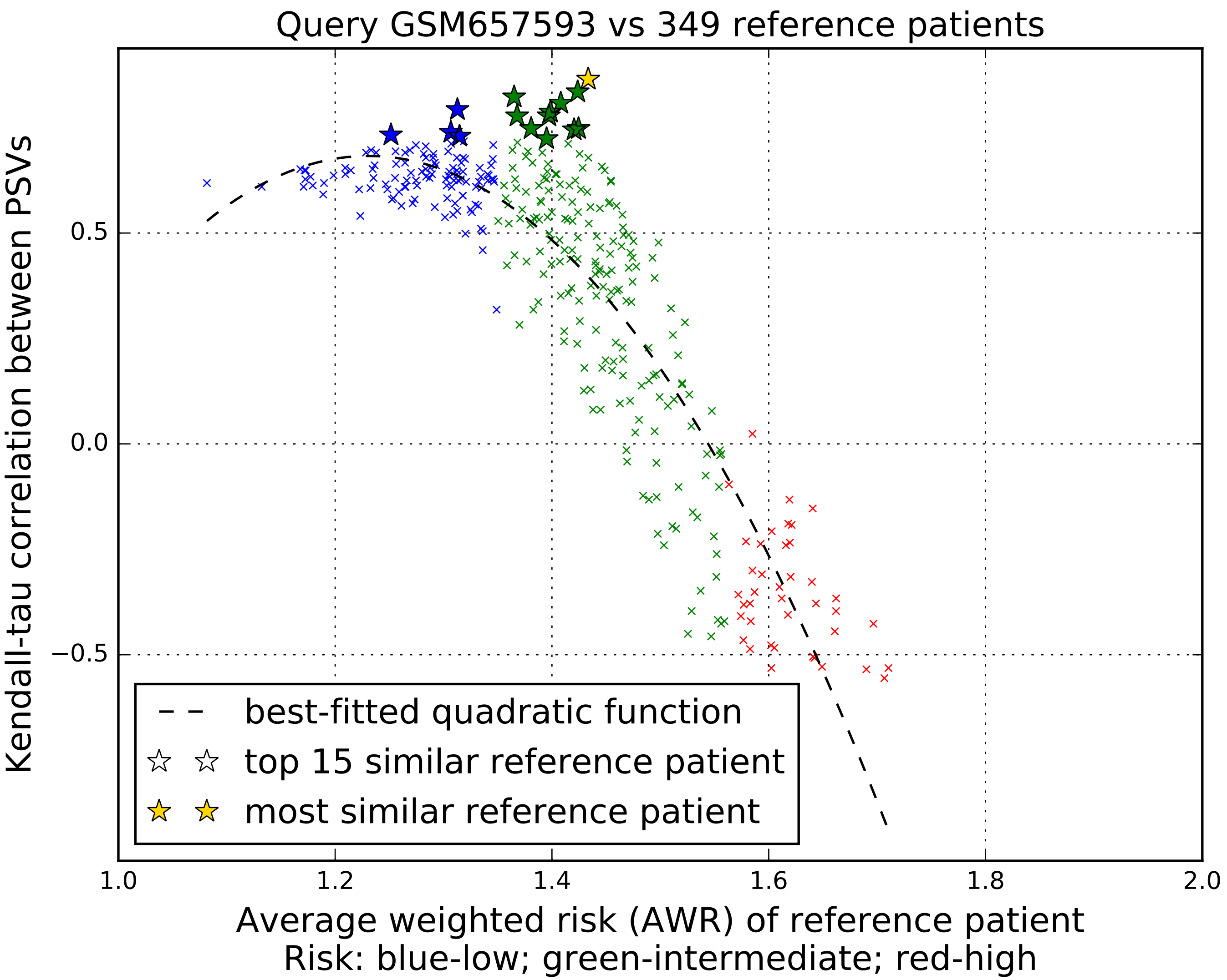

Query GSM657593 vs 349 reference patients
Kendall-tau correlation between PSVs
0.5
0.0
−0.5
1.0
1.2
1.4
1.6
1.8
2.0
best-fitted quadratic function
top 15 similar reference patient
most similar reference patient
Average weighted risk (AWR) of reference patient
Risk: blue-low; green-intermediate; red-high

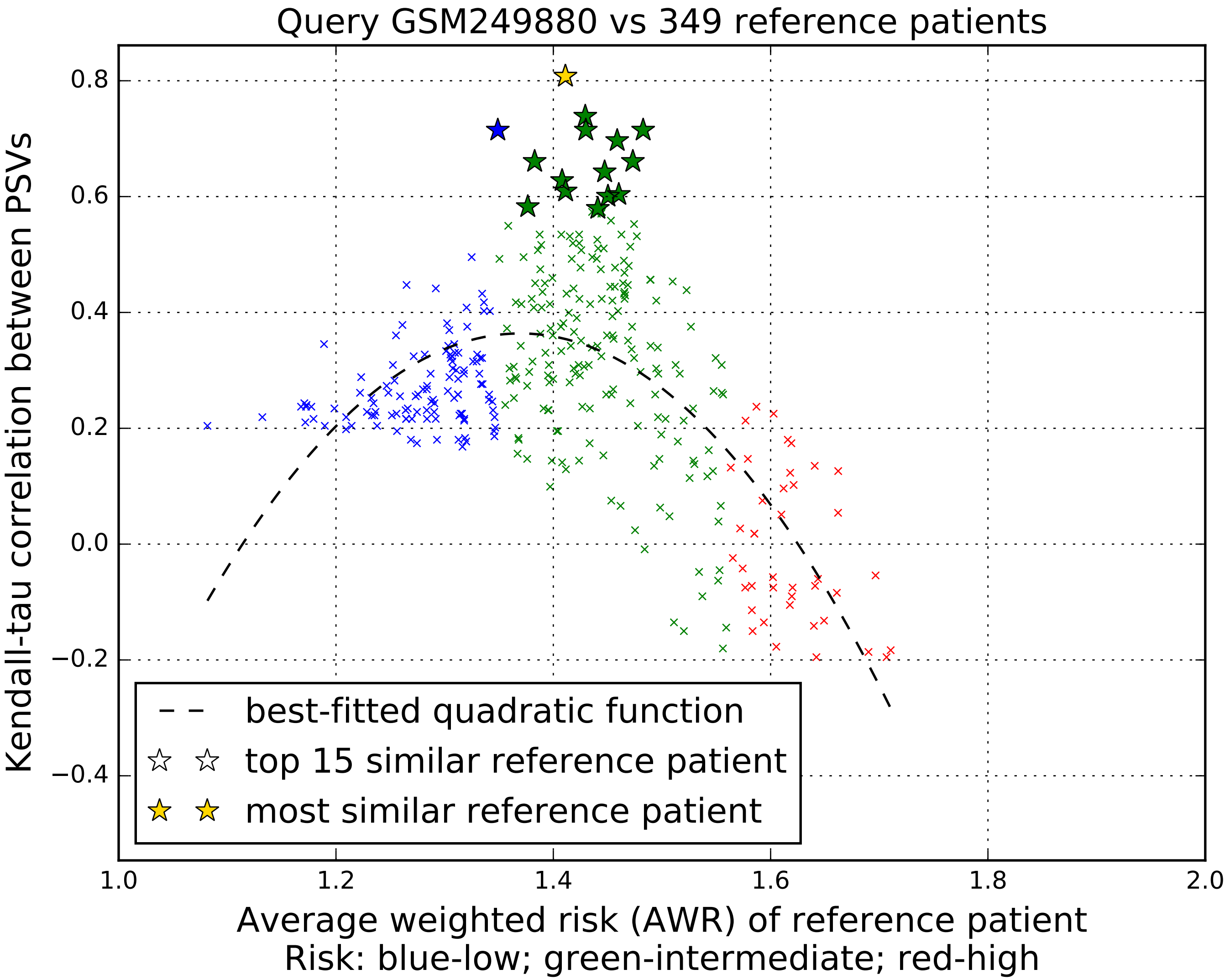
Query GSM249880 vs 349 reference patients
Kendall-tau correlation between PSVs
0.8
0.6
0.4
0.2
0.0
−0.2
−0.4
1.0
1.2
1.4
1.6
1.8
2.0
best-fitted quadratic function
top 15 similar reference patient
most similar reference patient
Average weighted risk (AWR) of reference patient
Risk: blue-low; green-intermediate; red-high

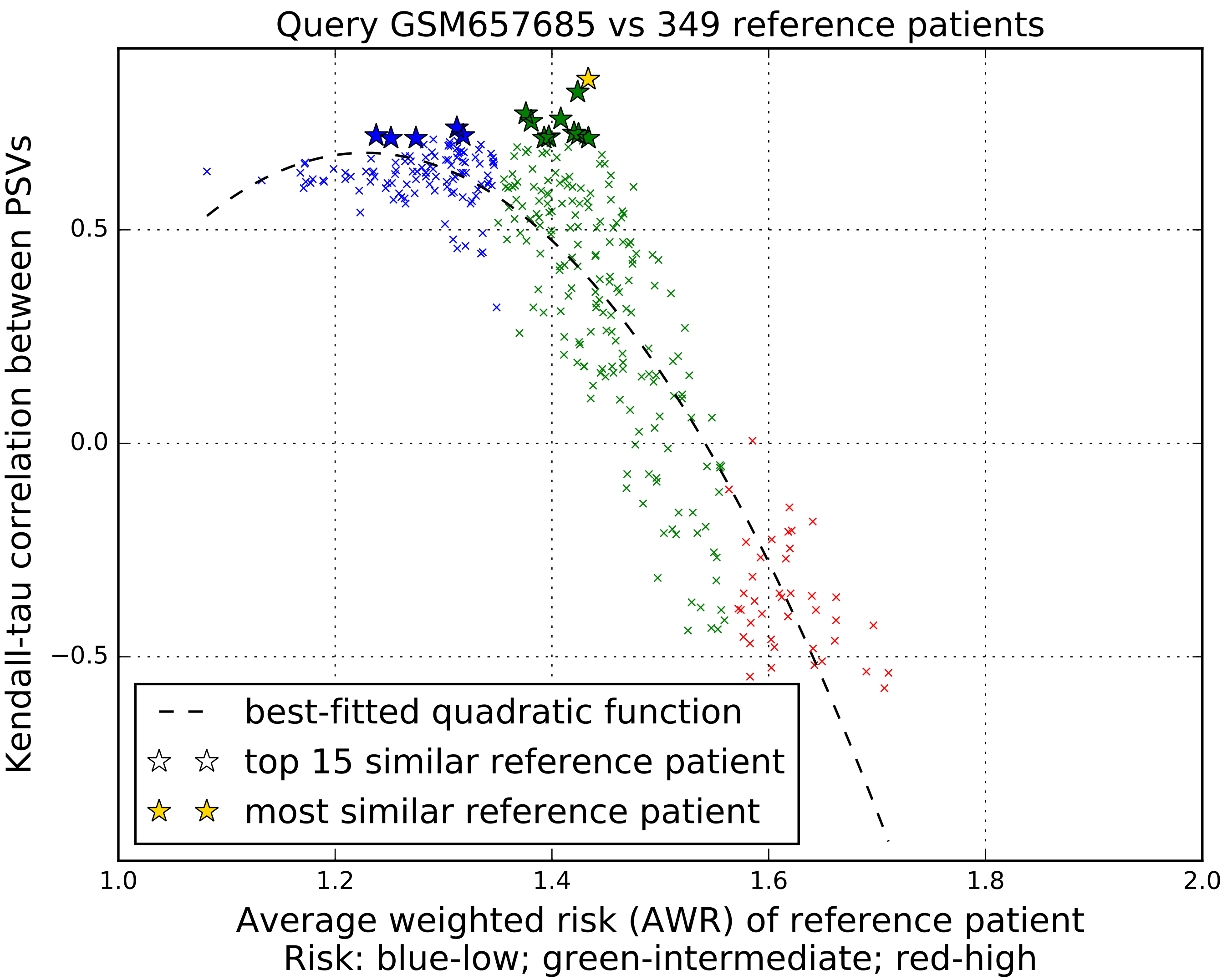
Query GSM657685 vs 349 reference patients
Kendall-tau correlation between PSVs
0.5
0.0
−0.5
1.0
1.2
1.4
1.6
1.8
2.0
best-fitted quadratic function
top 15 similar reference patient
most similar reference patient
Average weighted risk (AWR) of reference patient
Risk: blue-low; green-intermediate; red-high

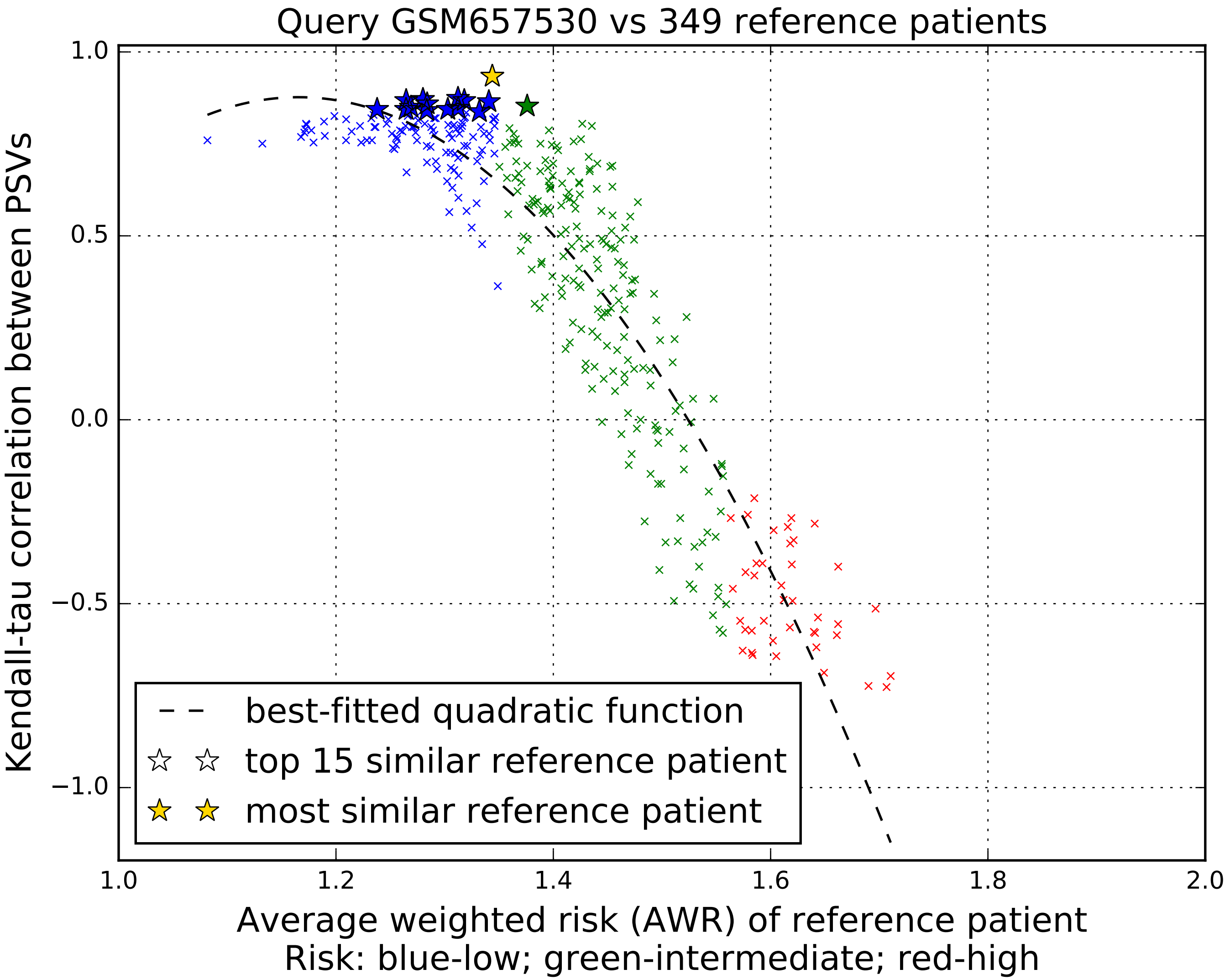
Query GSM657530 vs 349 reference patients
Kendall-tau correlation between PSVs
1.0
0.5
0.0
−0.5
−1.0
1.0
1.2
1.4
1.6
1.8
2.0
best-fitted quadratic function
top 15 similar reference patient
most similar reference patient
Average weighted risk (AWR) of reference patient
Risk: blue-low; green-intermediate; red-high

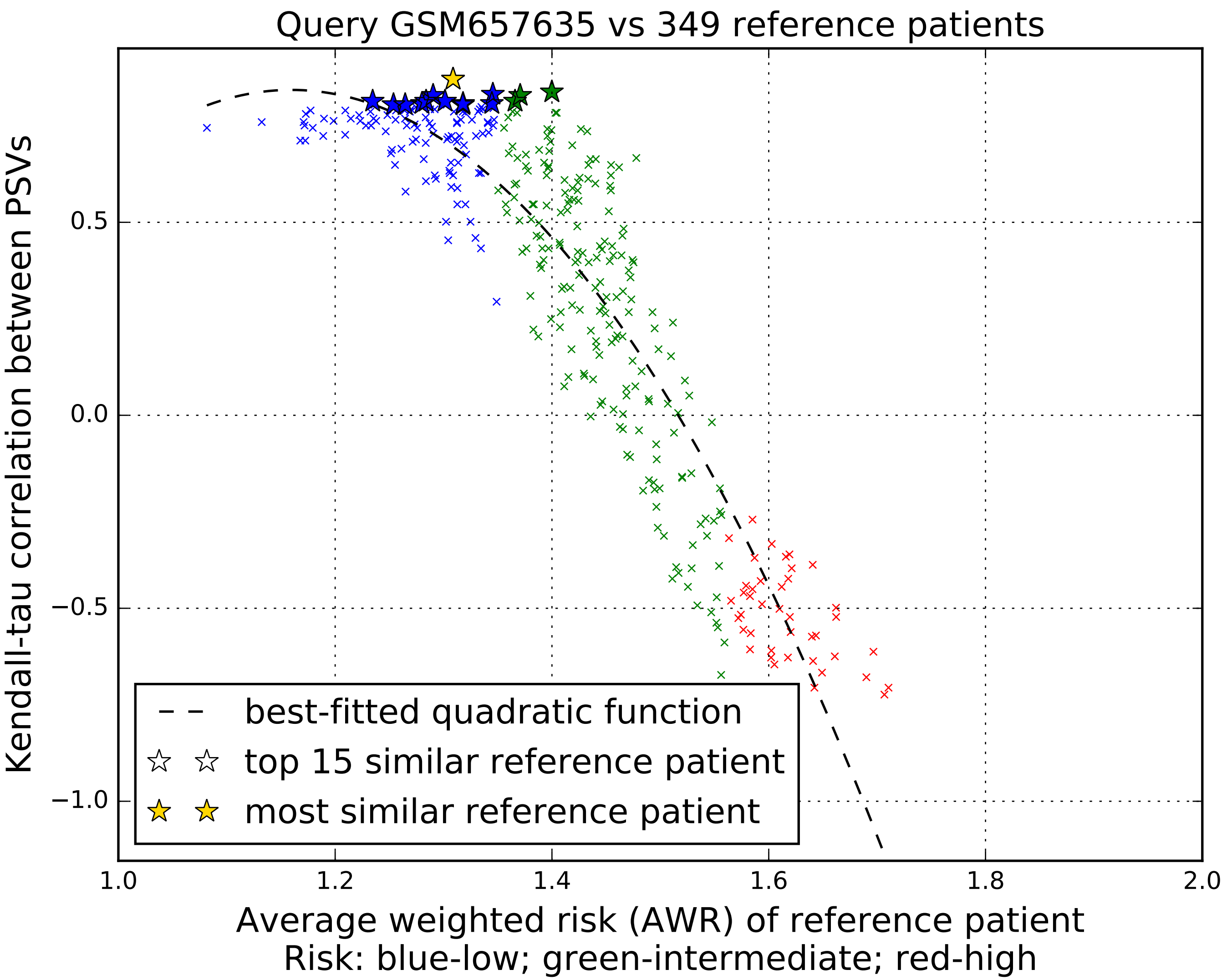
Query GSM657635 vs 349 reference patients
Kendall-tau correlation between PSVs
0.5
0.0
−0.5
−1.0
1.0
1.2
1.4
1.6
1.8
2.0
best-fitted quadratic function
top 15 similar reference patient
most similar reference patient
Average weighted risk (AWR) of reference patient
Risk: blue-low; green-intermediate; red-high

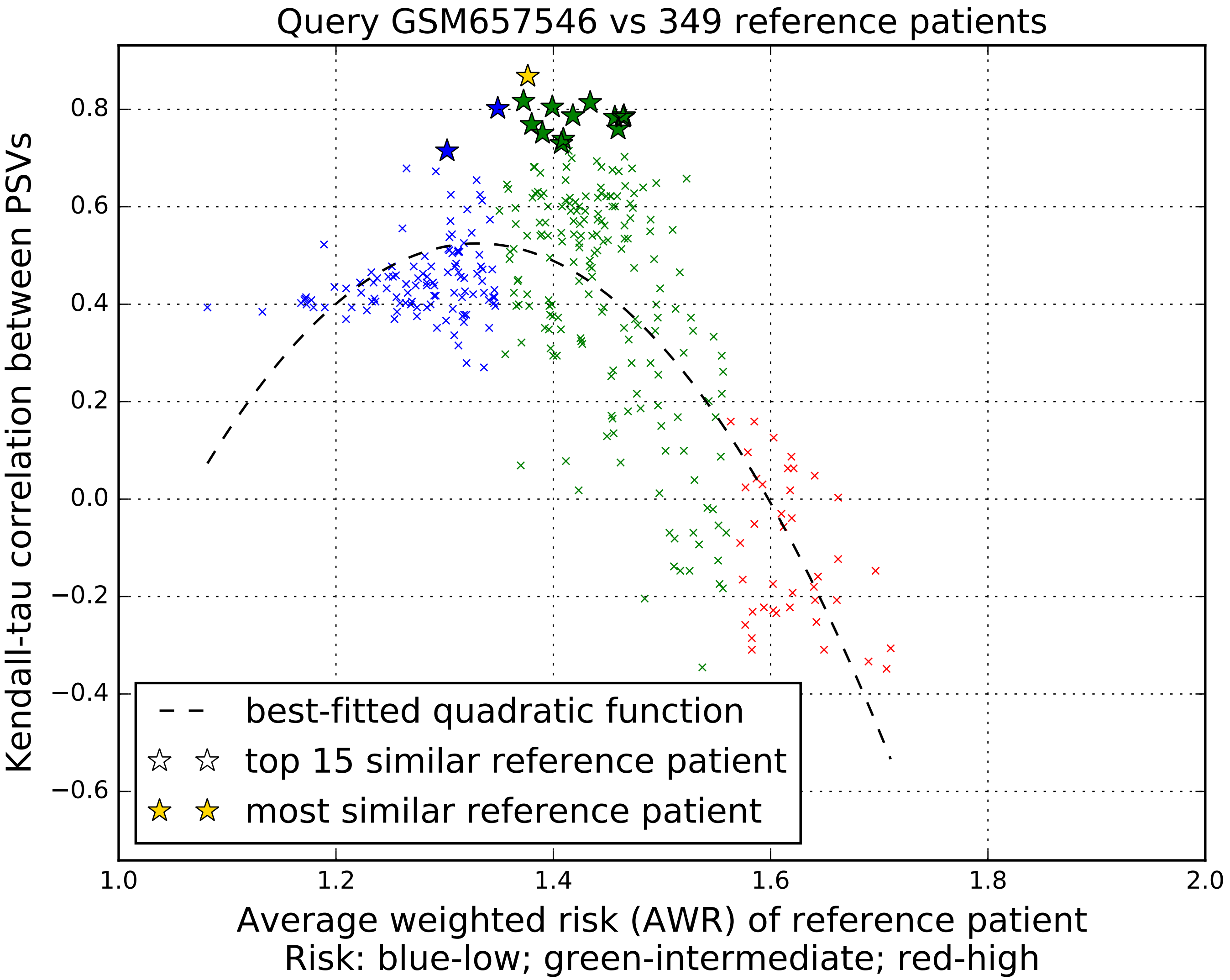

Query GSM657546 vs 349 reference patients
Kendall-tau correlation between PSVs
0.8
0.6
0.4
0.2
0.0
−0.2
−0.4
−0.6
1.0
1.2
1.4
1.6
1.8
2.0
best-fitted quadratic function
top 15 similar reference patient
most similar reference patient
Average weighted risk (AWR) of reference patient
Risk: blue-low; green-intermediate; red-high

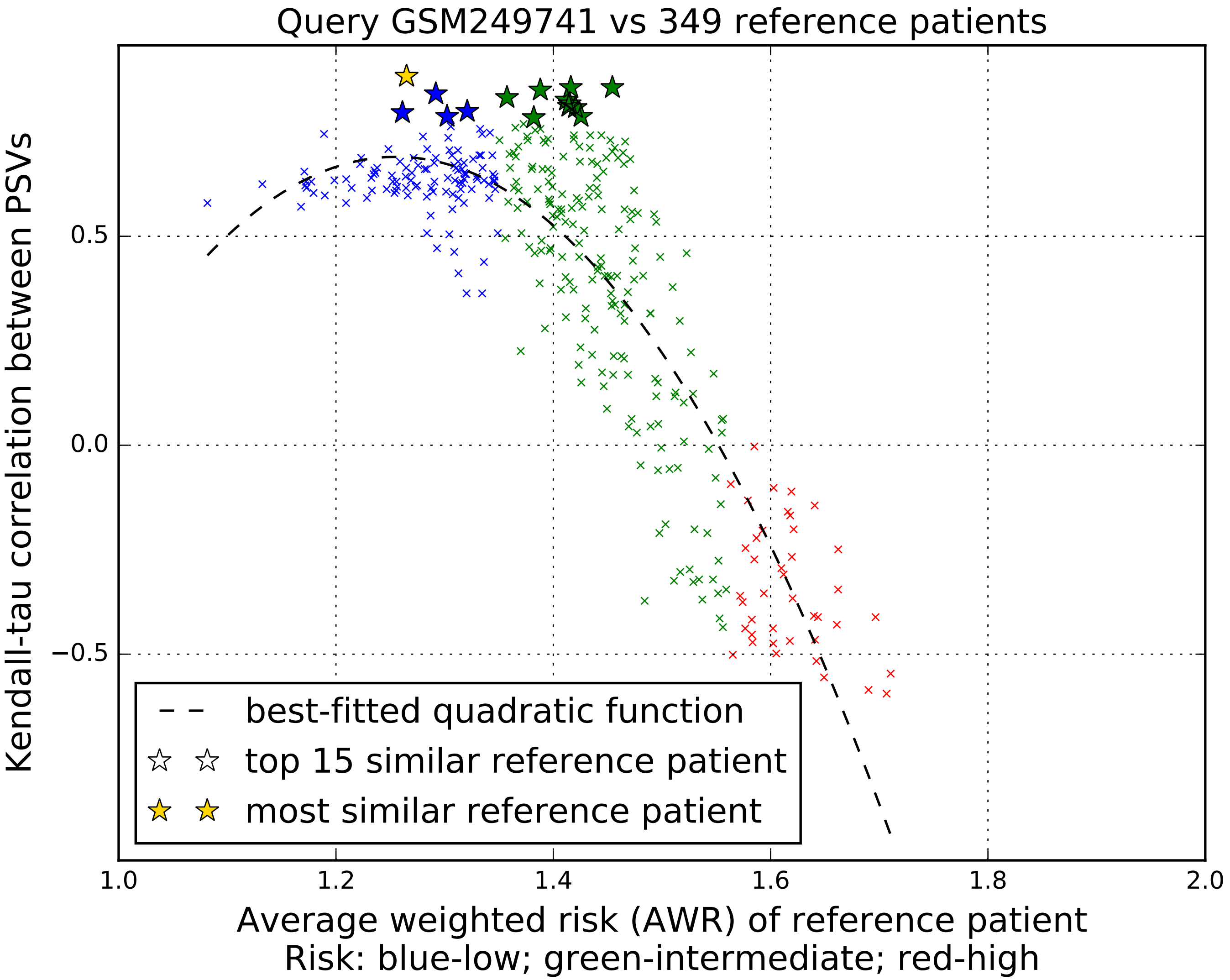
Query GSM249741 vs 349 reference patients
Kendall-tau correlation between PSVs
0.5
0.0
−0.5
1.0
1.2
1.4
1.6
1.8
2.0
best-fitted quadratic function
top 15 similar reference patient
most similar reference patient
Average weighted risk (AWR) of reference patient
Risk: blue-low; green-intermediate; red-high

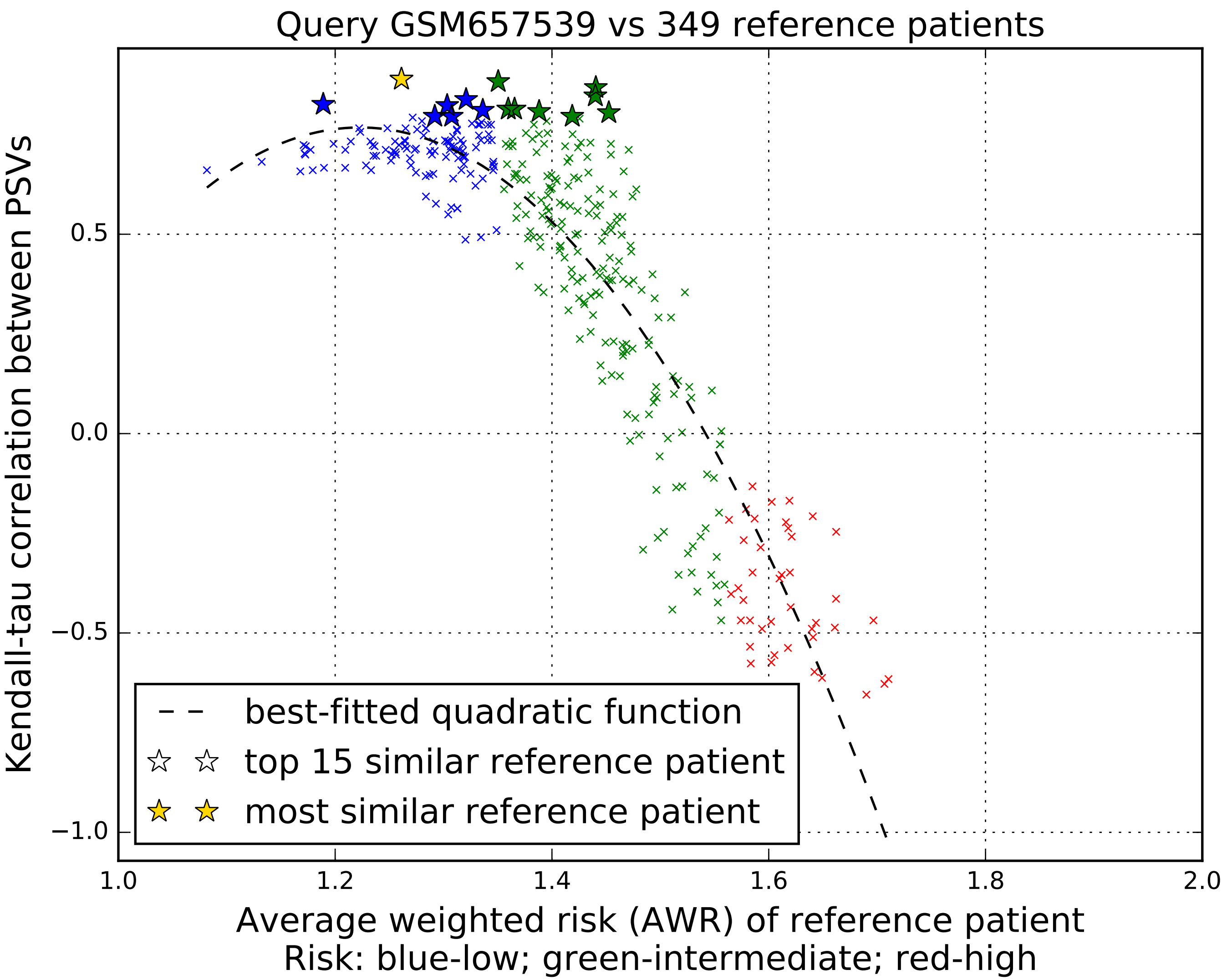

Query GSM657539 vs 349 reference patients
Kendall-tau correlation between PSVs
0.5
0.0
−0.5
−1.0
1.0
1.2
1.4
1.6
1.8
2.0
best-fitted quadratic function
top 15 similar reference patient
most similar reference patient
Average weighted risk (AWR) of reference patient
Risk: blue-low; green-intermediate; red-high

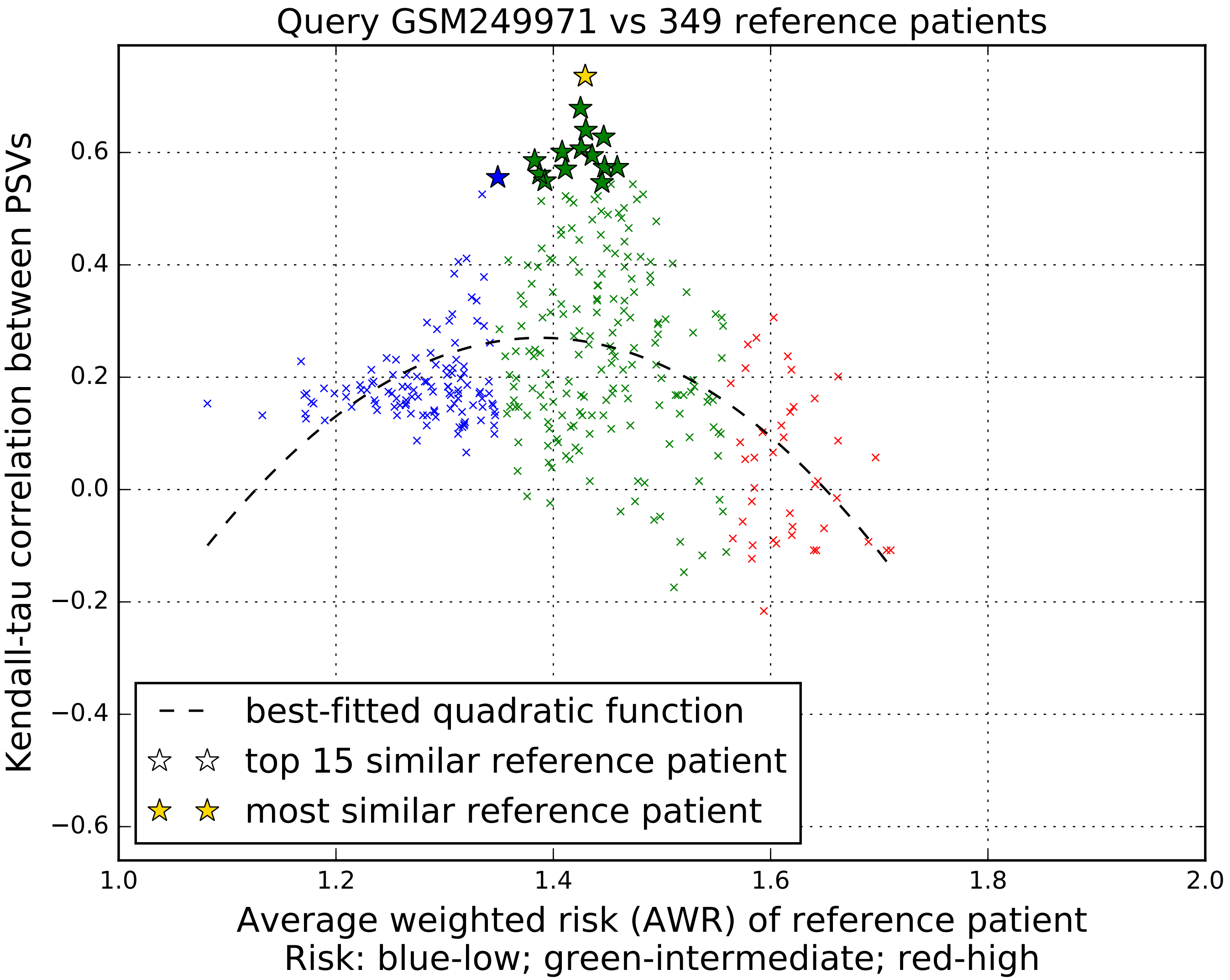
Query GSM249971 vs 349 reference patients
Kendall-tau correlation between PSVs
0.6
0.4
0.2
0.0
−0.2
−0.4
−0.6
1.0
1.2
1.4
1.6
1.8
2.0
best-fitted quadratic function
top 15 similar reference patient
most similar reference patient
Average weighted risk (AWR) of reference patient
Risk: blue-low; green-intermediate; red-high

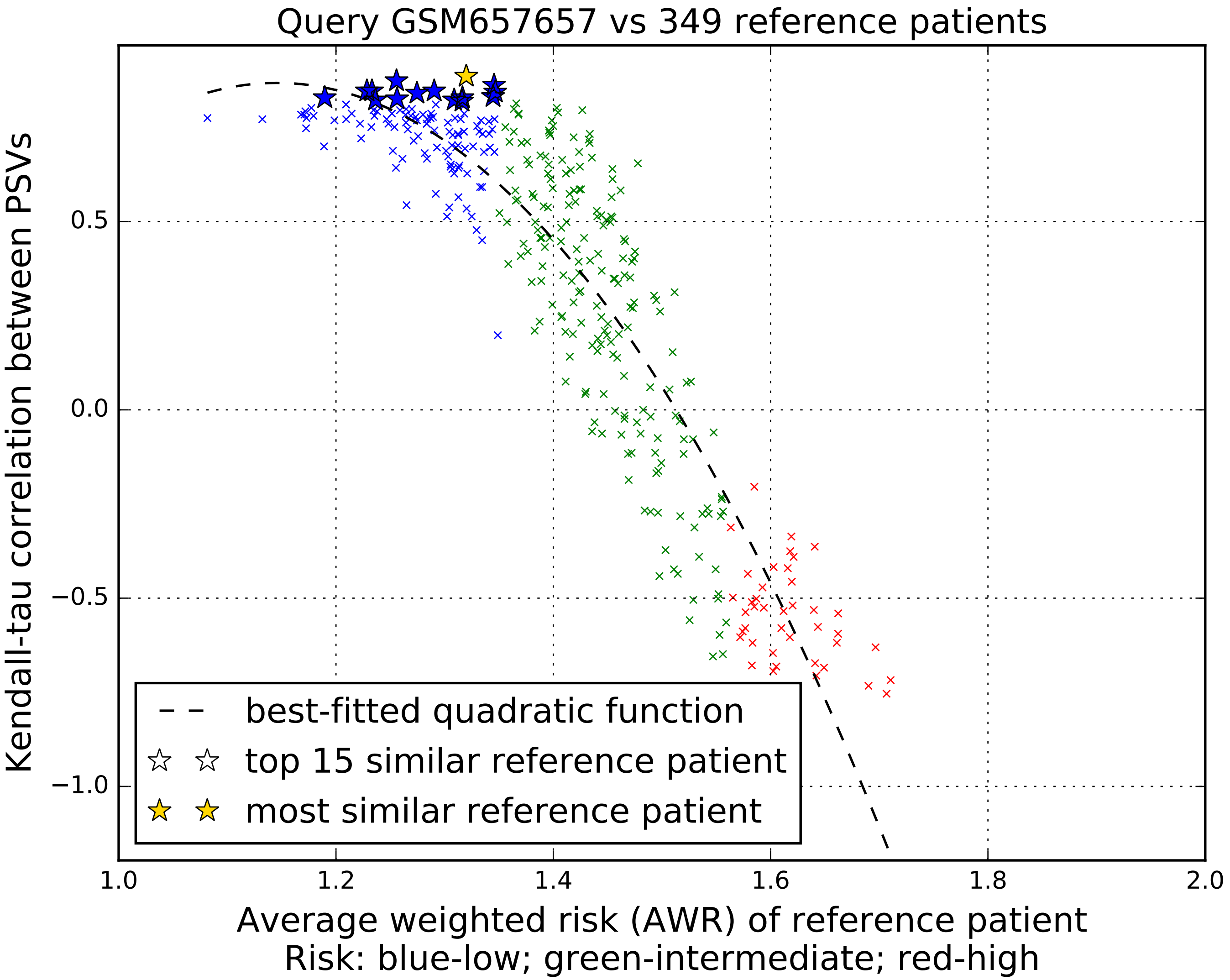

Query GSM657657 vs 349 reference patients
Kendall-tau correlation between PSVs
Average weighted risk (AWR) of reference patient
Risk: blue-low; green-intermediate; red-high
best-fitted quadratic function
top 15 similar reference patient
most similar reference patient
0.5
0.0
−0.5
−1.0
1.0
1.2
1.4
1.6
1.8
2.0

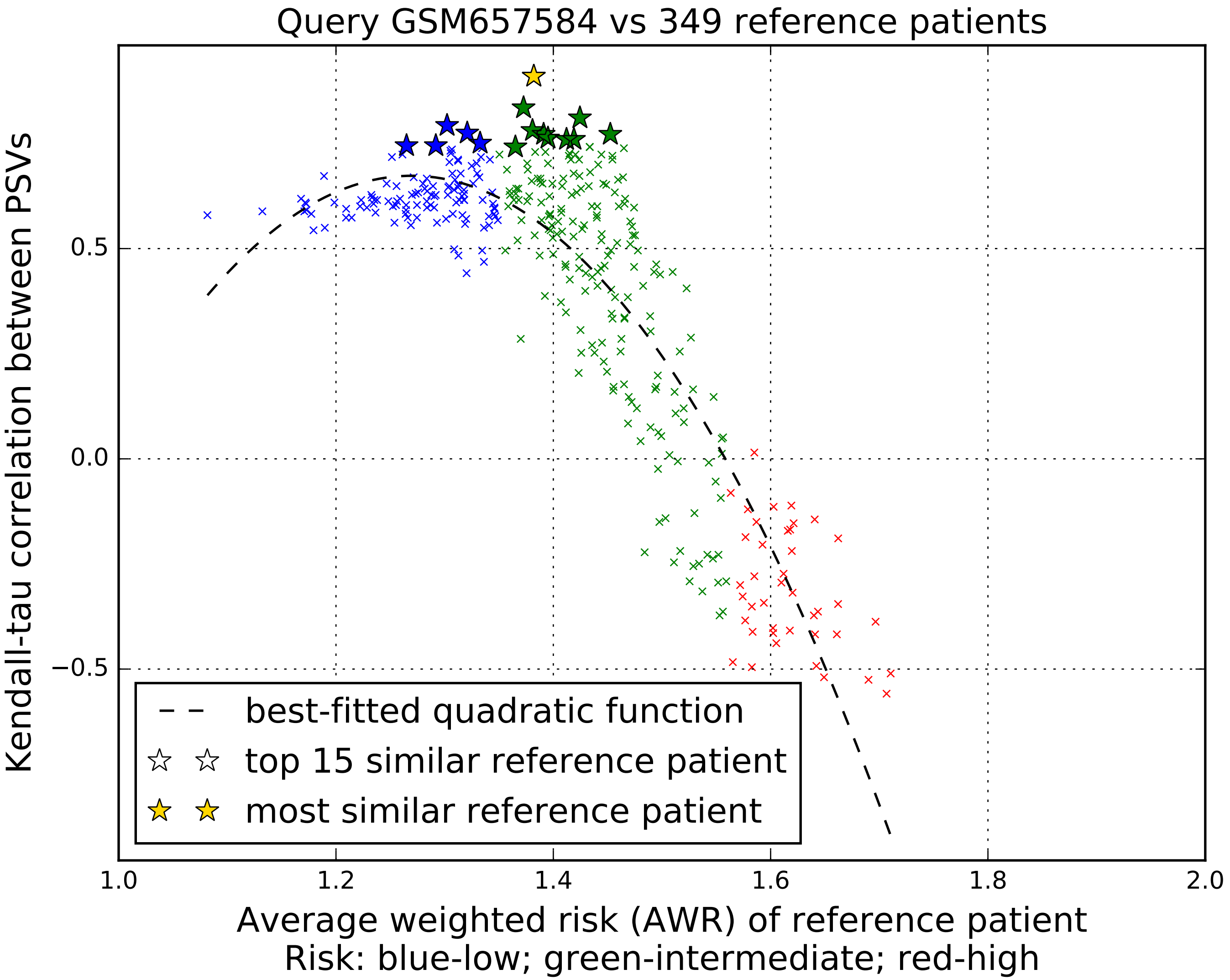
Query GSM657584 vs 349 reference patients
Kendall-tau correlation between PSVs
0.5
0.0
−0.5
1.0
1.2
1.4
1.6
1.8
2.0
best-fitted quadratic function
top 15 similar reference patient
most similar reference patient
Average weighted risk (AWR) of reference patient
Risk: blue-low; green-intermediate; red-high

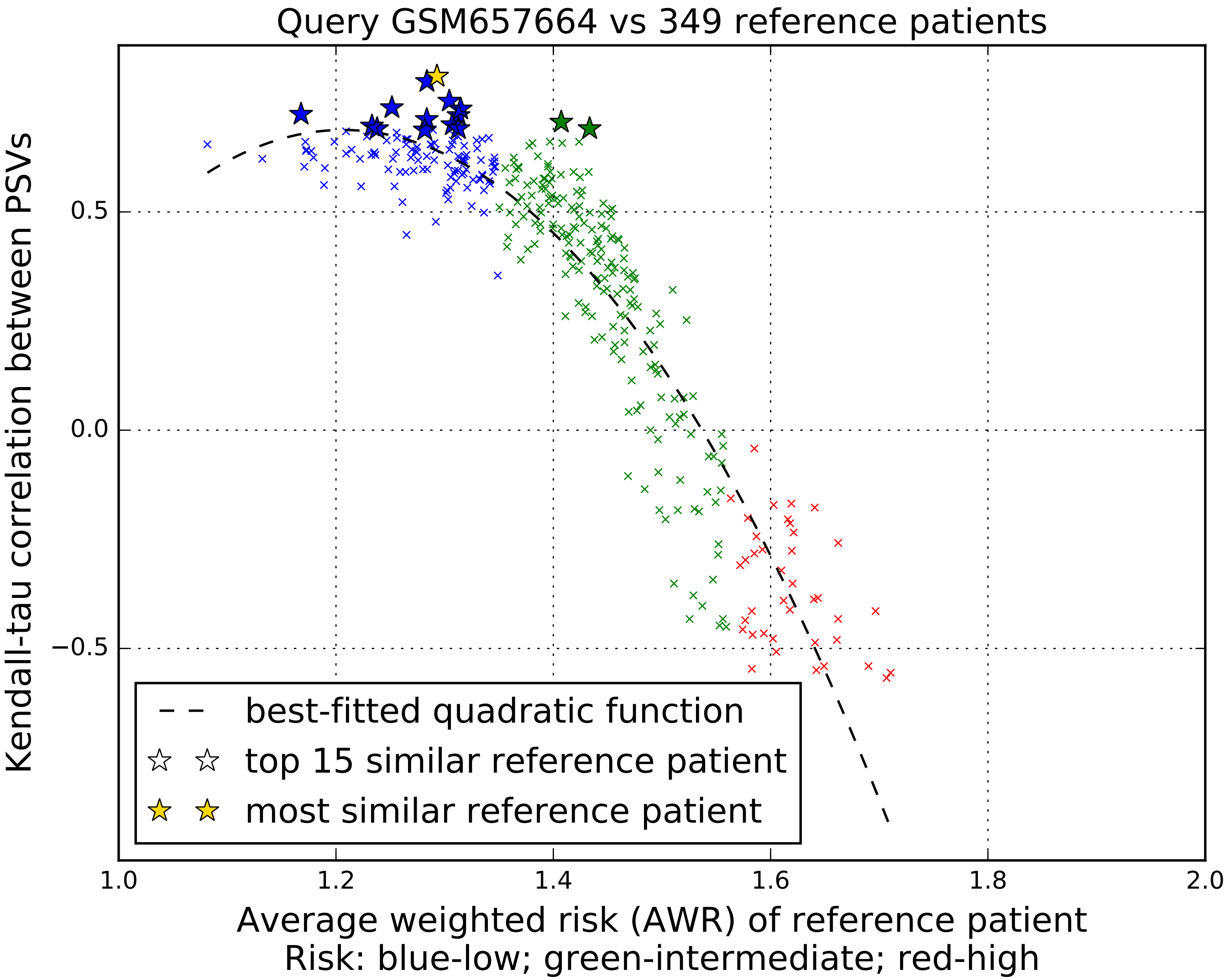

Query GSM657664 vs 349 reference patients
Kendall-tau correlation between PSVs
0.5
0.0
−0.5
1.0
1.2
1.4
1.6
1.8
2.0
best-fitted quadratic function
top 15 similar reference patient
most similar reference patient
Average weighted risk (AWR) of reference patient
Risk: blue-low; green-intermediate; red-high

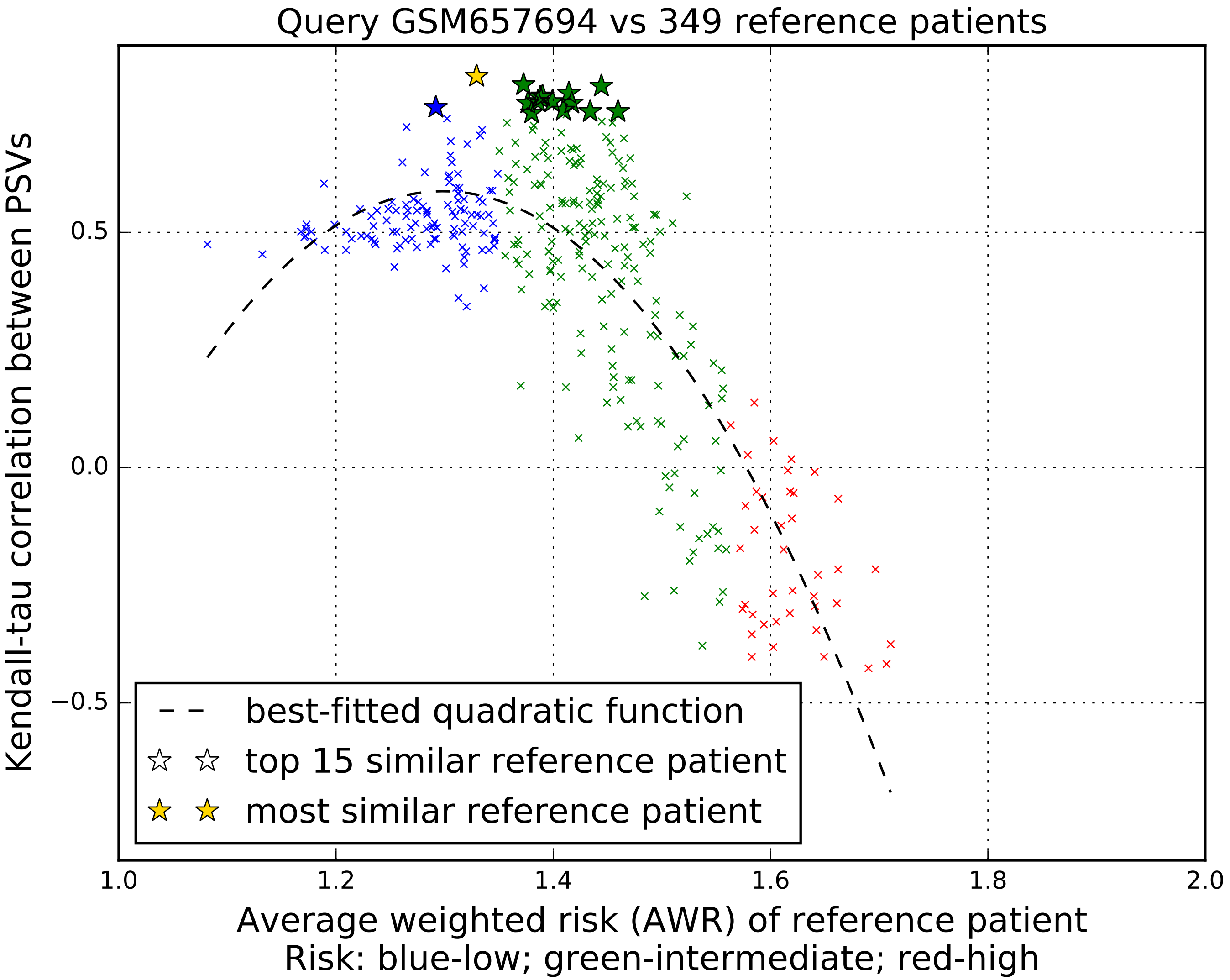

Query GSM657694 vs 349 reference patients
Kendall-tau correlation between PSVs
0.5
0.0
−0.5
1.0
1.2
1.4
1.6
1.8
2.0
best-fitted quadratic function
top 15 similar reference patient
most similar reference patient
Average weighted risk (AWR) of reference patient
Risk: blue-low; green-intermediate; red-high

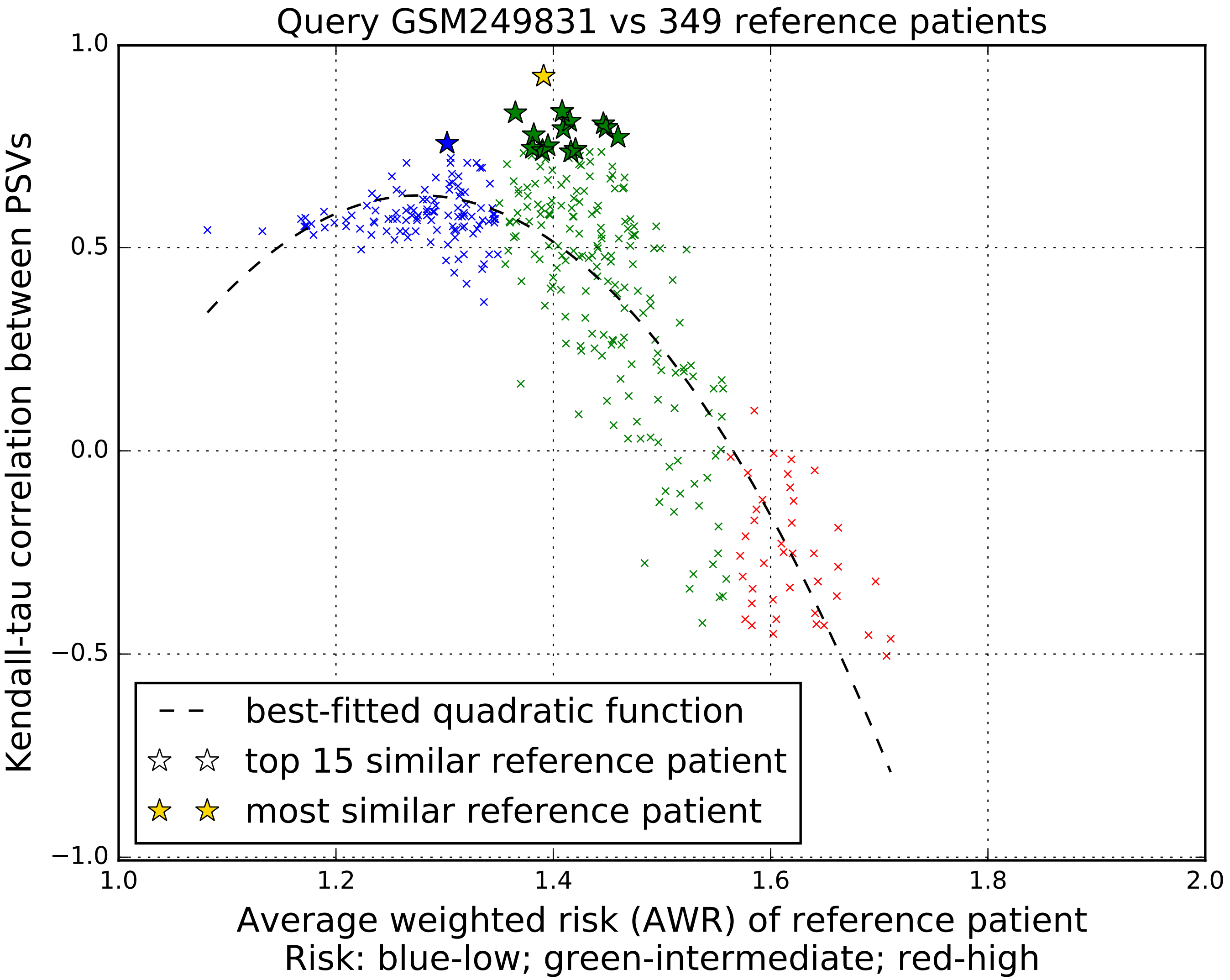
Query GSM249831 vs 349 reference patients
Kendall-tau correlation between PSVs
1.0
0.5
0.0
−0.5
−1.0
1.0
1.2
1.4
1.6
1.8
2.0
best-fitted quadratic function
top 15 similar reference patient
most similar reference patient
Average weighted risk (AWR) of reference patient
Risk: blue-low; green-intermediate; red-high

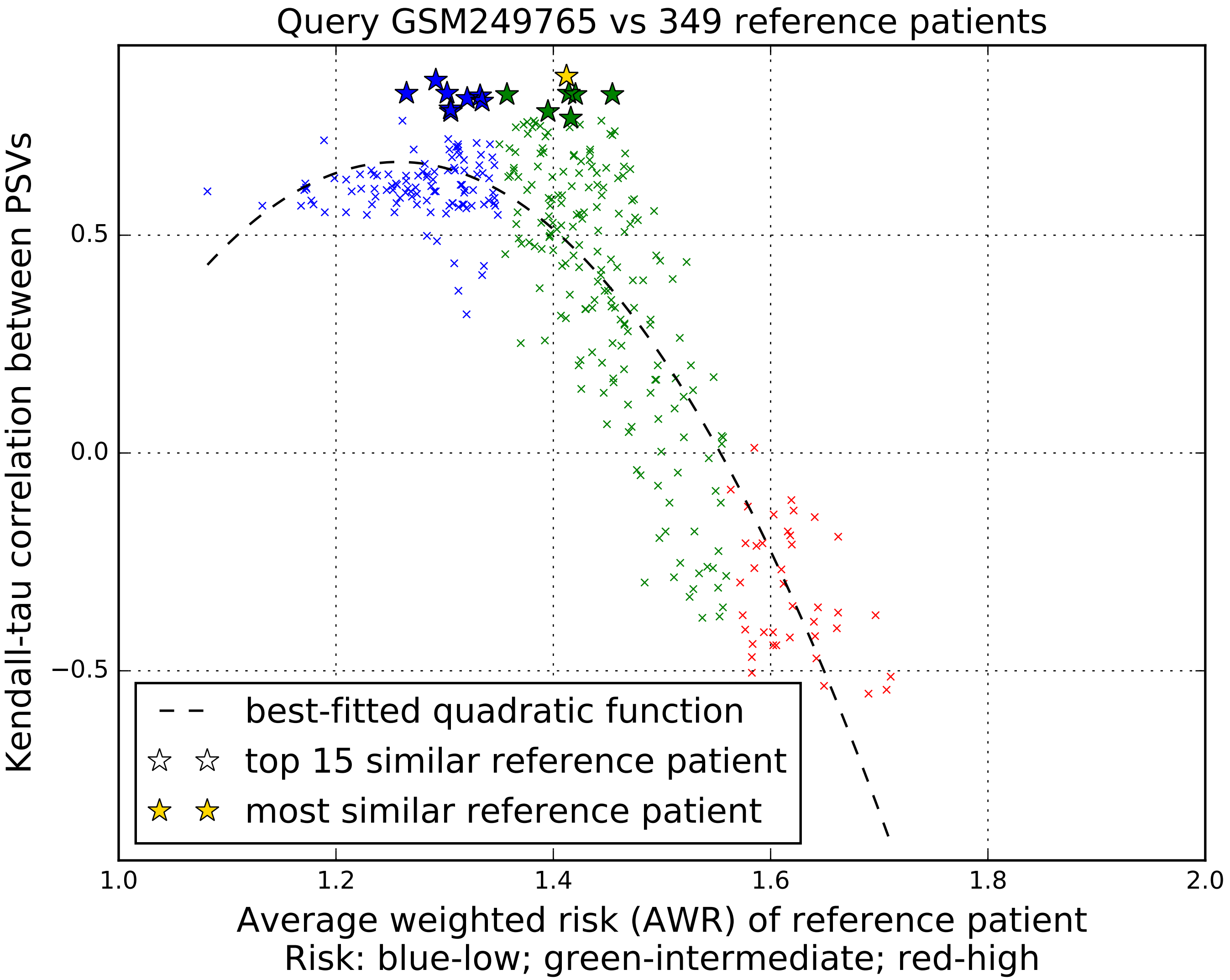

Query GSM249765 vs 349 reference patients
Kendall-tau correlation between PSVs
0.5
0.0
−0.5
1.0
1.2
1.4
1.6
1.8
2.0
best-fitted quadratic function
top 15 similar reference patient
most similar reference patient
Average weighted risk (AWR) of reference patient
Risk: blue-low; green-intermediate; red-high

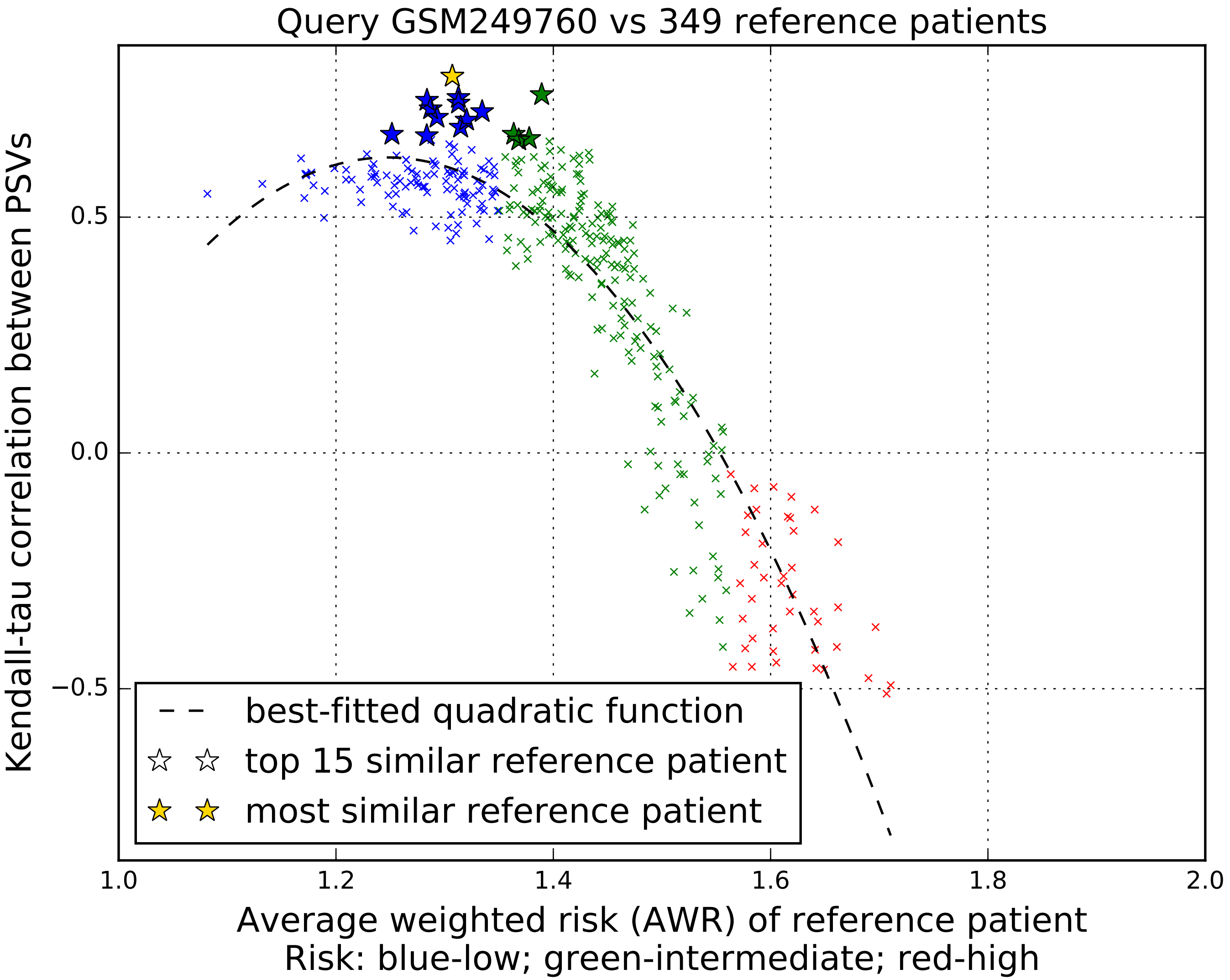
Query GSM249760 vs 349 reference patients
Kendall-tau correlation between PSVs
0.5
0.0
−0.5
1.0
1.2
1.4
1.6
1.8
2.0
best-fitted quadratic function
top 15 similar reference patient
most similar reference patient
Average weighted risk (AWR) of reference patient
Risk: blue-low; green-intermediate; red-high

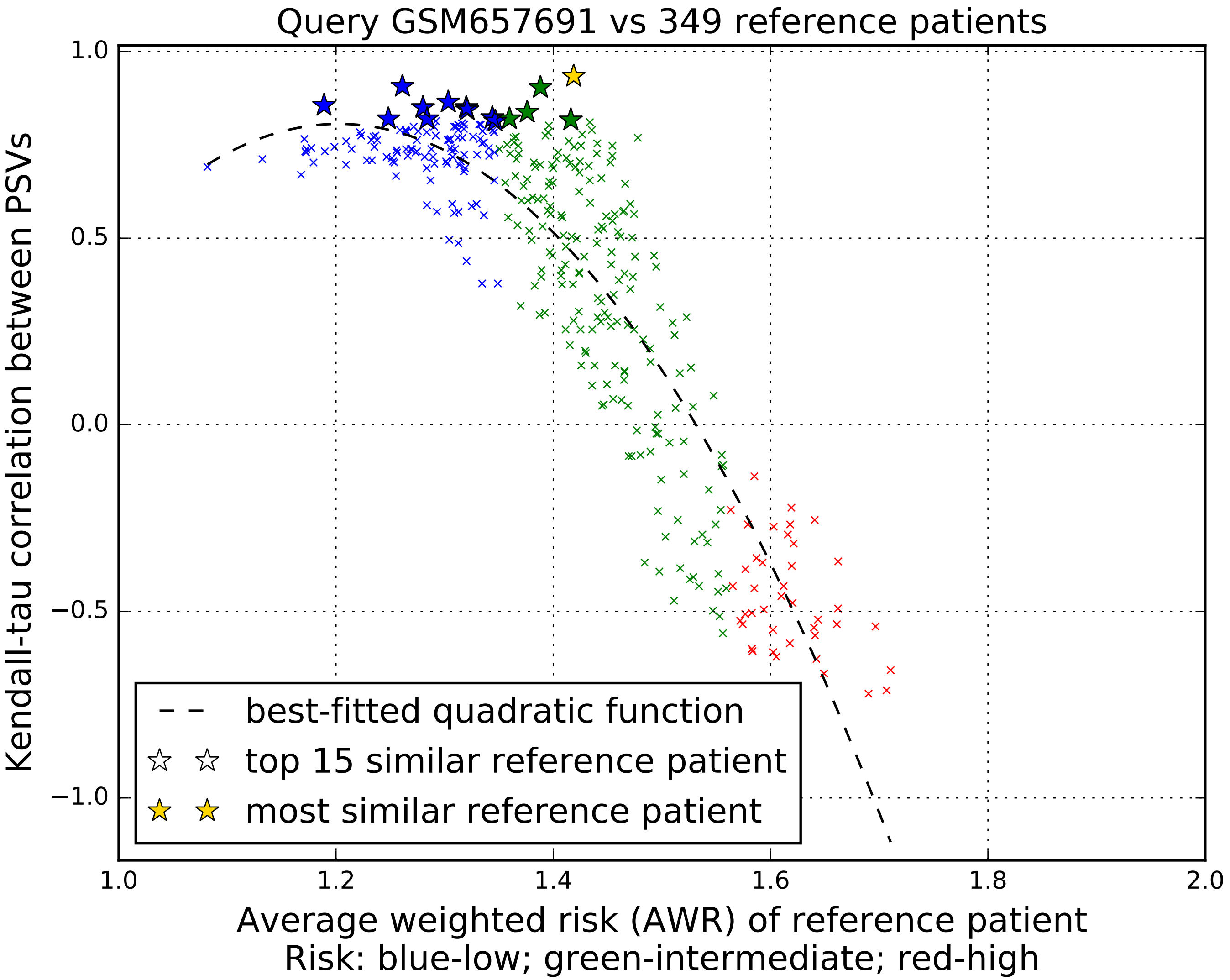

Query GSM657691 vs 349 reference patients
Kendall-tau correlation between PSVs
1.0
0.5
0.0
−0.5
−1.0
1.0
1.2
1.4
1.6
1.8
2.0
best-fitted quadratic function
top 15 similar reference patient
most similar reference patient
Average weighted risk (AWR) of reference patient
Risk: blue-low; green-intermediate; red-high

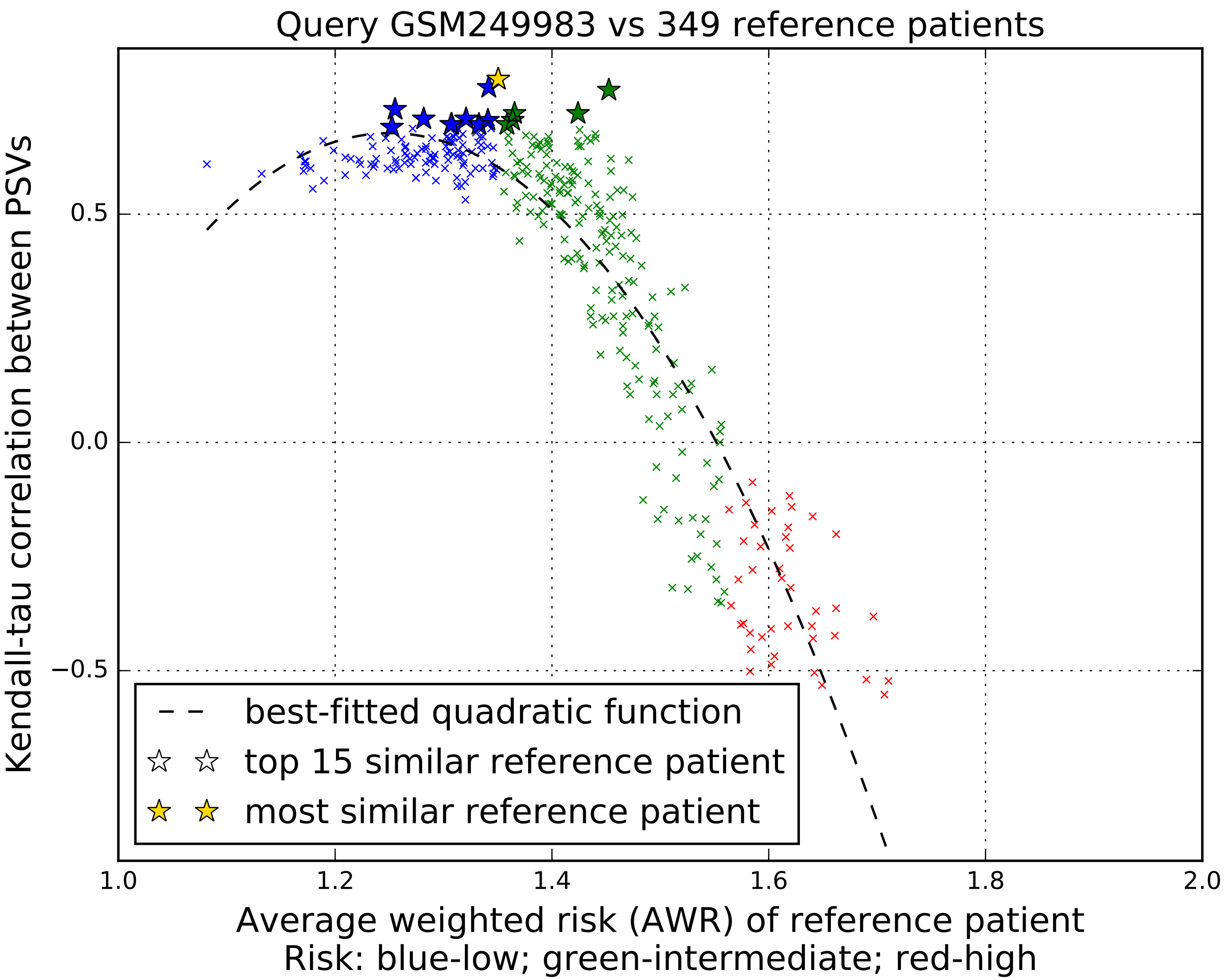
Query GSM249983 vs 349 reference patients
Kendall-tau correlation between PSVs
0.5
0.0
−0.5
1.0
1.2
1.4
1.6
1.8
2.0
best-fitted quadratic function
top 15 similar reference patient
most similar reference patient
Average weighted risk (AWR) of reference patient
Risk: blue-low; green-intermediate; red-high

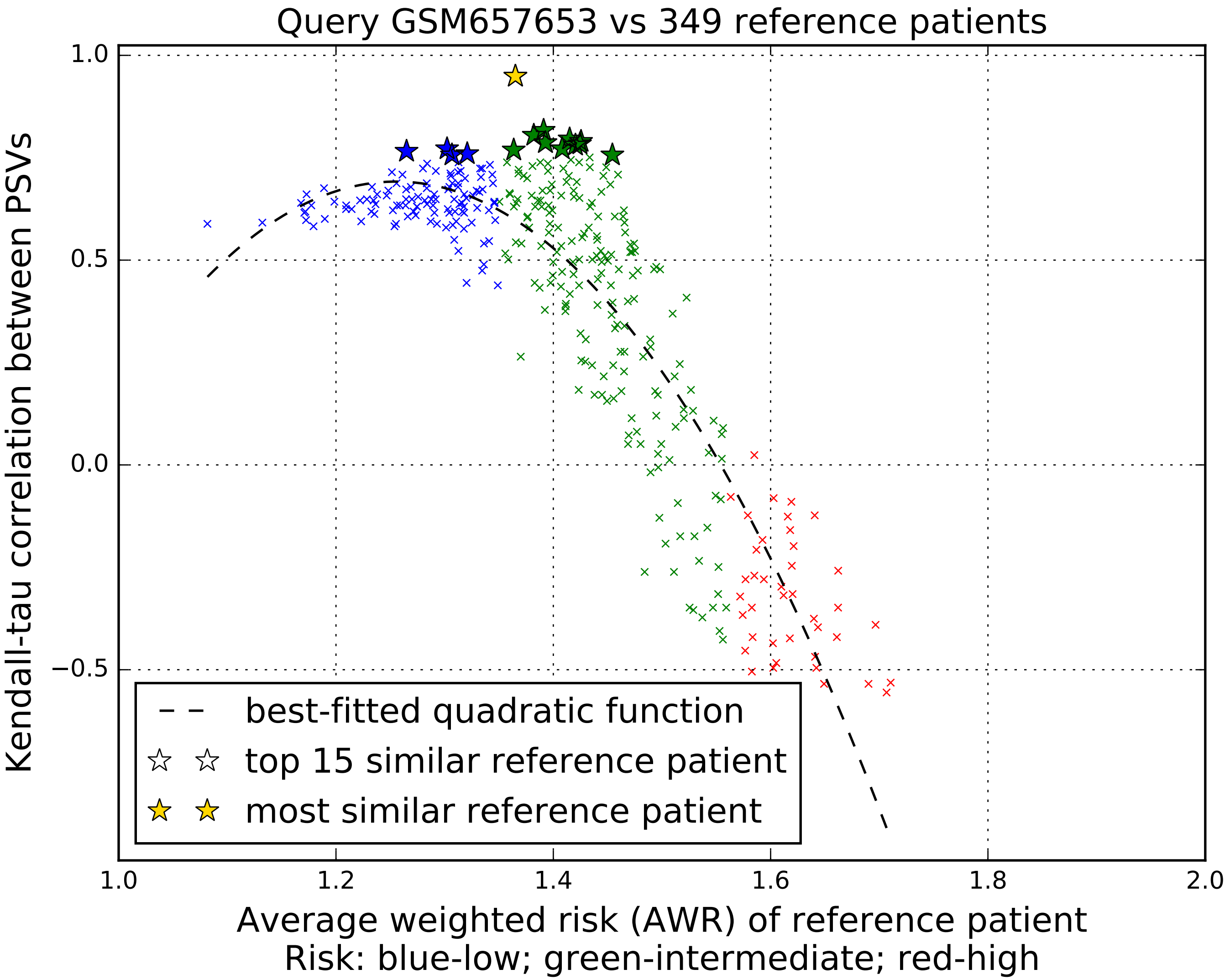
Query GSM657653 vs 349 reference patients
Kendall-tau correlation between PSVs
1.0
0.5
0.0
−0.5
1.0
1.2
1.4
1.6
1.8
2.0
best-fitted quadratic function
top 15 similar reference patient
most similar reference patient
Average weighted risk (AWR) of reference patient
Risk: blue-low; green-intermediate; red-high

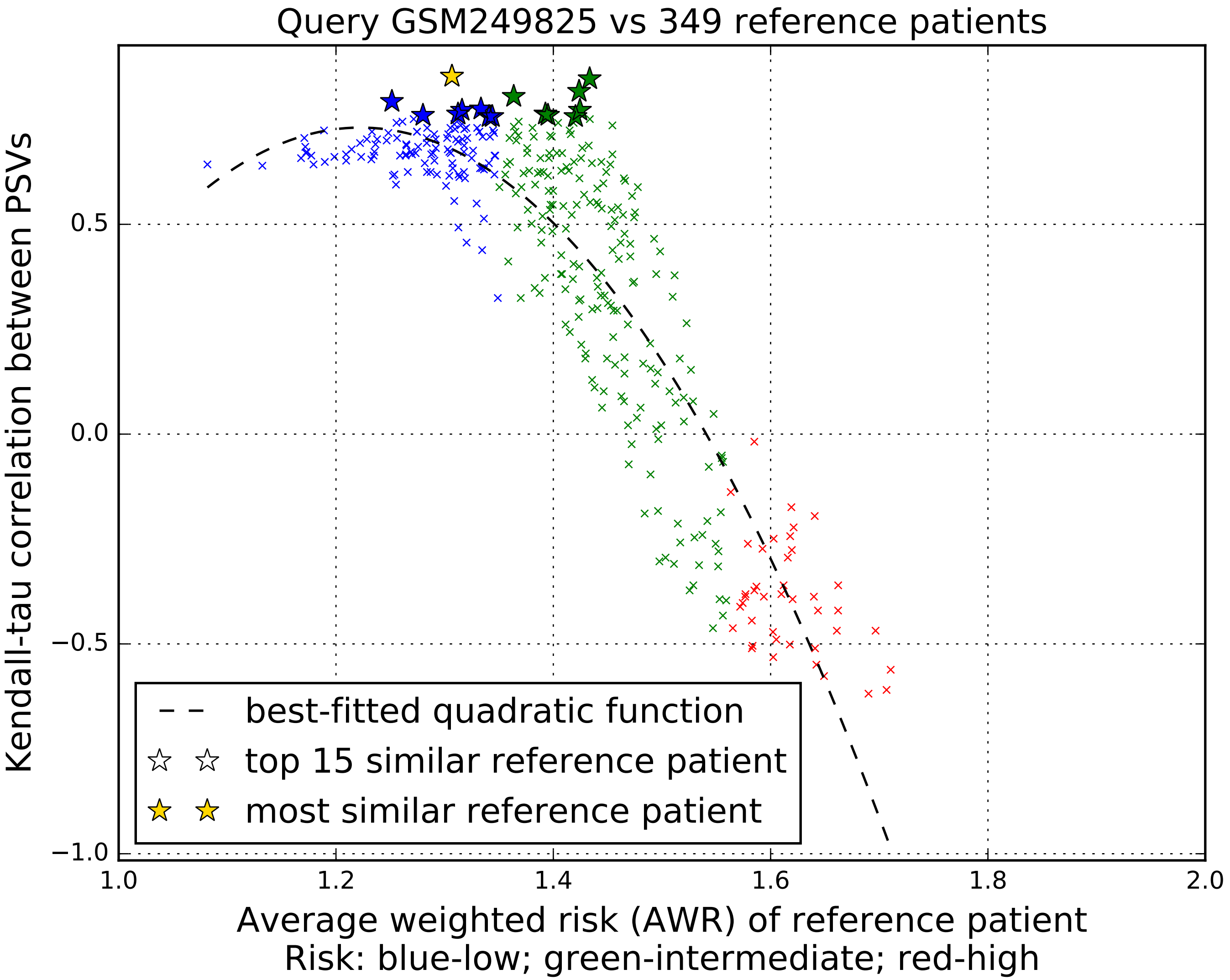

Query GSM249825 vs 349 reference patients
Kendall-tau correlation between PSVs
Average weighted risk (AWR) of reference patient
Risk: blue-low; green-intermediate; red-high
best-fitted quadratic function
top 15 similar reference patient
most similar reference patient
0.5
0.0
−0.5
−1.0
1.0
1.2
1.4
1.6
1.8
2.0

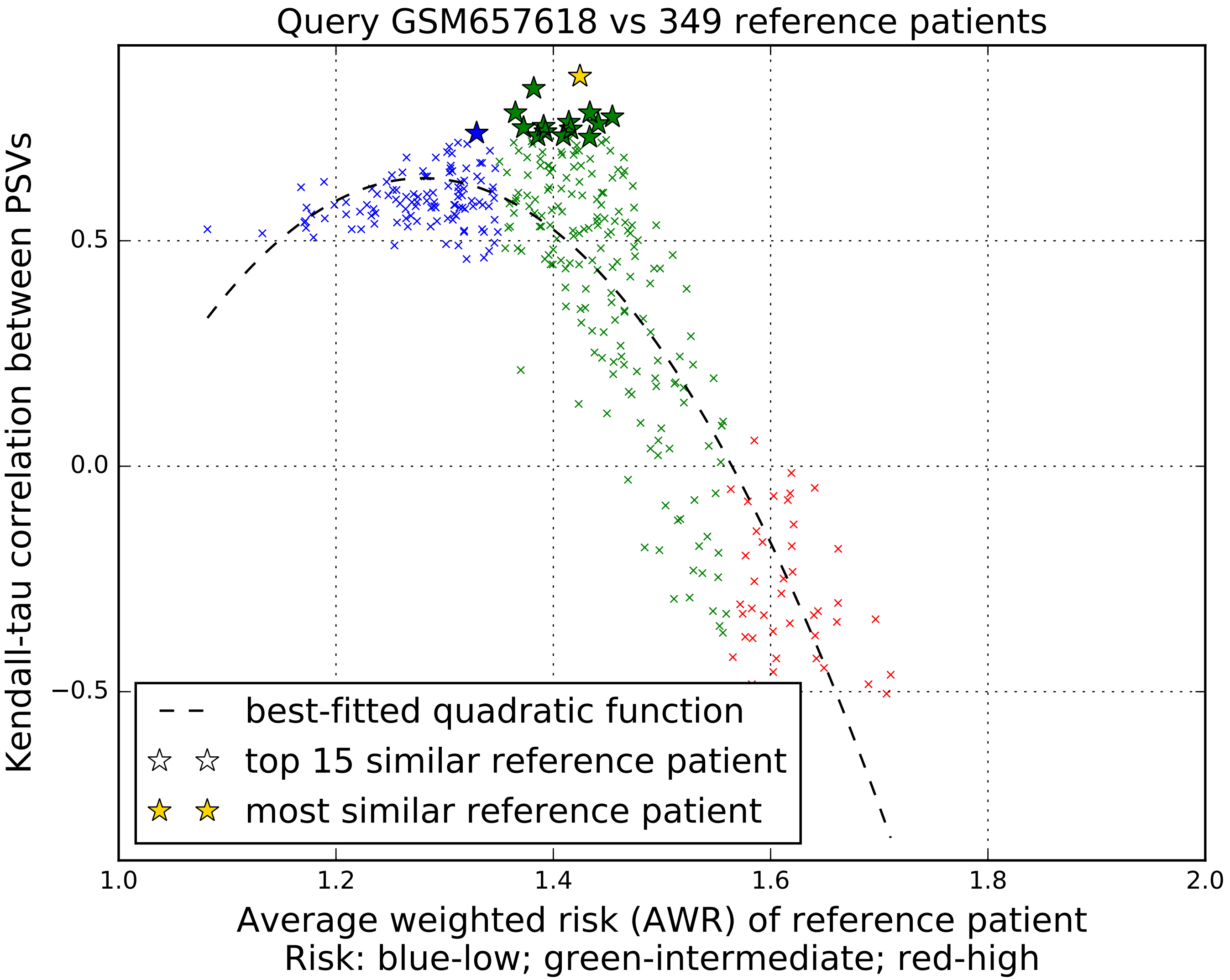

Query GSM657618 vs 349 reference patients
Kendall-tau correlation between PSVs
0.5
0.0
−0.5
1.0
1.2
1.4
1.6
1.8
2.0
best-fitted quadratic function
top 15 similar reference patient
most similar reference patient
Average weighted risk (AWR) of reference patient
Risk: blue-low; green-intermediate; red-high

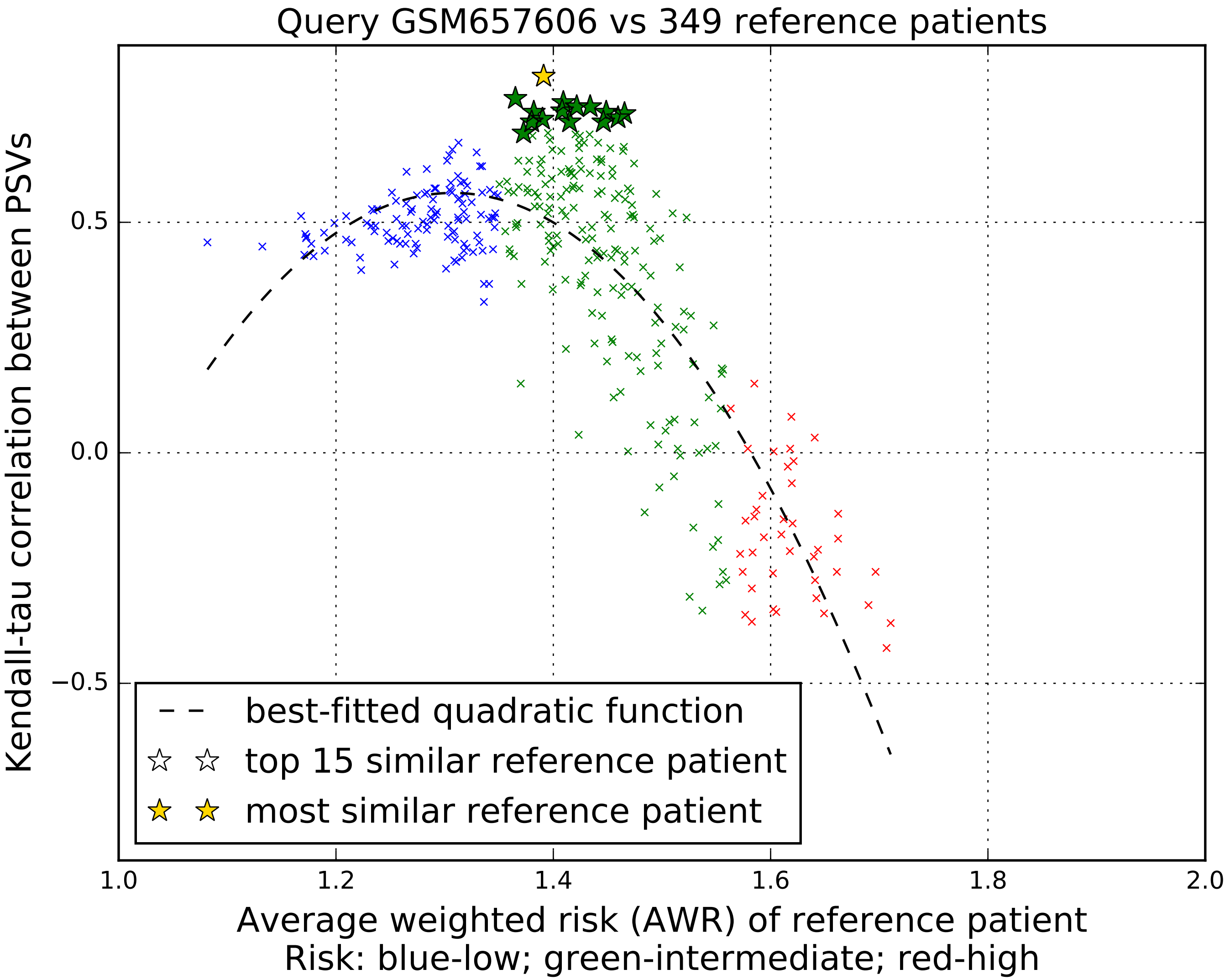

Query GSM657606 vs 349 reference patients
Kendall-tau correlation between PSVs
0.5
0.0
−0.5
1.0
1.2
1.4
1.6
1.8
2.0
best-fitted quadratic function
top 15 similar reference patient
most similar reference patient
Average weighted risk (AWR) of reference patient
Risk: blue-low; green-intermediate; red-high

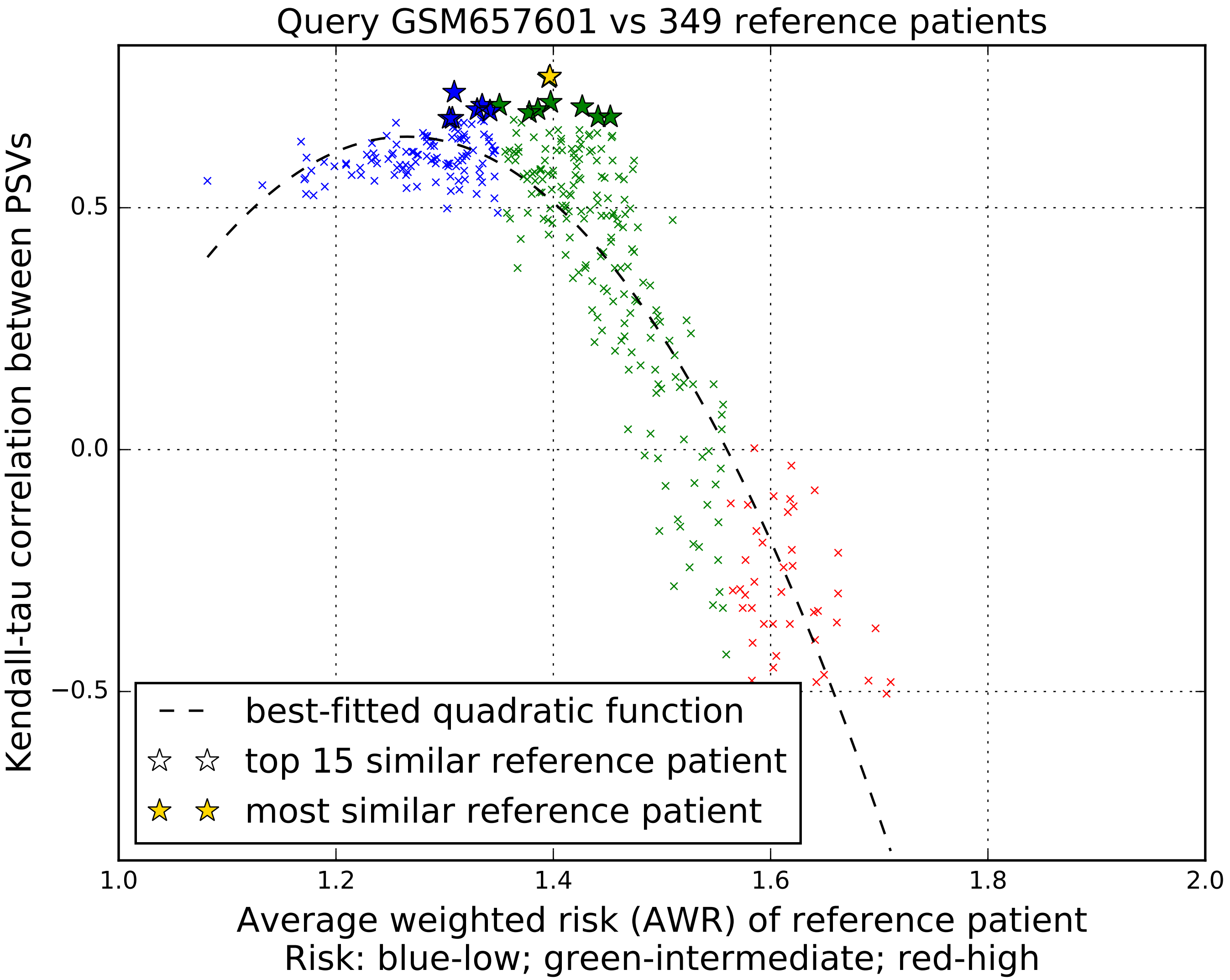
Query GSM657601 vs 349 reference patients
Kendall-tau correlation between PSVs
0.5
0.0
−0.5
1.0
1.2
1.4
1.6
1.8
2.0
best-fitted quadratic function
top 15 similar reference patient
most similar reference patient
Average weighted risk (AWR) of reference patient
Risk: blue-low; green-intermediate; red-high

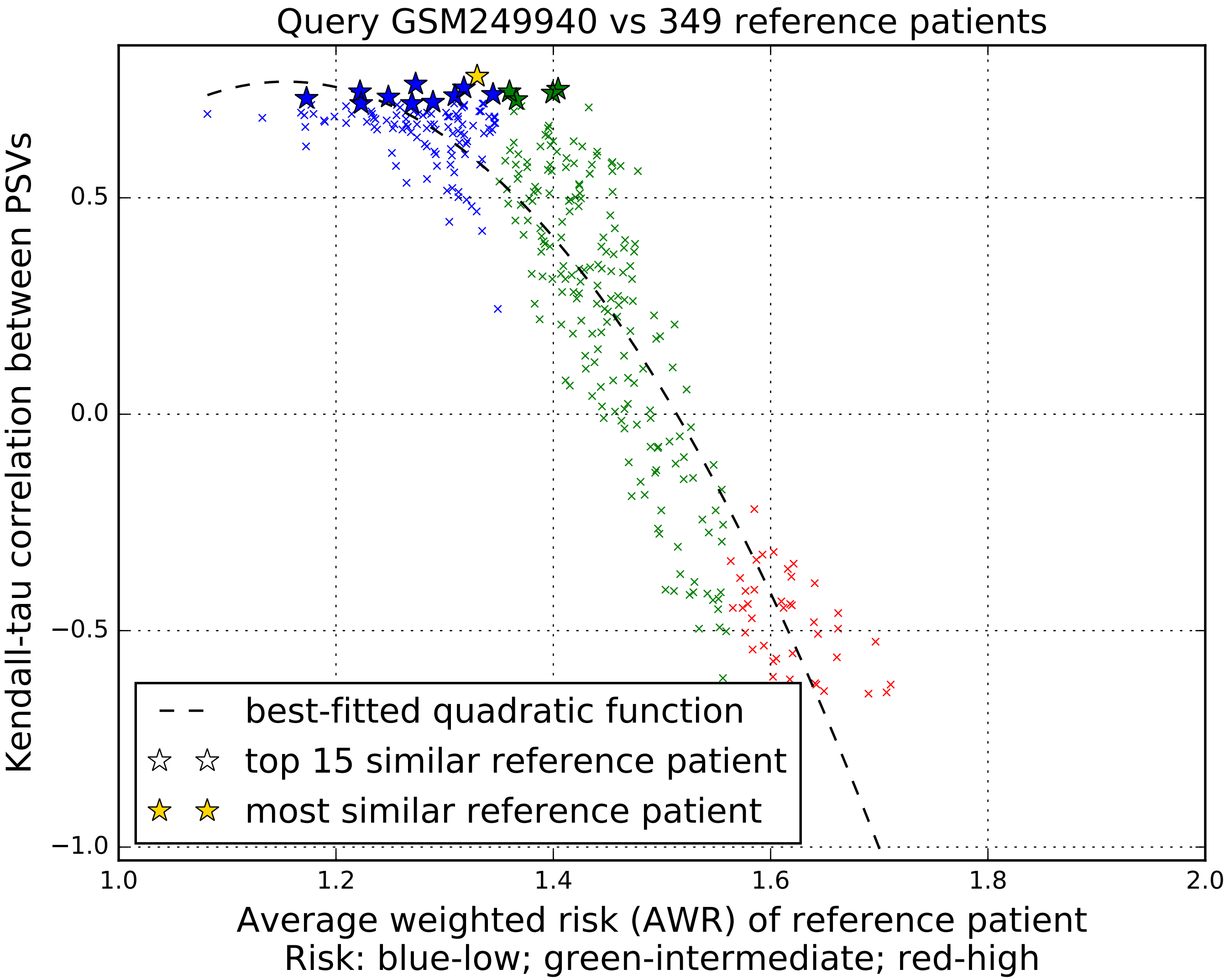
Query GSM249940 vs 349 reference patients
Kendall-tau correlation between PSVs
0.5
0.0
−0.5
−1.0
1.0
1.2
1.4
1.6
1.8
2.0
best-fitted quadratic function
top 15 similar reference patient
most similar reference patient
Average weighted risk (AWR) of reference patient
Risk: blue-low; green-intermediate; red-high

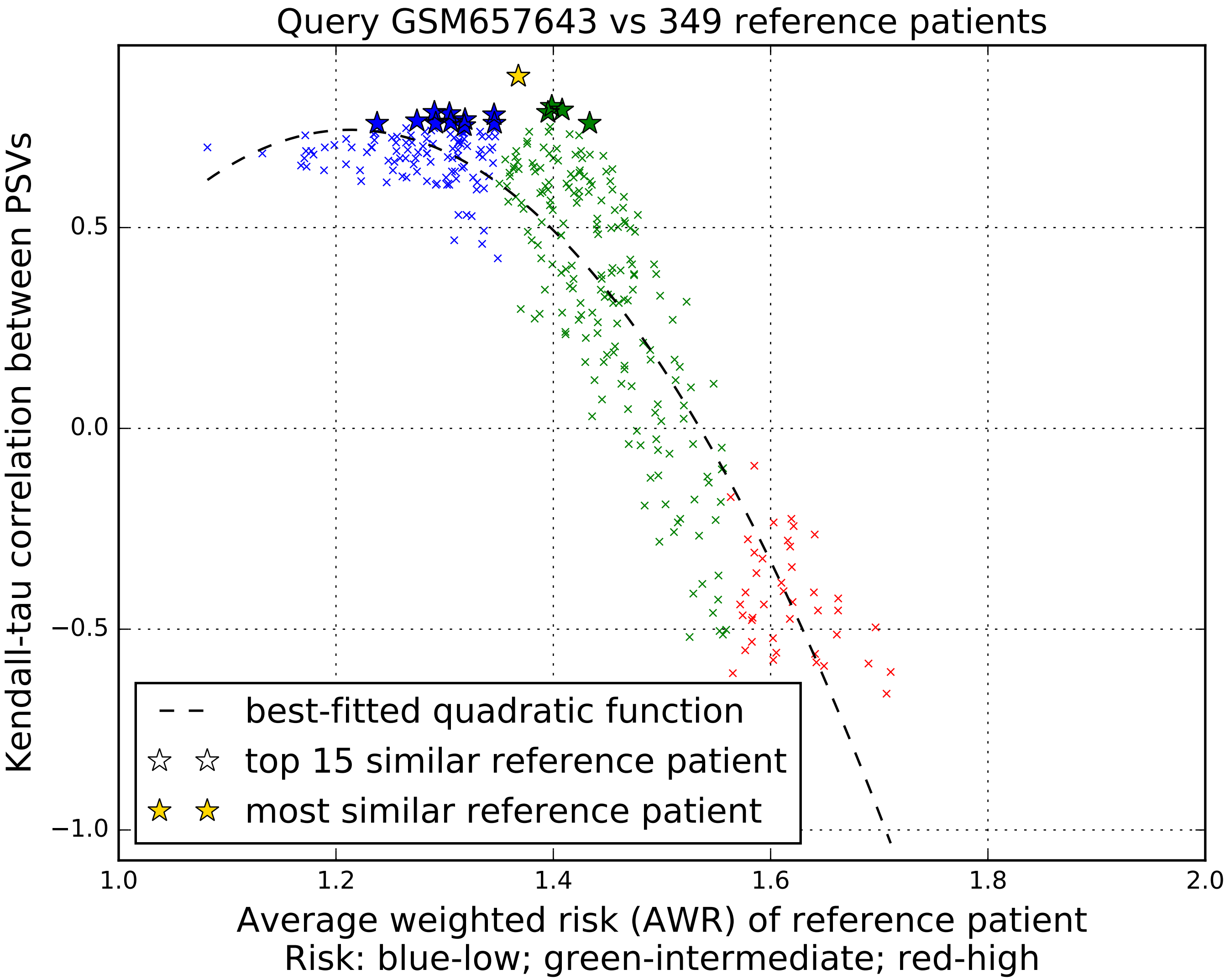

Query GSM657643 vs 349 reference patients
Kendall-tau correlation between PSVs
0.5
0.0
−0.5
−1.0
1.0
1.2
1.4
1.6
1.8
2.0
best-fitted quadratic function
top 15 similar reference patient
most similar reference patient
Average weighted risk (AWR) of reference patient
Risk: blue-low; green-intermediate; red-high

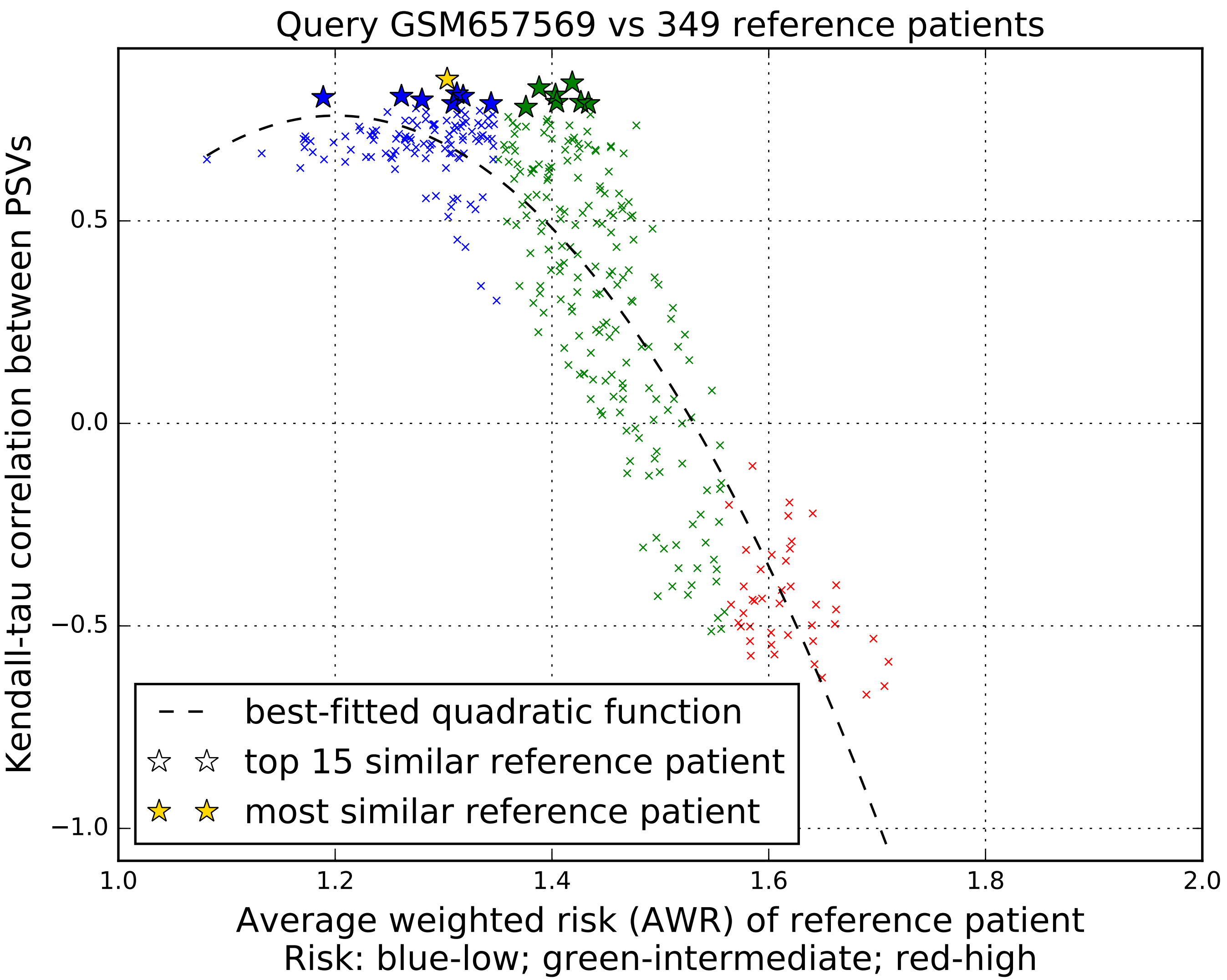

Query GSM657569 vs 349 reference patients
Kendall-tau correlation between PSVs
0.5
0.0
−0.5
−1.0
1.0
1.2
1.4
1.6
1.8
2.0
best-fitted quadratic function
top 15 similar reference patient
most similar reference patient
Average weighted risk (AWR) of reference patient
Risk: blue-low; green-intermediate; red-high

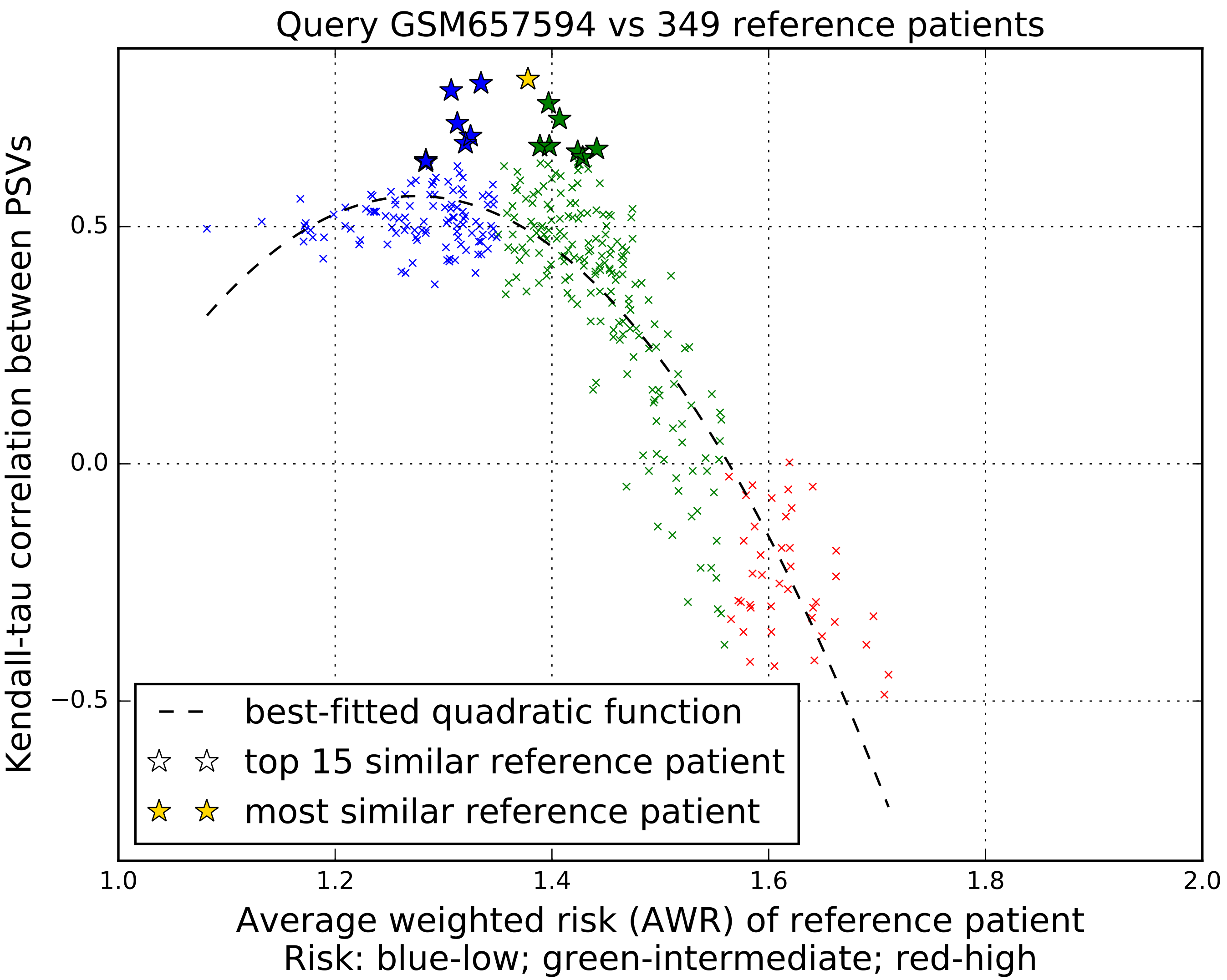

Query GSM657594 vs 349 reference patients
Kendall-tau correlation between PSVs
0.5
0.0
−0.5
1.0
1.2
1.4
1.6
1.8
2.0
best-fitted quadratic function
top 15 similar reference patient
most similar reference patient
Average weighted risk (AWR) of reference patient
Risk: blue-low; green-intermediate; red-high

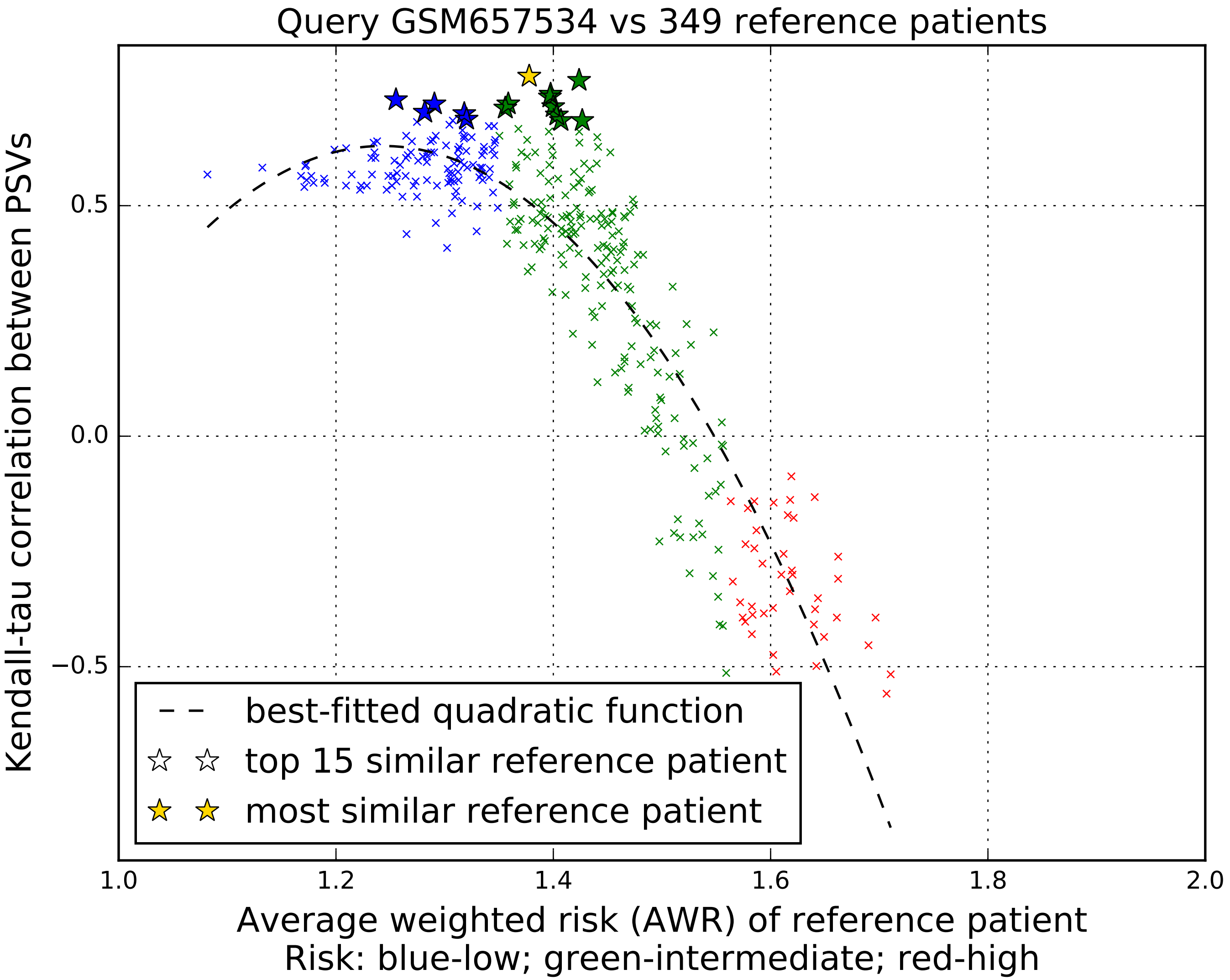
Query GSM657534 vs 349 reference patients
Kendall-tau correlation between PSVs
0.5
0.0
−0.5
1.0
1.2
1.4
1.6
1.8
2.0
best-fitted quadratic function
top 15 similar reference patient
most similar reference patient
Average weighted risk (AWR) of reference patient
Risk: blue-low; green-intermediate; red-high

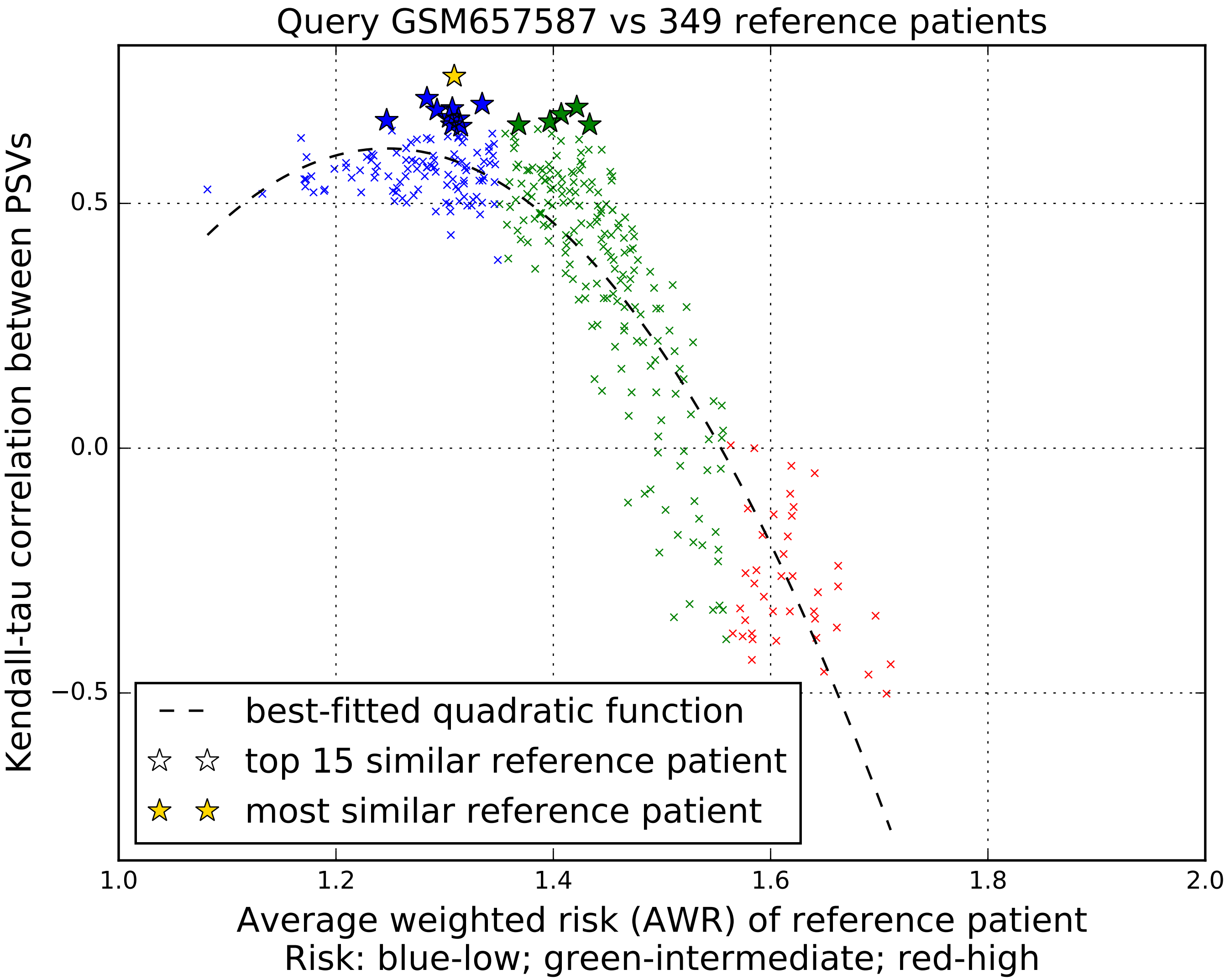

Query GSM657587 vs 349 reference patients
Kendall-tau correlation between PSVs
0.5
0.0
−0.5
1.0
1.2
1.4
1.6
1.8
2.0
best-fitted quadratic function
top 15 similar reference patient
most similar reference patient
Average weighted risk (AWR) of reference patient
Risk: blue-low; green-intermediate; red-high

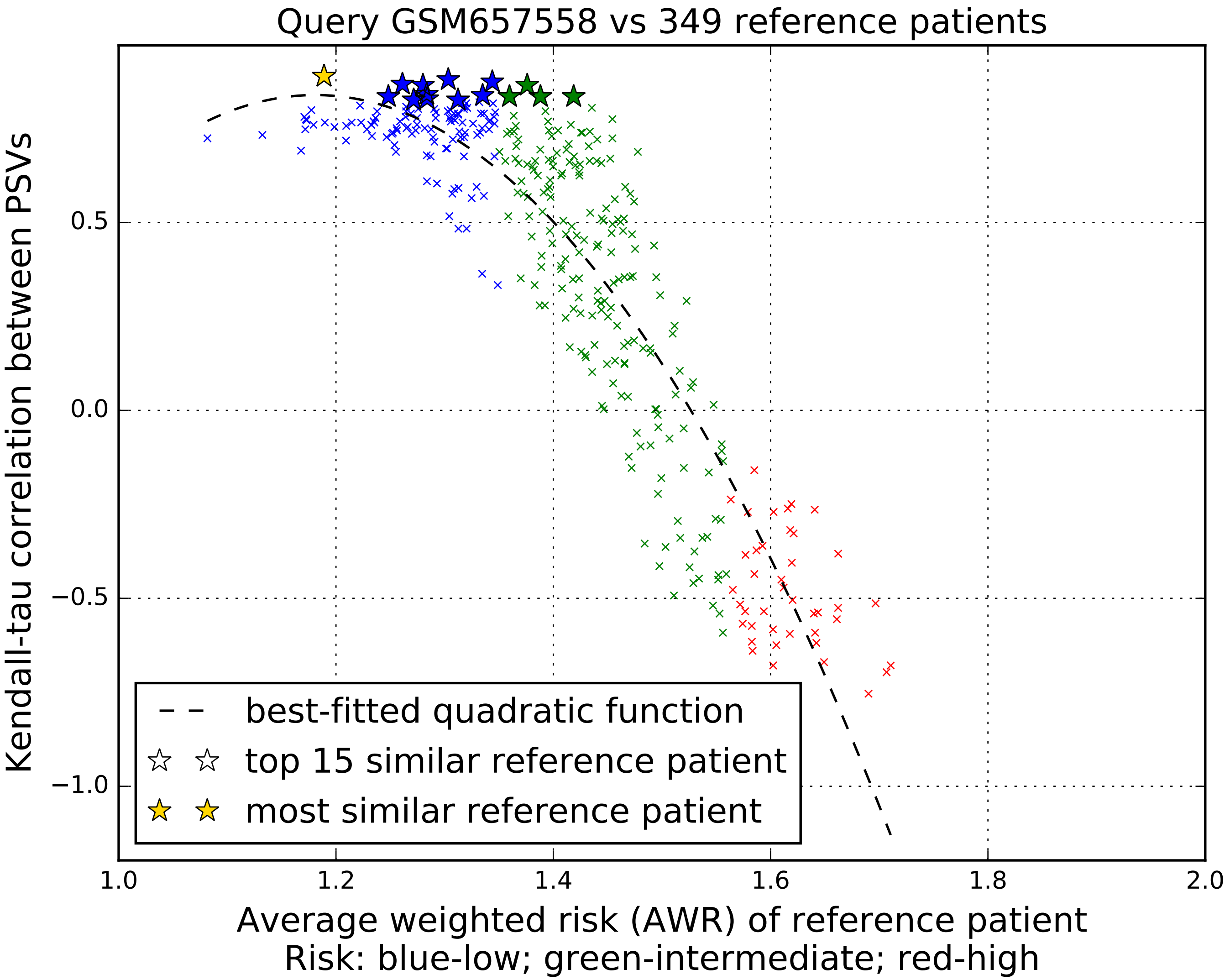

Query GSM657558 vs 349 reference patients
Kendall-tau correlation between PSVs
0.5
0.0
−0.5
−1.0
1.0
1.2
1.4
1.6
1.8
2.0
Average weighted risk (AWR) of reference patient
Risk: blue-low; green-intermediate; red-high
best-fitted quadratic function
top 15 similar reference patient
most similar reference patient

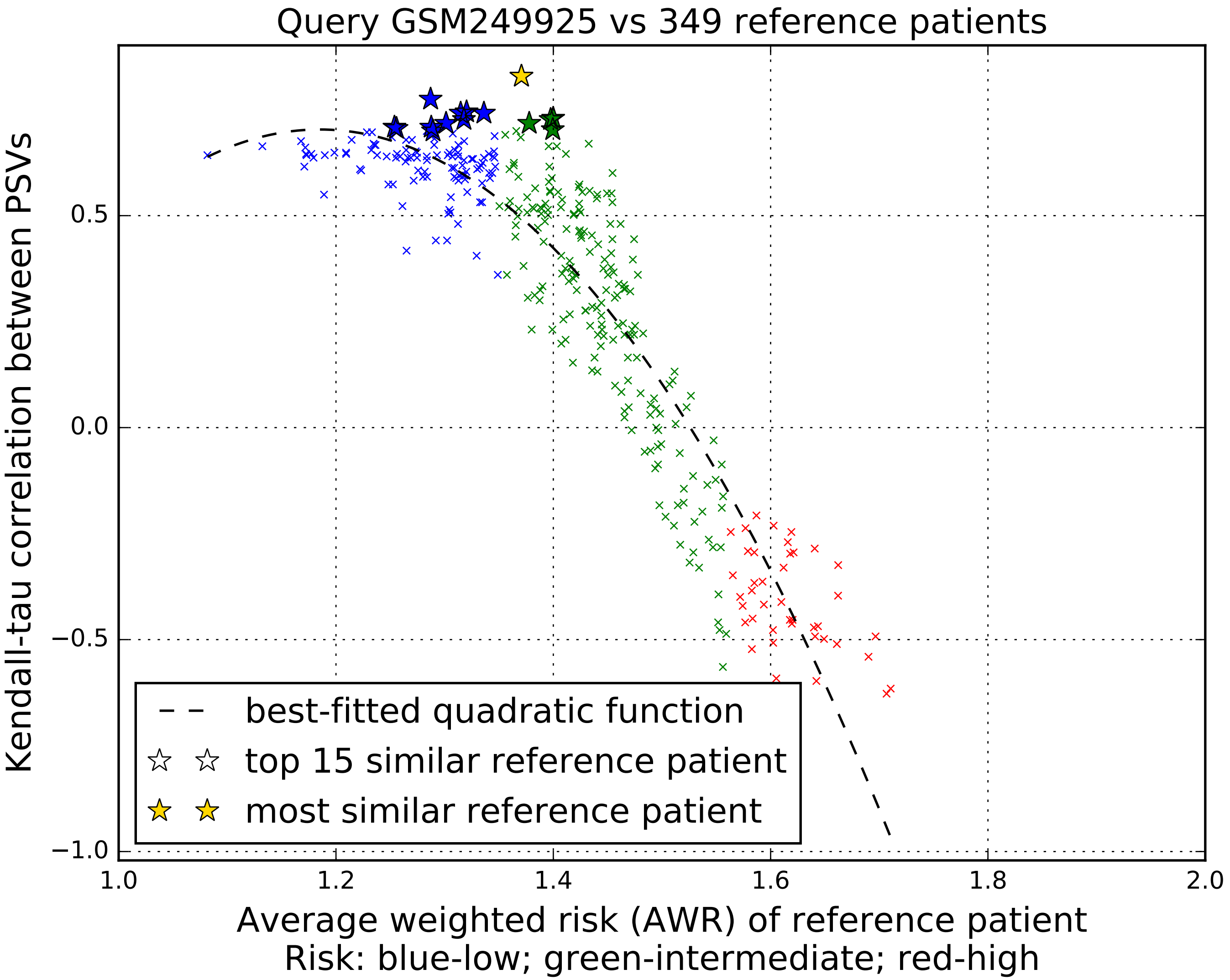
Query GSM249925 vs 349 reference patients
Kendall-tau correlation between PSVs
0.5
0.0
−0.5
−1.0
1.0
1.2
1.4
1.6
1.8
2.0
best-fitted quadratic function
top 15 similar reference patient
most similar reference patient
Average weighted risk (AWR) of reference patient
Risk: blue-low; green-intermediate; red-high

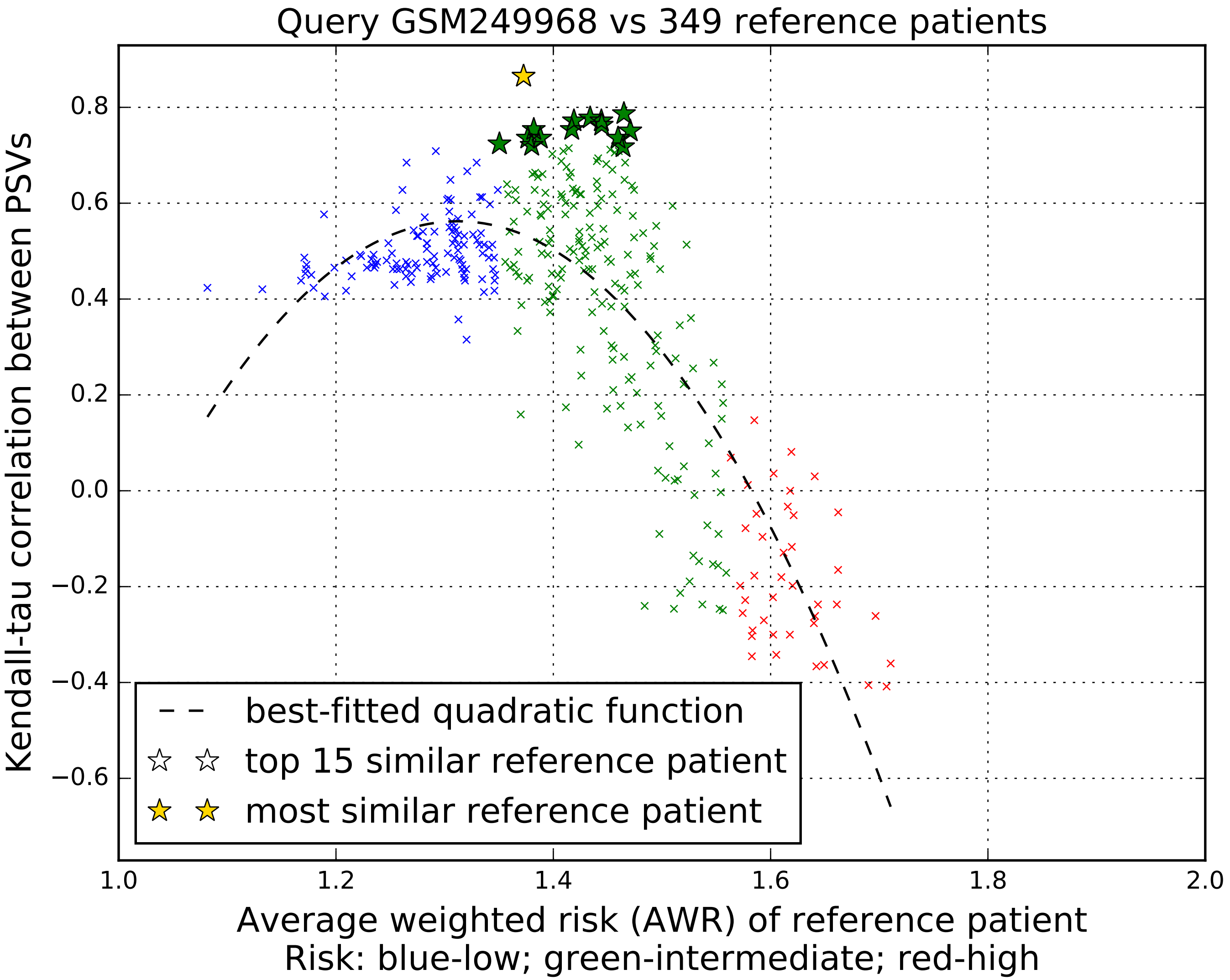

Query GSM249968 vs 349 reference patients
Kendall-tau correlation between PSVs
Average weighted risk (AWR) of reference patient
Risk: blue-low; green-intermediate; red-high
best-fitted quadratic function
top 15 similar reference patient
most similar reference patient
0.8
0.6
0.4
0.2
0.0
−0.2
−0.4
−0.6
1.0
1.2
1.4
1.6
1.8
2.0

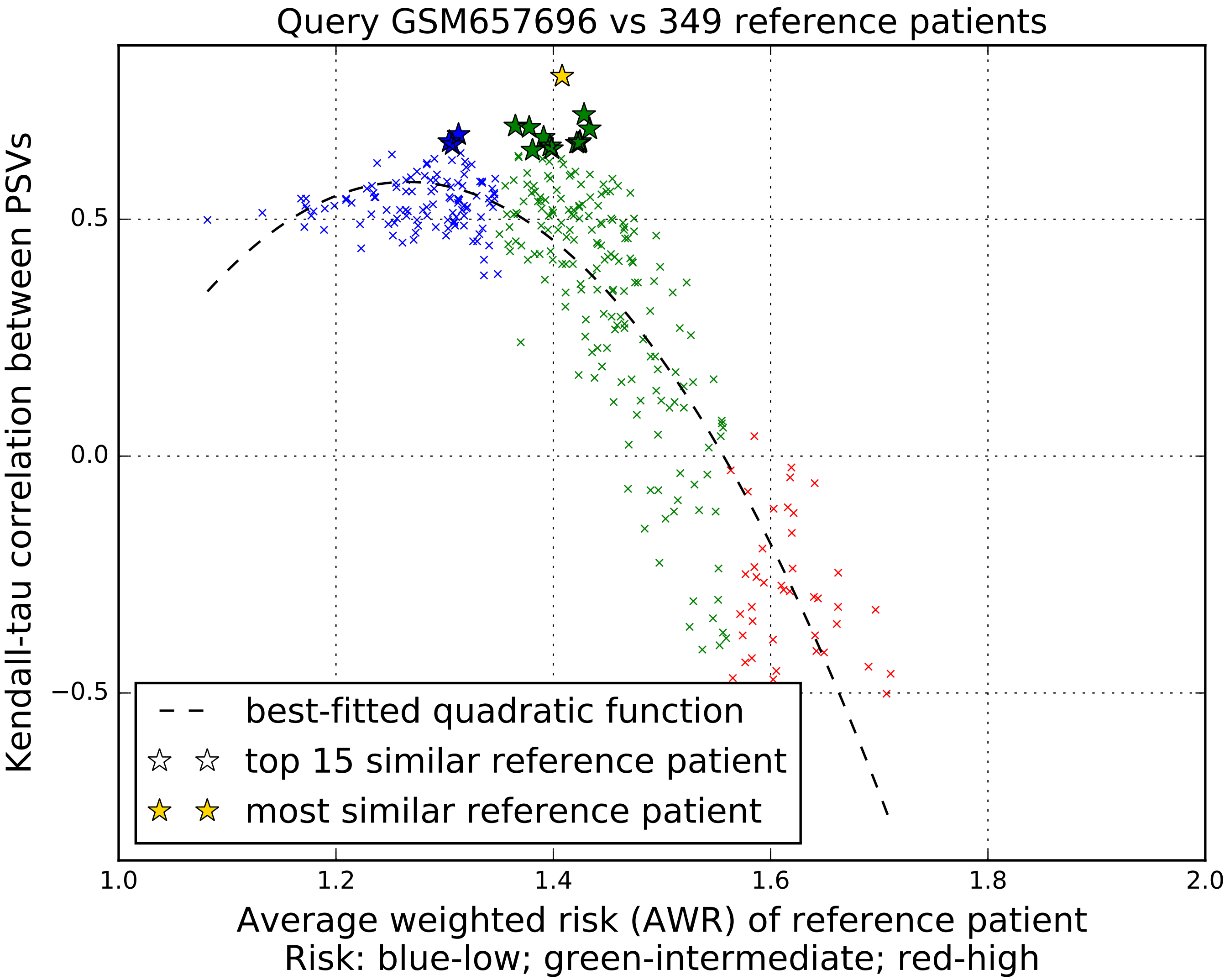

Query GSM657696 vs 349 reference patients
Kendall-tau correlation between PSVs
0.5
0.0
−0.5
1.0
1.2
1.4
1.6
1.8
2.0
best-fitted quadratic function
top 15 similar reference patient
most similar reference patient
Average weighted risk (AWR) of reference patient
Risk: blue-low; green-intermediate; red-high

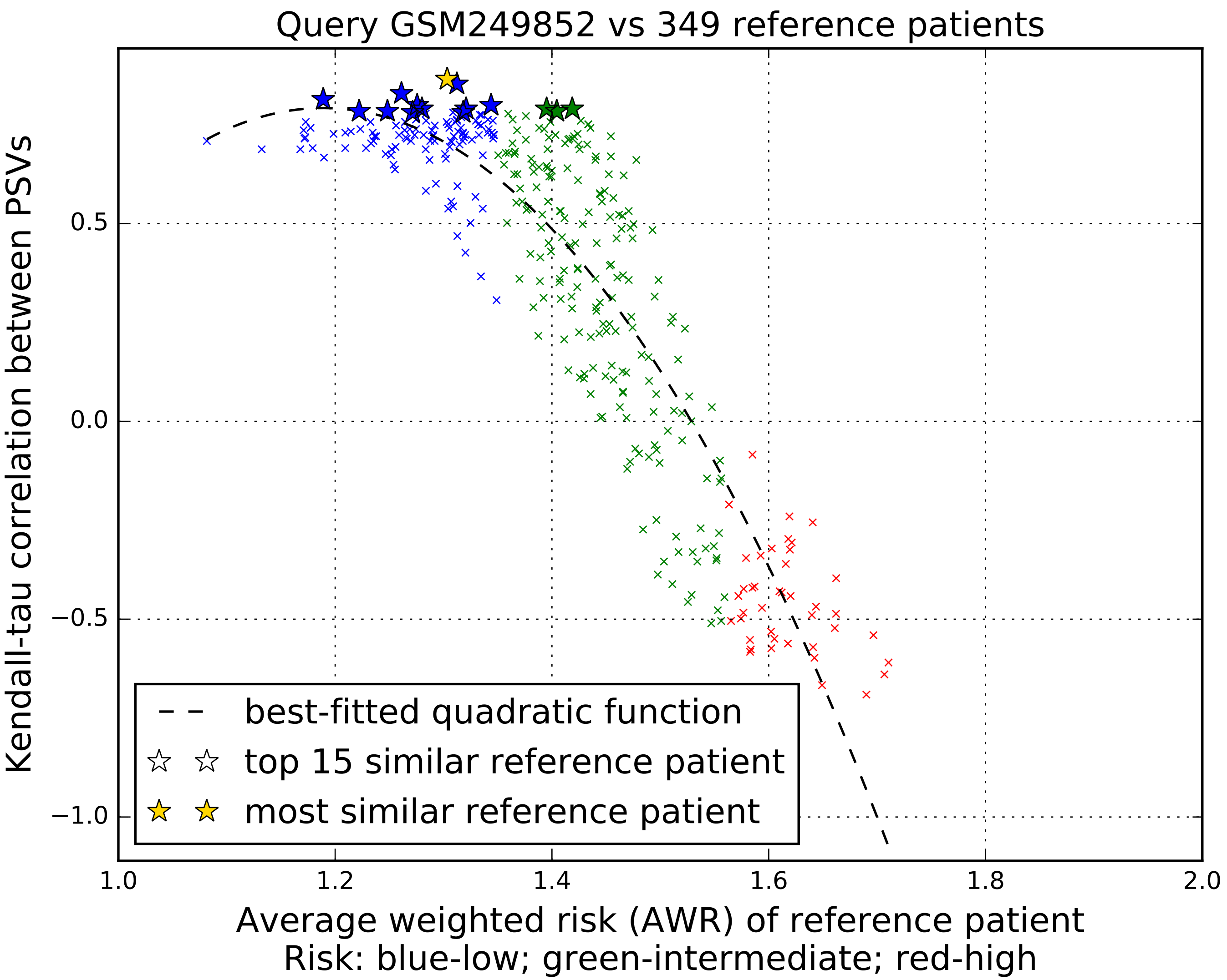
Query GSM249852 vs 349 reference patients
Kendall-tau correlation between PSVs
0.5
0.0
−0.5
−1.0
1.0
1.2
1.4
1.6
1.8
2.0
best-fitted quadratic function
top 15 similar reference patient
most similar reference patient
Average weighted risk (AWR) of reference patient
Risk: blue-low; green-intermediate; red-high

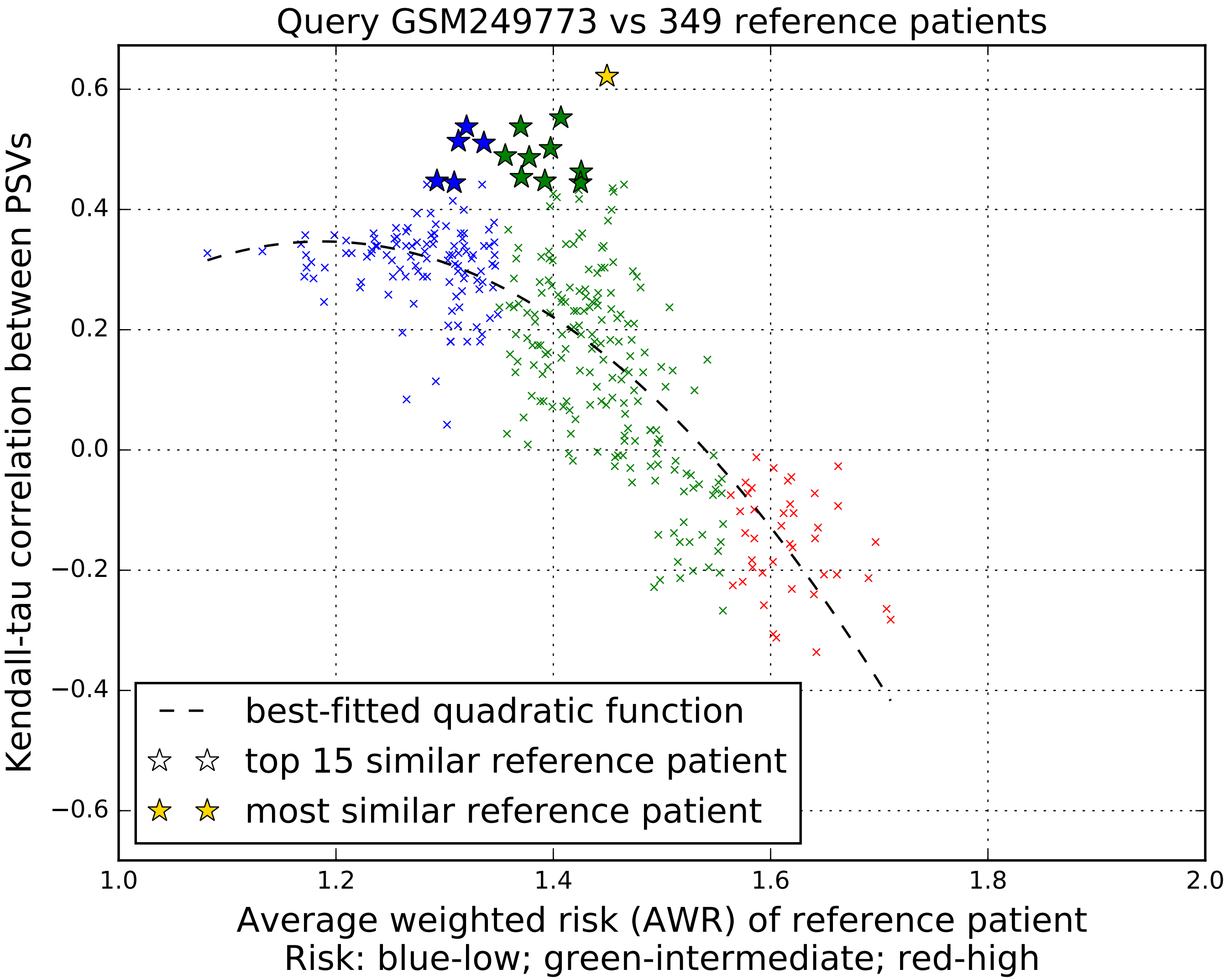

Query GSM249773 vs 349 reference patients
Kendall-tau correlation between PSVs
0.6
0.4
0.2
0.0
−0.2
−0.4
−0.6
1.0
1.2
1.4
1.6
1.8
2.0
best-fitted quadratic function
top 15 similar reference patient
most similar reference patient
Average weighted risk (AWR) of reference patient
Risk: blue-low; green-intermediate; red-high

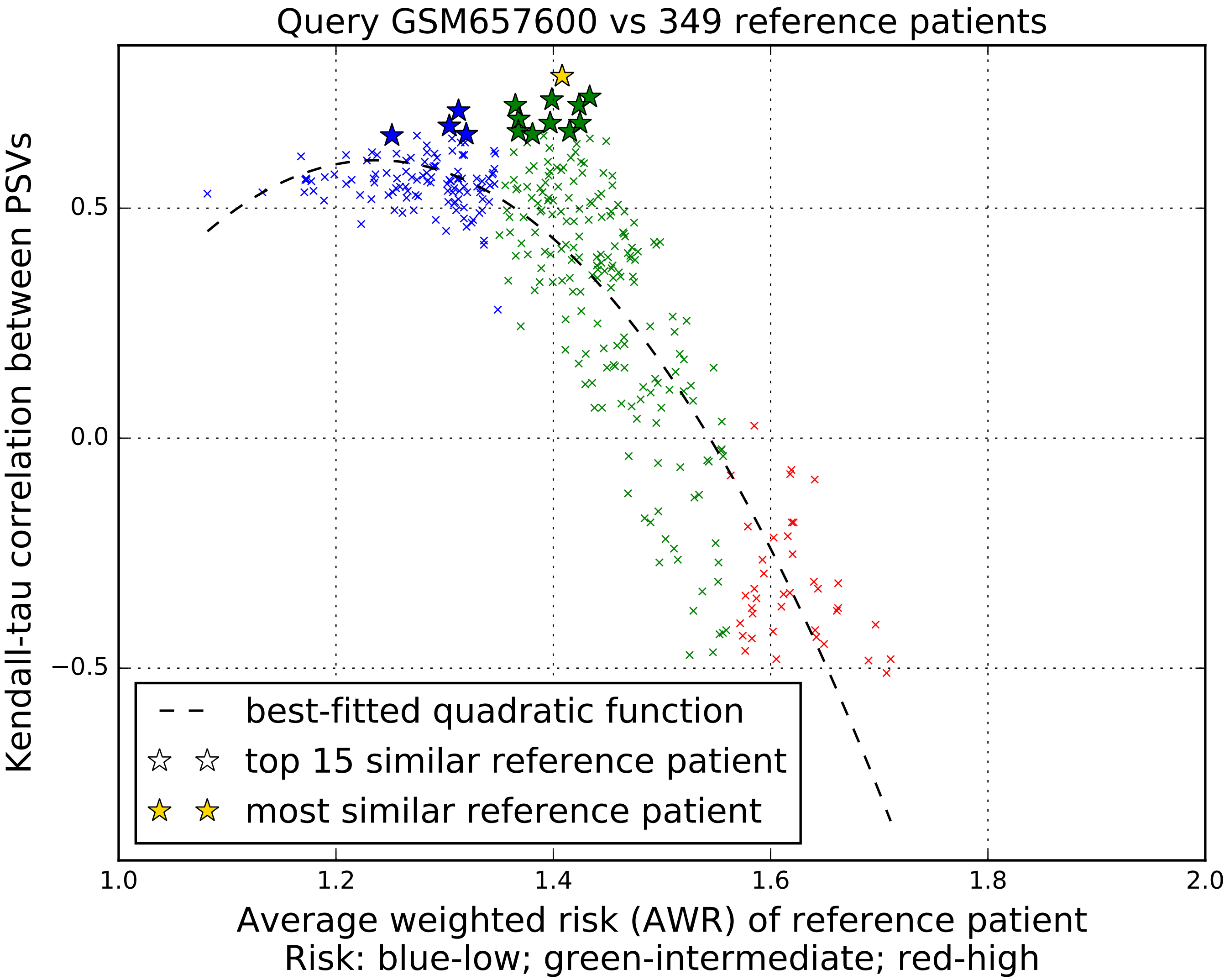

Query GSM657600 vs 349 reference patients
Kendall-tau correlation between PSVs
0.5
0.0
−0.5
1.0
1.2
1.4
1.6
1.8
2.0
best-fitted quadratic function
top 15 similar reference patient
most similar reference patient
Average weighted risk (AWR) of reference patient
Risk: blue-low; green-intermediate; red-high

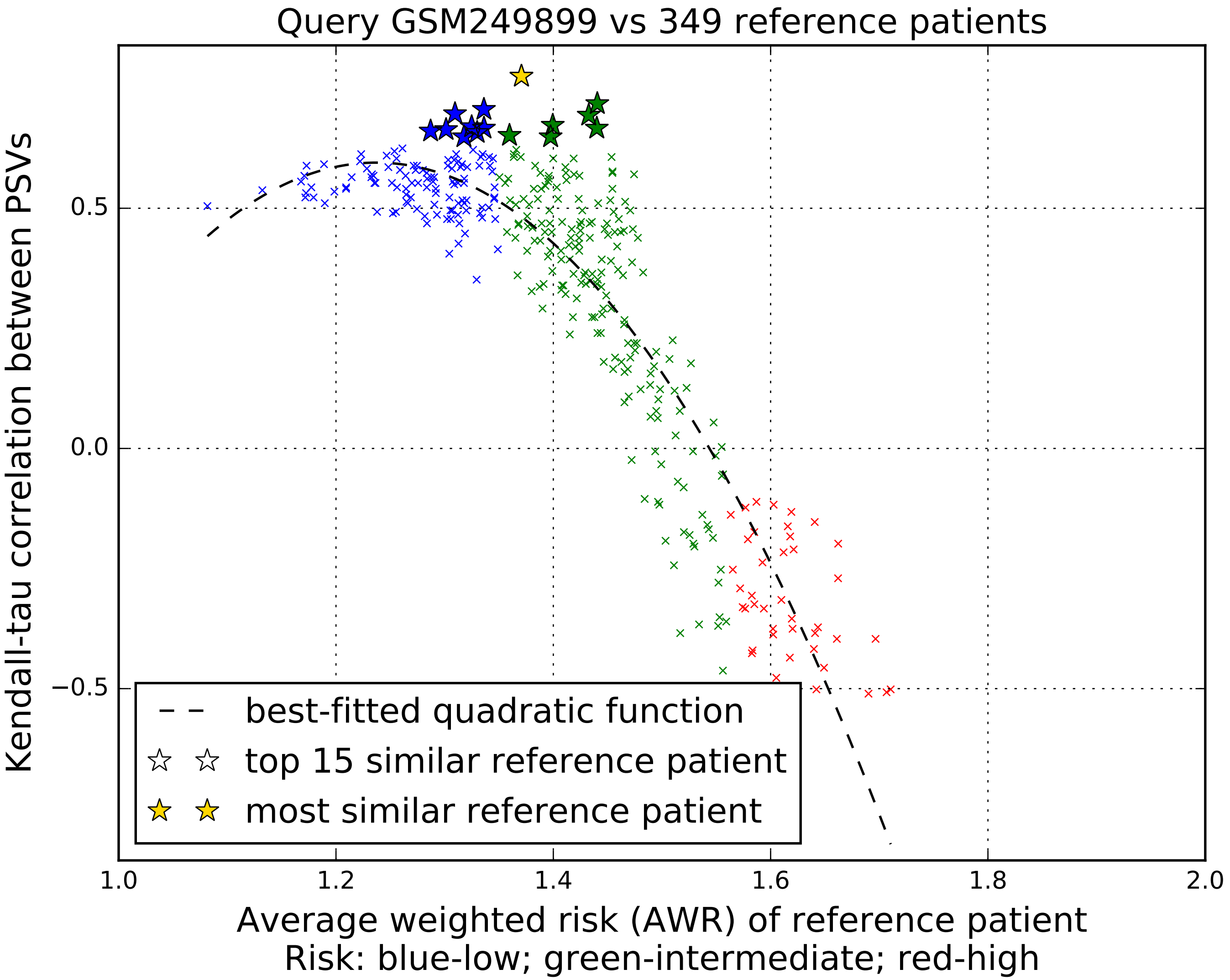

Query GSM249899 vs 349 reference patients
Kendall-tau correlation between PSVs
0.5
0.0
−0.5
1.0
1.2
1.4
1.6
1.8
2.0
best-fitted quadratic function
top 15 similar reference patient
most similar reference patient
Average weighted risk (AWR) of reference patient
Risk: blue-low; green-intermediate; red-high

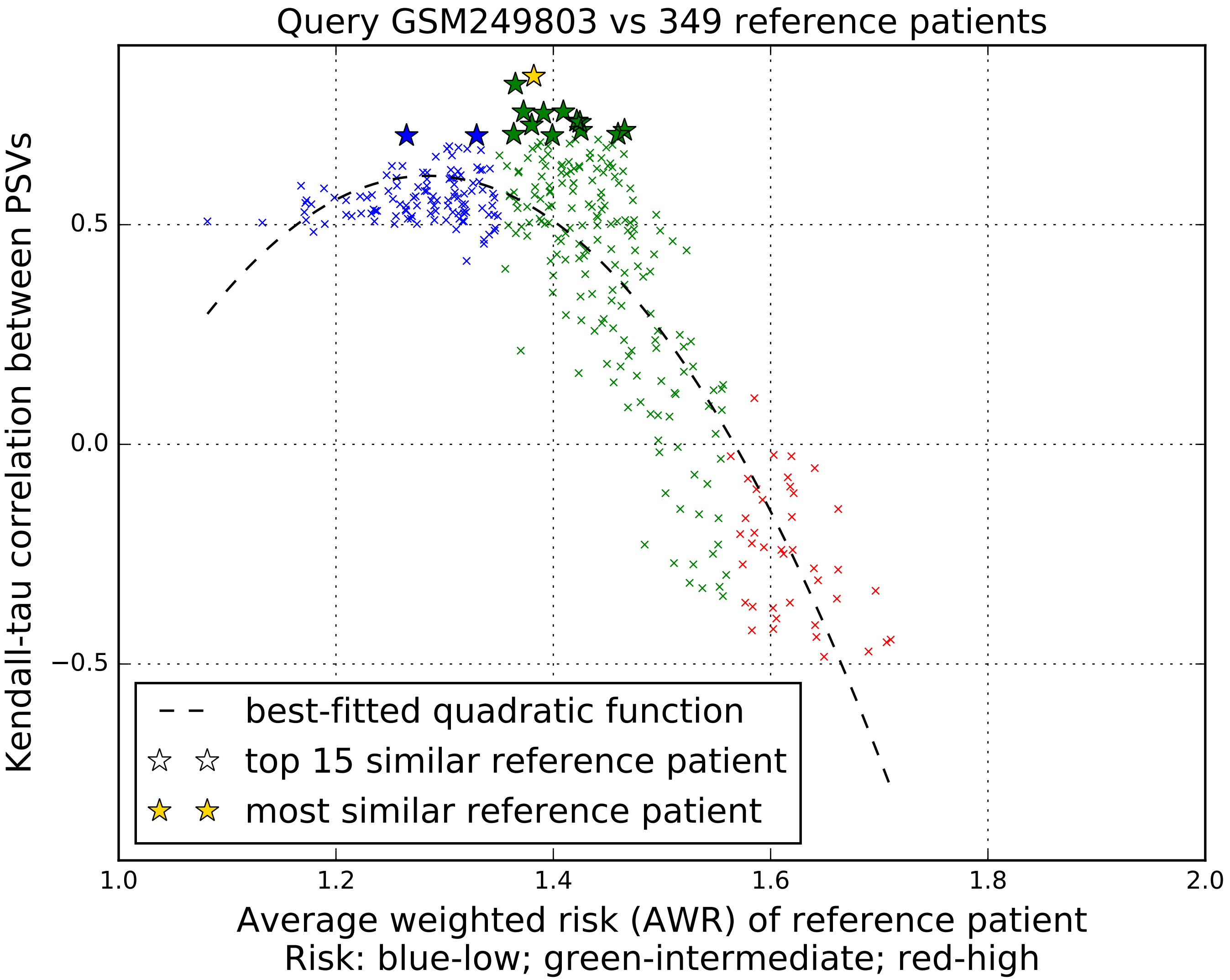

Query GSM249803 vs 349 reference patients
Kendall-tau correlation between PSVs
0.5
0.0
−0.5
1.0
1.2
1.4
1.6
1.8
2.0
Average weighted risk (AWR) of reference patient
Risk: blue-low; green-intermediate; red-high
best-fitted quadratic function
top 15 similar reference patient
most similar reference patient

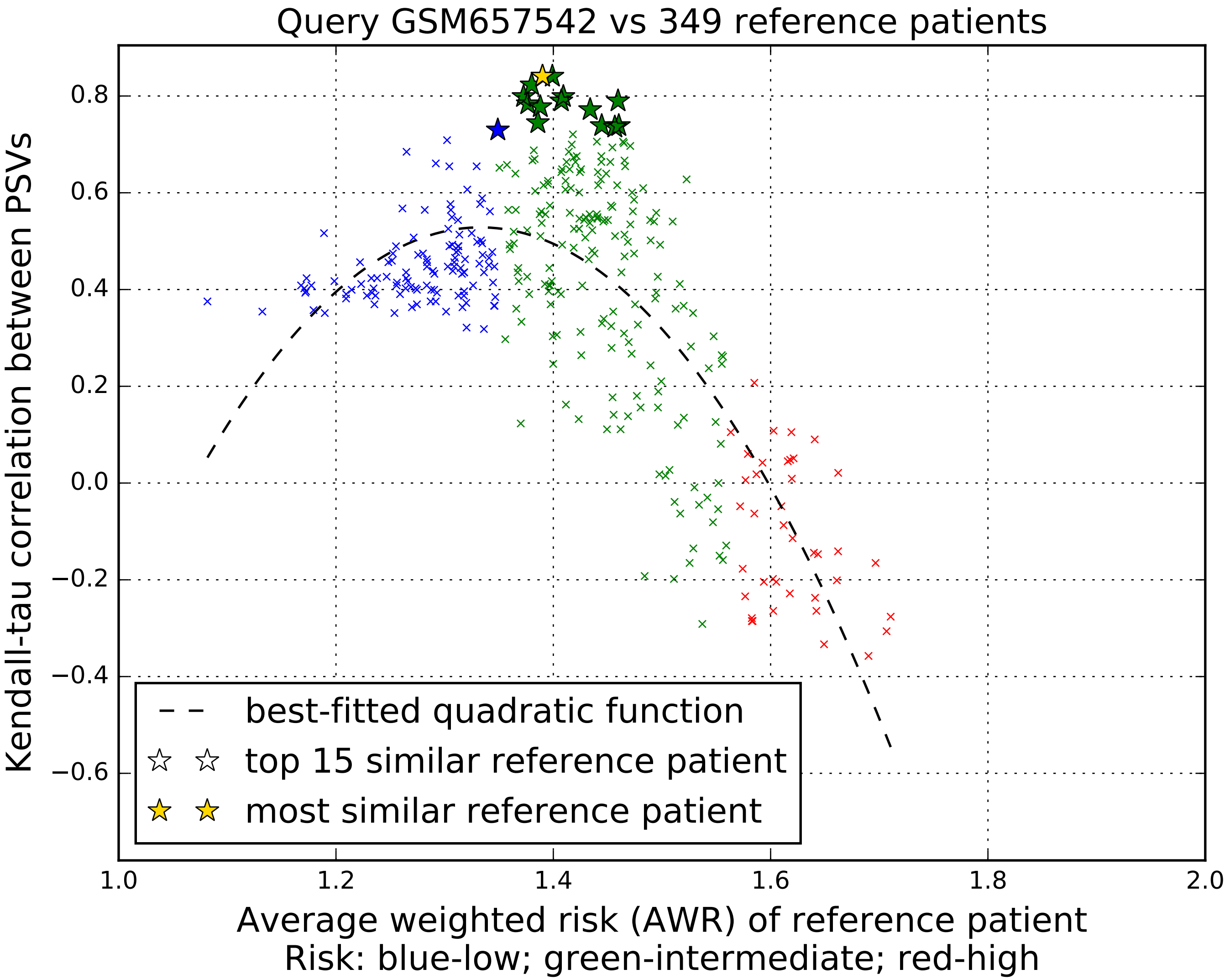
Query GSM657542 vs 349 reference patients
Kendall-tau correlation between PSVs
Average weighted risk (AWR) of reference patient
Risk: blue-low; green-intermediate; red-high
best-fitted quadratic function
top 15 similar reference patient
most similar reference patient
0.8
0.6
0.4
0.2
0.0
−0.2
−0.4
−0.6
1.0
1.2
1.4
1.6
1.8
2.0

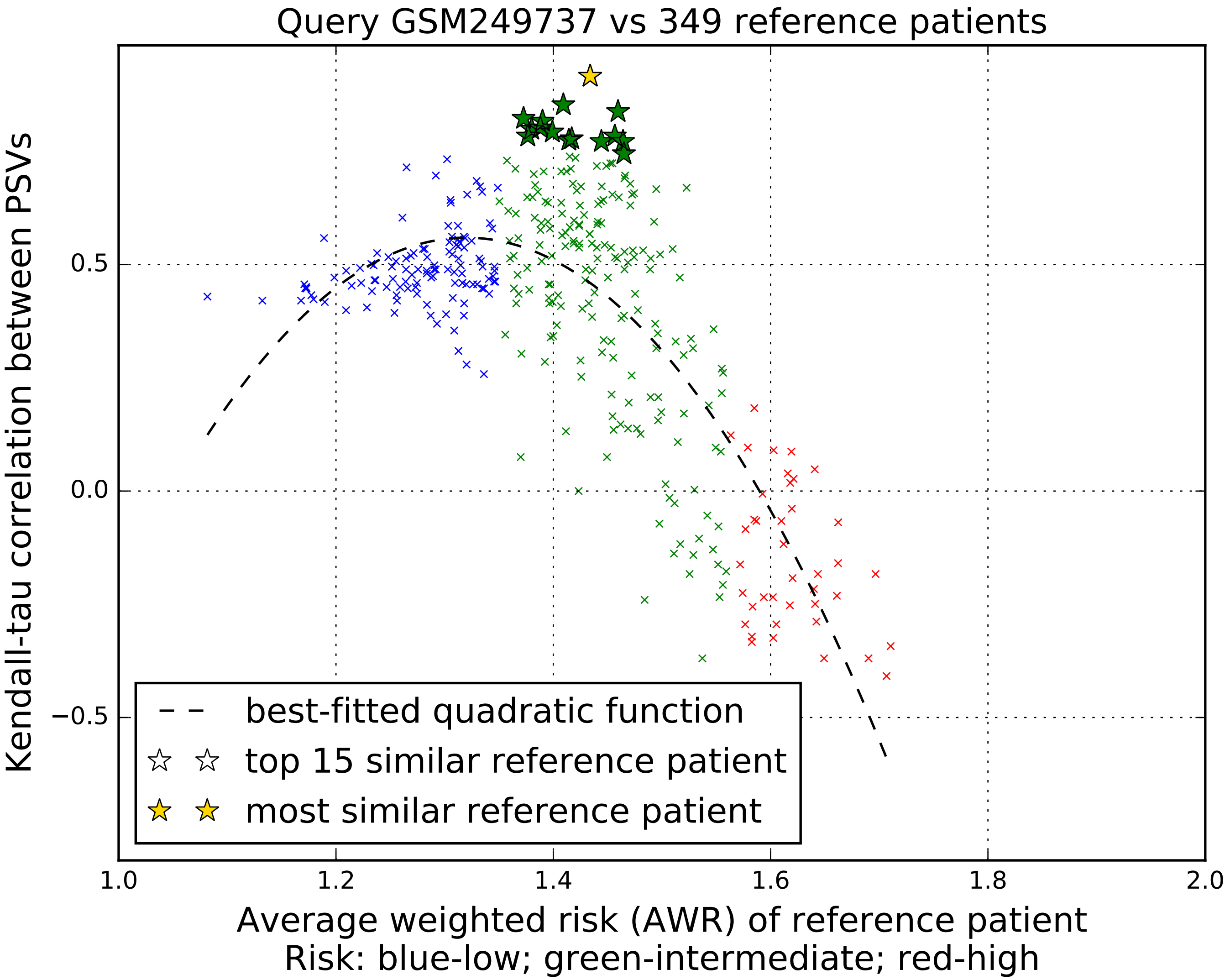

Query GSM249737 vs 349 reference patients
Kendall-tau correlation between PSVs
0.5
0.0
−0.5
1.0
1.2
1.4
1.6
1.8
2.0
best-fitted quadratic function
top 15 similar reference patient
most similar reference patient
Average weighted risk (AWR) of reference patient
Risk: blue-low; green-intermediate; red-high

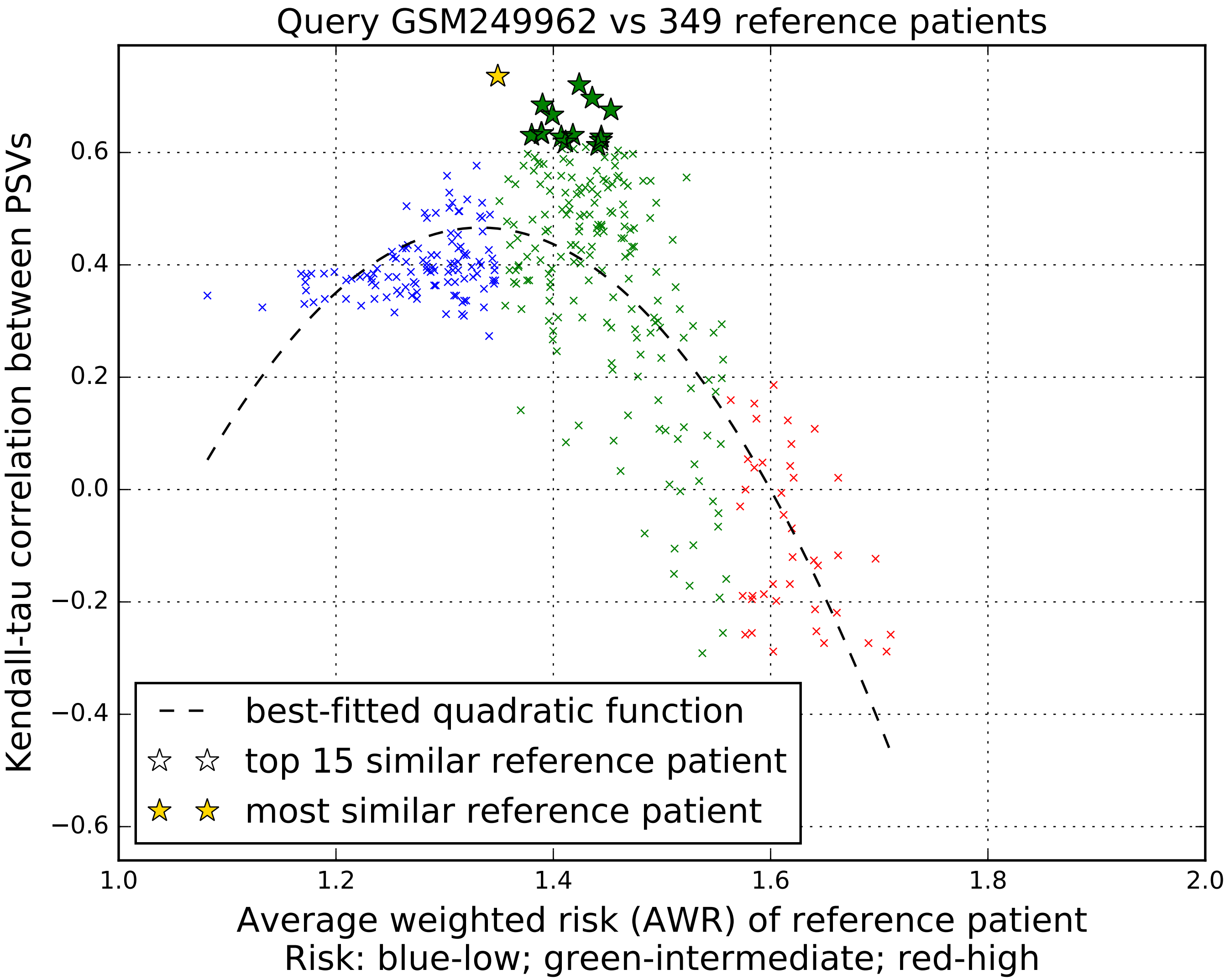

Query GSM249962 vs 349 reference patients
Kendall-tau correlation between PSVs
Average weighted risk (AWR) of reference patient
Risk: blue-low; green-intermediate; red-high
best-fitted quadratic function
top 15 similar reference patient
most similar reference patient
0.6
0.4
0.2
0.0
−0.2
−0.4
−0.6
1.0
1.2
1.4
1.6
1.8
2.0

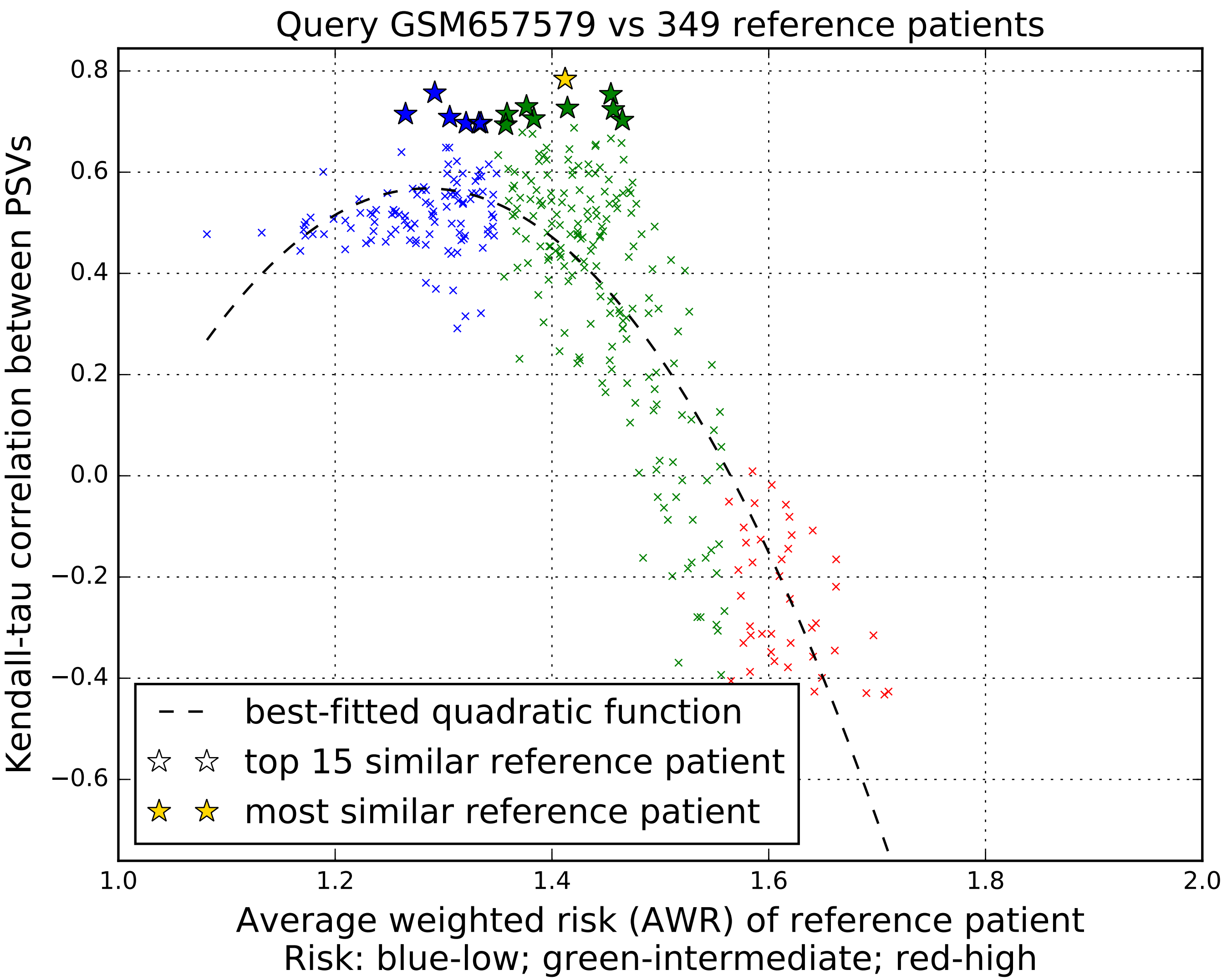
Query GSM657579 vs 349 reference patients
Kendall-tau correlation between PSVs
0.8
0.6
0.4
0.2
0.0
−0.2
−0.4
−0.6
1.0
1.2
1.4
1.6
1.8
2.0
best-fitted quadratic function
top 15 similar reference patient
most similar reference patient
Average weighted risk (AWR) of reference patient
Risk: blue-low; green-intermediate; red-high

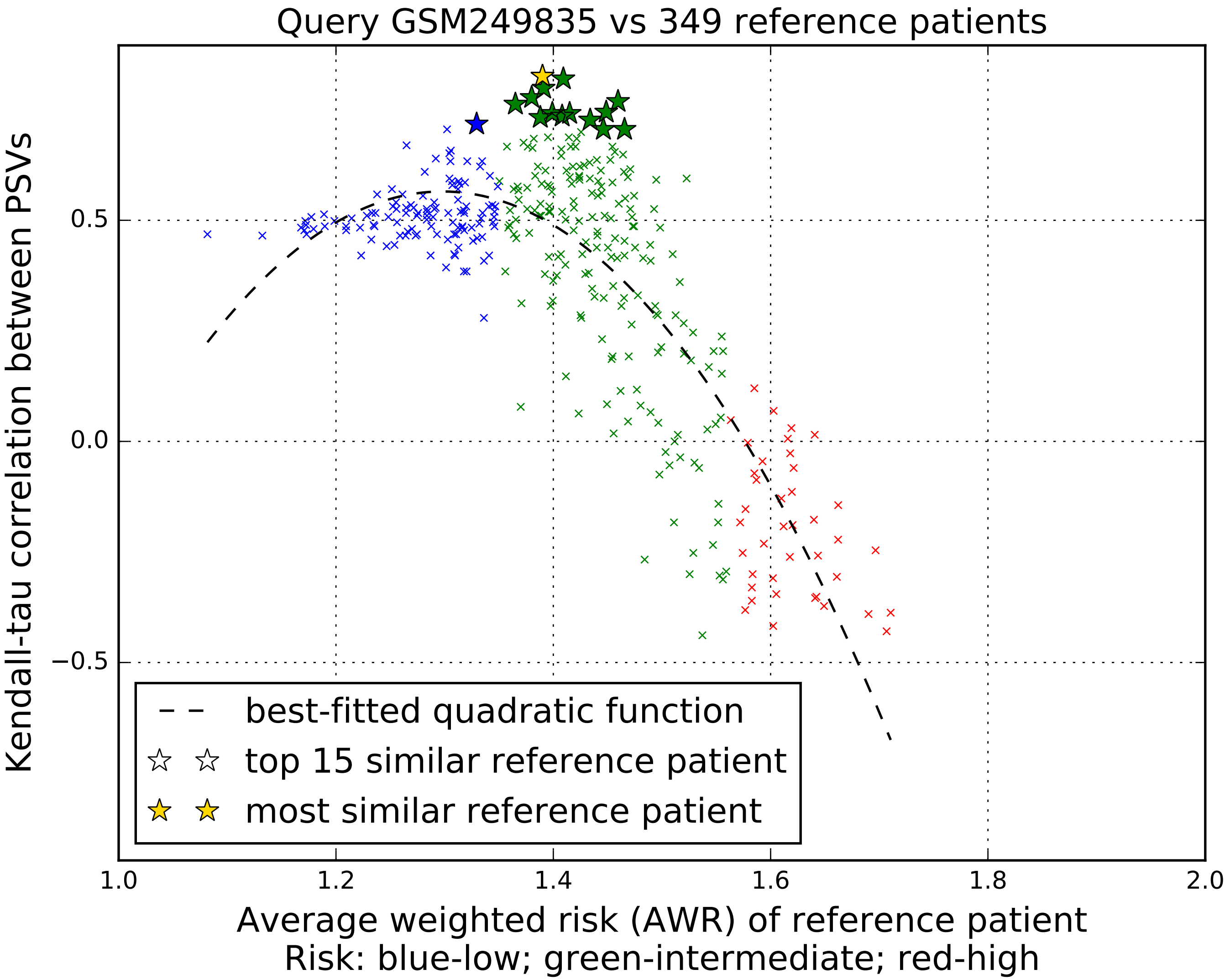

Query GSM249835 vs 349 reference patients
Kendall-tau correlation between PSVs
Average weighted risk (AWR) of reference patient
Risk: blue-low; green-intermediate; red-high
best-fitted quadratic function
top 15 similar reference patient
most similar reference patient
0.5
0.0
−0.5
1.0
1.2
1.4
1.6
1.8
2.0

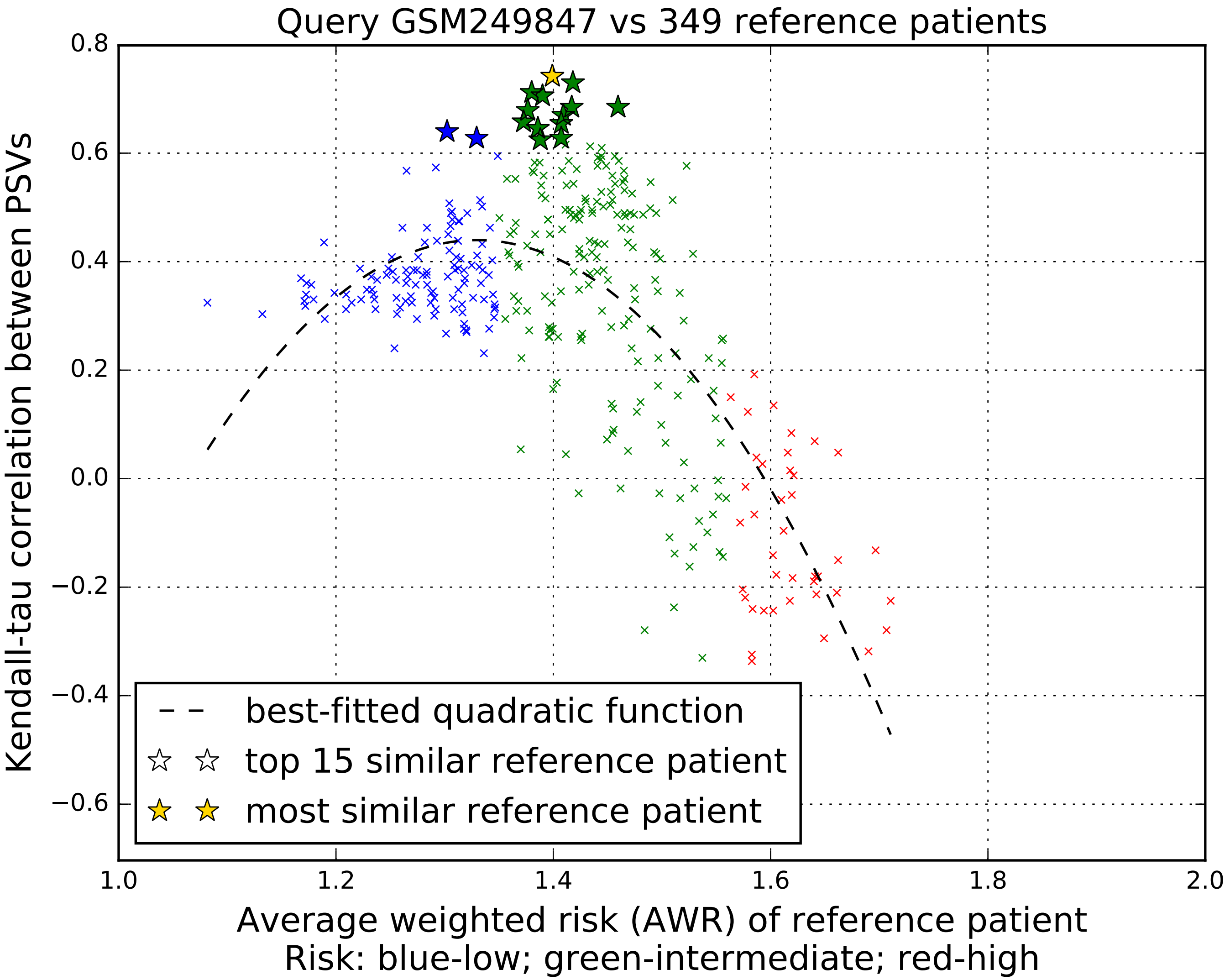

Query GSM249847 vs 349 reference patients
Kendall-tau correlation between PSVs
Average weighted risk (AWR) of reference patient
Risk: blue-low; green-intermediate; red-high
best-fitted quadratic function
top 15 similar reference patient
most similar reference patient
0.8
0.6
0.4
0.2
0.0
−0.2
−0.4
−0.6
1.0
1.2
1.4
1.6
1.8
2.0

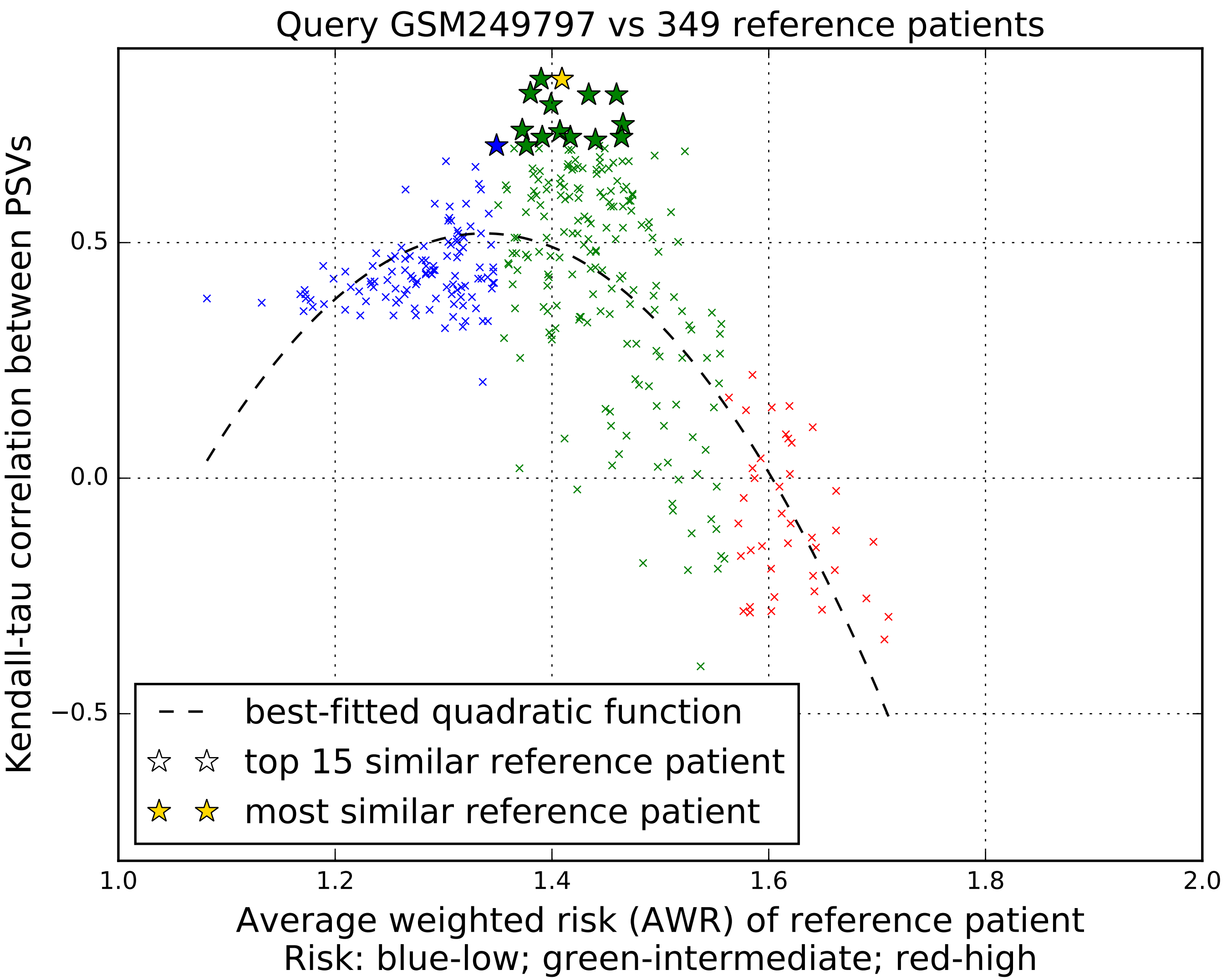
Query GSM249797 vs 349 reference patients
Kendall-tau correlation between PSVs
Average weighted risk (AWR) of reference patient
Risk: blue-low; green-intermediate; red-high
best-fitted quadratic function
top 15 similar reference patient
most similar reference patient
0.5
0.0
−0.5
1.0
1.2
1.4
1.6
1.8
2.0

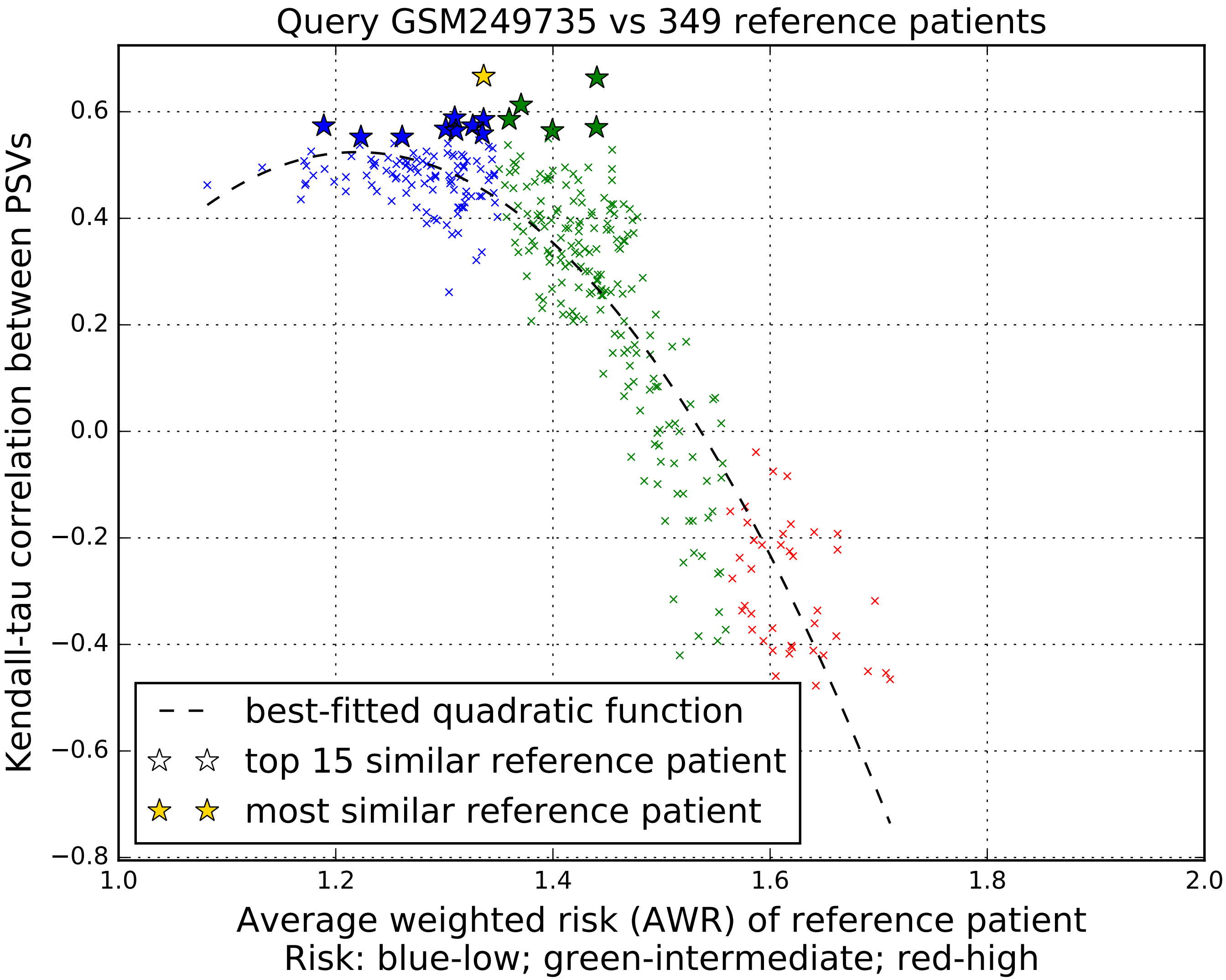

Query GSM249735 vs 349 reference patients
Kendall-tau correlation between PSVs
0.6
0.4
0.2
0.0
−0.2
−0.4
−0.6
−0.8
1.0
1.2
1.4
1.6
1.8
2.0
best-fitted quadratic function
top 15 similar reference patient
most similar reference patient
Average weighted risk (AWR) of reference patient
Risk: blue-low; green-intermediate; red-high

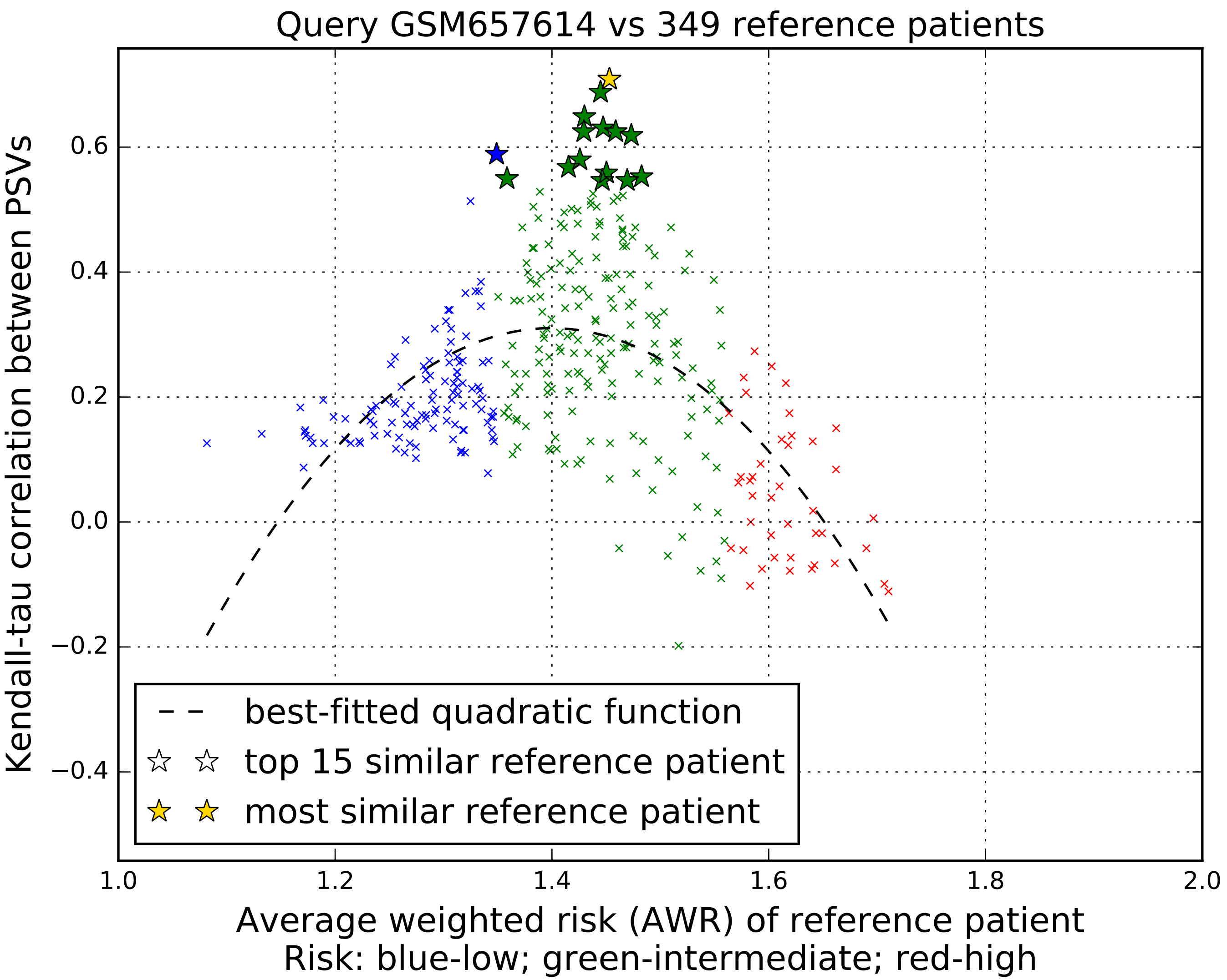

Query GSM657614 vs 349 reference patients
Kendall-tau correlation between PSVs
0.6
0.4
0.2
0.0
−0.2
−0.4
1.0
1.2
1.4
1.6
1.8
2.0
best-fitted quadratic function
top 15 similar reference patient
most similar reference patient
Average weighted risk (AWR) of reference patient
Risk: blue-low; green-intermediate; red-high

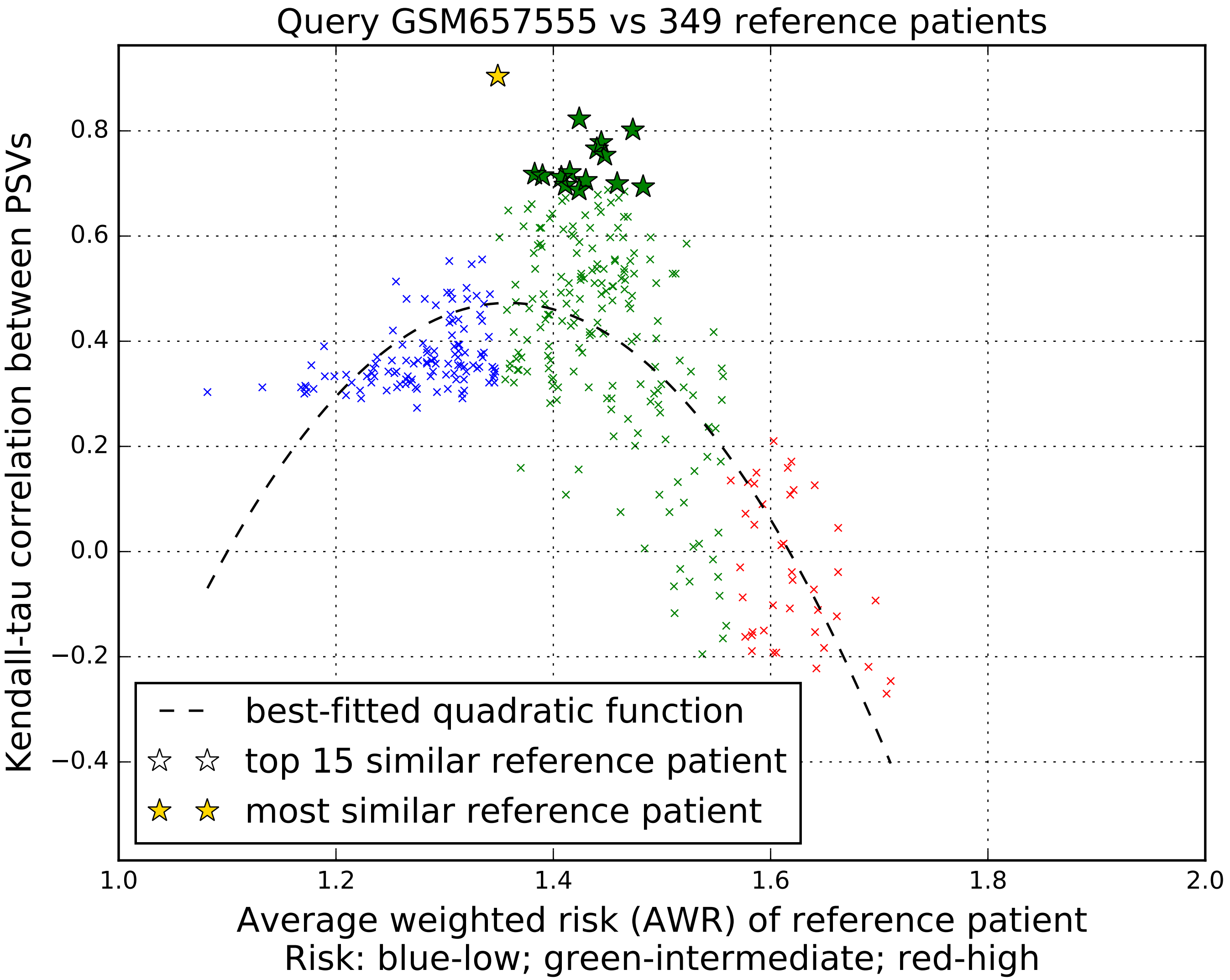

Query GSM657555 vs 349 reference patients
Kendall-tau correlation between PSVs
0.8
0.6
0.4
0.2
0.0
−0.2
−0.4
1.0
1.2
1.4
1.6
1.8
2.0
best-fitted quadratic function
top 15 similar reference patient
most similar reference patient
Average weighted risk (AWR) of reference patient
Risk: blue-low; green-intermediate; red-high

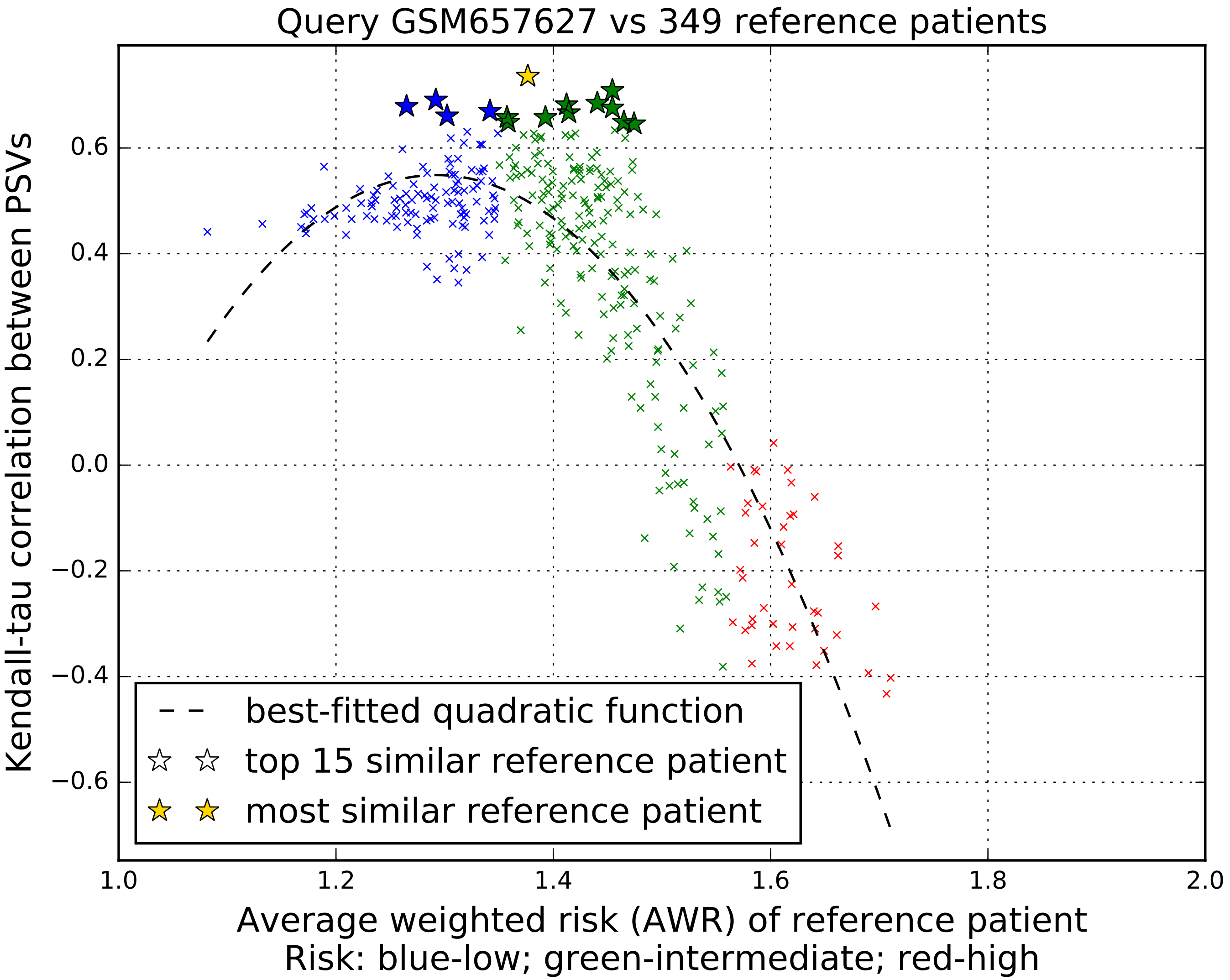

Query GSM657627 vs 349 reference patients
Kendall-tau correlation between PSVs
Average weighted risk (AWR) of reference patient
Risk: blue-low; green-intermediate; red-high
best-fitted quadratic function
top 15 similar reference patient
most similar reference patient
0.6
0.4
0.2
0.0
−0.2
−0.4
−0.6
1.0
1.2
1.4
1.6
1.8
2.0

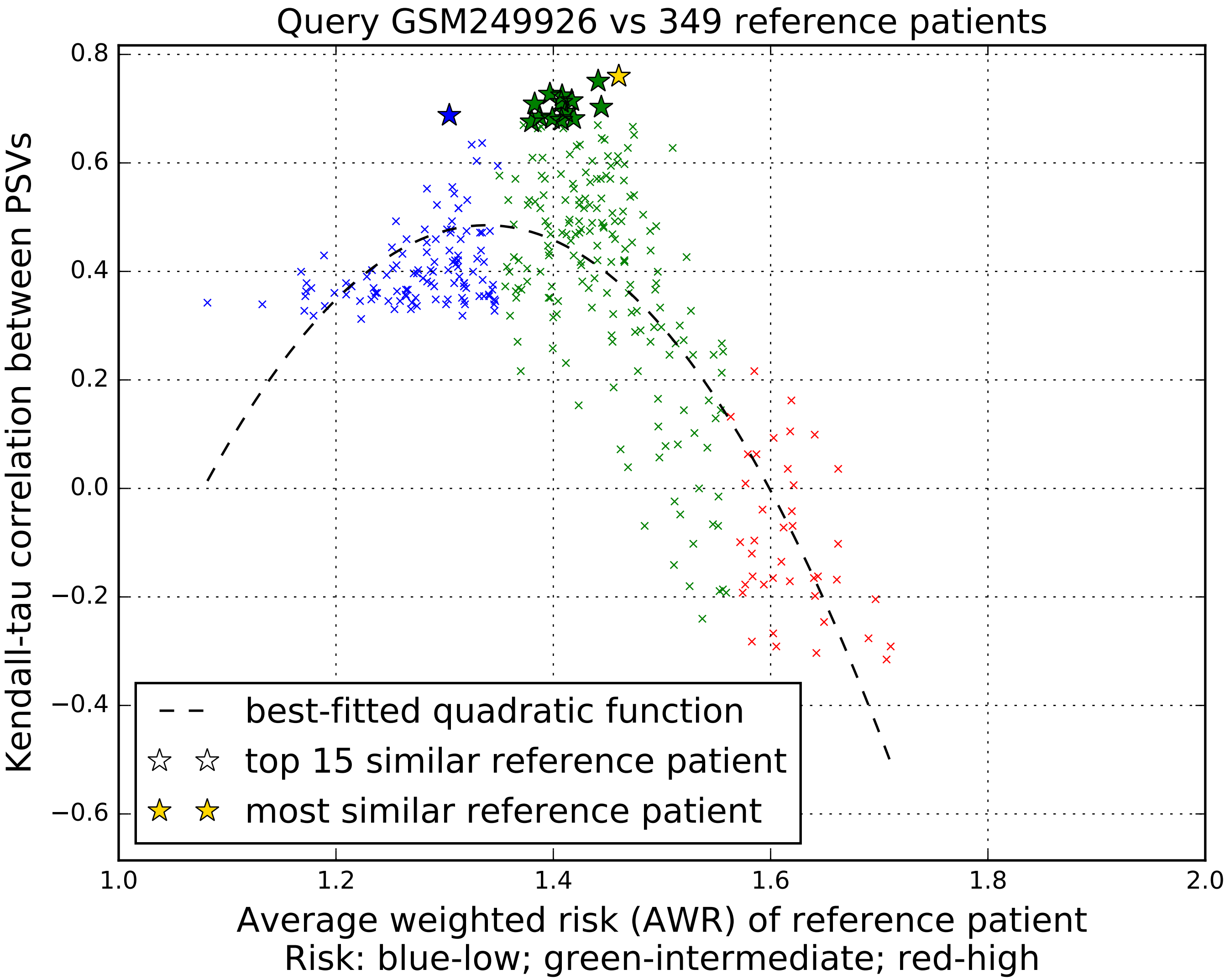
Query GSM249926 vs 349 reference patients
Kendall-tau correlation between PSVs
0.8
0.6
0.4
0.2
0.0
−0.2
−0.4
−0.6
1.0
1.2
1.4
1.6
1.8
2.0
best-fitted quadratic function
top 15 similar reference patient
most similar reference patient
Average weighted risk (AWR) of reference patient
Risk: blue-low; green-intermediate; red-high

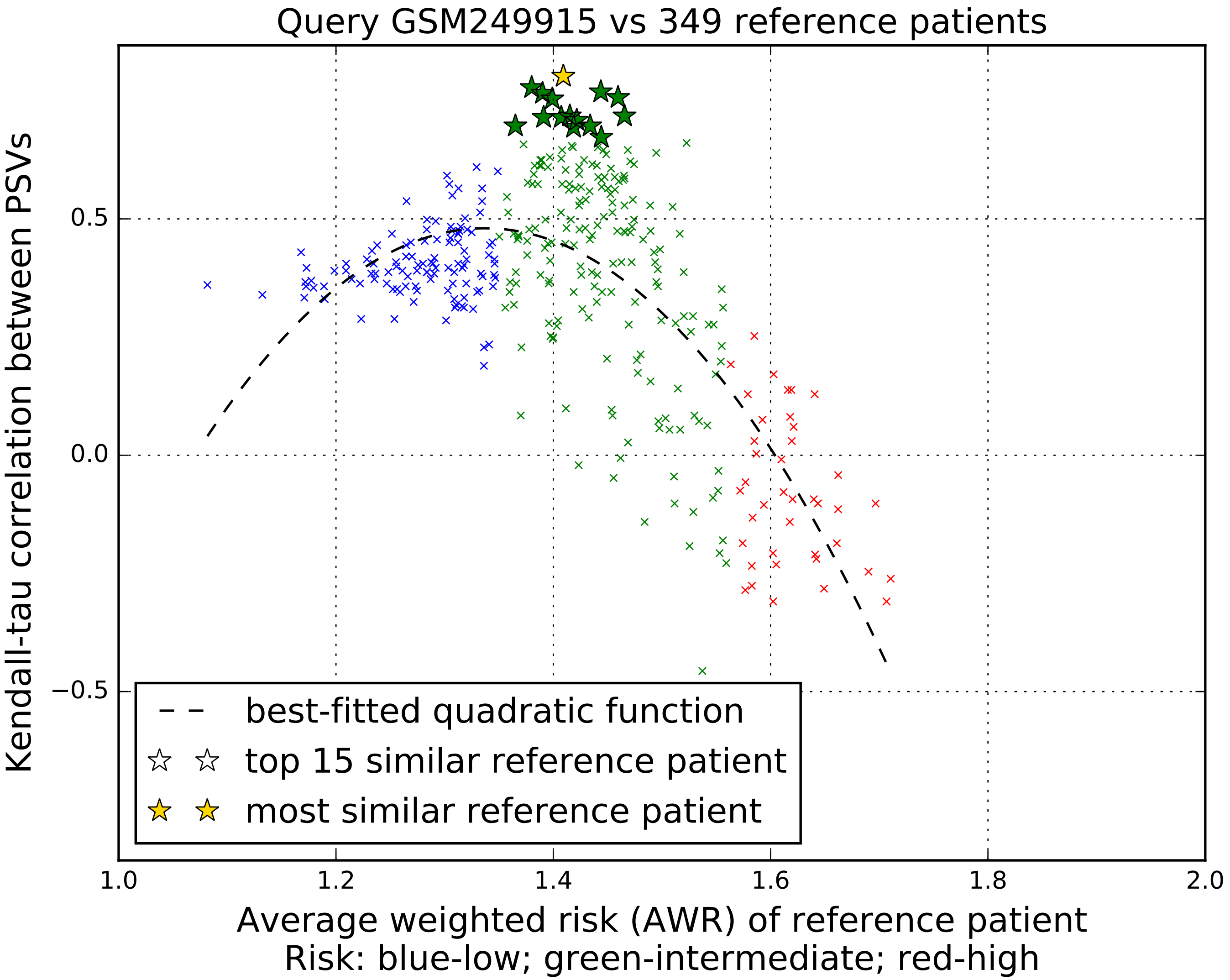

Query GSM249915 vs 349 reference patients
Kendall-tau correlation between PSVs
0.5
0.0
−0.5
1.0
1.2
1.4
1.6
1.8
2.0
best-fitted quadratic function
top 15 similar reference patient
most similar reference patient
Average weighted risk (AWR) of reference patient
Risk: blue-low; green-intermediate; red-high

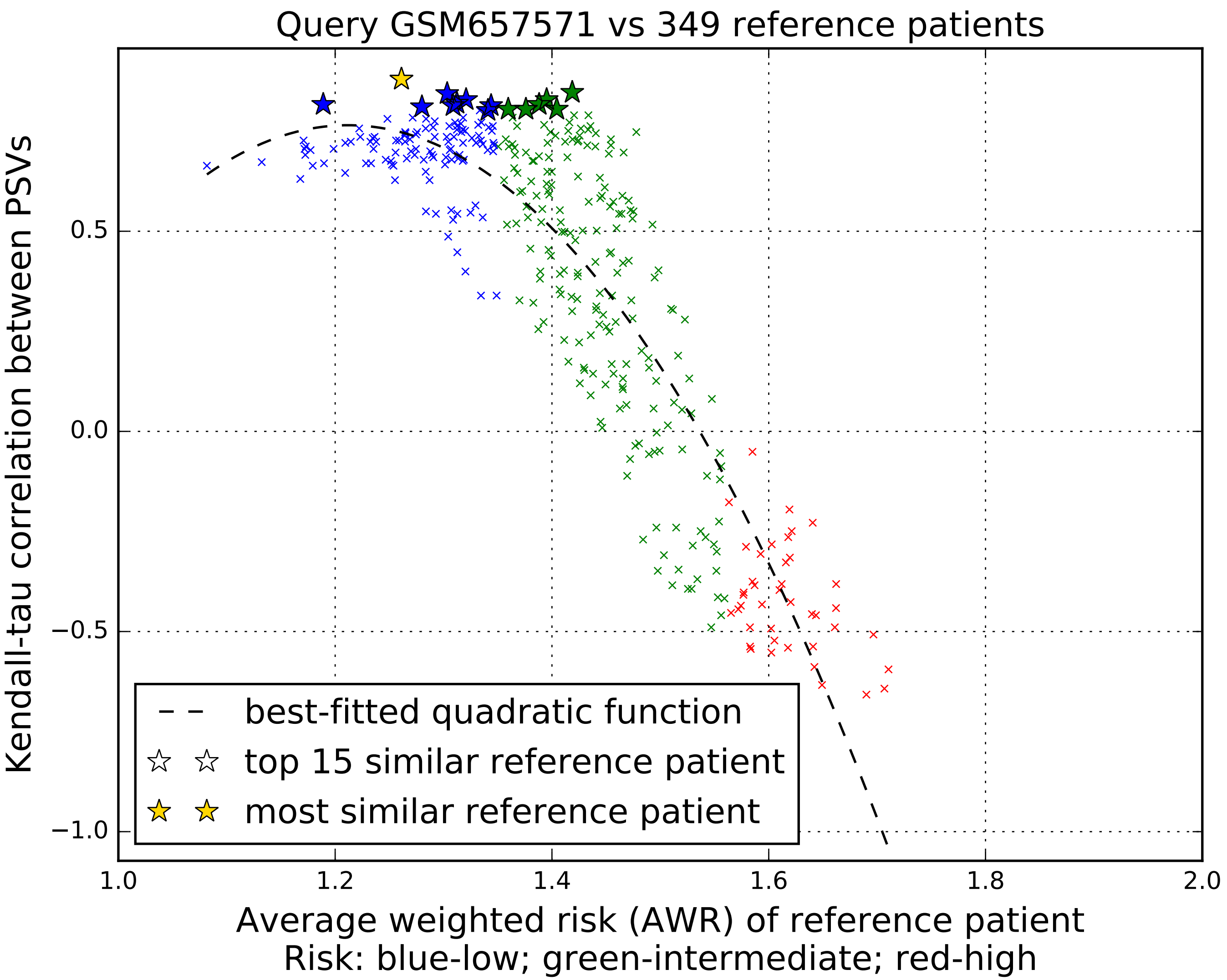
Query GSM657571 vs 349 reference patients
Kendall-tau correlation between PSVs
0.5
0.0
−0.5
−1.0
1.0
1.2
1.4
1.6
1.8
2.0
best-fitted quadratic function
top 15 similar reference patient
most similar reference patient
Average weighted risk (AWR) of reference patient
Risk: blue-low; green-intermediate; red-high

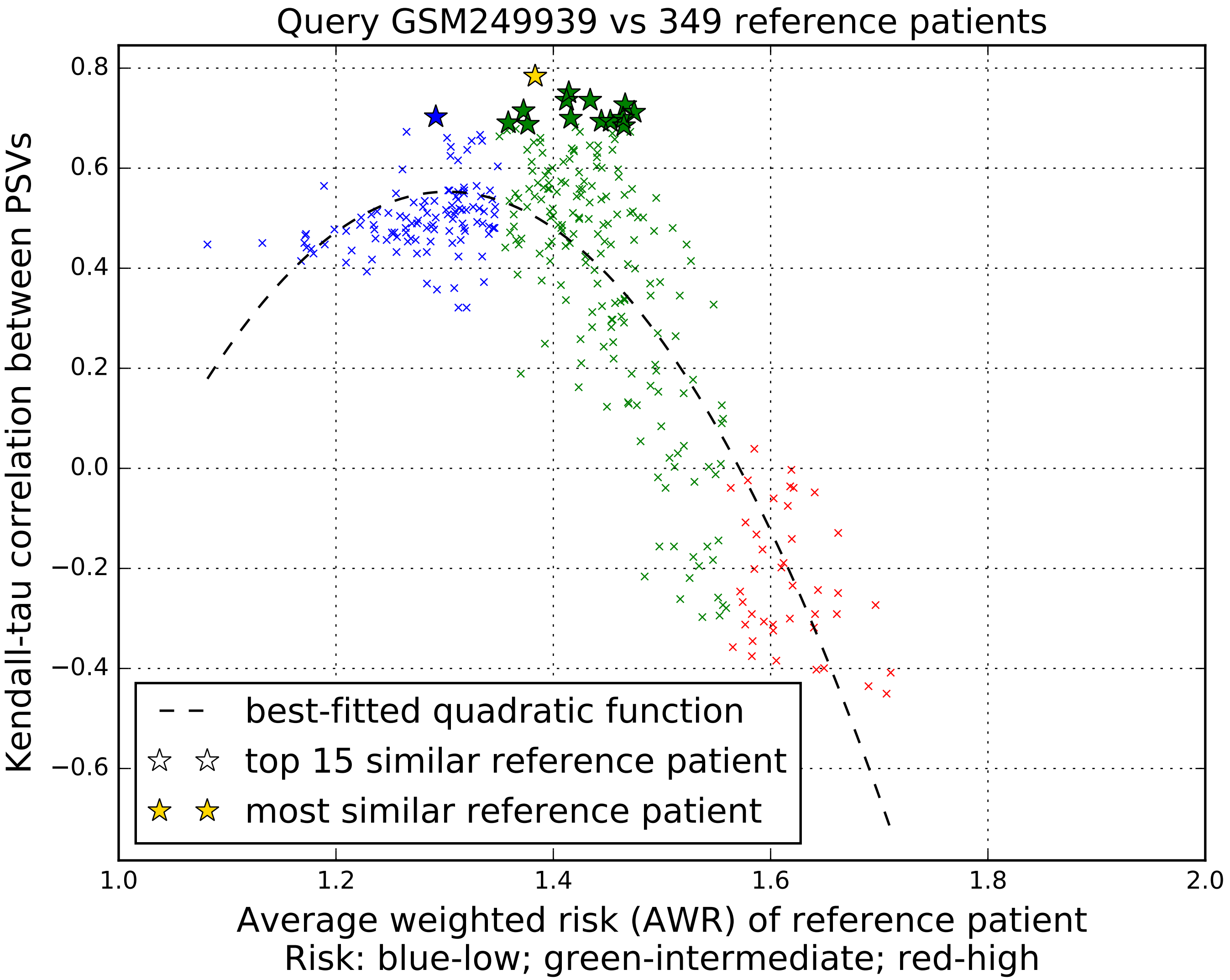

Query GSM249939 vs 349 reference patients
Kendall-tau correlation between PSVs
Average weighted risk (AWR) of reference patient
Risk: blue-low; green-intermediate; red-high
best-fitted quadratic function
top 15 similar reference patient
most similar reference patient
0.8
0.6
0.4
0.2
0.0
−0.2
−0.4
−0.6
1.0
1.2
1.4
1.6
1.8
2.0

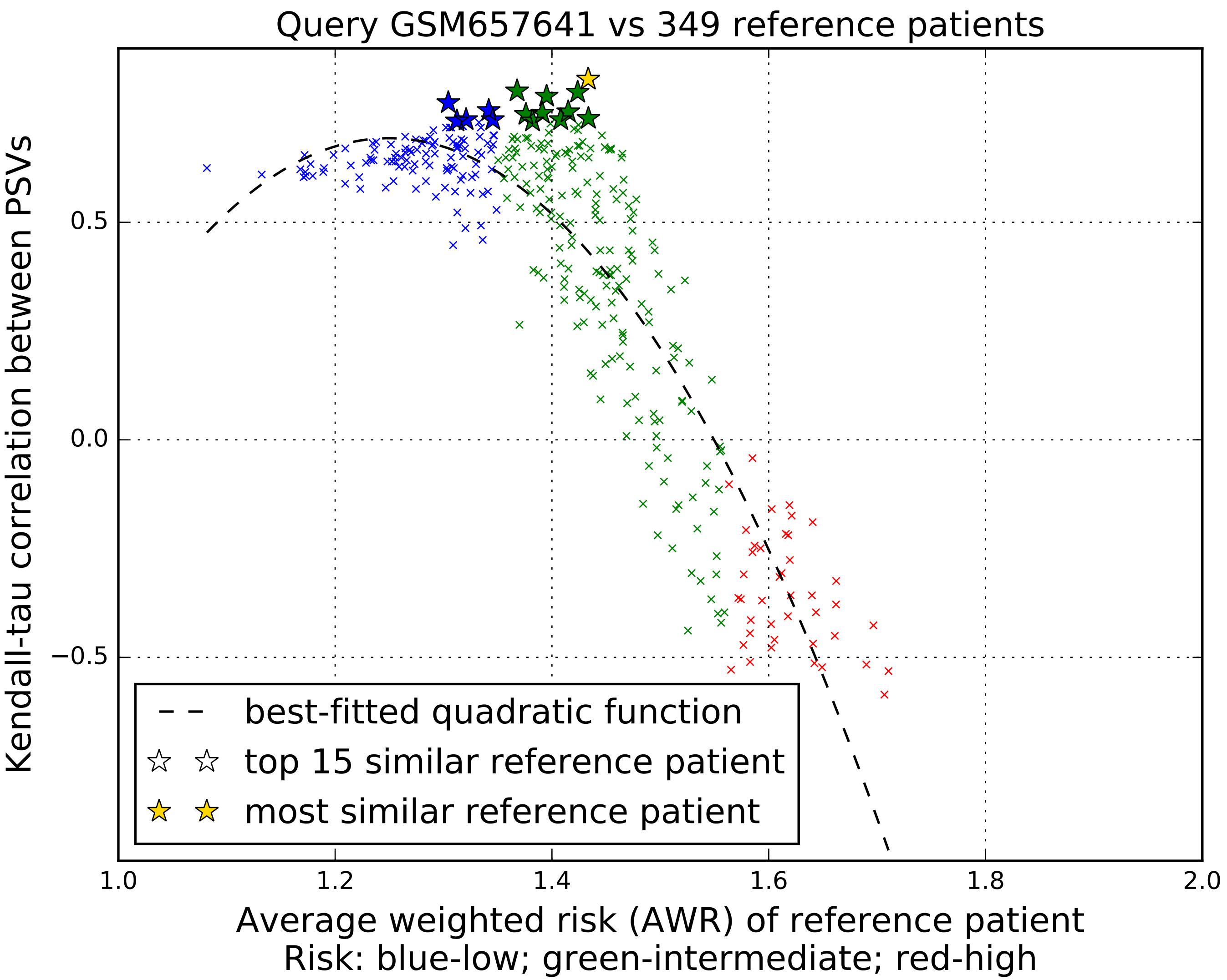
Query GSM657641 vs 349 reference patients
Kendall-tau correlation between PSVs
0.5
0.0
−0.5
1.0
1.2
1.4
1.6
1.8
2.0
Average weighted risk (AWR) of reference patient
Risk: blue-low; green-intermediate; red-high
best-fitted quadratic function
top 15 similar reference patient
most similar reference patient

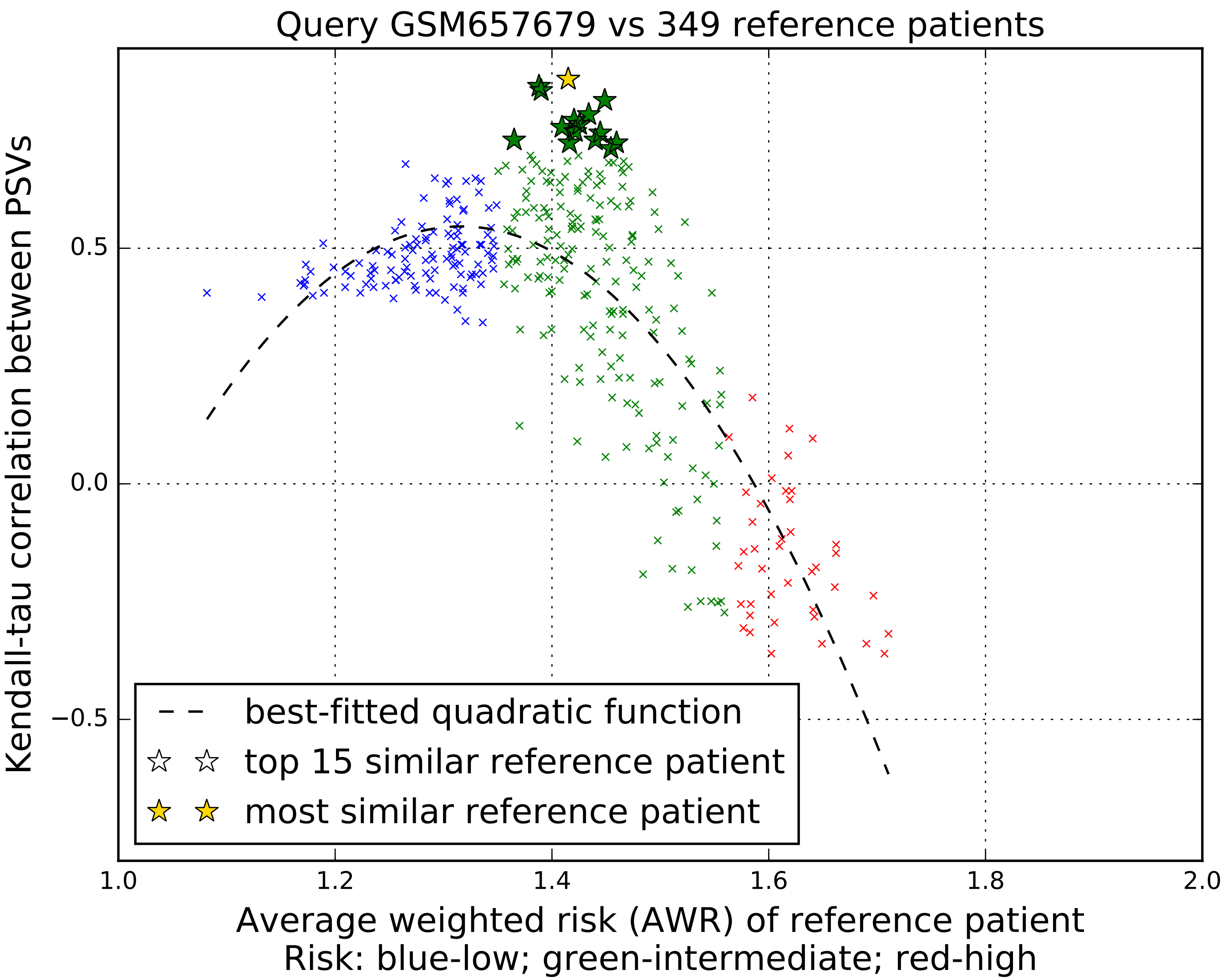
Query GSM657679 vs 349 reference patients
Kendall-tau correlation between PSVs
0.5
0.0
−0.5
1.0
1.2
1.4
1.6
1.8
2.0
best-fitted quadratic function
top 15 similar reference patient
most similar reference patient
Average weighted risk (AWR) of reference patient
Risk: blue-low; green-intermediate; red-high

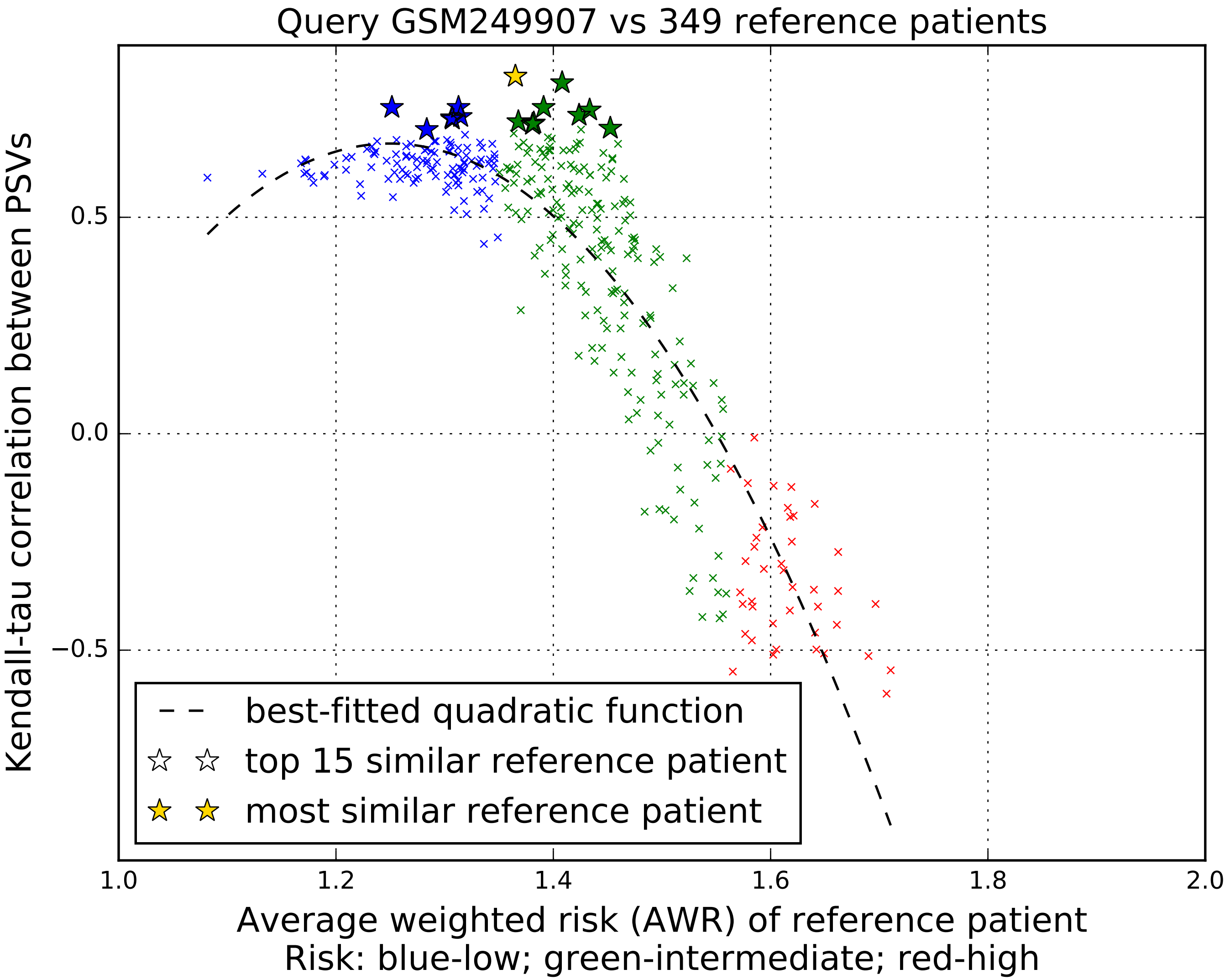

Query GSM249907 vs 349 reference patients
Kendall-tau correlation between PSVs
0.5
0.0
−0.5
1.0
1.2
1.4
1.6
1.8
2.0
best-fitted quadratic function
top 15 similar reference patient
most similar reference patient
Average weighted risk (AWR) of reference patient
Risk: blue-low; green-intermediate; red-high

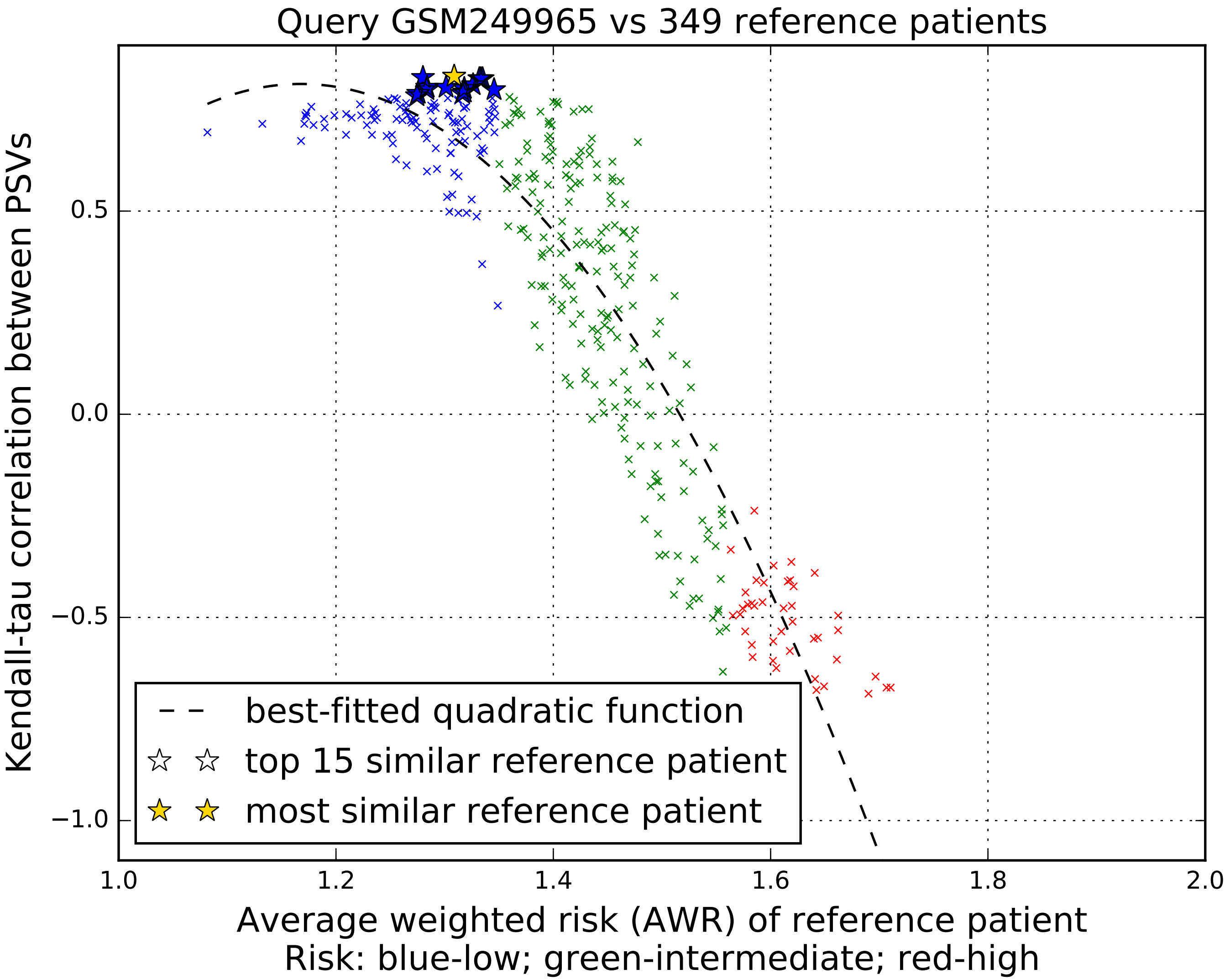

Query GSM249965 vs 349 reference patients
Kendall-tau correlation between PSVs
0.5
0.0
−0.5
−1.0
1.0
1.2
1.4
1.6
1.8
2.0
best-fitted quadratic function
top 15 similar reference patient
most similar reference patient
Average weighted risk (AWR) of reference patient
Risk: blue-low; green-intermediate; red-high

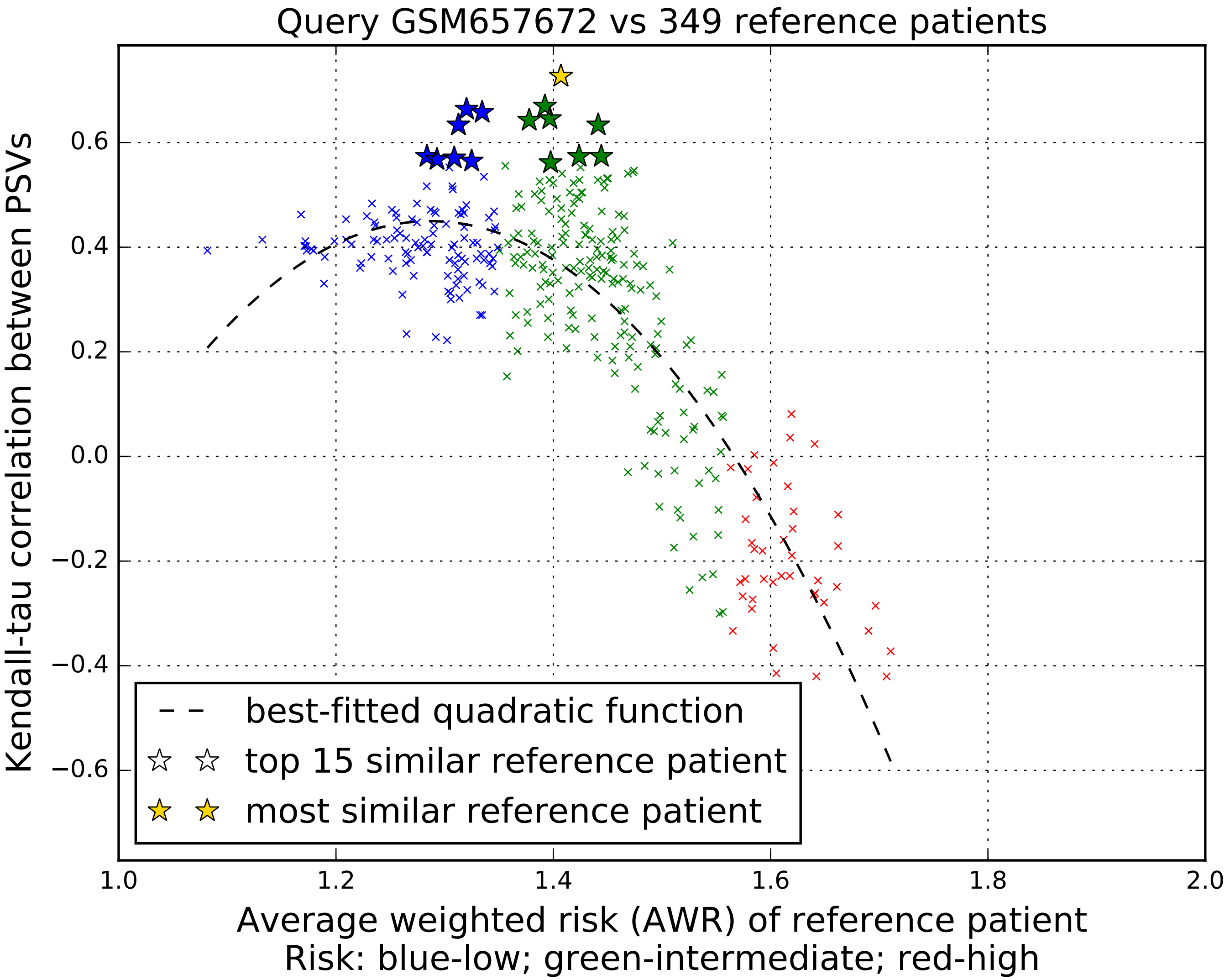

Query GSM657672 vs 349 reference patients
Kendall-tau correlation between PSVs
Average weighted risk (AWR) of reference patient
Risk: blue-low; green-intermediate; red-high
best-fitted quadratic function
top 15 similar reference patient
most similar reference patient
0.6
0.4
0.2
0.0
−0.2
−0.4
−0.6
1.0
1.2
1.4
1.6
1.8
2.0

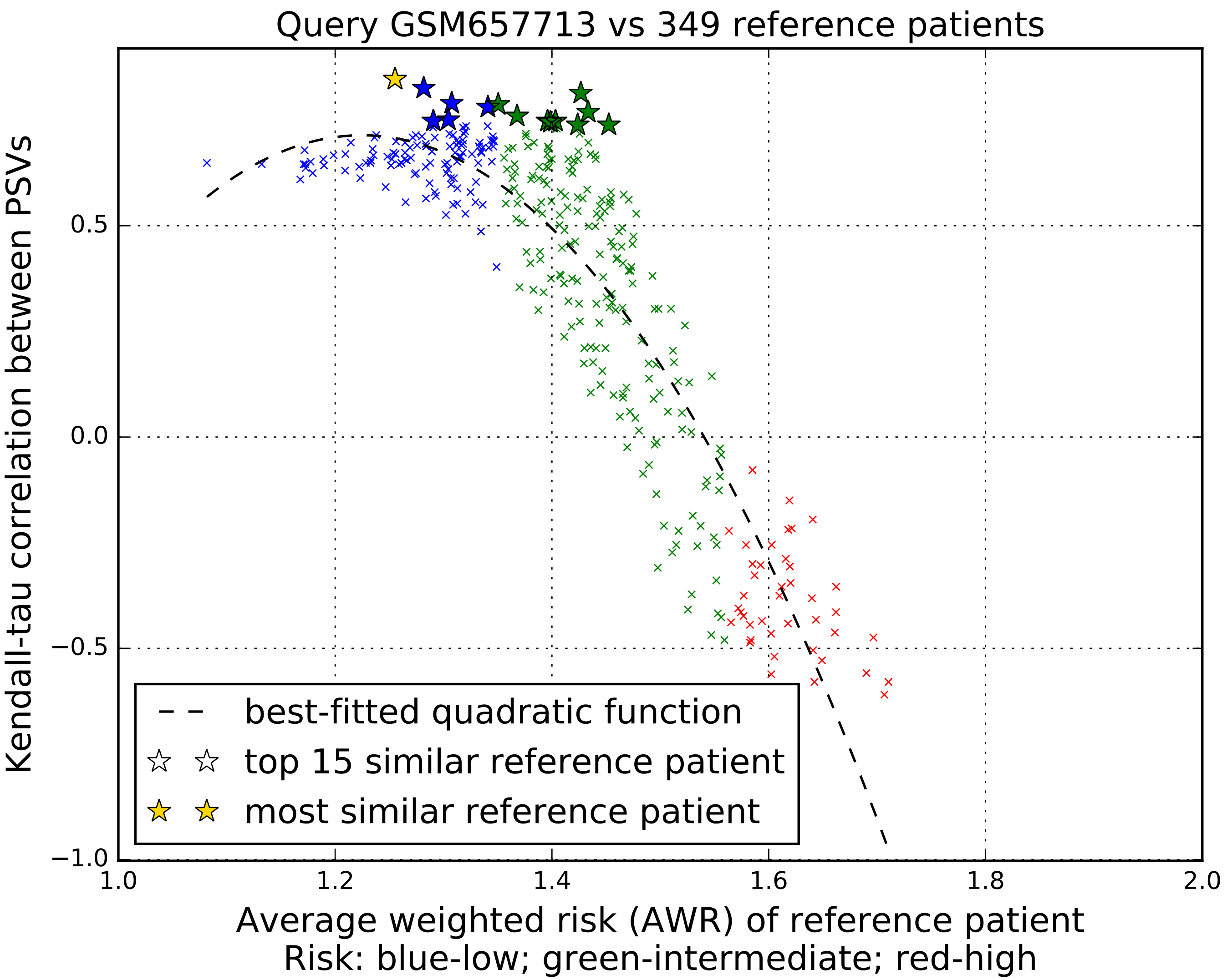
Query GSM657713 vs 349 reference patients
Kendall-tau correlation between PSVs
0.5
0.0
−0.5
−1.0
1.0
1.2
1.4
1.6
1.8
2.0
best-fitted quadratic function
top 15 similar reference patient
most similar reference patient
Average weighted risk (AWR) of reference patient
Risk: blue-low; green-intermediate; red-high

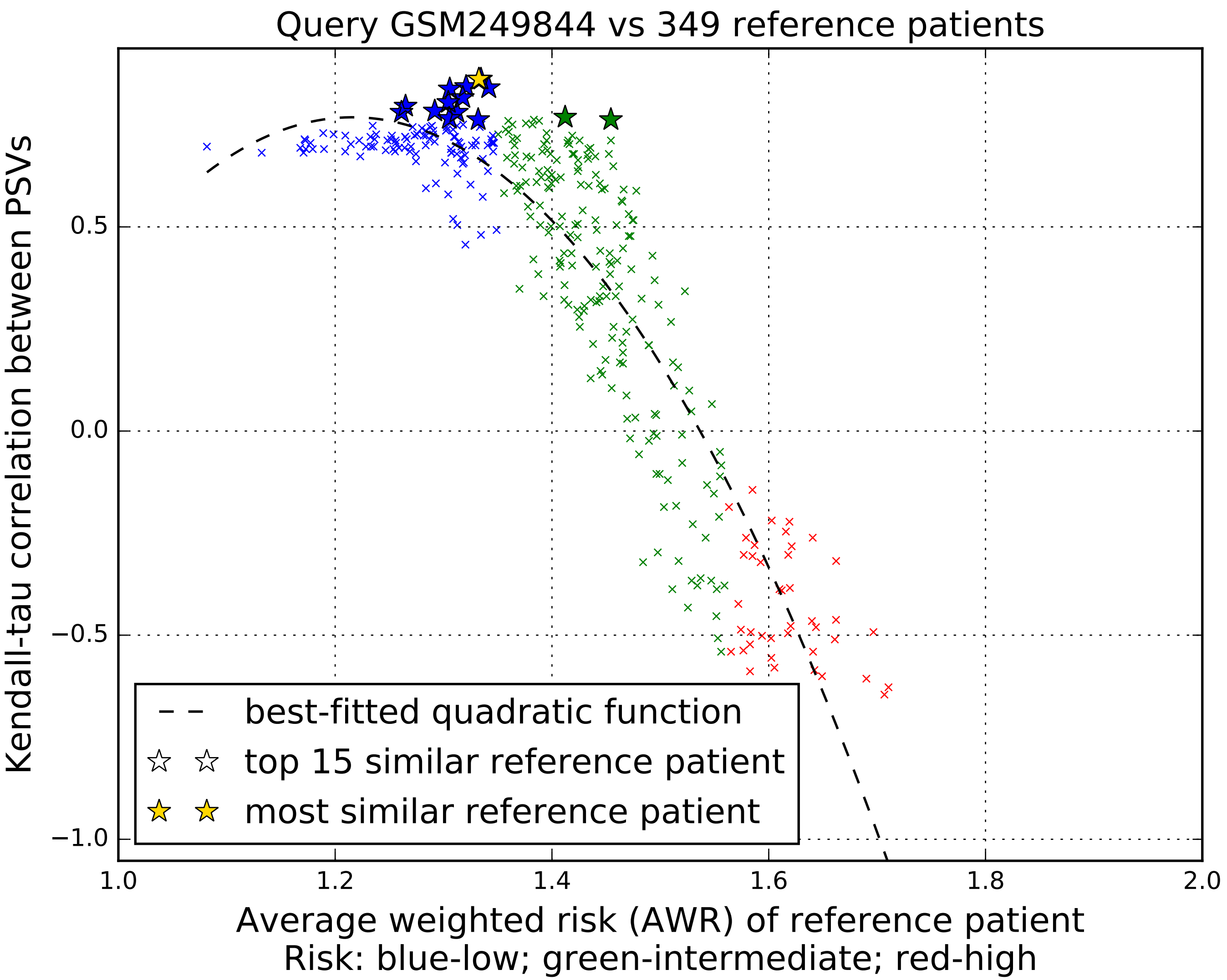

Query GSM249844 vs 349 reference patients
Kendall-tau correlation between PSVs
0.5
0.0
−0.5
−1.0
1.0
1.2
1.4
1.6
1.8
2.0
best-fitted quadratic function
top 15 similar reference patient
most similar reference patient
Average weighted risk (AWR) of reference patient
Risk: blue-low; green-intermediate; red-high

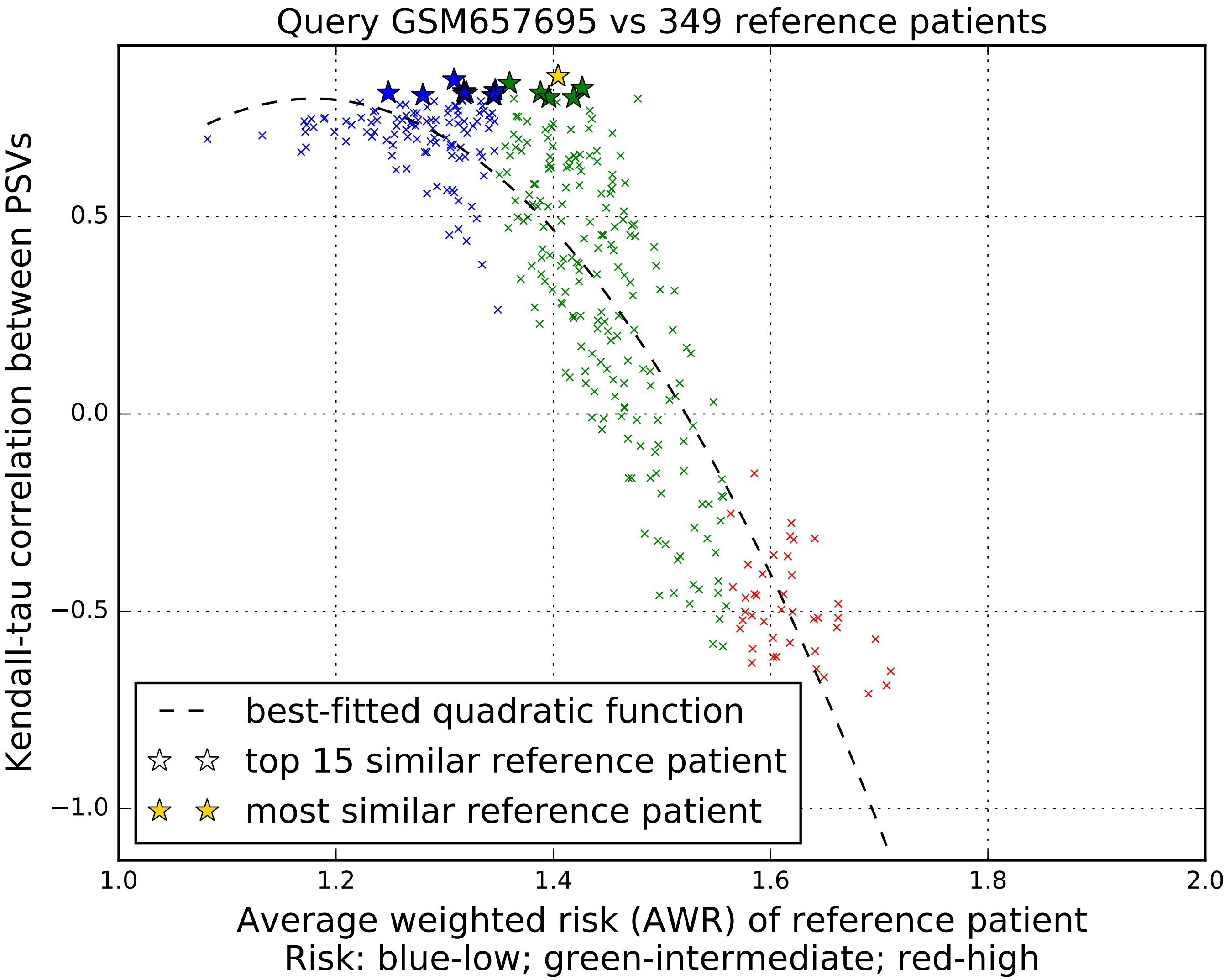
Query GSM657695 vs 349 reference patients
Kendall-tau correlation between PSVs
Average weighted risk (AWR) of reference patient
Risk: blue-low; green-intermediate; red-high
best-fitted quadratic function
top 15 similar reference patient
most similar reference patient
0.5
0.0
−0.5
−1.0
1.0
1.2
1.4
1.6
1.8
2.0

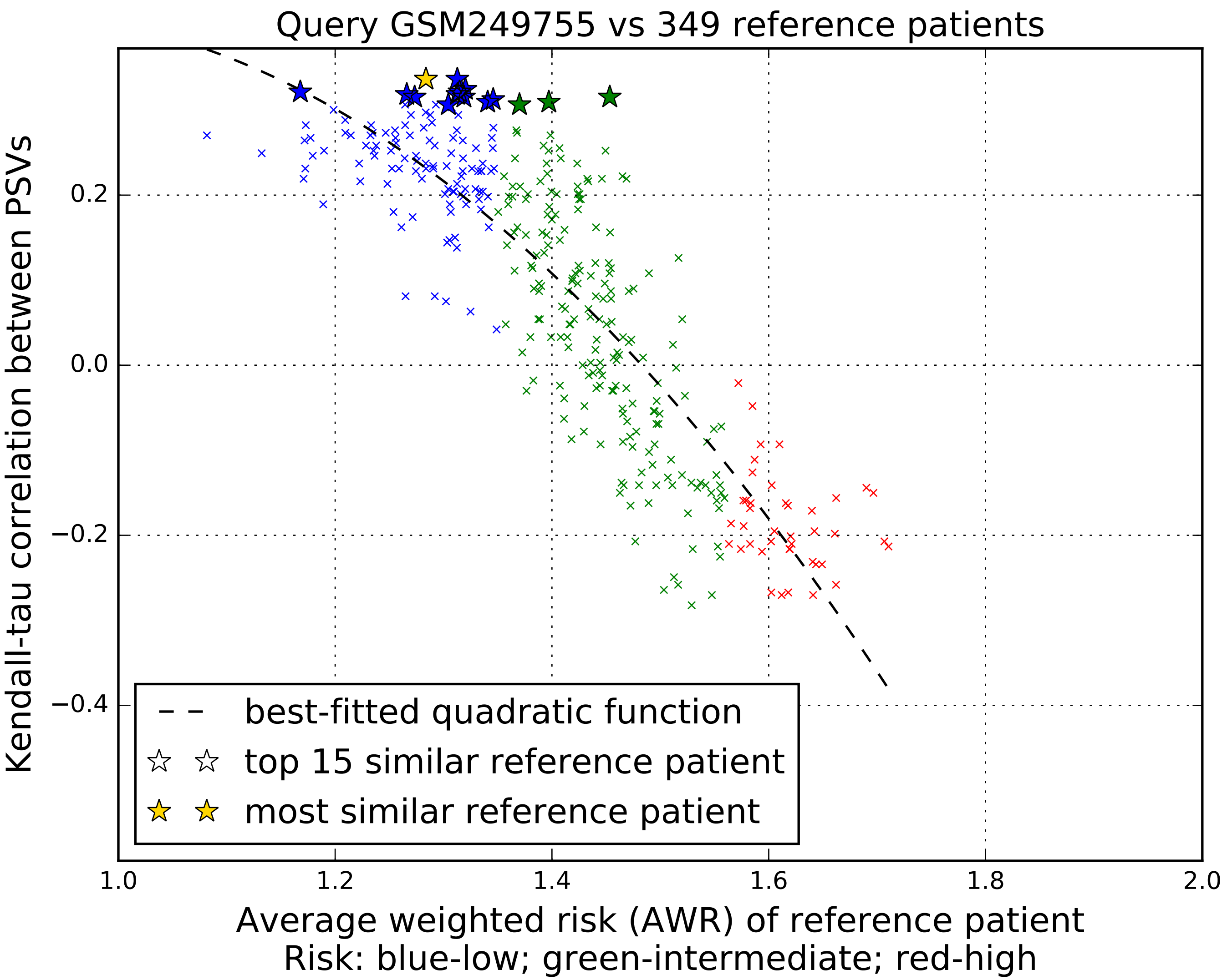
Query GSM249755 vs 349 reference patients
Kendall-tau correlation between PSVs
Average weighted risk (AWR) of reference patient
Risk: blue-low; green-intermediate; red-high
best-fitted quadratic function
top 15 similar reference patient
most similar reference patient
0.2
0.0
−0.2
−0.4
1.0
1.2
1.4
1.6
1.8
2.0

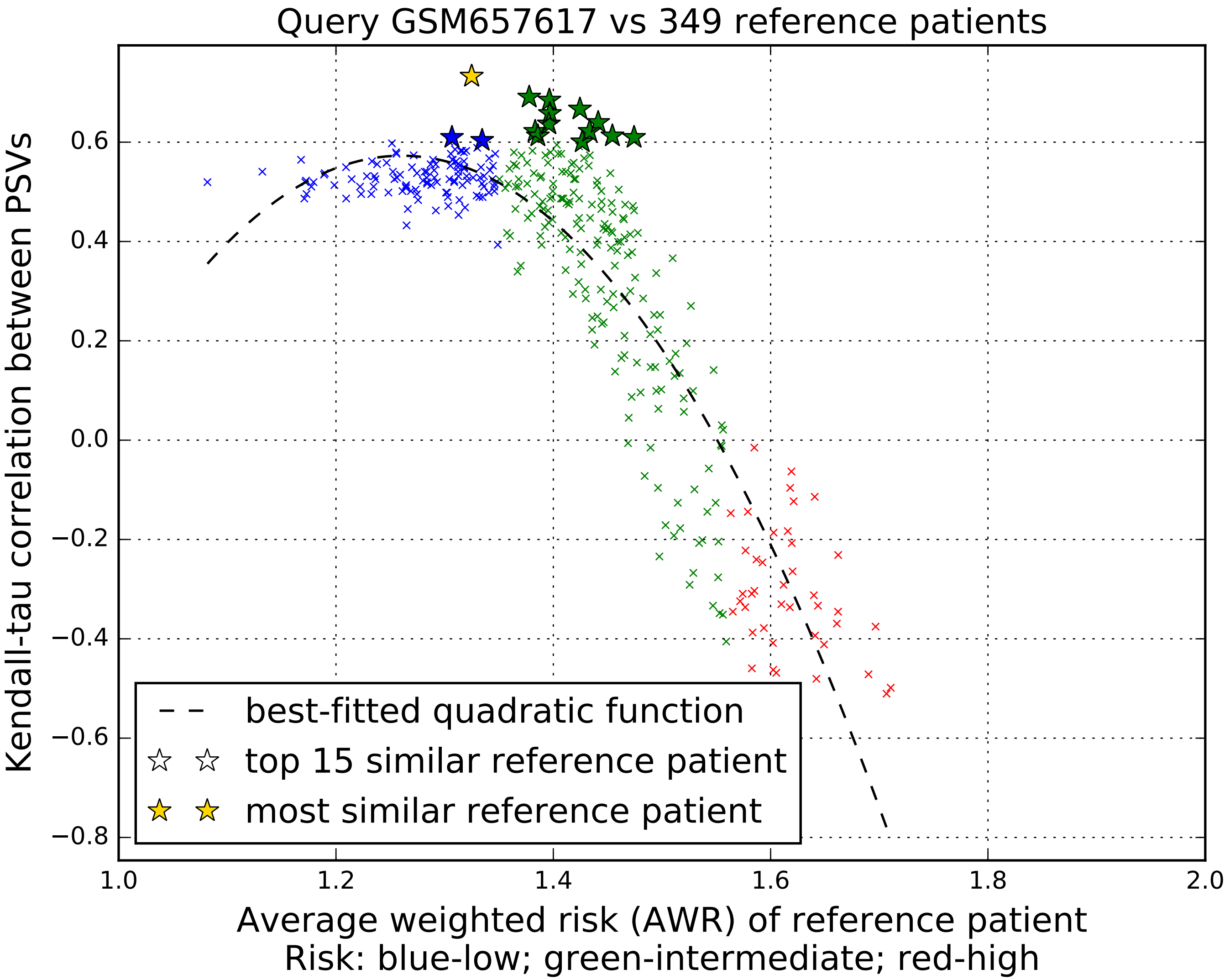

Query GSM657617 vs 349 reference patients
Kendall-tau correlation between PSVs
0.6
0.4
0.2
0.0
−0.2
−0.4
−0.6
−0.8
1.0
1.2
1.4
1.6
1.8
2.0
best-fitted quadratic function
top 15 similar reference patient
most similar reference patient
Average weighted risk (AWR) of reference patient
Risk: blue-low; green-intermediate; red-high

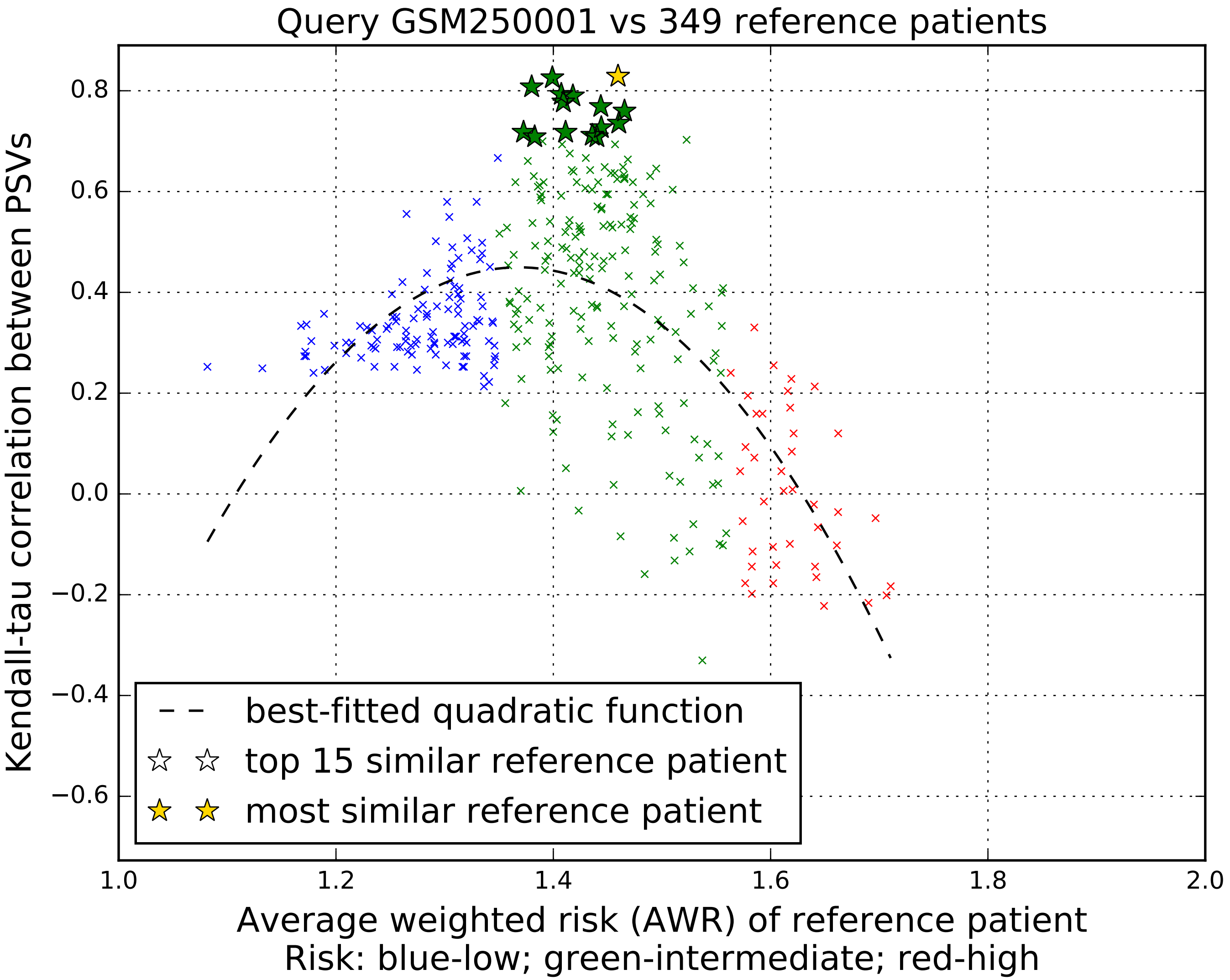

Query GSM250001 vs 349 reference patients
Kendall-tau correlation between PSVs
0.8
0.6
0.4
0.2
0.0
−0.2
−0.4
−0.6
1.0
1.2
1.4
1.6
1.8
2.0
best-fitted quadratic function
top 15 similar reference patient
most similar reference patient
Average weighted risk (AWR) of reference patient
Risk: blue-low; green-intermediate; red-high

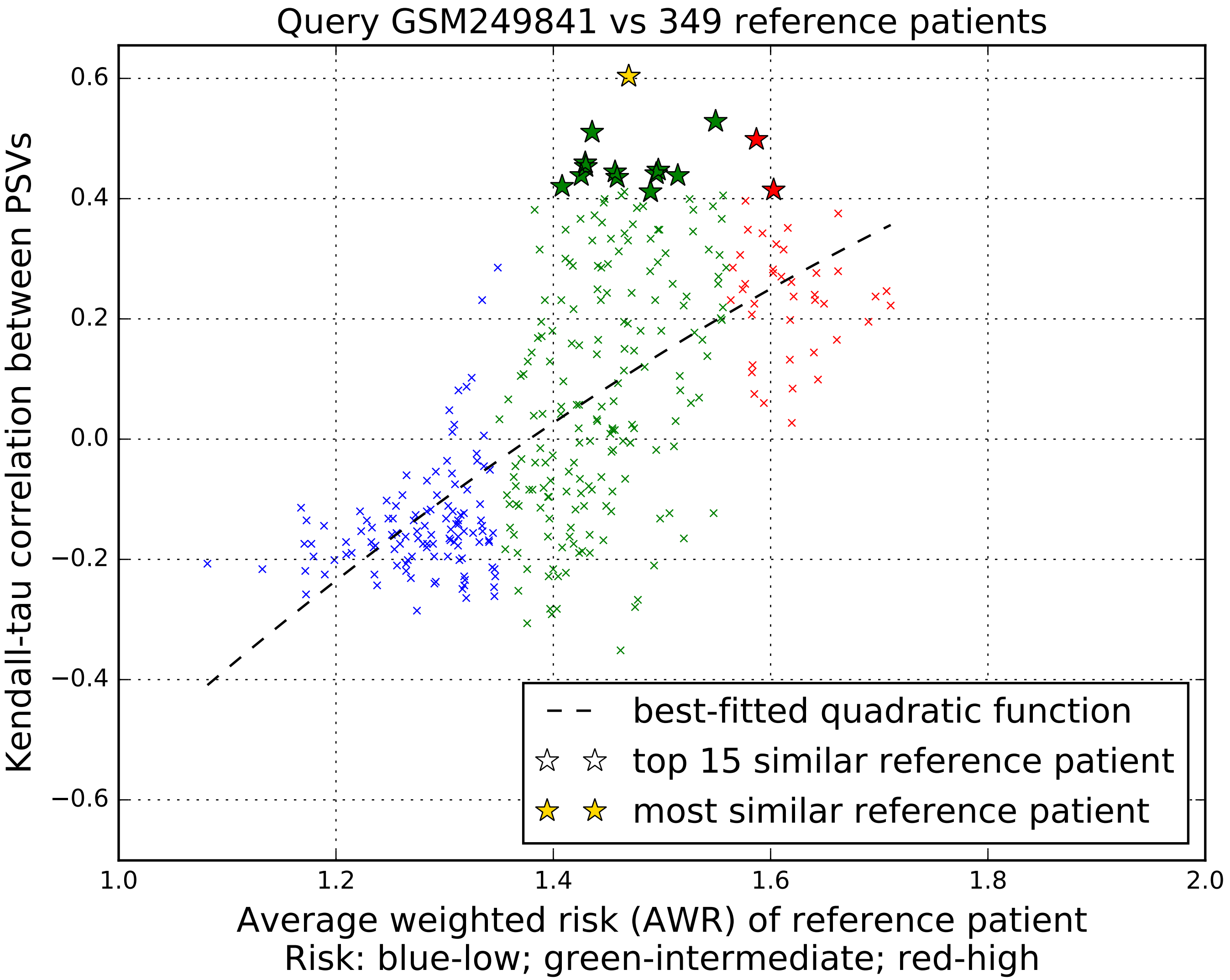
Query GSM249841 vs 349 reference patients
Kendall-tau correlation between PSVs
Average weighted risk (AWR) of reference patient
Risk: blue-low; green-intermediate; red-high
best-fitted quadratic function
top 15 similar reference patient
most similar reference patient
0.6
0.4
0.2
0.0
−0.2
−0.4
−0.6
1.0
1.2
1.4
1.6
1.8
2.0

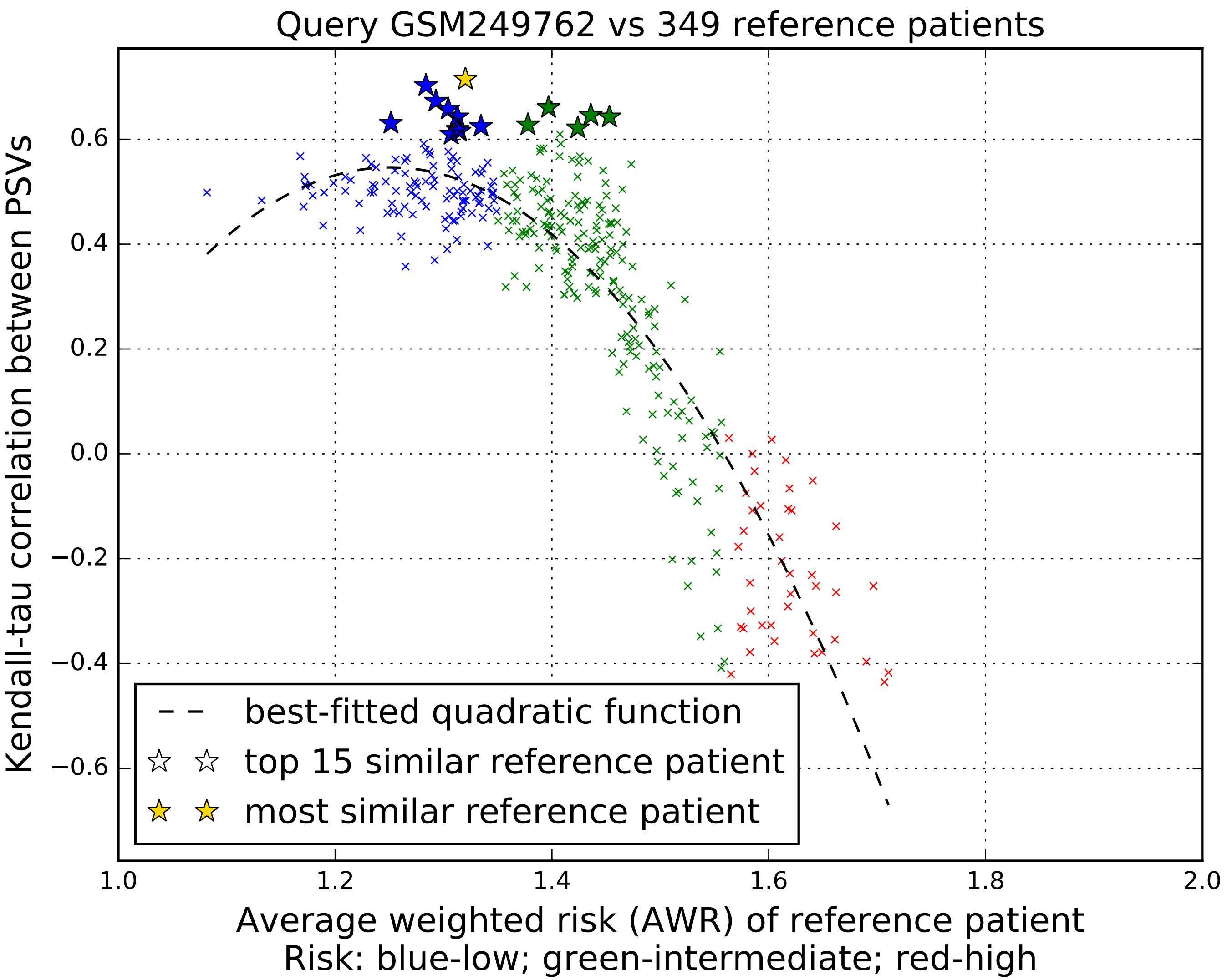

Query GSM249762 vs 349 reference patients
Kendall-tau correlation between PSVs
0.6
0.4
0.2
0.0
−0.2
−0.4
−0.6
1.0
1.2
1.4
1.6
1.8
2.0
best-fitted quadratic function
top 15 similar reference patient
most similar reference patient
Average weighted risk (AWR) of reference patient
Risk: blue-low; green-intermediate; red-high

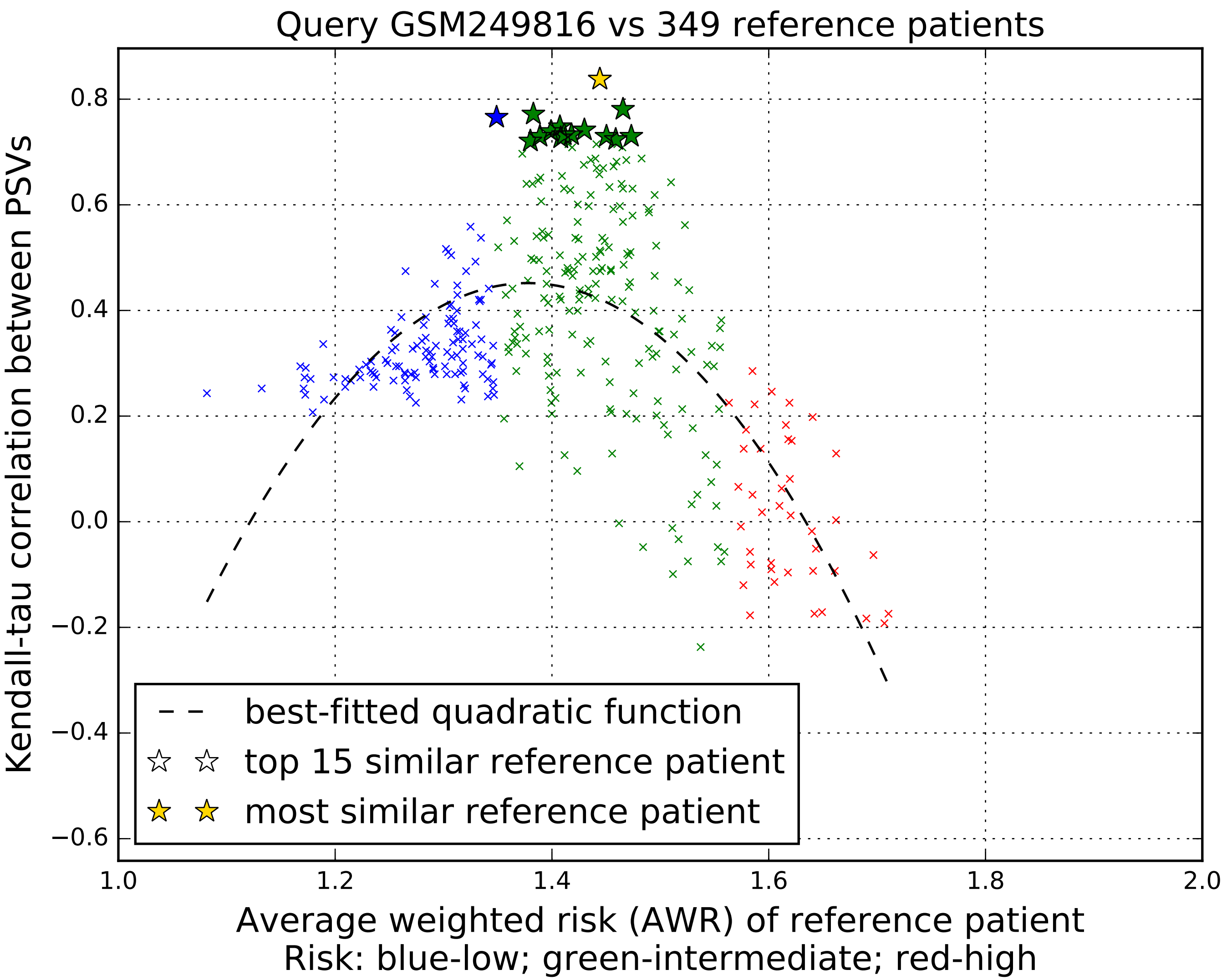
Query GSM249816 vs 349 reference patients
Kendall-tau correlation between PSVs
Average weighted risk (AWR) of reference patient
Risk: blue-low; green-intermediate; red-high
best-fitted quadratic function
top 15 similar reference patient
most similar reference patient
0.8
0.6
0.4
0.2
0.0
−0.2
−0.4
−0.6
1.0
1.2
1.4
1.6
1.8
2.0

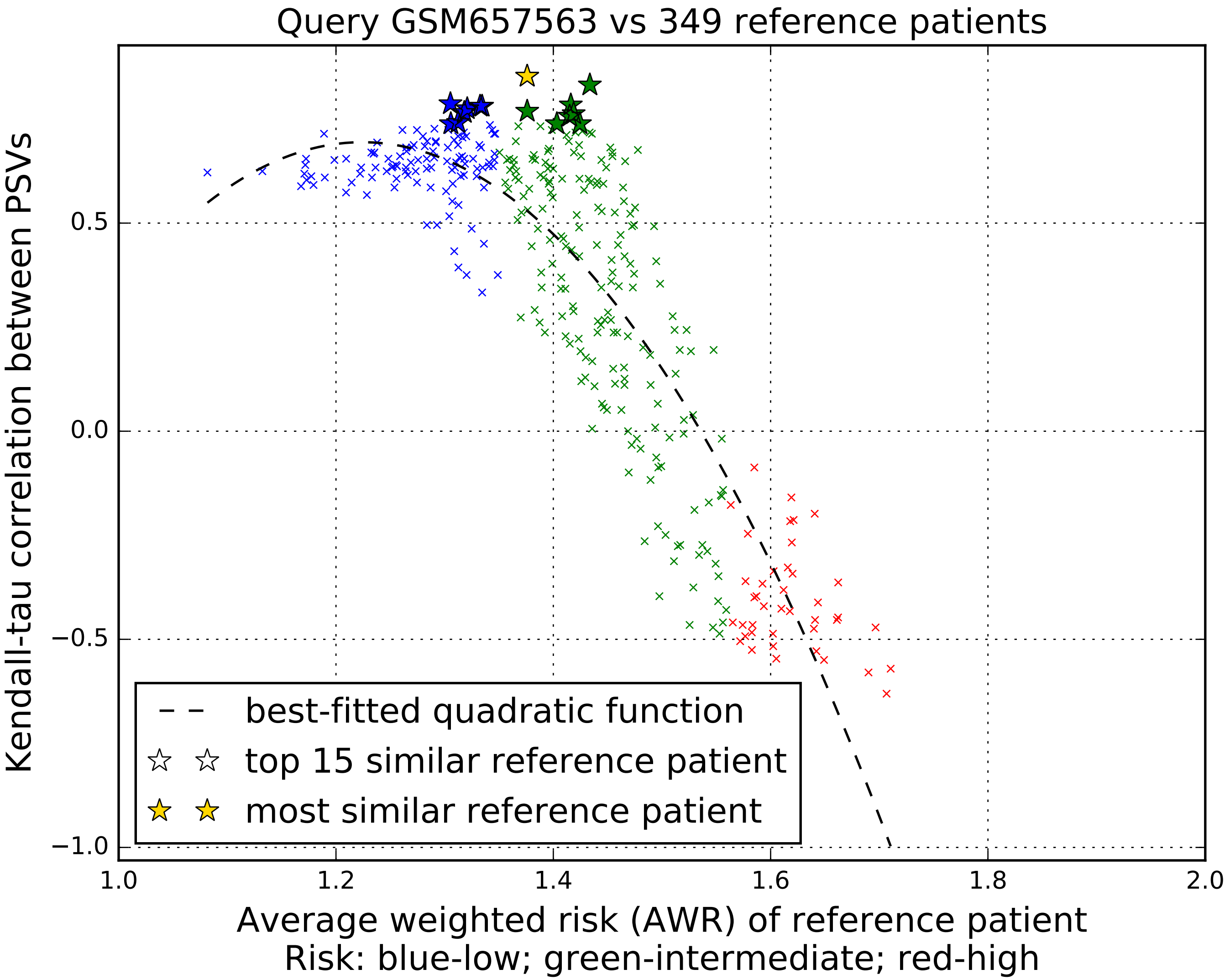
Query GSM657563 vs 349 reference patients
Kendall-tau correlation between PSVs
0.5
0.0
−0.5
−1.0
1.0
1.2
1.4
1.6
1.8
2.0
best-fitted quadratic function
top 15 similar reference patient
most similar reference patient
Average weighted risk (AWR) of reference patient
Risk: blue-low; green-intermediate; red-high

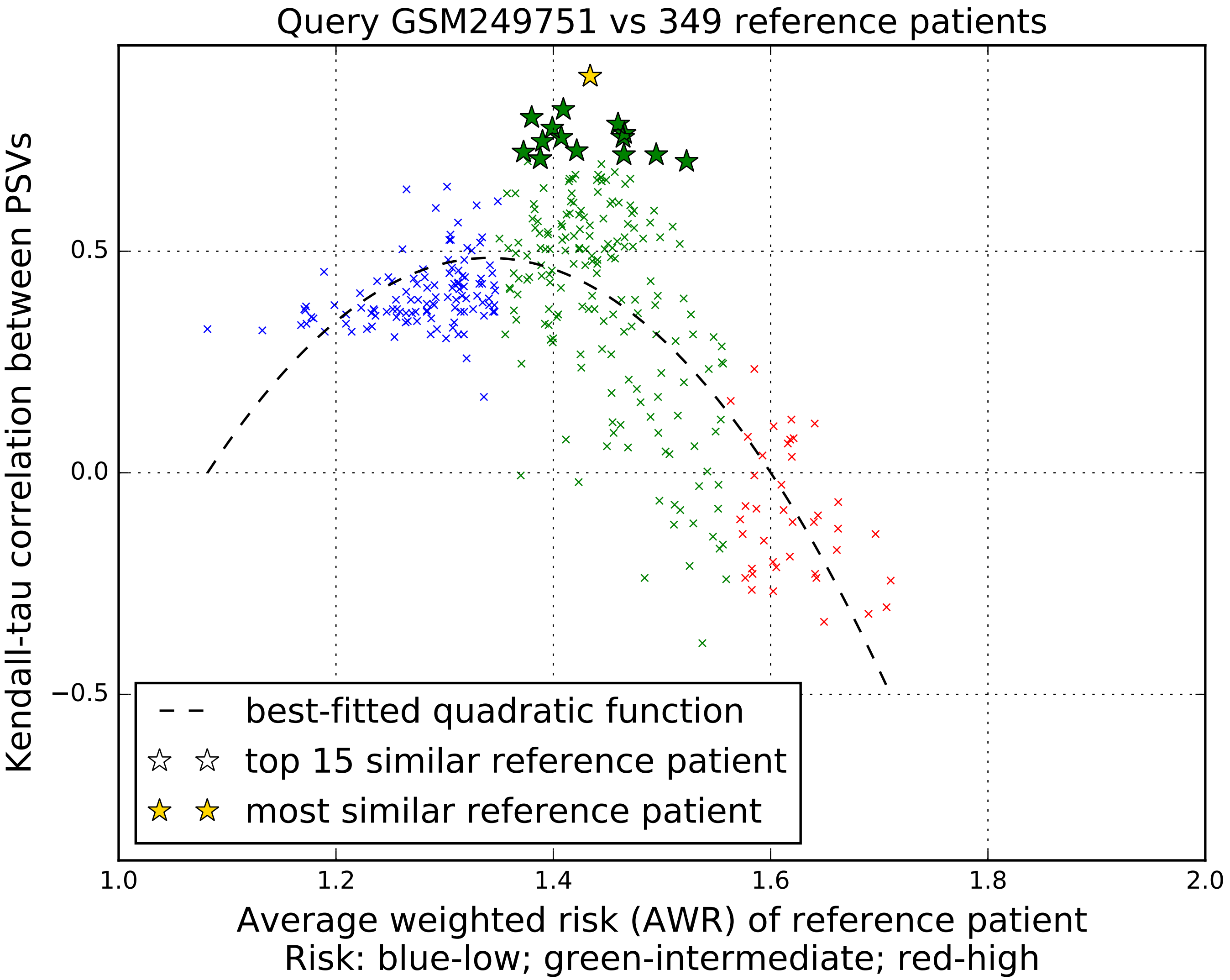
Query GSM249751 vs 349 reference patients
Kendall-tau correlation between PSVs
0.5
0.0
−0.5
1.0
1.2
1.4
1.6
1.8
2.0
best-fitted quadratic function
top 15 similar reference patient
most similar reference patient
Average weighted risk (AWR) of reference patient
Risk: blue-low; green-intermediate; red-high

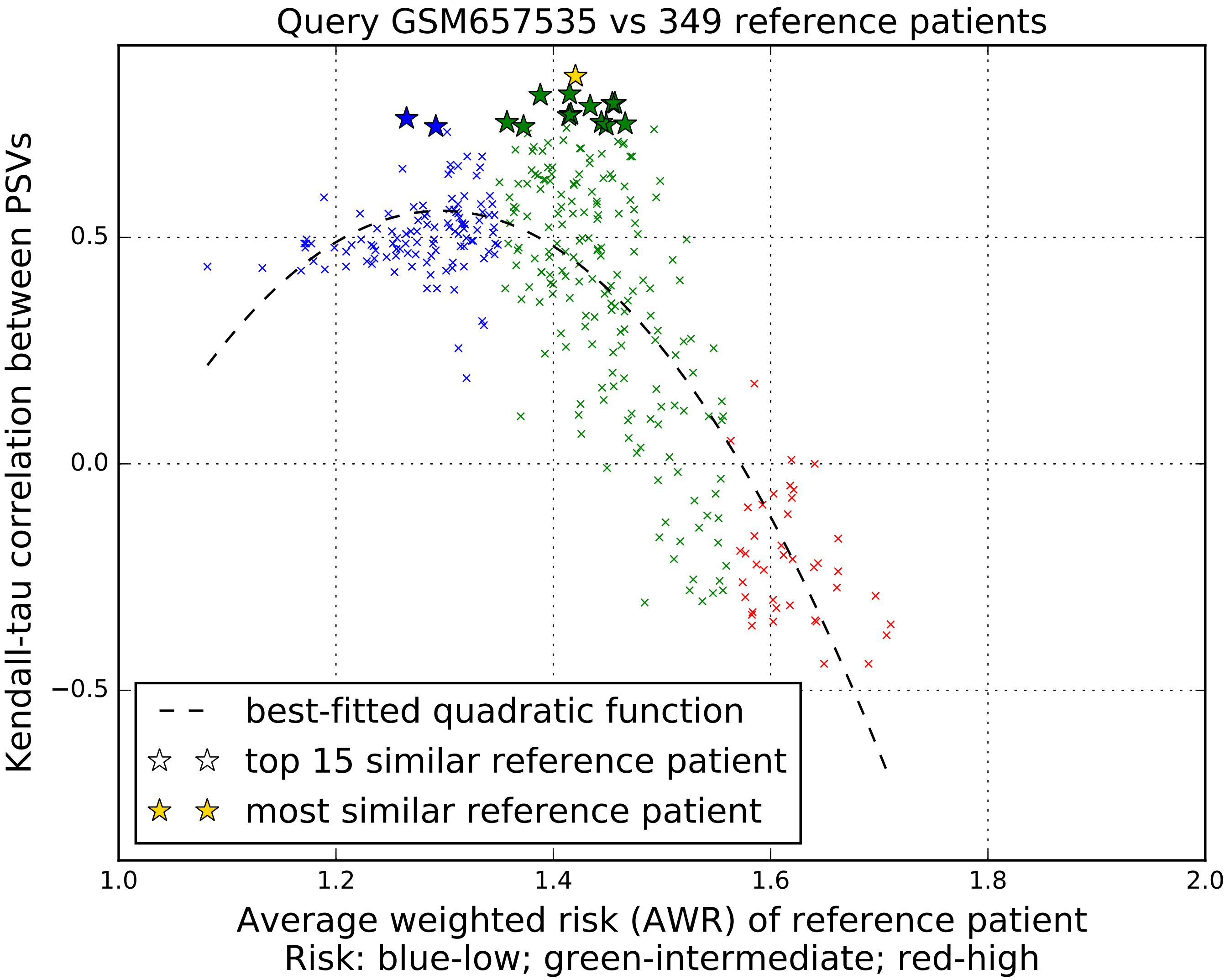
Query GSM657535 vs 349 reference patients
Kendall-tau correlation between PSVs
0.5
0.0
−0.5
1.0
1.2
1.4
1.6
1.8
2.0
best-fitted quadratic function
top 15 similar reference patient
most similar reference patient
Average weighted risk (AWR) of reference patient
Risk: blue-low; green-intermediate; red-high

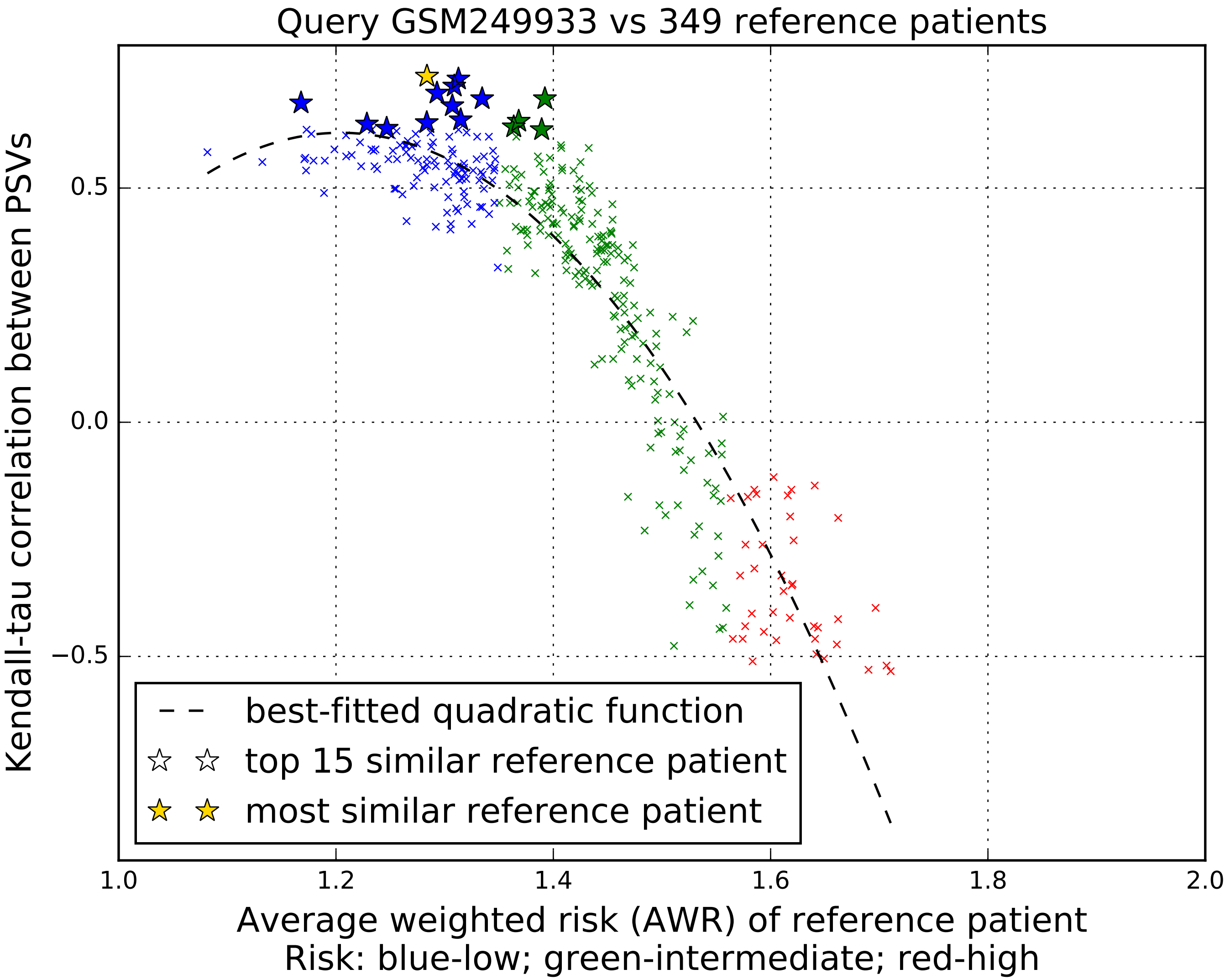

Query GSM249933 vs 349 reference patients
Kendall-tau correlation between PSVs
0.5
0.0
−0.5
1.0
1.2
1.4
1.6
1.8
2.0
best-fitted quadratic function
top 15 similar reference patient
most similar reference patient
Average weighted risk (AWR) of reference patient
Risk: blue-low; green-intermediate; red-high

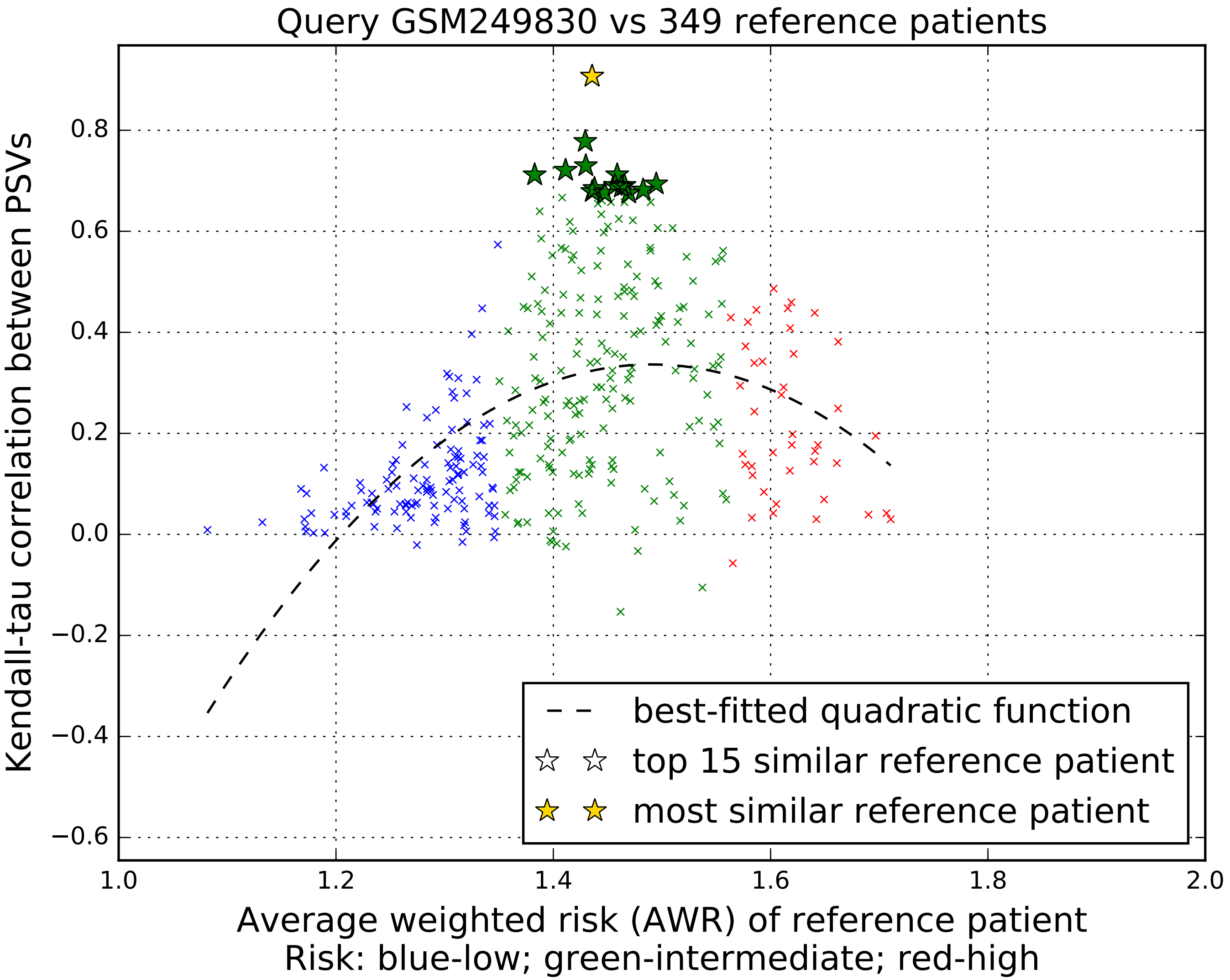

Query GSM249830 vs 349 reference patients
Kendall-tau correlation between PSVs
0.8
0.6
0.4
0.2
0.0
−0.2
−0.4
−0.6
1.0
1.2
1.4
1.6
1.8
2.0
best-fitted quadratic function
top 15 similar reference patient
most similar reference patient
Average weighted risk (AWR) of reference patient
Risk: blue-low; green-intermediate; red-high

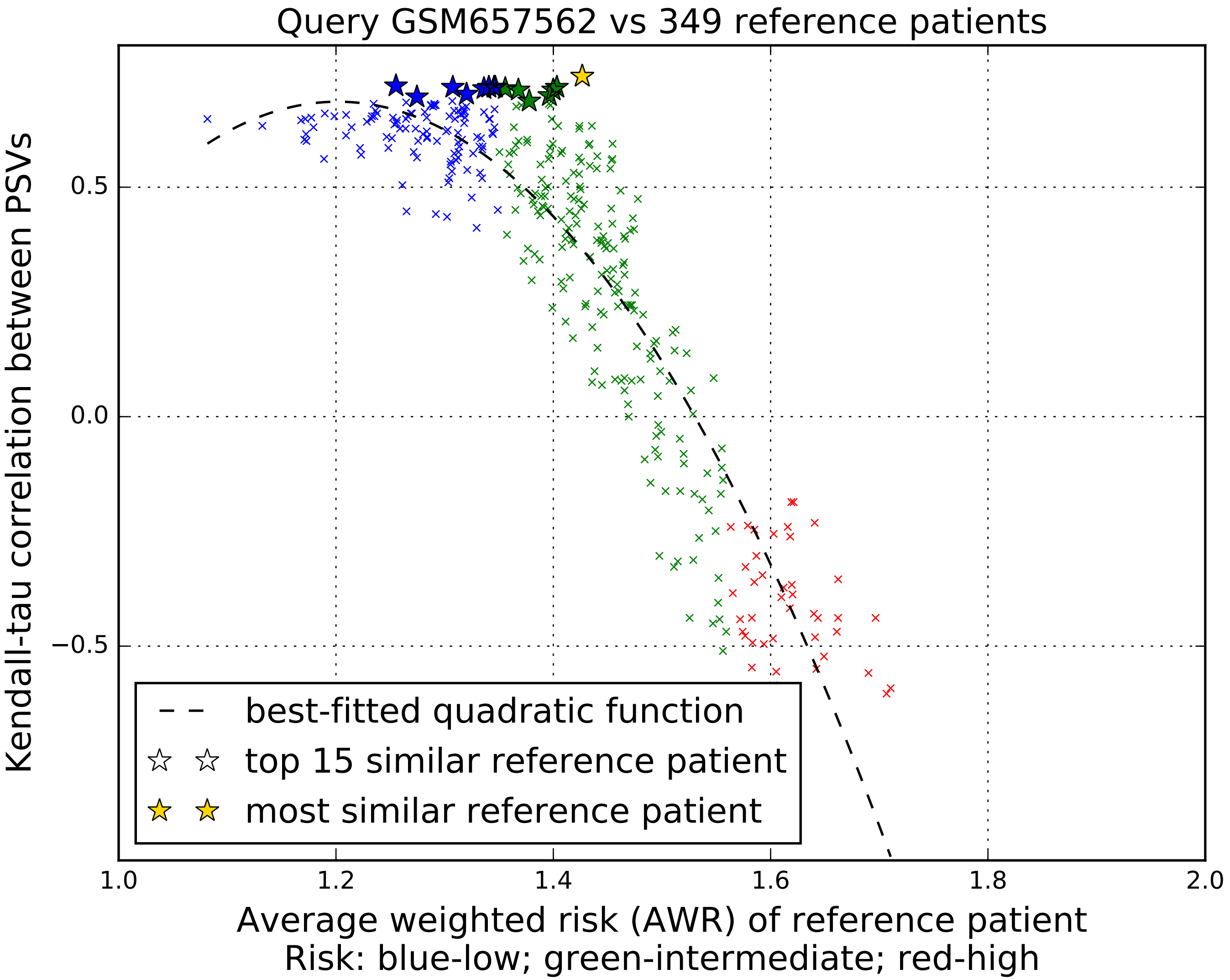

Query GSM657562 vs 349 reference patients
Kendall-tau correlation between PSVs
0.5
0.0
−0.5
1.0
1.2
1.4
1.6
1.8
2.0
best-fitted quadratic function
top 15 similar reference patient
most similar reference patient
Average weighted risk (AWR) of reference patient
Risk: blue-low; green-intermediate; red-high

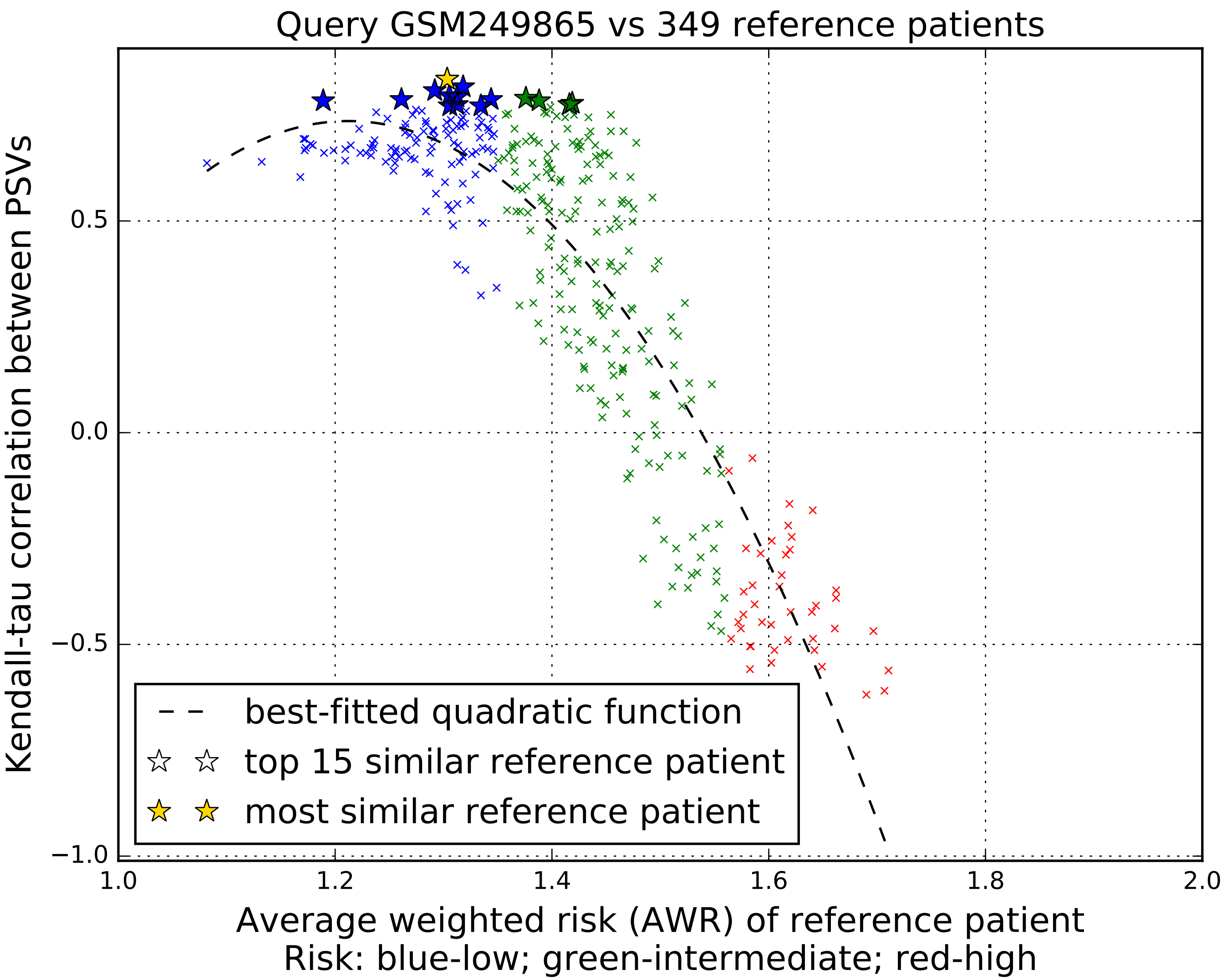

Query GSM249865 vs 349 reference patients
Kendall-tau correlation between PSVs
0.5
0.0
−0.5
−1.0
1.0
1.2
1.4
1.6
1.8
2.0
best-fitted quadratic function
top 15 similar reference patient
most similar reference patient
Average weighted risk (AWR) of reference patient
Risk: blue-low; green-intermediate; red-high

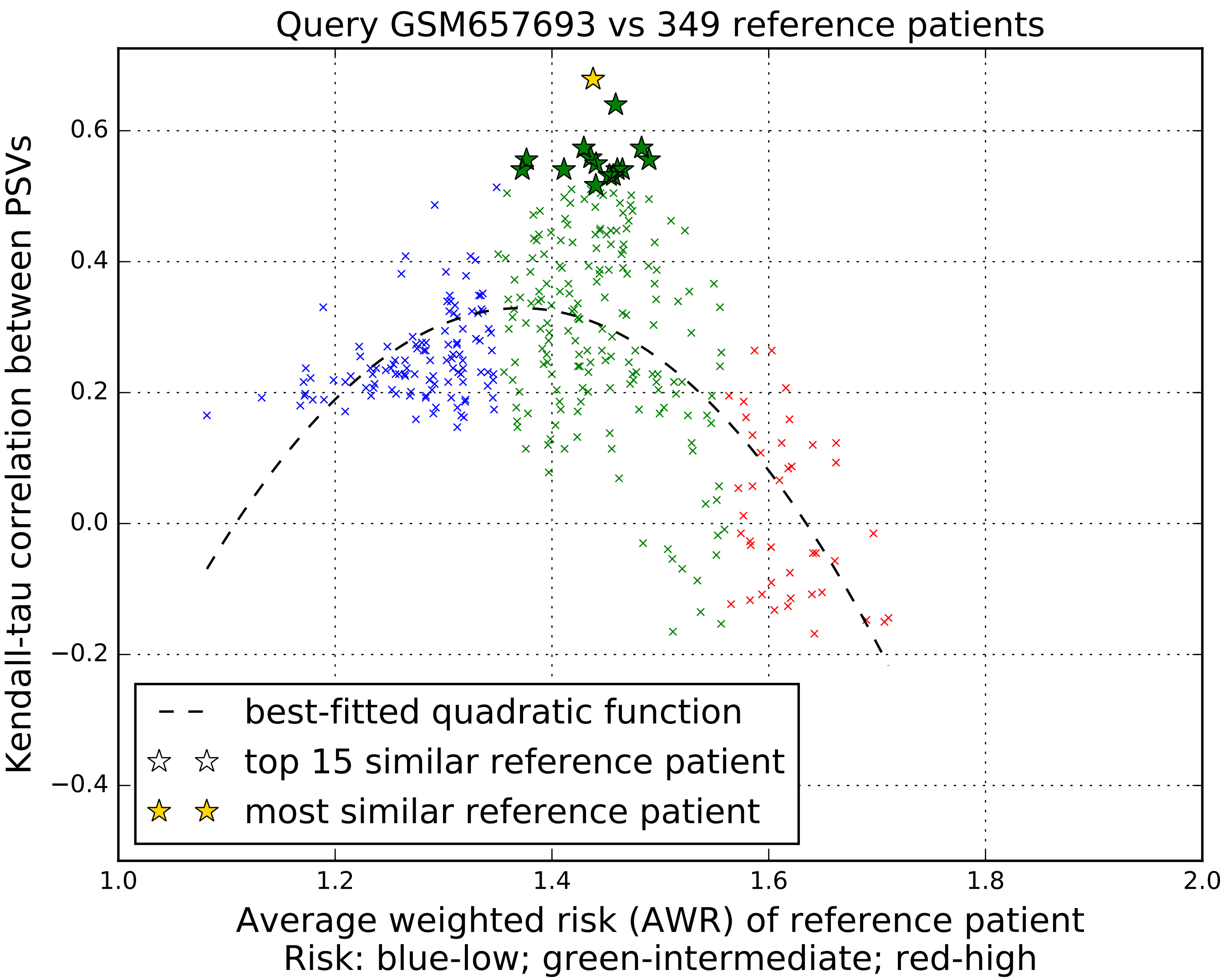

Query GSM657693 vs 349 reference patients
Kendall-tau correlation between PSVs
0.6
0.4
0.2
0.0
−0.2
−0.4
1.0
1.2
1.4
1.6
1.8
2.0
best-fitted quadratic function
top 15 similar reference patient
most similar reference patient
Average weighted risk (AWR) of reference patient
Risk: blue-low; green-intermediate; red-high

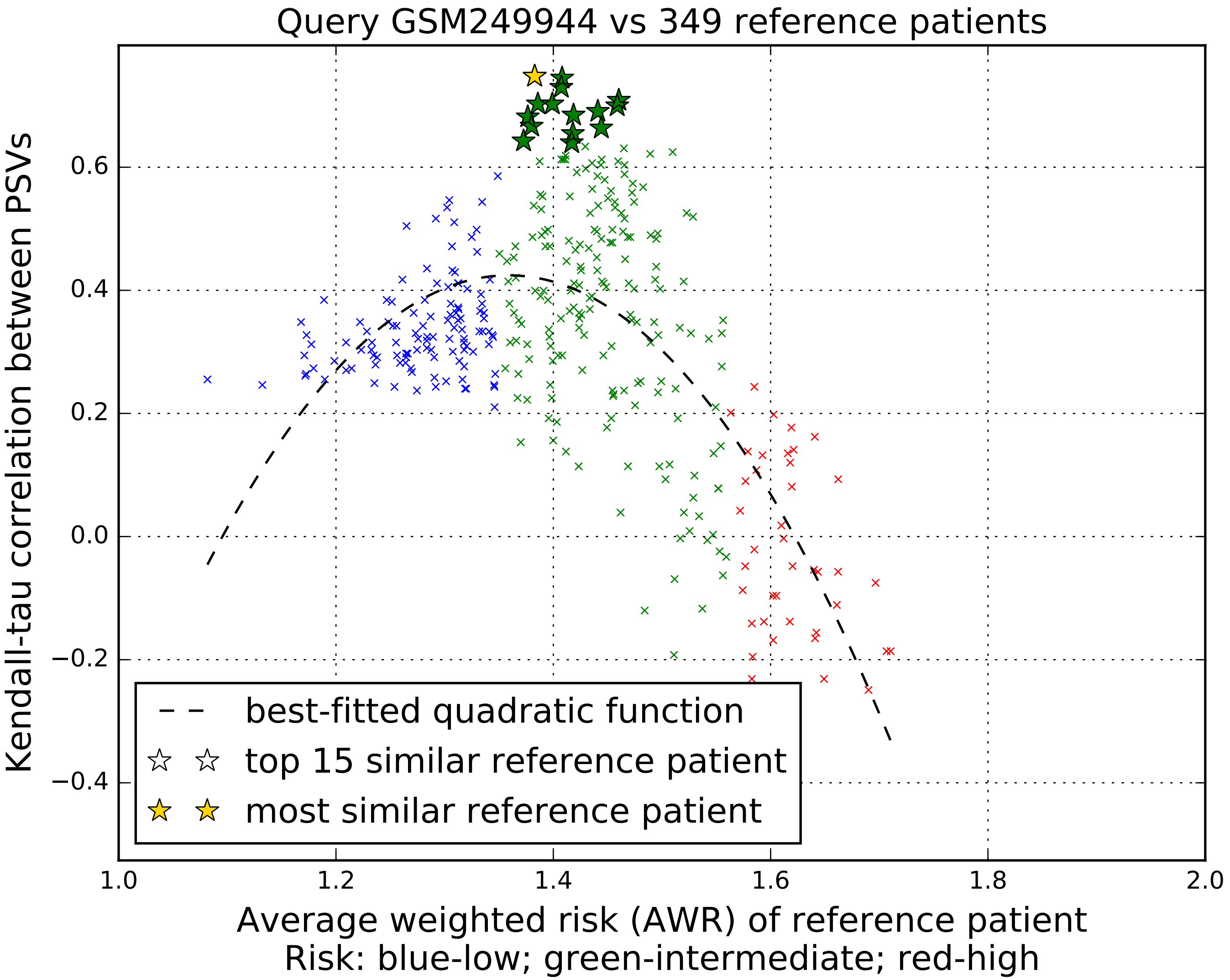
Query GSM249944 vs 349 reference patients
Kendall-tau correlation between PSVs
0.6
0.4
0.2
0.0
−0.2
−0.4
1.0
1.2
1.4
1.6
1.8
2.0
best-fitted quadratic function
top 15 similar reference patient
most similar reference patient
Average weighted risk (AWR) of reference patient
Risk: blue-low; green-intermediate; red-high

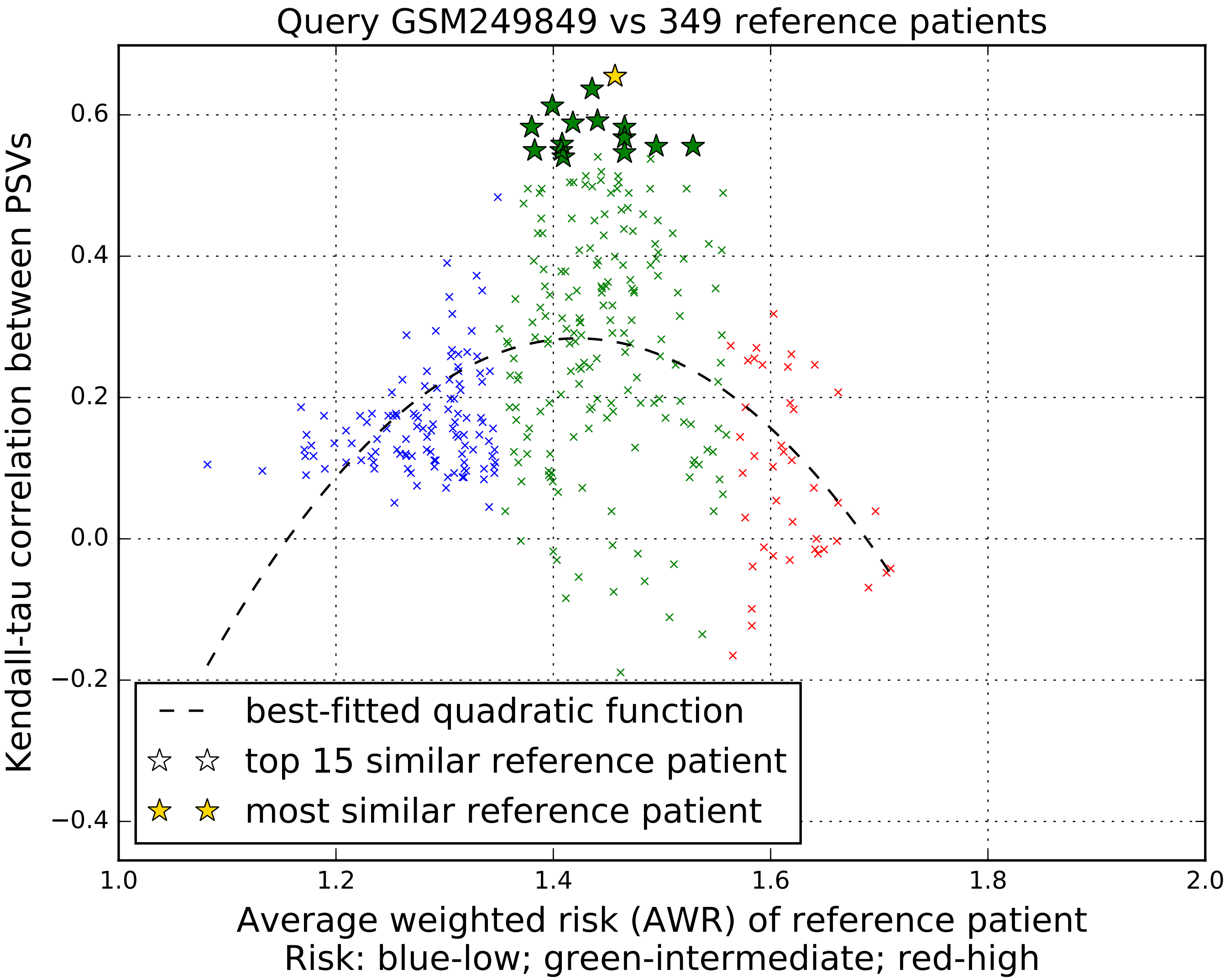

Query GSM249849 vs 349 reference patients
Kendall-tau correlation between PSVs
0.6
0.4
0.2
0.0
−0.2
−0.4
1.0
1.2
1.4
1.6
1.8
2.0
best-fitted quadratic function
top 15 similar reference patient
most similar reference patient
Average weighted risk (AWR) of reference patient
Risk: blue-low; green-intermediate; red-high

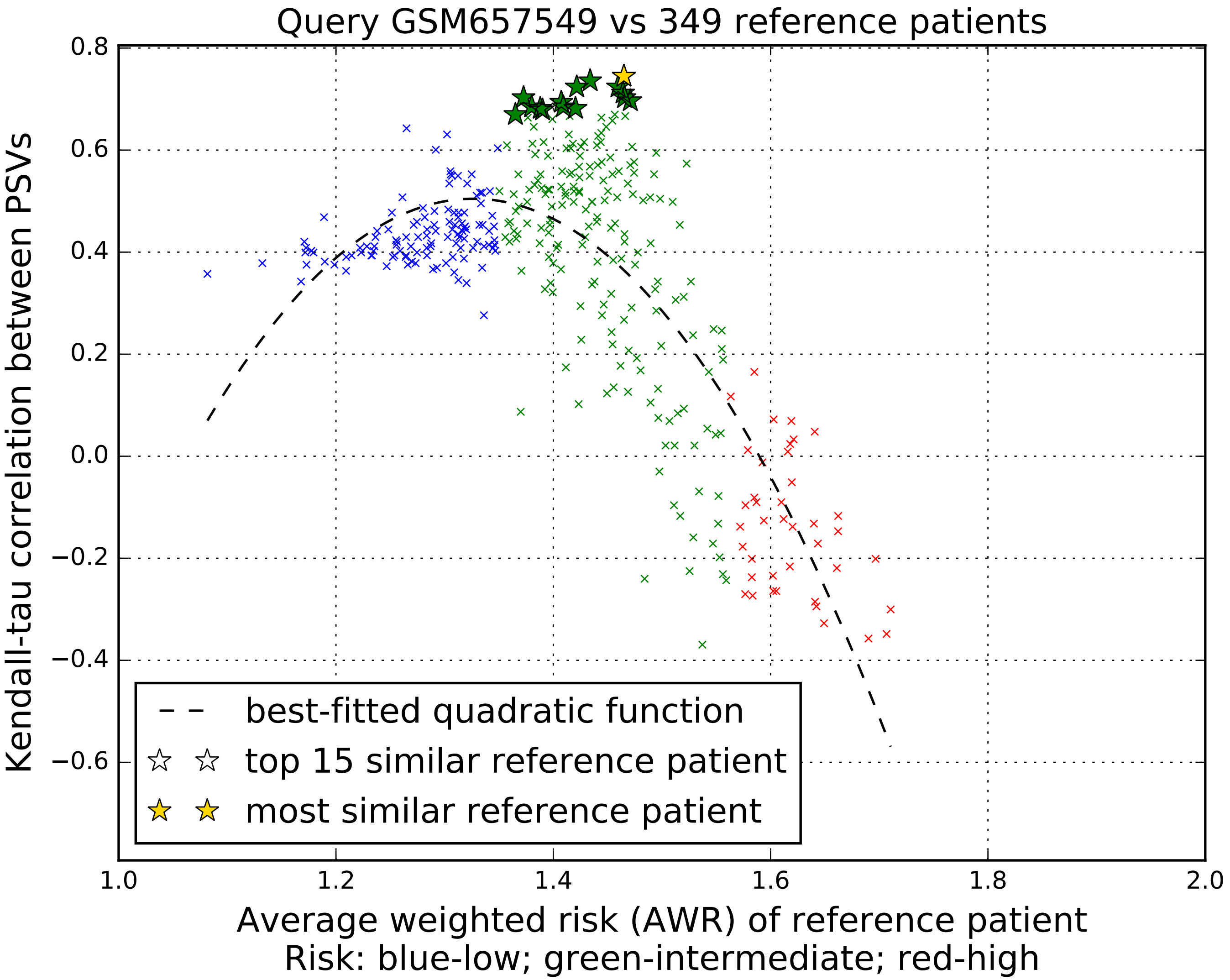

Query GSM657549 vs 349 reference patients
Kendall-tau correlation between PSVs
0.8
0.6
0.4
0.2
0.0
−0.2
−0.4
−0.6
1.0
1.2
1.4
1.6
1.8
2.0
best-fitted quadratic function
top 15 similar reference patient
most similar reference patient
Average weighted risk (AWR) of reference patient
Risk: blue-low; green-intermediate; red-high

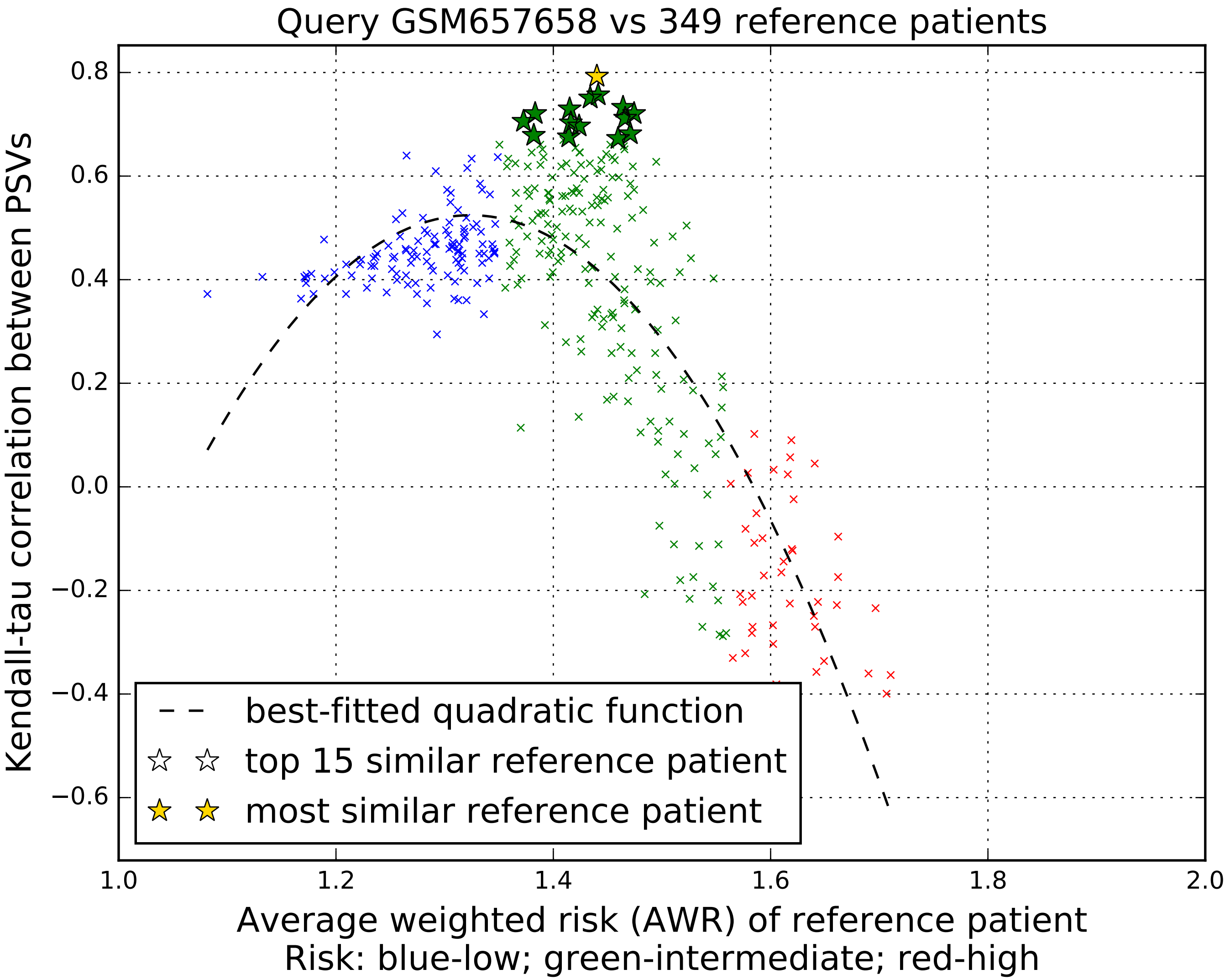

Query GSM657658 vs 349 reference patients
Kendall-tau correlation between PSVs
0.8
0.6
0.4
0.2
0.0
−0.2
−0.4
−0.6
1.0
1.2
1.4
1.6
1.8
2.0
best-fitted quadratic function
top 15 similar reference patient
most similar reference patient
Average weighted risk (AWR) of reference patient
Risk: blue-low; green-intermediate; red-high

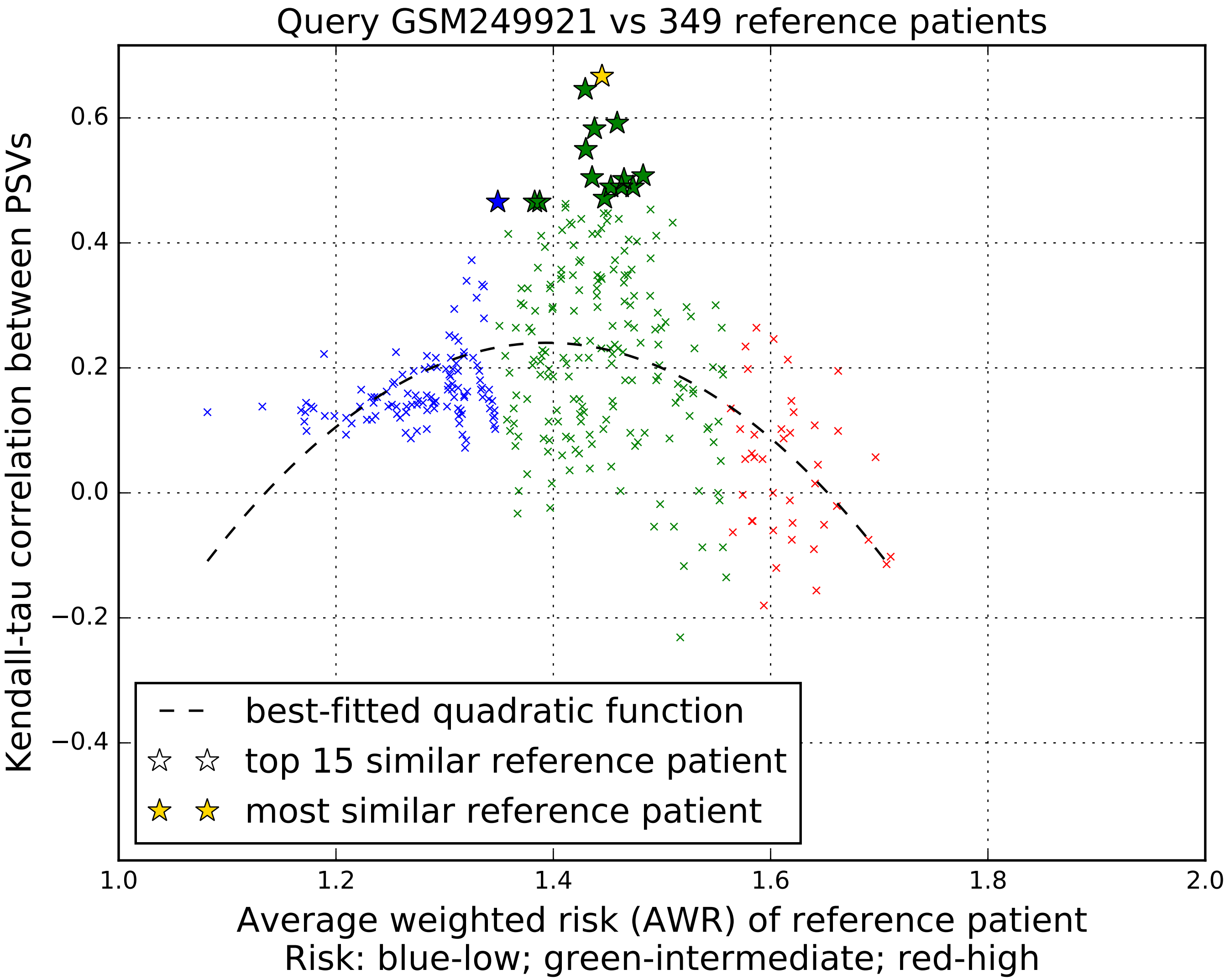

Query GSM249921 vs 349 reference patients
Kendall-tau correlation between PSVs
0.6
0.4
0.2
0.0
−0.2
−0.4
1.0
1.2
1.4
1.6
1.8
2.0
best-fitted quadratic function
top 15 similar reference patient
most similar reference patient
Average weighted risk (AWR) of reference patient
Risk: blue-low; green-intermediate; red-high

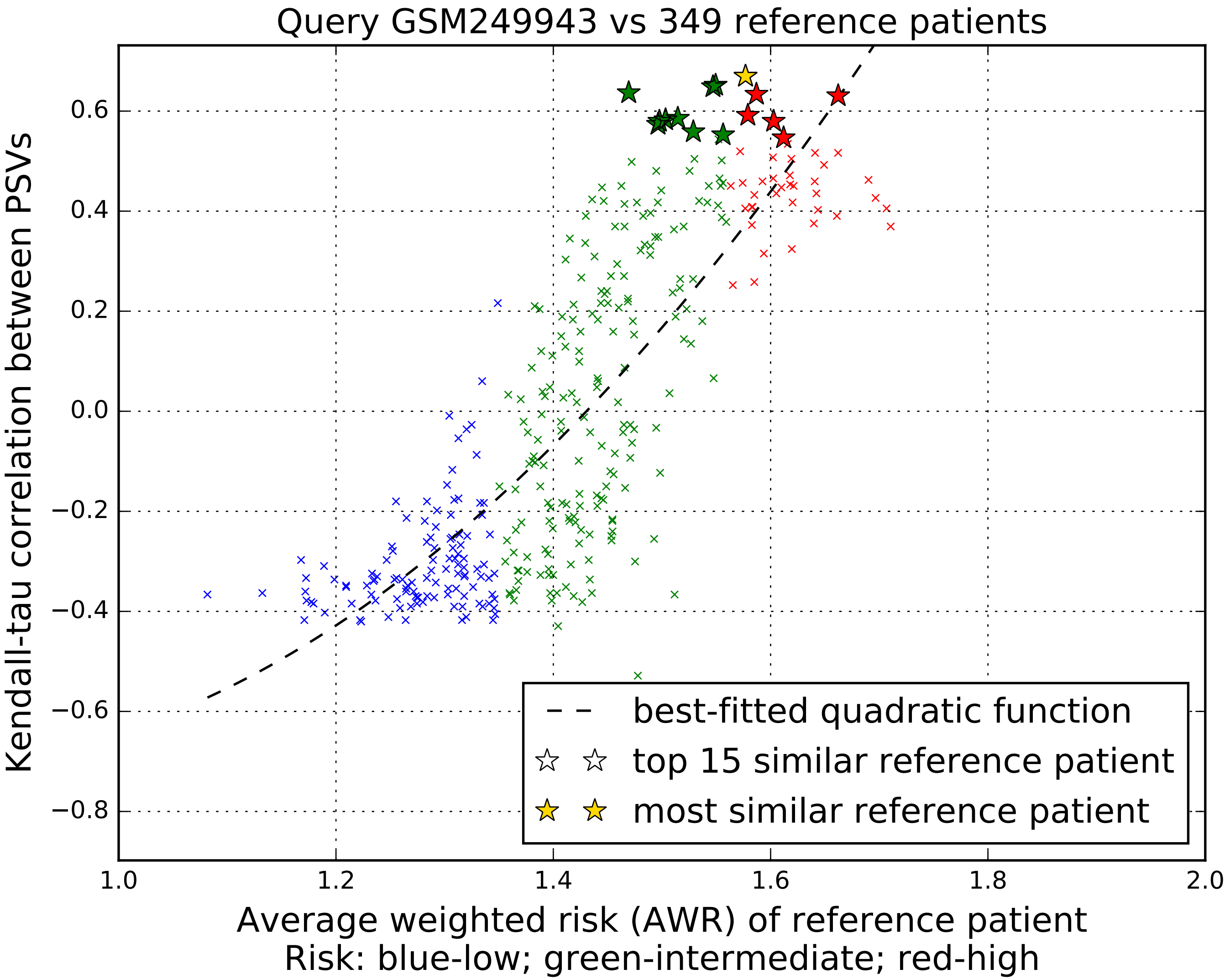
Query GSM249943 vs 349 reference patients
Kendall-tau correlation between PSVs
0.6
0.4
0.2
0.0
−0.2
−0.4
−0.6
−0.8
1.0
1.2
1.4
1.6
1.8
2.0
best-fitted quadratic function
top 15 similar reference patient
most similar reference patient
Average weighted risk (AWR) of reference patient
Risk: blue-low; green-intermediate; red-high

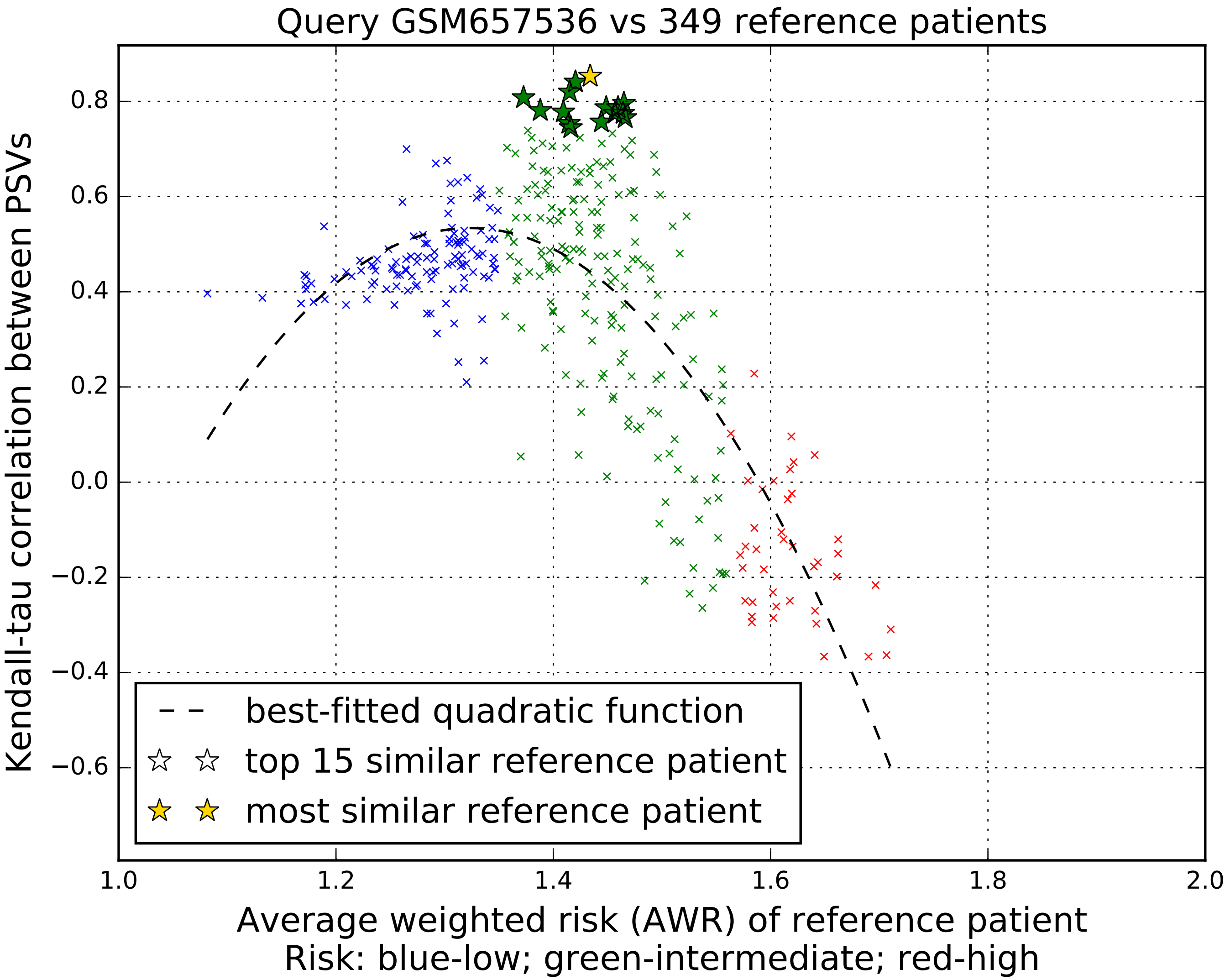

Query GSM657536 vs 349 reference patients
Kendall-tau correlation between PSVs
0.8
0.6
0.4
0.2
0.0
−0.2
−0.4
−0.6
1.0
1.2
1.4
1.6
1.8
2.0
best-fitted quadratic function
top 15 similar reference patient
most similar reference patient
Average weighted risk (AWR) of reference patient
Risk: blue-low; green-intermediate; red-high

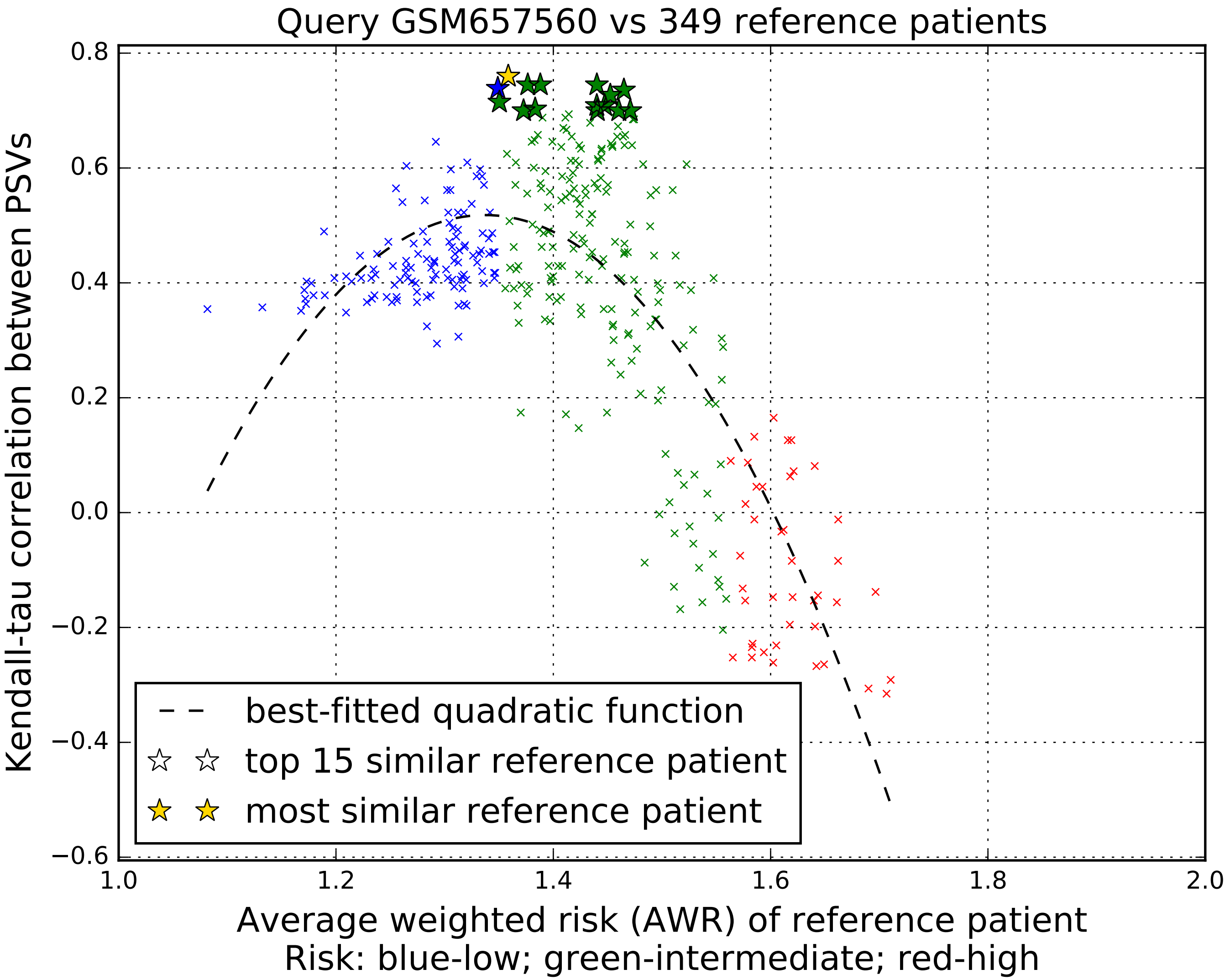
Query GSM657560 vs 349 reference patients
Kendall-tau correlation between PSVs
0.8
0.6
0.4
0.2
0.0
−0.2
−0.4
−0.6
1.0
1.2
1.4
1.6
1.8
2.0
best-fitted quadratic function
top 15 similar reference patient
most similar reference patient
Average weighted risk (AWR) of reference patient
Risk: blue-low; green-intermediate; red-high

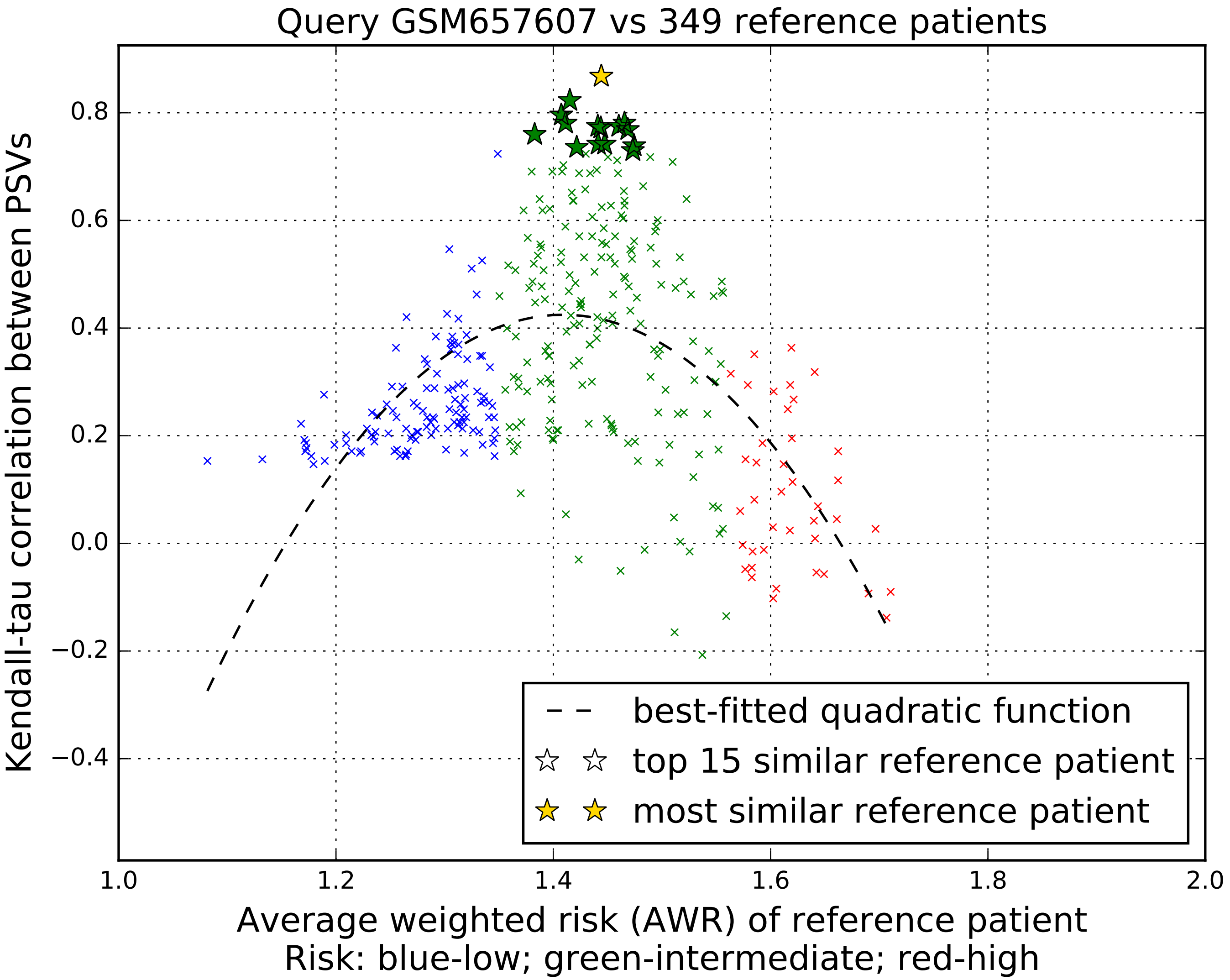
Query GSM657607 vs 349 reference patients
Kendall-tau correlation between PSVs
0.8
0.6
0.4
0.2
0.0
−0.2
−0.4
1.0
1.2
1.4
1.6
1.8
2.0
best-fitted quadratic function
top 15 similar reference patient
most similar reference patient
Average weighted risk (AWR) of reference patient
Risk: blue-low; green-intermediate; red-high

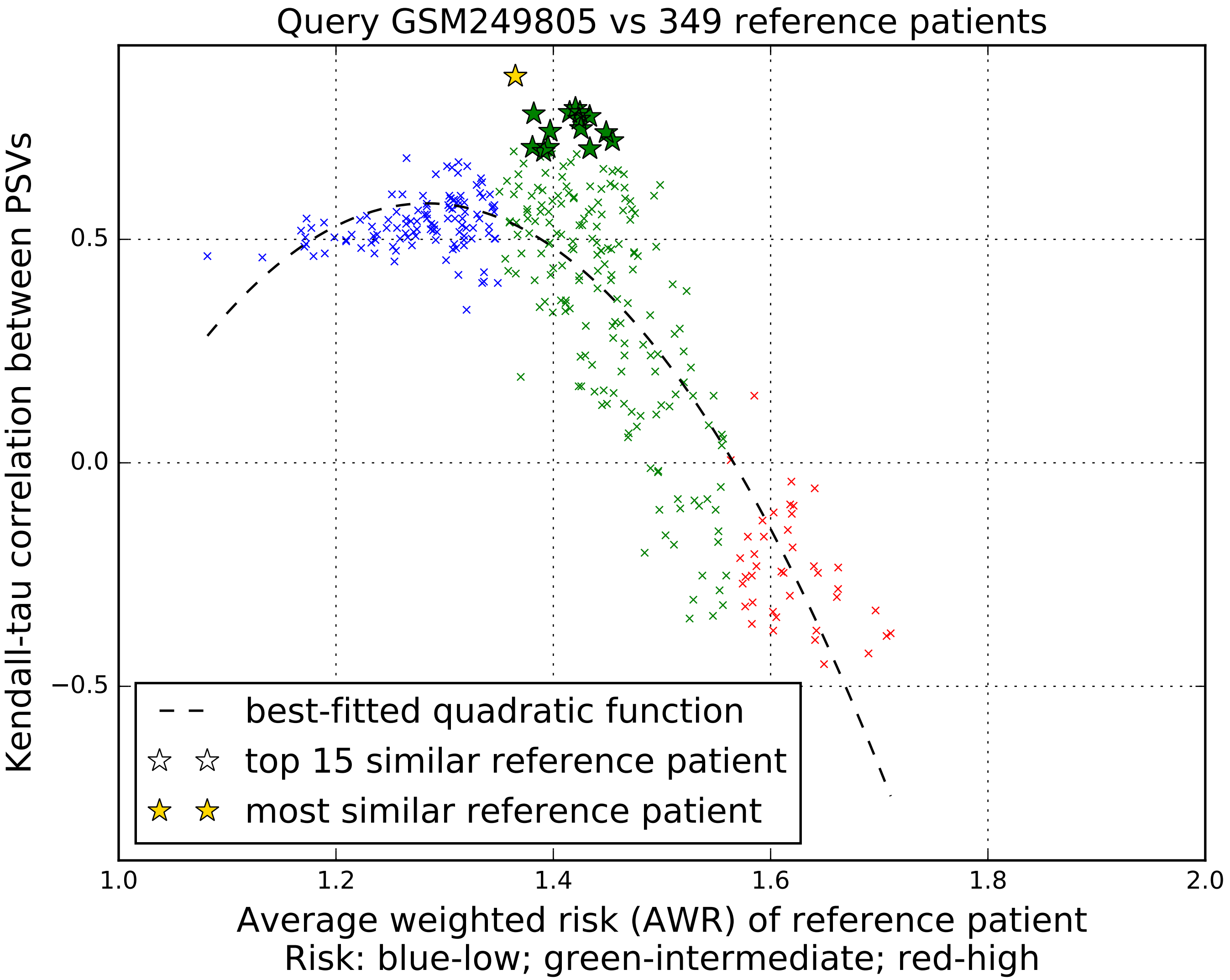

Query GSM249805 vs 349 reference patients
Kendall-tau correlation between PSVs
0.5
0.0
−0.5
1.0
1.2
1.4
1.6
1.8
2.0
best-fitted quadratic function
top 15 similar reference patient
most similar reference patient
Average weighted risk (AWR) of reference patient
Risk: blue-low; green-intermediate; red-high

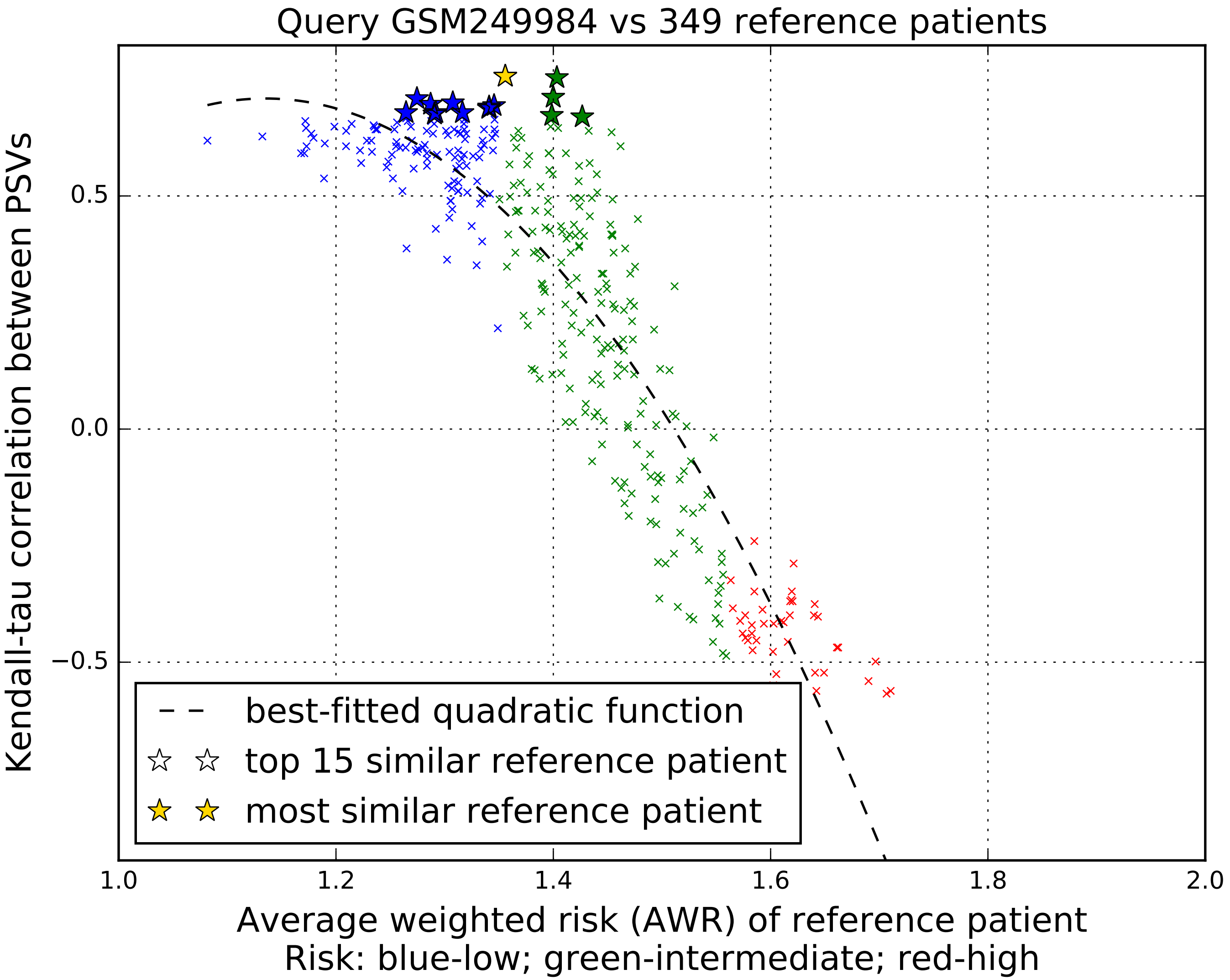

Query GSM249984 vs 349 reference patients
Kendall-tau correlation between PSVs
0.5
0.0
−0.5
1.0
1.2
1.4
1.6
1.8
2.0
best-fitted quadratic function
top 15 similar reference patient
most similar reference patient
Average weighted risk (AWR) of reference patient
Risk: blue-low; green-intermediate; red-high

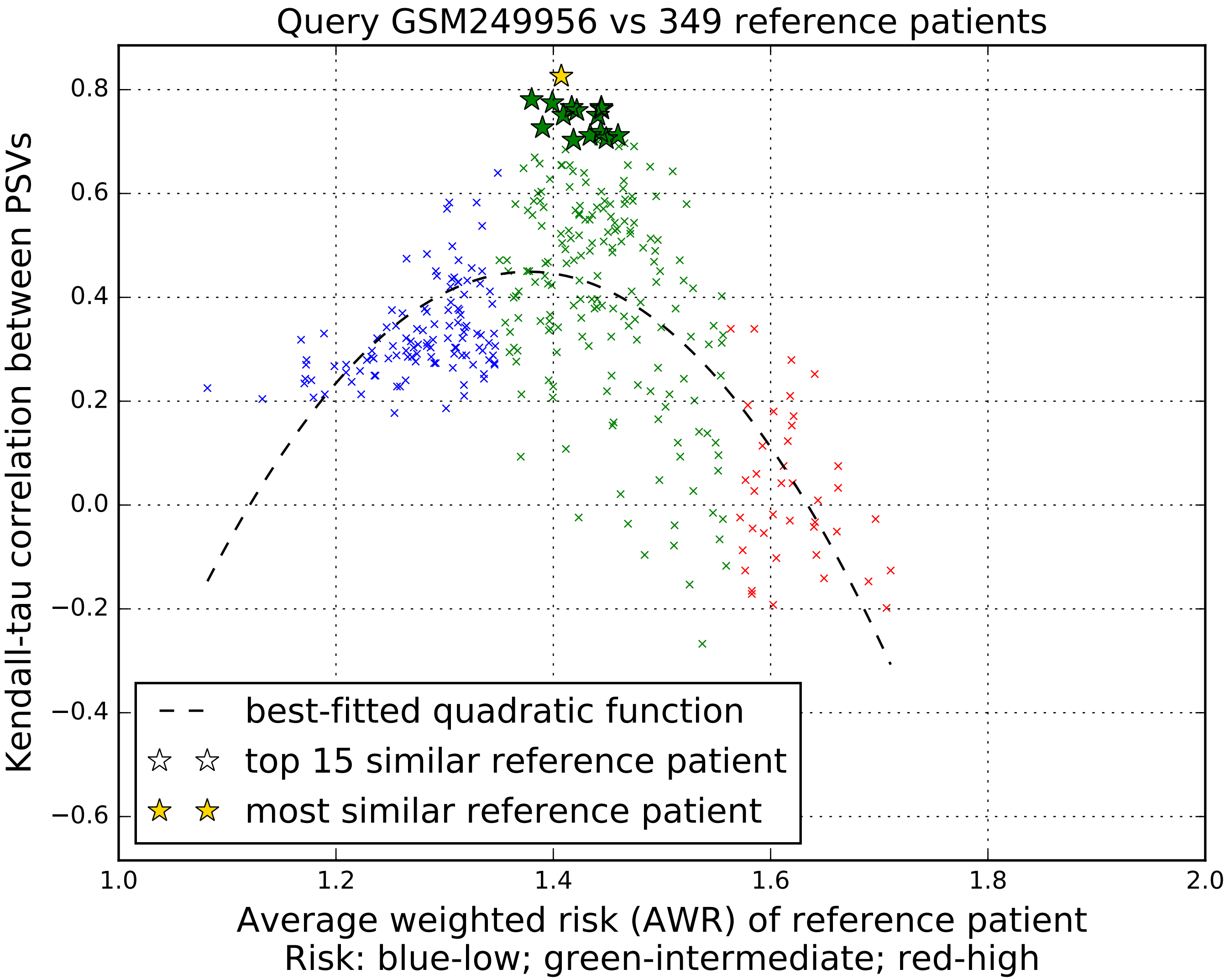
Query GSM249956 vs 349 reference patients
Kendall-tau correlation between PSVs
0.8
0.6
0.4
0.2
0.0
−0.2
−0.4
−0.6
1.0
1.2
1.4
1.6
1.8
2.0
best-fitted quadratic function
top 15 similar reference patient
most similar reference patient
Average weighted risk (AWR) of reference patient
Risk: blue-low; green-intermediate; red-high

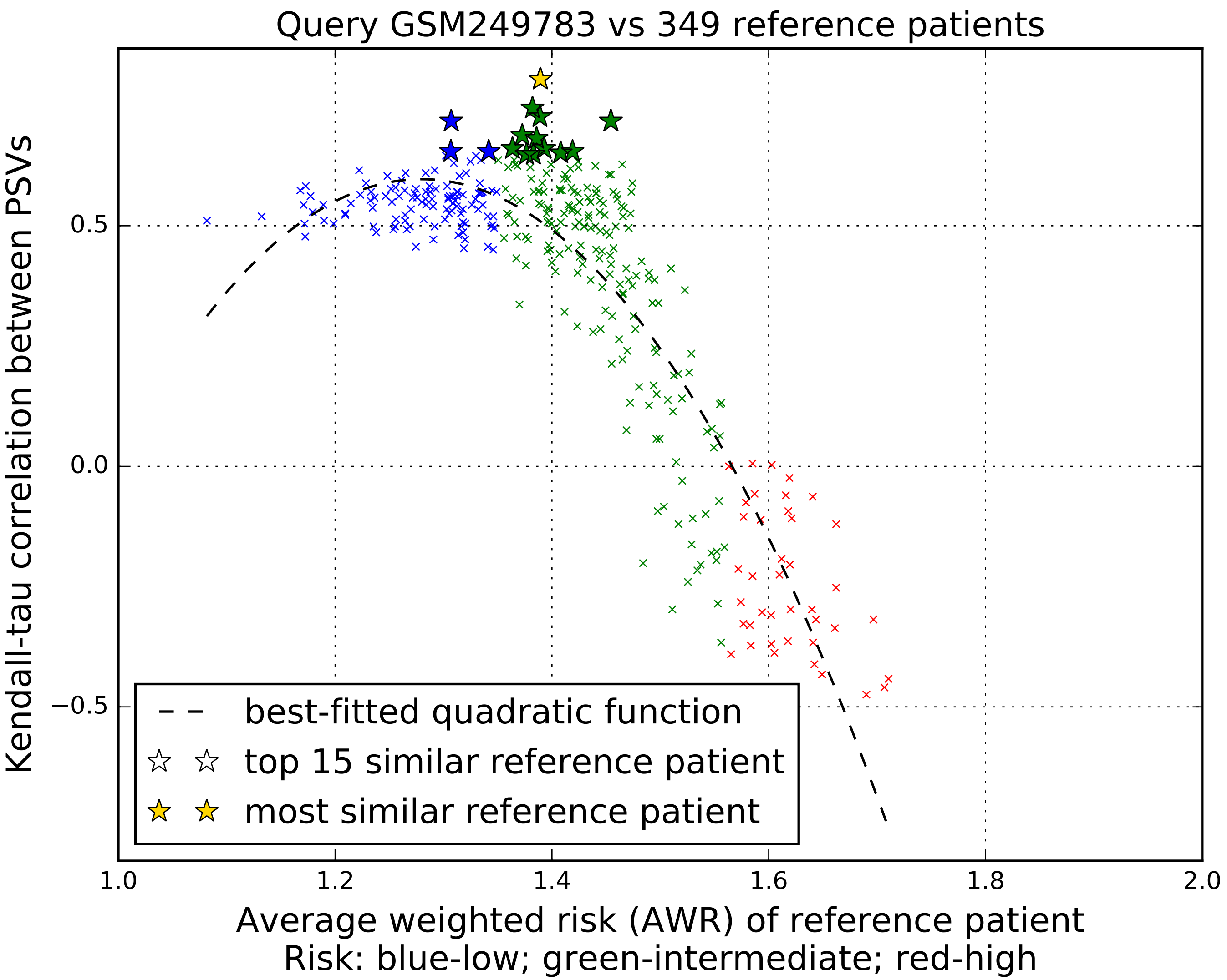
Query GSM249783 vs 349 reference patients
Kendall-tau correlation between PSVs
Average weighted risk (AWR) of reference patient
Risk: blue-low; green-intermediate; red-high
0.5
0.0
−0.5
1.0
1.2
1.4
1.6
1.8
2.0
best-fitted quadratic function
top 15 similar reference patient
most similar reference patient

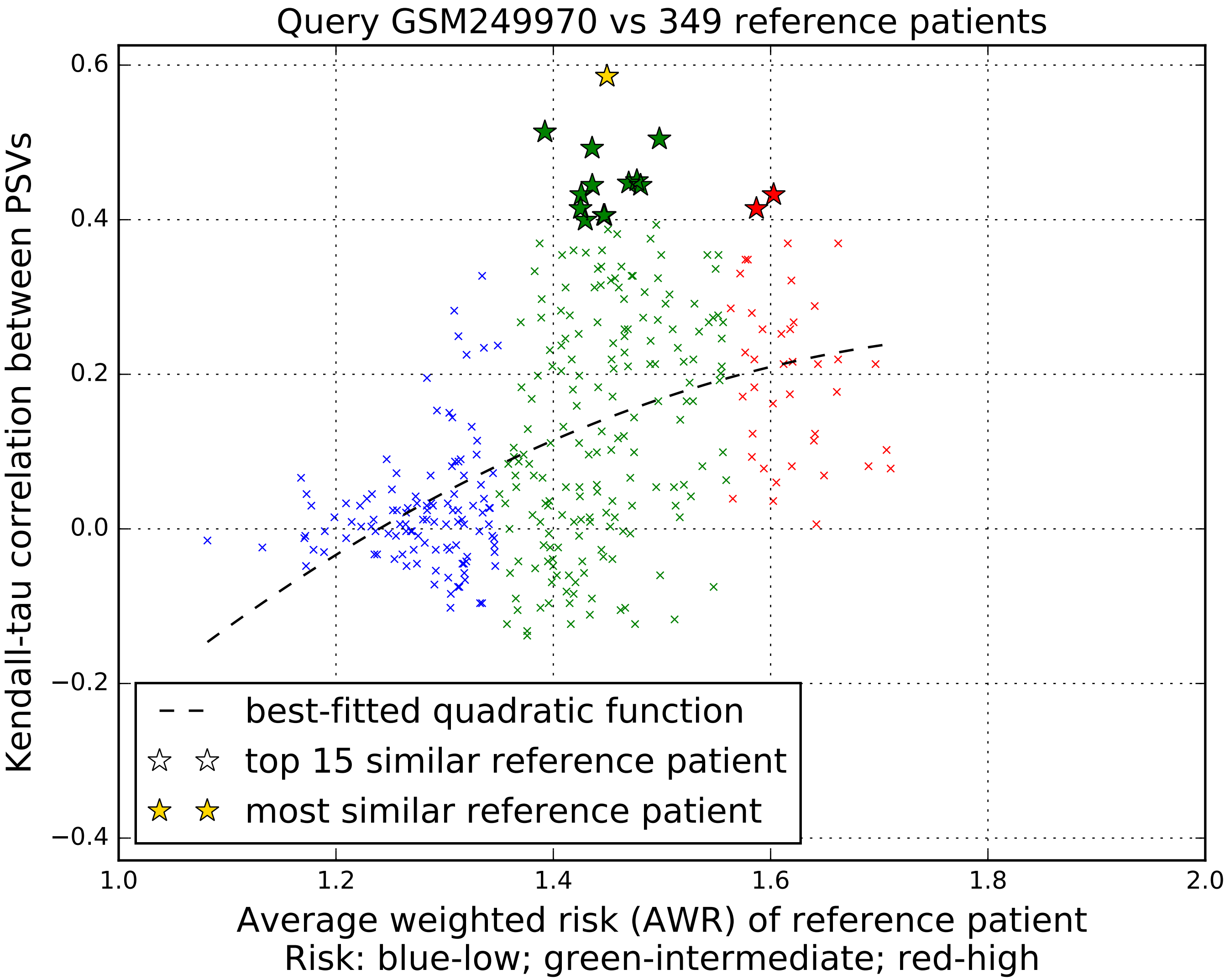

Query GSM249970 vs 349 reference patients
Kendall-tau correlation between PSVs
Average weighted risk (AWR) of reference patient
Risk: blue-low; green-intermediate; red-high
best-fitted quadratic function
top 15 similar reference patient
most similar reference patient
0.6
0.4
0.2
0.0
−0.2
−0.4
1.0
1.2
1.4
1.6
1.8
2.0

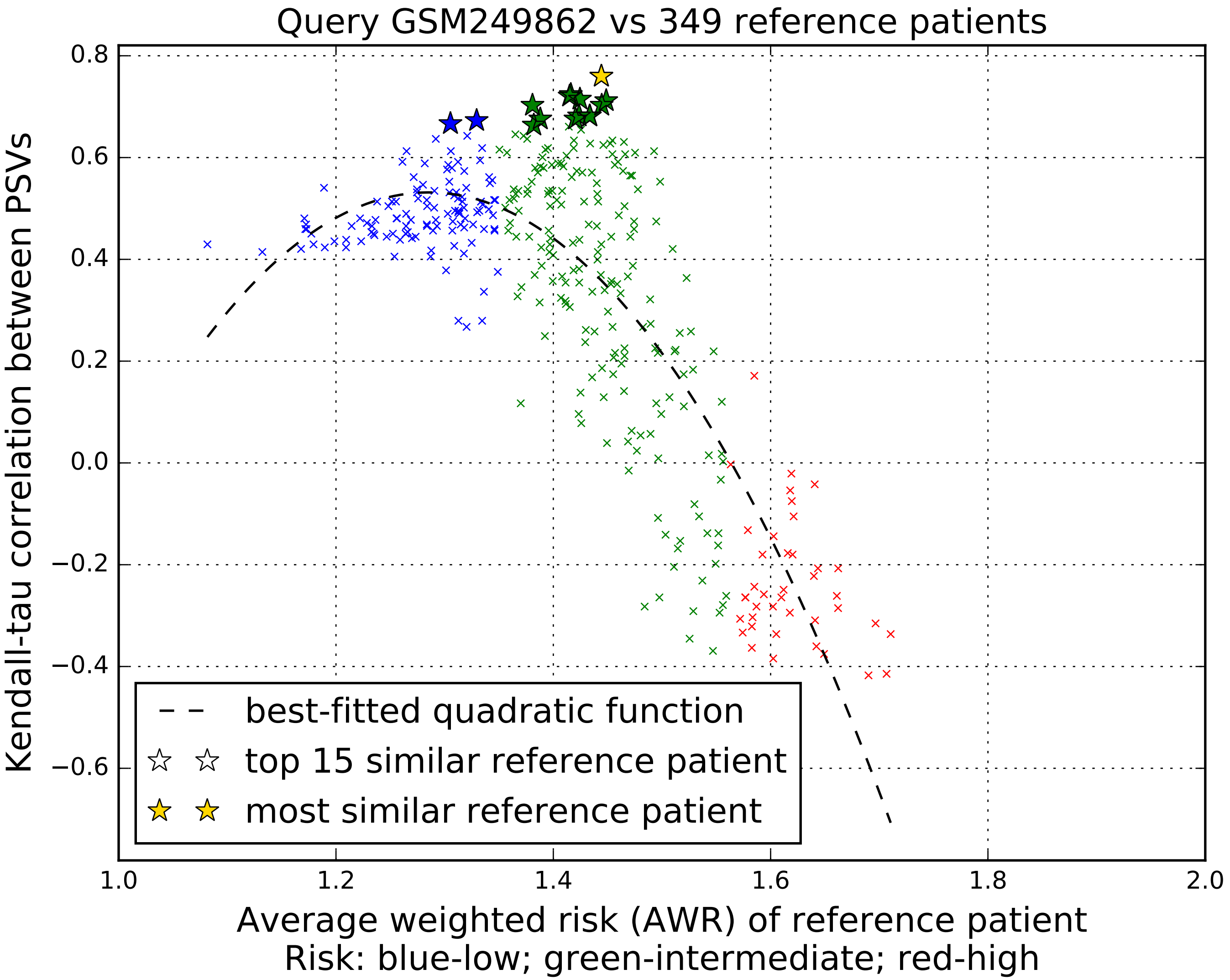

Query GSM249862 vs 349 reference patients
Kendall-tau correlation between PSVs
0.8
0.6
0.4
0.2
0.0
−0.2
−0.4
−0.6
1.0
1.2
1.4
1.6
1.8
2.0
best-fitted quadratic function
top 15 similar reference patient
most similar reference patient
Average weighted risk (AWR) of reference patient
Risk: blue-low; green-intermediate; red-high

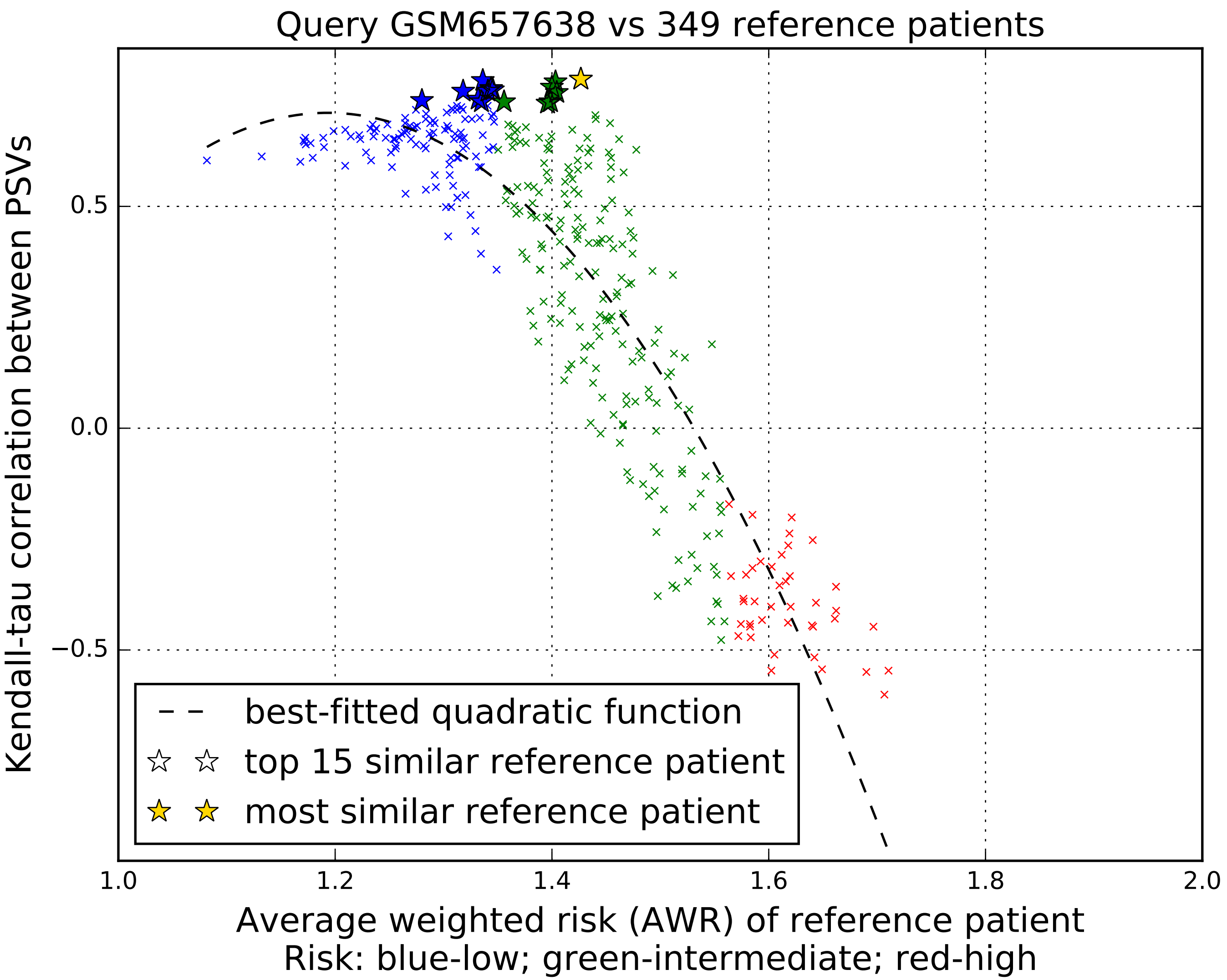
Query GSM657638 vs 349 reference patients
Kendall-tau correlation between PSVs
0.5
0.0
−0.5
1.0
1.2
1.4
1.6
1.8
2.0
best-fitted quadratic function
top 15 similar reference patient
most similar reference patient
Average weighted risk (AWR) of reference patient
Risk: blue-low; green-intermediate; red-high

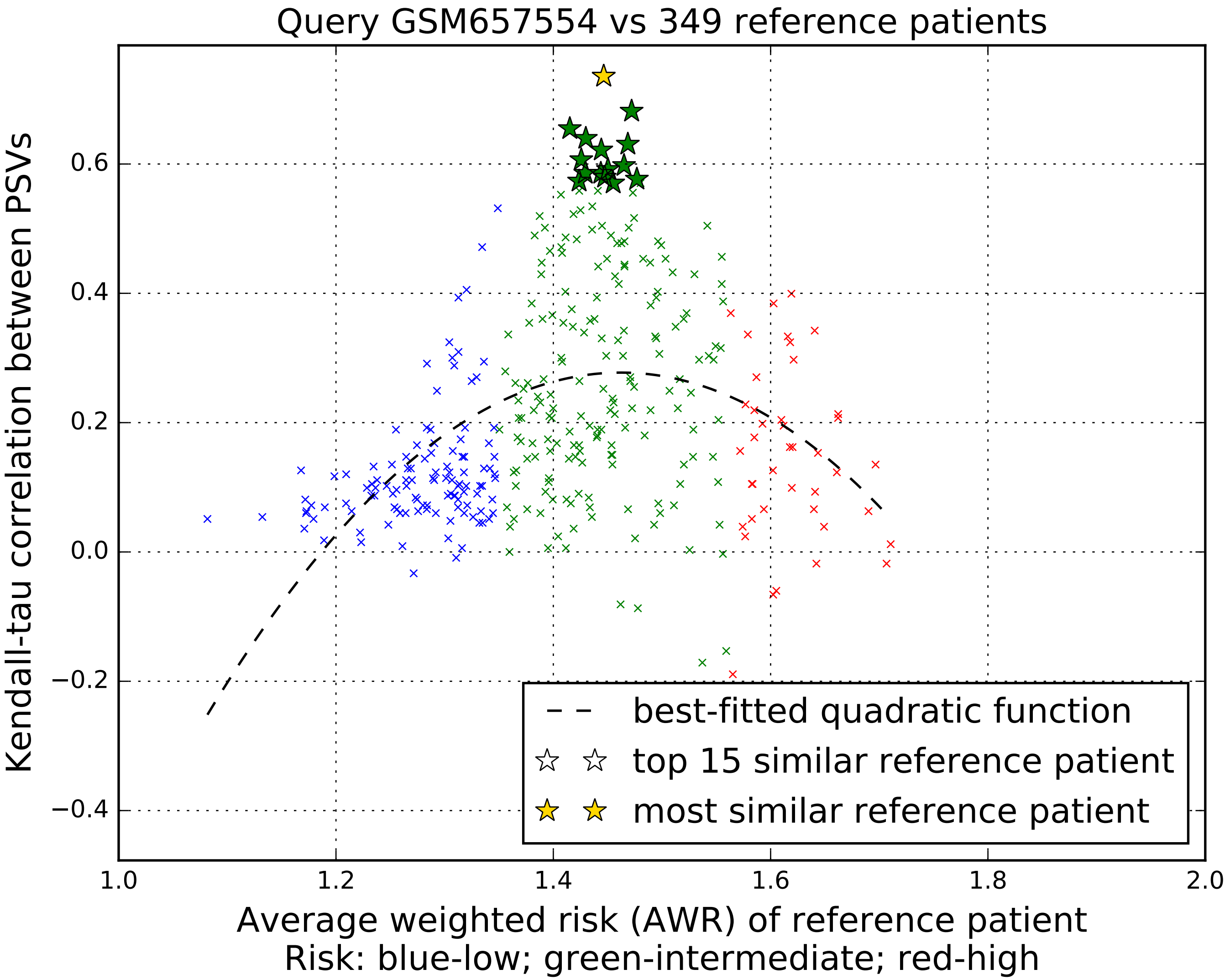

Query GSM657554 vs 349 reference patients
Kendall-tau correlation between PSVs
0.6
0.4
0.2
0.0
−0.2
−0.4
1.0
1.2
1.4
1.6
1.8
2.0
best-fitted quadratic function
top 15 similar reference patient
most similar reference patient
Average weighted risk (AWR) of reference patient
Risk: blue-low; green-intermediate; red-high

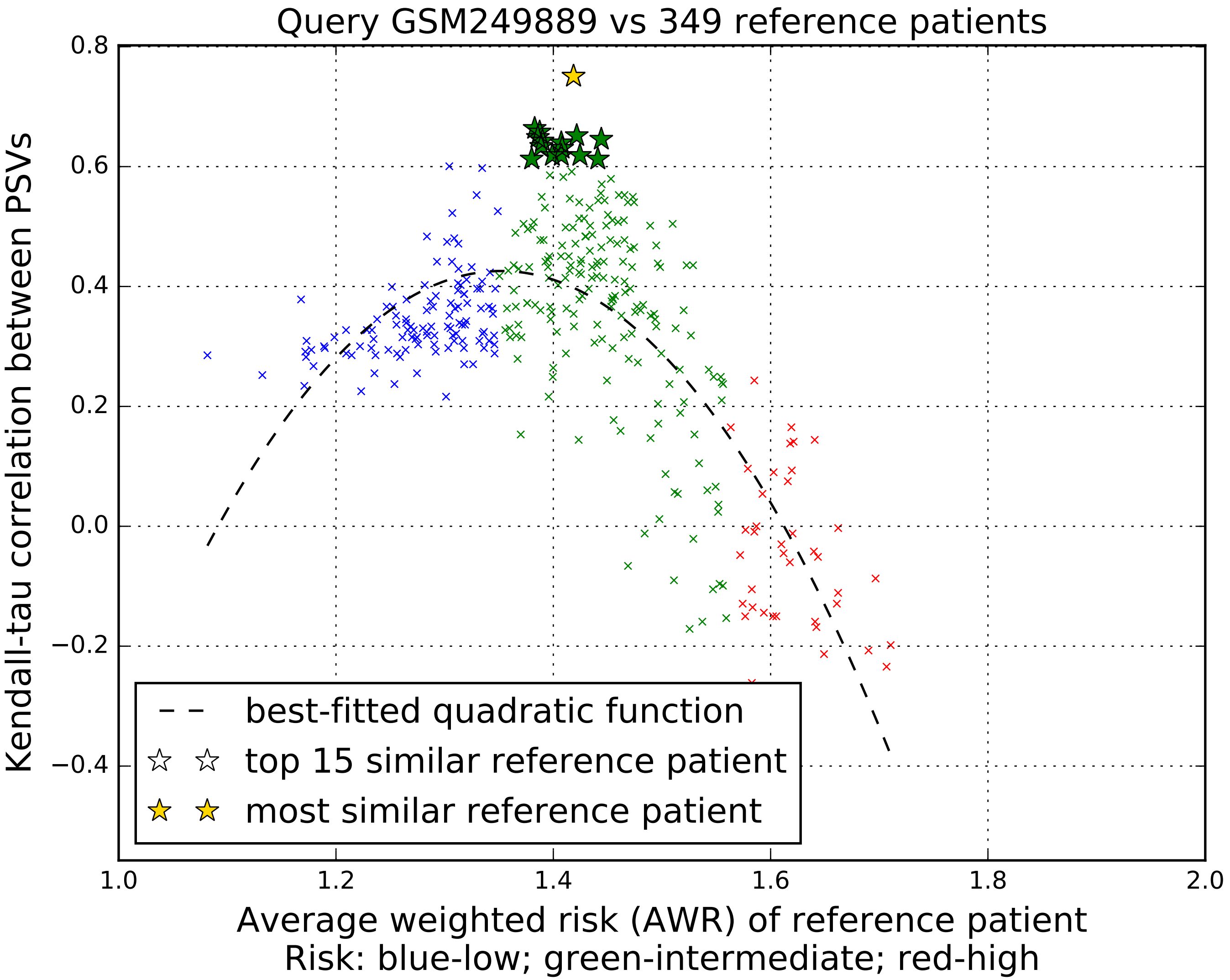

Query GSM249889 vs 349 reference patients
Kendall-tau correlation between PSVs
0.8
0.6
0.4
0.2
0.0
−0.2
−0.4
1.0
1.2
1.4
1.6
1.8
2.0
best-fitted quadratic function
top 15 similar reference patient
most similar reference patient
Average weighted risk (AWR) of reference patient
Risk: blue-low; green-intermediate; red-high

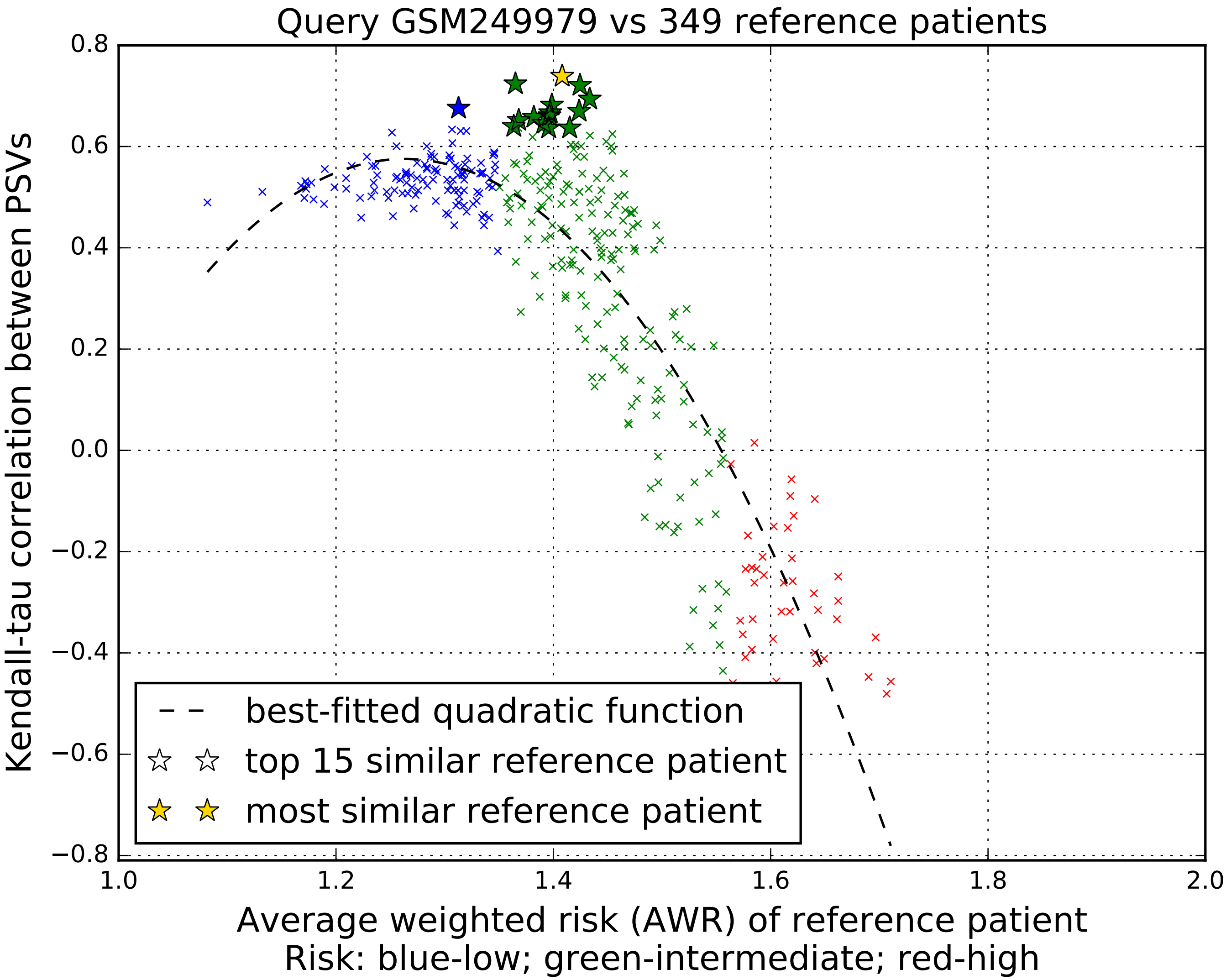
Query GSM249979 vs 349 reference patients
Kendall-tau correlation between PSVs
0.8
0.6
0.4
0.2
0.0
−0.2
−0.4
−0.6
−0.8
1.0
1.2
1.4
1.6
1.8
2.0
best-fitted quadratic function
top 15 similar reference patient
most similar reference patient
Average weighted risk (AWR) of reference patient
Risk: blue-low; green-intermediate; red-high

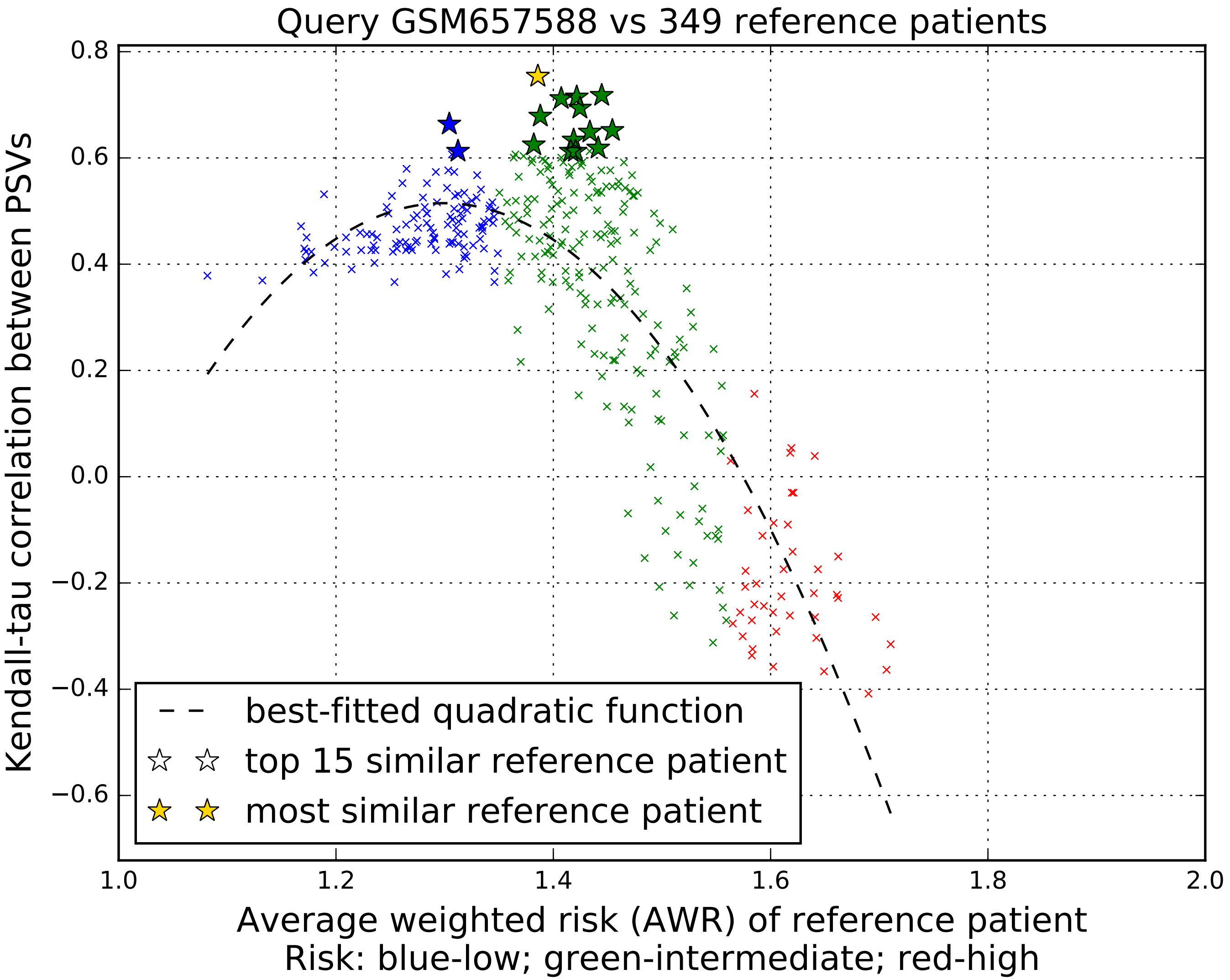

Query GSM657588 vs 349 reference patients
Kendall-tau correlation between PSVs
0.8
0.6
0.4
0.2
0.0
−0.2
−0.4
−0.6
1.0
1.2
1.4
1.6
1.8
2.0
best-fitted quadratic function
top 15 similar reference patient
most similar reference patient
Average weighted risk (AWR) of reference patient
Risk: blue-low; green-intermediate; red-high

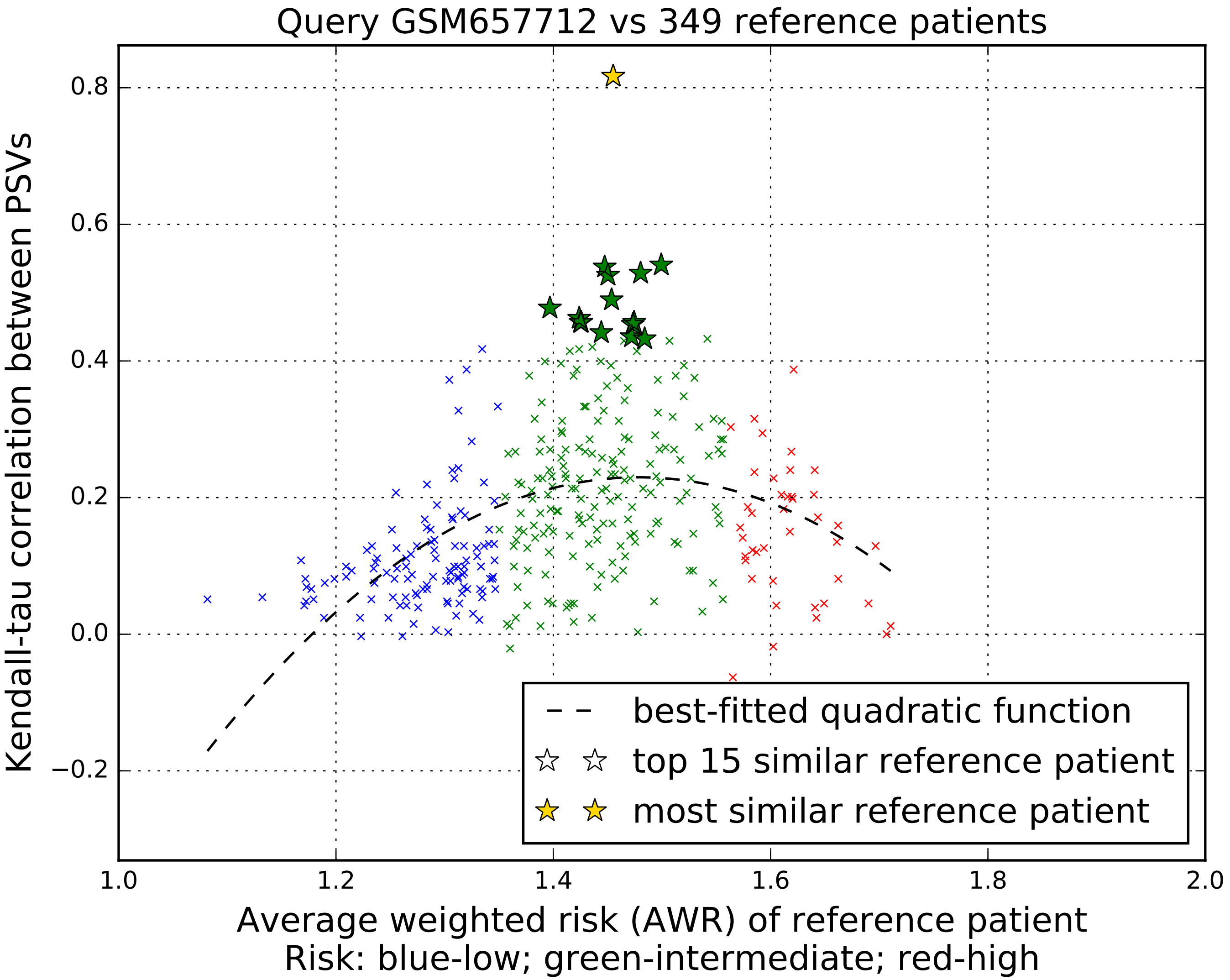

Query GSM657712 vs 349 reference patients
Kendall-tau correlation between PSVs
0.8
0.6
0.4
0.2
0.0
−0.2
1.0
1.2
1.4
1.6
1.8
2.0
best-fitted quadratic function
top 15 similar reference patient
most similar reference patient
Average weighted risk (AWR) of reference patient
Risk: blue-low; green-intermediate; red-high

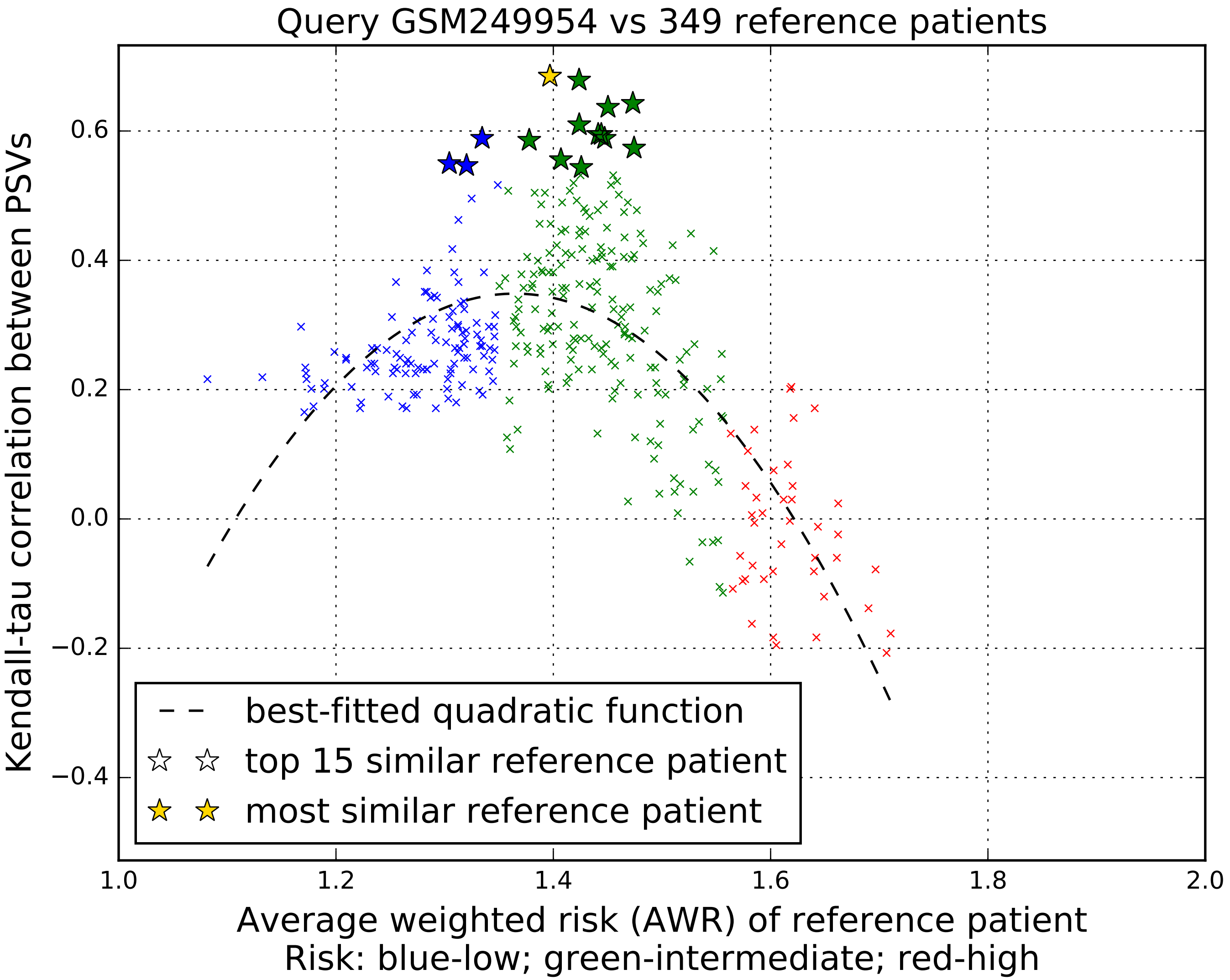
Query GSM249954 vs 349 reference patients
Kendall-tau correlation between PSVs
0.6
0.4
0.2
0.0
−0.2
−0.4
1.0
1.2
1.4
1.6
1.8
2.0
best-fitted quadratic function
top 15 similar reference patient
most similar reference patient
Average weighted risk (AWR) of reference patient
Risk: blue-low; green-intermediate; red-high

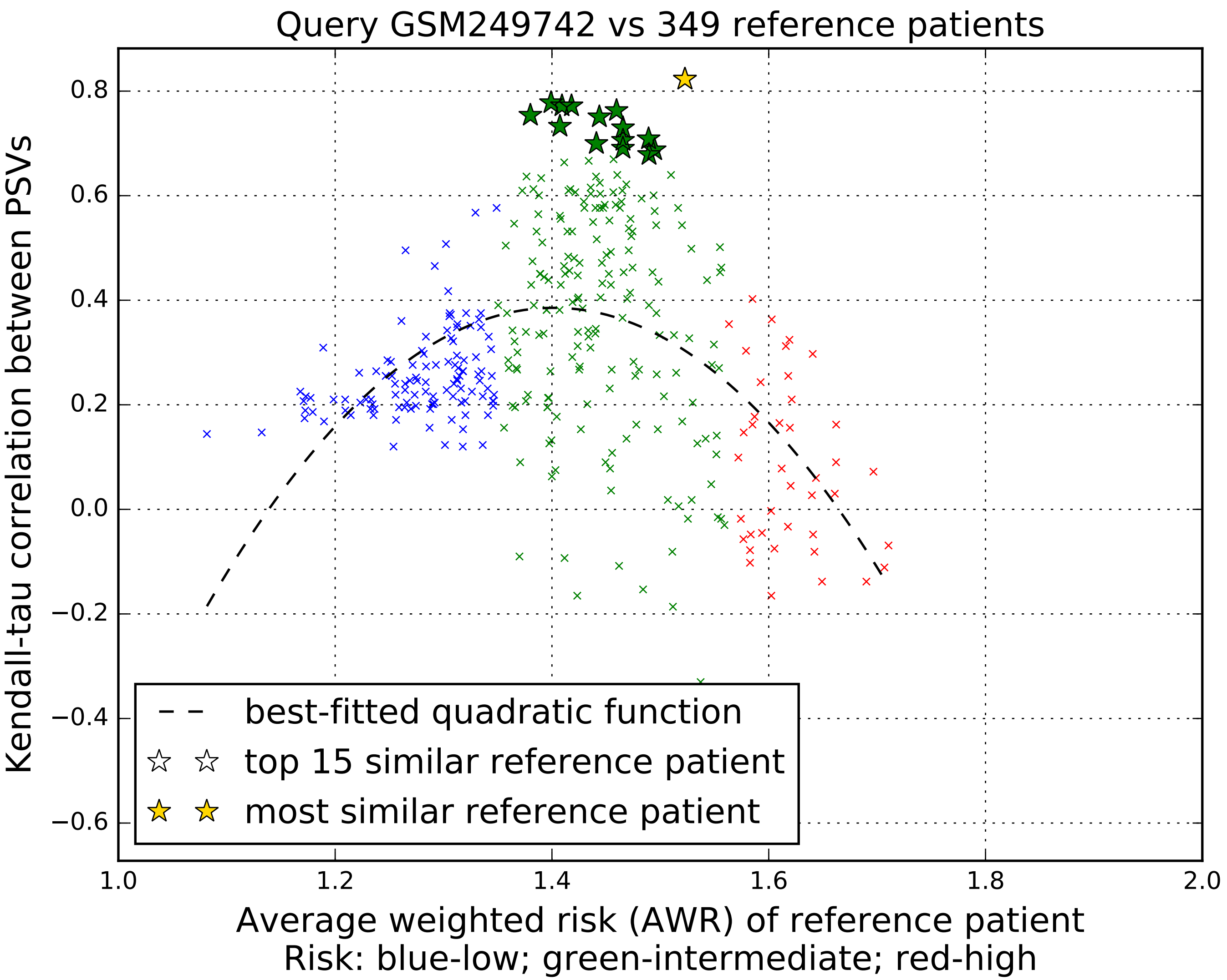

Query GSM249742 vs 349 reference patients
Kendall-tau correlation between PSVs
0.8
0.6
0.4
0.2
0.0
−0.2
−0.4
−0.6
1.0
1.2
1.4
1.6
1.8
2.0
best-fitted quadratic function
top 15 similar reference patient
most similar reference patient
Average weighted risk (AWR) of reference patient
Risk: blue-low; green-intermediate; red-high

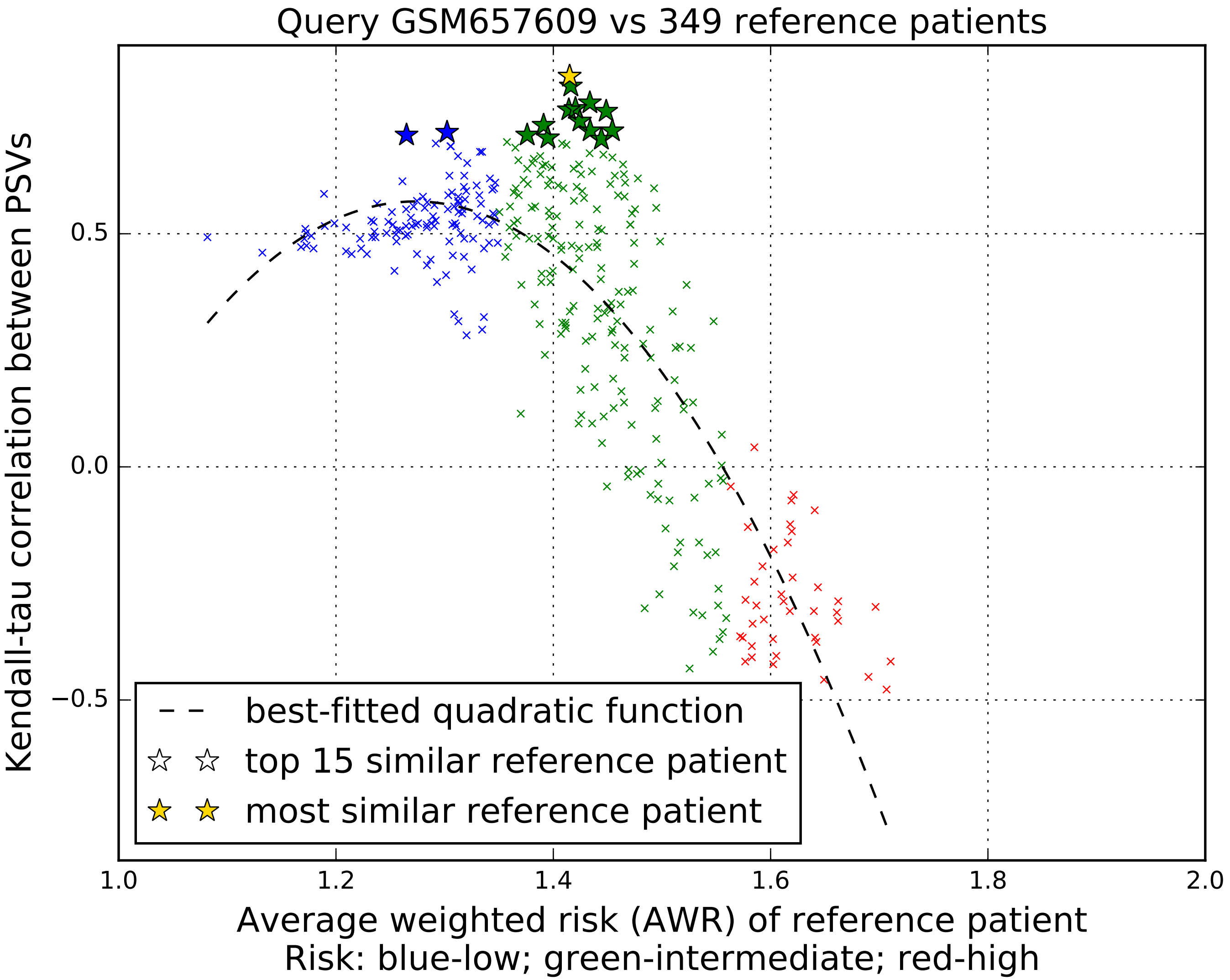

Query GSM657609 vs 349 reference patients
Kendall-tau correlation between PSVs
0.5
0.0
−0.5
1.0
1.2
1.4
1.6
1.8
2.0
best-fitted quadratic function
top 15 similar reference patient
most similar reference patient
Average weighted risk (AWR) of reference patient
Risk: blue-low; green-intermediate; red-high

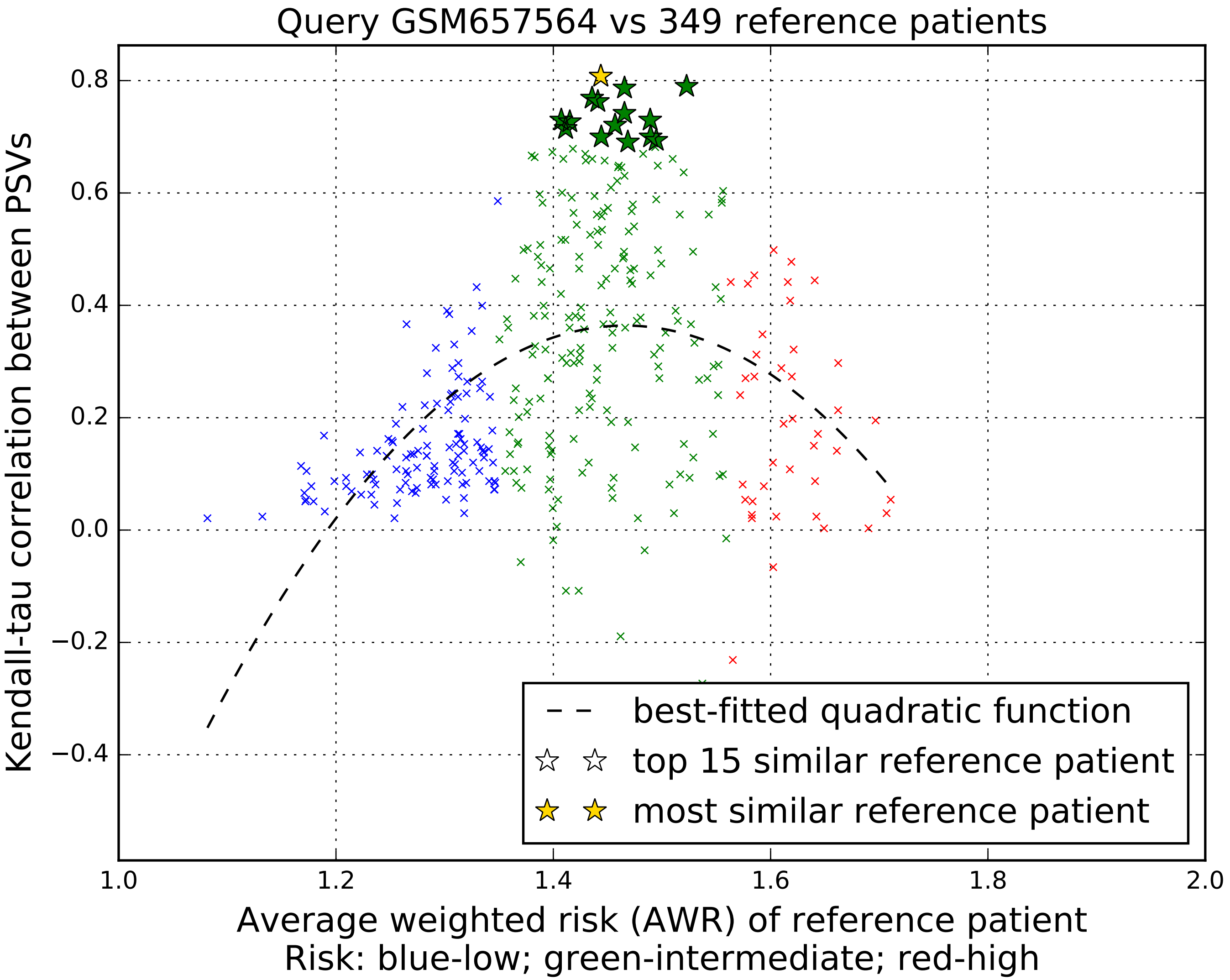
Query GSM657564 vs 349 reference patients
Kendall-tau correlation between PSVs
0.8
0.6
0.4
0.2
0.0
−0.2
−0.4
1.0
1.2
1.4
1.6
1.8
2.0
best-fitted quadratic function
top 15 similar reference patient
most similar reference patient
Average weighted risk (AWR) of reference patient
Risk: blue-low; green-intermediate; red-high

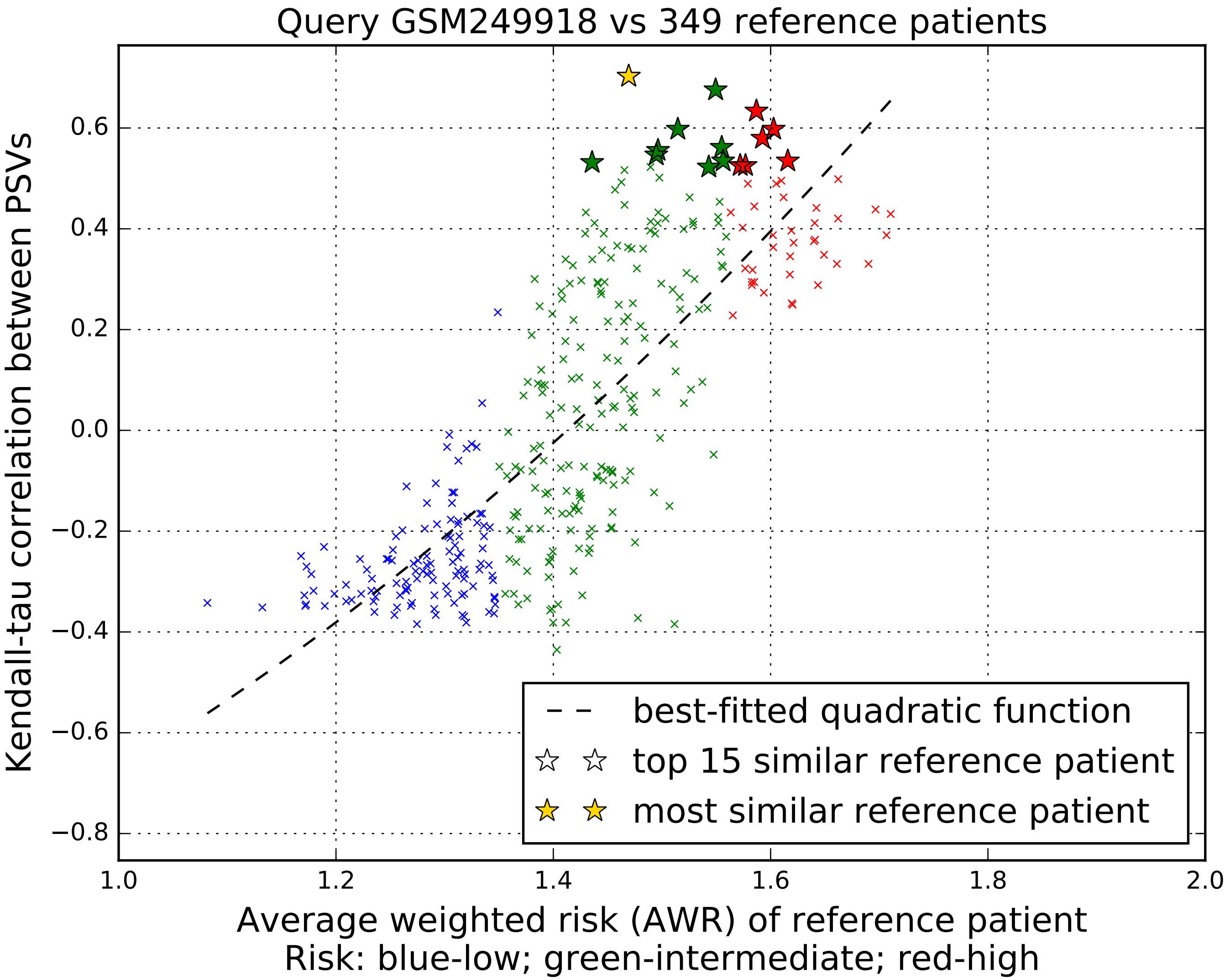

Query GSM249918 vs 349 reference patients
Kendall-tau correlation between PSVs
0.6
0.4
0.2
0.0
−0.2
−0.4
−0.6
−0.8
1.0
1.2
1.4
1.6
1.8
2.0
best-fitted quadratic function
top 15 similar reference patient
most similar reference patient
Average weighted risk (AWR) of reference patient
Risk: blue-low; green-intermediate; red-high

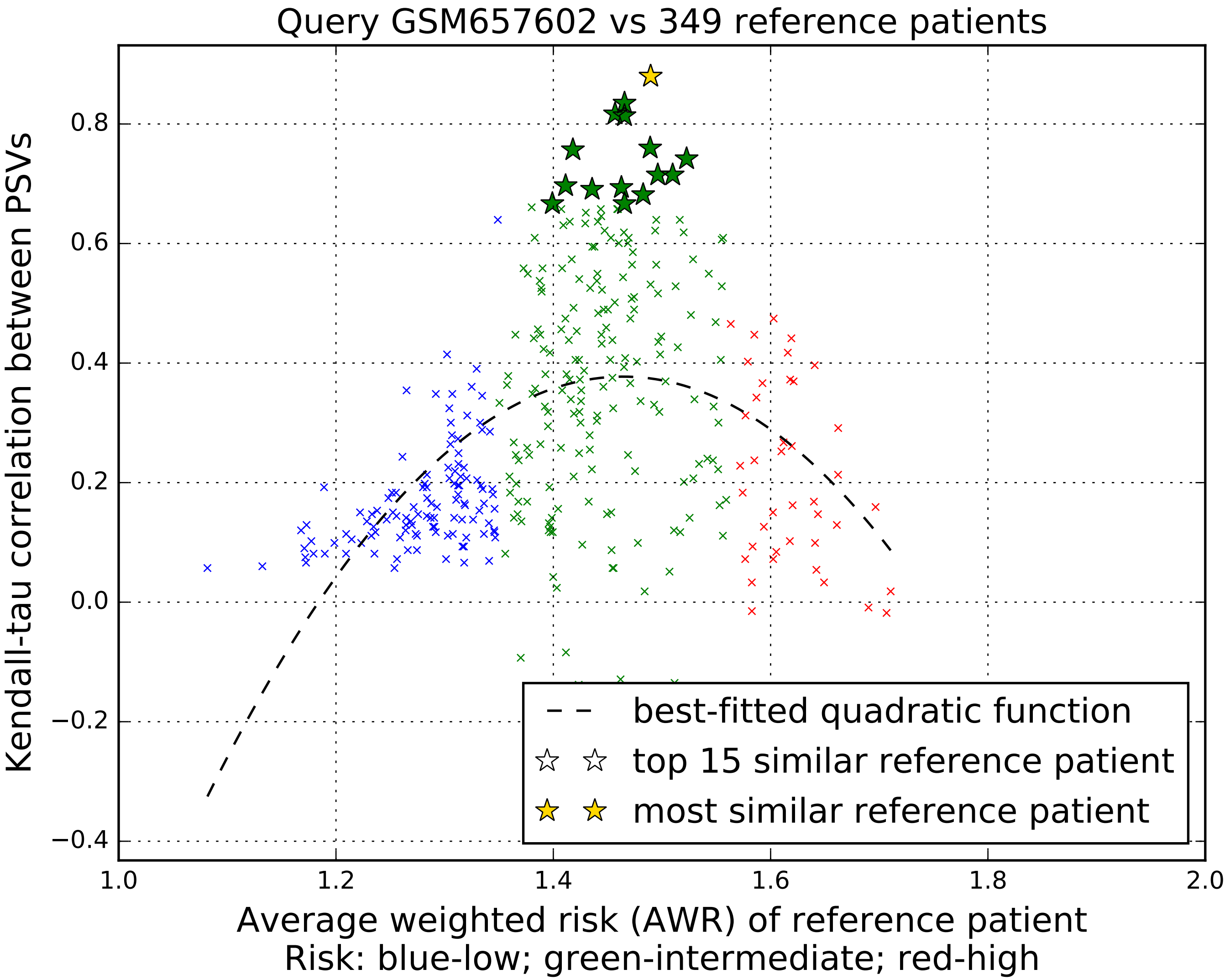

Query GSM657602 vs 349 reference patients
Kendall-tau correlation between PSVs
Average weighted risk (AWR) of reference patient
Risk: blue-low; green-intermediate; red-high
best-fitted quadratic function
top 15 similar reference patient
most similar reference patient
0.8
0.6
0.4
0.2
0.0
−0.2
−0.4
1.0
1.2
1.4
1.6
1.8
2.0

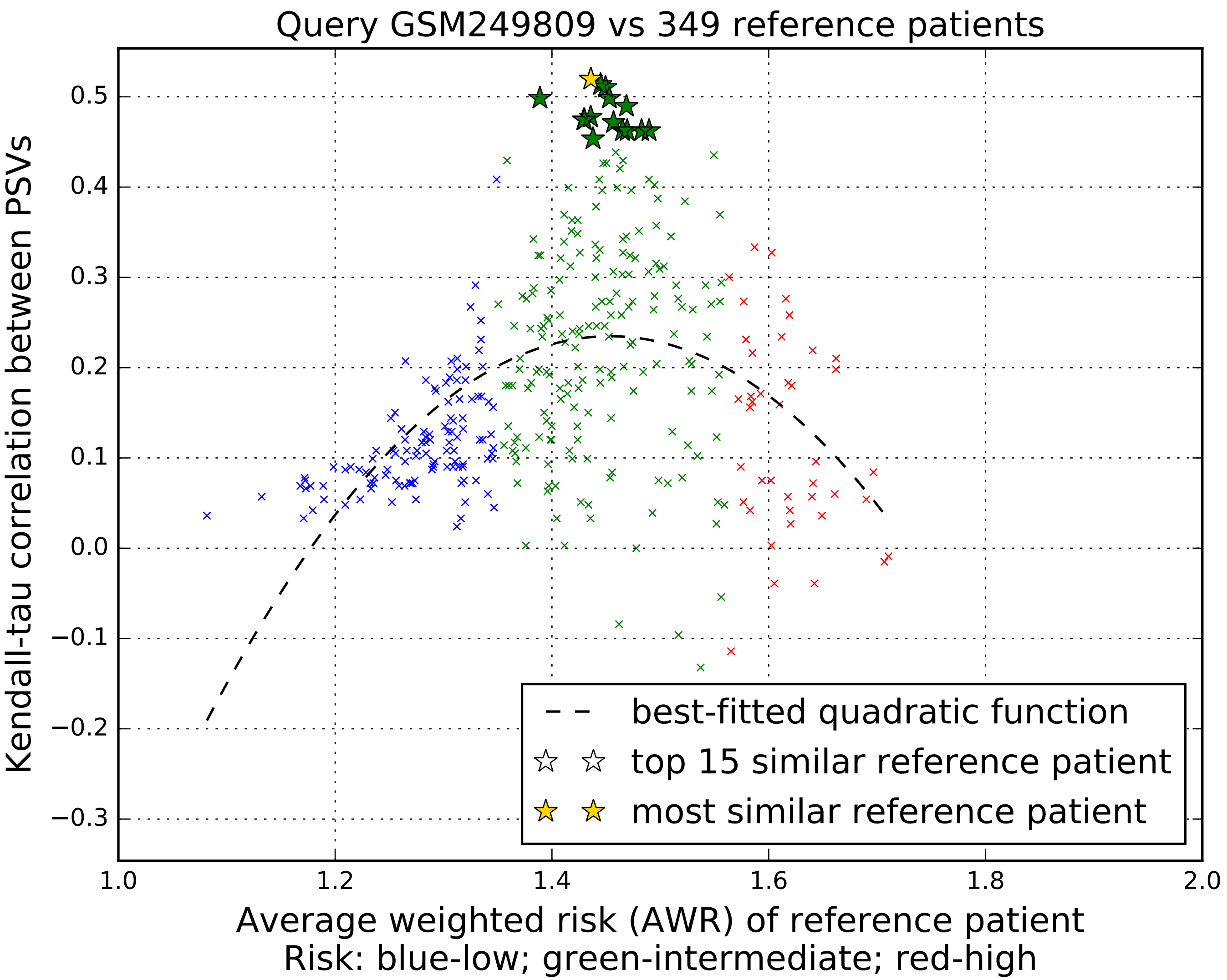
Query GSM249809 vs 349 reference patients
Kendall-tau correlation between PSVs
0.5
0.4
0.3
0.2
0.1
0.0
−0.1
−0.2
−0.3
1.0
1.2
1.4
1.6
1.8
2.0
best-fitted quadratic function
top 15 similar reference patient
most similar reference patient
Average weighted risk (AWR) of reference patient
Risk: blue-low; green-intermediate; red-high

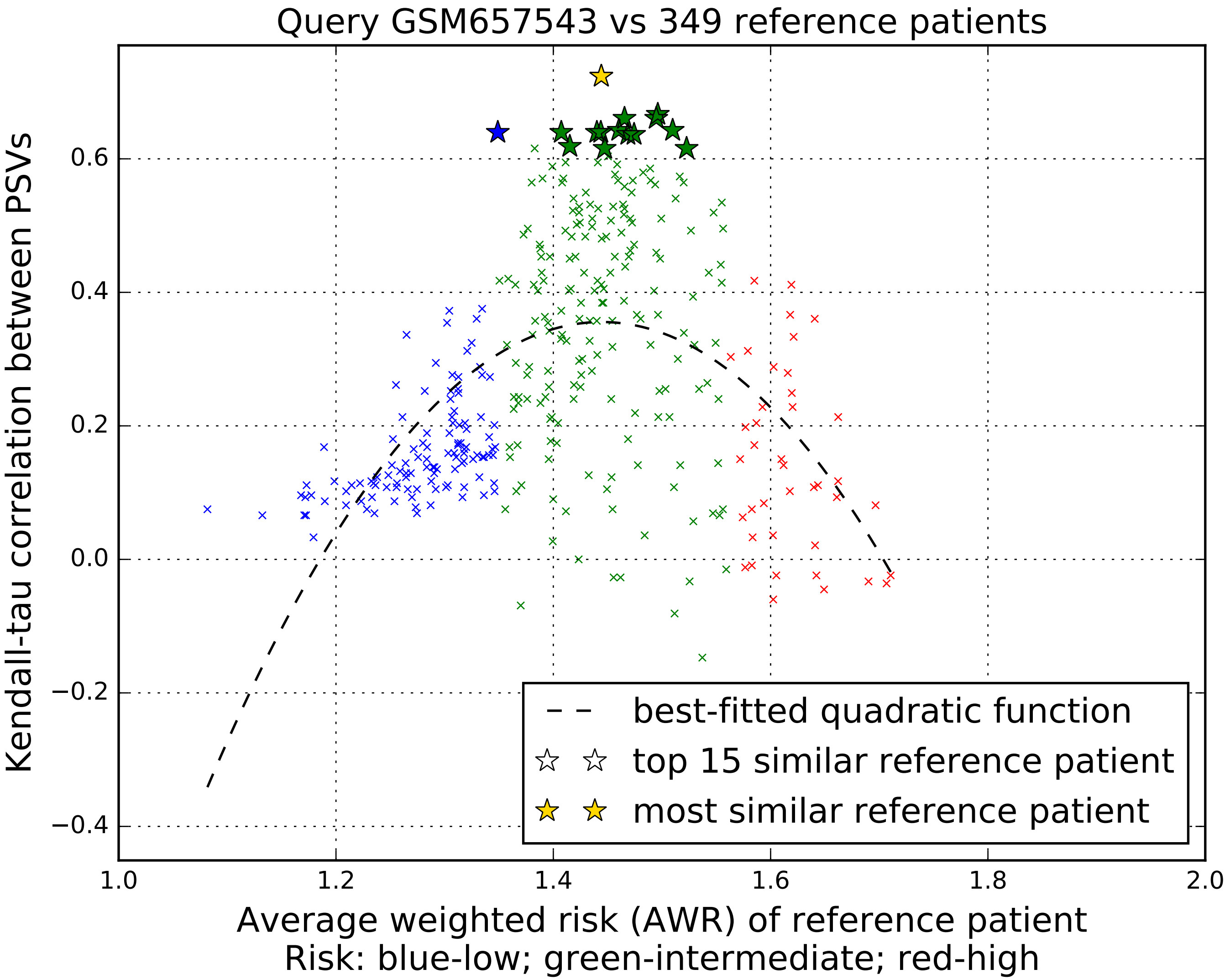

Query GSM657543 vs 349 reference patients
Kendall-tau correlation between PSVs
0.6
0.4
0.2
0.0
−0.2
−0.4
1.0
1.2
1.4
1.6
1.8
2.0
best-fitted quadratic function
top 15 similar reference patient
most similar reference patient
Average weighted risk (AWR) of reference patient
Risk: blue-low; green-intermediate; red-high

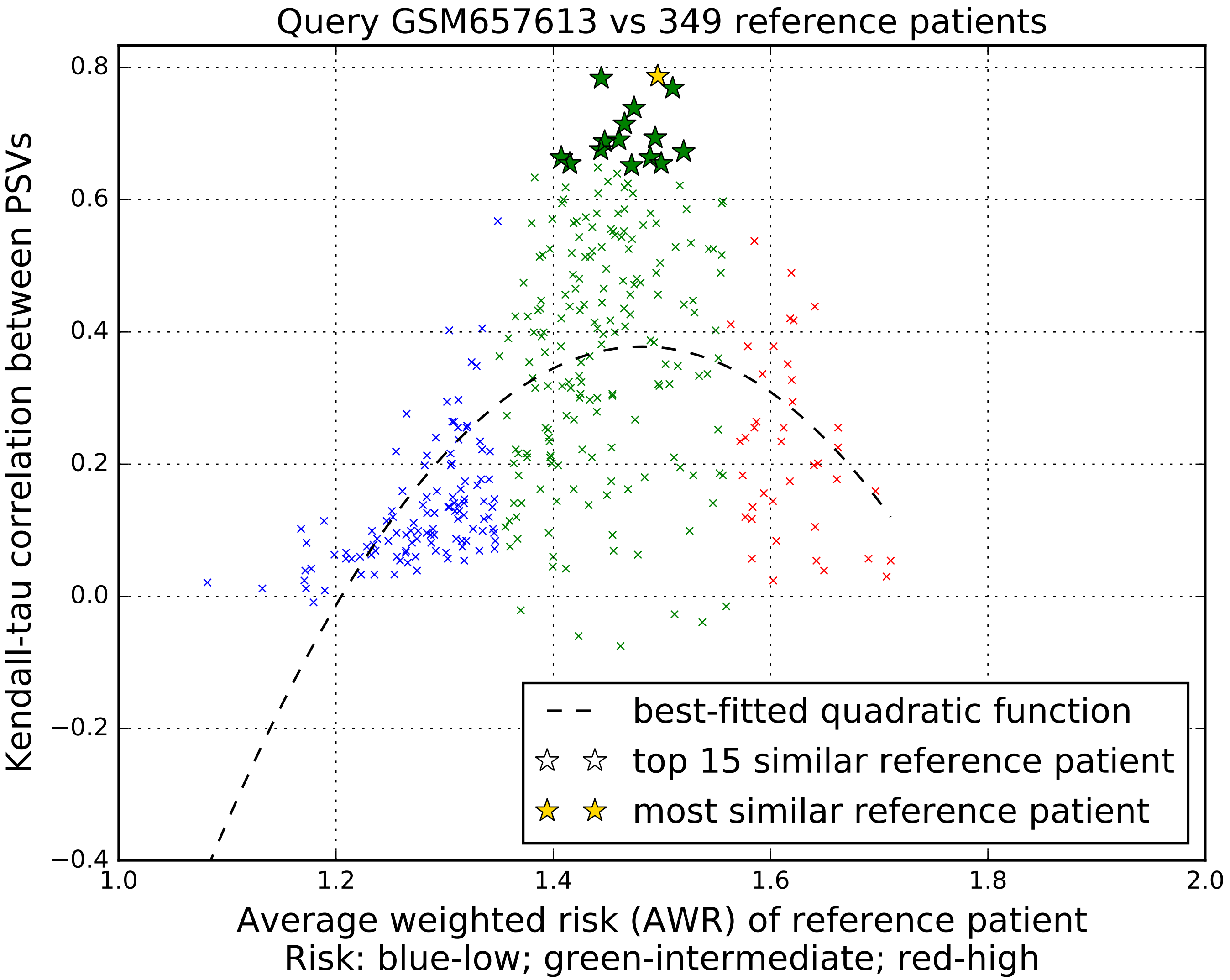

Query GSM657613 vs 349 reference patients
Kendall-tau correlation between PSVs
0.8
0.6
0.4
0.2
0.0
−0.2
−0.4
1.0
1.2
1.4
1.6
1.8
2.0
best-fitted quadratic function
top 15 similar reference patient
most similar reference patient
Average weighted risk (AWR) of reference patient
Risk: blue-low; green-intermediate; red-high

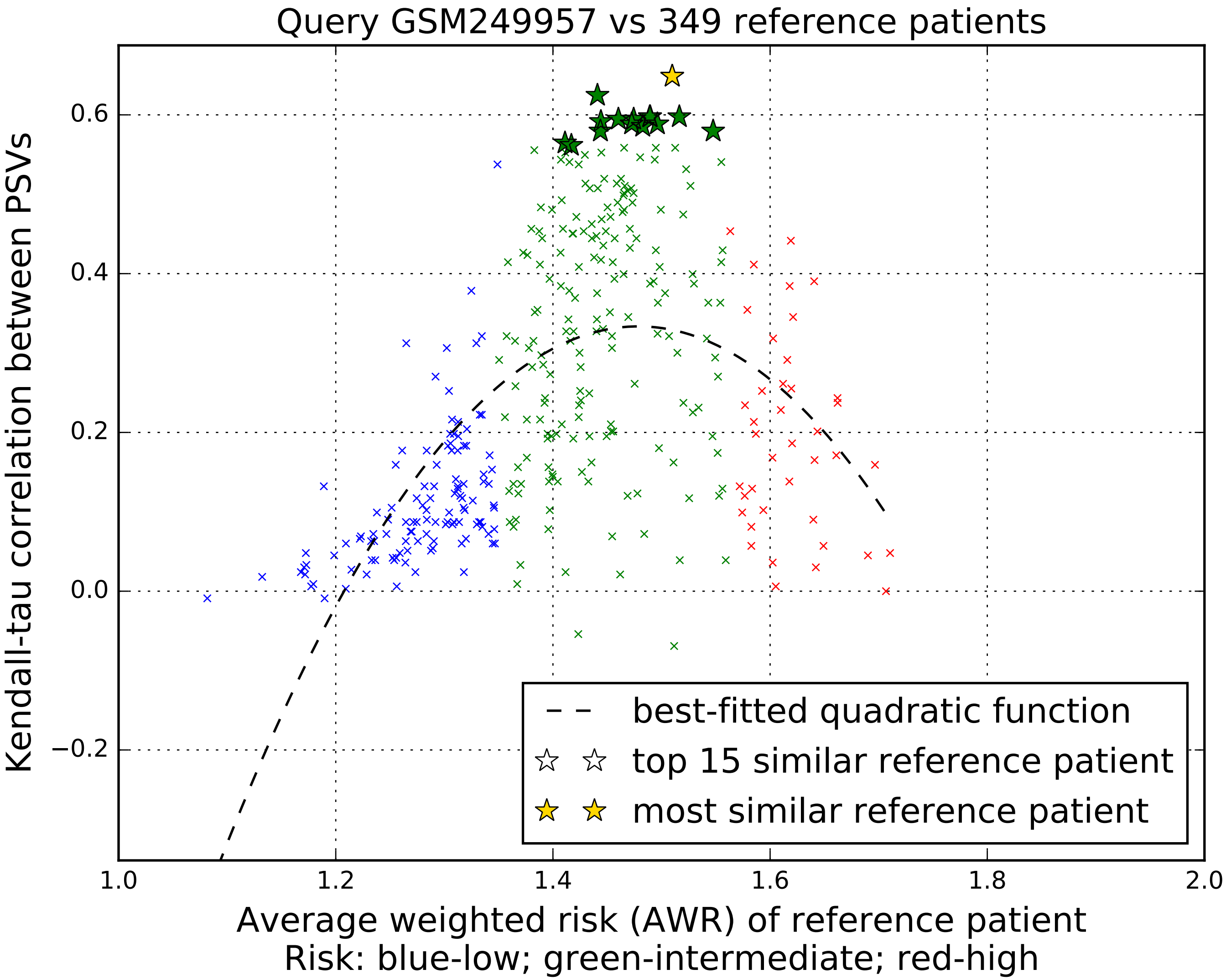

Query GSM249957 vs 349 reference patients
Kendall-tau correlation between PSVs
Average weighted risk (AWR) of reference patient
Risk: blue-low; green-intermediate; red-high
best-fitted quadratic function
top 15 similar reference patient
most similar reference patient
0.6
0.4
0.2
0.0
−0.2
1.0
1.2
1.4
1.6
1.8
2.0

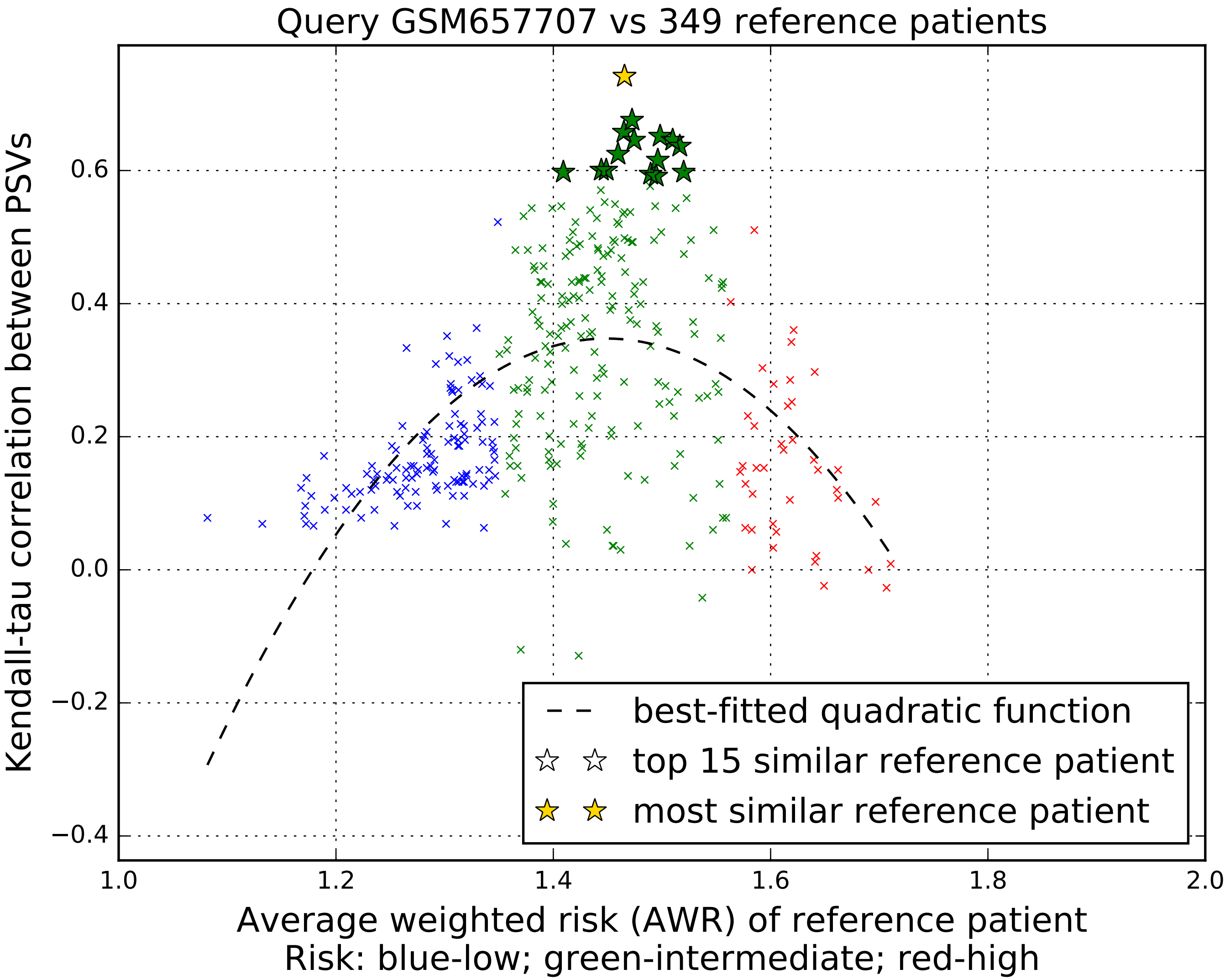

Query GSM657707 vs 349 reference patients
Kendall-tau correlation between PSVs
Average weighted risk (AWR) of reference patient
Risk: blue-low; green-intermediate; red-high
best-fitted quadratic function
top 15 similar reference patient
most similar reference patient
0.6
0.4
0.2
0.0
−0.2
−0.4
1.0
1.2
1.4
1.6
1.8
2.0

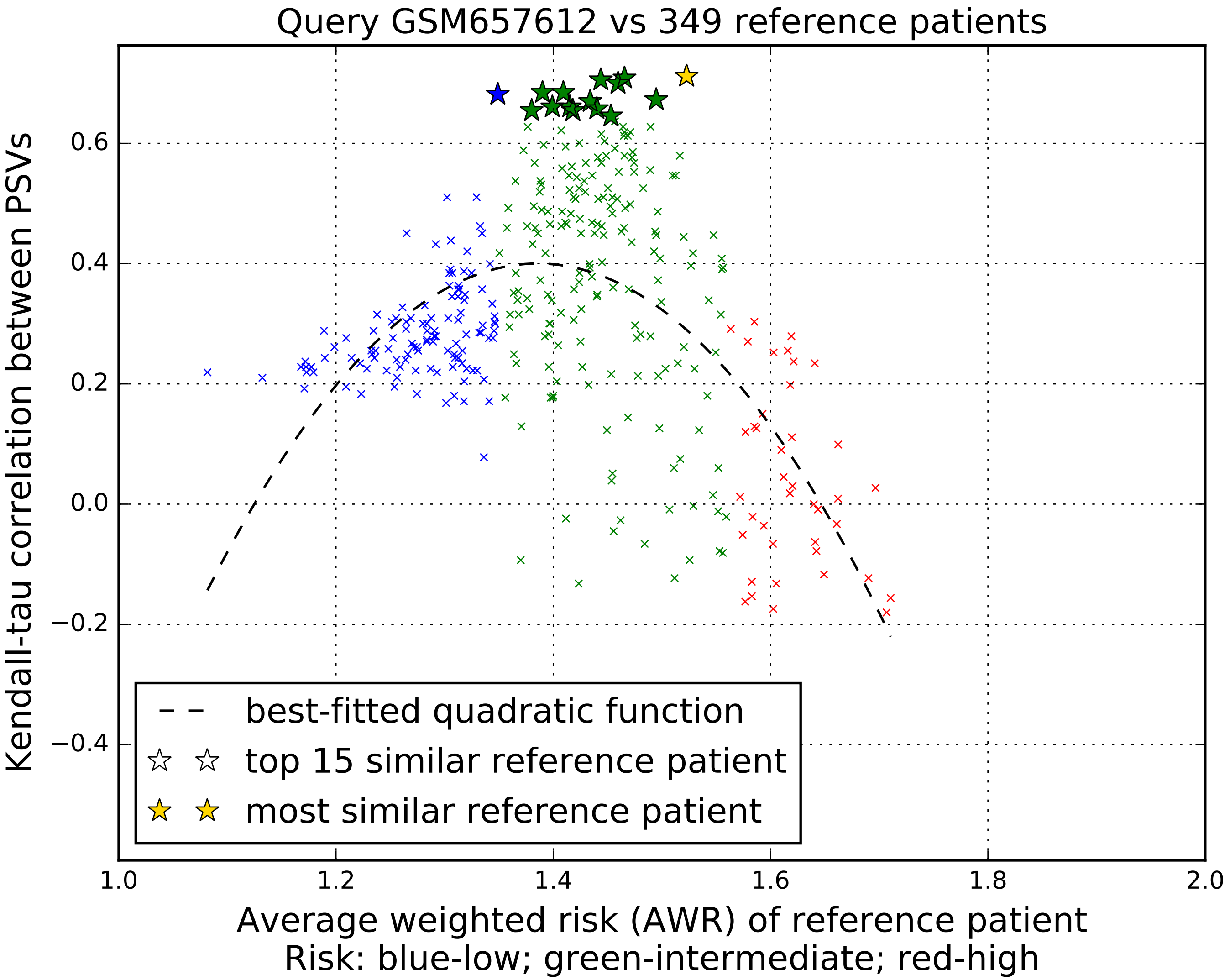

Query GSM657612 vs 349 reference patients
Kendall-tau correlation between PSVs
Average weighted risk (AWR) of reference patient
Risk: blue-low; green-intermediate; red-high
best-fitted quadratic function
top 15 similar reference patient
most similar reference patient
0.6
0.4
0.2
0.0
−0.2
−0.4
1.0
1.2
1.4
1.6
1.8
2.0

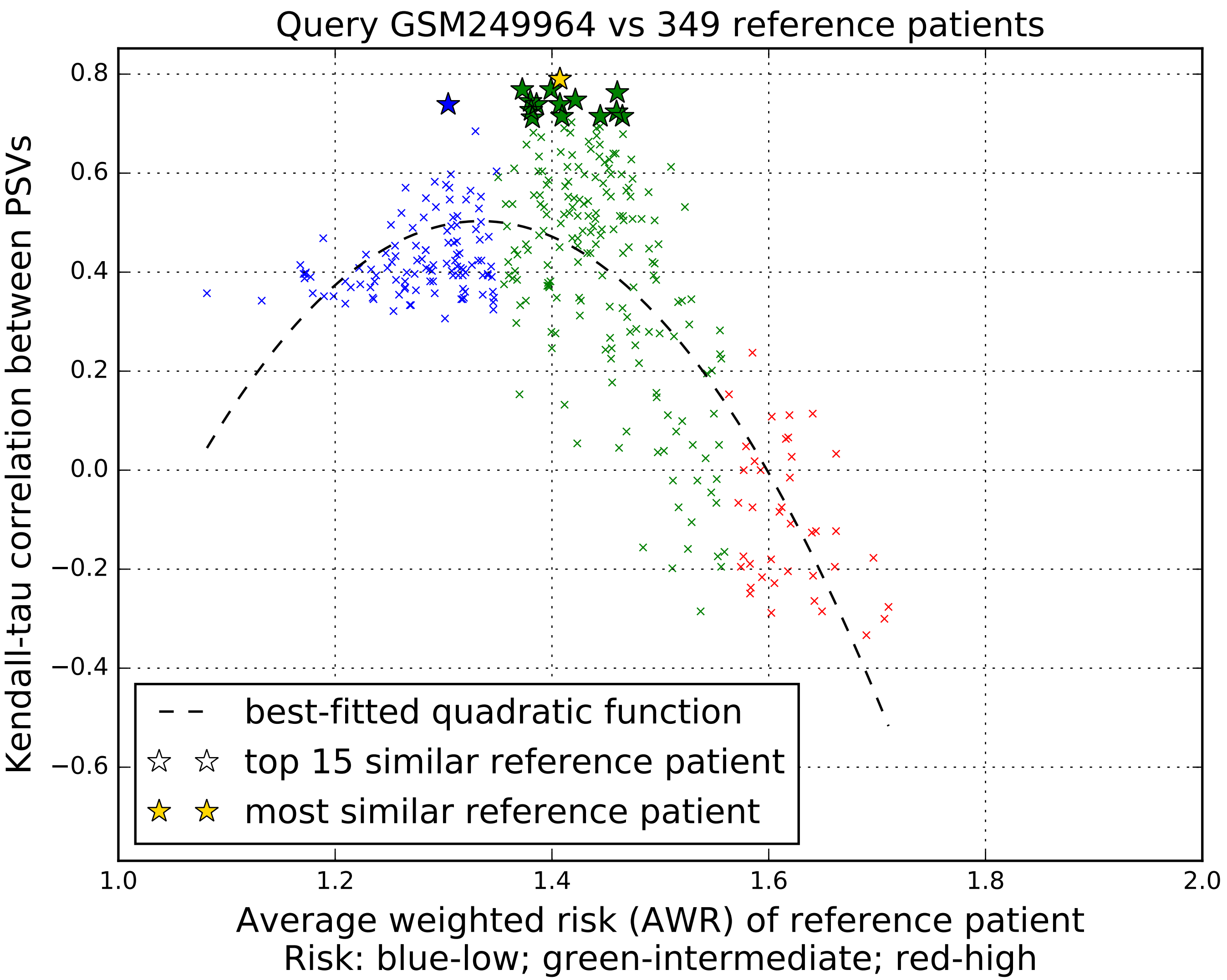
Query GSM249964 vs 349 reference patients
Kendall-tau correlation between PSVs
Average weighted risk (AWR) of reference patient
Risk: blue-low; green-intermediate; red-high
best-fitted quadratic function
top 15 similar reference patient
most similar reference patient
0.8
0.6
0.4
0.2
0.0
−0.2
−0.4
−0.6
1.0
1.2
1.4
1.6
1.8
2.0

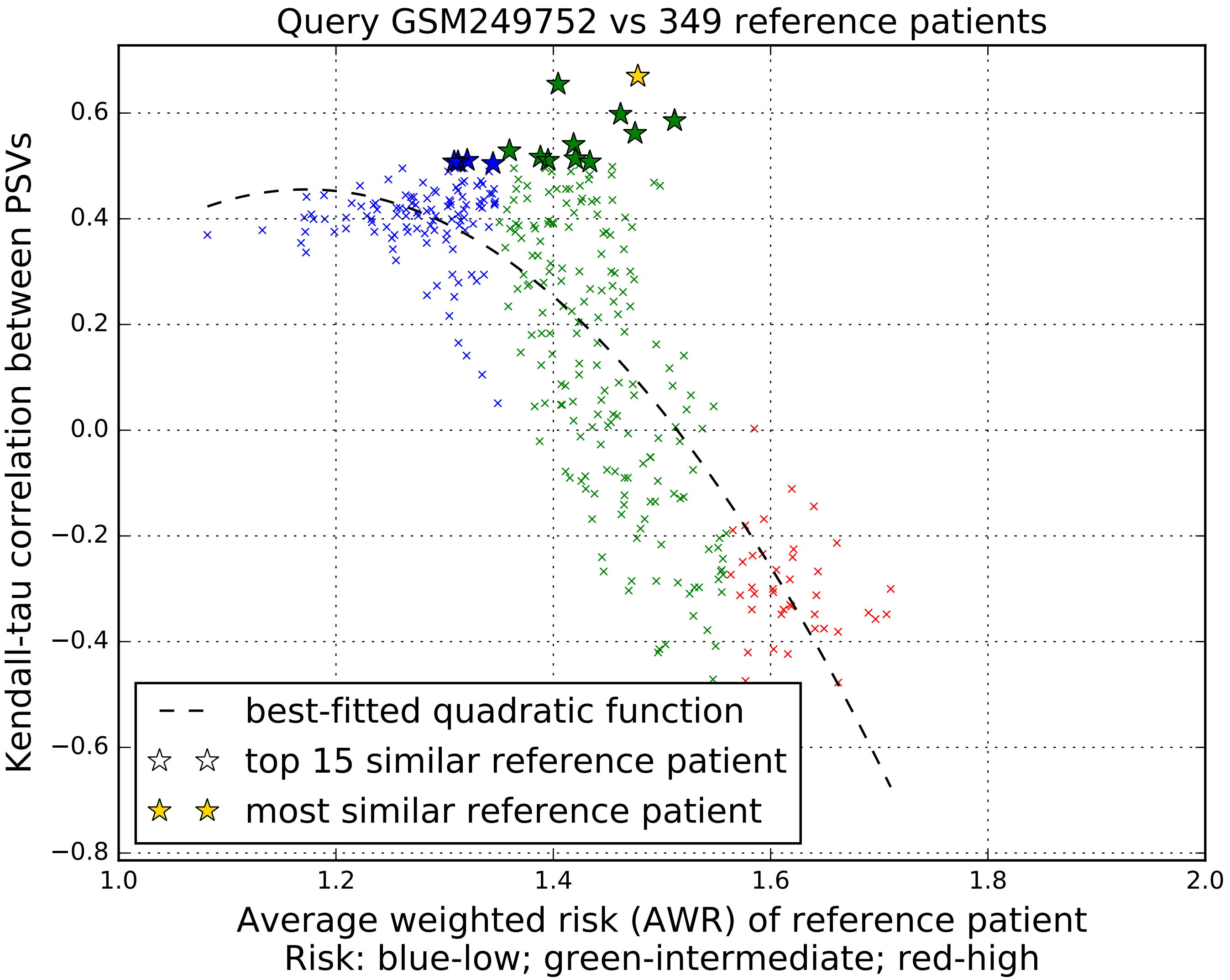
Query GSM249752 vs 349 reference patients
Kendall-tau correlation between PSVs
Average weighted risk (AWR) of reference patient
Risk: blue-low; green-intermediate; red-high
best-fitted quadratic function
top 15 similar reference patient
most similar reference patient
0.6
0.4
0.2
0.0
−0.2
−0.4
−0.6
−0.8
1.0
1.2
1.4
1.6
1.8
2.0

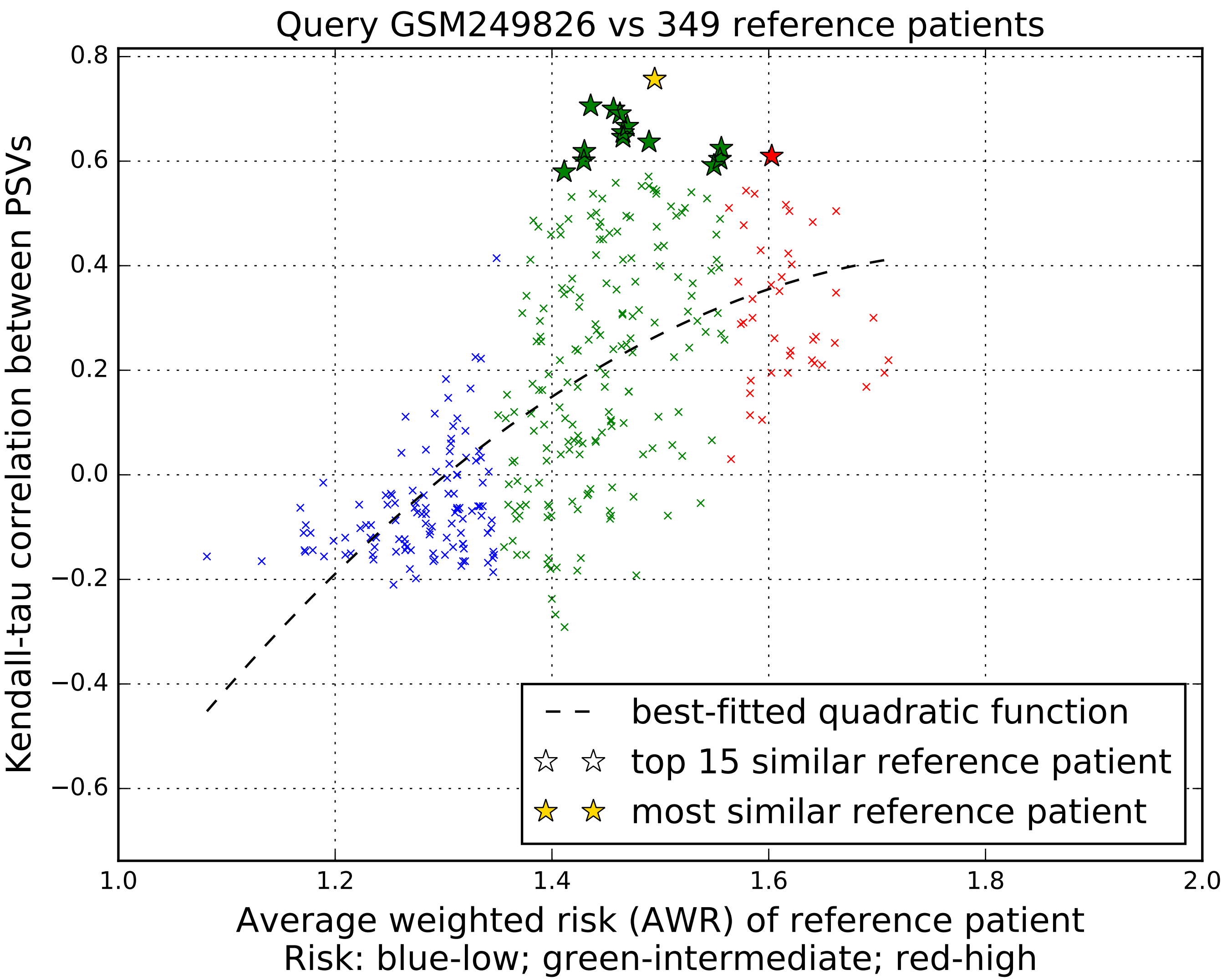

Query GSM249826 vs 349 reference patients
Kendall-tau correlation between PSVs
0.8
0.6
0.4
0.2
0.0
−0.2
−0.4
−0.6
1.0
1.2
1.4
1.6
1.8
2.0
best-fitted quadratic function
top 15 similar reference patient
most similar reference patient
Average weighted risk (AWR) of reference patient
Risk: blue-low; green-intermediate; red-high

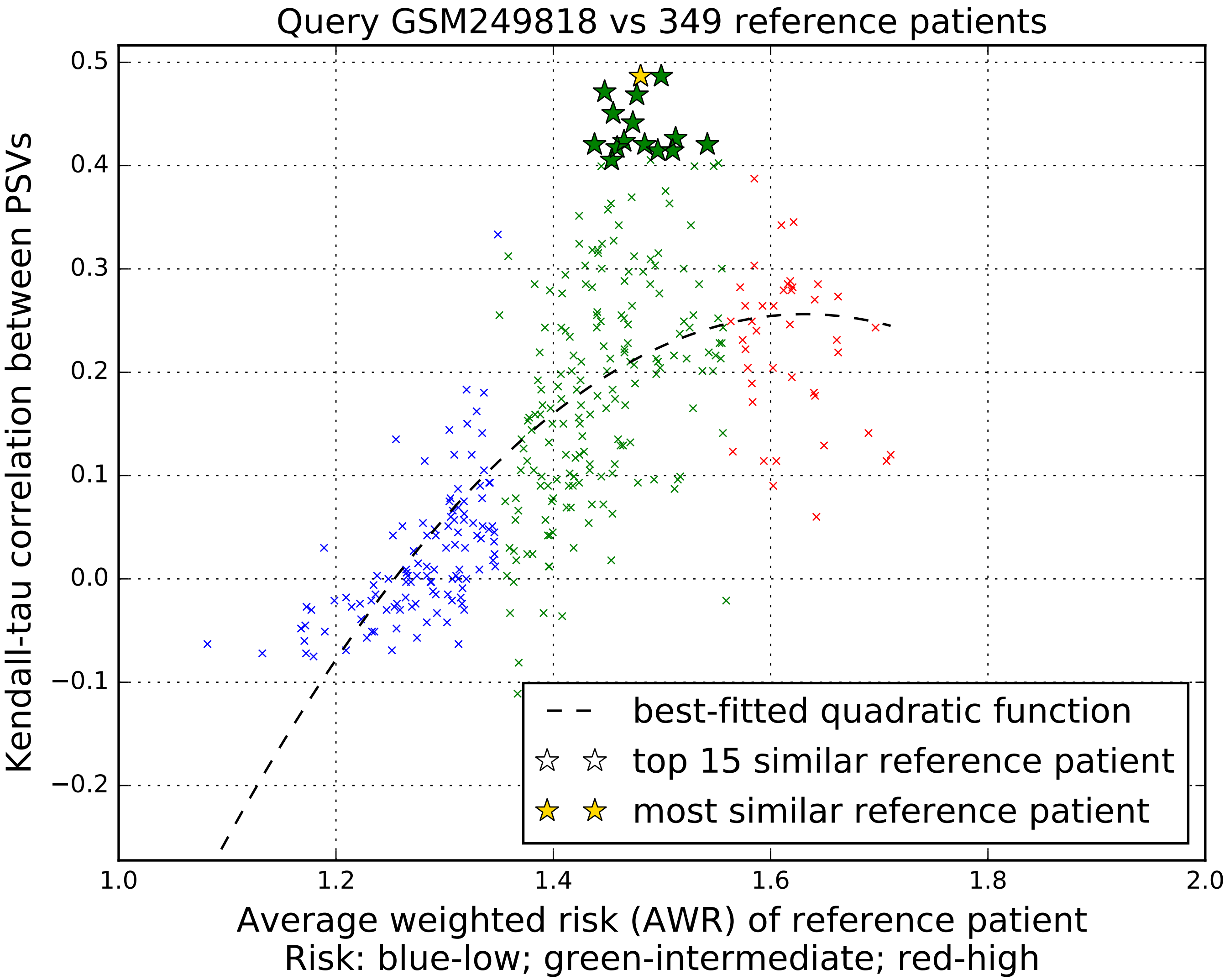

Query GSM249818 vs 349 reference patients
Kendall-tau correlation between PSVs
0.5
0.4
0.3
0.2
0.1
0.0
−0.1
−0.2
1.0
1.2
1.4
1.6
1.8
2.0
best-fitted quadratic function
top 15 similar reference patient
most similar reference patient
Average weighted risk (AWR) of reference patient
Risk: blue-low; green-intermediate; red-high

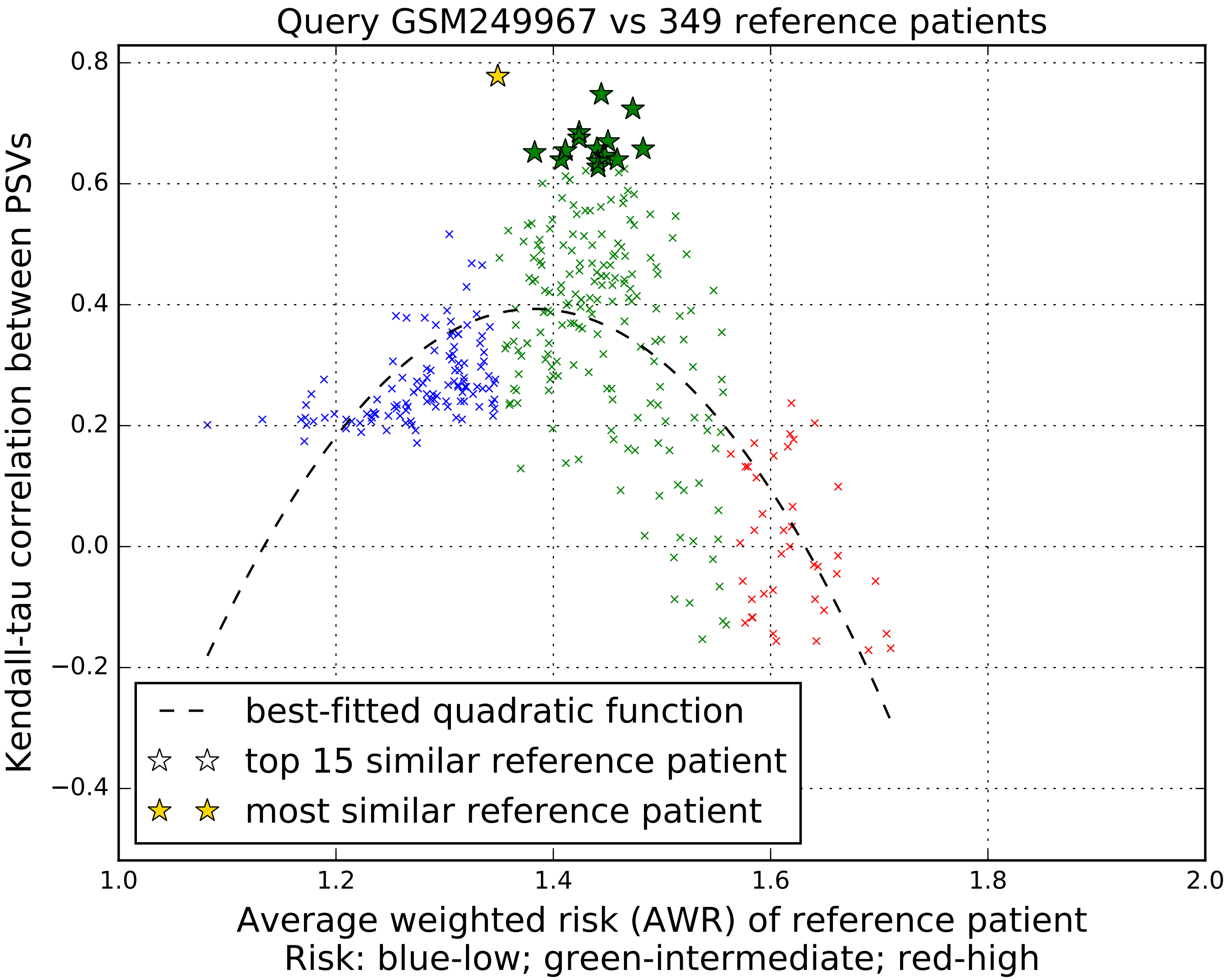

Query GSM249967 vs 349 reference patients
0.8
0.6
0.4
0.2
0.0
−0.2
−0.4
1.0
1.2
1.4
1.6
1.8
2.0
Kendall-tau correlation between PSVs
best-fitted quadratic function
top 15 similar reference patient
most similar reference patient
Average weighted risk (AWR) of reference patient
Risk: blue-low; green-intermediate; red-high

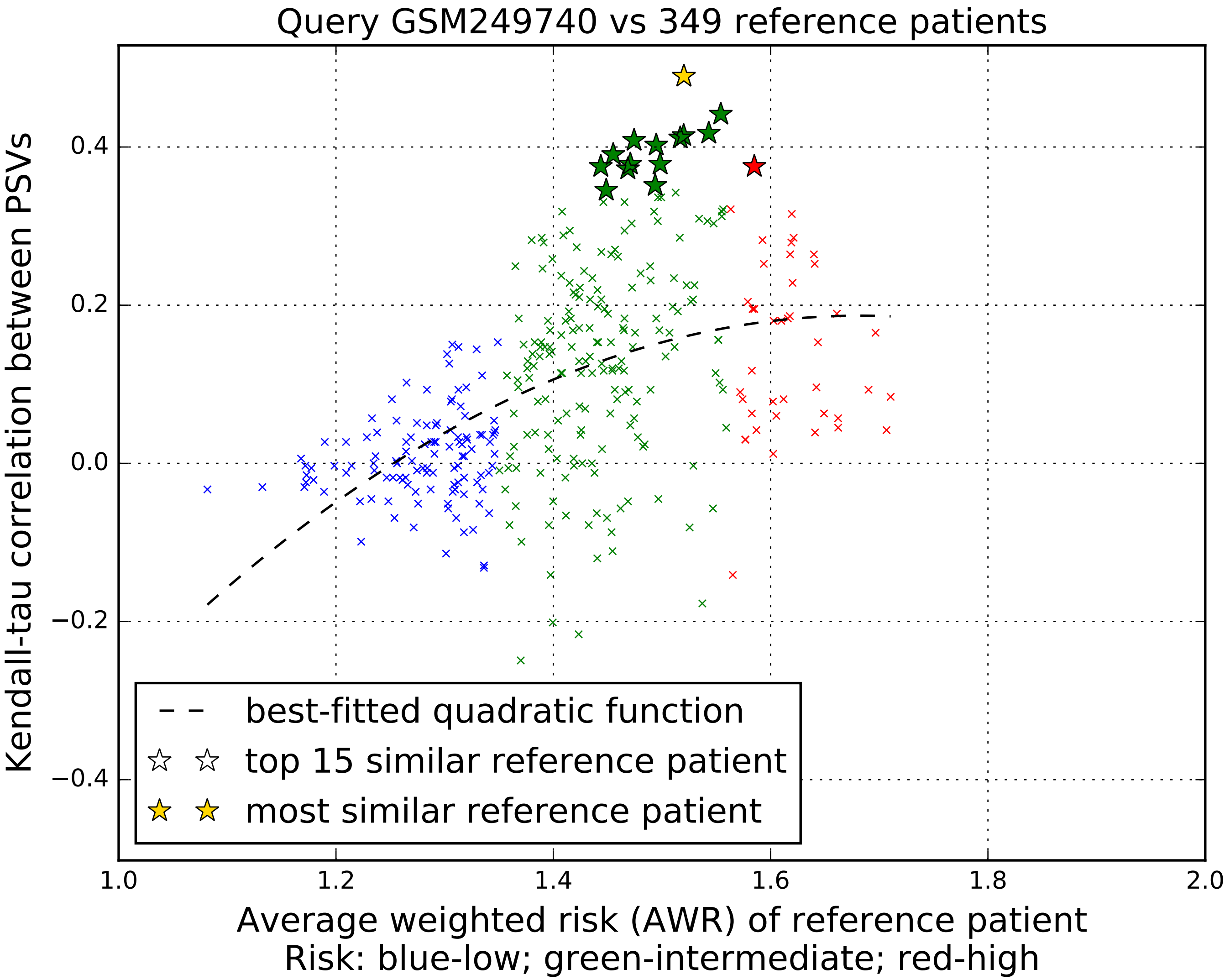
Query GSM249740 vs 349 reference patients
Kendall-tau correlation between PSVs
0.4
0.2
0.0
−0.2
−0.4
1.0
1.2
1.4
1.6
1.8
2.0
best-fitted quadratic function
top 15 similar reference patient
most similar reference patient
Average weighted risk (AWR) of reference patient
Risk: blue-low; green-intermediate; red-high

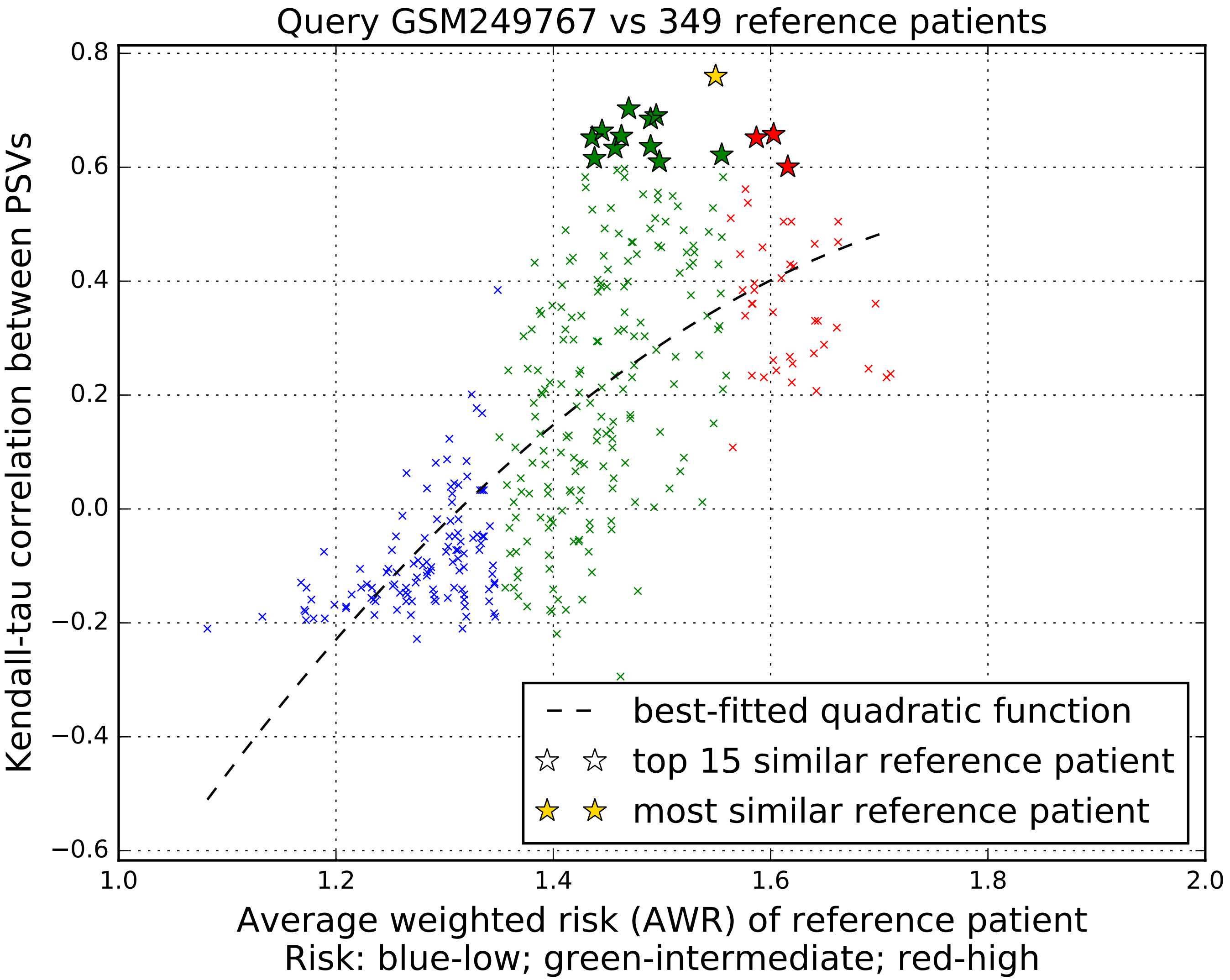
Query GSM249767 vs 349 reference patients
Kendall-tau correlation between PSVs
0.8
0.6
0.4
0.2
0.0
−0.2
−0.4
−0.6
1.0
1.2
1.4
1.6
1.8
2.0
best-fitted quadratic function
top 15 similar reference patient
most similar reference patient
Average weighted risk (AWR) of reference patient
Risk: blue-low; green-intermediate; red-high

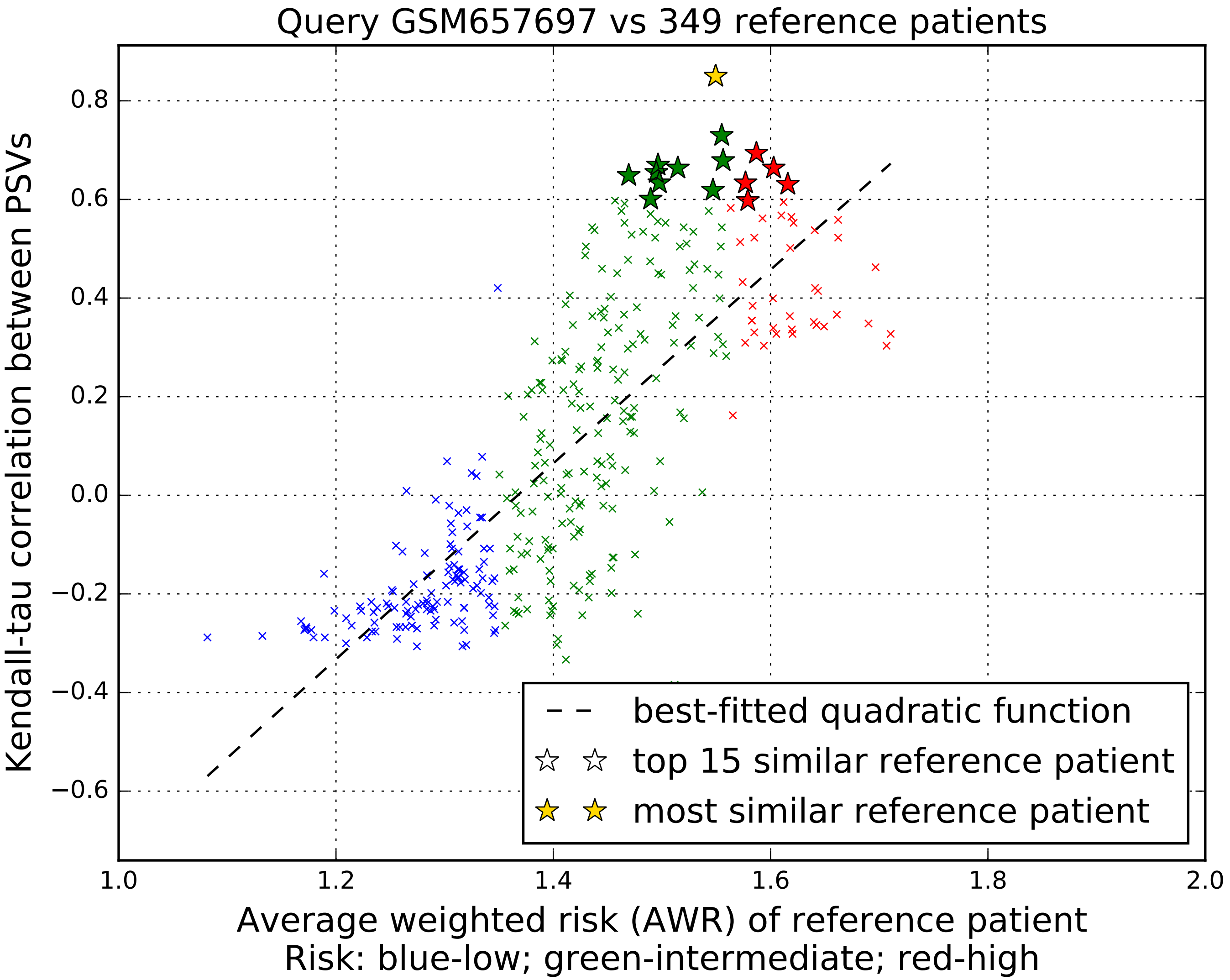

Query GSM657697 vs 349 reference patients
Kendall-tau correlation between PSVs
0.8
0.6
0.4
0.2
0.0
−0.2
−0.4
−0.6
1.0
1.2
1.4
1.6
1.8
2.0
best-fitted quadratic function
top 15 similar reference patient
most similar reference patient
Average weighted risk (AWR) of reference patient
Risk: blue-low; green-intermediate; red-high

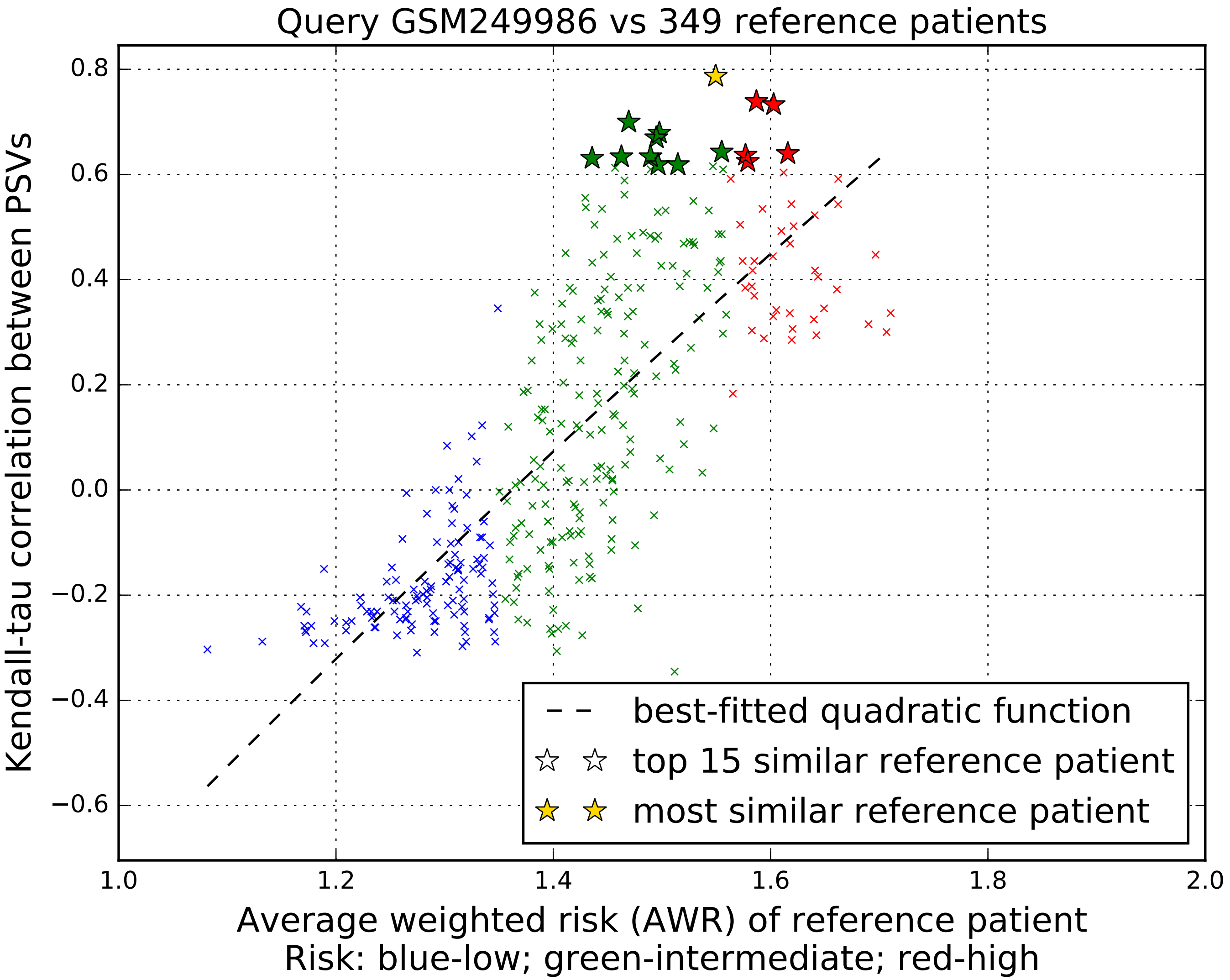

Query GSM249986 vs 349 reference patients
Kendall-tau correlation between PSVs
0.8
0.6
0.4
0.2
0.0
−0.2
−0.4
−0.6
1.0
1.2
1.4
1.6
1.8
2.0
best-fitted quadratic function
top 15 similar reference patient
most similar reference patient
Average weighted risk (AWR) of reference patient
Risk: blue-low; green-intermediate; red-high

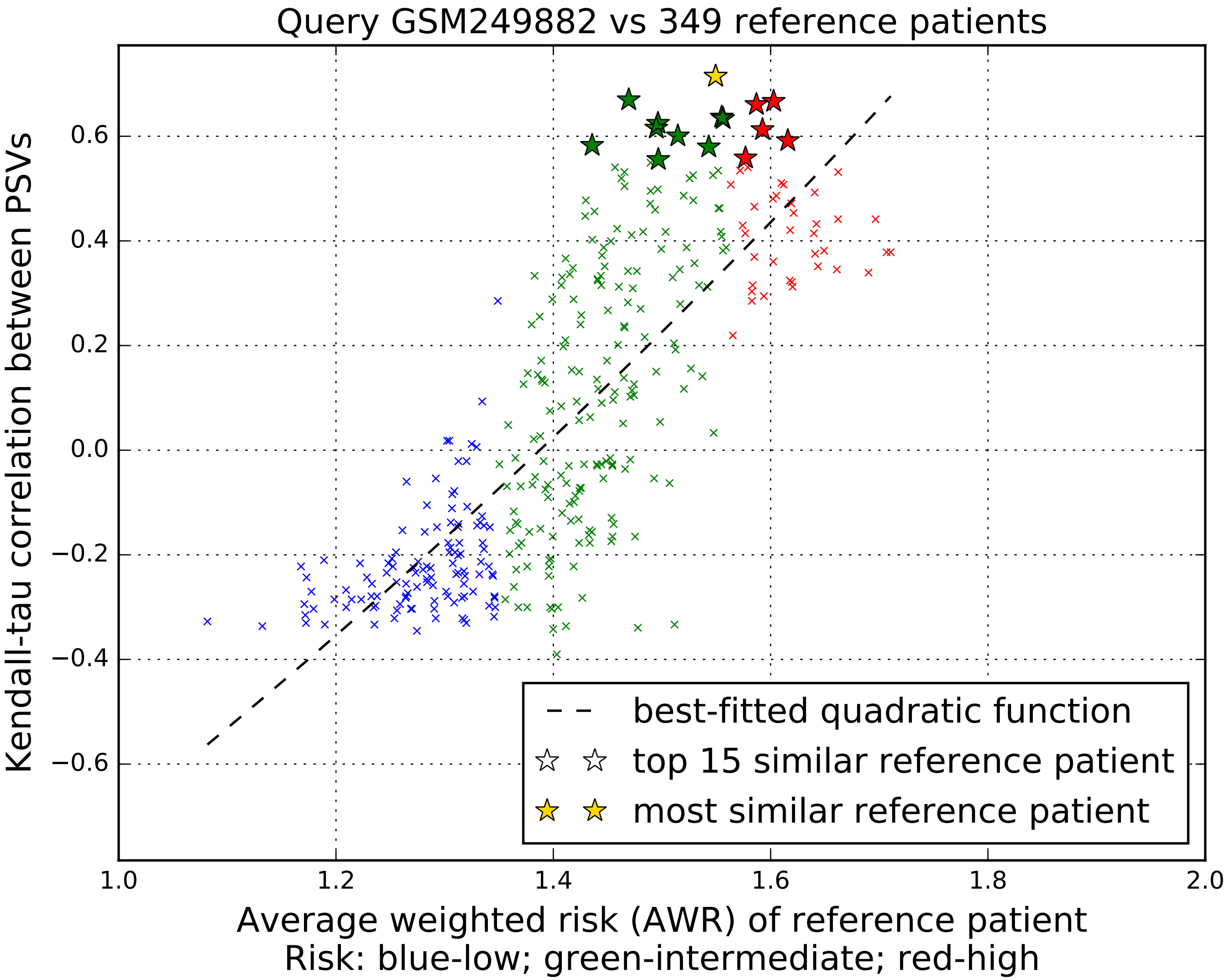
Query GSM249882 vs 349 reference patients
Kendall-tau correlation between PSVs
0.6
0.4
0.2
0.0
−0.2
−0.4
−0.6
1.0
1.2
1.4
1.6
1.8
2.0
Average weighted risk (AWR) of reference patient
Risk: blue-low; green-intermediate; red-high
best-fitted quadratic function
top 15 similar reference patient
most similar reference patient

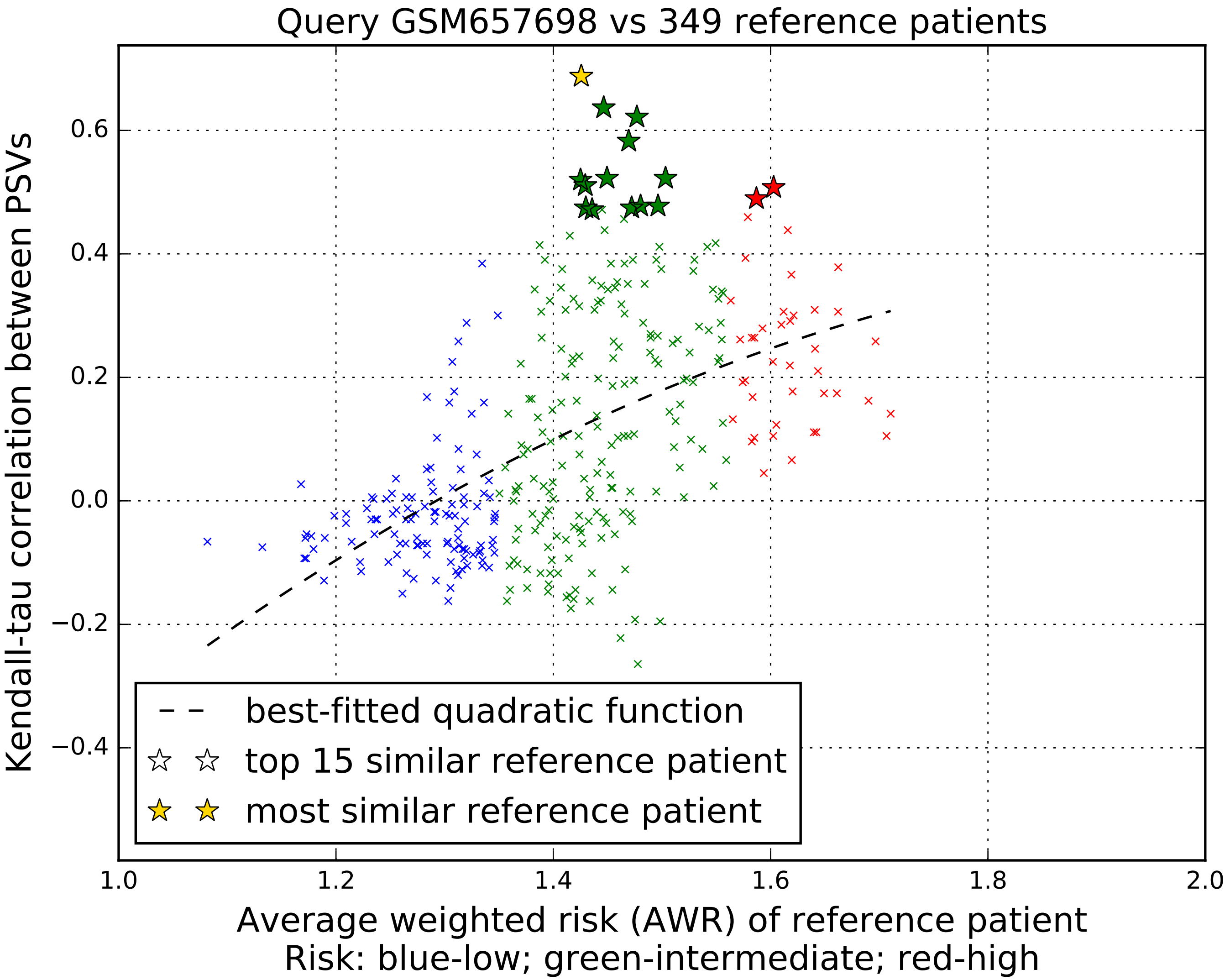

Query GSM657698 vs 349 reference patients
Kendall-tau correlation between PSVs
0.6
0.4
0.2
0.0
−0.2
−0.4
1.0
1.2
1.4
1.6
1.8
2.0
best-fitted quadratic function
top 15 similar reference patient
most similar reference patient
Average weighted risk (AWR) of reference patient
Risk: blue-low; green-intermediate; red-high

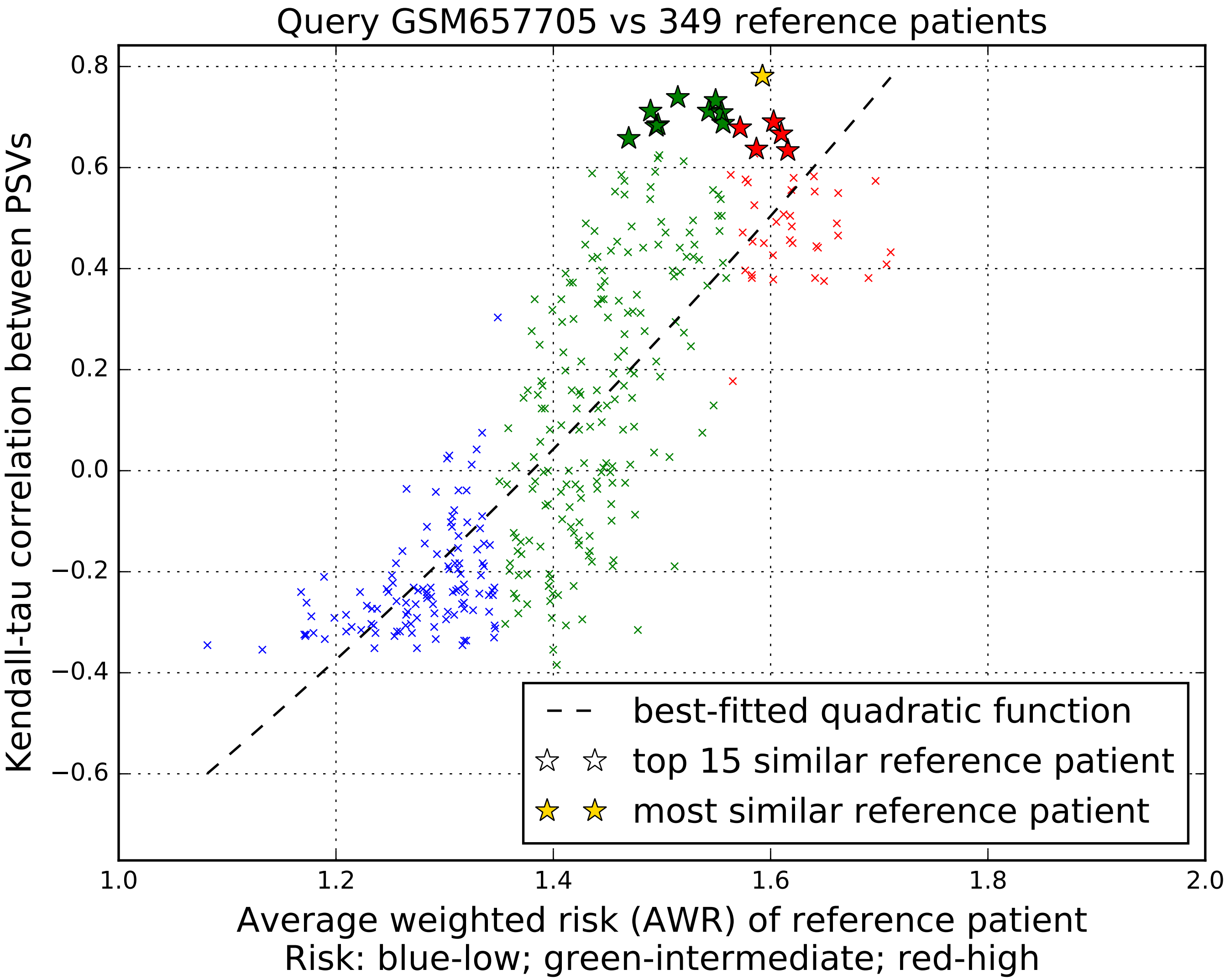

Query GSM657705 vs 349 reference patients
Kendall-tau correlation between PSVs
0.8
0.6
0.4
0.2
0.0
−0.2
−0.4
−0.6
1.0
1.2
1.4
1.6
1.8
2.0
best-fitted quadratic function
top 15 similar reference patient
most similar reference patient
Average weighted risk (AWR) of reference patient
Risk: blue-low; green-intermediate; red-high

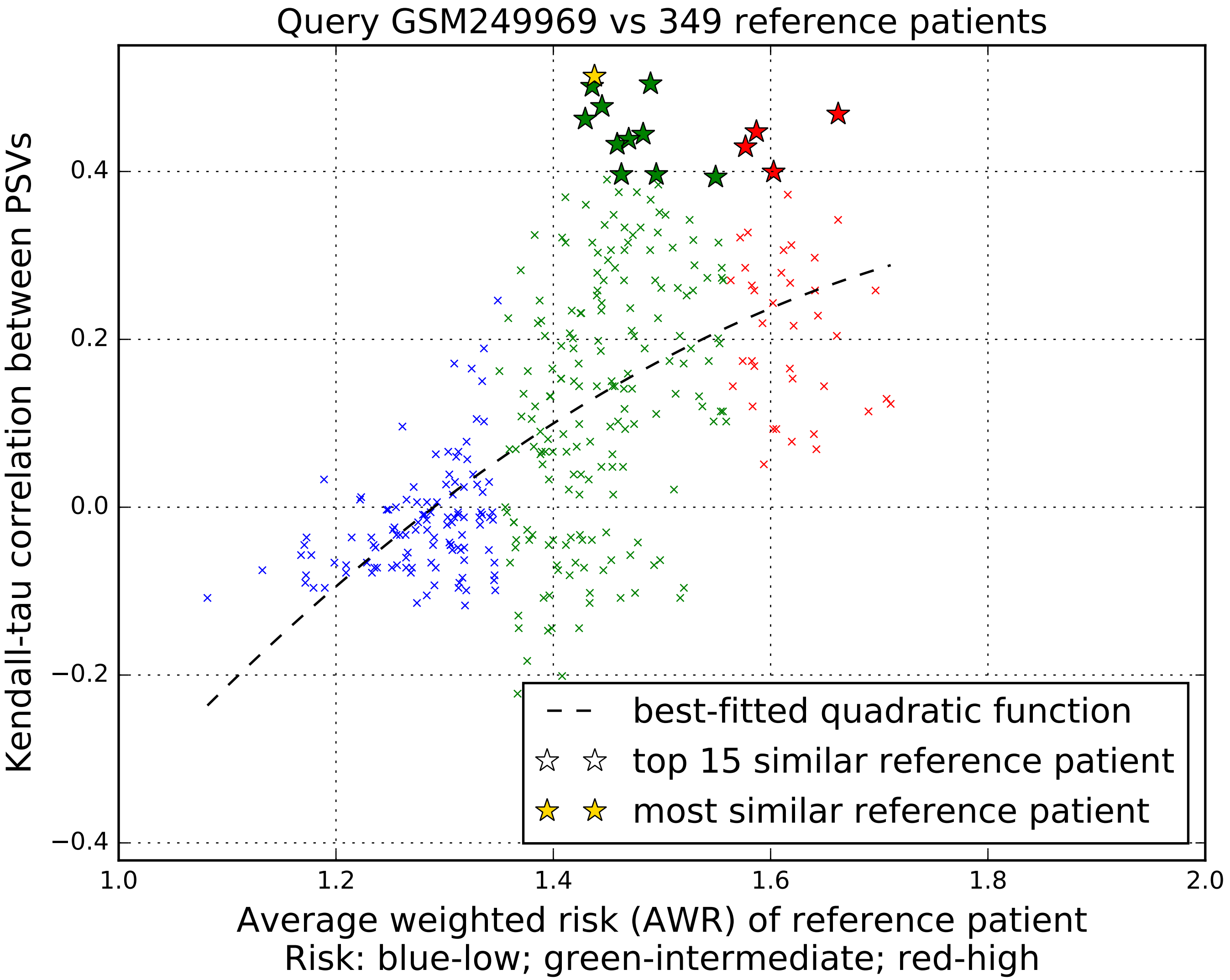

Query GSM249969 vs 349 reference patients
Kendall-tau correlation between PSVs
0.4
0.2
0.0
−0.2
−0.4
1.0
1.2
1.4
1.6
1.8
2.0
best-fitted quadratic function
top 15 similar reference patient
most similar reference patient
Average weighted risk (AWR) of reference patient
Risk: blue-low; green-intermediate; red-high

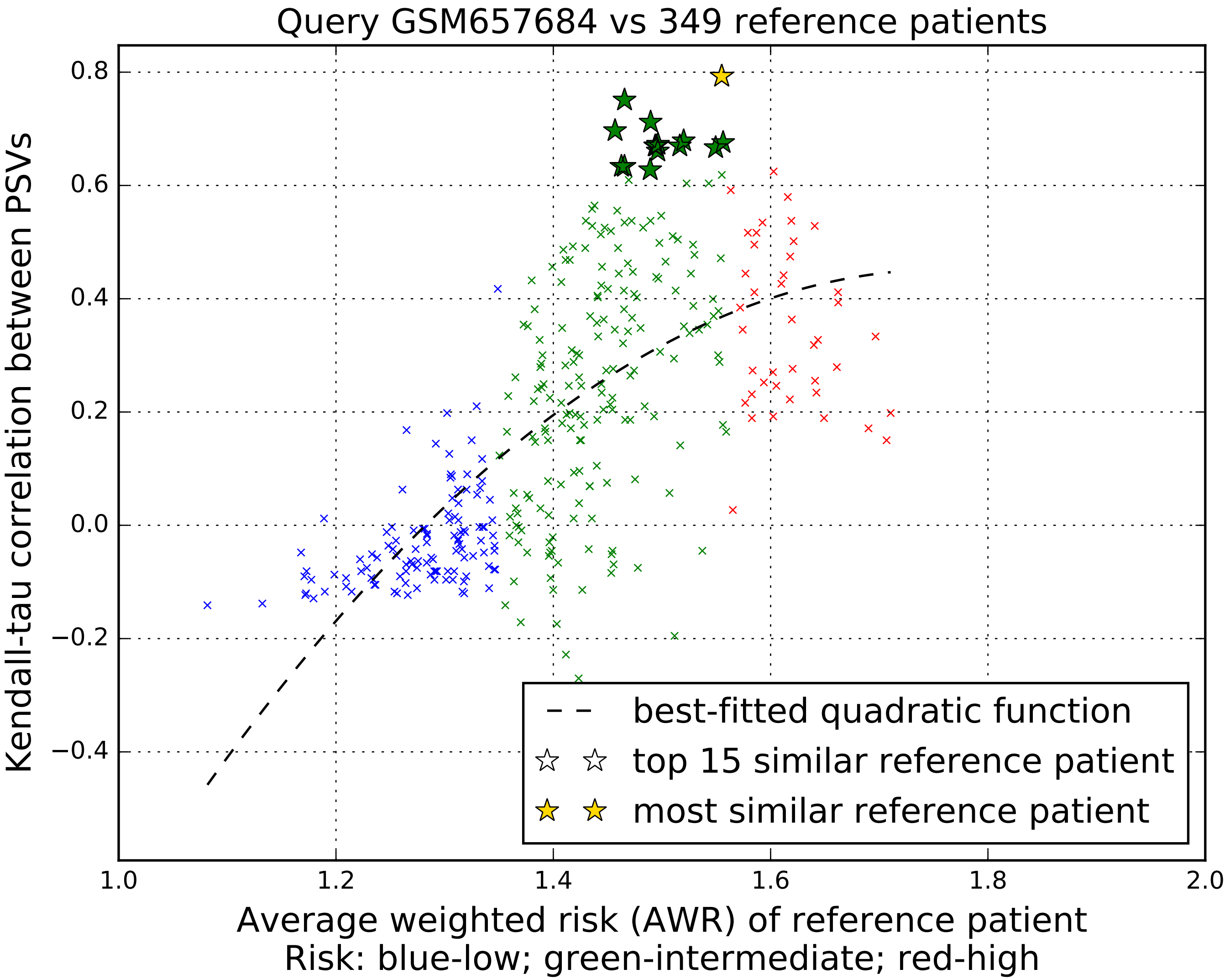
Query GSM657684 vs 349 reference patients
Kendall-tau correlation between PSVs
0.8
0.6
0.4
0.2
0.0
−0.2
−0.4
1.0
1.2
1.4
1.6
1.8
2.0
best-fitted quadratic function
top 15 similar reference patient
most similar reference patient
Average weighted risk (AWR) of reference patient
Risk: blue-low; green-intermediate; red-high

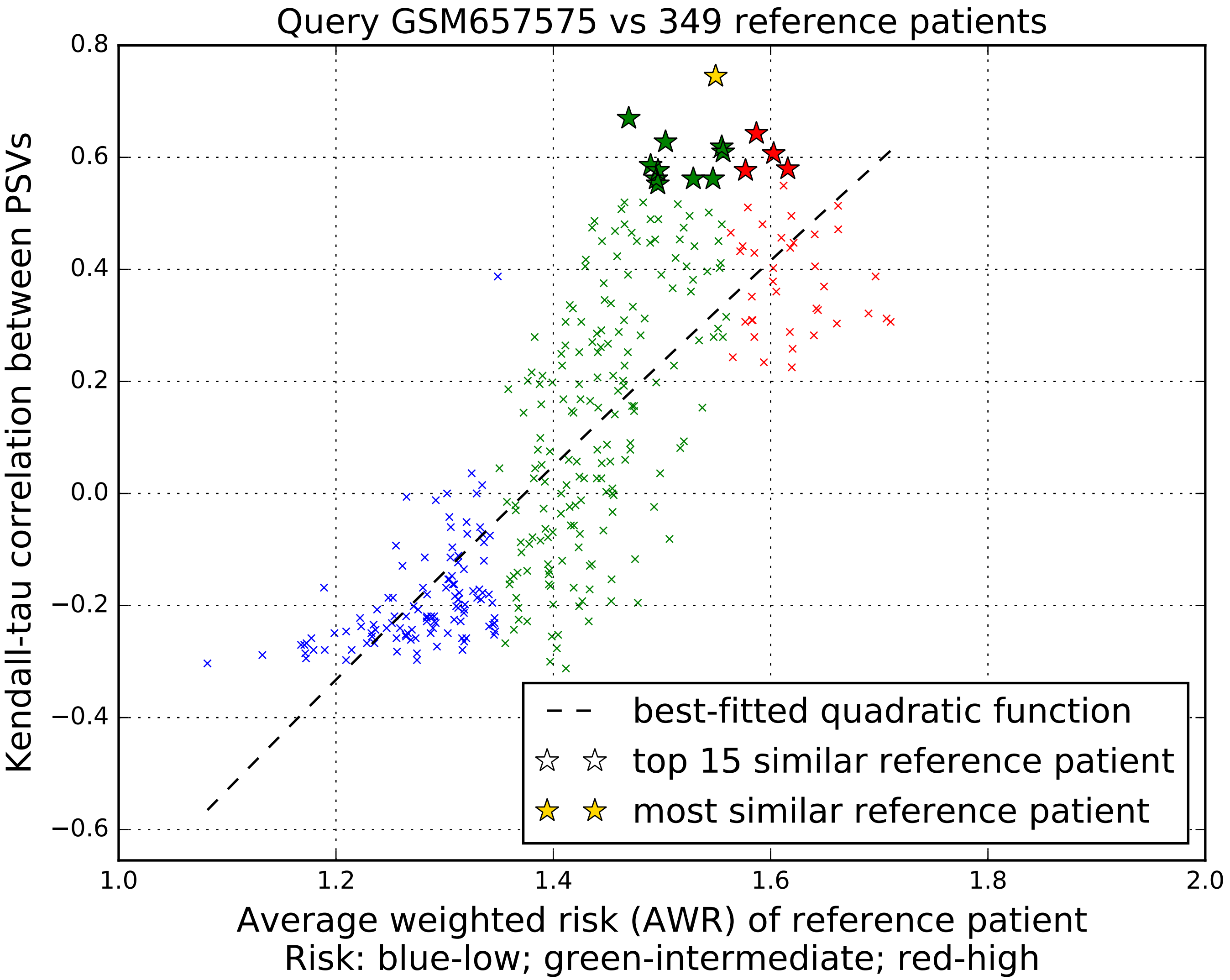

Query GSM657575 vs 349 reference patients
Kendall-tau correlation between PSVs
0.8
0.6
0.4
0.2
0.0
−0.2
−0.4
−0.6
1.0
1.2
1.4
1.6
1.8
2.0
best-fitted quadratic function
top 15 similar reference patient
most similar reference patient
Average weighted risk (AWR) of reference patient
Risk: blue-low; green-intermediate; red-high

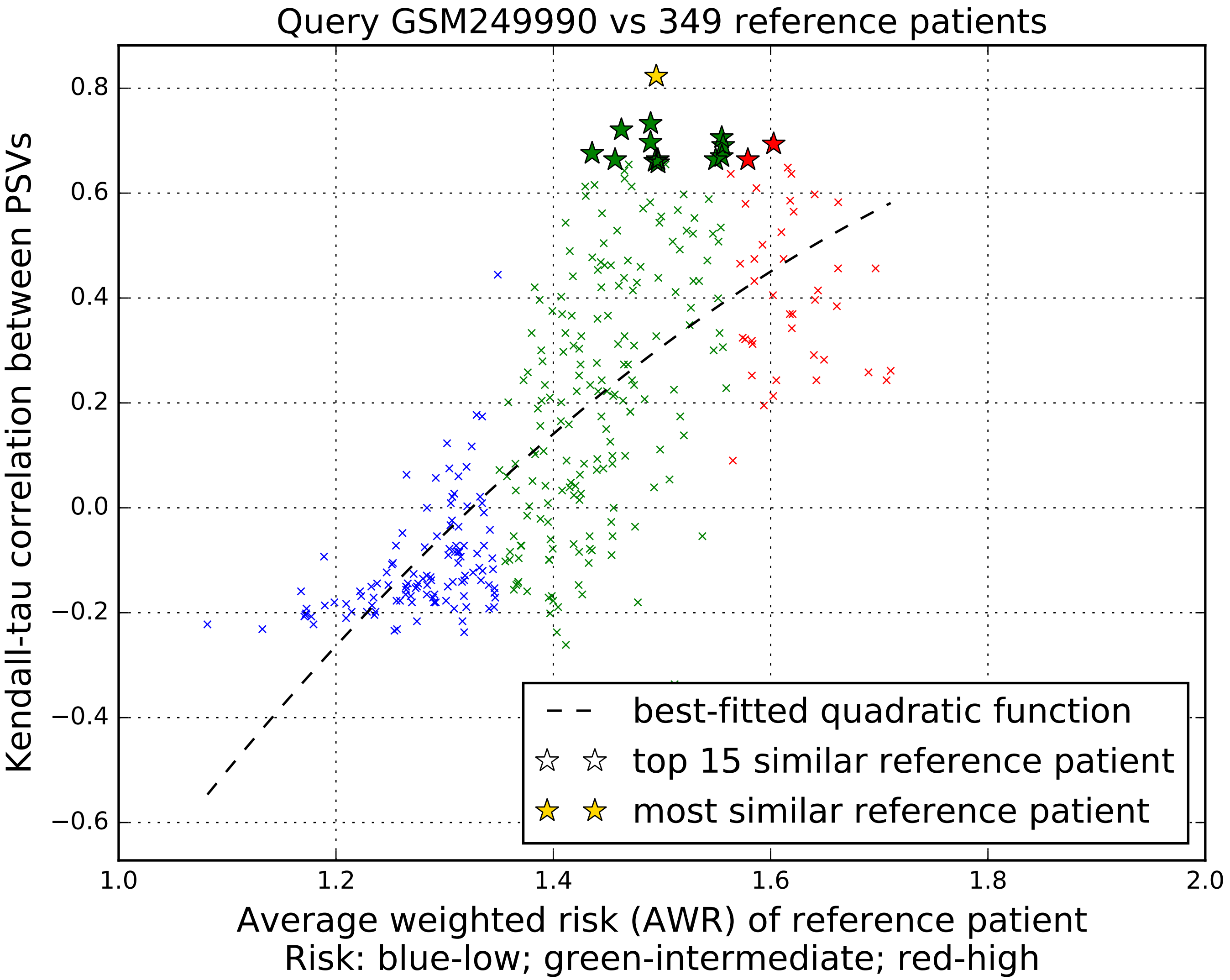

Query GSM249990 vs 349 reference patients
Kendall-tau correlation between PSVs
0.8
0.6
0.4
0.2
0.0
−0.2
−0.4
−0.6
1.0
1.2
1.4
1.6
1.8
2.0
best-fitted quadratic function
top 15 similar reference patient
most similar reference patient
Average weighted risk (AWR) of reference patient
Risk: blue-low; green-intermediate; red-high

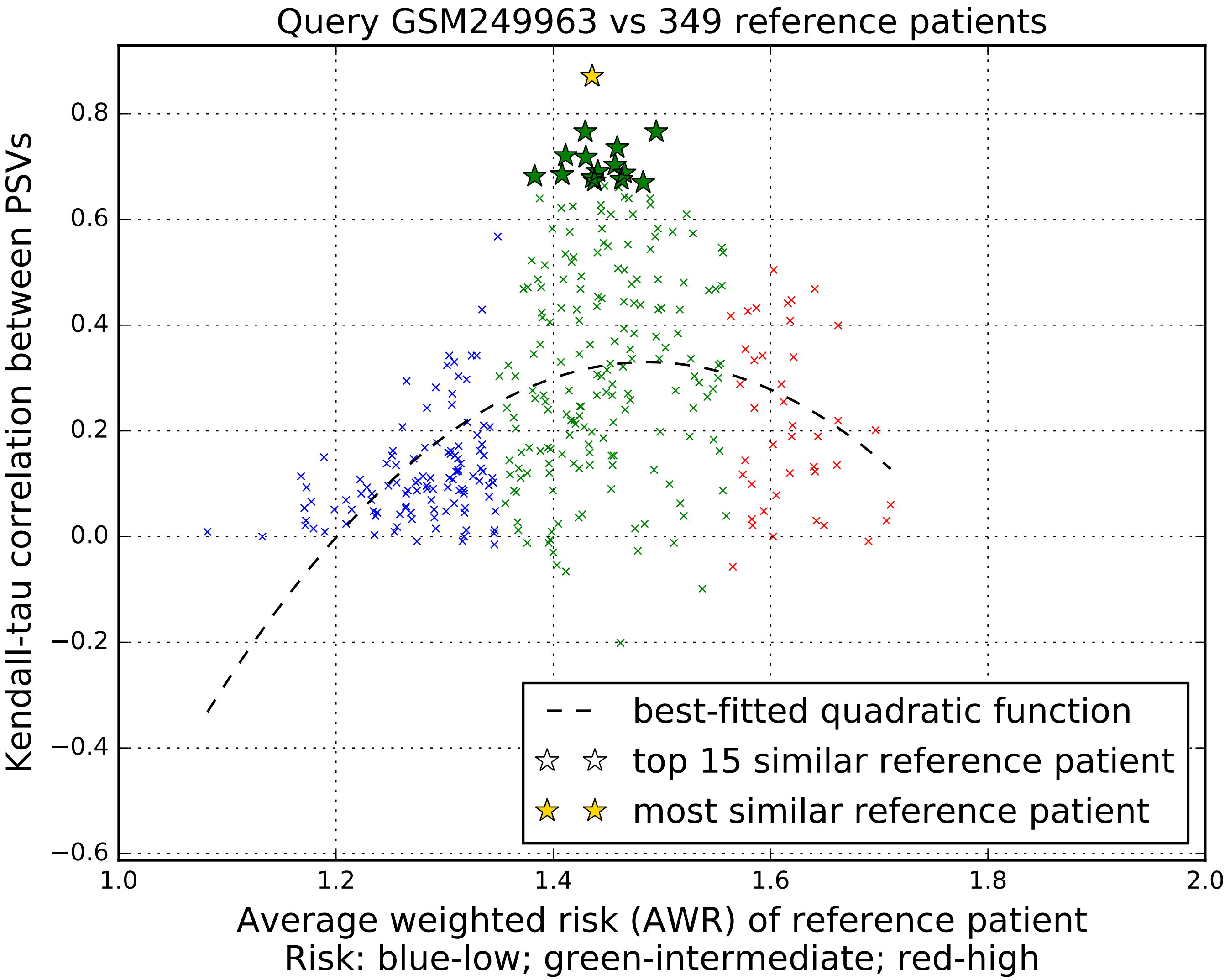
Query GSM249963 vs 349 reference patients
Kendall-tau correlation between PSVs
0.8
0.6
0.4
0.2
0.0
−0.2
−0.4
−0.6
1.0
1.2
1.4
1.6
1.8
2.0
best-fitted quadratic function
top 15 similar reference patient
most similar reference patient
Average weighted risk (AWR) of reference patient
Risk: blue-low; green-intermediate; red-high

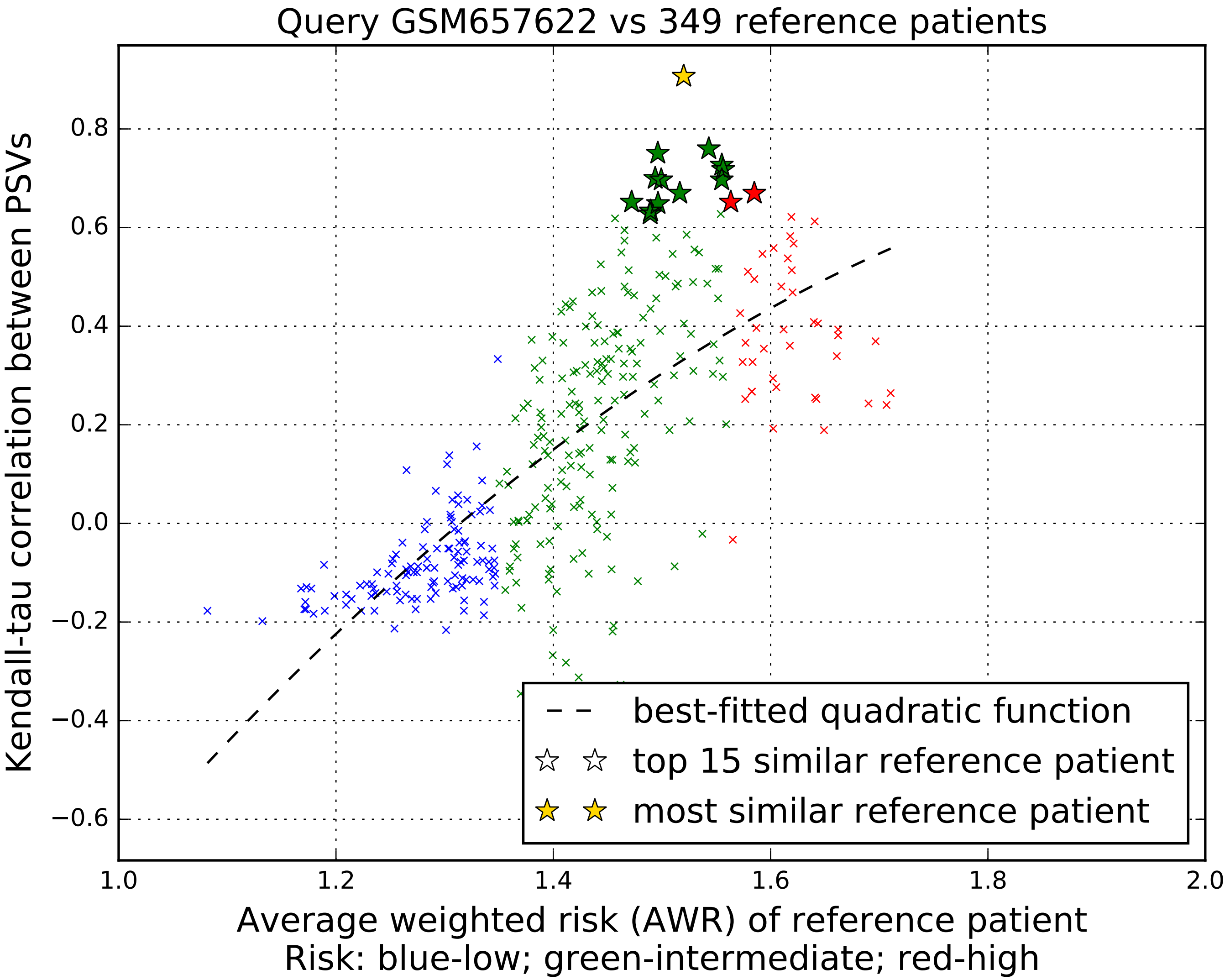
Query GSM657622 vs 349 reference patients
Kendall-tau correlation between PSVs
0.8
0.6
0.4
0.2
0.0
−0.2
−0.4
−0.6
1.0
1.2
1.4
1.6
1.8
2.0
best-fitted quadratic function
top 15 similar reference patient
most similar reference patient
Average weighted risk (AWR) of reference patient
Risk: blue-low; green-intermediate; red-high

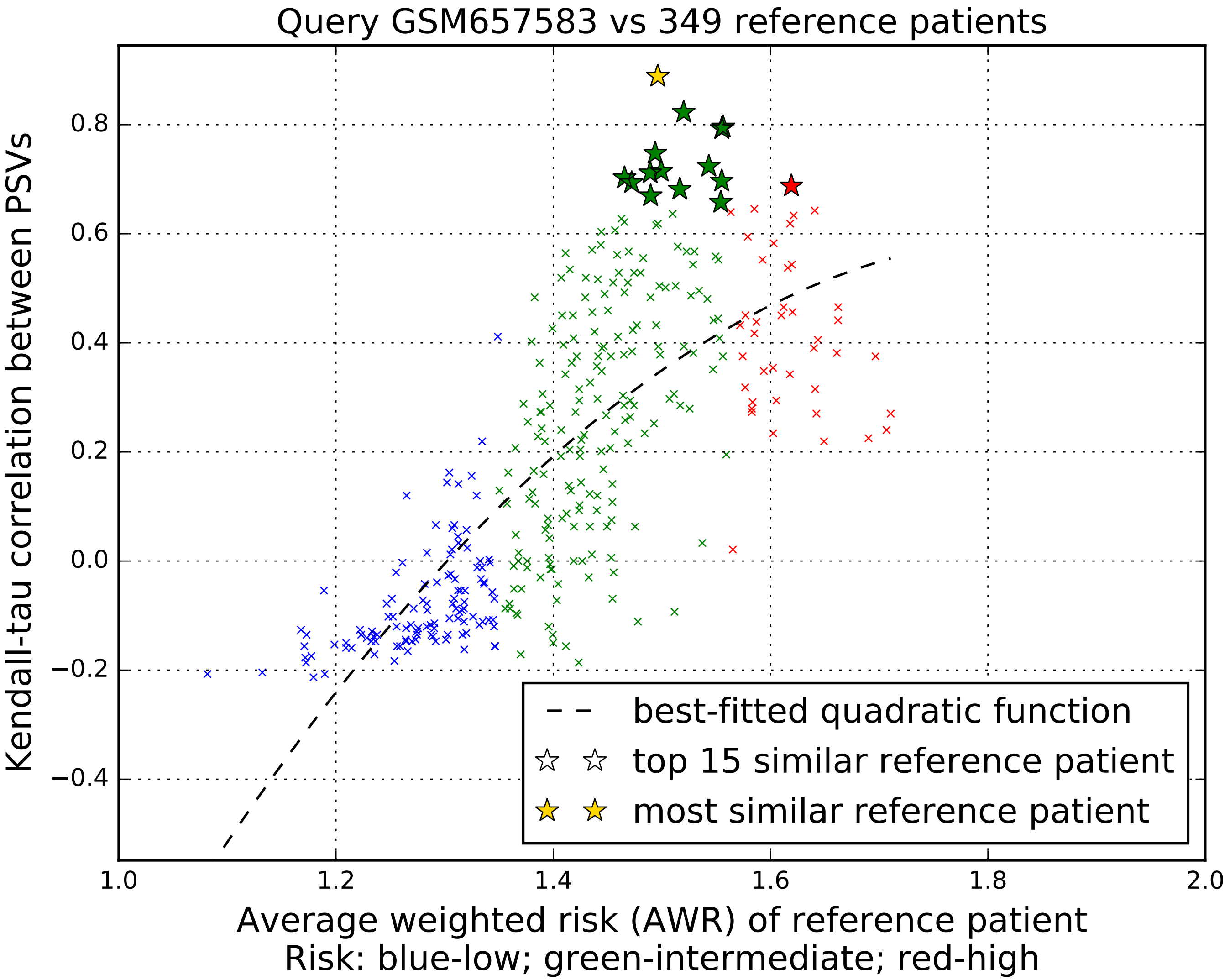
Query GSM657583 vs 349 reference patients
Kendall-tau correlation between PSVs
Average weighted risk (AWR) of reference patient
Risk: blue-low; green-intermediate; red-high
best-fitted quadratic function
top 15 similar reference patient
most similar reference patient
0.8
0.6
0.4
0.2
0.0
−0.2
−0.4
1.0
1.2
1.4
1.6
1.8
2.0

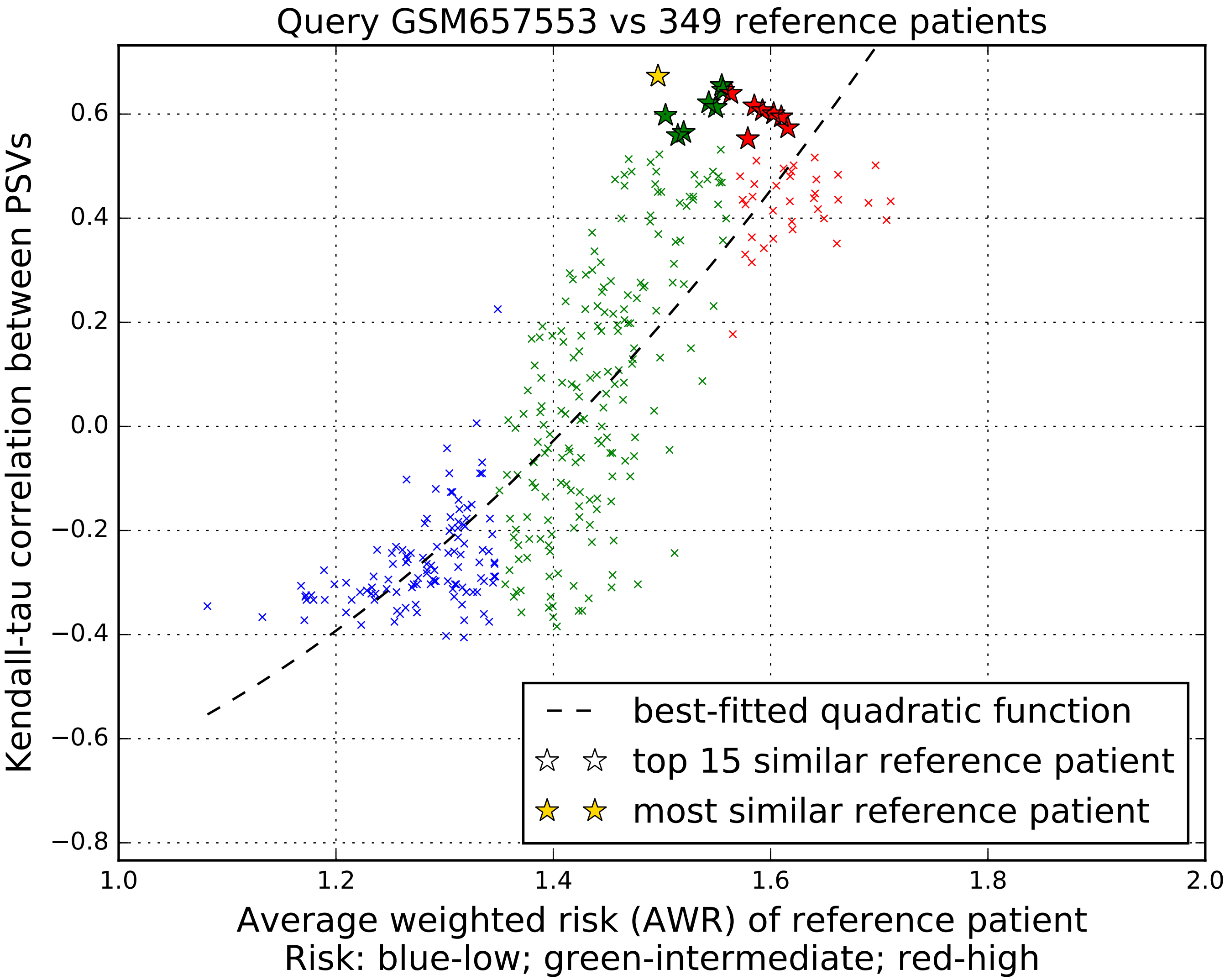

Query GSM657553 vs 349 reference patients
Kendall-tau correlation between PSVs
Average weighted risk (AWR) of reference patient
Risk: blue-low; green-intermediate; red-high
best-fitted quadratic function
top 15 similar reference patient
most similar reference patient
0.6
0.4
0.2
0.0
−0.2
−0.4
−0.6
−0.8
1.0
1.2
1.4
1.6
1.8
2.0

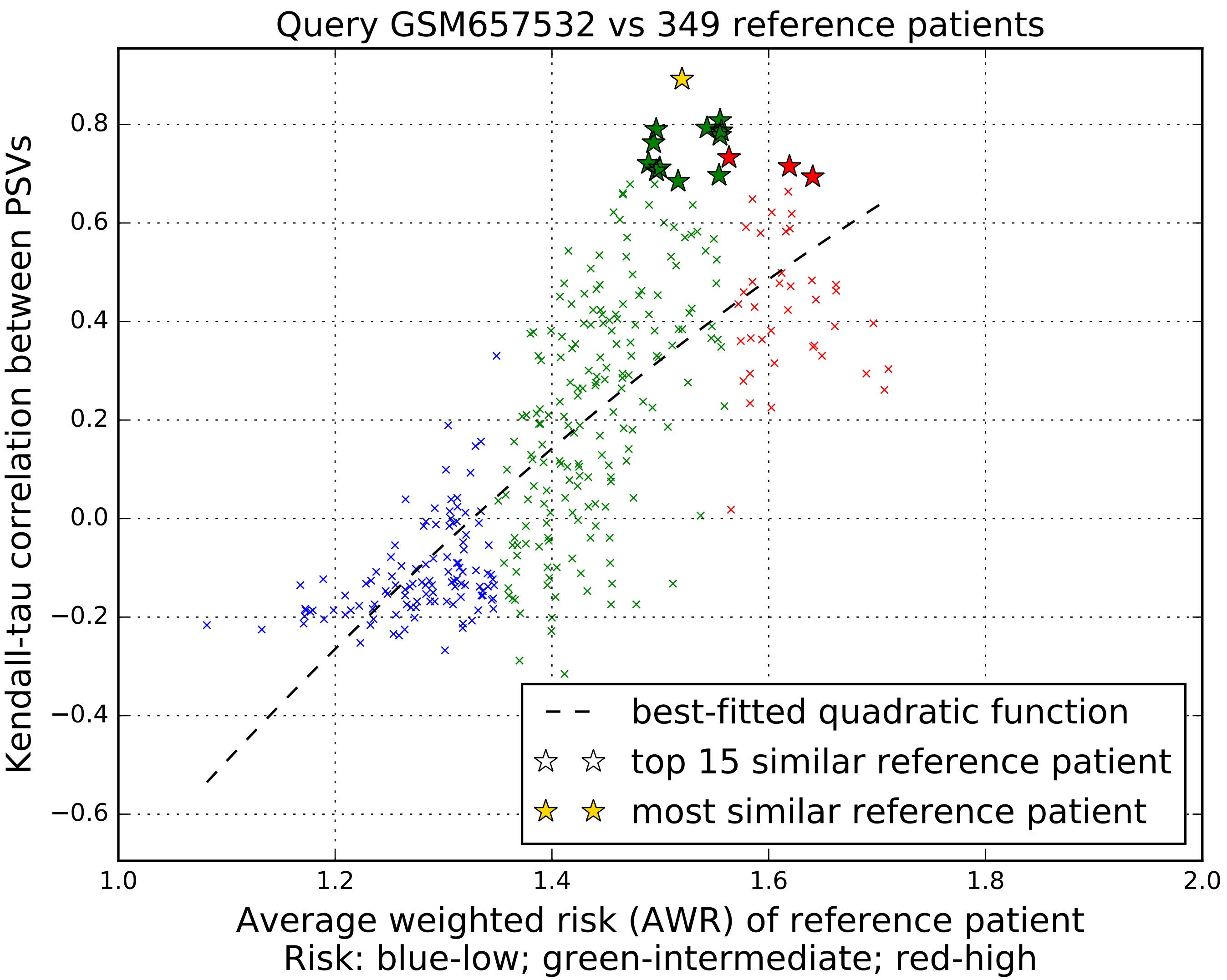

Query GSM657532 vs 349 reference patients
Kendall-tau correlation between PSVs
0.8
0.6
0.4
0.2
0.0
−0.2
−0.4
−0.6
1.0
1.2
1.4
1.6
1.8
2.0
best-fitted quadratic function
top 15 similar reference patient
most similar reference patient
Average weighted risk (AWR) of reference patient
Risk: blue-low; green-intermediate; red-high

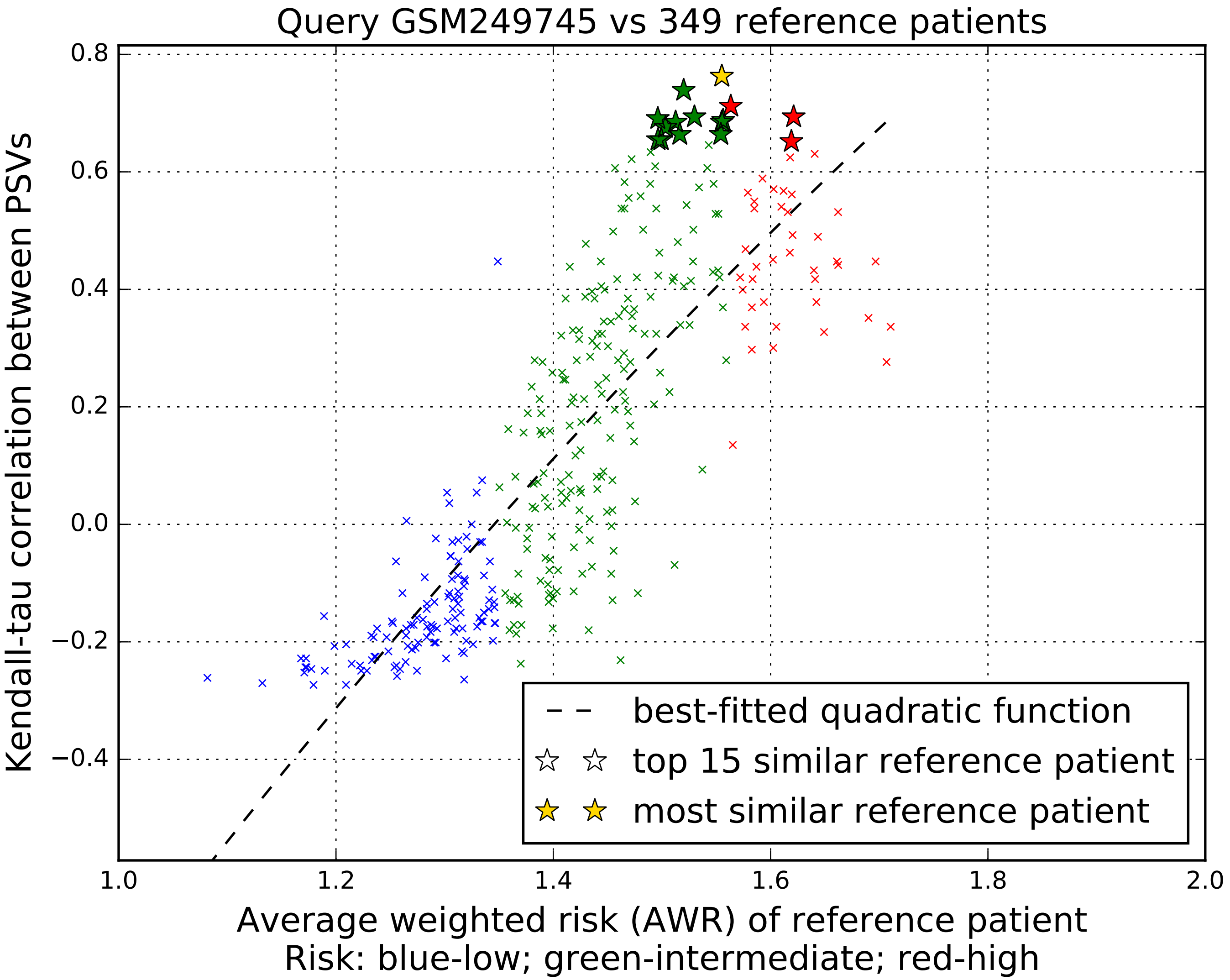

Query GSM249745 vs 349 reference patients
Kendall-tau correlation between PSVs
0.8
0.6
0.4
0.2
0.0
−0.2
−0.4
1.0
1.2
1.4
1.6
1.8
2.0
best-fitted quadratic function
top 15 similar reference patient
most similar reference patient
Average weighted risk (AWR) of reference patient
Risk: blue-low; green-intermediate; red-high

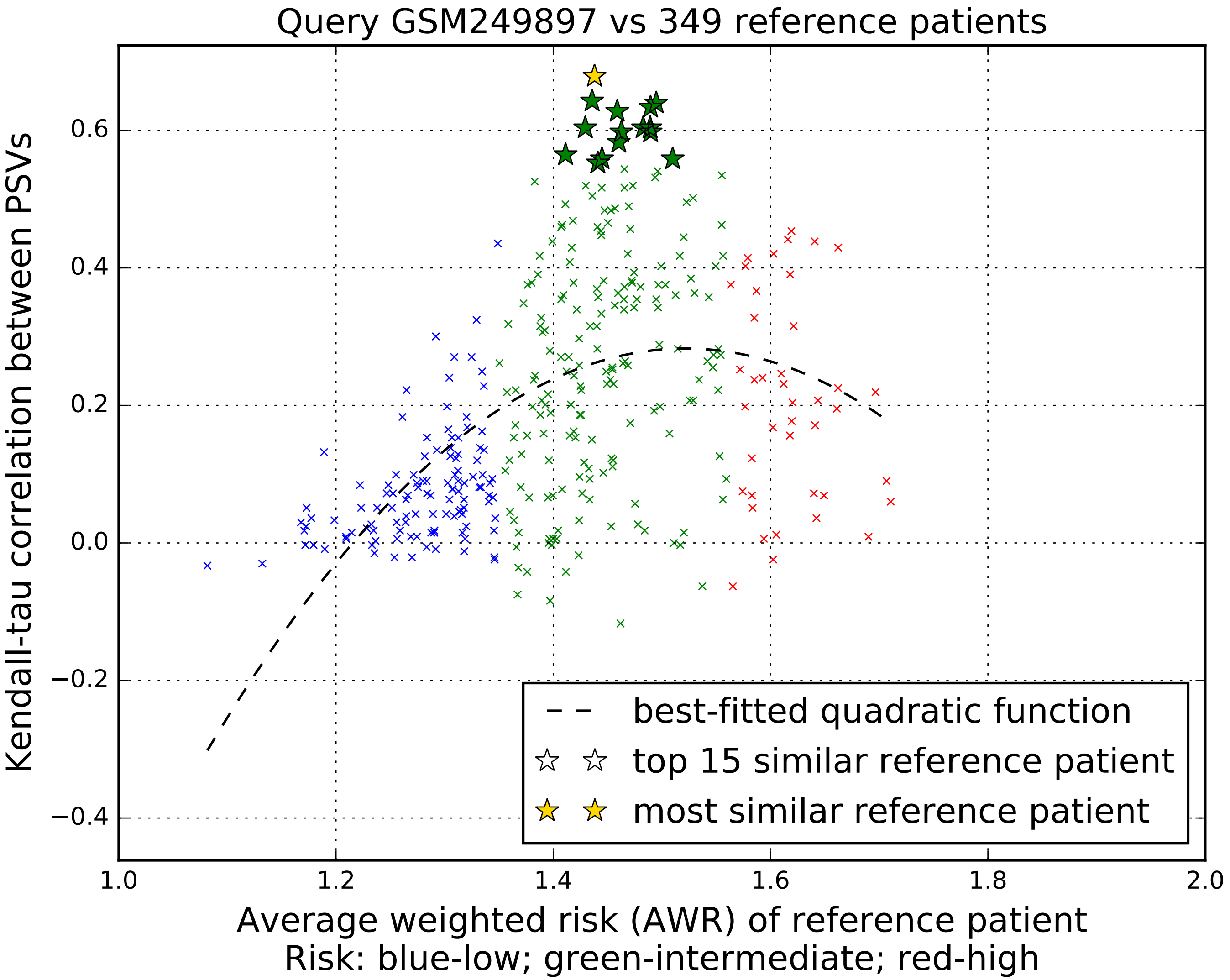

Query GSM249897 vs 349 reference patients
Kendall-tau correlation between PSVs
0.6
0.4
0.2
0.0
−0.2
−0.4
1.0
1.2
1.4
1.6
1.8
2.0
best-fitted quadratic function
top 15 similar reference patient
most similar reference patient
Average weighted risk (AWR) of reference patient
Risk: blue-low; green-intermediate; red-high

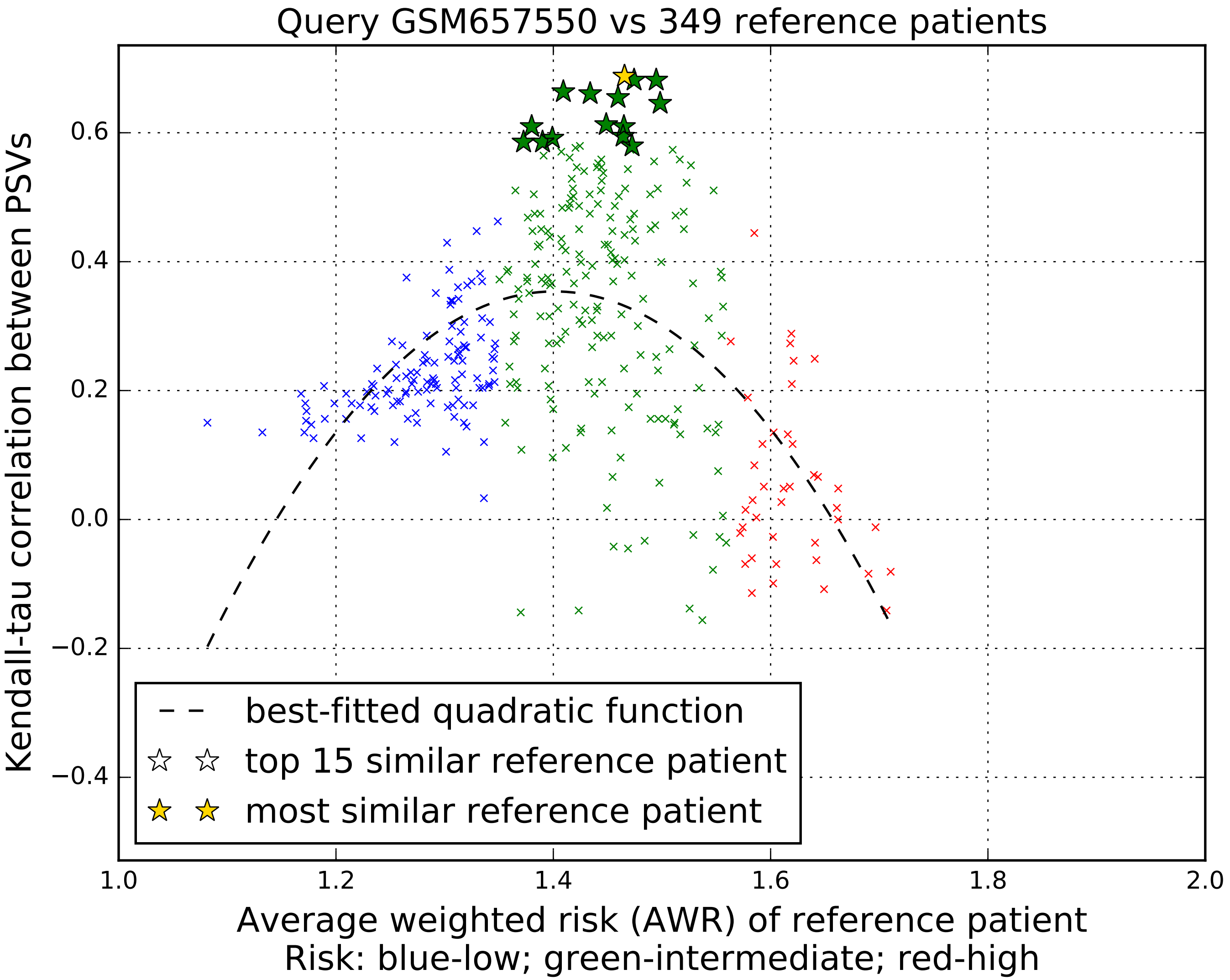

Query GSM657550 vs 349 reference patients
Kendall-tau correlation between PSVs
0.6
0.4
0.2
0.0
−0.2
−0.4
1.0
1.2
1.4
1.6
1.8
2.0
best-fitted quadratic function
top 15 similar reference patient
most similar reference patient
Average weighted risk (AWR) of reference patient
Risk: blue-low; green-intermediate; red-high

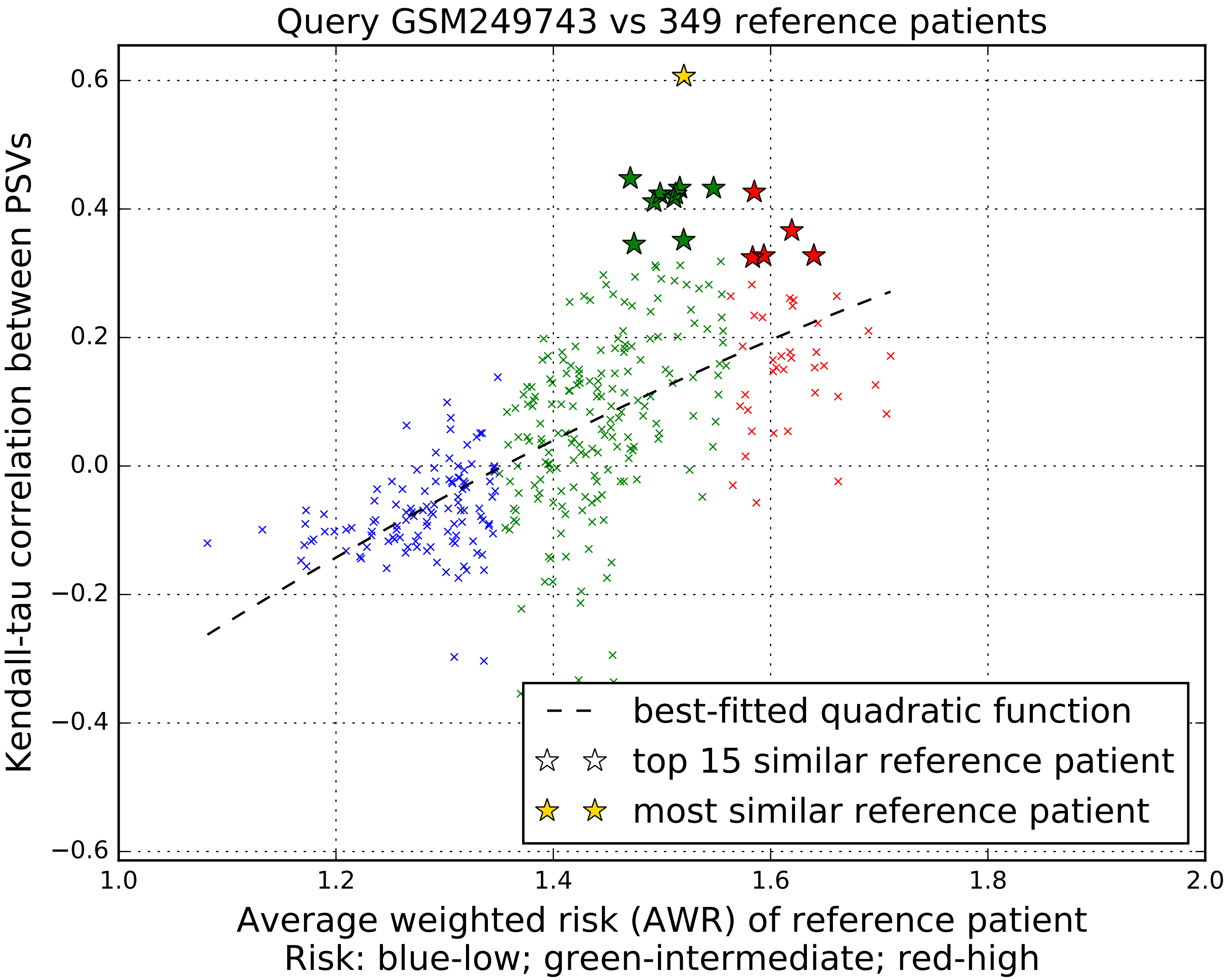
Query GSM249743 vs 349 reference patients
Kendall-tau correlation between PSVs
Average weighted risk (AWR) of reference patient
Risk: blue-low; green-intermediate; red-high
best-fitted quadratic function
top 15 similar reference patient
most similar reference patient
0.6
0.4
0.2
0.0
−0.2
−0.4
−0.6
1.0
1.2
1.4
1.6
1.8
2.0

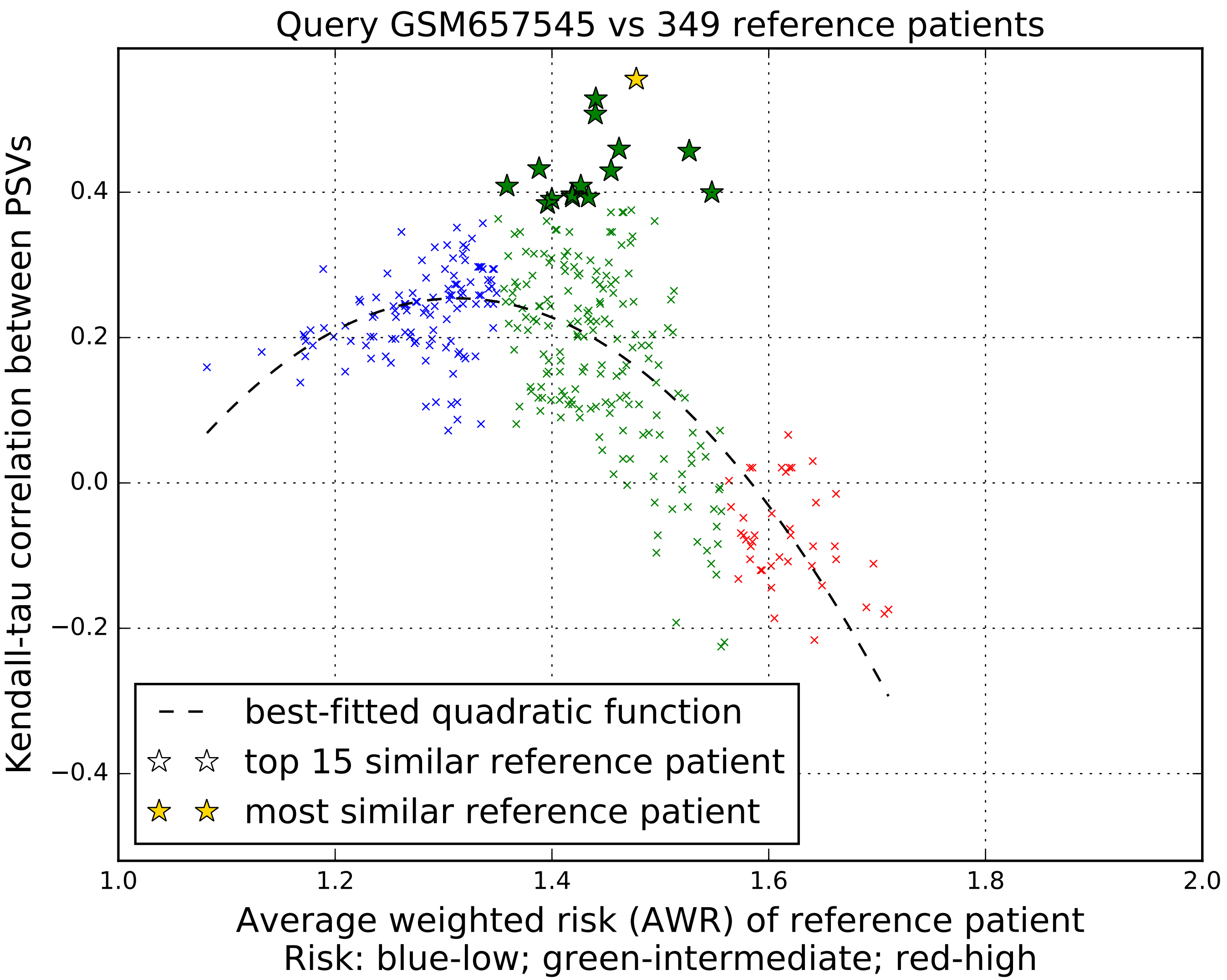

Query GSM657545 vs 349 reference patients
Kendall-tau correlation between PSVs
0.4
0.2
0.0
−0.2
−0.4
1.0
1.2
1.4
1.6
1.8
2.0
best-fitted quadratic function
top 15 similar reference patient
most similar reference patient
Average weighted risk (AWR) of reference patient
Risk: blue-low; green-intermediate; red-high

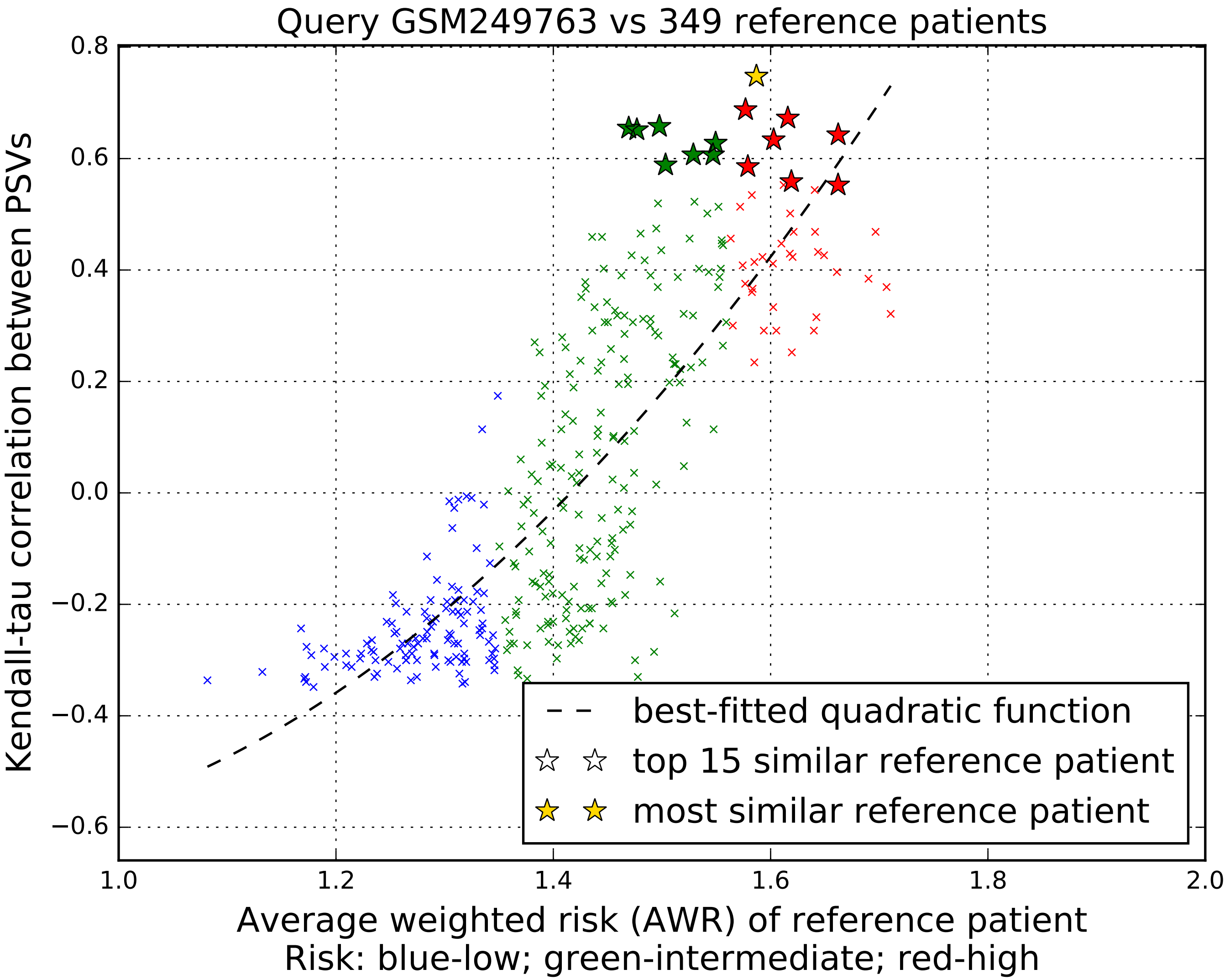
Query GSM249763 vs 349 reference patients
Kendall-tau correlation between PSVs
0.8
0.6
0.4
0.2
0.0
−0.2
−0.4
−0.6
1.0
1.2
1.4
1.6
1.8
2.0
best-fitted quadratic function
top 15 similar reference patient
most similar reference patient
Average weighted risk (AWR) of reference patient
Risk: blue-low; green-intermediate; red-high

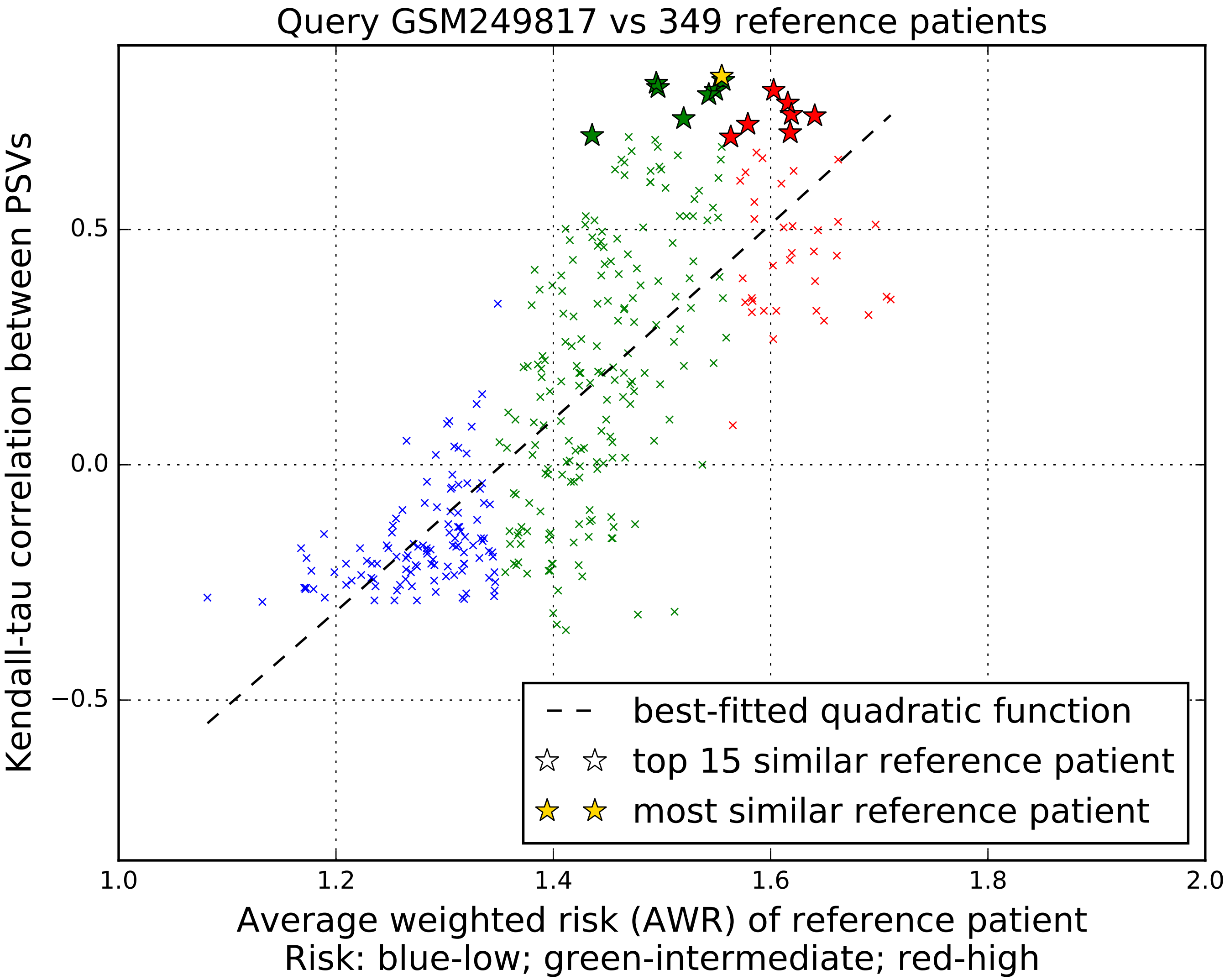

Query GSM249817 vs 349 reference patients
Kendall-tau correlation between PSVs
0.5
0.0
−0.5
1.0
1.2
1.4
1.6
1.8
2.0
best-fitted quadratic function
top 15 similar reference patient
most similar reference patient
Average weighted risk (AWR) of reference patient
Risk: blue-low; green-intermediate; red-high

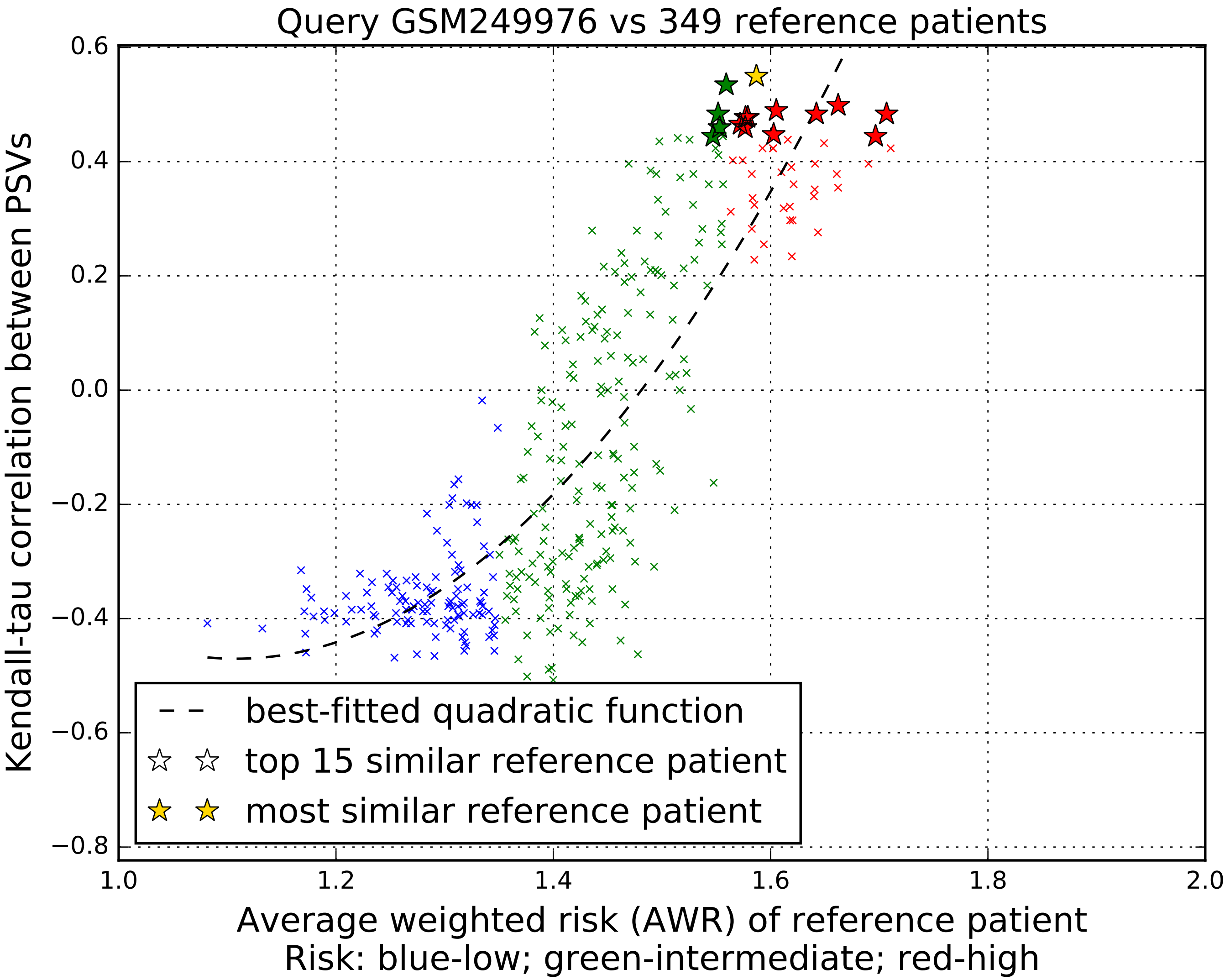
Query GSM249976 vs 349 reference patients
Kendall-tau correlation between PSVs
0.6
0.4
0.2
0.0
−0.2
−0.4
−0.6
−0.8
1.0
1.2
1.4
1.6
1.8
2.0
best-fitted quadratic function
top 15 similar reference patient
most similar reference patient
Average weighted risk (AWR) of reference patient
Risk: blue-low; green-intermediate; red-high

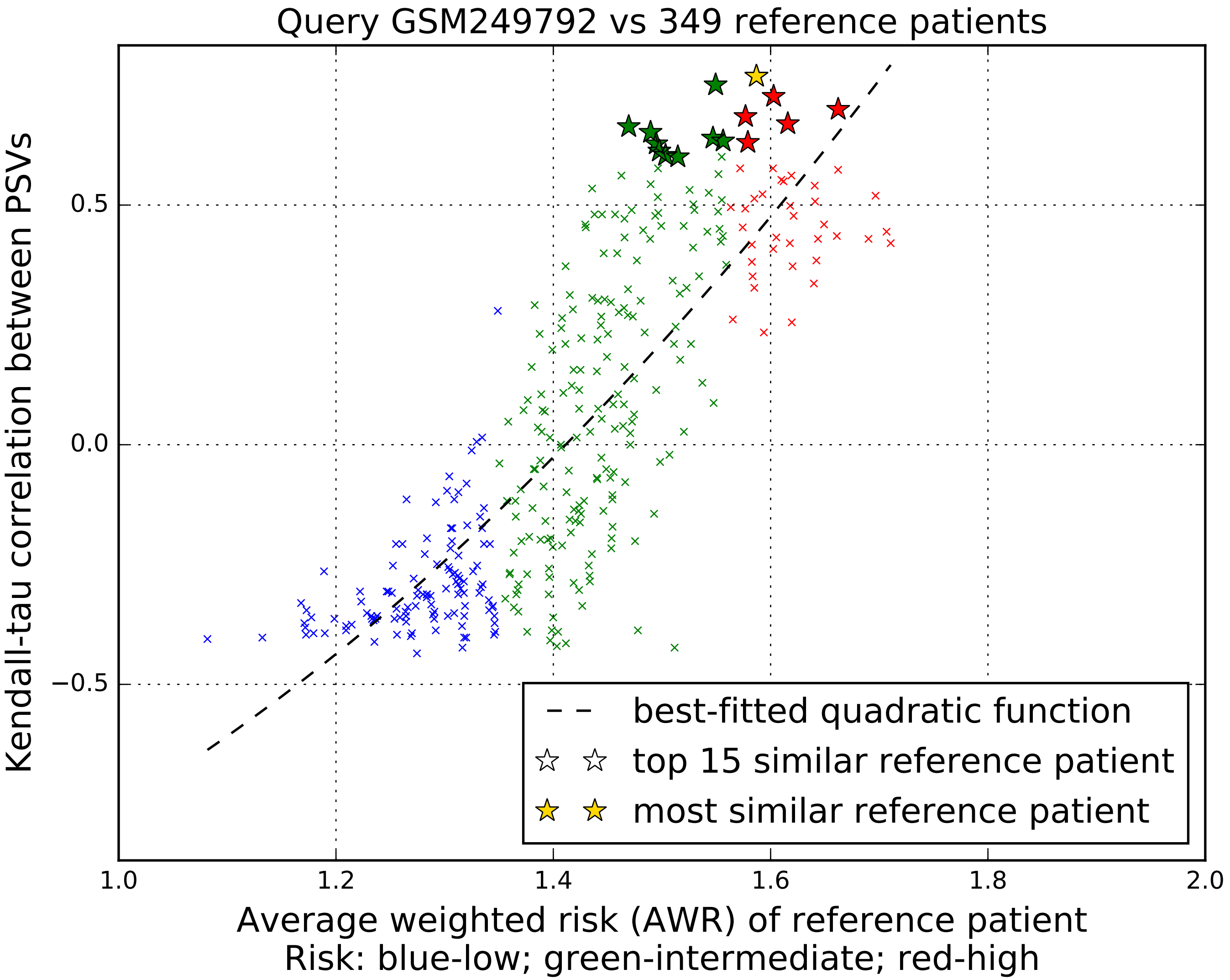
Query GSM249792 vs 349 reference patients
Kendall-tau correlation between PSVs
0.5
0.0
−0.5
1.0
1.2
1.4
1.6
1.8
2.0
best-fitted quadratic function
top 15 similar reference patient
most similar reference patient
Average weighted risk (AWR) of reference patient
Risk: blue-low; green-intermediate; red-high

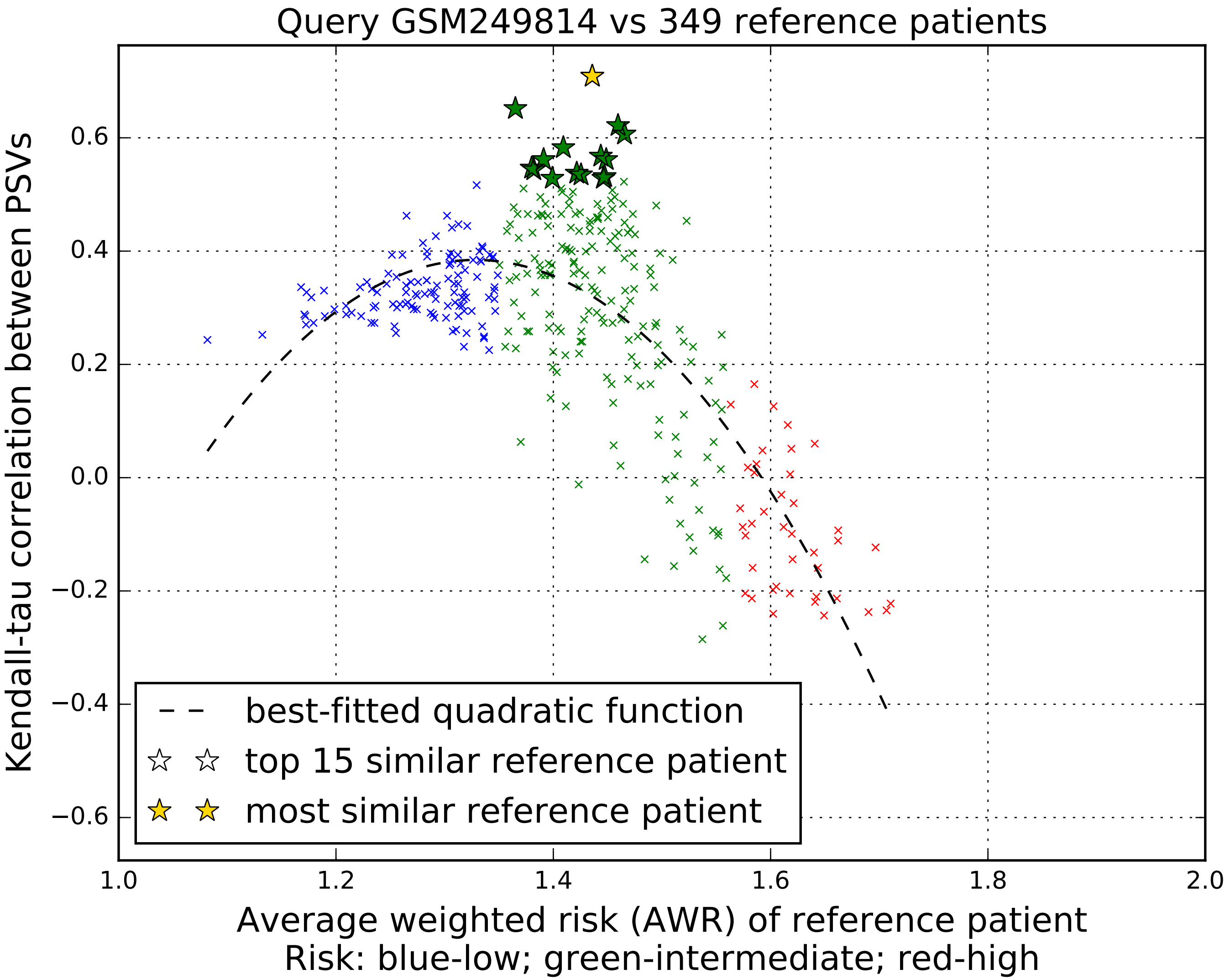
Query GSM249814 vs 349 reference patients
Kendall-tau correlation between PSVs
0.6
0.4
0.2
0.0
−0.2
−0.4
−0.6
1.0
1.2
1.4
1.6
1.8
2.0
best-fitted quadratic function
top 15 similar reference patient
most similar reference patient
Average weighted risk (AWR) of reference patient
Risk: blue-low; green-intermediate; red-high

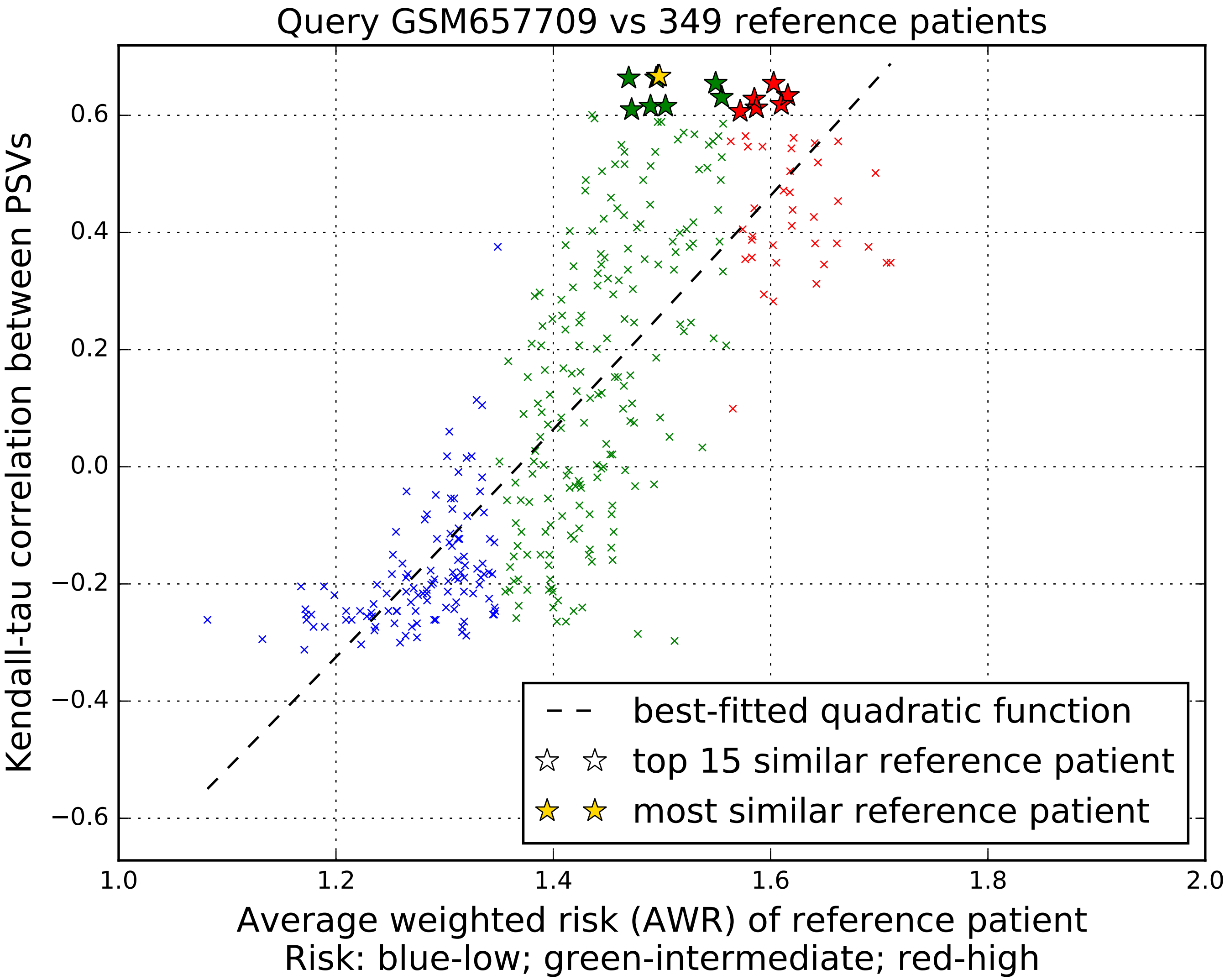
Query GSM657709 vs 349 reference patients
Kendall-tau correlation between PSVs
Average weighted risk (AWR) of reference patient
Risk: blue-low; green-intermediate; red-high
best-fitted quadratic function
top 15 similar reference patient
most similar reference patient
0.6
0.4
0.2
0.0
−0.2
−0.4
−0.6
1.0
1.2
1.4
1.6
1.8
2.0

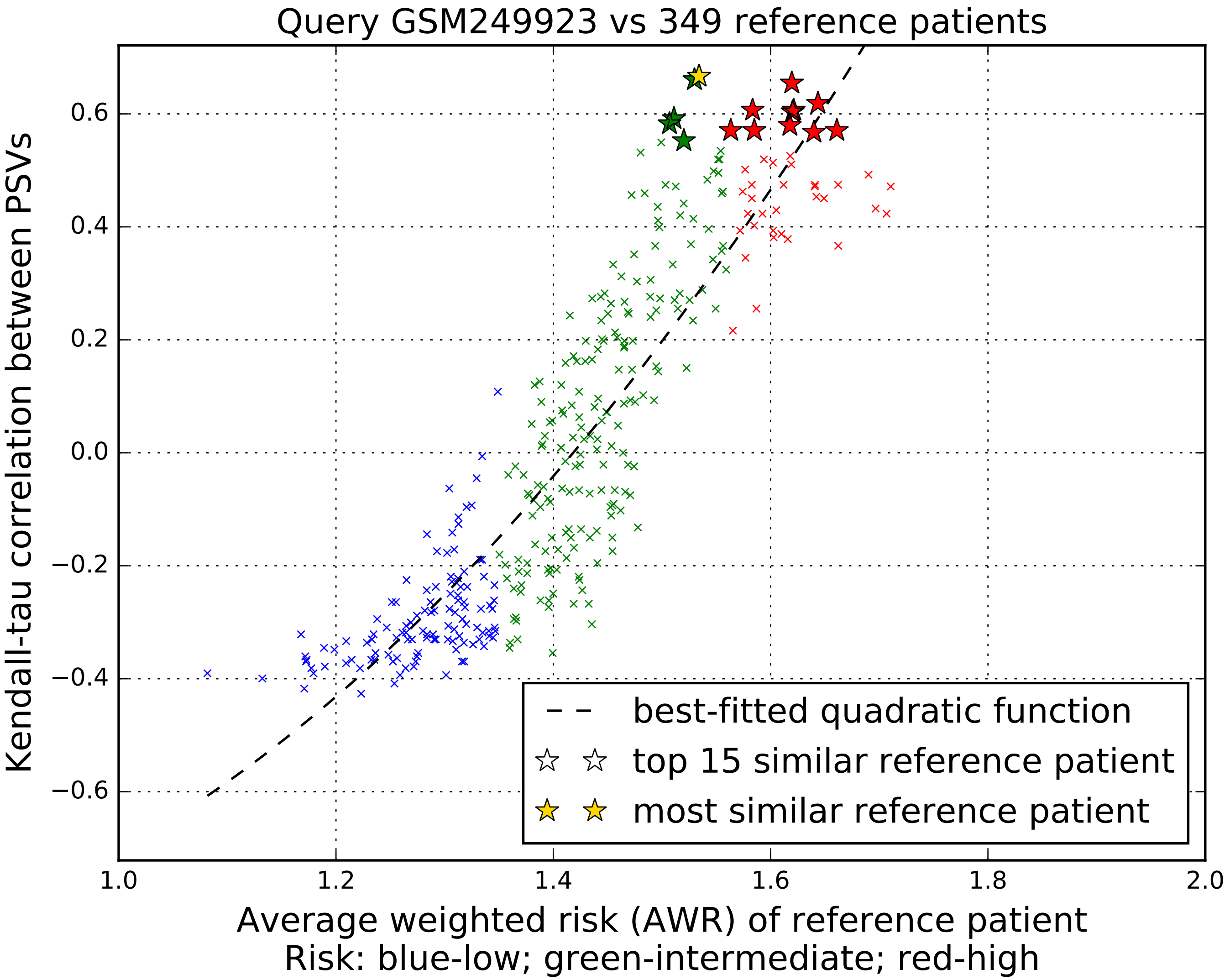

Query GSM249923 vs 349 reference patients
Kendall-tau correlation between PSVs
0.6
0.4
0.2
0.0
−0.2
−0.4
−0.6
1.0
1.2
1.4
1.6
1.8
2.0
best-fitted quadratic function
top 15 similar reference patient
most similar reference patient
Average weighted risk (AWR) of reference patient
Risk: blue-low; green-intermediate; red-high

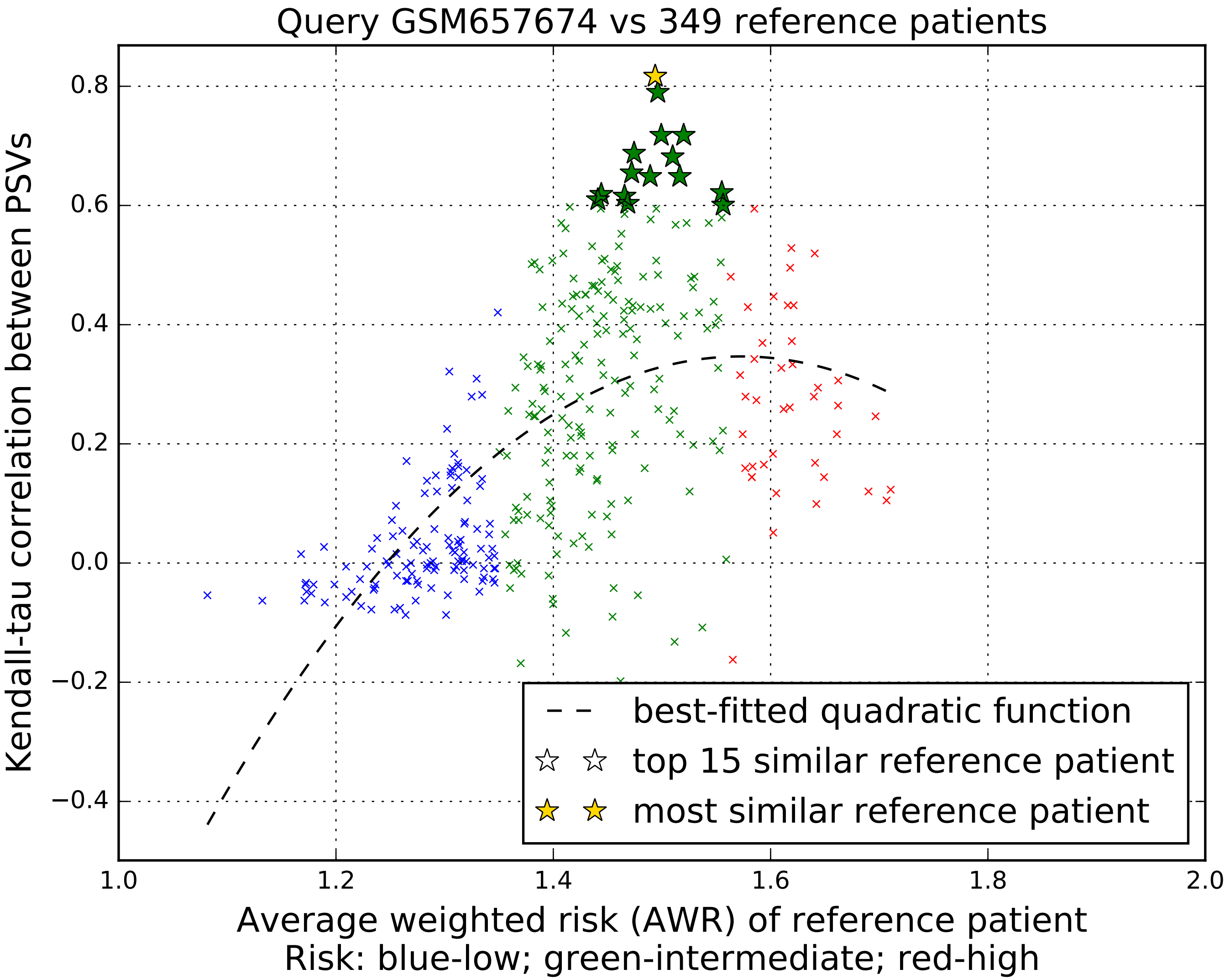
Query GSM657674 vs 349 reference patients
Kendall-tau correlation between PSVs
Average weighted risk (AWR) of reference patient
Risk: blue-low; green-intermediate; red-high
best-fitted quadratic function
top 15 similar reference patient
most similar reference patient
0.8
0.6
0.4
0.2
0.0
−0.2
−0.4
1.0
1.2
1.4
1.6
1.8
2.0

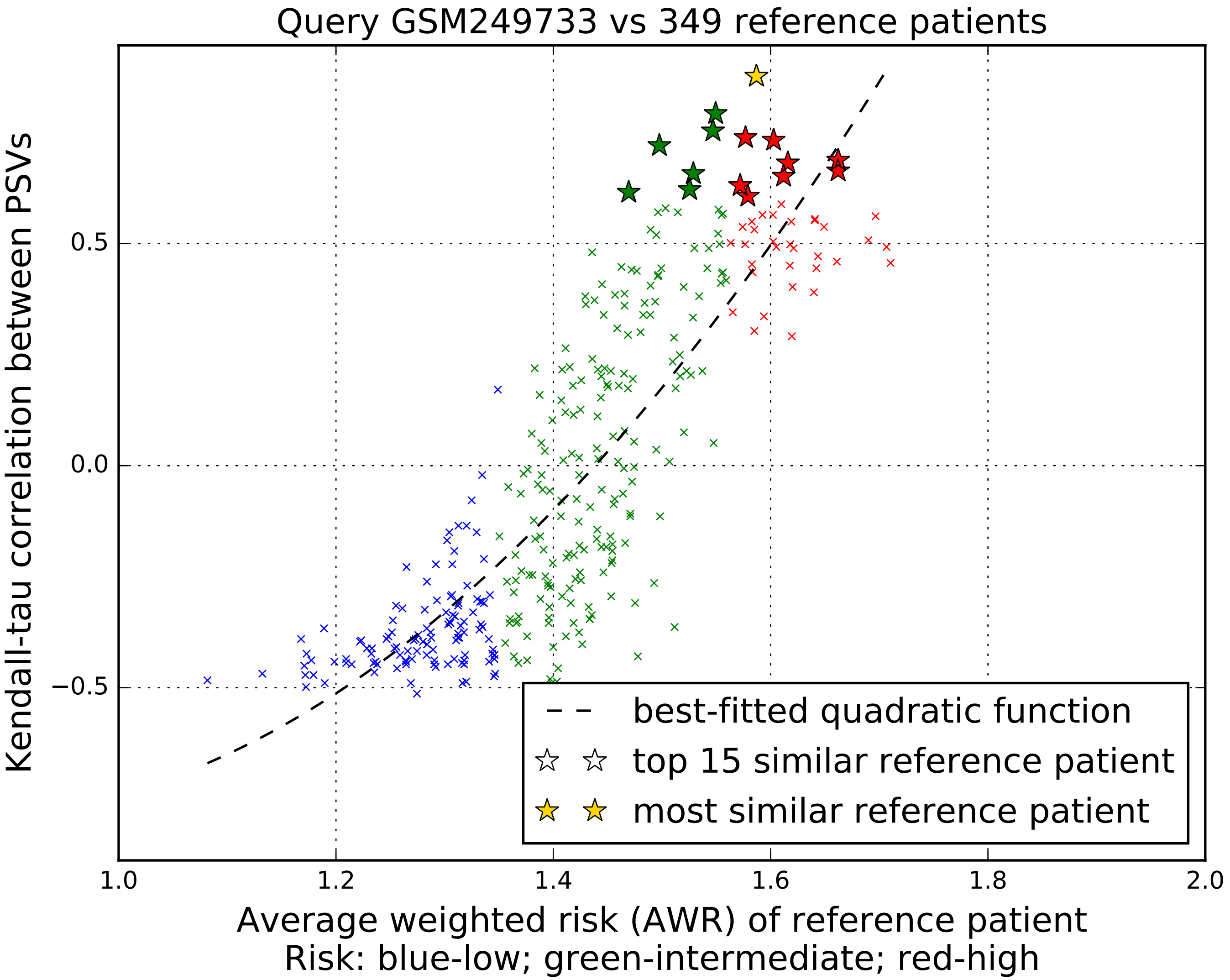
Query GSM249733 vs 349 reference patients
Kendall-tau correlation between PSVs
0.5
0.0
−0.5
1.0
1.2
1.4
1.6
1.8
2.0
best-fitted quadratic function
top 15 similar reference patient
most similar reference patient
Average weighted risk (AWR) of reference patient
Risk: blue-low; green-intermediate; red-high

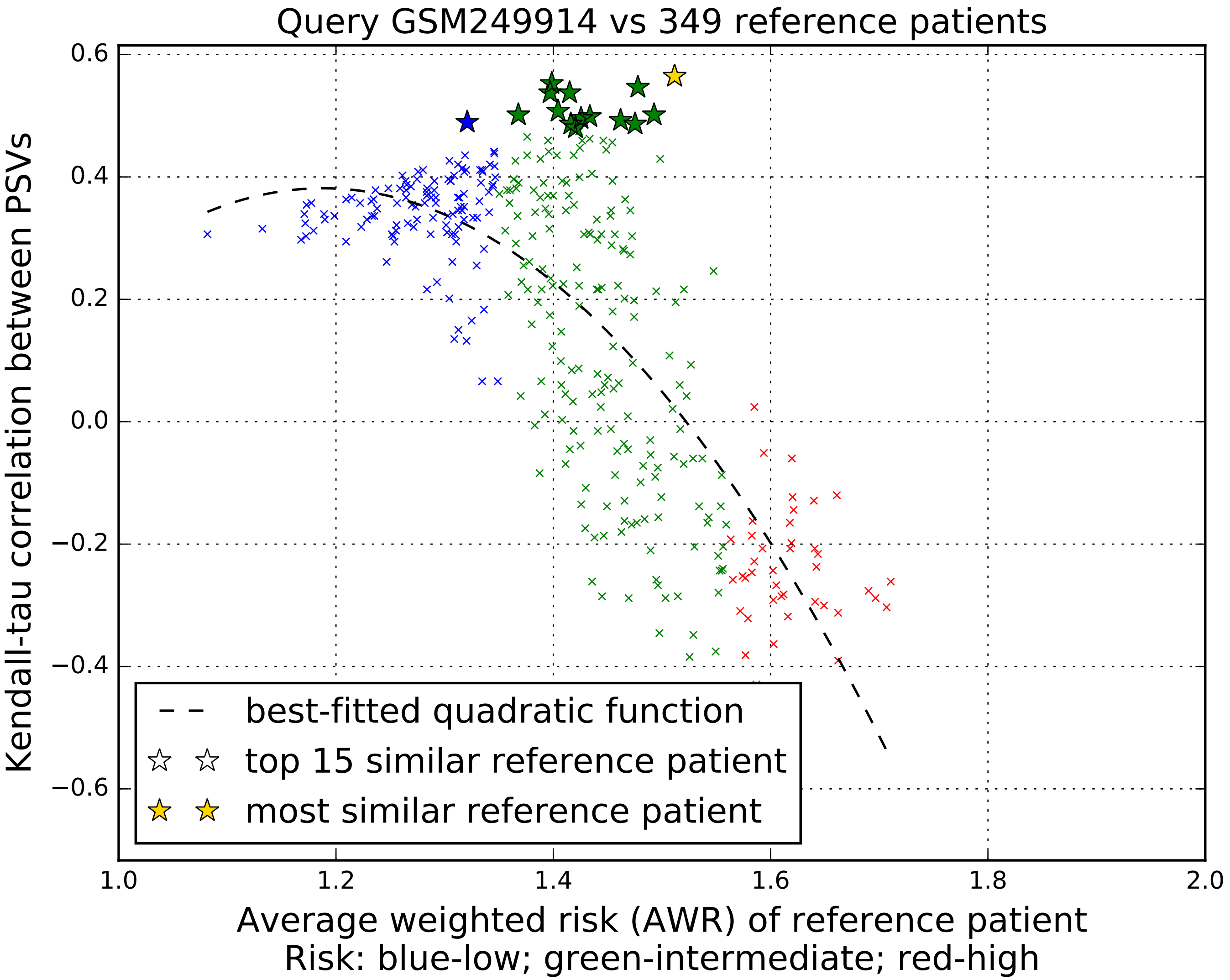

Query GSM249914 vs 349 reference patients
0.6
0.4
0.2
0.0
−0.2
−0.4
−0.6
1.0
1.2
1.4
1.6
1.8
2.0
Kendall-tau correlation between PSVs
Average weighted risk (AWR) of reference patient
Risk: blue-low; green-intermediate; red-high
best-fitted quadratic function
top 15 similar reference patient
most similar reference patient

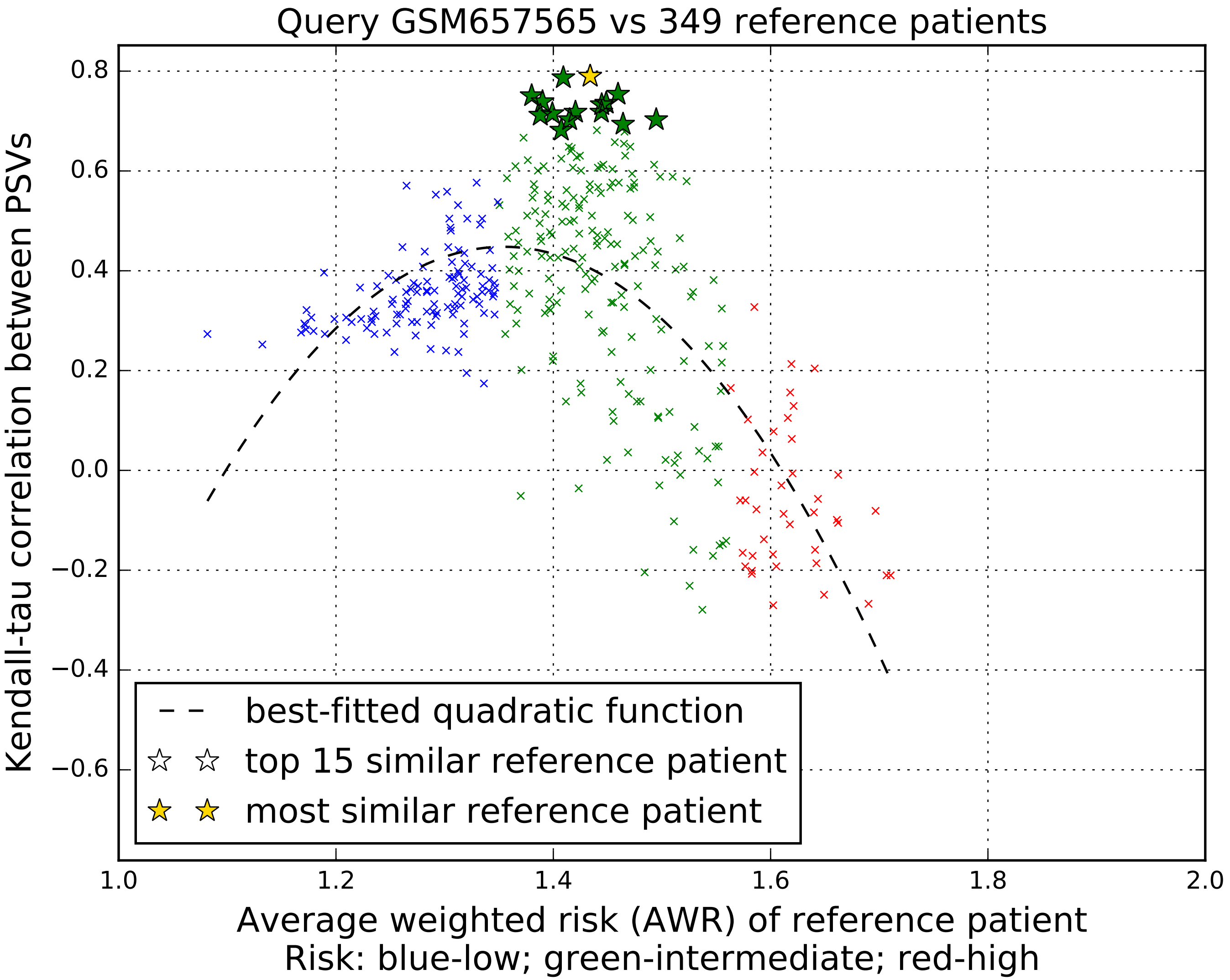

Query GSM657565 vs 349 reference patients
Kendall-tau correlation between PSVs
0.8
0.6
0.4
0.2
0.0
−0.2
−0.4
−0.6
1.0
1.2
1.4
1.6
1.8
2.0
best-fitted quadratic function
top 15 similar reference patient
most similar reference patient
Average weighted risk (AWR) of reference patient
Risk: blue-low; green-intermediate; red-high

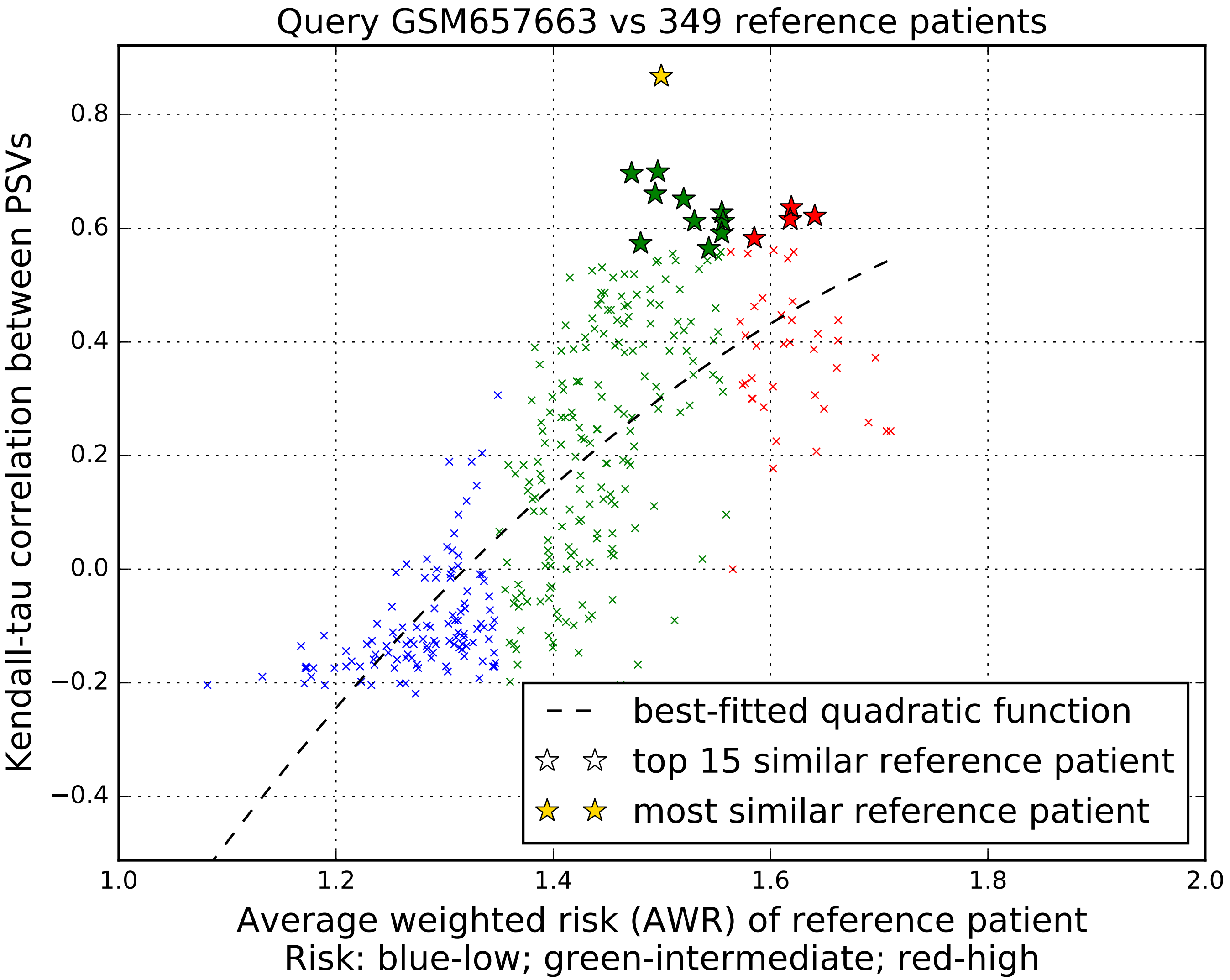
Query GSM657663 vs 349 reference patients
Kendall-tau correlation between PSVs
0.8
0.6
0.4
0.2
0.0
−0.2
−0.4
1.0
1.2
1.4
1.6
1.8
2.0
best-fitted quadratic function
top 15 similar reference patient
most similar reference patient
Average weighted risk (AWR) of reference patient
Risk: blue-low; green-intermediate; red-high

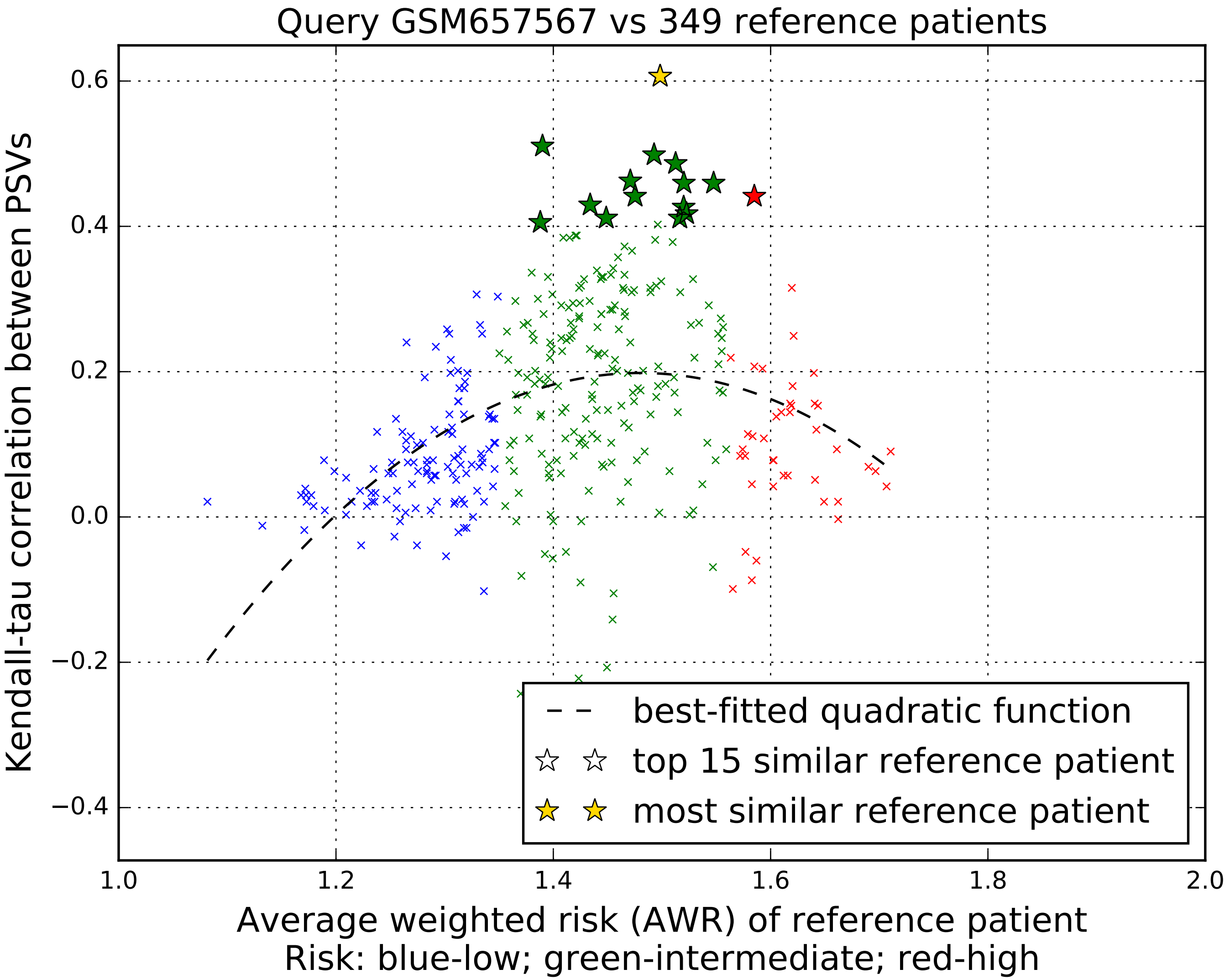

Query GSM657567 vs 349 reference patients
Kendall-tau correlation between PSVs
0.6
0.4
0.2
0.0
−0.2
−0.4
1.0
1.2
1.4
1.6
1.8
2.0
best-fitted quadratic function
top 15 similar reference patient
most similar reference patient
Average weighted risk (AWR) of reference patient
Risk: blue-low; green-intermediate; red-high

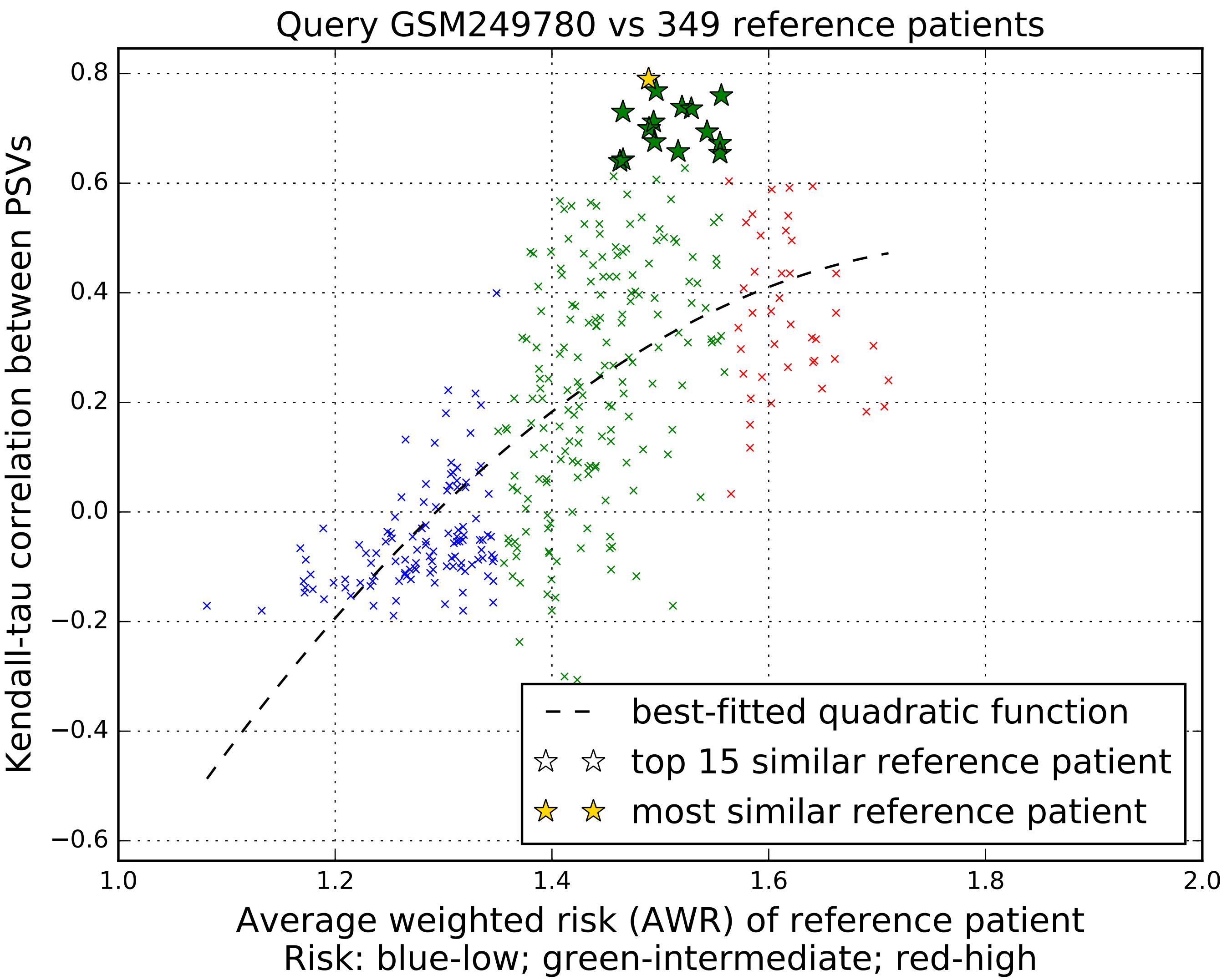

Query GSM249780 vs 349 reference patients
Kendall-tau correlation between PSVs
Average weighted risk (AWR) of reference patient
Risk: blue-low; green-intermediate; red-high
best-fitted quadratic function
top 15 similar reference patient
most similar reference patient
0.8
0.6
0.4
0.2
0.0
−0.2
−0.4
−0.6
1.0
1.2
1.4
1.6
1.8
2.0

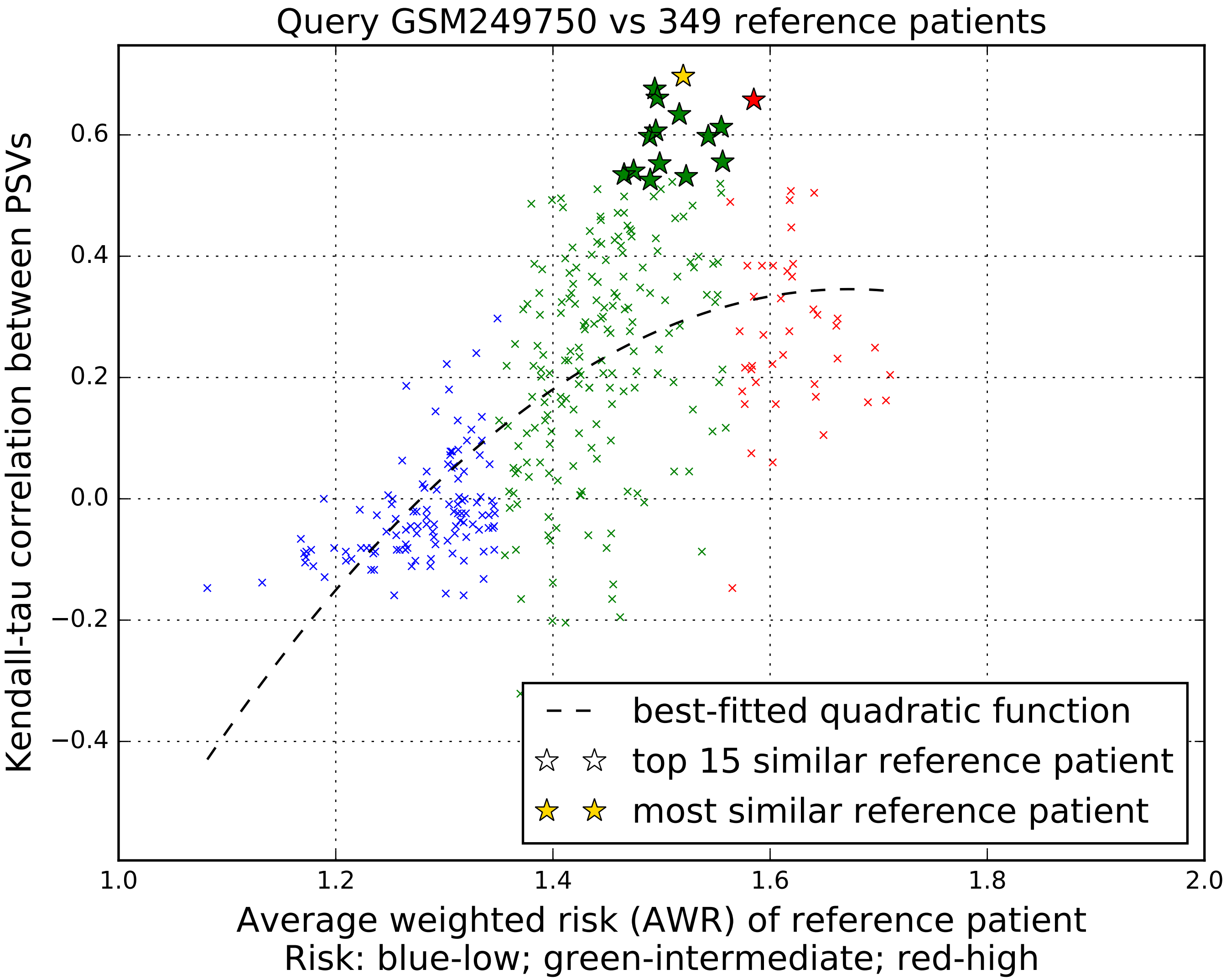

Query GSM249750 vs 349 reference patients
Kendall-tau correlation between PSVs
0.6
0.4
0.2
0.0
−0.2
−0.4
1.0
1.2
1.4
1.6
1.8
2.0
best-fitted quadratic function
top 15 similar reference patient
most similar reference patient
Average weighted risk (AWR) of reference patient
Risk: blue-low; green-intermediate; red-high

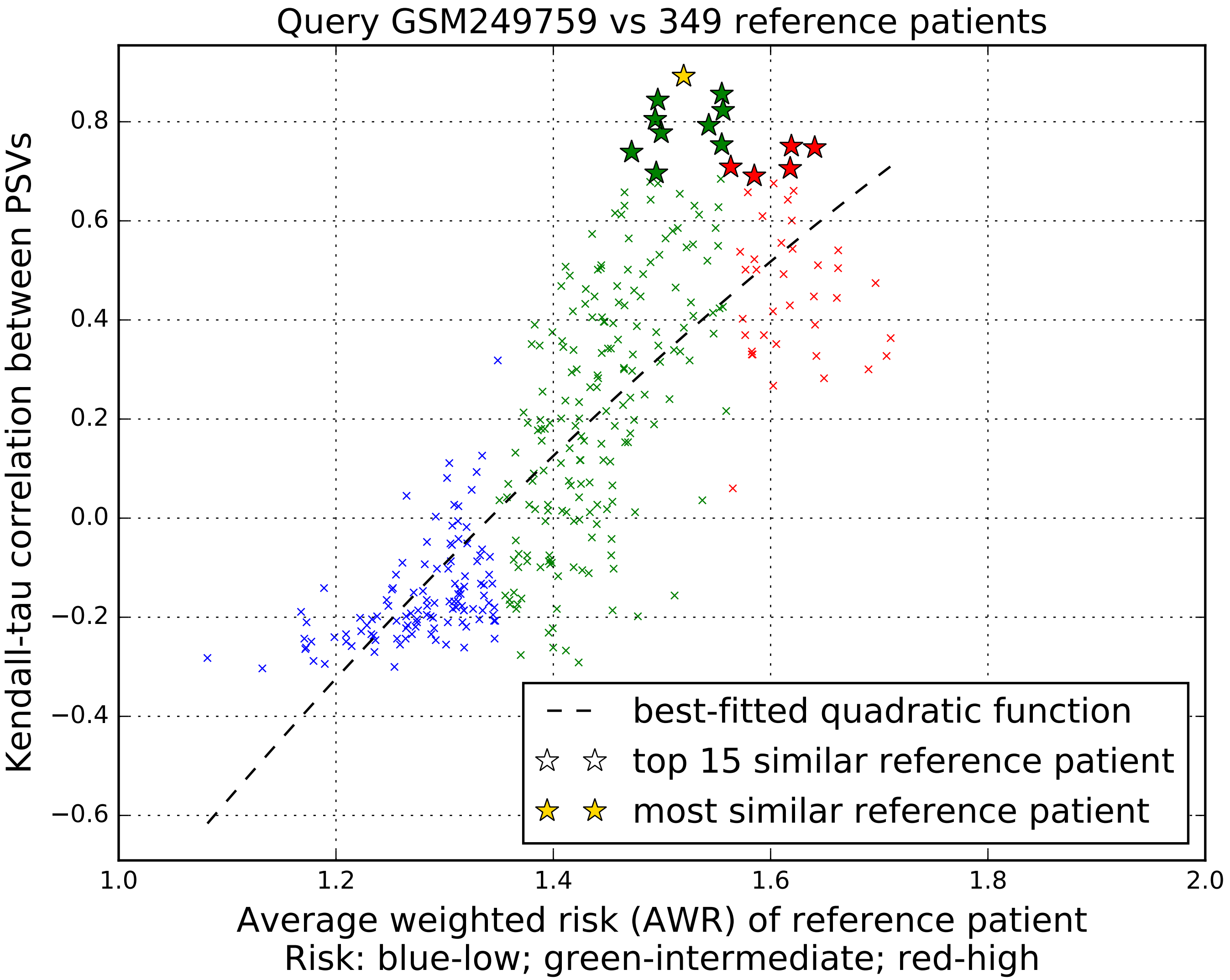
Query GSM249759 vs 349 reference patients
Kendall-tau correlation between PSVs
Average weighted risk (AWR) of reference patient
Risk: blue-low; green-intermediate; red-high
best-fitted quadratic function
top 15 similar reference patient
most similar reference patient
0.8
0.6
0.4
0.2
0.0
−0.2
−0.4
−0.6
1.0
1.2
1.4
1.6
1.8
2.0

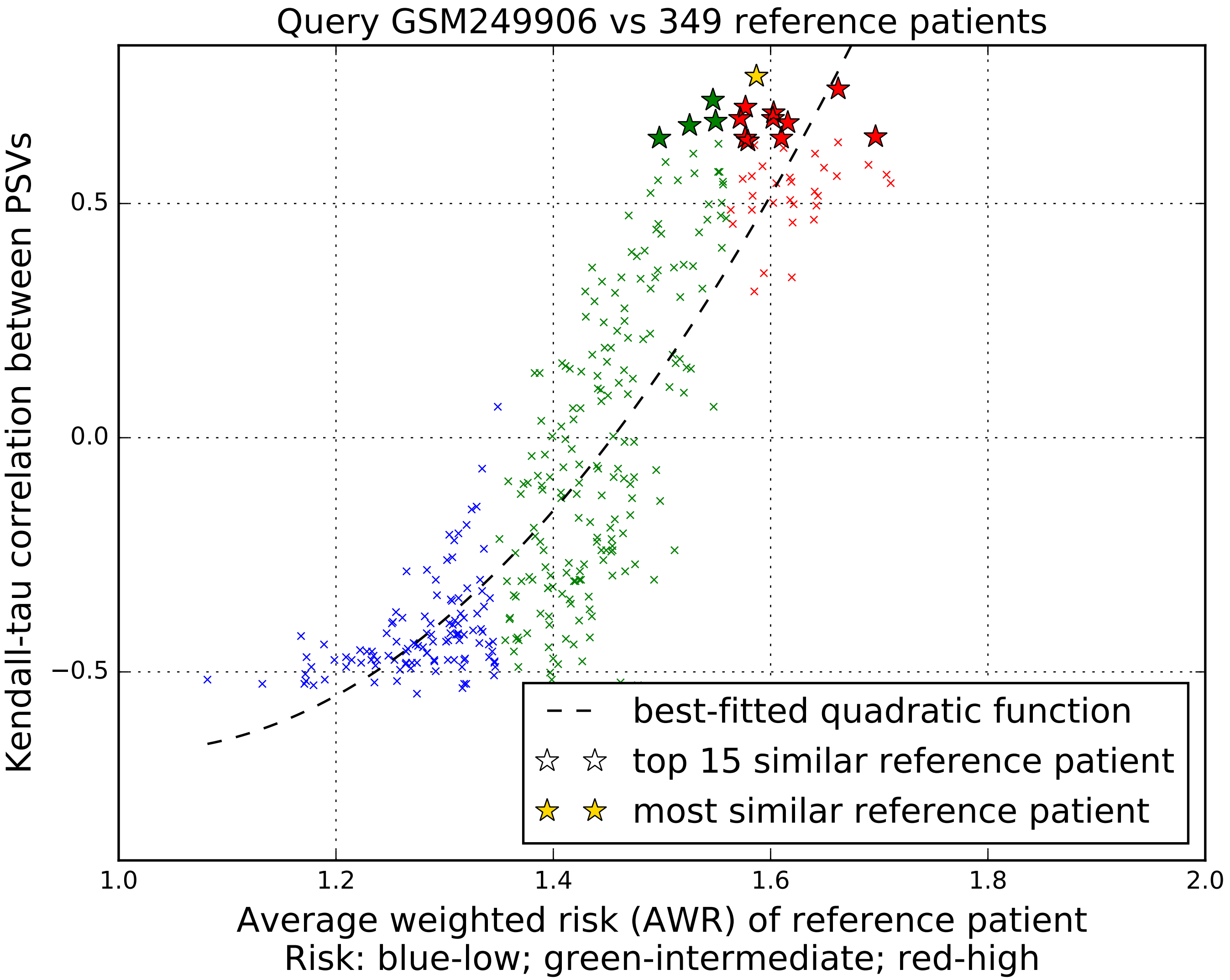

Query GSM249906 vs 349 reference patients
Kendall-tau correlation between PSVs
0.5
0.0
−0.5
1.0
1.2
1.4
1.6
1.8
2.0
best-fitted quadratic function
top 15 similar reference patient
most similar reference patient
Average weighted risk (AWR) of reference patient
Risk: blue-low; green-intermediate; red-high

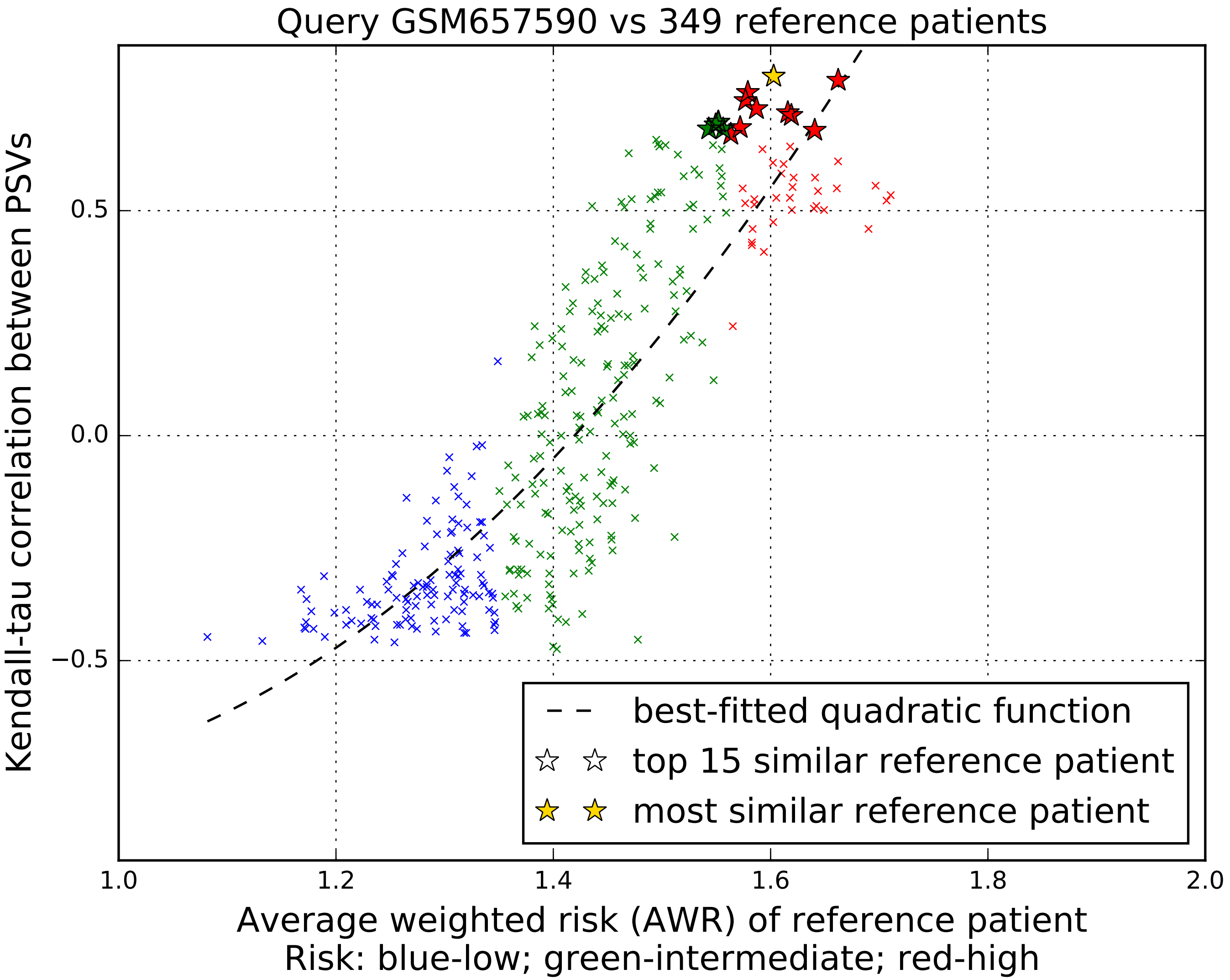
Query GSM657590 vs 349 reference patients
Kendall-tau correlation between PSVs
Average weighted risk (AWR) of reference patient
Risk: blue-low; green-intermediate; red-high
best-fitted quadratic function
top 15 similar reference patient
most similar reference patient
0.5
0.0
−0.5
1.0
1.2
1.4
1.6
1.8
2.0

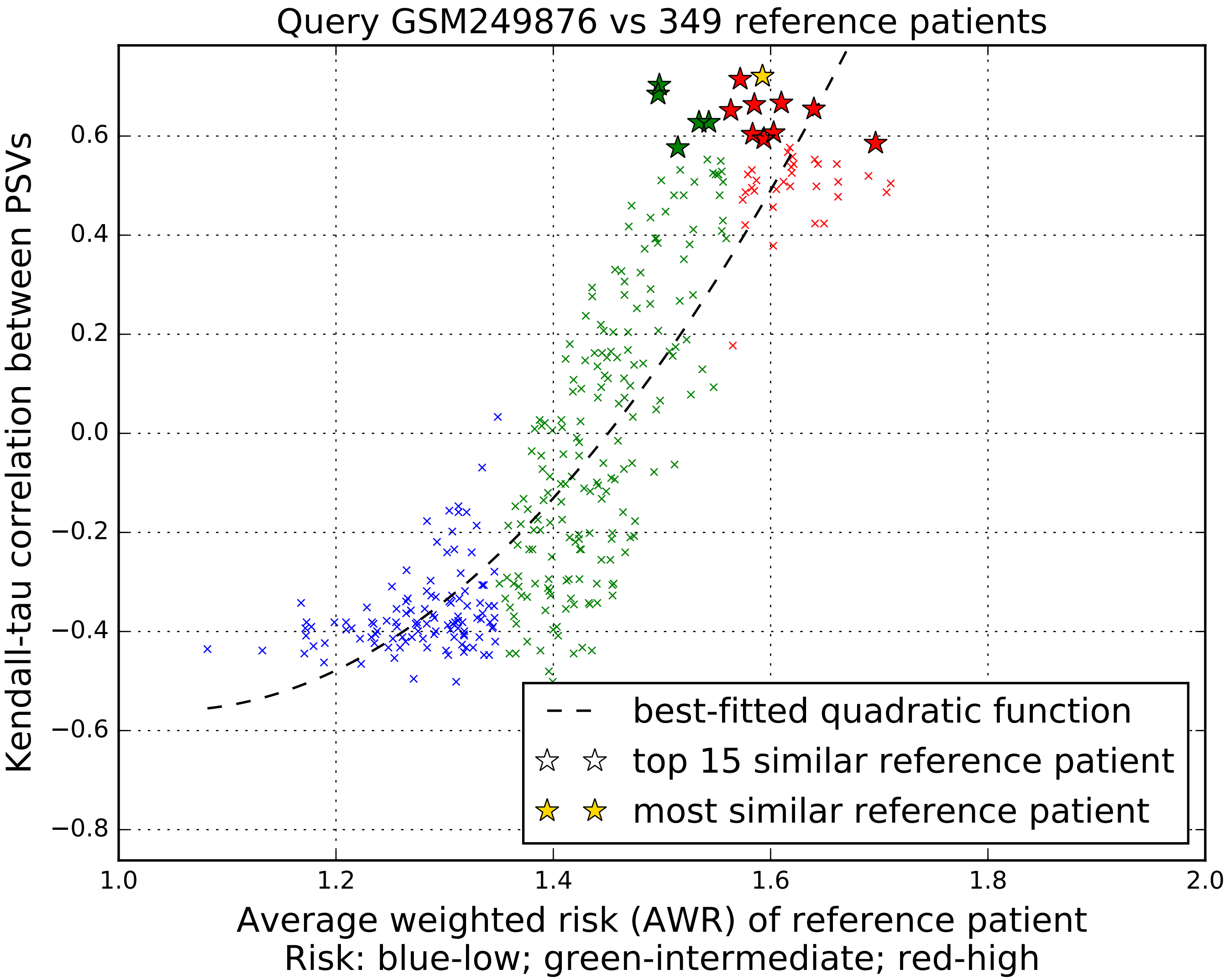

Query GSM249876 vs 349 reference patients
Kendall-tau correlation between PSVs
Average weighted risk (AWR) of reference patient
Risk: blue-low; green-intermediate; red-high
best-fitted quadratic function
top 15 similar reference patient
most similar reference patient
0.6
0.4
0.2
0.0
−0.2
−0.4
−0.6
−0.8
1.0
1.2
1.4
1.6
1.8
2.0

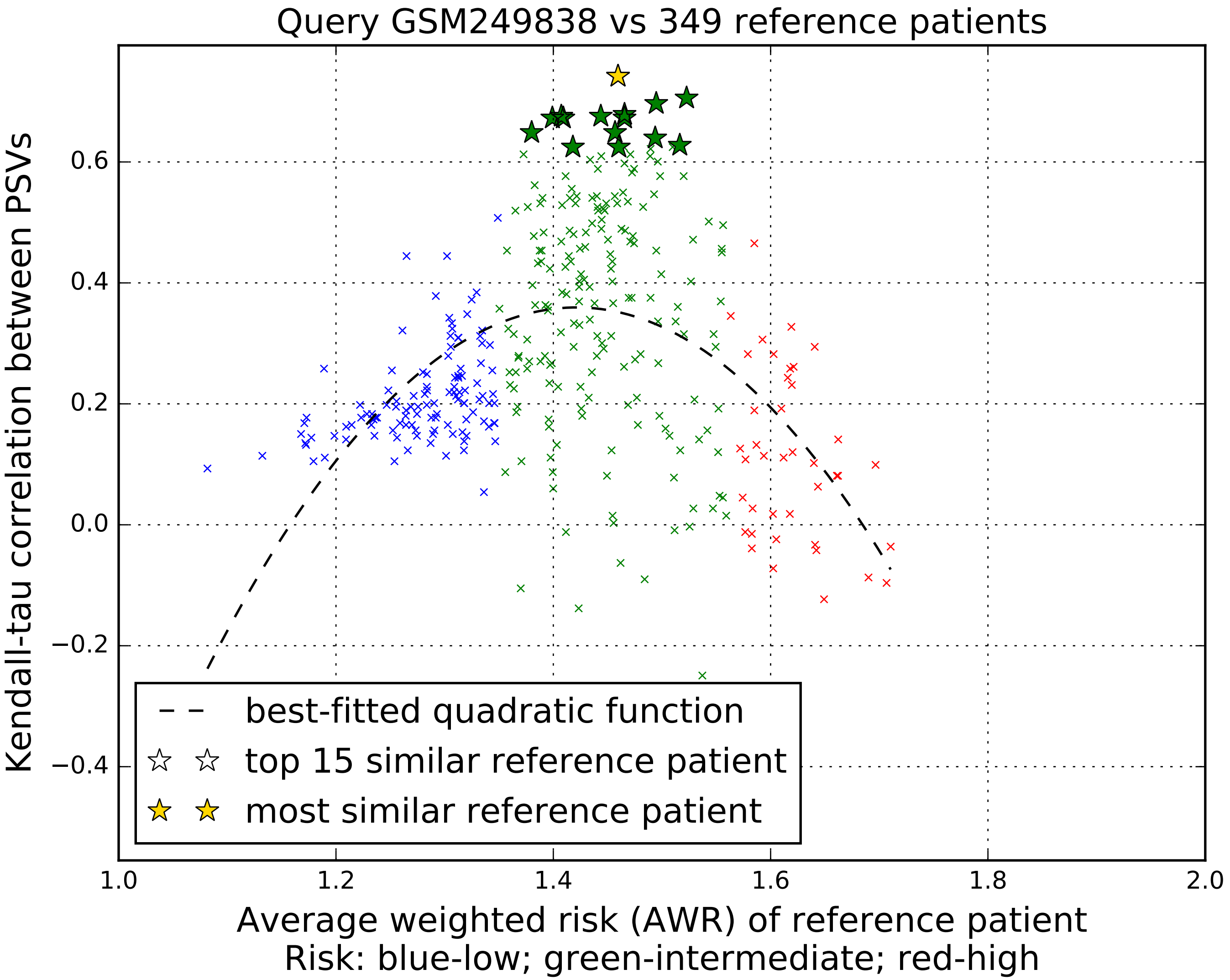

Query GSM249838 vs 349 reference patients
Kendall-tau correlation between PSVs
0.6
0.4
0.2
0.0
−0.2
−0.4
1.0
1.2
1.4
1.6
1.8
2.0
best-fitted quadratic function
top 15 similar reference patient
most similar reference patient
Average weighted risk (AWR) of reference patient
Risk: blue-low; green-intermediate; red-high

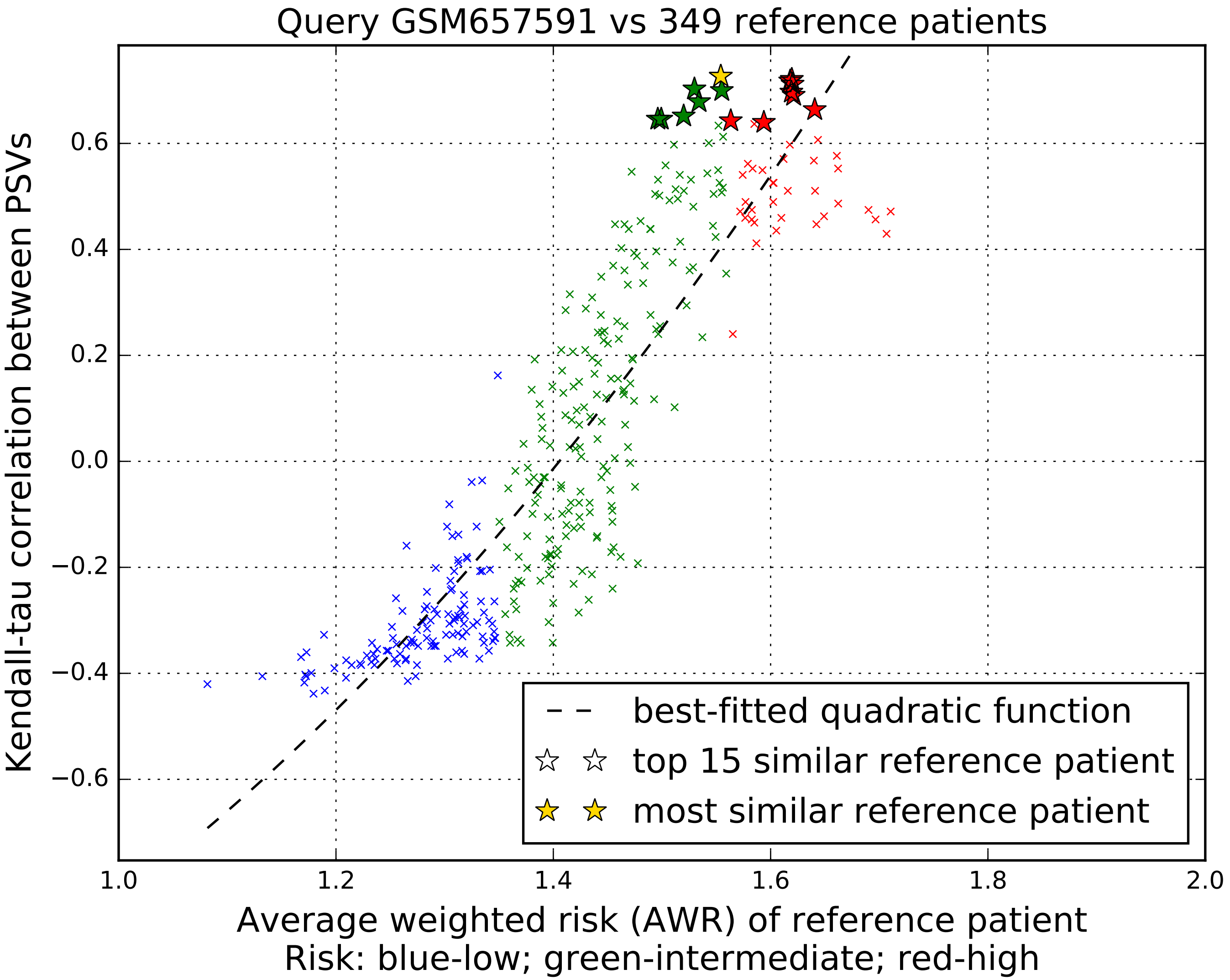

Query GSM657591 vs 349 reference patients
Kendall-tau correlation between PSVs
0.6
0.4
0.2
0.0
−0.2
−0.4
−0.6
1.0
1.2
1.4
1.6
1.8
2.0
best-fitted quadratic function
top 15 similar reference patient
most similar reference patient
Average weighted risk (AWR) of reference patient
Risk: blue-low; green-intermediate; red-high

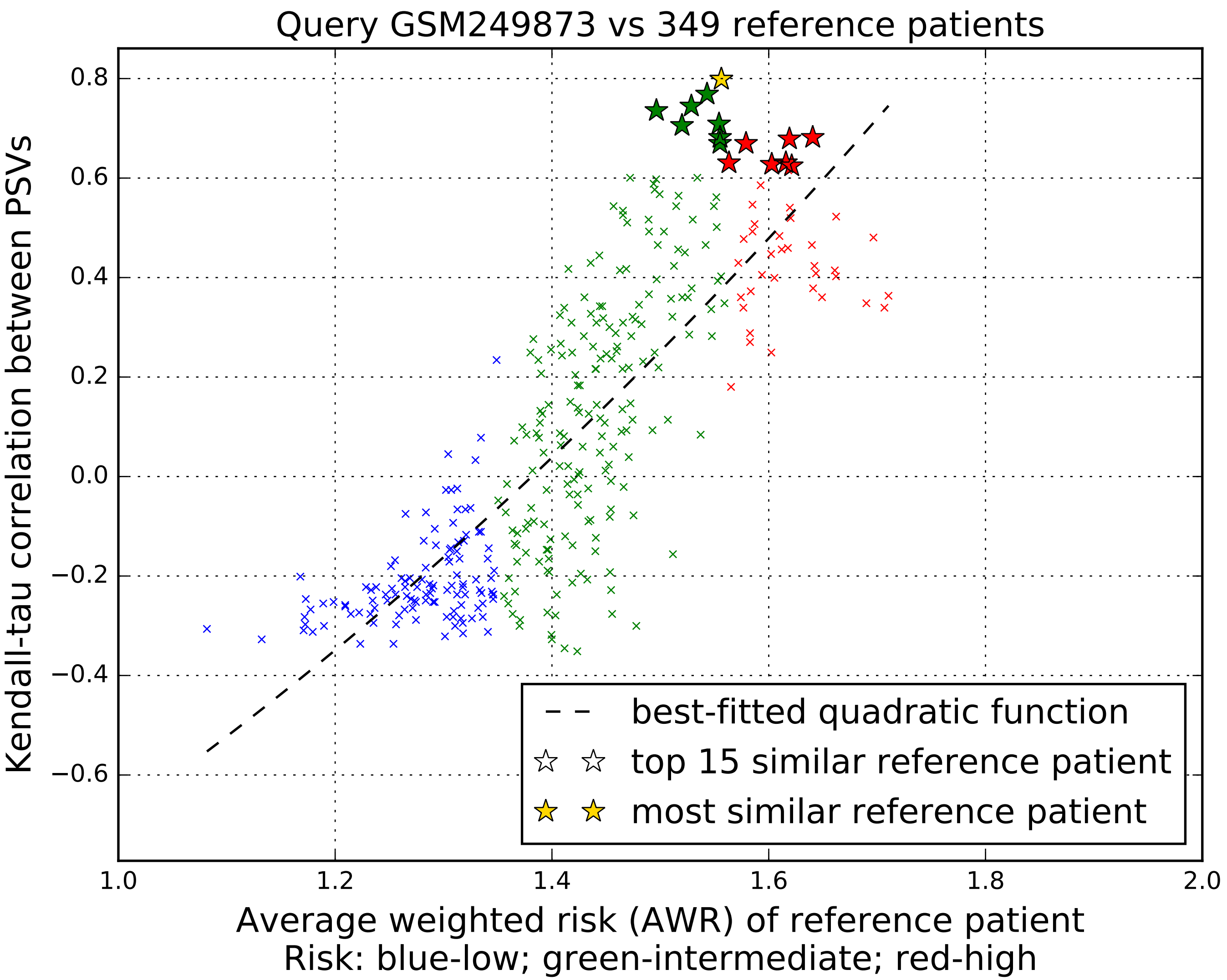
Query GSM249873 vs 349 reference patients
Kendall-tau correlation between PSVs
0.8
0.6
0.4
0.2
0.0
−0.2
−0.4
−0.6
1.0
1.2
1.4
1.6
1.8
2.0
best-fitted quadratic function
top 15 similar reference patient
most similar reference patient
Average weighted risk (AWR) of reference patient
Risk: blue-low; green-intermediate; red-high

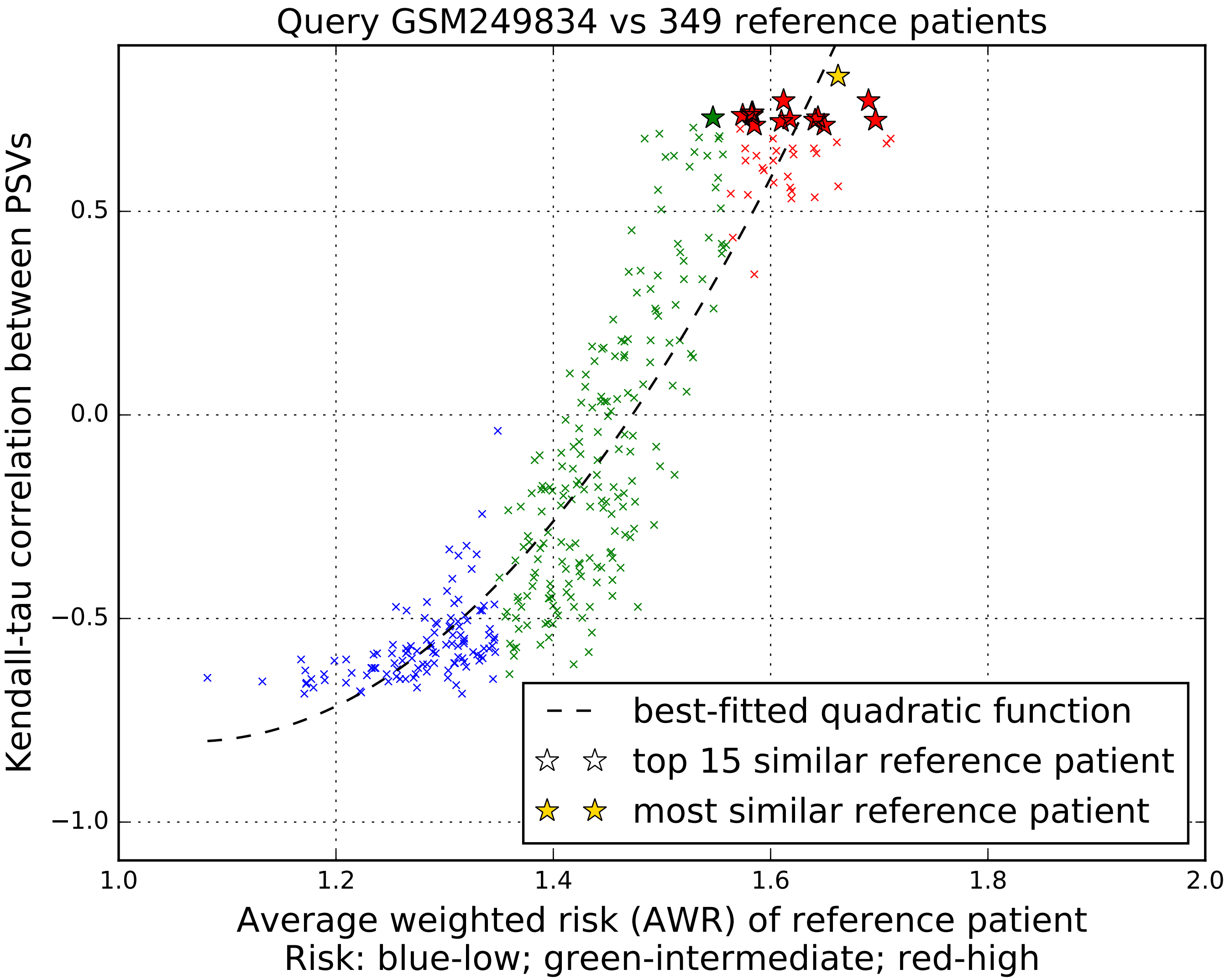

Query GSM249834 vs 349 reference patients
Kendall-tau correlation between PSVs
0.5
0.0
−0.5
−1.0
1.0
1.2
1.4
1.6
1.8
2.0
best-fitted quadratic function
top 15 similar reference patient
most similar reference patient
Average weighted risk (AWR) of reference patient
Risk: blue-low; green-intermediate; red-high

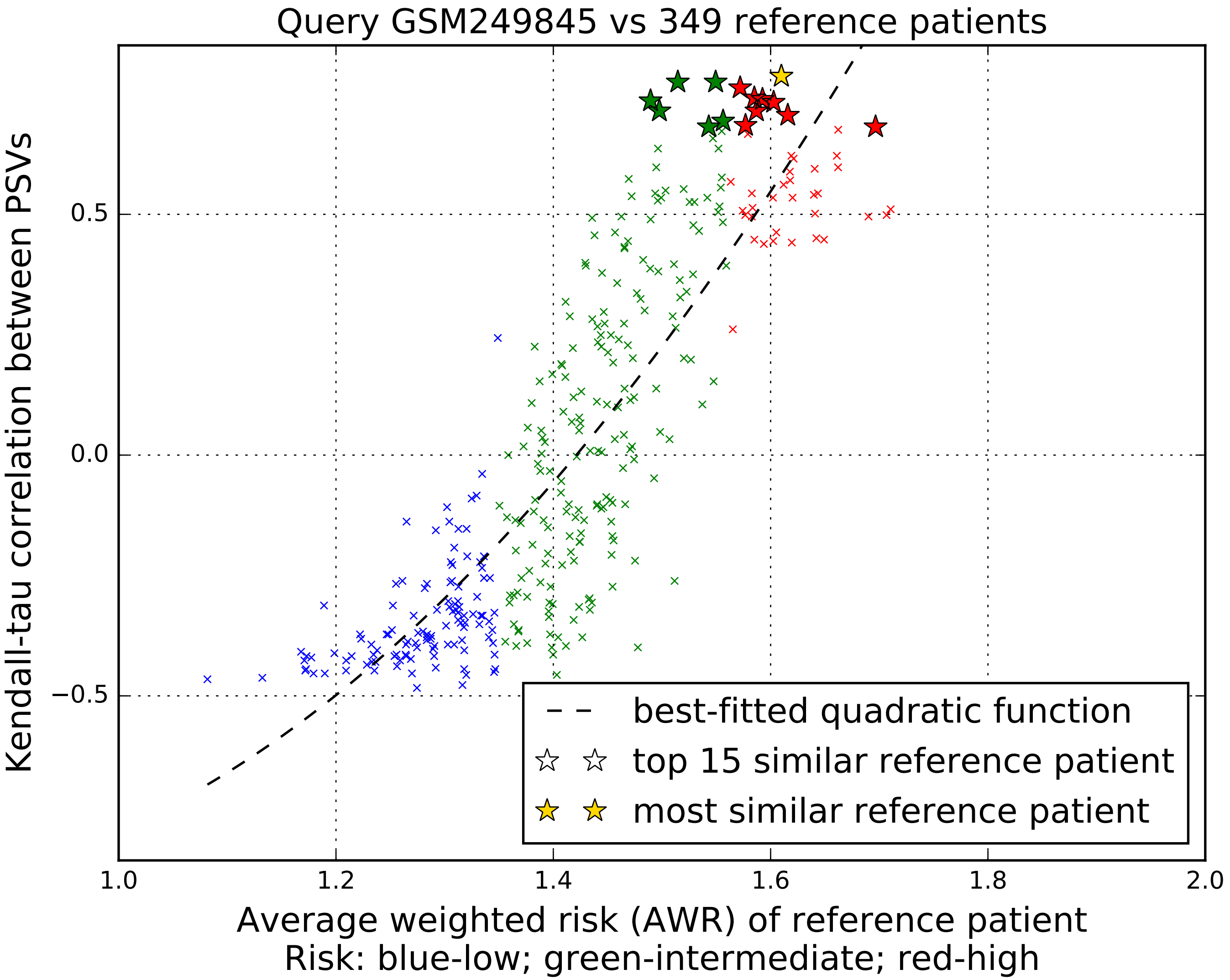
Query GSM249845 vs 349 reference patients
Kendall-tau correlation between PSVs
Average weighted risk (AWR) of reference patient
Risk: blue-low; green-intermediate; red-high
best-fitted quadratic function
top 15 similar reference patient
most similar reference patient
0.5
0.0
−0.5
1.0
1.2
1.4
1.6
1.8
2.0

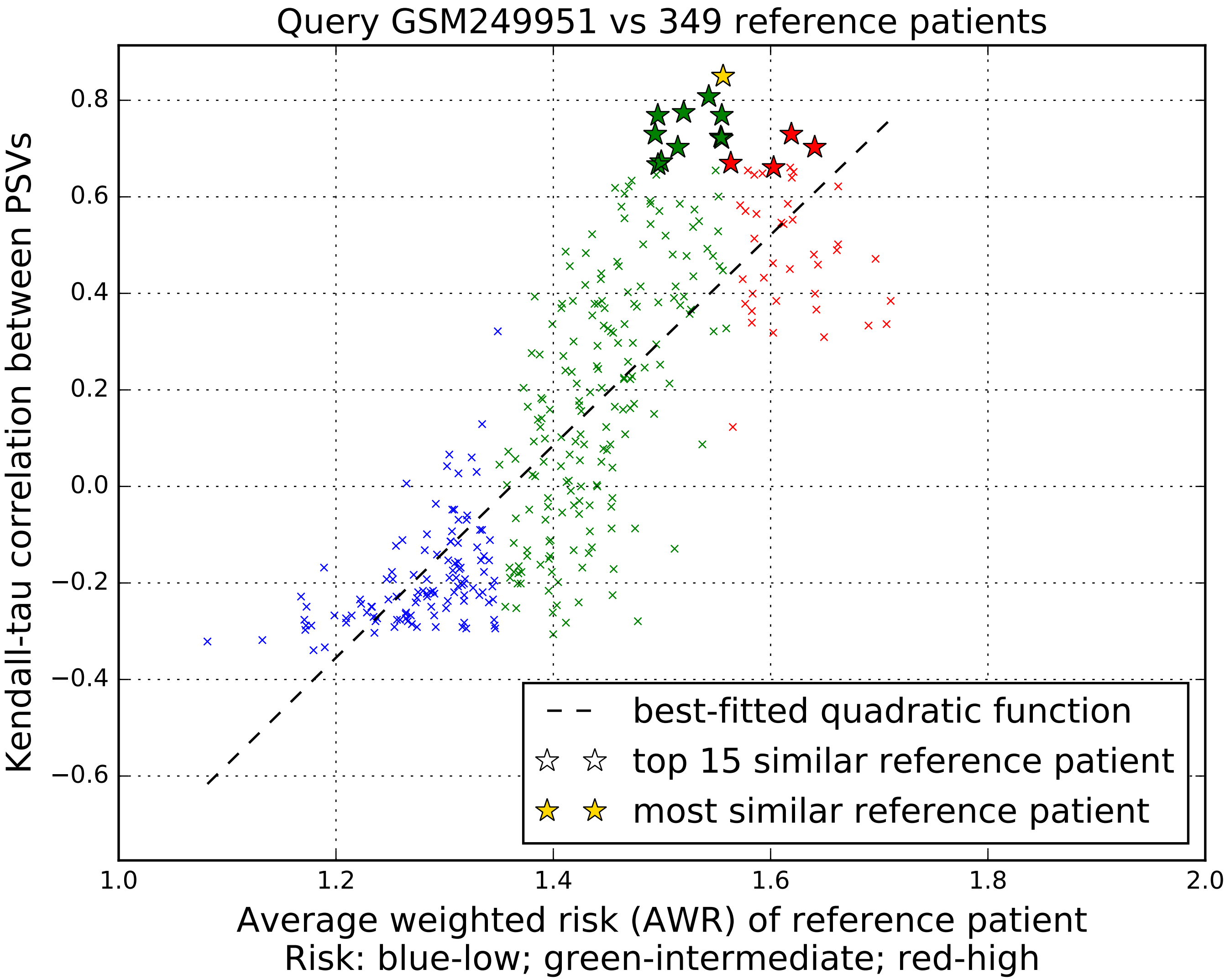
Query GSM249951 vs 349 reference patients
Kendall-tau correlation between PSVs
0.8
0.6
0.4
0.2
0.0
−0.2
−0.4
−0.6
1.0
1.2
1.4
1.6
1.8
2.0
best-fitted quadratic function
top 15 similar reference patient
most similar reference patient
Average weighted risk (AWR) of reference patient
Risk: blue-low; green-intermediate; red-high

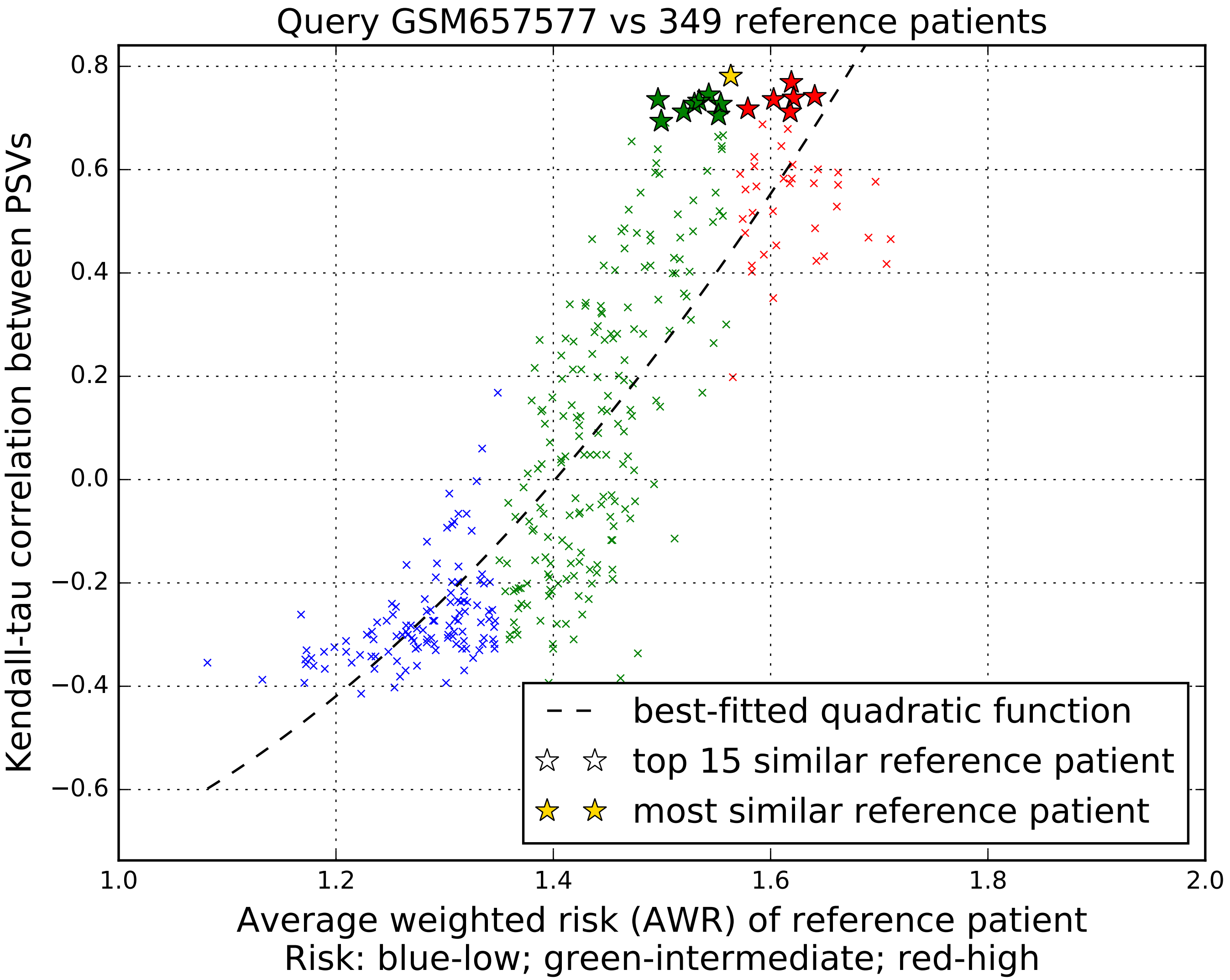
Query GSM657577 vs 349 reference patients
Kendall-tau correlation between PSVs
Average weighted risk (AWR) of reference patient
Risk: blue-low; green-intermediate; red-high
best-fitted quadratic function
top 15 similar reference patient
most similar reference patient
0.8
0.6
0.4
0.2
0.0
−0.2
−0.4
−0.6
1.0
1.2
1.4
1.6
1.8
2.0

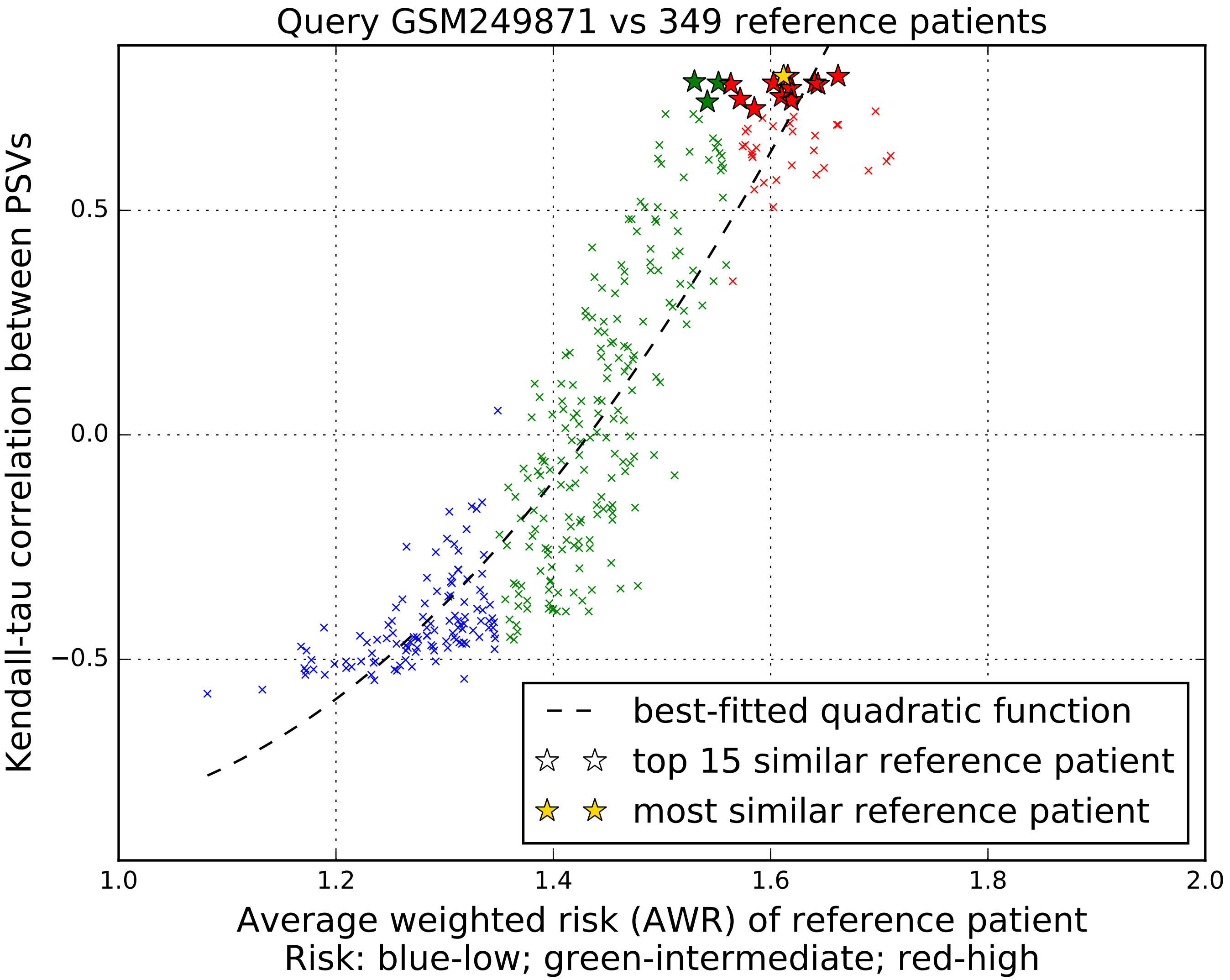
Query GSM249871 vs 349 reference patients
Kendall-tau correlation between PSVs
0.5
0.0
−0.5
1.0
1.2
1.4
1.6
1.8
2.0
best-fitted quadratic function
top 15 similar reference patient
most similar reference patient
Average weighted risk (AWR) of reference patient
Risk: blue-low; green-intermediate; red-high

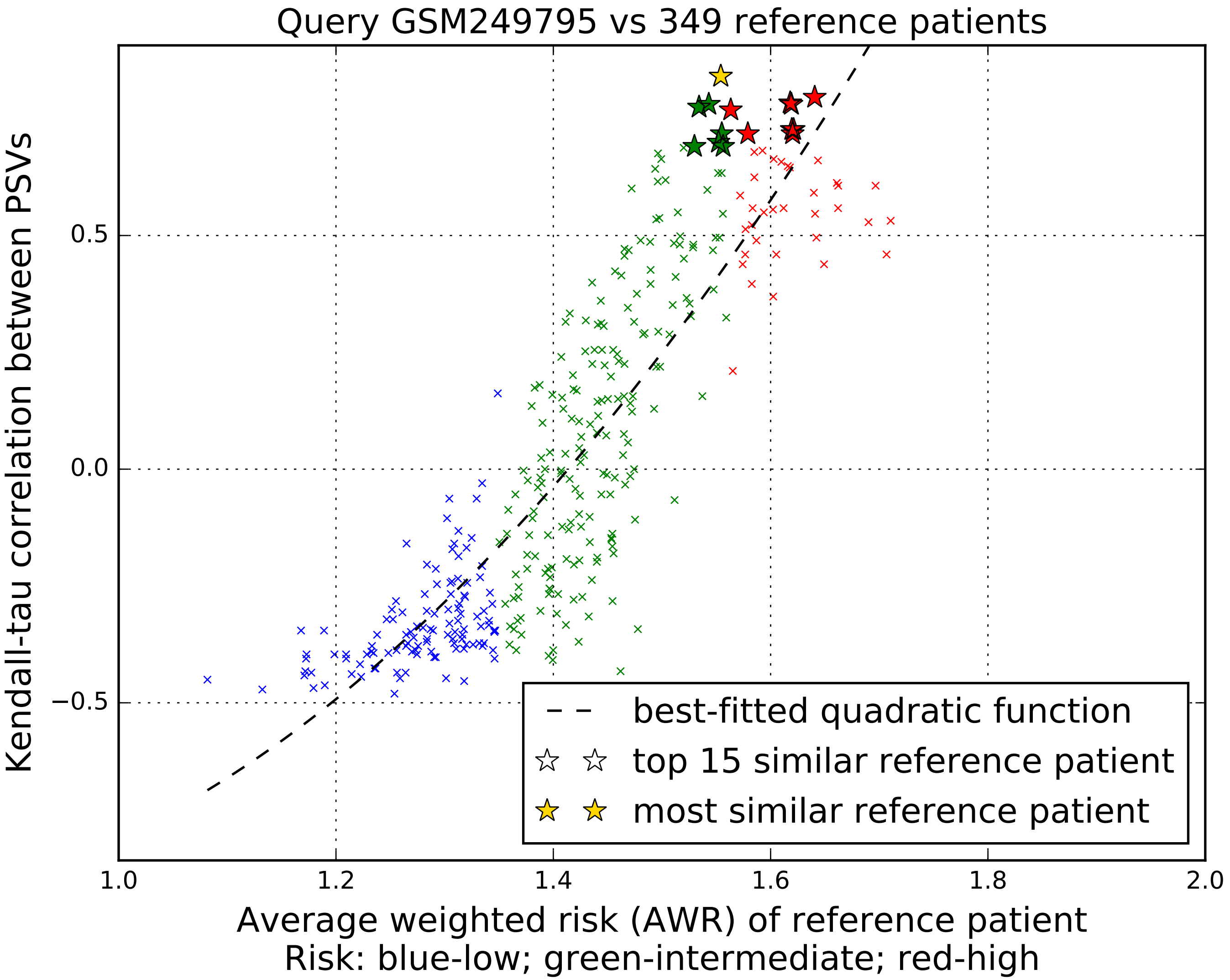
Query GSM249795 vs 349 reference patients
Kendall-tau correlation between PSVs
0.5
0.0
−0.5
1.0
1.2
1.4
1.6
1.8
2.0
best-fitted quadratic function
top 15 similar reference patient
most similar reference patient
Average weighted risk (AWR) of reference patient
Risk: blue-low; green-intermediate; red-high

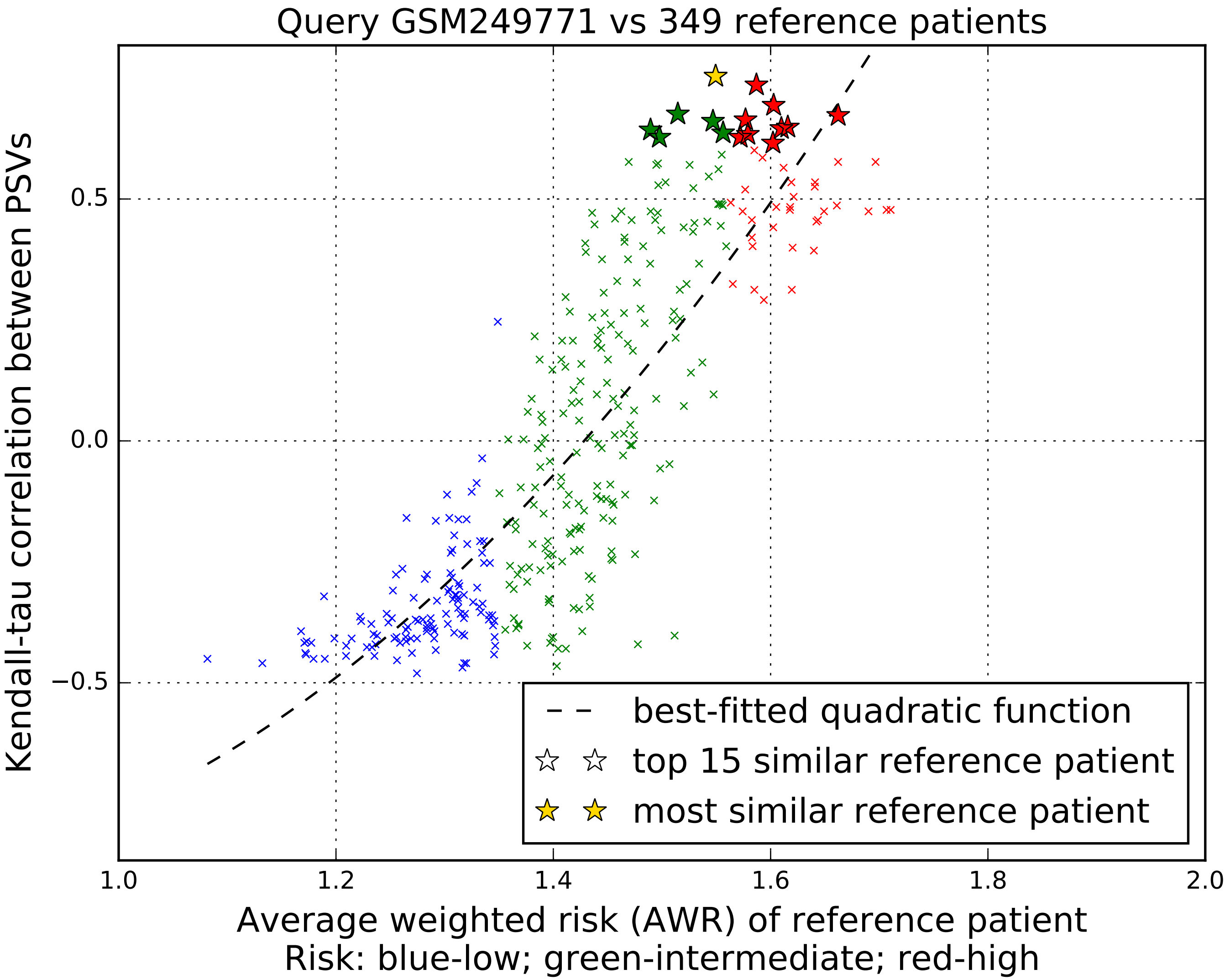
Query GSM249771 vs 349 reference patients
Kendall-tau correlation between PSVs
Average weighted risk (AWR) of reference patient
Risk: blue-low; green-intermediate; red-high
best-fitted quadratic function
top 15 similar reference patient
most similar reference patient
0.5
0.0
−0.5
1.0
1.2
1.4
1.6
1.8
2.0

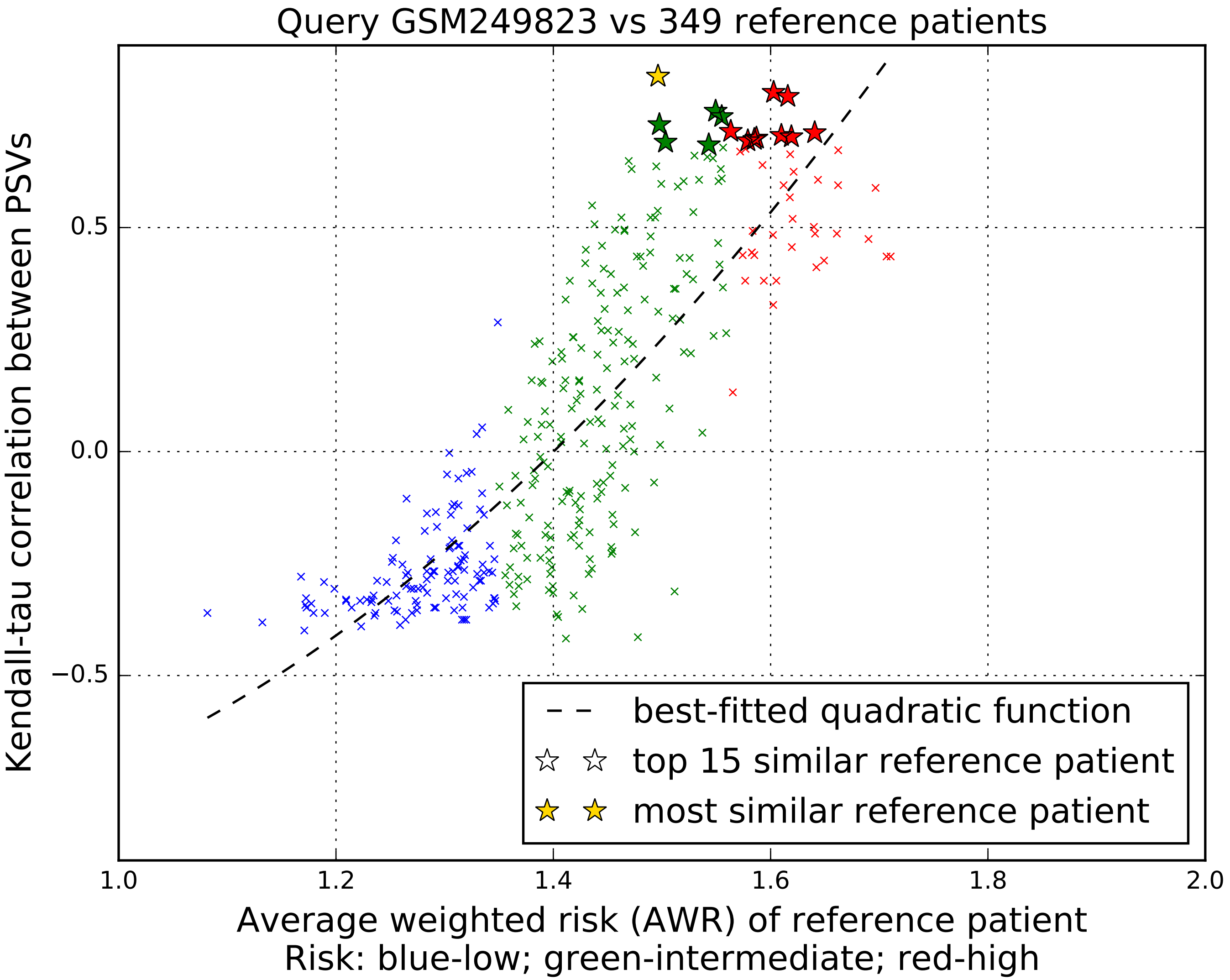
Query GSM249823 vs 349 reference patients
Kendall-tau correlation between PSVs
0.5
0.0
−0.5
1.0
1.2
1.4
1.6
1.8
2.0
best-fitted quadratic function
top 15 similar reference patient
most similar reference patient
Average weighted risk (AWR) of reference patient
Risk: blue-low; green-intermediate; red-high

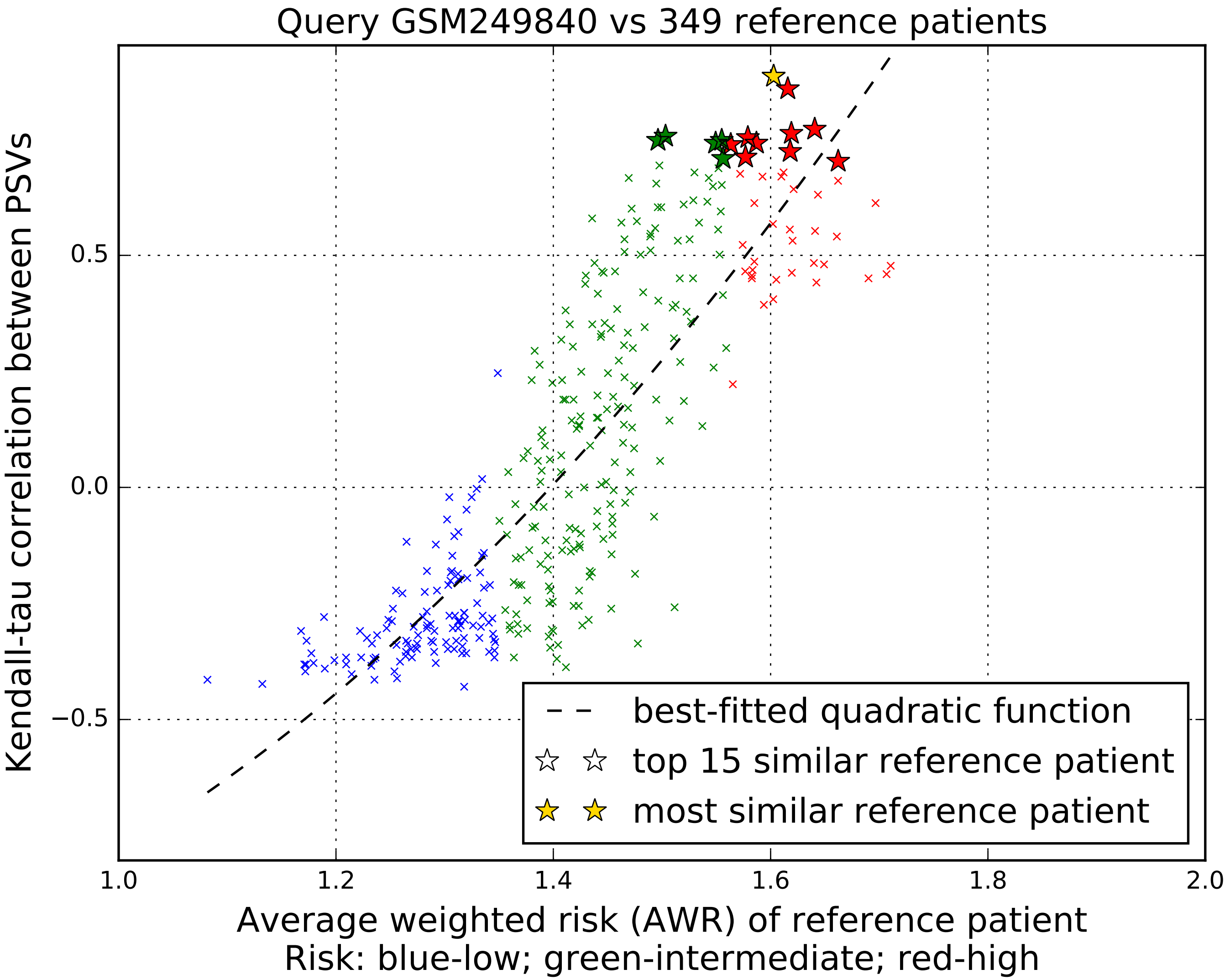
Query GSM249840 vs 349 reference patients
Kendall-tau correlation between PSVs
0.5
0.0
−0.5
1.0
1.2
1.4
1.6
1.8
2.0
best-fitted quadratic function
top 15 similar reference patient
most similar reference patient
Average weighted risk (AWR) of reference patient
Risk: blue-low; green-intermediate; red-high

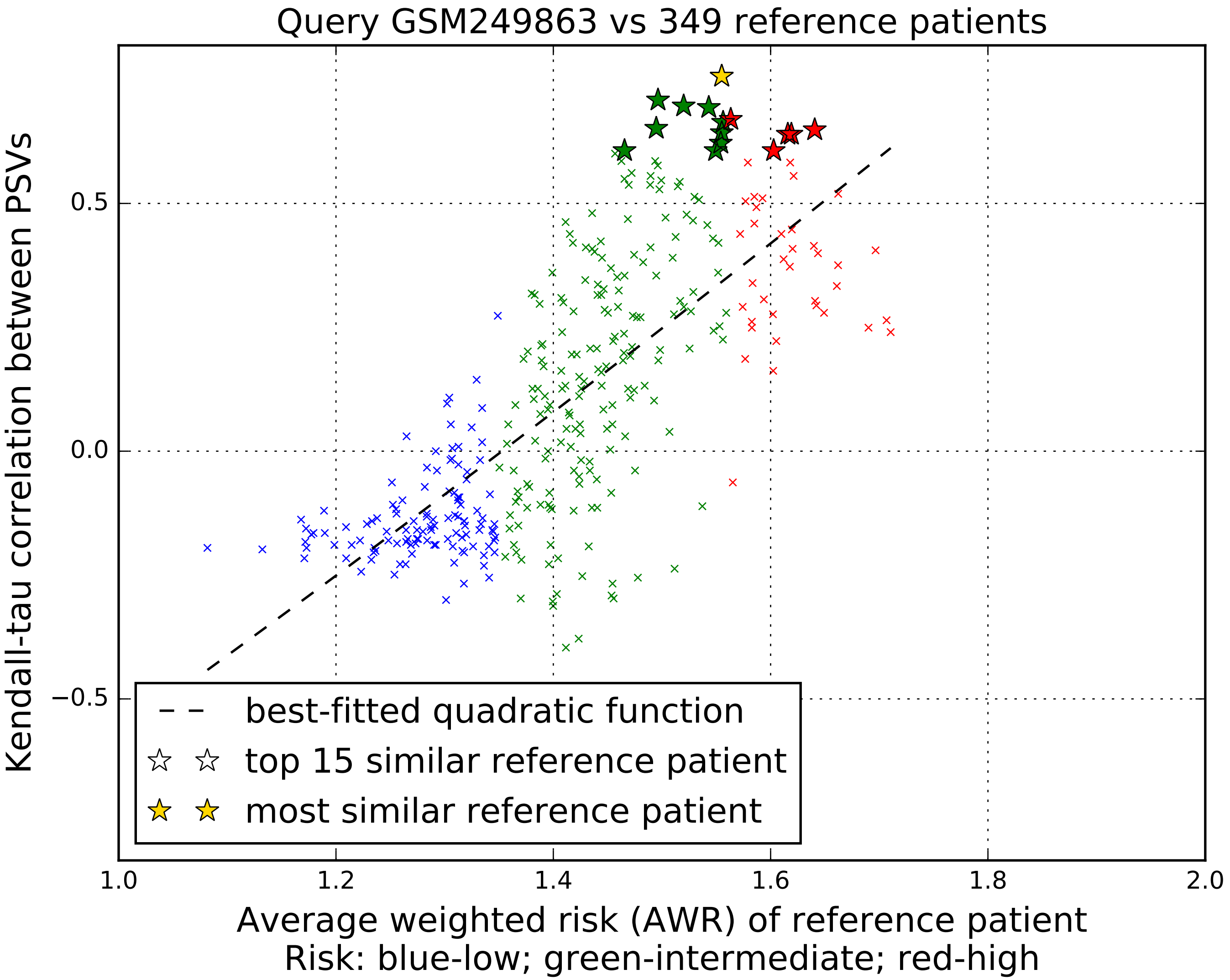
Query GSM249863 vs 349 reference patients
Kendall-tau correlation between PSVs
0.5
0.0
−0.5
1.0
1.2
1.4
1.6
1.8
2.0
best-fitted quadratic function
top 15 similar reference patient
most similar reference patient
Average weighted risk (AWR) of reference patient
Risk: blue-low; green-intermediate; red-high

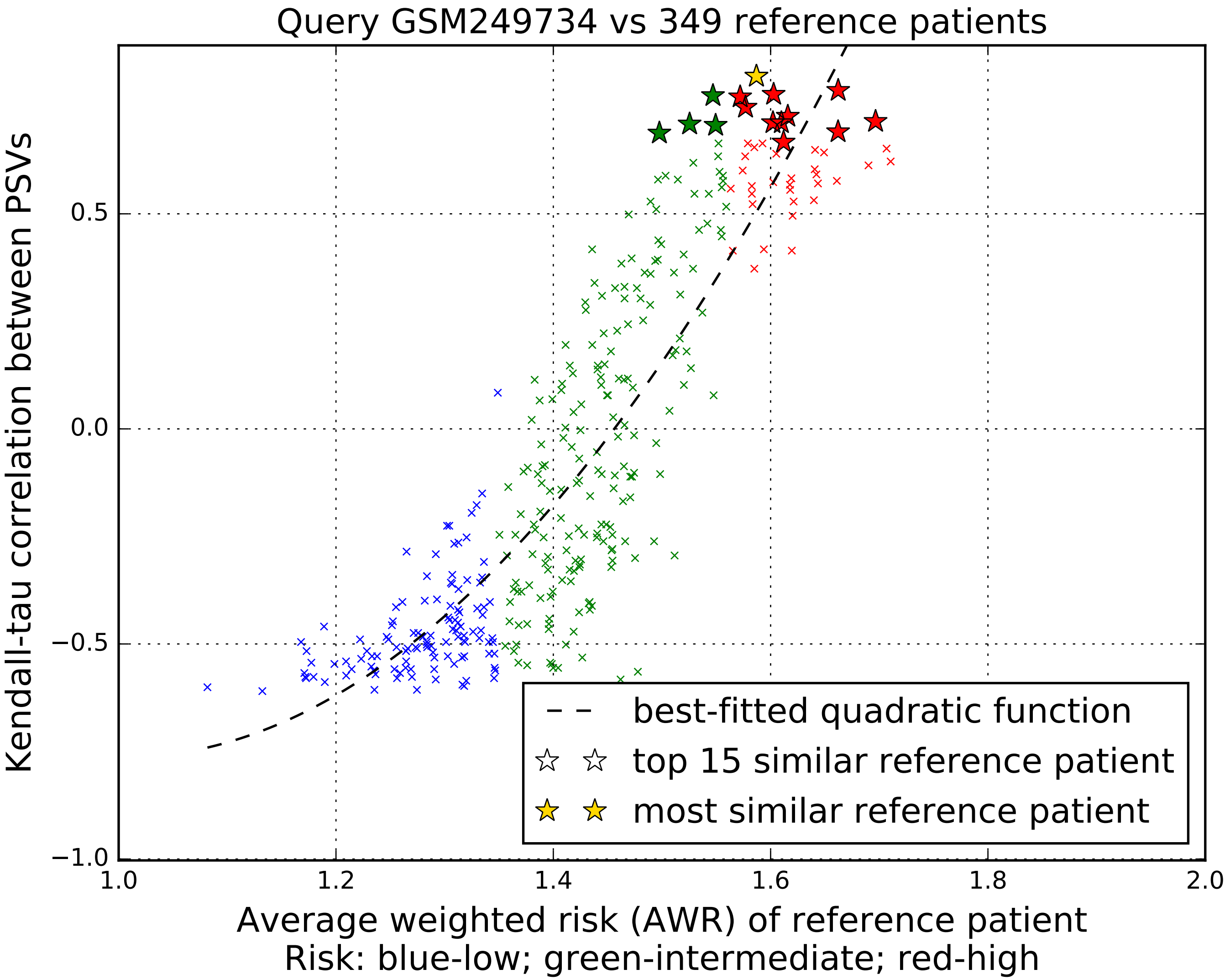
Query GSM249734 vs 349 reference patients
Kendall-tau correlation between PSVs
0.5
0.0
−0.5
−1.0
1.0
1.2
1.4
1.6
1.8
2.0
best-fitted quadratic function
top 15 similar reference patient
most similar reference patient
Average weighted risk (AWR) of reference patient
Risk: blue-low; green-intermediate; red-high

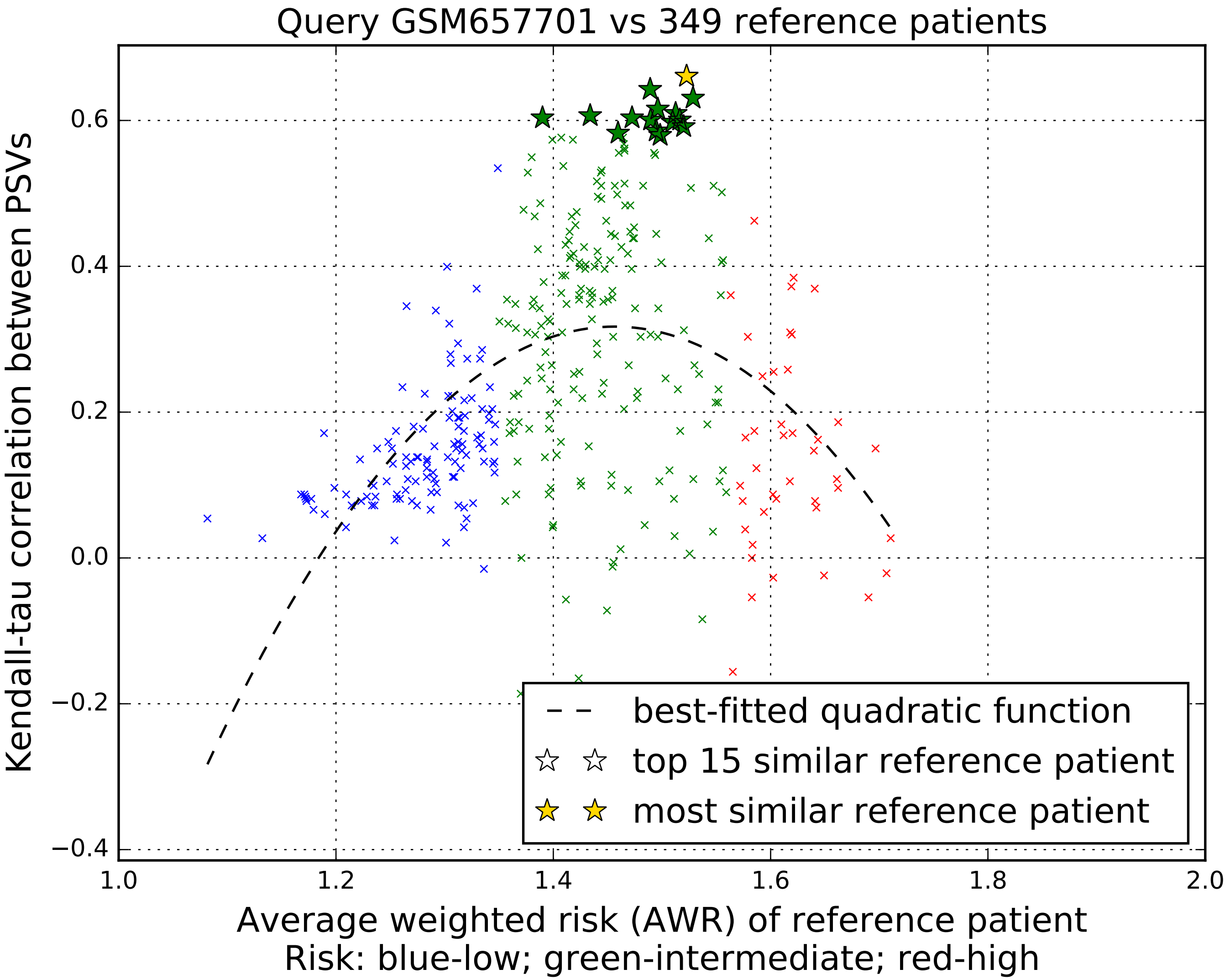

Query GSM657701 vs 349 reference patients
Kendall-tau correlation between PSVs
Average weighted risk (AWR) of reference patient
Risk: blue-low; green-intermediate; red-high
best-fitted quadratic function
top 15 similar reference patient
most similar reference patient
0.6
0.4
0.2
0.0
−0.2
−0.4
1.0
1.2
1.4
1.6
1.8
2.0

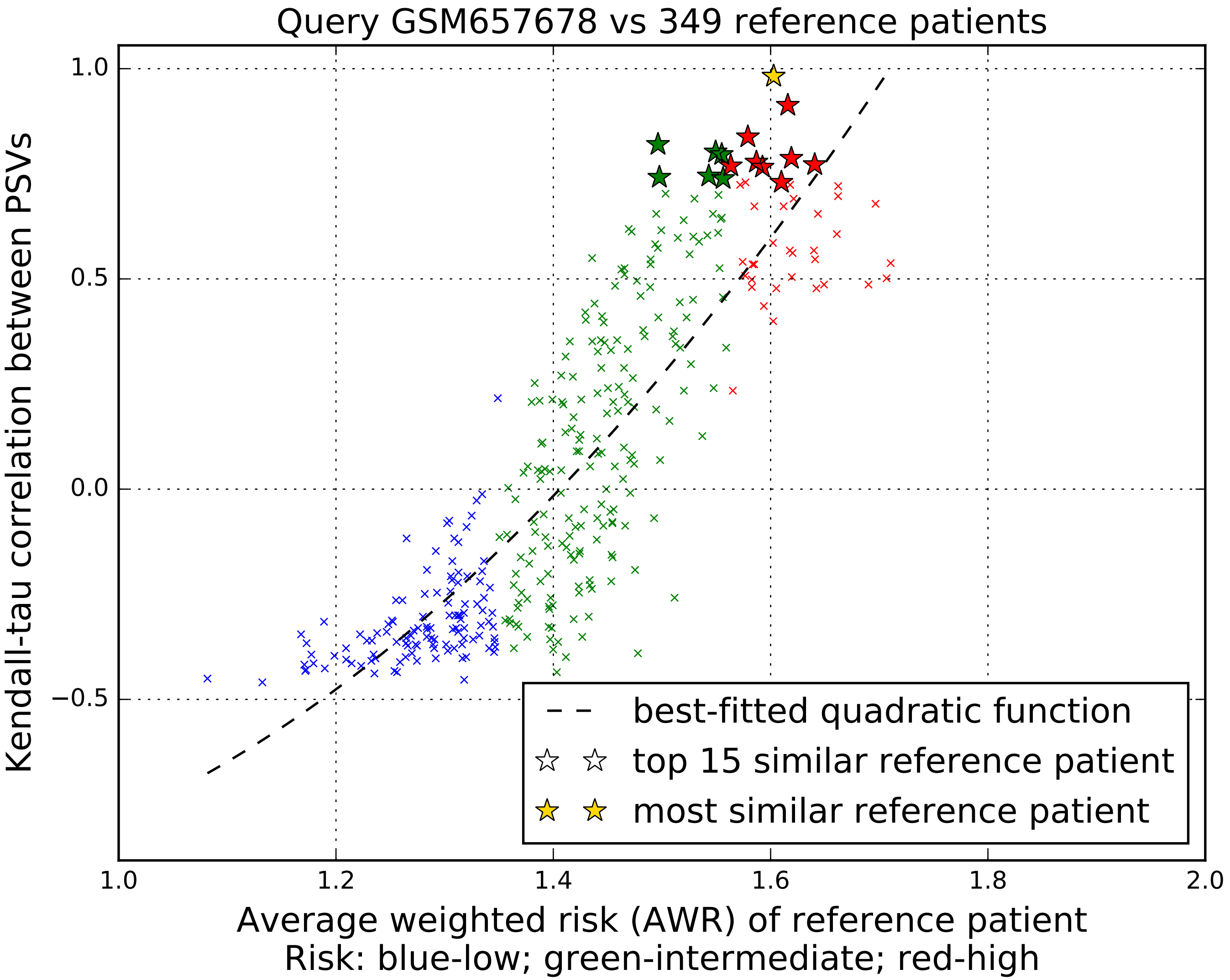

Query GSM657678 vs 349 reference patients
Kendall-tau correlation between PSVs
1.0
0.5
0.0
−0.5
1.0
1.2
1.4
1.6
1.8
2.0
best-fitted quadratic function
top 15 similar reference patient
most similar reference patient
Average weighted risk (AWR) of reference patient
Risk: blue-low; green-intermediate; red-high

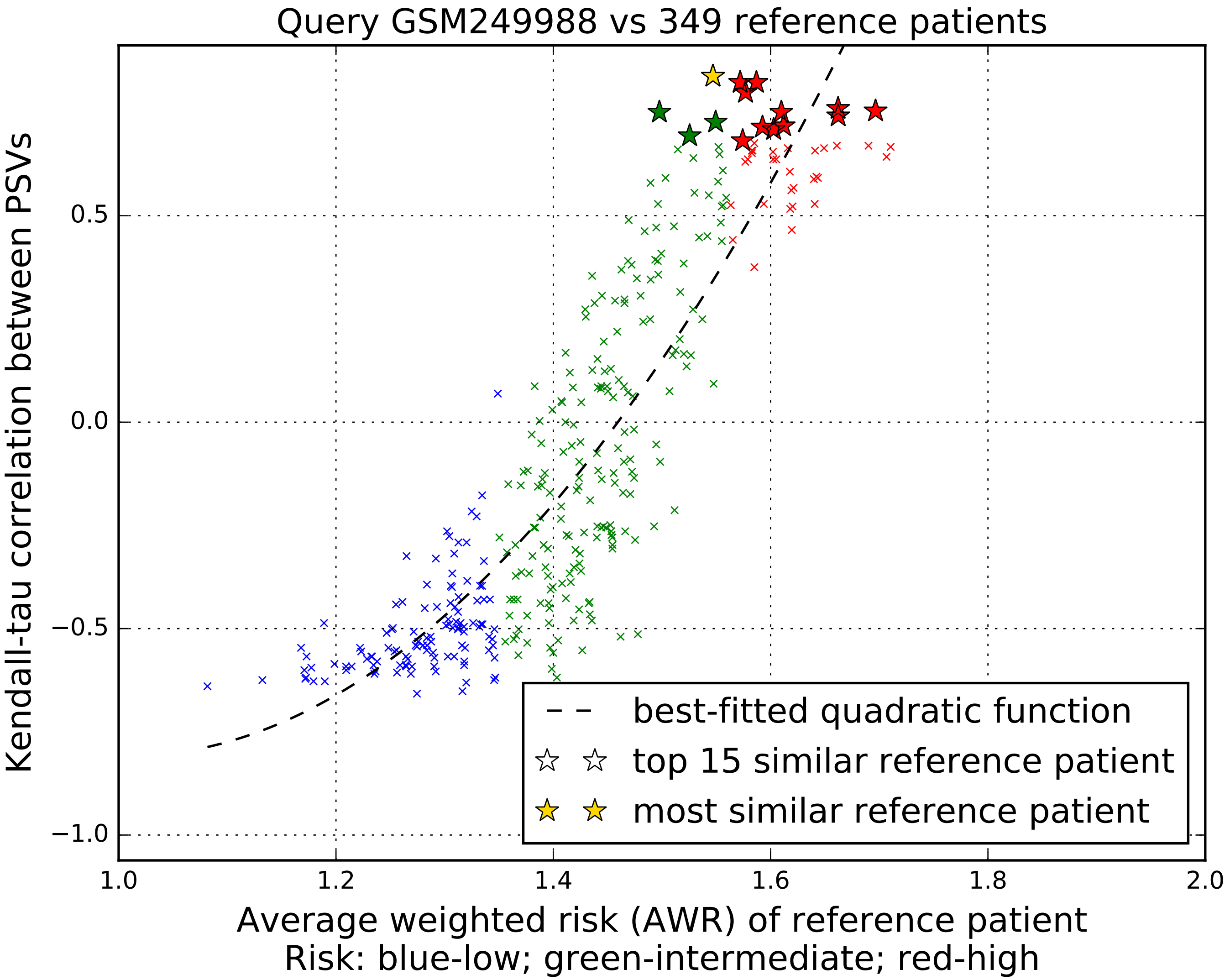

Query GSM249988 vs 349 reference patients
Kendall-tau correlation between PSVs
0.5
0.0
−0.5
−1.0
1.0
1.2
1.4
1.6
1.8
2.0
best-fitted quadratic function
top 15 similar reference patient
most similar reference patient
Average weighted risk (AWR) of reference patient
Risk: blue-low; green-intermediate; red-high

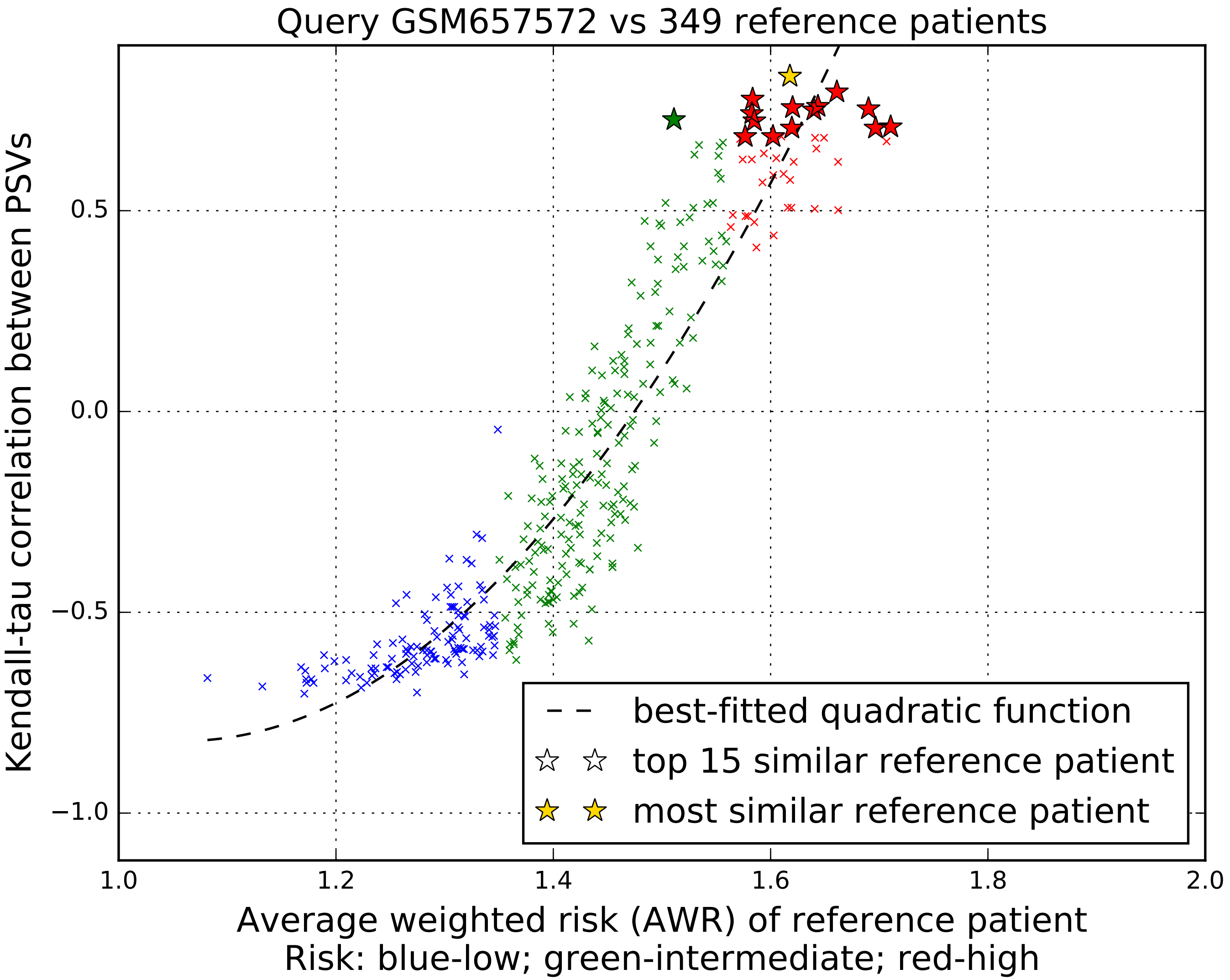

Query GSM657572 vs 349 reference patients
Kendall-tau correlation between PSVs
0.5
0.0
−0.5
−1.0
1.0
1.2
1.4
1.6
1.8
2.0
best-fitted quadratic function
top 15 similar reference patient
most similar reference patient
Average weighted risk (AWR) of reference patient
Risk: blue-low; green-intermediate; red-high

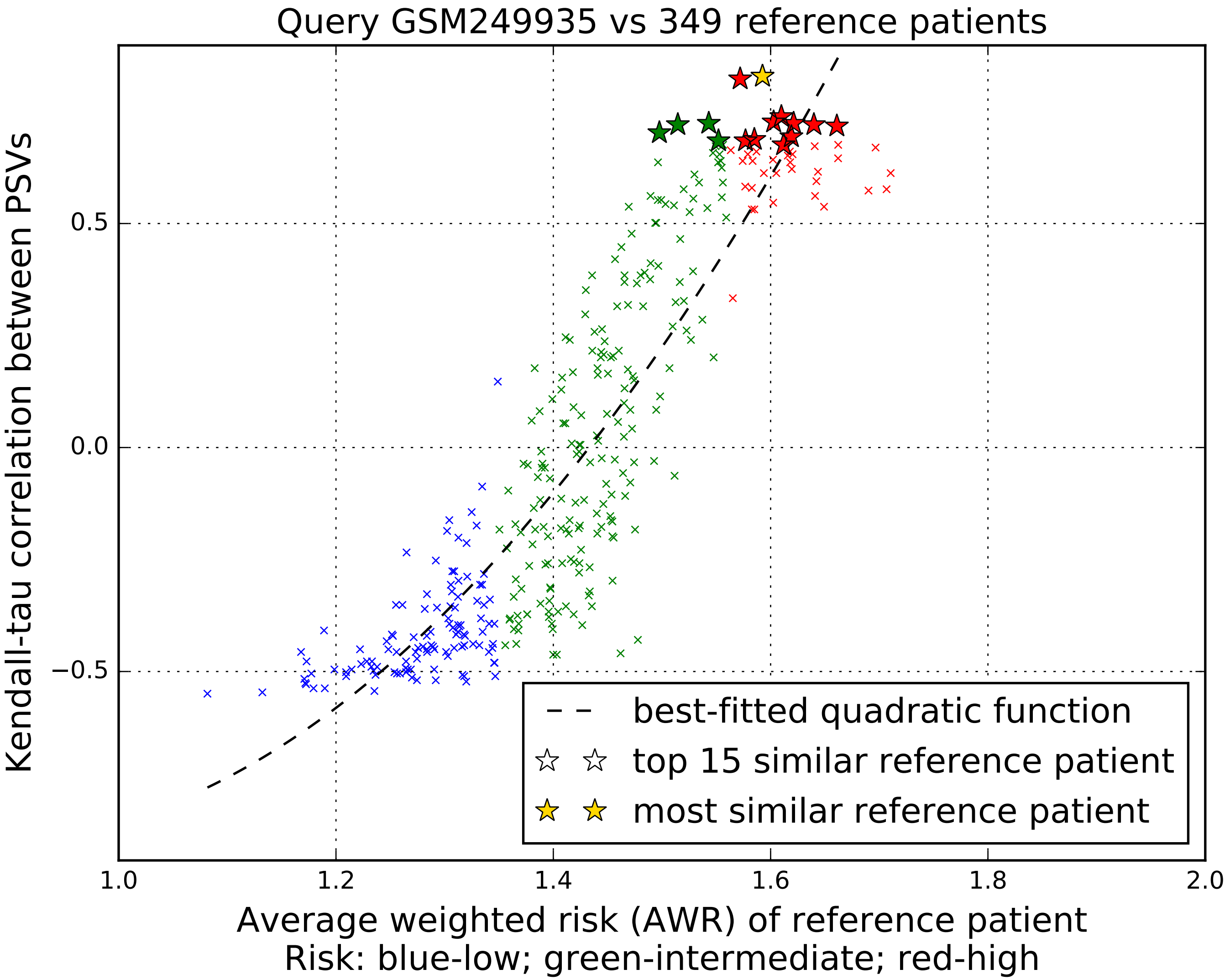
Query GSM249935 vs 349 reference patients
Kendall-tau correlation between PSVs
0.5
0.0
−0.5
1.0
1.2
1.4
1.6
1.8
2.0
best-fitted quadratic function
top 15 similar reference patient
most similar reference patient
Average weighted risk (AWR) of reference patient
Risk: blue-low; green-intermediate; red-high

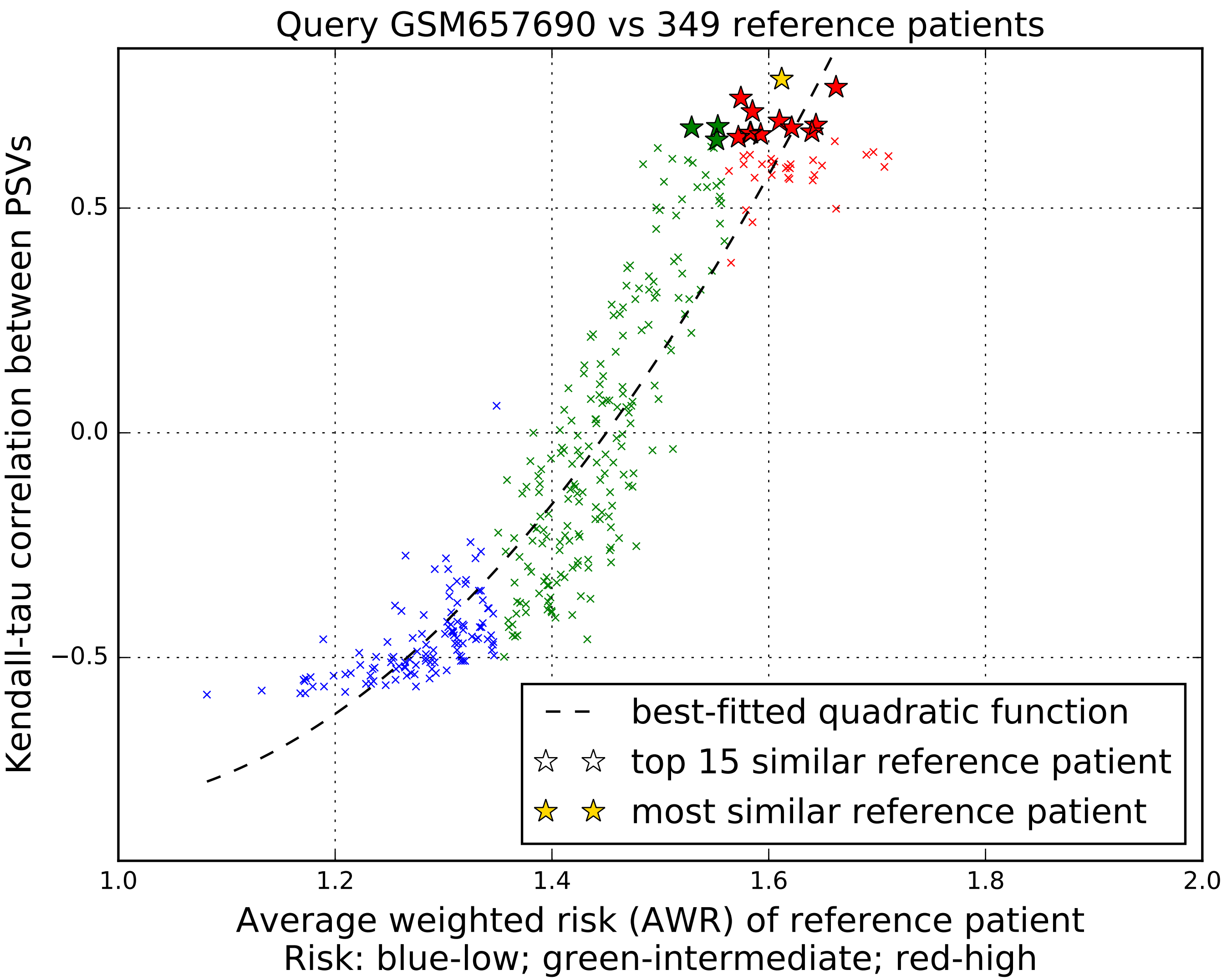

Query GSM657690 vs 349 reference patients
Kendall-tau correlation between PSVs
0.5
0.0
−0.5
1.0
1.2
1.4
1.6
1.8
2.0
best-fitted quadratic function
top 15 similar reference patient
most similar reference patient
Average weighted risk (AWR) of reference patient
Risk: blue-low; green-intermediate; red-high

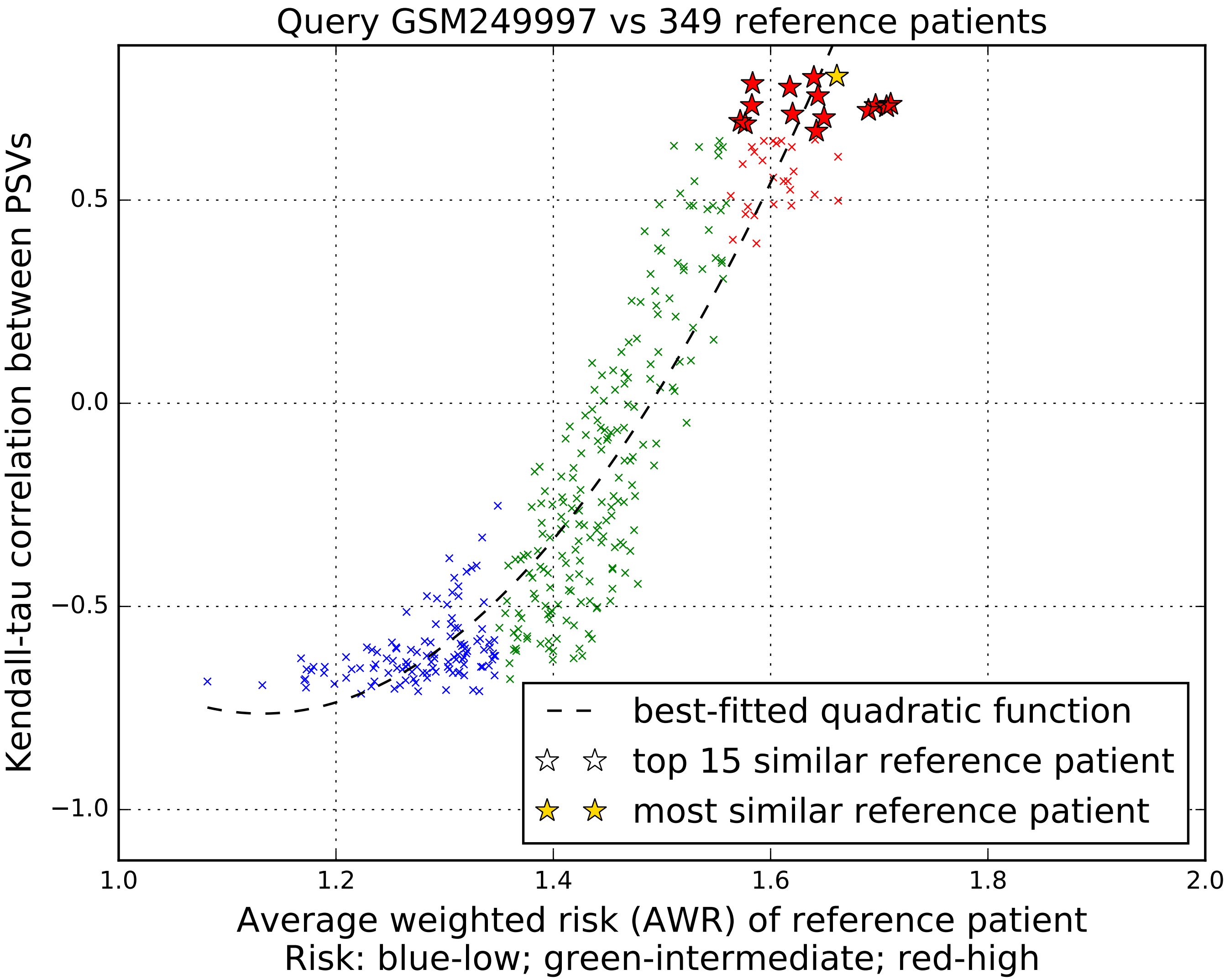

Query GSM249997 vs 349 reference patients
Kendall-tau correlation between PSVs
0.5
0.0
−0.5
−1.0
1.0
1.2
1.4
1.6
1.8
2.0
best-fitted quadratic function
top 15 similar reference patient
most similar reference patient
Average weighted risk (AWR) of reference patient
Risk: blue-low; green-intermediate; red-high

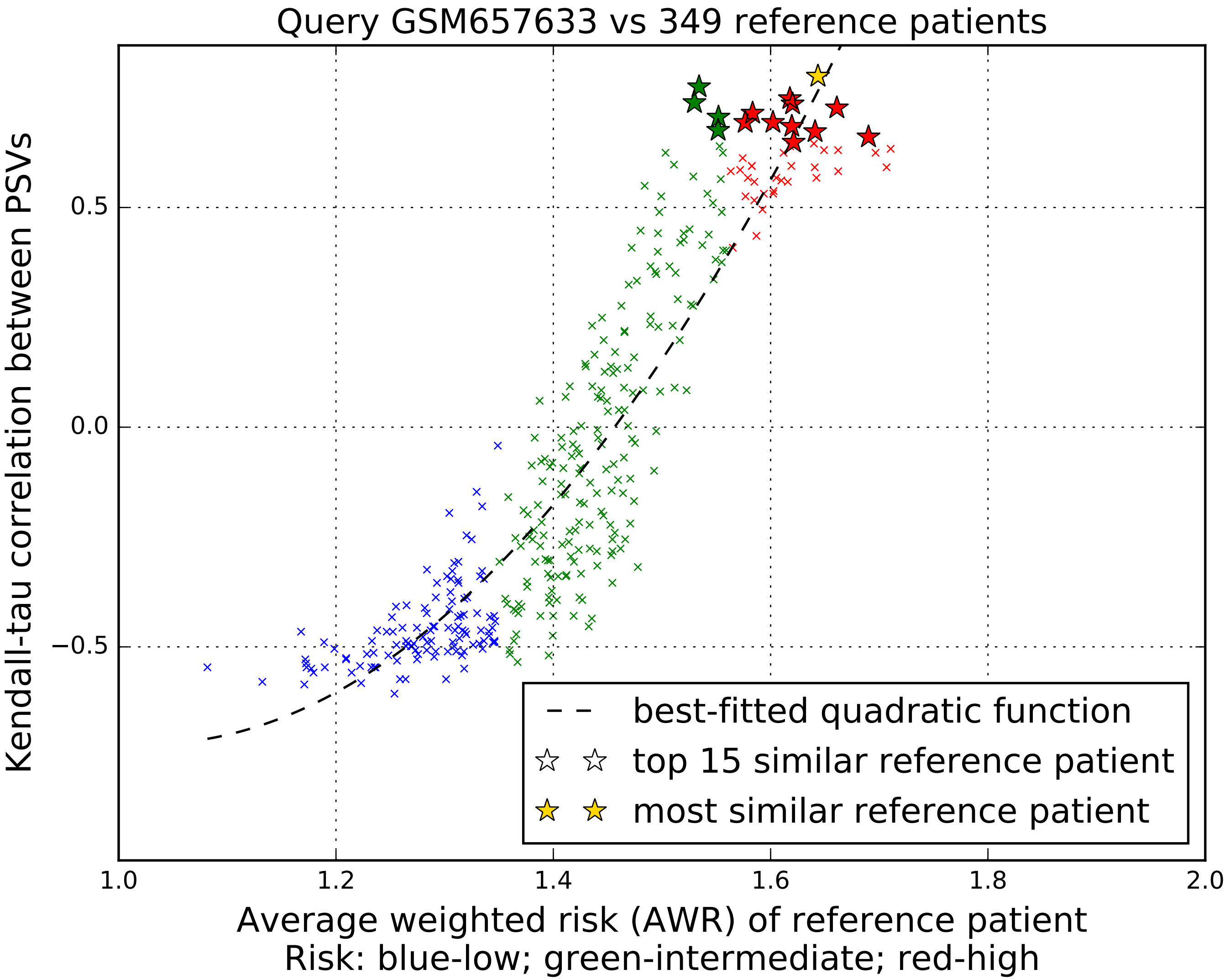

Query GSM657633 vs 349 reference patients
Kendall-tau correlation between PSVs
0.5
0.0
−0.5
1.0
1.2
1.4
1.6
1.8
2.0
best-fitted quadratic function
top 15 similar reference patient
most similar reference patient
Average weighted risk (AWR) of reference patient
Risk: blue-low; green-intermediate; red-high

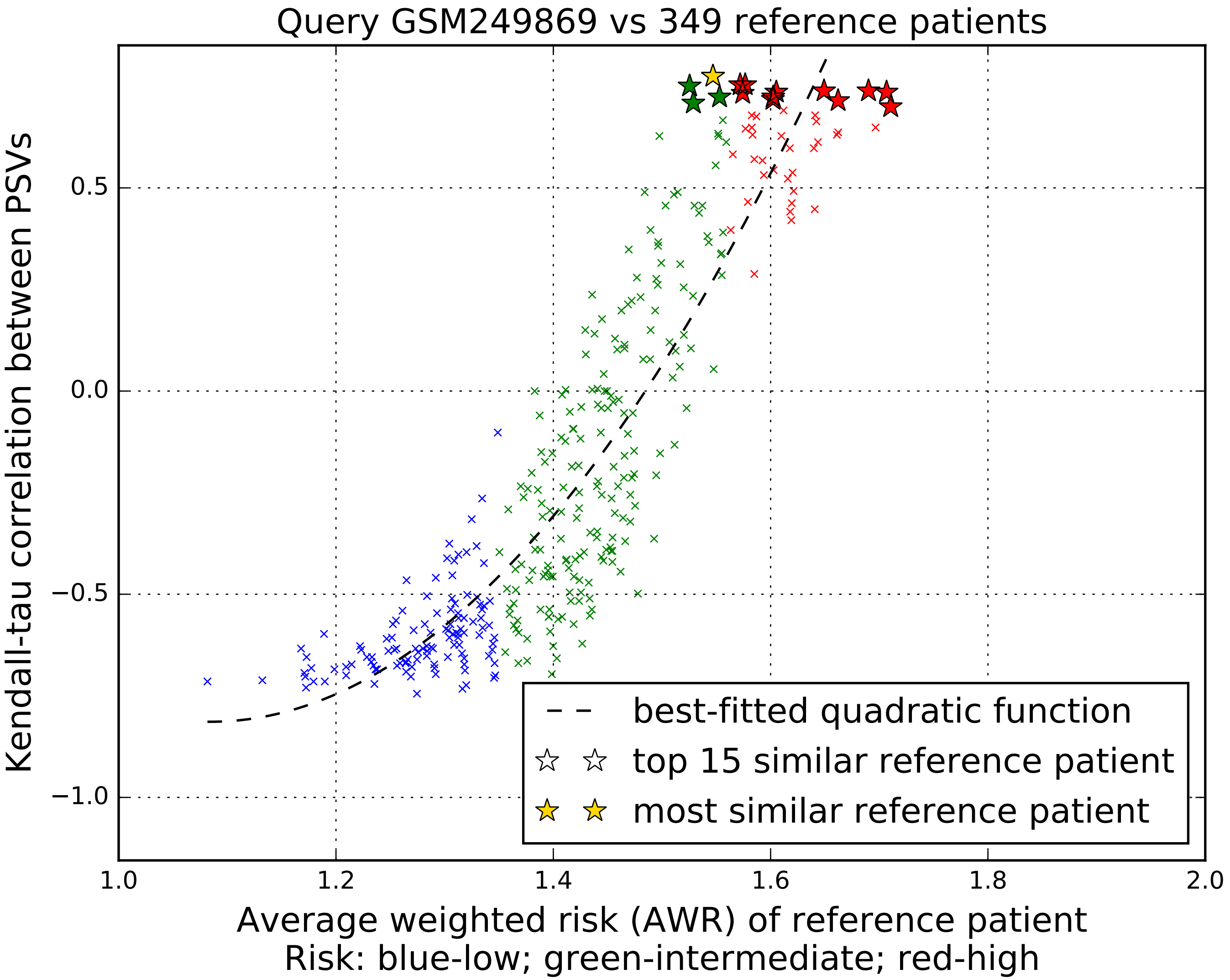
Query GSM249869 vs 349 reference patients
Kendall-tau correlation between PSVs
0.5
0.0
−0.5
−1.0
1.0
1.2
1.4
1.6
1.8
2.0
best-fitted quadratic function
top 15 similar reference patient
most similar reference patient
Average weighted risk (AWR) of reference patient
Risk: blue-low; green-intermediate; red-high

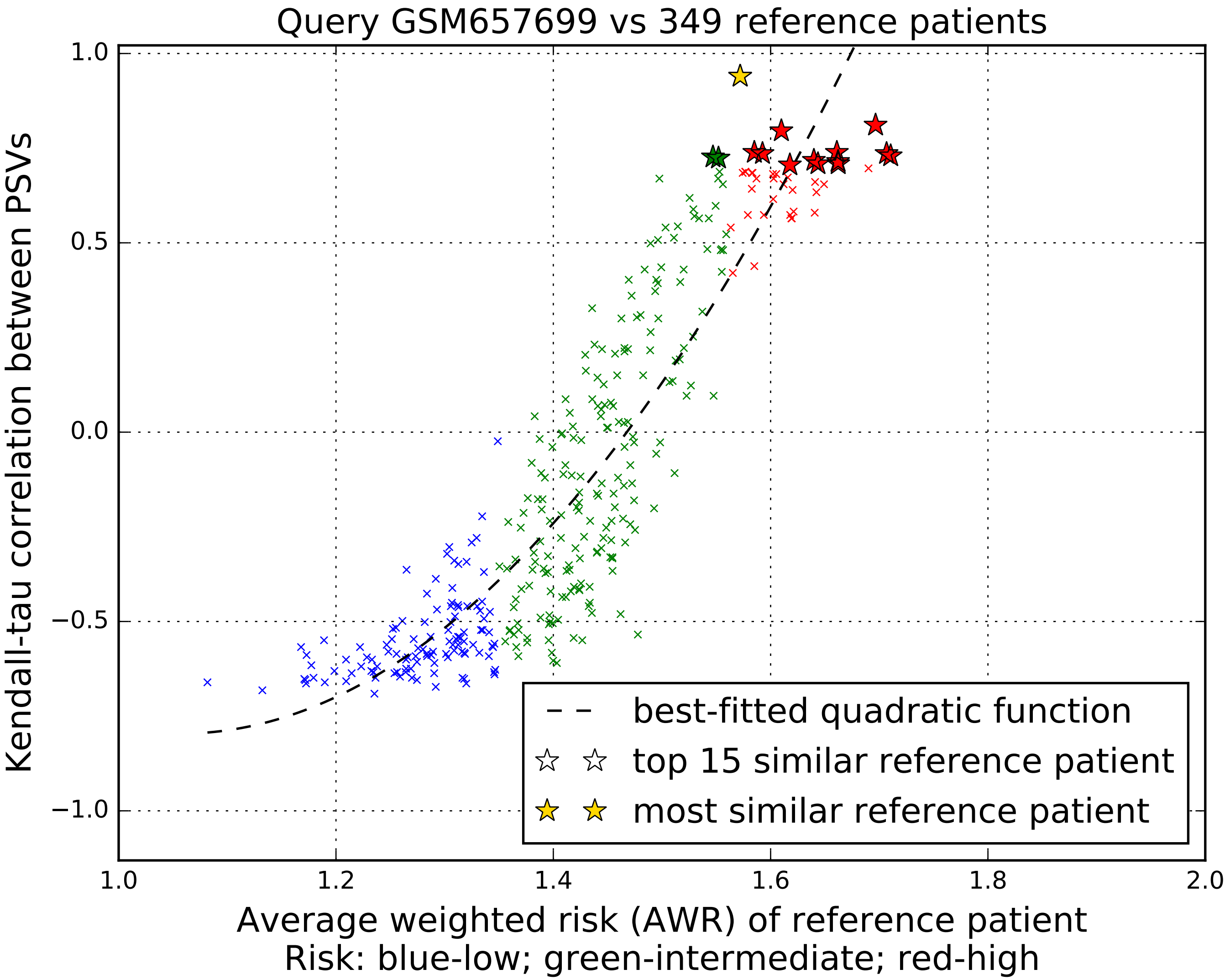

Query GSM657699 vs 349 reference patients
Kendall-tau correlation between PSVs
1.0
0.5
0.0
−0.5
−1.0
1.0
1.2
1.4
1.6
1.8
2.0
best-fitted quadratic function
top 15 similar reference patient
most similar reference patient
Average weighted risk (AWR) of reference patient
Risk: blue-low; green-intermediate; red-high

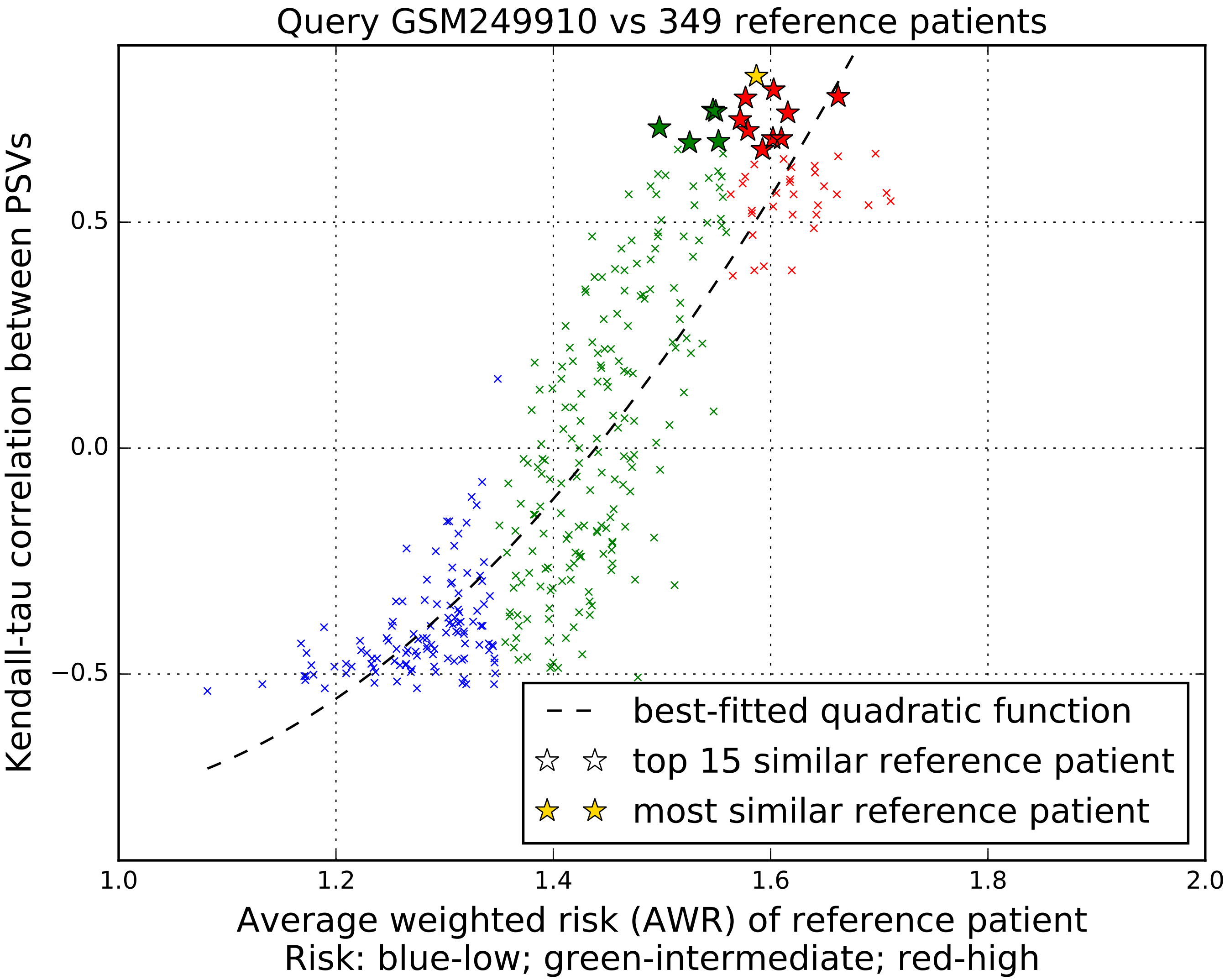

Query GSM249910 vs 349 reference patients
Kendall-tau correlation between PSVs
0.5
0.0
−0.5
1.0
1.2
1.4
1.6
1.8
2.0
best-fitted quadratic function
top 15 similar reference patient
most similar reference patient
Average weighted risk (AWR) of reference patient
Risk: blue-low; green-intermediate; red-high

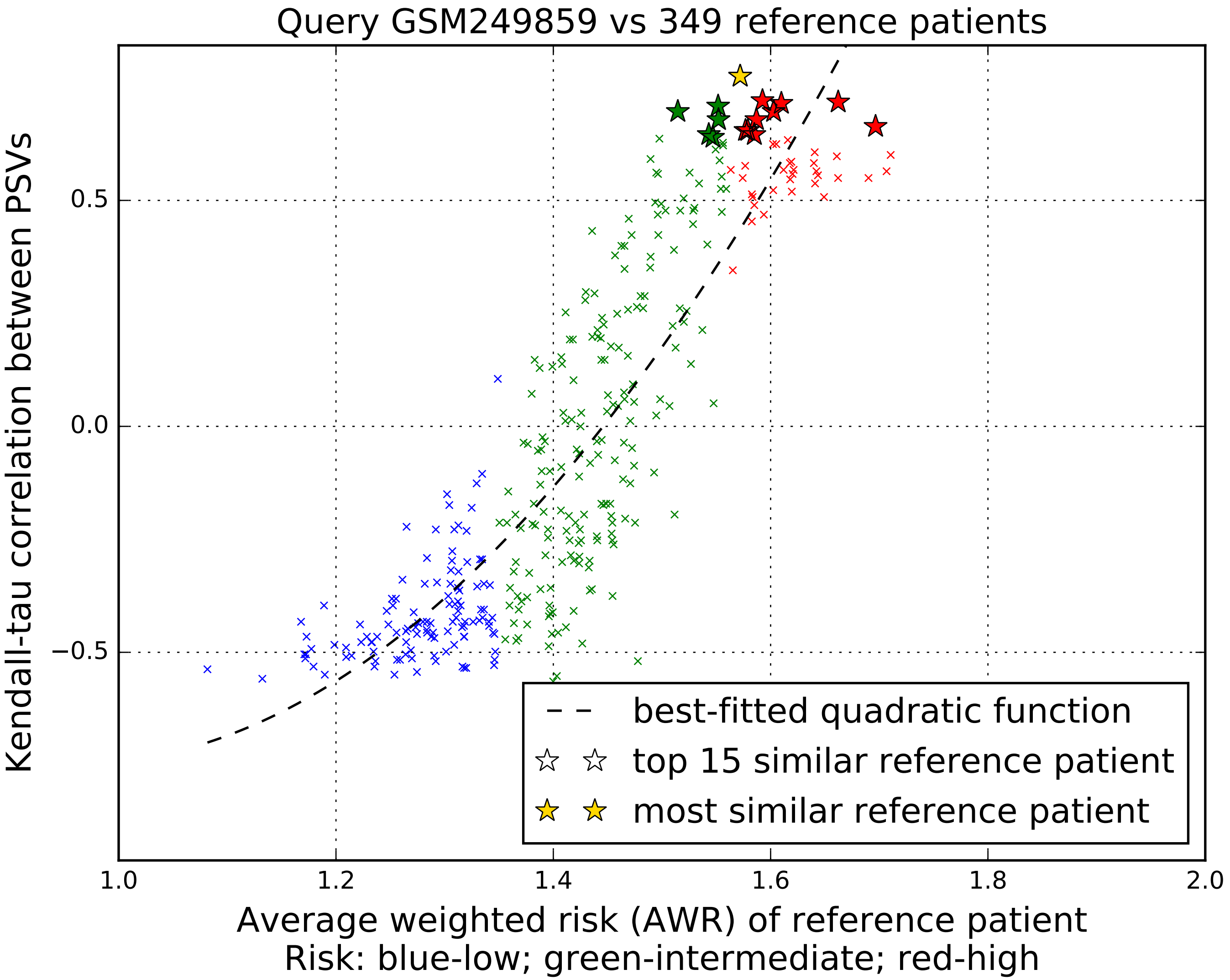

Query GSM249859 vs 349 reference patients
Kendall-tau correlation between PSVs
0.5
0.0
−0.5
1.0
1.2
1.4
1.6
1.8
2.0
best-fitted quadratic function
top 15 similar reference patient
most similar reference patient
Average weighted risk (AWR) of reference patient
Risk: blue-low; green-intermediate; red-high

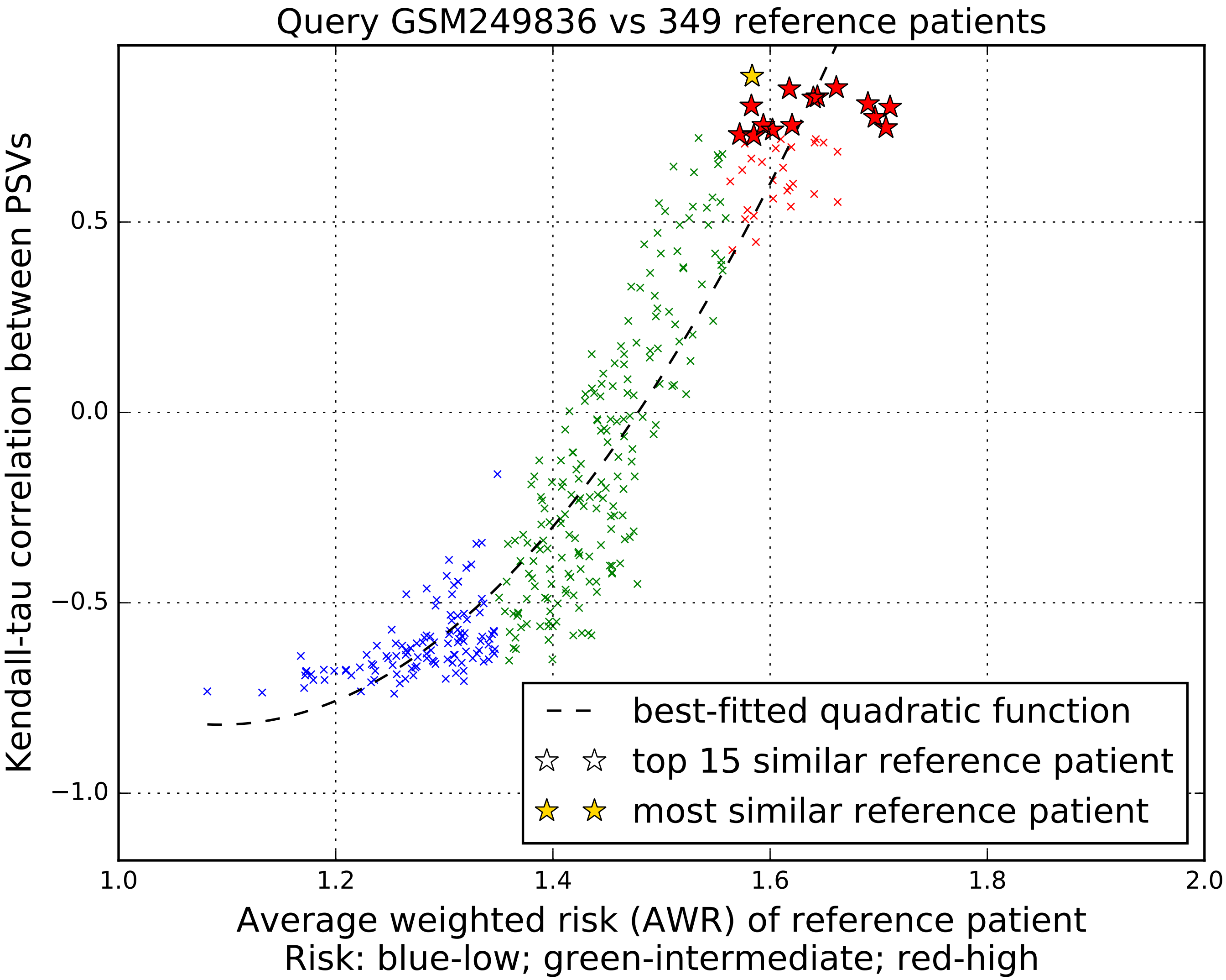

Query GSM249836 vs 349 reference patients
Kendall-tau correlation between PSVs
0.5
0.0
−0.5
−1.0
1.0
1.2
1.4
1.6
1.8
2.0
best-fitted quadratic function
top 15 similar reference patient
most similar reference patient
Average weighted risk (AWR) of reference patient
Risk: blue-low; green-intermediate; red-high

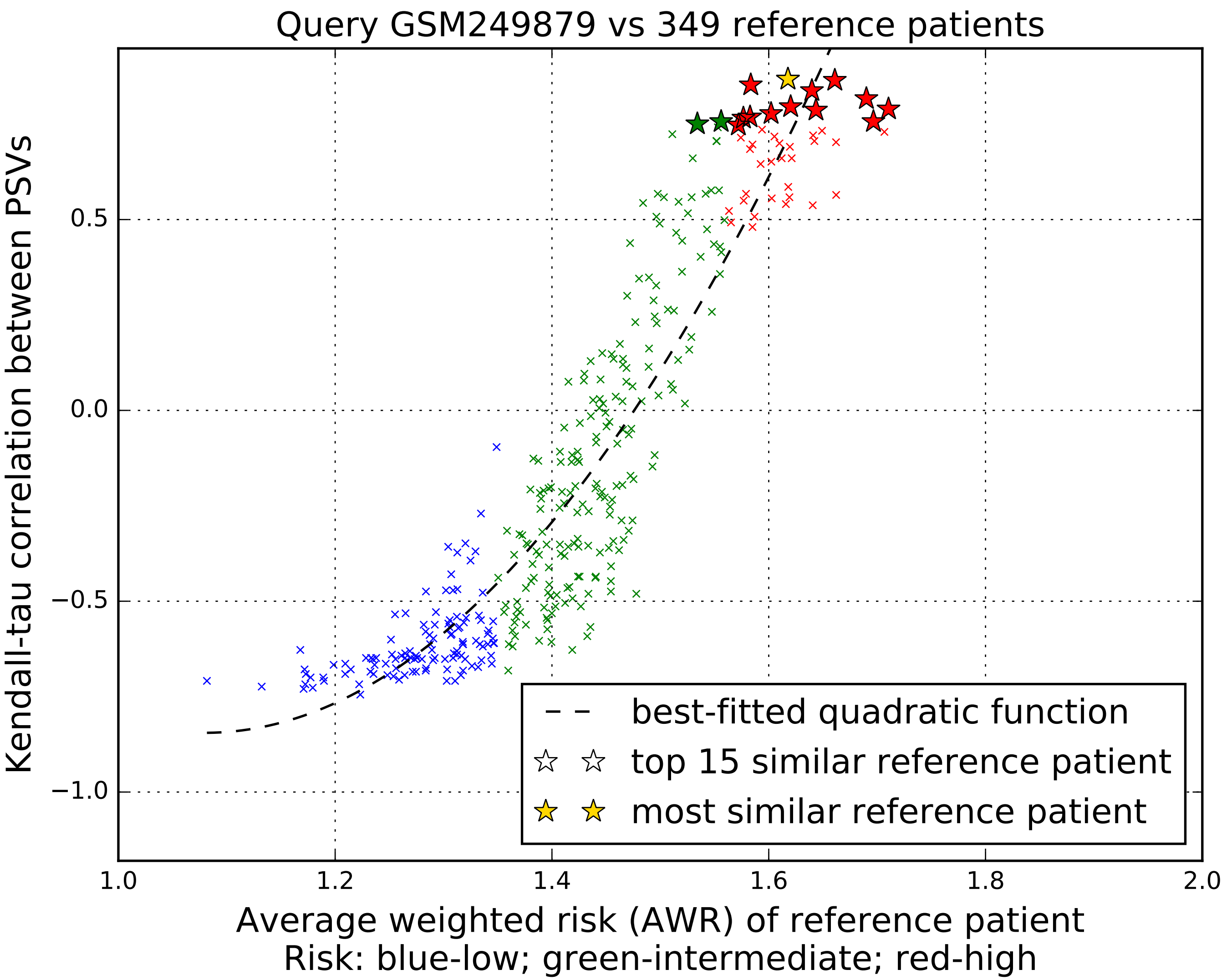

Query GSM249879 vs 349 reference patients
Kendall-tau correlation between PSVs
0.5
0.0
−0.5
−1.0
1.0
1.2
1.4
1.6
1.8
2.0
best-fitted quadratic function
top 15 similar reference patient
most similar reference patient
Average weighted risk (AWR) of reference patient
Risk: blue-low; green-intermediate; red-high

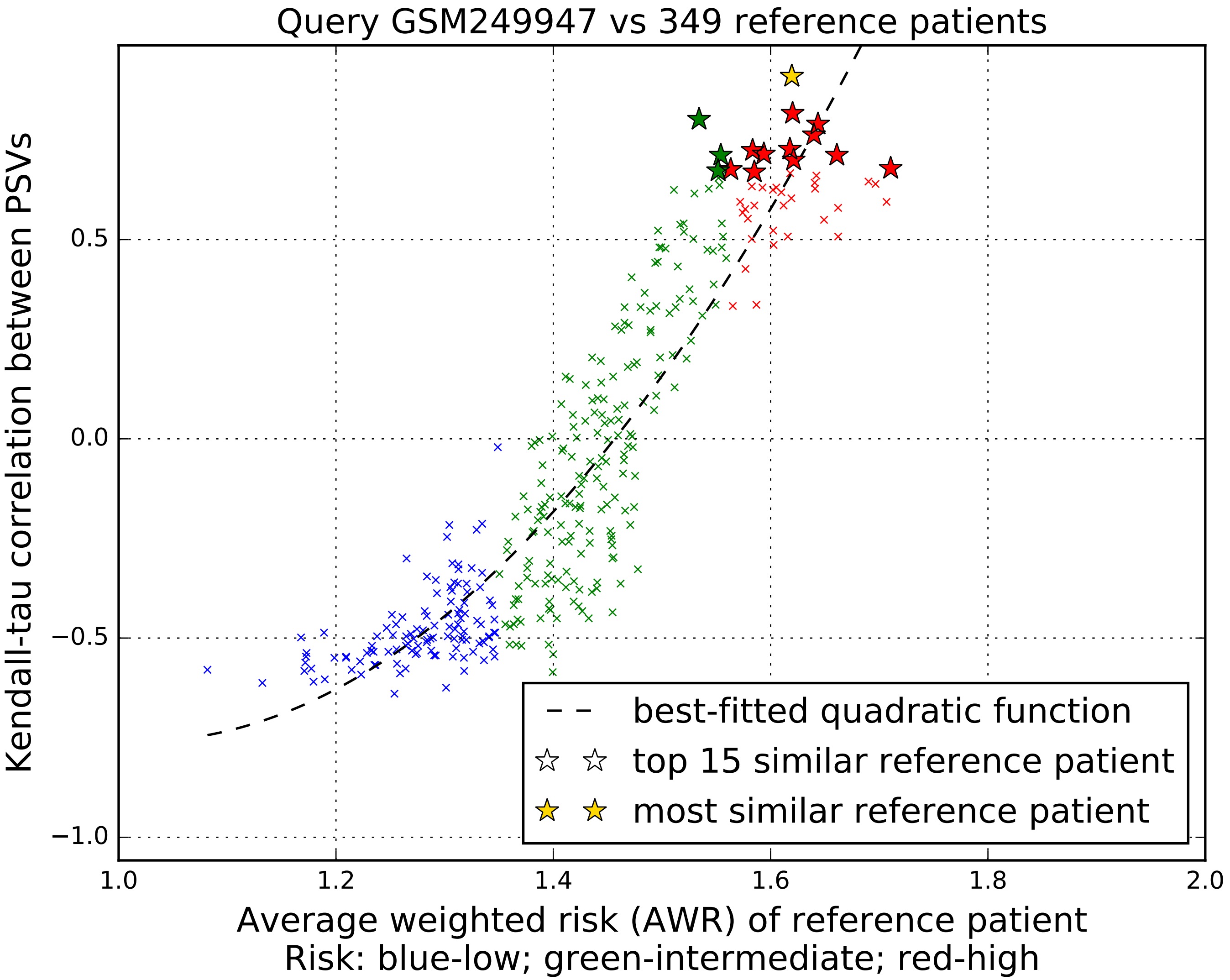
Query GSM249947 vs 349 reference patients
Kendall-tau correlation between PSVs
Average weighted risk (AWR) of reference patient
Risk: blue-low; green-intermediate; red-high
best-fitted quadratic function
top 15 similar reference patient
most similar reference patient
0.5
0.0
−0.5
−1.0
1.0
1.2
1.4
1.6
1.8
2.0

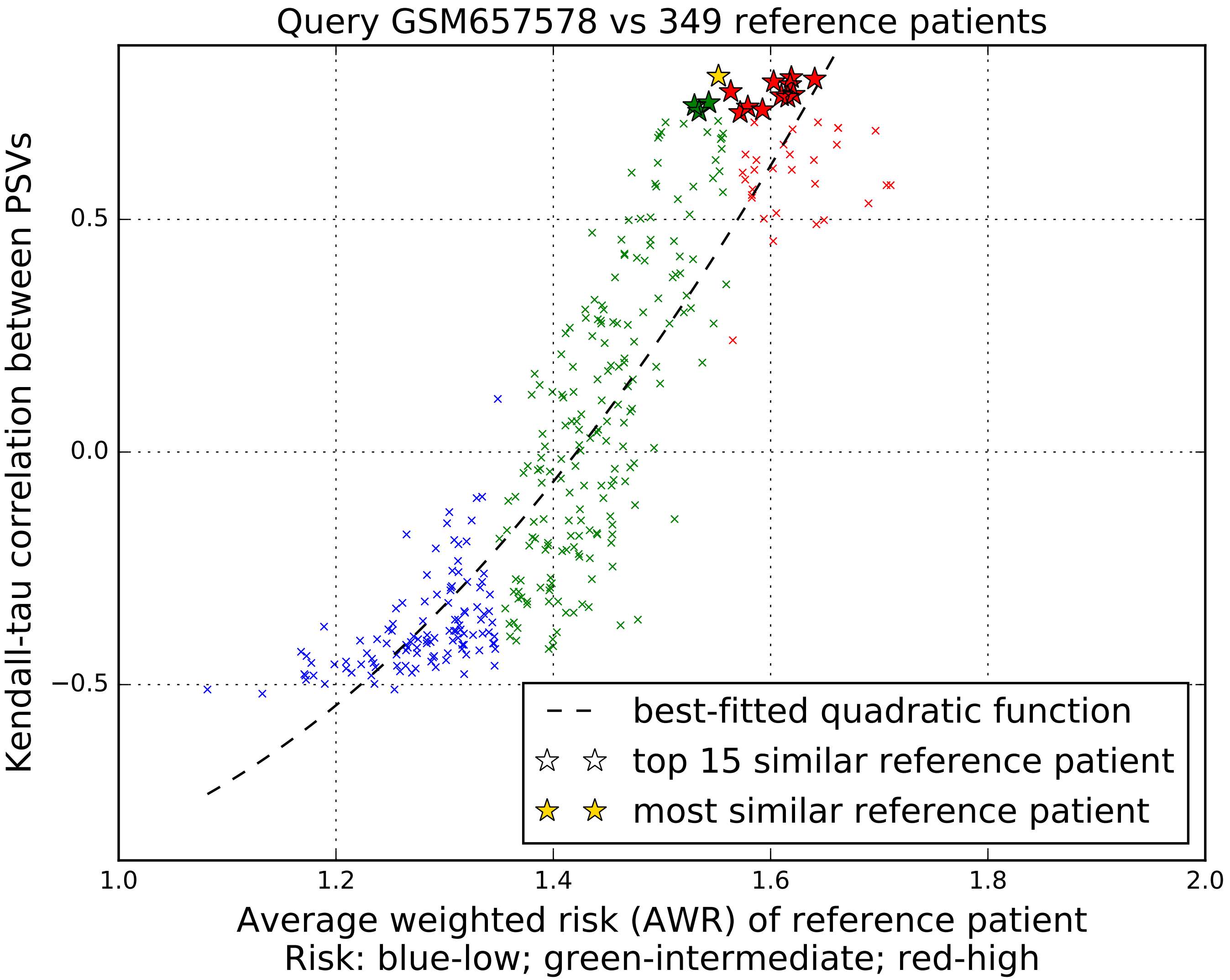

Query GSM657578 vs 349 reference patients
Kendall-tau correlation between PSVs
0.5
0.0
−0.5
1.0
1.2
1.4
1.6
1.8
2.0
best-fitted quadratic function
top 15 similar reference patient
most similar reference patient
Average weighted risk (AWR) of reference patient
Risk: blue-low; green-intermediate; red-high

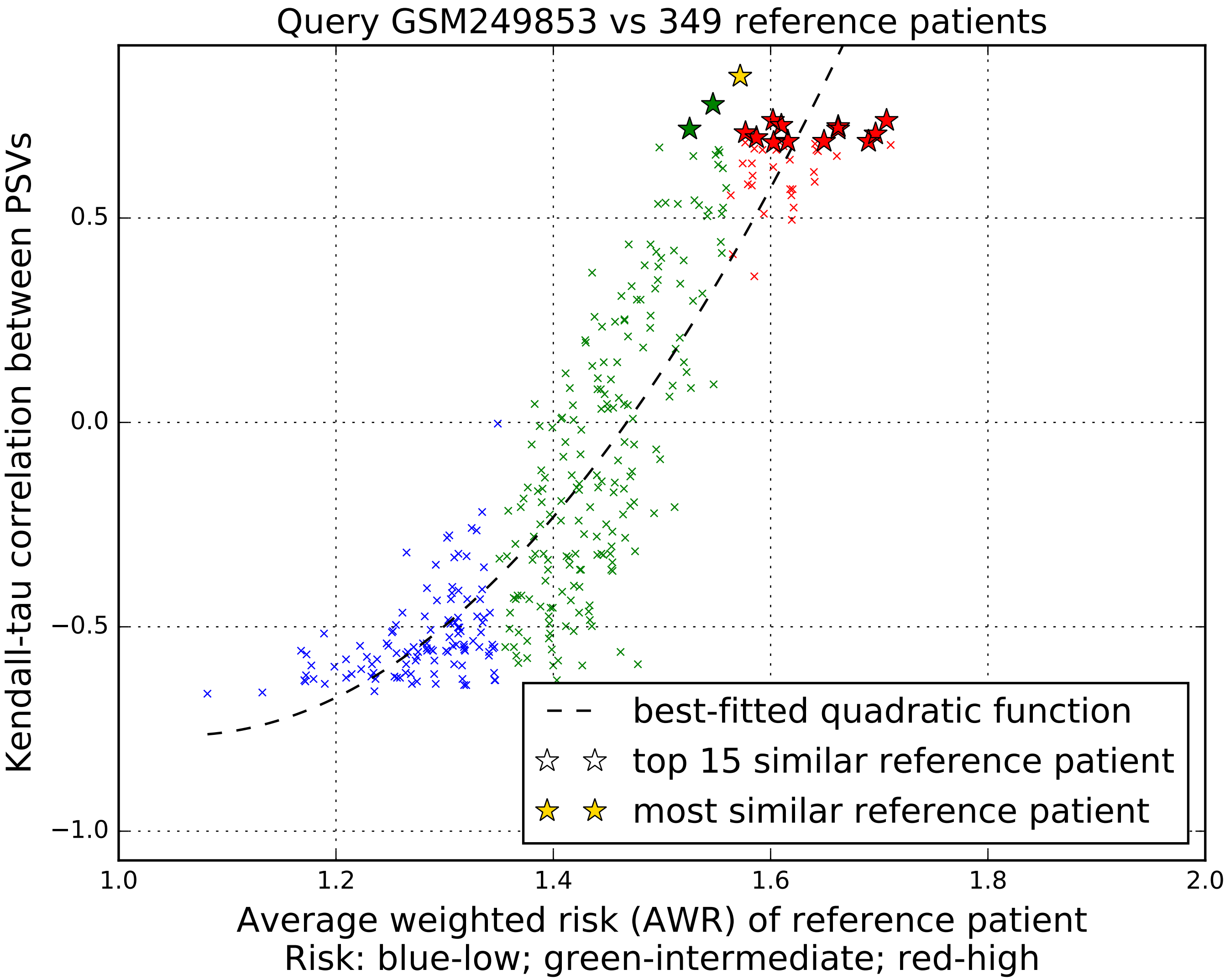
Query GSM249853 vs 349 reference patients
Kendall-tau correlation between PSVs
0.5
0.0
−0.5
−1.0
1.0
1.2
1.4
1.6
1.8
2.0
best-fitted quadratic function
top 15 similar reference patient
most similar reference patient
Average weighted risk (AWR) of reference patient
Risk: blue-low; green-intermediate; red-high

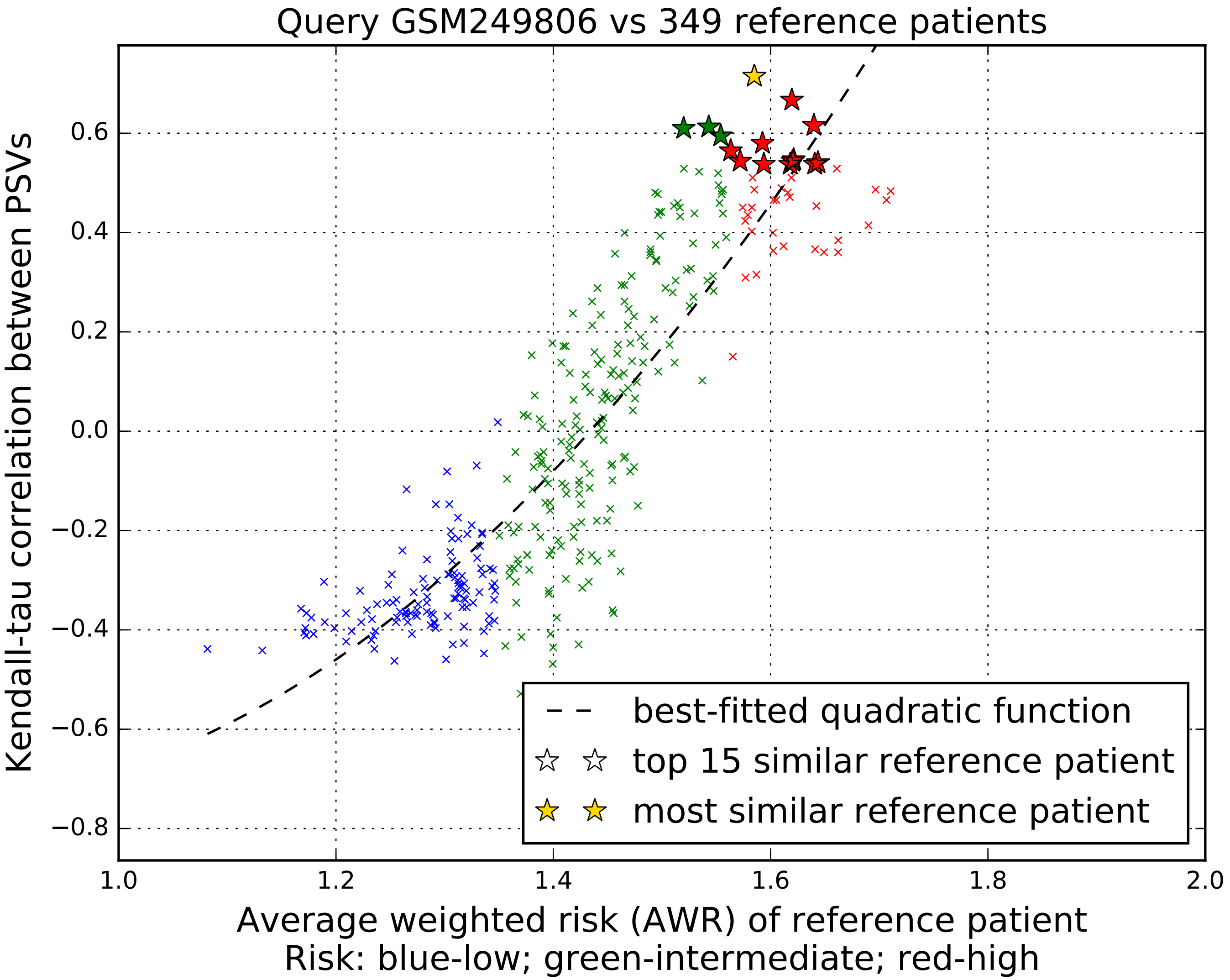

Query GSM249806 vs 349 reference patients
Kendall-tau correlation between PSVs
Average weighted risk (AWR) of reference patient
Risk: blue-low; green-intermediate; red-high
best-fitted quadratic function
top 15 similar reference patient
most similar reference patient
0.6
0.4
0.2
0.0
−0.2
−0.4
−0.6
−0.8
1.0
1.2
1.4
1.6
1.8
2.0

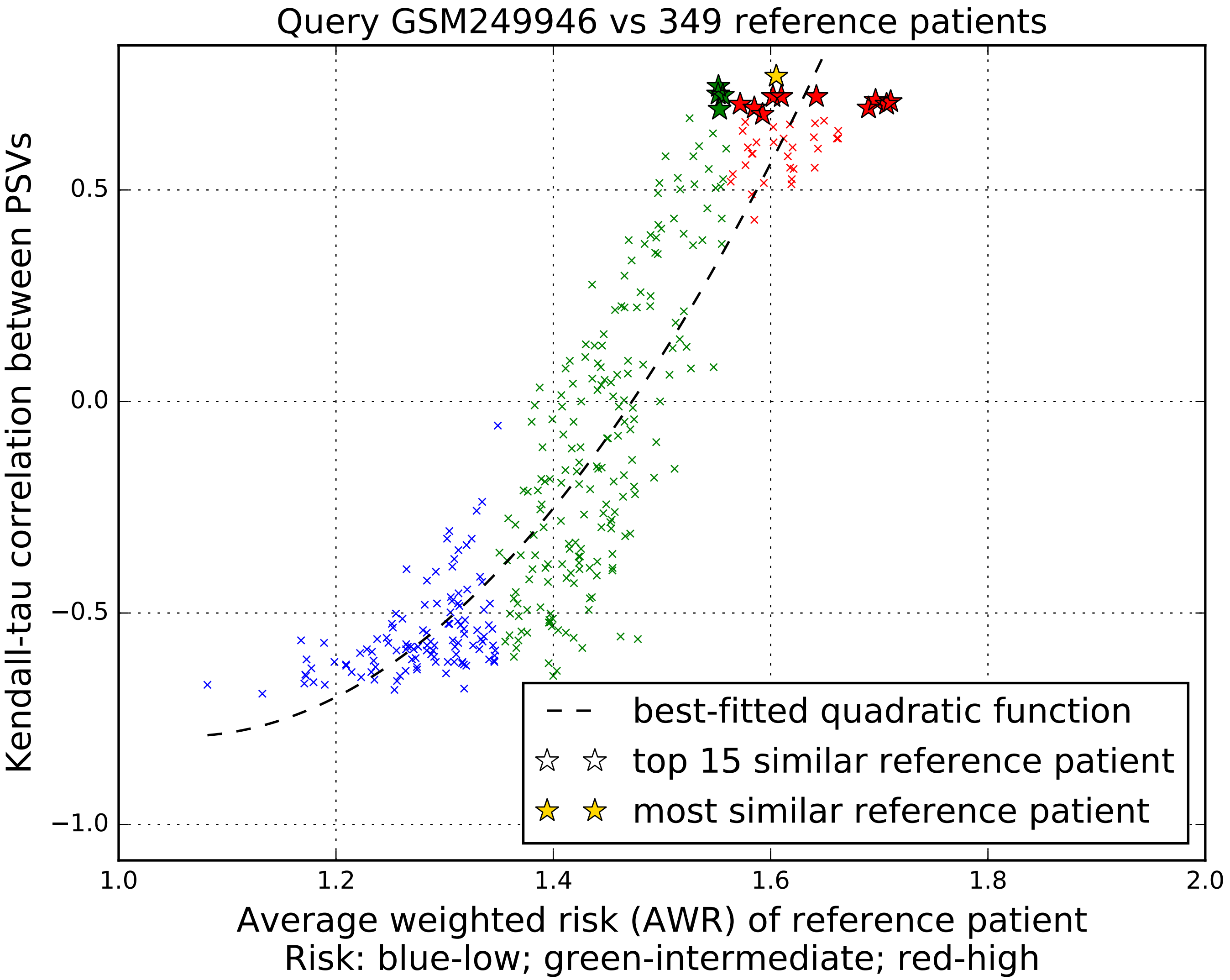
Query GSM249946 vs 349 reference patients
Kendall-tau correlation between PSVs
0.5
0.0
−0.5
−1.0
1.0
1.2
1.4
1.6
1.8
2.0
best-fitted quadratic function
top 15 similar reference patient
most similar reference patient
Average weighted risk (AWR) of reference patient
Risk: blue-low; green-intermediate; red-high

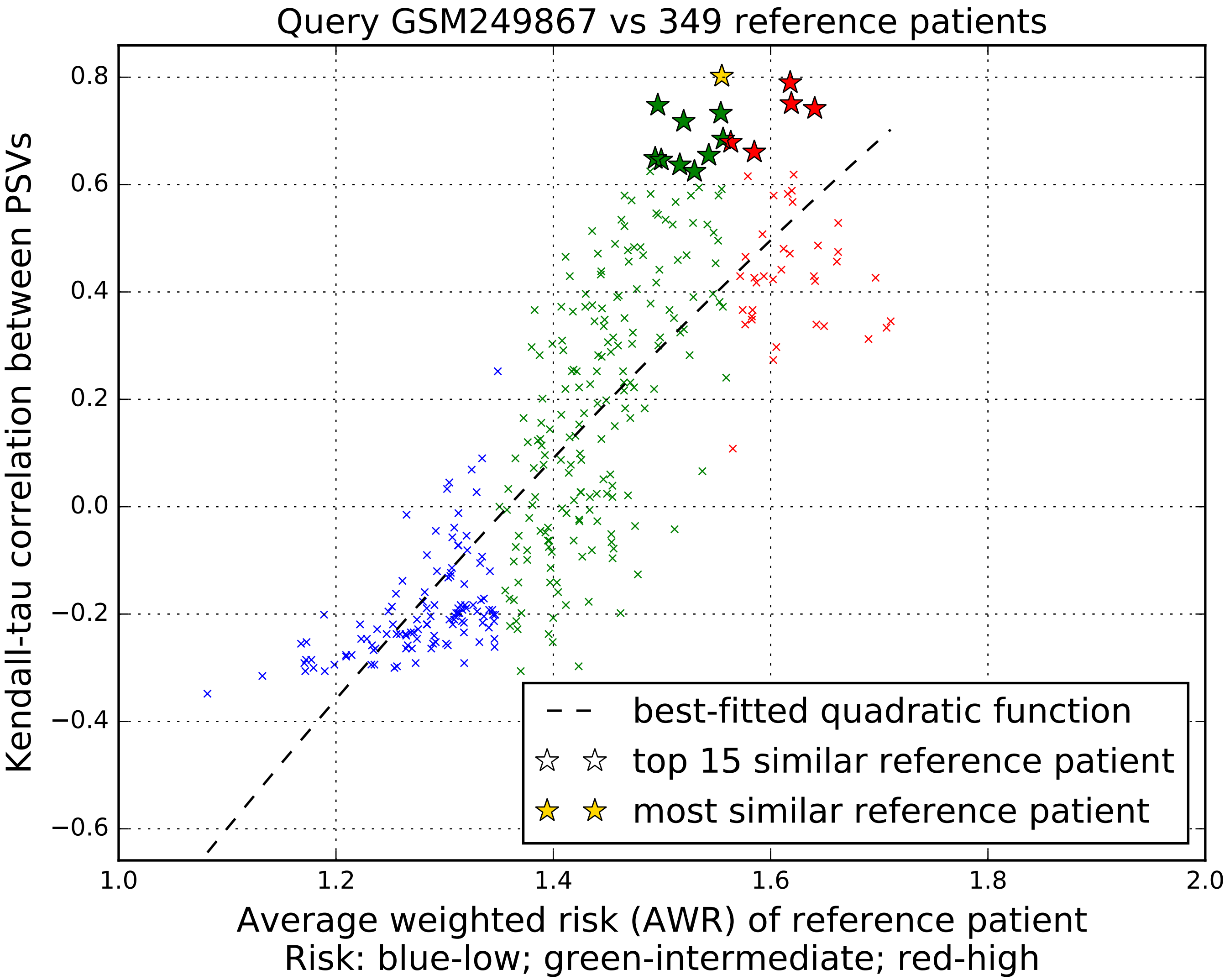
Query GSM249867 vs 349 reference patients
Kendall-tau correlation between PSVs
0.8
0.6
0.4
0.2
0.0
−0.2
−0.4
−0.6
1.0
1.2
1.4
1.6
1.8
2.0
best-fitted quadratic function
top 15 similar reference patient
most similar reference patient
Average weighted risk (AWR) of reference patient
Risk: blue-low; green-intermediate; red-high

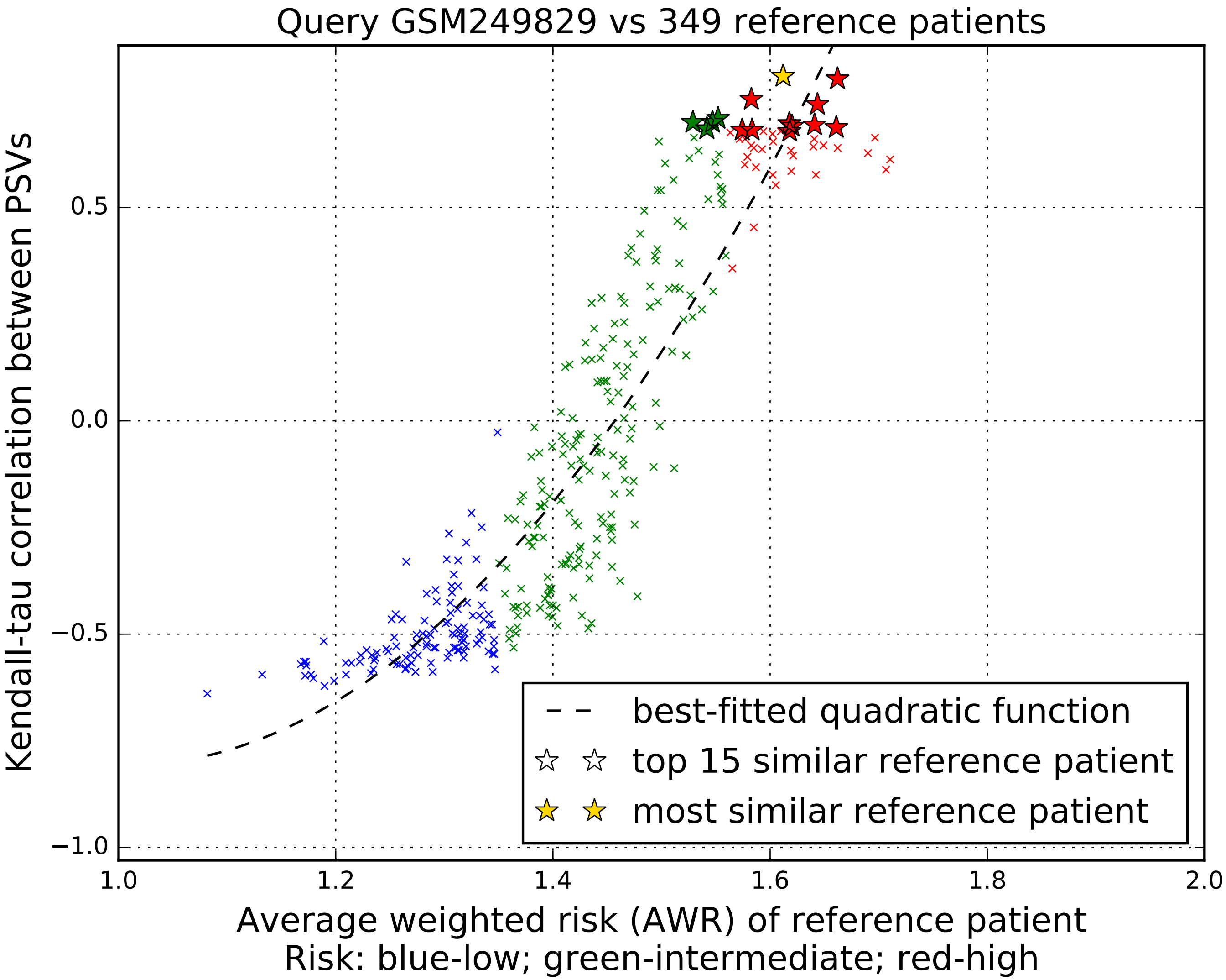

Query GSM249829 vs 349 reference patients
Kendall-tau correlation between PSVs
0.5
0.0
−0.5
−1.0
1.0
1.2
1.4
1.6
1.8
2.0
best-fitted quadratic function
top 15 similar reference patient
most similar reference patient
Average weighted risk (AWR) of reference patient
Risk: blue-low; green-intermediate; red-high

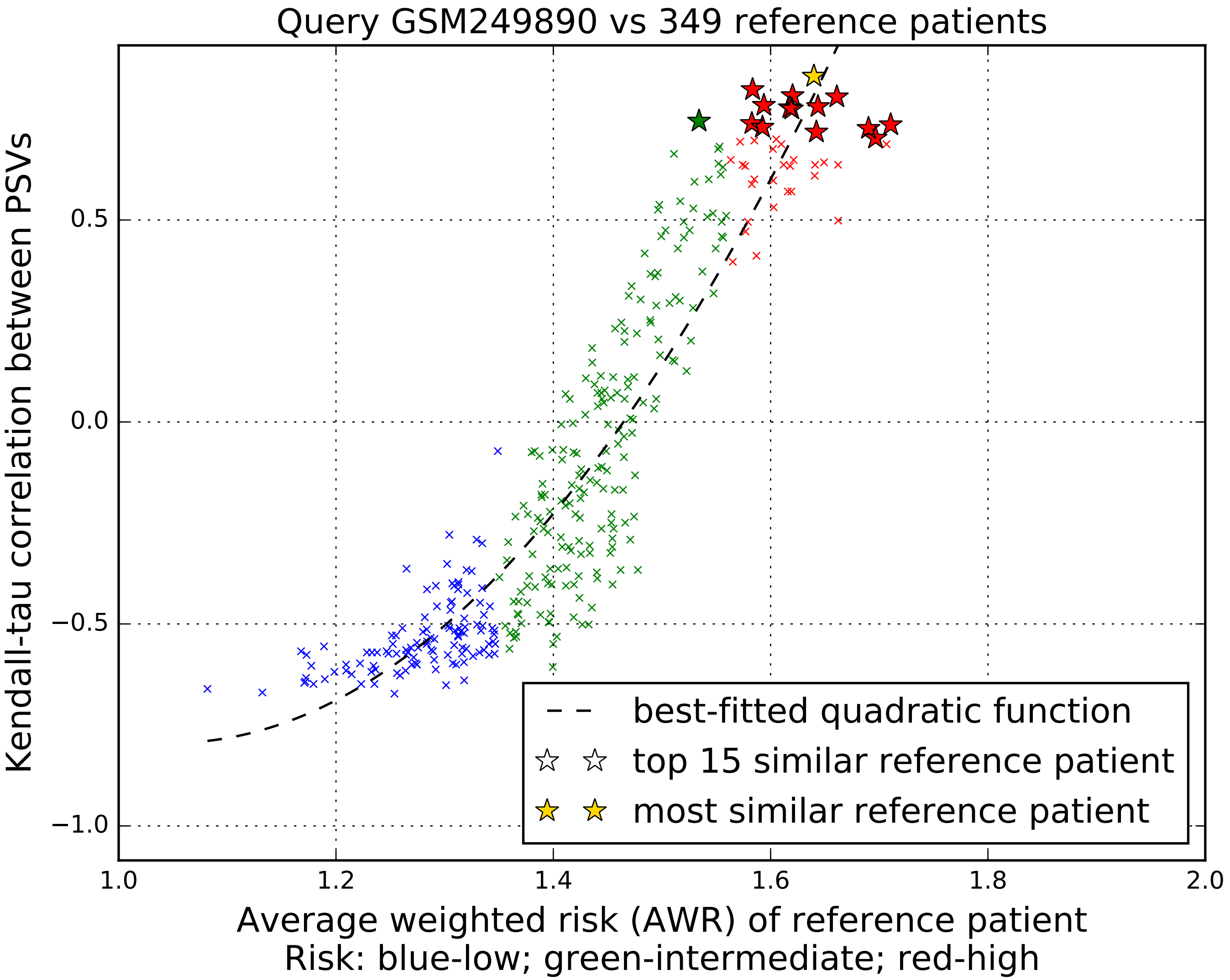

Query GSM249890 vs 349 reference patients
Kendall-tau correlation between PSVs
0.5
0.0
−0.5
−1.0
1.0
1.2
1.4
1.6
1.8
2.0
best-fitted quadratic function
top 15 similar reference patient
most similar reference patient
Average weighted risk (AWR) of reference patient
Risk: blue-low; green-intermediate; red-high

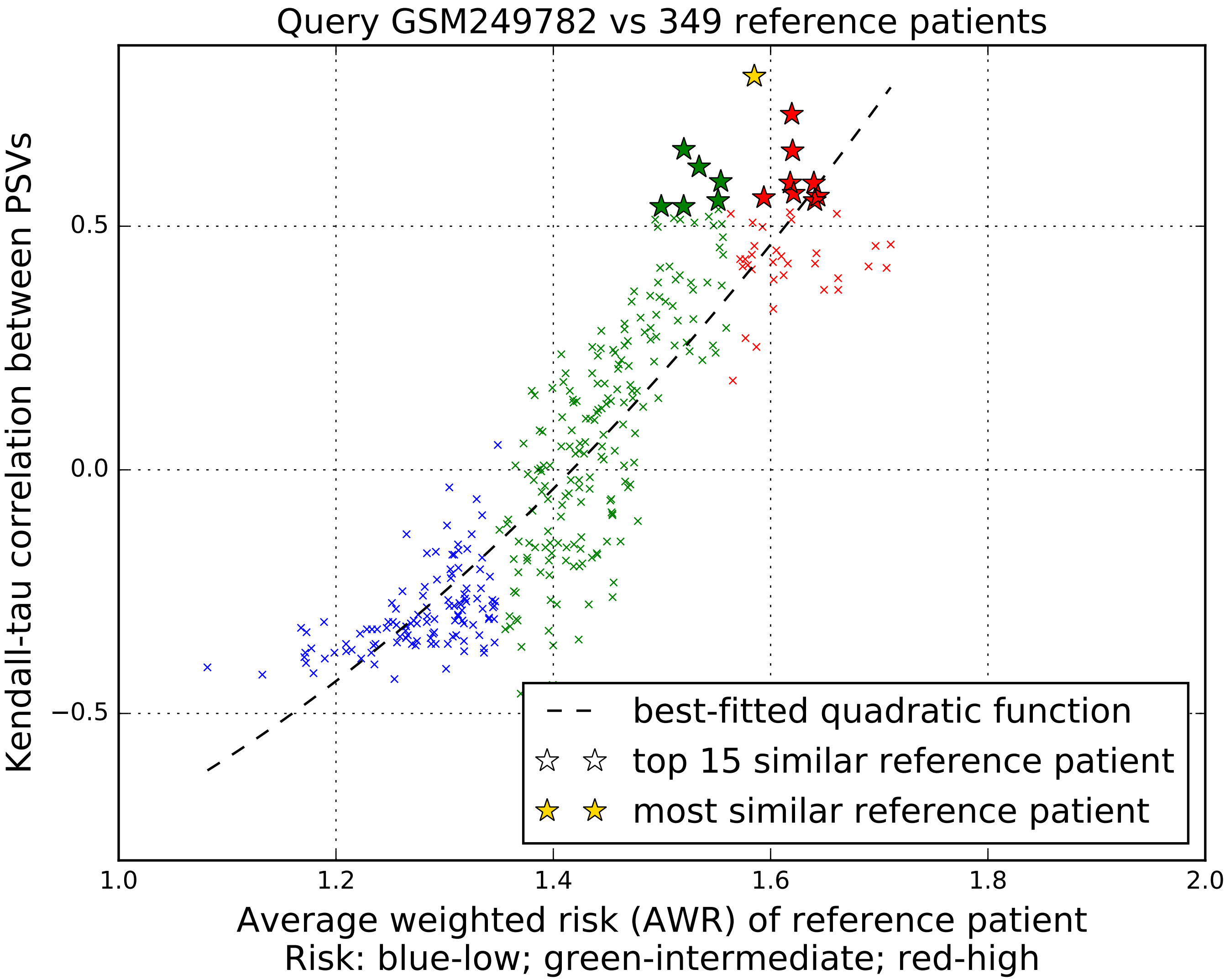
Query GSM249782 vs 349 reference patients
Kendall-tau correlation between PSVs
0.5
0.0
−0.5
1.0
1.2
1.4
1.6
1.8
2.0
Average weighted risk (AWR) of reference patient
Risk: blue-low; green-intermediate; red-high
best-fitted quadratic function
top 15 similar reference patient
most similar reference patient

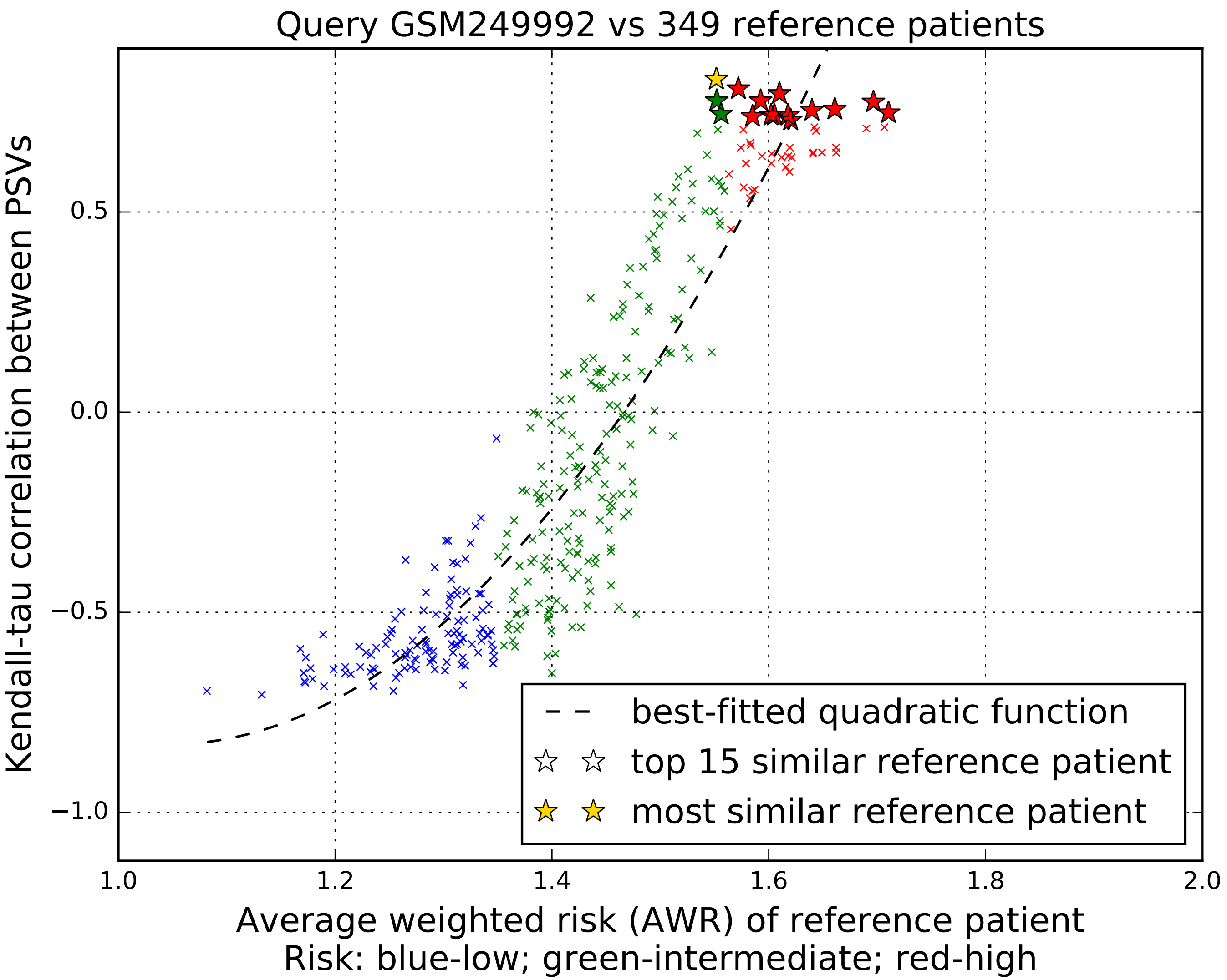
Query GSM249992 vs 349 reference patients
Kendall-tau correlation between PSVs
0.5
0.0
−0.5
−1.0
1.0
1.2
1.4
1.6
1.8
2.0
best-fitted quadratic function
top 15 similar reference patient
most similar reference patient
Average weighted risk (AWR) of reference patient
Risk: blue-low; green-intermediate; red-high

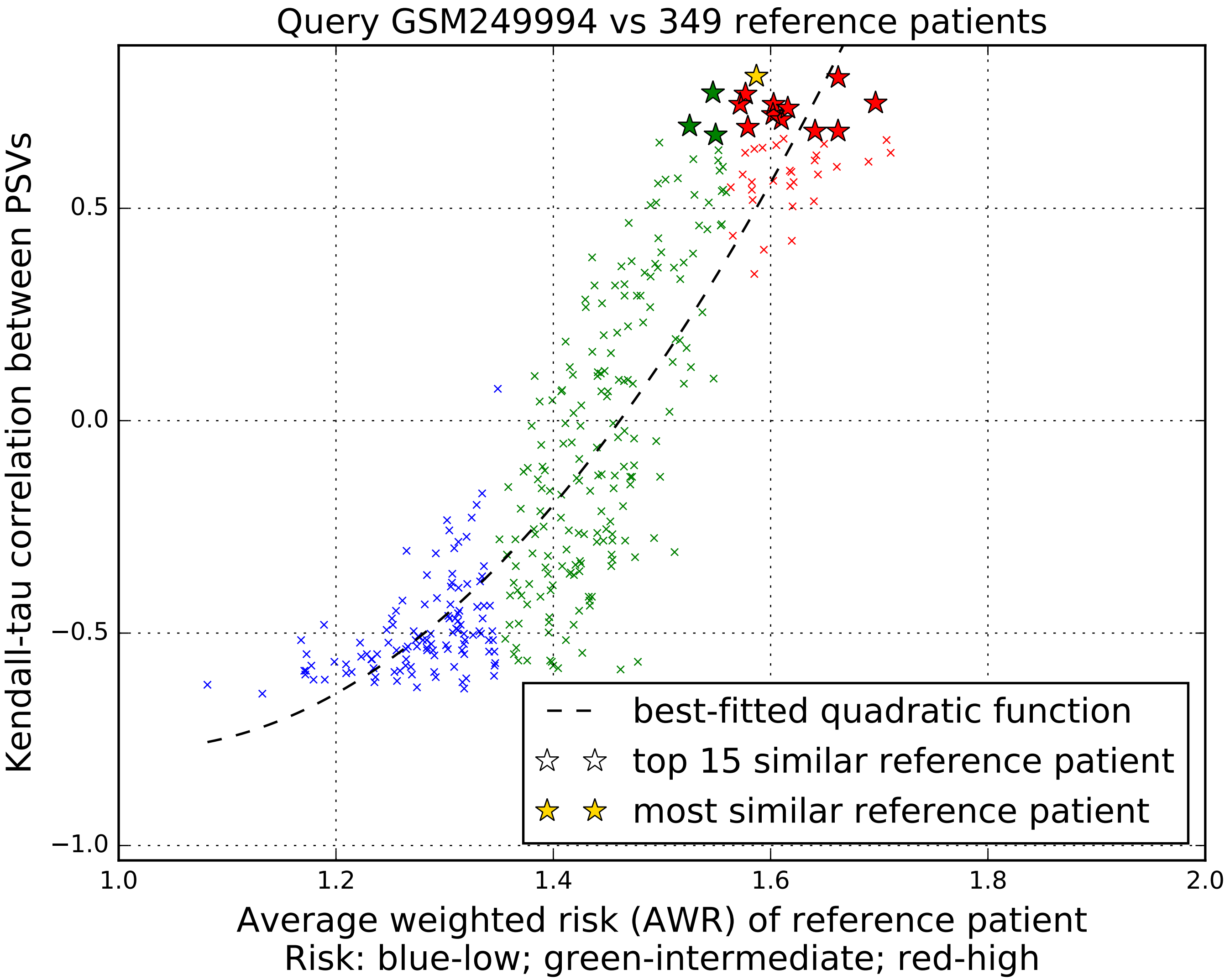

Query GSM249994 vs 349 reference patients
Kendall-tau correlation between PSVs
0.5
0.0
−0.5
−1.0
1.0
1.2
1.4
1.6
1.8
2.0
best-fitted quadratic function
top 15 similar reference patient
most similar reference patient
Average weighted risk (AWR) of reference patient
Risk: blue-low; green-intermediate; red-high

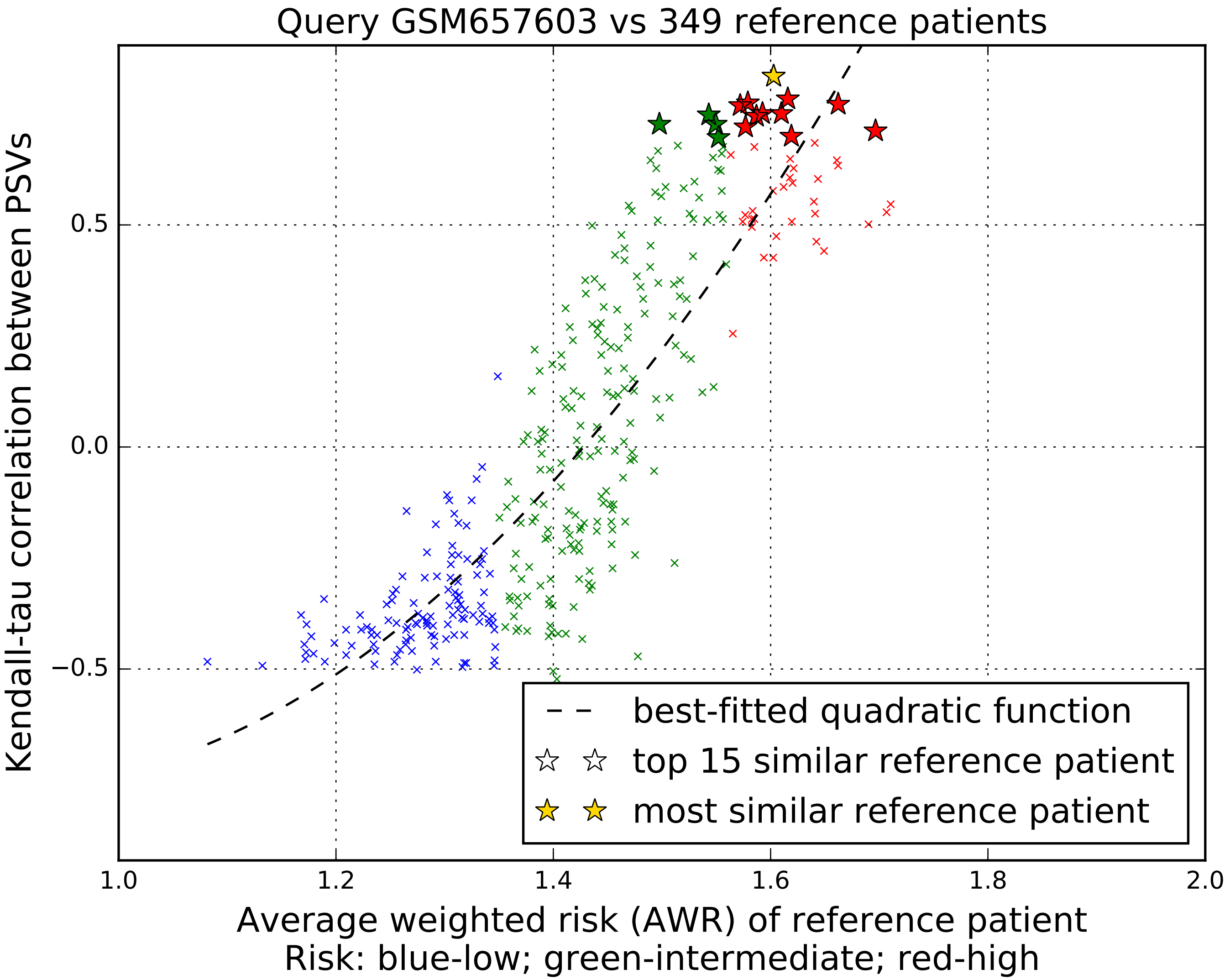

Query GSM657603 vs 349 reference patients
Kendall-tau correlation between PSVs
0.5
0.0
−0.5
1.0
1.2
1.4
1.6
1.8
2.0
best-fitted quadratic function
top 15 similar reference patient
most similar reference patient
Average weighted risk (AWR) of reference patient
Risk: blue-low; green-intermediate; red-high

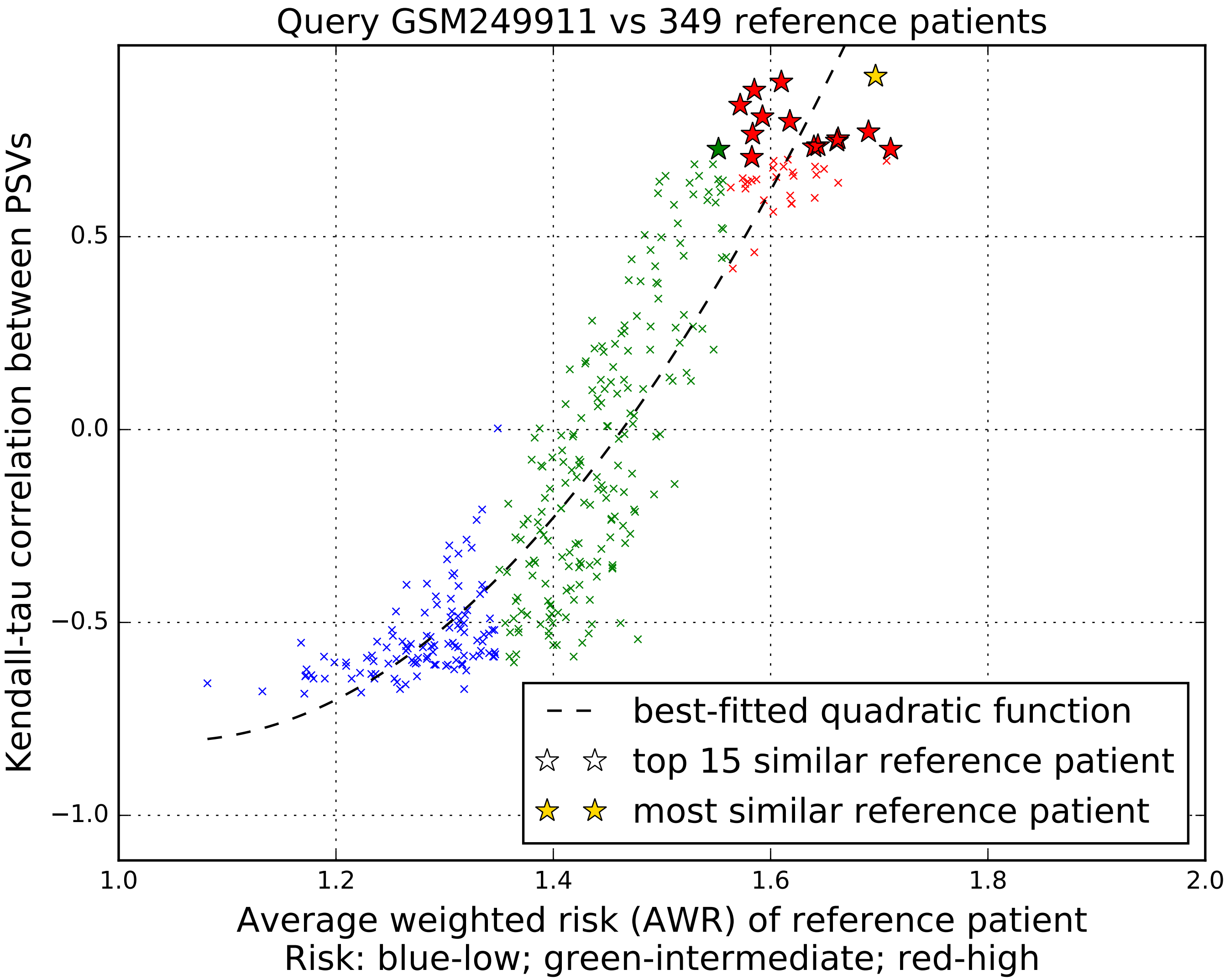

Query GSM249911 vs 349 reference patients
Kendall-tau correlation between PSVs
0.5
0.0
−0.5
−1.0
1.0
1.2
1.4
1.6
1.8
2.0
best-fitted quadratic function
top 15 similar reference patient
most similar reference patient
Average weighted risk (AWR) of reference patient
Risk: blue-low; green-intermediate; red-high

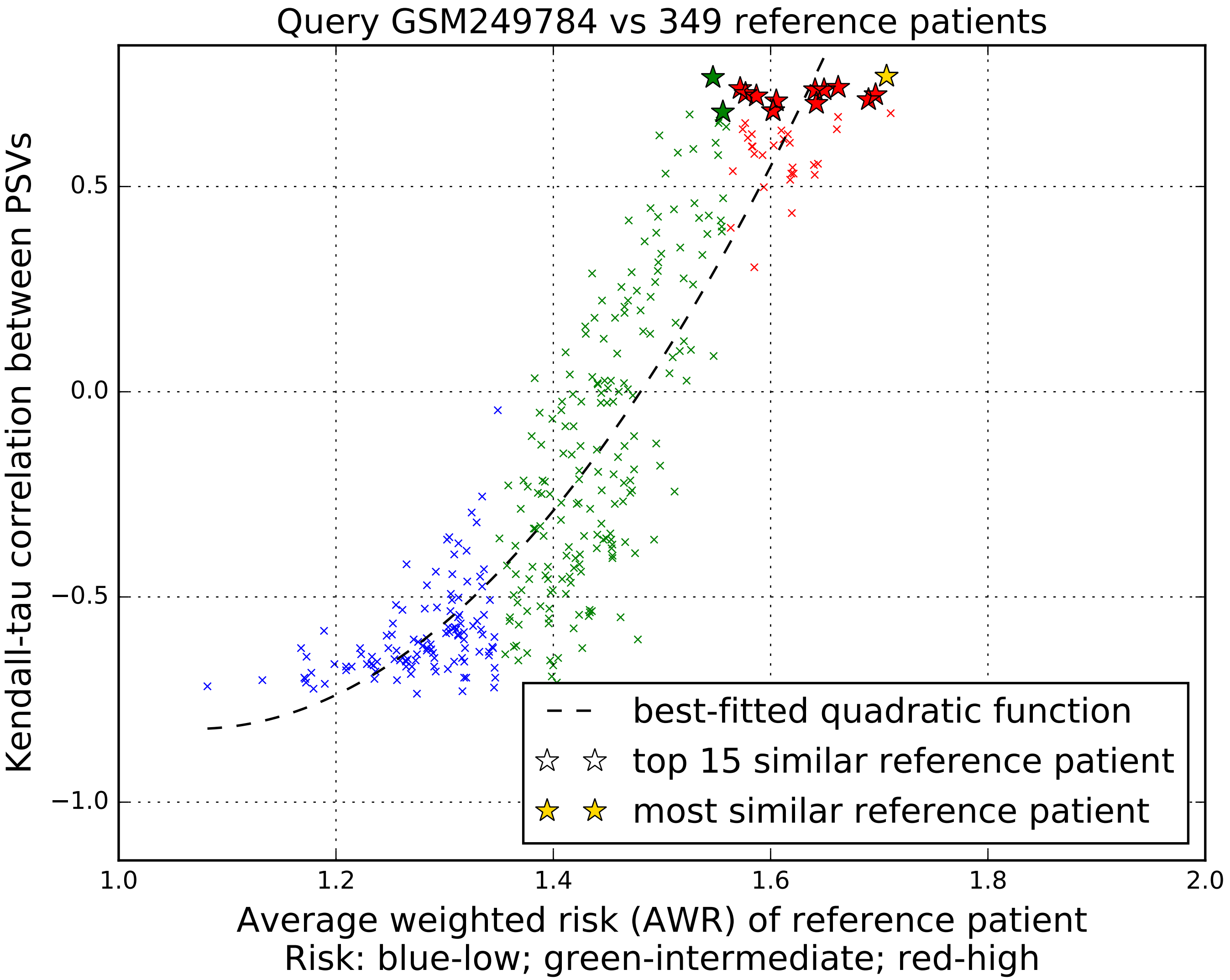
Query GSM249784 vs 349 reference patients
Kendall-tau correlation between PSVs
0.5
0.0
−0.5
−1.0
1.0
1.2
1.4
1.6
1.8
2.0
best-fitted quadratic function
top 15 similar reference patient
most similar reference patient
Average weighted risk (AWR) of reference patient
Risk: blue-low; green-intermediate; red-high

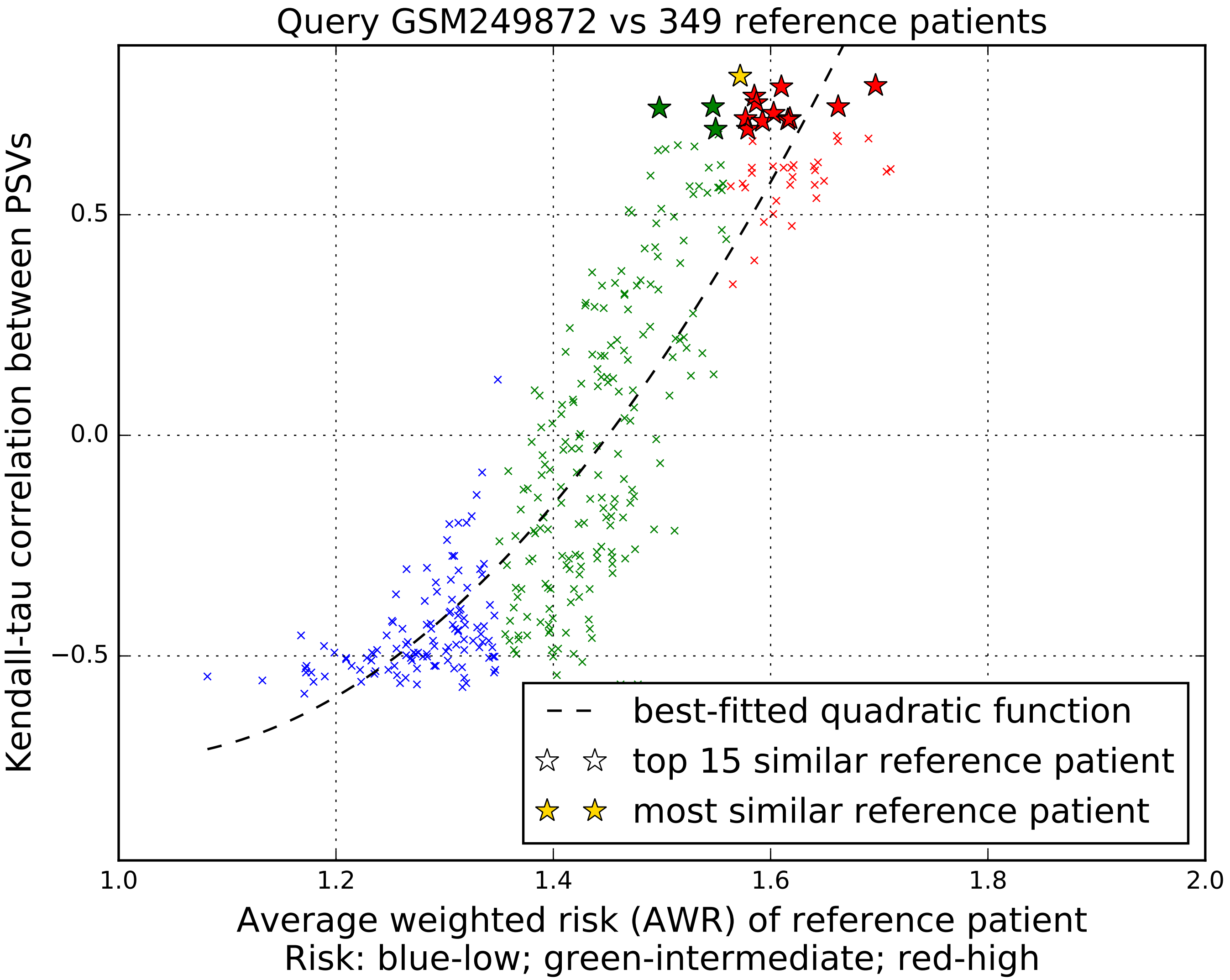
Query GSM249872 vs 349 reference patients
Kendall-tau correlation between PSVs
0.5
0.0
−0.5
1.0
1.2
1.4
1.6
1.8
2.0
best-fitted quadratic function
top 15 similar reference patient
most similar reference patient
Average weighted risk (AWR) of reference patient
Risk: blue-low; green-intermediate; red-high

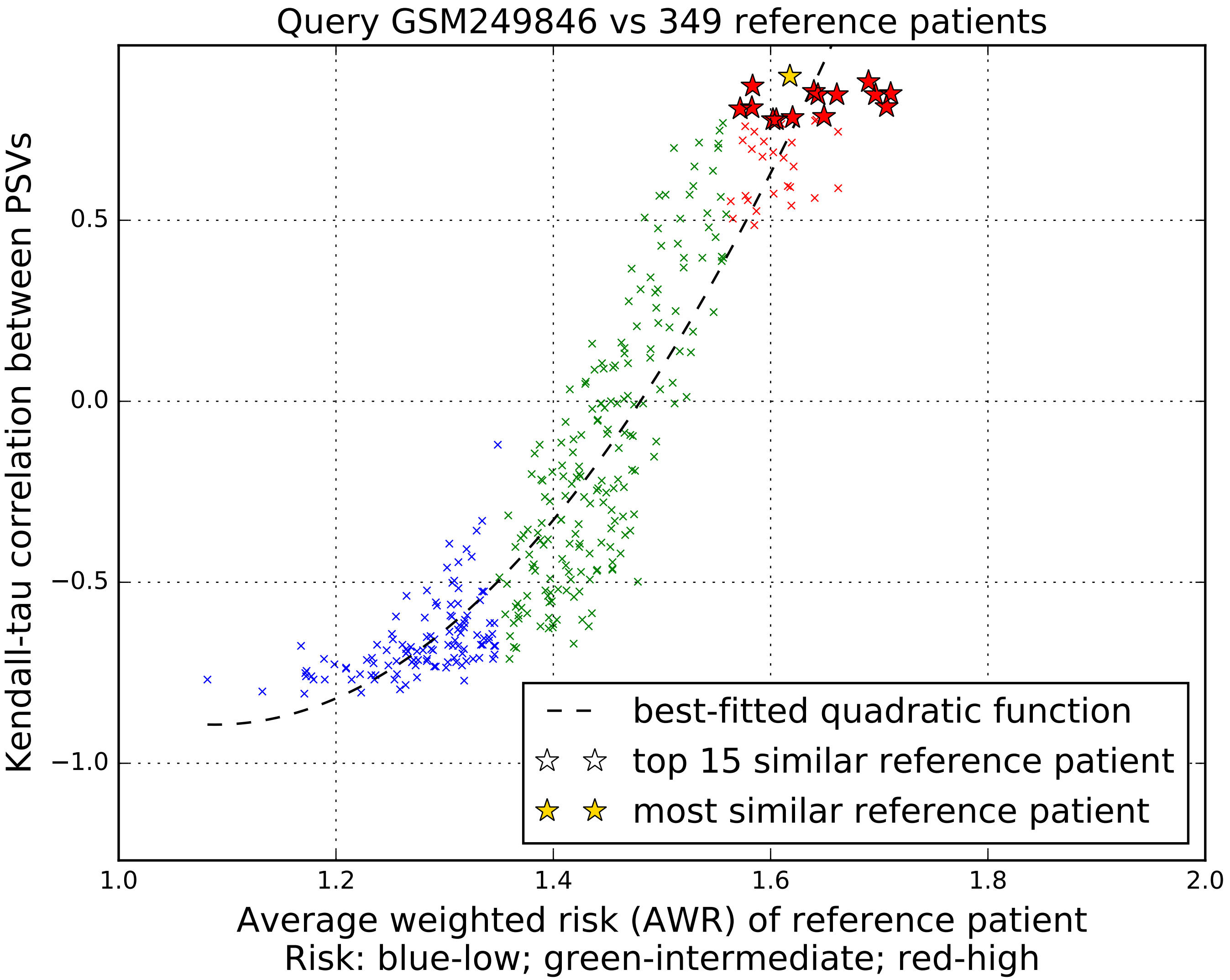

Query GSM249846 vs 349 reference patients
Kendall-tau correlation between PSVs
0.5
0.0
−0.5
−1.0
1.0
1.2
1.4
1.6
1.8
2.0
best-fitted quadratic function
top 15 similar reference patient
most similar reference patient
Average weighted risk (AWR) of reference patient
Risk: blue-low; green-intermediate; red-high

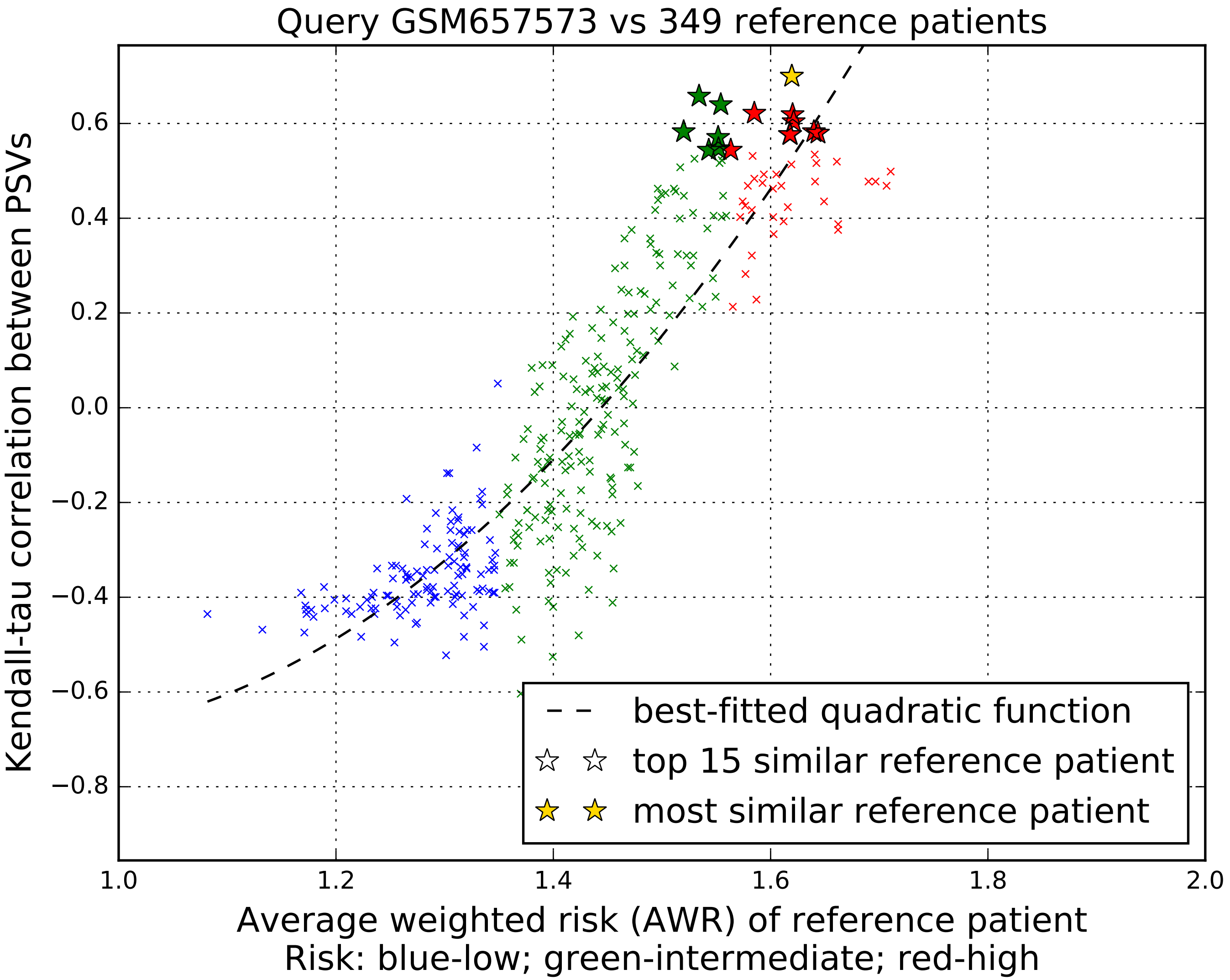

Query GSM657573 vs 349 reference patients
Kendall-tau correlation between PSVs
Average weighted risk (AWR) of reference patient
Risk: blue-low; green-intermediate; red-high
best-fitted quadratic function
top 15 similar reference patient
most similar reference patient
0.6
0.4
0.2
0.0
−0.2
−0.4
−0.6
−0.8
1.0
1.2
1.4
1.6
1.8
2.0

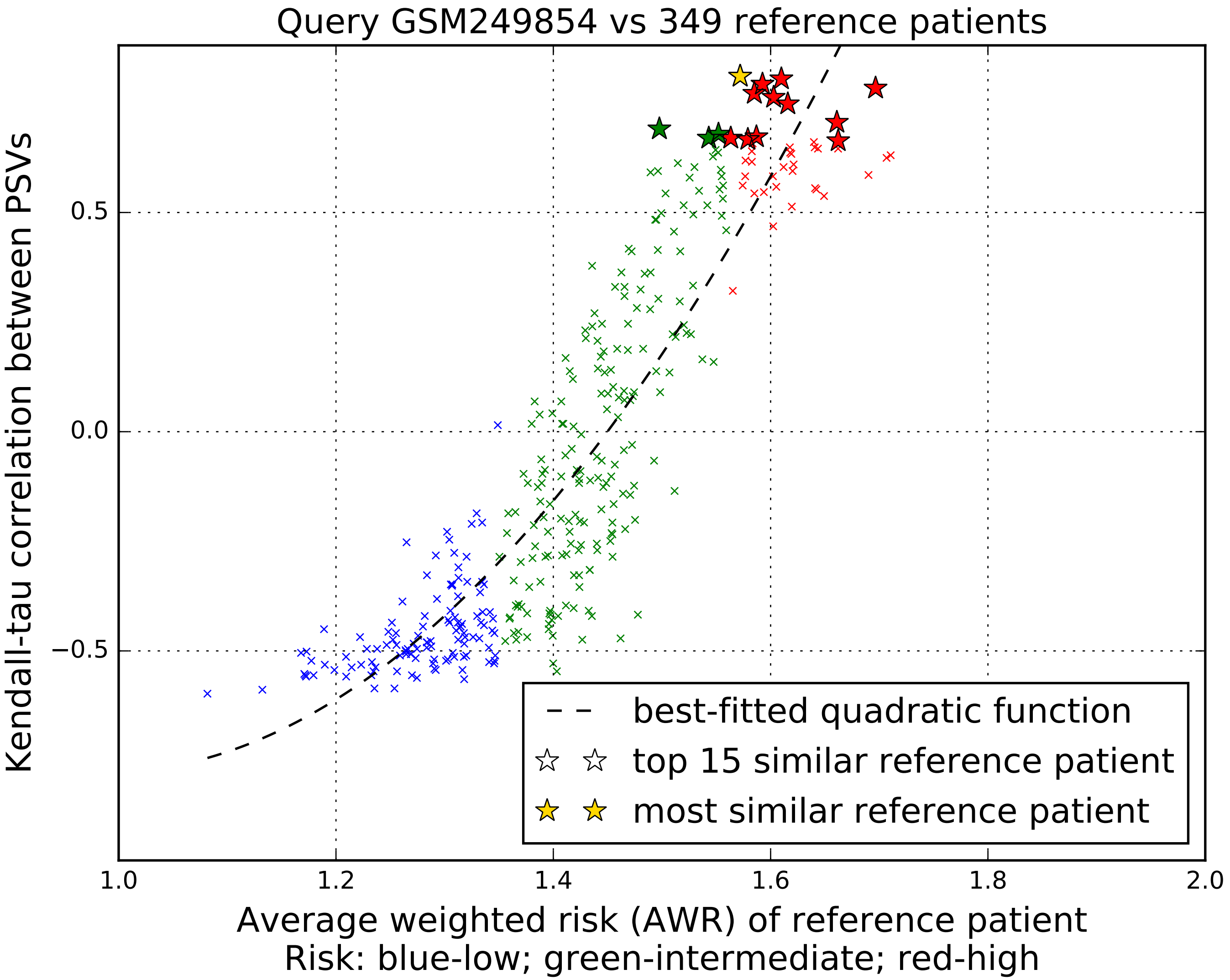

Query GSM249854 vs 349 reference patients
Kendall-tau correlation between PSVs
0.5
0.0
−0.5
1.0
1.2
1.4
1.6
1.8
2.0
best-fitted quadratic function
top 15 similar reference patient
most similar reference patient
Average weighted risk (AWR) of reference patient
Risk: blue-low; green-intermediate; red-high

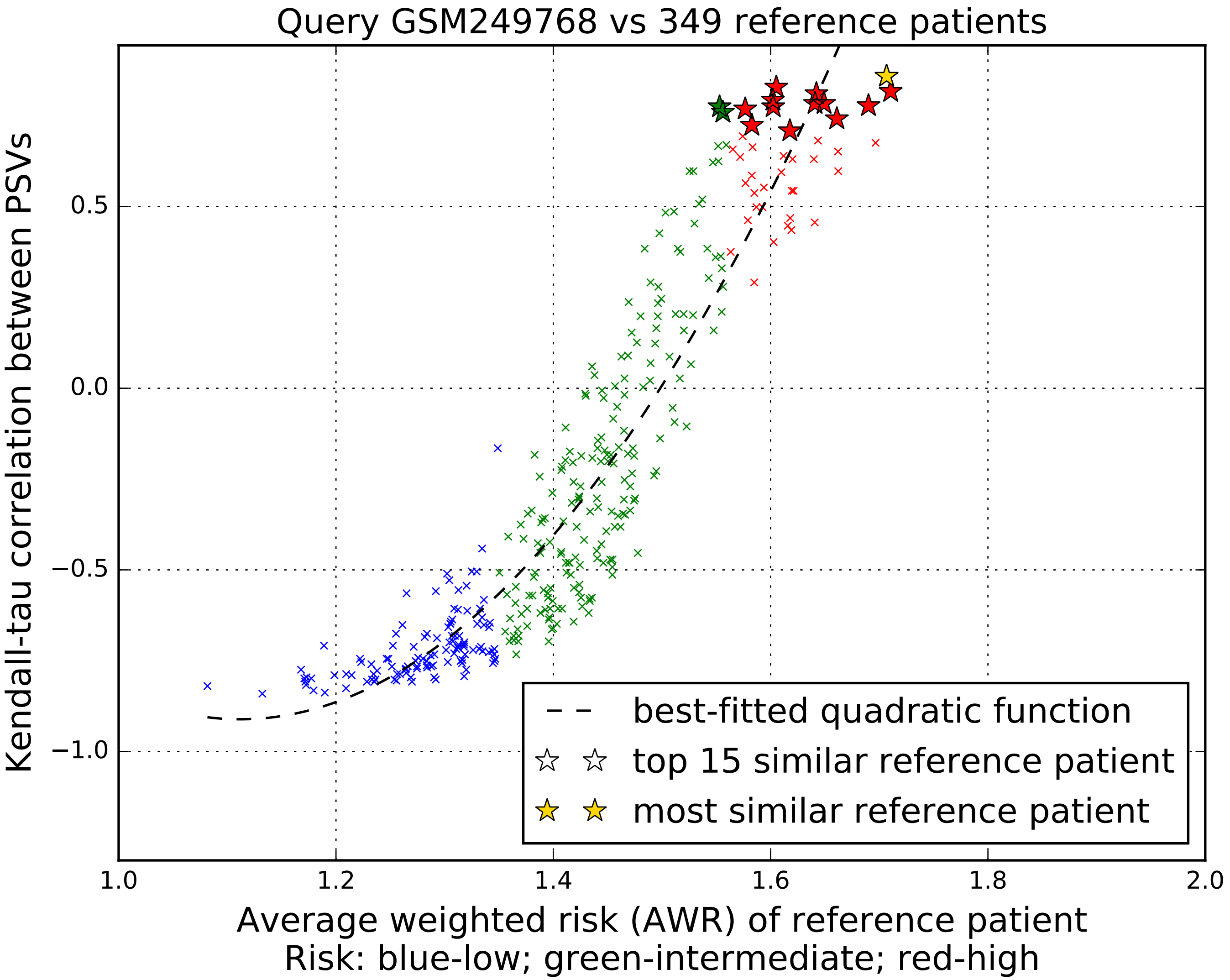

Query GSM249768 vs 349 reference patients
Kendall-tau correlation between PSVs
0.5
0.0
−0.5
−1.0
1.0
1.2
1.4
1.6
1.8
2.0
best-fitted quadratic function
top 15 similar reference patient
most similar reference patient
Average weighted risk (AWR) of reference patient
Risk: blue-low; green-intermediate; red-high

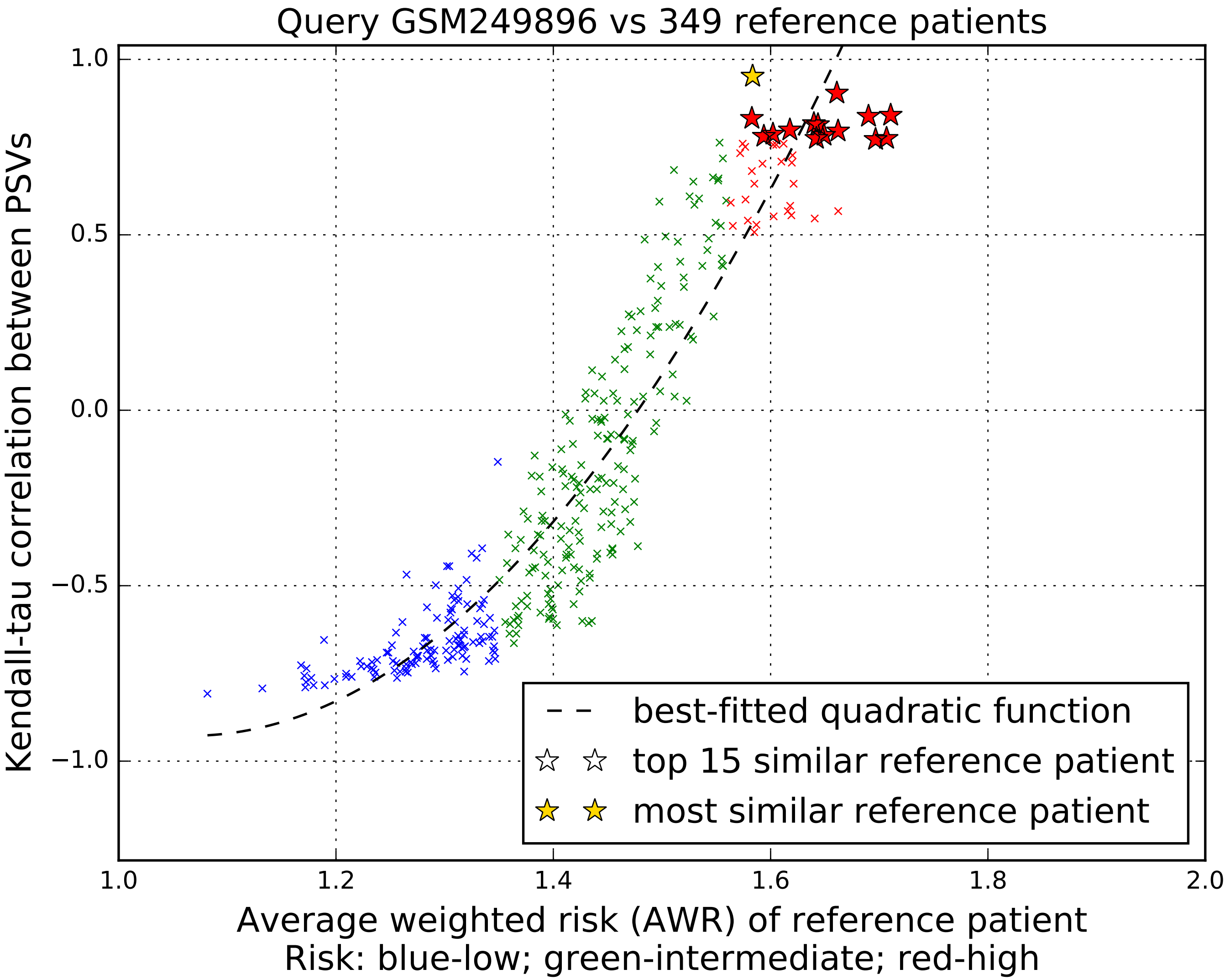

Query GSM249896 vs 349 reference patients
Kendall-tau correlation between PSVs
Average weighted risk (AWR) of reference patient
Risk: blue-low; green-intermediate; red-high
best-fitted quadratic function
top 15 similar reference patient
most similar reference patient
1.0
0.5
0.0
−0.5
−1.0
1.0
1.2
1.4
1.6
1.8
2.0

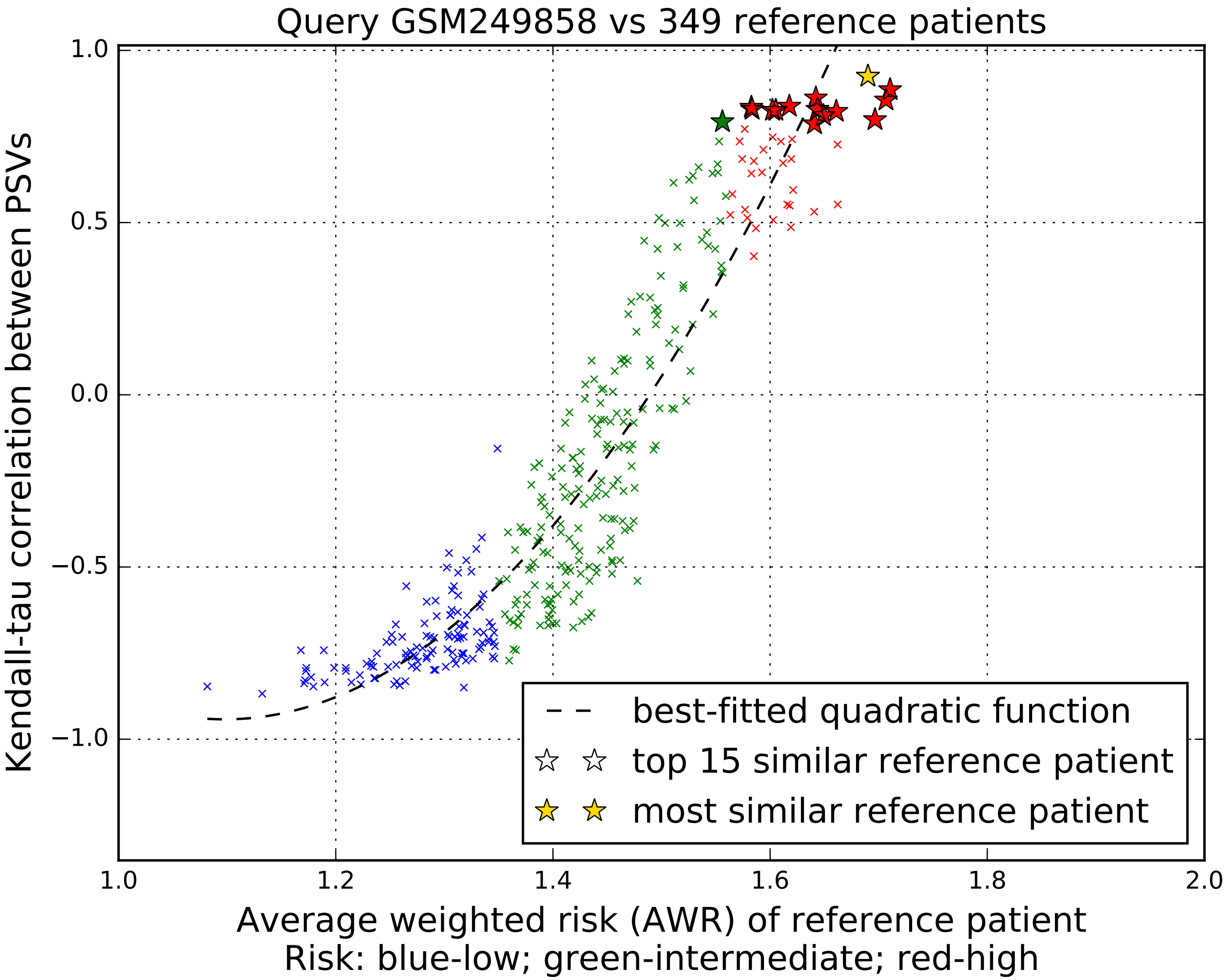

Query GSM249858 vs 349 reference patients
Kendall-tau correlation between PSVs
1.0
0.5
0.0
−0.5
−1.0
1.0
1.2
1.4
1.6
1.8
2.0
best-fitted quadratic function
top 15 similar reference patient
most similar reference patient
Average weighted risk (AWR) of reference patient
Risk: blue-low; green-intermediate; red-high

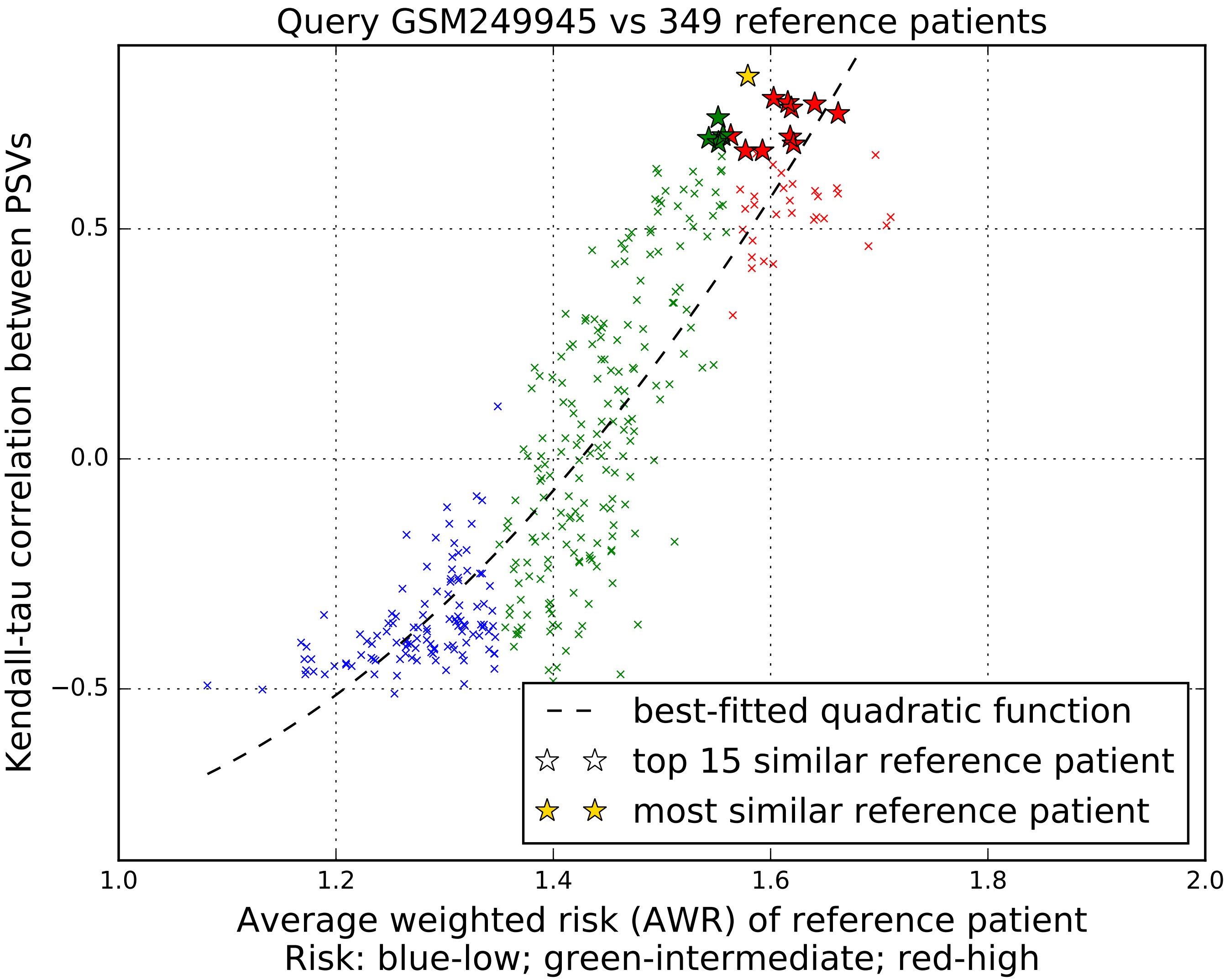

Query GSM249945 vs 349 reference patients
Kendall-tau correlation between PSVs
0.5
0.0
−0.5
1.0
1.2
1.4
1.6
1.8
2.0
Average weighted risk (AWR) of reference patient
Risk: blue-low; green-intermediate; red-high
best-fitted quadratic function
top 15 similar reference patient
most similar reference patient

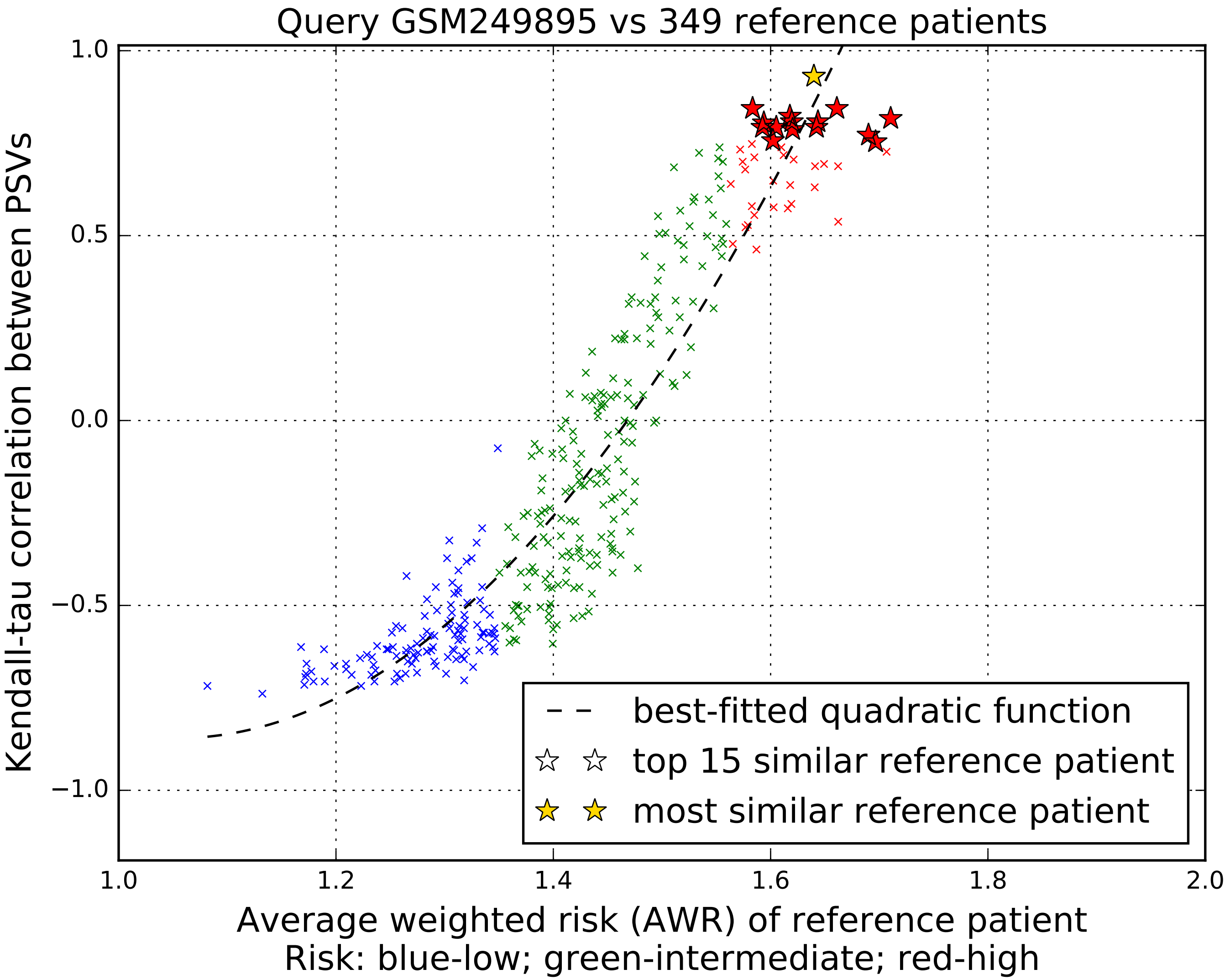

Query GSM249895 vs 349 reference patients
Kendall-tau correlation between PSVs
Average weighted risk (AWR) of reference patient
Risk: blue-low; green-intermediate; red-high
1.0
0.5
0.0
−0.5
−1.0
1.0
1.2
1.4
1.6
1.8
2.0
best-fitted quadratic function
top 15 similar reference patient
most similar reference patient

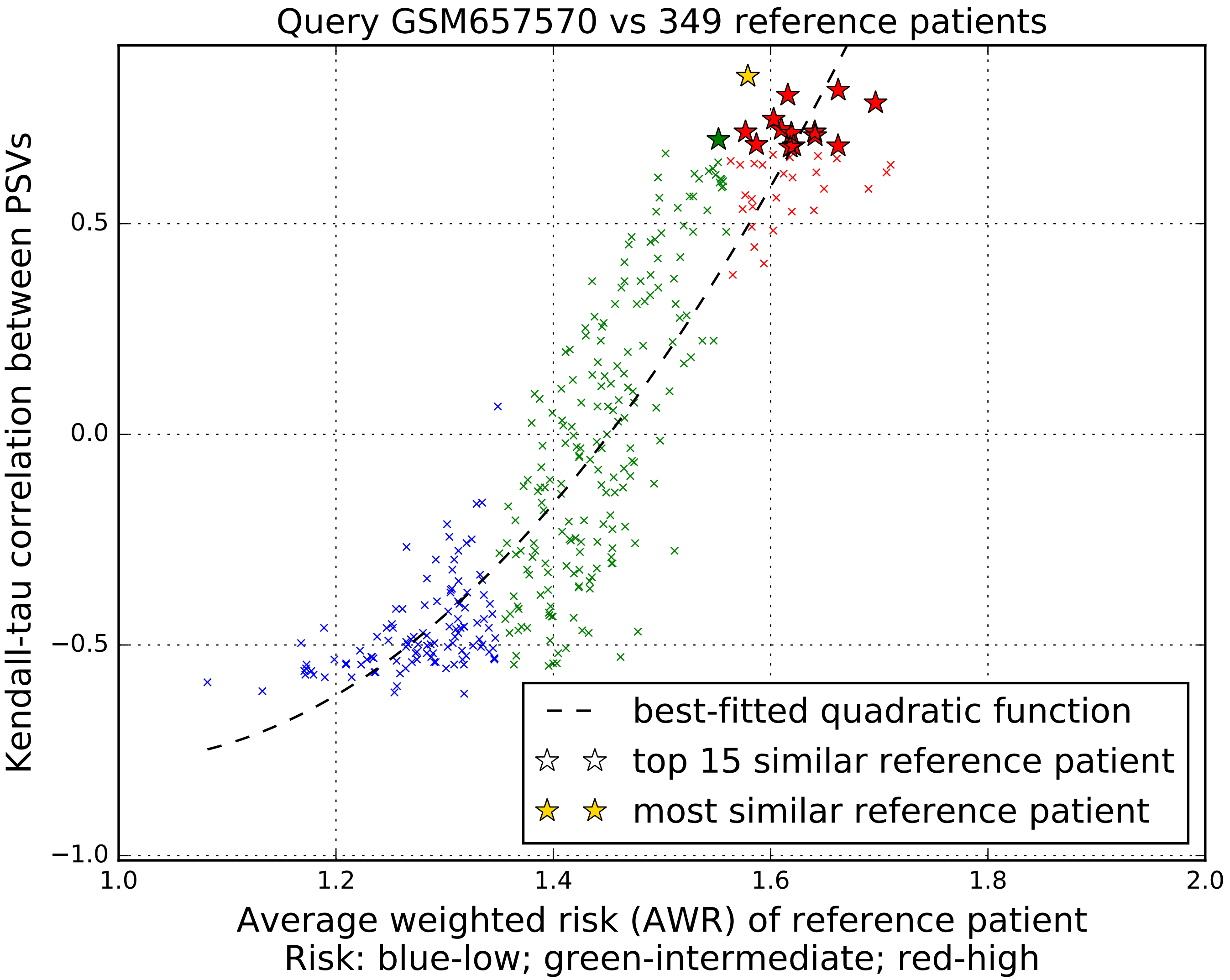
Query GSM657570 vs 349 reference patients
Kendall-tau correlation between PSVs
0.5
0.0
−0.5
−1.0
1.0
1.2
1.4
1.6
1.8
2.0
best-fitted quadratic function
top 15 similar reference patient
most similar reference patient
Average weighted risk (AWR) of reference patient
Risk: blue-low; green-intermediate; red-high

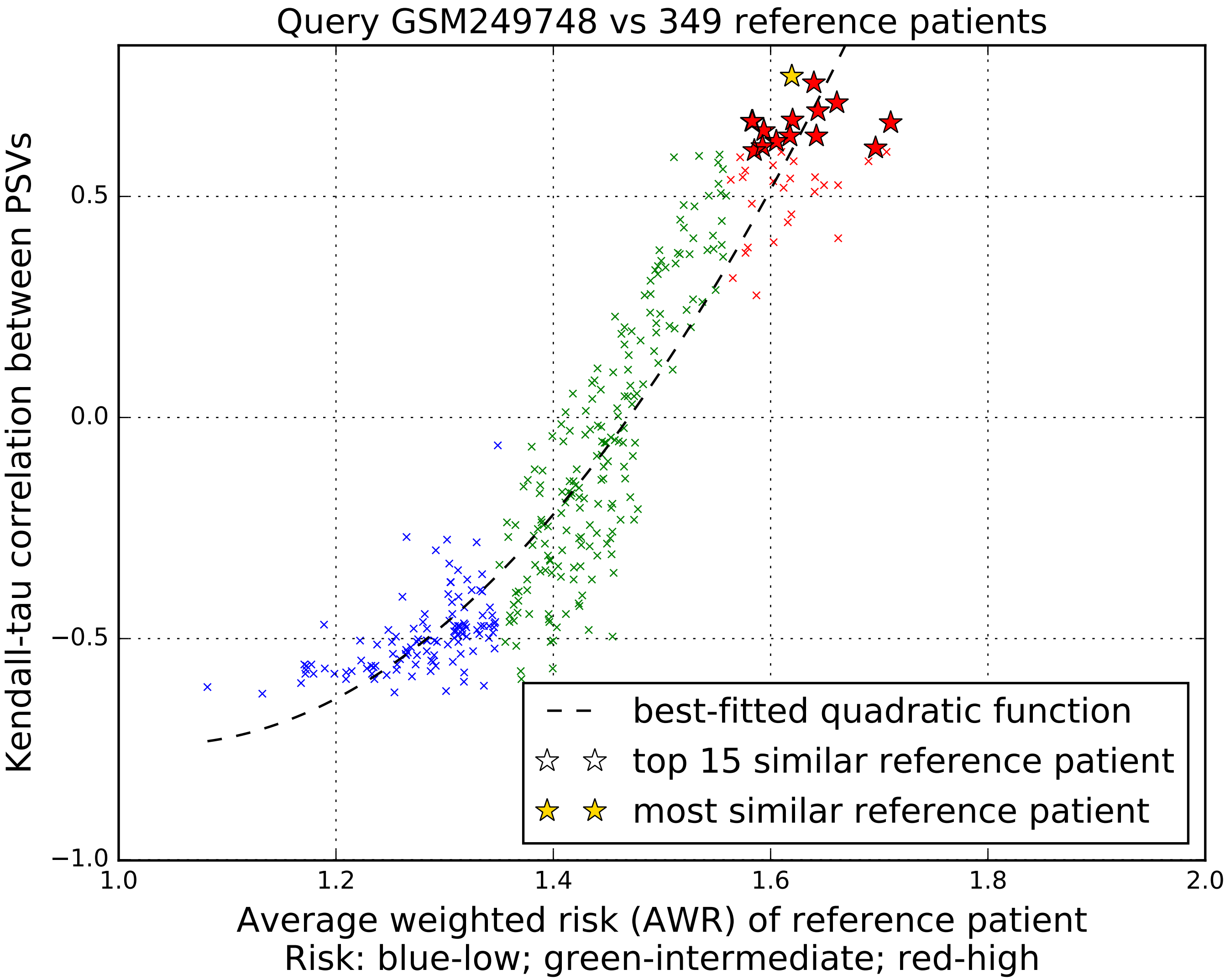
Query GSM249748 vs 349 reference patients
Kendall-tau correlation between PSVs
0.5
0.0
−0.5
−1.0
1.0
1.2
1.4
1.6
1.8
2.0
Average weighted risk (AWR) of reference patient
Risk: blue-low; green-intermediate; red-high
best-fitted quadratic function
top 15 similar reference patient
most similar reference patient

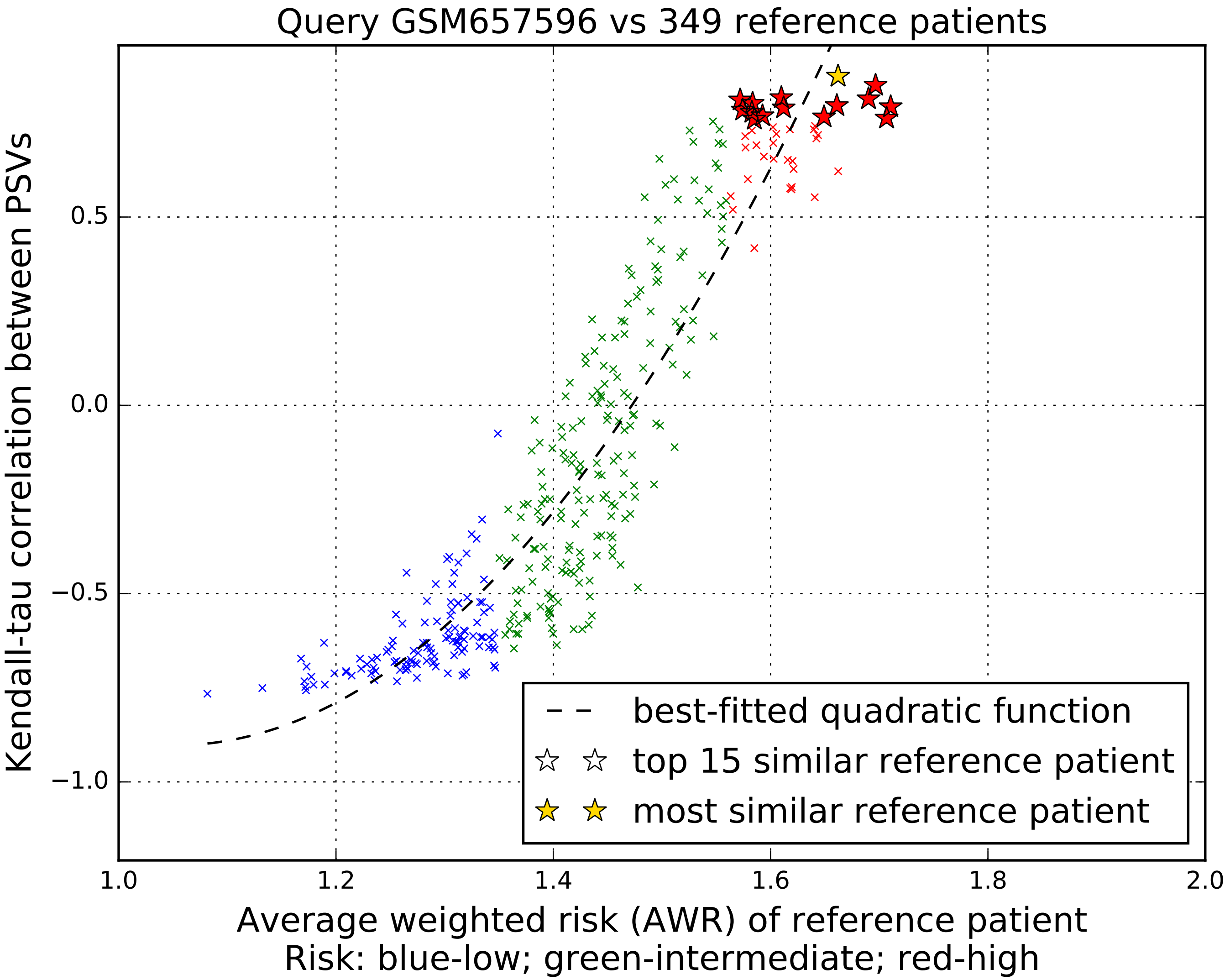
Query GSM657596 vs 349 reference patients
Kendall-tau correlation between PSVs
0.5
0.0
−0.5
−1.0
1.0
1.2
1.4
1.6
1.8
2.0
best-fitted quadratic function
top 15 similar reference patient
most similar reference patient
Average weighted risk (AWR) of reference patient
Risk: blue-low; green-intermediate; red-high

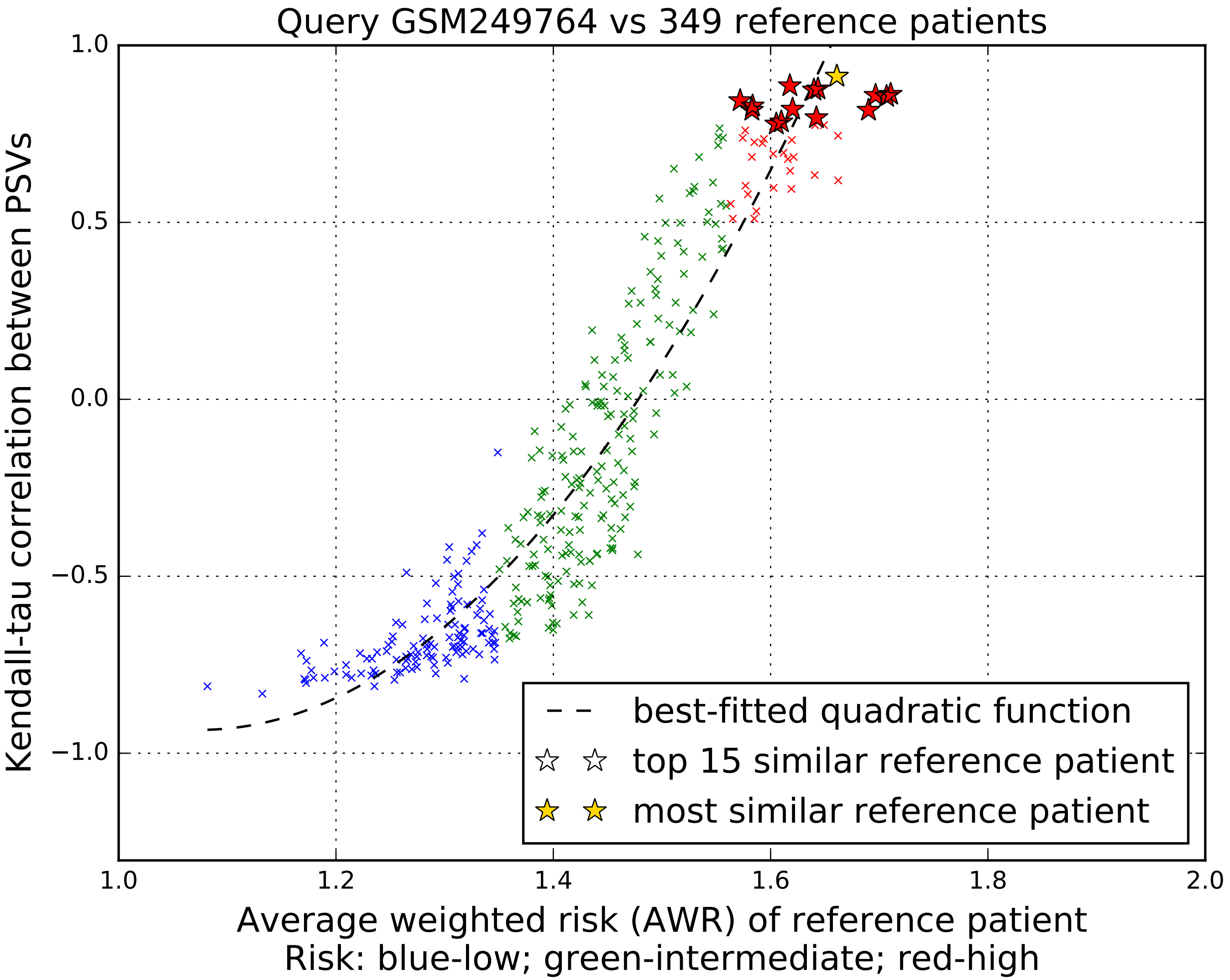

Query GSM249764 vs 349 reference patients
Kendall-tau correlation between PSVs
Average weighted risk (AWR) of reference patient
Risk: blue-low; green-intermediate; red-high
best-fitted quadratic function
top 15 similar reference patient
most similar reference patient
1.0
0.5
0.0
−0.5
−1.0
1.0
1.2
1.4
1.6
1.8
2.0

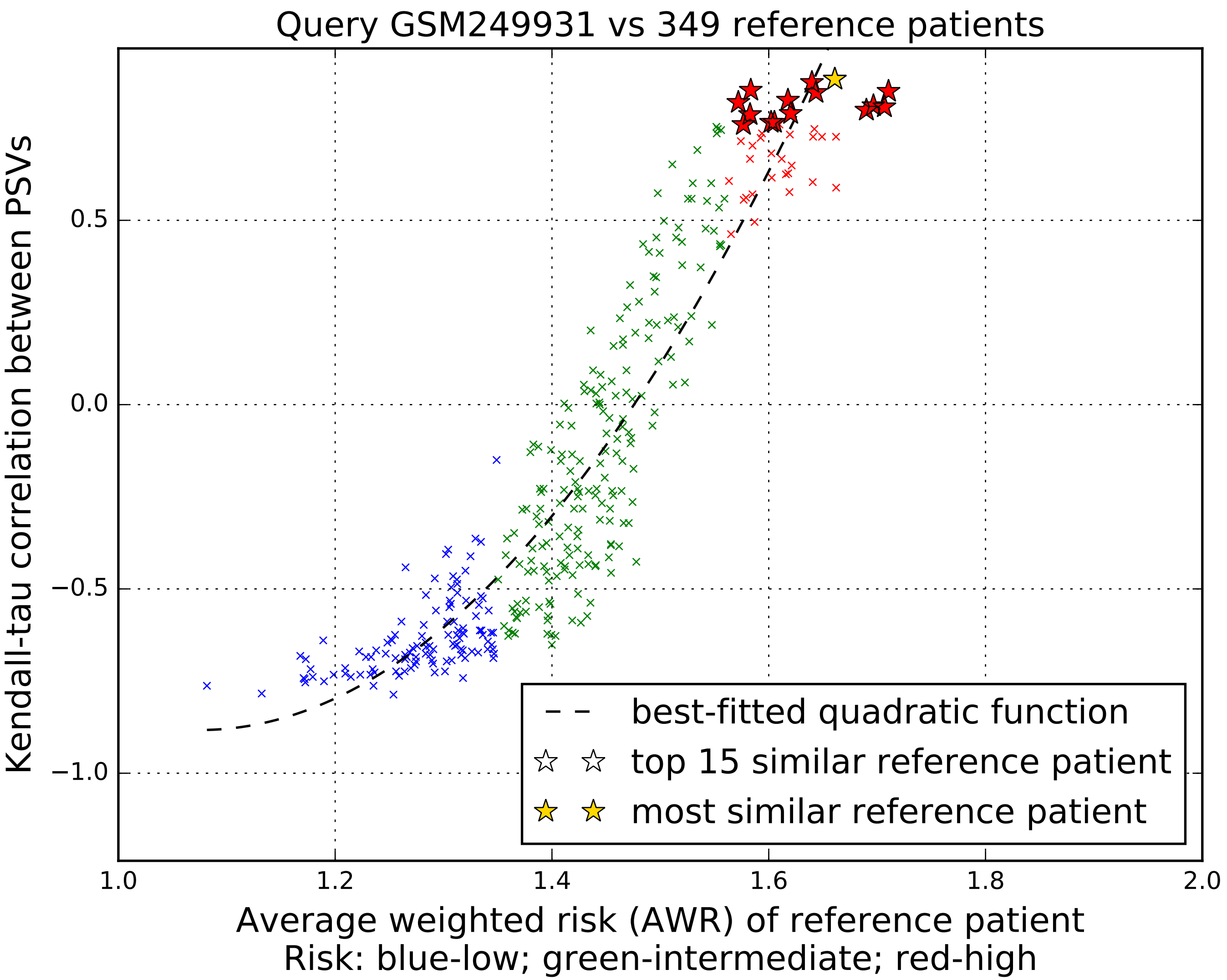
Query GSM249931 vs 349 reference patients
Kendall-tau correlation between PSVs
0.5
0.0
−0.5
−1.0
1.0
1.2
1.4
1.6
1.8
2.0
best-fitted quadratic function
top 15 similar reference patient
most similar reference patient
Average weighted risk (AWR) of reference patient
Risk: blue-low; green-intermediate; red-high

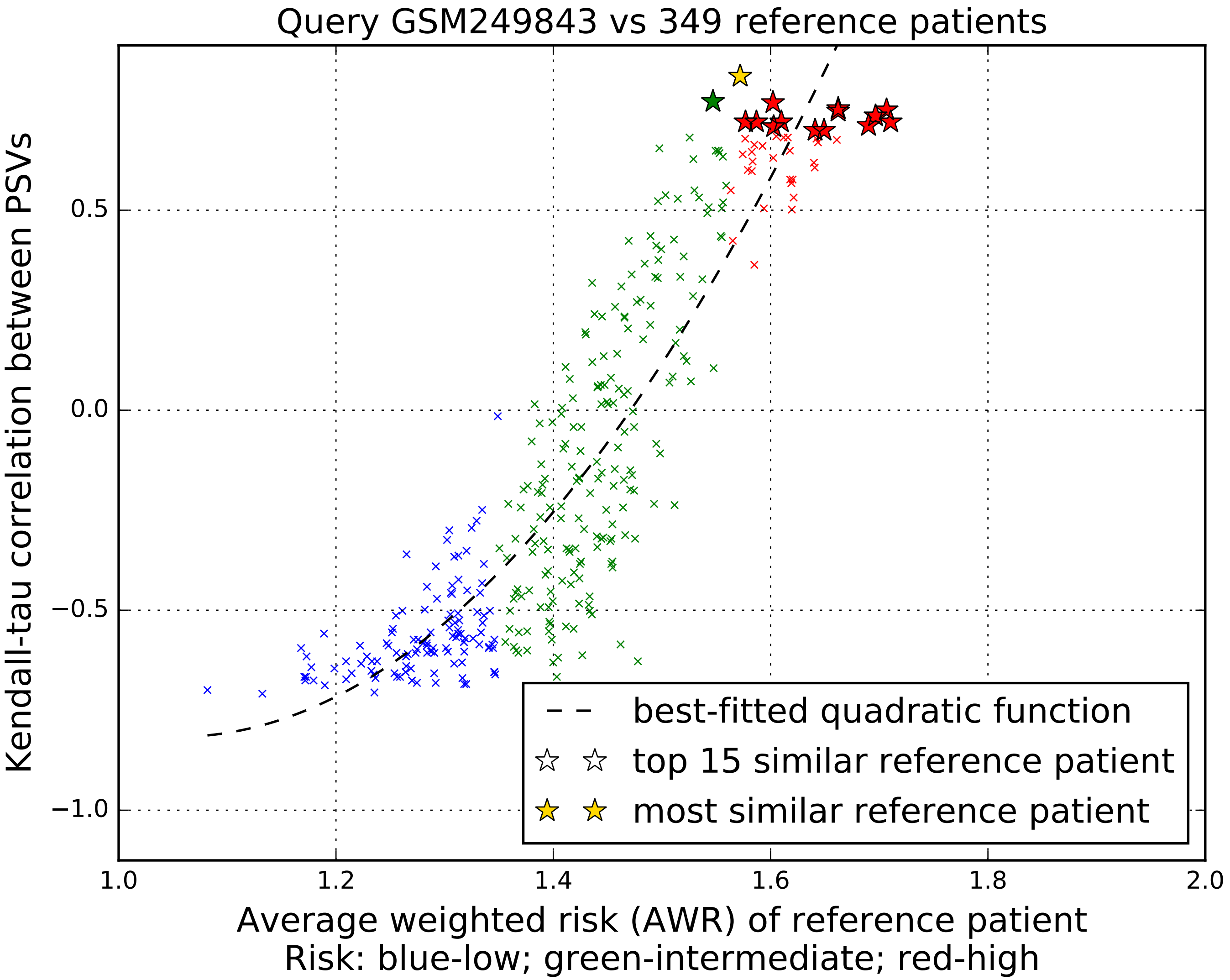

Query GSM249843 vs 349 reference patients
Kendall-tau correlation between PSVs
Average weighted risk (AWR) of reference patient
Risk: blue-low; green-intermediate; red-high
best-fitted quadratic function
top 15 similar reference patient
most similar reference patient
0.5
0.0
−0.5
−1.0
1.0
1.2
1.4
1.6
1.8
2.0

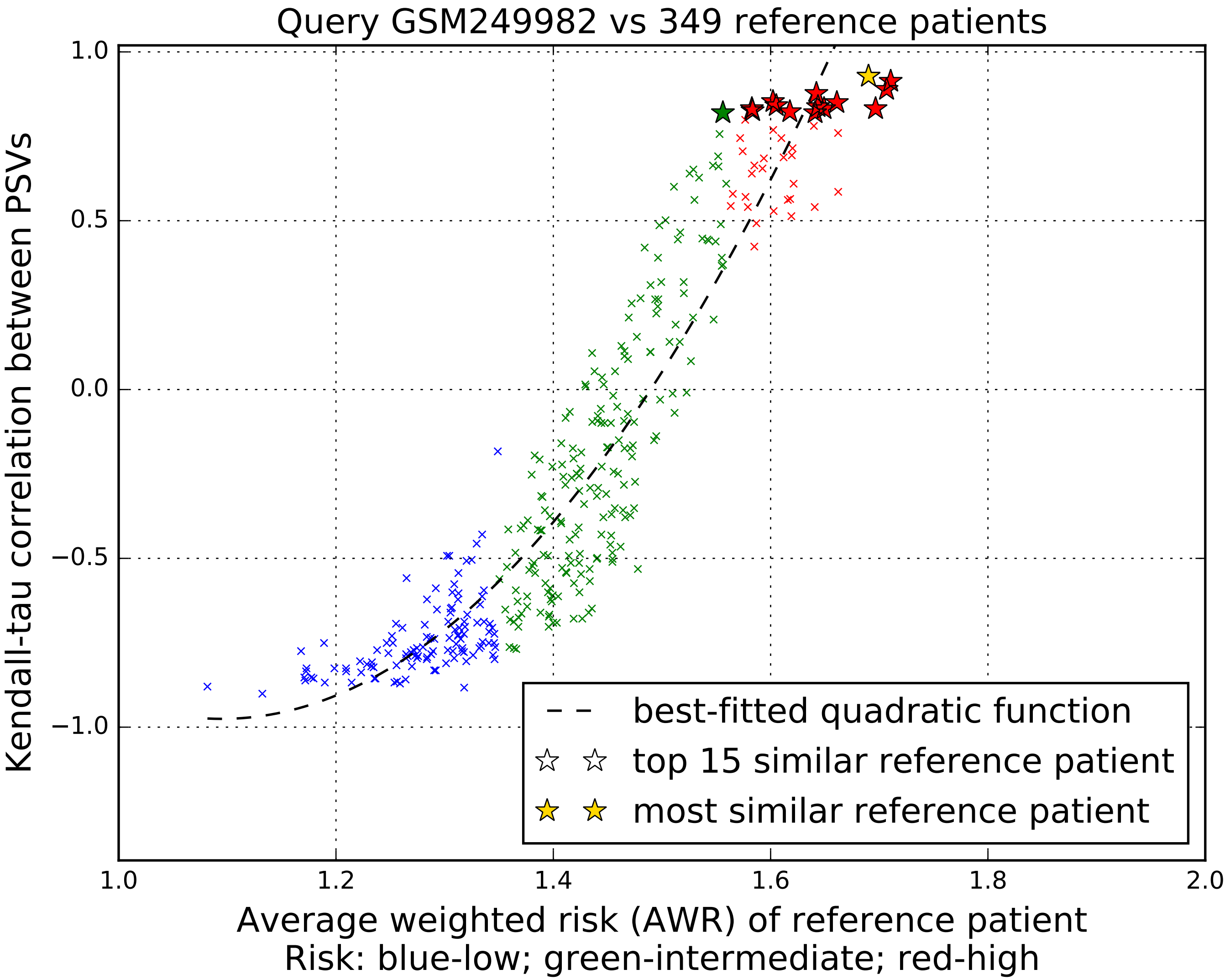

Query GSM249982 vs 349 reference patients
1.0
0.5
0.0
−0.5
−1.0
Kendall-tau correlation between PSVs
1.0
1.2
1.4
1.6
1.8
2.0
best-fitted quadratic function
top 15 similar reference patient
most similar reference patient
Average weighted risk (AWR) of reference patient
Risk: blue-low; green-intermediate; red-high

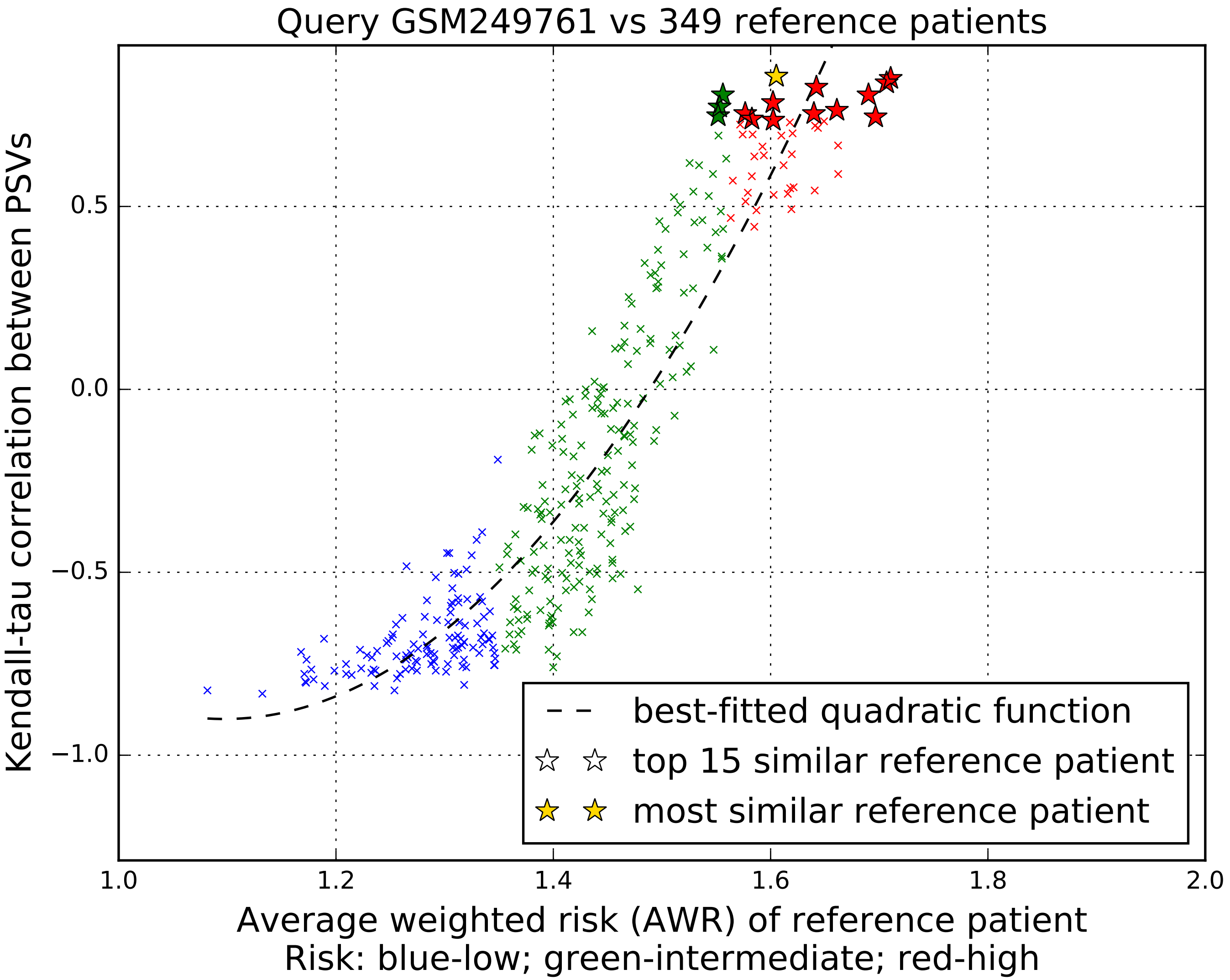
Query GSM249761 vs 349 reference patients
Kendall-tau correlation between PSVs
0.5
0.0
−0.5
−1.0
1.0
1.2
1.4
1.6
1.8
2.0
best-fitted quadratic function
top 15 similar reference patient
most similar reference patient
Average weighted risk (AWR) of reference patient
Risk: blue-low; green-intermediate; red-high

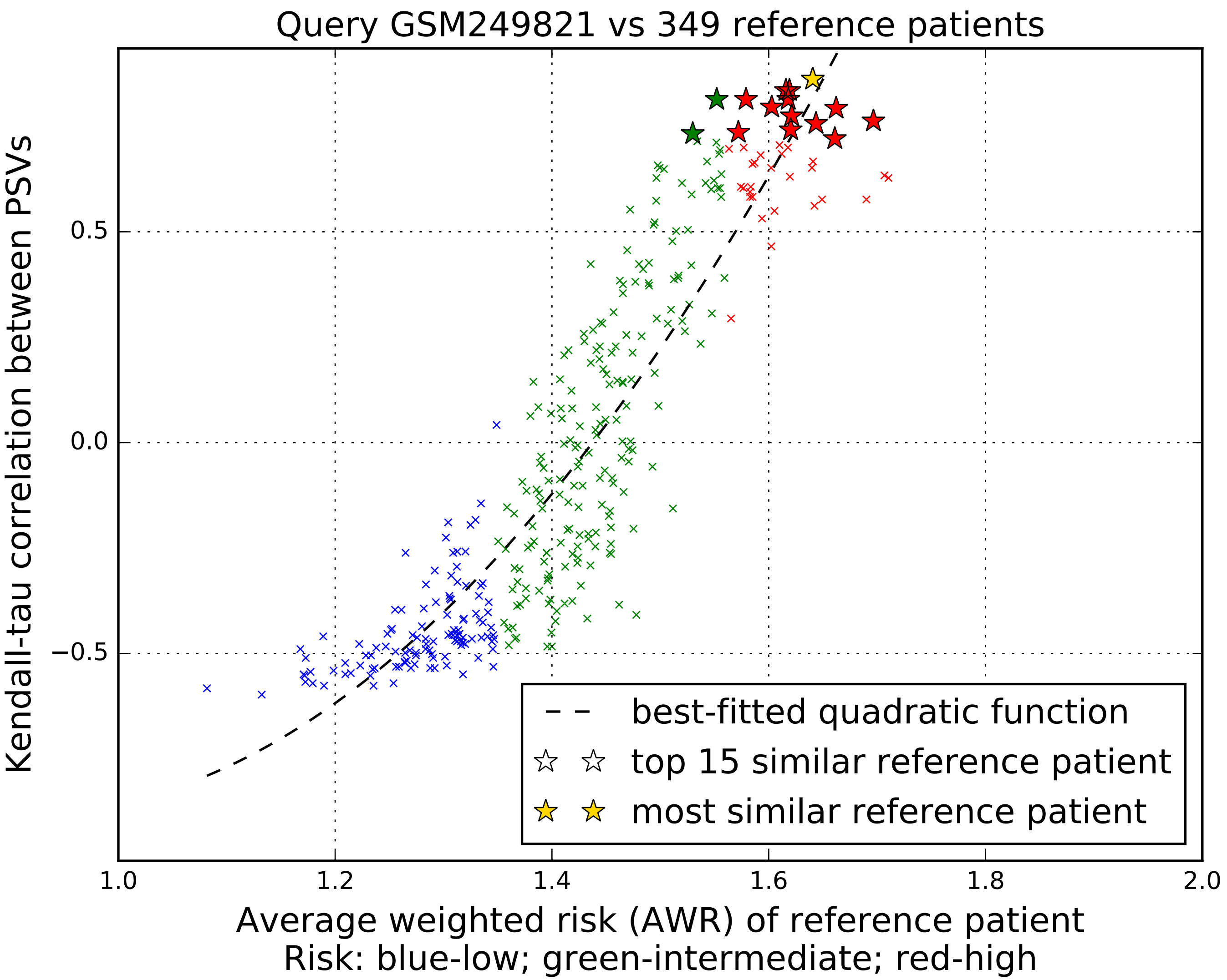

Query GSM249821 vs 349 reference patients
Kendall-tau correlation between PSVs
0.5
0.0
−0.5
1.0
1.2
1.4
1.6
1.8
2.0
best-fitted quadratic function
top 15 similar reference patient
most similar reference patient
Average weighted risk (AWR) of reference patient
Risk: blue-low; green-intermediate; red-high

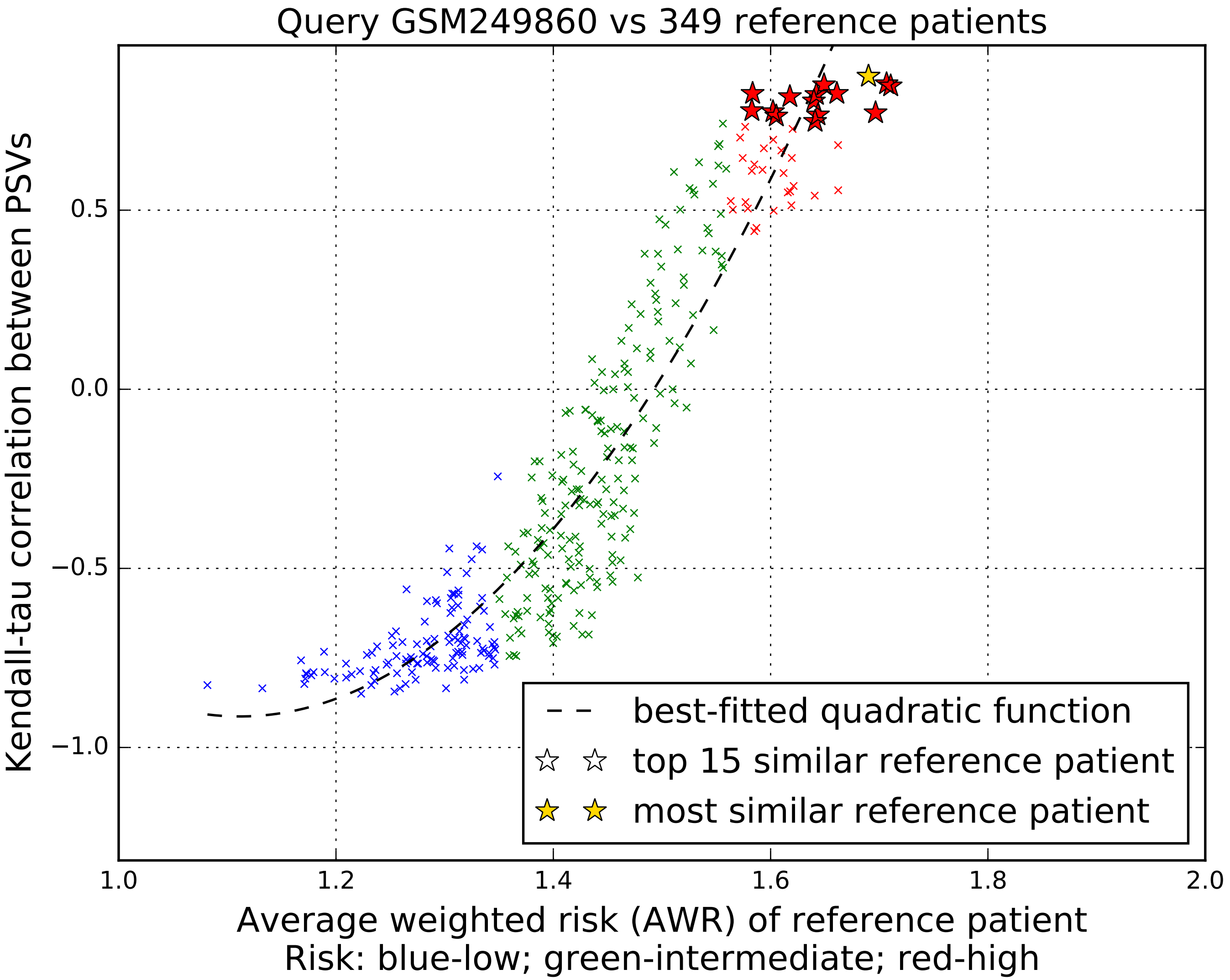

Query GSM249860 vs 349 reference patients
Kendall-tau correlation between PSVs
0.5
0.0
−0.5
−1.0
1.0
1.2
1.4
1.6
1.8
2.0
Average weighted risk (AWR) of reference patient
Risk: blue-low; green-intermediate; red-high
best-fitted quadratic function
top 15 similar reference patient
most similar reference patient
