# Supplementary material for: Big data and computational biology strategy for personalized prognosis
Source: Oncotarget. 2016 May 24;7(26):40200–20. doi: 10.18632/oncotarget.9571 (PMC5130003; doi:10.18632/oncotarget.9571)
Supplement: Supplementary file 14 [file oncotarget-07-40200-s014.pdf]

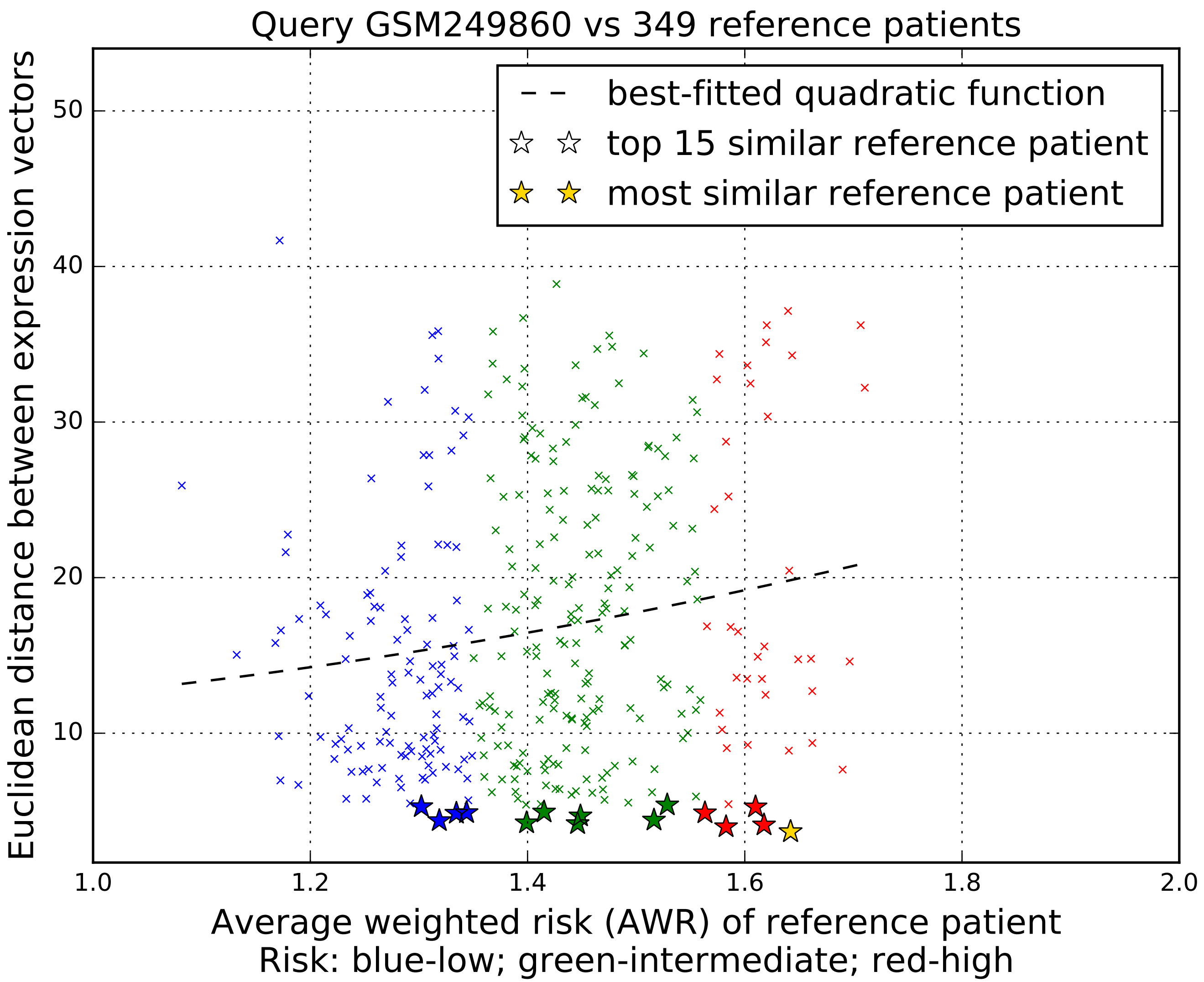
Query GSM249860 vs 349 reference patients
best-fitted quadratic function
top 15 similar reference patient
most similar reference patient
Euclidean distance between expression vectors
50
40
30
20
10
1.0
1.2
1.4
1.6
1.8
2.0
Average weighted risk (AWR) of reference patient
Risk: blue-low; green-intermediate; red-high

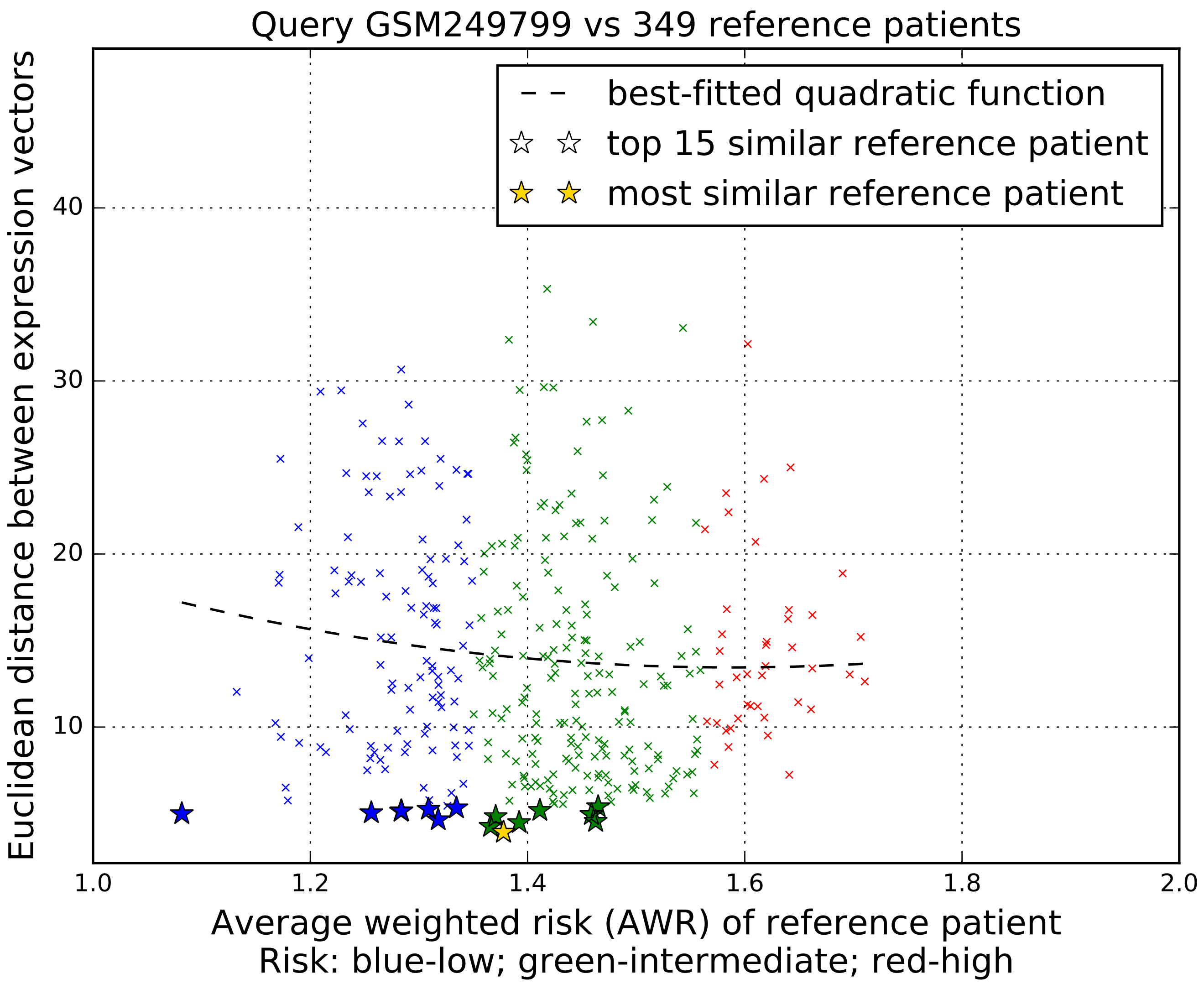
Query GSM249799 vs 349 reference patients
best-fitted quadratic function
top 15 similar reference patient
most similar reference patient
Euclidean distance between expression vectors
10
20
30
40
1.0
1.2
1.4
1.6
1.8
2.0
Average weighted risk (AWR) of reference patient
Risk: blue-low; green-intermediate; red-high

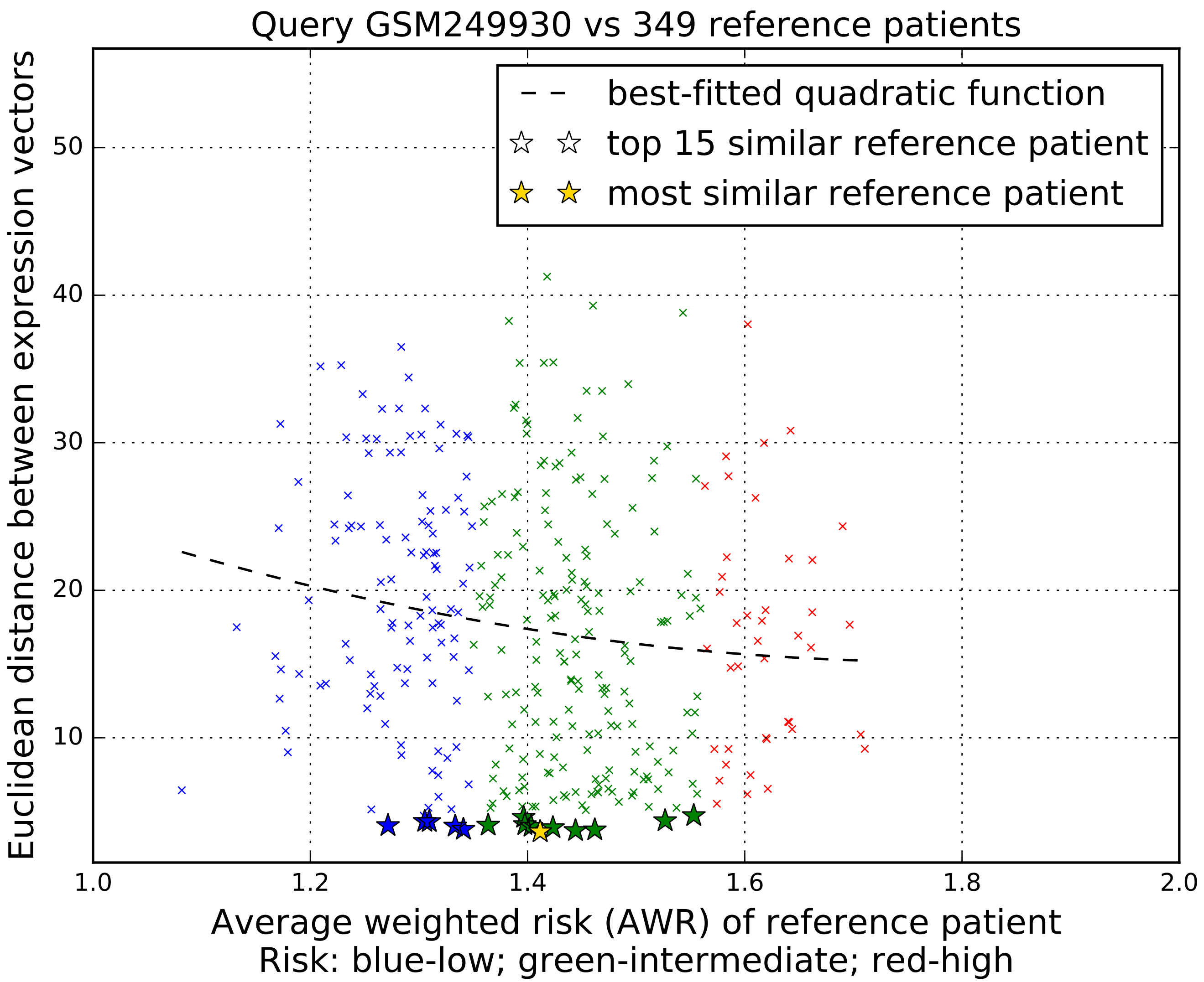
Query GSM249930 vs 349 reference patients
best-fitted quadratic function
top 15 similar reference patient
most similar reference patient
Euclidean distance between expression vectors
10
20
30
40
50
1.0
1.2
1.4
1.6
1.8
2.0
Average weighted risk (AWR) of reference patient
Risk: blue-low; green-intermediate; red-high

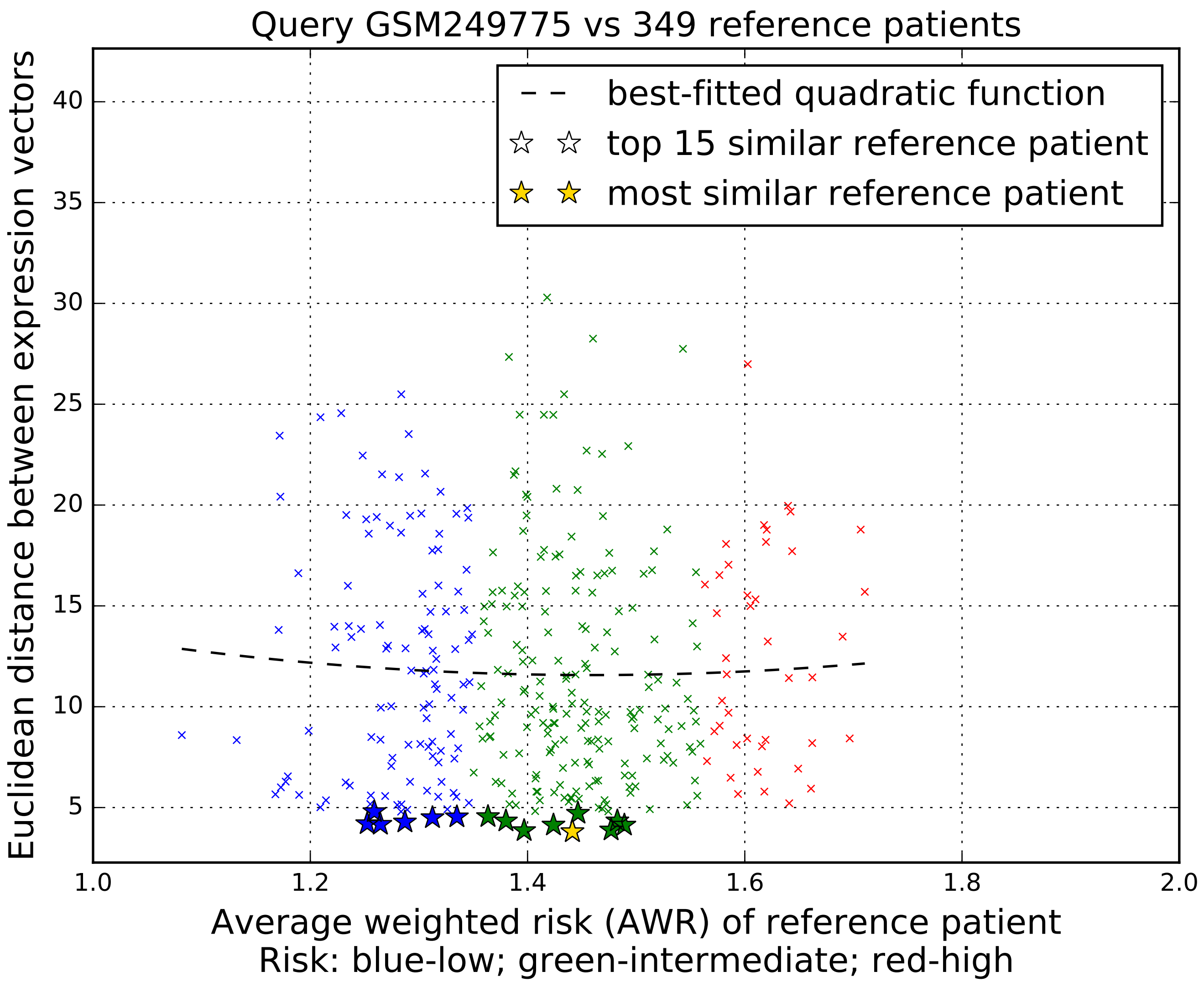

Query GSM249775 vs 349 reference patients
best-fitted quadratic function
top 15 similar reference patient
most similar reference patient
Euclidean distance between expression vectors
40
35
30
25
20
15
10
5
1.0
1.2
1.4
1.6
1.8
2.0
Average weighted risk (AWR) of reference patient
Risk: blue-low; green-intermediate; red-high

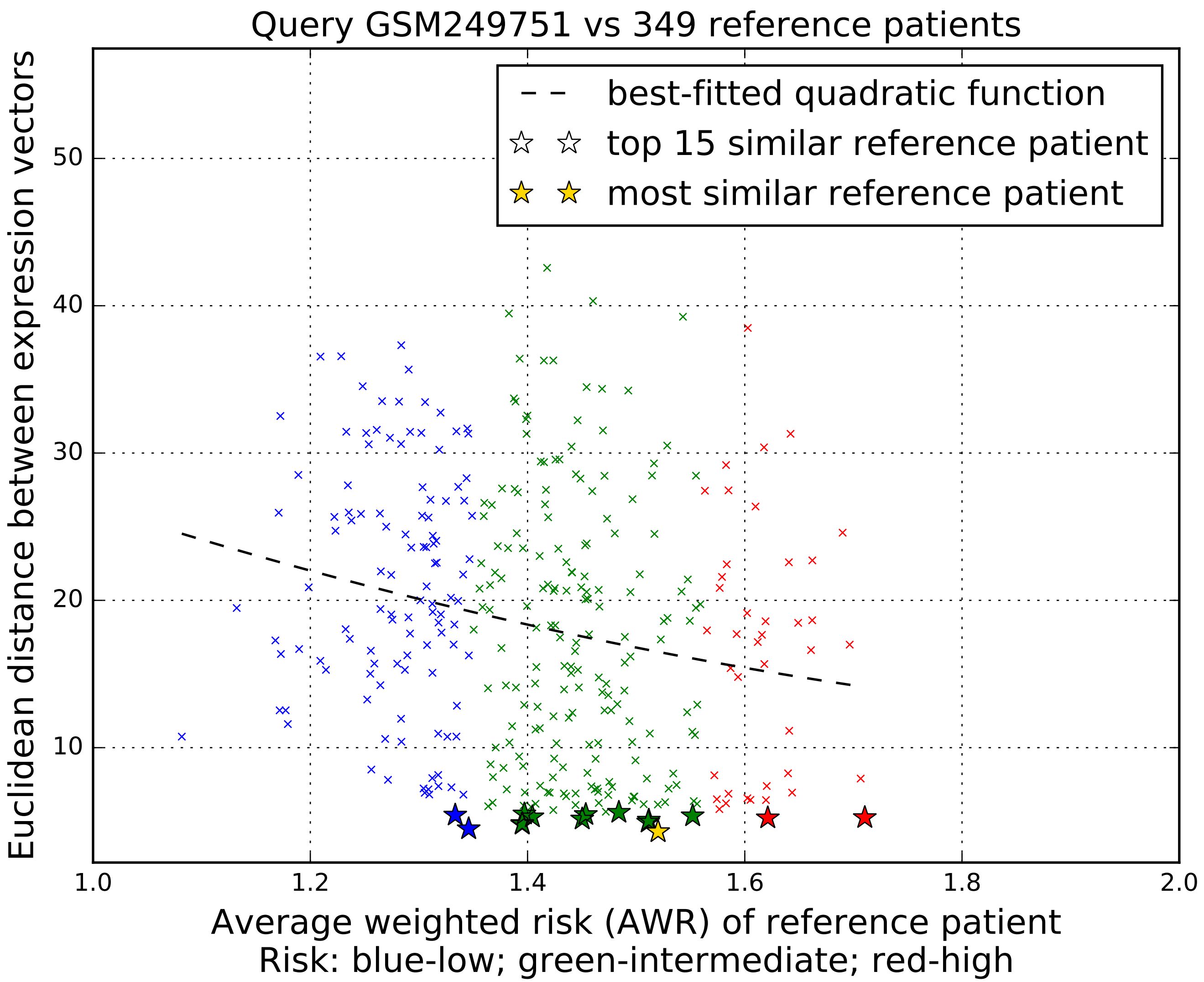

Query GSM249751 vs 349 reference patients
best-fitted quadratic function
top 15 similar reference patient
most similar reference patient
Euclidean distance between expression vectors
10
20
30
40
50
1.0
1.2
1.4
1.6
1.8
2.0
Average weighted risk (AWR) of reference patient
Risk: blue-low; green-intermediate; red-high

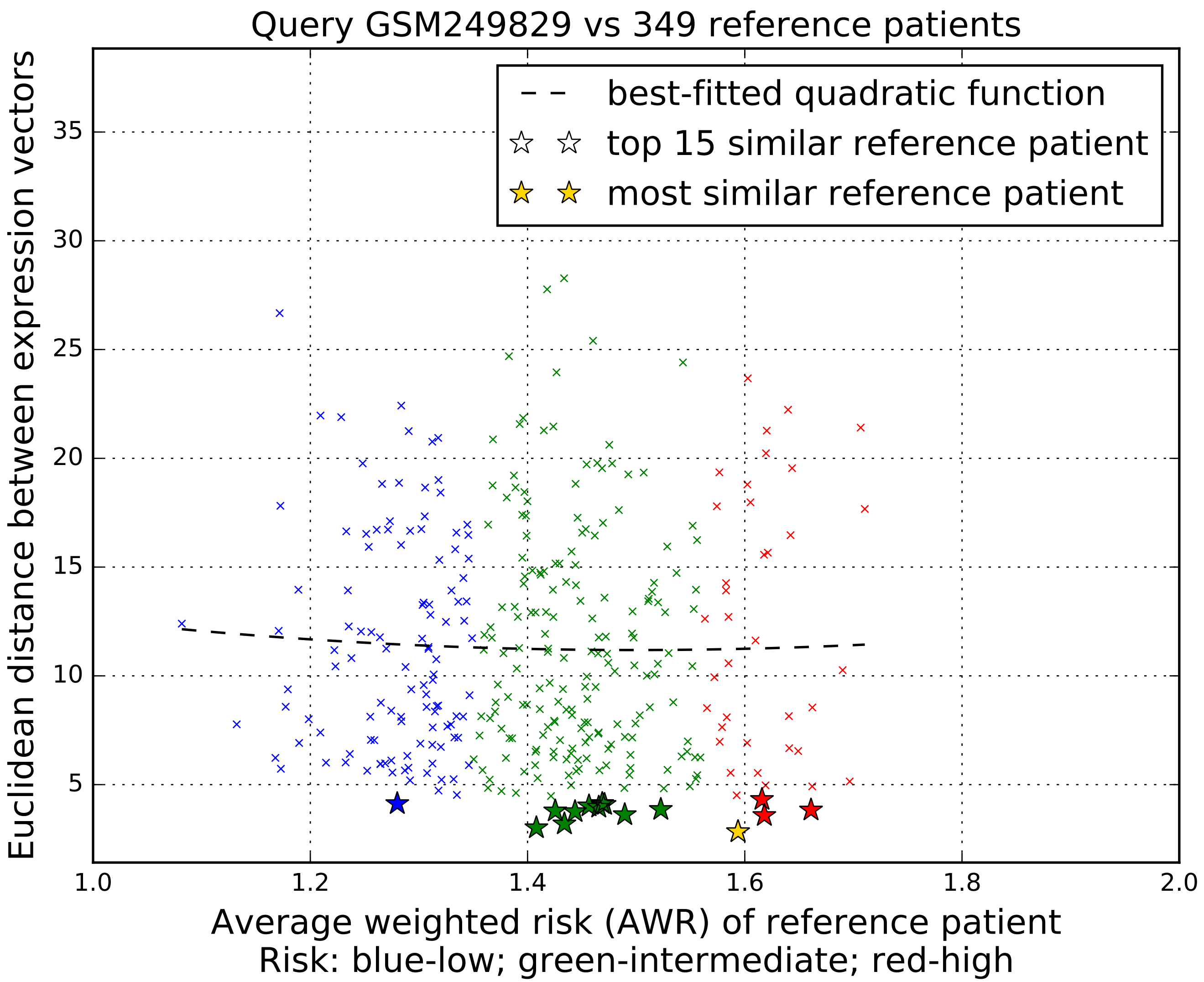

Query GSM249829 vs 349 reference patients
best-fitted quadratic function
top 15 similar reference patient
most similar reference patient
Euclidean distance between expression vectors
35
30
25
20
15
10
5
1.0
1.2
1.4
1.6
1.8
2.0
Average weighted risk (AWR) of reference patient
Risk: blue-low; green-intermediate; red-high

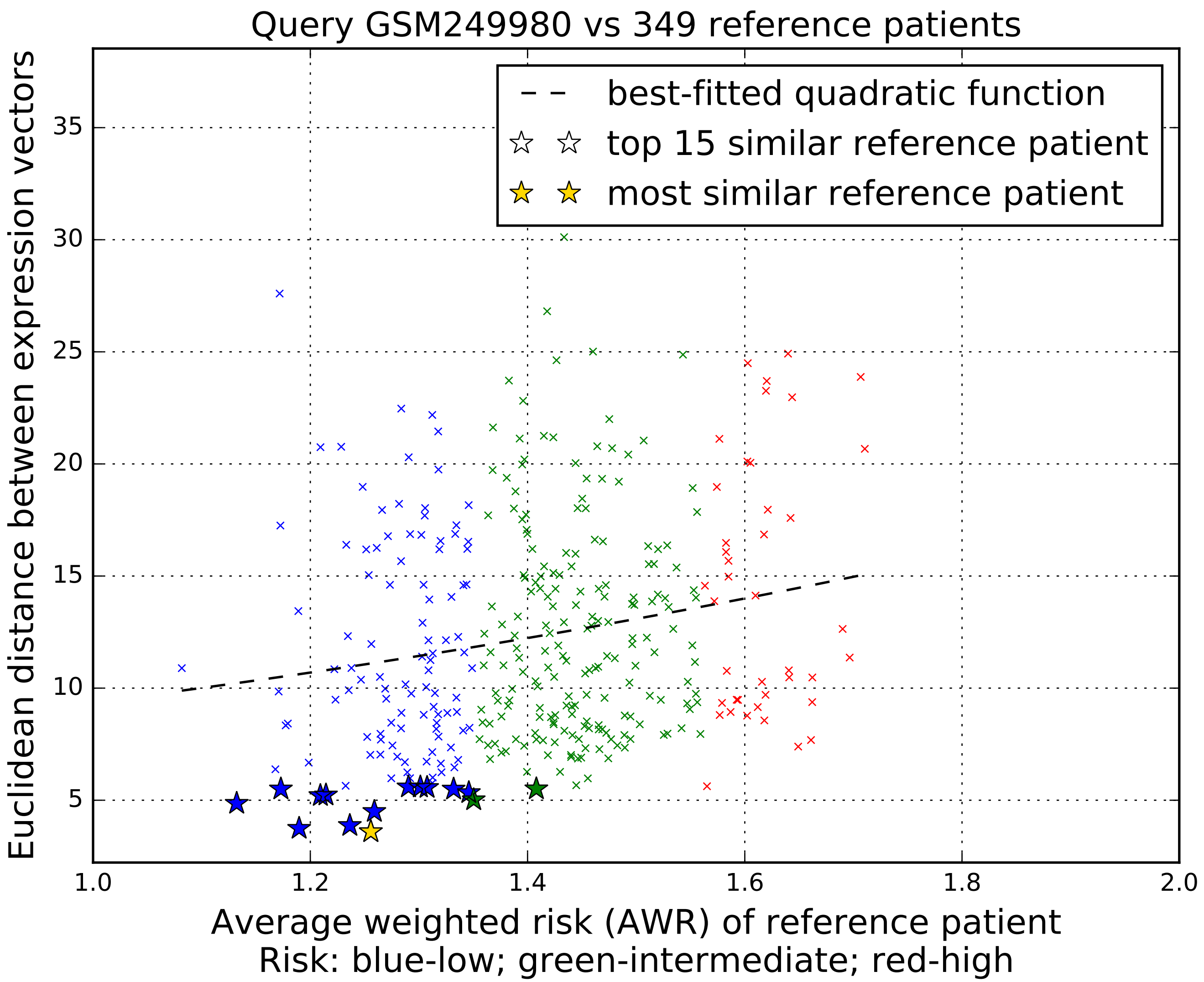

Query GSM249980 vs 349 reference patients
best-fitted quadratic function
top 15 similar reference patient
most similar reference patient
Euclidean distance between expression vectors
35
30
25
20
15
10
5
1.0
1.2
1.4
1.6
1.8
2.0
Average weighted risk (AWR) of reference patient
Risk: blue-low; green-intermediate; red-high

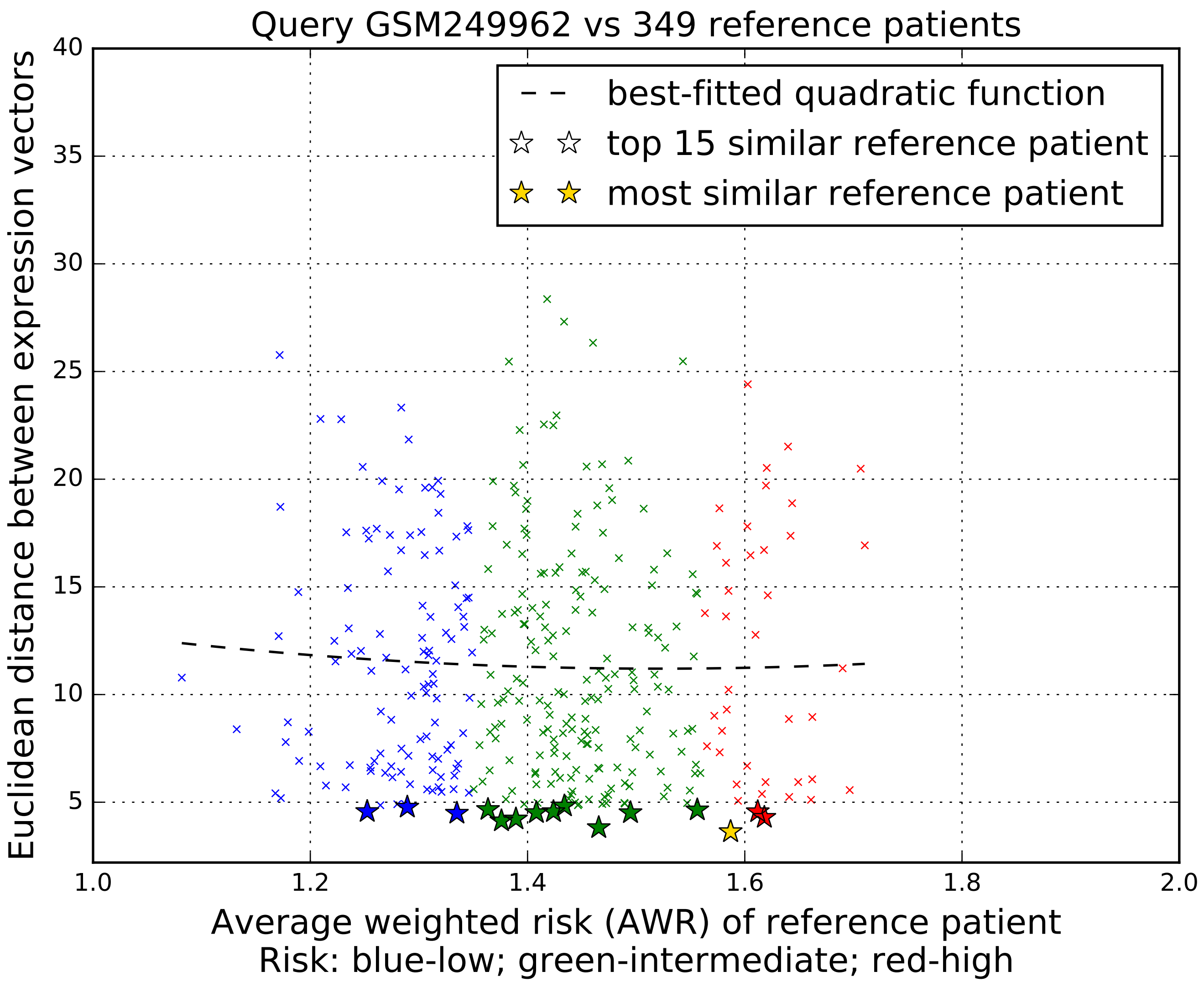

Query GSM249962 vs 349 reference patients
best-fitted quadratic function
top 15 similar reference patient
most similar reference patient
Euclidean distance between expression vectors
40
35
30
25
20
15
10
5
1.0
1.2
1.4
1.6
1.8
2.0
Average weighted risk (AWR) of reference patient
Risk: blue-low; green-intermediate; red-high

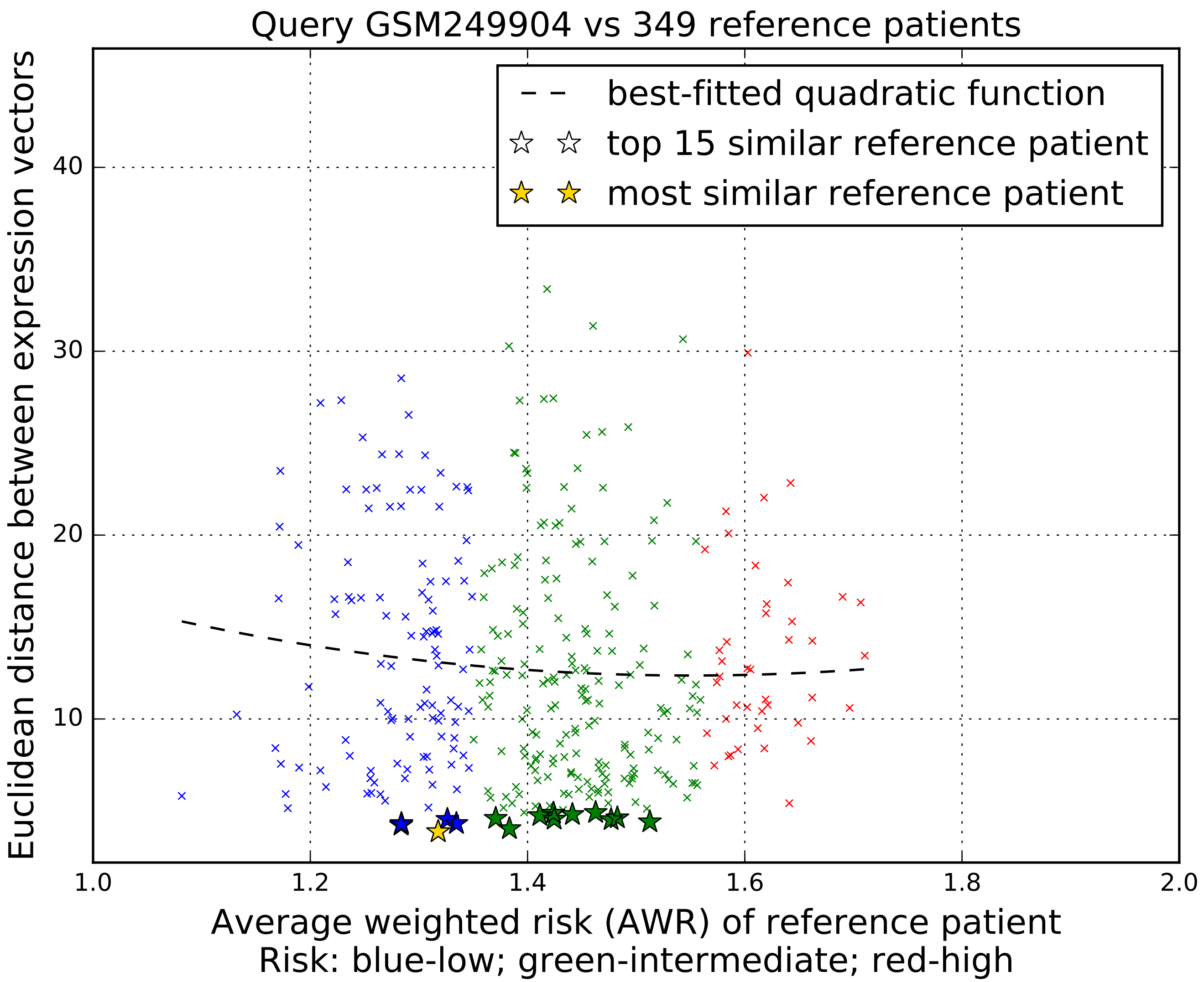
Query GSM249904 vs 349 reference patients
best-fitted quadratic function
top 15 similar reference patient
most similar reference patient
Euclidean distance between expression vectors
10
20
30
40
1.0
1.2
1.4
1.6
1.8
2.0
Average weighted risk (AWR) of reference patient
Risk: blue-low; green-intermediate; red-high

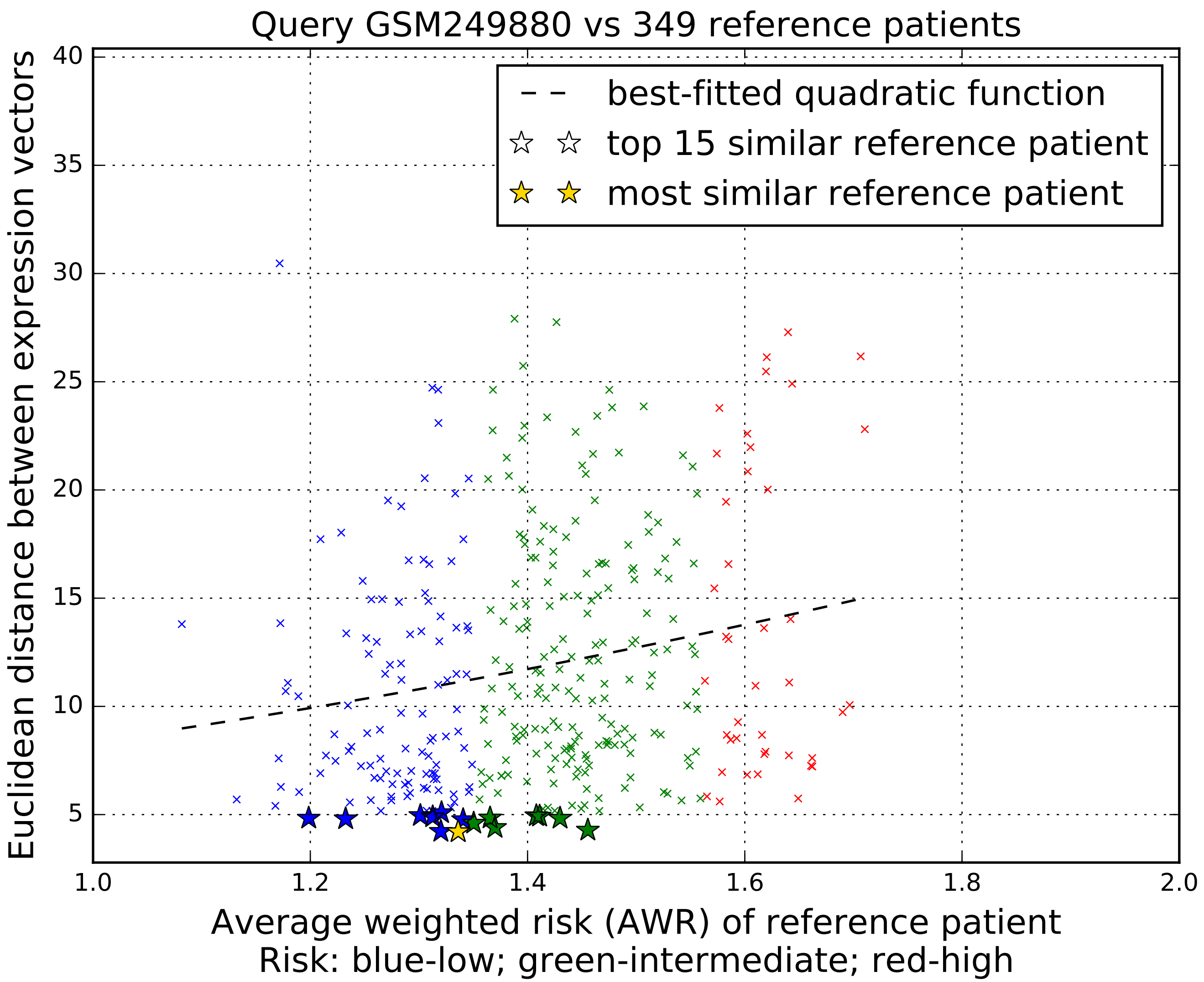

Query GSM249880 vs 349 reference patients
best-fitted quadratic function
top 15 similar reference patient
most similar reference patient
Euclidean distance between expression vectors
Average weighted risk (AWR) of reference patient
Risk: blue-low; green-intermediate; red-high
1.0
1.2
1.4
1.6
1.8
2.0
5
10
15
20
25
30
35
40

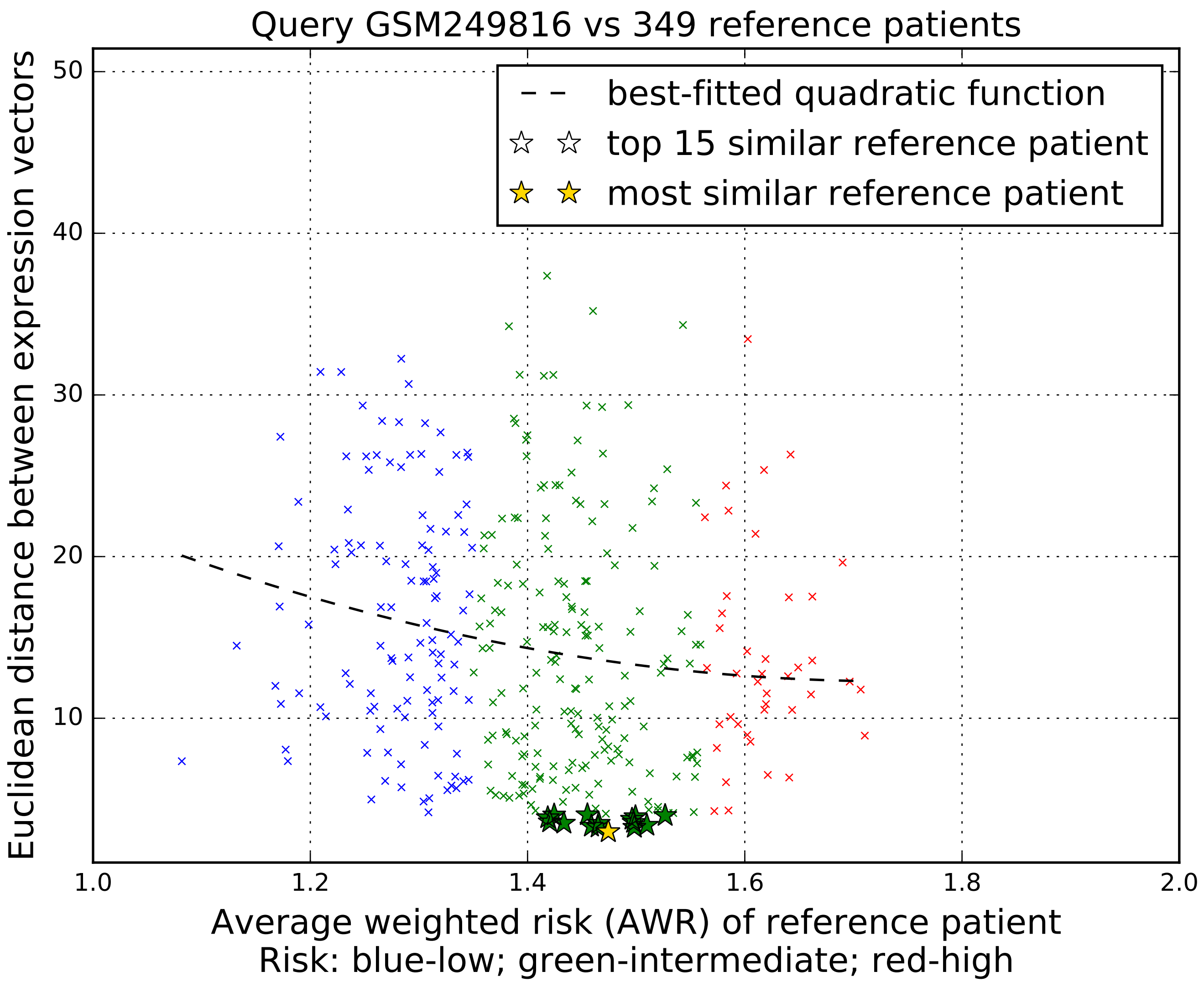

Query GSM249816 vs 349 reference patients
best-fitted quadratic function
top 15 similar reference patient
most similar reference patient
Euclidean distance between expression vectors
50
40
30
20
10
1.0
1.2
1.4
1.6
1.8
2.0
Average weighted risk (AWR) of reference patient
Risk: blue-low; green-intermediate; red-high

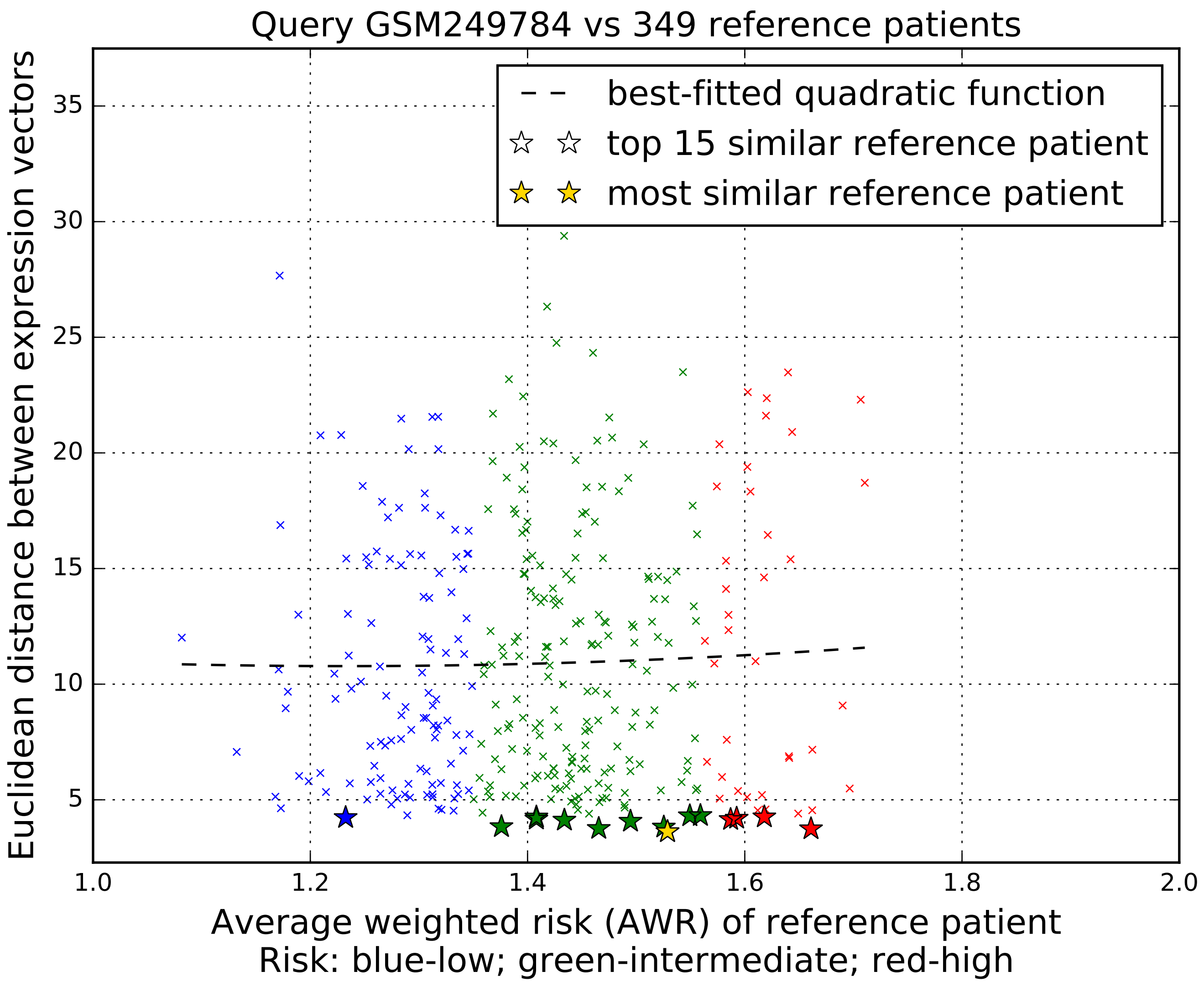
Query GSM249784 vs 349 reference patients
best-fitted quadratic function
top 15 similar reference patient
most similar reference patient
Euclidean distance between expression vectors
35
30
25
20
15
10
5
1.0
1.2
1.4
1.6
1.8
2.0
Average weighted risk (AWR) of reference patient
Risk: blue-low; green-intermediate; red-high

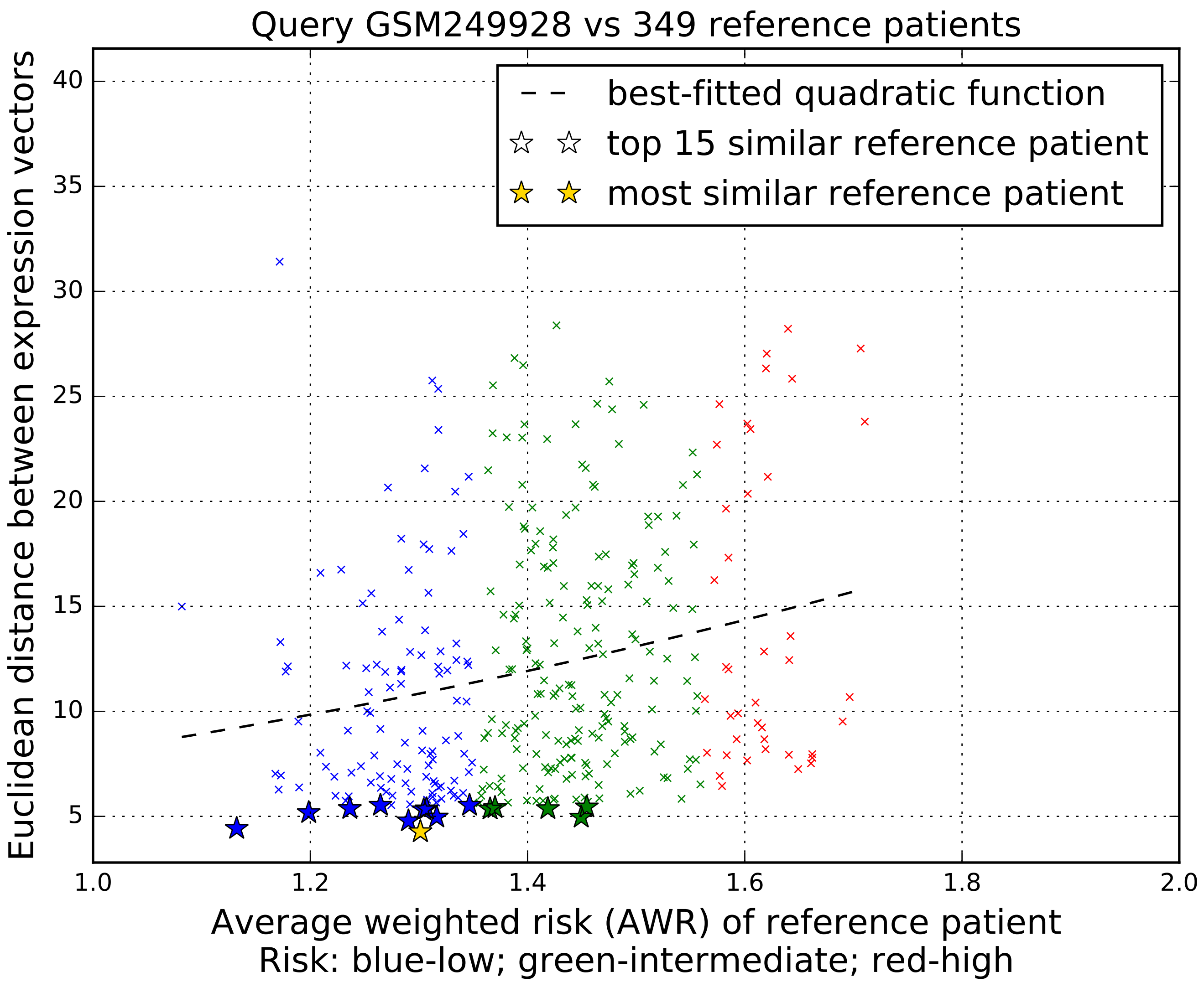

Query GSM249928 vs 349 reference patients
best-fitted quadratic function
top 15 similar reference patient
most similar reference patient
Euclidean distance between expression vectors
40
35
30
25
20
15
10
5
1.0
1.2
1.4
1.6
1.8
2.0
Average weighted risk (AWR) of reference patient
Risk: blue-low; green-intermediate; red-high

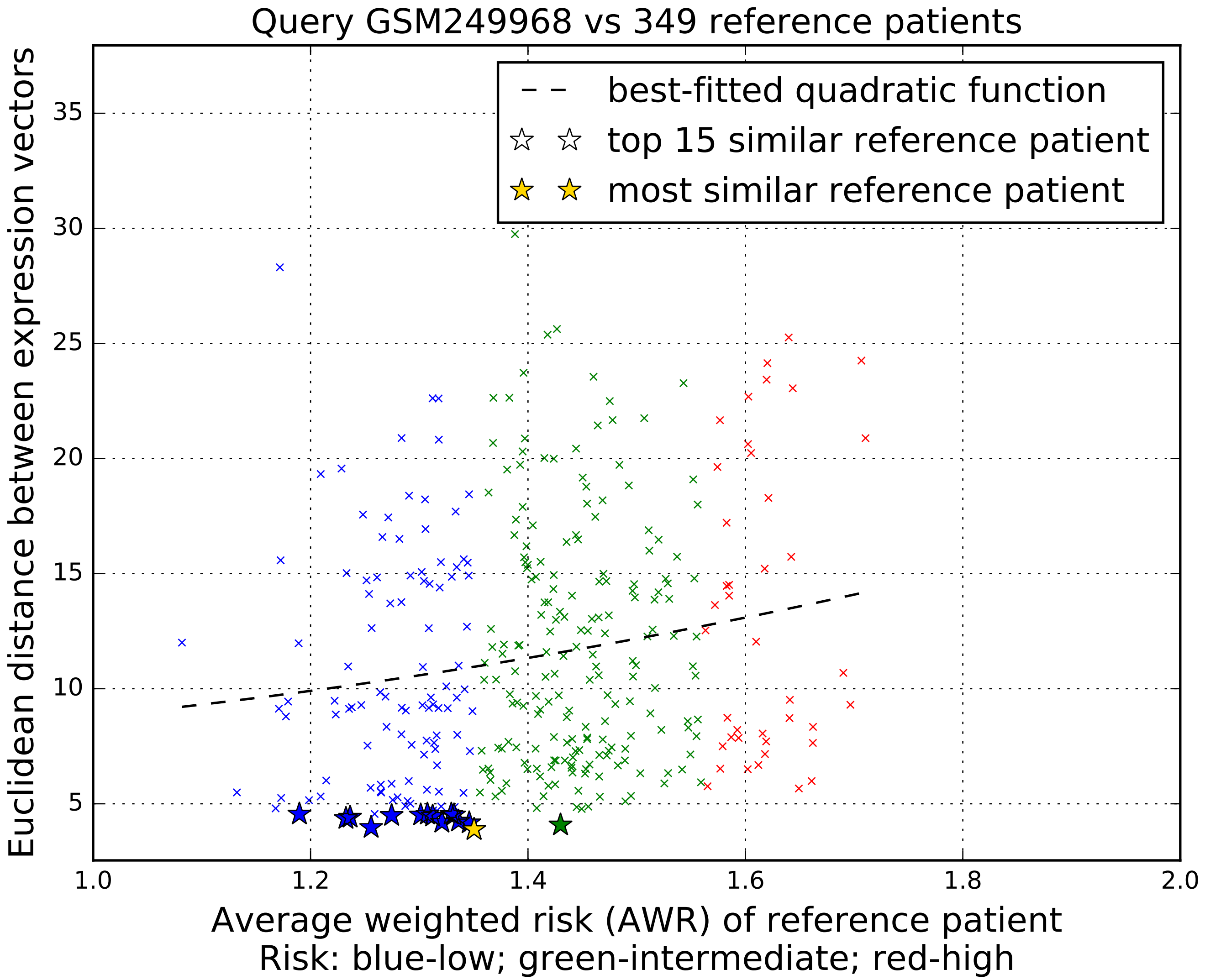
Query GSM249968 vs 349 reference patients
best-fitted quadratic function
top 15 similar reference patient
most similar reference patient
Euclidean distance between expression vectors
35
30
25
20
15
10
5
1.0
1.2
1.4
1.6
1.8
2.0
Average weighted risk (AWR) of reference patient
Risk: blue-low; green-intermediate; red-high

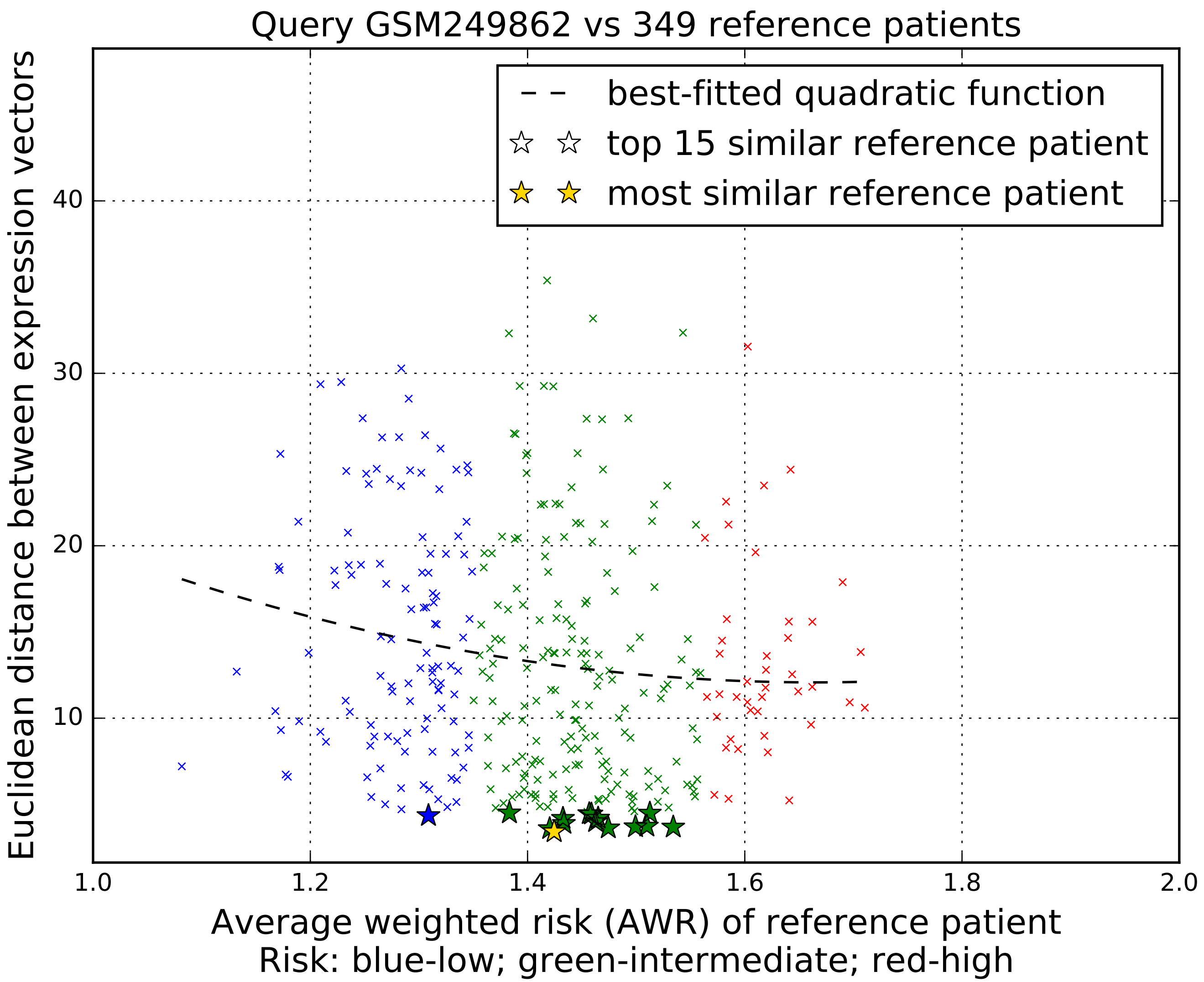

Query GSM249862 vs 349 reference patients
best-fitted quadratic function
top 15 similar reference patient
most similar reference patient
Euclidean distance between expression vectors
40
30
20
10
1.0
1.2
1.4
1.6
1.8
2.0
Average weighted risk (AWR) of reference patient
Risk: blue-low; green-intermediate; red-high

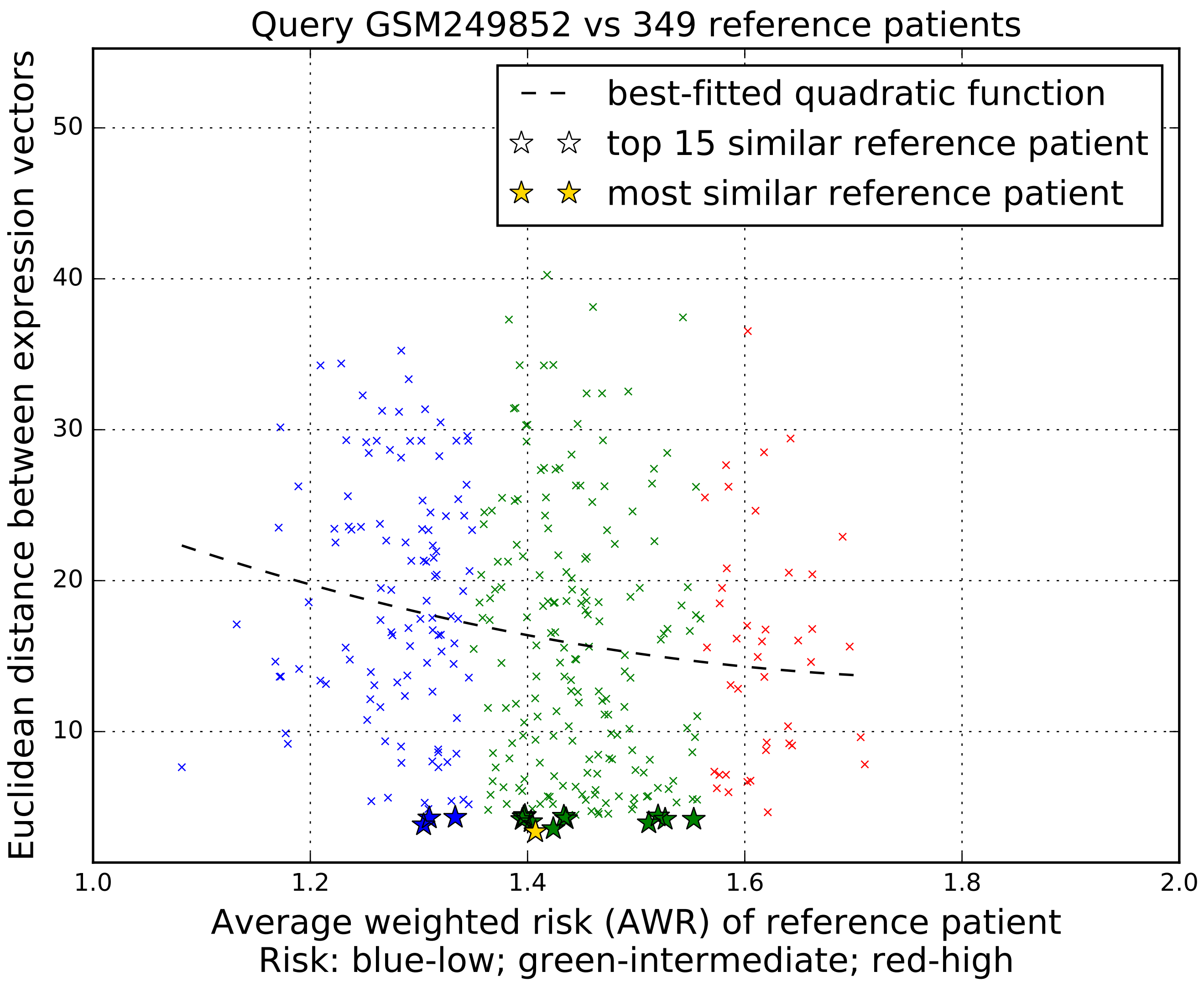
Query GSM249852 vs 349 reference patients
best-fitted quadratic function
top 15 similar reference patient
most similar reference patient
Euclidean distance between expression vectors
10
20
30
40
50
1.0
1.2
1.4
1.6
1.8
2.0
Average weighted risk (AWR) of reference patient
Risk: blue-low; green-intermediate; red-high

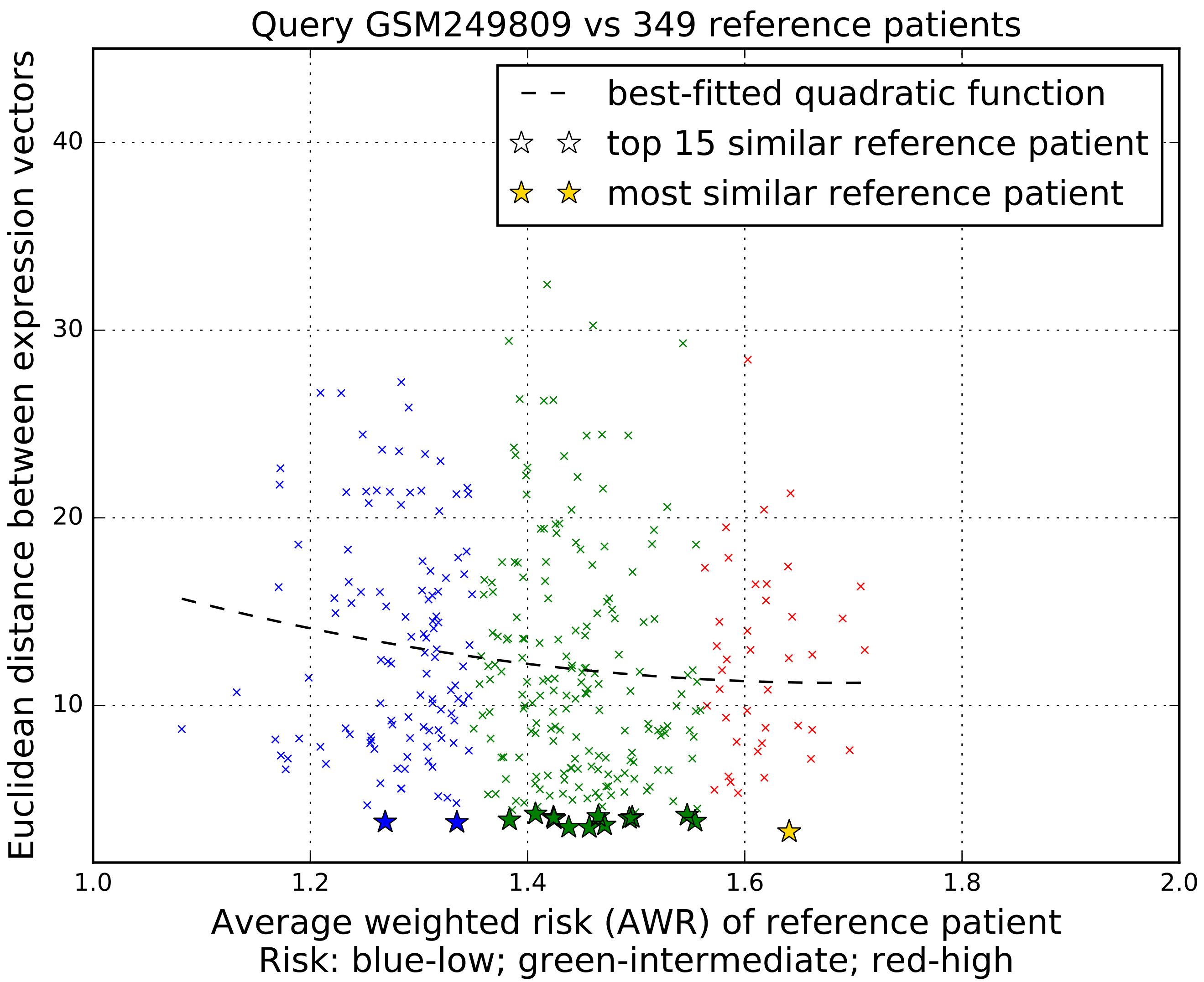

Query GSM249809 vs 349 reference patients
best-fitted quadratic function
top 15 similar reference patient
most similar reference patient
Euclidean distance between expression vectors
10
20
30
40
1.0
1.2
1.4
1.6
1.8
2.0
Average weighted risk (AWR) of reference patient
Risk: blue-low; green-intermediate; red-high

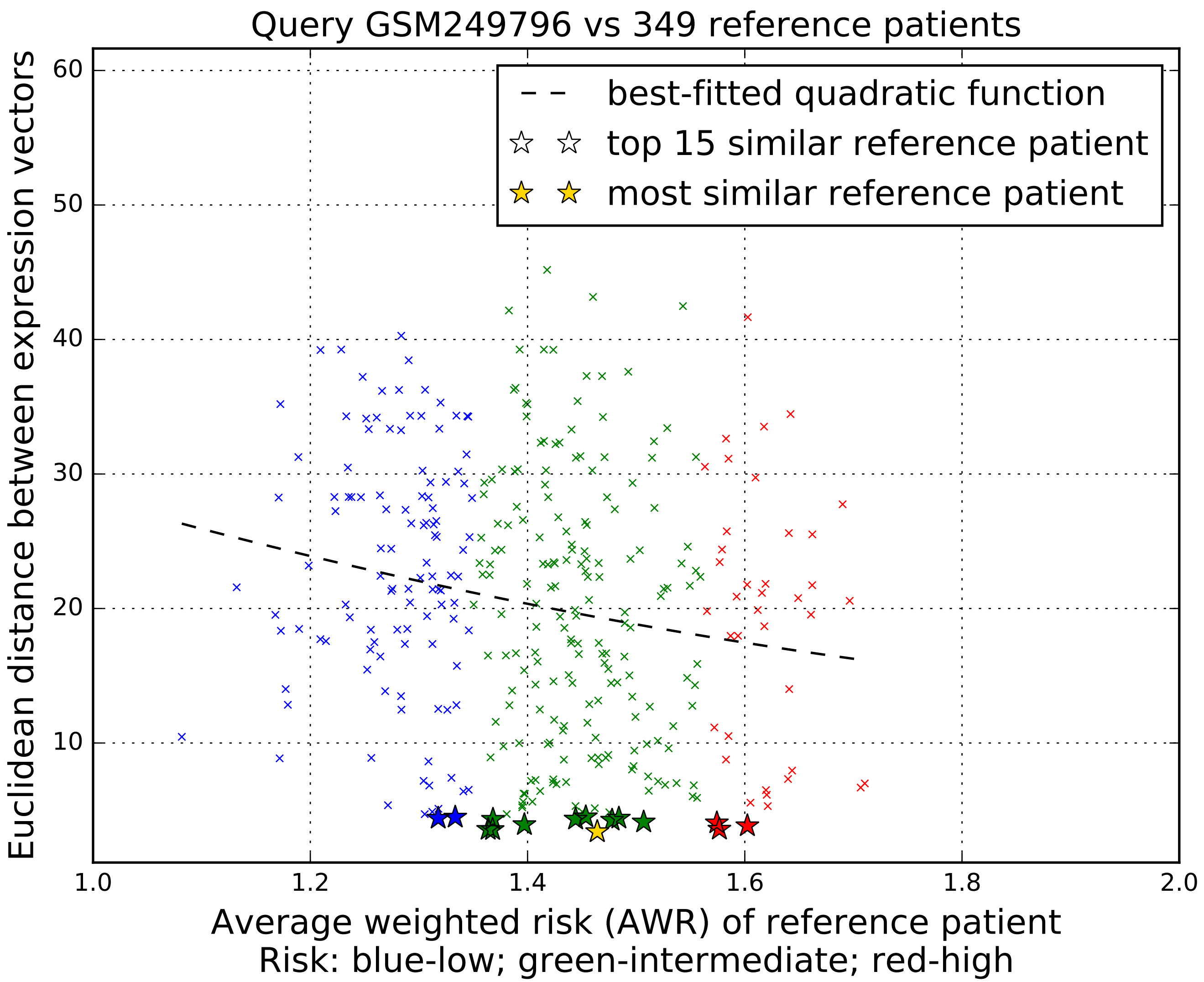

Query GSM249796 vs 349 reference patients
best-fitted quadratic function
top 15 similar reference patient
most similar reference patient
Euclidean distance between expression vectors
60
50
40
30
20
10
1.0
1.2
1.4
1.6
1.8
2.0
Average weighted risk (AWR) of reference patient
Risk: blue-low; green-intermediate; red-high

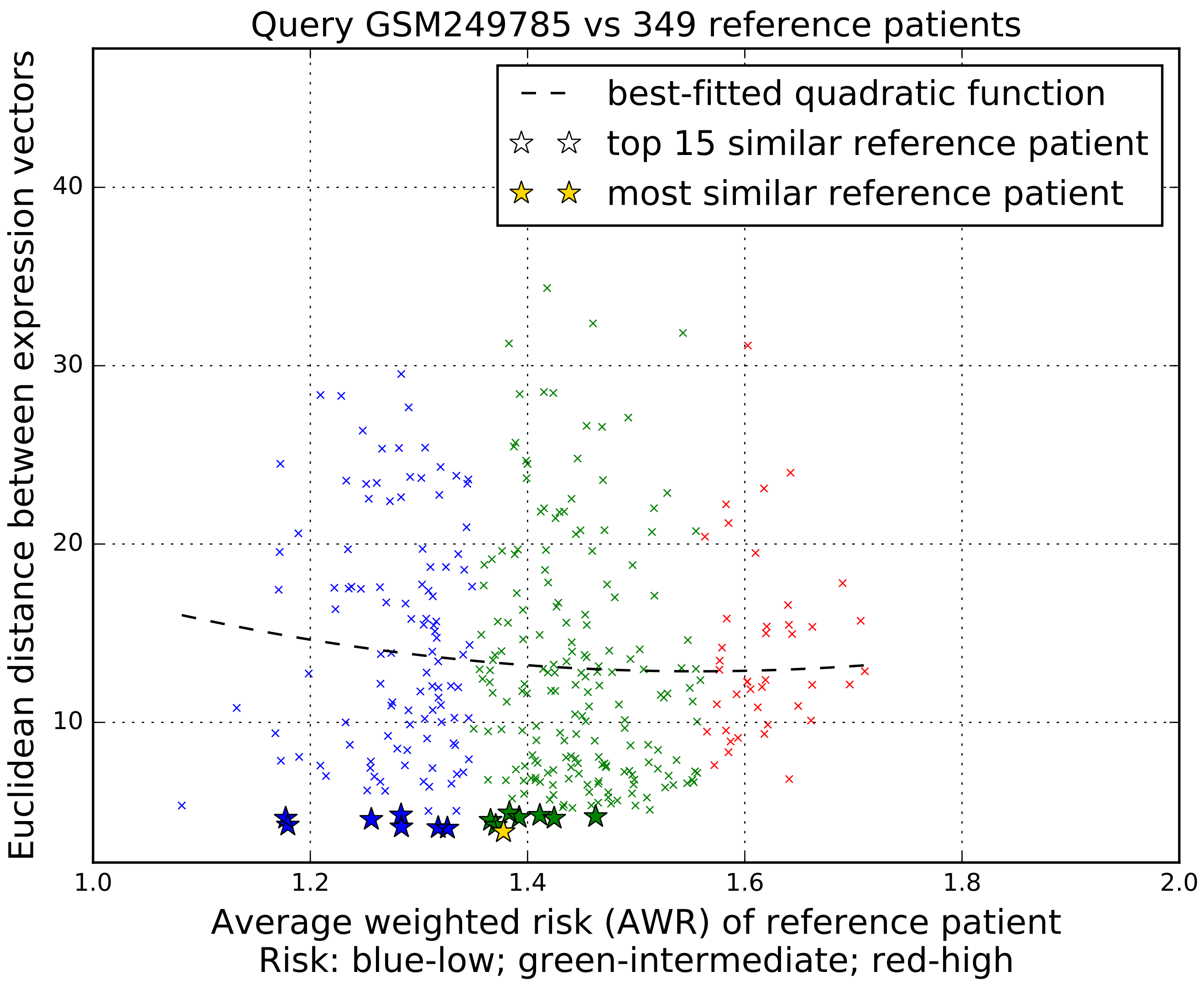
Query GSM249785 vs 349 reference patients
best-fitted quadratic function
top 15 similar reference patient
most similar reference patient
Euclidean distance between expression vectors
10
20
30
40
1.0
1.2
1.4
1.6
1.8
2.0
Average weighted risk (AWR) of reference patient
Risk: blue-low; green-intermediate; red-high

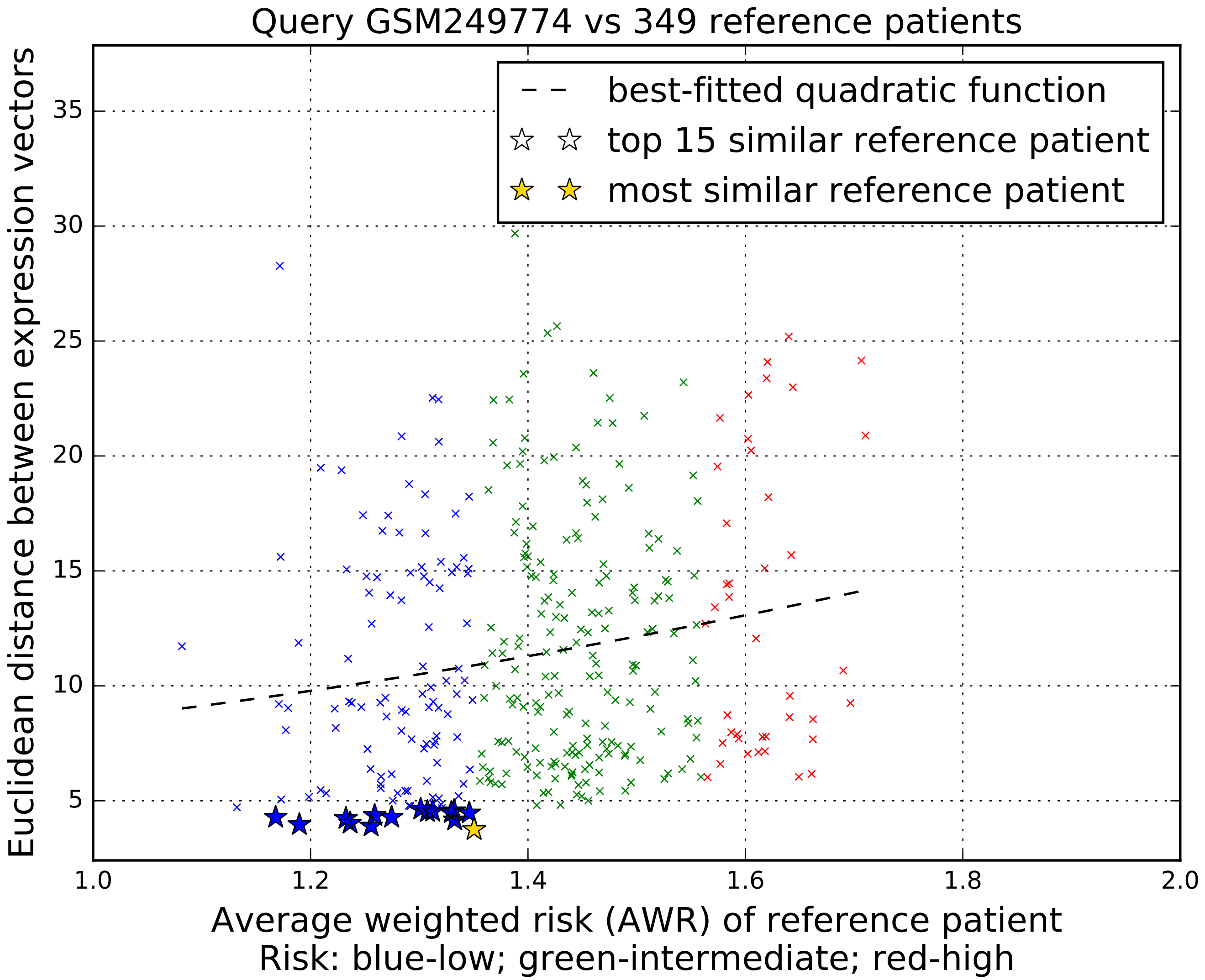
Query GSM249774 vs 349 reference patients
best-fitted quadratic function
top 15 similar reference patient
most similar reference patient
Euclidean distance between expression vectors
35
30
25
20
15
10
5
1.0
1.2
1.4
1.6
1.8
2.0
Average weighted risk (AWR) of reference patient
Risk: blue-low; green-intermediate; red-high

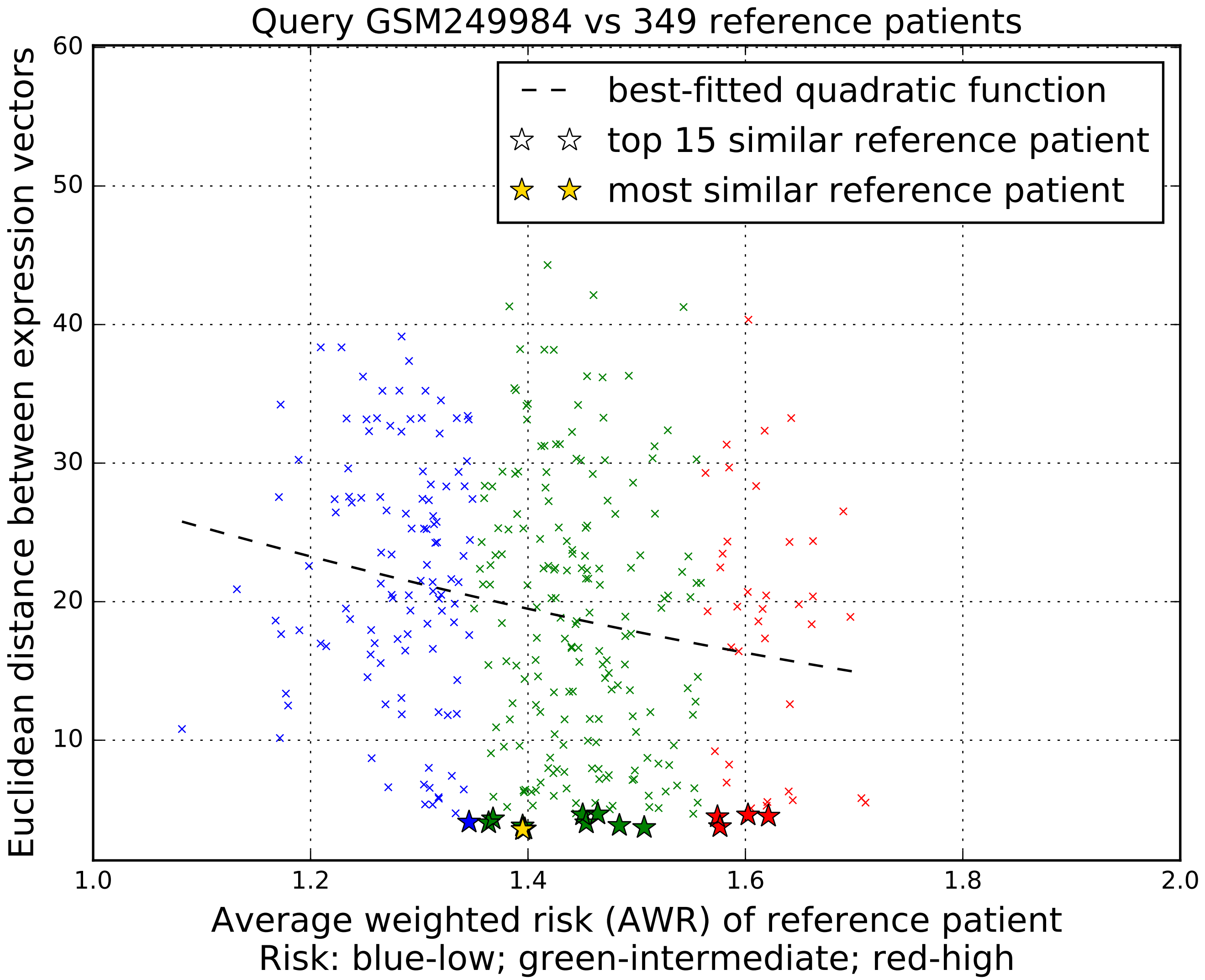
Query GSM249984 vs 349 reference patients
best-fitted quadratic function
top 15 similar reference patient
most similar reference patient
Euclidean distance between expression vectors
Average weighted risk (AWR) of reference patient
Risk: blue-low; green-intermediate; red-high
60
50
40
30
20
10
1.0
1.2
1.4
1.6
1.8
2.0

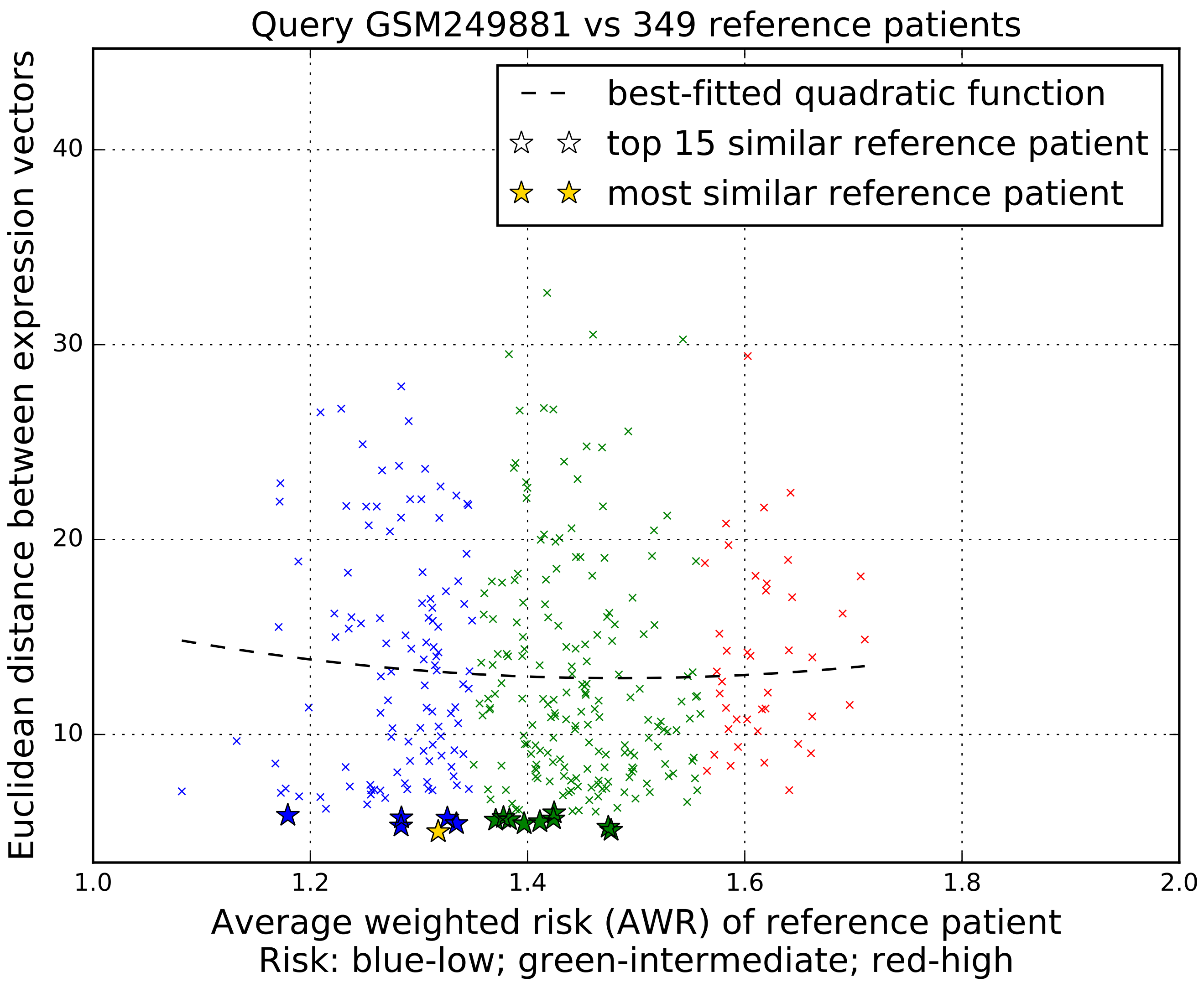

Query GSM249881 vs 349 reference patients
best-fitted quadratic function
top 15 similar reference patient
most similar reference patient
Euclidean distance between expression vectors
10
20
30
40
1.0
1.2
1.4
1.6
1.8
2.0
Average weighted risk (AWR) of reference patient
Risk: blue-low; green-intermediate; red-high

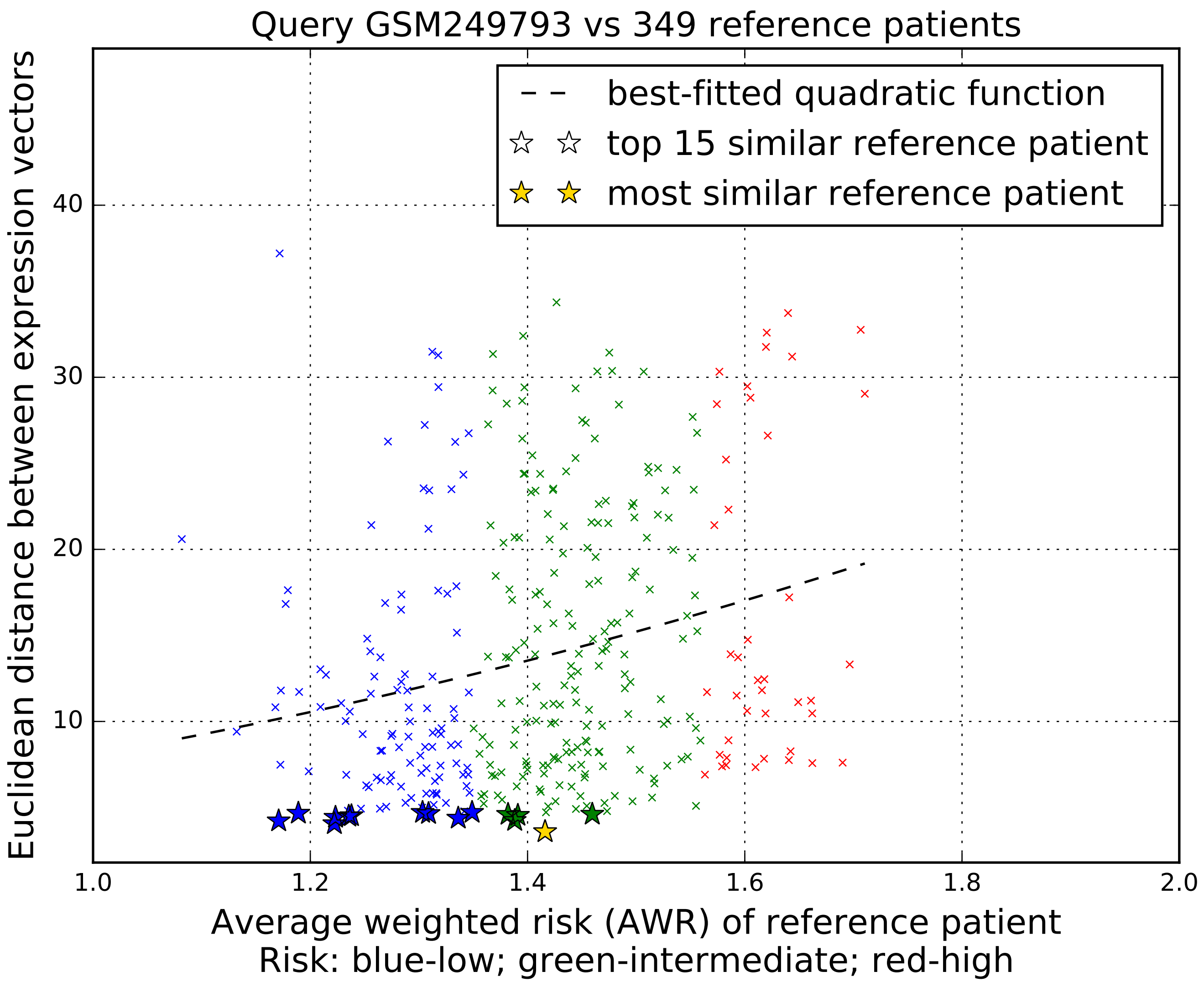

Query GSM249793 vs 349 reference patients
best-fitted quadratic function
top 15 similar reference patient
most similar reference patient
Euclidean distance between expression vectors
10
20
30
40
1.0
1.2
1.4
1.6
1.8
2.0
Average weighted risk (AWR) of reference patient
Risk: blue-low; green-intermediate; red-high

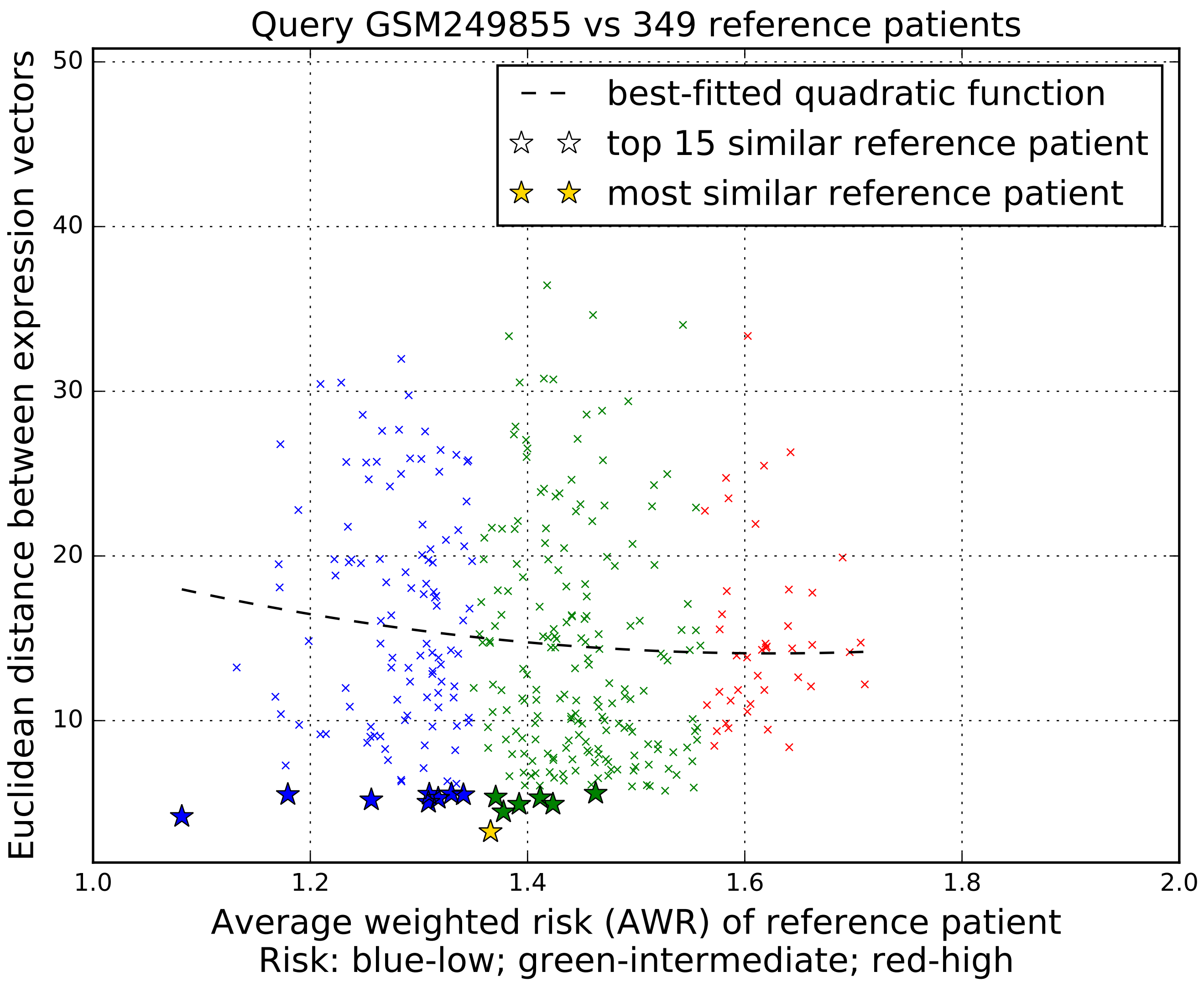
Query GSM249855 vs 349 reference patients
best-fitted quadratic function
top 15 similar reference patient
most similar reference patient
Euclidean distance between expression vectors
Average weighted risk (AWR) of reference patient
Risk: blue-low; green-intermediate; red-high
50
40
30
20
10
1.0
1.2
1.4
1.6
1.8
2.0

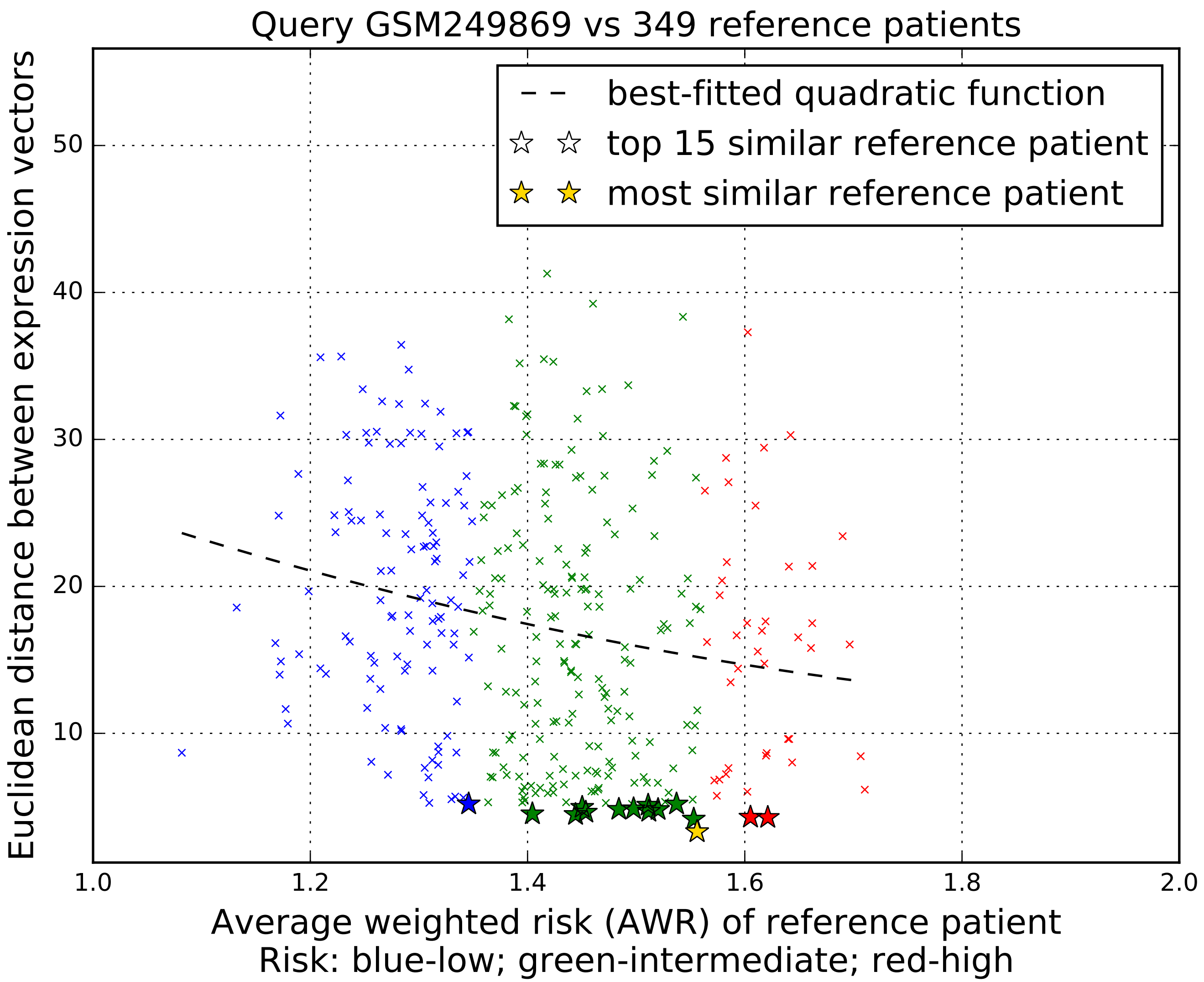

Query GSM249869 vs 349 reference patients
best-fitted quadratic function
top 15 similar reference patient
most similar reference patient
Euclidean distance between expression vectors
10
20
30
40
50
1.0
1.2
1.4
1.6
1.8
2.0
Average weighted risk (AWR) of reference patient
Risk: blue-low; green-intermediate; red-high

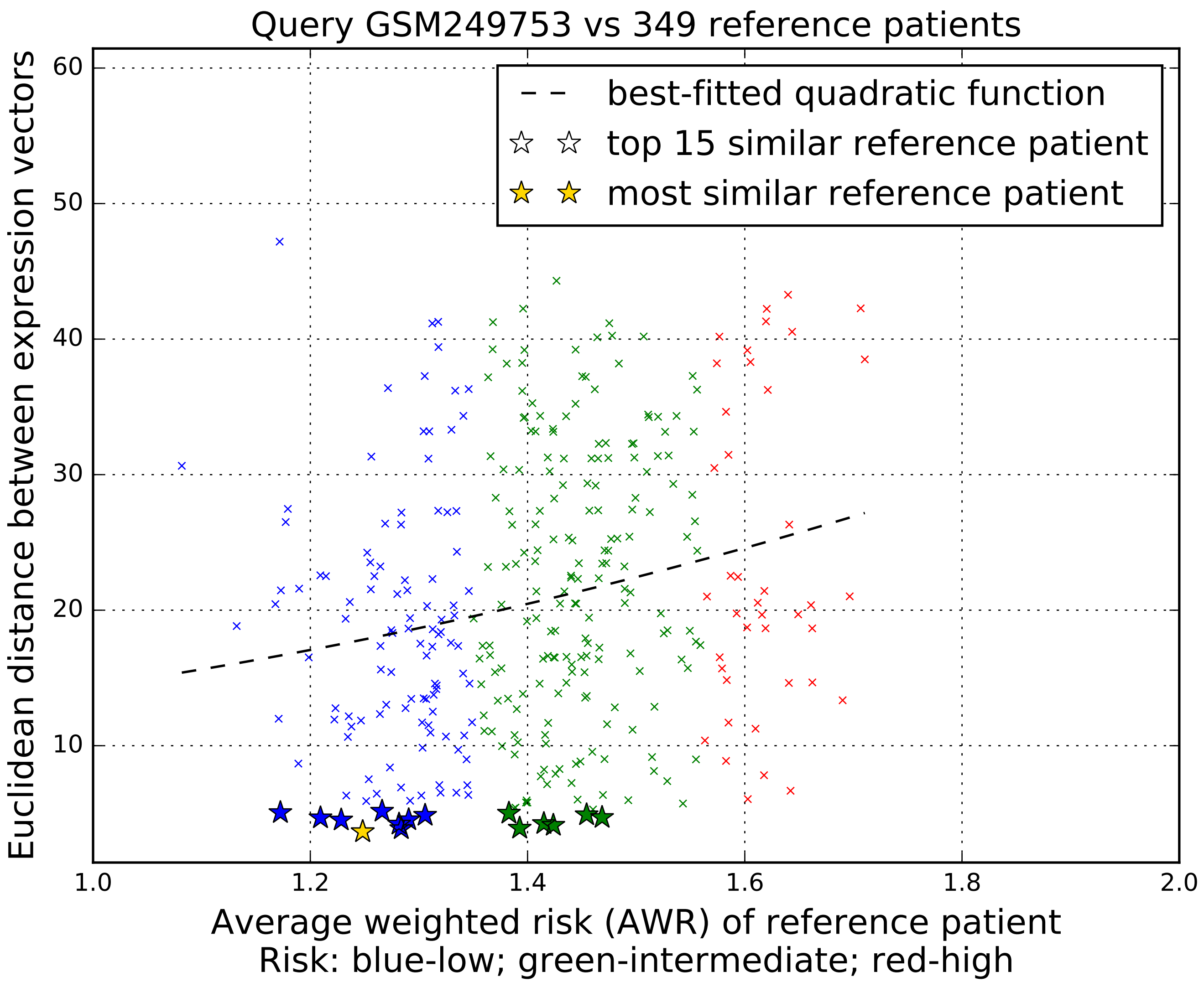

Query GSM249753 vs 349 reference patients
best-fitted quadratic function
top 15 similar reference patient
most similar reference patient
Euclidean distance between expression vectors
Average weighted risk (AWR) of reference patient
Risk: blue-low; green-intermediate; red-high
60
50
40
30
20
10
1.0
1.2
1.4
1.6
1.8
2.0

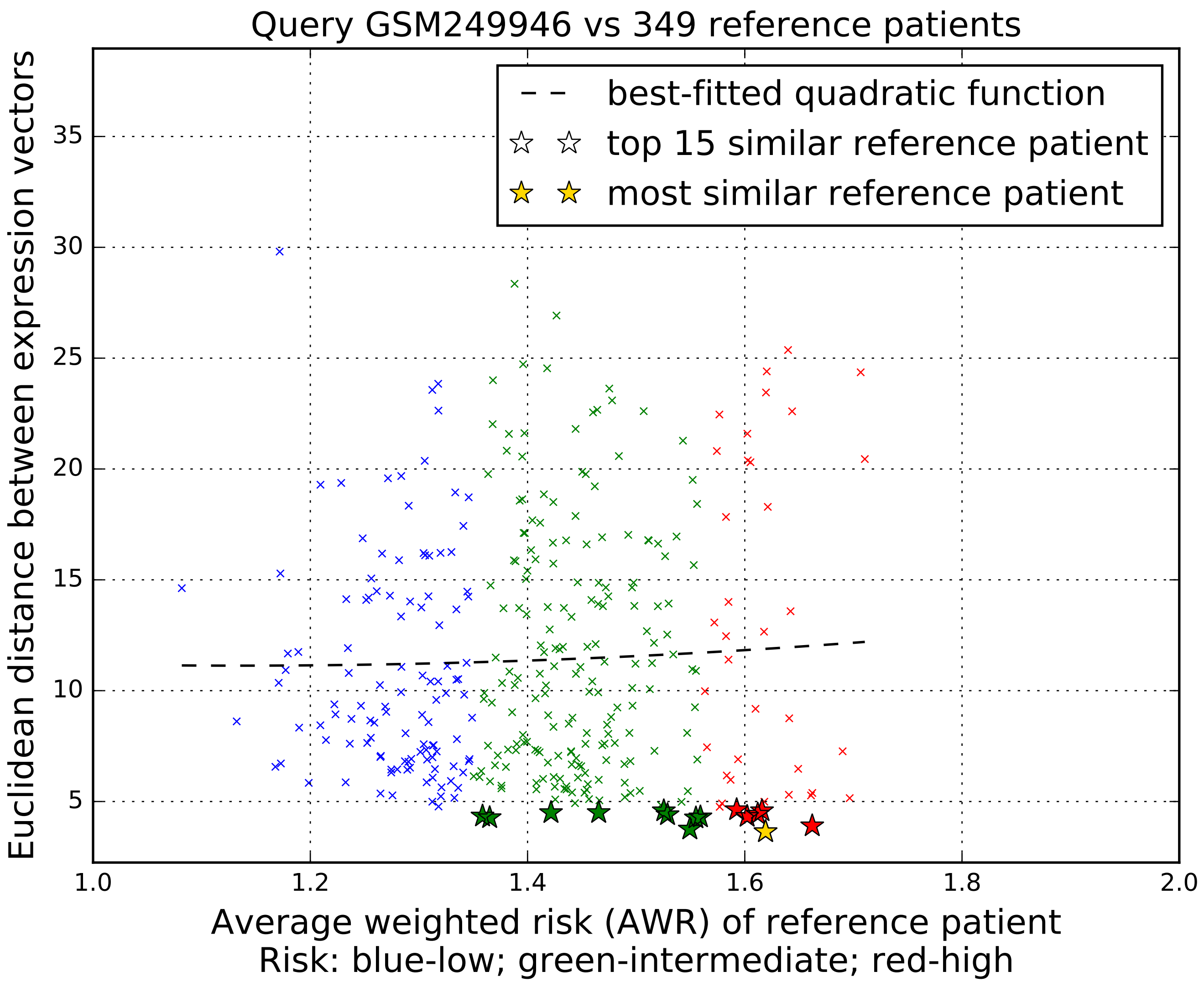

Query GSM249946 vs 349 reference patients
best-fitted quadratic function
top 15 similar reference patient
most similar reference patient
Euclidean distance between expression vectors
35
30
25
20
15
10
5
1.0
1.2
1.4
1.6
1.8
2.0
Average weighted risk (AWR) of reference patient
Risk: blue-low; green-intermediate; red-high

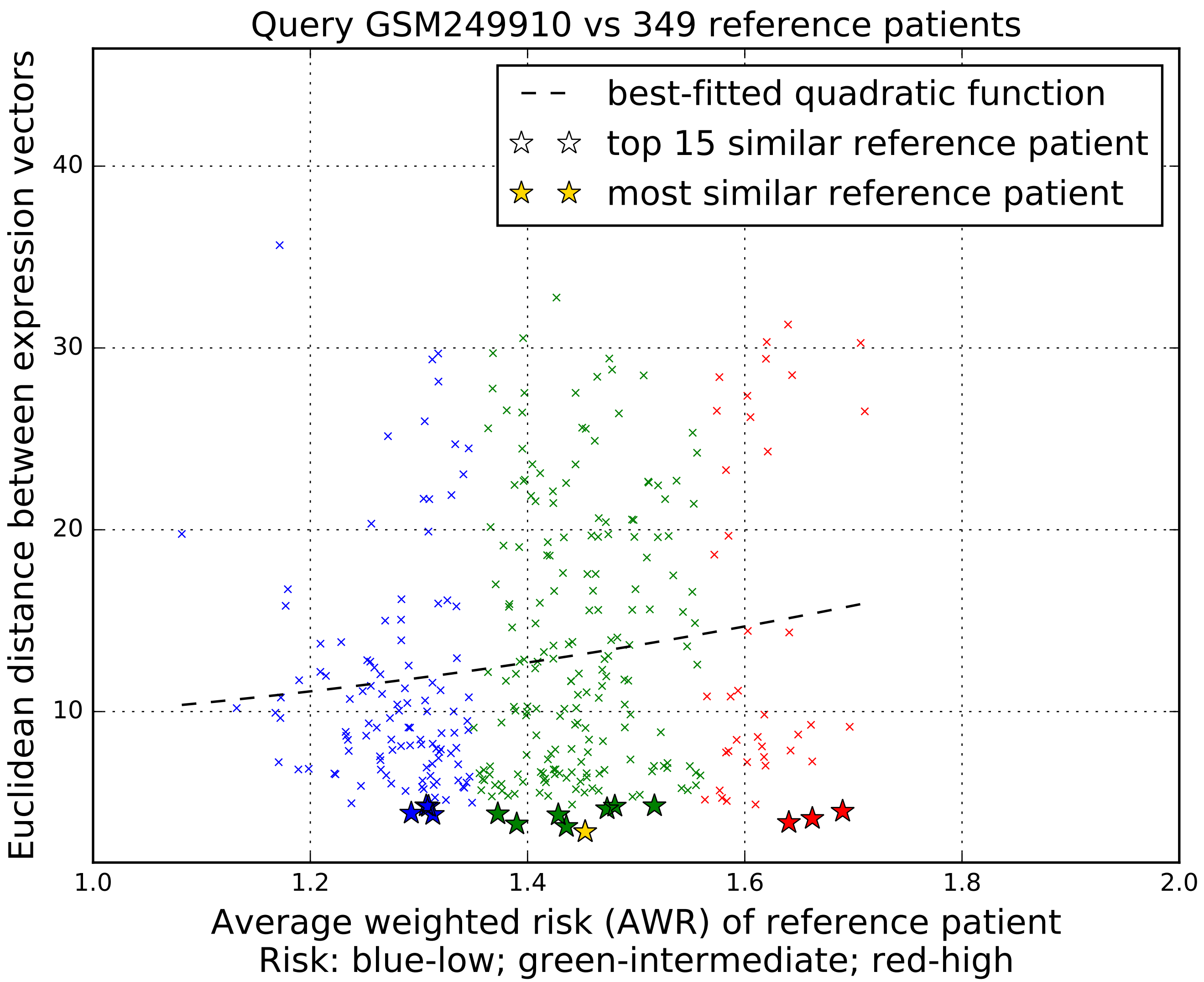

Query GSM249910 vs 349 reference patients
best-fitted quadratic function
top 15 similar reference patient
most similar reference patient
Euclidean distance between expression vectors
10
20
30
40
1.0
1.2
1.4
1.6
1.8
2.0
Average weighted risk (AWR) of reference patient
Risk: blue-low; green-intermediate; red-high

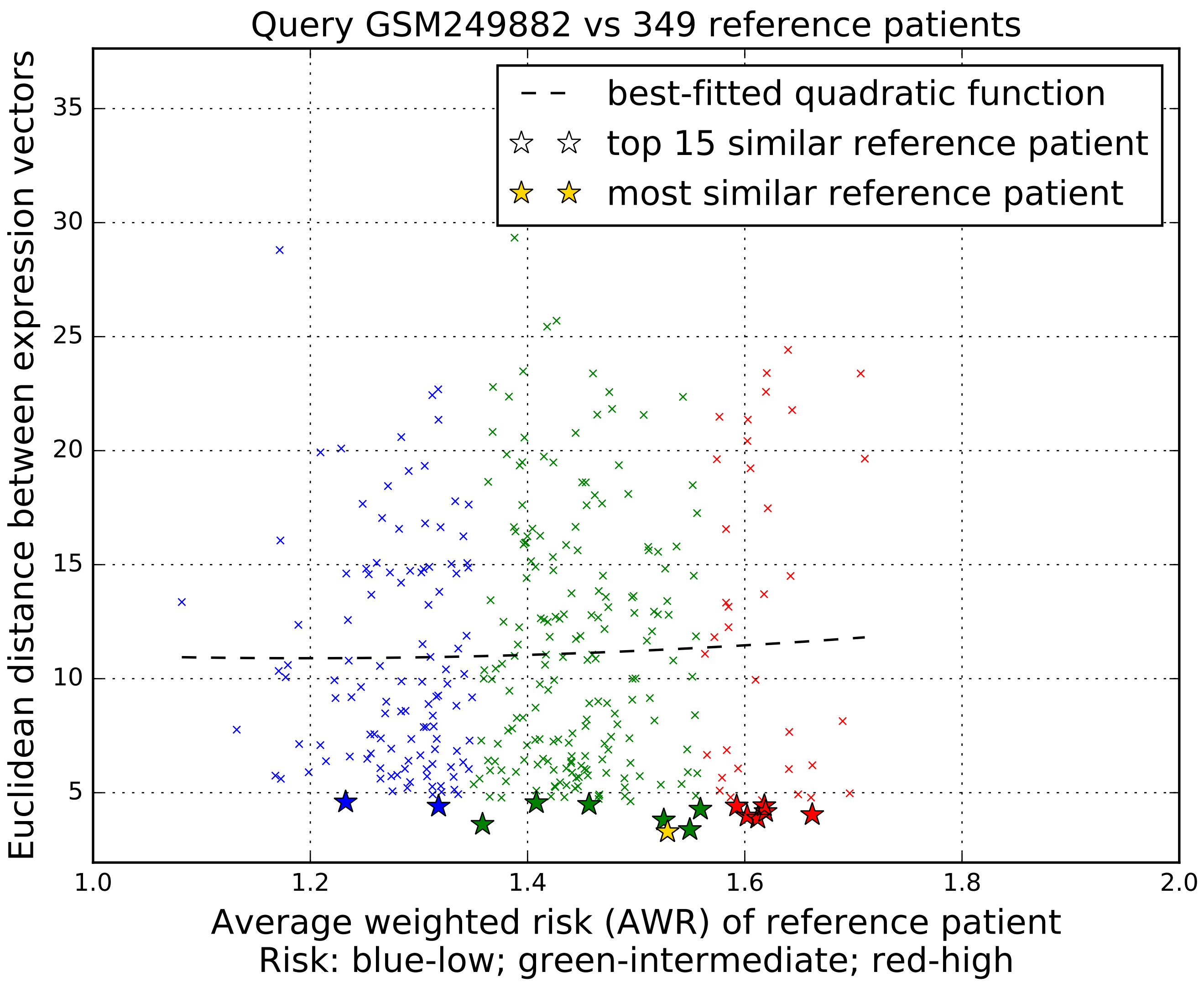
Query GSM249882 vs 349 reference patients
best-fitted quadratic function
top 15 similar reference patient
most similar reference patient
Euclidean distance between expression vectors
35
30
25
20
15
10
5
1.0
1.2
1.4
1.6
1.8
2.0
Average weighted risk (AWR) of reference patient
Risk: blue-low; green-intermediate; red-high

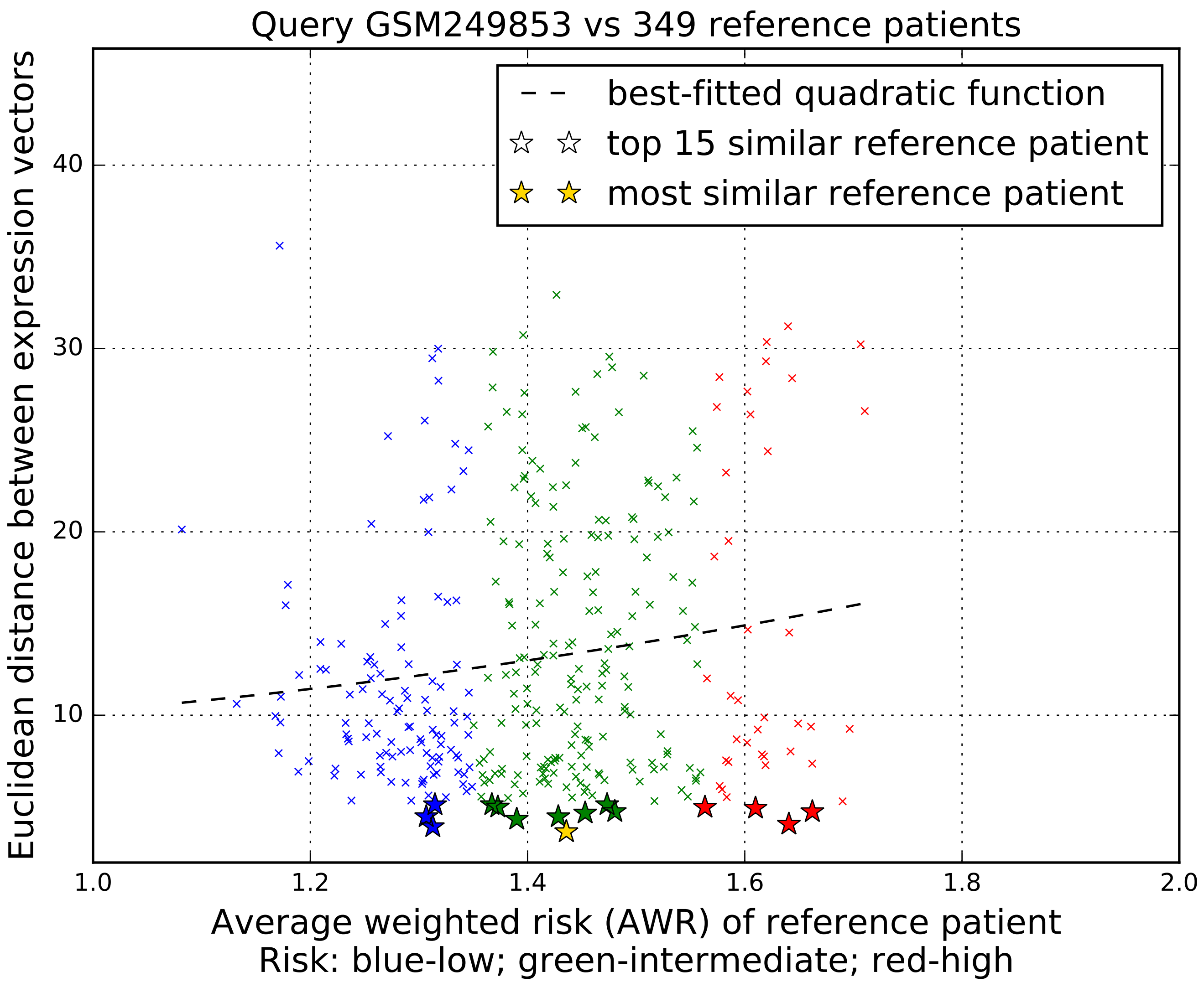

Query GSM249853 vs 349 reference patients
best-fitted quadratic function
top 15 similar reference patient
most similar reference patient
Euclidean distance between expression vectors
40
30
20
10
1.0
1.2
1.4
1.6
1.8
2.0
Average weighted risk (AWR) of reference patient
Risk: blue-low; green-intermediate; red-high

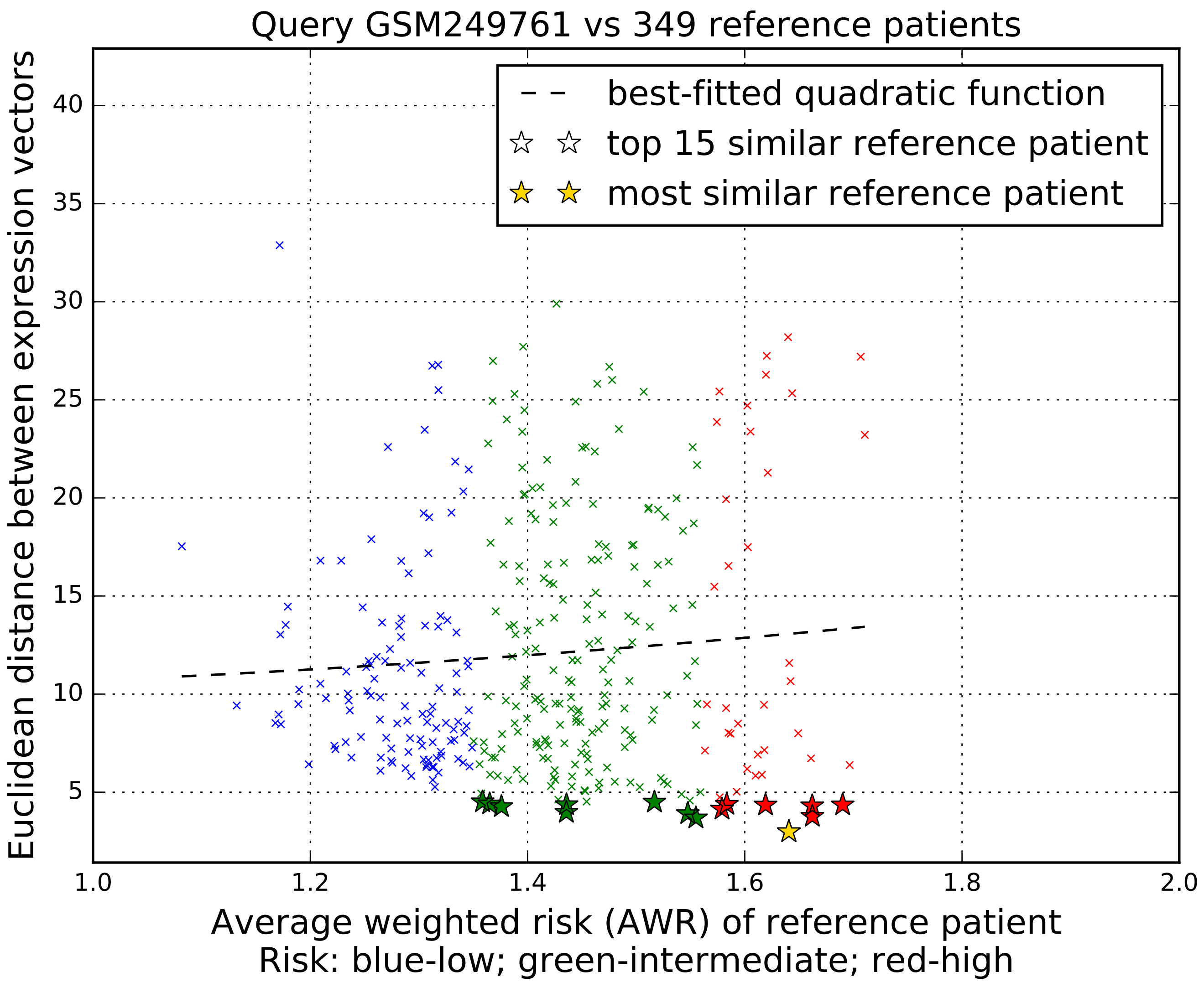
Query GSM249761 vs 349 reference patients
best-fitted quadratic function
top 15 similar reference patient
most similar reference patient
Euclidean distance between expression vectors
5
10
15
20
25
30
35
40
1.0
1.2
1.4
1.6
1.8
2.0
Average weighted risk (AWR) of reference patient
Risk: blue-low; green-intermediate; red-high

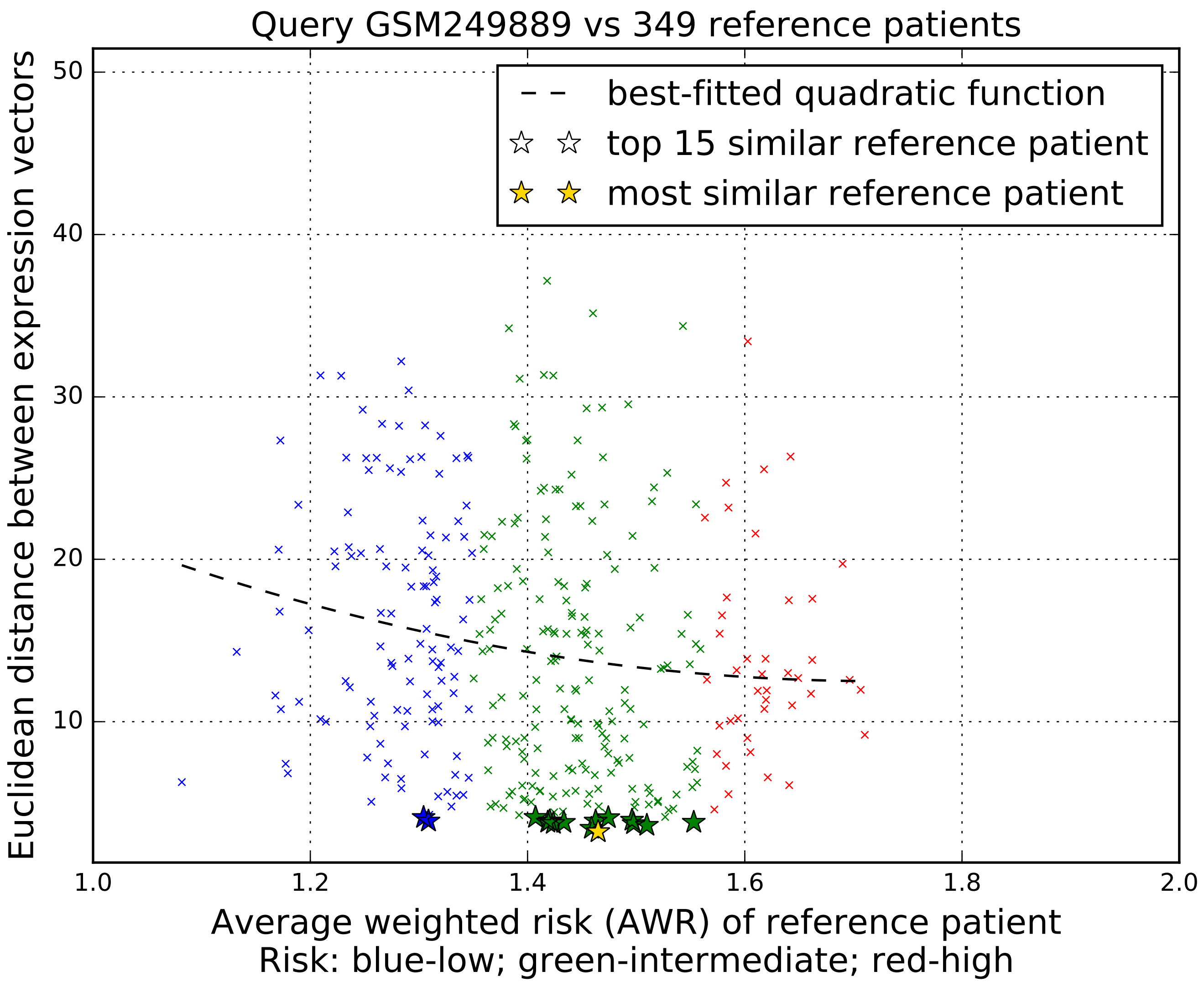

Query GSM249889 vs 349 reference patients
best-fitted quadratic function
top 15 similar reference patient
most similar reference patient
Euclidean distance between expression vectors
50
40
30
20
10
1.0
1.2
1.4
1.6
1.8
2.0
Average weighted risk (AWR) of reference patient
Risk: blue-low; green-intermediate; red-high

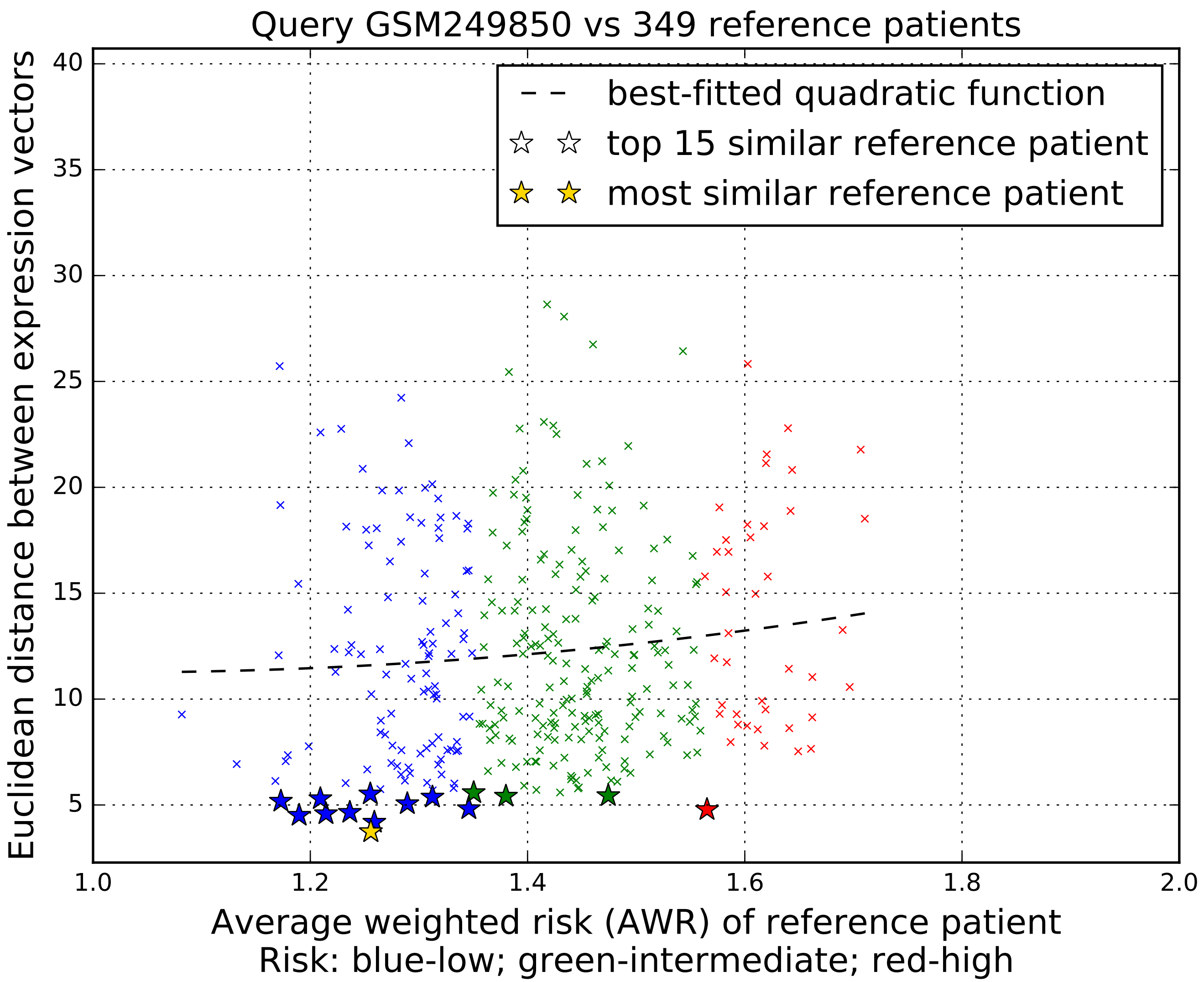

Query GSM249850 vs 349 reference patients
best-fitted quadratic function
top 15 similar reference patient
most similar reference patient
Euclidean distance between expression vectors
40
35
30
25
20
15
10
5
1.0
1.2
1.4
1.6
1.8
2.0
Average weighted risk (AWR) of reference patient
Risk: blue-low; green-intermediate; red-high

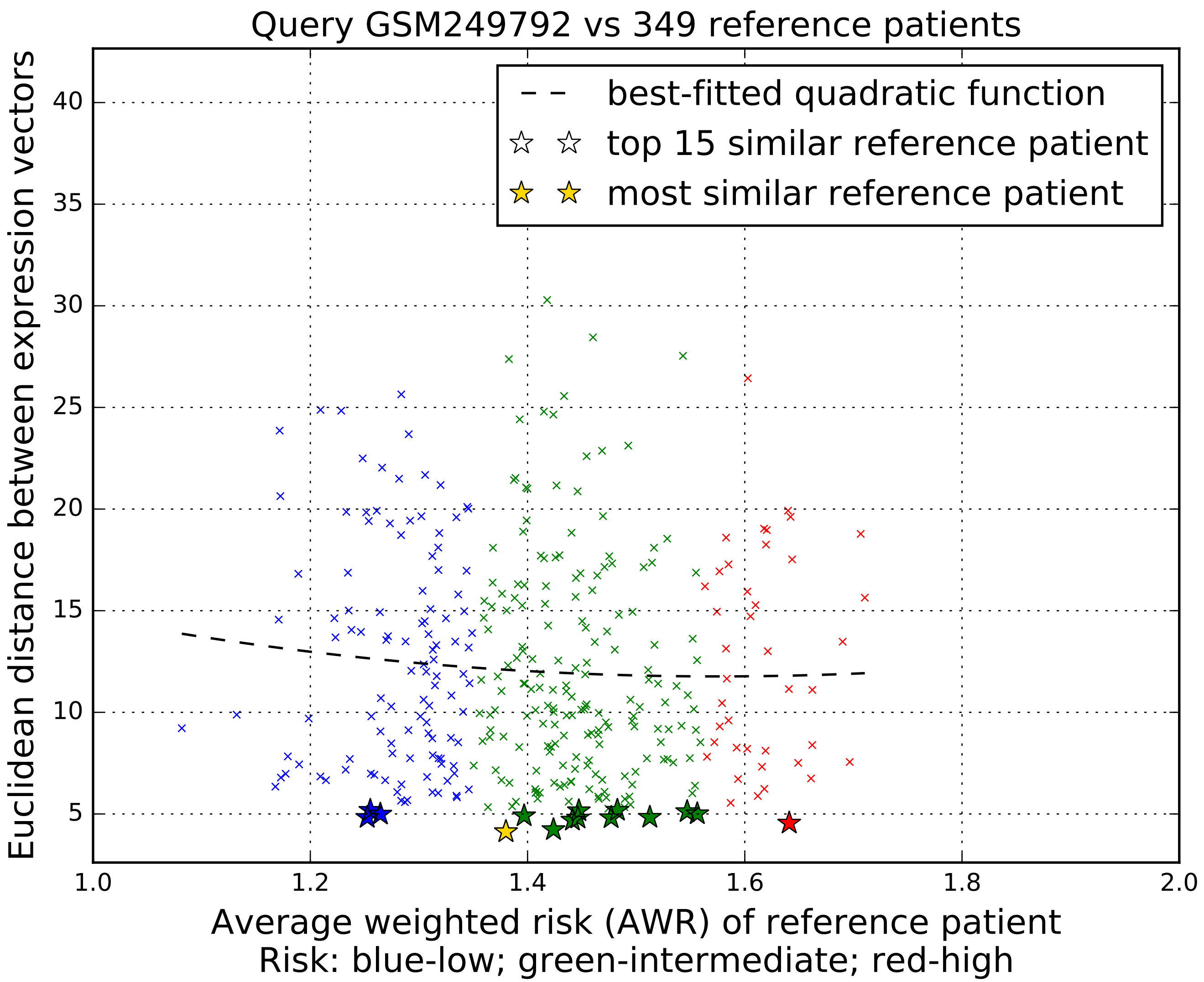
Query GSM249792 vs 349 reference patients
best-fitted quadratic function
top 15 similar reference patient
most similar reference patient
Euclidean distance between expression vectors
40
35
30
25
20
15
10
5
1.0
1.2
1.4
1.6
1.8
2.0
Average weighted risk (AWR) of reference patient
Risk: blue-low; green-intermediate; red-high

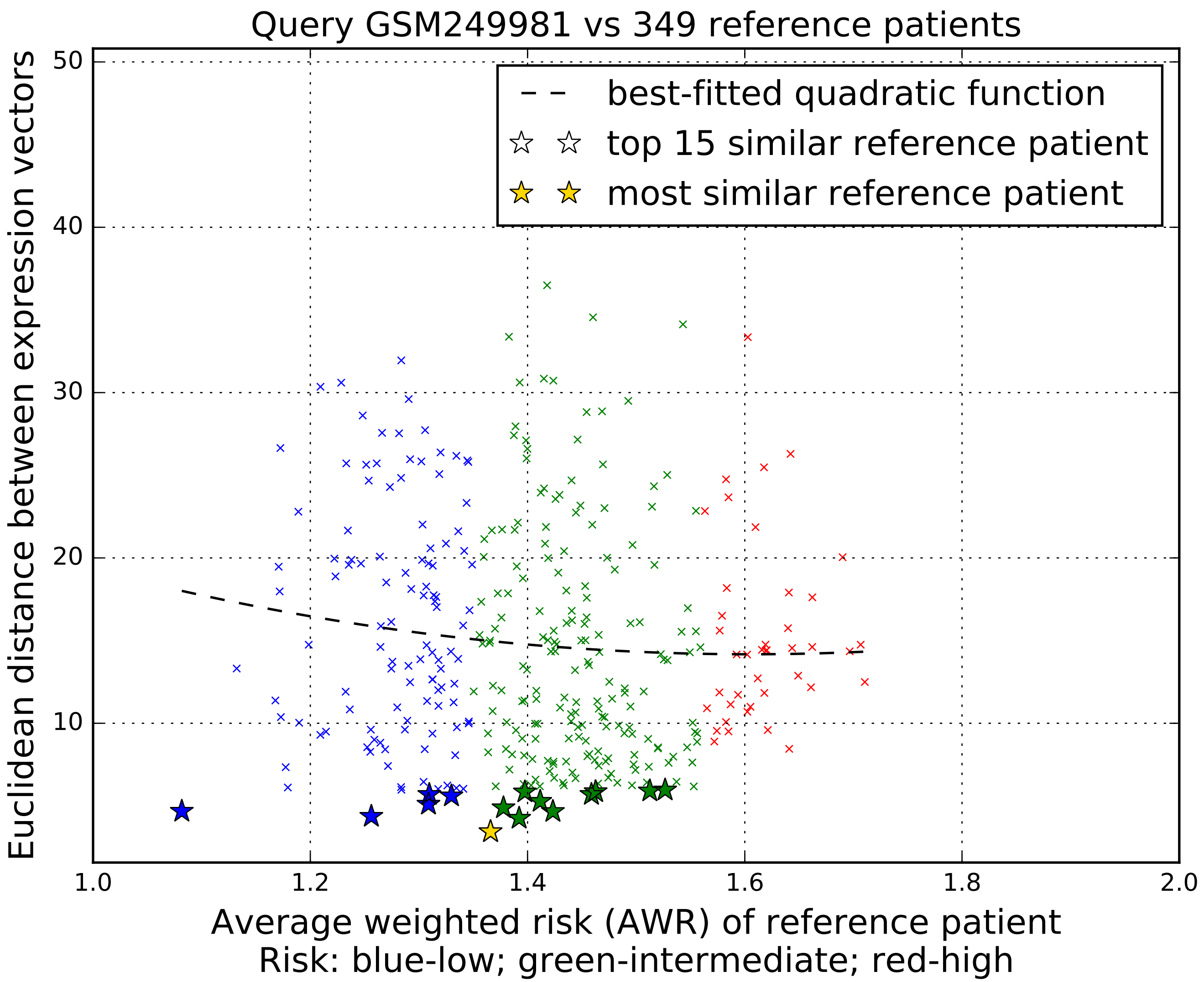

Query GSM249981 vs 349 reference patients
best-fitted quadratic function
top 15 similar reference patient
most similar reference patient
Euclidean distance between expression vectors
50
40
30
20
10
1.0
1.2
1.4
1.6
1.8
2.0
Average weighted risk (AWR) of reference patient
Risk: blue-low; green-intermediate; red-high

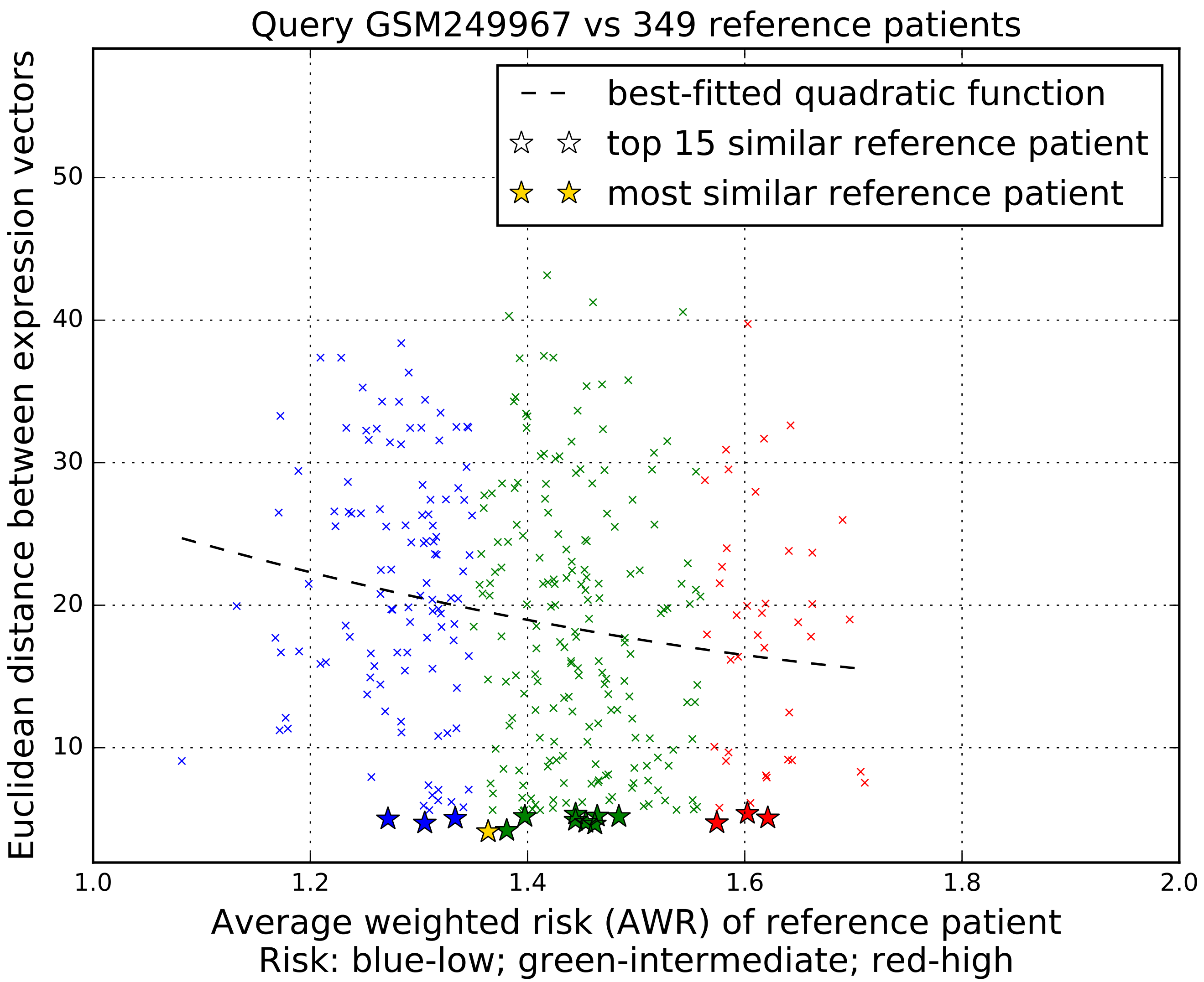
Query GSM249967 vs 349 reference patients
best-fitted quadratic function
top 15 similar reference patient
most similar reference patient
Euclidean distance between expression vectors
10
20
30
40
50
1.0
1.2
1.4
1.6
1.8
2.0
Average weighted risk (AWR) of reference patient
Risk: blue-low; green-intermediate; red-high

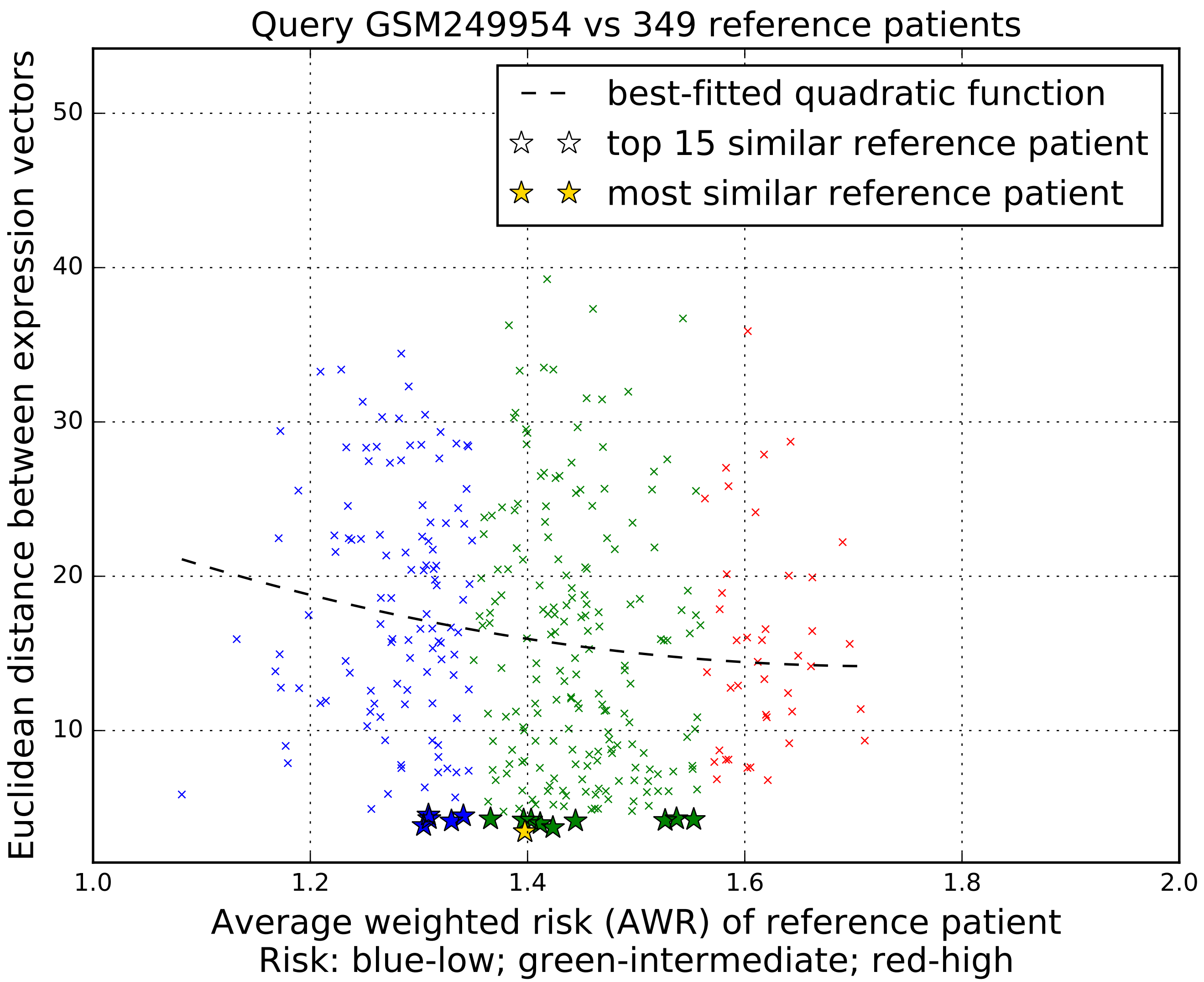
Query GSM249954 vs 349 reference patients
best-fitted quadratic function
top 15 similar reference patient
most similar reference patient
Euclidean distance between expression vectors
10
20
30
40
50
1.0
1.2
1.4
1.6
1.8
2.0
Average weighted risk (AWR) of reference patient
Risk: blue-low; green-intermediate; red-high

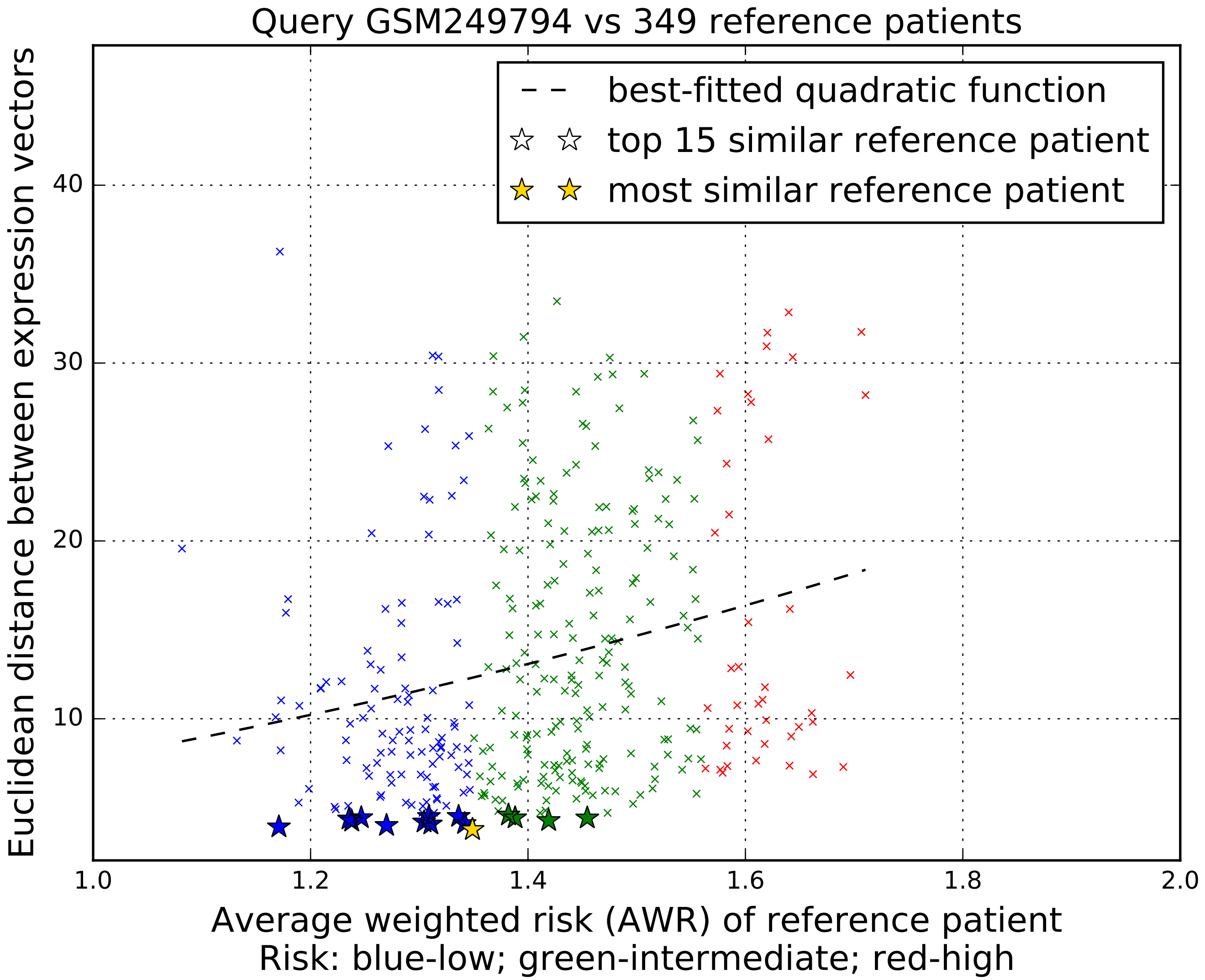
Query GSM249794 vs 349 reference patients
best-fitted quadratic function
top 15 similar reference patient
most similar reference patient
Euclidean distance between expression vectors
10
20
30
40
1.0
1.2
1.4
1.6
1.8
2.0
Average weighted risk (AWR) of reference patient
Risk: blue-low; green-intermediate; red-high

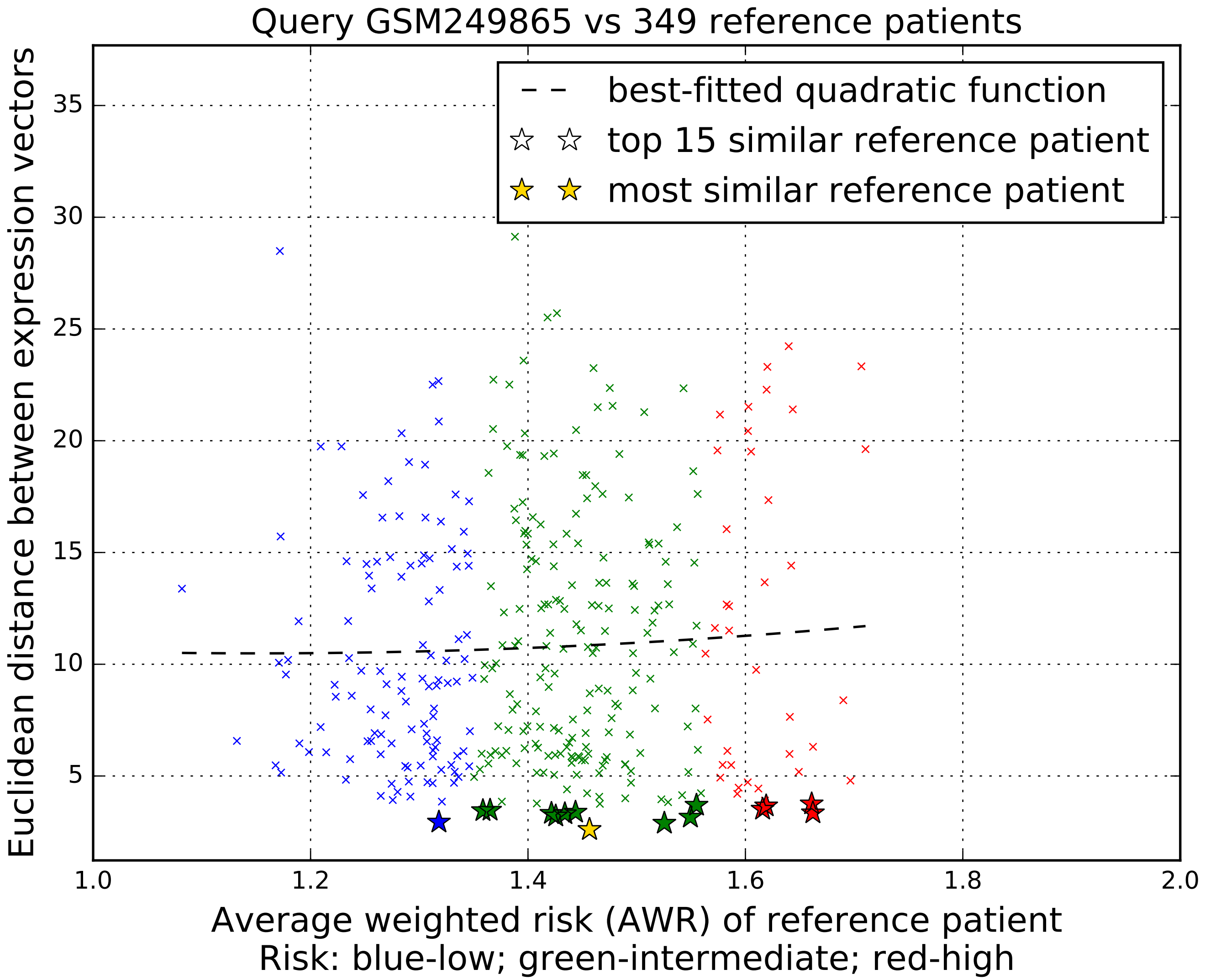
Query GSM249865 vs 349 reference patients
best-fitted quadratic function
top 15 similar reference patient
most similar reference patient
Euclidean distance between expression vectors
Average weighted risk (AWR) of reference patient
Risk: blue-low; green-intermediate; red-high
35
30
25
20
15
10
5
1.0
1.2
1.4
1.6
1.8
2.0

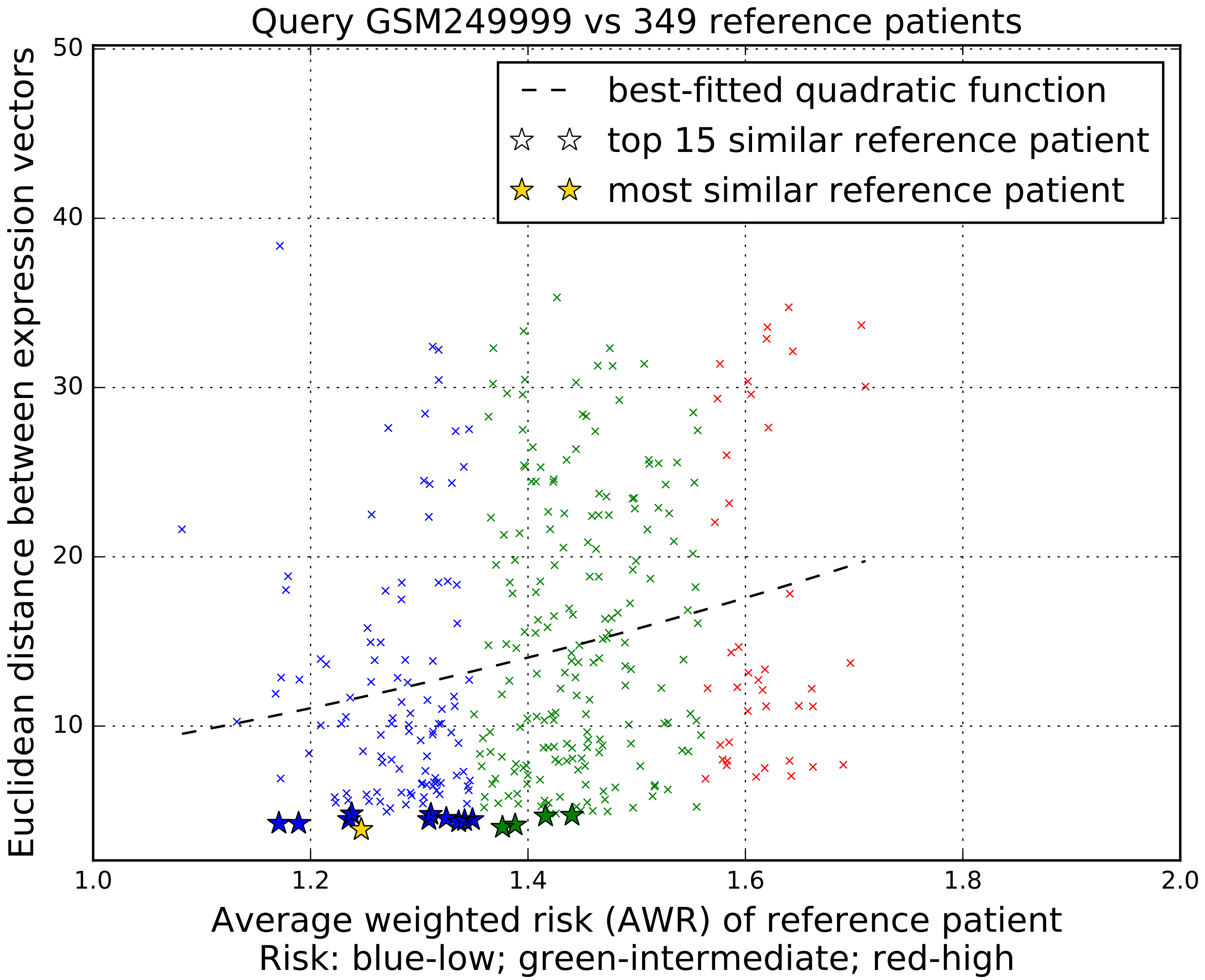
Query GSM249999 vs 349 reference patients
best-fitted quadratic function
top 15 similar reference patient
most similar reference patient
Euclidean distance between expression vectors
50
40
30
20
10
1.0
1.2
1.4
1.6
1.8
2.0
Average weighted risk (AWR) of reference patient
Risk: blue-low; green-intermediate; red-high

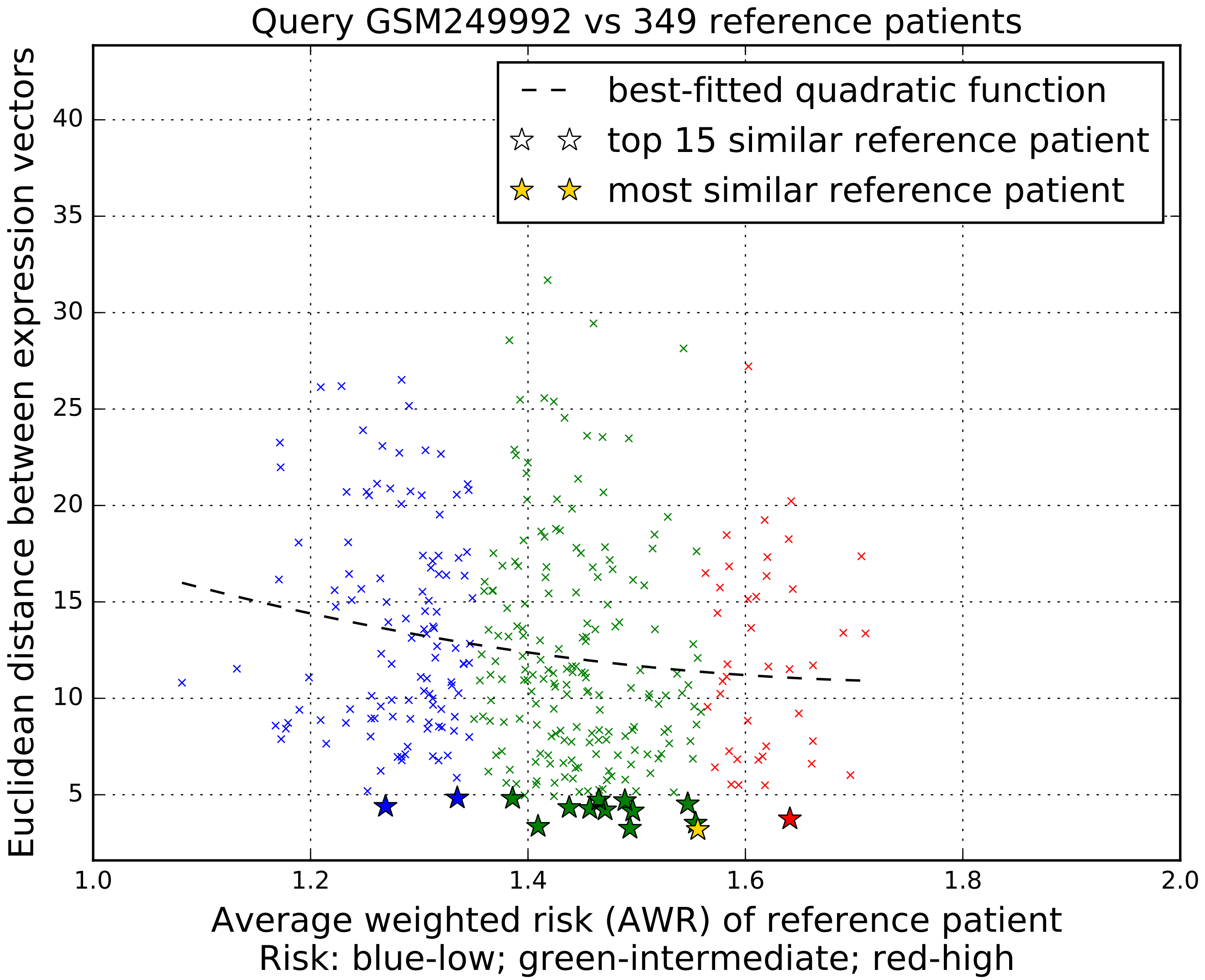

Query GSM249992 vs 349 reference patients
best-fitted quadratic function
top 15 similar reference patient
most similar reference patient
Euclidean distance between expression vectors
40
35
30
25
20
15
10
5
1.0
1.2
1.4
1.6
1.8
2.0
Average weighted risk (AWR) of reference patient
Risk: blue-low; green-intermediate; red-high

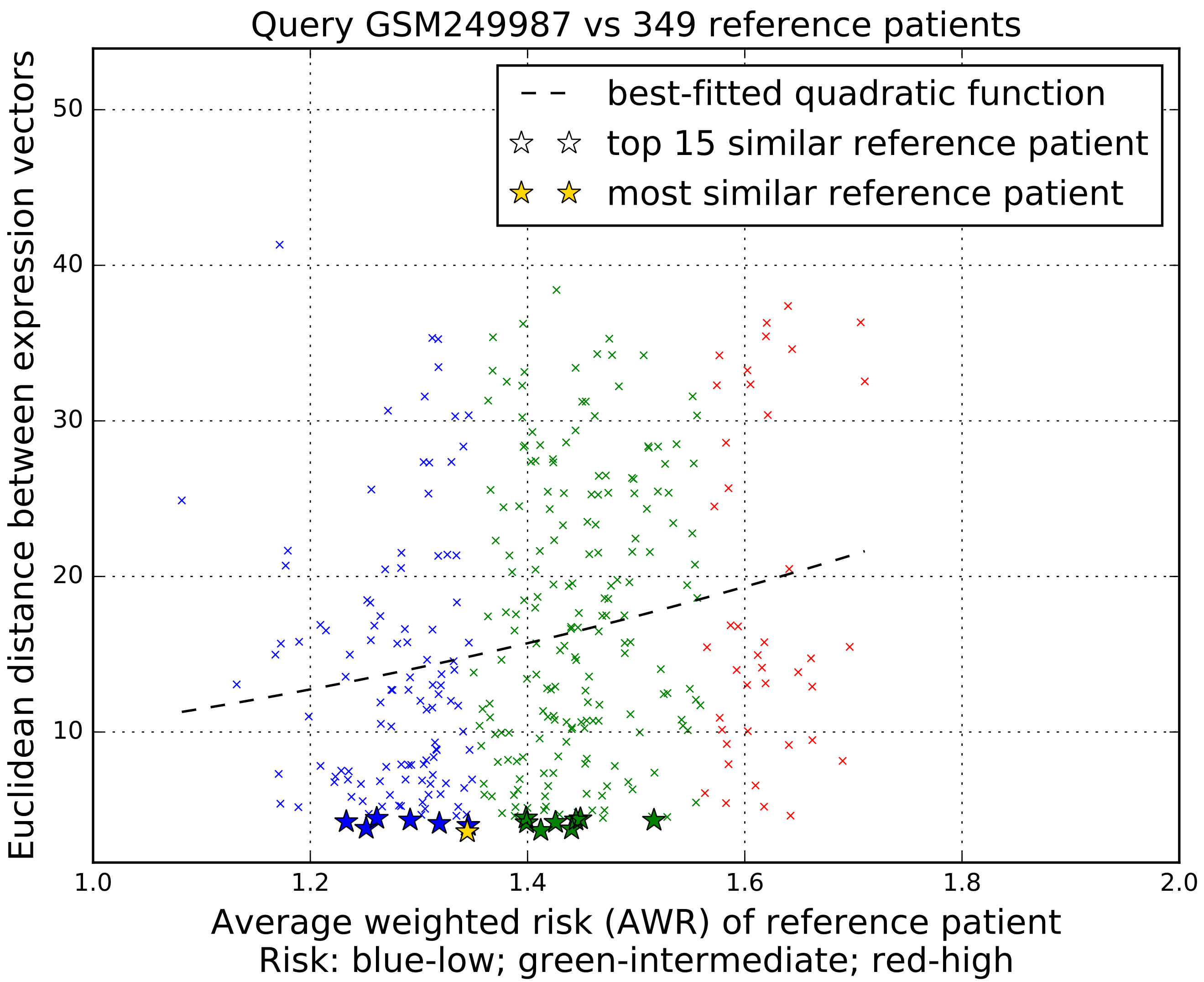

Query GSM249987 vs 349 reference patients
best-fitted quadratic function
top 15 similar reference patient
most similar reference patient
Euclidean distance between expression vectors
Average weighted risk (AWR) of reference patient
Risk: blue-low; green-intermediate; red-high
10
20
30
40
50
1.0
1.2
1.4
1.6
1.8
2.0

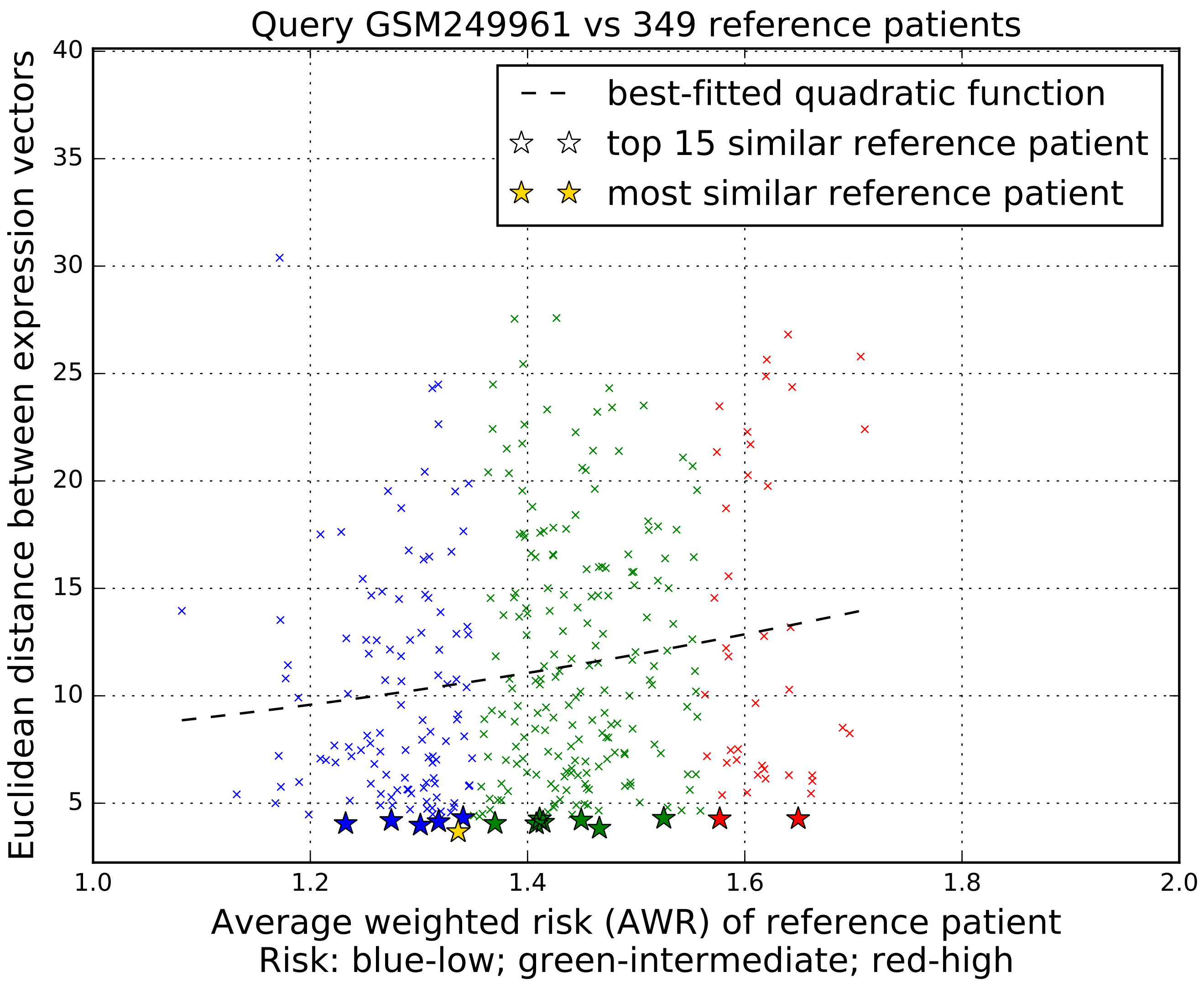
Query GSM249961 vs 349 reference patients
best-fitted quadratic function
top 15 similar reference patient
most similar reference patient
Euclidean distance between expression vectors
Average weighted risk (AWR) of reference patient
Risk: blue-low; green-intermediate; red-high
40
35
30
25
20
15
10
5
1.0
1.2
1.4
1.6
1.8
2.0

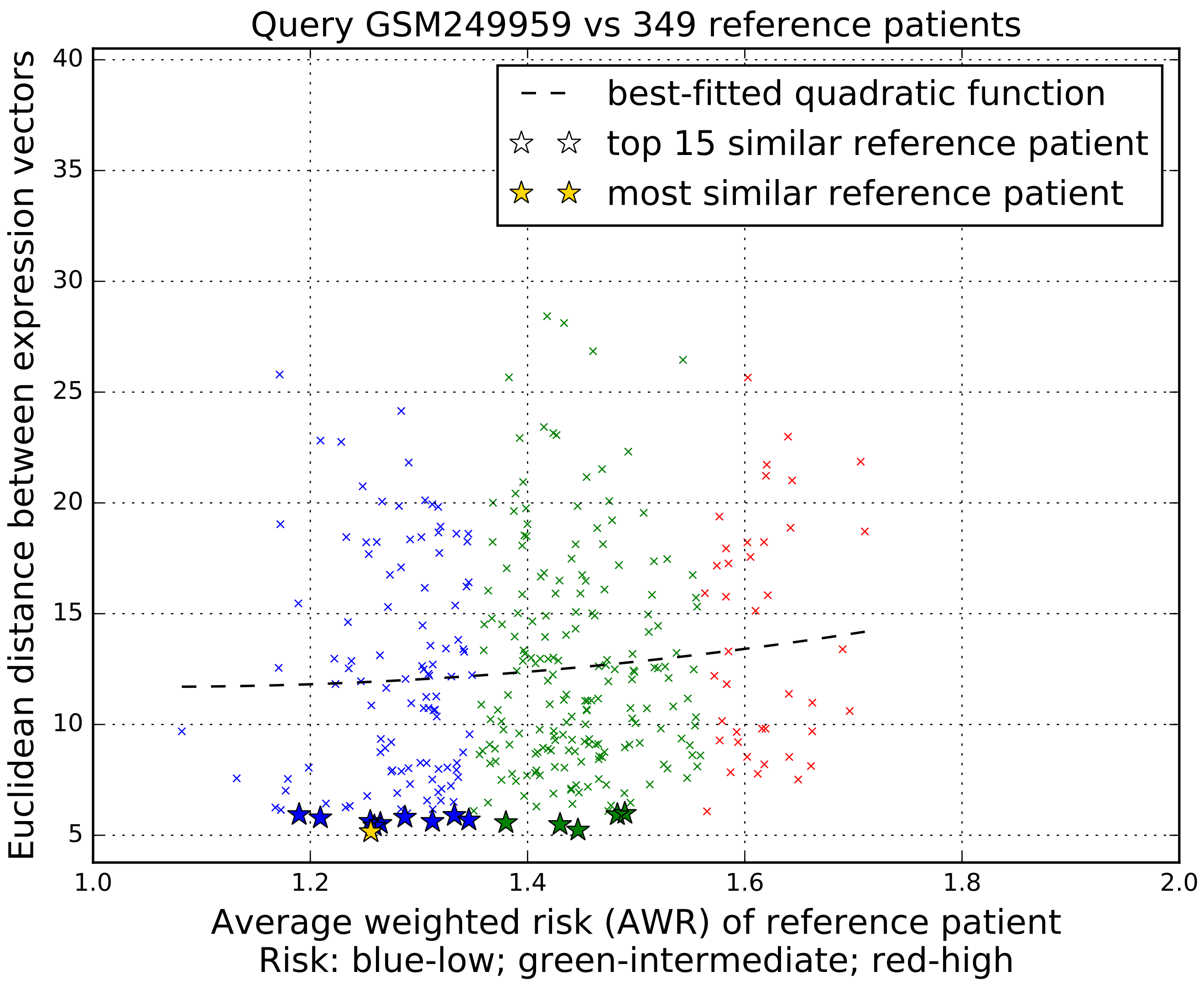

Query GSM249959 vs 349 reference patients
best-fitted quadratic function
top 15 similar reference patient
most similar reference patient
Euclidean distance between expression vectors
40
35
30
25
20
15
10
5
1.0
1.2
1.4
1.6
1.8
2.0
Average weighted risk (AWR) of reference patient
Risk: blue-low; green-intermediate; red-high

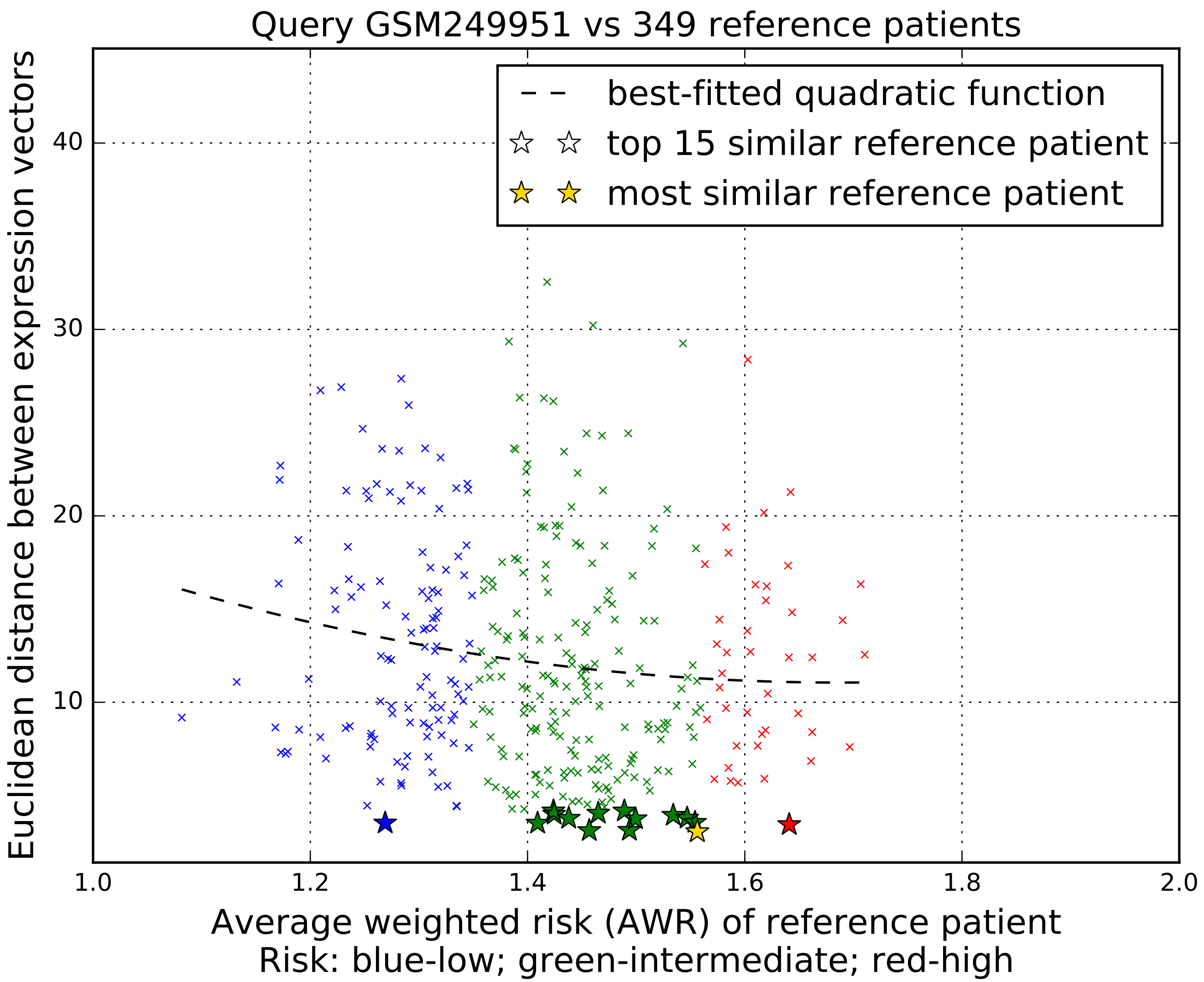
Query GSM249951 vs 349 reference patients
best-fitted quadratic function
top 15 similar reference patient
most similar reference patient
Euclidean distance between expression vectors
Average weighted risk (AWR) of reference patient
Risk: blue-low; green-intermediate; red-high
10
20
30
40
1.0
1.2
1.4
1.6
1.8
2.0

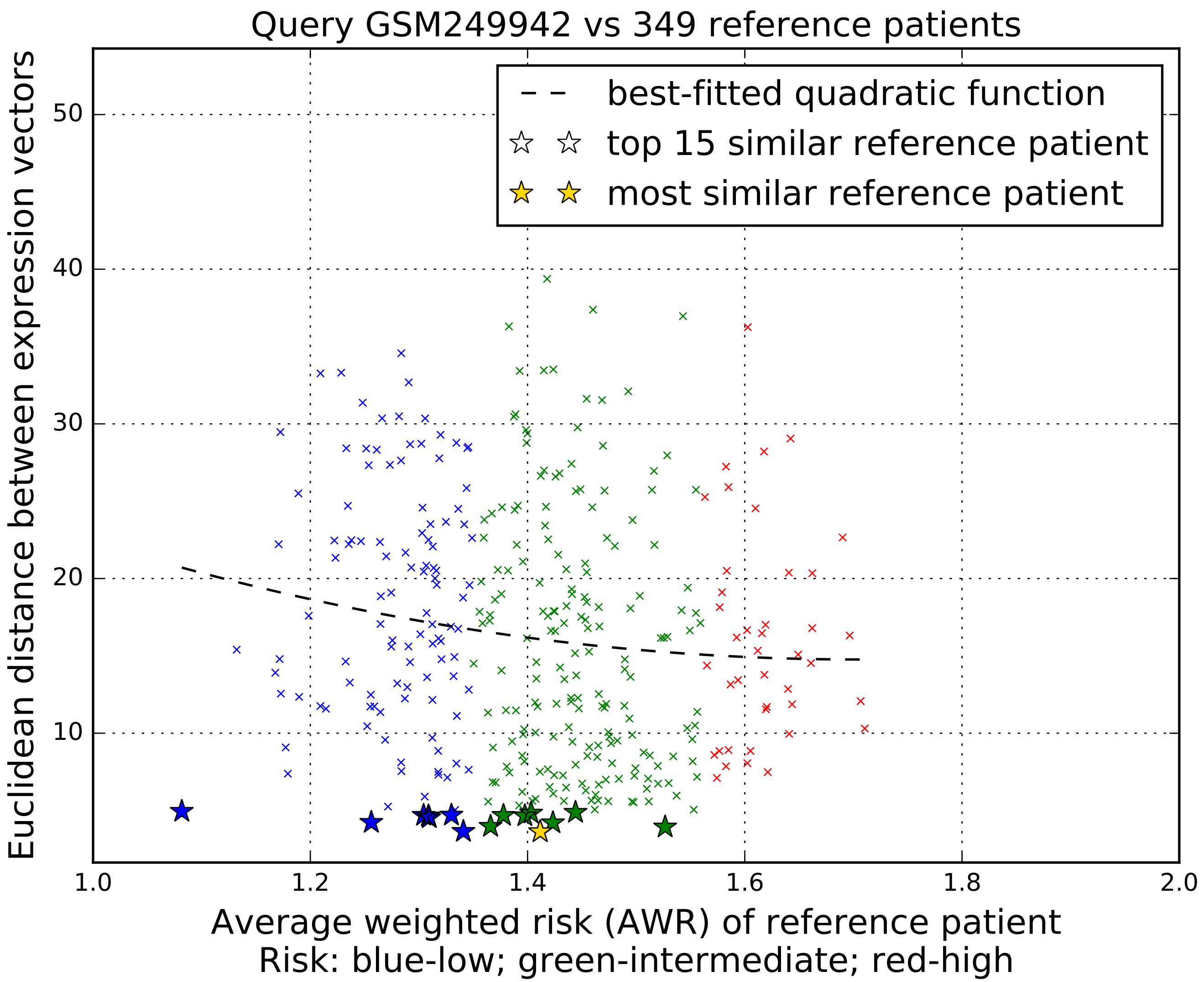

Query GSM249942 vs 349 reference patients
best-fitted quadratic function
top 15 similar reference patient
most similar reference patient
Euclidean distance between expression vectors
10
20
30
40
50
1.0
1.2
1.4
1.6
1.8
2.0
Average weighted risk (AWR) of reference patient
Risk: blue-low; green-intermediate; red-high

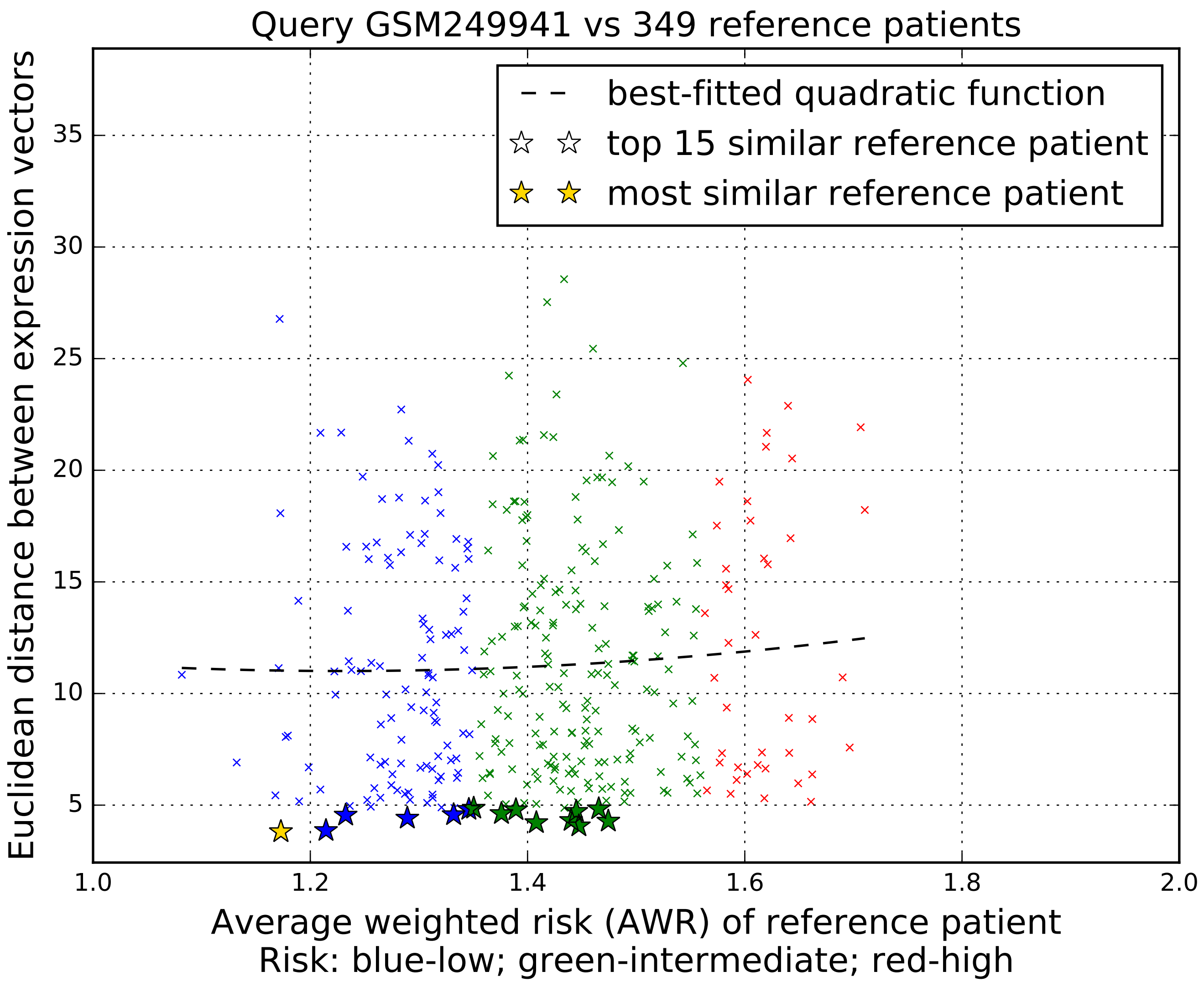
Query GSM249941 vs 349 reference patients
best-fitted quadratic function
top 15 similar reference patient
most similar reference patient
Euclidean distance between expression vectors
5
10
15
20
25
30
35
1.0
1.2
1.4
1.6
1.8
2.0
Average weighted risk (AWR) of reference patient
Risk: blue-low; green-intermediate; red-high

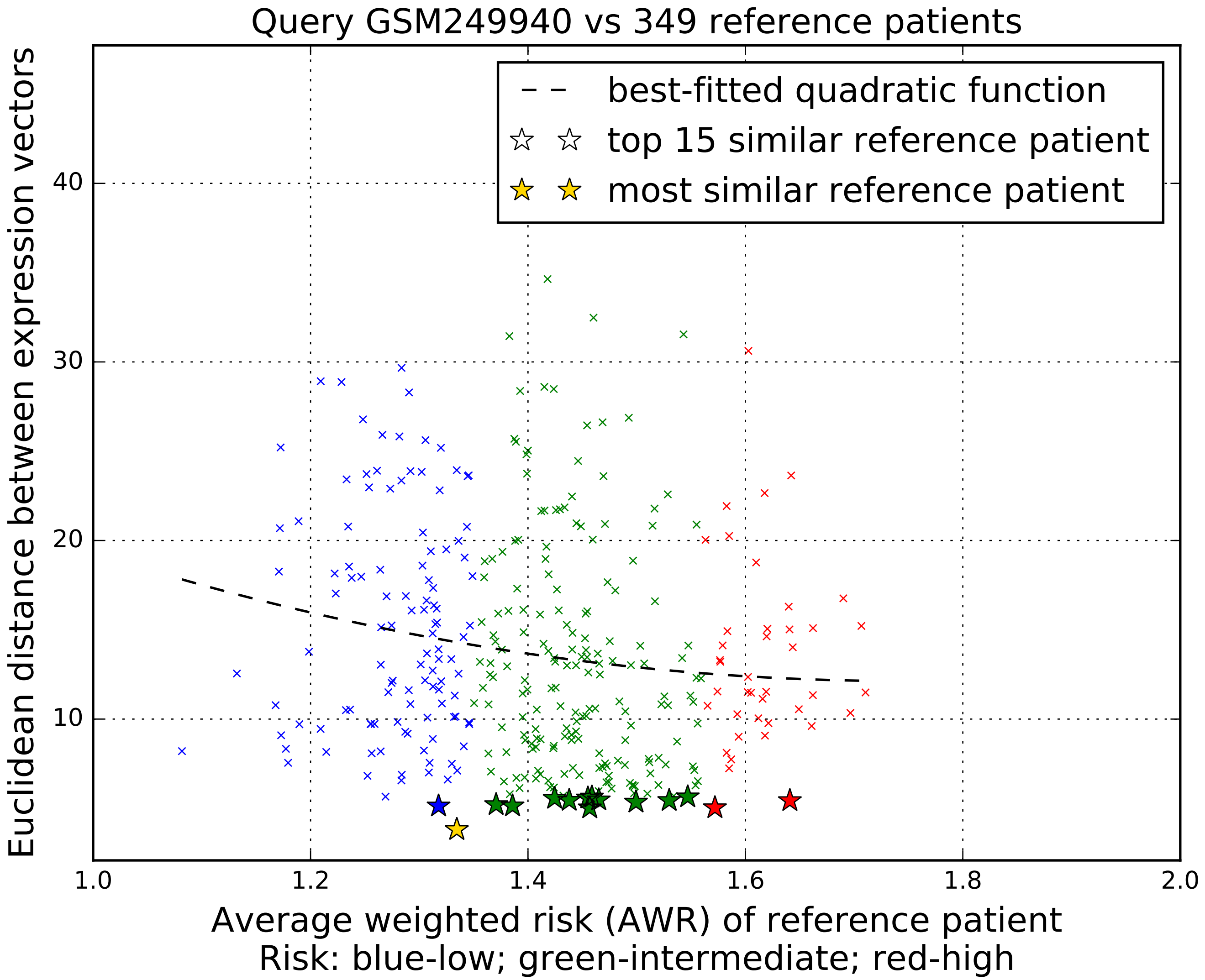
Query GSM249940 vs 349 reference patients
best-fitted quadratic function
top 15 similar reference patient
most similar reference patient
Euclidean distance between expression vectors
10
20
30
40
1.0
1.2
1.4
1.6
1.8
2.0
Average weighted risk (AWR) of reference patient
Risk: blue-low; green-intermediate; red-high

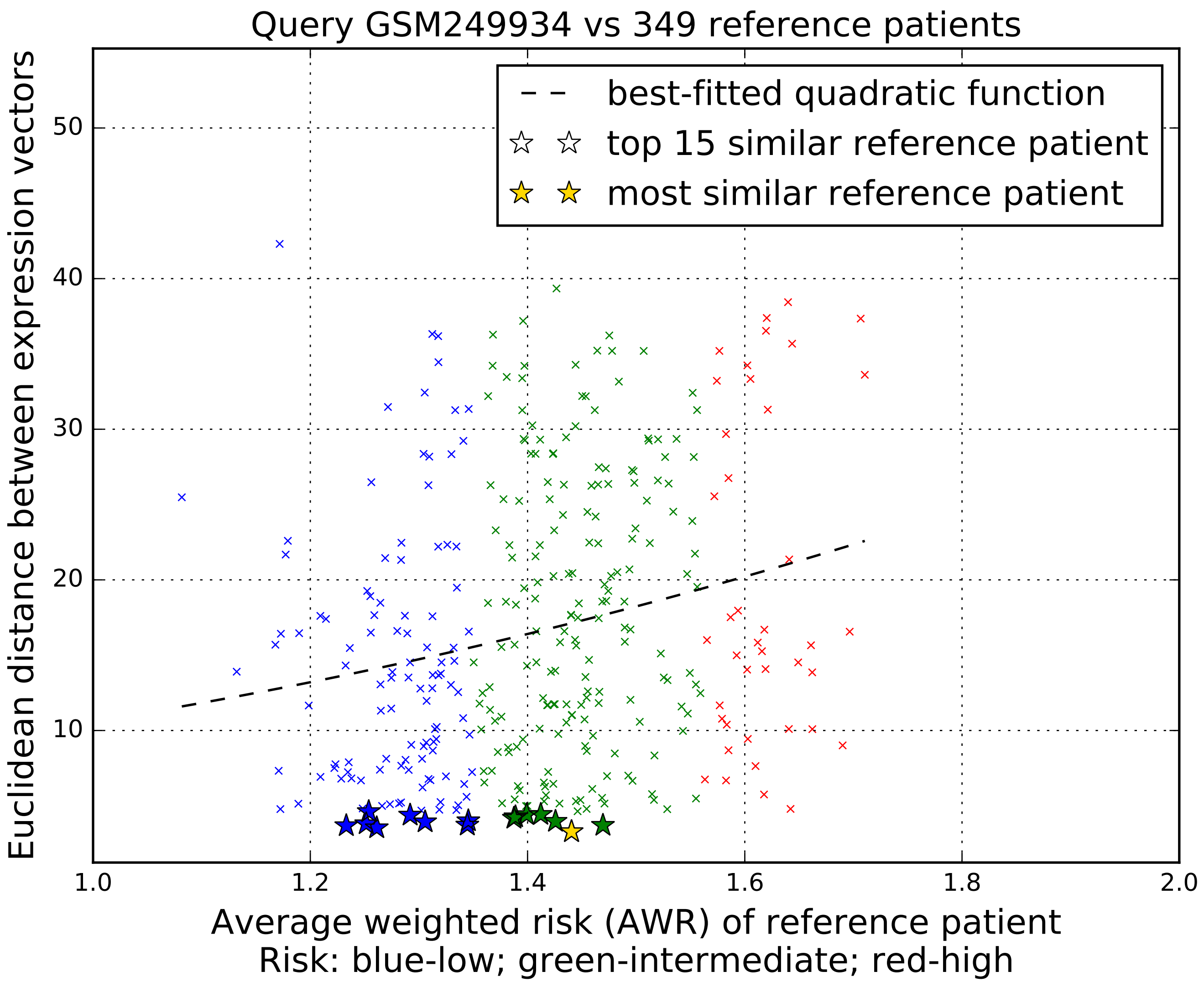
Query GSM249934 vs 349 reference patients
best-fitted quadratic function
top 15 similar reference patient
most similar reference patient
Euclidean distance between expression vectors
10
20
30
40
50
1.0
1.2
1.4
1.6
1.8
2.0
Average weighted risk (AWR) of reference patient
Risk: blue-low; green-intermediate; red-high

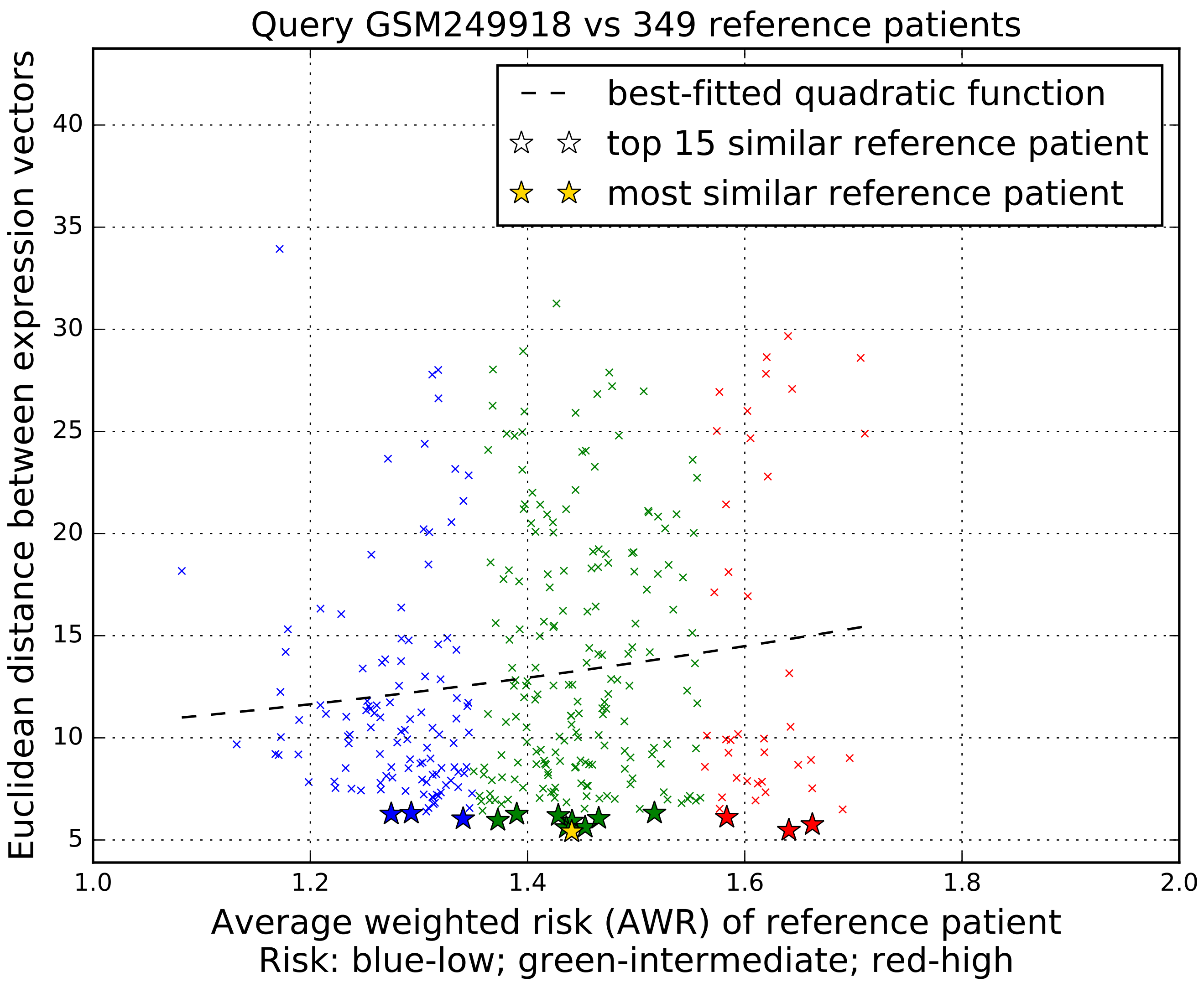

Query GSM249918 vs 349 reference patients
best-fitted quadratic function
top 15 similar reference patient
most similar reference patient
Euclidean distance between expression vectors
40
35
30
25
20
15
10
5
1.0
1.2
1.4
1.6
1.8
2.0
Average weighted risk (AWR) of reference patient
Risk: blue-low; green-intermediate; red-high

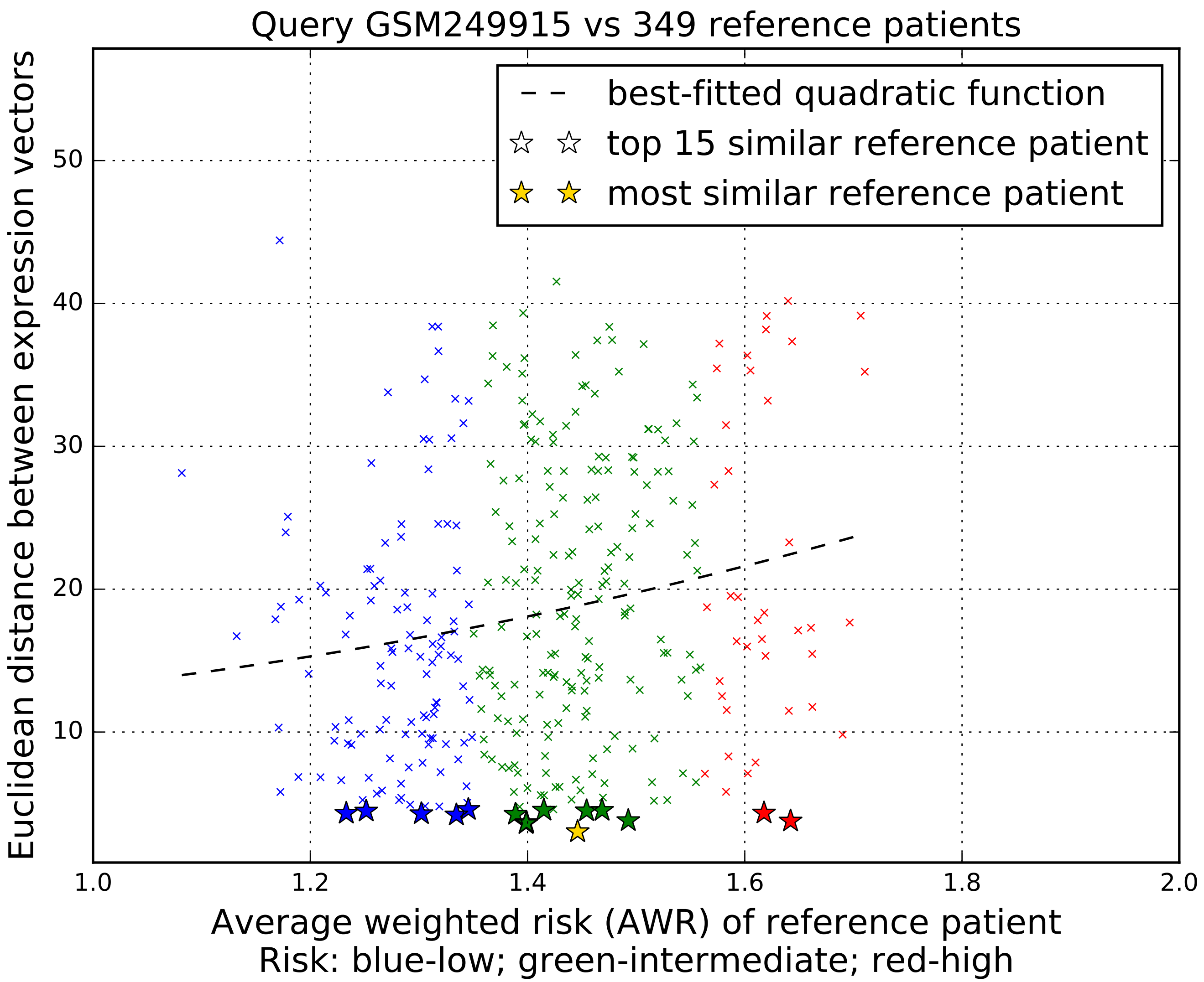

Query GSM249915 vs 349 reference patients
best-fitted quadratic function
top 15 similar reference patient
most similar reference patient
Euclidean distance between expression vectors
10
20
30
40
50
1.0
1.2
1.4
1.6
1.8
2.0
Average weighted risk (AWR) of reference patient
Risk: blue-low; green-intermediate; red-high

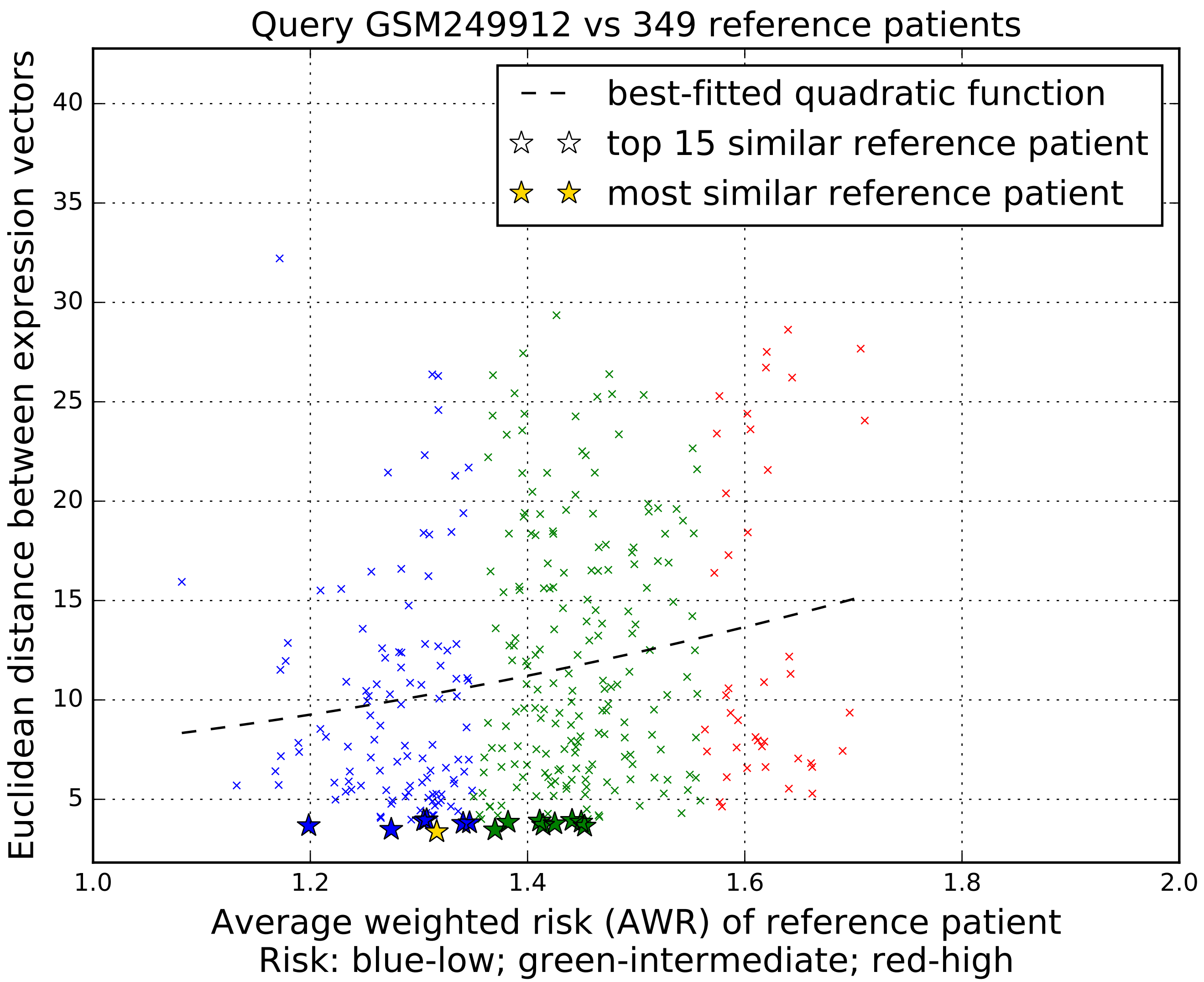

Query GSM249912 vs 349 reference patients
Euclidean distance between expression vectors
40
35
30
25
20
15
10
5
1.0
1.2
1.4
1.6
1.8
2.0
best-fitted quadratic function
top 15 similar reference patient
most similar reference patient
Average weighted risk (AWR) of reference patient
Risk: blue-low; green-intermediate; red-high

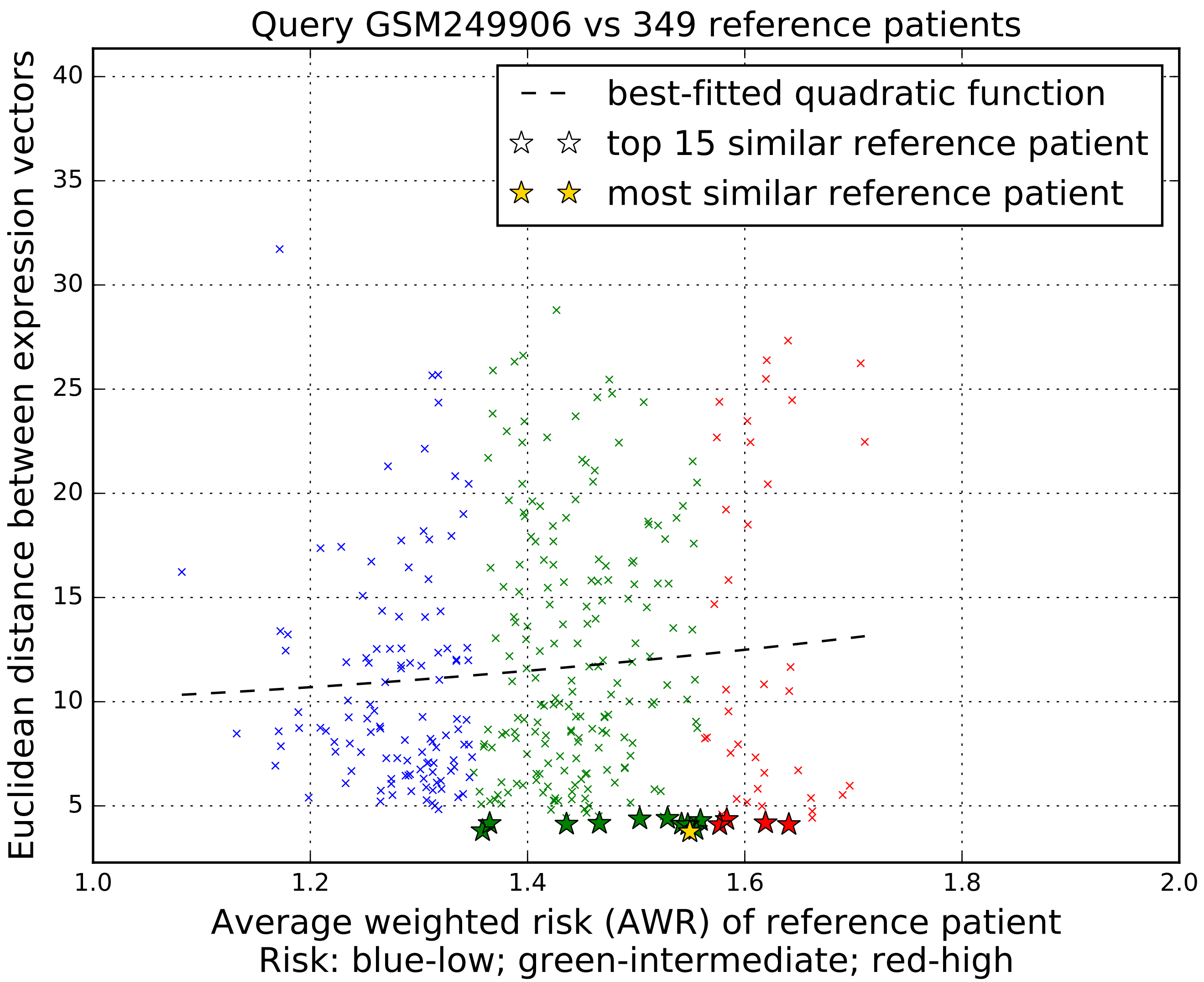
Query GSM249906 vs 349 reference patients
best-fitted quadratic function
top 15 similar reference patient
most similar reference patient
Euclidean distance between expression vectors
Average weighted risk (AWR) of reference patient
Risk: blue-low; green-intermediate; red-high
1.0
1.2
1.4
1.6
1.8
2.0
5
10
15
20
25
30
35
40

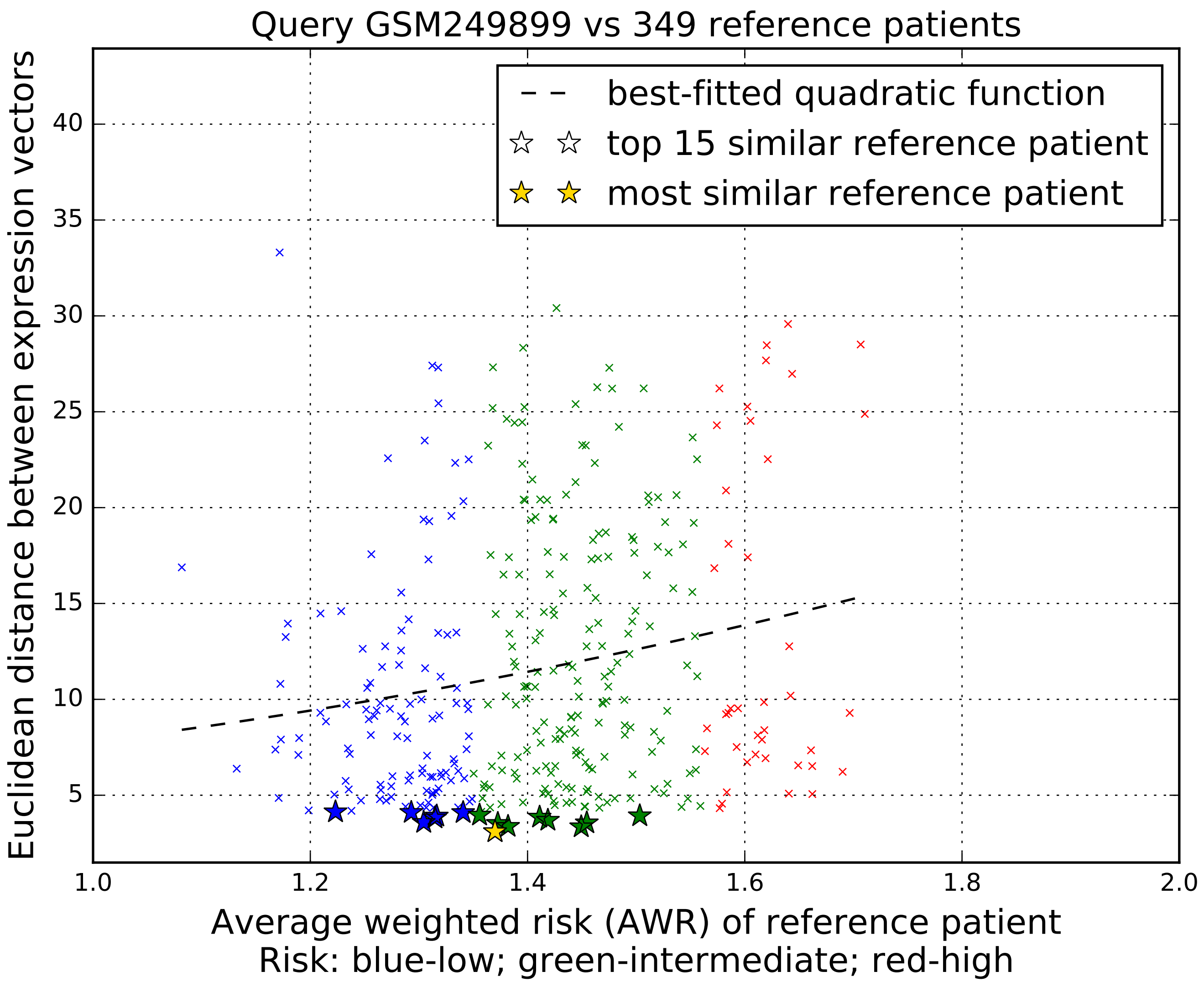

Query GSM249899 vs 349 reference patients
best-fitted quadratic function
top 15 similar reference patient
most similar reference patient
Euclidean distance between expression vectors
40
35
30
25
20
15
10
5
1.0
1.2
1.4
1.6
1.8
2.0
Average weighted risk (AWR) of reference patient
Risk: blue-low; green-intermediate; red-high

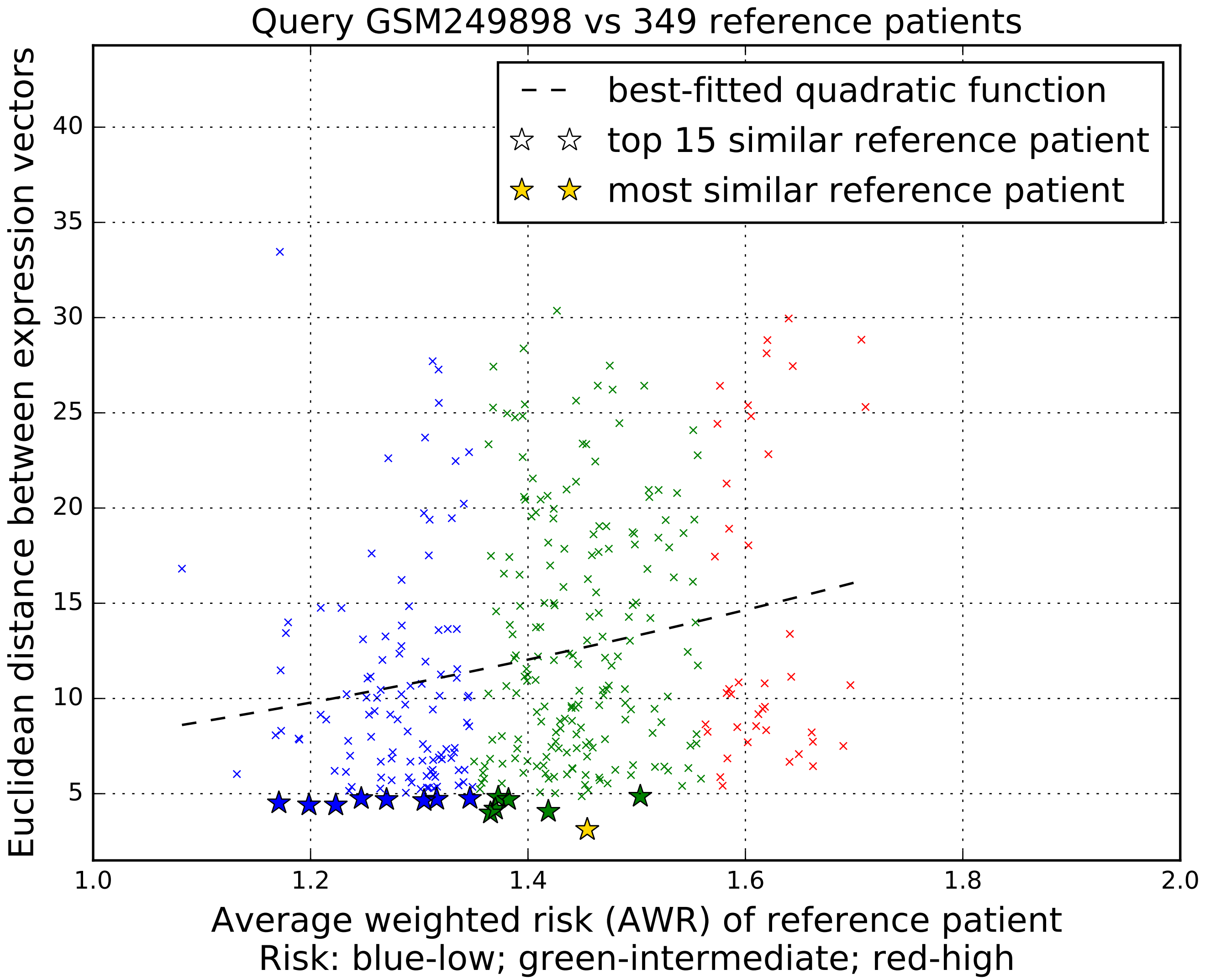
Query GSM249898 vs 349 reference patients
best-fitted quadratic function
top 15 similar reference patient
most similar reference patient
Euclidean distance between expression vectors
40
35
30
25
20
15
10
5
1.0
1.2
1.4
1.6
1.8
2.0
Average weighted risk (AWR) of reference patient
Risk: blue-low; green-intermediate; red-high

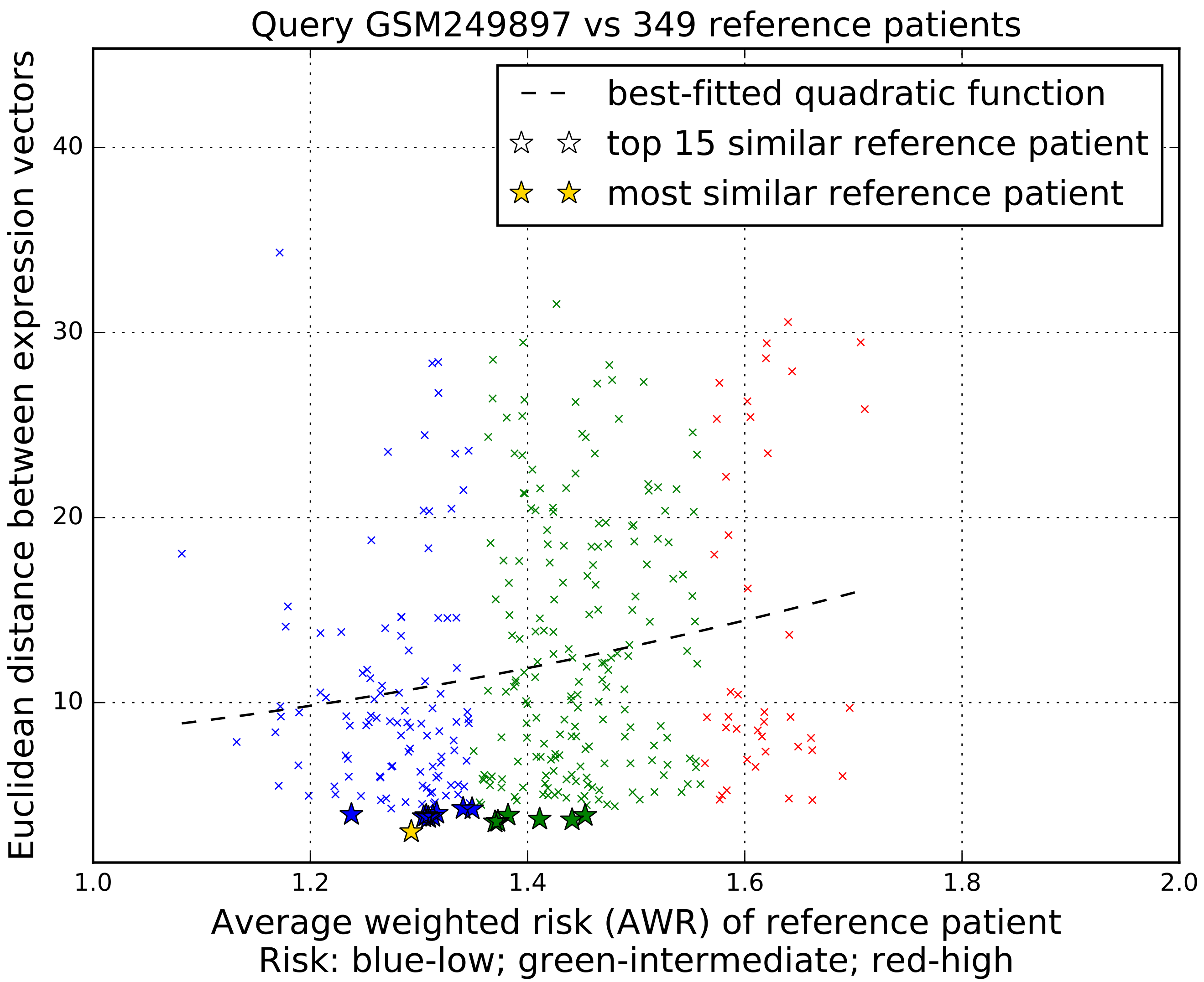

Query GSM249897 vs 349 reference patients
best-fitted quadratic function
top 15 similar reference patient
most similar reference patient
Euclidean distance between expression vectors
10
20
30
40
1.0
1.2
1.4
1.6
1.8
2.0
Average weighted risk (AWR) of reference patient
Risk: blue-low; green-intermediate; red-high

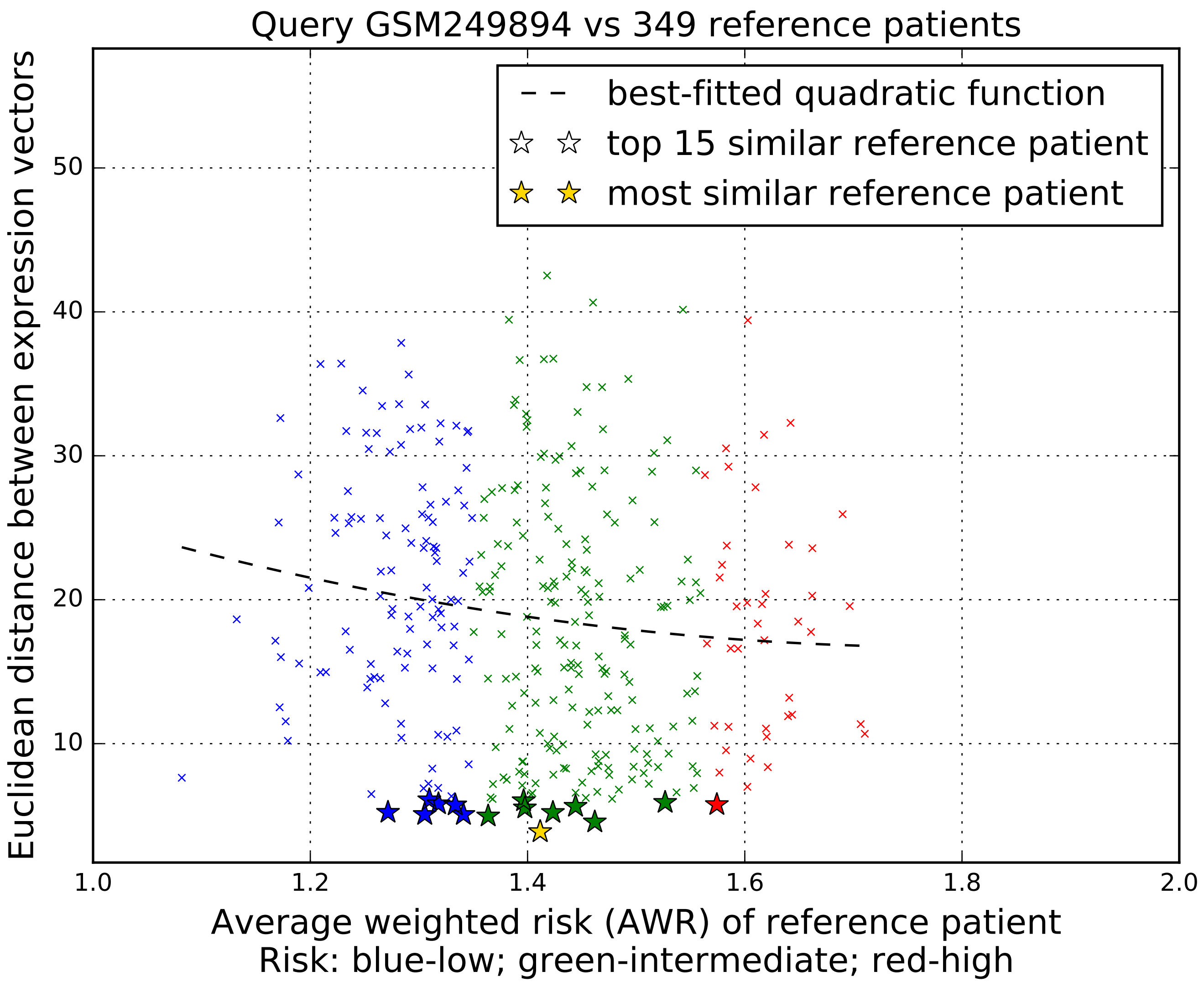
Query GSM249894 vs 349 reference patients
best-fitted quadratic function
top 15 similar reference patient
most similar reference patient
Euclidean distance between expression vectors
50
40
30
20
10
1.0
1.2
1.4
1.6
1.8
2.0
Average weighted risk (AWR) of reference patient
Risk: blue-low; green-intermediate; red-high

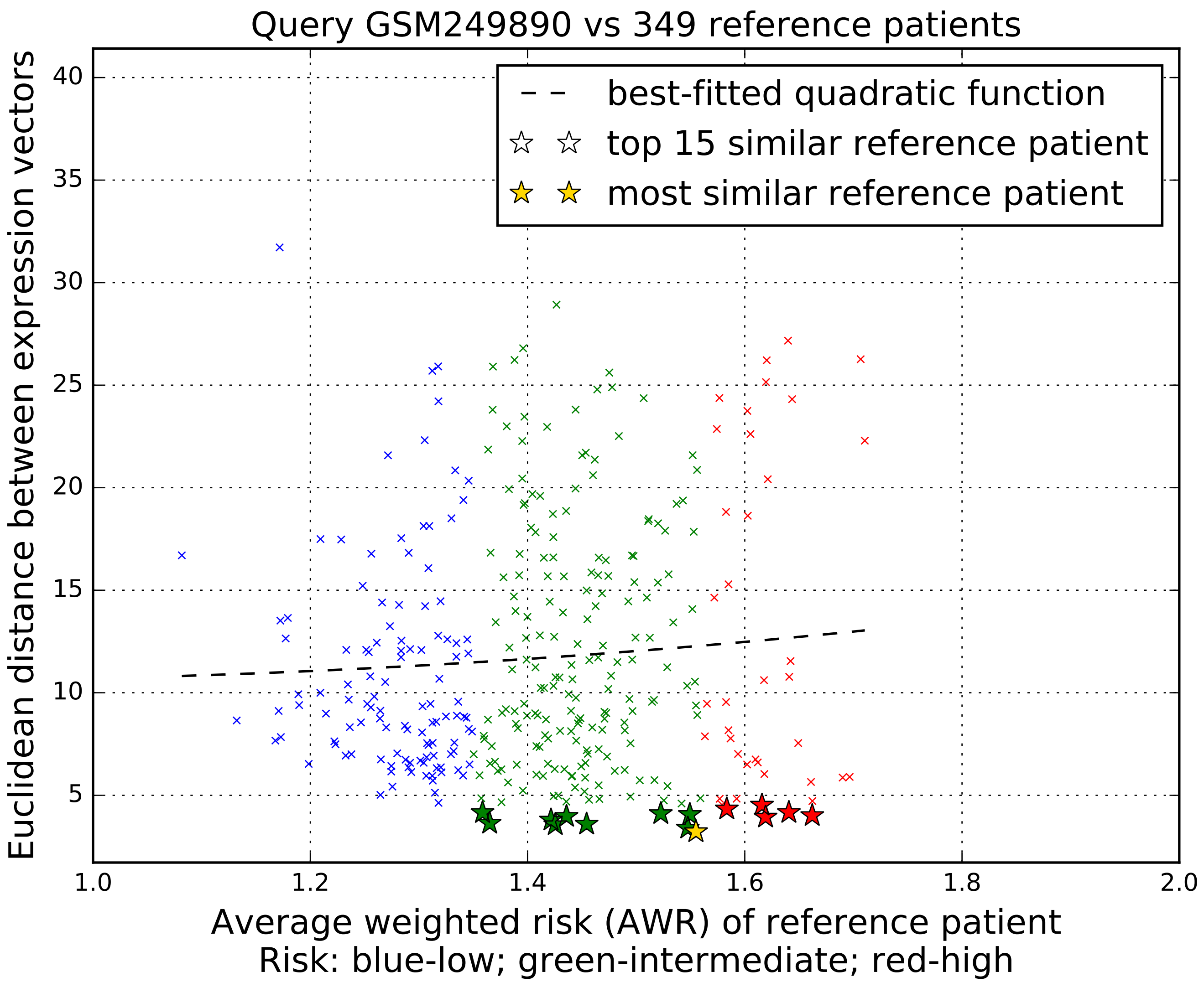

Query GSM249890 vs 349 reference patients
best-fitted quadratic function
top 15 similar reference patient
most similar reference patient
Euclidean distance between expression vectors
40
35
30
25
20
15
10
5
1.0
1.2
1.4
1.6
1.8
2.0
Average weighted risk (AWR) of reference patient
Risk: blue-low; green-intermediate; red-high

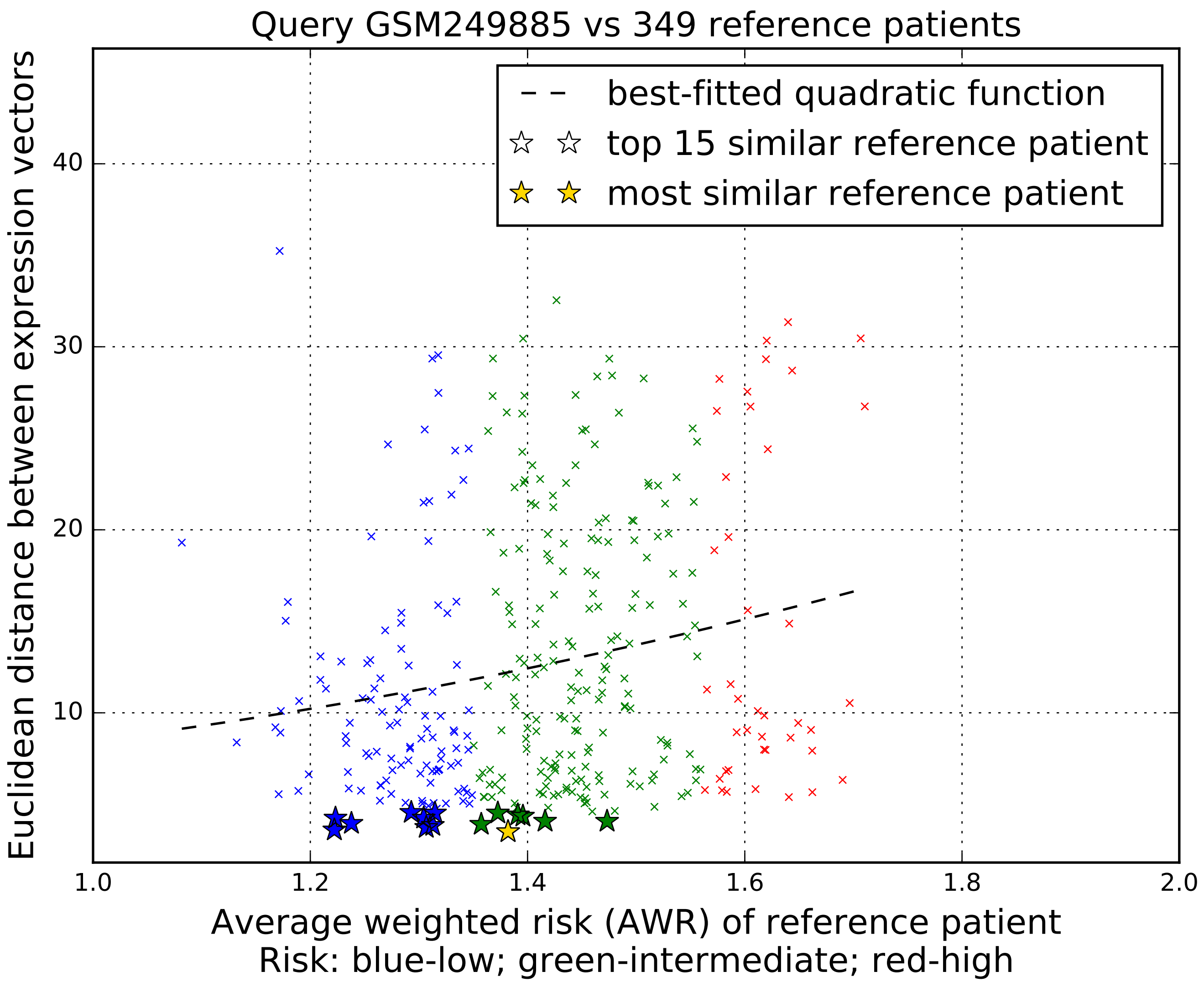
Query GSM249885 vs 349 reference patients
best-fitted quadratic function
top 15 similar reference patient
most similar reference patient
Euclidean distance between expression vectors
10
20
30
40
1.0
1.2
1.4
1.6
1.8
2.0
Average weighted risk (AWR) of reference patient
Risk: blue-low; green-intermediate; red-high

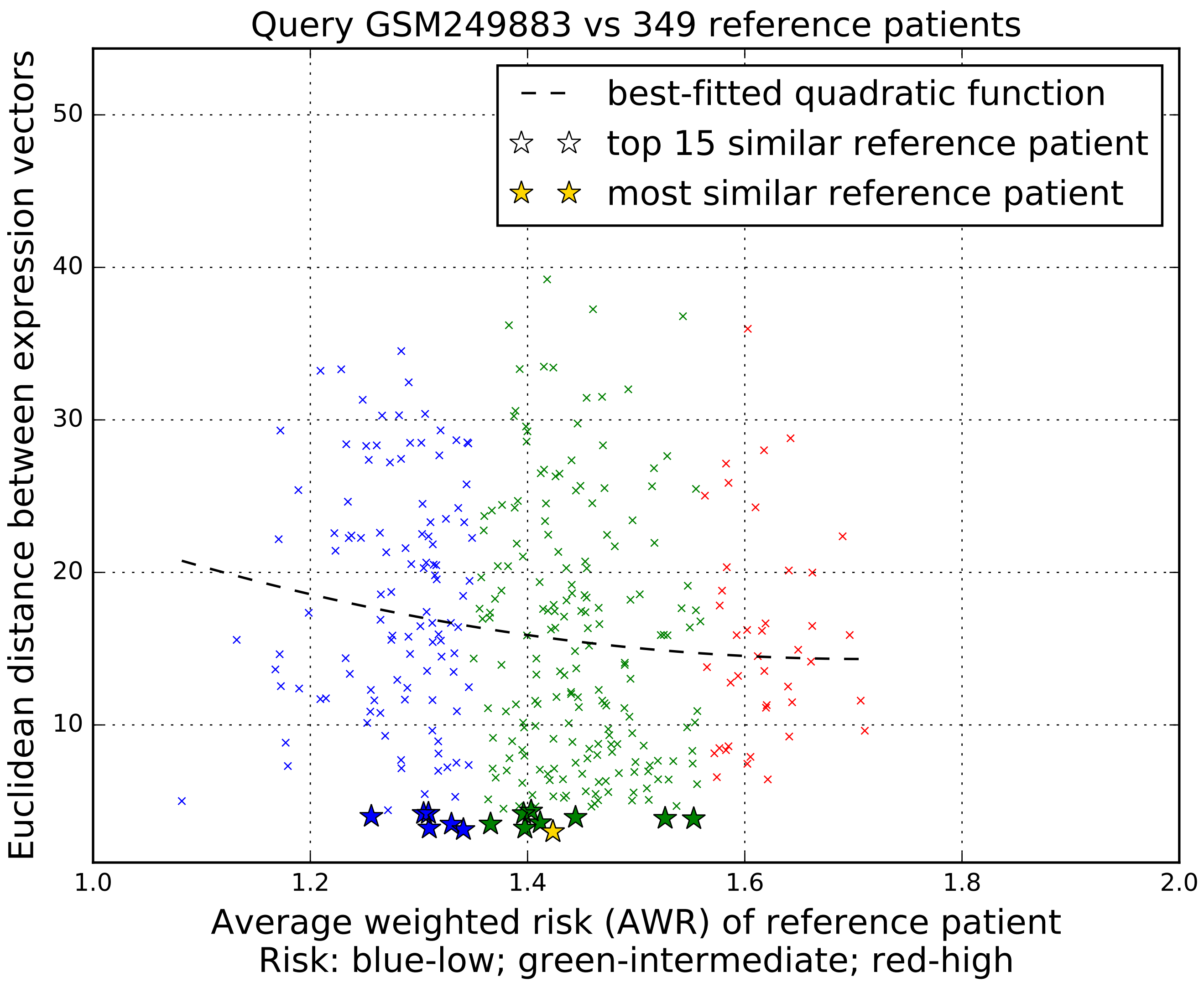

Query GSM249883 vs 349 reference patients
best-fitted quadratic function
top 15 similar reference patient
most similar reference patient
Euclidean distance between expression vectors
10
20
30
40
50
1.0
1.2
1.4
1.6
1.8
2.0
Average weighted risk (AWR) of reference patient
Risk: blue-low; green-intermediate; red-high

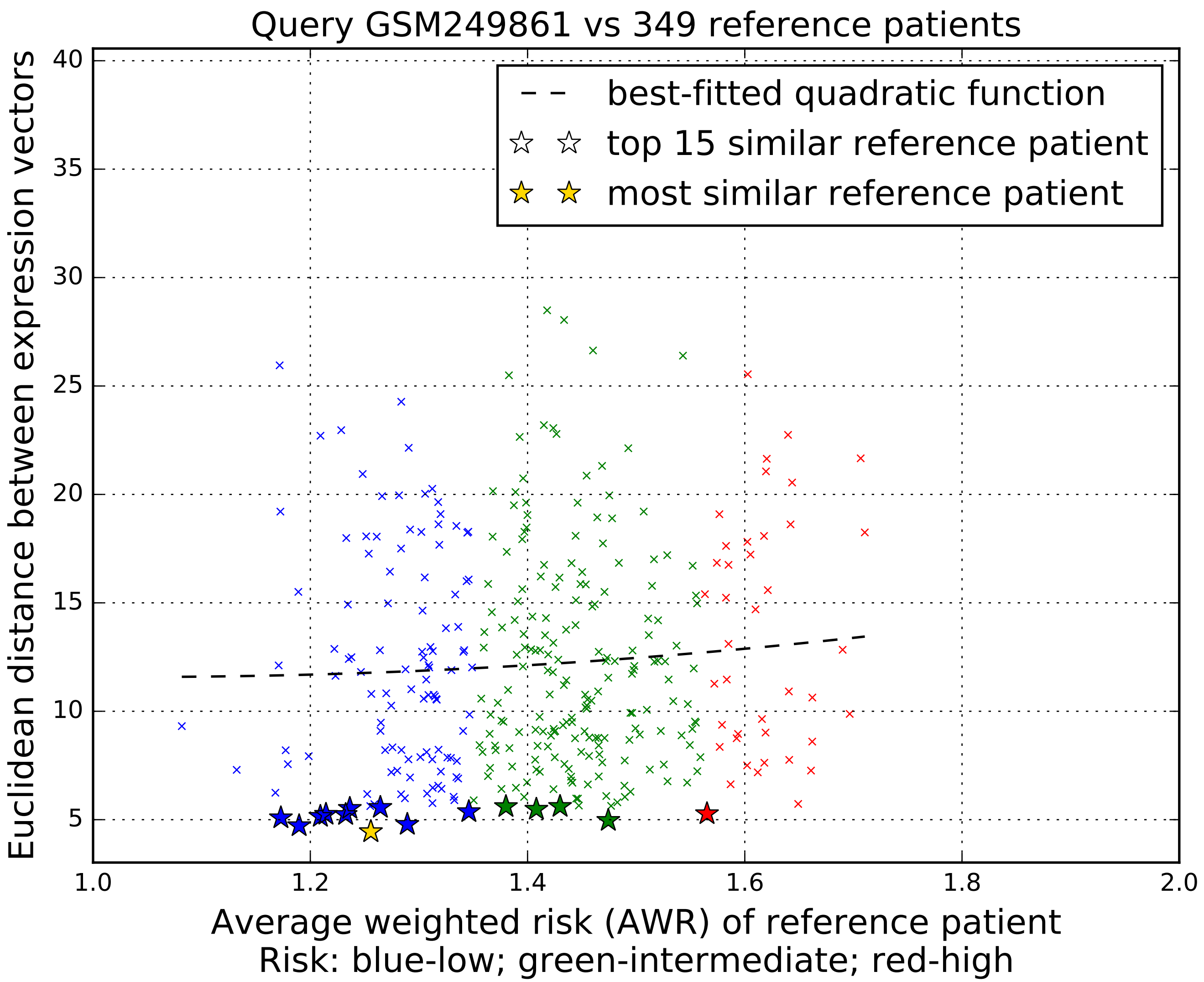
Query GSM249861 vs 349 reference patients
Euclidean distance between expression vectors
40
35
30
25
20
15
10
5
1.0
1.2
1.4
1.6
1.8
2.0
best-fitted quadratic function
top 15 similar reference patient
most similar reference patient
Average weighted risk (AWR) of reference patient
Risk: blue-low; green-intermediate; red-high

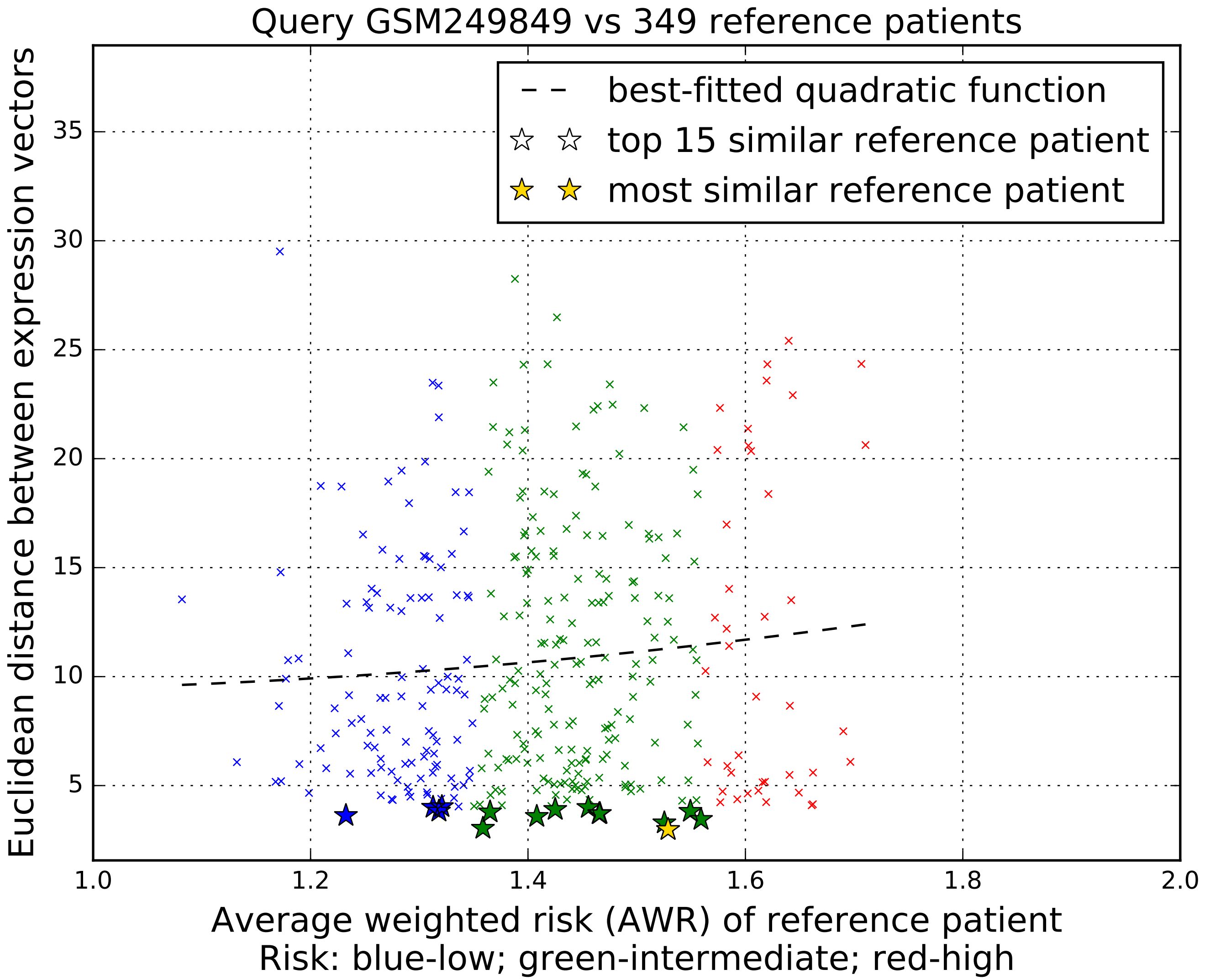

Query GSM249849 vs 349 reference patients
best-fitted quadratic function
top 15 similar reference patient
most similar reference patient
Euclidean distance between expression vectors
Average weighted risk (AWR) of reference patient
Risk: blue-low; green-intermediate; red-high
1.0
1.2
1.4
1.6
1.8
2.0
5
10
15
20
25
30
35

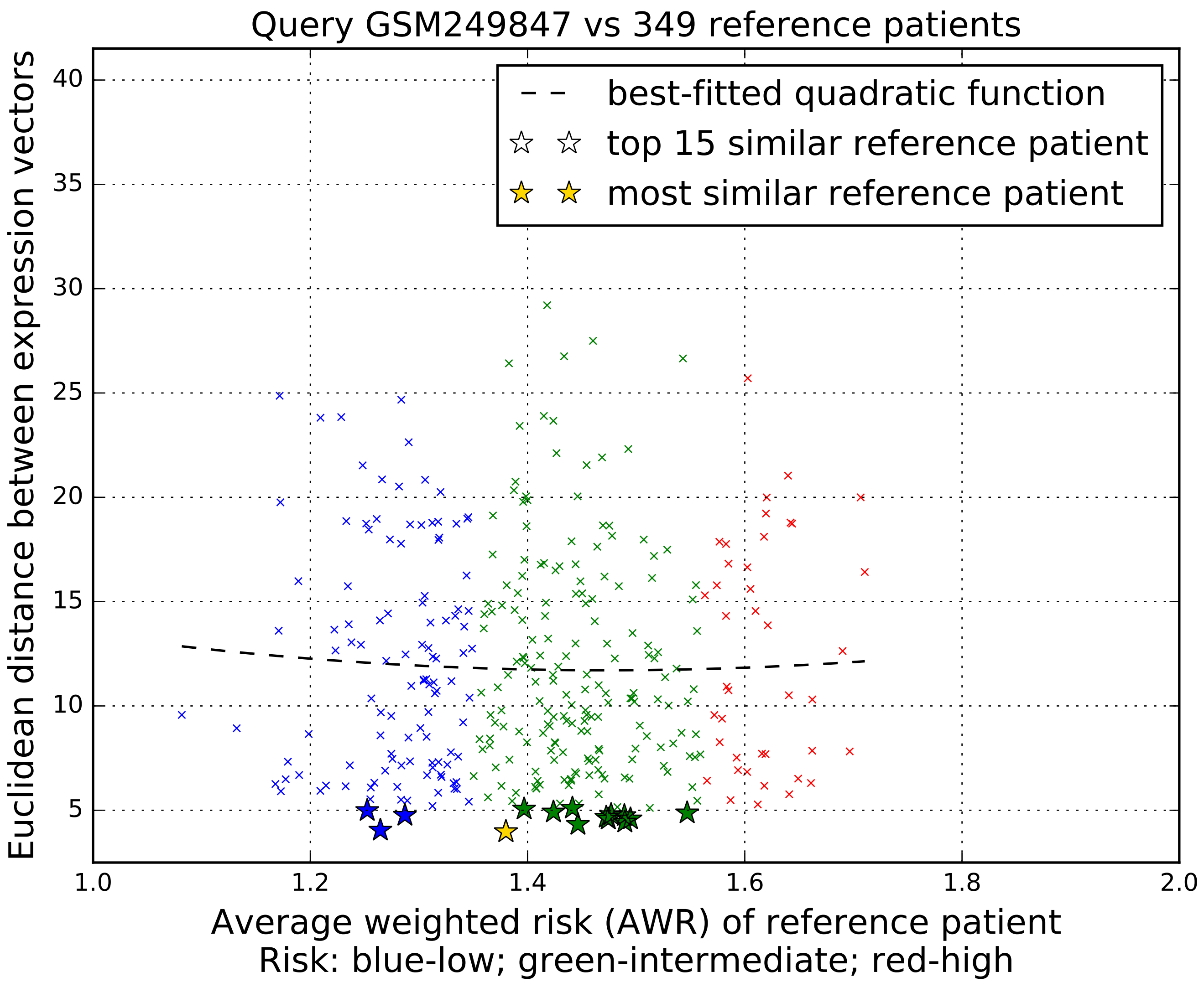

Query GSM249847 vs 349 reference patients
best-fitted quadratic function
top 15 similar reference patient
most similar reference patient
Euclidean distance between expression vectors
40
35
30
25
20
15
10
5
1.0
1.2
1.4
1.6
1.8
2.0
Average weighted risk (AWR) of reference patient
Risk: blue-low; green-intermediate; red-high

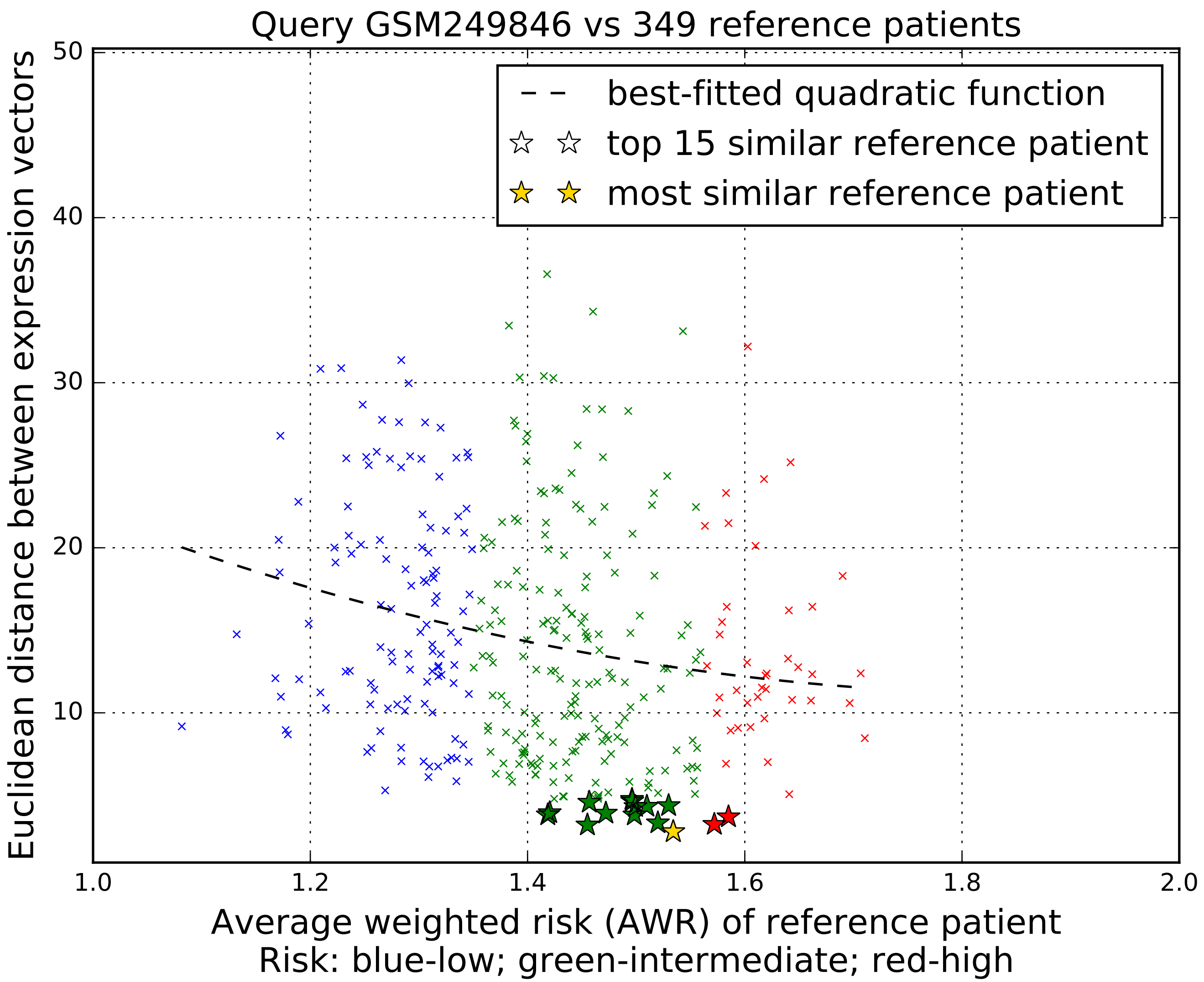

Query GSM249846 vs 349 reference patients
best-fitted quadratic function
top 15 similar reference patient
most similar reference patient
Euclidean distance between expression vectors
50
40
30
20
10
1.0
1.2
1.4
1.6
1.8
2.0
Average weighted risk (AWR) of reference patient
Risk: blue-low; green-intermediate; red-high

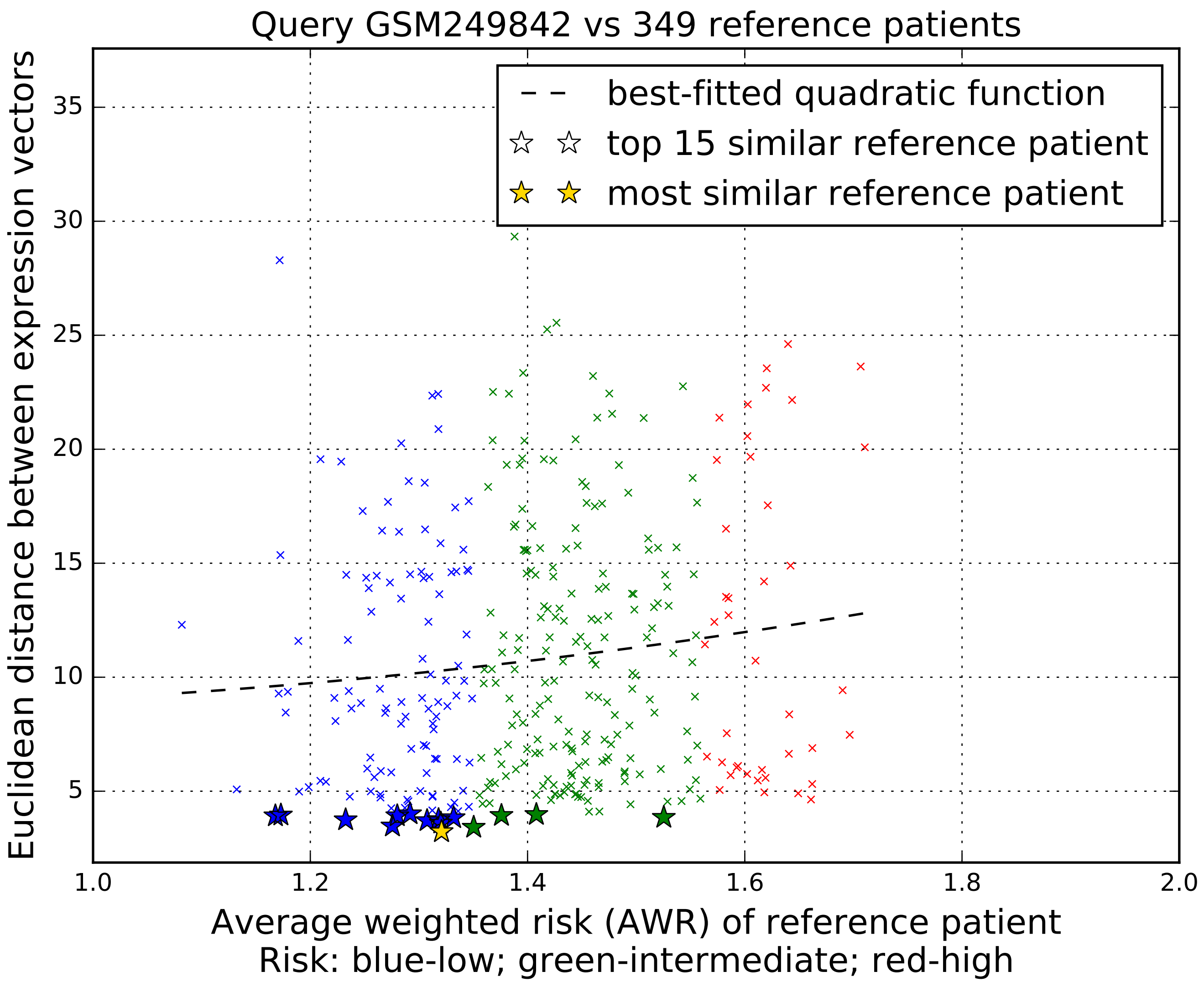
Query GSM249842 vs 349 reference patients
best-fitted quadratic function
top 15 similar reference patient
most similar reference patient
Euclidean distance between expression vectors
Average weighted risk (AWR) of reference patient
Risk: blue-low; green-intermediate; red-high
35
30
25
20
15
10
5
1.0
1.2
1.4
1.6
1.8
2.0

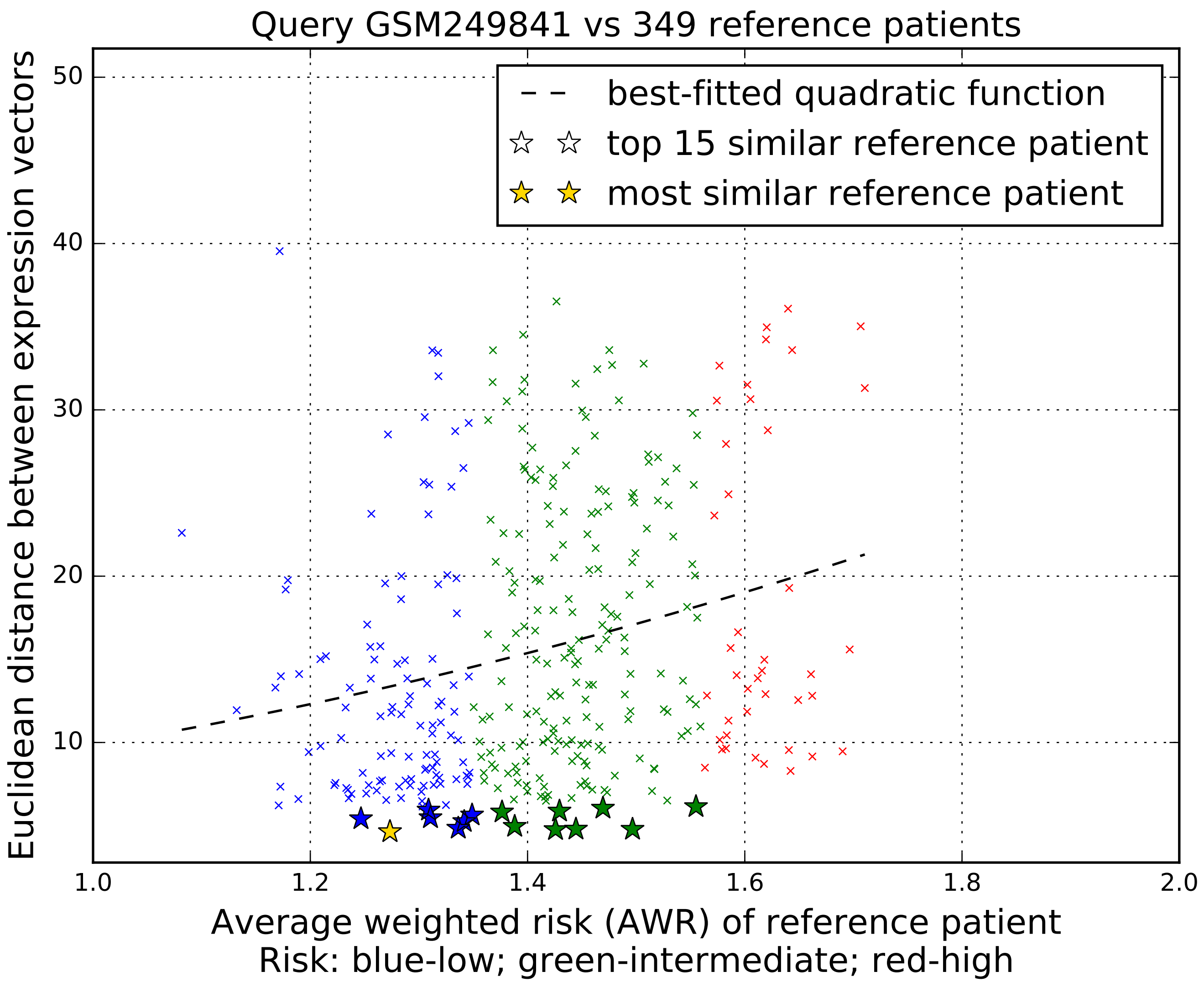

Query GSM249841 vs 349 reference patients
best-fitted quadratic function
top 15 similar reference patient
most similar reference patient
Euclidean distance between expression vectors
50
40
30
20
10
1.0
1.2
1.4
1.6
1.8
2.0
Average weighted risk (AWR) of reference patient
Risk: blue-low; green-intermediate; red-high

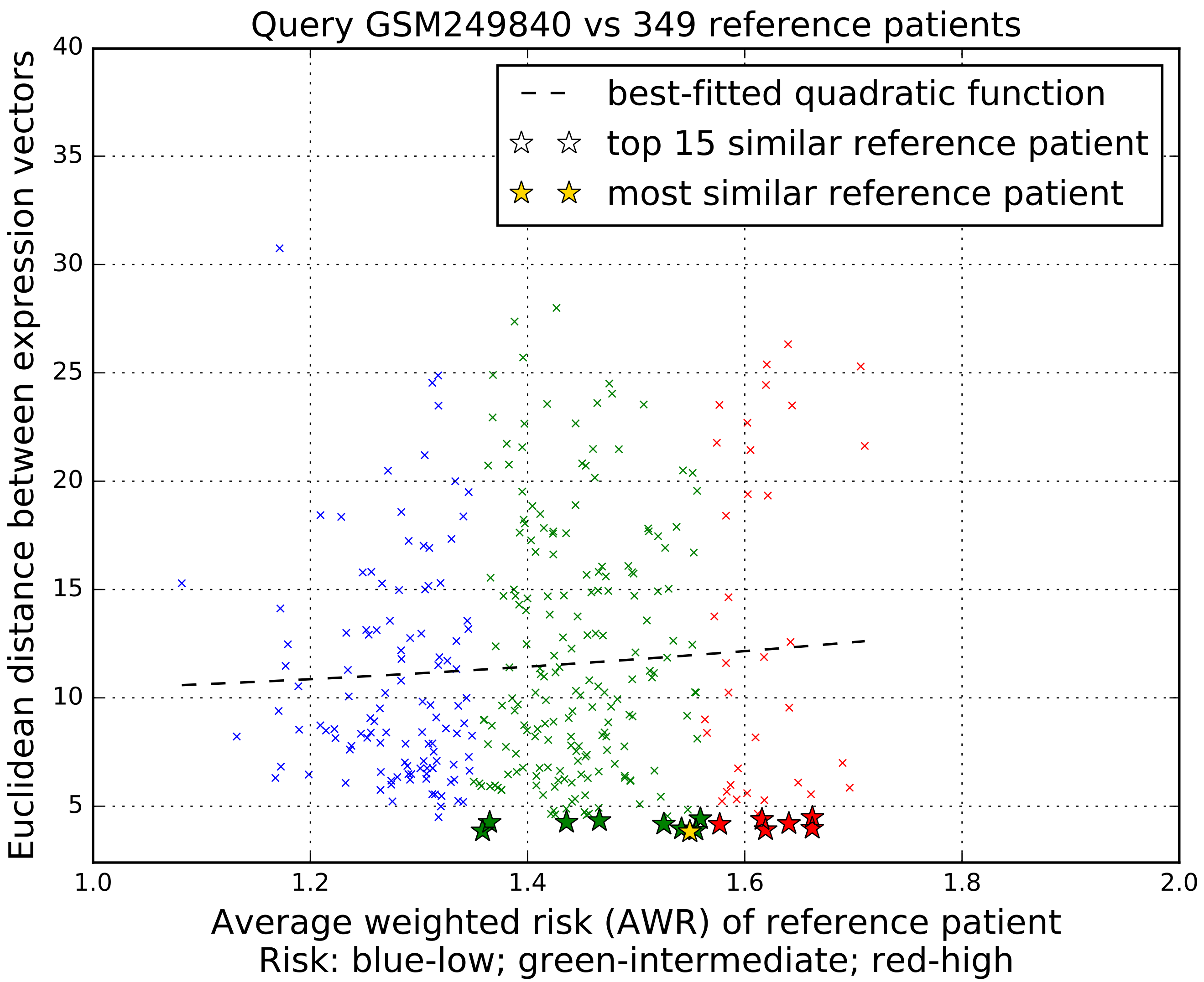
Query GSM249840 vs 349 reference patients
best-fitted quadratic function
top 15 similar reference patient
most similar reference patient
Euclidean distance between expression vectors
40
35
30
25
20
15
10
5
1.0
1.2
1.4
1.6
1.8
2.0
Average weighted risk (AWR) of reference patient
Risk: blue-low; green-intermediate; red-high

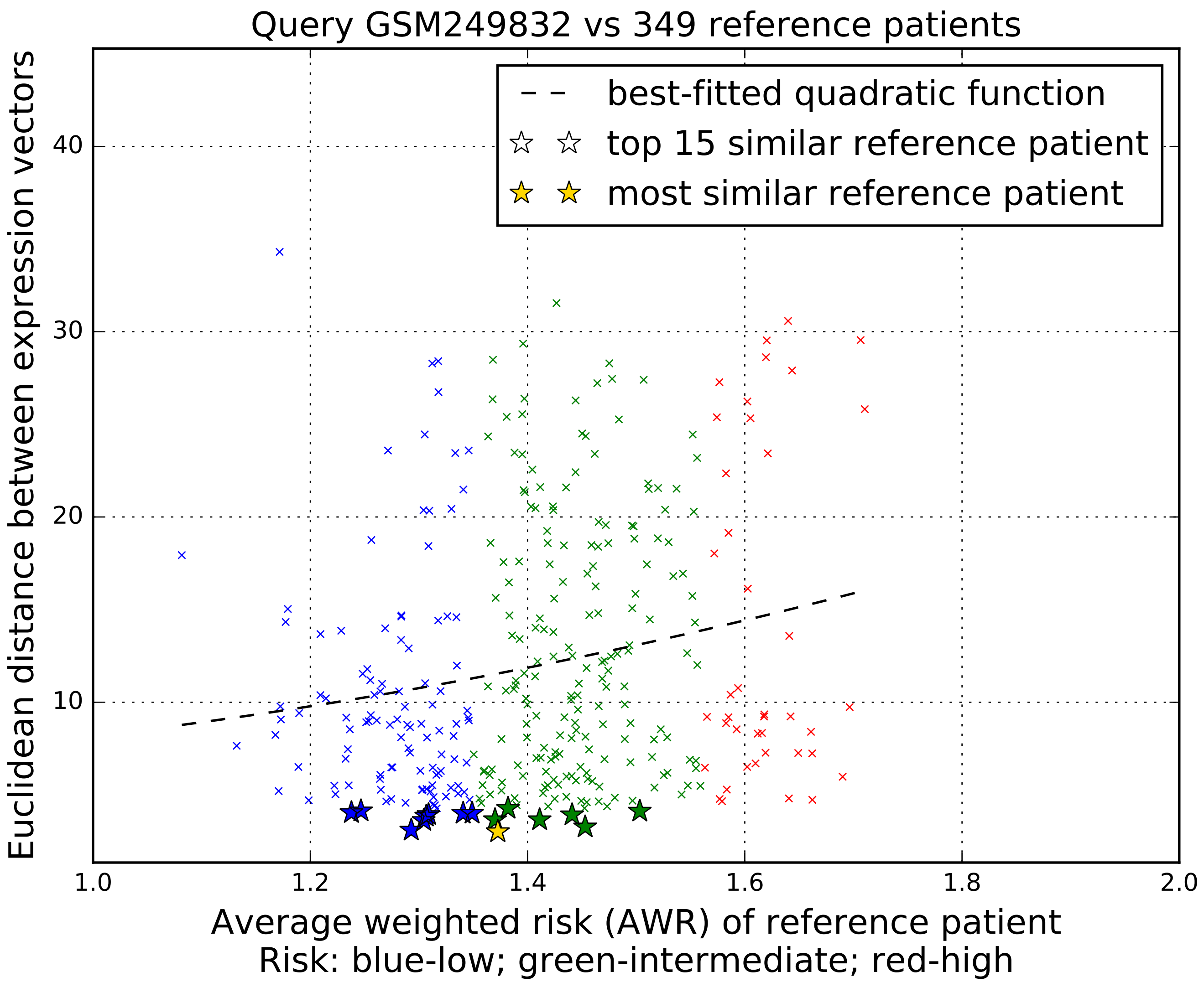
Query GSM249832 vs 349 reference patients
best-fitted quadratic function
top 15 similar reference patient
most similar reference patient
Euclidean distance between expression vectors
40
30
20
10
1.0
1.2
1.4
1.6
1.8
2.0
Average weighted risk (AWR) of reference patient
Risk: blue-low; green-intermediate; red-high

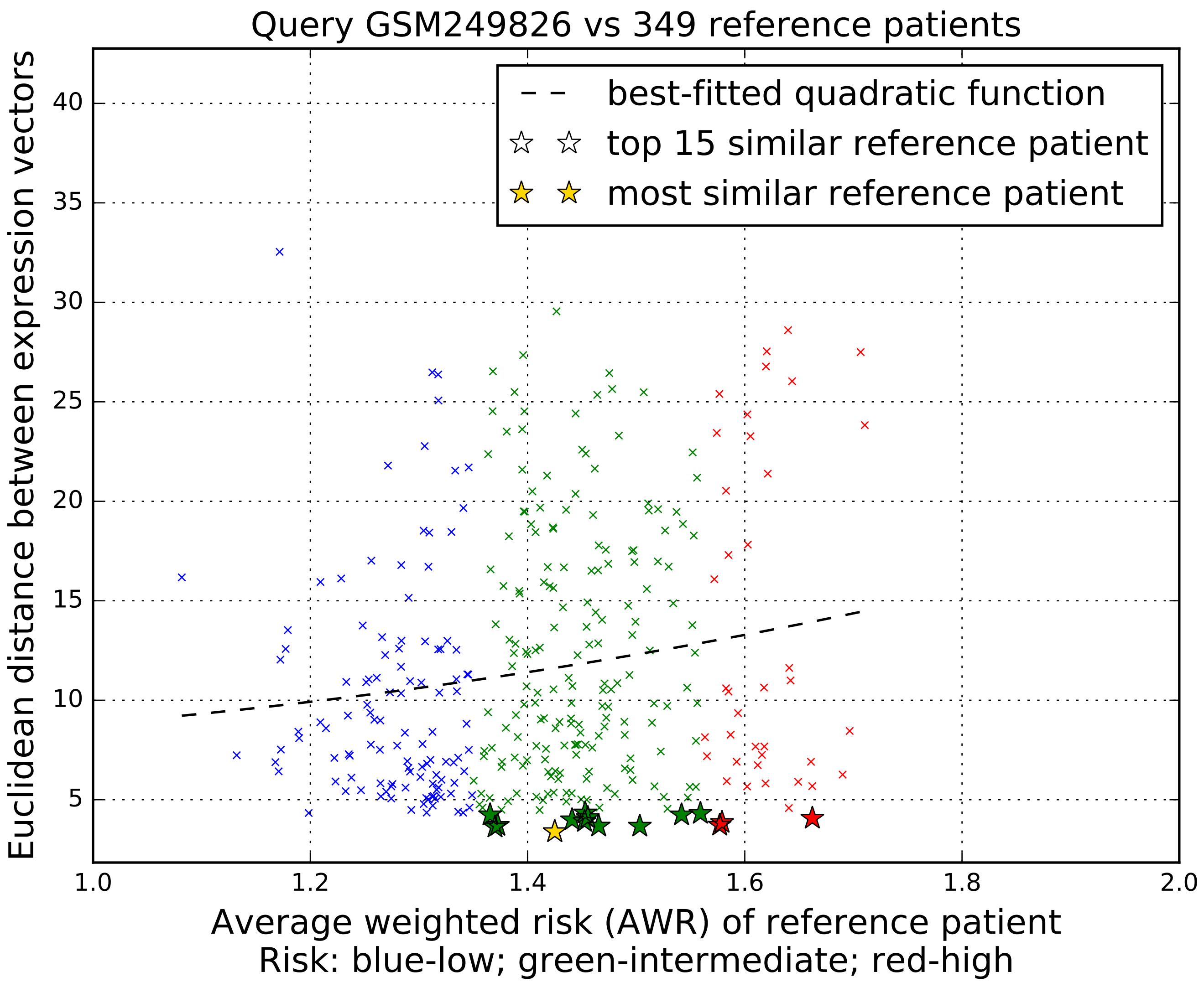
Query GSM249826 vs 349 reference patients
best-fitted quadratic function
top 15 similar reference patient
most similar reference patient
Euclidean distance between expression vectors
40
35
30
25
20
15
10
5
1.0
1.2
1.4
1.6
1.8
2.0
Average weighted risk (AWR) of reference patient
Risk: blue-low; green-intermediate; red-high

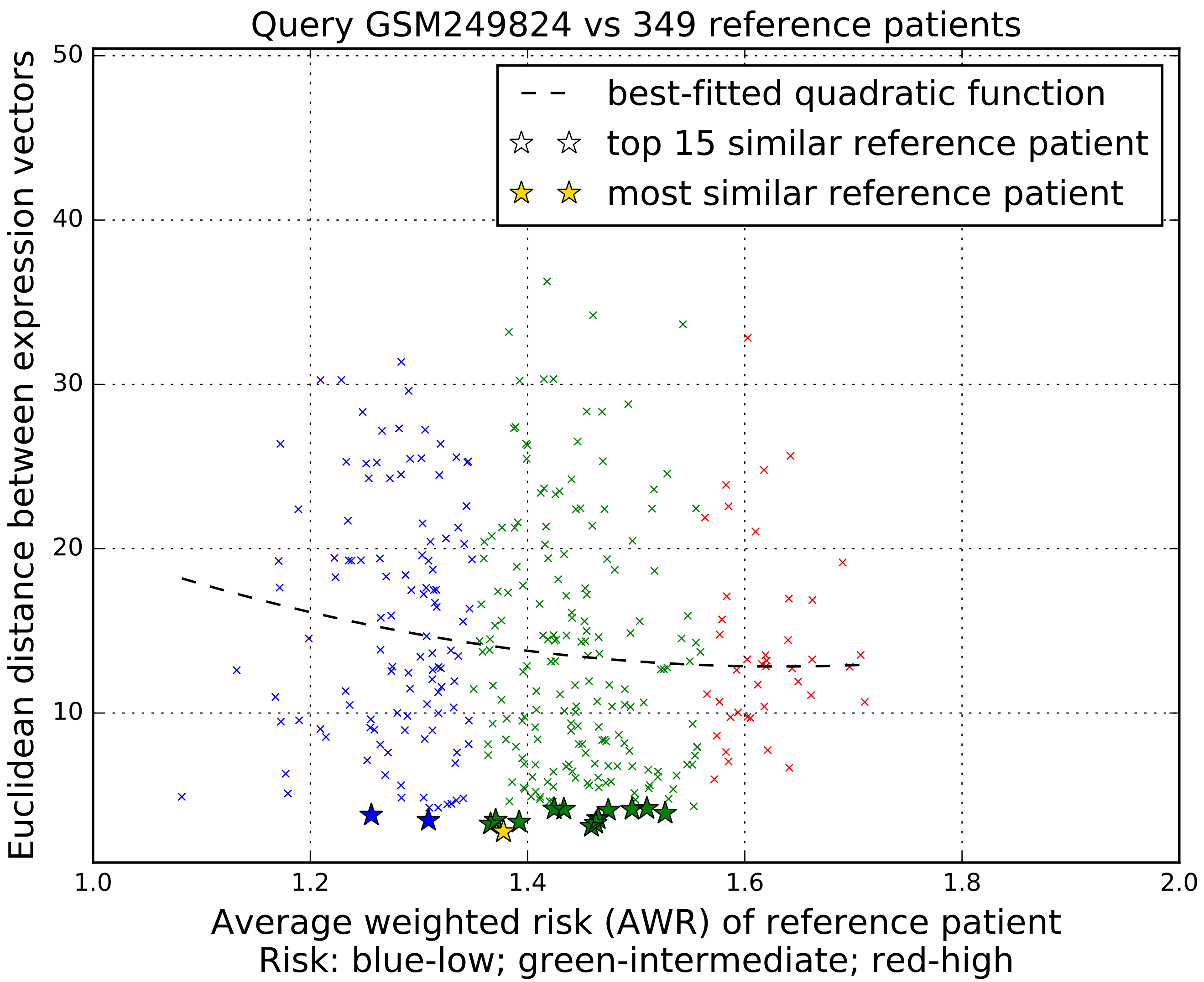

Query GSM249824 vs 349 reference patients
best-fitted quadratic function
top 15 similar reference patient
most similar reference patient
Euclidean distance between expression vectors
50
40
30
20
10
1.0
1.2
1.4
1.6
1.8
2.0
Average weighted risk (AWR) of reference patient
Risk: blue-low; green-intermediate; red-high

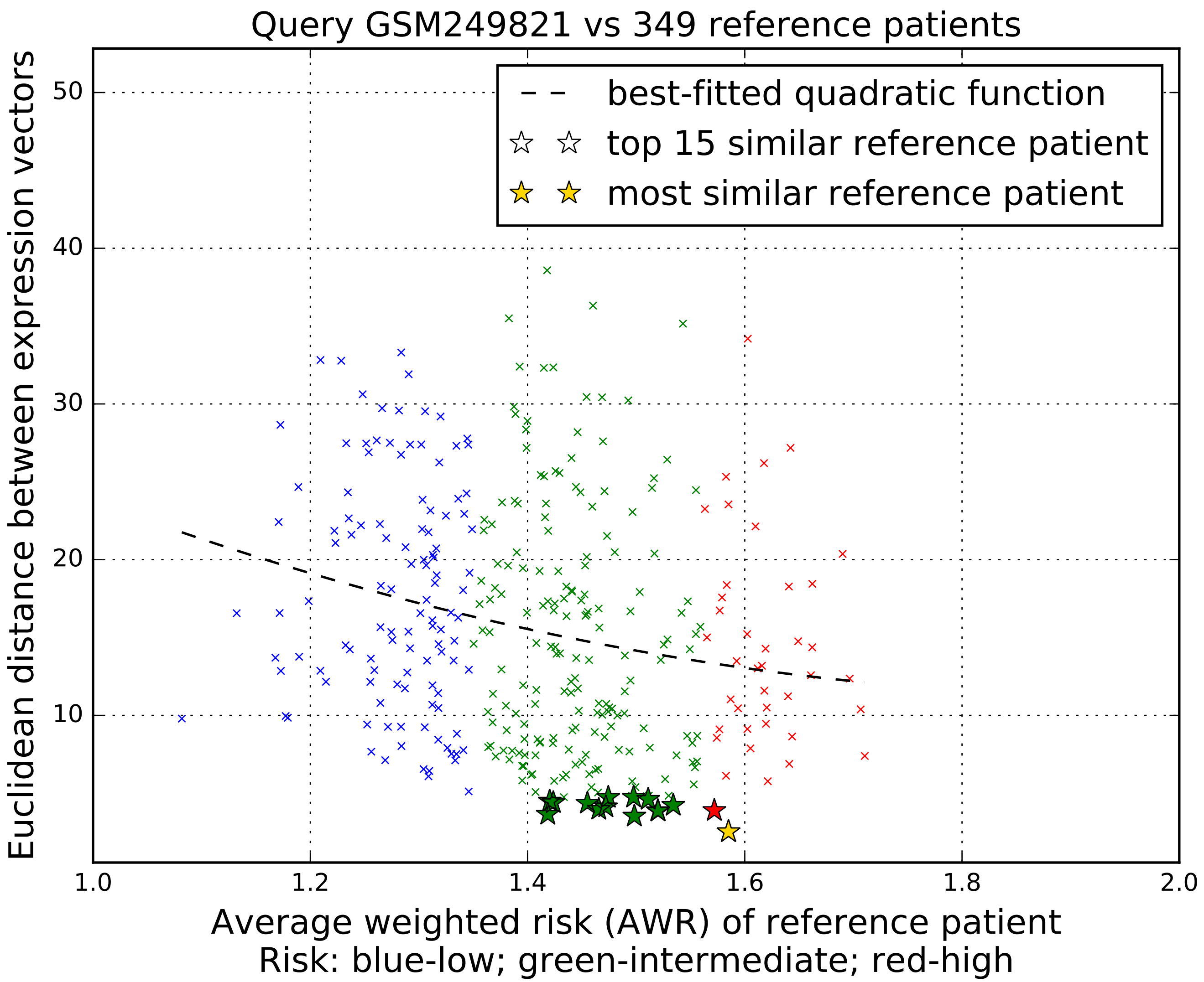

Query GSM249821 vs 349 reference patients
best-fitted quadratic function
top 15 similar reference patient
most similar reference patient
Euclidean distance between expression vectors
10
20
30
40
50
1.0
1.2
1.4
1.6
1.8
2.0
Average weighted risk (AWR) of reference patient
Risk: blue-low; green-intermediate; red-high

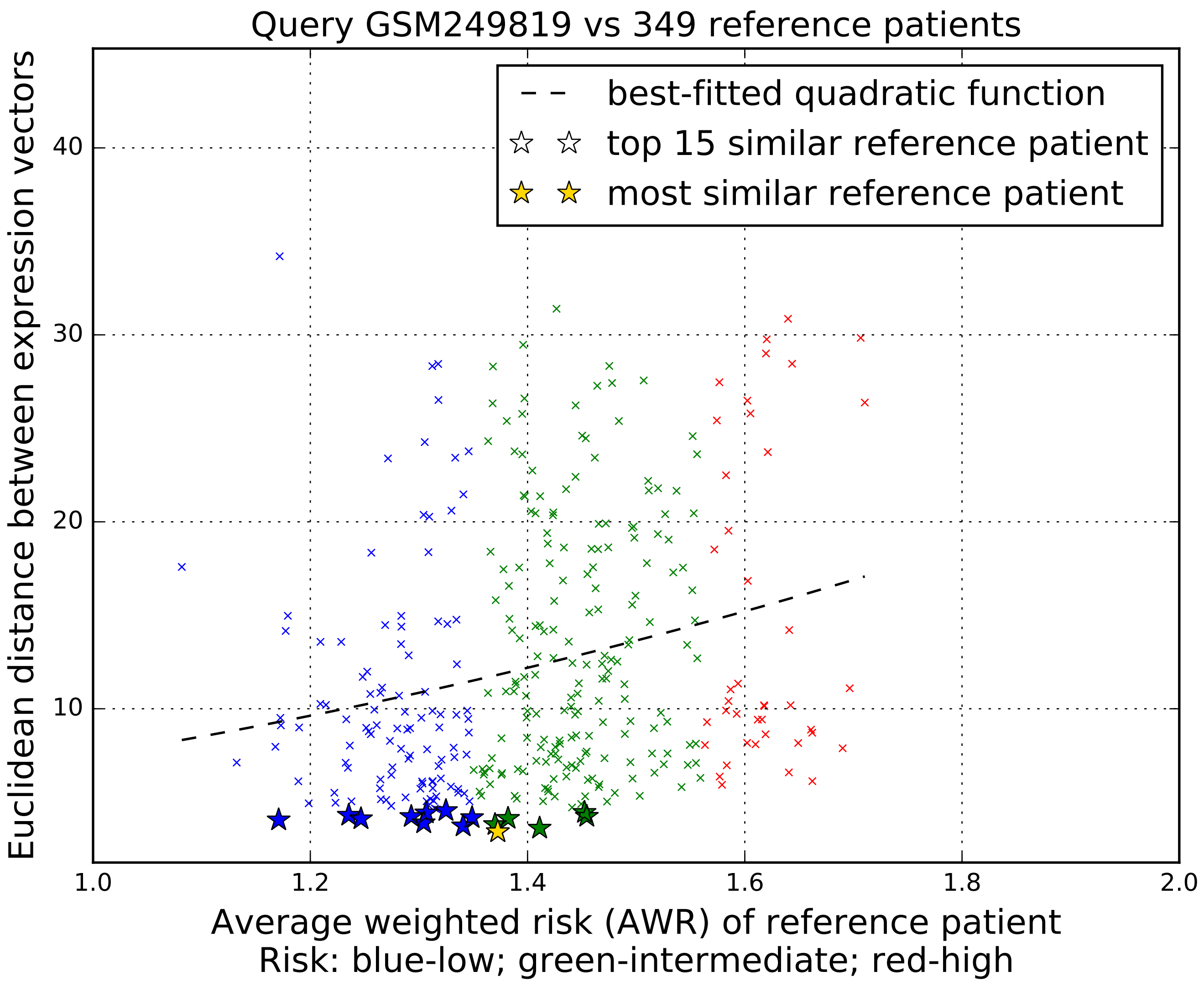

Query GSM249819 vs 349 reference patients
best-fitted quadratic function
top 15 similar reference patient
most similar reference patient
Euclidean distance between expression vectors
40
30
20
10
1.0
1.2
1.4
1.6
1.8
2.0
Average weighted risk (AWR) of reference patient
Risk: blue-low; green-intermediate; red-high

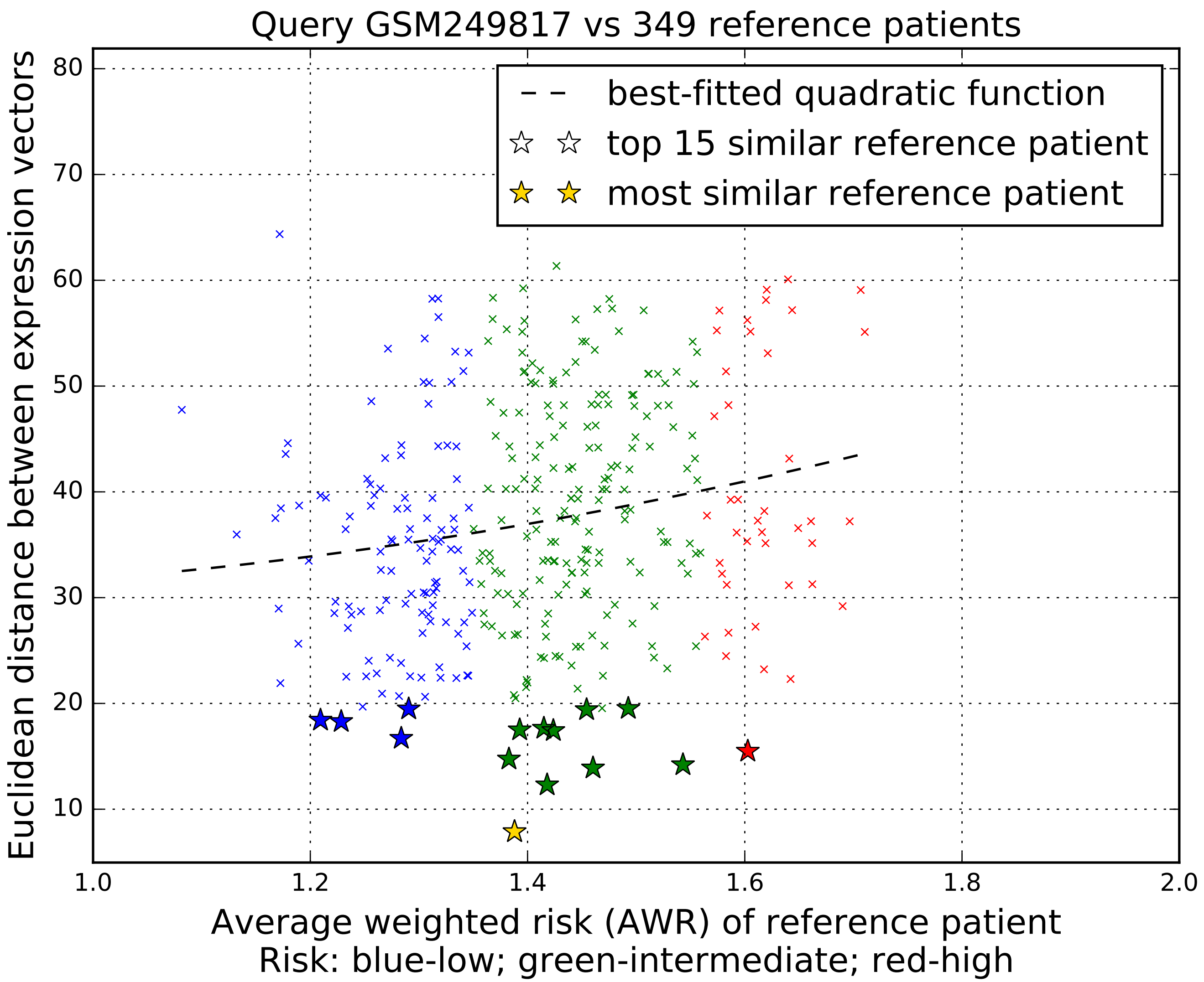
Query GSM249817 vs 349 reference patients
best-fitted quadratic function
top 15 similar reference patient
most similar reference patient
Euclidean distance between expression vectors
80
70
60
50
40
30
20
10
1.0
1.2
1.4
1.6
1.8
2.0
Average weighted risk (AWR) of reference patient
Risk: blue-low; green-intermediate; red-high

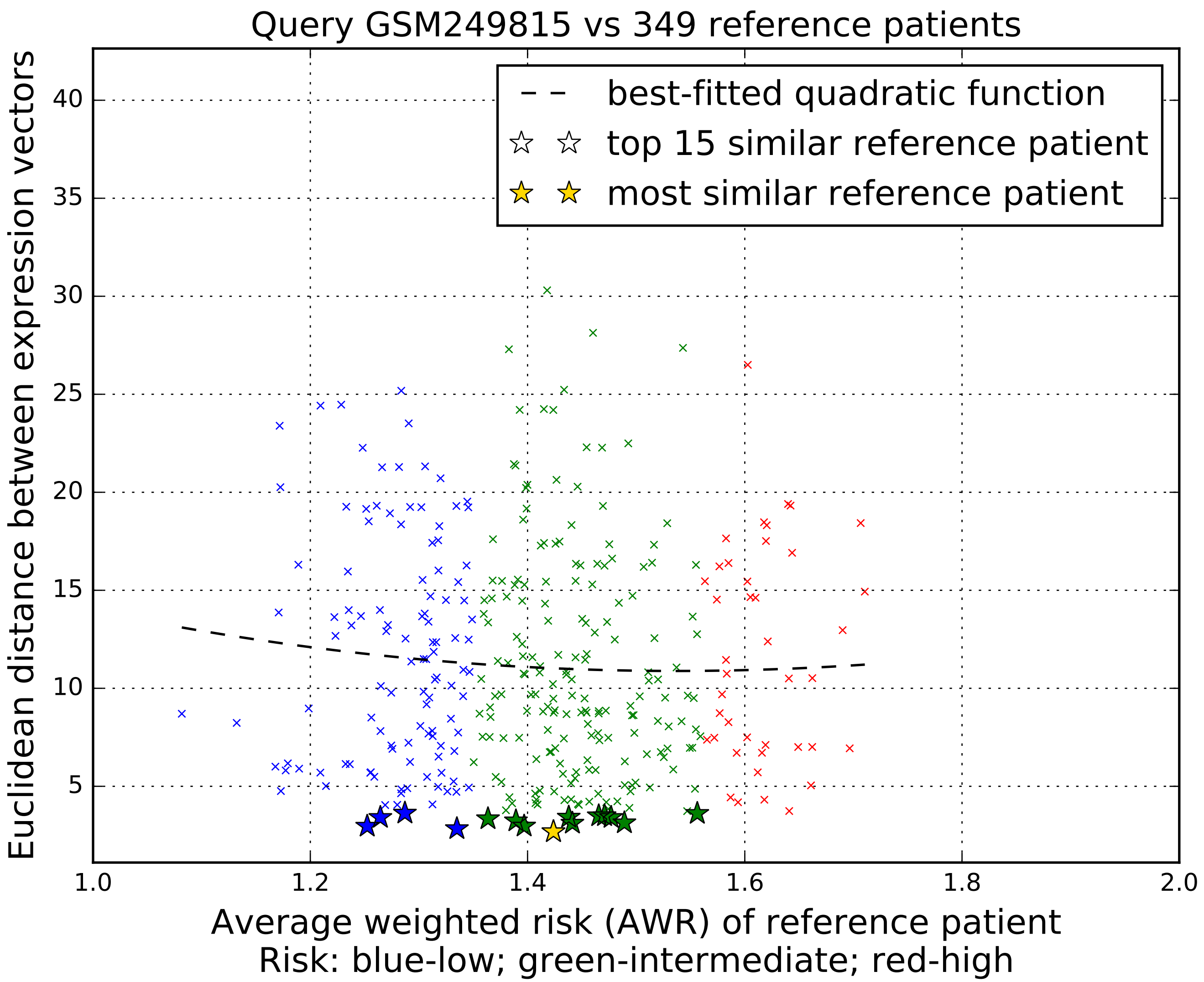
Query GSM249815 vs 349 reference patients
best-fitted quadratic function
top 15 similar reference patient
most similar reference patient
Euclidean distance between expression vectors
40
35
30
25
20
15
10
5
1.0
1.2
1.4
1.6
1.8
2.0
Average weighted risk (AWR) of reference patient
Risk: blue-low; green-intermediate; red-high

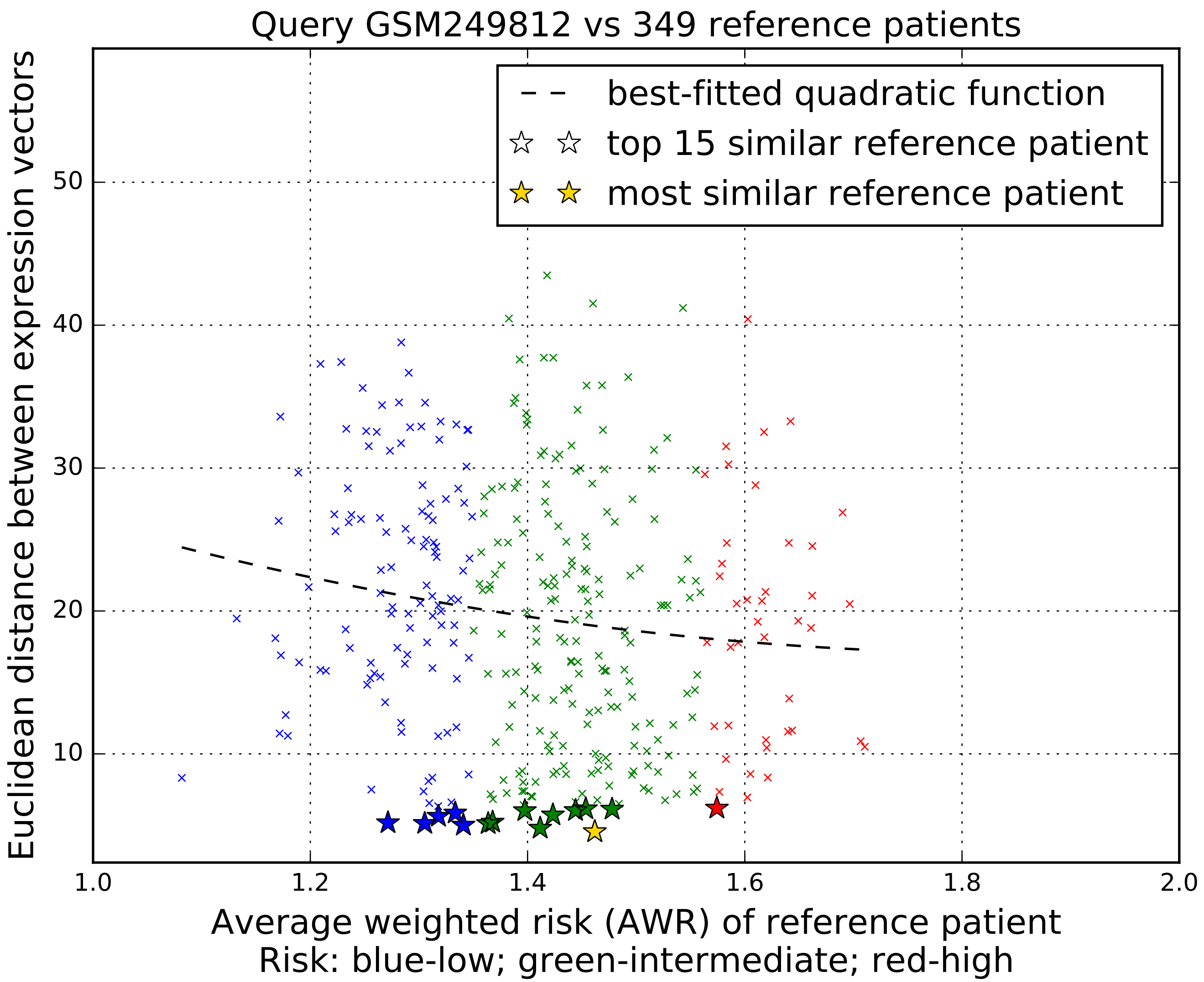

Query GSM249812 vs 349 reference patients
best-fitted quadratic function
top 15 similar reference patient
most similar reference patient
Euclidean distance between expression vectors
10
20
30
40
50
1.0
1.2
1.4
1.6
1.8
2.0
Average weighted risk (AWR) of reference patient
Risk: blue-low; green-intermediate; red-high

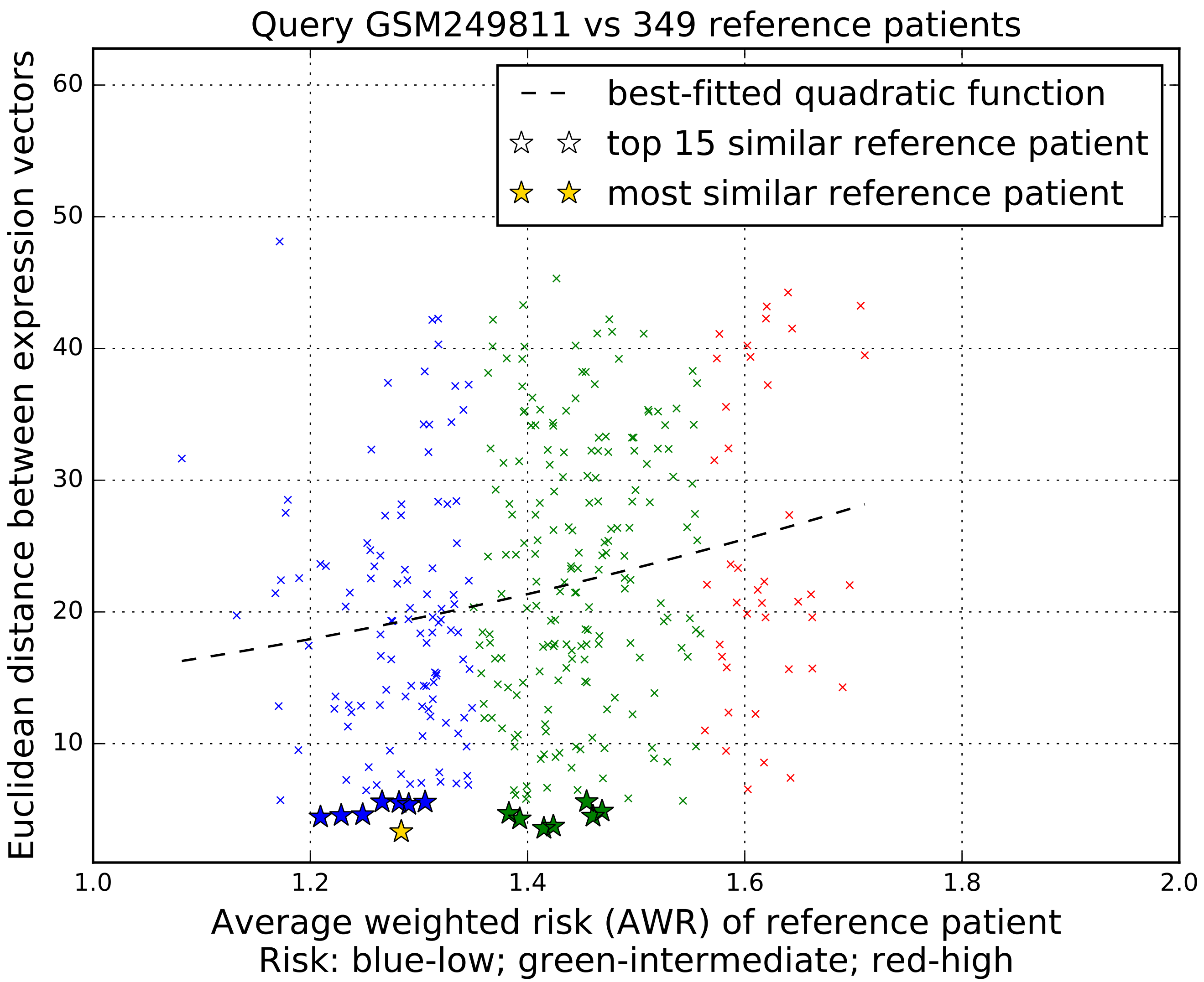
Query GSM249811 vs 349 reference patients
best-fitted quadratic function
top 15 similar reference patient
most similar reference patient
Euclidean distance between expression vectors
10
20
30
40
50
60
1.0
1.2
1.4
1.6
1.8
2.0
Average weighted risk (AWR) of reference patient
Risk: blue-low; green-intermediate; red-high

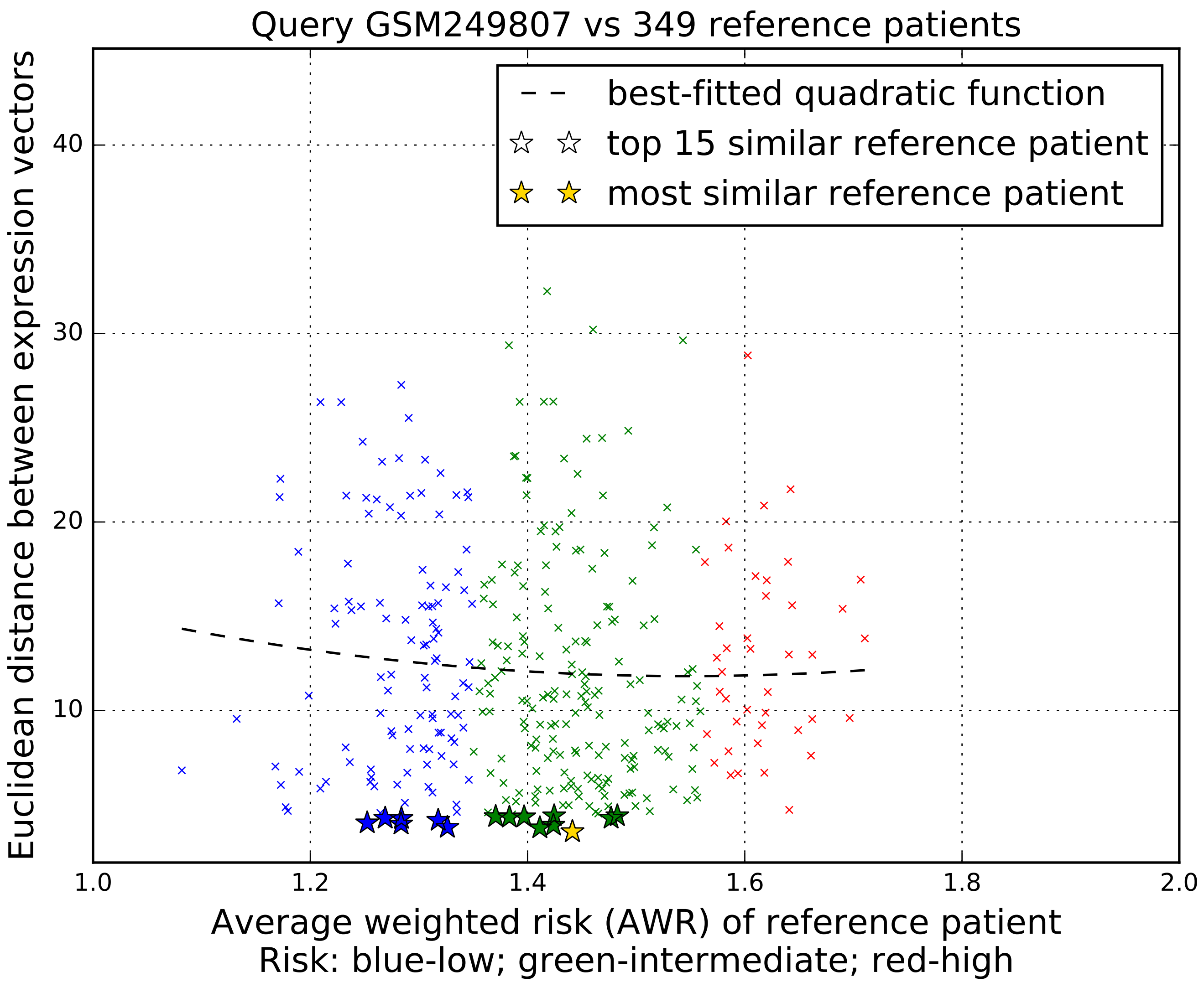
Query GSM249807 vs 349 reference patients
best-fitted quadratic function
top 15 similar reference patient
most similar reference patient
Euclidean distance between expression vectors
40
30
20
10
1.0
1.2
1.4
1.6
1.8
2.0
Average weighted risk (AWR) of reference patient
Risk: blue-low; green-intermediate; red-high

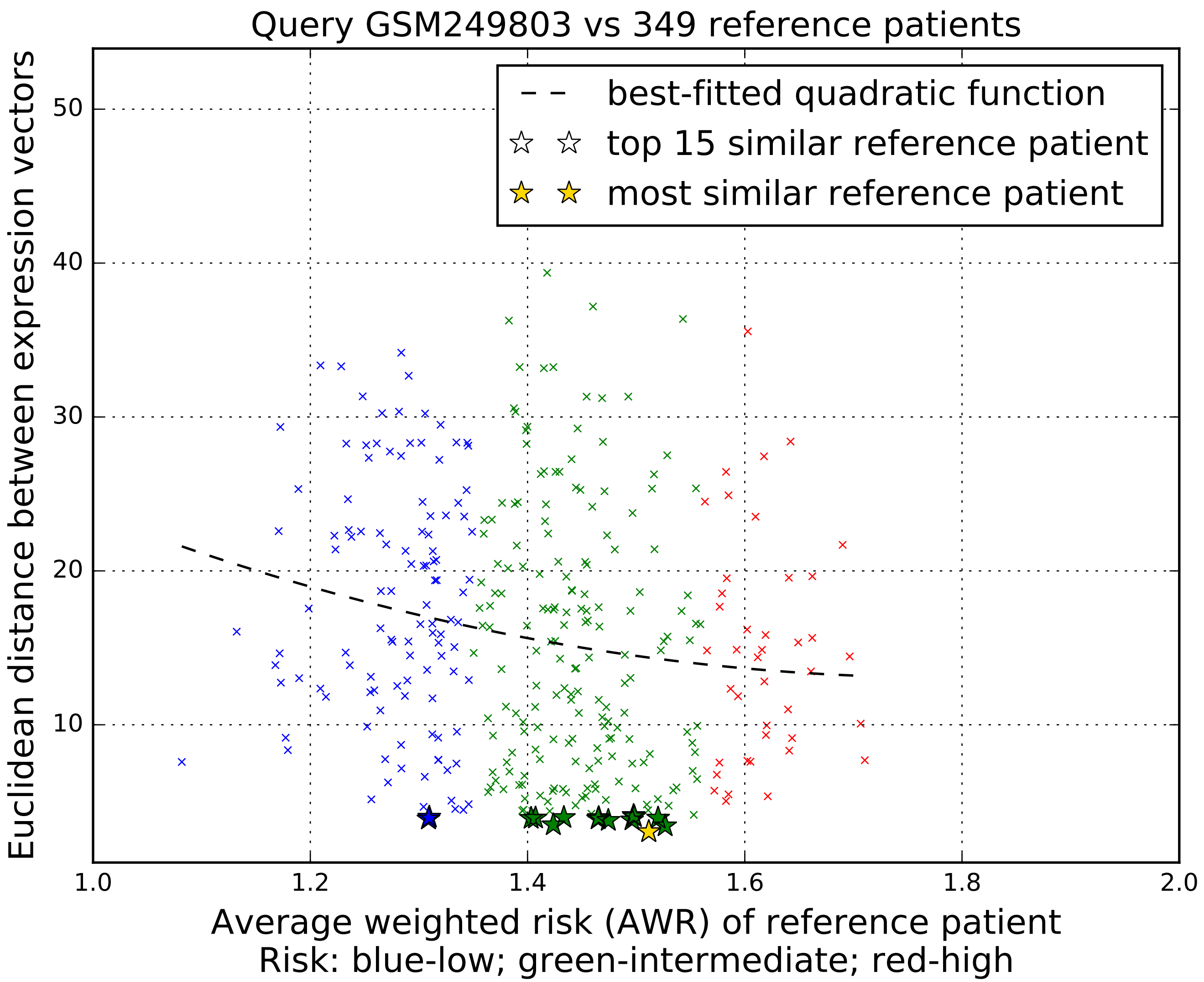
Query GSM249803 vs 349 reference patients
best-fitted quadratic function
top 15 similar reference patient
most similar reference patient
Euclidean distance between expression vectors
10
20
30
40
50
1.0
1.2
1.4
1.6
1.8
2.0
Average weighted risk (AWR) of reference patient
Risk: blue-low; green-intermediate; red-high

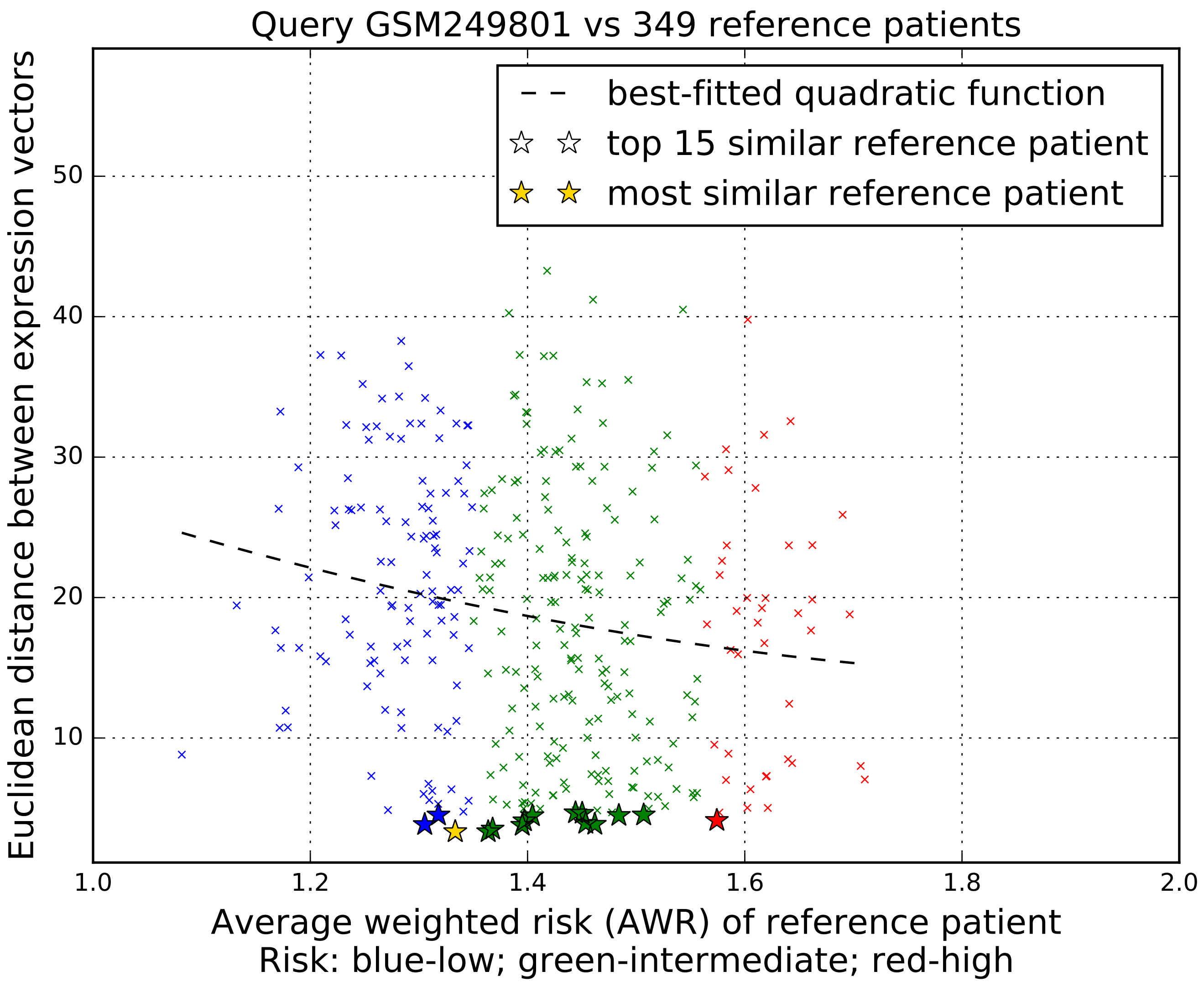
Query GSM249801 vs 349 reference patients
best-fitted quadratic function
top 15 similar reference patient
most similar reference patient
Euclidean distance between expression vectors
10
20
30
40
50
1.0
1.2
1.4
1.6
1.8
2.0
Average weighted risk (AWR) of reference patient
Risk: blue-low; green-intermediate; red-high

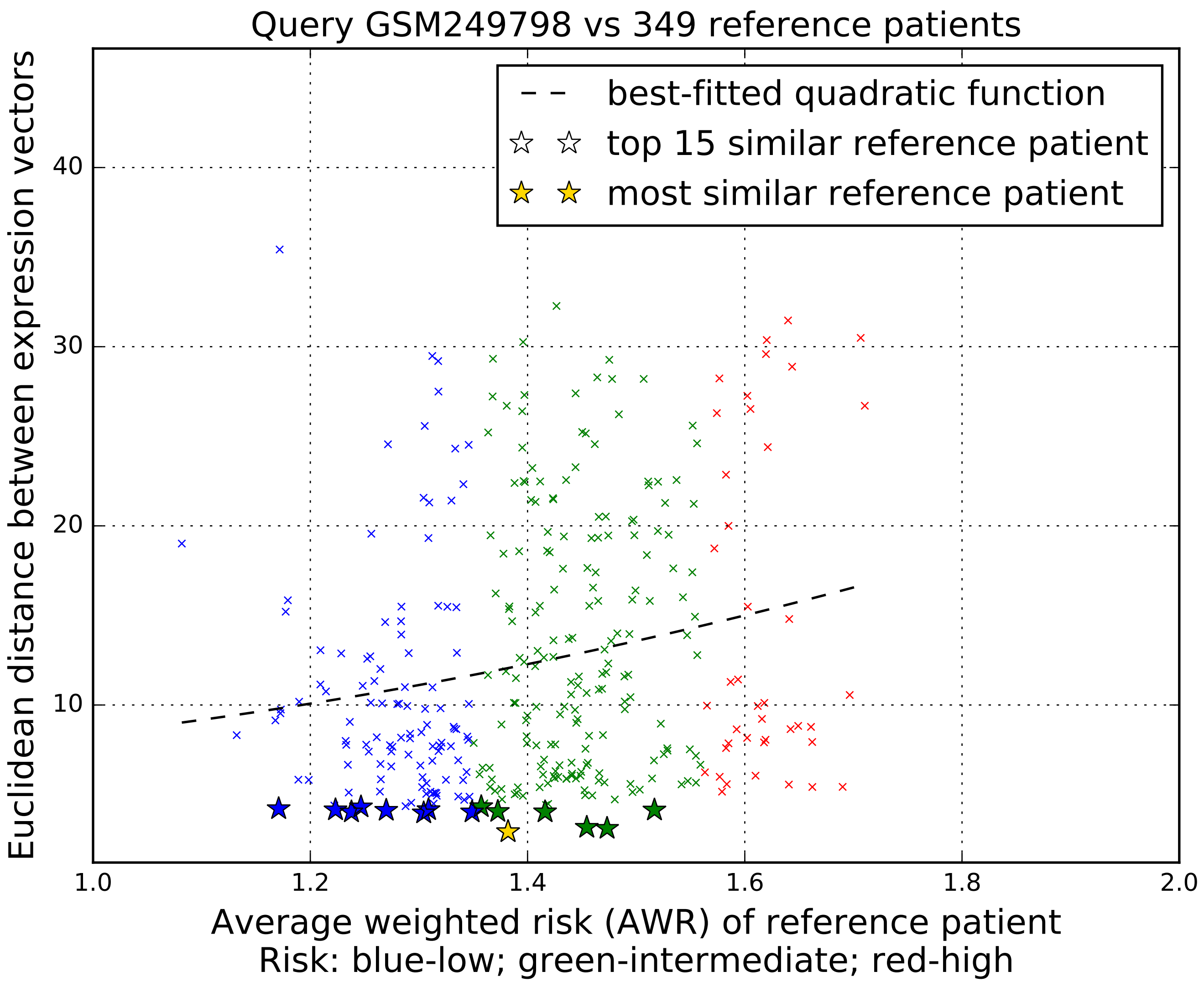

Query GSM249798 vs 349 reference patients
best-fitted quadratic function
top 15 similar reference patient
most similar reference patient
Euclidean distance between expression vectors
40
30
20
10
1.0
1.2
1.4
1.6
1.8
2.0
Average weighted risk (AWR) of reference patient
Risk: blue-low; green-intermediate; red-high

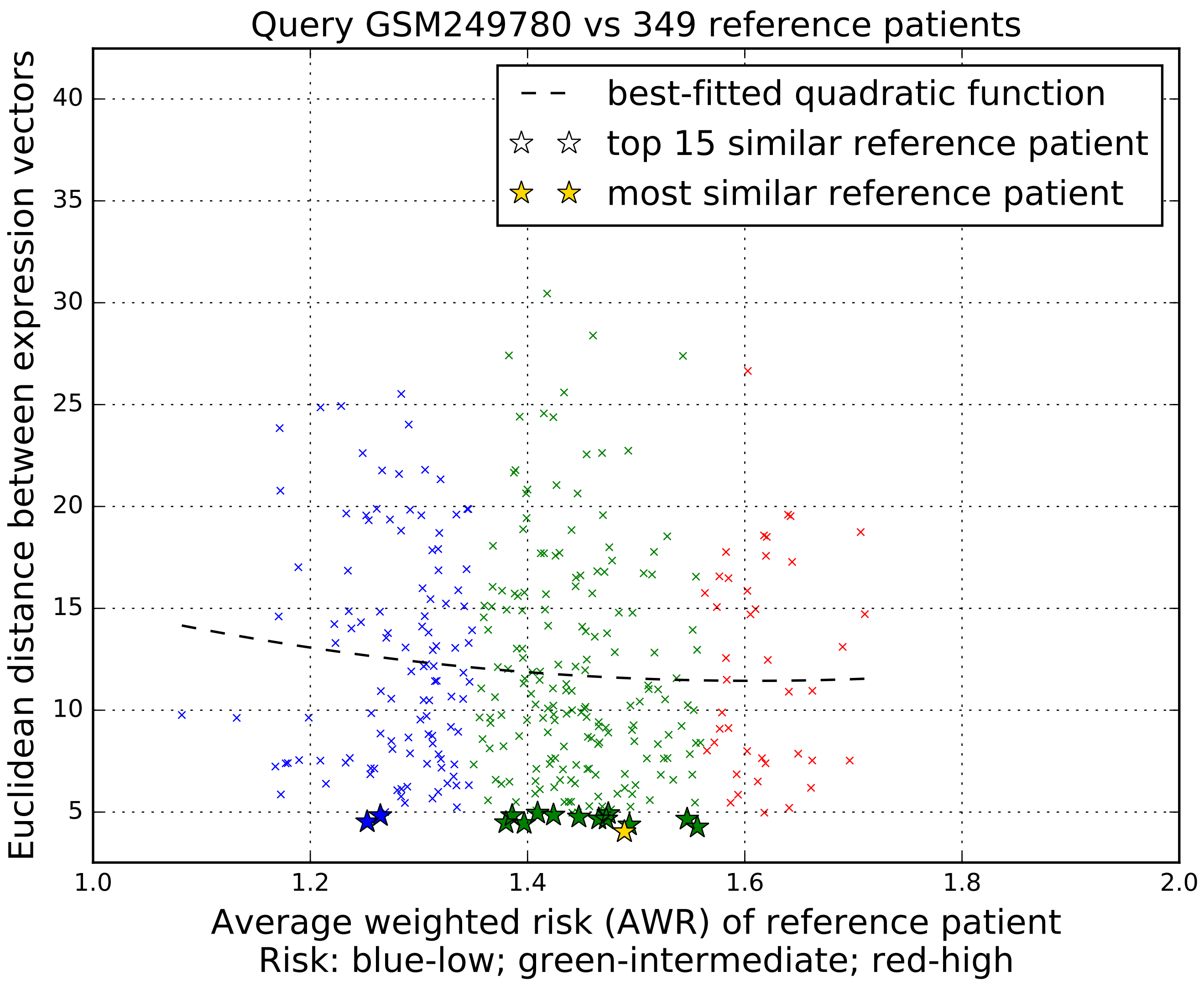

Query GSM249780 vs 349 reference patients
best-fitted quadratic function
top 15 similar reference patient
most similar reference patient
Euclidean distance between expression vectors
40
35
30
25
20
15
10
5
1.0
1.2
1.4
1.6
1.8
2.0
Average weighted risk (AWR) of reference patient
Risk: blue-low; green-intermediate; red-high

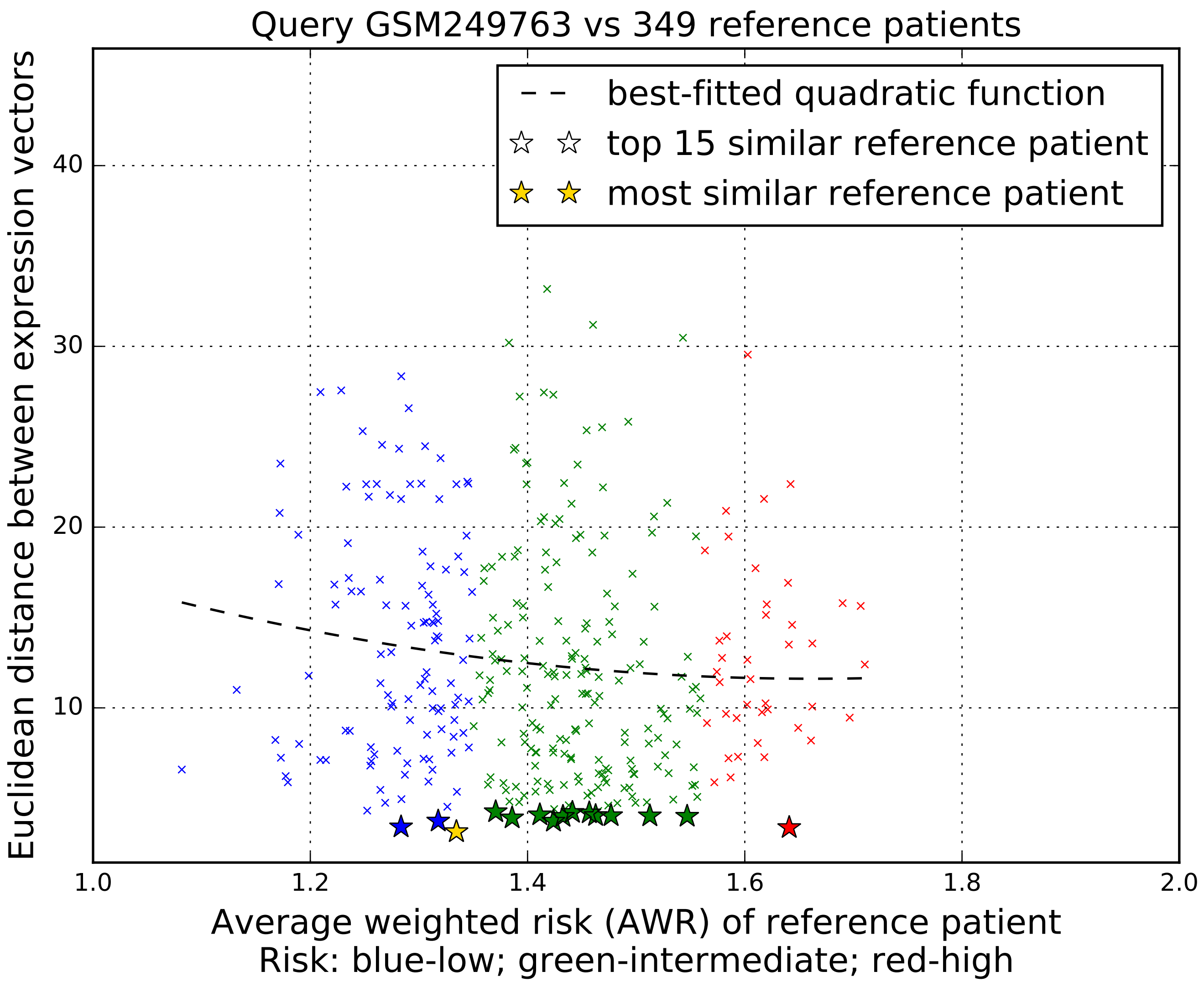
Query GSM249763 vs 349 reference patients
best-fitted quadratic function
top 15 similar reference patient
most similar reference patient
Euclidean distance between expression vectors
10
20
30
40
1.0
1.2
1.4
1.6
1.8
2.0
Average weighted risk (AWR) of reference patient
Risk: blue-low; green-intermediate; red-high

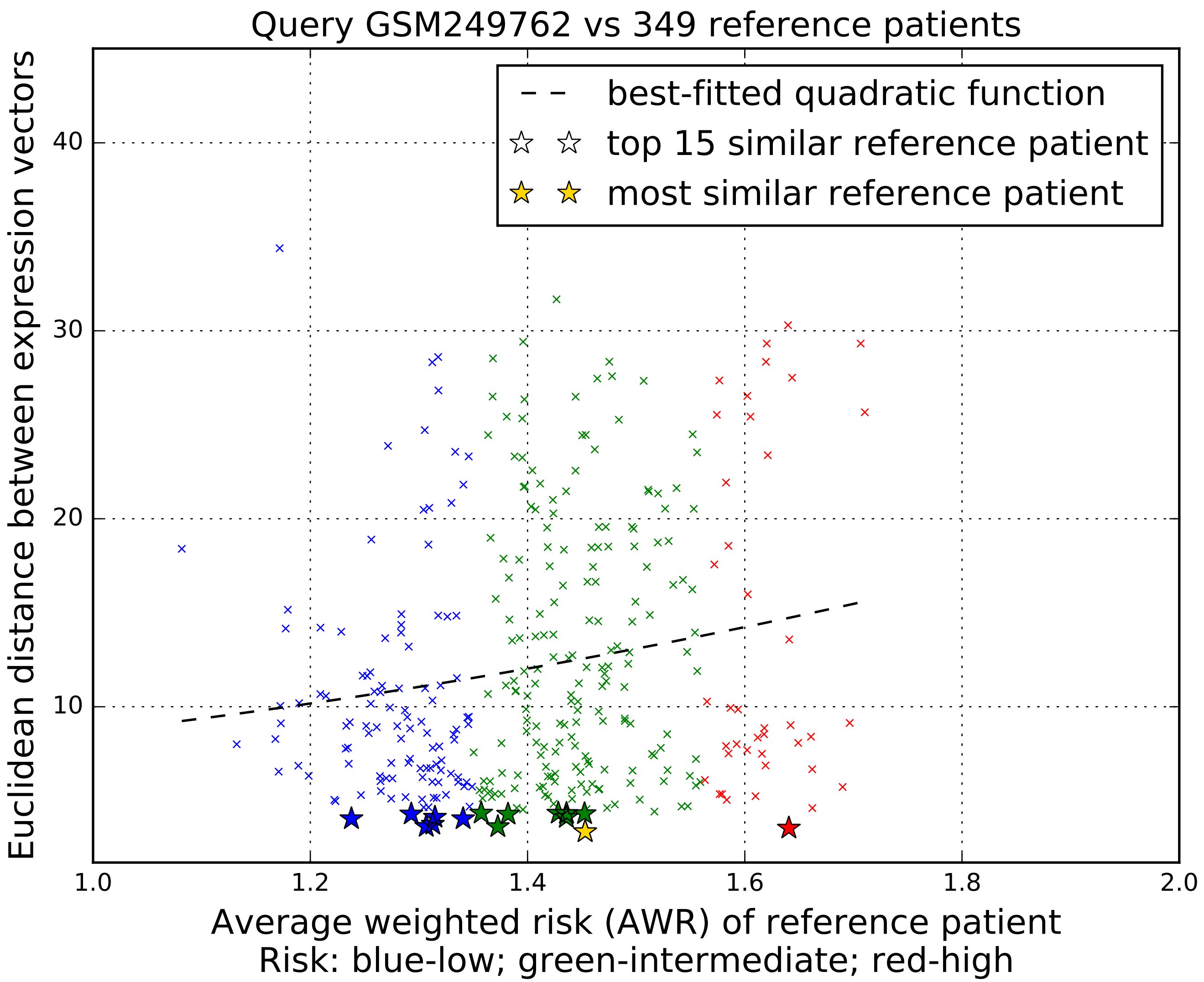
Query GSM249762 vs 349 reference patients
best-fitted quadratic function
top 15 similar reference patient
most similar reference patient
Euclidean distance between expression vectors
10
20
30
40
1.0
1.2
1.4
1.6
1.8
2.0
Average weighted risk (AWR) of reference patient
Risk: blue-low; green-intermediate; red-high

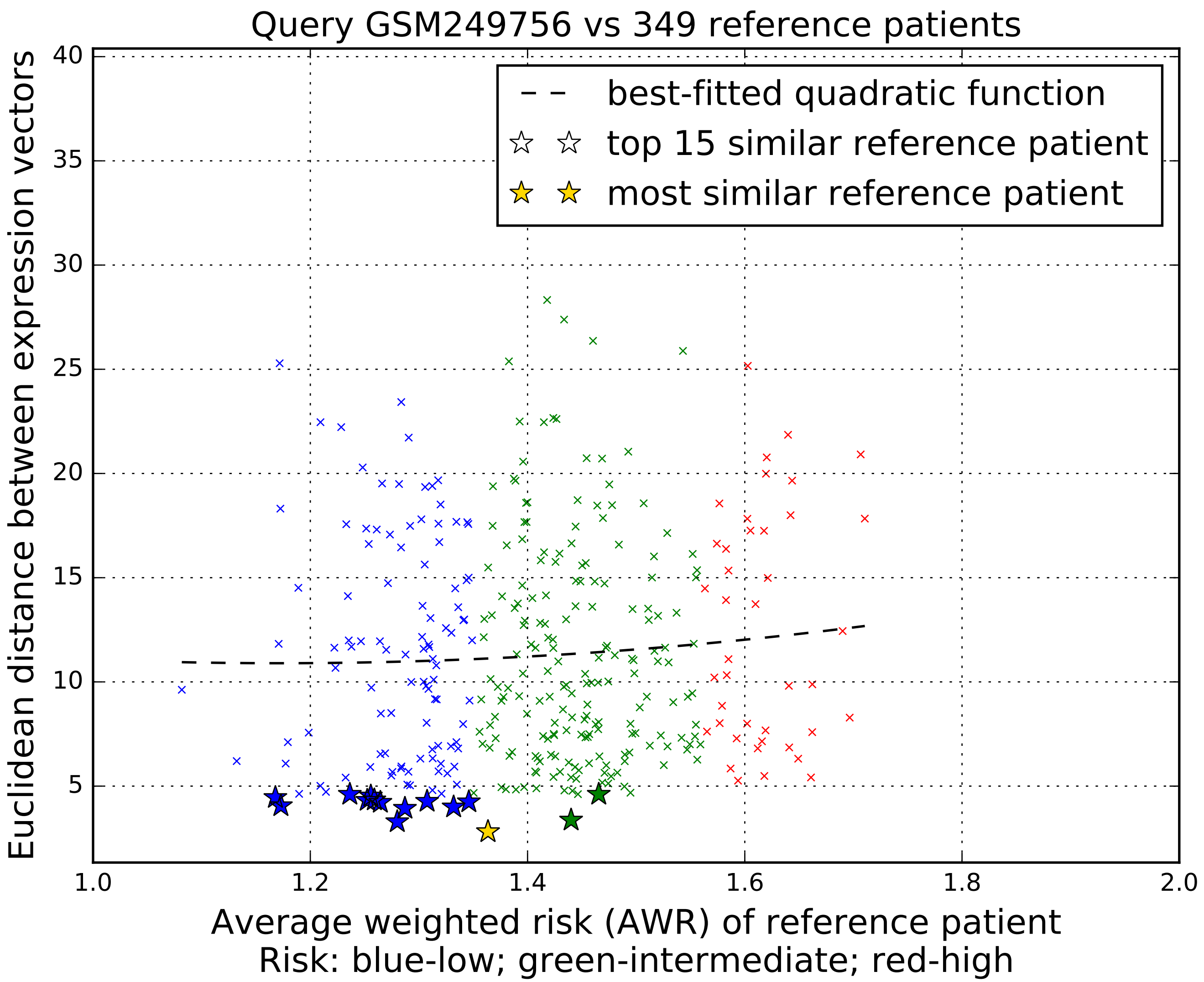
Query GSM249756 vs 349 reference patients
best-fitted quadratic function
top 15 similar reference patient
most similar reference patient
Euclidean distance between expression vectors
Average weighted risk (AWR) of reference patient
Risk: blue-low; green-intermediate; red-high
40
35
30
25
20
15
10
5
1.0
1.2
1.4
1.6
1.8
2.0

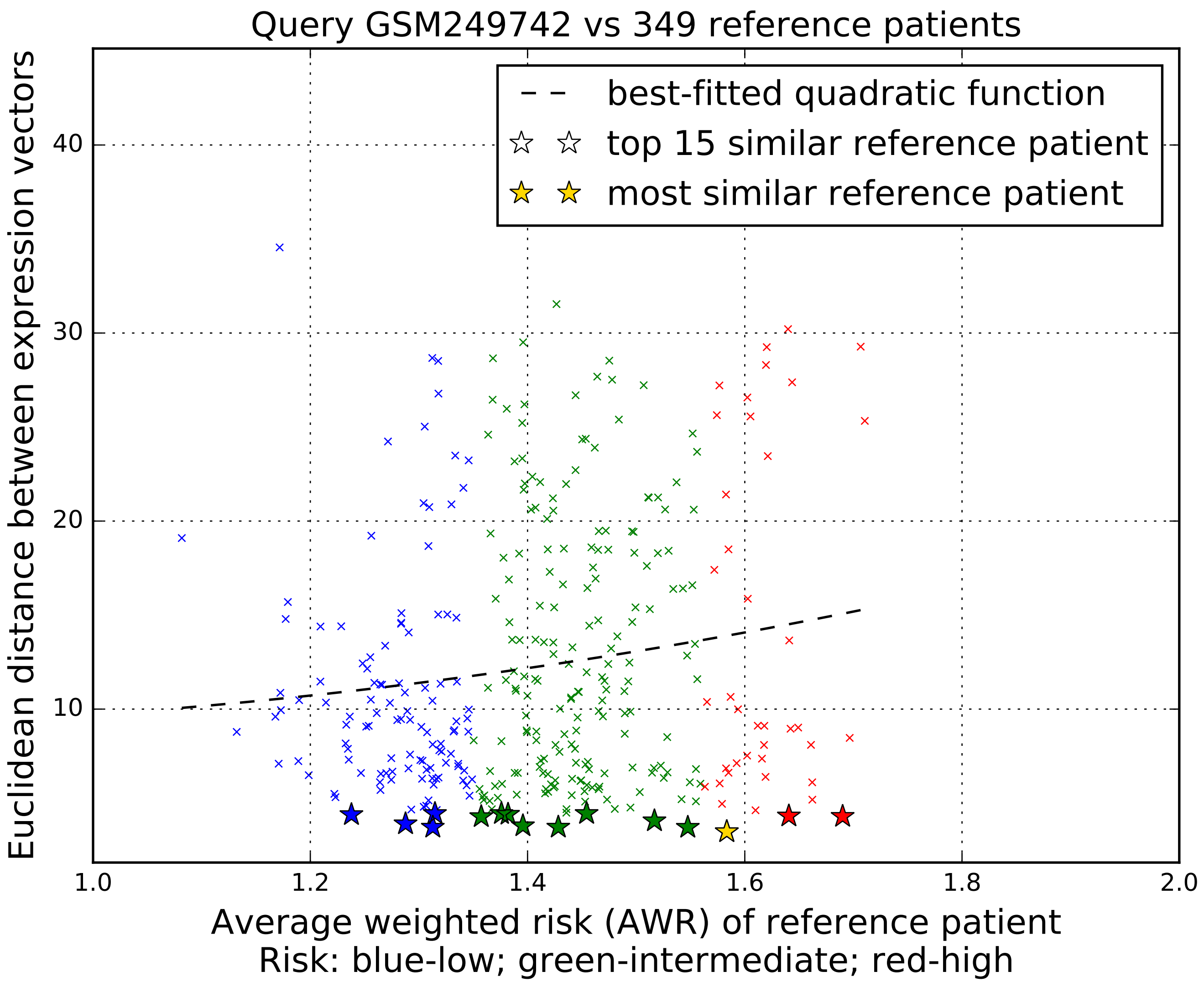

Query GSM249742 vs 349 reference patients
best-fitted quadratic function
top 15 similar reference patient
most similar reference patient
Euclidean distance between expression vectors
10
20
30
40
1.0
1.2
1.4
1.6
1.8
2.0
Average weighted risk (AWR) of reference patient
Risk: blue-low; green-intermediate; red-high

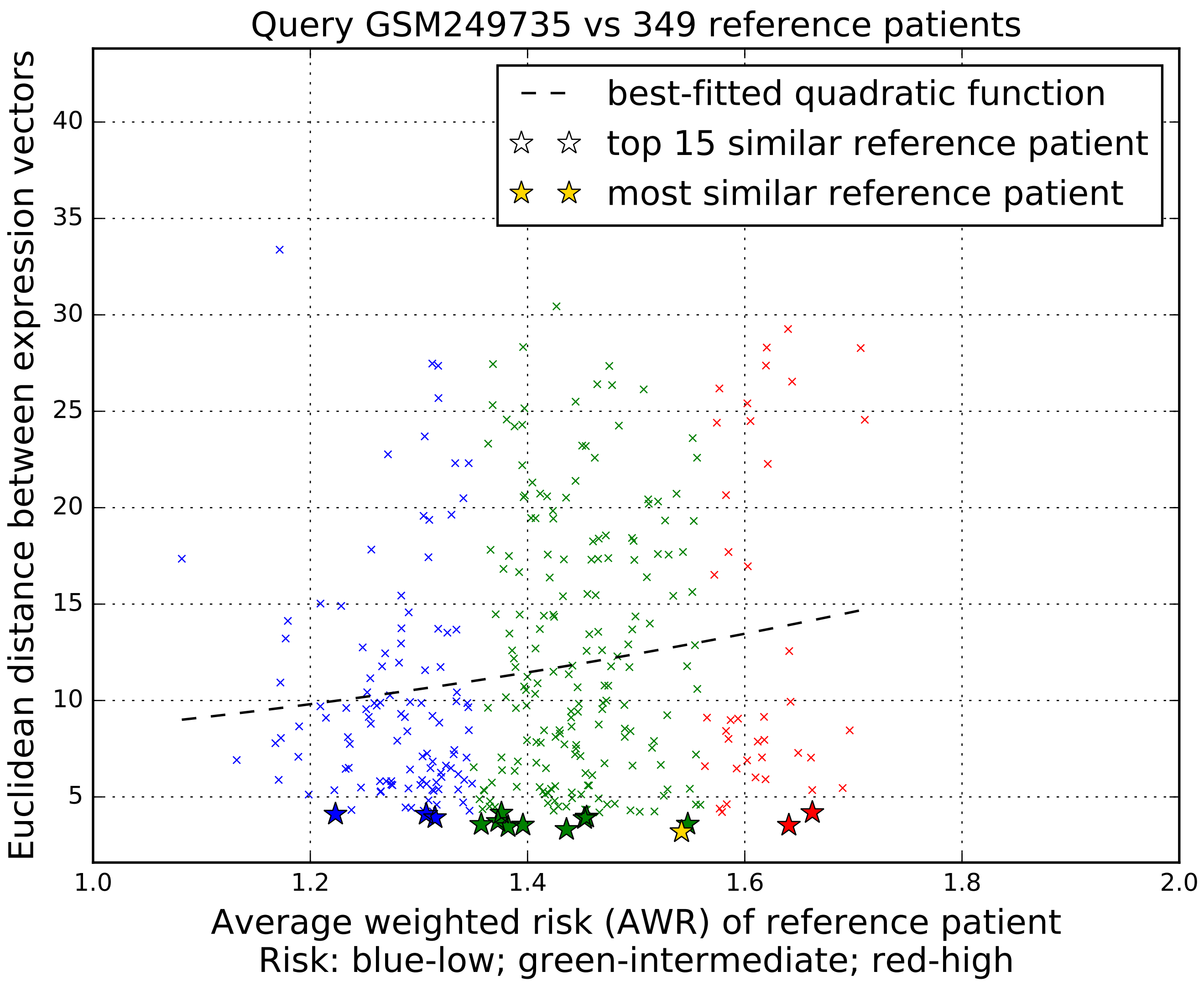
Query GSM249735 vs 349 reference patients
best-fitted quadratic function
top 15 similar reference patient
most similar reference patient
Euclidean distance between expression vectors
5
10
15
20
25
30
35
40
1.0
1.2
1.4
1.6
1.8
2.0
Average weighted risk (AWR) of reference patient
Risk: blue-low; green-intermediate; red-high

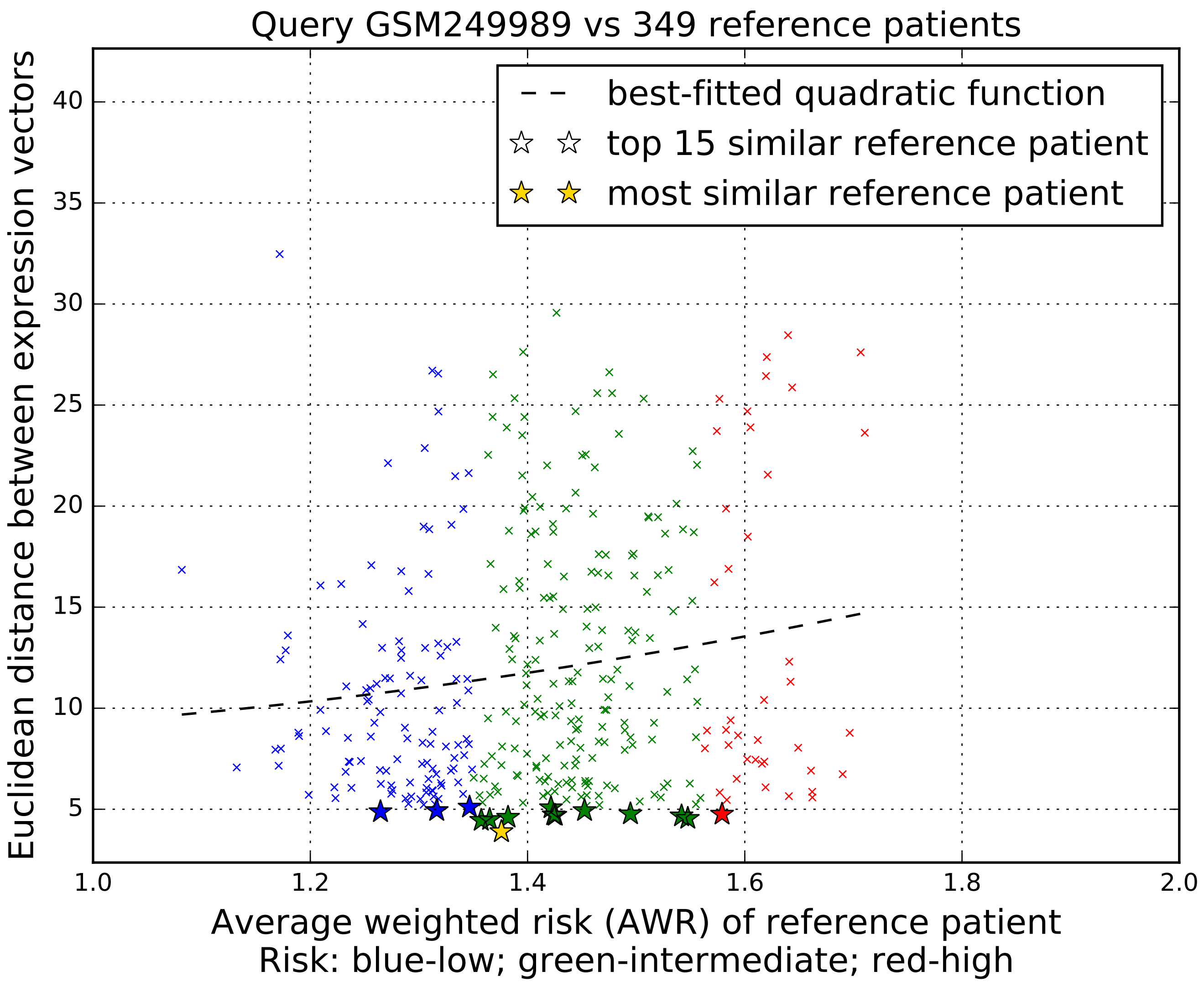

Query GSM249989 vs 349 reference patients
best-fitted quadratic function
top 15 similar reference patient
most similar reference patient
Euclidean distance between expression vectors
Average weighted risk (AWR) of reference patient
Risk: blue-low; green-intermediate; red-high

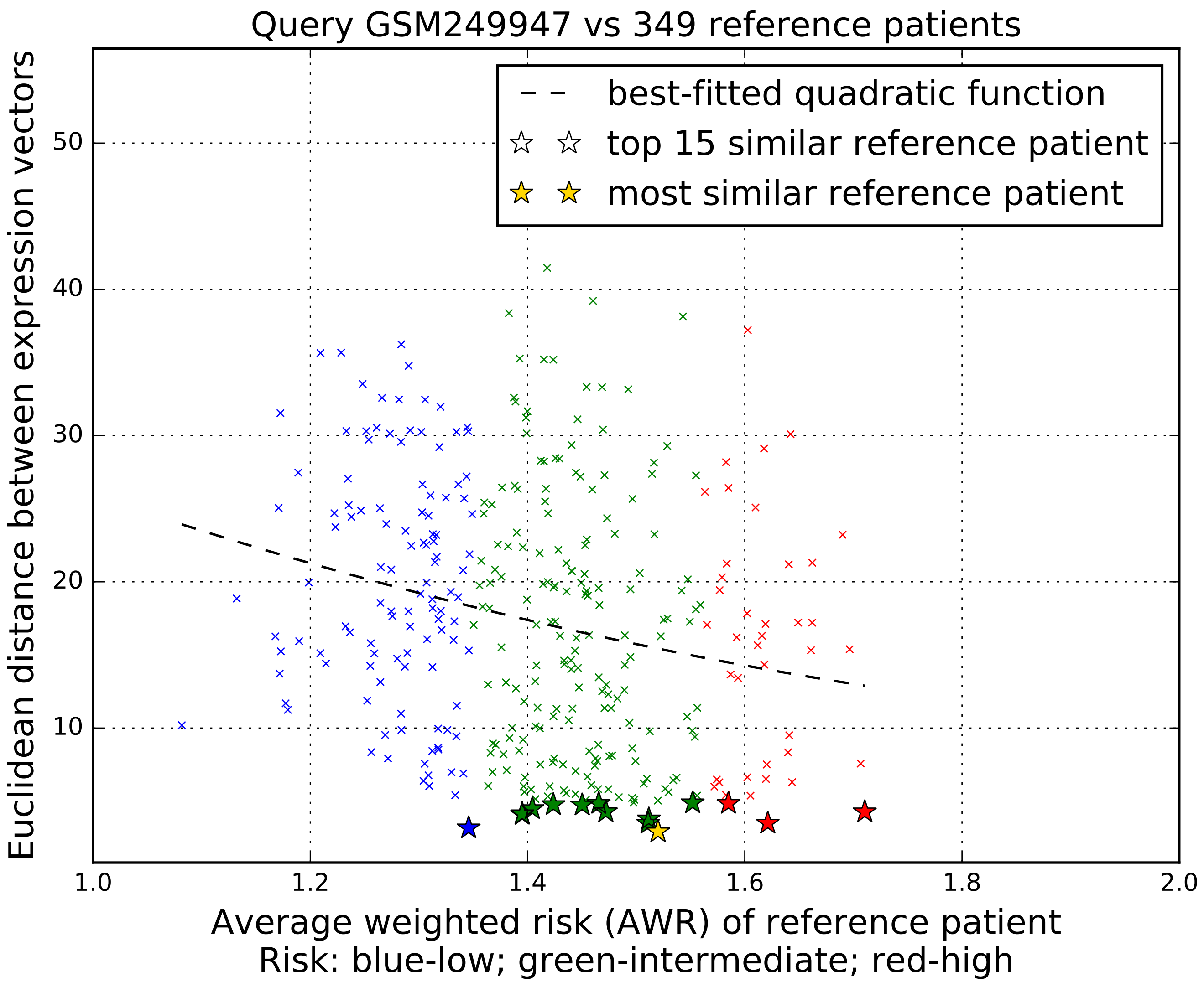

Query GSM249947 vs 349 reference patients
best-fitted quadratic function
top 15 similar reference patient
most similar reference patient
Euclidean distance between expression vectors
10
20
30
40
50
1.0
1.2
1.4
1.6
1.8
2.0
Average weighted risk (AWR) of reference patient
Risk: blue-low; green-intermediate; red-high

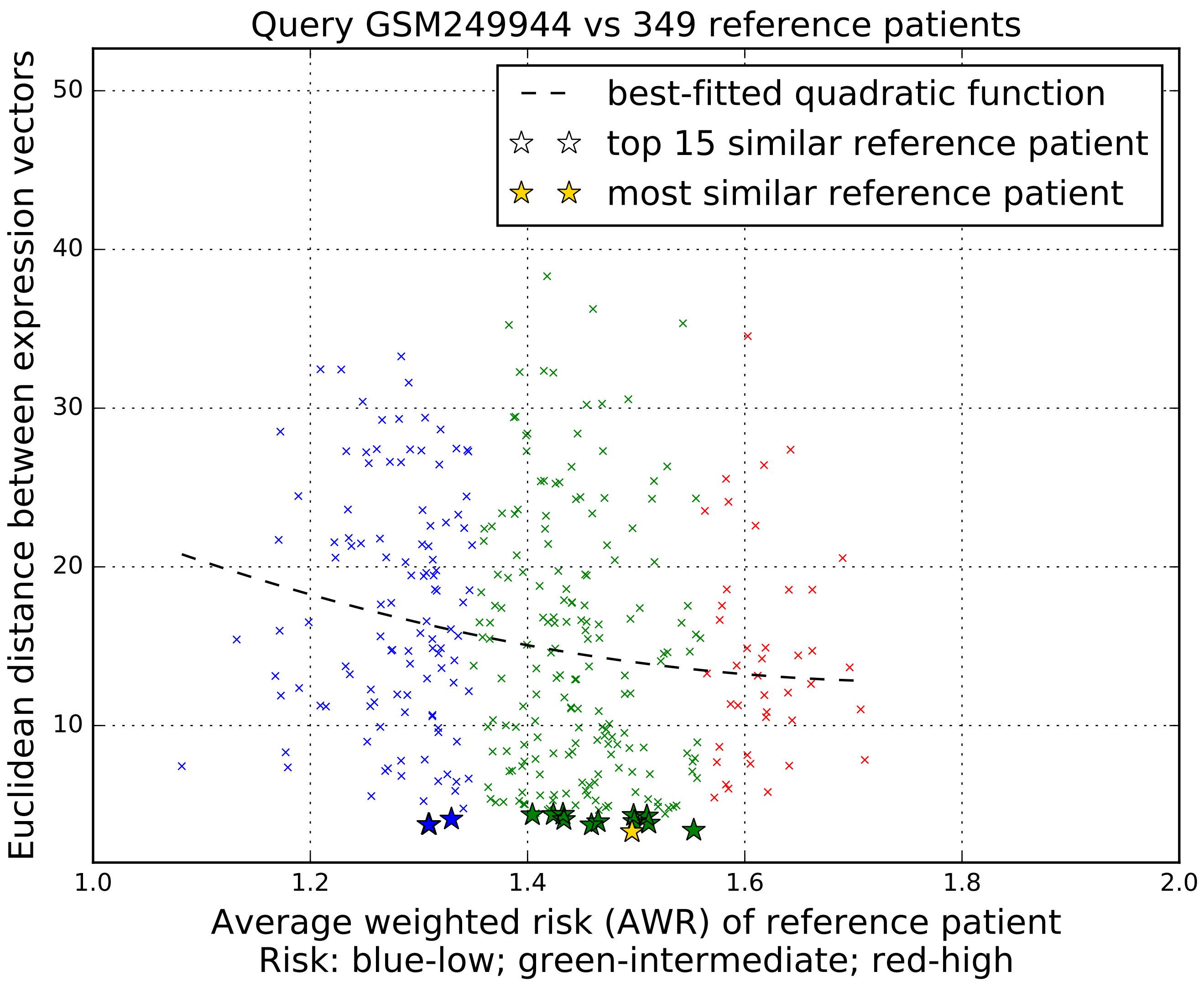
Query GSM249944 vs 349 reference patients
best-fitted quadratic function
top 15 similar reference patient
most similar reference patient
Euclidean distance between expression vectors
Average weighted risk (AWR) of reference patient
Risk: blue-low; green-intermediate; red-high
10
20
30
40
50
1.0
1.2
1.4
1.6
1.8
2.0

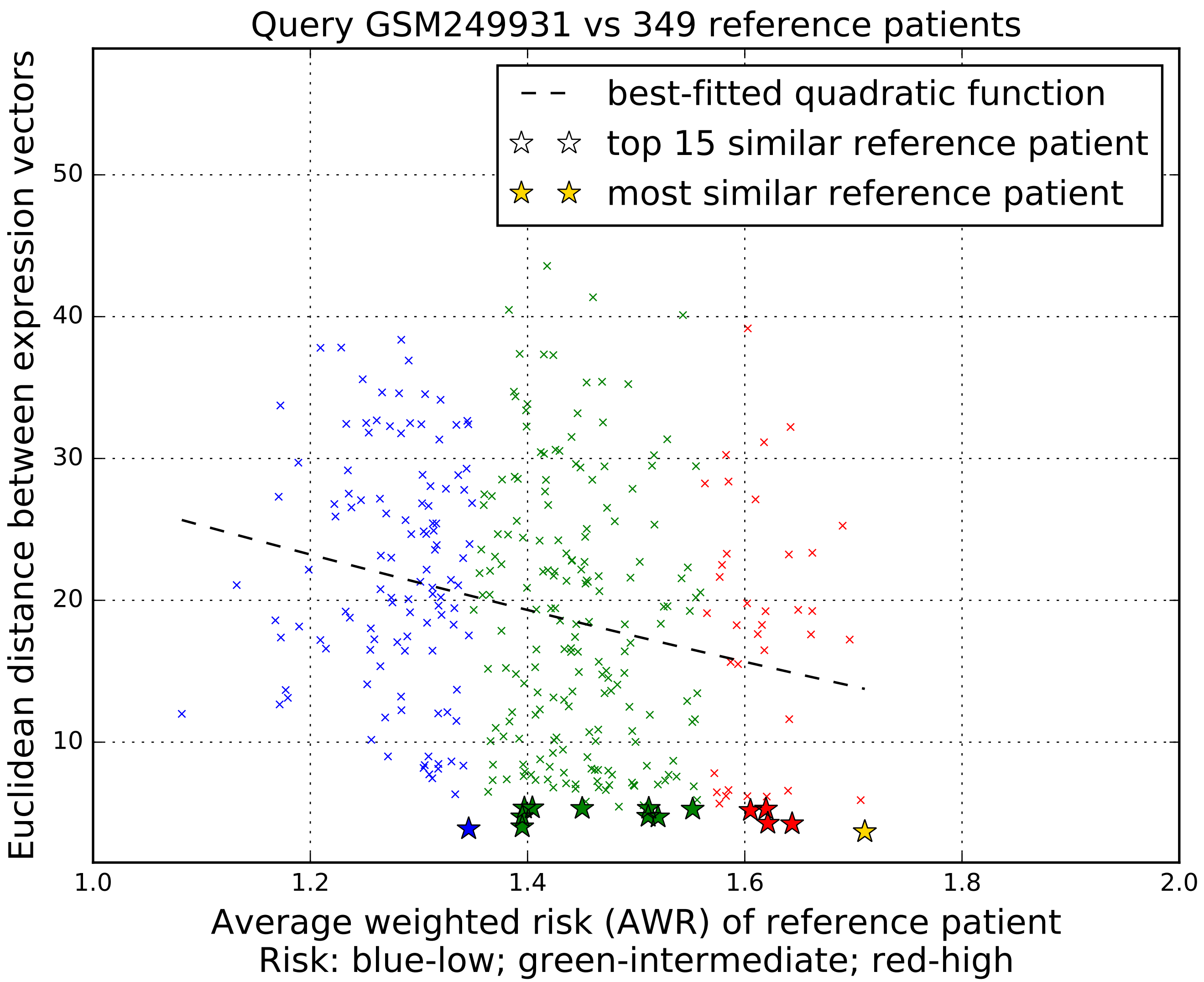
Query GSM249931 vs 349 reference patients
best-fitted quadratic function
top 15 similar reference patient
most similar reference patient
Euclidean distance between expression vectors
10
20
30
40
50
1.0
1.2
1.4
1.6
1.8
2.0
Average weighted risk (AWR) of reference patient
Risk: blue-low; green-intermediate; red-high

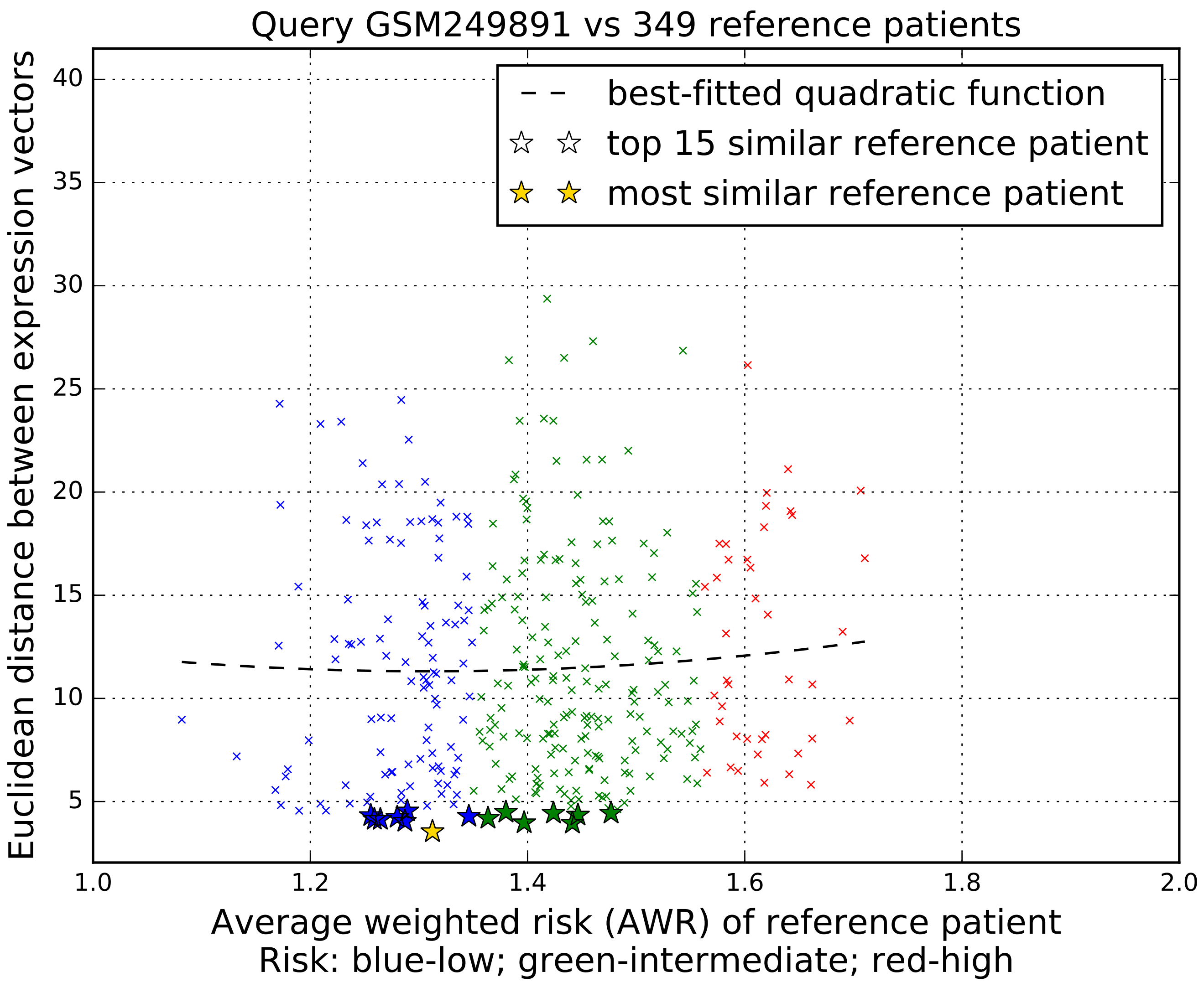
Query GSM249891 vs 349 reference patients
best-fitted quadratic function
top 15 similar reference patient
most similar reference patient
Euclidean distance between expression vectors
Average weighted risk (AWR) of reference patient
Risk: blue-low; green-intermediate; red-high
40
35
30
25
20
15
10
5
1.0
1.2
1.4
1.6
1.8
2.0

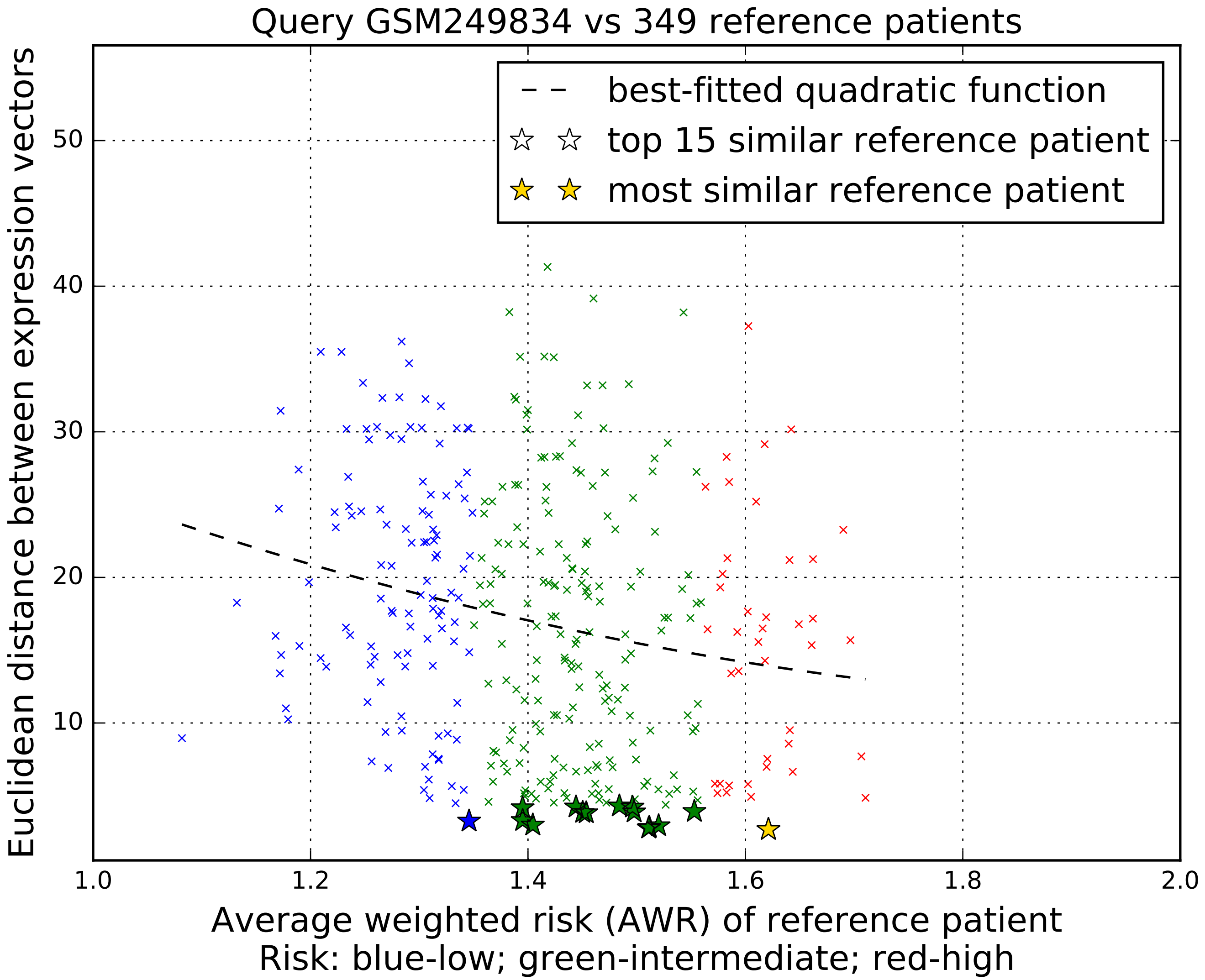
Query GSM249834 vs 349 reference patients
best-fitted quadratic function
top 15 similar reference patient
most similar reference patient
Euclidean distance between expression vectors
10
20
30
40
50
1.0
1.2
1.4
1.6
1.8
2.0
Average weighted risk (AWR) of reference patient
Risk: blue-low; green-intermediate; red-high

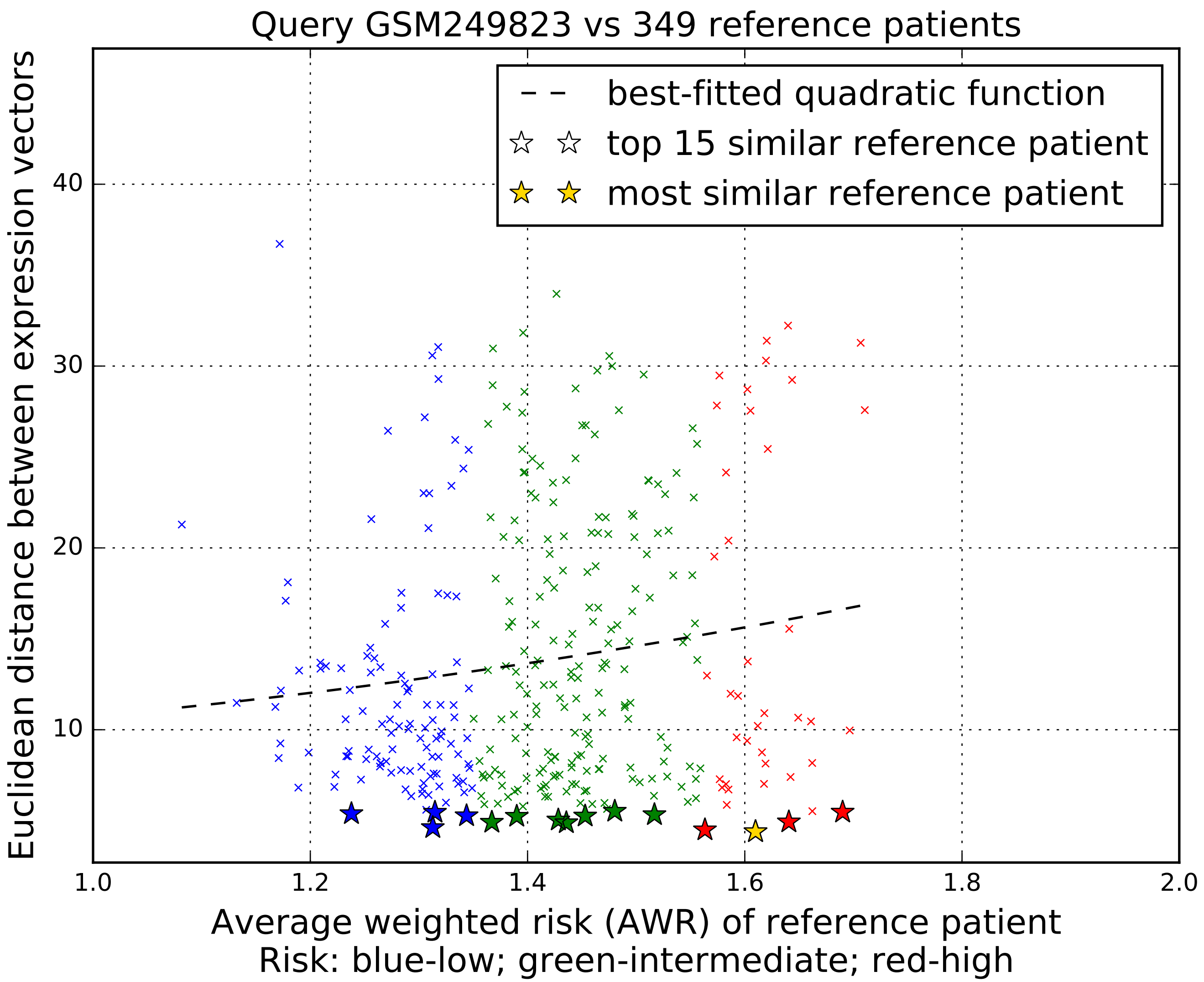
Query GSM249823 vs 349 reference patients
best-fitted quadratic function
top 15 similar reference patient
most similar reference patient
Euclidean distance between expression vectors
10
20
30
40
1.0
1.2
1.4
1.6
1.8
2.0
Average weighted risk (AWR) of reference patient
Risk: blue-low; green-intermediate; red-high

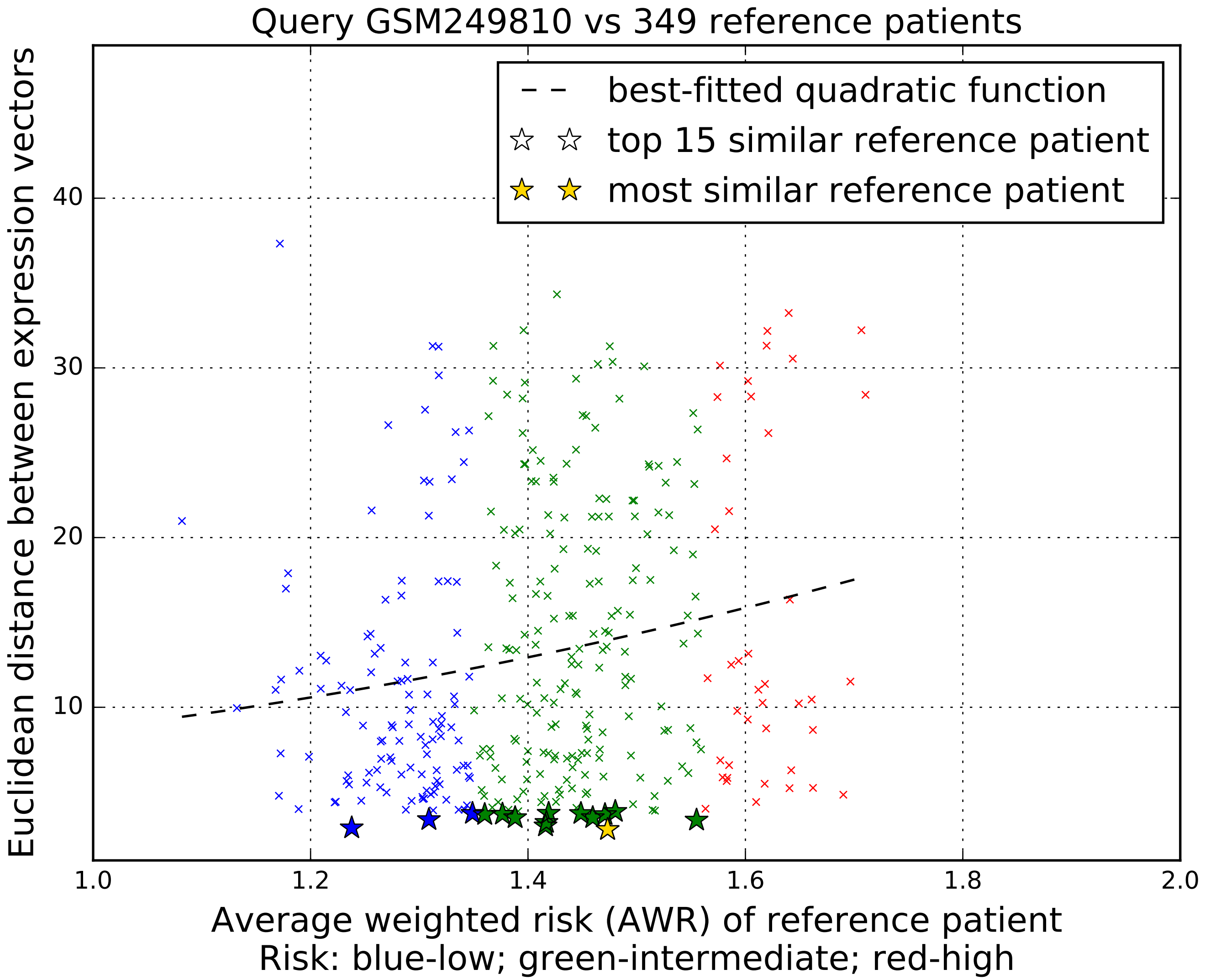
Query GSM249810 vs 349 reference patients
best-fitted quadratic function
top 15 similar reference patient
most similar reference patient
Euclidean distance between expression vectors
10
20
30
40
1.0
1.2
1.4
1.6
1.8
2.0
Average weighted risk (AWR) of reference patient
Risk: blue-low; green-intermediate; red-high

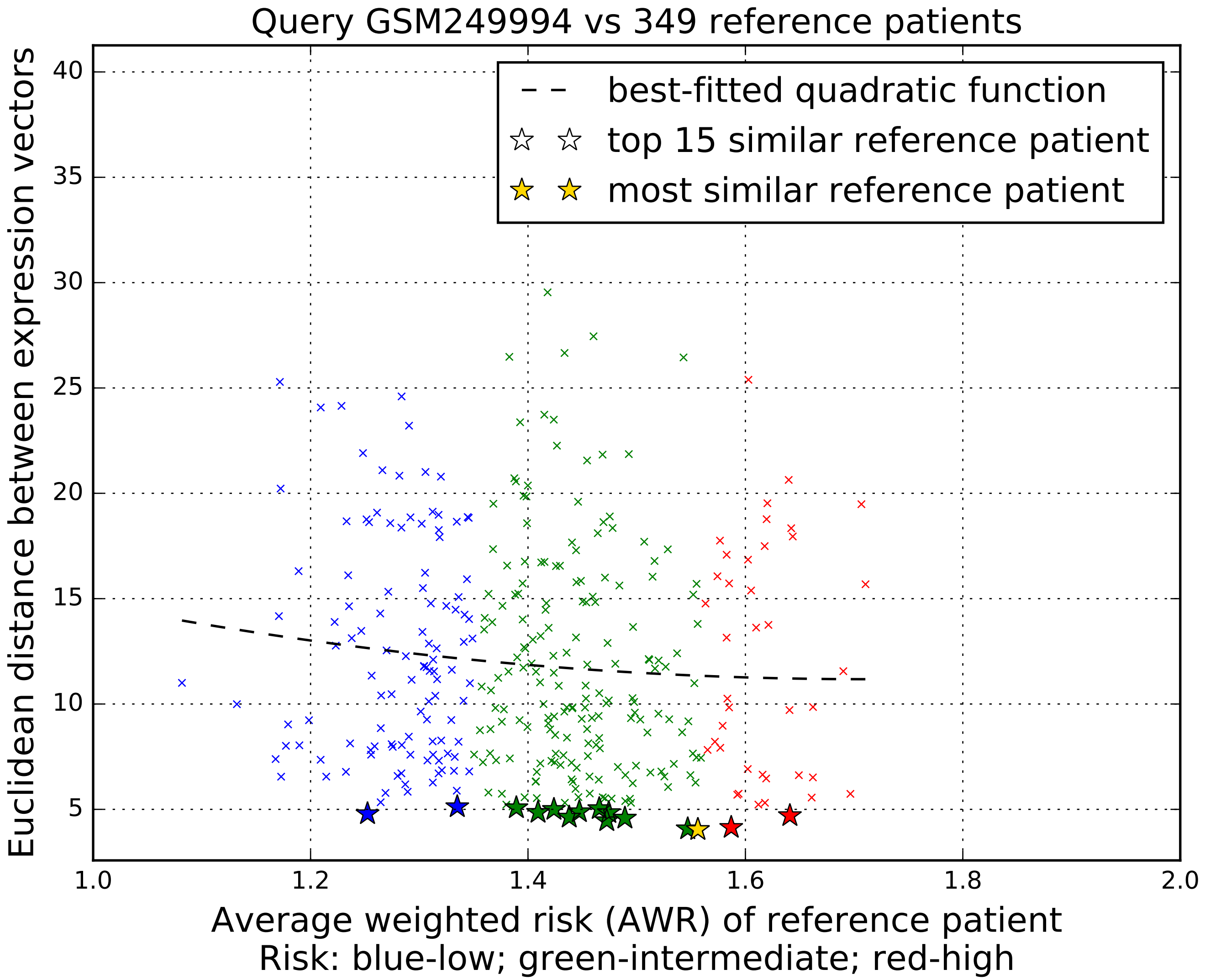

Query GSM249994 vs 349 reference patients
best-fitted quadratic function
top 15 similar reference patient
most similar reference patient
Euclidean distance between expression vectors
40
35
30
25
20
15
10
5
1.0
1.2
1.4
1.6
1.8
2.0
Average weighted risk (AWR) of reference patient
Risk: blue-low; green-intermediate; red-high

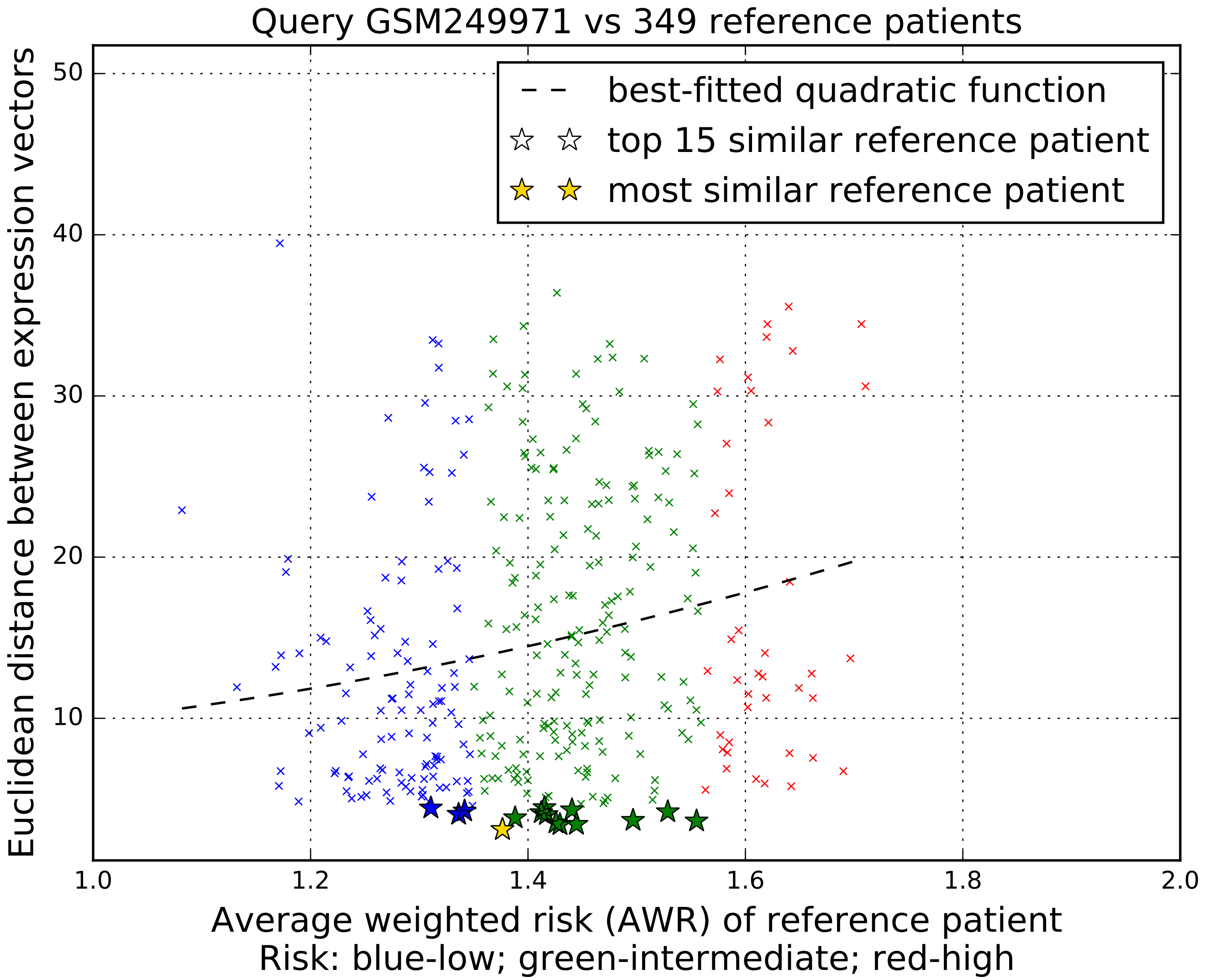
Query GSM249971 vs 349 reference patients
best-fitted quadratic function
top 15 similar reference patient
most similar reference patient
Euclidean distance between expression vectors
50
40
30
20
10
1.0
1.2
1.4
1.6
1.8
2.0
Average weighted risk (AWR) of reference patient
Risk: blue-low; green-intermediate; red-high

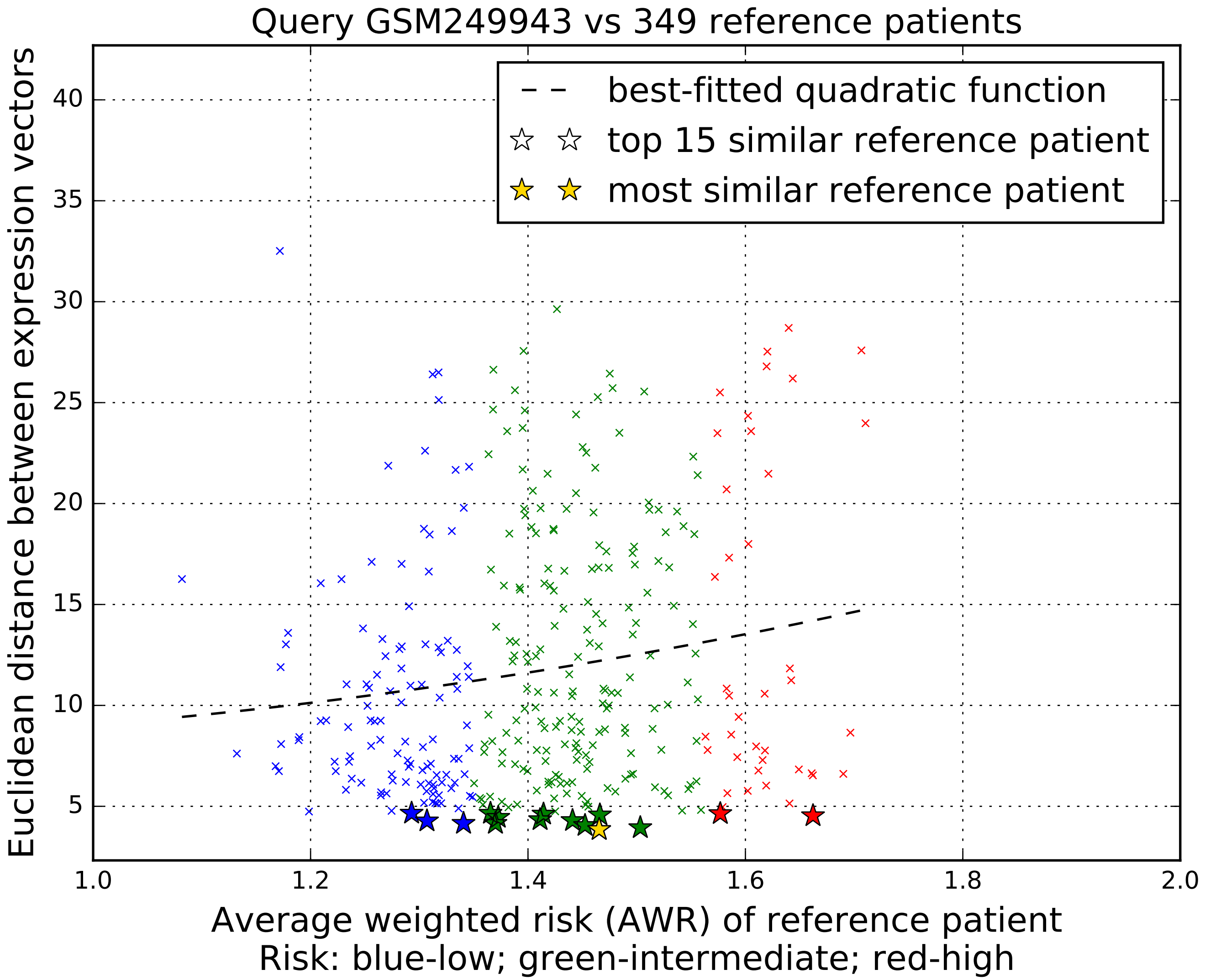
Query GSM249943 vs 349 reference patients
best-fitted quadratic function
top 15 similar reference patient
most similar reference patient
Euclidean distance between expression vectors
40
35
30
25
20
15
10
5
1.0
1.2
1.4
1.6
1.8
2.0
Average weighted risk (AWR) of reference patient
Risk: blue-low; green-intermediate; red-high

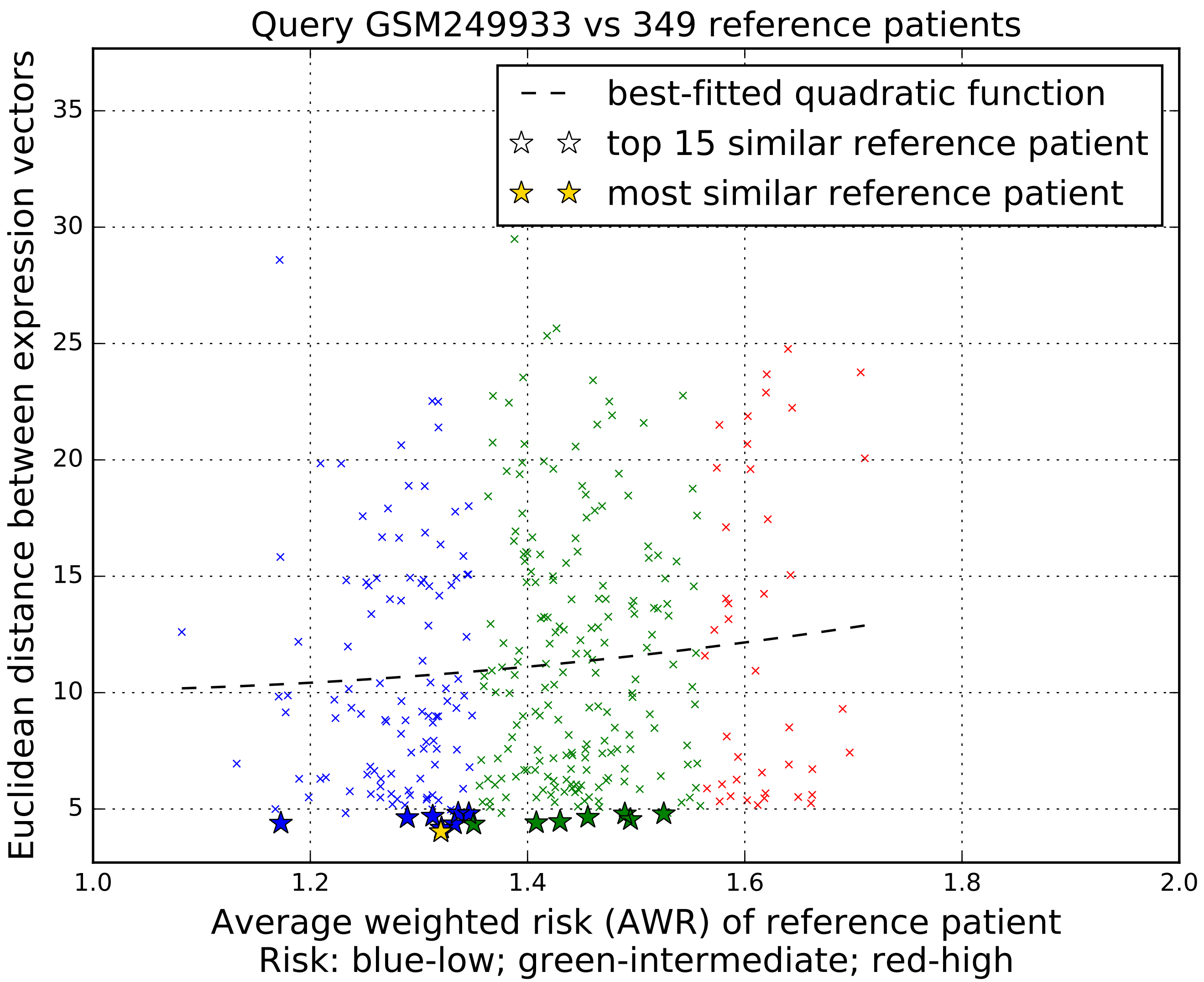
Query GSM249933 vs 349 reference patients
best-fitted quadratic function
top 15 similar reference patient
most similar reference patient
Euclidean distance between expression vectors
35
30
25
20
15
10
5
1.0
1.2
1.4
1.6
1.8
2.0
Average weighted risk (AWR) of reference patient
Risk: blue-low; green-intermediate; red-high

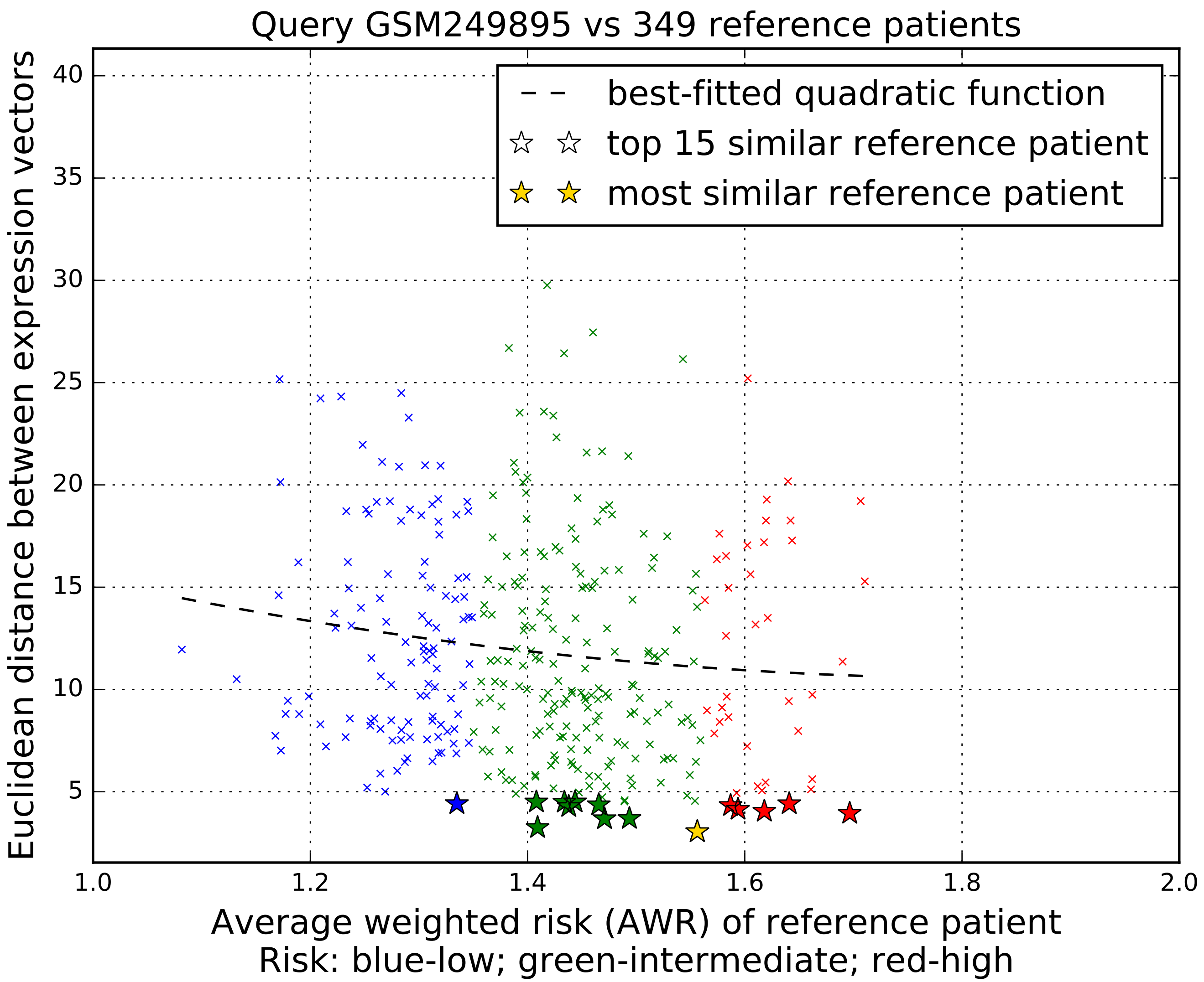
Query GSM249895 vs 349 reference patients
best-fitted quadratic function
top 15 similar reference patient
most similar reference patient
Euclidean distance between expression vectors
Average weighted risk (AWR) of reference patient
Risk: blue-low; green-intermediate; red-high
40
35
30
25
20
15
10
5
1.0
1.2
1.4
1.6
1.8
2.0

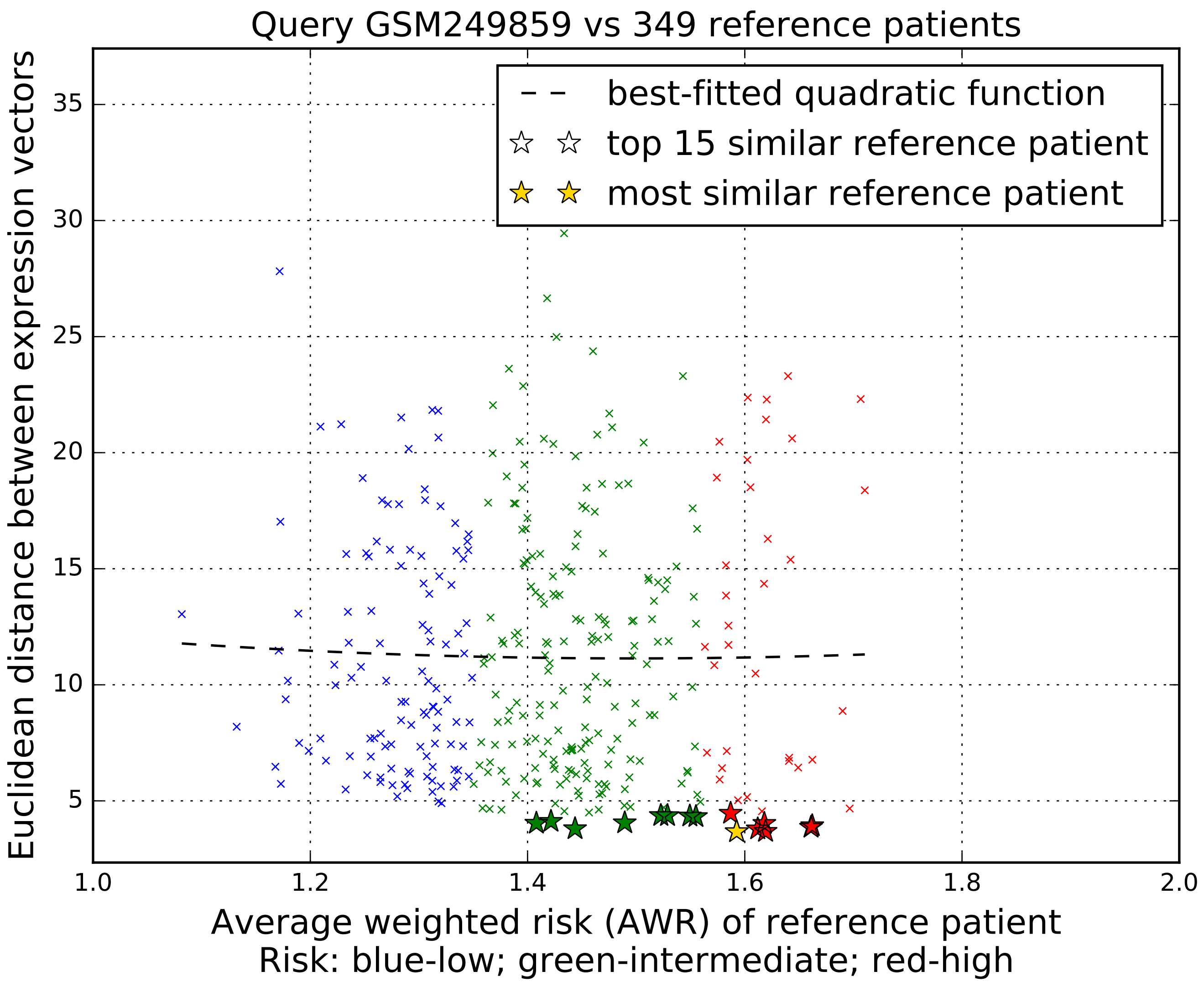

Query GSM249859 vs 349 reference patients
best-fitted quadratic function
top 15 similar reference patient
most similar reference patient
Euclidean distance between expression vectors
35
30
25
20
15
10
5
1.0
1.2
1.4
1.6
1.8
2.0
Average weighted risk (AWR) of reference patient
Risk: blue-low; green-intermediate; red-high

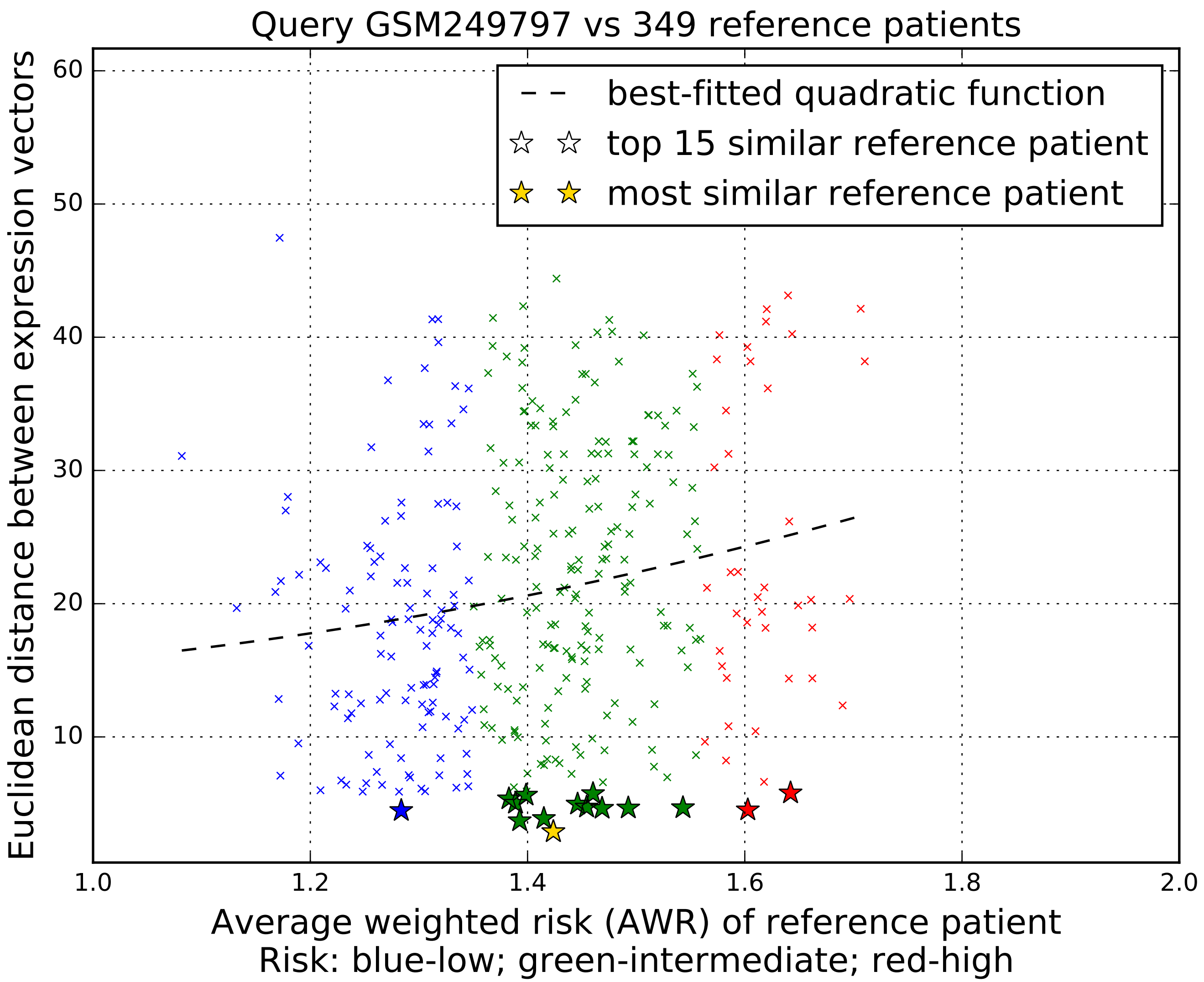
Query GSM249797 vs 349 reference patients
best-fitted quadratic function
top 15 similar reference patient
most similar reference patient
Euclidean distance between expression vectors
60
50
40
30
20
10
1.0
1.2
1.4
1.6
1.8
2.0
Average weighted risk (AWR) of reference patient
Risk: blue-low; green-intermediate; red-high

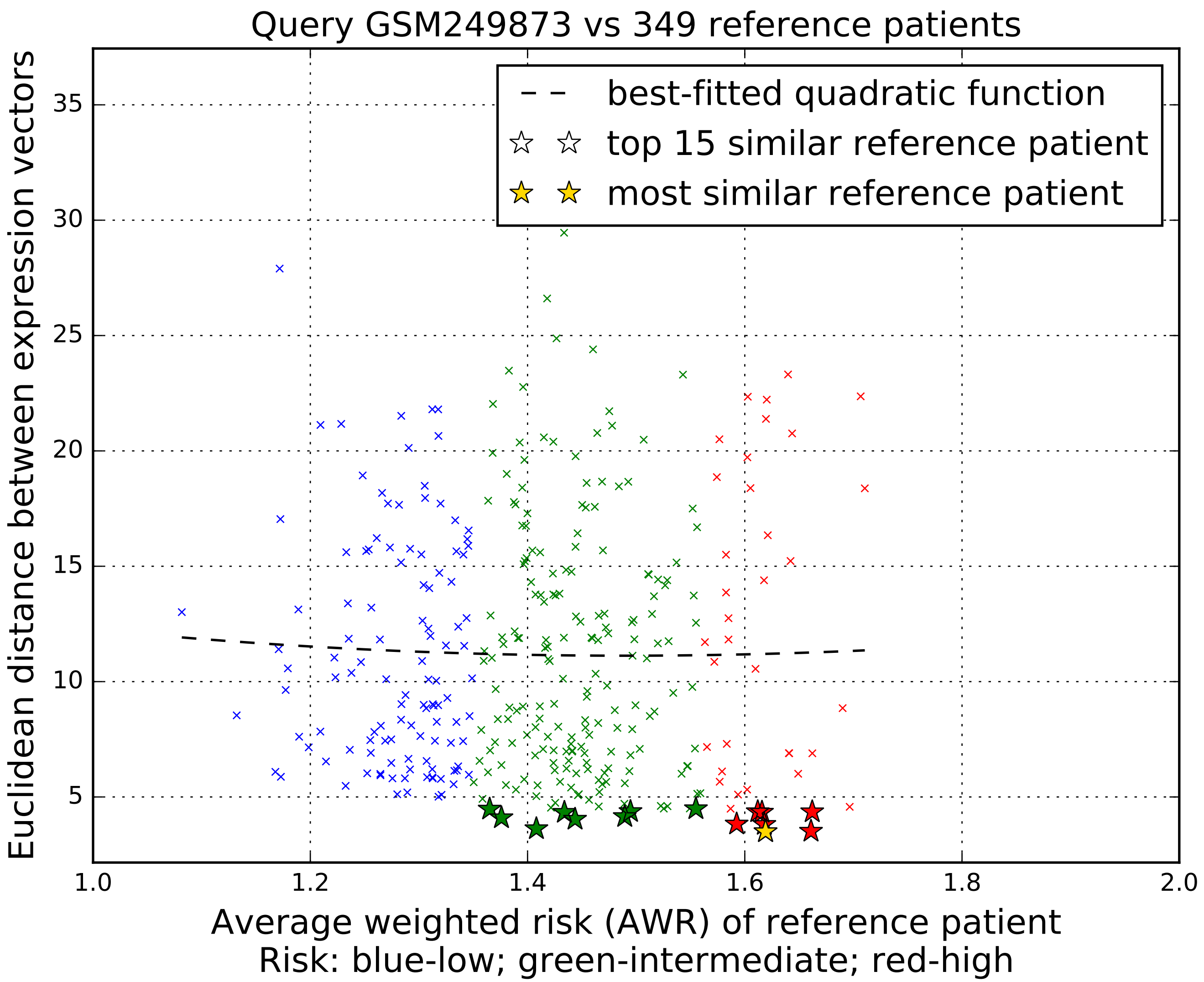

Query GSM249873 vs 349 reference patients
best-fitted quadratic function
top 15 similar reference patient
most similar reference patient
Euclidean distance between expression vectors
35
30
25
20
15
10
5
1.0
1.2
1.4
1.6
1.8
2.0
Average weighted risk (AWR) of reference patient
Risk: blue-low; green-intermediate; red-high

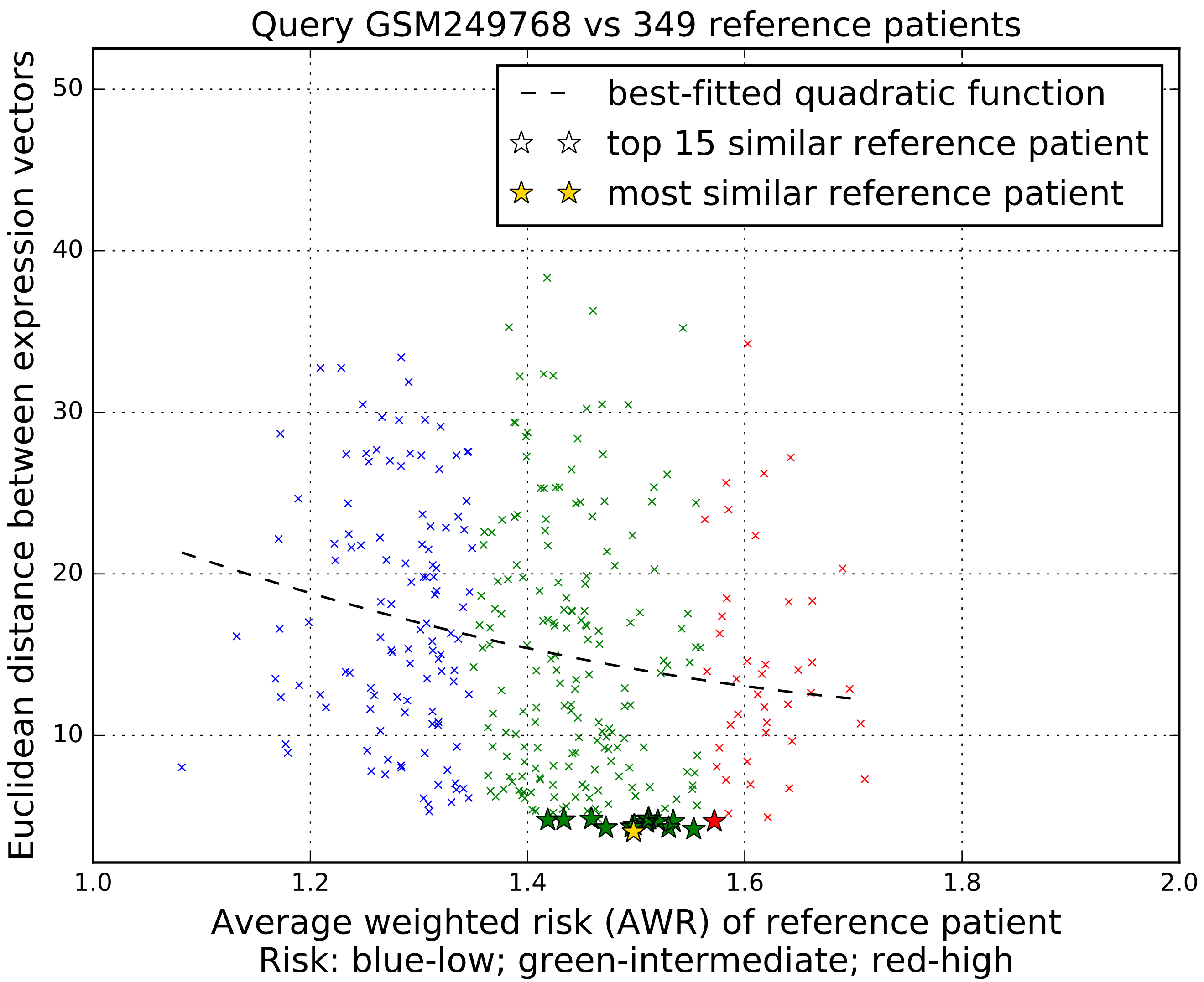
Query GSM249768 vs 349 reference patients
best-fitted quadratic function
top 15 similar reference patient
most similar reference patient
Euclidean distance between expression vectors
50
40
30
20
10
1.0
1.2
1.4
1.6
1.8
2.0
Average weighted risk (AWR) of reference patient
Risk: blue-low; green-intermediate; red-high

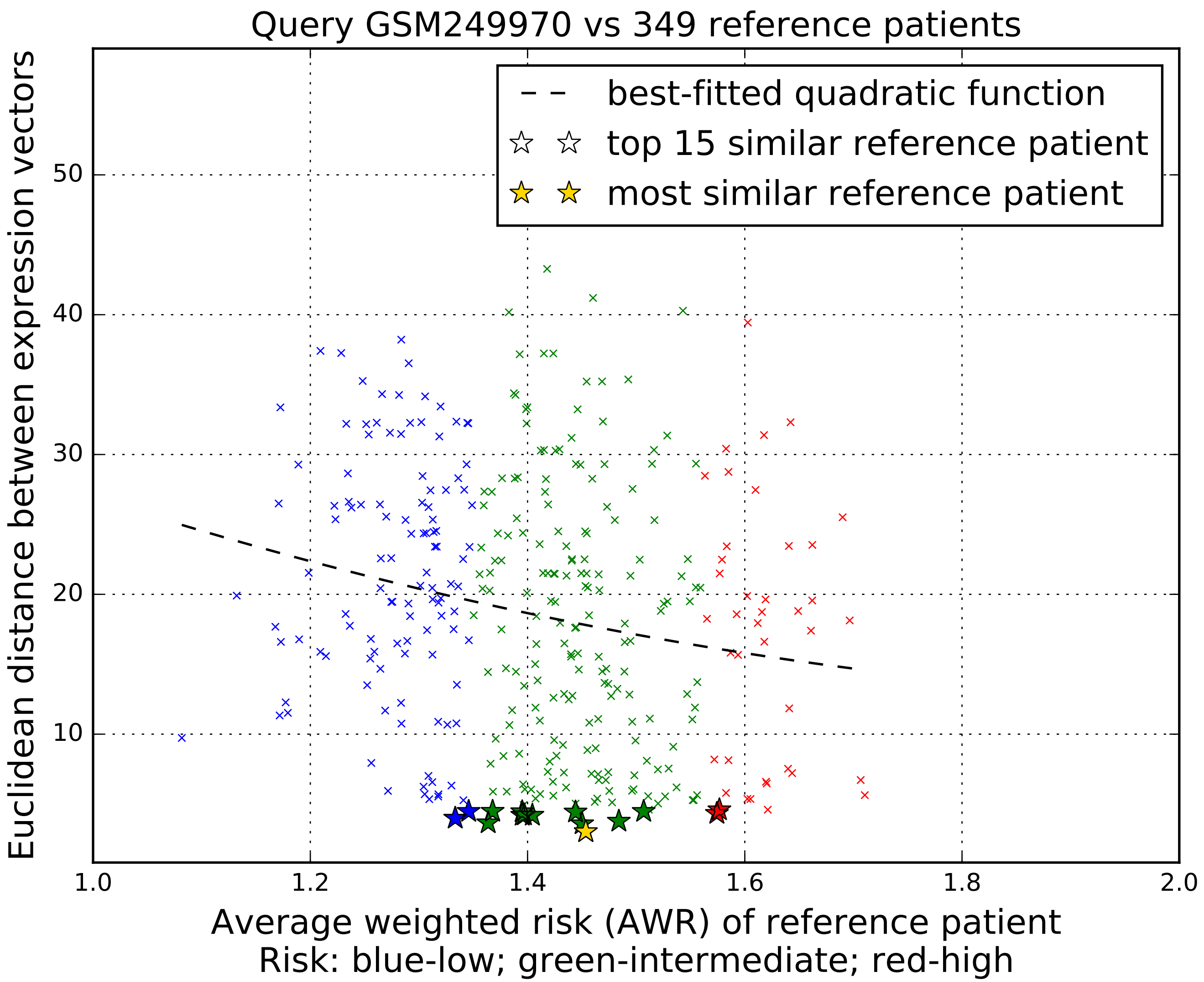
Query GSM249970 vs 349 reference patients
best-fitted quadratic function
top 15 similar reference patient
most similar reference patient
Euclidean distance between expression vectors
10
20
30
40
50
1.0
1.2
1.4
1.6
1.8
2.0
Average weighted risk (AWR) of reference patient
Risk: blue-low; green-intermediate; red-high

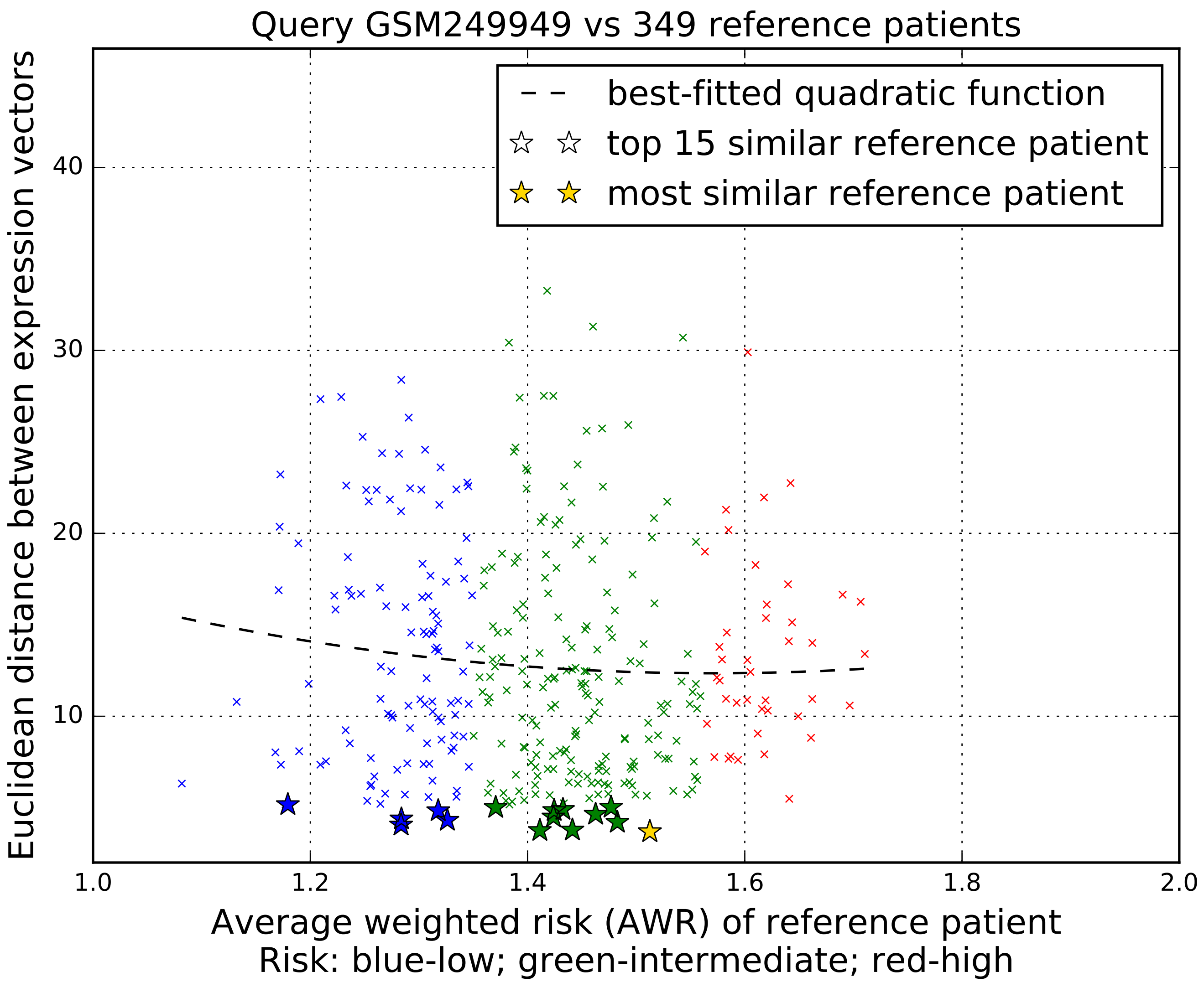

Query GSM249949 vs 349 reference patients
best-fitted quadratic function
top 15 similar reference patient
most similar reference patient
Euclidean distance between expression vectors
10
20
30
40
1.0
1.2
1.4
1.6
1.8
2.0
Average weighted risk (AWR) of reference patient
Risk: blue-low; green-intermediate; red-high

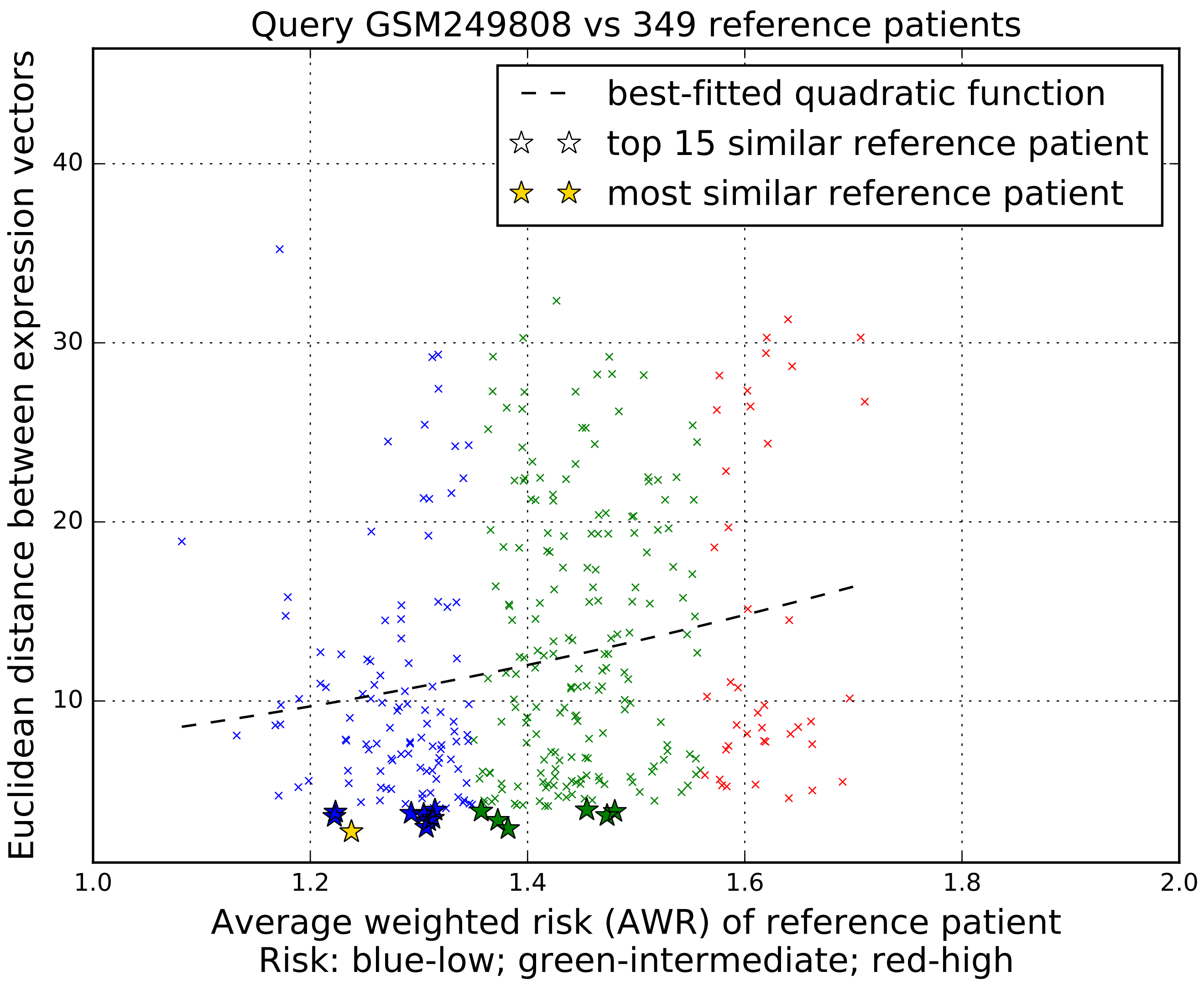
Query GSM249808 vs 349 reference patients
best-fitted quadratic function
top 15 similar reference patient
most similar reference patient
Euclidean distance between expression vectors
Average weighted risk (AWR) of reference patient
Risk: blue-low; green-intermediate; red-high
10
20
30
40
1.0
1.2
1.4
1.6
1.8
2.0

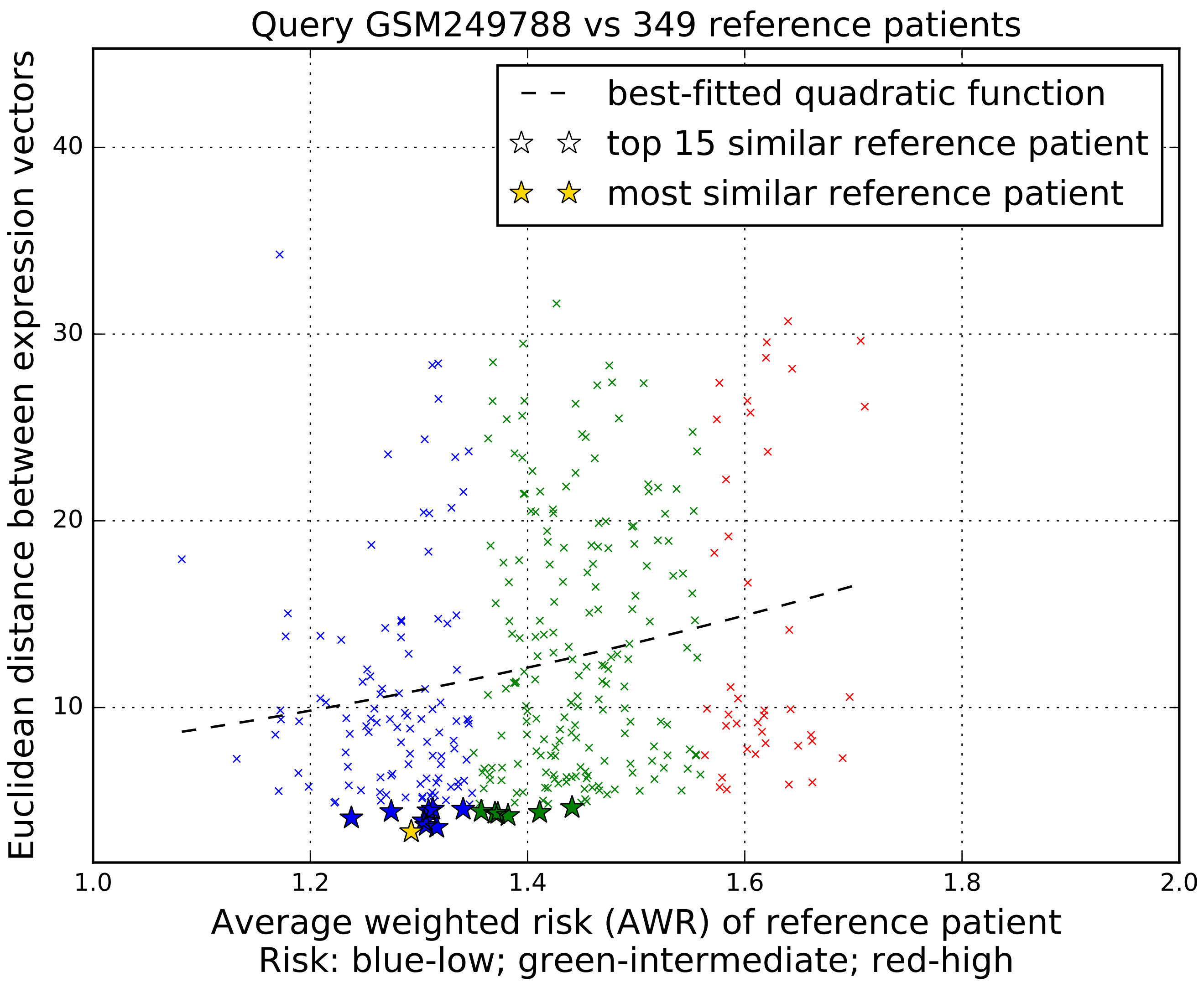

Query GSM249788 vs 349 reference patients
best-fitted quadratic function
top 15 similar reference patient
most similar reference patient
Euclidean distance between expression vectors
Average weighted risk (AWR) of reference patient
Risk: blue-low; green-intermediate; red-high
10
20
30
40
1.0
1.2
1.4
1.6
1.8
2.0

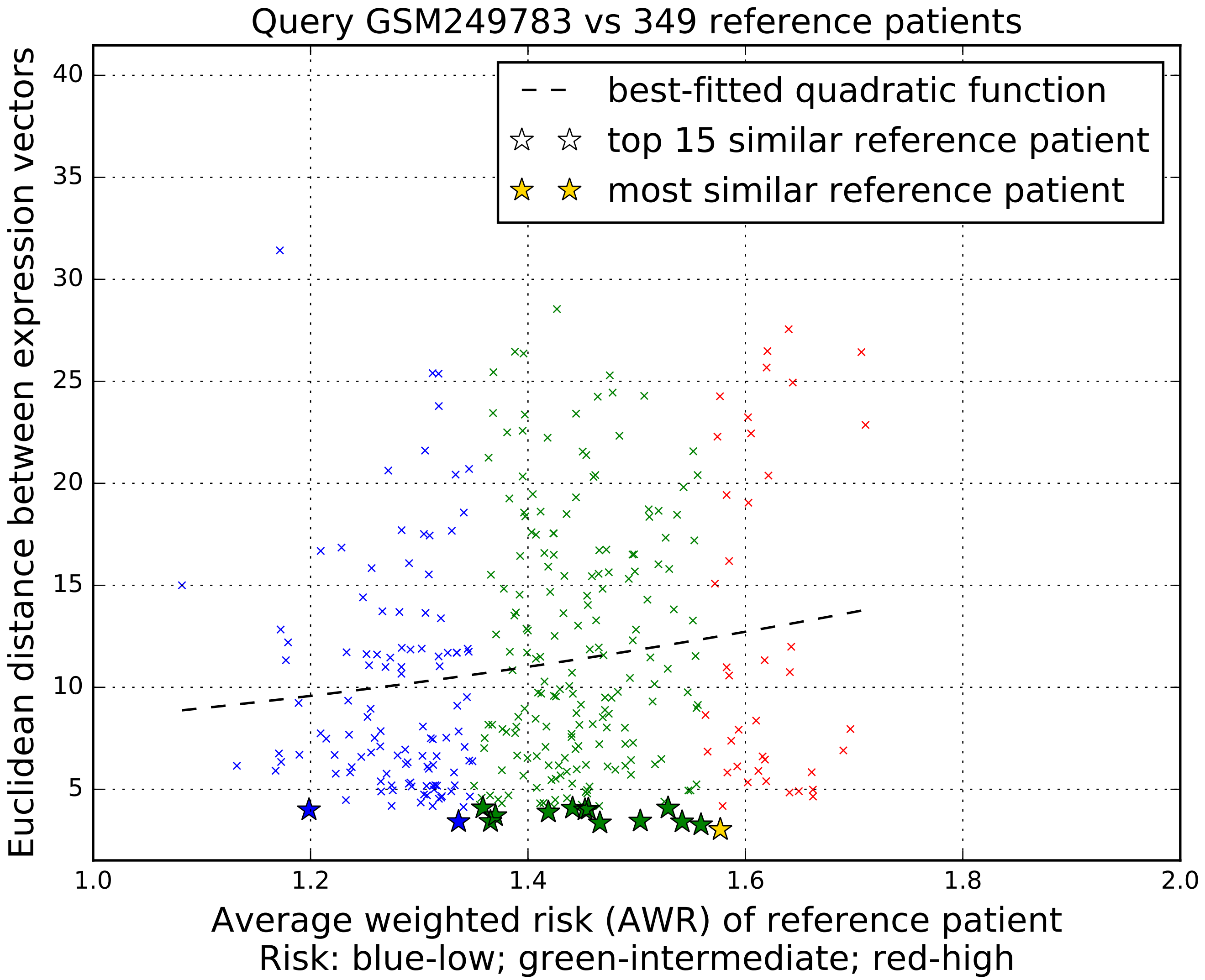
Query GSM249783 vs 349 reference patients
best-fitted quadratic function
top 15 similar reference patient
most similar reference patient
Euclidean distance between expression vectors
Average weighted risk (AWR) of reference patient
Risk: blue-low; green-intermediate; red-high
40
35
30
25
20
15
10
5
1.0
1.2
1.4
1.6
1.8
2.0

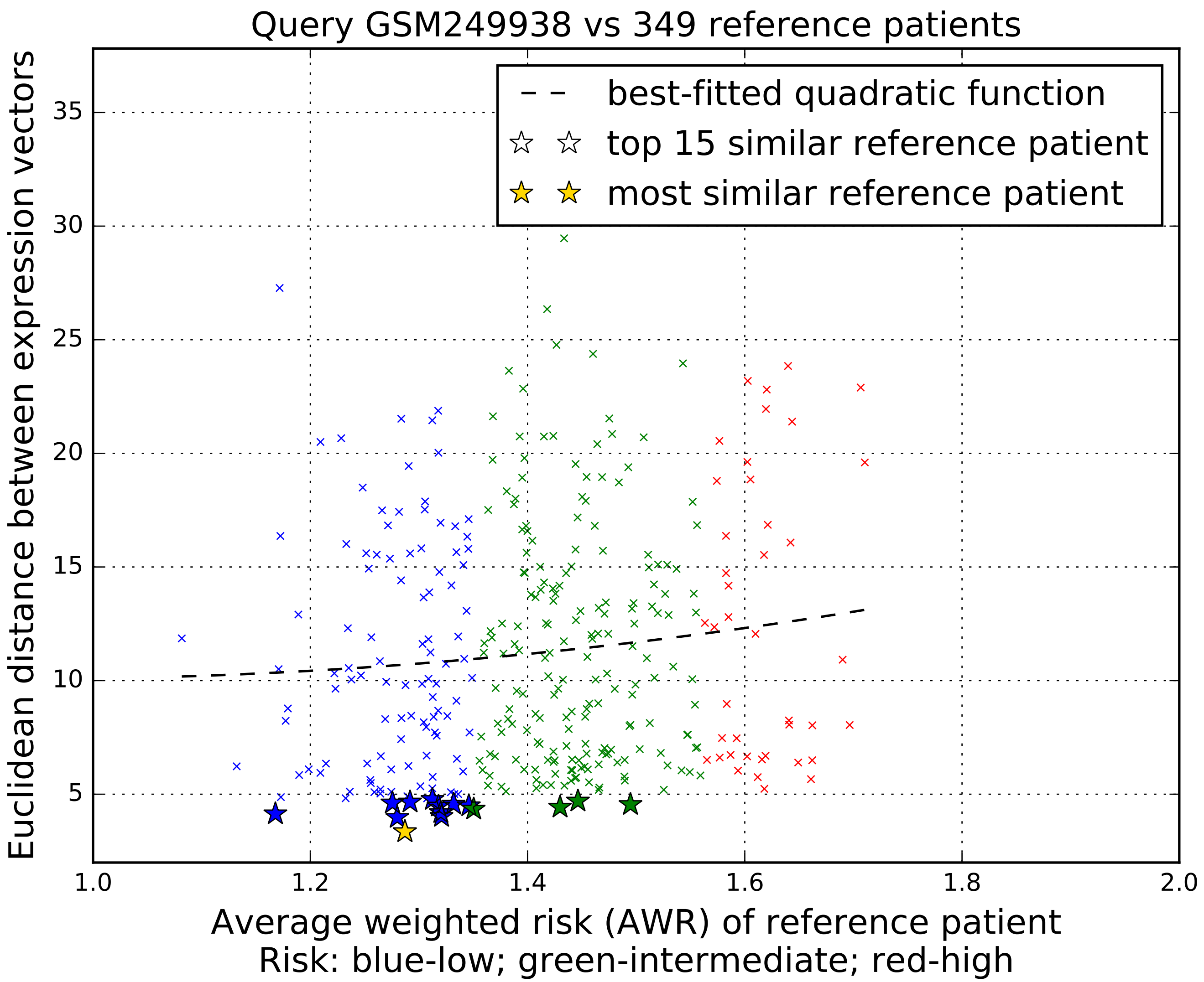

Query GSM249938 vs 349 reference patients
best-fitted quadratic function
top 15 similar reference patient
most similar reference patient
Euclidean distance between expression vectors
35
30
25
20
15
10
5
1.0
1.2
1.4
1.6
1.8
2.0
Average weighted risk (AWR) of reference patient
Risk: blue-low; green-intermediate; red-high

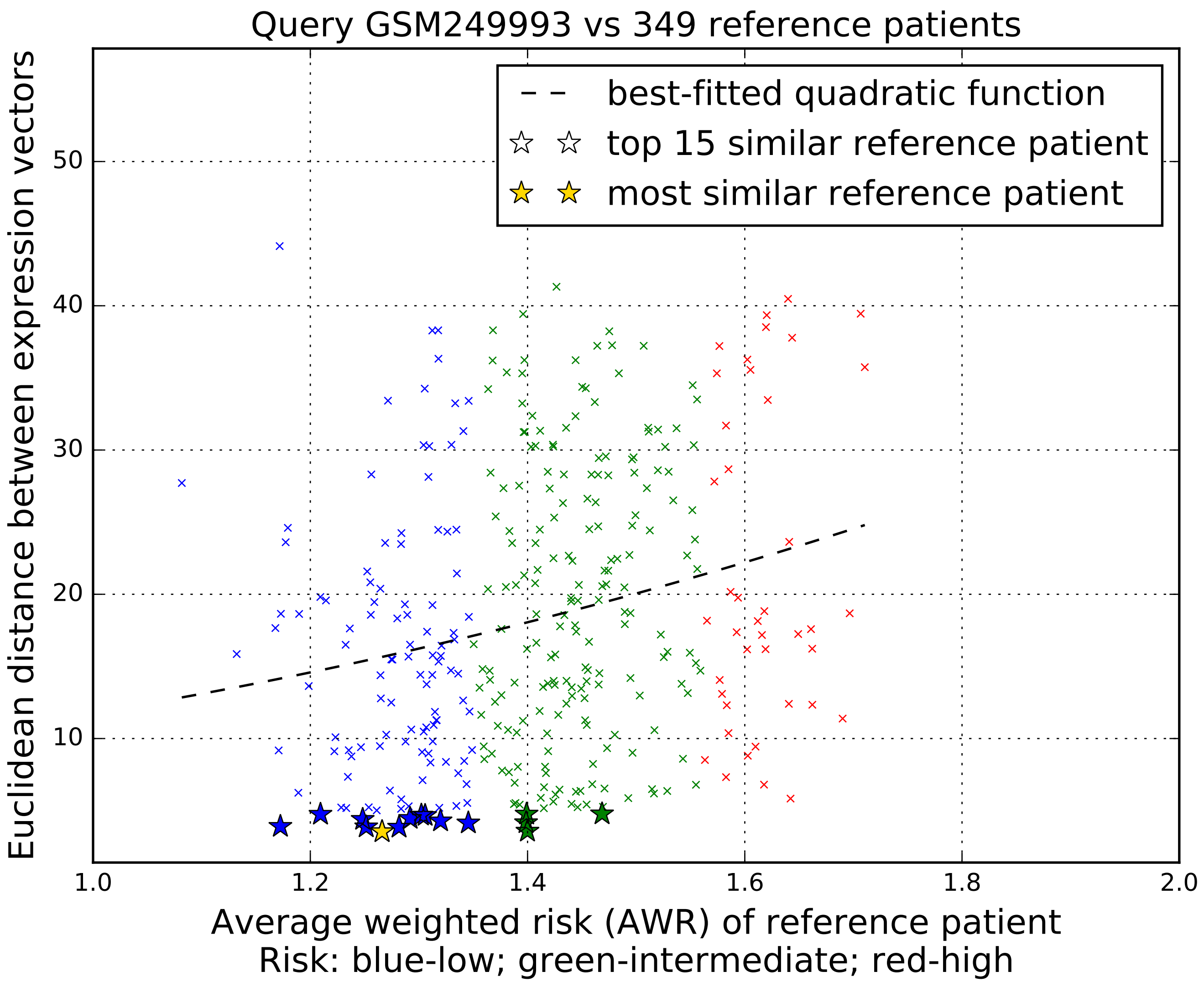

Query GSM249993 vs 349 reference patients
best-fitted quadratic function
top 15 similar reference patient
most similar reference patient
Euclidean distance between expression vectors
10
20
30
40
50
1.0
1.2
1.4
1.6
1.8
2.0
Average weighted risk (AWR) of reference patient
Risk: blue-low; green-intermediate; red-high

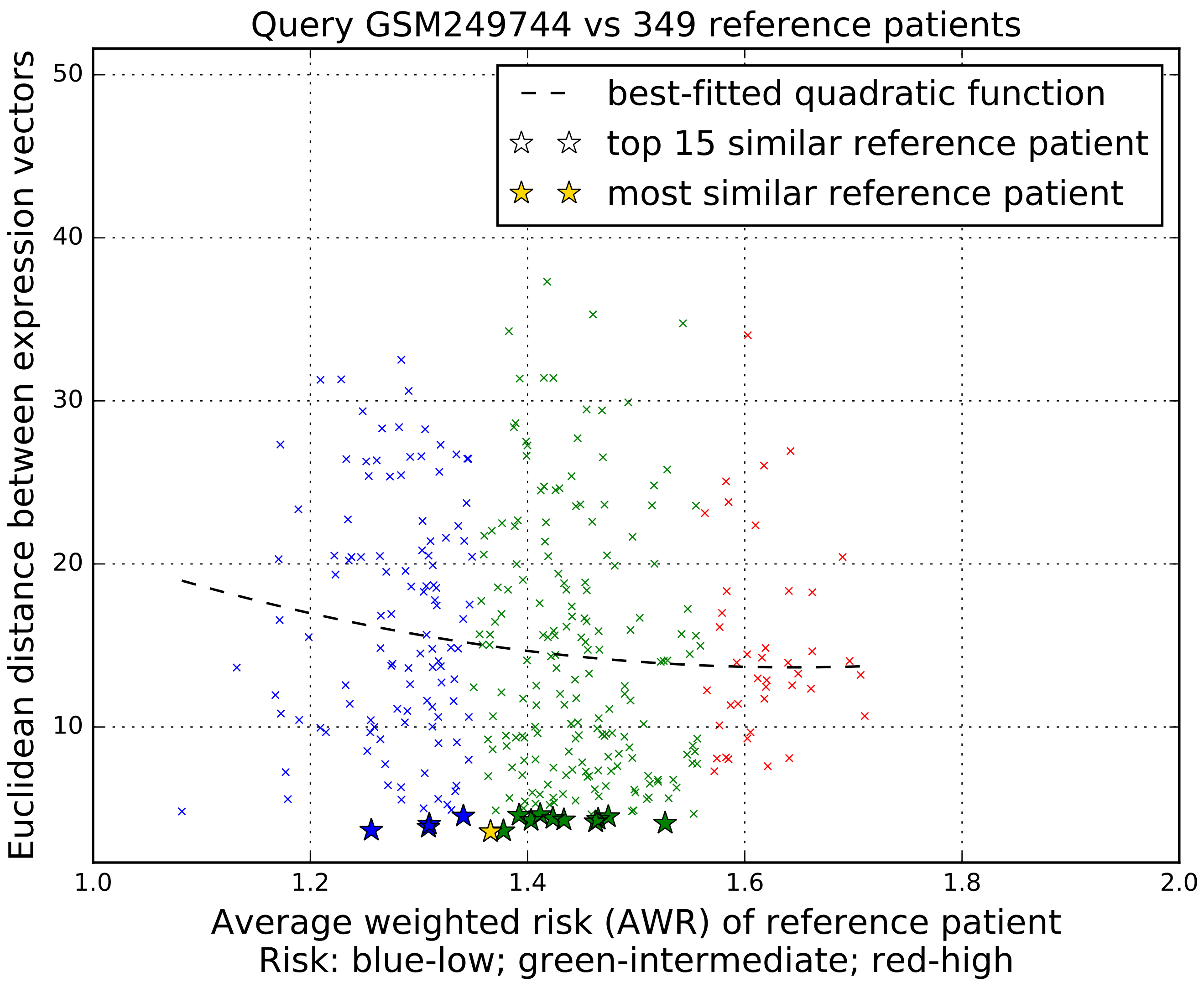

Query GSM249744 vs 349 reference patients
best-fitted quadratic function
top 15 similar reference patient
most similar reference patient
Euclidean distance between expression vectors
50
40
30
20
10
1.0
1.2
1.4
1.6
1.8
2.0
Average weighted risk (AWR) of reference patient
Risk: blue-low; green-intermediate; red-high

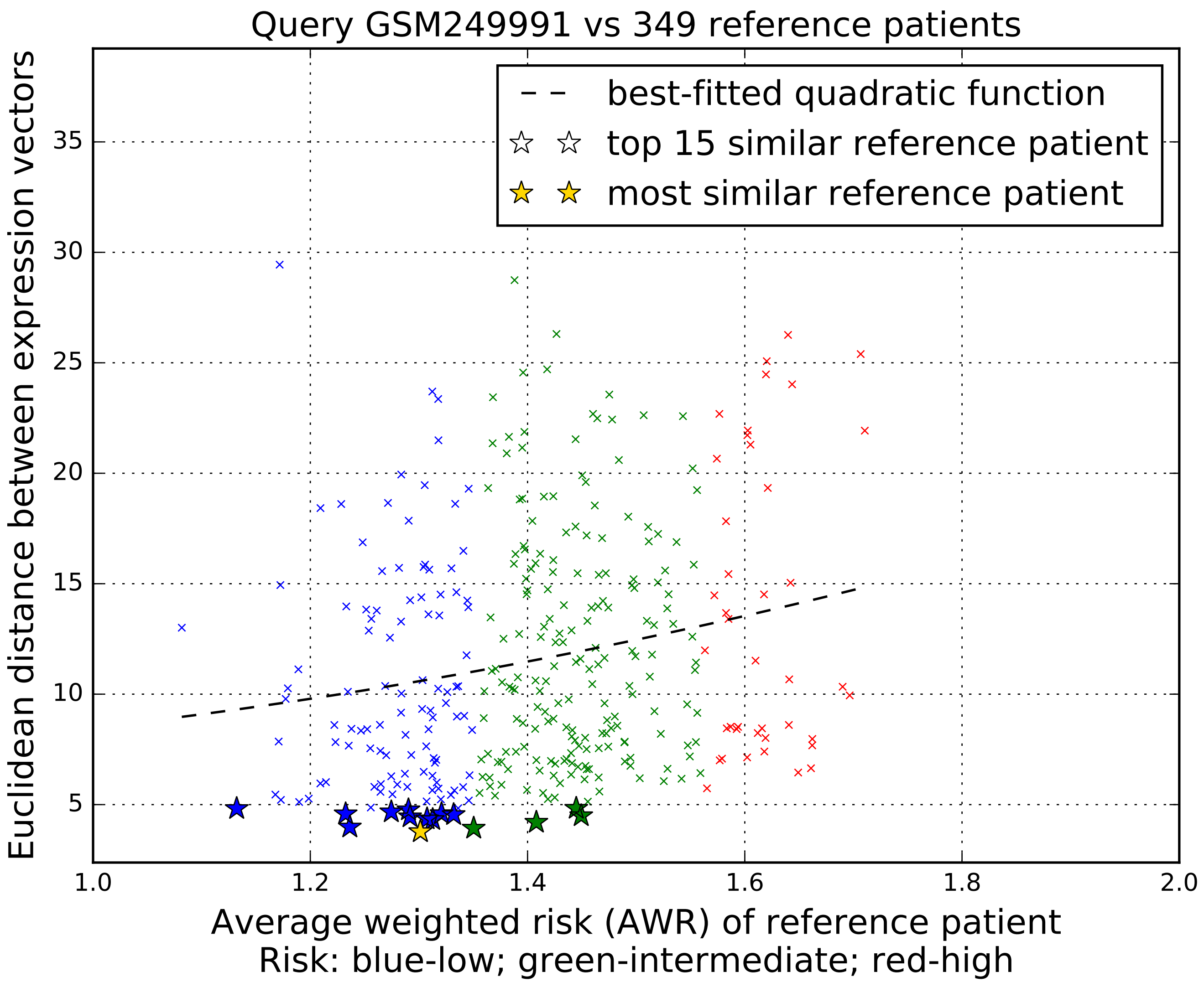

Query GSM249991 vs 349 reference patients
best-fitted quadratic function
top 15 similar reference patient
most similar reference patient
Euclidean distance between expression vectors
35
30
25
20
15
10
5
1.0
1.2
1.4
1.6
1.8
2.0
Average weighted risk (AWR) of reference patient
Risk: blue-low; green-intermediate; red-high

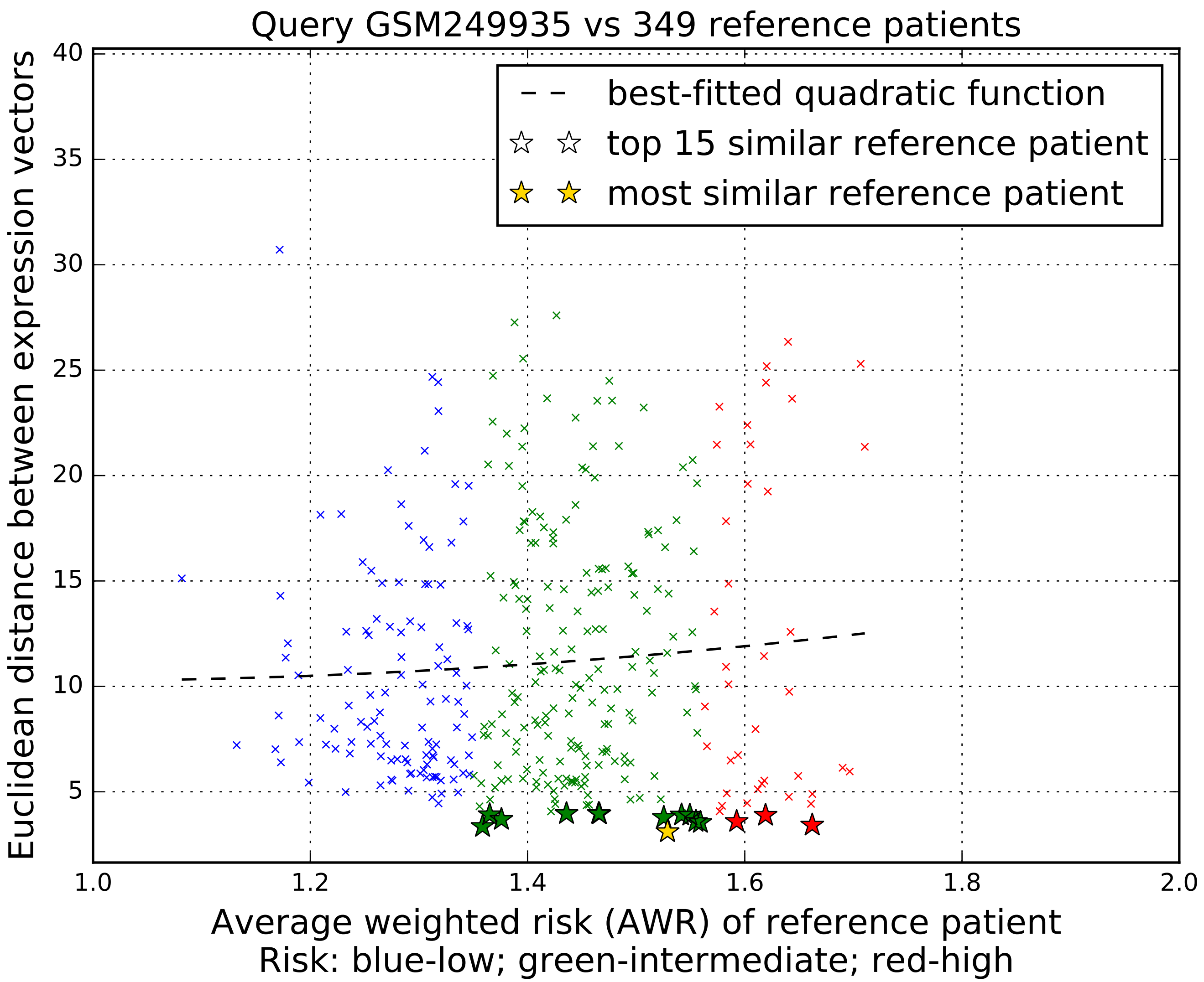
Query GSM249935 vs 349 reference patients
best-fitted quadratic function
top 15 similar reference patient
most similar reference patient
Euclidean distance between expression vectors
40
35
30
25
20
15
10
5
1.0
1.2
1.4
1.6
1.8
2.0
Average weighted risk (AWR) of reference patient
Risk: blue-low; green-intermediate; red-high

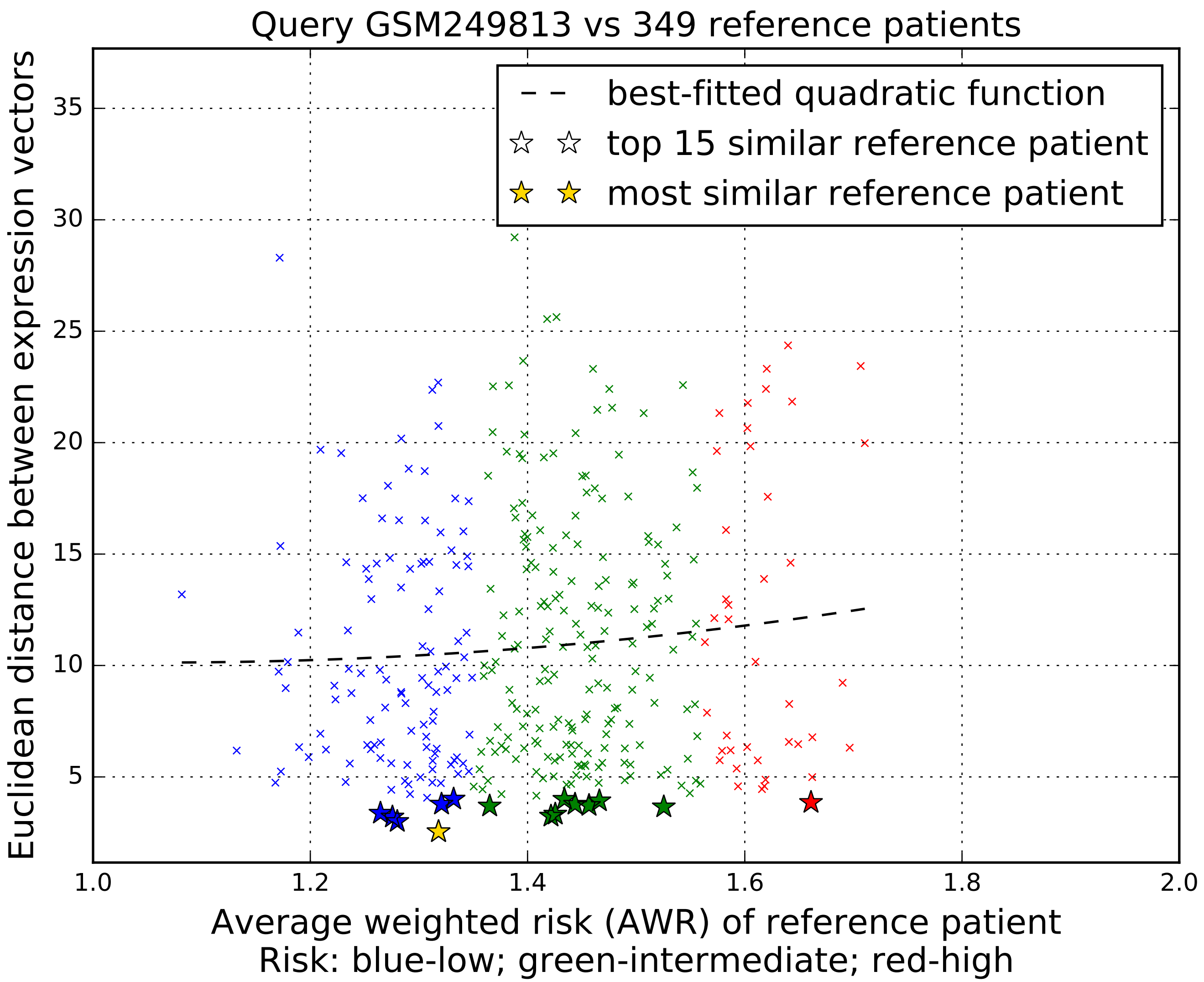
Query GSM249813 vs 349 reference patients
best-fitted quadratic function
top 15 similar reference patient
most similar reference patient
Euclidean distance between expression vectors
5
10
15
20
25
30
35
1.0
1.2
1.4
1.6
1.8
2.0
Average weighted risk (AWR) of reference patient
Risk: blue-low; green-intermediate; red-high

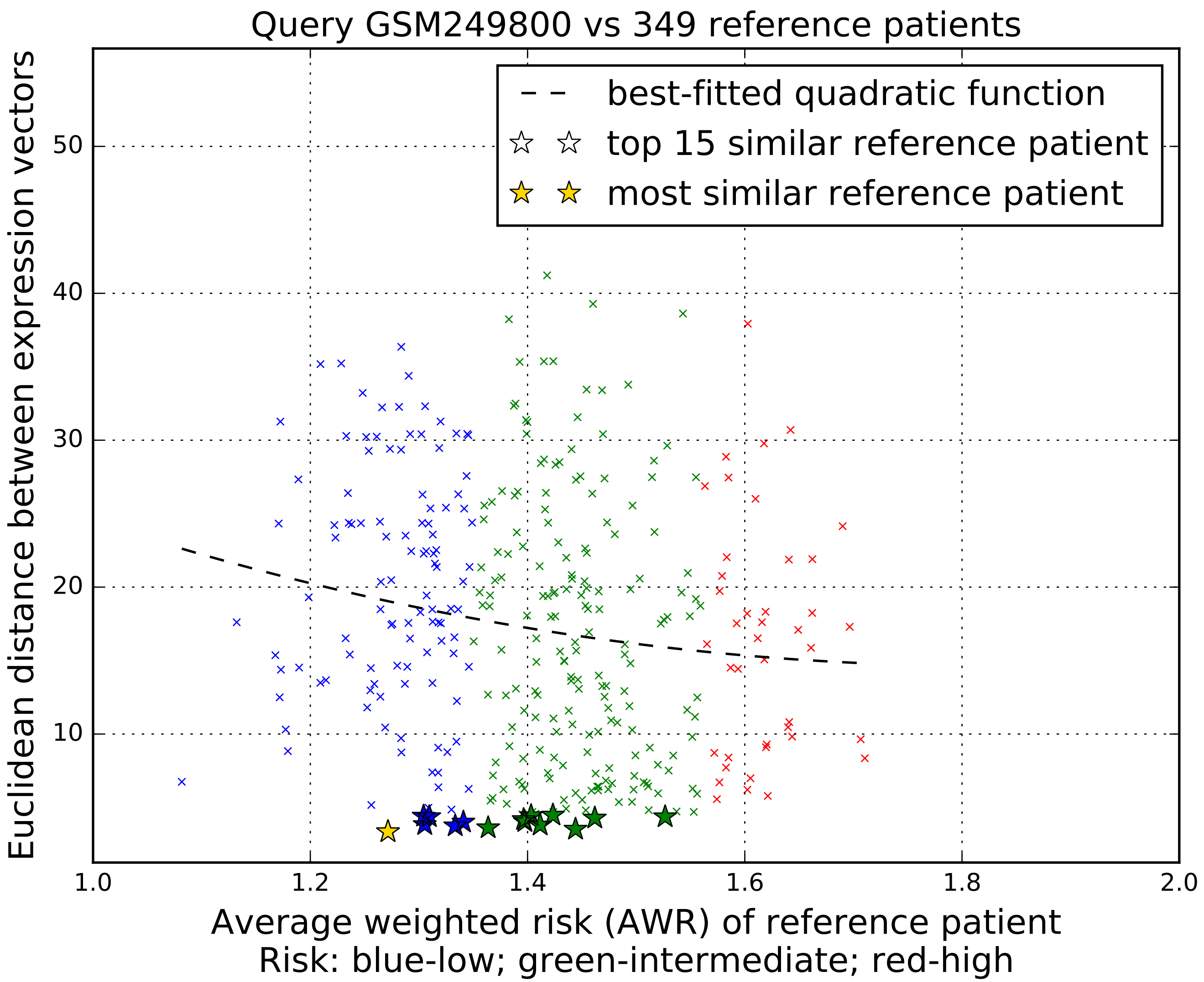
Query GSM249800 vs 349 reference patients
best-fitted quadratic function
top 15 similar reference patient
most similar reference patient
Euclidean distance between expression vectors
10
20
30
40
50
1.0
1.2
1.4
1.6
1.8
2.0
Average weighted risk (AWR) of reference patient
Risk: blue-low; green-intermediate; red-high

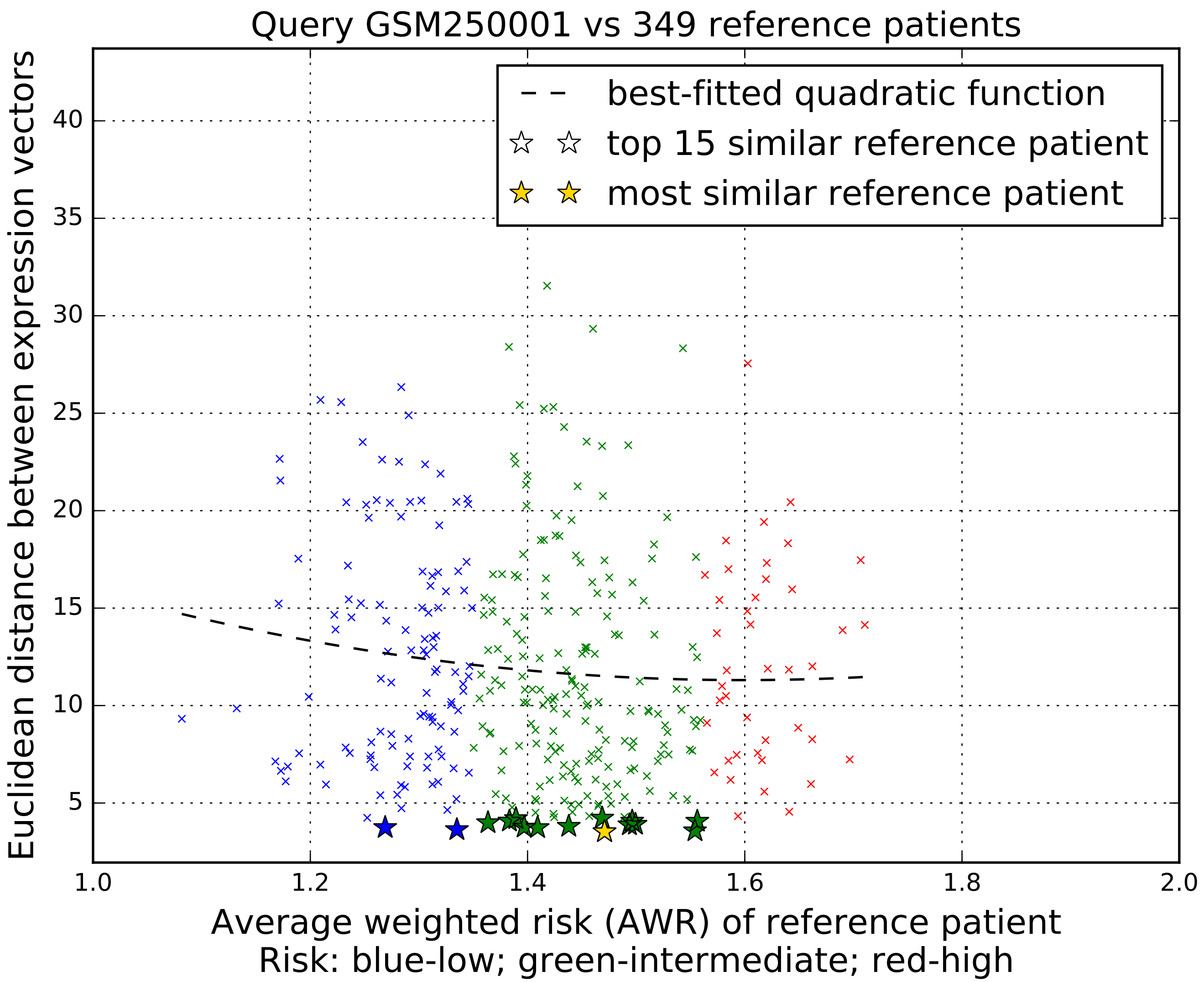

Query GSM250001 vs 349 reference patients
best-fitted quadratic function
top 15 similar reference patient
most similar reference patient
Euclidean distance between expression vectors
40
35
30
25
20
15
10
5
1.0
1.2
1.4
1.6
1.8
2.0
Average weighted risk (AWR) of reference patient
Risk: blue-low; green-intermediate; red-high

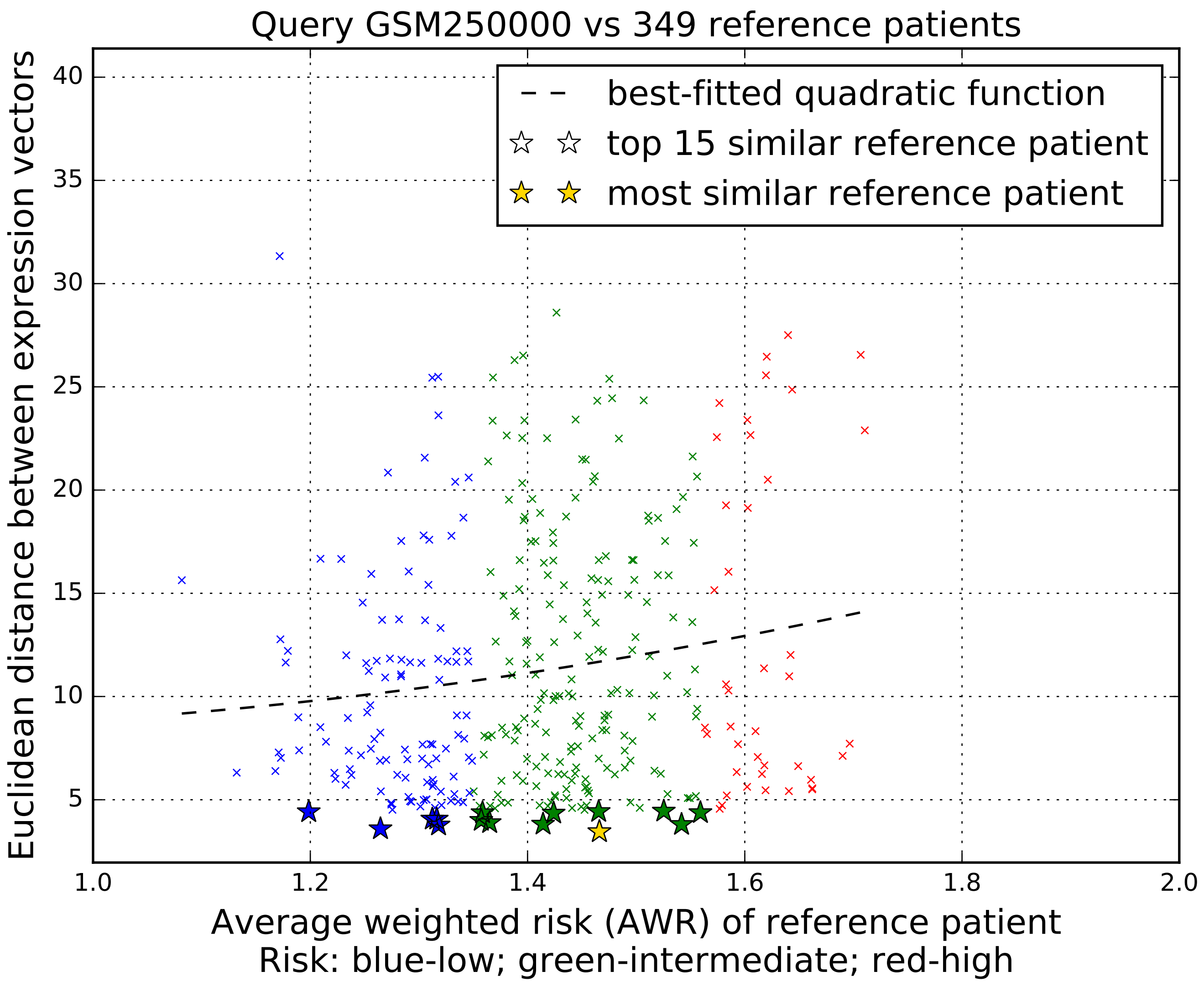
Query GSM250000 vs 349 reference patients
best-fitted quadratic function
top 15 similar reference patient
most similar reference patient
Euclidean distance between expression vectors
40
35
30
25
20
15
10
5
1.0
1.2
1.4
1.6
1.8
2.0
Average weighted risk (AWR) of reference patient
Risk: blue-low; green-intermediate; red-high

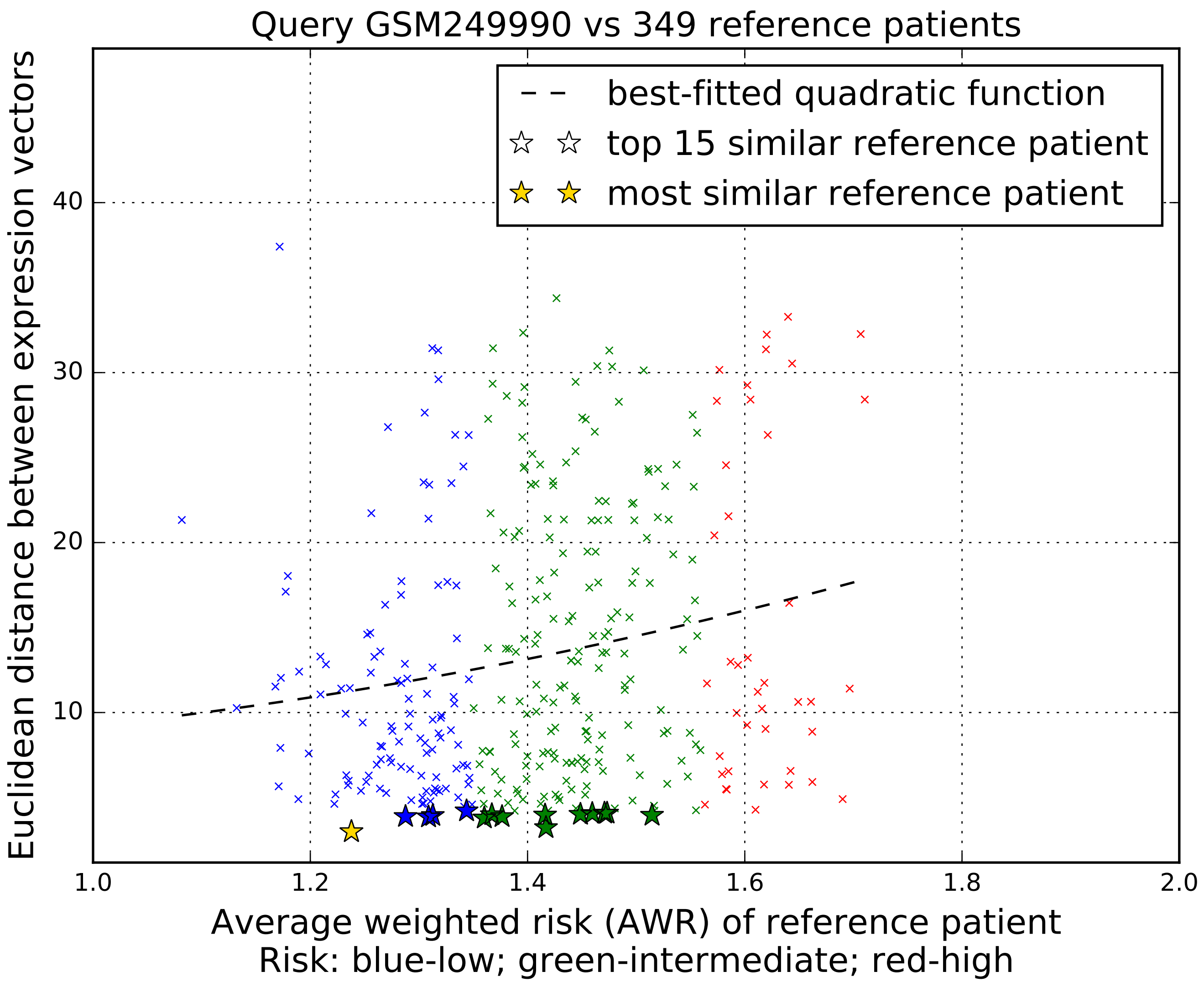
Query GSM249990 vs 349 reference patients
best-fitted quadratic function
top 15 similar reference patient
most similar reference patient
Euclidean distance between expression vectors
10
20
30
40
1.0
1.2
1.4
1.6
1.8
2.0
Average weighted risk (AWR) of reference patient
Risk: blue-low; green-intermediate; red-high

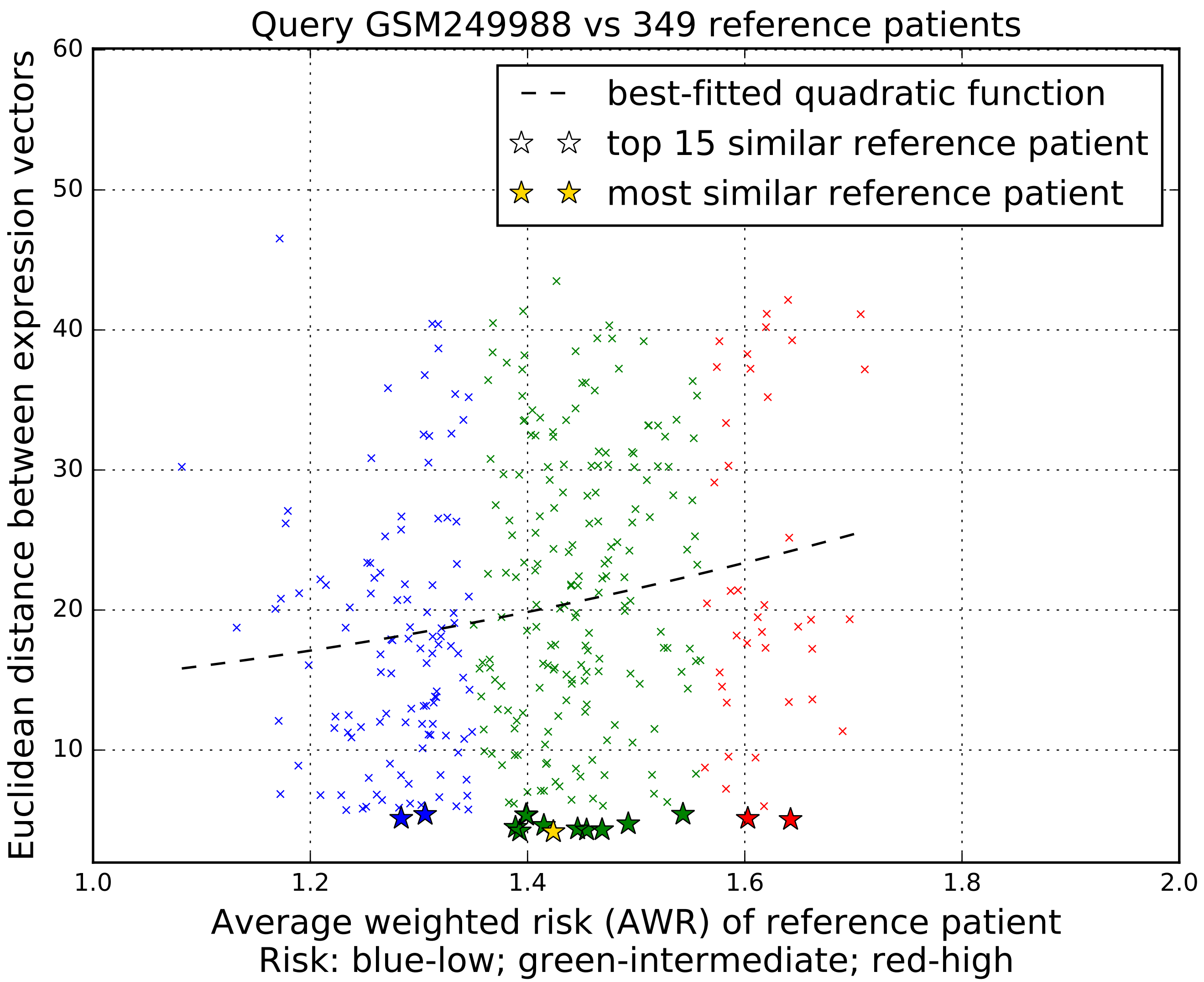

Query GSM249988 vs 349 reference patients
best-fitted quadratic function
top 15 similar reference patient
most similar reference patient
Euclidean distance between expression vectors
Average weighted risk (AWR) of reference patient
Risk: blue-low; green-intermediate; red-high
60
50
40
30
20
10
1.0
1.2
1.4
1.6
1.8
2.0

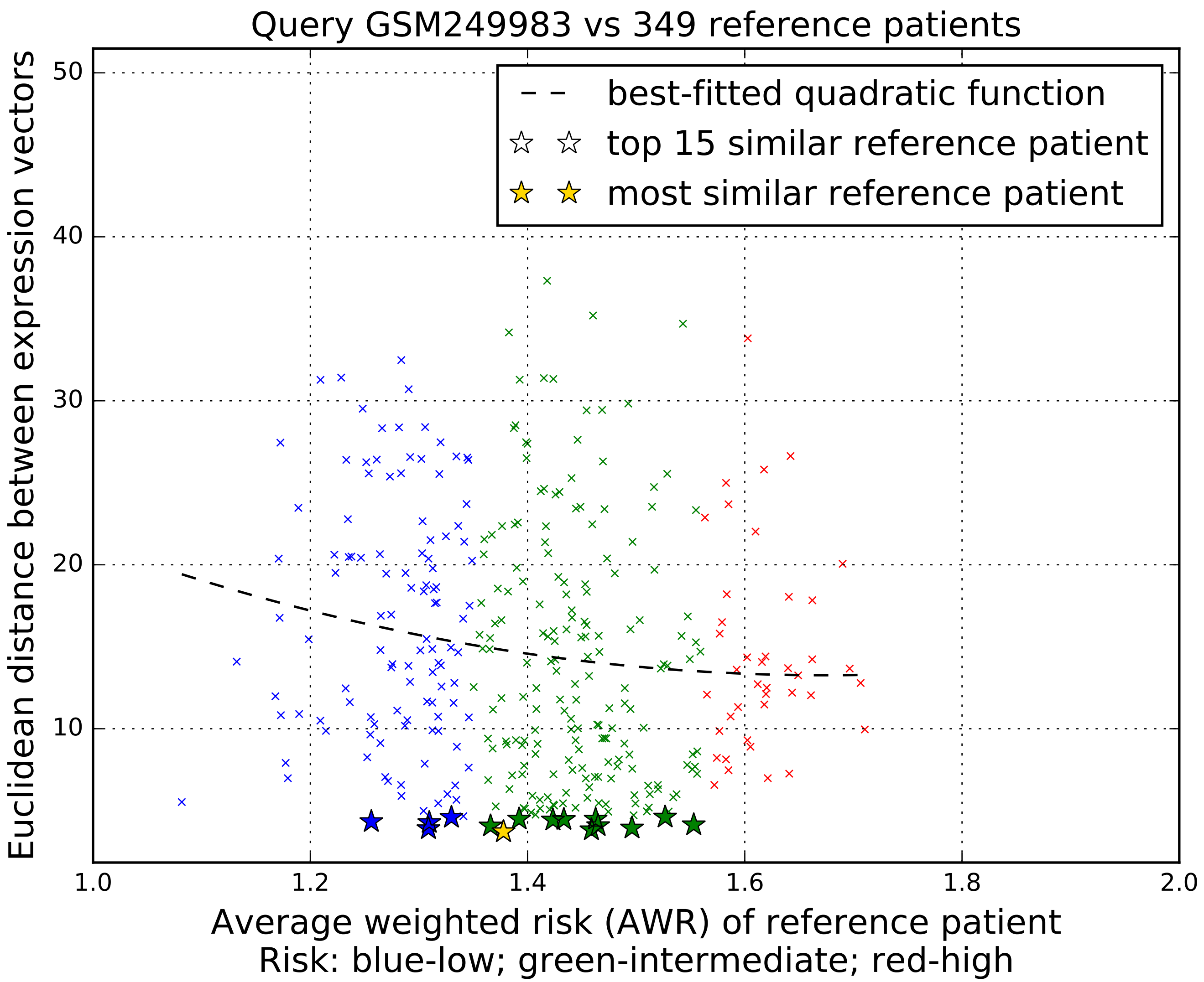
Query GSM249983 vs 349 reference patients
best-fitted quadratic function
top 15 similar reference patient
most similar reference patient
Euclidean distance between expression vectors
Average weighted risk (AWR) of reference patient
Risk: blue-low; green-intermediate; red-high
50
40
30
20
10
1.0
1.2
1.4
1.6
1.8
2.0

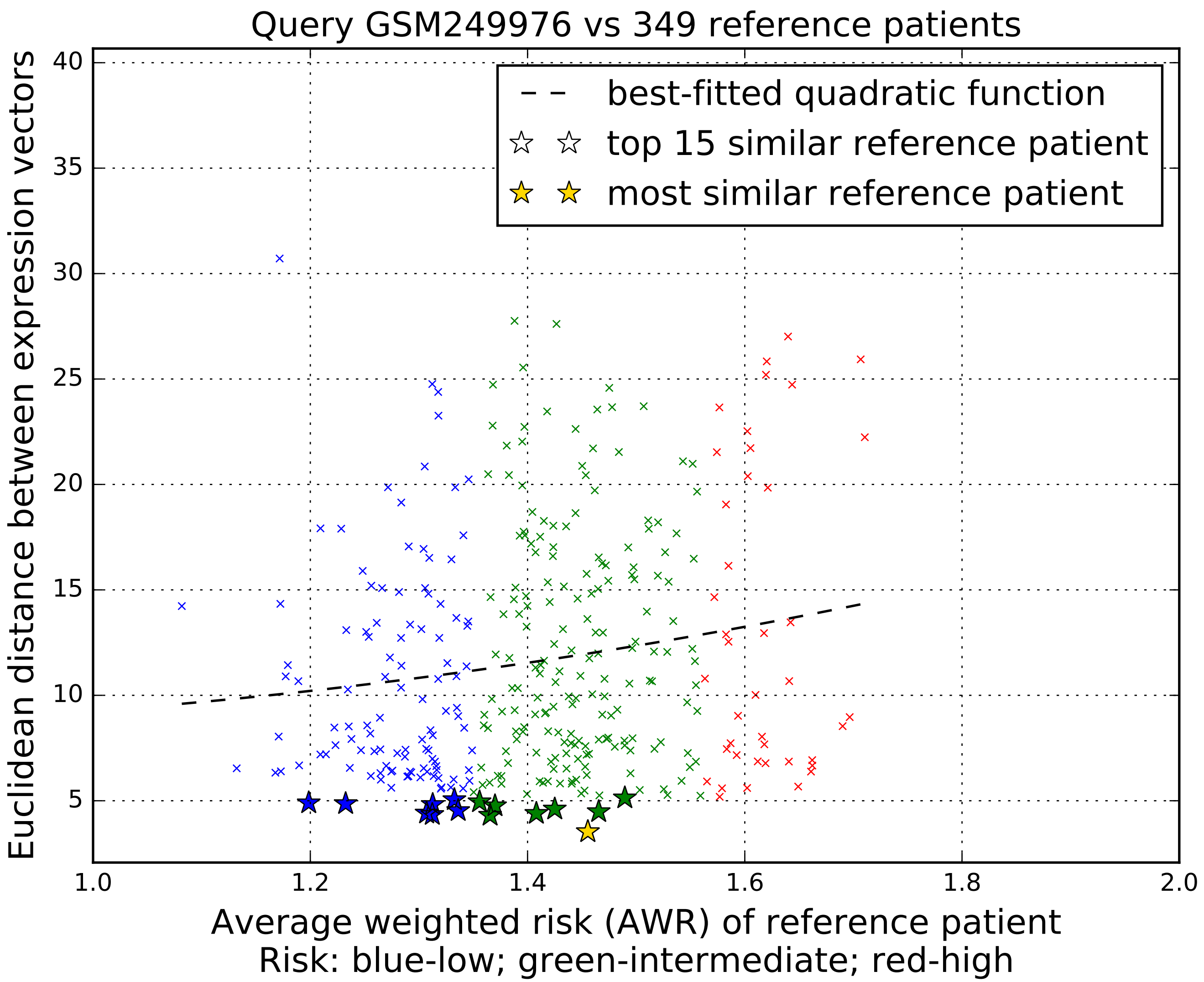

Query GSM249976 vs 349 reference patients
best-fitted quadratic function
top 15 similar reference patient
most similar reference patient
Euclidean distance between expression vectors
40
35
30
25
20
15
10
5
1.0
1.2
1.4
1.6
1.8
2.0
Average weighted risk (AWR) of reference patient
Risk: blue-low; green-intermediate; red-high

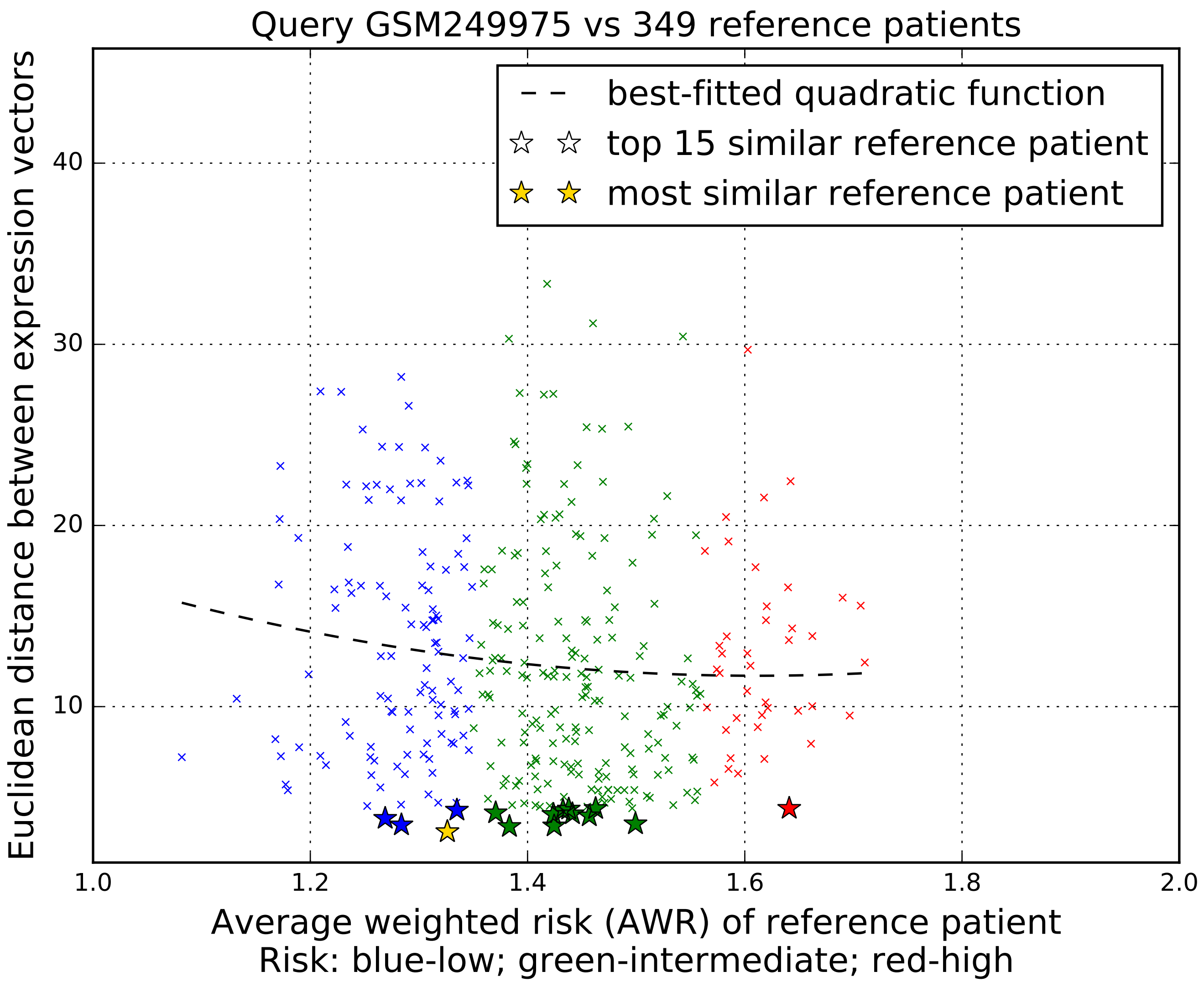

Query GSM249975 vs 349 reference patients
best-fitted quadratic function
top 15 similar reference patient
most similar reference patient
Euclidean distance between expression vectors
40
30
20
10
1.0
1.2
1.4
1.6
1.8
2.0
Average weighted risk (AWR) of reference patient
Risk: blue-low; green-intermediate; red-high

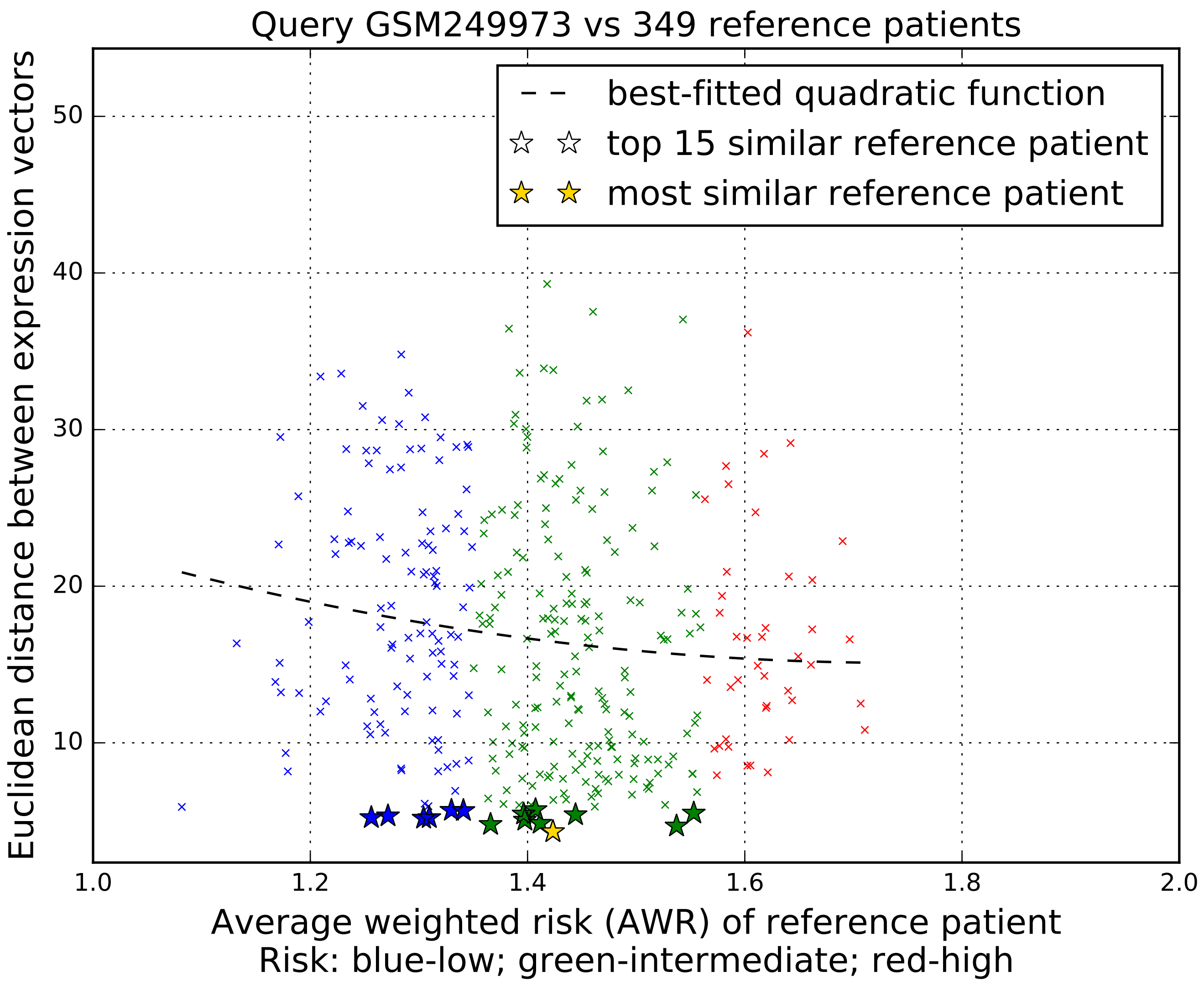

Query GSM249973 vs 349 reference patients
best-fitted quadratic function
top 15 similar reference patient
most similar reference patient
Euclidean distance between expression vectors
50
40
30
20
10
1.0
1.2
1.4
1.6
1.8
2.0
Average weighted risk (AWR) of reference patient
Risk: blue-low; green-intermediate; red-high

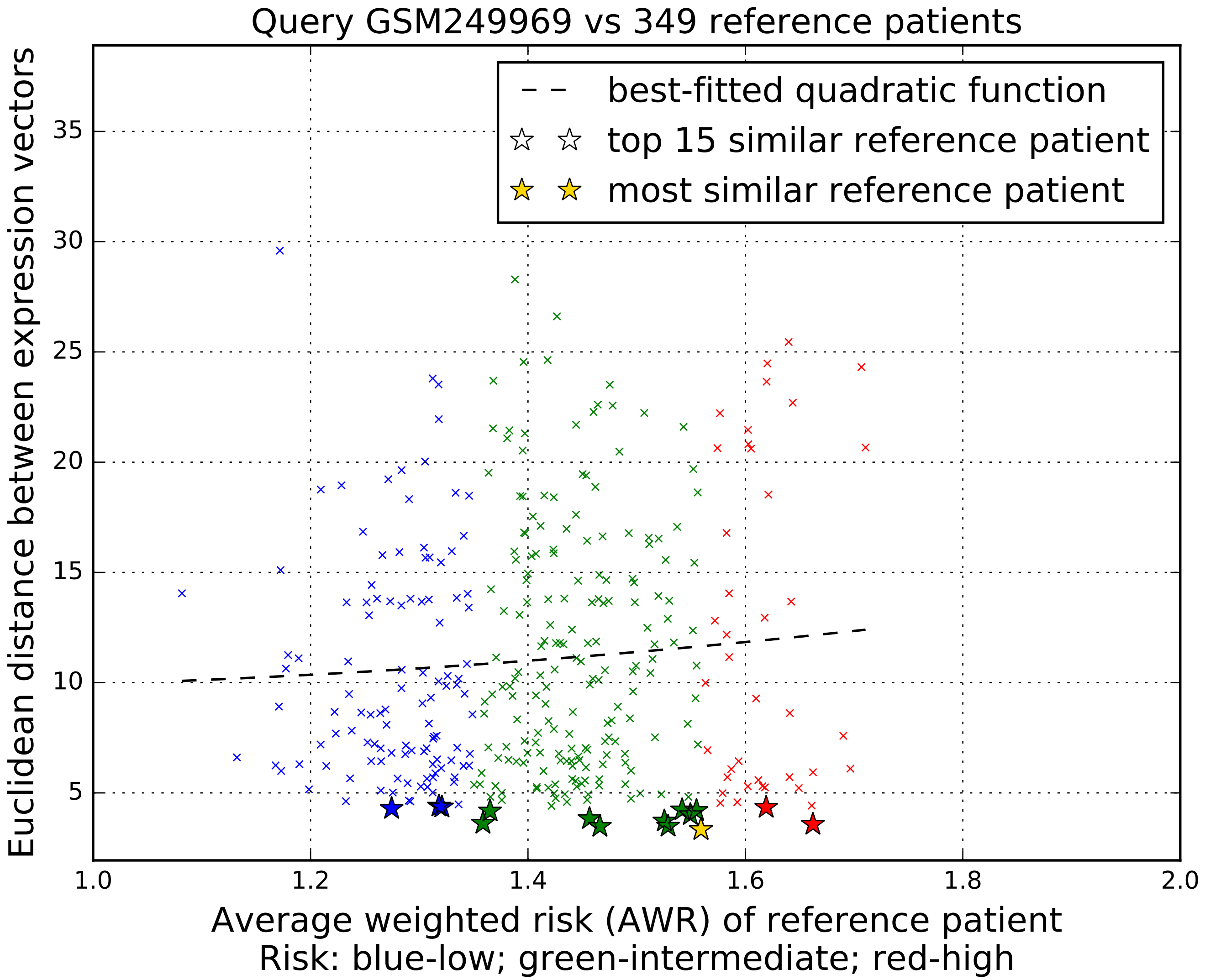

Query GSM249969 vs 349 reference patients
best-fitted quadratic function
top 15 similar reference patient
most similar reference patient
Euclidean distance between expression vectors
35
30
25
20
15
10
5
1.0
1.2
1.4
1.6
1.8
2.0
Average weighted risk (AWR) of reference patient
Risk: blue-low; green-intermediate; red-high

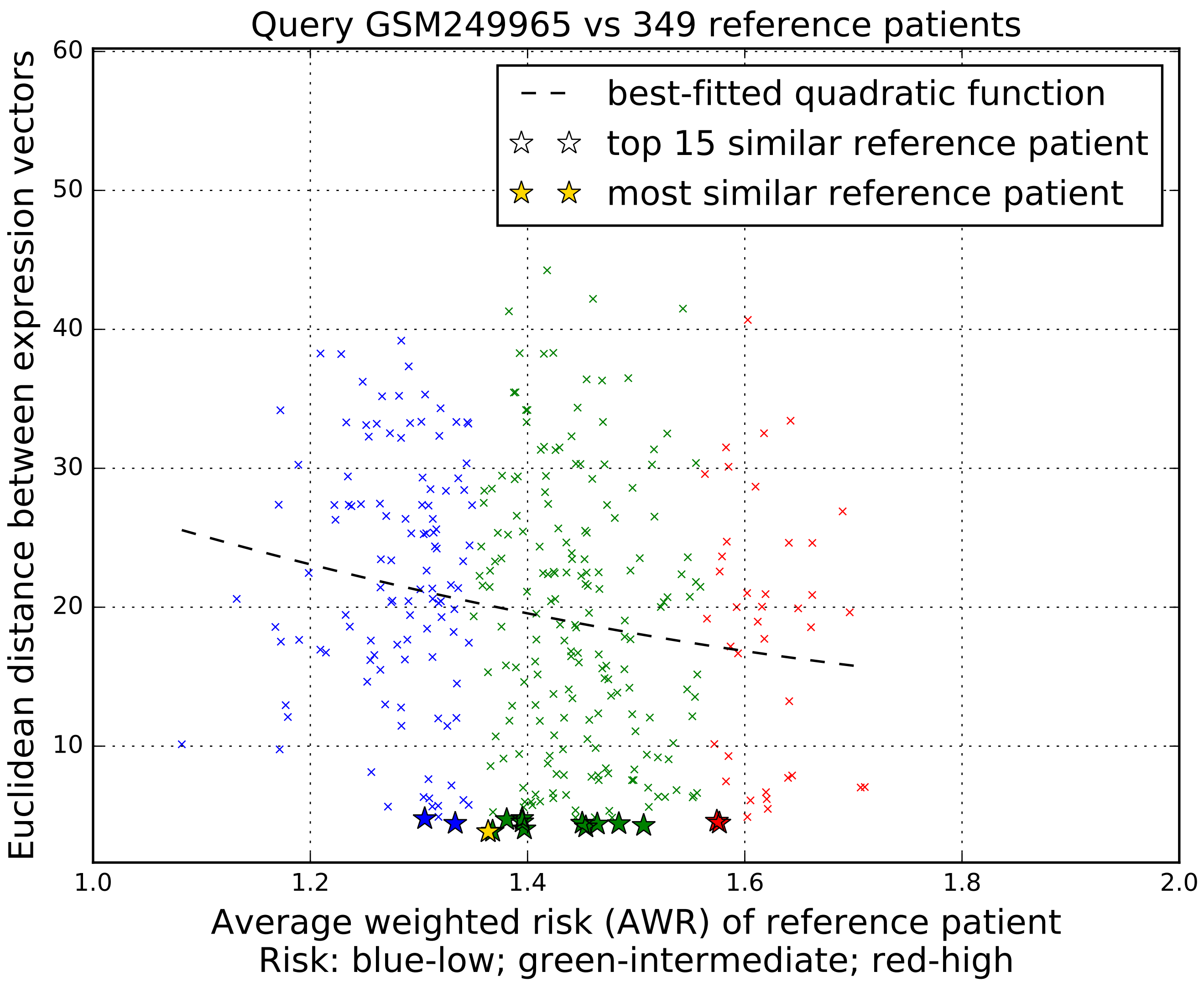
Query GSM249965 vs 349 reference patients
best-fitted quadratic function
top 15 similar reference patient
most similar reference patient
Euclidean distance between expression vectors
Average weighted risk (AWR) of reference patient
Risk: blue-low; green-intermediate; red-high
60
50
40
30
20
10
1.0
1.2
1.4
1.6
1.8
2.0

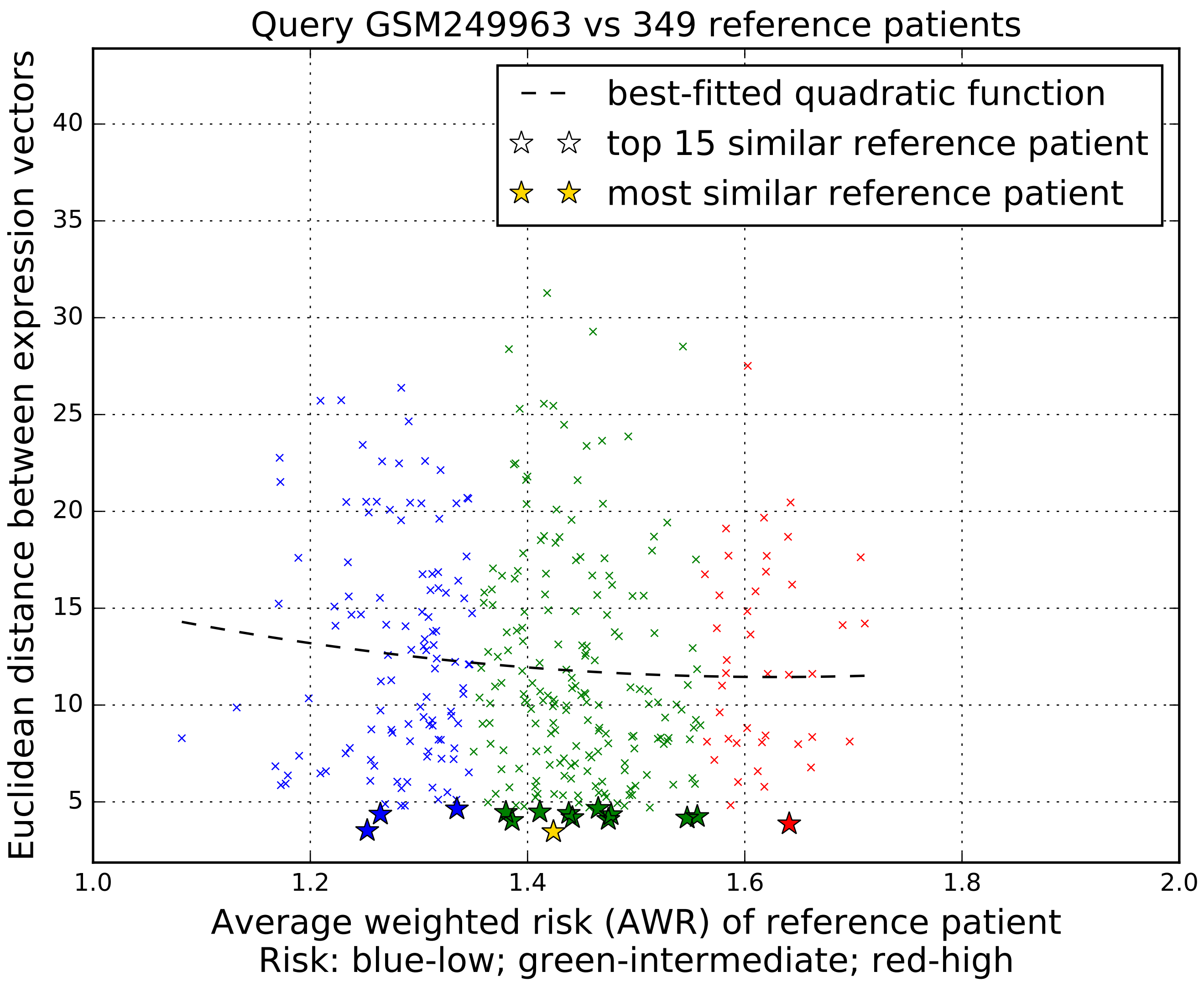

Query GSM249963 vs 349 reference patients
best-fitted quadratic function
top 15 similar reference patient
most similar reference patient
Euclidean distance between expression vectors
5
10
15
20
25
30
35
40
1.0
1.2
1.4
1.6
1.8
2.0
Average weighted risk (AWR) of reference patient
Risk: blue-low; green-intermediate; red-high

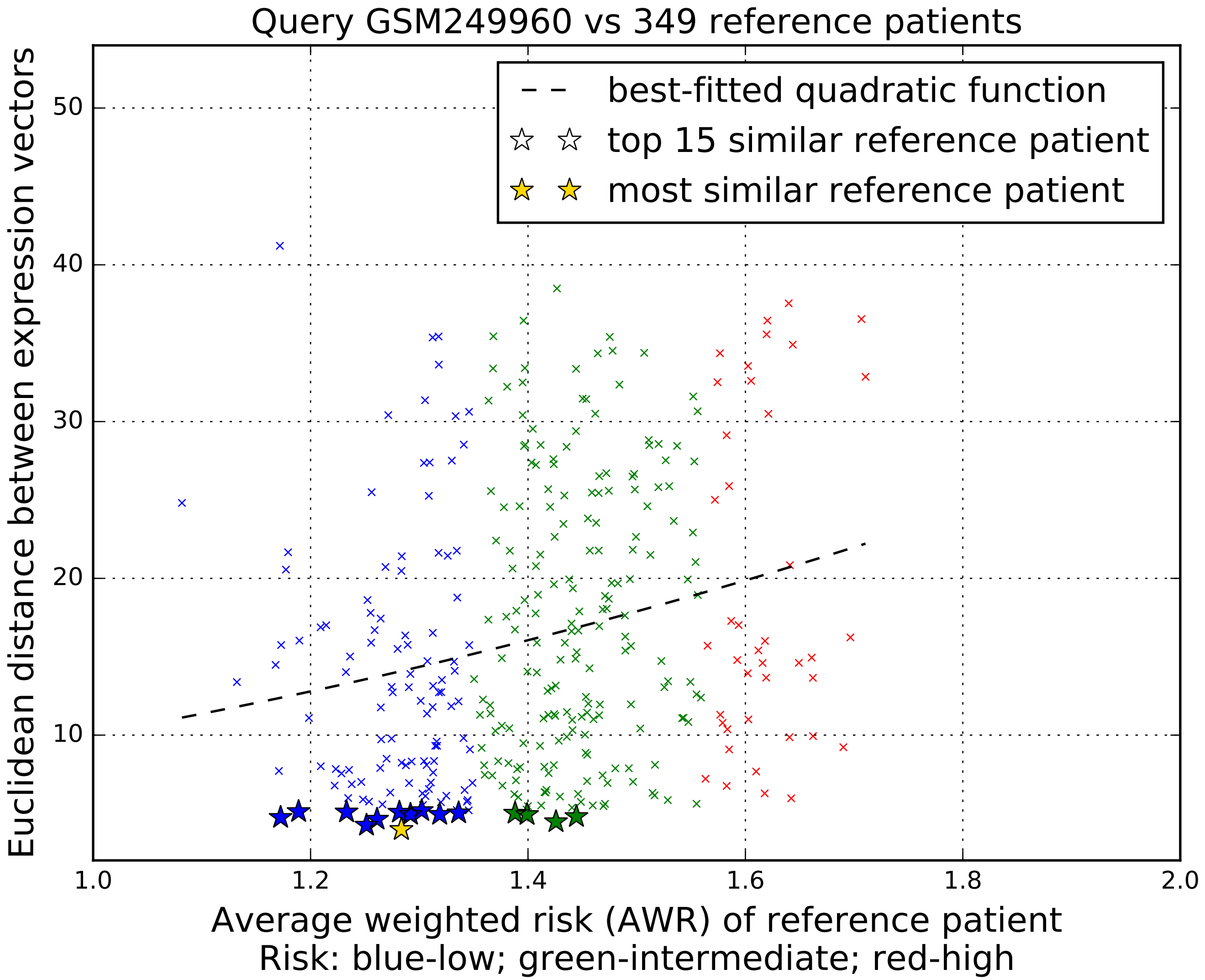

Query GSM249960 vs 349 reference patients
best-fitted quadratic function
top 15 similar reference patient
most similar reference patient
Euclidean distance between expression vectors
50
40
30
20
10
1.0
1.2
1.4
1.6
1.8
2.0
Average weighted risk (AWR) of reference patient
Risk: blue-low; green-intermediate; red-high

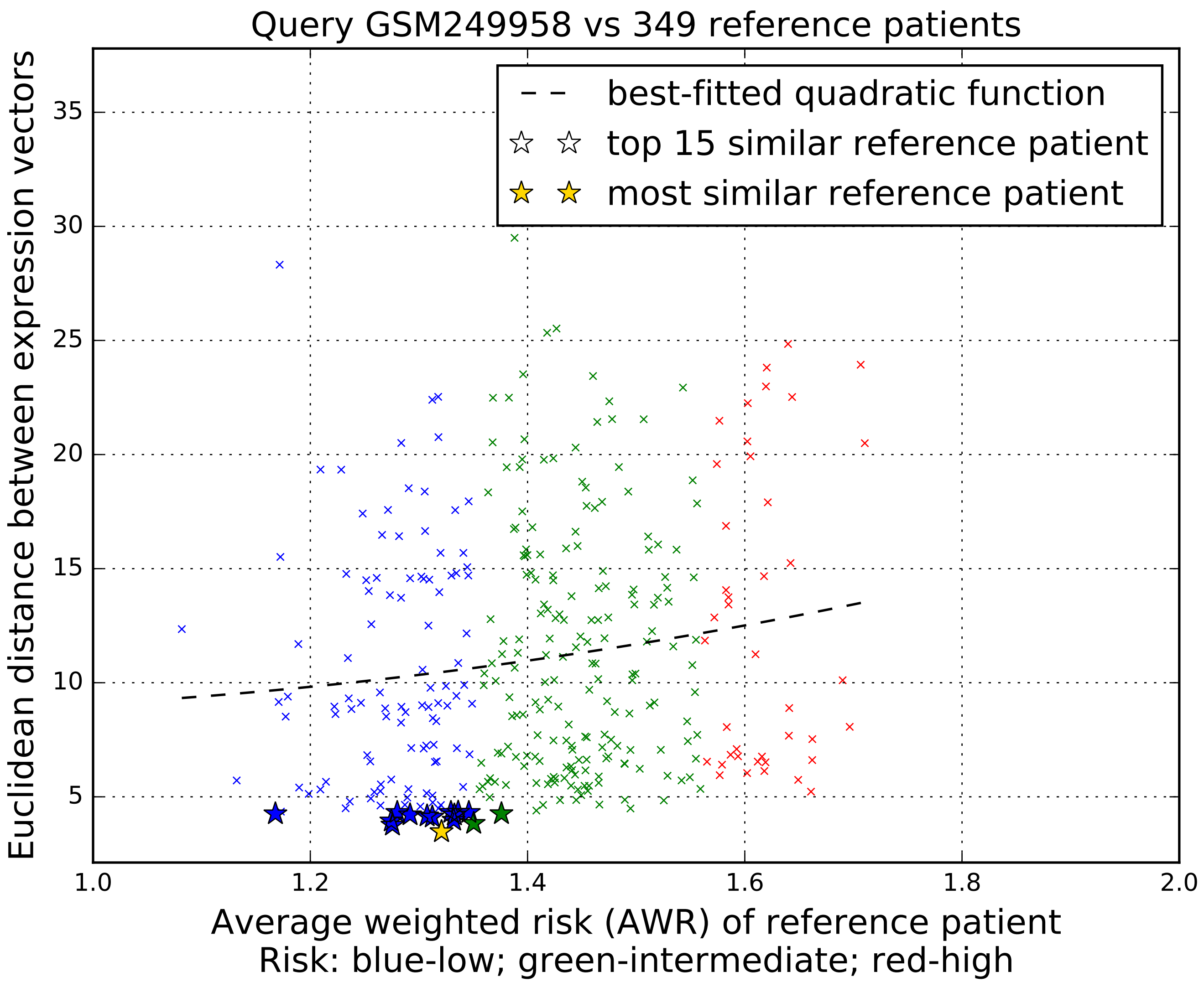
Query GSM249958 vs 349 reference patients
best-fitted quadratic function
top 15 similar reference patient
most similar reference patient
Euclidean distance between expression vectors
Average weighted risk (AWR) of reference patient
Risk: blue-low; green-intermediate; red-high
35
30
25
20
15
10
5
1.0
1.2
1.4
1.6
1.8
2.0

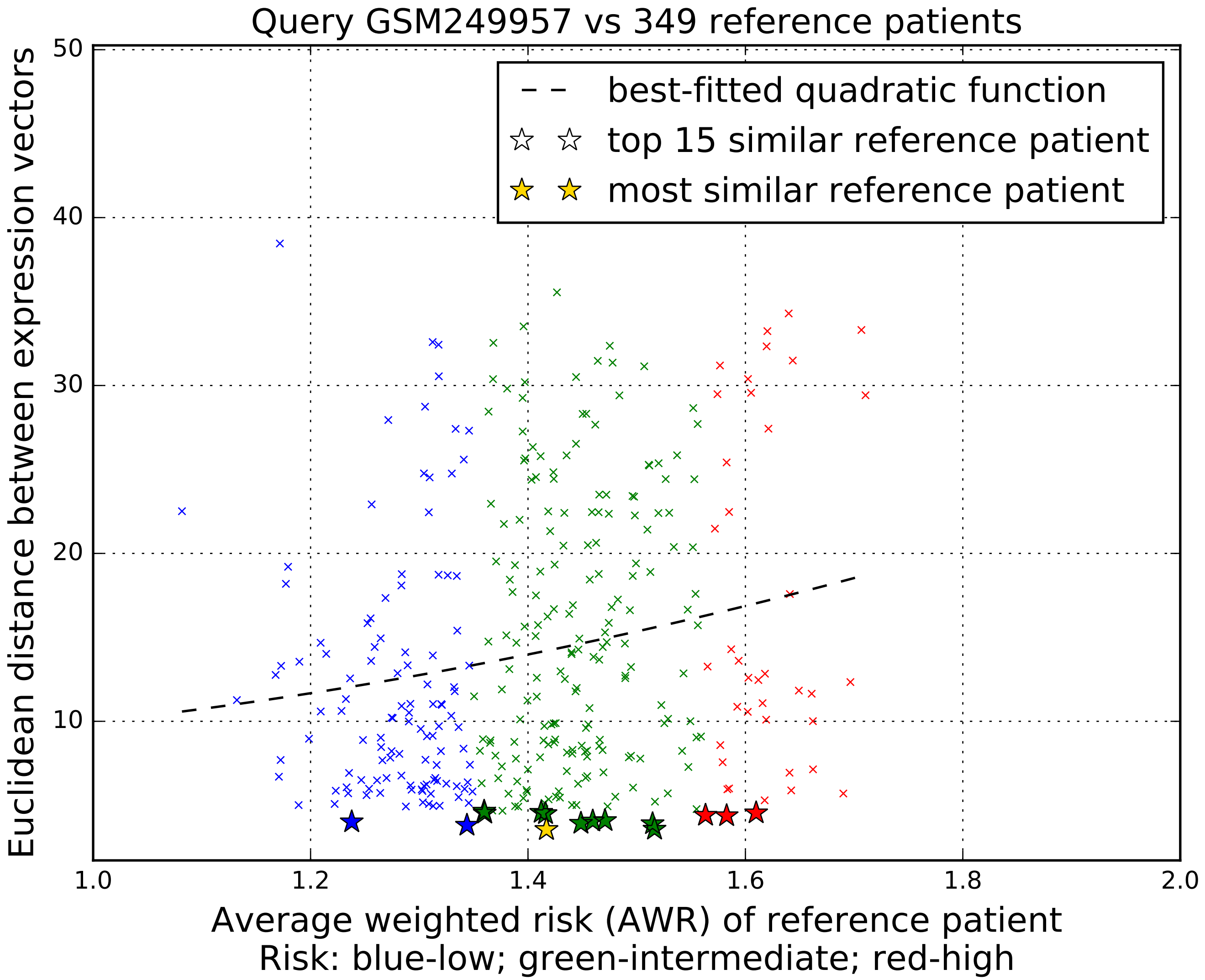
Query GSM249957 vs 349 reference patients
best-fitted quadratic function
top 15 similar reference patient
most similar reference patient
Euclidean distance between expression vectors
Average weighted risk (AWR) of reference patient
Risk: blue-low; green-intermediate; red-high
50
40
30
20
10
1.0
1.2
1.4
1.6
1.8
2.0

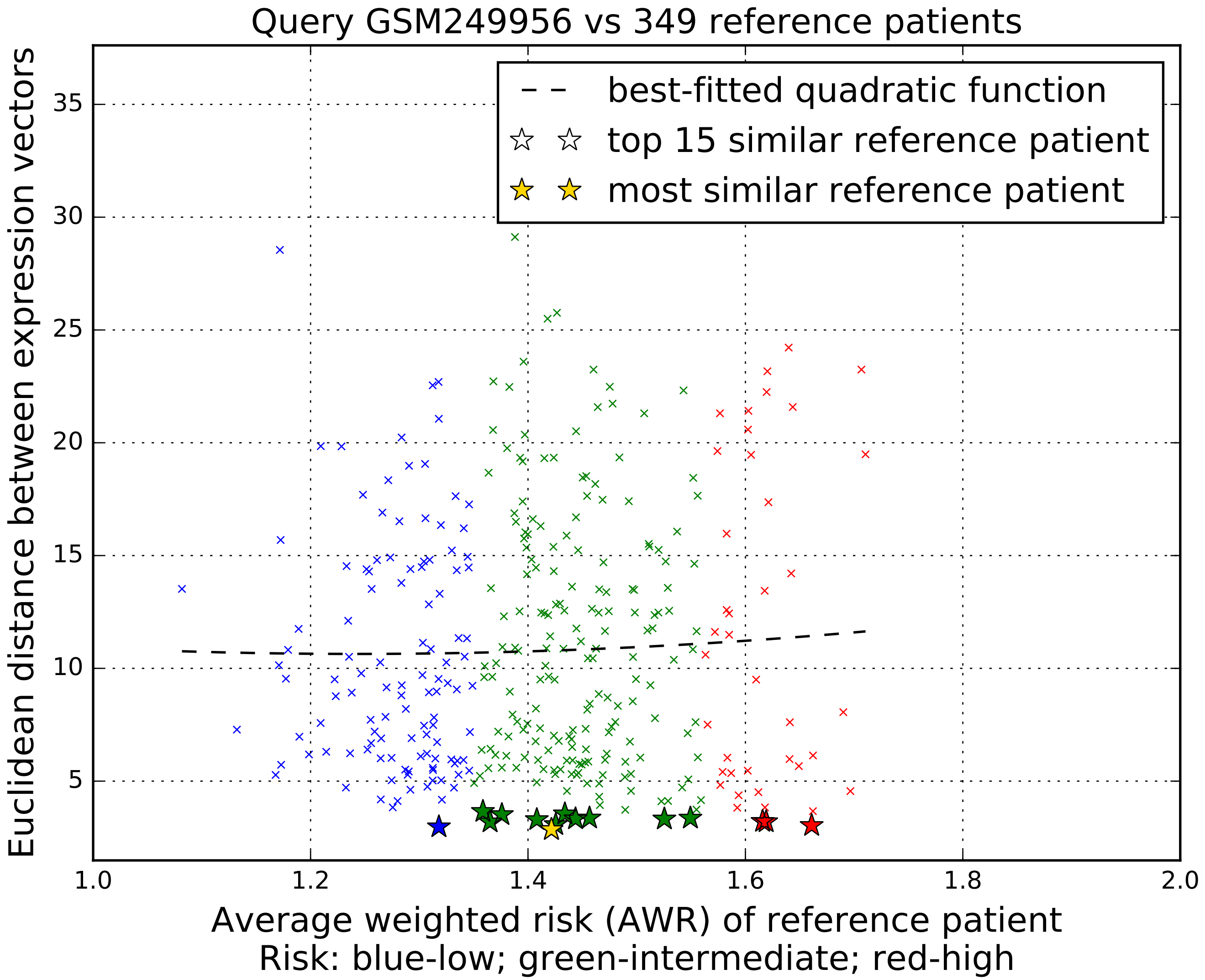

Query GSM249956 vs 349 reference patients
best-fitted quadratic function
top 15 similar reference patient
most similar reference patient
Euclidean distance between expression vectors
35
30
25
20
15
10
5
1.0
1.2
1.4
1.6
1.8
2.0
Average weighted risk (AWR) of reference patient
Risk: blue-low; green-intermediate; red-high

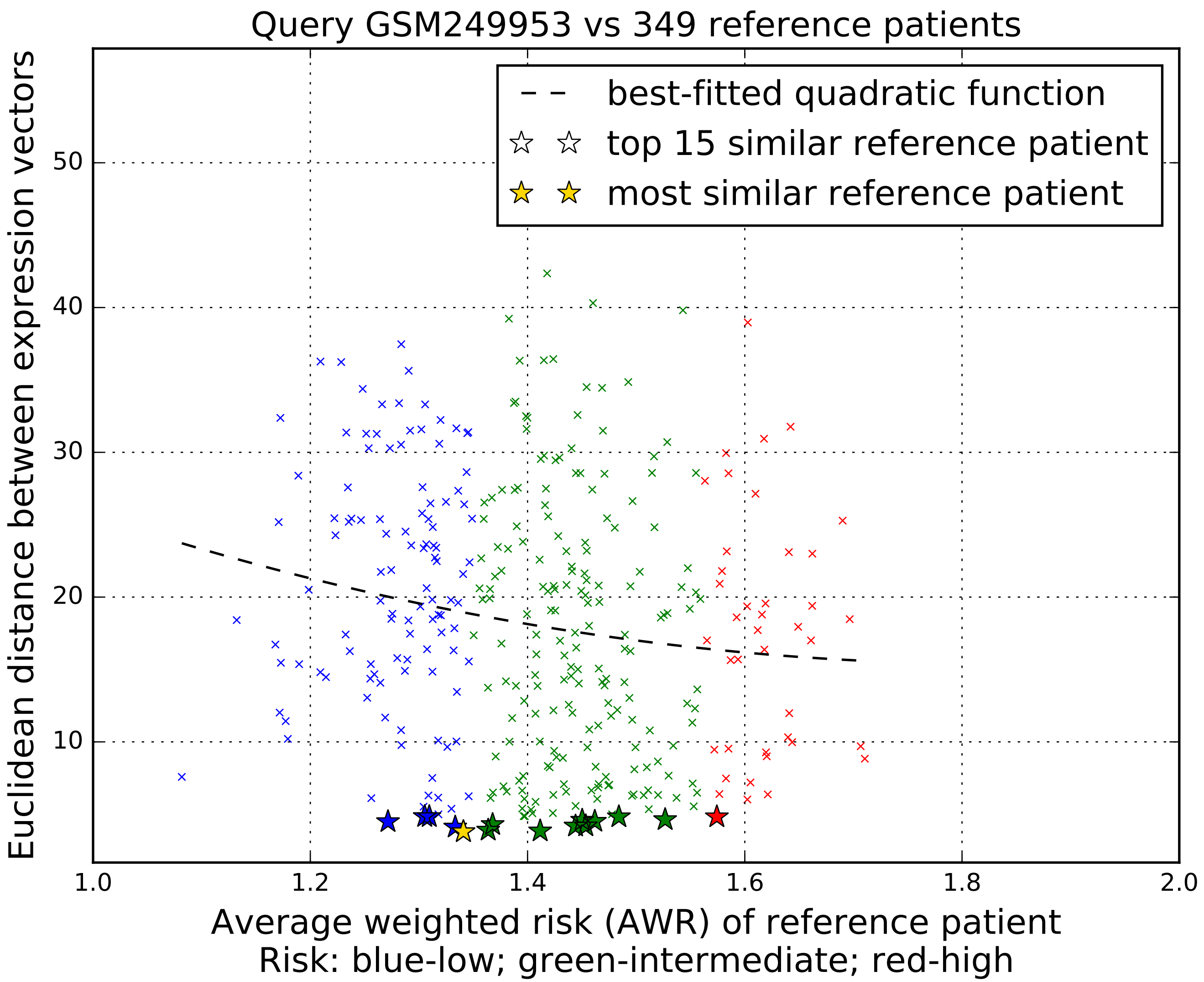
Query GSM249953 vs 349 reference patients
best-fitted quadratic function
top 15 similar reference patient
most similar reference patient
Euclidean distance between expression vectors
50
40
30
20
10
1.0
1.2
1.4
1.6
1.8
2.0
Average weighted risk (AWR) of reference patient
Risk: blue-low; green-intermediate; red-high

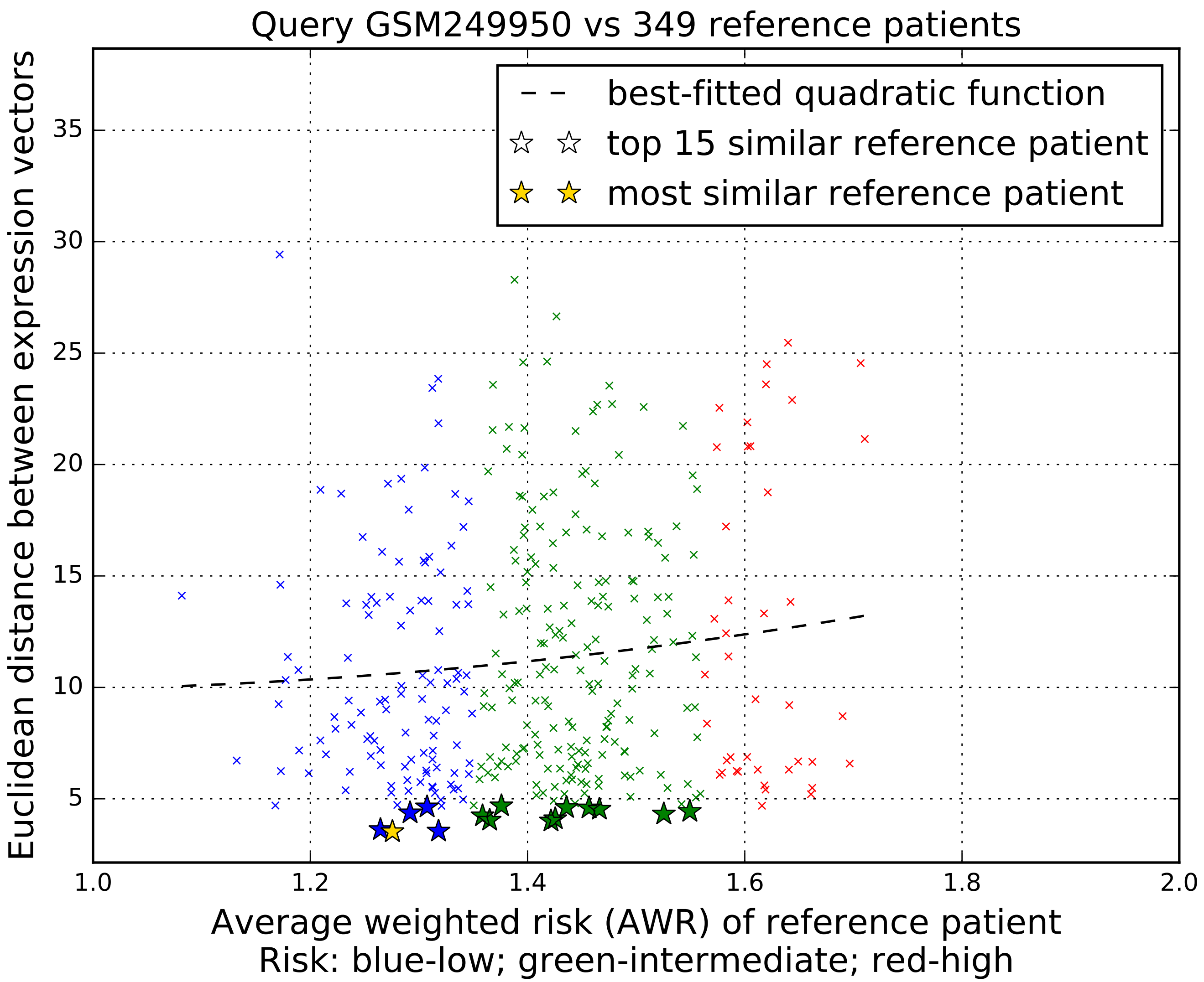
Query GSM249950 vs 349 reference patients
best-fitted quadratic function
top 15 similar reference patient
most similar reference patient
Euclidean distance between expression vectors
5
10
15
20
25
30
35
1.0
1.2
1.4
1.6
1.8
2.0
Average weighted risk (AWR) of reference patient
Risk: blue-low; green-intermediate; red-high

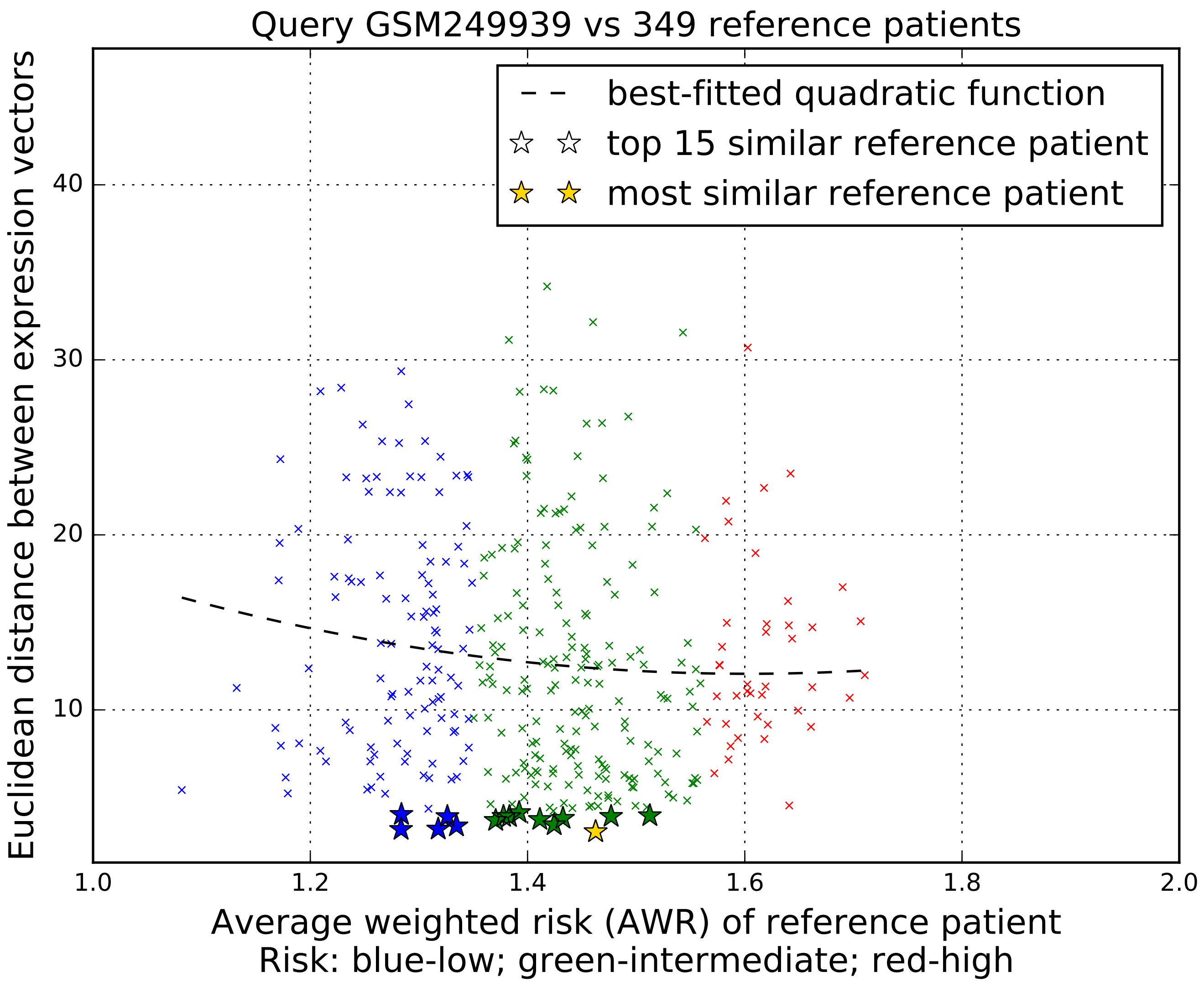
Query GSM249939 vs 349 reference patients
best-fitted quadratic function
top 15 similar reference patient
most similar reference patient
Euclidean distance between expression vectors
40
30
20
10
1.0
1.2
1.4
1.6
1.8
2.0
Average weighted risk (AWR) of reference patient
Risk: blue-low; green-intermediate; red-high

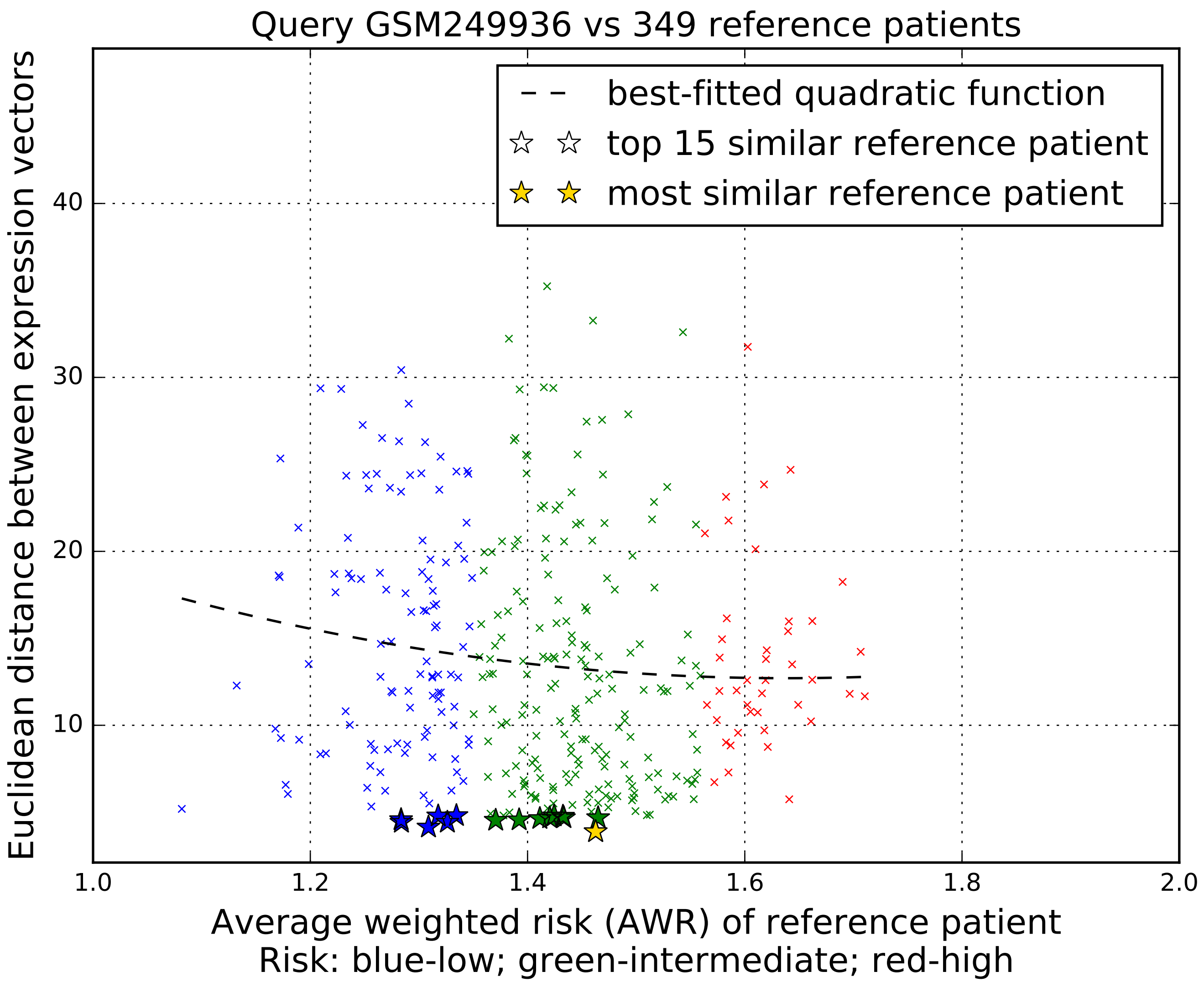
Query GSM249936 vs 349 reference patients
best-fitted quadratic function
top 15 similar reference patient
most similar reference patient
Euclidean distance between expression vectors
40
30
20
10
1.0
1.2
1.4
1.6
1.8
2.0
Average weighted risk (AWR) of reference patient
Risk: blue-low; green-intermediate; red-high

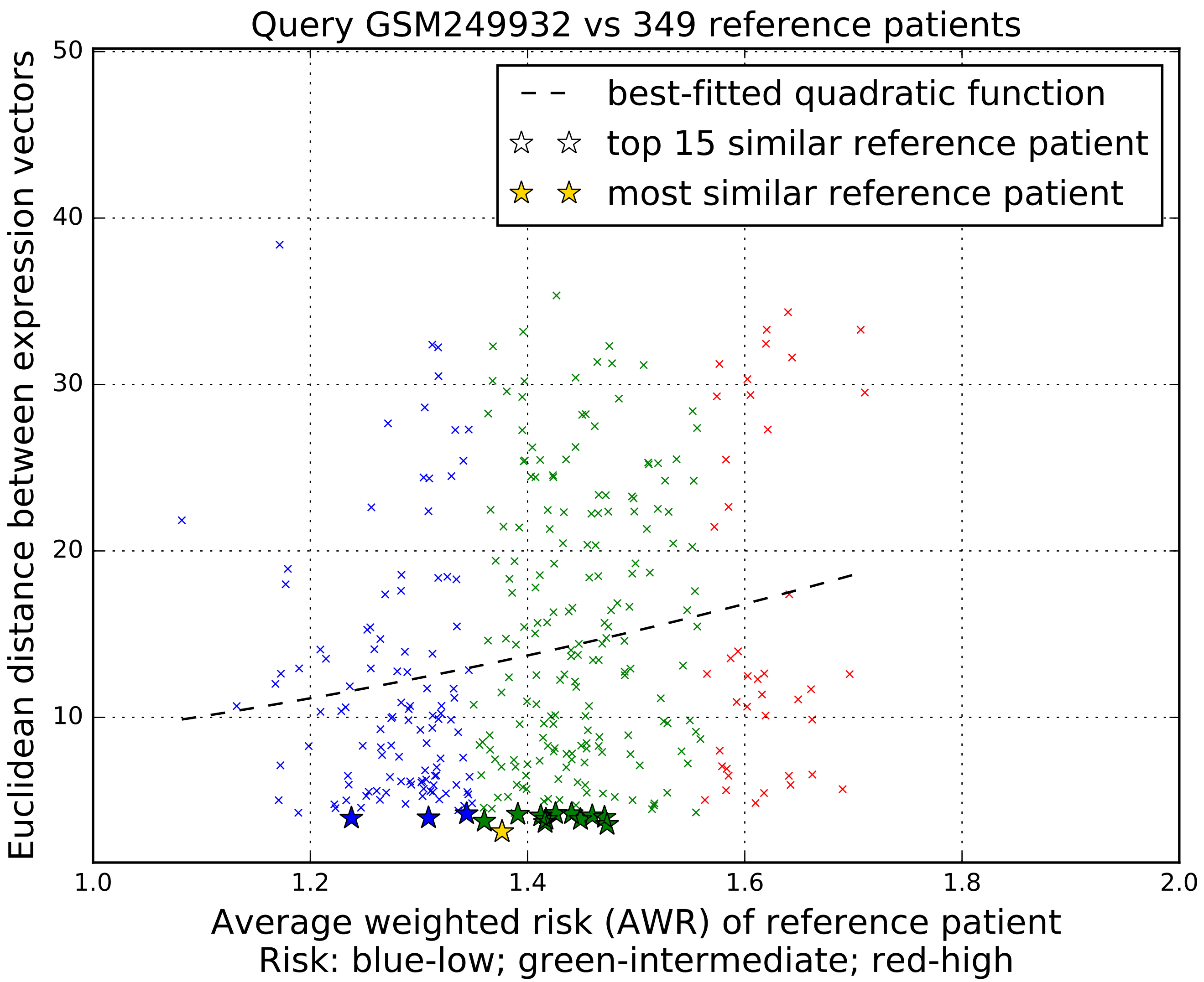
Query GSM249932 vs 349 reference patients
best-fitted quadratic function
top 15 similar reference patient
most similar reference patient
Euclidean distance between expression vectors
Average weighted risk (AWR) of reference patient
Risk: blue-low; green-intermediate; red-high
10
20
30
40
50
1.0
1.2
1.4
1.6
1.8
2.0

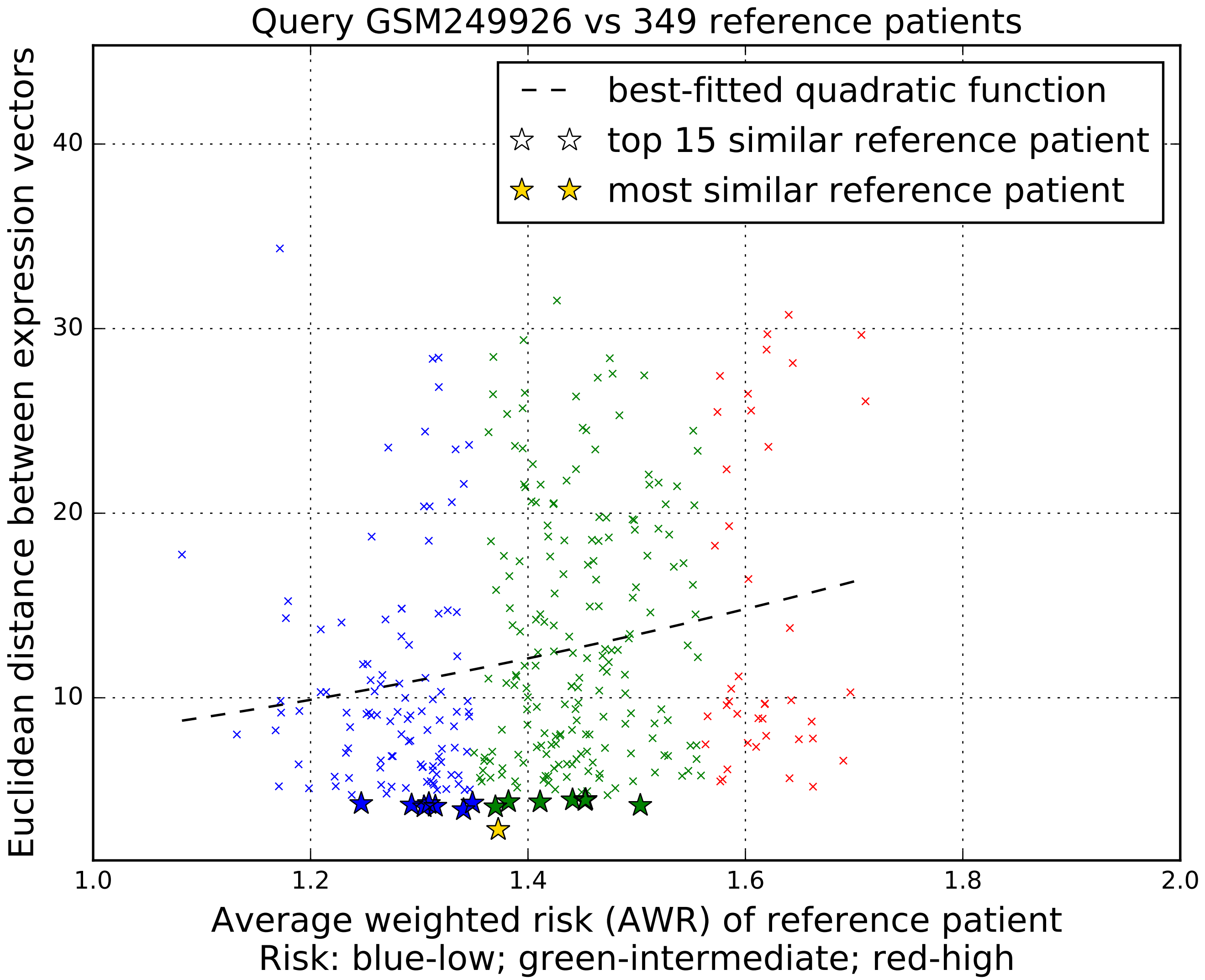

Query GSM249926 vs 349 reference patients
best-fitted quadratic function
top 15 similar reference patient
most similar reference patient
Euclidean distance between expression vectors
10
20
30
40
1.0
1.2
1.4
1.6
1.8
2.0
Average weighted risk (AWR) of reference patient
Risk: blue-low; green-intermediate; red-high

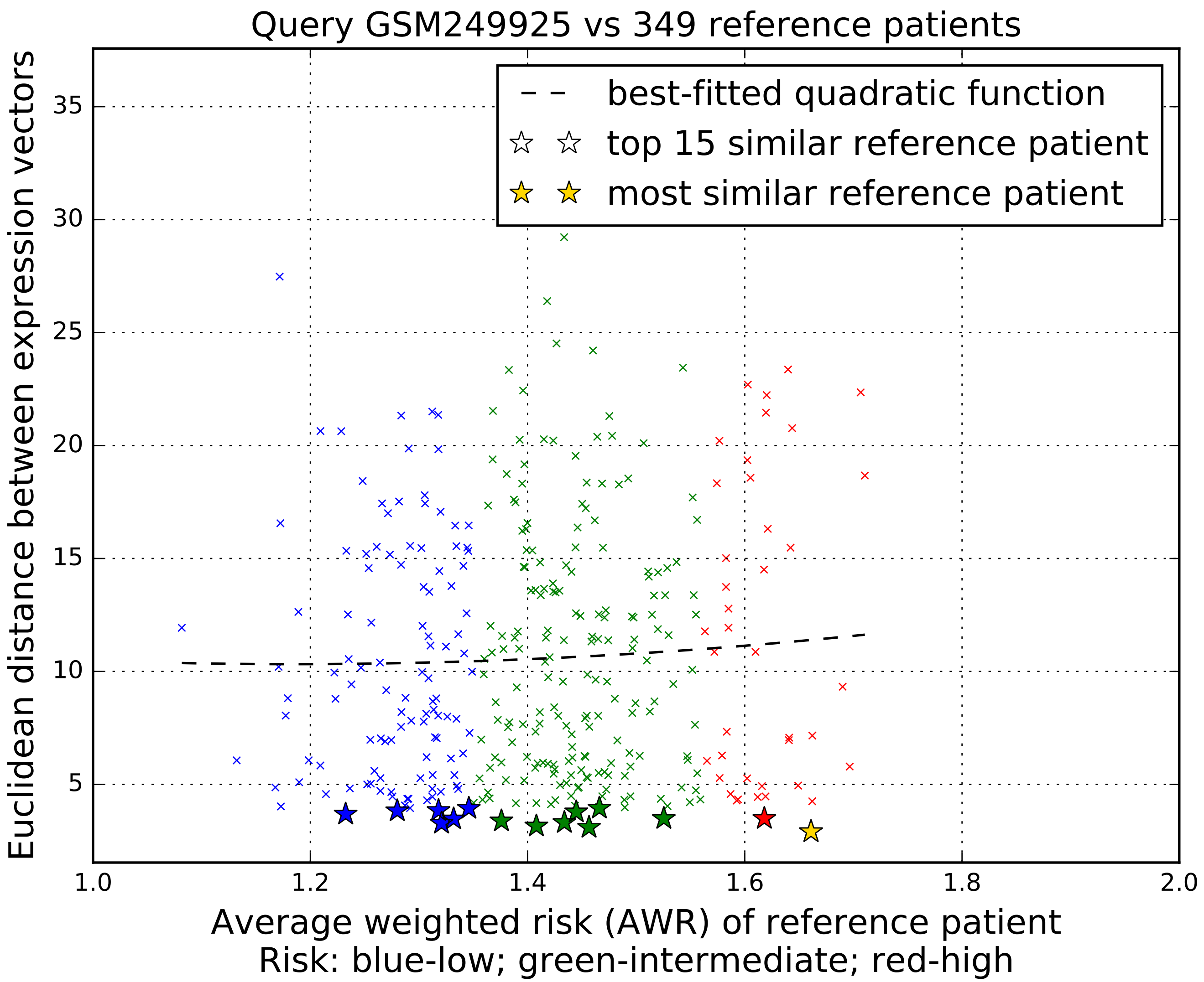
Query GSM249925 vs 349 reference patients
best-fitted quadratic function
top 15 similar reference patient
most similar reference patient
Euclidean distance between expression vectors
35
30
25
20
15
10
5
1.0
1.2
1.4
1.6
1.8
2.0
Average weighted risk (AWR) of reference patient
Risk: blue-low; green-intermediate; red-high

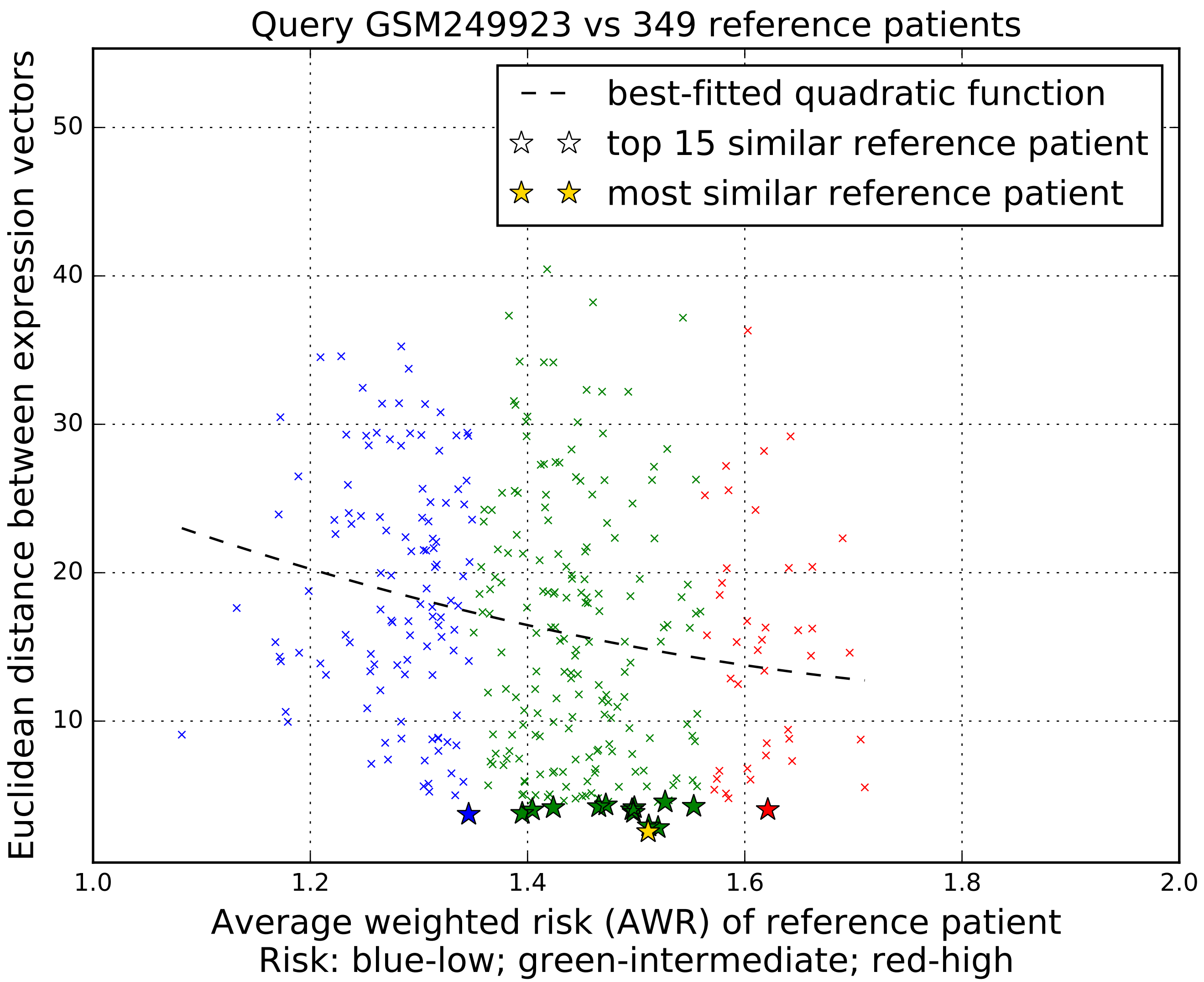
Query GSM249923 vs 349 reference patients
best-fitted quadratic function
top 15 similar reference patient
most similar reference patient
Euclidean distance between expression vectors
50
40
30
20
10
1.0
1.2
1.4
1.6
1.8
2.0
Average weighted risk (AWR) of reference patient
Risk: blue-low; green-intermediate; red-high

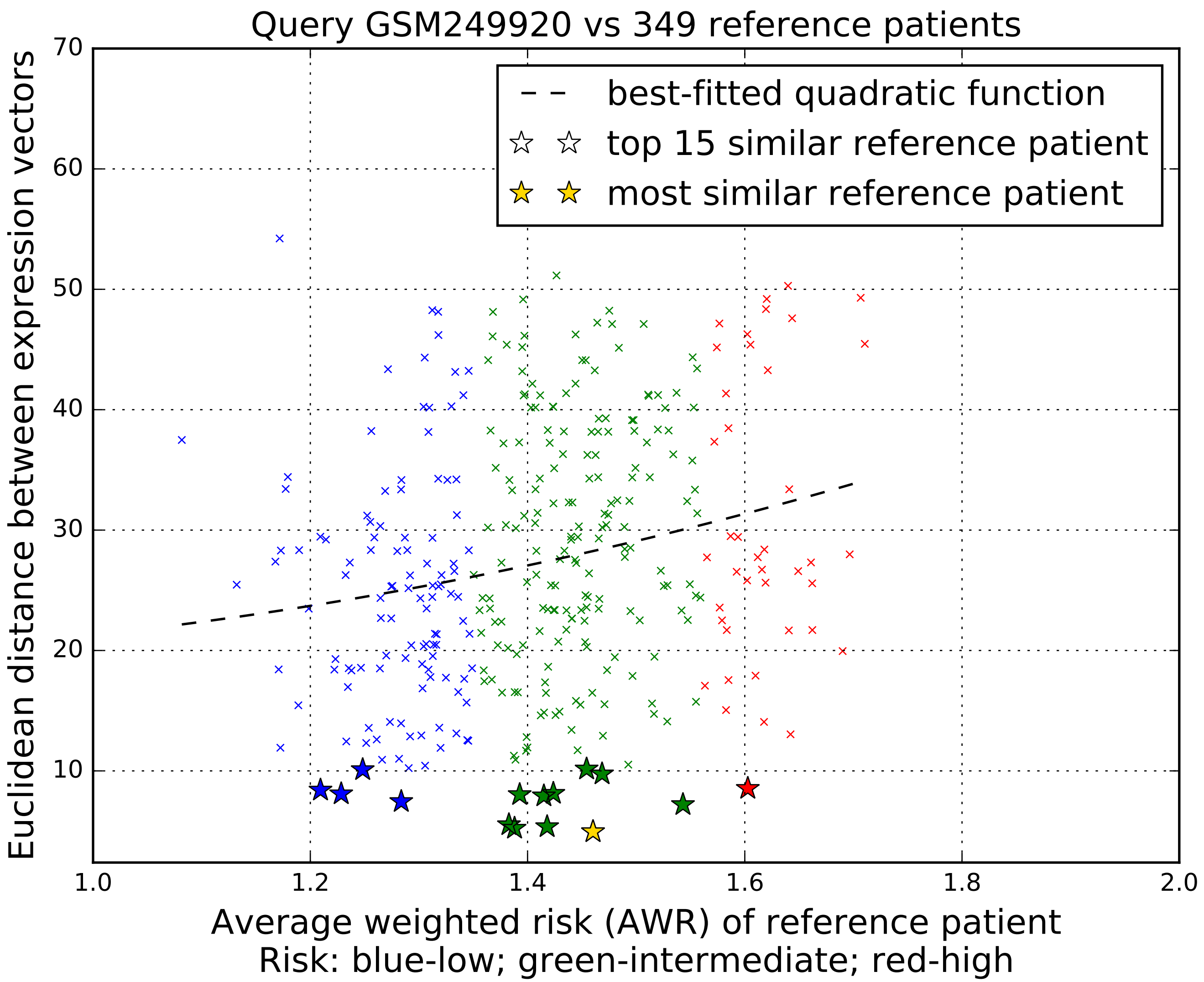

Query GSM249920 vs 349 reference patients
best-fitted quadratic function
top 15 similar reference patient
most similar reference patient
Euclidean distance between expression vectors
Average weighted risk (AWR) of reference patient
Risk: blue-low; green-intermediate; red-high
70
60
50
40
30
20
10
1.0
1.2
1.4
1.6
1.8
2.0

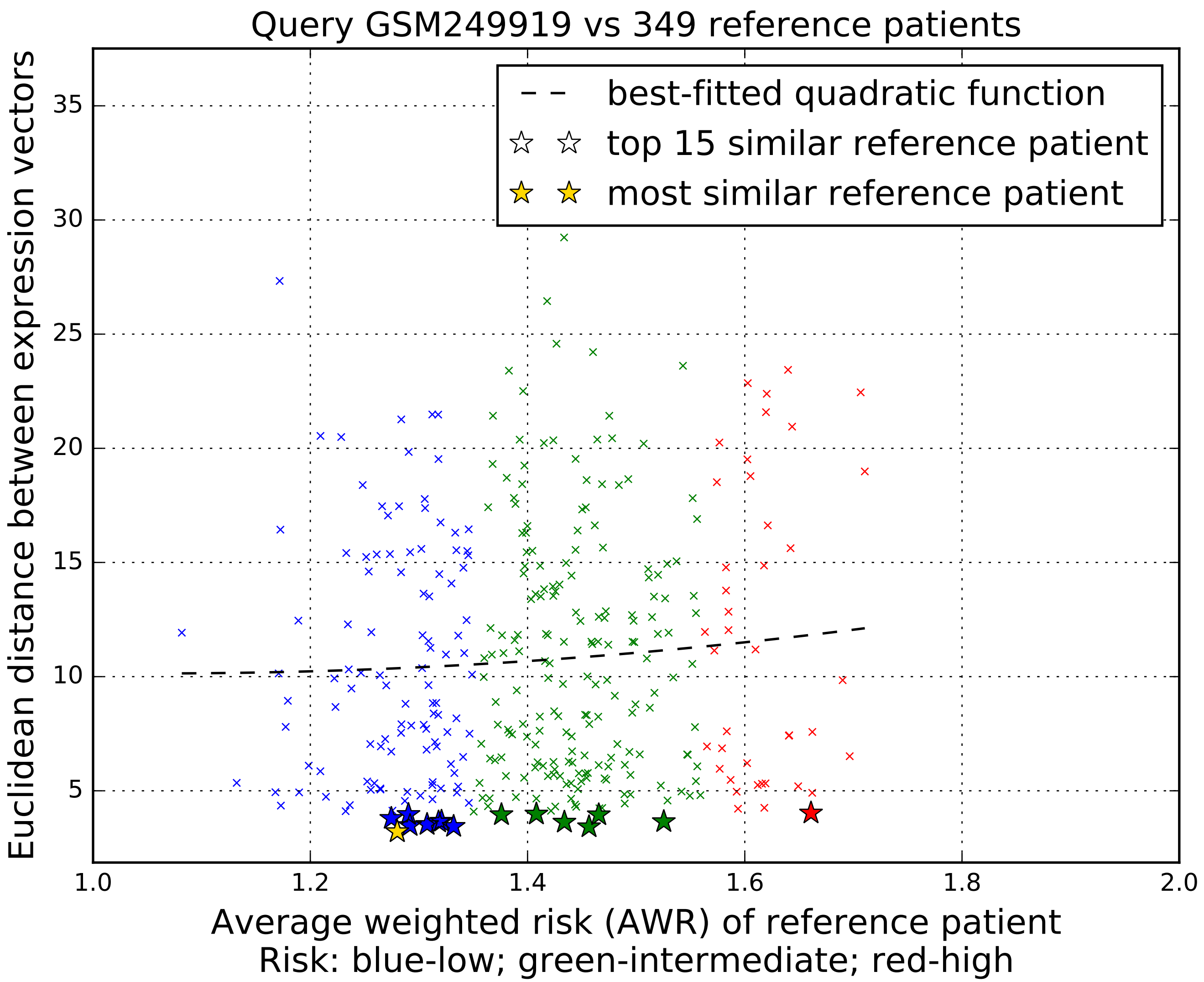

Query GSM249919 vs 349 reference patients
best-fitted quadratic function
top 15 similar reference patient
most similar reference patient
Euclidean distance between expression vectors
5
10
15
20
25
30
35
1.0
1.2
1.4
1.6
1.8
2.0
Average weighted risk (AWR) of reference patient
Risk: blue-low; green-intermediate; red-high

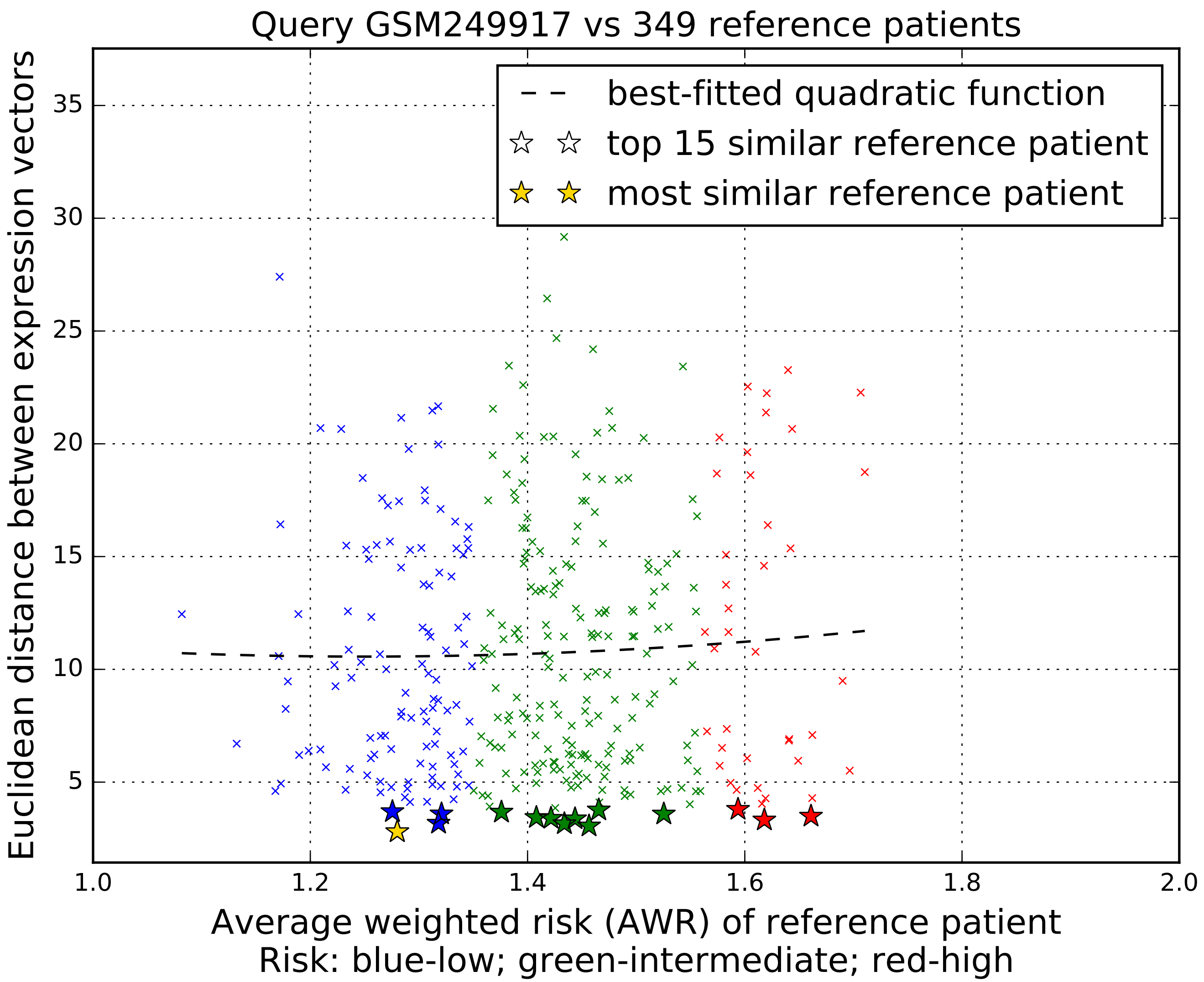

Query GSM249917 vs 349 reference patients
best-fitted quadratic function
top 15 similar reference patient
most similar reference patient
Euclidean distance between expression vectors
35
30
25
20
15
10
5
1.0
1.2
1.4
1.6
1.8
2.0
Average weighted risk (AWR) of reference patient
Risk: blue-low; green-intermediate; red-high

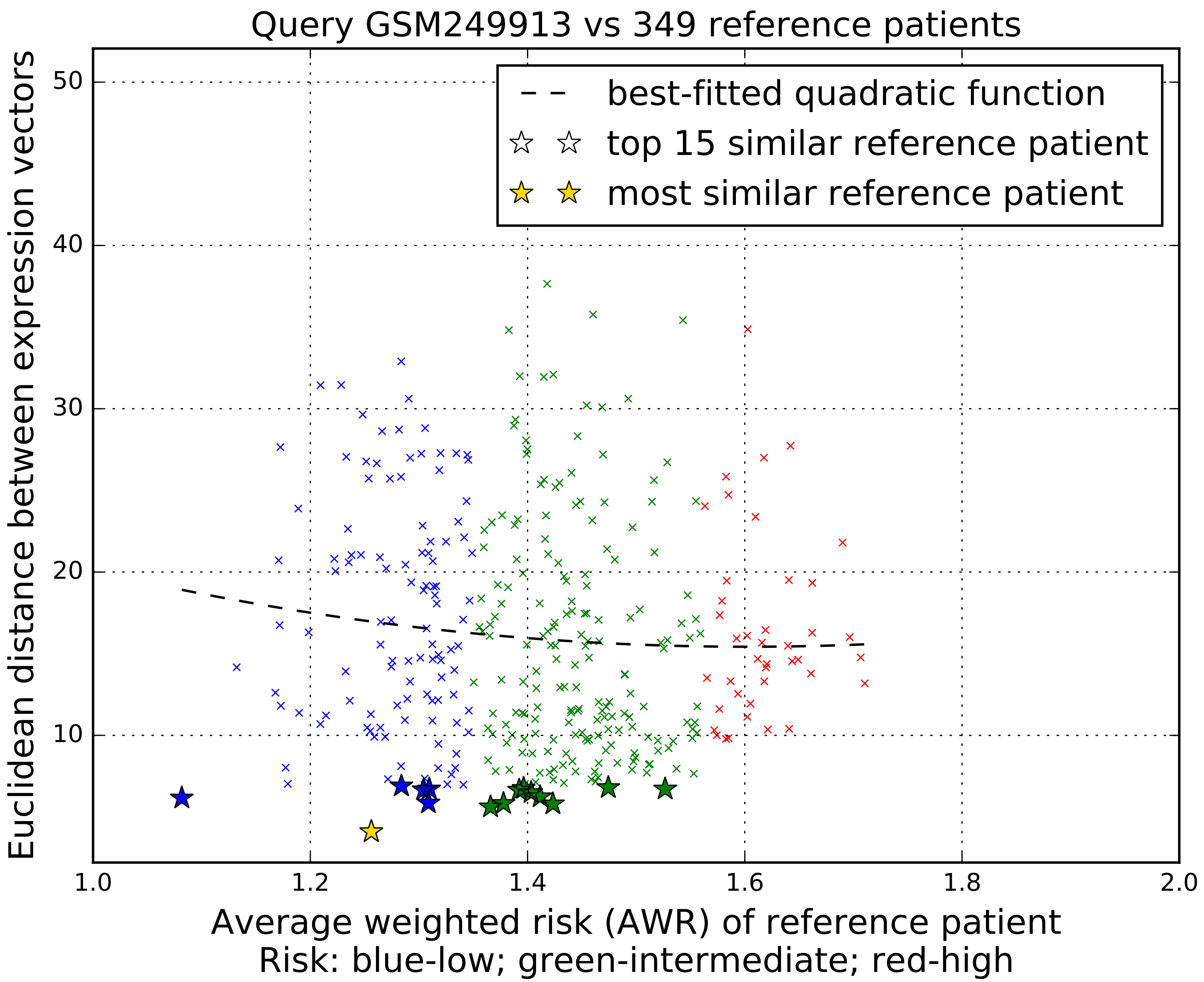

Query GSM249913 vs 349 reference patients
best-fitted quadratic function
top 15 similar reference patient
most similar reference patient
Euclidean distance between expression vectors
Average weighted risk (AWR) of reference patient
Risk: blue-low; green-intermediate; red-high
10
20
30
40
50
1.0
1.2
1.4
1.6
1.8
2.0

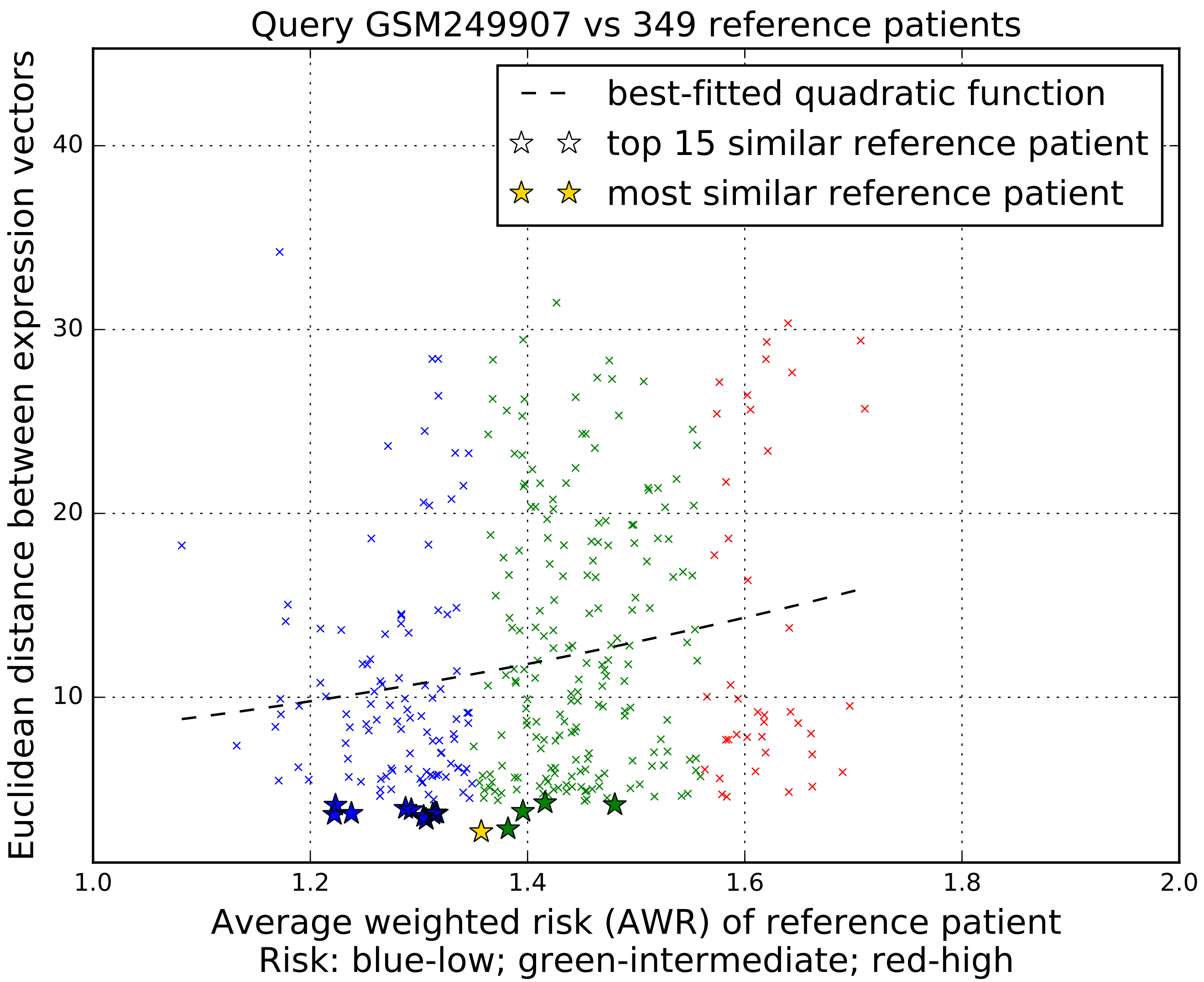
Query GSM249907 vs 349 reference patients
best-fitted quadratic function
top 15 similar reference patient
most similar reference patient
Euclidean distance between expression vectors
10
20
30
40
1.0
1.2
1.4
1.6
1.8
2.0
Average weighted risk (AWR) of reference patient
Risk: blue-low; green-intermediate; red-high

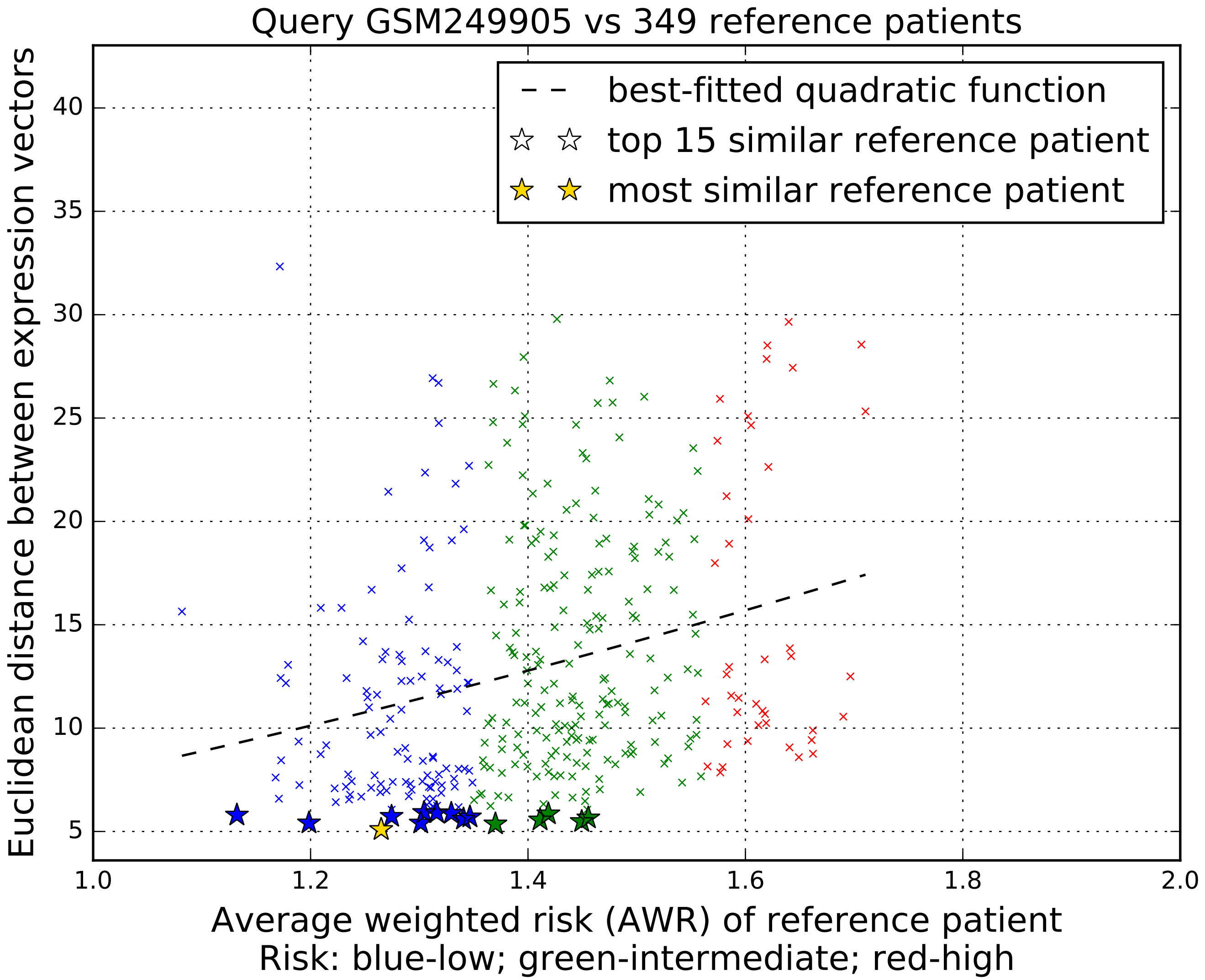

Query GSM249905 vs 349 reference patients
best-fitted quadratic function
top 15 similar reference patient
most similar reference patient
Euclidean distance between expression vectors
40
35
30
25
20
15
10
5
1.0
1.2
1.4
1.6
1.8
2.0
Average weighted risk (AWR) of reference patient
Risk: blue-low; green-intermediate; red-high

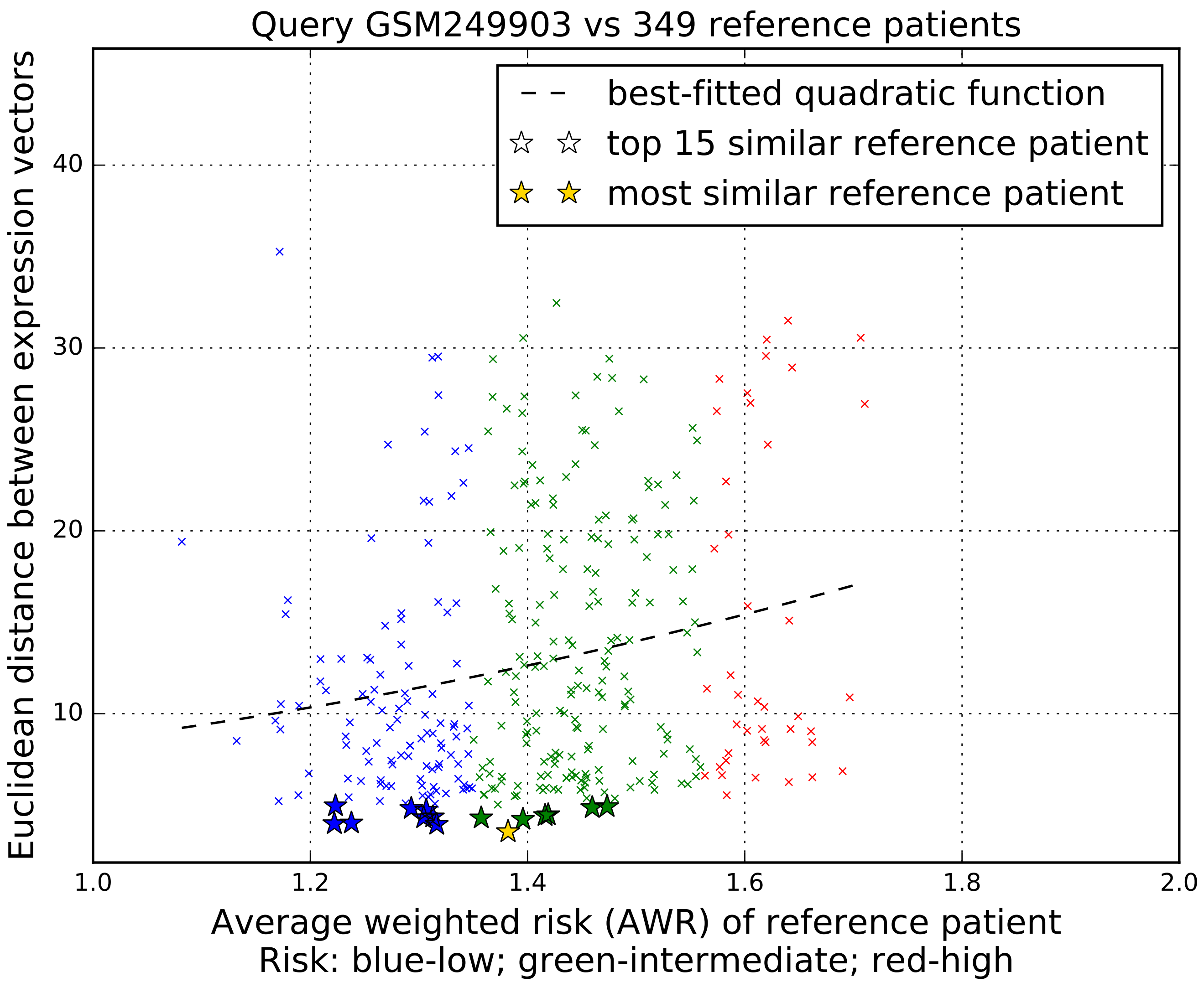

Query GSM249903 vs 349 reference patients
best-fitted quadratic function
top 15 similar reference patient
most similar reference patient
Euclidean distance between expression vectors
10
20
30
40
1.0
1.2
1.4
1.6
1.8
2.0
Average weighted risk (AWR) of reference patient
Risk: blue-low; green-intermediate; red-high

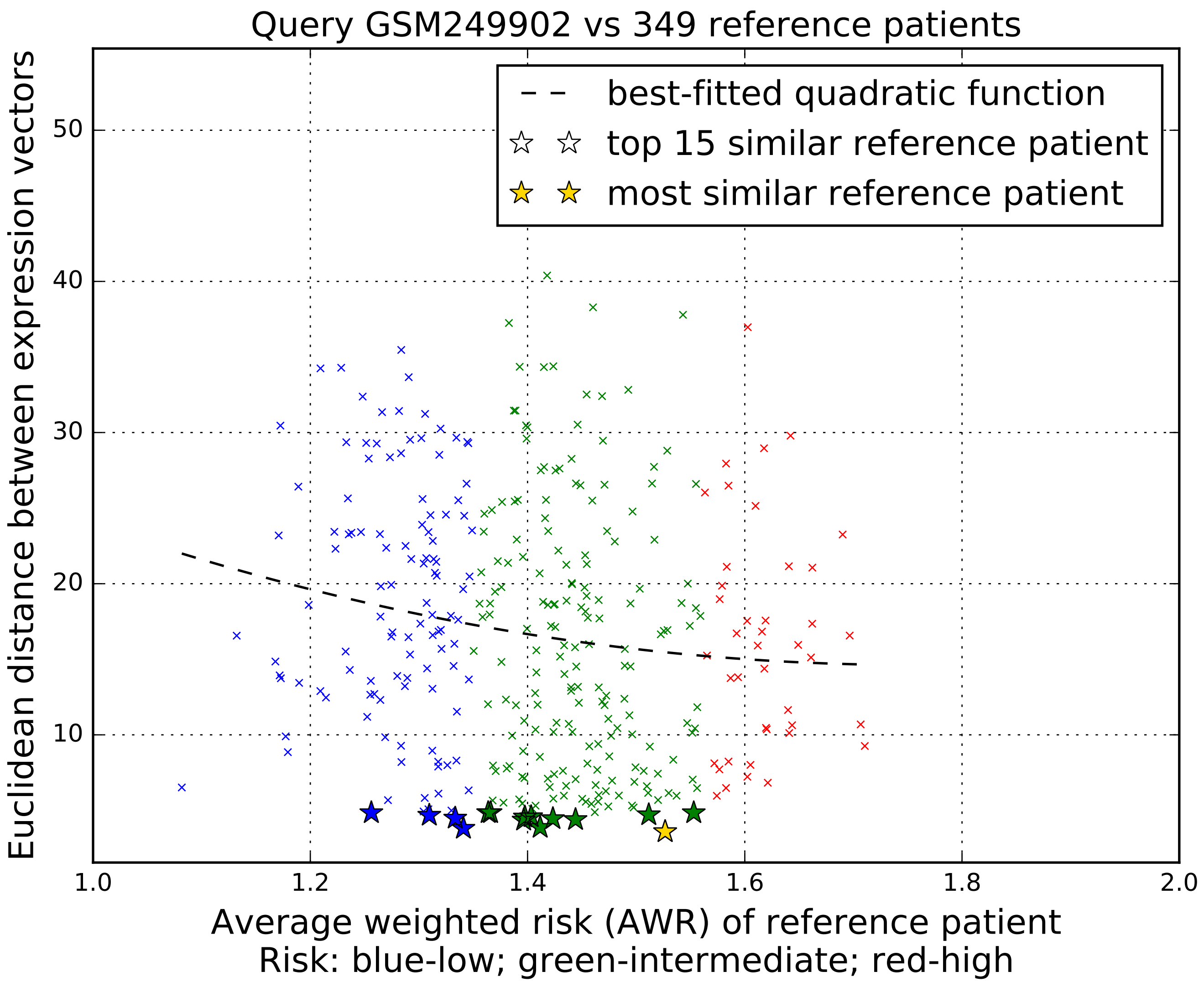
Query GSM249902 vs 349 reference patients
best-fitted quadratic function
top 15 similar reference patient
most similar reference patient
Euclidean distance between expression vectors
10
20
30
40
50
1.0
1.2
1.4
1.6
1.8
2.0
Average weighted risk (AWR) of reference patient
Risk: blue-low; green-intermediate; red-high

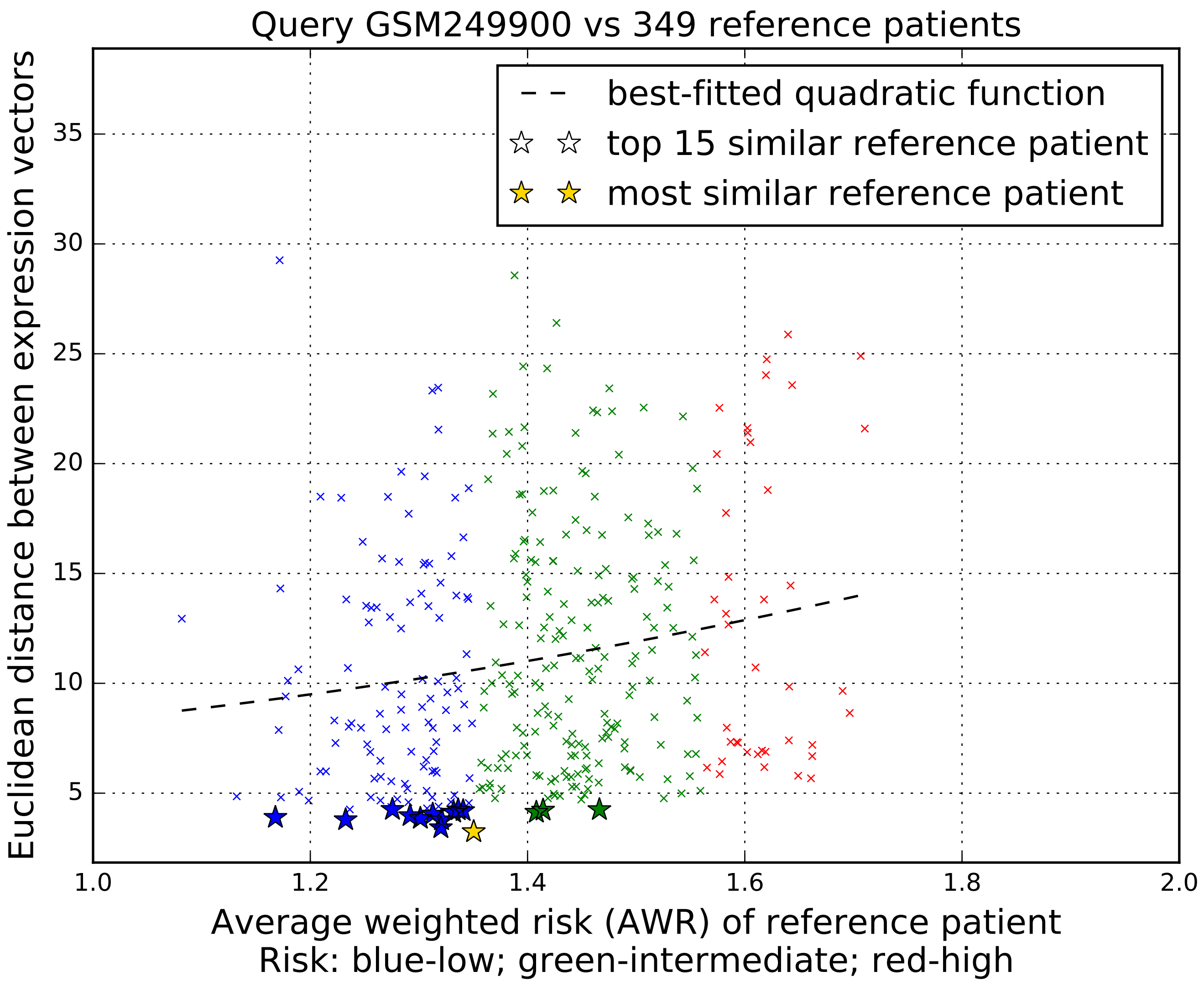
Query GSM249900 vs 349 reference patients
best-fitted quadratic function
top 15 similar reference patient
most similar reference patient
Euclidean distance between expression vectors
Average weighted risk (AWR) of reference patient
Risk: blue-low; green-intermediate; red-high
35
30
25
20
15
10
5
1.0
1.2
1.4
1.6
1.8
2.0

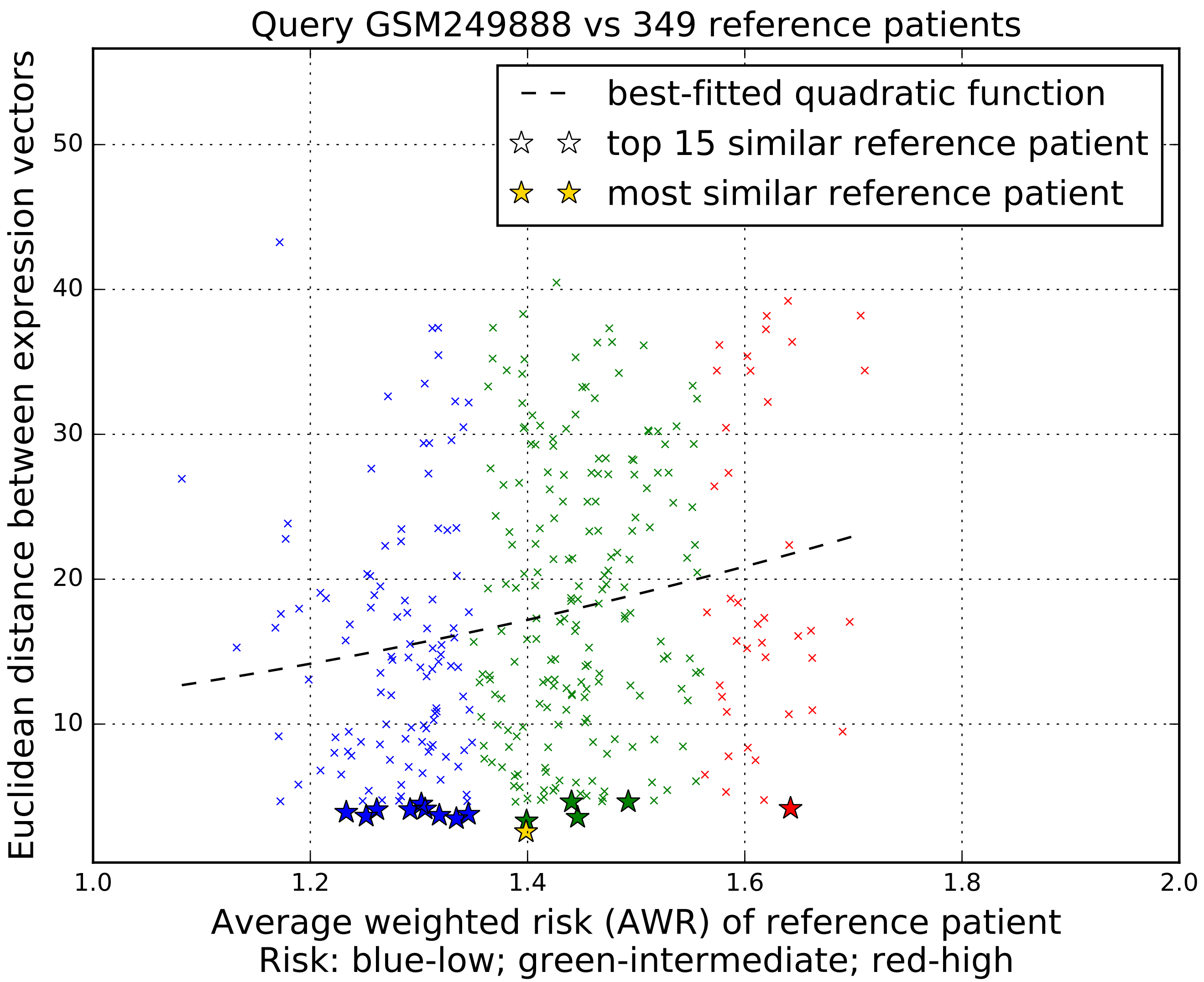
Query GSM249888 vs 349 reference patients
best-fitted quadratic function
top 15 similar reference patient
most similar reference patient
Euclidean distance between expression vectors
Average weighted risk (AWR) of reference patient
Risk: blue-low; green-intermediate; red-high
10
20
30
40
50
1.0
1.2
1.4
1.6
1.8
2.0

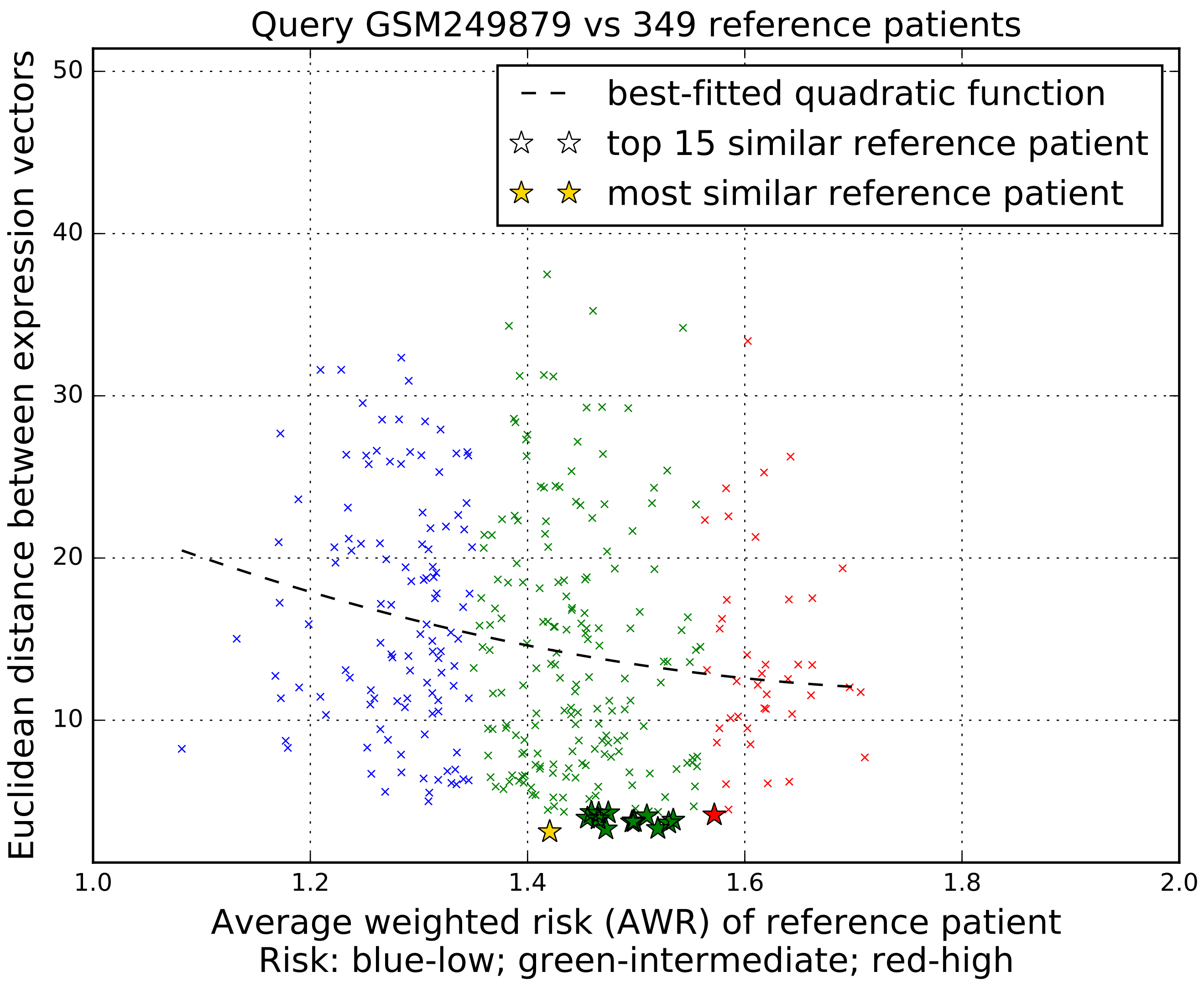

Query GSM249879 vs 349 reference patients
best-fitted quadratic function
top 15 similar reference patient
most similar reference patient
Euclidean distance between expression vectors
50
40
30
20
10
1.0
1.2
1.4
1.6
1.8
2.0
Average weighted risk (AWR) of reference patient
Risk: blue-low; green-intermediate; red-high

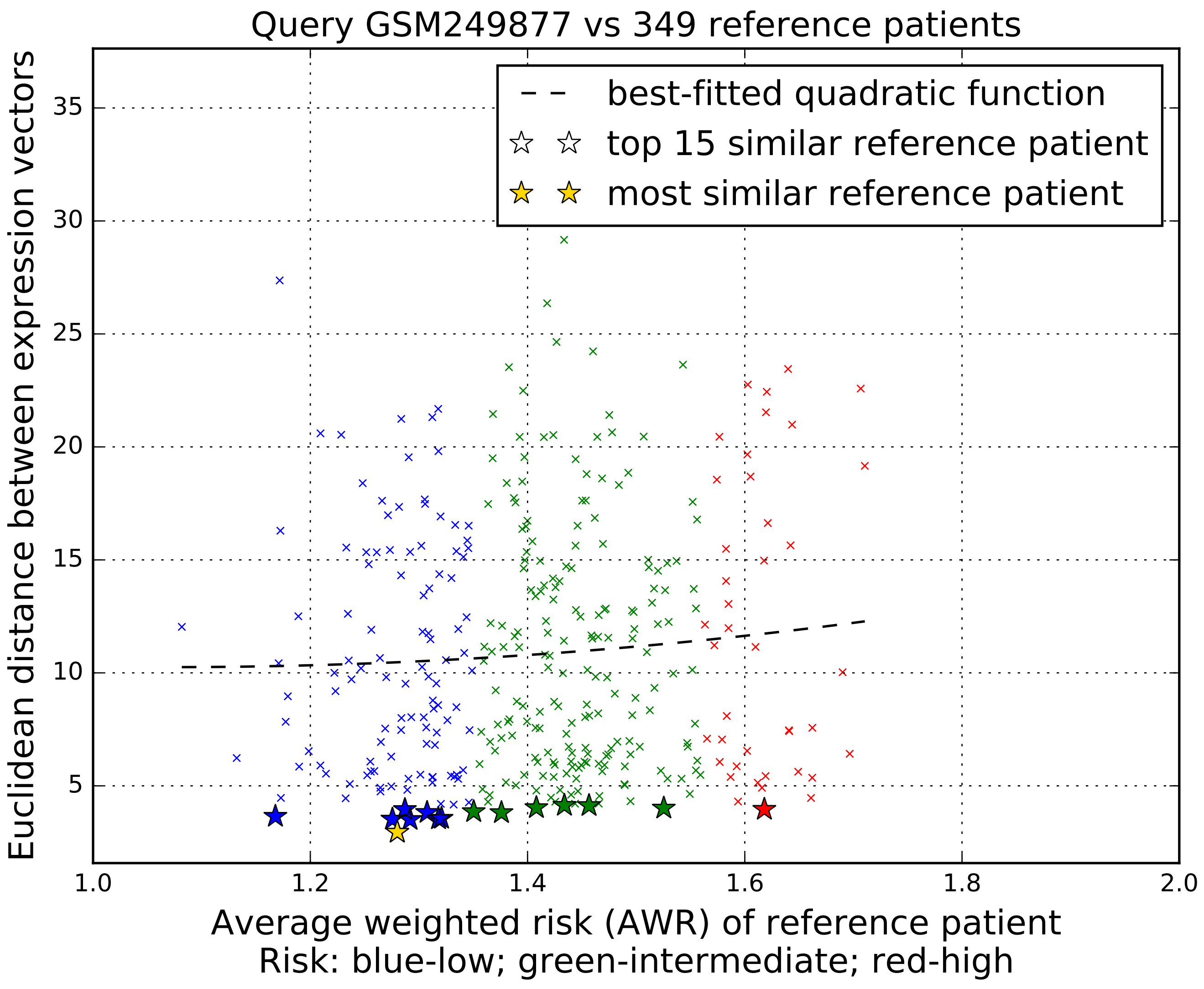
Query GSM249877 vs 349 reference patients
best-fitted quadratic function
top 15 similar reference patient
most similar reference patient
Euclidean distance between expression vectors
Average weighted risk (AWR) of reference patient
Risk: blue-low; green-intermediate; red-high
35
30
25
20
15
10
5
1.0
1.2
1.4
1.6
1.8
2.0

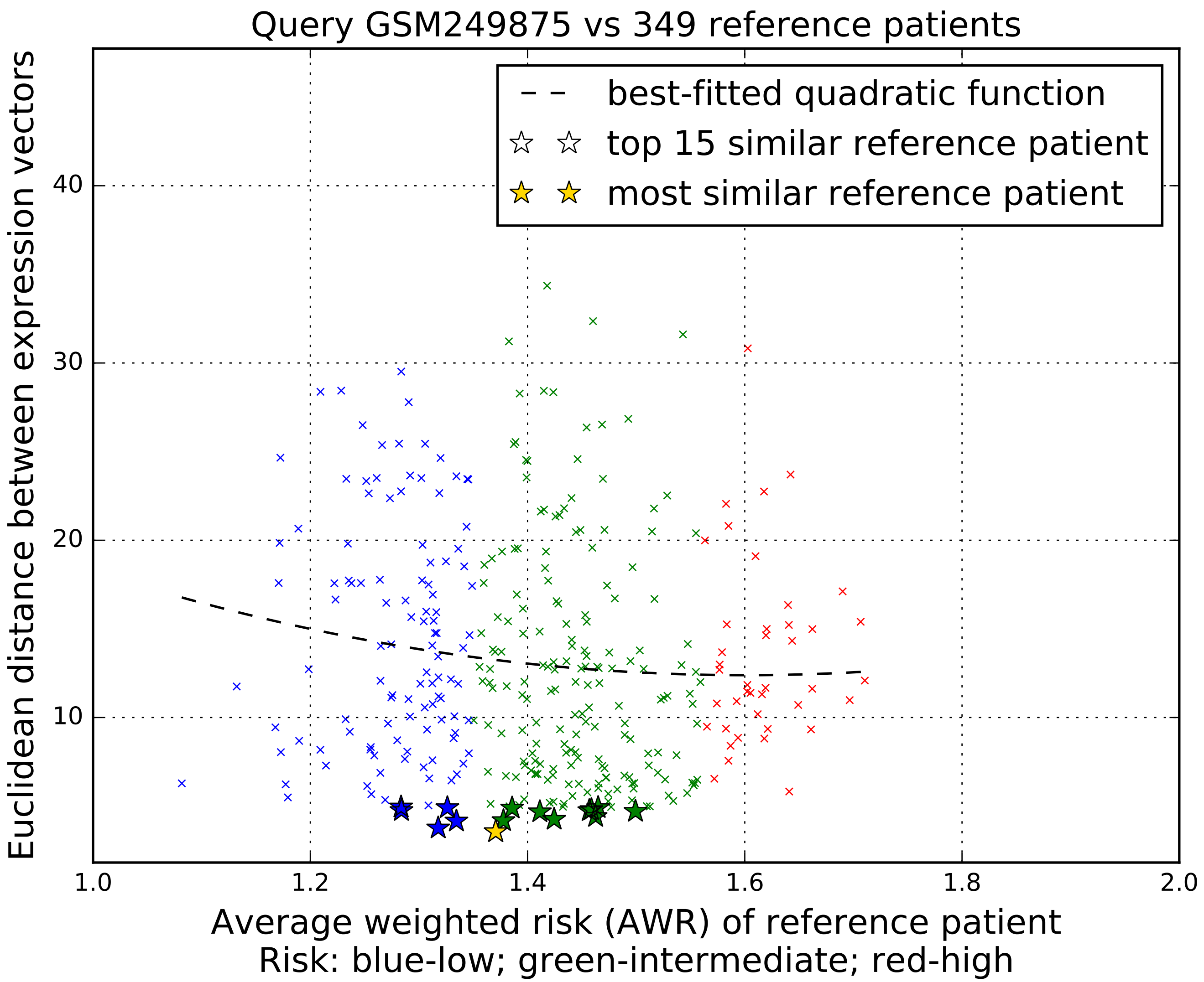

Query GSM249875 vs 349 reference patients
best-fitted quadratic function
top 15 similar reference patient
most similar reference patient
Euclidean distance between expression vectors
10
20
30
40
1.0
1.2
1.4
1.6
1.8
2.0
Average weighted risk (AWR) of reference patient
Risk: blue-low; green-intermediate; red-high

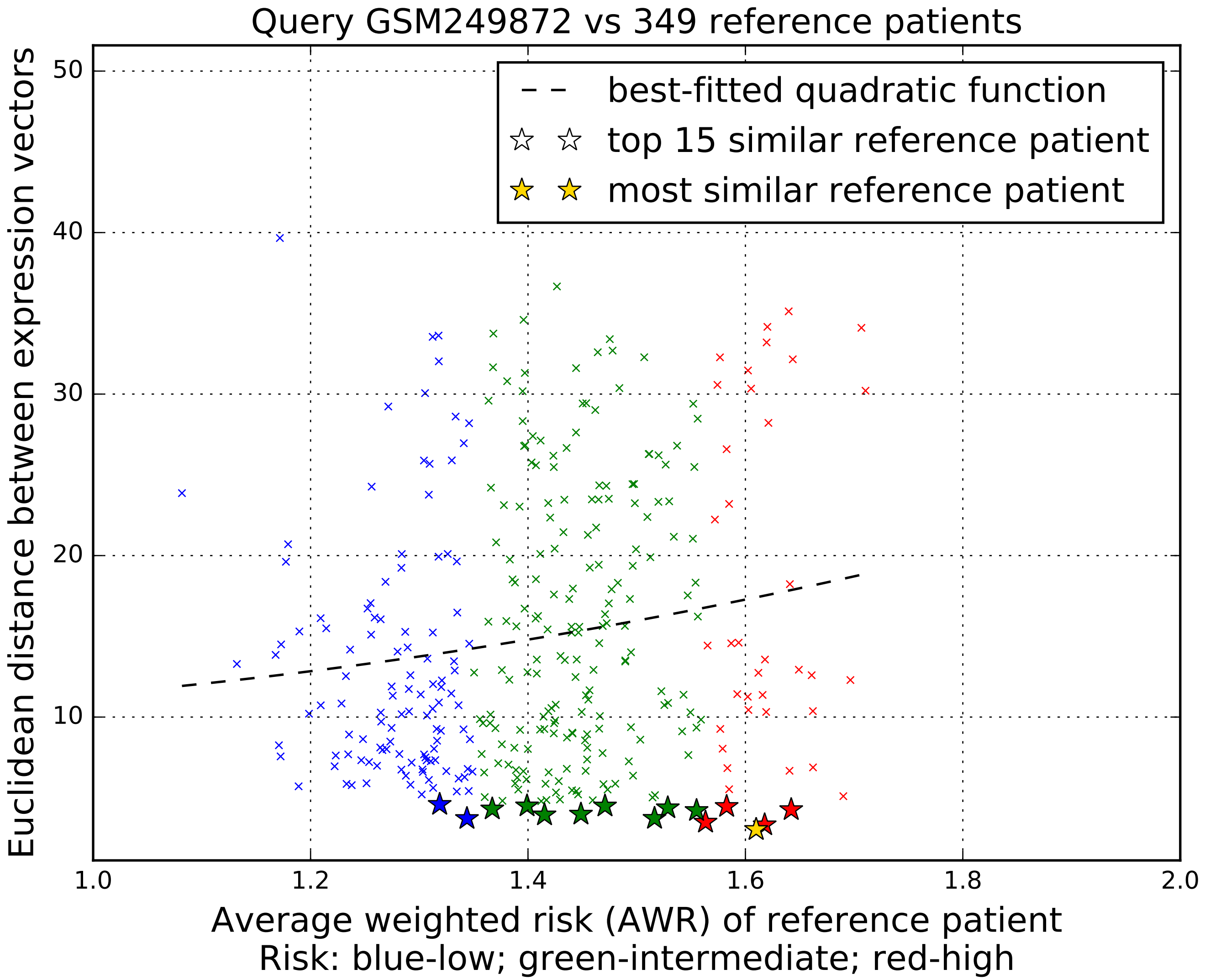
Query GSM249872 vs 349 reference patients
best-fitted quadratic function
top 15 similar reference patient
most similar reference patient
Euclidean distance between expression vectors
50
40
30
20
10
1.0
1.2
1.4
1.6
1.8
2.0
Average weighted risk (AWR) of reference patient
Risk: blue-low; green-intermediate; red-high

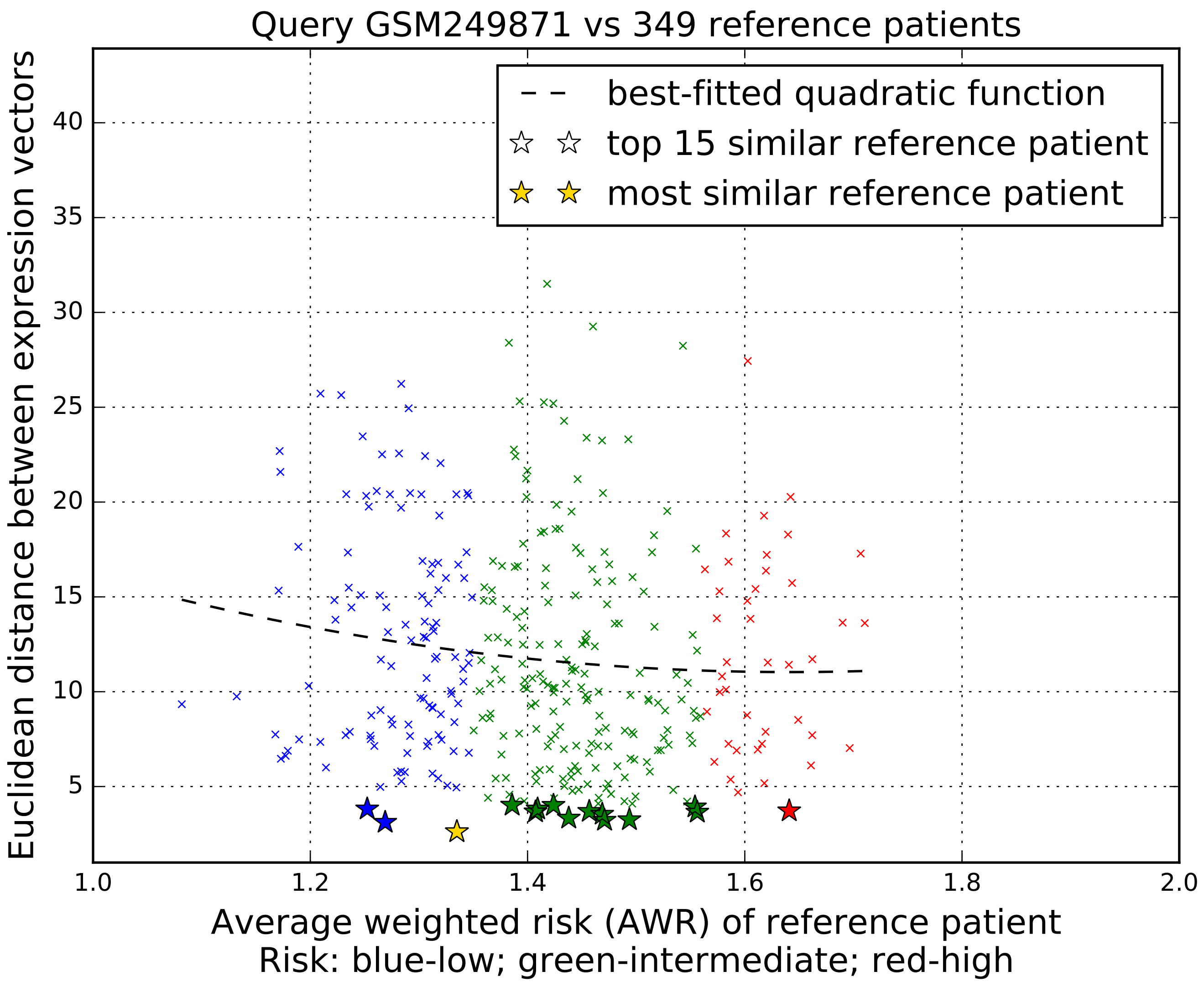
Query GSM249871 vs 349 reference patients
best-fitted quadratic function
top 15 similar reference patient
most similar reference patient
Euclidean distance between expression vectors
40
35
30
25
20
15
10
5
1.0
1.2
1.4
1.6
1.8
2.0
Average weighted risk (AWR) of reference patient
Risk: blue-low; green-intermediate; red-high

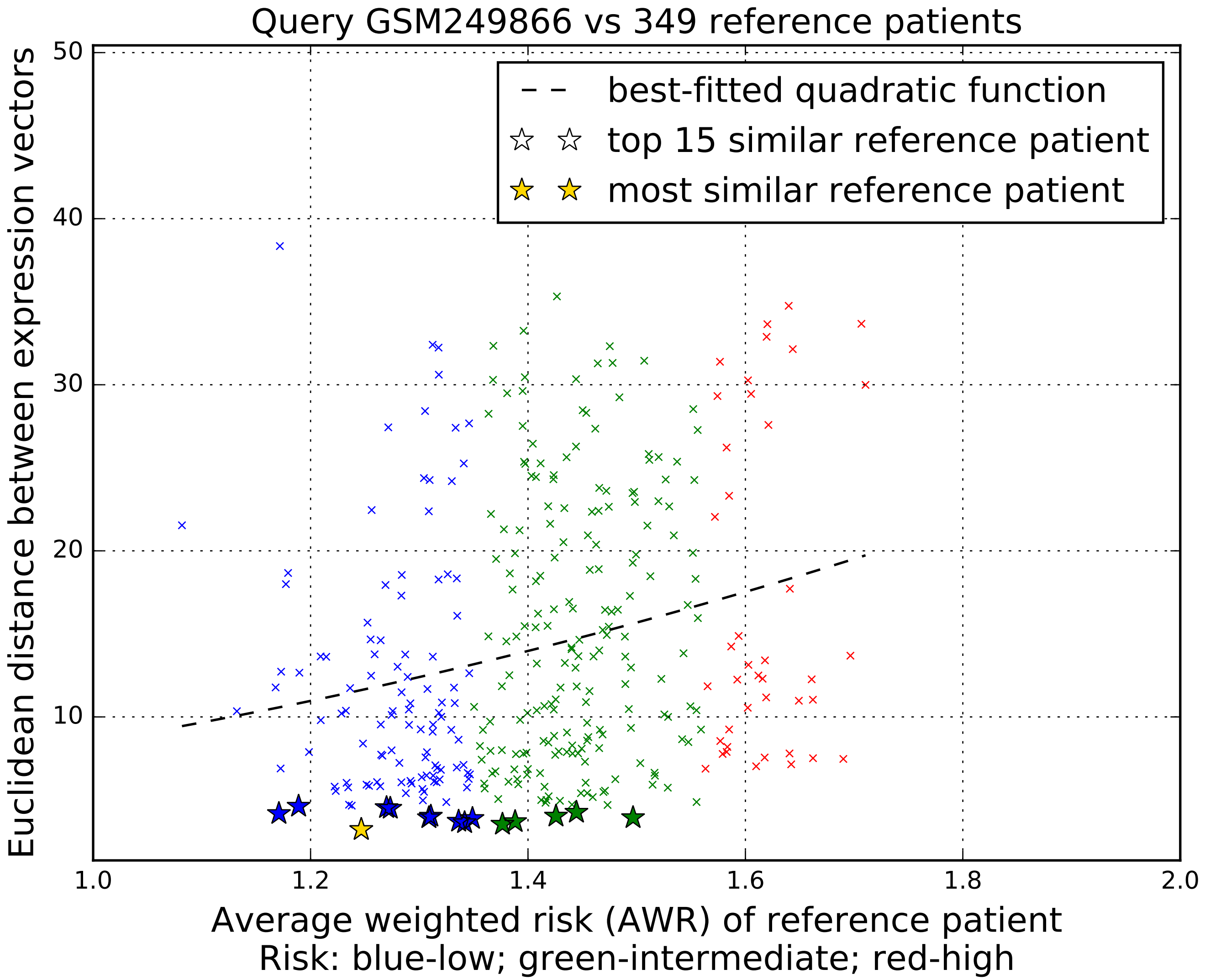
Query GSM249866 vs 349 reference patients
best-fitted quadratic function
top 15 similar reference patient
most similar reference patient
Euclidean distance between expression vectors
50
40
30
20
10
1.0
1.2
1.4
1.6
1.8
2.0
Average weighted risk (AWR) of reference patient
Risk: blue-low; green-intermediate; red-high

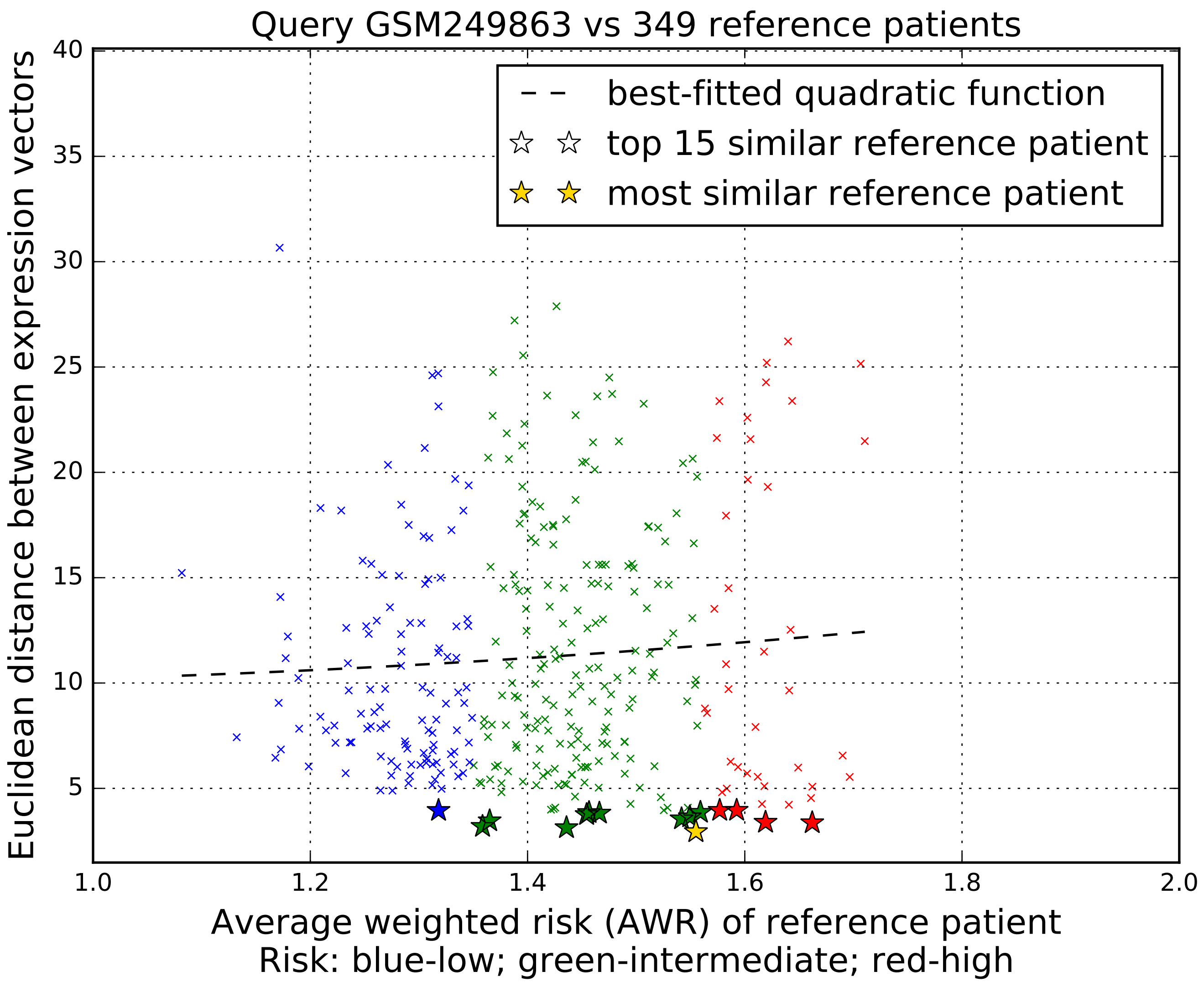

Query GSM249863 vs 349 reference patients
best-fitted quadratic function
top 15 similar reference patient
most similar reference patient
Euclidean distance between expression vectors
40
35
30
25
20
15
10
5
1.0
1.2
1.4
1.6
1.8
2.0
Average weighted risk (AWR) of reference patient
Risk: blue-low; green-intermediate; red-high

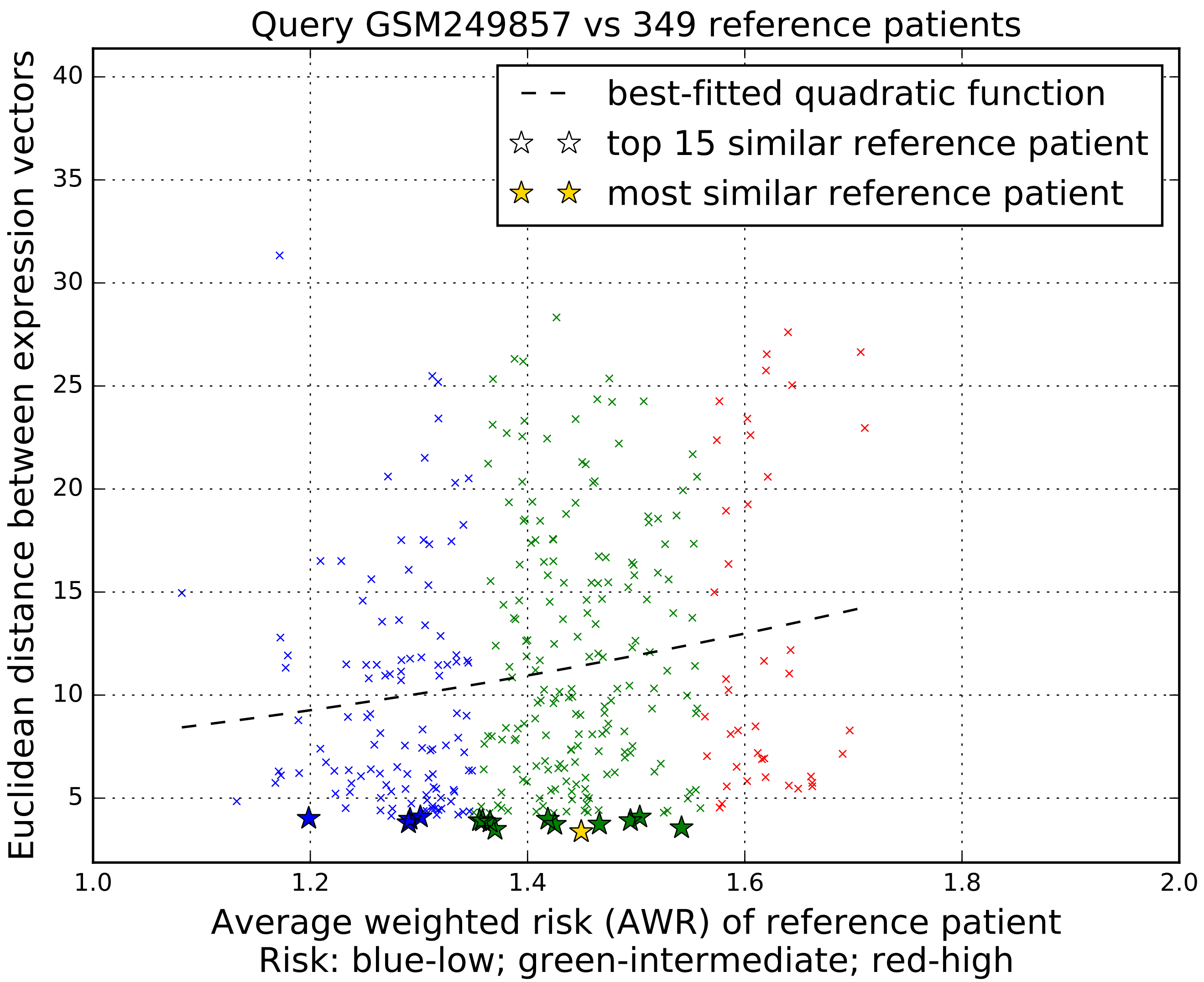

Query GSM249857 vs 349 reference patients
best-fitted quadratic function
top 15 similar reference patient
most similar reference patient
Euclidean distance between expression vectors
40
35
30
25
20
15
10
5
1.0
1.2
1.4
1.6
1.8
2.0
Average weighted risk (AWR) of reference patient
Risk: blue-low; green-intermediate; red-high

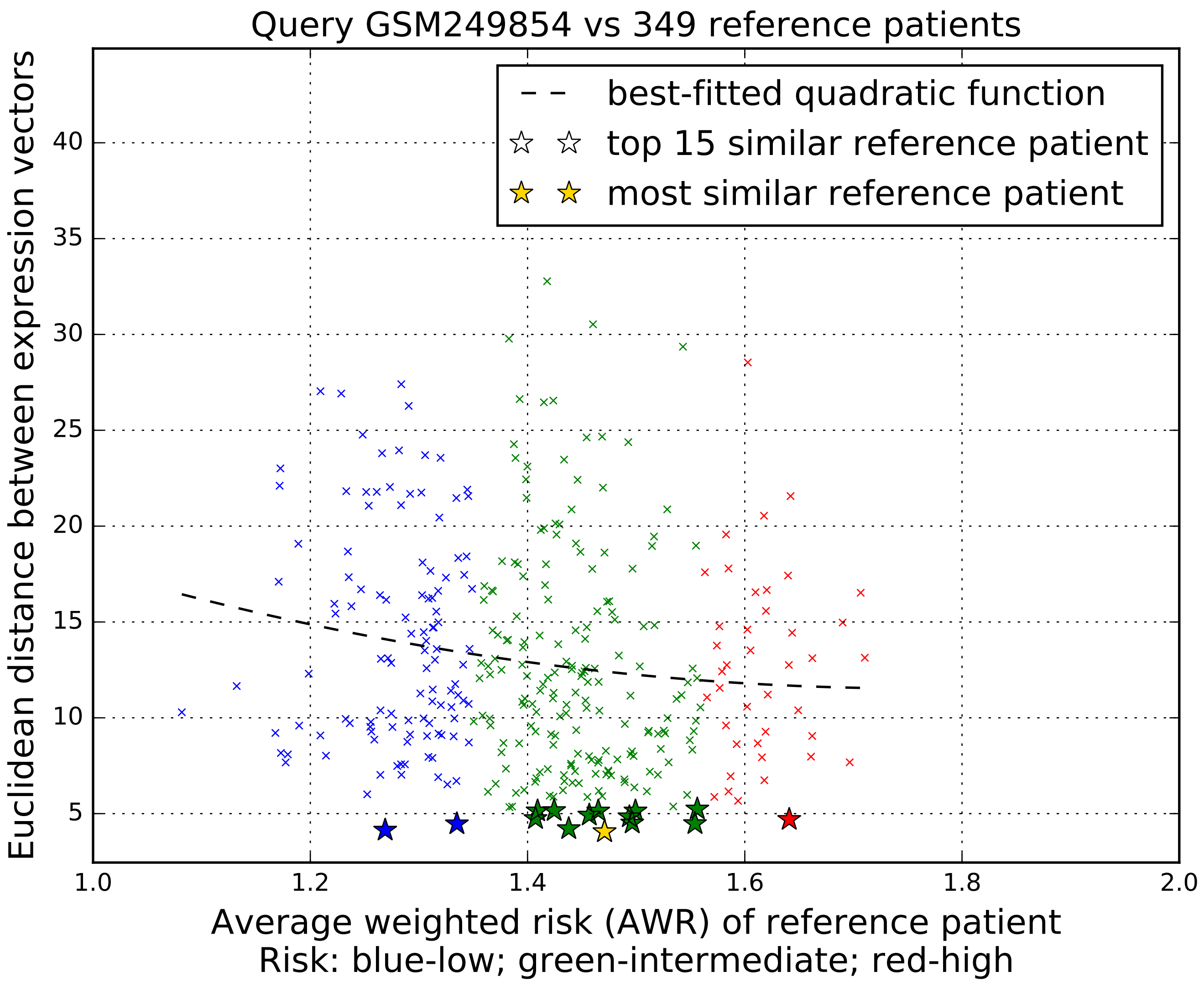

Query GSM249854 vs 349 reference patients
best-fitted quadratic function
top 15 similar reference patient
most similar reference patient
Euclidean distance between expression vectors
40
35
30
25
20
15
10
5
1.0
1.2
1.4
1.6
1.8
2.0
Average weighted risk (AWR) of reference patient
Risk: blue-low; green-intermediate; red-high

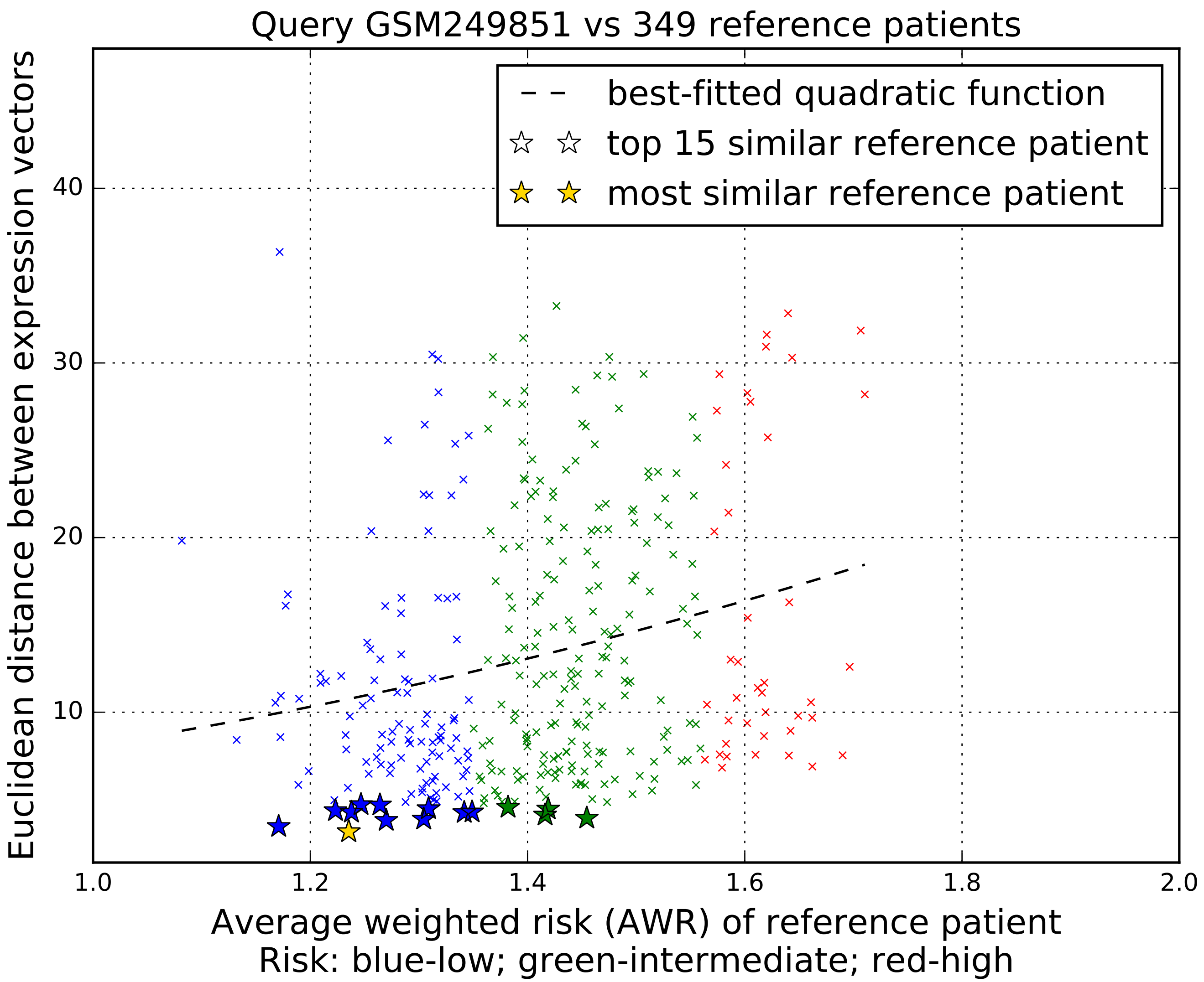
Query GSM249851 vs 349 reference patients
best-fitted quadratic function
top 15 similar reference patient
most similar reference patient
Euclidean distance between expression vectors
Average weighted risk (AWR) of reference patient
Risk: blue-low; green-intermediate; red-high
10
20
30
40
1.0
1.2
1.4
1.6
1.8
2.0

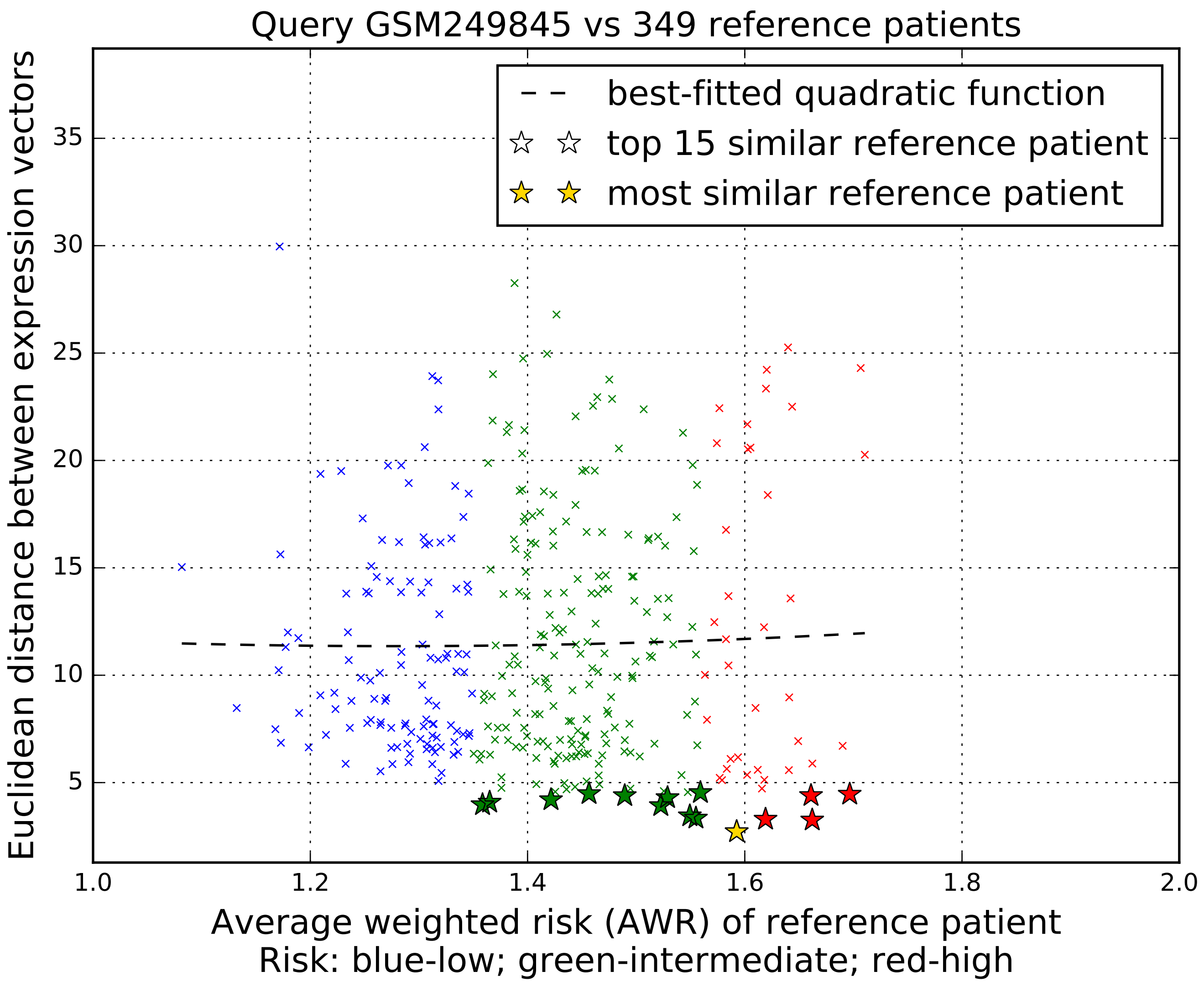

Query GSM249845 vs 349 reference patients
best-fitted quadratic function
top 15 similar reference patient
most similar reference patient
Euclidean distance between expression vectors
35
30
25
20
15
10
5
1.0
1.2
1.4
1.6
1.8
2.0
Average weighted risk (AWR) of reference patient
Risk: blue-low; green-intermediate; red-high

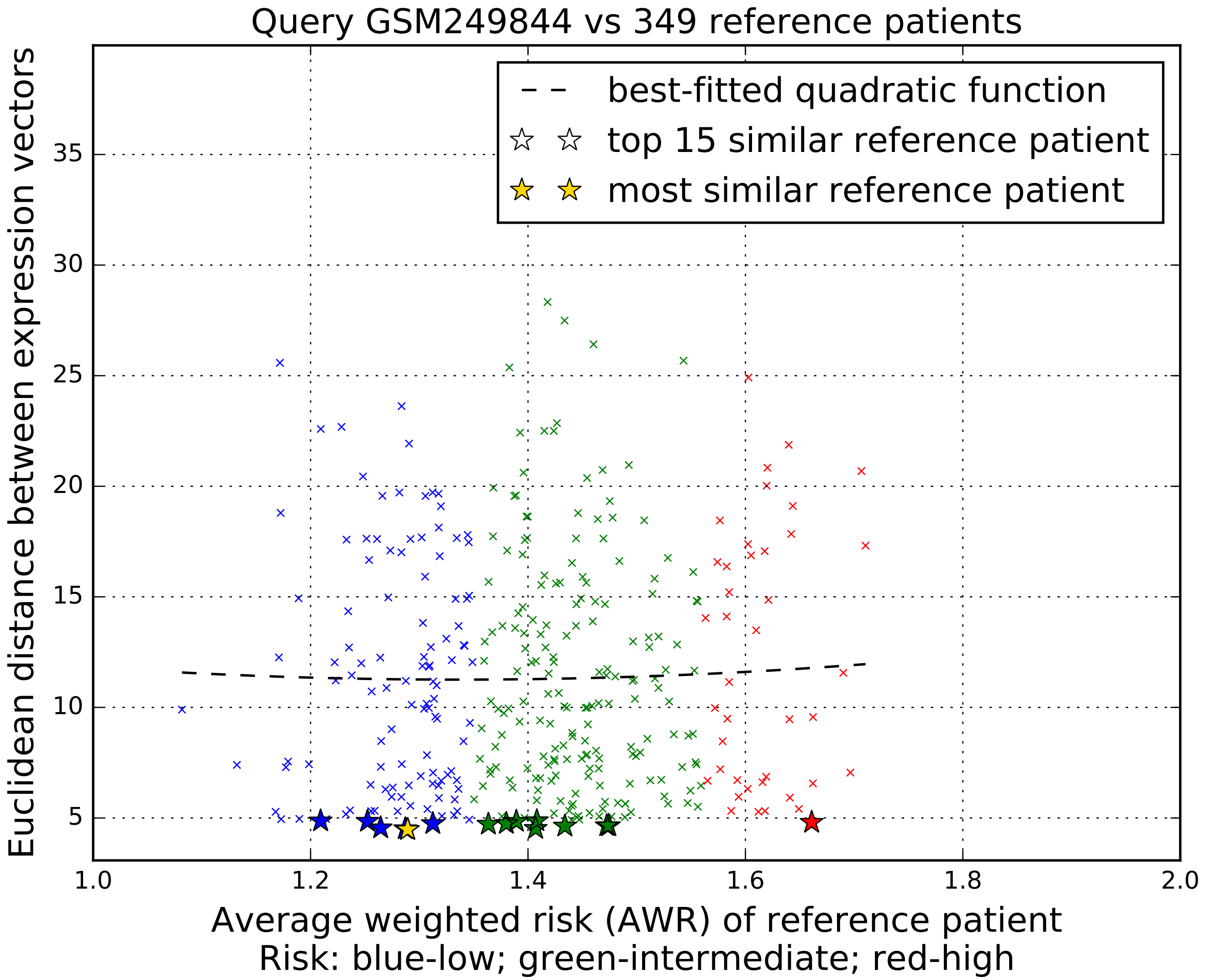

Query GSM249844 vs 349 reference patients
best-fitted quadratic function
top 15 similar reference patient
most similar reference patient
Euclidean distance between expression vectors
35
30
25
20
15
10
5
1.0
1.2
1.4
1.6
1.8
2.0
Average weighted risk (AWR) of reference patient
Risk: blue-low; green-intermediate; red-high

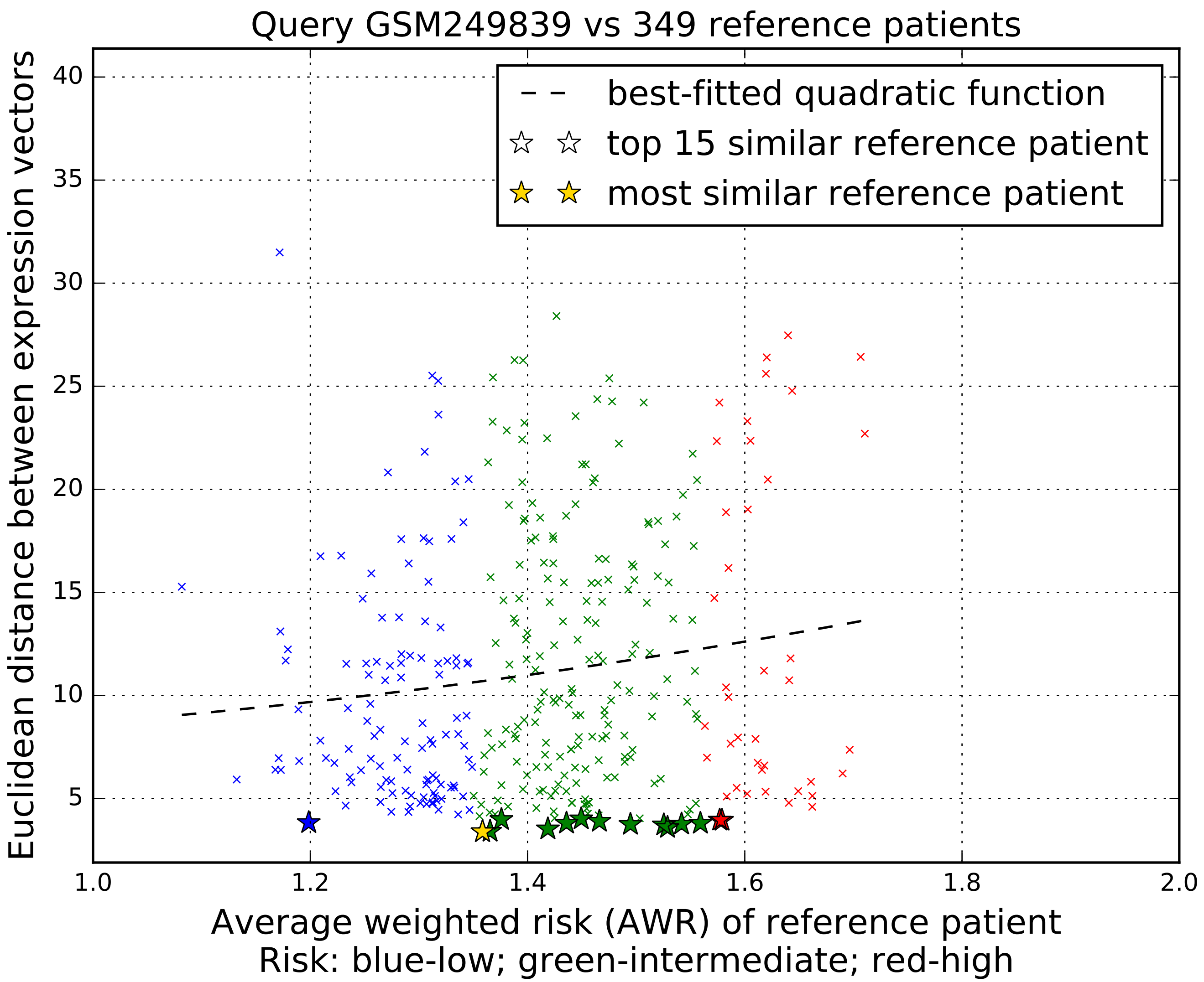

Query GSM249839 vs 349 reference patients
best-fitted quadratic function
top 15 similar reference patient
most similar reference patient
Euclidean distance between expression vectors
40
35
30
25
20
15
10
5
1.0
1.2
1.4
1.6
1.8
2.0
Average weighted risk (AWR) of reference patient
Risk: blue-low; green-intermediate; red-high

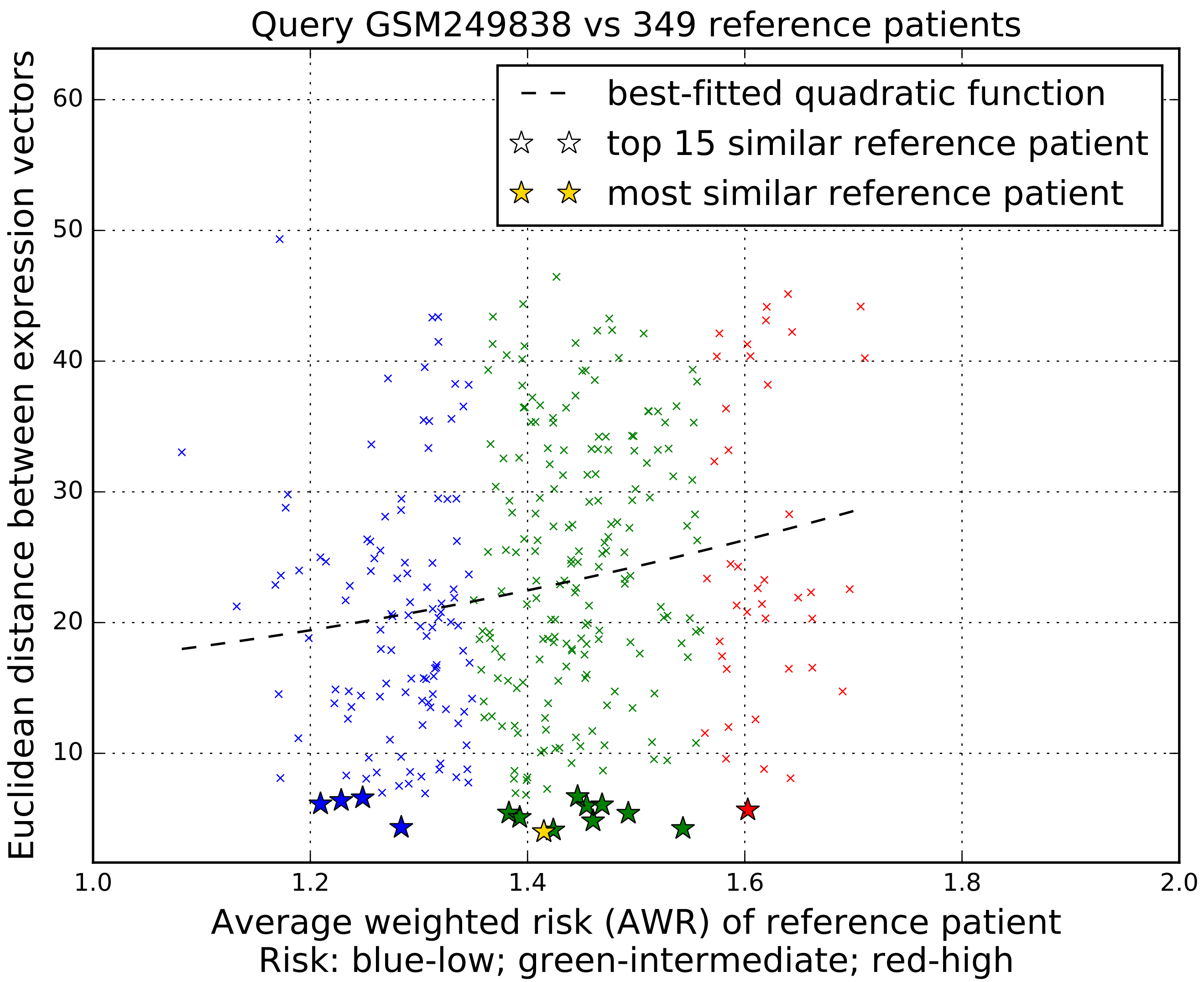

Query GSM249838 vs 349 reference patients
best-fitted quadratic function
top 15 similar reference patient
most similar reference patient
Euclidean distance between expression vectors
60
50
40
30
20
10
1.0
1.2
1.4
1.6
1.8
2.0
Average weighted risk (AWR) of reference patient
Risk: blue-low; green-intermediate; red-high

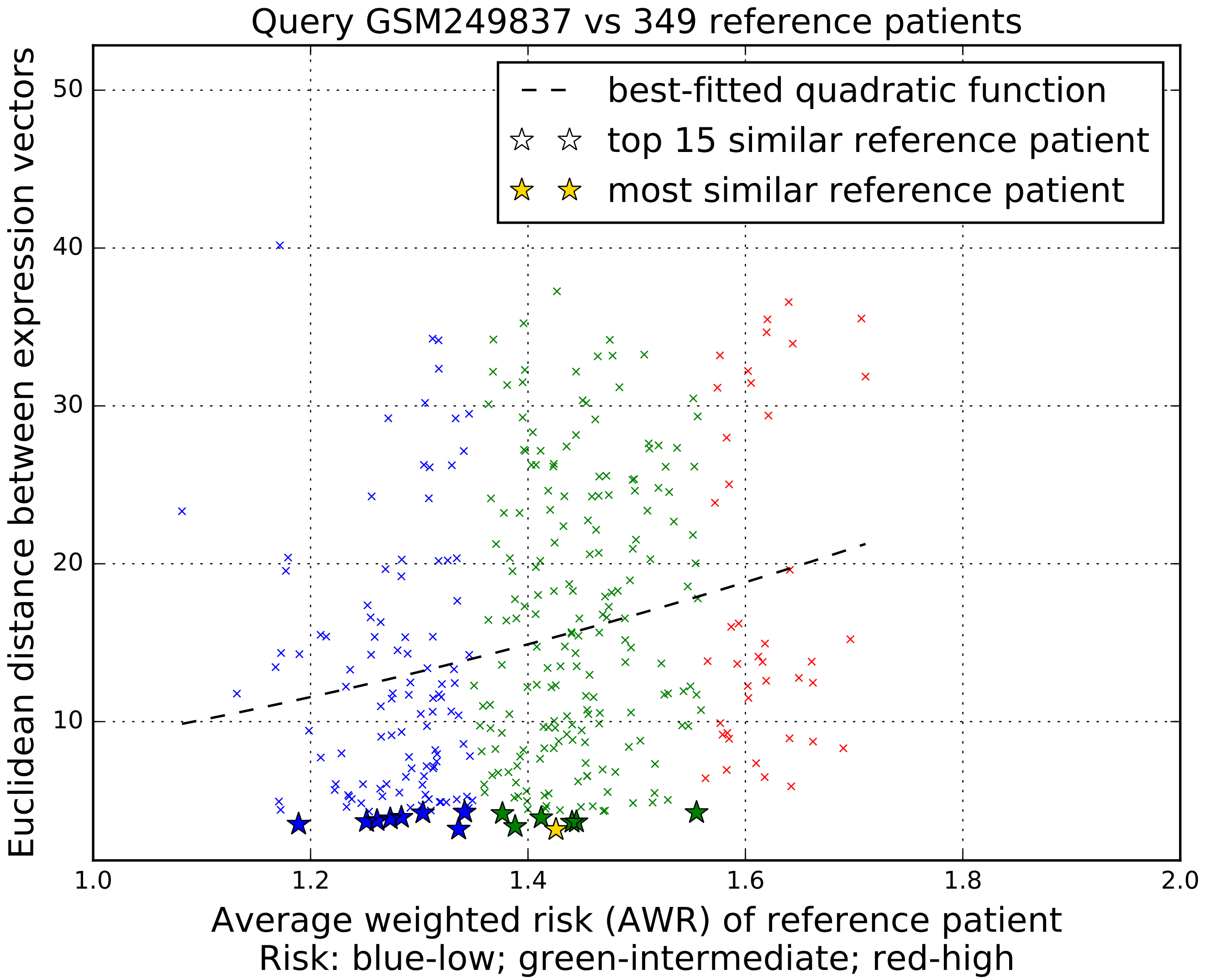

Query GSM249837 vs 349 reference patients
best-fitted quadratic function
top 15 similar reference patient
most similar reference patient
Euclidean distance between expression vectors
10
20
30
40
50
1.0
1.2
1.4
1.6
1.8
2.0
Average weighted risk (AWR) of reference patient
Risk: blue-low; green-intermediate; red-high

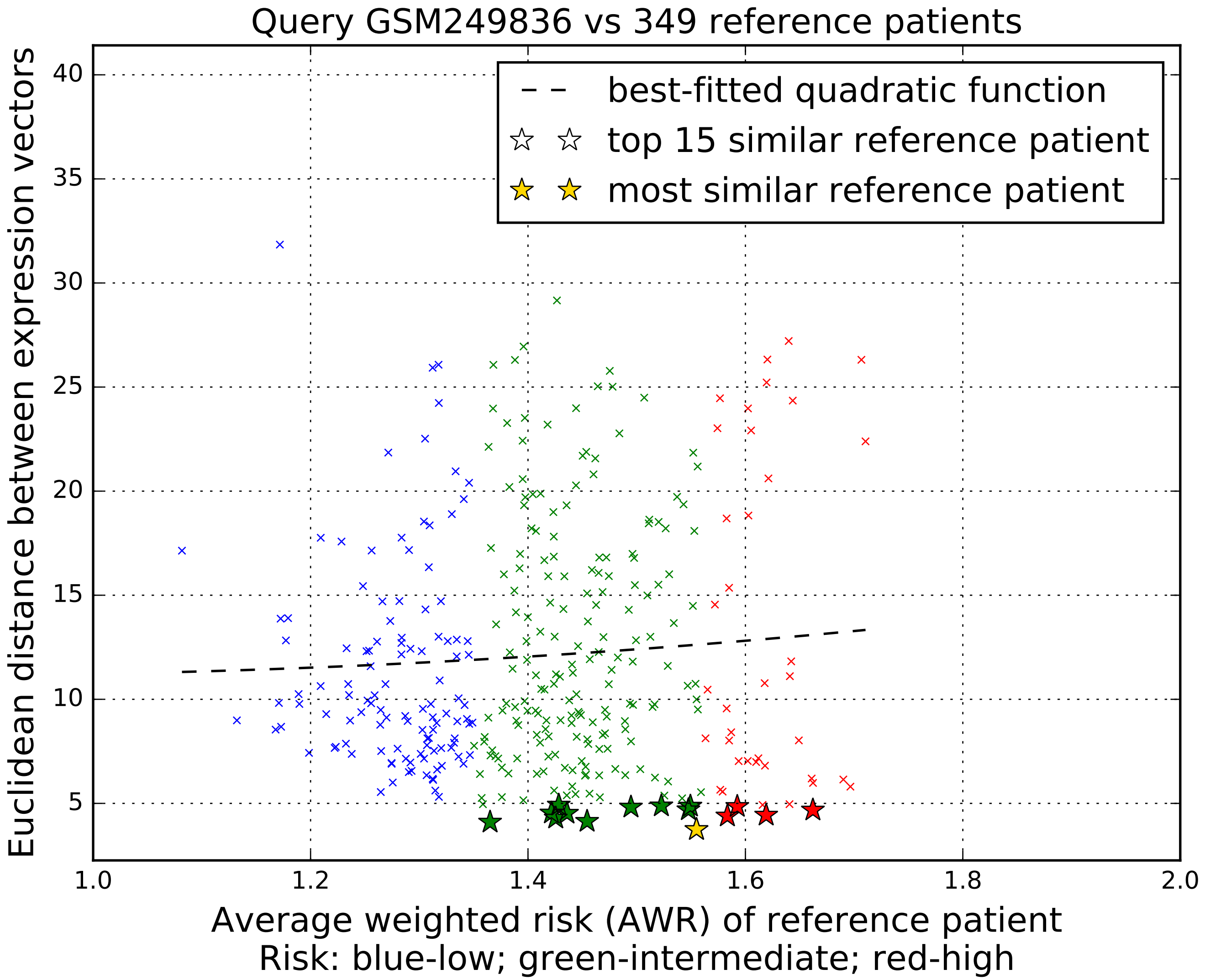
Query GSM249836 vs 349 reference patients
best-fitted quadratic function
top 15 similar reference patient
most similar reference patient
Euclidean distance between expression vectors
40
35
30
25
20
15
10
5
1.0
1.2
1.4
1.6
1.8
2.0
Average weighted risk (AWR) of reference patient
Risk: blue-low; green-intermediate; red-high

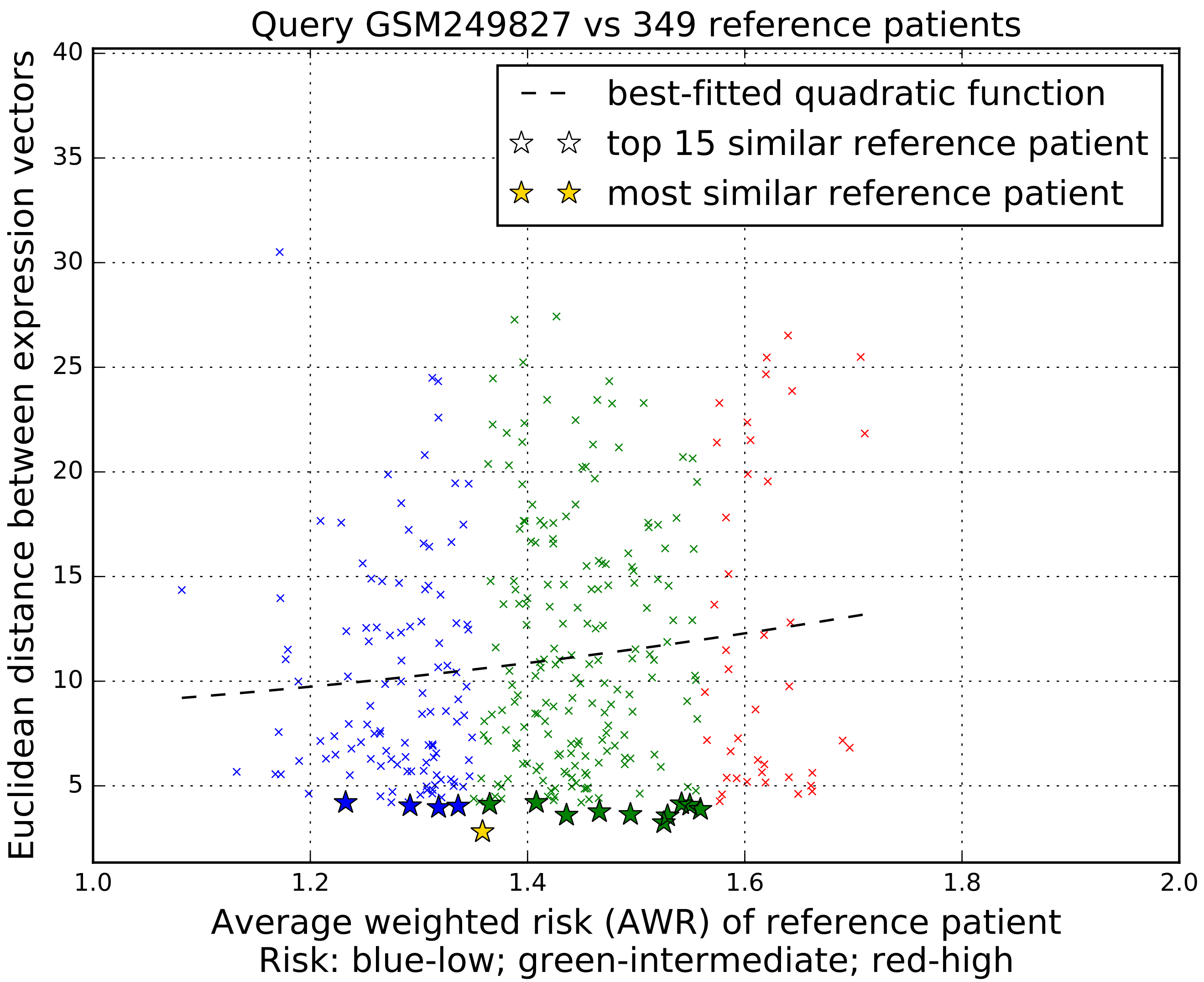
Query GSM249827 vs 349 reference patients
best-fitted quadratic function
top 15 similar reference patient
most similar reference patient
Euclidean distance between expression vectors
Average weighted risk (AWR) of reference patient
Risk: blue-low; green-intermediate; red-high
40
35
30
25
20
15
10
5
1.0
1.2
1.4
1.6
1.8
2.0

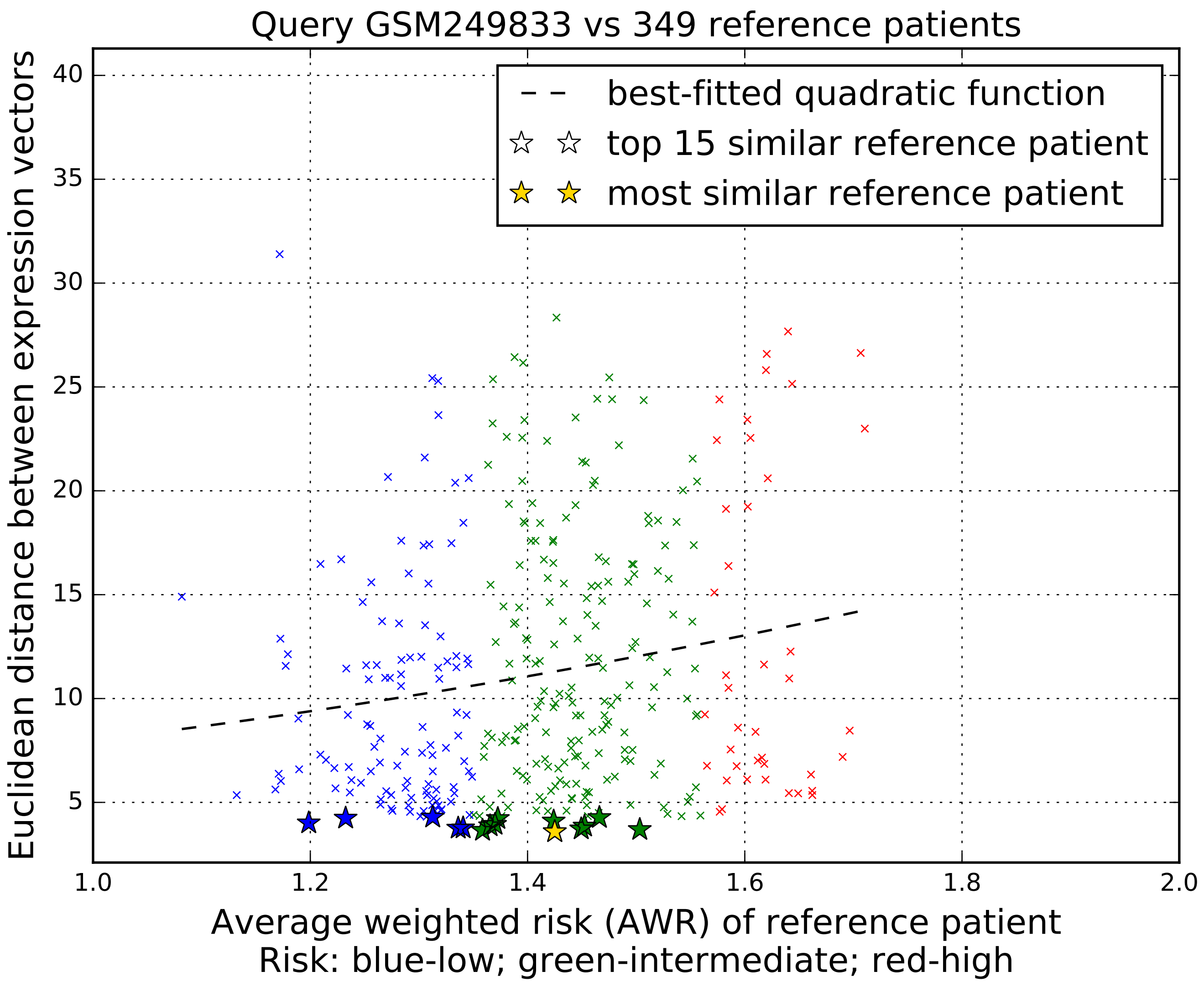

Query GSM249833 vs 349 reference patients
best-fitted quadratic function
top 15 similar reference patient
most similar reference patient
Euclidean distance between expression vectors
40
35
30
25
20
15
10
5
1.0
1.2
1.4
1.6
1.8
2.0
Average weighted risk (AWR) of reference patient
Risk: blue-low; green-intermediate; red-high

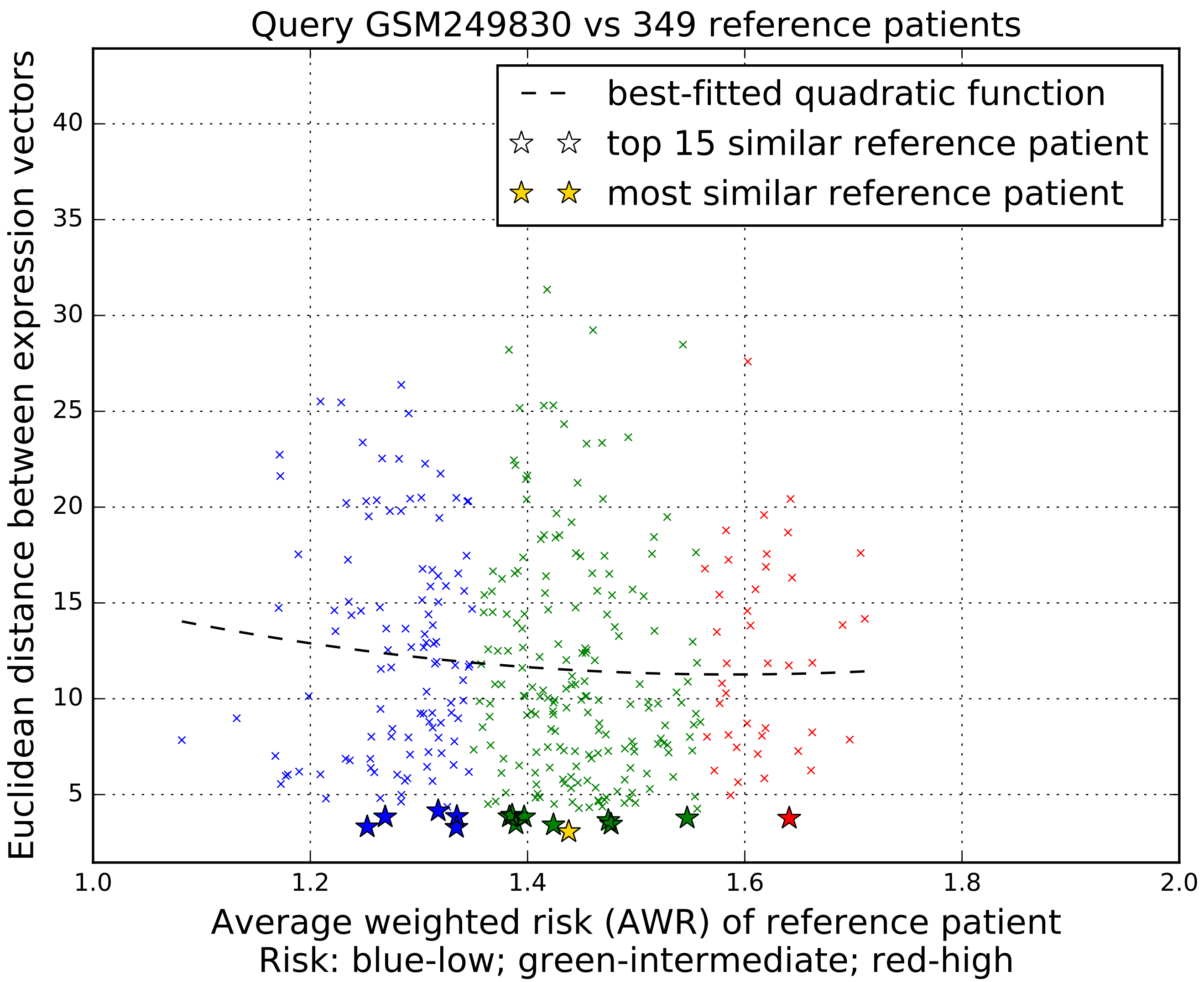

Query GSM249830 vs 349 reference patients
Euclidean distance between expression vectors
best-fitted quadratic function
top 15 similar reference patient
most similar reference patient
5
10
15
20
25
30
35
40
1.0
1.2
1.4
1.6
1.8
2.0
Average weighted risk (AWR) of reference patient
Risk: blue-low; green-intermediate; red-high

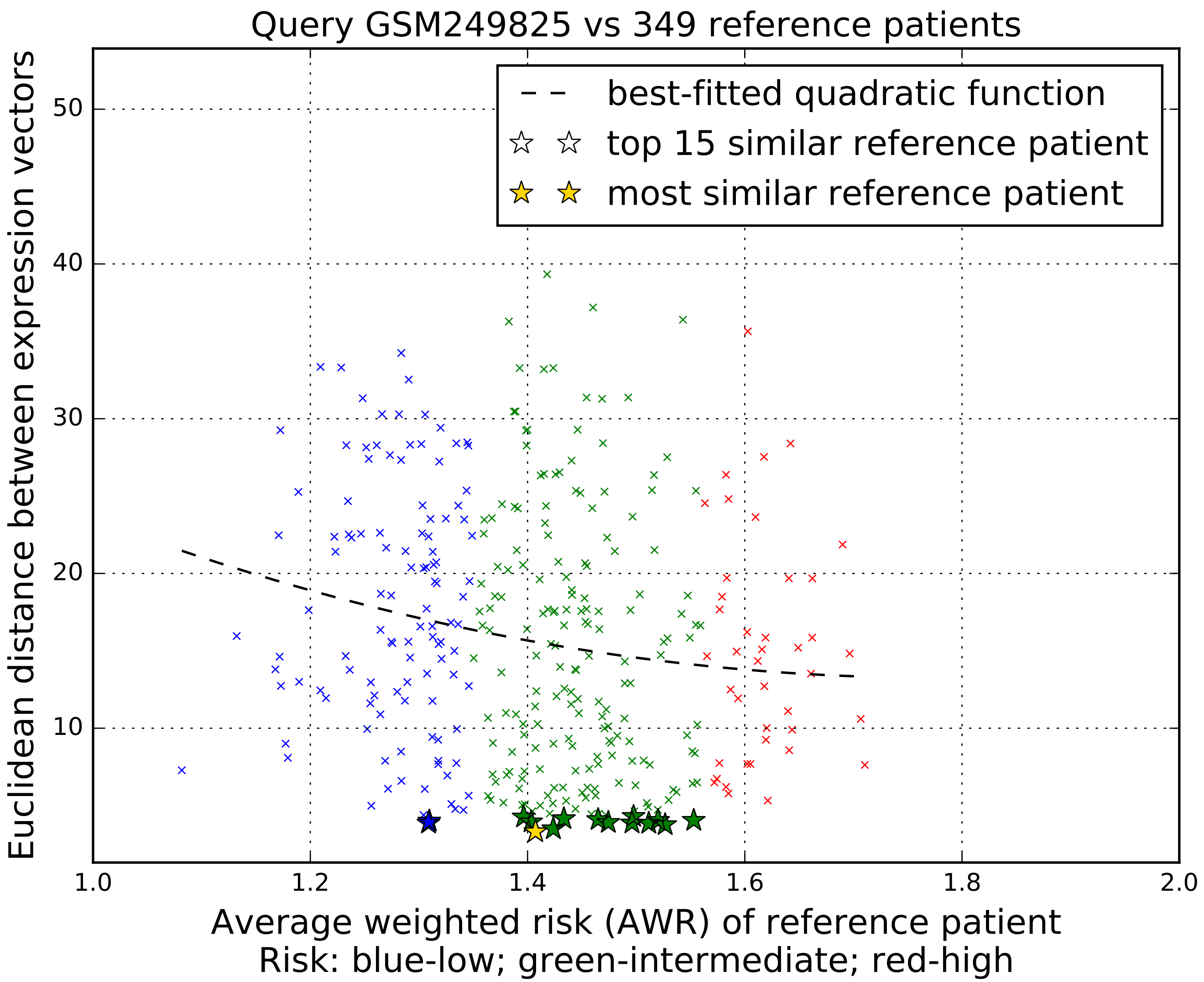

Query GSM249825 vs 349 reference patients
best-fitted quadratic function
top 15 similar reference patient
most similar reference patient
Euclidean distance between expression vectors
Average weighted risk (AWR) of reference patient
Risk: blue-low; green-intermediate; red-high
10
20
30
40
50
1.0
1.2
1.4
1.6
1.8
2.0

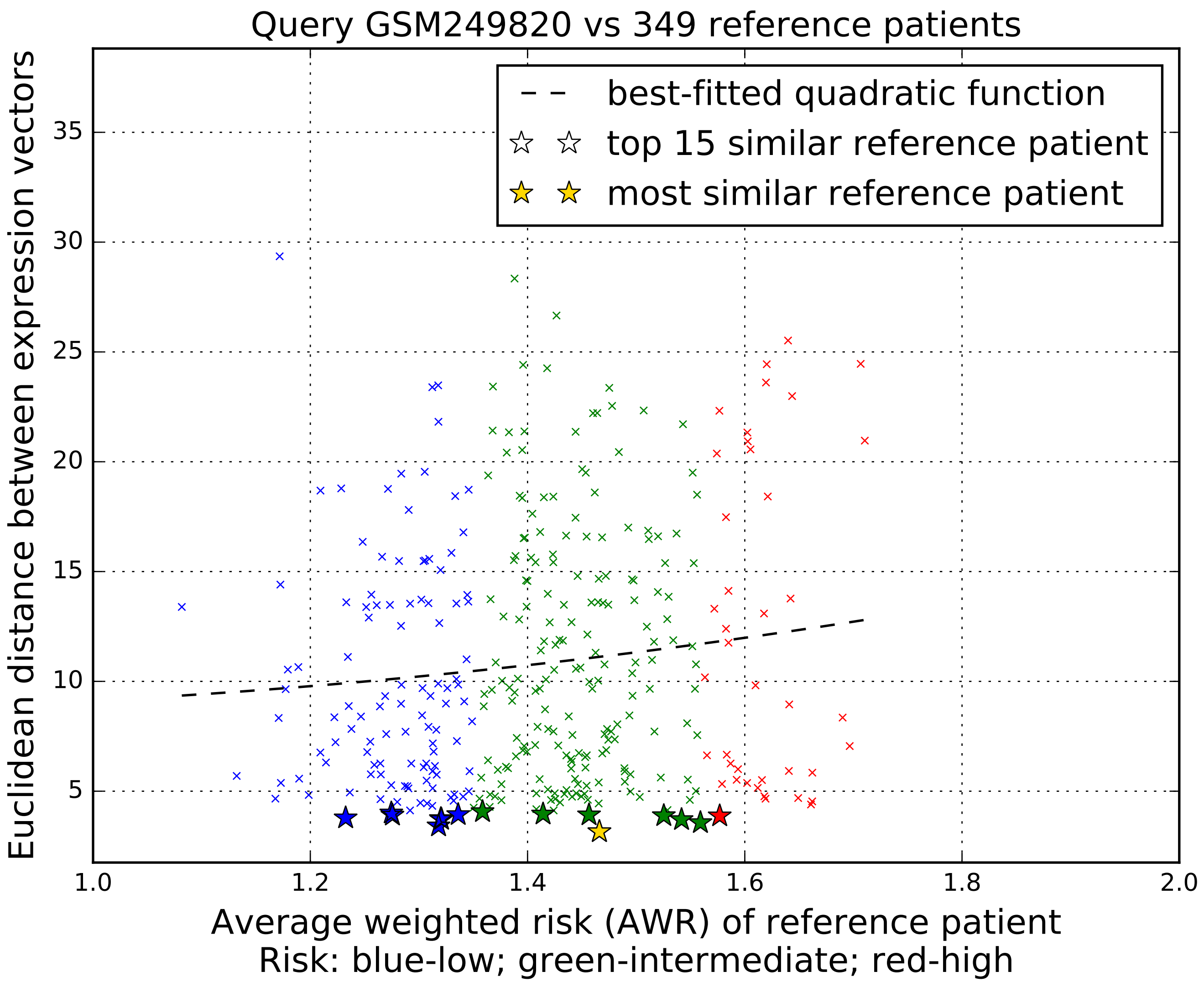

Query GSM249820 vs 349 reference patients
best-fitted quadratic function
top 15 similar reference patient
most similar reference patient
Euclidean distance between expression vectors
35
30
25
20
15
10
5
1.0
1.2
1.4
1.6
1.8
2.0
Average weighted risk (AWR) of reference patient
Risk: blue-low; green-intermediate; red-high

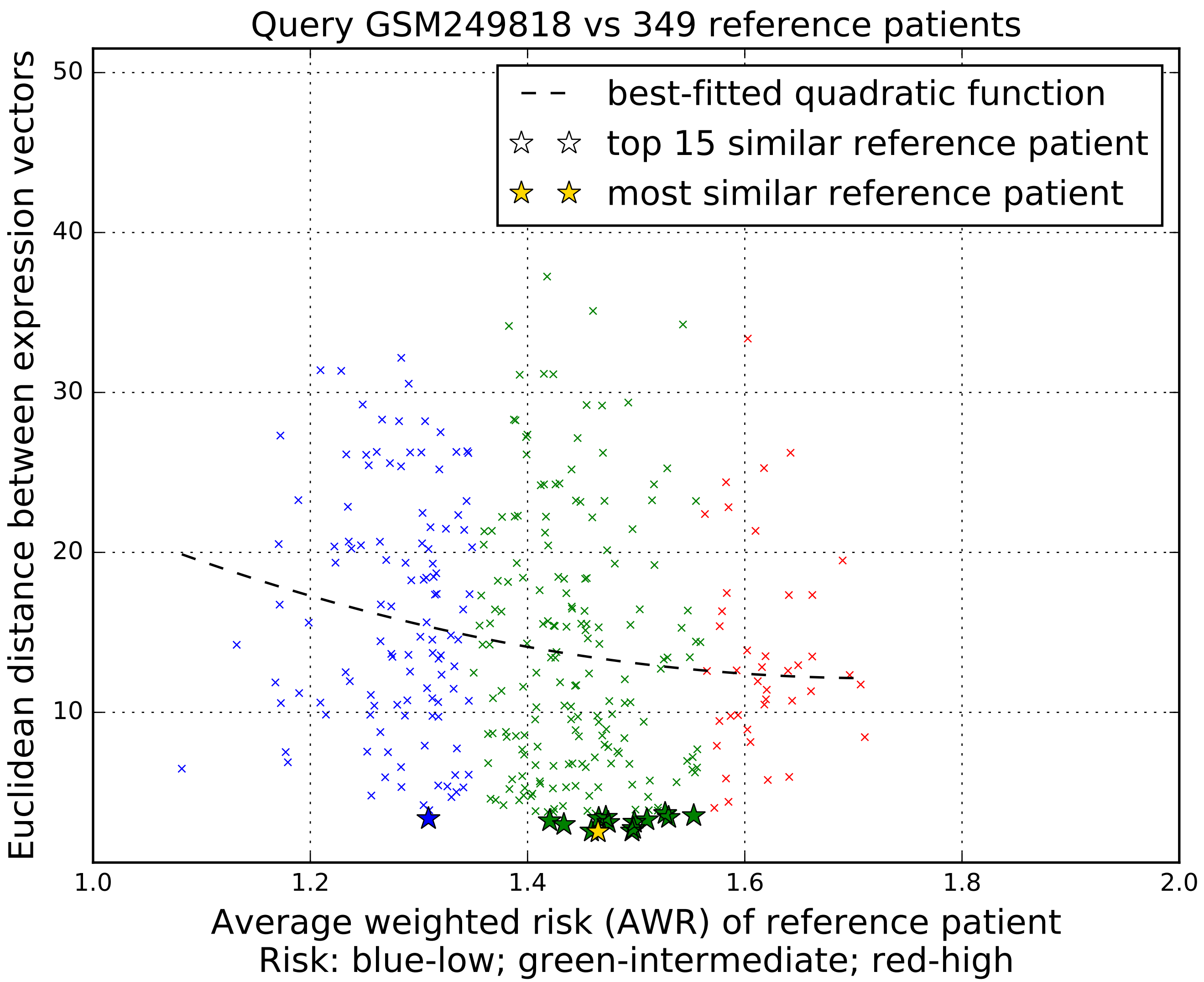

Query GSM249818 vs 349 reference patients
best-fitted quadratic function
top 15 similar reference patient
most similar reference patient
Euclidean distance between expression vectors
50
40
30
20
10
1.0
1.2
1.4
1.6
1.8
2.0
Average weighted risk (AWR) of reference patient
Risk: blue-low; green-intermediate; red-high

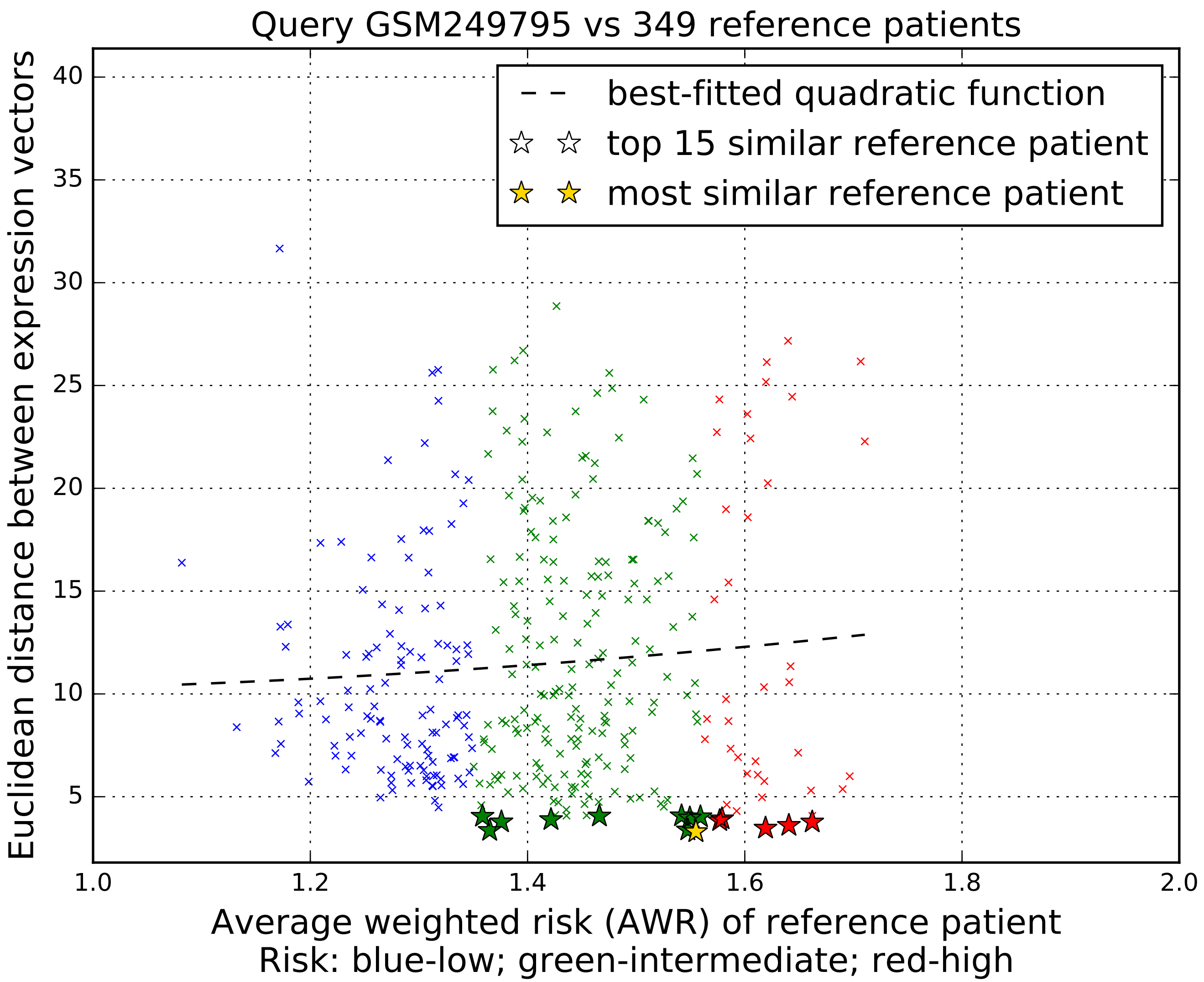

Query GSM249795 vs 349 reference patients
best-fitted quadratic function
top 15 similar reference patient
most similar reference patient
Euclidean distance between expression vectors
Average weighted risk (AWR) of reference patient
Risk: blue-low; green-intermediate; red-high
40
35
30
25
20
15
10
5
1.0
1.2
1.4
1.6
1.8
2.0

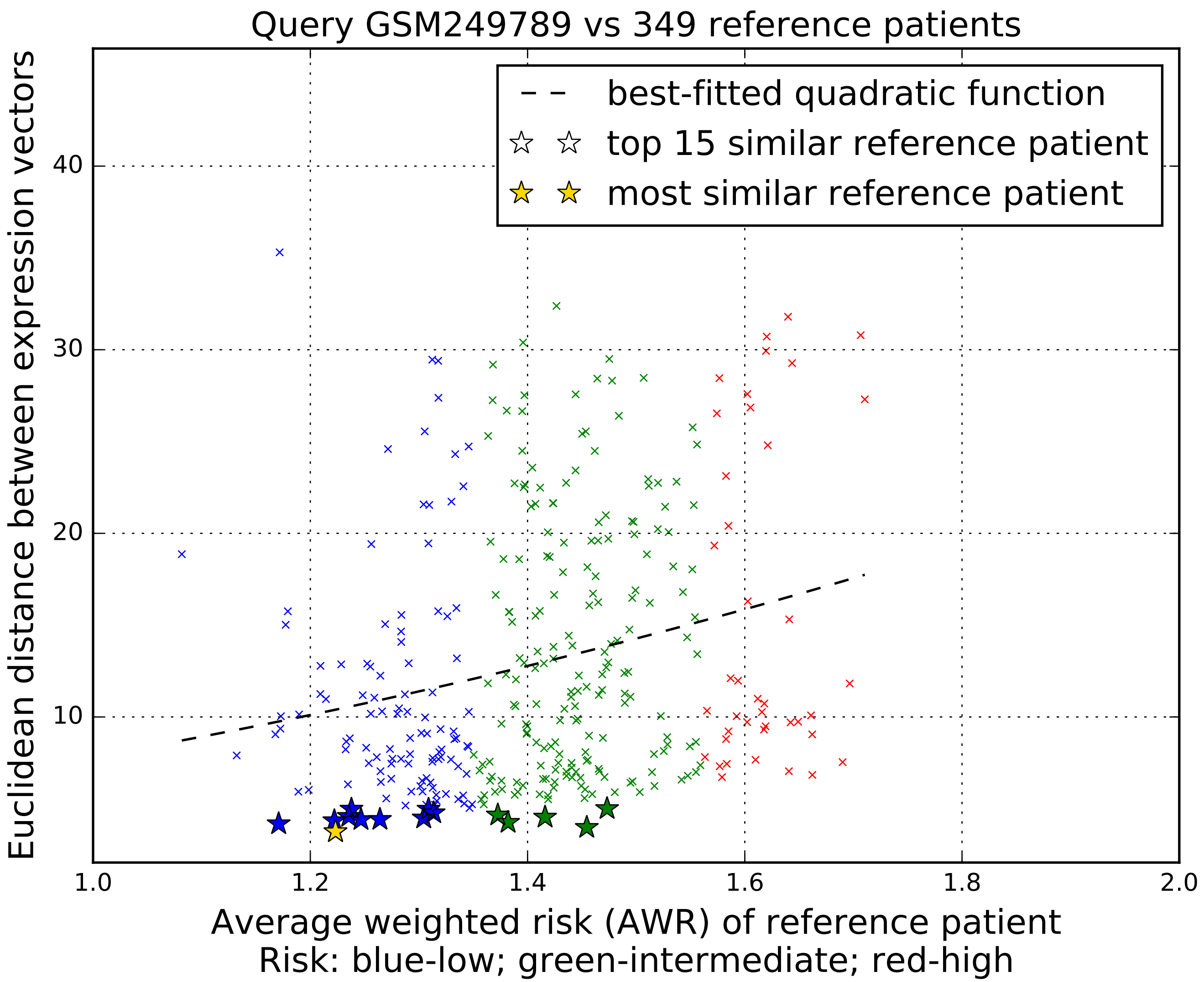
Query GSM249789 vs 349 reference patients
best-fitted quadratic function
top 15 similar reference patient
most similar reference patient
Euclidean distance between expression vectors
10
20
30
40
1.0
1.2
1.4
1.6
1.8
2.0
Average weighted risk (AWR) of reference patient
Risk: blue-low; green-intermediate; red-high

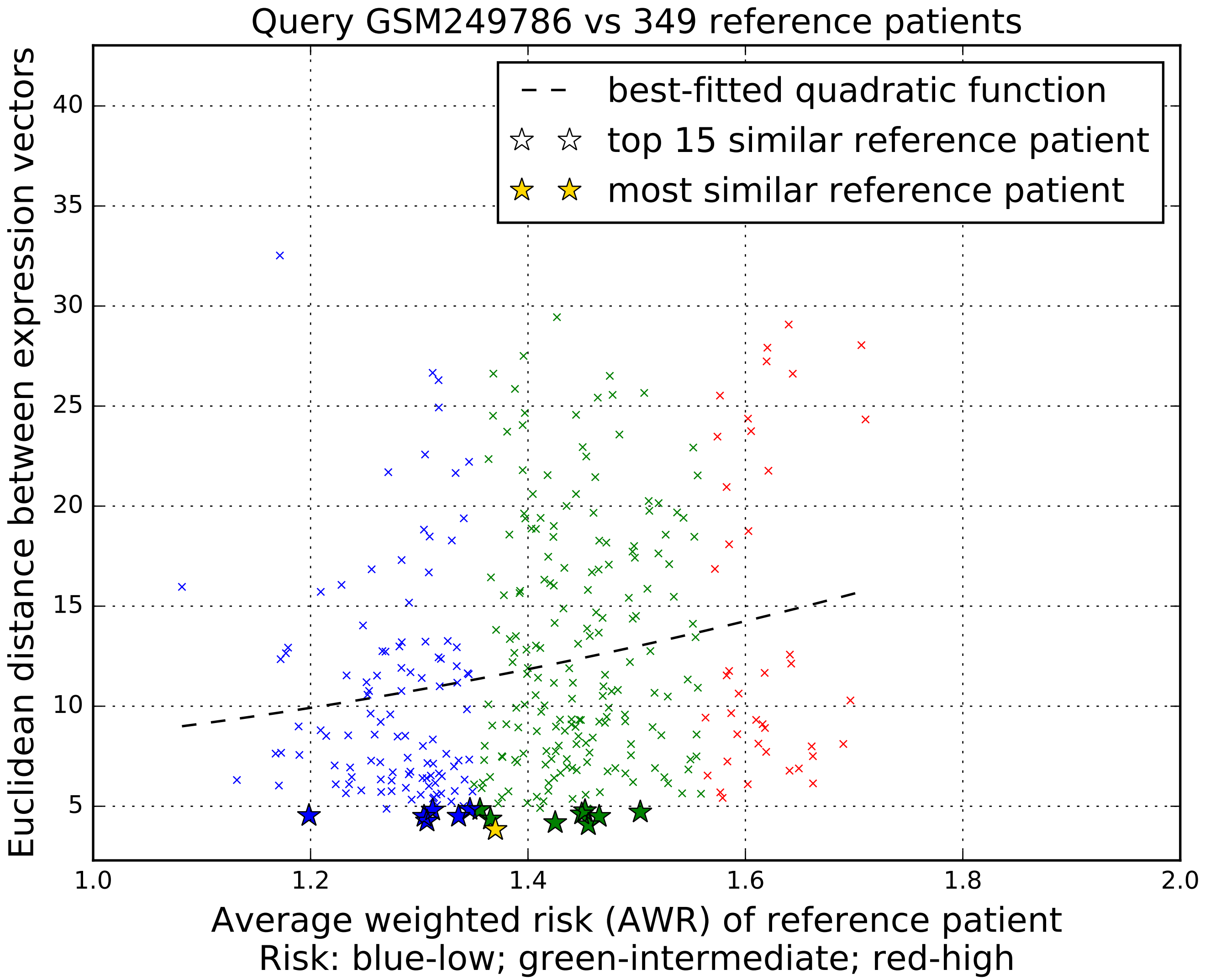
Query GSM249786 vs 349 reference patients
best-fitted quadratic function
top 15 similar reference patient
most similar reference patient
Euclidean distance between expression vectors
5
10
15
20
25
30
35
40
1.0
1.2
1.4
1.6
1.8
2.0
Average weighted risk (AWR) of reference patient
Risk: blue-low; green-intermediate; red-high

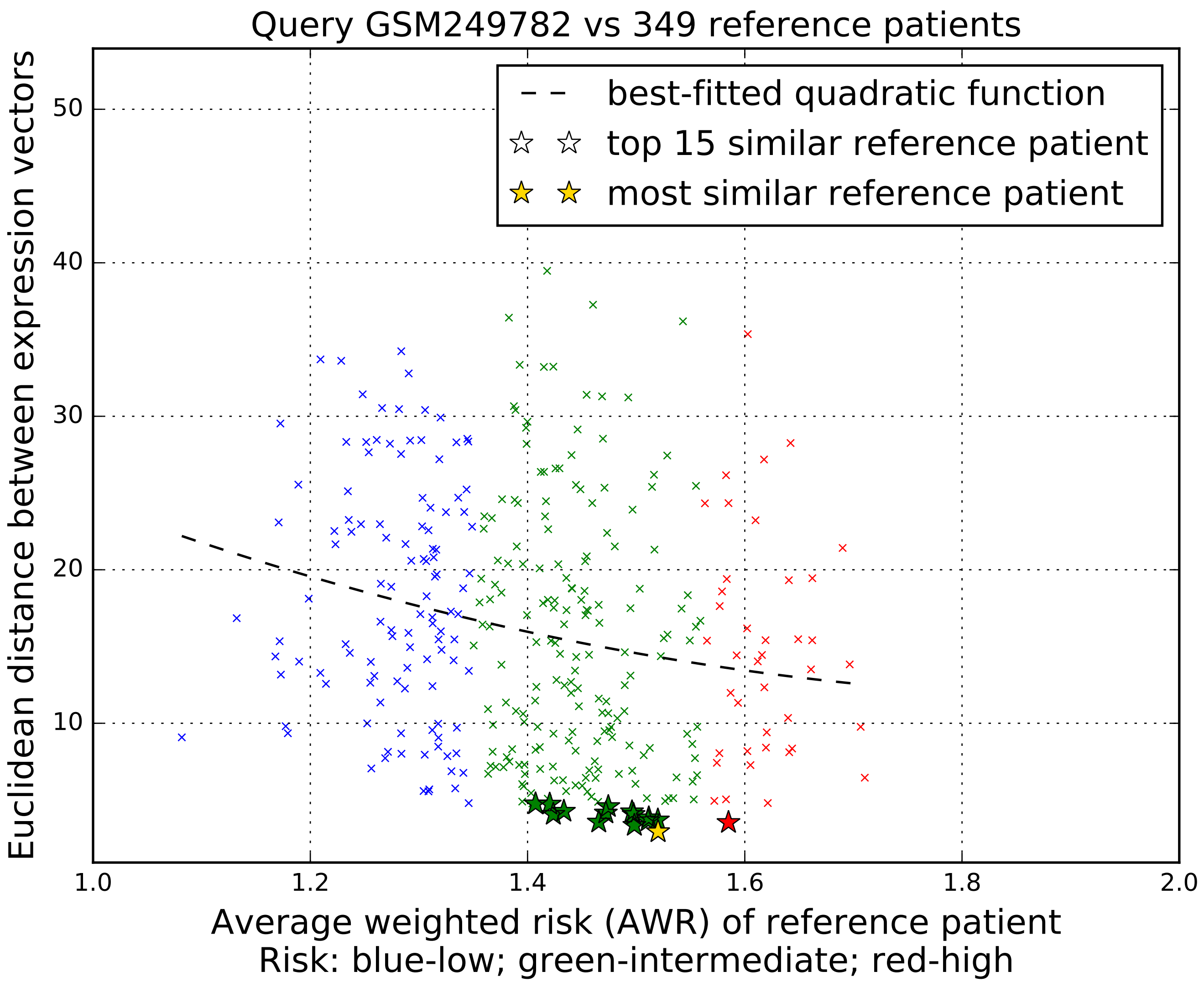

Query GSM249782 vs 349 reference patients
best-fitted quadratic function
top 15 similar reference patient
most similar reference patient
Euclidean distance between expression vectors
50
40
30
20
10
1.0
1.2
1.4
1.6
1.8
2.0
Average weighted risk (AWR) of reference patient
Risk: blue-low; green-intermediate; red-high

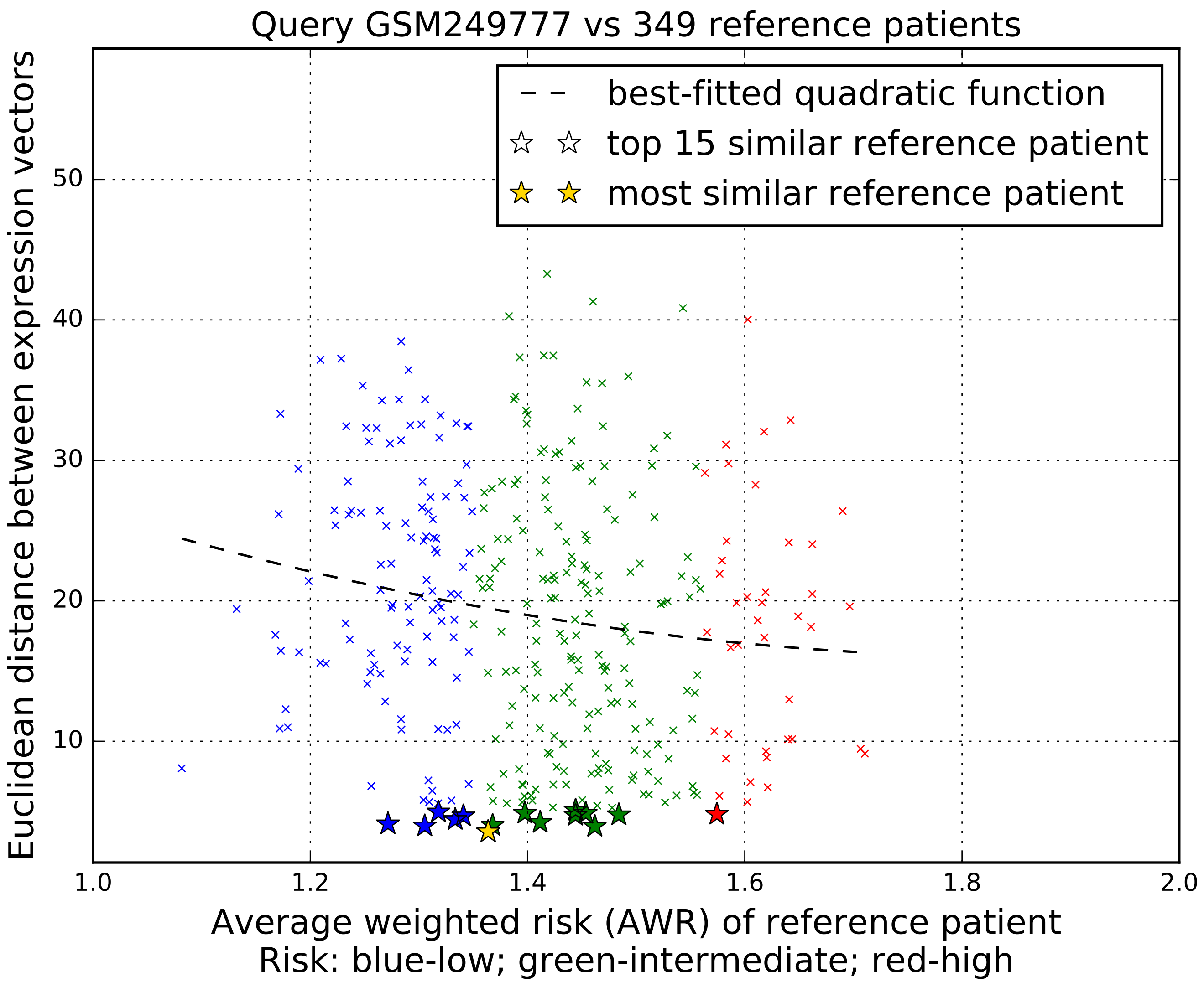
Query GSM249777 vs 349 reference patients
best-fitted quadratic function
top 15 similar reference patient
most similar reference patient
Euclidean distance between expression vectors
10
20
30
40
50
1.0
1.2
1.4
1.6
1.8
2.0
Average weighted risk (AWR) of reference patient
Risk: blue-low; green-intermediate; red-high

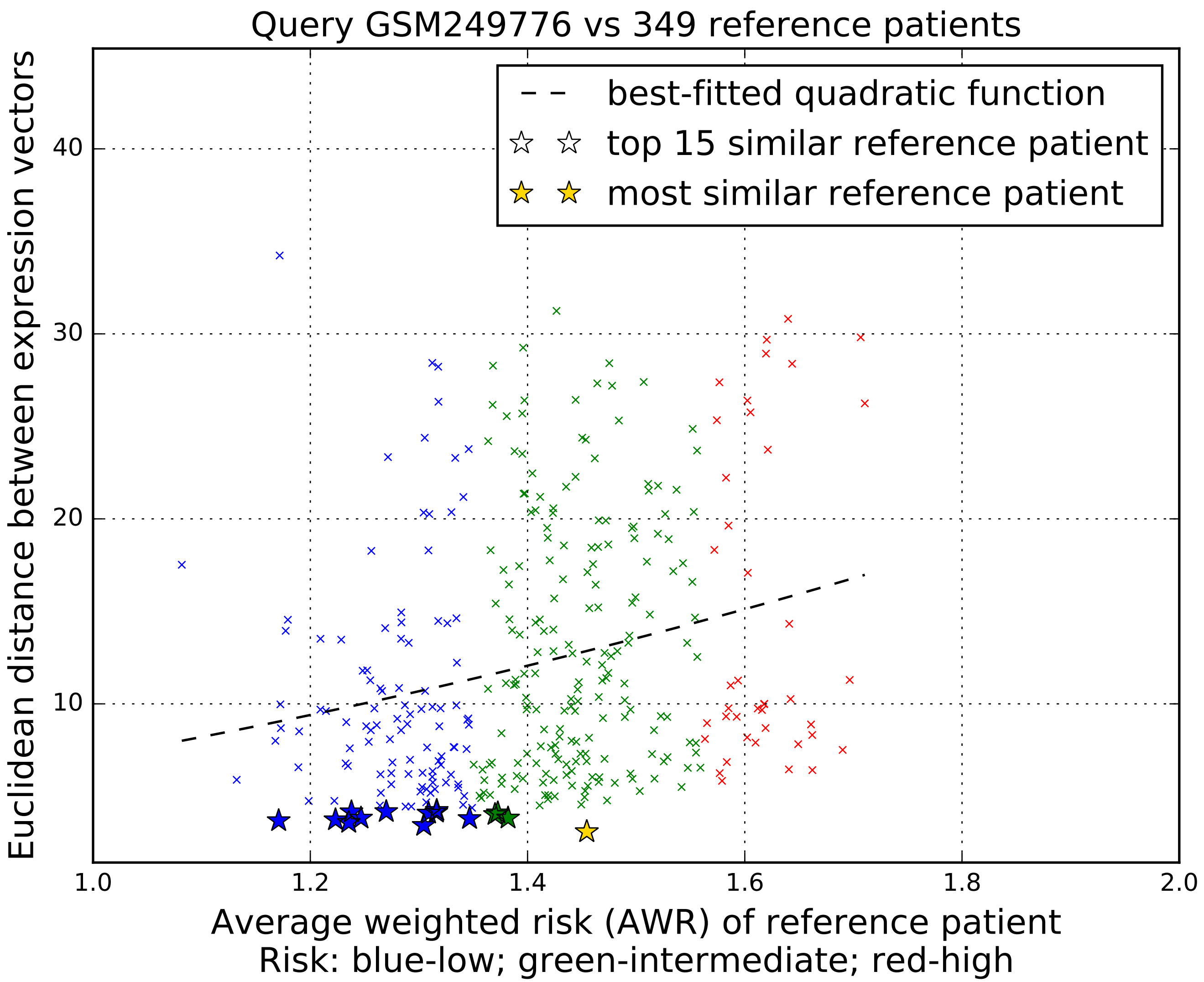

Query GSM249776 vs 349 reference patients
best-fitted quadratic function
top 15 similar reference patient
most similar reference patient
Euclidean distance between expression vectors
10
20
30
40
1.0
1.2
1.4
1.6
1.8
2.0
Average weighted risk (AWR) of reference patient
Risk: blue-low; green-intermediate; red-high

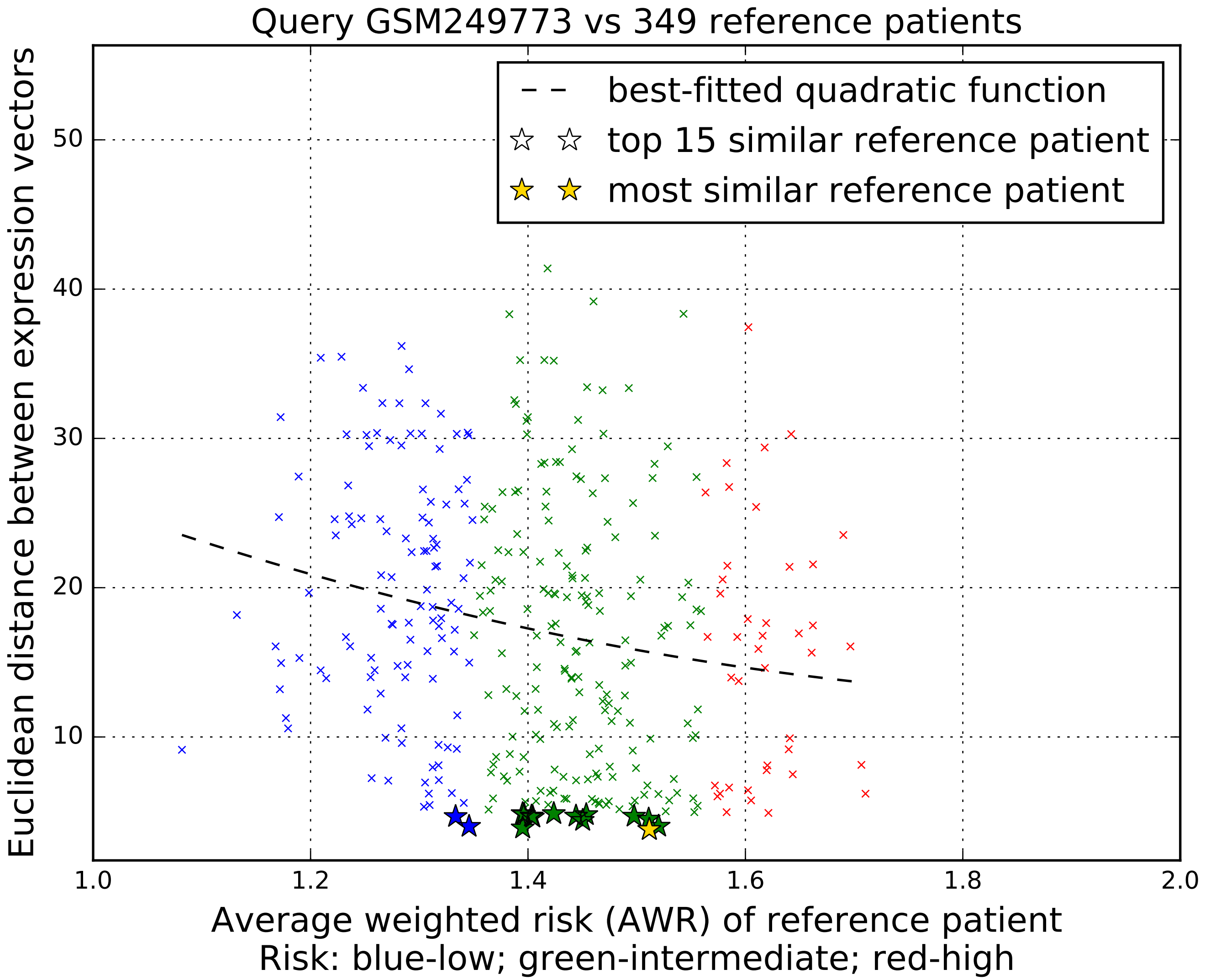
Query GSM249773 vs 349 reference patients
best-fitted quadratic function
top 15 similar reference patient
most similar reference patient
Euclidean distance between expression vectors
Average weighted risk (AWR) of reference patient
Risk: blue-low; green-intermediate; red-high
10
20
30
40
50
1.0
1.2
1.4
1.6
1.8
2.0

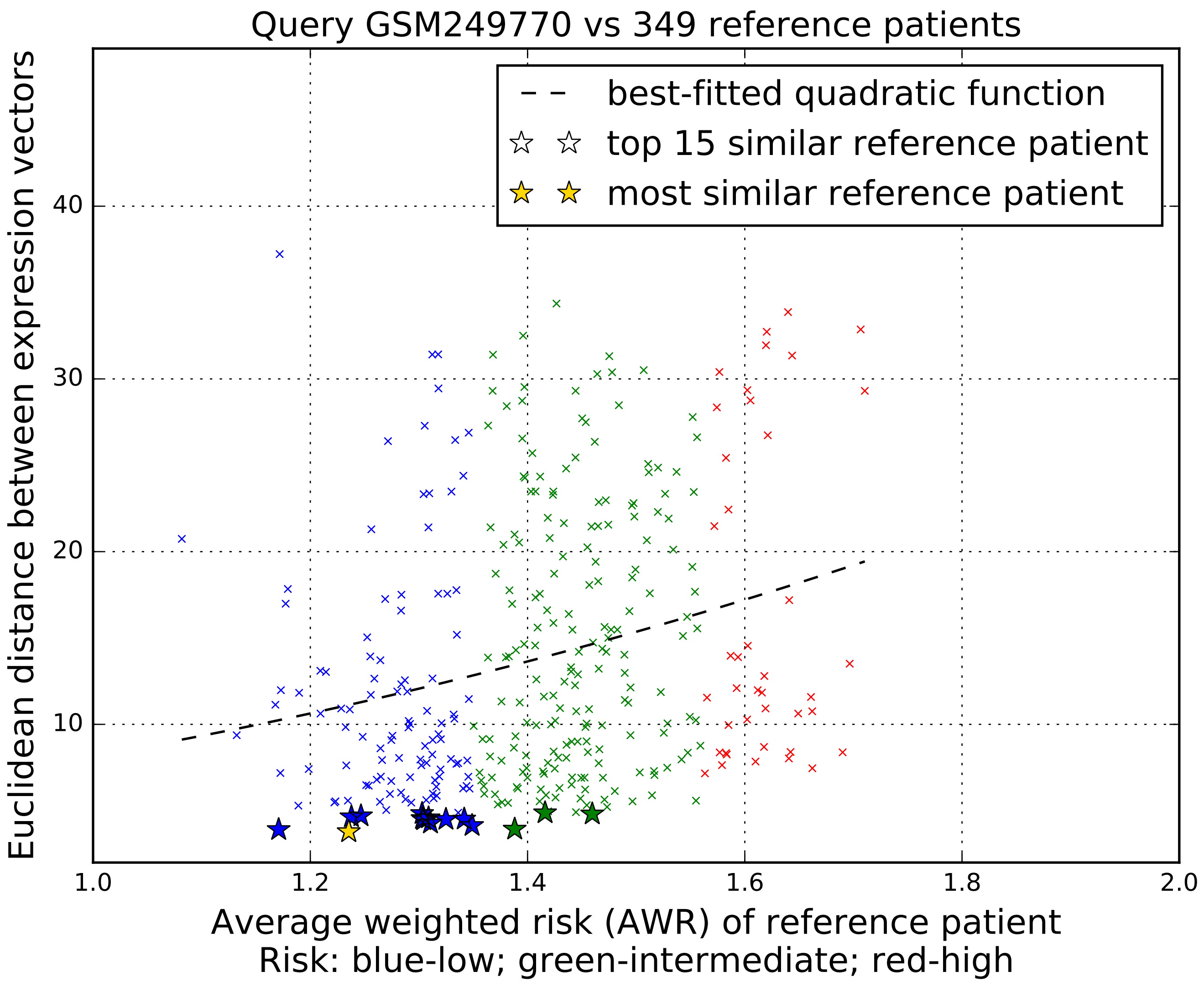
Query GSM249770 vs 349 reference patients
best-fitted quadratic function
top 15 similar reference patient
most similar reference patient
Euclidean distance between expression vectors
Average weighted risk (AWR) of reference patient
Risk: blue-low; green-intermediate; red-high
10
20
30
40
1.0
1.2
1.4
1.6
1.8
2.0

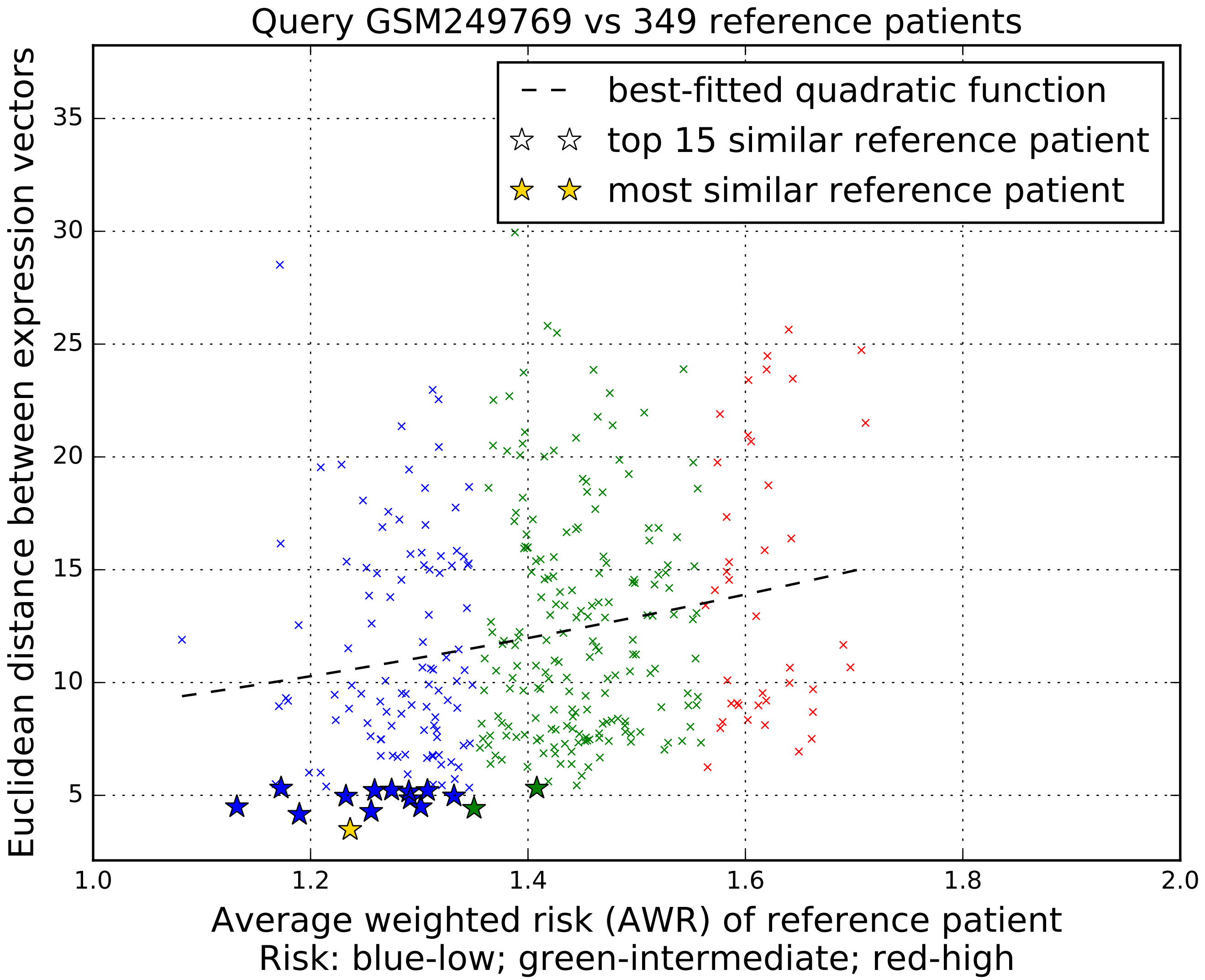

Query GSM249769 vs 349 reference patients
best-fitted quadratic function
top 15 similar reference patient
most similar reference patient
Euclidean distance between expression vectors
Average weighted risk (AWR) of reference patient
Risk: blue-low; green-intermediate; red-high
1.0
1.2
1.4
1.6
1.8
2.0
5
10
15
20
25
30
35

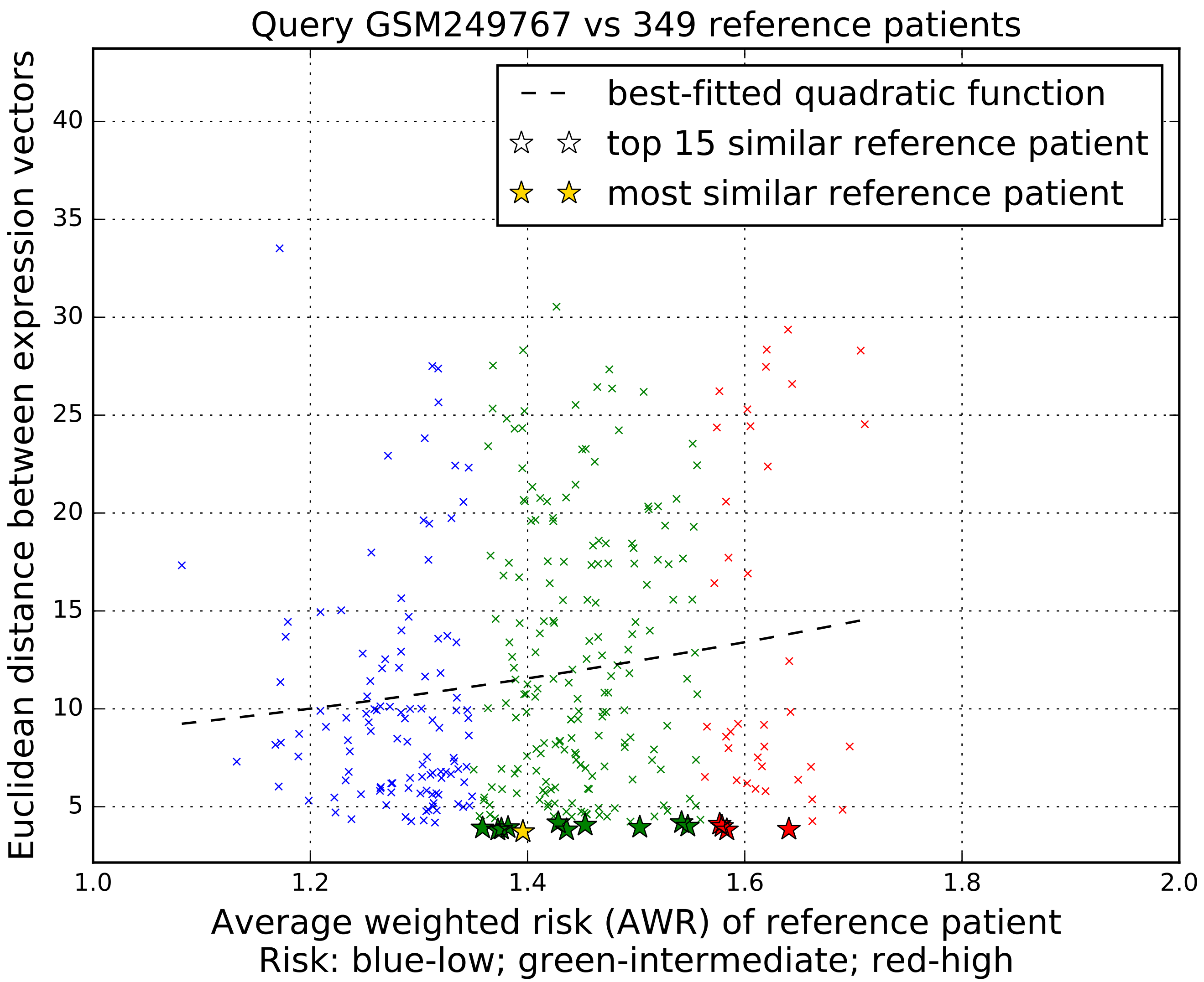

Query GSM249767 vs 349 reference patients
best-fitted quadratic function
top 15 similar reference patient
most similar reference patient
Euclidean distance between expression vectors
Average weighted risk (AWR) of reference patient
Risk: blue-low; green-intermediate; red-high
5
10
15
20
25
30
35
40
1.0
1.2
1.4
1.6
1.8
2.0

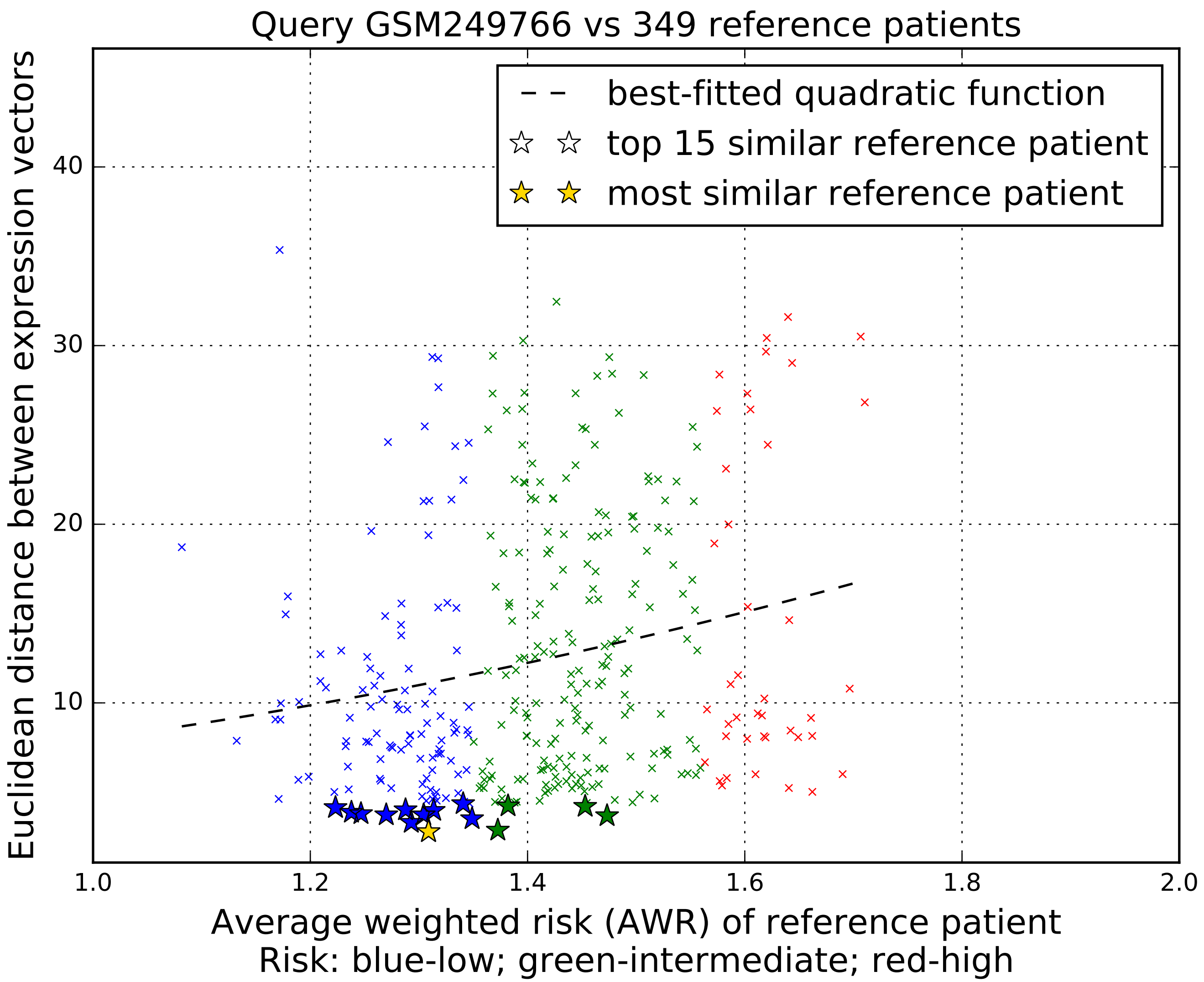

Query GSM249766 vs 349 reference patients
best-fitted quadratic function
top 15 similar reference patient
most similar reference patient
Euclidean distance between expression vectors
10
20
30
40
1.0
1.2
1.4
1.6
1.8
2.0
Average weighted risk (AWR) of reference patient
Risk: blue-low; green-intermediate; red-high

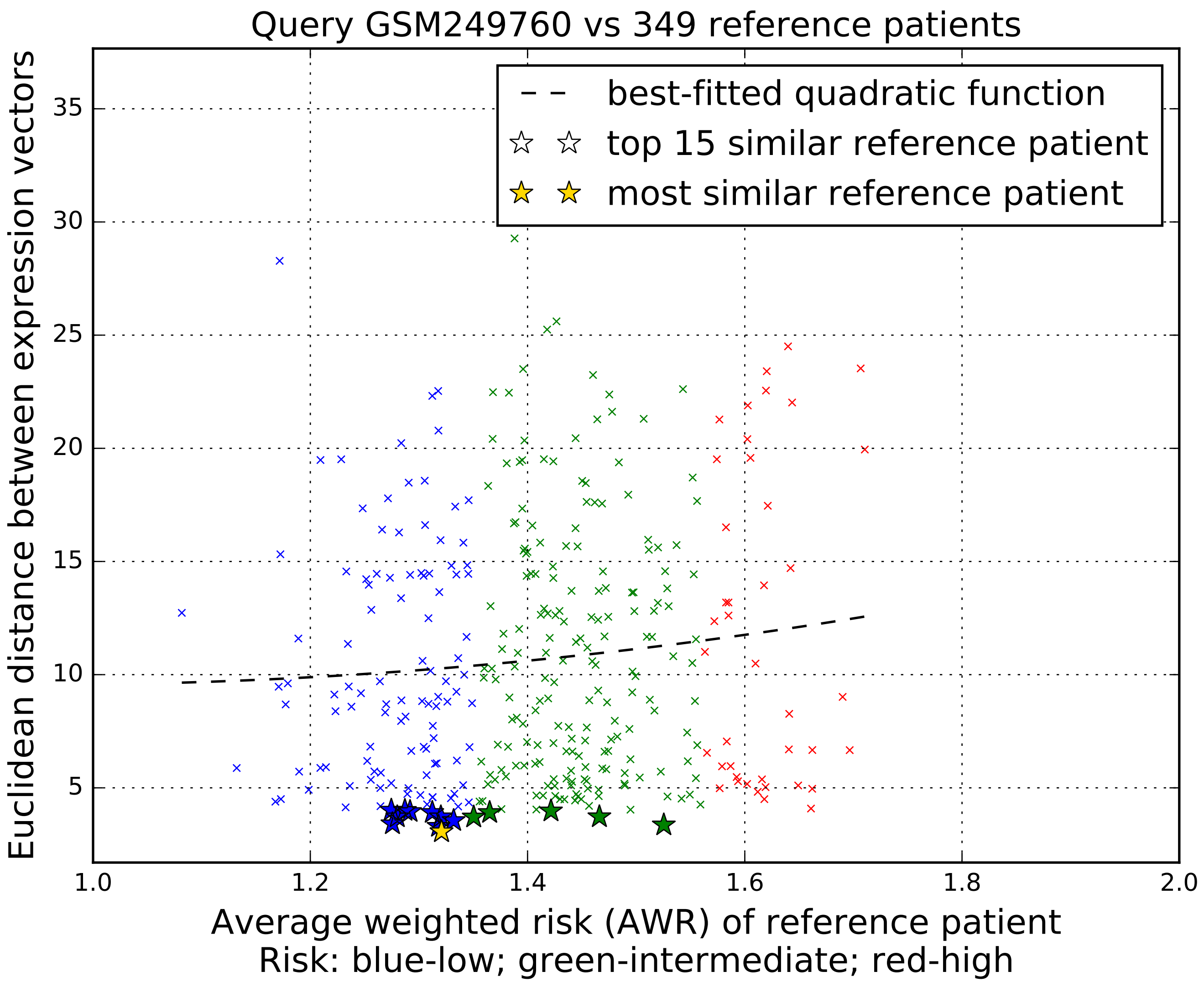
Query GSM249760 vs 349 reference patients
best-fitted quadratic function
top 15 similar reference patient
most similar reference patient
Euclidean distance between expression vectors
35
30
25
20
15
10
5
1.0
1.2
1.4
1.6
1.8
2.0
Average weighted risk (AWR) of reference patient
Risk: blue-low; green-intermediate; red-high

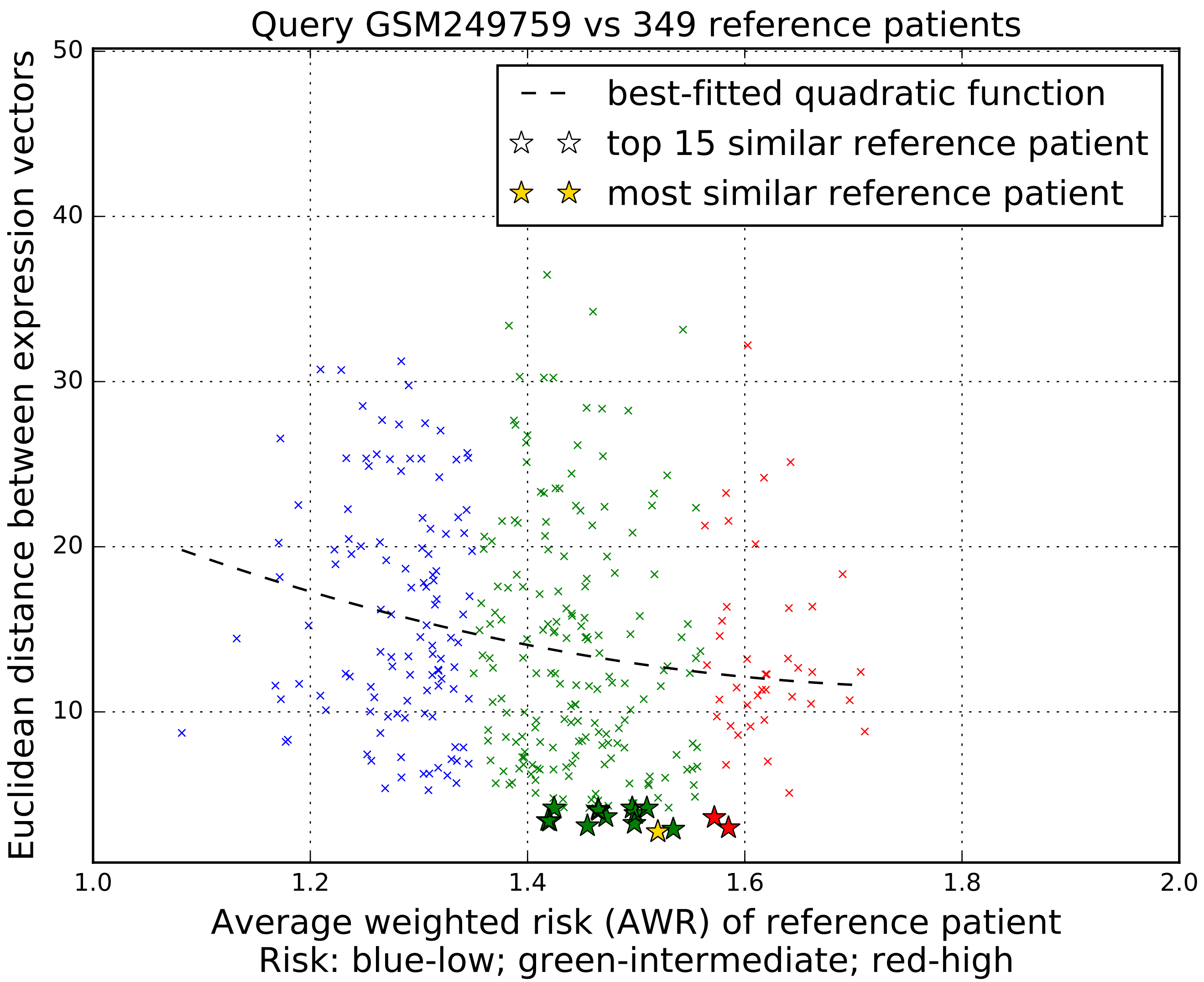

Query GSM249759 vs 349 reference patients
best-fitted quadratic function
top 15 similar reference patient
most similar reference patient
Euclidean distance between expression vectors
Average weighted risk (AWR) of reference patient
Risk: blue-low; green-intermediate; red-high
50
40
30
20
10
1.0
1.2
1.4
1.6
1.8
2.0

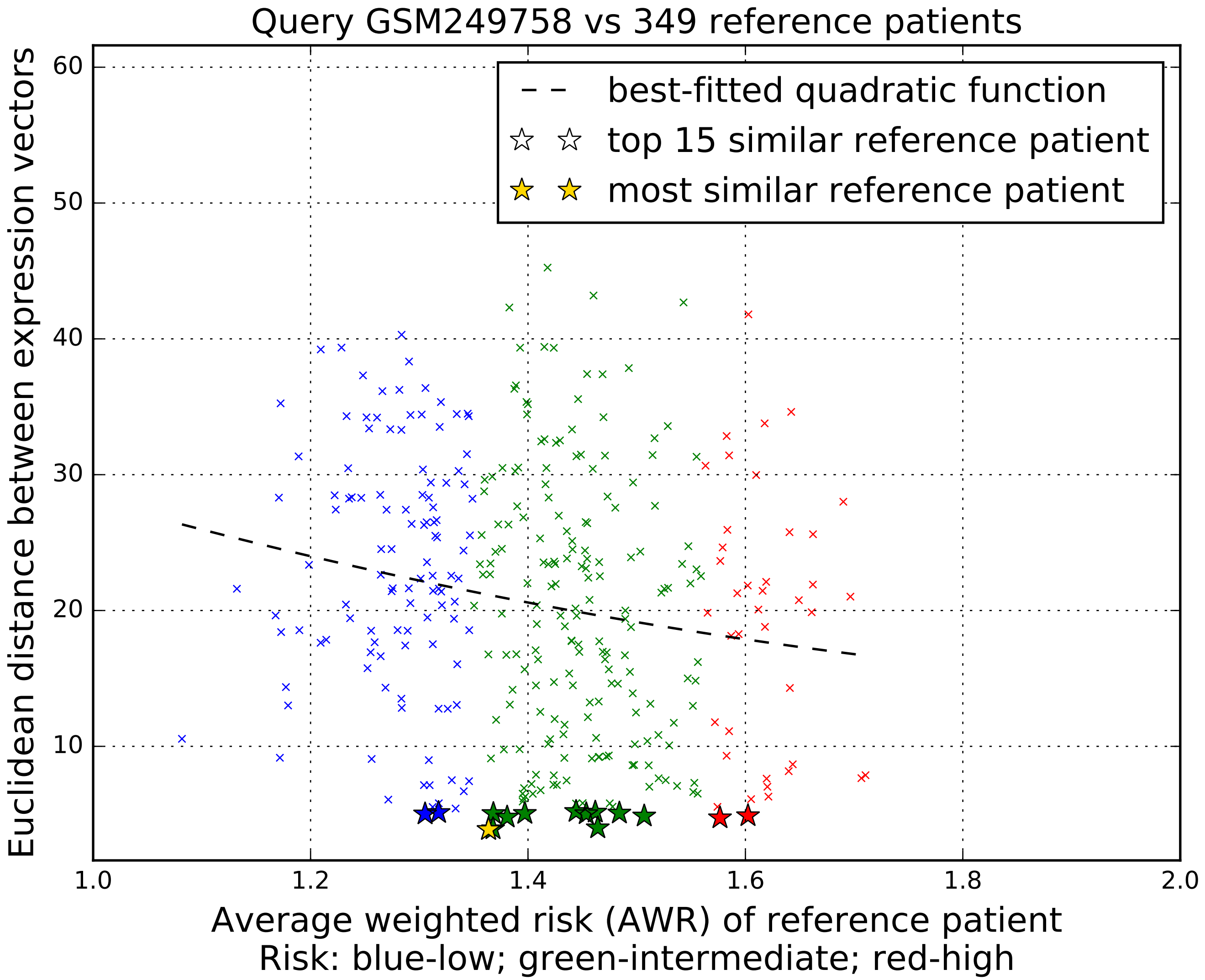

Query GSM249758 vs 349 reference patients
best-fitted quadratic function
top 15 similar reference patient
most similar reference patient
Euclidean distance between expression vectors
60
50
40
30
20
10
1.0
1.2
1.4
1.6
1.8
2.0
Average weighted risk (AWR) of reference patient
Risk: blue-low; green-intermediate; red-high

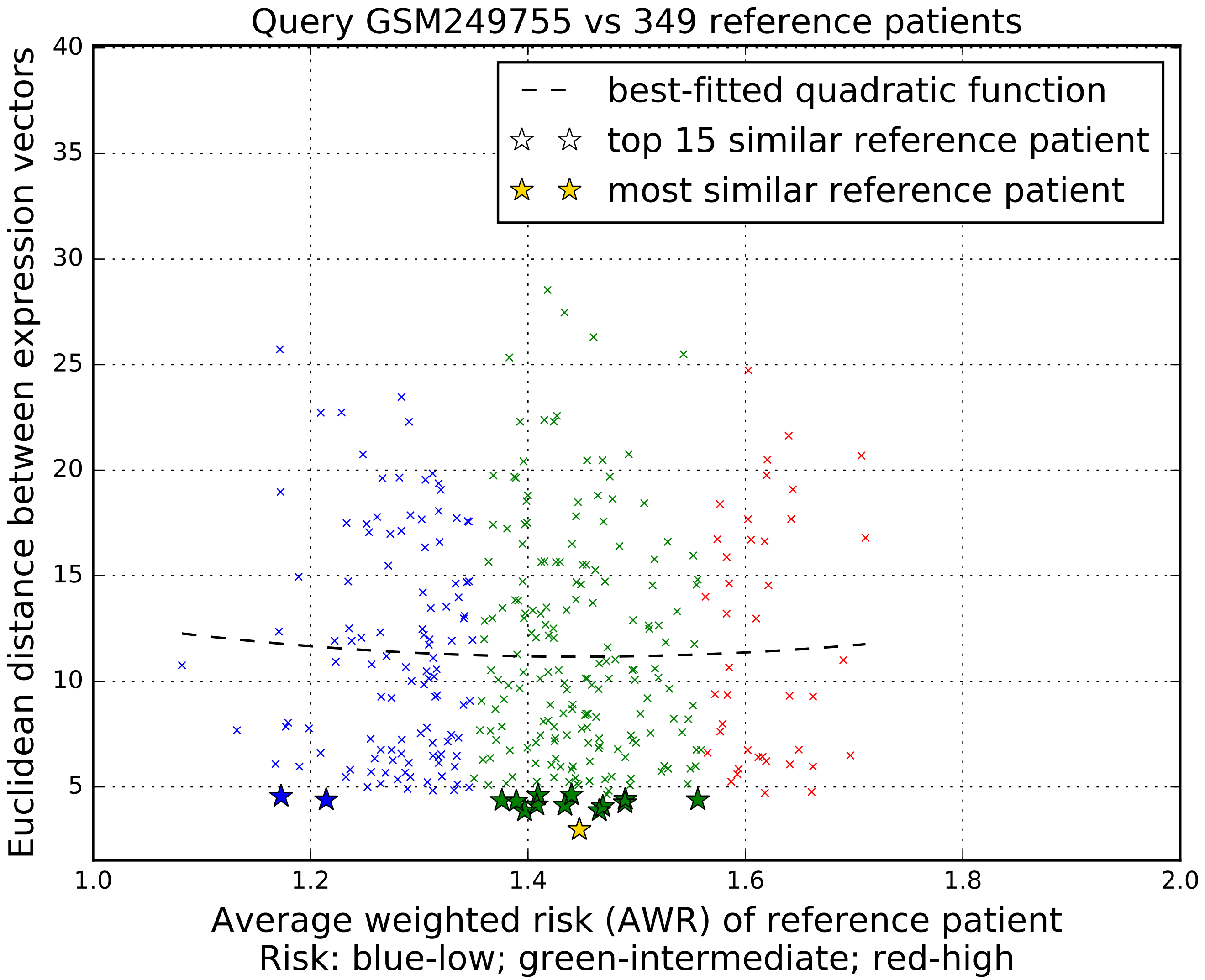
Query GSM249755 vs 349 reference patients
best-fitted quadratic function
top 15 similar reference patient
most similar reference patient
Euclidean distance between expression vectors
40
35
30
25
20
15
10
5
1.0
1.2
1.4
1.6
1.8
2.0
Average weighted risk (AWR) of reference patient
Risk: blue-low; green-intermediate; red-high

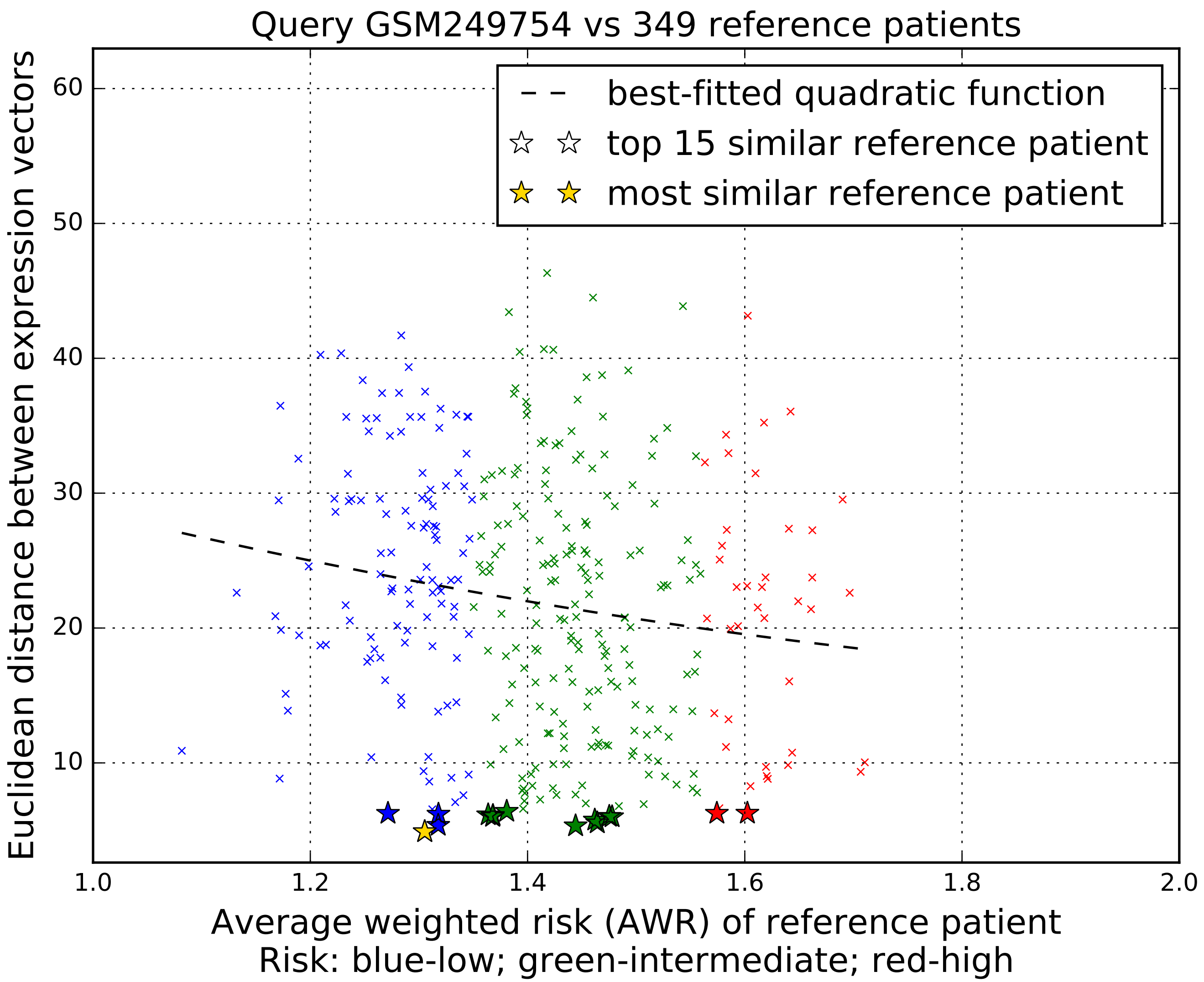

Query GSM249754 vs 349 reference patients
best-fitted quadratic function
top 15 similar reference patient
most similar reference patient
Euclidean distance between expression vectors
Average weighted risk (AWR) of reference patient
Risk: blue-low; green-intermediate; red-high
60
50
40
30
20
10
1.0
1.2
1.4
1.6
1.8
2.0

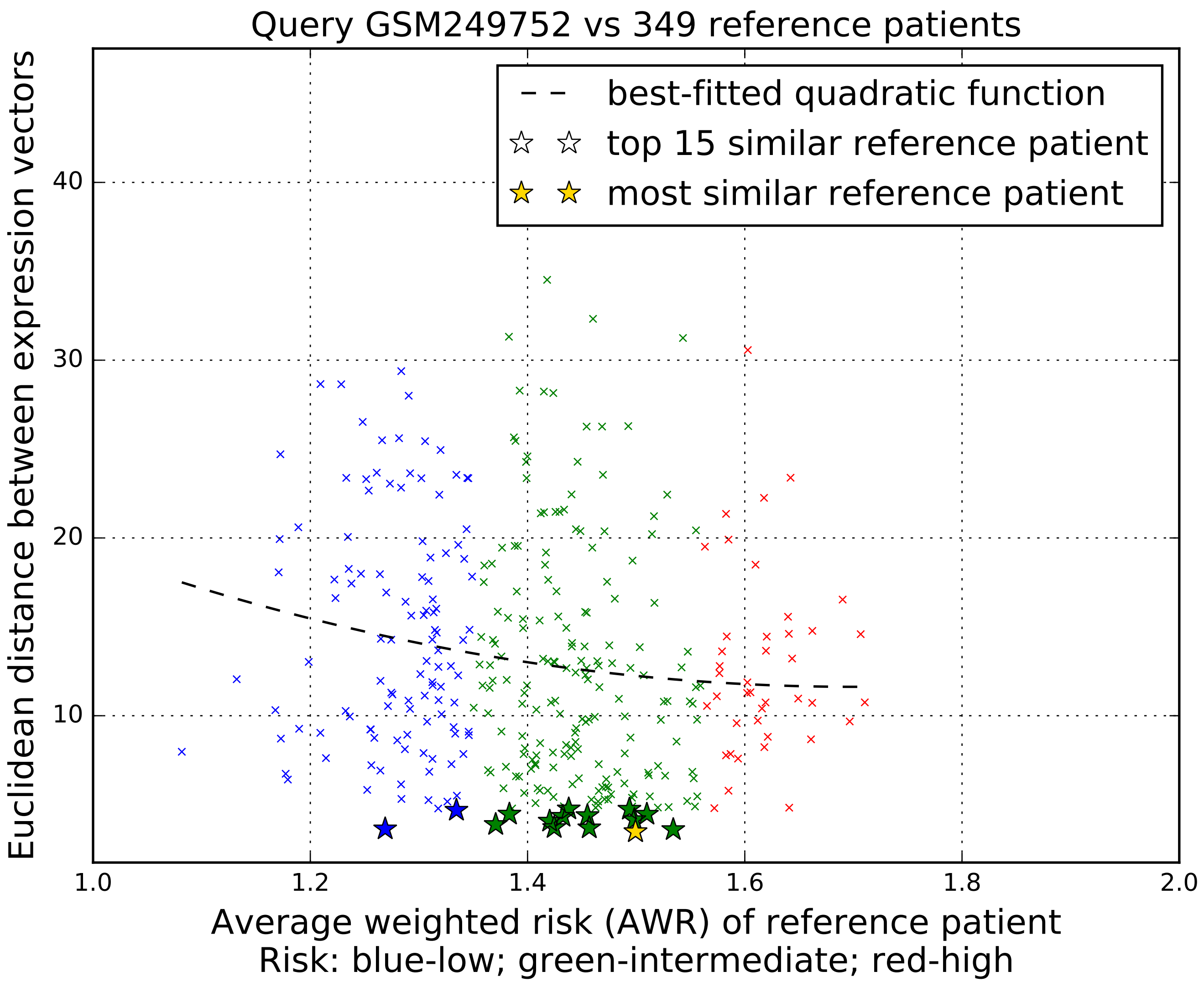
Query GSM249752 vs 349 reference patients
best-fitted quadratic function
top 15 similar reference patient
most similar reference patient
Euclidean distance between expression vectors
Average weighted risk (AWR) of reference patient
Risk: blue-low; green-intermediate; red-high
1.0
1.2
1.4
1.6
1.8
2.0
10
20
30
40

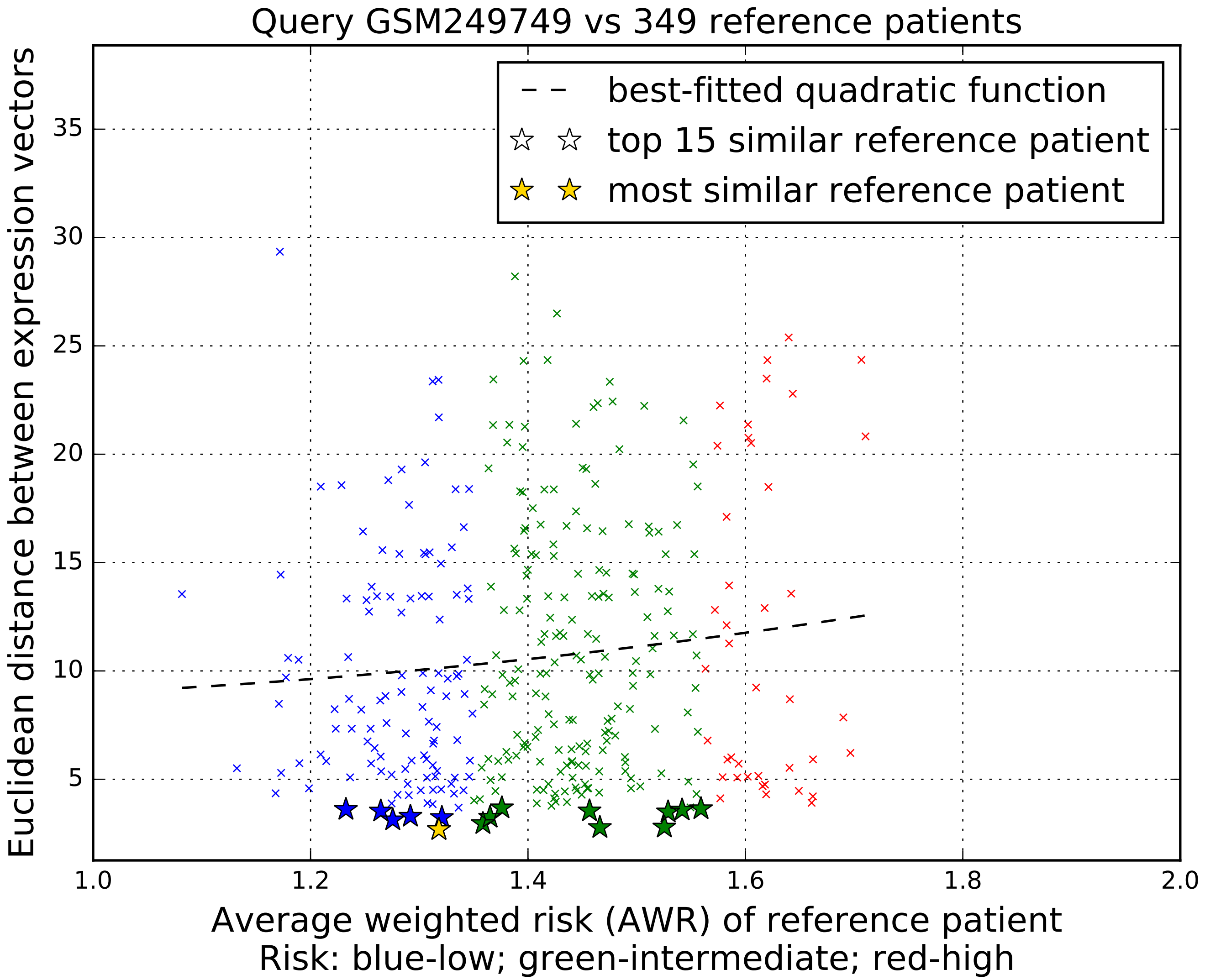

Query GSM249749 vs 349 reference patients
best-fitted quadratic function
top 15 similar reference patient
most similar reference patient
Euclidean distance between expression vectors
5
10
15
20
25
30
35
1.0
1.2
1.4
1.6
1.8
2.0
Average weighted risk (AWR) of reference patient
Risk: blue-low; green-intermediate; red-high

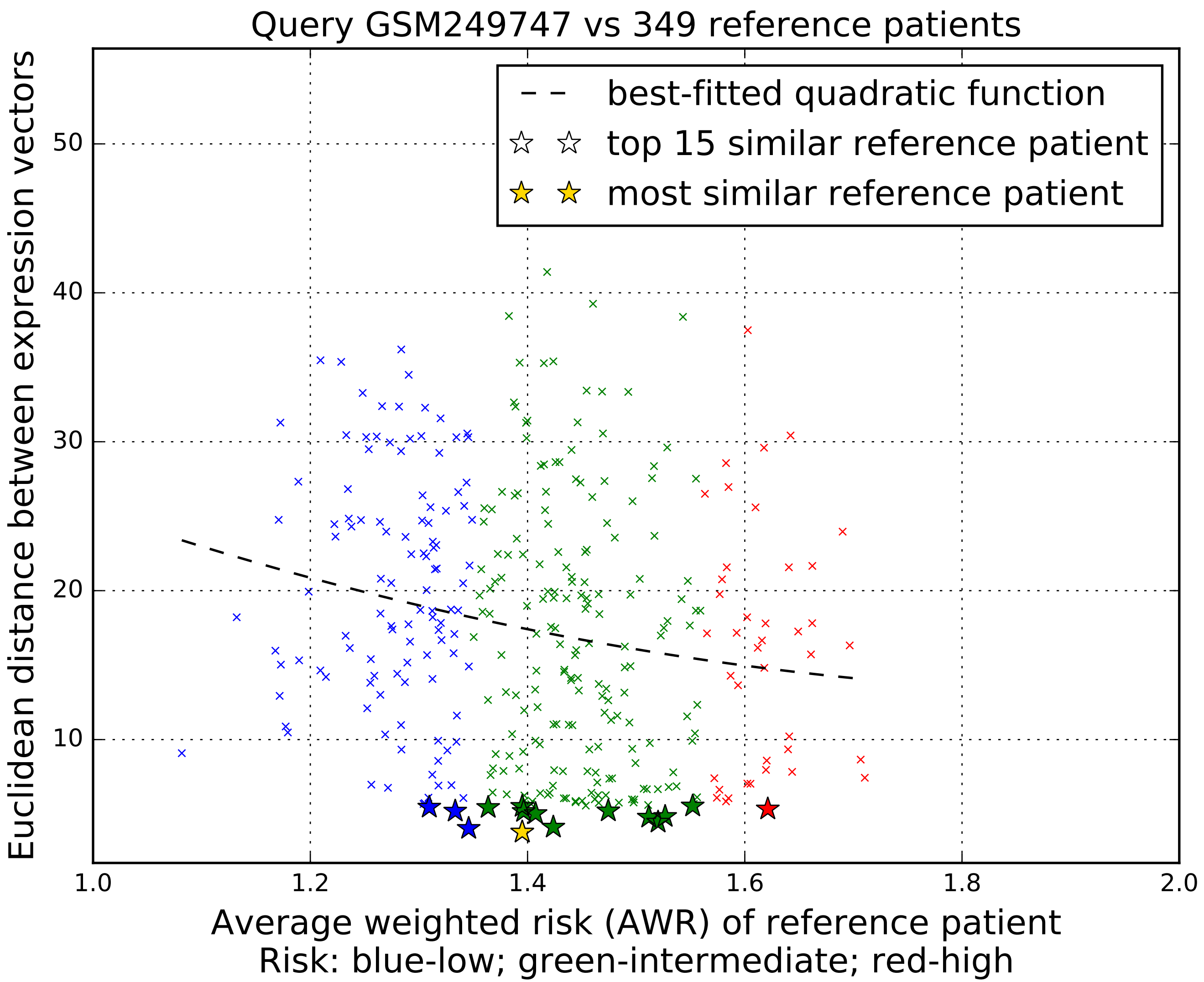

Query GSM249747 vs 349 reference patients
best-fitted quadratic function
top 15 similar reference patient
most similar reference patient
Euclidean distance between expression vectors
10
20
30
40
50
1.0
1.2
1.4
1.6
1.8
2.0
Average weighted risk (AWR) of reference patient
Risk: blue-low; green-intermediate; red-high

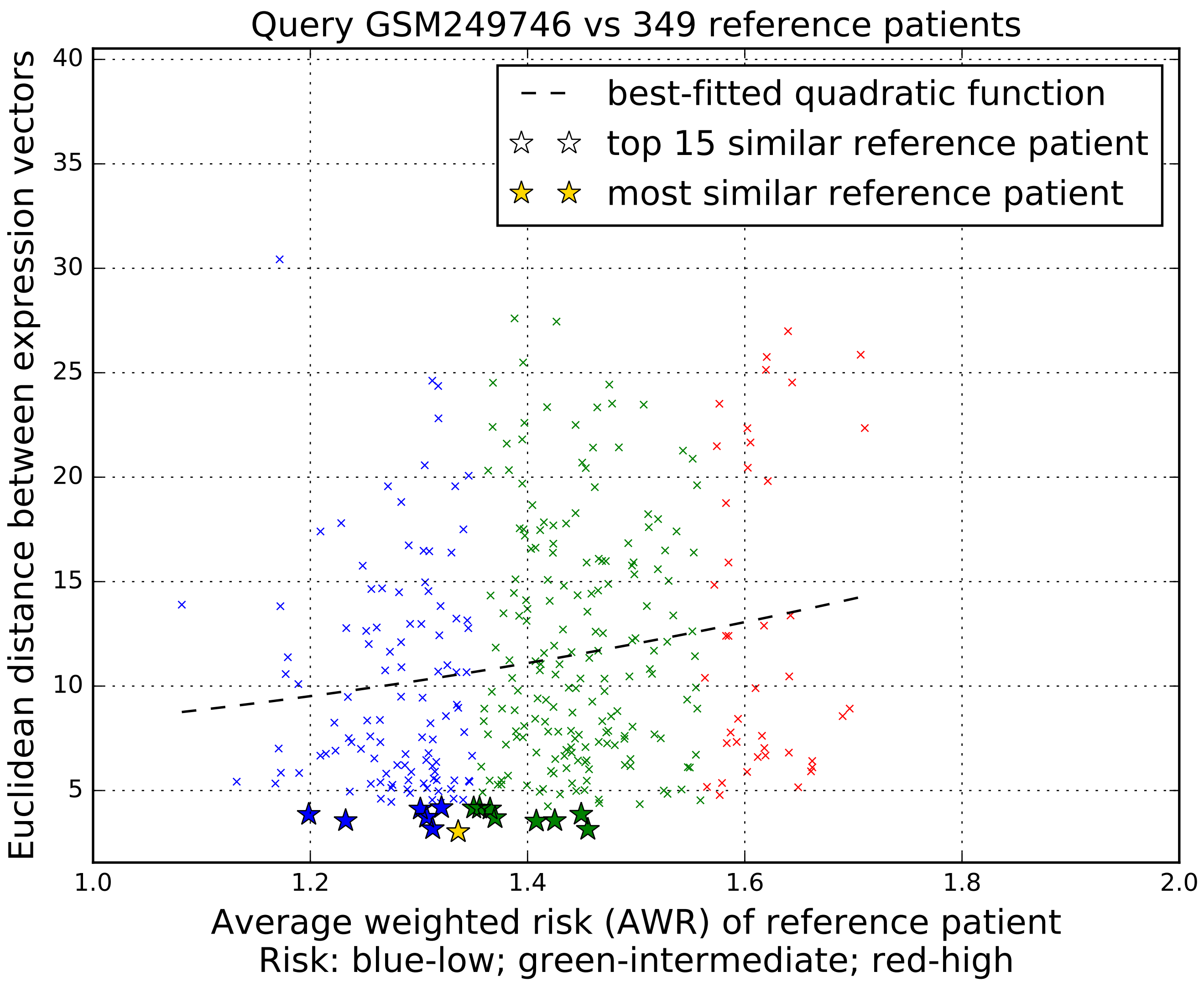

Query GSM249746 vs 349 reference patients
best-fitted quadratic function
top 15 similar reference patient
most similar reference patient
Euclidean distance between expression vectors
40
35
30
25
20
15
10
5
1.0
1.2
1.4
1.6
1.8
2.0
Average weighted risk (AWR) of reference patient
Risk: blue-low; green-intermediate; red-high

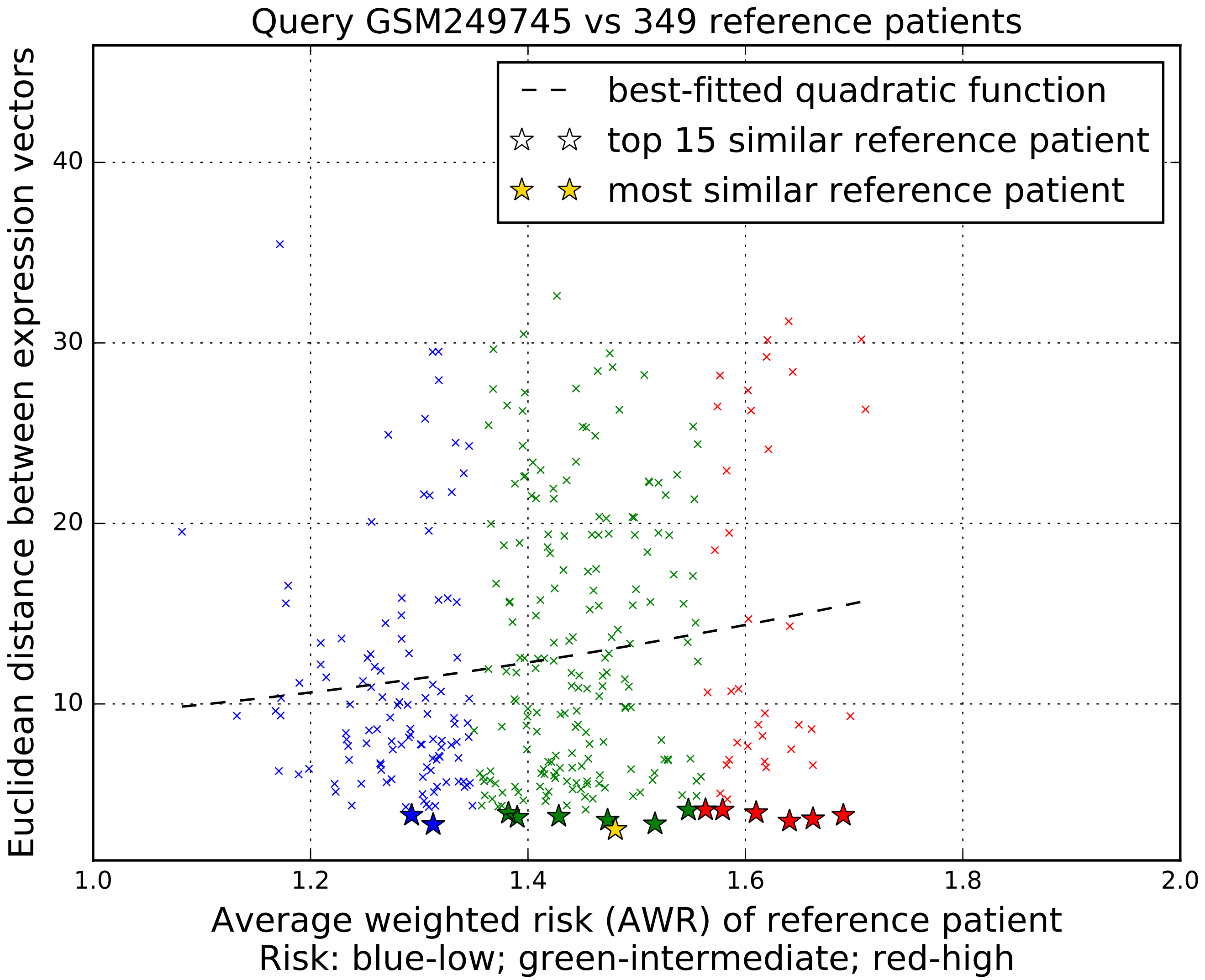

Query GSM249745 vs 349 reference patients
best-fitted quadratic function
top 15 similar reference patient
most similar reference patient
Euclidean distance between expression vectors
40
30
20
10
1.0
1.2
1.4
1.6
1.8
2.0
Average weighted risk (AWR) of reference patient
Risk: blue-low; green-intermediate; red-high

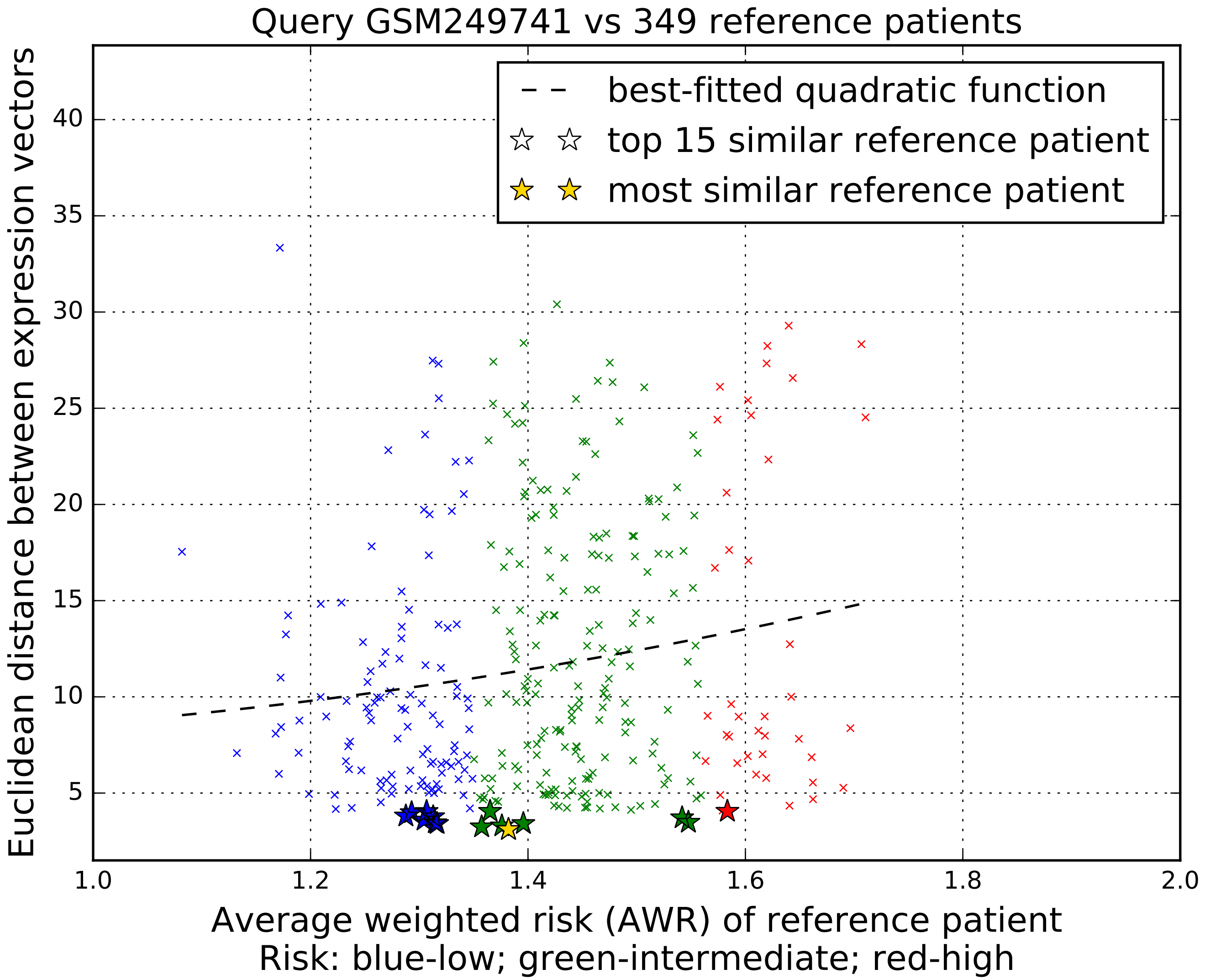

Query GSM249741 vs 349 reference patients
best-fitted quadratic function
top 15 similar reference patient
most similar reference patient
Euclidean distance between expression vectors
40
35
30
25
20
15
10
5
1.0
1.2
1.4
1.6
1.8
2.0
Average weighted risk (AWR) of reference patient
Risk: blue-low; green-intermediate; red-high

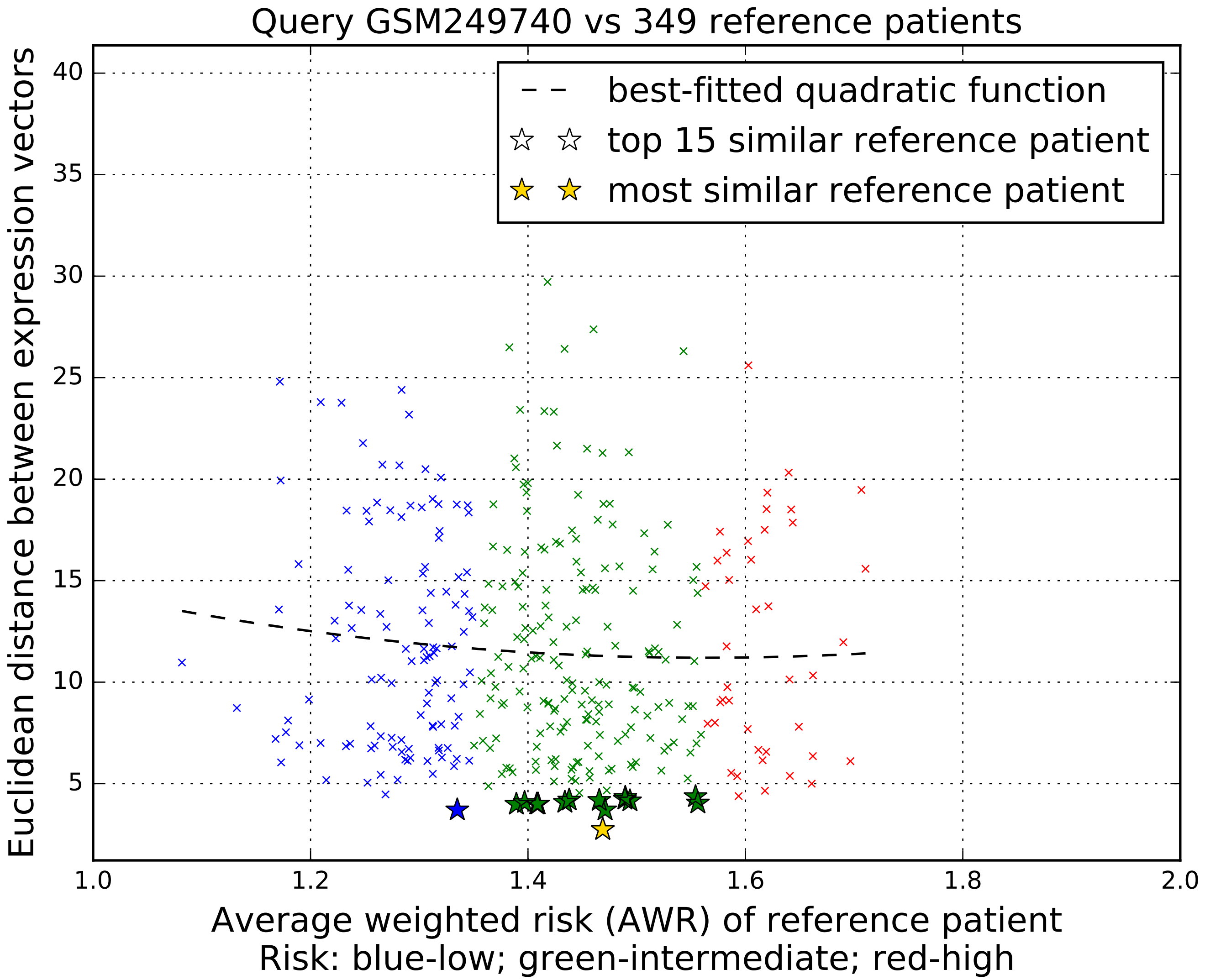

Query GSM249740 vs 349 reference patients
best-fitted quadratic function
top 15 similar reference patient
most similar reference patient
Euclidean distance between expression vectors
40
35
30
25
20
15
10
5
1.0
1.2
1.4
1.6
1.8
2.0
Average weighted risk (AWR) of reference patient
Risk: blue-low; green-intermediate; red-high

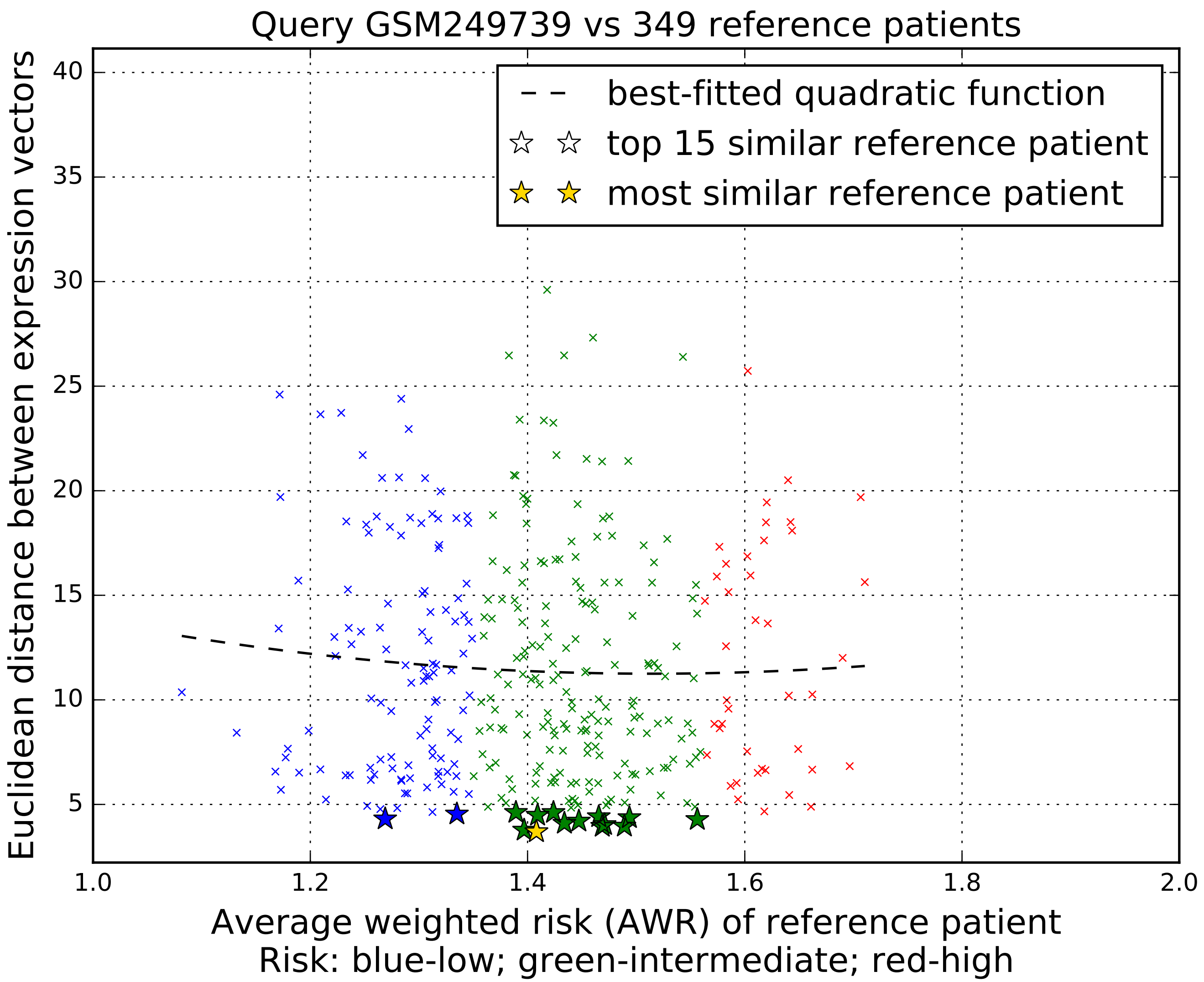
Query GSM249739 vs 349 reference patients
best-fitted quadratic function
top 15 similar reference patient
most similar reference patient
Euclidean distance between expression vectors
40
35
30
25
20
15
10
5
1.0
1.2
1.4
1.6
1.8
2.0
Average weighted risk (AWR) of reference patient
Risk: blue-low; green-intermediate; red-high

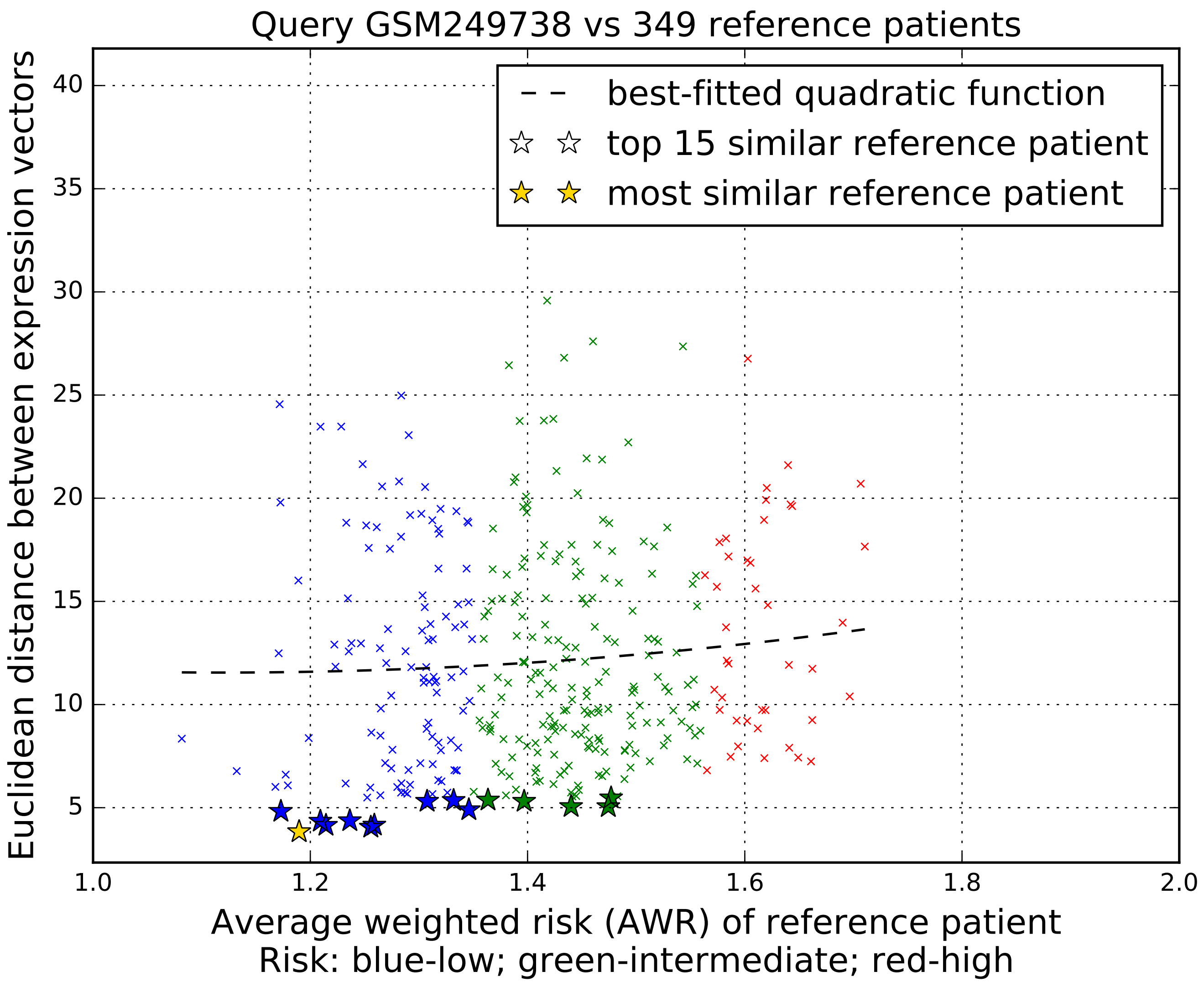

Query GSM249738 vs 349 reference patients
best-fitted quadratic function
top 15 similar reference patient
most similar reference patient
Euclidean distance between expression vectors
40
35
30
25
20
15
10
5
1.0
1.2
1.4
1.6
1.8
2.0
Average weighted risk (AWR) of reference patient
Risk: blue-low; green-intermediate; red-high

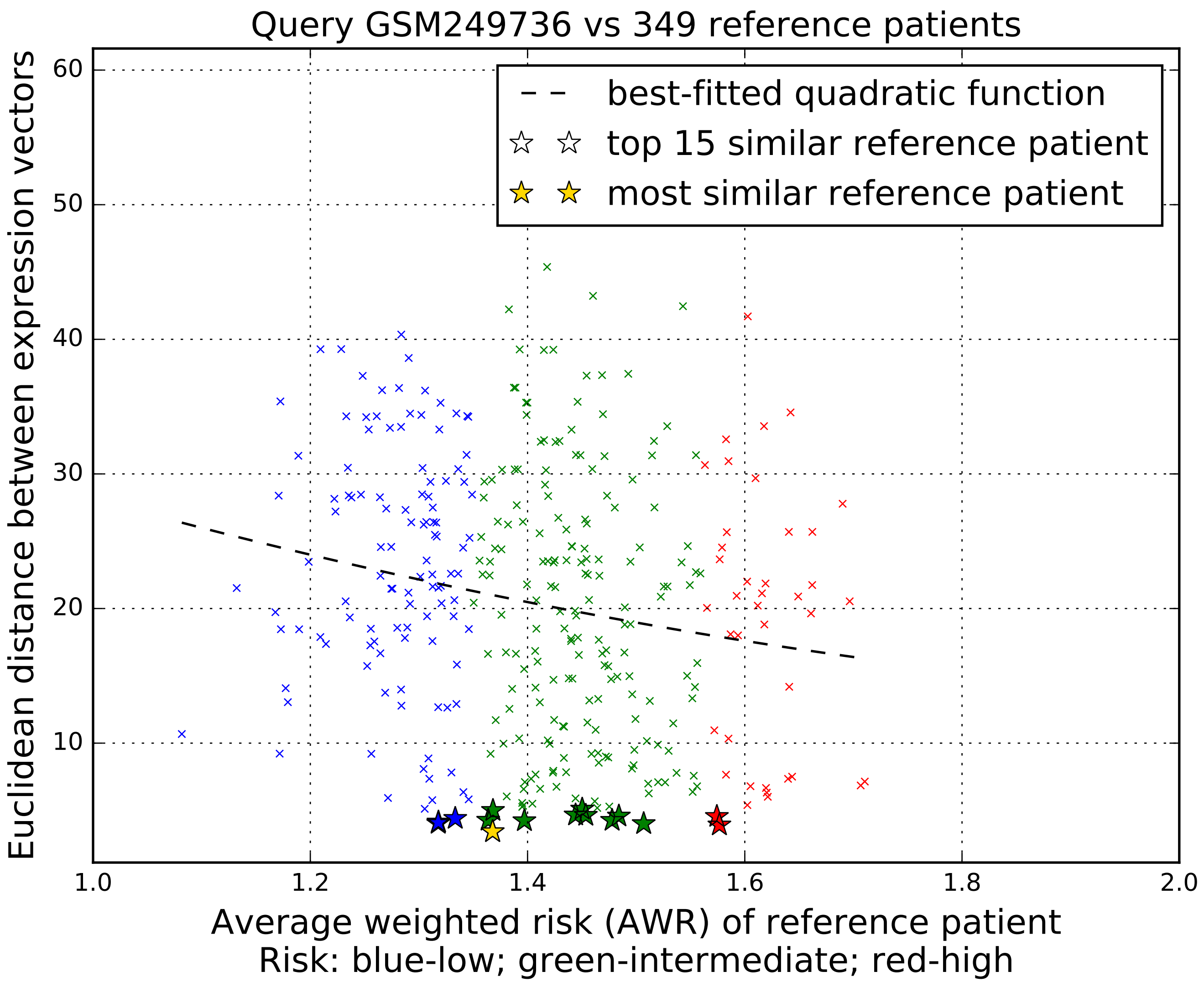

Query GSM249736 vs 349 reference patients
best-fitted quadratic function
top 15 similar reference patient
most similar reference patient
Euclidean distance between expression vectors
Average weighted risk (AWR) of reference patient
Risk: blue-low; green-intermediate; red-high
60
50
40
30
20
10
1.0
1.2
1.4
1.6
1.8
2.0

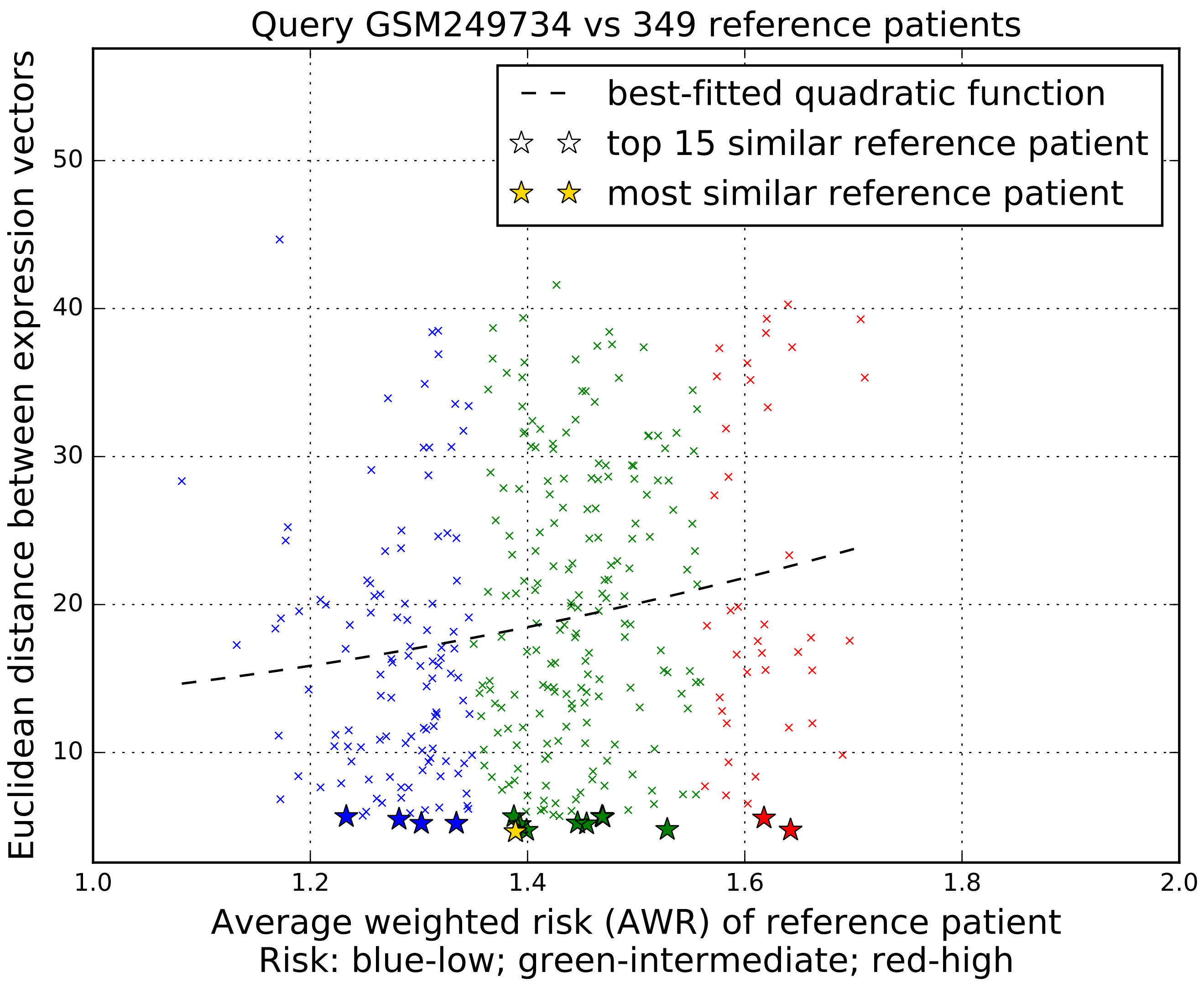

Query GSM249734 vs 349 reference patients
best-fitted quadratic function
top 15 similar reference patient
most similar reference patient
Euclidean distance between expression vectors
Average weighted risk (AWR) of reference patient
Risk: blue-low; green-intermediate; red-high
1.0
1.2
1.4
1.6
1.8
2.0
10
20
30
40
50

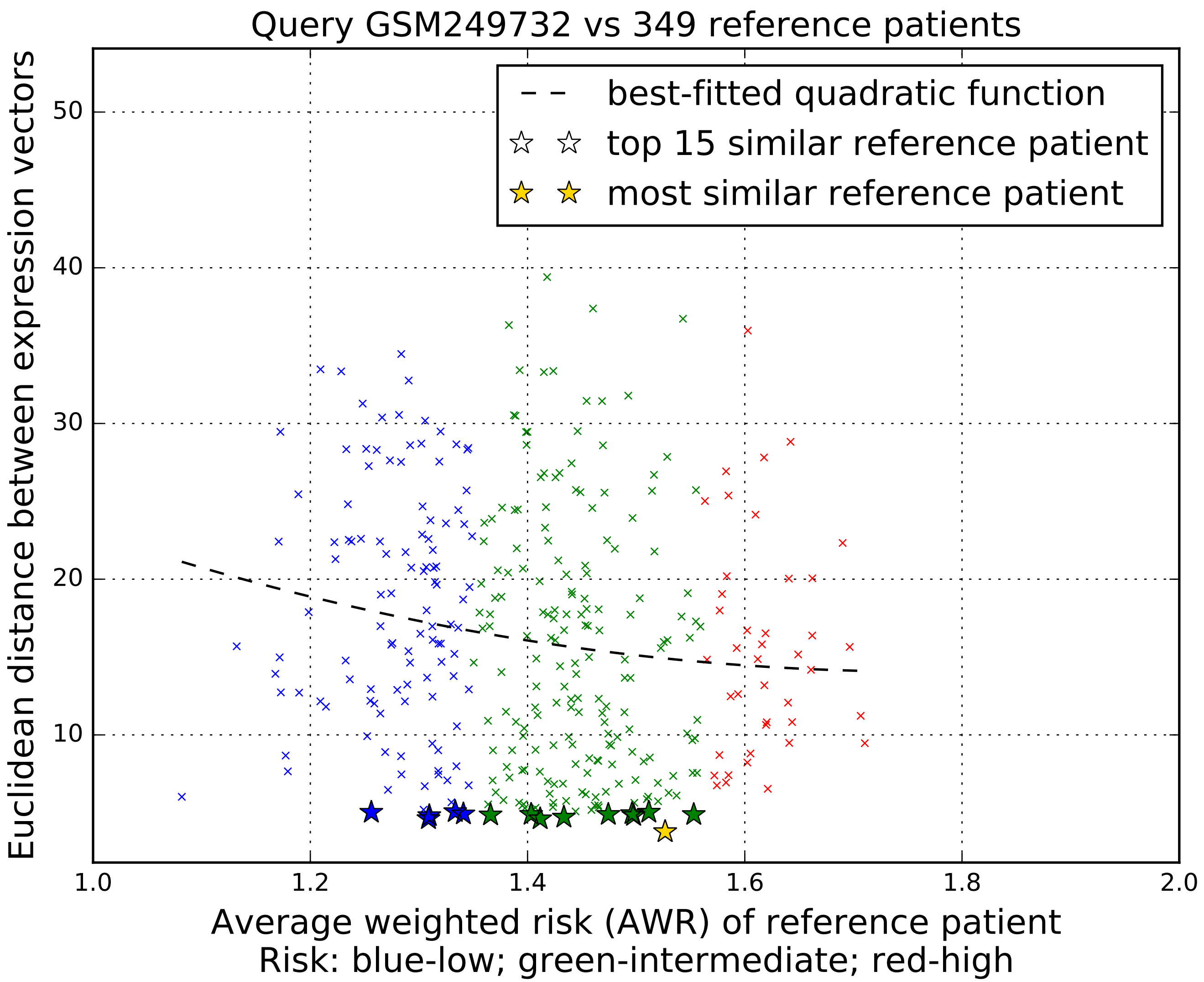

Query GSM249732 vs 349 reference patients
best-fitted quadratic function
top 15 similar reference patient
most similar reference patient
Euclidean distance between expression vectors
50
40
30
20
10
1.0
1.2
1.4
1.6
1.8
2.0
Average weighted risk (AWR) of reference patient
Risk: blue-low; green-intermediate; red-high

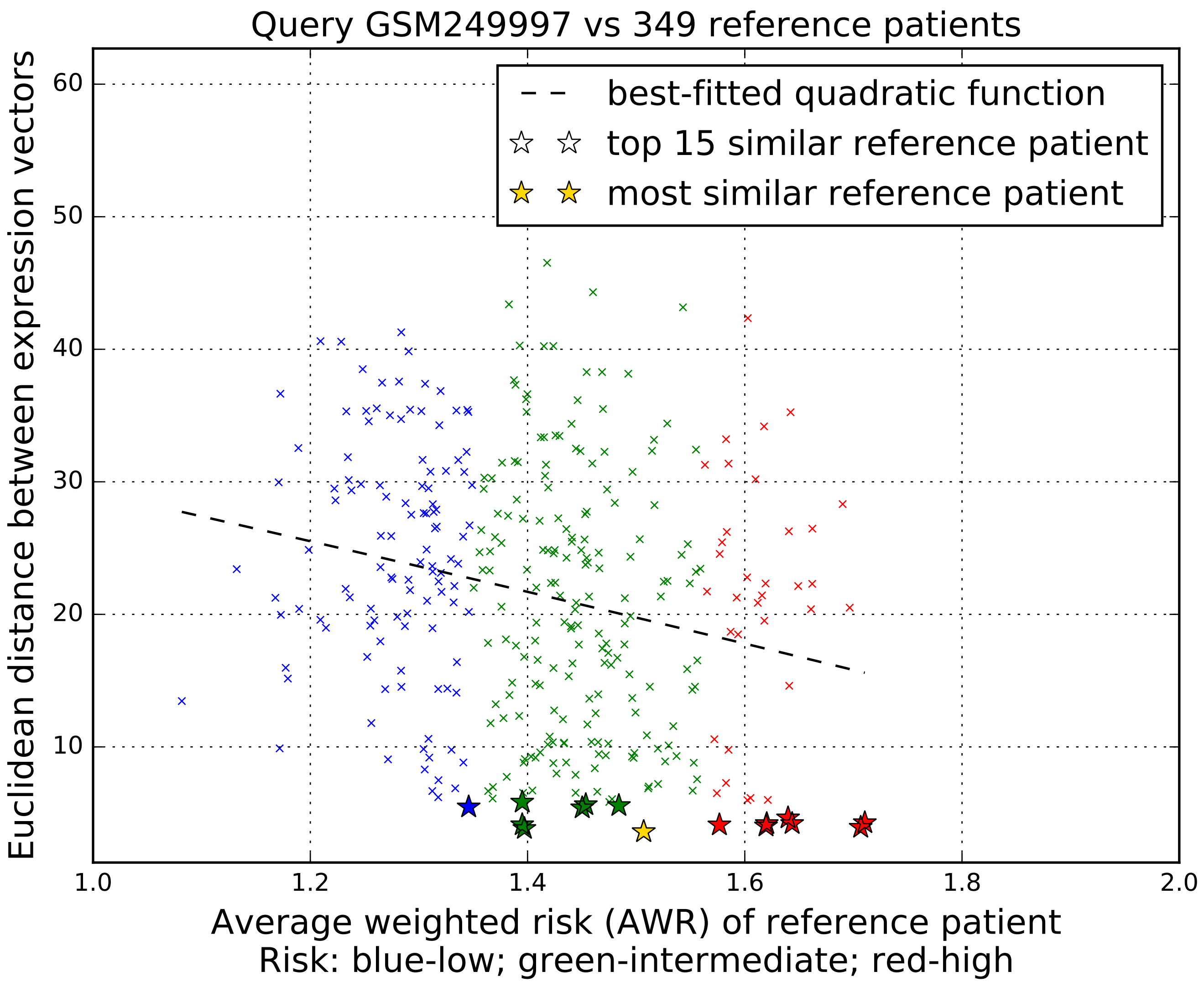

Query GSM249997 vs 349 reference patients
best-fitted quadratic function
top 15 similar reference patient
most similar reference patient
Euclidean distance between expression vectors
60
50
40
30
20
10
1.0
1.2
1.4
1.6
1.8
2.0
Average weighted risk (AWR) of reference patient
Risk: blue-low; green-intermediate; red-high

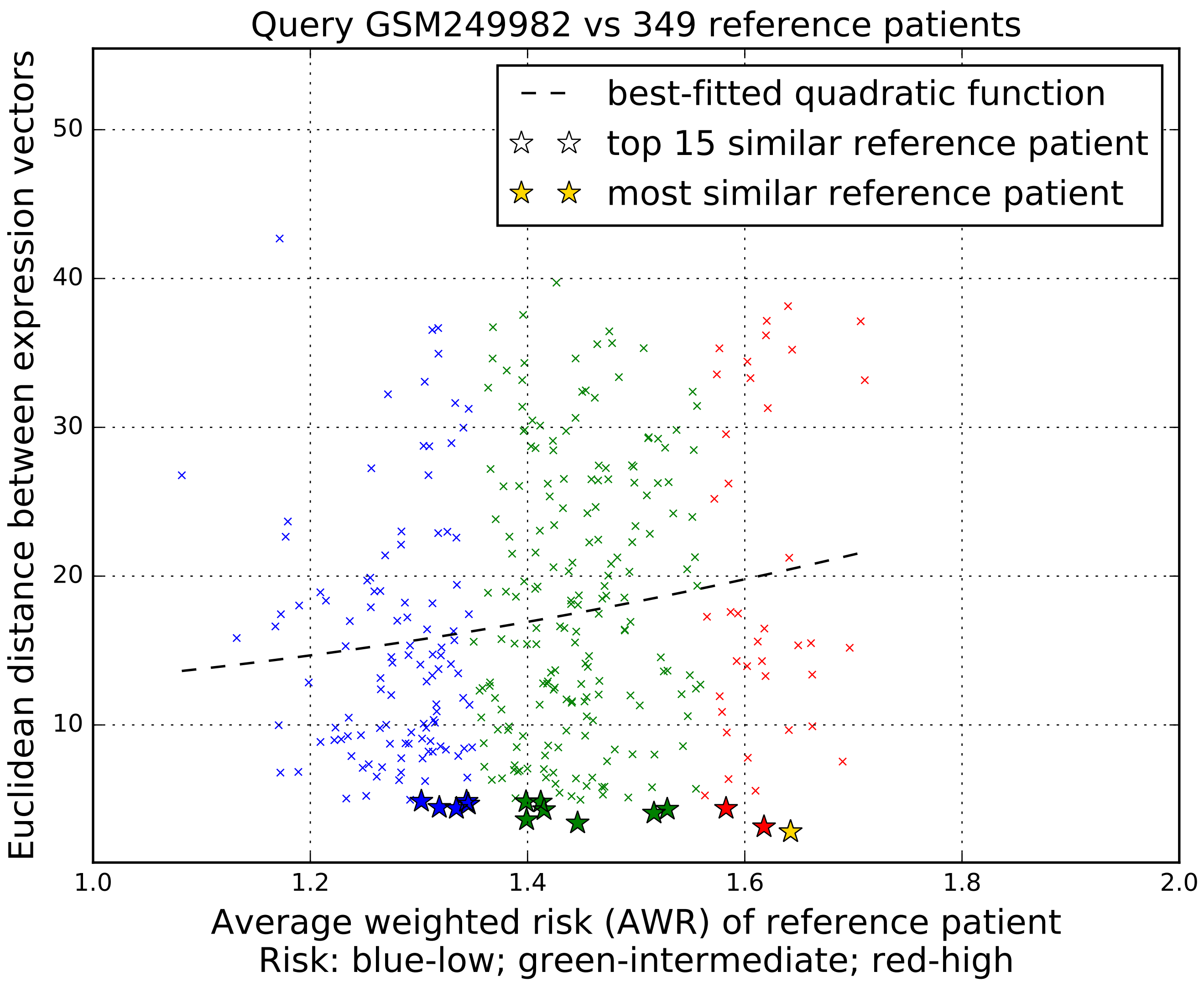
Query GSM249982 vs 349 reference patients
best-fitted quadratic function
top 15 similar reference patient
most similar reference patient
Euclidean distance between expression vectors
Average weighted risk (AWR) of reference patient
Risk: blue-low; green-intermediate; red-high
10
20
30
40
50
1.0
1.2
1.4
1.6
1.8
2.0

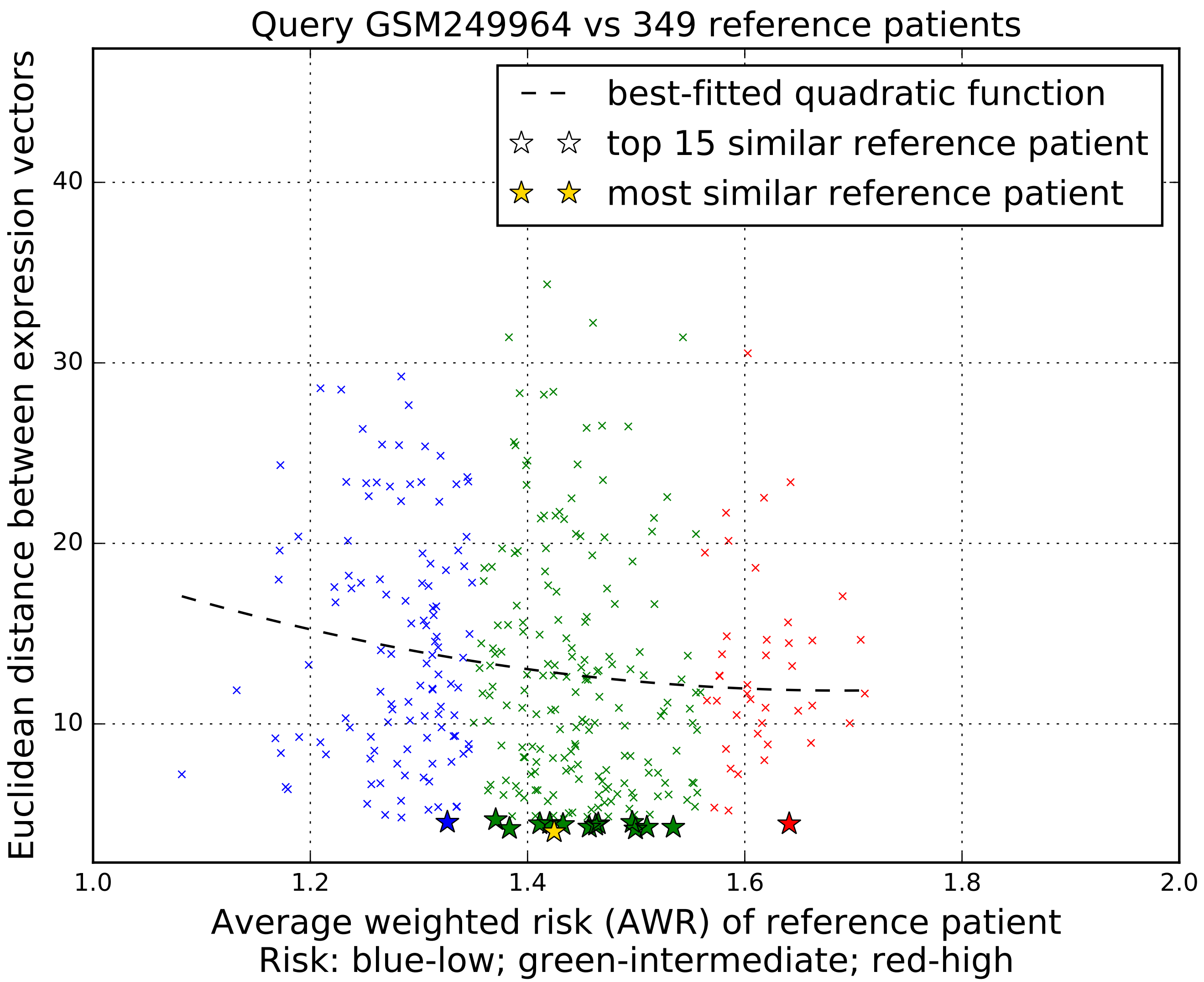

Query GSM249964 vs 349 reference patients
best-fitted quadratic function
top 15 similar reference patient
most similar reference patient
Euclidean distance between expression vectors
40
30
20
10
1.0
1.2
1.4
1.6
1.8
2.0
Average weighted risk (AWR) of reference patient
Risk: blue-low; green-intermediate; red-high

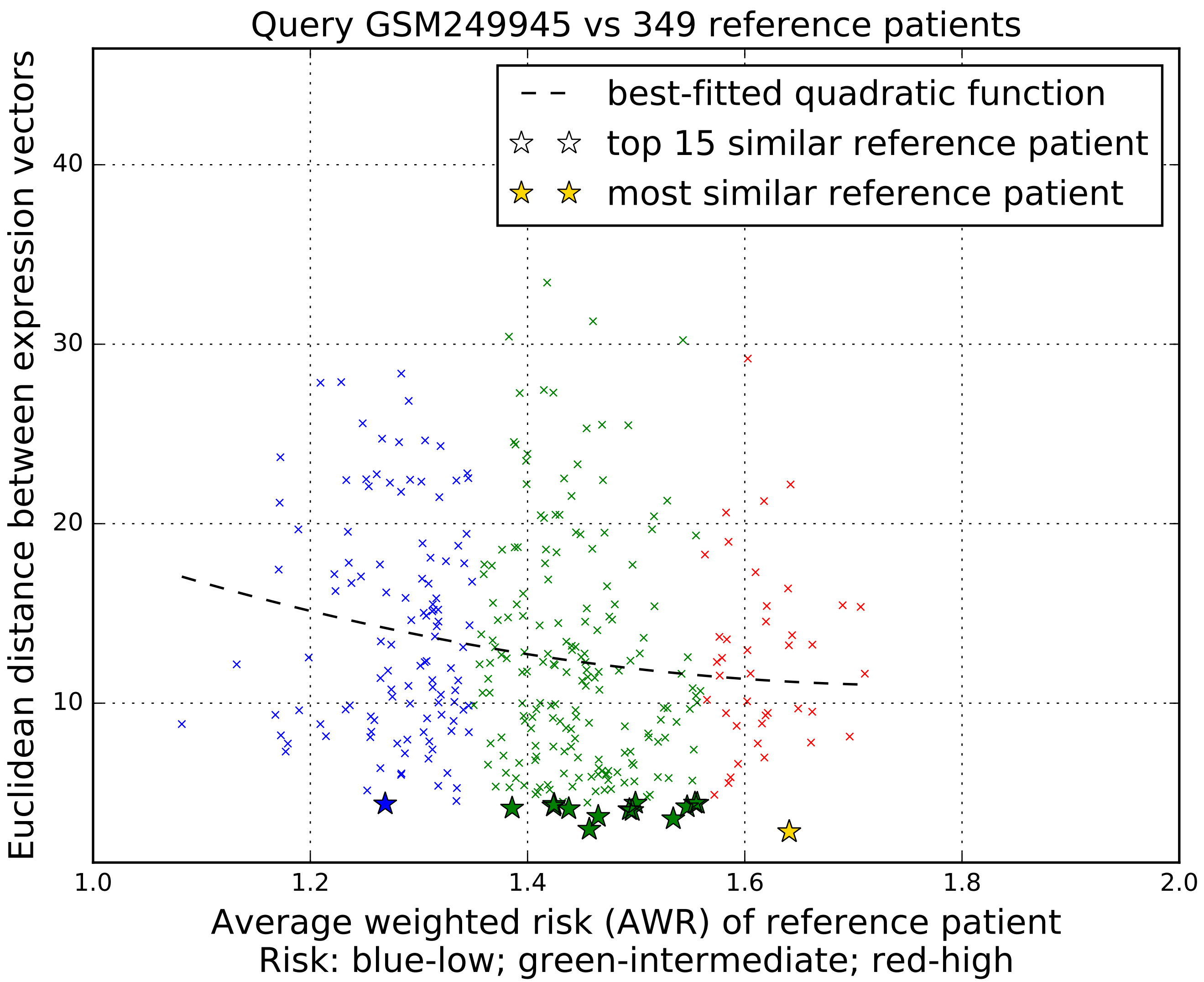
Query GSM249945 vs 349 reference patients
best-fitted quadratic function
top 15 similar reference patient
most similar reference patient
Euclidean distance between expression vectors
40
30
20
10
1.0
1.2
1.4
1.6
1.8
2.0
Average weighted risk (AWR) of reference patient
Risk: blue-low; green-intermediate; red-high

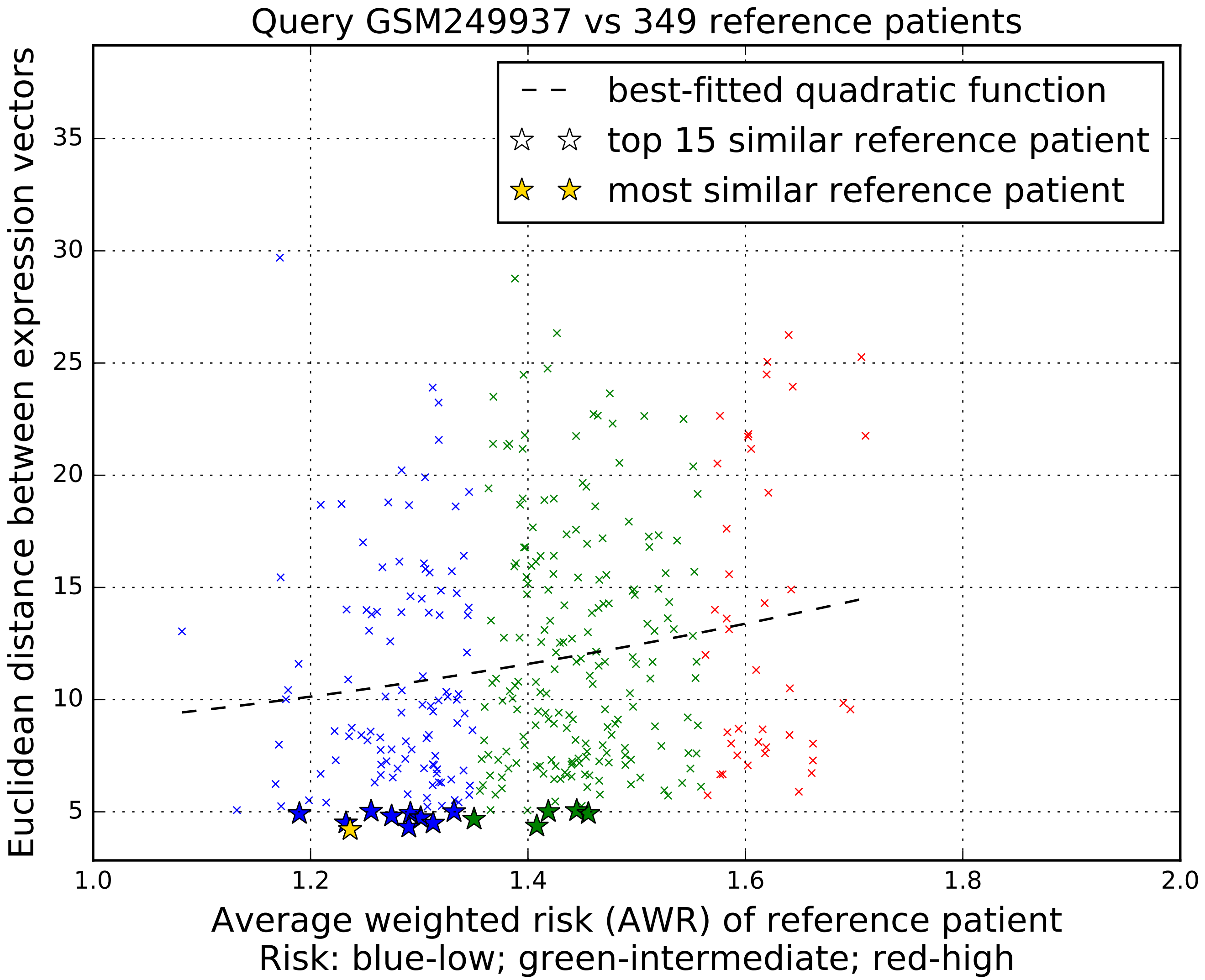
Query GSM249937 vs 349 reference patients
best-fitted quadratic function
top 15 similar reference patient
most similar reference patient
Euclidean distance between expression vectors
35
30
25
20
15
10
5
1.0
1.2
1.4
1.6
1.8
2.0
Average weighted risk (AWR) of reference patient
Risk: blue-low; green-intermediate; red-high

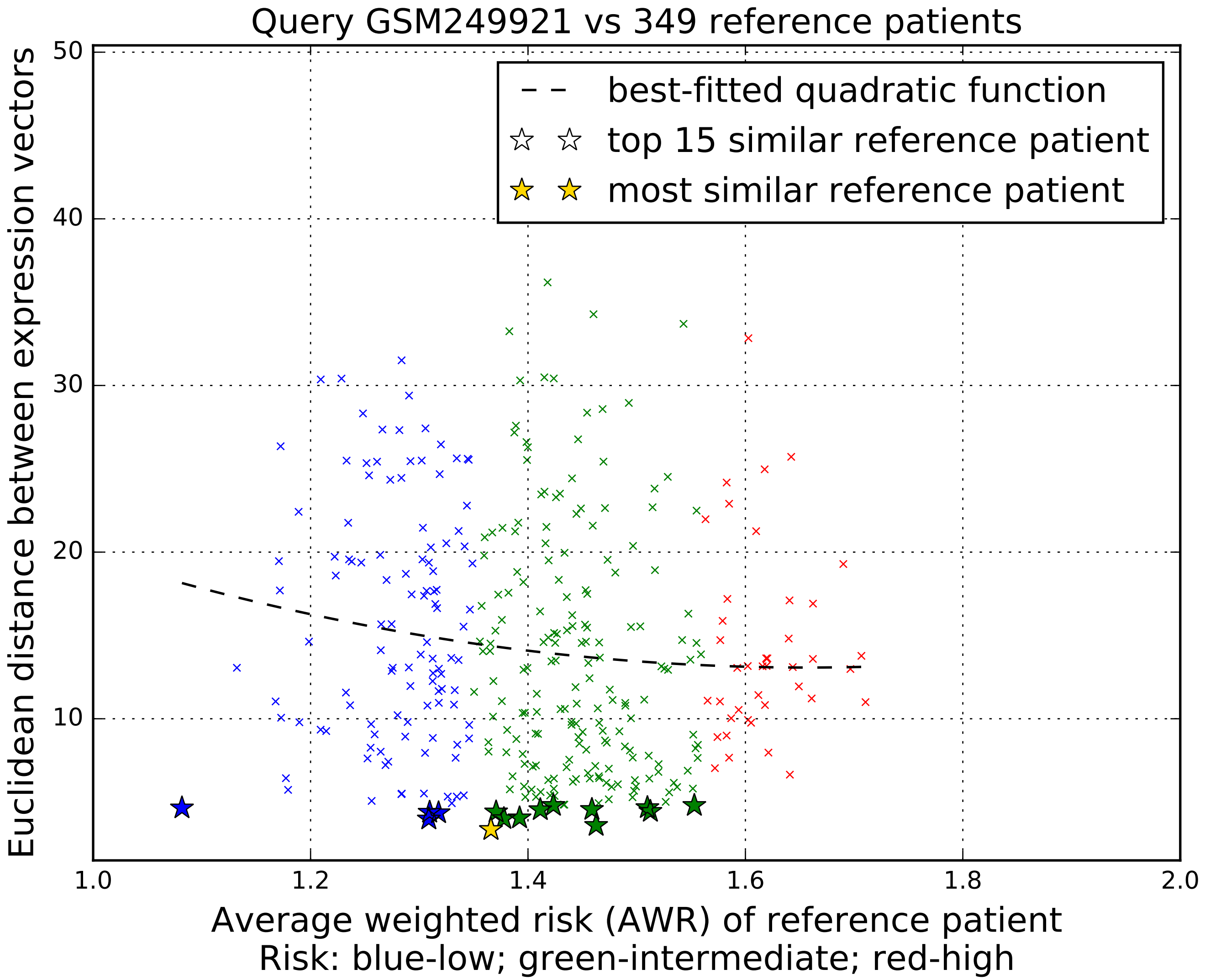

Query GSM249921 vs 349 reference patients
best-fitted quadratic function
top 15 similar reference patient
most similar reference patient
Euclidean distance between expression vectors
50
40
30
20
10
1.0
1.2
1.4
1.6
1.8
2.0
Average weighted risk (AWR) of reference patient
Risk: blue-low; green-intermediate; red-high

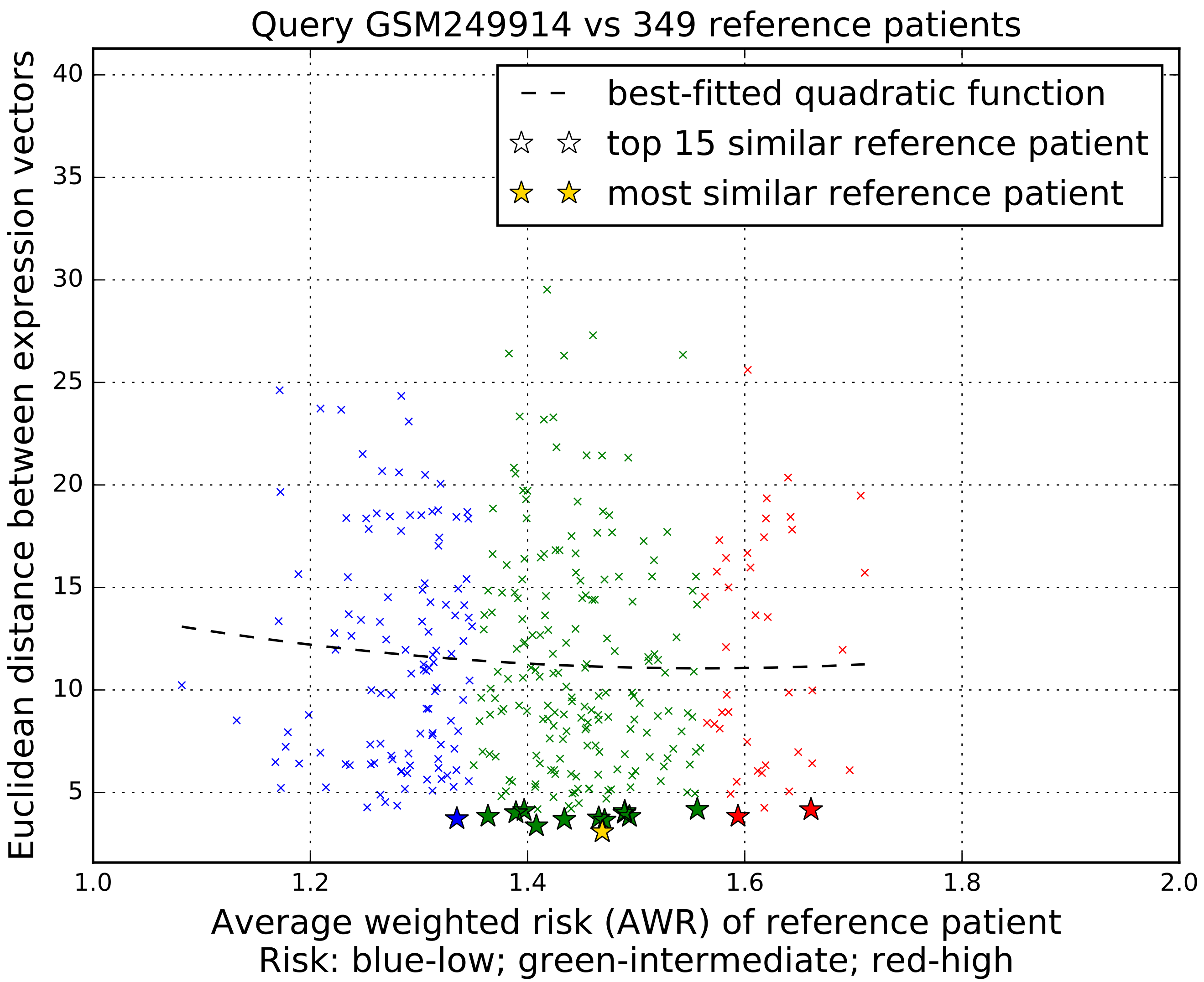

Query GSM249914 vs 349 reference patients
best-fitted quadratic function
top 15 similar reference patient
most similar reference patient
Euclidean distance between expression vectors
40
35
30
25
20
15
10
5
1.0
1.2
1.4
1.6
1.8
2.0
Average weighted risk (AWR) of reference patient
Risk: blue-low; green-intermediate; red-high

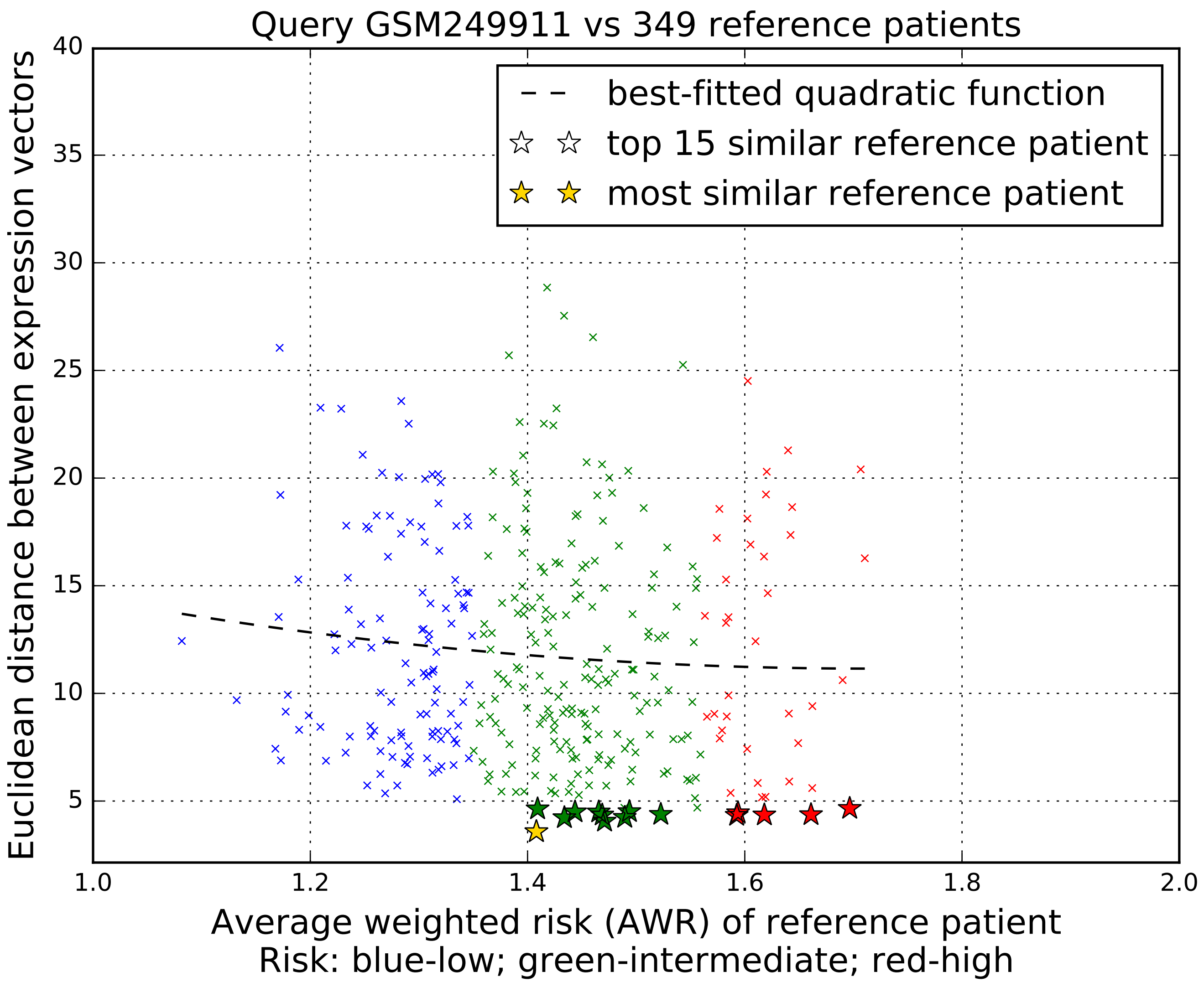

Query GSM249911 vs 349 reference patients
best-fitted quadratic function
top 15 similar reference patient
most similar reference patient
Euclidean distance between expression vectors
Average weighted risk (AWR) of reference patient
Risk: blue-low; green-intermediate; red-high
40
35
30
25
20
15
10
5
1.0
1.2
1.4
1.6
1.8
2.0

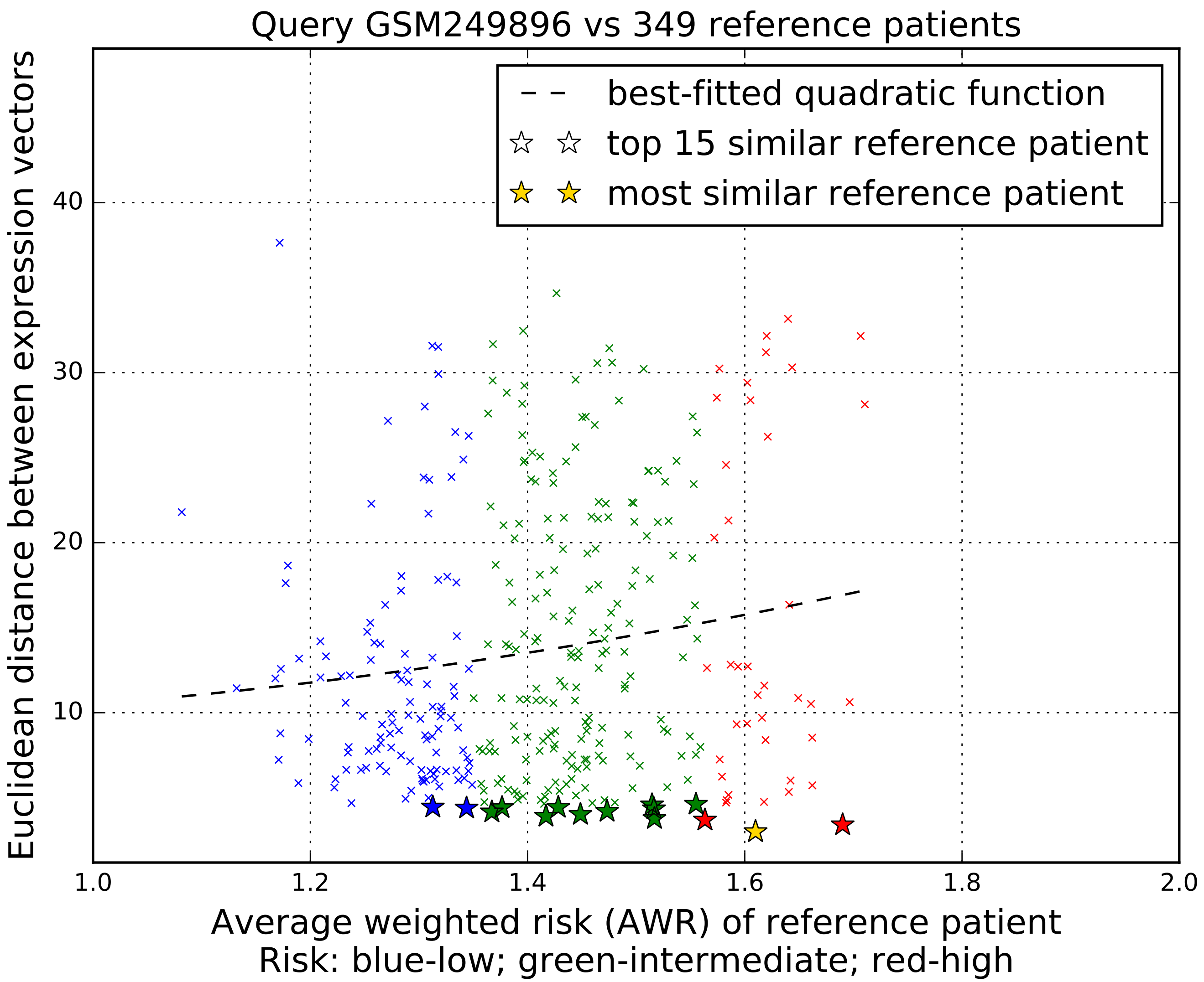
Query GSM249896 vs 349 reference patients
best-fitted quadratic function
top 15 similar reference patient
most similar reference patient
Euclidean distance between expression vectors
10
20
30
40
1.0
1.2
1.4
1.6
1.8
2.0
Average weighted risk (AWR) of reference patient
Risk: blue-low; green-intermediate; red-high

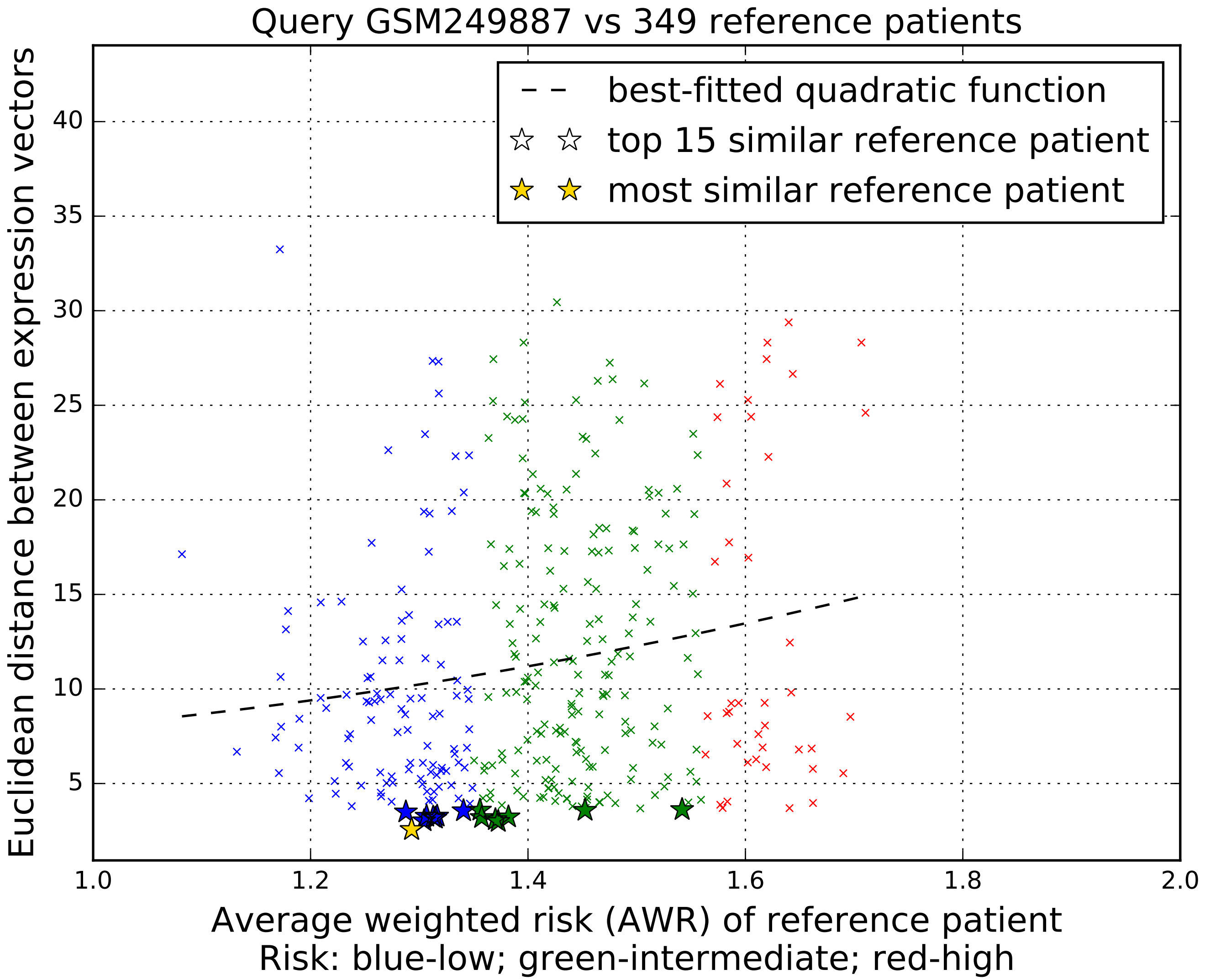

Query GSM249887 vs 349 reference patients
best-fitted quadratic function
top 15 similar reference patient
most similar reference patient
Euclidean distance between expression vectors
40
35
30
25
20
15
10
5
1.0
1.2
1.4
1.6
1.8
2.0
Average weighted risk (AWR) of reference patient
Risk: blue-low; green-intermediate; red-high

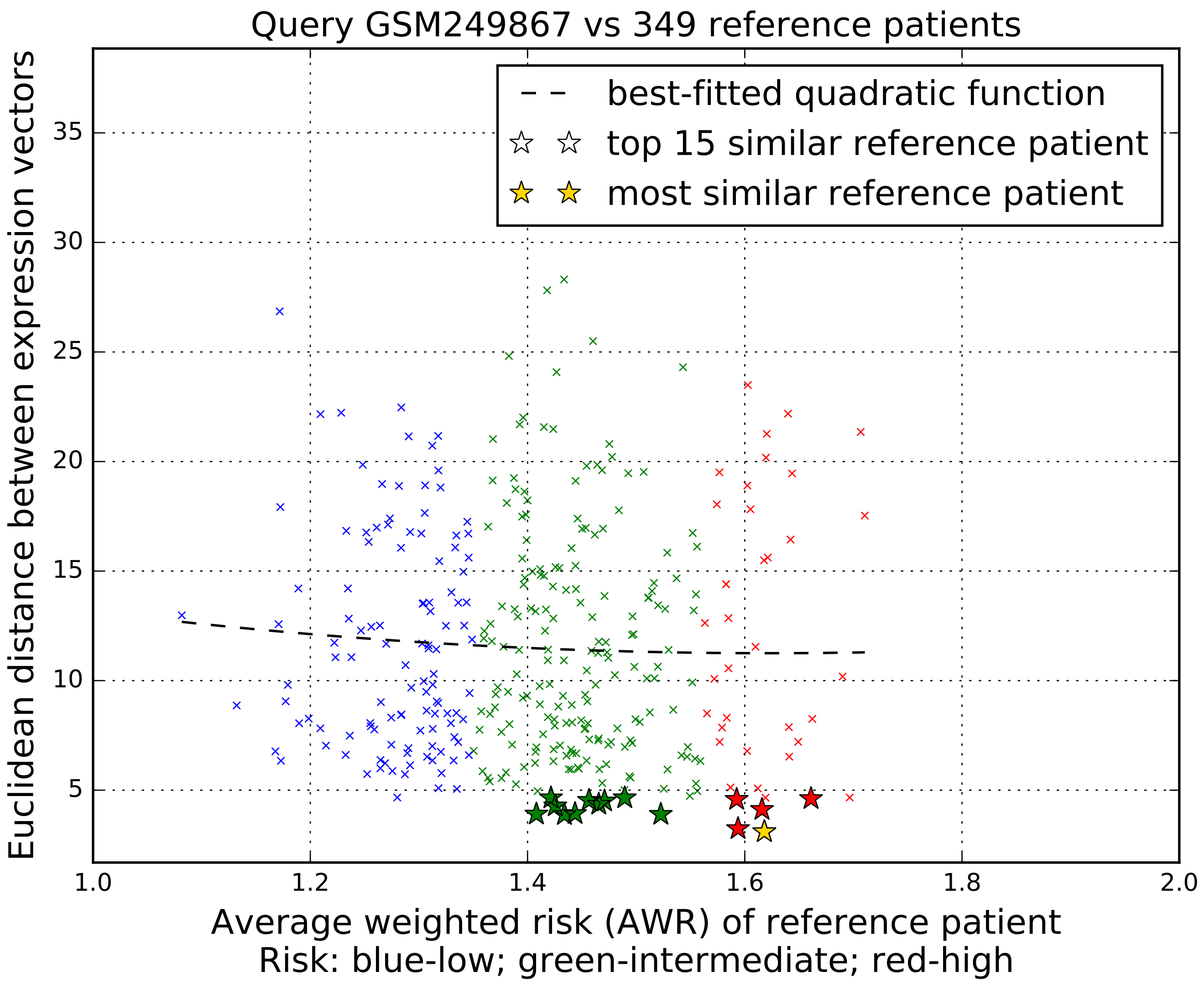

Query GSM249867 vs 349 reference patients
best-fitted quadratic function
top 15 similar reference patient
most similar reference patient
Euclidean distance between expression vectors
35
30
25
20
15
10
5
1.0
1.2
1.4
1.6
1.8
2.0
Average weighted risk (AWR) of reference patient
Risk: blue-low; green-intermediate; red-high

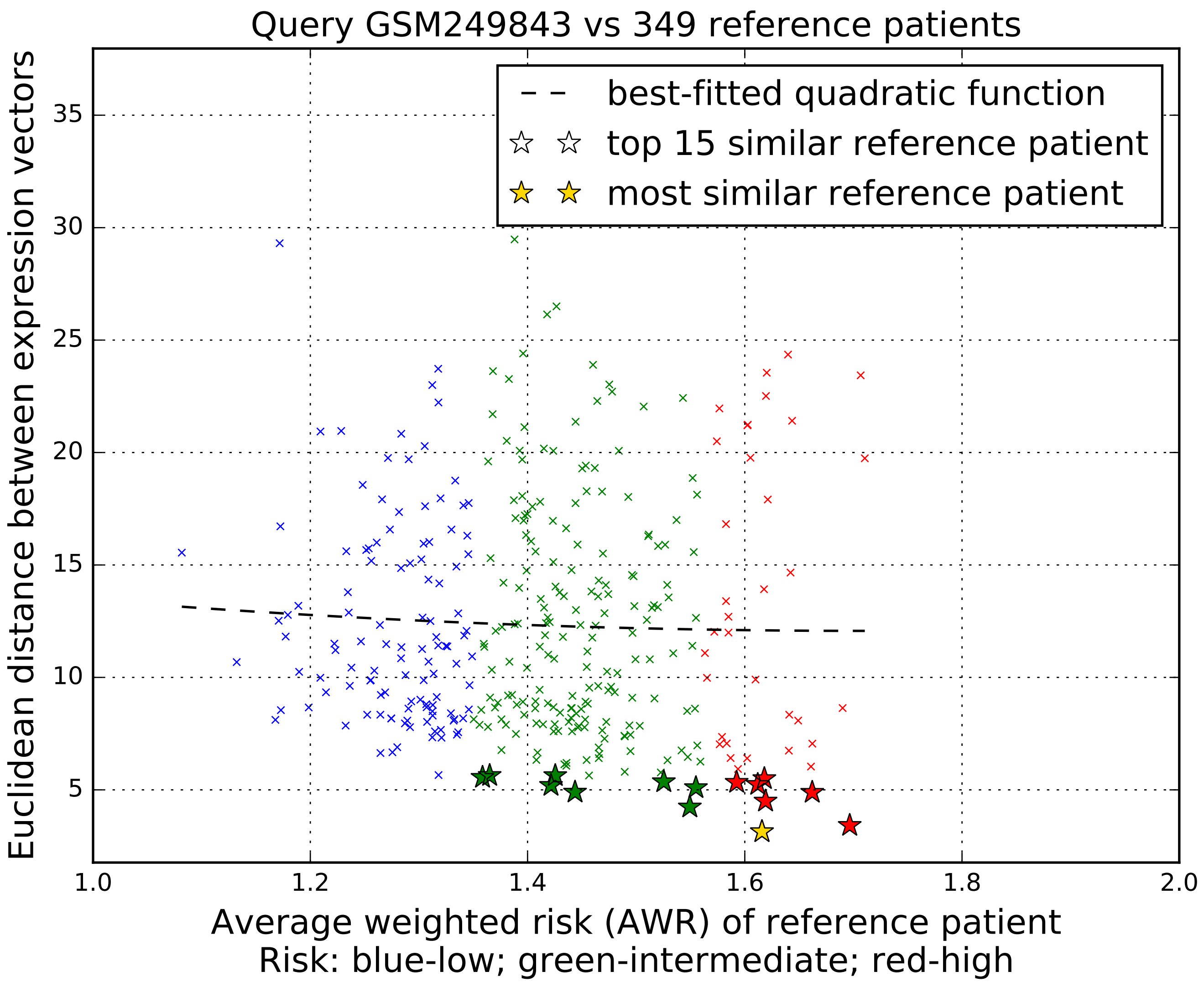

Query GSM249843 vs 349 reference patients
best-fitted quadratic function
top 15 similar reference patient
most similar reference patient
Euclidean distance between expression vectors
35
30
25
20
15
10
5
1.0
1.2
1.4
1.6
1.8
2.0
Average weighted risk (AWR) of reference patient
Risk: blue-low; green-intermediate; red-high

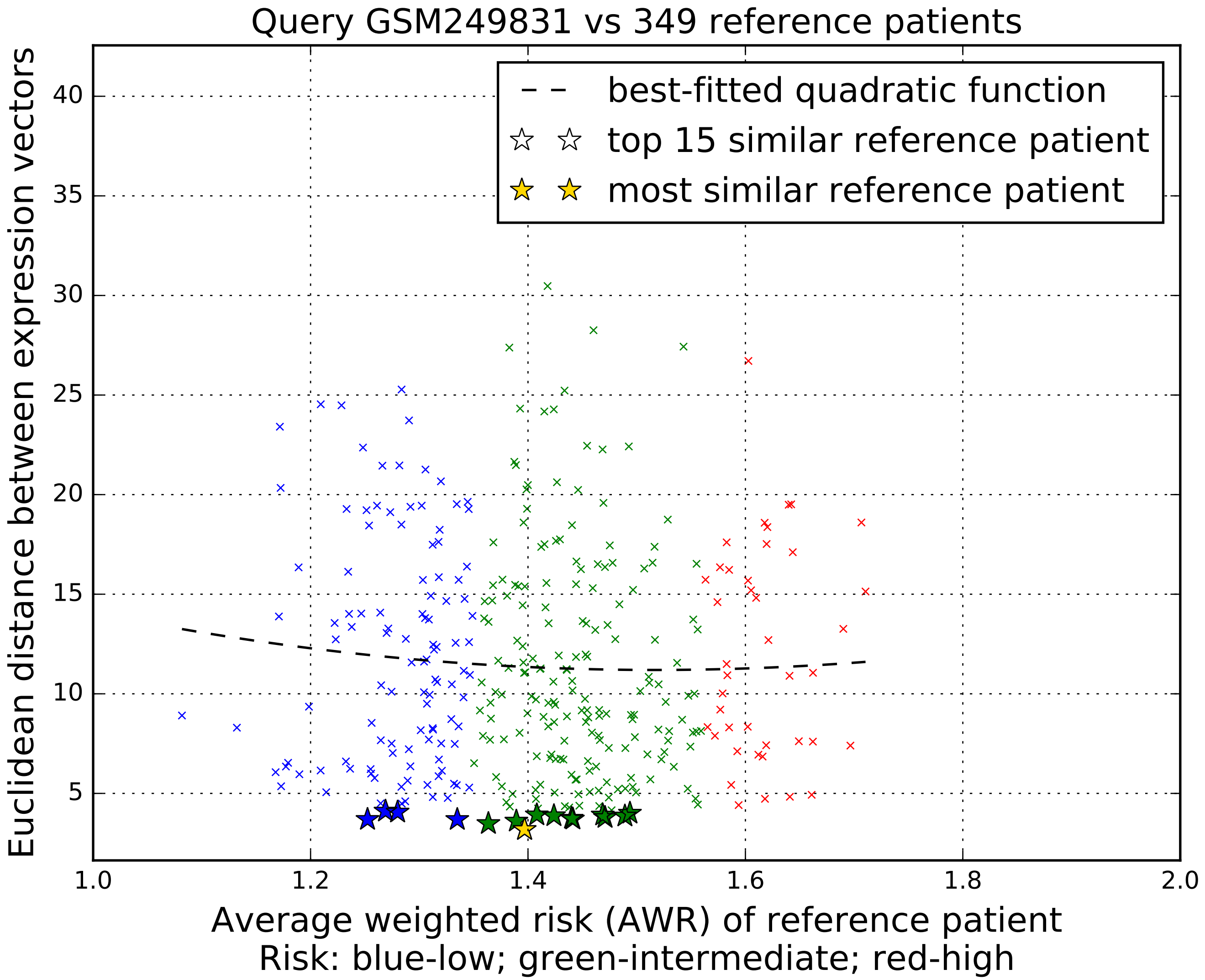

Query GSM249831 vs 349 reference patients
best-fitted quadratic function
top 15 similar reference patient
most similar reference patient
Euclidean distance between expression vectors
40
35
30
25
20
15
10
5
1.0
1.2
1.4
1.6
1.8
2.0
Average weighted risk (AWR) of reference patient
Risk: blue-low; green-intermediate; red-high

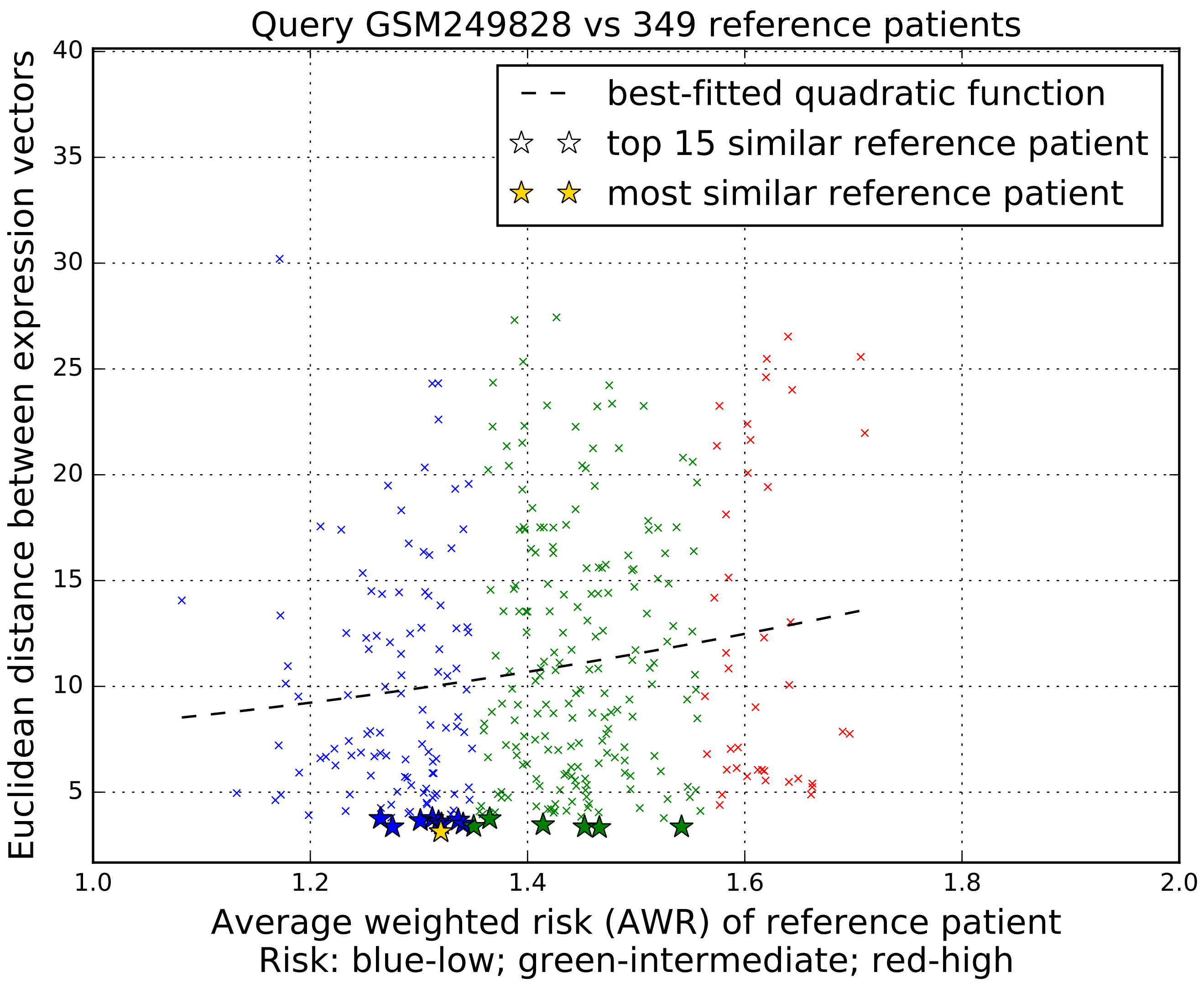

Query GSM249828 vs 349 reference patients
best-fitted quadratic function
top 15 similar reference patient
most similar reference patient
Euclidean distance between expression vectors
Average weighted risk (AWR) of reference patient
Risk: blue-low; green-intermediate; red-high
40
35
30
25
20
15
10
5
1.0
1.2
1.4
1.6
1.8
2.0

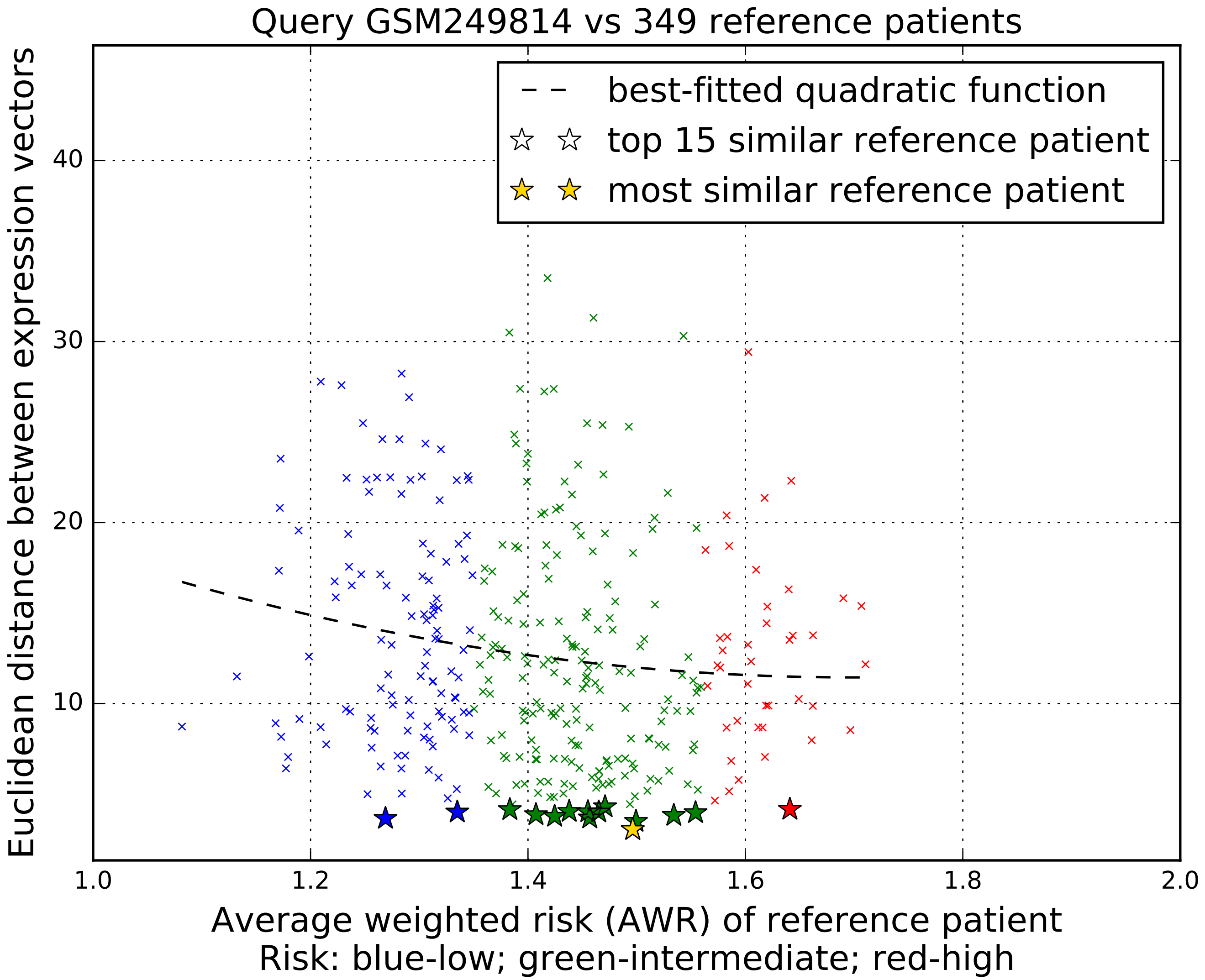
Query GSM249814 vs 349 reference patients
best-fitted quadratic function
top 15 similar reference patient
most similar reference patient
Euclidean distance between expression vectors
Average weighted risk (AWR) of reference patient
Risk: blue-low; green-intermediate; red-high
10
20
30
40
1.0
1.2
1.4
1.6
1.8
2.0

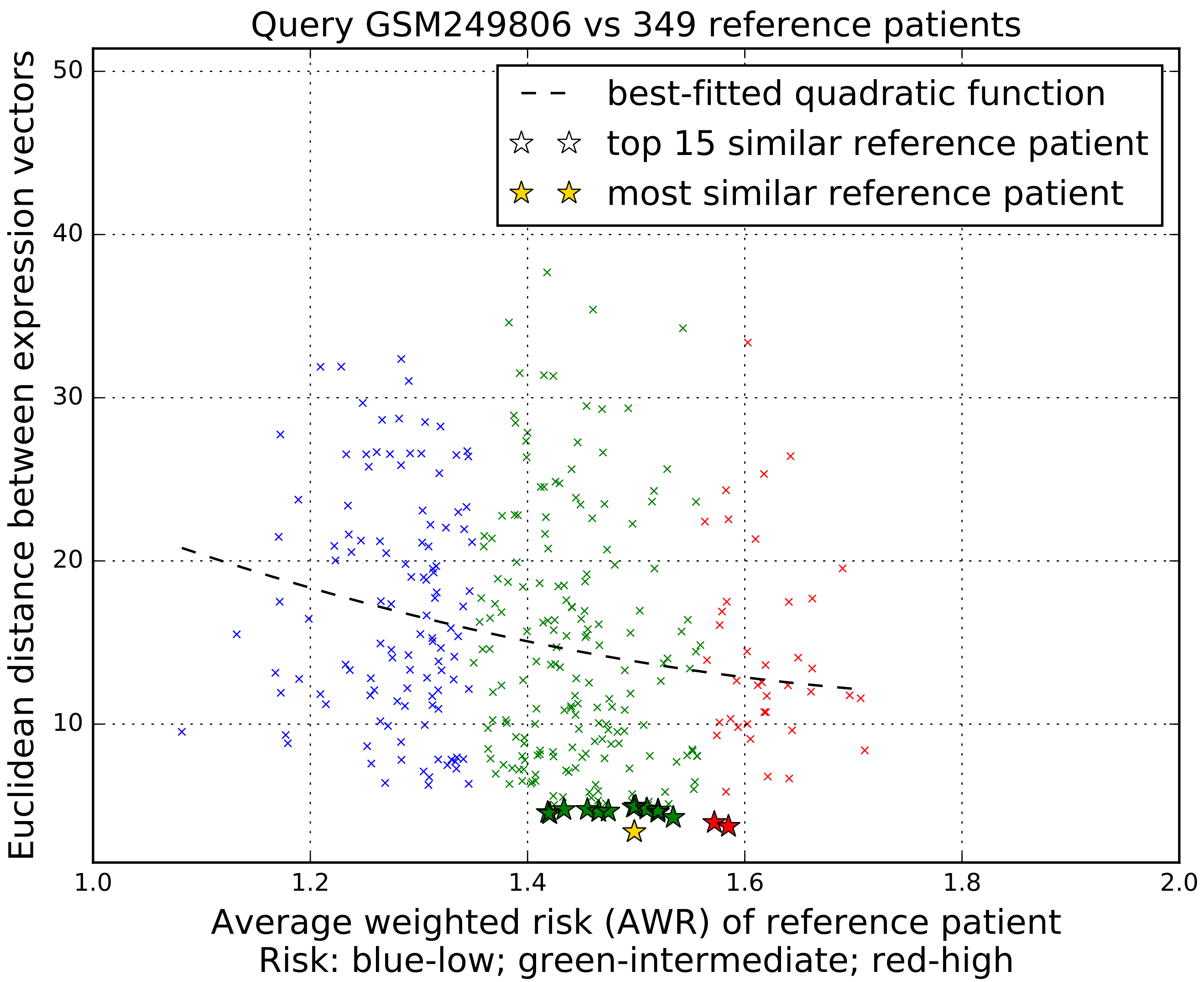

Query GSM249806 vs 349 reference patients
best-fitted quadratic function
top 15 similar reference patient
most similar reference patient
Euclidean distance between expression vectors
50
40
30
20
10
1.0
1.2
1.4
1.6
1.8
2.0
Average weighted risk (AWR) of reference patient
Risk: blue-low; green-intermediate; red-high

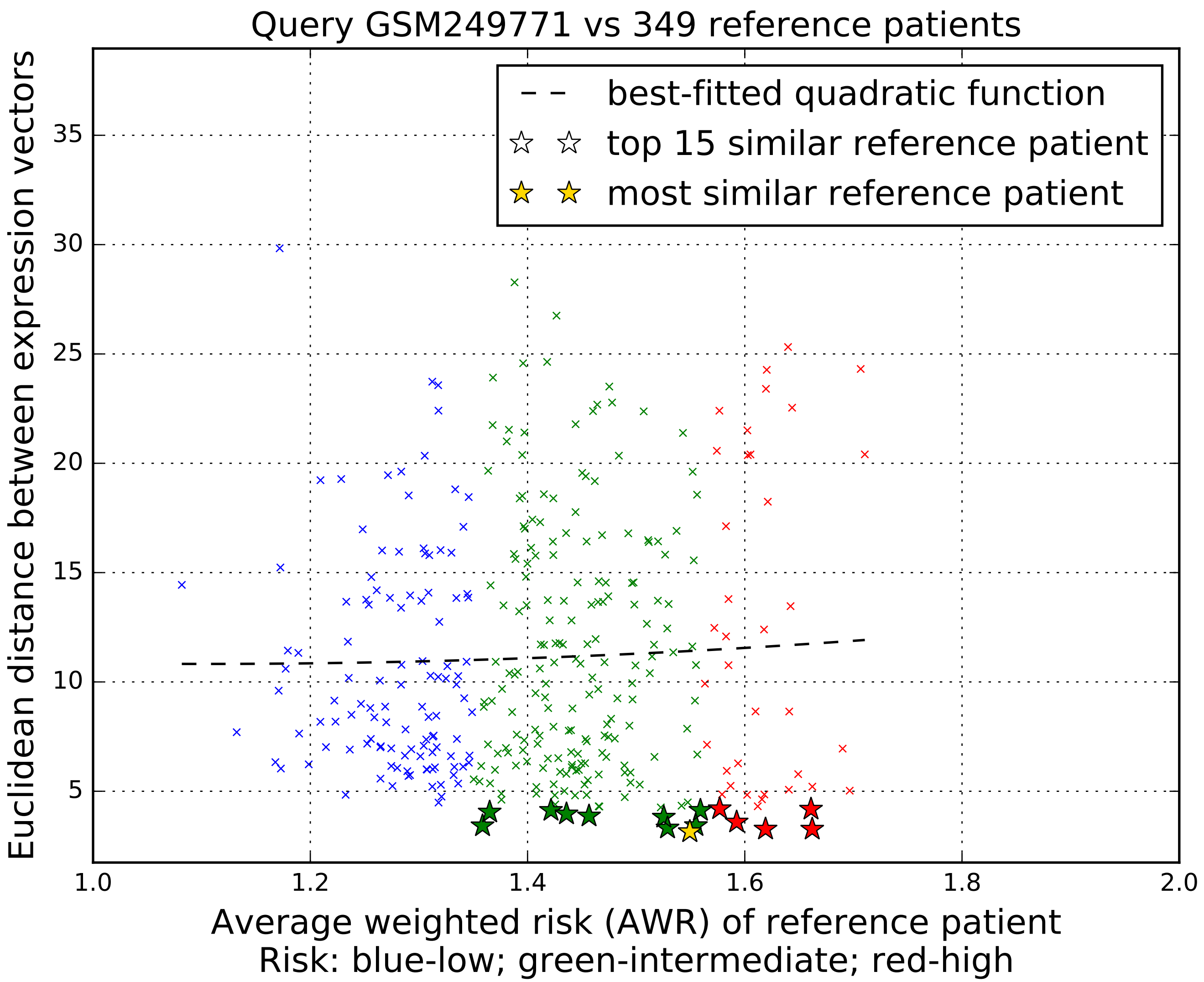
Query GSM249771 vs 349 reference patients
best-fitted quadratic function
top 15 similar reference patient
most similar reference patient
Euclidean distance between expression vectors
35
30
25
20
15
10
5
1.0
1.2
1.4
1.6
1.8
2.0
Average weighted risk (AWR) of reference patient
Risk: blue-low; green-intermediate; red-high

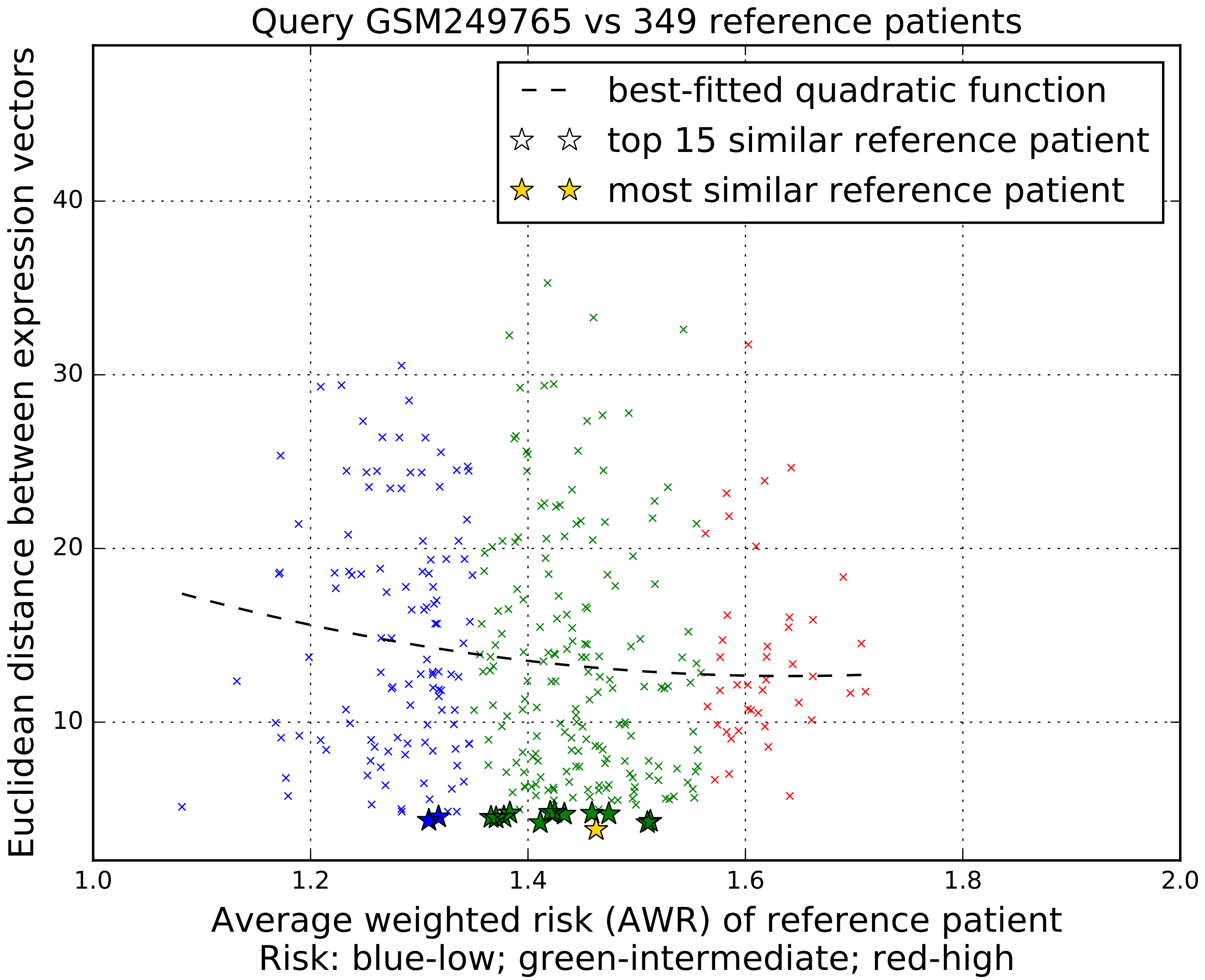
Query GSM249765 vs 349 reference patients
best-fitted quadratic function
top 15 similar reference patient
most similar reference patient
40
30
20
10
1.0
1.2
1.4
1.6
1.8
2.0
Euclidean distance between expression vectors
Average weighted risk (AWR) of reference patient
Risk: blue-low; green-intermediate; red-high

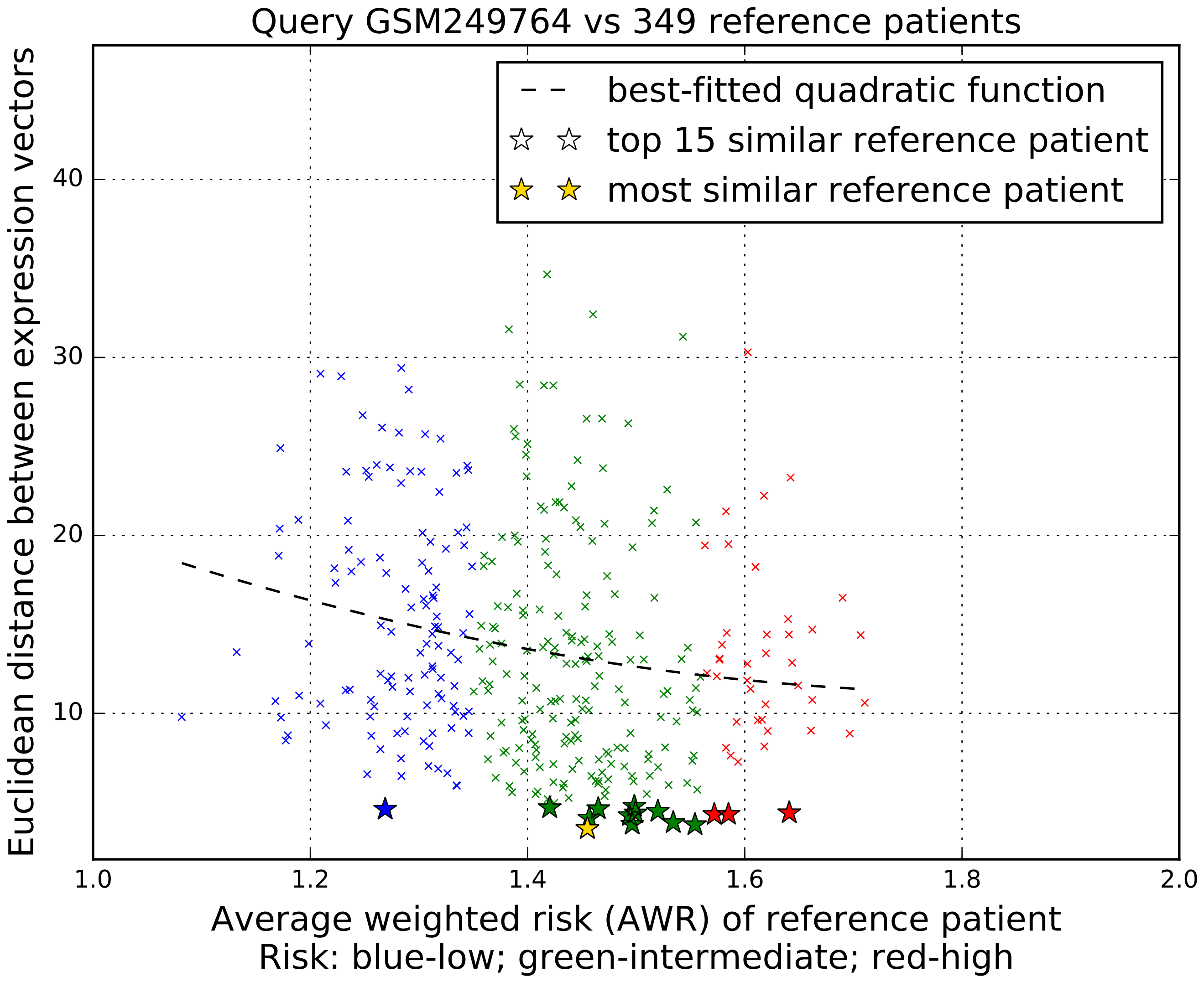
Query GSM249764 vs 349 reference patients
best-fitted quadratic function
top 15 similar reference patient
most similar reference patient
Euclidean distance between expression vectors
40
30
20
10
1.0
1.2
1.4
1.6
1.8
2.0
Average weighted risk (AWR) of reference patient
Risk: blue-low; green-intermediate; red-high

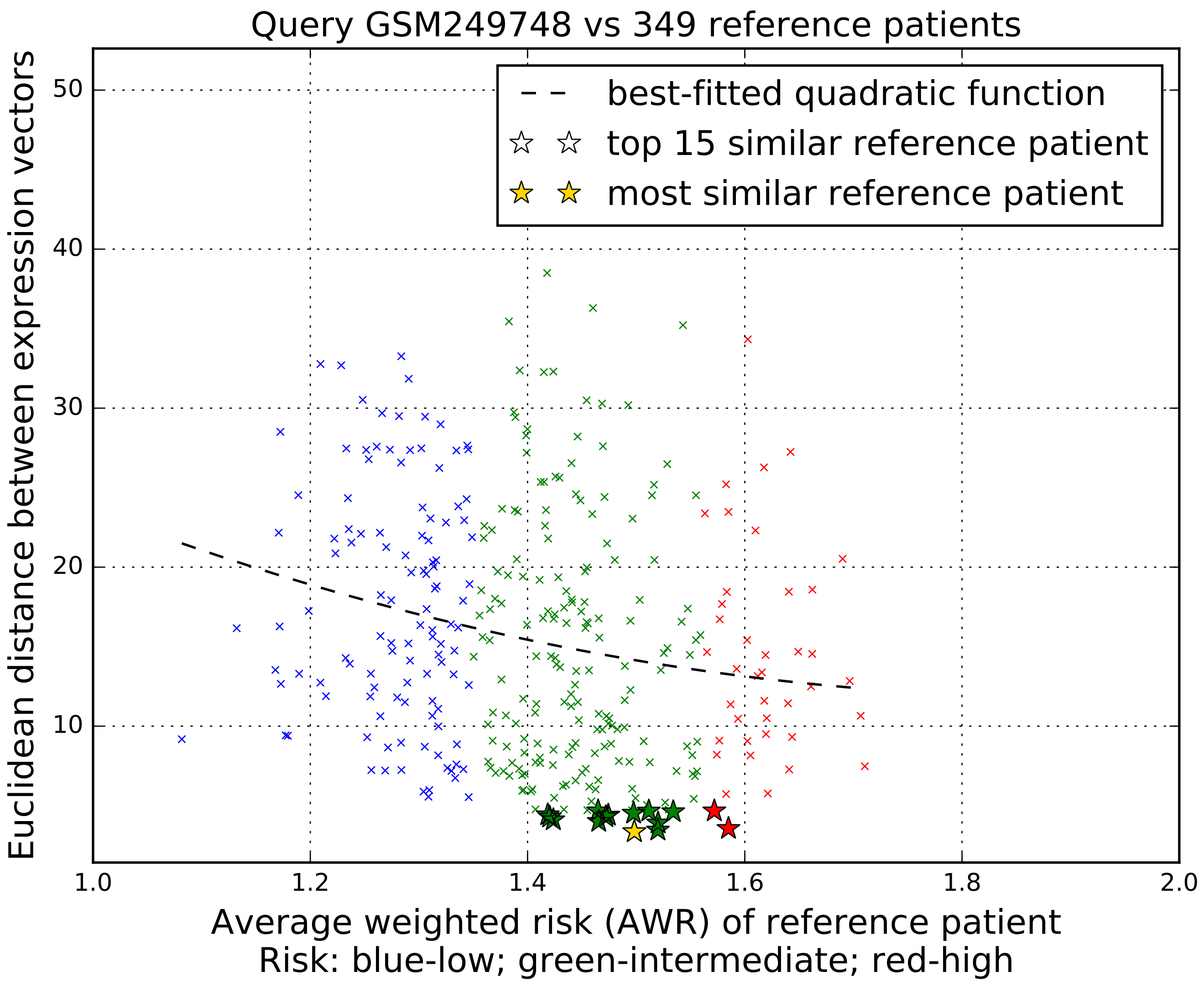
Query GSM249748 vs 349 reference patients
best-fitted quadratic function
top 15 similar reference patient
most similar reference patient
Euclidean distance between expression vectors
50
40
30
20
10
1.0
1.2
1.4
1.6
1.8
2.0
Average weighted risk (AWR) of reference patient
Risk: blue-low; green-intermediate; red-high

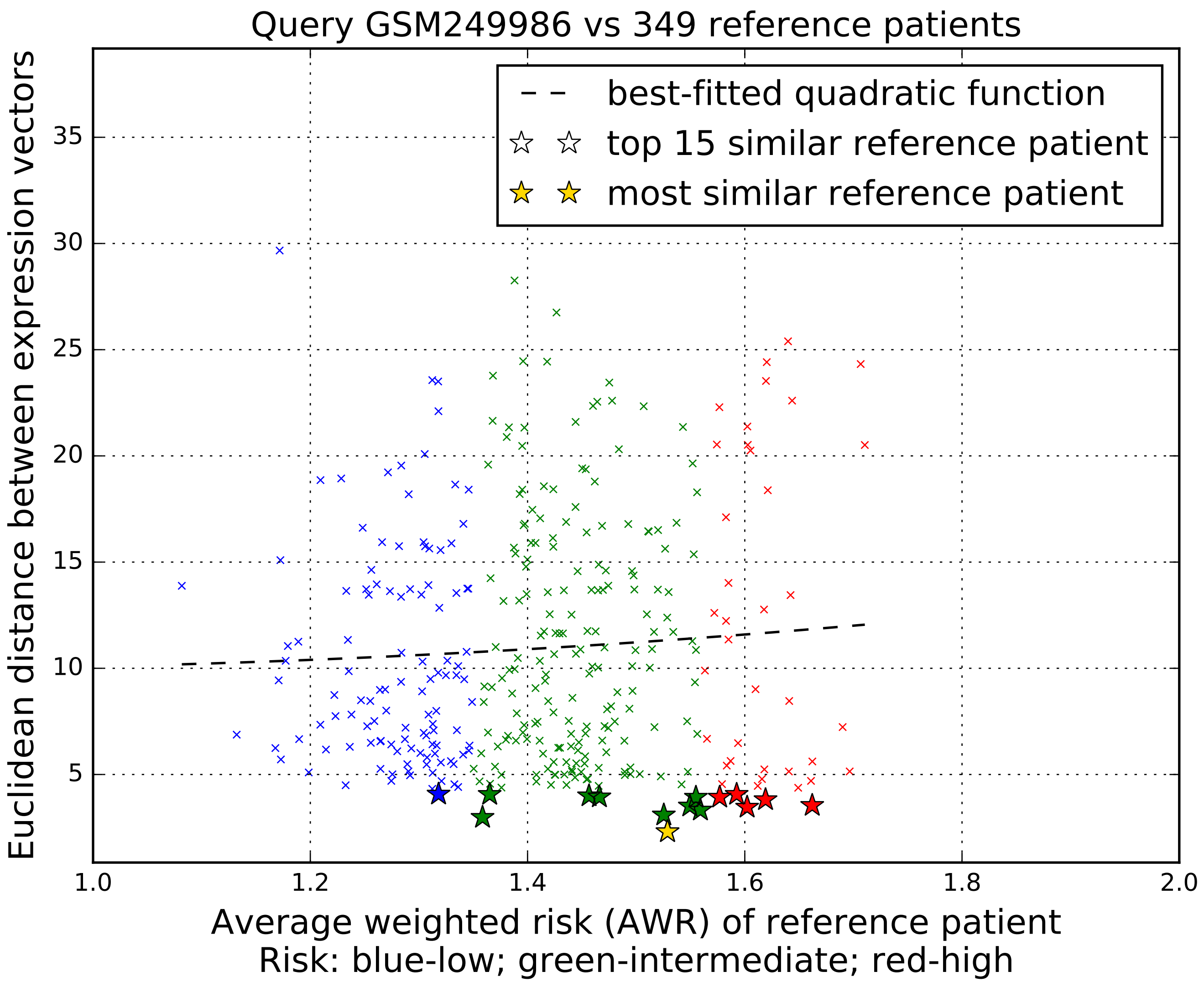
Query GSM249986 vs 349 reference patients
best-fitted quadratic function
top 15 similar reference patient
most similar reference patient
Euclidean distance between expression vectors
35
30
25
20
15
10
5
1.0
1.2
1.4
1.6
1.8
2.0
Average weighted risk (AWR) of reference patient
Risk: blue-low; green-intermediate; red-high

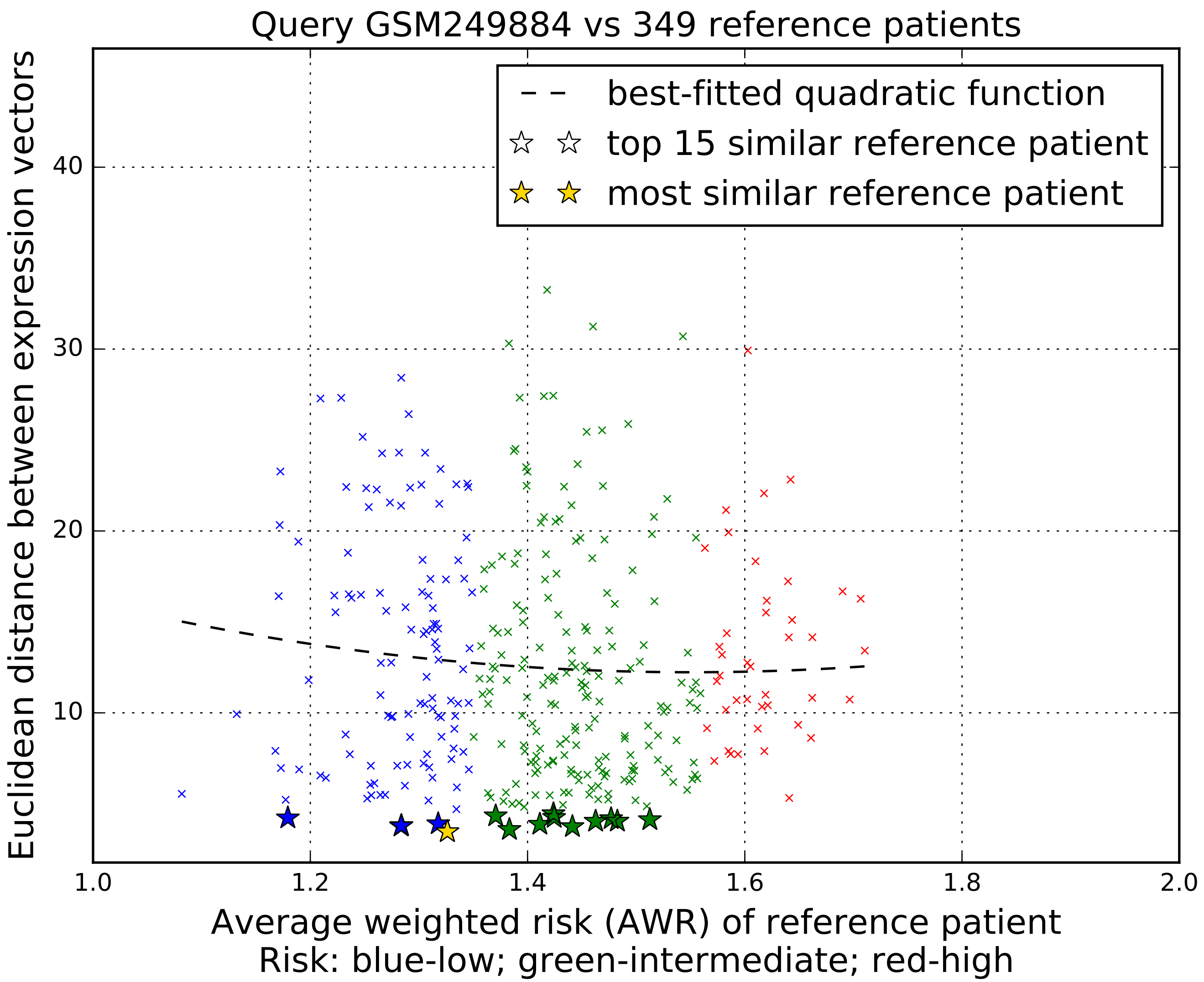
Query GSM249884 vs 349 reference patients
best-fitted quadratic function
top 15 similar reference patient
most similar reference patient
Euclidean distance between expression vectors
10
20
30
40
1.0
1.2
1.4
1.6
1.8
2.0
Average weighted risk (AWR) of reference patient
Risk: blue-low; green-intermediate; red-high

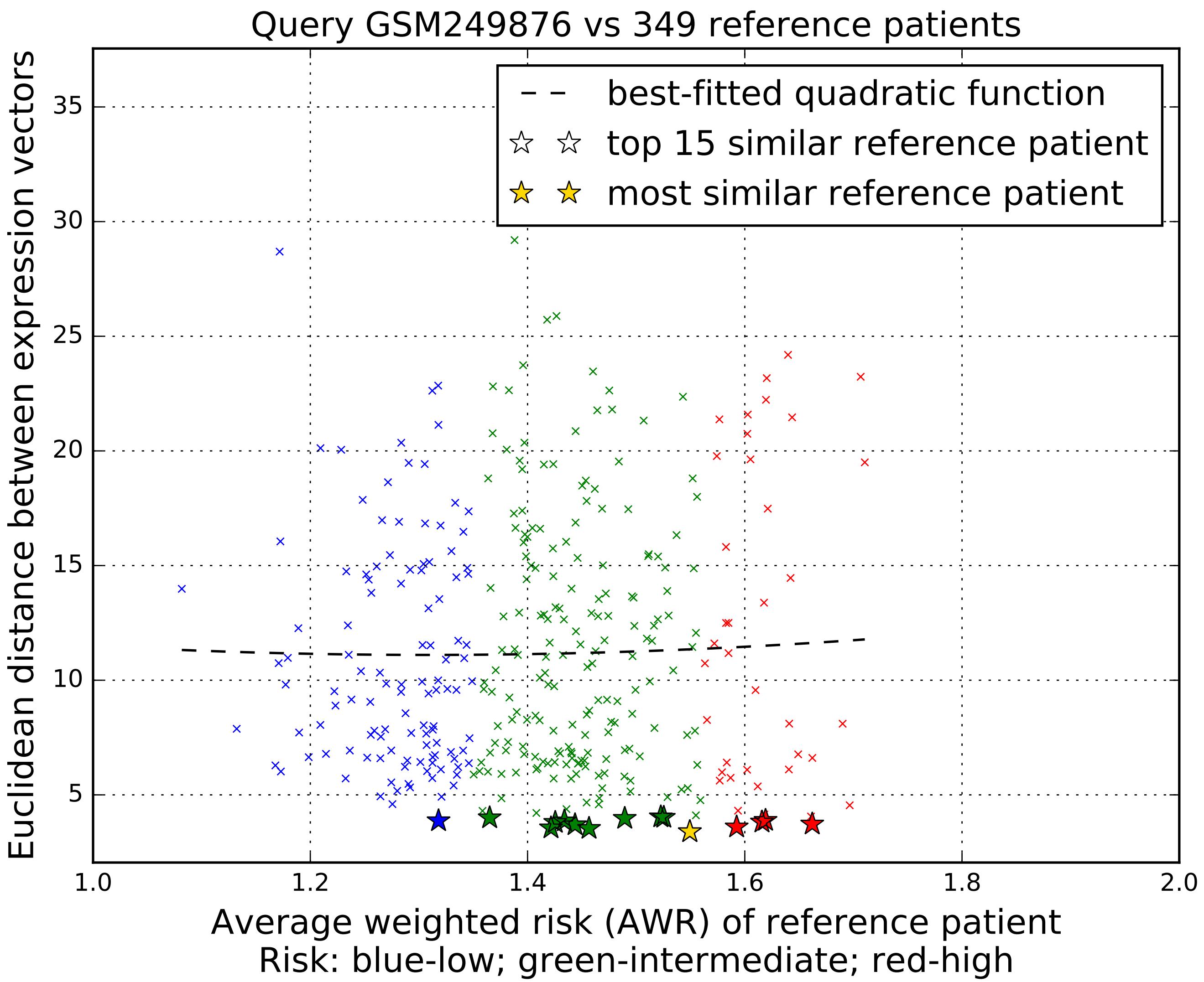

Query GSM249876 vs 349 reference patients
best-fitted quadratic function
top 15 similar reference patient
most similar reference patient
Euclidean distance between expression vectors
Average weighted risk (AWR) of reference patient
Risk: blue-low; green-intermediate; red-high
1.0
1.2
1.4
1.6
1.8
2.0
5
10
15
20
25
30
35

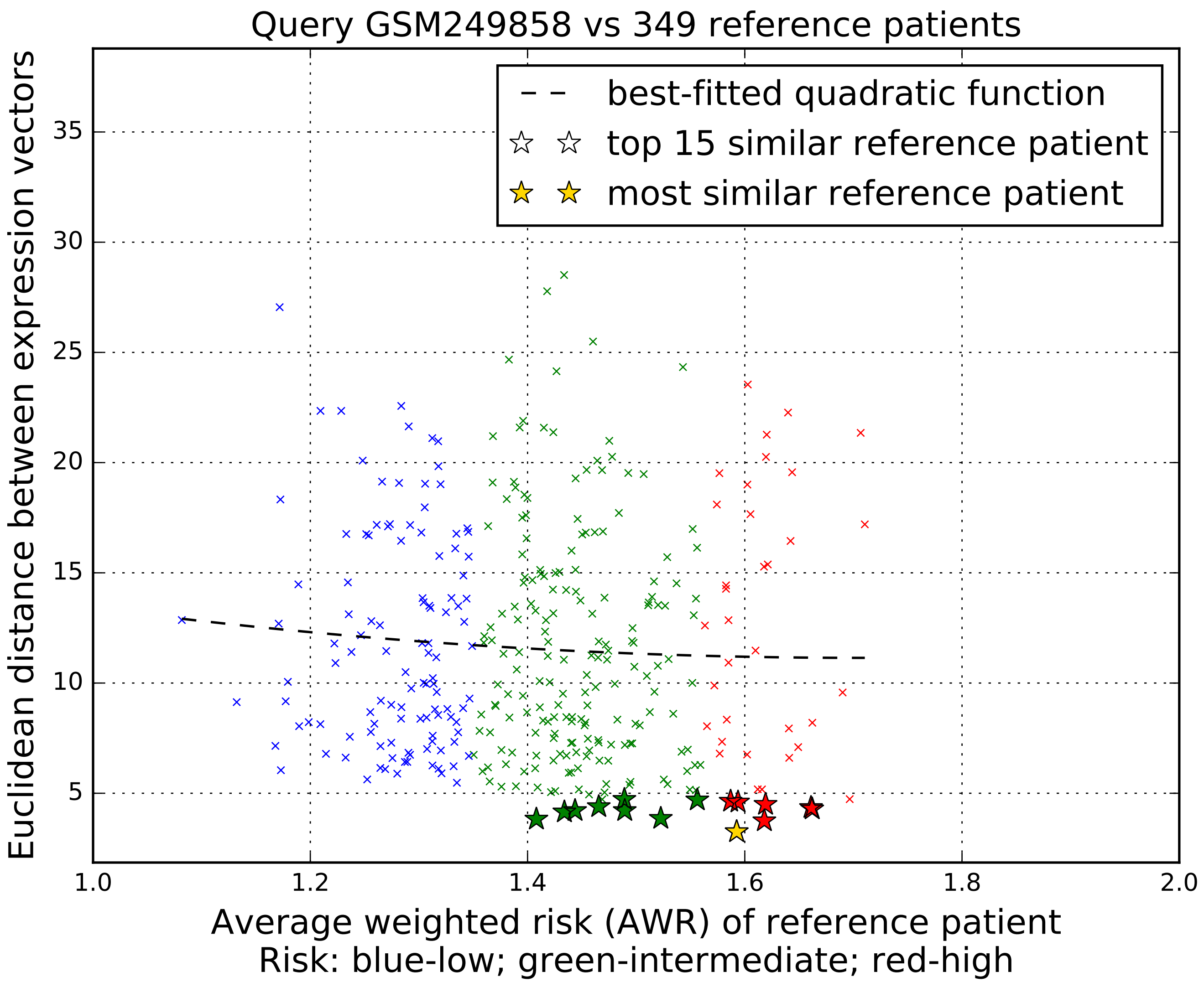
Query GSM249858 vs 349 reference patients
best-fitted quadratic function
top 15 similar reference patient
most similar reference patient
Euclidean distance between expression vectors
35
30
25
20
15
10
5
1.0
1.2
1.4
1.6
1.8
2.0
Average weighted risk (AWR) of reference patient
Risk: blue-low; green-intermediate; red-high

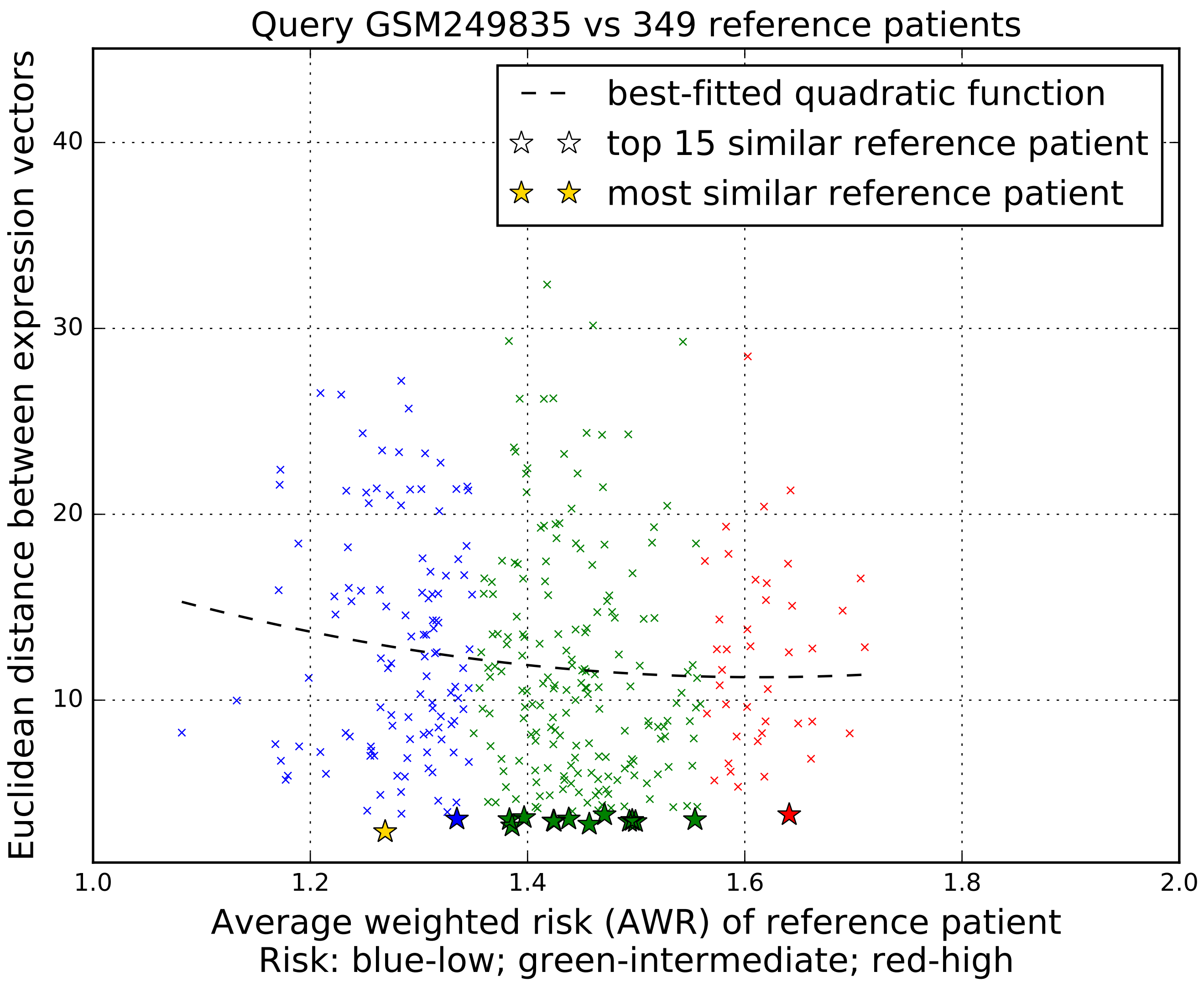

Query GSM249835 vs 349 reference patients
best-fitted quadratic function
top 15 similar reference patient
most similar reference patient
Euclidean distance between expression vectors
10
20
30
40
1.0
1.2
1.4
1.6
1.8
2.0
Average weighted risk (AWR) of reference patient
Risk: blue-low; green-intermediate; red-high

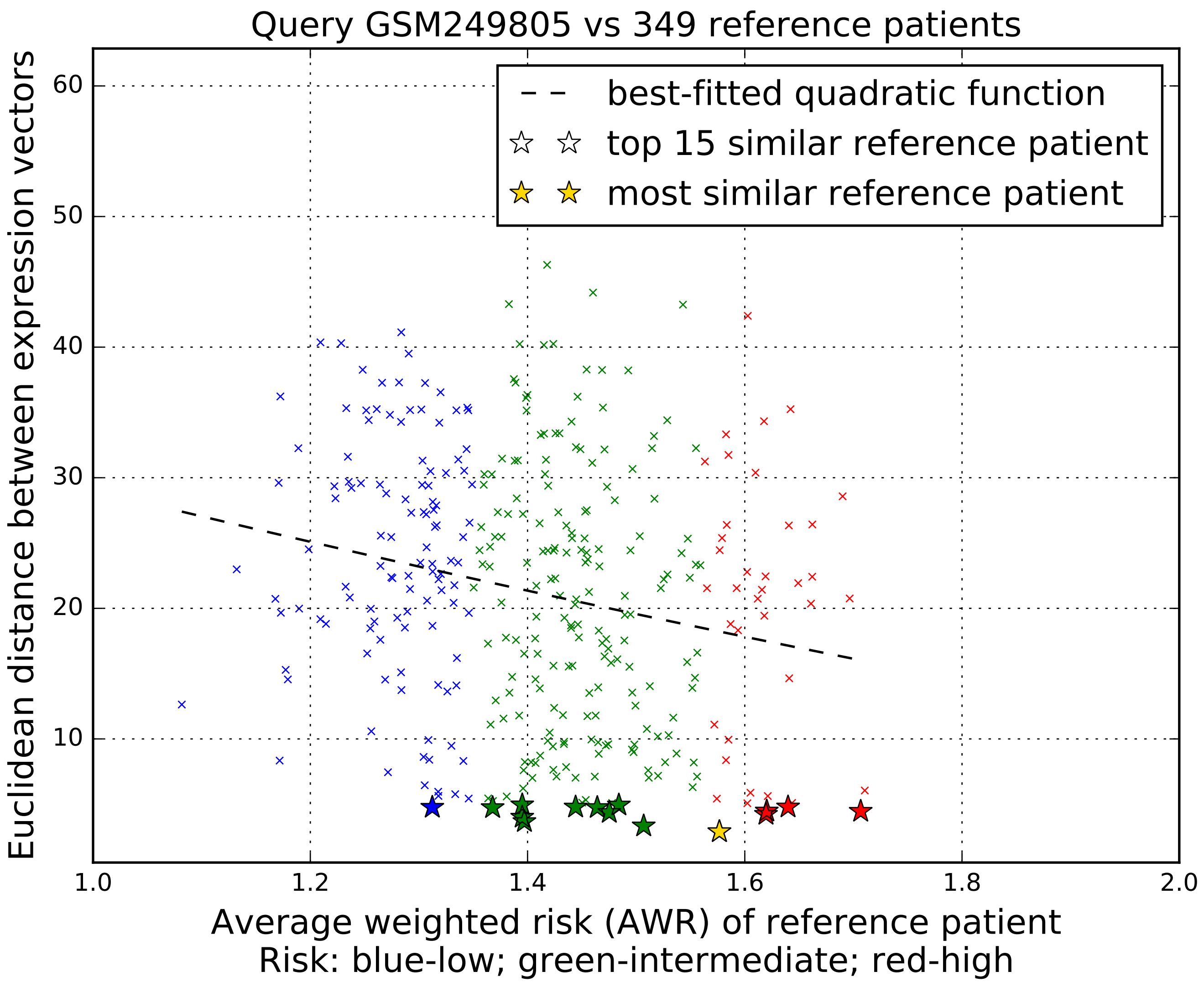
Query GSM249805 vs 349 reference patients
best-fitted quadratic function
top 15 similar reference patient
most similar reference patient
Euclidean distance between expression vectors
60
50
40
30
20
10
1.0
1.2
1.4
1.6
1.8
2.0
Average weighted risk (AWR) of reference patient
Risk: blue-low; green-intermediate; red-high

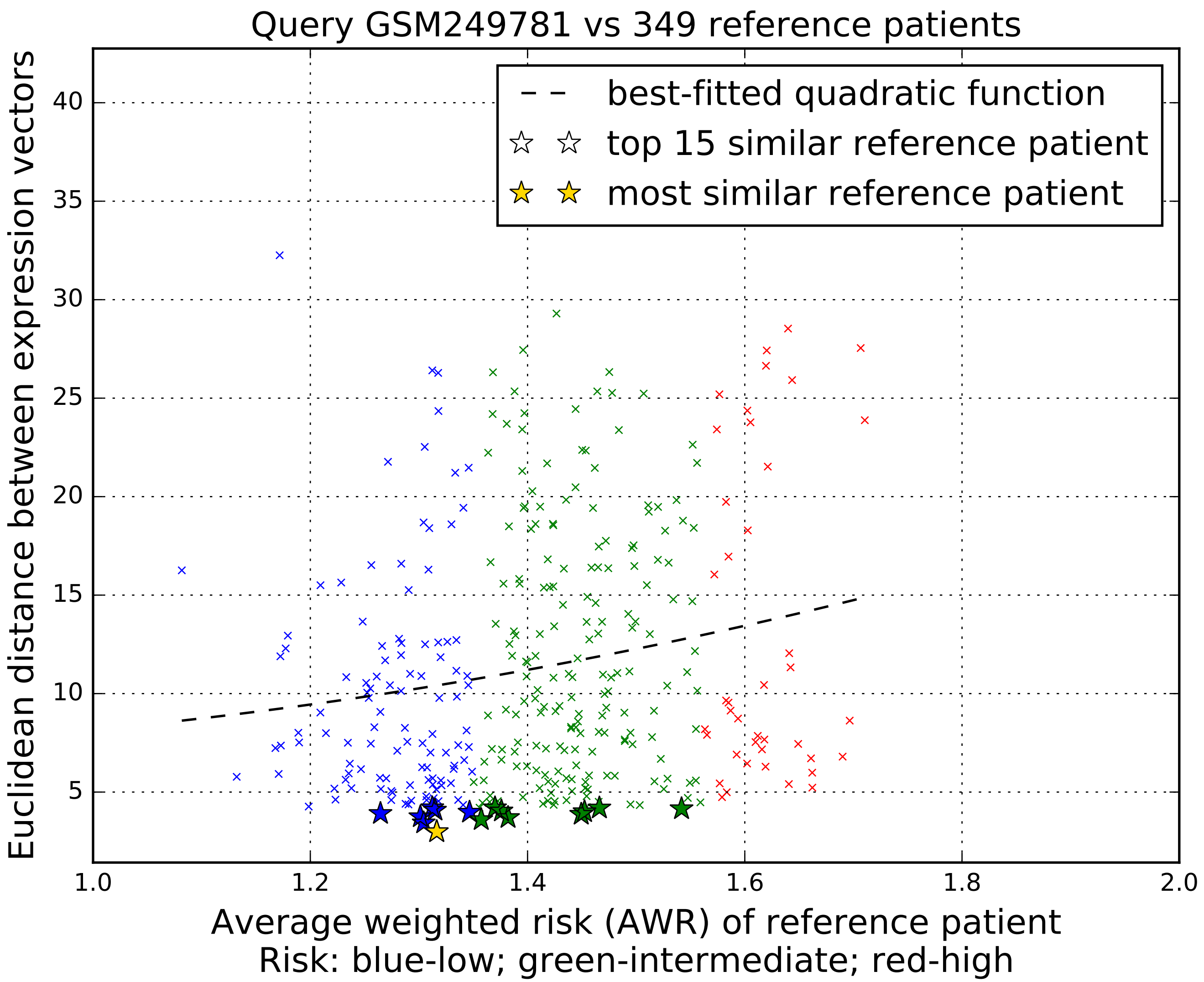

Query GSM249781 vs 349 reference patients
best-fitted quadratic function
top 15 similar reference patient
most similar reference patient
Euclidean distance between expression vectors
40
35
30
25
20
15
10
5
1.0
1.2
1.4
1.6
1.8
2.0
Average weighted risk (AWR) of reference patient
Risk: blue-low; green-intermediate; red-high

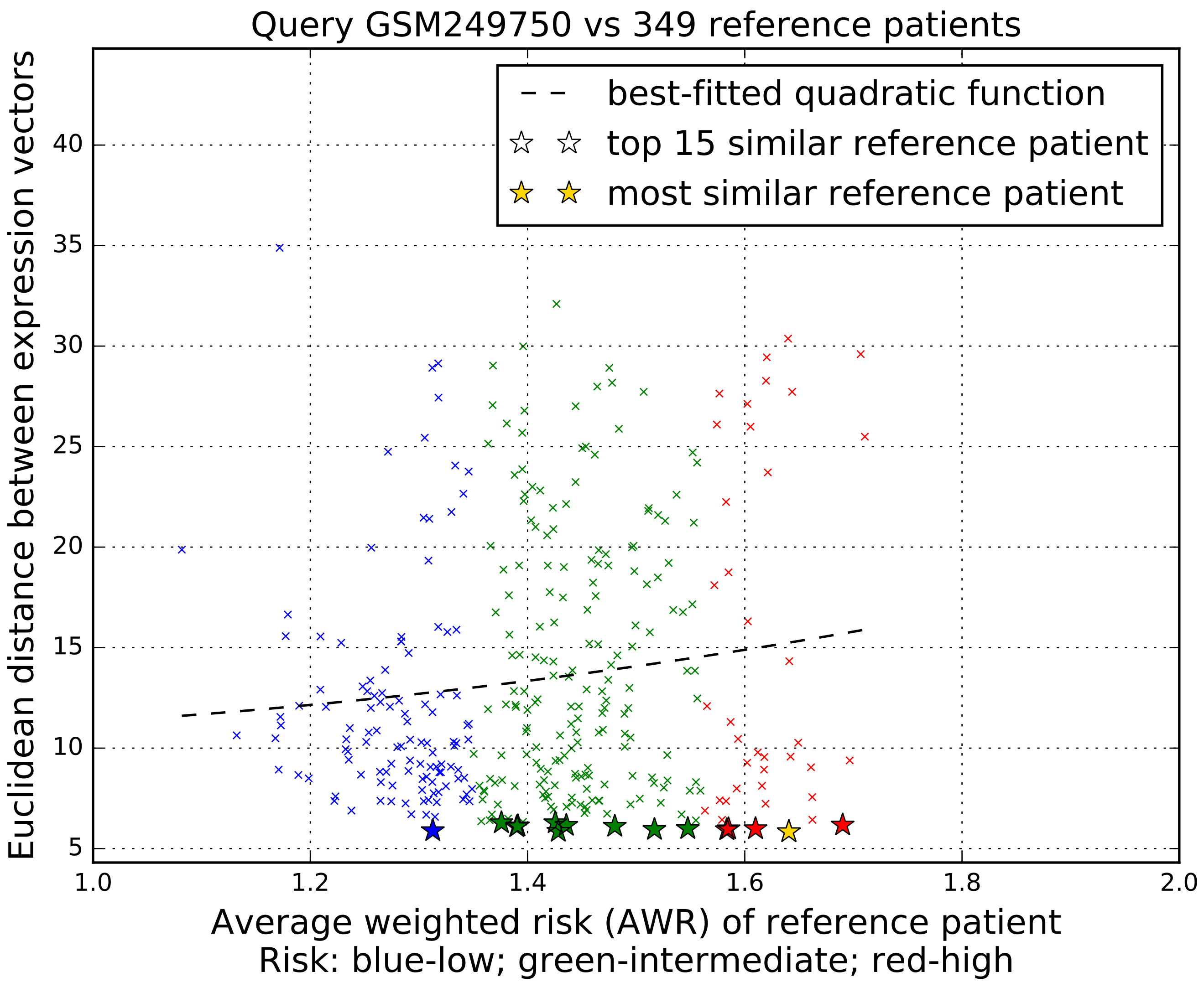

Query GSM249750 vs 349 reference patients
best-fitted quadratic function
top 15 similar reference patient
most similar reference patient
Euclidean distance between expression vectors
5
10
15
20
25
30
35
40
1.0
1.2
1.4
1.6
1.8
2.0
Average weighted risk (AWR) of reference patient
Risk: blue-low; green-intermediate; red-high

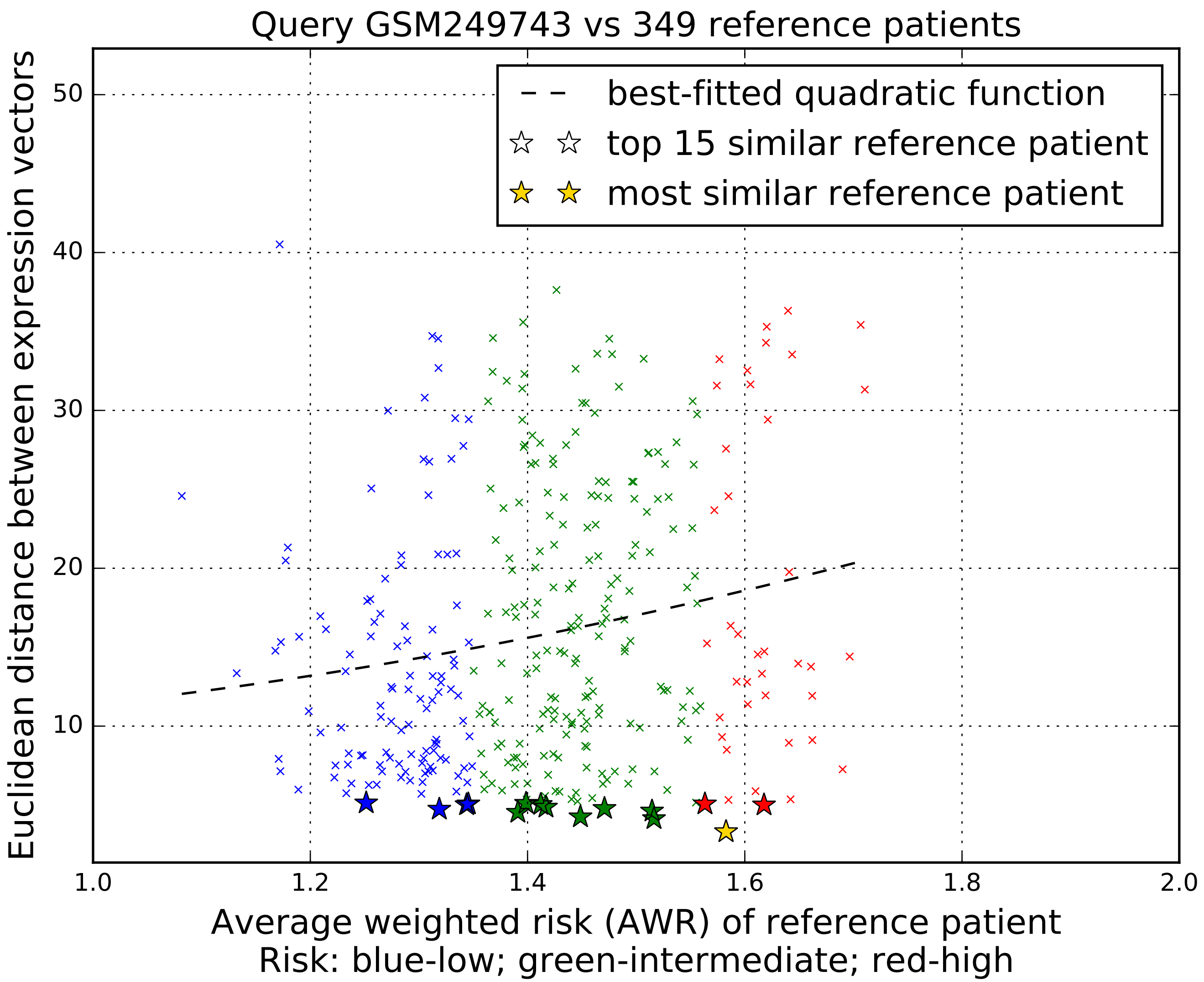

Query GSM249743 vs 349 reference patients
best-fitted quadratic function
top 15 similar reference patient
most similar reference patient
Euclidean distance between expression vectors
50
40
30
20
10
1.0
1.2
1.4
1.6
1.8
2.0
Average weighted risk (AWR) of reference patient
Risk: blue-low; green-intermediate; red-high

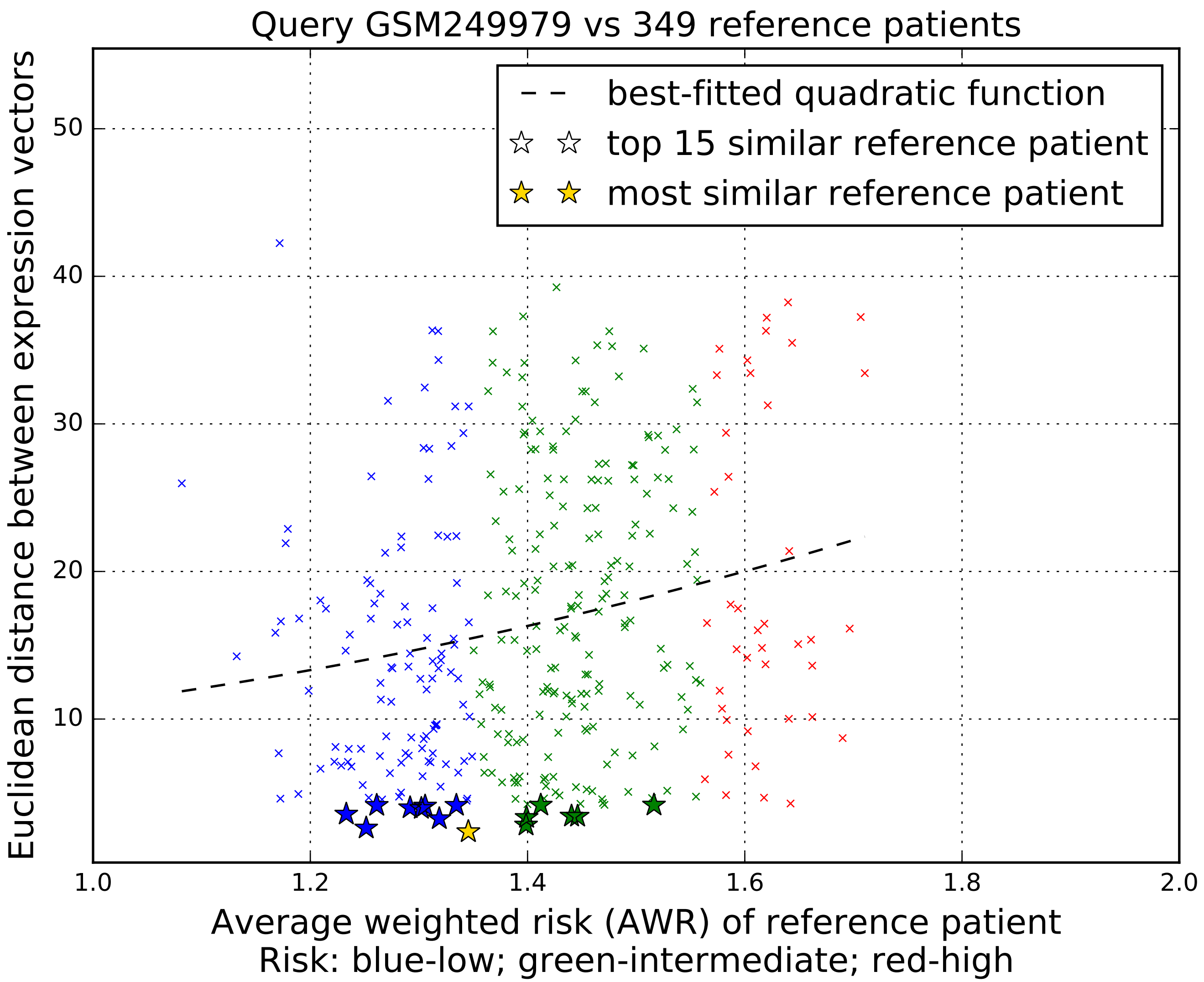

Query GSM249979 vs 349 reference patients
best-fitted quadratic function
top 15 similar reference patient
most similar reference patient
Euclidean distance between expression vectors
50
40
30
20
10
1.0
1.2
1.4
1.6
1.8
2.0
Average weighted risk (AWR) of reference patient
Risk: blue-low; green-intermediate; red-high

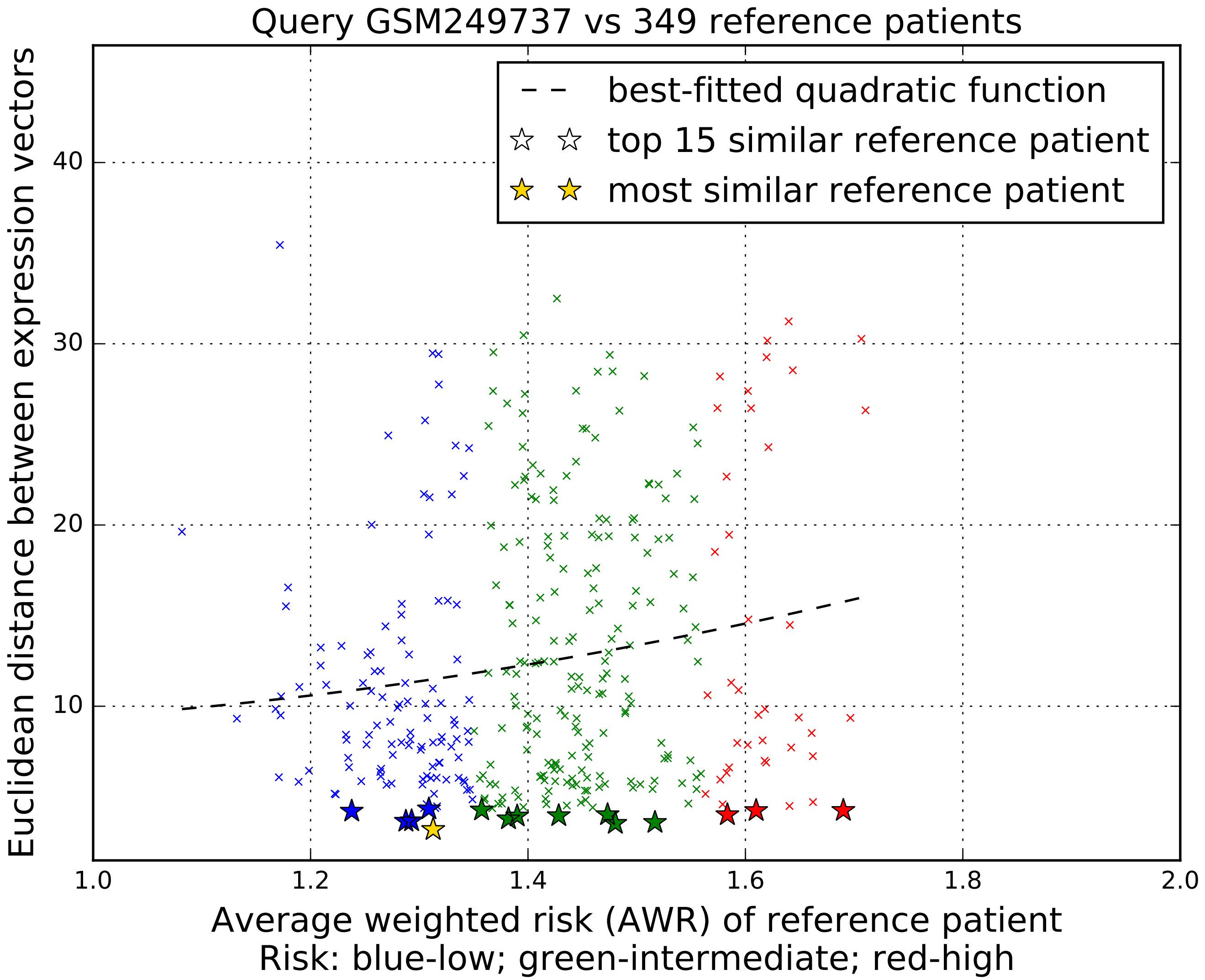
Query GSM249737 vs 349 reference patients
best-fitted quadratic function
top 15 similar reference patient
most similar reference patient
Euclidean distance between expression vectors
40
30
20
10
1.0
1.2
1.4
1.6
1.8
2.0
Average weighted risk (AWR) of reference patient
Risk: blue-low; green-intermediate; red-high

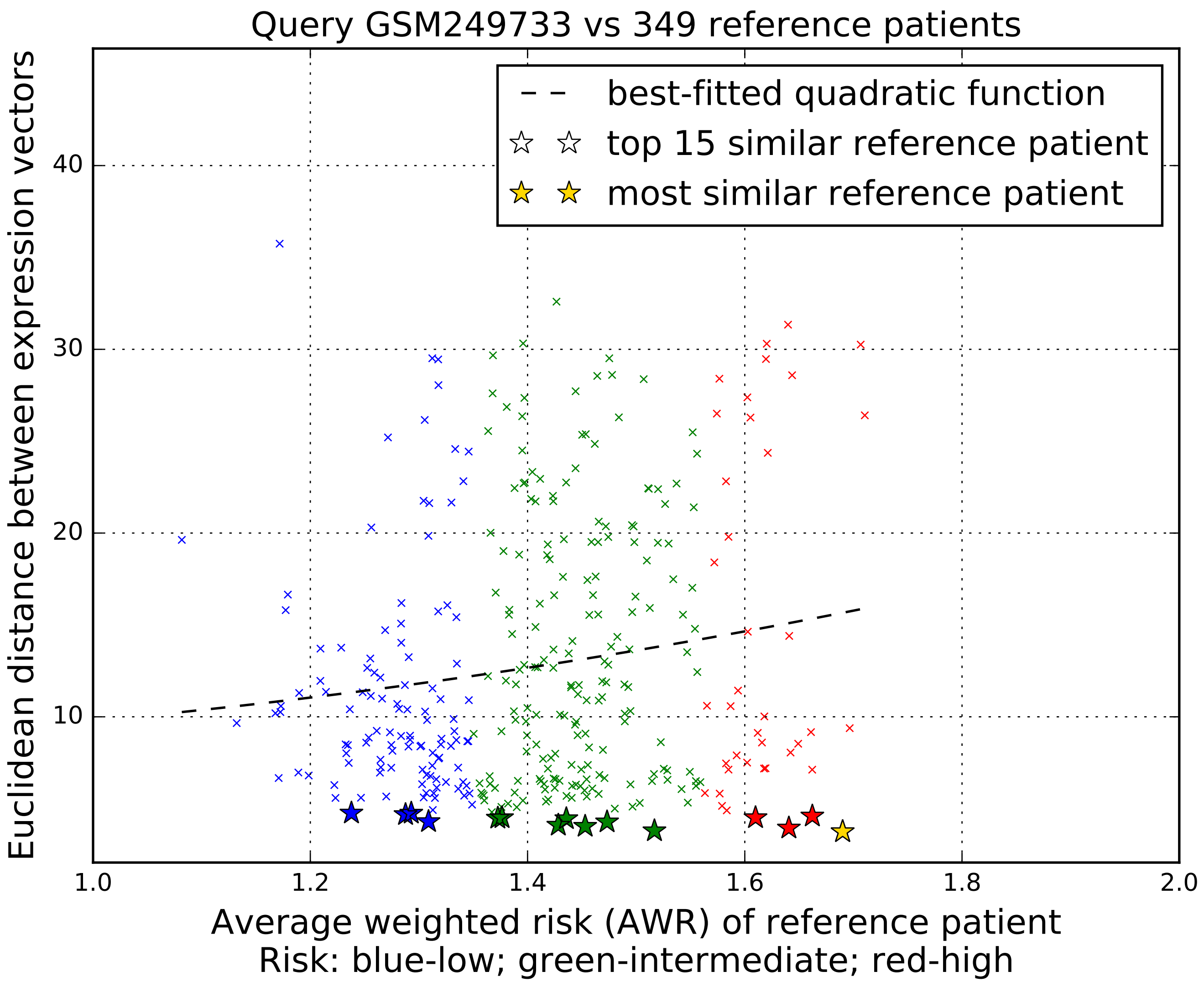

Query GSM249733 vs 349 reference patients
best-fitted quadratic function
top 15 similar reference patient
most similar reference patient
Euclidean distance between expression vectors
40
30
20
10
1.0
1.2
1.4
1.6
1.8
2.0
Average weighted risk (AWR) of reference patient
Risk: blue-low; green-intermediate; red-high

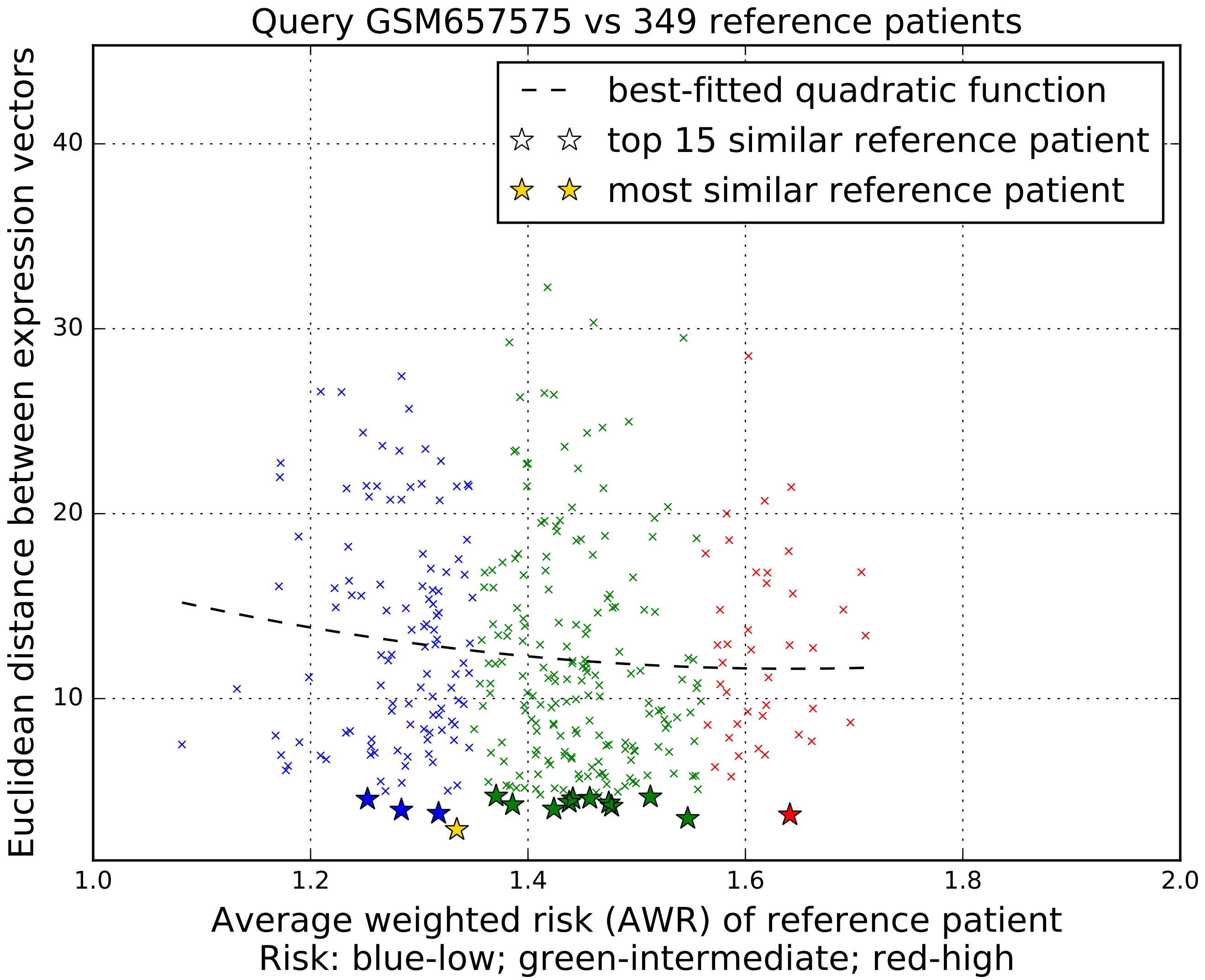
Query GSM657575 vs 349 reference patients
best-fitted quadratic function
top 15 similar reference patient
most similar reference patient
Euclidean distance between expression vectors
40
30
20
10
1.0
1.2
1.4
1.6
1.8
2.0
Average weighted risk (AWR) of reference patient
Risk: blue-low; green-intermediate; red-high

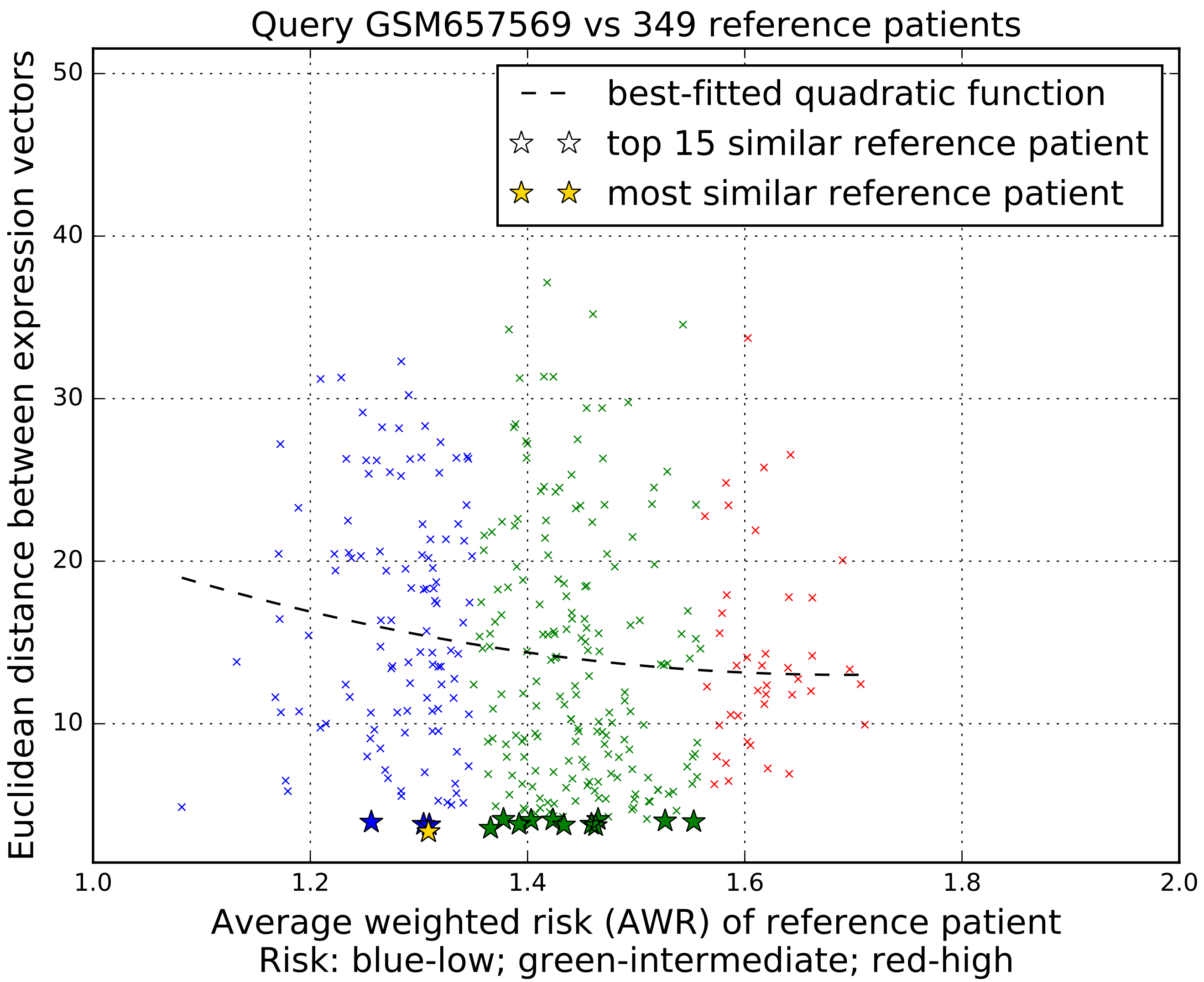

Query GSM657569 vs 349 reference patients
best-fitted quadratic function
top 15 similar reference patient
most similar reference patient
Euclidean distance between expression vectors
10
20
30
40
50
1.0
1.2
1.4
1.6
1.8
2.0
Average weighted risk (AWR) of reference patient
Risk: blue-low; green-intermediate; red-high

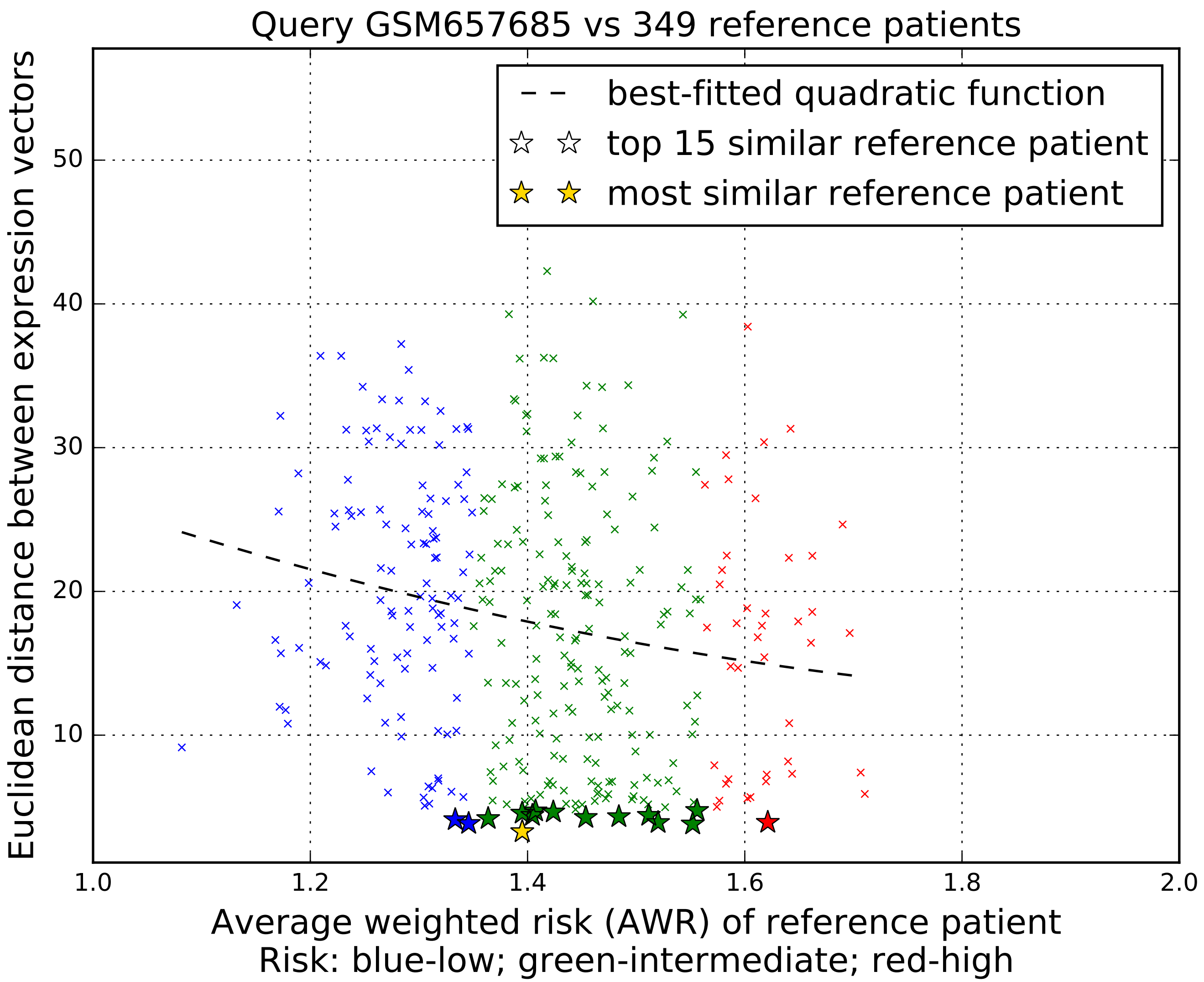

Query GSM657685 vs 349 reference patients
best-fitted quadratic function
top 15 similar reference patient
most similar reference patient
Euclidean distance between expression vectors
Average weighted risk (AWR) of reference patient
Risk: blue-low; green-intermediate; red-high
10
20
30
40
50
1.0
1.2
1.4
1.6
1.8
2.0

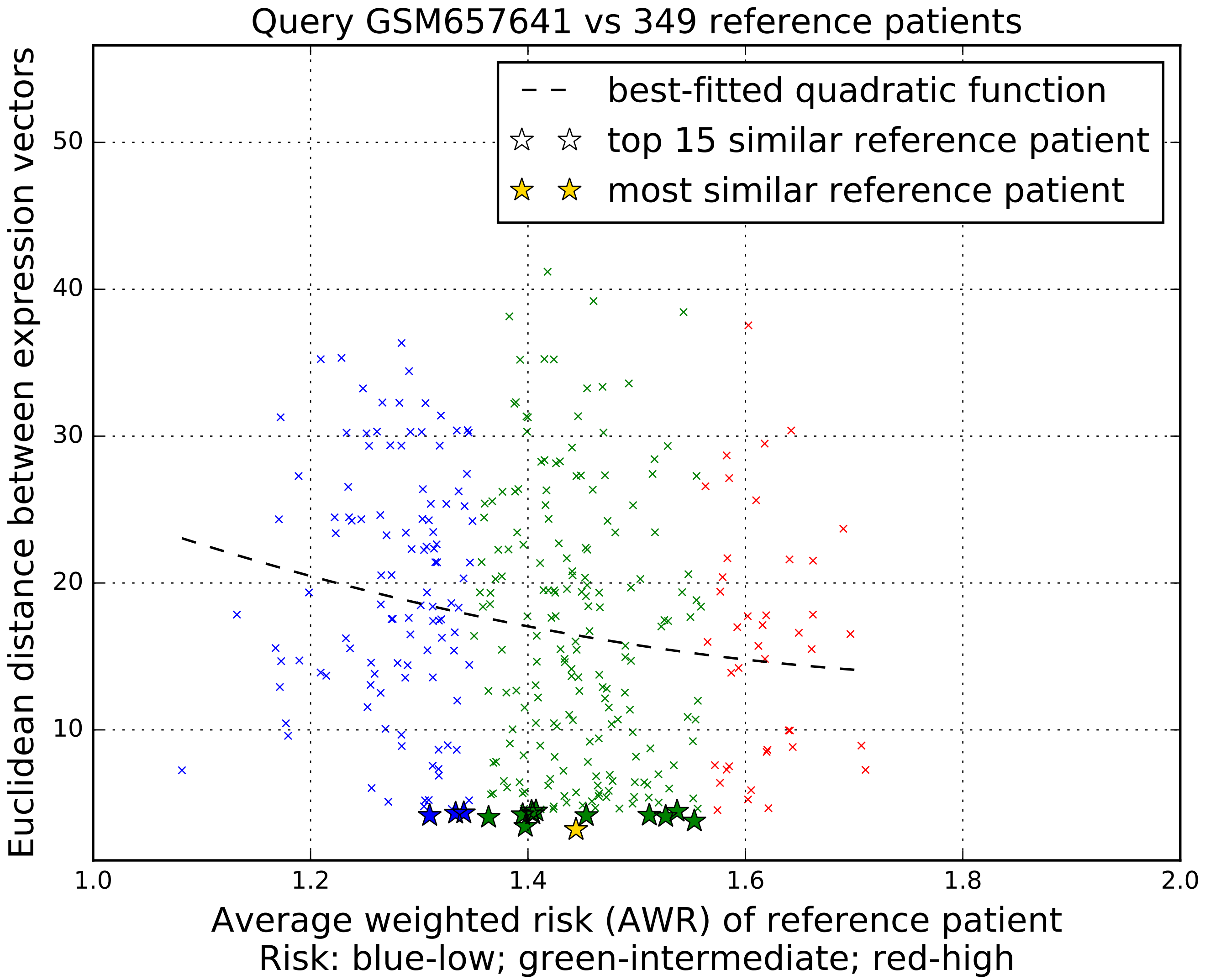
Query GSM657641 vs 349 reference patients
best-fitted quadratic function
top 15 similar reference patient
most similar reference patient
Euclidean distance between expression vectors
Average weighted risk (AWR) of reference patient
Risk: blue-low; green-intermediate; red-high
10
20
30
40
50
1.0
1.2
1.4
1.6
1.8
2.0

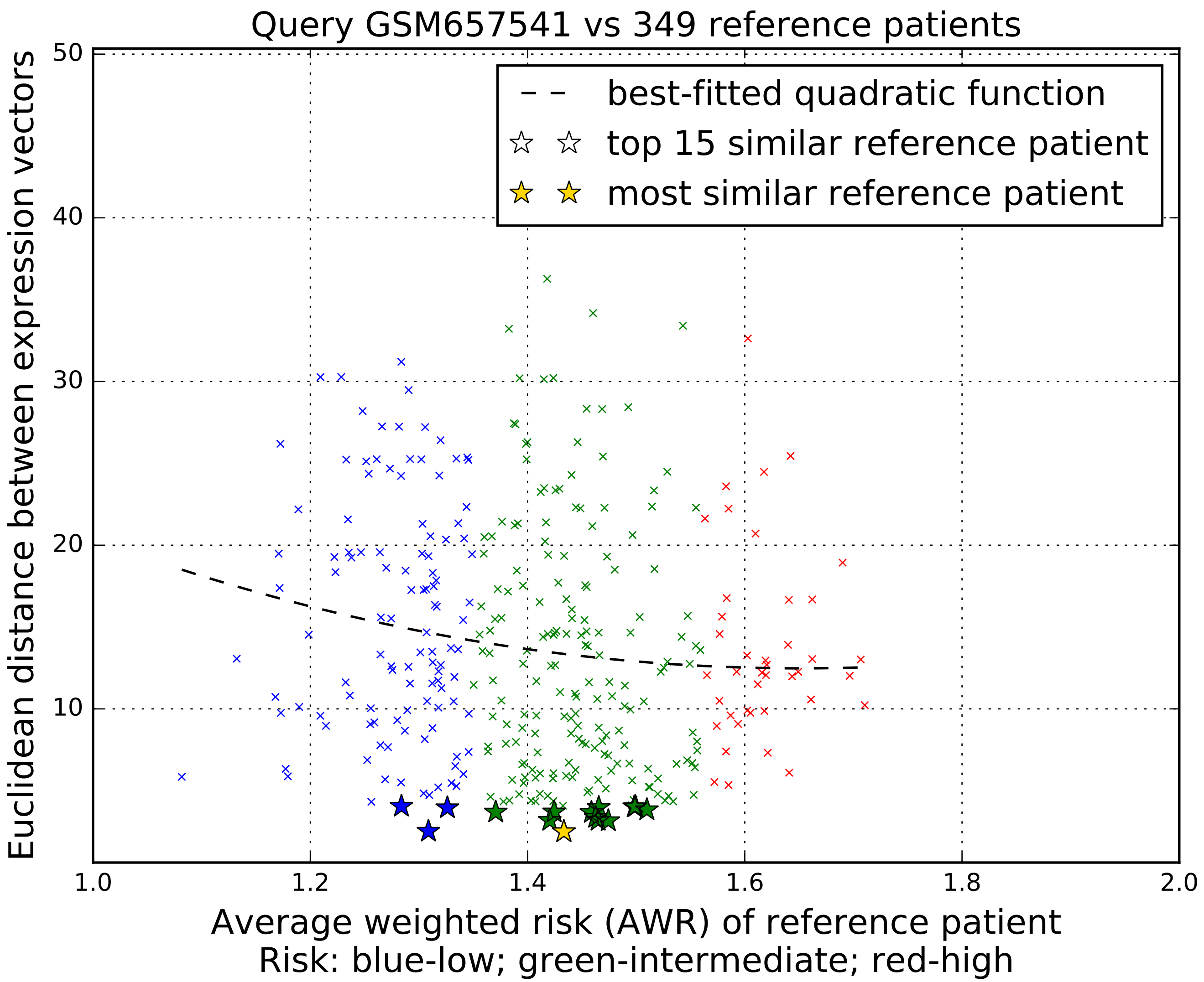

Query GSM657541 vs 349 reference patients
best-fitted quadratic function
top 15 similar reference patient
most similar reference patient
Euclidean distance between expression vectors
50
40
30
20
10
1.0
1.2
1.4
1.6
1.8
2.0
Average weighted risk (AWR) of reference patient
Risk: blue-low; green-intermediate; red-high

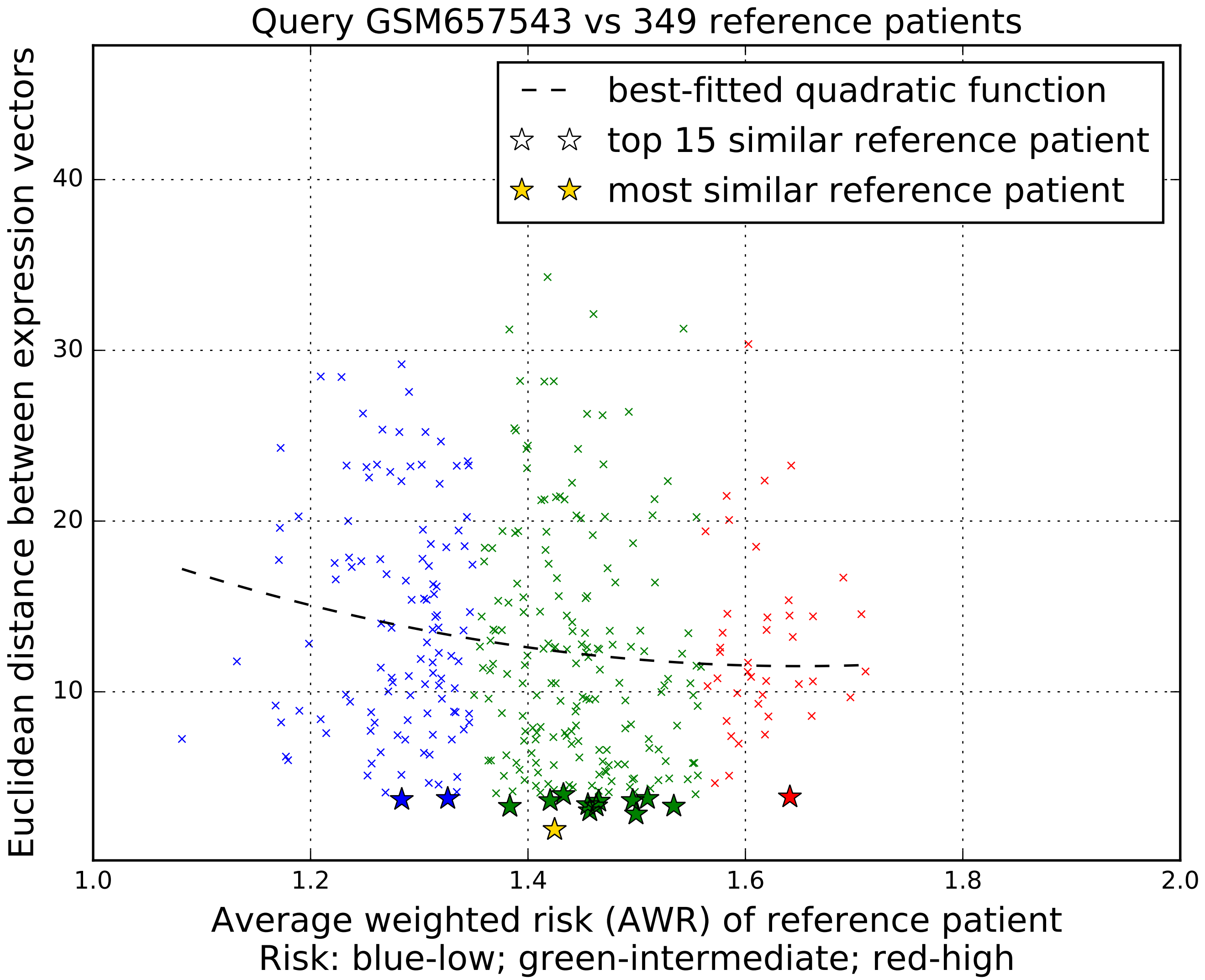

Query GSM657543 vs 349 reference patients
best-fitted quadratic function
top 15 similar reference patient
most similar reference patient
Euclidean distance between expression vectors
10
20
30
40
1.0
1.2
1.4
1.6
1.8
2.0
Average weighted risk (AWR) of reference patient
Risk: blue-low; green-intermediate; red-high

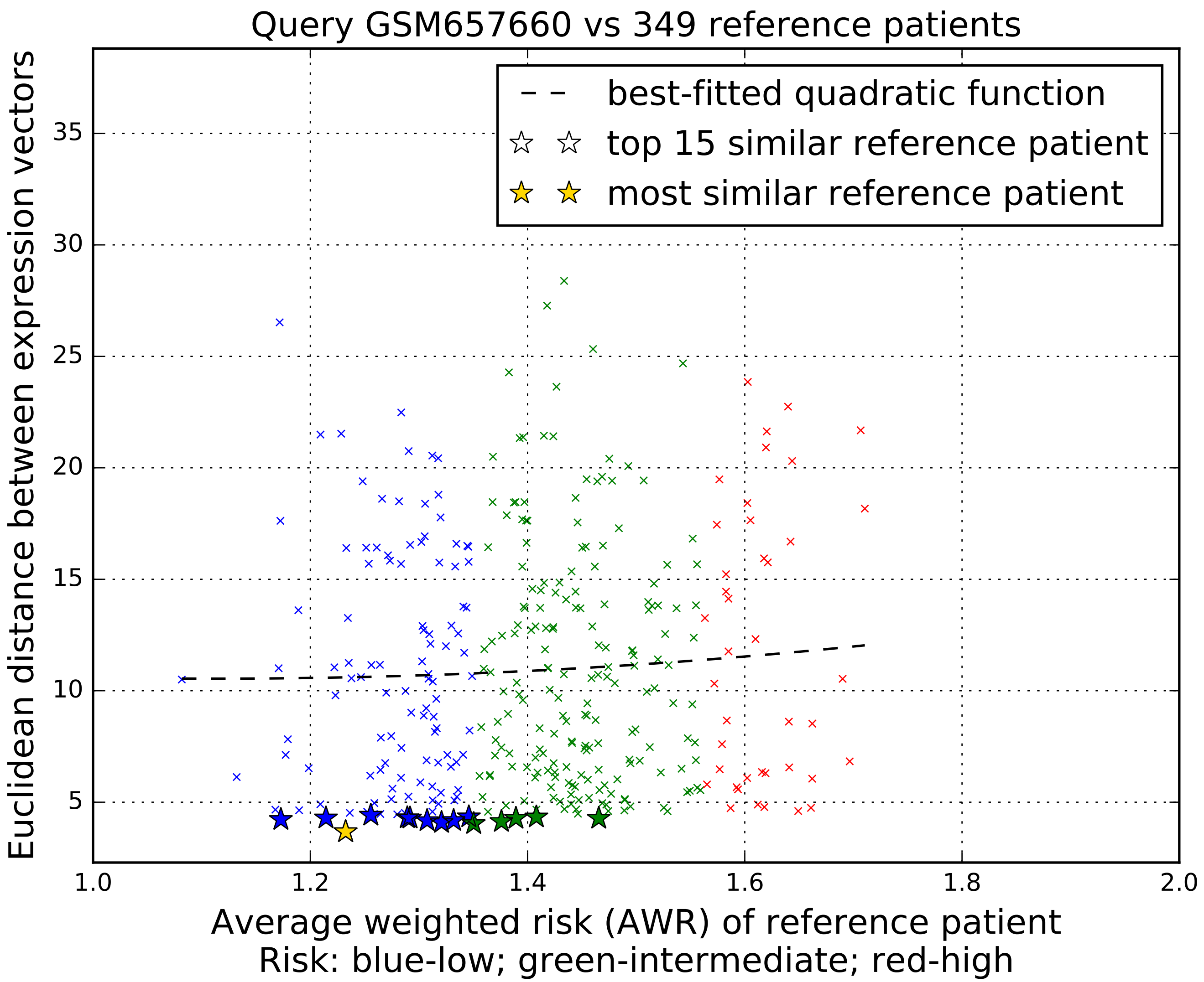
Query GSM657660 vs 349 reference patients
best-fitted quadratic function
top 15 similar reference patient
most similar reference patient
Euclidean distance between expression vectors
35
30
25
20
15
10
5
1.0
1.2
1.4
1.6
1.8
2.0
Average weighted risk (AWR) of reference patient
Risk: blue-low; green-intermediate; red-high

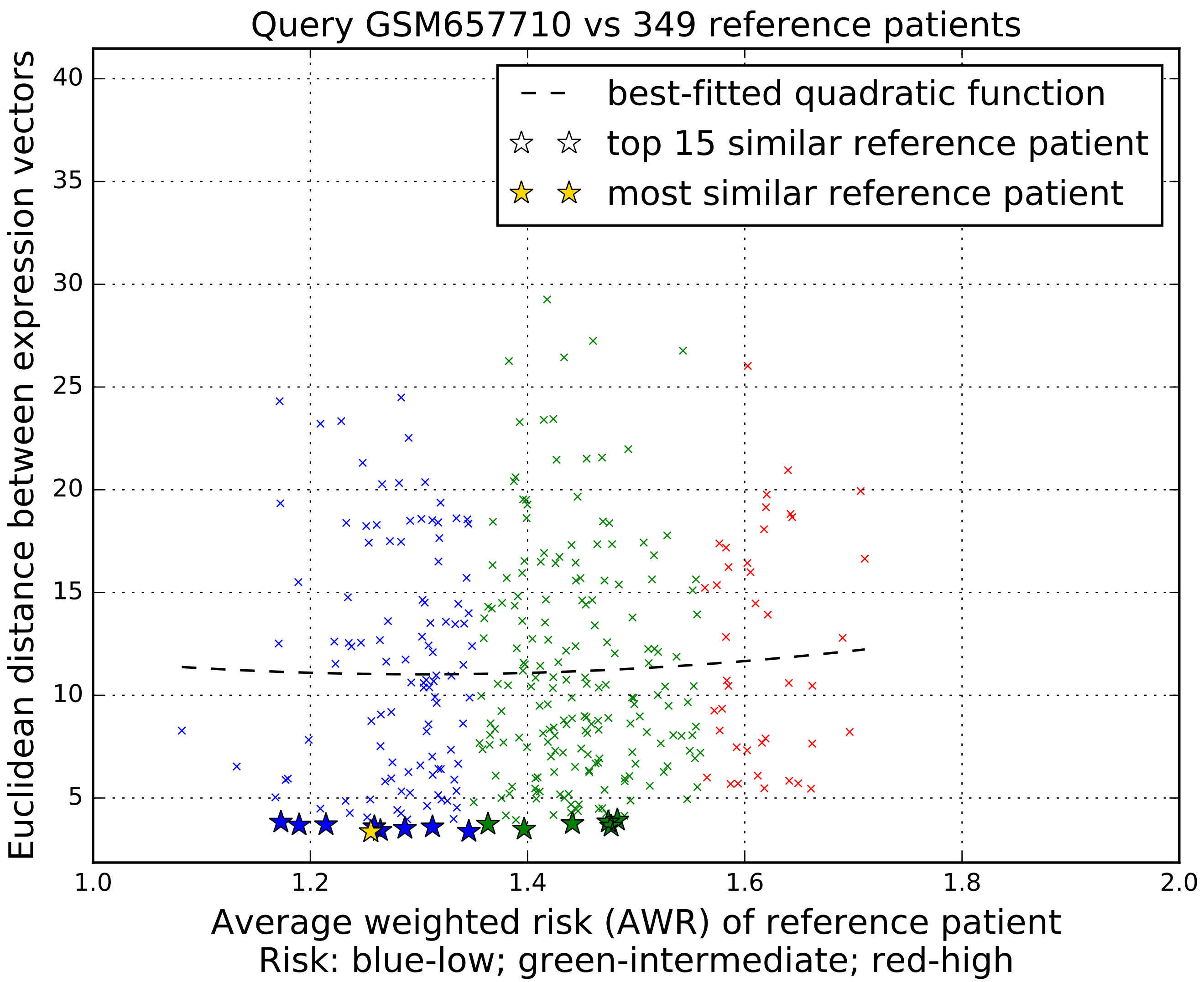

Query GSM657710 vs 349 reference patients
best-fitted quadratic function
top 15 similar reference patient
most similar reference patient
Euclidean distance between expression vectors
40
35
30
25
20
15
10
5
1.0
1.2
1.4
1.6
1.8
2.0
Average weighted risk (AWR) of reference patient
Risk: blue-low; green-intermediate; red-high

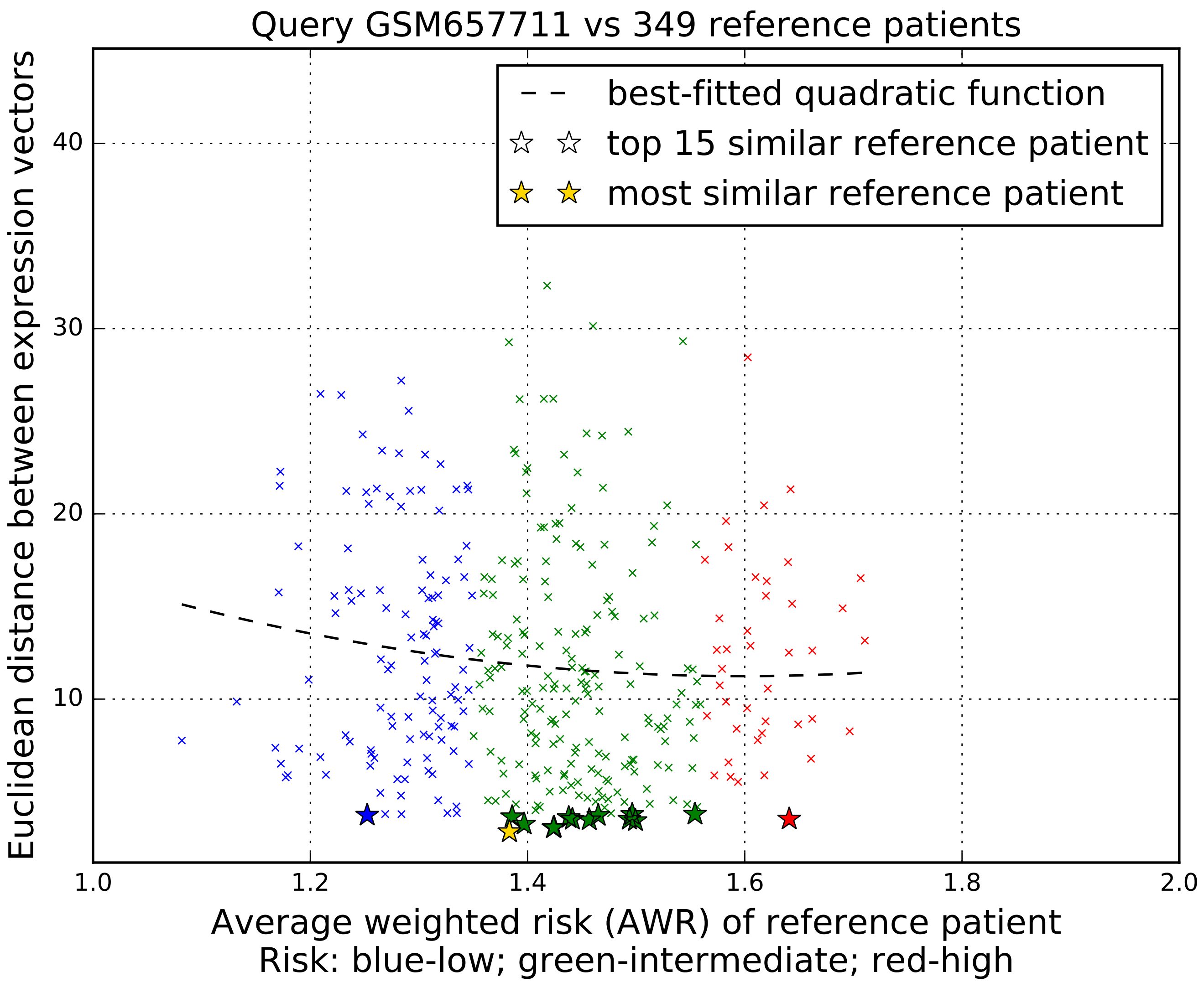

Query GSM657711 vs 349 reference patients
best-fitted quadratic function
top 15 similar reference patient
most similar reference patient
Euclidean distance between expression vectors
10
20
30
40
1.0
1.2
1.4
1.6
1.8
2.0
Average weighted risk (AWR) of reference patient
Risk: blue-low; green-intermediate; red-high

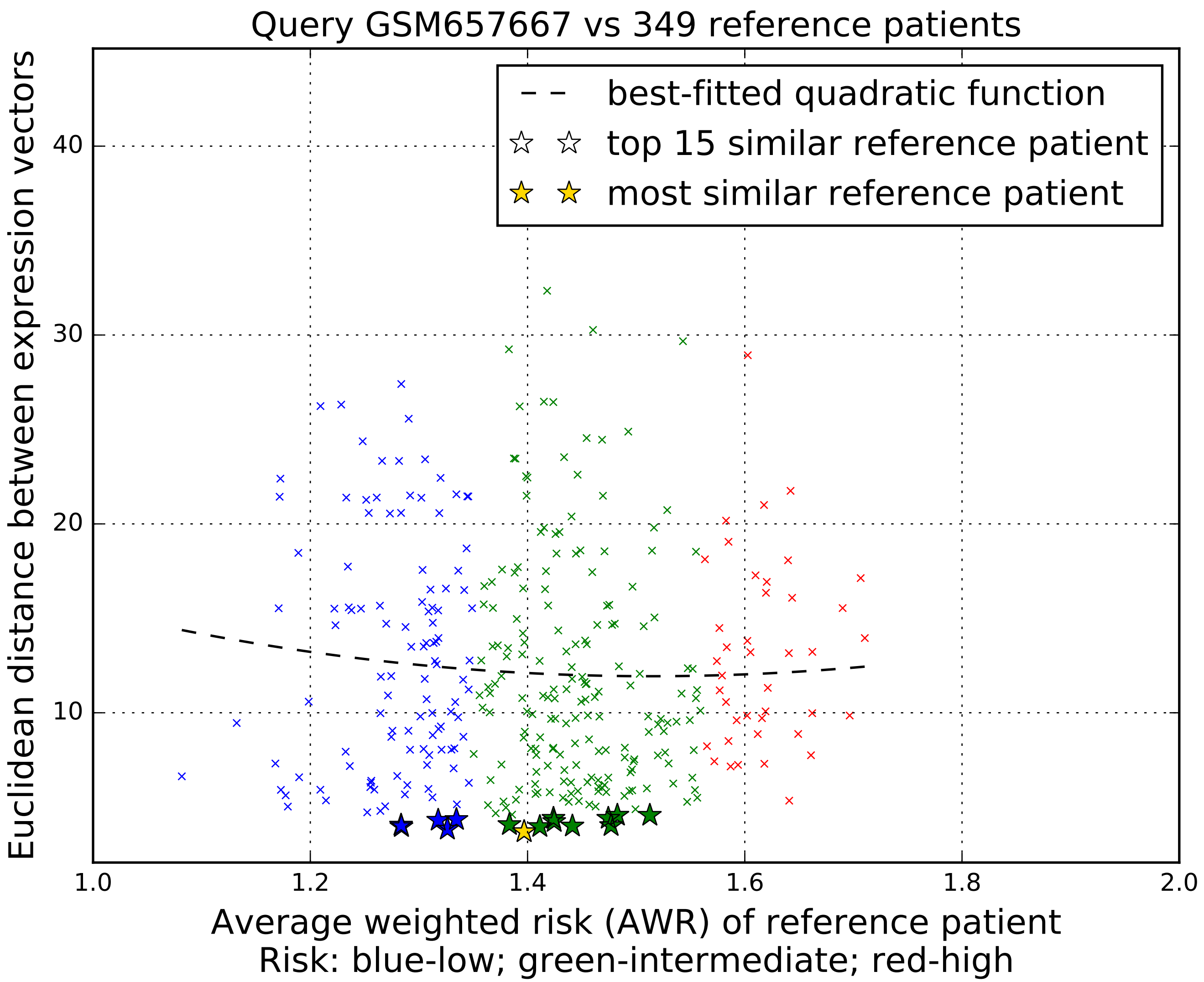
Query GSM657667 vs 349 reference patients
best-fitted quadratic function
top 15 similar reference patient
most similar reference patient
Euclidean distance between expression vectors
10
20
30
40
1.0
1.2
1.4
1.6
1.8
2.0
Average weighted risk (AWR) of reference patient
Risk: blue-low; green-intermediate; red-high

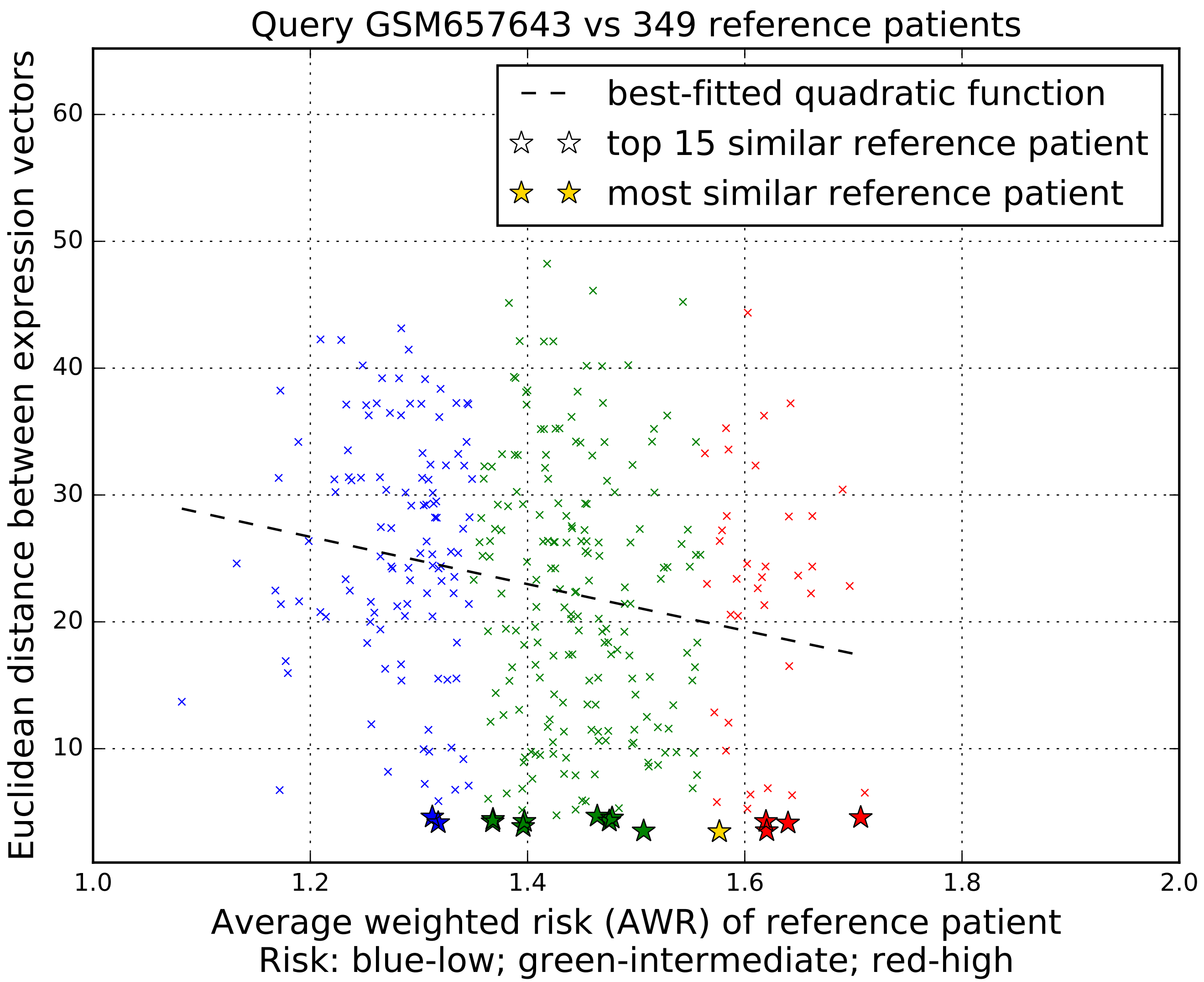

Query GSM657643 vs 349 reference patients
best-fitted quadratic function
top 15 similar reference patient
most similar reference patient
Euclidean distance between expression vectors
10
20
30
40
50
60
1.0
1.2
1.4
1.6
1.8
2.0
Average weighted risk (AWR) of reference patient
Risk: blue-low; green-intermediate; red-high

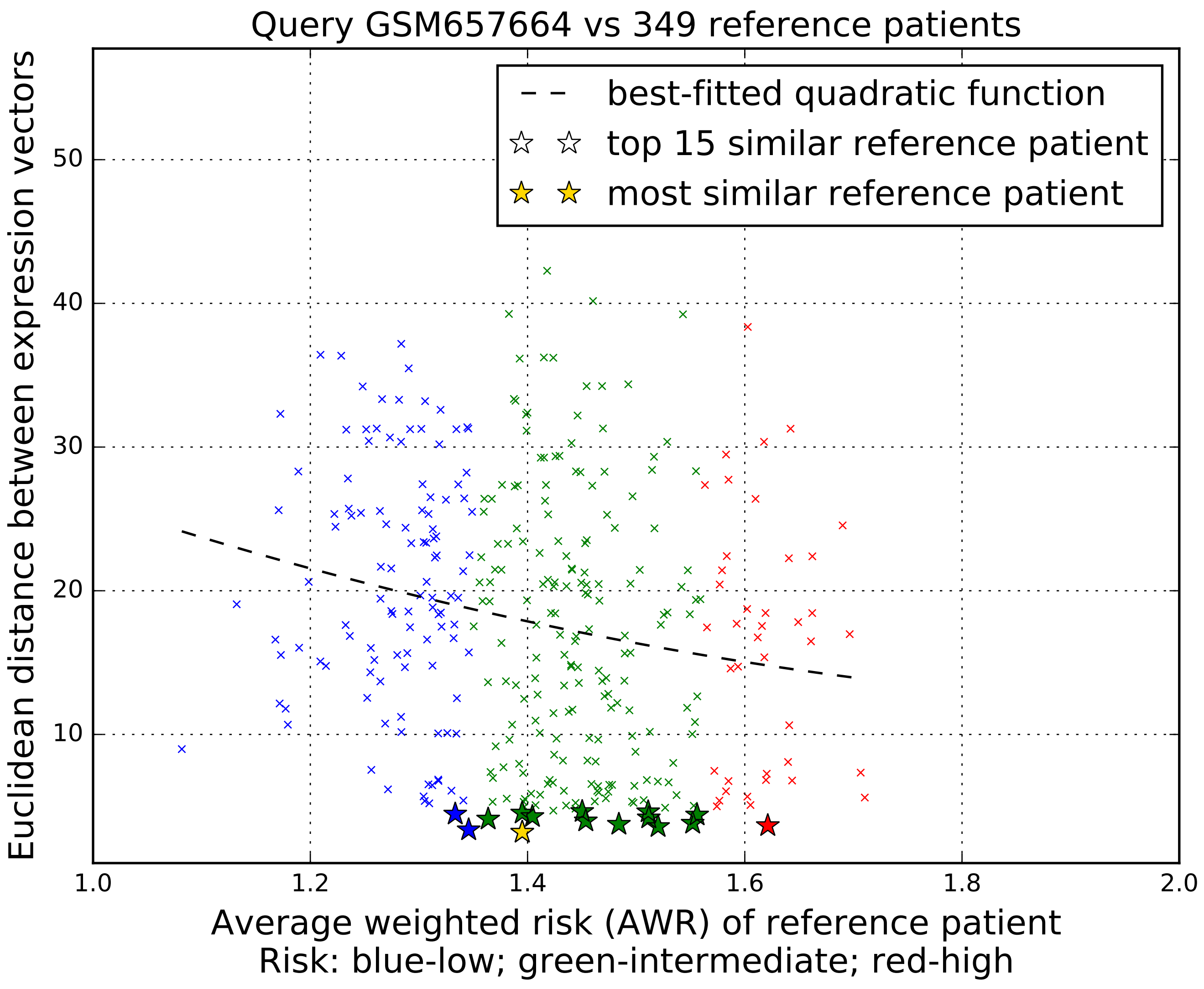

Query GSM657664 vs 349 reference patients
best-fitted quadratic function
top 15 similar reference patient
most similar reference patient
Euclidean distance between expression vectors
10
20
30
40
50
1.0
1.2
1.4
1.6
1.8
2.0
Average weighted risk (AWR) of reference patient
Risk: blue-low; green-intermediate; red-high

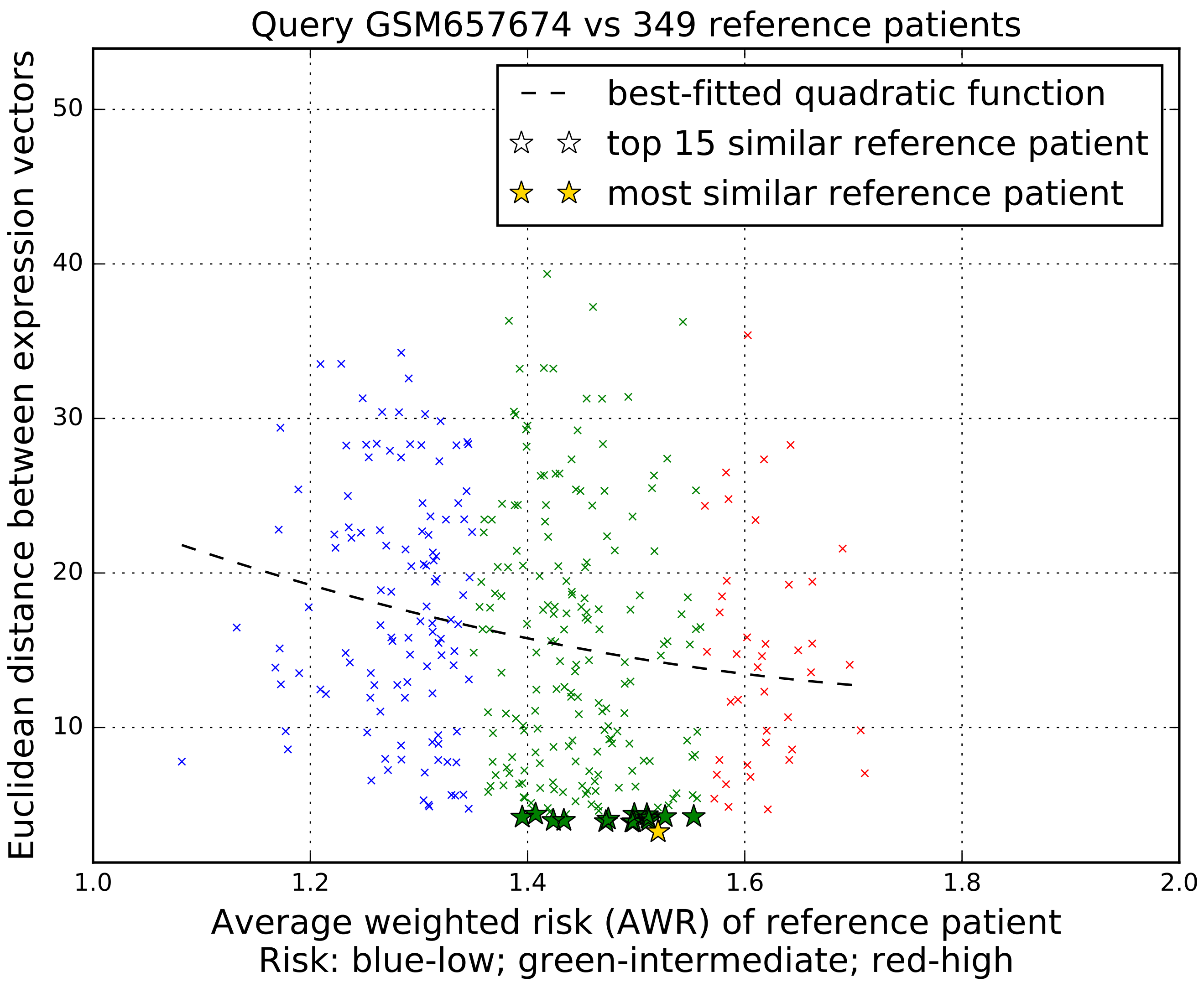
Query GSM657674 vs 349 reference patients
best-fitted quadratic function
top 15 similar reference patient
most similar reference patient
Euclidean distance between expression vectors
Average weighted risk (AWR) of reference patient
Risk: blue-low; green-intermediate; red-high
50
40
30
20
10
1.0
1.2
1.4
1.6
1.8
2.0

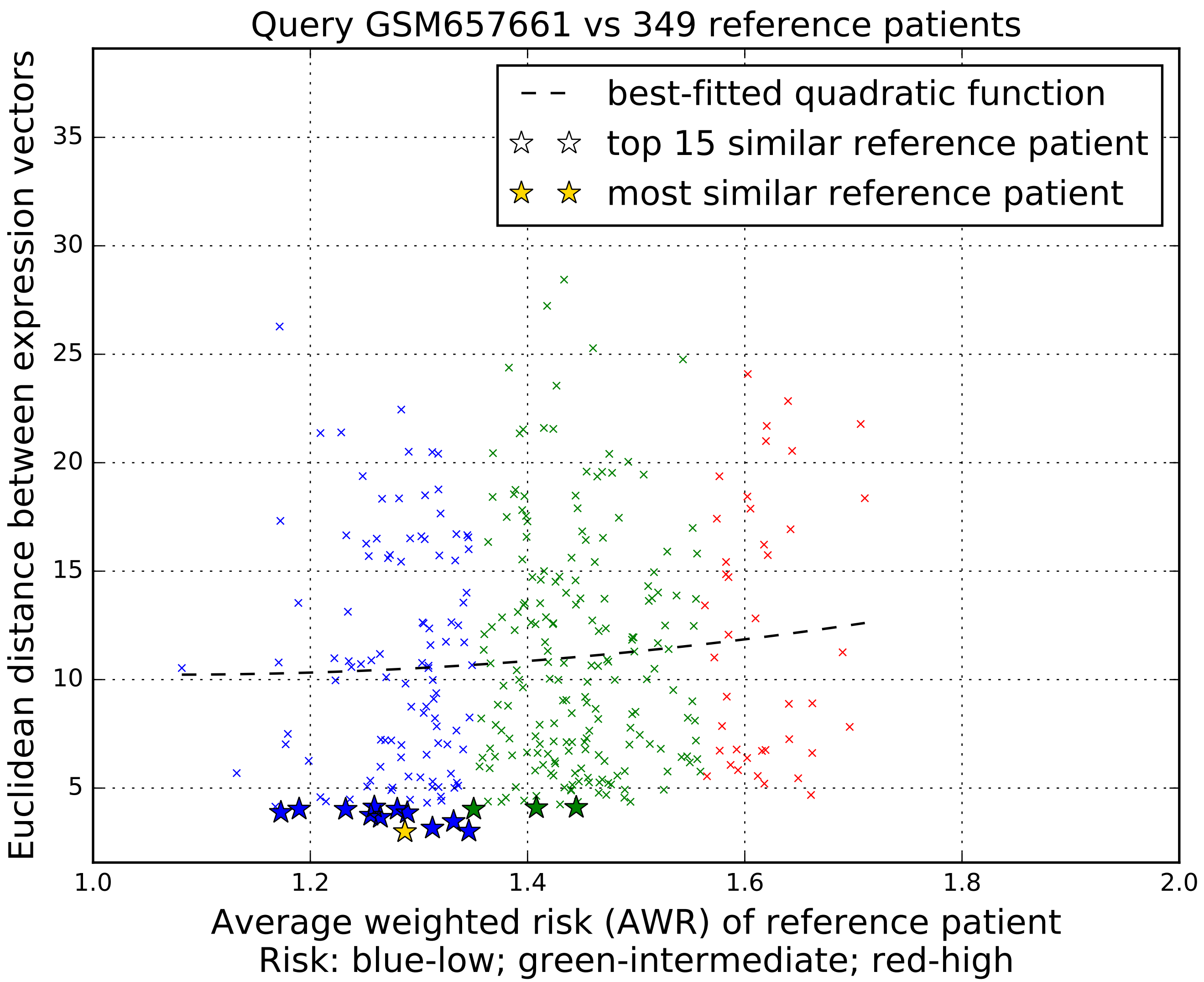

Query GSM657661 vs 349 reference patients
best-fitted quadratic function
top 15 similar reference patient
most similar reference patient
Euclidean distance between expression vectors
35
30
25
20
15
10
5
1.0
1.2
1.4
1.6
1.8
2.0
Average weighted risk (AWR) of reference patient
Risk: blue-low; green-intermediate; red-high

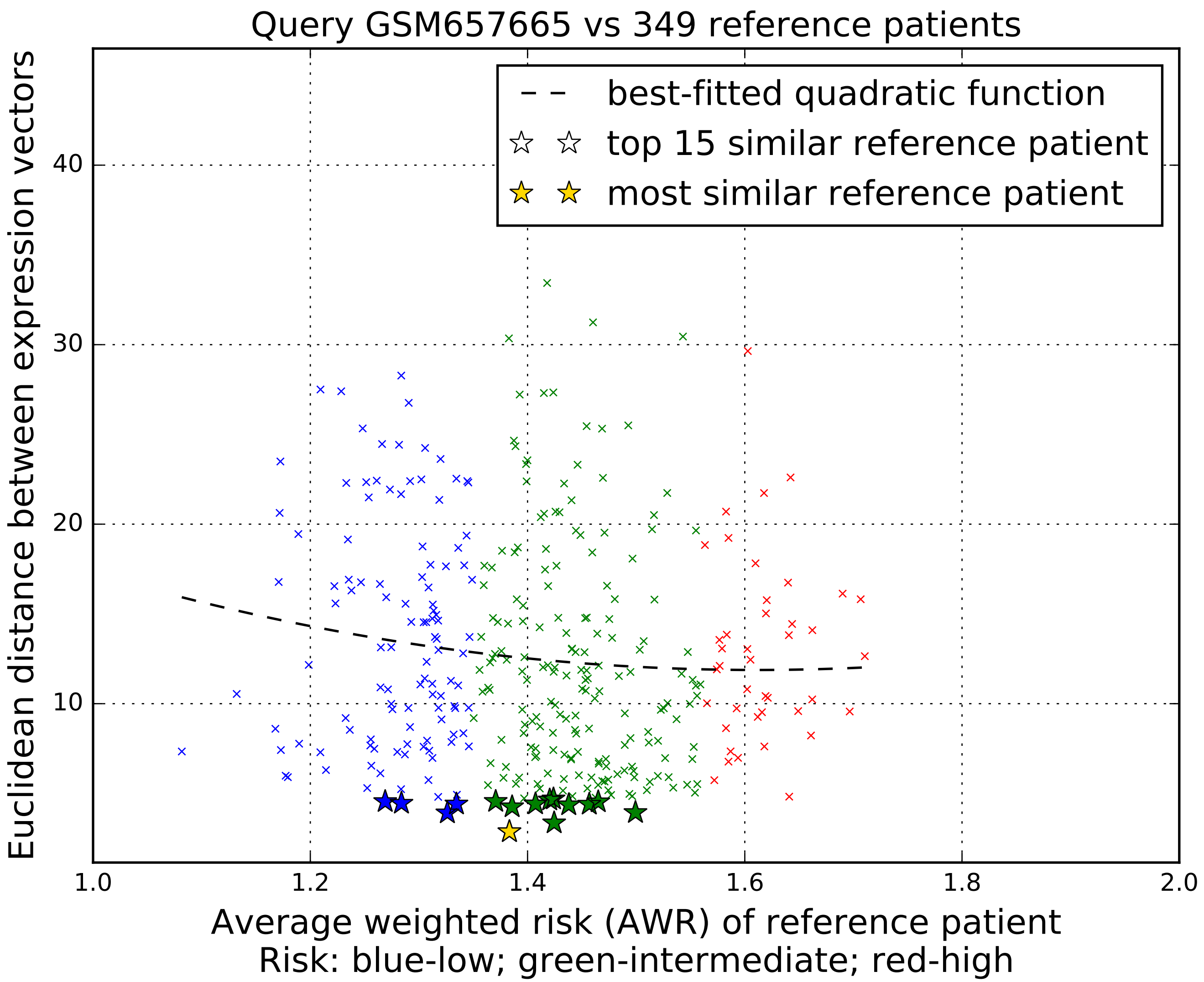
Query GSM657665 vs 349 reference patients
best-fitted quadratic function
top 15 similar reference patient
most similar reference patient
Euclidean distance between expression vectors
Average weighted risk (AWR) of reference patient
Risk: blue-low; green-intermediate; red-high
40
30
20
10
1.0
1.2
1.4
1.6
1.8
2.0

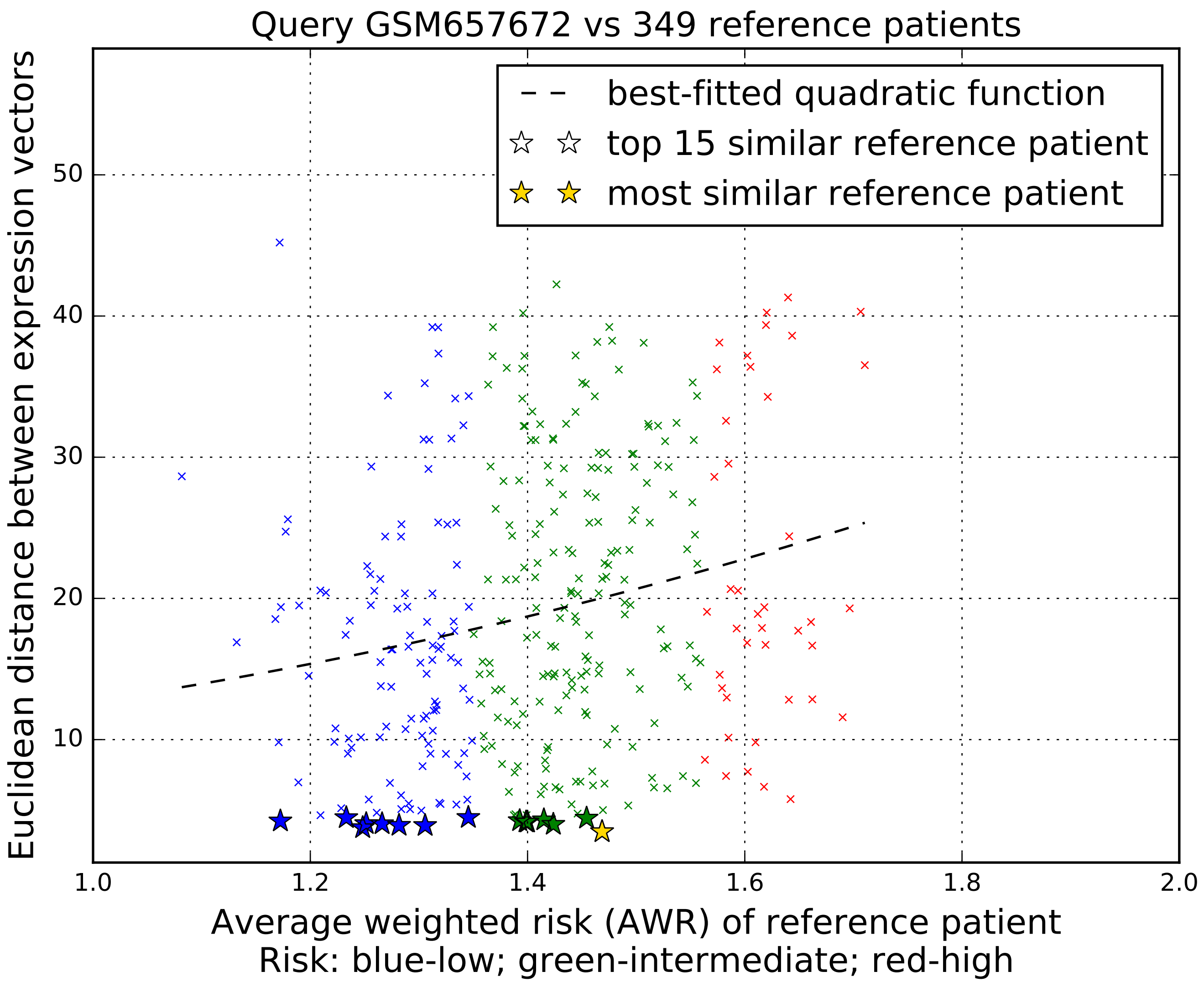

Query GSM657672 vs 349 reference patients
best-fitted quadratic function
top 15 similar reference patient
most similar reference patient
Euclidean distance between expression vectors
10
20
30
40
50
1.0
1.2
1.4
1.6
1.8
2.0
Average weighted risk (AWR) of reference patient
Risk: blue-low; green-intermediate; red-high

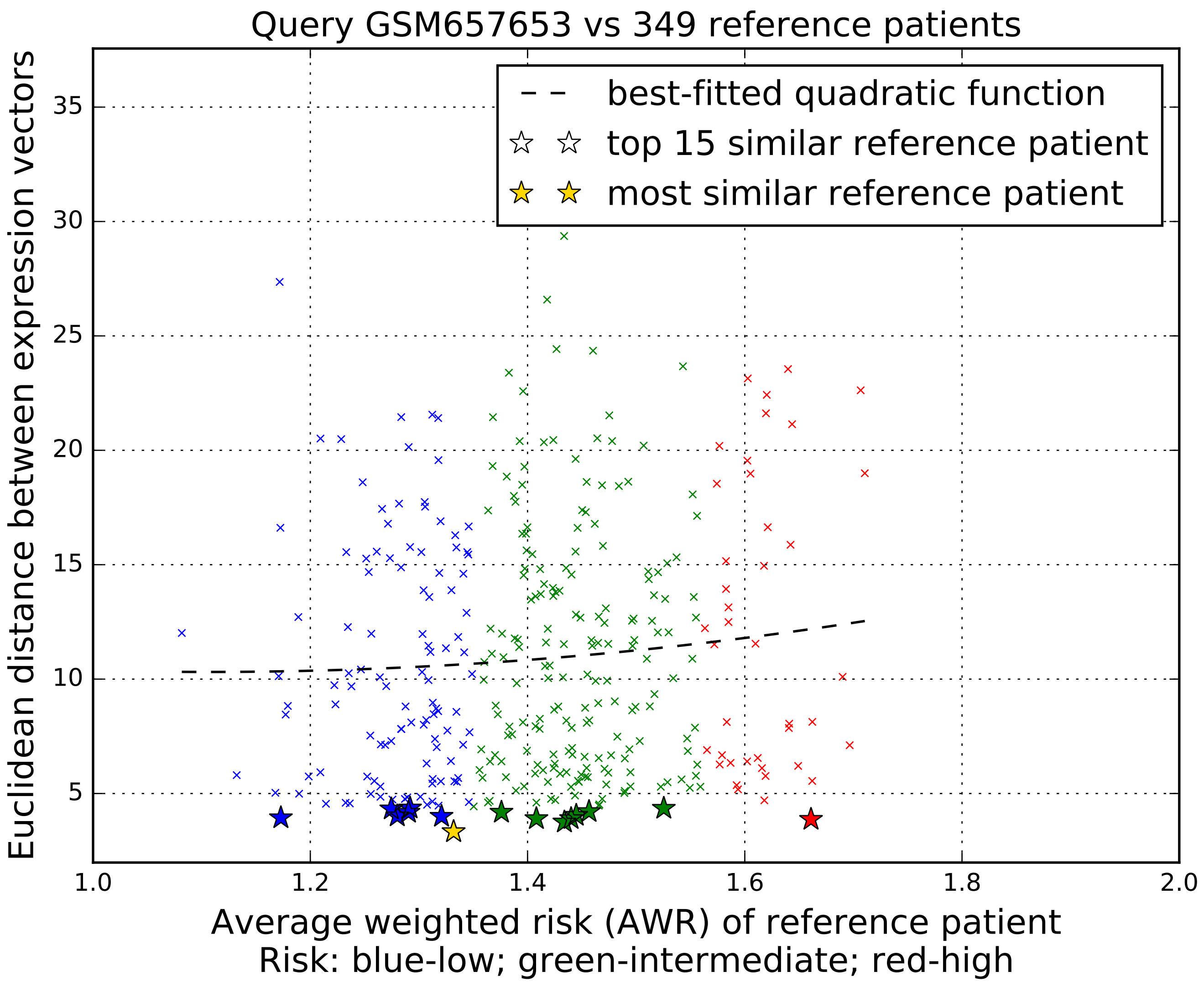
Query GSM657653 vs 349 reference patients
best-fitted quadratic function
top 15 similar reference patient
most similar reference patient
Euclidean distance between expression vectors
35
30
25
20
15
10
5
1.0
1.2
1.4
1.6
1.8
2.0
Average weighted risk (AWR) of reference patient
Risk: blue-low; green-intermediate; red-high

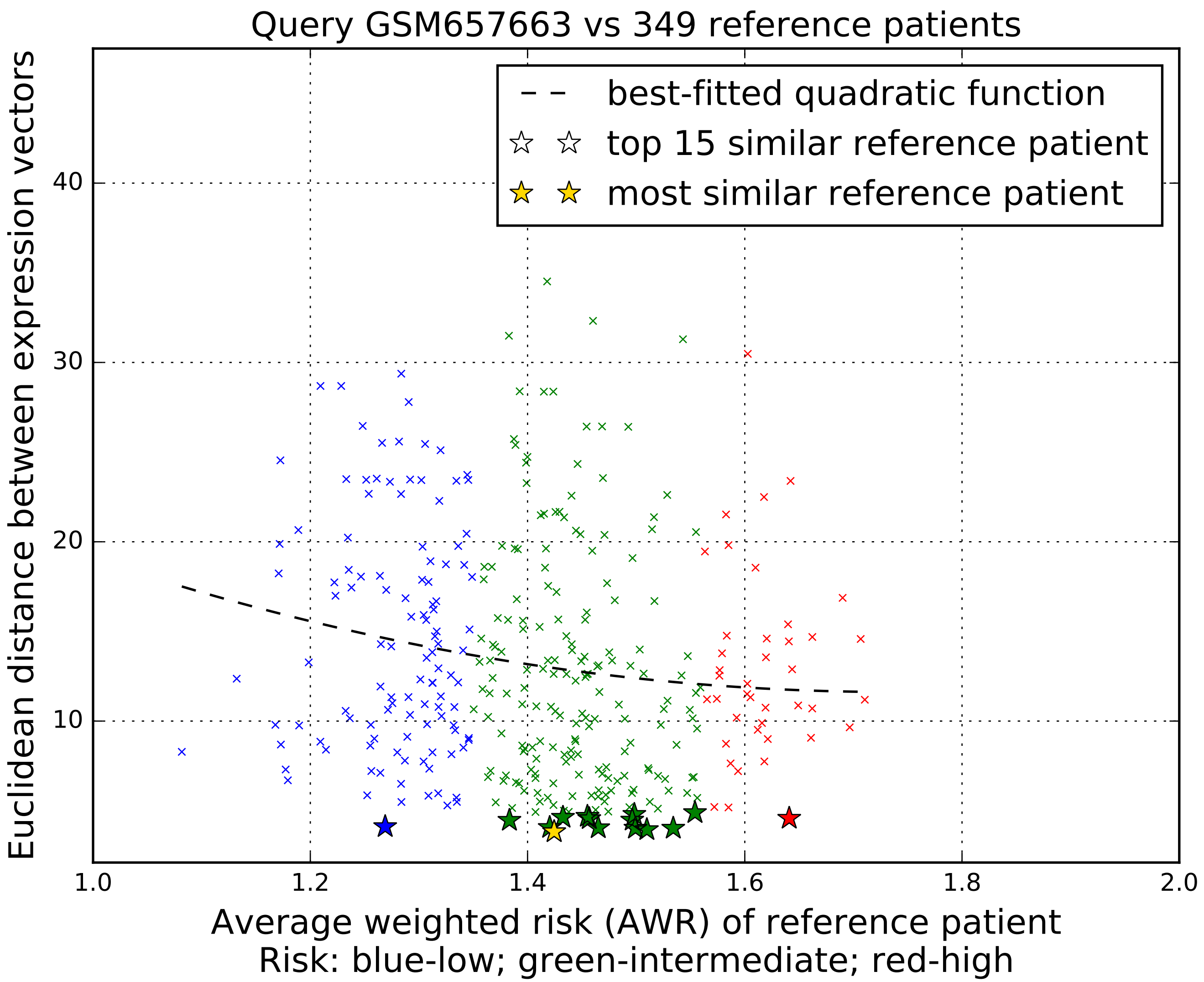

Query GSM657663 vs 349 reference patients
best-fitted quadratic function
top 15 similar reference patient
most similar reference patient
40
30
20
10
1.0
1.2
1.4
1.6
1.8
2.0
Euclidean distance between expression vectors
Average weighted risk (AWR) of reference patient
Risk: blue-low; green-intermediate; red-high

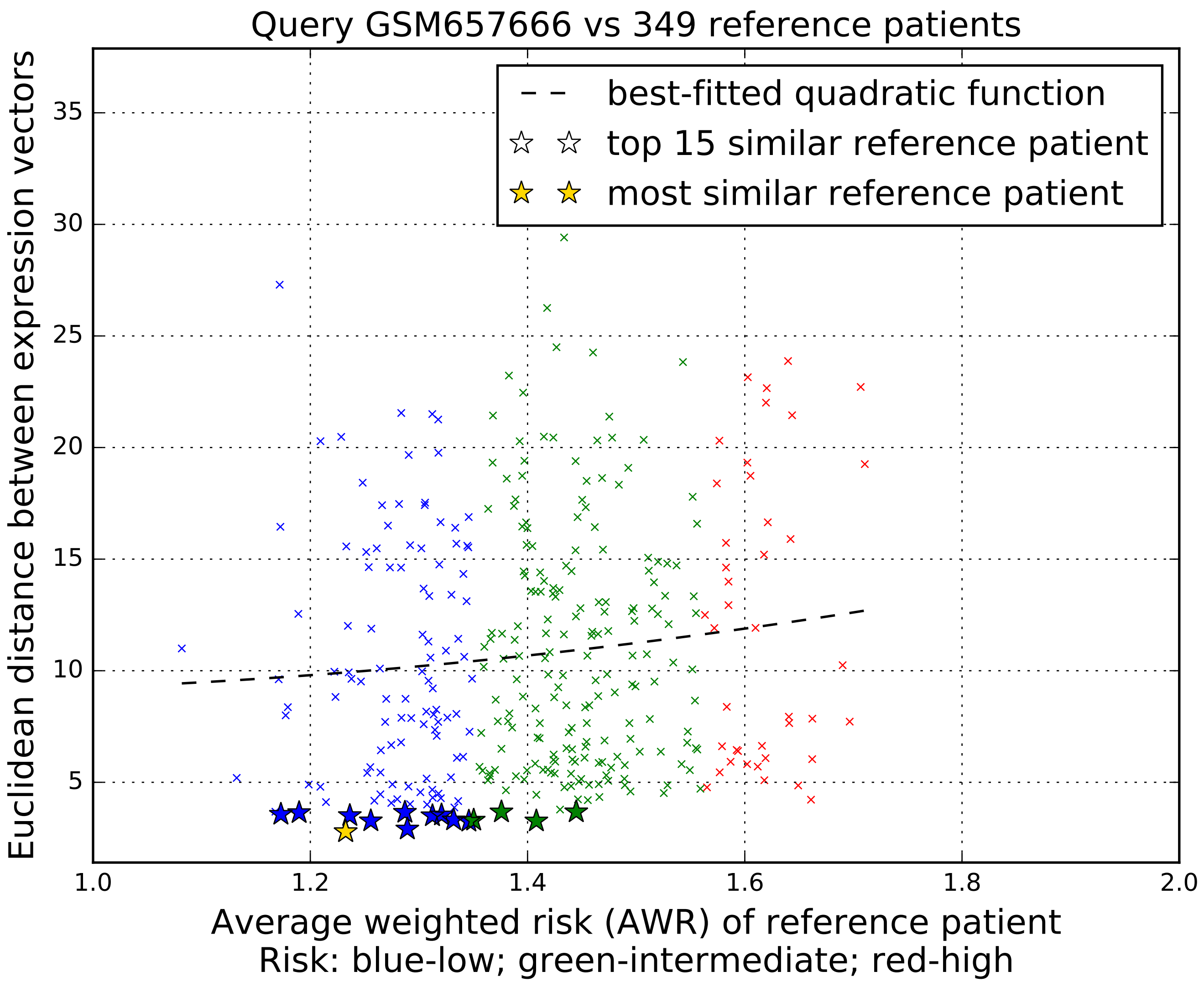
Query GSM657666 vs 349 reference patients
best-fitted quadratic function
top 15 similar reference patient
most similar reference patient
Euclidean distance between expression vectors
35
30
25
20
15
10
5
1.0
1.2
1.4
1.6
1.8
2.0
Average weighted risk (AWR) of reference patient
Risk: blue-low; green-intermediate; red-high

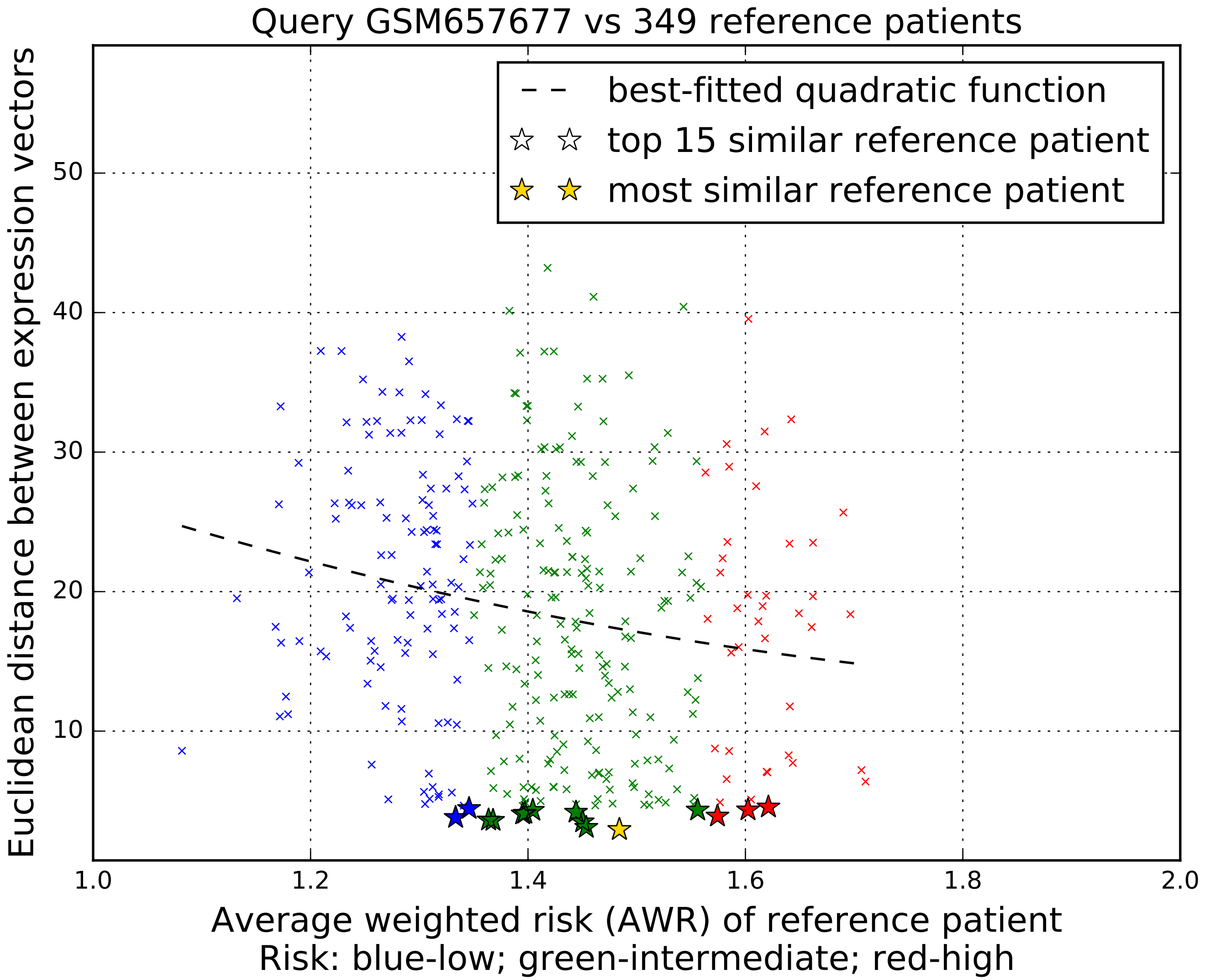
Query GSM657677 vs 349 reference patients
best-fitted quadratic function
top 15 similar reference patient
most similar reference patient
Euclidean distance between expression vectors
10
20
30
40
50
1.0
1.2
1.4
1.6
1.8
2.0
Average weighted risk (AWR) of reference patient
Risk: blue-low; green-intermediate; red-high

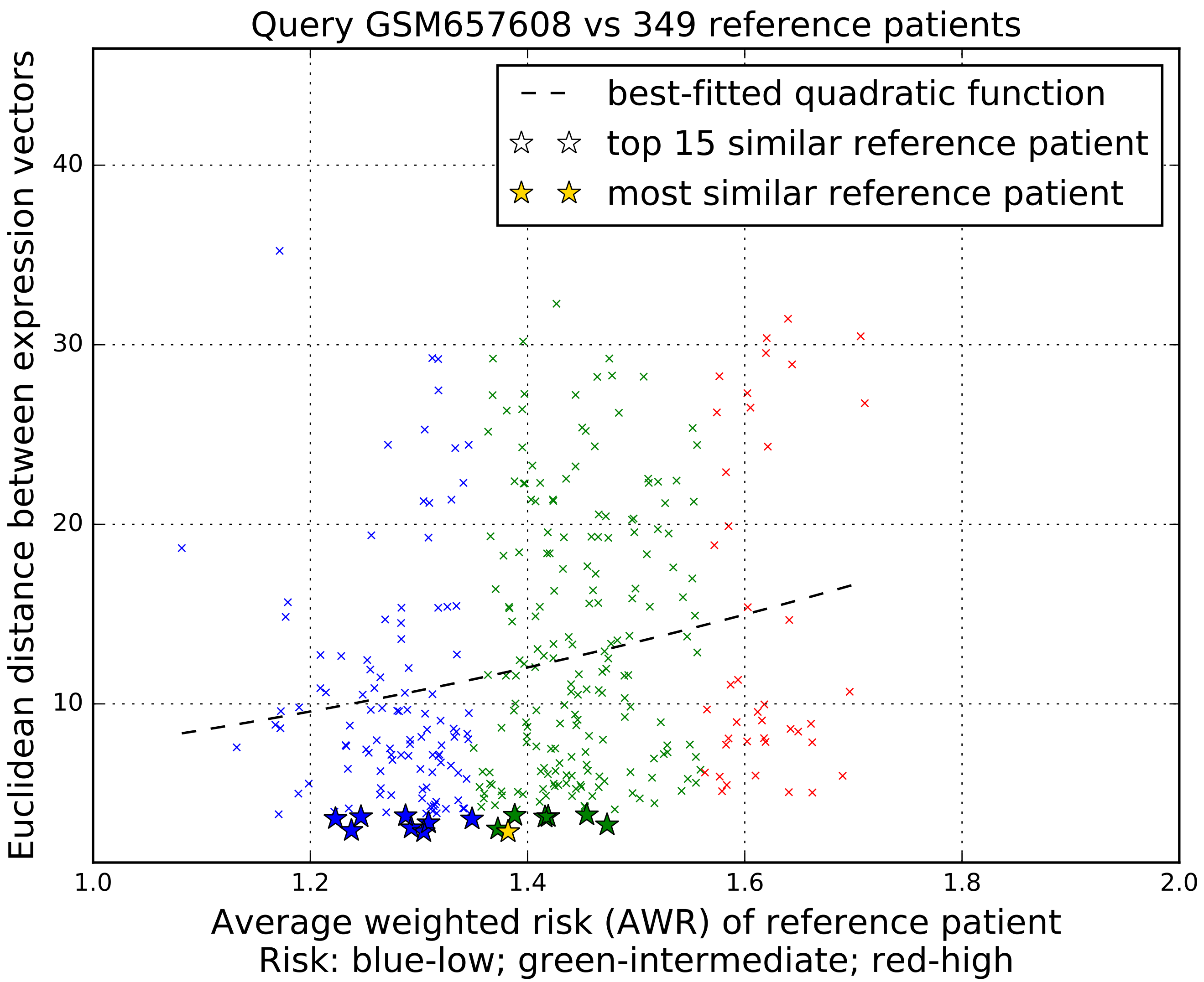

Query GSM657608 vs 349 reference patients
best-fitted quadratic function
top 15 similar reference patient
most similar reference patient
Euclidean distance between expression vectors
40
30
20
10
1.0
1.2
1.4
1.6
1.8
2.0
Average weighted risk (AWR) of reference patient
Risk: blue-low; green-intermediate; red-high

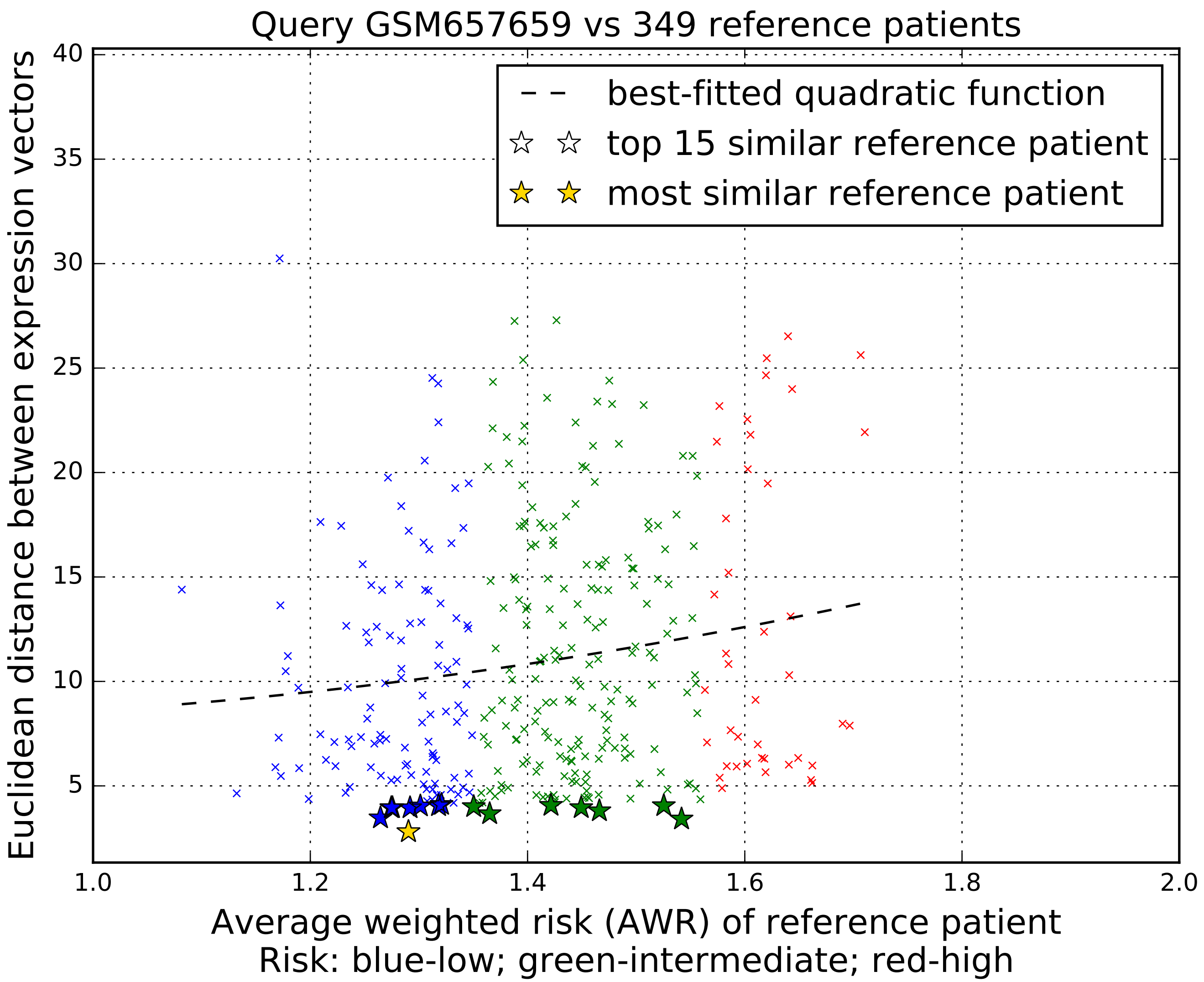
Query GSM657659 vs 349 reference patients
best-fitted quadratic function
top 15 similar reference patient
most similar reference patient
Euclidean distance between expression vectors
40
35
30
25
20
15
10
5
1.0
1.2
1.4
1.6
1.8
2.0
Average weighted risk (AWR) of reference patient
Risk: blue-low; green-intermediate; red-high

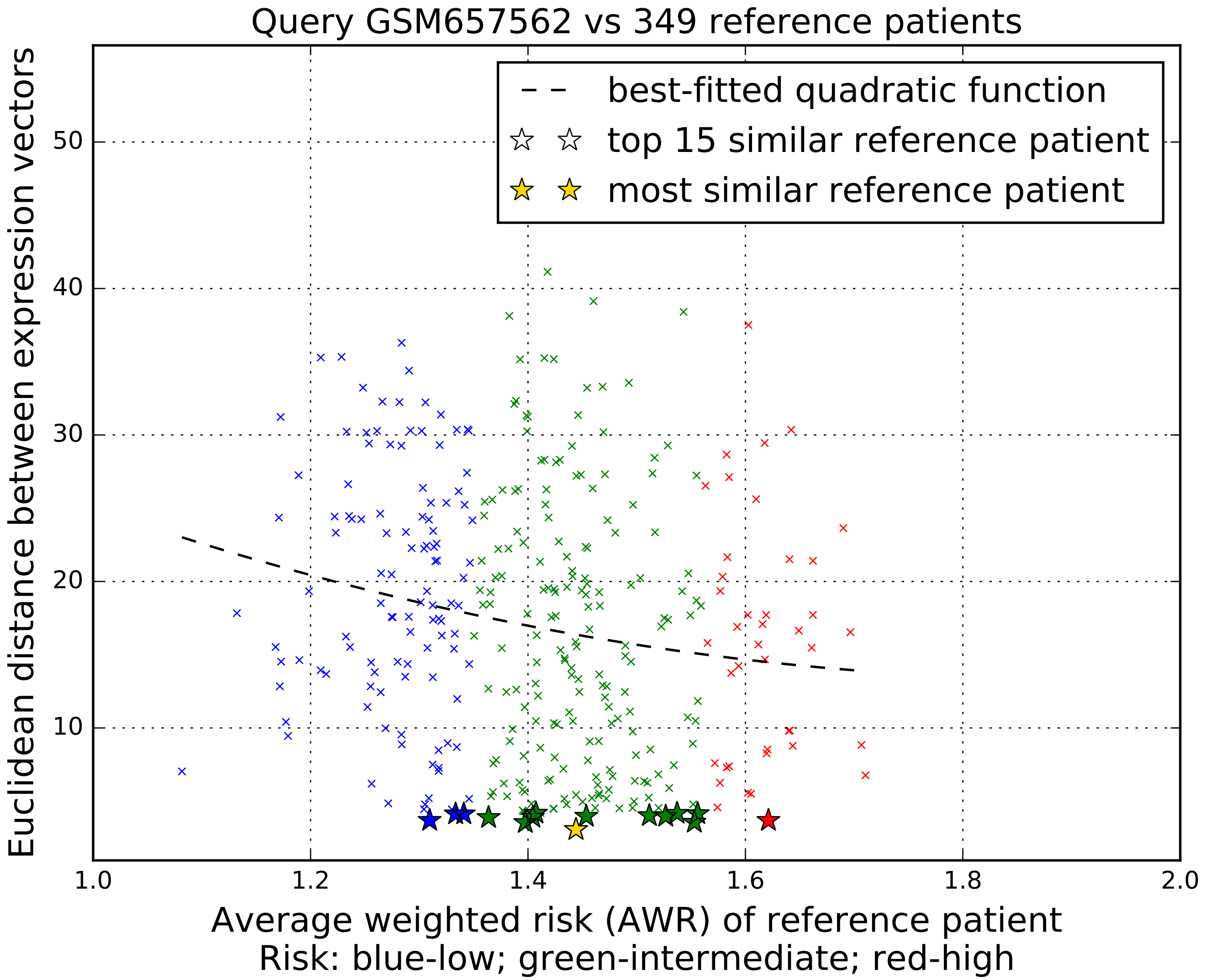
Query GSM657562 vs 349 reference patients
best-fitted quadratic function
top 15 similar reference patient
most similar reference patient
Euclidean distance between expression vectors
10
20
30
40
50
1.0
1.2
1.4
1.6
1.8
2.0
Average weighted risk (AWR) of reference patient
Risk: blue-low; green-intermediate; red-high

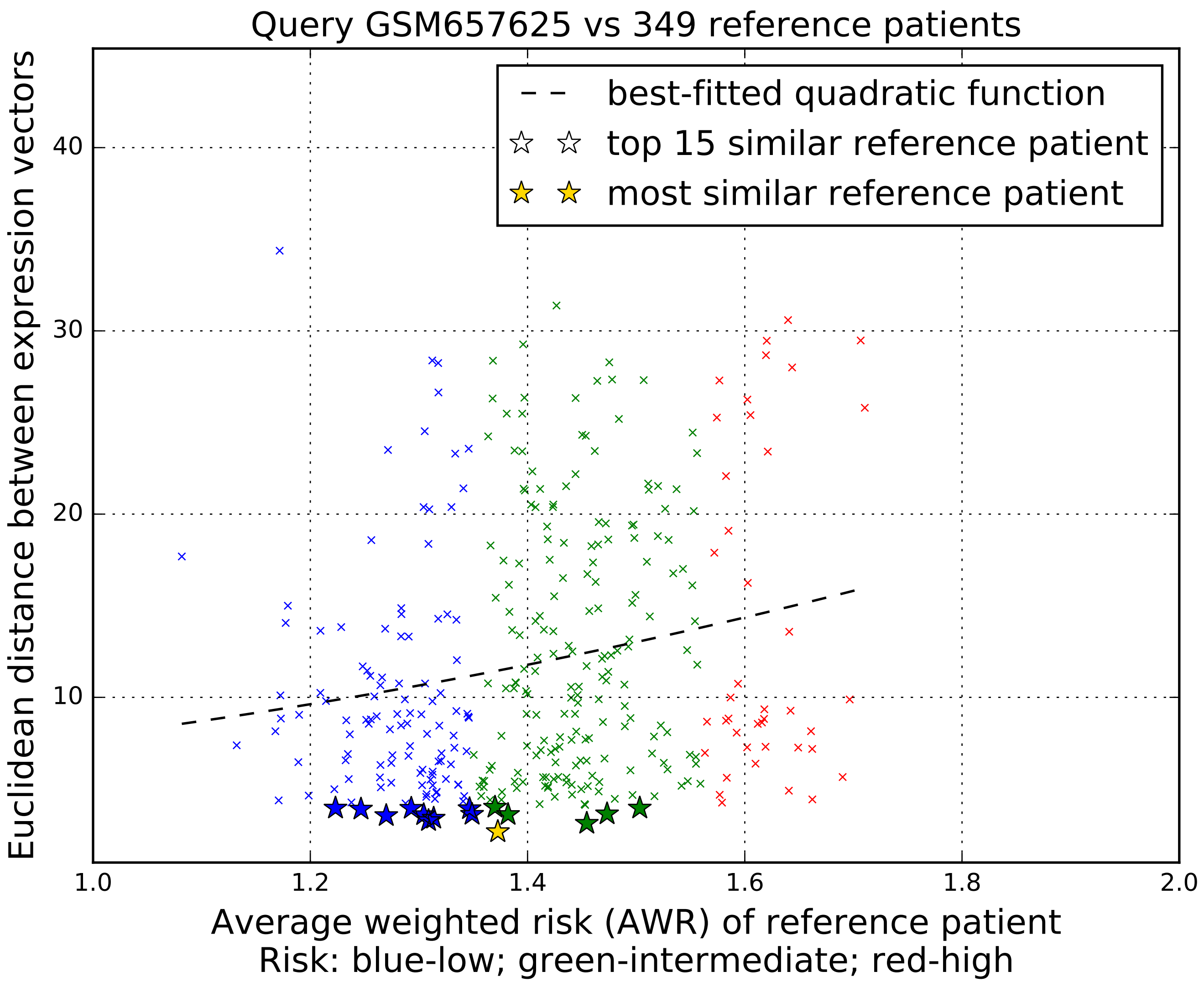

Query GSM657625 vs 349 reference patients
best-fitted quadratic function
top 15 similar reference patient
most similar reference patient
Euclidean distance between expression vectors
40
30
20
10
1.0
1.2
1.4
1.6
1.8
2.0
Average weighted risk (AWR) of reference patient
Risk: blue-low; green-intermediate; red-high

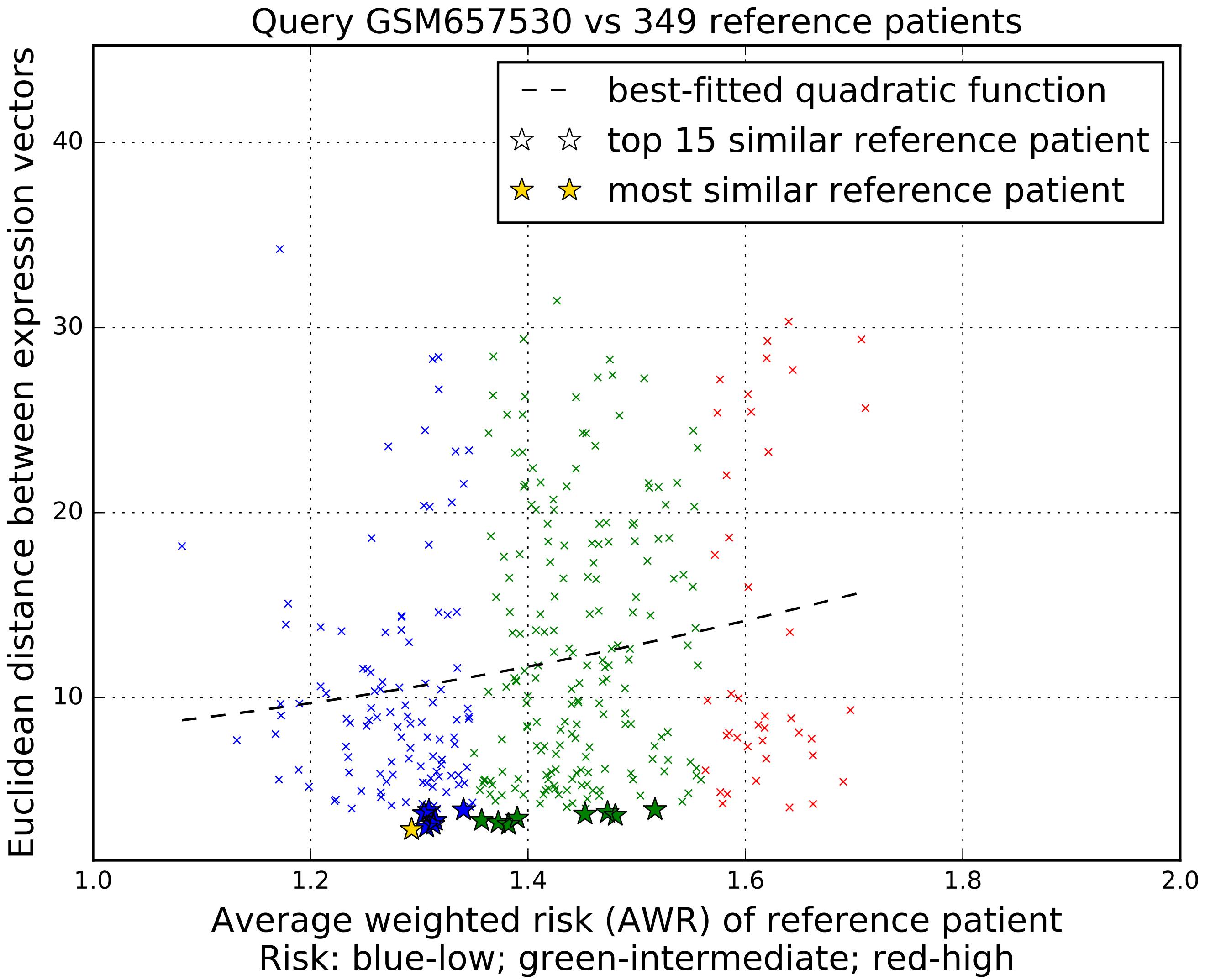
Query GSM657530 vs 349 reference patients
best-fitted quadratic function
top 15 similar reference patient
most similar reference patient
Euclidean distance between expression vectors
10
20
30
40
1.0
1.2
1.4
1.6
1.8
2.0
Average weighted risk (AWR) of reference patient
Risk: blue-low; green-intermediate; red-high

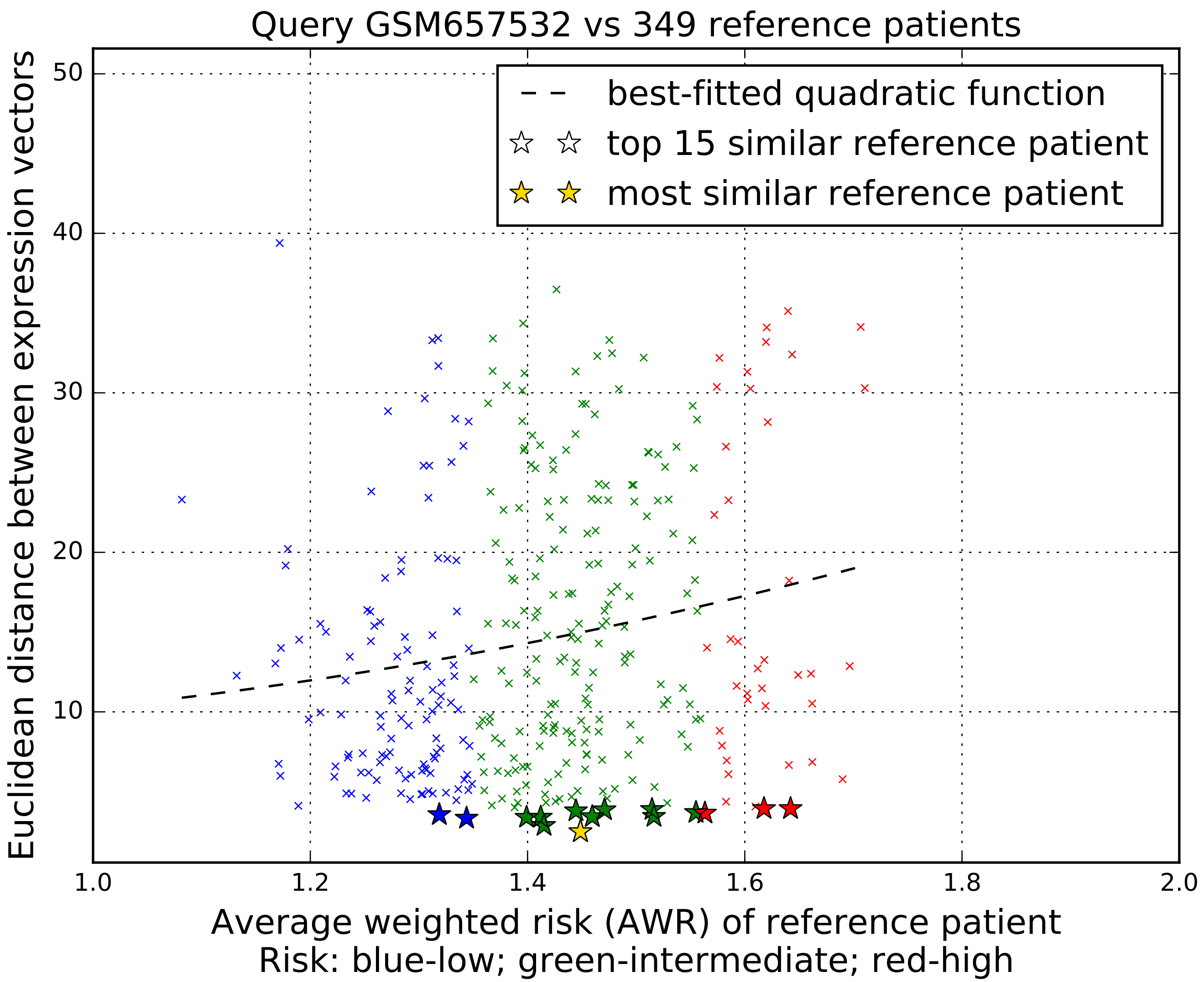

Query GSM657532 vs 349 reference patients
best-fitted quadratic function
top 15 similar reference patient
most similar reference patient
Euclidean distance between expression vectors
50
40
30
20
10
1.0
1.2
1.4
1.6
1.8
2.0
Average weighted risk (AWR) of reference patient
Risk: blue-low; green-intermediate; red-high

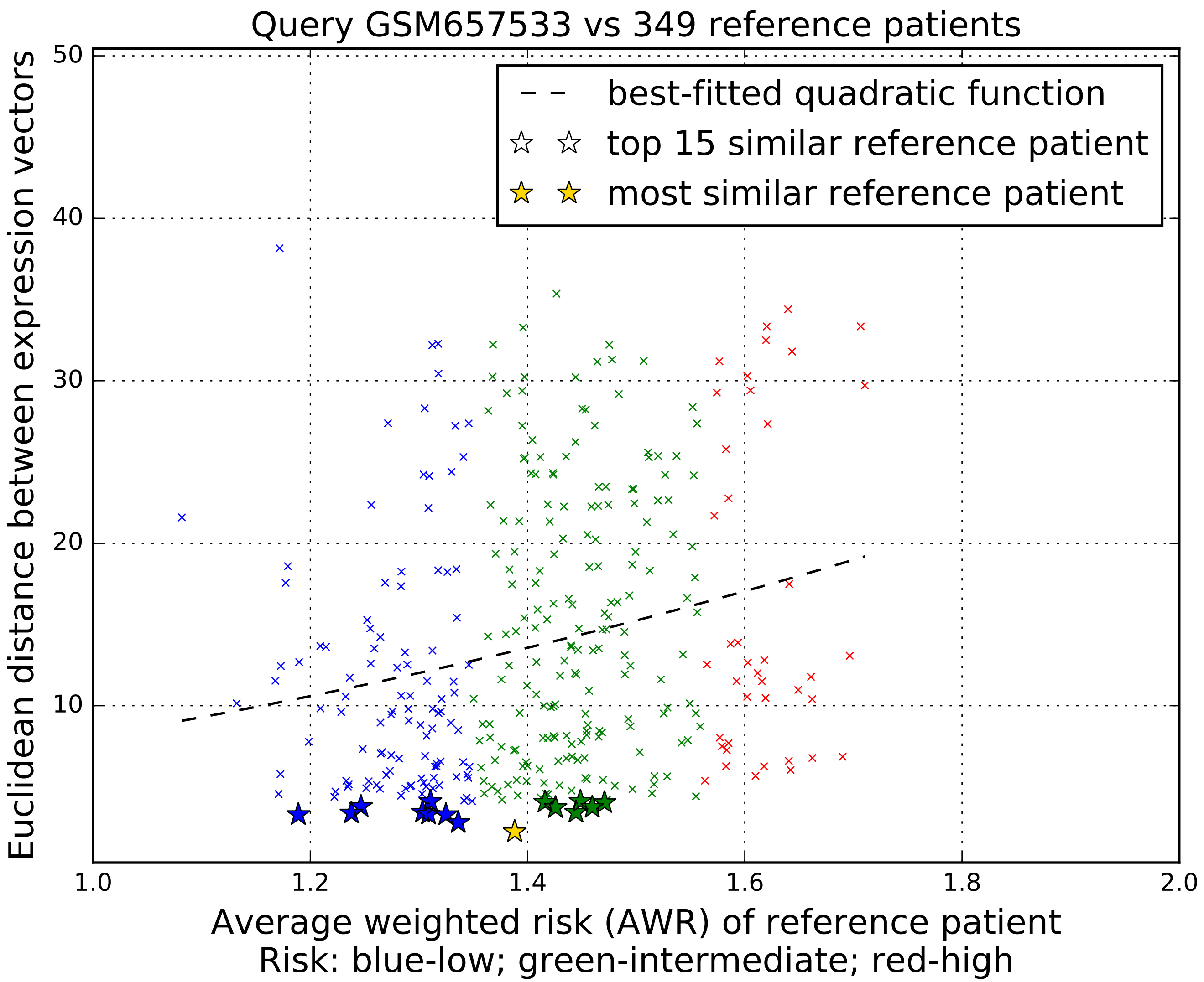
Query GSM657533 vs 349 reference patients
best-fitted quadratic function
top 15 similar reference patient
most similar reference patient
Euclidean distance between expression vectors
Average weighted risk (AWR) of reference patient
Risk: blue-low; green-intermediate; red-high
50
40
30
20
10
1.0
1.2
1.4
1.6
1.8
2.0

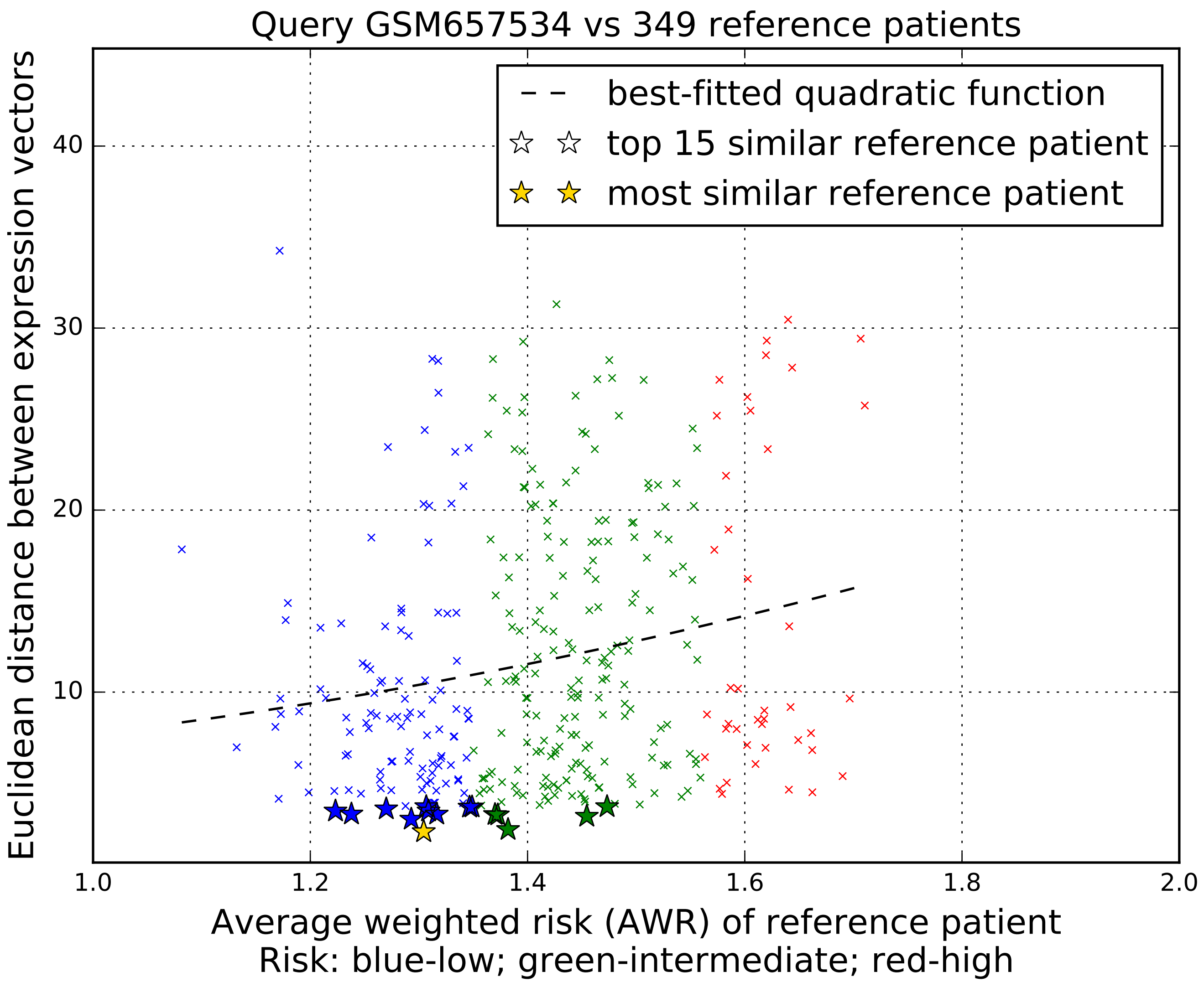

Query GSM657534 vs 349 reference patients
best-fitted quadratic function
top 15 similar reference patient
most similar reference patient
Euclidean distance between expression vectors
40
30
20
10
1.0
1.2
1.4
1.6
1.8
2.0
Average weighted risk (AWR) of reference patient
Risk: blue-low; green-intermediate; red-high

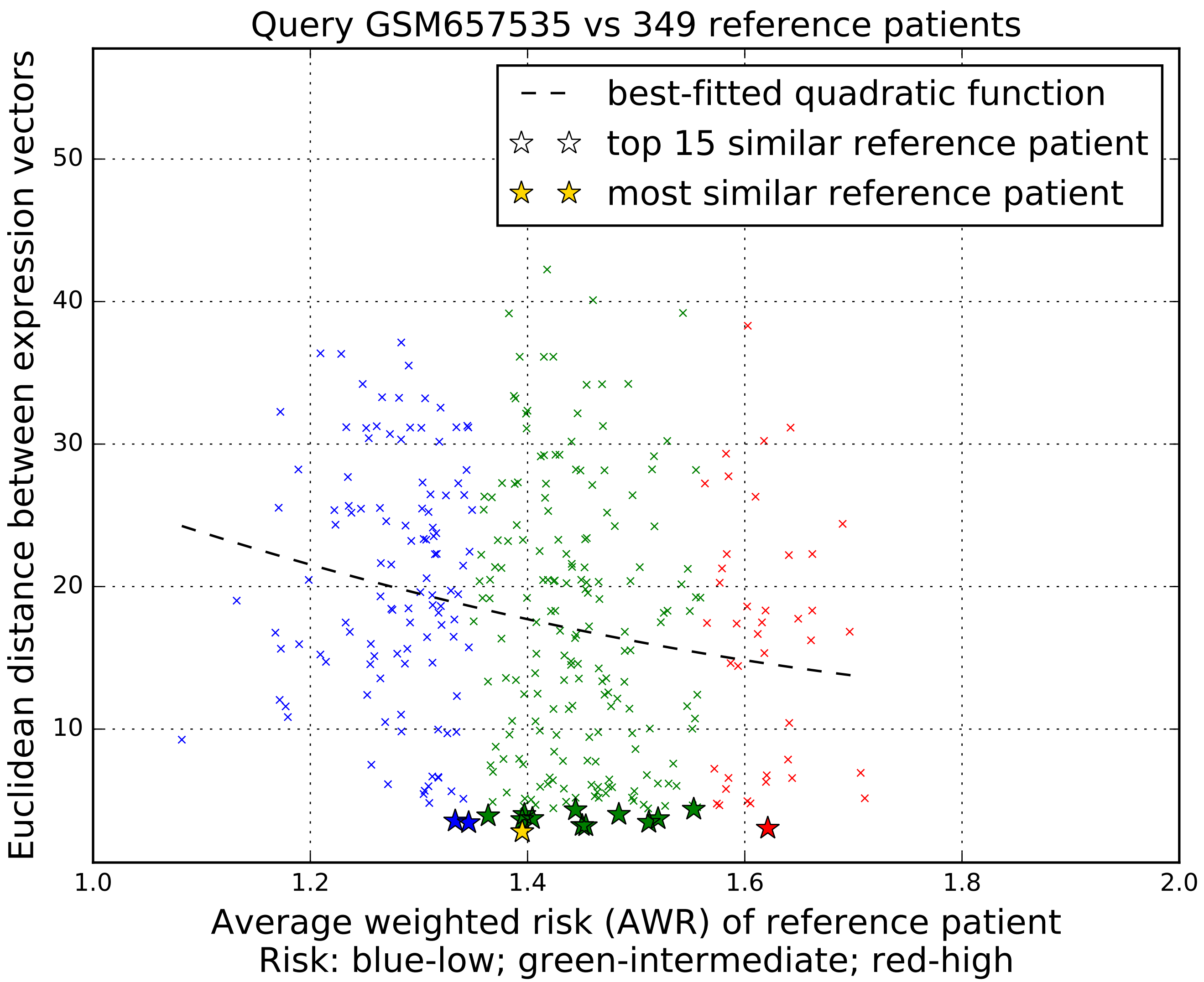
Query GSM657535 vs 349 reference patients
best-fitted quadratic function
top 15 similar reference patient
most similar reference patient
Euclidean distance between expression vectors
10
20
30
40
50
1.0
1.2
1.4
1.6
1.8
2.0
Average weighted risk (AWR) of reference patient
Risk: blue-low; green-intermediate; red-high

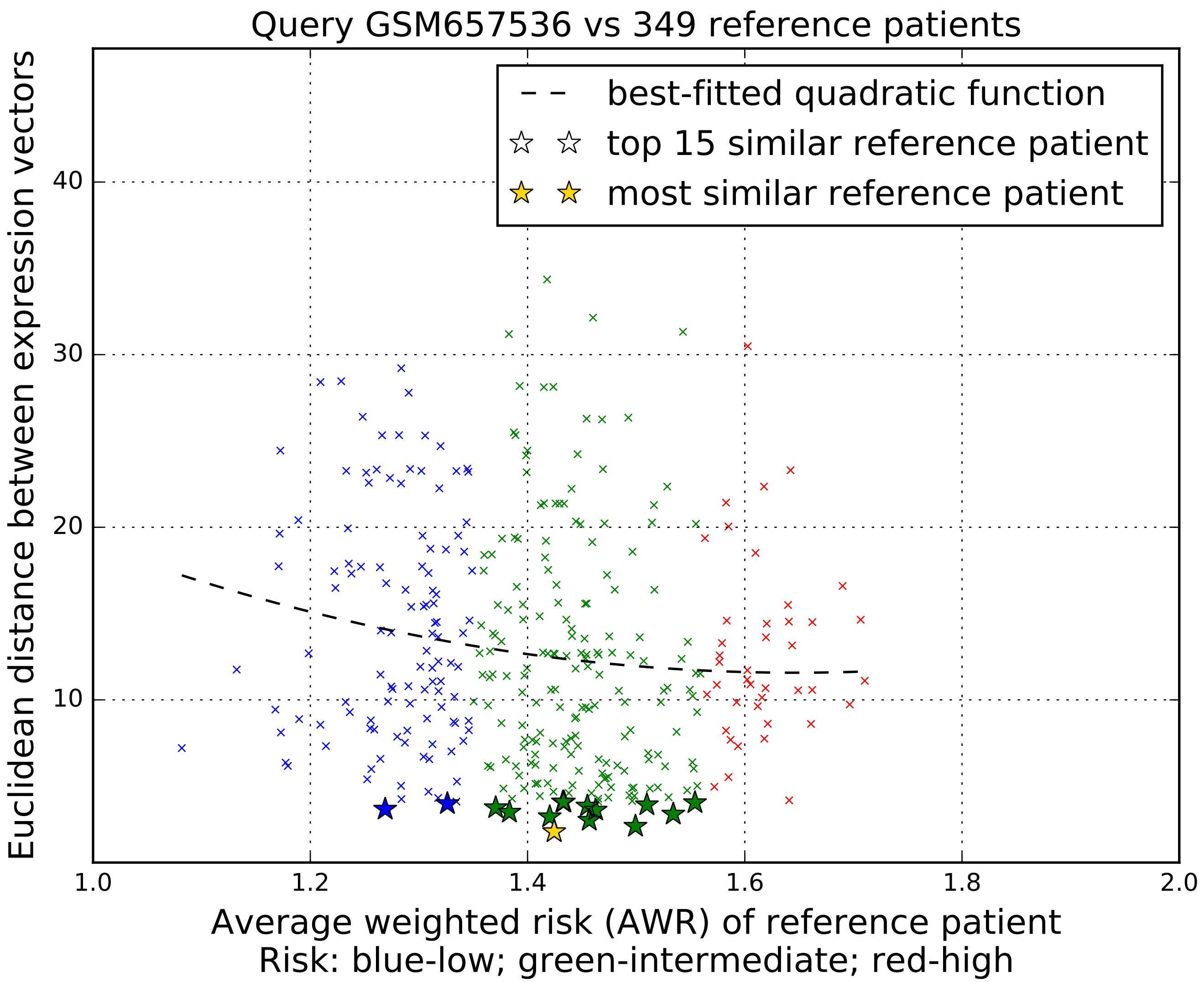

Query GSM657536 vs 349 reference patients
best-fitted quadratic function
top 15 similar reference patient
most similar reference patient
Euclidean distance between expression vectors
40
30
20
10
1.0
1.2
1.4
1.6
1.8
2.0
Average weighted risk (AWR) of reference patient
Risk: blue-low; green-intermediate; red-high

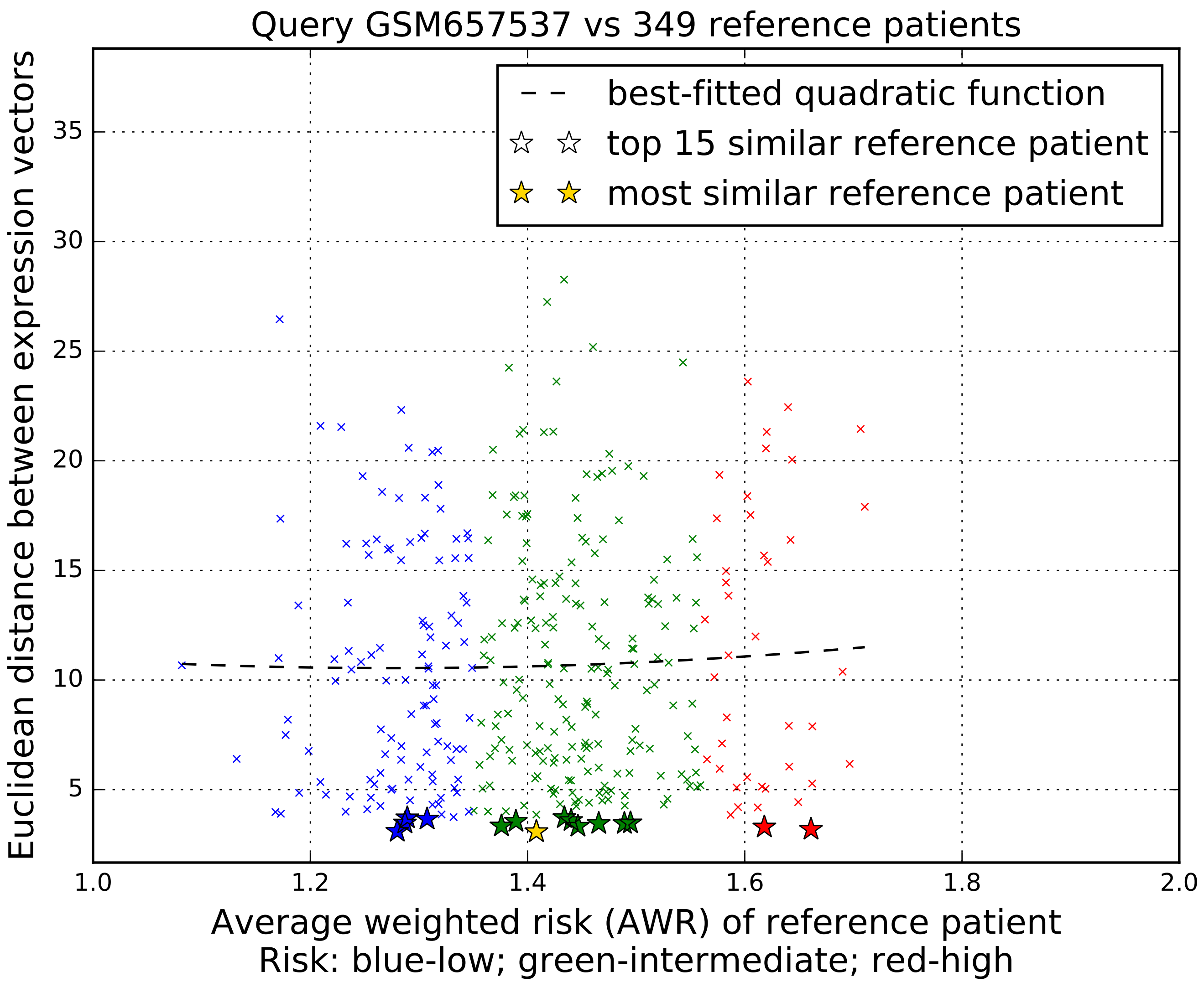

Query GSM657537 vs 349 reference patients
best-fitted quadratic function
top 15 similar reference patient
most similar reference patient
Euclidean distance between expression vectors
35
30
25
20
15
10
5
1.0
1.2
1.4
1.6
1.8
2.0
Average weighted risk (AWR) of reference patient
Risk: blue-low; green-intermediate; red-high

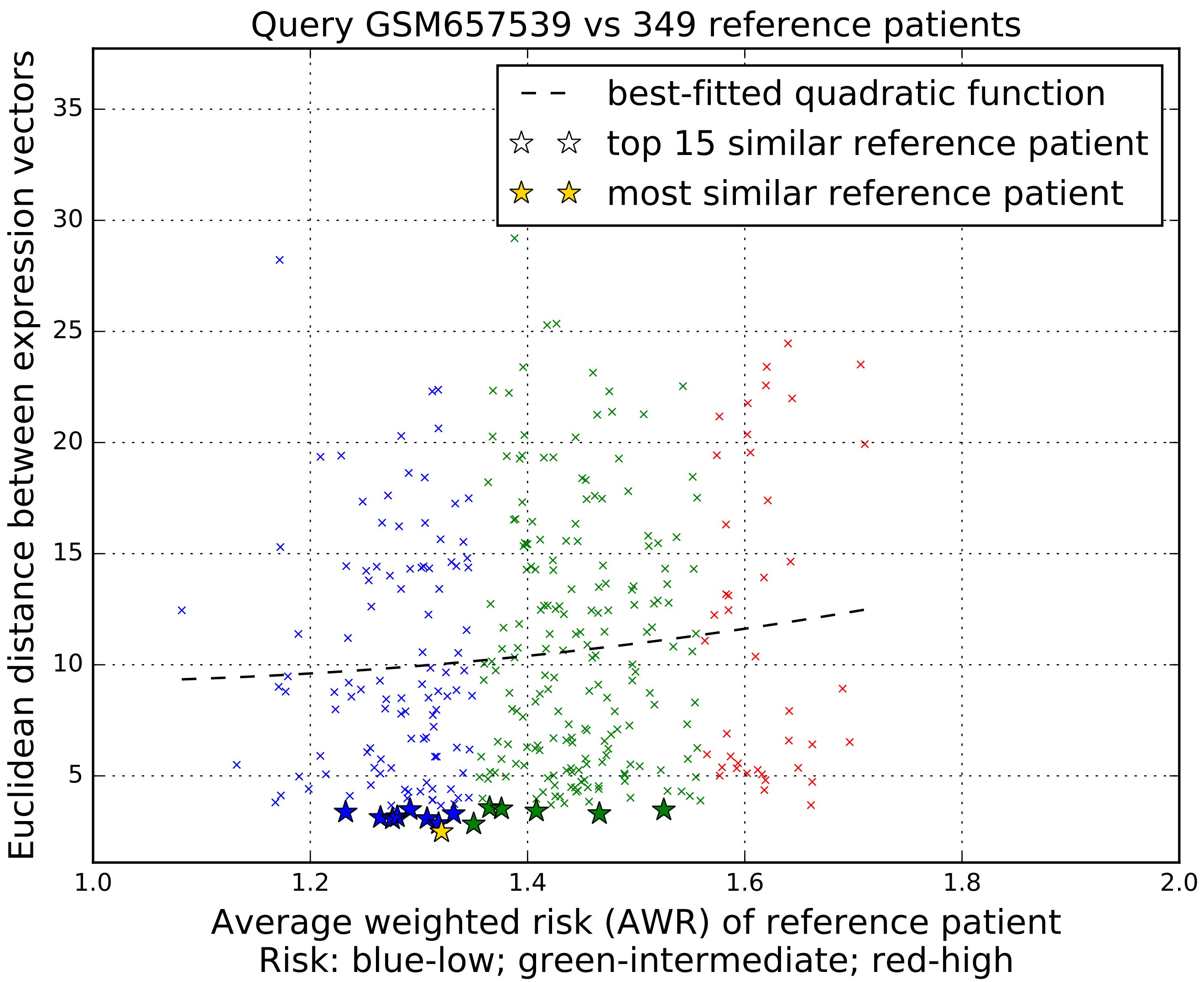
Query GSM657539 vs 349 reference patients
best-fitted quadratic function
top 15 similar reference patient
most similar reference patient
Euclidean distance between expression vectors
35
30
25
20
15
10
5
1.0
1.2
1.4
1.6
1.8
2.0
Average weighted risk (AWR) of reference patient
Risk: blue-low; green-intermediate; red-high

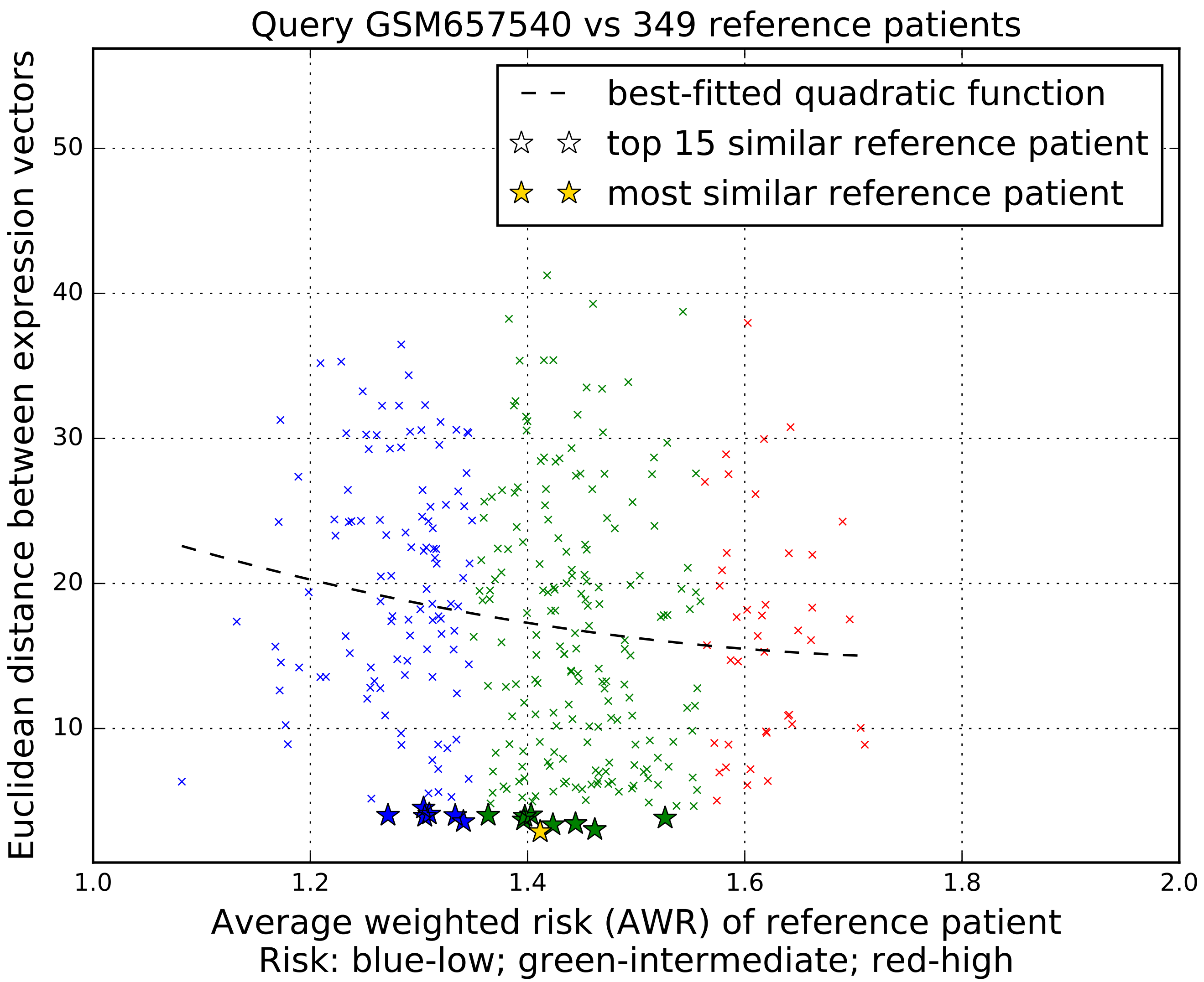
Query GSM657540 vs 349 reference patients
best-fitted quadratic function
top 15 similar reference patient
most similar reference patient
Euclidean distance between expression vectors
Average weighted risk (AWR) of reference patient
Risk: blue-low; green-intermediate; red-high
1.0
1.2
1.4
1.6
1.8
2.0
10
20
30
40
50

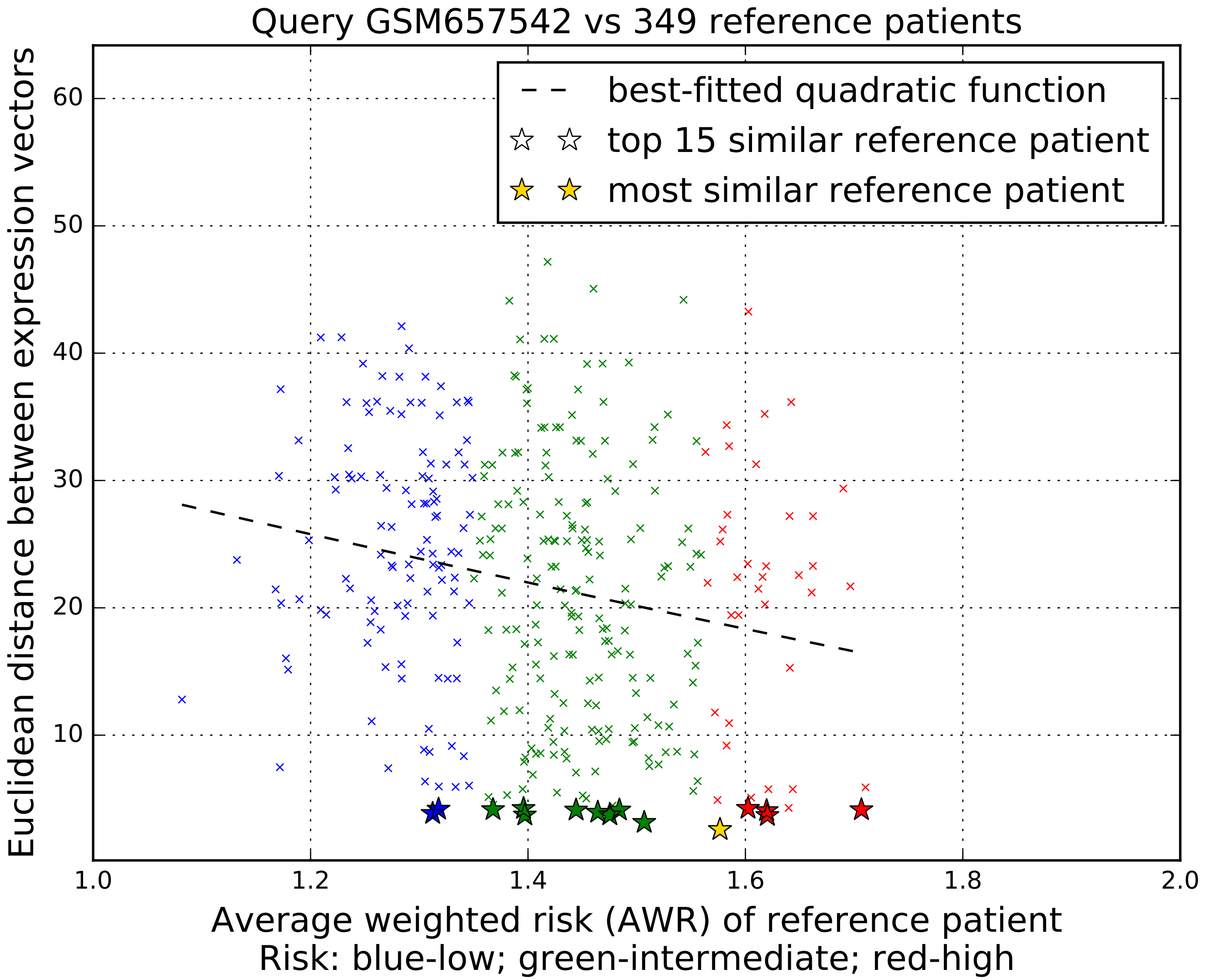

Query GSM657542 vs 349 reference patients
best-fitted quadratic function
top 15 similar reference patient
most similar reference patient
Euclidean distance between expression vectors
10
20
30
40
50
60
1.0
1.2
1.4
1.6
1.8
2.0
Average weighted risk (AWR) of reference patient
Risk: blue-low; green-intermediate; red-high

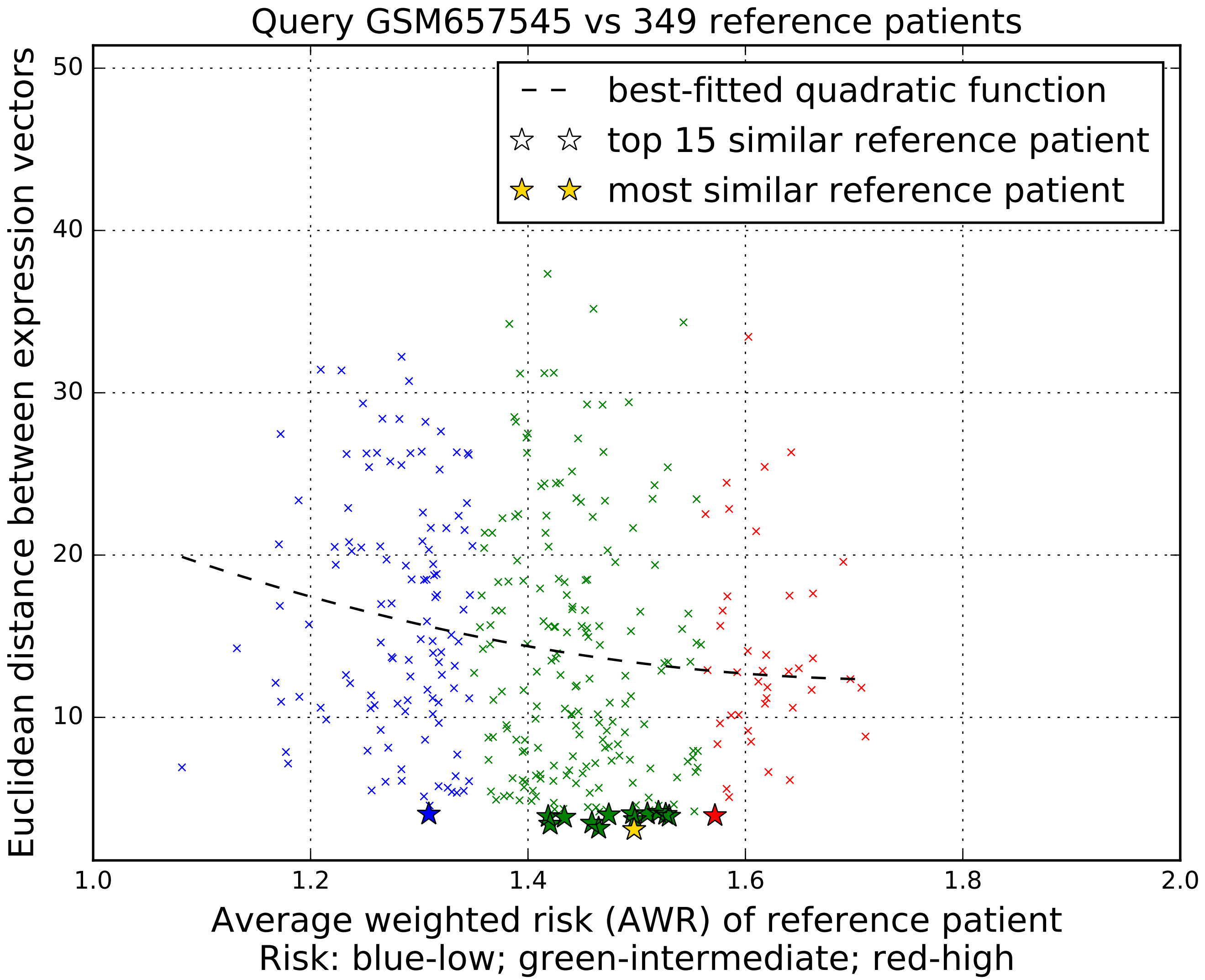

Query GSM657545 vs 349 reference patients
Euclidean distance between expression vectors
50
40
30
20
10
1.0
1.2
1.4
1.6
1.8
2.0
best-fitted quadratic function
top 15 similar reference patient
most similar reference patient
Average weighted risk (AWR) of reference patient
Risk: blue-low; green-intermediate; red-high

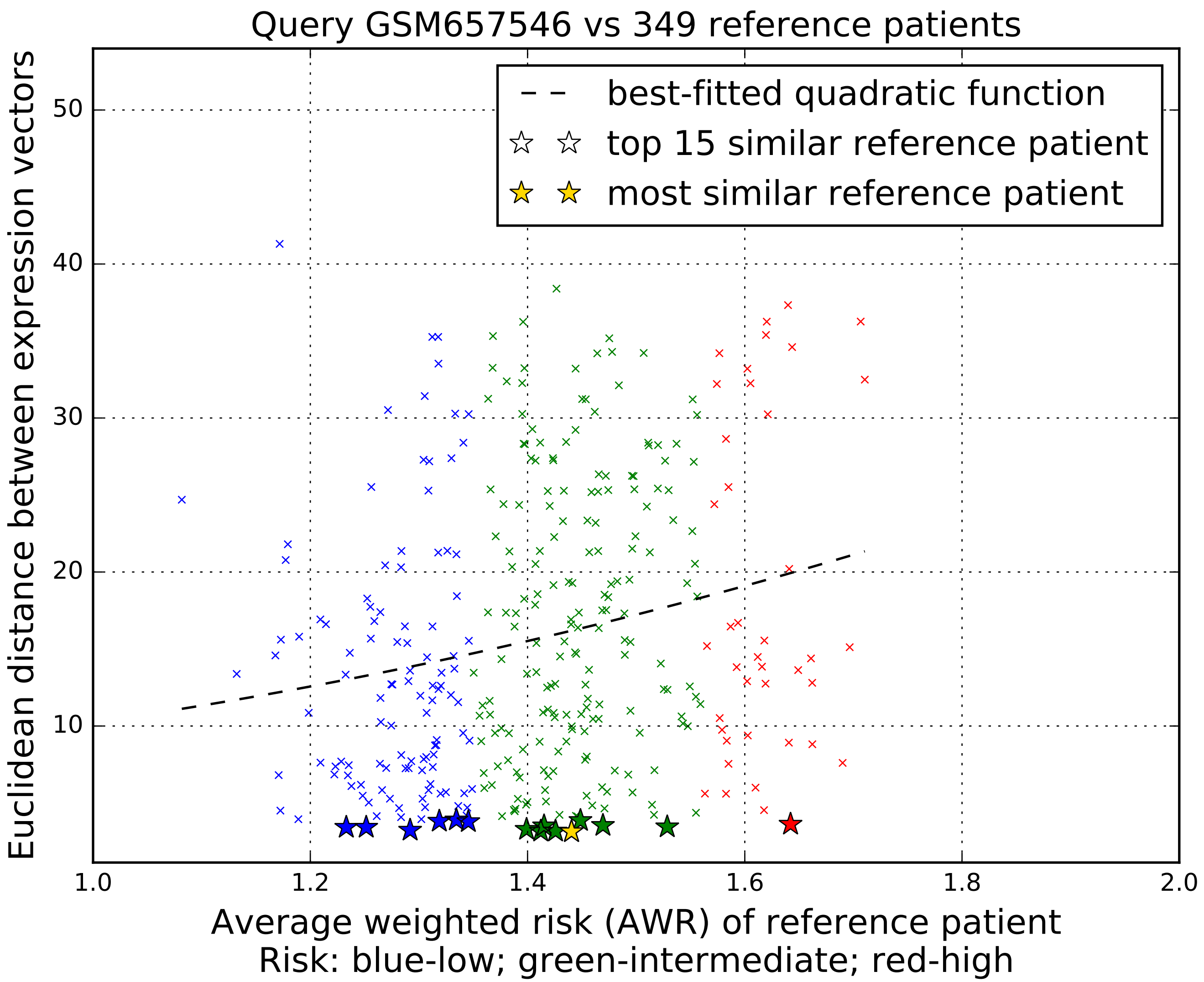
Query GSM657546 vs 349 reference patients
best-fitted quadratic function
top 15 similar reference patient
most similar reference patient
Euclidean distance between expression vectors
Average weighted risk (AWR) of reference patient
Risk: blue-low; green-intermediate; red-high
10
20
30
40
50
1.0
1.2
1.4
1.6
1.8
2.0

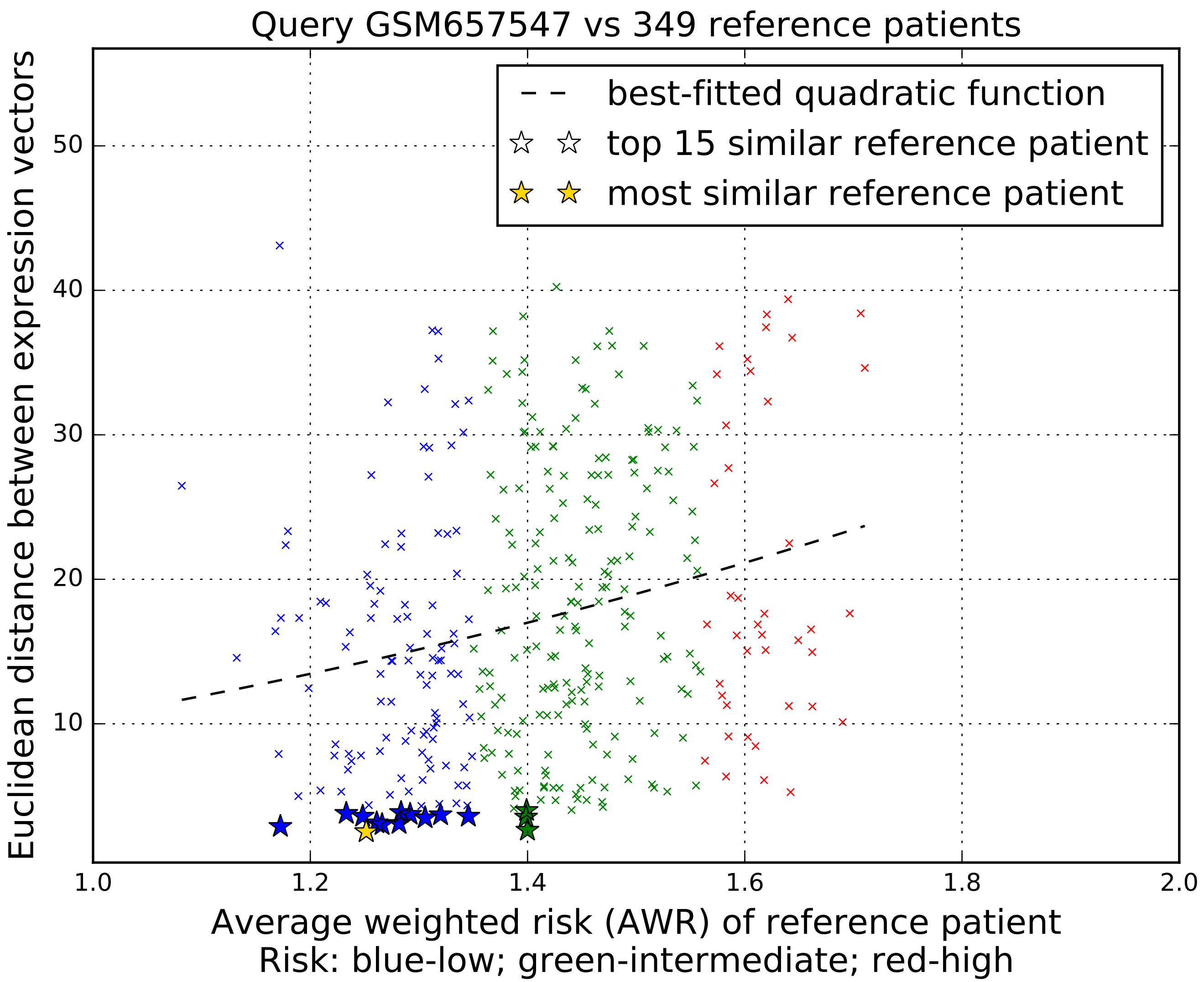
Query GSM657547 vs 349 reference patients
best-fitted quadratic function
top 15 similar reference patient
most similar reference patient
Euclidean distance between expression vectors
Average weighted risk (AWR) of reference patient
Risk: blue-low; green-intermediate; red-high
10
20
30
40
50
1.0
1.2
1.4
1.6
1.8
2.0

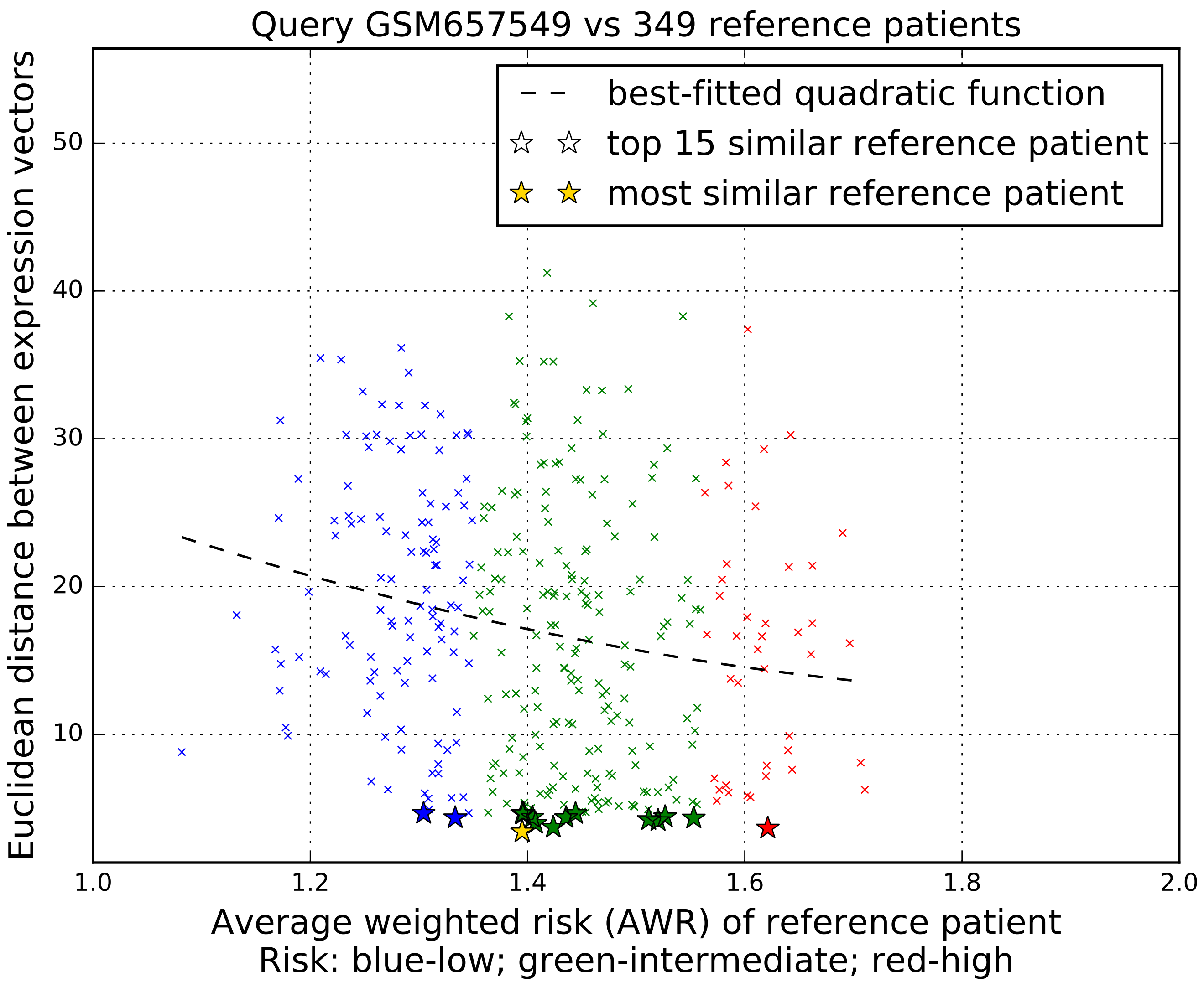

Query GSM657549 vs 349 reference patients
best-fitted quadratic function
top 15 similar reference patient
most similar reference patient
Euclidean distance between expression vectors
50
40
30
20
10
1.0
1.2
1.4
1.6
1.8
2.0
Average weighted risk (AWR) of reference patient
Risk: blue-low; green-intermediate; red-high

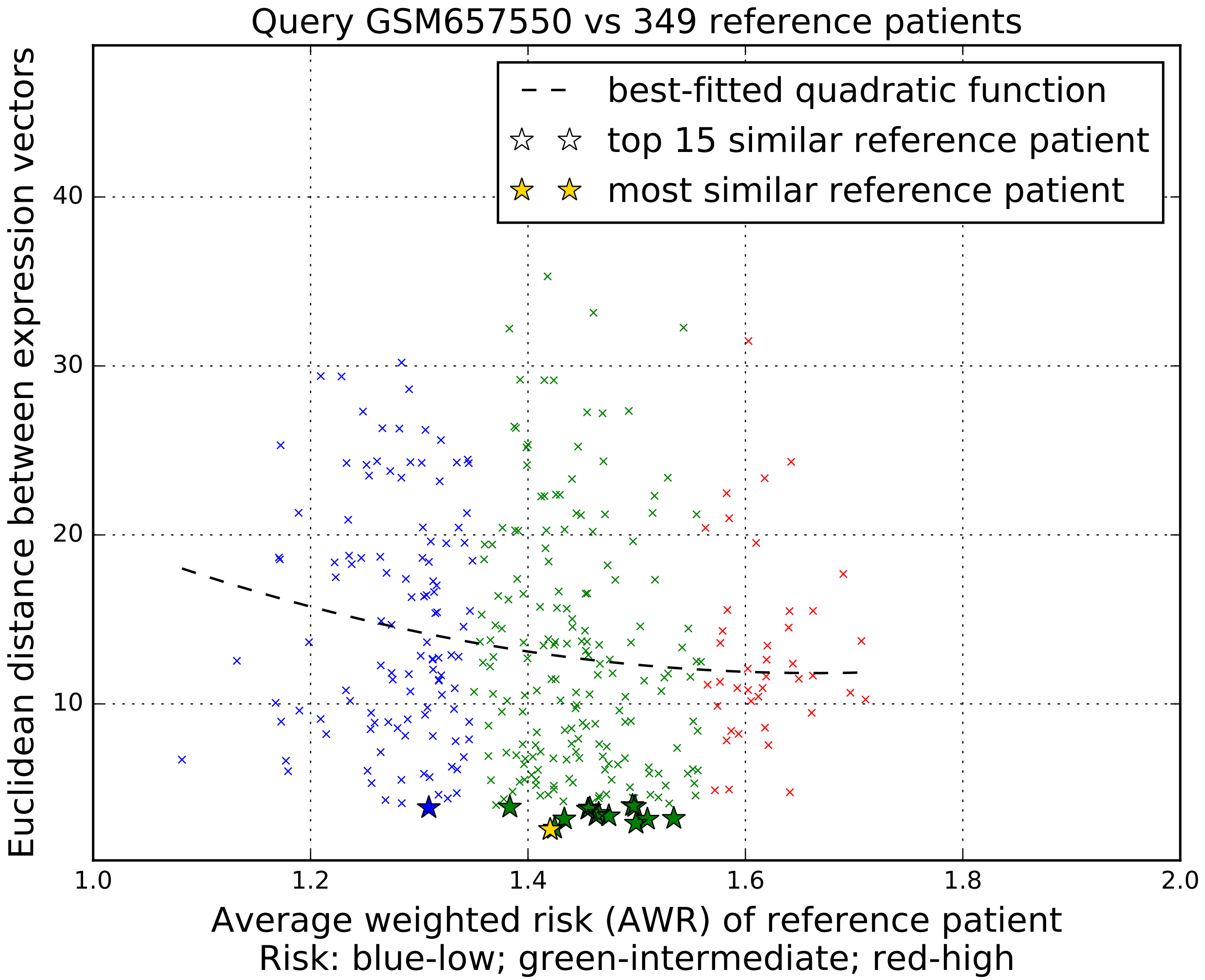

Query GSM657550 vs 349 reference patients
best-fitted quadratic function
top 15 similar reference patient
most similar reference patient
Euclidean distance between expression vectors
40
30
20
10
1.0
1.2
1.4
1.6
1.8
2.0
Average weighted risk (AWR) of reference patient
Risk: blue-low; green-intermediate; red-high

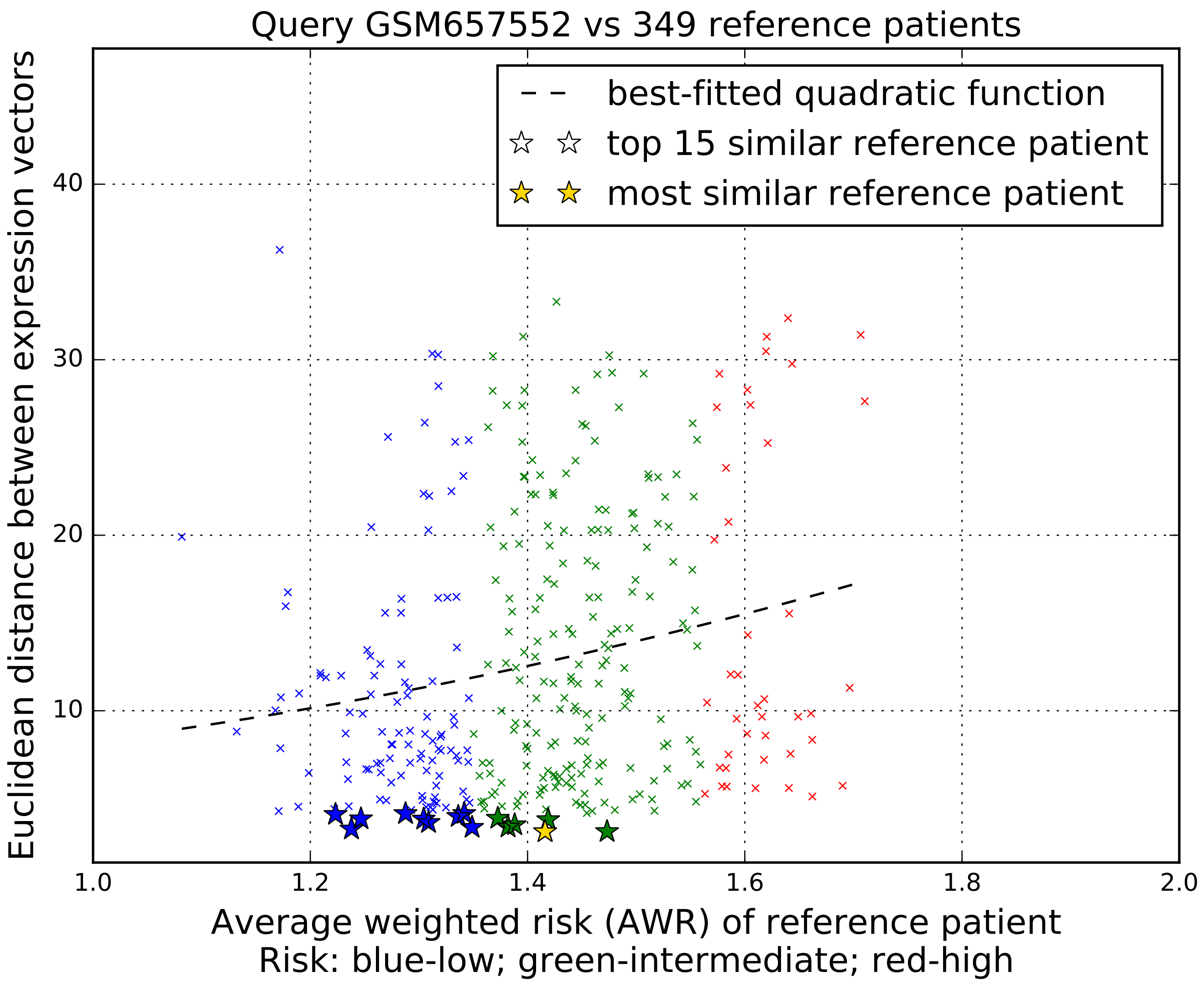
Query GSM657552 vs 349 reference patients
best-fitted quadratic function
top 15 similar reference patient
most similar reference patient
Euclidean distance between expression vectors
10
20
30
40
1.0
1.2
1.4
1.6
1.8
2.0
Average weighted risk (AWR) of reference patient
Risk: blue-low; green-intermediate; red-high

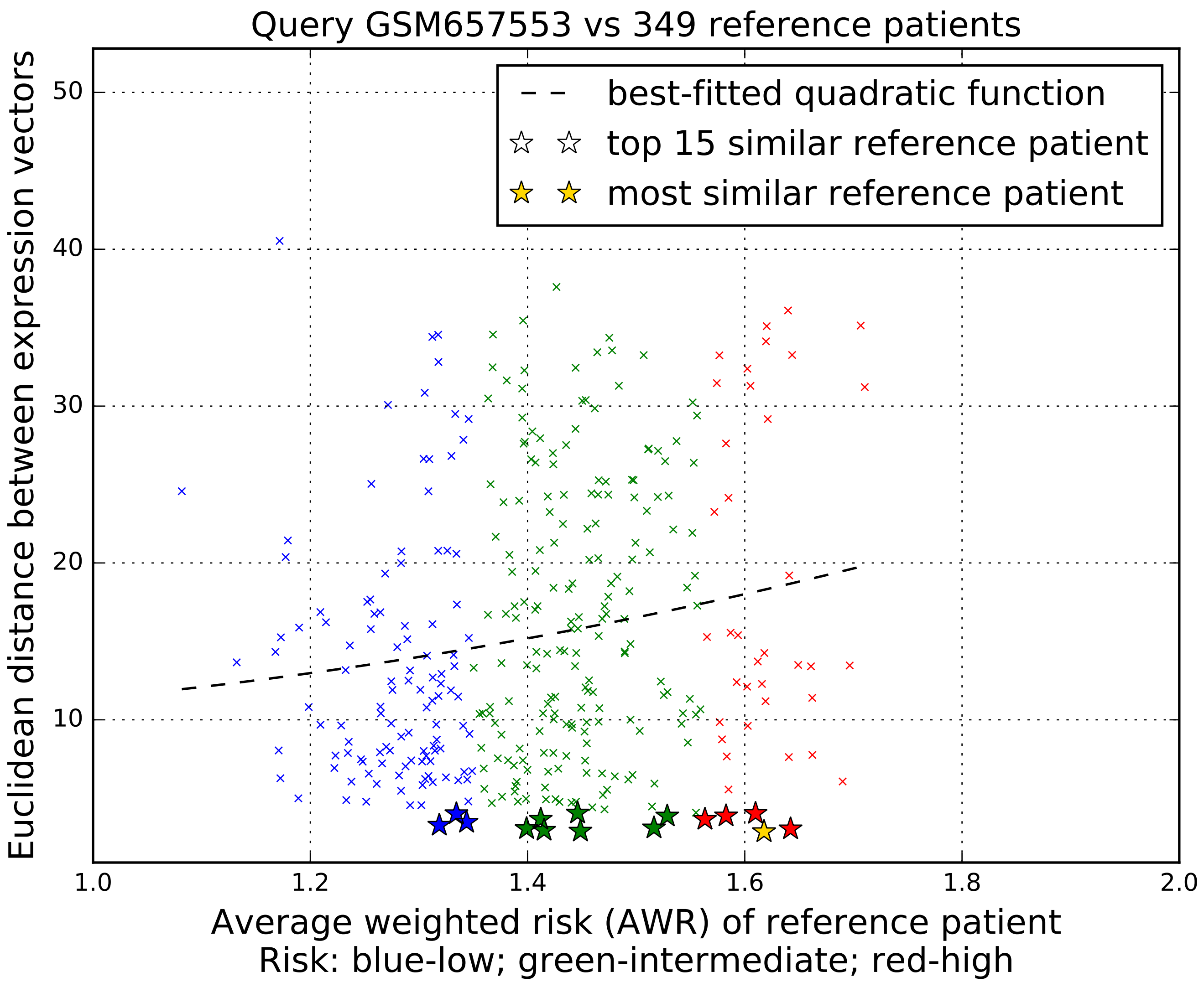

Query GSM657553 vs 349 reference patients
best-fitted quadratic function
top 15 similar reference patient
most similar reference patient
Euclidean distance between expression vectors
10
20
30
40
50
1.0
1.2
1.4
1.6
1.8
2.0
Average weighted risk (AWR) of reference patient
Risk: blue-low; green-intermediate; red-high

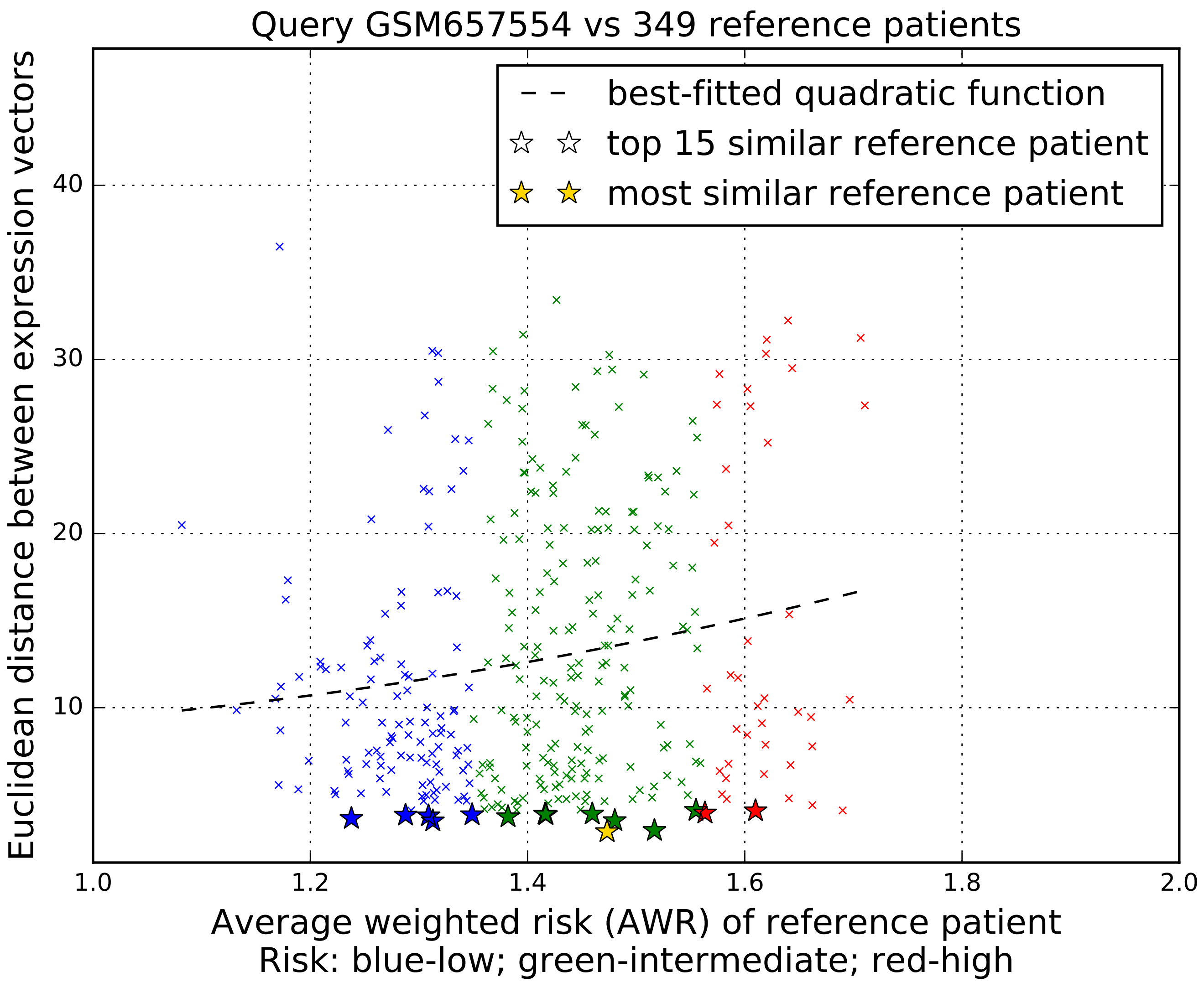

Query GSM657554 vs 349 reference patients
best-fitted quadratic function
top 15 similar reference patient
most similar reference patient
Euclidean distance between expression vectors
40
30
20
10
1.0
1.2
1.4
1.6
1.8
2.0
Average weighted risk (AWR) of reference patient
Risk: blue-low; green-intermediate; red-high

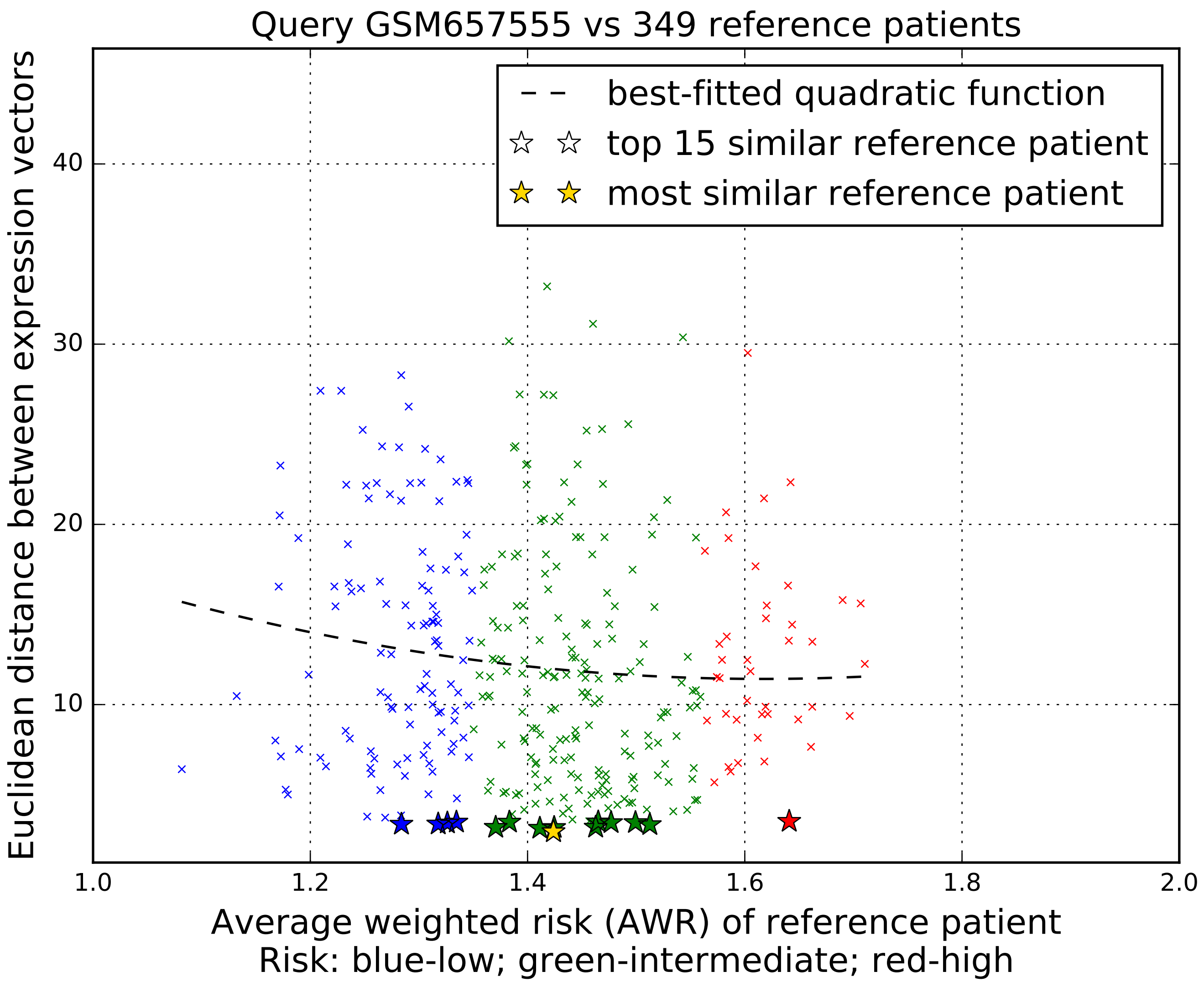

Query GSM657555 vs 349 reference patients
best-fitted quadratic function
top 15 similar reference patient
most similar reference patient
Euclidean distance between expression vectors
10
20
30
40
1.0
1.2
1.4
1.6
1.8
2.0
Average weighted risk (AWR) of reference patient
Risk: blue-low; green-intermediate; red-high

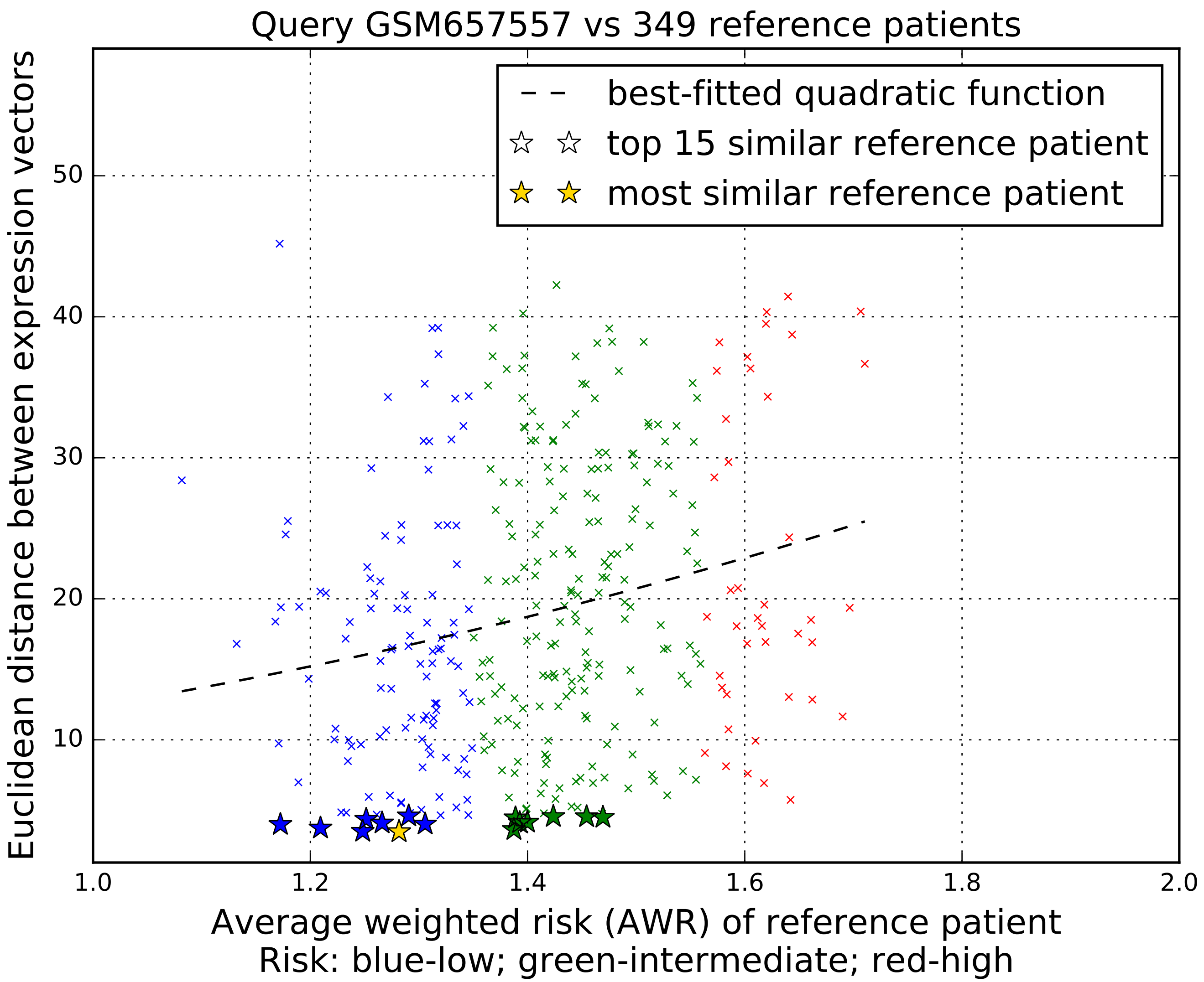
Query GSM657557 vs 349 reference patients
best-fitted quadratic function
top 15 similar reference patient
most similar reference patient
Euclidean distance between expression vectors
10
20
30
40
50
1.0
1.2
1.4
1.6
1.8
2.0
Average weighted risk (AWR) of reference patient
Risk: blue-low; green-intermediate; red-high

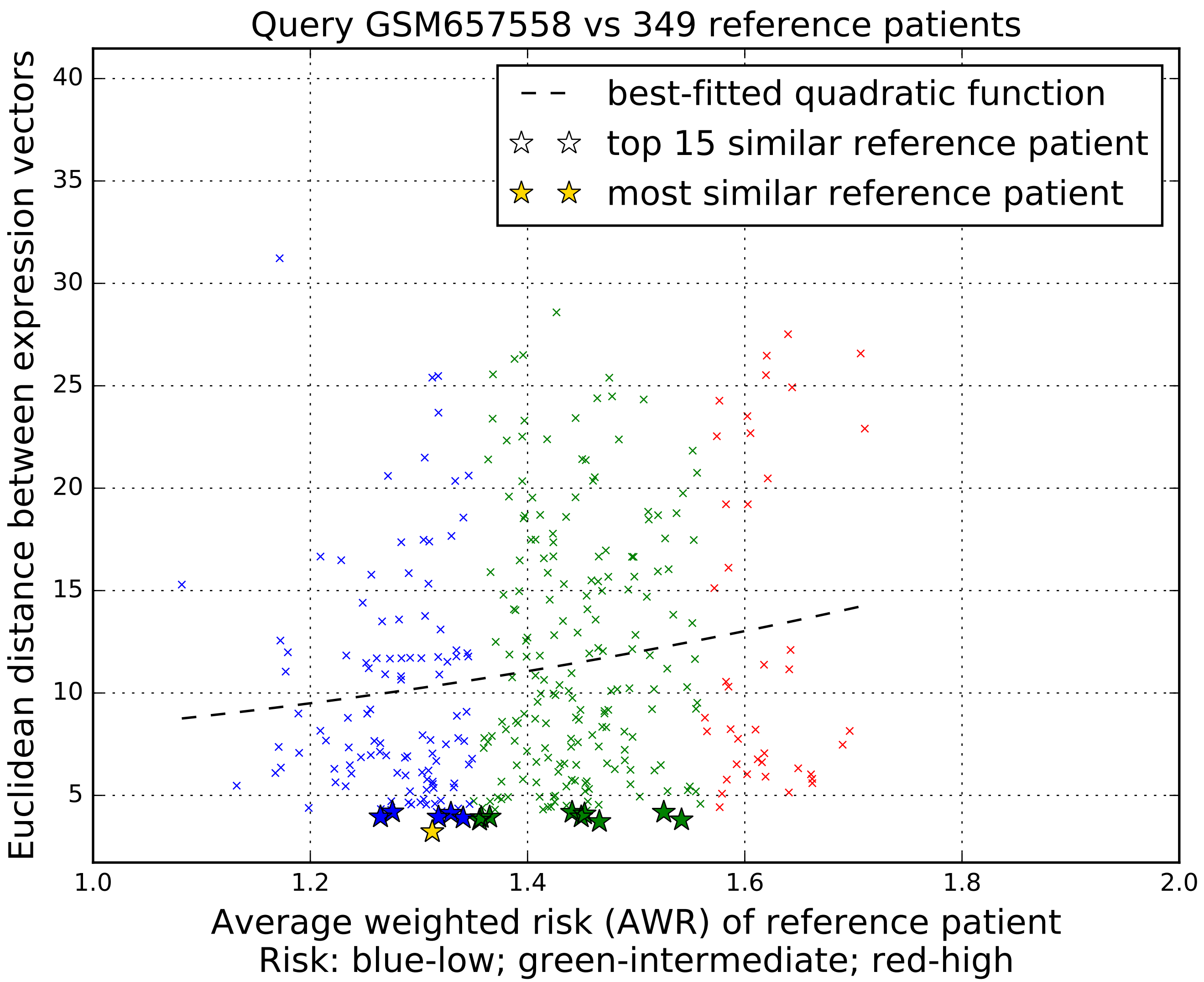
Query GSM657558 vs 349 reference patients
best-fitted quadratic function
top 15 similar reference patient
most similar reference patient
Euclidean distance between expression vectors
40
35
30
25
20
15
10
5
1.0
1.2
1.4
1.6
1.8
2.0
Average weighted risk (AWR) of reference patient
Risk: blue-low; green-intermediate; red-high

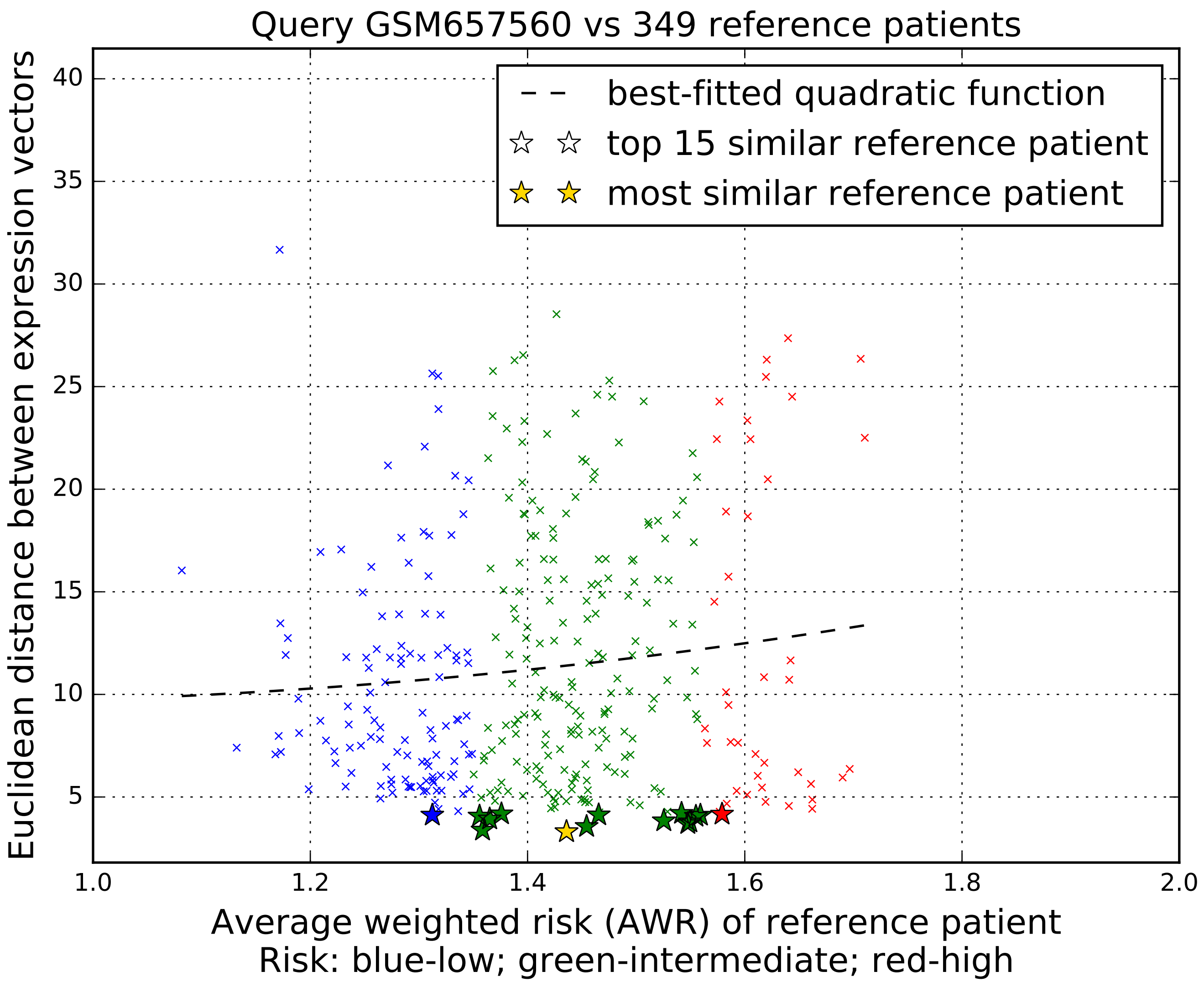

Query GSM657560 vs 349 reference patients
best-fitted quadratic function
top 15 similar reference patient
most similar reference patient
Euclidean distance between expression vectors
40
35
30
25
20
15
10
5
1.0
1.2
1.4
1.6
1.8
2.0
Average weighted risk (AWR) of reference patient
Risk: blue-low; green-intermediate; red-high

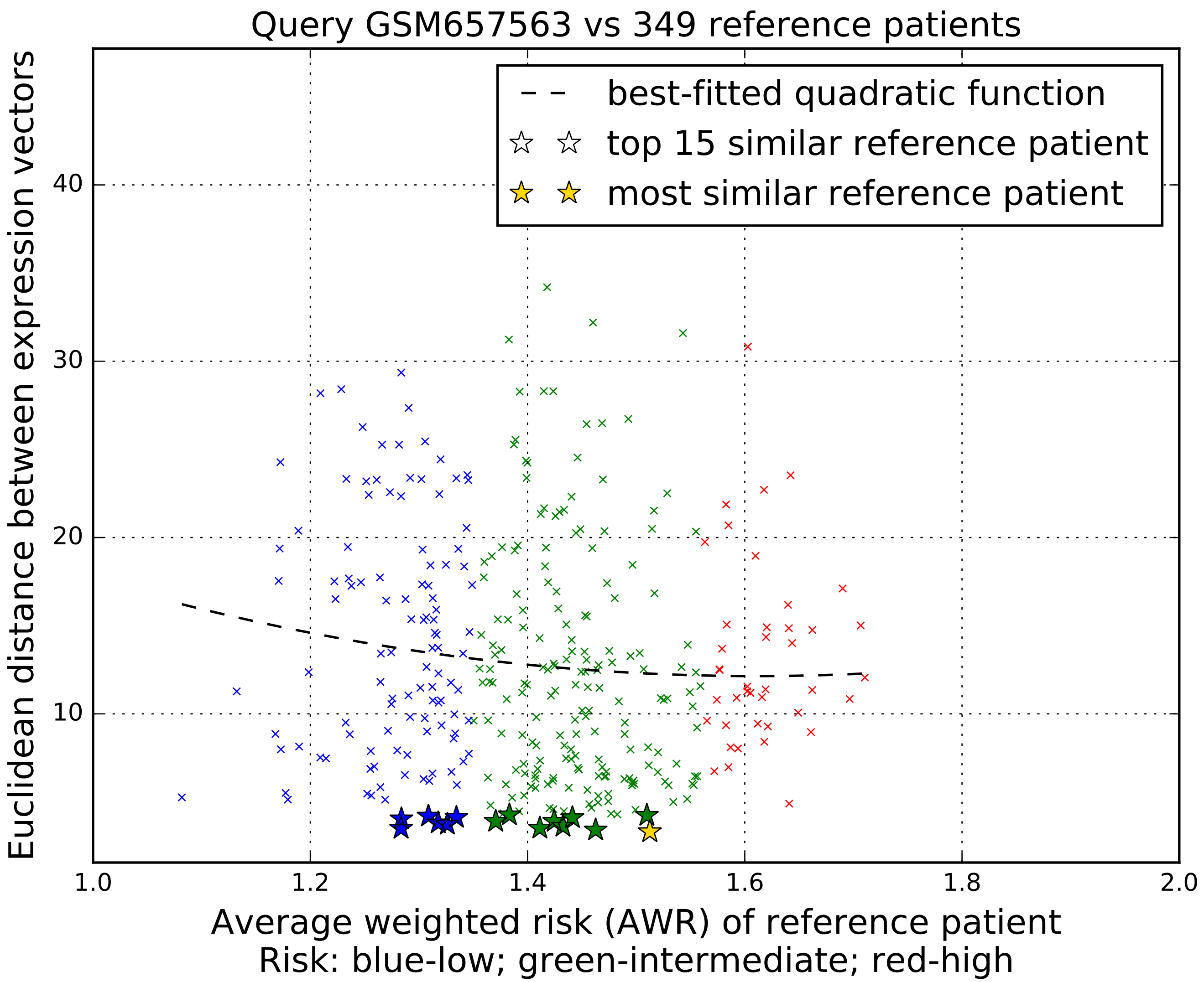
Query GSM657563 vs 349 reference patients
best-fitted quadratic function
top 15 similar reference patient
most similar reference patient
Euclidean distance between expression vectors
40
30
20
10
1.0
1.2
1.4
1.6
1.8
2.0
Average weighted risk (AWR) of reference patient
Risk: blue-low; green-intermediate; red-high

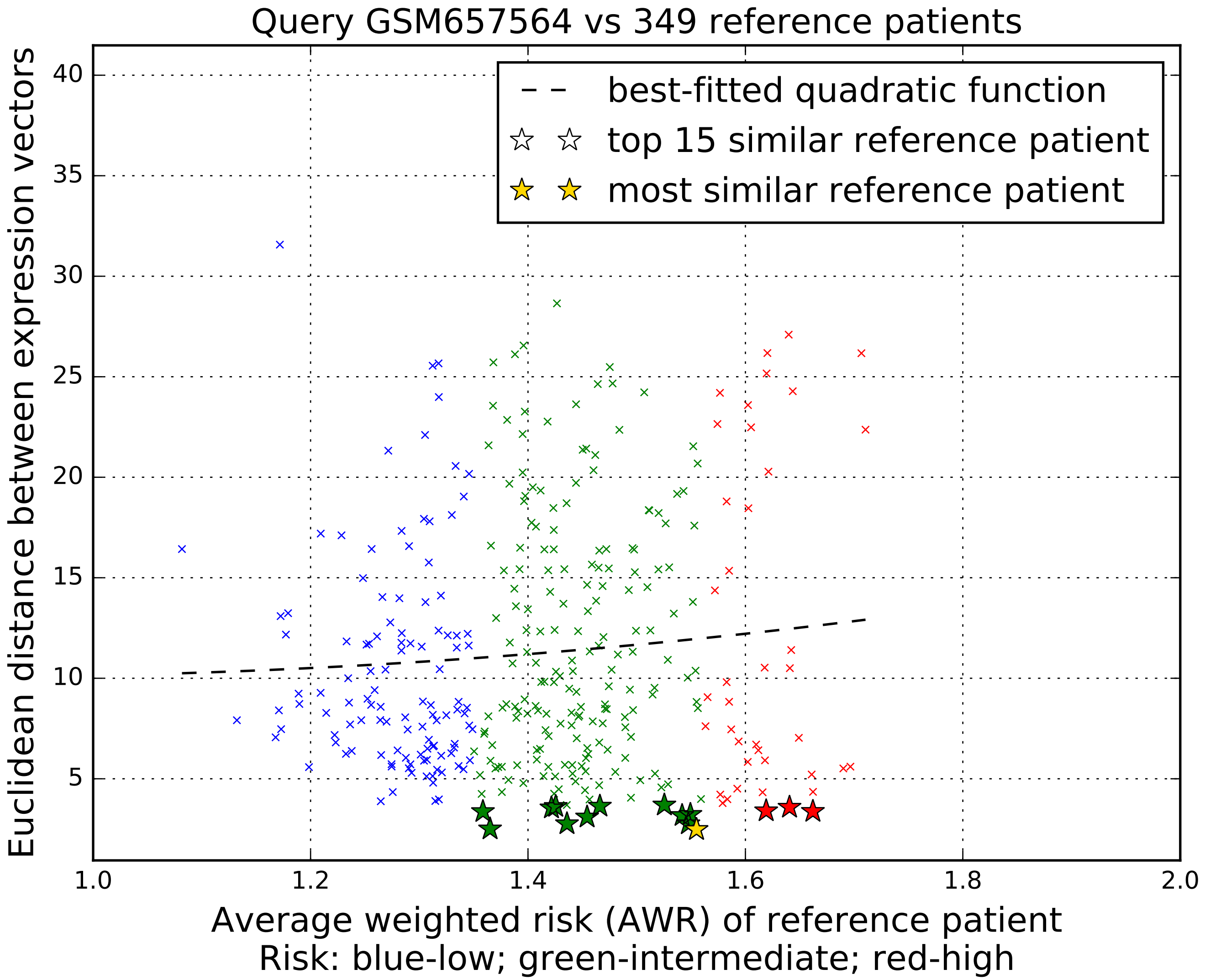

Query GSM657564 vs 349 reference patients
best-fitted quadratic function
top 15 similar reference patient
most similar reference patient
Euclidean distance between expression vectors
40
35
30
25
20
15
10
5
1.0
1.2
1.4
1.6
1.8
2.0
Average weighted risk (AWR) of reference patient
Risk: blue-low; green-intermediate; red-high

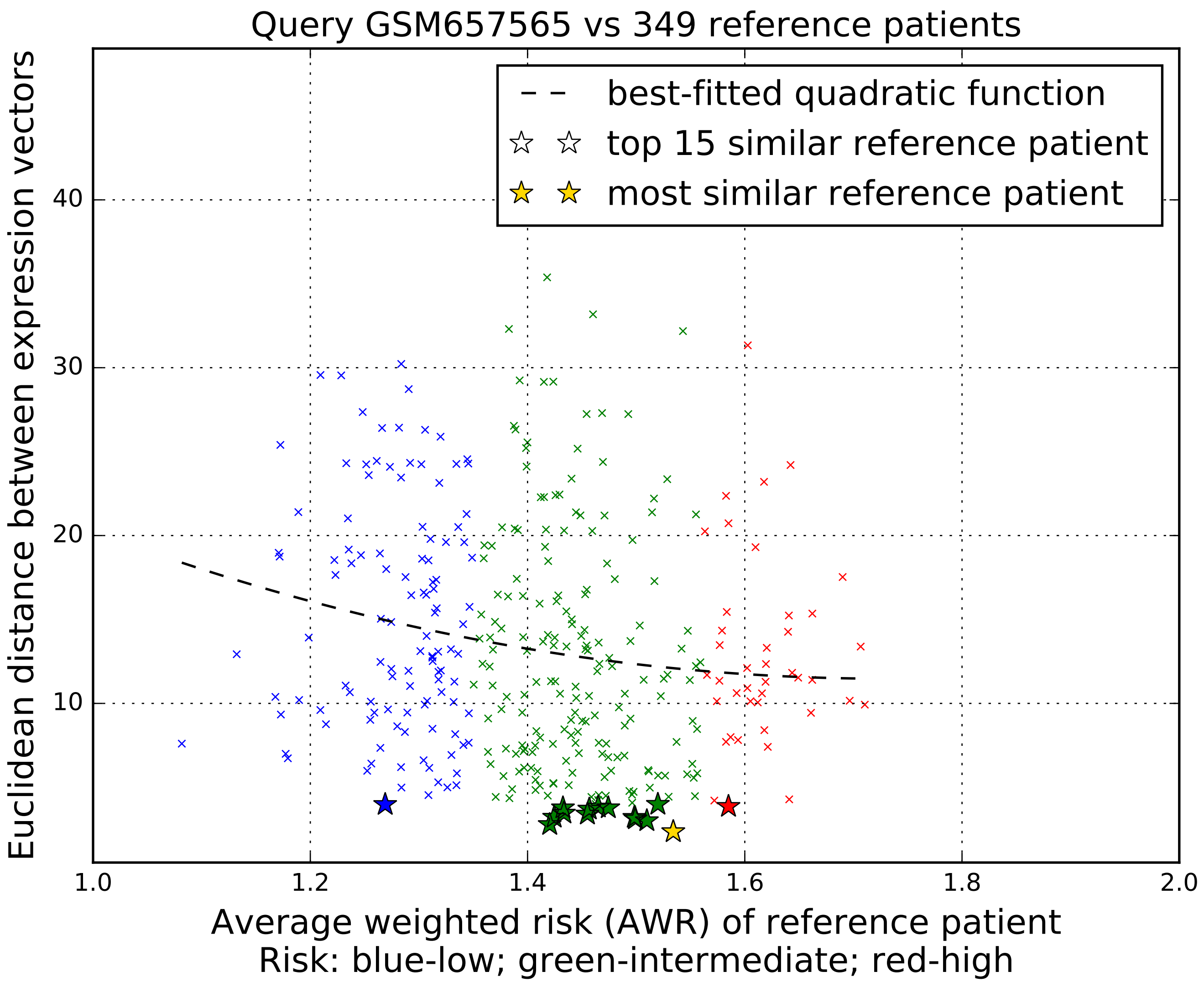

Query GSM657565 vs 349 reference patients
best-fitted quadratic function
top 15 similar reference patient
most similar reference patient
Euclidean distance between expression vectors
10
20
30
40
1.0
1.2
1.4
1.6
1.8
2.0
Average weighted risk (AWR) of reference patient
Risk: blue-low; green-intermediate; red-high

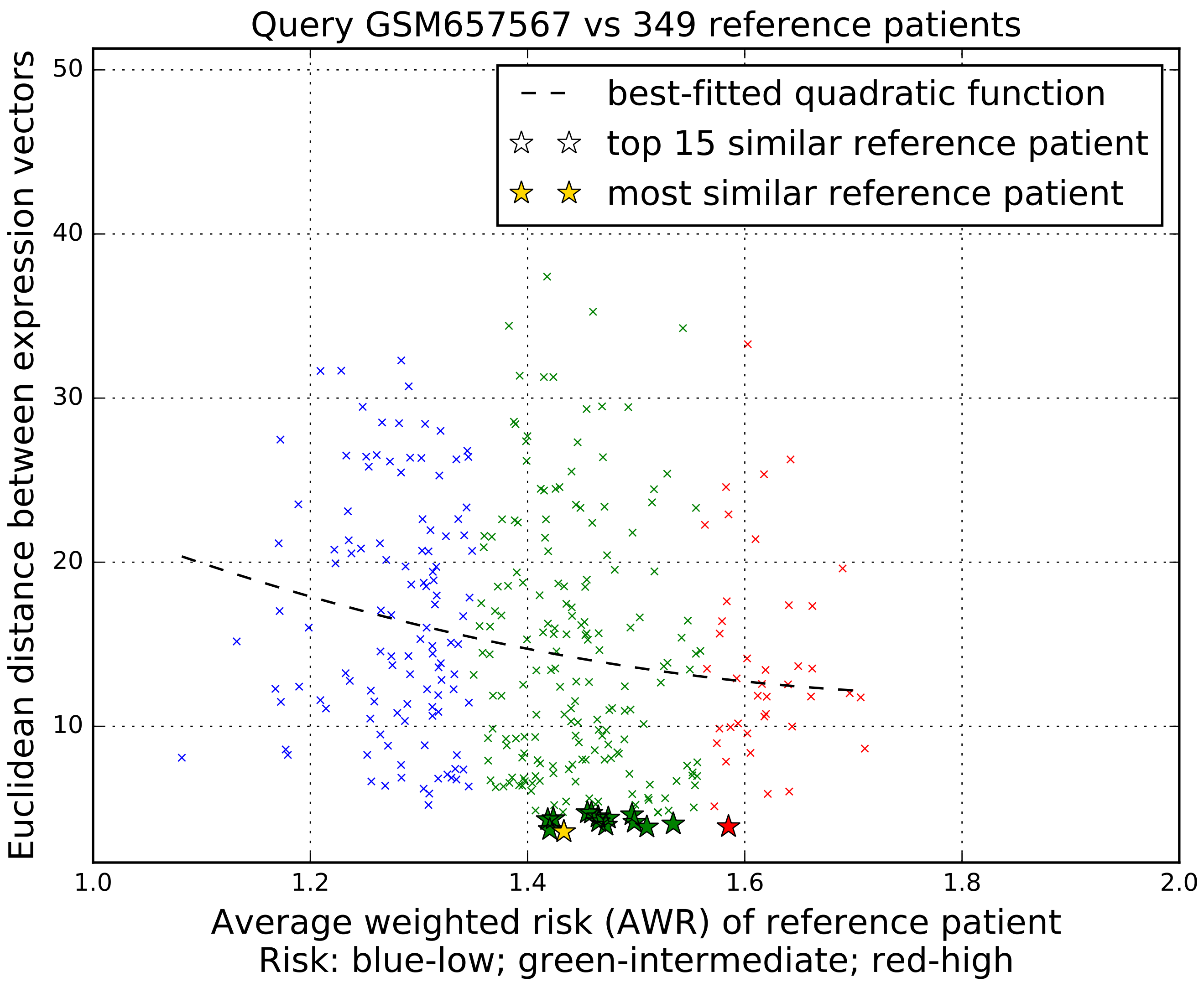

Query GSM657567 vs 349 reference patients
best-fitted quadratic function
top 15 similar reference patient
most similar reference patient
Euclidean distance between expression vectors
50
40
30
20
10
1.0
1.2
1.4
1.6
1.8
2.0
Average weighted risk (AWR) of reference patient
Risk: blue-low; green-intermediate; red-high

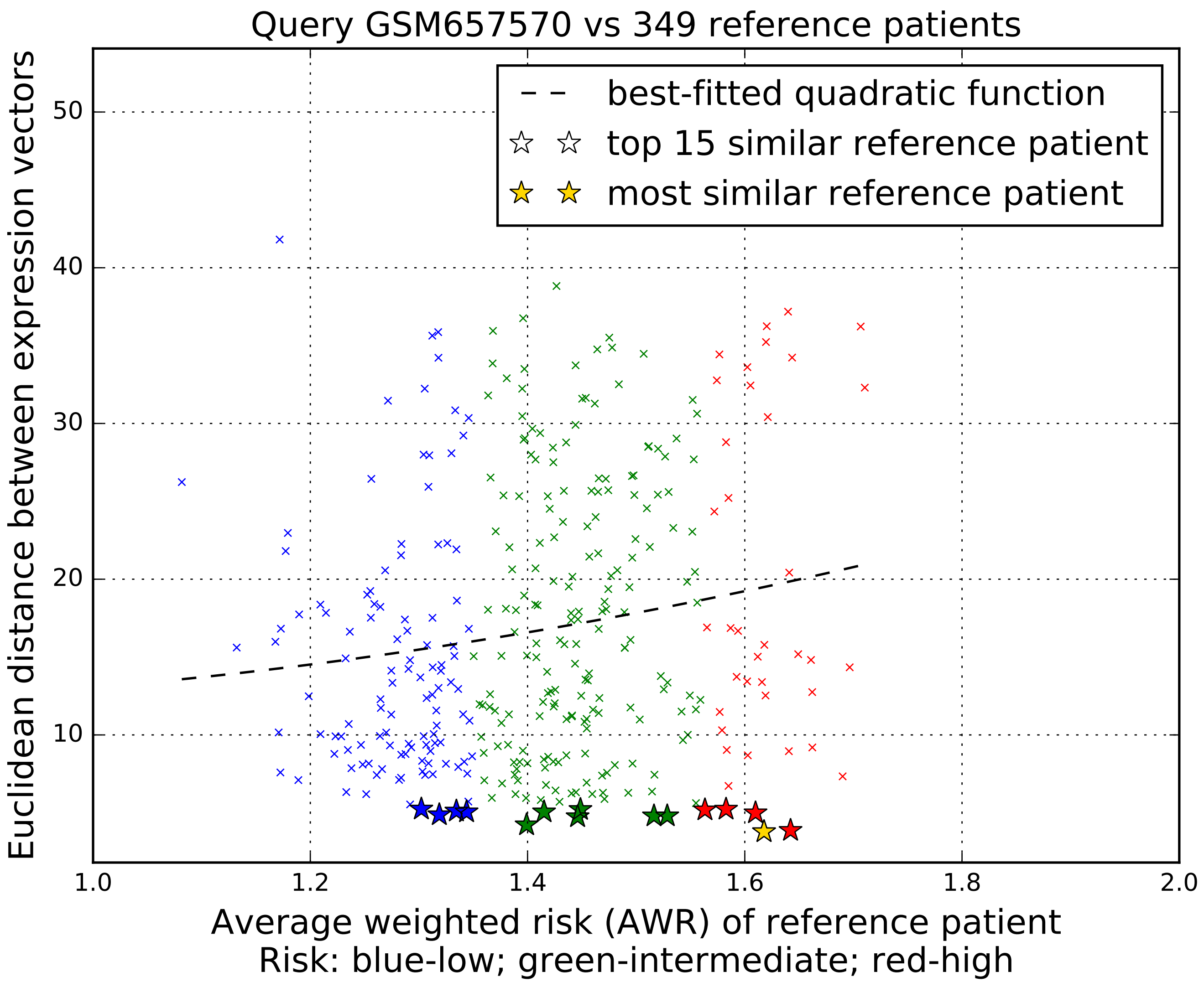
Query GSM657570 vs 349 reference patients
best-fitted quadratic function
top 15 similar reference patient
most similar reference patient
Euclidean distance between expression vectors
10
20
30
40
50
1.0
1.2
1.4
1.6
1.8
2.0
Average weighted risk (AWR) of reference patient
Risk: blue-low; green-intermediate; red-high

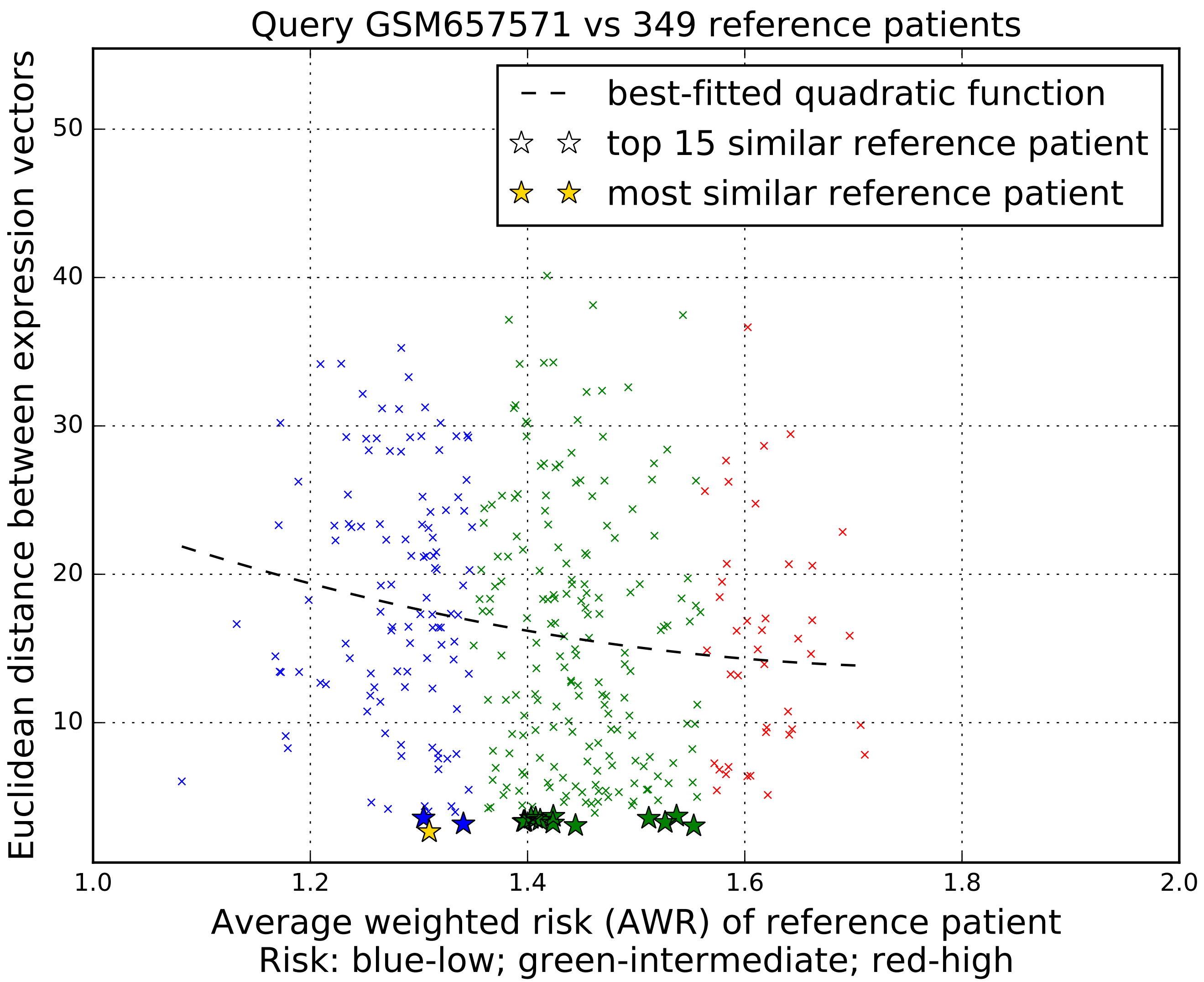

Query GSM657571 vs 349 reference patients
best-fitted quadratic function
top 15 similar reference patient
most similar reference patient
Euclidean distance between expression vectors
10
20
30
40
50
1.0
1.2
1.4
1.6
1.8
2.0
Average weighted risk (AWR) of reference patient
Risk: blue-low; green-intermediate; red-high

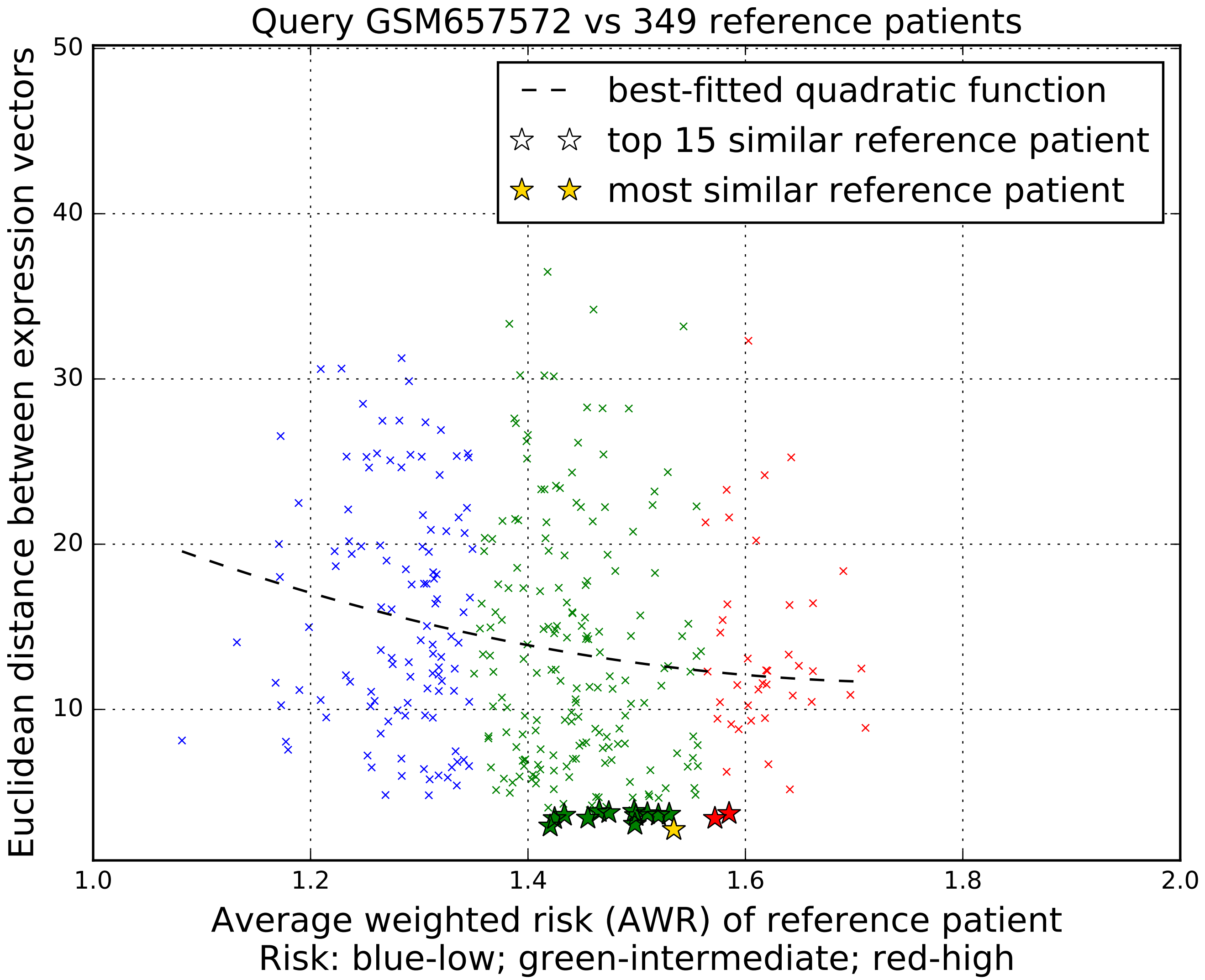

Query GSM657572 vs 349 reference patients
best-fitted quadratic function
top 15 similar reference patient
most similar reference patient
Euclidean distance between expression vectors
Average weighted risk (AWR) of reference patient
Risk: blue-low; green-intermediate; red-high
50
40
30
20
10
1.0
1.2
1.4
1.6
1.8
2.0

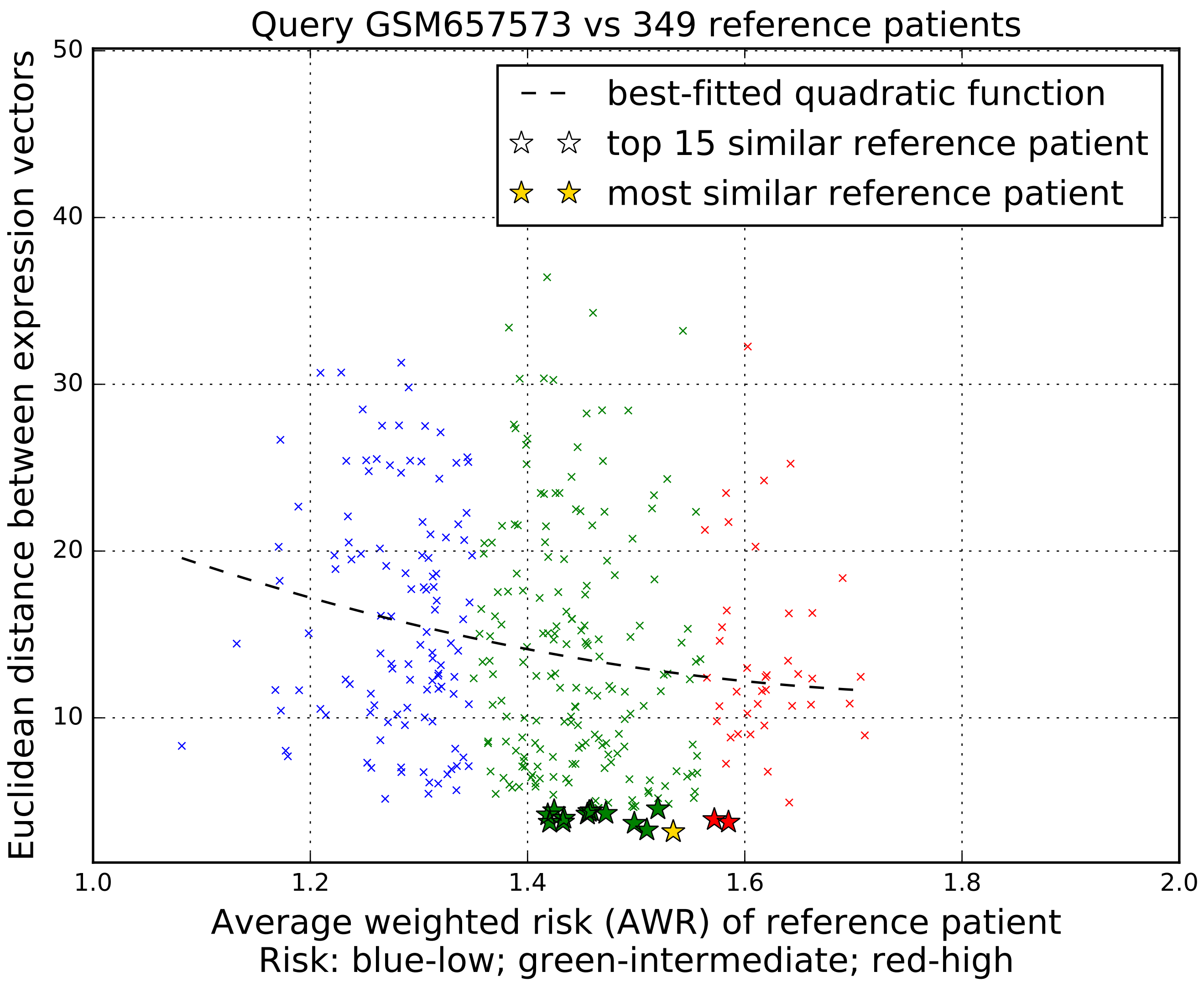

Query GSM657573 vs 349 reference patients
best-fitted quadratic function
top 15 similar reference patient
most similar reference patient
Euclidean distance between expression vectors
Average weighted risk (AWR) of reference patient
Risk: blue-low; green-intermediate; red-high
50
40
30
20
10
1.0
1.2
1.4
1.6
1.8
2.0

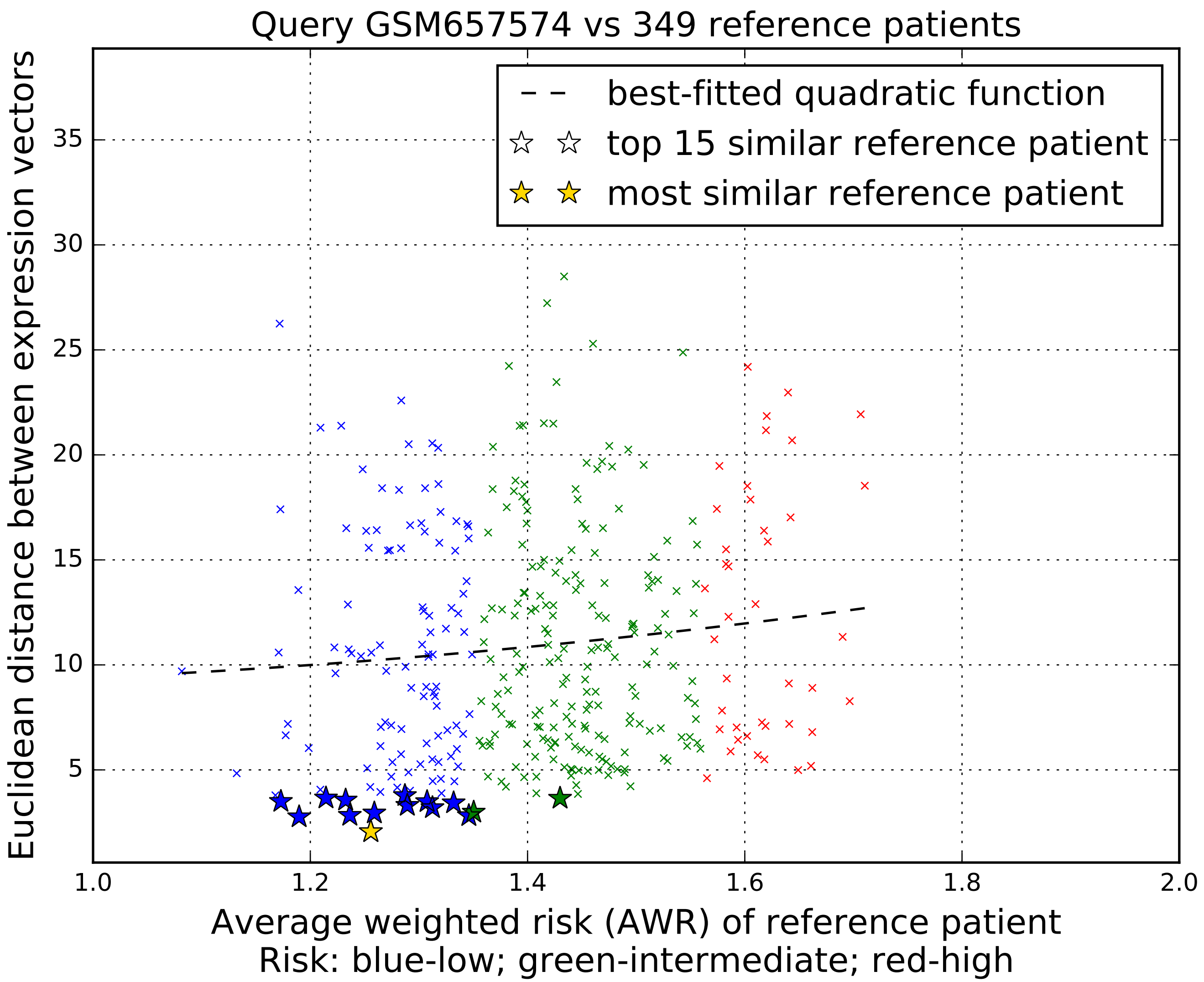
Query GSM657574 vs 349 reference patients
best-fitted quadratic function
top 15 similar reference patient
most similar reference patient
Euclidean distance between expression vectors
35
30
25
20
15
10
5
1.0
1.2
1.4
1.6
1.8
2.0
Average weighted risk (AWR) of reference patient
Risk: blue-low; green-intermediate; red-high

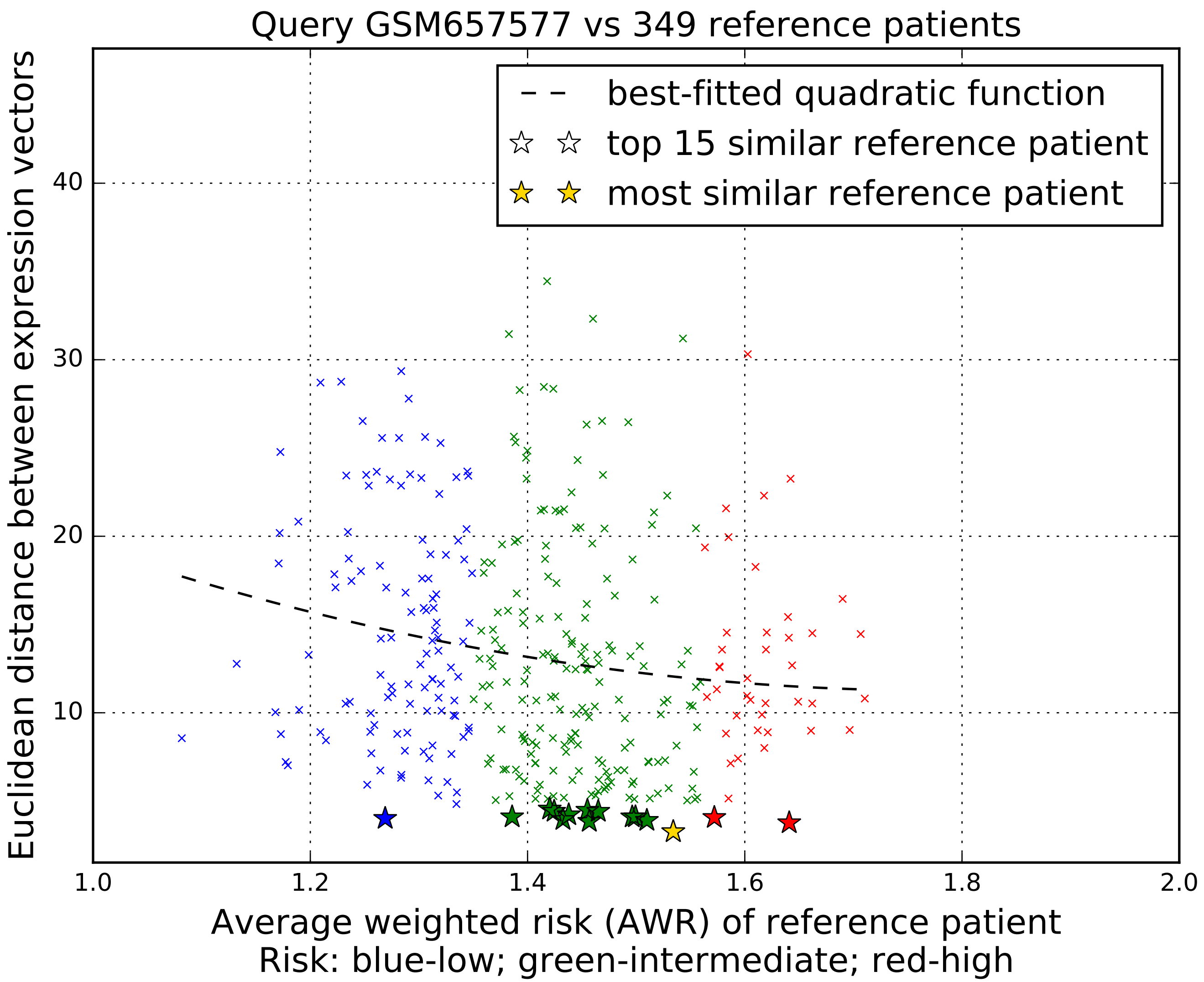

Query GSM657577 vs 349 reference patients
best-fitted quadratic function
top 15 similar reference patient
most similar reference patient
Euclidean distance between expression vectors
10
20
30
40
1.0
1.2
1.4
1.6
1.8
2.0
Average weighted risk (AWR) of reference patient
Risk: blue-low; green-intermediate; red-high

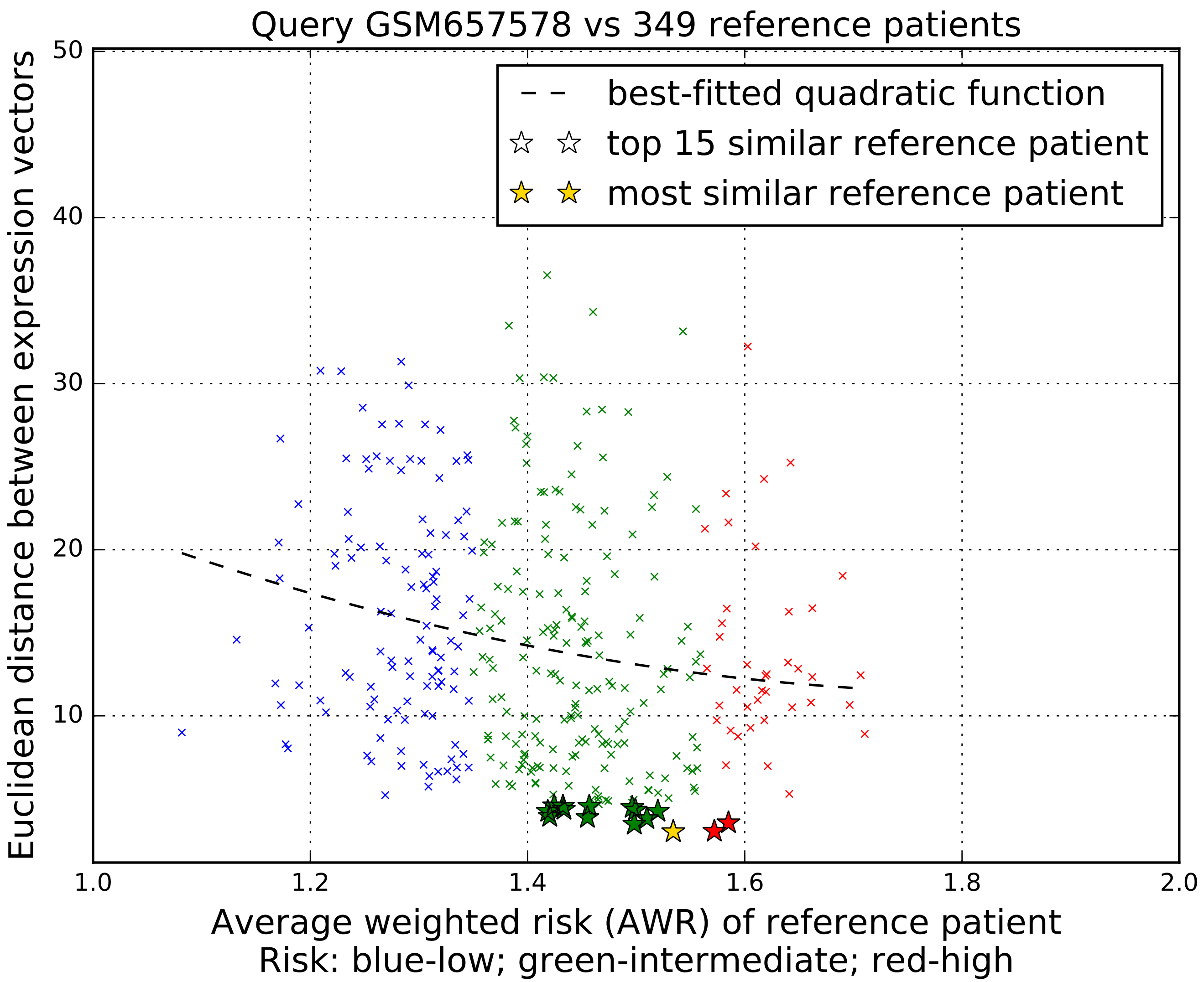

Query GSM657578 vs 349 reference patients
best-fitted quadratic function
top 15 similar reference patient
most similar reference patient
Euclidean distance between expression vectors
50
40
30
20
10
1.0
1.2
1.4
1.6
1.8
2.0
Average weighted risk (AWR) of reference patient
Risk: blue-low; green-intermediate; red-high

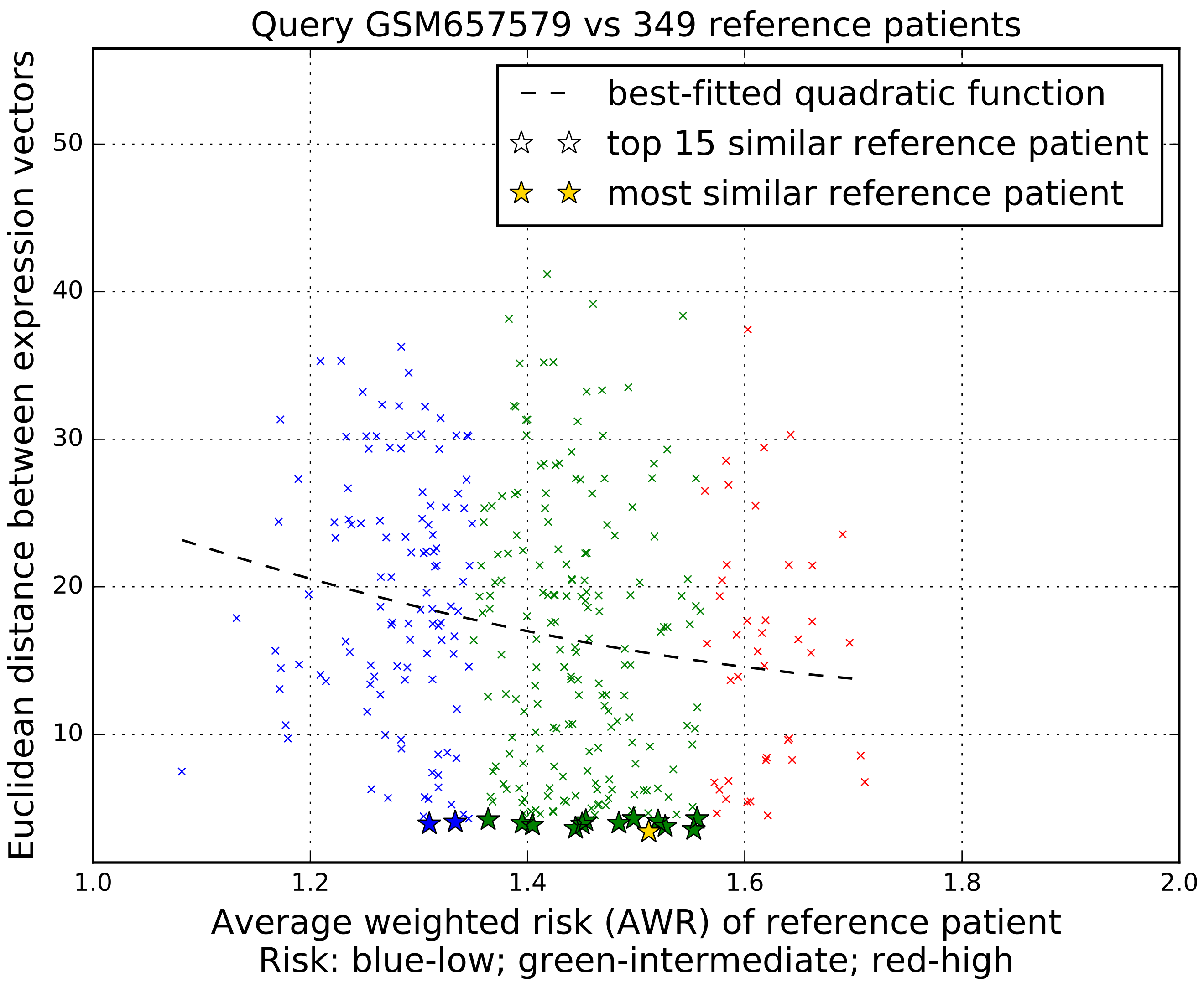

Query GSM657579 vs 349 reference patients
best-fitted quadratic function
top 15 similar reference patient
most similar reference patient
Euclidean distance between expression vectors
10
20
30
40
50
1.0
1.2
1.4
1.6
1.8
2.0
Average weighted risk (AWR) of reference patient
Risk: blue-low; green-intermediate; red-high

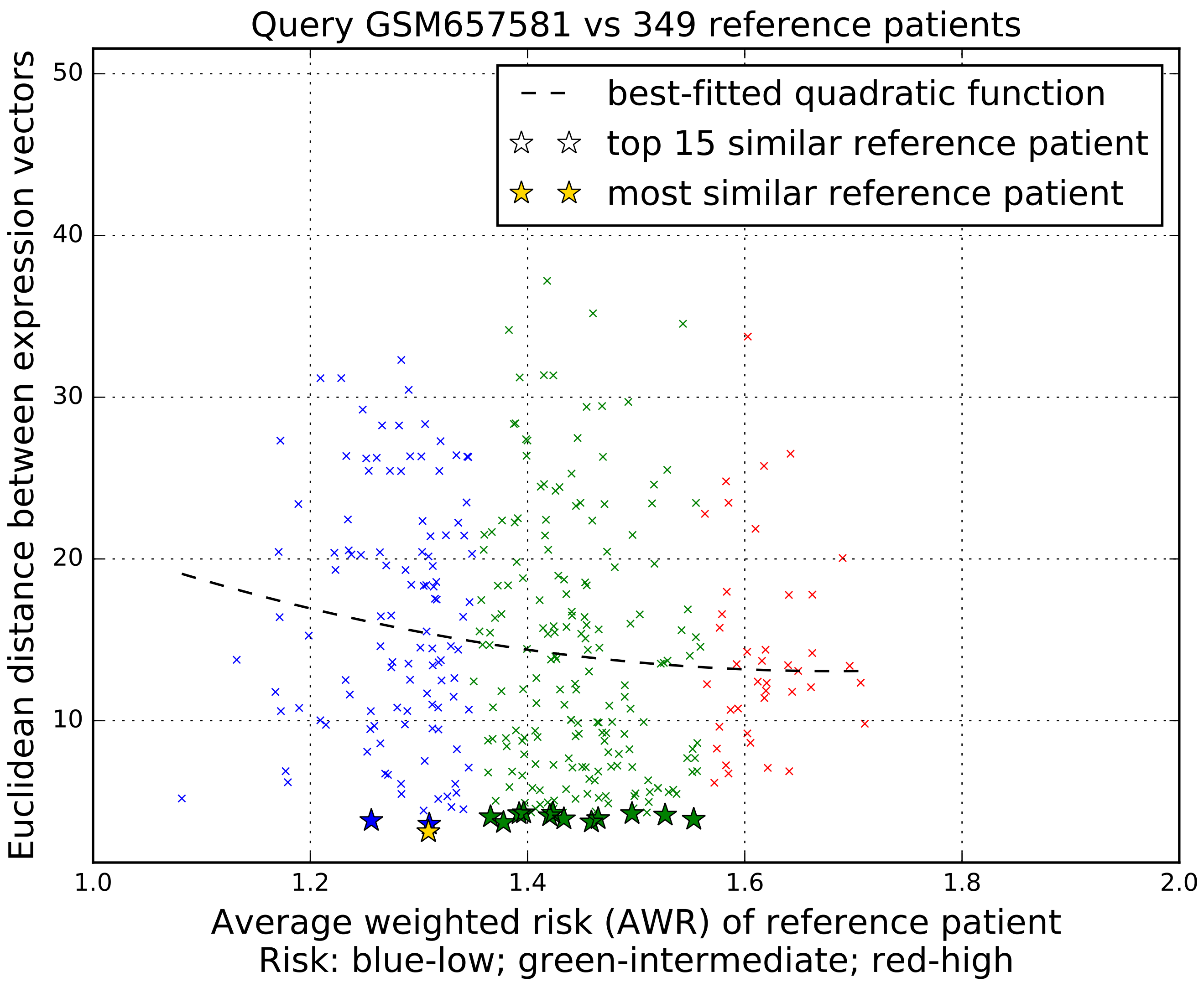
Query GSM657581 vs 349 reference patients
best-fitted quadratic function
top 15 similar reference patient
most similar reference patient
Euclidean distance between expression vectors
50
40
30
20
10
1.0
1.2
1.4
1.6
1.8
2.0
Average weighted risk (AWR) of reference patient
Risk: blue-low; green-intermediate; red-high

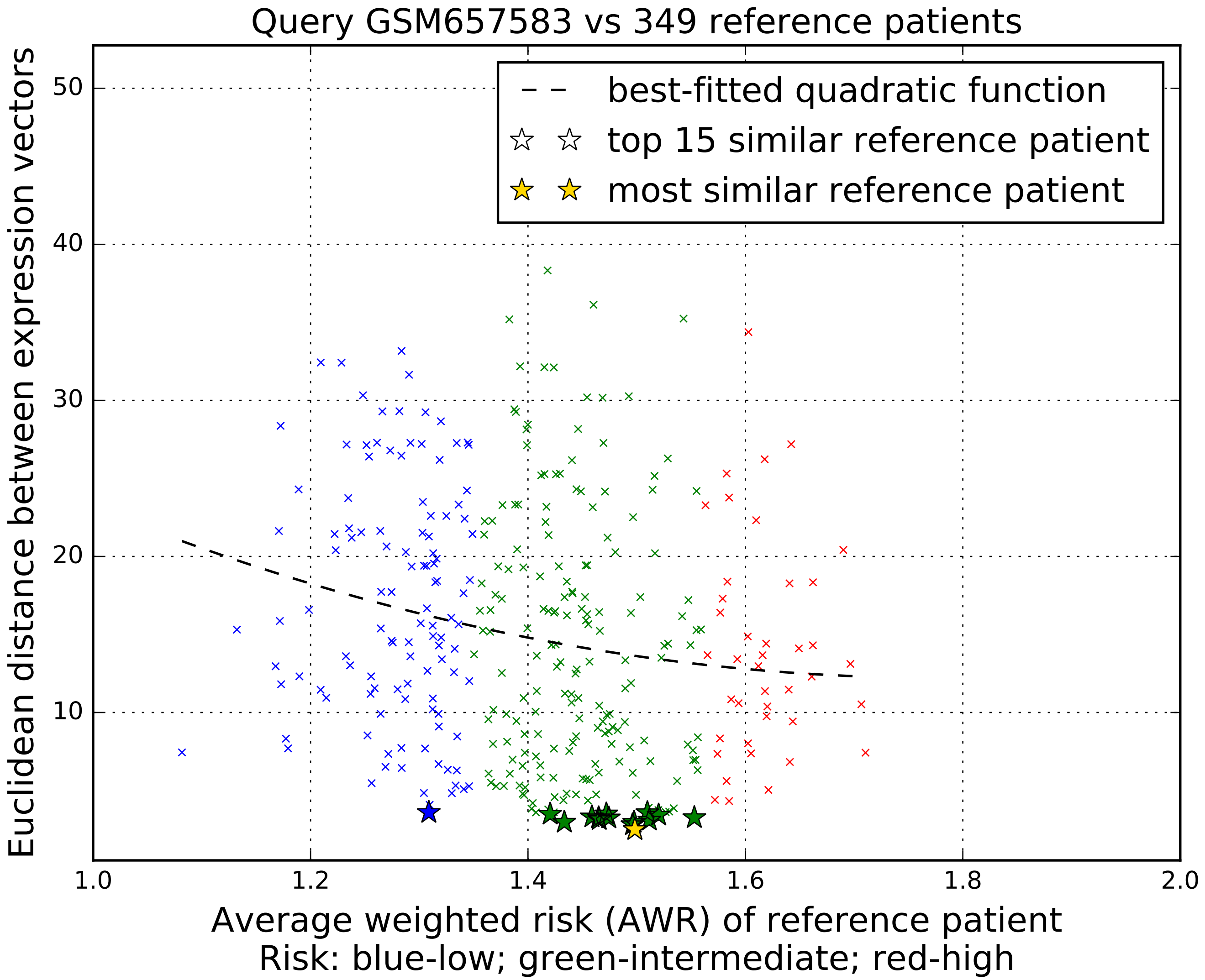

Query GSM657583 vs 349 reference patients
best-fitted quadratic function
top 15 similar reference patient
most similar reference patient
Euclidean distance between expression vectors
Average weighted risk (AWR) of reference patient
Risk: blue-low; green-intermediate; red-high
50
40
30
20
10
1.0
1.2
1.4
1.6
1.8
2.0

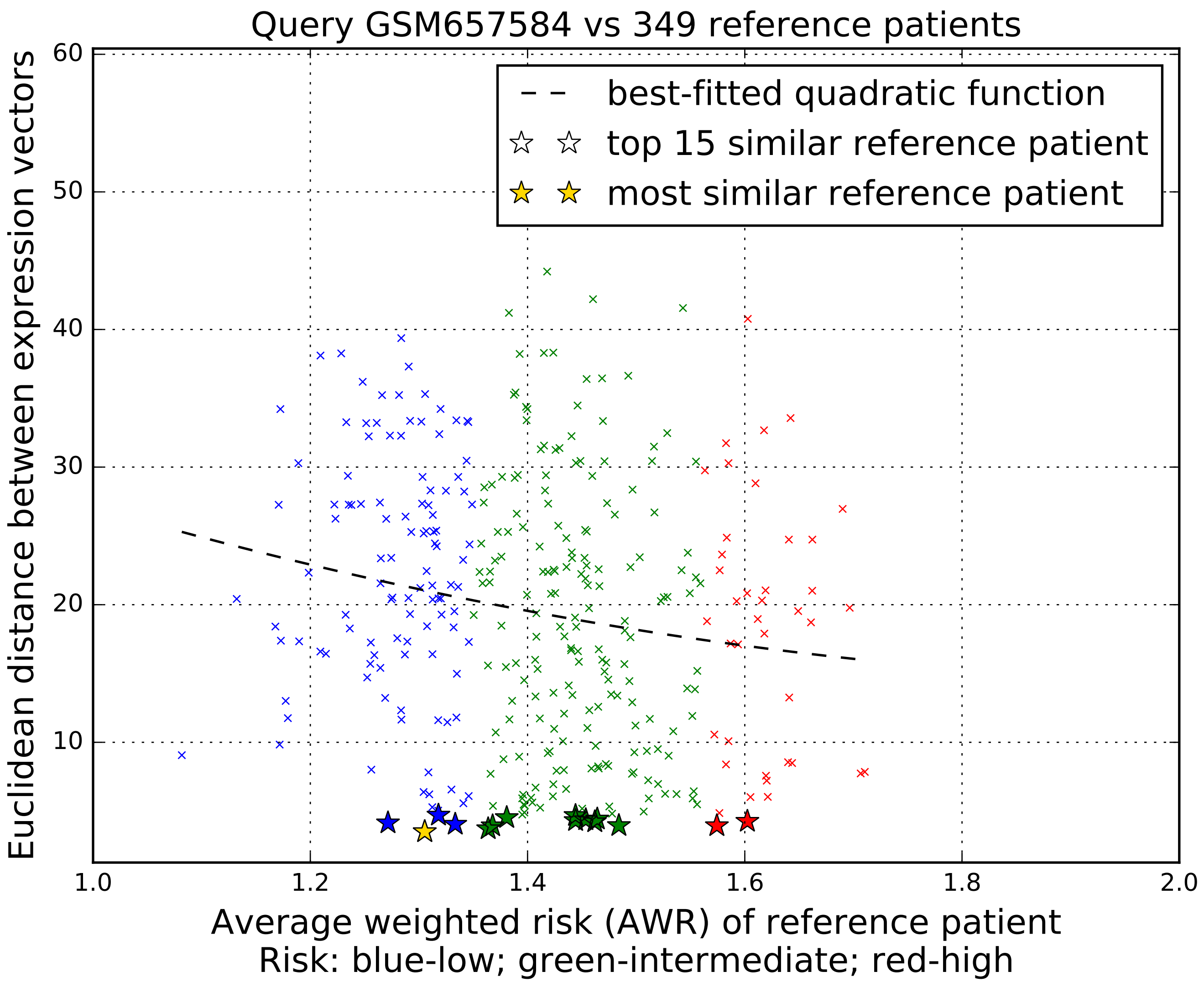
Query GSM657584 vs 349 reference patients
best-fitted quadratic function
top 15 similar reference patient
most similar reference patient
Euclidean distance between expression vectors
60
50
40
30
20
10
1.0
1.2
1.4
1.6
1.8
2.0
Average weighted risk (AWR) of reference patient
Risk: blue-low; green-intermediate; red-high

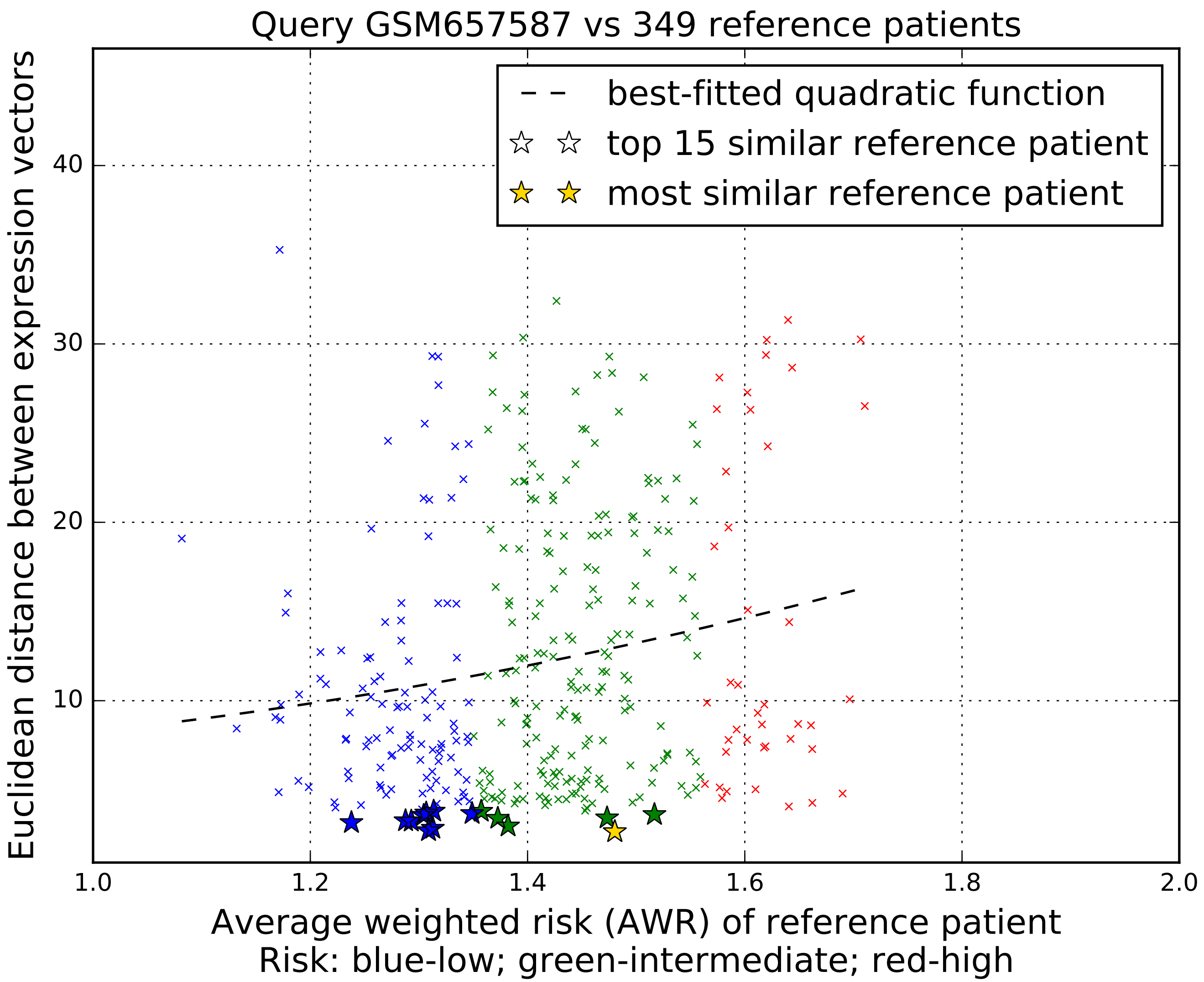

Query GSM657587 vs 349 reference patients
best-fitted quadratic function
top 15 similar reference patient
most similar reference patient
Euclidean distance between expression vectors
10
20
30
40
1.0
1.2
1.4
1.6
1.8
2.0
Average weighted risk (AWR) of reference patient
Risk: blue-low; green-intermediate; red-high

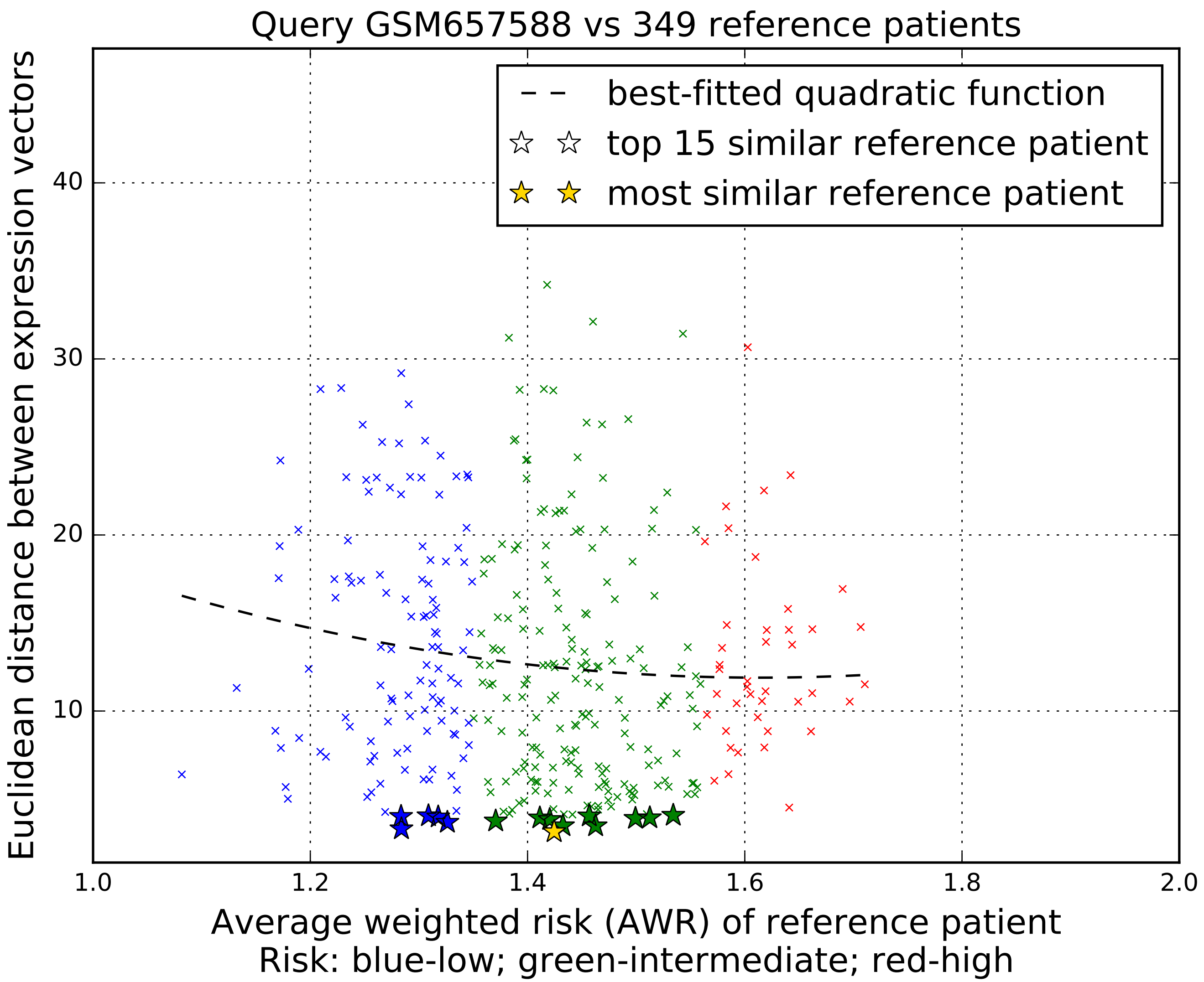
Query GSM657588 vs 349 reference patients
best-fitted quadratic function
top 15 similar reference patient
most similar reference patient
Euclidean distance between expression vectors
10
20
30
40
1.0
1.2
1.4
1.6
1.8
2.0
Average weighted risk (AWR) of reference patient
Risk: blue-low; green-intermediate; red-high

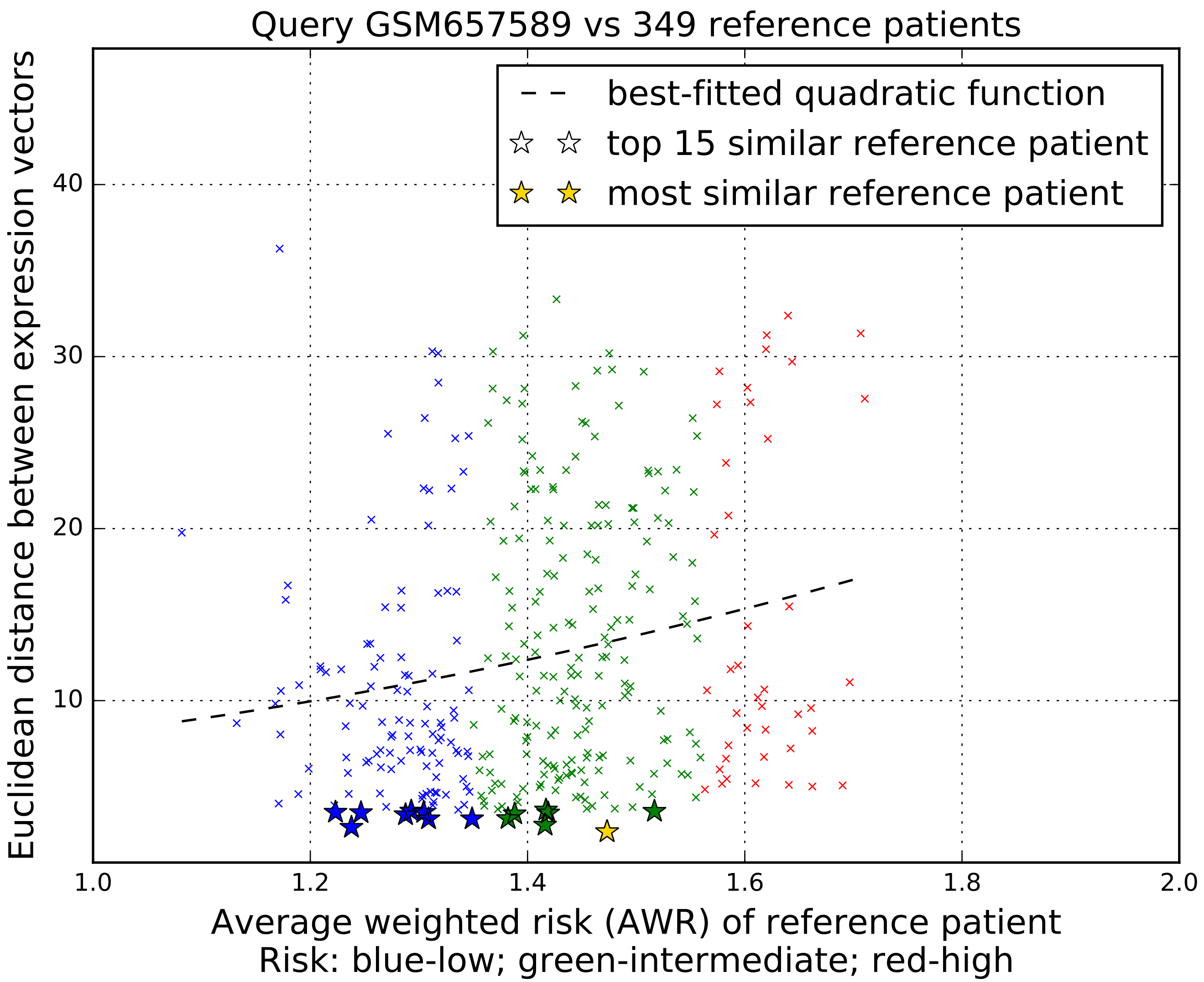
Query GSM657589 vs 349 reference patients
best-fitted quadratic function
top 15 similar reference patient
most similar reference patient
Euclidean distance between expression vectors
10
20
30
40
1.0
1.2
1.4
1.6
1.8
2.0
Average weighted risk (AWR) of reference patient
Risk: blue-low; green-intermediate; red-high

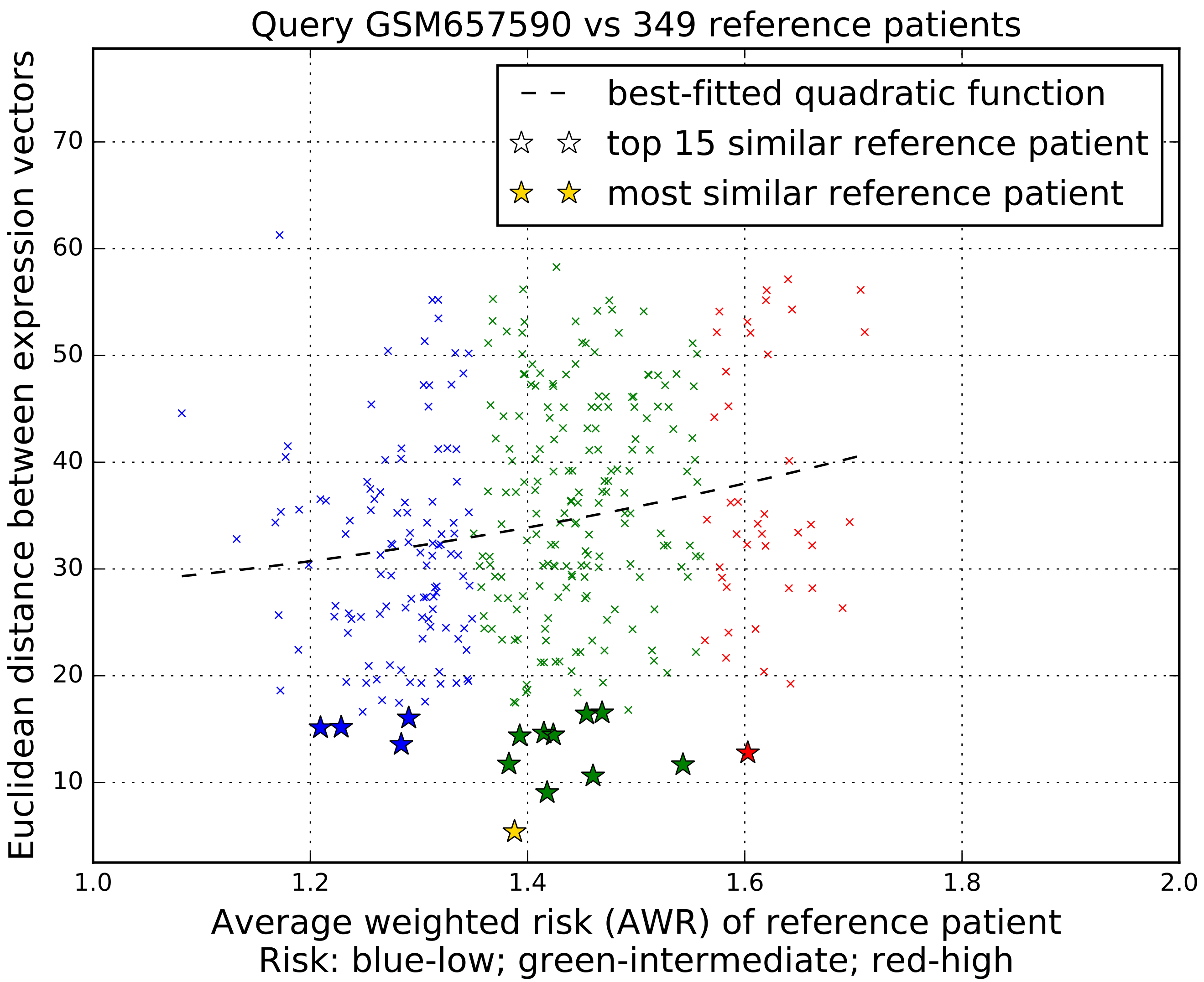

Query GSM657590 vs 349 reference patients
best-fitted quadratic function
top 15 similar reference patient
most similar reference patient
Euclidean distance between expression vectors
10
20
30
40
50
60
70
1.0
1.2
1.4
1.6
1.8
2.0
Average weighted risk (AWR) of reference patient
Risk: blue-low; green-intermediate; red-high

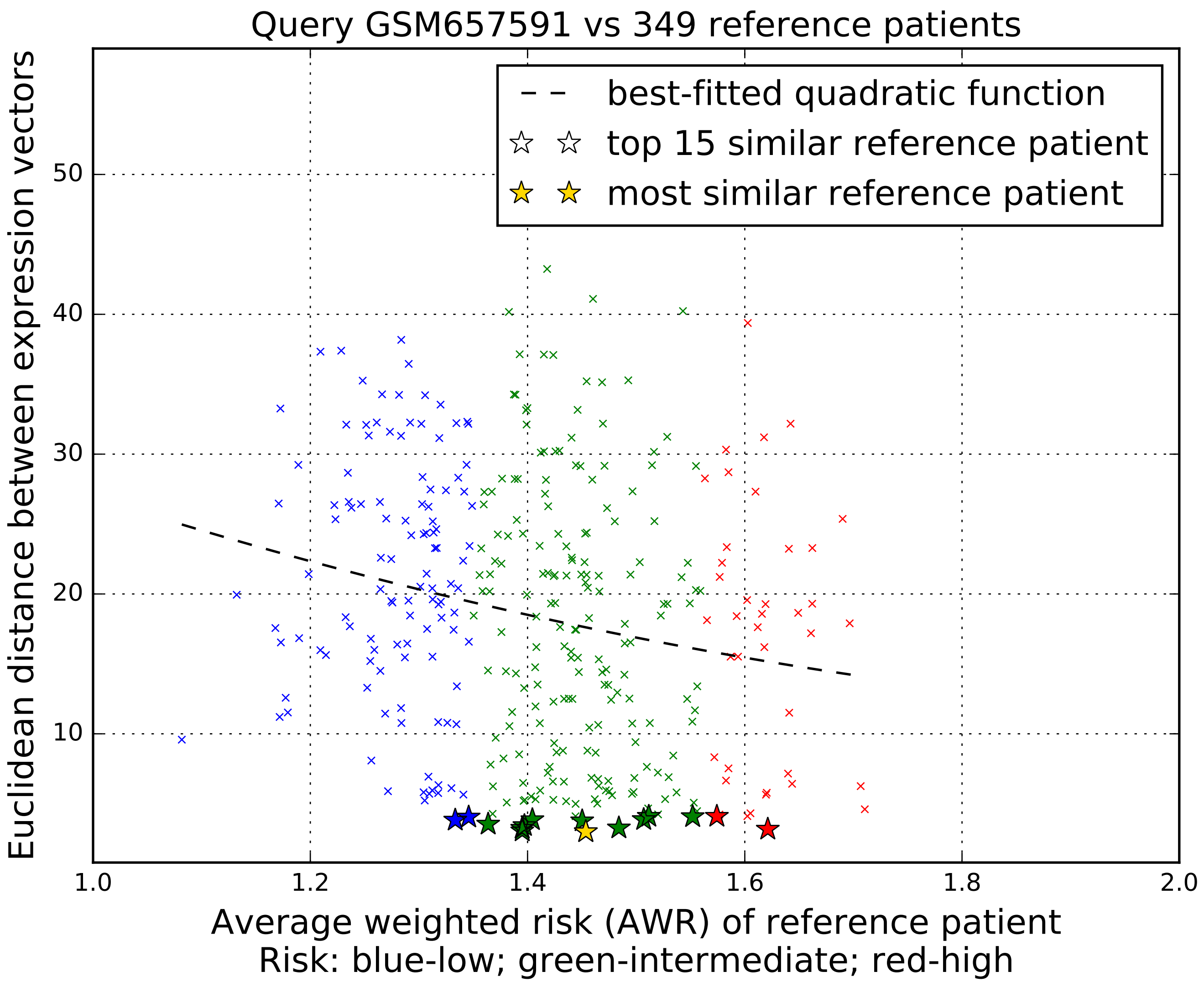

Query GSM657591 vs 349 reference patients
best-fitted quadratic function
top 15 similar reference patient
most similar reference patient
Euclidean distance between expression vectors
50
40
30
20
10
1.0
1.2
1.4
1.6
1.8
2.0
Average weighted risk (AWR) of reference patient
Risk: blue-low; green-intermediate; red-high

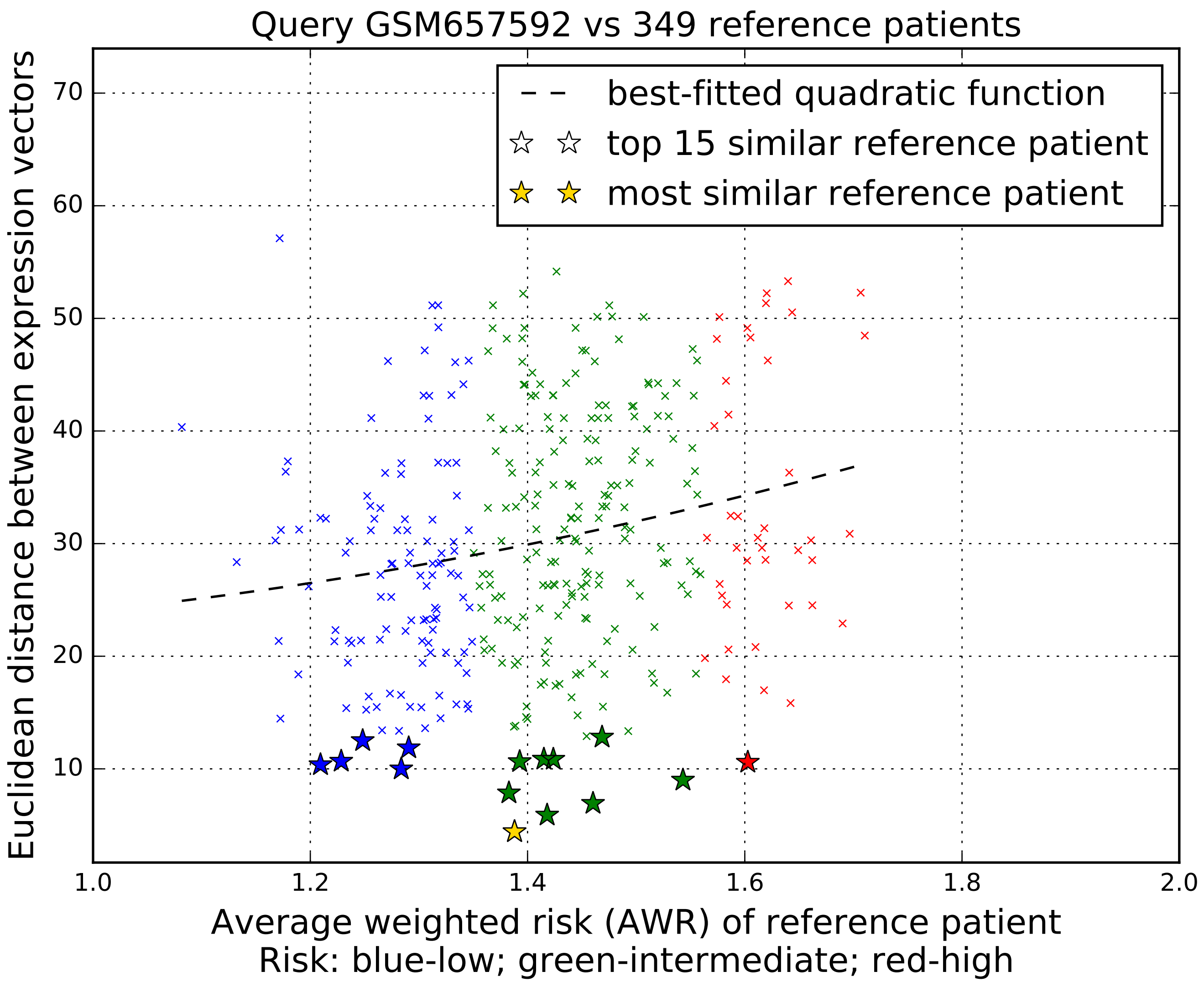
Query GSM657592 vs 349 reference patients
best-fitted quadratic function
top 15 similar reference patient
most similar reference patient
Euclidean distance between expression vectors
10
20
30
40
50
60
70
1.0
1.2
1.4
1.6
1.8
2.0
Average weighted risk (AWR) of reference patient
Risk: blue-low; green-intermediate; red-high

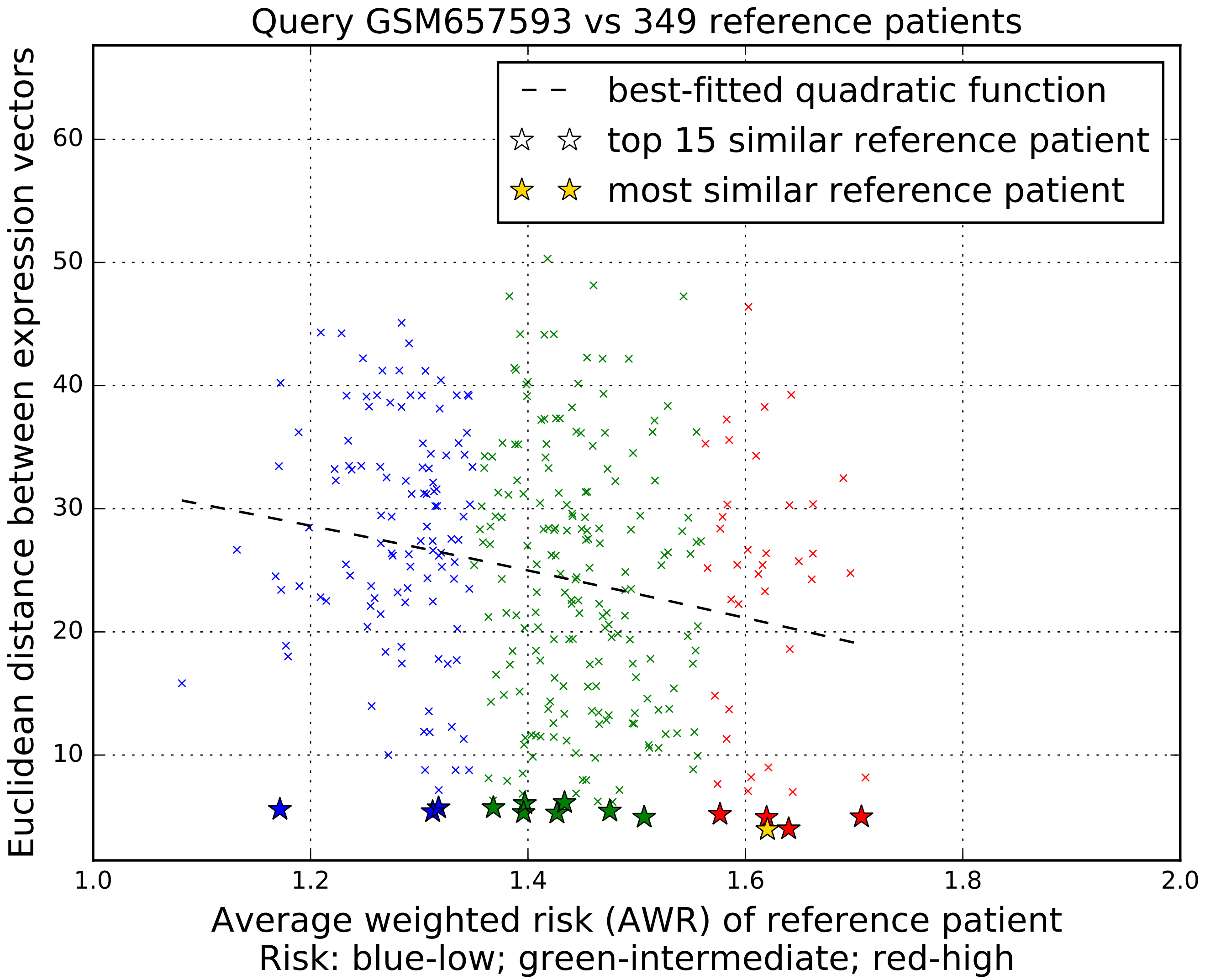
Query GSM657593 vs 349 reference patients
best-fitted quadratic function
top 15 similar reference patient
most similar reference patient
Euclidean distance between expression vectors
Average weighted risk (AWR) of reference patient
Risk: blue-low; green-intermediate; red-high
10
20
30
40
50
60
1.0
1.2
1.4
1.6
1.8
2.0

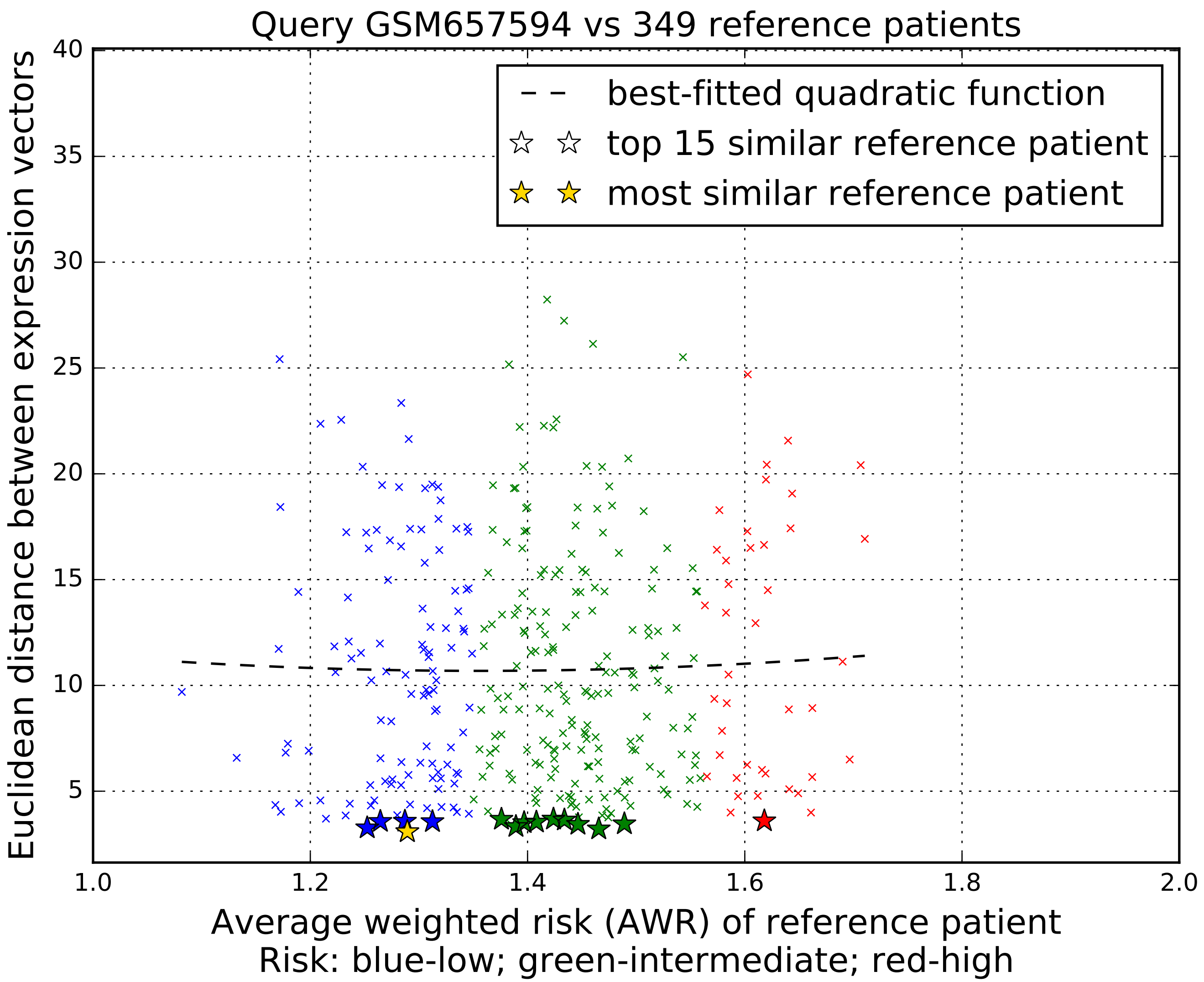

Query GSM657594 vs 349 reference patients
best-fitted quadratic function
top 15 similar reference patient
most similar reference patient
Euclidean distance between expression vectors
40
35
30
25
20
15
10
5
1.0
1.2
1.4
1.6
1.8
2.0
Average weighted risk (AWR) of reference patient
Risk: blue-low; green-intermediate; red-high

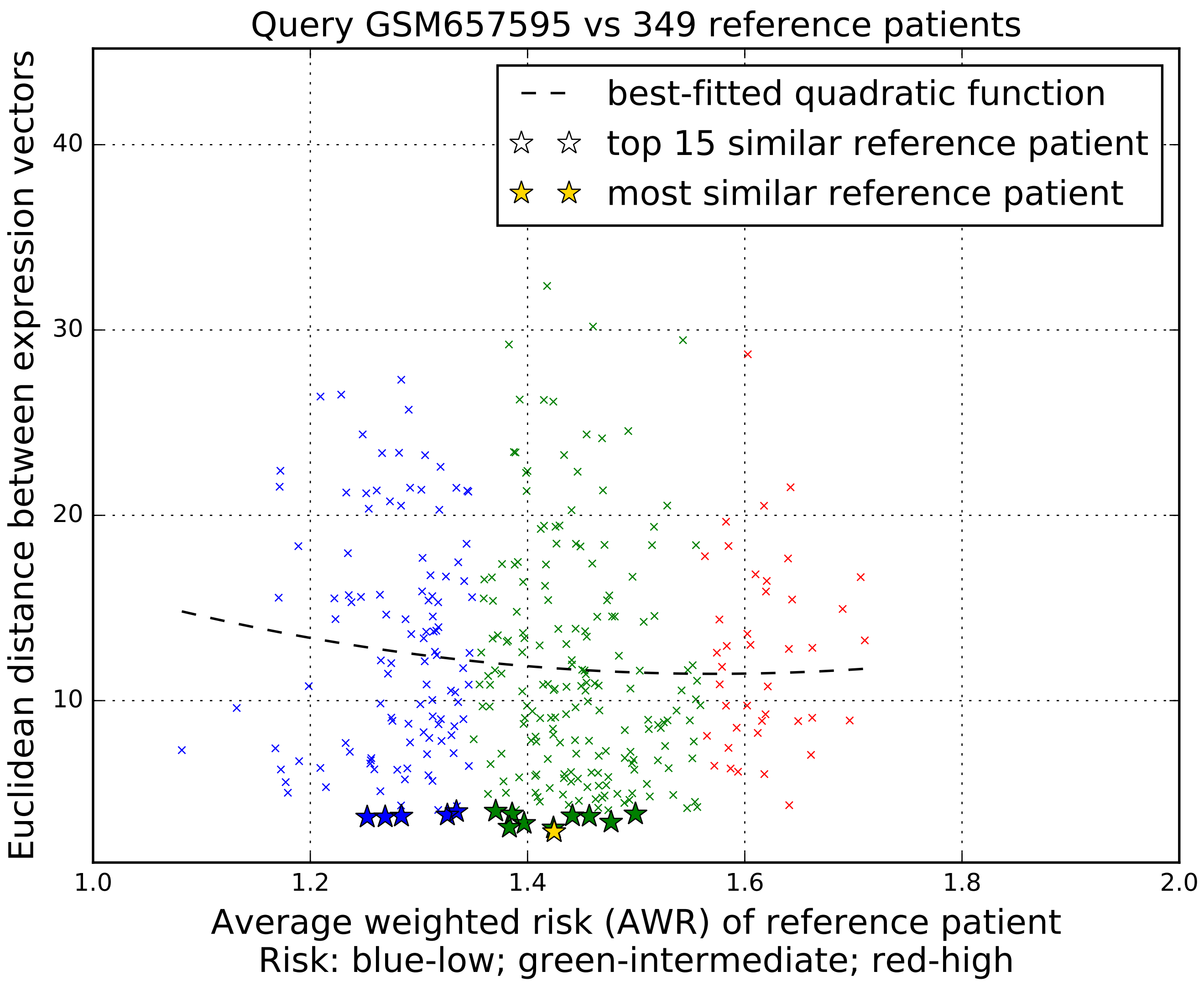

Query GSM657595 vs 349 reference patients
best-fitted quadratic function
top 15 similar reference patient
most similar reference patient
Euclidean distance between expression vectors
10
20
30
40
1.0
1.2
1.4
1.6
1.8
2.0
Average weighted risk (AWR) of reference patient
Risk: blue-low; green-intermediate; red-high

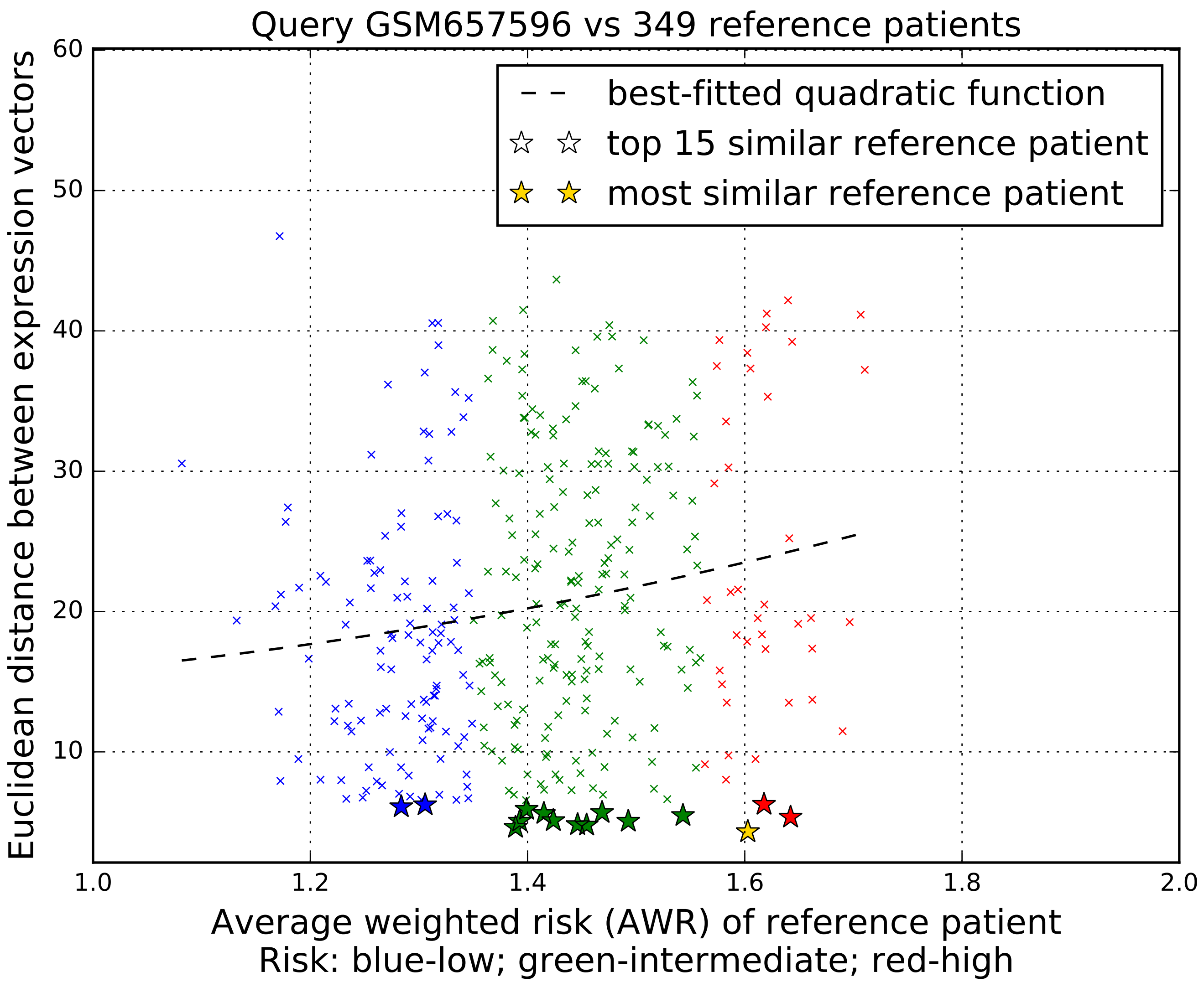

Query GSM657596 vs 349 reference patients
best-fitted quadratic function
top 15 similar reference patient
most similar reference patient
Euclidean distance between expression vectors
60
50
40
30
20
10
1.0
1.2
1.4
1.6
1.8
2.0
Average weighted risk (AWR) of reference patient
Risk: blue-low; green-intermediate; red-high

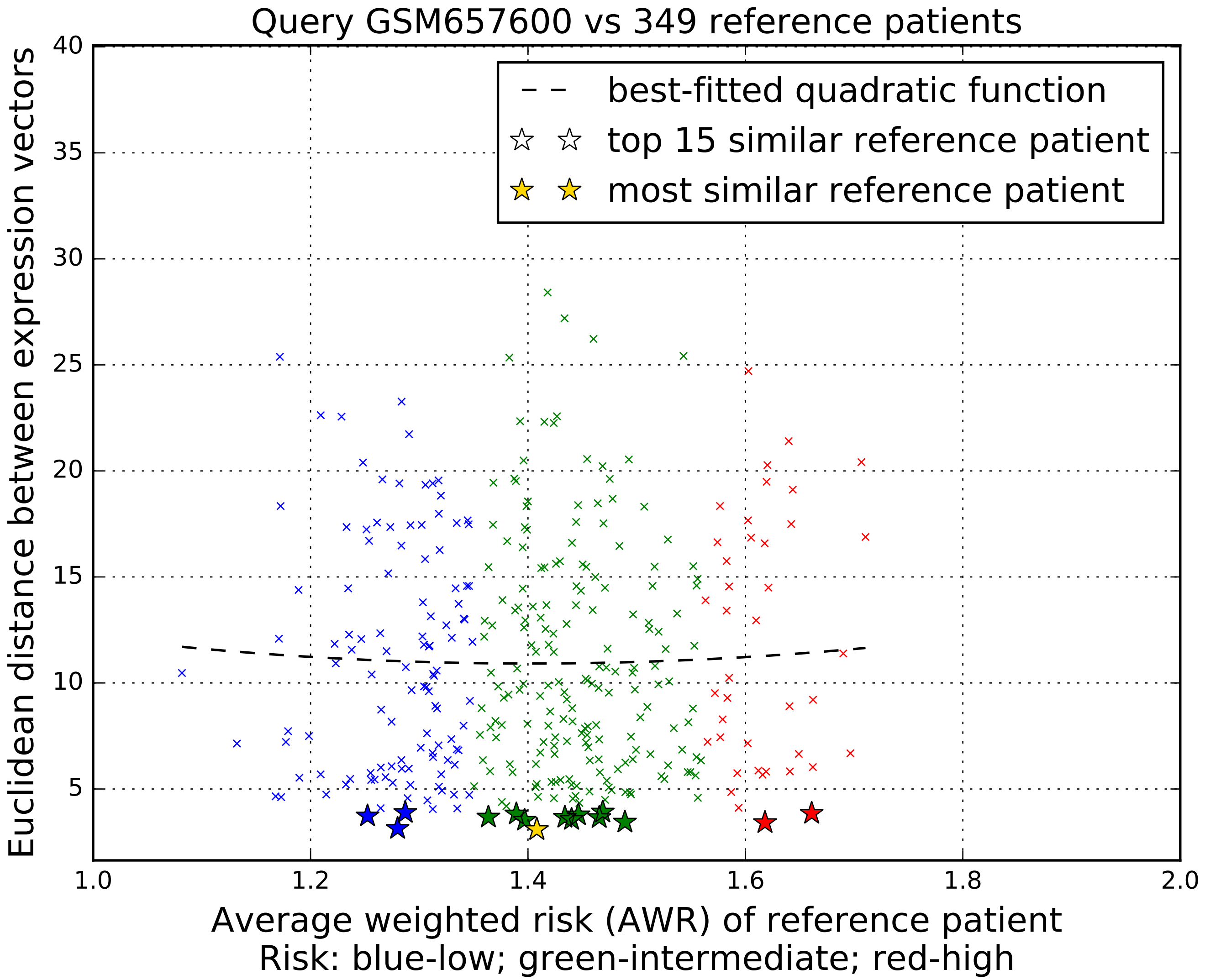
Query GSM657600 vs 349 reference patients
best-fitted quadratic function
top 15 similar reference patient
most similar reference patient
Euclidean distance between expression vectors
40
35
30
25
20
15
10
5
1.0
1.2
1.4
1.6
1.8
2.0
Average weighted risk (AWR) of reference patient
Risk: blue-low; green-intermediate; red-high

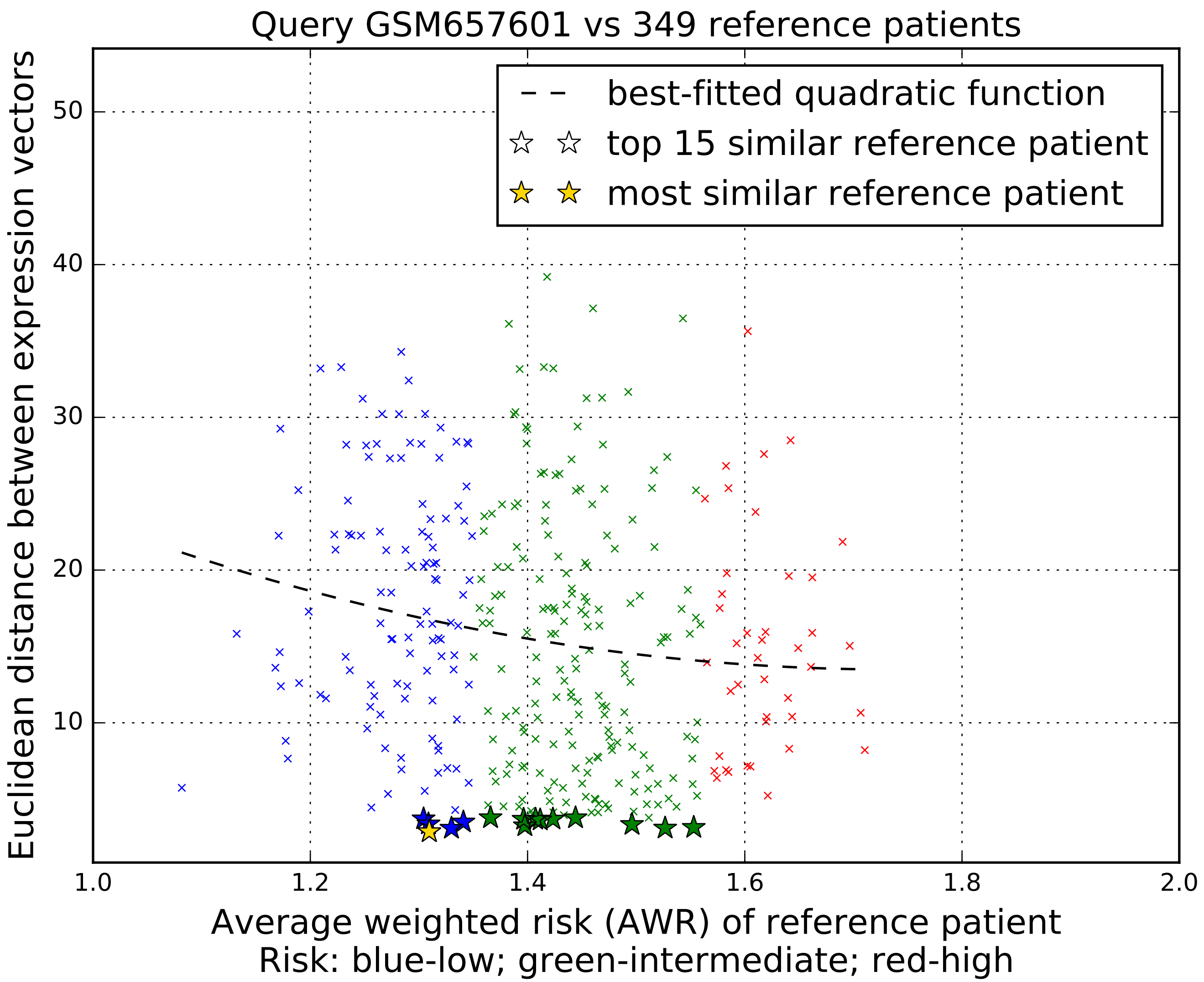
Query GSM657601 vs 349 reference patients
best-fitted quadratic function
top 15 similar reference patient
most similar reference patient
Euclidean distance between expression vectors
10
20
30
40
50
1.0
1.2
1.4
1.6
1.8
2.0
Average weighted risk (AWR) of reference patient
Risk: blue-low; green-intermediate; red-high

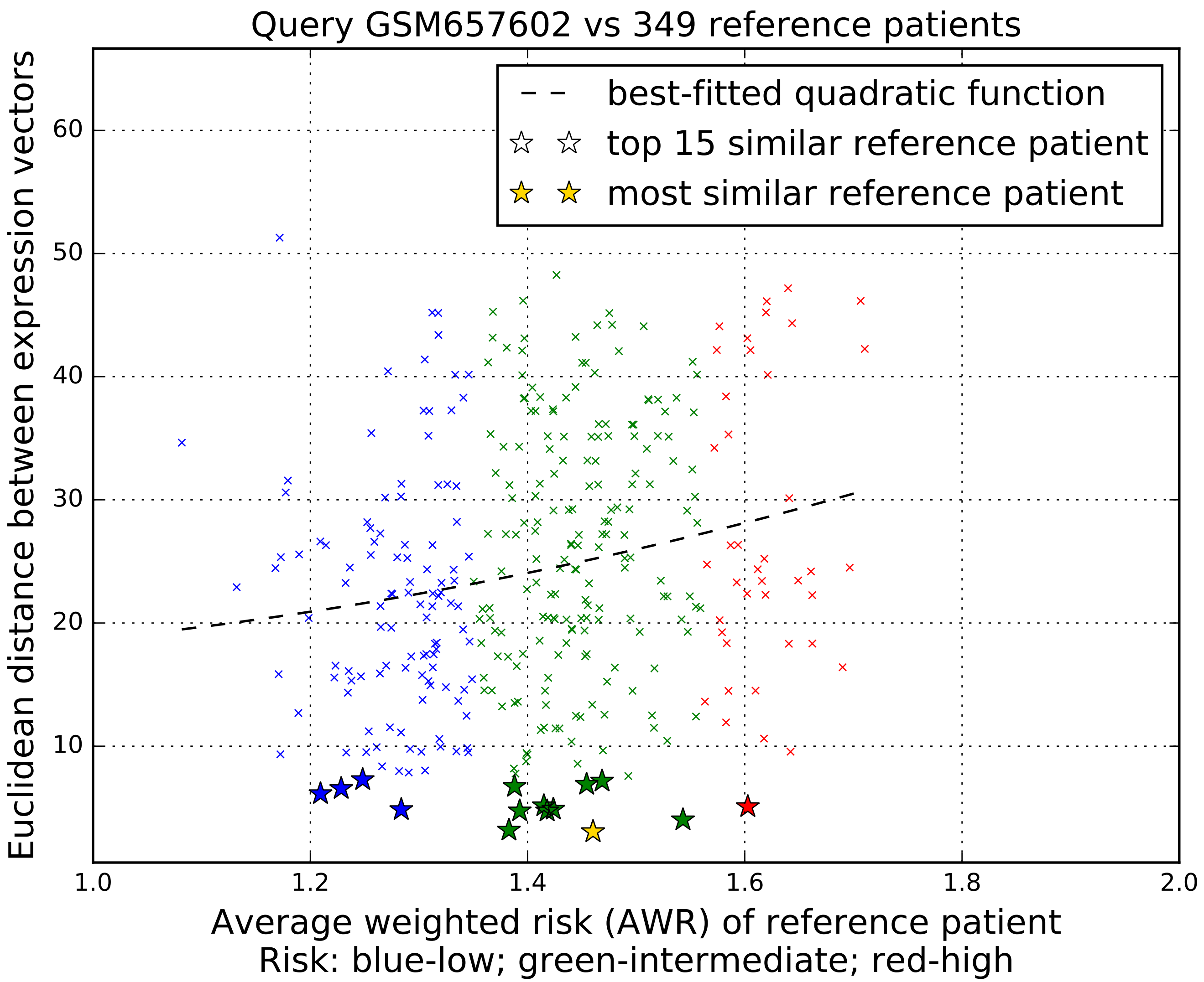
Query GSM657602 vs 349 reference patients
best-fitted quadratic function
top 15 similar reference patient
most similar reference patient
Euclidean distance between expression vectors
10
20
30
40
50
60
1.0
1.2
1.4
1.6
1.8
2.0
Average weighted risk (AWR) of reference patient
Risk: blue-low; green-intermediate; red-high

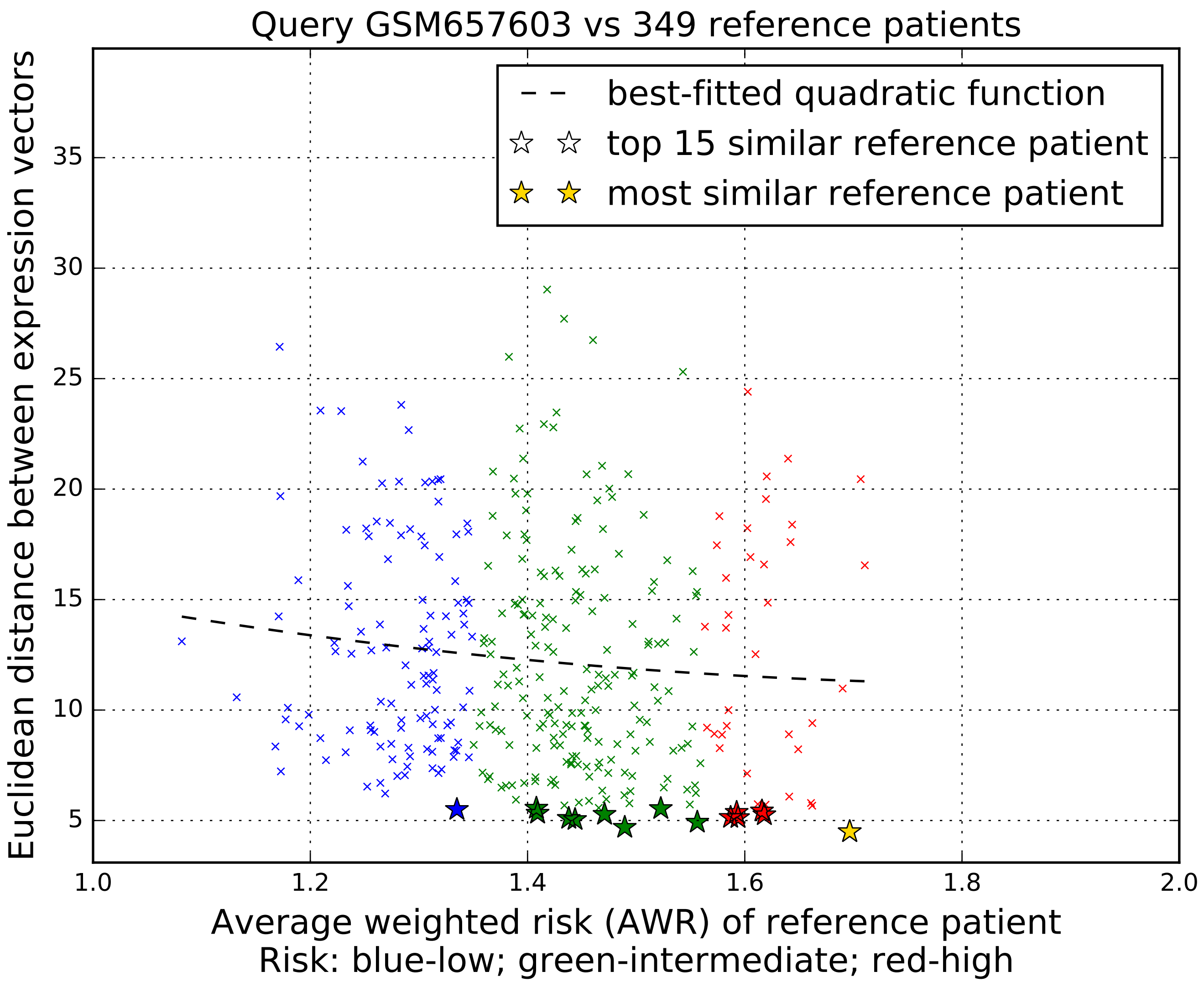
Query GSM657603 vs 349 reference patients
best-fitted quadratic function
top 15 similar reference patient
most similar reference patient
Euclidean distance between expression vectors
35
30
25
20
15
10
5
1.0
1.2
1.4
1.6
1.8
2.0
Average weighted risk (AWR) of reference patient
Risk: blue-low; green-intermediate; red-high

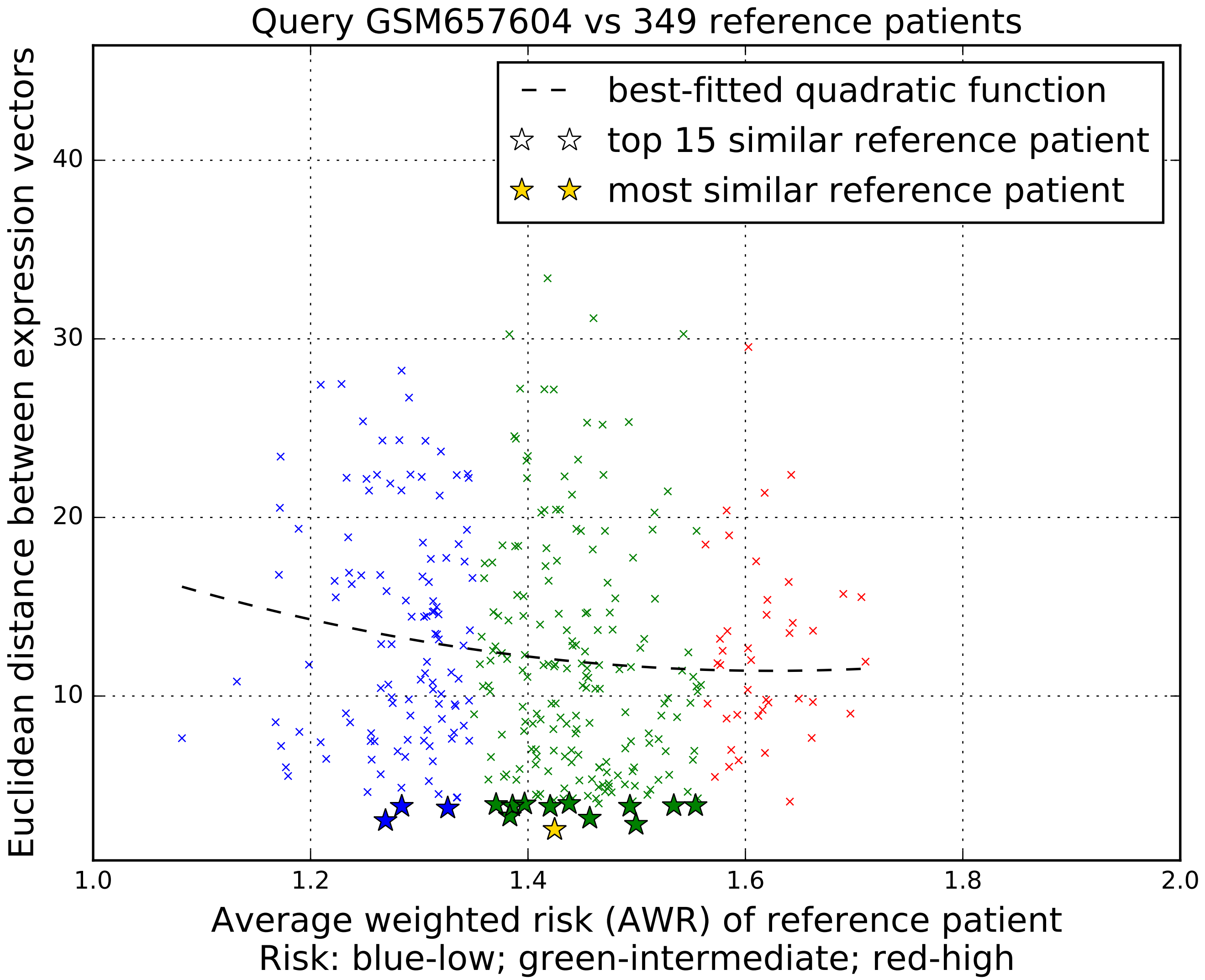
Query GSM657604 vs 349 reference patients
best-fitted quadratic function
top 15 similar reference patient
most similar reference patient
Euclidean distance between expression vectors
10
20
30
40
1.0
1.2
1.4
1.6
1.8
2.0
Average weighted risk (AWR) of reference patient
Risk: blue-low; green-intermediate; red-high

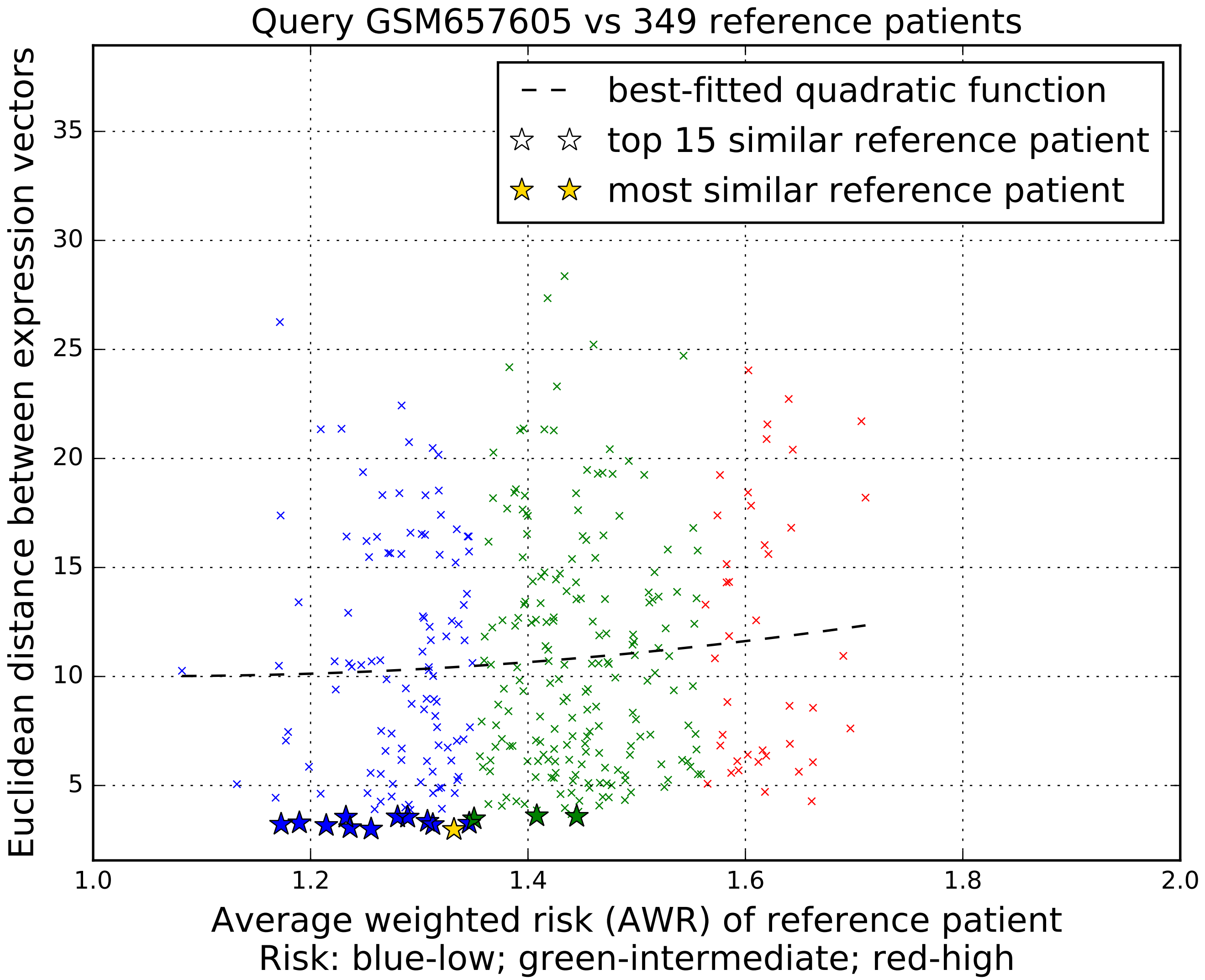
Query GSM657605 vs 349 reference patients
best-fitted quadratic function
top 15 similar reference patient
most similar reference patient
Euclidean distance between expression vectors
35
30
25
20
15
10
5
1.0
1.2
1.4
1.6
1.8
2.0
Average weighted risk (AWR) of reference patient
Risk: blue-low; green-intermediate; red-high

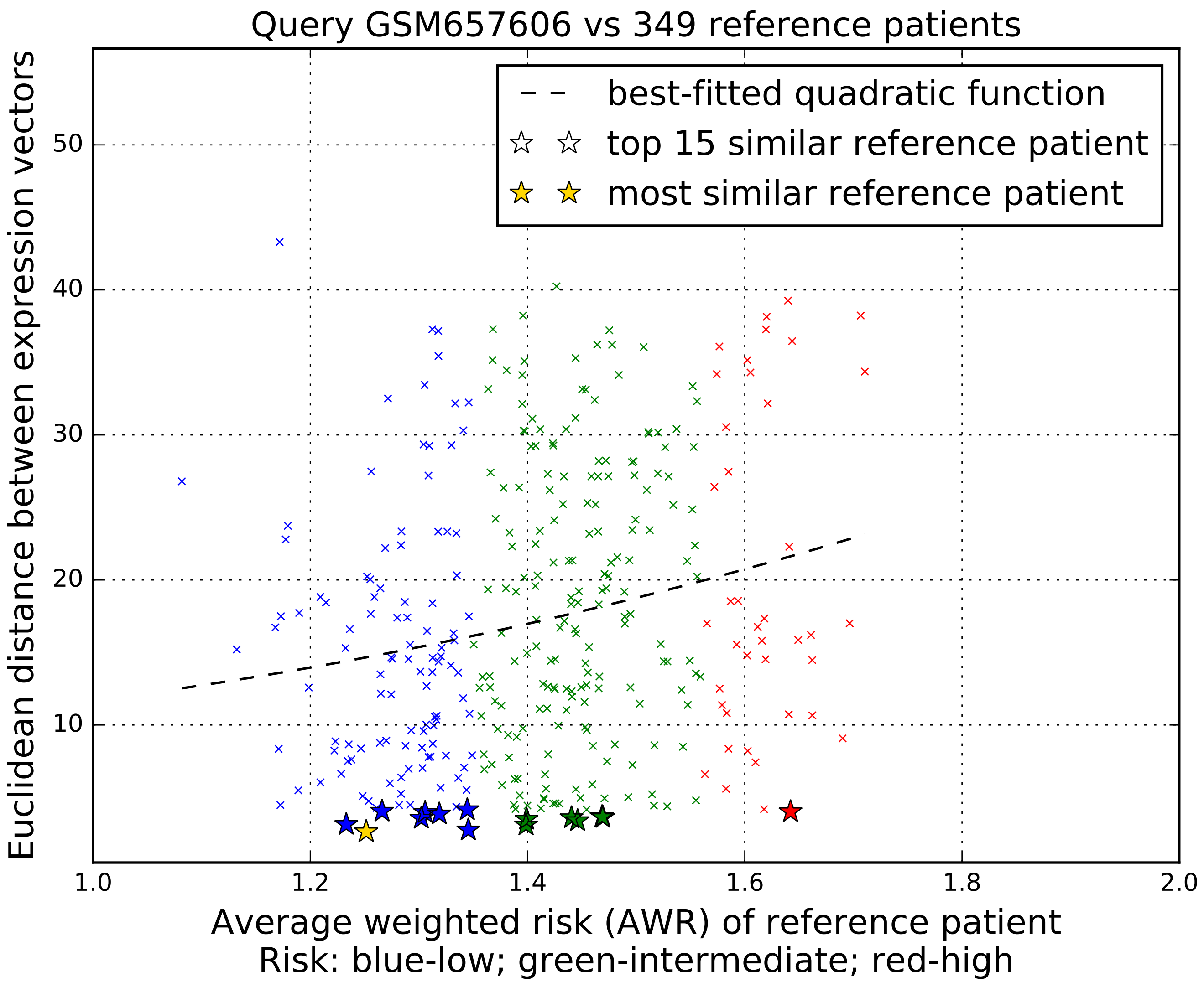
Query GSM657606 vs 349 reference patients
best-fitted quadratic function
top 15 similar reference patient
most similar reference patient
Euclidean distance between expression vectors
50
40
30
20
10
1.0
1.2
1.4
1.6
1.8
2.0
Average weighted risk (AWR) of reference patient
Risk: blue-low; green-intermediate; red-high

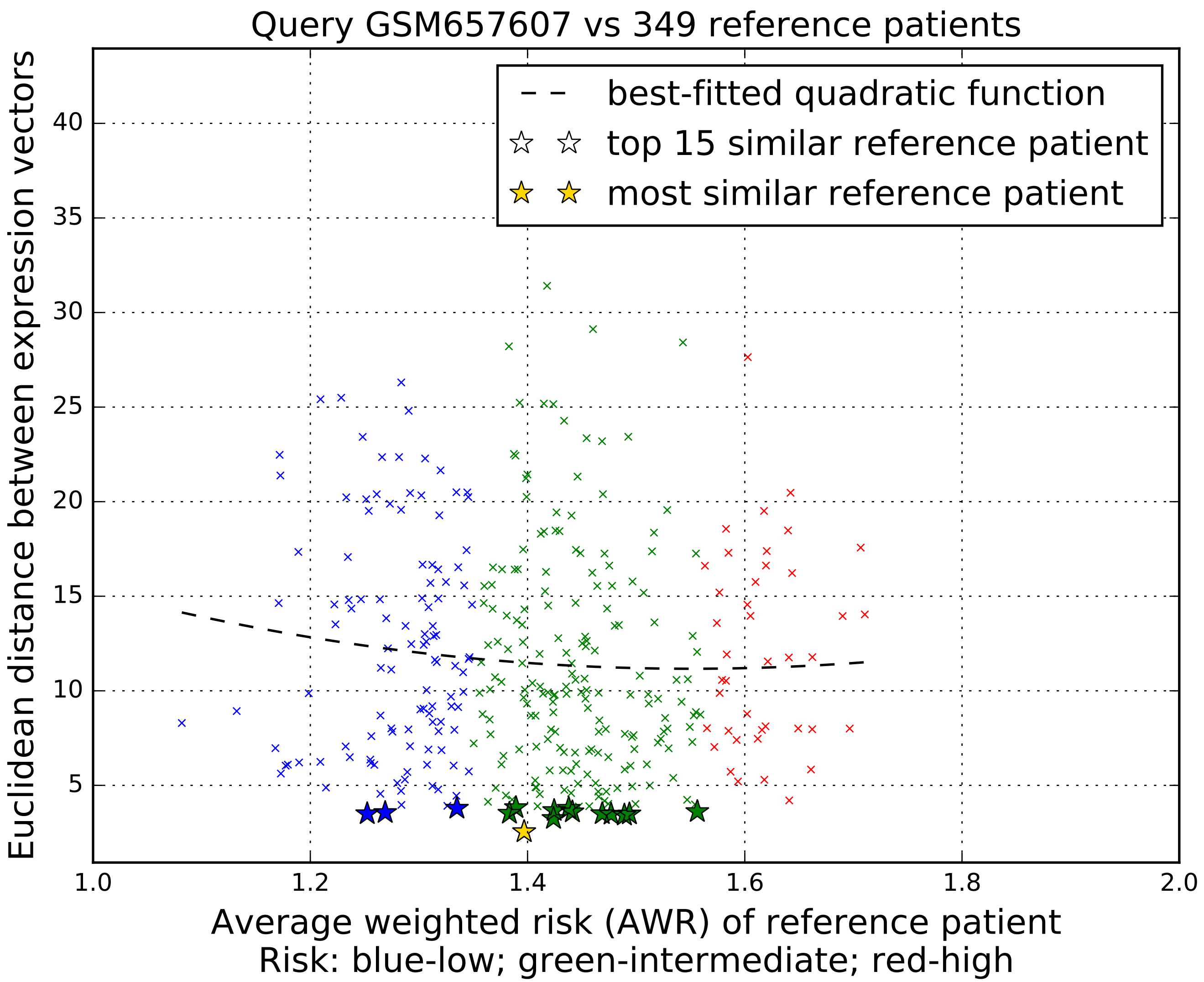

Query GSM657607 vs 349 reference patients
best-fitted quadratic function
top 15 similar reference patient
most similar reference patient
Euclidean distance between expression vectors
40
35
30
25
20
15
10
5
1.0
1.2
1.4
1.6
1.8
2.0
Average weighted risk (AWR) of reference patient
Risk: blue-low; green-intermediate; red-high

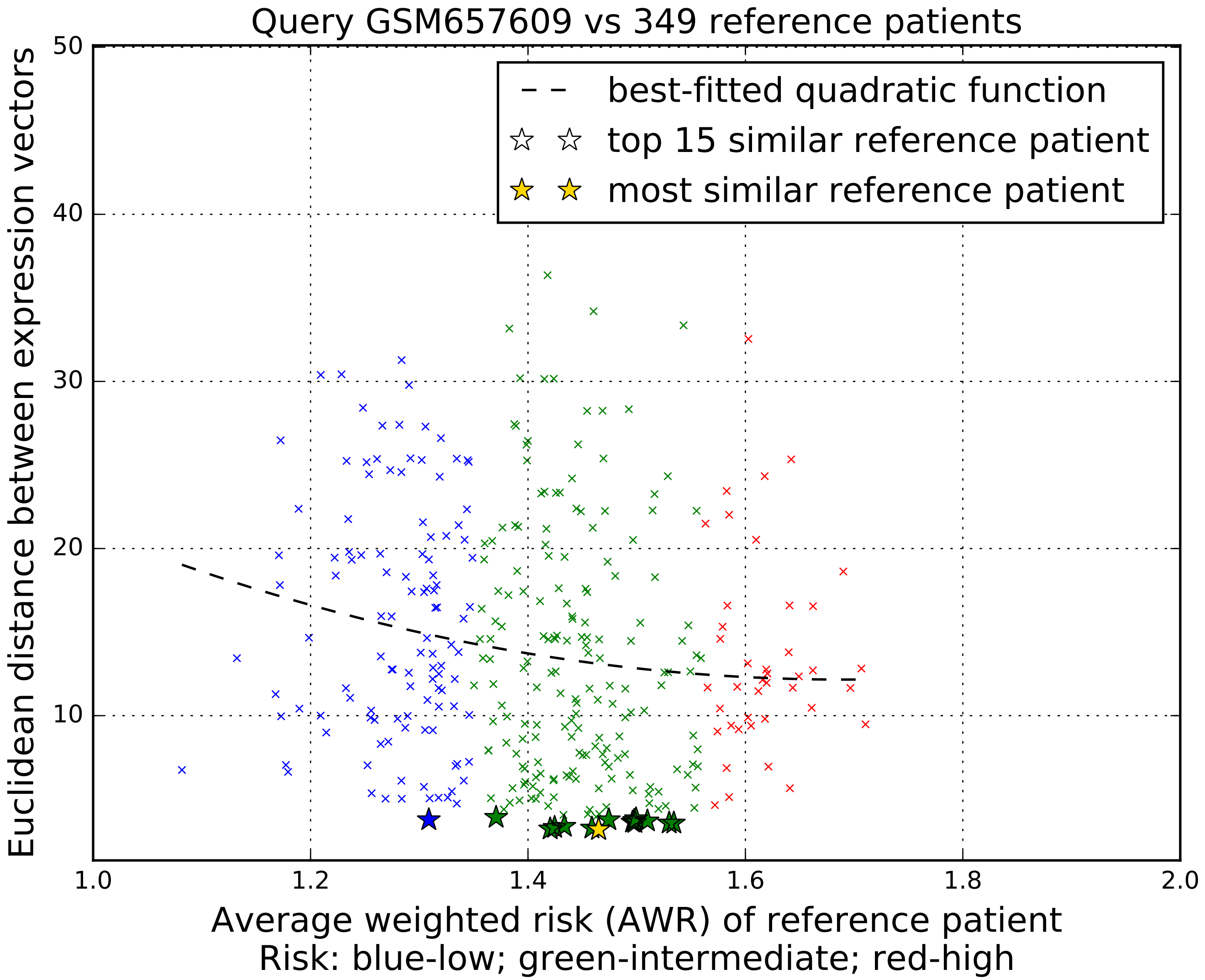
Query GSM657609 vs 349 reference patients
best-fitted quadratic function
top 15 similar reference patient
most similar reference patient
Euclidean distance between expression vectors
Average weighted risk (AWR) of reference patient
Risk: blue-low; green-intermediate; red-high
50
40
30
20
10
1.0
1.2
1.4
1.6
1.8
2.0

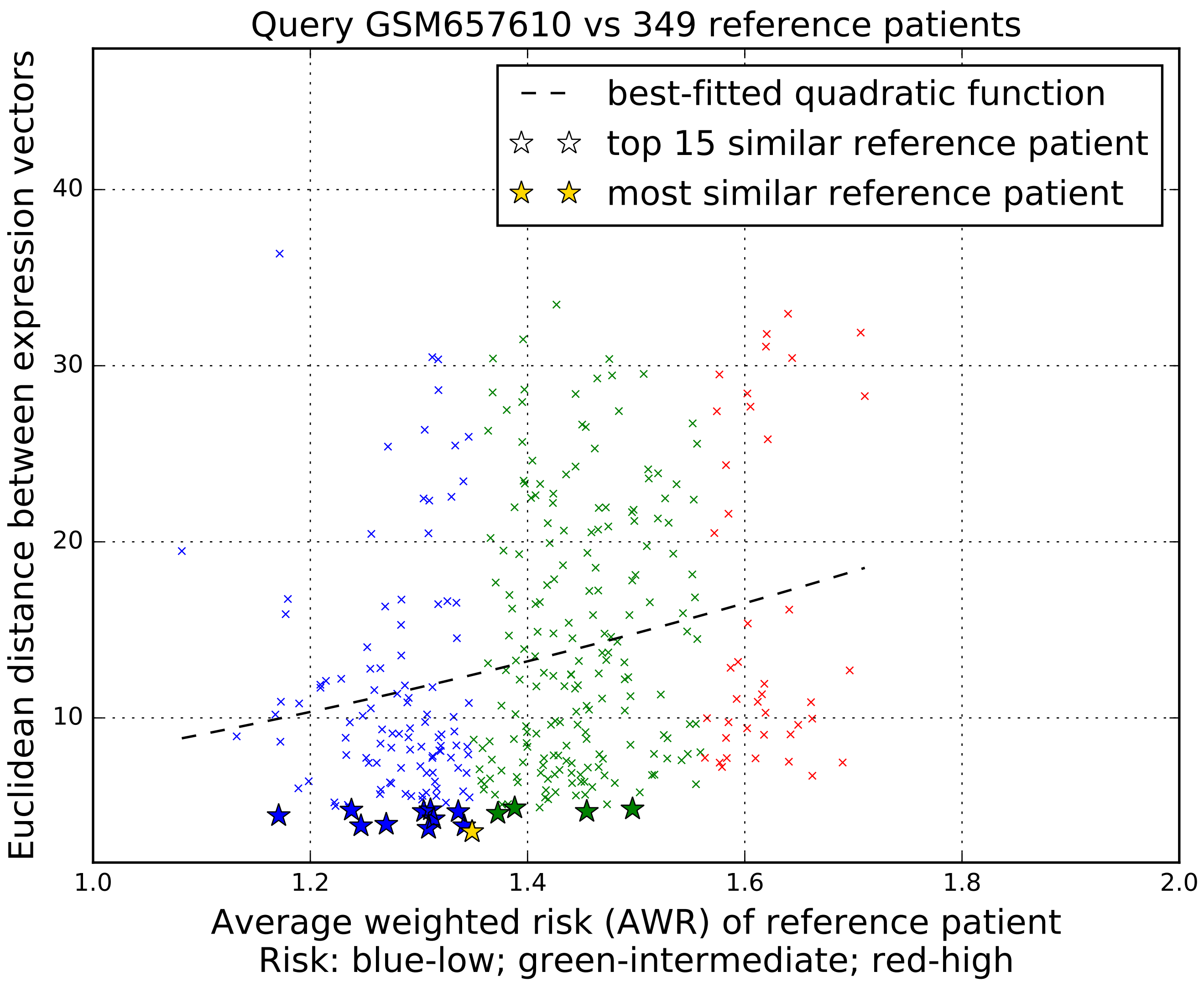

Query GSM657610 vs 349 reference patients
best-fitted quadratic function
top 15 similar reference patient
most similar reference patient
Euclidean distance between expression vectors
10
20
30
40
1.0
1.2
1.4
1.6
1.8
2.0
Average weighted risk (AWR) of reference patient
Risk: blue-low; green-intermediate; red-high

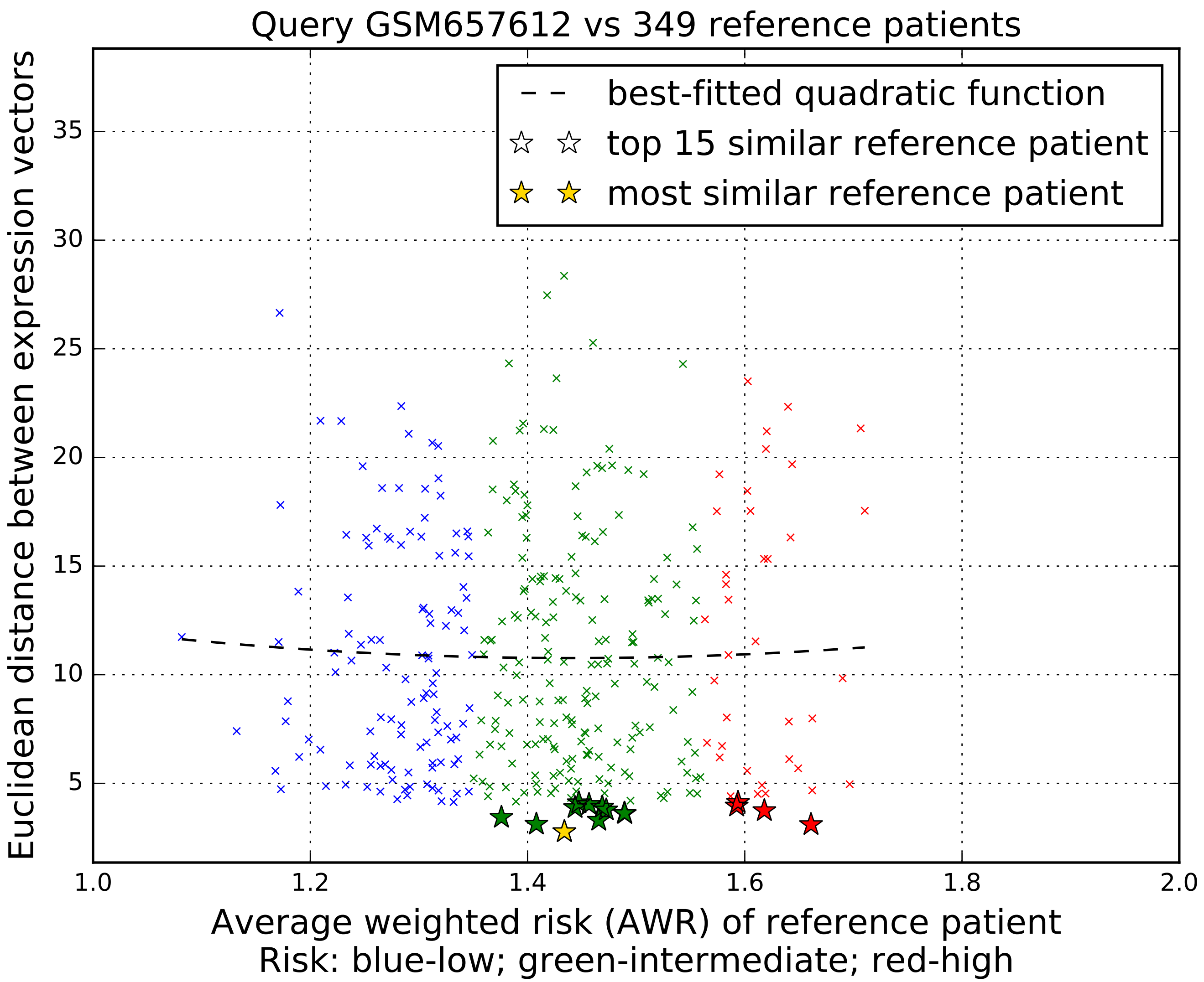

Query GSM657612 vs 349 reference patients
best-fitted quadratic function
top 15 similar reference patient
most similar reference patient
Euclidean distance between expression vectors
Average weighted risk (AWR) of reference patient
Risk: blue-low; green-intermediate; red-high
35
30
25
20
15
10
5
1.0
1.2
1.4
1.6
1.8
2.0

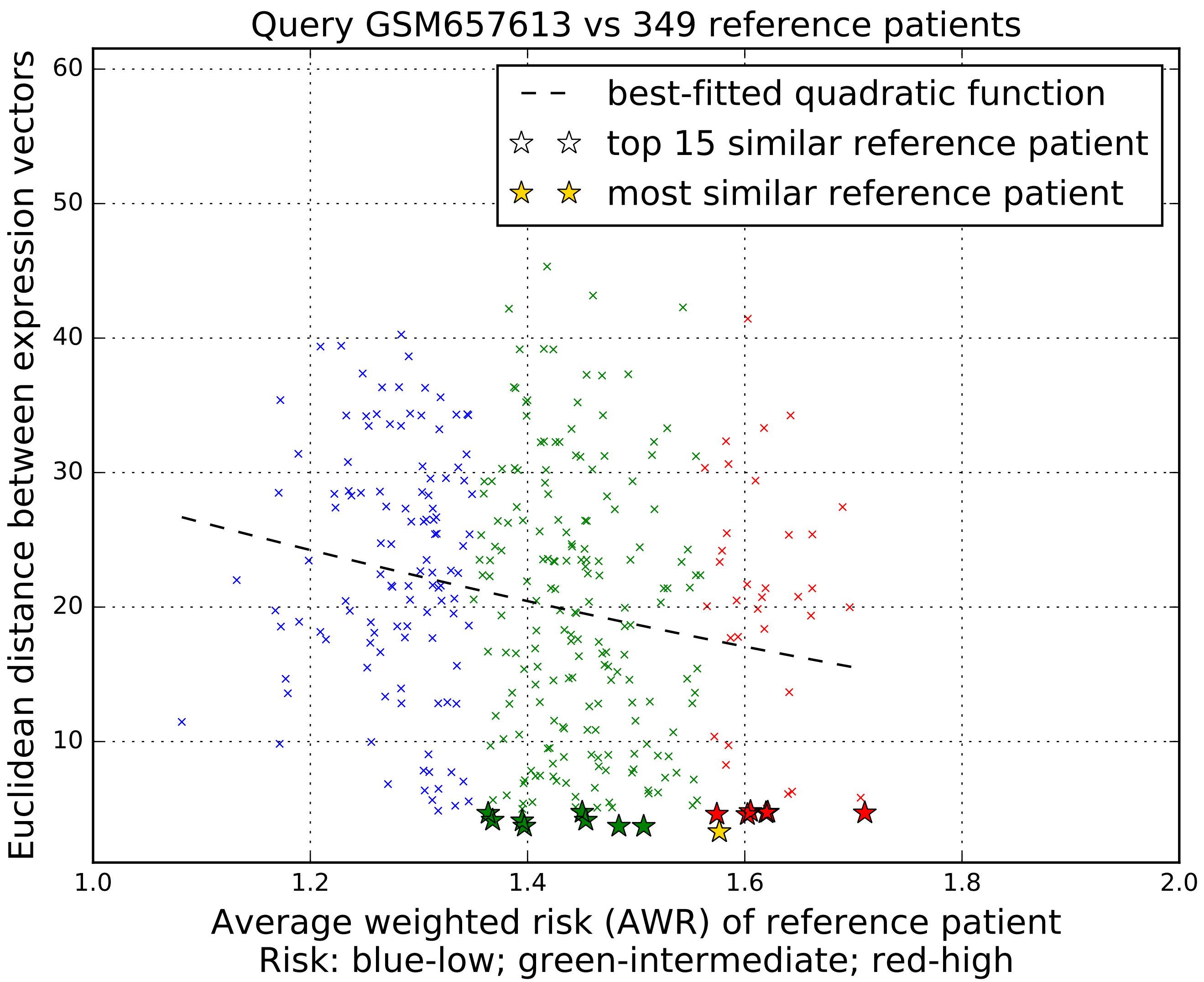

Query GSM657613 vs 349 reference patients
best-fitted quadratic function
top 15 similar reference patient
most similar reference patient
Euclidean distance between expression vectors
60
50
40
30
20
10
1.0
1.2
1.4
1.6
1.8
2.0
Average weighted risk (AWR) of reference patient
Risk: blue-low; green-intermediate; red-high

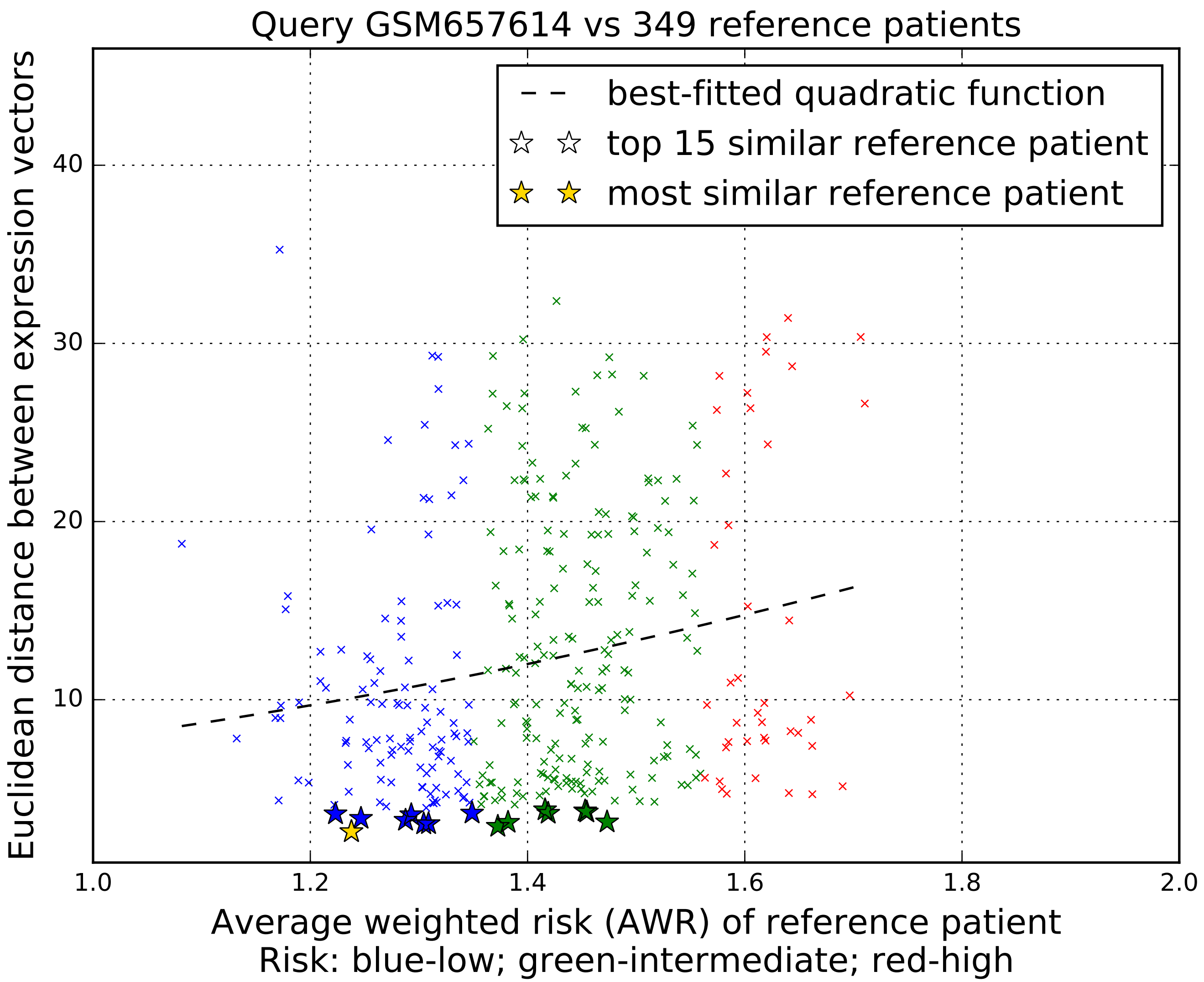
Query GSM657614 vs 349 reference patients
best-fitted quadratic function
top 15 similar reference patient
most similar reference patient
Euclidean distance between expression vectors
10
20
30
40
1.0
1.2
1.4
1.6
1.8
2.0
Average weighted risk (AWR) of reference patient
Risk: blue-low; green-intermediate; red-high

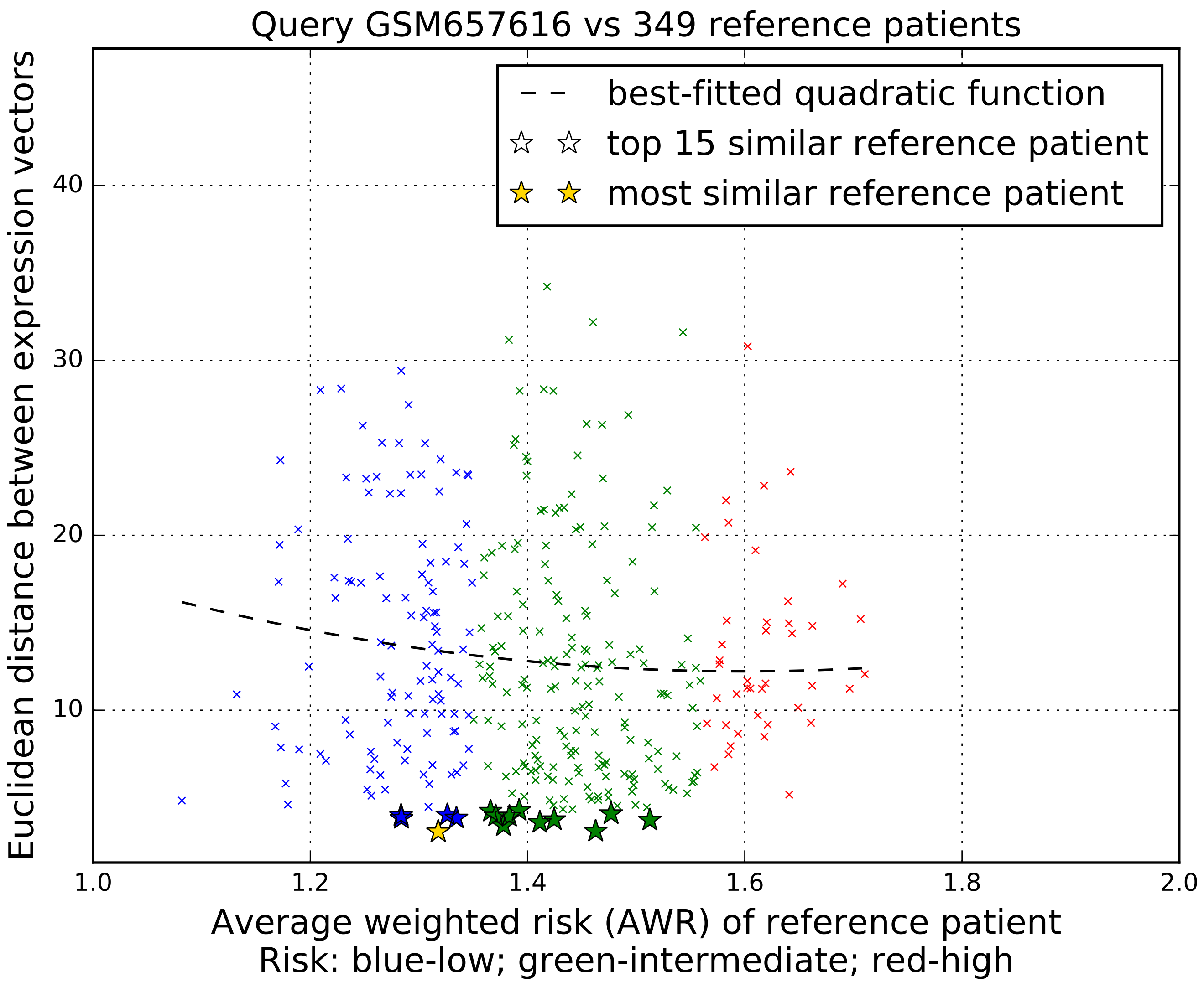

Query GSM657616 vs 349 reference patients
best-fitted quadratic function
top 15 similar reference patient
most similar reference patient
Euclidean distance between expression vectors
10
20
30
40
1.0
1.2
1.4
1.6
1.8
2.0
Average weighted risk (AWR) of reference patient
Risk: blue-low; green-intermediate; red-high

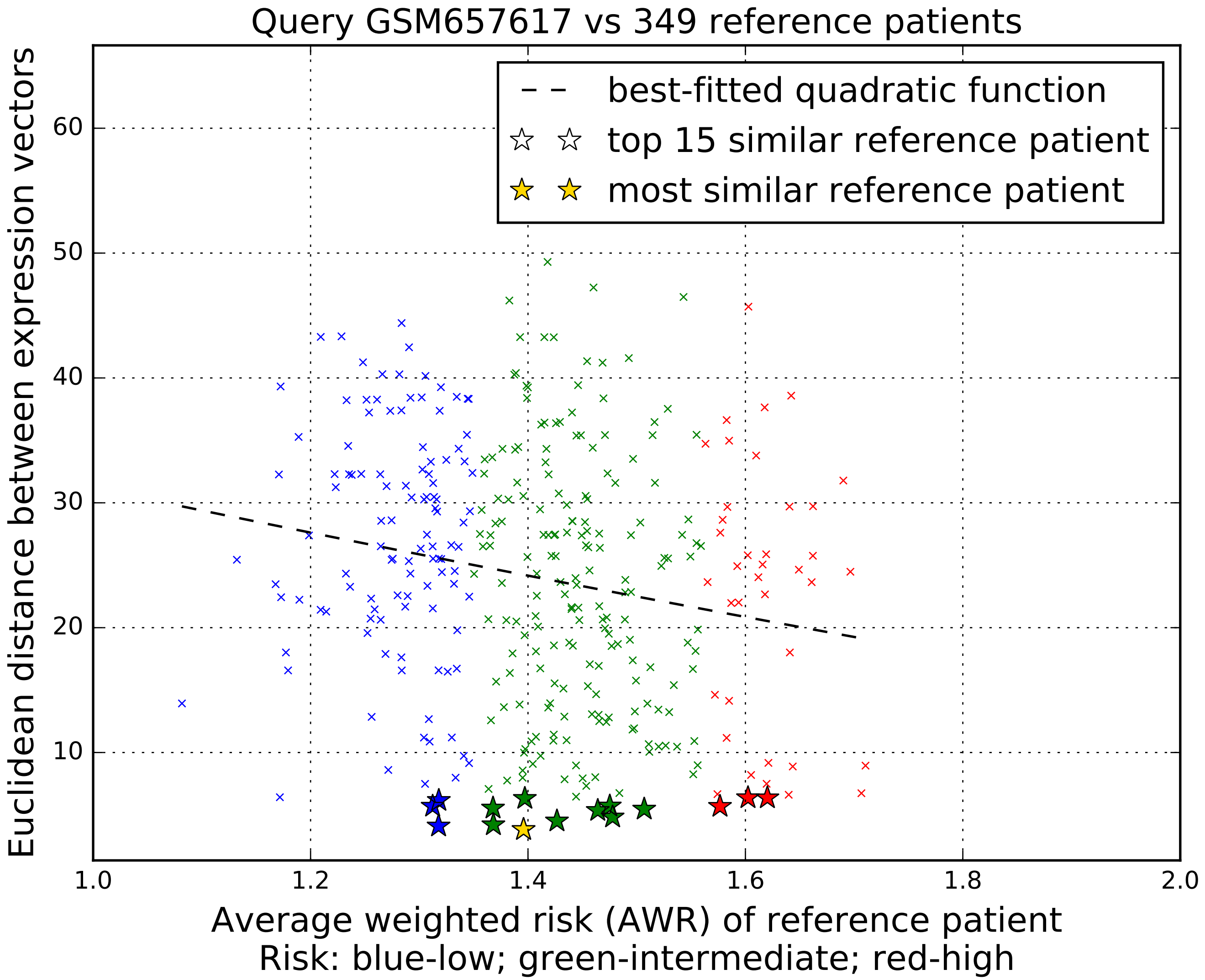
Query GSM657617 vs 349 reference patients
best-fitted quadratic function
top 15 similar reference patient
most similar reference patient
Euclidean distance between expression vectors
Average weighted risk (AWR) of reference patient
Risk: blue-low; green-intermediate; red-high
10
20
30
40
50
60
1.0
1.2
1.4
1.6
1.8
2.0

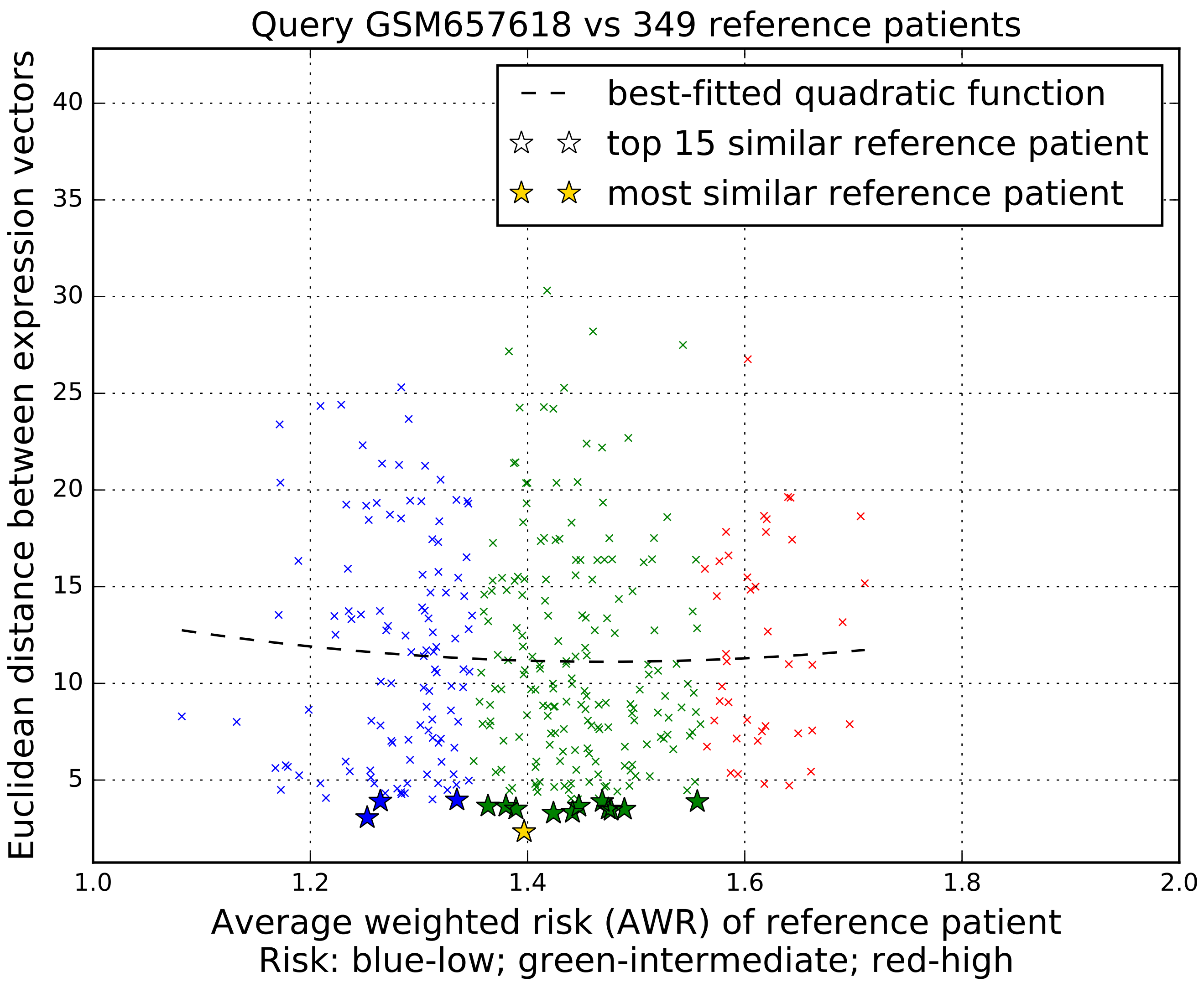
Query GSM657618 vs 349 reference patients
best-fitted quadratic function
top 15 similar reference patient
most similar reference patient
Euclidean distance between expression vectors
40
35
30
25
20
15
10
5
1.0
1.2
1.4
1.6
1.8
2.0
Average weighted risk (AWR) of reference patient
Risk: blue-low; green-intermediate; red-high

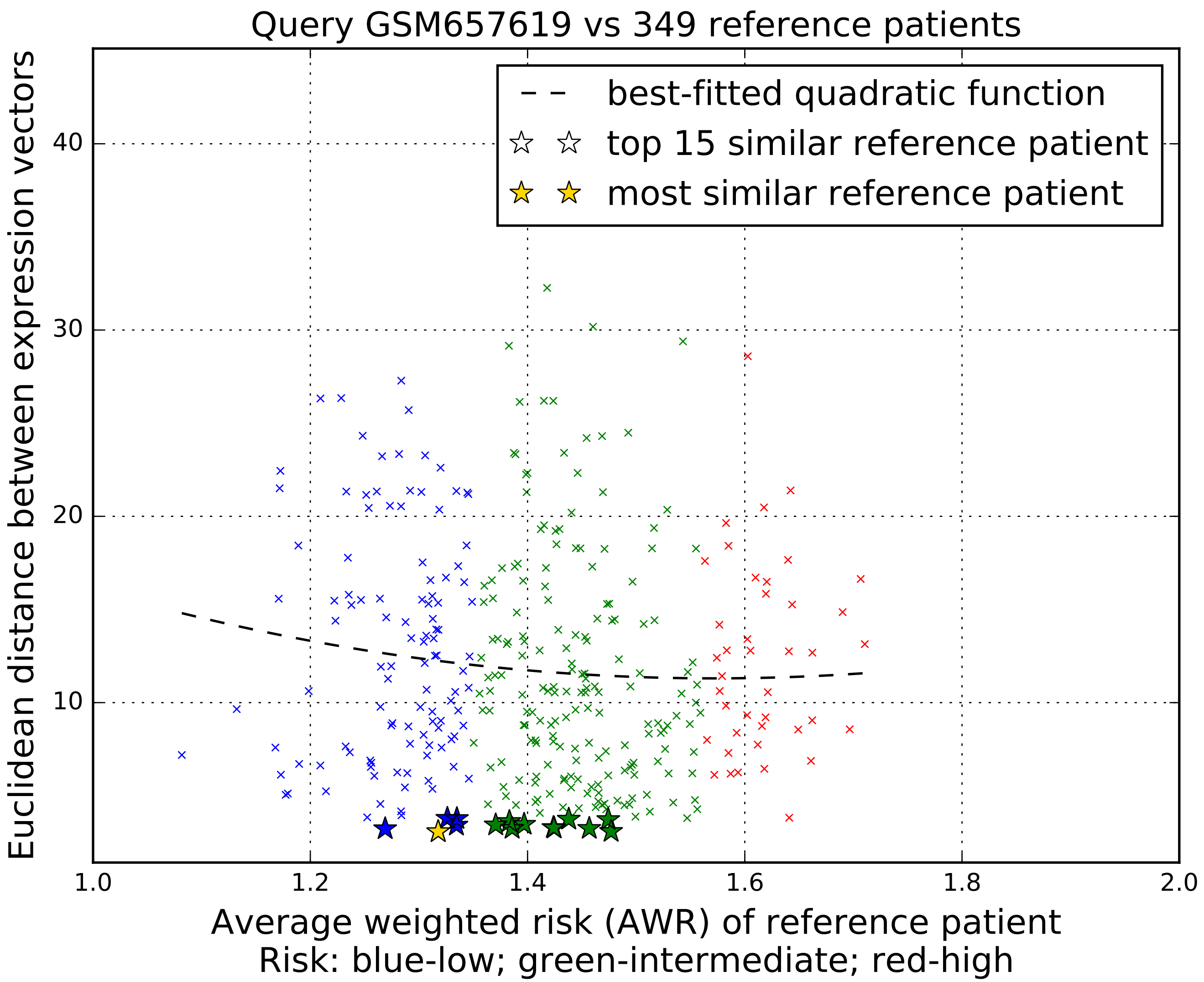
Query GSM657619 vs 349 reference patients
best-fitted quadratic function
top 15 similar reference patient
most similar reference patient
Euclidean distance between expression vectors
10
20
30
40
1.0
1.2
1.4
1.6
1.8
2.0
Average weighted risk (AWR) of reference patient
Risk: blue-low; green-intermediate; red-high

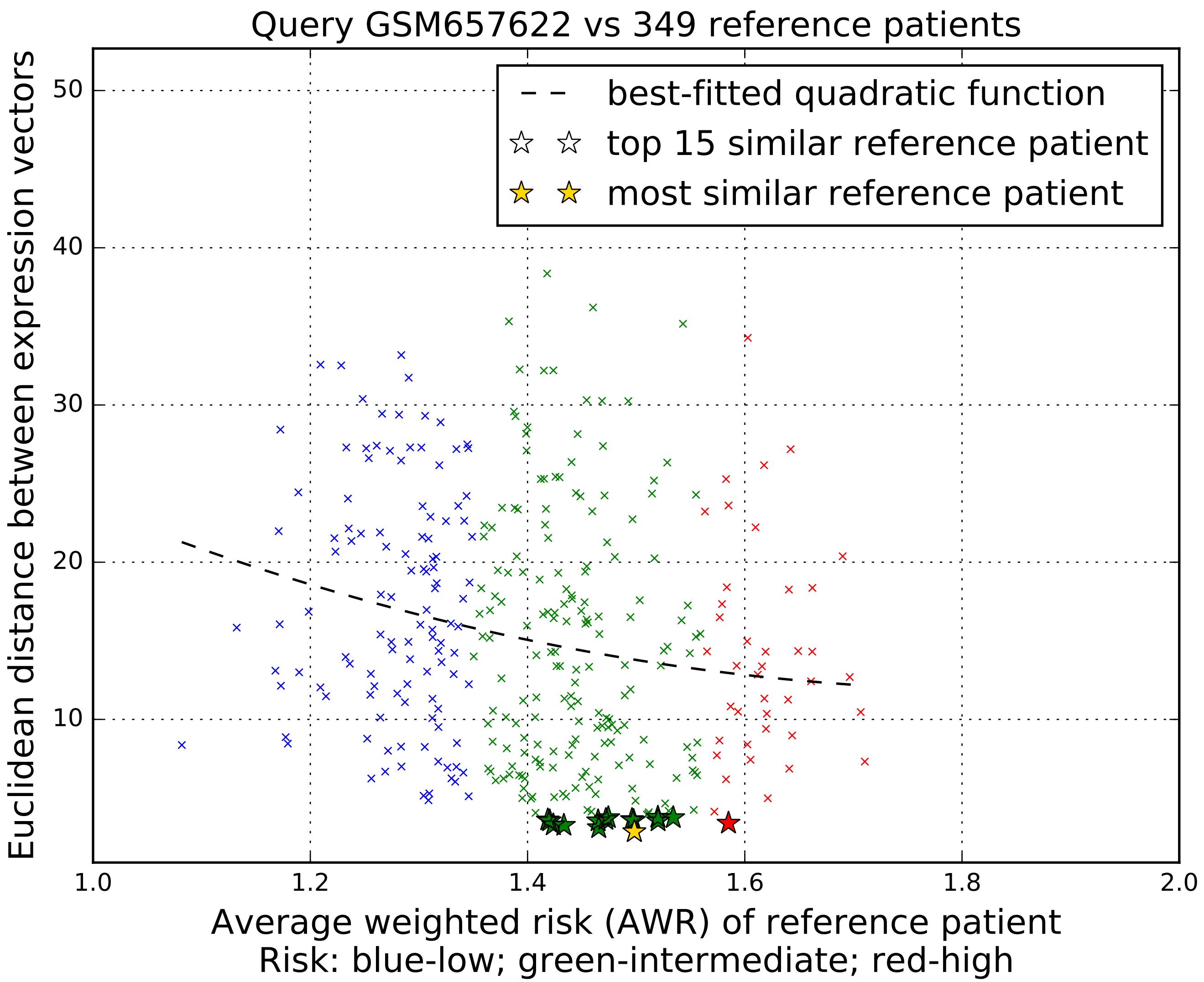
Query GSM657622 vs 349 reference patients
best-fitted quadratic function
top 15 similar reference patient
most similar reference patient
Euclidean distance between expression vectors
50
40
30
20
10
1.0
1.2
1.4
1.6
1.8
2.0
Average weighted risk (AWR) of reference patient
Risk: blue-low; green-intermediate; red-high

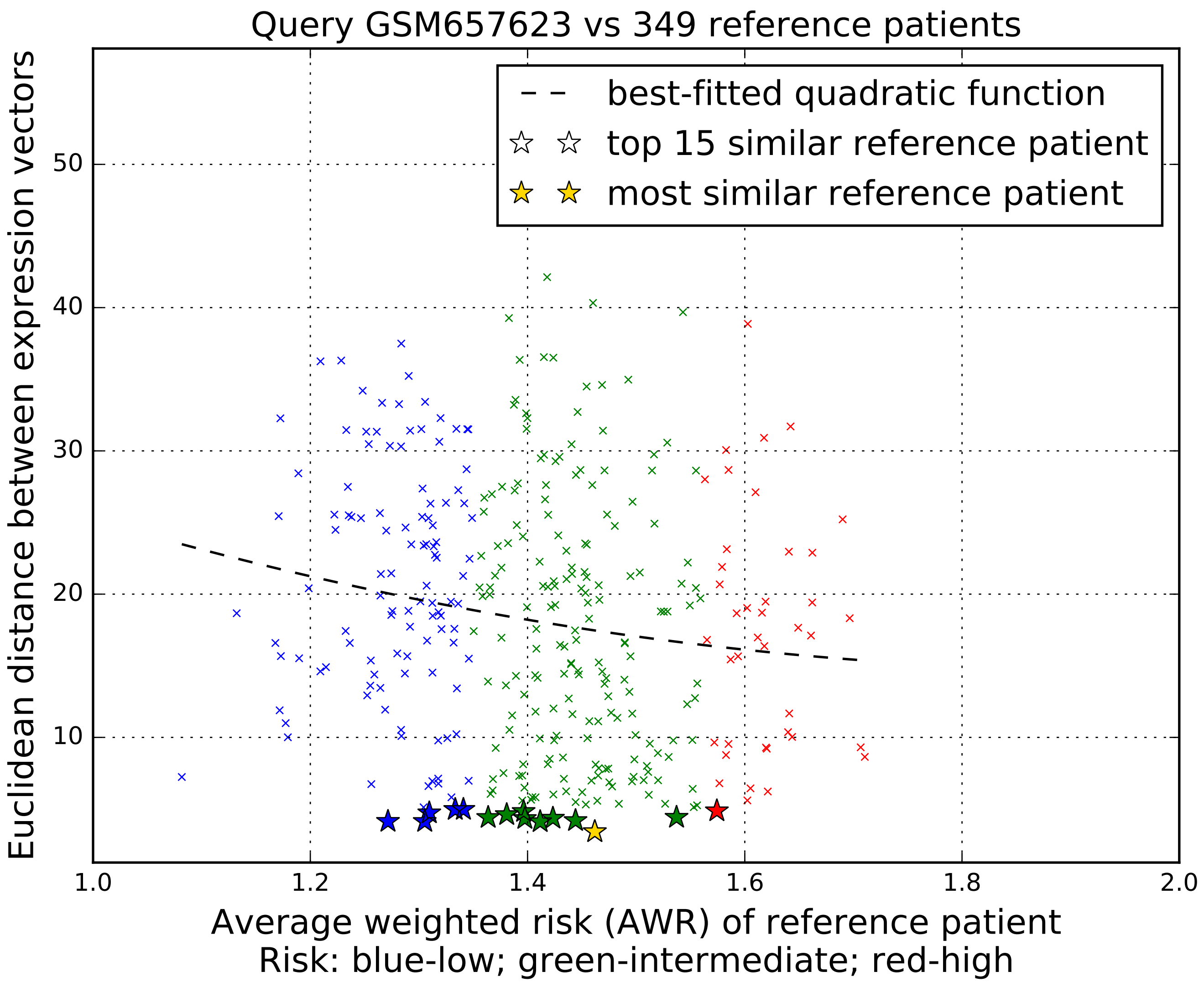
Query GSM657623 vs 349 reference patients
best-fitted quadratic function
top 15 similar reference patient
most similar reference patient
Euclidean distance between expression vectors
10
20
30
40
50
1.0
1.2
1.4
1.6
1.8
2.0
Average weighted risk (AWR) of reference patient
Risk: blue-low; green-intermediate; red-high

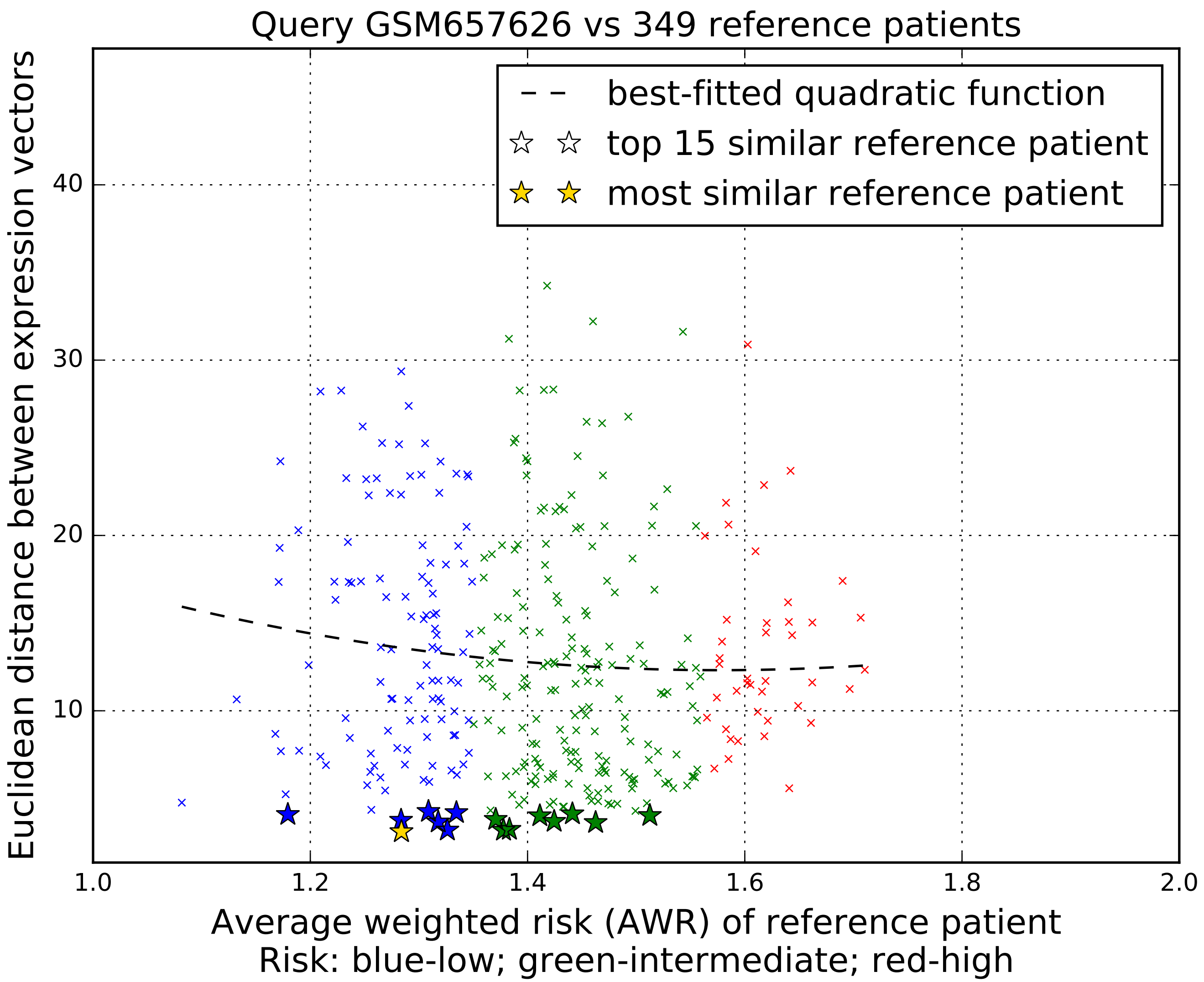
Query GSM657626 vs 349 reference patients
best-fitted quadratic function
top 15 similar reference patient
most similar reference patient
Euclidean distance between expression vectors
10
20
30
40
1.0
1.2
1.4
1.6
1.8
2.0
Average weighted risk (AWR) of reference patient
Risk: blue-low; green-intermediate; red-high

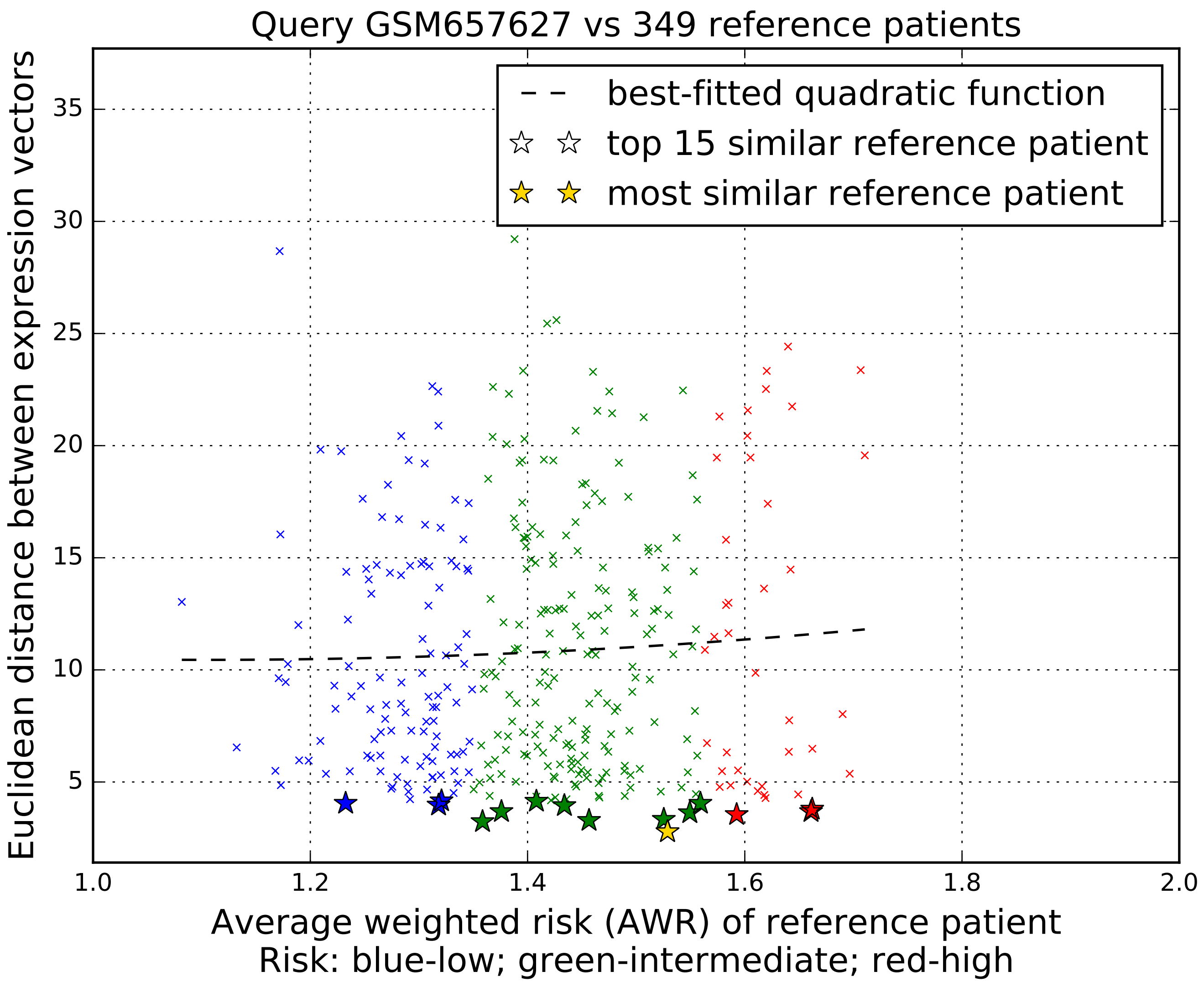
Query GSM657627 vs 349 reference patients
best-fitted quadratic function
top 15 similar reference patient
most similar reference patient
Euclidean distance between expression vectors
35
30
25
20
15
10
5
1.0
1.2
1.4
1.6
1.8
2.0
Average weighted risk (AWR) of reference patient
Risk: blue-low; green-intermediate; red-high

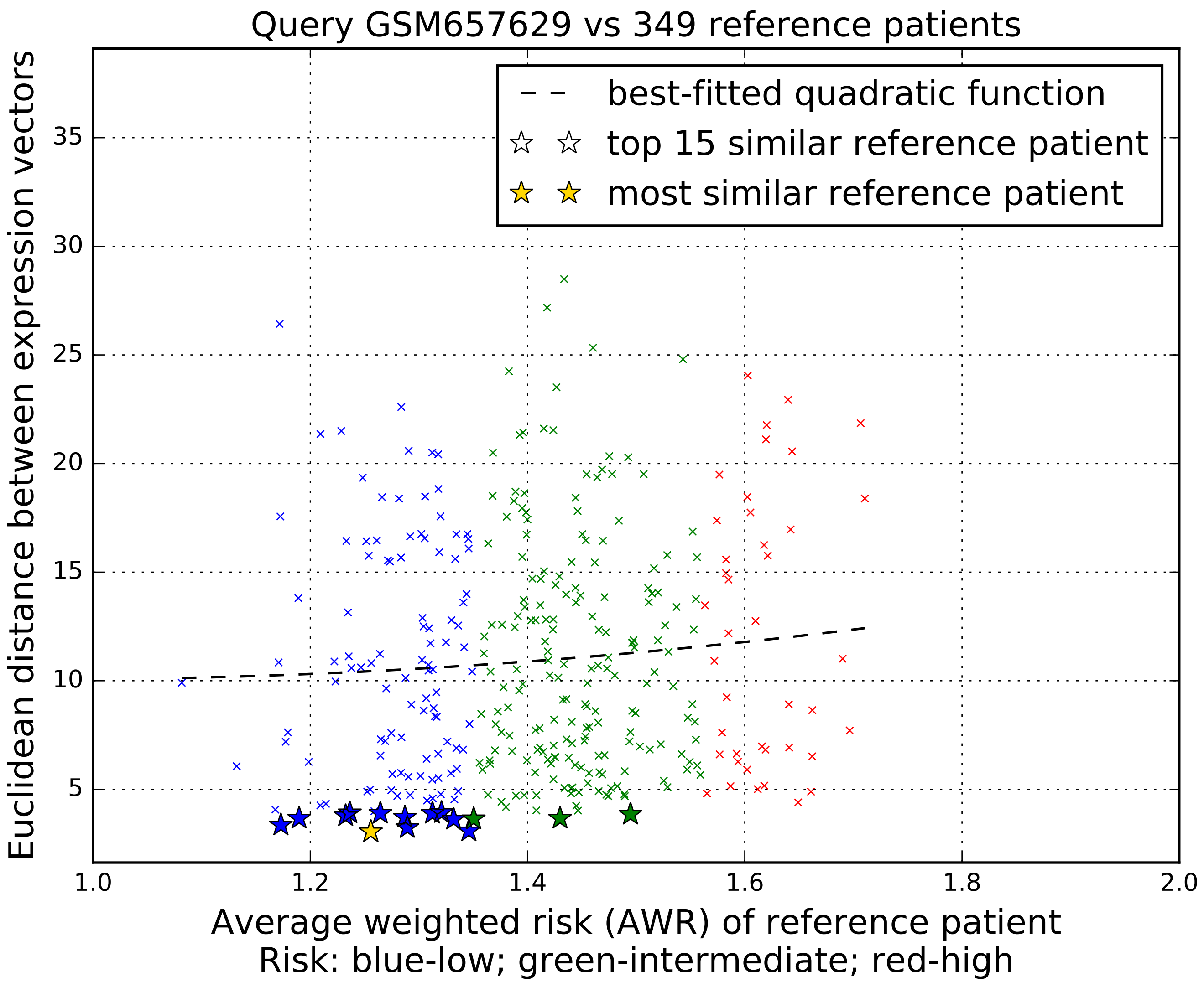
Query GSM657629 vs 349 reference patients
best-fitted quadratic function
top 15 similar reference patient
most similar reference patient
Euclidean distance between expression vectors
35
30
25
20
15
10
5
1.0
1.2
1.4
1.6
1.8
2.0
Average weighted risk (AWR) of reference patient
Risk: blue-low; green-intermediate; red-high

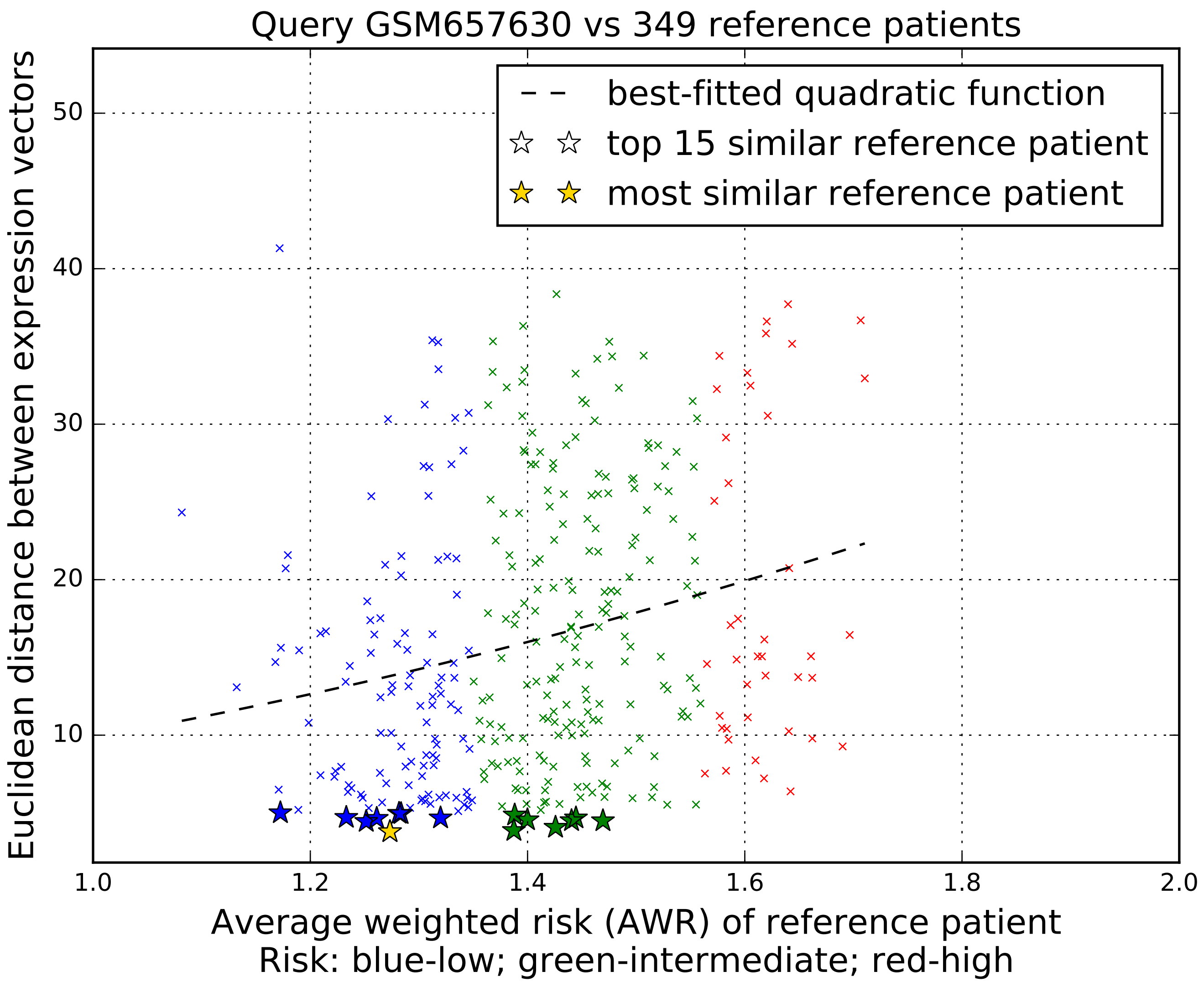

Query GSM657630 vs 349 reference patients
best-fitted quadratic function
top 15 similar reference patient
most similar reference patient
Euclidean distance between expression vectors
50
40
30
20
10
1.0
1.2
1.4
1.6
1.8
2.0
Average weighted risk (AWR) of reference patient
Risk: blue-low; green-intermediate; red-high

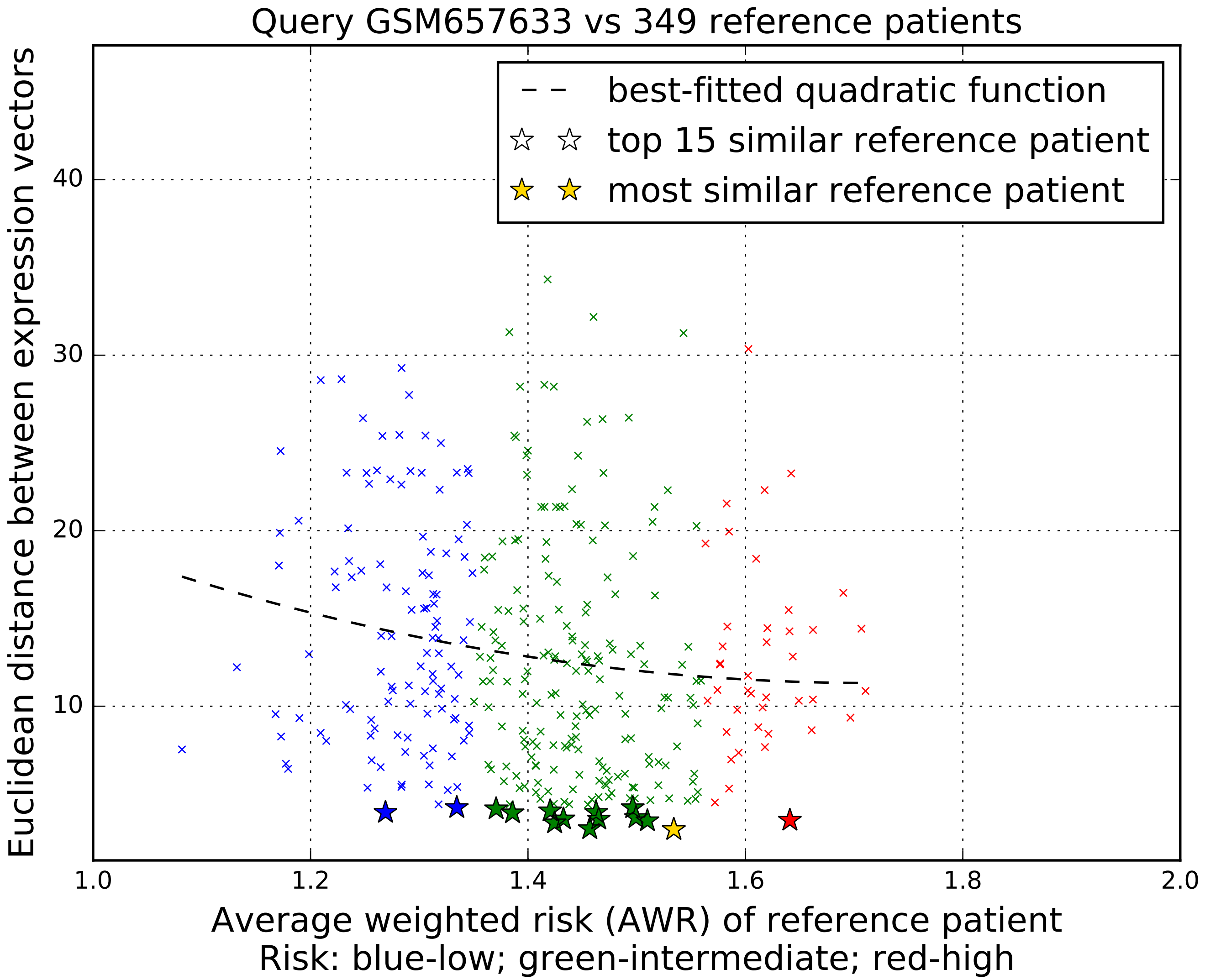

Query GSM657633 vs 349 reference patients
best-fitted quadratic function
top 15 similar reference patient
most similar reference patient
Euclidean distance between expression vectors
10
20
30
40
1.0
1.2
1.4
1.6
1.8
2.0
Average weighted risk (AWR) of reference patient
Risk: blue-low; green-intermediate; red-high

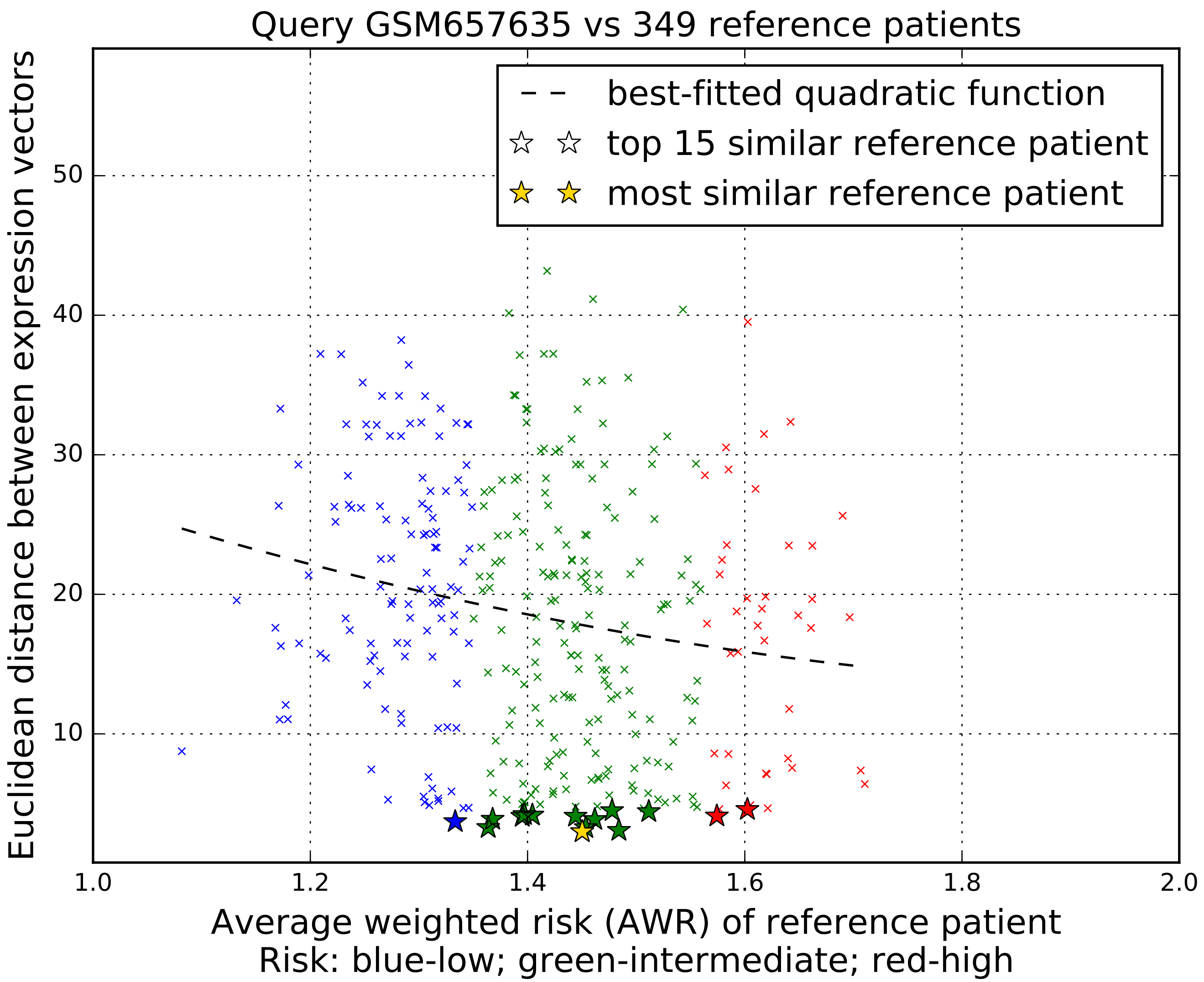

Query GSM657635 vs 349 reference patients
best-fitted quadratic function
top 15 similar reference patient
most similar reference patient
Euclidean distance between expression vectors
10
20
30
40
50
1.0
1.2
1.4
1.6
1.8
2.0
Average weighted risk (AWR) of reference patient
Risk: blue-low; green-intermediate; red-high

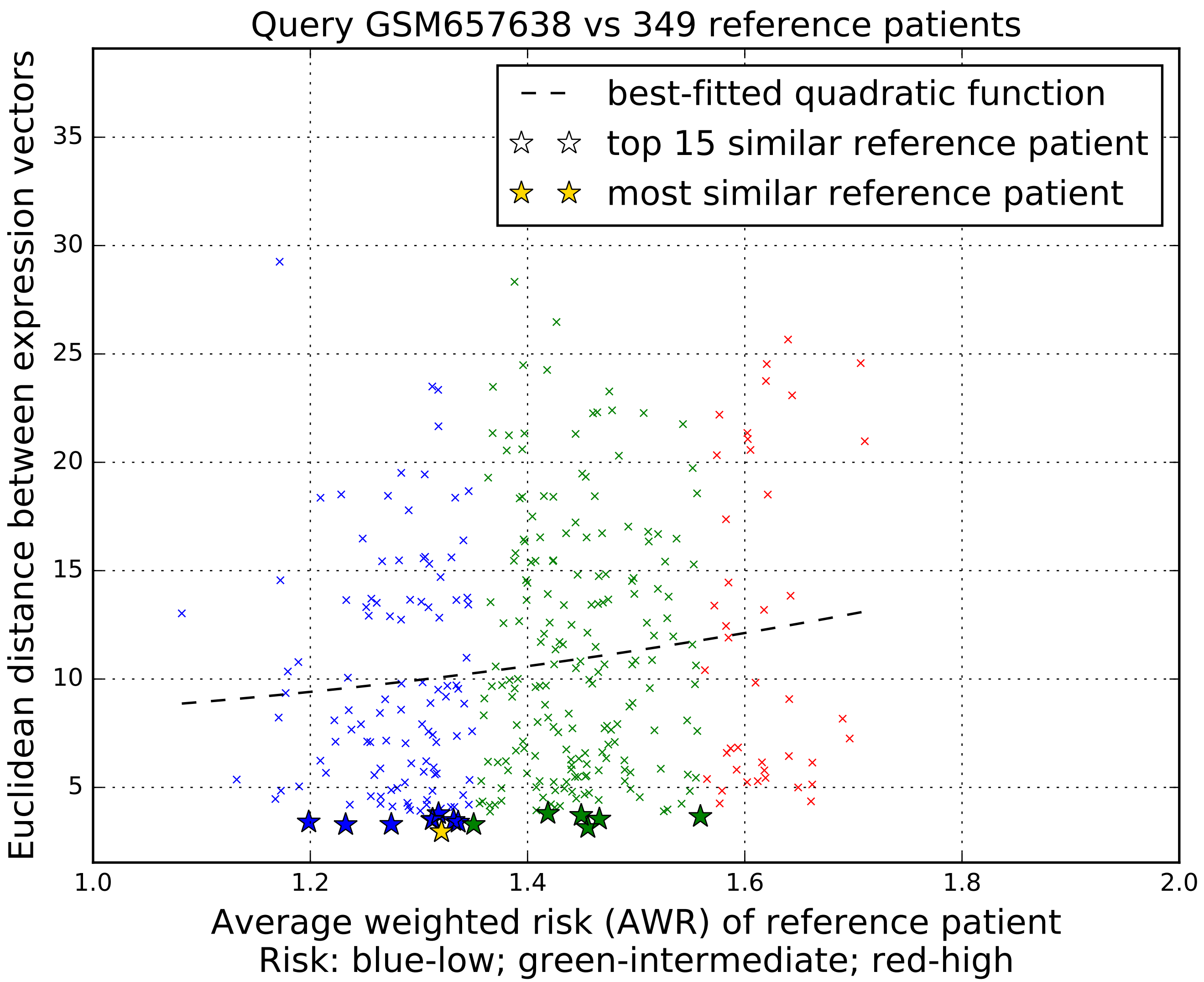

Query GSM657638 vs 349 reference patients
best-fitted quadratic function
top 15 similar reference patient
most similar reference patient
Euclidean distance between expression vectors
35
30
25
20
15
10
5
1.0
1.2
1.4
1.6
1.8
2.0
Average weighted risk (AWR) of reference patient
Risk: blue-low; green-intermediate; red-high

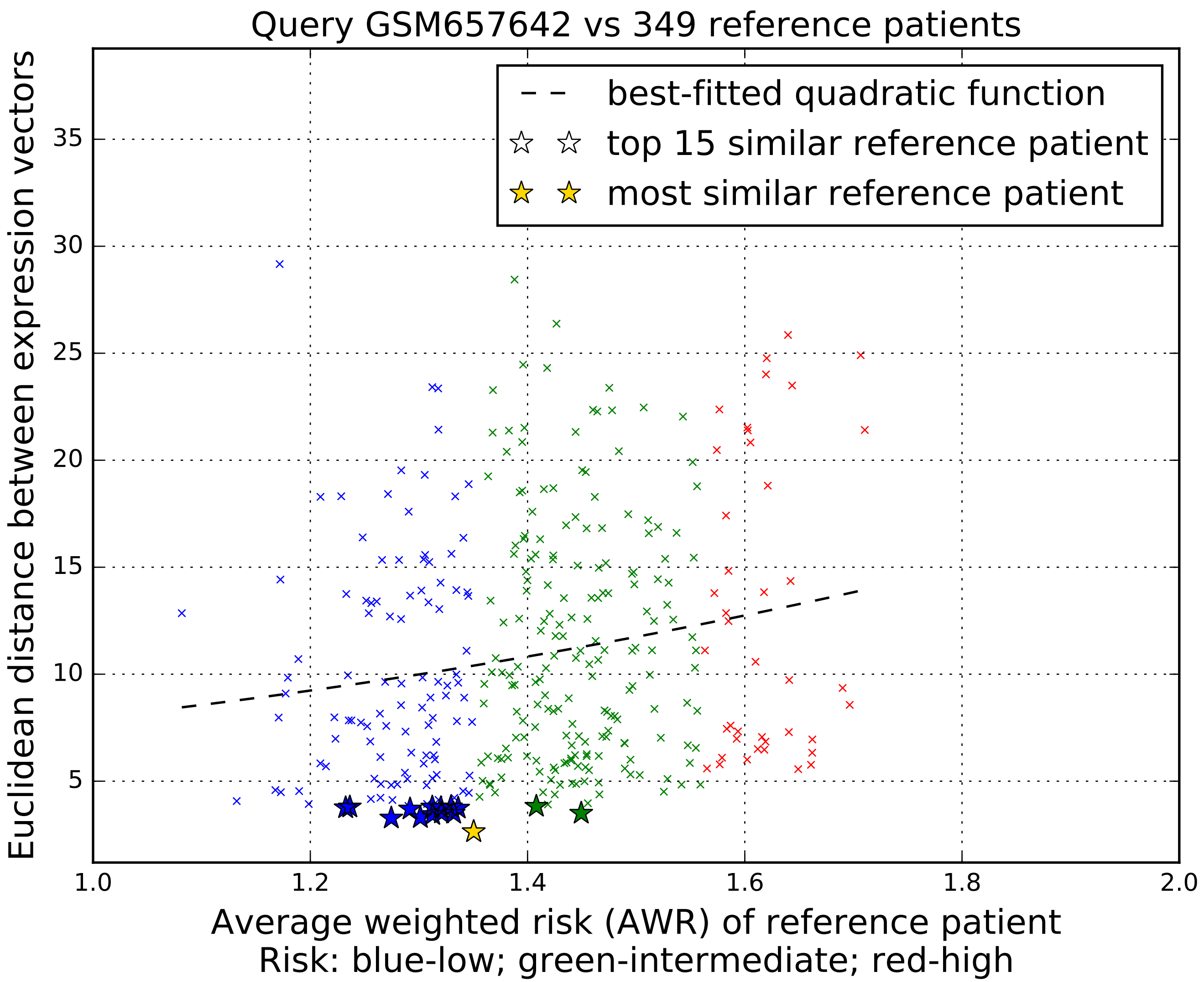

Query GSM657642 vs 349 reference patients
best-fitted quadratic function
top 15 similar reference patient
most similar reference patient
Euclidean distance between expression vectors
5
10
15
20
25
30
35
1.0
1.2
1.4
1.6
1.8
2.0
Average weighted risk (AWR) of reference patient
Risk: blue-low; green-intermediate; red-high

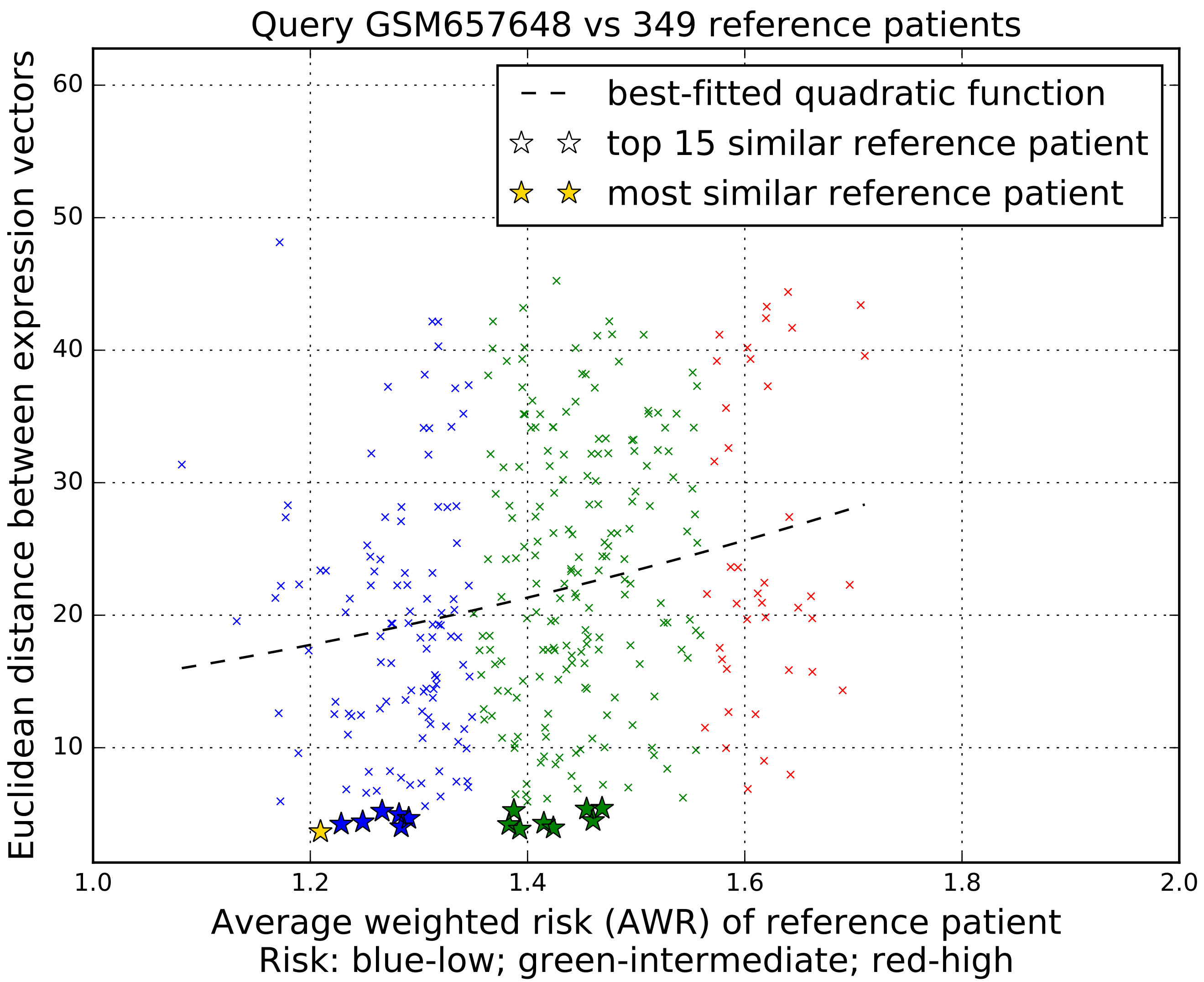
Query GSM657648 vs 349 reference patients
best-fitted quadratic function
top 15 similar reference patient
most similar reference patient
Euclidean distance between expression vectors
Average weighted risk (AWR) of reference patient
Risk: blue-low; green-intermediate; red-high
10
20
30
40
50
60
1.0
1.2
1.4
1.6
1.8
2.0

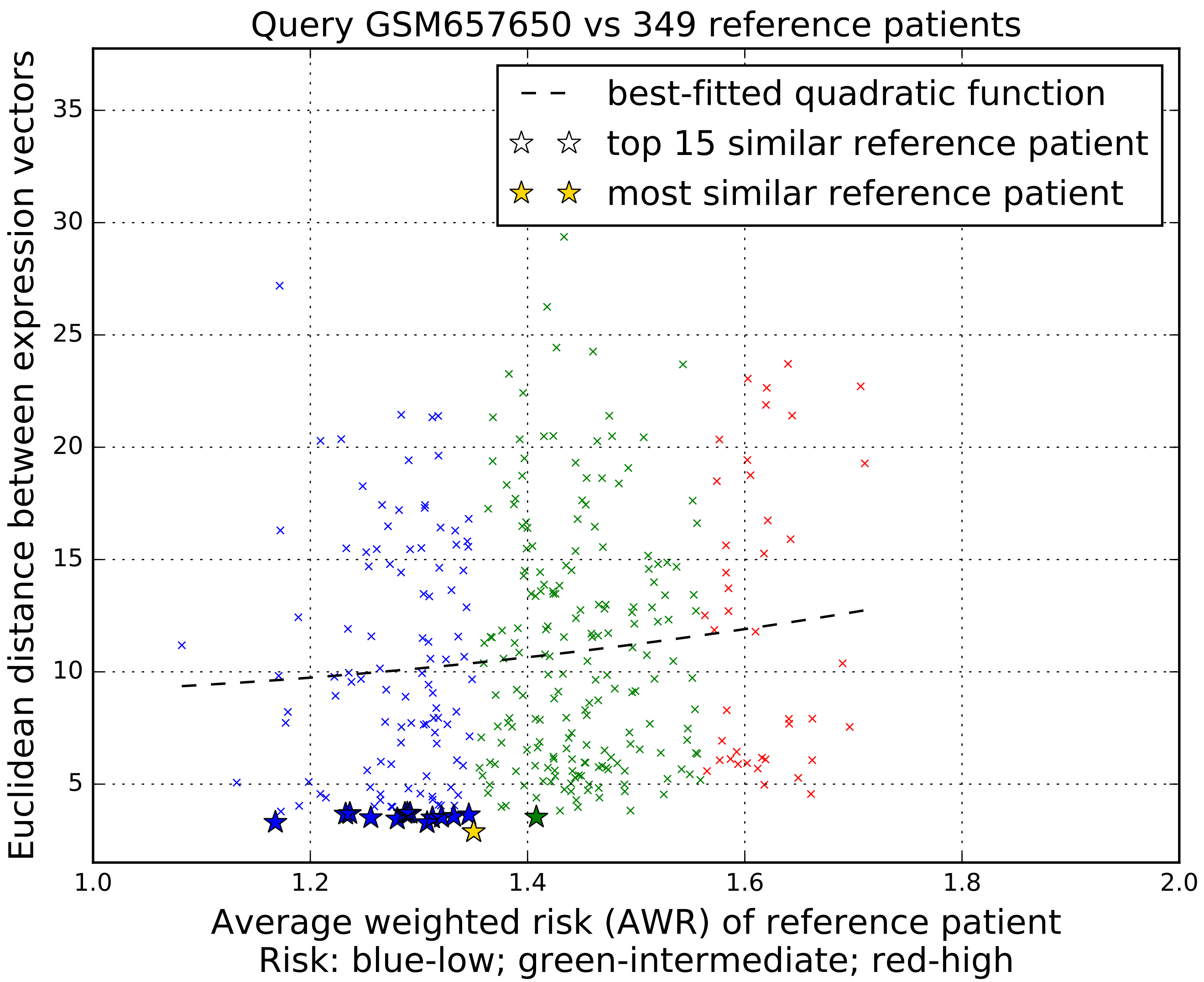

Query GSM657650 vs 349 reference patients
best-fitted quadratic function
top 15 similar reference patient
most similar reference patient
Euclidean distance between expression vectors
35
30
25
20
15
10
5
1.0
1.2
1.4
1.6
1.8
2.0
Average weighted risk (AWR) of reference patient
Risk: blue-low; green-intermediate; red-high

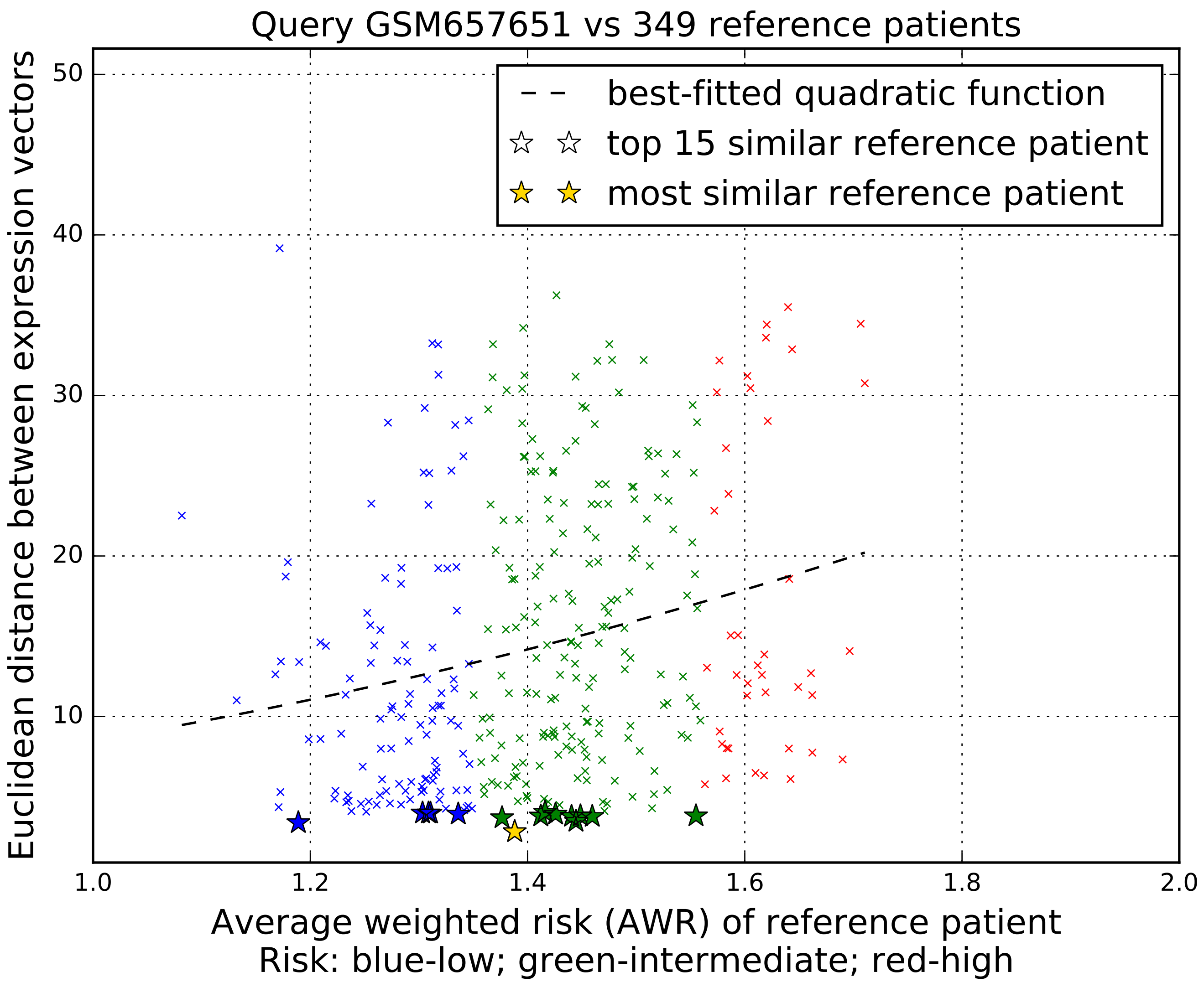

Query GSM657651 vs 349 reference patients
best-fitted quadratic function
top 15 similar reference patient
most similar reference patient
Euclidean distance between expression vectors
50
40
30
20
10
1.0
1.2
1.4
1.6
1.8
2.0
Average weighted risk (AWR) of reference patient
Risk: blue-low; green-intermediate; red-high

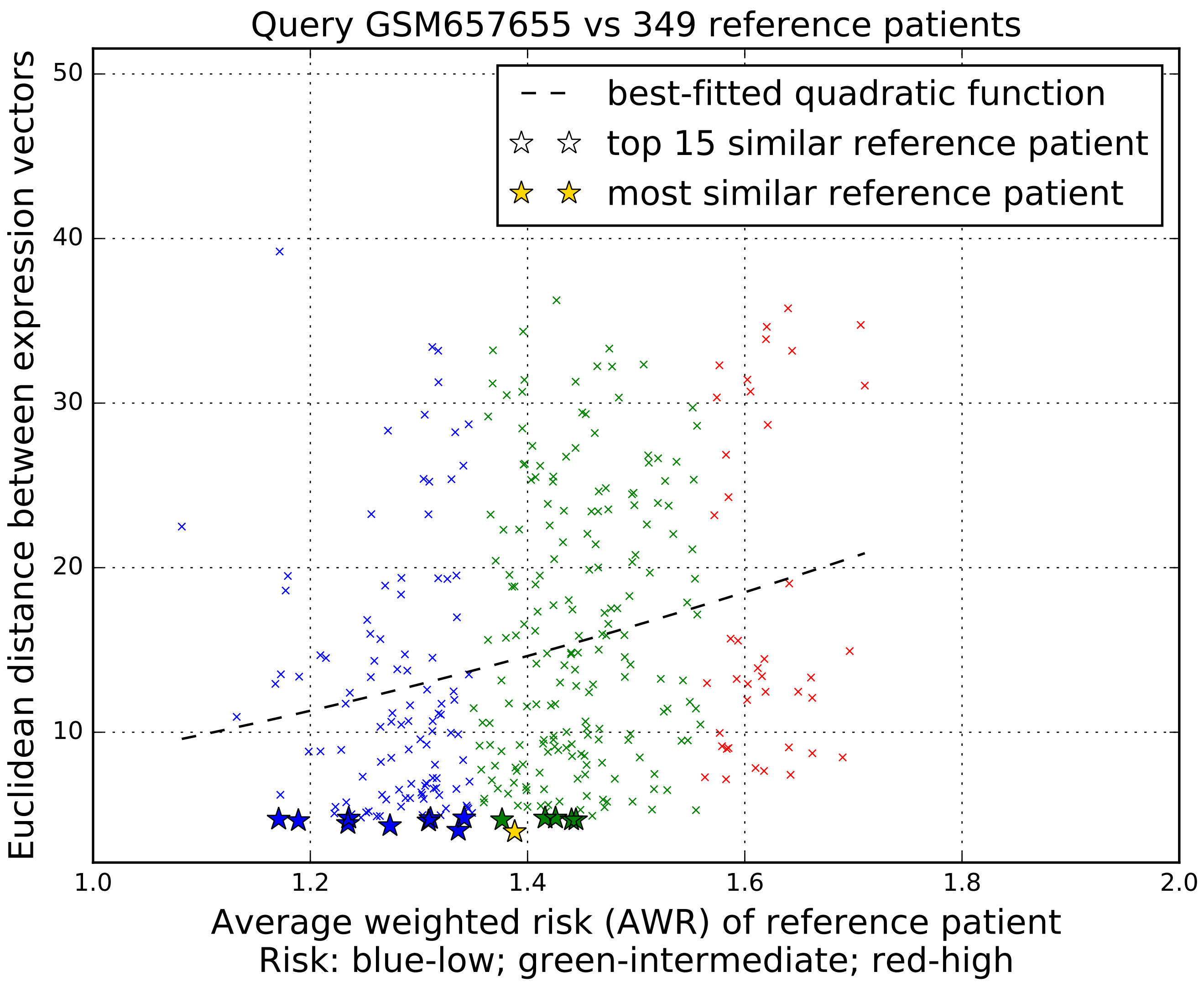

Query GSM657655 vs 349 reference patients
best-fitted quadratic function
top 15 similar reference patient
most similar reference patient
Euclidean distance between expression vectors
50
40
30
20
10
1.0
1.2
1.4
1.6
1.8
2.0
Average weighted risk (AWR) of reference patient
Risk: blue-low; green-intermediate; red-high

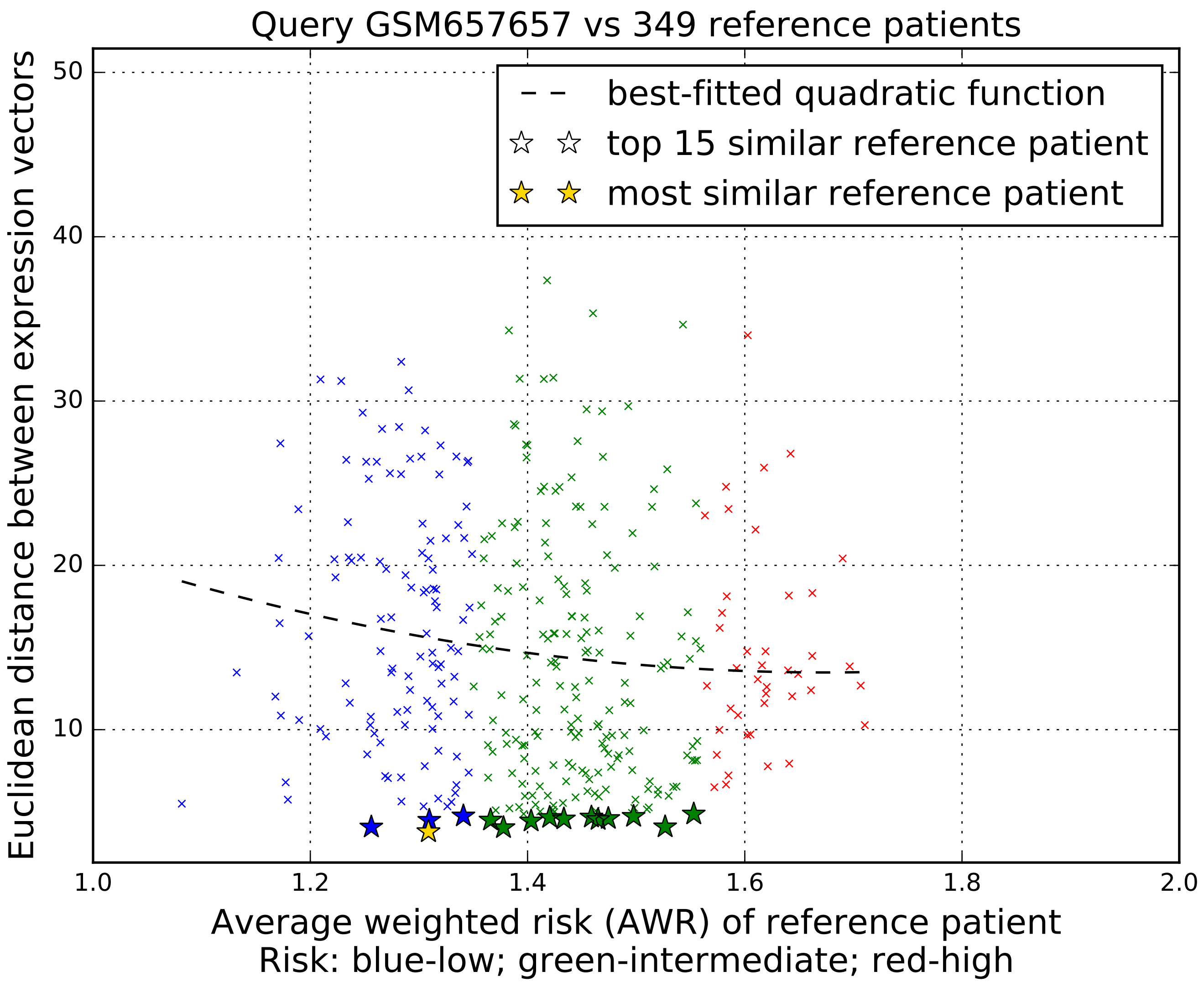

Query GSM657657 vs 349 reference patients
best-fitted quadratic function
top 15 similar reference patient
most similar reference patient
Euclidean distance between expression vectors
Average weighted risk (AWR) of reference patient
Risk: blue-low; green-intermediate; red-high
50
40
30
20
10
1.0
1.2
1.4
1.6
1.8
2.0

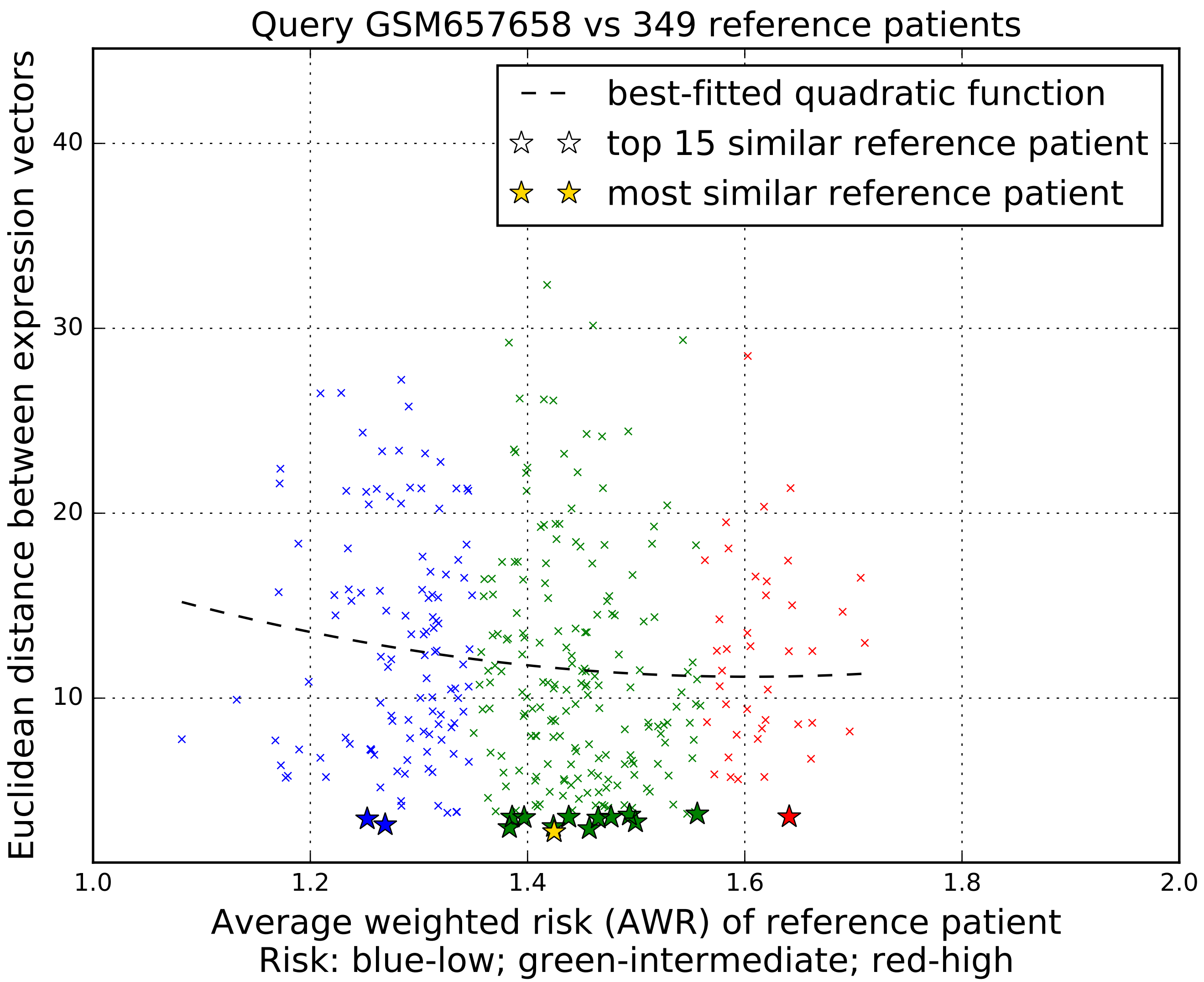
Query GSM657658 vs 349 reference patients
best-fitted quadratic function
top 15 similar reference patient
most similar reference patient
Euclidean distance between expression vectors
10
20
30
40
1.0
1.2
1.4
1.6
1.8
2.0
Average weighted risk (AWR) of reference patient
Risk: blue-low; green-intermediate; red-high

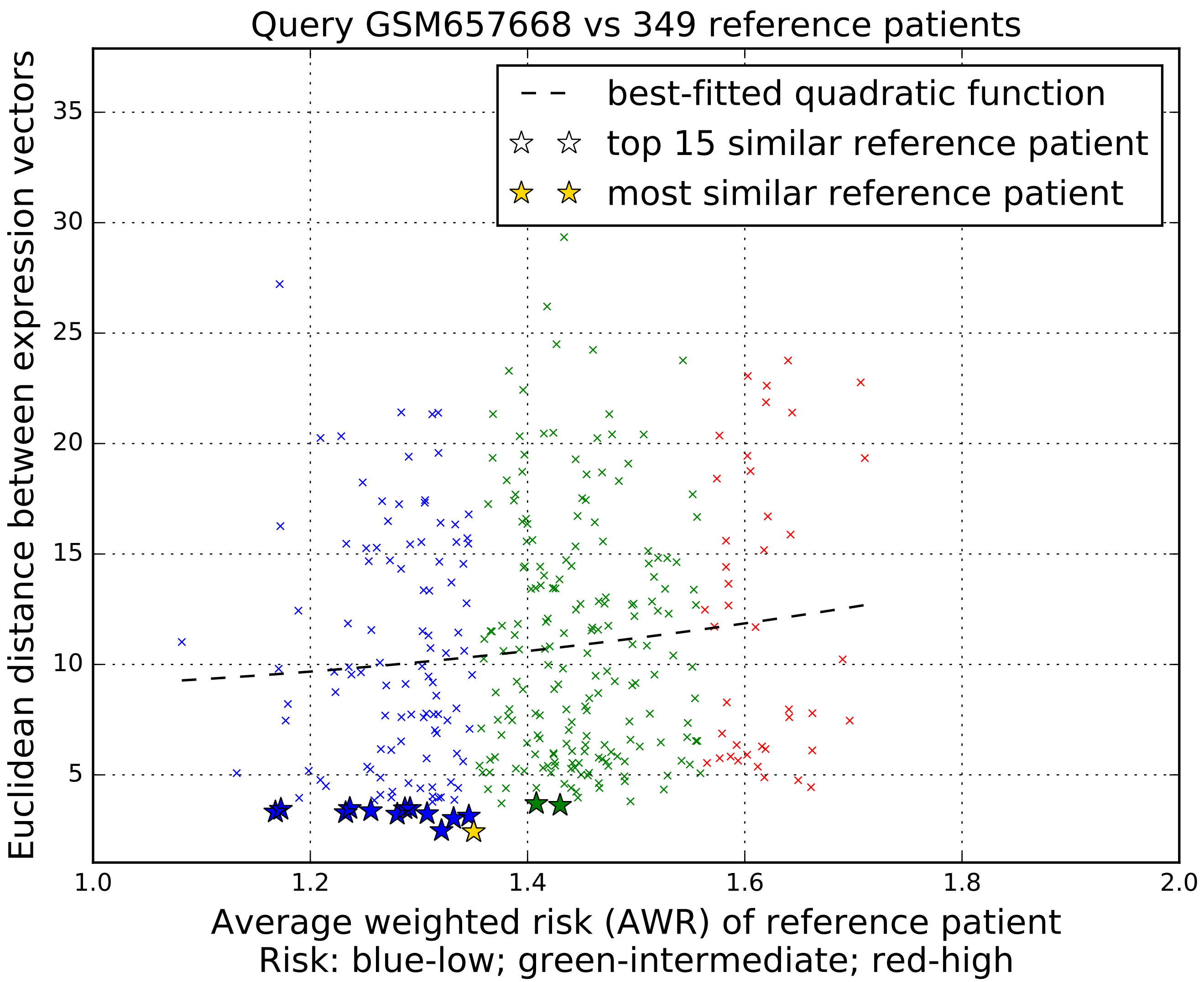

Query GSM657668 vs 349 reference patients
best-fitted quadratic function
top 15 similar reference patient
most similar reference patient
Euclidean distance between expression vectors
35
30
25
20
15
10
5
1.0
1.2
1.4
1.6
1.8
2.0
Average weighted risk (AWR) of reference patient
Risk: blue-low; green-intermediate; red-high

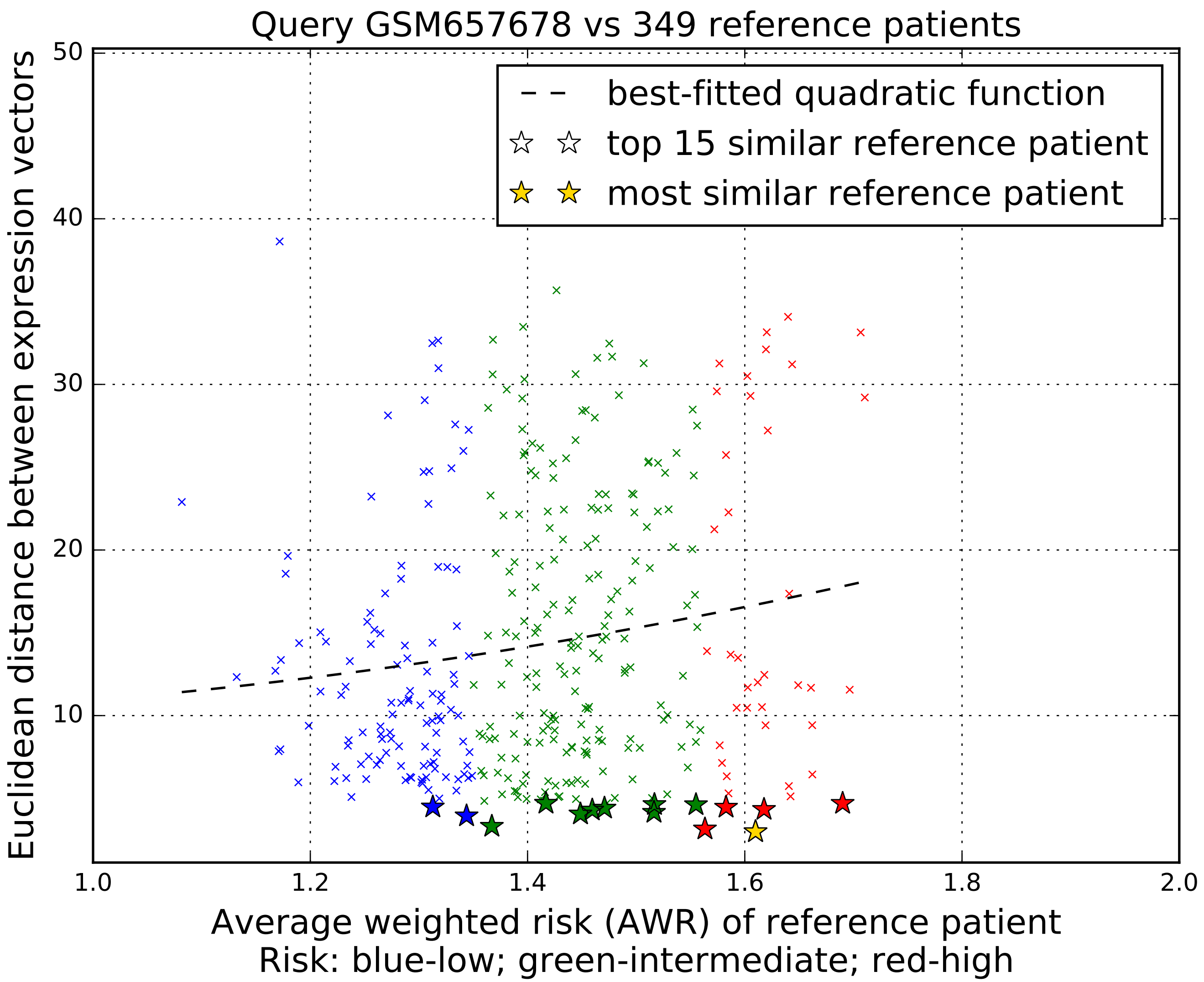

Query GSM657678 vs 349 reference patients
best-fitted quadratic function
top 15 similar reference patient
most similar reference patient
Euclidean distance between expression vectors
Average weighted risk (AWR) of reference patient
Risk: blue-low; green-intermediate; red-high
10
20
30
40
50
1.0
1.2
1.4
1.6
1.8
2.0

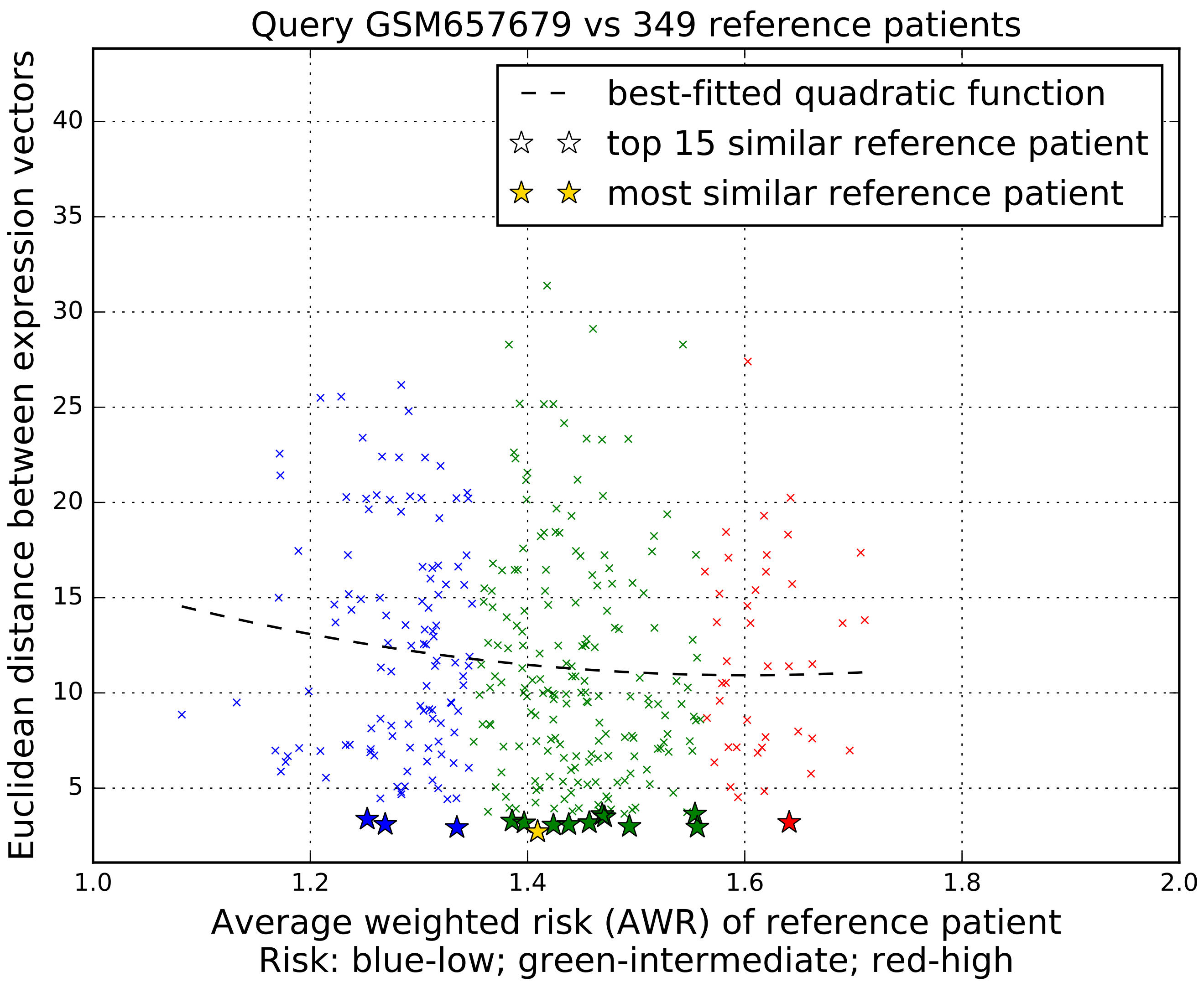

Query GSM657679 vs 349 reference patients
best-fitted quadratic function
top 15 similar reference patient
most similar reference patient
Euclidean distance between expression vectors
40
35
30
25
20
15
10
5
1.0
1.2
1.4
1.6
1.8
2.0
Average weighted risk (AWR) of reference patient
Risk: blue-low; green-intermediate; red-high

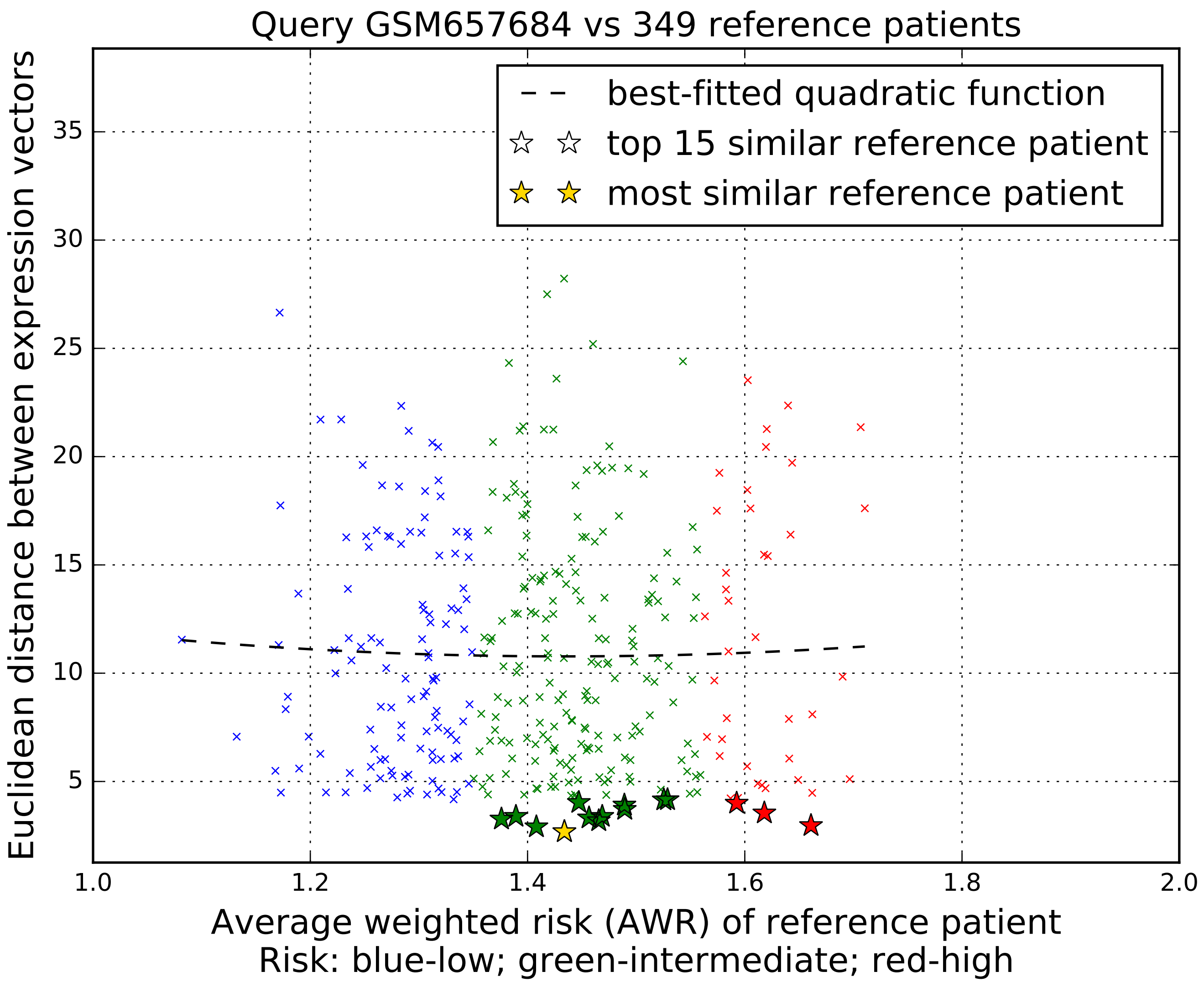

Query GSM657684 vs 349 reference patients
best-fitted quadratic function
top 15 similar reference patient
most similar reference patient
Euclidean distance between expression vectors
35
30
25
20
15
10
5
1.0
1.2
1.4
1.6
1.8
2.0
Average weighted risk (AWR) of reference patient
Risk: blue-low; green-intermediate; red-high

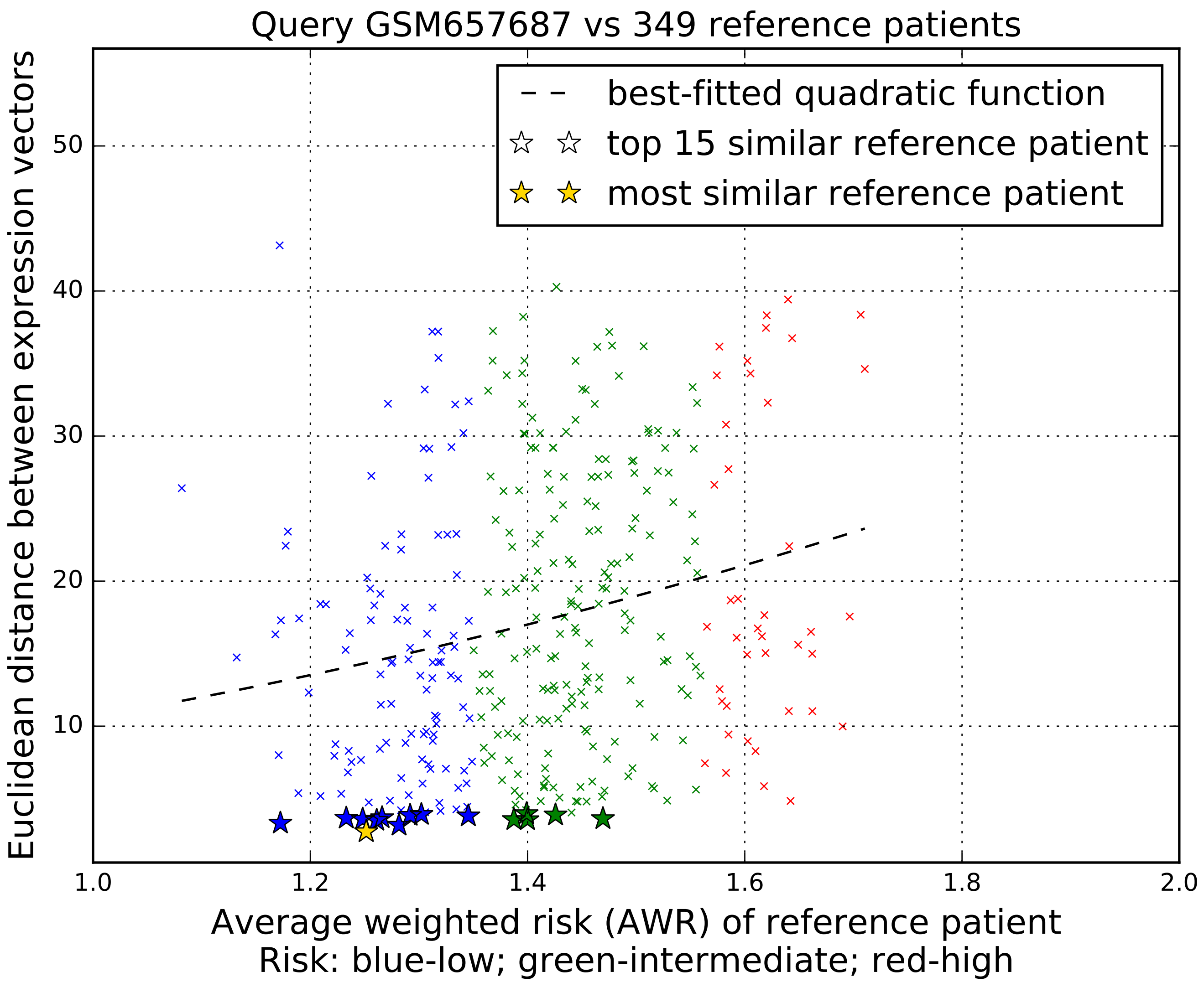

Query GSM657687 vs 349 reference patients
best-fitted quadratic function
top 15 similar reference patient
most similar reference patient
Euclidean distance between expression vectors
10
20
30
40
50
1.0
1.2
1.4
1.6
1.8
2.0
Average weighted risk (AWR) of reference patient
Risk: blue-low; green-intermediate; red-high

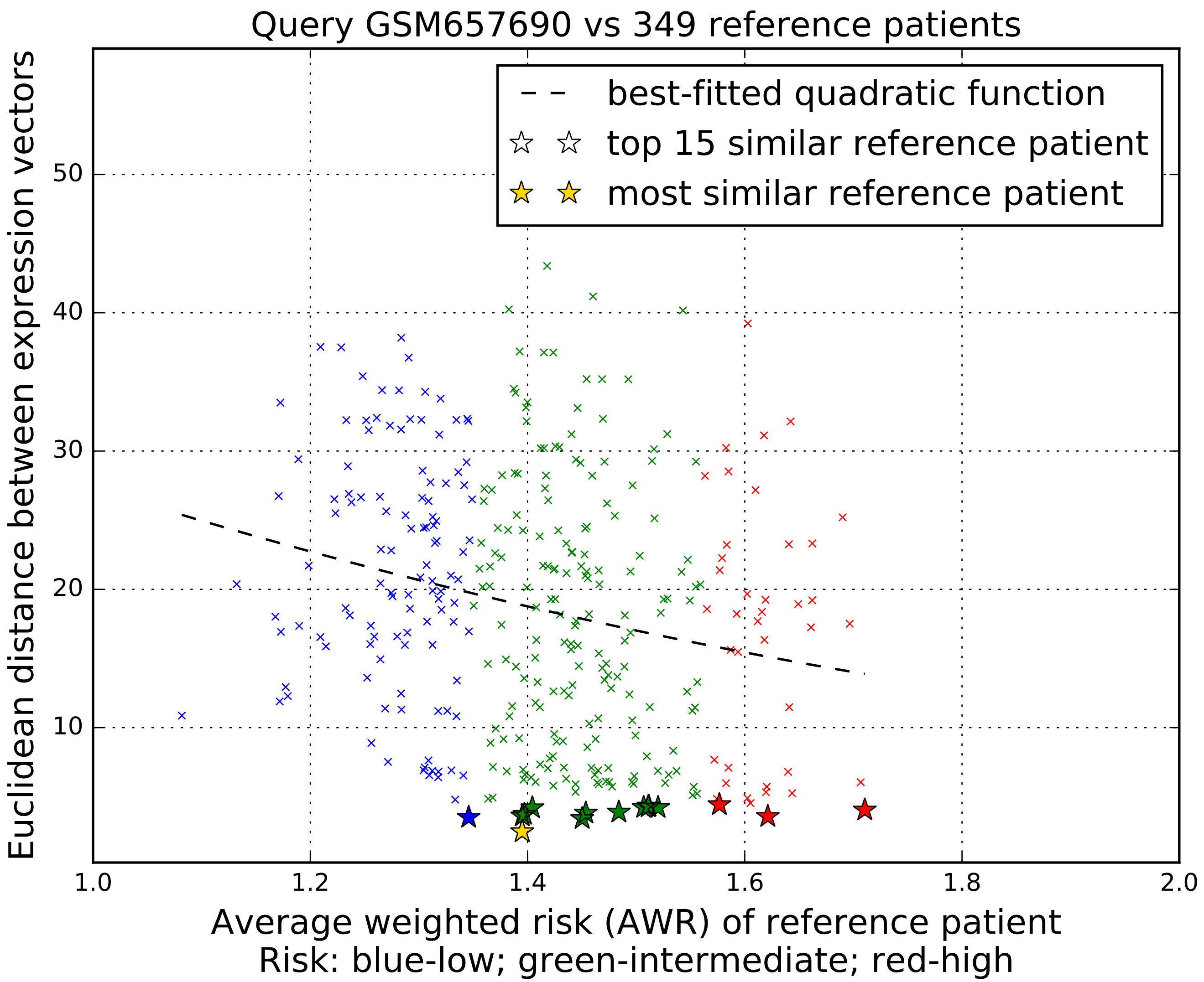

Query GSM657690 vs 349 reference patients
best-fitted quadratic function
top 15 similar reference patient
most similar reference patient
Euclidean distance between expression vectors
50
40
30
20
10
1.0
1.2
1.4
1.6
1.8
2.0
Average weighted risk (AWR) of reference patient
Risk: blue-low; green-intermediate; red-high

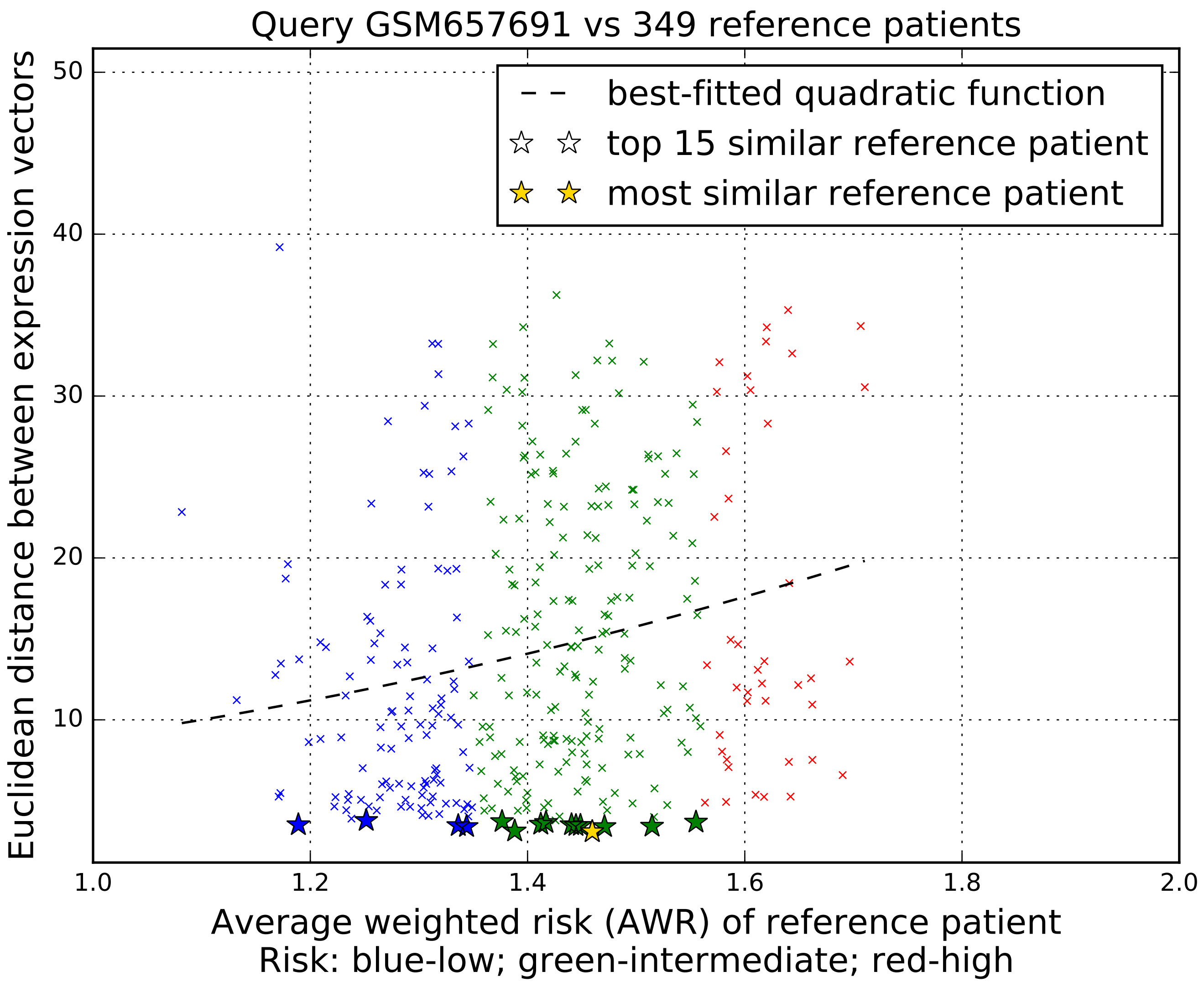
Query GSM657691 vs 349 reference patients
best-fitted quadratic function
top 15 similar reference patient
most similar reference patient
Euclidean distance between expression vectors
50
40
30
20
10
1.0
1.2
1.4
1.6
1.8
2.0
Average weighted risk (AWR) of reference patient
Risk: blue-low; green-intermediate; red-high

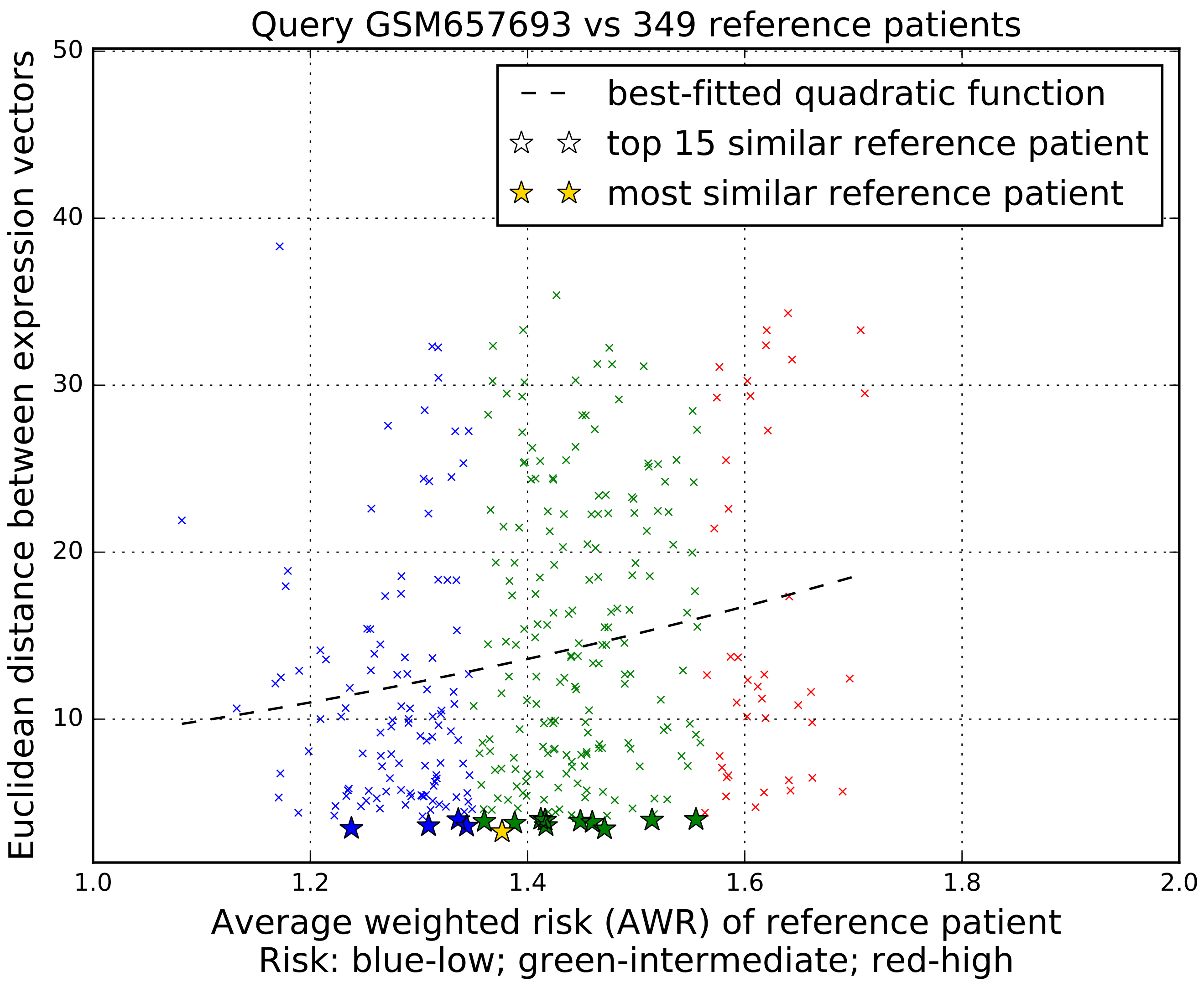
Query GSM657693 vs 349 reference patients
best-fitted quadratic function
top 15 similar reference patient
most similar reference patient
Euclidean distance between expression vectors
50
40
30
20
10
1.0
1.2
1.4
1.6
1.8
2.0
Average weighted risk (AWR) of reference patient
Risk: blue-low; green-intermediate; red-high

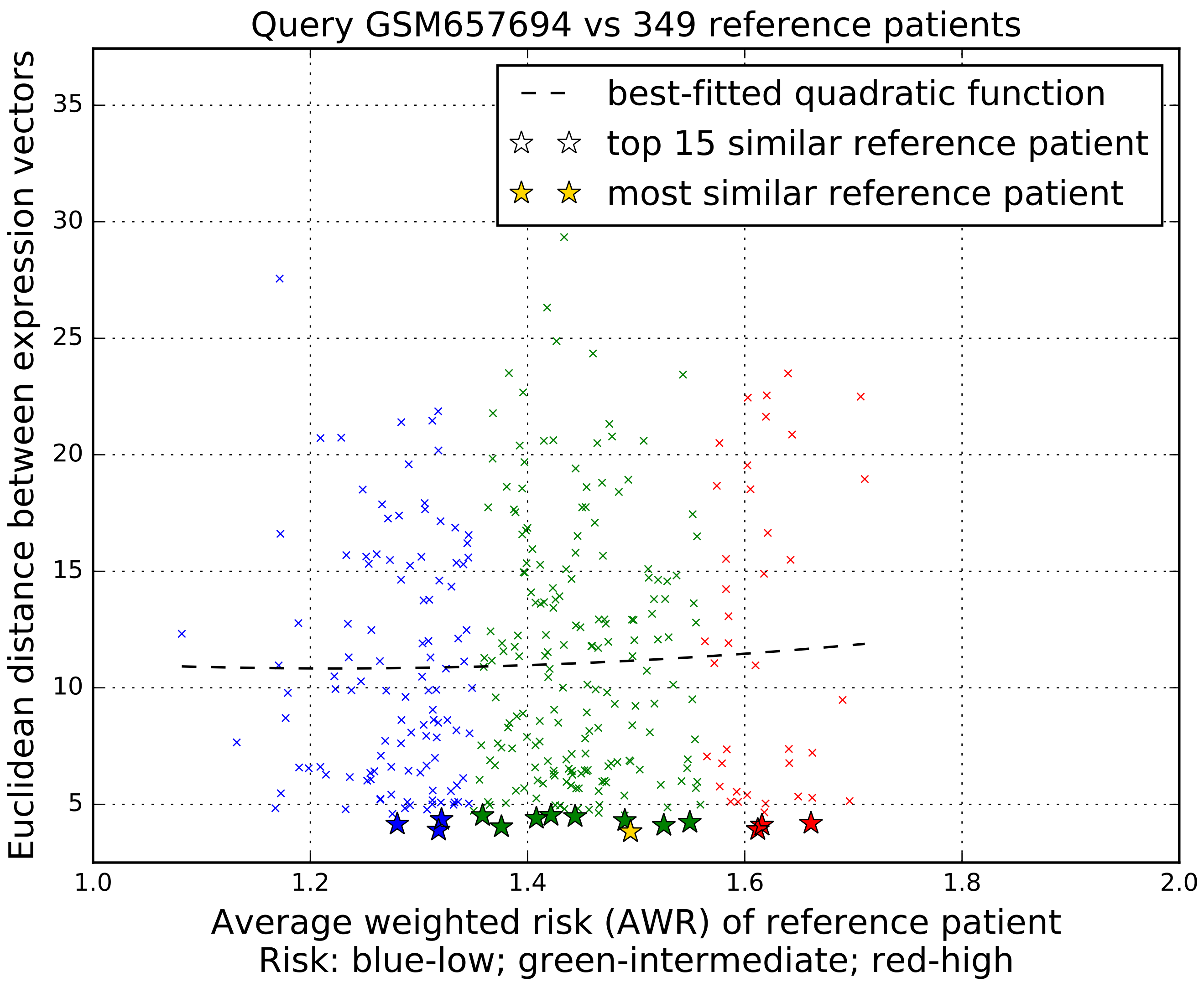
Query GSM657694 vs 349 reference patients
best-fitted quadratic function
top 15 similar reference patient
most similar reference patient
Euclidean distance between expression vectors
35
30
25
20
15
10
5
1.0
1.2
1.4
1.6
1.8
2.0
Average weighted risk (AWR) of reference patient
Risk: blue-low; green-intermediate; red-high

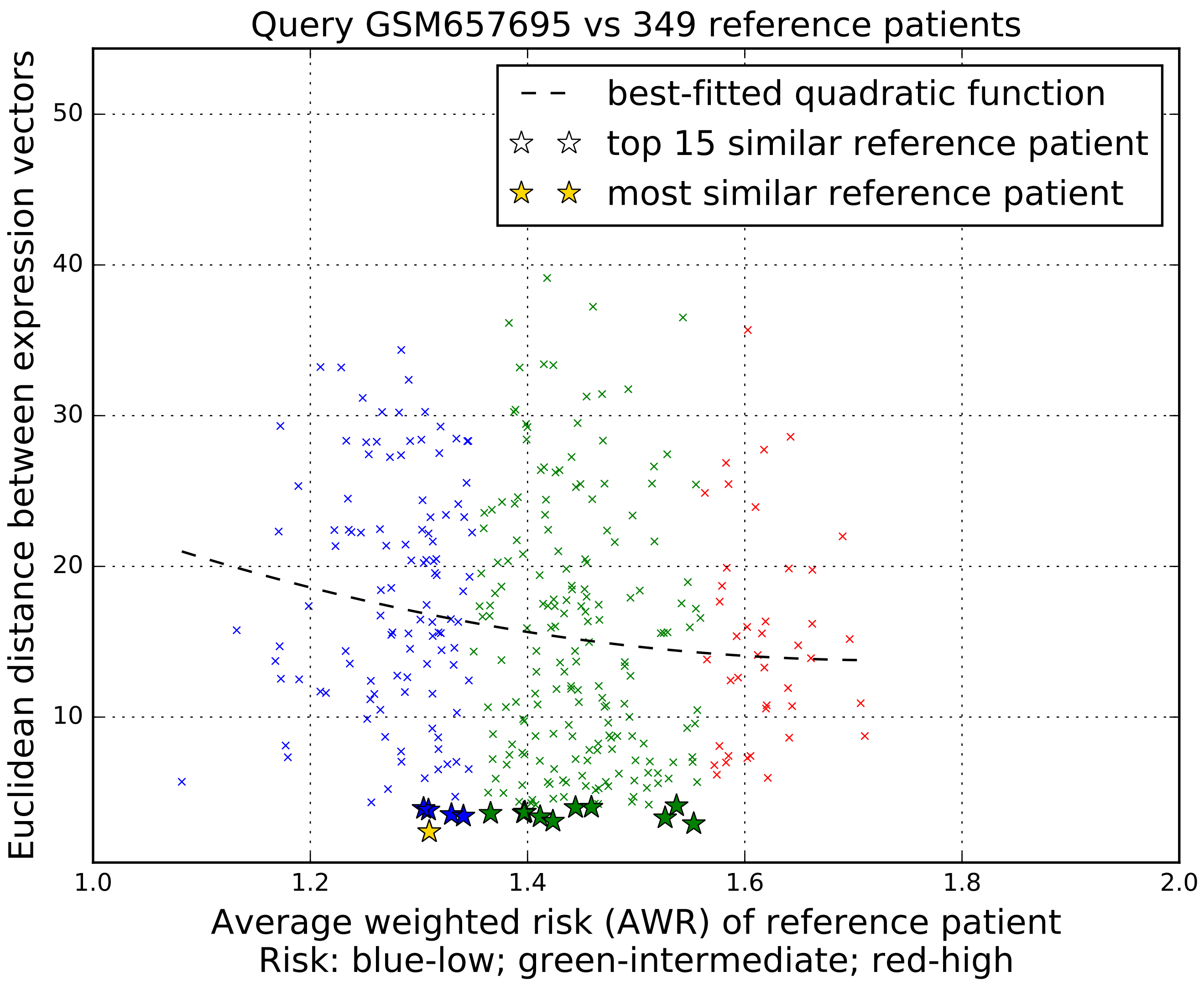

Query GSM657695 vs 349 reference patients
best-fitted quadratic function
top 15 similar reference patient
most similar reference patient
Euclidean distance between expression vectors
50
40
30
20
10
1.0
1.2
1.4
1.6
1.8
2.0
Average weighted risk (AWR) of reference patient
Risk: blue-low; green-intermediate; red-high

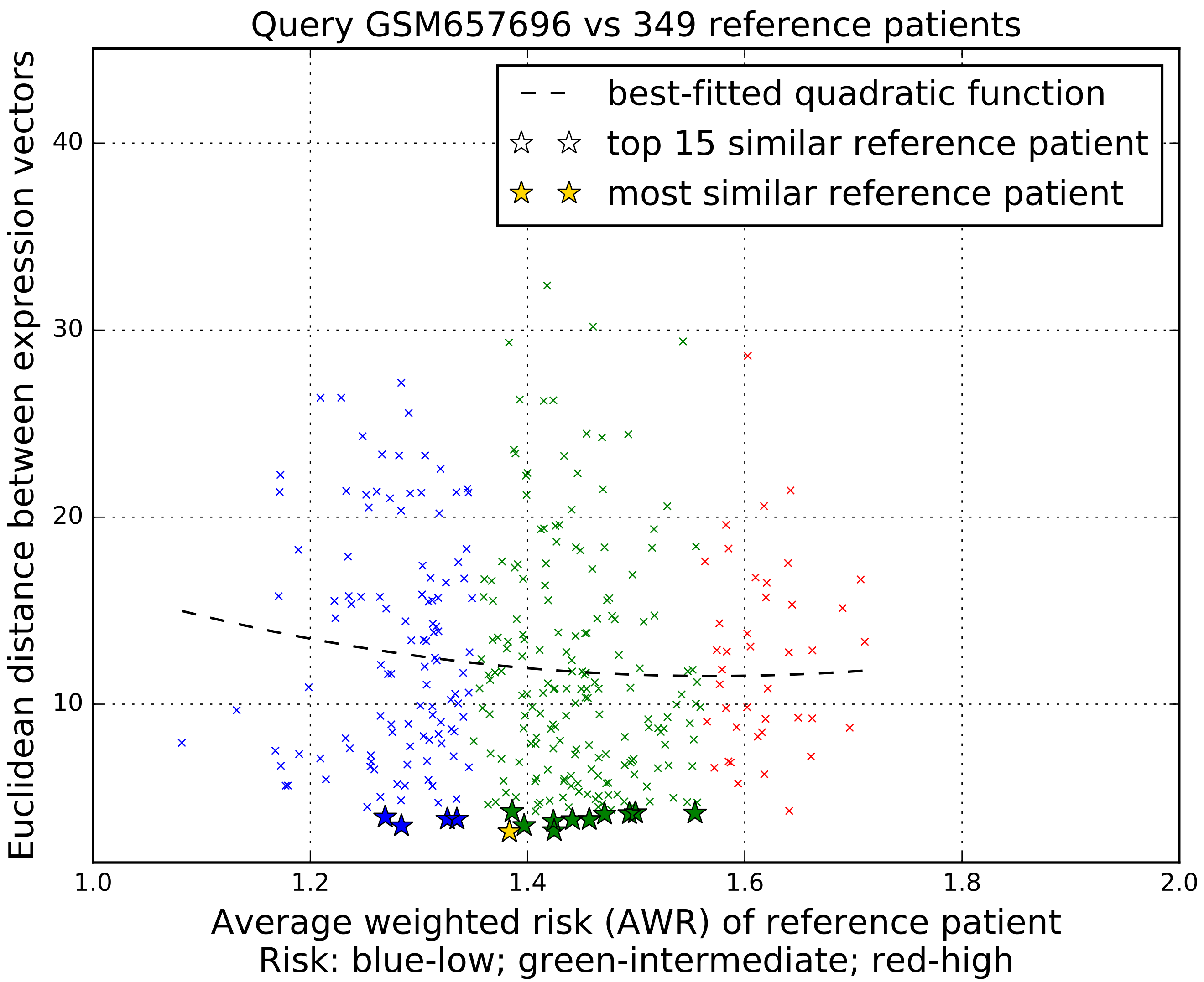

Query GSM657696 vs 349 reference patients
best-fitted quadratic function
top 15 similar reference patient
most similar reference patient
Euclidean distance between expression vectors
10
20
30
40
1.0
1.2
1.4
1.6
1.8
2.0
Average weighted risk (AWR) of reference patient
Risk: blue-low; green-intermediate; red-high

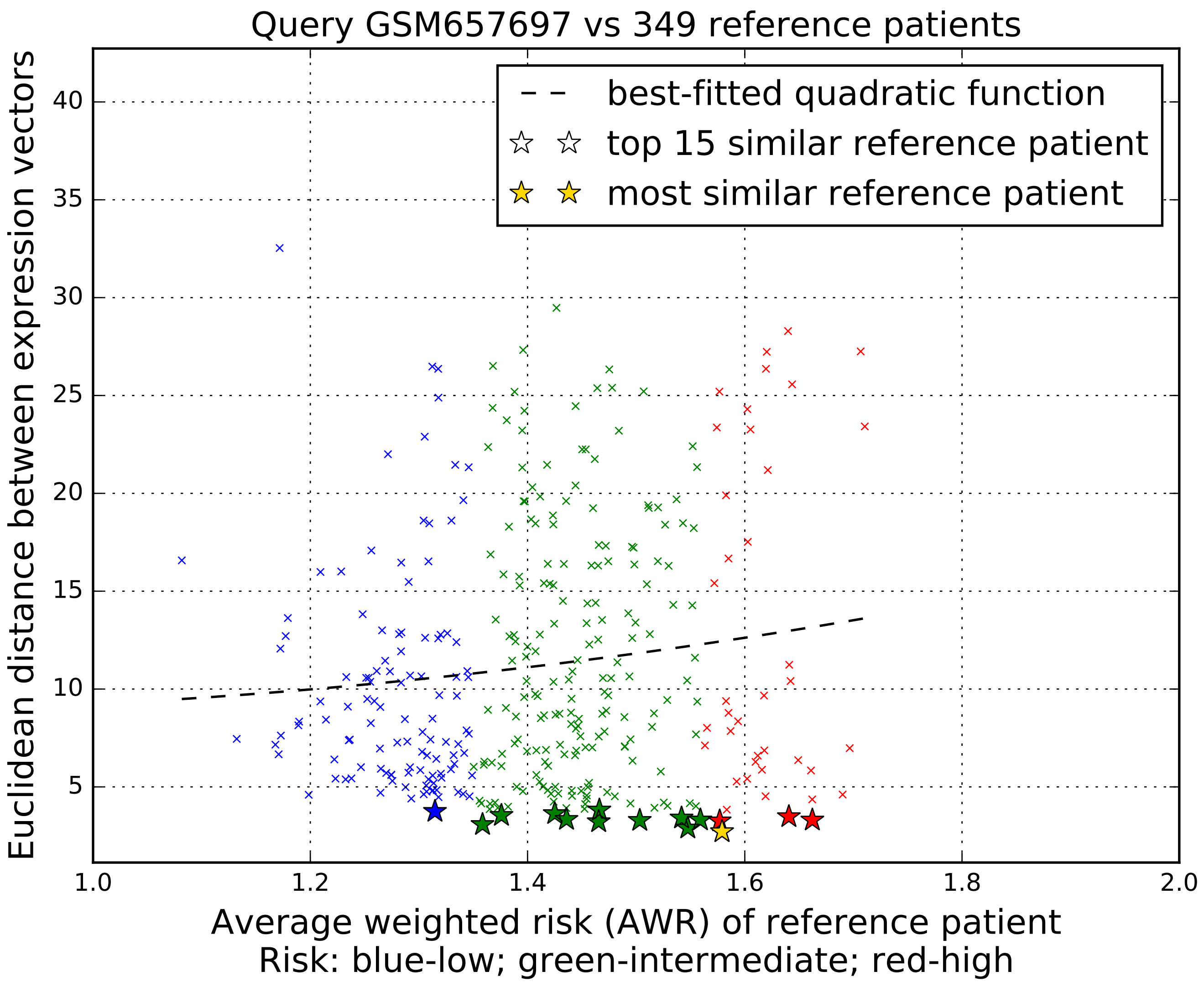

Query GSM657697 vs 349 reference patients
best-fitted quadratic function
top 15 similar reference patient
most similar reference patient
Euclidean distance between expression vectors
40
35
30
25
20
15
10
5
1.0
1.2
1.4
1.6
1.8
2.0
Average weighted risk (AWR) of reference patient
Risk: blue-low; green-intermediate; red-high

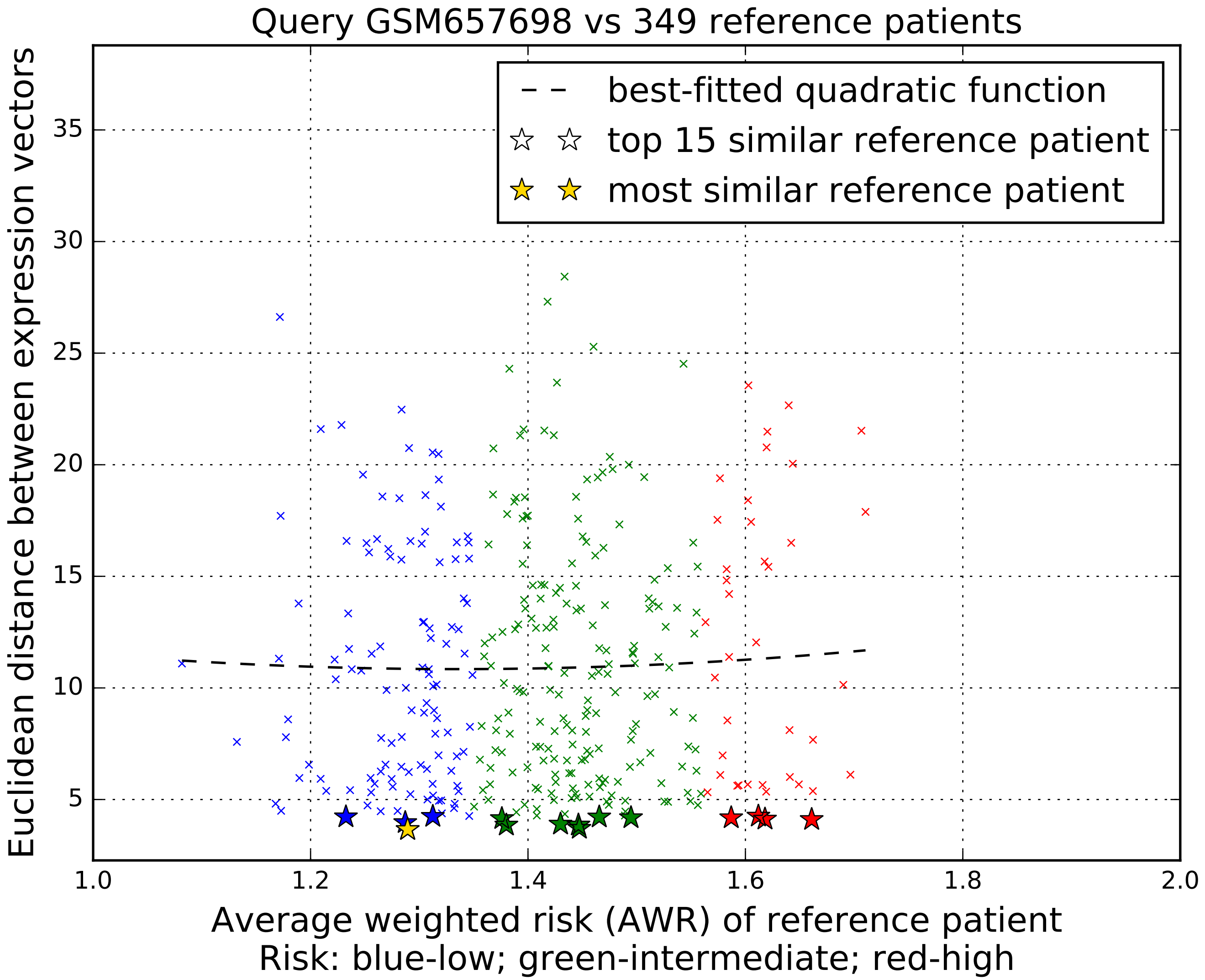
Query GSM657698 vs 349 reference patients
best-fitted quadratic function
top 15 similar reference patient
most similar reference patient
Euclidean distance between expression vectors
35
30
25
20
15
10
5
1.0
1.2
1.4
1.6
1.8
2.0
Average weighted risk (AWR) of reference patient
Risk: blue-low; green-intermediate; red-high

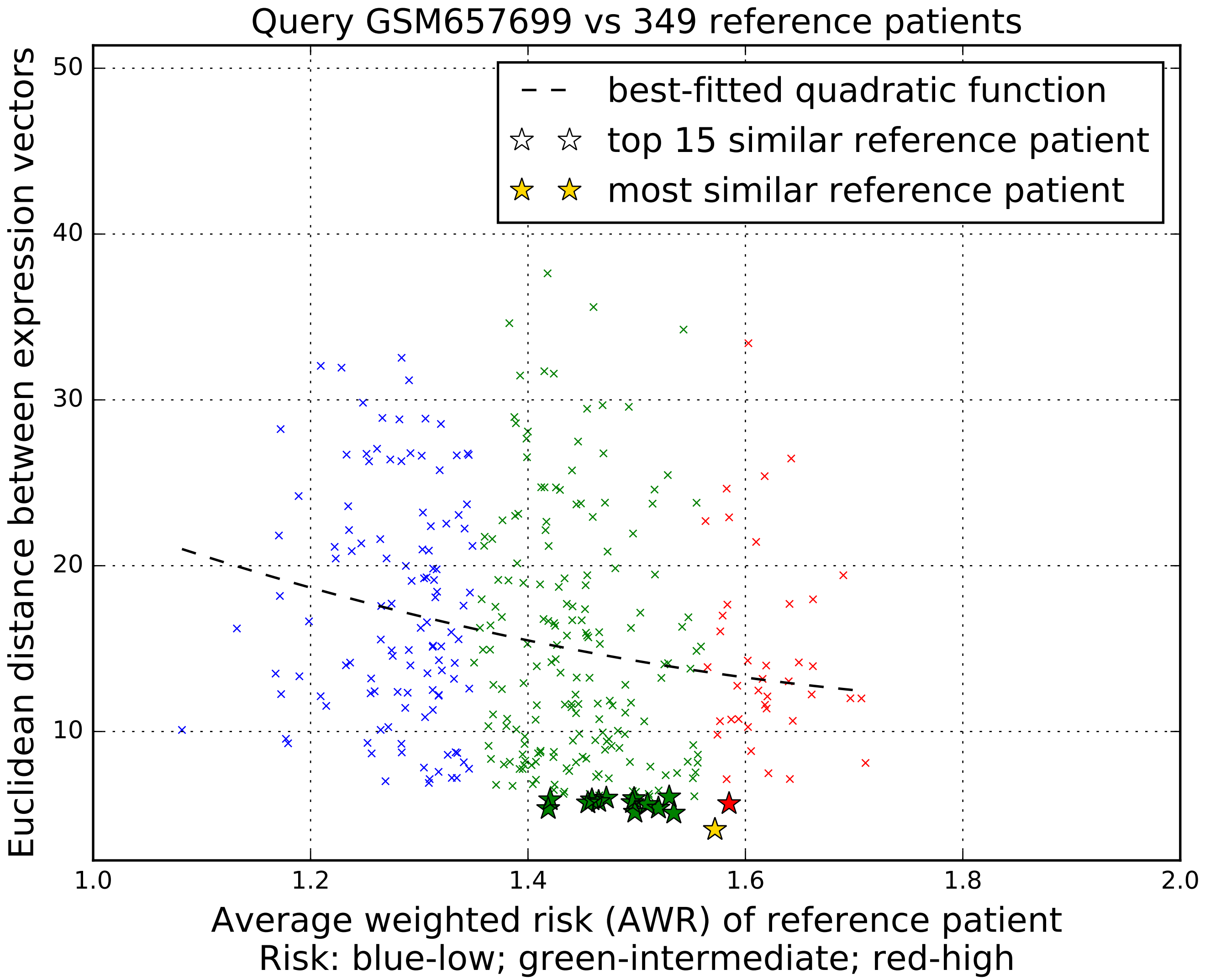
Query GSM657699 vs 349 reference patients
best-fitted quadratic function
top 15 similar reference patient
most similar reference patient
Euclidean distance between expression vectors
50
40
30
20
10
1.0
1.2
1.4
1.6
1.8
2.0
Average weighted risk (AWR) of reference patient
Risk: blue-low; green-intermediate; red-high

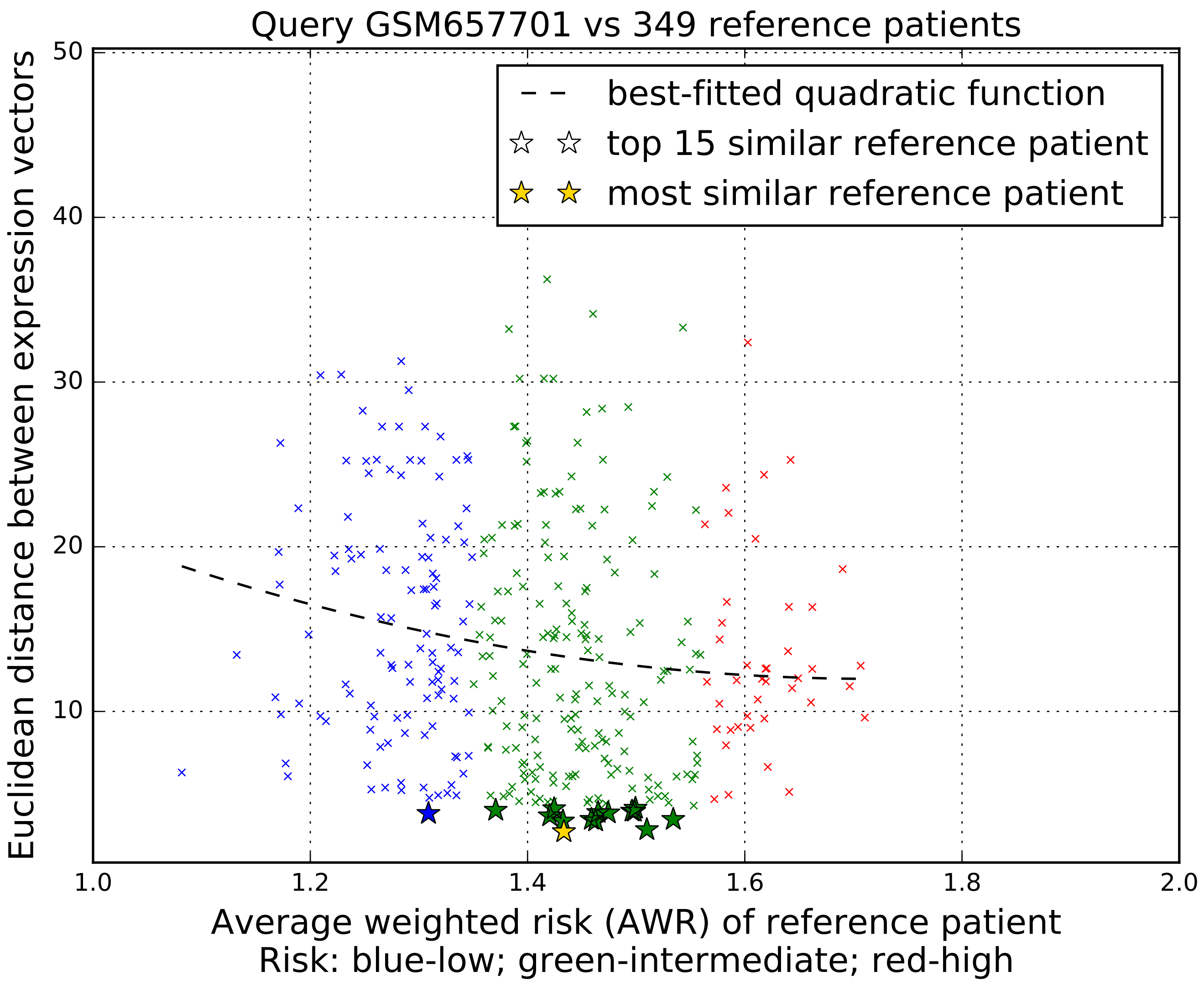
Query GSM657701 vs 349 reference patients
best-fitted quadratic function
top 15 similar reference patient
most similar reference patient
Euclidean distance between expression vectors
Average weighted risk (AWR) of reference patient
Risk: blue-low; green-intermediate; red-high
50
40
30
20
10
1.0
1.2
1.4
1.6
1.8
2.0

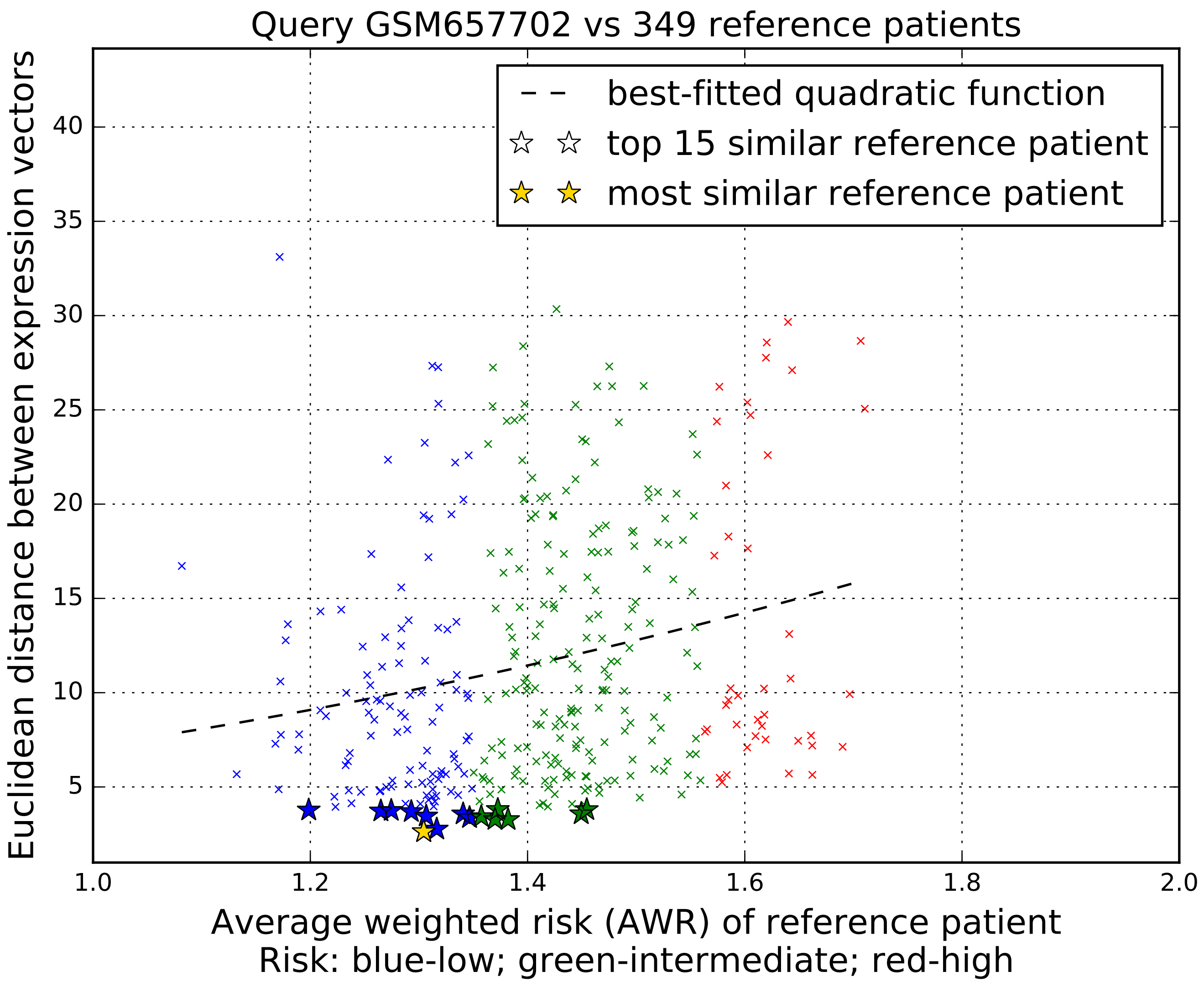
Query GSM657702 vs 349 reference patients
best-fitted quadratic function
top 15 similar reference patient
most similar reference patient
Euclidean distance between expression vectors
Average weighted risk (AWR) of reference patient
Risk: blue-low; green-intermediate; red-high
5
10
15
20
25
30
35
40
1.0
1.2
1.4
1.6
1.8
2.0

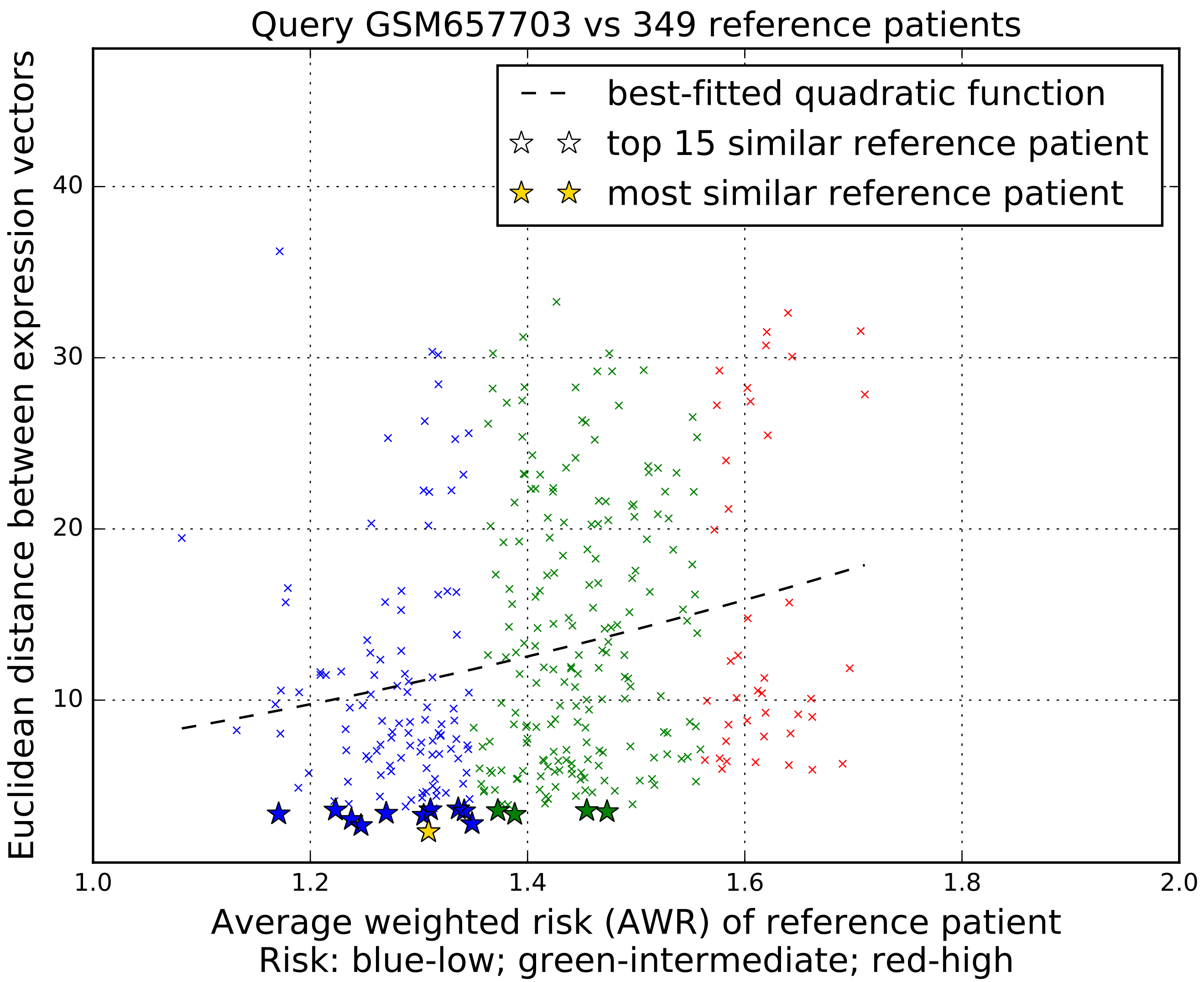
Query GSM657703 vs 349 reference patients
best-fitted quadratic function
top 15 similar reference patient
most similar reference patient
Euclidean distance between expression vectors
40
30
20
10
1.0
1.2
1.4
1.6
1.8
2.0
Average weighted risk (AWR) of reference patient
Risk: blue-low; green-intermediate; red-high

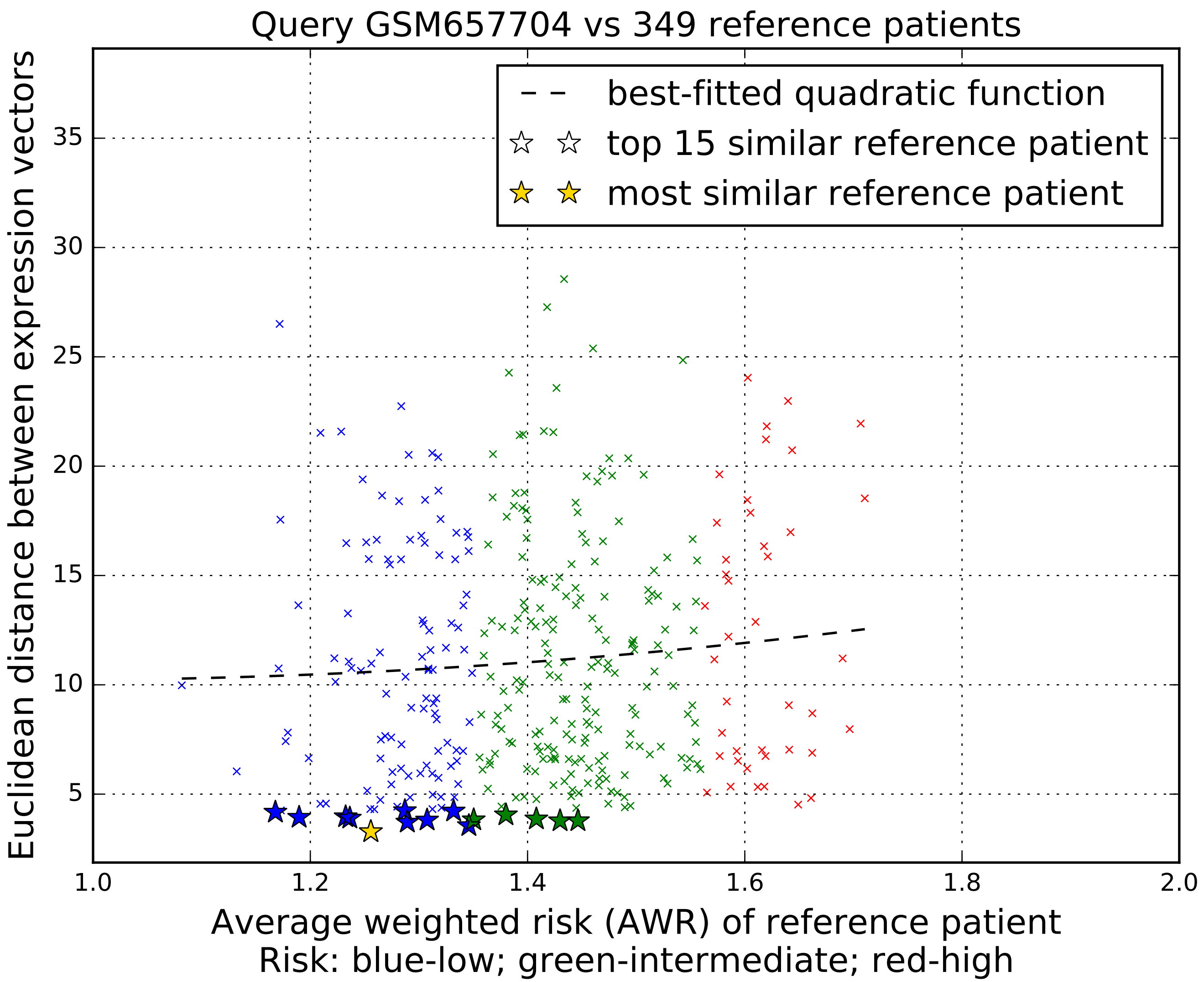

Query GSM657704 vs 349 reference patients
best-fitted quadratic function
top 15 similar reference patient
most similar reference patient
Euclidean distance between expression vectors
35
30
25
20
15
10
5
1.0
1.2
1.4
1.6
1.8
2.0
Average weighted risk (AWR) of reference patient
Risk: blue-low; green-intermediate; red-high

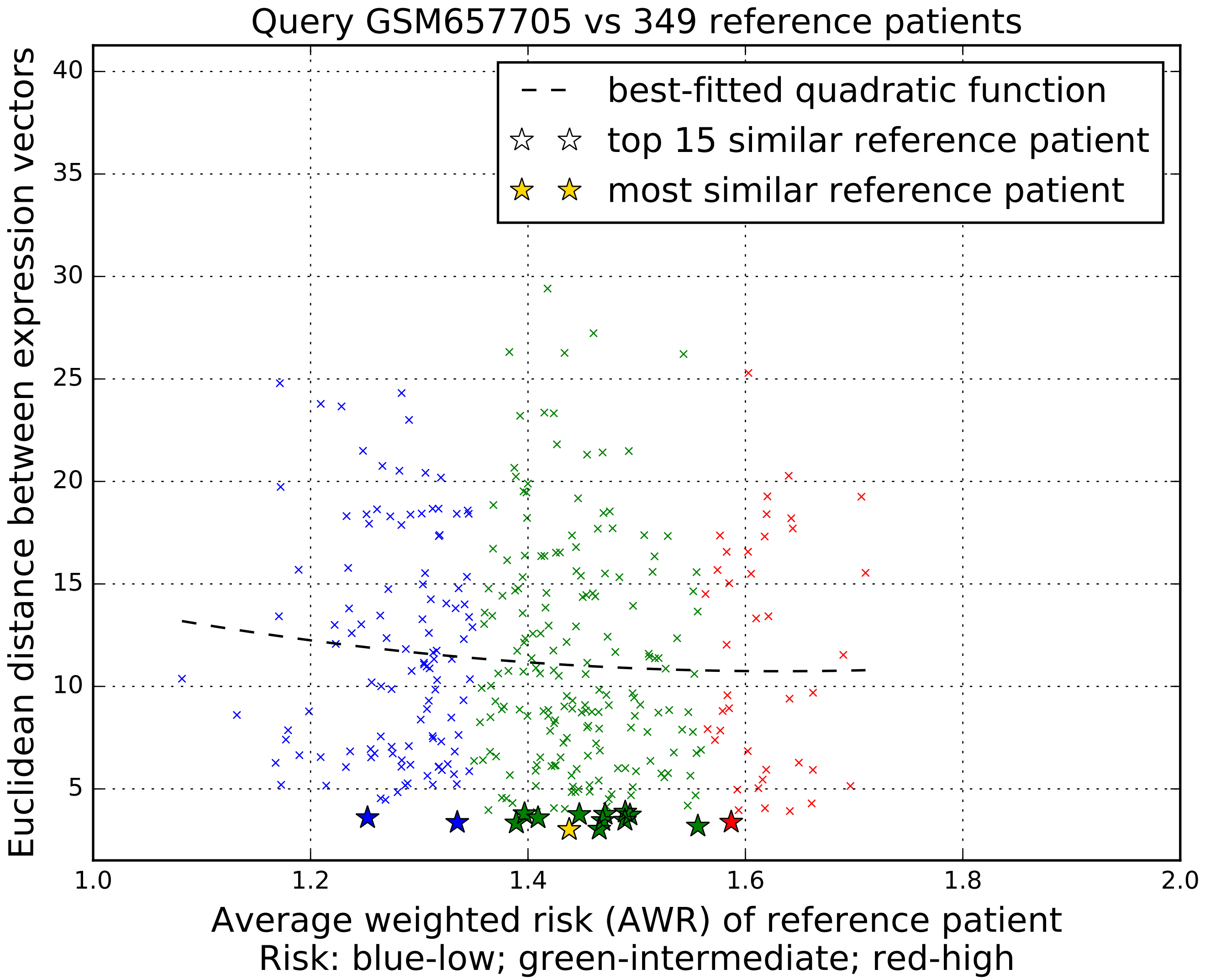
Query GSM657705 vs 349 reference patients
best-fitted quadratic function
top 15 similar reference patient
most similar reference patient
Euclidean distance between expression vectors
40
35
30
25
20
15
10
5
1.0
1.2
1.4
1.6
1.8
2.0
Average weighted risk (AWR) of reference patient
Risk: blue-low; green-intermediate; red-high

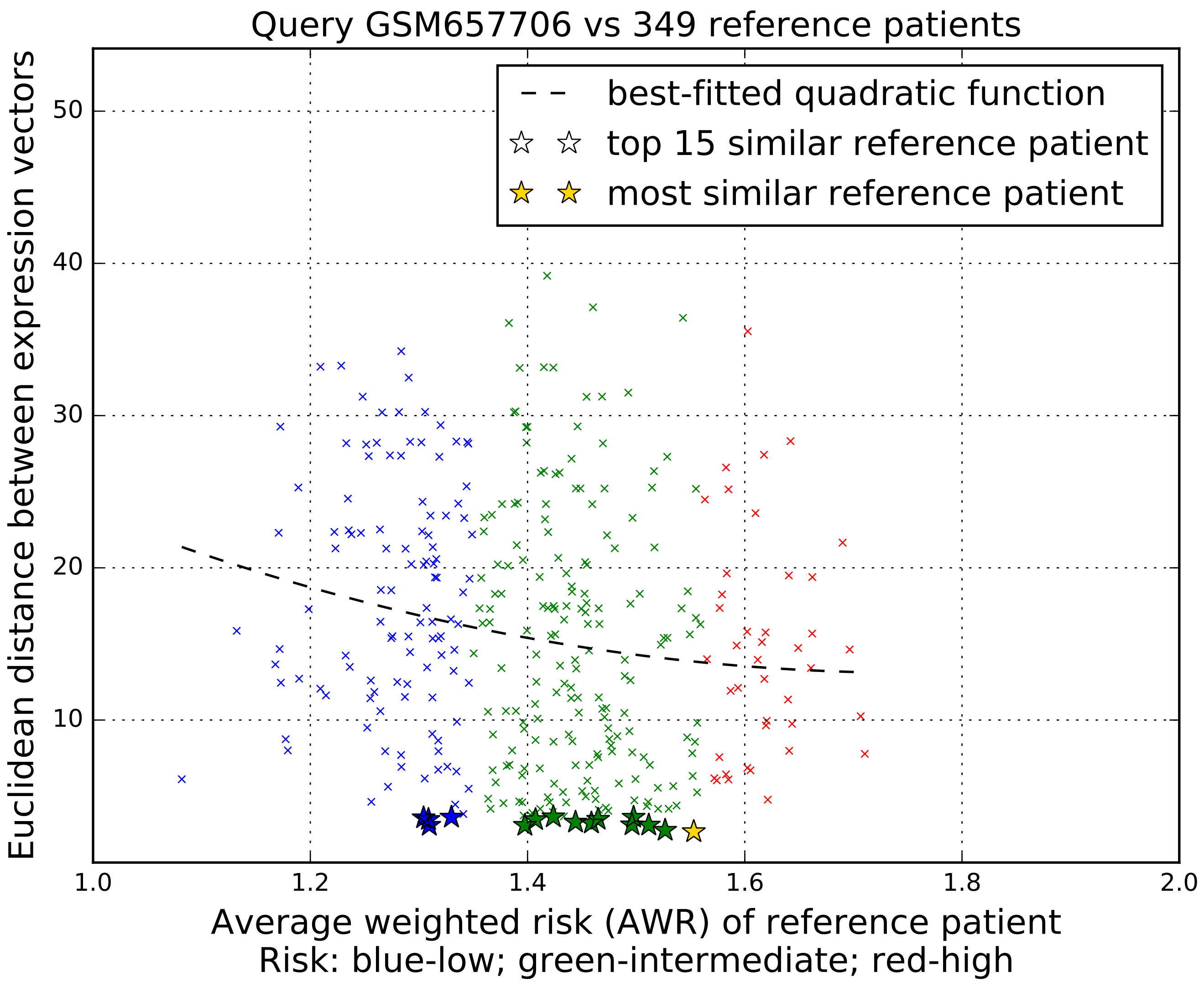

Query GSM657706 vs 349 reference patients
best-fitted quadratic function
top 15 similar reference patient
most similar reference patient
Euclidean distance between expression vectors
50
40
30
20
10
1.0
1.2
1.4
1.6
1.8
2.0
Average weighted risk (AWR) of reference patient
Risk: blue-low; green-intermediate; red-high

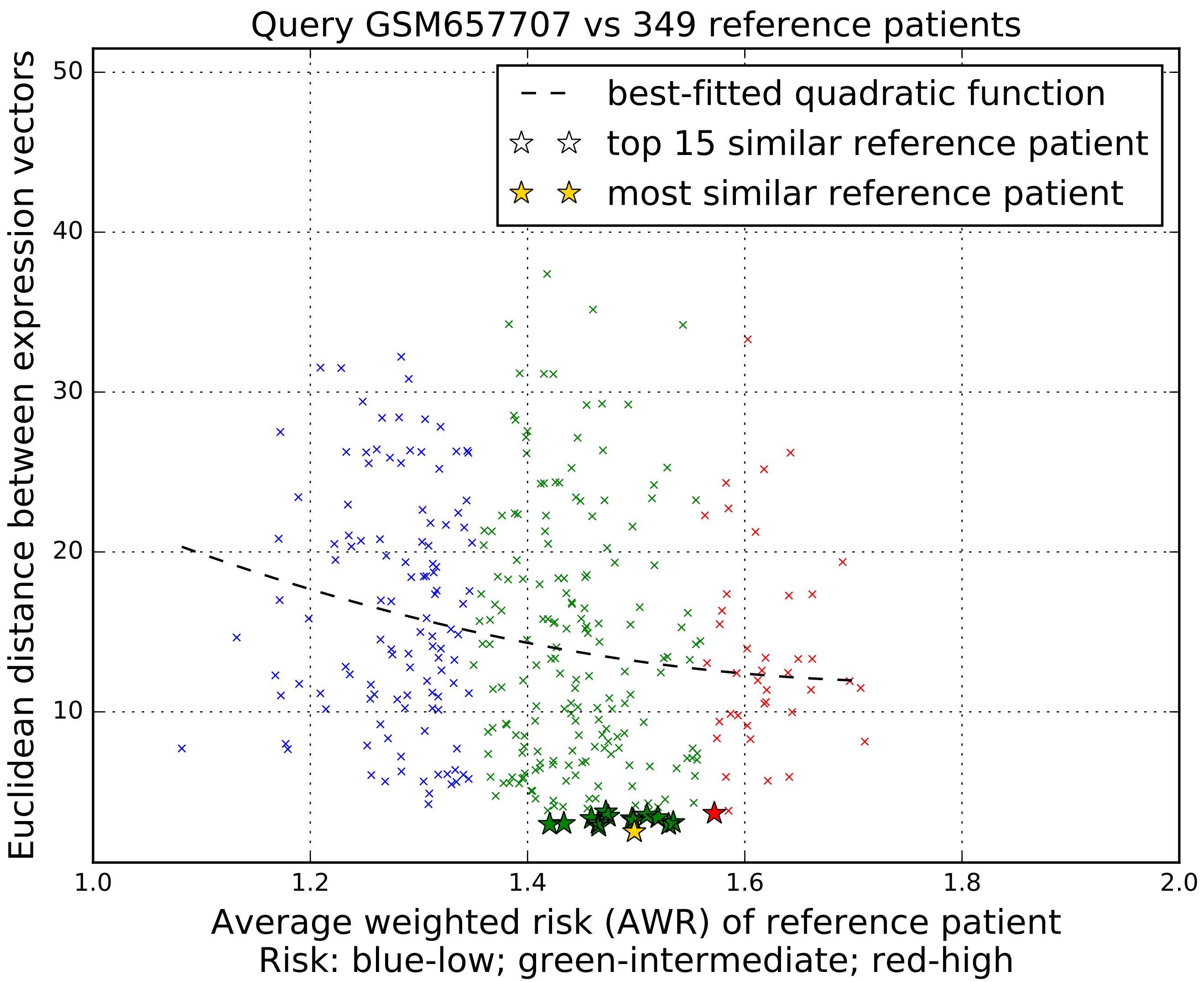

Query GSM657707 vs 349 reference patients
best-fitted quadratic function
top 15 similar reference patient
most similar reference patient
Euclidean distance between expression vectors
Average weighted risk (AWR) of reference patient
Risk: blue-low; green-intermediate; red-high
10
20
30
40
50
1.0
1.2
1.4
1.6
1.8
2.0

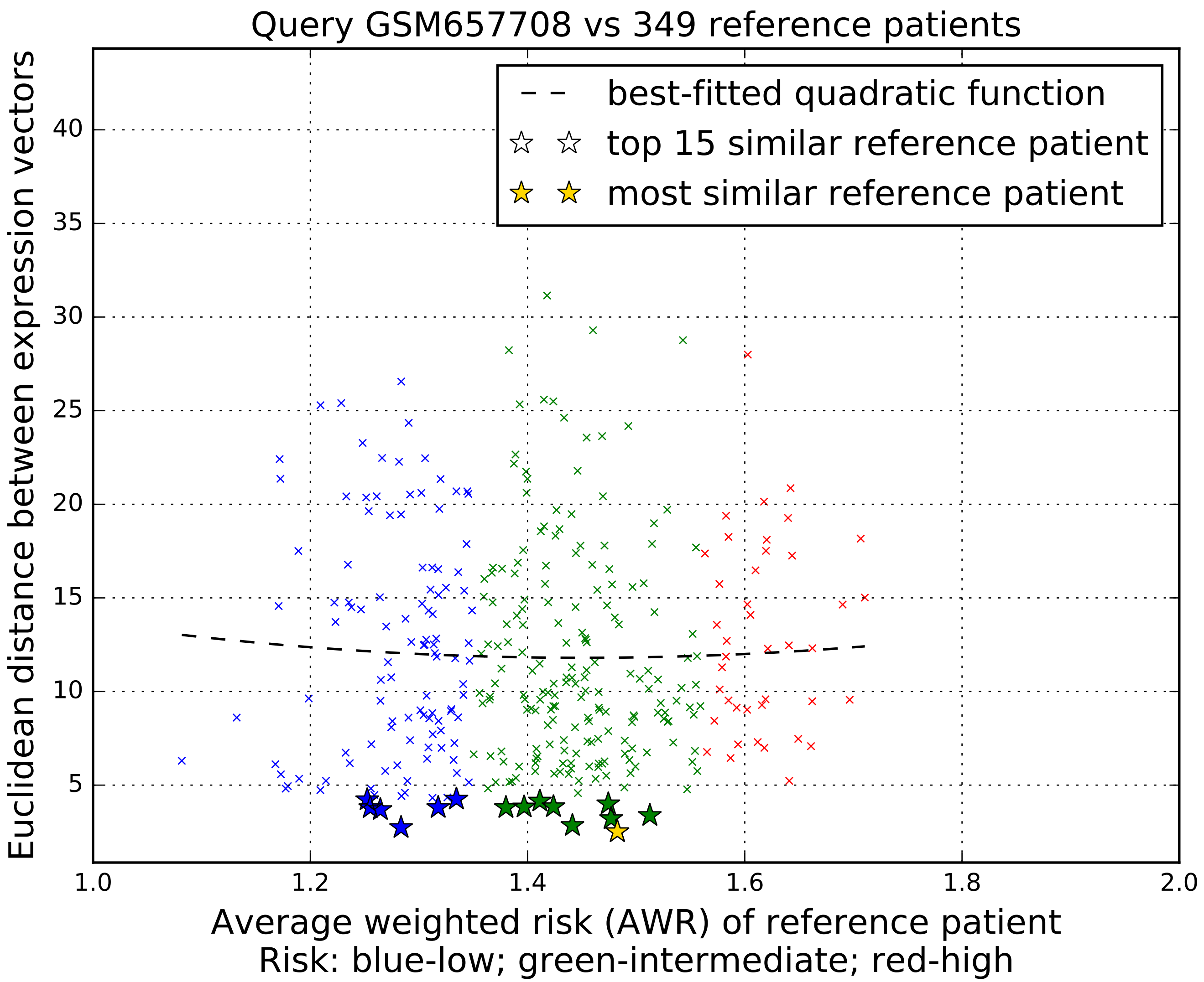
Query GSM657708 vs 349 reference patients
best-fitted quadratic function
top 15 similar reference patient
most similar reference patient
Euclidean distance between expression vectors
40
35
30
25
20
15
10
5
1.0
1.2
1.4
1.6
1.8
2.0
Average weighted risk (AWR) of reference patient
Risk: blue-low; green-intermediate; red-high

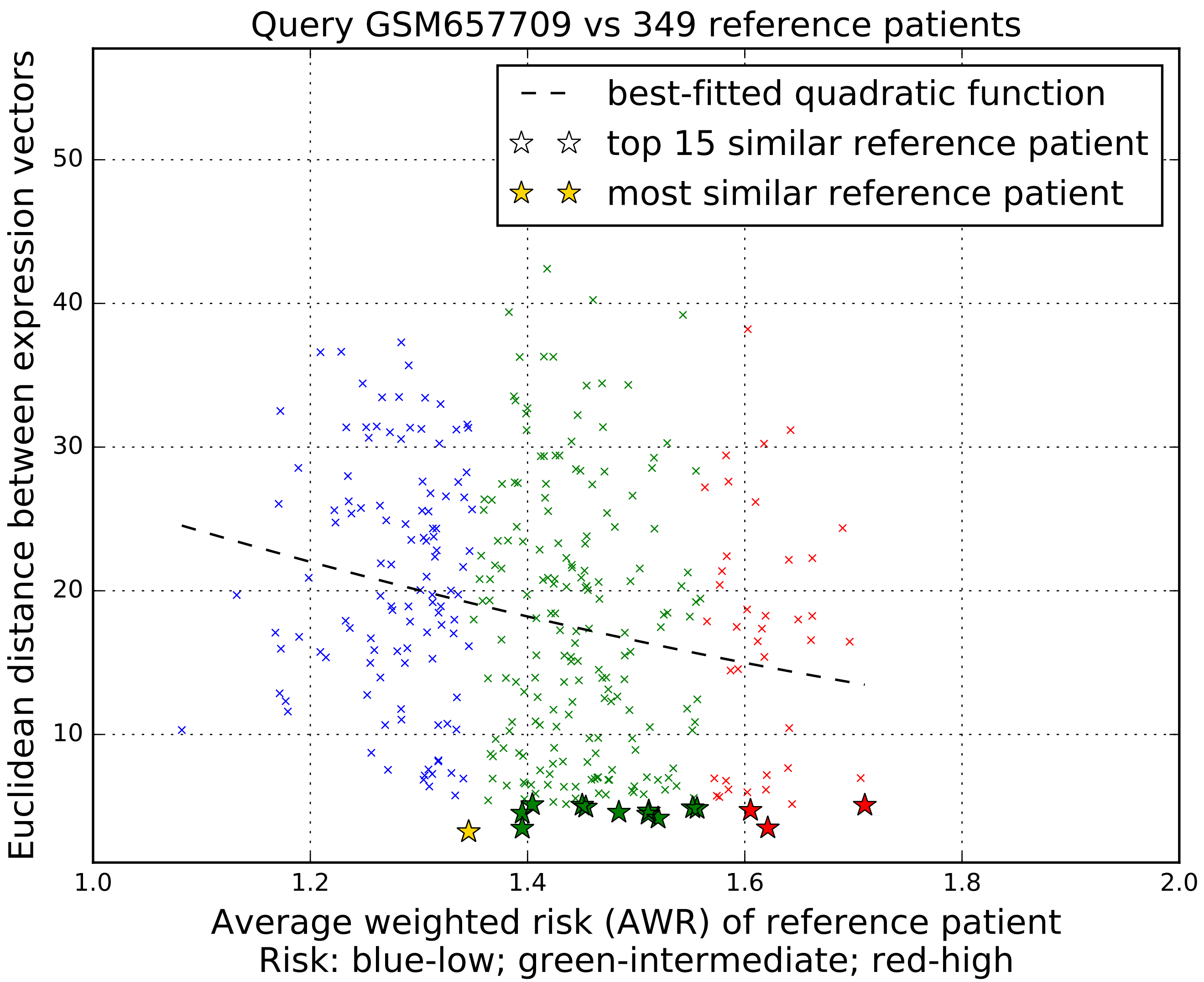
Query GSM657709 vs 349 reference patients
best-fitted quadratic function
top 15 similar reference patient
most similar reference patient
Euclidean distance between expression vectors
10
20
30
40
50
1.0
1.2
1.4
1.6
1.8
2.0
Average weighted risk (AWR) of reference patient
Risk: blue-low; green-intermediate; red-high

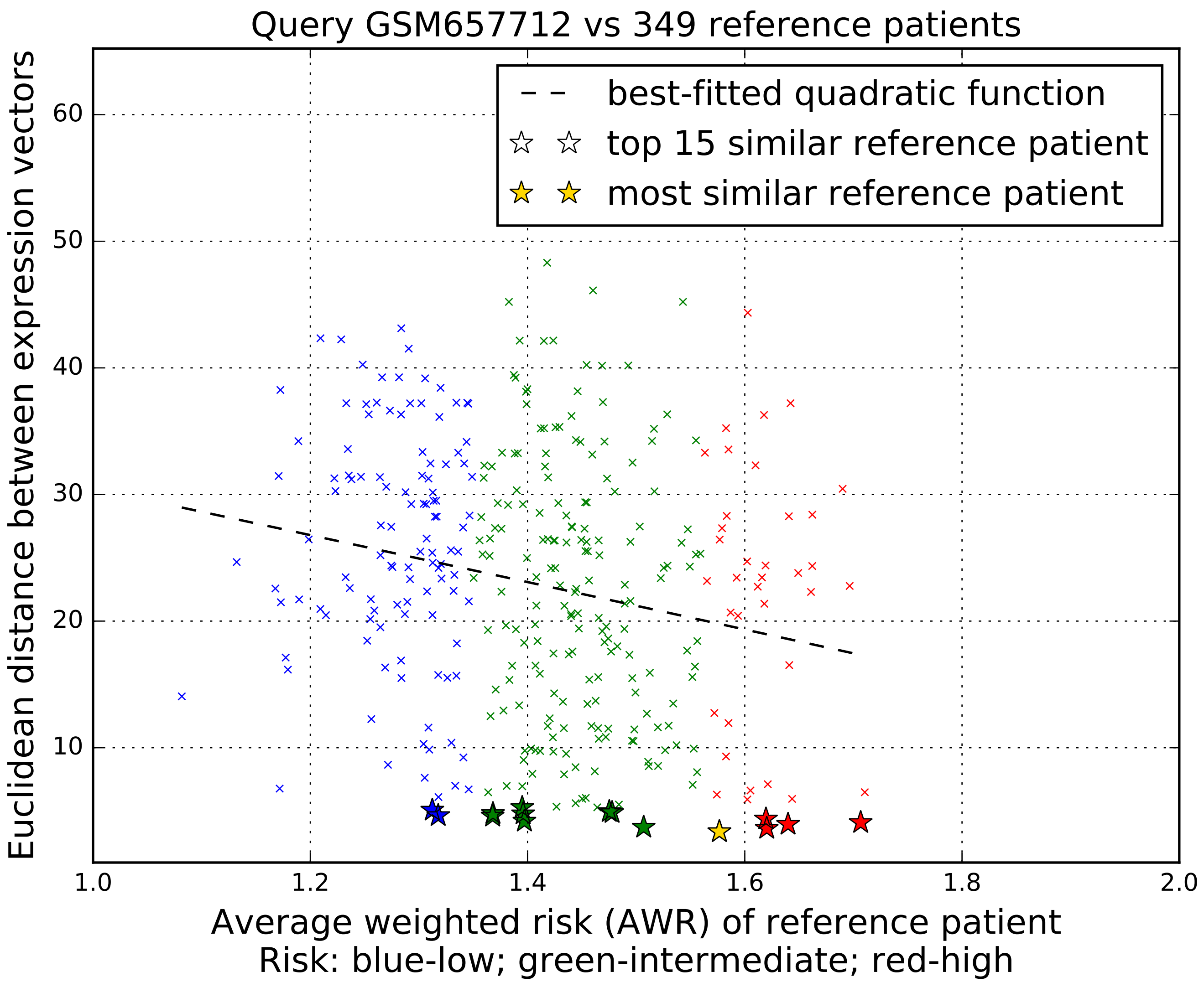
Query GSM657712 vs 349 reference patients
best-fitted quadratic function
top 15 similar reference patient
most similar reference patient
Euclidean distance between expression vectors
60
50
40
30
20
10
1.0
1.2
1.4
1.6
1.8
2.0
Average weighted risk (AWR) of reference patient
Risk: blue-low; green-intermediate; red-high

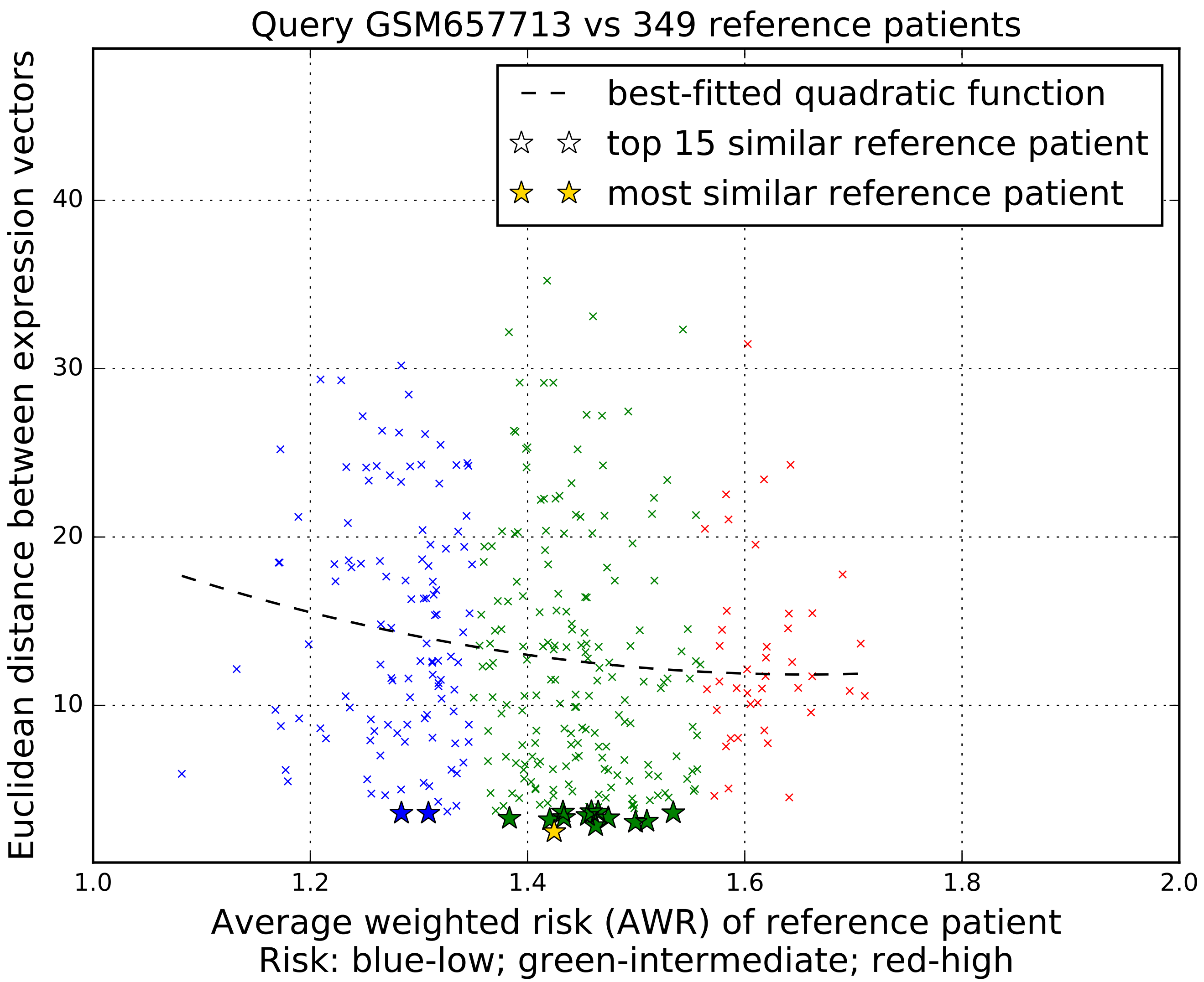

Query GSM657713 vs 349 reference patients
best-fitted quadratic function
top 15 similar reference patient
most similar reference patient
Euclidean distance between expression vectors
10
20
30
40
1.0
1.2
1.4
1.6
1.8
2.0
Average weighted risk (AWR) of reference patient
Risk: blue-low; green-intermediate; red-high
